# Global, regional, and national incidence, prevalence, and years lived with disability for 310 diseases and injuries, 1990–2015: a systematic analysis for the Global Burden of Disease Study 2015

**DOI:** 10.1016/S0140-6736(16)31678-6

**Published:** 2016-10-08

**Authors:** GBD 2015 Disease and Injury Incidence and Prevalence Collaborators, Theo Vos, Christine Allen, Megha Arora, Ryan M Barber, Zulfiqar A Bhutta, Alexandria Brown, Austin Carter, Daniel C Casey, Fiona J Charlson, Alan Z Chen, Megan Coggeshall, Leslie Cornaby, Lalit Dandona, Daniel J Dicker, Tina Dilegge, Holly E Erskine, Alize J Ferrari, Christina Fitzmaurice, Tom Fleming, Mohammad H Forouzanfar, Nancy Fullman, Peter W Gething, Ellen M Goldberg, Nicholas Graetz, Juanita A Haagsma, Catherine O Johnson, Nicholas J Kassebaum, Toana Kawashima, Laura Kemmer, Ibrahim A Khalil, Yohannes Kinfu, Hmwe H Kyu, Janni Leung, Xiaofeng Liang, Stephen S Lim, Alan D Lopez, Rafael Lozano, Laurie Marczak, George A Mensah, Ali H Mokdad, Mohsen Naghavi, Grant Nguyen, Elaine Nsoesie, Helen Olsen, David M Pigott, Christine Pinho, Zane Rankin, Nikolas Reinig, Joshua A Salomon, Logan Sandar, Alison Smith, Jeffrey Stanaway, Caitlyn Steiner, Stephanie Teeple, Bernadette A Thomas, Christopher Troeger, Joseph A Wagner, Haidong Wang, Valentine Wanga, Harvey A Whiteford, Leo Zoeckler, Amanuel Alemu Abajobir, Kalkidan Hassen Abate, Cristiana Abbafati, Kaja M Abbas, Foad Abd-Allah, Biju Abraham, Ibrahim Abubakar, Laith J Abu-Raddad, Niveen M E Abu-Rmeileh, Ilana N Ackerman, Akindele Olupelumi Adebiyi, Zanfina Ademi, Arsène Kouablan Adou, Kossivi Agbelenko Afanvi, Emilie Elisabet Agardh, Arnav Agarwal, Aliasghar Ahmad Kiadaliri, Hamid Ahmadieh, Oluremi N Ajala, Rufus Olusola Akinyemi, Nadia Akseer, Ziyad Al-Aly, Khurshid Alam, Noore K M Alam, Saleh Fahed Aldhahri, Miguel Angel Alegretti, Zewdie Aderaw Alemu, Lily T Alexander, Samia Alhabib, Raghib Ali, Ala'a Alkerwi, François Alla, Peter Allebeck, Rajaa Al-Raddadi, Ubai Alsharif, Khalid A Altirkawi, Nelson Alvis-Guzman, Azmeraw T Amare, Alemayehu Amberbir, Heresh Amini, Walid Ammar, Stephen Marc Amrock, Hjalte H Andersen, Gregory M Anderson, Benjamin O Anderson, Carl Abelardo T Antonio, Atsede Fantahun Aregay, Johan Ärnlöv, Al Artaman, Hamid Asayesh, Reza Assadi, Suleman Atique, Euripide Frinel G Arthur Avokpaho, Ashish Awasthi, Beatriz Paulina Ayala Quintanilla, Peter Azzopardi, Umar Bacha, Alaa Badawi, Kalpana Balakrishnan, Amitava Banerjee, Aleksandra Barac, Suzanne L Barker-Collo, Till Bärnighausen, Lars Barregard, Lope H Barrero, Arindam Basu, Shahrzad Bazargan-Hejazi, Brent Bell, Michelle L Bell, Derrick A Bennett, Isabela M Bensenor, Habib Benzian, Adugnaw Berhane, Eduardo Bernabé, Balem Demtsu Betsu, Addisu Shunu Beyene, Neeraj Bhala, Samir Bhatt, Sibhatu Biadgilign, Kelly Bienhoff, Boris Bikbov, Stan Biryukov, Donal Bisanzio, Espen Bjertness, Jed Blore, Rohan Borschmann, Soufiane Boufous, Michael Brainin, Alexandra Brazinova, Nicholas J K Breitborde, Jonathan Brown, Rachelle Buchbinder, Geoffrey Colin Buckle, Zahid A Butt, Bianca Calabria, Ismael Ricardo Campos-Nonato, Julio Cesar Campuzano, Hélène Carabin, Rosario Cárdenas, David O Carpenter, Juan Jesus Carrero, Carlos A Castañeda-Orjuela, Jacqueline Castillo Rivas, Ferrán Catalá-López, Jung-Chen Chang, Peggy Pei-Chia Chiang, Chioma Ezinne Chibueze, Vesper Hichilombwe Chisumpa, Jee-Young Jasmine Choi, Rajiv Chowdhury, Hanne Christensen, Devasahayam Jesudas Christopher, Liliana G Ciobanu, Massimo Cirillo, Matthew M Coates, Samantha M Colquhoun, Cyrus Cooper, Monica Cortinovis, John A Crump, Solomon Abrha Damtew, Rakhi Dandona, Farah Daoud, Paul I Dargan, José das Neves, Gail Davey, Adrian C Davis, Diego De Leo, Louisa Degenhardt, Liana C Del Gobbo, Robert P Dellavalle, Kebede Deribe, Amare Deribew, Sarah Derrett, Don C Des Jarlais, Samath D Dharmaratne, Preet K Dhillon, Cesar Diaz-Torné, Eric L Ding, Tim R Driscoll, Leilei Duan, Manisha Dubey, Bruce Bartholow Duncan, Hedyeh Ebrahimi, Richard G Ellenbogen, Iqbal Elyazar, Matthias Endres, Aman Yesuf Endries, Sergey Petrovich Ermakov, Babak Eshrati, Kara Estep, Talha A Farid, Carla Sofia e Sa Farinha, André Faro, Maryam S Farvid, Farshad Farzadfar, Valery L Feigin, David T Felson, Seyed-Mohammad Fereshtehnejad, Jefferson G Fernandes, Joao C Fernandes, Florian Fischer, Joseph R A Fitchett, Kyle Foreman, F Gerry R Fowkes, Jordan Fox, Richard C Franklin, Joseph Friedman, Joseph Frostad, Thomas Fürst, Neal D Futran, Belinda Gabbe, Parthasarathi Ganguly, Fortuné Gbètoho Gankpé, Teshome Gebre, Tsegaye Tewelde Gebrehiwot, Amanuel Tesfay Gebremedhin, Johanna M Geleijnse, Bradford D Gessner, Katherine B Gibney, Ibrahim Abdelmageem Mohamed Ginawi, Ababi Zergaw Giref, Maurice Giroud, Melkamu Dedefo Gishu, Elizabeth Glaser, William W Godwin, Hector Gomez-Dantes, Philimon Gona, Amador Goodridge, Sameer Vali Gopalani, Carolyn C Gotay, Atsushi Goto, Hebe N Gouda, Rebecca Grainger, Felix Greaves, Francis Guillemin, Yuming Guo, Rahul Gupta, Rajeev Gupta, Vipin Gupta, Reyna A Gutiérrez, Demewoz Haile, Alemayehu Desalegne Hailu, Gessessew Bugssa Hailu, Yara A Halasa, Randah Ribhi Hamadeh, Samer Hamidi, Mouhanad Hammami, Jamie Hancock, Alexis J Handal, Graeme J Hankey, Yuantao Hao, Hilda L Harb, Sivadasanpillai Harikrishnan, Josep Maria Haro, Rasmus Havmoeller, Roderick J Hay, Ileana Beatriz Heredia-Pi, Pouria Heydarpour, Hans W Hoek, Masako Horino, Nobuyuki Horita, H Dean Hosgood, Damian G Hoy, Aung Soe Htet, Hsiang Huang, John J Huang, Chantal Huynh, Marissa Iannarone, Kim Moesgaard Iburg, Kaire Innos, Manami Inoue, Veena J Iyer, Kathryn H Jacobsen, Nader Jahanmehr, Mihajlo B Jakovljevic, Mehdi Javanbakht, Achala Upendra Jayatilleke, Sun Ha Jee, Panniyammakal Jeemon, Paul N Jensen, Ying Jiang, Tariku Jibat, Aida Jimenez-Corona, Ye Jin, Jost B Jonas, Zubair Kabir, Yogeshwar Kalkonde, Ritul Kamal, Haidong Kan, André Karch, Corine Kakizi Karema, Chante Karimkhani, Amir Kasaeian, Anil Kaul, Norito Kawakami, Peter Njenga Keiyoro, Andrew Haddon Kemp, Andre Keren, Chandrasekharan Nair Kesavachandran, Yousef Saleh Khader, Abdur Rahman Khan, Ejaz Ahmad Khan, Young-Ho Khang, Sahil Khera, Tawfik Ahmed Muthafer Khoja, Jagdish Khubchandani, Christian Kieling, Pauline Kim, Cho-il Kim, Daniel Kim, Yun Jin Kim, Niranjan Kissoon, Luke D Knibbs, Ann Kristin Knudsen, Yoshihiro Kokubo, Dhaval Kolte, Jacek A Kopec, Soewarta Kosen, Georgios A Kotsakis, Parvaiz A Koul, Ai Koyanagi, Michael Kravchenko, Barthelemy Kuate Defo, Burcu Kucuk Bicer, Andreas A Kudom, Ernst J Kuipers, G Anil Kumar, Michael Kutz, Gene F Kwan, Aparna Lal, Ratilal Lalloo, Tea Lallukka, Hilton Lam, Jennifer O Lam, Sinead M Langan, Anders Larsson, Pablo M Lavados, Janet L Leasher, James Leigh, Ricky Leung, Miriam Levi, Yichong Li, Yongmei Li, Juan Liang, Shiwei Liu, Yang Liu, Belinda K Lloyd, Warren D Lo, Giancarlo Logroscino, Katharine J Looker, Paulo A Lotufo, Raimundas Lunevicius, Ronan A Lyons, Mark T Mackay, Mohammed Magdy, Abd El Razek, Mahdi Mahdavi, Marek Majdan, Azeem Majeed, Reza Malekzadeh, Wagner Marcenes, David Joel Margolis, Jose Martinez-Raga, Felix Masiye, João Massano, Stephen Theodore McGarvey, John J McGrath, Martin McKee, Brian J McMahon, Peter A Meaney, Alem Mehari, Fabiola Mejia-Rodriguez, Alemayehu B Mekonnen, Yohannes Adama Melaku, Peter Memiah, Ziad A Memish, Walter Mendoza, Atte Meretoja, Tuomo J Meretoja, Francis Apolinary Mhimbira, Ted R Miller, Edward J Mills, Mojde Mirarefin, Philip B Mitchell, Charles N Mock, Alireza Mohammadi, Shafiu Mohammed, Lorenzo Monasta, Julio Cesar Montañez Hernandez, Marcella Montico, Meghan D Mooney, Maziar Moradi-Lakeh, Lidia Morawska, Ulrich O Mueller, Erin Mullany, John Everett Mumford, Michele E Murdoch, Jean B Nachega, Gabriele Nagel, Aliya Naheed, Luigi Naldi, Vinay Nangia, John N Newton, Marie Ng, Frida Namnyak Ngalesoni, Quyen Le Nguyen, Muhammad Imran Nisar, Patrick Martial Nkamedjie Pete, Joan M Nolla, Ole F Norheim, Rosana E Norman, Bo Norrving, Bruno P Nunes, Felix Akpojene Ogbo, In-Hwan Oh, Takayoshi Ohkubo, Pedro R Olivares, Bolajoko Olubukunola Olusanya, Jacob Olusegun Olusanya, Alberto Ortiz, Majdi Osman, Erika Ota, Mahesh PA, Eun-Kee Park, Mahboubeh Parsaeian, Valéria Maria de Azeredo Passos, Angel J Paternina Caicedo, Scott B Patten, George C Patton, David M Pereira, Rogelio Perez-Padilla, Norberto Perico, Konrad Pesudovs, Max Petzold, Michael Robert Phillips, Frédéric B Piel, Julian David Pillay, Farhad Pishgar, Dietrich Plass, James A Platts-Mills, Suzanne Polinder, Constance D Pond, Svetlana Popova, Richie G Poulton, Farshad Pourmalek, Dorairaj Prabhakaran, Noela M Prasad, Mostafa Qorbani, Rynaz H S Rabiee, Amir Radfar, Anwar Rafay, Kazem Rahimi, Vafa Rahimi-Movaghar, Mahfuzar Rahman, Mohammad Hifz Ur Rahman, Sajjad Ur Rahman, Rajesh Kumar Rai, Sasa Rajsic, Usha Ram, Puja Rao, Amany H Refaat, Marissa B Reitsma, Giuseppe Remuzzi, Serge Resnikoff, Alex Reynolds, Antonio L Ribeiro, Maria Jesus Rios Blancas, Hirbo Shore Roba, David Rojas-Rueda, Luca Ronfani, Gholamreza Roshandel, Gregory A Roth, Dietrich Rothenbacher, Ambuj Roy, Rajesh Sagar, Ramesh Sahathevan, Juan R Sanabria, Maria Dolores Sanchez-Niño, Itamar S Santos, João Vasco Santos, Rodrigo Sarmiento-Suarez, Benn Sartorius, Maheswar Satpathy, Miloje Savic, Monika Sawhney, Michael P Schaub, Maria Inês Schmidt, Ione J C Schneider, Ben Schöttker, David C Schwebel, James G Scott, Soraya Seedat, Sadaf G Sepanlou, Edson E Servan-Mori, Katya A Shackelford, Amira Shaheen, Masood Ali Shaikh, Rajesh Sharma, Upasana Sharma, Jiabin Shen, Donald S Shepard, Kevin N Sheth, Kenji Shibuya, Min-Jeong Shin, Rahman Shiri, Ivy Shiue, Mark G Shrime, Inga Dora Sigfusdottir, Diego Augusto Santos Silva, Dayane Gabriele Alves Silveira, Abhishek Singh, Jasvinder A Singh, Om Prakash Singh, Prashant Kumar Singh, Anna Sivonda, Vegard Skirbekk, Jens Christoffer Skogen, Amber Sligar, Karen Sliwa, Michael Soljak, Kjetil Søreide, Joan B Soriano, Luciano A Sposato, Chandrashekhar T Sreeramareddy, Vasiliki Stathopoulou, Nicholas Steel, Dan J Stein, Timothy J Steiner, Sabine Steinke, Lars Stovner, Konstantinos Stroumpoulis, Bruno F Sunguya, Patrick Sur, Soumya Swaminathan, Bryan L Sykes, Cassandra E I Szoeke, Rafael Tabarés-Seisdedos, Jukka S Takala, Nikhil Tandon, David Tanne, Mohammad Tavakkoli, Bineyam Taye, Hugh R Taylor, Braden J Te Ao, Bemnet Amare Tedla, Abdullah Sulieman Terkawi, Alan J Thomson, Andrew L Thorne-Lyman, Amanda G Thrift, George D Thurston, Ruoyan Tobe-Gai, Marcello Tonelli, Roman Topor-Madry, Fotis Topouzis, Bach Xuan Tran, Zacharie Tsala Dimbuene, Miltiadis Tsilimbaris, Abera Kenay Tura, Emin Murat Tuzcu, Stefanos Tyrovolas, Kingsley N Ukwaja, Eduardo A Undurraga, Chigozie Jesse Uneke, Olalekan A Uthman, Coen H van Gool, Yuri Y Varakin, Tommi Vasankari, Narayanaswamy Venketasubramanian, Raj Kumar Verma, Francesco S Violante, Sergey K Vladimirov, Vasiliy Victorovich Vlassov, Stein Emil Vollset, Gregory R Wagner, Stephen G Waller, Linhong Wang, David A Watkins, Scott Weichenthal, Elisabete Weiderpass, Robert G Weintraub, Andrea Werdecker, Ronny Westerman, Richard A White, Hywel C Williams, Charles Shey Wiysonge, Charles D A Wolfe, Sungho Won, Rachel Woodbrook, Mamo Wubshet, Denis Xavier, Gelin Xu, Ajit Kumar Yadav, Lijing L Yan, Yuichiro Yano, Mehdi Yaseri, Pengpeng Ye, Henock Gebremedhin Yebyo, Paul Yip, Naohiro Yonemoto, Seok-Jun Yoon, Mustafa Z Younis, Chuanhua Yu, Zoubida Zaidi, Maysaa El Sayed Zaki, Hajo Zeeb, Maigeng Zhou, Sanjay Zodpey, Liesl Joanna Zuhlke, Christopher J L Murray

## Abstract

**Background:**

Non-fatal outcomes of disease and injury increasingly detract from the ability of the world's population to live in full health, a trend largely attributable to an epidemiological transition in many countries from causes affecting children, to non-communicable diseases (NCDs) more common in adults. For the Global Burden of Diseases, Injuries, and Risk Factors Study 2015 (GBD 2015), we estimated the incidence, prevalence, and years lived with disability for diseases and injuries at the global, regional, and national scale over the period of 1990 to 2015.

**Methods:**

We estimated incidence and prevalence by age, sex, cause, year, and geography with a wide range of updated and standardised analytical procedures. Improvements from GBD 2013 included the addition of new data sources, updates to literature reviews for 85 causes, and the identification and inclusion of additional studies published up to November, 2015, to expand the database used for estimation of non-fatal outcomes to 60 900 unique data sources. Prevalence and incidence by cause and sequelae were determined with DisMod-MR 2.1, an improved version of the DisMod-MR Bayesian meta-regression tool first developed for GBD 2010 and GBD 2013. For some causes, we used alternative modelling strategies where the complexity of the disease was not suited to DisMod-MR 2.1 or where incidence and prevalence needed to be determined from other data. For GBD 2015 we created a summary indicator that combines measures of income per capita, educational attainment, and fertility (the Socio-demographic Index [SDI]) and used it to compare observed patterns of health loss to the expected pattern for countries or locations with similar SDI scores.

**Findings:**

We generated 9·3 billion estimates from the various combinations of prevalence, incidence, and YLDs for causes, sequelae, and impairments by age, sex, geography, and year. In 2015, two causes had acute incidences in excess of 1 billion: upper respiratory infections (17·2 billion, 95% uncertainty interval [UI] 15·4–19·2 billion) and diarrhoeal diseases (2·39 billion, 2·30–2·50 billion). Eight causes of chronic disease and injury each affected more than 10% of the world's population in 2015: permanent caries, tension-type headache, iron-deficiency anaemia, age-related and other hearing loss, migraine, genital herpes, refraction and accommodation disorders, and ascariasis. The impairment that affected the greatest number of people in 2015 was anaemia, with 2·36 billion (2·35–2·37 billion) individuals affected. The second and third leading impairments by number of individuals affected were hearing loss and vision loss, respectively. Between 2005 and 2015, there was little change in the leading causes of years lived with disability (YLDs) on a global basis. NCDs accounted for 18 of the leading 20 causes of age-standardised YLDs on a global scale. Where rates were decreasing, the rate of decrease for YLDs was slower than that of years of life lost (YLLs) for nearly every cause included in our analysis. For low SDI geographies, Group 1 causes typically accounted for 20–30% of total disability, largely attributable to nutritional deficiencies, malaria, neglected tropical diseases, HIV/AIDS, and tuberculosis. Lower back and neck pain was the leading global cause of disability in 2015 in most countries. The leading cause was sense organ disorders in 22 countries in Asia and Africa and one in central Latin America; diabetes in four countries in Oceania; HIV/AIDS in three southern sub-Saharan African countries; collective violence and legal intervention in two north African and Middle Eastern countries; iron-deficiency anaemia in Somalia and Venezuela; depression in Uganda; onchoceriasis in Liberia; and other neglected tropical diseases in the Democratic Republic of the Congo.

**Interpretation:**

Ageing of the world's population is increasing the number of people living with sequelae of diseases and injuries. Shifts in the epidemiological profile driven by socioeconomic change also contribute to the continued increase in years lived with disability (YLDs) as well as the rate of increase in YLDs. Despite limitations imposed by gaps in data availability and the variable quality of the data available, the standardised and comprehensive approach of the GBD study provides opportunities to examine broad trends, compare those trends between countries or subnational geographies, benchmark against locations at similar stages of development, and gauge the strength or weakness of the estimates available.

**Funding:**

Bill & Melinda Gates Foundation.

## Introduction

Although substantial progress has been made toward reducing mortality and extending life expectancy throughout the world over the past few decades, the epidemiological transition is manifest in the growing importance of non-fatal diseases, outcomes, and injuries which pose, partly as a consequence of decreasing death rates, a rising challenge to the ability of the world's population to live in full health. Complementing information on deaths by age, sex, cause, geography, and time with equally detailed information on disease incidence, prevalence, and severity is key to a balanced debate in health policy. For this reason, the Global Burden of Disease (GBD) Study uses the disability-adjusted life-year (DALY), combining years of life lost (YLLs) due to mortality and years lived with disability (YLDs) in a single metric. One DALY can be thought of as one lost year of healthy life. The sum of DALYs in a population can be thought of as the gap between the population's present health status and an ideal situation where the entire population lives to an advanced age, free of disease. Assessments of how different diseases lead to multimorbidity and reductions in functional health status are important for both health system planning^1^ and a broader range of social policy issues such as the appropriate age for retirement in some countries.^2^, ^3^ Many challenges in making standardised estimates of non-fatal health outcomes are similar to those affecting mortality estimates (including variations in case definitions, data collection methods, variable quality of data collection, conflicting data, and missing data) but are compounded by more sparse and varied data sources, the need to characterise each disease by its disabling sequelae or consequence (s), and the need to quantify the severity of these consequences. The standardised approach of the annual GBD updates addresses these measurement problems to enhance comparability between causes by geography and over time.

The estimates from GBD 2013 drew attention to large increases in the number of YLDs over the previous decade, whereas rates of YLDs for most causes remained stable or showed only small decreases.^4^ The GBD 2013 assessment largely attributed increases in the number of YLDs to musculoskeletal disorders, mental and substance use disorders, neurological disorders, and chronic respiratory diseases, as well as population growth and ageing. GBD 2013 also brought attention to increased differences in trends between mortality and morbidity for many causes. YLDs as a proportion of DALYs increased globally, a manifestation of the continuing epidemiological transition in low-income and middle-income countries. Decreases in mortality from diseases such as pneumonia, diarrhoea, maternal and neonatal disorders, and an absence of progress in reducing YLD rates continued to drive a transition toward a greater global number of YLDs.

Along with broad recognition that data from some regions were sparse and that more and higher quality data in general would probably improve estimation, useful debates on the GBD results have been published. These debates have focused on the analysis or presentation of individual diseases, such as changes over time in GBD estimates of dementia,^5^ the accuracy of HIV incidence estimates,^6^, ^7^ the absence of sepsis as a disease,^8^, ^9^ the quality of some cancer registry data,^10^ and the absence of mental disorders as sequelae of neglected tropical diseases.^11^ The GBD empirical approach to measuring the public's view of health state severity has generated substantial interest with questions about the relative importance of different dimensions of health,^12^, ^13^ the quantification of health loss,^14^, ^15^ and discussions of the transferability of judgments about relative health to conventional notions of disability and dependence.^5^ In each cycle of the GBD, we seek to improve the estimates, reflecting published and unpublished critique through the acquisition of new data, expansion of the network of collaborators, changes in how data are corrected for bias, advances in modelling techniques, and the targeted expansion of the GBD cause list.

The primary objective of this component of the GBD was to use all available data of sufficient quality to generate reliable and valid assessments of disease and injury sequelae incidence, prevalence, and YLDs for all 310 causes in the GBD cause hierarchy for 591 locations in the GBD study during 1990–2015. We describe the change over time and between populations in relation to where countries fall on the development continuum.^16^ Continuing efforts to improve data and code transparency are an important part of the GBD cycle. These results thus supersede any previous publications about the GBD on disease incidence, prevalence, and YLDs.

## Methods

### Overall approach

**Figure 1:**
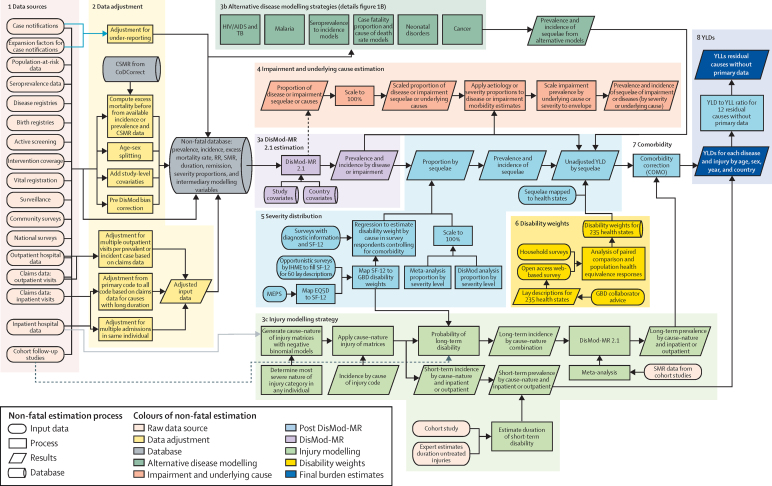
Analytical flow chart for the estimation of cause-specific YLDs by location, age, sex, and year for GBD 2015 Ovals represent data inputs, square boxes represent analytical steps, cylinders represent databases, and parallelograms represent intermediate and final results. The flow chart is colour-coded by major estimation component: raw data sources, in pink; data adjustments, in yellow; DisMod-MR 2.1 estimation, in purple; alternative modelling strategies, in light green; injury modelling strategy, in dark green; estimation of impairments and underlying causes, in brown; post-DisMod-MR and comorbidity correction, in blue; disability weights, in orange; and cause of death and demographic inputs, in grey. GBD=Global Burden of Disease. TB=tuberculosis. SF-12=Short Form 12 questions. MEPS=Medical Expenditure Panel Surveys. CSMR=cause-specific mortality rate. SMR=standardised mortality ratio. YLDs=years lived with disability. YLLs=years of life lost. IHME=Institute for Health Metrics and Evaluation.

We estimated incidence and prevalence by age, sex, cause, year, and geography using a wide range of updated and standardised analytical procedures. The overall logic of our analytical approach is shown for the entire non-fatal estimation process in figure 1. The appendix provides a single source for detail of inputs, analytical processes, and outputs and methods specific to each cause. This study complies with the Guidelines for Accurate and Transparent Health Estimates Reporting (GATHER) recommendations (methods appendix pp 1, 608–10).^17^

### Geographies in GBD 2015

The geographies included in GBD 2015 have been arranged into a set of hierarchical categories composed of seven super-regions and a further nested set of 21 regions containing 195 countries and territories. Eight additional subnational assessments were done for Brazil, China, India, Japan, Kenya, Saudi Arabia, South Africa, Sweden, and the USA (methods appendix pp 611–24). For this study we present data at the national and territory level.

### List of causes and sequelae

The GBD cause and sequelae list is organised hierarchically (methods appendix 625–53). At Level 1 there are three cause groups: communicable, maternal, neonatal, and nutritional diseases (Group 1 diseases); non-communicable diseases; and injuries. These Level 1 aggregates are subdivided at Level 2 of the hierarchy into 21 cause groupings. The disaggregation into Levels 3 and 4 contains the finest level of detail for causes captured in GBD 2015. Sequelae of diseases and injuries are organised at Levels 5 and 6 of the hierarchy. The finest detail for all sequelae estimated in GBD is at Level 6 and is aggregated into summary sequelae categories (Level 5) for causes with large numbers of sequelae. Sequelae in GBD are mutually exclusive and collectively exhaustive, and thus our YLD estimates at each level of the hierarchy sum to the total of the level above. Prevalence aggregations are estimated at the level of individuals who might have more than one sequela or disease and therefore are not additive.

The cause and sequelae list was expanded based upon feedback after the release of GBD 2013 and input from GBD 2015 collaborators. Nine causes for which non-fatal outcomes are estimated were added: Ebola virus disease, motor-neuron disease, environmental heat and cold exposure, four subtypes of leukaemia, and two subtypes of non-melanoma skin cancer (methods appendix pp 625–53). The incorporation of these changes expanded the cause list from the 301 causes with non-fatal estimates examined in GBD 2013, to 310 causes with non-fatal estimates and from 2337 to 2619 unique sequelae at Level 6 of the hierarchy. At the newly created Level 5 of the hierarchy there were 154 summary sequela categories. The methods appendix (pp 654–61) provides a list of International Classification of Diseases version 9 (ICD-9) and version 10 (ICD-10) codes used in the extraction of hospital and claims data, mapped to GBD 2015 non-fatal causes, impairments, and nature of injury categories.

### Period of analysis

A complete set of age-specific, sex-specific, cause-specific, and geography-specific incidence and prevalence numbers and rates were computed for the years 1990, 1995, 2000, 2005, 2010, and 2015. In this study we focus on trends for main and national results over the past decade, from 2005 to 2015, together with more detailed results for 2015. Online data visualisations at vizhub provide access to results for all GBD metrics.

Non-fatal modelling strategies vary substantially between causes. Figure 1 outlines the general process of non-fatal outcome estimation from data inputs to finalisation of YLD burden results; step 3b of that process identifies alternative modelling approaches used for specific causes (methods appendix pp 603, 604). The starting point for non-fatal estimation is the compilation of data sources identified through systematic analysis and extractions based on predetermined inclusion and exclusion criteria (methods appendix p 603). As part of the inclusion criteria, we defined disease-specific or injury-specific reference case definitions and study methods, as well as alternative allowable case definitions and study methods which were adjusted for if we detected a systematic bias. We used 15 types of primary data sources representing disease prevalence, incidence, mortality risk, duration, remission, or severity in the estimation process (oval shapes in figure 1).

### Data sources

For this iteration of the study, we updated data searches through systematic data and literature reviews for 85 causes published up to Oct 31, 2015. For other causes, input from GBD collaborators resulted in the identification and inclusion of a small number of additional studies published after January, 2013. Data were systematically screened from household surveys archived in the Global Health Data Exchange, sources suggested to us by in-country experts, and surveys identified in major multinational survey data catalogues and Ministry of Health and Central Statistical Office websites. Case notifications reported to WHO were updated up to and including 2015. Citations for all data sources used for non-fatal estimation in GBD 2015 are provided in searchable form through a new web tool. A description of the search terms used for cause-specific systematic reviews, inclusion and exclusion criteria, and the preferred and alternative case definitions and study methods are detailed by cause in the methods appendix (pp 26–601).

Hospital inpatient data were extracted from 284 country-year and 976 subnational-year combinations from 27 countries in North America, Latin America, Europe, and New Zealand. Outpatient encounter data were available from the USA, Norway, Sweden, and Canada for 48 country-years. For GBD 2015, we also accessed aggregate data derived from claims information in a database of US private and public insurance schemes for the years 2000, 2010, and 2012. From the linked claims data, we generated several correction factors to account for bias in health service encounter data from elsewhere, which were largely available to us aggregated by ICD code and by primary diagnosis only. First, for chronic disorders, we estimated the ratio between prevalence from primary diagnoses and prevalence from all diagnoses associated with a claim. Second, we used the claims data to generate the average number of outpatient visits per disorder. Similarly, we generated per person discharge rates from hospital inpatient data in the USA and New Zealand, the only sources with unique patient identifiers available for GBD 2015.

In GBD 2013, we calculated a geographical and temporal data representativeness index (DRI) of non-fatal data sources for each cause or impairment. The DRI represents the fraction of countries for which any incidence, prevalence, remission, or mortality risk data were available for a cause. This metric quantifies data availability, not data quality. The overall DRI and period-specific DRI measures for each cause and impairment are presented in the methods appendix (pp 662–68). DRI ranged from 90% for nine causes, including tuberculosis and measles, to less than 5% for acute hepatitis C and the category of other exposures to mechanical forces. Required case reporting resulted in high DRI values for notifiable infectious diseases; the network of population-based registries for cancers resulted in a DRI of above 50%. DRI values ranged from 6·1% in North Korea to 91·3% in the USA. Many high-income countries, as well as Brazil, India, and China, had DRI values above 63%; data availability was low in several countries, including Equatorial Guinea, Djibouti, and South Sudan.

### Non-fatal disease models

In addition to the corrections applied to claims and hospital data, a number of other adjustments were applied including age–sex splitting, bias correction, adjustments for under-reporting of notification data, and computing expected values of excess mortality. In GBD 2013, we estimated expected values of excess mortality from prevalence or incidence and cause-specific mortality rate data for a few causes only, including tuberculosis and chronic obstructive pulmonary disease. In order to achieve greater consistency between our cause of death and non-fatal data, we adopted this strategy systematically for GBD 2015. We matched every prevalence data point (or incidence datapoint for short duration disorders) with the cause-specific mortality rate value corresponding to the age range, sex, year, and location of the datapoint. The ratio of cause-specific mortality rate to prevalence is conceptually equivalent to an excess mortality rate.

To estimate non-fatal health outcomes in previous iterations of GBD, most diseases and impairments were modelled in DisMod-MR, a Bayesian meta-regression tool originally developed for GBD 2010 (step 3a in figure 1).^18^ DisMod-MR was designed to address statistical challenges in estimation of non-fatal health outcomes, and for synthesis of often sparse and heterogeneous epidemiological data. For GBD 2015, the computational engine of DisMod-MR 2.1 remained unchanged, but we substantially rewrote the code that organises the flow of data and settings at each level of the analytical cascade. The sequence of estimation occurs at five levels: global, super-region, region, country, and where applicable, subnational locations (appendix pp 611–24). At each level of the cascade, the DisMod-MR 2.1 computational engine enforces consistency between all disease parameters. For GBD 2015, we generated fits for the years 1990, 1995, 2000, 2005, 2010, and 2015. We log-linearly interpolated estimates for the intervening years in each 5-year period. Greater detail on DisMod-MR 2.1 is available at Global Health Data Exchange and the methods appendix (pp 7–11).

In previous iterations of GBD, custom models were created for a short list of causes for which the compartment model underpinning DisMod (susceptible, diseased, and dead) was insufficient to capture the complexity of the disease or for which incidence and prevalence needed to be derived from other data. Step 3b of figure 1 describes the development of custom models with greater detail shown in the methods appendix figure 1B (p 604, and for associated write-ups pp 26–601) for HIV/AIDS, tuberculosis, malaria, cancer, neonatal disorders, infectious diseases for which we derived incidence from seroprevalence data, and infectious diseases for which we derived incidence from cause of death rates and pooled estimates of the case fatality proportion.

In GBD 2013, we estimated the country–age–sex–year prevalence of nine impairments (step 4 of figure 1). Impairments in GBD are disorders or specific domains of functional health loss that are spread across many GBD causes as sequelae and for which there are better data to estimate the occurrence of the overall impairment than for each sequela based on the underlying cause. Overall impairment prevalence was estimated with DisMod-MR 2.1 except for anaemia, for which spatiotemporal Gaussian Process regression methods were applied. We constrained cause-specific estimates of impairments, such as in the 19 causes of blindness, to sum to the total prevalence estimated for that impairment. Anaemia, epilepsy, hearing loss, heart failure, and intellectual disability were estimated at different levels of severity.

### Severity distributions

In step 5, sequelae were further defined in terms of severity for 194 causes at Level 4 of the hierarchy (figure 1A). We generally followed the same approach for estimating the distribution of severity as in GBD 2013. For Ebola virus disease, we created a health state for the infectious disease episode with duration derived from average hospital admission times, and a health state for ongoing postinfection malaise and joint problems based on four follow-up studies^19^, ^20^, ^21^, ^22^ from which we derived an average duration. The health states for the subtypes of leukaemia and non-melanoma skin cancer were the same as the general cancer health states. For motor-neuron disease we accessed the Pooled Resource Open-Access ALS Clinical Trials (PROACT) database containing detailed information on symptoms and impairments for more than 8500 patients who took part in the trials.^23^

### Disability weights

We used the same disability weights as in GBD 2013 (see methods appendix pp 669–94 for a complete listing of the lay descriptions and values for the 235 health states used in GBD 2015).

### Comorbidity

In step 7, we estimated the co-occurrence of different diseases by simulating 40 000 individuals in each geography–age–sex–year combination as exposed to the independent probability of having any of the sequelae included in GBD 2015 based on disease prevalence. We tested the contribution of dependent and independent comorbidity in the US Medical Expenditure Panel Surveys (MEPS) data, and found that independent comorbidity was the dominant factor even though there are well known examples of dependent comorbidity. Age was the main predictor of comorbidity such that age-specific microsimulations accommodated most of the required comorbidity correction. Taking dependent comorbidity into account changed the overall YLDs estimated in the MEPS data by only 2·5% (and ranging from 0·6% to 3·4% depending on age) in comparison to assuming independent comorbidity (methods appendix pp 18–20).^24^

### YLD computation

We report 95% uncertainty intervals (95% UI) for each quantity in this analysis using 1000 samples from the posterior distribution of prevalence and 1000 samples of the disability weight to generate 1000 samples of the YLD distribution. The 95% UI is reported as the 25th and 975th values of the distribution. We report significant changes in disease estimates between countries or over time if the change was noted in more than 950 of the 1000 samples computed for each result. For GBD 2015, we computed age-standardised prevalence YLD rates from the updated world population age standard developed for GBD 2013.^25^ Less common diseases and their sequelae were included in 35 residual categories (methods appendix pp 695–97). For 22 of these residual categories, estimates were made from epidemiological data for incidence or prevalence. For 13 residual categories, we estimated YLDs by multiplying the residual YLL estimates by the ratio of YLDs to YLLs from the estimates for explicitly modelled Level 3 causes in the same disease category.

### Socio-demographic Index

In GBD 2013, a sociodemographic status variable was computed based on a principal components analysis of income per capita, educational attainment, average age of the population, and the total fertility rate.^26^ For GBD 2015, we excluded mean age of the population because it is directly affected by death rates. To improve interpretability for GBD 2015, we computed a Socio-demographic Index (SDI) similar to the computation of the human development index.^27^ In the SDI, each component was weighted equally and rescaled from zero (for the lowest value observed during 1980–2015) to one (for the highest value observed) for income per capita and average years of schooling, and the reverse for the total fertility rate. The final SDI score was computed as the geometric mean of each of the components. SDI ranged from 0·060 in Mozambique in 1987 to 0·978 in District of Columbia, USA, in 2015.

### Role of the funding source

The funder of the study had no role in study design, data collection, data analysis, data interpretation, or writing of the report. The corresponding author had full access to all the data in the study and had final responsibility for the decision to submit for publication.

## Results

### Global incidence and prevalence

We generated over 9·3 billion outcomes of incidence, prevalence, and YLDs for 310 diseases, injuries, and aggregate categories; 2619 unique and aggregate sequelae; nine impairments; 63 age–sex groups; 591 geographies; and 26 individual years from 1990 to 2015. Each of these 9·3 billion estimates was calculated 1000 times to determine uncertainty intervals. Here, we present key summary findings on global incidence of short duration diseases, global prevalence of long-term disorders, global prevalence of impairments, global numbers and rates of YLDs and changes from 2005 to 2015, global YLL and YLD rates of change, patterns of comorbidity, the expected changes in the composition of YLDs with SDI, and country findings of leading causes of YLDs.

Disorders of less than 3 months duration and injuries with incidence of more than 1 million cases per year in 2015 are listed in table 1. There were two disorders with incidence greater than 1 billion per year: upper respiratory infections (17·2 billion [95% UI 15·4–19·2 billion]), and diarrhoeal diseases (2·39 billion [2·30–2·50 billion]). A further 13 diseases and injuries caused between 100 million and 1 billion incident cases a year and 16 diseases and injuries had incident cases of between 10 million and 100 million per year (table 1).

The disease and injury sequelae with a duration of more than 3 months and a global prevalence of more than 1% in 2015 are presented in table 2, aggregated to the cause level. Prevalence for impairments is presented at the bottom of table 3. Eight out of 56 high-prevalence causes affected more than 10% of the world's population in 2015. A further 48 causes affected between 1% and 10% of the world's population (table 2). Although many of these causes are not among the dominant causes of YLDs because of comparatively low average disability weights, some causes, such as headaches, gynaecological diseases, oral disorders, and skin diseases, put great demands on health system resources by their sheer numbers.

Anaemia was the most common of our nine impairments, affecting 2·36 billion (2·35–2·37 billion) people in 2015. The next most common impairments were hearing loss of greater than 20 dB (1·33 billion [1·26–1·40 billion]), vision loss (940 million [905–974 million]), developmental intellectual disability (153 million [114–191 million]), infertility (113 million [93·4–136 million]), heart failure (40·0 million [38·6–41·4 million]), and epilepsy (39·2 million [34·3–43·7 million]; table 3; see results appendix (pp 740–48) for the prevalence estimates of the underlying causes of these impairments). Iron deficiency was the cause of anaemia in more than half of all cases. Over 90% of hearing loss was classified as age-related or other hearing loss. The largest number of people with vision loss had uncorrected refraction error. Idiopathic developmental intellectual disability, idiopathic female infertility, and idiopathic epilepsy were the most common causes of their impairments. Ischaemic heart disease was the most common cause of heart failure.

### Global causes of disability

#### Global trends in YLDs 2005 to 2015

**Figure 2:**
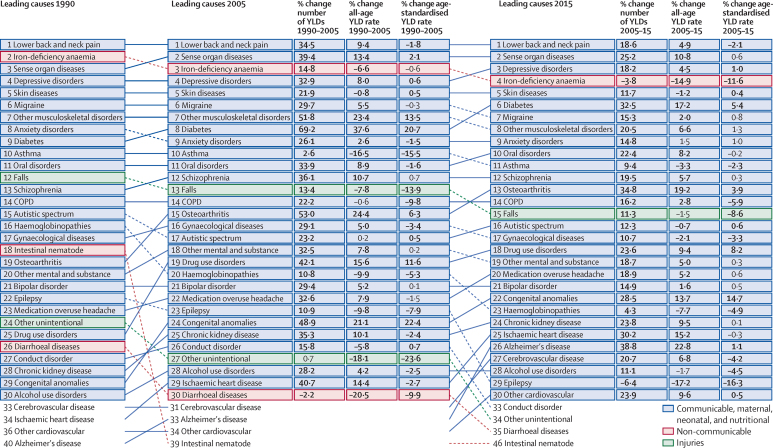
Leading 30 Level 3 causes of global YLDs for both sexes combined, 1990, 2005, and 2015, with percentage change in number of YLDs, and all-age and age-standardised rates Causes are connected by lines between time periods. For the time period of 1990 to 2005 and for 2005 to 2015, three measures of change are shown: percent change in the number of YLDs, percent change in the all-age YLD rate, and percent change in the age-standardised YLD rate. YLD=years lived with disability. COPD=chronic obstructive pulmonary disease.

GBD 2015 included the assessment of 2619 sequelae at Level 6 of the GBD cause hierarchy, including 1316 sequelae from injuries that contributed to the global burden of disability. Causes at Level 4 of the hierarchy that resulted in 30 million or more YLDs in 2015 included lower back pain, major depressive disorder, age-related and other hearing loss, and neck pain. Figure 2 compares the leading causes of global YLDs in 2005 and 2015, using the cause breakdowns at Level 3 of the GBD cause hierarchy. Among the ten leading causes of YLDs, iron-deficiency anaemia and depressive disorders switched ranks to positions three and four respectively, diabetes rose from the eighth to the sixth position, migraine dropped from position six to seven, and other musculoskeletal disorders dropped from rank seven to eight (figure 2).

Estimates of prevalence and YLDs at the global level for 2005 and 2015 for each cause are presented in table 3 (see results appendix pp 717–40 for full detail at the sequelae level). Prevalence and age-standardised YLDs for 21 Group 1 diseases decreased significantly and by more than 10%, including measles, African trypanosomiasis, diphtheria, lymphatic filariasis, and rabies.

Age-standardised YLD rates for all maternal causes and sequelae combined decreased between 2005 and 2015, whereas overall age-standardised YLDs for neonatal disorders increased by 3·11% (–0·32–6·79%) since 2005 to 144 YLDs per 100 000 (109–185 YLDs per 100 000) in 2015, and age-standardised YLD rates decreased and absolute numbers of cases increased for nutritional deficiencies. Age-standardised YLD rates attributable to hepatitis A, B, and C increased, and decreased for hepatitis E. The number of individuals with chronic hepatitis C infection increased from 121 million (108–133 million) in 2005 to 142 million (127–157 million) in 2015.

### Changes in age-standardised YLDs and YLLs over time

In 2005, non-communicable diseases (NCDs) accounted for 23 of the leading 25 causes of age-standardised YLDs worldwide and 23 of the 25 leading causes in 2015 (figure 2). Although diabetes rose only two ranks in the list of leading cause of YLDs, from position eight to six between 2005 and 2015, the increase in age-standardised rate was 5·4% (3·2–7·5%). Musculoskeletal disorders occupied three of the leading 25 causes of disability in both 2005 and 2015; lower back and neck pain were the single largest cause with little change in their rates. However, age-standardised rates of YLDs increased for osteoarthritis 3·90% (3·00–4·83%) by 2015. Depressive disorders were the fourth leading cause of disability in 2005 and the third leading cause of disability in 2015, and age-standardised YLD rates associated with the disorder increased marginally (1·0% [0·5–1·5%]). Age-standardised rates of YLDs from alcohol use disorders decreased after 2005 (4·5% [2·3–6·4%]) whereas disability due to drug use disorders increased by 8·2% (6·2–10·2%).

In contrast with global trends for NCDs, both relative ranks and age-standardised YLD rates decreased for most injuries. Falls, which were the 13th leading cause of disability in 2005, dropped to the 15th rank, and age-standardised YLD rates decreased (8·58% [5·23–12·2%]). Other unintentional injuries decreased in global rank from 27th in 2005 to 34th in 2015, and age-standardiSed YLD rates decreased by 16·7% (15·7–17·9%).

**Figure 3:**
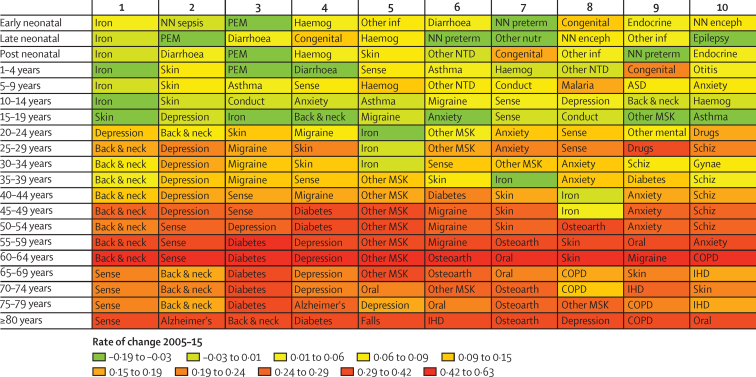
Leading ten Level 3 causes of global age-specific years lived with disability in 2015 Each cause is coloured by the percentage change in age-specific years lived with disability from 2005 to 2015. Alzheimer's=Alzheimer disease and other dementias. ASD=autism. Back & neck=low back and neck pain. Conduct=conduct disorders. Congenital=congenital anomalies. COPD=chronic obstructive pulmonary disease. Drugs=drug use disorders. Endocrine=endocrine, metabolic, blood, and immune disorders. Gyne=gynaecological disorders. Haemog=haemoglobinopathies and haemolytic anaemias. IHD=ischaemic heart disease. Iron=iron-deficiency anaemia. NN enceph=neonatal encephalopathy due to birth asphyxia and trauma. NN preterm=neonatal preterm birth complications. NN sepsis=neonatal sepsis and other neonatal infections. Other NTD=other neglected tropical diseases. Oral=oral disorders. Osteoarth=osteoarthritis. Other inf=other infectious diseases. Other mental=other mental and substance use disorders. Other MSK=other musculoskeletal disorders. Other nutr=other nutritional deficiencies. PEM=protein-energy malnutrition. Schiz=schizophrenia. Sense=sense organ disease. Skin=skin and subcutaneous diseases.

The leading causes of disability varied considerably with age (figure 3). The leading cause in children younger than 5 years was iron-deficiency anaemia followed by skin diseases, protein-energy malnutrition, and diarrhoea. In older children, iron-deficiency anaemia, skin diseases, asthma, and mental health disorders such as conduct, autistic spectrum, and anxiety disorders were top ten causes of disability. In adolescents and young adults (aged between 15 and 39 years), iron-deficiency anaemia, skin diseases, depression, lower back and neck pain, and migraine led the rankings. Other mental health disorders such as anxiety disorders and schizophrenia were in the top ten causes in this age group. In middle-aged adults, musculoskeletal disorders dominated the top rankings followed by mental health disorders, especially depression. Diabetes and sense organ disorders were more prominent causes of disability in middle-age. In older adults (older than 65 years), sense organ disorders were the top-ranked cause of disability. Musculoskeletal disorders remained a dominant source of disability, and chronic obstructive pulmonary disease entered the top ten. In the oldest age groups, ischaemic heart disease and Alzheimer's and other dementias made their first appearance in the top ten.

**Figure 4:**
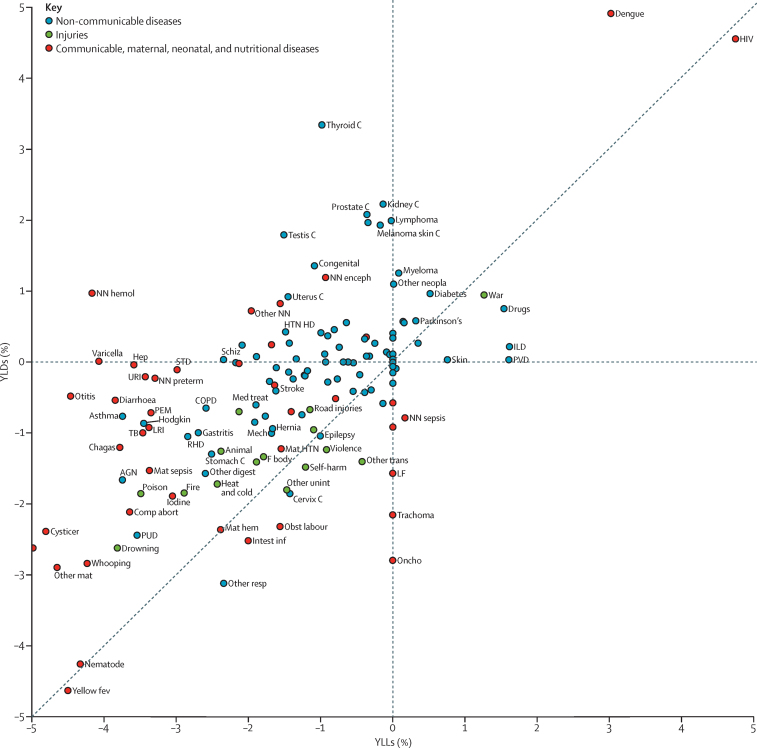
Global annualised rate of change in age-standardised years of life lost (YLLs) and years lived with disability (YLDs) for Level 3 causes between 1990 and 2015 TB=tuberculosis. HIV=HIV/AIDS. Diarrhoea=diarrhoeal diseases. Intest Inf=intestinal infectious diseases. LRI=lower respiratory infections. URI=upper respiratory infections. Otitis=otitis media. Whooping=whooping cough. Varicella=varicella and herpes zoster. Chagas=chagas disease. Cysticer=cysticercosis. LF=lymphatic filariasis. Oncho=onchocerciasis. Trachoma=trachoma. Dengue=dengue. Yellow Fev=yellow fever.Nematode=intestinal nematode infections. Mat hem=maternal haemorrhage. Mat sepsis=maternal sepsis and other maternal infections. Mat HTN=maternal hypertensive disorders. Obst labour=maternal obstructed labour and uterine rupture. Comp abort=maternal abortion, miscarriage, and ectopic pregnancy. Oth mat=other maternal disorders. NN preterm=neonatal preterm birth complications. NN enceph=neonatal encephalopathy due to birth asphyxia and trauma. NN sepsis=neonatal sepsis and other neonatal infections. NN haemol=haemolytic disease and other neonatal jaundice. Oth NN=other neonatal disorders. PEM=protein-energy malnutrition. Iodine=iodine deficiency. Oth nutr=other nutritional deficiencies. STD=sexually transmitted diseases excluding HIV. Hep=hepatitis. Stomach C=stomach cancer. Melanoma=malignant skin melanoma. Skin C=non-melanoma skin cancer. Cervix C=cervical cancer. Uterus C=uterine cancer. Prostate C=prostate cancer. Testis C=testicular cancer. Kidney C=kidney cancer. Thyroid C=thyroid cancer. Hodgkin=Hodgkin lymphoma. Lymphoma=non-Hodgkin lymphoma. Myeloma=multiple myeloma. Oth neopla=Other neoplasms. RHD=rheumatic heart disease. Stroke=cerebrovascular disease. HTN HD=hypertensive heart disease. PVD=peripheral vascular disease. COPD=chronic obstructive pulmonary disease. Asthma=asthma. ILD=interstitial lung disease and pulmonary sarcoidosis. Oth resp=other chronic respiratory diseases. PUD=peptic ulcer disease. Gastritis=gastritis and duodenitis. Hernia=inguinal, femoral, and abdominal hernia. Oth digest=other digestive diseases. Parkinson=Parkinson's disease. Schiz=schizophrenia. Drugs=drug use disorders. AGN=acute glomerulonephritis. Congenital=congenital anomalies. Skin=skin and subcutaneous diseases. Road inj=road injuries. Oth trans=other transport injuries. Drown=drowning. Fire=fire, heat, and hot substances. Poison=poisonings. Mech=exposure to mechanical forces. Med treat=adverse effects of medical treatment. Animal=animal contact. F body=foreign body. Heat & cold=environmental heat and cold exposure. Oth unint=other unintentional injuries. Violence=interpersonal violence. War=collective violence and legal intervention.

We examined the trends in YLDs and YLLs in a scatterplot (figure 4). YLLs decreased for the majority of causes. For Group 1 causes and injuries, the decrease in YLLs was accompanied by a decrease in YLDs, albeit at a slower pace. The exceptions were neonatal encephalopathy, haemolytic disease and other neonatal jaundice, leishmaniasis, meningitis, hepatitis, and sexually transmitted diseases with increasing YLD rates between 1990 and 2015. A few Group 1 disorders and injuries had a faster decrease in YLDs than in YLLs: intestinal infections, obstructed labour, and neonatal sepsis. The only NCDs with a faster decrease in YLDs compared with YLLs were epilepsy and cervical cancer. Another small number of NCDs saw an increase in YLDs and YLLs, including drug use disorders, diabetes, and Parkinson's disease. NCDs with decreasing YLLs but increasing YLDs included cancers of the prostate, testis, uterus, kidney, colorectum, and pancreas, melanoma, and congenital disorders. Some cancers (stomach and Hodgkin's lymphoma), rheumatic heart disease, asthma, chronic obstructive pulmonary disease, acute glomerulonephritis, peptic ulcer disease, gastritis, hernia, and gallbladder disease had decreasing YLLs and YLDs, but with a faster decrease in YLLs than in YLDs. The rate of change in YLDs for the main drivers of non-fatal health loss, musculoskeletal disorders, and mental and substance use disorders was small.

### Global distribution of disability weights across individuals

**Figure 5:**
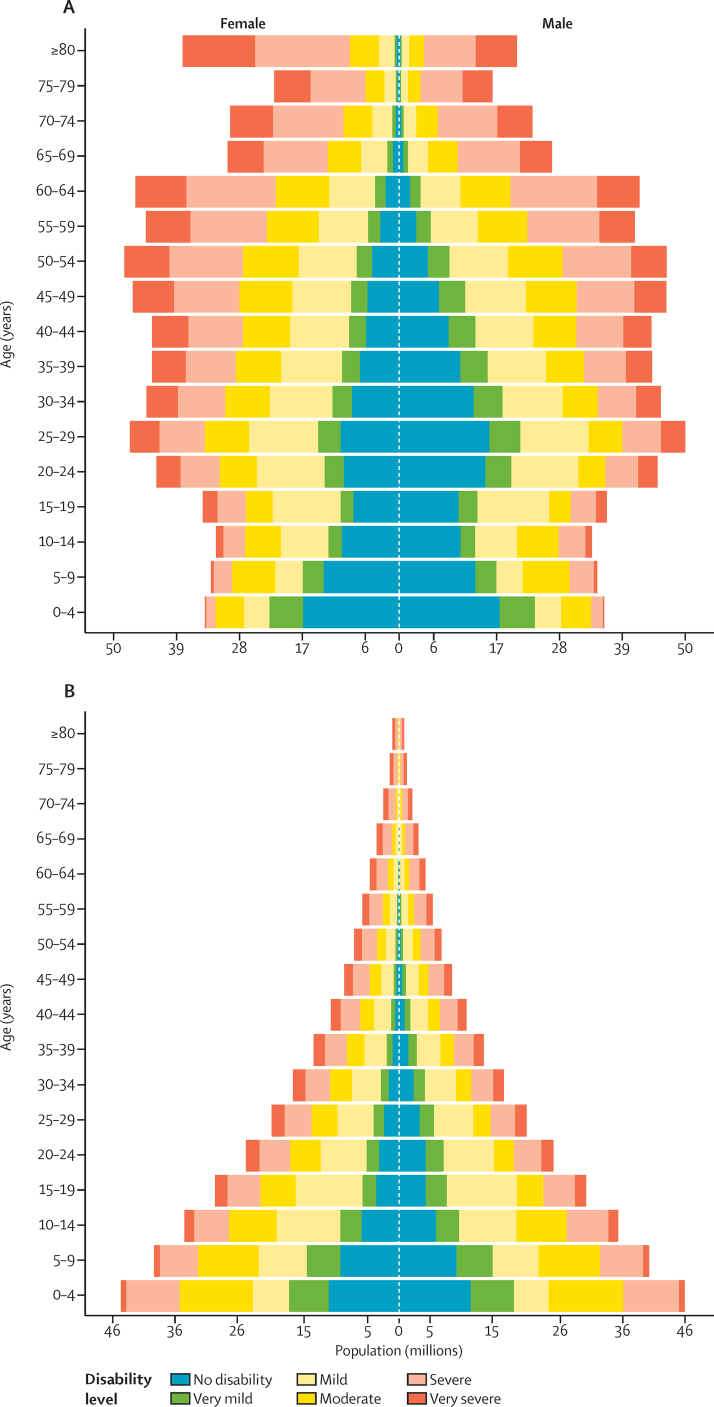
Population pyramids with the number of individuals, by age and sex, grouped by severity of their disability weight (DW) for all comorbid conditions combined into no disability, very mild disability (DW 0–0·01), mild disability (DW 0·01–0·05), moderate disability (DW 0·05–0·1), severe disability (DW 0·1–0·3), and very severe disability (DW >0·3) for geographies of high (A) and low (B) quintiles of Socio-demographic Index in 2015 Disability weights are combined multiplicatively as 1−(1−DW1)(1− DW2)…(1−DWn) for n comorbid sequelae. Socio-demographic Index (SDI) is calculated for each geography as a function of lag-dependent income per capita, average educational attainment in the population aged over 15 years, and the total fertility rate. SDI units are interpretable; a zero represents the lowest level of income per capita, educational attainment, and highest total fertility rate (TFR) observed from 1980 to 2015 and a one represents the highest income per capita, educational attainment, and lowest TFR observed in the same period. Cutoffs on the SDI scale for the quintiles have been selected based on examination of the entire distribution of geographies between 1980 and 2015.

Figure 5 shows the global distribution of individuals from our comorbidity microsimulation by six categories of disability, age, and sex for the highest and lowest SDI quintile. The six categories of disability are no disability, very mild disability (disability weight less than or equal to 0·01), mild disability (from 0·01 to 0·05 inclusive), moderate (from 0·05 to 0·1 inclusive), severe (from 0·1 to 0·3 inclusive), and profound (greater than 0·3). In 2015, most of the world's population experienced mild or greater disability. Having no disability at all was most common in children. After age 25 years, the proportion of the population having no disability became progressively smaller, and by age 55 years in low SDI countries and age 75 years in high SDI countries, nearly everyone had some form of disability. Very mild to moderate disability (ie, individuals with a disability weight of 0·1 or less) was common in childhood and young adults, but was replaced by more severe disability with increasing age. The patterns were similar for both sexes, apart from a much larger amount of disability in women older than 80 years in the top SDI quintiles, reflecting the much higher average age of women in this age category. For policy considerations around the age of retirement in ageing populations, it is noteworthy that from age 60 years onwards more than half of the population had severe or worse disability. The extent to which this loss of health limits or precludes the ability to work depends on the nature of the impairments and the type of employment in older workers with that level of disability.

### Expected changes in disease profile with higher Socio-demographic Index

**Figure 6:**
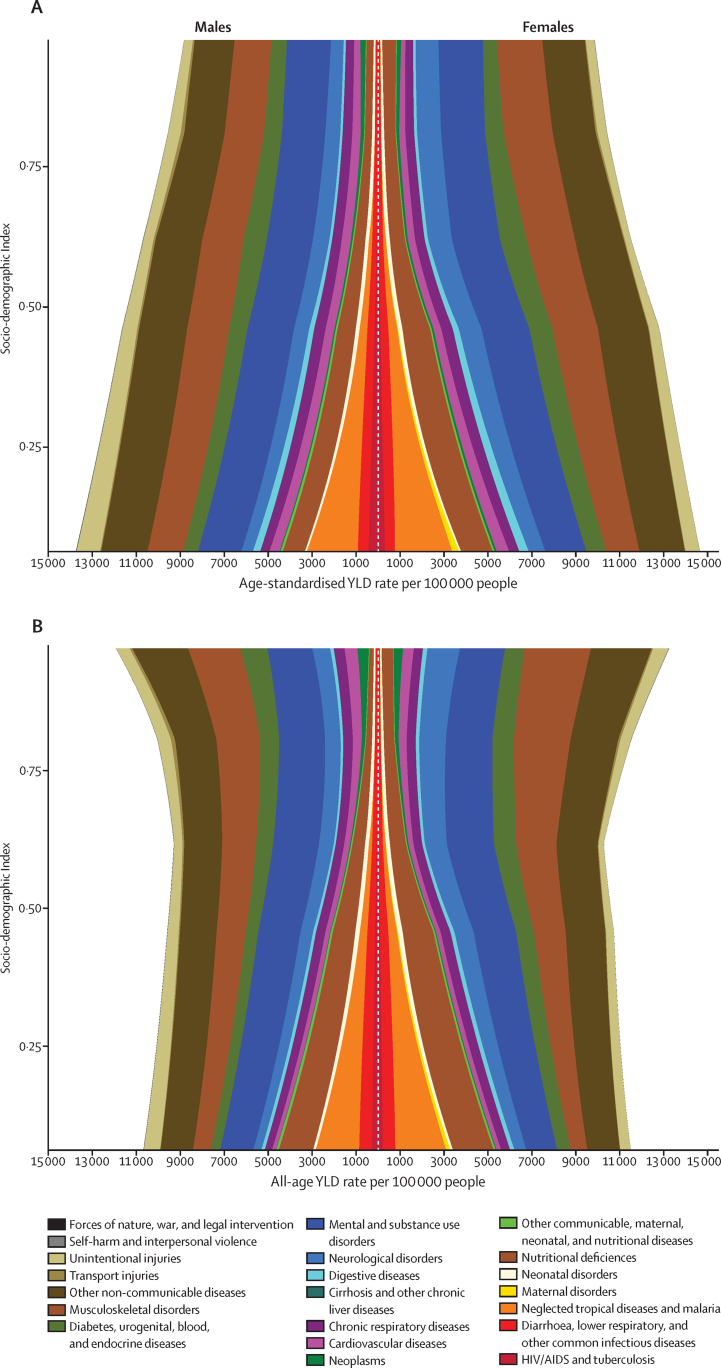
Expected relationship between age-standardised years lived with disability (YLD) rates per 100 000 people for the 21 GBD Level 2 causes and SDI (A) and the expected relationship between all-age years lived with disability (YLD) rates per 100 000 people for the 21 GBD Level 2 causes and SDI (B) by sex These stacked curves represent the average relationship between SDI and each cause of YLDs observed across all geographies over the time period 1990 to 2015. In each figure, the y-axis spans from lowest SDI up to highest SDI. To the left of the midline are male rates, and the female rates are to the right; higher rates are further from the midline. SDI=Socio-demographic Index.

Figure 6 depicts changes in patterns of disability by level of SDI for age-standardised and all-age YLD rates per 100 000. Age-standardised YLD rates gradually decreased with increasing SDI in both sexes (figure 6A). The cause composition of YLDs somewhat varied across levels of SDI; these differences were largely derived from absolute levels of disability due to communicable causes and nutritional deficiencies, and to a lesser extent, maternal and neonatal disorders. Age-standardised YLD rates due to NCDs and injuries were similar at all SDI levels.

Across levels of SDI, mental and substance use disorders, musculoskeletal disorders, and other NCDs were consistently among the leading causes of age-standardised YLD rates. Anaemia led to generally higher rates of age-standardised YLDs in women than in men across levels of SDI, but the largest imbalance occurred at SDI levels between 0·10 and 0·50. Disability from injuries exacted a larger burden for men than for women, particularly at lower levels of SDI.

Without adjustments for population age structure (figure 6B), the effect of ageing populations and causes of disability that disproportionately affect older individuals become prominent. At all levels of SDI, total YLDs per 100 000 did not notably differ by sex; instead, the cause composition of disability showed greater differences. Below an SDI score of 0·25, communicable causes accounted for 30–45% of total disability, primarily due to nutritional deficiencies, malaria, and neglected tropical diseases. YLDs per 100 000 due to musculoskeletal disorders, particularly lower back and neck pain and other musculoskeletal disorders, increased substantially from low to high SDI, with a more pronounced increase beginning at an SDI score of 0·6. This rise was particularly evident in women.

#### Trends in age-standardised YLDs per capita

Globally, age-standardised YLDs per capita (an indicator of overall disability experienced per person in a given place) moderately decreased for both sexes between 1990 and 2015 (results appendix pp 714–16). Age-standardised YLDs per capita were consistently higher for women than for men at the global level. For both sexes, YLDs per capita were generally higher for lower levels of SDI. YLDs per capita were noticeably larger for low SDI and low-middle SDI groups than for other SDI levels (ie, high SDI, high-middle SDI, and middle SDI), which were more similar to each other.

#### Leading causes of YLDs and deviations from expected levels based on Socio-demographic Index

**Figure 7:**
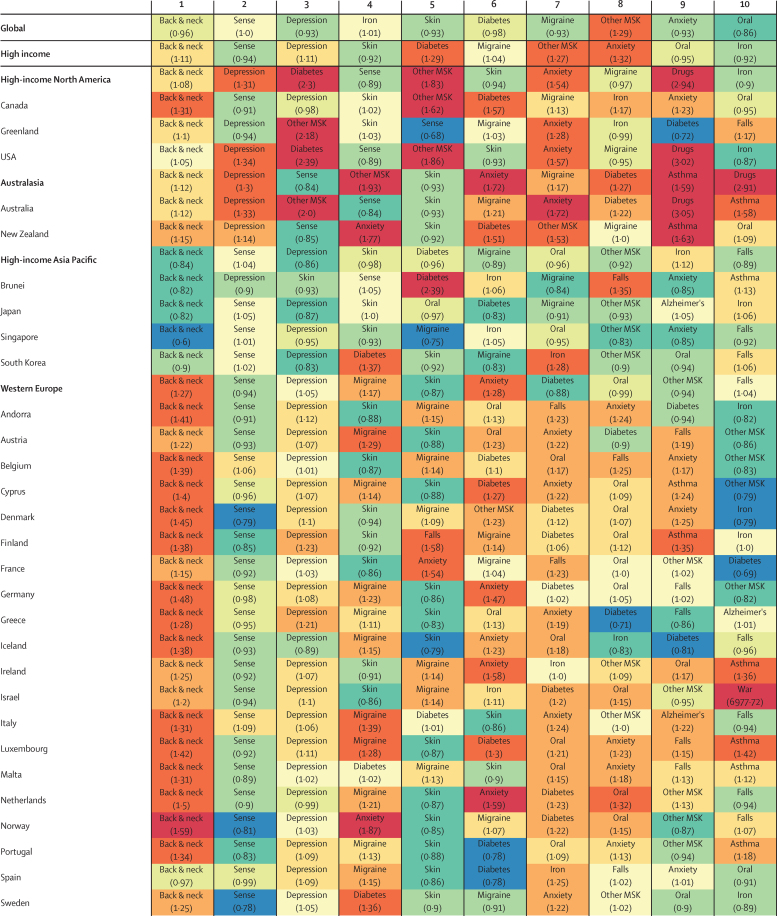

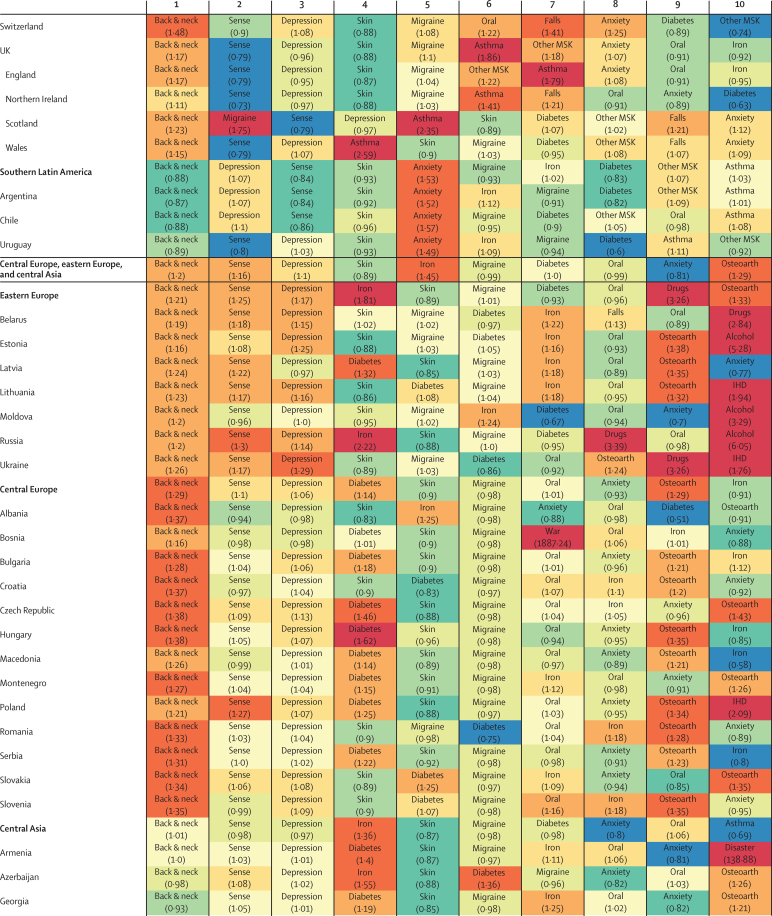

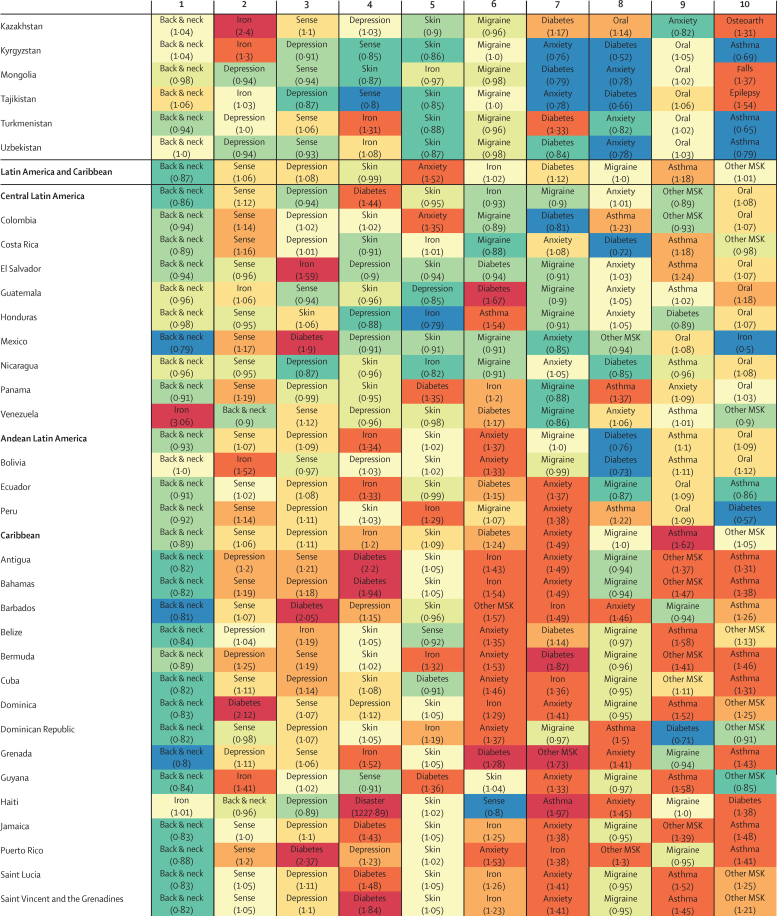

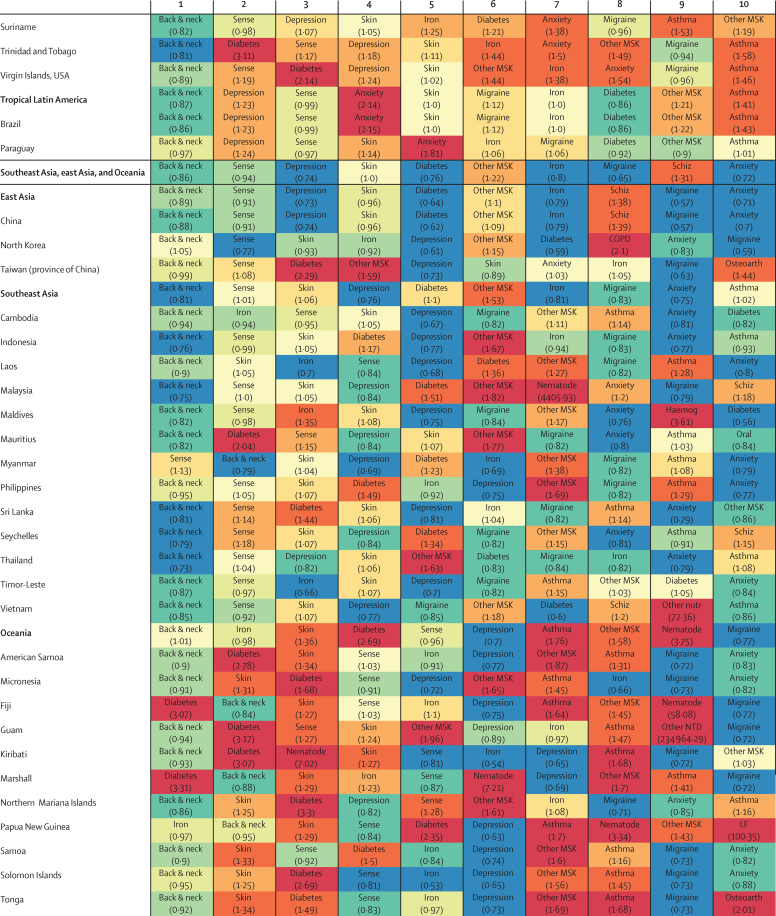

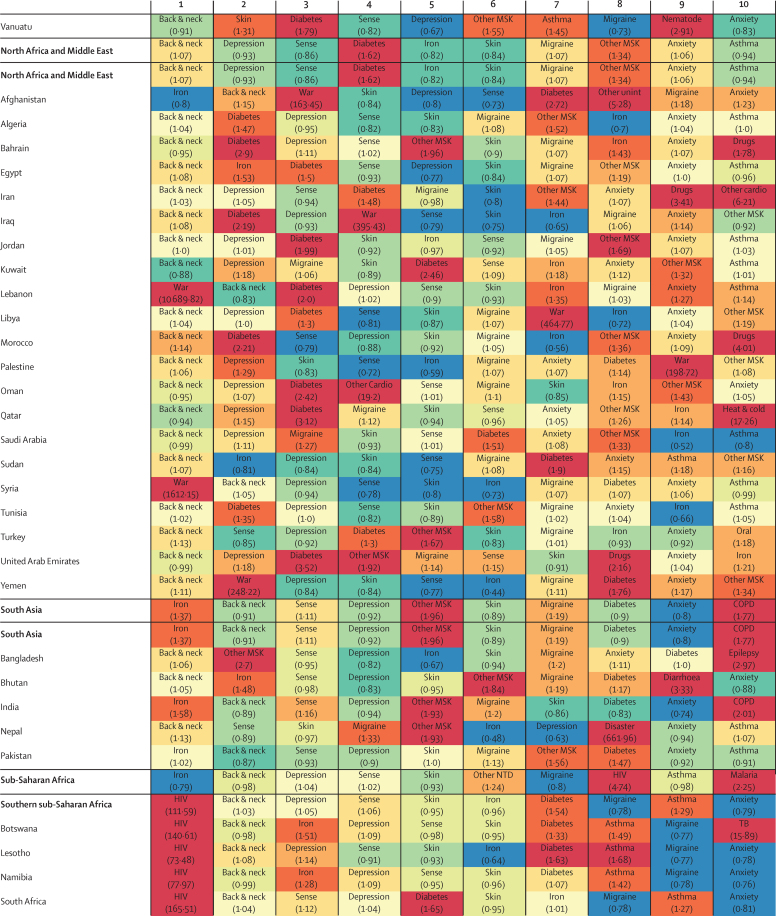

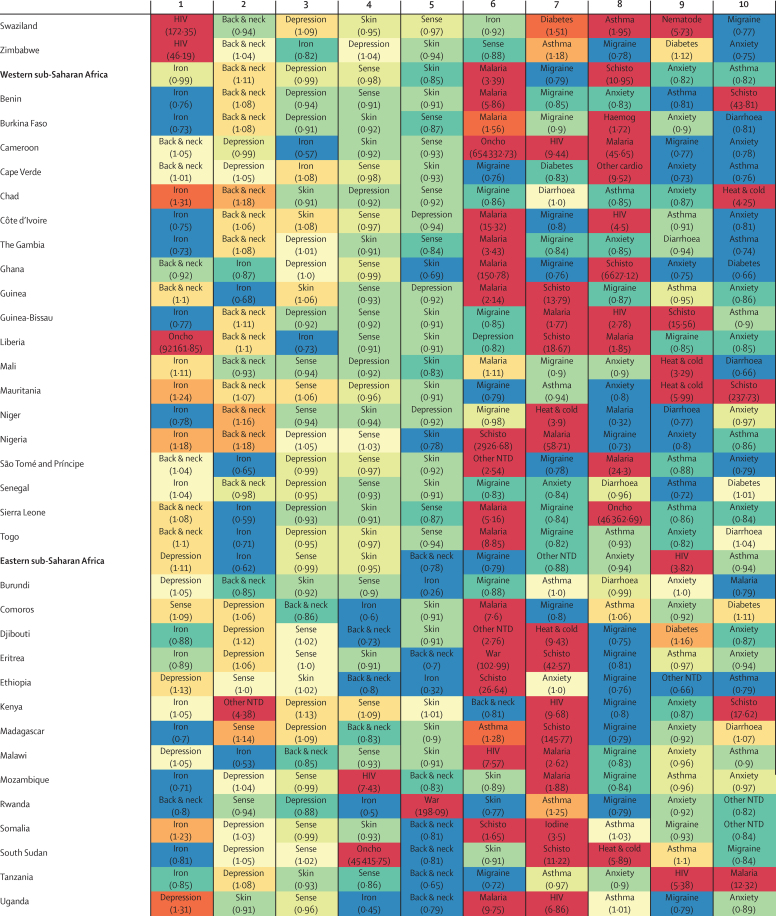

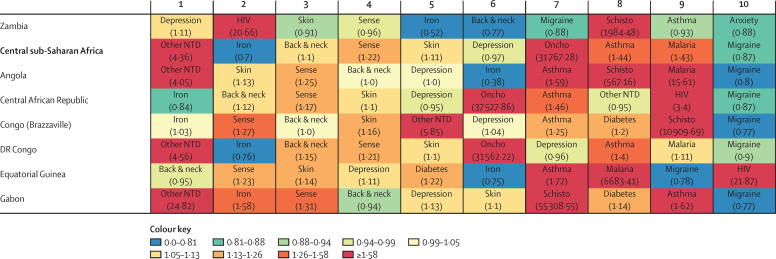
Leading ten causes of years lived with disability (YLDs) with the ratio of observed years lived with disability (YLDs) to years lived with disability (YLDs) expected on the basis of SDI in 2015, by location Shades of blue represent much lower observed YLDs than expected levels based on SDI, whereas red shows that observed YLDs exceed expected levels. Alzheimer's=Alzheimer's disease and other dementias. Back & neck=low back and neck pain. COPD=chronic obstructive pulmonary disease. Drugs=drug use disorders. Haemog=haemoglobinopathies and haemolytic anaemias. Heat & cold=environmental heat and cold exposure. IHD=ischaemic heart disease. Iron=iron-deficiency anaemia. NTD=neglected tropical diseases. Oncho=onchocerciasis. Oral=oral disorders. Osteoarth=osteoarthritis. Other cardio=other cardiovascular and circulatory diseases. Other MSK=other musculoskeletal disorders. Other nutr=other nutritional deficiencies. Other unint=other unintentional injuries. PEM=protein-energy malnutrition. Schitso=schistosomiasis. Schiz=schizophrenia. Sense=sense organ diseases. Skin=skin and subcutaneous diseases. TB=tuberculosis.

Clear, though varied, patterns emerged across and within GBD regions in comparison of observed levels of YLDs due to leading causes of disability with levels expected on the basis of SDI. Figure 7 displays ratios of observed and expected YLDs for the leading ten causes at level 3 of the GBD hierarchy in 2015, colour coded by the magnitude of differences between observed and expected YLDs.

Globally, lower back and neck pain was the leading cause of disability in 2015. Two mental disorders, major depressive and anxiety disorders, were the third and ninth leading causes of global disability, and diabetes was the sixth leading driver of disability. Iron-deficiency anaemia was the only Group 1 cause among the leading ten causes for global YLDs (ranked fourth). Sensory disorders ranked second and skin diseases ranked fifth. Lower back and neck pain was the leading global cause of disability in 2015 in most countries. The leading cause of disability in 2015 was iron-deficiency anaemia in 27 countries; HIV/AIDS in all six southern sub-Saharan African countries; depression in five eastern sub-Saharan Africa countries; other neglected tropical diseases in Angola, Democratic Republic of the Congo, and Gabon; sense organ disorders in Comoros and Myanmar; diabetes in Fiji and Marshall Islands; war in Lebanon and Syria; and onchocerciasis in Liberia.

#### Regional, country, territory, and selected subnational results

Lower back and neck pain was the leading cause of disability in all high-income countries in 2015. However, ratios of observed to expected YLDs from lower back and neck pain ranged from 0·60 for Singapore to more than 1·59 in Norway. Most high-income countries experienced higher than expected levels of disability due to depressive disorders. The USA and Australia were the only two high-income countries where drug use disorders were a top ten cause of disability, and observed levels of YLDs were much higher than expected. South Korea's ratio of observed-to-expected levels of YLDs due to diabetes exceeded 1·30, whereas Japan's diabetes-related disability was lower than expected on the basis of SDI. In the UK, observed disability due to asthma was well above expected levels.

In 2015, lower back and neck pain was the leading cause of YLDs for all but two countries in Latin America and the Caribbean; Haiti and Venezuela were the exceptions, with iron-deficiency anaemia as the leading causes of disability for both countries. Disability due to diabetes surpassed expected levels by at least a factor of two for six countries and territories: Antigua, Barbados, Dominica, Puerto Rico, Trinidad and Tobago, and the Virgin Islands. Peru, however, had a ratio of less than 0·6 for observed and expected YLDs for diabetes.

In 2015, lower back and neck pain was the leading cause of YLDs for 24 out of 28 countries and territories of southeast Asia, east Asia, and Oceania. Other leading causes of disability were sensory disorders in Myanmar, diabetes in Fiji and the Marshall Islands, and iron-deficiency anaemia in Papua New Guinea. Although lower back and neck pain was the primary cause of disability, many countries had far lower levels of this than expected given their SDI, including Thailand (0·73), Indonesia (0·76), and Malaysia (0·75). Observed YLDs due to depressive disorders were often lower than expected, with 22 countries recording ratios below 0·80. Conversely, numerous geographies recorded YLD ratios exceeding 2·00 for diabetes (eg, 2·29 in Taiwan).

Beyond lower back and neck pain, which was the leading cause of YLDs for three of five countries in south Asia in 2015 (with India and Pakistan being the exception), a mixture of causes accounted for the region's main causes of disability; this heterogeneity probably reflects the diversity of countries in the region and their places along the development spectrum. Iron-deficiency anaemia was the first leading cause and lower back and neck pain was the second leading cause of YLDs in both India and Pakistan, whereas sensory disorders, other musculoskeletal disorders, and iron-deficiency anaemia ranked second for disability in Nepal, Bangladesh, and Bhutan, respectively. YLDs due to anxiety were lower than expected in all countries in the region except for Bangladesh.

In the GBD super-region of central Europe, eastern Europe, and central Asia, lower back and neck pain was the leading cause of disability in every geographical region in 2015; Although all countries in eastern and central Europe recorded higher than expected YLD ratios for most of their top ten causes, central Asia mostly had lower than expected or as expected YLD ratios. Sense organ diseases were the second leading causes of disability and depressive disorders were the third leading causes in this super-region.

Sizeable discrepancies occurred for observed and expected YLDs based on SDI throughout north Africa and the Middle East, probably reflecting the uneven achievements in development found in this region. Lower back and neck pain was the primary driver of YLDs in the region. Exceptions to this were Lebanon and Syria, for which war caused the most disability in 2015, and Afghanistan for which iron-deficiency was the leading cause. Depression, sense organ diseases, diabetes ranked second, third, and fourth, respectively, for the region. Observed YLDs due to diabetes consistently exceeded expected levels, with two countries posting ratios higher than 3·00 (ie, Qatar [3·12], and the United Arab Emirates [3·52]). Iran and Morocco recorded much higher disability due to drug use disorders than expected based on SDI.

In comparison with the rest of the world, ranks of cause-specific disability, and their ratios of observed to expected values, were vastly different in sub-Saharan Africa. Of the 46 countries within the super-regions, nine had lower back and neck pain as the leading cause of YLDs in 2015. In southern Sub-Saharan Africa, HIV/AIDS was the leading cause of disability for all countries. Iron-deficiency anaemia ranked as the leading cause of YLDs for 11 countries in western sub-Saharan Africa. For the remaining countries, lower back and neck pain were primarily the leading cause of YLDs; Liberia, the exception, had onchocerciasis as its leading cause of disability. Malaria and various neglected tropical diseases caused more YLDs than expected in most west African countries. For eastern sub-Saharan Africa, sense organ diseases, iron deficiency, depressive disorders, and lower back and neck pain were leading causes of YLDs in 2015. In central sub-Saharan Africa, skin diseases were consistently among the leading three-to-four causes of disability in this region. HIV/AIDS resulted in far more YLDs than expected based on SDI, with Equatorial Guinea, and Central African Republic recording ratios of observed versus expected YLDs higher than 2·00.

## Discussion

We used 60 900 data sources to estimate the incidence and prevalence of 2619 sequelae of 310 causes for 591 geographical regions for the years 1990, 1995, 2000, 2005, 2010, and 2015. After accounting for variation over time and across regions in case definitions, disease assays, survey instruments, and coding practice, and using standardised modelling approaches to deal with missing data, conflicting data, and a range of sampling and non-sampling errors, we characterised the global and national patterns in non-fatal health outcomes. We found that global age-standardised YLDs per capita decreased slightly in the last 25 years from 0·114 (0·085–0·147) in 1990 to 0·110 (0·082–0·141) per capita in 2015, a total decrease of 3·69% (3·12–4·25%) over a generation (appendix pp 714–16). The ageing of the world's population and the general increase in YLDs per capita with age resulted in an increase in global YLDs per capita. Within this overall pattern, 133 out of 310 causes had statistically significant decreases in YLDs per capita between 2005 and 2015 compared with 82 causes with statistically significant increases in YLDs per capita over the same time period. The most important contributors to global YLDs were musculoskeletal disorders (18·5% [16·4–20·9%] of all YLDs in 2015), mental and substance use disorders (18·4% [15·6–21·2%]), and the category of other NCDs (17·9% [15·0–21·7%]), dominated by hearing loss, vision loss, and skin diseases.

### Typology of diseases based on YLDs and YLLs

The comparison of trends in age-standardised YLDs and YLLs provides insight into the drivers of differences in rates of increases and decreases for diseases and injuries. Diseases and injuries can be parsed into four groups. The first of these are a small set of disorders including causes such as drug use disorders and skin cancer for which age-standardised rates of both YLDs and YLLs increased at a rate of more than 0·4% per year from 2005 to 2015. A second category of diseases includes those in which YLDs are increasing but YLLs are decreasing, including disorders such as thyroid cancer, cirrhosis, and neonatal encephalopathy. One explanation for this pattern is that risk factors, broadly defined, are causing an increase in incidence, whereas access to effective treatment has improved enough to continue reducing mortality. For a third category of disorders, YLDs are essentially constant, but YLLs are decreasing. This grouping is quite large and includes many of the major causes of disability such as musculoskeletal disorders, various neurological disorders, and a number of cancers. For many infectious causes, decreased are occurring for both YLLs and YLDs but are much faster for YLLs; this pattern is to be expected if access to effective treatment is decreasing the number of deaths but not reducing prevalence or incidence. The last category includes disorders where decreases are equal, if not slightly higher, for YLDs including ischaemic heart disease, falls, cervical cancer, and other injuries. Examination of differential trends by region or country might provide insights into the role of access to treatment in reducing YLLs and, conversely, the role of increasing or decreasing risks and social determinants in driving changes in disease incidence and prevalence. More detailed exploration of these differential trends is warranted in future work.

#### Disability and retirement age

As life expectancy has steadily increased in most countries, there have been calls in some high SDI countries to extend retirement ages to reflect these changes in survival.^28^, ^29^, ^30^ A crucial factor in these debates is whether individuals are living for longer and have higher average levels of functional health at each age or not. In this study, we found that age-standardised YLDs per capita decreased, but rather slowly, over the period 2005 to 2015. Comparison of trends in age-standardised YLDs and YLLs by cause shows that the main causes of YLDs are not decreasing and in some settings might be increasing. Other than a somewhat slower rate of improvement for YLDs at oldest age (≥80 years), age discontinuities are not observable in these patterns. Ultimately, the debate on retirement age will hinge on the skill sets required for different types of work, whether work contributes to diminished or improved functional health status, and societal expectations for retirement. Our findings suggest that the burden from mental and substance use disorders, and musculoskeletal disorders, which are frequent causes of early retirement^31^, ^32^ in terms of age-specific prevalence, has not declined much over time; the continued prevalence of these and other disorders associated with increasing age might limit the capacities of an older workforce.

#### Epidemiological transition

The analysis of YLD rates by Level 2 causes as a function of SDI shows a very different picture than that reported for YLLs. At the lower end of the SDI spectrum, incremental increases in SDI are associated with reductions in both age-standardised YLDs and all-age YLD rates spurred by decreases in disability from neglected tropical diseases and anaemia as well as decreases in tuberculosis, diarrhoea, pneumonia, meningitis, and other infectious diseases. At SDI levels above 0·8 (see methods appendix pp 698–711 for SDI values for each country), age-standardised rates of health loss increase slightly, attributable to higher rates of mental and substance use disorders, musculoskeletal disorders, and neurological disorders. In this phase of the epidemiological transition, this increase in rates, although small, combined with population ageing, results in increases in YLDs per 100 000. The rising average YLD per capita rates have implications for health systems; a larger fraction of the population is likely to need care for many disorders. Some of these disorders are currently costly to manage.^33^ The increase in health-care costs per individual in the population that occurs once an SDI of 0·8 is exceeded is a predictable component of the epidemiological transition; these costs should be anticipated during the health planning processes of countries in that stage of the transition.

### Disease-specific issues

#### Musculoskeletal disorders

Musculoskeletal disorders continue to be a leading cause of disability worldwide and more so when taking into account that additional musculoskeletal burden from long-term sequelae of fractures and dislocations is classified under injuries in GBD. A key component of healthy ageing is to maintain mobility, and a key public health intervention recommended for improving health outcomes for all chronic diseases is physical activity. Painful musculoskeletal disorders increase with age and are a great threat to mobility, compromising health more broadly.^34^, ^35^, ^36^ Even if cures for musculoskeletal disorders are not yet available, the clinical goal of preventing disability is attainable.^37^, ^38^

### Mental and substance use disorders

Consistent with the findings of earlier GBD studies, GBD 2015 confirms the large contribution of mental and substance use disorders to global disability. For the first time, in GBD 2015 we found a positive association between conflict and depression and anxiety, albeit with wide uncertainty. This uncertainty is due to sparse and low-quality data for these disorders in post-conflict countries. Going forward, we plan to make separate estimates for post-traumatic stress disorder in GBD, which might add considerably more data from conflict settings and show a stronger association. GBD results have provided an evidence base to support global action such as a stated commitment by the World Bank and WHO to make mental health a global development priority^39^ and the consideration of mental health and substance use disorders in shaping the Sustainable Development Goals.^40^ Cost-effective interventions are able to reduce the burden imposed by mental and substance use disorders, including in low-income and middle-income countries.^41^

Increases in deaths attributable to drug use in the USA have resulted in considerable policy and media attention. In our assessment, the ten countries with the highest prevalence of opioid dependence in decreasing order in 2015 were Iran, United Arab Emirates, Russia, Morocco, the USA, Australia, Ukraine, Tunisia, Belarus, Canada, and Iraq. Among these countries, we estimated the highest excess mortality from opioid dependence in the eastern European countries at about twice the level of that in the USA, Canada, and the north African and Middle Eastern countries; the lowest rate among these highly prevalent countries was in Australia. Within the USA, prescription opioids have been estimated to account for 37% of drug overdose deaths in 2013.^42^, ^43^ The availability of overdose response treatments in the form of naloxone kits to laypersons has also accelerated.^44^ An alternative approach is opioid substitution treatment to reduce the risk of overdose. The intensity at which countries use harm-reduction strategies such as needle exchange and opioid substitution programmes follows an inverse pattern,^45^ with the most intense programmes in Australia, but very rare use of such strategies in eastern Europe, suggesting that embracing harm reduction is an effective means of reducing drug deaths.

### Diabetes

To obtain standard estimates for health loss due to diabetes for GBD 2015 using both fasting plasma glucose (FPG) means and standard deviations as well as diabetes prevalence, we re-extracted all available data from 1990 to 2015. Across studies, we found 20 different case definitions for diabetes. We also included, wherever possible, studies reporting on mean FPG but not diabetes prevalence, using a regression between mean FPG and diabetes prevalence from studies reporting on both. We have also made the assessment of diabetes prevalence more consistent with cause of death data for diabetes. Our improved efforts at measuring the prevalence of diabetes confirms the increase in global age-standardised incidence, prevalence, and YLDs. Of note, the increase in YLDs at the global scale is greater than the increase in YLLs, which reflects improved access to treatments that lower case fatality. The rise of diabetes prevalence, related to the global increase in body-mass index,^46^ given the costs of treating the disease^47^ and the related increases in cardiovascular risks, poses one of the more important challenges to health systems in the coming years. This is particularly the case in regions with high prevalence of diabetes such as central America, north Africa and the Middle East, and Oceania.

Ezzati and colleagues^48^ and the International Diabetes Federation (IDF)^49^ have estimated diabetes prevalence for many countries; the intra-class correlation coefficient for the estimates from Ezzati and colleagues and the GBD for 2015 is 0·74, and for the IDF estimates is 0·65. These large differences appear to stem from the inclusion of cause of death data in the modelling for GBD and also from the inclusion of self-reported diabetes prevalence in the study by Ezzati and colleagues.^48^ In GBD, these sources were excluded from our analyses because of changing patterns in the prevalence of known and unknown diabetes in surveys that vary with geography, making it difficult to crosswalk these studies.

### Dementia

Brayne and colleagues^50^, ^51^ reported that age-specific prevalence of Alzheimer's was decreasing in the UK. Of the four studies in the USA with similar measurements over time that were reviewed, one showed a decrease, whereas no change in prevalence was seen in the remaining studies.^52^, ^53^ Our assessment of age-standardised prevalence showed that Alzheimer's and other dementias decreased slowly in the UK, by 3·69% (2·53–4·85%) from 2005 to 2015, but remained constant in the rest of the world. The decrease might be due to reductions in vascular dementias rather than reductions in Alzheimer's and other dementias. Our assessment also suggests that the number of individuals with Alzheimer's disease increased from 21·7 million (18·9–24·8 million) worldwide in 1990 to 46·0 million (40·2–52·7 million) in 2015. Although it is useful to know that age-standardised rates might be starting to decrease, if only slightly, the rapid increase in the absolute number of cases points to the major challenge that dementia presents to societies with increasing life expectancy. This number is similar to the most recent estimates from the World Alzheimer Report 2015 that estimated 46·8 million people living with dementia in 2015. However, there is less agreement about new cases per year: The World Alzheimer Report estimated 9·9 million new cases of dementia per year in 2015 compared with 6·44 million (5·52–7·45 million) new cases in 2015 estimated in GBD 2015. The World Alzheimer Report separately analysed prevalence and incidence whereas we use DisMod-MR 2.1 to produce internally consistent prevalence and incidence estimates. We observed a fundamental disagreement in the underlying incidence and prevalence data in many countries and decided to exclude incidence data from our modelling approach, putting greater trust in prevalence studies than incidence studies, arguing that the point of incidence in dementia is more difficult to establish than prevalence when the diagnosis over time becomes more established.

### Hepatitis C

The availability of new medical treatments for chronic hepatitis C has driven considerable interest in the prevalence data for this disease. Available treatments are highly effective but costly.^54^ Some programmes have been launched in Egypt and other lower-SDI countries that offer treatment at lower cost. Good data on the number of courses of treatment that have been delivered are not widely available. Given the number of individuals with chronic infection and the potential to be cured, tracking the fraction of people treated each year should be undertaken. Even in high-income countries such as the USA, the fraction of those treated among those who would benefit from treatment is not yet available.

### Malaria

Our assessment of malaria prevalence and incidence in high burden sub-Saharan Africa was based on the Malaria Atlas Project spatial analysis of prevalence surveys done across Africa between 2000 and 2015.^55^, ^56^ The dynamic map of malaria prevalence was updated for GBD 2015 and extended back to 1980. Children younger than 5 years in sub-Saharan Africa showed a remarkable 33·5% (24·8–46·0%) decrease in incidence from the 2005 peak of 0·78 (0·56–0·96) cases per person per year to 0·52 (0·33–0·71) in 2015. Yet there were 81·7 million (52·0–111·7 million) incident cases in this age group in 2015. The decrease in children was more rapid than that recorded for older age groups. In adolescents and adults, incidence dropped by 30·0% (17·2–39·2%) and 21·1% (15·3–26·9%), respectively, and resulted in 58·7 million (37·7–91·5 million) and 53·3 million (40·7–67·5 million) incident cases, respectively, in 2015. The observed decreases in malaria incidence underscore the remarkable effect of the scale-up of antimalarial interventions^57^ across Africa in the past decade.^55^ Sustaining the levels of these interventions is crucial to continue to reduce malaria morbidity on the continent, avoid resurgence, and improve on these levels that are vital to global aspirations for malaria eradication.^58^ Overall, we estimate there were 286·9 million (219·7–377·3 million) cases of malaria worldwide in 2015, whereas the World Malaria Report (WMR) 2015 reports 214 million (149–303 million) cases in 2015.^59^ Of these cases, the WMR estimates that 88% of cases occurred in the WHO African region (188 million) whereas we estimate 189·4 million (151·7–239·4 million; 66·1%) cases for GBD 2015. Outside of Africa, the WMR reports 26 million cases versus our estimate of 97·3 million (50·2–181·8 million), but both sets of estimates feature identical regional rankings among the six WHO regions with malaria. Increasing convergence in estimates is expected as dynamic maps of malaria prevalence and incidence become available for all malaria-endemic countries outside of Africa.

### Tuberculosis

We added new data from tuberculosis prevalence surveys done in Indonesia, Ghana, and several subnational locations in India. Our analysis of tuberculosis relied on prevalence surveys, case notifications and cause-specific mortality estimates. We used the expert judgment values for the case-detection rates (CDRs) from WHO as an initial guide of how much notifications need to be increased to reflect incidence of all tuberculosis, but relied on DisMod-MR 2.1 to find an estimate that is consistent with available prevalence and cause-specific mortality rates. We found that, particularly in older age groups, our estimated CDR often falls below the all-age CDR of WHO. In GBD 2013, we used a relative risk approach to predict the proportion of HIV-infected individuals with tuberculosis. In GBD 2015, we improved our modelling strategy by making use of more abundantly available data on the proportions of HIV infected cases among all tuberculosis cases from the WHO case notifications to separate out combined HIV and tuberculosis from all forms of tuberculosis. In our modelling of tuberculosis, we have not separately estimated the incidence and prevalence related to multidrug-resistant tuberculosis. Given the policy interest in multidrug-resistant tuberculosis, we plan to include estimates for multidrug-resistant tuberculosis in future rounds of the GBD. Our global tuberculosis (all-forms) incidence estimate (10·2 million [9·2–11·5 million] cases in 2015) is slightly higher than that of WHO (9·6 million cases in 2014), and we estimate a slightly larger fraction of combined HIV and tuberculosis (13·0%) than WHO (12·0%). Our list of countries with high burden of tuberculosis is consistent with that of WHO, with a few exceptions. Afghanistan and Cambodia are lower in our estimates, and surpassed by Ukraine and Angola.

### Cardiovascular diseases

Ageing and population growth have led to a growing number of people living with atherosclerotic vascular disease worldwide, despite the decrease in incident myocardial infarction and ischaemic stroke in high-income regions. Rising levels of obesity and diabetes in many countries makes this an area of major concern for global health. Increased attention will need to be directed toward this highly treatable set of disorders. Health systems will need to improve the delivery of cost-effective treatments such as blood pressure and cholesterol-lowering drugs while efforts continue to focus on decreasing tobacco smoking, improving diet, and increasing physical activity. Investments in universal primary care and public health campaigns need to be balanced against the need to improve access to emergency care, including the strengthening of pre-hospital systems and expanded access to revascularisation treatments.

### Cancer

The International Agency for Research on Cancer last produced cancer estimates by country, age, sex, and cancer site for 2012 for the GLOBOCAN project.^60^ The total estimated cancer incidence from GLOBOCAN for 2012 was 14·1 million individuals. By comparison, GBD 2015 estimated 18·6 million (18·0–19·4 million) new cases of cancer in 2015, which includes cases of non-melanoma skin cancer. GLOBOCAN used nine different methods to estimate incidence, which precludes the calculation of uncertainty for those estimates. The GBD relies on the strength of the mortality estimates and transformation of the mortality estimates to incidence, taking into account uncertainty associated with both the mortality estimates and with the mortality to incidence ratios. Until high-quality cancer incidence is available in all countries, the GBD approach, which maximises the amount of data used to determine incidence estimates, is highly beneficial.

### Vision loss

Globally, 34·3 million (30·7–38·0 million) people are blind, an additional 24·3 million (21·6–27·6 million) have severe vision impairment, 214 million (193–237 million) have moderate vision impairment, and 663 million (638–690 million) have near vision impairment in 2015. Combined, vision loss accounts for 24·5 million (17·0–34·5 million) YLDs and is the third largest impairment after anaemia (77·9 million [52·4–111·4 million] YLDs) and hearing loss (46·2 million [31·6–63·2 million] YLDs). YLDs from vision loss are slightly lower than those for anxiety disorders, which rank ninth worldwide. Largely due to ageing, substantial increases occurred from 2005 to 2015 in the number of people with blindness (increasing by 23·3% [21·9–24·6%]), severe visual impairment (24·3% [22·4–26·0%]), moderate visual impairment (22·0% [20·1–23·7%]), and near vision impairment (22·5% [20·9–24·0%]). Most causes of vision loss can be prevented or cured using cost-effective interventions.^61^, ^62^

### Chronic kidney disease

Compared with our GBD 2013 estimates, we estimated global prevalence of chronic kidney disease to be lower by a third. Data for chronic kidney disease at younger ages are sparse. We increased the number of datapoints below the age of 30 years from 20 to 51, and that led to considerably lower prevalence estimates for these ages. We have used different strategies for estimating deaths versus YLDs secondary to chronic kidney disease due to other causes. In our cause of death analysis, deaths secondary to many of the causes typically categorised within the chronic kidney disease other category, such as cystic disease and other congenital renal diseases, were already counted under the primary disease following the principles of the ICD, and thus could not be recounted within the chronic kidney disease other category. In comparison, for the YLD analysis we counted chronic kidney disease from these causes in the chronic kidney disease other category. This difference in the classification of morbidity and mortality from chronic kidney disease other is driven by the underlying nature of the data on causes of death and prevalence. In future GBD versions, we plan to make explicit estimates of death and YLDs for polycystic kidney disease, an important component of the other category.

### Emerging infectious diseases

Emerging and re-emerging infectious diseases pose a persistent yet dynamic challenge to global health^63^ and to the GBD study. GBD 2015 has included Ebola virus disease in response to the devastating outbreak in west Africa starting in 2013,^64^ whereas we have not captured the recent expansions of chikungunya^65^ and Zika virus across the Americas,^66^ for instance. Quantifying the effect of newly emerging epidemic diseases is difficult because the formal reporting of the disease events might considerably lag the first presentation of cases. As a result, contemporary events such as the substantial deviation from baseline levels in reported congenital abnormalities in Brazil in 2015,^67^ now established to be associated with Zika virus,^68^ will need to be incorporated into GBD 2016.

### Advances in data and analysis

#### DisMod-MR

For GBD 2015, we improved the estimation for subnational units in DisMod-MR 2.1 compared to GBD 2013 and made substantive efforts to standardise the modelling approach across diseases. Three changes had an important effect on our estimates. First, the cascade was implemented such that national estimates were used to inform subnational estimation (in GBD 2013, each subnational unit was treated as a country within a GBD region). Second, wherever possible, we improved the linkage in the estimation between cause-specific mortality and disease incidence and prevalence by the systematic inclusion of estimated excess mortality data to provide more informative priors for each location in the geographical hierarchy. Third, in GBD 2010, and to a lesser degree in GBD 2013, many DisMod-MR models did not include fixed effects of country covariates for incidence or prevalence. Spatial heterogeneity was largely estimated from the random effects. For GBD 2015, we systematically tested the inclusion of more fixed effects in DisMod-MR 2.1 models including, where appropriate, variables related to the risk factor exposures we estimate for each disease.

#### Disability weights

For GBD 2015, we have not incorporated any new population-based data on disability weights; we have used the same disability weights as for GBD 2013. The present set of disability weights were based on population surveys in nine countries and an open internet survey. So far, these studies have not found systematic variation in disability weights across populations or within populations as a function of education, income per capita, or other variables. In future, we hope that more population surveys on disability weights will continue to be done to enrich the database for disability weight estimation. Given that we have not been able to collect disability weights in every location included in the GBD, it is possible that disability weights might vary systematically across communities. Even if this were to be confirmed empirically, for global standardised comparisons we would still use the global average disability weights; however, country analyses might use local disability weights. Until there is evidence of systematic variation across communities, this remains a theoretical and not a practical consideration. Given the close correlation between the internet survey and the population-based surveys, we are considering the implementation of an open rolling internet survey to collect more data for disability weights including new or revised health-state descriptions.

#### GATHER compliance

Providing all documentation for data sources, establishing access to the datasets used in modelling, and posting the code has been a labour-intensive activity; GBD 2015 is compliant with the new GATHER guidelines as a result.^17^ Posting code, we believe, will stimulate other researchers to explore the methods used in GBD estimation of non-fatal outcomes and hopefully lead to suggestions for improved estimation. With a steadily growing set of co-investigators and a widening community interested in global health estimation, the enhanced transparency of GBD will improve the efficacy of the overall effort.

#### Using claims data

For the USA we used claims data for selected disorders. These data were particularly useful in the estimation of state-by-state prevalence for some disorders. Claims data, however, have important potential biases. Because of exclusion of some disadvantaged groups from health insurance, disease rates might be different for individuals that are covered compared with those from the general population.^69^, ^70^ As the claims dataset included information from Medicaid and Medicare, the coverage schemes for low-income citizens and older adults (older than 65 years) in the USA, the bias toward underestimation might not be so great. Indeed, we found that for many diseases, such as asthma, diabetes, or rheumatoid arthritis, the claims data were consistent with high-quality survey estimates. A potential problem of overestimation exists because visits to rule out a diagnosis can be coded to the diagnosis. Others working with claims have counted diagnoses only if they had appeared at least twice during a year. Applying such a restriction would have led to rejection of 44–68% of skin disorders, such as acne, psoriasis, or scabies, where one would expect that even after a single visit a diagnosis can be reliably made. As we tend to adjust the claims data used in DisMod-MR 2.1 if a systematic bias is detected from population-based sources, such as the National Health and Nutrition Examination Survey or other quality surveys, we believe our estimates have not been influenced much by this problem. The potential for greater inclusion of claims data in the GBD analysis is large; in countries with universal health access, claims data might overcome the problems of non-responder bias in survey data, which tend to lead to underestimates of the true population prevalence or incidence.

#### Self-reported functional health status

We found only modest progress in high-SDI countries in reducing age-standardised YLDs per capita over the last 25 years. There are, however, reports in the literature of substantial improvements based on self-rated health.^71^, ^72^ In the USA, these improvements seem to be survey programme-specific and have not been reported in all data systems.^71^ Nevertheless, more work is needed to understand the different trends between our assessment of YLDs per capita and self-rated health. Item response theory has been used in some fields to identify changing response patterns for single items compared with the pool of all items.^73^ Item response theory cannot capture systematic changes that affect a set of items, and anchoring vignettes have been proposed as a strategy to deal with these challenges.^72^, ^74^ In the era of increased interest in measuring functional health status, more exploration of the reasons for the divergence from a sequelae-based approach to measuring functional health status used in GBD versus an overall self-rating will be an important avenue of future research.

#### Crosswalks

Because of the diversity of data sources available, we estimated the statistical association between measurements taken using different case definitions, different assays, different survey items, or different ascertainment methods to a reference category. We used these statistical associations to crosswalk each type of data to the reference category. Crosswalks are a crucial dimension in the GBD analysis; in most cases we estimate these crosswalks from within DisMod-MR 2.1. If crosswalks were believed to vary substantially by age, sex, or location, these crosswalks were estimated outside of DisMod-MR 2.1 and adjusted data were used as inputs. Where data are sparse, the estimated crosswalks can shift substantially when new studies are identified that inform the crosswalk. With each iteration of the GBD, we have recognised the crucial nature of the crosswalk analysis for data processing. We believe that more research is needed in future iterations of GBD to standardise the approaches used for estimating crosswalks across disorders and risk factors, and for propagating uncertainty in crosswalks into the final results.

#### Data gaps

We have described the availability of data by cause and geography over different time periods, highlighting very large gaps in information availability. The data availability by country ranged from 6·1% in North Korea to 91·3% in the USA (methods appendix p 606). Among the top five causes of YLDs, data availability was rather poor. Lower back pain, iron-deficiency anaemia, major depressive disorder, other hearing loss, and neck pain all have data representativeness indices below 50%. Because of the broad overview provided of major data sources for disease incidence and prevalence, GBD provides a framework to assist countries in prioritising the collection of new data to inform better monitoring of functional health status. By setting clear reference case definitions and data collection methods (methods appendix pp 4–7), GBD can also provide guidance on how to collect information most relevant to population health measurement.

### Future directions for GBD

With each cycle of the GBD we expect, given the present interest in subnational assessments, to report increasingly granular results. These subnational findings will be of important national policy interest, but the discipline of examining the evidence for each community and modelling at the more granular level will, we believe, improve the quality of the national estimation as well. Additions to the cause list will continue to be driven by policy interest, such as the need to incorporate Zika virus, to split diabetes into separate estimates for type 1 and type 2, and by adding diseases to our cause list that are main contributors to large residual categories, such as other cardiovascular disease, other musculoskeletal disorders, and other neurological disorders. We plan to continue to expand our networks of topic-specific and country collaborators to enhance the quality of our estimates through their feedback and by enhancing the amount of data we can bring to bear on GBD estimation. Our country collaborations to generate subnational estimates have shown us that these efforts greatly enhance access to valuable data sources, particularly administrative datasets on inpatient and outpatient episodes and unpublished surveys.

### Limitations

The GBD 2015 study has some key limitations. First, although we have sought to capture and include in our estimations many sources of uncertainty, we have not captured all sources. We have not been able to routinely capture uncertainty due to different model specifications in our results. We do not have the ability to reflect the uncertainty of data sources that exist but of which we are not aware. Subnational collaborations in China and Mexico revealed many data sources not captured in previous iterations of the GBD study. Inclusion of these data sources can lead to shifts in estimates that are outside the previously estimated 95% UIs.

Second, within DisMod-MR 2.1 we estimate the average association between different types of studies for reporting on a specific outcome such as the difference between 12-month recall and point prevalence in a survey. These estimated associations from within the DisMod-MR 2.1 likelihood estimation are used to adjust the data to a reference case definition or study design. These estimated adjustment factors are themselves uncertain, which increases the overall uncertainty in our estimation. More standardisation in data collection would be highly desirable and would reduce our dependence on the relatively challenging estimation process involved in crosswalks.

Third, for some disorders the data available to estimate excess mortality by age and sex and to capture how excess mortality changes with development status are very limited. There are probably many unpublished sources of information in some countries that could be usefully brought to bear on the challenge of estimating the age–sex–year–location levels of excess mortality.

Fourth, the microsimulation step of this study assumes that prevalence within an age–sex–location–year group of different sequelae is independent. Although this is clearly known not to be the case for some pairs of disorders, the independence assumption provides reasonable overall adjustments for comorbidity. Progress in incorporating dependent comorbidity has been difficult because information on the correlation structure of prevalence is extremely limited and only available for a minor fraction of all possible pairs of conditions in the GBD study.

Fifth, we estimated separate DisMod-MR 2.1 models for 1990, 1995, 2000, 2005, 2010, and 2015. Independent estimation of each time period implies that the uncertainty intervals for each period are also independent. Furthermore, compositional bias in the data available in different time periods might lead to spurious time trends. A more appealing strategy would be to simultaneously estimate the trends in incidence, excess mortality, and remission by age and sex consistent with all the available data for a geography on prevalence, incidence, remission, excess mortality, and cause-specific mortality by age and sex. DisMod-MR 2.1 does not allow for time-varying trends in incidence, excess mortality, and remission, but a prototype DisMod-MR AT (age and time) has been developed and is being tested. Allowing for all rates to change at different paces over age and time increases the number of parameters that need to be estimated by orders of magnitude.

Sixth, we have emphasised in the reporting of results the changes from calendar year 2005 to calendar year 2015, although we have estimated results for 1990, 1995, 2000, 2005, 2010, and 2015 and interpolated for the years in between. For some disorders, reporting the change from 2005 to 2015 (such as for Ebola virus disease) does not capture the major epidemic in west Africa in 2014. Likewise, disasters and wars are often concentrated in a single year; comparisons of any two years can provide misleading inferences about trends.

Seventh, for a few disorders (eg, tetanus, neonatal sepsis, rabies, and diphtheria) we estimate disease incidence from estimates of mortality and the inverse of the case-fatality rate estimated from available studies. When the case-fatality rate is very low, these estimates of incidence are highly sensitive to very small changes in the estimated case-fatality rate and have large uncertainty intervals.

Finally, although extraordinary efforts have gone into vetting the results by GBD researchers and the collaborator network, there are probably some findings that have not been scrutinised carefully enough. Making all results and underlying data available and having an increasing number of visualisation tools are effective strategies that we will keep expanding to meet our goal of producing the highest quality global health data to our growing audience of policy makers, researchers, the media, and the general population.

### Conclusion

The GBD studies seek to quantify the prevalence and incidence of the major sequelae for a comprehensive list of diseases and injuries; given the diversity of data sources, the range of biases in these sources, and the gaps in availability, this is a challenging task. Despite the limitations, the standardised and comprehensive approach of the GBD studies study provides useful insights. We believe users of the findings, including the wealth of country-specific and cause-specific detail available in the appendix, can usefully examine the broad trends for their country or subnational geography, benchmark against geographies at a similar level of development, and understand the strength or weakness of these estimates. Regular quantification of health is particularly important as the new health-related targets of the Sustainable Development Goals have broadened the health agenda throughout the world. At the same time, development and transformations in the risks to health experienced by different groups in the world are leading to some broad transformations in health. Everyone needs to have access to the timeliest, valid, reliable, and local information possible to enrich debates on how to accelerate health progress in all communities.

Correspondence to: Prof Theo Vos, Institute for Health Metrics and Evaluation, Seattle, WA 98121, USA tvos@uw.edu

For **Global Health Data Exchange** see http://ghdx.healthdata.org

For **data in GBD 2015** see http://ghdx.healthdata.org/global-burden-disease-study-2015

For **DisMod-MR 2.1 engine and the code** see http://ghdx.healthdata.org/global-burden-disease-study-2015

## Figures and Tables

**Table 1:** Global incidence of short duration (less than 3 months) sequelae in 2005 and 2015 for all ages and both sexes combined, with percentage change between 2005 and 2015 for level 4 causes with incidence greater than 1 million cases per year

	**Incidence (thousands)**		**Percentage change (%)**
	2005	2015	
Upper respiratory infections	15 624 257 (13 851 237–17 411 199)	17 230 659 (15 351 516–19 172 439)	10·3 (9·0 to 11·6)*
Diarrhoeal diseases	2 235 739 (2 139 050–2 348 407)	2 392 517 (2 301 101––2 503 094)	7·0 (6·0 to 8·0)*
Permanent caries	426 963 (371 216–484 332)	487 629 (423 507–552 895)	14·2 (13·1 to 15·4)*
Otitis media	429 820 (353 915–525 059)	471 027 (386 606–577 286)	9·6 (7·9 to 11·2)*
Lower respiratory infections	273 131 (257 286–288 078)	291 759 (276 244–307 004)	6·8 (5·6 to 8·1)*
Malaria	339 275 (261 417–447 605)	286 859 (219 712–377 332)	−15·4 (−23·0 to −8·1)*
Gastritis and duodenitis	185 250 (167 471–204 974)	213 729 (192 486–236 339)	15·4 (10·8 to 17·2%)*
Pyoderma	178 382 (172 199–184 147)	207 452 (200 498–213 936)	16·3 (15·6% to 17·0)*
Gonococcal infection	138 220 (107 106–184 385)	172 676 (129 731–235 737)	24·9 (17·9 to 31·0)*
Interstitial nephritis and urinary tract infections	127 380 (124 308–130 683)	152 295 (148 748–156 177)	19·6 (19·2 to 19·9)*
Varicella and herpes zoster	128 678 (124 298–133 090)	142 413 (137 804–147 181)	10·7 (10·0 to 11·4)*
Trichomoniasis	121 948 (104 825–141 284)	140 781 (121 207–163 163)	15·4 (14·5 to 16·5)*
Acute hepatitis A	109 609 (101 813–117 648)	114 212 (103 349–124 776)	4·2 (−7·2 to 17·0)
Hepatitis B	98 277 (86 507–112 527)	111 212 (97 410–126 251)	13·2 (−4·3 to 35·2)
Gallbladder and biliary diseases	88 215 (79 276–96 495)	104 322 (93 074–114 430)	18·3 (16·0 to 20·7)*
Peptic ulcer disease	83 388 (77 760–89 435)	87 410 (80 343–94 506)	4·8 (2·6 to 7·0)*
Dengue	32 749 (18 879–68 335)	79 609 (53 784–169 704)	143·1 (−0·3 to 564·7)
Other sense organ diseases	60 659 (58 721–62 579)	69 945 (67 856–72 080)	15·3 (14·6 to 16·0)*
Chlamydial infection	56 976 (45 489–70 839)	61 173 (48 871–76 698)	7·4 (5·5 to 9·4)*
Maternal abortion, miscarriage, and ectopic pregnancy	53 942 (43 630–67 168)	53 958 (43 224–67 417)	0·0 (−3·8 to 4·0)
Syphilis	43 515 (37 479–51 054)	45 413 (37 787–54 921)	4·4 (−0·3 to 8·1)
Deciduous caries	41 353 (28 723–58 131)	43 688 (29 630–62 543)	5·6 (2·1 to 8·1)*
Genital herpes	29 112 (25 131–33 432)	39 791 (35 569–44 572)	36·7 (32·4 to 41·6)*
Urolithiasis	17 875 (16 320–19 728)	22 080 (20 183–24 295)	23·5 (21·2 to 25·6)*
Cellulitis	17 312 (15 988–18 739)	21 211 (19 582–22 985)	22·5 (21·0 to 24·1)*
Maternal hypertensive disorders	20 416 (17 593–23 417)	20 731 (17 355–24 379)	1·5 (−3·0 to 6·3)
Acute hepatitis E	18 869 (17 340–20 580)	19 525 (18 011–21 273)	3·5 (2·2% to 4·7)*
Whooping cough	22 457 (17 322–28 268)	16 298 (12 599–20 445)	−27·4 (−29·5 to −25·2)*
Typhoid fever	15 341 (13 454–17 417)	12 538 (10 887–14 283)	−18·3 (−20·8 to −15·6)*
Maternal sepsis and other maternal infections	11 938 (9457–15 125)	11 817 (9300–15 009)	−1·0 (−4·6 to 2·8)
Appendicitis	10 159 (9549 to 10 840)	11 619 (10 918 to 12 407)	14·4 (13·5 to 15·2)*
Pancreatitis	7160 (6 660–7 694)	8 902 (8 212–9 643)	24·3 (21·9 to 26·6)*
Maternal haemorrhage	8438 (6614–10 620)	8740 (6774–11 061)	3·6 (−0·3 to 7·6)
Other sexually transmitted diseases	7569 (6511–8741)	7656 (6583–8853)	1·2 (−0·1 to 2·4)
Ischaemic heart disease	6308 (5918–6716)	7287 (6798–7808)	15·5 (14·3 to 16·8)*
Maternal obstructed labour and uterine rupture	6830 (5202–8933)	6521 (4977–8588)	−4·5 (−9·1 to −0·3)*
Hepatitis C	4609 (4316 to 4918)	5394 (5032 to 5778)	17·0 (15·7 to 18·4)*
Ischaemic stroke	4651 (4397 to 4912)	5385 (5017 to 5726)	15·8 (13·4 to 18·4)*
Measles	15 610 (6699–31 357)	4652 (2072–9206)	−70·2 (−73·6 to −66·0)*
Paratyphoid fever	5580 (4498–6896)	4587 (3738–5601)	−17·8 (−22·6 to −12·7)*
Haemorrhagic stroke	3144 (2973–3307)	3583 (3336–3822)	14·0 (11·4 to 16·2)*
Paralytic ileus and intestinal obstruction	2664 (2494–2855)	3167 (2946–3406)	18·9 (16·9 to 20·7)*
Encephalitis	1489 (1380–1608)	1603 (1472–1750)	7·7 (5·9 to 9·5)*
Acute glomerulonephritis	1528 (1395–1672)	1534 (1394–1685)	0·4 (−2·6 to 3·1)

Data in parentheses are 95% UIs.

* Percentage changes that are statistically significant.

**Table 2:** Global prevalence of longer duration (more than 3 months) sequelae in 2005 and 2015 for all ages and both sexes combined, with percentage change between 2005 and 2015 for Level 4 causes with prevalence greater than 1%

	**Prevalence (thousands)**		**Percentage change (%)**
	2005	2015	
Permanent caries	2 045 859 (1 909 845–2 170 355)	2 344 628 (2 193 751–2 488 741)	14·6 (13·7 to 15·5)*
Tension-type headache	1 306 390 (1 160 423–1 463 889)	1 505 892 (1 337 310–1 681 575)	15·3 (14·0 to 16·6)*
Iron-deficiency anaemia	1 456 387 (1 449 846–1 462 782)	1 477 531 (1 470 902–1 485 322)	1·5 (0·9 to 2·1)*
Age-related and other hearing loss	943 504 (886 325–995 403)	1 210 055 (1 140 224–1 274 665)	28·3 (27·4 to 29·2)*
Migraine	831 726 (755 991–918 968)	958 789 (872 109–1 055 631)	15·3 (14·0 to 16·6)*
Genital herpes	716 115 (626 987–817 387)	845 826 (736 724–968 066)	18·1 (16·4 to 19·9)*
Refraction and accommodation disorders	672 165 (649 969–694 024)	819 307 (789 917–848 059)	21·9 (20·5 to 23·2)*
Ascariasis	854 489 (790 000–924 895)	761 894 (682 558–861 031)	−10·8 (−22·5 to 2·5)
G6PD trait	663 704 (625 032–703 799)	728 549 (676 735–781 534)	9·8 (7·9 to 11·4)*
Acne vulgaris	605 008 (568 577–642 061)	632 741 (595 242–671 249)	4·6 (3·5 to 5·6)*
Other skin and subcutaneous diseases	492 883 (480 852–505 426)	605 036 (589 500–619 676)	22·8 (22·1 to 23·4)*
Deciduous caries	534 122 (449 525–635 866)	558 028 (462 649–669 027)	4·5 (2·3 to 6·1)*
Low back pain	460 164 (444 680–477 119)	539 907 (521 449–559 556)	17·3 (16·5 to 18·2)*
Periodontal diseases	428 784 (372 953–498 682)	537 506 (465 114–625 889)	25·4 (24·1 to 26·5)*
Fungal skin diseases	434 604 (395 512–475 904)	492 373 (448 951–538 232)	13·3 (12·5 to 14·1)*
Trichuriasis	473 399 (443 689–505 928)	463 652 (426 621–502 939)	−2·1 (−12·0 to 9·0)
Diabetes	333 325 (310 773–355 510)	435 328 (404 736–468 562)	30·6 (28·0 to 33·0)*
Premenstrual syndrome	391 207 (375 009–407 896)	430 697 (410 841–450 494)	10·1 (7·9 to 11·8)*
Hookworm disease	462 111 (430 885–495 596)	428 246 (394 486–468 292)	−7·3 (−16·9 to 3·5)
Sickle-cell trait	338 756 (318 736–378 582)	404 566 (381 223–448 155)	19·4 (18·3 to 20·3)*
Asthma	327 097 (296 406–358 060)	358 198 (323 134–393 466)	9·5 (7·6 to 11·6)*
Neck pain	295 532 (258 878–338 138)	358 007 (313 408–409 411)	21·1 (19·0 to 23·3)*
Hepatitis B	293 745 (284 478–303 036)	343 251 (330 541–357 195)	16·9 (15·3 to 18·4)*
Other musculoskeletal disorders	283 317 (254 135–315 519)	342 068 (305 431–385 147)	20·7 (17·5 to 24·0)*
Urolithiasis	259 567 (238 100–281 892)	318 763 (290 695 349 154)	22·8 (20·8 to 24·9)*
Thalassaemias trait	252 798 (247 008–259 972)	279 451 (272 819–287 357)	10·5 (10·1 to 11·0)*
Malaria	207 773 (186 530–230 942)	278 961 (240 158–320 921)	34·3 (26·8 to 42·3)*
Edentulism and severe tooth loss	216 473 (207 563–226 331)	275 619 (264 201–288 252)	27·3 (26·9 to 27·7)*
Anxiety disorders	232 597 (204 165–264 445)	267 202 (234 064–306 318)	14·9 (13·0 to 16·8)*
Other sense organ diseases	214 761 (207 401–222 622)	266 346 (257 047–276 383)	24·0 (23·1 to 24·9)*
Schistosomiasis	329 773 (297 093–367 878)	252 340 (211 032–321 081)	−23·5 (−32·5 to −4·4)*
G6PD deficiency	231 109 (205 067–259 769)	247 074 (209 307–286 713)	6·9 (1·4 to 11·9)*
Dermatitis	215 260 (199 590–230 536)	245 291 (227 283–262 752)	14·0 (13·2 to 14·8)*
Osteoarthritis	178 665 (173 558–184 053)	237 369 (230 336–244 648)	32·9 (31·9 to 33·8)*
Major depressive disorder	183 434 (163 947–206 420)	216 047 (192 863–243 319)	17·8 (16·6 to 19·0)*
Scabies	191 482 (166 101–223 739)	204 152 (177 534–237 466)	6·6 (4·0 to 9·5)*
Viral skin diseases	161 167 (152 218–170 651)	174 843 (165 156–185 072)	8·5 (8·0 to 9·0)*
Chronic obstructive pulmonary disease	149 115 (137 380–160 739)	174 483 (160 205–188 952)	17·0 (15·1 to 19·0)*
Genital prolapse	137 383 (121 623–154 875)	161 679 (142 335–182 566)	17·7 (15·2 to 20·0)*
Gastritis and duodenitis	135 993 (134 420–137 351)	157 060 (154 055–160 141)	15·5 (12·9 to 17·9)*
Peripheral vascular disease	115 109 (101 439–131 405)	154 651 (136 318–176 211)	34·4 (33·4 to 35·2)*
Uterine fibroids	126 797 (120 900–133 050)	151 115 (144 147–158 477)	19·2 (18·8 to 19·5)*
Hepatitis C	120 457 (108 080–133 129)	142 123 (126 978–157 045)	18·0 (16·7 to 19·2)*
Other mental and substance use disorders	107 895 (107 213–108 449)	128 178 (127 512–128 877)	18·8 (17·9 to 19·7)*
Iodine deficiency	103 701 (93 441–116 438)	110 920 (100 337–125 253)	7·0 (4·7 to 9·4)*
Ischaemic heart disease	87 511 (80 133–96 170)	110 193 (100 332–121 427)	25·9 (24·6 to 27·2)*
Benign prostatic hyperplasia	80 684 (70 338–90 853)	104 625 (90 730–118 244)	29·7 (27·5 to 32·0)*
Dysthymia	86 812 (74 974–99 103)	104 106 (90 398–118 969)	19·9 (18·4 to 21·5)*
Chronic kidney disease due to diabetes	79 184 (68 481–90 737)	100 824 (86 923–115 652)	27·3 (24·9 to 29·9)*
Chronic kidney disease due to other causes	74 917 (64 012–86 576)	94 553 (81 142–109 371)	26·2 (24·2 to 28·3)*
Idiopathic developmental intellectual disability	82 996 (47 588–117 109)	92 074 (52 280–130 411)	10·9 (9·5 to 11·9)*
Other cardiovascular and circulatory diseases	72 772 (68 090–77 763)	90 348 (84 711–96 659)	24·2 (22·7 to 25·5)*
Otitis media	83 022 (73 657–93 266)	86 393 (76 374–97 104)	4·1 (2·7% to 5·5)*
Psoriasis	67 753 (65 107–70 298)	79 700 (76 691–82 804)	17·6 (17·0 to 18·3)*
Other haemoglobinopathies and haemolytic anaemias	73 045 (72 600–73 470)	74 385 (73 898–74 862)	1·8 (0·9 to 2·7%)

Data in parentheses are 95% UIs.

* Percentage changes that are statistically significant.

**Table 3:** Global prevalence and YLDs for 2015, percentage change of counts, and percentage change of age-standardised rates between 2005 and 2015 for all causes, Level 5 sequelae, and nine impairments

						**Prevalence (thousands)**			**YLDs (thousands)**		
						2015	Percentage change in counts between 2005 and 2015	Percentage change in ASR between 2005 and 2015	2015	Percentage change in counts between 2005 and 2015	Percentage change in ASR between 2005 and 2015
**All causes**						**..**	**..**	**..**	**792 004·7 (588 538·7–1 019 955·2)**	15·1 (14·5 to 15·6)*	−2·1 (−2·5 to −1·7)*
	**Communicable, maternal, neonatal, and nutritional diseases**					**4 133 822·3 (4 108 132·5 to 4 161 262·6)**	8·0 (7·3 to 8·7)*	−4·2 (−4·8 to −3·6)*	**112 501·9 (80 347·4–156 270·9)**	**−0·5 (−2·4 to 2·5)**	−9·7 (−11·4 to −7·0)*
		HIV/AIDS and tuberculosis				46 005·2 (44 985·8 to 47 146·6)	18·6 (15·2 to 22·1)*	2·9 (−0·0 to 5·9)	6702·3 (4798·6–8863·5)	2·1 (−3·5 to 7·2)	−12·2 (−17·0 to −7·8)*
			Tuberculosis			8861·2 (8076·3 to 9707·2)	9·2 (6·3 to 12·2)*	−7·2 (−9·5 to −4·7)*	2712·4 (1829·9–3744·0)	9·4 (6·4 to 12·5)*	−6·8 (−9·2 to −4·2)*
			HIV/AIDS			37 277·5 (36 279·9 to 38 339·1)	21·1 (16·9 to 25·5)*	5·6 (2·0 to 9·6)*	3989·9 (2891·2–5159·5)	−2·4 (−10·5 to 5·5)	−15·6 (−23·0 to −8·5)*
			HIV/AIDS—tuberculosis			1258·0 (1142·5 to 1386·9)	−17·9 (−20·7 to −14·9)*	−28·3 (−30·7 to −25·7)*	471·9 (316·0–645·6)	−17·8 (−20·7 to −14·7)*	−28·1 (−30·6 to −25·5)*
				HIV/AIDS resulting in other diseases		37 543·3 (36 350·0 to 39 028·4)	23·6 (20·5 to 26·7)*	7·9 (5·1 to 10·6)*	3518·0 (2 555·4–4 558·9)	0·2 (−9·3 to 9·3)	−13·6 (−22·1 to −5·2)*
					HIV aggregate	21 149·0 (20 260·3 to 22 292·3)	−19·5 (−22·5 to −16·3)*	−29·7 (−32·3 to −26·9)*	1543·6 (1 107·2–2 025·2)	−20·4 (−23·4 to −17·2)*	−30·4 (−33·1 to −27·6)*
					AIDS aggregate	16 394·3 (15 747·0 to 17 154·2)	299·5 (251·4 to 355·5)*	232·9 (193·5 to 279·0)*	1974·4 (1432·0–2559·9)	25·5 (0·9 to 56·1)*	6·1 (−14·6 to 32·4)
	**Diarrhoea, lower respiratory infections, and other common infectious diseases**					**402 320·4 (393 746·5 to 408 994·2)**	8·0 (7·2 to 8·8)*	−2·9 (−3·7 to −2·3)*	**14 865·0 (10 397·0–20 283·4)**	6·2 (5·4 to 7·1)*	−4·0 (−4·7 to −3·3)*
		Diarrhoeal diseases				35 820·6 (34 342·1 to 37 537·8)	6·4 (5·5 to 7·3)*	−3·3 (−4·1 to −2·5)*	5731·7 (3 943·3–7 890·5)	6·4 (5·4 to 7·5)*	−3·1 (−4·0 to −2·2)*
			Diarrhoea episodes aggregate			35 816·3 (34 337·5 to 37 534·1)	6·4 (5·5 to 7·3)*	−3·3 (−4·1 to −2·5)*	5730·4 (3 942·2–7 889·0)	6·4 (5·4 to 7·5)*	−3·1 (−4·0 to −2·2)*
			Guillain-Barré syndrome due to diarrhoeal diseases			4·2 (3·2 to 5·5)	16·9 (14·7 to 19·5)*	−0·2 (−0·9 to 0·4)	1·3 (0·8–1·9)	16·9 (14·7 to 19·5)*	−0·2 (−0·9 to 0·4)
		Intestinal infectious diseases				1666·8 (1594·4 to 1 732·8)	−23·3 (−25·6 to −20·6)*	−28·2 (−30·4 to −25·8)*	220·3 (149·3–306·5)	−18·6 (−22·8 to −13·8)*	−24·2 (−28·1 to −19·8)*
			Typhoid fever			1446·7 (1256·2 to 1 648·1)	−18·3 (−20·8 to −15·6)*	−23·9 (−26·3 to −21·3)*	191·0 (129·5–267·8)	−17·7 (−22·1 to −12·6)*	−23·2 (−27·4 to −18·5)*
				Typhoid fever episodes aggregate		1197·9 (1041·3 to 1366·6)	−18·3 (−21·7 to −14·8)*	−23·9 (−27·1 to −20·5)*	113·3 (75·8–162·5)	−18·1 (−22·6 to −13·4)*	−23·7 (−27·8 to −19·2)*
				Complications of typhoid fever aggregate		248·8 (210·8 to 292·2)	−18·2 (−28·6 to −5·4)*	−23·8 (−33·5 to −12·1)*	77·8 (51·6–110·8)	−17·0 (−28·5 to −3·0)*	−22·6 (−33·1 to −9·9)*
			Paratyphoid fever			529·3 (431·3 to 646·3)	−17·8 (−22·6 to −12·7)*	−23·8 (−28·2 to −19·2)*	27·5 (17·5–40·7)	−17·5 (−24·7 to −9·2)*	−23·5 (−30·1 to −16·1)*
				Paratyphoid fever episodes aggregate		502·2 (409·6 to 612·7)	−17·8 (−22·6 to −12·4)*	−23·8 (−28·2 to −19·0)*	24·4 (15·5–36·4)	−17·5 (−24·9 to −8·7)*	−23·5 (−30·7 to −15·5)*
				Intestinal perforation due to paratyphoid		27·1 (20·9 to 34·5)	−17·6 (−33·0 to 1·4)	−23·7 (−37·8 to −6·1)*	3·1 (1·9–4·6)	−17·5 (−32·8 to 1·6)	−23·6 (−37·7 to −5·9)*
			Other intestinal infectious diseases			..	..	..	1·8 (0·6–4·1)	−67·0 (−78·8 to −45·8)*	−69·3 (−80·2 to −50·1)*
		Lower respiratory infections				8986·6 (8545·2 to 9393·7)	5·1 (4·0 to 6·2)*	−8·2 (−9·1 to −7·3)*	540·4 (365·3–760·3)	4·9 (3·5 to 6·3)*	−8·1 (−9·2 to −7·0)*
			Lower respiratory infection episodes aggregate			8982·2 (8539·8 to 9387·9)	5·1 (4·0 to 6·2)*	−8·2 (−9·1 to −7·3)*	539·1 (364·5–759·5)	4·9 (3·5 to 6·3)*	−8·1 (−9·2 to −7·0)*
			Guillain-Barré syndrome due to lower respiratory infections			4·4 (2·5 to 6·9)	16·9 (14·7 to 19·5)*	−0·2 (−0·9 to 0·4)	1·3 (0·7–2·2)	16·9 (14·7 to 19·5)*	−0·2 (−0·9 to 0·4)
		Upper respiratory infections				233 470·3 (208 009·7 to 259 076·8)	10·2 (8·9 to 11·5)*	−1·4 (−2·0 to −0·8)*	2738·4 (1538·6–4644·3)	10·1 (8·9 to 11·5)*	−1·3 (−1·9 to −0·6)*
			Upper respiratory infection episodes aggregate			233 458·2 (207 997·9 to 259 065·2)	10·2 (8·9 to 11·5)*	−1·4 (−2·0 to −0·8)*	2734·8 (1 533·9–4 641·4)	10·1 (8·9 to 11·5)*	−1·3 (−1·9 to −0·6)*
			Guillain-Barré syndrome due to upper respiratory infections			12·1 (9·6 to 15·2)	16·9 (14·7 to 19·5)*	−0·2 (−0·9 to 0·4)	3·6 (2·2–5·4)	17·0 (14·7 to 19·5)*	−0·2 (−0·9 to 0·4)
		Otitis media				112 089·1 (100 504·6 to 123 655·6)	5·2 (4·1 to 6·5)*	−4·4 (−5·5 to −3·4)*	3321·5 (2 114·2–4 906·3)	3·3 (0·6 to 6·0)*	−5·6 (−8·0 to −3·1)*
				Acute otitis media		25 630·8 (21 044·2 to 31 570·6)	9·4 (7·5 to 11·2)*	1·3 (−0·4 to 3·0)	334·9 (169·4–623·7)	9·4 (7·5 to 11·4)*	1·4 (−0·3 to 3·1)
				Chronic otitis media aggregate		86 458·3 (76 403·0 to 97 133·7)	4·1 (2·7 to 5·5)*	−6·0 (−7·2 to −4·8)*	2986·6 (1904·2–4 371·1)	2·7 (−0·2 to 5·6)	−6·3 (−8·9 to −3·6)*
			Meningitis			8733·5 (8320·6 to 9107·1)	16·9 (13·4 to 20·0)*	2·5 (−0·6 to 5·1)	1516·9 (1076·1–1986·0)	15·0 (13·7 to 16·5)*	1·9 (0·7 to 3·0)*
				Pneumococcal meningitis		7297·2 (6228·5 to 8635·3)	22·2 (19·7 to 25·1)*	6·9 (4·7 to 9·6)*	728·8 (513·1–959·9)	17·6 (15·5 to 19·7)*	3·9 (2·0 to 5·6)*
				Acute pneumococcal meningitis		40·3 (33·5 to 48·4)	25·3 (17·4 to 33·8)*	12·1 (5·2 to 19·5)*	5·3 (3·4–7·8)	25·1 (15·1 to 37·1)*	11·9 (3·2 to 22·3)*
				Complications of pneumococcal meningitis aggregate		7256·9 (6191·3 to 8591·7)	22·2 (19·7 to 25·1)*	6·9 (4·7 to 9·6)*	723·5 (509·2–952·4)	17·6 (15·4 to 19·6)*	3·8 (2·0 to 5·6)*
		*H influenzae* type b meningitis				2455·6 (1966·7 to 3032·6)	−8·3 (−12·2 to −3·9)*	−18·0 (−21·5 to −13·9)*	302·1 (209·4–407·1)	6·9 (3·4 to 10·6)*	−3·3 (−6·6 to 0·0)
			Acute H influenzae type b meningitis			22·0 (17·2 to 28·7)	−19·2 (−25·3 to −11·9)*	−26·0 (−31·8 to −19·2)*	2·9 (1·8–4·5)	−18·7 (−26·1 to −10·8)*	−25·6 (−32·4 to −18·1)*
			Complications of H influenzae type b meningitis aggregate			2433·5 (1947·4 to 3010·8)	−8·2 (−12·1 to −3·7)*	−17·9 (−21·5 to −13·7)*	299·1 (207·9–402·7)	7·3 (3·7 to 11·0)*	−3·0 (−6·3 to 0·3)
		Meningococcal meningitis				1720·8 (1327·7 to 2 180·5)	22·0 (17·9 to 26·2)*	7·5 (3·8 to 11·3)*	161·6 (111·7–215·6)	19·7 (17·5 to 22·1)*	5·1 (3·2 to 7·4)*
			Acute meningococcal meningitis			22·8 (18·9 to 27·7)	24·4 (14·4 to 34·9)*	12·7 (3·7 to 22·4)*	3·0 (1·8–4·5)	24·7 (10·9 to 39·4)*	12·9 (0·5 to 25·9)*
			Complications of meningococcal meningitis aggregate			1698·0 (1305·2 to 2155·2)	22·0 (17·9 to 26·2)*	7·4 (3·8 to 11·2)*	158·6 (109·4–211·9)	19·6 (17·4 to 22·0)*	5·0 (3·1 to 7·3)*
		Other meningitis				3015·2 (2438·5 to 3618·0)	19·0 (16·6 to 21·6)*	3·5 (1·4 to 5·9)*	324·5 (225·1–428·1)	15·3 (13·3 to 17·4)*	0·9 (−0·8 to 2·7)
			Other meningitis episodes aggregate			134·5 (120·0 to 151·7)	16·3 (10·2 to 22·5)*	5·7 (0·1 to 11·4)*	17·8 (11·5–25·3)	16·6 (8·6 to 24·0)*	5·9 (−1·1 to 12·5)
			Complications of other meningitis aggregate			2880·4 (2300·7 to 3474·9)	19·1 (16·5 to 21·9)*	3·4 (1·2 to 5·8)*	306·7 (213·3–406·6)	15·2 (13·2 to 17·5)*	0·6 (−1·1 to 2·6)
	Encephalitis					4315·8 (3145·8 to 5875·6)	4·8 (2·8 to 7·4)*	−9·3 (−11·3 to −6·7)*	457·0 (326·7–594·9)	6·7 (4·6 to 9·0)*	−6·3 (−8·2 to −4·3)*
		Acute encephalitis				81·8 (74·8 to 89·5)	7·1 (5·2 to 9·0)*	−4·1 (−5·7 to −2·4)*	10·8 (7·1–15·5)	7·5 (3·8 to 11·1)*	−3·7 (−6·9 to −0·5)*
		Complications of encephalitis aggregate				4233·6 (3069·6 to 5787·5)	4·8 (2·7 to 7·3)*	−9·4 (−11·4 to −6·8)*	446·2 (318·8–581·4)	6·7 (4·6 to 9·0)*	−6·4 (−8·3 to −4·4)*
	Diphtheria					0·6 (0·3 to 1·2)	−59·6 (−81·7 to −12·7)*	−62·8 (−83·1 to −19·5)*	0·0 (0·0–0·1)	−59·7 (−81·7 to −12·7)*	−62·8 (−83·1 to −19·5)*
	Whooping cough					2232·6 (1725·9 to 2800·7)	−27·4 (−29·5 to −25·2)*	−32·4 (−34·3 to −30·3)*	110·3 (65·6–167·0)	−27·3 (−29·6 to −24·8)*	−32·3 (−34·5 to −30·0)*
	Tetanus					209·3 (204·8 to 214·7)	8·4 (6·1 to 9·8)*	−2·7 (−5·0 to −1·4)*	9·1 (6·7–12·2)	−8·9 (−14·5 to −3·7)*	−18·1 (−23·3 to −13·3)*
		Severe tetanus				8·6 (6·5 to 13·0)	−46·3 (−54·0 to −36·0)*	−52·1 (−59·1 to −42·9)*	1·1 (0·7–1·9)	−45·9 (−53·8 to −35·7)*	−51·8 (−58·9 to −42·6)*
		Complications of tetanus aggregate				200·7 (196·7 to 204·5)	13·3 (12·8 to 13·9)*	1·8 (1·4 to 2·3)*	8·0 (5·8–10·6)	1·0 (−3·7 to 5·4)	−8·8 (−13·0 to −4·9)*
	Measles					127·4 (56·8 to 252·2)	−70·2 (−73·6 to −66·0)*	−72·3 (−75·4 to −68·4)*	11·5 (4·3–25·3)	−70·0 (−73·6 to −65·4)*	−72·0 (−75·4 to −67·7)*
	Varicella and herpes zoster					5907·7 (5489·4 to 6344·6)	15·2 (14·1 to 16·5)*	−0·4 (−0·8 to −0·0)*	207·8 (127·9–316·5)	18·5 (16·5 to 20·8)*	−0·4 (−1·4 to 0·7)
**Neglected tropical diseases and malaria**						**1 900 062·6 (1 863 148·8 to 1 940 058·5)**	**−0·4 (−2·5 to 1·9)**	−10·7 (−12·7 to −8·7)*	**20 763·1 (13 382·4–32 174·5)**	**−4·7 (−13·2 to 8·9)**	−14·5 (−22·2 to −2·4)*
	Malaria					295 717·3 (257 568·4 to 338 449·0)	29·9 (22·5 to 37·2)*	20·0 (12·9 to 27·2)*	3358·2 (2356·9–4703·9)	16·1 (12·8 to 19·3)*	8·5 (5·4 to 11·4)*
		Asymptomatic malaria parasitaemia (PfPR)				206 147·2 (168 670·7 to 246 888·7)	37·3 (27·0 to 48·1)*	26·4 (16·6 to 36·8)*	0·0 (0·0–0·0)	0·0 (0·0 to 0·0)	0·0 (0·0 to 0·0)
		Malaria episodes aggregate				16 756·8 (10 341·8 to 25 965·5)	−15·9 (−23·6 to −8·4)*	−21·1 (−28·4 to −14·1)*	346·6 (174·4–598·4)	−15·8 (−23·6 to −8·2)*	−20·9 (−28·2 to −13·7)*
		Complications of malaria aggregate				876·9 (798·1 to 957·9)	28·4 (26·5 to 30·5)*	18·4 (16·5 to 20·3)*	274·9 (209·7–344·8)	24·7 (20·5 to 29·1)*	15·0 (11·2 to 18·9)*
		Anaemia due to malaria parasitaemia (PfPR) aggregate				71 936·4 (70 137·5 to 73 504·2)	26·3 (24·4 to 28·0)*	17·0 (15·3 to 18·6)*	2736·7 (1848·0–3918·8)	21·1 (18·1 to 24·3)*	13·1 (10·5 to 16·1)*
	Chagas disease					6653·6 (5750·5 to 7575·6)	−7·1 (−9·7 to −4·1)*	−22·1 (−24·2 to −19·7)*	63·1 (42·1–90·8)	−1·8 (−5·1 to 1·7)	−21·0 (−23·6 to −18·3)*
		Chagas disease episodes aggregate				5634·7 (4850·5 to 6414·1)	−7·8 (−10·3 to −4·9)*	−22·2 (−24·3 to −19·8)*	0·0 (0·0–0·1)	−18·7 (−22·9 to −14·4)*	−27·6 (−31·1 to −24·1)*
		Complications of Chagas disease aggregate				666·0 (525·5 to 820·6)	−5·2 (−7·9 to −1·9)*	−22·0 (−24·3 to −19·6)*	44·8 (30·0–62·8)	−2·5 (−6·0 to 1·2)	−21·0 (−23·6 to −18·3)*
		Heart failure due to Chagas disease aggregate				353·0 (221·5 to 503·5)	0·3 (−2·9 to 4·0)	−20·9 (−23·4 to −18·2)*	18·2 (9·7–29·4)	0·2 (−3·6 to 4·4)	−20·9 (−23·8 to −17·8)*
	Leishmaniasis					3859·3 (3438·4 to 4570·4)	27·3 (5·9 to 55·0)*	11·4 (−7·0 to 35·1)	45·8 (22·8–86·7)	25·5 (21·9 to 28·9)*	10·5 (7·5 to 13·5)*
		Visceral leishmaniasis				60·8 (57·5 to 64·7)	10·6 (9·7 to 11·4)*	2·3 (1·6 to 3·0)*	4·3 (2·9–6·1)	10·8 (2·1 to 20·7)*	2·6 (−5·4 to 11·5)
		Cutaneous and mucocutaneous leishmaniasis				3895·9 (3 324·6 to 4 767·5)	27·0 (23·7 to 30·1)*	11·0 (8·3 to 13·8)*	41·5 (19·4–80·8)	27·3 (23·6 to 30·5)*	11·4 (8·3 to 14·5)*
	African trypanosomiasis					10·7 (6·0 to 17·0)	−68·7 (−70·4 to −67·3)*	−72·3 (−73·8 to −71·0)*	3·0 (1·4–5·3)	−67·4 (−72·6 to −62·1)*	−71·0 (−75·5 to −66·6)*
	Schistosomiasis					252 339·5 (211 032·5 to 321 081·3)	−23·5 (−32·5 to −4·4)*	−30·9 (−39·0 to −13·7)*	2472·6 (1275·0–4521·2)	−21·8 (−29·3 to −4·0)*	−29·1 (−36·0 to −13·1)*
		Schistosomiasis episodes aggregate				130 285·7 (114 433·9 to 156 900·0)	−26·3 (−34·5 to −13·6)*	−33·1 (−40·5 to −21·3)*	740·7 (292·3–1584·0)	−27·3 (−35·2 to −14·1)*	−34·0 (−41·0 to −22·0)*
		Complications of schistosomiasis aggregate				105 621·9 (76 641·0 to 160 339·4)	−19·6 (−32·6 to 14·1)	−27·7 (−39·4 to 3·0)	1188·8 (535·9–2 463·3)	−18·0 (−30·5 to 15·6)	−26·3 (−37·5 to 4·3)
		Anaemia due to schistosomiasis aggregate				16 331·3 (16 075·8 to 16 655·1)	−19·6 (−21·1 to −17·7)*	−27·0 (−28·3 to −25·3)*	543·1 (365·1–781·5)	−21·5 (−24·0 to −18·6)*	−27·8 (−30·0 to −25·1)*
	Cysticercosis					1931·0 (1597·8 to 2312·0)	−6·2 (−10·2 to −2·5)*	−20·8 (−24·3 to −17·6)*	286·7 (194·2–392·8)	−16·3 (−21·3 to −11·8)*	−29·2 (−33·3 to −25·3)*
	Cystic echinococcosis					1383·0 (1265·9 to 1498·6)	24·7 (22·6 to 27·0)*	6·7 (5·0 to 8·5)*	126·8 (86·7–174·6)	24·3 (21·1 to 27·5)*	6·6 (4·1 to 9·2)*
	Lymphatic filariasis					38 464·1 (31 328·2 to 46 783·0)	−44·9 (−49·9 to −39·9)*	−51·6 (−56·0 to −47·2)*	2075·0 (1120·6–3311·2)	−16·2 (−32·1 to −3·9)*	−27·7 (−41·5 to −17·1)*
		Prevalence of detectable microfiliaria due to lymphatic filariasis				19 707·9 (17 173·8 to 22 270·5)	−58·8 (−61·8 to −55·3)*	−63·5 (−66·1 to −60·3)*	0·0 (0·0–0·0)	0·0 (0·0 to 0·0)	0·0 (0·0 to 0·0)
		Complications of lymphatic filariasis aggregate				18 756·3 (12 420·1 to 26 397·1)	−14·8 (−29·9 to −3·5)*	−26·7 (−40·1 to −16·7)*	2075·0 (1120·6–3311·2)	−16·2 (−32·1 to −3·9)*	−27·7 (−41·5 to −17·1)*
	Onchocerciasis					15 531·5 (11 963·5 to 19 993·8)	−29·1 (−39·5 to −18·8)*	−36·8 (−46·0 to −27·5)*	1135·7 (546·2–2005·4)	−21·2 (−38·5 to −4·8)*	−31·2 (−46·7 to −16·2)*
		Asymptomatic onchocerciasis				2280·4 (1525·0 to 3173·2)	−62·4 (−66·1 to −59·6)*	−66·2 (−69·5 to −63·7)*	0·0 (0·0–0·0)	0·0 (0·0 to 0·0)	0·0 (0·0 to 0·0)
		Skin disease due to onchocerciasis aggregate				12 223·9 (8 474·7 to 16 585·2)	−17·4 (−31·0 to −2·7)*	−26·5 (−38·6 to −13·1)*	1055·6 (467·4–1894·7)	−22·2 (−41·8 to −3·2)*	−31·6 (−49·0 to −14·8)*
		Vision loss due to onchocerciasis aggregate				1025·4 (724·8 to 1452·1)	−2·3 (−15·0 to 11·8)	−22·3 (−32·3 to −11·0)*	80·1 (51·3–117·5)	−6·1 (−16·9 to 6·0)	−25·4 (−33·9 to −15·9)*
	Trachoma					3557·1 (2940·5 to 4321·8)	4·6 (0·4 to 9·2)*	−19·3 (−22·9 to −15·2)*	279·2 (192·8–396·0)	−1·2 (−5·6 to 3·0)	−23·9 (−27·5 to −20·3)*
	Dengue					4730·0 (2654·1 to 10 254·2)	143·6 (−0·3 to 564·7)	119·7 (−10·1 to 498·7)	764·1 (346·8–1 744·2)	140·8 (−0·1 to 558·4)	117·7 (−9·8 to 494·3)
		Dengue episodes aggregate				1409·2 (937·6 to 2943·8)	134·1 (−3·8 to 521·2)	110·8 (−13·2 to 459·0)	82·1 (45·0–183·9)	141·4 (0·4 to 549·2)*	117·9 (−9·4 to 486·7)
		Post-dengue chronic fatigue syndrome				3324·6 (1600·3 to 7347·9)	143·7 (−0·3 to 564·7)	119·8 (−10·1 to 498·7)	682·0 (295·2–1 608·9)	140·8 (−0·2 to 558·0)	117·6 (−9·8 to 494·1)
	Yellow fever					2·8 (0·8 to 7·7)	−25·7 (−31·6 to −19·2)*	−31·6 (−36·9 to −25·8)*	0·1 (0·0–0·3)	−25·7 (−31·6 to −19·2)*	−31·6 (−36·9 to −25·8)*
	Rabies					0·7 (0·6 to 0·8)	−43·4 (−51·5 to −33·8)*	−50·8 (−57·6 to −42·6)*	0·1 (0·1–0·1)	−43·4 (−51·5 to −33·8)*	−50·8 (−57·6 to −42·6)*
	Intestinal nematode infections					1 447 209·3 (1 414 995·0 to 1 481 332·3)	−2·5 (−5·1 to 0·2)	−12·8 (−15·2 to −10·4)*	3173·8 (1861·3–5090·8)	−22·8 (−27·3 to −17·3)*	−30·4 (−34·5 to −25·4)*
		Ascariasis				761 893·8 (682 557·5 to 861 031·5)	−10·8 (−22·5 to 2·5)	−20·3 (−30·7 to −8·2)*	871·0 (482·5–1 464·3)	−37·7 (−45·3 to −29·4)*	−43·9 (−50·8 to −36·4)*
			Complications of ascariasis aggregate			47 626·4 (44 087·5 to 51 711·9)	−35·6 (−41·9 to −28·4)*	−42·1 (−47·8 to −35·6)*	871·0 (482·5–1 464·3)	−37·7 (−45·3 to −29·4)*	−43·9 (−50·8 to −36·4)*
			Asymptomatic ascariasis			714 230·7 (634 201·9 to 814 009·5)	−8·5 (−20·9 to 6·4)	−18·2 (−29·3 to −4·9)*	0·0 (0·0–0·0)	0·0 (0·0 to 0·0)	0·0 (0·0 to 0·0)
		Trichuriasis				463 652·1 (426 621·2 to 502 939·4)	−2·1 (−12·0 to 9·0)	−12·2 (−21·1 to −2·1)*	544·1 (290·7–946·0)	−16·7 (−30·6 to 3·6)	−24·9 (−37·5 to −6·8)*
			Complications of trichuriasis aggregate			27 129·6 (24 188·7 to 31 890·8)	−15·6 (−27·8 to 2·1)	−24·1 (−35·0 to −8·2)*	544·1 (290·7–946·0)	−16·7 (−30·6 to 3·6)	−24·9 (−37·5 to −6·8)*
			Asymptomatic trichuriasis			436 525·4 (400 013·3 to 475 985·5)	−1·1 (−11·8 to 11·0)	−11·3 (−21·0 to −0·3)*	0·0 (0·0–0·0)	0·0 (0·0 to 0·0)	0·0 (0·0 to 0·0)
		Hookworm disease				428 245·9 (394 486·0 to 468 292·4)	−7·3 (−16·9 to 3·5)	−17·2 (−25·8 to −7·5)*	1758·8 (1088·5–2754·9)	−14·6 (−19·9 to −9·4)*	−22·9 (−27·7 to −18·2)*
			Complications of hookworm disease aggregate			51 012·9 (48 151·1 to 54 346·2)	−14·0 (−21·0 to −6·2)*	−23·2 (−29·5 to −16·3)*	1046·7 (572·8–1732·1)	−15·5 (−24·0 to −6·3)*	−24·4 (−32·1 to −16·3)*
			Anaemia due to hookworm disease aggregate			24 220·2 (24 078·2 to 24 365·2)	−6·2 (−7·3 to −5·1)	−15·5 (−16·5 to −14·6)*	712·1 (476·3–1031·1)	−13·2 (−15·6 to −11·1)*	−20·5 (−22·6 to −18·7)*
			Asymptomatic hookworm disease			352 948·6 (319 143·2 to 392 574·4)	−6·4 (−18·3 to 7·5)	−16·4 (−27·2 to −3·9)*	0·0 (0·0–0·0)	0·0 (0·0 to 0·0)	0·0 (0·0 to 0·0)
	Food-borne trematodiases					71 095·4 (67 365·5 to 75 246·9)	4·0 (1·5 to 6·6)*	−9·3 (−11·4 to −7·1)*	1686·5 (857·1–3066·8)	3·7 (−0·4 to 10·1)*	−10·0 (−13·4 to −5·2)*
		Asymptomatic food-borne trematodiases aggregate				56 131·7 (45 765·9 to 63 620·3)	3·8 (1·2 to 6·6)*	−9·3 (−11·6 to −6·9)*	0·0 (0·0–0·0)	0·0 (0·0–0·0)	0·0 (0·0–0·0)
		Symptomatic food-borne trematodiases aggregate				14 963·7 (8 632·7 to 25 256·6)	4·8 (0·5 to 11·4)*	−9·3 (−12·8 to −4·2)*	1686·5 (857·1–3066·8)	3·7 (−0·4 to 10·1)	−10·0 (−13·4 to −5·2)*
	Leprosy					514·2 (487·0 to 545·7)	−0·1 (−0·5 to 0·3)	−19·7 (−20·2 to −19·3)*	31·0 (20·9–43·5)	0·6 (−1·5 to 2·9)	−19·2 (−20·8 to −17·4)*
	Ebola					2·8 (1·2 to 5·2)	54 521·3 (46 386·2 to 68 346·9)*	49 817·9 (41 865·6 to 62 764·9)*	0·6 (0·2–1·1)	51 908·8 (42 739·0 to 66 884·3)*	47 453·0 (38 609·5 to 61 730·3)*
	Other neglected tropical diseases					61 452·6 (60 842·8 to 62 054·9)	0·1 (−1·0 to 1·3)	−7·7 (−8·8 to −6·6)*	5261·1 (2558·5–12 199·0)	7·4 (−18·9 to 50·6)	−1·2 (−25·5 to 38·0)
	Anaemia due to other neglected tropical diseases aggregate					61 452·6 (60 842·8 to 62 054·9)	0·1 (−1·0 to 1·3)	−7·7 (−8·8 to −6·6)*	2158·2 (1 446·3–3 073·1)	−6·0 (−8·2 to −3·8)*	−12·4 (−14·5 to −10·4)*
**Maternal disorders**						**11 372·9 (10 686·6 to 11 894·1)**	**2·1 (−4·2 to 9·2)**	−7·9 (−13·6 to −1·8)*	**898·8 (642·8–1222·9)**	**−5·1 (−18·7 to 11·2)**	−15·1 (−27·0 to −0·3)*
	Maternal haemorrhage					2304·8 (2231·6 to 2 393·5)	1·6 (−3·0 to 6·5)	−7·8 (−12·0 to −3·4)*	84·7 (57·3–119·5)	0·1 (−13·5 to 17·2)	−9·1 (−21·3 to 6·6)
		Maternal hemorrhage episodes aggregate				191·7 (127·8 to 277·5)	2·9 (−36·5 to 66·2)	−6·4 (−42·1 to 49·9)	31·3 (18·1–49·0)	3·3 (−30·6 to 54·9)	−6·0 (−36·3 to 41·1)*
		Anaemia due to maternal haemorrhage aggregate				2113·4 (2082·8 to 2141·5)	1·5 (−0·9 to 3·9)	−7·9 (−10·1 to −5·8)*	53·5 (35·0–78·6)	−1·8 (−5·9 to 2·6)	−10·8 (−14·5 to −6·9)*
	Maternal sepsis and other maternal infections					3140·8 (2658·0 to 3727·7)	3·7 (−15·2 to 27·8)	−7·8 (−24·2 to 13·1)	56·5 (29·5–102·4)	−1·0 (−42·3 to 70·4)	−9·7 (−47·1 to 55·2)
		Maternal sepsis and other maternal infections aggregate				900·0 (486·7 to 1462·3)	−2·4 (−50·3 to 93·7)	−10·2 (−54·4 to 78·1)	45·3 (22·2–82·7)	−2·7 (−49·0 to 91·0)	−10·5 (−53·2 to 73·6)
		Infertility due to puerperal sepsis				2242·1 (2 030·5 to 2483·0)	6·4 (4·4 to 8·9)*	−6·8 (−8·6 to −4·7)*	11·2 (4·2–24·3)	6·7 (4·0 to 9·5)*	−6·6 (−8·9 to −4·1)*
	Maternal hypertensive disorders					4624·4 (3030·8 to 6592·4)	2·7 (−37·9 to 69·8)*	−5·8 (−43·0 to 55·4)*	222·4 (118·4–365·4)	2·6 (−37·3 to 68·5)	−5·9 (−42·4 to 54·4)
		Maternal hypertensive disorders episodes aggregate				4629·2 (3031·5 to 6602·4)	2·8 (−37·8 to 70·0)	−5·7 (−42·9 to 55·7)	219·1 (115·4–363·3)	2·7 (−37·0 to 68·8)	−5·8 (−42·4 to 55·2)
		Eclampsia				5·4 (2·2 to 10·5)	−5·8 (−69·0 to 182·2)	−12·9 (−71·6 to 158·3)	3·3 (1·2–6·3)	−5·7 (−69·0 to 182·2)	−12·8 (−71·6 to 158·3)
	Maternal obstructed labour and uterine rupture					1077·2 (933·3 to 1255·2)	−13·1 (−17·5 to −7·9)*	−23·9 (−27·7 to −19·7)*	352·0 (234·6–499·9)	−12·6 (−17·3 to −7·3)*	−23·4 (−27·4 to −18·9)*
		Obstructed labour, acute event				89·6 (53·0 to 142·9)	−3·6 (−45·9 to 74·7)	−11·4 (−49·7 to 60·1)	27·7 (14·8–47·0)	−3·1 (−44·9 to 72·1)	−10·9 (−49·0 to 58·2)
		Fistula due to maternal obstructed labour and uterine rupture aggregate				987·6 (850·6 to 1156·0)	−13·9 (−16·3 to −11·4)*	−24·9 (−27·0 to −22·8)*	324·3 (216·2–457·4)	−13·3 (−16·6 to −10·1)*	−24·3 (−27·1 to −21·7)*
	Maternal abortion, miscarriage, and ectopic pregnancy					441·3 (285·5 to 630·6)	−0·4 (−37·2 to 58·1)	−9·4 (−42·4 to 44·2)	48·2 (27·2–78·5)	−0·4 (−36·9 to 60·5)	−9·3 (−42·4 to 45·6)
	Other maternal disorders					..	..	..	135·0 (90·6–190·2)	−2·2 (−19·6 to 17·5)	−12·2 (−27·7 to 5·5)
**Neonatal disorders**						52 961·9 (50 435·8 to 54 978·4)*	14·7 (11·9 to 17·8)*	3·9 (1·3 to 6·7)*	**10 710·5 (8113·9–13 786·1)**	13·3 (9·5 to 17·4)*	**3·1 (−0·3 to 6·8)**
	Neonatal preterm birth complications					41 855·4 (36 843·4 to 47 188·1)*	10·6 (6·2 to 15·2)*	−0·4 (−4·3 to 3·8)*	5090·9 (3864·5–6555·6)*	4·4 (−1·1 to 10·1)	−5·2 (−10·1 to 0·2)
		Vision loss due to retinopathy of prematurity aggregate				4245·1 (3386·2 to 5265·3)	11·1 (7·2 to 15·1)*	−2·3 (−5·8 to 1·3)	135·5 (83·0–208·8)	13·9 (9·5 to 18·4)	−0·8 (−4·5 to 3·2)
		Complications of preterm birth complications aggregate				37 609·0 (33 086·7 to 42 697·0)	10·5 (5·6 to 15·7)*	−0·1 (−4·6 to 4·5)	4955·4 (3756·9–6394·8)	4·1 (−1·4 to 10·0)	−5·3 (−10·4 to 0·2)
	Neonatal encephalopathy due to birth asphyxia and trauma					13 681·5 (9 797·3 to 19 759·3)	27·3 (24·6 to 30·0)*	16·2 (13·7 to 18·5)*	3769·4 (2451·7–5809·3)	25·1 (22·0 to 28·3)*	14·2 (11·5 to 17·0)*
	Neonatal sepsis and other neonatal infections					139·1 (79·9 to 216·0)	−1·6 (−3·0 to −0·0)*	−6·7 (−8·0 to −5·2)*	17·7 (9·2–31·0)	−1·8 (−3·5 to 0·1)	−6·9 (−8·5 to −5·1)*
		Severe infection due to neonatal sepsis and other neonatal infections				130·1 (71·1 to 207·6)	−2·2 (−3·8 to −0·5)*	−7·3 (−8·8 to −5·6)*	16·6 (8·1–30·0)	−2·1 (−3·7 to −0·3)*	−7·1 (−8·7 to −5·4)*
		Complications of neonatal sepsis and other neonatal infections aggregate				8·9 (7·5 to 10·4)	8·0 (4·3 to 12·7)*	2·4 (−1·1 to 6·9)	1·1 (0·6–1·6)	2·6 (−9·4 to 14·4)	−2·7 (−14·1 to 8·5)
	Haemolytic disease and other neonatal jaundice					2042·8 (1825·0 to 2309·0)	20·0 (18·8 to 21·4)*	9·2 (8·0 to 10·3)*	603·0 (451·9–770·8)	17·0 (14·8 to 19·4)*	6·5 (4·4 to 8·6)*
	Other neonatal disorders					..	..	..	1229·5 (836·5–1 647·2)	19·9 (0·8 to 41·1)*	8·5 (−8·5 to 27·6)
**Nutritional deficiencies**						**1 482 655·3 (1 477 085·9 to**	2·7 (2·2–3·2)*	−7·1 (−7·5––6·7)*	**54 632·4 (36 772·8–77 894·3)**	−3·5 (−4·9––2·1)*	−11·4 (−12·7––10·3)*
	Protein-energy malnutrition					22 834·3 (21 764·0 to 23 985·1)	−4·8 (−10·2 to 0·8)	−12·1 (−17·1 to −6·9)*	2823·3 (1820·3–3984·2)	−4·5 (−9·8 to 1·1)	−11·8 (−16·7 to −6·6)*
		Iodine deficiency				110 919·5 (100 337·2 to 125 252·8)	7·0 (4·7 to 9·4)*	−6·0 (−8·0 to −3·8)*	2386·9 (1 508·3–3 564·2)	7·7 (5·7 to 9·8)*	−4·4 (−6·4 to −2·4)*
		Visible goiter due to iodine deficiency aggregate				108 307·6 (97 705·7 to 122 260·0)	6·9 (4·5 to 9·4)*	−6·2 (−8·2 to −4·0)*	1927·5 (1192·0–3040·1)	6·9 (4·4 to 9·3)*	−6·0 (−8·2 to −3·9)*
		Visible goiter with heart failure due to iodine deficiency aggregate				0·3 (0·3 to 0·4)	39·6 (38·3 to 41·0)*	4·2 (3·3 to 5·2)*	0·0 (0·0–0·1)	39·6 (38·3 to 41·0)*	4·2 (3·3 to 5·2)*
	Intellectual disability due to iodine deficiency aggregate					2611·6 (1085·6 to 3621·3)	10·4 (6·1 to 12·6)*	2·0 (−2·0 to 4·3)	459·3 (163·4–752·8)	11·0 (6·4 to 13·6)*	2·8 (−1·6 to 5·2)
	Vitamin A deficiency					4901·1 (3975·6 to 5921·7)	9·3 (6·2 to 12·5)*	0·1 (−2·7 to 2·7)	232·4 (143·5–345·9)	10·9 (7·5 to 14·6)*	0·2 (−2·7 to 3·3)
		Iron-deficiency anaemia				1 477 530·5 (1 470 902·3 to 1 485 322·2)	1·5 (0·9 to 2·1)*	−8·0 (−8·5 to −7·4)*	48 529·2 (32 560·8–69 725·2)	−3·8 (−5·1 to −2·4)*	−11·6 (−12·8 to −10·5)*
		Iron-deficiency anaemia without heart failure aggregate				1 477 458·4 (1 470 824·4 to	39·7 (38·1–41·3)*	6·1 (4·7–7·4)*	8·1 (5·5–11·4)	39·6 (35·2–44·3)*	6·0 (2·5–9·9)*
	Iron-deficiency anaemia with heart failure aggregate					72·1 (64·6 to 80·7)	39·7 (38·1 to 41·3)*	6·1 (4·7 to 7·4)*	8·1 (5·5–11·4)	39·6 (35·2 to 44·3)*	6·0 (2·5 to 9·9)*
		Other nutritional deficiencies				..	..	..	660·7 (396·0–1026·9)	−16·7 (−36·8 to 5·8)	−22·9 (−41·5 to −2·1)*
**Other communicable, maternal, neonatal, and nutritional diseases**						**1 604 919·9 (1 563 786·8 to 1 636 531·0)**	16·1 (15·3 to 17·0)*	**−0·4 (−1·1 to 0·4)**	**3929·7 (2566·9–5786·6)**	6·5 (3·9 to 9·2)*	−4·0 (−6·2 to −1·7)*
		Sexually transmitted diseases excluding HIV				1 145 527·5 (1 100 971·2 to 1 176 685·1)	16·9 (15·7 to 18·3)*	−1·0 (−2·1 to 0·1)	1573·7 (974·0–2501·8)	16·9 (14·5 to 19·1)*	1·3 (−0·6 to 3·4)
				Syphilis		43 604·9 (37 160·3 to 50 658·3)	3·7 (−0·4 to 7·0)	−9·8 (−13·4 to −6·9)*	242·8 (166·1–333·7)	15·3 (12·2 to 18·5)*	−5·6 (−8·0 to −3·0)*
			Early syphilis aggregate			42 350·9 (35 898·4 to 49 441·3)	3·3 (−0·9 to 6·8)	−9·9 (−13·6 to −6·9)*	10·3 (3·0–25·3)	3·4 (−1·1 to 7·4)	−9·9 (−13·7 to −6·3)*
				Adult tertiary syphilis		1254·0 (1127·1 to 1385·8)	15·8 (13·2 to 18·3)*	−5·6 (−7·8 to −3·6)*	232·6 (155·4–322·8)	15·9 (12·6 to 19·1)*	−5·4 (−7·9 to −2·8)*
				Chlamydial infection		82 822·4 (66 426·4 to 104 328·5)	8·3 (6·1 to 10·7)*	−2·9 (−4·8 to −0·9)*	364·6 (210·6–589·5)	10·1 (6·9 to 13·4)*	−1·8 (−4·5 to 0·8)
				Chlamydial infection episodes aggregate		81 072·5 (64 735·8 to 102 635·8)	8·3 (6·0 to 10·7)*	−2·9 (−4·8 to −0·9)*	332·7 (185·2–546·0)	10·7 (7·1 to 14·4)*	−1·1 (−4·1 to 2·1)
				Pelvic inflammatory diseases due to chlamydial infection aggregate		1·73 (147·8 to 202·0)	1·4 (−0·8 to 3·4)	−11·5 (−13·2 to −9·8)*	23·1 (15·5 to 32·5)	1·8 (−2·2 to 5·7)	−11·1 (−14.5 to 7·8)*
				Infertility due to chlamydial infection aggregate		1576·6 (1450·8 to 1703·9)	11·1 (10·0 to 12·2)*	−3·0 (−3·9 to −2·0)	8·8 (3·6 to 18·4)	11·1 (9·0 to 13·2)*	−2·9 (−4·8 to −1·1)*
				Gonococcal infection		47 468·5 (35 848·1 to 62 099·7)	25·3 (18·3 to 31·2)*	12·2 (5·9 to 17·7)*	444·9 (260·8–691·8)	26·3 (20·0 to 31·4)*	12·6 (6·7 to 17·3)*
					Gonococcal infection episodes aggregate	46 561·5 (34 928·2 to 61 227·4)	25·6 (18·5 to 31·6)*	12·5 (6·1 to 18·1)*	430·3 (250·5–671·5)	27·3 (20·7 to 32·5)*	13·5 (7·4 to 18·3)*
					Pelvic inflammatory diseases due to gonococcal infection aggregate	907·0 (822·0 to 1 016·3)	10·0 (7·6 to 11·8)*	−3·8 (−6·0 to −2·2)*	14·6 (9·6–21·4)	3·5 (−0·8 to 7·1)	−8·7 (−12·4 to −5·7)*
				Trichomoniasis		167 618·6 (144 744·1 to 193 444·0)	16·2 (15·3 to 17·2)*	0·9 (0·4 to 1·3)*	194·3 (78·0–408·3)	16·1 (15·0 to 17·2)*	1·0 (0·3 to 1·8)*
				Genital herpes		885 169·4 (772 303·8 to 1 008 588·1)	18·1 (16·4 to 19·9)*	−1·3 (−2·7 to 0·1)	236·4 (74·3–554·2)	19·5 (17·4 to 23·0)*	0·7 (−1·4 to 5·0)
					Moderate infection due to initial genital herpes episode	389·0 (88·0 to 909·7)	38·1 (33·6 to 43·2)*	29·5 (25·4 to 34·3)*	19·0 (4·3–45·8)	37·6 (30·9 to 45·6)*	29·1 (22·9 to 36·7)*
					Complications of genital herpes aggregate	884 780·3 (771 914·6 to 1 008 400·6)	18·1 (16·4 to 19·9)*	−1·3 (−2·7 to 0·0)	217·3 (62·2–534·5)	18·1 (16·3 to 19·9)*	−1·2 (−2·7 to 0·3)
				Other sexually transmitted diseases		4821·8 (4452·8 to 5174·6)	9·6 (8·1 to 11·0)*	−4·7 (−5·9 to −3·6)*	90·7 (61·5–129·7)	3·7 (1·1 to 6·2)*	−10·0 (−12·2 to −7·8)*
					Pelvic inflammatory diseases due to other sexually transmitted diseases aggregate	507·2 (437·2 to 591·9)	1·2 (−0·1 to 2·6)	−12·2 (−13·1 to −11·2)*	66·8 (45·4–92·9)	1·3 (−1·2 to 4·2)	−12·0 (−14·3 to −9·8)*
					Infertility due to other sexually transmitted diseases aggregate	4314·5 (3943·8 to 4657·1)	10·7 (9·1 to 12·2)*	−3·8 (−5·1 to −2·4)*	23·9 (9·7–48·8)	10·8 (9·0 to 12·7)*	−3·6 (−5·2 to −2·0)*
					Other sexually transmitted diseases	..	..	..	329·4 (200·1–504·2)	19·5 (14·4 to 24·6)*	6·7 (2·2 to 11·3)*
	Hepatitis					497 901·2 (490 397·2 to 505 815·2)	16·7 (15·8 to 17·6)*	2·2 (1·4 to 2·9)*	406·4 (267·6–588·8)	12·6 (1·7 to 24·1)*	1·2 (−8·3 to 11·5)
		Acute hepatitis A				8785·5 (7 950·0 to 9 598·2)	4·2 (−7·2 to 17·0)	−2·8 (−13·5 to 9·1)	172·5 (111·5–249·7)	9·5 (−0·1 to 20·4)	1·9 (−6·9 to 12·0)
		Hepatitis B				356 083·4 (342 942·4 to 370 231·5)	16·7 (15·0 to 18·4)*	2·8 (1·3 to 4·3)*	190·2 (121·1–282·2)	16·9 (−4·5 to 40·2)	1·2 (−16·9 to 20·7)
			Acute hepatitis B aggregate			12 832·2 (11 239·6 to 14 567·4)	13·2 (−4·3 to 35·2)	1·0 (−14·6 to 20·0)	190·2 (121·1–282·2)	16·9 (−4·5 to 40·2)	1·2 (−16·9 to 20·7)
			Chronic hepatitis B			343 251·2 (330 541·3 to 357 194·6)	16·9 (15·3 to 18·4)*	2·8 (1·5 to 4·3)*	0·0 (0·0–0·0)	0·0 (0·0 to 0·0)	0·0 (0·0 to 0·0)
		Hepatitis C				142 745·3 (127 559·8 to 157 704·1)	18·0 (16·7 to 19·2)*	1·2 (0·1 to 2·2)*	8·7 (4·3–16·5)	17·0 (12·2 to 21·7)*	3·3 (−0·5 to 7·3)
			Acute hepatitis C aggregate			622·3 (580·4 to 666·6)	17·0 (15·7 to 18·4)*	3·4 (2·5 to 4·3)*	8·7 (4·3–16·5)	17·0 (12·2 to 21·7)*	3·3 (−0·5 to 7·3)
			Chronic hepatitis C			142 122·9 (126 977·7 to 157 044·9)	18·0 (16·7 to 19·2)*	1·2 (0·1 to 2·2)*	0·0 (0·0–0·0)	0·0 (0·0 to 0·0)	0·0 (0·0 to 0·0)
		Acute hepatitis E				1501·9 (1385·4 to 1636·4)	3·5 (2·2 to 4·7)*	−3·2 (−4·1 to −2·3)*	34·9 (22·4–51·7)	4·9 (−4·4 to 15·5)	−2·6 (−10·9 to 6·8)
	Other infectious diseases					54 835·4 (54 385·8 to 55 273·8)	0·4 (−1·6 to 2·3)	−8·0 (−9·8 to −6·2)*	1949·6 (1307·1–2797·1)	−1·6 (−5·1 to 2·0)	−8·7 (−12·0 to −5·5)*
**Non-communicable diseases**						**6 652 153·4 (6 633 099·8 to 6 668 805·2)**	13·7 (13·6 to 13·8)*	**0·0 (−0·0 to 0·1)**	**638 480·8 (478 716·6–819 498·9)**	18·8 (18·4 to 19·2)*	**−0·1 (−0·4 to 0·2)**
**Neoplasms**						**90 497·5 (89 215·7 to 91 896·1)**	44·4 (42·1 to 46·7)*	12·4 (10·6 to 14·2)*	**8569·3 (6265·5–11 079·2)**	36·6 (31·8 to 41·2)*	6·4 (2·7 to 9·7)*
	Lip and oral cavity cancer					2425·1 (2278·7 to 2582·3)	38·6 (30·3 to 47·1)*	7·9 (1·5 to 14·3)*	209·1 (150·2–271·4)	36·7 (28·6 to 45·3)*	6·2 (−0·1 to 12·7)
	Nasopharynx cancer					732·7 (580·5 to 883·9)	18·0 (0·2 to 39·8)*	−5·5 (−19·2 to 11·1)	69·7 (47·2–95·8)	16·8 (0·8 to 36·0)*	−6·8 (−18·7 to 7·7)
	Other pharynx cancer					945·5 (885·9 to 1015·0)	32·7 (23·8 to 41·5)*	2·2 (−4·5 to 9·0)	79·5 (57·8–104·3)	30·7 (21·9 to 39·2)*	0·6 (−6·2 to 7·2)
	Oesophageal cancer					746·0 (641·5 to 925·7)	19·6 (2·5 to 39·9)*	−8·2 (−21·2 to 7·1)	128·6 (92·0–167·5)	9·5 (−3·1 to 24·1)	−16·1 (−25·4 to −5·2)*
	Stomach cancer					3539·4 (3339·9 to 3776·2)	16·3 (8·9 to 24·4)*	−9·9 (−15·5 to −3·7)*	396·5 (292·4–504·2)	12·0 (4·8 to 20·2)*	−13·3 (−18·8 to −7·1)*
	Colon and rectum cancer					9399·0 (9059·3 to 9758·3)	42·6 (38·1 to 47·2)*	8·7 (5·4 to 12·2)*	762·8 (564·2–979·6)	38·4 (33·7 to 43·3)*	5·3 (1·9 to 8·9)*
		Colon and rectum cancer aggregate				9377·0 (9037·3 to 9736·4)	42·6 (38·1 to 47·3)*	8·7 (5·4 to 12·2)*	759·8 (561·9–975·3)	38·4 (33·7 to 43·3)*	5·4 (1·9 to 9·0)*
		Stoma due to colon and rectum cancer				22·0 (21·3 to 22·8)	26·1 (25·0 to 27·2)*	−4·9 (−5·7 to −4·1)*	3·0 (2·0–4·1)*	26·0 (20·7 to 31·6)*	−4·7 (−9·4 to −0·0)*
	Liver cancer					618·7 (550·7 to 688·0)	59·8 (38·2 to 82·2)*	23·6 (6·8 to 40·6)*	188·2 (130·4–245·6)	25·2 (9·9 to 45·0)*	−1·8 (−13·5 to 13·2)
		Liver cancer due to hepatitis B				305·9 (235·8 to 418·9)	20·6 (−9·1 to 63·2)	−3·6 (−26·5 to 29·1)	59·9 (40·2–80·4)	9·5 (−8·3 to 34·1)	−12·5 (−26·5 to 6·1)
		Liver cancer due to hepatitis C				268·7 (228·4 to 320·8)	80·1 (53·9 to 113·8)*	38·1 (17·1 to 65·1)*	44·3 (31·6–57·3)	47·7 (34·2 to 65·9)*	13·2 (2·7 to 27·7)*
		Liver cancer due to alcohol use				273·6 (229·0 to 350·7)	58·8 (28·9 to 94·8)*	22·1 (−0·3 to 49·1)	53·5 (37·3–70·1)	39·3 (22·1 to 61·6)*	7·2 (−5·8 to 23·5)
		Liver cancer due to other causes				151·0 (118·8 to 201·0)	24·9 (−5·6 to 63·5)	−2·0 (−26·3 to 29·5)	30·5 (20·7–40·6)	12·1 (−4·5 to 32·8)	−11·9 (−24·9 to 4·8)
	Gallbladder and biliary tract cancer					149·4 (138·5 to 158·9)	22·0 (14·4 to 30·1)*	−7·4 (−13·2 to −1·3)*	39·5 (27·7–51·5)	19·0 (11·8 to 26·8)*	−9·5 (−15·1 to −3·7)*
	Pancreatic cancer					393·8 (372·9 to 417·1)	46·2 (40·7 to 52·1)*	10·9 (6·7 to 15·4)*	86·6 (61·1–112·2)	38·4 (33·1 to 43·8)*	5·1 (1·1 to 9·3)*
	Larynx cancer					1412·6 (1340·0 to 1499·9)	26·2 (20·1 to 33·0)*	−2·3 (−7·0 to 2·9)	147·3 (105·7–194·3)	24·0 (18·1 to 30·0)*	−3·9 (−8·4 to 0·7)
		Larynx cancer aggregate				820·9 (749·1 to 911·1)	24·4 (14·2 to 35·8)*	−4·9 (−12·5 to 3·4)	93·6 (67·4–121·5)	21·5 (12·7 to 30·8)*	−6·8 (−13·1 to 0·2)
		Laryngectomy due to larynx cancer				591·0 (576·5 to 605·4)	28·7 (28·0 to 29·3)*	1·8 (1·3 to 2·4)*	53·7 (35·8–77·6)	28·6 (25·8 to 31·4)*	1·9 (−0·2 to 4·1)
	Tracheal, bronchus, and lung cancer					3299·7 (3095·1 to 3536·4)	37·7 (29·6 to 47·1)*	5·6 (−0·5 to 12·6)	513·6 (377·9–648·8)	31·1 (23·4 to 40·0)*	0·6 (−5·2 to 7·1)
	Malignant skin melanoma					3082·3 (2475·8 to 3901·7)	59·0 (50·7 to 67·0)*	26·2 (19·5 to 32·6)*	180·5 (120·8–257·6)	56·3 (47·9 to 64·0)*	23·7 (16·9 to 29·9)*
	Non-melanoma skin cancer					2558·2 (2494·8 to 2625·6)	76·8 (72·0 to 81·6)*	34·5 (30·8 to 38·2)*	148·0 (104·7–197·7)	82·9 (72·7 to 93·9)*	40·1 (31·9 to 48·7)*
		Non-melanoma skin cancer (squamous-cell carcinoma)				2155·9 (2019·5 to 2312·2)	93·5 (82·2 to 105·8)*	49·6 (40·5 to 59·2)*	142·0 (100·7–187·1)	86·4 (75·6 to 98·2)*	42·8 (34·2 to 52·1)*
		Non-melanoma skin cancer (basal-cell carcinoma)				578·1 (497·7 to 671·7)	27·0 (24·0 to 29·7)*	−3·9 (−6·0 to −1·8)*	6·0 (2·9–11·1)	26·9 (23·5 to 30·1)*	−3·8 (−6·4 to −1·2)*
	Breast cancer					21 361·8 (20 249·5 to 22 266·3)	41·5 (33·4 to 49·6)*	9·9 (4·1 to 15·7)*	1796·5 (1270·7–2411·6)	36·1 (29·2 to 42·7)*	5·3 (0·4 to 10·1)*
		Breast cancer aggregate				10 718·5 (9581·7 to 11 667·2)	62·7 (42·9 to 83·3)*	32·0 (16·2 to 48·1)*	989·8 (719·8–1271·0)	46·6 (32·7 to 61·5)*	15·8 (5·3 to 27·5)*
		Mastectomy due to breast cancer				10 638·5 (10 404·8 to 10 894·5)	25·1 (24·1 to 26·1)*	−4·7 (−5·4 to −3·9)*	806·8 (520·3–1171·8)	25·1 (24·0 to 26·2)*	−4·6 (−5·5 to −3·8)*
	Cervical cancer					3442·4 (3129·9 to 3765·0)	−1·6 (−11·4 to 9·8)	−20·5 (−28·2 to −11·5)*	264·0 (187·9–342·6)	−1·4 (−10·9 to 9·8)	−20·5 (−28·0 to −11·6)*
	Uterine cancer					3827·7 (3425·6 to 4285·2)	39·5 (22·9 to 55·9)*	8·2 (−4·0 to 20·0)	250·2 (172·4–341·0)	37·1 (21·1 to 52·9)*	6·1 (−5·4 to 17·6)
	Ovarian cancer					1174·2 (1107·4 to 1250·1)	28·3 (20·8 to 36·3)*	0·3 (−5·1 to 6·3)	150·7 (110·5–192·3)	25·8 (17·8 to 33·9)*	−1·7 (−7·4 to 4·3)
	Prostate cancer					14 434·4 (11 932·2 to 19 785·1)	70·6 (61·6 to 81·4)*	29·7 (22·5 to 38·4)*	1150·3 (804·9–1 643·3)	60·5 (51·7 to 70·6)*	21·5 (14·5 to 29·6)*
		Prostate cancer aggregate				13 492·3 (10 984·7 to 18 837·5)	74·6 (64·7 to 86·3)*	32·5 (24·6 to 42·1)*	1069·7 (739·5–1 543·7)	63·5 (53·7 to 74·9)*	23·5 (15·8 to 32·6)*
		Impotence and incontinence due to prostate cancer aggregate				942·0 (927·1 to 957·3)	29·0 (28·6 to 29·4)*	−0·9 (−1·2 to −0·5)*	80·6 (55·2–111·8)	28·9 (27·1 to 30·5)*	−1·0 (−2·4 to 0·2)
	Testicular cancer					685·8 (634·7 to 732·4)	41·9 (30·8 to 52·4)*	23·4 (13·9 to 32·2)*	42·1 (28·4–58·3)	40·3 (29·3 to 50·6)*	21·5 (11·9 to 30·4)*
	Kidney cancer					2870·3 (2728·0 to 3031·5)	57·9 (50·0 to 66·0)*	22·8 (16·8 to 29·1)*	202·7 (145·7–270·5)	54·4 (46·9 to 61·8)*	19·8 (14·0 to 25·6)*
	Bladder cancer					3407·9 (3240·0 to 3603·3)	35·7 (28·6 to 43·3)*	3·3 (−2·0 to 9·0)	267·0 (193·7–349·4)	32·1 (25·8 to 38·8)*	0·7 (−4·0 to 5·6)
		Bladder cancer episodes aggregate				3273·4 (3 105·3 to 3 470·7)	36·3 (28·9 to 44·3)*	3·7 (−1·8 to 9·6)	244·1 (176·2–317·4)	33·1 (26·1 to 40·4)*	1·2 (−3·9 to 6·7)
		Urinary incontinence due to bladder cancer				134·3 (129·7 to 139·4)	22·8 (22·0 to 23·7)*	−5·7 (−6·2 to −5·1)*	22·9 (15·9–31·2)	23·0 (18·7 to 27·1)*	−5·3 (−8·6 to −2·2)*
	Brain and nervous system cancer					1205·1 (1101·7 to 1322·9)	25·3 (14·9 to 37·6)*	4·2 (−3·9 to 13·8)	126·1 (90·3–164·5)	24·7 (15·0 to 36·3)*	2·8 (−4·7 to 12·0)
	Thyroid cancer					3166·5 (2936·7 to 3340·6)	101·1 (86·4 to 112·9)*	60·8 (48·9 to 70·2)*	190·0 (132·5–259·3)	96·8 (81·6 to 109·2)*	56·5 (44·3 to 66·3)*
	Mesothelioma					60·8 (58·1 to 63·6)	41·3 (35·1 to 47·7)*	9·5 (4·6 to 14·5)*	12·2 (8·7–15·8)	39·5 (32·1 to 46·8)*	7·9 (2·1 to 13·9)*
	Hodgkin's lymphoma					574·4 (519·0 to 672·9)	17·8 (10·5 to 25·3)*	0·0 (−6·1 to 6·4)	48·5 (33·7–65·8)	14·6 (7·4 to 21·7)*	−3·6 (−9·6 to 2·4)*
	Non-Hodgkin lymphoma					4292·3 (3741·0 to 4593·6)	63·5 (47·5 to 74·3)*	30·5 (19·1 to 38·4)*	312·3 (221·8–414·2)	57·9 (43·8 to 67·8)*	25·6 (15·1 to 33·1)*
	Multiple myeloma					488·2 (449·4 to 527·7)	54·6 (44·8 to 64·5)*	18·0 (10·7 to 25·4)*	104·0 (73·6–134·8)	49·0 (40·0 to 58·5)*	13·6 (6·9 to 21·0)*
	Leukaemia					2303·6 (2216·1 to 2384·5)	35·8 (29·7 to 41·8)*	10·2 (5·7 to 14·8)*	379·4 (276·2–488·9)	28·6 (21·2 to 36·9)*	4·3 (−1·1 to 10·4)
		Acute lymphoid leukaemia				875·5 (709·0 to 1072·3)	25·4 (7·3 to 46·4)*	10·2 (−5·0 to 27·8)	97·3 (68·0–129·8)	25·7 (11·5 to 40·8)*	8·1 (−3·1 to 20·0)
		Chronic lymphoid leukaemia				904·0 (833·2 to 974·4)	31·7 (22·2 to 42·9)*	4·5 (−2·2 to 12·4)	119·8 (87·2–152·9)	27·6 (18·9 to 37·4)*	0·5 (−6·0 to 7·7)
		Acute myeloid leukaemia				999·3 (882·5 to 1136·8)	38·4 (26·6 to 50·7)*	14·2 (5·7 to 23·5)*	120·9 (87·3–155·6)	36·5 (27·5 to 45·6)*	10·4 (3·7 to 17·4)*
		Chronic myeloid leukaemia				297·7 (271·8 to 324·7)	22·2 (14·3 to 31·0)*	−3·4 (−9·3 to 3·4)	41·4 (29·5–53·6)	17·7 (10·2 to 26·0)*	−7·5 (−13·3 to −1·2)*
	Other neoplasms					4577·5 (4145·5 to 4922·0)	36·1 (27·2 to 44·5)*	10·9 (4·1 to 17·6)*	323·2 (228·6–431·8)	34·4 (26·2 to 42·6)*	9·0 (2·4 to 15·4)*
**Cardiovascular diseases**						**422 738·4 (415 534·5 to 427 870·8)**	24·8 (24·0 to 25·6)*	−1·8 (−2·5 to −1·2)*	**25 620·1 (18 401·6–33 656·6)**	23·5 (21·7 to 24·5)*	−2·2 (−3·2 to −1·7)*
	Rheumatic heart disease					33 438·8 (29 725·7 to 43 119·8)	−4·7 (−11·0 to 0·1)	−16·3 (−22·5 to −12·1)*	1654·7 (1041·4–2530·9)	−3·4 (−9·9 to 1·4)	−15·4 (−21·5 to −11·1)*
	Rheumatic heart disease, without heart failure					32 236·8 (28 520·8 to 41 912·7)	−5·6 (−11·9 to −0·8)*	−16·9 (−23·1 to −12·7)*	1518·6 (941·1–2356·4)	−5·5 (−11·7 to −0·6)*	−16·8 (−22·9 to −12·3)*
		Heart failure due to rheumatic heart disease aggregate				1202·0 (1138·2 to 1269·0)	29·8 (28·5 to 31·2)*	2·2 (1·3 to 3·2)*	136·1 (94·5–187·6)	29·6 (27·2 to 32·1)*	2·4 (0·6 to 4·2)*
	Ischaemic heart disease					110 550·3 (100 675·9 to 121 798·7)	25·9 (24·6 to 27·2)*	−3·4 (−4·2 to −2·6)*	7274·7 (4958·6–9940·3)	30·2 (29·1 to 31·3)*	−0·3 (−1·0 to 0·3)
		Myocardial infarction episodes aggregate				15 930·1 (14 106·5 to 17 764·0)	6·4 (1·9 to 10·6)*	−18·1 (−21·6 to −14·8)*	33·0 (23·0–44·9)	15·2 (12·6 to 17·9)*	−13·1 (−15·1 to −11·1)*
		Angina due to ischaemic heart disease aggregate				72 344·6 (62 699·8 to 83 460·0)	29·3 (27·7 to 30·7)*	−0·3 (−1·2 to 0·5)	4794·5 (3100·7–6633·6)	29·5 (27·8 to 31·0)*	−0·0 (−1·0 to 0·8)
	Heart failure due to ischaemic heart disease aggregate					22 275·7 (21 272·0 to 23 320·4)	31·9 (31·1 to 32·7)*	−0·6 (−1·2 to −0·1)*	2447·2 (1736·8–3342·6)	31·9 (31·0 to 32·8)*	−0·5 (−1·1 to 0·1)
		Cerebrovascular disease				42 430·9 (42 068·2 to 42 767·1)	21·0 (20·4 to 21·5)*	−4·4 (−4·8 to −3·9)*	6455·2 (4487·0–8609·6)	20·7 (19·8 to 21·6)*	−4·2 (−4·9 to −3·5)*
			Ischaemic stroke			24 929·0 (24 362·2 to 25 610·0)	21·8 (21·0 to 22·6)*	−5·2 (−5·8 to −4·6)*	3660·0 (2559·3–4874·9)	22·0 (21·0 to 23·0)*	−4·9 (−5·7 to −4·2)*
			Chronic ischaemic stroke aggregate			24 663·2 (24 092·1 to 25 345·8)	21·9 (21·1 to 22·7)*	−5·1 (−5·8 to −4·5)*	3605·1 (2512·6–4815·3)	22·1 (21·1 to 23·1)*	−4·9 (−5·6 to −4·1)*
		Ischaemic stroke episodes aggregate				265·8 (246·6 to 285·1)	16·8 (14·2 to 19·7)*	−10·6 (−12·7 to −8·5)*	54·9 (36·3–74·5)	17·5 (13·8 to 20·9)*	−10·0 (−12·9 to −7·2)*
		Haemorrhagic stroke				18 669·6 (18 258·7 to 19 124·5)	19·2 (18·5 to 19·9)*	−3·3 (−3·8 to −2·7)*	2795·2 (1945·8–3749·6)	19·1 (18·1 to 20·2)*	−3·1 (−3·9 to −2·3)*
		Chronic haemorrhagic stroke aggregate				18 494·8 (18 088·5 to 18 959·5)	19·3 (18·6 to 19·9)*	−3·2 (−3·8 to −2·6)*	2757·3 (1918·2–3700·6)	19·2 (18·1 to 20·3)*	−3·0 (−3·8 to −2·2)*
	Acute haemorrhagic stroke aggregate					174·8 (162·3 to 187·8)	14·3 (11·6 to 16·6)*	−9·1 (−11·1 to −7·3)*	37·9 (24·7–51·2)	14·6 (11·8 to 17·0)*	−8·8 (−11·0 to −6·9)*
	Hypertensive heart disease					6086·2 (5732·7 to 6434·1)	37·1 (36·2 to 37·9)*	3·4 (2·9 to 3·9)*	670·0 (467·1–917·1)	37·2 (35·9 to 38·4)*	3·6 (2·8 to 4·4)*
		Cardiomyopathy and myocarditis				2536·8 (2404·8 to 2661·4)	26·7 (25·6 to 27·7)*	−1·3 (−2·0 to −0·7)*	275·1 (190·3–377·9)	26·3 (24·6 to 28·1)*	−1·3 (−2·5 to −0·2)*
		Acute myocarditis				156·3 (137·1 to 179·6)	21·4 (19·1 to 23·7)*	2·2 (1·3 to 3·1)*	7·8 (4·8–11·6)	21·1 (18·5 to 23·9)*	2·1 (0·3 to 3·8)*
	Heart failure due to cardiomyopathy aggregate					2380·4 (2254·3 to 2502·2)	27·0 (25·9 to 28·1)*	−1·5 (−2·2 to −0·9)*	267·3 (185·9–367·9)	26·5 (24·7 to 28·3)*	−1·4 (−2·6 to −0·2)*
	Atrial fibrillation and flutter					33 294·3 (29 959·8 to 37 202·0)	28·2 (27·2 to 29·1)*	−2·5 (−3·1 to −1·9)*	2634·6 (1782·6–3637·3)	28·2 (27·1 to 29·3)*	−2·4 (−3·1 to −1·7)*
	Peripheral vascular disease					154 650·6 (136 318·0 to 176 210·9)	34·4 (33·4 to 35·2)*	1·8 (1·3 to 2·3)*	572·8 (272·1–1056·2)	34·5 (32·4 to 36·8)*	2·0 (1·2 to 2·8)*
		Endocarditis				115·7 (108·1 to 124·8)	22·7 (20·9 to 24·5)*	−5·4 (−6·7 to −4·1)*	12·3 (8·5–17·1)	22·9 (18·6 to 27·6)*	−5·2 (−8·8 to −1·2)*
		Endocarditis episodes aggregate				16·9 (15·7 to 18·2)	22·8 (20·3 to 25·3)*	−0·7 (−2·7 to 1·2)	1·0 (0·6–1·4)	22·7 (20·0 to 25·3)*	−0·9 (−3·1 to 1·2)
	Heart failure due to endocarditis aggregate					98·8 (91·1 to 107·6)	22·7 (20·7 to 24·7)*	−6·1 (−7·6 to −4·6)*	11·4 (7·9–15·9)	22·9 (18·3 to 28·0)*	−5·5 (−9·3 to −1·2)*
		Heart failure due to other cardiovascular diseases aggregate				1126·5 (1045·9 to 1206·1)	34·4 (33·3 to 35·6)*	1·2 (0·4 to 2·0)*	123·7 (86·0–173·3)	34·4 (32·5 to 36·5)*	1·3 (−0·2 to 2·7)
		Other cardiovascular diseases episodes aggregate				89 221·1 (83 635·8 to 95 470·4)	24·0 (22·6 to 25·4)*	0·4 (−0·2 to 1·0)*	5947·0 (4074·6–8105·9)	23·7 (22·2 to 25·2)*	0·5 (−0·2 to 1·1)
**Chronic respiratory diseases**						**514 625·8 (503 322·3 to 527 617·0)**	12·1 (11·1 to 13·1)*	−3·3 (−4·1 to −2·4)*	**30 465·9 (23 341·4–38 294·2)**	11·8 (10·2 to 13·6)*	−4·6 (−6·2 to −3·1)*
	Chronic obstructive pulmonary disease					174 483·1 (160 204·9 to 188 951·7)	17·0 (15·1 to 19·0)*	−5·8 (−7·3 to −4·4)*	12 047·0 (10 206·8–13 725·4)	16·2 (13·4 to 18·8)*	−5·9 (−8·0 to −3·9)*
		Chronic obstructive pulmonary disease without heart failure aggregate				168 694·6 (154 443·1 to 182 957·1)	16·5 (14·5 to 18·5)*	−6·1 (−7·6 to −4·6)*	9671·6 (8183·6–11 080·9)	12·3 (9·2 to 15·3)*	−8·0 (−10·5 to −5·6)*
		Severe chronic obstructive pulmonary disease with heart failure aggregate				5876·4 (5073·7 to 6548·3)	34·9 (31·1 to 37·8)*	2·4 (−0·3 to 4·2)	2375·5 (1890·3–2747·2)	35·1 (31·4 to 38·2)*	2·5 (−0·0 to 4·5)
	Pneumoconiosis					2405·8 (2317·2 to 2486·3)	30·3 (27·8 to 32·9)*	8·2 (6·2 to 10·2)*	474·0 (305·6–682·6)	26·6 (23·6 to 29·3)*	6·0 (3·6 to 8·4)*
	Silicosis					402·9 (364·9 to 442·2)	18·1 (15·7 to 20·3)*	−4·3 (−6·1 to −2·6)*	65·7 (41·7–95·4)	18·5 (14·5 to 22·4)*	−3·9 (−7·0 to −0·9)*
		Silicosis without heart failure aggregate				389·0 (351·0 to 426·8)	17·6 (15·2 to 19·9)*	−4·6 (−6·4 to −2·8)*	59·2 (36·8–87·9)	17·1 (12·7 to 21·3)*	−4·7 (−8·0 to −1·5)*
		Severe silicosis with heart failure aggregate				13·9 (12·0 to 15·6)	32·2 (29·1 to 34·8)*	1·5 (−0·5 to 3·3)	6·4 (4·5–8·5)	33·1 (26·9 to 40·5)*	2·3 (−2·7 to 7·9)
	Asbestosis					157·3 (144·8 to 170·9)	30·6 (28·8 to 32·6)*	2·7 (1·3 to 4·1)*	24·8 (16·0–35·6)	30·2 (27·0 to 33·6)*	2·5 (−0·1 to 5·2)
		Asbestosis without heart failure aggregate				154·5 (141·8 to 168·1)	30·6 (28·7 to 32·5)*	2·7 (1·3 to 4·1)*	23·5 (15·0–34·0)	29·9 (26·4 to 33·4)*	2·5 (−0·2 to 5·4)
		Severe asbestosis with heart failure aggregate				2·8 (2·6 to 3·0)	35·6 (34·2 to 37·1)*	1·1 (0·1 to 2·1)*	1·3 (0·9–1·7)*	35·7 (31·3 to 40·0)*	1·3 (−2·2 to 4·7)
	Coal workers pneumoconiosis					84·2 (76·7 to 93·6)	37·0 (34·1 to 39·9)*	7·6 (5·2 to 10·0)*	13·3 (8·4–19·1)*	36·3 (29·4 to 43·4)*	7·0 (1·3 to 12·9)*
		Coal workers pneumoconiosis without heart failure aggregate				82·3 (74·9 to 91·7)	36·9 (34·0 to 39·8)*	7·5 (5·2 to 10·0)*	12·4 (7·8–17·9)*	36·0 (28·6 to 43·5)*	6·9 (0·9 to 13·2)*
		Severe coal workers pneumoconiosis with heart failure aggregate				1·9 (1·6 to 2·1)	41·6 (37·8 to 45·1)*	8·2 (5·4 to 10·9)*	0·9 (0·6–1·2)*	41·6 (37·7 to 45·2)*	8·2 (5·2 to 10·9)*
	Other pneumoconiosis					2388·6 (2164·7 to 2629·1)	27·9 (25·3 to 30·7)*	8·3 (6·0 to 10·5)*	370·2 (239·2–533·9)*	27·5 (23·9 to 31·0)*	8·3 (5·3 to 11·2)*
		Other pneumoconiosis without heart failure aggregate				2374·2 (2150·3 to 2615·1)	27·8 (25·2 to 30·7)*	8·3 (6·0 to 10·6)*	363·6 (234·0–524·9)*	27·3 (23·6 to 30·8)*	8·4 (5·3 to 11·4)*
		Severe other pneumoconiosis with heart failure aggregate				14·4 (13·1 to 15·6)	41·1 (39·0 to 43·0)*	4·5 (3·4 to 5·5)*	6·5 (4·6–8·5)	40·5 (35·1 to 45·7)*	4·3 (0·3 to 8·4)*
	Asthma					358 197·9 (323 133·7 to 393 465·6)	9·5 (7·6 to 11·6)*	−2·5 (−4·3 to −0·5)*	15 898·9 (10 371·0–22 344·1)	9·4 (7·4 to 11·5)*	−2·3 (−4·3 to −0·3)*
		Asymptomatic asthma				108 461·1 (92 842·5 to 126 250·5)	9·5 (7·6 to 11·6)*	−2·5 (−4·3 to −0·5)*	0·0 (0·0–0·0)	0·0 (0·0 to 0·0)	0·0 (0·0 to 0·0)
		Symptomatic asthma aggregate				249 736·8 (220 469·6 to 278 419·7)	9·5 (7·6 to 11·6)*	−2·5 (−4·3 to −0·5)*	15 898·9 (10 371·0–22 344·1)	9·4 (7·4 to 11·5)*	−2·3 (−4·3 to −0·3)*
	Interstitial lung disease and pulmonary sarcoidosis					1916·0 (1757·4 to 2075·5)	25·8 (23·8 to 27·7)*	0·6 (−0·9 to 2·1)	234·7 (147·5–334·2)	25·7 (23·5 to 28·1)*	0·7 (−1·1 to 2·6)
		Interstitial lung disease and pulmonary sarcoidosis without heart failure aggregate				1718·7 (1565·7 to 1881·0)	25·2 (23·1 to 27·2)*	0·5 (−1·1 to 2·1)	148·5 (88·8–221·4)	22·7 (19·6 to 25·9)*	0·2 (−2·2 to 2·6)
		Severe interstitial lung disease and pulmonary sarcoidosis with heart failure aggregate				197·4 (144·1 to 242·7)	31·5 (29·7 to 33·2)*	1·3 (−0·1 to 2·9)	86·2 (54·8–117·6)	31·3 (28·6 to 34·1)*	1·3 (−1·0 to 3·7)
	Other chronic respiratory diseases					..	..	..	1811·3 (1483·7–2123·4)	1·7 (−7·3 to 12·2)	−15·8 (−23·5 to −6·9)*
**Cirrhosis and other chronic liver diseases**						2828·3 (2791·3 to 2862·3)*	34·0 (32·4 to 35·8)*	8·2 (7·0 to 9·5)*	**501·0 (352·9–683·1)**	31·3 (29·8 to 32·9)*	7·3 (6·1 to 8·4)*
	Cirrhosis and other chronic liver diseases due to hepatitis B					796·5 (737·6 to 857·3)	30·5 (28·4 to 32·7)*	4·7 (3·2 to 6·2)*	130·6 (88·7–178·7)	30·5 (27·0 to 33·8)*	4·8 (2·2 to 7·3)*
	Cirrhosis and other chronic liver diseases due to hepatitis C					680·8 (628·3 to 734·2)	35·3 (33·0 to 37·5)*	7·6 (6·0 to 9·1)*	111·4 (78·2–152·6)	35·2 (31·6 to 38·6)*	7·6 (5·0 to 10·3)*
	Cirrhosis and other chronic liver diseases due to alcohol use					815·6 (758·7 to 877·2)	37·3 (35·4 to 39·3)*	9·8 (8·5 to 11·2)*	133·9 (92·5–185·1)	37·2 (33·9 to 40·5)*	9·9 (7·5 to 12·4)*
	Cirrhosis and other chronic liver diseases due to other causes					750·3 (714·3 to 785·9)	23·8 (22·5 to 25·1)*	6·8 (5·9 to 7·6)*	125·0 (87·5–172·2)	23·3 (20·1 to 26·8)*	6·8 (4·2 to 9·5)*
**Digestive diseases**						**258 248·2 (255 386·4 to 260 843·9)**	12·3 (10·9 to 13·7)*	−9·4 (−10·4 to −8·3)*	**12 142·8 (8 492·0–16 592·5)**	11·4 (9·8 to 13·2)*	−9·3 (−10·6 to −7·9)*
	Peptic ulcer disease					72 044·2 (71 302·7 to 72 763·7)	4·8 (3·7 to 6·0)*	−17·0 (−17·8 to −16·0)*	2341·7 (1605·6–3293·6)	1·1 (−0·2 to 2·5)	−19·5 (−20·6 to −18·4)*
		Peptic ulcer disease symptomatic episodes				4977·7 (4577·6 to 5379·5)	4·7 (2·4 to 6·9)*	−16·7 (−18·4 to −15·0)*	518·7 (357·9–703·2)	4·5 (2·2 to 6·8)*	−16·7 (−18·5 to −14·9)*
		Anaemia due to peptic ulcer disease aggregate				67 066·6 (66 465·4 to 67 616·9)	4·8 (3·7 to 6·1)*	−17·0 (−17·9 to −16·0)*	1823·1 (1225·8–2621·2)	0·2 (−1·4 to 1·9)	−20·3 (−21·6 to −19·0)*
	Gastritis and duodenitis					161 001·1 (157 928·3 to 164 071·1)	15·5 (13·0 to 17·9)*	−6·9 (−8·8 to −5·0)*	4913·0 (3347·3–6967·1)	11·2 (8·0 to 14·6)*	−9·5 (−11·9 to −6·9)*
		Gastritis and duodenitis, symptomatic episodes				3941·0 (3547·0 to 4358·9)	15·4 (10·9 to 17·3)*	−6·9 (−10·8 to −5·7)*	416·0 (277·1–567·9)	15·4 (11·2 to 17·5)*	−6·7 (−10·6 to −5·2)*
		Anaemia due to gastritis and duodenitis aggregate				157 060·1 (154 055·1 to 160 141·0)	15·5 (12·9 to 17·9)*	−6·9 (−8·9 to −5·0)*	4497·1 (3050·6–6393·0)	10·8 (7·4 to 14·5)*	−9·8 (−12·4 to −7·0)*
	Appendicitis					442·1 (414·6 to 472·3)	14·3 (13·4 to 15·2)*	2·8 (2·1 to 3·4)*	136·0 (92·0–184·6)	14·4 (10·6 to 18·0)*	3·0 (−0·2 to 6·1)
	Paralytic ileus and intestinal obstruction					119·7 (110·7 to 130·4)	18·9 (16·9 to 20·7)*	−1·3 (−2·8 to −0·0)*	37·3 (25·1–51·1)	18·6 (15·9 to 21·2)*	−1·1 (−3·4 to 1·2)
	Inguinal, femoral, and abdominal hernia					18 476·7 (16 577·2 to 20 339·8)	6·5 (4·2 to 8·6)*	−10·6 (−12·4 to −9·0)*	195·8 (97·0–363·1)	6·5 (4·3 to 8·7)*	−10·4 (−12·2 to −8·8)*
	Inflammatory bowel disease					11 223·5 (10 396·8 to 12 000·4)	15·7 (15·0 to 16·3)*	−3·7 (−4·1 to −3·4)*	2387·1 (1653·2–3 263·5)	15·6 (14·7 to 16·4)*	−3·6 (−4·2 to −3·0)*
	Vascular intestinal disorders					35·1 (32·4 to 38·1)	23·3 (21·0 to 25·8)*	−2·4 (−4·1 to −0·6)*	11·0 (7·4–14·8)	23·4 (19·7 to 27·2)*	−2·0 (−5·2 to 1·2)
	Gallbladder and biliary diseases					5656·5 (5199·3 to 6168·1)	16·9 (14·5 to 19·2)*	−4·1 (−5·9 to −2·2)*	601·0 (409·4–826·4)	16·8 (14·2 to 19·3)*	−3·9 (−5·9 to −1·9)*
	Pancreatitis					1018·2 (941·6 to 1 097·4)	24·2 (21·7 to 26·5)*	1·9 (−0·0 to 3·7)	300·0 (203·4–406·7)	24·0 (20·3 to 27·4)*	2·0 (−0·8 to 4·6)
	Other digestive diseases					..	..	..	1219·9 (838·5–1677·5)	21·9 (14·4 to 32·4)*	−1·0 (−7·1 to 7·4)
**Neurological disorders**						**2 261 316·4 (2 204 366·6 to 2 316 282·6)**	15·5 (14·6 to 16·4)*	**0·5 (−0·3 to 1·2)**	**59 325·6 (40 845·0–81 083·3)**	15·6 (14·3 to 16·9)*	−1·4 (−2·4 to −0·4)*
	Alzheimer's disease and other dementias					45 956·3 (40 178·5 to 52 655·9)	37·7 (36·2 to 39·0)*	1·1 (0·3 to 1·8)*	6851·2 (4883·9–9062·8)	38·8 (37·1 to 40·5)*	1·1 (0·2 to 1·8)*
	Parkinson's disease					6193·3 (5725·7 to 6777·2)	31·6 (28·2 to 35·5)*	0·6 (−1·9 to 3·5)	737·8 (516·1–990·1)	31·4 (27·8 to 35·3)*	0·7 (−2·1 to 3·7)
	Epilepsy					23 414·5 (21 549·6 to 25 419·4)	11·3 (7·0 to 15·5)*	−1·1 (−5·0 to 2·7)	6286·9 (4315·8–8250·2)	−6·4 (−11·5 to −1·2)*	−16·3 (−20·9 to −11·6)*
	Multiple sclerosis					2012·0 (1865·7 to 2166·9)	19·1 (17·0 to 21·3)*	−2·0 (−3·9 to −0·3)*	667·5 (472·0–855·6)	18·8 (16·2 to 21·5)*	−2·0 (−4·2 to 0·1)
	Motor neuron disease					202·4 (189·7 to 216·0)	22·3 (19·9 to 24·8)*	−1·3 (−3·3 to 0·7)	42·3 (30·5–55·1)	22·1 (19·5 to 24·8)*	−1·4 (−3·5 to 0·8)
	Migraine					958 789·2 (872 109·0 to 1 055 630·6)	15·3 (14·0 to 16·6)*	0·6 (−0·3 to 1·5)	32 898·8 (20 303·6–48 883·2)	15·3 (14·0 to 16·6)*	0·8 (−0·1 to 1·8)
	Tension-type headache					1 505 892·3 (1 337 310·0 to 1 681 575·0)	15·3 (14·0 to 16·6)*	0·5 (−0·1 to 1·1)	2260·5 (1058·1–4169·7)	15·3 (14·0 to 16·7)*	0·6 (−0·1 to 1·3)
	Medication overuse headache					58 454·5 (50 834·9 to 67 363·9)	19·0 (15·4 to 22·7)*	0·4 (−2·4 to 3·3)	9164·7 (6094·5–13 078·0)	18·9 (15·4 to 22·8)*	0·6 (−2·3 to 3·5)
	Other neurological disorders					11·5 (9·1 to 14·6)	17·0 (14·8 to 19·6)*	−0·2 (−0·9 to 0·4)	415·9 (301·2–547·5)	32·2 (27·5 to 36·3)*	−2·1 (−5·3 to 0·9)
**Mental and substance use disorders**						**1 058 903·8 (1 038 544·9 to 1 080 413·8)**	14·3 (13·6 to 14·9)*	**0·3 (−0·3 to 0·9)**	**149 977·9 (108 716·1–193 130·8)**	16·1 (15·5 to 16·8)*	1·1 (0·7 to 1·4)*
	Schizophrenia					23 383·0 (20 608·0 to 26 418·4)	19·5 (18·6 to 20·4)*	0·1 (−0·4 to 0·6)	15 020·5 (10 816·1–18 623·2)	19·5 (18·5 to 20·4)*	0·3 (−0·4 to 0·9)
	Alcohol use disorders					63 469·5 (57 507·8 to 69 863·5)	11·1 (8·9 to 13·5)*	−4·6 (−6·5 to −2·4)*	6321·3 (4205·6–8985·8)*	11·1 (9·0 to 13·6)*	−4·5 (−6·4 to −2·3)*
		Alcohol dependence aggregate				62 575·3 (56 621·7 to 68 944·3)	11·1 (8·9 to 13·5)*	−4·6 (−6·6 to −2·5)*	6276·2 (4167·7–8929·7)	11·1 (8·9 to 13·6)*	−4·5 (−6·5 to −2·3)*
		Fetal alcohol syndrome aggregate				894·2 (833·3 to 951·9)	11·1 (10·3 to 11·7)*	−0·5 (−1·1 to 0·2)	45·1 (29·2–65·1)	10·7 (8·2 to 13·1)*	−0·6 (−2·7 to 1·6)
	Drug use disorders					46 388·6 (45 252·4 to 47 446·6)	16·3 (15·0 to 17·6)*	3·9 (2·8 to 5·0)*	9849·4 (6959·0–12775·2)	23·6 (21·5 to 25·9)*	8·2 (6·2 to 10·2)*
		Opioid use disorders				16 746·5 (14 659·3 to 19 107·5)	23·3 (20·7 to 26·0)*	6·4 (4·2 to 8·6)*	6969·6 (4842·5–8999·8)	23·3 (20·6 to 26·2)*	6·5 (4·2 to 9·0)*
			Asymptomatic opioid dependence			2716·8 (1979·9 to 3704·4)	23·3 (20·7 to 26·0)*	6·4 (4·2 to 8·6)*	0·0 (0·0–0·0)	0·0 (0·0 to 0·0)	0·0 (0·0 to 0·0)*
			Symptomatic opioid dependence aggregate			14 029·7 (12 042·2 to 16 122·8)	23·3 (20·7 to 26·0)*	6·4 (4·2 to 8·6)*	6969·6 (4842·5–8999·8)	23·3 (20·6 to 26·2)*	6·5 (4·2 to 9·0)
		Cocaine use disorders				3846·3 (3401·6 to 4309·8)	31·1 (28·0 to 34·3)*	14·1 (12·0 to 16·4)*	521·5 (329·0–733·9)	30·6 (27·2 to 34·4)*	14·0 (11·4 to 17·0)*
			Asymptomatic cocaine dependence			1927·8 (1544·3 to 2357·8)	31·1 (28·0 to 34·3)*	14·1 (12·0 to 16·4)*	0·0 (0·0–0·0)	0·0 (0·0 to 0·0)	0·0 (0·0 to 0·0)
			Symptomatic cocaine dependence aggregate			1918·5 (1542·1 to 2341·9)	31·1 (28·0 to 34·3)*	14·1 (12·0 to 16·4)*	521·5 (329·0–733·9)	30·6 (27·2 to 34·4)*	14·0 (11·4 to 17·0)*
		Amphetamine use disorders				6599·8 (5295·7 to 8024·4)	30·4 (27·0 to 34·2)*	19·2 (16·1 to 22·5)*	874·7 (513·4–1308·7)	30·3 (26·1 to 34·8)*	19·1 (15·5 to 23·0)*
			Asymptomatic amphetamine dependence			3613·7 (2722·6 to 4630·7)	30·4 (27·0 to 34·2)*	19·2 (16·1 to 22·5)*	0·0 (0·0–0·0)	0·0 (0·0 to 0·0)	0·0 (0·0 to 0·0)
			Symptomatic amphetamine dependence aggregate			2986·1 (2186·5 to 3 893·0)	30·4 (27·0 to 34·2)*	19·2 (16·1 to 22·5)*	874·7 (513·4–1 308·7)	30·3 (26·1 to 34·8)*	19·1 (15·5 to 23·0)*
		Cannabis use disorders				19 762·5 (17 982·4 to 21 770·2)	5·3 (4·0 to 6·5)*	−3·8 (−4·7 to −2·9)*	577·2 (371·8–816·2)	5·3 (3·7 to 7·1)*	−3·7 (−5·0 to −2·3)*
			Asymptomatic cannabis dependence			11 430·9 (10 159·4 to 12 892·9)	5·3 (4·0 to 6·5)*	−3·8 (−4·7 to −2·9)*	0·0 (0·0–0·0)	0·0 (0·0 to 0·0)	0·0 (0·0 to 0·0)
			Symptomatic cannabis dependence aggregate			8331·6 (7244·3 to 9593·1)	5·3 (4·0 to 6·5)*	−3·8 (−4·7 to −2·9)*	577·2 (371·8–816·2)	5·3 (3·7 to 7·1)*	−3·7 (−5·0 to −2·3)*
		Other drug use disorders				..	..	..	906·4 (550·1–1 312·1)	30·5 (25·7 to 35·6)*	17·2 (12·9 to 21·7)*
	Depressive disorders					311 147·6 (300 016·8 to 320 544·3)	18·4 (17·2 to 19·5)*	0·7 (−0·3 to 1·6)	54 255·4 (37 569·9–72 943·3)	18·2 (17·2 to 19·2)*	1·0 (0·5 to 1·5)*
		Major depressive disorder				216 047·0 (192 863·4 to 243 319·4)	17·8 (16·6 to 19·0)*	0·8 (0·2 to 1·4)*	44 224·4 (29 672·5–60 297·0)	17·8 (16·6 to 19·0)*	1·1 (0·5 to 1·7)*
		Dysthymia				104 106·3 (90 398·1 to 118 968·9)	19·9 (18·4 to 21·5)*	0·6 (−0·3 to 1·4)	10 031·0 (6 605·9–14 267·1)	19·8 (18·3 to 21·5)*	0·7 (−0·2 to 1·6)
	Bipolar disorder					44 015·8 (38 150·4 to 50 912·5)	14·9 (14·1 to 15·8)*	0·3 (0·1 to 0·5)*	9004·7 (5501·7–13 388·0)	14·9 (13·9 to 15·9)*	0·5 (−0·0 to 0·9)
	Anxiety disorders					267 202·4 (234 064·3 to 306 318·0)	14·9 (13·0 to 16·8)*	0·8 (−0·5 to 2·1)	24 643·0 (16 813·7–33 647·8)	14·8 (12·8 to 16·6)*	1·0 (−0·4 to 2·3)
	Eating disorders					6468·4 (6170·6 to 6753·6)	19·1 (16·5 to 21·8)*	9·9 (7·5 to 12·4)*	1386·1 (915·9–1941·2)	19·0 (16·9 to 21·2)*	10·0 (8·1 to 11·9)*
		Anorexia nervosa				2912·1 (2357·7 to 3596·2)	12·0 (9·3 to 14·8)*	5·1 (2·6 to 7·6)*	620·5 (411·3–897·7)	12·1 (9·0 to 15·1)*	5·2 (2·3 to 8·0)*
		Bulimia nervosa				3629·4 (2937·5 to 4406·8)	25·3 (23·6 to 27·1)*	14·2 (12·5 to 15·8)*	765·6 (490·0–1108·5)	25·3 (23·2 to 27·6)*	14·2 (12·2 to 16·2)*
	Autistic spectrum disorders					62 212·4 (58 979·0 to 64 744·3)	12·3 (10·5 to 14·1)*	0·3 (−1·2 to 1·9)	10 051·5 (6740·1–13800·3)	12·3 (11·9 to 12·8)*	0·6 (0·2 to 0·9)*
		Autism				24 790·1 (20 957·6 to 29 393·1)	12·6 (12·2 to 12·9)*	0·5 (0·3 to 0·7)*	6335·9 (4112·0–8916·1)	12·5 (11·9 to 13·1)*	0·7 (0·2 to 1·2)*
		Asperger syndrome and other autistic spectrum disorders				37 245·4 (31 510·5 to 44 883·1)	12·1 (11·8 to 12·4)*	0·2 (0·1 to 0·3)*	3715·6 (2479·6–5424·6)	12·0 (11·5 to 12·5)*	0·3 (−0·1 to 0·7)
	Attention-deficit or hyperactivity disorder					51 094·3 (46 162·4 to 57 267·7)	0·3 (−0·4 to 1·0)	−3·6 (−4·2 to −3·0)*	620·1 (370·1–947·7)	0·4 (−0·6 to 1·2)	−3·5 (−4·4 to −2·8)*
	Conduct disorder					48 135·5 (39 315·2 to 58 151·6)	0·4 (−0·1 to 1·1)	1·3 (0·9 to 1·6)*	5770·5 (3485·2–8955·7)	0·5 (−0·3 to 1·4)	1·4 (0·7 to 2·0)*
	Idiopathic developmental intellectual disability					92 074·0 (52 280·1 to 130 411·2)	10·9 (9·5 to 11·9)*	1·0 (0·0 to 1·8)*	3442·1 (1506·4–5996·9)	10·3 (8·2 to 11·4)*	0·2 (−1·5 to 1·2)
	Other mental and substance use disorders					128 178·3 (127 512·5 to 128 877·2)	18·8 (17·9 to 19·7)*	0·2 (−0·6 to 0·9)	9613·4 (6699·7–12 951·9)	18·7 (17·7 to 19·8)*	0·3 (−0·6 to 1·1)
**Diabetes, urogenital, blood, and endocrine diseases**						**2 944 626·8 (2 925 016·2 to 2 968 511·3)**	15·5 (14·7 to 16·1)*	**−0·3 (−0·9 to 0·3)**	**66 092·7 (46 781·5–88 865·8)**	22·3 (20·8 to 24·0)*	1·2 (0·1 to 2·3)*
	Diabetes					435 328·4 (404 736·1 to 468 562·4)	30·6 (28·0 to 33·0)*	4·5 (2·4 to 6·4)*	33 360·8 (23 043·5–45 530·8)	32·5 (29·7 to 35·3)*	5·4 (3·2 to 7·5)*
		Uncomplicated diabetes				271 713·6 (241 414·2 to 302 381·7)	27·8 (24·9 to 30·4)*	3·2 (1·1 to 5·3)*	12 599·0 (7979·9–18 394·2)	27·7 (24·8 to 30·3)*	3·3 (1·2 to 5·4)*
		Neuropathy and other complications of diabetes aggregate				159 067·7 (134 666·7 to 187 174·0)	35·6 (32·2 to 38·8)*	6·6 (4·1 to 9·0)*	20 459·6 (13 572·0–28 749·6)	35·6 (32·3 to 38·8)*	6·7 (4·2 to 9·0)*
		Vision loss due to diabetes aggregate				4547·1 (3823·8 to 5416·0)	34·7 (32·4 to 36·9)*	5·0 (3·6 to 6·3)*	302·3 (210·1–415·5)	36·1 (33·6 to 38·7)*	5·7 (4·1 to 7·4)*
	Acute glomerulonephritis					100·5 (92·1 to 110·1)	−0·9 (−4·4 to 1·9)	−14·5 (−17·3 to −12·6)*	5·0 (3·2–7·5)	−0·6 (−4·6 to 2·8)	−14·1 (−17·4 to −11·4)*
	Chronic kidney disease					322 510·6 (312 718·2 to 330 351·1)	26·9 (25·8 to 28·0)*	0·9 (−0·0 to 1·8)	8172·8 (6118·6–10 229·3)	23·8 (22·9 to 24·8)*	0·1 (−0·4 to 0·6)
		Chronic kidney disease due to diabetes				100 823·9 (86 922·7 to 115 652·3)	27·3 (24·9 to 29·9)*	2·1 (0·5 to 3·7)*	2456·1 (1780·4–3151·2)	23·0 (20·7 to 25·4)*	0·6 (−0·7 to 2·0)
		Stage 3 chronic kidney disease due to diabetes aggregate				90 937·7 (77 906·5 to 104 617·0)	27·9 (25·4 to 30·5)*	2·3 (0·6 to 4·0)*	119·6 (79·7–175·5)	11·8 (6·5 to 16·7)*	−6·3 (−10·0 to −2·8)*
		Stage 4 chronic kidney disease due to diabetes aggregate				6441·3 (5521·3 to 7401·3)	21·6 (19·1 to 24·2)*	0·9 (−0·4 to 2·2)	787·7 (541·8–1 106·1)	20·2 (17·6 to 22·7)*	0·4 (−1·0 to 1·8)
		Stage 5 chronic kidney disease untreated due to diabetes				2694·4 (2331·9 to 3091·5)	25·6 (23·0 to 28·2)*	1·3 (−0·0 to 2·8)	1359·3 (920·5–1791·4)	25·3 (22·4 to 28·4)*	1·4 (−0·3 to 3·2)
		End-stage chronic kidney disease due to diabetes aggregate				750·5 (643·3 to 864·2)	19·8 (16·4 to 23·4)*	−3·0 (−5·2 to −0·7)*	189·5 (130·5–252·0)	26·6 (22·3 to 30·9)*	0·7 (−2·1 to 3·7)
	Chronic kidney disease due to hypertension					78 962·3 (67 846·7 to 91 021·2)	26·0 (23·6 to 28·6)*	0·2 (−1·4 to 1·9)	1508·0 (1108·7–1938·1)	28·5 (25·8 to 31·5)*	1·2 (−0·6 to 3·3)
		Stage 3 chronic kidney disease due to hypertension aggregate				72 772·4 (61 662·5 to 84 498·2)	25·9 (23·3 to 28·6)*	0·2 (−1·6 to 2·0)	80·8 (54·2–120·1)	22·1 (17·5 to 26·6)*	−4·4 (−8·0 to −0·9)*
		Stage 4 chronic kidney disease due to hypertension aggregate				4102·2 (3503·5 to 4772·6)	25·9 (22·4 to 29·5)*	0·7 (−1·6 to 2·8)	489·8 (341·4–696·7)	25·2 (22·1 to 28·7)*	0·5 (−1·5 to 2·5)
		Stage 5 chronic kidney disease untreated due to hypertension				1652·2 (1404·5 to 1913·7)	30·7 (27·5 to 34·2)*	1·8 (−0·5 to 4·1)	816·8 (565·2–1060·3)	30·5 (27·0 to 34·5)*	2·0 (−0·5 to 4·7)
		End-stage chronic kidney disease due to hypertension aggregate				435·5 (363·3 to 509·1)	27·5 (22·0 to 33·6)*	0·6 (−3·1 to 4·9)	120·6 (83·1–162·3)	33·2 (27·4 to 39·8)*	3·8 (−0·3 to 8·9)
	Chronic kidney disease due to glomerulonephritis					67 348·7 (58 198·9 to 77 439·5)	28·9 (25·8 to 32·3)*	1·1 (−1·1 to 3·6)	1951·4 (1432·2–2479·2)	22·2 (20·1 to 24·4)*	−0·7 (−2·1 to 0·7)
		Stage 3 chronic kidney disease due to glomerulonephritis aggregate				59 790·8 (50 844·6 to 69 692·7)	29·9 (26·4 to 33·6)*	1·3 (−1·1 to 4·1)	111·3 (73·5–165·2)	17·1 (13·1 to 20·7)*	−5·6 (−8·8 to −2·7)*
		Stage 4 chronic kidney disease due to glomerulonephritis aggregate				4872·4 (4297·2 to 5506·6)	21·3 (18·8 to 23·9)*	−0·5 (−2·2 to 1·6)	602·5 (418·4–845·6)	20·2 (17·7 to 22·9)*	−0·5 (−2·3 to 1·5)
		Stage 5 chronic kidney disease untreated due to glomerulonephritis				2228·1 (1895·7 to 2596·9)	23·6 (21·3 to 25·9)*	−0·6 (−2·0 to 0·7)	1122·6 (773·7–1461·3)	23·5 (20·5 to 26·3)*	−0·4 (−2·4 to 1·5)
		End-stage chronic kidney disease due to glomerulonephritis aggregate				457·4 (377·1 to 543·0)	20·5 (16·7 to 24·5)*	−2·6 (−5·1 to −0·0)*	115·0 (80·2–152·9)	26·0 (21·1 to 31·2)*	0·4 (−3·2 to 4·1)
	Chronic kidney disease due to other causes					94 553·5 (81 141·8 to 109 371·3)	26·2 (24·2 to 28·3)*	0·1 (−1·1 to 1·5)	2257·2 (1663·4–2892·2)	23·0 (21·1 to 25·1)*	−0·5 (−1·6 to 0·6)
		Stage 3 chronic kidney disease due to other causes aggregate				85 459·8 (72 471·6 to 99 605·4)	26·6 (24·6 to 28·8)*	0·1 (−1·2 to 1·6)*	132·2 (88·0–194·5)	17·6 (14·0 to 20·8)*	−6·2 (−8·8 to −3·8)*
		Stage 4 chronic kidney disease due to other causes aggregate				6058·4 (5257·9 to 6938·4)	21·3 (18·9 to 23·5)*	−0·2 (−1·1 to 0·8)	739·7 (513·5–1 029·6)	19·4 (17·0 to 21·6)*	−1·0 (−2·2 to 0·2)
		Stage 5 chronic kidney disease untreated due to other causes				2464·6 (2115·1 to 2835·9)	25·7 (23·8 to 27·5)*	0·1 (−0·9 to 1·1)	1233·0 (855·9–1604·1)	25·5 (23·0 to 28·0)*	0·3 (−1·1 to 1·8)
		End-stage chronic kidney disease due to other causes aggregate				570·6 (485·6 to 668·1)	21·6 (18·0 to 25·0)*	−2·2 (−4·2 to −0·2)*	152·4 (107·3–200·8)	26·3 (21·8 to 30·7)*	0·1 (−3·0 to 3·2)
	Urinary diseases and male infertility					436 081·7 (425 577·7 to 446 450·5)	24·2 (22·8 to 25·4)*	0·9 (−0·1 to 1·9)	4220·9 (2699·9–6045·2)	28·9 (26·8 to 31·0)*	−0·7 (−2·1 to 0·7)
		Interstitial nephritis and urinary tract infections				2901·2 (2831·9 to 2980·4)	19·3 (19·0 to 19·7)*	3·1 (2·9 to 3·3)	96·6 (59·9–144·0)	19·2 (17·5 to 20·9)*	3·3 (1·8 to 4·7)*
		Urolithiasis				319 600·2 (291 519·2 to 349 966·1)	22·8 (20·8 to 24·9)*	0·9 (−0·7 to 2·5)	89·7 (60·9–122·5)	23·4 (20·0 to 27·0)*	2·8 (0·1 to 5·5)*
		Benign prostatic hyperplasia				104 625·3 (90 729·8 to 118 244·0)	29·7 (27·5 to 32·0)*	−1·3 (−2·8 to 0·2)	3792·7 (2423·8–5437·3)	29·8 (27·6 to 32·2)*	−1·3 (−2·8 to 0·3)
		Male infertility				29 033·2 (23 972·5 to 34 766·8)	22·6 (19·3 to 25·6)*	9·6 (6·8 to 12·1)*	173·9 (70·1–365·3)*	22·7 (19·3 to 25·9)*	9·8 (6·9 to 12·5)*
		Other urinary diseases				..	..	..	67·9 (45·5–96·5)	17·2 (9·1 to 27·2)*	−0·6 (−7·3 to 7·7)*
	Gynaecological diseases					794 303·2 (786 497·9 to 801 169·7)	13·8 (13·2 to 14·5)*	−1·8 (−2·4 to −1·2)*	10 001·2 (6807·0–14 312·3)	10·7 (9·4 to 11·9)*	−3·3 (−4·3 to −2·5)*
		Uterine fibroids				151 115·0 (144 146·9 to 158 477·5)	19·2 (18·8 to 19·5)*	−0·4 (−0·6 to −0·2)*	2383·5 (1450·2–3834·4)	11·1 (8·6 to 13·2)*	−5·8 (−7·7 to −4·2)*
			Uterine fibroids cases aggregate			108 335·0 (101 618·4 to 115 233·6)	23·0 (22·3 to 23·7)*	1·7 (1·2 to 2·1)*	645·9 (314·9–1193·3)	26·1 (25·0 to 27·3)*	3·4 (2·7 to 4·3)*
			Mild abdominal pain with anaemia due to uterine fibroids aggregate			42 780·0 (41 634·5 to 43 881·4)	10·5 (9·5 to 11·5)*	−5·5 (−6·3 to −4·6)*	1737·5 (1125·7–2625·4)	6·4 (4·5 to 8·1)*	−8·9 (−10·5 to −7·4)*
		Polycystic ovarian syndrome				60 106·4 (46 139·3 to 75 963·4)	10·7 (10·1 to 11·4)*	−0·5 (−0·9 to −0·0)*	532·6 (238·9–1 044·6)	10·8 (9·9 to 11·5)*	−0·5 (−1·1 to 0·0)
			Polycystic ovarian syndrome cases aggregate			47 116·8 (35 880·1 to 60 919·9)	10·4 (9·8 to 11·1)*	−0·8 (−1·2 to −0·3)*	408·0 (181·1–797·7)	10·5 (9·7 to 11·3)*	−0·7 (−1·2 to −0·1)*
			Hirsutism and infertility due to polycystic ovarian syndrome aggregate			12 989·6 (8 635·6 to 18 975·6)	11·9 (10·9 to 12·7)*	0·4 (−0·4 to 1·1)	124·6 (47·2–278·0)	11·5 (9·6 to 12·9)*	0·1 (−1·5 to 1·3)
		Female infertility				61 300·1 (45 889·9 to 79 647·1)	24·6 (19·2 to 30·0)*	11·2 (6·5 to 15·8)*	344·5 (134·3–746·7)	25·0 (19·7 to 30·0)*	11·7 (7·2 to 16·2)*
		Endometriosis				10 758·2 (9 165·5 to 12 485·9)	11·6 (10·9 to 12·2)*	−2·6 (−3·0 to −2·2)*	996·4 (647·1–1 384·7)	11·7 (10·4 to 12·9)*	−2·4 (−3·5 to −1·5)*
		Endometriosis cases aggregate				10 167·5 (8 652·4 to 11 828·8)	11·5 (10·9 to 12·2)*	−2·7 (−3·1 to −2·3)*	937·3 (610·7–1 312·6)	11·6 (10·3 to 12·9)*	−2·5 (−3·6 to −1·6)*
		Abdominal pain and infertility due to endometriosis aggregate				590·7 (415·2 to 787·2)	12·3 (11·2 to 13·3)*	−1·0 (−1·8 to −0·3)*	59·1 (34·2–90·8)	12·3 (9·5 to 15·2)*	−0·9 (−3·4 to 1·5)
	Genital prolapse					161 679·1 (142 334·7 to 182 566·5)	17·7 (15·2 to 20·0)*	−6·6 (−8·5 to −4·8)*	503·0 (242·5–904·3)	17·8 (15·4 to 20·3)*	−6·5 (−8·4 to −4·6)*
	Premenstrual syndrome					430 696·9 (410 840·7 to 450 494·4)	10·1 (7·9 to 11·8)*	−2·1 (−3·9 to −0·7)*	3621·6 (2249·5–5428·5)	10·2 (8·0 to 12·0)*	−2·0 (−3·8 to −0·4)*
	Other gynaecological diseases					45 018·8 (44 057·3 to 46 031·2)	8·0 (7·3 to 8·6)*	−4·2 (−4·8 to −3·7)*	1619·8 (1129·8–2210·0)	6·2 (4·9 to 7·6)*	−5·7 (−6·8 to −4·6)*
		Other gynaecological diseases cases aggregate				23 908·1 (22 975·7 to 24 918·0)	13·8 (13·5 to 14·0)*	−0·4 (−0·5 to −0·2)*	936·8 (635·4–1 305·9)	13·8 (12·6 to 15·1)*	−0·2 (−1·2 to 0·8)
		Anaemia due to other gynaecological diseases aggregate				21 110·8 (20 900·6 to 21 328·2)	2·1 (0·9 to 3·4)*	−8·2 (−9·3 to −7·1)*	682·9 (461·0–978·0)	−2·7 (−4·6 to −0·7)*	−12·3 (−14·0 to −10·6)*
	Haemoglobinopathies and haemolytic anaemias					1 571 610·0 (1 545 204·5 to 1 605 099·8)	11·1 (9·5 to 12·6)*	−1·1 (−2·5 to 0·3)	8221·5 (5528·6–11 766·6)	4·3 (2·9 to 5·5)*	−4·9 (−6·0 to −3·9)*
		Thalassaemias				439·0 (405·7 to 496·3)	1·4 (−0·1 to 3·4)	−6·5 (−7·8 to −4·6)*	31·3 (21·2–44·4)	1·0 (−3·8 to 6·1)	−7·0 (−11·4 to −2·5)*
			β-thalassaemia major cases aggregate			229·2 (222·7 to 238·2)	4·4 (3·6 to 5·2)*	−4·2 (−4·9 to −3·4)*	16·1 (11·0–22·9)	4·1 (−2·5 to 11·5)	−4·7 (−10·6 to 1·9)
			Haemoglobin E or β-thalassaemia cases aggregate			67·4 (60·1 to 75·2)	5·4 (2·9 to 8·1)*	−2·5 (−4·8 to 0·1)	5·1 (3·4–7·3)	1·8 (−10·0 to 14·2)	−5·9 (−16·7 to 4·9)
			Haemoglobin H disease cases aggregate			139·0 (107·5 to 196·4)	−5·3 (−10·0 to 0·8)	−11·8 (−16·2 to −6·3)*	9·7 (6·4–14·1)	−4·9 (−12·3 to 4·9)	−11·4 (−18·2 to −2·4)*
			Heart failure due to thalassaemias aggregate			3·4 (3·2 to 3·6)	26·3 (24·3 to 28·4)*	0·1 (−1·1 to 1·4)*	0·4 (0·3–0·6)	26·4 (16·0 to 38·3)*	0·6 (−9·3 to 11·8)
		Thalassaemias trait				279 451·4 (272 818·9 to 287 357·4)	10·5 (10·1 to 11·0)*	−2·1 (−2·5 to −1·7)*	3922·2 (2607·9–5653·6)	6·1 (4·8 to 7·4)*	−3·6 (−4·6 to −2·6)*
			β-thalassaemia trait cases aggregate			226 029·6 (220 111·5 to 233 332·0)	10·1 (9·6 to 10·5)*	−2·5 (−3·0 to −2·1)*	3661·7 (2437·6–5270·8)	5·9 (4·7 to 7·2)*	−3·8 (−4·9 to −2·8)*
			Haemoglobin E trait cases aggregate			53 421·8 (51 122·3 to 55 882·5)	12·6 (11·5 to 13·7)*	−0·1 (−1·0 to 0·8)	260·5 (174·7–382·8)	8·4 (5·2 to 11·3)*	−0·5 (−3·2 to 2·1)
		Sickle-cell disorders				4449·9 (4293·7 to 4600·7)	8·2 (5·2 to 11·9)*	2·8 (−0·2 to 6·2)	371·4 (259·0–518·0)	7·7 (3·3 to 12·3)*	2·3 (−1·8 to 6·6)
			Homozygous sickle-cell and severe sickle-cell/β-thalassaemia cases aggregate			4035·1 (3876·0 to 4173·7)	6·5 (3·3 to 10·3)*	1·2 (−1·8 to 4·8)	339·4 (237·7–472·1)	6·5 (2·0 to 11·4)*	1·3 (−2·9 to 5·9)
			Haemoglobin SC disease cases aggregate			397·6 (368·2 to 427·4)	29·6 (18·3 to 45·6)*	21·5 (11·0 to 36·5)*	30·1 (21·1–42·1)	22·9 (9·6 to 37·4)*	15·2 (2·8 to 28·9)*
			Mild sickle-cell/β-thalassaemia cases aggregate			17·2 (15·9 to 18·6)	16·1 (10·1 to 21·8)*	6·4 (0·8 to 11·5)*	1·9 (1·3–2·6)	13·2 (4·7 to 21·9)*	2·7 (−4·9 to 10·5)
		Sickle-cell trait				404 565·9 (381 223·4 to 448 154·6)	19·4 (18·3 to 20·3)*	7·5 (6·5 to 8·3)*	1720·3 (1156·5–2459·0)	10·8 (7·3 to 13·4)*	1·4 (−1·8 to 3·6)
		G6PD deficiency				247 073·7 (209 306·7 to 286 712·7)	6·9 (1·4 to 11·9)*	−4·4 (−9·3 to 0·1)	28·4 (19·3–39·6)	1·8 (−2·1 to 6·0)	−7·7 (−11·2 to −4·0)*
			G6PD cases aggregate			247 068·5 (209 301·6 to 286 707·7)	6·9 (1·4 to 11·9)*	−4·4 (−9·3 to 0·1)	27·7 (18·9–38·7)	1·2 (−2·8 to 5·5)	−8·1 (−11·6 to −4·3)*
			Heart failure due to G6PD deficiency aggregate			5·2 (4·7 to 5·7)	38·1 (36·6 to 39·8)*	7·9 (6·8 to 9·1)*	0·6 (0·4–0·9)	38·1 (34·6 to 41·5)*	7·8 (5·0 to 10·6)*
		G6PD trait				728 549·2 (676 734·8 to 781 533·8)	9·8 (7·9 to 11·4)*	−2·7 (−4·3 to −1·2)*	28·0 (19·3–38·9)	4·3 (0·1 to 8·8)*	−5·3 (−9·0 to −1·2)*
		Other haemoglobinopathies and haemolytic anaemias				74 385·1 (73 897·6 to 74 861·6)	1·8 (0·9 to 2·7)*	−9·0 (−9·7 to −8·2)*	2119·8 (1423·9–3029·9)	−3·8 (−5·5 to −2·2)*	−12·3 (−13·8 to −11·0)*
			Other haemoglobinopathies and haemolytic anaemias cases aggregate			74 273·3 (73 786·0 to 74 751·1)	1·8 (0·9 to 2·7)*	−9·0 (−9·8 to −8·2)*	2067·4 (1384·3–2963·9)	−4·1 (−5·8 to −2·4)*	−12·6 (−14·1 to −11·2)*
			Heart failure due to other haemoglobinopathies and haemolytic anaemias aggregate			111·8 (103·8 to 119·6)	34·9 (33·9 to 36·0)*	2·0 (1·3 to 2·7)*	13·1 (8·9–18·2)	34·9 (31·2 to 38·7)*	2·1 (−0·8 to 5·0)
		Endocrine, metabolic, blood, and immune disorders				66 128·9 (65 597·1 to 66 663·8)	4·0 (2·2 to 5·9)*	−7·2 (−8·7 to −5·5)*	2110·5 (1431·5–2993·7)	1·3 (−1·1 to 3·8)	−8·3 (−10·4 to −6·1)*
		Endocrine metabolic blood and immune disorders cases aggregate				7577·2 (7382·6 to 7766·6)	19·8 (19·0 to 20·6)*	−0·6 (−1·2 to 0·0)	292·4 (200·2–401·8)	19·5 (18·1 to 21·0)*	−0·5 (−1·6 to 0·6)
						58 430·4 (57 936·0–58 946·7)	2·2 (0·3–4·3)*	−8·0 (−9·7––6·2)*	1804·4 (1216·0–2582·3)	−1·3 (−3·9–1·5)	−9·5 (−11·9––7·0)*
		Anaemia due to endocrine metabolic blood and immune disorders aggregate				58 430·4 (57 936·0 to 58 946·7)	2·2 (0·3 to 4·3)*	−8·0 (−9·7 to −6·2)*	1804·4 (1216·0–2582·3)	−1·3 (−3·9 to 1·5)	−9·5 (−11·9 to −7·0)*
**Heart failure due to endocrine metabolic blood and immune disorders aggregate**						**121·4 (113·3 to 130·0)**	30·7 (29·6 to 31·9)*	−1·9 (−2·6 to −1·2)*	**13·6 (9·4–19·1)**	30·7 (27·4 to 34·0)*	**−1·7 (−4·3 to 0·8)**
	Musculoskeletal disorders					1 304 100·4 (1 288 602·8 to 1 316 641·4)	20·7 (20·2 to 21·2)*	−0·7 (−1·1 to −0·3)*	146 783·8 (106 764·7–194 473·5)	20·5 (19·6 to 21·5)*	−0·7 (−1·3 to −0·0)*
	Rheumatoid arthritis					24 491·2 (22 552·0 to 26 750·7)	23·8 (21·1 to 26·7)*	0·6 (−1·5 to 2·9)	5777·8 (4016·1–7769·6)	23·6 (20·9 to 26·7)*	0·7 (−1·4 to 3·1)*
		Osteoarthritis				237 368·6 (230 335·9 to 244 648·1)	32·9 (31·9 to 33·8)*	2·2 (1·6 to 2·9)*	12 886·2 (8 999·7–17 540·0)	34·8 (33·6 to 36·0)*	3·9 (3·0 to 4·8)*
		Osteoarthritis of the hip cases aggregate				35 629·2 (32 482·6 to 38 970·1)	33·5 (32·4 to 34·6)*	1·8 (1·0 to 2·6)*	1776·2 (1224·9–2477·0)	35·8 (34·4 to 37·3)*	3·7 (2·6 to 5·0)*
	Osteoarthritis of the knee cases aggregate					201 739·4 (195 205·3 to 208 276·6)	32·7 (31·7 to 33·9)*	2·3 (1·5 to 3·1)*	11 110·0 (7742·1–15 123·2)	34·6 (33·3 to 35·9)*	3·9 (3·0 to 4·9)*
		Low back and neck pain				820 689·8 (803 467·4 to 837 808·9)	18·7 (17·9 to 19·4)*	−2·0 (−2·6 to −1·4)*	94 941·5 (67 825·3–128 035·0)	18·6 (17·6 to 19·6)*	−2·1 (−2·7 to −1·4)*
		Low back pain				539 907·4 (521 448·6 to 559 556·0)	17·3 (16·5 to 18·2)*	−2·8 (−3·4 to −2·2)*	60 074·8 (42 721·9–82 343·7)	17·2 (16·4 to 18·1)*	−2·6 (−3·2 to −2·0)*
	Neck pain					358 006·6 (313 408·4 to 409 411·0)	21·1 (19·0 to 23·3)*	−1·1 (−2·4 to 0·0)	34 866·7 (23 362·1–47 636·9)	21·0 (18·9 to 23·2)*	−1·1 (−2·3 to 0·1)
		Gout				42 214·2 (37 688·2 to 47 495·5)	26·4 (25·2 to 27·7)*	0·5 (0·1 to 1·0)*	1342·8 (910·0–1843·8)	26·3 (24·6 to 27·9)*	0·6 (−0·3 to 1·5)
		Asymptomatic gout				37 712·9 (33 598·4 to 42 467·0)	26·4 (25·2 to 27·7)*	0·5 (0·1 to 1·0)*	0·0 (0·0–0·0)	0·0 (0·0 to 0·0)	0·0 (0·0 to 0·0)
	Other musculoskeletal disorders					342 067·7 (305 430·7 to 385 146·7)	20·7 (17·5 to 24·0)*	1·2 (−1·2 to 3·7)	31 835·4 (21 489·1–44 268·4)	20·5 (17·2 to 23·7)*	1·3 (−1·1 to 3·8)
**Other non-communicable diseases**						**5 316 342·1 (5 283 701·8 to 5 355 928·3)**	14·6 (14·3 to 14·8)*	0·4 (0·2 to 0·6)*	**139 001·8 (95 459·0–197 704·3)**	20·4 (19·5 to 21·5)*	1·2 (0·6 to 2·0)*
	Congenital anomalies					95 706·4 (88 133·4 to 102 483·4)	22·5 (18·5 to 26·3)*	9·3 (5·8 to 12·8)*	8621·0 (5389·1–12 950·2)	28·5 (20·0 to 37·6)*	14·7 (7·1 to 22·7)*
		Neural tube defects				1449·6 (988·4 to 2091·8)	17·7 (14·5 to 20·3)*	7·2 (4·3 to 9·7)*	499·1 (285·3–823·7)	18·4 (14·1 to 22·2)*	8·1 (4·1 to 11·6)*
		Congenital heart anomalies				48 869·1 (34 325·7 to 72 829·2)	29·8 (25·3 to 34·1)*	15·4 (11·4 to 19·2)*	1688·2 (659·4–3116·9)	29·7 (25·6 to 33·7)*	15·6 (11·7 to 19·1)*
			Less severe heart anomalies cases aggregate			44 599·5 (29 800·6 to 68 498·5)	29·4 (24·5 to 34·0)*	15·2 (10·7 to 19·1)*	1431·0 (532·7–2747·7)	29·3 (24·2 to 34·0)*	15·2 (10·7 to 19·2)*
			Severe congenital heart anomalies			4008·6 (2671·1 to 6011·2)	33·7 (27·4 to 39·0)*	18·5 (12·9 to 23·3)*	235·0 (93·2–467·8)	33·5 (27·4 to 38·7)*	18·5 (13·2 to 23·2)*
			Critical congenital heart anomalies			155·8 (90·3 to 252·4)	42·0 (27·5 to 52·9)*	28·6 (15·5 to 38·5)*	9·2 (3·2–18·7)	42·0 (26·4 to 54·2)*	28·9 (14·9 to 40·1)*
			Heart failure due to congenital heart anomalies aggregate			105·2 (99·2 to 111·4)	11·6 (10·9 to 12·4)*	−1·0 (−1·5 to −0·5)*	12·9 (8·8–18·2)	11·6 (8·3 to 15·1)*	−1·0 (−3·9 to 2·0)
		Cleft lip and cleft palate				6882·6 (4029·2 to 11 483·5)	19·5 (7·7 to 27·5)*	6·6 (−3·9 to 13·9)	79·6 (39·5–143·1)	12·2 (2·6 to 19·9)*	0·9 (−7·9 to 7·7)
		Down syndrome				5361·8 (3424·6 to 8192·1)	17·9 (13·7 to 21·8)*	6·4 (2·3 to 10·0)*	507·0 (286·2–837·2)	20·3 (15·8 to 24·2)*	6·5 (2·2 to 10·2)*
		Turner syndrome				372·4 (174·6 to 677·1)	11·9 (9·8 to 13·9)*	0·1 (−1·7 to 1·9)	6·6 (2·4–14·4)	11·4 (8·7 to 14·0)*	−0·1 (−2·4 to 2·2)
		Klinefelter syndrome				220·5 (115·5 to 395·5)	12·8 (10·9 to 14·2)*	0·5 (−1·2 to 1·9)	1·3 (0·5–3·1)	13·5 (10·6 to 15·6)*	0·2 (−2·2 to 2·0)
		Other chromosomal abnormalities				4728·6 (2628·9 to 8303·2)	16·2 (11·8 to 19·8)*	4·3 (0·1 to 7·7)*	454·1 (233·5–852·1)	18·5 (13·6 to 22·3)*	4·2 (−0·5 to 7·9)
		Other congenital anomalies				32 363·0 (17 788·1 to 59 394·1)	14·5 (12·3 to 16·8)*	2·0 (−0·1 to 4·1)	5385·0 (3209·4–8562·1)	31·3 (18·8 to 45·9)*	17·1 (6·1 to 30·3)*
	Skin and subcutaneous diseases					2 239 493·4 (2 222 716·3 to 2 258 252·8)	12·5 (12·1 to 12·8)*	0·7 (0·4 to 1·1)*	44 896·0 (28 943·3–67 159·6)	11·7 (11·0 to 12·3)*	0·4 (0·1 to 0·7)*
		Dermatitis				245 290·6 (227 283·4 to 262 752·2)	14·0 (13·2 to 14·8)*	0·3 (0·1 to 0·7)*	8788·0 (5963·4–12 273·5)	13·6 (12·9 to 14·4)*	1·6 (1·0 to 2·2)*
			Eczema cases aggregate			85 585·6 (75 115·2 to 97 031·7)	13·0 (12·4 to 13·6)*	3·3 (2·8 to 3·8)*	5239·2 (3515·1 to 7348·6)	13·0 (12·2 13·9)*	3·4 (2·7 to 4·2)*
			Contact dermatitis cases aggregate			98 015·1 (85 097·0 to 110 396·7)	15·2 (13·3 to 16·9)*	−1·0 (−1·2 to −0·9)*	2632·0 (1625·8–3852·4)	15·0 (13·1 to 16·8)*	−0·9 (−1·4 to −0·5)*
			Seborrhoeic dermatitis cases aggregate			61 690·0 (54 642·8 to 68 788·8)	13·3 (12·0 to 14·7)*	−1·3 (−1·7 to −1·0)*	916·8 (521·6–1454·1)	13·2 (11·8 to 14·7)*	−1·3 (−1·8 to −0·8)*
		Psoriasis				79 699·7 (76 690·9 to 82 804·5)	17·6 (17·0 to 18·3)*	0·4 (−0·1 to 0·9)	6438·3 (4495·6–8734·5)	17·5 (16·6 to 18·4)*	0·6 (−0·0 to 1·2)
		Cellulitis				959·9 (887·6 to 1033·6)	24·0 (22·1 to 25·7)*	5·8 (4·3 to 7·2)*	69·0 (45·0–97·8)	23·6 (20·7 to 26·1)*	5·8 (3·5 to 8·1)*
		Pyoderma				5812·7 (5581·7 to 6019·0)	15·5 (14·7 to 16·3)*	1·4 (0·7 to 2·1)*	32·7 (13·2–68·4)	15·4 (14·0 to 16·8)*	1·5 (0·3 to 2·7)*
		Scabies				204 151·7 (177 533·7 to 237 466·2)	6·6 (4·0 to 9·5)*	−2·6 (−4·5 to −0·6)*	5268·9 (2966·9–8605·6)	6·6 (3·8 to 9·5)*	−2·5 (−4·5 to −0·5)*
		Fungal skin diseases				492 372·6 (448 950·9 to 538 232·5)	13·3 (12·5 to 14·1)*	1·6 (1·3 to 1·9)*	2783·3 (1105·6–5905·3)	13·3 (12·4 to 14·1)*	1·7 (1·4 to 2·0)*
		Viral skin diseases				174 843·1 (165 156·3 to 185 072·0)	8·5 (8·0 to 9·0)*	−1·1 (−1·4 to −0·8)*	5396·9 (3417·1–7959·9)	8·4 (7·9 to 9·0)*	−1·0 (−1·4 to −0·6)*
			Molluscum contagiosum cases aggregate			40 464·2 (35 685·6 to 46 233·4)	5·2 (4·7 to 5·8)*	−0·9 (−1·3 to −0·5)*	1263·0 (783·0–1932·3)	5·3 (4·6 to 6·1)*	−0·8 (−1·5 to −0·2)*
			Viral warts cases aggregate			134 378·9 (125 683·9 to 143 028·0)	9·5 (8·9 to 10·1)*	−1·1 (−1·4 to −0·7)*	4133·9 (2628·4–6153·4)	9·4 (8·8 to 10·1)*	−1·0 (−1·5 to −0·6)*
		Acne vulgaris				632 741·0 (595 241·9 to 671 248·9)	4·6 (3·5 to 5·6)*	0·8 (−0·1 to 1·7)	6854·0 (3275·1–12 713·9)	4·6 (3·5 to 5·6)*	0·8 (−0·1 to 1·8)
		Alopecia areata				20 594·9 (19 607·8 to 21 617·0)	14·0 (13·5 to 14·4)*	−1·1 (−1·3 to −1·0)*	695·6 (428·5–1035·6)	13·9 (13·1 to 14·7)*	−1·0 (−1·7 to −0·4)*
		Pruritus				69 582·5 (62 063·2 to 78 152·6)	17·6 (15·7 to 19·5)*	0·4 (−0·7 to 1·6)	741·6 (360·0–1348·6)	17·5 (15·6 to 19·4)*	0·5 (−0·7 to 1·7)
		Urticaria				67 749·8 (58 743·2 to 77 086·4)	10·7 (9·6 to 12·0)*	−0·1 (−0·3 to 0·0)	4115·7 (2567·2–5833·6)	10·7 (9·6 to 12·0)*	−0·0 (−0·5 to 0·4)
		Decubitus ulcer				1087·5 (991·2 to 1189·7)	35·4 (33·4 to 37·4)*	4·7 (3·3 to 6·0)*	161·3 (112·0–218·7)	34·5 (31·9 to 37·3)*	4·7 (2·6 to 6·9)*
		Other skin and subcutaneous diseases				605 036·3 (589 500·0 to 619 675·6)	22·8 (22·1 to 23·4)*	2·4 (1·9 to 2·9)*	3550·5 (1706·2–6514·4)	22·6 (22·0 to 23·3)*	2·5 (2·0 to 2·9)*
	Sense organ diseases					1 788 125·6 (1 771 367·0 to 1 808 438·8)	23·7 (23·3 to 24·1)*	0·6 (0·3 to 0·9)*	68 515·2 (47 798·2–93 894·8)	25·2 (24·2 to 26·4)*	0·6 (−0·0 to 1·3)
		Glaucoma				5954·5 (5077·5 to 6905·8)	39·1 (36·9 to 41·4)*	4·2 (2·8 to 5·5)*	541·3 (370·0–747·9)	39·4 (37·0 to 41·9)*	4·3 (2·6 to 5·8)*
		Cataract				59 727·0 (53 633·2 to 66 879·2)	26·9 (25·2 to 28·5)*	−3·2 (−4·5 to −2·0)*	3879·7 (2766·9–5229·2)	25·5 (24·1 to 26·9)*	−4·2 (−5·2 to −3·2)*
		Macular degeneration				6188·4 (5250·3 to 7273·2)	48·5 (46·1 to 50·8)*	8·2 (6·9 to 9·8)*	462·4 (327·4–633·0)	47·7 (45·2 to 49·9)*	6·9 (5·3 to 8·6)*
		Refraction and accommodation disorders				819 307·4 (789 917·4 to 848 059·2)	21·9 (20·5 to 23·2)*	−0·4 (−1·3 to 0·6)	14 593·8 (9392·9–22 901·1)	21·1 (20·2 to 22·1)*	−0·3 (−0·9 to 0·4)
		Age-related and other hearing loss				1 210 055·2 (1 140 224·2 to 1 274 664·8)	28·3 (27·4 to 29·2)*	1·4 (1·0 to 1·8)*	40 596·8 (27 898·4–56 075·0)	26·4 (24·6 to 28·3)*	1·1 (0·0 to 2·1)*
		Other vision loss				25 797·4 (22 675·6 to 28 504·6)	27·1 (24·6 to 29·5)*	5·5 (4·2 to 6·7)*	1 756·4 (1248·9–2392·7)	29·9 (27·8 to 32·0)*	5·6 (4·3 to 6·7)*
		Other sense organ diseases				267 689·7 (258 379·8 to 277 711·4)	24·0 (23·1 to 24·9)*	0·8 (0·1 to 1·4)*	6684·7 (4185·0–9714·7)	23·8 (22·9 to 24·8)*	0·9 (0·2 to 1·5)*
			Acute other sense organ diseases			1343·8 (1303·5 to 1385·9)	14·9 (14·2 to 15·7)*	0·9 (0·4 to 1·4)*	35·4 (21·6–51·6)	14·8 (12·7 to 16·9)*	0·9 (−0·9 to 2·7)
			Chronic other sense organ diseases			266 345·9 (257 047·4 to 276 383·0)	24·0 (23·1 to 24·9)*	0·8 (0·1 to 1·4)*	6649·3 (4162·3–9662·4)	23·9 (23·0 to 24·9)*	0·9 (0·2 to 1·5)*
	Oral disorders					3 521 901·1 (3 468 088·2 to 3 575 854·4)	14·5 (13·8 to 15·0)*	0·4 (−0·1 to 0·9)	16 969·6 (10 296·5–26 044·5)	22·4 (21·6 to 23·2)*	−0·2 (−0·5 to 0·1)
		Deciduous caries				572 694·1 (475 089·8 to 686 991·2)	4·5 (2·3 to 6·1)*	−2·3 (−4·2 to −0·7)*	147·2 (63·0–292·1)	4·1 (1·6 to 5·9)*	−2·7 (−4·9 to −0·8)*
		Permanent caries				2 521 197·8 (2 361 418·3 to 2 679 668·7)	14·5 (13·7 to 15·4)*	0·8 (0·3 to 1·4)*	1743·4 (776·7–3320·9)	13·5 (12·6 to 14·4)*	−0·4 (−1·2 to 0·4)
		Periodontal diseases				537 506·0 (465 113·9 to 625 888·8)	25·4 (24·1 to 26·5)*	1·1 (0·5 to 1·7)*	3520·7 (1359·3–7253·7)	25·4 (24·1 to 26·5)*	1·2 (0·6 to 1·8)*
		Edentulism and severe tooth loss				275 619·1 (264 200·8 to 288 252·3)	27·3 (26·9 to 27·7)*	−0·9 (−1·1 to −0·7)*	7640·3 (5096·6–10 562·3)	27·3 (26·9 to 27·7)*	−0·8 (−1·1 to −0·6)*
		Other oral disorders				133 719·0 (127 499·7 to 139 776·2)	15·9 (15·4 to 16·4)*	−0·1 (−0·2 to 0·1)	3918·1 (2432·7–5879·4)	15·8 (15·3 to 16·4)*	0·0 (−0·2 to 0·3)
**Injuries**						**1 019 157·4 (977 405·7 to 1 064 704·2)**	16·4 (15·5 to 17·4)*	−3·4 (−4·1 to −2·6)*	**41 022·0 (29 290·4–55 288·4)**	8·0 (4·6 to 11·1)*	−9·9 (−12·5 to −7·5)*
	**Transport injuries**					**115 981·0 (110 575·9 to 123 047·1)**	23·1 (22·5 to 23·7)*	**−0·0 (−0·4 to 0·4)**	**6444·8 (4500·6–8733·0)**	12·2 (8·7 to 15·8)*	−8·0 (−10·7 to −5·3)*
		Road injuries				107 722·4 (102 214·5 to 114 850·0)	23·4 (22·8 to 24·1)*	0·3 (−0·1 to 0·7)	5956·1 (4130·8–8076·3)	13·3 (9·9 to 16·7)*	−7·1 (−9·7 to −4·6)*
			Pedestrian road injuries			14 157·8 (12 664·5 to 16 095·9)	22·9 (21·7 to 24·1)*	−0·7 (−1·4 to 0·0)	770·8 (532·9–1060·8)	9·6 (5·5 to 13·6)*	−10·6 (−13·7 to −7·6)*
			Cyclist road injuries			18 700·6 (16 545·4 to 20 951·1)	21·4 (19·9 to 22·7)*	−0·4 (−1·2 to 0·4)	968·2 (655·2–1348·5)	9·9 (5·5 to 14·2)*	−9·0 (−12·5 to −5·9)*
			Motorcyclist road injuries			26 417·2 (23 496·7 to 29 722·0)	31·7 (30·3 to 33·0)*	7·3 (6·5 to 8·1)*	1397·9 (962·0–1919·8)	17·6 (13·0 to 22·1)*	−3·3 (−7·0 to 0·2)
			Motor vehicle road injuries			44 311·4 (40 212·6 to 49 496·3)	18·7 (17·7 to 19·8)*	−4·1 (−4·7 to −3·4)*	2585·4 (1811·2–3556·8)	12·2 (9·6 to 14·8)*	−8·4 (−10·4 to −6·6)*
				Other road injuries		4135·3 (3603·7 to 4692·7)	40·2 (38·6 to 42·1)*	15·2 (14·4 to 16·1)*	233·7 (158·3–320·9)	32·1 (28·3 to 35·5)*	9·5 (6·8 to 11·9)*
			Other transport injuries			8258·5 (7469·0 to 9141·6)	18·8 (17·9 to 19·7)*	−3·2 (−3·6 to −2·7)*	488·7 (348·6–660·5)	0·3 (−4·0 to 5·2)	−17·1 (−20·5 to −13·3)*
	**Unintentional injuries**					**790 101·8 (759 587·4 to 824 995·0)**	14·3 (13·8 to 14·8)*	−5·2 (−5·5 to −4·9)*	**30 679·5 (21 602·9–41 953·7)**	6·2 (3·1 to 9·0)*	−11·4 (−13·6 to −9·2)*
		Falls				225 736·9 (207 914·3 to 245 787·2)	25·1 (24·4 to 25·8)*	1·7 (1·3 to 2·1)*	11 770·2 (8257·6–16 166·4)	11·3 (6·7 to 15·7)*	−8·6 (−12·1 to −5·2)*
		Drowning				4455·5 (4041·2 to 4919·4)	7·0 (5·8 to 8·1)*	−13·1 (−13·5 to −12·7)*	247·3 (173·4–335·8)	−6·7 (−10·3 to −2·4)*	−23·4 (−26·2 to −20·2)*
		Fire, heat, and hot substances				69 666·6 (60 988·1 to 78 550·1)	10·8 (10·1 to 11·7)*	−8·3 (−8·8 to −7·9)*	2269·4 (1571·4–3131·8)	1·9 (−1·8 to 5·5)	−13·5 (−16·1 to −11·2)*
		Poisonings				5722·4 (4829·3 to 6854·1)	13·8 (12·7 to 14·9)*	−2·0 (−3·2 to −0·6)*	549·4 (367·8–791·1)	8·8 (6·7 to 10·8)*	−4·5 (−6·6 to −2·6)*
		Exposure to mechanical forces				183 582·4 (169 658·6 to 197 859·6)	13·9 (13·0 to 14·7)*	−3·5 (−4·0 to −2·9)*	3580·8 (2485·7–4922·9)	5·9 (2·9 to 8·9)*	−9·9 (−12·1 to −7·6)*
			Unintentional firearm injuries			2333·0 (2074·5 to 2625·1)	13·4 (12·4 to 14·4)*	−6·3 (−7·1 to −5·6)*	93·2 (65·4–126·2)	4·1 (0·6 to 7·4)*	−12·6 (−15·0 to −10·1)*
			Unintentional suffocation			9759·3 (8142·5 to 11 916·9)	9·7 (5·5 to 13·9)*	−7·7 (−10·3 to −5·1)*	353·7 (245·8–480·3)	11·4 (8·6 to 14·0)*	−6·8 (−8·6 to −5·1)*
			Other exposure to mechanical forces			171 490·1 (157 821·0 to 185 364·4)	14·1 (13·2 to 14·9)*	−3·2 (−3·7 to −2·6)*	3133·9 (2172·1–4356·6)	5·4 (2·2 to 8·5)*	−10·1 (−12·6 to −7·7)*
		Adverse effects of medical treatment				11 158·6 (8773·5 to 13 610·2)	3·1 (1·3 to 4·9)*	−13·9 (−15·4 to −12·4)*	1487·4 (926·9–2223·0)	3·1 (1·3 to 4·9)*	−13·9 (−15·4 to −12·4)*
		Animal contact				34 049·7 (30 774·2 to 37 569·0)	9·4 (8·0 to 10·6)*	−6·6 (−7·5 to −5·9)*	1100·9 (763·8–1486·9)	4·6 (3·0 to 6·2)*	−8·8 (−10·0 to −7·5)*
			Venomous animal contact			14 680·7 (13 173·4 to 16 375·1)	13·2 (12·2 to 14·2)*	−3·6 (−4·4 to −2·8)*	820·7 (558·1–1117·5)	5·3 (3·7 to 7·1)*	−7·8 (−9·2 to −6·2)*
			Non-venomous animal contact			19 369·1 (16 794·5 to 22 306·5)	6·7 (4·8 to 8·2)*	−8·8 (−10·1 to −7·7)*	280·3 (187·2–410·5)	2·5 (0·2 to 4·7)*	−11·4 (−13·1 to −9·8)*
		Foreign body				32 345·9 (28 877·3 to 36 026·4)	15·7 (14·1 to 17·1)*	−1·9 (−2·9 to −1·1)*	1263·3 (899·4–1722·5)	3·7 (0·2 to 7·5)*	−10·9 (−13·6 to −8·1)*
			Pulmonary aspiration and foreign body in airway			13 351·2 (11 069·0 to 16 798·7)	15·5 (12·5 to 18·4)*	−1·6 (−3·6 to 0·2)	690·2 (477·9–973·1)	−0·2 (−4·1 to 4·5)	−14·0 (−17·2 to −10·4)*
			Foreign body in eyes			1007·2 (444·0 to 1 593·4)	16·3 (15·0 to 18·2)*	1·4 (0·8 to 1·9)*	56·2 (24·0–100·6)	15·2 (13·8 to 16·7)*	0·5 (−0·9 to 1·3)
			Foreign body in other body part			17 987·4 (15 874·1 to 20 237·9)	15·9 (14·9 to 17·0)*	−2·3 (−3·0 to −1·6)*	517·0 (364·4–713·3)	8·0 (5·5 to 10·6)*	−7·7 (−9·6 to −5·8)*
		Environmental heat and cold exposure				61 942·3 (56 211·9 to 68 761·8)	15·9 (15·4 to 16·5)*	−4·2 (−4·6 to −3·8)*	2657·1 (1861·7–3636·2)	6·4 (3·7 to 9·4)*	−11·0 (−13·0 to −8·9)*
		Other unintentional injuries				161 441·6 (147 971·3 to 176 204·3)	4·7 (4·0 to 5·3)*	−13·7 (−14·1 to −13·3)*	5753·6 (3913·1–8024·1)	0·4 (−1·4 to 2·1)	−16·7 (−17·8 to −15·7)*
	**Self-harm and interpersonal violence**					**24 083·0 (22 279·7 to 25 744·4)**	15·2 (14·6 to 15·7)*	−5·5 (−5·9 to −5·2)*	**1077·5 (762·7–1 448·2)**	**1·9 (−1·6 to 5·8)**	−15·1 (−17·8 to −12·1)*
		Self-harm				6358·6 (5682·1 to 7154·5)	15·2 (14·5 to 15·8)*	−6·2 (−6·7 to −5·8)*	332·7 (232·4–446·9)	0·9 (−2·1 to 4·5)	−15·9 (−18·4 to −13·2)*
		Interpersonal violence				17 724·4 (16 186·6 to 19 268·4)	15·2 (14·5 to 15·9)*	−5·3 (−5·7 to −4·8)*	744·8 (521·9–1007·3)	2·4 (−1·4 to 6·4)	−14·6 (−17·5 to −11·6)*
			Assault by firearm			1043·6 (900·8 to 1213·2)	16·1 (14·9 to 17·3)*	−4·9 (−5·8 to −4·2)*	46·5 (32·4–64·9)	4·6 (0·7 to 8·1)*	−13·2 (−16·1 to −10·5)*
			Assault by sharp object			4019·5 (3330·4 to 4668·1)	12·5 (11·5 to 13·6)*	−7·0 (−7·7 to −6·3)*	123·4 (84·8–168·7)	−6·4 (−11·4 to −0·9)*	−21·1 (−24·8 to −16·8)*
			Assault by other means			12 661·3 (11 425·5 to 14 018·9)	16·0 (15·2 to 16·8)*	−4·7 (−5·3 to −4·3)*	574·8 (404·0–782·2)	4·3 (0·8 to 8·0)*	−13·3 (−15·9 to −10·4)*
	**Forces of nature, war, and legal intervention**					**88 991·7 (65 131·0 to 116 786·1)**	28·8 (18·4 to 40·5)*	11·7 (2·9 to 21·7)*	**2820·2 (1921·3 to 3850·1)**	23·4 (1·7 to 47·8)*	**7·2 (−11·4 to 28·3)**
		Exposure to forces of nature				25 293·7 (16 193·6 to 37 658·7)	13·5 (−2·1 to 32·2)	−1·3 (−14·5 to 14·1)	796·9 (541·4 to 1091·4)	−27·4 (−43·2 to −6·2)*	−35·9 (−49·6 to −17·8)*
		Collective violence and legal intervention				63 697·9 (45 248·7–83 186·4)	36·1 (20·9–54·9)*	18·1 (5·1–34·4)*	2023·3 (1303·8–2903·0)	70·5 (33·0–118·3)*	47·1 (15·3–88·8)*
	**Impairments**					**..**	**..**	**..**	**..**	**..**	**..**
		Anaemia				2 359 107·9 (2 349 938·3 to 2 369 065·8)	4·0 (3·5 to 4·5)*	−7·1 (−7·6 to −6·7)*	77 876·7 (52 436·9 to 111 360·0)	−0·7 (−2·0 to 0·4)	−10·2 (−11·2 to −9·2)*
		Developmental intellectual disability				152 664·0 (113 511·5 to 190 764·1)	12·6 (12·2 to 13·0)*	2·0 (1·6 to 2·4)*	16 875·1 (12 072·6 to 22 373·1)	15·0 (13·4 to 17·0)*	3·8 (2·2 to 5·7)*
		Epilepsy				39 160·5 (34 270·8 to 43 685·8)	15·8 (12·6 to 18·9)*	3·5 (0·5 to 6·4)*	11 770·0 (8 578·8 to 15 276·3)	5·8 (0·5 to 11·0)*	−4·9 (−9·8 to −0·1)*
		Guillain-Barré syndrome				35·0 (28·6 to 42·1)	17·0 (14·7 to 19·5)*	−0·2 (−0·9 to 0·4)	10·4 (6·6 to 15·0)	17·0 (14·7 to 19·5)	−0·2 (−0·9 to 0·4)
		Hearing loss				1 330 902·9 (1 261 401·0 to 1 397 100·6)	26·0 (25·2 to 26·7)*	0·9 (0·5 to 1·3)*	46 183·1 (31 566·7 to 63 209·9)	23·6 (22·1 to 25·1)*	0·6 (−0·4 to 1·5)
		Heart failure				40 048·6 (38 613·2 to 41 418·2)	32·3 (31·6 to 33·1)*	0·3 (−0·2 to 0·8)	6199·4 (4700·7 to 7785·5)	33·2 (31·7 to 34·6)*	1·1 (0·1 to 2·0)*
		Infertility				113 113·8 (93 429·4–136 497·5)	21·1 (17·6–24·5)*	8·0 (4·9–10·9)*	752·4 (327·7–1543·8)	19·9 (16·8–22·7)*	7·0 (4·3–9·4)*
		Pelvic inflammatory disease				754·1 (652·8 to 878·7)	1·1 (0·1 to 2·1)*	−12·0 (−12·7 to −11·2)*	99·9 (67·8 to 138·6)	1·3 (−0·7 to 3·4)	−11·7 (−13·4 to −10·0)*
		Vision loss				939 580·2 (905 009·5 to 974 112·3)	22·4 (21·2 to 23·6)*	−0·4 (−1·3 to 0·4)	24 462·7 (16 954·9 to 34 455·1)	22·4 (21·6 to 23·3)*	−0·2 (−0·8 to 0·3)

Data in parentheses are 95% UIs.

* Percentage changes that are statistically significant.
