# Department of Error

**DOI:** 10.1016/S0140-6736(16)32606-X

**Published:** 2017-01-07

*GBD 2015 Disease and Injury Incidence and Prevalence Collaborators. Global, regional, and national incidence, prevalence, and years lived with disability for 310 diseases and injuries, 1990–2015: a systematic analysis for the Global Burden of Disease Study 2015.* Lancet *2016; **388:** 1545–602*—In this Article, Simon I Hay, Sudha P Jayaraman, Thomas Truelsen, Reed J D Sorensen, Anoushka Millear, Giorgia Giussani, and Ettore Beghi should have been listed as authors. The funding statement for Simon I Hay has been added. These corrections have been made to the online version as of Jan 5, 2017.

