# Supplementary material for: Global, regional, and national incidence, prevalence, and years lived with disability for 310 diseases and injuries, 1990–2015: a systematic analysis for the Global Burden of Disease Study 2015
Source: Lancet. 2016 Oct 8;388(10053):1545–602. doi: 10.1016/S0140-6736(16)31678-6 (PMC5055577; doi:10.1016/S0140-6736(16)31678-6)
Supplement: Supplementary appendix [file mmc2.pdf]

Supplementary Results Appendix to Global, regional, and national incidence, prevalence, and YLDs for 310 diseases and injuries for 188 countries, 1990–2015: a systematic analysis for the Global Burden of Disease Study 2015

This component of the appendix provides supplementary results for incidence, prevalence, and years of life lived with disability (YLDs).

Supplementary Results: Tables & Figures

SR Figure 1. All-cause years lived with disability (YLD) rate per capita by geography and sex, 1990, 1995, 2000, 2010 and 2015, pg. 714

SR Table 1. Global sequela-level prevalence and YLDs for 2015, percent change, and percent change in age-standardized rates between 2005 and 2015 by cause & sequela, all ages and both sexes combined, pg. 717

SR Table 2. Prevalent cases and years lived with disability (YLDs) for 2015, percent change, and percent change in age-standardised rates between 2005 and 2015 of anemia by cause, all ages and both sexes combined, pg. 741

SR Table 3. Prevalent cases and years lived with disability (YLDs) for 2015, percent change, and percent change in age-standardised rates between 2005 and 2015 of epilepsy by cause, all ages and both sexes combined, pg. 742

SR Table 4. Prevalent cases and years lived with disability (YLDs) for 2015, percent change, and percent change in age-standardised rates between 2005 and 2015 of Guillain-Barre syndrome by cause, all ages and both sexes, combined, pg. 743

SR Table 5. Prevalent cases and years lived with disability (YLDs) for 2015, percent change, and percent change in age-standardised rates between 2005 and 2015 of hearing loss by cause, all ages and both sexes combined, pg. 744

SR Table 6. Prevalent cases and years lived with disability (YLDs) for 2015, percent change, and percent change in age-standardised rates between 2005 and 2015 of heart failure by cause, all ages and both sexes combined, pg. 745

SR Table 7. Prevalent cases and years lived with disability (YLDs) for 2015, percent change, and percent change in age-standardised rates between 2005 and 2015 of infertility by cause, all ages and both sexes combined, pg. 746

SR Table 8. Prevalent cases and years lived with disability (YLDs) for 2015, percent change, and percent change in age-standardised rates between 2005 and 2015 of developmental intellectual disability by cause, all ages and both sexes combined, pg. 747

SR Table 9. Prevalent cases and years lived with disability (YLDs) for 2015, percent change, and percent change in age-standardised rates between 2005 and 2015 of pelvic inflammatory disease by cause, all ages and both sexes combined, pg. 748

SR Table 10. Prevalent cases and years lived with disability (YLDs) for 2015, percent change, and percent change in age-standardised rates between 2005 and 2015 of vision loss by cause, all ages and both sexes combined, pg. 749

SR Table 11. Prevalent cases and YLDs for 2015, percent change, and change in age-standardised rates between 2005 and 2015 by cause, global, for each age group, separately pg. 750

SR Table 12.a-i. Prevalent cases and YLDs for 2015, percent change, and change in age-standardised rates between 2005 and 2015 by impairment and for each age group, separately pg. 930

SR Table 13. Prevalent cases and YLDs for 2015, percent change, and change in rates between 2005 and 2015 by cause, for all ages, for each location, separately, pg. 1110

SR Table 14.a-i. Prevalent cases and YLDs for 2015, percent change, and change in rates between 2005 and 2015 by impairment, for all ages, for each location, separately, pg. 2865

SR Figure 1. All-cause years lived with disability (YLD) rate per capita by geography and sex, 1990, 1995, 2000, 2010 and 2015

	Males						Females					
	1990	1995	2000	2005	2010	2015	1990	1995	2000	2005	2010	2015
**Global**	11.0%	10.9%	10.9%	10.8%	10.6%	10.5%	11.8%	11.8%	11.7%	11.6%	11.5%	11.4%
**High SDI**	10.2%	10.1%	10.1%	10.0%	9.9%	9.9%	11.2%	11.2%	11.1%	11.1%	11.0%	11.0%
**High-middle SDI**	10.4%	10.3%	10.2%	10.1%	10.0%	9.9%	11.0%	10.9%	10.8%	10.7%	10.6%	10.6%
**Middle SDI**	10.7%	10.5%	10.5%	10.4%	10.2%	10.2%	11.6%	11.4%	11.3%	11.2%	11.0%	11.0%
**Low-middle SDI**	12.5%	12.4%	12.4%	12.2%	11.9%	11.7%	13.6%	13.5%	13.4%	13.3%	13.0%	12.8%
**Low SDI**	13.2%	13.3%	13.2%	12.9%	12.5%	12.2%	14.0%	14.1%	14.1%	13.9%	13.5%	13.1%
**High-income**	10.0%	9.9%	9.9%	9.8%	9.7%	9.7%	11.1%	11.1%	11.1%	11.0%	11.0%	11.0%
**High-income North America**	10.7%	10.6%	10.7%	10.6%	10.7%	10.7%	11.8%	11.8%	11.9%	11.9%	12.0%	12.0%
Canada	10.3%	10.3%	10.3%	10.2%	10.1%	10.1%	11.1%	11.1%	11.2%	11.0%	10.9%	10.9%
Greenland	10.8%	10.7%	10.7%	10.5%	10.4%	10.4%	12.0%	12.0%	12.0%	11.9%	11.8%	11.7%
United States	10.7%	10.7%	10.7%	10.7%	10.7%	10.7%	11.9%	11.9%	12.0%	12.1%	12.1%	12.1%
**Australasia**	10.5%	10.5%	10.5%	10.5%	10.3%	10.3%	11.7%	11.7%	11.7%	11.6%	11.6%	11.5%
Australia	10.6%	10.6%	10.6%	10.5%	10.4%	10.4%	11.7%	11.7%	11.8%	11.7%	11.6%	11.5%
New Zealand	10.2%	10.1%	10.2%	10.1%	10.0%	10.0%	11.6%	11.5%	11.6%	11.5%	11.5%	11.4%
**High-income Asia Pacific**	8.8%	8.8%	8.7%	8.6%	8.4%	8.4%	9.7%	9.8%	9.6%	9.4%	9.3%	9.3%
Brunei	9.2%	9.2%	9.2%	9.1%	9.0%	9.0%	10.4%	10.3%	10.2%	10.1%	10.0%	9.9%
Japan	8.7%	8.7%	8.6%	8.5%	8.4%	8.4%	9.6%	9.7%	9.4%	9.3%	9.2%	9.2%
Singapore	8.3%	8.2%	8.1%	7.8%	7.6%	7.7%	9.4%	9.3%	9.1%	8.9%	8.7%	8.6%
South Korea	9.4%	9.3%	9.1%	8.9%	8.7%	8.6%	10.4%	10.4%	10.2%	10.0%	9.8%	9.7%
**Western Europe**	10.0%	9.9%	9.9%	9.8%	9.6%	9.5%	11.2%	11.2%	11.2%	11.1%	11.0%	10.9%
Andorra	9.9%	9.9%	9.8%	9.7%	9.6%	9.6%	11.1%	11.0%	10.9%	10.9%	10.8%	10.8%
Austria	10.2%	10.1%	10.1%	10.0%	9.8%	9.7%	11.3%	11.2%	11.2%	11.2%	11.1%	11.0%
Belgium	10.1%	10.1%	10.1%	10.0%	9.9%	9.8%	11.2%	11.2%	11.2%	11.1%	11.0%	10.9%
Cyprus	9.9%	9.9%	10.0%	9.9%	9.8%	9.8%	11.4%	11.3%	11.3%	11.2%	11.1%	11.0%
Denmark	10.0%	10.1%	10.0%	10.0%	9.8%	9.7%	11.3%	11.3%	11.3%	11.3%	11.2%	11.1%
Finland	10.3%	10.2%	10.3%	10.4%	10.2%	10.1%	11.5%	11.4%	11.4%	11.4%	11.3%	11.2%
France	9.9%	9.8%	9.7%	9.6%	9.5%	9.5%	11.1%	11.1%	11.0%	10.9%	10.8%	10.7%
Germany	10.2%	10.1%	10.0%	9.9%	9.8%	9.7%	11.6%	11.6%	11.5%	11.4%	11.3%	11.3%
Greece	9.6%	9.6%	9.6%	9.5%	9.4%	9.3%	11.0%	10.9%	10.8%	10.8%	10.7%	10.6%
Iceland	9.5%	10.1%	9.4%	9.4%	9.3%	9.3%	11.0%	12.8%	11.0%	11.0%	10.8%	10.7%
Ireland	10.2%	10.2%	10.2%	10.1%	9.9%	9.9%	11.3%	11.3%	11.3%	11.3%	11.1%	11.1%
Israel	9.7%	9.8%	9.8%	9.7%	9.6%	9.5%	11.0%	10.9%	11.0%	10.9%	10.8%	10.8%
Italy	9.7%	9.6%	9.7%	9.5%	9.3%	9.3%	11.3%	11.2%	11.2%	11.1%	10.9%	10.9%
Luxembourg	10.8%	10.7%	10.6%	10.4%	10.2%	10.0%	11.9%	11.8%	11.6%	11.5%	11.5%	11.4%
Malta	10.1%	10.1%	10.0%	9.9%	9.8%	9.8%	11.4%	11.3%	11.3%	11.2%	11.1%	11.1%
Netherlands	10.0%	10.0%	9.9%	9.9%	9.7%	9.7%	11.5%	11.5%	11.5%	11.4%	11.3%	11.2%
Norway	10.1%	10.1%	10.1%	10.1%	10.0%	9.8%	11.6%	11.5%	11.6%	11.6%	11.5%	11.2%
Portugal	10.4%	10.4%	10.2%	10.0%	9.8%	9.7%	11.6%	11.6%	11.5%	11.3%	11.2%	11.2%
Spain	9.8%	9.8%	9.7%	9.5%	9.2%	9.2%	10.9%	10.8%	10.7%	10.5%	10.5%	10.4%
Sweden	9.7%	9.7%	9.8%	9.7%	9.6%	9.6%	10.5%	10.6%	10.6%	10.6%	10.5%	10.5%
Switzerland	10.3%	10.2%	10.2%	10.0%	9.8%	9.6%	11.7%	11.5%	11.5%	11.4%	11.2%	11.0%
United Kingdom	10.0%	10.1%	10.0%	9.9%	9.8%	9.7%	11.0%	11.0%	11.0%	11.0%	10.9%	10.8%
England	10.0%	10.0%	9.9%	9.8%	9.7%	9.6%	11.0%	10.9%	10.9%	10.9%	10.7%	10.7%
East Midlands	9.9%	10.0%	10.0%	10.0%	9.9%	9.7%	10.8%	10.8%	10.9%	10.9%	11.0%	10.7%
East of England	10.6%	10.6%	10.6%	10.6%	10.4%	10.3%	11.8%	11.8%	11.8%	11.8%	11.7%	11.7%
Greater London	10.6%	10.6%	10.6%	10.6%	10.6%	10.5%	11.4%	11.4%	11.4%	11.3%	11.2%	11.2%
North East England	9.7%	9.7%	9.6%	9.5%	9.4%	9.4%	10.5%	10.5%	10.5%	10.4%	10.4%	10.4%
North West England	9.7%	9.8%	9.7%	9.6%	9.5%	9.5%	10.5%	10.5%	10.6%	10.5%	10.4%	10.4%
South East England	9.5%	9.5%	9.4%	9.3%	9.2%	9.2%	10.4%	10.4%	10.3%	10.3%	10.4%	10.3%
South West England	9.4%	9.4%	9.4%	9.3%	9.2%	9.2%	10.5%	10.4%	10.5%	10.4%	10.4%	10.3%
West Midlands	11.0%	11.1%	11.0%	10.8%	10.6%	10.6%	11.4%	11.4%	11.3%	11.2%	11.0%	11.0%
Yorkshire and the Humber	11.3%	11.4%	11.4%	11.2%	11.0%	10.9%	11.9%	11.9%	11.9%	11.7%	11.6%	11.5%
Northern Ireland	10.8%	10.9%	10.8%	10.8%	10.7%	10.7%	11.1%	11.1%	11.0%	10.9%	10.8%	10.8%
Scotland	11.0%	11.0%	10.9%	10.6%	10.3%	10.2%	11.4%	11.4%	11.1%	10.8%	10.6%	10.5%
Wales	11.2%	11.1%	11.0%	10.7%	10.5%	10.3%	11.3%	11.2%	11.0%	10.8%	10.6%	10.5%
**Southern Latin America**	11.0%	10.9%	10.9%	10.8%	10.6%	10.6%	11.2%	11.1%	11.0%	10.8%	10.7%	10.6%
Argentina	11.0%	10.9%	10.7%	10.5%	10.2%	10.2%	11.4%	11.3%	11.2%	10.9%	10.7%	10.7%
Chile	11.4%	11.5%	11.6%	11.4%	11.1%	11.0%	12.3%	12.3%	12.3%	12.1%	12.0%	11.9%
Uruguay	11.1%	11.2%	11.1%	10.9%	10.7%	10.7%	11.1%	11.1%	11.0%	10.8%	10.7%	10.7%
**Central Europe, Eastern Europe, and Central Asia**	10.6%	10.6%	10.4%	10.3%	10.1%	10.1%	10.6%	10.6%	10.5%	10.4%	10.2%	10.2%
**Eastern Europe**	10.8%	10.8%	10.7%	10.4%	10.1%	10.0%	10.9%	10.9%	10.8%	10.5%	10.3%	10.2%
Belarus	10.3%	13.6%	10.7%	10.5%	10.4%	10.4%	10.4%	12.6%	10.5%	10.3%	10.2%	10.3%
Estonia	10.5%	10.5%	10.4%	10.3%	10.1%	10.1%	10.5%	10.5%	10.4%	10.3%	10.2%	10.2%
Latvia	10.7%	10.7%	10.5%	10.3%	10.2%	10.2%	10.8%	10.7%	10.5%	10.4%	10.3%	10.3%
Lithuania	10.4%	10.3%	10.2%	10.2%	10.0%	10.0%	10.6%	10.5%	10.4%	10.4%	10.3%	10.2%
Moldova	10.7%	10.6%	10.5%	10.4%	10.3%	10.2%	11.0%	10.9%	10.8%	10.7%	10.6%	10.5%
Russia	10.3%	10.2%	10.0%	9.9%	9.8%	9.8%	10.3%	10.2%	10.0%	10.0%	9.9%	9.9%
Ukraine	10.4%	10.4%	10.3%	10.2%	10.1%	10.1%	10.3%	10.3%	10.2%	10.2%	10.0%	10.0%
**Central Europe**	10.6%	10.5%	10.4%	10.3%	10.1%	10.0%	10.5%	10.4%	10.3%	10.2%	10.1%	10.0%
Albania	10.7%	10.7%	10.6%	10.4%	10.2%	10.2%	10.8%	10.7%	10.6%	10.4%	10.2%	10.2%
Bosnia and Herzegovina	10.4%	10.3%	10.3%	10.2%	10.1%	10.1%	10.4%	10.3%	10.3%	10.3%	10.2%	10.2%
Bulgaria	10.7%	10.7%	10.6%	10.5%	10.3%	10.3%	10.4%	10.3%	10.3%	10.2%	10.1%	10.1%
Croatia	10.5%	10.4%	10.3%	10.2%	9.9%	9.9%	10.7%	10.6%	10.5%	10.4%	10.2%	10.1%
Czech Republic	10.5%	10.6%	10.5%	10.3%	10.2%	10.2%	11.1%	11.1%	11.0%	10.8%	10.7%	10.7%
Hungary	10.4%	10.3%	10.2%	10.1%	10.0%	10.0%	11.3%	11.1%	11.0%	10.9%	10.7%	10.6%
Macedonia	10.2%	10.2%	10.1%	10.0%	9.9%	10.0%	11.0%	11.0%	11.0%	10.8%	10.7%	10.8%
Montenegro	10.0%	9.8%	9.7%	9.8%	9.8%	10.0%	10.3%	10.2%	10.1%	10.1%	10.2%	10.3%
Poland	11.1%	11.3%	11.1%	11.0%	10.8%	10.7%	11.4%	11.5%	11.3%	11.2%	11.1%	11.0%
Romania	10.5%	10.5%	10.4%	10.3%	10.1%	10.0%	11.3%	11.3%	11.1%	11.0%	10.9%	10.9%
Serbia	10.9%	10.8%	10.8%	10.6%	10.7%	10.6%	10.7%	10.6%	10.5%	10.3%	10.2%	10.2%

Supplementary Results Table 1. All-cause years lived with disability (YLD) rate per capita by geography and sex, 1990-2015Fig

Slovakia	10.4%	10.5%	10.3%	10.2%	10.1%	10.1%	10.9%	11.0%	10.9%	10.7%	10.6%	10.5%
Slovenia	10.2%	10.2%	10.3%	10.2%	10.0%	10.0%	10.7%	10.7%	10.7%	10.5%	10.4%	10.4%
**Central Asia**	10.4%	10.4%	10.4%	10.2%	10.0%	10.1%	11.1%	11.1%	11.0%	10.7%	10.6%	10.6%
Armenia	10.4%	10.4%	10.4%	10.3%	10.3%	10.2%	11.3%	11.3%	11.3%	11.2%	11.3%	11.1%
Azerbaijan	10.0%	9.9%	9.9%	9.8%	9.8%	9.8%	10.8%	10.7%	10.7%	10.6%	10.6%	10.6%
Georgia	9.8%	9.7%	9.7%	9.6%	9.5%	9.5%	11.0%	10.8%	10.8%	10.7%	10.7%	10.7%
Kazakhstan	9.5%	9.5%	9.5%	9.5%	9.4%	9.5%	10.4%	10.4%	10.4%	10.4%	10.4%	10.4%
Kyrgyzstan	10.5%	10.4%	10.3%	10.2%	10.0%	9.9%	11.6%	11.4%	11.4%	11.4%	11.3%	11.3%
Mongolia	11.1%	10.9%	10.9%	11.0%	10.8%	10.7%	11.8%	11.6%	11.6%	11.6%	11.6%	11.5%
Tajikistan	10.4%	10.3%	10.2%	10.0%	9.8%	9.8%	11.5%	11.4%	11.3%	11.1%	11.0%	10.9%
Turkmenistan	9.9%	9.5%	9.4%	9.4%	9.4%	9.4%	10.5%	10.2%	10.2%	10.1%	10.1%	10.1%
Uzbekistan	10.2%	10.1%	10.0%	9.8%	9.6%	9.6%	11.1%	10.9%	10.7%	10.6%	10.5%	10.5%
**Latin America and Caribbean**	9.7%	9.8%	9.8%	9.8%	9.7%	9.8%	10.7%	10.7%	10.7%	10.7%	10.7%	10.8%
**Central Latin America**	10.2%	10.9%	11.5%	11.5%	11.4%	11.4%	11.0%	11.4%	11.7%	11.7%	11.6%	11.6%
Colombia	10.9%	10.7%	10.6%	10.4%	10.3%	10.3%	11.4%	11.2%	11.1%	10.9%	10.8%	10.8%
Costa Rica	10.9%	10.8%	10.7%	10.7%	10.6%	10.6%	11.6%	11.4%	11.3%	11.3%	11.4%	11.4%
El Salvador	10.8%	10.8%	10.6%	10.5%	10.3%	10.3%	11.4%	11.3%	11.1%	11.0%	10.9%	10.9%
Guatemala	11.0%	10.7%	10.5%	10.3%	10.2%	10.1%	11.4%	11.1%	10.9%	10.8%	10.6%	10.6%
Honduras	10.8%	10.8%	10.7%	10.6%	12.2%	10.6%	11.7%	11.6%	11.6%	11.5%	12.7%	11.6%
Mexico	10.6%	10.8%	10.7%	10.7%	10.5%	10.5%	11.0%	11.1%	11.1%	11.2%	11.2%	11.1%
Nicaragua	11.1%	11.1%	11.1%	10.9%	10.7%	10.6%	11.6%	11.6%	11.6%	11.6%	11.6%	11.5%
Panama	10.5%	10.6%	10.6%	10.5%	10.3%	10.4%	11.3%	11.4%	11.4%	11.4%	11.5%	11.5%
Venezuela	10.5%	10.6%	10.6%	10.6%	10.4%	10.4%	11.3%	11.3%	11.4%	11.5%	11.5%	11.5%
**Andean Latin America**	10.7%	10.6%	10.5%	10.4%	10.2%	10.3%	11.1%	11.1%	11.2%	11.1%	11.1%	11.1%
Bolivia	10.0%	10.0%	9.8%	9.8%	9.8%	9.8%	11.0%	11.0%	10.9%	11.0%	11.0%	11.0%
Ecuador	11.0%	11.1%	11.0%	11.1%	11.1%	11.1%	11.5%	11.5%	11.4%	11.4%	11.4%	11.5%
Peru	10.5%	10.5%	10.4%	10.3%	10.1%	10.1%	11.2%	11.1%	11.1%	11.0%	11.0%	10.9%
**Caribbean**	11.3%	11.0%	11.0%	11.1%	11.0%	11.0%	12.0%	11.6%	11.6%	11.7%	11.8%	11.8%
Antigua and Barbuda	11.8%	11.7%	11.6%	11.4%	11.2%	11.2%	12.2%	12.1%	12.1%	12.1%	12.0%	11.9%
The Bahamas	12.4%	12.2%	11.9%	11.8%	19.4%	12.0%	13.4%	13.3%	13.1%	12.8%	18.1%	13.1%
Barbados	10.4%	10.5%	10.6%	10.6%	10.6%	10.6%	11.4%	11.5%	11.5%	11.6%	11.7%	11.7%
Belize	10.7%	10.9%	10.8%	10.7%	10.6%	10.6%	11.2%	11.3%	11.3%	11.3%	11.3%	11.2%
Bermuda	11.7%	11.2%	11.0%	10.8%	10.7%	10.6%	12.3%	11.7%	11.5%	11.4%	11.4%	11.4%
Cuba	11.0%	11.1%	11.2%	11.0%	10.9%	10.9%	11.6%	11.7%	11.7%	11.6%	11.6%	11.6%
Dominica	10.7%	10.7%	10.7%	10.6%	10.5%	10.5%	11.6%	11.6%	11.6%	11.5%	11.4%	11.4%
Dominican Republic	10.9%	10.9%	10.9%	11.0%	11.0%	11.0%	11.6%	11.6%	11.6%	11.6%	11.7%	11.7%
Grenada	10.8%	10.9%	10.8%	10.8%	10.7%	10.7%	11.2%	11.3%	11.2%	11.2%	11.2%	11.2%
Guyana	10.8%	10.8%	10.8%	10.7%	10.6%	10.5%	11.8%	11.9%	12.0%	11.8%	11.8%	11.7%
Haiti	10.8%	10.8%	10.8%	10.7%	10.6%	10.5%	11.8%	11.9%	12.0%	11.9%	11.8%	11.7%
Jamaica	10.2%	10.3%	10.3%	10.4%	10.3%	10.4%	11.5%	11.5%	11.6%	11.6%	11.6%	11.7%
Puerto Rico	9.8%	9.7%	9.6%	9.5%	9.4%	9.3%	10.5%	10.4%	10.2%	10.1%	10.0%	9.9%
Saint Lucia	9.4%	9.3%	9.2%	9.1%	9.0%	8.9%	10.1%	9.9%	9.7%	9.6%	9.5%	9.4%
Saint Vincent and the Grenadines	9.5%	9.3%	9.2%	9.1%	9.0%	8.9%	10.1%	9.9%	9.7%	9.6%	9.4%	9.4%
Suriname	9.0%	9.2%	9.3%	9.0%	9.0%	9.0%	10.3%	10.5%	10.5%	10.3%	10.1%	10.0%
Trinidad and Tobago	9.3%	8.9%	8.9%	8.9%	8.9%	8.9%	10.6%	10.1%	10.1%	10.0%	10.0%	10.0%
Virgin Islands, U.S.	10.9%	10.8%	10.6%	10.5%	10.3%	10.2%	11.7%	11.5%	11.3%	11.2%	11.0%	10.9%
**Tropical Latin America**	11.8%	11.6%	11.4%	11.1%	10.6%	10.5%	13.1%	12.8%	12.5%	12.0%	11.5%	11.3%
Brazil	10.8%	10.6%	10.4%	10.4%	10.4%	10.3%	11.5%	11.3%	11.1%	11.1%	11.1%	11.0%
Paraguay	11.5%	11.4%	11.3%	11.1%	10.8%	10.7%	12.2%	12.0%	11.9%	11.6%	11.3%	11.2%
**Southeast Asia, East Asia, and Oceania**	10.3%	10.3%	10.3%	10.2%	10.1%	10.1%	11.0%	11.0%	10.9%	10.8%	10.7%	10.7%
**East Asia**	11.9%	11.5%	11.2%	10.6%	10.2%	10.0%	13.3%	12.8%	12.4%	11.7%	11.2%	11.0%
China	9.8%	9.8%	9.8%	9.9%	9.9%	10.0%	10.4%	10.3%	10.3%	10.4%	10.5%	10.5%
North Korea	11.7%	11.5%	11.5%	11.3%	11.1%	10.9%	12.2%	11.9%	11.9%	11.8%	11.5%	11.3%
Taiwan	11.6%	11.3%	11.3%	11.1%	10.9%	10.8%	12.4%	11.9%	12.0%	12.0%	11.8%	11.6%
**Southeast Asia**	11.0%	10.7%	10.5%	10.3%	10.4%	10.1%	11.4%	11.0%	10.8%	10.7%	10.7%	10.4%
Cambodia	10.0%	10.1%	10.2%	10.1%	10.0%	9.9%	10.3%	10.3%	10.3%	10.4%	10.3%	10.3%
Indonesia	10.7%	10.7%	10.5%	10.3%	10.1%	10.1%	11.2%	11.2%	11.0%	10.7%	10.5%	10.4%
Laos	11.2%	11.1%	11.8%	11.0%	10.9%	10.7%	11.4%	11.2%	11.4%	11.0%	10.9%	10.7%
Malaysia	10.4%	10.4%	10.2%	10.0%	9.6%	9.5%	11.6%	11.5%	11.2%	10.9%	10.4%	10.1%
Maldives	11.7%	11.7%	11.8%	11.7%	11.6%	11.5%	14.1%	14.0%	14.2%	14.2%	14.1%	14.1%
Mauritius	10.9%	11.0%	11.1%	11.0%	10.9%	10.9%	11.6%	11.6%	11.6%	11.6%	11.7%	11.6%
Myanmar	10.7%	10.6%	10.7%	10.7%	10.6%	10.6%	11.7%	11.7%	11.7%	11.6%	11.5%	11.5%
Philippines	11.8%	11.8%	12.1%	11.7%	11.5%	11.4%	12.6%	12.6%	13.0%	12.6%	12.5%	12.4%
Sri Lanka	9.8%	9.8%	9.9%	10.0%	10.1%	10.2%	11.2%	11.1%	11.2%	12.0%	12.5%	12.1%
Seychelles	11.9%	12.1%	12.2%	12.2%	12.0%	11.8%	12.9%	13.0%	12.9%	12.9%	12.8%	12.5%
Thailand	12.0%	12.1%	12.2%	12.2%	12.1%	12.2%	13.2%	13.3%	13.4%	13.4%	13.3%	13.3%
Timor-Leste	10.2%	10.1%	10.1%	10.0%	10.0%	10.0%	11.4%	11.2%	11.1%	11.0%	10.9%	10.9%
Vietnam	12.0%	11.9%	12.0%	12.0%	11.8%	11.8%	14.9%	14.9%	15.1%	15.1%	14.9%	14.8%
**Oceania**	10.8%	10.7%	10.8%	10.6%	10.6%	10.7%	11.2%	11.3%	11.4%	11.4%	11.5%	11.6%
American Samoa	10.8%	10.8%	10.8%	10.8%	10.7%	10.8%	12.4%	12.4%	12.4%	12.3%	12.3%	12.2%
Federated States of Micronesia	11.0%	10.6%	10.8%	10.7%	10.7%	10.6%	12.5%	12.0%	12.2%	12.0%	12.0%	11.9%
Fiji	10.6%	10.5%	10.6%	10.5%	10.6%	10.6%	11.5%	11.4%	11.5%	11.5%	11.6%	11.5%
Guam	11.8%	11.7%	11.5%	11.3%	11.2%	11.3%	12.7%	12.5%	12.4%	12.3%	12.1%	12.2%
Kiribati	11.8%	11.7%	11.5%	11.3%	11.2%	11.3%	12.7%	12.5%	12.4%	12.3%	12.1%	12.2%
Marshall Islands	14.8%	14.9%	14.5%	14.0%	13.7%	13.7%	14.8%	14.9%	14.7%	14.3%	14.2%	14.1%
Northern Mariana Islands	11.0%	11.0%	10.8%	10.6%	10.5%	10.6%	12.0%	12.0%	11.9%	11.7%	11.7%	11.7%
Papua New Guinea	11.5%	11.6%	11.5%	11.2%	11.0%	10.9%	13.1%	13.3%	13.2%	12.9%	12.8%	12.7%
Samoa	11.9%	11.6%	11.3%	11.2%	11.2%	11.3%	12.6%	12.3%	12.1%	12.4%	12.4%	12.4%
Solomon Islands	12.0%	11.7%	11.5%	11.2%	11.0%	11.0%	12.7%	12.4%	12.2%	12.0%	11.8%	11.7%
Tonga	13.2%	13.2%	13.1%	12.9%	12.6%	12.8%	13.1%	13.1%	13.1%	13.0%	12.7%	12.9%
Vanuatu	11.3%	11.1%	11.0%	10.9%	10.7%	10.7%	12.7%	12.6%	12.5%	12.6%	12.2%	12.1%
**North Africa and Middle East**	10.7%	10.5%	10.5%	10.3%	10.2%	10.1%	11.8%	11.7%	11.7%	11.6%	11.5%	11.4%
**North Africa and Middle East**	15.6%	14.3%	13.6%	12.8%	12.1%	11.9%	14.8%	14.0%	13.6%	13.1%	12.6%	12.4%

Supplementary Results Figure 1. All-cause years lived with disability (YLD) rate per capita by geography and sex, 1990-2015

Afghanistan	10.7%	10.6%	10.6%	10.6%	10.6%	11.1%	11.7%	11.7%	11.6%	11.7%	11.7%	11.9%
Algeria	12.3%	12.2%	12.1%	11.9%	11.8%	11.8%	12.6%	12.5%	12.4%	12.4%	12.3%	12.3%
Bahrain	12.4%	12.1%	12.1%	11.7%	11.4%	11.3%	12.0%	11.8%	11.7%	11.5%	11.3%	11.3%
Egypt	11.9%	11.8%	11.8%	11.7%	11.7%	11.7%	12.7%	12.6%	12.4%	12.4%	12.4%	12.3%
Iran	11.3%	11.2%	11.3%	11.0%	10.8%	10.8%	12.1%	12.1%	12.2%	12.0%	11.9%	11.9%
Iraq	10.4%	10.4%	10.3%	10.2%	10.0%	10.0%	11.6%	11.6%	11.5%	11.4%	11.2%	11.2%
Jordan	12.5%	12.5%	12.7%	12.5%	12.4%	12.2%	13.5%	13.5%	13.7%	13.5%	13.2%	13.0%
Kuwait	10.5%	10.3%	10.2%	10.0%	10.0%	12.9%	11.6%	11.4%	11.2%	11.1%	11.1%	12.9%
Lebanon	11.2%	11.1%	11.0%	10.9%	10.8%	10.8%	11.7%	11.6%	11.4%	11.3%	11.3%	11.3%
Libya	11.4%	11.2%	10.9%	10.5%	10.3%	10.3%	13.0%	12.7%	12.2%	11.7%	11.5%	11.5%
Morocco	12.1%	11.9%	11.7%	11.3%	11.1%	11.1%	12.6%	12.6%	12.4%	12.2%	12.0%	12.0%
Palestine	11.3%	11.3%	11.3%	11.2%	11.1%	11.6%	12.7%	12.6%	12.6%	12.5%	12.4%	12.6%
Oman	12.7%	12.6%	12.6%	12.4%	12.0%	11.8%	14.1%	13.9%	13.7%	13.6%	13.2%	12.9%
Qatar	12.7%	12.6%	12.6%	12.4%	12.0%	11.8%	14.1%	13.9%	13.7%	13.6%	13.2%	12.9%
Saudi Arabia	12.6%	12.2%	11.9%	11.7%	11.3%	11.2%	14.1%	13.8%	13.4%	13.2%	12.8%	12.6%
Sudan	12.0%	12.0%	12.2%	11.7%	11.6%	11.7%	13.3%	13.2%	13.3%	13.0%	12.9%	13.0%
Syria	12.8%	12.8%	12.8%	12.5%	12.1%	11.8%	14.2%	14.1%	13.9%	13.7%	13.3%	13.0%
Tunisia	11.8%	11.7%	11.6%	11.6%	11.2%	11.5%	12.5%	12.3%	12.2%	12.1%	11.9%	12.2%
Turkey	11.7%	11.7%	11.8%	12.0%	11.8%	11.8%	13.0%	12.8%	12.8%	13.2%	12.8%	12.7%
United Arab Emirates	13.0%	13.0%	13.1%	12.9%	12.5%	12.2%	13.7%	13.8%	13.9%	13.9%	13.6%	13.1%
Yemen	11.9%	12.3%	12.9%	13.2%	13.2%	12.8%	12.5%	12.9%	13.6%	14.1%	14.0%	13.5%
**South Asia**	12.6%	13.4%	14.3%	14.4%	14.0%	13.9%	12.9%	13.7%	14.6%	14.9%	14.4%	14.3%
**South Asia**	11.6%	12.0%	13.0%	13.7%	13.9%	14.0%	12.2%	12.6%	13.6%	14.5%	14.5%	14.4%
Bangladesh	11.9%	12.3%	12.9%	13.0%	12.5%	12.5%	12.6%	13.0%	13.5%	13.7%	13.2%	13.1%
Bhutan	11.9%	12.1%	12.7%	13.0%	13.1%	12.7%	12.6%	12.8%	13.4%	14.0%	14.1%	13.6%
India	12.2%	13.0%	14.1%	14.8%	14.9%	14.5%	12.7%	13.5%	14.6%	15.6%	15.6%	15.2%
Nepal	11.9%	13.0%	13.8%	13.9%	13.4%	12.5%	12.0%	13.0%	13.9%	14.2%	13.6%	12.8%
Pakistan	13.0%	12.8%	12.7%	12.6%	12.2%	11.9%	13.7%	13.6%	13.5%	13.4%	13.1%	12.7%
**Sub-Saharan Africa**	12.3%	12.1%	11.9%	11.5%	11.3%	10.9%	13.8%	13.6%	13.5%	13.1%	12.9%	12.6%
**Southern Sub-Saharan Africa**	13.1%	12.9%	12.6%	12.0%	11.2%	11.0%	13.7%	13.6%	13.4%	13.0%	12.3%	12.0%
Botswana	12.9%	12.9%	12.9%	12.9%	12.4%	11.9%	13.4%	13.4%	13.5%	13.5%	13.0%	12.6%
Lesotho	11.2%	11.3%	11.3%	11.1%	10.8%	10.7%	12.0%	11.9%	11.8%	11.6%	11.5%	11.4%
Namibia	13.1%	13.1%	13.2%	13.2%	13.0%	12.8%	13.5%	13.4%	13.4%	13.5%	13.3%	13.1%
South Africa	13.2%	13.2%	12.8%	12.5%	12.2%	11.8%	14.6%	14.3%	13.9%	13.6%	13.4%	13.0%
Swaziland	11.2%	11.2%	11.1%	11.1%	11.0%	10.9%	12.3%	12.3%	12.2%	12.1%	12.0%	11.9%
Zimbabwe	12.0%	11.8%	11.6%	11.3%	10.9%	10.5%	13.0%	12.7%	12.4%	12.4%	12.2%	11.7%
**Western Sub-Saharan Africa**	12.3%	12.2%	12.1%	11.9%	11.8%	11.6%	13.6%	13.4%	13.2%	13.1%	12.9%	12.7%
Benin	11.9%	11.9%	11.9%	11.8%	11.8%	11.8%	12.8%	12.8%	12.8%	12.8%	12.7%	12.7%
Burkina Faso	15.2%	15.3%	14.4%	14.4%	13.8%	13.2%	15.5%	15.6%	15.0%	14.9%	14.4%	13.8%
Cameroon	13.2%	13.0%	12.9%	12.6%	11.9%	11.6%	13.7%	13.6%	13.5%	13.4%	12.9%	12.6%
Cape Verde	13.0%	12.6%	12.3%	12.1%	11.8%	11.8%	13.9%	13.6%	13.4%	13.2%	12.8%	12.7%
Chad	11.9%	11.7%	11.8%	11.7%	11.4%	11.2%	12.6%	12.5%	12.5%	12.3%	12.0%	11.9%
Cote d'Ivoire	13.3%	13.1%	13.1%	13.1%	12.8%	12.5%	14.0%	13.8%	13.8%	13.8%	13.5%	13.1%
The Gambia	11.4%	11.4%	11.4%	11.4%	11.4%	11.4%	12.2%	12.3%	12.2%	12.3%	12.3%	12.3%
Ghana	11.3%	11.4%	11.3%	11.2%	11.1%	10.8%	12.7%	12.8%	12.7%	12.6%	12.4%	12.1%
Guinea	13.1%	13.6%	13.5%	13.2%	12.2%	11.7%	13.6%	13.8%	13.7%	13.6%	12.7%	12.2%
Guinea-Bissau	11.7%	11.8%	12.0%	11.9%	11.6%	11.2%	12.5%	12.6%	12.7%	12.7%	12.3%	11.9%
Liberia	12.8%	12.9%	12.9%	12.6%	12.1%	11.7%	13.3%	13.5%	13.6%	13.4%	12.9%	12.3%
Mali	12.3%	12.3%	12.2%	11.7%	10.9%	10.5%	12.7%	12.7%	12.7%	12.4%	11.5%	10.9%
Mauritania	12.3%	12.1%	12.0%	11.6%	11.2%	11.2%	12.7%	12.5%	12.3%	12.0%	11.7%	11.6%
Niger	11.7%	11.8%	12.0%	12.0%	12.0%	12.0%	12.4%	12.6%	12.8%	13.0%	13.0%	12.9%
Nigeria	12.7%	12.6%	18.8%	13.0%	12.7%	12.4%	13.3%	13.2%	15.6%	13.0%	12.8%	12.5%
Sao Tome and Principe	12.6%	12.3%	12.1%	11.8%	11.3%	11.2%	12.8%	12.7%	12.5%	12.1%	11.6%	11.4%
Senegal	12.3%	12.8%	13.1%	12.8%	12.3%	11.9%	13.3%	13.9%	14.5%	14.7%	14.5%	13.5%
Sierra Leone	13.5%	13.4%	13.3%	13.0%	12.5%	12.1%	13.8%	13.8%	13.6%	13.4%	12.9%	12.4%
Togo	12.9%	13.6%	13.8%	13.6%	12.5%	11.6%	13.5%	14.1%	14.3%	14.1%	13.2%	12.1%
**Eastern Sub-Saharan Africa**	13.2%	13.2%	13.4%	13.6%	13.5%	12.8%	14.2%	14.2%	14.5%	14.9%	14.9%	13.7%
Burundi	12.0%	13.9%	13.4%	12.7%	11.7%	11.4%	12.3%	14.0%	13.6%	13.0%	12.0%	11.8%
Comoros	12.4%	12.3%	12.3%	12.4%	12.5%	12.3%	14.7%	14.7%	14.7%	14.9%	14.8%	14.6%
Djibouti	14.4%	14.5%	14.3%	14.1%	13.7%	13.7%	15.0%	15.2%	15.0%	14.7%	14.3%	14.1%
Eritrea	12.8%	13.1%	13.0%	12.6%	12.1%	11.6%	13.1%	13.4%	13.3%	13.3%	12.8%	12.0%
Ethiopia	13.3%	13.5%	13.2%	12.7%	12.0%	11.7%	13.9%	14.0%	13.8%	13.7%	12.9%	12.2%
Kenya	12.3%	12.9%	13.2%	13.1%	12.6%	12.3%	12.4%	12.9%	13.3%	13.2%	12.8%	12.6%
Madagascar	14.7%	14.8%	14.8%	14.7%	14.2%	14.0%	16.2%	16.6%	16.9%	16.8%	16.6%	16.4%
Malawi	13.8%	13.7%	13.6%	13.3%	13.3%	13.0%	15.2%	15.3%	15.3%	15.2%	15.2%	14.7%
Mozambique	14.5%	14.6%	14.7%	14.6%	14.1%	13.7%	15.0%	15.1%	15.4%	15.6%	15.1%	14.6%
Rwanda	13.3%	13.3%	13.3%	12.9%	12.5%	12.5%	14.6%	14.7%	14.9%	14.9%	14.7%	14.8%
Somalia	15.3%	15.3%	15.4%	15.3%	14.7%	14.4%	16.8%	17.4%	17.7%	17.7%	17.4%	17.2%
South Sudan	13.1%	13.0%	12.8%	12.8%	12.6%	12.4%	13.9%	13.9%	13.7%	13.8%	13.7%	13.4%
Tanzania	13.2%	13.2%	13.4%	13.3%	13.3%	13.1%	14.6%	14.8%	15.0%	15.2%	15.5%	15.6%
Uganda	13.8%	13.9%	13.6%	13.0%	12.5%	12.1%	13.6%	13.7%	13.5%	13.1%	12.7%	12.5%
Zambia	13.3%	13.9%	14.2%	13.9%	13.4%	13.2%	13.2%	13.7%	14.1%	13.9%	13.5%	13.3%
**Central Sub-Saharan Africa**	15.0%	15.0%	14.9%	14.7%	14.2%	13.8%	15.3%	15.2%	15.2%	15.0%	14.7%	14.4%
Angola	14.6%	14.5%	14.1%	13.7%	13.3%	13.0%	14.6%	14.6%	14.4%	14.2%	13.9%	13.8%
Central African Republic	15.2%	15.2%	15.2%	15.1%	14.7%	14.3%	15.3%	15.4%	15.5%	15.5%	15.2%	15.0%
Congo	13.7%	13.7%	13.4%	12.9%	12.4%	12.3%	14.5%	14.6%	14.5%	14.3%	14.0%	14.0%
Democratic Republic of the Congo	15.3%	15.3%	15.3%	15.1%	14.5%	14.1%	15.5%	15.4%	15.5%	15.3%	14.9%	14.7%
Equatorial Guinea	14.0%	13.8%	13.4%	13.2%	12.9%	12.7%	14.5%	14.4%	14.1%	14.0%	13.8%	13.6%
Gabon	13.9%	13.7%	13.7%	13.4%	13.2%	12.9%	14.6%	14.6%	14.6%	14.5%	14.4%	14.3%

SR Table 1. Global sequelae-level prevalence and YLDs, all ages, 2005, 2015					
Cause	Sequela	Prevalence in thousands (95% confidence interval)		YLDs in thousands (95% confidence interval)	
		2005	2015	2005	2015
Tuberculosis	Tuberculosis	8,113.0 (7,441.0 to 8,850.3)	8,861.2 (8,076.3 to 9,707.2)	2,480.2 (1,675.7 to 3,409.6)	2,712.4 (1,829.9 to 3,744.0)
HIV/AIDS - Tuberculosis	HIV/AIDS resulting in mycobacterial infection	1,532.6 (1,403.6 to 1,685.9)	1,258.0 (1,142.5 to 1,386.9)	573.9 (384.6 to 777.3)	471.9 (316.0 to 645.6)
HIV/AIDS resulting in other diseases	Early HIV	19,561.9 (18,974.8 to 20,374.5)	15,844.5 (15,173.6 to 16,705.1)	235.5 (118.4 to 420.1)	191.1 (96.0 to 334.6)
HIV/AIDS resulting in other diseases	Symptomatic HIV	6,704.8 (6,509.7 to 6,990.0)	5,304.5 (5,084.0 to 5,586.7)	1,704.1 (1,170.3 to 2,301.2)	1,352.5 (924.9 to 1,819.9)
HIV/AIDS resulting in other diseases	AIDS with antiretroviral treatment	1,270.5 (1,096.1 to 1,505.6)	14,706.0 (14,143.4 to 15,317.4)	93.4 (61.5 to 134.1)	1,080.7 (733.2 to 1,508.5)
HIV/AIDS resulting in other diseases	AIDS without antiretroviral treatment	2,833.3 (2,315.1 to 3,464.0)	1,688.3 (1,464.9 to 1,984.1)	1,479.2 (1,008.1 to 1,991.4)	893.8 (622.6 to 1,174.5)
Diarrheal diseases	Mild diarrheal diseases	8,173.4 (7,691.3 to 8,686.9)	8,696.4 (8,234.2 to 9,238.0)	578.1 (382.0 to 820.5)	615.2 (407.1 to 872.9)
Diarrheal diseases	Moderate diarrheal diseases	20,776.8 (19,779.2 to 21,899.8)	22,106.3 (21,165.9 to 23,238.3)	3,706.2 (2,512.2 to 5,139.7)	3,943.7 (2,670.1 to 5,453.4)
Diarrheal diseases	Severe diarrheal diseases	4,712.1 (4,344.7 to 5,087.0)	5,013.6 (4,618.2 to 5,401.9)	1,100.3 (730.5 to 1,547.1)	1,171.6 (782.5 to 1,647.5)
Diarrheal diseases	Guillain-Barré syndrome due to diarrheal diseases	3.6 (2.7 to 4.7)	4.2 (3.2 to 5.5)	1.1 (0.7 to 1.6)	1.3 (0.8 to 1.9)
Typhoid fever	Acute typhoid infection	619.5 (537.9 to 717.2)	506.2 (433.2 to 584.6)	30.4 (19.2 to 45.4)	24.8 (15.5 to 36.9)
Typhoid fever	Severe typhoid fever	846.5 (739.2 to 972.5)	691.7 (600.5 to 798.3)	108.0 (72.5 to 156.3)	88.4 (59.0 to 127.6)
Typhoid fever	Intestinal perforation due to typhoid	300.0 (252.9 to 351.2)	245.4 (206.7 to 288.4)	92.3 (61.5 to 132.5)	76.7 (51.0 to 109.8)
Typhoid fever	Gastrointestinal bleeding due to typhoid	4.2 (1.3 to 10.0)	3.5 (1.0 to 7.9)	1.4 (0.4 to 3.4)	1.1 (0.3 to 2.7)
Paratyphoid fever	Acute paratyphoid infection	184.6 (142.6 to 234.2)	151.4 (118.9 to 192.4)	1.1 (0.4 to 2.3)	0.9 (0.3 to 1.8)
Paratyphoid fever	Moderate paratyphoid fever	333.2 (266.3 to 414.0)	274.4 (218.9 to 338.8)	16.3 (9.8 to 24.6)	13.5 (8.1 to 20.6)
Paratyphoid fever	Severe paratyphoid fever	93.1 (72.6 to 118.3)	76.5 (60.5 to 95.9)	12.2 (7.5 to 18.5)	10.1 (6.3 to 15.4)
Paratyphoid fever	Intestinal perforation due to paratyphoid	32.9 (25.2 to 42.0)	27.1 (20.9 to 34.5)	3.8 (2.4 to 5.5)	3.1 (1.9 to 4.6)
Lower respiratory infections	Moderate lower respiratory infections	7,256.6 (6,848.9 to 7,614.5)	7,627.5 (7,213.2 to 8,005.5)	351.6 (229.4 to 510.1)	368.7 (241.1 to 533.7)
Lower respiratory infections	Severe lower respiratory infections	1,288.8 (1,157.5 to 1,422.2)	1,354.7 (1,219.7 to 1,495.1)	162.5 (108.6 to 227.5)	170.4 (114.4 to 238.5)
Lower respiratory infections	Guillain-Barré syndrome due to lower respiratory infections	3.8 (2.1 to 5.9)	4.4 (2.5 to 6.9)	1.1 (0.6 to 1.9)	1.3 (0.7 to 2.2)
Upper respiratory infections	Mild upper respiratory infections	182,256.1 (162,006.3 to 202,694.7)	200,765.9 (178,590.9 to 222,754.1)	1,033.4 (410.9 to 2,205.3)	1,137.9 (451.7 to 2,427.6)
Upper respiratory infections	Moderate upper respiratory infections	29,677.8 (26,168.1 to 33,664.1)	32,692.2 (28,830.3 to 36,933.5)	1,450.3 (912.5 to 2,161.1)	1,596.9 (1,005.0 to 2,368.1)
Upper respiratory infections	Guillain-Barré syndrome due to upper respiratory infections	10.4 (8.2 to 13.0)	12.1 (9.6 to 15.2)	3.1 (1.9 to 4.6)	3.6 (2.2 to 5.4)
Otitis media	Acute otitis media	23,432.1 (19,301.9 to 28,894.6)	25,630.8 (21,044.2 to 31,570.6)	306.1 (153.7 to 572.5)	334.9 (169.4 to 623.7)
Otitis media	Severe infectious complications due to chronic otitis media	64.4 (2.6 to 231.9)	64.9 (2.6 to 233.6)	0.9 (0.0 to 3.4)	0.9 (0.0 to 3.4)
Otitis media	Vertigo with mild hearing loss due to chronic otitis media	3,551.0 (2,739.3 to 4,541.4)	3,575.8 (2,759.3 to 4,571.8)	414.6 (250.4 to 616.3)	417.7 (253.9 to 627.6)
Otitis media	Vertigo with mild hearing loss and ringing due to chronic otitis media	3,550.9 (2,739.3 to 4,540.4)	3,575.8 (2,759.3 to 4,571.1)	449.7 (271.9 to 664.7)	453.2 (273.6 to 676.4)
Otitis media	Mild hearing loss due to chronic otitis media	48,340.6 (42,283.6 to 54,802.9)	50,358.6 (44,224.5 to 56,921.4)	451.1 (199.5 to 850.7)	470.1 (210.3 to 892.5)
Otitis media	Mild hearing loss with ringing due to chronic otitis media	10,181.9 (8,639.1 to 11,909.4)	10,700.7 (9,077.8 to 12,504.6)	211.9 (113.5 to 341.1)	222.7 (117.9 to 355.2)
Otitis media	Vertigo with moderate hearing loss due to chronic otitis media	3,535.8 (2,737.4 to 4,485.4)	3,564.1 (2,757.3 to 4,531.3)	463.0 (278.2 to 689.0)	466.9 (280.3 to 693.4)
Otitis media	Vertigo with moderate hearing loss and ringing due to chronic otitis media	3,269.0 (2,632.7 to 3,964.8)	3,331.7 (2,681.6 to 4,064.2)	556.0 (353.0 to 794.8)	567.0 (356.1 to 808.2)
Otitis media	Moderate hearing loss due to chronic otitis media	8,602.0 (6,744.3 to 10,326.1)	9,123.7 (7,210.0 to 11,105.8)	221.5 (124.1 to 359.2)	235.2 (134.4 to 387.9)
Otitis media	Moderate hearing loss with ringing due to chronic otitis media	1,990.8 (1,184.2 to 2,792.9)	2,163.0 (1,370.1 to 3,040.0)	140.4 (74.7 to 231.9)	152.9 (82.2 to 247.0)
Pneumococcal meningitis	Acute pneumococcal meningitis	32.1 (27.4 to 37.4)	40.3 (33.5 to 48.4)	4.3 (2.8 to 6.1)	5.3 (3.4 to 7.8)
Pneumococcal meningitis	Mild behavioral problems due to pneumococcal meningitis	231.5 (133.5 to 366.5)	289.0 (165.3 to 454.9)	9.9 (4.9 to 18.2)	12.4 (6.2 to 22.9)
Pneumococcal meningitis	Mild motor impairment due to long term due to pneumococcal meningitis	638.4 (374.6 to 985.6)	797.4 (474.2 to 1,235.8)	6.3 (2.5 to 13.4)	7.8 (3.1 to 16.9)
Pneumococcal meningitis	Mild motor plus cognitive impairments due to pneumococcal meningitis	375.0 (221.3 to 597.9)	468.1 (274.1 to 744.3)	11.3 (5.4 to 20.8)	14.1 (6.8 to 25.8)
Pneumococcal meningitis	Borderline intellectual disability due to pneumococcal meningitis	469.2 (287.1 to 717.2)	586.0 (353.5 to 914.1)	4.9 (2.0 to 10.3)	6.1 (2.5 to 13.0)
Pneumococcal meningitis	Monocular distance vision loss due to pneumococcal meningitis	415.2 (242.7 to 649.1)	518.4 (304.4 to 806.3)	6.6 (2.9 to 12.5)	8.3 (3.6 to 15.6)
Pneumococcal meningitis	Mild intellectual disability due to pneumococcal meningitis	649.6 (521.4 to 787.9)	754.5 (606.2 to 914.0)	26.6 (16.1 to 41.2)	30.9 (18.7 to 47.7)
Pneumococcal meningitis	Moderate motor impairment due to pneumococcal meningitis	485.1 (382.5 to 633.8)	583.1 (455.2 to 776.8)	28.3 (17.2 to 42.6)	34.0 (20.9 to 51.1)
Pneumococcal meningitis	Severe motor impairment due to pneumococcal meningitis	664.8 (517.2 to 887.5)	787.7 (613.2 to 1,043.8)	248.3 (156.0 to 363.2)	294.0 (187.8 to 427.8)
Pneumococcal meningitis	Moderate motor plus cognitive impairments due to pneumococcal meningitis	384.1 (251.5 to 498.1)	436.1 (268.2 to 576.2)	73.6 (42.2 to 111.6)	83.6 (45.4 to 127.7)
Pneumococcal meningitis	Severe motor plus cognitive impairments due to pneumococcal meningitis	172.4 (10.8 to 288.7)	189.6 (9.6 to 321.3)	89.0 (5.4 to 157.5)	98.5 (4.9 to 177.0)
Pneumococcal meningitis	Epilepsy due to pneumococcal meningitis	204.7 (182.3 to 230.4)	244.4 (218.2 to 274.4)	36.2 (25.1 to 47.7)	38.8 (26.6 to 51.2)
Pneumococcal meningitis	Blindness due to pneumococcal meningitis	75.0 (53.4 to 105.0)	100.3 (70.8 to 142.4)	13.7 (7.9 to 21.5)	18.2 (10.6 to 28.4)
Pneumococcal meningitis	Mild hearing loss due to pneumococcal meningitis	348.0 (206.3 to 503.7)	441.2 (261.7 to 645.5)	3.2 (1.2 to 6.9)	4.1 (1.6 to 8.7)
Pneumococcal meningitis	Mild hearing loss with ringing due to pneumococcal meningitis	92.2 (54.5 to 134.4)	116.8 (69.6 to 172.3)	1.9 (0.9 to 3.6)	2.5 (1.1 to 4.6)
Pneumococcal meningitis	Moderate hearing loss due to pneumococcal meningitis	177.6 (15.7 to 252.5)	221.9 (20.0 to 313.1)	4.6 (0.4 to 8.5)	5.7 (0.4 to 10.6)
Pneumococcal meningitis	Moderate hearing loss with ringing due to pneumococcal meningitis	76.8 (6.9 to 108.9)	96.2 (8.5 to 137.4)	5.6 (0.5 to 9.3)	6.9 (0.5 to 11.7)
Pneumococcal meningitis	Moderately severe hearing loss due to pneumococcal meningitis	79.0 (7.0 to 116.0)	97.8 (8.7 to 142.5)	7.1 (0.6 to 12.0)	8.8 (0.8 to 14.8)
Pneumococcal meningitis	Moderately severe hearing loss with ringing due to pneumococcal meningitis	38.0 (3.3 to 55.7)	47.1 (4.2 to 69.8)	6.3 (0.5 to 10.4)	7.8 (0.7 to 13.0)
Pneumococcal meningitis	Severe hearing loss due to pneumococcal meningitis	13.8 (1.2 to 20.3)	17.1 (1.5 to 25.4)	2.2 (0.2 to 3.8)	2.7 (0.2 to 4.6)
Pneumococcal meningitis	Severe hearing loss with ringing due to pneumococcal meningitis	24.3 (2.2 to 36.3)	30.3 (2.7 to 45.5)	6.3 (0.5 to 10.7)	7.9 (0.7 to 13.3)
Pneumococcal meningitis	Profound hearing loss due to pneumococcal meningitis	14.6 (1.3 to 23.4)	17.9 (1.5 to 29.4)	3.0 (0.2 to 5.3)	3.6 (0.3 to 6.7)
Pneumococcal meningitis	Profound hearing loss with ringing due to pneumococcal meningitis	25.9 (2.2 to 41.1)	31.7 (2.8 to 50.7)	7.2 (0.6 to 12.9)	8.8 (0.7 to 15.8)
Pneumococcal meningitis	Complete hearing loss due to pneumococcal meningitis	3.0 (0.3 to 4.8)	3.8 (0.3 to 6.2)	0.7 (0.1 to 1.2)	0.8 (0.1 to 1.5)
Pneumococcal meningitis	Complete hearing loss with ringing due to pneumococcal meningitis	3.7 (0.3 to 5.9)	4.6 (0.4 to 7.3)	1.2 (0.1 to 2.0)	1.4 (0.1 to 2.6)

Cause	Sequela	Prevalence in thousands (95% confidence interval)		YLDs in thousands (95% confidence interval)	
		2005	2015	2005	2015
Pneumococcal meningitis	Moderate vision impairment due to pneumococcol meningitis	257.1 (195.8 to 334.3)	346.3 (259.2 to 452.5)	7.7 (4.3 to 12.5)	10.3 (5.8 to 16.7)
Pneumococcal meningitis	Severe vision impairment due to pneumococcol meningitis	21.9 (16.5 to 28.5)	29.6 (22.3 to 38.7)	4.0 (2.5 to 6.1)	5.4 (3.3 to 8.3)
H influenzae type B meningitis	Acute H influenzae type B meningitis	27.3 (22.2 to 33.2)	22.0 (17.2 to 28.7)	3.6 (2.2 to 5.4)	2.9 (1.8 to 4.5)
H influenzae type B meningitis	Mild behavioral problems due to H influenzae type B meningitis	66.1 (29.5 to 113.2)	50.4 (22.5 to 88.5)	2.9 (1.2 to 5.5)	2.2 (0.9 to 4.4)
H influenzae type B meningitis	Mild motor impairment due to long term due to H influenzae type B meningitis	246.4 (110.2 to 415.4)	187.8 (82.9 to 322.0)	2.4 (0.8 to 5.3)	1.9 (0.6 to 4.2)
H influenzae type B meningitis	Mild motor plus cognitive impairments due to H influenzae type B meningitis	146.0 (65.7 to 256.0)	111.4 (48.3 to 204.7)	4.5 (1.7 to 8.9)	3.4 (1.3 to 7.0)
H influenzae type B meningitis	Borderline intellectual disability due to H influenzae type B meningitis	440.3 (197.9 to 741.1)	336.0 (146.6 to 590.3)	4.6 (1.5 to 10.3)	3.5 (1.1 to 8.1)
H influenzae type B meningitis	Monocular distance vision loss due to H influenzae type B meningitis	263.9 (110.8 to 448.3)	201.2 (83.7 to 346.3)	4.2 (1.5 to 8.8)	3.2 (1.1 to 6.7)
H influenzae type B meningitis	Mild intellectual disability due to H influenzae type B meningitis	144.8 (111.3 to 181.6)	163.6 (125.9 to 204.9)	6.1 (3.4 to 9.7)	6.9 (3.9 to 10.9)
H influenzae type B meningitis	Moderate motor impairment due to H influenzae type B meningitis	182.5 (136.5 to 245.7)	211.7 (156.1 to 289.6)	10.9 (6.4 to 17.4)	12.7 (7.4 to 20.3)
H influenzae type B meningitis	Severe motor impairment due to H influenzae type B meningitis	279.2 (217.1 to 361.3)	320.6 (249.5 to 410.8)	106.8 (64.8 to 154.1)	122.8 (76.3 to 178.9)
H influenzae type B meningitis	Moderate motor plus cognitive impairments due to H influenzae type B meningitis	181.3 (119.7 to 233.2)	200.6 (126.6 to 262.3)	35.6 (20.3 to 55.1)	39.5 (21.7 to 61.4)
H influenzae type B meningitis	Severe motor plus cognitive impairments due to H influenzae type B meningitis	76.6 (5.4 to 129.2)	82.1 (4.7 to 139.8)	41.2 (2.9 to 75.6)	44.3 (2.6 to 82.4)
H influenzae type B meningitis	Epilepsy due to H influenzae type B meningitis	109.8 (94.8 to 127.0)	117.3 (101.1 to 136.5)	20.1 (13.5 to 26.7)	19.4 (12.8 to 26.2)
H influenzae type B meningitis	Blindness due to H influenzae type B meningitis	81.5 (56.5 to 113.4)	112.3 (77.2 to 157.7)	14.7 (8.3 to 23.2)	20.2 (11.3 to 32.0)
H influenzae type B meningitis	Mild hearing loss due to H influenzae type B meningitis	154.5 (94.3 to 226.7)	121.0 (72.2 to 182.0)	1.5 (0.5 to 3.1)	1.1 (0.4 to 2.5)
H influenzae type B meningitis	Mild hearing loss with ringing due to H influenzae type B meningitis	40.9 (24.7 to 59.8)	32.0 (19.2 to 48.4)	0.9 (0.4 to 1.7)	0.7 (0.3 to 1.3)
H influenzae type B meningitis	Moderate hearing loss due to H influenzae type B meningitis	82.1 (7.0 to 121.9)	65.8 (5.6 to 101.3)	2.2 (0.2 to 4.1)	1.7 (0.1 to 3.4)
H influenzae type B meningitis	Moderate hearing loss with ringing due to H influenzae type B meningitis	35.5 (3.1 to 52.9)	28.5 (2.4 to 43.9)	2.6 (0.2 to 4.6)	2.1 (0.2 to 3.8)
H influenzae type B meningitis	Moderately severe hearing loss due to H influenzae type B meningitis	37.8 (3.1 to 58.0)	29.1 (2.4 to 45.9)	3.5 (0.3 to 6.0)	2.7 (0.2 to 4.7)
H influenzae type B meningitis	Moderately severe hearing loss with ringing due to H influenzae type B meningitis	18.2 (1.5 to 28.0)	14.0 (1.1 to 22.2)	3.0 (0.2 to 5.2)	2.3 (0.2 to 4.0)
H influenzae type B meningitis	Severe hearing loss due to H influenzae type B meningitis	6.8 (0.6 to 10.8)	5.1 (0.4 to 8.1)	1.1 (0.1 to 1.9)	0.8 (0.1 to 1.5)
H influenzae type B meningitis	Severe hearing loss with ringing due to H influenzae type B meningitis	12.1 (1.0 to 18.7)	8.9 (0.7 to 14.3)	3.1 (0.2 to 5.6)	2.3 (0.2 to 4.3)
H influenzae type B meningitis	Profound hearing loss due to H influenzae type B meningitis	7.6 (0.6 to 12.8)	5.6 (0.4 to 9.6)	1.6 (0.1 to 2.9)	1.2 (0.1 to 2.2)
H influenzae type B meningitis	Profound hearing loss with ringing due to H influenzae type B meningitis	13.7 (1.2 to 22.5)	10.0 (0.8 to 17.0)	3.8 (0.3 to 6.8)	2.8 (0.2 to 5.3)
H influenzae type B meningitis	Complete hearing loss due to H influenzae type B meningitis	1.5 (0.1 to 2.6)	1.2 (0.1 to 2.1)	0.3 (0.0 to 0.6)	0.2 (0.0 to 0.5)
H influenzae type B meningitis	Complete hearing loss with ringing due to H influenzae type B meningitis	1.8 (0.1 to 3.1)	1.4 (0.1 to 2.4)	0.6 (0.0 to 1.1)	0.4 (0.0 to 0.8)
H influenzae type B meningitis	Moderate vision impairment due to H influenza type B meningitis	18.4 (13.2 to 24.7)	14.8 (10.3 to 20.7)	0.6 (0.3 to 1.0)	0.5 (0.2 to 0.8)
H influenzae type B meningitis	Severe vision impairment due to H influenza type B meningitis	1.5 (1.0 to 2.0)	1.2 (0.8 to 1.7)	0.3 (0.2 to 0.4)	0.2 (0.1 to 0.4)
Meningococcal meningitis	Acute meningococcal meningitis	18.3 (15.5 to 21.5)	22.8 (18.9 to 27.7)	2.4 (1.5 to 3.5)	3.0 (1.8 to 4.5)
Meningococcal meningitis	Mild behavioral problems due to meningococcal meningitis	24.5 (7.2 to 44.0)	29.2 (8.5 to 52.5)	1.1 (0.3 to 2.2)	1.3 (0.4 to 2.6)
Meningococcal meningitis	Mild motor impairment due to long term due to meningococcal meningitis	92.0 (29.1 to 163.3)	109.6 (34.8 to 196.2)	0.9 (0.2 to 2.1)	1.1 (0.3 to 2.5)
Meningococcal meningitis	Mild motor plus cognitive impairments due to meningococcal meningitis	54.2 (15.6 to 98.0)	64.6 (18.4 to 120.5)	1.7 (0.4 to 3.6)	2.0 (0.5 to 4.4)
Meningococcal meningitis	Borderline intellectual disability due to meningococcal meningitis	162.7 (48.1 to 290.2)	194.1 (55.7 to 348.9)	1.7 (0.4 to 3.9)	2.0 (0.5 to 4.7)
Meningococcal meningitis	Monocular distance vision loss due to meningococcal meningitis	97.1 (29.1 to 174.4)	115.7 (34.2 to 209.0)	1.6 (0.4 to 3.5)	1.9 (0.5 to 4.2)
Meningococcal meningitis	Mild intellectual disability due to meningococcal meningitis	64.7 (50.4 to 81.1)	77.4 (60.2 to 96.8)	2.7 (1.6 to 4.3)	3.3 (1.9 to 5.1)
Meningococcal meningitis	Moderate motor impairment due to meningococcal meningitis	81.8 (61.2 to 111.8)	99.8 (73.3 to 138.2)	4.9 (2.9 to 7.8)	6.0 (3.5 to 9.6)
Meningococcal meningitis	Severe motor impairment due to meningococcal meningitis	123.3 (93.5 to 167.8)	150.1 (114.7 to 202.5)	48.8 (29.6 to 73.4)	59.3 (36.4 to 88.7)
Meningococcal meningitis	Moderate motor plus cognitive impairments due to meningococcal meningitis	81.4 (51.5 to 108.0)	95.7 (57.7 to 129.0)	16.3 (9.0 to 25.7)	19.2 (10.0 to 30.6)
Meningococcal meningitis	Severe motor plus cognitive impairments due to meningococcal meningitis	38.3 (1.8 to 63.9)	43.4 (1.7 to 73.2)	20.5 (1.0 to 37.0)	23.3 (0.9 to 42.6)
Meningococcal meningitis	Epilepsy due to meningococcal meningitis	32.3 (27.2 to 38.4)	38.6 (32.5 to 45.9)	6.0 (3.9 to 8.2)	6.7 (4.4 to 9.2)
Meningococcal meningitis	Blindness due to meningococcal meningitis	5.8 (3.9 to 8.5)	4.9 (3.1 to 7.1)	1.1 (0.6 to 1.8)	0.9 (0.5 to 1.5)
Meningococcal meningitis	Mild hearing loss due to meningococcal meningitis	77.5 (44.6 to 110.2)	94.1 (54.0 to 133.3)	0.7 (0.3 to 1.5)	0.9 (0.3 to 1.9)
Meningococcal meningitis	Mild hearing loss with ringing due to meningococcal meningitis	20.5 (11.7 to 28.9)	24.9 (14.3 to 35.5)	0.4 (0.2 to 0.8)	0.5 (0.2 to 1.0)
Meningococcal meningitis	Moderate hearing loss due to meningococcal meningitis	35.8 (3.3 to 53.5)	42.7 (3.9 to 64.0)	0.9 (0.1 to 1.8)	1.1 (0.1 to 2.2)
Meningococcal meningitis	Moderate hearing loss with ringing due to meningococcal meningitis	15.5 (1.4 to 22.9)	18.5 (1.6 to 27.7)	1.1 (0.1 to 2.0)	1.4 (0.1 to 2.4)
Meningococcal meningitis	Moderately severe hearing loss due to meningococcal meningitis	17.1 (1.5 to 25.6)	20.3 (1.8 to 31.0)	1.6 (0.1 to 2.7)	1.9 (0.2 to 3.2)
Meningococcal meningitis	Moderately severe hearing loss with ringing due to meningococcal meningitis	8.2 (0.7 to 12.4)	9.8 (0.9 to 14.8)	1.4 (0.1 to 2.4)	1.6 (0.1 to 2.8)
Meningococcal meningitis	Severe hearing loss due to meningococcal meningitis	3.2 (0.3 to 5.0)	3.7 (0.3 to 5.9)	0.5 (0.0 to 0.9)	0.6 (0.0 to 1.1)
Meningococcal meningitis	Severe hearing loss with ringing due to meningococcal meningitis	5.6 (0.5 to 8.8)	6.6 (0.6 to 10.7)	1.5 (0.1 to 2.6)	1.7 (0.1 to 3.1)
Meningococcal meningitis	Profound hearing loss due to meningococcal meningitis	3.7 (0.3 to 6.2)	4.3 (0.4 to 7.2)	0.7 (0.1 to 1.4)	0.9 (0.1 to 1.7)
Meningococcal meningitis	Profound hearing loss with ringing due to meningococcal meningitis	6.5 (0.6 to 10.5)	7.7 (0.6 to 12.7)	1.8 (0.2 to 3.2)	2.1 (0.2 to 3.9)
Meningococcal meningitis	Complete hearing loss due to meningococcal meningitis	0.7 (0.1 to 1.2)	0.8 (0.1 to 1.3)	0.1 (0.0 to 0.3)	0.2 (0.0 to 0.3)
Meningococcal meningitis	Complete hearing loss with ringing due to meningococcal meningitis	0.8 (0.1 to 1.3)	0.9 (0.1 to 1.6)	0.3 (0.0 to 0.5)	0.3 (0.0 to 0.6)
Meningococcal meningitis	Moderate vision impairment due to meningococcal meningitis	312.0 (231.7 to 411.8)	405.9 (299.4 to 543.3)	9.4 (5.2 to 15.3)	12.2 (6.6 to 20.2)
Meningococcal meningitis	Severe vision impairment due to meningococcal meningitis	26.4 (19.6 to 34.5)	34.8 (25.3 to 46.4)	4.7 (2.8 to 7.3)	6.3 (3.7 to 9.8)
Other meningitis	Acute viral meningitis	86.2 (79.3 to 93.8)	97.1 (86.2 to 110.5)	11.3 (7.5 to 16.1)	12.8 (8.3 to 18.2)
Other meningitis	Other acute bacterial meningitis	29.6 (26.1 to 33.6)	37.8 (32.6 to 43.6)	3.9 (2.6 to 5.6)	5.0 (3.2 to 7.2)
Other meningitis	Mild behavioral problems due to other bacterial meningitis	47.7 (19.7 to 80.5)	56.8 (23.3 to 95.5)	2.1 (0.7 to 4.0)	2.5 (0.8 to 4.8)

Cause	Sequela	Prevalence in thousands (95% confidence interval)		YLDs in thousands (95% confidence interval)	
		2005	2015	2005	2015
Other meningitis	Mild motor impairment due to long term due to other bacterial meningitis	179.6 (74.5 to 300.3)	214.4 (89.6 to 362.3)	1.8 (0.5 to 4.1)	2.1 (0.6 to 5.0)
Other meningitis	Mild motor plus cognitive impairments due to other bacterial meningitis	103.8 (40.1 to 174.2)	123.2 (48.4 to 203.2)	3.2 (1.1 to 6.6)	3.8 (1.3 to 7.6)
Other meningitis	Borderline intellectual disability due to other bacterial meningitis	314.4 (126.2 to 505.9)	374.6 (150.5 to 611.3)	3.3 (1.0 to 6.9)	3.9 (1.2 to 8.3)
Other meningitis	Monocular distance vision loss due to other bacterial meningitis	189.0 (73.0 to 315.2)	225.1 (87.7 to 379.7)	3.1 (1.0 to 6.6)	3.6 (1.2 to 7.8)
Other meningitis	Mild intellectual disability due to other bacterial meningitis	138.8 (108.2 to 172.5)	157.5 (123.4 to 196.0)	5.8 (3.4 to 9.3)	6.6 (3.8 to 10.5)
Other meningitis	Moderate motor impairment due to other bacterial meningitis	181.9 (138.5 to 248.0)	215.4 (162.5 to 297.4)	10.8 (6.5 to 16.7)	12.8 (7.6 to 20.0)
Other meningitis	Severe motor impairment due to other bacterial meningitis	264.2 (202.6 to 355.7)	306.9 (236.2 to 408.3)	100.8 (62.5 to 146.8)	116.8 (72.3 to 168.8)
Other meningitis	Moderate motor plus cognitive impairments due to other bacterial meningitis	172.5 (111.3 to 223.6)	191.5 (115.3 to 249.5)	33.7 (19.4 to 50.6)	37.5 (20.8 to 57.2)
Other meningitis	Severe motor plus cognitive impairments due to other bacterial meningitis	82.1 (4.6 to 137.8)	88.7 (4.0 to 151.6)	43.6 (2.3 to 80.5)	47.3 (2.0 to 87.8)
Other meningitis	Epilepsy due to other meningitis	79.7 (69.3 to 90.8)	91.0 (79.6 to 103.2)	14.2 (9.7 to 19.1)	14.3 (9.7 to 19.6)
Other meningitis	Blindness due to other bacterial meningitis	84.5 (56.8 to 121.3)	111.5 (73.9 to 162.8)	15.2 (8.4 to 24.4)	19.9 (11.1 to 32.7)
Other meningitis	Mild hearing loss due to other bacterial meningitis	132.2 (79.9 to 184.9)	159.7 (97.4 to 224.2)	1.2 (0.5 to 2.5)	1.5 (0.6 to 3.0)
Other meningitis	Mild hearing loss due with ringing to other bacterial meningitis	35.0 (21.1 to 49.0)	42.3 (25.8 to 59.5)	0.7 (0.3 to 1.3)	0.9 (0.4 to 1.6)
Other meningitis	Moderate hearing loss due to other bacterial meningitis	67.2 (6.0 to 95.4)	81.6 (7.3 to 116.3)	1.8 (0.1 to 3.2)	2.1 (0.2 to 3.9)
Other meningitis	Moderate hearing loss with ringing due to other bacterial meningitis	29.1 (2.6 to 41.4)	35.3 (3.2 to 49.9)	2.1 (0.2 to 3.6)	2.6 (0.2 to 4.4)
Other meningitis	Moderately severe hearing loss due to other bacterial meningitis	29.4 (2.5 to 42.2)	35.4 (3.1 to 51.1)	2.7 (0.2 to 4.5)	3.2 (0.3 to 5.4)
Other meningitis	Moderately severe hearing loss with ringing due to other bacterial meningitis	14.2 (1.2 to 20.4)	17.0 (1.5 to 24.8)	2.4 (0.2 to 3.9)	2.8 (0.2 to 4.7)
Other meningitis	Severe hearing loss due to other bacterial meningitis	5.1 (0.5 to 7.9)	6.1 (0.5 to 9.5)	0.8 (0.1 to 1.4)	1.0 (0.1 to 1.7)
Other meningitis	Severe hearing loss with ringing due to other bacterial meningitis	9.0 (0.8 to 13.8)	10.8 (1.0 to 16.4)	2.4 (0.2 to 4.0)	2.8 (0.2 to 4.8)
Other meningitis	Profound hearing loss due to other bacterial meningitis	5.2 (0.4 to 8.3)	6.0 (0.5 to 9.8)	1.0 (0.1 to 1.9)	1.2 (0.1 to 2.2)
Other meningitis	Profound hearing loss with ringing due to other bacterial meningitis	9.3 (0.8 to 15.0)	10.8 (0.9 to 17.4)	2.6 (0.2 to 4.5)	3.0 (0.3 to 5.4)
Other meningitis	Complete hearing loss due to other bacterial meningitis	1.1 (0.1 to 1.8)	1.4 (0.1 to 2.2)	0.2 (0.0 to 0.4)	0.3 (0.0 to 0.6)
Other meningitis	Complete hearing loss with ringing due to other bacterial meningitis	1.3 (0.1 to 2.0)	1.6 (0.1 to 2.6)	0.4 (0.0 to 0.8)	0.5 (0.0 to 1.0)
Other meningitis	Moderate vision impairment due to other bacterial meningitis	222.3 (172.4 to 290.4)	290.6 (224.6 to 382.8)	6.7 (3.8 to 10.9)	8.7 (5.0 to 14.1)
Other meningitis	Severe vision impairment due to other bacterial meningitis	19.1 (14.9 to 24.6)	25.2 (19.4 to 32.3)	3.5 (2.2 to 5.2)	4.6 (2.9 to 6.9)
Encephalitis	Acute encephalitis	76.4 (70.4 to 83.0)	81.8 (74.8 to 89.5)	10.0 (6.6 to 14.3)	10.8 (7.1 to 15.5)
Encephalitis	Mild behavioral problems due to encephalitis	94.6 (16.3 to 211.3)	94.5 (16.6 to 210.9)	4.1 (0.7 to 9.8)	4.1 (0.7 to 9.8)
Encephalitis	Mild motor impairment due to long term due to encephalitis	353.8 (71.1 to 784.2)	354.0 (71.4 to 782.2)	3.5 (0.6 to 9.6)	3.5 (0.6 to 9.5)
Encephalitis	Mild motor plus cognitive impairments due to encephalitis	208.1 (39.6 to 453.6)	207.2 (39.8 to 451.9)	6.3 (1.1 to 15.5)	6.2 (1.1 to 15.4)
Encephalitis	Borderline intellectual disability due to encephalitis	624.1 (115.6 to 1,378.3)	624.1 (116.7 to 1,369.3)	6.5 (1.1 to 17.8)	6.5 (1.1 to 17.9)
Encephalitis	Monocular distance vision loss due to encephalitis	373.9 (72.5 to 819.1)	373.6 (72.3 to 821.1)	6.0 (1.0 to 15.4)	6.0 (1.0 to 15.3)
Encephalitis	Mild intellectual disability due to encephalitis	209.0 (159.4 to 262.4)	229.5 (173.7 to 290.5)	8.7 (5.0 to 14.1)	9.5 (5.5 to 15.4)
Encephalitis	Moderate motor impairment due to encephalitis	242.9 (175.8 to 336.0)	274.7 (193.7 to 391.6)	14.3 (8.2 to 22.3)	16.2 (9.1 to 25.6)
Encephalitis	Severe motor impairment due to encephalitis	397.8 (302.8 to 537.5)	447.0 (342.1 to 594.5)	150.3 (91.3 to 222.2)	169.0 (103.7 to 247.4)
Encephalitis	Moderate motor plus cognitive impairments due to encephalitis	271.1 (181.0 to 355.3)	290.3 (182.3 to 390.2)	52.5 (30.4 to 81.4)	56.3 (31.4 to 89.6)
Encephalitis	Severe motor plus cognitive impairments due to encephalitis	114.6 (8.9 to 196.6)	116.1 (7.7 to 202.4)	59.5 (4.4 to 110.6)	60.9 (3.6 to 113.2)
Encephalitis	Epilepsy due to encephalitis	209.0 (183.9 to 238.7)	227.3 (199.7 to 259.1)	35.4 (23.6 to 47.4)	32.8 (22.1 to 43.9)
Encephalitis	Blindness due to encephalitis	236.4 (175.2 to 312.8)	246.6 (183.2 to 335.5)	41.3 (25.0 to 64.5)	43.3 (26.2 to 67.1)
Encephalitis	Moderate vision impairment due to encephalitis	645.6 (535.3 to 780.7)	684.4 (563.9 to 830.3)	19.1 (11.3 to 30.2)	20.3 (11.9 to 32.1)
Encephalitis	Severe vision impairment due to encephalitis	59.8 (48.9 to 72.5)	64.6 (52.6 to 78.7)	10.6 (6.7 to 15.6)	11.6 (7.2 to 17.0)
Diphtheria	Moderate diphtheria	1.0 (0.6 to 1.9)	0.4 (0.2 to 0.8)	0.1 (0.0 to 0.1)	<0.05 (0.0 to <0.05)
Diphtheria	Severe diphtheria	0.4 (0.3 to 0.8)	0.2 (0.1 to 0.3)	0.1 (0.0 to 0.1)	<0.05 (0.0 to <0.05)
Whooping cough	Whooping cough	3,076.3 (2,372.9 to 3,872.3)	2,232.6 (1,725.9 to 2,800.7)	151.7 (89.9 to 232.2)	110.3 (65.6 to 167.0)
Tetanus	Severe tetanus	16.0 (12.5 to 23.7)	8.6 (6.5 to 13.0)	2.1 (1.3 to 3.4)	1.1 (0.7 to 1.9)
Tetanus	Mild motor impairment due to neonatal tetanus	81.4 (54.6 to 108.8)	93.7 (63.0 to 125.4)	0.8 (0.3 to 1.7)	0.9 (0.4 to 1.9)
Tetanus	Mild motor plus cognitive impairments due to neonatal tetanus	81.5 (53.4 to 107.8)	93.7 (61.5 to 124.4)	2.5 (1.3 to 4.2)	2.9 (1.4 to 4.8)
Tetanus	Moderate motor impairment due to neonatal tetanus	3.3 (2.7 to 4.4)	3.2 (2.6 to 4.3)	0.2 (0.1 to 0.3)	0.2 (0.1 to 0.3)
Tetanus	Moderate motor impairment with blindness due to neonatal tetanus	0.1 (0.1 to 0.1)	0.1 (0.1 to 0.1)	<0.05 (0.0 to <0.05)	<0.05 (0.0 to <0.05)
Tetanus	Moderate motor impairment with epilepsy due to neonatal tetanus	1.3 (1.2 to 1.5)	1.2 (1.0 to 1.4)	0.3 (0.2 to 0.4)	0.2 (0.2 to 0.3)
Tetanus	Moderate motor impairment with blindness and epilepsy due to neonatal tetanus	0.1 (0.1 to 0.1)	0.1 (0.1 to 0.1)	<0.05 (0.0 to 0.1)	<0.05 (0.0 to 0.1)
Tetanus	Moderate motor plus cognitive impairment with blindness due to neonatal tetanus	0.4 (0.2 to 0.5)	0.3 (0.2 to 0.4)	0.1 (0.1 to 0.2)	0.1 (0.1 to 0.2)
Tetanus	Moderate motor plus cognitive impairment with epilepsy due to neonatal tetanus	2.1 (1.3 to 2.5)	1.9 (1.2 to 2.4)	0.7 (0.4 to 1.0)	0.6 (0.3 to 0.9)
Tetanus	Moderate motor plus cognitive impairment with blindness and epilepsy due to neonatal tetanus	0.2 (0.1 to 0.2)	0.2 (0.1 to 0.2)	0.1 (0.1 to 0.1)	0.1 (0.0 to 0.1)
Tetanus	Severe motor impairment due to neonatal tetanus	4.6 (3.6 to 6.2)	4.5 (3.5 to 6.0)	1.9 (1.1 to 2.8)	1.8 (1.1 to 2.7)
Tetanus	Severe motor impairment with blindness due to neonatal tetanus	<0.05 (0.0 to <0.05)	<0.05 (0.0 to <0.05)	<0.05 (0.0 to <0.05)	<0.05 (0.0 to <0.05)
Tetanus	Severe motor impairment with epilepsy due to neonatal tetanus	0.4 (0.4 to 0.5)	0.4 (0.3 to 0.5)	0.2 (0.2 to 0.3)	0.2 (0.1 to 0.3)
Tetanus	Severe motor impairment with blindness and epilepsy due to neonatal tetanus	<0.05 (0.0 to <0.05)	<0.05 (0.0 to <0.05)	<0.05 (0.0 to <0.05)	<0.05 (0.0 to <0.05)
Tetanus	Severe motor plus cognitive impairment with blindness due to neonatal tetanus	0.2 (0.0 to 0.3)	0.2 (0.0 to 0.3)	0.1 (0.0 to 0.2)	0.1 (0.0 to 0.2)
Tetanus	Severe motor plus cognitive impairment with epilepsy due to neonatal tetanus	1.2 (0.0 to 1.9)	1.0 (0.0 to 1.7)	0.7 (0.0 to 1.3)	0.6 (0.0 to 1.1)

Cause	Sequela	Prevalence in thousands (95% confidence interval)		YLDs in thousands (95% confidence interval)	
		2005	2015	2005	2015
Tetanus	Severe motor plus cognitive impairment with blindness and epilepsy due to neonatal tetanus	0.1 (0.0 to 0.2)	0.1 (0.0 to 0.2)	0.1 (0.0 to 0.1)	0.1 (0.0 to 0.1)
Measles	Moderate measles	213.4 (89.0 to 437.5)	63.6 (28.1 to 127.5)	10.6 (3.8 to 25.2)	3.2 (1.2 to 7.3)
Measles	Severe measles	214.2 (89.3 to 433.4)	63.9 (27.9 to 128.9)	27.8 (9.9 to 62.6)	8.3 (3.1 to 18.6)
Varicella and herpes zoster	Chickenpox	2,144.5 (2,071.8 to 2,219.7)	2,345.5 (2,272.3 to 2,426.4)	12.2 (4.9 to 25.4)	13.3 (5.4 to 27.7)
Varicella and herpes zoster	Herpes zoster	2,983.1 (2,647.5 to 3,324.7)	3,562.2 (3,160.9 to 3,987.6)	163.1 (99.5 to 249.3)	194.4 (118.0 to 296.0)
Malaria	Asymptomatic malaria parasitemia (PfPR)	150,124.7 (129,878.9 to 172,703.0)	206,147.2 (168,670.7 to 246,888.7)	<0.05 (0.0 to <0.05)	<0.05 (0.0 to <0.05)
Malaria	Mild malaria	13,211.5 (8,341.2 to 20,774.1)	11,108.7 (6,865.3 to 17,218.3)	75.0 (26.1 to 171.8)	63.1 (21.6 to 142.1)
Malaria	Moderate malaria	6,618.3 (4,191.9 to 10,466.3)	5,564.8 (3,441.8 to 8,630.9)	323.7 (167.5 to 574.6)	272.6 (140.2 to 471.1)
Malaria	Severe malaria	99.1 (11.1 to 332.9)	83.3 (9.4 to 272.6)	12.8 (1.4 to 41.7)	10.8 (1.2 to 35.6)
Malaria	Moderate motor impairment due to malaria	135.9 (111.0 to 176.9)	181.3 (146.1 to 241.4)	8.0 (4.9 to 12.2)	10.7 (6.6 to 16.8)
Malaria	Moderate motor impairment with blindness due to malaria	6.7 (5.2 to 8.4)	8.3 (6.5 to 10.4)	1.6 (1.0 to 2.2)	2.0 (1.3 to 2.8)
Malaria	Moderate motor impairment with epilepsy due to malaria	67.3 (57.3 to 77.4)	85.5 (73.2 to 98.5)	15.5 (10.7 to 20.7)	18.4 (12.8 to 24.6)
Malaria	Moderate motor impairment with blindness and epilepsy due to malaria	6.3 (5.3 to 7.3)	8.0 (6.8 to 9.3)	2.4 (1.7 to 3.2)	3.0 (2.1 to 4.0)
Malaria	Moderate motor plus cognitive impairment with blindness due to malaria	20.4 (14.7 to 24.7)	25.5 (16.7 to 31.6)	7.2 (4.4 to 10.3)	8.9 (5.2 to 13.1)
Malaria	Moderate motor plus cognitive impairment with epilepsy due to malaria	115.7 (81.5 to 141.4)	144.2 (92.7 to 178.2)	38.8 (23.7 to 54.4)	46.6 (26.5 to 66.5)
Malaria	Moderate motor plus cognitive impairment with blindness and epilepsy due to malaria	10.8 (7.7 to 13.3)	13.5 (8.7 to 16.8)	5.1 (3.3 to 7.0)	6.2 (3.6 to 8.7)
Malaria	Severe motor impairment due to malaria	217.1 (171.4 to 291.4)	289.2 (230.5 to 378.4)	81.6 (49.4 to 122.5)	108.9 (68.8 to 160.8)
Malaria	Severe motor impairment with blindness due to malaria	2.2 (1.7 to 2.8)	2.7 (2.1 to 3.4)	1.1 (0.7 to 1.6)	1.4 (0.9 to 2.0)
Malaria	Severe motor impairment with epilepsy due to malaria	22.4 (18.9 to 26.0)	28.5 (24.2 to 33.0)	11.5 (8.1 to 15.4)	14.3 (10.0 to 19.1)
Malaria	Severe motor impairment with blindness and epilepsy due to malaria	2.1 (1.8 to 2.5)	2.7 (2.2 to 3.1)	1.3 (0.9 to 1.6)	1.6 (1.1 to 2.1)
Malaria	Severe motor plus cognitive impairment with blindness due to malaria	10.6 (0.7 to 16.7)	12.2 (0.6 to 20.0)	6.6 (0.4 to 11.4)	7.6 (0.4 to 13.4)
Malaria	Severe motor plus cognitive impairment with epilepsy due to malaria	59.8 (4.0 to 96.2)	69.0 (3.4 to 115.2)	35.8 (2.3 to 61.5)	40.9 (2.0 to 73.8)
Malaria	Severe motor plus cognitive impairment with blindness and epilepsy due to malaria	5.6 (0.4 to 9.2)	6.5 (0.3 to 11.0)	3.9 (0.2 to 6.8)	4.5 (0.2 to 8.1)
Malaria	Mild anemia due to malaria parasitemia (PfPR)	20,612.2 (20,083.4 to 21,025.9)	27,712.4 (27,117.0 to 28,260.1)	74.5 (26.9 to 163.0)	100.4 (35.7 to 220.7)
Malaria	Moderate anemia due to malaria parasitemia (PfPR)	32,308.2 (31,332.2 to 33,101.9)	39,612.7 (38,464.9 to 40,672.1)	1,611.5 (1,074.4 to 2,338.7)	1,980.2 (1,314.4 to 2,876.7)
Malaria	Severe anemia due to malaria parasitemia (PfPR)	4,045.1 (3,722.3 to 4,231.5)	4,611.3 (4,371.3 to 4,835.2)	574.1 (390.2 to 803.8)	656.1 (446.6 to 919.8)
Chagas disease	Acute Chagas disease	1.2 (0.6 to 2.0)	1.0 (0.5 to 1.7)	0.1 (0.0 to 0.1)	<0.05 (0.0 to 0.1)
Chagas disease	Mild chronic digestive disease due to Chagas disease	376.2 (296.4 to 466.9)	351.6 (275.6 to 438.1)	4.0 (1.9 to 7.5)	3.7 (1.8 to 7.0)
Chagas disease	Moderate chronic digestive disease due to Chagas disease	250.8 (197.6 to 311.3)	234.4 (183.7 to 292.1)	26.9 (17.4 to 39.6)	25.1 (16.2 to 37.2)
Chagas disease	Asymptomatic Chagas disease	6,107.5 (5,357.4 to 6,855.8)	5,633.7 (4,850.2 to 6,412.7)	<0.05 (0.0 to <0.05)	<0.05 (0.0 to <0.05)
Chagas disease	Atrial fibrillation and flutter due to Chagas disease	75.2 (57.0 to 98.2)	79.8 (60.5 to 104.8)	15.0 (9.6 to 22.6)	15.9 (10.1 to 23.8)
Chagas disease	Mild heart failure due to Chagas disease	292.1 (183.8 to 415.9)	293.0 (183.8 to 417.9)	11.4 (5.6 to 19.0)	11.4 (5.7 to 18.9)
Chagas disease	Moderate heart failure due to Chagas disease	31.7 (19.9 to 45.1)	31.8 (19.9 to 45.3)	2.1 (1.1 to 3.5)	2.1 (1.1 to 3.5)
Chagas disease	Severe heart failure due to Chagas disease	28.2 (17.7 to 40.1)	28.2 (17.7 to 40.3)	4.6 (2.5 to 7.4)	4.7 (2.5 to 7.5)
Visceral leishmaniasis	Moderate visceral leishmaniasis	41.3 (39.0 to 44.0)	45.6 (43.2 to 48.5)	2.0 (1.3 to 3.0)	2.3 (1.4 to 3.3)
Visceral leishmaniasis	Severe visceral leishmaniasis	13.8 (13.0 to 14.7)	15.2 (14.4 to 16.2)	1.8 (1.2 to 2.5)	2.0 (1.3 to 2.9)
Cutaneous and mucocutaneous leishmaniasis	Cutaneous and mucocutaneous leishmaniasis	3,067.5 (2,630.9 to 3,710.0)	3,895.9 (3,324.6 to 4,767.5)	32.6 (15.2 to 62.6)	41.5 (19.4 to 80.8)
African trypanosomiasis	Disfigurement due to African trypanosomiasis	18.1 (11.1 to 27.2)	5.6 (3.2 to 8.7)	1.2 (0.6 to 2.0)	0.4 (0.2 to 0.6)
African trypanosomiasis	Severe motor plus cognitive impairments due to African trypanosomiasis	16.0 (9.0 to 25.1)	5.1 (2.7 to 8.3)	8.0 (3.8 to 13.8)	2.6 (1.2 to 4.7)
Schistosomiasis	Mild schistosomiasis	177,618.9 (166,408.2 to 189,653.8)	129,391.4 (113,541.8 to 155,820.0)	1,001.1 (401.7 to 2,121.2)	727.2 (281.6 to 1,567.6)
Schistosomiasis	Mild diarrhea due to schistosomiasis	255.8 (103.3 to 566.2)	193.3 (74.6 to 435.5)	17.9 (6.1 to 40.3)	13.6 (4.6 to 33.5)
Schistosomiasis	Hematemesis due to schistosomiasis	6.2 (5.4 to 7.2)	7.6 (6.6 to 8.8)	2.0 (1.2 to 3.0)	2.5 (1.5 to 3.6)
Schistosomiasis	Hepatomegaly due to schistosomiasis	14,244.1 (11,705.5 to 17,820.8)	17,463.8 (14,264.2 to 21,711.3)	151.9 (73.1 to 283.9)	186.7 (88.4 to 350.4)
Schistosomiasis	Ascites due to schistosomiasis	442.0 (385.4 to 512.7)	538.7 (469.0 to 623.5)	47.2 (30.9 to 65.6)	57.7 (37.6 to 81.1)
Schistosomiasis	Dysuria due to schistosomiasis	9,427.1 (6,074.7 to 14,875.1)	6,616.6 (3,398.9 to 12,742.7)	100.6 (41.6 to 200.3)	70.3 (23.8 to 163.1)
Schistosomiasis	Bladder pathology due to schistosomiasis	90,753.2 (74,758.8 to 109,896.3)	68,929.9 (48,575.0 to 107,309.0)	969.4 (452.6 to 1,842.0)	734.5 (308.4 to 1,643.5)
Schistosomiasis	Hydronephrosis due to schistosomiasis	16,711.9 (14,268.4 to 19,737.5)	12,866.9 (9,486.9 to 19,366.7)	178.3 (83.6 to 339.1)	137.0 (59.2 to 301.8)
Schistosomiasis	Mild anemia due to schistosomiasis	9,026.3 (8,905.2 to 9,152.4)	7,454.4 (7,259.2 to 7,567.4)	32.5 (11.6 to 70.0)	26.9 (9.6 to 58.6)
Schistosomiasis	Moderate anemia due to schistosomiasis	10,176.3 (10,009.1 to 10,335.1)	8,047.9 (7,870.4 to 8,322.3)	503.4 (333.8 to 730.3)	399.3 (263.7 to 580.3)
Schistosomiasis	Severe anemia due to schistosomiasis	1,111.6 (1,062.4 to 1,157.3)	829.0 (784.9 to 877.6)	156.3 (106.6 to 216.9)	116.9 (80.7 to 160.3)
Cysticercosis	Neurocysticercosis with epilepsy	2,059.1 (1,743.3 to 2,449.5)	1,931.0 (1,597.8 to 2,312.0)	342.6 (234.3 to 462.5)	286.7 (194.2 to 392.8)
Cystic echinococcosis	Abdominal problems due to cystic echinococcosis	896.1 (814.5 to 979.8)	1,118.0 (1,014.0 to 1,226.9)	96.6 (65.7 to 133.9)	120.4 (81.9 to 166.5)
Cystic echinococcosis	Chronic respiratory disease due to cystic echinococcosis	203.2 (163.5 to 243.8)	253.5 (203.7 to 304.3)	3.9 (2.0 to 6.9)	4.8 (2.5 to 8.6)
Cystic echinococcosis	Epilepsy due to echinococcosis	9.3 (2.6 to 20.3)	11.5 (3.2 to 25.2)	1.5 (0.4 to 3.4)	1.6 (0.4 to 3.5)
Lymphatic filariasis	Prevalence of detectable microfiliaria due to lymphatic filariasis	47,828.5 (43,919.8 to 51,748.1)	19,707.9 (17,173.8 to 22,270.5)	<0.05 (0.0 to <0.05)	<0.05 (0.0 to <0.05)
Lymphatic filariasis	Lymphedema due to lymphatic filariasis	9,298.0 (6,037.4 to 12,871.1)	9,856.3 (6,401.6 to 13,670.2)	948.8 (519.6 to 1,504.3)	1,005.4 (553.4 to 1,599.7)
Lymphatic filariasis	Hydrocele due to lymphatic filariasis	12,708.1 (5,526.0 to 22,633.8)	8,899.9 (3,816.0 to 16,368.4)	1,527.8 (591.1 to 2,952.2)	1,069.6 (419.9 to 2,102.4)
Onchocerciasis	Asymptomatic onchocerciasis	6,072.1 (4,378.1 to 8,083.4)	2,280.4 (1,525.0 to 3,173.2)	<0.05 (0.0 to <0.05)	<0.05 (0.0 to <0.05)
Onchocerciasis	Mild skin disease due to onchocerciasis	1,460.2 (553.9 to 2,442.7)	1,523.7 (422.8 to 2,756.7)	37.3 (12.0 to 73.8)	39.1 (9.9 to 84.9)

Cause	Sequela	Prevalence in thousands (95% confidence interval)		YLDs in thousands (95% confidence interval)	
		2005	2015	2005	2015
Onchocerciasis	Mild skin disease without itch due to onchocerciasis	6,165.9 (3,501.9 to 9,402.4)	5,221.1 (2,159.8 to 9,763.8)	65.5 (26.3 to 137.4)	55.5 (16.5 to 130.4)
Onchocerciasis	Moderate skin disease due to onchocerciasis	4,954.8 (2,733.0 to 7,522.0)	3,703.5 (1,401.8 to 6,854.4)	859.2 (429.9 to 1,440.0)	642.7 (224.8 to 1,257.6)
Onchocerciasis	Severe skin disease due to onchocerciasis	2,159.1 (820.8 to 3,621.7)	1,733.6 (410.5 to 3,366.6)	375.4 (136.0 to 686.8)	302.4 (70.3 to 626.1)
Onchocerciasis	Severe skin disease without itch due to onchocerciasis	53.4 (5.7 to 117.0)	44.0 (4.0 to 100.3)	18.9 (1.8 to 44.4)	15.8 (1.2 to 38.5)
Onchocerciasis	Moderate vision impairment due to onchocerciasis	647.9 (448.7 to 889.4)	656.1 (352.7 to 1,084.6)	18.6 (10.3 to 30.5)	18.8 (8.6 to 35.1)
Onchocerciasis	Severe vision impairment due to onchocerciasis	110.1 (78.2 to 147.9)	112.7 (61.8 to 178.2)	18.1 (10.9 to 26.4)	18.5 (9.0 to 30.7)
Onchocerciasis	Blindness due to onchocerciasis	291.4 (201.4 to 402.6)	256.6 (135.9 to 427.6)	48.7 (30.1 to 74.9)	42.9 (21.2 to 75.2)
Trachoma	Moderate vision impairment due to trachoma	2,042.8 (1,605.2 to 2,586.0)	2,264.6 (1,728.8 to 2,905.6)	58.1 (34.4 to 91.3)	64.5 (37.4 to 102.3)
Trachoma	Severe vision impairment due to trachoma	280.0 (226.0 to 348.9)	309.7 (247.8 to 387.7)	45.5 (30.2 to 66.1)	50.4 (33.1 to 73.6)
Trachoma	Blindness due to trachoma	1,076.5 (836.0 to 1,378.2)	982.8 (759.4 to 1,280.5)	179.1 (113.9 to 267.4)	164.3 (102.7 to 243.9)
Dengue	Moderate dengue	508.7 (292.9 to 1,061.2)	1,236.1 (836.1 to 2,593.9)	25.0 (11.5 to 54.6)	60.3 (32.1 to 133.7)
Dengue	Severe dengue	69.2 (35.0 to 144.4)	169.3 (99.1 to 386.6)	9.0 (3.9 to 21.2)	21.8 (11.0 to 50.7)
Dengue	Post-dengue chronic fatigue syndrome	1,364.1 (584.5 to 2,777.3)	3,324.6 (1,600.3 to 7,347.9)	283.3 (104.9 to 648.4)	682.0 (295.2 to 1,608.9)
Yellow fever	Asymptomatic yellow fever	2.1 (0.5 to 6.2)	1.6 (0.4 to 4.6)	<0.05 (0.0 to <0.05)	<0.05 (0.0 to <0.05)
Yellow fever	Moderate yellow fever	1.2 (0.3 to 3.4)	0.9 (0.2 to 2.5)	0.1 (0.0 to 0.2)	<0.05 (0.0 to 0.1)
Yellow fever	Severe yellow fever	0.5 (0.1 to 1.4)	0.4 (0.1 to 1.0)	0.1 (0.0 to 0.2)	<0.05 (0.0 to 0.1)
Rabies	Rabies	1.2 (1.1 to 1.4)	0.7 (0.6 to 0.8)	0.2 (0.1 to 0.2)	0.1 (0.1 to 0.1)
Ascariasis	Heavy infestation of ascariasis	39,221.3 (34,914.8 to 44,750.5)	23,383.0 (20,814.5 to 26,751.5)	1,010.4 (549.8 to 1,651.9)	601.9 (328.2 to 986.8)
Ascariasis	Mild abdominopelvic problems due to ascariasis	34,627.8 (32,106.0 to 37,288.3)	24,205.0 (21,970.0 to 26,863.3)	371.7 (183.0 to 700.1)	259.7 (123.7 to 501.9)
Ascariasis	Severe wasting due to ascariasis	124.7 (93.6 to 162.2)	75.1 (55.4 to 97.1)	15.4 (8.7 to 23.8)	9.3 (5.5 to 14.5)
Ascariasis	Asymptomatic ascariasis	780,515.2 (715,516.1 to 851,522.7)	714,230.7 (634,201.9 to 814,009.5)	<0.05 (0.0 to <0.05)	<0.05 (0.0 to <0.05)
Trichuriasis	Heavy infestation of trichuriasis	20,234.1 (17,815.4 to 23,137.0)	16,708.9 (14,079.7 to 20,974.7)	521.0 (289.2 to 864.5)	430.1 (224.8 to 744.2)
Trichuriasis	Mild abdominopelvic problems due to trichuriasis	11,902.1 (10,744.4 to 13,142.8)	10,397.2 (9,202.9 to 11,959.9)	127.8 (62.4 to 244.6)	111.4 (52.9 to 210.6)
Trichuriasis	Severe wasting due to trichuriasis	35.2 (25.2 to 48.2)	20.7 (14.7 to 28.7)	4.4 (2.5 to 7.0)	2.6 (1.5 to 4.2)
Trichuriasis	Asymptomatic trichuriasis	441,228.0 (411,518.0 to 474,422.5)	436,525.4 (400,013.3 to 475,985.5)	<0.05 (0.0 to <0.05)	<0.05 (0.0 to <0.05)
Hookworm disease	Heavy infestation of hookworm	39,619.5 (36,285.9 to 43,240.4)	33,023.4 (30,344.7 to 35,992.7)	1,017.4 (555.5 to 1,662.5)	847.5 (463.6 to 1,385.5)
Hookworm disease	Mild abdominopelvic problems due to hookworm disease	19,661.3 (18,630.0 to 20,715.3)	17,999.6 (17,018.7 to 19,081.0)	210.7 (103.0 to 399.2)	192.5 (94.4 to 361.0)
Hookworm disease	Severe wasting due to hookworm disease	80.5 (61.9 to 100.5)	54.1 (42.0 to 67.1)	9.9 (5.8 to 14.9)	6.7 (3.9 to 10.3)
Hookworm disease	Mild anemia due to hookworm disease	12,463.3 (12,299.2 to 12,625.6)	12,350.7 (12,249.5 to 12,468.7)	45.0 (16.1 to 97.3)	44.5 (15.9 to 97.9)
Hookworm disease	Moderate anemia due to hookworm disease	12,160.3 (11,954.0 to 12,343.8)	11,030.2 (10,945.3 to 11,118.2)	604.3 (402.5 to 873.1)	548.6 (363.5 to 798.2)
Hookworm disease	Severe anemia due to hookworm disease	1,208.8 (1,157.2 to 1,263.4)	839.3 (791.7 to 879.5)	171.0 (117.6 to 238.8)	119.0 (81.5 to 166.4)
Hookworm disease	Asymptomatic hookworm disease	376,916.8 (345,843.7 to 410,037.9)	352,948.6 (319,143.2 to 392,574.4)	<0.05 (0.0 to <0.05)	<0.05 (0.0 to <0.05)
Food-borne trematodiases	Asymptomatic clonorchiasis	27,422.1 (24,991.9 to 30,199.2)	29,167.0 (26,611.9 to 32,253.4)	<0.05 (0.0 to <0.05)	<0.05 (0.0 to <0.05)
Food-borne trematodiases	Asymptomatic fascioliasis	661.7 (477.3 to 836.3)	790.0 (569.8 to 1,000.6)	<0.05 (0.0 to <0.05)	<0.05 (0.0 to <0.05)
Food-borne trematodiases	Asymptomatic intestinal fluke infection	1,229.6 (1,027.7 to 1,450.6)	1,603.0 (1,338.8 to 1,893.0)	<0.05 (0.0 to <0.05)	<0.05 (0.0 to <0.05)
Food-borne trematodiases	Asymptomatic opisthorchiasis	1,439.9 (941.7 to 1,962.9)	1,800.1 (1,172.2 to 2,343.3)	<0.05 (0.0 to <0.05)	<0.05 (0.0 to <0.05)
Food-borne trematodiases	Asymptomatic paragonimiasis	23,333.4 (13,242.2 to 30,251.8)	22,771.7 (12,990.5 to 29,225.4)	<0.05 (0.0 to <0.05)	<0.05 (0.0 to <0.05)
Food-borne trematodiases	Heavy opisthorchiasis due to food-borne trematodiases	491.3 (184.1 to 1,001.7)	614.9 (220.1 to 1,223.5)	51.2 (17.9 to 113.6)	64.1 (21.9 to 146.7)
Food-borne trematodiases	Heavy clonorchiasis due to food-borne trematodiases	5,895.1 (5,112.5 to 6,758.7)	6,594.2 (5,686.5 to 7,644.2)	645.1 (430.8 to 903.8)	720.5 (481.2 to 1,018.7)
Food-borne trematodiases	Heavy intestinal fluke infection due to food-borne trematodiases	133.0 (75.1 to 213.2)	182.2 (97.0 to 302.9)	14.3 (7.2 to 24.6)	19.4 (9.4 to 35.7)
Food-borne trematodiases	Heavy fascioliasis due to food-borne trematodiases	151.5 (34.4 to 327.2)	180.8 (41.0 to 389.5)	16.4 (3.5 to 38.1)	19.6 (4.3 to 44.5)
Food-borne trematodiases	Cerebral paragonimiasis	222.3 (64.1 to 449.5)	181.8 (59.4 to 353.0)	34.6 (9.5 to 71.4)	22.6 (6.8 to 45.9)
Food-borne trematodiases	Mild paragonimiasis due to food-borne trematodiases	4,861.0 (677.2 to 11,657.1)	4,745.4 (646.0 to 11,394.6)	90.8 (12.3 to 230.8)	88.4 (11.8 to 222.4)
Food-borne trematodiases	Moderate paragonimiasis due to food-borne trematodiases	1,141.1 (156.1 to 2,732.2)	1,113.9 (150.4 to 2,642.8)	243.1 (32.8 to 631.5)	236.4 (31.7 to 611.0)
Food-borne trematodiases	Severe paragonimiasis due to food-borne trematodiases	1,383.4 (194.0 to 3,270.3)	1,350.5 (189.4 to 3,207.8)	530.2 (74.4 to 1,391.1)	515.5 (71.3 to 1,351.0)
Leprosy	Disfigurement level 1 due to leprosy	24.9 (23.7 to 25.9)	20.9 (20.0 to 21.8)	0.3 (0.1 to 0.5)	0.2 (0.1 to 0.4)
Leprosy	Disfigurement level 2 due to leprosy	489.9 (463.4 to 520.5)	493.3 (466.5 to 524.3)	30.5 (20.4 to 42.6)	30.8 (20.7 to 43.2)
Ebola	Ebola cases	<0.05 (0.0 to <0.05)	0.3 (0.2 to 0.3)	<0.05 (0.0 to <0.05)	<0.05 (0.0 to 0.1)
Ebola	Post-Ebola chronic fatigue syndrome	<0.05 (0.0 to <0.05)	2.6 (1.0 to 4.9)	<0.05 (0.0 to <0.05)	0.5 (0.2 to 1.1)
Other neglected tropical diseases	Mild anemia due to other neglected tropical diseases	25,320.4 (25,108.8 to 25,513.0)	26,986.3 (26,621.7 to 27,358.0)	92.0 (32.9 to 201.0)	98.1 (35.1 to 214.9)
Other neglected tropical diseases	Moderate anemia due to other neglected tropical diseases	31,761.4 (31,366.7 to 32,149.9)	30,905.9 (30,394.9 to 31,393.5)	1,591.5 (1,051.7 to 2,309.7)	1,551.3 (1,020.3 to 2,246.1)
Other neglected tropical diseases	Severe anemia due to other neglected tropical diseases	4,294.1 (4,131.2 to 4,447.5)	3,560.3 (3,408.3 to 3,715.4)	611.5 (418.1 to 854.4)	508.8 (347.5 to 718.8)
Maternal hemorrhage	Maternal hemorrhage (< 1L blood lost)	138.0 (79.3 to 217.5)	141.0 (84.3 to 215.8)	15.1 (7.4 to 26.2)	15.4 (8.0 to 25.6)
Maternal hemorrhage	Maternal hemorrhage (> 1L blood lost)	48.2 (28.6 to 72.8)	50.3 (30.1 to 77.7)	15.2 (7.8 to 25.0)	15.8 (8.1 to 26.9)
Maternal hemorrhage	Mild anemia due to maternal hemorrhage	1,113.7 (1,087.1 to 1,143.2)	1,167.3 (1,149.9 to 1,186.8)	4.0 (1.4 to 8.8)	4.2 (1.5 to 9.3)
Maternal hemorrhage	Moderate anemia due to maternal hemorrhage	944.1 (912.9 to 972.8)	923.4 (900.5 to 946.0)	46.9 (31.0 to 68.2)	46.0 (30.4 to 67.2)
Maternal hemorrhage	Severe anemia due to maternal hemorrhage	24.0 (19.7 to 30.0)	22.8 (19.5 to 26.7)	3.4 (2.2 to 5.1)	3.3 (2.2 to 4.8)
Maternal sepsis and other maternal infections	Puerperal sepsis	19.0 (11.8 to 28.5)	17.8 (11.1 to 26.8)	2.5 (1.3 to 4.2)	2.4 (1.2 to 4.0)
Maternal sepsis and other maternal infections	Other maternal infections	902.6 (498.9 to 1,437.4)	881.0 (469.3 to 1,446.6)	44.1 (21.5 to 78.2)	42.9 (20.3 to 80.1)

Cause	Sequela	Prevalence in thousands (95% confidence interval)		YLDs in thousands (95% confidence interval)	
		2005	2015	2005	2015
Maternal sepsis and other maternal infections	Infertility due to puerperal sepsis	2,107.2 (1,913.3 to 2,321.8)	2,242.1 (2,030.5 to 2,483.0)	10.5 (4.0 to 22.7)	11.2 (4.2 to 24.3)
Maternal hypertensive disorders	Other hypertensive disorders of pregnancy	4,489.1 (2,958.5 to 6,381.0)	4,611.7 (3,016.7 to 6,579.0)	212.1 (118.1 to 360.3)	217.8 (114.8 to 361.6)
Maternal hypertensive disorders	Severe pre-eclampsia	6.9 (2.5 to 13.5)	7.2 (2.8 to 14.5)	1.2 (0.4 to 2.5)	1.2 (0.5 to 2.6)
Maternal hypertensive disorders	Long term sequelae of severe pre-eclampsia	<0.05 (0.0 to <0.05)	<0.05 (0.0 to <0.05)	<0.05 (0.0 to <0.05)	<0.05 (0.0 to <0.05)
Maternal hypertensive disorders	Eclampsia	5.7 (2.1 to 10.9)	5.4 (2.2 to 10.5)	3.5 (1.2 to 7.0)	3.3 (1.2 to 6.3)
Maternal hypertensive disorders	Long term sequelae of eclampsia	<0.05 (0.0 to <0.05)	<0.05 (0.0 to <0.05)	<0.05 (0.0 to <0.05)	<0.05 (0.0 to <0.05)
Maternal obstructed labor and uterine rupture	Obstructed labor, acute event	92.9 (56.8 to 145.0)	89.6 (53.0 to 142.9)	28.6 (15.1 to 47.6)	27.7 (14.8 to 47.0)
Maternal obstructed labor and uterine rupture	Rectovaginal fistula	106.1 (55.6 to 167.7)	91.4 (47.6 to 144.2)	48.6 (22.5 to 83.3)	42.3 (19.8 to 73.0)
Maternal obstructed labor and uterine rupture	Vesicovaginal fistula	1,040.3 (889.3 to 1,217.0)	896.2 (765.0 to 1,049.6)	325.5 (215.7 to 463.8)	282.0 (187.9 to 402.2)
Maternal abortion, miscarriage, and ectopic pregnancy	Maternal abortive outcome	443.2 (282.0 to 646.1)	441.3 (285.5 to 630.6)	48.4 (26.4 to 77.1)	48.2 (27.2 to 78.5)
Neonatal preterm birth complications	Asymptomatic retinopathy of prematurity	2,104.5 (1,480.2 to 2,819.3)	2,318.9 (1,613.4 to 3,098.8)	<0.05 (0.0 to <0.05)	<0.05 (0.0 to <0.05)
Neonatal preterm birth complications	Mild vision impairment due to retinopathy of prematurity	346.4 (114.5 to 703.9)	383.6 (124.8 to 790.9)	1.0 (0.2 to 3.0)	1.2 (0.2 to 3.5)
Neonatal preterm birth complications	Moderate vision impairment due to retinopathy of prematurity	851.0 (479.5 to 1,260.1)	944.2 (532.6 to 1,406.6)	25.3 (12.0 to 45.8)	28.1 (13.4 to 51.0)
Neonatal preterm birth complications	Severe vision impairment due to retinopathy of prematurity	283.3 (142.1 to 475.8)	320.5 (159.4 to 539.0)	50.0 (21.1 to 92.9)	56.6 (24.0 to 106.5)
Neonatal preterm birth complications	Blindness due to retinopathy of prematurity	239.2 (156.3 to 349.5)	279.2 (183.1 to 406.3)	42.6 (23.6 to 71.8)	49.6 (27.7 to 83.0)
Neonatal preterm birth complications	Mild motor impairment due to neonatal preterm birth complications <28wks	689.9 (414.6 to 1,129.1)	934.3 (548.6 to 1,534.5)	6.8 (2.6 to 15.3)	9.2 (3.5 to 20.7)
Neonatal preterm birth complications	Mild motor plus cognitive impairments due to neonatal preterm birth complications <28wks	535.9 (269.4 to 936.3)	727.6 (367.6 to 1,274.6)	16.2 (6.9 to 32.0)	22.0 (9.4 to 44.3)
Neonatal preterm birth complications	Mild motor impairment due to neonatal preterm birth complications 28-32wks	3,749.6 (2,373.4 to 5,700.9)	4,586.7 (2,822.9 to 7,014.3)	36.9 (15.0 to 75.5)	45.1 (18.1 to 94.6)
Neonatal preterm birth complications	Mild motor plus cognitive impairments due to neonatal preterm birth complications 28-32wks	3,083.9 (1,774.3 to 4,753.9)	3,787.2 (2,153.5 to 5,813.1)	92.7 (43.8 to 161.5)	113.8 (53.7 to 202.8)
Neonatal preterm birth complications	Mild motor impairment due to neonatal preterm birth complications 32-36wks	2,698.1 (1,826.0 to 3,731.0)	2,917.7 (2,013.7 to 3,996.7)	26.7 (11.5 to 56.1)	28.8 (11.9 to 57.3)
Neonatal preterm birth complications	Mild motor plus cognitive impairments due to neonatal preterm birth complications 32-36wks	2,169.4 (1,338.6 to 3,029.1)	2,325.3 (1,491.8 to 3,334.3)	65.5 (33.6 to 113.8)	70.1 (36.2 to 121.6)
Neonatal preterm birth complications	Moderate motor impairment due to neonatal preterm birth complications <28wks	1,020.8 (640.6 to 1,556.3)	1,200.9 (679.6 to 1,989.6)	60.1 (32.7 to 99.2)	70.6 (35.5 to 122.4)
Neonatal preterm birth complications	Moderate motor impairment with blindness due to neonatal preterm birth complications <28wks	26.3 (16.7 to 39.2)	33.5 (20.0 to 51.1)	6.2 (3.4 to 9.8)	7.9 (4.2 to 13.0)
Neonatal preterm birth complications	Moderate motor impairment with epilepsy due to neonatal preterm birth complications <28wks	371.1 (242.4 to 541.6)	428.0 (254.0 to 660.0)	74.4 (42.0 to 117.8)	75.2 (40.3 to 124.9)
Neonatal preterm birth complications	Moderate motor impairment with blindness and epilepsy due to neonatal preterm birth complications <28wks	34.8 (22.3 to 50.0)	40.1 (23.8 to 61.1)	12.4 (6.9 to 19.3)	13.4 (7.0 to 22.2)
Neonatal preterm birth complications	Moderate motor plus cognitive impairment with blindness due to neonatal preterm birth complications <28wks	79.0 (41.5 to 124.4)	94.2 (45.7 to 155.4)	27.6 (13.8 to 47.3)	32.9 (16.0 to 57.4)
Neonatal preterm birth complications	Moderate motor plus cognitive impairment with epilepsy due to neonatal preterm birth complications <28wks	446.4 (241.9 to 701.7)	532.1 (264.6 to 888.6)	140.7 (67.2 to 234.7)	156.1 (70.2 to 275.7)
Neonatal preterm birth complications	Moderate motor plus cognitive impairment with blindness and epilepsy due to neonatal preterm birth complications <28wks	42.2 (22.6 to 67.1)	50.3 (24.8 to 84.0)	19.2 (9.1 to 31.4)	21.9 (9.8 to 37.9)
Neonatal preterm birth complications	Severe motor impairment due to neonatal preterm birth complications <28wks	1,326.9 (806.2 to 2,049.9)	1,565.3 (862.6 to 2,564.8)	501.4 (277.8 to 847.2)	589.8 (307.9 to 1,041.6)
Neonatal preterm birth complications	Severe motor impairment with blindness due to neonatal preterm birth complications <28wks	8.8 (5.4 to 13.2)	11.2 (6.5 to 17.3)	4.5 (2.5 to 7.2)	5.7 (3.1 to 9.5)
Neonatal preterm birth complications	Severe motor impairment with epilepsy due to neonatal preterm birth complications <28wks	123.9 (79.5 to 182.5)	142.8 (85.3 to 219.2)	61.0 (34.0 to 95.6)	68.0 (35.3 to 113.5)
Neonatal preterm birth complications	Severe motor impairment with blindness and epilepsy due to neonatal preterm birth complications <28wks	11.6 (7.3 to 16.9)	13.4 (7.7 to 20.2)	6.8 (3.8 to 10.4)	7.7 (4.0 to 12.7)
Neonatal preterm birth complications	Severe motor plus cognitive impairment with blindness due to neonatal preterm birth complications <28wks	43.8 (1.6 to 81.0)	51.5 (1.8 to 99.1)	27.3 (1.0 to 52.4)	32.1 (1.0 to 63.1)
Neonatal preterm birth complications	Severe motor plus cognitive impairment with epilepsy due to neonatal preterm birth complications <28wks	248.6 (8.6 to 464.1)	292.2 (10.0 to 568.4)	147.7 (4.6 to 287.4)	169.9 (5.2 to 345.0)
Neonatal preterm birth complications	Severe motor plus cognitive impairment with blindness and epilepsy due to neonatal preterm birth complications <28wks	23.3 (0.8 to 44.0)	27.4 (0.9 to 54.0)	16.0 (0.5 to 31.7)	18.5 (0.6 to 38.2)
Neonatal preterm birth complications	Moderate motor impairment due to neonatal preterm birth complications 28-32wks	1,698.1 (1,291.6 to 2,282.2)	1,749.5 (1,250.2 to 2,430.2)	99.9 (61.2 to 155.1)	102.8 (60.4 to 166.9)
Neonatal preterm birth complications	Moderate motor impairment with blindness due to neonatal preterm birth complications 28-32wks	43.2 (32.3 to 56.6)	48.4 (33.9 to 65.2)	10.2 (6.2 to 15.1)	11.4 (6.7 to 17.5)
Neonatal preterm birth complications	Moderate motor impairment with epilepsy due to neonatal preterm birth complications 28-32wks	626.2 (501.4 to 769.1)	629.1 (466.6 to 812.0)	127.5 (87.7 to 175.7)	112.3 (73.9 to 160.7)
Neonatal preterm birth complications	Moderate motor impairment with blindness and epilepsy due to neonatal preterm birth complications 28-32wks	58.7 (47.3 to 71.8)	59.0 (45.0 to 76.7)	21.1 (14.3 to 29.2)	19.9 (12.8 to 28.7)
Neonatal preterm birth complications	Moderate motor plus cognitive impairment with blindness due to neonatal preterm birth complications 28-32wks	139.5 (82.2 to 187.7)	143.6 (81.7 to 199.5)	48.5 (25.7 to 73.7)	50.1 (25.2 to 79.6)
Neonatal preterm birth complications	Moderate motor plus cognitive impairment with epilepsy due to neonatal preterm birth complications 28-32wks	788.9 (465.6 to 1,055.9)	812.0 (465.1 to 1,129.3)	249.2 (129.1 to 373.8)	239.1 (121.2 to 367.2)
Neonatal preterm birth complications	Moderate motor plus cognitive impairment with blindness and epilepsy due to neonatal preterm birth complications 28-32wks	74.4 (45.1 to 102.9)	76.6 (43.9 to 109.7)	34.0 (17.8 to 51.1)	33.6 (17.1 to 52.1)
Neonatal preterm birth complications	Severe motor impairment due to neonatal preterm birth complications 28-32wks	2,223.2 (1,658.3 to 3,022.8)	2,294.3 (1,597.4 to 3,205.4)	841.0 (511.4 to 1,239.5)	867.5 (512.1 to 1,317.0)
Neonatal preterm birth complications	Severe motor impairment with blindness due to neonatal preterm birth complications 28-32wks	14.4 (10.7 to 19.1)	16.1 (11.4 to 21.8)	7.4 (4.5 to 10.5)	8.2 (5.0 to 11.9)
Neonatal preterm birth complications	Severe motor impairment with epilepsy due to neonatal preterm birth complications 28-32wks	209.1 (166.6 to 257.2)	210.0 (156.7 to 271.9)	102.1 (68.8 to 141.2)	99.3 (63.3 to 144.6)
Neonatal preterm birth complications	Severe motor impairment with blindness and epilepsy due to neonatal preterm birth complications 28-32wks	19.6 (15.6 to 24.1)	19.6 (14.5 to 25.5)	11.5 (8.0 to 15.8)	11.3 (7.4 to 16.0)
Neonatal preterm birth complications	Severe motor plus cognitive impairment with blindness due to neonatal preterm birth complications 28-32wks	78.4 (3.4 to 133.8)	79.1 (3.5 to 139.3)	48.9 (2.1 to 89.9)	49.4 (2.1 to 92.6)
Neonatal preterm birth complications	Severe motor plus cognitive impairment with epilepsy due to neonatal preterm birth complications 28-32wks	444.9 (18.4 to 742.3)	448.7 (19.3 to 777.0)	260.3 (10.1 to 481.4)	257.5 (9.6 to 486.5)
Neonatal preterm birth complications	Severe motor plus cognitive impairment with blindness and epilepsy due to neonatal preterm birth complications 28-32wks	41.8 (1.8 to 70.2)	42.1 (1.8 to 72.9)	28.7 (1.2 to 52.8)	28.5 (1.1 to 53.3)
Neonatal preterm birth complications	Moderate motor impairment due to neonatal preterm birth complications 32-36wks	1,367.2 (997.9 to 1,846.5)	1,390.8 (956.8 to 1,977.0)	80.6 (46.6 to 130.4)	81.9 (45.4 to 134.9)
Neonatal preterm birth complications	Moderate motor impairment with blindness due to neonatal preterm birth complications 32-36wks	34.7 (24.9 to 47.3)	38.1 (26.6 to 52.8)	8.2 (4.7 to 12.7)	9.0 (5.1 to 14.3)
Neonatal preterm birth complications	Moderate motor impairment with epilepsy due to neonatal preterm birth complications 32-36wks	502.3 (390.1 to 629.5)	500.0 (359.4 to 659.8)	102.9 (69.6 to 143.7)	90.5 (57.7 to 131.9)
Neonatal preterm birth complications	Moderate motor impairment with blindness and epilepsy due to neonatal preterm birth complications 32-36wks	47.1 (36.1 to 59.9)	46.9 (33.7 to 62.4)	17.0 (11.1 to 23.8)	15.9 (9.9 to 23.0)
Neonatal preterm birth complications	Moderate motor plus cognitive impairment with blindness due to neonatal preterm birth complications 32-36wks	111.4 (65.8 to 152.2)	113.9 (63.1 to 163.4)	39.0 (21.3 to 60.4)	39.9 (21.1 to 63.5)
Neonatal preterm birth complications	Moderate motor plus cognitive impairment with epilepsy due to neonatal preterm birth complications 32-36wks	629.4 (364.2 to 864.9)	643.6 (359.0 to 924.7)	199.5 (103.8 to 303.1)	190.5 (95.5 to 297.5)
Neonatal preterm birth complications	Moderate motor plus cognitive impairment with blindness and epilepsy due to neonatal preterm birth complications 32-36wks	59.4 (35.2 to 82.7)	60.7 (34.2 to 87.6)	27.1 (14.2 to 40.4)	26.7 (13.5 to 41.2)
Neonatal preterm birth complications	Severe motor impairment due to neonatal preterm birth complications 32-36wks	1,792.2 (1,286.2 to 2,436.0)	1,832.0 (1,244.0 to 2,548.0)	679.1 (399.2 to 1,027.6)	693.2 (392.4 to 1,060.6)
Neonatal preterm birth complications	Severe motor impairment with blindness due to neonatal preterm birth complications 32-36wks	11.6 (8.3 to 15.8)	12.7 (8.9 to 17.7)	5.9 (3.6 to 8.8)	6.5 (3.8 to 9.9)
Neonatal preterm birth complications	Severe motor impairment with epilepsy due to neonatal preterm birth complications 32-36wks	167.7 (126.5 to 212.2)	167.0 (119.5 to 220.7)	82.6 (54.1 to 119.5)	79.8 (50.7 to 119.2)

Cause	Sequela	Prevalence in thousands (95% confidence interval)		YLDs in thousands (95% confidence interval)	
		2005	2015	2005	2015
Neonatal preterm birth complications	Severe motor impairment with blindness and epilepsy due to neonatal preterm birth complications 32-36wks	15.7 (12.0 to 20.3)	15.6 (11.4 to 21.1)	9.2 (6.2 to 12.8)	9.0 (5.8 to 12.8)
Neonatal preterm birth complications	Severe motor plus cognitive impairment with blindness due to neonatal preterm birth complications 32-36wks	61.8 (2.4 to 104.2)	61.5 (2.6 to 107.7)	38.7 (1.5 to 70.8)	38.5 (1.4 to 71.0)
Neonatal preterm birth complications	Severe motor plus cognitive impairment with epilepsy due to neonatal preterm birth complications 32-36wks	350.9 (13.2 to 589.0)	349.0 (14.0 to 601.3)	206.4 (7.2 to 368.8)	201.9 (7.1 to 372.2)
Neonatal preterm birth complications	Severe motor plus cognitive impairment with blindness and epilepsy due to neonatal preterm birth complications 32-36wks	32.9 (1.2 to 54.9)	32.8 (1.3 to 57.1)	22.6 (0.8 to 40.8)	22.2 (0.7 to 40.7)
Neonatal preterm birth complications	Asymptomatic neonatla preterm birth <28wks	64.8 (39.8 to 105.1)	86.6 (50.6 to 142.8)	<0.05 (0.0 to <0.05)	<0.05 (0.0 to <0.05)
Neonatal preterm birth complications	Asymptomatic neonatal preterm birth 28-<32 wks	352.0 (234.9 to 506.5)	420.5 (279.3 to 604.7)	<0.05 (0.0 to <0.05)	<0.05 (0.0 to <0.05)
Neonatal preterm birth complications	Asymptomatic neonatal preterm birth 32-<37wks	5,235.9 (4,208.9 to 6,369.6)	5,447.3 (4,376.0 to 6,639.2)	<0.05 (0.0 to <0.05)	<0.05 (0.0 to <0.05)
Neonatal encephalopathy due to birth asphyxia and trauma	Mild motor plus cognitive impairments due to neonatal encephalopathy due to birth asphyxia and trauma	0.9 (0.3 to 1.7)	1.0 (0.3 to 1.8)	<0.05 (0.0 to 0.1)	<0.05 (0.0 to 0.1)
Neonatal encephalopathy due to birth asphyxia and trauma	Mild motor impairment due to neonatal encephalopathy due to birth asphyxia and trauma	3.0 (0.7 to 7.2)	3.4 (0.9 to 8.1)	<0.05 (0.0 to 0.1)	<0.05 (0.0 to 0.1)
Neonatal encephalopathy due to birth asphyxia and trauma	Moderate motor impairment due to neonatal encephalopathy due to birth asphyxia and trauma	2,572.9 (1,674.1 to 3,967.0)	3,288.9 (2,123.4 to 5,147.5)	150.9 (82.2 to 270.0)	192.8 (103.5 to 351.0)
Neonatal encephalopathy due to birth asphyxia and trauma	Moderate motor impairment with blindness due to neonatal encephalopathy due to birth asphyxia and trauma	75.8 (54.2 to 110.9)	101.5 (72.6 to 147.9)	17.9 (10.3 to 28.2)	23.9 (13.7 to 38.0)
Neonatal encephalopathy due to birth asphyxia and trauma	Moderate motor impairment with epilepsy due to neonatal encephalopathy due to birth asphyxia and trauma	992.4 (691.7 to 1,426.1)	1,250.2 (873.1 to 1,782.3)	211.9 (126.8 to 328.2)	241.6 (144.9 to 369.6)
Neonatal encephalopathy due to birth asphyxia and trauma	Moderate motor impairment with blindness and epilepsy due to neonatal encephalopathy due to birth asphyxia and trauma	93.1 (64.1 to 135.1)	117.3 (81.7 to 169.3)	34.3 (20.5 to 53.9)	41.1 (24.7 to 64.3)
Neonatal encephalopathy due to birth asphyxia and trauma	Moderate motor plus cognitive impairment with blindness due to neonatal encephalopathy due to birth asphyxia and trauma	235.4 (137.7 to 350.1)	303.2 (176.1 to 448.1)	79.3 (40.5 to 128.4)	101.7 (52.2 to 163.5)
Neonatal encephalopathy due to birth asphyxia and trauma	Moderate motor plus cognitive impairment with epilepsy due to neonatal encephalopathy due to birth asphyxia and trauma	1,330.9 (782.7 to 1,970.7)	1,714.0 (998.9 to 2,525.9)	429.0 (225.7 to 682.0)	521.5 (268.1 to 822.1)
Neonatal encephalopathy due to birth asphyxia and trauma	Moderate motor plus cognitive impairment with blindness and epilepsy due to neonatal encephalopathy due to birth asphyxia and trauma	125.6 (73.7 to 189.8)	161.8 (93.0 to 244.4)	57.8 (30.0 to 91.6)	71.4 (36.2 to 114.9)
Neonatal encephalopathy due to birth asphyxia and trauma	Severe motor impairment due to neonatal encephalopathy due to birth asphyxia and trauma	3,578.1 (2,316.3 to 5,637.3)	4,606.1 (3,002.2 to 7,231.3)	1,346.3 (746.3 to 2,260.0)	1,733.0 (954.9 to 2,862.0)
Neonatal encephalopathy due to birth asphyxia and trauma	Severe motor impairment with blindness due to neonatal encephalopathy due to birth asphyxia and trauma	25.3 (17.6 to 37.6)	33.8 (23.8 to 49.5)	12.9 (7.8 to 20.2)	17.3 (10.4 to 27.1)
Neonatal encephalopathy due to birth asphyxia and trauma	Severe motor impairment with epilepsy due to neonatal encephalopathy due to birth asphyxia and trauma	331.6 (231.0 to 491.6)	417.7 (291.6 to 613.1)	159.7 (95.6 to 254.7)	195.1 (118.1 to 317.3)
Neonatal encephalopathy due to birth asphyxia and trauma	Severe motor impairment with blindness and epilepsy due to neonatal encephalopathy due to birth asphyxia and trauma	31.0 (21.4 to 46.0)	39.1 (27.0 to 58.1)	18.4 (11.1 to 28.9)	22.8 (13.7 to 35.9)
Neonatal encephalopathy due to birth asphyxia and trauma	Severe motor plus cognitive impairment with blindness due to neonatal encephalopathy due to birth asphyxia and trauma	115.3 (6.8 to 207.0)	143.8 (8.2 to 258.0)	71.8 (4.1 to 133.8)	89.4 (4.8 to 165.2)
Neonatal encephalopathy due to birth asphyxia and trauma	Severe motor plus cognitive impairment with epilepsy due to neonatal encephalopathy due to birth asphyxia and trauma	654.9 (38.1 to 1,166.9)	816.3 (45.9 to 1,456.4)	380.2 (20.0 to 697.5)	465.3 (21.7 to 858.4)
Neonatal encephalopathy due to birth asphyxia and trauma	Severe motor plus cognitive impairment with blindness and epilepsy due to neonatal encephalopathy due to birth asphyxia and trauma	61.4 (3.6 to 108.0)	76.6 (4.1 to 133.7)	42.5 (2.4 to 78.6)	52.3 (2.7 to 97.1)
Neonatal encephalopathy due to birth asphyxia and trauma	Asymptomatic neonatal encephalopathy due to birth asphyxia and trauma	519.4 (346.0 to 738.3)	607.1 (401.6 to 870.4)	<0.05 (0.0 to <0.05)	<0.05 (0.0 to <0.05)
Neonatal sepsis and other neonatal infections	Severe infection due to neonatal sepsis and other neonatal infections	133.1 (73.0 to 210.4)	130.1 (71.1 to 207.6)	16.9 (8.2 to 30.3)	16.6 (8.1 to 30.0)
Neonatal sepsis and other neonatal infections	Asymptomatic neonatal sepsis and other neonatal infections	5.5 (4.4 to 6.6)	6.0 (4.9 to 7.1)	<0.05 (0.0 to <0.05)	<0.05 (0.0 to <0.05)
Neonatal sepsis and other neonatal infections	Mild motor plus cognitive impairments due to neonatal sepsis and other neonatal infections	0.2 (0.1 to 0.3)	0.2 (0.1 to 0.3)	<0.05 (0.0 to <0.05)	<0.05 (0.0 to <0.05)
Neonatal sepsis and other neonatal infections	Mild motor impairment due to neonatal sepsis and other neonatal infections	0.5 (0.4 to 0.6)	0.6 (0.4 to 0.7)	<0.05 (0.0 to <0.05)	<0.05 (0.0 to <0.05)
Neonatal sepsis and other neonatal infections	Moderate motor impairment due to neonatal sepsis and other neonatal infections	<0.05 (0.0 to <0.05)	<0.05 (0.0 to <0.05)	<0.05 (0.0 to <0.05)	<0.05 (0.0 to <0.05)
Neonatal sepsis and other neonatal infections	Moderate motor impairment with blindness due to neonatal sepsis and other neonatal infections	<0.05 (0.0 to <0.05)	<0.05 (0.0 to <0.05)	<0.05 (0.0 to <0.05)	<0.05 (0.0 to <0.05)
Neonatal sepsis and other neonatal infections	Moderate motor impairment with epilepsy due to neonatal sepsis and other neonatal infections	<0.05 (0.0 to <0.05)	<0.05 (0.0 to <0.05)	<0.05 (0.0 to <0.05)	<0.05 (0.0 to <0.05)
Neonatal sepsis and other neonatal infections	Moderate motor impairment with blindness and epilepsy due to neonatal sepsis and other neonatal infections	<0.05 (0.0 to <0.05)	<0.05 (0.0 to <0.05)	<0.05 (0.0 to <0.05)	<0.05 (0.0 to <0.05)
Neonatal sepsis and other neonatal infections	Moderate motor plus cognitive impairment with blindness due to neonatal sepsis and other neonatal infections	<0.05 (0.0 to <0.05)	<0.05 (0.0 to <0.05)	<0.05 (0.0 to <0.05)	<0.05 (0.0 to <0.05)
Neonatal sepsis and other neonatal infections	Moderate motor plus cognitive impairment with epilepsy due to neonatal sepsis and other neonatal infections	<0.05 (0.0 to <0.05)	<0.05 (0.0 to <0.05)	<0.05 (0.0 to <0.05)	<0.05 (0.0 to <0.05)
Neonatal sepsis and other neonatal infections	Moderate motor plus cognitive impairment with blindness and epilepsy due to neonatal sepsis and other neonatal infections	1.1 (0.8 to 1.5)	1.2 (0.8 to 1.7)	0.5 (0.3 to 0.7)	0.5 (0.3 to 0.7)
Neonatal sepsis and other neonatal infections	Severe motor impairment due to neonatal sepsis and other neonatal infections	<0.05 (0.0 to <0.05)	<0.05 (0.0 to <0.05)	<0.05 (0.0 to <0.05)	<0.05 (0.0 to <0.05)
Neonatal sepsis and other neonatal infections	Severe motor impairment with blindness due to neonatal sepsis and other neonatal infections	<0.05 (0.0 to <0.05)	<0.05 (0.0 to <0.05)	<0.05 (0.0 to <0.05)	<0.05 (0.0 to <0.05)
Neonatal sepsis and other neonatal infections	Severe motor impairment with epilepsy due to neonatal sepsis and other neonatal infections	<0.05 (0.0 to <0.05)	<0.05 (0.0 to <0.05)	<0.05 (0.0 to <0.05)	<0.05 (0.0 to <0.05)
Neonatal sepsis and other neonatal infections	Severe motor impairment with blindness and epilepsy due to neonatal sepsis and other neonatal infections	1.0 (0.7 to 1.4)	1.0 (0.7 to 1.5)	0.5 (0.3 to 0.8)	0.6 (0.3 to 0.9)
Neonatal sepsis and other neonatal infections	Severe motor plus cognitive impairment with blindness due to neonatal sepsis and other neonatal infections	<0.05 (0.0 to <0.05)	<0.05 (0.0 to <0.05)	<0.05 (0.0 to <0.05)	<0.05 (0.0 to <0.05)
Neonatal sepsis and other neonatal infections	Severe motor plus cognitive impairment with epilepsy due to neonatal sepsis and other neonatal infections	<0.05 (0.0 to <0.05)	<0.05 (0.0 to <0.05)	<0.05 (0.0 to <0.05)	<0.05 (0.0 to <0.05)
Neonatal sepsis and other neonatal infections	Severe motor plus cognitive impairment with blindness and epilepsy due to neonatal sepsis and other neonatal infections	<0.05 (0.0 to <0.05)	<0.05 (0.0 to <0.05)	<0.05 (0.0 to <0.05)	<0.05 (0.0 to <0.05)
Hemolytic disease and other neonatal jaundice	Moderate motor impairment due to hemolytic disease and other neonatal jaundice	428.8 (346.7 to 543.8)	517.0 (416.0 to 660.8)	25.4 (15.8 to 39.1)	30.6 (19.0 to 47.1)
Hemolytic disease and other neonatal jaundice	Moderate motor impairment with blindness due to hemolytic disease and other neonatal jaundice	13.0 (10.2 to 16.5)	16.4 (13.1 to 20.5)	3.1 (2.0 to 4.5)	3.9 (2.5 to 5.6)
Hemolytic disease and other neonatal jaundice	Moderate motor impairment with epilepsy due to hemolytic disease and other neonatal jaundice	164.8 (140.0 to 192.3)	194.5 (165.7 to 227.0)	36.5 (25.7 to 49.1)	38.3 (26.9 to 52.3)
Hemolytic disease and other neonatal jaundice	Moderate motor impairment with blindness and epilepsy due to hemolytic disease and other neonatal jaundice	15.5 (13.1 to 18.1)	18.2 (15.6 to 21.4)	5.7 (3.9 to 7.6)	6.4 (4.4 to 8.5)
Hemolytic disease and other neonatal jaundice	Moderate motor plus cognitive impairment with blindness due to hemolytic disease and other neonatal jaundice	39.1 (24.3 to 50.5)	47.1 (27.8 to 61.1)	13.7 (7.9 to 20.4)	16.5 (9.2 to 24.7)
Hemolytic disease and other neonatal jaundice	Moderate motor plus cognitive impairment with epilepsy due to hemolytic disease and other neonatal jaundice	221.0 (141.5 to 281.0)	265.9 (163.3 to 339.8)	73.4 (41.1 to 105.1)	82.7 (44.5 to 120.7)
Hemolytic disease and other neonatal jaundice	Moderate motor plus cognitive impairment with blindness and epilepsy due to hemolytic disease and other neonatal jaundice	20.9 (13.2 to 27.5)	25.1 (15.3 to 33.3)	9.7 (5.4 to 14.0)	11.2 (6.0 to 16.4)
Hemolytic disease and other neonatal jaundice	Severe motor impairment severe due to hemolytic disease and other neonatal jaundice	589.9 (465.9 to 753.2)	713.6 (566.8 to 904.8)	224.4 (139.2 to 324.6)	271.4 (168.2 to 386.8)
Hemolytic disease and other neonatal jaundice	Severe motor impairment with blindness due to hemolytic disease and other neonatal jaundice	4.3 (3.4 to 5.6)	5.5 (4.3 to 6.9)	2.2 (1.4 to 3.1)	2.8 (1.8 to 3.9)
Hemolytic disease and other neonatal jaundice	Severe motor impairment with epilepsy due to hemolytic disease and other neonatal jaundice	55.0 (46.9 to 65.1)	64.9 (55.3 to 76.4)	27.7 (18.9 to 37.5)	31.7 (21.6 to 43.3)
Hemolytic disease and other neonatal jaundice	Severe motor impairment with blindness and epilepsy due to hemolytic disease and other neonatal jaundice	5.1 (4.3 to 6.1)	6.1 (5.1 to 7.1)	3.1 (2.2 to 4.0)	3.5 (2.5 to 4.6)
Hemolytic disease and other neonatal jaundice	Severe motor plus cognitive impairment with blindness due to hemolytic disease and other neonatal jaundice	20.0 (1.1 to 34.1)	23.4 (1.1 to 40.2)	12.5 (0.7 to 22.7)	14.6 (0.7 to 27.0)
Hemolytic disease and other neonatal jaundice	Severe motor plus cognitive impairment with epilepsy due to hemolytic disease and other neonatal jaundice	113.5 (5.8 to 192.3)	132.8 (6.3 to 227.7)	70.5 (3.6 to 124.6)	80.8 (3.7 to 144.1)
Hemolytic disease and other neonatal jaundice	Severe motor plus cognitive impairment with blindness and epilepsy due to hemolytic disease and other neonatal jaundice	10.7 (0.6 to 18.0)	12.5 (0.6 to 21.2)	7.4 (0.4 to 13.1)	8.5 (0.4 to 15.3)
Protein-energy malnutrition	Kwashiokor due to protein-energy malnutrition	36.7 (20.8 to 58.3)	27.9 (16.3 to 45.3)	1.8 (0.8 to 3.4)	1.4 (0.6 to 2.6)
Protein-energy malnutrition	Marasmus due to protein-energy malnutrition	1,025.7 (630.1 to 1,575.5)	891.3 (553.5 to 1,398.5)	122.5 (63.8 to 206.7)	106.1 (55.5 to 184.5)
Protein-energy malnutrition	Severe wasting due to protein-energy malnutrition	22,910.6 (22,139.2 to 23,783.1)	21,915.0 (20,815.3 to 23,113.4)	2,832.4 (1,841.4 to 4,025.2)	2,715.8 (1,752.0 to 3,813.4)

Cause	Sequela	Prevalence in thousands (95% confidence interval)		YLDs in thousands (95% confidence interval)	
		2005	2015	2005	2015
Iodine deficiency	Visible goiter without symptoms	8,614.4 (7,739.3 to 9,713.3)	9,207.2 (8,272.1 to 10,379.1)	549.0 (361.0 to 781.1)	586.9 (385.8 to 834.3)
Iodine deficiency	Visible goiter without heart failure due to iodine deficiency	91,203.1 (82,065.5 to 102,333.9)	97,478.7 (87,920.6 to 110,017.6)	973.0 (469.7 to 1,798.8)	1,040.0 (503.4 to 1,909.0)
Iodine deficiency	Visible goiter with mild heart failure due to iodine deficiency	0.1 (0.0 to 0.1)	0.1 (0.1 to 0.1)	<0.05 (0.0 to <0.05)	<0.05 (0.0 to <0.05)
Iodine deficiency	Visible goiter with moderate heart failure due to iodine deficiency	<0.05 (0.0 to 0.1)	0.1 (0.0 to 0.1)	<0.05 (0.0 to <0.05)	<0.05 (0.0 to <0.05)
Iodine deficiency	Visible goiter with severe heart failure due to iodine deficiency	0.1 (0.1 to 0.2)	0.2 (0.2 to 0.2)	<0.05 (0.0 to <0.05)	<0.05 (0.0 to 0.1)
Iodine deficiency	Visible goiter with signs and symptoms	1,517.3 (1,335.3 to 1,735.1)	1,621.7 (1,430.1 to 1,866.9)	281.2 (186.9 to 389.6)	300.6 (199.6 to 420.0)
Iodine deficiency	Severe intellectual disability due to iodine deficiency	919.0 (106.2 to 1,451.2)	969.9 (97.1 to 1,542.1)	139.5 (15.5 to 260.9)	147.6 (14.4 to 277.7)
Iodine deficiency	Profound intellectual disability due to iodine deficiency	1,446.9 (216.9 to 2,212.3)	1,641.7 (233.2 to 2,504.8)	274.1 (36.2 to 481.8)	311.7 (41.6 to 552.2)
Vitamin A deficiency	Moderate vision impairment loss due to vitamin A deficiency	3,973.3 (3,170.2 to 4,962.0)	4,317.5 (3,446.3 to 5,322.5)	118.9 (69.4 to 190.9)	129.3 (75.5 to 207.1)
Vitamin A deficiency	Severe vision impairment loss due to vitamin A deficiency	206.4 (100.5 to 306.7)	234.9 (119.2 to 343.8)	36.6 (15.4 to 59.8)	41.6 (18.3 to 67.3)
Vitamin A deficiency	Blindness due to vitamin A deficiency	306.3 (148.4 to 515.8)	348.7 (168.5 to 569.7)	53.9 (23.3 to 100.1)	61.5 (26.2 to 113.2)
Iron-deficiency anemia	Mild iron-deficiency anemia	657,412.4 (654,535.0 to 660,480.1)	700,896.0 (697,496.4 to 704,645.5)	2,379.3 (849.3 to 5,182.2)	2,536.4 (907.3 to 5,541.2)
Iron-deficiency anemia	Moderate iron-deficiency anemia	711,044.8 (705,810.8 to 716,007.0)	699,197.6 (693,413.6 to 705,586.7)	35,587.3 (23,579.8 to 51,577.8)	35,007.3 (23,177.2 to 50,767.0)
Iron-deficiency anemia	Severe iron-deficiency anemia	87,878.1 (86,041.3 to 89,612.6)	77,364.8 (75,763.4 to 79,035.7)	12,452.4 (8,519.9 to 17,376.5)	10,977.4 (7,522.0 to 15,306.7)
Iron-deficiency anemia	Mild heart failure due to iron-deficiency anemia	12.7 (8.2 to 17.4)	17.7 (11.4 to 24.3)	0.5 (0.3 to 0.8)	0.7 (0.4 to 1.1)
Iron-deficiency anemia	Moderate heart failure due to iron-deficiency anemia	10.5 (7.4 to 13.7)	14.6 (10.3 to 19.1)	0.7 (0.4 to 1.1)	1.0 (0.6 to 1.6)
Iron-deficiency anemia	Severe heart failure due to iron-deficiency anemia	28.5 (23.8 to 34.2)	39.8 (33.0 to 47.5)	4.6 (3.0 to 6.6)	6.4 (4.2 to 9.1)
Syphilis	Adult tertiary syphilis	1,082.8 (980.6 to 1,186.7)	1,254.0 (1,127.1 to 1,385.8)	200.6 (134.9 to 277.9)	232.6 (155.4 to 322.8)
Syphilis	Asymptomatic early syphilis infection	39,209.2 (34,246.3 to 44,489.6)	40,518.9 (34,257.2 to 47,358.5)	<0.05 (0.0 to <0.05)	<0.05 (0.0 to <0.05)
Syphilis	Mild early syphilis infection	1,773.3 (780.0 to 3,093.6)	1,832.0 (807.2 to 3,232.5)	9.9 (2.9 to 24.7)	10.3 (3.0 to 25.3)
Chlamydial infection	Asymptomatic chlamydial infection	52,227.4 (41,586.3 to 66,109.5)	56,168.0 (44,845.4 to 70,982.7)	<0.05 (0.0 to <0.05)	<0.05 (0.0 to <0.05)
Chlamydial infection	Mild chlamydial infection	21,185.9 (16,930.9 to 26,731.3)	23,282.7 (18,689.8 to 29,481.0)	120.9 (45.6 to 271.8)	132.9 (49.5 to 300.3)
Chlamydial infection	Epididymo-orchitis due to chlamydial infection	1,460.2 (1,023.6 to 1,983.1)	1,621.8 (1,154.9 to 2,177.9)	179.7 (108.4 to 278.5)	199.8 (122.8 to 308.4)
Chlamydial infection	Moderate pelvic inflammatory diseases due to chlamydial infection	152.2 (129.8 to 177.5)	154.2 (132.0 to 180.2)	16.6 (11.1 to 23.3)	17.0 (11.3 to 24.1)
Chlamydial infection	Severe pelvic inflammatory diseases due to chlamydial infection	18.8 (15.3 to 22.9)	19.1 (15.6 to 23.2)	6.1 (4.0 to 8.8)	6.2 (4.0 to 8.9)
Chlamydial infection	Primary infertility due to chlamydial infection	349.0 (273.6 to 441.5)	384.3 (292.8 to 495.8)	2.6 (1.1 to 5.2)	2.8 (1.2 to 5.9)
Chlamydial infection	Secondary infertility due to chlamydial infection	1,069.7 (940.1 to 1,200.0)	1,192.2 (1,042.1 to 1,348.4)	5.4 (2.1 to 11.7)	6.0 (2.3 to 13.2)
Gonococcal infection	Asymptomatic gonococcal infection	17,406.8 (13,286.9 to 22,650.4)	21,764.9 (16,359.1 to 29,215.0)	<0.05 (0.0 to <0.05)	<0.05 (0.0 to <0.05)
Gonococcal infection	Mild gonococcal infection	17,702.3 (13,631.9 to 22,775.0)	22,309.8 (16,847.5 to 29,337.8)	100.2 (39.5 to 214.1)	126.4 (49.0 to 274.9)
Gonococcal infection	Epididymo-orchitis due to gonococcal infection	1,950.2 (1,386.8 to 2,644.5)	2,486.8 (1,733.1 to 3,392.7)	237.8 (141.3 to 366.9)	303.9 (176.8 to 470.9)
Gonococcal infection	Moderate pelvic inflammatory diseases due to gonococcal infection	65.7 (54.7 to 77.6)	65.5 (54.2 to 77.9)	7.3 (4.8 to 10.6)	7.3 (4.7 to 10.6)
Gonococcal infection	Severe pelvic inflammatory diseases due to gonococcal infection	8.1 (6.5 to 10.1)	8.1 (6.4 to 10.2)	2.6 (1.7 to 3.9)	2.6 (1.7 to 3.9)
Gonococcal infection	Primary infertility due to gonococcal infection	185.0 (143.1 to 237.4)	202.3 (152.9 to 261.6)	1.4 (0.6 to 2.8)	1.5 (0.6 to 3.1)
Gonococcal infection	Secondary infertility due to gonococcal infection	565.5 (480.4 to 658.2)	631.1 (536.4 to 726.9)	2.8 (1.1 to 6.3)	3.2 (1.2 to 7.0)
Trichomoniasis	Asymptomatic trichomoniasis infection	114,499.3 (98,756.2 to 132,246.4)	133,100.2 (114,905.6 to 153,696.5)	<0.05 (0.0 to <0.05)	<0.05 (0.0 to <0.05)
Trichomoniasis	Acute trichomoniasis infection	29,756.3 (25,249.0 to 35,264.3)	34,518.3 (29,287.6 to 40,777.0)	167.4 (67.2 to 352.8)	194.3 (78.0 to 408.3)
Genital herpes	Asymptomatic genital herpes	716,114.7 (626,987.0 to 817,387.1)	845,826.2 (736,724.3 to 968,066.4)	<0.05 (0.0 to <0.05)	<0.05 (0.0 to <0.05)
Genital herpes	Symptomatic genital herpes	32,980.1 (14,773.1 to 56,972.2)	38,954.1 (17,407.0 to 67,048.5)	184.0 (52.7 to 457.6)	217.3 (62.2 to 534.5)
Genital herpes	Moderate infection due to initial genital herpes episode	281.7 (62.7 to 653.2)	389.0 (88.0 to 909.7)	13.8 (3.1 to 33.6)	19.0 (4.3 to 45.8)
Other sexually transmitted diseases	Other sexually transmitted diseases	--	--	275.7 (168.6 to 420.0)	329.4 (200.1 to 504.2)
Other sexually transmitted diseases	Moderate pelvic inflammatory diseases due to other sexually transmitted diseases	445.9 (385.0 to 518.9)	451.3 (390.2 to 525.5)	48.3 (32.2 to 67.7)	48.9 (32.7 to 69.1)
Other sexually transmitted diseases	Severe pelvic inflammatory diseases due to other sexually transmitted diseases	55.2 (45.4 to 67.6)	55.9 (45.9 to 68.4)	17.7 (11.6 to 25.9)	18.0 (11.6 to 26.2)
Other sexually transmitted diseases	Primary infertility due to other sexually transmitted diseases	853.0 (653.5 to 1,091.9)	950.9 (707.9 to 1,244.0)	6.3 (2.6 to 12.8)	7.0 (2.9 to 14.4)
Other sexually transmitted diseases	Secondary infertility due to other sexually transmitted diseases	3,045.4 (2,667.2 to 3,379.3)	3,363.6 (2,915.4 to 3,755.7)	15.3 (5.9 to 32.9)	16.9 (6.5 to 36.0)
Acute hepatitis A	Asymptomatic acute hepatitis A	5,259.6 (4,690.6 to 5,856.8)	5,310.9 (4,565.9 to 6,102.2)	<0.05 (0.0 to <0.05)	<0.05 (0.0 to <0.05)
Acute hepatitis A	Moderate acute hepatitis A	3,149.2 (2,948.3 to 3,364.2)	3,449.9 (3,225.3 to 3,690.0)	154.6 (99.7 to 224.8)	169.2 (108.8 to 245.5)
Acute hepatitis A	Severe acute hepatitis A	22.7 (19.7 to 25.8)	24.8 (21.8 to 28.2)	3.0 (2.0 to 4.3)	3.3 (2.1 to 4.8)
Hepatitis B	Asymptomatic acute hepatitis B	8,999.2 (7,910.1 to 10,343.7)	10,095.1 (8,812.5 to 11,490.6)	<0.05 (0.0 to <0.05)	<0.05 (0.0 to <0.05)
Hepatitis B	Moderate acute hepatitis B	1,706.2 (1,230.4 to 2,180.3)	1,996.6 (1,393.2 to 2,591.1)	82.6 (47.8 to 127.4)	96.8 (54.6 to 150.9)
Hepatitis B	Severe acute hepatitis B	634.2 (284.4 to 1,112.8)	740.5 (335.3 to 1,288.8)	80.0 (31.6 to 155.1)	93.4 (38.1 to 177.2)
Hepatitis B	Chronic hepatitis B	293,744.7 (284,478.3 to 303,036.4)	343,251.2 (330,541.3 to 357,194.6)	<0.05 (0.0 to <0.05)	<0.05 (0.0 to <0.05)
Hepatitis C	Asymptomatic acute hepatitis C	393.4 (288.7 to 459.2)	460.4 (338.9 to 537.1)	<0.05 (0.0 to <0.05)	<0.05 (0.0 to <0.05)
Hepatitis C	Moderate acute hepatitis C	132.8 (74.1 to 236.7)	155.4 (86.1 to 277.4)	6.7 (3.1 to 13.0)	7.8 (3.6 to 15.0)
Hepatitis C	Severe acute hepatitis C	5.6 (2.7 to 11.0)	6.6 (3.2 to 12.9)	0.8 (0.3 to 1.6)	0.9 (0.4 to 1.9)
Hepatitis C	Chronic hepatitis C	120,457.5 (108,080.0 to 133,129.1)	142,122.9 (126,977.7 to 157,044.9)	<0.05 (0.0 to <0.05)	<0.05 (0.0 to <0.05)
Acute hepatitis E	Asymptomatic acute hepatitis E	811.1 (733.3 to 895.6)	829.8 (752.3 to 923.1)	<0.05 (0.0 to <0.05)	<0.05 (0.0 to <0.05)
Acute hepatitis E	Moderate acute hepatitis E	619.0 (552.9 to 698.2)	649.8 (579.6 to 729.7)	30.4 (19.3 to 44.9)	32.0 (20.3 to 48.0)
Acute hepatitis E	Severe acute hepatitis E	21.4 (18.1 to 25.3)	22.4 (18.9 to 26.2)	2.9 (1.8 to 4.2)	3.0 (1.9 to 4.4)
Other infectious diseases	Mild anemia due to other infectious diseases	25,652.0 (25,039.0 to 26,562.4)	25,867.8 (25,629.5 to 26,086.1)	93.1 (33.0 to 203.8)	93.9 (33.4 to 204.5)

Cause	Sequela	Prevalence in thousands (95% confidence interval)		YLDs in thousands (95% confidence interval)	
		2005	2015	2005	2015
Other infectious diseases	Moderate anemia due to other infectious diseases	25,788.4 (25,015.5 to 26,486.0)	26,306.0 (25,967.2 to 26,664.5)	1,290.6 (845.4 to 1,861.3)	1,318.3 (872.4 to 1,906.4)
Other infectious diseases	Severe anemia due to other infectious diseases	3,194.8 (3,056.4 to 3,310.2)	2,659.0 (2,512.4 to 2,789.5)	454.7 (311.5 to 633.4)	379.8 (258.4 to 534.9)
Other infectious diseases	Guillain-Barré syndrome due to other infectious diseases	2.3 (1.6 to 3.1)	2.7 (1.9 to 3.7)	0.7 (0.4 to 1.1)	0.8 (0.5 to 1.2)
Lip and oral cavity cancer	Diagnosis and primary therapy phase of mouth cancer	104.2 (99.8 to 109.0)	143.2 (135.0 to 152.0)	27.6 (19.0 to 37.7)	37.8 (25.7 to 51.5)
Lip and oral cavity cancer	Controlled phase of mouth cancer	1,512.2 (1,439.6 to 1,589.6)	2,103.1 (1,973.9 to 2,242.4)	69.2 (44.7 to 101.0)	96.2 (62.2 to 140.8)
Lip and oral cavity cancer	Metastatic phase of mouth cancer	118.0 (113.4 to 123.1)	158.5 (150.1 to 167.6)	48.0 (32.6 to 63.4)	64.3 (44.4 to 84.1)
Lip and oral cavity cancer	Terminal phase of mouth cancer	15.2 (14.5 to 15.8)	20.3 (19.3 to 21.5)	8.1 (5.6 to 10.5)	10.8 (7.5 to 14.0)
Nasopharynx cancer	Diagnosis and primary therapy phase of nasopharynx cancer	34.7 (29.3 to 39.8)	40.8 (32.5 to 48.6)	9.8 (6.3 to 13.7)	11.5 (7.2 to 16.5)
Nasopharynx cancer	Controlled phase of nasopharynx cancer	529.0 (437.4 to 616.6)	626.0 (494.1 to 759.1)	24.6 (15.3 to 36.6)	29.1 (17.4 to 45.1)
Nasopharynx cancer	Metastatic phase of nasopharynx cancer	52.1 (44.2 to 58.0)	60.0 (48.8 to 69.2)	22.5 (14.8 to 30.4)	25.9 (17.1 to 35.1)
Nasopharynx cancer	Terminal phase of nasopharynx cancer	5.2 (4.4 to 5.7)	5.9 (4.8 to 6.8)	2.8 (1.9 to 3.7)	3.2 (2.1 to 4.3)
Other pharynx cancer	Diagnosis and primary therapy phase of other pharynx cancer	41.5 (39.5 to 43.9)	54.7 (51.3 to 58.6)	11.3 (7.6 to 15.5)	14.8 (10.0 to 20.3)
Other pharynx cancer	Controlled phase of other pharynx cancer	622.4 (591.6 to 657.9)	829.2 (776.4 to 891.5)	28.5 (18.4 to 41.3)	37.9 (24.2 to 55.1)
Other pharynx cancer	Metastatic phase of other pharynx cancer	42.1 (40.3 to 44.2)	53.7 (50.7 to 57.2)	17.7 (12.0 to 23.3)	22.5 (15.3 to 29.6)
Other pharynx cancer	Terminal phase of other pharynx cancer	6.3 (6.0 to 6.6)	7.9 (7.5 to 8.4)	3.4 (2.3 to 4.3)	4.3 (3.0 to 5.5)
Esophageal cancer	Diagnosis and primary therapy phase of esophageal cancer	56.1 (49.4 to 66.6)	66.4 (57.4 to 82.3)	14.9 (9.9 to 21.0)	17.7 (11.6 to 24.9)
Esophageal cancer	Controlled phase of esophageal cancer	362.5 (305.9 to 454.8)	466.5 (388.4 to 606.5)	16.6 (10.3 to 25.5)	21.3 (13.3 to 32.7)
Esophageal cancer	Metastatic phase of esophageal cancer	168.6 (157.7 to 182.5)	175.0 (159.0 to 197.3)	67.6 (47.3 to 87.8)	70.3 (48.5 to 91.7)
Esophageal cancer	Terminal phase of esophageal cancer	36.6 (34.3 to 39.7)	38.0 (34.6 to 42.9)	18.4 (13.1 to 23.6)	19.4 (13.7 to 24.7)
Stomach cancer	Diagnosis and primary therapy phase of stomach cancer	279.1 (269.1 to 289.3)	316.4 (298.4 to 337.8)	71.8 (49.8 to 97.6)	81.1 (56.2 to 109.9)
Stomach cancer	Controlled phase of stomach cancer	2,342.0 (2,254.6 to 2,432.9)	2,765.9 (2,609.4 to 2,950.7)	106.8 (69.3 to 155.2)	125.8 (81.4 to 182.1)
Stomach cancer	Metastatic phase of stomach cancer	335.7 (323.5 to 348.3)	363.5 (342.7 to 389.1)	133.3 (92.7 to 172.8)	144.0 (100.4 to 187.0)
Stomach cancer	Terminal phase of stomach cancer	86.5 (83.4 to 89.8)	93.7 (88.3 to 100.3)	42.0 (30.0 to 53.0)	45.5 (32.3 to 57.0)
Colon and rectum cancer	Diagnosis and primary therapy phase of colon and rectum cancers	289.5 (283.7 to 296.3)	405.4 (391.0 to 420.3)	74.0 (51.1 to 99.8)	103.5 (71.4 to 139.5)
Colon and rectum cancer	Controlled phase of colon and rectum cancers	5,755.9 (5,621.1 to 5,908.5)	8,273.6 (7,970.3 to 8,597.9)	261.2 (169.8 to 375.1)	375.3 (242.8 to 543.2)
Colon and rectum cancer	Metastatic phase of colon and rectum cancers	468.3 (459.6 to 478.1)	616.6 (596.9 to 638.6)	183.5 (128.1 to 237.0)	241.4 (167.7 to 312.4)
Colon and rectum cancer	Terminal phase of colon and rectum cancers	62.2 (61.0 to 63.5)	81.3 (78.6 to 84.3)	30.3 (21.6 to 38.0)	39.6 (28.0 to 49.8)
Colon and rectum cancer	Stoma due to colon and rectum cancer	17.5 (17.0 to 18.0)	22.0 (21.3 to 22.8)	2.4 (1.6 to 3.2)	3.0 (2.0 to 4.1)
Liver cancer due to hepatitis B	Diagnosis and primary therapy phase of liver cancer due to hepatitis B	57.0 (47.4 to 67.3)	61.3 (52.3 to 74.5)	15.5 (10.1 to 22.1)	16.8 (10.9 to 23.5)
Liver cancer due to hepatitis B	Controlled phase of liver cancer due to hepatitis B	123.3 (77.6 to 202.0)	167.6 (114.6 to 255.0)	5.8 (3.0 to 10.2)	7.8 (4.4 to 13.1)
Liver cancer due to hepatitis B	Metastatic phase of liver cancer due to hepatitis B	52.4 (43.9 to 60.1)	55.0 (47.7 to 65.4)	22.3 (14.6 to 29.9)	23.6 (15.9 to 31.7)
Liver cancer due to hepatitis B	Terminal phase of liver cancer due to hepatitis B	20.9 (17.5 to 23.9)	21.9 (19.0 to 26.1)	11.0 (7.3 to 14.6)	11.7 (8.0 to 15.3)
Liver cancer due to hepatitis C	Diagnosis and primary therapy phase of liver cancer due to hepatitis C	31.3 (28.5 to 33.6)	45.6 (41.1 to 51.2)	8.5 (5.7 to 11.8)	12.2 (8.2 to 16.7)
Liver cancer due to hepatitis C	Controlled phase of liver cancer due to hepatitis C	78.3 (67.7 to 94.1)	168.4 (136.0 to 210.0)	3.6 (2.3 to 5.4)	7.7 (4.9 to 11.9)
Liver cancer due to hepatitis C	Metastatic phase of liver cancer due to hepatitis C	28.3 (25.9 to 30.2)	39.1 (35.4 to 43.1)	12.0 (8.2 to 15.9)	16.4 (11.3 to 21.5)
Liver cancer due to hepatitis C	Terminal phase of liver cancer due to hepatitis C	11.3 (10.3 to 12.0)	15.6 (14.1 to 17.2)	5.9 (4.1 to 7.6)	8.0 (5.6 to 10.3)
Liver cancer due to alcohol use	Diagnosis and primary therapy phase of liver cancer due to alcohol use	40.7 (35.4 to 45.7)	56.0 (49.4 to 65.0)	11.0 (7.2 to 15.1)	15.0 (9.8 to 20.4)
Liver cancer due to alcohol use	Controlled phase of liver cancer due to alcohol use	78.8 (62.0 to 118.1)	147.1 (113.3 to 208.5)	3.6 (2.1 to 6.1)	6.8 (4.0 to 10.9)
Liver cancer due to alcohol use	Metastatic phase of liver cancer due to alcohol use	37.8 (32.8 to 41.5)	50.4 (44.6 to 58.0)	15.9 (10.6 to 20.9)	21.2 (14.2 to 28.3)
Liver cancer due to alcohol use	Terminal phase of liver cancer due to alcohol use	15.0 (13.1 to 16.5)	20.1 (17.8 to 23.1)	7.9 (5.4 to 10.2)	10.5 (7.3 to 13.7)
Liver cancer due to other causes	Diagnosis and primary therapy phase of liver cancer due to other causes	28.1 (23.8 to 32.7)	30.8 (26.7 to 36.2)	7.8 (5.2 to 11.0)	8.6 (5.6 to 12.0)
Liver cancer due to other causes	Controlled phase of liver cancer due to other causes	56.5 (35.5 to 93.0)	81.4 (57.8 to 121.2)	2.7 (1.3 to 4.7)	3.8 (2.1 to 6.2)
Liver cancer due to other causes	Metastatic phase of liver cancer due to other causes	26.0 (22.2 to 29.3)	27.7 (24.2 to 31.8)	11.3 (7.5 to 15.2)	12.2 (8.0 to 16.3)
Liver cancer due to other causes	Terminal phase of liver cancer due to other causes	10.3 (8.8 to 11.7)	11.1 (9.7 to 12.7)	5.5 (3.8 to 7.3)	5.9 (4.0 to 7.8)
Gallbladder and biliary tract cancer	Diagnosis and primary therapy phase of gallbladder and biliary tract cancer	24.4 (23.5 to 25.4)	29.3 (27.2 to 31.0)	6.7 (4.5 to 9.3)	8.0 (5.4 to 11.0)
Gallbladder and biliary tract cancer	Controlled phase of gallbladder and biliary tract cancer	42.4 (39.3 to 45.8)	54.5 (49.6 to 60.1)	2.0 (1.3 to 2.9)	2.5 (1.6 to 3.7)
Gallbladder and biliary tract cancer	Metastatic phase of gallbladder and biliary tract cancer	43.2 (41.7 to 44.7)	50.9 (47.5 to 53.7)	18.0 (12.4 to 23.5)	21.2 (14.6 to 28.0)
Gallbladder and biliary tract cancer	Terminal phase of gallbladder and biliary tract cancer	12.5 (12.0 to 12.9)	14.7 (13.7 to 15.5)	6.6 (4.6 to 8.4)	7.7 (5.4 to 9.9)
Pancreatic cancer	Diagnosis and primary therapy phase of pancreatic cancer	68.9 (67.7 to 70.2)	95.4 (92.3 to 98.8)	18.0 (12.4 to 24.4)	24.9 (17.0 to 34.0)
Pancreatic cancer	Controlled phase of pancreatic cancer	110.1 (101.0 to 120.5)	175.1 (158.2 to 194.4)	5.0 (3.3 to 7.4)	8.0 (5.1 to 11.8)
Pancreatic cancer	Metastatic phase of pancreatic cancer	64.8 (63.6 to 65.9)	88.4 (85.6 to 91.3)	26.4 (18.3 to 34.4)	35.9 (24.6 to 46.7)
Pancreatic cancer	Terminal phase of pancreatic cancer	25.5 (25.1 to 25.9)	34.8 (33.7 to 35.9)	13.1 (9.2 to 16.7)	17.8 (12.5 to 22.4)
Larynx cancer	Diagnosis and primary therapy phase of larynx cancer	60.9 (58.6 to 63.5)	74.2 (69.6 to 79.9)	16.2 (11.0 to 21.9)	19.7 (13.2 to 27.0)
Larynx cancer	Controlled phase of larynx cancer	510.5 (481.7 to 542.3)	641.7 (580.8 to 718.6)	23.2 (15.0 to 33.4)	29.2 (18.6 to 42.7)
Larynx cancer	Metastatic phase of larynx cancer	78.5 (75.9 to 81.6)	93.3 (88.0 to 99.8)	32.0 (22.0 to 41.8)	38.1 (26.1 to 49.9)
Larynx cancer	Terminal phase of larynx cancer	10.5 (10.1 to 10.9)	12.4 (11.7 to 13.2)	5.6 (3.9 to 7.2)	6.7 (4.7 to 8.6)
Larynx cancer	Laryngectomy due to larynx cancer	459.4 (447.5 to 471.0)	591.0 (576.5 to 605.4)	41.8 (27.9 to 60.6)	53.7 (35.8 to 77.6)
Tracheal, bronchus, and lung cancer	Diagnosis and primary therapy phase of lung, bronchus, and trachea cancer	161.9 (156.1 to 169.2)	218.5 (205.9 to 233.7)	41.7 (28.9 to 56.6)	56.1 (39.0 to 75.7)
Tracheal, bronchus, and lung cancer	Controlled phase of lung, bronchus, and trachea cancer	1,550.0 (1,477.8 to 1,635.9)	2,206.9 (2,060.4 to 2,379.7)	70.4 (45.5 to 102.9)	100.2 (64.7 to 147.2)

Cause	Sequela	Prevalence in thousands (95% confidence interval)		YLDs in thousands (95% confidence interval)	
		2005	2015	2005	2015
Tracheal, bronchus, and lung cancer	Metastatic phase of lung, bronchus, and trachea cancer	559.5 (542.5 to 580.2)	715.7 (676.5 to 762.2)	220.3 (151.9 to 284.0)	281.5 (196.5 to 363.6)
Tracheal, bronchus, and lung cancer	Terminal phase of lung, bronchus, and trachea cancer	124.1 (120.3 to 128.7)	158.7 (150.0 to 169.0)	59.3 (42.6 to 74.4)	75.7 (53.9 to 94.4)
Malignant skin melanoma	Diagnosis and primary therapy phase of malignant skin melanoma	51.0 (42.2 to 65.4)	80.3 (64.4 to 101.5)	13.7 (9.2 to 20.2)	21.3 (14.0 to 31.7)
Malignant skin melanoma	Controlled phase of malignant skin melanoma	1,848.8 (1,537.8 to 2,367.5)	2,945.2 (2,364.0 to 3,730.5)	85.0 (52.9 to 127.7)	134.8 (83.5 to 201.6)
Malignant skin melanoma	Metastatic phase of malignant skin melanoma	33.6 (27.1 to 42.3)	49.4 (38.2 to 60.8)	14.1 (9.4 to 19.7)	20.4 (13.4 to 28.4)
Malignant skin melanoma	Terminal phase of malignant skin melanoma	5.0 (4.1 to 6.3)	7.4 (5.7 to 9.1)	2.7 (1.8 to 3.7)	3.9 (2.6 to 5.3)
Non-melanoma skin cancer (squamous-cell carcinoma)	Diagnosis and primary therapy phase of cutaneous squamous cell carcinoma	28.3 (27.2 to 29.7)	54.4 (50.9 to 58.2)	7.7 (5.2 to 10.6)	14.5 (9.8 to 19.8)
Non-melanoma skin cancer (squamous-cell carcinoma)	Control phase of cutaneous squamous cell carcinoma	1,032.9 (982.1 to 1,090.4)	2,010.7 (1,879.2 to 2,160.5)	47.0 (30.7 to 68.0)	91.1 (58.4 to 131.7)
Non-melanoma skin cancer (squamous-cell carcinoma)	Metastatic phase of cutaneous squamous cell carcinoma	49.4 (45.8 to 53.7)	85.0 (78.2 to 93.0)	19.6 (13.4 to 25.4)	33.3 (23.0 to 43.4)
Non-melanoma skin cancer (squamous-cell carcinoma)	Terminal phase of cutaneous squamous cell carcinoma	3.4 (3.1 to 3.8)	5.8 (5.3 to 6.4)	1.8 (1.2 to 2.4)	3.0 (2.1 to 4.0)
Non-melanoma skin cancer (basal-cell carcinoma)	Disfigurement due to basal cell carcinoma	455.2 (394.0 to 528.0)	578.1 (497.7 to 671.7)	4.7 (2.2 to 8.7)	6.0 (2.9 to 11.1)
Breast cancer	Diagnosis and primary therapy phase of breast cancer	371.9 (352.3 to 393.4)	541.8 (509.8 to 568.8)	96.0 (66.0 to 131.8)	139.7 (95.5 to 189.9)
Breast cancer	Controlled phase of breast cancer	5,402.7 (4,777.4 to 6,112.5)	9,116.2 (8,066.4 to 9,974.9)	251.0 (155.7 to 374.9)	422.3 (260.9 to 615.0)
Breast cancer	Metastatic phase of breast cancer	765.2 (725.0 to 816.9)	999.9 (940.1 to 1,052.4)	303.0 (210.6 to 396.7)	395.4 (274.4 to 515.1)
Breast cancer	Terminal phase of breast cancer	50.6 (47.8 to 54.0)	65.4 (61.4 to 69.0)	25.3 (17.8 to 32.3)	32.5 (22.7 to 41.2)
Breast cancer	Mastectomy due to breast cancer	8,506.3 (8,333.9 to 8,695.1)	10,638.5 (10,404.8 to 10,894.5)	645.0 (414.7 to 928.5)	806.8 (520.3 to 1,171.8)
Cervical cancer	Diagnosis and primary therapy phase of cervical cancer	173.1 (159.9 to 186.0)	170.3 (155.7 to 185.5)	45.2 (30.3 to 61.4)	44.6 (30.3 to 61.2)
Cervical cancer	Controlled phase of cervical cancer	3,138.5 (2,881.6 to 3,388.8)	3,089.4 (2,804.6 to 3,390.3)	144.9 (92.7 to 211.4)	142.6 (91.9 to 206.4)
Cervical cancer	Metastatic phase of cervical cancer	163.7 (152.2 to 175.7)	161.3 (149.0 to 174.0)	66.2 (44.5 to 87.3)	65.5 (44.7 to 85.7)
Cervical cancer	Terminal phase of cervical cancer	21.6 (20.0 to 23.2)	21.3 (19.6 to 23.0)	11.4 (7.8 to 14.7)	11.3 (7.9 to 14.6)
Uterine cancer	Diagnosis and primary therapy phase of uterine cancer	115.3 (105.3 to 128.4)	159.2 (143.0 to 177.6)	30.1 (20.3 to 40.5)	41.3 (27.7 to 56.8)
Uterine cancer	Controlled phase of uterine cancer	2,542.5 (2,309.3 to 2,846.9)	3,558.4 (3,181.3 to 3,988.9)	116.6 (74.9 to 171.5)	163.1 (103.8 to 244.2)
Uterine cancer	Metastatic phase of uterine cancer	78.0 (72.7 to 84.8)	99.7 (91.2 to 109.9)	31.5 (21.6 to 41.0)	40.1 (27.3 to 52.9)
Uterine cancer	Terminal phase of uterine cancer	8.2 (7.6 to 8.9)	10.5 (9.6 to 11.5)	4.4 (3.0 to 5.6)	5.6 (3.8 to 7.3)
Ovarian cancer	Diagnosis and primary therapy phase of ovarian cancer	30.1 (28.8 to 32.0)	38.5 (36.3 to 40.9)	8.3 (5.6 to 11.5)	10.6 (7.1 to 14.8)
Ovarian cancer	Controlled phase of ovarian cancer	691.0 (656.4 to 734.9)	894.4 (841.1 to 954.7)	31.8 (20.5 to 46.2)	41.1 (26.1 to 59.5)
Ovarian cancer	Metastatic phase of ovarian cancer	182.3 (175.3 to 191.8)	226.6 (215.8 to 239.3)	73.3 (50.4 to 95.5)	91.0 (62.8 to 118.5)
Ovarian cancer	Terminal phase of ovarian cancer	12.0 (11.5 to 12.6)	14.8 (14.1 to 15.6)	6.4 (4.4 to 8.3)	8.0 (5.5 to 10.3)
Prostate cancer	Diagnosis and primary therapy phase of prostate cancer	264.5 (213.8 to 360.8)	457.6 (372.5 to 637.8)	66.4 (43.1 to 100.2)	114.8 (75.9 to 175.2)
Prostate cancer	Controlled phase of prostate cancer	6,725.1 (5,390.7 to 9,322.9)	11,954.1 (9,726.4 to 16,766.2)	302.9 (187.0 to 465.1)	538.6 (328.8 to 828.9)
Prostate cancer	Metastatic phase of prostate cancer	708.0 (579.5 to 925.2)	1,036.3 (849.0 to 1,376.2)	270.3 (180.7 to 384.3)	395.6 (261.9 to 568.6)
Prostate cancer	Terminal phase of prostate cancer	31.0 (25.4 to 40.3)	44.4 (36.4 to 58.7)	14.5 (9.8 to 20.0)	20.6 (14.1 to 28.6)
Prostate cancer	Impotence due to prostate cancer	550.2 (541.9 to 558.7)	709.7 (698.5 to 721.2)	33.2 (20.9 to 48.7)	42.8 (27.1 to 63.2)
Prostate cancer	Incontinence due to prostate cancer	180.1 (177.4 to 182.8)	232.3 (228.6 to 236.0)	29.4 (20.8 to 39.0)	37.8 (26.7 to 50.4)
Testicular cancer	Diagnosis and primary therapy phase of testicular cancer	14.5 (13.9 to 15.2)	20.6 (19.0 to 22.0)	4.1 (2.8 to 5.8)	5.9 (3.9 to 8.2)
Testicular cancer	Controlled phase of testicular cancer	460.0 (438.7 to 482.2)	653.5 (604.5 to 698.1)	21.8 (14.2 to 32.0)	31.0 (19.8 to 45.4)
Testicular cancer	Metastatic phase of testicular cancer	8.2 (7.8 to 8.7)	10.9 (10.0 to 11.8)	3.7 (2.5 to 4.9)	4.9 (3.3 to 6.6)
Testicular cancer	Terminal phase of testicular cancer	0.6 (0.6 to 0.7)	0.8 (0.7 to 0.9)	0.3 (0.2 to 0.4)	0.4 (0.3 to 0.6)
Kidney cancer	Diagnosis and primary therapy phase of kidney cancer	94.4 (91.5 to 97.9)	147.4 (140.2 to 155.5)	24.7 (17.1 to 33.7)	38.4 (26.2 to 51.7)
Kidney cancer	Controlled phase of kidney cancer	1,651.1 (1,595.3 to 1,716.2)	2,617.8 (2,486.4 to 2,766.7)	75.6 (48.5 to 109.3)	119.6 (77.1 to 174.8)
Kidney cancer	Metastatic phase of kidney cancer	60.6 (59.0 to 62.6)	88.2 (84.4 to 92.8)	24.8 (16.9 to 32.6)	35.9 (24.5 to 46.5)
Kidney cancer	Terminal phase of kidney cancer	11.7 (11.3 to 12.0)	16.9 (16.2 to 17.8)	6.2 (4.3 to 7.8)	8.9 (6.2 to 11.3)
Bladder cancer	Diagnosis and primary therapy phase of bladder cancer	140.5 (136.9 to 144.5)	186.2 (177.6 to 195.8)	36.1 (24.7 to 48.5)	47.8 (32.8 to 64.6)
Bladder cancer	Controlled phase of bladder cancer	2,138.8 (2,074.9 to 2,210.0)	2,933.0 (2,779.1 to 3,112.8)	96.6 (62.9 to 140.2)	132.3 (86.2 to 192.8)
Bladder cancer	Metastatic phase of bladder cancer	103.8 (101.2 to 106.4)	130.7 (125.0 to 136.5)	41.3 (28.8 to 53.7)	52.0 (36.5 to 67.4)
Bladder cancer	Terminal phase of bladder cancer	18.8 (18.4 to 19.3)	23.7 (22.7 to 24.8)	9.6 (6.7 to 12.1)	12.1 (8.5 to 15.4)
Bladder cancer	Urinary incontinence due to bladder cancer	109.4 (105.9 to 113.2)	134.3 (129.7 to 139.4)	18.6 (13.0 to 25.2)	22.9 (15.9 to 31.2)
Brain and nervous system cancer	Diagnosis and primary therapy phase of brain and nervous system cancers	55.5 (50.4 to 60.9)	69.9 (63.9 to 76.6)	15.8 (10.5 to 22.3)	19.8 (13.2 to 27.7)
Brain and nervous system cancer	Controlled phase of brain and nervous system cancers	799.5 (726.2 to 886.5)	1,001.6 (915.3 to 1,100.2)	37.4 (23.8 to 54.7)	46.7 (30.0 to 68.2)
Brain and nervous system cancer	Metastatic phase of brain and nervous system cancers	90.9 (82.5 to 97.7)	113.6 (102.4 to 122.9)	39.3 (26.5 to 52.2)	48.8 (32.9 to 64.1)
Brain and nervous system cancer	Terminal phase of brain and nervous system cancers	16.0 (14.5 to 17.2)	19.9 (18.0 to 21.5)	8.6 (5.9 to 11.1)	10.8 (7.4 to 13.9)
Thyroid cancer	Diagnosis and primary therapy phase of thyroid cancer	39.3 (37.4 to 41.7)	78.9 (73.2 to 83.3)	11.0 (7.5 to 15.1)	21.3 (14.4 to 29.1)
Thyroid cancer	Controlled phase of thyroid cancer	1,497.1 (1,427.2 to 1,587.8)	3,013.8 (2,793.8 to 3,177.5)	69.0 (44.0 to 100.1)	138.1 (90.1 to 199.9)
Thyroid cancer	Metastatic phase of thyroid cancer	35.9 (34.0 to 39.1)	69.4 (63.0 to 75.0)	15.2 (10.5 to 20.0)	28.3 (19.4 to 36.9)
Thyroid cancer	Terminal phase of thyroid cancer	2.4 (2.3 to 2.6)	4.5 (4.0 to 4.8)	1.3 (0.9 to 1.7)	2.4 (1.7 to 3.1)
Mesothelioma	Diagnosis and primary therapy phase of mesothelioma	3.5 (3.4 to 3.6)	5.0 (4.8 to 5.2)	1.0 (0.7 to 1.4)	1.4 (1.0 to 1.9)
Mesothelioma	Controlled phase of mesothelioma	24.6 (23.8 to 25.5)	34.9 (33.5 to 36.6)	1.2 (0.7 to 1.7)	1.6 (1.0 to 2.4)
Mesothelioma	Metastatic phase of mesothelioma	12.8 (12.3 to 13.2)	17.9 (17.0 to 18.8)	5.5 (3.7 to 7.2)	7.6 (5.2 to 10.0)
Mesothelioma	Terminal phase of mesothelioma	2.1 (2.0 to 2.2)	3.0 (2.8 to 3.1)	1.1 (0.8 to 1.5)	1.6 (1.1 to 2.0)

Cause	Sequela	Prevalence in thousands (95% confidence interval)		YLDs in thousands (95% confidence interval)	
		2005	2015	2005	2015
Hodgkin lymphoma	Diagnosis and primary therapy phase of Hodgkin disease	15.5 (14.0 to 17.8)	18.3 (16.6 to 21.5)	4.5 (2.9 to 6.4)	5.3 (3.4 to 7.5)
Hodgkin lymphoma	Controlled phase of Hodgkin disease	433.6 (390.0 to 496.6)	513.8 (463.9 to 600.9)	20.4 (12.7 to 30.0)	24.1 (15.1 to 36.4)
Hodgkin lymphoma	Metastatic phase of Hodgkin disease	35.9 (32.3 to 41.2)	39.7 (35.9 to 47.4)	16.1 (10.7 to 22.1)	17.7 (11.8 to 24.2)
Hodgkin lymphoma	Terminal phase of Hodgkin disease	2.4 (2.2 to 2.8)	2.6 (2.3 to 3.1)	1.3 (0.9 to 1.7)	1.4 (1.0 to 1.8)
Non-Hodgkin lymphoma	Diagnosis and primary therapy phase of non-Hodgkin lymphoma	96.4 (87.2 to 104.2)	155.6 (135.4 to 166.5)	26.1 (17.8 to 36.1)	41.7 (28.4 to 57.5)
Non-Hodgkin lymphoma	Controlled phase of non-Hodgkin lymphoma	2,384.9 (2,159.1 to 2,600.1)	3,925.1 (3,420.4 to 4,203.2)	109.8 (70.4 to 158.8)	180.4 (114.0 to 259.5)
Non-Hodgkin lymphoma	Metastatic phase of non-Hodgkin lymphoma	124.5 (112.7 to 132.2)	183.1 (159.0 to 195.3)	51.5 (35.5 to 68.5)	75.1 (51.2 to 99.9)
Non-Hodgkin lymphoma	Terminal phase of non-Hodgkin lymphoma	19.6 (17.7 to 20.8)	28.5 (24.8 to 30.4)	10.4 (7.2 to 13.4)	15.0 (10.4 to 19.5)
Multiple myeloma	Diagnosis and primary therapy phase of multiple myeloma	19.0 (17.7 to 20.5)	29.9 (27.3 to 32.7)	5.2 (3.5 to 7.1)	8.1 (5.4 to 11.3)
Multiple myeloma	Controlled phase of multiple myeloma	156.7 (144.9 to 169.8)	251.6 (228.1 to 276.7)	7.2 (4.6 to 10.6)	11.5 (7.3 to 16.7)
Multiple myeloma	Metastatic phase of multiple myeloma	131.9 (125.9 to 138.3)	195.5 (182.6 to 208.1)	53.1 (36.8 to 69.5)	78.3 (53.9 to 102.3)
Multiple myeloma	Terminal phase of multiple myeloma	8.1 (7.8 to 8.4)	11.3 (10.7 to 11.8)	4.3 (3.0 to 5.6)	6.1 (4.2 to 7.8)
Acute lymphoid leukemia	Diagnosis and primary therapy phase of acute lymphoid leukemia	27.1 (21.5 to 34.5)	34.1 (27.4 to 41.9)	7.7 (4.9 to 11.6)	9.7 (6.2 to 14.2)
Acute lymphoid leukemia	Controlled phase of acute lymphoid leukemia	574.7 (450.6 to 747.6)	719.6 (569.4 to 899.2)	27.3 (16.8 to 43.1)	34.1 (20.4 to 53.0)
Acute lymphoid leukemia	Metastatic phase of acute lymphoid leukemia	89.8 (80.2 to 102.8)	114.1 (101.7 to 128.0)	39.0 (25.9 to 52.6)	49.3 (32.3 to 64.9)
Acute lymphoid leukemia	Terminal phase of acute lymphoid leukemia	6.4 (5.8 to 7.2)	7.7 (7.0 to 8.5)	3.4 (2.3 to 4.5)	4.2 (2.8 to 5.4)
Chronic lymphoid leukemia	Diagnosis and primary therapy phase of chronic lymphoid leukemia	26.7 (24.8 to 28.7)	35.4 (32.6 to 38.3)	7.5 (5.0 to 10.4)	9.8 (6.7 to 13.7)
Chronic lymphoid leukemia	Controlled phase of chronic lymphoid leukemia	508.3 (469.0 to 547.4)	678.5 (621.3 to 734.5)	23.4 (14.9 to 34.3)	31.2 (20.0 to 45.0)
Chronic lymphoid leukemia	Metastatic phase of chronic lymphoid leukemia	142.4 (135.1 to 150.3)	179.3 (167.9 to 191.4)	58.3 (40.1 to 76.6)	73.0 (50.7 to 95.4)
Chronic lymphoid leukemia	Terminal phase of chronic lymphoid leukemia	8.8 (8.4 to 9.4)	10.8 (10.2 to 11.5)	4.7 (3.3 to 6.0)	5.8 (4.0 to 7.5)
Acute myeloid leukemia	Diagnosis and primary therapy phase of acute myeloid leukemia	28.3 (24.5 to 32.3)	39.3 (34.5 to 44.9)	8.0 (5.2 to 11.4)	11.1 (7.4 to 15.6)
Acute myeloid leukemia	Controlled phase of acute myeloid leukemia	565.2 (483.2 to 658.1)	784.7 (684.8 to 905.4)	26.4 (16.8 to 39.5)	36.5 (23.0 to 53.4)
Acute myeloid leukemia	Metastatic phase of acute myeloid leukemia	121.0 (110.8 to 132.0)	165.4 (152.3 to 181.7)	50.1 (33.8 to 66.3)	68.0 (45.9 to 89.4)
Acute myeloid leukemia	Terminal phase of acute myeloid leukemia	7.5 (6.9 to 8.1)	9.8 (9.1 to 10.7)	4.0 (2.8 to 5.3)	5.3 (3.6 to 6.9)
Chronic myeloid leukemia	Diagnosis and primary therapy phase of chronic myeloid leukemia	9.5 (8.5 to 10.4)	11.7 (10.6 to 12.8)	2.7 (1.8 to 3.7)	3.3 (2.2 to 4.6)
Chronic myeloid leukemia	Controlled phase of chronic myeloid leukemia	178.1 (160.1 to 196.8)	221.4 (200.9 to 242.4)	8.3 (5.3 to 12.2)	10.3 (6.7 to 15.1)
Chronic myeloid leukemia	Metastatic phase of chronic myeloid leukemia	52.8 (49.3 to 56.7)	60.9 (56.8 to 65.4)	22.4 (15.3 to 29.7)	25.8 (17.5 to 33.8)
Chronic myeloid leukemia	Terminal phase of chronic myeloid leukemia	3.4 (3.1 to 3.7)	3.7 (3.5 to 4.0)	1.8 (1.3 to 2.3)	2.0 (1.4 to 2.5)
Other neoplasms	Diagnosis and primary therapy phase of other neoplasms	66.7 (62.5 to 70.8)	90.4 (81.6 to 97.0)	18.5 (12.6 to 25.7)	24.9 (16.9 to 34.8)
Other neoplasms	Controlled phase of other neoplasms	3,115.7 (2,924.3 to 3,331.8)	4,247.3 (3,854.8 to 4,573.1)	144.2 (92.3 to 210.3)	196.1 (124.4 to 289.2)
Other neoplasms	Metastatic phase of other neoplasms	156.2 (144.4 to 164.2)	206.4 (184.2 to 221.5)	64.3 (45.0 to 85.3)	84.5 (57.5 to 111.7)
Other neoplasms	Terminal phase of other neoplasms	25.4 (23.4 to 26.7)	33.4 (29.8 to 35.9)	13.5 (9.4 to 17.3)	17.7 (12.3 to 23.0)
Rheumatic heart disease	Rheumatic heart disease, without heart failure	34,150.0 (30,128.4 to 47,038.7)	32,236.8 (28,520.8 to 41,912.7)	1,607.7 (978.3 to 2,569.2)	1,518.6 (941.1 to 2,356.4)
Rheumatic heart disease	Mild heart failure due to rheumatic heart disease	227.6 (149.6 to 307.8)	295.3 (194.1 to 401.4)	8.9 (4.9 to 14.4)	11.5 (6.2 to 19.0)
Rheumatic heart disease	Moderate heart failure due to rheumatic heart disease	187.9 (136.1 to 246.2)	243.7 (176.6 to 320.9)	12.7 (7.5 to 19.5)	16.4 (9.7 to 25.1)
Rheumatic heart disease	Severe heart failure due to rheumatic heart disease	511.0 (436.6 to 585.9)	663.0 (566.8 to 763.9)	83.4 (55.9 to 116.4)	108.2 (72.8 to 150.9)
Ischemic heart disease	Asymptomatic ischemic heart disease following myocardial infarction	14,668.0 (13,311.8 to 15,975.6)	15,573.2 (13,753.5 to 17,416.6)	<0.05 (0.0 to <0.05)	<0.05 (0.0 to <0.05)
Ischemic heart disease	Acute myocardial infarction first 2 days	22.2 (20.4 to 24.3)	25.5 (23.3 to 28.0)	9.1 (6.1 to 12.2)	10.6 (7.0 to 14.3)
Ischemic heart disease	Acute myocardial infarction 3 to 28 days	288.6 (269.5 to 310.0)	331.4 (307.9 to 358.9)	19.5 (13.0 to 27.5)	22.5 (15.0 to 31.9)
Ischemic heart disease	Asymptomatic angina due to ischemic heart disease	17,012.0 (14,095.2 to 20,297.6)	21,996.3 (18,093.9 to 26,287.6)	<0.05 (0.0 to <0.05)	<0.05 (0.0 to <0.05)
Ischemic heart disease	Mild angina due to ischemic heart disease	13,465.8 (10,070.2 to 17,127.5)	17,410.1 (12,944.0 to 22,165.7)	416.7 (246.4 to 674.8)	539.6 (318.3 to 872.2)
Ischemic heart disease	Moderate angina due to ischemic heart disease	7,039.5 (4,954.1 to 9,394.8)	9,101.8 (6,389.4 to 12,200.0)	514.4 (291.2 to 789.8)	666.2 (375.9 to 1,026.0)
Ischemic heart disease	Severe angina due to ischemic heart disease	18,435.0 (14,861.6 to 22,572.2)	23,836.4 (19,166.7 to 29,305.9)	2,770.2 (1,744.8 to 3,955.6)	3,588.7 (2,248.7 to 5,151.2)
Ischemic heart disease	Mild heart failure due to ischemic heart disease	4,148.9 (2,709.8 to 5,582.5)	5,471.7 (3,570.8 to 7,348.0)	157.5 (88.8 to 255.1)	207.7 (116.8 to 339.4)
Ischemic heart disease	Moderate heart failure due to ischemic heart disease	3,425.3 (2,473.3 to 4,462.8)	4,517.2 (3,259.6 to 5,876.3)	223.2 (135.3 to 342.5)	294.3 (178.7 to 450.9)
Ischemic heart disease	Severe heart failure due to ischemic heart disease	9,316.4 (7,999.6 to 10,706.8)	12,286.7 (10,569.9 to 14,141.7)	1,475.1 (997.2 to 2,047.6)	1,945.1 (1,311.1 to 2,696.0)
Ischemic stroke	Asymptomatic chronic ischemic stroke	4,825.7 (4,218.2 to 5,461.9)	5,880.3 (5,144.4 to 6,656.4)	<0.05 (0.0 to <0.05)	<0.05 (0.0 to <0.05)
Ischemic stroke	Chronic ischemic stroke severity level 1	3,615.3 (2,480.4 to 4,855.0)	4,405.4 (3,028.5 to 5,915.8)	64.4 (32.0 to 111.4)	78.6 (38.9 to 136.3)
Ischemic stroke	Chronic ischemic stroke severity level 2	5,236.1 (4,220.2 to 6,287.2)	6,380.5 (5,140.2 to 7,636.4)	337.4 (218.5 to 484.9)	411.7 (267.1 to 594.0)
Ischemic stroke	Chronic ischemic stroke severity level 4	1,228.0 (872.1 to 1,669.8)	1,496.4 (1,061.4 to 2,026.8)	589.4 (365.5 to 891.2)	719.2 (443.7 to 1,084.2)
Ischemic stroke	Chronic ischemic stroke severity level 3	3,262.6 (2,700.8 to 3,896.7)	3,975.8 (3,288.9 to 4,746.6)	909.6 (596.4 to 1,287.6)	1,110.6 (732.1 to 1,574.3)
Ischemic stroke	Chronic ischemic stroke severity level 5	2,072.0 (1,083.2 to 3,367.0)	2,524.9 (1,323.8 to 4,107.4)	1,052.5 (507.5 to 1,797.0)	1,285.1 (619.8 to 2,197.1)
Ischemic stroke	Acute ischemic stroke severity level 1	53.3 (37.2 to 70.7)	62.3 (43.3 to 82.7)	1.0 (0.5 to 1.7)	1.1 (0.6 to 2.0)
Ischemic stroke	Acute ischemic stroke severity level 2	77.3 (61.2 to 93.4)	90.4 (71.4 to 109.8)	5.1 (3.2 to 7.4)	5.9 (3.7 to 8.7)
Ischemic stroke	Acute ischemic stroke severity level 4	18.1 (12.5 to 25.4)	21.2 (14.6 to 29.9)	9.7 (5.8 to 14.8)	11.4 (6.7 to 17.5)
Ischemic stroke	Acute ischemic stroke severity level 3	48.3 (39.0 to 59.3)	56.4 (45.5 to 69.4)	14.1 (8.9 to 19.9)	16.5 (10.4 to 23.9)
Ischemic stroke	Acute ischemic stroke severity level 5	30.5 (15.5 to 47.9)	35.6 (18.2 to 55.9)	16.9 (7.9 to 28.0)	19.8 (9.2 to 33.4)
Hemorrhagic stroke	Asymptomatic chronic hemorrhagic stroke	3,697.5 (3,243.1 to 4,168.2)	4,409.6 (3,867.9 to 4,983.0)	<0.05 (0.0 to <0.05)	<0.05 (0.0 to <0.05)
Hemorrhagic stroke	Chronic hemorrhagic stroke severity level 1	2,770.3 (1,893.1 to 3,728.4)	3,303.8 (2,267.0 to 4,440.0)	50.0 (24.7 to 86.8)	59.6 (29.4 to 103.6)

Cause	Sequela	Prevalence in thousands (95% confidence interval)		YLDs in thousands (95% confidence interval)	
		2005	2015	2005	2015
Hemorrhagic stroke	Chronic hemorrhagic stroke severity level 2	4,011.9 (3,239.2 to 4,812.8)	4,784.5 (3,850.4 to 5,731.5)	262.6 (170.2 to 381.1)	313.0 (203.3 to 454.4)
Hemorrhagic stroke	Chronic hemorrhagic stroke severity level 4	940.9 (665.8 to 1,273.1)	1,122.1 (792.9 to 1,520.9)	461.9 (283.2 to 698.7)	550.4 (338.2 to 832.1)
Hemorrhagic stroke	Chronic hemorrhagic stroke severity level 3	2,499.8 (2,066.5 to 2,982.6)	2,981.3 (2,460.6 to 3,551.4)	712.4 (463.5 to 1,008.6)	849.2 (552.7 to 1,203.9)
Hemorrhagic stroke	Chronic hemorrhagic stroke severity level 5	1,587.6 (826.4 to 2,577.2)	1,893.5 (984.4 to 3,075.3)	826.7 (398.8 to 1,425.9)	985.0 (477.1 to 1,698.8)
Hemorrhagic stroke	Acute hemorrhagic stroke severity level 1	35.8 (24.9 to 47.4)	40.9 (28.4 to 54.6)	0.7 (0.3 to 1.2)	0.8 (0.4 to 1.4)
Hemorrhagic stroke	Acute hemorrhagic stroke severity level 2	52.0 (41.7 to 63.5)	59.4 (47.2 to 72.5)	3.5 (2.2 to 5.2)	4.1 (2.6 to 6.0)
Hemorrhagic stroke	Acute hemorrhagic stroke severity level 4	12.2 (8.6 to 17.2)	13.9 (9.7 to 19.6)	6.7 (3.9 to 10.2)	7.7 (4.5 to 11.7)
Hemorrhagic stroke	Acute hemorrhagic stroke severity level 3	32.5 (26.3 to 39.6)	37.1 (29.8 to 45.2)	10.1 (6.4 to 14.5)	11.6 (7.3 to 16.7)
Hemorrhagic stroke	Acute hemorrhagic stroke severity level 5	20.5 (10.5 to 31.8)	23.4 (12.1 to 36.4)	12.0 (5.5 to 20.0)	13.8 (6.3 to 23.0)
Hypertensive heart disease	Mild heart failure due to hypertensive heart disease	1,090.4 (714.2 to 1,467.0)	1,494.6 (981.0 to 2,012.8)	41.5 (23.2 to 66.6)	56.9 (31.9 to 90.9)
Hypertensive heart disease	Moderate heart failure due to hypertensive heart disease	900.4 (648.9 to 1,170.8)	1,234.2 (887.0 to 1,607.1)	58.8 (35.3 to 89.4)	80.7 (48.6 to 122.5)
Hypertensive heart disease	Severe heart failure due to hypertensive heart disease	2,449.6 (2,085.6 to 2,847.9)	3,357.5 (2,858.4 to 3,915.0)	388.2 (258.3 to 540.5)	532.4 (356.1 to 739.3)
Cardiomyopathy and myocarditis	Acute myocarditis	128.8 (112.5 to 148.6)	156.3 (137.1 to 179.6)	6.4 (4.0 to 9.6)	7.8 (4.8 to 11.6)
Cardiomyopathy and myocarditis	Mild heart failure due to cardiomyopathy and myocarditis	460.2 (303.3 to 616.9)	584.7 (383.9 to 782.3)	17.9 (10.0 to 28.7)	22.6 (12.6 to 36.3)
Cardiomyopathy and myocarditis	Moderate heart failure due to cardiomyopathy and myocarditis	380.0 (271.9 to 495.2)	482.7 (345.7 to 629.7)	25.4 (15.2 to 38.4)	32.2 (19.1 to 48.2)
Cardiomyopathy and myocarditis	Severe heart failure due to cardiomyopathy and myocarditis	1,033.5 (887.0 to 1,187.1)	1,313.0 (1,126.7 to 1,514.9)	168.0 (112.8 to 236.3)	212.5 (142.8 to 300.3)
Atrial fibrillation and flutter	Asymptomatic atrial fibrillation and flutter	15,582.2 (13,988.7 to 17,461.0)	19,974.7 (17,898.0 to 22,463.3)	<0.05 (0.0 to <0.05)	<0.05 (0.0 to <0.05)
Atrial fibrillation and flutter	Symptomatic atrial fibrillation and flutter	10,390.7 (9,295.2 to 11,663.5)	13,319.7 (11,871.9 to 14,969.5)	2,054.5 (1,384.5 to 2,848.8)	2,634.6 (1,782.6 to 3,637.3)
Peripheral vascular disease	Asymptomatic peripheral vascular disease	82,714.9 (71,489.1 to 95,074.2)	111,131.9 (96,353.3 to 127,669.6)	<0.05 (0.0 to <0.05)	<0.05 (0.0 to <0.05)
Peripheral vascular disease	Symptomatic claudication due to peripheral vascular disease	32,394.3 (26,198.5 to 40,442.0)	43,518.7 (35,435.1 to 53,838.6)	426.0 (200.8 to 793.8)	572.8 (272.1 to 1,056.2)
Endocarditis	Moderate endocarditis	12.8 (12.0 to 13.6)	15.7 (14.6 to 16.9)	0.6 (0.4 to 1.0)	0.8 (0.5 to 1.2)
Endocarditis	Severe endocarditis	1.0 (0.9 to 1.0)	1.2 (1.1 to 1.3)	0.1 (0.1 to 0.2)	0.2 (0.1 to 0.2)
Endocarditis	Mild heart failure due to endocarditis	19.8 (12.9 to 26.9)	24.3 (15.9 to 32.9)	0.8 (0.4 to 1.3)	1.0 (0.5 to 1.5)
Endocarditis	Moderate heart failure due to endocarditis	16.3 (11.8 to 21.1)	20.0 (14.4 to 26.0)	1.1 (0.7 to 1.7)	1.4 (0.8 to 2.1)
Endocarditis	Severe heart failure due to endocarditis	44.4 (37.8 to 51.8)	54.5 (46.0 to 63.7)	7.4 (4.9 to 10.4)	9.0 (6.0 to 12.8)
Other cardiovascular and circulatory diseases	Mild heart failure due to other cardiovascular diseases	205.8 (136.2 to 278.6)	276.7 (182.5 to 373.7)	7.8 (4.4 to 12.6)	10.5 (5.9 to 16.9)
Other cardiovascular and circulatory diseases	Moderate heart failure due to other cardiovascular diseases	169.9 (121.9 to 221.9)	228.4 (164.3 to 298.4)	11.1 (6.7 to 17.0)	14.9 (9.0 to 22.8)
Other cardiovascular and circulatory diseases	Severe heart failure due to other cardiovascular diseases	462.3 (392.9 to 540.8)	621.4 (527.2 to 729.3)	73.1 (48.8 to 102.1)	98.3 (65.3 to 137.6)
Other cardiovascular and circulatory diseases	Asymptomatic other cardiovascular diseases	19,847.9 (17,533.4 to 22,483.2)	24,617.8 (21,803.1 to 27,883.7)	<0.05 (0.0 to <0.05)	<0.05 (0.0 to <0.05)
Other cardiovascular and circulatory diseases	Mild other cardiovascular diseases	21,705.3 (17,330.6 to 26,232.4)	26,922.2 (21,564.7 to 32,524.6)	848.1 (522.3 to 1,296.1)	1,050.0 (648.1 to 1,608.4)
Other cardiovascular and circulatory diseases	Moderate other cardiovascular diseases	10,554.4 (7,742.6 to 13,797.0)	13,090.5 (9,595.5 to 17,079.4)	705.6 (420.4 to 1,070.8)	873.3 (522.7 to 1,325.0)
Other cardiovascular and circulatory diseases	Severe other cardiovascular diseases	19,826.2 (16,415.4 to 23,355.2)	24,590.7 (20,434.5 to 28,868.3)	3,252.5 (2,130.9 to 4,572.5)	4,023.7 (2,649.3 to 5,654.7)
Chronic obstructive pulmonary disease	Asymptomatic chronic obstructive pulmonary disease	64,892.5 (58,153.8 to 72,063.6)	76,989.9 (68,925.8 to 87,086.2)	<0.05 (0.0 to <0.05)	<0.05 (0.0 to <0.05)
Chronic obstructive pulmonary disease	Mild chronic obstructive pulmonary disease	53,192.6 (44,949.4 to 60,971.1)	61,532.6 (51,825.2 to 71,082.8)	980.0 (519.1 to 1,622.9)	1,132.0 (595.4 to 1,865.6)
Chronic obstructive pulmonary disease	Moderate chronic obstructive pulmonary disease	12,487.2 (9,788.4 to 15,286.4)	14,446.0 (11,187.2 to 17,759.2)	2,579.6 (1,507.8 to 3,949.3)	2,978.2 (1,718.2 to 4,550.2)
Chronic obstructive pulmonary disease	Severe chronic obstructive pulmonary disease without heart failure	14,186.1 (8,517.9 to 21,055.9)	15,638.2 (9,190.9 to 23,714.2)	5,049.9 (3,976.2 to 6,024.0)	5,561.3 (4,296.8 to 6,686.0)
Chronic obstructive pulmonary disease	Mild heart failure due to severe chronic obstructive pulmonary disease	1,070.0 (675.2 to 1,461.0)	1,443.3 (907.7 to 1,991.1)	390.8 (238.3 to 562.4)	527.8 (323.6 to 747.0)
Chronic obstructive pulmonary disease	Moderate heart failure due to severe chronic obstructive pulmonary disease	883.0 (636.0 to 1,163.3)	1,190.9 (854.2 to 1,579.6)	335.6 (219.8 to 466.1)	453.5 (298.8 to 627.6)
Chronic obstructive pulmonary disease	Severe heart failure due to severe chronic obstructive pulmonary disease	2,403.4 (1,963.3 to 2,873.2)	3,242.1 (2,591.9 to 3,909.1)	1,032.2 (789.5 to 1,245.6)	1,394.2 (1,082.2 to 1,675.6)
Silicosis	Asymptomatic silicosis	77.2 (50.4 to 108.3)	91.1 (59.4 to 128.3)	<0.05 (0.0 to <0.05)	<0.05 (0.0 to <0.05)
Silicosis	Mild silicosis	109.5 (78.0 to 145.1)	129.3 (91.8 to 170.9)	2.1 (1.0 to 3.8)	2.4 (1.2 to 4.6)
Silicosis	Moderate silicosis	43.4 (16.8 to 74.3)	51.2 (19.9 to 88.2)	9.5 (3.5 to 17.9)	11.2 (4.2 to 21.2)
Silicosis	Severe silicosis without heart failure	100.7 (63.8 to 138.9)	117.4 (74.1 to 162.9)	39.0 (21.6 to 61.7)	45.6 (24.9 to 72.0)
Silicosis	Mild heart failure due to severe silicosis	2.6 (1.7 to 3.5)	3.4 (2.2 to 4.7)	1.1 (0.6 to 1.7)	1.5 (0.8 to 2.2)
Silicosis	Moderate heart failure due to severe silicosis	2.1 (1.5 to 2.8)	2.8 (2.0 to 3.8)	0.9 (0.6 to 1.4)	1.3 (0.7 to 1.9)
Silicosis	Severe heart failure due to severe silicosis	5.8 (4.8 to 6.9)	7.7 (6.2 to 9.2)	2.8 (1.9 to 3.8)	3.7 (2.5 to 5.0)
Asbestosis	Asymptomatic asbestosis	27.2 (18.0 to 38.9)	35.6 (23.6 to 50.9)	<0.05 (0.0 to <0.05)	<0.05 (0.0 to <0.05)
Asbestosis	Mild asbestosis	38.6 (27.9 to 51.3)	50.5 (36.3 to 66.9)	0.7 (0.4 to 1.3)	0.9 (0.5 to 1.7)
Asbestosis	Moderate asbestosis	15.3 (5.9 to 26.5)	20.0 (7.7 to 34.7)	3.3 (1.2 to 6.3)	4.3 (1.5 to 8.2)
Asbestosis	Severe asbestosis without heart failure	37.2 (23.9 to 50.1)	48.5 (31.1 to 65.4)	14.1 (8.0 to 21.6)	18.3 (10.5 to 27.9)
Asbestosis	Mild heart failure due to severe asbestosis	0.5 (0.3 to 0.7)	0.7 (0.4 to 0.9)	0.2 (0.1 to 0.3)	0.3 (0.2 to 0.4)
Asbestosis	Moderate heart failure due to severe asbestosis	0.4 (0.3 to 0.5)	0.6 (0.4 to 0.7)	0.2 (0.1 to 0.3)	0.3 (0.2 to 0.4)
Asbestosis	Severe heart failure due to severe asbestosis	1.1 (1.0 to 1.3)	1.5 (1.3 to 1.8)	0.6 (0.4 to 0.7)	0.8 (0.5 to 1.0)
Coal workers pneumoconiosis	Asymptomatic coal workers pneumoconiosis	13.9 (9.1 to 19.9)	19.0 (12.4 to 27.1)	<0.05 (0.0 to <0.05)	<0.05 (0.0 to <0.05)
Coal workers pneumoconiosis	Mild coal workers pneumoconiosis	19.7 (14.0 to 26.4)	27.0 (19.2 to 36.2)	0.4 (0.2 to 0.7)	0.5 (0.2 to 0.9)
Coal workers pneumoconiosis	Moderate coal workers pneumoconiosis	7.8 (3.1 to 13.4)	10.7 (4.2 to 18.4)	1.7 (0.6 to 3.2)	2.3 (0.9 to 4.4)
Coal workers pneumoconiosis	Severe coal workers pneumoconiosis without heart failure	18.7 (12.0 to 25.7)	25.6 (16.4 to 35.4)	7.1 (3.9 to 10.8)	9.6 (5.3 to 14.7)
Coal workers pneumoconiosis	Mild heart failure due to severe coal workers pneumoconiosis	0.3 (0.2 to 0.4)	0.5 (0.3 to 0.6)	0.1 (0.1 to 0.2)	0.2 (0.1 to 0.3)
Coal workers pneumoconiosis	Moderate heart failure due to severe coal workers pneumoconiosis	0.3 (0.2 to 0.4)	0.4 (0.3 to 0.5)	0.1 (0.1 to 0.2)	0.2 (0.1 to 0.3)

Cause	Sequela	Prevalence in thousands (95% confidence interval)		YLDs in thousands (95% confidence interval)	
		2005	2015	2005	2015
Coal workers pneumoconiosis	Severe heart failure due to severe coal workers pneumoconiosis	0.7 (0.6 to 0.9)	1.0 (0.8 to 1.2)	0.4 (0.2 to 0.5)	0.5 (0.4 to 0.7)
Other pneumoconiosis	Asymptomatic other pneumoconiosis	422.3 (275.4 to 599.1)	540.2 (348.6 to 774.8)	<0.05 (0.0 to <0.05)	<0.05 (0.0 to <0.05)
Other pneumoconiosis	Mild other pneumoconiosis	599.1 (423.8 to 801.2)	766.2 (541.4 to 1,023.3)	11.2 (5.5 to 21.1)	14.3 (7.0 to 27.0)
Other pneumoconiosis	Moderate other pneumoconiosis	237.6 (93.2 to 407.4)	303.9 (119.7 to 522.8)	50.7 (19.2 to 96.3)	64.5 (23.9 to 121.9)
Other pneumoconiosis	Severe other pneumoconiosis without heart failure	598.6 (390.0 to 818.8)	764.0 (493.0 to 1,034.4)	223.7 (131.0 to 340.5)	284.9 (167.9 to 434.8)
Other pneumoconiosis	Mild heart failure due to severe other pneumoconiosis	2.5 (1.6 to 3.4)	3.5 (2.3 to 4.8)	1.1 (0.6 to 1.6)	1.5 (0.9 to 2.3)
Other pneumoconiosis	Moderate heart failure due to severe other pneumoconiosis	2.1 (1.5 to 2.7)	2.9 (2.1 to 3.8)	0.9 (0.5 to 1.4)	1.3 (0.8 to 1.9)
Other pneumoconiosis	Severe heart failure due to severe other pneumoconiosis	5.6 (4.7 to 6.5)	7.9 (6.6 to 9.2)	2.7 (1.9 to 3.6)	3.8 (2.6 to 5.0)
Asthma	Asymptomatic asthma	99,044.0 (85,042.6 to 114,899.5)	108,461.1 (92,842.5 to 126,250.5)	<0.05 (0.0 to <0.05)	<0.05 (0.0 to <0.05)
Asthma	Controlled asthma	66,396.4 (46,053.0 to 87,675.9)	72,707.7 (50,401.0 to 96,100.2)	940.7 (436.4 to 1,779.3)	1,028.9 (475.3 to 1,940.9)
Asthma	Partially controlled asthma	73,432.4 (56,853.3 to 92,586.1)	80,416.6 (62,120.9 to 101,597.8)	2,511.7 (1,494.6 to 4,006.2)	2,747.6 (1,627.6 to 4,403.9)
Asthma	Uncontrolled asthma	88,224.1 (69,165.8 to 109,344.1)	96,612.5 (75,998.9 to 119,370.3)	11,085.3 (6,916.2 to 16,343.9)	12,122.3 (7,546.0 to 17,836.4)
Interstitial lung disease and pulmonary sarcoidosis	Asymptomatic interstitial lung disease and pulmonary sarcoidosis	303.6 (208.0 to 422.4)	381.8 (262.3 to 530.9)	<0.05 (0.0 to <0.05)	<0.05 (0.0 to <0.05)
Interstitial lung disease and pulmonary sarcoidosis	Mild interstitial lung disease and pulmonary sarcoidosis	693.7 (560.2 to 842.7)	872.6 (703.8 to 1,060.0)	12.8 (6.7 to 22.5)	16.0 (8.4 to 28.4)
Interstitial lung disease and pulmonary sarcoidosis	Moderate interstitial lung disease and pulmonary sarcoidosis	201.8 (104.3 to 309.3)	253.9 (131.8 to 390.5)	42.1 (19.2 to 71.9)	52.8 (24.0 to 90.3)
Interstitial lung disease and pulmonary sarcoidosis	Severe interstitial lung disease and pulmonary sarcoidosis without heart failure	174.0 (84.6 to 278.2)	210.4 (100.1 to 343.2)	66.2 (28.0 to 114.1)	79.7 (33.3 to 138.9)
Interstitial lung disease and pulmonary sarcoidosis	Mild heart failure due to severe interstitial lung disease and pulmonary sarcoidosis	36.9 (22.2 to 54.2)	48.5 (29.0 to 71.2)	14.8 (7.6 to 23.6)	19.5 (10.0 to 31.3)
Interstitial lung disease and pulmonary sarcoidosis	Moderate heart failure due to severe interstitial lung disease and pulmonary sarcoidosis	30.4 (19.9 to 42.8)	40.0 (26.2 to 56.1)	12.9 (7.3 to 19.6)	16.9 (9.5 to 25.8)
Interstitial lung disease and pulmonary sarcoidosis	Severe heart failure due to severe interstitial lung disease and pulmonary sarcoidosis	82.8 (58.7 to 106.6)	108.9 (77.6 to 140.9)	37.9 (24.0 to 52.9)	49.8 (31.4 to 69.4)
Cirrhosis and other chronic liver diseases due to hepatitis B	Cirrhosis and other chronic liver diseases due to hepatitis B	610.1 (569.1 to 648.8)	796.5 (737.6 to 857.3)	100.1 (68.5 to 137.3)	130.6 (88.7 to 178.7)
Cirrhosis and other chronic liver diseases due to hepatitis C	Cirrhosis and other chronic liver diseases due to hepatitis C	503.1 (468.2 to 538.8)	680.8 (628.3 to 734.2)	82.4 (57.8 to 112.2)	111.4 (78.2 to 152.6)
Cirrhosis and other chronic liver diseases due to alcohol use	Cirrhosis and other chronic liver diseases due to alcohol	594.1 (557.1 to 634.6)	815.6 (758.7 to 877.2)	97.6 (68.1 to 134.4)	133.9 (92.5 to 185.1)
Cirrhosis and other chronic liver diseases due to other causes	Cirrhosis and other chronic liver diseases due to other cause	606.2 (578.5 to 633.0)	750.3 (714.3 to 785.9)	101.4 (70.6 to 139.7)	125.0 (87.5 to 172.2)
Peptic ulcer disease	Peptic ulcer disease, symptomatic episodes	4,754.2 (4,427.6 to 5,091.2)	4,977.7 (4,577.6 to 5,379.5)	496.5 (343.1 to 671.4)	518.7 (357.9 to 703.2)
Peptic ulcer disease	Mild anemia due to peptic ulcer disease	32,802.6 (32,258.7 to 33,247.5)	35,234.2 (34,768.0 to 35,659.6)	114.9 (41.6 to 248.1)	123.2 (44.3 to 266.4)
Peptic ulcer disease	Moderate anemia due to peptic ulcer disease	28,726.6 (28,420.6 to 29,087.8)	29,718.3 (29,383.2 to 30,046.2)	1,374.2 (921.0 to 1,969.0)	1,418.4 (953.0 to 2,031.6)
Peptic ulcer disease	Severe anemia due to peptic ulcer disease	2,469.6 (2,419.1 to 2,537.2)	2,114.0 (2,069.2 to 2,166.0)	329.8 (230.7 to 450.6)	281.4 (196.9 to 383.0)
Gastritis and duodenitis	Gastritis and duodenitis, symptomatic episodes	3,415.3 (3,082.0 to 3,783.4)	3,941.0 (3,547.0 to 4,358.9)	360.3 (243.2 to 492.3)	416.0 (277.1 to 567.9)
Gastritis and duodenitis	Mild anemia due to gastritis and duodenitis	68,616.6 (67,197.6 to 69,725.6)	81,086.0 (78,782.2 to 82,964.5)	242.5 (86.8 to 528.9)	286.3 (102.8 to 619.3)
Gastritis and duodenitis	Moderate anemia due to gastritis and duodenitis	61,057.1 (60,317.2 to 61,870.6)	69,952.4 (68,250.9 to 72,034.6)	2,953.8 (1,978.0 to 4,224.5)	3,386.3 (2,256.4 to 4,859.4)
Gastritis and duodenitis	Severe anemia due to gastritis and duodenitis	6,319.6 (6,006.4 to 6,740.3)	6,021.7 (5,683.1 to 6,492.7)	861.6 (598.7 to 1,173.7)	824.4 (559.2 to 1,133.9)
Appendicitis	Appendicitis	386.7 (362.5 to 412.5)	442.1 (414.6 to 472.3)	118.9 (80.3 to 161.2)	136.0 (92.0 to 184.6)
Paralytic ileus and intestinal obstruction	Paralytic ileus and intestinal obstruction	100.7 (93.8 to 109.0)	119.7 (110.7 to 130.4)	31.5 (21.3 to 42.7)	37.3 (25.1 to 51.1)
Inguinal, femoral, and abdominal hernia	Inguinal, femoral, and abdominal hernia cases	17,346.1 (15,617.0 to 19,113.5)	18,476.7 (16,577.2 to 20,339.8)	183.8 (91.0 to 344.4)	195.8 (97.0 to 363.1)
Inflammatory bowel disease	Ulcerative colitis	3,813.4 (3,451.1 to 4,191.0)	4,481.7 (4,052.4 to 4,921.8)	808.5 (552.2 to 1,125.2)	948.8 (649.5 to 1,322.8)
Inflammatory bowel disease	Crohn's disease	5,888.1 (5,299.9 to 6,452.0)	6,741.7 (6,073.0 to 7,386.6)	1,257.2 (851.0 to 1,745.2)	1,438.3 (975.2 to 1,992.5)
Vascular intestinal disorders	Vascular intestinal disorders	28.4 (26.5 to 30.5)	35.1 (32.4 to 38.1)	8.9 (6.0 to 12.0)	11.0 (7.4 to 14.8)
Gallbladder and biliary diseases	Gallbladder and biliary diseases	4,836.9 (4,469.2 to 5,242.8)	5,656.5 (5,199.3 to 6,168.1)	514.4 (349.4 to 711.4)	601.0 (409.4 to 826.4)
Pancreatitis	Pancreatitis cases	819.7 (763.4 to 877.9)	1,018.2 (941.6 to 1,097.4)	242.0 (163.5 to 326.2)	300.0 (203.4 to 406.7)
Alzheimer disease and other dementias	Mild Alzheimer disease and other dementias	22,910.5 (19,892.9 to 26,139.2)	31,362.7 (27,125.4 to 35,930.5)	1,426.9 (943.6 to 2,041.2)	1,954.7 (1,289.6 to 2,796.4)
Alzheimer disease and other dementias	Moderate Alzheimer disease and other dementias	7,327.7 (6,162.3 to 8,824.2)	10,212.5 (8,582.4 to 12,265.0)	2,334.3 (1,589.2 to 3,151.9)	3,256.4 (2,214.7 to 4,423.3)
Alzheimer disease and other dementias	Severe Alzheimer disease and other dementias	3,141.7 (2,298.4 to 4,116.1)	4,381.0 (3,166.4 to 5,802.8)	1,175.4 (761.2 to 1,702.7)	1,640.1 (1,053.5 to 2,384.0)
Parkinson disease	Mild Parkinson disease	2,733.6 (2,426.4 to 3,077.9)	3,597.4 (3,158.4 to 4,087.9)	25.4 (12.3 to 48.1)	33.4 (16.3 to 62.2)
Parkinson disease	Moderate Parkinson disease	1,686.9 (1,408.8 to 1,986.9)	2,219.9 (1,852.3 to 2,638.9)	394.2 (268.7 to 562.7)	518.3 (346.6 to 745.7)
Parkinson disease	Severe Parkinson disease	285.7 (179.8 to 413.6)	376.0 (236.5 to 547.0)	141.7 (79.7 to 218.0)	186.1 (103.9 to 287.2)
Epilepsy	Seizure-free, treated epilepsy	3,227.3 (2,417.3 to 3,959.7)	4,964.3 (3,837.3 to 5,991.2)	151.8 (90.0 to 232.8)	233.0 (141.8 to 353.9)
Epilepsy	Less severe epilepsy	9,571.0 (8,592.3 to 10,634.6)	12,711.8 (11,320.7 to 14,267.6)	2,355.9 (1,558.6 to 3,294.9)	3,123.0 (2,074.2 to 4,359.9)
Epilepsy	Severe epilepsy	8,234.9 (7,565.8 to 8,949.3)	5,738.4 (5,172.4 to 6,364.1)	4,208.2 (2,890.0 to 5,417.1)	2,930.9 (2,016.3 to 3,829.3)
Multiple sclerosis	Mild multiple sclerosis	903.6 (799.7 to 1,017.3)	1,076.0 (949.9 to 1,215.3)	153.2 (103.5 to 214.5)	182.1 (123.2 to 256.7)
Multiple sclerosis	Moderate multiple sclerosis	463.7 (379.4 to 552.2)	552.2 (451.8 to 661.6)	196.7 (134.3 to 269.3)	233.6 (156.8 to 319.6)
Multiple sclerosis	Severe multiple sclerosis	322.4 (243.4 to 408.3)	383.9 (291.7 to 488.3)	212.2 (143.2 to 285.1)	251.9 (168.6 to 343.9)
Motor neuron disease	Mild motor impairment, mild respiratory problems, and speech problems due to motor neuron disease	3.0 (2.7 to 3.2)	3.6 (3.3 to 4.0)	0.2 (0.1 to 0.4)	0.3 (0.2 to 0.4)
Motor neuron disease	Mild motor impairment, moderate respiratory problems, and speech problems due to motor neuron disease	0.4 (0.4 to 0.5)	0.5 (0.4 to 0.7)	0.1 (0.1 to 0.2)	0.1 (0.1 to 0.2)
Motor neuron disease	Mild motor impairment, severe respiratory problems and speech problems due to motor neuron disease	0.1 (0.1 to 0.2)	0.2 (0.1 to 0.2)	0.1 (0.0 to 0.1)	0.1 (0.0 to 0.1)
Motor neuron disease	Mild motor impairment and speech problems due to motor neuron disease	3.4 (3.1 to 3.7)	4.2 (3.8 to 4.5)	0.2 (0.1 to 0.3)	0.3 (0.2 to 0.4)
Motor neuron disease	Moderate motor impairment, mild respiratory problems, and speech problems due to motor neuron disease	5.6 (5.2 to 6.0)	6.9 (6.3 to 7.4)	0.7 (0.5 to 1.0)	0.9 (0.6 to 1.3)
Motor neuron disease	Moderate motor impairment, moderate respiratory problems, and speech problems due to motor neuron disease	1.2 (1.1 to 1.3)	1.4 (1.3 to 1.6)	0.4 (0.3 to 0.5)	0.4 (0.3 to 0.6)
Motor neuron disease	Moderate motor impairment, severe respiratory problems, and speech problems due to motor neuron disease	0.5 (0.4 to 0.6)	0.6 (0.5 to 0.7)	0.2 (0.2 to 0.3)	0.3 (0.2 to 0.4)
Motor neuron disease	Severe motor impairment, mild respiratory problems, and speech problems due to motor neuron disease	8.7 (8.1 to 9.3)	10.7 (9.9 to 11.5)	3.9 (2.7 to 5.1)	4.7 (3.4 to 6.2)

Cause	Sequela	Prevalence in thousands (95% confidence interval)		YLDs in thousands (95% confidence interval)	
		2005	2015	2005	2015
Motor neuron disease	Severe motor impairment, moderate respiratory problems, and speech problems due to motor neuron disease	3.7<br>(3.4 to 4.0)	4.6<br>(4.2 to 5.0)	2.1<br>(1.5 to 2.7)	2.5<br>(1.9 to 3.3)
Motor neuron disease	Severe motor impairment, severe respiratory problems, and speech problems due to motor neuron disease	2.3<br>(2.1 to 2.5)	2.8<br>(2.5 to 3.1)	1.5<br>(1.1 to 1.9)	1.8<br>(1.4 to 2.3)
Motor neuron disease	Mild motor impairment and mild respiratory problems due to motor neuron disease	3.7<br>(3.4 to 4.1)	4.6<br>(4.2 to 5.0)	0.3<br>(0.2 to 0.4)	0.4<br>(0.2 to 0.5)
Motor neuron disease	Mild motor impairment and severe respiratory problems due to motor neuron disease	0.1<br>(0.1 to 0.2)	0.1<br>(0.1 to 0.2)	0.1<br>(0.0 to 0.1)	0.1<br>(0.0 to 0.1)
Motor neuron disease	Moderate motor impairment and mild respiratory problems due to motor neuron disease	11.2<br>(10.4 to 12.0)	13.7<br>(12.7 to 14.7)	0.3<br>(0.2 to 0.6)	0.4<br>(0.2 to 0.7)
Motor neuron disease	Moderate motor impairment and moderate respiratory problems due to motor neuron disease	2.2<br>(1.9 to 2.4)	2.6<br>(2.4 to 2.9)	0.5<br>(0.3 to 0.7)	0.6<br>(0.4 to 0.9)
Motor neuron disease	Moderate motor impairment and severe respiratory problems due to motor neuron disease	0.7<br>(0.6 to 0.8)	0.8<br>(0.7 to 1.0)	0.3<br>(0.2 to 0.4)	0.3<br>(0.2 to 0.5)
Motor neuron disease	Severe motor impairment and mild respiratory problems due to motor neuron disease	11.5<br>(10.7 to 12.2)	14.0<br>(13.0 to 15.1)	4.7<br>(3.3 to 6.3)	5.8<br>(4.0 to 7.7)
Motor neuron disease	Severe motor impairment and moderate respiratory problems due to motor neuron disease	3.3<br>(3.0 to 3.6)	4.1<br>(3.7 to 4.4)	1.8<br>(1.3 to 2.3)	2.2<br>(1.5 to 2.8)
Motor neuron disease	Severe motor impairment and severe respiratory problems due to motor neuron disease	1.8<br>(1.6 to 2.0)	2.2<br>(1.9 to 2.4)	1.1<br>(0.8 to 1.4)	1.4<br>(1.0 to 1.8)
Motor neuron disease	Moderate motor impairment and speech problems due to motor neuron disease	5.0<br>(4.7 to 5.4)	6.2<br>(5.7 to 6.7)	0.5<br>(0.4 to 0.8)	0.7<br>(0.4 to 1.0)
Motor neuron disease	Severe motor impairment and speech problems due to motor neuron disease	7.9<br>(7.4 to 8.5)	9.7<br>(8.9 to 10.5)	3.4<br>(2.4 to 4.6)	4.2<br>(2.9 to 5.6)
Motor neuron disease	Mild motor impairment due to motor neuron disease	17.2<br>(16.2 to 18.4)	21.1<br>(19.6 to 22.6)	0.2<br>(0.1 to 0.3)	0.2<br>(0.1 to 0.4)
Motor neuron disease	Moderate motor impairment due to motor neuron disease	33.4<br>(31.5 to 35.5)	40.9<br>(38.2 to 43.9)	2.0<br>(1.3 to 2.9)	2.4<br>(1.6 to 3.5)
Motor neuron disease	Severe motor impairment due to motor neuron disease	25.0<br>(23.4 to 26.5)	30.5<br>(28.4 to 32.7)	9.6<br>(6.5 to 13.0)	11.7<br>(7.9 to 15.7)
Motor neuron disease	Mild respiratory problems and speech problems due to motor neuron disease	1.9<br>(1.7 to 2.1)	2.4<br>(2.1 to 2.6)	0.1<br>(0.1 to 0.2)	0.2<br>(0.1 to 0.3)
Motor neuron disease	Moderate respiratory problems and speech problems due to motor neuron disease	0.2<br>(0.2 to 0.3)	0.3<br>(0.2 to 0.4)	0.1<br>(0.0 to 0.1)	0.1<br>(0.0 to 0.1)
Motor neuron disease	Severe respiratory problems and speech problems due to motor neuron disease	<0.05<br>(0.0 to 0.1)	<0.05<br>(0.0 to 0.1)	<0.05<br>(0.0 to <0.05)	<0.05<br>(0.0 to <0.05)
Motor neuron disease	Mild respiratory problems due to motor neuron disease	1.1<br>(0.9 to 1.2)	1.3<br>(1.1 to 1.5)	<0.05<br>(0.0 to <0.05)	<0.05<br>(0.0 to <0.05)
Motor neuron disease	Moderate respiratory problems due to motor neuron disease	0.1<br>(0.0 to 0.1)	0.1<br>(0.1 to 0.1)	<0.05<br>(0.0 to <0.05)	<0.05<br>(0.0 to <0.05)
Motor neuron disease	Severe respiratory problems due to motor neuron disease	<0.05<br>(0.0 to <0.05)	<0.05<br>(0.0 to <0.05)	<0.05<br>(0.0 to <0.05)	<0.05<br>(0.0 to <0.05)
Motor neuron disease	Speech problems due to motor neuron disease	4.1<br>(3.7 to 4.4)	5.0<br>(4.5 to 5.4)	0.2<br>(0.1 to 0.3)	0.3<br>(0.2 to 0.4)
Motor neuron disease	Diagnosis of motor neuron disease	6.2<br>(5.7 to 6.7)	7.6<br>(7.0 to 8.2)	0.1<br>(0.0 to 0.1)	0.1<br>(0.0 to 0.2)
Migraine	Asymptomatic migraine	761,576.7<br>(689,095.7 to 847,054.7)	877,922.4<br>(793,979.8 to 971,652.3)	<0.05<br>(0.0 to <0.05)	<0.05<br>(0.0 to <0.05)
Migraine	Symptomatic migraine	70,148.9<br>(51,093.9 to 90,985.5)	80,866.8<br>(58,781.1 to 104,710.8)	28,538.7<br>(17,586.8 to 42,402.1)	32,898.8<br>(20,303.6 to 48,883.2)
Tension-type headache	Asymptomatic tension-type headache	1,251,733.8<br>(1,112,665.8 to 1,401,102.3)	1,442,889.6<br>(1,285,206.3 to 1,609,466.0)	<0.05<br>(0.0 to <0.05)	<0.05<br>(0.0 to <0.05)
Tension-type headache	Symptomatic tension-type headache	54,655.8<br>(31,397.8 to 84,372.6)	63,002.7<br>(36,334.3 to 97,098.7)	1,961.3<br>(916.1 to 3,621.9)	2,260.5<br>(1,058.1 to 4,169.7)
Medication overuse headache	Asymptomatic medication overuse headache	11,842.3<br>(10,018.5 to 13,963.8)	14,088.7<br>(11,851.9 to 16,582.2)	<0.05<br>(0.0 to <0.05)	<0.05<br>(0.0 to <0.05)
Medication overuse headache	Symptomatic medication overuse headache	37,291.9<br>(32,278.1 to 42,678.9)	44,365.9<br>(38,344.4 to 50,808.8)	7,705.4<br>(5,121.0 to 10,956.3)	9,164.7<br>(6,094.5 to 13,078.0)
Other neurological disorders	Guillain-Barré syndrome due to other neurological disorders	9.9<br>(7.7 to 12.4)	11.5<br>(9.1 to 14.6)	2.9<br>(1.8 to 4.3)	3.4<br>(2.1 to 5.0)
Schizophrenia	Schizophrenia residual state	7,254.4<br>(2,821.4 to 12,032.0)	8,670.6<br>(3,381.4 to 14,350.8)	3,891.5<br>(1,417.9 to 6,928.8)	4,649.2<br>(1,692.4 to 8,277.1)
Schizophrenia	Schizophrenia acute state	12,308.7<br>(7,483.7 to 17,072.9)	14,712.5<br>(8,914.3 to 20,369.1)	8,680.6<br>(4,950.0 to 12,488.5)	10,371.3<br>(5,893.0 to 14,953.7)
Alcohol use disorders	Asymptomatic alcohol dependence	17,950.2<br>(14,341.7 to 21,534.1)	19,941.6<br>(15,834.4 to 24,036.6)	<0.05<br>(0.0 to <0.05)	<0.05<br>(0.0 to <0.05)
Alcohol use disorders	Very mild alcohol dependence	33,126.9<br>(29,082.8 to 37,435.0)	36,801.0<br>(32,314.8 to 41,978.7)	3,856.0<br>(2,567.4 to 5,581.3)	4,283.2<br>(2,867.5 to 6,214.7)
Alcohol use disorders	Mild alcohol dependence	2,086.7<br>(978.1 to 3,529.6)	2,317.7<br>(1,070.5 to 3,963.4)	459.4<br>(194.6 to 859.5)	510.2<br>(216.9 to 953.7)
Alcohol use disorders	Moderate alcohol dependence	1,823.5<br>(717.9 to 3,455.7)	2,026.2<br>(798.7 to 3,859.1)	631.0<br>(218.1 to 1,272.2)	701.1<br>(241.7 to 1,401.5)
Alcohol use disorders	Severe alcohol dependence	1,339.8<br>(269.4 to 2,943.4)	1,488.7<br>(296.7 to 3,254.1)	703.3<br>(161.3 to 1,560.8)	781.7<br>(180.7 to 1,744.2)
Alcohol use disorders	Asymptomatic fetal alcohol syndrome	93.7<br>(67.8 to 123.6)	104.4<br>(75.6 to 137.8)	<0.05<br>(0.0 to <0.05)	<0.05<br>(0.0 to <0.05)
Alcohol use disorders	Mild fetal alcohol syndrome	214.9<br>(161.4 to 278.0)	239.6<br>(179.9 to 310.0)	3.5<br>(1.7 to 6.6)	3.9<br>(1.8 to 7.4)
Alcohol use disorders	Moderate fetal alcohol syndrome	413.6<br>(337.4 to 484.8)	458.7<br>(374.9 to 539.2)	22.4<br>(13.8 to 33.7)	24.8<br>(15.1 to 37.9)
Alcohol use disorders	Severe fetal alcohol syndrome	83.1<br>(47.4 to 120.1)	91.6<br>(50.2 to 133.5)	14.9<br>(7.6 to 24.3)	16.4<br>(8.1 to 27.1)
Opioid use disorders	Asymptomatic opioid dependence	2,203.1<br>(1,608.7 to 2,969.2)	2,716.8<br>(1,979.9 to 3,704.4)	<0.05<br>(0.0 to <0.05)	<0.05<br>(0.0 to <0.05)
Opioid use disorders	Mild opioid dependence	4,942.5<br>(3,151.2 to 6,889.8)	6,095.5<br>(3,866.1 to 8,497.4)	1,541.4<br>(854.1 to 2,378.0)	1,899.9<br>(1,034.5 to 2,960.1)
Opioid use disorders	Severe opioid dependence	6,434.1<br>(4,588.3 to 8,493.7)	7,934.2<br>(5,664.8 to 10,469.3)	4,112.1<br>(2,680.5 to 5,707.5)	5,069.7<br>(3,294.9 to 7,103.9)
Cocaine use disorders	Asymptomatic cocaine dependence	1,470.8<br>(1,160.1 to 1,823.6)	1,927.8<br>(1,544.3 to 2,357.8)	<0.05<br>(0.0 to <0.05)	<0.05<br>(0.0 to <0.05)
Cocaine use disorders	Mild cocaine dependence	747.6<br>(530.5 to 999.6)	979.8<br>(702.4 to 1,298.5)	82.1<br>(48.0 to 127.6)	107.3<br>(62.5 to 163.9)
Cocaine use disorders	Severe cocaine dependence	716.1<br>(503.9 to 953.3)	938.7<br>(663.6 to 1,242.1)	317.3<br>(188.3 to 467.7)	414.1<br>(247.0 to 608.2)
Amphetamine use disorders	Asymptomatic amphetamine dependence	2,771.3<br>(2,086.0 to 3,561.2)	3,613.7<br>(2,722.6 to 4,630.7)	<0.05<br>(0.0 to <0.05)	<0.05<br>(0.0 to <0.05)
Amphetamine use disorders	Mild amphetamine dependence	998.8<br>(622.6 to 1,453.4)	1,302.1<br>(818.6 to 1,892.1)	76.6<br>(39.4 to 126.3)	99.9<br>(51.5 to 163.0)
Amphetamine use disorders	Severe amphetamine dependence	1,291.5<br>(844.8 to 1,846.9)	1,684.0<br>(1,107.1 to 2,396.0)	594.6<br>(325.9 to 918.5)	774.8<br>(429.6 to 1,195.6)
Cannabis use disorders	Asymptomatic cannabis dependence	10,858.1<br>(9,655.3 to 12,213.5)	11,430.9<br>(10,159.4 to 12,892.9)	<0.05<br>(0.0 to <0.05)	<0.05<br>(0.0 to <0.05)
Cannabis use disorders	Mild cannabis dependence	6,766.5<br>(5,820.6 to 7,958.1)	7,123.4<br>(6,129.2 to 8,357.7)	259.4<br>(155.4 to 397.5)	273.1<br>(164.6 to 418.7)
Cannabis use disorders	Severe cannabis dependence	1,147.6<br>(730.2 to 1,654.1)	1,208.2<br>(770.0 to 1,742.8)	288.7<br>(157.5 to 458.7)	304.1<br>(164.8 to 483.5)
Major depressive disorder	Major depressive disorder, currently without symptoms	23,714.0<br>(17,440.1 to 31,212.4)	27,929.9<br>(20,516.9 to 36,823.1)	<0.05<br>(0.0 to <0.05)	<0.05<br>(0.0 to <0.05)
Major depressive disorder	Mild major depressive disorder	109,347.2<br>(91,700.1 to 128,543.4)	128,789.0<br>(107,960.5 to 151,557.8)	14,924.1<br>(9,790.1 to 21,220.5)	17,578.3<br>(11,583.5 to 24,947.1)
Major depressive disorder	Moderate major depressive disorder	31,874.4<br>(23,437.3 to 41,178.0)	37,541.0<br>(27,650.9 to 48,522.8)	11,575.6<br>(7,059.6 to 16,822.9)	13,634.7<br>(8,300.9 to 19,846.0)
Major depressive disorder	Severe major depressive disorder	18,498.3<br>(6,778.3 to 33,220.9)	21,787.1<br>(7,950.6 to 39,136.6)	11,045.1<br>(3,948.8 to 20,389.4)	13,011.4<br>(4,614.2 to 23,901.5)
Dysthymia	Dysthymia, currently without symptoms	25,229.9<br>(19,982.8 to 31,232.4)	30,256.9<br>(24,007.5 to 37,502.9)	<0.05<br>(0.0 to <0.05)	<0.05<br>(0.0 to <0.05)
Dysthymia	Symptomatic dysthymia	61,582.2<br>(52,237.9 to 71,076.2)	73,849.4<br>(62,480.6 to 85,155.8)	8,371.3<br>(5,544.5 to 11,973.4)	10,031.0<br>(6,605.9 to 14,267.1)

Cause	Sequela	Prevalence in thousands (95% confidence interval)		YLDs in thousands (95% confidence interval)	
		2005	2015	2005	2015
Bipolar disorder	Bipolar disorder residual state	20,678.2 (14,250.1 to 27,554.6)	23,756.2 (16,272.2 to 31,584.1)	638.8 (340.2 to 1,094.3)	733.8 (391.0 to 1,254.0)
Bipolar disorder	Bipolar disorder depressive state	9,213.0 (4,747.9 to 15,114.1)	10,585.0 (5,460.1 to 17,362.1)	3,379.9 (1,605.9 to 6,124.0)	3,882.5 (1,842.5 to 7,055.3)
Bipolar disorder	Bipolar disorder manic state	8,421.0 (4,599.4 to 13,458.4)	9,674.6 (5,292.8 to 15,474.3)	3,820.1 (1,874.4 to 6,458.3)	4,388.4 (2,161.3 to 7,423.8)
Anxiety disorders	Anxiety disorders, currently without symptoms	46,663.5 (38,027.4 to 56,399.8)	53,604.2 (43,582.7 to 65,082.8)	<0.05 (0.0 to <0.05)	<0.05 (0.0 to <0.05)
Anxiety disorders	Mild anxiety disorders	115,112.2 (96,847.6 to 136,393.8)	132,238.2 (110,915.7 to 156,505.9)	3,290.2 (1,941.2 to 5,295.6)	3,777.0 (2,219.6 to 6,064.2)
Anxiety disorders	Moderate anxiety disorders	44,709.8 (33,745.0 to 56,955.2)	51,362.4 (39,078.4 to 65,497.7)	5,633.7 (3,523.8 to 8,299.8)	6,466.3 (4,086.8 to 9,574.7)
Anxiety disorders	Severe anxiety disorders	26,111.4 (16,340.0 to 37,520.0)	29,997.7 (18,834.2 to 43,252.8)	12,550.9 (7,193.7 to 19,359.2)	14,399.7 (8,247.3 to 22,292.7)
Anorexia nervosa	Anorexia nervosa	2,599.7 (2,095.4 to 3,179.8)	2,912.1 (2,357.7 to 3,596.2)	553.7 (366.8 to 796.7)	620.5 (411.3 to 897.7)
Bulimia nervosa	Bulimia nervosa	2,897.3 (2,338.8 to 3,500.7)	3,629.4 (2,937.5 to 4,406.8)	610.9 (393.1 to 887.0)	765.6 (490.0 to 1,108.5)
Autism	Autism without intellectual disability	3,677.6 (1,816.3 to 5,381.3)	4,139.4 (2,041.8 to 6,061.7)	742.0 (258.8 to 1,286.2)	834.8 (290.2 to 1,440.5)
Autism	Autism with borderline intellectual disability	3,661.6 (1,804.5 to 5,671.6)	4,121.3 (2,024.4 to 6,385.3)	769.8 (307.7 to 1,362.3)	865.9 (349.5 to 1,533.6)
Autism	Autism with mild intellectual disability	8,591.8 (4,696.9 to 11,410.8)	9,670.5 (5,299.6 to 12,849.4)	2,066.8 (858.0 to 3,208.2)	2,324.5 (971.1 to 3,607.4)
Autism	Autism with moderate intellectual disability	4,327.0 (2,268.9 to 6,209.1)	4,870.3 (2,551.2 to 6,983.7)	1,334.3 (583.4 to 2,122.4)	1,500.7 (661.3 to 2,383.9)
Autism	Autism with severe intellectual disability	1,457.7 (0.0 to 9,544.7)	1,640.9 (0.0 to 10,759.0)	567.6 (0.0 to 3,581.6)	638.7 (0.0 to 4,035.1)
Autism	Autism with profound intellectual disability	308.9 (0.0 to 6,189.2)	347.7 (0.0 to 6,969.2)	152.3 (0.0 to 3,057.0)	171.4 (0.0 to 3,423.8)
Asperger syndrome and other autistic spectrum disorders	Asperger syndrome and other autistic spectrum disorders	33,230.9 (28,105.5 to 40,079.8)	37,245.4 (31,510.5 to 44,883.1)	3,317.1 (2,215.1 to 4,847.9)	3,715.6 (2,479.6 to 5,424.6)
Attention-deficit/hyperactivity disorder	Attention-deficit/hyperactivity disorder, currently without symptoms	36,719.0 (32,502.4 to 41,852.7)	36,829.5 (32,478.9 to 41,990.4)	<0.05 (0.0 to <0.05)	<0.05 (0.0 to <0.05)
Attention-deficit/hyperactivity disorder	Symptomatic attention-deficit/hyperactivity disorder	14,221.7 (11,248.7 to 17,204.1)	14,264.7 (11,255.4 to 17,248.1)	617.9 (369.2 to 942.9)	620.1 (370.1 to 947.7)
Conduct disorder	Conduct disorder, currently without symptoms	23,106.8 (17,235.8 to 29,853.8)	23,203.7 (17,307.5 to 29,925.1)	<0.05 (0.0 to <0.05)	<0.05 (0.0 to <0.05)
Conduct disorder	Symptomatic conduct disorder	24,827.5 (18,460.8 to 31,830.7)	24,931.7 (18,550.8 to 31,925.2)	5,740.5 (3,467.9 to 8,920.1)	5,770.5 (3,485.2 to 8,955.7)
Idiopathic developmental intellectual disability	Borderline idiopathic developmental intellectual disability	25,131.2 (18,582.7 to 31,998.3)	27,626.9 (20,454.6 to 35,444.4)	265.4 (121.7 to 505.1)	291.8 (132.7 to 560.6)
Idiopathic developmental intellectual disability	Mild idiopathic developmental intellectual disability	52,934.8 (18,009.4 to 85,732.2)	59,372.7 (20,104.2 to 96,126.8)	2,187.5 (654.4 to 4,113.7)	2,453.4 (740.0 to 4,616.9)
Idiopathic developmental intellectual disability	Moderate idiopathic developmental intellectual disability	2,725.0 (816.9 to 5,995.2)	2,687.4 (760.8 to 6,282.4)	260.1 (70.5 to 586.5)	256.9 (66.1 to 613.0)
Idiopathic developmental intellectual disability	Severe idiopathic developmental intellectual disability	330.3 (76.3 to 489.5)	361.3 (84.6 to 516.4)	51.2 (11.4 to 87.6)	56.1 (12.5 to 94.5)
Idiopathic developmental intellectual disability	Profound idiopathic developmental intellectual disability	1,874.8 (142.3 to 3,578.5)	2,025.7 (147.5 to 3,964.9)	355.7 (24.8 to 730.3)	383.8 (23.5 to 804.3)
Other mental and substance use disorders	Other mental disorders, currently without symptoms	14,907.6 (11,805.8 to 18,338.5)	17,710.0 (14,032.6 to 21,733.8)	<0.05 (0.0 to <0.05)	<0.05 (0.0 to <0.05)
Other mental and substance use disorders	Mild other mental disorders	67,946.5 (63,356.0 to 72,509.7)	80,720.4 (75,188.5 to 86,321.0)	1,936.7 (1,171.2 to 3,007.1)	2,300.0 (1,389.5 to 3,579.6)
Other mental and substance use disorders	Moderate other mental disorders	16,495.9 (13,419.0 to 19,879.6)	19,597.0 (15,949.3 to 23,589.3)	2,071.6 (1,373.3 to 3,024.4)	2,460.0 (1,633.9 to 3,575.7)
Other mental and substance use disorders	Severe other mental disorders	8,544.8 (5,253.7 to 12,785.1)	10,151.0 (6,237.5 to 15,215.0)	4,087.6 (2,237.5 to 6,458.0)	4,853.4 (2,667.5 to 7,668.7)
Diabetes mellitus	Uncomplicated diabetes mellitus	212,661.3 (190,101.0 to 234,507.1)	271,713.6 (241,414.2 to 302,381.7)	9,867.8 (6,226.1 to 14,448.4)	12,599.0 (7,979.9 to 18,394.2)
Diabetes mellitus	Diabetic neuropathy	95,833.2 (78,783.9 to 115,739.1)	130,136.8 (105,433.6 to 157,265.0)	11,750.1 (7,771.7 to 17,002.6)	15,957.1 (10,412.8 to 23,176.6)
Diabetes mellitus	Diabetic foot due to neuropathy	16,047.2 (13,121.7 to 19,609.8)	21,620.7 (17,708.3 to 26,415.6)	2,181.2 (1,452.1 to 3,128.7)	2,938.7 (1,967.2 to 4,202.3)
Diabetes mellitus	Diabetic neuropathy and amputation with treatment	2,070.6 (1,717.5 to 2,445.8)	2,756.8 (2,297.0 to 3,262.8)	313.1 (208.4 to 435.5)	416.1 (278.3 to 576.4)
Diabetes mellitus	Diabetic neuropathy and amputation without treatment	3,337.7 (2,903.1 to 3,783.8)	4,553.4 (3,963.4 to 5,177.9)	841.5 (594.6 to 1,140.2)	1,147.6 (807.6 to 1,556.4)
Diabetes mellitus	Moderate vision impairment due to diabetes mellitus	2,481.9 (2,013.5 to 3,075.0)	3,319.3 (2,682.9 to 4,100.4)	71.9 (43.1 to 111.5)	96.2 (57.7 to 149.0)
Diabetes mellitus	Severe vision impairment due to diabetes mellitus	328.9 (264.4 to 402.6)	435.4 (349.4 to 535.3)	54.9 (35.8 to 80.2)	72.6 (47.2 to 104.7)
Diabetes mellitus	Blindness due to diabetes mellitus	564.6 (449.6 to 690.8)	792.3 (635.8 to 975.0)	95.3 (61.5 to 139.3)	133.5 (86.4 to 192.9)
Acute glomerulonephritis	Acute glomerulonephritis	101.5 (93.1 to 110.2)	100.5 (92.1 to 110.1)	5.1 (3.2 to 7.5)	5.0 (3.2 to 7.5)
Chronic kidney disease due to diabetes mellitus	Stage III chronic kidney disease without anemia due to diabetes mellitus	64,974.2 (55,004.5 to 75,879.0)	83,256.8 (70,254.0 to 96,941.9)	<0.05 (0.0 to <0.05)	<0.05 (0.0 to <0.05)
Chronic kidney disease due to diabetes mellitus	Stage III chronic kidney disease and mild anemia due to diabetes mellitus	4,897.4 (4,843.8 to 4,946.3)	6,290.7 (6,231.0 to 6,351.9)	17.3 (6.2 to 37.6)	22.2 (8.0 to 48.1)
Chronic kidney disease due to diabetes mellitus	Stage III chronic kidney disease and moderate anemia due to diabetes mellitus	925.3 (889.5 to 959.9)	1,064.8 (1,026.4 to 1,104.9)	45.0 (30.2 to 64.9)	51.6 (34.2 to 73.5)
Chronic kidney disease due to diabetes mellitus	Stage III chronic kidney disease and severe anemia due to diabetes mellitus	318.3 (304.4 to 333.1)	325.4 (308.5 to 342.7)	44.8 (30.6 to 61.7)	45.8 (31.2 to 63.6)
Chronic kidney disease due to diabetes mellitus	Stage IV chronic kidney disease without anemia due to diabetes mellitus	942.7 (557.1 to 1,467.0)	1,167.8 (699.8 to 1,802.7)	92.4 (47.2 to 160.9)	114.1 (59.9 to 194.2)
Chronic kidney disease due to diabetes mellitus	Stage IV chronic kidney disease and mild anemia due to diabetes mellitus	1,585.9 (1,439.5 to 1,728.3)	1,977.3 (1,786.0 to 2,178.5)	157.8 (109.3 to 218.9)	196.4 (135.8 to 272.1)
Chronic kidney disease due to diabetes mellitus	Stage IV chronic kidney disease and moderate anemia due to diabetes mellitus	2,535.8 (2,303.1 to 2,741.3)	3,071.7 (2,798.3 to 3,331.9)	353.2 (248.3 to 478.9)	426.6 (299.8 to 578.9)
Chronic kidney disease due to diabetes mellitus	Stage IV chronic kidney disease and severe anemia due to diabetes mellitus	231.9 (211.7 to 248.9)	224.5 (205.1 to 240.5)	52.0 (37.0 to 70.3)	50.5 (35.2 to 68.8)
Chronic kidney disease due to diabetes mellitus	Stage V chronic kidney disease untreated due to diabetes mellitus	2,145.9 (1,852.9 to 2,474.4)	2,694.4 (2,331.9 to 3,091.5)	1,085.2 (739.4 to 1,432.1)	1,359.3 (920.5 to 1,791.4)
Chronic kidney disease due to diabetes mellitus	End-stage renal disease after transplant due to diabetes mellitus	350.9 (290.8 to 419.7)	397.5 (327.0 to 476.8)	8.3 (4.5 to 13.6)	9.4 (5.0 to 15.3)
Chronic kidney disease due to diabetes mellitus	End-stage renal disease on dialysis due to diabetes mellitus	275.6 (233.4 to 324.0)	353.0 (296.7 to 417.2)	141.4 (95.8 to 187.3)	180.2 (120.8 to 242.8)
Chronic kidney disease due to hypertension	Stage III chronic kidney disease without anemia due to hypertension	53,882.2 (45,340.5 to 63,314.7)	67,748.1 (56,661.0 to 79,494.8)	<0.05 (0.0 to <0.05)	<0.05 (0.0 to <0.05)
Chronic kidney disease due to hypertension	Stage III chronic kidney disease and mild anemia due to hypertension	2,902.1 (2,868.4 to 2,937.3)	3,781.7 (3,739.7 to 3,828.4)	10.1 (3.6 to 22.0)	13.2 (4.8 to 28.5)
Chronic kidney disease due to hypertension	Stage III chronic kidney disease and moderate anemia due to hypertension	916.8 (885.7 to 945.4)	1,151.1 (1,107.9 to 1,189.5)	43.7 (29.2 to 62.9)	54.8 (36.9 to 79.0)
Chronic kidney disease due to hypertension	Stage III chronic kidney disease and severe anemia due to hypertension	88.2 (83.2 to 92.9)	91.5 (86.4 to 96.5)	12.4 (8.6 to 17.3)	12.8 (8.9 to 18.0)
Chronic kidney disease due to hypertension	Stage IV chronic kidney disease without anemia due to hypertension	796.8 (455.6 to 1,237.4)	945.3 (537.7 to 1,487.8)	77.7 (40.6 to 137.8)	92.3 (47.8 to 162.8)
Chronic kidney disease due to hypertension	Stage IV chronic kidney disease and mild anemia due to hypertension	962.4 (864.9 to 1,047.6)	1,239.4 (1,110.5 to 1,351.3)	94.9 (66.1 to 134.8)	122.0 (85.0 to 169.8)
Chronic kidney disease due to hypertension	Stage IV chronic kidney disease and moderate anemia due to hypertension	1,331.5 (1,185.5 to 1,456.0)	1,745.0 (1,551.3 to 1,917.6)	180.8 (128.1 to 247.3)	236.6 (167.7 to 323.7)
Chronic kidney disease due to hypertension	Stage IV chronic kidney disease and severe anemia due to hypertension	167.8 (152.0 to 180.9)	172.4 (155.6 to 184.7)	37.8 (26.7 to 51.8)	38.9 (27.6 to 53.2)
Chronic kidney disease due to hypertension	Stage V chronic kidney disease untreated due to hypertension	1,264.5 (1,078.5 to 1,457.3)	1,652.2 (1,404.5 to 1,913.7)	625.8 (437.3 to 813.8)	816.8 (565.2 to 1,060.3)
Chronic kidney disease due to hypertension	End-stage renal disease after transplant due to hypertension	170.8 (137.9 to 209.6)	206.3 (166.0 to 253.1)	4.0 (2.2 to 6.6)	4.8 (2.6 to 7.9)

Cause	Sequela	Prevalence in thousands (95% confidence interval)		YLDs in thousands (95% confidence interval)	
		2005	2015	2005	2015
Chronic kidney disease due to hypertension	End-stage renal disease on dialysis due to hypertension	170.8 (140.8 to 204.0)	229.1 (186.1 to 276.6)	86.5 (57.9 to 116.9)	115.7 (78.4 to 157.9)
Chronic kidney disease due to glomerulonephritis	Stage III chronic kidney disease without anemia due to glomerulonephritis	41,012.7 (34,203.4 to 48,798.1)	53,602.3 (44,631.9 to 63,539.5)	<0.05 (0.0 to <0.05)	<0.05 (0.0 to <0.05)
Chronic kidney disease due to glomerulonephritis	Stage III chronic kidney disease and mild anemia due to glomerulonephritis	3,489.3 (3,434.9 to 3,544.0)	4,374.7 (4,323.0 to 4,439.0)	12.3 (4.4 to 26.9)	15.4 (5.6 to 33.5)
Chronic kidney disease due to glomerulonephritis	Stage III chronic kidney disease and moderate anemia due to glomerulonephritis	1,440.5 (1,400.5 to 1,480.3)	1,725.1 (1,672.6 to 1,765.6)	69.5 (46.7 to 99.5)	83.3 (55.6 to 119.5)
Chronic kidney disease due to glomerulonephritis	Stage III chronic kidney disease and severe anemia due to glomerulonephritis	93.2 (85.7 to 102.8)	88.6 (80.6 to 96.7)	13.2 (8.9 to 18.6)	12.6 (8.5 to 17.6)
Chronic kidney disease due to glomerulonephritis	Stage IV chronic kidney disease without anemia due to glomerulonephritis	507.4 (264.8 to 846.0)	643.0 (332.2 to 1,095.4)	49.0 (23.8 to 90.8)	62.0 (29.6 to 118.0)
Chronic kidney disease due to glomerulonephritis	Stage IV chronic kidney disease and mild anemia due to glomerulonephritis	1,416.1 (1,281.0 to 1,545.6)	1,661.6 (1,495.6 to 1,812.6)	141.5 (97.0 to 195.2)	165.7 (113.0 to 228.4)
Chronic kidney disease due to glomerulonephritis	Stage IV chronic kidney disease and moderate anemia due to glomerulonephritis	1,857.9 (1,661.9 to 2,030.9)	2,345.9 (2,114.5 to 2,553.6)	257.4 (179.7 to 352.7)	324.6 (225.0 to 443.6)
Chronic kidney disease due to glomerulonephritis	Stage IV chronic kidney disease and severe anemia due to glomerulonephritis	236.3 (212.0 to 254.3)	221.9 (198.1 to 240.8)	53.3 (37.2 to 72.8)	50.2 (35.4 to 68.8)
Chronic kidney disease due to glomerulonephritis	Stage V chronic kidney disease untreated due to glomerulonephritis	1,802.8 (1,540.4 to 2,101.9)	2,228.1 (1,895.7 to 2,596.9)	909.3 (626.2 to 1,188.8)	1,122.6 (773.7 to 1,461.3)
Chronic kidney disease due to glomerulonephritis	End-stage renal disease after transplant due to glomerulonephritis	213.1 (168.9 to 265.1)	244.9 (193.3 to 306.2)	5.0 (2.7 to 8.5)	5.8 (3.2 to 9.9)
Chronic kidney disease due to glomerulonephritis	End-stage renal disease on dialysis due to glomerulonephritis	166.7 (138.7 to 202.2)	212.5 (175.8 to 260.5)	86.2 (57.9 to 113.7)	109.2 (74.2 to 146.0)
Chronic kidney disease due to other causes	Stage III chronic kidney disease without anemia due to other causes	61,522.5 (51,553.5 to 72,508.1)	78,030.8 (65,081.6 to 92,183.7)	<0.05 (0.0 to <0.05)	<0.05 (0.0 to <0.05)
Chronic kidney disease due to other causes	Stage III chronic kidney disease and mild anemia due to other causes	4,186.5 (4,142.2 to 4,237.6)	5,309.5 (5,242.8 to 5,383.6)	14.7 (5.3 to 31.9)	18.7 (6.7 to 40.2)
Chronic kidney disease due to other causes	Stage III chronic kidney disease and moderate anemia due to other causes	1,650.2 (1,607.8 to 1,693.3)	1,989.7 (1,931.4 to 2,049.0)	79.2 (53.1 to 114.1)	95.4 (64.2 to 136.6)
Chronic kidney disease due to other causes	Stage III chronic kidney disease and severe anemia due to other causes	132.6 (125.4 to 139.4)	129.8 (122.9 to 136.5)	18.5 (12.7 to 25.5)	18.1 (12.4 to 24.9)
Chronic kidney disease due to other causes	Stage IV chronic kidney disease without anemia due to other causes	920.0 (553.4 to 1,390.2)	1,110.5 (669.5 to 1,692.0)	89.6 (47.1 to 151.9)	108.0 (57.0 to 182.5)
Chronic kidney disease due to other causes	Stage IV chronic kidney disease and mild anemia due to other causes	1,487.2 (1,363.1 to 1,612.4)	1,901.4 (1,734.8 to 2,062.3)	148.0 (101.5 to 204.7)	189.0 (131.1 to 259.2)
Chronic kidney disease due to other causes	Stage IV chronic kidney disease and moderate anemia due to other causes	2,292.5 (2,086.2 to 2,475.7)	2,768.5 (2,528.9 to 2,993.5)	315.6 (221.9 to 424.6)	380.0 (268.8 to 511.9)
Chronic kidney disease due to other causes	Stage IV chronic kidney disease and severe anemia due to other causes	295.5 (268.6 to 314.7)	278.0 (251.4 to 298.7)	66.4 (47.0 to 89.7)	62.6 (43.9 to 85.8)
Chronic kidney disease due to other causes	Stage V chronic kidney disease untreated due to other causes	1,960.3 (1,686.3 to 2,267.7)	2,464.6 (2,115.1 to 2,835.9)	982.1 (682.8 to 1,287.1)	1,233.0 (855.9 to 1,604.1)
Chronic kidney disease due to other causes	End-stage renal disease after transplant due to other causes	244.6 (198.6 to 298.7)	284.2 (232.9 to 345.3)	5.8 (3.1 to 9.5)	6.7 (3.6 to 10.9)
Chronic kidney disease due to other causes	End-stage renal disease on dialysis due to other causes	224.7 (189.8 to 270.4)	286.4 (241.0 to 346.0)	114.9 (78.2 to 153.6)	145.7 (99.5 to 194.7)
Interstitial nephritis and urinary tract infections	Mild interstitial nephritis and urinary tract infections	854.1 (670.6 to 1,033.9)	1,019.1 (799.3 to 1,233.7)	4.8 (1.9 to 10.2)	5.7 (2.2 to 12.1)
Interstitial nephritis and urinary tract infections	Moderate interstitial nephritis and urinary tract infections	1,577.4 (1,387.9 to 1,766.3)	1,882.1 (1,658.2 to 2,106.0)	76.2 (47.5 to 113.5)	90.9 (56.4 to 134.8)
Urolithiasis	Acute urolithiasis	677.3 (619.0 to 746.7)	837.4 (765.2 to 920.2)	72.7 (49.5 to 99.6)	89.7 (60.9 to 122.5)
Urolithiasis	Chronic urolithiasis	259,566.7 (238,099.5 to 281,891.6)	318,762.7 (290,694.9 to 349,153.9)	<0.05 (0.0 to <0.05)	<0.05 (0.0 to <0.05)
Benign prostatic hyperplasia	Asymptomatic benign prostatic hyperplasia	33,439.3 (28,891.7 to 38,029.1)	43,362.4 (37,330.8 to 49,556.1)	<0.05 (0.0 to <0.05)	<0.05 (0.0 to <0.05)
Benign prostatic hyperplasia	Symptomatic benign prostatic hyperplasia	47,244.2 (41,192.8 to 53,779.9)	61,263.0 (53,229.3 to 69,777.8)	2,922.0 (1,868.6 to 4,183.7)	3,792.7 (2,423.8 to 5,437.3)
Male infertility	Primary male infertility	9,323.7 (7,412.2 to 11,689.1)	11,409.4 (8,772.8 to 14,594.9)	69.2 (30.2 to 138.9)	84.7 (36.6 to 172.1)
Male infertility	Secondary male infertility	14,350.4 (11,200.3 to 18,320.9)	17,623.8 (13,655.7 to 22,418.3)	72.6 (27.6 to 166.8)	89.2 (33.7 to 202.6)
Uterine fibroids	Asymptomatic uterine fibroids	39,840.1 (34,252.0 to 45,337.8)	47,480.9 (40,845.4 to 54,124.8)	<0.05 (0.0 to <0.05)	<0.05 (0.0 to <0.05)
Uterine fibroids	Mild abdominal pain due to uterine fibroids, without anemia	48,253.8 (43,040.1 to 54,125.0)	60,854.0 (54,482.9 to 67,901.0)	512.3 (250.2 to 943.9)	645.9 (314.9 to 1,193.3)
Uterine fibroids	Mild abdominal pain due to uterine fibroids, with mild anemia	18,391.6 (17,932.8 to 18,848.5)	21,229.3 (20,729.2 to 21,733.4)	260.6 (120.3 to 508.1)	301.0 (138.0 to 585.1)
Uterine fibroids	Mild abdominal pain due to uterine fibroids, with moderate anemia	18,283.2 (17,683.9 to 18,899.4)	19,653.0 (19,034.1 to 20,267.0)	1,075.5 (701.8 to 1,594.4)	1,157.8 (756.3 to 1,711.8)
Uterine fibroids	Mild abdominal pain due to uterine fibroids, with severe anemia	2,028.5 (1,940.8 to 2,110.8)	1,897.8 (1,818.2 to 1,978.5)	297.0 (206.2 to 402.8)	278.8 (194.8 to 378.6)
Polycystic ovarian syndrome	Asymptomatic polycystic ovarian syndrome	8,386.4 (3,683.7 to 15,109.0)	9,261.2 (4,063.8 to 16,731.9)	<0.05 (0.0 to <0.05)	<0.05 (0.0 to <0.05)
Polycystic ovarian syndrome	Hirsutism due to polycystic ovarian syndrome	34,278.6 (25,419.4 to 44,924.2)	37,855.6 (28,072.9 to 49,637.4)	369.2 (164.2 to 725.2)	408.0 (181.1 to 797.7)
Polycystic ovarian syndrome	Hirsutism and primary infertility due to polycystic ovarian syndrome	2,706.4 (1,719.3 to 3,962.3)	2,980.5 (1,856.6 to 4,421.7)	48.7 (20.2 to 102.2)	53.7 (21.9 to 111.7)
Polycystic ovarian syndrome	Primary infertility due to polycystic ovarian syndrome	631.6 (50.6 to 1,822.0)	695.9 (55.4 to 1,996.9)	4.7 (0.3 to 15.2)	5.1 (0.4 to 17.0)
Polycystic ovarian syndrome	Hirsutism and secondary infertility due to polycystic ovarian syndrome	6,703.3 (4,417.6 to 9,667.3)	7,546.4 (4,953.3 to 10,916.9)	33.8 (11.9 to 77.8)	38.1 (13.5 to 87.8)
Polycystic ovarian syndrome	Secondary infertility due to polycystic ovarian syndrome	1,568.1 (130.0 to 4,708.6)	1,766.9 (146.2 to 5,296.7)	24.5 (1.7 to 80.6)	27.7 (2.0 to 92.3)
Female infertility	Idiopathic primary female infertility	12,054.8 (8,982.0 to 15,849.5)	15,357.7 (11,323.3 to 20,154.7)	88.8 (37.9 to 180.7)	113.3 (48.1 to 234.0)
Female infertility	Idiopathic secondary female infertility	37,134.6 (25,835.4 to 51,728.5)	45,942.5 (32,132.8 to 63,119.2)	186.7 (68.6 to 431.0)	231.2 (86.1 to 525.6)
Endometriosis	Asymptomatic endometriosis	2,791.7 (1,991.0 to 3,779.4)	3,113.5 (2,221.7 to 4,203.7)	<0.05 (0.0 to <0.05)	<0.05 (0.0 to <0.05)
Endometriosis	Mild abdominal pain due to endometriosis	511.5 (327.3 to 742.8)	570.4 (364.6 to 832.6)	5.5 (2.3 to 10.9)	6.1 (2.6 to 12.3)
Endometriosis	Moderate abdominal pain due to endometriosis	4,749.8 (3,594.9 to 5,986.6)	5,297.3 (4,030.5 to 6,685.3)	514.2 (322.9 to 741.6)	574.0 (360.4 to 825.9)
Endometriosis	Severe endometriosis	1,063.7 (698.1 to 1,493.2)	1,186.3 (778.2 to 1,662.2)	319.8 (177.5 to 507.2)	357.1 (198.6 to 562.1)
Endometriosis	Primary infertility due to endometriosis	45.8 (28.5 to 67.6)	50.7 (31.5 to 75.8)	0.3 (0.1 to 0.7)	0.4 (0.2 to 0.8)
Endometriosis	Mild abdominal pain and primary infertility due to endometriosis	8.6 (5.1 to 13.2)	9.5 (5.7 to 14.8)	0.2 (0.1 to 0.3)	0.2 (0.1 to 0.4)
Endometriosis	Moderate abdominal pain and primary infertility due to endometriosis	78.9 (47.1 to 117.9)	87.4 (52.3 to 132.6)	9.2 (4.8 to 15.6)	10.2 (5.3 to 17.3)
Endometriosis	Severe abdominal pain and primary infertility due to endometriosis	17.5 (10.1 to 27.2)	19.4 (11.0 to 30.2)	5.7 (3.0 to 9.9)	6.4 (3.3 to 11.0)
Endometriosis	Secondary infertility due to endometriosis	114.1 (73.9 to 168.1)	128.8 (83.3 to 189.4)	0.6 (0.2 to 1.3)	0.6 (0.2 to 1.5)
Endometriosis	Mild abdominal pain and secondary infertility due to endometriosis	21.3 (13.2 to 31.5)	24.0 (14.9 to 35.3)	0.3 (0.1 to 0.7)	0.4 (0.2 to 0.9)
Endometriosis	Moderate abdominal pain and secondary infertility due to endometriosis	196.4 (123.6 to 288.5)	221.7 (138.9 to 325.8)	22.2 (11.7 to 37.1)	25.1 (13.1 to 42.5)
Endometriosis	Severe abdominal pain and secondary infertility due to endometriosis	43.6 (27.0 to 64.8)	49.2 (30.3 to 73.2)	14.0 (7.4 to 22.9)	15.9 (8.5 to 26.1)
Genital prolapse	Asymptomatic genital prolapse	111,085.0 (94,557.4 to 127,795.1)	130,730.0 (111,648.1 to 149,996.0)	<0.05 (0.0 to <0.05)	<0.05 (0.0 to <0.05)
Genital prolapse	Abdominal pain due to genital prolapse	9,887.4 (2,020.8 to 21,794.2)	11,634.4 (2,435.7 to 25,899.7)	103.1 (17.5 to 266.4)	121.5 (20.8 to 311.6)
Genital prolapse	Stress incontinence due to genital prolapse	15,023.2 (9,103.7 to 22,685.3)	17,681.8 (10,783.0 to 26,761.4)	283.6 (129.9 to 534.4)	334.1 (153.2 to 627.0)

Cause	Sequela	Prevalence in thousands (95% confidence interval)		YLDs in thousands (95% confidence interval)	
		2005	2015	2005	2015
Genital prolapse	Abdominal pain and stress incontinence due to genital prolapse	1,387.9 (337.5 to 3,105.3)	1,633.0 (398.9 to 3,642.3)	40.2 (8.5 to 102.3)	47.4 (10.1 to 122.1)
Premenstrual syndrome	Asymptomatic premenstrual syndrome	347,642.8 (331,048.3 to 364,839.7)	382,735.6 (362,359.5 to 402,107.6)	<0.05 (0.0 to <0.05)	<0.05 (0.0 to <0.05)
Premenstrual syndrome	Abdominal pain and depression due to premenstrual syndrome	15,680.6 (11,652.1 to 20,509.9)	17,262.8 (12,831.6 to 22,441.3)	2,291.1 (1,396.1 to 3,459.8)	2,523.7 (1,544.2 to 3,836.8)
Premenstrual syndrome	Abdominal pain due to premenstrual syndrome	22,389.8 (16,000.7 to 29,662.8)	24,649.0 (17,562.7 to 32,690.4)	240.4 (106.6 to 479.4)	264.7 (117.0 to 527.3)
Premenstrual syndrome	Depression due to premenstrual syndrome	5,493.7 (1,737.3 to 11,158.6)	6,049.5 (1,915.7 to 12,224.2)	756.3 (218.6 to 1,650.2)	833.1 (238.3 to 1,855.8)
Other gynecological diseases	Asymptomatic other gynecological disorders	7,207.7 (6,470.8 to 8,046.4)	8,199.0 (7,365.9 to 9,158.7)	<0.05 (0.0 to <0.05)	<0.05 (0.0 to <0.05)
Other gynecological diseases	Mild other gynecological disorders	9,612.3 (8,492.4 to 10,766.0)	10,934.3 (9,665.7 to 12,236.3)	102.8 (50.0 to 192.1)	117.0 (57.1 to 218.1)
Other gynecological diseases	Moderate other gynecological disorders	2,814.5 (1,777.5 to 3,988.1)	3,201.5 (2,025.8 to 4,537.4)	304.7 (171.4 to 477.1)	346.9 (195.5 to 542.9)
Other gynecological diseases	Severe other gynecological disorders	1,383.0 (970.7 to 1,871.9)	1,573.2 (1,104.4 to 2,132.8)	415.4 (254.5 to 622.5)	472.9 (288.1 to 714.8)
Other gynecological diseases	Mild anemia due to other gynecological diseases	9,310.2 (9,224.5 to 9,393.6)	9,893.6 (9,740.0 to 10,017.9)	33.6 (12.0 to 73.5)	35.7 (12.8 to 77.4)
Other gynecological diseases	Moderate anemia due to other gynecological diseases	10,165.3 (10,043.7 to 10,293.2)	10,192.5 (10,043.1 to 10,365.0)	502.0 (332.9 to 728.9)	504.1 (336.4 to 730.2)
Other gynecological diseases	Severe anemia due to other gynecological diseases	1,192.8 (1,160.7 to 1,221.4)	1,024.6 (994.2 to 1,054.4)	166.1 (114.3 to 228.8)	143.1 (98.5 to 198.2)
Thalassemias	Beta-thalassemia major, with mild anemia	21.3 (13.5 to 29.7)	20.8 (13.4 to 29.3)	0.3 (0.1 to 0.7)	0.3 (0.1 to 0.7)
Thalassemias	Beta-thalassemia major, with moderate anemia	137.6 (126.6 to 147.5)	141.8 (132.6 to 152.3)	8.5 (5.4 to 12.6)	8.8 (5.7 to 12.9)
Thalassemias	Beta-thalassemia major, with severe anemia	39.3 (36.1 to 42.9)	41.5 (37.8 to 45.4)	6.1 (4.1 to 8.6)	6.4 (4.3 to 9.1)
Thalassemias	Beta-thalassemia major, severe infection with severe anemia	0.9 (0.3 to 1.8)	0.9 (0.3 to 1.9)	0.2 (0.1 to 0.5)	0.3 (0.1 to 0.5)
Thalassemias	Hemoglobin E/beta-thalassemia, with mild anemia	4.2 (2.5 to 6.1)	5.9 (3.6 to 8.7)	0.1 (0.0 to 0.1)	0.1 (0.0 to 0.2)
Thalassemias	Hemoglobin E/beta-thalassemia, with moderate anemia	36.7 (32.9 to 40.4)	39.1 (35.2 to 42.9)	2.3 (1.4 to 3.5)	2.4 (1.5 to 3.7)
Thalassemias	Hemoglobin E/beta-thalassemia, with severe anemia	15.7 (14.0 to 17.8)	15.2 (13.0 to 17.5)	2.5 (1.6 to 3.4)	2.4 (1.6 to 3.4)
Thalassemias	Hemoglobin E/beta-thalassemia, severe infection with severe anemia	0.3 (0.1 to 0.5)	0.3 (0.1 to 0.5)	0.1 (0.0 to 0.1)	0.1 (0.0 to 0.1)
Thalassemias	Hemoglobin H disease, with mild anemia	17.9 (10.9 to 28.7)	19.7 (9.8 to 33.0)	0.3 (0.1 to 0.7)	0.3 (0.1 to 0.7)
Thalassemias	Hemoglobin H disease, with moderate anemia	82.1 (65.1 to 100.0)	72.1 (56.4 to 92.3)	5.1 (3.1 to 7.8)	4.5 (2.7 to 7.0)
Thalassemias	Hemoglobin H disease, with severe anemia	28.2 (25.9 to 30.8)	28.9 (26.0 to 34.0)	4.4 (3.1 to 6.1)	4.5 (3.1 to 6.4)
Thalassemias	Hemoglobin H disease, severe infection with severe anemia	0.7 (0.2 to 1.3)	0.7 (0.2 to 1.3)	0.2 (0.1 to 0.4)	0.2 (0.1 to 0.4)
Thalassemias	Mild heart failure due to thalassemias	0.7 (0.4 to 0.9)	0.8 (0.5 to 1.1)	<0.05 (0.0 to <0.05)	<0.05 (0.0 to 0.1)
Thalassemias	Moderate heart failure due to thalassemias	0.5 (0.4 to 0.7)	0.7 (0.5 to 0.9)	<0.05 (0.0 to 0.1)	<0.05 (0.0 to 0.1)
Thalassemias	Severe heart failure due to thalassemias	1.5 (1.2 to 1.7)	1.9 (1.6 to 2.2)	0.3 (0.2 to 0.4)	0.3 (0.2 to 0.5)
Thalassemias	Hemoglobin H disease, without anemia	17.8 (3.8 to 60.6)	17.6 (3.7 to 64.0)	0.2 (0.0 to 0.8)	0.2 (0.0 to 0.9)
Thalassemias	Beta-thalassemia major, without anemia	20.4 (11.3 to 33.2)	24.1 (14.5 to 35.6)	0.3 (0.1 to 0.5)	0.3 (0.1 to 0.6)
Thalassemias	Hemoglobin E/Beta-thalassemia, without anemia	7.0 (3.2 to 12.5)	6.9 (2.7 to 12.7)	0.1 (0.0 to 0.2)	0.1 (0.0 to 0.2)
Thalassemias trait	Asymptomatic B-thalassemia trait	95,425.5 (90,371.4 to 102,236.4)	106,085.9 (99,993.8 to 113,474.2)	<0.05 (0.0 to <0.05)	<0.05 (0.0 to <0.05)
Thalassemias trait	Mild anemia due to B-thalassemia trait	52,766.4 (52,344.7 to 53,214.9)	59,227.7 (58,689.7 to 59,772.3)	190.9 (68.0 to 415.0)	214.0 (76.3 to 466.6)
Thalassemias trait	Moderate anemia due to B-thalassemia trait	52,651.1 (52,100.3 to 53,186.0)	56,151.2 (55,632.7 to 56,692.6)	2,625.4 (1,736.9 to 3,799.6)	2,797.5 (1,855.6 to 4,033.0)
Thalassemias trait	Severe anemia due to B-thalassemia trait	4,493.0 (4,423.1 to 4,564.8)	4,564.8 (4,493.5 to 4,641.6)	639.7 (441.2 to 887.1)	650.2 (446.7 to 905.9)
Thalassemias trait	Asymptomatic hemoglobin E trait	39,188.0 (37,105.2 to 41,302.4)	43,992.3 (41,674.1 to 46,457.2)	<0.05 (0.0 to <0.05)	<0.05 (0.0 to <0.05)
Thalassemias trait	Mild anemia due to hemoglobin E trait	4,315.3 (4,269.0 to 4,359.5)	5,160.1 (5,083.2 to 5,246.2)	15.6 (5.6 to 33.9)	18.6 (6.6 to 40.3)
Thalassemias trait	Moderate anemia due to hemoglobin E trait	3,674.8 (3,624.3 to 3,725.7)	3,970.2 (3,887.1 to 4,047.7)	183.7 (122.1 to 266.0)	198.5 (131.5 to 288.4)
Thalassemias trait	Severe anemia due to hemoglobin E trait	284.3 (268.0 to 302.7)	299.2 (277.8 to 322.0)	41.0 (27.9 to 57.2)	43.3 (29.4 to 60.4)
Sickle cell disorders	Homozygous sickle cell and severe sickle cell/beta-thalassemia, without anemia	<0.05 (0.0 to <0.05)	<0.05 (0.0 to <0.05)	<0.05 (0.0 to <0.05)	<0.05 (0.0 to <0.05)
Sickle cell disorders	Homozygous sickle cell and severe sickle cell/beta-thalassemia, with vaso-occlusive crisis, without anemia	50.0 (33.8 to 83.7)	54.8 (37.2 to 87.5)	16.0 (9.1 to 27.8)	17.5 (10.1 to 29.8)
Sickle cell disorders	Homozygous sickle cell and severe sickle cell/beta-thalassemia, with stroke, without anemia	38.4 (24.2 to 55.5)	48.9 (31.4 to 70.0)	11.9 (6.5 to 19.2)	15.1 (8.2 to 24.4)
Sickle cell disorders	Homozygous sickle cell and severe sickle cell/beta-thalassemia, with vaso-occlusive crisis and stroke, without anemia	3.8 (2.3 to 5.8)	5.0 (3.0 to 7.7)	2.0 (1.0 to 3.4)	2.6 (1.4 to 4.4)
Sickle cell disorders	Homozygous sickle cell and severe sickle cell/beta-thalassemia, with mild anemia	448.6 (344.4 to 564.5)	525.5 (346.9 to 647.5)	7.1 (3.0 to 13.9)	8.3 (3.5 to 16.8)
Sickle cell disorders	Homozygous sickle cell and severe sickle cell/beta-thalassemia, with moderate anemia	2,758.3 (2,667.7 to 2,856.7)	2,904.5 (2,788.6 to 3,024.4)	169.8 (108.8 to 250.7)	179.1 (116.7 to 261.9)
Sickle cell disorders	Homozygous sickle cell and severe sickle cell/beta-thalassemia, with severe anemia	347.3 (272.0 to 444.1)	339.9 (261.7 to 481.0)	53.1 (33.5 to 78.7)	52.2 (32.8 to 78.7)
Sickle cell disorders	Homozygous sickle cell and severe sickle cell/beta-thalassemia, with vaso-occlusive crisis and severe anemia	124.0 (80.6 to 172.0)	137.3 (87.2 to 191.1)	51.0 (29.0 to 80.4)	56.5 (31.7 to 89.1)
Sickle cell disorders	Homozygous sickle cell and severe sickle cell/beta-thalassemia, with stroke and severe anemia	17.1 (13.0 to 21.1)	17.3 (12.5 to 23.6)	6.8 (4.4 to 9.9)	7.0 (4.3 to 10.9)
Sickle cell disorders	Homozygous sickle cell and severe sickle cell/beta-thalassemia, with vaso-occlusive crisis, stroke, and severe anemia	1.9 (1.1 to 2.8)	1.9 (1.2 to 3.0)	1.1 (0.6 to 1.8)	1.1 (0.6 to 2.0)
Sickle cell disorders	Hemoglobin SC disease, without anemia	<0.05 (0.0 to <0.05)	<0.05 (0.0 to <0.05)	<0.05 (0.0 to <0.05)	<0.05 (0.0 to <0.05)
Sickle cell disorders	Hemoglobin SC disease, with vaso-occlusive crisis, without anemia	6.3 (3.3 to 10.5)	8.2 (4.9 to 13.5)	2.0 (0.9 to 3.8)	2.6 (1.3 to 4.8)
Sickle cell disorders	Hemoglobin SC disease, with stroke, without anemia	3.5 (2.2 to 5.2)	4.3 (2.7 to 6.3)	1.1 (0.5 to 1.9)	1.4 (0.7 to 2.4)
Sickle cell disorders	Hemoglobin SC disease, with vaso-occlusive crisis and stroke, without anemia	0.4 (0.2 to 0.6)	0.5 (0.2 to 0.7)	0.2 (0.1 to 0.3)	0.2 (0.1 to 0.4)
Sickle cell disorders	Hemoglobin SC disease, with mild anemia	56.9 (18.9 to 74.1)	89.8 (63.4 to 104.5)	0.9 (0.2 to 1.9)	1.4 (0.6 to 2.8)
Sickle cell disorders	Hemoglobin SC disease, with moderate anemia	196.7 (176.3 to 228.5)	248.6 (226.7 to 270.9)	12.0 (7.5 to 18.2)	15.3 (9.7 to 23.0)
Sickle cell disorders	Hemoglobin SC disease, with severe anemia	36.9 (31.4 to 46.2)	38.5 (29.5 to 50.4)	5.6 (3.8 to 8.4)	5.9 (3.6 to 8.7)
Sickle cell disorders	Hemoglobin SC disease, with vaso-occlusive crisis and severe anemia	4.4 (2.4 to 7.9)	5.7 (2.9 to 9.0)	1.9 (0.9 to 3.7)	2.4 (1.1 to 4.2)
Sickle cell disorders	Hemoglobin SC disease, with stroke and severe anemia	1.5 (0.9 to 2.3)	1.8 (1.2 to 3.1)	0.6 (0.3 to 1.0)	0.8 (0.4 to 1.4)
Sickle cell disorders	Hemoglobin SC disease, with vaso-occlusive crisis, stroke, and severe anemia	0.2 (0.1 to 0.3)	0.3 (0.2 to 0.5)	0.1 (0.1 to 0.2)	0.2 (0.1 to 0.3)
Sickle cell disorders	Mild sickle cell/beta-thalassemia, without anemia	<0.05 (0.0 to <0.05)	<0.05 (0.0 to <0.05)	<0.05 (0.0 to <0.05)	<0.05 (0.0 to <0.05)

Cause	Sequela	Prevalence in thousands (95% confidence interval)		YLDs in thousands (95% confidence interval)	
		2005	2015	2005	2015
Sickle cell disorders	Mild sickle cell/beta-thalassemia, with vaso-occlusive crisis, without anemia	0.1 (0.0 to 0.1)	<0.05 (0.0 to <0.05)	<0.05 (0.0 to <0.05)	<0.05 (0.0 to <0.05)
Sickle cell disorders	Mild sickle cell/beta-thalassemia, with stroke, without anemia	0.1 (0.0 to 0.1)	<0.05 (0.0 to <0.05)	<0.05 (0.0 to <0.05)	<0.05 (0.0 to <0.05)
Sickle cell disorders	Mild sickle cell/beta-thalassemia, with vaso-occlusive crisis and stroke, without anemia	<0.05 (0.0 to <0.05)	<0.05 (0.0 to <0.05)	<0.05 (0.0 to <0.05)	<0.05 (0.0 to <0.05)
Sickle cell disorders	Mild sickle cell/beta-thalassemia, with mild anemia	1.6 (1.1 to 2.3)	2.0 (1.3 to 2.8)	<0.05 (0.0 to 0.1)	<0.05 (0.0 to 0.1)
Sickle cell disorders	Mild sickle cell/beta-thalassemia, with moderate anemia	6.8 (6.2 to 7.4)	8.0 (7.1 to 9.0)	0.4 (0.3 to 0.6)	0.5 (0.3 to 0.8)
Sickle cell disorders	Mild sickle cell/beta-thalassemia, with severe anemia	5.6 (5.0 to 6.1)	6.4 (5.7 to 7.1)	0.9 (0.6 to 1.3)	1.0 (0.7 to 1.4)
Sickle cell disorders	Mild sickle cell/beta-thalassemia, with vaso-occlusive crisis and severe anemia	0.3 (0.3 to 0.4)	0.4 (0.3 to 0.5)	0.1 (0.1 to 0.2)	0.2 (0.1 to 0.2)
Sickle cell disorders	Mild sickle cell/beta-thalassemia, with stroke and severe anemia	0.4 (0.3 to 0.5)	0.4 (0.3 to 0.5)	0.2 (0.1 to 0.2)	0.2 (0.1 to 0.3)
Sickle cell disorders	Mild sickle cell/beta-thalassemia, with vaso-occlusive crisis, stroke, and severe anemia	<0.05 (0.0 to <0.05)	<0.05 (0.0 to <0.05)	<0.05 (0.0 to <0.05)	<0.05 (0.0 to <0.05)
Sickle cell trait	Asymptomatic sickle cell trait	296,022.1 (276,020.4 to 336,115.9)	353,682.8 (330,395.2 to 397,433.5)	<0.05 (0.0 to <0.05)	<0.05 (0.0 to <0.05)
Sickle cell trait	Mild anemia due to sickle cell trait	17,960.4 (17,836.3 to 18,073.9)	23,062.4 (22,795.7 to 23,604.6)	64.7 (23.0 to 141.3)	83.1 (29.7 to 180.7)
Sickle cell trait	Moderate anemia due to sickle cell trait	21,933.7 (21,734.2 to 22,167.6)	25,047.8 (24,367.5 to 25,358.3)	1,088.1 (723.6 to 1,572.9)	1,244.8 (829.0 to 1,807.0)
Sickle cell trait	Severe anemia due to sickle cell trait	2,839.4 (2,751.2 to 2,930.6)	2,772.9 (2,647.0 to 2,878.4)	400.2 (273.3 to 549.7)	392.4 (268.0 to 542.5)
G6PD deficiency	Mild anemia due to G6PD deficiency	193.0 (189.5 to 196.6)	209.4 (201.8 to 220.9)	0.7 (0.3 to 1.5)	0.8 (0.3 to 1.7)
G6PD deficiency	Moderate anemia due to G6PD deficiency	158.0 (155.5 to 160.6)	162.7 (155.2 to 169.3)	8.0 (5.3 to 11.9)	8.3 (5.5 to 12.0)
G6PD deficiency	Severe anemia due to G6PD deficiency	127.7 (125.3 to 129.9)	127.3 (122.6 to 130.9)	18.7 (12.7 to 26.1)	18.7 (12.5 to 26.2)
G6PD deficiency	Mild heart failure due to G6PD deficiency	0.9 (0.6 to 1.2)	1.3 (0.8 to 1.7)	<0.05 (0.0 to 0.1)	0.1 (0.0 to 0.1)
G6PD deficiency	Moderate heart failure due to G6PD deficiency	0.8 (0.5 to 1.0)	1.0 (0.7 to 1.4)	0.1 (0.0 to 0.1)	0.1 (0.0 to 0.1)
G6PD deficiency	Severe heart failure due to G6PD deficiency	2.1 (1.7 to 2.4)	2.8 (2.4 to 3.3)	0.4 (0.2 to 0.5)	0.5 (0.3 to 0.7)
G6PD deficiency	Asymptomatic G6PD deficiency	230,626.2 (204,585.0 to 259,288.0)	246,569.1 (208,806.5 to 286,203.3)	<0.05 (0.0 to <0.05)	<0.05 (0.0 to <0.05)
G6PD trait	Asymptomatic hemizygous G6PD deficiency	663,098.8 (624,421.0 to 703,191.7)	727,908.7 (676,092.6 to 780,889.7)	<0.05 (0.0 to <0.05)	<0.05 (0.0 to <0.05)
G6PD trait	Mild anemia due to hemizygous G6PD deficiency	298.4 (292.9 to 304.8)	325.5 (317.5 to 333.6)	1.1 (0.4 to 2.4)	1.2 (0.4 to 2.6)
G6PD trait	Moderate anemia due to hemizygous G6PD deficiency	195.3 (191.1 to 199.6)	196.8 (189.4 to 202.9)	9.8 (6.5 to 14.4)	9.9 (6.5 to 14.4)
G6PD trait	Severe anemia due to hemizygous G6PD deficiency	111.4 (108.7 to 113.8)	118.2 (115.2 to 121.1)	16.0 (11.0 to 22.4)	17.0 (11.8 to 23.4)
Other hemoglobinopathies and hemolytic anemias	Mild anemia due to other hemoglobinopathies and hemolytic anemias	39,205.9 (38,898.2 to 39,513.0)	41,658.0 (41,354.3 to 41,989.0)	141.4 (50.6 to 307.8)	150.1 (53.7 to 327.3)
Other hemoglobinopathies and hemolytic anemias	Moderate anemia due to other hemoglobinopathies and hemolytic anemias	30,072.7 (29,761.7 to 30,382.2)	29,383.7 (29,037.9 to 29,741.5)	1,494.5 (993.1 to 2,161.0)	1,461.0 (972.4 to 2,113.2)
Other hemoglobinopathies and hemolytic anemias	Severe anemia due to other hemoglobinopathies and hemolytic anemias	3,684.1 (3,591.9 to 3,779.5)	3,231.6 (3,142.5 to 3,322.3)	519.7 (357.7 to 721.5)	456.2 (310.6 to 633.4)
Other hemoglobinopathies and hemolytic anemias	Mild heart failure due to other hemoglobinopathies and hemolytic anemias	20.3 (13.3 to 27.5)	27.5 (17.9 to 37.2)	0.8 (0.5 to 1.4)	1.1 (0.6 to 1.8)
Other hemoglobinopathies and hemolytic anemias	Moderate heart failure due to other hemoglobinopathies and hemolytic anemias	16.8 (12.1 to 21.9)	22.7 (16.4 to 29.6)	1.2 (0.7 to 1.8)	1.6 (0.9 to 2.5)
Other hemoglobinopathies and hemolytic anemias	Severe heart failure due to other hemoglobinopathies and hemolytic anemias	45.7 (38.9 to 53.4)	61.7 (52.5 to 71.8)	7.7 (5.0 to 10.9)	10.4 (6.8 to 14.8)
Endocrine, metabolic, blood, and immune disorders	Asymptomatic endocrine, metabolic, blood, and immune disorders	2,269.2 (2,101.6 to 2,457.2)	2,718.0 (2,519.7 to 2,947.3)	<0.05 (0.0 to <0.05)	<0.05 (0.0 to <0.05)
Endocrine, metabolic, blood, and immune disorders	Mild endocrine, metabolic, blood, and immune disorders	2,692.1 (2,436.4 to 2,943.2)	3,224.5 (2,919.1 to 3,529.6)	48.0 (25.3 to 80.7)	57.4 (30.5 to 96.4)
Endocrine, metabolic, blood, and immune disorders	Moderate endocrine, metabolic, blood, and immune disorders	427.2 (255.8 to 632.6)	511.6 (307.7 to 756.4)	58.3 (30.9 to 94.0)	69.6 (36.8 to 113.3)
Endocrine, metabolic, blood, and immune disorders	Severe endocrine, metabolic, blood, and immune disorders	937.7 (779.6 to 1,109.3)	1,123.1 (932.0 to 1,330.3)	138.5 (87.7 to 198.8)	165.5 (105.0 to 237.5)
Endocrine, metabolic, blood, and immune disorders	Mild anemia due to endocrine, metabolic, blood, and immune disorders	28,586.4 (28,050.9 to 29,331.7)	29,904.3 (29,624.5 to 30,259.8)	103.2 (36.9 to 225.5)	107.9 (38.5 to 235.4)
Endocrine, metabolic, blood, and immune disorders	Moderate anemia due to endocrine, metabolic, blood, and immune disorders	25,317.3 (24,590.8 to 25,979.3)	25,530.6 (25,101.3 to 25,902.2)	1,261.4 (833.2 to 1,821.2)	1,272.5 (840.2 to 1,840.7)
Endocrine, metabolic, blood, and immune disorders	Severe anemia due to endocrine, metabolic, blood, and immune disorders	3,279.1 (3,123.0 to 3,449.2)	2,995.4 (2,909.3 to 3,090.7)	463.1 (316.3 to 642.1)	424.0 (291.0 to 588.7)
Endocrine, metabolic, blood, and immune disorders	Mild heart failure due to endocrine, metabolic, blood, and immune disorders	22.8 (15.1 to 30.8)	29.8 (19.7 to 40.2)	0.9 (0.5 to 1.4)	1.2 (0.6 to 1.9)
Endocrine, metabolic, blood, and immune disorders	Moderate heart failure due to endocrine, metabolic, blood, and immune disorders	18.8 (13.6 to 24.5)	24.6 (17.7 to 32.0)	1.3 (0.8 to 2.0)	1.7 (1.0 to 2.6)
Endocrine, metabolic, blood, and immune disorders	Severe heart failure due to endocrine, metabolic, blood, and immune disorders	51.2 (43.4 to 59.6)	67.0 (56.7 to 78.1)	8.3 (5.5 to 11.7)	10.8 (7.1 to 15.1)
Rheumatoid arthritis	Mild rheumatoid arthritis	9,223.6 (7,895.9 to 10,539.5)	11,420.9 (9,707.3 to 13,074.0)	1,002.1 (681.2 to 1,397.9)	1,239.3 (842.1 to 1,736.6)
Rheumatoid arthritis	Moderate rheumatoid arthritis	7,782.8 (6,691.8 to 8,988.6)	9,636.9 (8,269.3 to 11,212.6)	2,237.1 (1,500.6 to 3,088.2)	2,764.9 (1,845.3 to 3,831.2)
Rheumatoid arthritis	Severe rheumatoid arthritis	2,772.9 (1,909.8 to 3,765.7)	3,433.4 (2,350.4 to 4,671.8)	1,435.1 (874.5 to 2,125.7)	1,773.6 (1,080.1 to 2,636.6)
Osteoarthritis	Mild osteoarthritis of the hip	15,898.9 (14,276.8 to 17,562.3)	20,913.3 (18,861.3 to 23,116.5)	335.7 (195.9 to 552.9)	441.6 (257.5 to 723.9)
Osteoarthritis	Moderate osteoarthritis of the hip	8,187.0 (7,087.8 to 9,288.9)	11,058.1 (9,612.4 to 12,506.0)	587.7 (396.9 to 828.4)	794.5 (540.9 to 1,114.6)
Osteoarthritis	Severe osteoarthritis of the hip	2,607.8 (2,266.9 to 2,970.8)	3,657.8 (3,178.9 to 4,159.5)	384.4 (260.4 to 535.2)	540.1 (367.0 to 750.8)
Osteoarthritis	Mild osteoarthritis of the knee	83,131.5 (78,916.9 to 87,409.0)	108,888.0 (103,460.7 to 114,533.3)	1,775.4 (1,023.6 to 2,865.4)	2,326.5 (1,339.8 to 3,759.7)
Osteoarthritis	Moderate osteoarthritis of the knee	49,738.7 (46,081.4 to 53,674.3)	66,649.1 (61,824.3 to 71,607.7)	3,619.5 (2,501.0 to 4,955.8)	4,854.5 (3,343.3 to 6,653.5)
Osteoarthritis	Severe osteoarthritis of the knee	19,101.1 (16,759.3 to 21,441.9)	26,202.3 (22,929.5 to 29,360.9)	2,859.8 (1,952.5 to 3,954.2)	3,928.9 (2,677.9 to 5,423.5)
Low back pain	Mild low back pain without leg pain	141,950.2 (108,957.2 to 174,840.2)	166,547.5 (128,259.8 to 204,547.1)	2,712.9 (1,397.4 to 4,636.2)	3,181.2 (1,636.0 to 5,430.8)
Low back pain	Mild low back pain with leg pain	25,110.3 (4,495.1 to 65,223.6)	29,461.7 (5,270.5 to 76,819.5)	482.5 (67.5 to 1,319.7)	565.9 (79.3 to 1,553.7)
Low back pain	Moderate low back pain without leg pain	130,030.0 (101,474.4 to 160,223.9)	152,565.6 (119,045.2 to 188,152.0)	6,652.4 (4,091.9 to 9,952.3)	7,800.6 (4,796.0 to 11,629.9)
Low back pain	Moderate low back pain with leg pain	34,578.7 (13,921.1 to 63,269.2)	40,572.6 (16,343.1 to 74,479.5)	1,768.8 (650.7 to 3,440.5)	2,074.2 (761.3 to 4,070.1)
Low back pain	Severe low back pain without leg pain	39,522.3 (31,613.8 to 48,999.9)	46,370.5 (37,151.7 to 57,516.6)	9,855.8 (6,416.8 to 14,237.4)	11,554.0 (7,544.8 to 16,691.1)
Low back pain	Severe low back pain with leg pain	13,124.7 (4,140.6 to 25,738.2)	15,398.1 (4,838.0 to 30,129.7)	3,902.9 (1,095.9 to 8,017.0)	4,574.4 (1,284.4 to 9,372.6)
Low back pain	Most severe low back pain without leg pain	54,384.4 (36,360.6 to 75,565.4)	63,808.3 (42,601.1 to 88,829.5)	18,419.8 (10,571.1 to 28,429.5)	21,593.6 (12,412.2 to 33,323.6)
Low back pain	Most severe low back pain with leg pain	21,463.7 (3,823.4 to 52,516.5)	25,183.1 (4,501.5 to 61,405.0)	7,447.8 (1,308.9 to 18,623.9)	8,731.0 (1,533.9 to 21,865.1)
Neck pain	Mild neck pain	203,457.1 (172,978.7 to 237,470.0)	246,466.0 (209,082.4 to 287,683.2)	10,187.6 (6,290.7 to 15,296.7)	12,330.8 (7,606.1 to 18,551.3)
Neck pain	Moderate neck pain	33,303.5 (18,479.6 to 51,042.4)	40,342.2 (22,204.8 to 61,823.7)	3,548.7 (1,737.9 to 6,154.7)	4,294.3 (2,095.5 to 7,451.4)

Cause	Sequela	Prevalence in thousands (95% confidence interval)		YLDs in thousands (95% confidence interval)	
		2005	2015	2005	2015
Neck pain	Severe neck pain	18,046.2 (12,211.6 to 25,502.6)	21,862.8 (14,802.8 to 30,792.9)	3,797.6 (2,143.3 to 6,074.0)	4,595.6 (2,581.0 to 7,329.3)
Neck pain	Most severe neck pain	40,725.4 (26,835.9 to 57,184.7)	49,335.6 (32,836.9 to 69,408.1)	11,276.7 (6,461.8 to 17,858.4)	13,646.0 (7,830.5 to 21,623.8)
Gout	Asymptomatic gout	29,827.2 (26,630.6 to 33,609.5)	37,712.9 (33,598.4 to 42,467.0)	<0.05 (0.0 to <0.05)	<0.05 (0.0 to <0.05)
Gout	Symptomatic episodes of gout	3,101.9 (2,571.2 to 3,686.2)	3,922.0 (3,245.7 to 4,636.3)	825.5 (545.9 to 1,146.8)	1,043.0 (689.6 to 1,446.7)
Gout	Polyarticular gout	458.2 (409.0 to 513.8)	579.3 (517.6 to 647.8)	237.6 (167.4 to 307.7)	299.8 (211.4 to 384.9)
Other musculoskeletal disorders	Asymptomatic other musculoskeletal disorders	70,708.4 (60,641.0 to 82,142.4)	85,371.1 (72,964.7 to 99,477.0)	<0.05 (0.0 to <0.05)	<0.05 (0.0 to <0.05)
Other musculoskeletal disorders	Other musculoskeletal disorders severity level 2	60,107.4 (45,721.3 to 76,763.7)	72,570.3 (55,026.3 to 93,834.8)	1,628.9 (898.3 to 2,751.3)	1,964.7 (1,084.2 to 3,305.8)
Other musculoskeletal disorders	Other musculoskeletal disorders severity level 3	30,765.8 (19,643.3 to 44,651.0)	37,144.9 (23,548.3 to 54,012.9)	3,369.0 (1,842.0 to 5,309.5)	4,062.5 (2,217.9 to 6,493.9)
Other musculoskeletal disorders	Other musculoskeletal disorders severity level 5	22,697.2 (17,767.2 to 28,055.6)	27,402.6 (21,470.4 to 33,969.4)	6,592.8 (4,245.1 to 9,426.0)	7,946.0 (5,098.0 to 11,433.0)
Other musculoskeletal disorders	Other musculoskeletal disorders severity level 6	20,639.6 (11,282.5 to 32,408.4)	24,918.9 (13,536.1 to 39,182.3)	10,815.4 (5,453.9 to 17,540.9)	13,032.7 (6,523.7 to 21,096.7)
Other musculoskeletal disorders	Other musculoskeletal disorders severity level 1	60,921.5 (41,663.8 to 82,805.9)	73,558.4 (49,885.4 to 100,275.9)	1,325.2 (660.8 to 2,312.9)	1,598.4 (799.1 to 2,787.6)
Other musculoskeletal disorders	Other musculoskeletal disorders severity level 4	17,476.7 (12,951.0 to 22,822.2)	21,101.5 (15,567.2 to 27,809.4)	2,679.7 (1,639.8 to 4,116.7)	3,231.1 (1,979.2 to 4,965.8)
Neural tube defects	Moderate motor impairment due to moderate neural tube defects	365.0 (231.9 to 599.7)	421.2 (264.9 to 702.5)	21.7 (11.2 to 38.9)	25.0 (12.9 to 46.0)
Neural tube defects	Severe motor impairment due to severe neural tube defects	141.1 (86.2 to 219.1)	167.0 (101.0 to 262.2)	55.9 (29.9 to 95.6)	66.3 (35.1 to 114.3)
Neural tube defects	Severe motor impairment with incontinence due to severe neural tube defects	564.7 (348.9 to 887.0)	668.3 (405.9 to 1,063.1)	258.9 (145.0 to 430.2)	305.7 (169.4 to 516.6)
Neural tube defects	Severe motor impairment with mild intellectual disability and incontinence due to severe neural tube defects	43.9 (25.6 to 71.5)	52.4 (30.7 to 85.4)	22.2 (12.1 to 37.8)	26.5 (14.5 to 45.7)
Neural tube defects	Severe motor impairment with moderate intellectual disability and incontinence due to severe neural tube defects	30.5 (14.9 to 50.7)	37.3 (18.2 to 61.8)	16.3 (7.5 to 29.7)	19.9 (9.3 to 35.9)
Neural tube defects	Severe motor impairment with severe intellectual disability and incontinence due to severe neural tube defects	13.9 (0.5 to 28.5)	16.8 (0.6 to 34.3)	7.8 (0.3 to 16.7)	9.5 (0.3 to 20.3)
Neural tube defects	Severe motor impairment with profound intellectual disability and incontinence due to severe neural tube defects	40.2 (6.1 to 67.8)	47.9 (7.3 to 82.0)	23.5 (3.4 to 41.1)	28.0 (4.1 to 49.6)
Neural tube defects	Severe motor impairment with mild intellectual disability due to severe neural tube defects	11.0 (4.1 to 23.4)	13.1 (4.9 to 27.7)	4.7 (1.7 to 10.3)	5.6 (2.0 to 12.2)
Neural tube defects	Severe motor impairment with moderate intellectual disability due to severe neural tube defects	7.7 (2.5 to 16.5)	9.4 (3.1 to 20.1)	3.5 (1.1 to 8.1)	4.3 (1.3 to 10.0)
Neural tube defects	Severe motor impairment with severe intellectual disability due to severe neural tube defects	3.4 (0.1 to 8.3)	4.2 (0.1 to 10.1)	1.7 (0.0 to 4.3)	2.1 (0.1 to 5.1)
Neural tube defects	Severe motor impairment with profound intellectual disability due to severe neural tube defects	10.1 (1.5 to 21.9)	12.0 (1.8 to 26.2)	5.2 (0.6 to 12.0)	6.2 (0.7 to 14.3)
Congenital heart anomalies	Asymptomatic less severe congenital heart anomalies	15,506.5 (10,614.1 to 23,064.8)	20,070.9 (13,265.6 to 30,495.2)	<0.05 (0.0 to <0.05)	<0.05 (0.0 to <0.05)
Congenital heart anomalies	Symptomatic less severe congenital heart anomalies	18,950.3 (12,909.7 to 28,786.0)	24,528.6 (16,490.2 to 38,298.0)	1,107.1 (419.3 to 2,098.7)	1,431.0 (532.7 to 2,747.7)
Congenital heart anomalies	Severe congenital heart anomalies	2,998.0 (2,042.4 to 4,443.6)	4,008.6 (2,671.1 to 6,011.2)	176.0 (69.3 to 353.1)	235.0 (93.2 to 467.8)
Congenital heart anomalies	Critical congenital heart anomalies	109.7 (68.5 to 168.0)	155.8 (90.3 to 252.4)	6.5 (2.3 to 13.0)	9.2 (3.2 to 18.7)
Congenital heart anomalies	Mild heart failure due to congenital heart anomalies	23.1 (15.3 to 31.2)	25.8 (17.1 to 34.9)	1.0 (0.5 to 1.6)	1.1 (0.6 to 1.8)
Congenital heart anomalies	Moderate heart failure due to congenital heart anomalies	19.1 (13.8 to 25.1)	21.3 (15.4 to 28.0)	1.4 (0.8 to 2.2)	1.5 (0.9 to 2.4)
Congenital heart anomalies	Severe heart failure due to congenital heart anomalies	52.0 (44.3 to 60.6)	58.0 (49.6 to 67.5)	9.2 (6.1 to 13.3)	10.3 (6.8 to 14.9)
Cleft lip and cleft palate	Asymptomatic orofacial clefts	4,619.4 (2,915.0 to 7,406.3)	5,601.4 (3,293.1 to 9,320.9)	<0.05 (0.0 to <0.05)	<0.05 (0.0 to <0.05)
Cleft lip and cleft palate	Disfigurement level 1 due to orofacial clefts	380.7 (234.6 to 622.1)	427.0 (245.3 to 716.9)	4.1 (1.7 to 8.4)	4.6 (1.8 to 9.8)
Cleft lip and cleft palate	Disfigurement level 2 due to orofacial clefts	380.8 (236.0 to 621.3)	427.0 (247.4 to 716.7)	24.8 (13.0 to 43.5)	27.8 (14.0 to 49.9)
Cleft lip and cleft palate	Disfigurement level 2 and speech problems due to orofacial clefts	380.7 (234.9 to 623.5)	427.2 (247.7 to 720.1)	42.1 (22.0 to 72.1)	47.2 (23.5 to 84.4)
Down syndrome	Asymptomatic Down syndrome	122.3 (77.4 to 189.1)	141.8 (87.6 to 224.0)	<0.05 (0.0 to <0.05)	<0.05 (0.0 to <0.05)
Down syndrome	Isolated congenital heart disease due to Down syndrome	88.7 (56.2 to 135.8)	102.8 (64.0 to 161.2)	5.4 (1.9 to 10.9)	6.3 (2.3 to 12.7)
Down syndrome	Borderline intellectual disability due to Down syndrome	293.9 (186.0 to 436.1)	340.7 (213.4 to 518.0)	3.1 (1.3 to 6.4)	3.6 (1.5 to 7.5)
Down syndrome	Mild intellectual disability due to Down syndrome	775.7 (500.9 to 1,168.2)	899.3 (569.9 to 1,384.4)	32.1 (16.9 to 57.4)	37.2 (19.4 to 66.9)
Down syndrome	Moderate intellectual disability due to Down syndrome	632.5 (401.0 to 962.4)	733.3 (454.6 to 1,136.7)	61.0 (33.7 to 101.9)	70.7 (38.8 to 117.7)
Down syndrome	Severe intellectual disability due to Down syndrome	408.0 (255.7 to 625.0)	473.1 (292.2 to 733.3)	63.0 (33.9 to 106.1)	72.9 (38.5 to 124.1)
Down syndrome	Profound intellectual disability due to Down syndrome	215.3 (137.5 to 327.6)	249.6 (156.9 to 389.8)	42.2 (22.9 to 73.1)	48.9 (26.0 to 86.9)
Down syndrome	Borderline intellectual disability with congenital heart disease due to Down syndrome	213.4 (137.6 to 328.1)	247.4 (155.6 to 388.7)	2.3 (0.9 to 4.7)	2.6 (1.1 to 5.6)
Down syndrome	Mild intellectual disability with congenital heart disease due to Down syndrome	562.9 (359.1 to 868.4)	652.6 (402.1 to 1,023.9)	23.3 (12.3 to 41.5)	27.0 (14.1 to 48.2)
Down syndrome	Moderate intellectual disability with congenital heart disease due to Down syndrome	457.7 (294.0 to 706.5)	530.6 (332.0 to 831.8)	44.3 (24.2 to 73.9)	51.3 (27.5 to 87.4)
Down syndrome	Severe intellectual disability with congenital heart disease due to Down syndrome	296.6 (189.6 to 456.4)	343.9 (214.9 to 535.8)	46.1 (24.9 to 76.9)	53.4 (28.6 to 91.9)
Down syndrome	Profound intellectual disability with congenital heart disease due to Down syndrome	155.9 (101.2 to 238.5)	180.8 (114.3 to 279.6)	30.8 (16.5 to 51.6)	35.7 (18.7 to 61.2)
Down syndrome	Mild dementia due to Down syndrome	10.5 (6.9 to 15.6)	15.0 (9.5 to 23.2)	0.7 (0.4 to 1.2)	1.0 (0.6 to 1.8)
Down syndrome	Moderate dementia due to Down syndrome	2.5 (1.7 to 3.8)	3.6 (2.3 to 5.6)	1.0 (0.5 to 1.6)	1.4 (0.7 to 2.4)
Down syndrome	Severe dementia due to Down syndrome	0.9 (0.6 to 1.3)	1.2 (0.8 to 1.9)	0.4 (0.2 to 0.6)	0.6 (0.3 to 0.9)
Down syndrome	Congenital heart disease and mild dementia due to Down syndrome	7.6 (5.0 to 11.3)	10.9 (6.9 to 16.8)	0.5 (0.3 to 0.9)	0.8 (0.4 to 1.3)
Down syndrome	Congenital heart disease and moderate dementia due to Down syndrome	1.8 (1.2 to 2.7)	2.6 (1.7 to 4.0)	0.7 (0.4 to 1.1)	1.0 (0.5 to 1.7)
Down syndrome	Congenital heart disease and severe dementia due to Down syndrome	0.6 (0.4 to 0.9)	0.9 (0.6 to 1.4)	0.3 (0.2 to 0.5)	0.4 (0.2 to 0.7)
Down syndrome	Borderline intellectual disability, mild dementia, and congenital heart disease due to Down syndrome	18.2 (11.9 to 27.0)	26.1 (16.4 to 40.0)	1.4 (0.8 to 2.5)	2.1 (1.1 to 3.7)
Down syndrome	Mild intellectual disability, mild dementia, and congenital heart disease due to Down syndrome	34.6 (22.4 to 51.7)	50.6 (32.1 to 77.2)	3.6 (2.0 to 6.1)	5.3 (2.8 to 9.1)
Down syndrome	Moderate intellectual disability, mild dementia, and congenital heart disease due to Down syndrome	21.5 (12.0 to 34.2)	30.7 (16.4 to 49.7)	3.4 (1.7 to 5.7)	4.9 (2.4 to 8.4)
Down syndrome	Severe intellectual disability, mild dementia, and congenital heart disease due to Down syndrome	9.2 (0.1 to 16.4)	12.8 (0.2 to 23.7)	2.0 (0.0 to 4.0)	2.8 (0.0 to 5.7)
Down syndrome	Profound intellectual disability, mild dementia, and congenital heart disease due to Down syndrome	12.3 (0.7 to 18.8)	17.7 (1.1 to 27.4)	3.1 (0.2 to 5.2)	4.5 (0.2 to 7.6)
Down syndrome	Borderline intellectual disability, moderate dementia, and congenital heart disease due to Down syndrome	4.4 (2.9 to 6.5)	6.3 (3.9 to 9.7)	1.7 (0.9 to 2.8)	2.4 (1.3 to 4.2)
Down syndrome	Mild intellectual disability, moderate dementia, and congenital heart disease due to Down syndrome	8.0 (5.2 to 12.3)	11.8 (7.5 to 18.4)	3.2 (1.9 to 5.2)	4.7 (2.6 to 7.8)

Cause	Sequela	Prevalence in thousands (95% confidence interval)		YLDs in thousands (95% confidence interval)	
		2005	2015	2005	2015
Down syndrome	Moderate intellectual disability, moderate dementia, and congenital heart disease due to Down syndrome	5.0 (2.7 to 8.0)	7.2 (3.8 to 11.6)	2.2 (1.1 to 3.8)	3.1 (1.5 to 5.5)
Down syndrome	Severe intellectual disability, moderate dementia, and congenital heart disease due to Down syndrome	2.2 (0.0 to 3.9)	3.1 (0.0 to 5.7)	1.1 (0.0 to 2.0)	1.5 (0.0 to 2.9)
Down syndrome	Profound intellectual disability, moderate dementia, and congenital heart disease due to Down syndrome	3.0 (0.1 to 4.5)	4.3 (0.2 to 6.6)	1.5 (0.1 to 2.4)	2.1 (0.1 to 3.6)
Down syndrome	Borderline intellectual disability, severe dementia, and congenital heart disease due to Down syndrome	1.5 (1.0 to 2.3)	2.1 (1.4 to 3.3)	0.7 (0.4 to 1.1)	1.0 (0.5 to 1.7)
Down syndrome	Mild intellectual disability, severe dementia, and congenital heart disease due to Down syndrome	2.6 (1.7 to 3.9)	3.8 (2.4 to 5.9)	1.2 (0.7 to 2.0)	1.8 (1.0 to 3.0)
Down syndrome	Moderate intellectual disability, severe dementia, and congenital heart disease due to Down syndrome	1.6 (0.9 to 2.6)	2.3 (1.2 to 3.8)	0.8 (0.4 to 1.4)	1.2 (0.6 to 2.1)
Down syndrome	Severe intellectual disability, severe dementia, and congenital heart disease due to Down syndrome	0.8 (0.0 to 1.3)	1.0 (0.0 to 1.9)	0.4 (0.0 to 0.8)	0.6 (0.0 to 1.1)
Down syndrome	Profound intellectual disability, severe dementia, and congenital heart disease due to Down syndrome	1.0 (0.0 to 1.5)	1.5 (0.1 to 2.2)	0.6 (0.0 to 0.9)	0.8 (0.0 to 1.3)
Down syndrome	Profound intellectual disability and mild dementia due to Down syndrome	17.0 (0.9 to 25.6)	24.4 (1.5 to 37.6)	4.3 (0.2 to 7.2)	6.2 (0.3 to 10.7)
Down syndrome	Severe intellectual disability and mild dementia due to Down syndrome	12.6 (0.2 to 22.8)	17.7 (0.3 to 32.4)	2.8 (0.0 to 5.4)	3.9 (0.1 to 7.6)
Down syndrome	Moderate intellectual disability and mild dementia due to Down syndrome	29.6 (16.4 to 47.3)	42.4 (22.6 to 68.3)	4.7 (2.3 to 7.9)	6.6 (3.2 to 11.4)
Down syndrome	Mild intellectual disability and mild dementia due to Down syndrome	47.8 (31.2 to 72.5)	69.8 (44.4 to 108.4)	5.0 (2.8 to 8.4)	7.2 (3.9 to 12.4)
Down syndrome	Borderline intellectual disability and mild dementia due to Down syndrome	25.1 (16.3 to 37.8)	36.0 (22.5 to 55.9)	2.0 (1.1 to 3.4)	2.8 (1.5 to 4.9)
Down syndrome	Profound intellectual disability and moderate dementia due to Down syndrome	4.1 (0.2 to 6.2)	5.9 (0.3 to 9.1)	2.1 (0.1 to 3.4)	2.9 (0.1 to 4.9)
Down syndrome	Severe intellectual disability and moderate dementia due to Down syndrome	3.0 (0.0 to 5.5)	4.3 (0.1 to 7.7)	1.5 (0.0 to 2.8)	2.0 (0.0 to 4.0)
Down syndrome	Moderate intellectual disability and moderate dementia due to Down syndrome	6.9 (3.8 to 11.1)	9.9 (5.2 to 16.2)	3.0 (1.5 to 5.2)	4.3 (2.1 to 7.6)
Down syndrome	Mild intellectual disability and moderate dementia due to Down syndrome	11.1 (7.1 to 16.8)	16.2 (10.1 to 25.4)	4.5 (2.5 to 7.1)	6.5 (3.6 to 10.8)
Down syndrome	Borderline intellectual disability and moderate dementia due to Down syndrome	6.1 (4.0 to 9.0)	8.7 (5.5 to 13.4)	2.3 (1.3 to 3.9)	3.3 (1.8 to 5.7)
Down syndrome	Profound intellectual disability and severe dementia due to Down syndrome	1.4 (0.1 to 2.1)	2.0 (0.1 to 3.1)	0.8 (0.0 to 1.2)	1.1 (0.0 to 1.8)
Down syndrome	Severe intellectual disability and severe dementia due to Down syndrome	1.0 (0.0 to 1.9)	1.4 (0.0 to 2.7)	0.6 (0.0 to 1.1)	0.8 (0.0 to 1.5)
Down syndrome	Moderate intellectual disability and severe dementia due to Down syndrome	2.3 (1.2 to 3.7)	3.2 (1.7 to 5.3)	1.1 (0.6 to 2.0)	1.6 (0.8 to 2.9)
Down syndrome	Mild intellectual disability and severe dementia due to Down syndrome	3.6 (2.3 to 5.5)	5.3 (3.3 to 8.2)	1.7 (1.0 to 2.8)	2.5 (1.3 to 4.1)
Down syndrome	Borderline intellectual disability and severe dementia due to Down syndrome	2.1 (1.3 to 3.1)	3.0 (1.9 to 4.6)	0.9 (0.5 to 1.5)	1.3 (0.7 to 2.3)
Turner syndrome	Asymptomatic Turner syndrome	114.3 (53.8 to 207.8)	131.4 (61.7 to 237.4)	<0.05 (0.0 to <0.05)	<0.05 (0.0 to <0.05)
Turner syndrome	Congenital heart disease due to Turner syndrome	33.7 (15.8 to 61.4)	38.8 (18.4 to 71.5)	2.0 (0.6 to 4.7)	2.4 (0.7 to 5.4)
Turner syndrome	Primary infertility due to Turner syndrome	142.7 (67.2 to 257.2)	156.1 (73.2 to 282.7)	1.1 (0.3 to 2.6)	1.2 (0.4 to 2.9)
Turner syndrome	Congenital heart disease with infertility due to Turner syndrome	42.1 (19.8 to 77.3)	46.0 (21.7 to 85.3)	2.8 (1.0 to 6.4)	3.1 (1.0 to 7.0)
Klinefelter syndrome	Asymptomatic Klinefelter syndrome	87.5 (45.7 to 157.2)	98.0 (51.0 to 175.8)	<0.05 (0.0 to <0.05)	<0.05 (0.0 to <0.05)
Klinefelter syndrome	Borderline intellectual disability due to Klinefelter syndrome	20.8 (9.6 to 41.5)	23.2 (10.7 to 46.2)	0.2 (0.1 to 0.6)	0.2 (0.1 to 0.6)
Klinefelter syndrome	Borderline intellectual disability with infertility due to Klinefelter syndrome	16.2 (7.0 to 31.8)	18.4 (8.0 to 36.6)	0.3 (0.1 to 0.7)	0.3 (0.1 to 0.8)
Klinefelter syndrome	Mild intellectual disability due to Klinefelter syndrome	1.7 (0.8 to 3.5)	2.0 (0.9 to 3.9)	0.1 (0.0 to 0.2)	0.1 (0.0 to 0.2)
Klinefelter syndrome	Mild intellectual disability with infertility due to Klinefelter syndrome	1.4 (0.5 to 2.9)	1.6 (0.6 to 3.3)	0.1 (0.0 to 0.2)	0.1 (0.0 to 0.2)
Klinefelter syndrome	Primary infertility due to Klinefelter syndrome	67.8 (35.2 to 122.6)	77.3 (39.7 to 140.6)	0.5 (0.2 to 1.2)	0.6 (0.2 to 1.4)
Other chromosomal abnormalities	Asymptomatic other chromosomal abnormalities	108.2 (59.7 to 185.4)	123.7 (67.3 to 217.8)	<0.05 (0.0 to <0.05)	<0.05 (0.0 to <0.05)
Other chromosomal abnormalities	Isolated congenital heart disease due to other chromosomal abnormalities	78.4 (44.6 to 137.2)	89.7 (49.7 to 160.9)	4.8 (1.5 to 10.5)	5.5 (1.7 to 12.2)
Other chromosomal abnormalities	Borderline intellectual disability due to other chromosomal abnormalities	260.2 (145.5 to 454.3)	297.6 (162.6 to 531.9)	2.8 (1.0 to 6.1)	3.2 (1.2 to 7.1)
Other chromosomal abnormalities	Mild intellectual disability due to other chromosomal abnormalities	686.8 (391.6 to 1,213.7)	785.6 (436.6 to 1,418.2)	28.4 (13.7 to 54.9)	32.5 (15.4 to 63.9)
Other chromosomal abnormalities	Moderate intellectual disability due to other chromosomal abnormalities	559.9 (313.1 to 983.3)	640.4 (348.5 to 1,145.2)	54.0 (27.6 to 104.0)	61.8 (30.6 to 121.5)
Other chromosomal abnormalities	Severe intellectual disability due to other chromosomal abnormalities	361.3 (200.9 to 652.9)	413.3 (224.4 to 758.0)	55.9 (27.6 to 105.9)	63.9 (31.1 to 124.0)
Other chromosomal abnormalities	Profound intellectual disability due to other chromosomal abnormalities	190.5 (108.6 to 334.4)	217.9 (121.8 to 390.1)	37.7 (18.2 to 70.8)	43.2 (20.6 to 81.8)
Other chromosomal abnormalities	Borderline intellectual disability with congenital heart disease due to other chromosomal abnormalities	188.8 (105.9 to 328.9)	215.9 (117.3 to 385.5)	2.0 (0.8 to 4.5)	2.3 (0.9 to 5.2)
Other chromosomal abnormalities	Mild intellectual disability with congenital heart disease due to other chromosomal abnormalities	498.4 (275.0 to 853.9)	570.1 (307.0 to 996.7)	20.7 (9.7 to 40.1)	23.6 (10.8 to 46.6)
Other chromosomal abnormalities	Moderate intellectual disability with congenital heart disease due to other chromosomal abnormalities	405.2 (225.6 to 715.4)	463.5 (252.7 to 836.9)	39.3 (19.6 to 75.3)	44.9 (21.9 to 87.8)
Other chromosomal abnormalities	Severe intellectual disability with congenital heart disease due to other chromosomal abnormalities	262.4 (146.5 to 467.1)	300.1 (163.3 to 545.6)	41.1 (20.2 to 75.6)	46.9 (22.5 to 88.6)
Other chromosomal abnormalities	Profound intellectual disability with congenital heart disease due to other chromosomal abnormalities	138.2 (76.6 to 240.2)	158.0 (85.3 to 281.3)	27.5 (13.4 to 51.4)	31.5 (15.1 to 60.0)
Other chromosomal abnormalities	Mild dementia due to other chromosomal abnormalities	10.8 (6.3 to 18.4)	14.6 (8.0 to 26.0)	0.7 (0.4 to 1.4)	1.0 (0.5 to 2.0)
Other chromosomal abnormalities	Moderate dementia due to other chromosomal abnormalities	2.6 (1.5 to 4.5)	3.6 (2.0 to 6.4)	1.0 (0.5 to 1.8)	1.3 (0.6 to 2.6)
Other chromosomal abnormalities	Severe dementia due to other chromosomal abnormalities	0.9 (0.5 to 1.5)	1.2 (0.7 to 2.2)	0.4 (0.2 to 0.7)	0.5 (0.3 to 1.0)
Other chromosomal abnormalities	Congenital heart disease and mild dementia due to other chromosomal abnormalities	7.8 (4.6 to 13.4)	10.6 (5.9 to 18.9)	0.5 (0.3 to 1.0)	0.7 (0.4 to 1.4)
Other chromosomal abnormalities	Congenital heart disease and moderate dementia due to other chromosomal abnormalities	1.9 (1.1 to 3.2)	2.6 (1.4 to 4.5)	0.7 (0.4 to 1.3)	1.0 (0.5 to 1.8)
Other chromosomal abnormalities	Congenital heart disease and severe dementia due to other chromosomal abnormalities	0.7 (0.4 to 1.1)	0.9 (0.5 to 1.6)	0.3 (0.1 to 0.5)	0.4 (0.2 to 0.7)
Other chromosomal abnormalities	Borderline intellectual disability, mild dementia, and congenital heart disease due to other chromosomal abnormalities	18.7 (10.8 to 31.5)	25.3 (13.9 to 44.6)	1.5 (0.7 to 2.7)	2.0 (1.0 to 3.9)
Other chromosomal abnormalities	Mild intellectual disability, mild dementia, and congenital heart disease due to other chromosomal abnormalities	34.3 (20.1 to 57.7)	47.9 (27.1 to 83.8)	3.6 (1.8 to 6.7)	5.1 (2.5 to 9.7)
Other chromosomal abnormalities	Moderate intellectual disability, mild dementia, and congenital heart disease due to other chromosomal abnormalities	22.3 (11.5 to 37.3)	30.7 (14.8 to 53.6)	3.6 (1.7 to 6.5)	4.9 (2.2 to 9.3)
Other chromosomal abnormalities	Severe intellectual disability, mild dementia, and congenital heart disease due to other chromosomal abnormalities	9.6 (0.2 to 18.4)	12.9 (0.2 to 25.5)	2.1 (0.0 to 4.3)	2.8 (0.0 to 5.9)
Other chromosomal abnormalities	Profound intellectual disability, mild dementia, and congenital heart disease due to other chromosomal abnormalities	12.5 (0.6 to 20.8)	17.0 (1.0 to 30.3)	3.2 (0.2 to 5.8)	4.3 (0.3 to 8.1)
Other chromosomal abnormalities	Borderline intellectual disability, moderate dementia, and congenital heart disease due to other chromosomal abnormalities	4.5 (2.6 to 7.7)	6.1 (3.4 to 10.8)	1.7 (0.9 to 3.2)	2.4 (1.2 to 4.4)
Other chromosomal abnormalities	Mild intellectual disability, moderate dementia, and congenital heart disease due to other chromosomal abnormalities	8.0 (4.7 to 13.3)	11.2 (6.3 to 19.7)	3.2 (1.7 to 5.7)	4.5 (2.3 to 8.5)
Other chromosomal abnormalities	Moderate intellectual disability, moderate dementia, and congenital heart disease due to other chromosomal abnormalities	5.3 (2.7 to 8.8)	7.2 (3.5 to 12.4)	2.3 (1.1 to 4.2)	3.2 (1.4 to 6.0)

Cause	Sequela	Prevalence in thousands (95% confidence interval)		YLDs in thousands (95% confidence interval)	
		2005	2015	2005	2015
Other chromosomal abnormalities	Severe intellectual disability, moderate dementia, and congenital heart disease due to other chromosomal abnormalities	2.3 (0.0 to 4.4)	3.1 (0.0 to 6.1)	1.1 (0.0 to 2.2)	1.5 (0.0 to 3.1)
Other chromosomal abnormalities	Profound intellectual disability, moderate dementia, and congenital heart disease due to other chromosomal abnormalities	3.0 (0.1 to 4.9)	4.1 (0.2 to 7.1)	1.5 (0.1 to 2.6)	2.1 (0.1 to 3.8)
Other chromosomal abnormalities	Borderline intellectual disability, severe dementia, and congenital heart disease due to other chromosomal abnormalities	1.6 (0.9 to 2.7)	2.1 (1.2 to 3.8)	0.7 (0.4 to 1.3)	1.0 (0.5 to 1.8)
Other chromosomal abnormalities	Mild intellectual disability, severe dementia, and congenital heart disease due to other chromosomal abnormalities	2.6 (1.5 to 4.3)	3.6 (2.0 to 6.2)	1.2 (0.6 to 2.2)	1.7 (0.9 to 3.1)
Other chromosomal abnormalities	Moderate intellectual disability, severe dementia, and congenital heart disease due to other chromosomal abnormalities	1.7 (0.9 to 2.9)	2.4 (1.1 to 4.1)	0.9 (0.4 to 1.6)	1.2 (0.5 to 2.2)
Other chromosomal abnormalities	Severe intellectual disability, severe dementia, and congenital heart disease due to other chromosomal abnormalities	0.8 (0.0 to 1.5)	1.1 (0.0 to 2.1)	0.4 (0.0 to 0.8)	0.6 (0.0 to 1.2)
Other chromosomal abnormalities	Profound intellectual disability, severe dementia, and congenital heart disease due to other chromosomal abnormalities	1.0 (0.0 to 1.7)	1.4 (0.1 to 2.4)	0.6 (0.0 to 1.0)	0.8 (0.0 to 1.5)
Other chromosomal abnormalities	Profound intellectual disability and mild dementia due to other chromosomal abnormalities	17.2 (0.9 to 28.0)	23.5 (1.4 to 40.9)	4.3 (0.2 to 7.9)	6.0 (0.4 to 11.2)
Other chromosomal abnormalities	Severe intellectual disability and mild dementia due to other chromosomal abnormalities	13.2 (0.2 to 25.2)	17.7 (0.3 to 34.9)	2.9 (0.0 to 5.9)	3.9 (0.1 to 8.2)
Other chromosomal abnormalities	Moderate intellectual disability and mild dementia due to other chromosomal abnormalities	30.8 (15.8 to 51.1)	42.4 (20.3 to 73.2)	4.8 (2.3 to 9.0)	6.7 (2.9 to 12.9)
Other chromosomal abnormalities	Mild intellectual disability and mild dementia due to other chromosomal abnormalities	47.2 (27.9 to 79.9)	66.1 (37.6 to 116.2)	4.9 (2.5 to 9.1)	6.9 (3.4 to 13.0)
Other chromosomal abnormalities	Borderline intellectual disability and mild dementia due to other chromosomal abnormalities	25.8 (14.9 to 43.6)	34.9 (19.2 to 62.2)	2.0 (1.0 to 3.7)	2.7 (1.3 to 5.2)
Other chromosomal abnormalities	Profound intellectual disability and moderate dementia due to other chromosomal abnormalities	4.2 (0.2 to 6.8)	5.7 (0.3 to 9.9)	2.1 (0.1 to 3.7)	2.9 (0.2 to 5.3)
Other chromosomal abnormalities	Severe intellectual disability and moderate dementia due to other chromosomal abnormalities	3.2 (0.0 to 6.0)	4.3 (0.1 to 8.4)	1.5 (0.0 to 3.0)	2.0 (0.0 to 4.2)
Other chromosomal abnormalities	Moderate intellectual disability and moderate dementia due to other chromosomal abnormalities	7.3 (3.6 to 12.0)	9.9 (4.7 to 17.5)	3.2 (1.6 to 5.8)	4.4 (2.0 to 8.1)
Other chromosomal abnormalities	Mild intellectual disability and moderate dementia due to other chromosomal abnormalities	11.0 (6.5 to 17.9)	15.4 (8.7 to 26.3)	4.4 (2.3 to 8.0)	6.2 (3.2 to 11.6)
Other chromosomal abnormalities	Borderline intellectual disability and moderate dementia due to other chromosomal abnormalities	6.3 (3.6 to 10.6)	8.5 (4.7 to 15.2)	2.4 (1.2 to 4.4)	3.2 (1.6 to 6.2)
Other chromosomal abnormalities	Profound intellectual disability and severe dementia due to other chromosomal abnormalities	1.4 (0.1 to 2.3)	2.0 (0.1 to 3.4)	0.8 (0.0 to 1.4)	1.1 (0.1 to 2.0)
Other chromosomal abnormalities	Severe intellectual disability and severe dementia due to other chromosomal abnormalities	1.1 (0.0 to 2.1)	1.5 (0.0 to 2.9)	0.6 (0.0 to 1.2)	0.8 (0.0 to 1.6)
Other chromosomal abnormalities	Moderate intellectual disability and severe dementia due to other chromosomal abnormalities	2.4 (1.2 to 4.0)	3.3 (1.5 to 5.7)	1.2 (0.6 to 2.2)	1.6 (0.7 to 3.1)
Other chromosomal abnormalities	Mild intellectual disability and severe dementia due to other chromosomal abnormalities	3.6 (2.1 to 6.1)	5.0 (2.8 to 8.9)	1.7 (0.9 to 3.0)	2.4 (1.2 to 4.4)
Other chromosomal abnormalities	Borderline intellectual disability and severe dementia due to other chromosomal abnormalities	2.2 (1.3 to 3.7)	2.9 (1.6 to 5.2)	1.0 (0.5 to 1.8)	1.3 (0.7 to 2.5)
Other congenital anomalies	Mild hearing loss due to other congenital anomalies	12,252.7 (5,118.1 to 24,766.8)	14,185.0 (5,977.6 to 28,377.9)	114.7 (34.7 to 292.4)	132.7 (39.9 to 336.1)
Other congenital anomalies	Mild hearing loss with ringing due to other congenital anomalies	3,243.8 (1,373.6 to 6,470.7)	3,754.7 (1,586.9 to 7,537.2)	67.8 (23.4 to 162.2)	78.5 (27.1 to 186.8)
Other congenital anomalies	Moderate hearing loss due to other congenital anomalies	1,224.0 (444.6 to 2,700.2)	1,531.5 (555.1 to 3,413.1)	31.5 (10.2 to 74.6)	39.4 (12.7 to 94.1)
Other congenital anomalies	Moderate hearing loss with ringing due to other congenital anomalies	530.4 (192.2 to 1,176.6)	662.2 (242.2 to 1,454.3)	37.8 (12.6 to 82.9)	47.2 (15.9 to 101.9)
Other congenital anomalies	Moderately severe hearing loss due to other congenital anomalies	2,857.4 (1,274.6 to 6,012.4)	3,186.3 (1,433.2 to 6,697.9)	251.7 (101.7 to 538.5)	280.7 (112.6 to 609.1)
Other congenital anomalies	Moderately severe hearing loss with ringing due to other congenital anomalies	1,374.3 (618.9 to 2,876.3)	1,535.2 (684.6 to 3,174.3)	217.2 (88.5 to 461.5)	242.6 (99.2 to 515.8)
Other congenital anomalies	Severe hearing loss with ringing due to other congenital anomalies	805.5 (339.1 to 1,649.6)	883.7 (375.5 to 1,891.4)	197.1 (74.2 to 410.5)	216.3 (81.4 to 471.7)
Other congenital anomalies	Severe hearing loss due to other congenital anomalies	1,424.3 (595.1 to 2,979.1)	1,556.8 (667.6 to 3,279.7)	213.3 (81.1 to 455.6)	233.1 (88.6 to 493.2)
Other congenital anomalies	Profound hearing loss due to other congenital anomalies	1,172.2 (496.7 to 2,354.9)	1,314.7 (564.3 to 2,646.9)	225.0 (86.6 to 454.8)	252.3 (97.6 to 533.0)
Other congenital anomalies	Profound hearing loss with ringing due to other congenital anomalies	2,089.4 (893.8 to 4,217.4)	2,337.7 (989.5 to 4,717.7)	541.2 (207.6 to 1,164.4)	605.2 (237.0 to 1,262.8)
Other congenital anomalies	Complete hearing loss due to other congenital anomalies	584.1 (236.3 to 1,252.8)	641.9 (257.4 to 1,396.9)	118.6 (43.2 to 261.0)	130.0 (48.3 to 282.8)
Other congenital anomalies	Complete hearing loss with ringing due to other congenital anomalies	698.1 (283.7 to 1,507.4)	773.2 (301.8 to 1,697.3)	206.1 (77.8 to 438.5)	228.1 (84.2 to 498.3)
Dermatitis	Mild eczema	65,399.1 (56,457.9 to 74,497.6)	73,913.7 (63,901.3 to 84,026.7)	1,687.8 (948.5 to 2,690.7)	1,907.4 (1,067.6 to 3,049.6)
Dermatitis	Moderate eczema	7,278.3 (4,508.8 to 10,619.5)	8,225.7 (5,096.8 to 12,015.0)	1,303.5 (700.0 to 2,166.6)	1,473.2 (791.8 to 2,449.0)
Dermatitis	Severe eczema	3,049.1 (1,705.7 to 4,707.8)	3,446.1 (1,927.9 to 5,347.7)	1,644.6 (864.5 to 2,703.9)	1,858.6 (974.1 to 3,046.3)
Dermatitis	Asymptomatic contact dermatitis	35,636.5 (30,721.1 to 40,817.5)	41,053.4 (35,415.9 to 46,942.5)	<0.05 (0.0 to <0.05)	<0.05 (0.0 to <0.05)
Dermatitis	Mild contact dermatitis	42,620.1 (36,450.8 to 48,804.7)	49,097.9 (41,982.4 to 56,034.6)	1,087.6 (610.9 to 1,737.5)	1,251.5 (704.6 to 2,024.8)
Dermatitis	Severe contact dermatitis	6,826.4 (4,437.6 to 9,769.2)	7,863.8 (5,112.8 to 11,270.8)	1,200.2 (615.8 to 1,948.0)	1,380.5 (714.1 to 2,247.9)
Dermatitis	Asymptomatic seborrhoeic dermatitis	22,776.6 (20,157.8 to 25,540.9)	25,805.1 (22,922.0 to 28,952.0)	<0.05 (0.0 to <0.05)	<0.05 (0.0 to <0.05)
Dermatitis	Symptomatic seborrhoeic dermatitis	31,673.4 (28,004.8 to 35,432.9)	35,884.9 (31,748.5 to 40,079.9)	810.1 (460.2 to 1,279.2)	916.8 (521.6 to 1,454.1)
Psoriasis	Mild psoriasis	55,059.9 (50,974.6 to 59,048.8)	64,768.1 (59,935.9 to 69,397.6)	1,404.6 (809.8 to 2,228.6)	1,651.0 (947.8 to 2,611.2)
Psoriasis	Moderate psoriasis	7,417.8 (4,723.3 to 10,652.7)	8,726.0 (5,543.8 to 12,534.2)	1,305.4 (698.8 to 2,185.9)	1,534.0 (821.1 to 2,566.8)
Psoriasis	Severe psoriasis	5,275.5 (3,386.7 to 7,512.6)	6,205.6 (3,985.9 to 8,806.2)	2,768.9 (1,627.5 to 4,255.0)	3,253.3 (1,912.9 to 4,962.1)
Cellulitis	Mild cellulitis	542.0 (482.5 to 604.6)	672.0 (598.4 to 750.9)	13.9 (7.8 to 22.6)	17.1 (9.6 to 27.9)
Cellulitis	Severe cellulitis	232.2 (187.6 to 283.0)	287.9 (232.3 to 351.0)	42.0 (26.3 to 62.8)	51.9 (32.5 to 77.6)
Pyoderma	Impetigo	331.0 (321.1 to 341.7)	356.9 (346.4 to 367.5)	1.9 (0.8 to 4.0)	2.0 (0.8 to 4.3)
Pyoderma	Abscess and other bacterial skin diseases	4,701.4 (4,497.1 to 4,891.4)	5,455.8 (5,227.9 to 5,664.7)	26.5 (10.7 to 55.4)	30.7 (12.3 to 64.1)
Scabies	Scabies	191,481.8 (166,101.3 to 223,739.2)	204,151.7 (177,533.7 to 237,466.2)	4,944.3 (2,789.1 to 8,047.0)	5,268.9 (2,966.9 to 8,605.6)
Fungal skin diseases	Tinea capitis	106,899.4 (85,997.5 to 134,431.3)	118,151.7 (94,680.4 to 148,908.5)	609.8 (225.9 to 1,274.7)	674.6 (250.7 to 1,415.2)
Fungal skin diseases	Other fungal skin diseases	327,704.8 (297,660.1 to 360,984.6)	374,220.9 (340,511.9 to 411,019.2)	1,847.7 (737.0 to 3,870.9)	2,108.7 (835.3 to 4,424.0)
Viral skin diseases	Mild molluscum contagiosum	21,982.0 (18,156.2 to 26,497.9)	23,130.5 (19,108.9 to 27,873.1)	125.7 (51.4 to 270.7)	132.3 (54.0 to 285.7)
Viral skin diseases	Severe molluscum contagiosum	16,473.3 (12,883.3 to 20,439.4)	17,333.8 (13,527.5 to 21,537.2)	1,073.7 (656.5 to 1,622.7)	1,130.7 (695.1 to 1,706.3)
Viral skin diseases	Mild viral warts	70,146.4 (59,256.1 to 81,271.9)	76,815.4 (64,647.2 to 88,935.7)	396.9 (158.0 to 833.1)	434.3 (173.1 to 914.5)
Viral skin diseases	Severe viral warts	52,565.2 (41,859.8 to 64,200.8)	57,563.5 (45,780.5 to 70,370.9)	3,381.1 (2,142.5 to 4,965.8)	3,699.5 (2,351.2 to 5,419.7)
Acne vulgaris	Acne vulgaris	605,008.2 (568,576.9 to 642,060.7)	632,741.0 (595,241.9 to 671,248.9)	6,553.4 (3,140.3 to 12,150.3)	6,854.0 (3,275.1 to 12,713.9)
Alopecia areata	Mild alopecia areata	10,266.4 (8,209.7 to 12,240.5)	11,699.2 (9,358.7 to 13,975.6)	110.1 (51.1 to 211.1)	125.5 (58.1 to 241.4)
Alopecia areata	Severe alopecia areata	7,806.1 (5,846.7 to 9,785.2)	8,895.7 (6,662.8 to 11,154.7)	500.4 (305.1 to 750.0)	570.1 (346.6 to 855.8)

Cause	Sequela	Prevalence in thousands (95% confidence interval)		YLDs in thousands (95% confidence interval)	
		2005	2015	2005	2015
Pruritus	Pruritus	59,184.2 (52,738.9 to 66,499.9)	69,582.5 (62,063.2 to 78,152.6)	631.4 (306.7 to 1,149.2)	741.6 (360.0 to 1,348.6)
Urticaria	Mild urticaria	47,230.5 (39,496.9 to 54,932.5)	52,286.8 (43,939.5 to 60,740.8)	1,218.8 (675.7 to 1,983.8)	1,349.0 (748.7 to 2,189.5)
Urticaria	Severe urticaria	13,968.3 (9,657.7 to 18,986.1)	15,463.0 (10,679.8 to 20,945.5)	2,499.7 (1,443.7 to 3,896.0)	2,766.8 (1,611.4 to 4,279.6)
Decubitus ulcer	Mild decubitus ulcer	218.2 (161.0 to 275.1)	295.5 (217.2 to 373.2)	2.3 (1.0 to 4.4)	3.1 (1.4 to 5.9)
Decubitus ulcer	Moderate decubitus ulcer	313.9 (259.0 to 379.8)	425.1 (350.2 to 514.9)	19.6 (12.7 to 28.6)	26.4 (17.1 to 38.7)
Decubitus ulcer	Severe decubitus ulcer	271.0 (218.2 to 331.8)	367.0 (294.1 to 448.4)	98.0 (63.7 to 137.5)	131.8 (85.7 to 185.2)
Other skin and subcutaneous diseases	Asymptomatic other skin and subcutaneous diseases	219,898.0 (212,799.7 to 227,141.9)	269,934.6 (261,283.9 to 278,730.7)	<0.05 (0.0 to <0.05)	<0.05 (0.0 to <0.05)
Other skin and subcutaneous diseases	Symptomatic other skin and subcutaneous diseases	272,984.5 (265,119.4 to 281,303.3)	335,101.7 (325,331.2 to 345,173.8)	2,895.3 (1,393.9 to 5,314.6)	3,550.5 (1,706.2 to 6,514.4)
Glaucoma	Moderate vision impairment due to glaucoma	2,329.0 (1,845.4 to 2,879.3)	3,239.1 (2,561.5 to 4,032.9)	66.7 (38.5 to 106.7)	92.9 (53.4 to 151.4)
Glaucoma	Severe vision impairment due to glaucoma	324.2 (257.1 to 408.7)	443.5 (351.3 to 559.1)	53.0 (35.0 to 76.9)	72.6 (48.0 to 106.3)
Glaucoma	Blindness due to glaucoma	1,627.9 (1,312.6 to 2,029.3)	2,271.9 (1,832.9 to 2,819.0)	268.8 (172.7 to 387.7)	375.9 (241.9 to 540.9)
Cataract	Moderate vision impairment due to cataract	34,163.6 (30,103.6 to 39,032.3)	43,693.8 (38,136.5 to 50,310.5)	971.5 (612.0 to 1,506.1)	1,244.2 (767.6 to 1,918.2)
Cataract	Severe vision impairment due to cataract	4,597.9 (3,951.8 to 5,462.8)	5,870.5 (5,024.3 to 7,002.0)	743.9 (505.1 to 1,044.8)	950.8 (645.3 to 1,350.0)
Cataract	Blindness due to cataract	8,307.8 (7,260.4 to 9,512.5)	10,162.6 (8,853.6 to 11,756.2)	1,375.9 (928.3 to 1,903.4)	1,684.8 (1,138.6 to 2,339.6)
Macular degeneration	Moderate vision impairment due to macular degeneration	2,723.9 (2,192.0 to 3,381.3)	4,066.4 (3,281.3 to 5,024.0)	77.5 (46.6 to 124.4)	115.8 (69.7 to 185.6)
Macular degeneration	Severe vision impairment due to macular degeneration	402.5 (316.5 to 501.7)	589.0 (463.0 to 734.1)	65.3 (42.1 to 94.9)	95.5 (61.4 to 137.6)
Macular degeneration	Blindness due to macular degeneration	1,040.7 (845.9 to 1,278.8)	1,533.0 (1,242.7 to 1,878.9)	170.4 (111.1 to 241.3)	251.1 (164.8 to 357.7)
Refraction and accommodation disorders	Moderate vision impairment due to uncorrected refractive error	111,337.1 (100,524.6 to 123,114.3)	132,845.4 (119,390.5 to 147,033.9)	3,269.5 (1,997.7 to 5,051.5)	3,898.6 (2,394.3 to 6,053.9)
Refraction and accommodation disorders	Severe vision impairment due to uncorrected refractive error	11,526.0 (10,314.1 to 12,852.7)	14,046.2 (12,467.5 to 15,839.6)	1,933.8 (1,316.1 to 2,682.8)	2,355.2 (1,606.5 to 3,282.0)
Refraction and accommodation disorders	Blindness due to uncorrected refractive error	7,875.3 (7,029.5 to 8,771.0)	9,432.5 (8,374.3 to 10,549.5)	1,351.8 (918.7 to 1,894.4)	1,619.5 (1,093.9 to 2,265.5)
Refraction and accommodation disorders	Near vision impairment	541,427.0 (521,986.5 to 561,189.2)	662,983.4 (637,817.9 to 689,865.6)	5,491.8 (2,582.6 to 10,276.1)	6,720.5 (3,176.2 to 12,491.5)
Age-related and other hearing loss	Mild hearing loss due to age-related and other hearing loss	474,863.8 (434,365.6 to 513,204.4)	609,203.4 (560,100.4 to 658,035.0)	4,333.3 (1,946.8 to 8,338.8)	5,560.0 (2,476.4 to 10,731.2)
Age-related and other hearing loss	Mild hearing loss with ringing due to age-related and other hearing loss	125,806.8 (115,102.7 to 136,444.7)	161,291.4 (147,458.7 to 175,122.5)	2,557.7 (1,400.2 to 4,183.6)	3,279.6 (1,807.0 to 5,363.4)
Age-related and other hearing loss	Moderate hearing loss due to age-related and other hearing loss	154,340.7 (137,627.0 to 172,004.4)	200,412.7 (178,561.3 to 223,095.8)	3,853.4 (2,259.4 to 6,138.4)	5,006.2 (2,915.9 to 7,968.8)
Age-related and other hearing loss	Moderate hearing loss with ringing due to age-related and other hearing loss	66,882.6 (58,979.1 to 74,751.7)	86,695.9 (76,805.2 to 96,990.4)	4,570.1 (3,022.7 to 6,708.2)	5,926.2 (3,843.3 to 8,636.3)
Age-related and other hearing loss	Moderately severe hearing loss due to age-related and other hearing loss	54,639.2 (47,145.2 to 62,474.8)	69,829.9 (60,748.4 to 79,552.3)	4,640.9 (3,069.3 to 6,481.9)	5,926.7 (3,951.2 to 8,293.3)
Age-related and other hearing loss	Moderately severe hearing loss with ringing due to age-related and other hearing loss	26,277.3 (22,678.7 to 30,166.8)	33,602.5 (29,082.6 to 38,733.0)	3,973.1 (2,709.8 to 5,451.5)	5,078.7 (3,476.0 to 6,939.2)
Age-related and other hearing loss	Severe hearing loss with ringing due to age-related and other hearing loss	8,614.8 (6,883.6 to 10,365.3)	10,606.2 (8,563.3 to 12,833.1)	2,021.7 (1,322.0 to 2,915.7)	2,483.2 (1,637.5 to 3,570.4)
Age-related and other hearing loss	Severe hearing loss due to age-related and other hearing loss	15,236.6 (12,387.2 to 18,170.7)	18,700.5 (15,209.8 to 22,234.1)	2,200.5 (1,442.6 to 3,194.2)	2,696.8 (1,761.0 to 3,943.1)
Age-related and other hearing loss	Profound hearing loss due to age-related and other hearing loss	4,732.2 (3,202.2 to 6,208.5)	5,481.2 (3,783.6 to 7,116.5)	900.2 (524.1 to 1,363.2)	1,041.0 (617.6 to 1,590.7)
Age-related and other hearing loss	Profound hearing loss with ringing due to age-related and other hearing loss	8,416.1 (5,661.9 to 11,064.4)	9,745.9 (6,729.2 to 12,546.5)	2,163.1 (1,250.6 to 3,221.5)	2,501.8 (1,489.5 to 3,696.1)
Age-related and other hearing loss	Complete hearing loss due to age-related and other hearing loss	1,678.0 (1,030.9 to 2,260.5)	2,038.5 (1,290.9 to 2,779.4)	329.1 (185.7 to 528.1)	399.2 (229.5 to 627.2)
Age-related and other hearing loss	Complete hearing loss with ringing due to age-related and other hearing loss	2,015.7 (1,277.6 to 2,733.9)	2,447.1 (1,572.2 to 3,271.4)	575.9 (325.8 to 903.5)	697.4 (390.9 to 1,090.4)
Other vision loss	Moderate vision impairment due to other vision loss	14,912.2 (12,786.9 to 16,873.6)	18,659.1 (15,998.7 to 21,094.6)	439.6 (266.7 to 683.3)	548.9 (332.6 to 849.2)
Other vision loss	Severe vision impairment due to other vision loss	1,488.2 (1,266.5 to 1,725.5)	1,925.4 (1,640.9 to 2,228.1)	250.7 (166.2 to 355.7)	323.5 (214.1 to 455.8)
Other vision loss	Blindness due to other vision loss	3,902.0 (3,241.9 to 4,608.1)	5,212.9 (4,324.0 to 6,146.3)	661.7 (437.6 to 941.1)	884.0 (590.0 to 1,260.0)
Other sense organ diseases	Asymptomatic acute sense organ diseases	456.2 (405.2 to 513.4)	524.4 (466.8 to 589.4)	<0.05 (0.0 to <0.05)	<0.05 (0.0 to <0.05)
Other sense organ diseases	Mild acute sense organ diseases	333.4 (273.4 to 396.7)	383.3 (313.6 to 454.9)	1.9 (0.7 to 4.0)	2.2 (0.9 to 4.6)
Other sense organ diseases	Moderate acute sense organ diseases	132.4 (73.1 to 198.0)	152.2 (83.7 to 227.9)	1.5 (0.5 to 3.1)	1.7 (0.6 to 3.6)
Other sense organ diseases	Severe acute sense organ diseases	247.0 (184.6 to 306.6)	283.9 (212.2 to 352.5)	27.5 (15.9 to 40.7)	31.6 (18.3 to 46.6)
Other sense organ diseases	Asymptomatic chronic sense organ diseases	83,812.3 (74,368.7 to 94,110.3)	103,943.6 (92,276.1 to 116,454.2)	<0.05 (0.0 to <0.05)	<0.05 (0.0 to <0.05)
Other sense organ diseases	Mild chronic sense organ diseases	61,253.7 (49,934.9 to 72,590.8)	75,970.4 (61,938.2 to 89,765.1)	339.3 (135.3 to 721.0)	420.4 (167.9 to 890.8)
Other sense organ diseases	Moderate chronic sense organ diseases	24,326.8 (13,254.7 to 36,855.5)	30,169.7 (16,519.8 to 45,499.3)	257.1 (96.4 to 524.5)	318.5 (119.6 to 647.7)
Other sense organ diseases	Severe chronic sense organ diseases	45,367.8 (34,168.2 to 56,651.9)	56,262.2 (42,447.8 to 70,475.8)	4,770.5 (2,810.2 to 6,982.2)	5,910.3 (3,498.0 to 8,600.4)
Deciduous caries	Asymptomatic deciduous caries	534,122.1 (449,524.6 to 635,865.6)	558,028.4 (462,649.3 to 669,027.3)	<0.05 (0.0 to <0.05)	<0.05 (0.0 to <0.05)
Deciduous caries	Tooth pain due to deciduous caries	14,106.5 (11,335.5 to 17,250.6)	14,665.8 (11,606.9 to 18,113.6)	141.4 (61.6 to 279.4)	147.2 (63.0 to 292.1)
Permanent caries	Asymptomatic permanent caries	2,045,859.5 (1,909,845.1 to 2,170,355.5)	2,344,628.4 (2,193,751.0 to 2,488,741.5)	<0.05 (0.0 to <0.05)	<0.05 (0.0 to <0.05)
Permanent caries	Tooth pain due to permanent caries	155,509.3 (136,303.6 to 174,154.4)	176,569.4 (154,592.8 to 197,727.4)	1,536.3 (682.4 to 2,935.8)	1,743.4 (776.7 to 3,320.9)
Periodontal diseases	Chronic periodontal diseases	428,784.1 (372,953.4 to 498,682.1)	537,506.0 (465,113.9 to 625,888.8)	2,808.4 (1,085.7 to 5,816.4)	3,520.7 (1,359.3 to 7,253.7)
Edentulism and severe tooth loss	Asymptomatic edentulism and severe tooth loss	120,351.6 (115,005.1 to 125,900.6)	153,234.9 (146,353.0 to 160,537.3)	<0.05 (0.0 to <0.05)	<0.05 (0.0 to <0.05)
Edentulism and severe tooth loss	Difficulty eating due to edentulism and severe tooth loss	96,121.2 (91,955.7 to 101,031.7)	122,384.2 (117,005.4 to 128,597.4)	5,999.5 (4,012.7 to 8,286.4)	7,640.3 (5,096.6 to 10,562.3)
Other oral disorders	Mild other oral disorders	51,539.4 (42,858.8 to 60,427.3)	59,735.1 (49,699.3 to 69,853.4)	289.9 (112.2 to 621.1)	335.8 (130.2 to 719.2)
Other oral disorders	Severe other oral disorders	63,834.1 (55,120.1 to 73,046.1)	73,984.0 (63,942.2 to 84,649.6)	3,092.8 (1,911.6 to 4,651.9)	3,582.3 (2,215.2 to 5,403.2)
**Injuries by cause**		875,575.3 (841,264.9 to 913,846.3)	1,019,157.4 (977,405.7 to 1,064,704.2)	37,974.5 (27,541.3 to 50,476.0)	41,022.0 (29,290.4 to 55,288.4)
Transport injuries		94,217.8 (89,871.8 to 99,731.0)	115,981.0 (110,575.9 to 123,047.1)	5,742.0 (4,089.8 to 7,705.8)	6,444.8 (4,500.6 to 8,733.0)
Road injuries		87,268.1 (82,797.8 to 92,816.5)	107,722.4 (102,214.5 to 114,850.0)	5,254.7 (3,709.8 to 7,084.4)	5,956.1 (4,130.8 to 8,076.3)
Pedestrian road injuries		11,520.3 (10,326.8 to 13,116.0)	14,157.8 (12,664.5 to 16,095.9)	703.6 (494.6 to 950.9)	770.8 (532.9 to 1,060.8)
Cyclist road injuries		15,405.8 (13,657.8 to 17,252.4)	18,700.6 (16,545.4 to 20,951.1)	880.8 (609.3 to 1,202.9)	968.2 (655.2 to 1,348.5)

Cause	Sequela	Prevalence in thousands (95% confidence interval)		YLDs in thousands (95% confidence interval)	
		2005	2015	2005	2015
Motorcyclist road injuries		20,062.3 (17,839.8 to 22,608.8)	26,417.2 (23,496.7 to 29,722.0)	1,188.4 (829.2 to 1,594.6)	1,397.9 (962.0 to 1,919.8)
Motor vehicle road injuries		37,330.9 (33,902.6 to 41,623.6)	44,311.4 (40,212.6 to 49,496.3)	2,305.0 (1,626.1 to 3,122.4)	2,585.4 (1,811.2 to 3,556.8)
Other road injuries		2,948.9 (2,581.9 to 3,334.6)	4,135.3 (3,603.7 to 4,692.7)	176.9 (122.1 to 241.1)	233.7 (158.3 to 320.9)
Other transport injuries		6,949.6 (6,298.1 to 7,679.2)	8,258.5 (7,469.0 to 9,141.6)	487.3 (348.1 to 648.0)	488.7 (348.6 to 660.5)
Unintentional injuries		691,349.3 (665,389.4 to 721,217.6)	790,101.8 (759,587.4 to 824,995.0)	28,890.4 (20,522.1 to 38,745.7)	30,679.5 (21,602.9 to 41,953.7)
Falls		180,475.1 (166,648.8 to 196,362.2)	225,736.9 (207,914.3 to 245,787.2)	10,573.9 (7,588.7 to 14,259.5)	11,770.2 (8,257.6 to 16,166.4)
Drowning		4,165.5 (3,764.1 to 4,613.3)	4,455.5 (4,041.2 to 4,919.4)	265.0 (186.7 to 357.9)	247.3 (173.4 to 335.8)
Fire, heat, and hot substances		62,866.6 (54,975.7 to 70,866.6)	69,666.6 (60,988.1 to 78,550.1)	2,227.0 (1,587.7 to 3,010.3)	2,269.4 (1,571.4 to 3,131.8)
Poisonings		5,028.1 (4,255.5 to 5,993.8)	5,722.4 (4,829.3 to 6,854.1)	504.8 (339.0 to 721.0)	549.4 (367.8 to 791.1)
Exposure to mechanical forces		161,248.2 (149,147.0 to 173,791.4)	183,582.4 (169,658.6 to 197,859.6)	3,381.0 (2,398.8 to 4,634.3)	3,580.8 (2,485.7 to 4,922.9)
Unintentional firearm injuries		2,056.6 (1,833.4 to 2,303.9)	2,333.0 (2,074.5 to 2,625.1)	89.5 (64.0 to 120.5)	93.2 (65.4 to 126.2)
Unintentional suffocation		8,896.4 (7,214.5 to 11,186.5)	9,759.3 (8,142.5 to 11,916.9)	317.6 (218.9 to 432.9)	353.7 (245.8 to 480.3)
Other exposure to mechanical forces		150,295.1 (138,177.3 to 162,533.2)	171,490.1 (157,821.0 to 185,364.4)	2,973.9 (2,110.8 to 4,096.7)	3,133.9 (2,172.1 to 4,356.6)
Adverse effects of medical treatment		10,823.1 (8,564.9 to 13,168.8)	11,158.6 (8,773.5 to 13,610.2)	1,442.7 (909.3 to 2,130.6)	1,487.4 (926.9 to 2,223.0)
Animal contact		31,130.7 (28,082.8 to 34,542.0)	34,049.7 (30,774.2 to 37,569.0)	1,053.0 (732.6 to 1,429.1)	1,100.9 (763.8 to 1,486.9)
Venomous animal contact		12,969.8 (11,662.8 to 14,401.3)	14,680.7 (13,173.4 to 16,375.1)	779.6 (532.9 to 1,059.1)	820.7 (558.1 to 1,117.5)
Non-venomous animal contact		18,160.9 (15,697.6 to 21,059.7)	19,369.1 (16,794.5 to 22,306.5)	273.4 (184.9 to 397.2)	280.3 (187.2 to 410.5)
Foreign body		27,944.7 (24,798.5 to 31,384.1)	32,345.9 (28,877.3 to 36,026.4)	1,218.6 (871.3 to 1,663.7)	1,263.3 (899.4 to 1,722.5)
Pulmonary aspiration and foreign body in airway		11,557.3 (9,408.9 to 14,973.1)	13,351.2 (11,069.0 to 16,798.7)	691.4 (476.7 to 969.6)	690.2 (477.9 to 973.1)
Foreign body in eyes		865.7 (377.4 to 1,372.9)	1,007.2 (444.0 to 1,593.4)	48.8 (20.9 to 87.8)	56.2 (24.0 to 100.6)
Foreign body in other body part		15,521.6 (13,732.5 to 17,476.1)	17,987.4 (15,874.1 to 20,237.9)	478.5 (340.2 to 651.9)	517.0 (364.4 to 713.3)
Environmental heat and cold exposure		53,425.5 (48,496.8 to 59,357.0)	61,942.3 (56,211.9 to 68,761.8)	2,496.4 (1,758.6 to 3,371.4)	2,657.1 (1,861.7 to 3,636.2)
Other unintentional injuries		154,242.0 (141,498.2 to 168,273.1)	161,441.6 (147,971.3 to 176,204.3)	5,728.2 (3,926.1 to 7,931.6)	5,753.6 (3,913.1 to 8,024.1)
Self-harm and interpersonal violence		20,904.7 (19,372.0 to 22,333.9)	24,083.0 (22,279.7 to 25,744.4)	1,057.1 (759.9 to 1,404.5)	1,077.5 (762.7 to 1,448.2)
Self-harm		5,519.9 (4,934.6 to 6,208.1)	6,358.6 (5,682.1 to 7,154.5)	329.6 (234.1 to 441.0)	332.7 (232.4 to 446.9)
Interpersonal violence		15,384.8 (14,065.0 to 16,689.5)	17,724.4 (16,186.6 to 19,268.4)	727.5 (518.5 to 973.0)	744.8 (521.9 to 1,007.3)
Assault by firearm		898.9 (777.8 to 1,045.1)	1,043.6 (900.8 to 1,213.2)	44.5 (31.3 to 60.8)	46.5 (32.4 to 64.9)
Assault by sharp object		3,571.9 (2,994.0 to 4,124.8)	4,019.5 (3,330.4 to 4,668.1)	131.8 (92.0 to 177.1)	123.4 (84.8 to 168.7)
Assault by other means		10,914.0 (9,879.2 to 12,054.5)	12,661.3 (11,425.5 to 14,018.9)	551.2 (395.3 to 743.7)	574.8 (404.0 to 782.2)
Forces of nature, war, and legal intervention		69,103.5 (50,798.3 to 90,506.6)	88,991.7 (65,131.0 to 116,786.1)	2,284.9 (1,643.9 to 3,064.0)	2,820.2 (1,921.3 to 3,850.1)
Exposure to forces of nature		22,294.6 (14,541.1 to 31,952.5)	25,293.7 (16,193.6 to 37,658.7)	1,097.9 (689.5 to 1,600.8)	796.9 (541.4 to 1,091.4)
Collective violence and legal intervention		46,808.9 (32,994.8 to 62,217.3)	63,697.9 (45,248.7 to 83,186.4)	1,187.0 (828.0 to 1,595.9)	2,023.3 (1,303.8 to 2,903.0)
**Injuries by sequela**					
Injuries	Amputation of lower limbs, bilateral	166.2 (148.6 to 186.1)	195.0 (174.3 to 218.5)	43.2 (29.5 to 59.3)	37.7 (25.3 to 52.4)
Injuries	Amputation of upper limbs, bilateral	2,155.8 (2,027.2 to 2,299.0)	2,497.7 (2,349.3 to 2,667.0)	602.2 (416.3 to 792.6)	496.8 (349.7 to 652.9)
Injuries	Amputation of fingers (excluding thumb)	8,310.2 (7,889.8 to 8,751.6)	9,572.6 (9,090.7 to 10,107.4)	38.1 (14.5 to 81.7)	43.9 (16.6 to 93.8)
Injuries	Amputation of lower limb, unilateral	21,124.7 (19,345.2 to 23,006.9)	24,909.9 (22,864.0 to 27,042.9)	2,354.7 (1,641.9 to 3,218.9)	1,987.2 (1,396.6 to 2,686.4)
Injuries	Amputation of upper limb, unilateral	2,192.6 (2,084.9 to 2,313.6)	2,543.8 (2,421.4 to 2,684.5)	179.3 (124.7 to 246.0)	160.6 (109.0 to 220.2)
Injuries	Amputation of thumb	3,075.9 (1,723.8 to 4,504.9)	3,697.5 (2,056.5 to 5,425.0)	34.1 (13.6 to 67.2)	40.9 (15.8 to 81.0)
Injuries	Amputation of toe/toes	27,656.8 (23,780.8 to 32,258.6)	32,352.7 (27,629.5 to 37,692.1)	156.5 (63.4 to 323.6)	182.9 (74.2 to 376.6)
Injuries	Burns, <20% total burned surface area without lower airway burns	99,901.0 (90,668.1 to 109,561.3)	112,362.0 (101,946.0 to 123,898.2)	1,842.1 (1,067.4 to 2,929.6)	1,954.7 (1,118.0 to 3,098.2)
Injuries	Burns, >=20% total burned surface area or >= 10% burned surface area if head/neck or hands/wrist involved w/o lower airway burns	4,202.8 (3,823.9 to 4,609.4)	5,326.2 (4,895.1 to 5,823.3)	1,516.4 (1,048.7 to 2,008.2)	1,392.4 (976.2 to 1,832.4)
Injuries	Lower airway burns	--	--	1.3 (0.6 to 2.7)	0.9 (0.5 to 1.4)
Injuries	Dislocation of hip	1,999.2 (1,849.6 to 2,171.1)	2,373.6 (2,197.1 to 2,576.7)	34.6 (18.2 to 57.7)	40.6 (21.3 to 68.0)
Injuries	Dislocation of knee	161.7 (150.3 to 174.9)	196.8 (182.5 to 212.8)	52.4 (34.1 to 75.7)	60.2 (38.6 to 87.4)
Injuries	Dislocation of shoulder	--	--	55.2 (13.5 to 108.1)	65.9 (16.2 to 129.6)
Injuries	Muscle and tendon injuries, including sprains and strains lesser dislocations	39,110.5 (36,822.5 to 41,872.1)	45,609.8 (42,984.0 to 48,812.9)	381.3 (167.2 to 750.8)	437.8 (191.6 to 861.9)
Injuries	Fracture of clavicle, scapula, or humerus	1,990.7 (1,858.7 to 2,142.8)	2,430.5 (2,272.2 to 2,624.3)	175.7 (104.2 to 280.1)	205.6 (121.8 to 320.3)
Injuries	Fracture of face bones	429.2 (401.0 to 468.0)	519.6 (486.3 to 562.2)	99.3 (61.3 to 153.5)	154.2 (91.8 to 242.0)
Injuries	Fracture of foot bones except ankle	8,240.2 (7,796.0 to 8,746.3)	9,793.4 (9,266.6 to 10,394.5)	234.1 (138.6 to 365.9)	275.9 (163.8 to 430.6)
Injuries	Fracture of hand (wrist and other distal part of hand)	10,208.8 (9,616.4 to 10,914.2)	12,229.6 (11,532.0 to 13,093.1)	163.1 (84.1 to 291.6)	190.5 (98.2 to 340.8)
Injuries	Fracture of hip	12,695.5 (11,653.8 to 13,881.8)	15,958.1 (14,617.2 to 17,397.6)	4,112.1 (2,750.6 to 5,677.0)	3,145.0 (2,054.6 to 4,420.2)
Injuries	Fracture of patella, tibia or fibula, or ankle	222,636.3 (211,766.5 to 235,285.7)	267,732.5 (254,641.9 to 282,666.4)	12,138.0 (8,169.4 to 17,441.3)	14,513.1 (9,772.7 to 20,809.2)
Injuries	Fracture of pelvis	9,530.8 (8,914.5 to 10,325.8)	11,254.1 (10,507.3 to 12,244.4)	1,856.2 (1,295.7 to 2,489.7)	2,129.1 (1,521.9 to 2,852.3)
Injuries	Fracture of radius and/or ulna	1,418.2 (1,330.9 to 1,516.7)	1,743.2 (1,632.2 to 1,867.7)	231.5 (132.9 to 384.9)	232.1 (138.1 to 376.6)
Injuries	Fracture of skull	1,171.4 (1,102.0 to 1,251.4)	1,348.1 (1,267.5 to 1,442.3)	119.5 (80.0 to 169.0)	135.8 (90.0 to 192.1)
Injuries	Fracture of sternum and/or fracture of one or more ribs	766.0 (719.9 to 809.9)	880.3 (828.0 to 935.2)	104.1 (69.7 to 144.7)	114.8 (77.4 to 161.0)
Injuries	Fracture of vertebral column	1,869.5 (1,759.4 to 1,994.2)	2,220.4 (2,083.9 to 2,379.4)	389.1 (254.5 to 568.1)	433.5 (287.8 to 618.1)

Cause	Sequela	Prevalence in thousands (95% confidence interval)		YLDs in thousands (95% confidence interval)	
		2005	2015	2005	2015
Injuries	Fracture of femur, other than femoral neck	12,928.5 (12,322.2 to 13,595.5)	15,609.2 (14,867.4 to 16,415.4)	968.5 (595.9 to 1,453.5)	1,056.2 (659.4 to 1,560.5)
Injuries	Minor TBI	4,880.0 (4,674.3 to 5,114.0)	5,788.9 (5,534.6 to 6,083.2)	697.3 (488.7 to 947.8)	816.5 (574.1 to 1,101.1)
Injuries	Moderate TBI	16,424.0 (15,653.2 to 17,223.7)	20,745.6 (19,792.6 to 21,739.7)	2,712.1 (1,917.2 to 3,664.8)	3,390.9 (2,413.2 to 4,544.9)
Injuries	Severe TBI	--	--	<0.05 (0.0 to <0.05)	<0.05 (0.0 to <0.05)
Injuries	Foreign body in ear	--	--	0.2 (0.0 to 0.6)	0.2 (0.0 to 0.6)
Injuries	Foreign body in respiratory system	--	--	37.1 (19.2 to 61.2)	37.1 (19.6 to 60.5)
Injuries	Foreign body in GI and urogenital system	--	--	18.7 (9.2 to 31.8)	18.5 (9.0 to 31.0)
Injuries	Spinal cord lesion at neck level A	1,403.8 (1,286.2 to 1,523.6)	1,695.2 (1,551.3 to 1,838.1)	847.5 (610.8 to 1,045.9)	953.0 (669.1 to 1,199.1)
Injuries	Spinal cord lesion at neck level B	206.3 (156.5 to 257.4)	249.1 (188.8 to 311.2)	113.3 (71.6 to 157.7)	120.3 (73.3 to 168.5)
Injuries	Spinal cord lesion at neck level C	390.8 (322.8 to 462.0)	472.0 (388.7 to 558.9)	209.1 (146.4 to 275.3)	222.5 (151.3 to 299.5)
Injuries	Spinal cord lesion at neck level D	753.3 (664.7 to 841.5)	909.7 (799.4 to 1,017.1)	43.8 (28.7 to 64.1)	52.8 (34.8 to 77.9)
Injuries	Spinal cord lesion below neck level A	1,285.1 (1,173.6 to 1,395.2)	1,545.1 (1,409.6 to 1,676.7)	550.1 (385.4 to 707.0)	523.2 (361.4 to 697.8)
Injuries	Spinal cord lesion below neck level B	188.9 (142.7 to 236.0)	227.1 (171.5 to 284.6)	49.1 (31.4 to 69.5)	32.4 (21.0 to 45.9)
Injuries	Spinal cord lesion below neck level C	357.8 (294.4 to 423.9)	430.2 (353.4 to 509.8)	90.5 (61.5 to 123.3)	60.0 (41.4 to 80.8)
Injuries	Spinal cord lesion below neck level D	689.7 (605.3 to 774.4)	829.2 (726.9 to 932.2)	40.0 (26.1 to 58.9)	48.0 (31.2 to 70.4)
Injuries	Drowning and nonfatal submersion	102.6 (94.3 to 112.6)	114.4 (104.8 to 125.3)	25.0 (16.6 to 34.8)	27.6 (18.6 to 38.2)
Injuries	Asphyxiation	--	--	0.5 (0.2 to 0.9)	0.5 (0.2 to 0.9)
Injuries	Crush injury	1,496.1 (1,420.1 to 1,585.1)	1,812.8 (1,720.2 to 1,922.2)	194.4 (135.1 to 269.7)	234.8 (165.9 to 326.6)
Injuries	Nerve injury	1,763.4 (1,667.9 to 1,877.0)	2,081.5 (1,964.9 to 2,219.2)	219.8 (150.6 to 298.1)	279.7 (184.8 to 384.5)
Injuries	Injury to eyes	--	--	175.9 (61.0 to 320.7)	217.9 (76.1 to 407.5)
Injuries	Open wound(s)	49,304.2 (46,662.7 to 52,287.5)	57,860.0 (54,783.0 to 61,243.1)	582.4 (278.7 to 1,063.9)	674.2 (323.9 to 1,222.9)
Injuries	Poisoning requiring urgent care	7,764.7 (7,044.2 to 8,574.8)	8,603.5 (7,774.0 to 9,538.8)	1,259.9 (852.3 to 1,755.2)	1,390.2 (943.5 to 1,916.1)
Injuries	Severe chest Injury	421.1 (404.3 to 440.3)	488.5 (468.8 to 511.8)	337.4 (193.3 to 524.9)	418.9 (239.5 to 642.3)
Injuries	Internal hemorrhage in abdomen and pelvis	2,548.5 (2,452.5 to 2,663.1)	2,987.6 (2,872.7 to 3,120.1)	756.7 (463.3 to 1,189.3)	916.8 (562.3 to 1,368.3)
Injuries	Contusion in any part of the body	17,154.5 (16,049.4 to 18,418.1)	19,027.7 (17,748.8 to 20,477.8)	132.8 (58.0 to 257.7)	146.4 (64.3 to 283.4)
Injuries	Effect of different environmental factors	2,909.3 (2,737.0 to 3,136.2)	3,476.1 (3,258.1 to 3,777.5)	278.1 (175.9 to 428.8)	235.4 (162.2 to 326.8)
Injuries	Complications following therapeutic procedures	<0.05 (0.0 to <0.05)	<0.05 (0.0 to <0.05)	1,713.6 (1,072.6 to 2,557.5)	2,027.8 (1,254.4 to 3,042.6)
Injuries	Superficial injury of any part of the body	--	--	11.8 (5.0 to 24.5)	16.3 (6.7 to 34.2)
Injuries	Multiple fractures, dislocations, crashs, wounds , prains, and strains	3,615.2 (3,393.8 to 3,834.7)	4,413.0 (4,163.1 to 4,679.9)	2,118.5 (1,276.1 to 3,250.5)	1,600.9 (970.8 to 2,309.7)

SR Table 2. Prevalent cases and years lived with disability (YLDs) for 2015, percent change, and percent change in age-standardised rates between 2005 and 2015 of anemia by cause, for all ages, both sexes combined

Cause	Prevalence in thousands (95% confidence interval)			YLDs in thousands (95% confidence interval)		
	2015	Percent change in counts between 2005 and 2015	Percent change in ASR between 2005 and 2015	2015	Percent change in counts between 2005 and 2015	Percent change in ASR between 2005 and 2015
Iron-deficiency anemia	1,477,458.4 (1,470,824.4 to 1,485,250.1)	1.5 (0.9 to 2.1)*	-8.0 (-8.5 to -7.4)*	48,521.1 (32,555.6 to 69,714.9)	-3.8 (-5.1 to -2.5)*	-11.6 (-12.8 to -10.5)*
Gastritis and duodenitis	157,060.1 (154,055.1 to 160,141.0)	15.5 (12.9 to 17.9)*	-6.9 (-8.9 to -5.0)*	4,497.1 (3,050.6 to 6,393.0)	10.8 (7.4 to 14.5)*	-9.8 (-12.4 to -7.0)*
Thalassemias trait	129,373.2 (128,629.5 to 130,209.0)	9.5 (8.6 to 10.2)*	-2.3 (-3.1 to -1.6)*	3,922.2 (2,607.9 to 5,653.6)	6.1 (4.8 to 7.4)*	-3.6 (-4.6 to -2.6)*
Malaria	71,936.4 (70,137.5 to 73,504.2)	26.3 (24.4 to 28.0)*	17.0 (15.3 to 18.6)*	2,736.7 (1,848.0 to 3,918.8)	21.1 (18.1 to 24.3)*	13.1 (10.5 to 16.1)*
Other neglected tropical diseases	61,452.6 (60,842.8 to 62,054.9)	0.1 (-1.0 to 1.3)	-7.7 (-8.8 to -6.6)*	2,158.2 (1,446.3 to 3,073.1)	-6.0 (-8.2 to -3.8)*	-12.4 (-14.5 to -10.4)*
Other hemoglobinopathies and hemolytic anemias	74,273.3 (73,786.0 to 74,751.1)	1.8 (0.9 to 2.7)*	-9.0 (-9.8 to -8.2)*	2,067.4 (1,384.3 to 2,963.9)	-4.1 (-5.8 to -2.4)*	-12.6 (-14.1 to -11.2)*
Peptic ulcer disease	67,066.6 (66,465.4 to 67,616.9)	4.8 (3.7 to 6.1)*	-17.0 (-17.9 to -16.0)*	1,823.1 (1,225.8 to 2,621.2)	0.2 (-1.4 to 1.9)	-20.3 (-21.6 to -19.0)*
Endocrine, metabolic, blood, and immune disorders	58,430.4 (57,936.0 to 58,946.7)	2.2 (0.3 to 4.3)*	-8.0 (-9.7 to -6.2)*	1,804.4 (1,216.0 to 2,582.3)	-1.3 (-3.9 to 1.5)	-9.5 (-11.9 to -7.0)*
Other infectious diseases	54,832.7 (54,383.6 to 55,271.4)	0.4 (-1.6 to 2.3)	-8.0 (-9.8 to -6.2)*	1,792.0 (1,199.2 to 2,569.5)	-2.5 (-5.4 to 0.3)	-9.3 (-11.9 to -6.7)*
Uterine fibroids	42,780.0 (41,634.5 to 43,881.4)	10.5 (9.5 to 11.5)*	-5.5 (-6.3 to -4.6)*	1,737.5 (1,125.7 to 2,625.4)	6.4 (4.5 to 8.1)*	-8.9 (-10.5 to -7.4)*
Sickle cell trait	50,883.1 (50,136.5 to 51,518.3)	19.1 (16.7 to 20.8)*	7.4 (5.3 to 9.0)*	1,720.3 (1,156.5 to 2,459.0)	10.8 (7.3 to 13.4)*	1.4 (-1.8 to 3.6)
Chronic kidney disease due to diabetes mellitus	12,954.4 (12,474.8 to 13,404.9)	23.4 (22.2 to 24.7)*	-0.2 (-1.1 to 0.6)	793.1 (554.4 to 1,079.2)	18.4 (16.3 to 20.4)*	-1.3 (-2.7 to 0.1)
Chronic kidney disease due to other causes	12,377.0 (11,952.9 to 12,766.2)	23.2 (21.9 to 24.6)*	-1.3 (-2.4 to -0.2)*	763.8 (537.2 to 1,037.4)	18.9 (16.9 to 20.9)*	-2.4 (-3.7 to -1.0)*
Hookworm disease	24,220.2 (24,078.2 to 24,365.2)	-6.2 (-7.3 to -5.1)*	-15.5 (-16.5 to -14.6)*	712.1 (476.3 to 1,031.1)	-13.2 (-15.6 to -11.1)*	-20.5 (-22.6 to -18.7)*
Other gynecological diseases	21,110.8 (20,900.6 to 21,328.2)	2.1 (0.9 to 3.4)*	-8.2 (-9.3 to -7.1)*	682.9 (461.0 to 978.0)	-2.7 (-4.6 to -0.7)*	-12.3 (-14.0 to -10.6)*
Chronic kidney disease due to glomerulonephritis	10,417.9 (9,993.1 to 10,775.5)	22.1 (20.6 to 23.6)*	-1.5 (-2.7 to -0.3)*	651.8 (454.1 to 897.7)	19.1 (17.0 to 21.3)*	-1.7 (-3.3 to 0.1)
Schistosomiasis	16,331.3 (16,075.8 to 16,655.1)	-19.6 (-21.1 to -17.7)*	-27.0 (-28.3 to -25.3)*	543.1 (365.1 to 781.5)	-21.5 (-24.0 to -18.6)*	-27.8 (-30.0 to -25.1)*
Chronic kidney disease due to hypertension	8,181.1 (7,844.7 to 8,463.3)	28.5 (27.0 to 30.1)*	0.1 (-1.1 to 1.3)	478.3 (337.3 to 656.8)	26.0 (23.6 to 28.6)*	-0.4 (-2.1 to 1.3)
Sickle cell disorders	4,328.2 (4,175.5 to 4,461.7)	8.0 (4.8 to 11.7)*	2.4 (-0.5 to 6.0)	331.9 (228.4 to 469.2)	6.5 (1.5 to 11.8)*	0.9 (-3.9 to 5.9)
Maternal hemorrhage	2,113.4 (2,082.8 to 2,141.5)	1.5 (-0.9 to 3.9)	-7.9 (-10.1 to -5.8)*	53.5 (35.0 to 78.6)	-1.8 (-5.9 to 2.6)	-10.8 (-14.5 to -6.9)*
Thalassemias	387.0 (360.5 to 416.4)	0.5 (-4.1 to 5.5)	-7.1 (-11.3 to -2.5)*	30.3 (20.5 to 42.9)	0.6 (-4.4 to 6.3)	-7.2 (-11.9 to -2.2)*
G6PD trait	640.4 (629.6 to 651.1)	5.8 (3.8 to 7.8)*	-5.8 (-7.6 to -4.1)*	28.0 (19.3 to 38.9)	4.3 (0.1 to 8.8)*	-5.3 (-9.0 to -1.2)*
G6PD deficiency	499.4 (487.1 to 513.4)	4.3 (1.6 to 7.4)*	-5.7 (-8.2 to -2.9)*	27.7 (18.9 to 38.7)	1.2 (-2.8 to 5.5)	-8.1 (-11.6 to -4.3)*
**All causes**	**2,359,107.9 (2,349,938.3 to 2,369,065.8)**	**4.0 (3.5 to 4.5)***	**-7.1 (-7.6 to -6.7)***	**77,876.7 (52,436.9 to 111,360.0)**	**-0.7 (-2.0 to 0.4)**	**-10.2 (-11.2 to -9.2)***

SR Table 3. Prevalent cases and years lived with disability (YLDs) for 2015, percent change, and percent change in age-standardised rates between 2005 and 2015 of epilepsy by cause, for all ages, both sexes combined

Cause	Prevalence in thousands (95% confidence interval)			YLDs in thousands (95% confidence interval)		
	**2015**	**Percent change in counts between 2005 and 2015**	**Percent change in ASR between 2005 and 2015**	**2015**	**Percent change in counts between 2005 and 2015**	**Percent change in ASR between 2005 and 2015**
Epilepsy	23,414.5 (21,549.6 to 25,419.4)	11.3 (7.0 to 15.5)*	-1.1 (-5.0 to 2.7)	6,286.9 (4,315.8 to 8,250.2)	-6.4 (-11.5 to -1.2)*	-16.3 (-20.9 to -11.6)*
Neonatal preterm birth complications	7,110.9 (4,668.1 to 9,554.0)	14.2 (8.2 to 20.3)*	4.0 (-1.4 to 9.8)	2,702.9 (1,384.0 to 4,096.7)	15.9 (9.8 to 22.4)*	5.5 (0.0 to 11.7)*
Neonatal encephalopathy due to birth asphyxia and trauma	5,676.9 (3,482.3 to 8,334.2)	37.6 (33.2 to 42.4)*	25.3 (21.5 to 29.8)*	2,070.9 (1,066.5 to 3,333.9)	37.2 (32.1 to 42.4)*	25.0 (20.5 to 29.6)*
Hemolytic disease and other neonatal jaundice	902.8 (616.6 to 1,168.5)	29.3 (26.0 to 32.5)*	17.5 (14.4 to 20.5)*	346.2 (181.0 to 526.2)	29.4 (25.1 to 33.5)*	17.4 (13.4 to 21.1)*
Malaria	446.6 (307.9 to 570.0)	30.7 (23.1 to 37.6)*	20.4 (13.4 to 26.9)*	165.7 (87.0 to 246.4)	28.0 (19.6 to 36.0)*	17.9 (10.1 to 25.3)*
Cysticercosis	1,086.9 (898.8 to 1,315.8)	11.2 (5.3 to 17.0)*	-6.2 (-11.3 to -1.3)*	129.1 (84.8 to 184.5)	11.7 (5.2 to 17.9)*	-5.7 (-11.1 to -0.7)*
Pneumococcal meningitis	127.7 (113.3 to 145.2)	41.8 (36.1 to 47.5)*	28.2 (23.2 to 33.1)*	15.8 (10.5 to 22.1)	41.9 (34.5 to 49.9)*	28.4 (21.7 to 35.4)*
Encephalitis	125.0 (108.6 to 143.1)	33.4 (27.9 to 38.6)*	18.0 (13.5 to 22.6)*	15.3 (9.9 to 21.2)	33.8 (26.7 to 41.1)*	18.5 (12.4 to 24.8)*
Food-borne trematodiases	121.5 (40.2 to 233.5)	-2.7 (-21.6 to 19.5)	-15.1 (-31.2 to 3.9)	14.9 (4.4 to 31.1)	-1.6 (-20.5 to 21.2)	-14.0 (-30.5 to 5.4)
H influenzae type B meningitis	61.0 (52.1 to 71.9)	27.4 (21.6 to 33.5)*	18.1 (13.1 to 23.7)*	7.6 (5.0 to 10.9)	27.9 (19.7 to 37.3)*	18.6 (11.1 to 27.5)*
Other meningitis	48.9 (42.6 to 56.1)	35.5 (30.3 to 41.0)*	21.6 (17.0 to 25.9)*	6.2 (4.0 to 8.7)	35.5 (28.4 to 42.3)*	21.5 (15.3 to 27.4)*
Neonatal sepsis and other neonatal infections	5.6 (3.7 to 8.0)	9.0 (-3.5 to 20.5)	3.4 (-8.5 to 14.3)	2.6 (1.6 to 4.0)	9.0 (-4.0 to 21.0)	3.3 (-8.9 to 14.7)
Tetanus	6.7 (4.4 to 8.6)	-2.1 (-7.2 to 3.6)	-11.6 (-16.3 to -6.4)*	2.6 (1.3 to 4.0)	-1.6 (-7.4 to 4.2)	-11.2 (-16.5 to -5.9)*
Meningococcal meningitis	19.0 (15.8 to 22.5)	37.9 (30.5 to 45.2)*	26.6 (19.7 to 33.0)*	2.4 (1.5 to 3.5)	37.9 (26.1 to 50.0)*	26.6 (15.8 to 37.3)*
Cystic echinococcosis	6.4 (1.9 to 13.9)	44.0 (37.3 to 50.1)*	23.1 (17.6 to 28.2)*	0.8 (0.2 to 1.9)	44.4 (37.6 to 50.7)*	23.3 (17.7 to 28.5)*
**All causes**	**39,160.5 (34,270.8 to 43,685.8)**	**15.8 (12.6 to 18.9)***	**3.5 (0.5 to 6.4)***	**11,770.0 (8,578.8 to 15,276.3)**	**5.8 (0.5 to 11.0)***	**-4.9 (-9.8 to -0.1)***

SR Table 4. Prevalent cases and years lived with disability (YLDs) for 2015, percent change, and percent change in age-standardized rates between 2005 and 2015 of Guillain-Barré syndrome by cause, all ages and both sexes combined

Cause	Prevalence in thousands (95% confidence interval)			YLDs in thousands (95% confidence interval)		
	**2015**	**Percent change in counts between 2005 and 2015**	**Percent change in ASR between 2005 and 2015**	**2015**	**Percent change in counts between 2005 and 2015**	**Percent change in ASR between 2005 and 2015**
Upper respiratory infections	12.1 (9.6 to 15.2)	16.9 (14.7 to 19.5)*	-0.2 (-0.9 to 0.4)	3.6 (2.2 to 5.4)	17.0 (14.7 to 19.5)*	-0.2 (-0.9 to 0.4)
Other neurological disorders	11.5 (9.1 to 14.6)	17.0 (14.8 to 19.6)*	-0.2 (-0.9 to 0.4)	3.4 (2.1 to 5.0)	17.0 (14.8 to 19.6)*	-0.2 (-0.9 to 0.4)
Lower respiratory infections	4.4 (2.5 to 6.9)	16.9 (14.7 to 19.5)*	-0.2 (-0.9 to 0.4)	1.3 (0.7 to 2.2)	16.9 (14.7 to 19.5)*	-0.2 (-0.9 to 0.4)
Diarrheal diseases	4.2 (3.2 to 5.5)	16.9 (14.7 to 19.5)*	-0.2 (-0.9 to 0.4)	1.3 (0.8 to 1.9)	16.9 (14.7 to 19.5)*	-0.2 (-0.9 to 0.4)
Other infectious diseases	2.7 (1.9 to 3.7)	16.9 (14.7 to 19.5)*	-0.2 (-0.9 to 0.4)	0.8 (0.5 to 1.2)	16.9 (14.7 to 19.5)*	-0.2 (-0.9 to 0.4)
**All causes**	**35.0 (28.6 to 42.1)**	**17.0 (14.7 to 19.5)***	**-0.2 (-0.9 to 0.4)**	**10.4 (6.6 to 15.0)**	**17.0 (14.7 to 19.5)***	**-0.2 (-0.9 to 0.4)**

SR Table 5. Prevalent cases and years lived with disability (YLDs) for 2015, percent change, and percent change in age-standardised rates between 2005 and 2015 of hearing loss by cause, for all ages, both sexes combined

Cause	Prevalence in thousands (95% confidence interval)			YLDs in thousands (95% confidence interval)		
	**2015**	**Percent change in counts between 2005 and 2015**	**Percent change in ASR between 2005 and 2015**	**2015**	**Percent change in counts between 2005 and 2015**	**Percent change in ASR between 2005 and 2015**
Age-related and other hearing loss	1,210,055.2 (1,140,224.2 to 1,274,664.8)	28.3 (27.4 to 29.2)*	1.4 (1.0 to 1.8)*	40,596.8 (27,898.4 to 56,075.0)	26.4 (24.6 to 28.3)*	1.1 (0.0 to 2.1)*
Otitis media	86,393.5 (76,373.6 to 97,104.5)	4.1 (2.7 to 5.5)*	-6.0 (-7.2 to -4.8)*	2,985.7 (1,903.3 to 4,369.7)	2.7 (-0.2 to 5.6)	-6.3 (-8.9 to -3.6)*
Other congenital anomalies	32,363.0 (17,788.1 to 59,394.1)	14.5 (12.3 to 16.8)*	2.0 (-0.1 to 4.1)	2,485.9 (1,123.1 to 4,998.5)	11.9 (9.3 to 14.5)*	-0.3 (-2.6 to 2.1)
Pneumococcal meningitis	1,126.3 (553.1 to 1,511.0)	25.6 (20.5 to 31.2)*	8.7 (4.1 to 13.6)*	61.0 (10.0 to 96.3)	24.3 (18.7 to 30.3)*	10.3 (5.0 to 15.6)*
Other meningitis	408.0 (198.7 to 529.1)	20.7 (16.1 to 25.5)*	3.7 (-0.3 to 8.1)	22.0 (3.8 to 35.2)	19.8 (14.8 to 25.2)*	5.5 (0.7 to 10.3)*
H influenzae type B meningitis	322.5 (146.9 to 446.7)	-21.8 (-27.6 to -16.1)*	-30.9 (-36.5 to -25.6)*	18.4 (2.7 to 31.0)	-23.5 (-29.5 to -17.4)*	-30.7 (-36.2 to -25.4)*
Meningococcal meningitis	234.3 (116.2 to 314.9)	20.1 (13.8 to 26.4)*	4.9 (-0.8 to 10.6)	13.2 (2.3 to 21.4)	18.6 (11.8 to 25.3)*	6.4 (0.3 to 12.5)*
**All causes**	**1,330,902.9 (1,261,401.0 to 1,397,100.6)**	**26.0 (25.2 to 26.7)***	**0.9 (0.5 to 1.3)***	**46,183.1 (31,566.7 to 63,209.9)**	**23.6 (22.1 to 25.1)***	**0.6 (-0.4 to 1.5)**

SR Table 6. Prevalent cases and years lived with disability (YLDs) for 2015, percent change, and percent change in age-standardised rates between 2005 and 2015 of heart failure by cause, for all ages, both sexes combined

Cause	Prevalence in thousands (95% confidence interval)			YLDs in thousands (95% confidence interval)		
	**2015**	**Percent change in counts between 2005 and 2015**	**Percent change in ASR between 2005 and 2015**	**2015**	**Percent change in counts between 2005 and 2015**	**Percent change in ASR between 2005 and 2015**
Ischemic heart disease	22,275.7 (21,272.0 to 23,320.4)	31.9 (31.1 to 32.7)*	-0.6 (-1.2 to -0.1)*	2,447.2 (1,736.8 to 3,342.6)	31.9 (31.0 to 32.8)*	-0.5 (-1.1 to 0.1)
Chronic obstructive pulmonary disease	5,876.4 (5,073.7 to 6,548.3)	34.9 (31.1 to 37.8)*	2.4 (-0.3 to 4.2)	2,375.5 (1,890.3 to 2,747.2)	35.1 (31.4 to 38.2)*	2.5 (-0.0 to 4.5)
Hypertensive heart disease	6,086.2 (5,732.7 to 6,434.1)	37.1 (36.2 to 37.9)*	3.4 (2.9 to 3.9)*	670.0 (467.1 to 917.1)	37.2 (35.9 to 38.4)*	3.6 (2.8 to 4.4)*
Cardiomyopathy and myocarditis	2,380.4 (2,254.3 to 2,502.2)	27.0 (25.9 to 28.1)*	-1.5 (-2.2 to -0.9)*	267.3 (185.9 to 367.9)	26.5 (24.7 to 28.3)*	-1.4 (-2.6 to -0.2)*
Rheumatic heart disease	1,202.0 (1,138.2 to 1,269.0)	29.8 (28.5 to 31.2)*	2.2 (1.3 to 3.2)*	136.1 (94.5 to 187.6)	29.6 (27.2 to 32.1)*	2.4 (0.6 to 4.2)*
Other cardiovascular and circulatory diseases	1,126.5 (1,045.9 to 1,206.1)	34.4 (33.3 to 35.6)*	1.2 (0.4 to 2.0)*	123.7 (86.0 to 173.3)	34.4 (32.5 to 36.5)*	1.3 (-0.2 to 2.7)
Interstitial lung disease and pulmonary sarcoidosis	197.4 (144.1 to 242.7)	31.5 (29.7 to 33.2)*	1.3 (-0.1 to 2.9)	86.2 (54.8 to 117.6)	31.3 (28.6 to 34.1)*	1.3 (-1.0 to 3.7)
Chagas disease	353.0 (221.5 to 503.5)	0.3 (-2.9 to 4.0)	-20.9 (-23.4 to -18.2)*	18.2 (9.7 to 29.4)	0.2 (-3.6 to 4.4)	-20.9 (-23.8 to -17.8)*
Endocrine, metabolic, blood, and immune disorders	121.4 (113.3 to 130.0)	30.7 (29.6 to 31.9)*	-1.9 (-2.6 to -1.2)*	13.6 (9.4 to 19.1)	30.7 (27.4 to 34.0)*	-1.7 (-4.3 to 0.8)
Other hemoglobinopathies and hemolytic anemias	111.8 (103.8 to 119.6)	34.9 (33.9 to 36.0)*	2.0 (1.3 to 2.7)*	13.1 (8.9 to 18.2)	34.9 (31.2 to 38.7)*	2.1 (-0.8 to 5.0)
Congenital heart anomalies	105.2 (99.2 to 111.4)	11.6 (10.9 to 12.4)*	-1.0 (-1.5 to -0.5)*	12.9 (8.8 to 18.2)	11.6 (8.3 to 15.1)*	-1.0 (-3.9 to 2.0)
Endocarditis	98.8 (91.1 to 107.6)	22.7 (20.7 to 24.7)*	-6.1 (-7.6 to -4.6)*	11.4 (7.9 to 15.9)	22.9 (18.3 to 28.0)*	-5.5 (-9.3 to -1.2)*
Iron-deficiency anemia	72.1 (64.6 to 80.7)	39.7 (38.1 to 41.3)*	6.1 (4.7 to 7.4)*	8.1 (5.5 to 11.4)	39.6 (35.2 to 44.3)*	6.0 (2.5 to 9.9)*
Other pneumoconiosis	14.4 (13.1 to 15.6)	41.1 (39.0 to 43.0)*	4.5 (3.4 to 5.5)*	6.5 (4.6 to 8.5)	40.5 (35.1 to 45.7)*	4.3 (0.3 to 8.4)*
Silicosis	13.9 (12.0 to 15.6)	32.2 (29.1 to 34.8)*	1.5 (-0.5 to 3.3)	6.4 (4.5 to 8.5)	33.1 (26.9 to 40.5)*	2.3 (-2.7 to 7.9)
Asbestosis	2.8 (2.6 to 3.0)	35.6 (34.2 to 37.1)*	1.1 (0.1 to 2.1)*	1.3 (0.9 to 1.7)	35.7 (31.3 to 40.0)*	1.3 (-2.2 to 4.7)
Coal workers pneumoconiosis	1.9 (1.6 to 2.1)	41.6 (37.8 to 45.1)*	8.2 (5.4 to 10.9)*	0.9 (0.6 to 1.2)	41.6 (37.7 to 45.2)*	8.2 (5.2 to 10.9)*
G6PD deficiency	5.2 (4.7 to 5.7)	38.1 (36.6 to 39.8)*	7.9 (6.8 to 9.1)*	0.6 (0.4 to 0.9)	38.1 (34.6 to 41.5)*	7.8 (5.0 to 10.6)*
Thalassemias	3.4 (3.2 to 3.6)	26.3 (24.3 to 28.4)*	0.1 (-1.1 to 1.4)	0.4 (0.3 to 0.6)	26.4 (16.0 to 38.3)*	0.6 (-9.3 to 11.8)
Iodine deficiency	0.3 (0.3 to 0.4)	39.6 (38.3 to 41.0)*	4.2 (3.3 to 5.2)*	0.0 (0.0 to 0.1)	39.6 (38.3 to 41.0)*	4.2 (3.3 to 5.2)*
**All causes**	**40,048.6 (38,613.2 to 41,418.2)**	**32.3 (31.6 to 33.1)***	**0.3 (-0.2 to 0.8)**	**6,199.4 (4,700.7 to 7,785.5)**	**33.2 (31.7 to 34.6)***	**1.1 (0.1 to 2.0)***

SR Table 7. Prevalent cases and years lived with disability (YLDs) for 2015, percent change, and percent change in age-standardised rates between 2005 and 2015 of infertility by cause, for all ages, both sexes combined

Cause	Prevalence in thousands (95% confidence interval)			YLDs in thousands (95% confidence interval)		
	2015	Percent change in counts between 2005 and 2015	Percent change in ASR between 2005 and 2015	2015	Percent change in counts between 2005 and 2015	Percent change in ASR between 2005 and 2015
Female infertility	61,300.1 (45,889.9 to 79,647.1)	24.6 (19.2 to 30.0)*	11.2 (6.5 to 15.8)*	344.5 (134.3 to 746.7)	25.0 (19.7 to 30.0)*	11.7 (7.2 to 16.2)*
Male infertility	29,033.2 (23,972.5 to 34,766.8)	22.6 (19.3 to 25.6)*	9.6 (6.8 to 12.1)*	173.9 (70.1 to 365.3)	22.7 (19.3 to 25.9)*	9.8 (6.9 to 12.5)*
Polycystic ovarian syndrome	12,989.6 (8,635.6 to 18,975.6)	11.9 (10.9 to 12.7)*	0.4 (-0.4 to 1.1)	124.6 (47.2 to 278.0)	11.5 (9.6 to 12.9)*	0.1 (-1.5 to 1.3)
Endometriosis	590.7 (415.2 to 787.2)	12.3 (11.2 to 13.3)*	-1.0 (-1.8 to -0.3)*	59.1 (34.2 to 90.8)	12.3 (9.5 to 15.2)*	-0.9 (-3.4 to 1.5)
Other sexually transmitted diseases	4,314.5 (3,943.8 to 4,657.1)	10.7 (9.1 to 12.2)*	-3.8 (-5.1 to -2.4)*	23.9 (9.7 to 48.8)	10.8 (9.0 to 12.7)*	-3.6 (-5.2 to -2.0)*
Maternal sepsis and other maternal infections	2,242.1 (2,030.5 to 2,483.0)	6.4 (4.4 to 8.9)*	-6.8 (-8.6 to -4.7)*	11.2 (4.2 to 24.3)	6.7 (4.0 to 9.5)*	-6.6 (-8.9 to -4.1)*
Chlamydial infection	1,576.6 (1,450.8 to 1,703.9)	11.1 (10.0 to 12.2)*	-3.0 (-3.9 to -2.0)*	8.8 (3.6 to 18.4)	11.1 (9.0 to 13.2)*	-2.9 (-4.8 to -1.1)*
Gonococcal infection	833.5 (749.4 to 935.4)	11.1 (8.3 to 12.9)*	-3.0 (-5.5 to -1.3)*	4.7 (1.9 to 9.9)	11.1 (7.5 to 14.3)*	-3.0 (-6.0 to -0.1)*
Turner syndrome	156.1 (73.2 to 282.7)	9.4 (7.3 to 11.3)*	-1.0 (-2.9 to 0.7)	1.2 (0.4 to 2.9)	9.4 (3.4 to 16.3)*	-0.9 (-6.4 to 5.1)
Klinefelter syndrome	77.3 (39.7 to 140.6)	14.0 (12.0 to 15.4)*	-0.0 (-1.9 to 1.2)	0.6 (0.2 to 1.4)	14.0 (9.3 to 17.6)*	-0.1 (-4.1 to 3.1)
**All causes**	**113,113.8 (93,429.4 to 136,497.5)**	**21.1 (17.6 to 24.5)***	**8.0 (4.9 to 10.9)***	**752.4 (327.7 to 1,543.8)**	**19.9 (16.8 to 22.7)***	**7.0 (4.3 to 9.4)***

SR Table 8. Prevalent cases and years lived with disability (YLDs) for 2015, percent change, and percent change in age-standardised rates between 2005 and 2015 of intellectual disability by cause, for all ages, both sexes combined

Cause	Prevalence in thousands (95% confidence interval)			YLDs in thousands (95% confidence interval)		
	**2015**	**Percent change in counts between 2005 and 2015**	**Percent change in ASR between 2005 and 2015**	**2015**	**Percent change in counts between 2005 and 2015**	**Percent change in ASR between 2005 and 2015**
Autism	20,650.8 (16,751.3 to 24,631.1)	12.6 (12.2 to 12.9)*	0.5 (0.3 to 0.7)*	5,501.2 (3,451.8 to 8,150.3)	12.5 (11.9 to 13.2)*	0.7 (0.2 to 1.2)*
Idiopathic developmental intellectual disability	92,074.0 (52,280.1 to 130,411.2)	10.9 (9.5 to 11.9)*	1.0 (0.0 to 1.8)*	3,442.1 (1,506.4 to 5,996.9)	10.3 (8.2 to 11.4)*	0.2 (-1.5 to 1.2)
Neonatal preterm birth complications	13,547.2 (9,936.3 to 16,980.2)	14.7 (8.8 to 21.0)*	3.2 (-2.1 to 8.8)	2,982.7 (1,487.4 to 4,487.2)	12.5 (7.2 to 17.9)*	2.3 (-2.5 to 7.4)
Neonatal encephalopathy due to birth asphyxia and trauma	5,421.8 (3,085.1 to 8,166.0)	34.0 (28.6 to 38.6)*	22.1 (17.4 to 26.3)*	2,152.8 (1,059.3 to 3,507.3)	33.2 (27.3 to 38.4)*	21.3 (16.0 to 25.9)*
Down syndrome	5,082.8 (3,253.9 to 7,748.4)	17.9 (13.6 to 21.7)*	6.4 (2.4 to 10.0)*	495.6 (279.8 to 814.7)	20.2 (15.7 to 24.0)*	6.5 (2.2 to 10.1)*
Iodine deficiency	2,611.6 (1,085.6 to 3,621.3)	10.4 (6.1 to 12.6)*	2.0 (-2.0 to 4.3)	459.3 (163.4 to 752.8)	11.0 (6.4 to 13.6)*	2.8 (-1.6 to 5.2)
Other chromosomal abnormalities	4,481.7 (2,490.6 to 7,876.6)	16.2 (11.8 to 19.8)*	4.3 (0.1 to 7.7)*	443.7 (226.2 to 828.5)	18.3 (13.5 to 22.2)*	4.2 (-0.5 to 7.9)
Hemolytic disease and other neonatal jaundice	860.7 (523.9 to 1,165.1)	25.7 (21.3 to 28.8)*	14.2 (10.1 to 17.1)*	358.7 (174.8 to 549.5)	25.4 (20.4 to 29.3)*	13.8 (9.3 to 17.3)*
Malaria	590.9 (320.9 to 817.4)	25.0 (16.9 to 31.8)*	15.1 (7.5 to 21.6)*	262.1 (101.8 to 404.2)	23.3 (14.8 to 31.5)*	13.4 (5.5 to 21.0)*
Pneumococcal meningitis	2,434.3 (1,937.0 to 3,023.5)	18.7 (15.9 to 21.8)*	4.5 (2.0 to 7.2)*	233.2 (128.0 to 351.5)	13.6 (9.5 to 16.8)*	0.2 (-3.4 to 3.3)
Encephalitis	1,467.1 (770.2 to 2,431.2)	2.8 (0.3 to 6.3)*	-10.7 (-13.3 to -6.5)*	139.5 (70.7 to 211.3)	4.5 (-0.6 to 8.5)	-7.6 (-12.3 to -4.1)*
Neural tube defects	193.1 (110.7 to 307.7)	20.1 (15.6 to 23.9)*	9.9 (5.6 to 13.5)*	102.0 (55.4 to 170.1)	20.1 (15.6 to 23.9)*	9.9 (5.6 to 13.5)*
Other meningitis	935.5 (615.3 to 1,244.8)	15.3 (12.0 to 18.3)*	0.4 (-2.4 to 3.1)	99.2 (48.0 to 152.4)	10.6 (6.6 to 13.7)*	-3.6 (-7.1 to -0.4)*
H influenzae type B meningitis	893.7 (615.1 to 1,228.2)	-9.6 (-15.2 to -2.3)*	-18.8 (-23.9 to -11.9)*	97.6 (49.8 to 149.3)	6.2 (-0.7 to 10.3)	-3.9 (-10.0 to -0.0)*
Meningococcal meningitis	475.1 (292.6 to 679.2)	18.4 (14.3 to 22.1)*	3.9 (0.4 to 7.4)*	49.8 (24.5 to 77.4)	15.9 (11.3 to 19.5)*	0.8 (-3.1 to 4.3)
Alcohol use disorders	789.8 (725.4 to 850.5)	11.0 (10.2 to 11.8)*	-0.5 (-1.2 to 0.2)	45.1 (29.2 to 65.1)	10.7 (8.2 to 13.1)*	-0.6 (-2.7 to 1.6)
Tetanus	102.4 (70.9 to 133.0)	12.8 (11.3 to 14.1)*	1.4 (0.1 to 2.5)*	7.0 (3.8 to 10.3)	2.5 (-2.4 to 9.0)	-7.7 (-12.2 to -1.7)*
Neonatal sepsis and other neonatal infections	6.4 (4.5 to 8.8)	9.0 (-1.8 to 19.4)	3.4 (-6.9 to 13.2)	2.7 (1.6 to 4.1)	9.0 (-3.9 to 20.9)	3.3 (-8.8 to 14.7)
Klinefelter syndrome	45.2 (22.1 to 85.6)	12.9 (10.2 to 15.3)*	0.5 (-1.3 to 1.9)	0.7 (0.3 to 1.8)	13.1 (10.7 to 15.0)*	0.4 (-1.4 to 1.8)
**All causes**	**152,664.0 (113,511.5 to 190,764.1)**	**12.6 (12.2 to 13.0)***	**2.0 (1.6 to 2.4)***	**16,875.1 (12,072.6 to 22,373.1)**	**15.0 (13.4 to 17.0)***	**3.8 (2.2 to 5.7)***

SR Table 9. Prevalent cases and years lived with disability (YLDs) for 2015, percent change, and percent change in age-standardised rates between 2005 and 2015 of pelvic inflammatory disease by cause, for all ages, both sexes combined

Cause	Prevalence in thousands (95% confidence interval)			YLDs in thousands (95% confidence interval)		
	2015	Percent change in counts between 2005 and 2015	Percent change in ASR between 2005 and 2015	2015	Percent change in counts between 2005 and 2015	Percent change in ASR between 2005 and 2015
Other sexually transmitted diseases	507.2 (437.2 to 591.9)	1.2 (-0.1 to 2.6)	-12.2 (-13.1 to -11.2)*	66.8 (45.4 to 92.9)	1.3 (-1.2 to 4.2)	-12.0 (-14.3 to -9.8)*
Chlamydial infection	173.3 (147.8 to 202.0)	1.4 (-0.8 to 3.4)	-11.5 (-13.2 to -9.8)*	23.1 (15.5 to 32.5)	1.8 (-2.2 to 5.7)	-11.1 (-14.4 to -7.8)*
Gonococcal infection	73.6 (61.0 to 87.8)	-0.4 (-3.5 to 2.6)	-11.8 (-14.4 to -9.1)*	10.0 (6.6 to 14.3)	0.3 (-4.4 to 4.7)	-11.2 (-15.3 to -7.3)*
**All causes**	**754.1 (652.8 to 878.7)**	**1.1 (0.1 to 2.1)***	**-12.0 (-12.7 to -11.2)***	**99.9 (67.8 to 138.6)**	**1.3 (-0.7 to 3.4)**	**-11.7 (-13.4 to -10.0)***

SR Table 10. Prevalent cases and years lived with disability (YLDs) for 2015, percent change, and percent change in age-standardised rates between 2005 and 2015 of vision loss by cause, for all ages, both sexes combined

Cause	Prevalence in thousands (95% confidence interval)			YLDs in thousands (95% confidence interval)		
	**2015**	**Percent change in counts between 2005 and 2015**	**Percent change in ASR between 2005 and 2015**	**2015**	**Percent change in counts between 2005 and 2015**	**Percent change in ASR between 2005 and 2015**
Refraction and accommodation disorders	819,307.4<br>(789,917.4 to 848,059.2)	21.9<br>(20.5 to 23.2)*	-0.4<br>(-1.3 to 0.6)	14,593.8<br>(9,392.9 to 22,901.1)	21.1<br>(20.2 to 22.1)*	-0.3<br>(-0.9 to 0.4)
Near vision impairment	662,983.4<br>(637,817.9 to 689,865.6)	22.5<br>(20.9 to 24.0)*	-0.5<br>(-1.7 to 0.8)	6,720.5<br>(3,176.2 to 12,491.5)	22.4<br>(20.8 to 24.0)*	-0.4<br>(-1.5 to 0.9)
Cataract	59,727.0<br>(53,633.2 to 66,879.2)	26.9<br>(25.2 to 28.5)*	-3.2<br>(-4.5 to -2.0)*	3,879.7<br>(2,766.9 to 5,229.2)	25.5<br>(24.1 to 26.9)*	-4.2<br>(-5.2 to -3.2)*
Other vision loss	25,797.4<br>(22,675.6 to 28,504.6)	27.1<br>(24.6 to 29.5)*	5.5<br>(4.2 to 6.7)*	1,756.4<br>(1,248.9 to 2,392.7)	29.9<br>(27.8 to 32.0)*	5.6<br>(4.3 to 6.7)*
Neonatal preterm birth complications	4,229.6<br>(3,308.1 to 5,174.9)	10.0<br>(6.3 to 13.9)*	-1.8<br>(-5.0 to 1.7)	1,179.1<br>(712.7 to 1,645.5)	9.1<br>(4.3 to 14.1)*	-1.4<br>(-5.7 to 3.2)
Neonatal encephalopathy due to birth asphyxia and trauma	1,878.2<br>(1,174.3 to 2,749.2)	30.3<br>(26.3 to 34.4)*	18.3<br>(14.7 to 21.9)*	825.1<br>(466.0 to 1,310.8)	29.4<br>(25.1 to 33.8)*	17.5<br>(13.7 to 21.4)*
Glaucoma	5,954.5<br>(5,077.5 to 6,905.8)	39.1<br>(36.9 to 41.4)*	4.2<br>(2.8 to 5.5)*	541.3<br>(370.0 to 747.9)	39.4<br>(37.0 to 41.9)*	4.3<br>(2.6 to 5.8)*
Macular degeneration	6,188.4<br>(5,250.3 to 7,273.2)	48.5<br>(46.1 to 50.8)*	8.2<br>(6.9 to 9.8)*	462.4<br>(327.4 to 633.0)	47.7<br>(45.2 to 49.9)*	6.9<br>(5.3 to 8.6)*
Diabetes mellitus	4,547.1<br>(3,823.8 to 5,416.0)	34.7<br>(32.4 to 36.9)*	5.0<br>(3.6 to 6.3)*	302.3<br>(210.1 to 415.5)	36.1<br>(33.6 to 38.7)*	5.7<br>(4.1 to 7.4)*
Trachoma	3,557.1<br>(2,940.5 to 4,321.8)	4.6<br>(0.4 to 9.2)*	-19.3<br>(-22.9 to -15.2)*	279.2<br>(192.8 to 396.0)	-1.2<br>(-5.6 to 3.0)	-23.9<br>(-27.5 to -20.3)*
Vitamin A deficiency	4,901.1<br>(3,975.6 to 5,921.7)	9.3<br>(6.2 to 12.5)*	0.1<br>(-2.7 to 2.7)	232.4<br>(143.5 to 345.9)	10.9<br>(7.5 to 14.6)*	0.2<br>(-2.7 to 3.3)
Hemolytic disease and other neonatal jaundice	296.9<br>(202.7 to 384.2)	22.1<br>(19.1 to 24.6)*	10.5<br>(7.7 to 12.8)*	133.3<br>(77.3 to 194.3)	21.7<br>(18.5 to 24.5)*	10.2<br>(7.3 to 12.8)*
Onchocerciasis	1,025.4<br>(724.8 to 1,452.1)	-2.3<br>(-15.0 to 11.8)	-22.3<br>(-32.3 to -11.0)*	80.1<br>(51.3 to 117.5)	-6.1<br>(-16.9 to 6.0)	-25.4<br>(-33.9 to -15.9)*
Malaria	152.5<br>(106.7 to 194.4)	24.2<br>(17.3 to 29.4)*	14.1<br>(7.7 to 19.0)*	68.3<br>(40.3 to 97.6)	22.6<br>(16.1 to 28.7)*	12.7<br>(6.7 to 18.2)*
Encephalitis	620.3<br>(306.5 to 1,075.3)	1.6<br>(-1.1 to 5.2)	-14.3<br>(-16.9 to -11.6)*	49.3<br>(30.0 to 74.0)	4.1<br>(-1.1 to 9.2)	-13.5<br>(-18.0 to -8.9)*
Pneumococcal meningitis	618.7<br>(397.7 to 903.7)	26.2<br>(21.7 to 31.8)*	9.3<br>(5.3 to 13.8)*	26.5<br>(15.9 to 40.3)	30.6<br>(24.2 to 36.7)*	10.4<br>(5.3 to 15.6)*
Other meningitis	336.7<br>(194.7 to 495.8)	23.1<br>(18.3 to 29.8)*	5.7<br>(1.6 to 10.9)*	23.6<br>(13.8 to 37.3)	29.4<br>(21.0 to 38.5)*	9.7<br>(2.5 to 17.4)*
H influenzae type B meningitis	313.6<br>(188.5 to 465.5)	-9.2<br>(-16.3 to 1.8)	-20.4<br>(-26.4 to -11.5)*	23.5<br>(13.4 to 36.0)	23.8<br>(12.0 to 34.1)*	5.2<br>(-3.9 to 13.7)
Meningococcal meningitis	120.6<br>(38.7 to 214.4)	17.2<br>(10.2 to 23.4)*	3.0<br>(-3.4 to 8.7)	2.8<br>(1.2 to 5.2)	4.5<br>(-8.3 to 13.6)	-9.1<br>(-21.2 to -0.7)*
Neonatal sepsis and other neonatal infections	5.6<br>(3.7 to 8.0)	8.9<br>(-3.6 to 20.4)	3.3<br>(-8.6 to 14.2)	2.6<br>(1.6 to 4.0)	8.9<br>(-4.0 to 20.9)	3.3<br>(-9.0 to 14.7)
Tetanus	2.1<br>(1.4 to 2.8)	-7.1<br>(-11.9 to -2.3)*	-16.4<br>(-20.8 to -12.0)*	1.0<br>(0.6 to 1.4)	-7.5<br>(-12.4 to -2.5)*	-16.7<br>(-21.1 to -12.1)*
**All causes**	**939,580.2<br>(905,009.5 to 974,112.3)**	**22.4<br>(21.2 to 23.6)***	**-0.4<br>(-1.3 to 0.4)**	**24,462.7<br>(16,954.9 to 34,455.1)**	**22.4<br>(21.6 to 23.3)***	**-0.2<br>(-0.8 to 0.3)**

SR Table 11. Prevalent cases and YLDs for 2015, percent change, and change in age-standardised rates between 2005 and 2015 by cause, global, ages 0-6 days						
**Cause**	**Prevalence in thousands (95% confidence interval)**			**YLDs in thousands (95% confidence interval)**		
	**2015**	**Percent change in counts between 2005 and 2015**	**Percent change in rates between 2005 and 2015**	**2015**	**Percent change in counts between 2005 and 2015**	**Percent change in rates between 2005 and 2015**
**All causes**	--	--	--	**209.7 (146.4 to 287.0)**	**0.2 (-1.4 to 1.6)**	**-4.9 (-6.5 to -3.6)***
Communicable, maternal, neonatal, and nutritional diseases	1,963.3 (1,953.4 to 1,973.0)	4.3 (3.7 to 4.9)*	-1.1 (-1.7 to -0.5)*	180.1 (124.9 to 248.2)	-0.4 (-2.1 to 1.0)	-5.6 (-7.2 to -4.2)*
HIV/AIDS and tuberculosis	0.0 (0.0 to 0.0)	-92.7 (-96.4 to -86.8)*	-93.1 (-96.5 to -87.5)*	0.0 (0.0 to 0.0)	-41.7 (-50.1 to -32.5)*	-44.7 (-52.7 to -36.0)*
Tuberculosis	0.0 (0.0 to 0.0)	-42.3 (-49.6 to -33.4)*	-45.3 (-52.2 to -36.9)*	0.0 (0.0 to 0.0)	-41.7 (-50.1 to -32.5)*	-44.7 (-52.7 to -36.0)*
HIV/AIDS	0.0 (0.0 to 0.0)	0.0 (0.0 to 0.0)	0.0 (0.0 to 0.0)	0.0 (0.0 to 0.0)	0.0 (0.0 to 0.0)	0.0 (0.0 to 0.0)
HIV/AIDS - Tuberculosis	0.0 (0.0 to 0.0)	0.0 (0.0 to 0.0)	0.0 (0.0 to 0.0)	0.0 (0.0 to 0.0)	0.0 (0.0 to 0.0)	0.0 (0.0 to 0.0)
HIV/AIDS resulting in other diseases	0.0 (0.0 to 0.0)	0.0 (0.0 to 0.0)	0.0 (0.0 to 0.0)	0.0 (0.0 to 0.0)	0.0 (0.0 to 0.0)	0.0 (0.0 to 0.0)
HIV aggregate	0.0 (0.0 to 0.0)	0.0 (0.0 to 0.0)	0.0 (0.0 to 0.0)	0.0 (0.0 to 0.0)	0.0 (0.0 to 0.0)	0.0 (0.0 to 0.0)
AIDS aggregate	0.0 (0.0 to 0.0)	0.0 (0.0 to 0.0)	0.0 (0.0 to 0.0)	0.0 (0.0 to 0.0)	0.0 (0.0 to 0.0)	0.0 (0.0 to 0.0)
Diarrhea, lower respiratory, and other common infectious diseases	167.4 (159.0 to 175.0)	-4.8 (-6.4 to -3.3)*	-9.8 (-11.2 to -8.3)*	11.5 (8.0 to 15.8)	-4.9 (-6.9 to -3.0)*	-9.8 (-11.7 to -8.0)*
Diarrheal diseases	42.4 (39.2 to 46.0)	5.0 (3.8 to 6.2)*	-0.5 (-1.5 to 0.7)	6.8 (4.6 to 9.3)	5.2 (3.7 to 6.8)*	-0.2 (-1.7 to 1.3)
Diarrhoea episodes aggregate	42.4 (39.2 to 46.0)	5.0 (3.8 to 6.2)*	-0.5 (-1.5 to 0.7)	6.8 (4.6 to 9.3)	5.2 (3.7 to 6.8)*	-0.2 (-1.7 to 1.3)
Guillain-Barré syndrome due to diarrheal diseases	0.0 (0.0 to 0.0)	6.5 (5.8 to 7.1)*	1.0 (0.3 to 1.5)*	0.0 (0.0 to 0.0)	6.5 (5.8 to 7.1)*	1.0 (0.3 to 1.5)*
Intestinal infectious diseases	0.0 (0.0 to 0.0)	0.0 (0.0 to 0.0)	0.0 (0.0 to 0.0)	0.0 (0.0 to 0.0)	0.0 (0.0 to 0.0)	0.0 (0.0 to 0.0)
Typhoid fever	0.0 (0.0 to 0.0)	0.0 (0.0 to 0.0)	0.0 (0.0 to 0.0)	0.0 (0.0 to 0.0)	0.0 (0.0 to 0.0)	0.0 (0.0 to 0.0)
Typhoid fever episodes aggregate	0.0 (0.0 to 0.0)	0.0 (0.0 to 0.0)	0.0 (0.0 to 0.0)	0.0 (0.0 to 0.0)	0.0 (0.0 to 0.0)	0.0 (0.0 to 0.0)
Complications of typhoid fever aggregate	0.0 (0.0 to 0.0)	0.0 (0.0 to 0.0)	0.0 (0.0 to 0.0)	0.0 (0.0 to 0.0)	0.0 (0.0 to 0.0)	0.0 (0.0 to 0.0)
Paratyphoid fever	0.0 (0.0 to 0.0)	0.0 (0.0 to 0.0)	0.0 (0.0 to 0.0)	0.0 (0.0 to 0.0)	0.0 (0.0 to 0.0)	0.0 (0.0 to 0.0)
Paratyphoid fever episodes aggregate	0.0 (0.0 to 0.0)	0.0 (0.0 to 0.0)	0.0 (0.0 to 0.0)	0.0 (0.0 to 0.0)	0.0 (0.0 to 0.0)	0.0 (0.0 to 0.0)
Intestinal perforation due to paratyphoid	0.0 (0.0 to 0.0)	0.0 (0.0 to 0.0)	0.0 (0.0 to 0.0)	0.0 (0.0 to 0.0)	0.0 (0.0 to 0.0)	0.0 (0.0 to 0.0)
Other intestinal infectious diseases	--	--	--	0.0 (0.0 to 0.0)	0.0 (0.0 to 0.0)	0.0 (0.0 to 0.0)
Lower respiratory infections	63.0 (52.1 to 75.6)	-17.4 (-21.2 to -13.6)*	-21.7 (-25.2 to -18.1)*	3.8 (2.5 to 5.5)	-17.4 (-21.2 to -13.5)*	-21.6 (-25.3 to -18.0)*
Lower respiratory infection episodes aggregate	63.0 (52.1 to 75.6)	-17.4 (-21.2 to -13.6)*	-21.7 (-25.2 to -18.1)*	3.8 (2.5 to 5.5)	-17.4 (-21.2 to -13.5)*	-21.6 (-25.3 to -18.0)*
Guillain-Barré syndrome due to lower respiratory infections	0.0 (0.0 to 0.0)	6.5 (5.8 to 7.1)*	1.0 (0.3 to 1.5)*	0.0 (0.0 to 0.0)	6.5 (5.8 to 7.1)*	1.0 (0.3 to 1.5)*
Upper respiratory infections	50.6 (39.4 to 66.1)	6.8 (4.8 to 8.3)*	1.3 (-0.7 to 2.7)	0.6 (0.3 to 1.0)	6.9 (4.6 to 8.9)*	1.4 (-0.8 to 3.2)
Upper respiratory infection episodes aggregate	50.6 (39.4 to 66.1)	6.8 (4.8 to 8.3)*	1.3 (-0.7 to 2.7)	0.6 (0.3 to 1.0)	6.9 (4.6 to 8.9)*	1.4 (-0.8 to 3.2)
Guillain-Barré syndrome due to upper respiratory infections	0.0 (0.0 to 0.0)	6.5 (5.8 to 7.1)*	1.0 (0.3 to 1.5)*	0.0 (0.0 to 0.0)	6.5 (5.8 to 7.1)*	1.0 (0.3 to 1.5)*
Otitis media	4.0 (3.0 to 5.2)	3.4 (0.8 to 5.6)*	-2.0 (-4.4 to 0.1)	0.1 (0.0 to 0.1)	2.2 (-1.2 to 5.4)	-3.1 (-6.3 to -0.1)*
Acute otitis media	3.4 (2.4 to 4.5)	4.4 (1.4 to 7.0)*	-1.0 (-3.9 to 1.4)	0.0 (0.0 to 0.1)	4.5 (0.1 to 8.6)*	-0.9 (-5.1 to 2.9)
Chronic otitis media aggregate	0.7 (0.5 to 0.9)	-1.4 (-4.8 to 2.2)	-6.5 (-9.7 to -3.1)*	0.0 (0.0 to 0.0)	-1.6 (-5.9 to 3.1)	-6.7 (-10.7 to -2.2)*
Meningitis	0.2 (0.1 to 0.2)	-8.9 (-24.9 to 12.2)	-13.7 (-28.8 to 6.4)	0.0 (0.0 to 0.1)	-1.3 (-14.6 to 12.0)	-6.4 (-19.0 to 6.2)
Pneumococcal meningitis	0.1 (0.0 to 0.1)	14.9 (3.9 to 26.9)*	9.0 (-1.5 to 20.3)	0.0 (0.0 to 0.0)	14.2 (-8.6 to 40.7)	8.3 (-13.4 to 33.4)
Acute pneumococcal meningitis	0.0 (0.0 to 0.1)	15.9 (0.4 to 33.0)*	9.9 (-4.8 to 26.1)	0.0 (0.0 to 0.0)	16.1 (-16.6 to 54.8)	10.1 (-21.0 to 46.8)
Complications of pneumococcal meningitis aggregate	0.0 (0.0 to 0.0)	12.6 (9.9 to 15.4)*	6.7 (4.2 to 9.4)*	0.0 (0.0 to 0.0)	10.0 (8.3 to 11.6)*	4.3 (2.6 to 5.8)*
H influenzae type B meningitis	0.1 (0.0 to 0.1)	-28.9 (-36.0 to -21.2)*	-32.5 (-39.3 to -25.3)*	0.0 (0.0 to 0.0)	-26.1 (-38.8 to -12.2)*	-29.9 (-42.0 to -16.7)*
Acute H influenzae type B meningitis	0.0 (0.0 to 0.1)	-31.7 (-40.8 to -22.4)*	-35.2 (-43.8 to -26.4)*	0.0 (0.0 to 0.0)	-30.2 (-45.7 to -14.0)*	-33.8 (-48.5 to -18.5)*
Complications of of H influenzae type B meningitis aggregate	0.0 (0.0 to 0.0)	-13.0 (-16.7 to -9.0)*	-17.5 (-21.0 to -13.7)*	0.0 (0.0 to 0.0)	-5.1 (-8.3 to -1.9)*	-10.0 (-13.0 to -7.0)*
Meningococcal meningitis	0.0 (0.0 to 0.1)	7.6 (-6.6 to 23.1)	2.0 (-11.5 to 16.7)	0.0 (0.0 to 0.0)	8.0 (-22.6 to 48.0)	2.4 (-26.6 to 40.3)
Acute meningococcal meningitis	0.0 (0.0 to 0.1)	7.3 (-9.2 to 24.8)	1.7 (-13.9 to 18.3)	0.0 (0.0 to 0.0)	7.5 (-26.5 to 54.7)	1.9 (-30.3 to 46.7)
Complications of meningococcal meningitis aggregate	0.0 (0.0 to 0.0)	9.5 (5.0 to 14.2)*	3.8 (-0.4 to 8.3)	0.0 (0.0 to 0.0)	12.2 (9.5 to 14.7)*	6.3 (3.8 to 8.8)*
Other meningitis	0.2 (0.2 to 0.2)	1.8 (-9.4 to 12.3)	-3.5 (-14.1 to 6.5)	0.0 (0.0 to 0.0)	1.8 (-14.9 to 18.4)	-3.5 (-19.3 to 12.3)
Other meningitis episodes aggregate	0.2 (0.1 to 0.2)	0.5 (-10.9 to 11.4)	-4.7 (-15.5 to 5.6)	0.0 (0.0 to 0.0)	1.7 (-15.5 to 19.2)	-3.5 (-19.8 to 13.0)
Complications of other meningitis aggregate	0.0 (0.0 to 0.0)	6.2 (2.5 to 9.7)*	0.7 (-2.9 to 4.0)	0.0 (0.0 to 0.0)	2.4 (-0.7 to 4.8)	-2.9 (-5.8 to -0.7)*
Encephalitis	0.0 (0.0 to 0.1)	-11.5 (-16.3 to -7.2)*	-16.1 (-20.7 to -12.0)*	0.0 (0.0 to 0.0)	-11.6 (-19.6 to -2.2)*	-16.1 (-23.7 to -7.3)*
Acute encephalitis	0.0 (0.0 to 0.0)	-11.0 (-16.2 to -6.5)*	-15.6 (-20.6 to -11.4)*	0.0 (0.0 to 0.0)	-10.9 (-19.7 to -0.3)*	-15.5 (-23.9 to -5.4)*
Complications of encephalitis aggregate	0.0 (0.0 to 0.0)	-16.0 (-18.5 to -13.4)*	-20.3 (-22.8 to -17.9)*	0.0 (0.0 to 0.0)	-18.0 (-20.4 to -15.8)*	-22.2 (-24.6 to -20.2)*
Diphtheria	0.0 (0.0 to 0.0)	0.0 (0.0 to 0.0)	0.0 (0.0 to 0.0)	0.0 (0.0 to 0.0)	0.0 (0.0 to 0.0)	0.0 (0.0 to 0.0)
Whooping cough	0.0 (0.0 to 0.0)	0.0 (0.0 to 0.0)	0.0 (0.0 to 0.0)	0.0 (0.0 to 0.0)	0.0 (0.0 to 0.0)	0.0 (0.0 to 0.0)
Tetanus	1.0 (0.8 to 1.3)	-56.2 (-65.6 to -44.4)*	-58.5 (-67.3 to -47.3)*	0.1 (0.1 to 0.2)	-56.3 (-65.5 to -44.3)*	-58.5 (-67.3 to -47.2)*
Severe tetanus	1.0 (0.8 to 1.3)	-56.6 (-65.9 to -44.7)*	-58.8 (-67.7 to -47.6)*	0.1 (0.1 to 0.2)	-56.4 (-65.6 to -44.4)*	-58.6 (-67.4 to -47.2)*
Complications of tetanus aggregate	0.0 (0.0 to 0.0)	-2.0 (-2.5 to -1.6)*	-7.1 (-7.6 to -6.6)*	0.0 (0.0 to 0.0)	-9.6 (-12.0 to -7.1)*	-14.3 (-16.5 to -11.9)*
Measles	0.0 (0.0 to 0.0)	0.0 (0.0 to 0.0)	0.0 (0.0 to 0.0)	0.0 (0.0 to 0.0)	0.0 (0.0 to 0.0)	0.0 (0.0 to 0.0)
Varicella and herpes zoster	9.9 (6.7 to 13.8)	4.8 (4.2 to 5.6)*	-0.6 (-1.2 to 0.1)	0.1 (0.0 to 0.1)	5.0 (2.9 to 7.0)*	-0.5 (-2.4 to 1.4)
Neglected tropical diseases and malaria	59.9 (52.2 to 67.9)	19.9 (3.2 to 38.9)*	13.7 (-2.1 to 31.7)	1.2 (0.9 to 1.7)	20.4 (13.9 to 27.2)*	14.1 (8.0 to 20.6)*
Malaria	61.8 (44.4 to 83.1)	20.4 (8.0 to 33.6)*	14.2 (2.4 to 26.7)*	1.2 (0.9 to 1.7)	20.4 (13.9 to 27.2)*	14.1 (8.0 to 20.6)*
Asymptomatic malaria parasitemia(PfPR)	40.1 (23.5 to 60.5)	19.2 (-0.2 to 41.7)	13.1 (-5.3 to 34.4)	0.0 (0.0 to 0.0)	0.0 (0.0 to 0.0)	0.0 (0.0 to 0.0)
Malaria episodes aggregate	0.0 (0.0 to 0.0)	0.0 (0.0 to 0.0)	0.0 (0.0 to 0.0)	0.0 (0.0 to 0.0)	0.0 (0.0 to 0.0)	0.0 (0.0 to 0.0)
Complications of malaria aggregate	0.0 (0.0 to 0.0)	8.6 (4.3 to 12.9)*	3.0 (-1.1 to 7.1)	0.0 (0.0 to 0.0)	7.9 (3.5 to 12.6)*	2.3 (-1.9 to 6.8)
Anemia due to malaria parasitemia(PfPR) aggregate	21.7 (20.5 to 23.0)	22.6 (16.1 to 29.7)*	16.3 (10.1 to 23.0)*	1.2 (0.9 to 1.7)	20.4 (13.9 to 27.2)*	14.2 (8.0 to 20.6)*
Chagas disease	0.0 (0.0 to 0.0)	0.0 (0.0 to 0.0)	0.0 (0.0 to 0.0)	0.0 (0.0 to 0.0)	0.0 (0.0 to 0.0)	0.0 (0.0 to 0.0)
Chagas disease episodes aggregate	0.0 (0.0 to 0.0)	0.0 (0.0 to 0.0)	0.0 (0.0 to 0.0)	0.0 (0.0 to 0.0)	0.0 (0.0 to 0.0)	0.0 (0.0 to 0.0)
Complications of Chagas disease aggregate	0.0 (0.0 to 0.0)	0.0 (0.0 to 0.0)	0.0 (0.0 to 0.0)	0.0 (0.0 to 0.0)	0.0 (0.0 to 0.0)	0.0 (0.0 to 0.0)

Cause	Prevalence in thousands (95% confidence interval)			YLDs in thousands (95% confidence interval)		
	2015	Percent change in counts between 2005 and 2015	Percent change in rates between 2005 and 2015	2015	Percent change in counts between 2005 and 2015	Percent change in rates between 2005 and 2015
Heart failure due to Chagas disease aggregate	0.0<br>(0.0 to 0.0)	0.0<br>(0.0 to 0.0)	0.0<br>(0.0 to 0.0)	0.0<br>(0.0 to 0.0)	0.0<br>(0.0 to 0.0)	0.0<br>(0.0 to 0.0)
Leishmaniasis	0.0<br>(0.0 to 0.0)	0.0<br>(0.0 to 0.0)	0.0<br>(0.0 to 0.0)	0.0<br>(0.0 to 0.0)	0.0<br>(0.0 to 0.0)	0.0<br>(0.0 to 0.0)
Visceral leishmaniasis	0.0<br>(0.0 to 0.0)	0.0<br>(0.0 to 0.0)	0.0<br>(0.0 to 0.0)	0.0<br>(0.0 to 0.0)	0.0<br>(0.0 to 0.0)	0.0<br>(0.0 to 0.0)
Cutaneous and mucocutaneous leishmaniasis	0.0<br>(0.0 to 0.0)	0.0<br>(0.0 to 0.0)	0.0<br>(0.0 to 0.0)	0.0<br>(0.0 to 0.0)	0.0<br>(0.0 to 0.0)	0.0<br>(0.0 to 0.0)
African trypanosomiasis	0.0<br>(0.0 to 0.0)	0.0<br>(0.0 to 0.0)	0.0<br>(0.0 to 0.0)	0.0<br>(0.0 to 0.0)	0.0<br>(0.0 to 0.0)	0.0<br>(0.0 to 0.0)
Schistosomiasis	0.0<br>(0.0 to 0.0)	0.0<br>(0.0 to 0.0)	0.0<br>(0.0 to 0.0)	0.0<br>(0.0 to 0.0)	0.0<br>(0.0 to 0.0)	0.0<br>(0.0 to 0.0)
Schistosomiasis episodes aggregate	0.0<br>(0.0 to 0.0)	0.0<br>(0.0 to 0.0)	0.0<br>(0.0 to 0.0)	0.0<br>(0.0 to 0.0)	0.0<br>(0.0 to 0.0)	0.0<br>(0.0 to 0.0)
Complications of schistosomiasis aggregate	0.0<br>(0.0 to 0.0)	0.0<br>(0.0 to 0.0)	0.0<br>(0.0 to 0.0)	0.0<br>(0.0 to 0.0)	0.0<br>(0.0 to 0.0)	0.0<br>(0.0 to 0.0)
Anemia due to schistosomiasis aggregate	0.0<br>(0.0 to 0.0)	0.0<br>(0.0 to 0.0)	0.0<br>(0.0 to 0.0)	0.0<br>(0.0 to 0.0)	0.0<br>(0.0 to 0.0)	0.0<br>(0.0 to 0.0)
Cysticercosis	0.0<br>(0.0 to 0.0)	0.0<br>(0.0 to 0.0)	0.0<br>(0.0 to 0.0)	0.0<br>(0.0 to 0.0)	0.0<br>(0.0 to 0.0)	0.0<br>(0.0 to 0.0)
Cystic echinococcosis	0.0<br>(0.0 to 0.0)	0.0<br>(0.0 to 0.0)	0.0<br>(0.0 to 0.0)	0.0<br>(0.0 to 0.0)	0.0<br>(0.0 to 0.0)	0.0<br>(0.0 to 0.0)
Lymphatic filariasis	0.0<br>(0.0 to 0.0)	0.0<br>(0.0 to 0.0)	0.0<br>(0.0 to 0.0)	0.0<br>(0.0 to 0.0)	0.0<br>(0.0 to 0.0)	0.0<br>(0.0 to 0.0)
Prevalence of detectable microfiliaria due to lymphatic filariasis	0.0<br>(0.0 to 0.0)	0.0<br>(0.0 to 0.0)	0.0<br>(0.0 to 0.0)	0.0<br>(0.0 to 0.0)	0.0<br>(0.0 to 0.0)	0.0<br>(0.0 to 0.0)
Complications of lymphatic filariasis aggregate	0.0<br>(0.0 to 0.0)	0.0<br>(0.0 to 0.0)	0.0<br>(0.0 to 0.0)	0.0<br>(0.0 to 0.0)	0.0<br>(0.0 to 0.0)	0.0<br>(0.0 to 0.0)
Onchocerciasis	0.0<br>(0.0 to 0.0)	0.0<br>(0.0 to 0.0)	0.0<br>(0.0 to 0.0)	0.0<br>(0.0 to 0.0)	0.0<br>(0.0 to 0.0)	0.0<br>(0.0 to 0.0)
Asymptomatic onchocerciasis	0.0<br>(0.0 to 0.0)	0.0<br>(0.0 to 0.0)	0.0<br>(0.0 to 0.0)	0.0<br>(0.0 to 0.0)	0.0<br>(0.0 to 0.0)	0.0<br>(0.0 to 0.0)
Skin disease due to onchocerciasis aggregate	0.0<br>(0.0 to 0.0)	0.0<br>(0.0 to 0.0)	0.0<br>(0.0 to 0.0)	0.0<br>(0.0 to 0.0)	0.0<br>(0.0 to 0.0)	0.0<br>(0.0 to 0.0)
Vision loss due to onchocerciasis aggregate	0.0<br>(0.0 to 0.0)	0.0<br>(0.0 to 0.0)	0.0<br>(0.0 to 0.0)	0.0<br>(0.0 to 0.0)	0.0<br>(0.0 to 0.0)	0.0<br>(0.0 to 0.0)
Trachoma	0.0<br>(0.0 to 0.0)	0.0<br>(0.0 to 0.0)	0.0<br>(0.0 to 0.0)	0.0<br>(0.0 to 0.0)	0.0<br>(0.0 to 0.0)	0.0<br>(0.0 to 0.0)
Dengue	0.0<br>(0.0 to 0.0)	0.0<br>(0.0 to 0.0)	0.0<br>(0.0 to 0.0)	0.0<br>(0.0 to 0.0)	0.0<br>(0.0 to 0.0)	0.0<br>(0.0 to 0.0)
Dengue episodes aggregate	0.0<br>(0.0 to 0.0)	0.0<br>(0.0 to 0.0)	0.0<br>(0.0 to 0.0)	0.0<br>(0.0 to 0.0)	0.0<br>(0.0 to 0.0)	0.0<br>(0.0 to 0.0)
Post-dengue chronic fatigue syndrome	0.0<br>(0.0 to 0.0)	0.0<br>(0.0 to 0.0)	0.0<br>(0.0 to 0.0)	0.0<br>(0.0 to 0.0)	0.0<br>(0.0 to 0.0)	0.0<br>(0.0 to 0.0)
Yellow fever	0.0<br>(0.0 to 0.0)	0.0<br>(0.0 to 0.0)	0.0<br>(0.0 to 0.0)	0.0<br>(0.0 to 0.0)	0.0<br>(0.0 to 0.0)	0.0<br>(0.0 to 0.0)
Rabies	0.0<br>(0.0 to 0.0)	0.0<br>(0.0 to 0.0)	0.0<br>(0.0 to 0.0)	0.0<br>(0.0 to 0.0)	0.0<br>(0.0 to 0.0)	0.0<br>(0.0 to 0.0)
Intestinal nematode infections	0.0<br>(0.0 to 0.0)	0.0<br>(0.0 to 0.0)	0.0<br>(0.0 to 0.0)	0.0<br>(0.0 to 0.0)	0.0<br>(0.0 to 0.0)	0.0<br>(0.0 to 0.0)
Ascariasis	0.0<br>(0.0 to 0.0)	0.0<br>(0.0 to 0.0)	0.0<br>(0.0 to 0.0)	0.0<br>(0.0 to 0.0)	0.0<br>(0.0 to 0.0)	0.0<br>(0.0 to 0.0)
Complications of ascariasis aggregate	0.0<br>(0.0 to 0.0)	0.0<br>(0.0 to 0.0)	0.0<br>(0.0 to 0.0)	0.0<br>(0.0 to 0.0)	0.0<br>(0.0 to 0.0)	0.0<br>(0.0 to 0.0)
Asymptomatic ascariasis	0.0<br>(0.0 to 0.0)	0.0<br>(0.0 to 0.0)	0.0<br>(0.0 to 0.0)	0.0<br>(0.0 to 0.0)	0.0<br>(0.0 to 0.0)	0.0<br>(0.0 to 0.0)
Trichuriasis	0.0<br>(0.0 to 0.0)	0.0<br>(0.0 to 0.0)	0.0<br>(0.0 to 0.0)	0.0<br>(0.0 to 0.0)	0.0<br>(0.0 to 0.0)	0.0<br>(0.0 to 0.0)
Complications of trichuriasis aggregate	0.0<br>(0.0 to 0.0)	0.0<br>(0.0 to 0.0)	0.0<br>(0.0 to 0.0)	0.0<br>(0.0 to 0.0)	0.0<br>(0.0 to 0.0)	0.0<br>(0.0 to 0.0)
Asymptomatic trichuriasis	0.0<br>(0.0 to 0.0)	0.0<br>(0.0 to 0.0)	0.0<br>(0.0 to 0.0)	0.0<br>(0.0 to 0.0)	0.0<br>(0.0 to 0.0)	0.0<br>(0.0 to 0.0)
Hookworm disease	0.0<br>(0.0 to 0.0)	0.0<br>(0.0 to 0.0)	0.0<br>(0.0 to 0.0)	0.0<br>(0.0 to 0.0)	0.0<br>(0.0 to 0.0)	0.0<br>(0.0 to 0.0)
Complications of hookworm disease aggregate	0.0<br>(0.0 to 0.0)	0.0<br>(0.0 to 0.0)	0.0<br>(0.0 to 0.0)	0.0<br>(0.0 to 0.0)	0.0<br>(0.0 to 0.0)	0.0<br>(0.0 to 0.0)
Anemia due to hookworm disease aggregate	0.0<br>(0.0 to 0.0)	0.0<br>(0.0 to 0.0)	0.0<br>(0.0 to 0.0)	0.0<br>(0.0 to 0.0)	0.0<br>(0.0 to 0.0)	0.0<br>(0.0 to 0.0)
Asymptomatic hookworm disease	0.0<br>(0.0 to 0.0)	0.0<br>(0.0 to 0.0)	0.0<br>(0.0 to 0.0)	0.0<br>(0.0 to 0.0)	0.0<br>(0.0 to 0.0)	0.0<br>(0.0 to 0.0)
Food-borne trematodiases	0.0<br>(0.0 to 0.0)	0.0<br>(0.0 to 0.0)	0.0<br>(0.0 to 0.0)	0.0<br>(0.0 to 0.0)	0.0<br>(0.0 to 0.0)	0.0<br>(0.0 to 0.0)
Asymptomatic food-borne trematodiases aggregate	0.0<br>(0.0 to 0.0)	0.0<br>(0.0 to 0.0)	0.0<br>(0.0 to 0.0)	0.0<br>(0.0 to 0.0)	0.0<br>(0.0 to 0.0)	0.0<br>(0.0 to 0.0)
Symptomatic food-borne trematodiases aggregate	0.0<br>(0.0 to 0.0)	0.0<br>(0.0 to 0.0)	0.0<br>(0.0 to 0.0)	0.0<br>(0.0 to 0.0)	0.0<br>(0.0 to 0.0)	0.0<br>(0.0 to 0.0)
Leprosy	0.0<br>(0.0 to 0.0)	0.0<br>(0.0 to 0.0)	0.0<br>(0.0 to 0.0)	0.0<br>(0.0 to 0.0)	0.0<br>(0.0 to 0.0)	0.0<br>(0.0 to 0.0)
Ebola	0.0<br>(0.0 to 0.0)	0.0<br>(0.0 to 0.0)	0.0<br>(0.0 to 0.0)	0.0<br>(0.0 to 0.0)	0.0<br>(0.0 to 0.0)	0.0<br>(0.0 to 0.0)
Other neglected tropical diseases	0.0<br>(0.0 to 0.0)	0.0<br>(0.0 to 0.0)	0.0<br>(0.0 to 0.0)	0.0<br>(0.0 to 0.0)	0.0<br>(0.0 to 0.0)	0.0<br>(0.0 to 0.0)
Anemia due to other neglected tropical diseases aggregate	0.0<br>(0.0 to 0.0)	0.0<br>(0.0 to 0.0)	0.0<br>(0.0 to 0.0)	0.0<br>(0.0 to 0.0)	0.0<br>(0.0 to 0.0)	0.0<br>(0.0 to 0.0)
Maternal disorders	0.0<br>(0.0 to 0.0)	0.0<br>(0.0 to 0.0)	0.0<br>(0.0 to 0.0)	0.0<br>(0.0 to 0.0)	0.0<br>(0.0 to 0.0)	0.0<br>(0.0 to 0.0)
Maternal hemorrhage	0.0<br>(0.0 to 0.0)	0.0<br>(0.0 to 0.0)	0.0<br>(0.0 to 0.0)	0.0<br>(0.0 to 0.0)	0.0<br>(0.0 to 0.0)	0.0<br>(0.0 to 0.0)
Maternal hemorrhage episodes aggregate	0.0<br>(0.0 to 0.0)	0.0<br>(0.0 to 0.0)	0.0<br>(0.0 to 0.0)	0.0<br>(0.0 to 0.0)	0.0<br>(0.0 to 0.0)	0.0<br>(0.0 to 0.0)
Anemia due to maternal hemorrhage aggregate	0.0<br>(0.0 to 0.0)	0.0<br>(0.0 to 0.0)	0.0<br>(0.0 to 0.0)	0.0<br>(0.0 to 0.0)	0.0<br>(0.0 to 0.0)	0.0<br>(0.0 to 0.0)
Maternal sepsis and other maternal infections	0.0<br>(0.0 to 0.0)	0.0<br>(0.0 to 0.0)	0.0<br>(0.0 to 0.0)	0.0<br>(0.0 to 0.0)	0.0<br>(0.0 to 0.0)	0.0<br>(0.0 to 0.0)
Maternal sepsis and other maternal infections aggregate	0.0<br>(0.0 to 0.0)	0.0<br>(0.0 to 0.0)	0.0<br>(0.0 to 0.0)	0.0<br>(0.0 to 0.0)	0.0<br>(0.0 to 0.0)	0.0<br>(0.0 to 0.0)
Infertility due to puerperal sepsis	0.0<br>(0.0 to 0.0)	0.0<br>(0.0 to 0.0)	0.0<br>(0.0 to 0.0)	0.0<br>(0.0 to 0.0)	0.0<br>(0.0 to 0.0)	0.0<br>(0.0 to 0.0)
Maternal hypertensive disorders	0.0<br>(0.0 to 0.0)	0.0<br>(0.0 to 0.0)	0.0<br>(0.0 to 0.0)	0.0<br>(0.0 to 0.0)	0.0<br>(0.0 to 0.0)	0.0<br>(0.0 to 0.0)
Maternal hypertensive disorders episodes aggregate	0.0<br>(0.0 to 0.0)	0.0<br>(0.0 to 0.0)	0.0<br>(0.0 to 0.0)	0.0<br>(0.0 to 0.0)	0.0<br>(0.0 to 0.0)	0.0<br>(0.0 to 0.0)
Eclampsia	0.0<br>(0.0 to 0.0)	0.0<br>(0.0 to 0.0)	0.0<br>(0.0 to 0.0)	0.0<br>(0.0 to 0.0)	0.0<br>(0.0 to 0.0)	0.0<br>(0.0 to 0.0)
Complications of maternal hypertensive disorders aggregate	0.0<br>(0.0 to 0.0)	0.0<br>(0.0 to 0.0)	0.0<br>(0.0 to 0.0)	0.0<br>(0.0 to 0.0)	0.0<br>(0.0 to 0.0)	0.0<br>(0.0 to 0.0)
Maternal obstructed labor and uterine rupture	0.0<br>(0.0 to 0.0)	0.0<br>(0.0 to 0.0)	0.0<br>(0.0 to 0.0)	0.0<br>(0.0 to 0.0)	0.0<br>(0.0 to 0.0)	0.0<br>(0.0 to 0.0)
Obstructed labor, acute event	0.0<br>(0.0 to 0.0)	0.0<br>(0.0 to 0.0)	0.0<br>(0.0 to 0.0)	0.0<br>(0.0 to 0.0)	0.0<br>(0.0 to 0.0)	0.0<br>(0.0 to 0.0)
Fistula due to maternal obstructed labor and uterine rupture aggregate	0.0<br>(0.0 to 0.0)	0.0<br>(0.0 to 0.0)	0.0<br>(0.0 to 0.0)	0.0<br>(0.0 to 0.0)	0.0<br>(0.0 to 0.0)	0.0<br>(0.0 to 0.0)
Maternal abortion, miscarriage, and ectopic pregnancy	0.0<br>(0.0 to 0.0)	0.0<br>(0.0 to 0.0)	0.0<br>(0.0 to 0.0)	0.0<br>(0.0 to 0.0)	0.0<br>(0.0 to 0.0)	0.0<br>(0.0 to 0.0)
Other maternal disorders	--	--	--	0.0<br>(0.0 to 0.0)	0.0<br>(0.0 to 0.0)	0.0<br>(0.0 to 0.0)
Neonatal disorders	307.3<br>(282.2 to 326.6)	1.6<br>(-1.4 to 4.7)	-3.7<br>(-6.5 to -0.7)*	31.4<br>(20.3 to 47.8)	-0.9<br>(-4.4 to 2.7)	-6.1<br>(-9.3 to -2.7)*
Neonatal preterm birth complications	145.5<br>(123.5 to 169.1)	2.5<br>(1.0 to 4.0)*	-2.8<br>(-4.2 to -1.4)*	5.4<br>(3.9 to 7.2)	-7.5<br>(-12.8 to -0.9)*	-12.3<br>(-17.4 to -6.0)*
Vision loss due to retinopathy of prematurity aggregate	1.4<br>(1.1 to 1.8)	3.1<br>(-0.9 to 7.4)	-2.2<br>(-6.0 to 1.8)	0.0<br>(0.0 to 0.1)	3.5<br>(-7.5 to 15.1)	-1.8<br>(-12.3 to 9.1)
Complications of preterm birth complications aggregate	144.0<br>(122.0 to 167.6)	2.5<br>(1.0 to 4.0)*	-2.8<br>(-4.3 to -1.4)*	5.4<br>(3.8 to 7.2)	-7.6<br>(-13.0 to -0.9)*	-12.4<br>(-17.5 to -6.0)*
Neonatal encephalopathy due to birth asphyxia and trauma	34.6<br>(26.6 to 45.4)	11.3<br>(7.3 to 15.6)*	5.5<br>(1.7 to 9.6)*	4.3<br>(2.8 to 6.3)	8.4<br>(5.6 to 11.6)*	2.8<br>(0.1 to 5.8)*

Cause	Prevalence in thousands (95% confidence interval)			YLDs in thousands (95% confidence interval)		
	2015	Percent change in counts between 2005 and 2015	Percent change in rates between 2005 and 2015	2015	Percent change in counts between 2005 and 2015	Percent change in rates between 2005 and 2015
Neonatal sepsis and other neonatal infections	139.1<br>(79.9 to 216.0)	-1.6<br>(-3.0 to -0.0)*	-6.7<br>(-8.0 to -5.2)*	17.7<br>(9.2 to 31.0)	-1.8<br>(-3.5 to 0.1)	-6.9<br>(-8.5 to -5.1)*
Severe infection due to neonatal sepsis and other neonatal infections	130.1<br>(71.1 to 207.6)	-2.2<br>(-3.8 to -0.5)*	-7.3<br>(-8.8 to -5.6)*	16.6<br>(8.1 to 30.0)	-2.1<br>(-3.7 to -0.3)*	-7.1<br>(-8.7 to -5.4)*
Complications of neonatal sepsis and other neonatal infections aggregate	8.9<br>(7.5 to 10.4)	8.0<br>(4.3 to 12.7)*	2.4<br>(-1.1 to 6.9)	1.1<br>(0.6 to 1.6)	2.6<br>(-9.4 to 14.4)	-2.7<br>(-14.1 to 8.5)
Hemolytic disease and other neonatal jaundice	2.5<br>(2.2 to 3.0)	3.6<br>(1.3 to 6.0)*	-1.8<br>(-4.0 to 0.5)	0.6<br>(0.4 to 0.8)	3.0<br>(-2.2 to 8.1)	-2.4<br>(-7.2 to 2.5)
Other neonatal disorders	--	--	--	3.5<br>(2.0 to 5.7)	3.4<br>(-22.5 to 36.3)	-1.9<br>(-26.5 to 29.2)
Nutritional deficiencies	1,732.0<br>(1,721.0 to 1,742.2)	4.2<br>(3.3 to 5.0)*	-1.2<br>(-2.1 to -0.4)*	128.7<br>(88.3 to 180.1)	-0.5<br>(-2.5 to 1.4)	-5.6<br>(-7.6 to -3.9)*
Protein-energy malnutrition	130.7<br>(123.1 to 139.1)	-7.4<br>(-12.8 to -1.8)*	-12.2<br>(-17.3 to -6.9)*	15.9<br>(10.3 to 22.4)	-7.2<br>(-12.7 to -1.5)*	-12.0<br>(-17.2 to -6.6)*
Iodine deficiency	0.0<br>(0.0 to 0.0)	0.0<br>(0.0 to 0.0)	0.0<br>(0.0 to 0.0)	0.0<br>(0.0 to 0.0)	0.0<br>(0.0 to 0.0)	0.0<br>(0.0 to 0.0)
Visible goiter due to iodine deficiency aggregate	0.0<br>(0.0 to 0.0)	0.0<br>(0.0 to 0.0)	0.0<br>(0.0 to 0.0)	0.0<br>(0.0 to 0.0)	0.0<br>(0.0 to 0.0)	0.0<br>(0.0 to 0.0)
Visible goiter with heart failure due to iodine deficiency aggregate	0.0<br>(0.0 to 0.0)	0.0<br>(0.0 to 0.0)	0.0<br>(0.0 to 0.0)	0.0<br>(0.0 to 0.0)	0.0<br>(0.0 to 0.0)	0.0<br>(0.0 to 0.0)
Intellectual disability due to iodine deficiency aggregate	0.0<br>(0.0 to 0.0)	0.0<br>(0.0 to 0.0)	0.0<br>(0.0 to 0.0)	0.0<br>(0.0 to 0.0)	0.0<br>(0.0 to 0.0)	0.0<br>(0.0 to 0.0)
Vitamin A deficiency	0.0<br>(0.0 to 0.0)	0.0<br>(0.0 to 0.0)	0.0<br>(0.0 to 0.0)	0.0<br>(0.0 to 0.0)	0.0<br>(0.0 to 0.0)	0.0<br>(0.0 to 0.0)
Iron-deficiency anemia	2,060.1<br>(2,040.6 to 2,080.6)	4.6<br>(3.4 to 5.9)*	-0.9<br>(-2.0 to 0.4)	109.1<br>(73.7 to 154.5)	1.4<br>(-0.1 to 3.0)	-3.8<br>(-5.3 to -2.4)*
Iron-deficiency anemia without heart failure aggregate	2,060.1<br>(2,040.6 to 2,080.6)	4.6<br>(3.4 to 5.9)*	-0.9<br>(-2.0 to 0.4)	109.1<br>(73.7 to 154.5)	1.4<br>(-0.1 to 3.0)	-3.8<br>(-5.3 to -2.4)*
Iron-deficiency anemia with heart failure aggregate	0.0<br>(0.0 to 0.0)	10.2<br>(7.6 to 12.8)*	4.5<br>(2.0 to 7.0)*	0.0<br>(0.0 to 0.0)	10.2<br>(7.6 to 12.8)*	4.5<br>(2.0 to 7.0)*
Other nutritional deficiencies	--	--	--	3.7<br>(2.2 to 5.7)	-19.5<br>(-39.2 to 2.3)	-23.7<br>(-42.3 to -3.0)*
Other communicable, maternal, neonatal, and nutritional diseases	208.6<br>(205.2 to 212.0)	8.0<br>(5.2 to 10.6)*	2.4<br>(-0.2 to 4.9)	7.3<br>(5.1 to 10.2)	7.3<br>(3.4 to 11.5)*	1.8<br>(-2.0 to 5.8)
Sexually transmitted diseases excluding HIV	0.0<br>(0.0 to 0.0)	0.0<br>(0.0 to 0.0)	0.0<br>(0.0 to 0.0)	0.0<br>(0.0 to 0.0)	0.0<br>(0.0 to 0.0)	0.0<br>(0.0 to 0.0)
Syphilis	0.0<br>(0.0 to 0.0)	0.0<br>(0.0 to 0.0)	0.0<br>(0.0 to 0.0)	0.0<br>(0.0 to 0.0)	0.0<br>(0.0 to 0.0)	0.0<br>(0.0 to 0.0)
Early syphilis aggregate	0.0<br>(0.0 to 0.0)	0.0<br>(0.0 to 0.0)	0.0<br>(0.0 to 0.0)	0.0<br>(0.0 to 0.0)	0.0<br>(0.0 to 0.0)	0.0<br>(0.0 to 0.0)
Adult tertiary syphilis	0.0<br>(0.0 to 0.0)	0.0<br>(0.0 to 0.0)	0.0<br>(0.0 to 0.0)	0.0<br>(0.0 to 0.0)	0.0<br>(0.0 to 0.0)	0.0<br>(0.0 to 0.0)
Chlamydial infection	0.0<br>(0.0 to 0.0)	0.0<br>(0.0 to 0.0)	0.0<br>(0.0 to 0.0)	0.0<br>(0.0 to 0.0)	0.0<br>(0.0 to 0.0)	0.0<br>(0.0 to 0.0)
Chlamydial infection episodes aggregate	0.0<br>(0.0 to 0.0)	0.0<br>(0.0 to 0.0)	0.0<br>(0.0 to 0.0)	0.0<br>(0.0 to 0.0)	0.0<br>(0.0 to 0.0)	0.0<br>(0.0 to 0.0)
Pelvic inflammatory diseases due to chlamydial infection aggregate	0.0<br>(0.0 to 0.0)	0.0<br>(0.0 to 0.0)	0.0<br>(0.0 to 0.0)	0.0<br>(0.0 to 0.0)	0.0<br>(0.0 to 0.0)	0.0<br>(0.0 to 0.0)
Infertility due to clamydial infection aggregate	0.0<br>(0.0 to 0.0)	0.0<br>(0.0 to 0.0)	0.0<br>(0.0 to 0.0)	0.0<br>(0.0 to 0.0)	0.0<br>(0.0 to 0.0)	0.0<br>(0.0 to 0.0)
Gonococcal infection	0.0<br>(0.0 to 0.0)	0.0<br>(0.0 to 0.0)	0.0<br>(0.0 to 0.0)	0.0<br>(0.0 to 0.0)	0.0<br>(0.0 to 0.0)	0.0<br>(0.0 to 0.0)
Gonococcal infection episodes aggregate	0.0<br>(0.0 to 0.0)	0.0<br>(0.0 to 0.0)	0.0<br>(0.0 to 0.0)	0.0<br>(0.0 to 0.0)	0.0<br>(0.0 to 0.0)	0.0<br>(0.0 to 0.0)
Pelvic inflammatory diseases due to gonococcal infection aggregate	0.0<br>(0.0 to 0.0)	0.0<br>(0.0 to 0.0)	0.0<br>(0.0 to 0.0)	0.0<br>(0.0 to 0.0)	0.0<br>(0.0 to 0.0)	0.0<br>(0.0 to 0.0)
Trichomoniasis	0.0<br>(0.0 to 0.0)	0.0<br>(0.0 to 0.0)	0.0<br>(0.0 to 0.0)	0.0<br>(0.0 to 0.0)	0.0<br>(0.0 to 0.0)	0.0<br>(0.0 to 0.0)
Genital herpes	0.0<br>(0.0 to 0.0)	0.0<br>(0.0 to 0.0)	0.0<br>(0.0 to 0.0)	0.0<br>(0.0 to 0.0)	0.0<br>(0.0 to 0.0)	0.0<br>(0.0 to 0.0)
Moderate infection due to initial genital herpes episode	0.0<br>(0.0 to 0.0)	0.0<br>(0.0 to 0.0)	0.0<br>(0.0 to 0.0)	0.0<br>(0.0 to 0.0)	0.0<br>(0.0 to 0.0)	0.0<br>(0.0 to 0.0)
Complications of genital herpes aggregate	0.0<br>(0.0 to 0.0)	0.0<br>(0.0 to 0.0)	0.0<br>(0.0 to 0.0)	0.0<br>(0.0 to 0.0)	0.0<br>(0.0 to 0.0)	0.0<br>(0.0 to 0.0)
Other sexually transmitted diseases	0.0<br>(0.0 to 0.0)	0.0<br>(0.0 to 0.0)	0.0<br>(0.0 to 0.0)	0.0<br>(0.0 to 0.0)	0.0<br>(0.0 to 0.0)	0.0<br>(0.0 to 0.0)
Pelvic inflammatory diseases due to other sexually transmitted diseases aggregate	0.0<br>(0.0 to 0.0)	0.0<br>(0.0 to 0.0)	0.0<br>(0.0 to 0.0)	0.0<br>(0.0 to 0.0)	0.0<br>(0.0 to 0.0)	0.0<br>(0.0 to 0.0)
Infertility due to other sexually transmitted diseases aggregate	0.0<br>(0.0 to 0.0)	0.0<br>(0.0 to 0.0)	0.0<br>(0.0 to 0.0)	0.0<br>(0.0 to 0.0)	0.0<br>(0.0 to 0.0)	0.0<br>(0.0 to 0.0)
Other sexually transmitted diseases	--	--	--	0.0<br>(0.0 to 0.0)	0.0<br>(0.0 to 0.0)	0.0<br>(0.0 to 0.0)
Hepatitis	84.3<br>(80.8 to 87.2)	13.8<br>(10.1 to 17.3)*	7.9<br>(4.4 to 11.2)*	0.0<br>(0.0 to 0.0)	7.7<br>(-32.3 to 69.2)	2.1<br>(-35.8 to 60.5)
Acute hepatitis A	0.0<br>(0.0 to 0.0)	0.0<br>(0.0 to 0.0)	0.0<br>(0.0 to 0.0)	0.0<br>(0.0 to 0.0)	0.0<br>(0.0 to 0.0)	0.0<br>(0.0 to 0.0)
Hepatitis B	84.8<br>(75.6 to 95.7)	13.9<br>(8.5 to 19.0)*	8.0<br>(2.9 to 12.8)*	0.0<br>(0.0 to 0.0)	7.7<br>(-32.3 to 69.2)	2.1<br>(-35.8 to 60.5)
Acute Hepatitis B aggregate	0.5<br>(0.5 to 0.6)	7.4<br>(0.9 to 13.9)*	1.8<br>(-4.3 to 8.0)	0.0<br>(0.0 to 0.0)	7.7<br>(-32.3 to 69.2)	2.1<br>(-35.8 to 60.5)
Chronic hepatitis B	84.2<br>(75.0 to 95.2)	13.9<br>(8.5 to 19.0)*	8.0<br>(2.9 to 12.9)*	0.0<br>(0.0 to 0.0)	0.0<br>(0.0 to 0.0)	0.0<br>(0.0 to 0.0)
Hepatitis C	0.0<br>(0.0 to 0.0)	0.0<br>(0.0 to 0.0)	0.0<br>(0.0 to 0.0)	0.0<br>(0.0 to 0.0)	0.0<br>(0.0 to 0.0)	0.0<br>(0.0 to 0.0)
Acute Hepatitis C aggregate	0.0<br>(0.0 to 0.0)	0.0<br>(0.0 to 0.0)	0.0<br>(0.0 to 0.0)	0.0<br>(0.0 to 0.0)	0.0<br>(0.0 to 0.0)	0.0<br>(0.0 to 0.0)
Chronic hepatitis C	0.0<br>(0.0 to 0.0)	0.0<br>(0.0 to 0.0)	0.0<br>(0.0 to 0.0)	0.0<br>(0.0 to 0.0)	0.0<br>(0.0 to 0.0)	0.0<br>(0.0 to 0.0)
Acute hepatitis E	0.0<br>(0.0 to 0.0)	0.0<br>(0.0 to 0.0)	0.0<br>(0.0 to 0.0)	0.0<br>(0.0 to 0.0)	0.0<br>(0.0 to 0.0)	0.0<br>(0.0 to 0.0)
Other infectious diseases	130.0<br>(127.9 to 132.2)	4.6<br>(0.9 to 8.6)*	-0.8<br>(-4.3 to 3.0)	7.3<br>(5.1 to 10.2)	7.3<br>(3.4 to 11.5)*	1.8<br>(-2.0 to 5.8)
Non-communicable diseases	887.1<br>(875.3 to 898.5)	3.3<br>(2.4 to 4.4)*	-2.0<br>(-2.9 to -1.1)*	29.5<br>(21.3 to 39.4)	4.6<br>(2.3 to 7.1)*	-0.8<br>(-3.0 to 1.6)
Neoplasms	0.0<br>(0.0 to 0.0)	0.0<br>(0.0 to 0.0)	0.0<br>(0.0 to 0.0)	0.0<br>(0.0 to 0.0)	0.0<br>(0.0 to 0.0)	0.0<br>(0.0 to 0.0)
Lip and oral cavity cancer	0.0<br>(0.0 to 0.0)	0.0<br>(0.0 to 0.0)	0.0<br>(0.0 to 0.0)	0.0<br>(0.0 to 0.0)	0.0<br>(0.0 to 0.0)	0.0<br>(0.0 to 0.0)
Nasopharynx cancer	0.0<br>(0.0 to 0.0)	0.0<br>(0.0 to 0.0)	0.0<br>(0.0 to 0.0)	0.0<br>(0.0 to 0.0)	0.0<br>(0.0 to 0.0)	0.0<br>(0.0 to 0.0)
Other pharynx cancer	0.0<br>(0.0 to 0.0)	0.0<br>(0.0 to 0.0)	0.0<br>(0.0 to 0.0)	0.0<br>(0.0 to 0.0)	0.0<br>(0.0 to 0.0)	0.0<br>(0.0 to 0.0)
Esophageal cancer	0.0<br>(0.0 to 0.0)	0.0<br>(0.0 to 0.0)	0.0<br>(0.0 to 0.0)	0.0<br>(0.0 to 0.0)	0.0<br>(0.0 to 0.0)	0.0<br>(0.0 to 0.0)
Stomach cancer	0.0<br>(0.0 to 0.0)	0.0<br>(0.0 to 0.0)	0.0<br>(0.0 to 0.0)	0.0<br>(0.0 to 0.0)	0.0<br>(0.0 to 0.0)	0.0<br>(0.0 to 0.0)
Colon and rectum cancer	0.0<br>(0.0 to 0.0)	0.0<br>(0.0 to 0.0)	0.0<br>(0.0 to 0.0)	0.0<br>(0.0 to 0.0)	0.0<br>(0.0 to 0.0)	0.0<br>(0.0 to 0.0)
Colon and rectum cancer aggregate	0.0<br>(0.0 to 0.0)	0.0<br>(0.0 to 0.0)	0.0<br>(0.0 to 0.0)	0.0<br>(0.0 to 0.0)	0.0<br>(0.0 to 0.0)	0.0<br>(0.0 to 0.0)
Stoma due to colon and rectum cancer	0.0<br>(0.0 to 0.0)	0.0<br>(0.0 to 0.0)	0.0<br>(0.0 to 0.0)	0.0<br>(0.0 to 0.0)	0.0<br>(0.0 to 0.0)	0.0<br>(0.0 to 0.0)
Liver cancer	0.0<br>(0.0 to 0.0)	0.0<br>(0.0 to 0.0)	0.0<br>(0.0 to 0.0)	0.0<br>(0.0 to 0.0)	0.0<br>(0.0 to 0.0)	0.0<br>(0.0 to 0.0)
Liver cancer due to hepatitis B	0.0<br>(0.0 to 0.0)	0.0<br>(0.0 to 0.0)	0.0<br>(0.0 to 0.0)	0.0<br>(0.0 to 0.0)	0.0<br>(0.0 to 0.0)	0.0<br>(0.0 to 0.0)
Liver cancer due to hepatitis C	0.0<br>(0.0 to 0.0)	0.0<br>(0.0 to 0.0)	0.0<br>(0.0 to 0.0)	0.0<br>(0.0 to 0.0)	0.0<br>(0.0 to 0.0)	0.0<br>(0.0 to 0.0)
Liver cancer due to alcohol use	0.0<br>(0.0 to 0.0)	0.0<br>(0.0 to 0.0)	0.0<br>(0.0 to 0.0)	0.0<br>(0.0 to 0.0)	0.0<br>(0.0 to 0.0)	0.0<br>(0.0 to 0.0)
Liver cancer due to other causes	0.0<br>(0.0 to 0.0)	0.0<br>(0.0 to 0.0)	0.0<br>(0.0 to 0.0)	0.0<br>(0.0 to 0.0)	0.0<br>(0.0 to 0.0)	0.0<br>(0.0 to 0.0)
Gallbladder and biliary tract cancer	0.0<br>(0.0 to 0.0)	0.0<br>(0.0 to 0.0)	0.0<br>(0.0 to 0.0)	0.0<br>(0.0 to 0.0)	0.0<br>(0.0 to 0.0)	0.0<br>(0.0 to 0.0)
Pancreatic cancer	0.0<br>(0.0 to 0.0)	0.0<br>(0.0 to 0.0)	0.0<br>(0.0 to 0.0)	0.0<br>(0.0 to 0.0)	0.0<br>(0.0 to 0.0)	0.0<br>(0.0 to 0.0)

Cause	Prevalence in thousands (95% confidence interval)			YLDs in thousands (95% confidence interval)		
	2015	Percent change in counts between 2005 and 2015	Percent change in rates between 2005 and 2015	2015	Percent change in counts between 2005 and 2015	Percent change in rates between 2005 and 2015
Larynx cancer	0.0 (0.0 to 0.0)	0.0 (0.0 to 0.0)	0.0 (0.0 to 0.0)	0.0 (0.0 to 0.0)	0.0 (0.0 to 0.0)	0.0 (0.0 to 0.0)
Larynx cancer aggregate	0.0 (0.0 to 0.0)	0.0 (0.0 to 0.0)	0.0 (0.0 to 0.0)	0.0 (0.0 to 0.0)	0.0 (0.0 to 0.0)	0.0 (0.0 to 0.0)
Laryngectomy due to larynx cancer	0.0 (0.0 to 0.0)	0.0 (0.0 to 0.0)	0.0 (0.0 to 0.0)	0.0 (0.0 to 0.0)	0.0 (0.0 to 0.0)	0.0 (0.0 to 0.0)
Tracheal, bronchus, and lung cancer	0.0 (0.0 to 0.0)	0.0 (0.0 to 0.0)	0.0 (0.0 to 0.0)	0.0 (0.0 to 0.0)	0.0 (0.0 to 0.0)	0.0 (0.0 to 0.0)
Malignant skin melanoma	0.0 (0.0 to 0.0)	0.0 (0.0 to 0.0)	0.0 (0.0 to 0.0)	0.0 (0.0 to 0.0)	0.0 (0.0 to 0.0)	0.0 (0.0 to 0.0)
Non-melanoma skin cancer	0.0 (0.0 to 0.0)	0.0 (0.0 to 0.0)	0.0 (0.0 to 0.0)	0.0 (0.0 to 0.0)	0.0 (0.0 to 0.0)	0.0 (0.0 to 0.0)
Non-melanoma skin cancer(squamous-cell carcinoma)	0.0 (0.0 to 0.0)	0.0 (0.0 to 0.0)	0.0 (0.0 to 0.0)	0.0 (0.0 to 0.0)	0.0 (0.0 to 0.0)	0.0 (0.0 to 0.0)
Non-melanoma skin cancer(basal-cell carcinoma)	0.0 (0.0 to 0.0)	0.0 (0.0 to 0.0)	0.0 (0.0 to 0.0)	0.0 (0.0 to 0.0)	0.0 (0.0 to 0.0)	0.0 (0.0 to 0.0)
Breast cancer	0.0 (0.0 to 0.0)	0.0 (0.0 to 0.0)	0.0 (0.0 to 0.0)	0.0 (0.0 to 0.0)	0.0 (0.0 to 0.0)	0.0 (0.0 to 0.0)
Breast cancer aggregate	0.0 (0.0 to 0.0)	0.0 (0.0 to 0.0)	0.0 (0.0 to 0.0)	0.0 (0.0 to 0.0)	0.0 (0.0 to 0.0)	0.0 (0.0 to 0.0)
Mastectomy due to breast cancer	0.0 (0.0 to 0.0)	0.0 (0.0 to 0.0)	0.0 (0.0 to 0.0)	0.0 (0.0 to 0.0)	0.0 (0.0 to 0.0)	0.0 (0.0 to 0.0)
Cervical cancer	0.0 (0.0 to 0.0)	0.0 (0.0 to 0.0)	0.0 (0.0 to 0.0)	0.0 (0.0 to 0.0)	0.0 (0.0 to 0.0)	0.0 (0.0 to 0.0)
Uterine cancer	0.0 (0.0 to 0.0)	0.0 (0.0 to 0.0)	0.0 (0.0 to 0.0)	0.0 (0.0 to 0.0)	0.0 (0.0 to 0.0)	0.0 (0.0 to 0.0)
Ovarian cancer	0.0 (0.0 to 0.0)	0.0 (0.0 to 0.0)	0.0 (0.0 to 0.0)	0.0 (0.0 to 0.0)	0.0 (0.0 to 0.0)	0.0 (0.0 to 0.0)
Prostate cancer	0.0 (0.0 to 0.0)	0.0 (0.0 to 0.0)	0.0 (0.0 to 0.0)	0.0 (0.0 to 0.0)	0.0 (0.0 to 0.0)	0.0 (0.0 to 0.0)
Prostate cancer aggregate	0.0 (0.0 to 0.0)	0.0 (0.0 to 0.0)	0.0 (0.0 to 0.0)	0.0 (0.0 to 0.0)	0.0 (0.0 to 0.0)	0.0 (0.0 to 0.0)
Impotence and incontinence due to prostate cancer aggregate	0.0 (0.0 to 0.0)	0.0 (0.0 to 0.0)	0.0 (0.0 to 0.0)	0.0 (0.0 to 0.0)	0.0 (0.0 to 0.0)	0.0 (0.0 to 0.0)
Testicular cancer	0.0 (0.0 to 0.0)	0.0 (0.0 to 0.0)	0.0 (0.0 to 0.0)	0.0 (0.0 to 0.0)	0.0 (0.0 to 0.0)	0.0 (0.0 to 0.0)
Kidney cancer	0.0 (0.0 to 0.0)	0.0 (0.0 to 0.0)	0.0 (0.0 to 0.0)	0.0 (0.0 to 0.0)	0.0 (0.0 to 0.0)	0.0 (0.0 to 0.0)
Bladder cancer	0.0 (0.0 to 0.0)	0.0 (0.0 to 0.0)	0.0 (0.0 to 0.0)	0.0 (0.0 to 0.0)	0.0 (0.0 to 0.0)	0.0 (0.0 to 0.0)
Bladder cancer episodes aggregate	0.0 (0.0 to 0.0)	0.0 (0.0 to 0.0)	0.0 (0.0 to 0.0)	0.0 (0.0 to 0.0)	0.0 (0.0 to 0.0)	0.0 (0.0 to 0.0)
Urinary incontinence due to bladder cancer	0.0 (0.0 to 0.0)	0.0 (0.0 to 0.0)	0.0 (0.0 to 0.0)	0.0 (0.0 to 0.0)	0.0 (0.0 to 0.0)	0.0 (0.0 to 0.0)
Brain and nervous system cancer	0.0 (0.0 to 0.0)	0.0 (0.0 to 0.0)	0.0 (0.0 to 0.0)	0.0 (0.0 to 0.0)	0.0 (0.0 to 0.0)	0.0 (0.0 to 0.0)
Thyroid cancer	0.0 (0.0 to 0.0)	0.0 (0.0 to 0.0)	0.0 (0.0 to 0.0)	0.0 (0.0 to 0.0)	0.0 (0.0 to 0.0)	0.0 (0.0 to 0.0)
Mesothelioma	0.0 (0.0 to 0.0)	0.0 (0.0 to 0.0)	0.0 (0.0 to 0.0)	0.0 (0.0 to 0.0)	0.0 (0.0 to 0.0)	0.0 (0.0 to 0.0)
Hodgkin lymphoma	0.0 (0.0 to 0.0)	0.0 (0.0 to 0.0)	0.0 (0.0 to 0.0)	0.0 (0.0 to 0.0)	0.0 (0.0 to 0.0)	0.0 (0.0 to 0.0)
Non-Hodgkin lymphoma	0.0 (0.0 to 0.0)	0.0 (0.0 to 0.0)	0.0 (0.0 to 0.0)	0.0 (0.0 to 0.0)	0.0 (0.0 to 0.0)	0.0 (0.0 to 0.0)
Multiple myeloma	0.0 (0.0 to 0.0)	0.0 (0.0 to 0.0)	0.0 (0.0 to 0.0)	0.0 (0.0 to 0.0)	0.0 (0.0 to 0.0)	0.0 (0.0 to 0.0)
Leukemia	0.0 (0.0 to 0.0)	0.0 (0.0 to 0.0)	0.0 (0.0 to 0.0)	0.0 (0.0 to 0.0)	0.0 (0.0 to 0.0)	0.0 (0.0 to 0.0)
Acute lymphoid leukemia	0.0 (0.0 to 0.0)	0.0 (0.0 to 0.0)	0.0 (0.0 to 0.0)	0.0 (0.0 to 0.0)	0.0 (0.0 to 0.0)	0.0 (0.0 to 0.0)
Chronic lymphoid leukemia	0.0 (0.0 to 0.0)	0.0 (0.0 to 0.0)	0.0 (0.0 to 0.0)	0.0 (0.0 to 0.0)	0.0 (0.0 to 0.0)	0.0 (0.0 to 0.0)
Acute myeloid leukemia	0.0 (0.0 to 0.0)	0.0 (0.0 to 0.0)	0.0 (0.0 to 0.0)	0.0 (0.0 to 0.0)	0.0 (0.0 to 0.0)	0.0 (0.0 to 0.0)
Chronic myeloid leukemia	0.0 (0.0 to 0.0)	0.0 (0.0 to 0.0)	0.0 (0.0 to 0.0)	0.0 (0.0 to 0.0)	0.0 (0.0 to 0.0)	0.0 (0.0 to 0.0)
Other neoplasms	0.0 (0.0 to 0.0)	0.0 (0.0 to 0.0)	0.0 (0.0 to 0.0)	0.0 (0.0 to 0.0)	0.0 (0.0 to 0.0)	0.0 (0.0 to 0.0)
Cardiovascular diseases	0.0 (0.0 to 0.0)	0.0 (0.0 to 0.0)	0.0 (0.0 to 0.0)	0.0 (0.0 to 0.0)	0.6 (-4.0 to 5.3)	-4.6 (-8.9 to -0.1)*
Rheumatic heart disease	0.0 (0.0 to 0.0)	0.0 (0.0 to 0.0)	0.0 (0.0 to 0.0)	0.0 (0.0 to 0.0)	0.0 (0.0 to 0.0)	0.0 (0.0 to 0.0)
Rheumatic heart disease, without heart failure	0.0 (0.0 to 0.0)	0.0 (0.0 to 0.0)	0.0 (0.0 to 0.0)	0.0 (0.0 to 0.0)	0.0 (0.0 to 0.0)	0.0 (0.0 to 0.0)
Heart failure fue to rheumatic heart disease aggregate	0.0 (0.0 to 0.0)	0.0 (0.0 to 0.0)	0.0 (0.0 to 0.0)	0.0 (0.0 to 0.0)	0.0 (0.0 to 0.0)	0.0 (0.0 to 0.0)
Ischemic heart disease	0.0 (0.0 to 0.0)	0.0 (0.0 to 0.0)	0.0 (0.0 to 0.0)	0.0 (0.0 to 0.0)	0.0 (0.0 to 0.0)	0.0 (0.0 to 0.0)
Myocardial infarction episodes aggregate	0.0 (0.0 to 0.0)	0.0 (0.0 to 0.0)	0.0 (0.0 to 0.0)	0.0 (0.0 to 0.0)	0.0 (0.0 to 0.0)	0.0 (0.0 to 0.0)
Angina due to ischemic heart disease aggregate	0.0 (0.0 to 0.0)	0.0 (0.0 to 0.0)	0.0 (0.0 to 0.0)	0.0 (0.0 to 0.0)	0.0 (0.0 to 0.0)	0.0 (0.0 to 0.0)
Heart failure fue to ischemic heart disease aggregate	0.0 (0.0 to 0.0)	0.0 (0.0 to 0.0)	0.0 (0.0 to 0.0)	0.0 (0.0 to 0.0)	0.0 (0.0 to 0.0)	0.0 (0.0 to 0.0)
Cerebrovascular disease	0.0 (0.0 to 0.0)	0.0 (0.0 to 0.0)	0.0 (0.0 to 0.0)	0.0 (0.0 to 0.0)	0.0 (0.0 to 0.0)	0.0 (0.0 to 0.0)
Ischemic stroke	0.0 (0.0 to 0.0)	0.0 (0.0 to 0.0)	0.0 (0.0 to 0.0)	0.0 (0.0 to 0.0)	0.0 (0.0 to 0.0)	0.0 (0.0 to 0.0)
Chronic ischemic stroke aggregate	0.0 (0.0 to 0.0)	0.0 (0.0 to 0.0)	0.0 (0.0 to 0.0)	0.0 (0.0 to 0.0)	0.0 (0.0 to 0.0)	0.0 (0.0 to 0.0)
Ischemic stroke episodes aggregate	0.0 (0.0 to 0.0)	0.0 (0.0 to 0.0)	0.0 (0.0 to 0.0)	0.0 (0.0 to 0.0)	0.0 (0.0 to 0.0)	0.0 (0.0 to 0.0)
Hemorrhagic stroke	0.0 (0.0 to 0.0)	0.0 (0.0 to 0.0)	0.0 (0.0 to 0.0)	0.0 (0.0 to 0.0)	0.0 (0.0 to 0.0)	0.0 (0.0 to 0.0)
Chronic hemorrhagic stroke aggregate	0.0 (0.0 to 0.0)	0.0 (0.0 to 0.0)	0.0 (0.0 to 0.0)	0.0 (0.0 to 0.0)	0.0 (0.0 to 0.0)	0.0 (0.0 to 0.0)
Acute hemorrhagic stroke aggregate	0.0 (0.0 to 0.0)	0.0 (0.0 to 0.0)	0.0 (0.0 to 0.0)	0.0 (0.0 to 0.0)	0.0 (0.0 to 0.0)	0.0 (0.0 to 0.0)
Hypertensive heart disease	0.0 (0.0 to 0.0)	0.0 (0.0 to 0.0)	0.0 (0.0 to 0.0)	0.0 (0.0 to 0.0)	0.0 (0.0 to 0.0)	0.0 (0.0 to 0.0)
Cardiomyopathy and myocarditis	0.0 (0.0 to 0.0)	0.0 (0.0 to 0.0)	0.0 (0.0 to 0.0)	0.0 (0.0 to 0.0)	0.0 (0.0 to 0.0)	0.0 (0.0 to 0.0)
Acute myocarditis	0.0 (0.0 to 0.0)	0.0 (0.0 to 0.0)	0.0 (0.0 to 0.0)	0.0 (0.0 to 0.0)	0.0 (0.0 to 0.0)	0.0 (0.0 to 0.0)
Heart failure due to cardiomyopathy aggregate	0.0 (0.0 to 0.0)	0.0 (0.0 to 0.0)	0.0 (0.0 to 0.0)	0.0 (0.0 to 0.0)	0.0 (0.0 to 0.0)	0.0 (0.0 to 0.0)
Atrial fibrillation and flutter	0.0 (0.0 to 0.0)	0.0 (0.0 to 0.0)	0.0 (0.0 to 0.0)	0.0 (0.0 to 0.0)	0.0 (0.0 to 0.0)	0.0 (0.0 to 0.0)
Peripheral vascular disease	0.0 (0.0 to 0.0)	0.0 (0.0 to 0.0)	0.0 (0.0 to 0.0)	0.0 (0.0 to 0.0)	0.0 (0.0 to 0.0)	0.0 (0.0 to 0.0)
Endocarditis	0.0 (0.0 to 0.0)	0.7 (-4.0 to 5.4)	-4.5 (-8.9 to -0.0)*	0.0 (0.0 to 0.0)	0.6 (-4.0 to 5.3)	-4.6 (-8.9 to -0.1)*
Endocarditis episodes aggregate	0.0 (0.0 to 0.0)	0.7 (-4.0 to 5.4)	-4.5 (-8.9 to -0.0)*	0.0 (0.0 to 0.0)	0.6 (-4.0 to 5.3)	-4.6 (-8.9 to -0.1)*
Heart failure due to endocarditis aggregate	0.0 (0.0 to 0.0)	0.0 (0.0 to 0.0)	0.0 (0.0 to 0.0)	0.0 (0.0 to 0.0)	0.0 (0.0 to 0.0)	0.0 (0.0 to 0.0)
Other cardiovascular and circulatory diseases	0.0 (0.0 to 0.0)	0.0 (0.0 to 0.0)	0.0 (0.0 to 0.0)	0.0 (0.0 to 0.0)	0.0 (0.0 to 0.0)	0.0 (0.0 to 0.0)
Heart failure due to other cardiovascular diseases aggregate	0.0 (0.0 to 0.0)	0.0 (0.0 to 0.0)	0.0 (0.0 to 0.0)	0.0 (0.0 to 0.0)	0.0 (0.0 to 0.0)	0.0 (0.0 to 0.0)
Other cardiovascular diseases episodes aggregate	0.0 (0.0 to 0.0)	0.0 (0.0 to 0.0)	0.0 (0.0 to 0.0)	0.0 (0.0 to 0.0)	0.0 (0.0 to 0.0)	0.0 (0.0 to 0.0)
Chronic respiratory diseases	0.0 (0.0 to 0.0)	0.0 (0.0 to 0.0)	0.0 (0.0 to 0.0)	0.0 (0.0 to 0.0)	0.0 (0.0 to 0.0)	0.0 (0.0 to 0.0)
Chronic obstructive pulmonary disease	0.0 (0.0 to 0.0)	0.0 (0.0 to 0.0)	0.0 (0.0 to 0.0)	0.0 (0.0 to 0.0)	0.0 (0.0 to 0.0)	0.0 (0.0 to 0.0)

Cause	Prevalence in thousands (95% confidence interval)			YLDs in thousands (95% confidence interval)		
	2015	Percent change in counts between 2005 and 2015	Percent change in rates between 2005 and 2015	2015	Percent change in counts between 2005 and 2015	Percent change in rates between 2005 and 2015
Chronic obstructive pulmonary disease without heart failure aggregate	0.0 (0.0 to 0.0)	0.0 (0.0 to 0.0)	0.0 (0.0 to 0.0)	0.0 (0.0 to 0.0)	0.0 (0.0 to 0.0)	0.0 (0.0 to 0.0)
Severe chronic obstructive pulmonary disease with heart failure aggregate	0.0 (0.0 to 0.0)	0.0 (0.0 to 0.0)	0.0 (0.0 to 0.0)	0.0 (0.0 to 0.0)	0.0 (0.0 to 0.0)	0.0 (0.0 to 0.0)
Pneumoconiosis	0.0 (0.0 to 0.0)	0.0 (0.0 to 0.0)	0.0 (0.0 to 0.0)	0.0 (0.0 to 0.0)	0.0 (0.0 to 0.0)	0.0 (0.0 to 0.0)
Silicosis	0.0 (0.0 to 0.0)	0.0 (0.0 to 0.0)	0.0 (0.0 to 0.0)	0.0 (0.0 to 0.0)	0.0 (0.0 to 0.0)	0.0 (0.0 to 0.0)
Silicosis without heart failure aggregate	0.0 (0.0 to 0.0)	0.0 (0.0 to 0.0)	0.0 (0.0 to 0.0)	0.0 (0.0 to 0.0)	0.0 (0.0 to 0.0)	0.0 (0.0 to 0.0)
Severe silicosis with heart failure aggregate	0.0 (0.0 to 0.0)	0.0 (0.0 to 0.0)	0.0 (0.0 to 0.0)	0.0 (0.0 to 0.0)	0.0 (0.0 to 0.0)	0.0 (0.0 to 0.0)
Asbestosis	0.0 (0.0 to 0.0)	0.0 (0.0 to 0.0)	0.0 (0.0 to 0.0)	0.0 (0.0 to 0.0)	0.0 (0.0 to 0.0)	0.0 (0.0 to 0.0)
Asbestosis without heart failure aggregate	0.0 (0.0 to 0.0)	0.0 (0.0 to 0.0)	0.0 (0.0 to 0.0)	0.0 (0.0 to 0.0)	0.0 (0.0 to 0.0)	0.0 (0.0 to 0.0)
Severe asbestosis with heart failure aggregate	0.0 (0.0 to 0.0)	0.0 (0.0 to 0.0)	0.0 (0.0 to 0.0)	0.0 (0.0 to 0.0)	0.0 (0.0 to 0.0)	0.0 (0.0 to 0.0)
Coal workers pneumoconiosis	0.0 (0.0 to 0.0)	0.0 (0.0 to 0.0)	0.0 (0.0 to 0.0)	0.0 (0.0 to 0.0)	0.0 (0.0 to 0.0)	0.0 (0.0 to 0.0)
Coal workers pneumoconiosis without heart failure aggregate	0.0 (0.0 to 0.0)	0.0 (0.0 to 0.0)	0.0 (0.0 to 0.0)	0.0 (0.0 to 0.0)	0.0 (0.0 to 0.0)	0.0 (0.0 to 0.0)
Severe coal workers pneumoconiosis with heart failure aggregate	0.0 (0.0 to 0.0)	0.0 (0.0 to 0.0)	0.0 (0.0 to 0.0)	0.0 (0.0 to 0.0)	0.0 (0.0 to 0.0)	0.0 (0.0 to 0.0)
Other pneumoconiosis	0.0 (0.0 to 0.0)	0.0 (0.0 to 0.0)	0.0 (0.0 to 0.0)	0.0 (0.0 to 0.0)	0.0 (0.0 to 0.0)	0.0 (0.0 to 0.0)
Other pneumoconiosis without heart failure aggregate	0.0 (0.0 to 0.0)	0.0 (0.0 to 0.0)	0.0 (0.0 to 0.0)	0.0 (0.0 to 0.0)	0.0 (0.0 to 0.0)	0.0 (0.0 to 0.0)
Severe other pneumoconiosis with heart failure aggregate	0.0 (0.0 to 0.0)	0.0 (0.0 to 0.0)	0.0 (0.0 to 0.0)	0.0 (0.0 to 0.0)	0.0 (0.0 to 0.0)	0.0 (0.0 to 0.0)
Asthma	0.0 (0.0 to 0.0)	0.0 (0.0 to 0.0)	0.0 (0.0 to 0.0)	0.0 (0.0 to 0.0)	0.0 (0.0 to 0.0)	0.0 (0.0 to 0.0)
Asymptomatic asthma	0.0 (0.0 to 0.0)	0.0 (0.0 to 0.0)	0.0 (0.0 to 0.0)	0.0 (0.0 to 0.0)	0.0 (0.0 to 0.0)	0.0 (0.0 to 0.0)
Symptomatic asthma aggregate	0.0 (0.0 to 0.0)	0.0 (0.0 to 0.0)	0.0 (0.0 to 0.0)	0.0 (0.0 to 0.0)	0.0 (0.0 to 0.0)	0.0 (0.0 to 0.0)
Interstitial lung disease and pulmonary sarcoidosis	0.0 (0.0 to 0.0)	0.0 (0.0 to 0.0)	0.0 (0.0 to 0.0)	0.0 (0.0 to 0.0)	0.0 (0.0 to 0.0)	0.0 (0.0 to 0.0)
Interstitial lung disease and pulmonary sarcoidosis without heart failure aggregate	0.0 (0.0 to 0.0)	0.0 (0.0 to 0.0)	0.0 (0.0 to 0.0)	0.0 (0.0 to 0.0)	0.0 (0.0 to 0.0)	0.0 (0.0 to 0.0)
Severe interstitial lung disease and pulmonary sarcoidosis with heart failure aggregate	0.0 (0.0 to 0.0)	0.0 (0.0 to 0.0)	0.0 (0.0 to 0.0)	0.0 (0.0 to 0.0)	0.0 (0.0 to 0.0)	0.0 (0.0 to 0.0)
Other chronic respiratory diseases	--	--	--	0.0 (0.0 to 0.0)	0.0 (0.0 to 0.0)	0.0 (0.0 to 0.0)
Cirrhosis and other chronic liver diseases	0.0 (0.0 to 0.0)	0.0 (0.0 to 0.0)	0.0 (0.0 to 0.0)	0.0 (0.0 to 0.0)	6.0 (5.1 to 6.7)*	0.5 (-0.3 to 1.2)
Cirrhosis and other chronic liver diseases due to hepatitis B	0.0 (0.0 to 0.0)	7.1 (3.9 to 10.0)*	1.5 (-1.5 to 4.3)	0.0 (0.0 to 0.0)	7.1 (3.9 to 10.0)*	1.5 (-1.5 to 4.3)
Cirrhosis and other chronic liver diseases due to hepatitis C	0.0 (0.0 to 0.0)	7.0 (2.4 to 11.1)*	1.5 (-2.9 to 5.3)	0.0 (0.0 to 0.0)	7.0 (2.4 to 11.1)*	1.4 (-2.9 to 5.3)
Cirrhosis and other chronic liver diseases due to alcohol use	0.0 (0.0 to 0.0)	0.0 (0.0 to 0.0)	0.0 (0.0 to 0.0)	0.0 (0.0 to 0.0)	0.0 (0.0 to 0.0)	0.0 (0.0 to 0.0)
Cirrhosis and other chronic liver diseases due to other causes	0.0 (0.0 to 0.0)	6.0 (5.1 to 6.7)*	0.5 (-0.3 to 1.2)	0.0 (0.0 to 0.0)	6.0 (5.1 to 6.7)*	0.5 (-0.3 to 1.2)
Digestive diseases	0.4 (0.3 to 0.4)	5.7 (-5.7 to 19.0)	0.3 (-10.6 to 12.9)	0.1 (0.1 to 0.3)	6.4 (-3.9 to 17.2)	0.9 (-8.9 to 11.1)
Peptic ulcer disease	0.0 (0.0 to 0.0)	0.0 (0.0 to 0.0)	0.0 (0.0 to 0.0)	0.0 (0.0 to 0.0)	0.0 (0.0 to 0.0)	0.0 (0.0 to 0.0)
Peptic ulcer disease, symptomatic episodes	0.0 (0.0 to 0.0)	0.0 (0.0 to 0.0)	0.0 (0.0 to 0.0)	0.0 (0.0 to 0.0)	0.0 (0.0 to 0.0)	0.0 (0.0 to 0.0)
Anemia due to peptic ulcer disease aggregate	0.0 (0.0 to 0.0)	0.0 (0.0 to 0.0)	0.0 (0.0 to 0.0)	0.0 (0.0 to 0.0)	0.0 (0.0 to 0.0)	0.0 (0.0 to 0.0)
Gastritis and duodenitis	0.0 (0.0 to 0.0)	0.0 (0.0 to 0.0)	0.0 (0.0 to 0.0)	0.0 (0.0 to 0.0)	0.0 (0.0 to 0.0)	0.0 (0.0 to 0.0)
Gastritis and duodenitis, symptomatic episodes	0.0 (0.0 to 0.0)	0.0 (0.0 to 0.0)	0.0 (0.0 to 0.0)	0.0 (0.0 to 0.0)	0.0 (0.0 to 0.0)	0.0 (0.0 to 0.0)
Anemia due to gastritis and duodenitis aggregate	0.0 (0.0 to 0.0)	0.0 (0.0 to 0.0)	0.0 (0.0 to 0.0)	0.0 (0.0 to 0.0)	0.0 (0.0 to 0.0)	0.0 (0.0 to 0.0)
Appendicitis	0.0 (0.0 to 0.0)	0.0 (0.0 to 0.0)	0.0 (0.0 to 0.0)	0.0 (0.0 to 0.0)	0.0 (0.0 to 0.0)	0.0 (0.0 to 0.0)
Paralytic ileus and intestinal obstruction	0.4 (0.2 to 0.7)	5.5 (2.8 to 7.0)*	0.0 (-2.5 to 1.5)	0.1 (0.0 to 0.2)	5.7 (-4.8 to 16.5)	0.2 (-9.7 to 10.5)
Inguinal, femoral, and abdominal hernia	0.0 (0.0 to 0.0)	0.0 (0.0 to 0.0)	0.0 (0.0 to 0.0)	0.0 (0.0 to 0.0)	0.0 (0.0 to 0.0)	0.0 (0.0 to 0.0)
Inflammatory bowel disease	0.0 (0.0 to 0.0)	0.0 (0.0 to 0.0)	0.0 (0.0 to 0.0)	0.0 (0.0 to 0.0)	0.0 (0.0 to 0.0)	0.0 (0.0 to 0.0)
Vascular intestinal disorders	0.0 (0.0 to 0.0)	0.0 (0.0 to 0.0)	0.0 (0.0 to 0.0)	0.0 (0.0 to 0.0)	0.0 (0.0 to 0.0)	0.0 (0.0 to 0.0)
Gallbladder and biliary diseases	0.0 (0.0 to 0.0)	0.0 (0.0 to 0.0)	0.0 (0.0 to 0.0)	0.0 (0.0 to 0.0)	0.0 (0.0 to 0.0)	0.0 (0.0 to 0.0)
Pancreatitis	0.0 (0.0 to 0.0)	0.0 (0.0 to 0.0)	0.0 (0.0 to 0.0)	0.0 (0.0 to 0.0)	0.0 (0.0 to 0.0)	0.0 (0.0 to 0.0)
Other digestive diseases	--	--	--	0.0 (0.0 to 0.0)	12.2 (-1.7 to 29.2)	6.4 (-6.8 to 22.5)
Neurological disorders	8.1 (7.3 to 9.2)	0.1 (-6.4 to 6.3)	-5.1 (-11.2 to 0.8)	2.4 (1.5 to 3.5)	-12.7 (-21.9 to -3.8)*	-17.2 (-25.9 to -8.8)*
Alzheimer disease and other dementias	0.0 (0.0 to 0.0)	0.0 (0.0 to 0.0)	0.0 (0.0 to 0.0)	0.0 (0.0 to 0.0)	0.0 (0.0 to 0.0)	0.0 (0.0 to 0.0)
Parkinson disease	0.0 (0.0 to 0.0)	0.0 (0.0 to 0.0)	0.0 (0.0 to 0.0)	0.0 (0.0 to 0.0)	0.0 (0.0 to 0.0)	0.0 (0.0 to 0.0)
Epilepsy	8.1 (6.0 to 10.7)	0.6 (-8.7 to 9.5)	-4.6 (-13.4 to 3.8)	2.4 (1.5 to 3.5)	-12.7 (-21.9 to -3.8)*	-17.2 (-25.9 to -8.8)*
Multiple sclerosis	0.0 (0.0 to 0.0)	0.0 (0.0 to 0.0)	0.0 (0.0 to 0.0)	0.0 (0.0 to 0.0)	0.0 (0.0 to 0.0)	0.0 (0.0 to 0.0)
Motor neuron disease	0.0 (0.0 to 0.0)	-7.3 (-9.9 to -5.1)*	-12.1 (-14.6 to -10.0)*	0.0 (0.0 to 0.0)	-7.3 (-9.9 to -5.1)*	-12.1 (-14.6 to -10.0)*
Migraine	0.0 (0.0 to 0.0)	0.0 (0.0 to 0.0)	0.0 (0.0 to 0.0)	0.0 (0.0 to 0.0)	0.0 (0.0 to 0.0)	0.0 (0.0 to 0.0)
Tension-type headache	0.0 (0.0 to 0.0)	0.0 (0.0 to 0.0)	0.0 (0.0 to 0.0)	0.0 (0.0 to 0.0)	0.0 (0.0 to 0.0)	0.0 (0.0 to 0.0)
Medication overuse headache	0.0 (0.0 to 0.0)	0.0 (0.0 to 0.0)	0.0 (0.0 to 0.0)	0.0 (0.0 to 0.0)	0.0 (0.0 to 0.0)	0.0 (0.0 to 0.0)
Other neurological disorders	0.0 (0.0 to 0.0)	0.0 (0.0 to 0.0)	0.0 (0.0 to 0.0)	0.0 (0.0 to 0.0)	0.0 (0.0 to 0.0)	0.0 (0.0 to 0.0)
Mental and substance use disorders	18.9 (17.0 to 20.9)	3.6 (-1.2 to 8.8)	-1.7 (-6.3 to 3.2)	2.0 (1.2 to 3.0)	5.1 (3.4 to 6.7)*	-0.4 (-2.0 to 1.1)
Schizophrenia	0.0 (0.0 to 0.0)	0.0 (0.0 to 0.0)	0.0 (0.0 to 0.0)	0.0 (0.0 to 0.0)	0.0 (0.0 to 0.0)	0.0 (0.0 to 0.0)
Alcohol use disorders	0.3 (0.2 to 0.3)	3.6 (1.1 to 6.0)*	-1.8 (-4.1 to 0.5)	0.0 (0.0 to 0.0)	2.8 (-9.7 to 15.8)	-2.6 (-14.4 to 9.8)
Alcohol dependence aggregate	0.0 (0.0 to 0.0)	0.0 (0.0 to 0.0)	0.0 (0.0 to 0.0)	0.0 (0.0 to 0.0)	0.0 (0.0 to 0.0)	0.0 (0.0 to 0.0)
Fetal alcohol syndrome aggregate	0.3 (0.2 to 0.3)	3.6 (1.1 to 6.0)*	-1.8 (-4.1 to 0.5)	0.0 (0.0 to 0.0)	2.8 (-9.7 to 15.8)	-2.6 (-14.4 to 9.8)
Drug use disorders	0.0 (0.0 to 0.0)	0.0 (0.0 to 0.0)	0.0 (0.0 to 0.0)	0.0 (0.0 to 0.0)	0.0 (0.0 to 0.0)	0.0 (0.0 to 0.0)
Opioid use disorders	0.0 (0.0 to 0.0)	0.0 (0.0 to 0.0)	0.0 (0.0 to 0.0)	0.0 (0.0 to 0.0)	0.0 (0.0 to 0.0)	0.0 (0.0 to 0.0)
Asymptomatic opioid dependence	0.0 (0.0 to 0.0)	0.0 (0.0 to 0.0)	0.0 (0.0 to 0.0)	0.0 (0.0 to 0.0)	0.0 (0.0 to 0.0)	0.0 (0.0 to 0.0)
Symptomatic opioid dependence aggregate	0.0 (0.0 to 0.0)	0.0 (0.0 to 0.0)	0.0 (0.0 to 0.0)	0.0 (0.0 to 0.0)	0.0 (0.0 to 0.0)	0.0 (0.0 to 0.0)
Cocaine use disorders	0.0 (0.0 to 0.0)	0.0 (0.0 to 0.0)	0.0 (0.0 to 0.0)	0.0 (0.0 to 0.0)	0.0 (0.0 to 0.0)	0.0 (0.0 to 0.0)
Asymptomatic cocaine dependence	0.0 (0.0 to 0.0)	0.0 (0.0 to 0.0)	0.0 (0.0 to 0.0)	0.0 (0.0 to 0.0)	0.0 (0.0 to 0.0)	0.0 (0.0 to 0.0)

Cause	Prevalence in thousands (95% confidence interval)			YLDs in thousands (95% confidence interval)		
	2015	Percent change in counts between 2005 and 2015	Percent change in rates between 2005 and 2015	2015	Percent change in counts between 2005 and 2015	Percent change in rates between 2005 and 2015
Symptomatic cocaine dependence aggregate	0.0 (0.0 to 0.0)	0.0 (0.0 to 0.0)	0.0 (0.0 to 0.0)	0.0 (0.0 to 0.0)	0.0 (0.0 to 0.0)	0.0 (0.0 to 0.0)
Amphetamine use disorders	0.0 (0.0 to 0.0)	0.0 (0.0 to 0.0)	0.0 (0.0 to 0.0)	0.0 (0.0 to 0.0)	0.0 (0.0 to 0.0)	0.0 (0.0 to 0.0)
Asymptomatic amphetamine dependence	0.0 (0.0 to 0.0)	0.0 (0.0 to 0.0)	0.0 (0.0 to 0.0)	0.0 (0.0 to 0.0)	0.0 (0.0 to 0.0)	0.0 (0.0 to 0.0)
Symptomatic amphetamine dependence aggregate	0.0 (0.0 to 0.0)	0.0 (0.0 to 0.0)	0.0 (0.0 to 0.0)	0.0 (0.0 to 0.0)	0.0 (0.0 to 0.0)	0.0 (0.0 to 0.0)
Cannabis use disorders	0.0 (0.0 to 0.0)	0.0 (0.0 to 0.0)	0.0 (0.0 to 0.0)	0.0 (0.0 to 0.0)	0.0 (0.0 to 0.0)	0.0 (0.0 to 0.0)
Asymptomatic cannabis dependence	0.0 (0.0 to 0.0)	0.0 (0.0 to 0.0)	0.0 (0.0 to 0.0)	0.0 (0.0 to 0.0)	0.0 (0.0 to 0.0)	0.0 (0.0 to 0.0)
Symptomatic cannabis dependence aggregate	0.0 (0.0 to 0.0)	0.0 (0.0 to 0.0)	0.0 (0.0 to 0.0)	0.0 (0.0 to 0.0)	0.0 (0.0 to 0.0)	0.0 (0.0 to 0.0)
Other drug use disorders	--	--	--	0.0 (0.0 to 0.0)	0.0 (0.0 to 0.0)	0.0 (0.0 to 0.0)
Depressive disorders	0.0 (0.0 to 0.0)	0.0 (0.0 to 0.0)	0.0 (0.0 to 0.0)	0.0 (0.0 to 0.0)	0.0 (0.0 to 0.0)	0.0 (0.0 to 0.0)
Major depressive disorder	0.0 (0.0 to 0.0)	0.0 (0.0 to 0.0)	0.0 (0.0 to 0.0)	0.0 (0.0 to 0.0)	0.0 (0.0 to 0.0)	0.0 (0.0 to 0.0)
Dysthymia	0.0 (0.0 to 0.0)	0.0 (0.0 to 0.0)	0.0 (0.0 to 0.0)	0.0 (0.0 to 0.0)	0.0 (0.0 to 0.0)	0.0 (0.0 to 0.0)
Bipolar disorder	0.0 (0.0 to 0.0)	0.0 (0.0 to 0.0)	0.0 (0.0 to 0.0)	0.0 (0.0 to 0.0)	0.0 (0.0 to 0.0)	0.0 (0.0 to 0.0)
Anxiety disorders	0.0 (0.0 to 0.0)	0.0 (0.0 to 0.0)	0.0 (0.0 to 0.0)	0.0 (0.0 to 0.0)	0.0 (0.0 to 0.0)	0.0 (0.0 to 0.0)
Eating disorders	0.0 (0.0 to 0.0)	0.0 (0.0 to 0.0)	0.0 (0.0 to 0.0)	0.0 (0.0 to 0.0)	0.0 (0.0 to 0.0)	0.0 (0.0 to 0.0)
Anorexia nervosa	0.0 (0.0 to 0.0)	0.0 (0.0 to 0.0)	0.0 (0.0 to 0.0)	0.0 (0.0 to 0.0)	0.0 (0.0 to 0.0)	0.0 (0.0 to 0.0)
Bulimia nervosa	0.0 (0.0 to 0.0)	0.0 (0.0 to 0.0)	0.0 (0.0 to 0.0)	0.0 (0.0 to 0.0)	0.0 (0.0 to 0.0)	0.0 (0.0 to 0.0)
Autistic spectrum disorders	11.1 (9.7 to 12.1)	5.4 (0.8 to 10.2)*	-0.0 (-4.4 to 4.5)	1.7 (1.0 to 2.6)	5.5 (3.6 to 7.3)*	0.0 (-1.8 to 1.7)
Autism	3.5 (2.0 to 5.2)	5.1 (4.6 to 5.6)*	-0.3 (-0.8 to 0.2)	0.9 (0.5 to 1.5)	5.3 (1.9 to 8.4)*	-0.1 (-3.3 to 2.8)
Asperger syndrome and other autistic spectrum disorders	7.8 (4.9 to 11.4)	5.4 (5.1 to 5.8)*	-0.0 (-0.3 to 0.3)	0.8 (0.4 to 1.3)	5.7 (3.5 to 7.9)*	0.2 (-1.8 to 2.3)
Attention-deficit/hyperactivity disorder	0.0 (0.0 to 0.0)	0.0 (0.0 to 0.0)	0.0 (0.0 to 0.0)	0.0 (0.0 to 0.0)	0.0 (0.0 to 0.0)	0.0 (0.0 to 0.0)
Conduct disorder	0.0 (0.0 to 0.0)	0.0 (0.0 to 0.0)	0.0 (0.0 to 0.0)	0.0 (0.0 to 0.0)	0.0 (0.0 to 0.0)	0.0 (0.0 to 0.0)
Idiopathic developmental intellectual disability	7.2 (2.8 to 12.1)	1.7 (-2.4 to 3.9)	-3.6 (-7.5 to -1.4)*	0.3 (0.1 to 0.6)	2.8 (-0.6 to 5.7)	-2.5 (-5.7 to 0.2)
Other mental and substance use disorders	0.0 (0.0 to 0.0)	0.0 (0.0 to 0.0)	0.0 (0.0 to 0.0)	0.0 (0.0 to 0.0)	0.0 (0.0 to 0.0)	0.0 (0.0 to 0.0)
Diabetes, urogenital, blood, and endocrine diseases	752.9 (742.7 to 764.5)	3.2 (1.9 to 4.5)*	-2.1 (-3.3 to -0.9)*	17.2 (11.7 to 24.2)	5.3 (3.3 to 7.4)*	-0.1 (-2.0 to 1.8)
Diabetes mellitus	0.0 (0.0 to 0.0)	0.0 (0.0 to 0.0)	0.0 (0.0 to 0.0)	0.0 (0.0 to 0.0)	0.0 (0.0 to 0.0)	0.0 (0.0 to 0.0)
Uncomplicated diabetes mellitus	0.0 (0.0 to 0.0)	0.0 (0.0 to 0.0)	0.0 (0.0 to 0.0)	0.0 (0.0 to 0.0)	0.0 (0.0 to 0.0)	0.0 (0.0 to 0.0)
Neuropathy and other complications of diabetes mellitus aggregate	0.0 (0.0 to 0.0)	0.0 (0.0 to 0.0)	0.0 (0.0 to 0.0)	0.0 (0.0 to 0.0)	0.0 (0.0 to 0.0)	0.0 (0.0 to 0.0)
Vision loss due to diabetes mellitus aggregate	0.0 (0.0 to 0.0)	0.0 (0.0 to 0.0)	0.0 (0.0 to 0.0)	0.0 (0.0 to 0.0)	0.0 (0.0 to 0.0)	0.0 (0.0 to 0.0)
Acute glomerulonephritis	0.0 (0.0 to 0.0)	-9.3 (-13.9 to -5.7)*	-14.0 (-18.4 to -10.6)*	0.0 (0.0 to 0.0)	-9.3 (-13.9 to -5.7)*	-14.0 (-18.4 to -10.6)*
Chronic kidney disease	0.0 (0.0 to 0.0)	0.0 (0.0 to 0.0)	0.0 (0.0 to 0.0)	0.0 (0.0 to 0.0)	5.0 (3.8 to 6.2)*	-0.4 (-1.5 to 0.7)
Chronic kidney disease due to diabetes mellitus	0.0 (0.0 to 0.0)	4.8 (1.7 to 7.8)*	-0.6 (-3.6 to 2.2)	0.0 (0.0 to 0.0)	4.8 (1.8 to 7.9)*	-0.6 (-3.5 to 2.3)
Stage III chronic kidney disease due to diabetes mellitus aggregate	0.0 (0.0 to 0.0)	5.0 (1.8 to 8.0)*	-0.4 (-3.5 to 2.4)	0.0 (0.0 to 0.0)	8.9 (7.9 to 10.0)*	3.3 (2.3 to 4.3)*
Stage IV chronic kidney disease due to diabetes mellitus aggregate	0.0 (0.0 to 0.0)	0.0 (0.0 to 0.0)	0.0 (0.0 to 0.0)	0.0 (0.0 to 0.0)	0.0 (0.0 to 0.0)	0.0 (0.0 to 0.0)
Stage V chronic kidney disease untreated due to diabetes mellitus	0.0 (0.0 to 0.0)	4.7 (1.5 to 7.9)*	-0.7 (-3.7 to 2.3)	0.0 (0.0 to 0.0)	4.7 (1.5 to 7.9)*	-0.7 (-3.7 to 2.3)
End-stage chronic kidney disease due to diabetes aggregate	0.0 (0.0 to 0.0)	-1.3 (-7.2 to 4.4)	-6.4 (-12.0 to -1.0)*	0.0 (0.0 to 0.0)	2.3 (-2.1 to 6.9)	-3.0 (-7.2 to 1.4)
Chronic kidney disease due to hypertension	0.0 (0.0 to 0.0)	4.0 (0.5 to 6.9)*	-1.4 (-4.7 to 1.4)	0.0 (0.0 to 0.0)	4.5 (-1.3 to 9.6)	-0.9 (-6.4 to 3.9)
Stage III chronic kidney disease due to hypertension aggregate	0.0 (0.0 to 0.0)	4.0 (0.2 to 7.2)*	-1.4 (-5.0 to 1.7)	0.0 (0.0 to 0.0)	8.8 (7.7 to 10.2)*	3.2 (2.1 to 4.5)*
Stage IV chronic kidney disease due to hypertension aggregate	0.0 (0.0 to 0.0)	0.0 (0.0 to 0.0)	0.0 (0.0 to 0.0)	0.0 (0.0 to 0.0)	0.0 (0.0 to 0.0)	0.0 (0.0 to 0.0)
Stage V chronic kidney disease untreated due to hypertension	0.0 (0.0 to 0.0)	4.3 (-1.6 to 9.6)	-1.1 (-6.7 to 4.0)	0.0 (0.0 to 0.0)	4.3 (-1.6 to 9.6)	-1.1 (-6.7 to 4.0)
End-stage chronic kidney disease due to hypertension aggregate	0.0 (0.0 to 0.0)	-2.6 (-12.5 to 8.9)	-7.7 (-17.1 to 3.3)	0.0 (0.0 to 0.0)	0.4 (-7.6 to 9.3)	-4.8 (-12.3 to 3.6)
Chronic kidney disease due to glomerulonephritis	0.0 (0.0 to 0.0)	5.2 (3.1 to 7.1)*	-0.2 (-2.2 to 1.6)	0.0 (0.0 to 0.0)	4.6 (1.7 to 7.1)*	-0.9 (-3.6 to 1.6)
Stage III chronic kidney disease due to glomerulonephritis aggregate	0.0 (0.0 to 0.0)	7.3 (5.4 to 9.2)*	1.8 (-0.1 to 3.5)	0.0 (0.0 to 0.0)	6.8 (5.6 to 8.0)*	1.3 (0.2 to 2.4)*
Stage IV chronic kidney disease due to glomerulonephritis aggregate	0.0 (0.0 to 0.0)	0.0 (0.0 to 0.0)	0.0 (0.0 to 0.0)	0.0 (0.0 to 0.0)	0.0 (0.0 to 0.0)	0.0 (0.0 to 0.0)
Stage V chronic kidney disease untreated due to glomerulonephritis	0.0 (0.0 to 0.0)	4.5 (1.4 to 7.1)*	-0.9 (-3.8 to 1.6)	0.0 (0.0 to 0.0)	4.5 (1.4 to 7.1)*	-0.9 (-3.8 to 1.6)
End-stage chronic kidney disease due to glomerulonephritis aggregate	0.0 (0.0 to 0.0)	-0.4 (-5.8 to 5.0)	-5.6 (-10.7 to -0.5)*	0.0 (0.0 to 0.0)	3.9 (-1.4 to 9.5)	-1.5 (-6.6 to 3.8)
Chronic kidney disease due to other causes	0.0 (0.0 to 0.0)	5.3 (3.8 to 6.9)*	-0.1 (-1.6 to 1.3)	0.0 (0.0 to 0.0)	5.3 (3.6 to 7.0)*	-0.1 (-1.7 to 1.5)
Stage III chronic kidney disease due to other causes aggregate	0.0 (0.0 to 0.0)	5.5 (3.9 to 7.2)*	0.0 (-1.5 to 1.6)	0.0 (0.0 to 0.0)	10.0 (8.6 to 11.9)*	4.3 (3.0 to 6.1)*
Stage IV chronic kidney disease due to other causes aggregate	0.0 (0.0 to 0.0)	0.0 (0.0 to 0.0)	0.0 (0.0 to 0.0)	0.0 (0.0 to 0.0)	0.0 (0.0 to 0.0)	0.0 (0.0 to 0.0)
Stage V chronic kidney disease untreated due to other causes	0.0 (0.0 to 0.0)	5.1 (3.4 to 7.0)*	-0.3 (-2.0 to 1.4)	0.0 (0.0 to 0.0)	5.1 (3.4 to 7.0)*	-0.3 (-2.0 to 1.4)
End-stage chronic kidney disease due to other causes aggregate	0.0 (0.0 to 0.0)	-0.7 (-3.9 to 2.5)	-5.9 (-8.9 to -2.8)*	0.0 (0.0 to 0.0)	3.3 (-1.0 to 7.1)	-2.1 (-6.1 to 1.5)
Urinary diseases and male infertility	0.7 (0.6 to 0.7)	11.0 (3.5 to 20.0)*	5.2 (-1.9 to 13.7)	0.0 (0.0 to 0.0)	10.6 (1.0 to 21.2)*	4.9 (-4.2 to 14.9)
Interstitial nephritis and urinary tract infections	0.7 (0.6 to 0.7)	11.3 (10.7 to 12.0)*	5.6 (5.0 to 6.2)*	0.0 (0.0 to 0.0)	11.5 (2.2 to 21.6)*	5.8 (-3.1 to 15.3)
Urolithiasis	0.0 (0.0 to 0.0)	0.0 (0.0 to 0.0)	0.0 (0.0 to 0.0)	0.0 (0.0 to 0.0)	0.0 (0.0 to 0.0)	0.0 (0.0 to 0.0)
Benign prostatic hyperplasia	0.0 (0.0 to 0.0)	0.0 (0.0 to 0.0)	0.0 (0.0 to 0.0)	0.0 (0.0 to 0.0)	0.0 (0.0 to 0.0)	0.0 (0.0 to 0.0)
Male infertility	0.0 (0.0 to 0.0)	0.0 (0.0 to 0.0)	0.0 (0.0 to 0.0)	0.0 (0.0 to 0.0)	0.0 (0.0 to 0.0)	0.0 (0.0 to 0.0)
Other urinary diseases	--	--	--	0.0 (0.0 to 0.0)	7.9 (-4.7 to 23.1)	2.3 (-9.7 to 16.7)
Gynecological diseases	0.0 (0.0 to 0.0)	0.0 (0.0 to 0.0)	0.0 (0.0 to 0.0)	0.0 (0.0 to 0.0)	0.0 (0.0 to 0.0)	0.0 (0.0 to 0.0)
Uterine fibroids	0.0 (0.0 to 0.0)	0.0 (0.0 to 0.0)	0.0 (0.0 to 0.0)	0.0 (0.0 to 0.0)	0.0 (0.0 to 0.0)	0.0 (0.0 to 0.0)
Uterine fibroids cases aggregate	0.0 (0.0 to 0.0)	0.0 (0.0 to 0.0)	0.0 (0.0 to 0.0)	0.0 (0.0 to 0.0)	0.0 (0.0 to 0.0)	0.0 (0.0 to 0.0)
Mild abdominal pain with anemia due to uterine fibroids aggregate	0.0 (0.0 to 0.0)	0.0 (0.0 to 0.0)	0.0 (0.0 to 0.0)	0.0 (0.0 to 0.0)	0.0 (0.0 to 0.0)	0.0 (0.0 to 0.0)
Polycystic ovarian syndrome	0.0 (0.0 to 0.0)	0.0 (0.0 to 0.0)	0.0 (0.0 to 0.0)	0.0 (0.0 to 0.0)	0.0 (0.0 to 0.0)	0.0 (0.0 to 0.0)
Polycystic ovarian syndrome cases aggregate	0.0 (0.0 to 0.0)	0.0 (0.0 to 0.0)	0.0 (0.0 to 0.0)	0.0 (0.0 to 0.0)	0.0 (0.0 to 0.0)	0.0 (0.0 to 0.0)
Hirsutism and infertility due to polycystic ovarian syndrome aggregate	0.0 (0.0 to 0.0)	0.0 (0.0 to 0.0)	0.0 (0.0 to 0.0)	0.0 (0.0 to 0.0)	0.0 (0.0 to 0.0)	0.0 (0.0 to 0.0)

Cause	Prevalence in thousands (95% confidence interval)			YLDs in thousands (95% confidence interval)		
	2015	Percent change in counts between 2005 and 2015	Percent change in rates between 2005 and 2015	2015	Percent change in counts between 2005 and 2015	Percent change in rates between 2005 and 2015
Female infertility	0.0 (0.0 to 0.0)	0.0 (0.0 to 0.0)	0.0 (0.0 to 0.0)	0.0 (0.0 to 0.0)	0.0 (0.0 to 0.0)	0.0 (0.0 to 0.0)
Endometriosis	0.0 (0.0 to 0.0)	0.0 (0.0 to 0.0)	0.0 (0.0 to 0.0)	0.0 (0.0 to 0.0)	0.0 (0.0 to 0.0)	0.0 (0.0 to 0.0)
Endometriosis cases aggregate	0.0 (0.0 to 0.0)	0.0 (0.0 to 0.0)	0.0 (0.0 to 0.0)	0.0 (0.0 to 0.0)	0.0 (0.0 to 0.0)	0.0 (0.0 to 0.0)
Abdominal pain and infertility due to endometriosis aggregate	0.0 (0.0 to 0.0)	0.0 (0.0 to 0.0)	0.0 (0.0 to 0.0)	0.0 (0.0 to 0.0)	0.0 (0.0 to 0.0)	0.0 (0.0 to 0.0)
Genital prolapse	0.0 (0.0 to 0.0)	0.0 (0.0 to 0.0)	0.0 (0.0 to 0.0)	0.0 (0.0 to 0.0)	0.0 (0.0 to 0.0)	0.0 (0.0 to 0.0)
Premenstrual syndrome	0.0 (0.0 to 0.0)	0.0 (0.0 to 0.0)	0.0 (0.0 to 0.0)	0.0 (0.0 to 0.0)	0.0 (0.0 to 0.0)	0.0 (0.0 to 0.0)
Other gynecological diseases	0.0 (0.0 to 0.0)	0.0 (0.0 to 0.0)	0.0 (0.0 to 0.0)	0.0 (0.0 to 0.0)	0.0 (0.0 to 0.0)	0.0 (0.0 to 0.0)
Other gynecological diseases cases aggregate	0.0 (0.0 to 0.0)	0.0 (0.0 to 0.0)	0.0 (0.0 to 0.0)	0.0 (0.0 to 0.0)	0.0 (0.0 to 0.0)	0.0 (0.0 to 0.0)
Anemia due to other gynecological diseases aggregate	0.0 (0.0 to 0.0)	0.0 (0.0 to 0.0)	0.0 (0.0 to 0.0)	0.0 (0.0 to 0.0)	0.0 (0.0 to 0.0)	0.0 (0.0 to 0.0)
Hemoglobinopathies and hemolytic anemias	681.6 (671.0 to 693.1)	2.9 (1.5 to 4.3)*	-2.4 (-3.7 to -1.1)*	12.2 (8.3 to 17.2)	5.5 (3.5 to 7.5)*	0.0 (-1.9 to 1.9)
Thalassemias	2.2 (2.0 to 2.5)	1.2 (-0.1 to 3.3)	-4.1 (-5.3 to -2.0)*	0.2 (0.2 to 0.3)	3.4 (-2.8 to 9.9)	-1.9 (-7.9 to 4.2)
Beta-thalassemia major cases aggregate	1.3 (1.2 to 1.3)	3.7 (2.9 to 4.6)*	-1.6 (-2.4 to -0.8)*	0.1 (0.1 to 0.2)	6.7 (-1.2 to 15.6)	1.2 (-6.3 to 9.6)
Hemoglobin E/beta-thalassemia cases aggregate	0.4 (0.3 to 0.4)	3.4 (0.4 to 6.4)*	-1.9 (-4.8 to 0.9)	0.0 (0.0 to 0.0)	3.2 (-11.1 to 17.6)	-2.2 (-15.7 to 11.5)
Hemoglobin H disease cases aggregate	0.5 (0.4 to 0.9)	-5.6 (-10.3 to 2.2)	-10.5 (-15.0 to -3.1)*	0.1 (0.0 to 0.1)	-4.1 (-15.3 to 10.0)	-9.1 (-19.7 to 4.3)
Heart failure due to thalassemias aggregate	0.0 (0.0 to 0.0)	0.0 (0.0 to 0.0)	0.0 (0.0 to 0.0)	0.0 (0.0 to 0.0)	0.0 (0.0 to 0.0)	0.0 (0.0 to 0.0)
Thalassemias trait	92.7 (90.5 to 95.3)	5.6 (4.9 to 6.3)*	0.1 (-0.6 to 0.8)	4.5 (3.0 to 6.2)	8.8 (6.5 to 11.0)*	3.1 (1.0 to 5.3)*
B-thalassemia trait cases aggregate	75.6 (73.7 to 78.0)	6.3 (5.4 to 7.1)*	0.7 (-0.1 to 1.6)	4.1 (2.8 to 5.8)	9.0 (6.8 to 11.1)*	3.4 (1.3 to 5.4)*
Hemoglobin E trait cases aggregate	17.1 (16.3 to 17.9)	2.7 (1.5 to 3.9)*	-2.6 (-3.8 to -1.5)*	0.3 (0.2 to 0.4)	5.9 (-2.6 to 13.8)	0.4 (-7.6 to 7.9)
Sickle cell disorders	12.0 (11.5 to 12.5)	3.7 (0.2 to 7.9)*	-1.6 (-5.0 to 2.3)	1.1 (0.7 to 1.5)	1.1 (-4.6 to 6.3)	-4.1 (-9.5 to 0.8)
Homozygous sickle cell and severe sickle cell/beta-thalassemia cases aggregate	9.8 (9.4 to 10.3)	0.3 (-3.6 to 4.9)	-4.9 (-8.6 to -0.5)*	0.9 (0.6 to 1.2)	-1.9 (-8.2 to 3.2)	-7.0 (-12.9 to -2.1)*
Hemoglobin SC disease cases aggregate	2.1 (1.9 to 2.3)	22.5 (16.2 to 28.8)*	16.2 (10.2 to 22.2)*	0.2 (0.1 to 0.2)	19.7 (7.9 to 33.2)*	13.5 (2.3 to 26.3)*
Mild sickle cell/beta-thalassemia cases aggregate	0.1 (0.1 to 0.1)	20.4 (14.1 to 26.6)*	14.2 (8.2 to 20.1)*	0.0 (0.0 to 0.0)	18.1 (-3.1 to 44.4)	12.0 (-8.1 to 37.0)
Sickle cell trait	202.6 (192.5 to 219.7)	6.9 (5.2 to 8.1)*	1.3 (-0.2 to 2.5)	2.4 (1.7 to 3.4)	6.3 (0.3 to 11.0)*	0.8 (-4.9 to 5.2)
G6PD deficiency	107.9 (91.1 to 125.6)	-2.8 (-8.7 to 2.3)	-7.9 (-13.5 to -3.0)*	0.0 (0.0 to 0.0)	-2.3 (-14.2 to 9.6)	-7.4 (-18.6 to 4.0)
G6PD cases aggregate	107.9 (91.1 to 125.6)	-2.8 (-8.7 to 2.3)	-7.9 (-13.5 to -3.0)*	0.0 (0.0 to 0.0)	-2.3 (-14.2 to 9.6)	-7.4 (-18.6 to 4.0)
Heart failure due to G6PD deficiency aggregate	0.0 (0.0 to 0.0)	0.0 (0.0 to 0.0)	0.0 (0.0 to 0.0)	0.0 (0.0 to 0.0)	0.0 (0.0 to 0.0)	0.0 (0.0 to 0.0)
G6PD trait	283.6 (264.1 to 303.5)	1.2 (-0.6 to 2.9)	-4.0 (-5.8 to -2.4)*	0.0 (0.0 to 0.0)	3.5 (-7.5 to 14.9)	-1.9 (-12.3 to 8.9)
Other hemoglobinopathies and hemolytic anemias	75.0 (74.2 to 75.9)	4.7 (2.3 to 6.9)*	-0.8 (-3.0 to 1.4)	4.0 (2.7 to 5.6)	2.9 (0.3 to 5.4)*	-2.4 (-4.9 to -0.1)*
Other hemoglobinopathies and hemolytic anemias cases aggregate	75.0 (74.2 to 75.9)	4.7 (2.3 to 6.9)*	-0.8 (-3.0 to 1.4)	3.9 (2.7 to 5.5)	3.0 (0.3 to 5.5)*	-2.3 (-4.9 to 0.0)
Heart failure due to other hemoglobinopathies and hemolytic anemias aggregate	0.0 (0.0 to 0.0)	0.0 (0.0 to 0.0)	0.0 (0.0 to 0.0)	0.0 (0.0 to 0.0)	0.0 (0.0 to 0.0)	0.0 (0.0 to 0.0)
Endocrine, metabolic, blood, and immune disorders	95.9 (94.0 to 97.9)	4.9 (-0.2 to 10.2)	-0.6 (-5.4 to 4.5)	4.9 (3.4 to 6.9)	4.8 (1.1 to 9.3)*	-0.6 (-4.2 to 3.6)
Endocrine, metabolic, blood, and immune disorders cases aggregate	0.8 (0.8 to 0.9)	9.4 (7.8 to 10.8)*	3.7 (2.2 to 5.0)*	0.0 (0.0 to 0.0)	9.4 (4.3 to 15.2)*	3.7 (-1.1 to 9.2)
Anemia due to endocrine, metabolic, blood, and immune disorders aggregate	95.1 (93.1 to 97.1)	4.8 (-0.3 to 10.2)	-0.6 (-5.5 to 4.5)	4.9 (3.3 to 6.9)	4.8 (1.0 to 9.3)*	-0.6 (-4.2 to 3.6)
Heart failure due to endocrine, metabolic, blood, and immune disorders aggregate	0.0 (0.0 to 0.0)	4.1 (3.0 to 5.2)*	-1.3 (-2.4 to -0.3)*	0.0 (0.0 to 0.0)	4.1 (3.0 to 5.2)*	-1.3 (-2.4 to -0.3)*
Musculoskeletal disorders	0.0 (0.0 to 0.0)	0.0 (0.0 to 0.0)	0.0 (0.0 to 0.0)	0.0 (0.0 to 0.0)	0.0 (0.0 to 0.0)	0.0 (0.0 to 0.0)
Rheumatoid arthritis	0.0 (0.0 to 0.0)	0.0 (0.0 to 0.0)	0.0 (0.0 to 0.0)	0.0 (0.0 to 0.0)	0.0 (0.0 to 0.0)	0.0 (0.0 to 0.0)
Osteoarthritis	0.0 (0.0 to 0.0)	0.0 (0.0 to 0.0)	0.0 (0.0 to 0.0)	0.0 (0.0 to 0.0)	0.0 (0.0 to 0.0)	0.0 (0.0 to 0.0)
Osteoarthritis of the hip cases aggregate	0.0 (0.0 to 0.0)	0.0 (0.0 to 0.0)	0.0 (0.0 to 0.0)	0.0 (0.0 to 0.0)	0.0 (0.0 to 0.0)	0.0 (0.0 to 0.0)
Osteoarthritis of the knee cases aggregate	0.0 (0.0 to 0.0)	0.0 (0.0 to 0.0)	0.0 (0.0 to 0.0)	0.0 (0.0 to 0.0)	0.0 (0.0 to 0.0)	0.0 (0.0 to 0.0)
Low back and neck pain	0.0 (0.0 to 0.0)	0.0 (0.0 to 0.0)	0.0 (0.0 to 0.0)	0.0 (0.0 to 0.0)	0.0 (0.0 to 0.0)	0.0 (0.0 to 0.0)
Low back pain	0.0 (0.0 to 0.0)	0.0 (0.0 to 0.0)	0.0 (0.0 to 0.0)	0.0 (0.0 to 0.0)	0.0 (0.0 to 0.0)	0.0 (0.0 to 0.0)
Neck pain	0.0 (0.0 to 0.0)	0.0 (0.0 to 0.0)	0.0 (0.0 to 0.0)	0.0 (0.0 to 0.0)	0.0 (0.0 to 0.0)	0.0 (0.0 to 0.0)
Gout	0.0 (0.0 to 0.0)	0.0 (0.0 to 0.0)	0.0 (0.0 to 0.0)	0.0 (0.0 to 0.0)	0.0 (0.0 to 0.0)	0.0 (0.0 to 0.0)
Asymptomatic gout	0.0 (0.0 to 0.0)	0.0 (0.0 to 0.0)	0.0 (0.0 to 0.0)	0.0 (0.0 to 0.0)	0.0 (0.0 to 0.0)	0.0 (0.0 to 0.0)
Symptomatic gout cases aggregate	0.0 (0.0 to 0.0)	0.0 (0.0 to 0.0)	0.0 (0.0 to 0.0)	0.0 (0.0 to 0.0)	0.0 (0.0 to 0.0)	0.0 (0.0 to 0.0)
Other musculoskeletal disorders	0.0 (0.0 to 0.0)	0.0 (0.0 to 0.0)	0.0 (0.0 to 0.0)	0.0 (0.0 to 0.0)	0.0 (0.0 to 0.0)	0.0 (0.0 to 0.0)
Other non-communicable diseases	164.5 (158.1 to 170.1)	3.3 (1.6 to 4.9)*	-2.1 (-3.7 to -0.5)*	7.7 (5.2 to 10.9)	9.5 (3.0 to 16.5)*	3.9 (-2.3 to 10.5)
Congenital anomalies	52.1 (48.3 to 55.6)	5.9 (2.9 to 9.3)*	0.5 (-2.5 to 3.6)	5.2 (3.2 to 7.9)	12.7 (3.0 to 23.0)*	6.9 (-2.3 to 16.6)
Neural tube defects	1.4 (1.0 to 2.1)	2.5 (1.4 to 3.7)*	-2.8 (-3.9 to -1.7)*	0.5 (0.3 to 0.9)	3.0 (-2.7 to 8.3)	-2.4 (-7.8 to 2.7)
Congenital heart anomalies	29.6 (21.8 to 42.7)	6.7 (5.3 to 7.9)*	1.1 (-0.2 to 2.3)	1.1 (0.4 to 2.0)	6.3 (4.5 to 8.0)*	0.8 (-0.9 to 2.4)
Less severe heart anomalies cases aggregate	23.4 (15.5 to 36.5)	7.6 (5.8 to 9.0)*	2.0 (0.4 to 3.4)*	0.8 (0.3 to 1.5)	7.8 (5.5 to 10.0)*	2.2 (0.0 to 4.3)*
Severe congenital heart anomalies	5.2 (3.5 to 7.7)	3.2 (1.9 to 4.6)*	-2.2 (-3.4 to -0.8)*	0.3 (0.1 to 0.6)	3.2 (0.1 to 6.2)*	-2.1 (-5.0 to 0.7)
Critical congenital heart anomalies	1.0 (0.6 to 1.6)	4.4 (3.1 to 5.4)*	-1.0 (-2.2 to -0.1)*	0.1 (0.0 to 0.1)	4.4 (-1.5 to 10.6)	-1.0 (-6.6 to 4.8)
Heart failure due to congenital heart anomalies aggregate	0.0 (0.0 to 0.0)	5.7 (4.6 to 6.9)*	0.3 (-0.8 to 1.3)	0.0 (0.0 to 0.0)	5.7 (4.6 to 6.9)*	0.3 (-0.8 to 1.3)
Cleft lip and cleft palate	3.9 (2.2 to 6.5)	3.8 (-0.5 to 6.9)	-1.6 (-5.7 to 1.4)	0.1 (0.0 to 0.1)	-2.0 (-9.5 to 5.2)	-7.1 (-14.2 to -0.3)*
Down syndrome	2.8 (1.8 to 4.3)	4.0 (1.8 to 5.8)*	-1.4 (-3.5 to 0.3)	0.2 (0.1 to 0.4)	4.1 (-0.1 to 8.2)	-1.3 (-5.3 to 2.6)
Turner syndrome	0.2 (0.1 to 0.3)	2.5 (1.0 to 3.9)*	-2.8 (-4.3 to -1.5)*	0.0 (0.0 to 0.0)	2.5 (-0.7 to 5.6)	-2.8 (-5.9 to 0.2)
Klinefelter syndrome	0.1 (0.0 to 0.2)	4.0 (2.4 to 5.2)*	-1.4 (-2.9 to -0.3)*	0.0 (0.0 to 0.0)	4.0 (2.4 to 5.2)*	-1.4 (-2.9 to -0.3)*
Other chromosomal abnormalities	2.4 (1.3 to 4.1)	5.2 (3.2 to 6.6)*	-0.2 (-2.1 to 1.1)	0.2 (0.1 to 0.4)	5.4 (0.8 to 9.2)*	-0.0 (-4.4 to 3.5)
Other congenital anomalies	13.1 (7.2 to 23.8)	6.2 (4.6 to 7.8)*	0.7 (-0.8 to 2.2)	3.1 (1.8 to 4.9)	18.7 (2.1 to 38.2)*	12.6 (-3.2 to 31.1)
Skin and subcutaneous diseases	15.1 (14.5 to 15.6)	6.1 (4.0 to 8.3)*	0.6 (-1.4 to 2.7)	0.2 (0.1 to 0.4)	5.2 (1.7 to 8.6)*	-0.3 (-3.6 to 3.0)
Dermatitis	0.0 (0.0 to 0.0)	0.0 (0.0 to 0.0)	0.0 (0.0 to 0.0)	0.0 (0.0 to 0.0)	0.0 (0.0 to 0.0)	0.0 (0.0 to 0.0)
Eczema cases aggregate	0.0 (0.0 to 0.0)	0.0 (0.0 to 0.0)	0.0 (0.0 to 0.0)	0.0 (0.0 to 0.0)	0.0 (0.0 to 0.0)	0.0 (0.0 to 0.0)

Cause	Prevalence in thousands (95% confidence interval)			YLDs in thousands (95% confidence interval)		
	2015	Percent change in counts between 2005 and 2015	Percent change in rates between 2005 and 2015	2015	Percent change in counts between 2005 and 2015	Percent change in rates between 2005 and 2015
Contact dermatitis cases aggregate	0.0 (0.0 to 0.0)	0.0 (0.0 to 0.0)	0.0 (0.0 to 0.0)	0.0 (0.0 to 0.0)	0.0 (0.0 to 0.0)	0.0 (0.0 to 0.0)
Seborrhoeic dermatitis cases aggregate	0.0 (0.0 to 0.0)	0.0 (0.0 to 0.0)	0.0 (0.0 to 0.0)	0.0 (0.0 to 0.0)	0.0 (0.0 to 0.0)	0.0 (0.0 to 0.0)
Psoriasis	0.0 (0.0 to 0.0)	0.0 (0.0 to 0.0)	0.0 (0.0 to 0.0)	0.0 (0.0 to 0.0)	0.0 (0.0 to 0.0)	0.0 (0.0 to 0.0)
Cellulitis	0.1 (0.1 to 0.1)	8.3 (5.8 to 10.7)*	2.7 (0.3 to 4.9)*	0.0 (0.0 to 0.0)	8.3 (5.8 to 10.6)*	2.7 (0.3 to 4.9)*
Pyoderma	0.9 (0.8 to 1.0)	8.5 (7.1 to 9.9)*	2.9 (1.5 to 4.2)*	0.0 (0.0 to 0.0)	8.4 (2.0 to 15.8)*	2.8 (-3.3 to 9.8)
Scabies	2.4 (1.8 to 3.2)	2.8 (-0.8 to 6.2)	-2.5 (-6.0 to 0.7)	0.1 (0.0 to 0.1)	3.0 (-2.3 to 8.1)	-2.4 (-7.3 to 2.5)
Fungal skin diseases	7.3 (5.7 to 9.1)	6.1 (5.8 to 6.4)*	0.6 (0.3 to 0.9)*	0.0 (0.0 to 0.1)	6.1 (3.5 to 8.7)*	0.6 (-1.8 to 3.0)
Viral skin diseases	0.0 (0.0 to 0.0)	0.0 (0.0 to 0.0)	0.0 (0.0 to 0.0)	0.0 (0.0 to 0.0)	0.0 (0.0 to 0.0)	0.0 (0.0 to 0.0)
Molluscum contagiosum cases aggregate	0.0 (0.0 to 0.0)	0.0 (0.0 to 0.0)	0.0 (0.0 to 0.0)	0.0 (0.0 to 0.0)	0.0 (0.0 to 0.0)	0.0 (0.0 to 0.0)
Viral warts cases aggregate	0.0 (0.0 to 0.0)	0.0 (0.0 to 0.0)	0.0 (0.0 to 0.0)	0.0 (0.0 to 0.0)	0.0 (0.0 to 0.0)	0.0 (0.0 to 0.0)
Acne vulgaris	0.0 (0.0 to 0.0)	0.0 (0.0 to 0.0)	0.0 (0.0 to 0.0)	0.0 (0.0 to 0.0)	0.0 (0.0 to 0.0)	0.0 (0.0 to 0.0)
Alopecia areata	0.0 (0.0 to 0.0)	0.0 (0.0 to 0.0)	0.0 (0.0 to 0.0)	0.0 (0.0 to 0.0)	0.0 (0.0 to 0.0)	0.0 (0.0 to 0.0)
Pruritus	0.2 (0.1 to 0.3)	5.8 (3.1 to 8.8)*	0.4 (-2.3 to 3.2)	0.0 (0.0 to 0.0)	6.0 (-7.5 to 21.1)	0.6 (-12.3 to 14.9)
Urticaria	1.6 (1.2 to 2.0)	5.1 (4.8 to 5.3)*	-0.4 (-0.7 to -0.1)*	0.1 (0.1 to 0.1)	5.3 (-1.6 to 12.5)	-0.1 (-6.7 to 6.7)
Decubitus ulcer	0.0 (0.0 to 0.0)	0.0 (0.0 to 0.0)	0.0 (0.0 to 0.0)	0.0 (0.0 to 0.0)	0.0 (0.0 to 0.0)	0.0 (0.0 to 0.0)
Other skin and subcutaneous diseases	3.0 (2.8 to 3.2)	8.0 (6.5 to 9.6)*	2.4 (1.0 to 3.9)*	0.0 (0.0 to 0.0)	8.0 (3.6 to 12.8)*	2.4 (-1.8 to 7.0)
Sense organ diseases	100.1 (95.9 to 103.5)	1.5 (-0.4 to 3.4)	-3.8 (-5.6 to -2.0)*	2.2 (1.4 to 3.5)	3.1 (1.3 to 5.1)*	-2.2 (-4.0 to -0.4)*
Glaucoma	0.0 (0.0 to 0.0)	0.0 (0.0 to 0.0)	0.0 (0.0 to 0.0)	0.0 (0.0 to 0.0)	0.0 (0.0 to 0.0)	0.0 (0.0 to 0.0)
Cataract	0.0 (0.0 to 0.0)	0.0 (0.0 to 0.0)	0.0 (0.0 to 0.0)	0.0 (0.0 to 0.0)	0.0 (0.0 to 0.0)	0.0 (0.0 to 0.0)
Macular degeneration	0.0 (0.0 to 0.0)	0.0 (0.0 to 0.0)	0.0 (0.0 to 0.0)	0.0 (0.0 to 0.0)	0.0 (0.0 to 0.0)	0.0 (0.0 to 0.0)
Refraction and accommodation disorders	91.8 (82.7 to 102.3)	1.0 (-0.5 to 2.5)	-4.2 (-5.7 to -2.8)*	1.7 (1.0 to 2.8)	2.5 (0.7 to 4.5)*	-2.8 (-4.6 to -0.9)*
Age-related and other hearing loss	6.2 (3.4 to 11.7)	7.1 (4.8 to 9.5)*	1.6 (-0.6 to 3.9)	0.5 (0.2 to 0.9)	4.4 (-0.7 to 9.9)	-1.0 (-5.8 to 4.2)
Other vision loss	2.9 (1.5 to 4.7)	5.7 (2.4 to 9.2)*	0.2 (-2.9 to 3.6)	0.1 (0.1 to 0.2)	6.4 (1.0 to 12.3)*	0.9 (-4.3 to 6.5)
Other sense organ diseases	0.0 (0.0 to 0.0)	0.0 (0.0 to 0.0)	0.0 (0.0 to 0.0)	0.0 (0.0 to 0.0)	0.0 (0.0 to 0.0)	0.0 (0.0 to 0.0)
Acute other sense organ diseases	0.0 (0.0 to 0.0)	0.0 (0.0 to 0.0)	0.0 (0.0 to 0.0)	0.0 (0.0 to 0.0)	0.0 (0.0 to 0.0)	0.0 (0.0 to 0.0)
Chronic other sense organ diseases	0.0 (0.0 to 0.0)	0.0 (0.0 to 0.0)	0.0 (0.0 to 0.0)	0.0 (0.0 to 0.0)	0.0 (0.0 to 0.0)	0.0 (0.0 to 0.0)
Oral disorders	0.0 (0.0 to 0.0)	0.0 (0.0 to 0.0)	0.0 (0.0 to 0.0)	0.0 (0.0 to 0.0)	0.0 (0.0 to 0.0)	0.0 (0.0 to 0.0)
Deciduous caries	0.0 (0.0 to 0.0)	0.0 (0.0 to 0.0)	0.0 (0.0 to 0.0)	0.0 (0.0 to 0.0)	0.0 (0.0 to 0.0)	0.0 (0.0 to 0.0)
Permanent caries	0.0 (0.0 to 0.0)	0.0 (0.0 to 0.0)	0.0 (0.0 to 0.0)	0.0 (0.0 to 0.0)	0.0 (0.0 to 0.0)	0.0 (0.0 to 0.0)
Periodontal diseases	0.0 (0.0 to 0.0)	0.0 (0.0 to 0.0)	0.0 (0.0 to 0.0)	0.0 (0.0 to 0.0)	0.0 (0.0 to 0.0)	0.0 (0.0 to 0.0)
Edentulism and severe tooth loss	0.0 (0.0 to 0.0)	0.0 (0.0 to 0.0)	0.0 (0.0 to 0.0)	0.0 (0.0 to 0.0)	0.0 (0.0 to 0.0)	0.0 (0.0 to 0.0)
Other oral disorders	0.0 (0.0 to 0.0)	0.0 (0.0 to 0.0)	0.0 (0.0 to 0.0)	0.0 (0.0 to 0.0)	0.0 (0.0 to 0.0)	0.0 (0.0 to 0.0)
Injuries	1.6 (1.4 to 1.8)	-8.9 (-10.4 to -7.5)*	-13.6 (-15.0 to -12.3)*	0.1 (0.1 to 0.2)	-10.7 (-12.9 to -8.9)*	-15.3 (-17.4 to -13.6)*
Transport injuries	0.0 (0.0 to 0.0)	-0.3 (-3.6 to 2.9)	-5.5 (-8.6 to -2.4)*	0.0 (0.0 to 0.0)	-4.0 (-7.4 to -0.5)*	-9.0 (-12.2 to -5.6)*
Road injuries	0.0 (0.0 to 0.0)	4.2 (-0.6 to 9.5)	-1.2 (-5.8 to 3.9)	0.0 (0.0 to 0.0)	1.1 (-4.2 to 6.7)	-4.1 (-9.2 to 1.2)
Pedestrian road injuries	0.0 (0.0 to 0.0)	-2.1 (-8.2 to 4.3)	-7.2 (-13.0 to -1.1)*	0.0 (0.0 to 0.0)	-5.4 (-11.9 to 1.2)	-10.3 (-16.5 to -4.1)*
Cyclist road injuries	0.0 (0.0 to 0.0)	10.2 (3.7 to 17.1)*	4.5 (-1.7 to 11.0)	0.0 (0.0 to 0.0)	6.6 (-0.1 to 13.8)	1.1 (-5.3 to 7.9)
Motorcyclist road injuries	0.0 (0.0 to 0.0)	4.8 (-1.3 to 11.4)	-0.6 (-6.4 to 5.7)	0.0 (0.0 to 0.0)	1.9 (-4.8 to 8.8)	-3.4 (-9.7 to 3.1)
Motor vehicle road injuries	0.0 (0.0 to 0.0)	2.9 (-2.3 to 8.2)	-2.4 (-7.3 to 2.6)	0.0 (0.0 to 0.0)	0.3 (-5.4 to 6.0)	-4.9 (-10.3 to 0.5)
Other road injuries	0.0 (0.0 to 0.0)	16.0 (8.6 to 23.8)*	10.0 (3.0 to 17.4)*	0.0 (0.0 to 0.0)	13.1 (5.5 to 21.2)*	7.2 (0.0 to 14.9)*
Other transport injuries	0.0 (0.0 to 0.0)	-4.6 (-8.3 to -1.3)*	-9.5 (-13.0 to -6.4)*	0.0 (0.0 to 0.0)	-8.0 (-11.8 to -4.3)*	-12.8 (-16.4 to -9.2)*
Unintentional injuries	1.6 (1.4 to 1.8)	-8.9 (-10.3 to -7.5)*	-13.6 (-15.0 to -12.2)*	0.1 (0.1 to 0.2)	-10.0 (-11.7 to -8.5)*	-14.7 (-16.2 to -13.2)*
Falls	0.2 (0.1 to 0.2)	7.4 (4.1 to 10.5)*	1.8 (-1.3 to 4.8)	0.0 (0.0 to 0.0)	5.0 (1.4 to 8.4)*	-0.4 (-3.8 to 2.8)
Drowning	0.0 (0.0 to 0.0)	-8.5 (-11.7 to -5.8)*	-13.3 (-16.3 to -10.7)*	0.0 (0.0 to 0.0)	-11.5 (-14.9 to -8.4)*	-16.1 (-19.3 to -13.1)*
Fire, heat, and hot substances	0.1 (0.1 to 0.2)	-4.4 (-7.5 to -1.6)*	-9.4 (-12.3 to -6.7)*	0.0 (0.0 to 0.0)	-7.8 (-11.8 to -3.9)*	-12.6 (-16.4 to -8.9)*
Poisonings	0.0 (0.0 to 0.0)	4.4 (2.0 to 6.6)*	-1.0 (-3.3 to 1.1)	0.0 (0.0 to 0.0)	2.9 (0.3 to 5.2)*	-2.4 (-4.8 to -0.2)*
Exposure to mechanical forces	0.3 (0.3 to 0.4)	-12.4 (-15.1 to -9.8)*	-17.0 (-19.5 to -14.4)*	0.0 (0.0 to 0.0)	-14.3 (-17.0 to -11.3)*	-18.7 (-21.3 to -15.9)*
Unintentional firearm injuries	0.0 (0.0 to 0.0)	-9.2 (-16.0 to -3.1)*	-13.9 (-20.3 to -8.1)*	0.0 (0.0 to 0.0)	-10.1 (-17.5 to -3.3)*	-14.7 (-21.8 to -8.3)*
Unintentional suffocation	0.1 (0.1 to 0.1)	-9.9 (-13.4 to -6.3)*	-14.5 (-17.9 to -11.1)*	0.0 (0.0 to 0.0)	-11.1 (-14.7 to -7.3)*	-15.7 (-19.1 to -12.1)*
Other exposure to mechanical forces	0.2 (0.2 to 0.3)	-13.4 (-16.4 to -10.6)*	-17.9 (-20.7 to -15.3)*	0.0 (0.0 to 0.0)	-15.7 (-18.7 to -12.6)*	-20.1 (-22.9 to -17.2)*
Adverse effects of medical treatment	0.2 (0.2 to 0.3)	-11.6 (-14.7 to -8.7)*	-16.2 (-19.1 to -13.4)*	0.0 (0.0 to 0.0)	-11.6 (-14.7 to -8.7)*	-16.2 (-19.1 to -13.4)*
Animal contact	0.1 (0.1 to 0.1)	-11.2 (-15.6 to -6.7)*	-15.8 (-20.0 to -11.5)*	0.0 (0.0 to 0.0)	-11.3 (-15.4 to -7.2)*	-15.9 (-19.8 to -12.0)*
Venomous animal contact	0.0 (0.0 to 0.0)	-11.3 (-15.9 to -6.5)*	-15.9 (-20.2 to -11.3)*	0.0 (0.0 to 0.0)	-11.7 (-16.1 to -7.2)*	-16.3 (-20.5 to -12.0)*
Non-venomous animal contact	0.1 (0.0 to 0.1)	-11.2 (-15.9 to -6.4)*	-15.8 (-20.2 to -11.2)*	0.0 (0.0 to 0.0)	-11.0 (-15.7 to -6.4)*	-15.6 (-20.1 to -11.2)*
Foreign body	0.1 (0.1 to 0.2)	-7.1 (-9.0 to -5.1)*	-11.9 (-13.7 to -10.1)*	0.0 (0.0 to 0.0)	-10.4 (-12.7 to -8.1)*	-15.0 (-17.2 to -12.8)*
Pulmonary aspiration and foreign body in airway	0.1 (0.0 to 0.1)	-9.9 (-12.4 to -7.8)*	-14.5 (-16.9 to -12.6)*	0.0 (0.0 to 0.0)	-14.1 (-16.8 to -11.5)*	-18.6 (-21.1 to -16.1)*
Foreign body in eyes	0.0 (0.0 to 0.0)	3.8 (3.1 to 4.4)*	-1.6 (-2.3 to -1.0)*	0.0 (0.0 to 0.0)	3.3 (1.7 to 4.0)*	-2.1 (-3.5 to -1.4)*
Foreign body in other body part	0.1 (0.1 to 0.1)	-5.7 (-8.7 to -2.8)*	-10.6 (-13.5 to -7.8)*	0.0 (0.0 to 0.0)	-6.0 (-9.3 to -3.0)*	-10.9 (-14.0 to -8.1)*
Environmental heat and cold exposure	0.1 (0.1 to 0.1)	-1.0 (-4.7 to 2.9)	-6.1 (-9.7 to -2.4)*	0.0 (0.0 to 0.0)	-2.5 (-6.4 to 1.8)	-7.5 (-11.3 to -3.5)*
Other unintentional injuries	0.5 (0.4 to 0.5)	-13.1 (-16.6 to -9.5)*	-17.6 (-20.9 to -14.1)*	0.0 (0.0 to 0.1)	-14.7 (-18.4 to -10.6)*	-19.1 (-22.6 to -15.2)*
Self-harm and interpersonal violence	0.0 (0.0 to 0.0)	-18.8 (-22.7 to -14.6)*	-23.0 (-26.7 to -19.0)*	0.0 (0.0 to 0.0)	-19.6 (-23.9 to -15.1)*	-23.7 (-27.8 to -19.5)*
Self-harm	0.0 (0.0 to 0.0)	1.0 (-1.4 to 2.7)	-4.3 (-6.5 to -2.6)*	0.0 (0.0 to 0.0)	-1.8 (-3.9 to -0.0)*	-6.9 (-8.9 to -5.2)*
Interpersonal violence	0.0 (0.0 to 0.0)	-19.4 (-23.5 to -15.0)*	-23.5 (-27.5 to -19.4)*	0.0 (0.0 to 0.0)	-20.3 (-24.9 to -15.7)*	-24.4 (-28.8 to -20.0)*

Cause	Prevalence in thousands (95% confidence interval)			YLDs in thousands (95% confidence interval)		
	**2015**	**Percent change in counts between 2005 and 2015**	**Percent change in rates between 2005 and 2015**	**2015**	**Percent change in counts between 2005 and 2015**	**Percent change in rates between 2005 and 2015**
Assault by firearm	0.0 (0.0 to 0.0)	-15.8 (-21.6 to -10.0)*	-20.1 (-25.7 to -14.7)*	0.0 (0.0 to 0.0)	-17.7 (-24.7 to -10.7)*	-21.9 (-28.6 to -15.3)*
Assault by sharp object	0.0 (0.0 to 0.0)	-18.5 (-24.6 to -12.1)*	-22.7 (-28.5 to -16.7)*	0.0 (0.0 to 0.0)	-19.9 (-26.6 to -13.3)*	-24.1 (-30.4 to -17.8)*
Assault by other means	0.0 (0.0 to 0.0)	-19.5 (-23.6 to -15.2)*	-23.7 (-27.6 to -19.6)*	0.0 (0.0 to 0.0)	-20.4 (-25.0 to -15.9)*	-24.5 (-28.8 to -20.2)*
Forces of nature, war, and legal intervention	0.0 (0.0 to 0.0)	0.0 (0.0 to 0.0)	0.0 (0.0 to 0.0)	0.0 (0.0 to 0.0)	-100.0 (-100.0 to 0.0)	-100.0 (-100.0 to 0.0)
Exposure to forces of nature	0.0 (0.0 to 0.0)	0.0 (0.0 to 0.0)	0.0 (0.0 to 0.0)	0.0 (0.0 to 0.0)	0.0 (0.0 to 0.0)	0.0 (0.0 to 0.0)
Collective violence and legal intervention	0.0 (0.0 to 0.0)	0.0 (0.0 to 0.0)	0.0 (0.0 to 0.0)	0.0 (0.0 to 0.0)	-100.0 (-100.0 to 0.0)	-100.0 (-100.0 to 0.0)
**Anemia**	**2,514.1 (2,490.4 to 2,538.9)**	**5.0 (3.8 to 6.2)***	**-0.5 (-1.5 to 0.7)**	**134.2 (90.7 to 189.8)**	**2.3 (0.9 to 3.9)***	**-3.0 (-4.4 to -1.5)***
**Developmental intellectual disability**	**24.6 (19.6 to 30.3)**	**5.0 (2.3 to 8.1)***	**-0.4 (-3.0 to 2.5)**	**4.6 (3.2 to 6.2)**	**7.1 (-0.0 to 14.9)**	**1.5 (-5.2 to 8.9)**
**Epilepsy**	**18.2 (15.0 to 21.8)**	**6.5 (0.6 to 12.2)***	**0.9 (-4.6 to 6.4)**	**6.3 (4.6 to 8.2)**	**0.8 (-6.4 to 8.2)**	**-4.5 (-11.3 to 2.6)**
**Guillain-Barré syndrome**	**0.0 (0.0 to 0.0)**	**6.5 (5.8 to 7.1)***	**1.0 (0.3 to 1.5)***	**0.0 (0.0 to 0.0)**	**6.5 (5.8 to 7.1)***	**1.0 (0.3 to 1.5)***
**Hearing loss**	**19.9 (11.3 to 36.2)**	**6.2 (4.9 to 7.5)***	**0.7 (-0.5 to 2.0)**	**1.5 (0.7 to 2.9)**	**3.8 (0.5 to 7.1)***	**-1.6 (-4.7 to 1.6)**
**Heart failure**	**0.0 (0.0 to 0.0)**	**5.7 (4.6 to 6.9)***	**0.3 (-0.8 to 1.3)**	**0.0 (0.0 to 0.0)**	**5.7 (4.6 to 6.9)***	**0.3 (-0.8 to 1.3)**
**Infertility**	**0.0 (0.0 to 0.0)**	**0.0 (0.0 to 0.0)**	**0.0 (0.0 to 0.0)**	**0.0 (0.0 to 0.0)**	**0.0 (0.0 to 0.0)**	**0.0 (0.0 to 0.0)**
**Pelvic inflammatory disease**	**0.0 (0.0 to 0.0)**	**0.0 (0.0 to 0.0)**	**0.0 (0.0 to 0.0)**	**0.0 (0.0 to 0.0)**	**0.0 (0.0 to 0.0)**	**0.0 (0.0 to 0.0)**
**Vision loss**	**102.0 (91.8 to 113.4)**	**1.6 (0.1 to 3.2)***	**-3.7 (-5.1 to -2.1)***	**4.9 (3.4 to 6.6)**	**6.2 (-0.3 to 13.5)**	**0.7 (-5.5 to 7.7)**

SR Table 11. Prevalent cases and YLDs for 2015, percent change, and change in age-standardised rates between 2005 and 2015 by cause, global, ages 7-27 days

Cause	Prevalence in thousands (95% confidence interval)			YLDs in thousands (95% confidence interval)		
	2015	Percent change in counts between 2005 and 2015	Percent change in rates between 2005 and 2015	2015	Percent change in counts between 2005 and 2015	Percent change in rates between 2005 and 2015
**All causes**	--	--	--	**339.1 (241.2 to 459.6)**	**-0.4 (-2.3 to 1.3)**	**-5.9 (-7.7 to -4.3)***
Communicable, maternal, neonatal, and nutritional diseases	4,310.3 (4,279.6 to 4,341.9)	3.6 (2.8 to 4.3)*	-2.1 (-2.9 to -1.4)*	278.6 (196.0 to 379.7)	-1.4 (-3.5 to 0.6)	-6.8 (-8.8 to -4.9)*
HIV/AIDS and tuberculosis	0.1 (0.0 to 0.1)	-56.8 (-67.4 to -42.0)*	-59.2 (-69.2 to -45.2)*	0.0 (0.0 to 0.1)	-35.5 (-47.3 to -22.0)*	-39.0 (-50.2 to -26.3)*
Tuberculosis	0.1 (0.1 to 0.2)	-36.1 (-45.2 to -26.5)*	-39.6 (-48.2 to -30.5)*	0.0 (0.0 to 0.1)	-35.5 (-47.3 to -22.0)*	-39.0 (-50.2 to -26.3)*
HIV/AIDS	0.0 (0.0 to 0.0)	0.0 (0.0 to 0.0)	0.0 (0.0 to 0.0)	0.0 (0.0 to 0.0)	0.0 (0.0 to 0.0)	0.0 (0.0 to 0.0)
HIV/AIDS - Tuberculosis	0.0 (0.0 to 0.0)	0.0 (0.0 to 0.0)	0.0 (0.0 to 0.0)	0.0 (0.0 to 0.0)	0.0 (0.0 to 0.0)	0.0 (0.0 to 0.0)
HIV/AIDS resulting in other diseases	0.0 (0.0 to 0.0)	0.0 (0.0 to 0.0)	0.0 (0.0 to 0.0)	0.0 (0.0 to 0.0)	0.0 (0.0 to 0.0)	0.0 (0.0 to 0.0)
HIV aggregate	0.0 (0.0 to 0.0)	0.0 (0.0 to 0.0)	0.0 (0.0 to 0.0)	0.0 (0.0 to 0.0)	0.0 (0.0 to 0.0)	0.0 (0.0 to 0.0)
AIDS aggregate	0.0 (0.0 to 0.0)	0.0 (0.0 to 0.0)	0.0 (0.0 to 0.0)	0.0 (0.0 to 0.0)	0.0 (0.0 to 0.0)	0.0 (0.0 to 0.0)
Diarrhea, lower respiratory, and other common infectious diseases	728.0 (692.5 to 758.6)	2.9 (1.4 to 4.4)*	-2.7 (-4.2 to -1.3)*	54.6 (37.7 to 74.8)	2.5 (1.4 to 3.6)*	-3.1 (-4.2 to -2.0)*
Diarrheal diseases	265.9 (248.1 to 285.8)	5.2 (4.3 to 6.3)*	-0.5 (-1.5 to 0.5)	43.6 (29.5 to 60.5)	5.4 (4.1 to 6.6)*	-0.4 (-1.6 to 0.8)
Diarrhoea episodes aggregate	265.9 (248.1 to 285.8)	5.2 (4.3 to 6.3)*	-0.5 (-1.5 to 0.5)	43.6 (29.5 to 60.5)	5.4 (4.1 to 6.6)*	-0.4 (-1.6 to 0.8)
Guillain-Barré syndrome due to diarrheal diseases	0.0 (0.0 to 0.0)	6.9 (6.2 to 7.4)*	1.0 (0.3 to 1.5)*	0.0 (0.0 to 0.0)	6.9 (6.2 to 7.4)*	1.0 (0.3 to 1.5)*
Intestinal infectious diseases	0.0 (0.0 to 0.0)	0.0 (0.0 to 0.0)	0.0 (0.0 to 0.0)	0.0 (0.0 to 0.0)	0.0 (0.0 to 0.0)	0.0 (0.0 to 0.0)
Typhoid fever	0.0 (0.0 to 0.0)	0.0 (0.0 to 0.0)	0.0 (0.0 to 0.0)	0.0 (0.0 to 0.0)	0.0 (0.0 to 0.0)	0.0 (0.0 to 0.0)
Typhoid fever episodes aggregate	0.0 (0.0 to 0.0)	0.0 (0.0 to 0.0)	0.0 (0.0 to 0.0)	0.0 (0.0 to 0.0)	0.0 (0.0 to 0.0)	0.0 (0.0 to 0.0)
Complications of typhoid fever aggregate	0.0 (0.0 to 0.0)	0.0 (0.0 to 0.0)	0.0 (0.0 to 0.0)	0.0 (0.0 to 0.0)	0.0 (0.0 to 0.0)	0.0 (0.0 to 0.0)
Paratyphoid fever	0.0 (0.0 to 0.0)	0.0 (0.0 to 0.0)	0.0 (0.0 to 0.0)	0.0 (0.0 to 0.0)	0.0 (0.0 to 0.0)	0.0 (0.0 to 0.0)
Paratyphoid fever episodes aggregate	0.0 (0.0 to 0.0)	0.0 (0.0 to 0.0)	0.0 (0.0 to 0.0)	0.0 (0.0 to 0.0)	0.0 (0.0 to 0.0)	0.0 (0.0 to 0.0)
Intestinal perforation due to paratyphoid	0.0 (0.0 to 0.0)	0.0 (0.0 to 0.0)	0.0 (0.0 to 0.0)	0.0 (0.0 to 0.0)	0.0 (0.0 to 0.0)	0.0 (0.0 to 0.0)
Other intestinal infectious diseases	--	--	--	0.0 (0.0 to 0.0)	0.0 (0.0 to 0.0)	0.0 (0.0 to 0.0)
Lower respiratory infections	91.1 (80.7 to 103.1)	-14.0 (-16.8 to -11.1)*	-18.7 (-21.4 to -16.0)*	5.6 (3.7 to 8.1)	-13.9 (-16.9 to -11.0)*	-18.7 (-21.4 to -15.9)*
Lower respiratory infection episodes aggregate	91.1 (80.7 to 103.1)	-14.0 (-16.8 to -11.1)*	-18.7 (-21.4 to -16.0)*	5.6 (3.7 to 8.1)	-13.9 (-16.9 to -11.0)*	-18.7 (-21.4 to -15.9)*
Guillain-Barré syndrome due to lower respiratory infections	0.0 (0.0 to 0.0)	6.9 (6.2 to 7.4)*	1.0 (0.3 to 1.5)*	0.0 (0.0 to 0.0)	6.9 (6.2 to 7.4)*	1.0 (0.3 to 1.5)*
Upper respiratory infections	319.5 (251.8 to 411.8)	7.1 (5.1 to 8.6)*	1.2 (-0.6 to 2.6)	3.8 (2.1 to 6.6)	7.2 (4.9 to 9.0)*	1.3 (-0.9 to 3.0)
Upper respiratory infection episodes aggregate	319.5 (251.8 to 411.8)	7.1 (5.1 to 8.6)*	1.2 (-0.6 to 2.6)	3.8 (2.1 to 6.6)	7.2 (4.9 to 9.0)*	1.3 (-0.9 to 3.0)
Guillain-Barré syndrome due to upper respiratory infections	0.0 (0.0 to 0.0)	6.9 (6.2 to 7.4)*	1.0 (0.3 to 1.5)*	0.0 (0.0 to 0.0)	6.9 (6.2 to 7.4)*	1.0 (0.3 to 1.5)*
Otitis media	44.2 (34.1 to 56.1)	4.0 (1.7 to 6.1)*	-1.8 (-3.9 to 0.2)	0.8 (0.5 to 1.3)	2.8 (-0.9 to 6.1)	-2.8 (-6.3 to 0.3)
Acute otitis media	36.0 (26.0 to 47.5)	4.8 (2.2 to 7.4)*	-1.0 (-3.5 to 1.5)	0.5 (0.2 to 0.9)	4.8 (1.8 to 7.9)*	-0.9 (-3.8 to 2.0)
Chronic otitis media aggregate	8.2 (6.4 to 10.2)	0.4 (-2.8 to 3.7)	-5.1 (-8.2 to -2.0)*	0.3 (0.2 to 0.5)	-0.0 (-6.4 to 6.5)	-5.5 (-11.5 to 0.7)
Meningitis	2.4 (2.2 to 2.7)	-4.3 (-13.5 to 5.9)	-9.6 (-18.3 to 0.1)	0.5 (0.3 to 0.7)	-0.2 (-10.4 to 10.1)	-5.7 (-15.3 to 4.1)
Pneumococcal meningitis	0.7 (0.5 to 0.9)	15.4 (6.1 to 26.0)*	9.0 (0.2 to 19.1)*	0.1 (0.1 to 0.1)	14.0 (-0.3 to 30.2)	7.7 (-5.7 to 23.1)
Acute pneumococcal meningitis	0.4 (0.3 to 0.6)	16.9 (1.4 to 34.1)*	10.5 (-4.1 to 26.7)	0.1 (0.0 to 0.1)	16.5 (-7.9 to 45.4)	10.1 (-12.9 to 37.4)
Complications of pneumococcal meningitis aggregate	0.3 (0.2 to 0.3)	13.1 (10.6 to 15.9)*	6.9 (4.5 to 9.5)*	0.0 (0.0 to 0.0)	10.3 (8.7 to 11.9)*	4.3 (2.7 to 5.7)*
H influenzae type B meningitis	0.5 (0.4 to 0.7)	-26.9 (-32.9 to -19.6)*	-30.9 (-36.6 to -24.0)*	0.1 (0.0 to 0.1)	-24.3 (-34.8 to -13.2)*	-28.5 (-38.4 to -17.9)*
Acute H influenzae type B meningitis	0.4 (0.3 to 0.6)	-31.0 (-40.2 to -21.6)*	-34.8 (-43.5 to -25.9)*	0.1 (0.0 to 0.1)	-30.4 (-44.3 to -16.0)*	-34.2 (-47.4 to -20.6)*
Complications of of H influenzae type B meningitis aggregate	0.1 (0.1 to 0.2)	-12.6 (-16.3 to -8.6)*	-17.4 (-20.9 to -13.7)*	0.0 (0.0 to 0.0)	-4.6 (-7.7 to -1.6)*	-9.8 (-12.8 to -7.0)*
Meningococcal meningitis	0.4 (0.3 to 0.6)	8.6 (-4.4 to 23.3)	2.6 (-9.7 to 16.5)	0.1 (0.0 to 0.1)	9.1 (-11.4 to 33.3)	3.1 (-16.2 to 26.0)
Acute meningococcal meningitis	0.3 (0.2 to 0.5)	8.3 (-8.1 to 26.3)	2.3 (-13.2 to 19.4)	0.0 (0.0 to 0.1)	8.4 (-17.0 to 38.2)	2.5 (-21.6 to 30.6)
Complications of meningococcal meningitis aggregate	0.1 (0.1 to 0.1)	9.8 (5.4 to 14.6)*	3.8 (-0.4 to 8.3)	0.0 (0.0 to 0.0)	12.4 (9.7 to 15.0)*	6.2 (3.6 to 8.6)*
Other meningitis	1.8 (1.5 to 2.2)	2.7 (-8.4 to 13.0)	-3.0 (-13.4 to 6.8)	0.2 (0.1 to 0.4)	2.8 (-10.1 to 15.6)	-2.9 (-15.0 to 9.2)
Other meningitis episodes aggregate	1.7 (1.3 to 2.1)	1.5 (-10.1 to 12.3)	-4.1 (-15.0 to 6.1)	0.2 (0.1 to 0.3)	2.8 (-10.9 to 16.2)	-2.9 (-15.8 to 9.8)
Complications of other meningitis aggregate	0.1 (0.1 to 0.1)	6.7 (3.0 to 10.4)*	0.8 (-2.7 to 4.3)	0.0 (0.0 to 0.0)	2.6 (-0.2 to 5.0)	-3.0 (-5.7 to -0.7)*
Encephalitis	0.4 (0.4 to 0.5)	-10.9 (-15.5 to -6.7)*	-15.8 (-20.1 to -11.9)*	0.1 (0.0 to 0.1)	-11.1 (-21.7 to 1.7)	-16.0 (-26.0 to -3.9)*
Acute encephalitis	0.4 (0.3 to 0.4)	-10.1 (-15.4 to -5.5)*	-15.0 (-20.0 to -10.7)*	0.0 (0.0 to 0.1)	-10.0 (-22.3 to 4.6)	-14.9 (-26.5 to -1.1)*
Complications of encephalitis aggregate	0.1 (0.0 to 0.1)	-15.5 (-18.0 to -12.9)*	-20.2 (-22.5 to -17.7)*	0.0 (0.0 to 0.0)	-17.6 (-19.9 to -15.5)*	-22.1 (-24.3 to -20.1)*
Diphtheria	0.0 (0.0 to 0.0)	0.0 (0.0 to 0.0)	0.0 (0.0 to 0.0)	0.0 (0.0 to 0.0)	0.0 (0.0 to 0.0)	0.0 (0.0 to 0.0)
Whooping cough	0.0 (0.0 to 0.0)	0.0 (0.0 to 0.0)	0.0 (0.0 to 0.0)	0.0 (0.0 to 0.0)	0.0 (0.0 to 0.0)	0.0 (0.0 to 0.0)
Tetanus	1.4 (1.1 to 1.7)	-59.0 (-66.9 to -49.6)*	-61.3 (-68.8 to -52.3)*	0.2 (0.1 to 0.3)	-60.9 (-69.4 to -50.8)*	-63.1 (-71.1 to -53.5)*
Severe tetanus	1.2 (0.9 to 1.6)	-62.1 (-70.3 to -52.5)*	-64.1 (-71.9 to -55.1)*	0.2 (0.1 to 0.2)	-61.9 (-70.3 to -51.6)*	-64.0 (-72.0 to -54.2)*
Complications of tetanus aggregate	0.2 (0.2 to 0.2)	-1.5 (-2.0 to -1.0)*	-6.9 (-7.4 to -6.5)*	0.0 (0.0 to 0.0)	-8.9 (-16.2 to -0.4)*	-13.9 (-20.8 to -5.9)*
Measles	0.0 (0.0 to 0.0)	0.0 (0.0 to 0.0)	0.0 (0.0 to 0.0)	0.0 (0.0 to 0.0)	0.0 (0.0 to 0.0)	0.0 (0.0 to 0.0)
Varicella and herpes zoster	28.9 (20.2 to 40.1)	5.1 (4.5 to 5.9)*	-0.6 (-1.2 to 0.1)	0.2 (0.1 to 0.4)	5.2 (3.2 to 7.1)*	-0.6 (-2.5 to 1.2)
Neglected tropical diseases and malaria	187.1 (163.5 to 209.7)	20.2 (4.0 to 38.2)*	13.6 (-1.7 to 30.6)	1.7 (1.1 to 2.4)	-2.4 (-14.3 to 10.3)	-7.8 (-19.0 to 4.2)
Malaria	192.1 (141.8 to 255.2)	19.8 (7.1 to 32.9)*	13.3 (1.2 to 25.6)*	1.7 (1.1 to 2.4)	-2.4 (-14.3 to 10.3)	-7.8 (-19.0 to 4.2)
Asymptomatic malaria parasitemia(PfPR)	137.0 (88.5 to 200.3)	38.6 (16.2 to 78.5)*	31.0 (9.8 to 68.7)*	0.0 (0.0 to 0.0)	0.0 (0.0 to 0.0)	0.0 (0.0 to 0.0)
Malaria episodes aggregate	14.7 (8.1 to 23.4)	-49.8 (-59.0 to -42.8)*	-52.6 (-61.3 to -45.9)*	0.3 (0.1 to 0.6)	-49.8 (-59.0 to -42.2)*	-52.6 (-61.3 to -45.4)*
Complications of malaria aggregate	0.0 (0.0 to 0.0)	13.7 (8.1 to 21.3)*	7.4 (2.1 to 14.6)*	0.0 (0.0 to 0.0)	12.7 (6.9 to 20.6)*	6.5 (1.0 to 13.9)*
Anemia due to malaria parasitemia(PfPR) aggregate	40.4 (36.8 to 43.5)	26.0 (13.4 to 36.7)*	19.1 (7.2 to 29.2)*	1.4 (0.9 to 2.0)	23.7 (5.9 to 35.9)*	16.9 (0.1 to 28.4)*
Chagas disease	0.0 (0.0 to 0.0)	0.0 (0.0 to 0.0)	0.0 (0.0 to 0.0)	0.0 (0.0 to 0.0)	0.0 (0.0 to 0.0)	0.0 (0.0 to 0.0)
Chagas disease episodes aggregate	0.0 (0.0 to 0.0)	0.0 (0.0 to 0.0)	0.0 (0.0 to 0.0)	0.0 (0.0 to 0.0)	0.0 (0.0 to 0.0)	0.0 (0.0 to 0.0)
Complications of Chagas disease aggregate	0.0 (0.0 to 0.0)	0.0 (0.0 to 0.0)	0.0 (0.0 to 0.0)	0.0 (0.0 to 0.0)	0.0 (0.0 to 0.0)	0.0 (0.0 to 0.0)

Cause	Prevalence in thousands (95% confidence interval)			YLDs in thousands (95% confidence interval)		
	2015	Percent change in counts between 2005 and 2015	Percent change in rates between 2005 and 2015	2015	Percent change in counts between 2005 and 2015	Percent change in rates between 2005 and 2015
Heart failure due to Chagas disease aggregate	0.0 (0.0 to 0.0)	0.0 (0.0 to 0.0)	0.0 (0.0 to 0.0)	0.0 (0.0 to 0.0)	0.0 (0.0 to 0.0)	0.0 (0.0 to 0.0)
Leishmaniasis	0.0 (0.0 to 0.0)	0.0 (0.0 to 0.0)	0.0 (0.0 to 0.0)	0.0 (0.0 to 0.0)	0.0 (0.0 to 0.0)	0.0 (0.0 to 0.0)
Visceral leishmaniasis	0.0 (0.0 to 0.0)	0.0 (0.0 to 0.0)	0.0 (0.0 to 0.0)	0.0 (0.0 to 0.0)	0.0 (0.0 to 0.0)	0.0 (0.0 to 0.0)
Cutaneous and mucocutaneous leishmaniasis	0.0 (0.0 to 0.0)	0.0 (0.0 to 0.0)	0.0 (0.0 to 0.0)	0.0 (0.0 to 0.0)	0.0 (0.0 to 0.0)	0.0 (0.0 to 0.0)
African trypanosomiasis	0.0 (0.0 to 0.0)	0.0 (0.0 to 0.0)	0.0 (0.0 to 0.0)	0.0 (0.0 to 0.0)	0.0 (0.0 to 0.0)	0.0 (0.0 to 0.0)
Schistosomiasis	0.0 (0.0 to 0.0)	0.0 (0.0 to 0.0)	0.0 (0.0 to 0.0)	0.0 (0.0 to 0.0)	0.0 (0.0 to 0.0)	0.0 (0.0 to 0.0)
Schistosomiasis episodes aggregate	0.0 (0.0 to 0.0)	0.0 (0.0 to 0.0)	0.0 (0.0 to 0.0)	0.0 (0.0 to 0.0)	0.0 (0.0 to 0.0)	0.0 (0.0 to 0.0)
Complications of schistosomiasis aggregate	0.0 (0.0 to 0.0)	0.0 (0.0 to 0.0)	0.0 (0.0 to 0.0)	0.0 (0.0 to 0.0)	0.0 (0.0 to 0.0)	0.0 (0.0 to 0.0)
Anemia due to schistosomiasis aggregate	0.0 (0.0 to 0.0)	0.0 (0.0 to 0.0)	0.0 (0.0 to 0.0)	0.0 (0.0 to 0.0)	0.0 (0.0 to 0.0)	0.0 (0.0 to 0.0)
Cysticercosis	0.0 (0.0 to 0.0)	0.0 (0.0 to 0.0)	0.0 (0.0 to 0.0)	0.0 (0.0 to 0.0)	0.0 (0.0 to 0.0)	0.0 (0.0 to 0.0)
Cystic echinococcosis	0.0 (0.0 to 0.0)	0.0 (0.0 to 0.0)	0.0 (0.0 to 0.0)	0.0 (0.0 to 0.0)	0.0 (0.0 to 0.0)	0.0 (0.0 to 0.0)
Lymphatic filariasis	0.0 (0.0 to 0.0)	0.0 (0.0 to 0.0)	0.0 (0.0 to 0.0)	0.0 (0.0 to 0.0)	0.0 (0.0 to 0.0)	0.0 (0.0 to 0.0)
Prevalence of detectable microfiliaria due to lymphatic filariasis	0.0 (0.0 to 0.0)	0.0 (0.0 to 0.0)	0.0 (0.0 to 0.0)	0.0 (0.0 to 0.0)	0.0 (0.0 to 0.0)	0.0 (0.0 to 0.0)
Complications of lymphatic filariasis aggregate	0.0 (0.0 to 0.0)	0.0 (0.0 to 0.0)	0.0 (0.0 to 0.0)	0.0 (0.0 to 0.0)	0.0 (0.0 to 0.0)	0.0 (0.0 to 0.0)
Onchocerciasis	0.0 (0.0 to 0.0)	0.0 (0.0 to 0.0)	0.0 (0.0 to 0.0)	0.0 (0.0 to 0.0)	0.0 (0.0 to 0.0)	0.0 (0.0 to 0.0)
Asymptomatic onchocerciasis	0.0 (0.0 to 0.0)	0.0 (0.0 to 0.0)	0.0 (0.0 to 0.0)	0.0 (0.0 to 0.0)	0.0 (0.0 to 0.0)	0.0 (0.0 to 0.0)
Skin disease due to onchocerciasis aggregate	0.0 (0.0 to 0.0)	0.0 (0.0 to 0.0)	0.0 (0.0 to 0.0)	0.0 (0.0 to 0.0)	0.0 (0.0 to 0.0)	0.0 (0.0 to 0.0)
Vision loss due to onchocerciasis aggregate	0.0 (0.0 to 0.0)	0.0 (0.0 to 0.0)	0.0 (0.0 to 0.0)	0.0 (0.0 to 0.0)	0.0 (0.0 to 0.0)	0.0 (0.0 to 0.0)
Trachoma	0.0 (0.0 to 0.0)	0.0 (0.0 to 0.0)	0.0 (0.0 to 0.0)	0.0 (0.0 to 0.0)	0.0 (0.0 to 0.0)	0.0 (0.0 to 0.0)
Dengue	0.0 (0.0 to 0.0)	0.0 (0.0 to 0.0)	0.0 (0.0 to 0.0)	0.0 (0.0 to 0.0)	0.0 (0.0 to 0.0)	0.0 (0.0 to 0.0)
Dengue episodes aggregate	0.0 (0.0 to 0.0)	0.0 (0.0 to 0.0)	0.0 (0.0 to 0.0)	0.0 (0.0 to 0.0)	0.0 (0.0 to 0.0)	0.0 (0.0 to 0.0)
Post-dengue chronic fatigue syndrome	0.0 (0.0 to 0.0)	0.0 (0.0 to 0.0)	0.0 (0.0 to 0.0)	0.0 (0.0 to 0.0)	0.0 (0.0 to 0.0)	0.0 (0.0 to 0.0)
Yellow fever	0.0 (0.0 to 0.0)	-33.0 (-46.7 to -14.2)*	-36.7 (-49.6 to -18.9)*	0.0 (0.0 to 0.0)	-33.0 (-46.7 to -14.2)*	-36.7 (-49.6 to -18.9)*
Rabies	0.0 (0.0 to 0.0)	0.0 (0.0 to 0.0)	0.0 (0.0 to 0.0)	0.0 (0.0 to 0.0)	0.0 (0.0 to 0.0)	0.0 (0.0 to 0.0)
Intestinal nematode infections	0.0 (0.0 to 0.0)	0.0 (0.0 to 0.0)	0.0 (0.0 to 0.0)	0.0 (0.0 to 0.0)	0.0 (0.0 to 0.0)	0.0 (0.0 to 0.0)
Ascariasis	0.0 (0.0 to 0.0)	0.0 (0.0 to 0.0)	0.0 (0.0 to 0.0)	0.0 (0.0 to 0.0)	0.0 (0.0 to 0.0)	0.0 (0.0 to 0.0)
Complications of ascariasis aggregate	0.0 (0.0 to 0.0)	0.0 (0.0 to 0.0)	0.0 (0.0 to 0.0)	0.0 (0.0 to 0.0)	0.0 (0.0 to 0.0)	0.0 (0.0 to 0.0)
Asymptomatic ascariasis	0.0 (0.0 to 0.0)	0.0 (0.0 to 0.0)	0.0 (0.0 to 0.0)	0.0 (0.0 to 0.0)	0.0 (0.0 to 0.0)	0.0 (0.0 to 0.0)
Trichuriasis	0.0 (0.0 to 0.0)	0.0 (0.0 to 0.0)	0.0 (0.0 to 0.0)	0.0 (0.0 to 0.0)	0.0 (0.0 to 0.0)	0.0 (0.0 to 0.0)
Complications of trichuriasis aggregate	0.0 (0.0 to 0.0)	0.0 (0.0 to 0.0)	0.0 (0.0 to 0.0)	0.0 (0.0 to 0.0)	0.0 (0.0 to 0.0)	0.0 (0.0 to 0.0)
Asymptomatic trichuriasis	0.0 (0.0 to 0.0)	0.0 (0.0 to 0.0)	0.0 (0.0 to 0.0)	0.0 (0.0 to 0.0)	0.0 (0.0 to 0.0)	0.0 (0.0 to 0.0)
Hookworm disease	0.0 (0.0 to 0.0)	0.0 (0.0 to 0.0)	0.0 (0.0 to 0.0)	0.0 (0.0 to 0.0)	0.0 (0.0 to 0.0)	0.0 (0.0 to 0.0)
Complications of hookworm disease aggregate	0.0 (0.0 to 0.0)	0.0 (0.0 to 0.0)	0.0 (0.0 to 0.0)	0.0 (0.0 to 0.0)	0.0 (0.0 to 0.0)	0.0 (0.0 to 0.0)
Anemia due to hookworm disease aggregate	0.0 (0.0 to 0.0)	0.0 (0.0 to 0.0)	0.0 (0.0 to 0.0)	0.0 (0.0 to 0.0)	0.0 (0.0 to 0.0)	0.0 (0.0 to 0.0)
Asymptomatic hookworm disease	0.0 (0.0 to 0.0)	0.0 (0.0 to 0.0)	0.0 (0.0 to 0.0)	0.0 (0.0 to 0.0)	0.0 (0.0 to 0.0)	0.0 (0.0 to 0.0)
Food-borne trematodiases	0.0 (0.0 to 0.0)	0.0 (0.0 to 0.0)	0.0 (0.0 to 0.0)	0.0 (0.0 to 0.0)	0.0 (0.0 to 0.0)	0.0 (0.0 to 0.0)
Asymptomatic food-borne trematodiases aggregate	0.0 (0.0 to 0.0)	0.0 (0.0 to 0.0)	0.0 (0.0 to 0.0)	0.0 (0.0 to 0.0)	0.0 (0.0 to 0.0)	0.0 (0.0 to 0.0)
Symptomatic food-borne trematodiases aggregate	0.0 (0.0 to 0.0)	0.0 (0.0 to 0.0)	0.0 (0.0 to 0.0)	0.0 (0.0 to 0.0)	0.0 (0.0 to 0.0)	0.0 (0.0 to 0.0)
Leprosy	0.0 (0.0 to 0.0)	0.0 (0.0 to 0.0)	0.0 (0.0 to 0.0)	0.0 (0.0 to 0.0)	0.0 (0.0 to 0.0)	0.0 (0.0 to 0.0)
Ebola	0.0 (0.0 to 0.0)	0.0 (0.0 to 0.0)	0.0 (0.0 to 0.0)	0.0 (0.0 to 0.0)	0.0 (0.0 to 0.0)	0.0 (0.0 to 0.0)
Other neglected tropical diseases	0.0 (0.0 to 0.0)	0.0 (0.0 to 0.0)	0.0 (0.0 to 0.0)	0.0 (0.0 to 0.0)	-33.1 (-66.5 to 29.3)	-36.8 (-68.3 to 22.2)
Anemia due to other neglected tropical diseases aggregate	0.0 (0.0 to 0.0)	0.0 (0.0 to 0.0)	0.0 (0.0 to 0.0)	0.0 (0.0 to 0.0)	0.0 (0.0 to 0.0)	0.0 (0.0 to 0.0)
Maternal disorders	0.0 (0.0 to 0.0)	0.0 (0.0 to 0.0)	0.0 (0.0 to 0.0)	0.0 (0.0 to 0.0)	0.0 (0.0 to 0.0)	0.0 (0.0 to 0.0)
Maternal hemorrhage	0.0 (0.0 to 0.0)	0.0 (0.0 to 0.0)	0.0 (0.0 to 0.0)	0.0 (0.0 to 0.0)	0.0 (0.0 to 0.0)	0.0 (0.0 to 0.0)
Maternal hemorrhage episodes aggregate	0.0 (0.0 to 0.0)	0.0 (0.0 to 0.0)	0.0 (0.0 to 0.0)	0.0 (0.0 to 0.0)	0.0 (0.0 to 0.0)	0.0 (0.0 to 0.0)
Anemia due to maternal hemorrhage aggregate	0.0 (0.0 to 0.0)	0.0 (0.0 to 0.0)	0.0 (0.0 to 0.0)	0.0 (0.0 to 0.0)	0.0 (0.0 to 0.0)	0.0 (0.0 to 0.0)
Maternal sepsis and other maternal infections	0.0 (0.0 to 0.0)	0.0 (0.0 to 0.0)	0.0 (0.0 to 0.0)	0.0 (0.0 to 0.0)	0.0 (0.0 to 0.0)	0.0 (0.0 to 0.0)
Maternal sepsis and other maternal infections aggregate	0.0 (0.0 to 0.0)	0.0 (0.0 to 0.0)	0.0 (0.0 to 0.0)	0.0 (0.0 to 0.0)	0.0 (0.0 to 0.0)	0.0 (0.0 to 0.0)
Infertility due to puerperal sepsis	0.0 (0.0 to 0.0)	0.0 (0.0 to 0.0)	0.0 (0.0 to 0.0)	0.0 (0.0 to 0.0)	0.0 (0.0 to 0.0)	0.0 (0.0 to 0.0)
Maternal hypertensive disorders	0.0 (0.0 to 0.0)	0.0 (0.0 to 0.0)	0.0 (0.0 to 0.0)	0.0 (0.0 to 0.0)	0.0 (0.0 to 0.0)	0.0 (0.0 to 0.0)
Maternal hypertensive disorders episodes aggregate	0.0 (0.0 to 0.0)	0.0 (0.0 to 0.0)	0.0 (0.0 to 0.0)	0.0 (0.0 to 0.0)	0.0 (0.0 to 0.0)	0.0 (0.0 to 0.0)
Eclampsia	0.0 (0.0 to 0.0)	0.0 (0.0 to 0.0)	0.0 (0.0 to 0.0)	0.0 (0.0 to 0.0)	0.0 (0.0 to 0.0)	0.0 (0.0 to 0.0)
Complications of maternal hypertensive disorders aggregate	0.0 (0.0 to 0.0)	0.0 (0.0 to 0.0)	0.0 (0.0 to 0.0)	0.0 (0.0 to 0.0)	0.0 (0.0 to 0.0)	0.0 (0.0 to 0.0)
Maternal obstructed labor and uterine rupture	0.0 (0.0 to 0.0)	0.0 (0.0 to 0.0)	0.0 (0.0 to 0.0)	0.0 (0.0 to 0.0)	0.0 (0.0 to 0.0)	0.0 (0.0 to 0.0)
Obstructed labor, acute event	0.0 (0.0 to 0.0)	0.0 (0.0 to 0.0)	0.0 (0.0 to 0.0)	0.0 (0.0 to 0.0)	0.0 (0.0 to 0.0)	0.0 (0.0 to 0.0)
Fistula due to maternal obstructed labor and uterine rupture aggregate	0.0 (0.0 to 0.0)	0.0 (0.0 to 0.0)	0.0 (0.0 to 0.0)	0.0 (0.0 to 0.0)	0.0 (0.0 to 0.0)	0.0 (0.0 to 0.0)
Maternal abortion, miscarriage, and ectopic pregnancy	0.0 (0.0 to 0.0)	0.0 (0.0 to 0.0)	0.0 (0.0 to 0.0)	0.0 (0.0 to 0.0)	0.0 (0.0 to 0.0)	0.0 (0.0 to 0.0)
Other maternal disorders	--	--	--	0.0 (0.0 to 0.0)	0.0 (0.0 to 0.0)	0.0 (0.0 to 0.0)
Neonatal disorders	494.3 (467.4 to 514.3)	4.7 (2.3 to 6.9)*	-1.1 (-3.3 to 1.0)	28.3 (20.3 to 38.1)	-0.1 (-4.8 to 4.7)	-5.6 (-10.1 to -1.0)*
Neonatal preterm birth complications	419.0 (353.9 to 489.8)	3.2 (1.7 to 4.7)*	-2.4 (-3.9 to -1.1)*	13.3 (9.5 to 17.9)	-7.2 (-12.8 to -0.7)*	-12.3 (-17.6 to -6.1)*
Vision loss due to retinopathy of prematurity aggregate	4.3 (3.3 to 5.4)	3.5 (-0.6 to 7.7)	-2.2 (-6.0 to 1.8)	0.1 (0.1 to 0.2)	3.9 (-6.6 to 15.4)	-1.8 (-11.8 to 9.1)
Complications of preterm birth complications aggregate	414.7 (349.6 to 485.8)	3.2 (1.7 to 4.7)*	-2.4 (-3.9 to -1.1)*	13.2 (9.4 to 17.7)	-7.2 (-12.9 to -0.7)*	-12.3 (-17.7 to -6.2)*
Neonatal encephalopathy due to birth asphyxia and trauma	78.2 (59.0 to 104.0)	12.2 (7.4 to 17.4)*	6.1 (1.5 to 11.0)*	10.2 (6.6 to 15.2)	8.6 (5.8 to 11.7)*	2.6 (-0.0 to 5.5)

Cause	Prevalence in thousands (95% confidence interval)			YLDs in thousands (95% confidence interval)		
	**2015**	**Percent change in counts between 2005 and 2015**	**Percent change in rates between 2005 and 2015**	**2015**	**Percent change in counts between 2005 and 2015**	**Percent change in rates between 2005 and 2015**
Neonatal sepsis and other neonatal infections	0.0 (0.0 to 0.0)	0.0 (0.0 to 0.0)	0.0 (0.0 to 0.0)	0.0 (0.0 to 0.0)	0.0 (0.0 to 0.0)	0.0 (0.0 to 0.0)
Severe infection due to neonatal sepsis and other neonatal infections	0.0 (0.0 to 0.0)	0.0 (0.0 to 0.0)	0.0 (0.0 to 0.0)	0.0 (0.0 to 0.0)	0.0 (0.0 to 0.0)	0.0 (0.0 to 0.0)
Complications of neonatal sepsis and other neonatal infections aggregate	0.0 (0.0 to 0.0)	0.0 (0.0 to 0.0)	0.0 (0.0 to 0.0)	0.0 (0.0 to 0.0)	0.0 (0.0 to 0.0)	0.0 (0.0 to 0.0)
Hemolytic disease and other neonatal jaundice	7.2 (6.1 to 8.5)	4.5 (2.3 to 6.7)*	-1.3 (-3.3 to 0.8)	1.7 (1.2 to 2.3)	3.8 (-0.8 to 8.9)	-1.9 (-6.3 to 2.9)
Other neonatal disorders	--	--	--	3.1 (1.9 to 4.8)	4.1 (-23.0 to 39.6)	-1.6 (-27.2 to 31.9)
Nutritional deficiencies	3,352.1 (3,322.5 to 3,382.6)	2.3 (1.2 to 3.5)*	-3.3 (-4.4 to -2.2)*	185.8 (126.6 to 260.0)	-2.9 (-5.9 to -0.2)*	-8.2 (-11.1 to -5.7)*
Protein-energy malnutrition	387.1 (364.6 to 411.7)	-7.0 (-12.4 to -1.4)*	-12.1 (-17.2 to -6.8)*	48.3 (31.0 to 68.0)	-6.9 (-12.3 to -1.3)*	-12.0 (-17.2 to -6.8)*
Iodine deficiency	0.0 (0.0 to 0.0)	0.0 (0.0 to 0.0)	0.0 (0.0 to 0.0)	0.0 (0.0 to 0.0)	0.0 (0.0 to 0.0)	0.0 (0.0 to 0.0)
Visible goiter due to iodine deficiency aggregate	0.0 (0.0 to 0.0)	0.0 (0.0 to 0.0)	0.0 (0.0 to 0.0)	0.0 (0.0 to 0.0)	0.0 (0.0 to 0.0)	0.0 (0.0 to 0.0)
Visible goiter with heart failure due to iodine deficiency aggregate	0.0 (0.0 to 0.0)	0.0 (0.0 to 0.0)	0.0 (0.0 to 0.0)	0.0 (0.0 to 0.0)	0.0 (0.0 to 0.0)	0.0 (0.0 to 0.0)
Intellectual disability due to iodine deficiency aggregate	0.0 (0.0 to 0.0)	0.0 (0.0 to 0.0)	0.0 (0.0 to 0.0)	0.0 (0.0 to 0.0)	0.0 (0.0 to 0.0)	0.0 (0.0 to 0.0)
Vitamin A deficiency	0.0 (0.0 to 0.0)	0.0 (0.0 to 0.0)	0.0 (0.0 to 0.0)	0.0 (0.0 to 0.0)	0.0 (0.0 to 0.0)	0.0 (0.0 to 0.0)
Iron-deficiency anemia	3,620.9 (3,581.8 to 3,664.7)	2.2 (1.0 to 3.5)*	-3.4 (-4.5 to -2.2)*	126.4 (84.7 to 183.0)	0.6 (-1.2 to 2.3)	-5.0 (-6.6 to -3.3)*
Iron-deficiency anemia without heart failure aggregate	3,620.9 (3,581.8 to 3,664.7)	2.2 (1.0 to 3.5)*	-3.4 (-4.5 to -2.2)*	126.4 (84.7 to 183.0)	0.6 (-1.2 to 2.3)	-5.0 (-6.6 to -3.3)*
Iron-deficiency anemia with heart failure aggregate	0.0 (0.0 to 0.0)	10.6 (7.9 to 13.1)*	4.5 (2.0 to 6.9)*	0.0 (0.0 to 0.0)	10.6 (7.9 to 13.1)*	4.5 (2.0 to 6.9)*
Other nutritional deficiencies	--	--	--	11.1 (6.6 to 17.3)	-19.3 (-38.8 to 2.4)	-23.8 (-42.2 to -3.3)*
Other communicable, maternal, neonatal, and nutritional diseases	467.9 (459.4 to 476.8)	8.1 (5.7 to 10.7)*	2.2 (-0.1 to 4.6)	8.2 (5.5 to 11.8)	5.6 (0.9 to 10.4)*	-0.2 (-4.6 to 4.4)
Sexually transmitted diseases excluding HIV	0.0 (0.0 to 0.0)	0.0 (0.0 to 0.0)	0.0 (0.0 to 0.0)	0.0 (0.0 to 0.0)	0.0 (0.0 to 0.0)	0.0 (0.0 to 0.0)
Syphilis	0.0 (0.0 to 0.0)	0.0 (0.0 to 0.0)	0.0 (0.0 to 0.0)	0.0 (0.0 to 0.0)	0.0 (0.0 to 0.0)	0.0 (0.0 to 0.0)
Early syphilis aggregate	0.0 (0.0 to 0.0)	0.0 (0.0 to 0.0)	0.0 (0.0 to 0.0)	0.0 (0.0 to 0.0)	0.0 (0.0 to 0.0)	0.0 (0.0 to 0.0)
Adult tertiary syphilis	0.0 (0.0 to 0.0)	0.0 (0.0 to 0.0)	0.0 (0.0 to 0.0)	0.0 (0.0 to 0.0)	0.0 (0.0 to 0.0)	0.0 (0.0 to 0.0)
Chlamydial infection	0.0 (0.0 to 0.0)	0.0 (0.0 to 0.0)	0.0 (0.0 to 0.0)	0.0 (0.0 to 0.0)	0.0 (0.0 to 0.0)	0.0 (0.0 to 0.0)
Chlamydial infection episodes aggregate	0.0 (0.0 to 0.0)	0.0 (0.0 to 0.0)	0.0 (0.0 to 0.0)	0.0 (0.0 to 0.0)	0.0 (0.0 to 0.0)	0.0 (0.0 to 0.0)
Pelvic inflammatory diseases due to chlamydial infection aggregate	0.0 (0.0 to 0.0)	0.0 (0.0 to 0.0)	0.0 (0.0 to 0.0)	0.0 (0.0 to 0.0)	0.0 (0.0 to 0.0)	0.0 (0.0 to 0.0)
Infertility due to clamydial infection aggregate	0.0 (0.0 to 0.0)	0.0 (0.0 to 0.0)	0.0 (0.0 to 0.0)	0.0 (0.0 to 0.0)	0.0 (0.0 to 0.0)	0.0 (0.0 to 0.0)
Gonococcal infection	0.0 (0.0 to 0.0)	0.0 (0.0 to 0.0)	0.0 (0.0 to 0.0)	0.0 (0.0 to 0.0)	0.0 (0.0 to 0.0)	0.0 (0.0 to 0.0)
Gonococcal infection episodes aggregate	0.0 (0.0 to 0.0)	0.0 (0.0 to 0.0)	0.0 (0.0 to 0.0)	0.0 (0.0 to 0.0)	0.0 (0.0 to 0.0)	0.0 (0.0 to 0.0)
Pelvic inflammatory diseases due to gonococcal infection aggregate	0.0 (0.0 to 0.0)	0.0 (0.0 to 0.0)	0.0 (0.0 to 0.0)	0.0 (0.0 to 0.0)	0.0 (0.0 to 0.0)	0.0 (0.0 to 0.0)
Trichomoniasis	0.0 (0.0 to 0.0)	0.0 (0.0 to 0.0)	0.0 (0.0 to 0.0)	0.0 (0.0 to 0.0)	0.0 (0.0 to 0.0)	0.0 (0.0 to 0.0)
Genital herpes	0.0 (0.0 to 0.0)	0.0 (0.0 to 0.0)	0.0 (0.0 to 0.0)	0.0 (0.0 to 0.0)	0.0 (0.0 to 0.0)	0.0 (0.0 to 0.0)
Moderate infection due to initial genital herpes episode	0.0 (0.0 to 0.0)	0.0 (0.0 to 0.0)	0.0 (0.0 to 0.0)	0.0 (0.0 to 0.0)	0.0 (0.0 to 0.0)	0.0 (0.0 to 0.0)
Complications of genital herpes aggregate	0.0 (0.0 to 0.0)	0.0 (0.0 to 0.0)	0.0 (0.0 to 0.0)	0.0 (0.0 to 0.0)	0.0 (0.0 to 0.0)	0.0 (0.0 to 0.0)
Other sexually transmitted diseases	0.0 (0.0 to 0.0)	0.0 (0.0 to 0.0)	0.0 (0.0 to 0.0)	0.0 (0.0 to 0.0)	0.0 (0.0 to 0.0)	0.0 (0.0 to 0.0)
Pelvic inflammatory diseases due to other sexually transmitted diseases aggregate	0.0 (0.0 to 0.0)	0.0 (0.0 to 0.0)	0.0 (0.0 to 0.0)	0.0 (0.0 to 0.0)	0.0 (0.0 to 0.0)	0.0 (0.0 to 0.0)
Infertility due to other sexually transmitted diseases aggregate	0.0 (0.0 to 0.0)	0.0 (0.0 to 0.0)	0.0 (0.0 to 0.0)	0.0 (0.0 to 0.0)	0.0 (0.0 to 0.0)	0.0 (0.0 to 0.0)
Other sexually transmitted diseases	--	--	--	0.0 (0.0 to 0.0)	0.0 (0.0 to 0.0)	0.0 (0.0 to 0.0)
Hepatitis	249.5 (239.3 to 259.2)	14.2 (10.7 to 18.1)*	7.9 (4.7 to 11.6)*	0.0 (0.0 to 0.0)	7.0 (-0.8 to 15.2)	1.1 (-6.2 to 8.8)
Acute hepatitis A	0.0 (0.0 to 0.0)	0.0 (0.0 to 0.0)	0.0 (0.0 to 0.0)	0.0 (0.0 to 0.0)	0.0 (0.0 to 0.0)	0.0 (0.0 to 0.0)
Hepatitis B	251.1 (223.8 to 283.5)	14.3 (8.9 to 19.3)*	8.0 (2.9 to 12.8)*	0.0 (0.0 to 0.0)	7.0 (-0.8 to 15.2)	1.1 (-6.2 to 8.8)
Acute Hepatitis B aggregate	1.6 (1.4 to 1.7)	6.9 (0.5 to 13.2)*	1.0 (-5.0 to 7.0)	0.0 (0.0 to 0.0)	7.0 (-0.8 to 15.2)	1.1 (-6.2 to 8.8)
Chronic hepatitis B	249.5 (222.2 to 281.9)	14.3 (8.9 to 19.4)*	8.0 (2.9 to 12.9)*	0.0 (0.0 to 0.0)	0.0 (0.0 to 0.0)	0.0 (0.0 to 0.0)
Hepatitis C	0.0 (0.0 to 0.0)	0.0 (0.0 to 0.0)	0.0 (0.0 to 0.0)	0.0 (0.0 to 0.0)	0.0 (0.0 to 0.0)	0.0 (0.0 to 0.0)
Acute Hepatitis C aggregate	0.0 (0.0 to 0.0)	0.0 (0.0 to 0.0)	0.0 (0.0 to 0.0)	0.0 (0.0 to 0.0)	0.0 (0.0 to 0.0)	0.0 (0.0 to 0.0)
Chronic hepatitis C	0.0 (0.0 to 0.0)	0.0 (0.0 to 0.0)	0.0 (0.0 to 0.0)	0.0 (0.0 to 0.0)	0.0 (0.0 to 0.0)	0.0 (0.0 to 0.0)
Acute hepatitis E	0.0 (0.0 to 0.0)	0.0 (0.0 to 0.0)	0.0 (0.0 to 0.0)	0.0 (0.0 to 0.0)	0.0 (0.0 to 0.0)	0.0 (0.0 to 0.0)
Other infectious diseases	228.1 (223.3 to 232.7)	2.2 (-1.3 to 5.6)	-3.4 (-6.7 to -0.2)*	8.1 (5.5 to 11.8)	5.6 (0.9 to 10.4)*	-0.2 (-4.6 to 4.4)
Non-communicable diseases	2,591.6 (2,553.2 to 2,627.5)	3.5 (2.5 to 4.5)*	-2.2 (-3.1 to -1.2)*	59.2 (42.6 to 79.5)	4.7 (1.8 to 8.0)*	-1.1 (-3.8 to 2.0)
Neoplasms	0.0 (0.0 to 0.0)	0.0 (0.0 to 0.0)	0.0 (0.0 to 0.0)	0.0 (0.0 to 0.0)	0.0 (0.0 to 0.0)	0.0 (0.0 to 0.0)
Lip and oral cavity cancer	0.0 (0.0 to 0.0)	0.0 (0.0 to 0.0)	0.0 (0.0 to 0.0)	0.0 (0.0 to 0.0)	0.0 (0.0 to 0.0)	0.0 (0.0 to 0.0)
Nasopharynx cancer	0.0 (0.0 to 0.0)	0.0 (0.0 to 0.0)	0.0 (0.0 to 0.0)	0.0 (0.0 to 0.0)	0.0 (0.0 to 0.0)	0.0 (0.0 to 0.0)
Other pharynx cancer	0.0 (0.0 to 0.0)	0.0 (0.0 to 0.0)	0.0 (0.0 to 0.0)	0.0 (0.0 to 0.0)	0.0 (0.0 to 0.0)	0.0 (0.0 to 0.0)
Esophageal cancer	0.0 (0.0 to 0.0)	0.0 (0.0 to 0.0)	0.0 (0.0 to 0.0)	0.0 (0.0 to 0.0)	0.0 (0.0 to 0.0)	0.0 (0.0 to 0.0)
Stomach cancer	0.0 (0.0 to 0.0)	0.0 (0.0 to 0.0)	0.0 (0.0 to 0.0)	0.0 (0.0 to 0.0)	0.0 (0.0 to 0.0)	0.0 (0.0 to 0.0)
Colon and rectum cancer	0.0 (0.0 to 0.0)	0.0 (0.0 to 0.0)	0.0 (0.0 to 0.0)	0.0 (0.0 to 0.0)	0.0 (0.0 to 0.0)	0.0 (0.0 to 0.0)
Colon and rectum cancer aggregate	0.0 (0.0 to 0.0)	0.0 (0.0 to 0.0)	0.0 (0.0 to 0.0)	0.0 (0.0 to 0.0)	0.0 (0.0 to 0.0)	0.0 (0.0 to 0.0)
Stoma due to colon and rectum cancer	0.0 (0.0 to 0.0)	0.0 (0.0 to 0.0)	0.0 (0.0 to 0.0)	0.0 (0.0 to 0.0)	0.0 (0.0 to 0.0)	0.0 (0.0 to 0.0)
Liver cancer	0.0 (0.0 to 0.0)	0.0 (0.0 to 0.0)	0.0 (0.0 to 0.0)	0.0 (0.0 to 0.0)	0.0 (0.0 to 0.0)	0.0 (0.0 to 0.0)
Liver cancer due to hepatitis B	0.0 (0.0 to 0.0)	0.0 (0.0 to 0.0)	0.0 (0.0 to 0.0)	0.0 (0.0 to 0.0)	0.0 (0.0 to 0.0)	0.0 (0.0 to 0.0)
Liver cancer due to hepatitis C	0.0 (0.0 to 0.0)	0.0 (0.0 to 0.0)	0.0 (0.0 to 0.0)	0.0 (0.0 to 0.0)	0.0 (0.0 to 0.0)	0.0 (0.0 to 0.0)
Liver cancer due to alcohol use	0.0 (0.0 to 0.0)	0.0 (0.0 to 0.0)	0.0 (0.0 to 0.0)	0.0 (0.0 to 0.0)	0.0 (0.0 to 0.0)	0.0 (0.0 to 0.0)
Liver cancer due to other causes	0.0 (0.0 to 0.0)	0.0 (0.0 to 0.0)	0.0 (0.0 to 0.0)	0.0 (0.0 to 0.0)	0.0 (0.0 to 0.0)	0.0 (0.0 to 0.0)
Gallbladder and biliary tract cancer	0.0 (0.0 to 0.0)	0.0 (0.0 to 0.0)	0.0 (0.0 to 0.0)	0.0 (0.0 to 0.0)	0.0 (0.0 to 0.0)	0.0 (0.0 to 0.0)
Pancreatic cancer	0.0 (0.0 to 0.0)	0.0 (0.0 to 0.0)	0.0 (0.0 to 0.0)	0.0 (0.0 to 0.0)	0.0 (0.0 to 0.0)	0.0 (0.0 to 0.0)

Cause	Prevalence in thousands (95% confidence interval)			YLDs in thousands (95% confidence interval)		
	2015	Percent change in counts between 2005 and 2015	Percent change in rates between 2005 and 2015	2015	Percent change in counts between 2005 and 2015	Percent change in rates between 2005 and 2015
Larynx cancer	0.0 (0.0 to 0.0)	0.0 (0.0 to 0.0)	0.0 (0.0 to 0.0)	0.0 (0.0 to 0.0)	0.0 (0.0 to 0.0)	0.0 (0.0 to 0.0)
Larynx cancer aggregate	0.0 (0.0 to 0.0)	0.0 (0.0 to 0.0)	0.0 (0.0 to 0.0)	0.0 (0.0 to 0.0)	0.0 (0.0 to 0.0)	0.0 (0.0 to 0.0)
Laryngectomy due to larynx cancer	0.0 (0.0 to 0.0)	0.0 (0.0 to 0.0)	0.0 (0.0 to 0.0)	0.0 (0.0 to 0.0)	0.0 (0.0 to 0.0)	0.0 (0.0 to 0.0)
Tracheal, bronchus, and lung cancer	0.0 (0.0 to 0.0)	0.0 (0.0 to 0.0)	0.0 (0.0 to 0.0)	0.0 (0.0 to 0.0)	0.0 (0.0 to 0.0)	0.0 (0.0 to 0.0)
Malignant skin melanoma	0.0 (0.0 to 0.0)	0.0 (0.0 to 0.0)	0.0 (0.0 to 0.0)	0.0 (0.0 to 0.0)	0.0 (0.0 to 0.0)	0.0 (0.0 to 0.0)
Non-melanoma skin cancer	0.0 (0.0 to 0.0)	0.0 (0.0 to 0.0)	0.0 (0.0 to 0.0)	0.0 (0.0 to 0.0)	0.0 (0.0 to 0.0)	0.0 (0.0 to 0.0)
Non-melanoma skin cancer(squamous-cell carcinoma)	0.0 (0.0 to 0.0)	0.0 (0.0 to 0.0)	0.0 (0.0 to 0.0)	0.0 (0.0 to 0.0)	0.0 (0.0 to 0.0)	0.0 (0.0 to 0.0)
Non-melanoma skin cancer(basal-cell carcinoma)	0.0 (0.0 to 0.0)	0.0 (0.0 to 0.0)	0.0 (0.0 to 0.0)	0.0 (0.0 to 0.0)	0.0 (0.0 to 0.0)	0.0 (0.0 to 0.0)
Breast cancer	0.0 (0.0 to 0.0)	0.0 (0.0 to 0.0)	0.0 (0.0 to 0.0)	0.0 (0.0 to 0.0)	0.0 (0.0 to 0.0)	0.0 (0.0 to 0.0)
Breast cancer aggregate	0.0 (0.0 to 0.0)	0.0 (0.0 to 0.0)	0.0 (0.0 to 0.0)	0.0 (0.0 to 0.0)	0.0 (0.0 to 0.0)	0.0 (0.0 to 0.0)
Mastectomy due to breast cancer	0.0 (0.0 to 0.0)	0.0 (0.0 to 0.0)	0.0 (0.0 to 0.0)	0.0 (0.0 to 0.0)	0.0 (0.0 to 0.0)	0.0 (0.0 to 0.0)
Cervical cancer	0.0 (0.0 to 0.0)	0.0 (0.0 to 0.0)	0.0 (0.0 to 0.0)	0.0 (0.0 to 0.0)	0.0 (0.0 to 0.0)	0.0 (0.0 to 0.0)
Uterine cancer	0.0 (0.0 to 0.0)	0.0 (0.0 to 0.0)	0.0 (0.0 to 0.0)	0.0 (0.0 to 0.0)	0.0 (0.0 to 0.0)	0.0 (0.0 to 0.0)
Ovarian cancer	0.0 (0.0 to 0.0)	0.0 (0.0 to 0.0)	0.0 (0.0 to 0.0)	0.0 (0.0 to 0.0)	0.0 (0.0 to 0.0)	0.0 (0.0 to 0.0)
Prostate cancer	0.0 (0.0 to 0.0)	0.0 (0.0 to 0.0)	0.0 (0.0 to 0.0)	0.0 (0.0 to 0.0)	0.0 (0.0 to 0.0)	0.0 (0.0 to 0.0)
Prostate cancer aggregate	0.0 (0.0 to 0.0)	0.0 (0.0 to 0.0)	0.0 (0.0 to 0.0)	0.0 (0.0 to 0.0)	0.0 (0.0 to 0.0)	0.0 (0.0 to 0.0)
Impotence and incontinence due to prostate cancer aggregate	0.0 (0.0 to 0.0)	0.0 (0.0 to 0.0)	0.0 (0.0 to 0.0)	0.0 (0.0 to 0.0)	0.0 (0.0 to 0.0)	0.0 (0.0 to 0.0)
Testicular cancer	0.0 (0.0 to 0.0)	0.0 (0.0 to 0.0)	0.0 (0.0 to 0.0)	0.0 (0.0 to 0.0)	0.0 (0.0 to 0.0)	0.0 (0.0 to 0.0)
Kidney cancer	0.0 (0.0 to 0.0)	0.0 (0.0 to 0.0)	0.0 (0.0 to 0.0)	0.0 (0.0 to 0.0)	0.0 (0.0 to 0.0)	0.0 (0.0 to 0.0)
Bladder cancer	0.0 (0.0 to 0.0)	0.0 (0.0 to 0.0)	0.0 (0.0 to 0.0)	0.0 (0.0 to 0.0)	0.0 (0.0 to 0.0)	0.0 (0.0 to 0.0)
Bladder cancer episodes aggregate	0.0 (0.0 to 0.0)	0.0 (0.0 to 0.0)	0.0 (0.0 to 0.0)	0.0 (0.0 to 0.0)	0.0 (0.0 to 0.0)	0.0 (0.0 to 0.0)
Urinary incontinence due to bladder cancer	0.0 (0.0 to 0.0)	0.0 (0.0 to 0.0)	0.0 (0.0 to 0.0)	0.0 (0.0 to 0.0)	0.0 (0.0 to 0.0)	0.0 (0.0 to 0.0)
Brain and nervous system cancer	0.0 (0.0 to 0.0)	0.0 (0.0 to 0.0)	0.0 (0.0 to 0.0)	0.0 (0.0 to 0.0)	0.0 (0.0 to 0.0)	0.0 (0.0 to 0.0)
Thyroid cancer	0.0 (0.0 to 0.0)	0.0 (0.0 to 0.0)	0.0 (0.0 to 0.0)	0.0 (0.0 to 0.0)	0.0 (0.0 to 0.0)	0.0 (0.0 to 0.0)
Mesothelioma	0.0 (0.0 to 0.0)	0.0 (0.0 to 0.0)	0.0 (0.0 to 0.0)	0.0 (0.0 to 0.0)	0.0 (0.0 to 0.0)	0.0 (0.0 to 0.0)
Hodgkin lymphoma	0.0 (0.0 to 0.0)	0.0 (0.0 to 0.0)	0.0 (0.0 to 0.0)	0.0 (0.0 to 0.0)	0.0 (0.0 to 0.0)	0.0 (0.0 to 0.0)
Non-Hodgkin lymphoma	0.0 (0.0 to 0.0)	0.0 (0.0 to 0.0)	0.0 (0.0 to 0.0)	0.0 (0.0 to 0.0)	0.0 (0.0 to 0.0)	0.0 (0.0 to 0.0)
Multiple myeloma	0.0 (0.0 to 0.0)	0.0 (0.0 to 0.0)	0.0 (0.0 to 0.0)	0.0 (0.0 to 0.0)	0.0 (0.0 to 0.0)	0.0 (0.0 to 0.0)
Leukemia	0.0 (0.0 to 0.0)	0.0 (0.0 to 0.0)	0.0 (0.0 to 0.0)	0.0 (0.0 to 0.0)	0.0 (0.0 to 0.0)	0.0 (0.0 to 0.0)
Acute lymphoid leukemia	0.0 (0.0 to 0.0)	0.0 (0.0 to 0.0)	0.0 (0.0 to 0.0)	0.0 (0.0 to 0.0)	0.0 (0.0 to 0.0)	0.0 (0.0 to 0.0)
Chronic lymphoid leukemia	0.0 (0.0 to 0.0)	0.0 (0.0 to 0.0)	0.0 (0.0 to 0.0)	0.0 (0.0 to 0.0)	0.0 (0.0 to 0.0)	0.0 (0.0 to 0.0)
Acute myeloid leukemia	0.0 (0.0 to 0.0)	0.0 (0.0 to 0.0)	0.0 (0.0 to 0.0)	0.0 (0.0 to 0.0)	0.0 (0.0 to 0.0)	0.0 (0.0 to 0.0)
Chronic myeloid leukemia	0.0 (0.0 to 0.0)	0.0 (0.0 to 0.0)	0.0 (0.0 to 0.0)	0.0 (0.0 to 0.0)	0.0 (0.0 to 0.0)	0.0 (0.0 to 0.0)
Other neoplasms	0.0 (0.0 to 0.0)	0.0 (0.0 to 0.0)	0.0 (0.0 to 0.0)	0.0 (0.0 to 0.0)	0.0 (0.0 to 0.0)	0.0 (0.0 to 0.0)
Cardiovascular diseases	0.0 (0.0 to 0.0)	0.0 (0.0 to 0.0)	0.0 (0.0 to 0.0)	0.0 (0.0 to 0.0)	1.5 (-3.0 to 6.4)	-4.1 (-8.3 to 0.6)
Rheumatic heart disease	0.0 (0.0 to 0.0)	0.0 (0.0 to 0.0)	0.0 (0.0 to 0.0)	0.0 (0.0 to 0.0)	0.0 (0.0 to 0.0)	0.0 (0.0 to 0.0)
Rheumatic heart disease, without heart failure	0.0 (0.0 to 0.0)	0.0 (0.0 to 0.0)	0.0 (0.0 to 0.0)	0.0 (0.0 to 0.0)	0.0 (0.0 to 0.0)	0.0 (0.0 to 0.0)
Heart failure fue to rheumatic heart disease aggregate	0.0 (0.0 to 0.0)	0.0 (0.0 to 0.0)	0.0 (0.0 to 0.0)	0.0 (0.0 to 0.0)	0.0 (0.0 to 0.0)	0.0 (0.0 to 0.0)
Ischemic heart disease	0.0 (0.0 to 0.0)	0.0 (0.0 to 0.0)	0.0 (0.0 to 0.0)	0.0 (0.0 to 0.0)	0.0 (0.0 to 0.0)	0.0 (0.0 to 0.0)
Myocardial infarction episodes aggregate	0.0 (0.0 to 0.0)	0.0 (0.0 to 0.0)	0.0 (0.0 to 0.0)	0.0 (0.0 to 0.0)	0.0 (0.0 to 0.0)	0.0 (0.0 to 0.0)
Angina due to ischemic heart disease aggregate	0.0 (0.0 to 0.0)	0.0 (0.0 to 0.0)	0.0 (0.0 to 0.0)	0.0 (0.0 to 0.0)	0.0 (0.0 to 0.0)	0.0 (0.0 to 0.0)
Heart failure fue to ischemic heart disease aggregate	0.0 (0.0 to 0.0)	0.0 (0.0 to 0.0)	0.0 (0.0 to 0.0)	0.0 (0.0 to 0.0)	0.0 (0.0 to 0.0)	0.0 (0.0 to 0.0)
Cerebrovascular disease	0.0 (0.0 to 0.0)	0.0 (0.0 to 0.0)	0.0 (0.0 to 0.0)	0.0 (0.0 to 0.0)	0.0 (0.0 to 0.0)	0.0 (0.0 to 0.0)
Ischemic stroke	0.0 (0.0 to 0.0)	0.0 (0.0 to 0.0)	0.0 (0.0 to 0.0)	0.0 (0.0 to 0.0)	0.0 (0.0 to 0.0)	0.0 (0.0 to 0.0)
Chronic ischemic stroke aggregate	0.0 (0.0 to 0.0)	0.0 (0.0 to 0.0)	0.0 (0.0 to 0.0)	0.0 (0.0 to 0.0)	0.0 (0.0 to 0.0)	0.0 (0.0 to 0.0)
Ischemic stroke episodes aggregate	0.0 (0.0 to 0.0)	0.0 (0.0 to 0.0)	0.0 (0.0 to 0.0)	0.0 (0.0 to 0.0)	0.0 (0.0 to 0.0)	0.0 (0.0 to 0.0)
Hemorrhagic stroke	0.0 (0.0 to 0.0)	0.0 (0.0 to 0.0)	0.0 (0.0 to 0.0)	0.0 (0.0 to 0.0)	0.0 (0.0 to 0.0)	0.0 (0.0 to 0.0)
Chronic hemorrhagic stroke aggregate	0.0 (0.0 to 0.0)	0.0 (0.0 to 0.0)	0.0 (0.0 to 0.0)	0.0 (0.0 to 0.0)	0.0 (0.0 to 0.0)	0.0 (0.0 to 0.0)
Acute hemorrhagic stroke aggregate	0.0 (0.0 to 0.0)	0.0 (0.0 to 0.0)	0.0 (0.0 to 0.0)	0.0 (0.0 to 0.0)	0.0 (0.0 to 0.0)	0.0 (0.0 to 0.0)
Hypertensive heart disease	0.0 (0.0 to 0.0)	0.0 (0.0 to 0.0)	0.0 (0.0 to 0.0)	0.0 (0.0 to 0.0)	0.0 (0.0 to 0.0)	0.0 (0.0 to 0.0)
Cardiomyopathy and myocarditis	0.0 (0.0 to 0.0)	0.0 (0.0 to 0.0)	0.0 (0.0 to 0.0)	0.0 (0.0 to 0.0)	0.0 (0.0 to 0.0)	0.0 (0.0 to 0.0)
Acute myocarditis	0.0 (0.0 to 0.0)	0.0 (0.0 to 0.0)	0.0 (0.0 to 0.0)	0.0 (0.0 to 0.0)	0.0 (0.0 to 0.0)	0.0 (0.0 to 0.0)
Heart failure due to cardiomyopathy aggregate	0.0 (0.0 to 0.0)	0.0 (0.0 to 0.0)	0.0 (0.0 to 0.0)	0.0 (0.0 to 0.0)	0.0 (0.0 to 0.0)	0.0 (0.0 to 0.0)
Atrial fibrillation and flutter	0.0 (0.0 to 0.0)	0.0 (0.0 to 0.0)	0.0 (0.0 to 0.0)	0.0 (0.0 to 0.0)	0.0 (0.0 to 0.0)	0.0 (0.0 to 0.0)
Peripheral vascular disease	0.0 (0.0 to 0.0)	0.0 (0.0 to 0.0)	0.0 (0.0 to 0.0)	0.0 (0.0 to 0.0)	0.0 (0.0 to 0.0)	0.0 (0.0 to 0.0)
Endocarditis	0.0 (0.0 to 0.0)	1.5 (-2.9 to 6.5)	-4.1 (-8.3 to 0.7)	0.0 (0.0 to 0.0)	1.5 (-3.0 to 6.4)	-4.1 (-8.3 to 0.6)
Endocarditis episodes aggregate	0.0 (0.0 to 0.0)	1.5 (-2.9 to 6.5)	-4.1 (-8.3 to 0.7)	0.0 (0.0 to 0.0)	1.5 (-3.0 to 6.4)	-4.1 (-8.3 to 0.6)
Heart failure due to endocarditis aggregate	0.0 (0.0 to 0.0)	0.0 (0.0 to 0.0)	0.0 (0.0 to 0.0)	0.0 (0.0 to 0.0)	0.0 (0.0 to 0.0)	0.0 (0.0 to 0.0)
Other cardiovascular and circulatory diseases	0.0 (0.0 to 0.0)	0.0 (0.0 to 0.0)	0.0 (0.0 to 0.0)	0.0 (0.0 to 0.0)	0.0 (0.0 to 0.0)	0.0 (0.0 to 0.0)
Heart failure due to other cardiovascular diseases aggregate	0.0 (0.0 to 0.0)	0.0 (0.0 to 0.0)	0.0 (0.0 to 0.0)	0.0 (0.0 to 0.0)	0.0 (0.0 to 0.0)	0.0 (0.0 to 0.0)
Other cardiovascular diseases episodes aggregate	0.0 (0.0 to 0.0)	0.0 (0.0 to 0.0)	0.0 (0.0 to 0.0)	0.0 (0.0 to 0.0)	0.0 (0.0 to 0.0)	0.0 (0.0 to 0.0)
Chronic respiratory diseases	0.0 (0.0 to 0.0)	0.0 (0.0 to 0.0)	0.0 (0.0 to 0.0)	0.0 (0.0 to 0.0)	0.0 (0.0 to 0.0)	0.0 (0.0 to 0.0)
Chronic obstructive pulmonary disease	0.0 (0.0 to 0.0)	0.0 (0.0 to 0.0)	0.0 (0.0 to 0.0)	0.0 (0.0 to 0.0)	0.0 (0.0 to 0.0)	0.0 (0.0 to 0.0)

Cause	Prevalence in thousands (95% confidence interval)			YLDs in thousands (95% confidence interval)		
	2015	Percent change in counts between 2005 and 2015	Percent change in rates between 2005 and 2015	2015	Percent change in counts between 2005 and 2015	Percent change in rates between 2005 and 2015
Chronic obstructive pulmonary disease without heart failure aggregate	0.0 (0.0 to 0.0)	0.0 (0.0 to 0.0)	0.0 (0.0 to 0.0)	0.0 (0.0 to 0.0)	0.0 (0.0 to 0.0)	0.0 (0.0 to 0.0)
Severe chronic obstructive pulmonary disease with heart failure aggregate	0.0 (0.0 to 0.0)	0.0 (0.0 to 0.0)	0.0 (0.0 to 0.0)	0.0 (0.0 to 0.0)	0.0 (0.0 to 0.0)	0.0 (0.0 to 0.0)
Pneumoconiosis	0.0 (0.0 to 0.0)	0.0 (0.0 to 0.0)	0.0 (0.0 to 0.0)	0.0 (0.0 to 0.0)	0.0 (0.0 to 0.0)	0.0 (0.0 to 0.0)
Silicosis	0.0 (0.0 to 0.0)	0.0 (0.0 to 0.0)	0.0 (0.0 to 0.0)	0.0 (0.0 to 0.0)	0.0 (0.0 to 0.0)	0.0 (0.0 to 0.0)
Silicosis without heart failure aggregate	0.0 (0.0 to 0.0)	0.0 (0.0 to 0.0)	0.0 (0.0 to 0.0)	0.0 (0.0 to 0.0)	0.0 (0.0 to 0.0)	0.0 (0.0 to 0.0)
Severe silicosis with heart failure aggregate	0.0 (0.0 to 0.0)	0.0 (0.0 to 0.0)	0.0 (0.0 to 0.0)	0.0 (0.0 to 0.0)	0.0 (0.0 to 0.0)	0.0 (0.0 to 0.0)
Asbestosis	0.0 (0.0 to 0.0)	0.0 (0.0 to 0.0)	0.0 (0.0 to 0.0)	0.0 (0.0 to 0.0)	0.0 (0.0 to 0.0)	0.0 (0.0 to 0.0)
Asbestosis without heart failure aggregate	0.0 (0.0 to 0.0)	0.0 (0.0 to 0.0)	0.0 (0.0 to 0.0)	0.0 (0.0 to 0.0)	0.0 (0.0 to 0.0)	0.0 (0.0 to 0.0)
Severe asbestosis with heart failure aggregate	0.0 (0.0 to 0.0)	0.0 (0.0 to 0.0)	0.0 (0.0 to 0.0)	0.0 (0.0 to 0.0)	0.0 (0.0 to 0.0)	0.0 (0.0 to 0.0)
Coal workers pneumoconiosis	0.0 (0.0 to 0.0)	0.0 (0.0 to 0.0)	0.0 (0.0 to 0.0)	0.0 (0.0 to 0.0)	0.0 (0.0 to 0.0)	0.0 (0.0 to 0.0)
Coal workers pneumoconiosis without heart failure aggregate	0.0 (0.0 to 0.0)	0.0 (0.0 to 0.0)	0.0 (0.0 to 0.0)	0.0 (0.0 to 0.0)	0.0 (0.0 to 0.0)	0.0 (0.0 to 0.0)
Severe coal workers pneumoconiosis with heart failure aggregate	0.0 (0.0 to 0.0)	0.0 (0.0 to 0.0)	0.0 (0.0 to 0.0)	0.0 (0.0 to 0.0)	0.0 (0.0 to 0.0)	0.0 (0.0 to 0.0)
Other pneumoconiosis	0.0 (0.0 to 0.0)	0.0 (0.0 to 0.0)	0.0 (0.0 to 0.0)	0.0 (0.0 to 0.0)	0.0 (0.0 to 0.0)	0.0 (0.0 to 0.0)
Other pneumoconiosis without heart failure aggregate	0.0 (0.0 to 0.0)	0.0 (0.0 to 0.0)	0.0 (0.0 to 0.0)	0.0 (0.0 to 0.0)	0.0 (0.0 to 0.0)	0.0 (0.0 to 0.0)
Severe other pneumoconiosis with heart failure aggregate	0.0 (0.0 to 0.0)	0.0 (0.0 to 0.0)	0.0 (0.0 to 0.0)	0.0 (0.0 to 0.0)	0.0 (0.0 to 0.0)	0.0 (0.0 to 0.0)
Asthma	0.0 (0.0 to 0.0)	0.0 (0.0 to 0.0)	0.0 (0.0 to 0.0)	0.0 (0.0 to 0.0)	0.0 (0.0 to 0.0)	0.0 (0.0 to 0.0)
Asymptomatic asthma	0.0 (0.0 to 0.0)	0.0 (0.0 to 0.0)	0.0 (0.0 to 0.0)	0.0 (0.0 to 0.0)	0.0 (0.0 to 0.0)	0.0 (0.0 to 0.0)
Symptomatic asthma aggregate	0.0 (0.0 to 0.0)	0.0 (0.0 to 0.0)	0.0 (0.0 to 0.0)	0.0 (0.0 to 0.0)	0.0 (0.0 to 0.0)	0.0 (0.0 to 0.0)
Interstitial lung disease and pulmonary sarcoidosis	0.0 (0.0 to 0.0)	0.0 (0.0 to 0.0)	0.0 (0.0 to 0.0)	0.0 (0.0 to 0.0)	0.0 (0.0 to 0.0)	0.0 (0.0 to 0.0)
Interstitial lung disease and pulmonary sarcoidosis without heart failure aggregate	0.0 (0.0 to 0.0)	0.0 (0.0 to 0.0)	0.0 (0.0 to 0.0)	0.0 (0.0 to 0.0)	0.0 (0.0 to 0.0)	0.0 (0.0 to 0.0)
Severe interstitial lung disease and pulmonary sarcoidosis with heart failure aggregate	0.0 (0.0 to 0.0)	0.0 (0.0 to 0.0)	0.0 (0.0 to 0.0)	0.0 (0.0 to 0.0)	0.0 (0.0 to 0.0)	0.0 (0.0 to 0.0)
Other chronic respiratory diseases	--	--	--	0.0 (0.0 to 0.0)	0.0 (0.0 to 0.0)	0.0 (0.0 to 0.0)
Cirrhosis and other chronic liver diseases	0.0 (0.0 to 0.0)	0.0 (0.0 to 0.0)	0.0 (0.0 to 0.0)	0.0 (0.0 to 0.0)	6.4 (5.6 to 7.2)*	0.6 (-0.2 to 1.3)
Cirrhosis and other chronic liver diseases due to hepatitis B	0.0 (0.0 to 0.0)	7.5 (4.3 to 10.4)*	1.6 (-1.4 to 4.3)	0.0 (0.0 to 0.0)	7.5 (4.3 to 10.4)*	1.6 (-1.4 to 4.3)
Cirrhosis and other chronic liver diseases due to hepatitis C	0.0 (0.0 to 0.0)	7.5 (2.8 to 11.5)*	1.6 (-2.8 to 5.4)	0.0 (0.0 to 0.0)	7.5 (2.8 to 11.5)*	1.5 (-2.8 to 5.4)
Cirrhosis and other chronic liver diseases due to alcohol use	0.0 (0.0 to 0.0)	0.0 (0.0 to 0.0)	0.0 (0.0 to 0.0)	0.0 (0.0 to 0.0)	0.0 (0.0 to 0.0)	0.0 (0.0 to 0.0)
Cirrhosis and other chronic liver diseases due to other causes	0.0 (0.0 to 0.0)	6.4 (5.6 to 7.2)*	0.6 (-0.2 to 1.3)	0.0 (0.0 to 0.0)	6.4 (5.6 to 7.2)*	0.6 (-0.2 to 1.3)
Digestive diseases	0.7 (0.6 to 0.8)	6.7 (-5.9 to 20.5)	0.8 (-11.1 to 13.9)	0.2 (0.1 to 0.4)	6.6 (-6.0 to 20.1)	0.7 (-11.1 to 13.5)
Peptic ulcer disease	0.0 (0.0 to 0.0)	0.0 (0.0 to 0.0)	0.0 (0.0 to 0.0)	0.0 (0.0 to 0.0)	0.0 (0.0 to 0.0)	0.0 (0.0 to 0.0)
Peptic ulcer disease, symptomatic episodes	0.0 (0.0 to 0.0)	0.0 (0.0 to 0.0)	0.0 (0.0 to 0.0)	0.0 (0.0 to 0.0)	0.0 (0.0 to 0.0)	0.0 (0.0 to 0.0)
Anemia due to peptic ulcer disease aggregate	0.0 (0.0 to 0.0)	0.0 (0.0 to 0.0)	0.0 (0.0 to 0.0)	0.0 (0.0 to 0.0)	0.0 (0.0 to 0.0)	0.0 (0.0 to 0.0)
Gastritis and duodenitis	0.0 (0.0 to 0.0)	0.0 (0.0 to 0.0)	0.0 (0.0 to 0.0)	0.0 (0.0 to 0.0)	0.0 (0.0 to 0.0)	0.0 (0.0 to 0.0)
Gastritis and duodenitis, symptomatic episodes	0.0 (0.0 to 0.0)	0.0 (0.0 to 0.0)	0.0 (0.0 to 0.0)	0.0 (0.0 to 0.0)	0.0 (0.0 to 0.0)	0.0 (0.0 to 0.0)
Anemia due to gastritis and duodenitis aggregate	0.0 (0.0 to 0.0)	0.0 (0.0 to 0.0)	0.0 (0.0 to 0.0)	0.0 (0.0 to 0.0)	0.0 (0.0 to 0.0)	0.0 (0.0 to 0.0)
Appendicitis	0.0 (0.0 to 0.0)	0.0 (0.0 to 0.0)	0.0 (0.0 to 0.0)	0.0 (0.0 to 0.0)	0.0 (0.0 to 0.0)	0.0 (0.0 to 0.0)
Paralytic ileus and intestinal obstruction	0.7 (0.4 to 1.2)	5.5 (3.2 to 7.2)*	-0.3 (-2.5 to 1.3)	0.2 (0.1 to 0.4)	6.0 (-6.7 to 19.7)	0.1 (-11.8 to 13.1)
Inguinal, femoral, and abdominal hernia	0.0 (0.0 to 0.0)	0.0 (0.0 to 0.0)	0.0 (0.0 to 0.0)	0.0 (0.0 to 0.0)	0.0 (0.0 to 0.0)	0.0 (0.0 to 0.0)
Inflammatory bowel disease	0.0 (0.0 to 0.0)	0.0 (0.0 to 0.0)	0.0 (0.0 to 0.0)	0.0 (0.0 to 0.0)	0.0 (0.0 to 0.0)	0.0 (0.0 to 0.0)
Vascular intestinal disorders	0.0 (0.0 to 0.0)	0.0 (0.0 to 0.0)	0.0 (0.0 to 0.0)	0.0 (0.0 to 0.0)	0.0 (0.0 to 0.0)	0.0 (0.0 to 0.0)
Gallbladder and biliary diseases	0.0 (0.0 to 0.0)	0.0 (0.0 to 0.0)	0.0 (0.0 to 0.0)	0.0 (0.0 to 0.0)	0.0 (0.0 to 0.0)	0.0 (0.0 to 0.0)
Pancreatitis	0.0 (0.0 to 0.0)	0.0 (0.0 to 0.0)	0.0 (0.0 to 0.0)	0.0 (0.0 to 0.0)	0.0 (0.0 to 0.0)	0.0 (0.0 to 0.0)
Other digestive diseases	--	--	--	0.0 (0.0 to 0.0)	12.3 (-3.2 to 31.9)	6.2 (-8.5 to 24.7)
Neurological disorders	24.2 (21.6 to 27.1)	0.6 (-5.6 to 6.3)	-4.9 (-10.8 to 0.4)	7.4 (4.6 to 10.8)	-12.6 (-21.9 to -3.7)*	-17.4 (-26.2 to -9.0)*
Alzheimer disease and other dementias	0.0 (0.0 to 0.0)	0.0 (0.0 to 0.0)	0.0 (0.0 to 0.0)	0.0 (0.0 to 0.0)	0.0 (0.0 to 0.0)	0.0 (0.0 to 0.0)
Parkinson disease	0.0 (0.0 to 0.0)	0.0 (0.0 to 0.0)	0.0 (0.0 to 0.0)	0.0 (0.0 to 0.0)	0.0 (0.0 to 0.0)	0.0 (0.0 to 0.0)
Epilepsy	24.1 (17.9 to 31.8)	1.0 (-8.2 to 9.8)	-4.6 (-13.3 to 3.8)	7.4 (4.6 to 10.8)	-12.6 (-21.9 to -3.7)*	-17.4 (-26.2 to -9.0)*
Multiple sclerosis	0.0 (0.0 to 0.0)	0.0 (0.0 to 0.0)	0.0 (0.0 to 0.0)	0.0 (0.0 to 0.0)	0.0 (0.0 to 0.0)	0.0 (0.0 to 0.0)
Motor neuron disease	0.0 (0.0 to 0.0)	-5.7 (-8.0 to -3.5)*	-10.9 (-13.1 to -8.8)*	0.0 (0.0 to 0.0)	-5.7 (-8.0 to -3.5)*	-10.9 (-13.1 to -8.8)*
Migraine	0.0 (0.0 to 0.0)	0.0 (0.0 to 0.0)	0.0 (0.0 to 0.0)	0.0 (0.0 to 0.0)	0.0 (0.0 to 0.0)	0.0 (0.0 to 0.0)
Tension-type headache	0.0 (0.0 to 0.0)	0.0 (0.0 to 0.0)	0.0 (0.0 to 0.0)	0.0 (0.0 to 0.0)	0.0 (0.0 to 0.0)	0.0 (0.0 to 0.0)
Medication overuse headache	0.0 (0.0 to 0.0)	0.0 (0.0 to 0.0)	0.0 (0.0 to 0.0)	0.0 (0.0 to 0.0)	0.0 (0.0 to 0.0)	0.0 (0.0 to 0.0)
Other neurological disorders	0.0 (0.0 to 0.0)	0.0 (0.0 to 0.0)	0.0 (0.0 to 0.0)	0.0 (0.0 to 0.0)	0.0 (0.0 to 0.0)	0.0 (0.0 to 0.0)
Mental and substance use disorders	58.6 (52.8 to 63.6)	3.9 (-0.5 to 8.8)	-1.8 (-6.0 to 2.8)	6.2 (3.8 to 9.3)	5.4 (3.7 to 6.8)*	-0.4 (-2.0 to 1.0)
Schizophrenia	0.0 (0.0 to 0.0)	0.0 (0.0 to 0.0)	0.0 (0.0 to 0.0)	0.0 (0.0 to 0.0)	0.0 (0.0 to 0.0)	0.0 (0.0 to 0.0)
Alcohol use disorders	0.9 (0.7 to 1.0)	4.9 (2.6 to 7.1)*	-0.8 (-3.0 to 1.2)	0.0 (0.0 to 0.0)	4.8 (-7.3 to 18.1)	-0.9 (-12.4 to 11.6)
Alcohol dependence aggregate	0.0 (0.0 to 0.0)	0.0 (0.0 to 0.0)	0.0 (0.0 to 0.0)	0.0 (0.0 to 0.0)	0.0 (0.0 to 0.0)	0.0 (0.0 to 0.0)
Fetal alcohol syndrome aggregate	0.9 (0.7 to 1.0)	4.9 (2.6 to 7.1)*	-0.8 (-3.0 to 1.2)	0.0 (0.0 to 0.0)	4.8 (-7.3 to 18.1)	-0.9 (-12.4 to 11.6)
Drug use disorders	0.0 (0.0 to 0.0)	0.0 (0.0 to 0.0)	0.0 (0.0 to 0.0)	0.0 (0.0 to 0.0)	0.0 (0.0 to 0.0)	0.0 (0.0 to 0.0)
Opioid use disorders	0.0 (0.0 to 0.0)	0.0 (0.0 to 0.0)	0.0 (0.0 to 0.0)	0.0 (0.0 to 0.0)	0.0 (0.0 to 0.0)	0.0 (0.0 to 0.0)
Asymptomatic opioid dependence	0.0 (0.0 to 0.0)	0.0 (0.0 to 0.0)	0.0 (0.0 to 0.0)	0.0 (0.0 to 0.0)	0.0 (0.0 to 0.0)	0.0 (0.0 to 0.0)
Symptomatic opioid dependence aggregate	0.0 (0.0 to 0.0)	0.0 (0.0 to 0.0)	0.0 (0.0 to 0.0)	0.0 (0.0 to 0.0)	0.0 (0.0 to 0.0)	0.0 (0.0 to 0.0)
Cocaine use disorders	0.0 (0.0 to 0.0)	0.0 (0.0 to 0.0)	0.0 (0.0 to 0.0)	0.0 (0.0 to 0.0)	0.0 (0.0 to 0.0)	0.0 (0.0 to 0.0)
Asymptomatic cocaine dependence	0.0 (0.0 to 0.0)	0.0 (0.0 to 0.0)	0.0 (0.0 to 0.0)	0.0 (0.0 to 0.0)	0.0 (0.0 to 0.0)	0.0 (0.0 to 0.0)

Cause	Prevalence in thousands (95% confidence interval)			YLDs in thousands (95% confidence interval)		
	2015	Percent change in counts between 2005 and 2015	Percent change in rates between 2005 and 2015	2015	Percent change in counts between 2005 and 2015	Percent change in rates between 2005 and 2015
Symptomatic cocaine dependence aggregate	0.0 (0.0 to 0.0)	0.0 (0.0 to 0.0)	0.0 (0.0 to 0.0)	0.0 (0.0 to 0.0)	0.0 (0.0 to 0.0)	0.0 (0.0 to 0.0)
Amphetamine use disorders	0.0 (0.0 to 0.0)	0.0 (0.0 to 0.0)	0.0 (0.0 to 0.0)	0.0 (0.0 to 0.0)	0.0 (0.0 to 0.0)	0.0 (0.0 to 0.0)
Asymptomatic amphetamine dependence	0.0 (0.0 to 0.0)	0.0 (0.0 to 0.0)	0.0 (0.0 to 0.0)	0.0 (0.0 to 0.0)	0.0 (0.0 to 0.0)	0.0 (0.0 to 0.0)
Symptomatic amphetamine dependence aggregate	0.0 (0.0 to 0.0)	0.0 (0.0 to 0.0)	0.0 (0.0 to 0.0)	0.0 (0.0 to 0.0)	0.0 (0.0 to 0.0)	0.0 (0.0 to 0.0)
Cannabis use disorders	0.0 (0.0 to 0.0)	0.0 (0.0 to 0.0)	0.0 (0.0 to 0.0)	0.0 (0.0 to 0.0)	0.0 (0.0 to 0.0)	0.0 (0.0 to 0.0)
Asymptomatic cannabis dependence	0.0 (0.0 to 0.0)	0.0 (0.0 to 0.0)	0.0 (0.0 to 0.0)	0.0 (0.0 to 0.0)	0.0 (0.0 to 0.0)	0.0 (0.0 to 0.0)
Symptomatic cannabis dependence aggregate	0.0 (0.0 to 0.0)	0.0 (0.0 to 0.0)	0.0 (0.0 to 0.0)	0.0 (0.0 to 0.0)	0.0 (0.0 to 0.0)	0.0 (0.0 to 0.0)
Other drug use disorders	--	--	--	0.0 (0.0 to 0.0)	0.0 (0.0 to 0.0)	0.0 (0.0 to 0.0)
Depressive disorders	0.0 (0.0 to 0.0)	0.0 (0.0 to 0.0)	0.0 (0.0 to 0.0)	0.0 (0.0 to 0.0)	0.0 (0.0 to 0.0)	0.0 (0.0 to 0.0)
Major depressive disorder	0.0 (0.0 to 0.0)	0.0 (0.0 to 0.0)	0.0 (0.0 to 0.0)	0.0 (0.0 to 0.0)	0.0 (0.0 to 0.0)	0.0 (0.0 to 0.0)
Dysthymia	0.0 (0.0 to 0.0)	0.0 (0.0 to 0.0)	0.0 (0.0 to 0.0)	0.0 (0.0 to 0.0)	0.0 (0.0 to 0.0)	0.0 (0.0 to 0.0)
Bipolar disorder	0.0 (0.0 to 0.0)	0.0 (0.0 to 0.0)	0.0 (0.0 to 0.0)	0.0 (0.0 to 0.0)	0.0 (0.0 to 0.0)	0.0 (0.0 to 0.0)
Anxiety disorders	0.0 (0.0 to 0.0)	0.0 (0.0 to 0.0)	0.0 (0.0 to 0.0)	0.0 (0.0 to 0.0)	0.0 (0.0 to 0.0)	0.0 (0.0 to 0.0)
Eating disorders	0.0 (0.0 to 0.0)	0.0 (0.0 to 0.0)	0.0 (0.0 to 0.0)	0.0 (0.0 to 0.0)	0.0 (0.0 to 0.0)	0.0 (0.0 to 0.0)
Anorexia nervosa	0.0 (0.0 to 0.0)	0.0 (0.0 to 0.0)	0.0 (0.0 to 0.0)	0.0 (0.0 to 0.0)	0.0 (0.0 to 0.0)	0.0 (0.0 to 0.0)
Bulimia nervosa	0.0 (0.0 to 0.0)	0.0 (0.0 to 0.0)	0.0 (0.0 to 0.0)	0.0 (0.0 to 0.0)	0.0 (0.0 to 0.0)	0.0 (0.0 to 0.0)
Autistic spectrum disorders	33.1 (29.0 to 36.0)	5.7 (1.3 to 10.4)*	-0.1 (-4.3 to 4.3)	5.2 (3.1 to 7.9)	5.8 (3.8 to 7.5)*	-0.0 (-1.9 to 1.6)
Autism	10.6 (6.1 to 15.6)	5.5 (5.0 to 6.0)*	-0.3 (-0.8 to 0.2)	2.8 (1.4 to 4.7)	5.6 (2.6 to 8.5)*	-0.2 (-3.1 to 2.5)
Asperger syndrome and other autistic spectrum disorders	23.4 (14.7 to 34.0)	5.8 (5.5 to 6.2)*	0.0 (-0.3 to 0.3)	2.4 (1.3 to 3.9)	6.0 (3.8 to 8.1)*	0.1 (-1.9 to 2.2)
Attention-deficit/hyperactivity disorder	0.0 (0.0 to 0.0)	0.0 (0.0 to 0.0)	0.0 (0.0 to 0.0)	0.0 (0.0 to 0.0)	0.0 (0.0 to 0.0)	0.0 (0.0 to 0.0)
Conduct disorder	0.0 (0.0 to 0.0)	0.0 (0.0 to 0.0)	0.0 (0.0 to 0.0)	0.0 (0.0 to 0.0)	0.0 (0.0 to 0.0)	0.0 (0.0 to 0.0)
Idiopathic developmental intellectual disability	23.6 (10.1 to 37.8)	2.1 (-1.9 to 4.3)	-3.5 (-7.3 to -1.5)*	0.9 (0.3 to 1.8)	3.1 (-0.5 to 5.9)	-2.5 (-6.0 to 0.0)
Other mental and substance use disorders	0.0 (0.0 to 0.0)	0.0 (0.0 to 0.0)	0.0 (0.0 to 0.0)	0.0 (0.0 to 0.0)	0.0 (0.0 to 0.0)	0.0 (0.0 to 0.0)
Diabetes, urogenital, blood, and endocrine diseases	2,081.8 (2,051.3 to 2,117.8)	3.1 (1.8 to 4.4)*	-2.6 (-3.8 to -1.4)*	20.5 (13.7 to 29.4)	6.0 (3.5 to 8.9)*	0.2 (-2.2 to 2.9)
Diabetes mellitus	0.0 (0.0 to 0.0)	0.0 (0.0 to 0.0)	0.0 (0.0 to 0.0)	0.0 (0.0 to 0.0)	0.0 (0.0 to 0.0)	0.0 (0.0 to 0.0)
Uncomplicated diabetes mellitus	0.0 (0.0 to 0.0)	0.0 (0.0 to 0.0)	0.0 (0.0 to 0.0)	0.0 (0.0 to 0.0)	0.0 (0.0 to 0.0)	0.0 (0.0 to 0.0)
Neuropathy and other complications of diabetes mellitus aggregate	0.0 (0.0 to 0.0)	0.0 (0.0 to 0.0)	0.0 (0.0 to 0.0)	0.0 (0.0 to 0.0)	0.0 (0.0 to 0.0)	0.0 (0.0 to 0.0)
Vision loss due to diabetes mellitus aggregate	0.0 (0.0 to 0.0)	0.0 (0.0 to 0.0)	0.0 (0.0 to 0.0)	0.0 (0.0 to 0.0)	0.0 (0.0 to 0.0)	0.0 (0.0 to 0.0)
Acute glomerulonephritis	0.0 (0.0 to 0.0)	-9.2 (-13.8 to -5.4)*	-14.2 (-18.5 to -10.6)*	0.0 (0.0 to 0.0)	-9.2 (-13.8 to -5.4)*	-14.2 (-18.5 to -10.6)*
Chronic kidney disease	0.0 (0.0 to 0.0)	0.0 (0.0 to 0.0)	0.0 (0.0 to 0.0)	0.0 (0.0 to 0.0)	5.2 (4.0 to 6.4)*	-0.6 (-1.7 to 0.6)
Chronic kidney disease due to diabetes mellitus	0.0 (0.0 to 0.0)	5.1 (2.1 to 8.0)*	-0.6 (-3.5 to 2.1)	0.0 (0.0 to 0.0)	5.0 (2.0 to 8.1)*	-0.7 (-3.6 to 2.1)
Stage III chronic kidney disease due to diabetes mellitus aggregate	0.0 (0.0 to 0.0)	5.3 (2.1 to 8.3)*	-0.5 (-3.5 to 2.3)	0.0 (0.0 to 0.0)	9.0 (7.2 to 10.7)*	3.0 (1.3 to 4.6)*
Stage IV chronic kidney disease due to diabetes mellitus aggregate	0.0 (0.0 to 0.0)	0.0 (0.0 to 0.0)	0.0 (0.0 to 0.0)	0.0 (0.0 to 0.0)	0.0 (0.0 to 0.0)	0.0 (0.0 to 0.0)
Stage V chronic kidney disease untreated due to diabetes mellitus	0.0 (0.0 to 0.0)	5.0 (1.9 to 8.1)*	-0.8 (-3.7 to 2.2)	0.0 (0.0 to 0.0)	5.0 (1.9 to 8.1)*	-0.8 (-3.7 to 2.2)
End-stage chronic kidney disease due to diabetes aggregate	0.0 (0.0 to 0.0)	-1.0 (-6.7 to 4.6)	-6.4 (-11.9 to -1.1)*	0.0 (0.0 to 0.0)	2.5 (-2.0 to 7.0)	-3.2 (-7.4 to 1.1)
Chronic kidney disease due to hypertension	0.0 (0.0 to 0.0)	4.9 (1.9 to 7.5)*	-0.9 (-3.7 to 1.6)	0.0 (0.0 to 0.0)	4.6 (-1.1 to 9.8)	-1.1 (-6.5 to 3.8)
Stage III chronic kidney disease due to hypertension aggregate	0.0 (0.0 to 0.0)	4.9 (1.6 to 7.7)*	-0.9 (-4.0 to 1.8)	0.0 (0.0 to 0.0)	8.7 (6.8 to 10.5)*	2.8 (1.0 to 4.5)*
Stage IV chronic kidney disease due to hypertension aggregate	0.0 (0.0 to 0.0)	0.0 (0.0 to 0.0)	0.0 (0.0 to 0.0)	0.0 (0.0 to 0.0)	0.0 (0.0 to 0.0)	0.0 (0.0 to 0.0)
Stage V chronic kidney disease untreated due to hypertension	0.0 (0.0 to 0.0)	4.6 (-1.3 to 9.9)	-1.1 (-6.7 to 3.9)	0.0 (0.0 to 0.0)	4.6 (-1.3 to 9.9)	-1.2 (-6.7 to 3.9)
End-stage chronic kidney disease due to hypertension aggregate	0.0 (0.0 to 0.0)	-2.3 (-12.0 to 8.8)	-7.7 (-16.8 to 2.9)	0.0 (0.0 to 0.0)	0.6 (-7.2 to 9.4)	-4.9 (-12.3 to 3.4)
Chronic kidney disease due to glomerulonephritis	0.0 (0.0 to 0.0)	5.1 (2.9 to 7.0)*	-0.7 (-2.7 to 1.1)	0.0 (0.0 to 0.0)	4.8 (1.9 to 7.3)*	-1.0 (-3.7 to 1.4)
Stage III chronic kidney disease due to glomerulonephritis aggregate	0.0 (0.0 to 0.0)	6.6 (3.6 to 9.3)*	0.7 (-2.1 to 3.3)	0.0 (0.0 to 0.0)	6.1 (4.2 to 7.9)*	0.3 (-1.6 to 2.0)
Stage IV chronic kidney disease due to glomerulonephritis aggregate	0.0 (0.0 to 0.0)	0.0 (0.0 to 0.0)	0.0 (0.0 to 0.0)	0.0 (0.0 to 0.0)	0.0 (0.0 to 0.0)	0.0 (0.0 to 0.0)
Stage V chronic kidney disease untreated due to glomerulonephritis	0.0 (0.0 to 0.0)	4.7 (1.7 to 7.4)*	-1.0 (-3.8 to 1.5)	0.0 (0.0 to 0.0)	4.8 (1.7 to 7.4)*	-1.0 (-3.8 to 1.5)
End-stage chronic kidney disease due to glomerulonephritis aggregate	0.0 (0.0 to 0.0)	-0.1 (-5.4 to 5.0)	-5.6 (-10.6 to -0.8)*	0.0 (0.0 to 0.0)	4.0 (-1.3 to 9.5)	-1.7 (-6.7 to 3.5)
Chronic kidney disease due to other causes	0.1 (0.0 to 0.1)	5.6 (4.1 to 7.2)*	-0.2 (-1.6 to 1.3)	0.0 (0.0 to 0.0)	5.5 (3.8 to 7.2)*	-0.3 (-1.9 to 1.3)
Stage III chronic kidney disease due to other causes aggregate	0.0 (0.0 to 0.1)	5.9 (4.3 to 7.5)*	0.0 (-1.4 to 1.6)	0.0 (0.0 to 0.0)	10.3 (8.6 to 12.4)*	4.3 (2.6 to 6.2)*
Stage IV chronic kidney disease due to other causes aggregate	0.0 (0.0 to 0.0)	0.0 (0.0 to 0.0)	0.0 (0.0 to 0.0)	0.0 (0.0 to 0.0)	0.0 (0.0 to 0.0)	0.0 (0.0 to 0.0)
Stage V chronic kidney disease untreated due to other causes	0.0 (0.0 to 0.0)	5.4 (3.6 to 7.2)*	-0.4 (-2.1 to 1.3)	0.0 (0.0 to 0.0)	5.4 (3.6 to 7.2)*	-0.4 (-2.1 to 1.3)
End-stage chronic kidney disease due to other causes aggregate	0.0 (0.0 to 0.0)	-0.4 (-3.5 to 2.7)	-5.9 (-8.8 to -2.9)*	0.0 (0.0 to 0.0)	3.5 (-0.7 to 7.2)	-2.2 (-6.1 to 1.3)
Urinary diseases and male infertility	4.9 (4.7 to 5.1)	11.9 (6.9 to 16.9)*	5.7 (1.0 to 10.5)*	0.2 (0.1 to 0.3)	11.0 (4.7 to 17.9)*	4.9 (-1.1 to 11.5)
Interstitial nephritis and urinary tract infections	4.9 (4.6 to 5.2)	11.7 (11.1 to 12.5)*	5.6 (5.0 to 6.3)*	0.2 (0.1 to 0.3)	11.9 (6.4 to 18.3)*	5.7 (0.6 to 11.8)*
Urolithiasis	0.0 (0.0 to 0.0)	0.0 (0.0 to 0.0)	0.0 (0.0 to 0.0)	0.0 (0.0 to 0.0)	0.0 (0.0 to 0.0)	0.0 (0.0 to 0.0)
Benign prostatic hyperplasia	0.0 (0.0 to 0.0)	0.0 (0.0 to 0.0)	0.0 (0.0 to 0.0)	0.0 (0.0 to 0.0)	0.0 (0.0 to 0.0)	0.0 (0.0 to 0.0)
Male infertility	0.0 (0.0 to 0.0)	0.0 (0.0 to 0.0)	0.0 (0.0 to 0.0)	0.0 (0.0 to 0.0)	0.0 (0.0 to 0.0)	0.0 (0.0 to 0.0)
Other urinary diseases	--	--	--	0.1 (0.0 to 0.1)	8.4 (-2.1 to 20.8)	2.5 (-7.4 to 14.1)
Gynecological diseases	0.0 (0.0 to 0.0)	0.0 (0.0 to 0.0)	0.0 (0.0 to 0.0)	0.0 (0.0 to 0.0)	0.0 (0.0 to 0.0)	0.0 (0.0 to 0.0)
Uterine fibroids	0.0 (0.0 to 0.0)	0.0 (0.0 to 0.0)	0.0 (0.0 to 0.0)	0.0 (0.0 to 0.0)	0.0 (0.0 to 0.0)	0.0 (0.0 to 0.0)
Uterine fibroids cases aggregate	0.0 (0.0 to 0.0)	0.0 (0.0 to 0.0)	0.0 (0.0 to 0.0)	0.0 (0.0 to 0.0)	0.0 (0.0 to 0.0)	0.0 (0.0 to 0.0)
Mild abdominal pain with anemia due to uterine fibroids aggregate	0.0 (0.0 to 0.0)	0.0 (0.0 to 0.0)	0.0 (0.0 to 0.0)	0.0 (0.0 to 0.0)	0.0 (0.0 to 0.0)	0.0 (0.0 to 0.0)
Polycystic ovarian syndrome	0.0 (0.0 to 0.0)	0.0 (0.0 to 0.0)	0.0 (0.0 to 0.0)	0.0 (0.0 to 0.0)	0.0 (0.0 to 0.0)	0.0 (0.0 to 0.0)
Polycystic ovarian syndrome cases aggregate	0.0 (0.0 to 0.0)	0.0 (0.0 to 0.0)	0.0 (0.0 to 0.0)	0.0 (0.0 to 0.0)	0.0 (0.0 to 0.0)	0.0 (0.0 to 0.0)
Hirsutism and infertility due to polycystic ovarian syndrome aggregate	0.0 (0.0 to 0.0)	0.0 (0.0 to 0.0)	0.0 (0.0 to 0.0)	0.0 (0.0 to 0.0)	0.0 (0.0 to 0.0)	0.0 (0.0 to 0.0)

Cause	Prevalence in thousands (95% confidence interval)			YLDs in thousands (95% confidence interval)		
	2015	Percent change in counts between 2005 and 2015	Percent change in rates between 2005 and 2015	2015	Percent change in counts between 2005 and 2015	Percent change in rates between 2005 and 2015
Female infertility	0.0 (0.0 to 0.0)	0.0 (0.0 to 0.0)	0.0 (0.0 to 0.0)	0.0 (0.0 to 0.0)	0.0 (0.0 to 0.0)	0.0 (0.0 to 0.0)
Endometriosis	0.0 (0.0 to 0.0)	0.0 (0.0 to 0.0)	0.0 (0.0 to 0.0)	0.0 (0.0 to 0.0)	0.0 (0.0 to 0.0)	0.0 (0.0 to 0.0)
Endometriosis cases aggregate	0.0 (0.0 to 0.0)	0.0 (0.0 to 0.0)	0.0 (0.0 to 0.0)	0.0 (0.0 to 0.0)	0.0 (0.0 to 0.0)	0.0 (0.0 to 0.0)
Abdominal pain and infertility due to endometriosis aggregate	0.0 (0.0 to 0.0)	0.0 (0.0 to 0.0)	0.0 (0.0 to 0.0)	0.0 (0.0 to 0.0)	0.0 (0.0 to 0.0)	0.0 (0.0 to 0.0)
Genital prolapse	0.0 (0.0 to 0.0)	0.0 (0.0 to 0.0)	0.0 (0.0 to 0.0)	0.0 (0.0 to 0.0)	0.0 (0.0 to 0.0)	0.0 (0.0 to 0.0)
Premenstrual syndrome	0.0 (0.0 to 0.0)	0.0 (0.0 to 0.0)	0.0 (0.0 to 0.0)	0.0 (0.0 to 0.0)	0.0 (0.0 to 0.0)	0.0 (0.0 to 0.0)
Other gynecological diseases	0.0 (0.0 to 0.0)	0.0 (0.0 to 0.0)	0.0 (0.0 to 0.0)	0.0 (0.0 to 0.0)	0.0 (0.0 to 0.0)	0.0 (0.0 to 0.0)
Other gynecological diseases cases aggregate	0.0 (0.0 to 0.0)	0.0 (0.0 to 0.0)	0.0 (0.0 to 0.0)	0.0 (0.0 to 0.0)	0.0 (0.0 to 0.0)	0.0 (0.0 to 0.0)
Anemia due to other gynecological diseases aggregate	0.0 (0.0 to 0.0)	0.0 (0.0 to 0.0)	0.0 (0.0 to 0.0)	0.0 (0.0 to 0.0)	0.0 (0.0 to 0.0)	0.0 (0.0 to 0.0)
Hemoglobinopathies and hemolytic anemias	1,948.4 (1,917.7 to 1,985.6)	3.0 (1.6 to 4.5)*	-2.6 (-4.0 to -1.3)*	14.2 (9.5 to 20.4)	6.8 (4.2 to 9.5)*	0.9 (-1.5 to 3.4)
Thalassemias	6.5 (5.9 to 7.5)	1.5 (0.2 to 3.7)*	-4.0 (-5.3 to -2.0)*	0.5 (0.3 to 0.6)	3.8 (-1.2 to 9.5)	-1.9 (-6.7 to 3.5)
Beta-thalassemia major cases aggregate	3.7 (3.6 to 3.9)	4.1 (3.2 to 5.0)*	-1.6 (-2.5 to -0.8)*	0.3 (0.2 to 0.4)	6.9 (0.2 to 14.9)*	1.1 (-5.3 to 8.6)
Hemoglobin E/beta-thalassemia cases aggregate	1.1 (1.0 to 1.2)	4.0 (1.0 to 7.0)*	-1.7 (-4.5 to 1.1)	0.1 (0.0 to 0.1)	6.6 (-4.8 to 19.6)	0.7 (-10.0 to 13.1)
Hemoglobin H disease cases aggregate	1.6 (1.1 to 2.6)	-5.3 (-10.1 to 2.5)	-10.5 (-15.0 to -3.2)*	0.1 (0.1 to 0.2)	-5.4 (-15.5 to 7.6)	-10.6 (-20.2 to 1.6)
Heart failure due to thalassemias aggregate	0.0 (0.0 to 0.0)	0.0 (0.0 to 0.0)	0.0 (0.0 to 0.0)	0.0 (0.0 to 0.0)	0.0 (0.0 to 0.0)	0.0 (0.0 to 0.0)
Thalassemias trait	271.0 (263.4 to 280.4)	5.6 (5.1 to 6.1)*	-0.2 (-0.7 to 0.3)	4.7 (3.1 to 6.8)	10.9 (8.4 to 13.6)*	4.8 (2.5 to 7.4)*
B-thalassemia trait cases aggregate	220.3 (213.3 to 228.6)	6.3 (5.7 to 6.8)*	0.4 (-0.1 to 0.9)	4.4 (2.9 to 6.3)	11.5 (9.0 to 14.1)*	5.3 (3.0 to 7.8)*
Hemoglobin E trait cases aggregate	50.8 (48.4 to 53.3)	3.0 (1.8 to 4.2)*	-2.7 (-3.8 to -1.5)*	0.3 (0.2 to 0.5)	3.6 (-5.6 to 15.3)	-2.1 (-10.8 to 9.0)
Sickle cell disorders	33.0 (31.4 to 34.5)	4.1 (-0.8 to 9.2)	-1.7 (-6.3 to 3.2)	1.7 (1.1 to 2.5)	4.9 (-1.1 to 11.1)	-0.8 (-6.5 to 5.0)
Homozygous sickle cell and severe sickle cell/beta-thalassemia cases aggregate	27.7 (26.3 to 29.1)	1.0 (-4.0 to 5.9)	-4.6 (-9.2 to 0.0)	1.4 (0.9 to 2.1)	2.5 (-3.5 to 8.5)	-3.2 (-8.8 to 2.6)
Hemoglobin SC disease cases aggregate	5.0 (4.4 to 5.5)	24.0 (3.7 to 58.4)*	17.2 (-2.0 to 49.7)	0.3 (0.2 to 0.4)	20.1 (-3.2 to 39.3)	13.5 (-8.5 to 31.7)
Mild sickle cell/beta-thalassemia cases aggregate	0.3 (0.3 to 0.3)	20.4 (11.4 to 29.2)*	13.8 (5.3 to 22.1)*	0.0 (0.0 to 0.0)	18.3 (-9.9 to 50.6)	11.8 (-14.8 to 42.3)
Sickle cell trait	598.5 (568.7 to 649.4)	7.3 (5.6 to 8.5)*	1.4 (-0.2 to 2.6)	2.5 (1.7 to 3.6)	9.1 (2.2 to 14.8)*	3.1 (-3.4 to 8.5)
G6PD deficiency	319.6 (269.7 to 372.1)	-2.5 (-8.4 to 2.7)	-7.8 (-13.4 to -3.0)*	0.0 (0.0 to 0.1)	0.6 (-9.7 to 10.8)	-4.9 (-14.7 to 4.7)
G6PD cases aggregate	319.6 (269.7 to 372.1)	-2.5 (-8.4 to 2.7)	-7.8 (-13.4 to -3.0)*	0.0 (0.0 to 0.1)	0.6 (-9.7 to 10.8)	-4.9 (-14.7 to 4.7)
Heart failure due to G6PD deficiency aggregate	0.0 (0.0 to 0.0)	0.0 (0.0 to 0.0)	0.0 (0.0 to 0.0)	0.0 (0.0 to 0.0)	0.0 (0.0 to 0.0)	0.0 (0.0 to 0.0)
G6PD trait	842.3 (784.4 to 901.5)	1.5 (-0.3 to 3.2)	-4.0 (-5.8 to -2.5)*	0.0 (0.0 to 0.0)	3.8 (-5.7 to 14.0)	-1.9 (-10.9 to 7.7)
Other hemoglobinopathies and hemolytic anemias	131.7 (130.3 to 133.3)	2.2 (-0.6 to 5.3)	-3.4 (-6.0 to -0.5)*	4.8 (3.2 to 6.8)	2.9 (-1.5 to 7.8)	-2.7 (-6.9 to 1.9)
Other hemoglobinopathies and hemolytic anemias cases aggregate	131.7 (130.3 to 133.3)	2.2 (-0.6 to 5.3)	-3.4 (-6.0 to -0.5)*	4.6 (3.1 to 6.6)	2.8 (-1.5 to 7.7)	-2.8 (-7.0 to 1.8)
Heart failure due to other hemoglobinopathies and hemolytic anemias aggregate	0.0 (0.0 to 0.0)	0.0 (0.0 to 0.0)	0.0 (0.0 to 0.0)	0.0 (0.0 to 0.0)	0.0 (0.0 to 0.0)	0.0 (0.0 to 0.0)
Endocrine, metabolic, blood, and immune disorders	176.6 (171.6 to 181.2)	2.9 (-2.3 to 7.9)	-2.8 (-7.6 to 1.9)	6.0 (4.0 to 8.5)	4.1 (-2.4 to 10.4)	-1.6 (-7.8 to 4.4)
Endocrine, metabolic, blood, and immune disorders cases aggregate	9.6 (9.0 to 10.2)	9.8 (8.4 to 11.2)*	3.8 (2.4 to 5.1)*	0.4 (0.3 to 0.5)	9.8 (4.4 to 16.1)*	3.8 (-1.3 to 9.7)
Anemia due to endocrine, metabolic, blood, and immune disorders aggregate	167.0 (161.9 to 171.7)	2.5 (-2.9 to 7.8)	-3.1 (-8.2 to 1.9)	5.6 (3.7 to 8.0)	3.7 (-3.1 to 10.5)	-2.0 (-8.4 to 4.4)
Heart failure due to endocrine, metabolic, blood, and immune disorders aggregate	0.0 (0.0 to 0.0)	4.4 (3.3 to 5.4)*	-1.4 (-2.4 to -0.4)*	0.0 (0.0 to 0.0)	4.4 (3.3 to 5.4)*	-1.4 (-2.4 to -0.4)*
Musculoskeletal disorders	0.0 (0.0 to 0.0)	0.0 (0.0 to 0.0)	0.0 (0.0 to 0.0)	0.0 (0.0 to 0.0)	0.0 (0.0 to 0.0)	0.0 (0.0 to 0.0)
Rheumatoid arthritis	0.0 (0.0 to 0.0)	0.0 (0.0 to 0.0)	0.0 (0.0 to 0.0)	0.0 (0.0 to 0.0)	0.0 (0.0 to 0.0)	0.0 (0.0 to 0.0)
Osteoarthritis	0.0 (0.0 to 0.0)	0.0 (0.0 to 0.0)	0.0 (0.0 to 0.0)	0.0 (0.0 to 0.0)	0.0 (0.0 to 0.0)	0.0 (0.0 to 0.0)
Osteoarthritis of the hip cases aggregate	0.0 (0.0 to 0.0)	0.0 (0.0 to 0.0)	0.0 (0.0 to 0.0)	0.0 (0.0 to 0.0)	0.0 (0.0 to 0.0)	0.0 (0.0 to 0.0)
Osteoarthritis of the knee cases aggregate	0.0 (0.0 to 0.0)	0.0 (0.0 to 0.0)	0.0 (0.0 to 0.0)	0.0 (0.0 to 0.0)	0.0 (0.0 to 0.0)	0.0 (0.0 to 0.0)
Low back and neck pain	0.0 (0.0 to 0.0)	0.0 (0.0 to 0.0)	0.0 (0.0 to 0.0)	0.0 (0.0 to 0.0)	0.0 (0.0 to 0.0)	0.0 (0.0 to 0.0)
Low back pain	0.0 (0.0 to 0.0)	0.0 (0.0 to 0.0)	0.0 (0.0 to 0.0)	0.0 (0.0 to 0.0)	0.0 (0.0 to 0.0)	0.0 (0.0 to 0.0)
Neck pain	0.0 (0.0 to 0.0)	0.0 (0.0 to 0.0)	0.0 (0.0 to 0.0)	0.0 (0.0 to 0.0)	0.0 (0.0 to 0.0)	0.0 (0.0 to 0.0)
Gout	0.0 (0.0 to 0.0)	0.0 (0.0 to 0.0)	0.0 (0.0 to 0.0)	0.0 (0.0 to 0.0)	0.0 (0.0 to 0.0)	0.0 (0.0 to 0.0)
Asymptomatic gout	0.0 (0.0 to 0.0)	0.0 (0.0 to 0.0)	0.0 (0.0 to 0.0)	0.0 (0.0 to 0.0)	0.0 (0.0 to 0.0)	0.0 (0.0 to 0.0)
Symptomatic gout cases aggregate	0.0 (0.0 to 0.0)	0.0 (0.0 to 0.0)	0.0 (0.0 to 0.0)	0.0 (0.0 to 0.0)	0.0 (0.0 to 0.0)	0.0 (0.0 to 0.0)
Other musculoskeletal disorders	0.0 (0.0 to 0.0)	0.0 (0.0 to 0.0)	0.0 (0.0 to 0.0)	0.0 (0.0 to 0.0)	0.0 (0.0 to 0.0)	0.0 (0.0 to 0.0)
Other non-communicable diseases	623.3 (602.6 to 637.8)	4.4 (3.1 to 5.8)*	-1.3 (-2.6 to -0.1)*	24.9 (17.3 to 34.7)	9.8 (4.5 to 15.9)*	3.8 (-1.2 to 9.5)
Congenital anomalies	148.9 (137.7 to 158.2)	7.2 (3.8 to 10.5)*	1.3 (-1.9 to 4.4)	15.2 (9.3 to 22.5)	13.7 (4.9 to 23.5)*	7.5 (-0.9 to 16.7)
Neural tube defects	4.2 (2.8 to 6.1)	3.3 (2.1 to 4.5)*	-2.4 (-3.5 to -1.2)*	1.5 (0.9 to 2.6)	3.6 (-1.5 to 8.5)	-2.1 (-6.9 to 2.5)
Congenital heart anomalies	82.5 (60.3 to 120.2)	8.3 (6.8 to 9.6)*	2.4 (1.0 to 3.6)*	3.1 (1.3 to 5.7)	8.1 (6.1 to 10.0)*	2.2 (0.3 to 4.0)*
Less severe heart anomalies cases aggregate	66.6 (43.9 to 103.7)	8.9 (7.2 to 10.4)*	2.9 (1.3 to 4.4)*	2.2 (0.8 to 4.3)	9.1 (6.8 to 11.4)*	3.1 (0.9 to 5.3)*
Severe congenital heart anomalies	13.3 (8.8 to 19.8)	6.0 (4.4 to 8.2)*	0.1 (-1.3 to 2.3)	0.8 (0.3 to 1.6)	6.0 (2.4 to 9.4)*	0.2 (-3.2 to 3.4)
Critical congenital heart anomalies	2.5 (1.5 to 4.0)	5.7 (4.3 to 7.0)*	-0.1 (-1.4 to 1.1)	0.2 (0.1 to 0.3)	5.7 (-1.1 to 13.5)	-0.1 (-6.5 to 7.2)
Heart failure due to congenital heart anomalies aggregate	0.1 (0.1 to 0.1)	6.0 (4.9 to 7.2)*	0.2 (-0.8 to 1.3)	0.0 (0.0 to 0.0)	5.7 (-2.2 to 14.4)	-0.2 (-7.6 to 8.2)
Cleft lip and cleft palate	11.4 (6.5 to 19.0)	4.6 (0.1 to 7.8)*	-1.1 (-5.4 to 1.9)	0.2 (0.1 to 0.3)	-1.1 (-9.6 to 6.3)	-6.6 (-14.6 to 0.5)
Down syndrome	8.2 (5.2 to 12.7)	4.5 (2.3 to 6.4)*	-1.2 (-3.3 to 0.5)	0.7 (0.4 to 1.2)	4.6 (0.6 to 8.5)*	-1.1 (-5.0 to 2.6)
Turner syndrome	0.5 (0.2 to 0.9)	2.9 (1.4 to 4.3)*	-2.7 (-4.2 to -1.4)*	0.0 (0.0 to 0.0)	2.9 (-0.1 to 5.7)	-2.8 (-5.6 to -0.1)*
Klinefelter syndrome	0.3 (0.1 to 0.5)	4.4 (2.8 to 5.6)*	-1.4 (-2.8 to -0.2)*	0.0 (0.0 to 0.0)	4.4 (2.8 to 5.6)*	-1.4 (-2.8 to -0.2)*
Other chromosomal abnormalities	6.9 (3.8 to 12.1)	5.8 (3.7 to 7.2)*	-0.1 (-2.0 to 1.3)	0.6 (0.3 to 1.1)	5.8 (1.4 to 9.7)*	-0.0 (-4.2 to 3.6)
Other congenital anomalies	38.8 (21.4 to 70.5)	6.6 (5.0 to 8.2)*	0.8 (-0.8 to 2.3)	9.1 (5.2 to 14.0)	19.6 (4.0 to 38.5)*	13.0 (-1.7 to 30.9)
Skin and subcutaneous diseases	197.2 (190.2 to 203.9)	6.3 (4.7 to 7.9)*	0.5 (-1.1 to 2.0)	3.1 (1.9 to 5.0)	5.4 (3.5 to 7.5)*	-0.3 (-2.2 to 1.6)
Dermatitis	0.0 (0.0 to 0.0)	0.0 (0.0 to 0.0)	0.0 (0.0 to 0.0)	0.0 (0.0 to 0.0)	0.0 (0.0 to 0.0)	0.0 (0.0 to 0.0)
Eczema cases aggregate	0.0 (0.0 to 0.0)	0.0 (0.0 to 0.0)	0.0 (0.0 to 0.0)	0.0 (0.0 to 0.0)	0.0 (0.0 to 0.0)	0.0 (0.0 to 0.0)

Cause	Prevalence in thousands (95% confidence interval)			YLDs in thousands (95% confidence interval)		
	2015	Percent change in counts between 2005 and 2015	Percent change in rates between 2005 and 2015	2015	Percent change in counts between 2005 and 2015	Percent change in rates between 2005 and 2015
Contact dermatitis cases aggregate	0.0 (0.0 to 0.0)	0.0 (0.0 to 0.0)	0.0 (0.0 to 0.0)	0.0 (0.0 to 0.0)	0.0 (0.0 to 0.0)	0.0 (0.0 to 0.0)
Seborrhoeic dermatitis cases aggregate	0.0 (0.0 to 0.0)	0.0 (0.0 to 0.0)	0.0 (0.0 to 0.0)	0.0 (0.0 to 0.0)	0.0 (0.0 to 0.0)	0.0 (0.0 to 0.0)
Psoriasis	0.0 (0.0 to 0.0)	0.0 (0.0 to 0.0)	0.0 (0.0 to 0.0)	0.0 (0.0 to 0.0)	0.0 (0.0 to 0.0)	0.0 (0.0 to 0.0)
Cellulitis	1.0 (0.8 to 1.3)	8.7 (6.2 to 11.1)*	2.7 (0.4 to 5.0)*	0.1 (0.0 to 0.1)	8.8 (2.4 to 15.3)*	2.8 (-3.2 to 9.0)
Pyoderma	7.8 (7.3 to 8.3)	8.7 (7.2 to 10.3)*	2.7 (1.3 to 4.2)*	0.0 (0.0 to 0.1)	8.8 (5.0 to 12.7)*	2.8 (-0.7 to 6.5)
Scabies	33.2 (25.7 to 42.6)	3.2 (-0.3 to 6.4)	-2.5 (-5.8 to 0.5)	0.9 (0.4 to 1.5)	3.3 (-0.6 to 7.2)	-2.4 (-6.0 to 1.3)
Fungal skin diseases	93.8 (74.8 to 114.5)	6.5 (6.2 to 6.8)*	0.6 (0.3 to 0.9)*	0.5 (0.2 to 1.2)	6.5 (5.3 to 7.6)*	0.7 (-0.5 to 1.7)
Viral skin diseases	0.0 (0.0 to 0.0)	0.0 (0.0 to 0.0)	0.0 (0.0 to 0.0)	0.0 (0.0 to 0.0)	0.0 (0.0 to 0.0)	0.0 (0.0 to 0.0)
Molluscum contagiosum cases aggregate	0.0 (0.0 to 0.0)	0.0 (0.0 to 0.0)	0.0 (0.0 to 0.0)	0.0 (0.0 to 0.0)	0.0 (0.0 to 0.0)	0.0 (0.0 to 0.0)
Viral warts cases aggregate	0.0 (0.0 to 0.0)	0.0 (0.0 to 0.0)	0.0 (0.0 to 0.0)	0.0 (0.0 to 0.0)	0.0 (0.0 to 0.0)	0.0 (0.0 to 0.0)
Acne vulgaris	0.0 (0.0 to 0.0)	0.0 (0.0 to 0.0)	0.0 (0.0 to 0.0)	0.0 (0.0 to 0.0)	0.0 (0.0 to 0.0)	0.0 (0.0 to 0.0)
Alopecia areata	0.0 (0.0 to 0.0)	0.0 (0.0 to 0.0)	0.0 (0.0 to 0.0)	0.0 (0.0 to 0.0)	0.0 (0.0 to 0.0)	0.0 (0.0 to 0.0)
Pruritus	2.7 (2.1 to 3.6)	6.2 (3.5 to 9.1)*	0.4 (-2.1 to 3.1)	0.0 (0.0 to 0.1)	6.4 (-0.4 to 13.0)	0.5 (-5.9 to 6.8)
Urticaria	21.6 (16.9 to 27.6)	5.4 (5.1 to 5.7)*	-0.4 (-0.6 to -0.1)*	1.3 (0.8 to 2.0)	5.6 (2.4 to 8.9)*	-0.2 (-3.2 to 2.9)
Decubitus ulcer	0.0 (0.0 to 0.0)	0.0 (0.0 to 0.0)	0.0 (0.0 to 0.0)	0.0 (0.0 to 0.0)	0.0 (0.0 to 0.0)	0.0 (0.0 to 0.0)
Other skin and subcutaneous diseases	41.1 (38.2 to 44.6)	8.3 (6.9 to 9.9)*	2.4 (1.0 to 3.8)*	0.2 (0.1 to 0.5)	8.4 (5.8 to 11.3)*	2.4 (-0.0 to 5.2)
Sense organ diseases	293.6 (283.8 to 301.8)	1.9 (0.1 to 3.7)*	-3.7 (-5.4 to -2.0)*	6.6 (4.1 to 10.3)	3.5 (1.7 to 5.6)*	-2.1 (-3.9 to -0.2)*
Glaucoma	0.0 (0.0 to 0.0)	0.0 (0.0 to 0.0)	0.0 (0.0 to 0.0)	0.0 (0.0 to 0.0)	0.0 (0.0 to 0.0)	0.0 (0.0 to 0.0)
Cataract	0.0 (0.0 to 0.0)	0.0 (0.0 to 0.0)	0.0 (0.0 to 0.0)	0.0 (0.0 to 0.0)	0.0 (0.0 to 0.0)	0.0 (0.0 to 0.0)
Macular degeneration	0.0 (0.0 to 0.0)	0.0 (0.0 to 0.0)	0.0 (0.0 to 0.0)	0.0 (0.0 to 0.0)	0.0 (0.0 to 0.0)	0.0 (0.0 to 0.0)
Refraction and accommodation disorders	273.3 (246.0 to 303.7)	1.4 (-0.1 to 2.9)	-4.2 (-5.6 to -2.8)*	5.1 (2.9 to 8.4)	2.8 (1.1 to 4.8)*	-2.8 (-4.5 to -1.0)*
Age-related and other hearing loss	13.4 (8.5 to 19.8)	10.9 (7.2 to 15.2)*	4.8 (1.3 to 8.9)*	1.2 (0.6 to 1.9)	5.9 (0.3 to 13.1)*	0.1 (-5.3 to 6.8)
Other vision loss	8.6 (4.6 to 14.0)	6.1 (2.9 to 9.5)*	0.3 (-2.7 to 3.4)	0.3 (0.2 to 0.7)	6.6 (1.2 to 13.0)*	0.8 (-4.4 to 6.8)
Other sense organ diseases	0.0 (0.0 to 0.0)	0.0 (0.0 to 0.0)	0.0 (0.0 to 0.0)	0.0 (0.0 to 0.0)	0.0 (0.0 to 0.0)	0.0 (0.0 to 0.0)
Acute other sense organ diseases	0.0 (0.0 to 0.0)	0.0 (0.0 to 0.0)	0.0 (0.0 to 0.0)	0.0 (0.0 to 0.0)	0.0 (0.0 to 0.0)	0.0 (0.0 to 0.0)
Chronic other sense organ diseases	0.0 (0.0 to 0.0)	0.0 (0.0 to 0.0)	0.0 (0.0 to 0.0)	0.0 (0.0 to 0.0)	0.0 (0.0 to 0.0)	0.0 (0.0 to 0.0)
Oral disorders	0.0 (0.0 to 0.0)	0.0 (0.0 to 0.0)	0.0 (0.0 to 0.0)	0.0 (0.0 to 0.0)	0.0 (0.0 to 0.0)	0.0 (0.0 to 0.0)
Deciduous caries	0.0 (0.0 to 0.0)	0.0 (0.0 to 0.0)	0.0 (0.0 to 0.0)	0.0 (0.0 to 0.0)	0.0 (0.0 to 0.0)	0.0 (0.0 to 0.0)
Permanent caries	0.0 (0.0 to 0.0)	0.0 (0.0 to 0.0)	0.0 (0.0 to 0.0)	0.0 (0.0 to 0.0)	0.0 (0.0 to 0.0)	0.0 (0.0 to 0.0)
Periodontal diseases	0.0 (0.0 to 0.0)	0.0 (0.0 to 0.0)	0.0 (0.0 to 0.0)	0.0 (0.0 to 0.0)	0.0 (0.0 to 0.0)	0.0 (0.0 to 0.0)
Edentulism and severe tooth loss	0.0 (0.0 to 0.0)	0.0 (0.0 to 0.0)	0.0 (0.0 to 0.0)	0.0 (0.0 to 0.0)	0.0 (0.0 to 0.0)	0.0 (0.0 to 0.0)
Other oral disorders	0.0 (0.0 to 0.0)	0.0 (0.0 to 0.0)	0.0 (0.0 to 0.0)	0.0 (0.0 to 0.0)	0.0 (0.0 to 0.0)	0.0 (0.0 to 0.0)
Injuries	15.5 (13.9 to 17.0)	-7.9 (-9.3 to -6.6)*	-13.0 (-14.3 to -11.7)*	1.2 (0.9 to 1.7)	-9.5 (-11.1 to -7.9)*	-14.4 (-15.9 to -13.0)*
Transport injuries	0.1 (0.1 to 0.1)	0.8 (-2.1 to 3.8)	-4.7 (-7.4 to -1.9)*	0.0 (0.0 to 0.0)	-3.6 (-6.9 to -0.2)*	-8.9 (-12.0 to -5.7)*
Road injuries	0.1 (0.0 to 0.1)	5.5 (1.0 to 10.5)*	-0.3 (-4.6 to 4.5)	0.0 (0.0 to 0.0)	2.0 (-3.1 to 7.6)	-3.6 (-8.4 to 1.6)
Pedestrian road injuries	0.0 (0.0 to 0.0)	-0.6 (-5.9 to 5.2)	-6.1 (-11.0 to -0.5)*	0.0 (0.0 to 0.0)	-4.8 (-10.7 to 1.3)	-10.0 (-15.7 to -4.2)*
Cyclist road injuries	0.0 (0.0 to 0.0)	11.7 (5.6 to 18.1)*	5.5 (-0.2 to 11.6)	0.0 (0.0 to 0.0)	7.6 (1.0 to 14.5)*	1.7 (-4.6 to 8.2)
Motorcyclist road injuries	0.0 (0.0 to 0.0)	6.4 (0.8 to 12.4)*	0.5 (-4.7 to 6.3)	0.0 (0.0 to 0.0)	2.8 (-3.4 to 9.5)	-2.9 (-8.7 to 3.5)
Motor vehicle road injuries	0.0 (0.0 to 0.0)	4.1 (-0.8 to 9.2)	-1.6 (-6.3 to 3.2)	0.0 (0.0 to 0.0)	1.2 (-4.2 to 6.7)	-4.4 (-9.5 to 0.8)
Other road injuries	0.0 (0.0 to 0.0)	17.7 (10.8 to 24.8)*	11.2 (4.7 to 18.0)*	0.0 (0.0 to 0.0)	14.4 (6.9 to 22.0)*	8.1 (1.1 to 15.3)*
Other transport injuries	0.1 (0.0 to 0.1)	-3.2 (-6.4 to -0.3)*	-8.5 (-11.6 to -5.8)*	0.0 (0.0 to 0.0)	-7.8 (-11.3 to -4.3)*	-12.8 (-16.1 to -9.5)*
Unintentional injuries	15.3 (13.7 to 16.7)	-7.9 (-9.3 to -6.5)*	-13.0 (-14.3 to -11.7)*	1.2 (0.9 to 1.7)	-9.4 (-11.0 to -7.9)*	-14.4 (-15.9 to -12.9)*
Falls	1.7 (1.4 to 2.1)	8.4 (5.4 to 11.4)*	2.5 (-0.4 to 5.3)	0.1 (0.1 to 0.2)	5.5 (2.1 to 8.8)*	-0.3 (-3.5 to 2.8)
Drowning	0.0 (0.0 to 0.0)	-8.1 (-10.5 to -5.9)*	-13.1 (-15.4 to -11.1)*	0.0 (0.0 to 0.0)	-12.2 (-15.1 to -9.3)*	-17.0 (-19.7 to -14.3)*
Fire, heat, and hot substances	1.4 (1.1 to 1.7)	-3.9 (-6.7 to -1.5)*	-9.1 (-11.8 to -6.9)*	0.2 (0.1 to 0.2)	-7.8 (-11.5 to -4.1)*	-12.9 (-16.4 to -9.4)*
Poisonings	0.1 (0.1 to 0.2)	5.6 (3.7 to 7.4)*	-0.2 (-2.0 to 1.5)	0.0 (0.0 to 0.0)	4.0 (2.0 to 5.9)*	-1.7 (-3.6 to 0.0)
Exposure to mechanical forces	3.2 (2.7 to 3.8)	-11.3 (-13.7 to -8.9)*	-16.2 (-18.5 to -13.9)*	0.1 (0.1 to 0.2)	-13.7 (-16.3 to -10.8)*	-18.5 (-20.9 to -15.7)*
Unintentional firearm injuries	0.0 (0.0 to 0.0)	-8.3 (-14.3 to -2.7)*	-13.3 (-19.0 to -8.1)*	0.0 (0.0 to 0.0)	-9.9 (-16.9 to -3.4)*	-14.8 (-21.5 to -8.7)*
Unintentional suffocation	0.8 (0.7 to 1.1)	-9.2 (-12.6 to -5.8)*	-14.2 (-17.4 to -11.0)*	0.0 (0.0 to 0.1)	-10.5 (-14.0 to -6.8)*	-15.4 (-18.7 to -11.9)*
Other exposure to mechanical forces	2.3 (1.9 to 2.8)	-12.0 (-14.6 to -9.6)*	-16.9 (-19.3 to -14.5)*	0.1 (0.1 to 0.1)	-15.1 (-18.0 to -12.2)*	-19.8 (-22.5 to -17.0)*
Adverse effects of medical treatment	1.8 (1.4 to 2.3)	-11.1 (-14.1 to -8.1)*	-16.0 (-18.8 to -13.1)*	0.2 (0.1 to 0.4)	-11.1 (-14.1 to -8.1)*	-16.0 (-18.8 to -13.1)*
Animal contact	0.7 (0.5 to 0.9)	-10.3 (-14.5 to -5.8)*	-15.2 (-19.2 to -11.0)*	0.0 (0.0 to 0.0)	-10.3 (-14.2 to -6.3)*	-15.2 (-18.9 to -11.5)*
Venomous animal contact	0.1 (0.1 to 0.1)	-9.8 (-13.9 to -5.5)*	-14.8 (-18.7 to -10.7)*	0.0 (0.0 to 0.0)	-10.3 (-14.3 to -6.3)*	-15.3 (-19.0 to -11.4)*
Non-venomous animal contact	0.6 (0.4 to 0.8)	-10.4 (-14.9 to -5.6)*	-15.3 (-19.6 to -10.8)*	0.0 (0.0 to 0.0)	-10.3 (-15.0 to -5.7)*	-15.2 (-19.7 to -10.9)*
Foreign body	1.3 (1.1 to 1.6)	-6.4 (-8.1 to -4.6)*	-11.5 (-13.2 to -9.8)*	0.1 (0.1 to 0.1)	-10.3 (-12.5 to -8.1)*	-15.2 (-17.3 to -13.1)*
Pulmonary aspiration and foreign body in airway	0.6 (0.5 to 0.8)	-9.1 (-11.2 to -7.2)*	-14.1 (-16.1 to -12.3)*	0.1 (0.0 to 0.1)	-14.1 (-16.5 to -11.6)*	-18.8 (-21.1 to -16.4)*
Foreign body in eyes	0.1 (0.0 to 0.1)	4.5 (3.8 to 5.1)*	-1.2 (-1.9 to -0.6)*	0.0 (0.0 to 0.0)	4.0 (2.5 to 4.7)*	-1.7 (-3.2 to -1.0)*
Foreign body in other body part	0.7 (0.5 to 0.9)	-4.7 (-7.6 to -1.9)*	-9.9 (-12.7 to -7.3)*	0.0 (0.0 to 0.1)	-5.3 (-8.6 to -2.5)*	-10.5 (-13.6 to -7.8)*
Environmental heat and cold exposure	0.7 (0.6 to 0.9)	0.1 (-3.3 to 3.7)	-5.4 (-8.7 to -2.0)*	0.1 (0.0 to 0.1)	-1.9 (-5.7 to 2.2)	-7.3 (-10.9 to -3.4)*
Other unintentional injuries	4.3 (3.6 to 5.1)	-12.3 (-15.6 to -8.7)*	-17.1 (-20.2 to -13.7)*	0.3 (0.2 to 0.5)	-14.1 (-17.7 to -10.0)*	-18.8 (-22.2 to -15.0)*
Self-harm and interpersonal violence	0.1 (0.1 to 0.1)	-17.7 (-21.3 to -13.7)*	-22.2 (-25.6 to -18.5)*	0.0 (0.0 to 0.0)	-19.0 (-23.2 to -14.7)*	-23.5 (-27.4 to -19.4)*
Self-harm	0.0 (0.0 to 0.0)	2.1 (0.7 to 3.4)*	-3.5 (-4.8 to -2.3)*	0.0 (0.0 to 0.0)	-1.2 (-2.8 to 0.3)	-6.7 (-8.1 to -5.2)*
Interpersonal violence	0.1 (0.1 to 0.1)	-18.4 (-22.3 to -14.3)*	-22.9 (-26.6 to -19.0)*	0.0 (0.0 to 0.0)	-19.9 (-24.3 to -15.4)*	-24.3 (-28.5 to -20.1)*

Cause	Prevalence in thousands (95% confidence interval)			YLDs in thousands (95% confidence interval)		
	2015	Percent change in counts between 2005 and 2015	Percent change in rates between 2005 and 2015	2015	Percent change in counts between 2005 and 2015	Percent change in rates between 2005 and 2015
Assault by firearm	0.0 (0.0 to 0.0)	-14.8 (-20.1 to -9.4)*	-19.4 (-24.5 to -14.4)*	0.0 (0.0 to 0.0)	-17.4 (-24.1 to -10.5)*	-21.9 (-28.3 to -15.4)*
Assault by sharp object	0.0 (0.0 to 0.0)	-17.2 (-22.9 to -11.4)*	-21.7 (-27.1 to -16.2)*	0.0 (0.0 to 0.0)	-19.7 (-26.1 to -13.3)*	-24.1 (-30.2 to -18.0)*
Assault by other means	0.1 (0.1 to 0.1)	-18.6 (-22.6 to -14.5)*	-23.1 (-26.8 to -19.2)*	0.0 (0.0 to 0.0)	-19.9 (-24.3 to -15.5)*	-24.3 (-28.5 to -20.2)*
Forces of nature, war, and legal intervention	0.0 (0.0 to 0.0)	0.0 (0.0 to 0.0)	0.0 (0.0 to 0.0)	0.0 (0.0 to 0.0)	0.0 (0.0 to 0.0)	0.0 (0.0 to 0.0)
Exposure to forces of nature	0.0 (0.0 to 0.0)	0.0 (0.0 to 0.0)	0.0 (0.0 to 0.0)	0.0 (0.0 to 0.0)	0.0 (0.0 to 0.0)	0.0 (0.0 to 0.0)
Collective violence and legal intervention	0.0 (0.0 to 0.0)	0.0 (0.0 to 0.0)	0.0 (0.0 to 0.0)	0.0 (0.0 to 0.0)	0.0 (0.0 to 0.0)	0.0 (0.0 to 0.0)
**Anemia**	**4,435.6 (4,386.7 to 4,490.1)**	**2.8 (1.7 to 4.2)***	**-2.8 (-3.9 to -1.6)***	**155.3 (104.1 to 224.6)**	**1.6 (-0.1 to 3.4)**	**-4.0 (-5.6 to -2.3)***
**Developmental intellectual disability**	**54.5 (40.4 to 70.4)**	**4.1 (3.2 to 4.9)***	**-1.6 (-2.4 to -0.8)***	**5.9 (4.0 to 8.6)**	**5.0 (3.2 to 7.0)***	**-0.8 (-2.5 to 1.1)**
**Epilepsy**	**35.4 (28.4 to 43.8)**	**5.1 (-1.3 to 11.3)**	**-0.7 (-6.7 to 5.2)**	**10.6 (7.3 to 14.3)**	**-5.3 (-12.4 to 1.8)**	**-10.5 (-17.2 to -3.8)***
**Guillain-Barré syndrome**	**0.0 (0.0 to 0.0)**	**6.9 (6.2 to 7.4)***	**1.0 (0.3 to 1.5)***	**0.0 (0.0 to 0.0)**	**6.9 (6.2 to 7.4)***	**1.0 (0.3 to 1.5)***
**Hearing loss**	**60.3 (38.3 to 96.4)**	**6.6 (5.5 to 7.6)***	**0.8 (-0.3 to 1.7)**	**4.5 (2.3 to 7.8)**	**4.1 (1.0 to 7.2)***	**-1.6 (-4.5 to 1.3)**
**Heart failure**	**0.1 (0.1 to 0.1)**	**6.0 (4.9 to 7.2)***	**0.2 (-0.8 to 1.3)**	**0.0 (0.0 to 0.0)**	**5.7 (-2.2 to 14.4)**	**-0.1 (-7.6 to 8.1)**
**Infertility**	**0.0 (0.0 to 0.0)**	**0.0 (0.0 to 0.0)**	**0.0 (0.0 to 0.0)**	**0.0 (0.0 to 0.0)**	**0.0 (0.0 to 0.0)**	**0.0 (0.0 to 0.0)**
**Pelvic inflammatory disease**	**0.0 (0.0 to 0.0)**	**0.0 (0.0 to 0.0)**	**0.0 (0.0 to 0.0)**	**0.0 (0.0 to 0.0)**	**0.0 (0.0 to 0.0)**	**0.0 (0.0 to 0.0)**
**Vision loss**	**286.8 (257.4 to 321.1)**	**1.5 (0.1 to 3.0)***	**-4.0 (-5.4 to -2.7)***	**6.7 (4.3 to 10.4)**	**3.4 (1.7 to 5.0)***	**-2.3 (-3.9 to -0.8)***

SR Table 11. Prevalent cases and YLDs for 2015, percent change, and change in age-standardised rates between 2005 and 2015 by cause, global, ages 28-364 days						
Cause	Prevalence in thousands (95% confidence interval)			YLDs in thousands (95% confidence interval)		
	2015	Percent change in counts between 2005 and 2015	Percent change in rates between 2005 and 2015	2015	Percent change in counts between 2005 and 2015	Percent change in rates between 2005 and 2015
**All causes**	--	--	--	**6,988.0 (4,922.4 to 9,554.5)**	**-1.6 (-5.1 to 2.3)**	**-7.7 (-10.9 to -3.9)***
Communicable, maternal, neonatal, and nutritional diseases	81,269.7 (80,683.0 to 81,828.3)	2.8 (1.9 to 3.8)*	-3.5 (-4.3 to -2.6)*	5,314.4 (3,718.7 to 7,267.8)	-2.8 (-6.8 to 1.9)	-8.7 (-12.5 to -4.3)*
HIV/AIDS and tuberculosis	215.2 (207.6 to 223.1)	-32.2 (-35.2 to -29.4)*	-36.3 (-39.1 to -33.7)*	30.2 (21.3 to 40.3)	-31.4 (-35.9 to -27.3)*	-35.6 (-39.8 to -31.7)*
Tuberculosis	32.3 (24.6 to 41.3)	-37.5 (-44.6 to -30.8)*	-41.3 (-48.0 to -35.1)*	10.3 (6.4 to 15.4)	-37.1 (-44.8 to -29.2)*	-41.0 (-48.2 to -33.5)*
HIV/AIDS	184.1 (176.9 to 191.5)	-31.2 (-34.4 to -28.1)*	-35.4 (-38.5 to -32.5)*	19.9 (14.4 to 26.0)	-28.0 (-32.7 to -23.3)*	-32.4 (-36.9 to -28.0)*
HIV/AIDS - Tuberculosis	5.9 (5.3 to 6.5)	-54.6 (-56.2 to -52.9)*	-57.4 (-58.9 to -55.8)*	2.3 (1.5 to 3.2)	-54.0 (-58.0 to -50.2)*	-56.8 (-60.5 to -53.2)*
HIV/AIDS resulting in other diseases	183.5 (175.7 to 192.2)	-29.0 (-31.5 to -26.5)*	-33.4 (-35.7 to -31.0)*	17.6 (12.8 to 22.9)	-22.2 (-28.0 to -16.4)*	-27.0 (-32.4 to -21.5)*
HIV aggregate	130.1 (123.7 to 137.1)	-47.7 (-49.8 to -45.6)*	-50.9 (-52.8 to -48.9)*	9.5 (6.8 to 12.7)	-47.5 (-50.8 to -44.0)*	-50.7 (-53.8 to -47.4)*
AIDS aggregate	53.5 (50.8 to 56.0)	440.8 (360.2 to 548.1)*	407.6 (332.0 to 508.4)*	8.1 (5.9 to 10.3)	81.5 (43.3 to 137.8)*	70.3 (34.6 to 123.2)*
Diarrhea, lower respiratory, and other common infectious diseases	13,644.8 (13,087.0 to 14,115.9)	0.9 (-0.4 to 2.2)	-5.3 (-6.5 to -4.0)*	855.8 (586.7 to 1,180.6)	-1.6 (-3.2 to -0.1)*	-7.7 (-9.1 to -6.2)*
Diarrheal diseases	3,690.9 (3,309.2 to 4,065.7)	1.6 (0.1 to 3.4)*	-4.6 (-6.1 to -3.0)*	599.2 (398.5 to 825.6)	1.9 (0.2 to 3.8)*	-4.4 (-6.0 to -2.6)*
Diarrhoea episodes aggregate	3,690.8 (3,309.1 to 4,065.7)	1.6 (0.1 to 3.4)*	-4.6 (-6.1 to -3.0)*	599.1 (398.5 to 825.6)	1.9 (0.2 to 3.8)*	-4.4 (-6.0 to -2.6)*
Guillain-Barré syndrome due to diarrheal diseases	0.0 (0.0 to 0.1)	7.6 (6.9 to 8.1)*	1.0 (0.4 to 1.5)*	0.0 (0.0 to 0.0)	7.6 (6.9 to 8.1)*	1.0 (0.4 to 1.5)*
Intestinal infectious diseases	51.3 (46.5 to 56.4)	-29.4 (-34.6 to -23.8)*	-33.7 (-38.6 to -28.4)*	6.5 (4.3 to 9.8)	-27.7 (-37.2 to -17.1)*	-32.1 (-41.1 to -22.2)*
Typhoid fever	43.4 (34.2 to 54.6)	-27.2 (-31.5 to -22.4)*	-31.7 (-35.7 to -27.2)*	5.7 (3.7 to 8.5)	-26.9 (-37.8 to -14.5)*	-31.4 (-41.6 to -19.7)*
Typhoid fever episodes aggregate	35.9 (28.1 to 45.6)	-27.2 (-33.8 to -19.5)*	-31.7 (-37.8 to -24.4)*	3.4 (2.2 to 5.2)	-27.1 (-37.0 to -16.8)*	-31.5 (-40.8 to -21.9)*
Complications of typhoid fever aggregate	7.5 (5.1 to 10.4)	-27.2 (-51.2 to 6.6)	-31.7 (-54.2 to 0.1)	2.3 (1.4 to 3.7)	-26.6 (-51.5 to 11.2)	-31.1 (-54.5 to 4.3)
Paratyphoid fever	14.2 (9.4 to 20.1)	-26.6 (-35.0 to -17.6)*	-31.1 (-39.0 to -22.7)*	0.7 (0.4 to 1.2)	-25.9 (-40.9 to -5.0)*	-30.4 (-44.5 to -10.9)*
Paratyphoid fever episodes aggregate	13.5 (8.9 to 19.0)	-26.6 (-35.0 to -17.4)*	-31.1 (-39.0 to -22.5)*	0.7 (0.4 to 1.1)	-26.0 (-41.7 to -3.8)*	-30.6 (-45.2 to -9.7)*
Intestinal perforation due to paratyphoid	0.7 (0.4 to 1.2)	-25.0 (-58.7 to 36.2)	-29.6 (-61.2 to 27.8)	0.1 (0.0 to 0.2)	-24.9 (-58.7 to 36.2)	-29.5 (-61.2 to 27.8)
Other intestinal infectious diseases	--	--	--	0.1 (0.0 to 0.1)	-70.6 (-82.0 to -51.6)*	-72.4 (-83.1 to -54.6)*
Lower respiratory infections	826.8 (752.2 to 915.5)	-9.1 (-10.8 to -7.4)*	-14.7 (-16.3 to -13.1)*	50.7 (34.0 to 73.1)	-9.0 (-11.1 to -6.9)*	-14.6 (-16.6 to -12.6)*
Lower respiratory infection episodes aggregate	826.8 (752.2 to 915.4)	-9.1 (-10.8 to -7.4)*	-14.7 (-16.3 to -13.1)*	50.7 (34.0 to 73.1)	-9.0 (-11.2 to -6.9)*	-14.6 (-16.6 to -12.6)*
Guillain-Barré syndrome due to lower respiratory infections	0.0 (0.0 to 0.1)	7.6 (6.9 to 8.1)*	1.0 (0.4 to 1.5)*	0.0 (0.0 to 0.0)	7.6 (6.9 to 8.1)*	1.0 (0.4 to 1.5)*
Upper respiratory infections	5,728.9 (4,731.9 to 7,173.0)	7.8 (6.1 to 9.2)*	1.2 (-0.4 to 2.5)	68.1 (36.7 to 117.5)	7.9 (6.0 to 9.6)*	1.3 (-0.5 to 2.9)
Upper respiratory infection episodes aggregate	5,728.8 (4,731.8 to 7,172.8)	7.8 (6.1 to 9.2)*	1.2 (-0.4 to 2.5)	68.1 (36.6 to 117.4)	7.9 (6.0 to 9.6)*	1.3 (-0.5 to 2.9)
Guillain-Barré syndrome due to upper respiratory infections	0.1 (0.1 to 0.2)	7.6 (6.9 to 8.1)*	1.0 (0.4 to 1.5)*	0.0 (0.0 to 0.1)	7.6 (6.9 to 8.1)*	1.0 (0.4 to 1.5)*
Otitis media	2,562.8 (2,087.6 to 3,174.8)	5.7 (3.6 to 7.6)*	-0.8 (-2.7 to 1.0)	60.6 (37.0 to 94.3)	3.7 (1.1 to 6.2)*	-2.7 (-5.1 to -0.3)*
Acute otitis media	1,720.2 (1,272.6 to 2,305.6)	6.4 (3.6 to 9.1)*	-0.2 (-2.8 to 2.4)	22.6 (10.7 to 41.6)	6.4 (3.5 to 9.4)*	-0.1 (-2.9 to 2.6)
Chronic otitis media aggregate	842.7 (706.9 to 1,007.3)	4.3 (2.3 to 6.1)*	-2.1 (-3.9 to -0.4)*	38.0 (23.8 to 55.5)	2.1 (-1.6 to 5.7)	-4.1 (-7.6 to -0.7)*
Meningitis	69.5 (65.6 to 73.9)	6.0 (-1.1 to 13.4)	-0.5 (-7.2 to 6.4)	15.3 (10.6 to 20.8)	5.6 (-0.1 to 12.0)	-0.9 (-6.2 to 5.1)
Pneumococcal meningitis	43.9 (37.3 to 51.7)	15.7 (12.0 to 19.9)*	8.6 (5.2 to 12.6)*	5.2 (3.6 to 7.0)	12.9 (3.6 to 23.2)*	6.0 (-2.7 to 15.6)
Acute pneumococcal meningitis	6.5 (4.3 to 9.1)	23.8 (8.8 to 40.4)*	16.2 (2.2 to 31.8)*	0.8 (0.5 to 1.3)	23.7 (0.8 to 51.4)*	16.1 (-5.4 to 42.1)
Complications of pneumococcal meningitis aggregate	37.4 (32.0 to 44.0)	14.4 (11.7 to 17.5)*	7.4 (4.9 to 10.3)*	4.4 (3.0 to 5.8)	11.0 (0.9 to 21.5)*	4.2 (-5.3 to 14.0)
H influenzae type B meningitis	24.8 (20.5 to 30.3)	-17.1 (-20.9 to -12.8)*	-22.2 (-25.7 to -18.1)*	3.4 (2.3 to 4.7)	-9.4 (-17.4 to -1.2)*	-15.0 (-22.4 to -7.3)*
Acute H influenzae type B meningitis	5.8 (4.0 to 8.5)	-26.4 (-35.5 to -16.6)*	-30.9 (-39.5 to -21.7)*	0.8 (0.4 to 1.3)	-25.4 (-40.3 to -10.3)*	-30.0 (-43.9 to -15.8)*
Complications of of H influenzae type B meningitis aggregate	19.0 (15.9 to 22.8)	-13.7 (-17.6 to -9.2)*	-19.0 (-22.6 to -14.7)*	2.6 (1.8 to 3.6)	-3.4 (-12.6 to 6.5)	-9.3 (-18.0 to -0.0)*
Meningococcal meningitis	15.2 (11.8 to 19.2)	13.2 (6.4 to 20.9)*	6.3 (-0.1 to 13.5)	1.8 (1.2 to 2.5)	14.5 (-2.7 to 32.6)	7.5 (-8.6 to 24.5)
Acute meningococcal meningitis	5.1 (3.5 to 6.9)	15.8 (-0.1 to 32.9)	8.7 (-6.3 to 24.7)	0.7 (0.4 to 1.1)	16.3 (-9.7 to 44.8)	9.1 (-15.2 to 36.0)
Complications of meningococcal meningitis aggregate	10.1 (7.7 to 12.9)	11.9 (7.5 to 16.5)*	5.1 (0.9 to 9.3)*	1.1 (0.8 to 1.5)	13.4 (-5.4 to 38.7)	6.5 (-11.2 to 30.2)
Other meningitis	39.5 (33.0 to 47.1)	8.8 (1.7 to 16.4)*	2.2 (-4.5 to 9.2)	5.0 (3.2 to 7.1)	7.4 (-2.3 to 18.0)	0.9 (-8.3 to 10.8)
Other meningitis episodes aggregate	25.3 (20.1 to 32.0)	8.1 (-3.4 to 19.3)	1.5 (-9.3 to 12.0)	3.3 (2.0 to 5.0)	9.2 (-5.6 to 24.8)	2.5 (-11.4 to 17.1)
Complications of other meningitis aggregate	14.0 (11.2 to 17.3)	9.0 (5.8 to 12.2)*	2.3 (-0.6 to 5.3)	1.7 (1.1 to 2.2)	4.2 (-2.6 to 11.1)	-2.2 (-8.5 to 4.3)
Encephalitis	14.5 (12.0 to 17.4)	-9.7 (-12.1 to -6.9)*	-15.2 (-17.4 to -12.6)*	1.8 (1.2 to 2.5)	-10.6 (-16.3 to -4.3)*	-16.0 (-21.4 to -10.2)*
Acute encephalitis	6.5 (5.4 to 7.7)	-5.2 (-9.0 to -1.2)*	-11.0 (-14.5 to -7.3)*	0.8 (0.5 to 1.3)	-5.1 (-17.8 to 9.4)	-10.9 (-22.9 to 2.7)
Complications of encephalitis aggregate	8.0 (6.0 to 10.4)	-13.0 (-14.9 to -11.0)*	-18.4 (-20.1 to -16.5)*	1.0 (0.7 to 1.3)	-14.7 (-17.6 to -12.0)*	-20.0 (-22.7 to -17.4)*
Diphtheria	0.1 (0.0 to 0.3)	-61.5 (-88.9 to 33.6)	-63.9 (-89.6 to 25.4)	0.0 (0.0 to 0.0)	-61.6 (-88.9 to 33.6)	-63.9 (-89.6 to 25.4)
Whooping cough	981.8 (759.1 to 1,231.8)	-28.2 (-30.2 to -26.0)*	-32.6 (-34.5 to -30.5)*	48.4 (28.8 to 73.8)	-28.1 (-30.3 to -25.6)*	-32.5 (-34.5 to -30.2)*
Tetanus	4.2 (4.1 to 4.4)	-11.3 (-15.1 to -7.7)*	-16.7 (-20.3 to -13.4)*	0.2 (0.1 to 0.3)	-28.7 (-37.5 to -19.6)*	-33.1 (-41.3 to -24.6)*
Severe tetanus	0.3 (0.2 to 0.4)	-61.6 (-71.5 to -48.1)*	-64.0 (-73.3 to -51.3)*	0.0 (0.0 to 0.1)	-61.5 (-71.3 to -47.4)*	-63.8 (-73.1 to -50.7)*
Complications of tetanus aggregate	3.9 (3.8 to 4.0)	-0.7 (-1.1 to -0.2)*	-6.8 (-7.2 to -6.3)*	0.2 (0.1 to 0.2)	-7.6 (-15.8 to 2.3)	-13.2 (-21.0 to -4.0)*
Measles	29.8 (13.3 to 59.1)	-71.0 (-74.4 to -66.9)*	-72.8 (-75.9 to -68.9)*	2.7 (1.0 to 5.8)	-70.9 (-74.9 to -65.8)*	-72.7 (-76.5 to -67.9)*
Varicella and herpes zoster	373.7 (271.3 to 499.1)	5.3 (4.5 to 6.1)*	-1.1 (-1.9 to -0.4)*	2.2 (0.8 to 4.6)	5.4 (3.3 to 7.5)*	-1.1 (-3.0 to 0.9)
Neglected tropical diseases and malaria	26,268.9 (25,571.1 to 27,016.9)	-7.4 (-10.6 to -4.3)*	-13.1 (-16.1 to -10.2)*	401.6 (275.8 to 566.2)	-2.8 (-8.6 to 6.2)	-8.7 (-14.2 to -0.3)*
Malaria	4,686.6 (3,704.1 to 5,979.8)	15.6 (6.3 to 26.0)*	8.5 (-0.2 to 18.3)	86.0 (58.0 to 122.9)	3.3 (-7.0 to 22.8)	-3.1 (-12.7 to 15.3)
Asymptomatic malaria parasitemia(PfPR)	2,574.8 (1,618.6 to 3,828.0)	39.4 (17.7 to 81.2)*	30.8 (10.5 to 70.1)*	0.0 (0.0 to 0.0)	0.0 (0.0 to 0.0)	0.0 (0.0 to 0.0)
Malaria episodes aggregate	698.6 (372.0 to 1,134.8)	-36.0 (-47.3 to -27.6)*	-39.9 (-50.5 to -32.0)*	14.5 (6.3 to 26.9)	-35.9 (-47.2 to -27.4)*	-39.8 (-50.4 to -31.8)*
Complications of malaria aggregate	0.6 (0.5 to 0.7)	17.5 (10.6 to 24.7)*	10.3 (3.8 to 17.0)*	0.1 (0.1 to 0.2)	16.3 (8.9 to 23.9)*	9.2 (2.2 to 16.3)*
Anemia due to malaria parasitemia(PfPR) aggregate	1,412.6 (1,328.3 to 1,518.2)	26.8 (18.0 to 38.2)*	19.0 (10.8 to 29.7)*	71.4 (47.3 to 100.8)	17.8 (4.7 to 44.3)*	10.6 (-1.7 to 35.4)
Chagas disease	23.9 (19.9 to 28.5)	8.5 (1.2 to 16.0)*	1.9 (-5.0 to 8.9)	0.0 (0.0 to 0.0)	-35.6 (-43.4 to -24.4)*	-39.6 (-46.9 to -29.0)*
Chagas disease episodes aggregate	23.9 (19.9 to 28.4)	8.5 (1.2 to 16.0)*	1.9 (-5.0 to 8.9)	0.0 (0.0 to 0.0)	-46.6 (-54.8 to -38.9)*	-49.9 (-57.5 to -42.7)*
Complications of Chagas disease aggregate	0.0 (0.0 to 0.0)	8.5 (1.2 to 16.0)*	1.9 (-5.0 to 8.9)	0.0 (0.0 to 0.0)	8.5 (1.2 to 16.0)*	1.9 (-5.0 to 8.9)

Cause	Prevalence in thousands (95% confidence interval)			YLDs in thousands (95% confidence interval)		
	2015	Percent change in counts between 2005 and 2015	Percent change in rates between 2005 and 2015	2015	Percent change in counts between 2005 and 2015	Percent change in rates between 2005 and 2015
Heart failure due to Chagas disease aggregate	0.0 (0.0 to 0.0)	9.4 (2.0 to 17.3)*	2.7 (-4.3 to 10.1)	0.0 (0.0 to 0.0)	9.4 (2.0 to 17.3)*	2.7 (-4.3 to 10.1)
Leishmaniasis	18.3 (16.1 to 21.6)	5.5 (-11.9 to 27.4)	-1.0 (-17.3 to 19.6)	0.3 (0.2 to 0.6)	4.0 (-5.8 to 14.6)	-2.4 (-11.6 to 7.5)
Visceral leishmaniasis	1.9 (1.7 to 2.2)	2.0 (0.4 to 3.7)*	-4.3 (-5.7 to -2.6)*	0.1 (0.1 to 0.2)	1.8 (-17.6 to 26.1)	-4.5 (-22.6 to 18.4)
Cutaneous and mucocutaneous leishmaniasis	19.2 (16.1 to 23.8)	5.3 (2.1 to 9.4)*	-1.1 (-4.2 to 2.7)	0.2 (0.1 to 0.4)	5.4 (-2.4 to 14.3)	-1.0 (-8.4 to 7.3)
African trypanosomiasis	0.0 (0.0 to 0.0)	0.0 (0.0 to 0.0)	0.0 (0.0 to 0.0)	0.0 (0.0 to 0.0)	0.0 (0.0 to 0.0)	0.0 (0.0 to 0.0)
Schistosomiasis	0.0 (0.0 to 0.0)	0.0 (0.0 to 0.0)	0.0 (0.0 to 0.0)	0.0 (0.0 to 0.0)	0.0 (0.0 to 0.0)	0.0 (0.0 to 0.0)
Schistosomiasis episodes aggregate	0.0 (0.0 to 0.0)	0.0 (0.0 to 0.0)	0.0 (0.0 to 0.0)	0.0 (0.0 to 0.0)	0.0 (0.0 to 0.0)	0.0 (0.0 to 0.0)
Complications of schistosomiasis aggregate	0.0 (0.0 to 0.0)	0.0 (0.0 to 0.0)	0.0 (0.0 to 0.0)	0.0 (0.0 to 0.0)	0.0 (0.0 to 0.0)	0.0 (0.0 to 0.0)
Anemia due to schistosomiasis aggregate	0.0 (0.0 to 0.0)	0.0 (0.0 to 0.0)	0.0 (0.0 to 0.0)	0.0 (0.0 to 0.0)	0.0 (0.0 to 0.0)	0.0 (0.0 to 0.0)
Cysticercosis	0.0 (0.0 to 0.0)	0.0 (0.0 to 0.0)	0.0 (0.0 to 0.0)	0.0 (0.0 to 0.0)	0.0 (0.0 to 0.0)	0.0 (0.0 to 0.0)
Cystic echinococcosis	0.0 (0.0 to 0.0)	0.0 (0.0 to 0.0)	0.0 (0.0 to 0.0)	0.0 (0.0 to 0.0)	0.0 (0.0 to 0.0)	0.0 (0.0 to 0.0)
Lymphatic filariasis	0.0 (0.0 to 0.0)	0.0 (0.0 to 0.0)	0.0 (0.0 to 0.0)	0.0 (0.0 to 0.0)	0.0 (0.0 to 0.0)	0.0 (0.0 to 0.0)
Prevalence of detectable microfiliaria due to lymphatic filariasis	0.0 (0.0 to 0.0)	0.0 (0.0 to 0.0)	0.0 (0.0 to 0.0)	0.0 (0.0 to 0.0)	0.0 (0.0 to 0.0)	0.0 (0.0 to 0.0)
Complications of lymphatic filariasis aggregate	0.0 (0.0 to 0.0)	0.0 (0.0 to 0.0)	0.0 (0.0 to 0.0)	0.0 (0.0 to 0.0)	0.0 (0.0 to 0.0)	0.0 (0.0 to 0.0)
Onchocerciasis	0.0 (0.0 to 0.0)	0.0 (0.0 to 0.0)	0.0 (0.0 to 0.0)	0.0 (0.0 to 0.0)	0.0 (0.0 to 0.0)	0.0 (0.0 to 0.0)
Asymptomatic onchocerciasis	0.0 (0.0 to 0.0)	0.0 (0.0 to 0.0)	0.0 (0.0 to 0.0)	0.0 (0.0 to 0.0)	0.0 (0.0 to 0.0)	0.0 (0.0 to 0.0)
Skin disease due to onchocerciasis aggregate	0.0 (0.0 to 0.0)	0.0 (0.0 to 0.0)	0.0 (0.0 to 0.0)	0.0 (0.0 to 0.0)	0.0 (0.0 to 0.0)	0.0 (0.0 to 0.0)
Vision loss due to onchocerciasis aggregate	0.0 (0.0 to 0.0)	0.0 (0.0 to 0.0)	0.0 (0.0 to 0.0)	0.0 (0.0 to 0.0)	0.0 (0.0 to 0.0)	0.0 (0.0 to 0.0)
Trachoma	0.0 (0.0 to 0.0)	0.0 (0.0 to 0.0)	0.0 (0.0 to 0.0)	0.0 (0.0 to 0.0)	0.0 (0.0 to 0.0)	0.0 (0.0 to 0.0)
Dengue	73.6 (40.9 to 160.2)	123.1 (-8.8 to 501.8)	109.4 (-14.4 to 464.9)	12.1 (5.6 to 27.3)	121.6 (-10.8 to 507.6)	108.0 (-16.2 to 470.3)
Dengue episodes aggregate	22.1 (14.3 to 47.3)	120.5 (-10.7 to 494.4)	107.0 (-16.2 to 458.0)	1.3 (0.7 to 2.9)	121.2 (-9.4 to 523.5)	107.6 (-15.0 to 485.3)
Post-dengue chronic fatigue syndrome	51.7 (24.6 to 114.7)	123.2 (-8.8 to 501.8)	109.5 (-14.4 to 464.9)	10.8 (4.8 to 25.1)	121.7 (-10.4 to 505.3)	108.1 (-15.9 to 468.2)
Yellow fever	0.0 (0.0 to 0.1)	-32.7 (-46.8 to -14.2)*	-36.8 (-50.1 to -19.5)*	0.0 (0.0 to 0.0)	-32.8 (-46.8 to -14.2)*	-36.9 (-50.1 to -19.5)*
Rabies	0.0 (0.0 to 0.0)	-42.9 (-68.0 to -1.5)*	-46.4 (-69.9 to -7.5)*	0.0 (0.0 to 0.0)	-42.9 (-68.0 to -1.5)*	-46.4 (-69.9 to -7.5)*
Intestinal nematode infections	18,405.1 (17,665.3 to 19,107.9)	-15.0 (-19.1 to -10.9)*	-20.2 (-24.0 to -16.4)*	46.2 (28.7 to 71.1)	-28.4 (-34.1 to -22.5)*	-32.8 (-38.2 to -27.2)*
Ascariasis	9,293.9 (8,141.9 to 10,630.1)	-21.7 (-33.8 to -7.2)*	-26.5 (-37.9 to -12.8)*	14.7 (8.5 to 24.5)	-36.7 (-46.7 to -24.5)*	-40.6 (-50.0 to -29.2)*
Complications of ascariasis aggregate	743.2 (674.3 to 819.5)	-35.5 (-44.1 to -26.6)*	-39.4 (-47.5 to -31.1)*	14.7 (8.5 to 24.5)	-36.7 (-46.7 to -24.5)*	-40.6 (-50.0 to -29.2)*
Asymptomatic ascariasis	8,544.7 (7,412.4 to 9,862.6)	-20.2 (-33.5 to -3.8)*	-25.1 (-37.6 to -9.7)*	0.0 (0.0 to 0.0)	0.0 (0.0 to 0.0)	0.0 (0.0 to 0.0)
Trichuriasis	6,039.6 (5,439.8 to 6,697.0)	-15.3 (-26.1 to -2.2)*	-20.5 (-30.6 to -8.2)*	5.0 (2.8 to 8.7)	-30.4 (-47.8 to -7.5)*	-34.7 (-51.0 to -13.2)*
Complications of trichuriasis aggregate	236.7 (204.2 to 286.2)	-28.9 (-43.9 to -9.9)*	-33.2 (-47.3 to -15.4)*	5.0 (2.8 to 8.7)	-30.4 (-47.8 to -7.5)*	-34.7 (-51.0 to -13.2)*
Asymptomatic trichuriasis	5,800.6 (5,202.8 to 6,459.0)	-14.6 (-25.9 to -0.4)*	-19.8 (-30.5 to -6.5)*	0.0 (0.0 to 0.0)	0.0 (0.0 to 0.0)	0.0 (0.0 to 0.0)
Hookworm disease	4,708.5 (4,267.6 to 5,141.0)	-19.5 (-29.0 to -9.1)*	-24.4 (-33.4 to -14.6)*	26.5 (17.4 to 38.8)	-22.3 (-29.0 to -15.1)*	-27.1 (-33.4 to -20.3)*
Complications of hookworm disease aggregate	478.7 (448.2 to 512.8)	-23.3 (-30.9 to -15.9)*	-28.0 (-35.1 to -21.1)*	10.8 (6.3 to 17.2)	-24.7 (-34.0 to -14.6)*	-29.3 (-38.1 to -19.9)*
Anemia due to hookworm disease aggregate	381.7 (365.9 to 397.1)	-15.8 (-20.9 to -10.3)*	-21.0 (-25.8 to -15.8)*	15.8 (10.7 to 22.6)	-20.6 (-29.0 to -11.3)*	-25.5 (-33.4 to -16.7)*
Asymptomatic hookworm disease	3,844.2 (3,410.9 to 4,278.6)	-19.4 (-30.9 to -6.0)*	-24.3 (-35.2 to -11.7)*	0.0 (0.0 to 0.0)	0.0 (0.0 to 0.0)	0.0 (0.0 to 0.0)
Food-borne trematodiases	0.0 (0.0 to 0.0)	0.0 (0.0 to 0.0)	0.0 (0.0 to 0.0)	0.0 (0.0 to 0.0)	0.0 (0.0 to 0.0)	0.0 (0.0 to 0.0)
Asymptomatic food-borne trematodiases aggregate	0.0 (0.0 to 0.0)	0.0 (0.0 to 0.0)	0.0 (0.0 to 0.0)	0.0 (0.0 to 0.0)	0.0 (0.0 to 0.0)	0.0 (0.0 to 0.0)
Symptomatic food-borne trematodiases aggregate	0.0 (0.0 to 0.0)	0.0 (0.0 to 0.0)	0.0 (0.0 to 0.0)	0.0 (0.0 to 0.0)	0.0 (0.0 to 0.0)	0.0 (0.0 to 0.0)
Leprosy	0.0 (0.0 to 0.0)	0.0 (0.0 to 0.0)	0.0 (0.0 to 0.0)	0.0 (0.0 to 0.0)	0.0 (0.0 to 0.0)	0.0 (0.0 to 0.0)
Ebola	0.0 (0.0 to 0.1)	0.0 (0.0 to 0.0)	0.0 (0.0 to 0.0)	0.0 (0.0 to 0.0)	0.0 (0.0 to 0.0)	0.0 (0.0 to 0.0)
Other neglected tropical diseases	5,380.2 (5,244.8 to 5,532.5)	3.9 (0.5 to 7.7)*	-2.5 (-5.7 to 1.1)	256.8 (176.9 to 366.7)	-1.0 (-7.6 to 6.4)	-7.0 (-13.2 to -0.1)*
Anemia due to other neglected tropical diseases aggregate	5,380.2 (5,244.8 to 5,532.5)	3.9 (0.5 to 7.7)*	-2.5 (-5.7 to 1.1)	254.2 (174.5 to 362.1)	-1.6 (-8.1 to 5.8)	-7.6 (-13.7 to -0.7)*
Maternal disorders	0.0 (0.0 to 0.0)	0.0 (0.0 to 0.0)	0.0 (0.0 to 0.0)	0.0 (0.0 to 0.0)	0.0 (0.0 to 0.0)	0.0 (0.0 to 0.0)
Maternal hemorrhage	0.0 (0.0 to 0.0)	0.0 (0.0 to 0.0)	0.0 (0.0 to 0.0)	0.0 (0.0 to 0.0)	0.0 (0.0 to 0.0)	0.0 (0.0 to 0.0)
Maternal hemorrhage episodes aggregate	0.0 (0.0 to 0.0)	0.0 (0.0 to 0.0)	0.0 (0.0 to 0.0)	0.0 (0.0 to 0.0)	0.0 (0.0 to 0.0)	0.0 (0.0 to 0.0)
Anemia due to maternal hemorrhage aggregate	0.0 (0.0 to 0.0)	0.0 (0.0 to 0.0)	0.0 (0.0 to 0.0)	0.0 (0.0 to 0.0)	0.0 (0.0 to 0.0)	0.0 (0.0 to 0.0)
Maternal sepsis and other maternal infections	0.0 (0.0 to 0.0)	0.0 (0.0 to 0.0)	0.0 (0.0 to 0.0)	0.0 (0.0 to 0.0)	0.0 (0.0 to 0.0)	0.0 (0.0 to 0.0)
Maternal sepsis and other maternal infections aggregate	0.0 (0.0 to 0.0)	0.0 (0.0 to 0.0)	0.0 (0.0 to 0.0)	0.0 (0.0 to 0.0)	0.0 (0.0 to 0.0)	0.0 (0.0 to 0.0)
Infertility due to puerperal sepsis	0.0 (0.0 to 0.0)	0.0 (0.0 to 0.0)	0.0 (0.0 to 0.0)	0.0 (0.0 to 0.0)	0.0 (0.0 to 0.0)	0.0 (0.0 to 0.0)
Maternal hypertensive disorders	0.0 (0.0 to 0.0)	0.0 (0.0 to 0.0)	0.0 (0.0 to 0.0)	0.0 (0.0 to 0.0)	0.0 (0.0 to 0.0)	0.0 (0.0 to 0.0)
Maternal hypertensive disorders episodes aggregate	0.0 (0.0 to 0.0)	0.0 (0.0 to 0.0)	0.0 (0.0 to 0.0)	0.0 (0.0 to 0.0)	0.0 (0.0 to 0.0)	0.0 (0.0 to 0.0)
Eclampsia	0.0 (0.0 to 0.0)	0.0 (0.0 to 0.0)	0.0 (0.0 to 0.0)	0.0 (0.0 to 0.0)	0.0 (0.0 to 0.0)	0.0 (0.0 to 0.0)
Complications of maternal hypertensive disorders aggregate	0.0 (0.0 to 0.0)	0.0 (0.0 to 0.0)	0.0 (0.0 to 0.0)	0.0 (0.0 to 0.0)	0.0 (0.0 to 0.0)	0.0 (0.0 to 0.0)
Maternal obstructed labor and uterine rupture	0.0 (0.0 to 0.0)	0.0 (0.0 to 0.0)	0.0 (0.0 to 0.0)	0.0 (0.0 to 0.0)	0.0 (0.0 to 0.0)	0.0 (0.0 to 0.0)
Obstructed labor, acute event	0.0 (0.0 to 0.0)	0.0 (0.0 to 0.0)	0.0 (0.0 to 0.0)	0.0 (0.0 to 0.0)	0.0 (0.0 to 0.0)	0.0 (0.0 to 0.0)
Fistula due to maternal obstructed labor and uterine rupture aggregate	0.0 (0.0 to 0.0)	0.0 (0.0 to 0.0)	0.0 (0.0 to 0.0)	0.0 (0.0 to 0.0)	0.0 (0.0 to 0.0)	0.0 (0.0 to 0.0)
Maternal abortion, miscarriage, and ectopic pregnancy	0.0 (0.0 to 0.0)	0.0 (0.0 to 0.0)	0.0 (0.0 to 0.0)	0.0 (0.0 to 0.0)	0.0 (0.0 to 0.0)	0.0 (0.0 to 0.0)
Other maternal disorders	--	--	--	0.0 (0.0 to 0.0)	0.0 (0.0 to 0.0)	0.0 (0.0 to 0.0)
Neonatal disorders	7,393.1 (7,010.7 to 7,736.6)	6.0 (3.6 to 8.4)*	-0.5 (-2.7 to 1.8)	318.4 (229.5 to 424.9)	3.0 (-1.5 to 7.7)	-3.3 (-7.5 to 1.1)
Neonatal preterm birth complications	6,388.1 (5,385.4 to 7,508.5)	4.7 (3.1 to 6.1)*	-1.8 (-3.2 to -0.4)*	144.4 (104.5 to 191.2)	-4.7 (-10.3 to 1.4)	-10.6 (-15.8 to -4.8)*
Vision loss due to retinopathy of prematurity aggregate	68.3 (53.3 to 85.6)	4.1 (0.1 to 8.3)*	-2.3 (-6.0 to 1.7)	1.9 (1.1 to 3.1)	4.7 (-5.8 to 14.8)	-1.7 (-11.5 to 7.8)
Complications of preterm birth complications aggregate	6,319.9 (5,317.8 to 7,436.6)	4.7 (3.1 to 6.1)*	-1.7 (-3.2 to -0.4)*	142.5 (102.8 to 188.8)	-4.8 (-10.6 to 1.3)	-10.7 (-16.1 to -4.9)*
Neonatal encephalopathy due to birth asphyxia and trauma	1,061.6 (797.4 to 1,409.1)	14.9 (9.2 to 20.7)*	7.8 (2.5 to 13.3)*	119.8 (76.6 to 180.2)	11.8 (8.6 to 15.5)*	5.0 (1.9 to 8.4)*

Cause	Prevalence in thousands (95% confidence interval)			YLDs in thousands (95% confidence interval)		
	2015	Percent change in counts between 2005 and 2015	Percent change in rates between 2005 and 2015	2015	Percent change in counts between 2005 and 2015	Percent change in rates between 2005 and 2015
Neonatal sepsis and other neonatal infections	0.0<br>(0.0 to 0.0)	0.0<br>(0.0 to 0.0)	0.0<br>(0.0 to 0.0)	0.0<br>(0.0 to 0.0)	0.0<br>(0.0 to 0.0)	0.0<br>(0.0 to 0.0)
Severe infection due to neonatal sepsis and other neonatal infections	0.0<br>(0.0 to 0.0)	0.0<br>(0.0 to 0.0)	0.0<br>(0.0 to 0.0)	0.0<br>(0.0 to 0.0)	0.0<br>(0.0 to 0.0)	0.0<br>(0.0 to 0.0)
Complications of neonatal sepsis and other neonatal infections aggregate	0.0<br>(0.0 to 0.0)	0.0<br>(0.0 to 0.0)	0.0<br>(0.0 to 0.0)	0.0<br>(0.0 to 0.0)	0.0<br>(0.0 to 0.0)	0.0<br>(0.0 to 0.0)
Hemolytic disease and other neonatal jaundice	79.5<br>(69.4 to 91.4)	7.8<br>(6.1 to 9.4)*	1.2<br>(-0.4 to 2.7)	19.2<br>(13.8 to 25.3)	6.8<br>(1.3 to 12.4)*	0.3<br>(-4.9 to 5.5)
Other neonatal disorders	--	--	--	35.0<br>(21.7 to 52.7)	8.2<br>(-17.9 to 40.9)	1.5<br>(-23.0 to 32.3)
Nutritional deficiencies	55,032.7<br>(54,347.4 to 55,743.6)	3.5<br>(1.6 to 5.4)*	-2.9<br>(-4.6 to -1.1)*	3,527.2<br>(2,442.0 to 4,925.6)	-3.4<br>(-8.5 to 2.5)	-9.3<br>(-14.1 to -3.7)*
Protein-energy malnutrition	4,715.1<br>(4,487.7 to 4,958.2)	-6.1<br>(-11.6 to -0.5)*	-11.8<br>(-17.0 to -6.6)*	581.4<br>(375.7 to 819.4)	-5.9<br>(-11.2 to -0.3)*	-11.6<br>(-16.6 to -6.4)*
Iodine deficiency	0.0<br>(0.0 to 0.0)	0.0<br>(0.0 to 0.0)	0.0<br>(0.0 to 0.0)	0.0<br>(0.0 to 0.0)	0.0<br>(0.0 to 0.0)	0.0<br>(0.0 to 0.0)
Visible goiter due to iodine deficiency aggregate	0.0<br>(0.0 to 0.0)	0.0<br>(0.0 to 0.0)	0.0<br>(0.0 to 0.0)	0.0<br>(0.0 to 0.0)	0.0<br>(0.0 to 0.0)	0.0<br>(0.0 to 0.0)
Visible goiter with heart failure due to iodine deficiency aggregate	0.0<br>(0.0 to 0.0)	0.0<br>(0.0 to 0.0)	0.0<br>(0.0 to 0.0)	0.0<br>(0.0 to 0.0)	0.0<br>(0.0 to 0.0)	0.0<br>(0.0 to 0.0)
Intellectual disability due to iodine deficiency aggregate	0.0<br>(0.0 to 0.0)	0.0<br>(0.0 to 0.0)	0.0<br>(0.0 to 0.0)	0.0<br>(0.0 to 0.0)	0.0<br>(0.0 to 0.0)	0.0<br>(0.0 to 0.0)
Vitamin A deficiency	0.0<br>(0.0 to 0.0)	0.0<br>(0.0 to 0.0)	0.0<br>(0.0 to 0.0)	0.0<br>(0.0 to 0.0)	0.0<br>(0.0 to 0.0)	0.0<br>(0.0 to 0.0)
Iron-deficiency anemia	60,768.9<br>(59,420.7 to 62,273.3)	3.5<br>(0.5 to 6.8)*	-2.9<br>(-5.7 to 0.3)	2,810.5<br>(1,913.6 to 3,980.4)	-2.0<br>(-8.0 to 5.1)	-8.0<br>(-13.6 to -1.4)*
Iron-deficiency anemia without heart failure aggregate	60,768.9<br>(59,420.7 to 62,273.3)	3.5<br>(0.5 to 6.8)*	-2.9<br>(-5.7 to 0.3)	2,810.5<br>(1,913.6 to 3,980.4)	-2.0<br>(-8.0 to 5.1)	-8.0<br>(-13.6 to -1.4)*
Iron-deficiency anemia with heart failure aggregate	0.0<br>(0.0 to 0.0)	11.7<br>(9.2 to 14.2)*	4.8<br>(2.5 to 7.2)*	0.0<br>(0.0 to 0.0)	11.7<br>(9.2 to 14.2)*	4.8<br>(2.5 to 7.2)*
Other nutritional deficiencies	--	--	--	135.2<br>(80.4 to 211.3)	-17.6<br>(-37.5 to 4.7)	-22.7<br>(-41.4 to -1.7)*
Other communicable, maternal, neonatal, and nutritional diseases	9,077.4<br>(8,822.0 to 9,263.6)	7.0<br>(3.8 to 10.2)*	0.5<br>(-2.6 to 3.5)	181.2<br>(122.9 to 257.2)	1.1<br>(-5.5 to 9.2)	-5.1<br>(-11.3 to 2.5)
Sexually transmitted diseases excluding HIV	0.0<br>(0.0 to 0.0)	0.0<br>(0.0 to 0.0)	0.0<br>(0.0 to 0.0)	0.0<br>(0.0 to 0.0)	0.0<br>(0.0 to 0.0)	0.0<br>(0.0 to 0.0)
Syphilis	0.0<br>(0.0 to 0.0)	0.0<br>(0.0 to 0.0)	0.0<br>(0.0 to 0.0)	0.0<br>(0.0 to 0.0)	0.0<br>(0.0 to 0.0)	0.0<br>(0.0 to 0.0)
Early syphilis aggregate	0.0<br>(0.0 to 0.0)	0.0<br>(0.0 to 0.0)	0.0<br>(0.0 to 0.0)	0.0<br>(0.0 to 0.0)	0.0<br>(0.0 to 0.0)	0.0<br>(0.0 to 0.0)
Adult tertiary syphilis	0.0<br>(0.0 to 0.0)	0.0<br>(0.0 to 0.0)	0.0<br>(0.0 to 0.0)	0.0<br>(0.0 to 0.0)	0.0<br>(0.0 to 0.0)	0.0<br>(0.0 to 0.0)
Chlamydial infection	0.0<br>(0.0 to 0.0)	0.0<br>(0.0 to 0.0)	0.0<br>(0.0 to 0.0)	0.0<br>(0.0 to 0.0)	0.0<br>(0.0 to 0.0)	0.0<br>(0.0 to 0.0)
Chlamydial infection episodes aggregate	0.0<br>(0.0 to 0.0)	0.0<br>(0.0 to 0.0)	0.0<br>(0.0 to 0.0)	0.0<br>(0.0 to 0.0)	0.0<br>(0.0 to 0.0)	0.0<br>(0.0 to 0.0)
Pelvic inflammatory diseases due to chlamydial infection aggregate	0.0<br>(0.0 to 0.0)	0.0<br>(0.0 to 0.0)	0.0<br>(0.0 to 0.0)	0.0<br>(0.0 to 0.0)	0.0<br>(0.0 to 0.0)	0.0<br>(0.0 to 0.0)
Infertility due to clamydial infection aggregate	0.0<br>(0.0 to 0.0)	0.0<br>(0.0 to 0.0)	0.0<br>(0.0 to 0.0)	0.0<br>(0.0 to 0.0)	0.0<br>(0.0 to 0.0)	0.0<br>(0.0 to 0.0)
Gonococcal infection	0.0<br>(0.0 to 0.0)	0.0<br>(0.0 to 0.0)	0.0<br>(0.0 to 0.0)	0.0<br>(0.0 to 0.0)	0.0<br>(0.0 to 0.0)	0.0<br>(0.0 to 0.0)
Gonococcal infection episodes aggregate	0.0<br>(0.0 to 0.0)	0.0<br>(0.0 to 0.0)	0.0<br>(0.0 to 0.0)	0.0<br>(0.0 to 0.0)	0.0<br>(0.0 to 0.0)	0.0<br>(0.0 to 0.0)
Pelvic inflammatory diseases due to gonococcal infection aggregate	0.0<br>(0.0 to 0.0)	0.0<br>(0.0 to 0.0)	0.0<br>(0.0 to 0.0)	0.0<br>(0.0 to 0.0)	0.0<br>(0.0 to 0.0)	0.0<br>(0.0 to 0.0)
Trichomoniasis	0.0<br>(0.0 to 0.0)	0.0<br>(0.0 to 0.0)	0.0<br>(0.0 to 0.0)	0.0<br>(0.0 to 0.0)	0.0<br>(0.0 to 0.0)	0.0<br>(0.0 to 0.0)
Genital herpes	0.0<br>(0.0 to 0.0)	0.0<br>(0.0 to 0.0)	0.0<br>(0.0 to 0.0)	0.0<br>(0.0 to 0.0)	0.0<br>(0.0 to 0.0)	0.0<br>(0.0 to 0.0)
Moderate infection due to initial genital herpes episode	0.0<br>(0.0 to 0.0)	0.0<br>(0.0 to 0.0)	0.0<br>(0.0 to 0.0)	0.0<br>(0.0 to 0.0)	0.0<br>(0.0 to 0.0)	0.0<br>(0.0 to 0.0)
Complications of genital herpes aggregate	0.0<br>(0.0 to 0.0)	0.0<br>(0.0 to 0.0)	0.0<br>(0.0 to 0.0)	0.0<br>(0.0 to 0.0)	0.0<br>(0.0 to 0.0)	0.0<br>(0.0 to 0.0)
Other sexually transmitted diseases	0.0<br>(0.0 to 0.0)	0.0<br>(0.0 to 0.0)	0.0<br>(0.0 to 0.0)	0.0<br>(0.0 to 0.0)	0.0<br>(0.0 to 0.0)	0.0<br>(0.0 to 0.0)
Pelvic inflammatory diseases due to other sexually transmitted diseases aggregate	0.0<br>(0.0 to 0.0)	0.0<br>(0.0 to 0.0)	0.0<br>(0.0 to 0.0)	0.0<br>(0.0 to 0.0)	0.0<br>(0.0 to 0.0)	0.0<br>(0.0 to 0.0)
Infertility due to other sexually transmitted diseases aggregate	0.0<br>(0.0 to 0.0)	0.0<br>(0.0 to 0.0)	0.0<br>(0.0 to 0.0)	0.0<br>(0.0 to 0.0)	0.0<br>(0.0 to 0.0)	0.0<br>(0.0 to 0.0)
Other sexually transmitted diseases	--	--	--	0.0<br>(0.0 to 0.0)	0.0<br>(0.0 to 0.0)	0.0<br>(0.0 to 0.0)
Hepatitis	5,456.6<br>(5,185.1 to 5,653.0)	9.7<br>(4.3 to 13.8)*	3.0<br>(-2.1 to 6.8)	1.5<br>(0.8 to 2.5)	10.3<br>(-4.8 to 26.2)	3.5<br>(-10.6 to 18.4)
Acute hepatitis A	1,140.1<br>(693.2 to 1,718.8)	-7.3<br>(-47.8 to 57.5)	-13.0<br>(-51.0 to 47.9)	0.2<br>(0.1 to 0.4)	-7.1<br>(-57.9 to 104.6)	-12.8<br>(-60.5 to 92.0)
Hepatitis B	4,020.3<br>(3,605.2 to 4,518.4)	15.1<br>(9.7 to 20.0)*	8.0<br>(3.0 to 12.7)*	0.3<br>(0.2 to 0.4)	7.6<br>(-5.0 to 20.8)	1.0<br>(-10.8 to 13.4)
Acute Hepatitis B aggregate	35.2<br>(31.1 to 40.0)	7.5<br>(-4.2 to 20.0)	0.9<br>(-10.1 to 12.6)	0.3<br>(0.2 to 0.4)	7.6<br>(-5.0 to 20.8)	1.0<br>(-10.8 to 13.4)
Chronic hepatitis B	3,985.1<br>(3,568.4 to 4,484.3)	15.1<br>(9.8 to 20.2)*	8.1<br>(3.1 to 12.8)*	0.0<br>(0.0 to 0.0)	0.0<br>(0.0 to 0.0)	0.0<br>(0.0 to 0.0)
Hepatitis C	370.9<br>(324.4 to 422.3)	14.0<br>(12.1 to 15.9)*	7.0<br>(5.2 to 8.8)*	1.0<br>(0.5 to 2.0)	15.0<br>(4.9 to 27.6)*	8.0<br>(-1.5 to 19.8)
Acute Hepatitis C aggregate	74.8<br>(66.0 to 84.9)	15.0<br>(12.8 to 17.0)*	7.9<br>(5.8 to 9.8)*	1.0<br>(0.5 to 2.0)	15.0<br>(4.9 to 27.6)*	8.0<br>(-1.5 to 19.8)
Chronic hepatitis C	296.2<br>(257.1 to 339.3)	13.8<br>(11.9 to 15.7)*	6.8<br>(5.1 to 8.6)*	0.0<br>(0.0 to 0.0)	0.0<br>(0.0 to 0.0)	0.0<br>(0.0 to 0.0)
Acute hepatitis E	6.0<br>(4.1 to 9.0)	4.8<br>(1.5 to 8.0)*	-1.7<br>(-4.7 to 1.4)	0.0<br>(0.0 to 0.0)	4.6<br>(-35.5 to 67.5)	-1.8<br>(-39.4 to 57.3)
Other infectious diseases	3,830.2<br>(3,731.3 to 3,940.3)	3.5<br>(-0.5 to 8.0)	-2.8<br>(-6.6 to 1.4)	179.7<br>(122.0 to 254.9)	1.0<br>(-5.6 to 9.1)	-5.2<br>(-11.4 to 2.5)
Non-communicable diseases	51,241.9<br>(50,641.7 to 51,762.8)	5.1<br>(4.3 to 5.8)*	-1.4<br>(-2.1 to -0.6)*	1,371.4<br>(974.5 to 1,879.4)	4.8<br>(2.1 to 7.7)*	-1.7<br>(-4.2 to 1.1)
Neoplasms	147.7<br>(133.4 to 166.6)	12.0<br>(-0.9 to 27.4)	5.1<br>(-7.0 to 19.6)	8.8<br>(5.3 to 13.7)	14.2<br>(-4.6 to 38.9)	7.2<br>(-10.4 to 30.4)
Lip and oral cavity cancer	0.0<br>(0.0 to 0.0)	0.0<br>(0.0 to 0.0)	0.0<br>(0.0 to 0.0)	0.0<br>(0.0 to 0.0)	0.0<br>(0.0 to 0.0)	0.0<br>(0.0 to 0.0)
Nasopharynx cancer	0.0<br>(0.0 to 0.0)	0.0<br>(0.0 to 0.0)	0.0<br>(0.0 to 0.0)	0.0<br>(0.0 to 0.0)	0.0<br>(0.0 to 0.0)	0.0<br>(0.0 to 0.0)
Other pharynx cancer	0.0<br>(0.0 to 0.0)	0.0<br>(0.0 to 0.0)	0.0<br>(0.0 to 0.0)	0.0<br>(0.0 to 0.0)	0.0<br>(0.0 to 0.0)	0.0<br>(0.0 to 0.0)
Esophageal cancer	0.0<br>(0.0 to 0.0)	0.0<br>(0.0 to 0.0)	0.0<br>(0.0 to 0.0)	0.0<br>(0.0 to 0.0)	0.0<br>(0.0 to 0.0)	0.0<br>(0.0 to 0.0)
Stomach cancer	0.0<br>(0.0 to 0.0)	0.0<br>(0.0 to 0.0)	0.0<br>(0.0 to 0.0)	0.0<br>(0.0 to 0.0)	0.0<br>(0.0 to 0.0)	0.0<br>(0.0 to 0.0)
Colon and rectum cancer	0.0<br>(0.0 to 0.0)	0.0<br>(0.0 to 0.0)	0.0<br>(0.0 to 0.0)	0.0<br>(0.0 to 0.0)	0.0<br>(0.0 to 0.0)	0.0<br>(0.0 to 0.0)
Colon and rectum cancer aggregate	0.0<br>(0.0 to 0.0)	0.0<br>(0.0 to 0.0)	0.0<br>(0.0 to 0.0)	0.0<br>(0.0 to 0.0)	0.0<br>(0.0 to 0.0)	0.0<br>(0.0 to 0.0)
Stoma due to colon and rectum cancer	0.0<br>(0.0 to 0.0)	0.0<br>(0.0 to 0.0)	0.0<br>(0.0 to 0.0)	0.0<br>(0.0 to 0.0)	0.0<br>(0.0 to 0.0)	0.0<br>(0.0 to 0.0)
Liver cancer	0.0<br>(0.0 to 0.0)	0.0<br>(0.0 to 0.0)	0.0<br>(0.0 to 0.0)	0.0<br>(0.0 to 0.0)	0.0<br>(0.0 to 0.0)	0.0<br>(0.0 to 0.0)
Liver cancer due to hepatitis B	0.0<br>(0.0 to 0.0)	0.0<br>(0.0 to 0.0)	0.0<br>(0.0 to 0.0)	0.0<br>(0.0 to 0.0)	0.0<br>(0.0 to 0.0)	0.0<br>(0.0 to 0.0)
Liver cancer due to hepatitis C	0.0<br>(0.0 to 0.0)	0.0<br>(0.0 to 0.0)	0.0<br>(0.0 to 0.0)	0.0<br>(0.0 to 0.0)	0.0<br>(0.0 to 0.0)	0.0<br>(0.0 to 0.0)
Liver cancer due to alcohol use	0.0<br>(0.0 to 0.0)	0.0<br>(0.0 to 0.0)	0.0<br>(0.0 to 0.0)	0.0<br>(0.0 to 0.0)	0.0<br>(0.0 to 0.0)	0.0<br>(0.0 to 0.0)
Liver cancer due to other causes	0.0<br>(0.0 to 0.0)	0.0<br>(0.0 to 0.0)	0.0<br>(0.0 to 0.0)	0.0<br>(0.0 to 0.0)	0.0<br>(0.0 to 0.0)	0.0<br>(0.0 to 0.0)
Gallbladder and biliary tract cancer	0.0<br>(0.0 to 0.0)	0.0<br>(0.0 to 0.0)	0.0<br>(0.0 to 0.0)	0.0<br>(0.0 to 0.0)	0.0<br>(0.0 to 0.0)	0.0<br>(0.0 to 0.0)
Pancreatic cancer	0.0<br>(0.0 to 0.0)	0.0<br>(0.0 to 0.0)	0.0<br>(0.0 to 0.0)	0.0<br>(0.0 to 0.0)	0.0<br>(0.0 to 0.0)	0.0<br>(0.0 to 0.0)

Cause	Prevalence in thousands (95% confidence interval)			YLDs in thousands (95% confidence interval)		
	2015	Percent change in counts between 2005 and 2015	Percent change in rates between 2005 and 2015	2015	Percent change in counts between 2005 and 2015	Percent change in rates between 2005 and 2015
Larynx cancer	0.0 (0.0 to 0.0)	0.0 (0.0 to 0.0)	0.0 (0.0 to 0.0)	0.0 (0.0 to 0.0)	0.0 (0.0 to 0.0)	0.0 (0.0 to 0.0)
Larynx cancer aggregate	0.0 (0.0 to 0.0)	0.0 (0.0 to 0.0)	0.0 (0.0 to 0.0)	0.0 (0.0 to 0.0)	0.0 (0.0 to 0.0)	0.0 (0.0 to 0.0)
Laryngectomy due to larynx cancer	0.0 (0.0 to 0.0)	0.0 (0.0 to 0.0)	0.0 (0.0 to 0.0)	0.0 (0.0 to 0.0)	0.0 (0.0 to 0.0)	0.0 (0.0 to 0.0)
Tracheal, bronchus, and lung cancer	0.0 (0.0 to 0.0)	0.0 (0.0 to 0.0)	0.0 (0.0 to 0.0)	0.0 (0.0 to 0.0)	0.0 (0.0 to 0.0)	0.0 (0.0 to 0.0)
Malignant skin melanoma	0.0 (0.0 to 0.0)	0.0 (0.0 to 0.0)	0.0 (0.0 to 0.0)	0.0 (0.0 to 0.0)	0.0 (0.0 to 0.0)	0.0 (0.0 to 0.0)
Non-melanoma skin cancer	0.0 (0.0 to 0.0)	0.0 (0.0 to 0.0)	0.0 (0.0 to 0.0)	0.0 (0.0 to 0.0)	0.0 (0.0 to 0.0)	0.0 (0.0 to 0.0)
Non-melanoma skin cancer(squamous-cell carcinoma)	0.0 (0.0 to 0.0)	0.0 (0.0 to 0.0)	0.0 (0.0 to 0.0)	0.0 (0.0 to 0.0)	0.0 (0.0 to 0.0)	0.0 (0.0 to 0.0)
Non-melanoma skin cancer(basal-cell carcinoma)	0.0 (0.0 to 0.0)	0.0 (0.0 to 0.0)	0.0 (0.0 to 0.0)	0.0 (0.0 to 0.0)	0.0 (0.0 to 0.0)	0.0 (0.0 to 0.0)
Breast cancer	0.0 (0.0 to 0.0)	0.0 (0.0 to 0.0)	0.0 (0.0 to 0.0)	0.0 (0.0 to 0.0)	0.0 (0.0 to 0.0)	0.0 (0.0 to 0.0)
Breast cancer aggregate	0.0 (0.0 to 0.0)	0.0 (0.0 to 0.0)	0.0 (0.0 to 0.0)	0.0 (0.0 to 0.0)	0.0 (0.0 to 0.0)	0.0 (0.0 to 0.0)
Mastectomy due to breast cancer	0.0 (0.0 to 0.0)	0.0 (0.0 to 0.0)	0.0 (0.0 to 0.0)	0.0 (0.0 to 0.0)	0.0 (0.0 to 0.0)	0.0 (0.0 to 0.0)
Cervical cancer	0.0 (0.0 to 0.0)	0.0 (0.0 to 0.0)	0.0 (0.0 to 0.0)	0.0 (0.0 to 0.0)	0.0 (0.0 to 0.0)	0.0 (0.0 to 0.0)
Uterine cancer	0.0 (0.0 to 0.0)	0.0 (0.0 to 0.0)	0.0 (0.0 to 0.0)	0.0 (0.0 to 0.0)	0.0 (0.0 to 0.0)	0.0 (0.0 to 0.0)
Ovarian cancer	0.0 (0.0 to 0.0)	0.0 (0.0 to 0.0)	0.0 (0.0 to 0.0)	0.0 (0.0 to 0.0)	0.0 (0.0 to 0.0)	0.0 (0.0 to 0.0)
Prostate cancer	0.0 (0.0 to 0.0)	0.0 (0.0 to 0.0)	0.0 (0.0 to 0.0)	0.0 (0.0 to 0.0)	0.0 (0.0 to 0.0)	0.0 (0.0 to 0.0)
Prostate cancer aggregate	0.0 (0.0 to 0.0)	0.0 (0.0 to 0.0)	0.0 (0.0 to 0.0)	0.0 (0.0 to 0.0)	0.0 (0.0 to 0.0)	0.0 (0.0 to 0.0)
Impotence and incontinence due to prostate cancer aggregate	0.0 (0.0 to 0.0)	0.0 (0.0 to 0.0)	0.0 (0.0 to 0.0)	0.0 (0.0 to 0.0)	0.0 (0.0 to 0.0)	0.0 (0.0 to 0.0)
Testicular cancer	0.0 (0.0 to 0.0)	0.0 (0.0 to 0.0)	0.0 (0.0 to 0.0)	0.0 (0.0 to 0.0)	0.0 (0.0 to 0.0)	0.0 (0.0 to 0.0)
Kidney cancer	0.0 (0.0 to 0.0)	0.0 (0.0 to 0.0)	0.0 (0.0 to 0.0)	0.0 (0.0 to 0.0)	0.0 (0.0 to 0.0)	0.0 (0.0 to 0.0)
Bladder cancer	0.0 (0.0 to 0.0)	0.0 (0.0 to 0.0)	0.0 (0.0 to 0.0)	0.0 (0.0 to 0.0)	0.0 (0.0 to 0.0)	0.0 (0.0 to 0.0)
Bladder cancer episodes aggregate	0.0 (0.0 to 0.0)	0.0 (0.0 to 0.0)	0.0 (0.0 to 0.0)	0.0 (0.0 to 0.0)	0.0 (0.0 to 0.0)	0.0 (0.0 to 0.0)
Urinary incontinence due to bladder cancer	0.0 (0.0 to 0.0)	0.0 (0.0 to 0.0)	0.0 (0.0 to 0.0)	0.0 (0.0 to 0.0)	0.0 (0.0 to 0.0)	0.0 (0.0 to 0.0)
Brain and nervous system cancer	0.0 (0.0 to 0.0)	0.0 (0.0 to 0.0)	0.0 (0.0 to 0.0)	0.0 (0.0 to 0.0)	0.0 (0.0 to 0.0)	0.0 (0.0 to 0.0)
Thyroid cancer	0.0 (0.0 to 0.0)	0.0 (0.0 to 0.0)	0.0 (0.0 to 0.0)	0.0 (0.0 to 0.0)	0.0 (0.0 to 0.0)	0.0 (0.0 to 0.0)
Mesothelioma	0.0 (0.0 to 0.0)	0.0 (0.0 to 0.0)	0.0 (0.0 to 0.0)	0.0 (0.0 to 0.0)	0.0 (0.0 to 0.0)	0.0 (0.0 to 0.0)
Hodgkin lymphoma	3.7 (2.8 to 4.5)	-27.1 (-39.4 to -10.1)*	-31.6 (-43.1 to -15.6)*	0.2 (0.1 to 0.3)	-26.9 (-41.8 to -6.1)*	-31.4 (-45.3 to -11.9)*
Non-Hodgkin lymphoma	0.0 (0.0 to 0.0)	0.0 (0.0 to 0.0)	0.0 (0.0 to 0.0)	0.0 (0.0 to 0.0)	0.0 (0.0 to 0.0)	0.0 (0.0 to 0.0)
Multiple myeloma	0.0 (0.0 to 0.0)	0.0 (0.0 to 0.0)	0.0 (0.0 to 0.0)	0.0 (0.0 to 0.0)	0.0 (0.0 to 0.0)	0.0 (0.0 to 0.0)
Leukemia	52.1 (43.5 to 62.1)	11.1 (-7.7 to 33.7)	4.3 (-13.3 to 25.5)	3.4 (1.8 to 6.0)	15.9 (-16.3 to 62.9)	8.8 (-21.4 to 52.9)
Acute lymphoid leukemia	38.1 (23.5 to 57.5)	14.2 (-16.3 to 60.5)	7.2 (-21.4 to 50.7)	2.2 (1.1 to 3.8)	14.5 (-17.9 to 61.1)	7.5 (-22.9 to 51.2)
Chronic lymphoid leukemia	0.0 (0.0 to 0.0)	0.0 (0.0 to 0.0)	0.0 (0.0 to 0.0)	0.0 (0.0 to 0.0)	0.0 (0.0 to 0.0)	0.0 (0.0 to 0.0)
Acute myeloid leukemia	21.9 (13.0 to 34.9)	18.3 (-16.7 to 71.7)	11.0 (-21.8 to 61.2)	1.3 (0.6 to 2.3)	18.6 (-17.2 to 71.5)	11.3 (-22.3 to 61.0)
Chronic myeloid leukemia	0.0 (0.0 to 0.0)	0.0 (0.0 to 0.0)	0.0 (0.0 to 0.0)	0.0 (0.0 to 0.0)	0.0 (0.0 to 0.0)	0.0 (0.0 to 0.0)
Other neoplasms	99.2 (77.6 to 124.9)	15.6 (-4.5 to 39.3)	8.5 (-10.3 to 30.7)	5.1 (3.0 to 7.8)	15.7 (-5.1 to 39.5)	8.6 (-10.9 to 31.0)
Cardiovascular diseases	0.0 (0.0 to 0.0)	0.0 (0.0 to 0.0)	0.0 (0.0 to 0.0)	0.0 (0.0 to 0.0)	3.1 (-1.5 to 8.2)	-3.2 (-7.6 to 1.6)
Rheumatic heart disease	0.0 (0.0 to 0.0)	0.0 (0.0 to 0.0)	0.0 (0.0 to 0.0)	0.0 (0.0 to 0.0)	0.0 (0.0 to 0.0)	0.0 (0.0 to 0.0)
Rheumatic heart disease, without heart failure	0.0 (0.0 to 0.0)	0.0 (0.0 to 0.0)	0.0 (0.0 to 0.0)	0.0 (0.0 to 0.0)	0.0 (0.0 to 0.0)	0.0 (0.0 to 0.0)
Heart failure fue to rheumatic heart disease aggregate	0.0 (0.0 to 0.0)	0.0 (0.0 to 0.0)	0.0 (0.0 to 0.0)	0.0 (0.0 to 0.0)	0.0 (0.0 to 0.0)	0.0 (0.0 to 0.0)
Ischemic heart disease	0.0 (0.0 to 0.0)	0.0 (0.0 to 0.0)	0.0 (0.0 to 0.0)	0.0 (0.0 to 0.0)	0.0 (0.0 to 0.0)	0.0 (0.0 to 0.0)
Myocardial infarction episodes aggregate	0.0 (0.0 to 0.0)	0.0 (0.0 to 0.0)	0.0 (0.0 to 0.0)	0.0 (0.0 to 0.0)	0.0 (0.0 to 0.0)	0.0 (0.0 to 0.0)
Angina due to ischemic heart disease aggregate	0.0 (0.0 to 0.0)	0.0 (0.0 to 0.0)	0.0 (0.0 to 0.0)	0.0 (0.0 to 0.0)	0.0 (0.0 to 0.0)	0.0 (0.0 to 0.0)
Heart failure fue to ischemic heart disease aggregate	0.0 (0.0 to 0.0)	0.0 (0.0 to 0.0)	0.0 (0.0 to 0.0)	0.0 (0.0 to 0.0)	0.0 (0.0 to 0.0)	0.0 (0.0 to 0.0)
Cerebrovascular disease	0.0 (0.0 to 0.0)	0.0 (0.0 to 0.0)	0.0 (0.0 to 0.0)	0.0 (0.0 to 0.0)	0.0 (0.0 to 0.0)	0.0 (0.0 to 0.0)
Ischemic stroke	0.0 (0.0 to 0.0)	0.0 (0.0 to 0.0)	0.0 (0.0 to 0.0)	0.0 (0.0 to 0.0)	0.0 (0.0 to 0.0)	0.0 (0.0 to 0.0)
Chronic ischemic stroke aggregate	0.0 (0.0 to 0.0)	0.0 (0.0 to 0.0)	0.0 (0.0 to 0.0)	0.0 (0.0 to 0.0)	0.0 (0.0 to 0.0)	0.0 (0.0 to 0.0)
Ischemic stroke episodes aggregate	0.0 (0.0 to 0.0)	0.0 (0.0 to 0.0)	0.0 (0.0 to 0.0)	0.0 (0.0 to 0.0)	0.0 (0.0 to 0.0)	0.0 (0.0 to 0.0)
Hemorrhagic stroke	0.0 (0.0 to 0.0)	0.0 (0.0 to 0.0)	0.0 (0.0 to 0.0)	0.0 (0.0 to 0.0)	0.0 (0.0 to 0.0)	0.0 (0.0 to 0.0)
Chronic hemorrhagic stroke aggregate	0.0 (0.0 to 0.0)	0.0 (0.0 to 0.0)	0.0 (0.0 to 0.0)	0.0 (0.0 to 0.0)	0.0 (0.0 to 0.0)	0.0 (0.0 to 0.0)
Acute hemorrhagic stroke aggregate	0.0 (0.0 to 0.0)	0.0 (0.0 to 0.0)	0.0 (0.0 to 0.0)	0.0 (0.0 to 0.0)	0.0 (0.0 to 0.0)	0.0 (0.0 to 0.0)
Hypertensive heart disease	0.0 (0.0 to 0.0)	0.0 (0.0 to 0.0)	0.0 (0.0 to 0.0)	0.0 (0.0 to 0.0)	0.0 (0.0 to 0.0)	0.0 (0.0 to 0.0)
Cardiomyopathy and myocarditis	0.0 (0.0 to 0.0)	0.0 (0.0 to 0.0)	0.0 (0.0 to 0.0)	0.0 (0.0 to 0.0)	0.0 (0.0 to 0.0)	0.0 (0.0 to 0.0)
Acute myocarditis	0.0 (0.0 to 0.0)	0.0 (0.0 to 0.0)	0.0 (0.0 to 0.0)	0.0 (0.0 to 0.0)	0.0 (0.0 to 0.0)	0.0 (0.0 to 0.0)
Heart failure due to cardiomyopathy aggregate	0.0 (0.0 to 0.0)	0.0 (0.0 to 0.0)	0.0 (0.0 to 0.0)	0.0 (0.0 to 0.0)	0.0 (0.0 to 0.0)	0.0 (0.0 to 0.0)
Atrial fibrillation and flutter	0.0 (0.0 to 0.0)	0.0 (0.0 to 0.0)	0.0 (0.0 to 0.0)	0.0 (0.0 to 0.0)	0.0 (0.0 to 0.0)	0.0 (0.0 to 0.0)
Peripheral vascular disease	0.0 (0.0 to 0.0)	0.0 (0.0 to 0.0)	0.0 (0.0 to 0.0)	0.0 (0.0 to 0.0)	0.0 (0.0 to 0.0)	0.0 (0.0 to 0.0)
Endocarditis	0.3 (0.2 to 0.3)	3.1 (-1.5 to 8.2)	-3.2 (-7.5 to 1.6)	0.0 (0.0 to 0.0)	3.1 (-1.5 to 8.2)	-3.2 (-7.6 to 1.6)
Endocarditis episodes aggregate	0.3 (0.2 to 0.3)	3.1 (-1.5 to 8.2)	-3.2 (-7.5 to 1.6)	0.0 (0.0 to 0.0)	3.1 (-1.5 to 8.2)	-3.2 (-7.6 to 1.6)
Heart failure due to endocarditis aggregate	0.0 (0.0 to 0.0)	0.0 (0.0 to 0.0)	0.0 (0.0 to 0.0)	0.0 (0.0 to 0.0)	0.0 (0.0 to 0.0)	0.0 (0.0 to 0.0)
Other cardiovascular and circulatory diseases	0.0 (0.0 to 0.0)	0.0 (0.0 to 0.0)	0.0 (0.0 to 0.0)	0.0 (0.0 to 0.0)	0.0 (0.0 to 0.0)	0.0 (0.0 to 0.0)
Heart failure due to other cardiovascular diseases aggregate	0.0 (0.0 to 0.0)	0.0 (0.0 to 0.0)	0.0 (0.0 to 0.0)	0.0 (0.0 to 0.0)	0.0 (0.0 to 0.0)	0.0 (0.0 to 0.0)
Other cardiovascular diseases episodes aggregate	0.0 (0.0 to 0.0)	0.0 (0.0 to 0.0)	0.0 (0.0 to 0.0)	0.0 (0.0 to 0.0)	0.0 (0.0 to 0.0)	0.0 (0.0 to 0.0)
Chronic respiratory diseases	0.0 (0.0 to 0.0)	0.0 (0.0 to 0.0)	0.0 (0.0 to 0.0)	0.0 (0.0 to 0.0)	0.0 (0.0 to 0.0)	0.0 (0.0 to 0.0)
Chronic obstructive pulmonary disease	0.0 (0.0 to 0.0)	0.0 (0.0 to 0.0)	0.0 (0.0 to 0.0)	0.0 (0.0 to 0.0)	0.0 (0.0 to 0.0)	0.0 (0.0 to 0.0)

Cause	Prevalence in thousands (95% confidence interval)			YLDs in thousands (95% confidence interval)		
	2015	Percent change in counts between 2005 and 2015	Percent change in rates between 2005 and 2015	2015	Percent change in counts between 2005 and 2015	Percent change in rates between 2005 and 2015
Chronic obstructive pulmonary disease without heart failure aggregate	0.0 (0.0 to 0.0)	0.0 (0.0 to 0.0)	0.0 (0.0 to 0.0)	0.0 (0.0 to 0.0)	0.0 (0.0 to 0.0)	0.0 (0.0 to 0.0)
Severe chronic obstructive pulmonary disease with heart failure aggregate	0.0 (0.0 to 0.0)	0.0 (0.0 to 0.0)	0.0 (0.0 to 0.0)	0.0 (0.0 to 0.0)	0.0 (0.0 to 0.0)	0.0 (0.0 to 0.0)
Pneumoconiosis	0.0 (0.0 to 0.0)	0.0 (0.0 to 0.0)	0.0 (0.0 to 0.0)	0.0 (0.0 to 0.0)	0.0 (0.0 to 0.0)	0.0 (0.0 to 0.0)
Silicosis	0.0 (0.0 to 0.0)	0.0 (0.0 to 0.0)	0.0 (0.0 to 0.0)	0.0 (0.0 to 0.0)	0.0 (0.0 to 0.0)	0.0 (0.0 to 0.0)
Silicosis without heart failure aggregate	0.0 (0.0 to 0.0)	0.0 (0.0 to 0.0)	0.0 (0.0 to 0.0)	0.0 (0.0 to 0.0)	0.0 (0.0 to 0.0)	0.0 (0.0 to 0.0)
Severe silicosis with heart failure aggregate	0.0 (0.0 to 0.0)	0.0 (0.0 to 0.0)	0.0 (0.0 to 0.0)	0.0 (0.0 to 0.0)	0.0 (0.0 to 0.0)	0.0 (0.0 to 0.0)
Asbestosis	0.0 (0.0 to 0.0)	0.0 (0.0 to 0.0)	0.0 (0.0 to 0.0)	0.0 (0.0 to 0.0)	0.0 (0.0 to 0.0)	0.0 (0.0 to 0.0)
Asbestosis without heart failure aggregate	0.0 (0.0 to 0.0)	0.0 (0.0 to 0.0)	0.0 (0.0 to 0.0)	0.0 (0.0 to 0.0)	0.0 (0.0 to 0.0)	0.0 (0.0 to 0.0)
Severe asbestosis with heart failure aggregate	0.0 (0.0 to 0.0)	0.0 (0.0 to 0.0)	0.0 (0.0 to 0.0)	0.0 (0.0 to 0.0)	0.0 (0.0 to 0.0)	0.0 (0.0 to 0.0)
Coal workers pneumoconiosis	0.0 (0.0 to 0.0)	0.0 (0.0 to 0.0)	0.0 (0.0 to 0.0)	0.0 (0.0 to 0.0)	0.0 (0.0 to 0.0)	0.0 (0.0 to 0.0)
Coal workers pneumoconiosis without heart failure aggregate	0.0 (0.0 to 0.0)	0.0 (0.0 to 0.0)	0.0 (0.0 to 0.0)	0.0 (0.0 to 0.0)	0.0 (0.0 to 0.0)	0.0 (0.0 to 0.0)
Severe coal workers pneumoconiosis with heart failure aggregate	0.0 (0.0 to 0.0)	0.0 (0.0 to 0.0)	0.0 (0.0 to 0.0)	0.0 (0.0 to 0.0)	0.0 (0.0 to 0.0)	0.0 (0.0 to 0.0)
Other pneumoconiosis	0.0 (0.0 to 0.0)	0.0 (0.0 to 0.0)	0.0 (0.0 to 0.0)	0.0 (0.0 to 0.0)	0.0 (0.0 to 0.0)	0.0 (0.0 to 0.0)
Other pneumoconiosis without heart failure aggregate	0.0 (0.0 to 0.0)	0.0 (0.0 to 0.0)	0.0 (0.0 to 0.0)	0.0 (0.0 to 0.0)	0.0 (0.0 to 0.0)	0.0 (0.0 to 0.0)
Severe other pneumoconiosis with heart failure aggregate	0.0 (0.0 to 0.0)	0.0 (0.0 to 0.0)	0.0 (0.0 to 0.0)	0.0 (0.0 to 0.0)	0.0 (0.0 to 0.0)	0.0 (0.0 to 0.0)
Asthma	0.0 (0.0 to 0.0)	0.0 (0.0 to 0.0)	0.0 (0.0 to 0.0)	0.0 (0.0 to 0.0)	0.0 (0.0 to 0.0)	0.0 (0.0 to 0.0)
Asymptomatic asthma	0.0 (0.0 to 0.0)	0.0 (0.0 to 0.0)	0.0 (0.0 to 0.0)	0.0 (0.0 to 0.0)	0.0 (0.0 to 0.0)	0.0 (0.0 to 0.0)
Symptomatic asthma aggregate	0.0 (0.0 to 0.0)	0.0 (0.0 to 0.0)	0.0 (0.0 to 0.0)	0.0 (0.0 to 0.0)	0.0 (0.0 to 0.0)	0.0 (0.0 to 0.0)
Interstitial lung disease and pulmonary sarcoidosis	0.0 (0.0 to 0.0)	0.0 (0.0 to 0.0)	0.0 (0.0 to 0.0)	0.0 (0.0 to 0.0)	0.0 (0.0 to 0.0)	0.0 (0.0 to 0.0)
Interstitial lung disease and pulmonary sarcoidosis without heart failure aggregate	0.0 (0.0 to 0.0)	0.0 (0.0 to 0.0)	0.0 (0.0 to 0.0)	0.0 (0.0 to 0.0)	0.0 (0.0 to 0.0)	0.0 (0.0 to 0.0)
Severe interstitial lung disease and pulmonary sarcoidosis with heart failure aggregate	0.0 (0.0 to 0.0)	0.0 (0.0 to 0.0)	0.0 (0.0 to 0.0)	0.0 (0.0 to 0.0)	0.0 (0.0 to 0.0)	0.0 (0.0 to 0.0)
Other chronic respiratory diseases	--	--	--	0.0 (0.0 to 0.0)	0.0 (0.0 to 0.0)	0.0 (0.0 to 0.0)
Cirrhosis and other chronic liver diseases	1.1 (0.8 to 1.5)	-4.4 (-38.2 to 41.9)	-10.3 (-42.0 to 33.2)	0.7 (0.5 to 1.0)	7.7 (-5.4 to 22.5)	1.1 (-11.2 to 15.0)
Cirrhosis and other chronic liver diseases due to hepatitis B	0.0 (0.0 to 0.0)	8.2 (5.0 to 11.1)*	1.6 (-1.4 to 4.3)	0.0 (0.0 to 0.0)	8.2 (5.0 to 11.1)*	1.6 (-1.4 to 4.3)
Cirrhosis and other chronic liver diseases due to hepatitis C	0.0 (0.0 to 0.0)	8.5 (3.9 to 12.8)*	1.9 (-2.5 to 5.9)	0.0 (0.0 to 0.0)	8.5 (3.9 to 12.8)*	1.9 (-2.5 to 5.9)
Cirrhosis and other chronic liver diseases due to alcohol use	0.0 (0.0 to 0.0)	0.0 (0.0 to 0.0)	0.0 (0.0 to 0.0)	0.0 (0.0 to 0.0)	0.0 (0.0 to 0.0)	0.0 (0.0 to 0.0)
Cirrhosis and other chronic liver diseases due to other causes	4.0 (3.7 to 4.2)	7.3 (6.6 to 8.0)*	0.7 (0.0 to 1.3)*	0.7 (0.5 to 1.0)	7.7 (-5.4 to 22.6)	1.1 (-11.2 to 15.1)
Digestive diseases	3.5 (2.9 to 4.3)	2.9 (-20.6 to 31.3)	-3.4 (-25.5 to 23.3)	2.1 (1.3 to 3.1)	5.2 (-8.7 to 21.3)	-1.3 (-14.3 to 13.9)
Peptic ulcer disease	0.0 (0.0 to 0.0)	0.0 (0.0 to 0.0)	0.0 (0.0 to 0.0)	0.0 (0.0 to 0.0)	0.0 (0.0 to 0.0)	0.0 (0.0 to 0.0)
Peptic ulcer disease, symptomatic episodes	0.0 (0.0 to 0.0)	0.0 (0.0 to 0.0)	0.0 (0.0 to 0.0)	0.0 (0.0 to 0.0)	0.0 (0.0 to 0.0)	0.0 (0.0 to 0.0)
Anemia due to peptic ulcer disease aggregate	0.0 (0.0 to 0.0)	0.0 (0.0 to 0.0)	0.0 (0.0 to 0.0)	0.0 (0.0 to 0.0)	0.0 (0.0 to 0.0)	0.0 (0.0 to 0.0)
Gastritis and duodenitis	0.0 (0.0 to 0.0)	0.0 (0.0 to 0.0)	0.0 (0.0 to 0.0)	0.0 (0.0 to 0.0)	0.0 (0.0 to 0.0)	0.0 (0.0 to 0.0)
Gastritis and duodenitis, symptomatic episodes	0.0 (0.0 to 0.0)	0.0 (0.0 to 0.0)	0.0 (0.0 to 0.0)	0.0 (0.0 to 0.0)	0.0 (0.0 to 0.0)	0.0 (0.0 to 0.0)
Anemia due to gastritis and duodenitis aggregate	0.0 (0.0 to 0.0)	0.0 (0.0 to 0.0)	0.0 (0.0 to 0.0)	0.0 (0.0 to 0.0)	0.0 (0.0 to 0.0)	0.0 (0.0 to 0.0)
Appendicitis	0.0 (0.0 to 0.0)	0.0 (0.0 to 0.0)	0.0 (0.0 to 0.0)	0.0 (0.0 to 0.0)	0.0 (0.0 to 0.0)	0.0 (0.0 to 0.0)
Paralytic ileus and intestinal obstruction	6.1 (4.8 to 7.8)	4.2 (1.9 to 6.4)*	-2.2 (-4.3 to -0.2)*	1.9 (1.2 to 2.8)	4.5 (-9.5 to 20.9)	-1.9 (-15.0 to 13.5)
Inguinal, femoral, and abdominal hernia	0.0 (0.0 to 0.0)	0.0 (0.0 to 0.0)	0.0 (0.0 to 0.0)	0.0 (0.0 to 0.0)	0.0 (0.0 to 0.0)	0.0 (0.0 to 0.0)
Inflammatory bowel disease	0.0 (0.0 to 0.0)	0.0 (0.0 to 0.0)	0.0 (0.0 to 0.0)	0.0 (0.0 to 0.0)	0.0 (0.0 to 0.0)	0.0 (0.0 to 0.0)
Vascular intestinal disorders	0.0 (0.0 to 0.0)	0.0 (0.0 to 0.0)	0.0 (0.0 to 0.0)	0.0 (0.0 to 0.0)	0.0 (0.0 to 0.0)	0.0 (0.0 to 0.0)
Gallbladder and biliary diseases	0.0 (0.0 to 0.0)	0.0 (0.0 to 0.0)	0.0 (0.0 to 0.0)	0.0 (0.0 to 0.0)	0.0 (0.0 to 0.0)	0.0 (0.0 to 0.0)
Pancreatitis	0.0 (0.0 to 0.0)	0.0 (0.0 to 0.0)	0.0 (0.0 to 0.0)	0.0 (0.0 to 0.0)	0.0 (0.0 to 0.0)	0.0 (0.0 to 0.0)
Other digestive diseases	--	--	--	0.2 (0.1 to 0.3)	11.7 (-5.0 to 32.8)	4.8 (-10.9 to 24.6)
Neurological disorders	386.7 (348.1 to 427.8)	2.4 (-3.3 to 8.2)	-3.9 (-9.2 to 1.6)	117.1 (74.3 to 166.9)	-11.2 (-19.2 to -3.0)*	-16.6 (-24.2 to -8.9)*
Alzheimer disease and other dementias	0.0 (0.0 to 0.0)	0.0 (0.0 to 0.0)	0.0 (0.0 to 0.0)	0.0 (0.0 to 0.0)	0.0 (0.0 to 0.0)	0.0 (0.0 to 0.0)
Parkinson disease	0.0 (0.0 to 0.0)	0.0 (0.0 to 0.0)	0.0 (0.0 to 0.0)	0.0 (0.0 to 0.0)	0.0 (0.0 to 0.0)	0.0 (0.0 to 0.0)
Epilepsy	386.0 (299.3 to 496.9)	2.7 (-5.6 to 10.7)	-3.6 (-11.4 to 3.9)	117.1 (74.2 to 166.8)	-11.2 (-19.2 to -3.0)*	-16.6 (-24.2 to -8.9)*
Multiple sclerosis	0.0 (0.0 to 0.0)	0.0 (0.0 to 0.0)	0.0 (0.0 to 0.0)	0.0 (0.0 to 0.0)	0.0 (0.0 to 0.0)	0.0 (0.0 to 0.0)
Motor neuron disease	0.4 (0.3 to 0.4)	-0.4 (-3.2 to 2.0)	-6.5 (-9.1 to -4.3)*	0.1 (0.1 to 0.1)	-0.4 (-3.2 to 2.0)	-6.5 (-9.1 to -4.3)*
Migraine	0.0 (0.0 to 0.0)	0.0 (0.0 to 0.0)	0.0 (0.0 to 0.0)	0.0 (0.0 to 0.0)	0.0 (0.0 to 0.0)	0.0 (0.0 to 0.0)
Tension-type headache	0.0 (0.0 to 0.0)	0.0 (0.0 to 0.0)	0.0 (0.0 to 0.0)	0.0 (0.0 to 0.0)	0.0 (0.0 to 0.0)	0.0 (0.0 to 0.0)
Medication overuse headache	0.0 (0.0 to 0.0)	0.0 (0.0 to 0.0)	0.0 (0.0 to 0.0)	0.0 (0.0 to 0.0)	0.0 (0.0 to 0.0)	0.0 (0.0 to 0.0)
Other neurological disorders	0.0 (0.0 to 0.0)	0.0 (0.0 to 0.0)	0.0 (0.0 to 0.0)	0.0 (0.0 to 0.0)	0.0 (0.0 to 0.0)	0.0 (0.0 to 0.0)
Mental and substance use disorders	1,029.2 (927.5 to 1,107.1)	4.7 (0.0 to 9.6)*	-1.7 (-6.1 to 2.8)	108.9 (67.4 to 160.8)	6.4 (4.7 to 8.0)*	-0.2 (-1.7 to 1.4)
Schizophrenia	0.0 (0.0 to 0.0)	0.0 (0.0 to 0.0)	0.0 (0.0 to 0.0)	0.0 (0.0 to 0.0)	0.0 (0.0 to 0.0)	0.0 (0.0 to 0.0)
Alcohol use disorders	14.3 (11.9 to 16.3)	5.6 (3.4 to 7.5)*	-0.9 (-3.0 to 0.9)	0.5 (0.3 to 0.8)	5.6 (-6.1 to 18.5)	-0.9 (-11.9 to 11.3)
Alcohol dependence aggregate	0.0 (0.0 to 0.0)	0.0 (0.0 to 0.0)	0.0 (0.0 to 0.0)	0.0 (0.0 to 0.0)	0.0 (0.0 to 0.0)	0.0 (0.0 to 0.0)
Fetal alcohol syndrome aggregate	14.3 (11.9 to 16.3)	5.6 (3.4 to 7.5)*	-0.9 (-3.0 to 0.9)	0.5 (0.3 to 0.8)	5.6 (-6.1 to 18.5)	-0.9 (-11.9 to 11.3)
Drug use disorders	0.0 (0.0 to 0.0)	0.0 (0.0 to 0.0)	0.0 (0.0 to 0.0)	0.0 (0.0 to 0.0)	0.0 (0.0 to 0.0)	0.0 (0.0 to 0.0)
Opioid use disorders	0.0 (0.0 to 0.0)	0.0 (0.0 to 0.0)	0.0 (0.0 to 0.0)	0.0 (0.0 to 0.0)	0.0 (0.0 to 0.0)	0.0 (0.0 to 0.0)
Asymptomatic opioid dependence	0.0 (0.0 to 0.0)	0.0 (0.0 to 0.0)	0.0 (0.0 to 0.0)	0.0 (0.0 to 0.0)	0.0 (0.0 to 0.0)	0.0 (0.0 to 0.0)
Symptomatic opioid dependence aggregate	0.0 (0.0 to 0.0)	0.0 (0.0 to 0.0)	0.0 (0.0 to 0.0)	0.0 (0.0 to 0.0)	0.0 (0.0 to 0.0)	0.0 (0.0 to 0.0)
Cocaine use disorders	0.0 (0.0 to 0.0)	0.0 (0.0 to 0.0)	0.0 (0.0 to 0.0)	0.0 (0.0 to 0.0)	0.0 (0.0 to 0.0)	0.0 (0.0 to 0.0)
Asymptomatic cocaine dependence	0.0 (0.0 to 0.0)	0.0 (0.0 to 0.0)	0.0 (0.0 to 0.0)	0.0 (0.0 to 0.0)	0.0 (0.0 to 0.0)	0.0 (0.0 to 0.0)

Cause	Prevalence in thousands (95% confidence interval)			YLDs in thousands (95% confidence interval)		
	2015	Percent change in counts between 2005 and 2015	Percent change in rates between 2005 and 2015	2015	Percent change in counts between 2005 and 2015	Percent change in rates between 2005 and 2015
Symptomatic cocaine dependence aggregate	0.0 (0.0 to 0.0)	0.0 (0.0 to 0.0)	0.0 (0.0 to 0.0)	0.0 (0.0 to 0.0)	0.0 (0.0 to 0.0)	0.0 (0.0 to 0.0)
Amphetamine use disorders	0.0 (0.0 to 0.0)	0.0 (0.0 to 0.0)	0.0 (0.0 to 0.0)	0.0 (0.0 to 0.0)	0.0 (0.0 to 0.0)	0.0 (0.0 to 0.0)
Asymptomatic amphetamine dependence	0.0 (0.0 to 0.0)	0.0 (0.0 to 0.0)	0.0 (0.0 to 0.0)	0.0 (0.0 to 0.0)	0.0 (0.0 to 0.0)	0.0 (0.0 to 0.0)
Symptomatic amphetamine dependence aggregate	0.0 (0.0 to 0.0)	0.0 (0.0 to 0.0)	0.0 (0.0 to 0.0)	0.0 (0.0 to 0.0)	0.0 (0.0 to 0.0)	0.0 (0.0 to 0.0)
Cannabis use disorders	0.0 (0.0 to 0.0)	0.0 (0.0 to 0.0)	0.0 (0.0 to 0.0)	0.0 (0.0 to 0.0)	0.0 (0.0 to 0.0)	0.0 (0.0 to 0.0)
Asymptomatic cannabis dependence	0.0 (0.0 to 0.0)	0.0 (0.0 to 0.0)	0.0 (0.0 to 0.0)	0.0 (0.0 to 0.0)	0.0 (0.0 to 0.0)	0.0 (0.0 to 0.0)
Symptomatic cannabis dependence aggregate	0.0 (0.0 to 0.0)	0.0 (0.0 to 0.0)	0.0 (0.0 to 0.0)	0.0 (0.0 to 0.0)	0.0 (0.0 to 0.0)	0.0 (0.0 to 0.0)
Other drug use disorders	--	--	--	0.0 (0.0 to 0.0)	0.0 (0.0 to 0.0)	0.0 (0.0 to 0.0)
Depressive disorders	0.0 (0.0 to 0.0)	0.0 (0.0 to 0.0)	0.0 (0.0 to 0.0)	0.0 (0.0 to 0.0)	0.0 (0.0 to 0.0)	0.0 (0.0 to 0.0)
Major depressive disorder	0.0 (0.0 to 0.0)	0.0 (0.0 to 0.0)	0.0 (0.0 to 0.0)	0.0 (0.0 to 0.0)	0.0 (0.0 to 0.0)	0.0 (0.0 to 0.0)
Dysthymia	0.0 (0.0 to 0.0)	0.0 (0.0 to 0.0)	0.0 (0.0 to 0.0)	0.0 (0.0 to 0.0)	0.0 (0.0 to 0.0)	0.0 (0.0 to 0.0)
Bipolar disorder	0.0 (0.0 to 0.0)	0.0 (0.0 to 0.0)	0.0 (0.0 to 0.0)	0.0 (0.0 to 0.0)	0.0 (0.0 to 0.0)	0.0 (0.0 to 0.0)
Anxiety disorders	0.0 (0.0 to 0.0)	0.0 (0.0 to 0.0)	0.0 (0.0 to 0.0)	0.0 (0.0 to 0.0)	0.0 (0.0 to 0.0)	0.0 (0.0 to 0.0)
Eating disorders	0.0 (0.0 to 0.0)	0.0 (0.0 to 0.0)	0.0 (0.0 to 0.0)	0.0 (0.0 to 0.0)	0.0 (0.0 to 0.0)	0.0 (0.0 to 0.0)
Anorexia nervosa	0.0 (0.0 to 0.0)	0.0 (0.0 to 0.0)	0.0 (0.0 to 0.0)	0.0 (0.0 to 0.0)	0.0 (0.0 to 0.0)	0.0 (0.0 to 0.0)
Bulimia nervosa	0.0 (0.0 to 0.0)	0.0 (0.0 to 0.0)	0.0 (0.0 to 0.0)	0.0 (0.0 to 0.0)	0.0 (0.0 to 0.0)	0.0 (0.0 to 0.0)
Autistic spectrum disorders	579.6 (526.6 to 628.9)	6.3 (2.2 to 10.7)*	-0.2 (-4.1 to 3.9)	92.1 (55.8 to 138.6)	6.7 (4.7 to 8.7)*	0.2 (-1.7 to 2.0)
Autism	197.5 (130.6 to 276.2)	6.3 (5.9 to 6.7)*	-0.2 (-0.6 to 0.2)	51.9 (29.4 to 83.2)	6.6 (3.4 to 9.6)*	0.1 (-2.9 to 2.9)
Asperger syndrome and other autistic spectrum disorders	396.5 (263.7 to 562.9)	6.6 (6.3 to 6.9)*	0.0 (-0.3 to 0.3)	40.2 (22.8 to 63.8)	6.8 (4.7 to 8.9)*	0.2 (-1.7 to 2.2)
Attention-deficit/hyperactivity disorder	0.0 (0.0 to 0.0)	0.0 (0.0 to 0.0)	0.0 (0.0 to 0.0)	0.0 (0.0 to 0.0)	0.0 (0.0 to 0.0)	0.0 (0.0 to 0.0)
Conduct disorder	0.0 (0.0 to 0.0)	0.0 (0.0 to 0.0)	0.0 (0.0 to 0.0)	0.0 (0.0 to 0.0)	0.0 (0.0 to 0.0)	0.0 (0.0 to 0.0)
Idiopathic developmental intellectual disability	422.5 (183.1 to 666.0)	3.1 (-0.4 to 5.1)	-3.2 (-6.5 to -1.3)*	16.3 (5.4 to 30.9)	4.5 (1.3 to 7.0)*	-1.9 (-4.9 to 0.5)
Other mental and substance use disorders	0.0 (0.0 to 0.0)	0.0 (0.0 to 0.0)	0.0 (0.0 to 0.0)	0.0 (0.0 to 0.0)	0.0 (0.0 to 0.0)	0.0 (0.0 to 0.0)
Diabetes, urogenital, blood, and endocrine diseases	33,288.3 (32,793.5 to 33,876.8)	3.8 (2.5 to 5.2)*	-2.5 (-3.8 to -1.3)*	495.0 (338.7 to 700.7)	3.2 (-2.7 to 10.5)	-3.1 (-8.7 to 3.8)
Diabetes mellitus	0.0 (0.0 to 0.0)	0.0 (0.0 to 0.0)	0.0 (0.0 to 0.0)	0.0 (0.0 to 0.0)	0.0 (0.0 to 0.0)	0.0 (0.0 to 0.0)
Uncomplicated diabetes mellitus	0.0 (0.0 to 0.0)	0.0 (0.0 to 0.0)	0.0 (0.0 to 0.0)	0.0 (0.0 to 0.0)	0.0 (0.0 to 0.0)	0.0 (0.0 to 0.0)
Neuropathy and other complications of diabetes mellitus aggregate	0.0 (0.0 to 0.0)	0.0 (0.0 to 0.0)	0.0 (0.0 to 0.0)	0.0 (0.0 to 0.0)	0.0 (0.0 to 0.0)	0.0 (0.0 to 0.0)
Vision loss due to diabetes mellitus aggregate	0.0 (0.0 to 0.0)	0.0 (0.0 to 0.0)	0.0 (0.0 to 0.0)	0.0 (0.0 to 0.0)	0.0 (0.0 to 0.0)	0.0 (0.0 to 0.0)
Acute glomerulonephritis	1.1 (0.9 to 1.4)	-9.4 (-14.3 to -5.2)*	-14.9 (-19.5 to -11.0)*	0.1 (0.0 to 0.1)	-9.4 (-14.3 to -5.2)*	-14.9 (-19.5 to -11.0)*
Chronic kidney disease	2.0 (1.6 to 2.5)	17.1 (-9.9 to 51.0)	9.9 (-15.4 to 41.8)	1.5 (1.0 to 2.1)	5.8 (4.7 to 6.8)*	-0.7 (-1.7 to 0.2)
Chronic kidney disease due to diabetes mellitus	3.4 (2.1 to 5.5)	5.7 (2.8 to 8.4)*	-0.8 (-3.5 to 1.8)	0.3 (0.2 to 0.5)	5.6 (3.0 to 8.1)*	-0.8 (-3.3 to 1.5)
Stage III chronic kidney disease due to diabetes mellitus aggregate	2.9 (1.7 to 4.7)	5.9 (2.9 to 8.6)*	-0.6 (-3.4 to 2.0)	0.0 (0.0 to 0.0)	9.7 (6.6 to 13.1)*	3.0 (0.0 to 6.2)*
Stage IV chronic kidney disease due to diabetes mellitus aggregate	0.0 (0.0 to 0.1)	6.4 (3.5 to 9.3)*	-0.1 (-2.8 to 2.6)	0.0 (0.0 to 0.0)	7.1 (4.1 to 9.9)*	0.5 (-2.3 to 3.2)
Stage V chronic kidney disease untreated due to diabetes mellitus	0.5 (0.3 to 0.7)	5.3 (2.3 to 8.0)*	-1.2 (-3.9 to 1.4)	0.3 (0.2 to 0.4)	5.3 (2.3 to 8.0)*	-1.2 (-3.9 to 1.4)
End-stage chronic kidney disease due to diabetes aggregate	0.0 (0.0 to 0.1)	-0.5 (-6.1 to 4.1)	-6.6 (-11.8 to -2.3)*	0.0 (0.0 to 0.0)	2.5 (-1.7 to 6.5)	-3.7 (-7.7 to -0.1)*
Chronic kidney disease due to hypertension	3.2 (1.8 to 5.2)	5.4 (2.7 to 8.0)*	-1.0 (-3.6 to 1.4)	0.0 (0.0 to 0.1)	5.1 (0.0 to 9.5)*	-1.3 (-6.1 to 2.8)
Stage III chronic kidney disease due to hypertension aggregate	3.1 (1.8 to 5.1)	5.4 (2.5 to 8.0)*	-1.0 (-3.8 to 1.4)	0.0 (0.0 to 0.0)	9.0 (5.6 to 12.4)*	2.3 (-0.9 to 5.5)
Stage IV chronic kidney disease due to hypertension aggregate	0.0 (0.0 to 0.0)	6.1 (0.4 to 11.2)*	-0.4 (-5.7 to 4.4)	0.0 (0.0 to 0.0)	6.7 (1.0 to 12.0)*	0.2 (-5.2 to 5.1)
Stage V chronic kidney disease untreated due to hypertension	0.1 (0.0 to 0.1)	4.8 (-0.7 to 9.6)	-1.6 (-6.8 to 2.9)	0.0 (0.0 to 0.0)	4.8 (-0.7 to 9.6)	-1.6 (-6.8 to 2.9)
End-stage chronic kidney disease due to hypertension aggregate	0.0 (0.0 to 0.0)	-1.6 (-10.9 to 8.0)	-7.7 (-16.4 to 1.3)	0.0 (0.0 to 0.0)	0.8 (-6.5 to 8.2)	-5.3 (-12.2 to 1.6)
Chronic kidney disease due to glomerulonephritis	1.5 (1.2 to 1.8)	5.8 (4.1 to 7.5)*	-0.7 (-2.3 to 0.9)	0.4 (0.2 to 0.6)	5.2 (3.0 to 7.2)*	-1.2 (-3.3 to 0.6)
Stage III chronic kidney disease due to glomerulonephritis aggregate	0.8 (0.6 to 1.0)	6.8 (4.4 to 9.2)*	0.2 (-2.0 to 2.5)	0.0 (0.0 to 0.0)	8.2 (5.4 to 11.2)*	1.5 (-1.1 to 4.4)
Stage IV chronic kidney disease due to glomerulonephritis aggregate	0.0 (0.0 to 0.1)	6.6 (4.1 to 9.1)*	0.1 (-2.3 to 2.5)	0.0 (0.0 to 0.0)	6.9 (4.4 to 9.4)*	0.4 (-2.0 to 2.7)
Stage V chronic kidney disease untreated due to glomerulonephritis	0.6 (0.4 to 0.9)	5.0 (2.5 to 7.2)*	-1.5 (-3.7 to 0.6)	0.3 (0.2 to 0.5)	5.0 (2.5 to 7.2)*	-1.5 (-3.7 to 0.6)
End-stage chronic kidney disease due to glomerulonephritis aggregate	0.1 (0.0 to 0.1)	0.0 (-4.6 to 4.8)	-6.1 (-10.5 to -1.6)*	0.0 (0.0 to 0.0)	3.6 (-1.4 to 8.1)	-2.8 (-7.5 to 1.5)
Chronic kidney disease due to other causes	8.1 (5.5 to 11.4)	6.3 (4.9 to 7.8)*	-0.2 (-1.5 to 1.2)	0.7 (0.5 to 1.1)	6.1 (4.7 to 7.6)*	-0.4 (-1.7 to 1.0)
Stage III chronic kidney disease due to other causes aggregate	6.8 (4.5 to 10.0)	6.5 (5.0 to 8.1)*	-0.0 (-1.4 to 1.4)	0.1 (0.0 to 0.1)	10.4 (6.9 to 14.4)*	3.6 (0.4 to 7.4)*
Stage IV chronic kidney disease due to other causes aggregate	0.1 (0.1 to 0.1)	7.2 (5.7 to 8.9)*	0.6 (-0.8 to 2.3)	0.0 (0.0 to 0.0)	7.4 (5.6 to 9.2)*	0.9 (-0.8 to 2.6)
Stage V chronic kidney disease untreated due to other causes	1.1 (0.8 to 1.5)	5.8 (4.1 to 7.5)*	-0.7 (-2.3 to 0.9)	0.6 (0.4 to 0.9)	5.8 (4.1 to 7.5)*	-0.7 (-2.3 to 0.9)
End-stage chronic kidney disease due to other causes aggregate	0.1 (0.1 to 0.1)	0.2 (-2.7 to 3.3)	-5.9 (-8.6 to -3.1)*	0.0 (0.0 to 0.0)	3.8 (-0.3 to 7.5)	-2.5 (-6.4 to 1.0)
Urinary diseases and male infertility	89.6 (86.5 to 92.7)	12.3 (7.7 to 16.9)*	5.4 (1.1 to 9.7)*	4.1 (2.5 to 6.3)	11.8 (6.4 to 17.8)*	5.0 (-0.1 to 10.6)
Interstitial nephritis and urinary tract infections	90.0 (84.9 to 95.5)	12.6 (12.0 to 13.4)*	5.7 (5.1 to 6.4)*	3.1 (1.9 to 4.7)	12.7 (8.0 to 18.1)*	5.8 (1.4 to 10.8)*
Urolithiasis	0.0 (0.0 to 0.0)	0.0 (0.0 to 0.0)	0.0 (0.0 to 0.0)	0.0 (0.0 to 0.0)	0.0 (0.0 to 0.0)	0.0 (0.0 to 0.0)
Benign prostatic hyperplasia	0.0 (0.0 to 0.0)	0.0 (0.0 to 0.0)	0.0 (0.0 to 0.0)	0.0 (0.0 to 0.0)	0.0 (0.0 to 0.0)	0.0 (0.0 to 0.0)
Male infertility	0.0 (0.0 to 0.0)	0.0 (0.0 to 0.0)	0.0 (0.0 to 0.0)	0.0 (0.0 to 0.0)	0.0 (0.0 to 0.0)	0.0 (0.0 to 0.0)
Other urinary diseases	--	--	--	1.0 (0.6 to 1.6)	9.2 (-0.7 to 20.9)	2.5 (-6.8 to 13.5)
Gynecological diseases	0.0 (0.0 to 0.0)	0.0 (0.0 to 0.0)	0.0 (0.0 to 0.0)	0.0 (0.0 to 0.0)	0.0 (0.0 to 0.0)	0.0 (0.0 to 0.0)
Uterine fibroids	0.0 (0.0 to 0.0)	0.0 (0.0 to 0.0)	0.0 (0.0 to 0.0)	0.0 (0.0 to 0.0)	0.0 (0.0 to 0.0)	0.0 (0.0 to 0.0)
Uterine fibroids cases aggregate	0.0 (0.0 to 0.0)	0.0 (0.0 to 0.0)	0.0 (0.0 to 0.0)	0.0 (0.0 to 0.0)	0.0 (0.0 to 0.0)	0.0 (0.0 to 0.0)
Mild abdominal pain with anemia due to uterine fibroids aggregate	0.0 (0.0 to 0.0)	0.0 (0.0 to 0.0)	0.0 (0.0 to 0.0)	0.0 (0.0 to 0.0)	0.0 (0.0 to 0.0)	0.0 (0.0 to 0.0)
Polycystic ovarian syndrome	0.0 (0.0 to 0.0)	0.0 (0.0 to 0.0)	0.0 (0.0 to 0.0)	0.0 (0.0 to 0.0)	0.0 (0.0 to 0.0)	0.0 (0.0 to 0.0)
Polycystic ovarian syndrome cases aggregate	0.0 (0.0 to 0.0)	0.0 (0.0 to 0.0)	0.0 (0.0 to 0.0)	0.0 (0.0 to 0.0)	0.0 (0.0 to 0.0)	0.0 (0.0 to 0.0)
Hirsutism and infertility due to polycystic ovarian syndrome aggregate	0.0 (0.0 to 0.0)	0.0 (0.0 to 0.0)	0.0 (0.0 to 0.0)	0.0 (0.0 to 0.0)	0.0 (0.0 to 0.0)	0.0 (0.0 to 0.0)

Cause	Prevalence in thousands (95% confidence interval)			YLDs in thousands (95% confidence interval)		
	2015	Percent change in counts between 2005 and 2015	Percent change in rates between 2005 and 2015	2015	Percent change in counts between 2005 and 2015	Percent change in rates between 2005 and 2015
Female infertility	0.0<br>(0.0 to 0.0)	0.0<br>(0.0 to 0.0)	0.0<br>(0.0 to 0.0)	0.0<br>(0.0 to 0.0)	0.0<br>(0.0 to 0.0)	0.0<br>(0.0 to 0.0)
Endometriosis	0.0<br>(0.0 to 0.0)	0.0<br>(0.0 to 0.0)	0.0<br>(0.0 to 0.0)	0.0<br>(0.0 to 0.0)	0.0<br>(0.0 to 0.0)	0.0<br>(0.0 to 0.0)
Endometriosis cases aggregate	0.0<br>(0.0 to 0.0)	0.0<br>(0.0 to 0.0)	0.0<br>(0.0 to 0.0)	0.0<br>(0.0 to 0.0)	0.0<br>(0.0 to 0.0)	0.0<br>(0.0 to 0.0)
Abdominal pain and infertility due to endometriosis aggregate	0.0<br>(0.0 to 0.0)	0.0<br>(0.0 to 0.0)	0.0<br>(0.0 to 0.0)	0.0<br>(0.0 to 0.0)	0.0<br>(0.0 to 0.0)	0.0<br>(0.0 to 0.0)
Genital prolapse	0.0<br>(0.0 to 0.0)	0.0<br>(0.0 to 0.0)	0.0<br>(0.0 to 0.0)	0.0<br>(0.0 to 0.0)	0.0<br>(0.0 to 0.0)	0.0<br>(0.0 to 0.0)
Premenstrual syndrome	0.0<br>(0.0 to 0.0)	0.0<br>(0.0 to 0.0)	0.0<br>(0.0 to 0.0)	0.0<br>(0.0 to 0.0)	0.0<br>(0.0 to 0.0)	0.0<br>(0.0 to 0.0)
Other gynecological diseases	0.0<br>(0.0 to 0.0)	0.0<br>(0.0 to 0.0)	0.0<br>(0.0 to 0.0)	0.0<br>(0.0 to 0.0)	0.0<br>(0.0 to 0.0)	0.0<br>(0.0 to 0.0)
Other gynecological diseases cases aggregate	0.0<br>(0.0 to 0.0)	0.0<br>(0.0 to 0.0)	0.0<br>(0.0 to 0.0)	0.0<br>(0.0 to 0.0)	0.0<br>(0.0 to 0.0)	0.0<br>(0.0 to 0.0)
Anemia due to other gynecological diseases aggregate	0.0<br>(0.0 to 0.0)	0.0<br>(0.0 to 0.0)	0.0<br>(0.0 to 0.0)	0.0<br>(0.0 to 0.0)	0.0<br>(0.0 to 0.0)	0.0<br>(0.0 to 0.0)
Hemoglobinopathies and hemolytic anemias	30,948.3<br>(30,439.3 to 31,531.2)	3.7<br>(2.2 to 5.1)*	-2.7<br>(-4.1 to -1.3)*	350.6<br>(239.6 to 500.4)	3.1<br>(-3.3 to 10.8)	-3.2<br>(-9.2 to 4.0)
Thalassemias	89.1<br>(81.8 to 103.5)	2.0<br>(0.7 to 4.2)*	-4.2<br>(-5.5 to -2.2)*	6.4<br>(4.4 to 9.3)	2.4<br>(-4.6 to 10.4)	-3.9<br>(-10.4 to 3.7)
Beta-thalassemia major cases aggregate	50.9<br>(49.2 to 53.3)	4.6<br>(3.8 to 5.5)*	-1.8<br>(-2.6 to -1.0)*	3.6<br>(2.4 to 5.3)	6.0<br>(-3.6 to 17.2)	-0.5<br>(-9.5 to 10.0)
Hemoglobin E/beta-thalassemia cases aggregate	14.8<br>(12.9 to 16.9)	4.5<br>(1.5 to 7.5)*	-1.9<br>(-4.7 to 0.9)	1.0<br>(0.7 to 1.5)	3.5<br>(-11.6 to 21.1)	-2.9<br>(-17.0 to 13.7)
Hemoglobin H disease cases aggregate	23.4<br>(16.7 to 36.7)	-4.6<br>(-9.4 to 3.0)	-10.4<br>(-15.0 to -3.4)*	1.8<br>(1.1 to 2.6)	-5.0<br>(-17.9 to 14.5)	-10.8<br>(-22.9 to 7.5)
Heart failure due to thalassemias aggregate	0.0<br>(0.0 to 0.0)	0.0<br>(0.0 to 0.0)	0.0<br>(0.0 to 0.0)	0.0<br>(0.0 to 0.0)	0.0<br>(0.0 to 0.0)	0.0<br>(0.0 to 0.0)
Thalassemias trait	4,404.0<br>(4,274.4 to 4,555.3)	6.7<br>(4.9 to 8.8)*	0.1<br>(-1.5 to 2.2)	119.6<br>(80.3 to 173.0)	8.0<br>(0.0 to 16.6)*	1.3<br>(-6.1 to 9.5)
B-thalassemia trait cases aggregate	3,593.2<br>(3,473.3 to 3,732.6)	7.4<br>(5.2 to 10.0)*	0.8<br>(-1.2 to 3.2)	111.6<br>(74.9 to 161.8)	8.1<br>(0.2 to 16.9)*	1.5<br>(-5.9 to 9.7)
Hemoglobin E trait cases aggregate	810.8<br>(772.1 to 851.1)	3.5<br>(2.3 to 4.8)*	-2.9<br>(-3.9 to -1.7)*	8.0<br>(5.3 to 11.6)	5.9<br>(-7.1 to 20.4)	-0.6<br>(-12.8 to 13.0)
Sickle cell disorders	480.9<br>(443.2 to 516.7)	4.7<br>(-4.4 to 18.1)	-1.7<br>(-10.2 to 10.8)	41.4<br>(27.7 to 58.6)	2.3<br>(-14.5 to 17.6)	-3.9<br>(-19.7 to 10.4)
Homozygous sickle cell and severe sickle cell/beta-thalassemia cases aggregate	396.5<br>(361.5 to 429.4)	1.3<br>(-8.5 to 16.8)	-4.9<br>(-14.1 to 9.6)	34.8<br>(23.1 to 49.6)	-0.5<br>(-17.5 to 13.8)	-6.6<br>(-22.6 to 6.8)
Hemoglobin SC disease cases aggregate	80.6<br>(72.4 to 90.0)	24.8<br>(12.6 to 40.1)*	17.2<br>(5.7 to 31.5)*	6.2<br>(4.0 to 9.1)	20.8<br>(0.4 to 57.0)*	13.4<br>(-5.8 to 47.3)
Mild sickle cell/beta-thalassemia cases aggregate	3.8<br>(3.0 to 4.4)	20.2<br>(-0.0 to 43.5)	12.8<br>(-6.1 to 34.7)	0.3<br>(0.2 to 0.5)	19.5<br>(-14.9 to 72.5)	12.1<br>(-20.1 to 61.9)
Sickle cell trait	9,435.5<br>(8,963.0 to 10,241.7)	8.0<br>(6.3 to 9.3)*	1.4<br>(-0.2 to 2.6)	78.4<br>(52.8 to 111.3)	3.7<br>(-7.3 to 18.8)	-2.6<br>(-13.0 to 11.5)
G6PD deficiency	5,057.2<br>(4,267.7 to 5,886.8)	-1.8<br>(-7.7 to 3.4)	-7.8<br>(-13.3 to -3.0)*	0.9<br>(0.6 to 1.3)	-2.2<br>(-17.1 to 14.4)	-8.2<br>(-22.2 to 7.4)
G6PD cases aggregate	5,057.2<br>(4,267.7 to 5,886.8)	-1.8<br>(-7.7 to 3.4)	-7.8<br>(-13.3 to -3.0)*	0.9<br>(0.6 to 1.3)	-2.2<br>(-17.1 to 14.4)	-8.2<br>(-22.2 to 7.4)
Heart failure due to G6PD deficiency aggregate	0.0<br>(0.0 to 0.0)	0.0<br>(0.0 to 0.0)	0.0<br>(0.0 to 0.0)	0.0<br>(0.0 to 0.0)	0.0<br>(0.0 to 0.0)	0.0<br>(0.0 to 0.0)
G6PD trait	13,352.8<br>(12,433.7 to 14,291.9)	2.1<br>(0.2 to 3.8)*	-4.2<br>(-5.9 to -2.6)*	0.8<br>(0.6 to 1.2)	4.5<br>(-8.2 to 18.7)	-1.9<br>(-13.8 to 11.4)
Other hemoglobinopathies and hemolytic anemias	2,210.6<br>(2,154.7 to 2,269.6)	3.6<br>(-0.1 to 7.4)	-2.8<br>(-6.2 to 0.8)	103.1<br>(70.0 to 145.6)	-2.0<br>(-8.2 to 5.2)	-8.0<br>(-13.8 to -1.2)*
Other hemoglobinopathies and hemolytic anemias cases aggregate	2,210.6<br>(2,154.7 to 2,269.6)	3.6<br>(-0.1 to 7.4)	-2.8<br>(-6.2 to 0.8)	99.7<br>(67.6 to 140.9)	-2.0<br>(-8.4 to 5.4)	-8.0<br>(-14.0 to -1.1)*
Heart failure due to other hemoglobinopathies and hemolytic anemias aggregate	0.0<br>(0.0 to 0.0)	0.0<br>(0.0 to 0.0)	0.0<br>(0.0 to 0.0)	0.0<br>(0.0 to 0.0)	0.0<br>(0.0 to 0.0)	0.0<br>(0.0 to 0.0)
Endocrine, metabolic, blood, and immune disorders	3,075.9<br>(2,998.1 to 3,152.8)	4.3<br>(-0.6 to 9.5)	-2.1<br>(-6.7 to 2.8)	138.7<br>(94.8 to 197.0)	3.2<br>(-3.8 to 11.4)	-3.1<br>(-9.7 to 4.6)
Endocrine, metabolic, blood, and immune disorders cases aggregate	276.7<br>(260.7 to 293.8)	12.1<br>(10.7 to 13.8)*	5.3<br>(3.9 to 6.8)*	11.1<br>(7.4 to 15.4)	12.2<br>(7.7 to 16.5)*	5.3<br>(1.1 to 9.4)*
Anemia due to endocrine, metabolic, blood, and immune disorders aggregate	2,799.2<br>(2,724.7 to 2,873.5)	3.6<br>(-1.8 to 9.2)	-2.7<br>(-7.8 to 2.5)	127.6<br>(87.0 to 180.4)	2.5<br>(-5.1 to 11.4)	-3.8<br>(-10.9 to 4.5)
Heart failure due to endocrine, metabolic, blood, and immune disorders aggregate	0.0<br>(0.0 to 0.0)	5.3<br>(4.3 to 6.4)*	-1.1<br>(-2.1 to -0.1)*	0.0<br>(0.0 to 0.0)	5.3<br>(4.3 to 6.4)*	-1.1<br>(-2.1 to -0.1)*
Musculoskeletal disorders	0.0<br>(0.0 to 0.0)	0.0<br>(0.0 to 0.0)	0.0<br>(0.0 to 0.0)	0.0<br>(0.0 to 0.0)	0.0<br>(0.0 to 0.0)	0.0<br>(0.0 to 0.0)
Rheumatoid arthritis	0.0<br>(0.0 to 0.0)	0.0<br>(0.0 to 0.0)	0.0<br>(0.0 to 0.0)	0.0<br>(0.0 to 0.0)	0.0<br>(0.0 to 0.0)	0.0<br>(0.0 to 0.0)
Osteoarthritis	0.0<br>(0.0 to 0.0)	0.0<br>(0.0 to 0.0)	0.0<br>(0.0 to 0.0)	0.0<br>(0.0 to 0.0)	0.0<br>(0.0 to 0.0)	0.0<br>(0.0 to 0.0)
Osteoarthritis of the hip cases aggregate	0.0<br>(0.0 to 0.0)	0.0<br>(0.0 to 0.0)	0.0<br>(0.0 to 0.0)	0.0<br>(0.0 to 0.0)	0.0<br>(0.0 to 0.0)	0.0<br>(0.0 to 0.0)
Osteoarthritis of the knee cases aggregate	0.0<br>(0.0 to 0.0)	0.0<br>(0.0 to 0.0)	0.0<br>(0.0 to 0.0)	0.0<br>(0.0 to 0.0)	0.0<br>(0.0 to 0.0)	0.0<br>(0.0 to 0.0)
Low back and neck pain	0.0<br>(0.0 to 0.0)	0.0<br>(0.0 to 0.0)	0.0<br>(0.0 to 0.0)	0.0<br>(0.0 to 0.0)	0.0<br>(0.0 to 0.0)	0.0<br>(0.0 to 0.0)
Low back pain	0.0<br>(0.0 to 0.0)	0.0<br>(0.0 to 0.0)	0.0<br>(0.0 to 0.0)	0.0<br>(0.0 to 0.0)	0.0<br>(0.0 to 0.0)	0.0<br>(0.0 to 0.0)
Neck pain	0.0<br>(0.0 to 0.0)	0.0<br>(0.0 to 0.0)	0.0<br>(0.0 to 0.0)	0.0<br>(0.0 to 0.0)	0.0<br>(0.0 to 0.0)	0.0<br>(0.0 to 0.0)
Gout	0.0<br>(0.0 to 0.0)	0.0<br>(0.0 to 0.0)	0.0<br>(0.0 to 0.0)	0.0<br>(0.0 to 0.0)	0.0<br>(0.0 to 0.0)	0.0<br>(0.0 to 0.0)
Asymptomatic gout	0.0<br>(0.0 to 0.0)	0.0<br>(0.0 to 0.0)	0.0<br>(0.0 to 0.0)	0.0<br>(0.0 to 0.0)	0.0<br>(0.0 to 0.0)	0.0<br>(0.0 to 0.0)
Symptomatic gout cases aggregate	0.0<br>(0.0 to 0.0)	0.0<br>(0.0 to 0.0)	0.0<br>(0.0 to 0.0)	0.0<br>(0.0 to 0.0)	0.0<br>(0.0 to 0.0)	0.0<br>(0.0 to 0.0)
Other musculoskeletal disorders	0.0<br>(0.0 to 0.0)	0.0<br>(0.0 to 0.0)	0.0<br>(0.0 to 0.0)	0.0<br>(0.0 to 0.0)	0.0<br>(0.0 to 0.0)	0.0<br>(0.0 to 0.0)
Other non-communicable diseases	23,126.4<br>(22,548.9 to 23,768.2)	6.5<br>(5.5 to 7.5)*	-0.0<br>(-1.0 to 0.9)	638.7<br>(436.6 to 908.8)	9.2<br>(6.7 to 12.8)*	2.5<br>(0.1 to 5.8)*
Congenital anomalies	1,959.0<br>(1,798.0 to 2,089.9)	12.1<br>(8.6 to 15.8)*	5.2<br>(2.0 to 8.7)*	190.3<br>(121.7 to 280.6)	18.1<br>(9.3 to 26.5)*	10.8<br>(2.6 to 18.8)*
Neural tube defects	50.7<br>(34.1 to 73.2)	8.4<br>(6.4 to 10.2)*	1.7<br>(-0.1 to 3.5)	18.0<br>(10.1 to 29.9)	9.1<br>(2.9 to 15.2)*	2.4<br>(-3.4 to 8.2)
Congenital heart anomalies	965.8<br>(673.6 to 1,443.1)	16.7<br>(14.5 to 19.0)*	9.6<br>(7.5 to 11.7)*	35.5<br>(14.7 to 64.5)	16.8<br>(13.9 to 19.4)*	9.6<br>(7.0 to 12.1)*
Less severe heart anomalies cases aggregate	845.7<br>(553.8 to 1,317.8)	16.4<br>(13.8 to 18.9)*	9.3<br>(6.9 to 11.6)*	27.6<br>(10.1 to 53.5)	16.5<br>(13.4 to 19.5)*	9.4<br>(6.4 to 12.2)*
Severe congenital heart anomalies	95.5<br>(64.2 to 145.0)	20.3<br>(16.2 to 25.8)*	12.9<br>(9.1 to 18.0)*	5.7<br>(2.2 to 11.3)	20.2<br>(14.4 to 26.6)*	12.9<br>(7.4 to 18.9)*
Critical congenital heart anomalies	12.3<br>(7.1 to 19.4)	21.8<br>(17.4 to 25.7)*	14.3<br>(10.2 to 18.0)*	0.7<br>(0.3 to 1.5)	21.6<br>(12.0 to 30.5)*	14.1<br>(5.1 to 22.5)*
Heart failure due to congenital heart anomalies aggregate	12.3<br>(11.1 to 13.5)	7.0<br>(6.0 to 8.0)*	0.5<br>(-0.4 to 1.4)	1.5<br>(1.0 to 2.1)	7.0<br>(-1.8 to 16.6)	0.4<br>(-7.8 to 9.5)
Cleft lip and cleft palate	150.5<br>(86.1 to 252.6)	8.6<br>(2.7 to 12.4)*	2.0<br>(-3.6 to 5.5)	2.1<br>(1.0 to 3.8)	2.9<br>(-6.5 to 10.6)	-3.4<br>(-12.2 to 3.8)
Down syndrome	121.7<br>(77.3 to 188.6)	6.9<br>(4.5 to 9.1)*	0.4<br>(-1.9 to 2.4)	10.2<br>(5.7 to 17.2)	7.1<br>(2.6 to 10.8)*	0.5<br>(-3.7 to 4.0)
Turner syndrome	7.6<br>(3.6 to 13.7)	4.6<br>(3.0 to 6.1)*	-1.8<br>(-3.3 to -0.4)*	0.1<br>(0.0 to 0.2)	4.6<br>(1.1 to 7.7)*	-1.8<br>(-5.1 to 1.1)
Klinefelter syndrome	4.1<br>(2.1 to 7.3)	5.3<br>(3.7 to 6.5)*	-1.2<br>(-2.6 to -0.0)*	0.0<br>(0.0 to 0.0)	5.3<br>(3.7 to 6.5)*	-1.2<br>(-2.6 to -0.0)*
Other chromosomal abnormalities	102.9<br>(56.9 to 182.0)	8.2<br>(6.0 to 9.9)*	1.6<br>(-0.5 to 3.2)	8.7<br>(4.4 to 16.5)	8.3<br>(3.6 to 12.6)*	1.6<br>(-2.8 to 5.7)
Other congenital anomalies	614.7<br>(338.8 to 1,117.8)	7.3<br>(5.7 to 9.0)*	0.8<br>(-0.8 to 2.3)	115.7<br>(69.1 to 180.0)	22.4<br>(8.8 to 38.6)*	14.9<br>(2.1 to 30.1)*
Skin and subcutaneous diseases	17,289.4<br>(16,723.1 to 17,860.4)	6.9<br>(5.6 to 8.2)*	0.3<br>(-0.9 to 1.6)	324.9<br>(192.2 to 507.2)	6.0<br>(4.7 to 7.2)*	-0.5<br>(-1.7 to 0.6)
Dermatitis	0.0<br>(0.0 to 0.0)	0.0<br>(0.0 to 0.0)	0.0<br>(0.0 to 0.0)	0.0<br>(0.0 to 0.0)	0.0<br>(0.0 to 0.0)	0.0<br>(0.0 to 0.0)
Eczema cases aggregate	0.0<br>(0.0 to 0.0)	0.0<br>(0.0 to 0.0)	0.0<br>(0.0 to 0.0)	0.0<br>(0.0 to 0.0)	0.0<br>(0.0 to 0.0)	0.0<br>(0.0 to 0.0)

Cause	Prevalence in thousands (95% confidence interval)			YLDs in thousands (95% confidence interval)		
	2015	Percent change in counts between 2005 and 2015	Percent change in rates between 2005 and 2015	2015	Percent change in counts between 2005 and 2015	Percent change in rates between 2005 and 2015
Contact dermatitis cases aggregate	0.0 (0.0 to 0.0)	0.0 (0.0 to 0.0)	0.0 (0.0 to 0.0)	0.0 (0.0 to 0.0)	0.0 (0.0 to 0.0)	0.0 (0.0 to 0.0)
Seborrhoeic dermatitis cases aggregate	0.0 (0.0 to 0.0)	0.0 (0.0 to 0.0)	0.0 (0.0 to 0.0)	0.0 (0.0 to 0.0)	0.0 (0.0 to 0.0)	0.0 (0.0 to 0.0)
Psoriasis	0.0 (0.0 to 0.0)	0.0 (0.0 to 0.0)	0.0 (0.0 to 0.0)	0.0 (0.0 to 0.0)	0.0 (0.0 to 0.0)	0.0 (0.0 to 0.0)
Cellulitis	25.3 (20.0 to 31.8)	11.3 (8.4 to 14.3)*	4.5 (1.8 to 7.3)*	1.9 (1.1 to 2.9)	11.3 (0.9 to 22.9)*	4.4 (-5.3 to 15.4)
Pyoderma	159.1 (147.7 to 171.5)	9.6 (8.1 to 11.0)*	2.8 (1.5 to 4.2)*	0.9 (0.4 to 1.9)	9.6 (6.3 to 13.2)*	2.9 (-0.2 to 6.2)
Scabies	3,567.2 (2,736.4 to 4,514.3)	3.6 (0.3 to 6.8)*	-2.8 (-5.9 to 0.3)	93.2 (47.3 to 158.1)	3.7 (0.3 to 7.1)*	-2.6 (-5.9 to 0.5)
Fungal skin diseases	7,313.9 (5,950.7 to 8,873.6)	7.3 (6.9 to 7.8)*	0.7 (0.3 to 1.2)*	41.8 (16.7 to 91.2)	7.4 (6.8 to 8.0)*	0.8 (0.2 to 1.4)*
Viral skin diseases	0.0 (0.0 to 0.0)	0.0 (0.0 to 0.0)	0.0 (0.0 to 0.0)	0.0 (0.0 to 0.0)	0.0 (0.0 to 0.0)	0.0 (0.0 to 0.0)
Molluscum contagiosum cases aggregate	0.0 (0.0 to 0.0)	0.0 (0.0 to 0.0)	0.0 (0.0 to 0.0)	0.0 (0.0 to 0.0)	0.0 (0.0 to 0.0)	0.0 (0.0 to 0.0)
Viral warts cases aggregate	0.0 (0.0 to 0.0)	0.0 (0.0 to 0.0)	0.0 (0.0 to 0.0)	0.0 (0.0 to 0.0)	0.0 (0.0 to 0.0)	0.0 (0.0 to 0.0)
Acne vulgaris	0.0 (0.0 to 0.0)	0.0 (0.0 to 0.0)	0.0 (0.0 to 0.0)	0.0 (0.0 to 0.0)	0.0 (0.0 to 0.0)	0.0 (0.0 to 0.0)
Alopecia areata	0.0 (0.0 to 0.0)	0.0 (0.0 to 0.0)	0.0 (0.0 to 0.0)	0.0 (0.0 to 0.0)	0.0 (0.0 to 0.0)	0.0 (0.0 to 0.0)
Pruritus	374.4 (293.1 to 475.4)	6.9 (4.8 to 9.3)*	0.3 (-1.6 to 2.6)	4.1 (1.8 to 7.4)	7.0 (4.1 to 10.0)*	0.4 (-2.3 to 3.3)
Urticaria	2,527.5 (1,953.8 to 3,216.5)	6.1 (5.9 to 6.4)*	-0.4 (-0.6 to -0.2)*	155.9 (91.7 to 238.2)	6.4 (5.1 to 7.5)*	-0.2 (-1.3 to 0.9)
Decubitus ulcer	0.0 (0.0 to 0.0)	0.0 (0.0 to 0.0)	0.0 (0.0 to 0.0)	0.0 (0.0 to 0.0)	0.0 (0.0 to 0.0)	0.0 (0.0 to 0.0)
Other skin and subcutaneous diseases	4,503.8 (4,242.3 to 4,795.6)	9.1 (7.8 to 10.4)*	2.4 (1.2 to 3.6)*	27.2 (13.2 to 51.3)	9.3 (7.7 to 10.7)*	2.6 (1.1 to 3.9)*
Sense organ diseases	4,880.6 (4,736.6 to 5,019.6)	3.1 (1.4 to 4.8)*	-3.2 (-4.8 to -1.6)*	123.5 (76.7 to 188.2)	5.3 (3.3 to 7.3)*	-1.2 (-3.1 to 0.7)
Glaucoma	0.0 (0.0 to 0.0)	0.0 (0.0 to 0.0)	0.0 (0.0 to 0.0)	0.0 (0.0 to 0.0)	0.0 (0.0 to 0.0)	0.0 (0.0 to 0.0)
Cataract	0.0 (0.0 to 0.0)	0.0 (0.0 to 0.0)	0.0 (0.0 to 0.0)	0.0 (0.0 to 0.0)	0.0 (0.0 to 0.0)	0.0 (0.0 to 0.0)
Macular degeneration	0.0 (0.0 to 0.0)	0.0 (0.0 to 0.0)	0.0 (0.0 to 0.0)	0.0 (0.0 to 0.0)	0.0 (0.0 to 0.0)	0.0 (0.0 to 0.0)
Refraction and accommodation disorders	4,498.3 (4,092.1 to 4,924.9)	2.2 (0.8 to 3.6)*	-4.1 (-5.4 to -2.7)*	85.3 (50.1 to 140.6)	3.7 (2.2 to 5.4)*	-2.6 (-4.1 to -1.1)*
Age-related and other hearing loss	326.2 (27.8 to 559.2)	15.6 (7.8 to 47.2)*	8.5 (1.2 to 38.2)*	35.7 (8.5 to 64.0)	8.8 (2.7 to 17.4)*	2.1 (-3.6 to 10.2)
Other vision loss	63.1 (37.0 to 99.8)	11.8 (8.7 to 15.3)*	5.0 (2.0 to 8.2)*	2.4 (1.1 to 4.6)	12.1 (6.4 to 18.6)*	5.2 (-0.1 to 11.3)
Other sense organ diseases	0.0 (0.0 to 0.0)	0.0 (0.0 to 0.0)	0.0 (0.0 to 0.0)	0.0 (0.0 to 0.0)	0.0 (0.0 to 0.0)	0.0 (0.0 to 0.0)
Acute other sense organ diseases	0.0 (0.0 to 0.0)	0.0 (0.0 to 0.0)	0.0 (0.0 to 0.0)	0.0 (0.0 to 0.0)	0.0 (0.0 to 0.0)	0.0 (0.0 to 0.0)
Chronic other sense organ diseases	0.0 (0.0 to 0.0)	0.0 (0.0 to 0.0)	0.0 (0.0 to 0.0)	0.0 (0.0 to 0.0)	0.0 (0.0 to 0.0)	0.0 (0.0 to 0.0)
Oral disorders	0.0 (0.0 to 0.0)	0.0 (0.0 to 0.0)	0.0 (0.0 to 0.0)	0.0 (0.0 to 0.0)	0.0 (0.0 to 0.0)	0.0 (0.0 to 0.0)
Deciduous caries	0.0 (0.0 to 0.0)	0.0 (0.0 to 0.0)	0.0 (0.0 to 0.0)	0.0 (0.0 to 0.0)	0.0 (0.0 to 0.0)	0.0 (0.0 to 0.0)
Permanent caries	0.0 (0.0 to 0.0)	0.0 (0.0 to 0.0)	0.0 (0.0 to 0.0)	0.0 (0.0 to 0.0)	0.0 (0.0 to 0.0)	0.0 (0.0 to 0.0)
Periodontal diseases	0.0 (0.0 to 0.0)	0.0 (0.0 to 0.0)	0.0 (0.0 to 0.0)	0.0 (0.0 to 0.0)	0.0 (0.0 to 0.0)	0.0 (0.0 to 0.0)
Edentulism and severe tooth loss	0.0 (0.0 to 0.0)	0.0 (0.0 to 0.0)	0.0 (0.0 to 0.0)	0.0 (0.0 to 0.0)	0.0 (0.0 to 0.0)	0.0 (0.0 to 0.0)
Other oral disorders	0.0 (0.0 to 0.0)	0.0 (0.0 to 0.0)	0.0 (0.0 to 0.0)	0.0 (0.0 to 0.0)	0.0 (0.0 to 0.0)	0.0 (0.0 to 0.0)
Injuries	3,616.8 (3,235.5 to 3,990.7)	-6.9 (-8.4 to -5.5)*	-12.6 (-14.0 to -11.3)*	302.2 (208.1 to 415.6)	-8.2 (-9.9 to -6.6)*	-13.9 (-15.4 to -12.3)*
Transport injuries	24.7 (20.3 to 29.9)	2.3 (-0.9 to 5.5)	-4.0 (-7.0 to -0.9)*	2.4 (1.6 to 3.5)	-1.7 (-5.2 to 1.9)	-7.7 (-11.0 to -4.4)*
Road injuries	12.5 (9.4 to 16.4)	6.9 (1.9 to 12.3)*	0.4 (-4.4 to 5.4)	1.1 (0.7 to 1.7)	3.6 (-1.8 to 9.4)	-2.7 (-7.8 to 2.7)
Pedestrian road injuries	1.4 (1.0 to 2.0)	0.4 (-5.5 to 6.8)	-5.7 (-11.3 to 0.3)	0.1 (0.1 to 0.2)	-3.3 (-9.8 to 3.2)	-9.2 (-15.3 to -3.1)*
Cyclist road injuries	2.0 (1.4 to 3.0)	13.5 (7.1 to 20.4)*	6.6 (0.5 to 13.0)*	0.2 (0.1 to 0.3)	9.7 (2.7 to 17.0)*	3.0 (-3.6 to 9.9)
Motorcyclist road injuries	3.2 (2.3 to 4.3)	7.7 (1.6 to 14.2)*	1.1 (-4.6 to 7.2)	0.3 (0.2 to 0.4)	4.4 (-2.4 to 11.5)	-2.0 (-8.3 to 4.7)
Motor vehicle road injuries	5.6 (4.1 to 7.8)	5.5 (0.1 to 10.9)*	-1.0 (-6.1 to 4.1)	0.6 (0.3 to 0.9)	2.7 (-3.0 to 8.5)	-3.6 (-8.9 to 1.8)
Other road injuries	0.3 (0.2 to 0.5)	19.4 (12.0 to 27.1)*	12.0 (5.1 to 19.3)*	0.0 (0.0 to 0.0)	16.2 (8.5 to 24.4)*	9.1 (1.8 to 16.7)*
Other transport injuries	12.2 (9.3 to 15.2)	-2.1 (-5.5 to 1.0)	-8.1 (-11.3 to -5.2)*	1.3 (0.8 to 1.9)	-5.9 (-9.7 to -2.0)*	-11.6 (-15.2 to -8.0)*
Unintentional injuries	3,566.8 (3,192.3 to 3,937.1)	-6.9 (-8.4 to -5.4)*	-12.6 (-14.0 to -11.2)*	297.5 (205.1 to 409.3)	-8.2 (-9.9 to -6.6)*	-13.8 (-15.4 to -12.3)*
Falls	411.6 (328.5 to 504.2)	9.7 (6.5 to 12.9)*	3.0 (-0.0 to 6.0)	35.3 (21.7 to 52.8)	7.1 (3.5 to 10.5)*	0.5 (-2.8 to 3.7)
Drowning	4.1 (3.3 to 4.9)	-7.4 (-10.1 to -4.9)*	-13.1 (-15.6 to -10.8)*	0.4 (0.2 to 0.5)	-10.9 (-14.1 to -7.8)*	-16.3 (-19.3 to -13.5)*
Fire, heat, and hot substances	294.9 (235.7 to 359.5)	-3.2 (-6.4 to -0.4)*	-9.2 (-12.1 to -6.5)*	35.9 (22.6 to 53.7)	-6.4 (-10.3 to -2.6)*	-12.2 (-15.8 to -8.6)*
Poisonings	30.1 (24.5 to 37.6)	6.7 (4.6 to 8.7)*	0.2 (-1.8 to 2.0)	3.7 (2.5 to 5.5)	5.4 (1.4 to 9.3)*	-1.1 (-4.8 to 2.6)
Exposure to mechanical forces	729.6 (612.0 to 879.1)	-10.3 (-12.9 to -7.8)*	-15.8 (-18.2 to -13.5)*	32.3 (21.8 to 45.9)	-12.7 (-15.4 to -9.6)*	-18.0 (-20.6 to -15.1)*
Unintentional firearm injuries	3.2 (2.4 to 4.2)	-7.1 (-13.5 to -1.2)*	-12.8 (-18.8 to -7.3)*	0.3 (0.2 to 0.5)	-8.4 (-15.7 to -1.7)*	-14.0 (-20.9 to -7.7)*
Unintentional suffocation	199.5 (159.4 to 248.8)	-8.2 (-11.9 to -4.6)*	-13.9 (-17.3 to -10.5)*	9.6 (6.0 to 14.1)	-9.4 (-13.2 to -5.5)*	-14.9 (-18.5 to -11.3)*
Other exposure to mechanical forces	526.9 (441.5 to 637.1)	-11.1 (-13.8 to -8.5)*	-16.6 (-19.1 to -14.2)*	22.4 (14.8 to 32.2)	-14.1 (-17.1 to -11.1)*	-19.3 (-22.1 to -16.5)*
Adverse effects of medical treatment	443.3 (340.7 to 566.6)	-9.8 (-12.9 to -6.7)*	-15.3 (-18.2 to -12.4)*	59.1 (35.5 to 89.6)	-9.8 (-12.9 to -6.7)*	-15.3 (-18.2 to -12.4)*
Animal contact	172.8 (128.6 to 227.4)	-9.4 (-13.8 to -4.6)*	-14.9 (-19.1 to -10.5)*	7.1 (4.7 to 10.0)	-9.3 (-13.6 to -5.0)*	-14.9 (-18.9 to -10.8)*
Venomous animal contact	27.5 (21.7 to 34.5)	-9.0 (-13.7 to -4.2)*	-14.6 (-19.0 to -10.1)*	3.2 (2.1 to 4.5)	-9.4 (-14.1 to -4.4)*	-15.0 (-19.4 to -10.3)*
Non-venomous animal contact	145.3 (103.3 to 195.7)	-9.4 (-14.1 to -4.5)*	-15.0 (-19.3 to -10.4)*	3.9 (2.5 to 5.7)	-9.2 (-14.1 to -4.4)*	-14.8 (-19.3 to -10.3)*
Foreign body	305.4 (248.2 to 369.3)	-5.2 (-7.2 to -3.3)*	-11.0 (-12.8 to -9.2)*	23.8 (16.3 to 33.0)	-8.7 (-11.2 to -6.3)*	-14.3 (-16.6 to -12.1)*
Pulmonary aspiration and foreign body in airway	124.5 (101.3 to 152.4)	-8.5 (-10.7 to -6.3)*	-14.1 (-16.1 to -12.0)*	13.1 (8.9 to 18.6)	-12.7 (-15.5 to -9.9)*	-18.1 (-20.7 to -15.5)*
Foreign body in eyes	13.7 (4.6 to 24.7)	6.1 (5.3 to 6.7)*	-0.4 (-1.1 to 0.2)	0.8 (0.2 to 1.5)	5.6 (3.9 to 6.3)*	-0.9 (-2.4 to -0.2)*
Foreign body in other body part	167.2 (123.4 to 222.1)	-3.5 (-6.6 to -0.5)*	-9.4 (-12.4 to -6.6)*	9.9 (6.4 to 14.2)	-3.9 (-7.2 to -0.9)*	-9.8 (-12.9 to -7.0)*
Environmental heat and cold exposure	169.6 (140.1 to 205.4)	1.4 (-2.4 to 5.2)	-4.9 (-8.4 to -1.2)*	16.3 (10.4 to 23.5)	-0.5 (-4.5 to 3.8)	-6.6 (-10.4 to -2.6)*
Other unintentional injuries	1,005.4 (839.7 to 1,192.5)	-11.2 (-14.6 to -7.4)*	-16.6 (-19.9 to -13.1)*	83.6 (53.9 to 121.2)	-12.9 (-16.7 to -8.7)*	-18.2 (-21.8 to -14.3)*
Self-harm and interpersonal violence	25.4 (20.9 to 30.7)	-16.9 (-20.9 to -12.7)*	-22.0 (-25.7 to -18.1)*	2.3 (1.5 to 3.3)	-18.0 (-22.4 to -13.5)*	-23.0 (-27.2 to -18.8)*
Self-harm	1.0 (0.7 to 1.3)	2.8 (1.2 to 4.2)*	-3.5 (-5.0 to -2.1)*	0.1 (0.1 to 0.2)	-0.1 (-3.0 to 3.1)	-6.2 (-9.0 to -3.2)*
Interpersonal violence	24.4 (20.0 to 29.7)	-17.6 (-21.8 to -13.2)*	-22.6 (-26.6 to -18.5)*	2.2 (1.4 to 3.1)	-18.7 (-23.4 to -14.1)*	-23.7 (-28.1 to -19.4)*

Cause	Prevalence in thousands (95% confidence interval)			YLDs in thousands (95% confidence interval)		
	2015	Percent change in counts between 2005 and 2015	Percent change in rates between 2005 and 2015	2015	Percent change in counts between 2005 and 2015	Percent change in rates between 2005 and 2015
Assault by firearm	0.4 (0.3 to 0.5)	-14.2 (-20.0 to -8.2)*	-19.5 (-24.9 to -13.8)*	0.1 (0.0 to 0.1)	-16.3 (-23.4 to -9.1)*	-21.4 (-28.1 to -14.6)*
Assault by sharp object	2.1 (1.6 to 2.8)	-16.6 (-22.7 to -10.2)*	-21.7 (-27.4 to -15.7)*	0.3 (0.2 to 0.4)	-18.5 (-25.2 to -11.9)*	-23.5 (-29.7 to -17.3)*
Assault by other means	21.9 (17.8 to 26.6)	-17.7 (-21.9 to -13.3)*	-22.8 (-26.7 to -18.7)*	1.9 (1.2 to 2.7)	-18.9 (-23.5 to -14.2)*	-23.8 (-28.2 to -19.4)*
Forces of nature, war, and legal intervention	0.0 (0.0 to 0.0)	0.0 (0.0 to 0.0)	0.0 (0.0 to 0.0)	0.0 (0.0 to 0.0)	0.0 (0.0 to 0.0)	0.0 (0.0 to 0.0)
Exposure to forces of nature	0.0 (0.0 to 0.0)	0.0 (0.0 to 0.0)	0.0 (0.0 to 0.0)	0.0 (0.0 to 0.0)	0.0 (0.0 to 0.0)	0.0 (0.0 to 0.0)
Collective violence and legal intervention	0.0 (0.0 to 0.0)	0.0 (0.0 to 0.0)	0.0 (0.0 to 0.0)	0.0 (0.0 to 0.0)	0.0 (0.0 to 0.0)	0.0 (0.0 to 0.0)
**Anemia**	**81,997.0 (80,200.6 to 83,975.3)**	**4.1 (1.1 to 7.5)***	**-2.2 (-5.1 to 0.9)**	**3,801.6 (2,597.9 to 5,387.2)**	**-1.0 (-6.9 to 6.3)**	**-7.1 (-12.6 to -0.2)***
**Developmental intellectual disability**	**957.1 (706.9 to 1,218.4)**	**5.1 (4.4 to 5.9)***	**-1.3 (-2.0 to -0.6)***	**105.3 (72.0 to 150.0)**	**6.0 (3.9 to 8.0)***	**-0.5 (-2.5 to 1.4)**
**Epilepsy**	**537.5 (438.0 to 655.4)**	**6.1 (0.1 to 12.1)***	**-0.4 (-6.0 to 5.3)**	**161.4 (112.5 to 216.1)**	**-4.9 (-11.4 to 1.8)**	**-10.7 (-16.8 to -4.4)***
**Guillain-Barré syndrome**	**0.2 (0.2 to 0.3)**	**7.6 (6.9 to 8.1)***	**1.0 (0.4 to 1.5)***	**0.1 (0.0 to 0.1)**	**7.6 (6.9 to 8.1)***	**1.0 (0.4 to 1.5)***
**Hearing loss**	**1,787.4 (1,470.1 to 2,190.0)**	**7.2 (6.3 to 8.2)***	**0.7 (-0.2 to 1.5)**	**120.8 (78.9 to 174.5)**	**5.0 (2.3 to 7.6)***	**-1.4 (-4.0 to 1.0)**
**Heart failure**	**12.3 (11.1 to 13.6)**	**7.0 (6.1 to 8.0)***	**0.5 (-0.4 to 1.4)**	**1.5 (1.0 to 2.1)**	**7.0 (-1.8 to 16.6)**	**0.4 (-7.8 to 9.5)**
**Infertility**	**0.0 (0.0 to 0.0)**	**0.0 (0.0 to 0.0)**	**0.0 (0.0 to 0.0)**	**0.0 (0.0 to 0.0)**	**0.0 (0.0 to 0.0)**	**0.0 (0.0 to 0.0)**
**Pelvic inflammatory disease**	**0.0 (0.0 to 0.0)**	**0.0 (0.0 to 0.0)**	**0.0 (0.0 to 0.0)**	**0.0 (0.0 to 0.0)**	**0.0 (0.0 to 0.0)**	**0.0 (0.0 to 0.0)**
**Vision loss**	**4,638.5 (4,220.9 to 5,093.7)**	**2.3 (1.0 to 3.8)***	**-3.9 (-5.2 to -2.6)***	**106.5 (69.5 to 163.3)**	**4.1 (2.7 to 5.6)***	**-2.2 (-3.6 to -0.8)***

SR Table 11. Prevalent cases and YLDs for 2015, percent change, and change in age-standardised rates between 2005 and 2015 by cause, global, ages 1-4 years						
**Cause**	**Prevalence in thousands (95% confidence interval)**			**YLDs in thousands (95% confidence interval)**		
	**2015**	**Percent change in counts between 2005 and 2015**	**Percent change in rates between 2005 and 2015**	**2015**	**Percent change in counts between 2005 and 2015**	**Percent change in rates between 2005 and 2015**
**All causes**	**--**	**--**	**--**	**25,785.8 (18,209.0 to 34,888.1)**	**0.3 (-2.2 to 3.0)**	**-7.5 (-9.7 to -5.0)***
Communicable, maternal, neonatal, and nutritional diseases	296,077.4 (293,013.5 to 298,983.5)	2.6 (1.5 to 3.7)*	-5.3 (-6.3 to -4.3)*	15,165.5 (10,632.7 to 21,167.7)	-4.7 (-8.0 to -1.1)*	-12.1 (-15.1 to -8.7)*
HIV/AIDS and tuberculosis	831.7 (799.8 to 865.1)	-26.7 (-29.8 to -23.6)*	-32.4 (-35.2 to -29.5)*	109.1 (76.2 to 146.5)	-20.7 (-25.6 to -15.9)*	-26.8 (-31.3 to -22.3)*
Tuberculosis	85.9 (57.4 to 123.4)	-2.9 (-11.9 to 6.0)	-10.3 (-18.7 to -2.2)*	27.6 (15.6 to 44.2)	-2.4 (-15.1 to 10.1)	-9.9 (-21.6 to 1.6)
HIV/AIDS	748.4 (717.8 to 780.0)	-28.7 (-32.0 to -25.4)*	-34.2 (-37.3 to -31.2)*	81.5 (58.5 to 107.1)	-25.4 (-30.1 to -20.5)*	-31.2 (-35.5 to -26.6)*
HIV/AIDS - Tuberculosis	23.9 (21.7 to 26.0)	-49.7 (-52.6 to -46.3)*	-53.6 (-56.2 to -50.5)*	9.5 (6.3 to 13.0)	-49.1 (-54.1 to -43.6)*	-53.1 (-57.6 to -48.0)*
HIV/AIDS resulting in other diseases	746.9 (714.7 to 781.4)	-26.8 (-29.2 to -24.2)*	-32.4 (-34.7 to -30.1)*	72.0 (51.6 to 95.4)	-20.5 (-25.9 to -14.6)*	-26.7 (-31.6 to -21.2)*
HIV aggregate	528.7 (503.3 to 556.9)	-45.9 (-48.1 to -43.8)*	-50.1 (-52.1 to -48.2)*	38.9 (27.1 to 52.1)	-45.8 (-49.1 to -42.3)*	-50.0 (-53.1 to -46.8)*
AIDS aggregate	218.2 (207.5 to 228.5)	425.1 (356.3 to 525.0)*	384.6 (321.1 to 476.9)*	33.0 (24.2 to 42.8)	76.2 (41.0 to 124.7)*	62.6 (30.2 to 107.4)*
Diarrhea, lower respiratory, and other common infectious diseases	56,787.7 (54,667.7 to 58,847.7)	4.0 (2.7 to 5.3)*	-4.0 (-5.2 to -2.8)*	3,042.1 (2,149.9 to 4,189.5)	-2.8 (-4.3 to -1.1)*	-10.2 (-11.7 to -8.7)*
Diarrheal diseases	10,994.7 (9,781.3 to 12,309.6)	-5.0 (-6.5 to -3.4)*	-12.3 (-13.7 to -10.9)*	1,793.1 (1,211.7 to 2,552.8)	-4.7 (-6.4 to -3.0)*	-12.0 (-13.6 to -10.5)*
Diarrhoea episodes aggregate	10,994.4 (9,781.1 to 12,309.3)	-5.0 (-6.5 to -3.4)*	-12.3 (-13.7 to -10.9)*	1,793.0 (1,211.7 to 2,552.7)	-4.7 (-6.4 to -3.0)*	-12.0 (-13.6 to -10.5)*
Guillain-Barré syndrome due to diarrheal diseases	0.3 (0.2 to 0.4)	9.4 (8.6 to 10.0)*	0.9 (0.2 to 1.6)*	0.1 (0.0 to 0.1)	9.4 (8.6 to 10.0)*	0.9 (0.2 to 1.6)*
Intestinal infectious diseases	295.4 (271.1 to 318.7)	-29.1 (-33.8 to -24.1)*	-34.6 (-38.9 to -30.0)*	36.1 (23.6 to 53.0)	-28.2 (-37.4 to -17.9)*	-33.7 (-42.3 to -24.2)*
Typhoid fever	238.9 (183.6 to 303.2)	-28.0 (-33.5 to -22.4)*	-33.5 (-38.6 to -28.4)*	31.7 (20.5 to 46.8)	-27.4 (-37.7 to -15.9)*	-33.0 (-42.5 to -22.4)*
Typhoid fever episodes aggregate	197.8 (151.7 to 255.4)	-28.0 (-35.4 to -20.2)*	-33.5 (-40.3 to -26.3)*	18.9 (12.2 to 28.4)	-27.6 (-37.1 to -16.0)*	-33.2 (-42.0 to -22.4)*
Complications of typhoid fever aggregate	41.1 (27.3 to 57.7)	-28.0 (-51.2 to 5.0)	-33.5 (-55.0 to -3.1)*	12.9 (7.2 to 20.5)	-27.1 (-52.1 to 8.1)	-32.7 (-55.8 to -0.3)*
Paratyphoid fever	79.1 (54.7 to 110.9)	-27.6 (-36.4 to -18.9)*	-33.2 (-41.3 to -25.2)*	4.1 (2.4 to 6.4)	-27.0 (-42.3 to -6.7)*	-32.7 (-46.7 to -13.9)*
Paratyphoid fever episodes aggregate	75.0 (52.0 to 107.0)	-27.6 (-36.6 to -18.0)*	-33.2 (-41.5 to -24.3)*	3.6 (2.1 to 5.7)	-27.1 (-43.1 to -5.3)*	-32.7 (-47.5 to -12.5)*
Intestinal perforation due to paratyphoid	4.1 (2.3 to 6.6)	-26.5 (-58.5 to 30.7)	-32.2 (-61.7 to 20.7)	0.5 (0.2 to 0.8)	-26.3 (-58.1 to 30.6)	-32.0 (-61.3 to 20.5)
Other intestinal infectious diseases	--	--	--	0.3 (0.1 to 0.7)	-71.0 (-82.4 to -49.5)*	-73.3 (-83.7 to -53.4)*
Lower respiratory infections	2,283.4 (1,984.0 to 2,591.6)	-8.5 (-10.8 to -6.1)*	-15.5 (-17.6 to -13.3)*	140.5 (92.6 to 200.3)	-8.3 (-11.1 to -5.3)*	-15.3 (-17.9 to -12.6)*
Lower respiratory infection episodes aggregate	2,283.1 (1,983.8 to 2,591.5)	-8.5 (-10.8 to -6.1)*	-15.5 (-17.6 to -13.3)*	140.5 (92.5 to 200.2)	-8.3 (-11.1 to -5.3)*	-15.3 (-18.0 to -12.6)*
Guillain-Barré syndrome due to lower respiratory infections	0.3 (0.1 to 0.5)	9.4 (8.6 to 10.0)*	0.9 (0.2 to 1.6)*	0.1 (0.0 to 0.2)	9.4 (8.6 to 10.0)*	0.9 (0.2 to 1.6)*
Upper respiratory infections	23,505.2 (18,174.0 to 29,716.4)	9.6 (7.6 to 11.2)*	1.1 (-0.7 to 2.7)	280.2 (148.0 to 494.7)	9.7 (7.5 to 11.6)*	1.2 (-0.7 to 3.0)
Upper respiratory infection episodes aggregate	23,504.4 (18,172.9 to 29,715.8)	9.6 (7.6 to 11.2)*	1.1 (-0.7 to 2.7)	280.0 (147.8 to 494.5)	9.7 (7.5 to 11.6)*	1.2 (-0.7 to 3.0)
Guillain-Barré syndrome due to upper respiratory infections	0.8 (0.5 to 1.3)	9.4 (8.6 to 10.0)*	0.9 (0.2 to 1.6)*	0.2 (0.1 to 0.4)	9.4 (8.6 to 10.0)*	0.9 (0.2 to 1.6)*
Otitis media	20,264.3 (16,388.6 to 25,200.3)	8.1 (6.4 to 9.9)*	-0.2 (-1.8 to 1.4)	600.2 (374.7 to 915.2)	5.7 (2.6 to 8.3)*	-2.5 (-5.3 to -0.0)*
Acute otitis media	9,595.5 (6,360.0 to 13,785.3)	9.3 (6.1 to 12.4)*	0.9 (-2.1 to 3.7)	125.8 (57.3 to 245.9)	9.4 (6.1 to 12.7)*	0.9 (-2.0 to 4.0)
Chronic otitis media aggregate	10,668.8 (8,767.9 to 12,938.5)	7.1 (5.5 to 8.6)*	-1.2 (-2.6 to 0.2)	474.4 (298.4 to 710.7)	4.7 (1.1 to 7.9)*	-3.3 (-6.6 to -0.4)*
Meningitis	552.6 (525.7 to 580.6)	10.8 (5.4 to 16.1)*	2.2 (-2.7 to 7.1)	108.0 (76.7 to 143.1)	8.8 (4.6 to 13.0)*	0.4 (-3.5 to 4.3)
Pneumococcal meningitis	417.5 (357.1 to 494.2)	18.1 (15.3 to 21.2)*	9.0 (6.4 to 11.9)*	48.4 (33.6 to 65.1)	13.5 (7.1 to 20.6)*	4.7 (-1.1 to 11.3)
Acute pneumococcal meningitis	9.8 (6.1 to 14.5)	32.6 (13.7 to 52.9)*	22.4 (4.9 to 41.1)*	1.3 (0.7 to 2.2)	31.8 (-0.3 to 68.5)	21.6 (-7.9 to 55.5)
Complications of pneumococcal meningitis aggregate	407.7 (348.2 to 483.6)	17.8 (15.0 to 21.0)*	8.7 (6.1 to 11.7)*	47.2 (32.9 to 63.2)	13.1 (6.7 to 20.3)*	4.3 (-1.5 to 11.0)
H influenzae type B meningitis	205.3 (171.9 to 247.5)	-10.3 (-14.2 to -5.7)*	-17.2 (-20.8 to -12.9)*	28.5 (19.6 to 38.5)	0.0 (-7.8 to 8.7)	-7.7 (-14.9 to 0.3)
Acute H influenzae type B meningitis	7.3 (4.6 to 11.4)	-17.0 (-28.5 to -5.4)*	-23.4 (-34.0 to -12.7)*	1.0 (0.5 to 1.7)	-16.7 (-29.7 to -3.3)*	-23.1 (-35.1 to -10.8)*
Complications of of H influenzae type B meningitis aggregate	198.0 (164.6 to 238.5)	-10.0 (-14.1 to -5.3)*	-17.0 (-20.7 to -12.6)*	27.5 (18.9 to 37.3)	0.7 (-7.4 to 9.8)	-7.0 (-14.6 to 1.3)
Meningococcal meningitis	98.6 (72.4 to 131.0)	17.3 (12.0 to 23.0)*	8.2 (3.3 to 13.5)*	9.1 (6.1 to 12.5)	15.7 (4.7 to 28.0)*	6.7 (-3.4 to 18.2)
Acute meningococcal meningitis	6.4 (4.1 to 9.3)	34.0 (12.3 to 56.2)*	23.6 (3.7 to 44.2)*	0.8 (0.4 to 1.4)	34.5 (-4.4 to 77.1)	24.1 (-11.8 to 63.5)
Complications of meningococcal meningitis aggregate	92.2 (66.9 to 124.5)	16.3 (10.9 to 21.6)*	7.3 (2.4 to 12.2)*	8.2 (5.5 to 11.3)	14.0 (2.7 to 27.1)*	5.2 (-5.2 to 17.3)
Other meningitis	183.2 (150.5 to 219.5)	13.8 (10.0 to 17.4)*	5.1 (1.6 to 8.4)*	22.0 (15.1 to 29.5)	8.7 (1.4 to 16.4)*	0.3 (-6.5 to 7.4)
Other meningitis episodes aggregate	33.5 (25.2 to 44.1)	20.7 (6.0 to 35.2)*	11.4 (-2.2 to 24.8)	4.4 (2.6 to 6.9)	20.5 (0.5 to 40.9)*	11.2 (-7.2 to 30.1)
Complications of other meningitis aggregate	149.6 (119.1 to 185.1)	12.4 (9.5 to 15.7)*	3.7 (1.1 to 6.8)*	17.6 (12.0 to 24.0)	6.0 (-1.7 to 14.4)	-2.1 (-9.2 to 5.6)
Encephalitis	125.7 (96.0 to 163.3)	-6.0 (-8.3 to -3.5)*	-13.3 (-15.4 to -11.0)*	15.3 (10.8 to 20.5)	-7.0 (-15.1 to 1.7)	-14.2 (-21.7 to -6.1)*
Acute encephalitis	13.7 (10.4 to 18.2)	3.2 (-0.6 to 7.3)	-4.7 (-8.3 to -1.0)*	1.8 (1.1 to 2.8)	3.4 (-11.8 to 21.0)	-4.6 (-18.6 to 11.7)
Complications of encephalitis aggregate	112.0 (83.9 to 147.6)	-7.0 (-9.1 to -4.6)*	-14.2 (-16.1 to -11.9)*	13.5 (9.4 to 18.0)	-8.2 (-16.8 to 1.3)	-15.3 (-23.2 to -6.5)*
Diphtheria	0.2 (0.1 to 0.7)	-61.9 (-91.2 to 68.6)	-64.8 (-91.8 to 55.6)	0.0 (0.0 to 0.1)	-62.0 (-91.2 to 68.6)	-64.9 (-91.8 to 55.6)
Whooping cough	1,093.7 (845.4 to 1,372.0)	-26.8 (-28.9 to -24.6)*	-32.5 (-34.4 to -30.4)*	54.1 (32.3 to 82.0)	-26.7 (-29.6 to -23.5)*	-32.4 (-35.0 to -29.4)*
Tetanus	16.8 (16.4 to 17.3)	-1.5 (-4.3 to 0.7)	-9.1 (-11.7 to -7.1)*	0.7 (0.5 to 1.0)	-12.0 (-21.8 to -1.6)*	-18.8 (-27.8 to -9.2)*
Severe tetanus	0.4 (0.2 to 0.8)	-50.5 (-70.6 to -13.5)*	-54.3 (-72.9 to -20.1)*	0.1 (0.0 to 0.1)	-50.4 (-70.6 to -13.5)*	-54.2 (-72.9 to -20.1)*
Complications of tetanus aggregate	16.4 (16.1 to 16.7)	1.2 (0.8 to 1.7)*	-6.6 (-7.0 to -6.1)*	0.6 (0.5 to 0.9)	-5.3 (-13.4 to 4.7)	-12.6 (-20.0 to -3.4)*
Measles	79.2 (35.3 to 156.8)	-70.1 (-73.5 to -65.9)*	-72.4 (-75.6 to -68.5)*	7.1 (2.7 to 15.8)	-69.9 (-74.1 to -64.8)*	-72.2 (-76.1 to -67.5)*
Varicella and herpes zoster	806.7 (626.9 to 985.2)	6.8 (5.4 to 7.8)*	-1.4 (-2.8 to -0.5)*	6.7 (3.3 to 12.5)	7.2 (1.7 to 12.7)*	-1.0 (-6.1 to 4.1)
Neglected tropical diseases and malaria	125,930.7 (122,722.0 to 129,214.4)	-4.8 (-7.3 to -2.1)*	-12.1 (-14.4 to -9.6)*	1,503.1 (980.4 to 2,289.6)	-5.6 (-12.4 to 3.8)	-12.9 (-19.2 to -4.2)*
Malaria	44,129.1 (34,792.4 to 55,936.5)	15.4 (9.7 to 22.3)*	6.5 (1.2 to 12.9)*	525.1 (344.2 to 759.9)	-2.0 (-10.2 to 7.6)	-9.5 (-17.1 to -0.7)*
Asymptomatic malaria parasitemia(PfPR)	28,231.3 (19,244.0 to 40,103.6)	26.9 (16.7 to 41.8)*	17.1 (7.8 to 30.9)*	0.0 (0.0 to 0.0)	0.0 (0.0 to 0.0)	0.0 (0.0 to 0.0)
Malaria episodes aggregate	5,327.5 (2,859.3 to 8,673.4)	-19.4 (-34.2 to -8.9)*	-25.6 (-39.3 to -15.9)*	110.8 (48.6 to 209.1)	-19.2 (-34.0 to -8.5)*	-25.4 (-39.1 to -15.5)*
Complications of malaria aggregate	20.9 (17.7 to 24.8)	16.8 (12.2 to 21.2)*	7.8 (3.5 to 11.9)*	5.7 (4.1 to 7.7)	13.4 (-3.6 to 33.2)	4.7 (-11.0 to 22.9)
Anemia due to malaria parasitemia(PfPR) aggregate	10,549.3 (9,781.4 to 11,258.9)	12.7 (5.6 to 19.7)*	4.0 (-2.5 to 10.5)	408.6 (274.7 to 586.9)	3.8 (-6.1 to 16.4)	-4.2 (-13.3 to 7.4)
Chagas disease	113.9 (96.7 to 134.1)	-3.2 (-9.0 to 2.5)	-10.7 (-16.0 to -5.4)*	0.0 (0.0 to 0.0)	-21.6 (-29.3 to -12.8)*	-27.6 (-34.7 to -19.5)*
Chagas disease episodes aggregate	113.8 (96.7 to 134.0)	-3.2 (-9.0 to 2.5)	-10.7 (-16.0 to -5.4)*	0.0 (0.0 to 0.0)	-49.2 (-56.2 to -42.4)*	-53.2 (-59.6 to -46.8)*
Complications of Chagas disease aggregate	0.1 (0.0 to 0.1)	-3.2 (-9.0 to 2.5)	-10.7 (-16.0 to -5.4)*	0.0 (0.0 to 0.0)	-3.2 (-9.0 to 2.5)	-10.7 (-16.0 to -5.4)*

Cause	Prevalence in thousands (95% confidence interval)			YLDs in thousands (95% confidence interval)		
	2015	Percent change in counts between 2005 and 2015	Percent change in rates between 2005 and 2015	2015	Percent change in counts between 2005 and 2015	Percent change in rates between 2005 and 2015
Heart failure due to Chagas disease aggregate	0.0 (0.0 to 0.0)	-2.7 (-9.0 to 3.5)	-10.2 (-16.0 to -4.5)*	0.0 (0.0 to 0.0)	-2.7 (-9.0 to 3.5)	-10.2 (-16.1 to -4.5)*
Leishmaniasis	134.6 (112.8 to 173.3)	11.6 (-11.3 to 44.9)	3.0 (-18.1 to 33.8)	2.1 (1.1 to 3.6)	9.3 (0.0 to 19.7)*	0.9 (-7.7 to 10.5)
Visceral leishmaniasis	8.4 (7.4 to 9.4)	4.8 (3.5 to 6.4)*	-3.3 (-4.5 to -1.8)*	0.6 (0.4 to 0.9)	4.9 (-15.2 to 31.4)	-3.2 (-21.8 to 21.3)
Cutaneous and mucocutaneous leishmaniasis	134.1 (108.4 to 176.9)	11.0 (6.5 to 16.4)*	2.5 (-1.7 to 7.4)	1.5 (0.7 to 2.9)	11.2 (2.4 to 21.4)*	2.6 (-5.5 to 12.0)
African trypanosomiasis	0.9 (0.5 to 1.5)	-69.9 (-71.6 to -68.5)*	-72.3 (-73.8 to -70.9)*	0.3 (0.1 to 0.5)	-68.6 (-77.8 to -53.3)*	-71.0 (-79.5 to -56.9)*
Schistosomiasis	6,015.4 (5,058.5 to 7,521.8)	-27.4 (-35.3 to -12.4)*	-33.0 (-40.3 to -19.2)*	45.4 (21.6 to 86.7)	-30.6 (-39.5 to -18.9)*	-35.9 (-44.2 to -25.1)*
Schistosomiasis episodes aggregate	5,585.2 (4,644.4 to 7,056.9)	-27.5 (-35.9 to -11.4)*	-33.0 (-40.9 to -18.2)*	32.0 (12.5 to 68.7)	-27.4 (-35.8 to -11.0)*	-33.0 (-40.7 to -17.9)*
Complications of schistosomiasis aggregate	26.1 (12.9 to 52.7)	-29.3 (-44.6 to 6.4)	-34.8 (-48.9 to -1.8)*	0.3 (0.1 to 0.7)	-28.5 (-49.7 to 12.8)	-34.0 (-53.6 to 4.1)
Anemia due to schistosomiasis aggregate	380.7 (333.4 to 424.7)	-28.2 (-37.9 to -19.0)*	-33.8 (-42.7 to -25.2)*	13.1 (8.2 to 19.8)	-37.3 (-51.7 to -21.4)*	-42.1 (-55.4 to -27.4)*
Cysticercosis	0.0 (0.0 to 0.0)	0.0 (0.0 to 0.0)	0.0 (0.0 to 0.0)	0.0 (0.0 to 0.0)	0.0 (0.0 to 0.0)	0.0 (0.0 to 0.0)
Cystic echinococcosis	39.3 (32.1 to 47.9)	17.9 (15.1 to 20.9)*	8.8 (6.2 to 11.5)*	3.7 (2.4 to 5.5)	17.8 (3.1 to 33.0)*	8.7 (-4.9 to 22.7)
Lymphatic filariasis	1,135.6 (918.1 to 1,375.6)	-57.5 (-60.6 to -53.8)*	-60.7 (-63.6 to -57.3)*	0.0 (0.0 to 0.0)	0.0 (0.0 to 0.0)	0.0 (0.0 to 0.0)
Prevalence of detectable microfiliaria due to lymphatic filariasis	1,135.6 (918.1 to 1,375.6)	-57.5 (-60.6 to -53.8)*	-60.7 (-63.6 to -57.3)*	0.0 (0.0 to 0.0)	0.0 (0.0 to 0.0)	0.0 (0.0 to 0.0)
Complications of lymphatic filariasis aggregate	0.0 (0.0 to 0.0)	0.0 (0.0 to 0.0)	0.0 (0.0 to 0.0)	0.0 (0.0 to 0.0)	0.0 (0.0 to 0.0)	0.0 (0.0 to 0.0)
Onchocerciasis	0.0 (0.0 to 0.0)	0.0 (0.0 to 0.0)	0.0 (0.0 to 0.0)	0.0 (0.0 to 0.0)	0.0 (0.0 to 0.0)	0.0 (0.0 to 0.0)
Asymptomatic onchocerciasis	0.0 (0.0 to 0.0)	0.0 (0.0 to 0.0)	0.0 (0.0 to 0.0)	0.0 (0.0 to 0.0)	0.0 (0.0 to 0.0)	0.0 (0.0 to 0.0)
Skin disease due to onchocerciasis aggregate	0.0 (0.0 to 0.0)	0.0 (0.0 to 0.0)	0.0 (0.0 to 0.0)	0.0 (0.0 to 0.0)	0.0 (0.0 to 0.0)	0.0 (0.0 to 0.0)
Vision loss due to onchocerciasis aggregate	0.0 (0.0 to 0.0)	0.0 (0.0 to 0.0)	0.0 (0.0 to 0.0)	0.0 (0.0 to 0.0)	0.0 (0.0 to 0.0)	0.0 (0.0 to 0.0)
Trachoma	0.0 (0.0 to 0.0)	0.0 (0.0 to 0.0)	0.0 (0.0 to 0.0)	0.0 (0.0 to 0.0)	0.0 (0.0 to 0.0)	0.0 (0.0 to 0.0)
Dengue	255.3 (141.6 to 556.5)	128.7 (-6.1 to 513.7)	111.1 (-13.3 to 466.5)	42.3 (19.3 to 97.4)	126.7 (-4.5 to 500.7)	109.2 (-11.8 to 454.5)
Dengue episodes aggregate	75.6 (49.7 to 158.5)	123.2 (-7.7 to 486.3)	106.0 (-14.8 to 441.2)	4.5 (2.4 to 10.5)	125.9 (-8.5 to 538.3)	108.5 (-15.5 to 489.1)
Post-dengue chronic fatigue syndrome	179.4 (85.2 to 398.9)	128.8 (-6.1 to 513.7)	111.2 (-13.3 to 466.4)	37.8 (16.1 to 87.7)	126.8 (-6.0 to 501.8)	109.3 (-13.2 to 455.5)
Yellow fever	0.3 (0.1 to 0.7)	-30.1 (-45.2 to -11.2)*	-35.5 (-49.4 to -18.0)*	0.0 (0.0 to 0.0)	-30.2 (-45.2 to -11.2)*	-35.5 (-49.4 to -18.0)*
Rabies	0.1 (0.1 to 0.1)	-39.0 (-62.5 to 1.1)	-43.7 (-65.3 to -6.7)*	0.0 (0.0 to 0.0)	-38.9 (-62.5 to 1.1)	-43.7 (-65.3 to -6.7)*
Intestinal nematode infections	77,240.0 (74,288.0 to 80,166.7)	-13.7 (-17.8 to -9.6)*	-20.4 (-24.1 to -16.5)*	156.5 (94.0 to 251.5)	-30.3 (-36.6 to -23.9)*	-35.7 (-41.5 to -29.7)*
Ascariasis	39,081.3 (34,211.6 to 44,714.9)	-20.5 (-32.9 to -5.6)*	-26.6 (-38.0 to -12.8)*	61.1 (35.2 to 101.1)	-36.2 (-46.6 to -23.3)*	-41.1 (-50.7 to -29.2)*
Complications of ascariasis aggregate	3,125.7 (2,829.4 to 3,455.2)	-34.6 (-43.6 to -25.1)*	-39.7 (-47.9 to -30.9)*	61.1 (35.2 to 101.1)	-36.2 (-46.6 to -23.3)*	-41.1 (-50.7 to -29.2)*
Asymptomatic ascariasis	35,943.7 (31,118.1 to 41,547.3)	-19.0 (-32.7 to -2.0)*	-25.3 (-37.8 to -9.5)*	0.0 (0.0 to 0.0)	0.0 (0.0 to 0.0)	0.0 (0.0 to 0.0)
Trichuriasis	25,372.8 (22,835.7 to 28,141.1)	-14.0 (-25.2 to -0.6)*	-20.7 (-31.0 to -8.3)*	20.7 (11.6 to 36.3)	-30.1 (-48.1 to -6.6)*	-35.5 (-52.1 to -13.8)*
Complications of trichuriasis aggregate	999.9 (862.2 to 1,211.6)	-28.2 (-43.5 to -8.7)*	-33.7 (-47.9 to -15.7)*	20.7 (11.6 to 36.3)	-30.1 (-48.1 to -6.6)*	-35.5 (-52.1 to -13.8)*
Asymptomatic trichuriasis	24,369.0 (21,838.9 to 27,116.7)	-13.3 (-25.0 to 0.7)	-20.0 (-30.8 to -7.1)*	0.0 (0.0 to 0.0)	0.0 (0.0 to 0.0)	0.0 (0.0 to 0.0)
Hookworm disease	19,598.7 (17,759.9 to 21,412.8)	-18.1 (-28.0 to -7.3)*	-24.4 (-33.5 to -14.5)*	74.7 (46.3 to 115.3)	-24.8 (-32.4 to -17.8)*	-30.6 (-37.6 to -24.1)*
Complications of hookworm disease aggregate	1,994.9 (1,863.9 to 2,139.0)	-21.9 (-29.9 to -14.1)*	-27.9 (-35.3 to -20.7)*	44.3 (25.9 to 70.3)	-23.6 (-33.7 to -12.9)*	-29.4 (-38.8 to -19.6)*
Anemia due to hookworm disease aggregate	1,010.8 (960.0 to 1,057.6)	-18.8 (-23.8 to -13.4)*	-25.1 (-29.6 to -20.1)*	30.4 (19.9 to 45.3)	-26.5 (-35.3 to -17.8)*	-32.2 (-40.3 to -24.2)*
Asymptomatic hookworm disease	16,586.0 (14,764.0 to 18,396.8)	-17.6 (-29.1 to -4.5)*	-23.9 (-34.5 to -11.8)*	0.0 (0.0 to 0.0)	0.0 (0.0 to 0.0)	0.0 (0.0 to 0.0)
Food-borne trematodiases	712.4 (605.5 to 849.0)	3.7 (0.2 to 7.2)*	-4.3 (-7.5 to -1.1)*	6.1 (3.4 to 9.8)	7.8 (-2.8 to 18.7)	-0.5 (-10.2 to 9.5)
Asymptomatic food-borne trematodiases aggregate	658.8 (553.2 to 794.2)	3.4 (-0.1 to 6.9)	-4.6 (-7.8 to -1.3)*	0.0 (0.0 to 0.0)	0.0 (0.0 to 0.0)	0.0 (0.0 to 0.0)
Symptomatic food-borne trematodiases aggregate	53.6 (35.2 to 76.6)	7.3 (1.6 to 13.7)*	-1.0 (-6.3 to 5.0)	6.1 (3.4 to 9.8)	7.8 (-2.8 to 18.7)	-0.5 (-10.2 to 9.5)
Leprosy	0.3 (0.2 to 0.4)	-34.4 (-36.3 to -32.5)*	-39.4 (-41.2 to -37.7)*	0.0 (0.0 to 0.0)	-34.5 (-36.5 to -32.4)*	-39.5 (-41.4 to -37.6)*
Ebola	0.2 (0.1 to 0.3)	0.0 (0.0 to 0.0)	0.0 (0.0 to 0.0)	0.0 (0.0 to 0.1)	0.0 (0.0 to 0.0)	0.0 (0.0 to 0.0)
Other neglected tropical diseases	15,808.0 (15,324.6 to 16,302.8)	0.6 (-2.8 to 4.3)	-7.1 (-10.3 to -3.7)*	721.6 (448.8 to 1,169.3)	-2.0 (-11.9 to 13.3)	-9.6 (-18.7 to 4.5)
Anemia due to other neglected tropical diseases aggregate	15,808.0 (15,324.6 to 16,302.8)	0.6 (-2.8 to 4.3)	-7.1 (-10.3 to -3.7)*	580.2 (386.9 to 827.9)	-6.6 (-12.4 to -0.3)*	-13.8 (-19.1 to -8.0)*
Maternal disorders	0.0 (0.0 to 0.0)	0.0 (0.0 to 0.0)	0.0 (0.0 to 0.0)	0.0 (0.0 to 0.0)	0.0 (0.0 to 0.0)	0.0 (0.0 to 0.0)
Maternal hemorrhage	0.0 (0.0 to 0.0)	0.0 (0.0 to 0.0)	0.0 (0.0 to 0.0)	0.0 (0.0 to 0.0)	0.0 (0.0 to 0.0)	0.0 (0.0 to 0.0)
Maternal hemorrhage episodes aggregate	0.0 (0.0 to 0.0)	0.0 (0.0 to 0.0)	0.0 (0.0 to 0.0)	0.0 (0.0 to 0.0)	0.0 (0.0 to 0.0)	0.0 (0.0 to 0.0)
Anemia due to maternal hemorrhage aggregate	0.0 (0.0 to 0.0)	0.0 (0.0 to 0.0)	0.0 (0.0 to 0.0)	0.0 (0.0 to 0.0)	0.0 (0.0 to 0.0)	0.0 (0.0 to 0.0)
Maternal sepsis and other maternal infections	0.0 (0.0 to 0.0)	0.0 (0.0 to 0.0)	0.0 (0.0 to 0.0)	0.0 (0.0 to 0.0)	0.0 (0.0 to 0.0)	0.0 (0.0 to 0.0)
Maternal sepsis and other maternal infections aggregate	0.0 (0.0 to 0.0)	0.0 (0.0 to 0.0)	0.0 (0.0 to 0.0)	0.0 (0.0 to 0.0)	0.0 (0.0 to 0.0)	0.0 (0.0 to 0.0)
Infertility due to puerperal sepsis	0.0 (0.0 to 0.0)	0.0 (0.0 to 0.0)	0.0 (0.0 to 0.0)	0.0 (0.0 to 0.0)	0.0 (0.0 to 0.0)	0.0 (0.0 to 0.0)
Maternal hypertensive disorders	0.0 (0.0 to 0.0)	0.0 (0.0 to 0.0)	0.0 (0.0 to 0.0)	0.0 (0.0 to 0.0)	0.0 (0.0 to 0.0)	0.0 (0.0 to 0.0)
Maternal hypertensive disorders episodes aggregate	0.0 (0.0 to 0.0)	0.0 (0.0 to 0.0)	0.0 (0.0 to 0.0)	0.0 (0.0 to 0.0)	0.0 (0.0 to 0.0)	0.0 (0.0 to 0.0)
Eclampsia	0.0 (0.0 to 0.0)	0.0 (0.0 to 0.0)	0.0 (0.0 to 0.0)	0.0 (0.0 to 0.0)	0.0 (0.0 to 0.0)	0.0 (0.0 to 0.0)
Complications of maternal hypertensive disorders aggregate	0.0 (0.0 to 0.0)	0.0 (0.0 to 0.0)	0.0 (0.0 to 0.0)	0.0 (0.0 to 0.0)	0.0 (0.0 to 0.0)	0.0 (0.0 to 0.0)
Maternal obstructed labor and uterine rupture	0.0 (0.0 to 0.0)	0.0 (0.0 to 0.0)	0.0 (0.0 to 0.0)	0.0 (0.0 to 0.0)	0.0 (0.0 to 0.0)	0.0 (0.0 to 0.0)
Obstructed labor, acute event	0.0 (0.0 to 0.0)	0.0 (0.0 to 0.0)	0.0 (0.0 to 0.0)	0.0 (0.0 to 0.0)	0.0 (0.0 to 0.0)	0.0 (0.0 to 0.0)
Fistula due to maternal obstructed labor and uterine rupture aggregate	0.0 (0.0 to 0.0)	0.0 (0.0 to 0.0)	0.0 (0.0 to 0.0)	0.0 (0.0 to 0.0)	0.0 (0.0 to 0.0)	0.0 (0.0 to 0.0)
Maternal abortion, miscarriage, and ectopic pregnancy	0.0 (0.0 to 0.0)	0.0 (0.0 to 0.0)	0.0 (0.0 to 0.0)	0.0 (0.0 to 0.0)	0.0 (0.0 to 0.0)	0.0 (0.0 to 0.0)
Other maternal disorders	--	--	--	0.0 (0.0 to 0.0)	0.0 (0.0 to 0.0)	0.0 (0.0 to 0.0)
Neonatal disorders	4,486.8 (4,228.9 to 4,664.2)	10.3 (6.9 to 13.8)*	1.8 (-1.3 to 5.1)	1,044.8 (771.4 to 1,368.3)	8.4 (4.0 to 13.1)*	0.0 (-4.0 to 4.4)
Neonatal preterm birth complications	3,086.8 (2,688.5 to 3,530.5)	5.7 (0.4 to 10.9)*	-2.5 (-7.3 to 2.3)	478.4 (353.4 to 624.8)	0.4 (-5.3 to 6.5)	-7.4 (-12.6 to -1.7)*
Vision loss due to retinopathy of prematurity aggregate	288.3 (226.0 to 362.8)	5.6 (1.5 to 9.9)*	-2.5 (-6.3 to 1.4)	8.0 (4.5 to 12.9)	5.7 (-4.9 to 16.6)	-2.4 (-12.2 to 7.7)
Complications of preterm birth complications aggregate	2,798.5 (2,426.4 to 3,242.3)	5.7 (0.0 to 11.5)*	-2.5 (-7.7 to 2.9)	470.4 (346.5 to 613.6)	0.3 (-5.4 to 6.6)	-7.5 (-12.7 to -1.6)*
Neonatal encephalopathy due to birth asphyxia and trauma	1,507.1 (1,038.8 to 2,251.0)	19.6 (16.6 to 22.7)*	10.4 (7.6 to 13.2)*	394.1 (251.4 to 598.3)	17.5 (13.1 to 22.0)*	8.4 (4.4 to 12.6)*

Cause	Prevalence in thousands (95% confidence interval)			YLDs in thousands (95% confidence interval)		
	2015	Percent change in counts between 2005 and 2015	Percent change in rates between 2005 and 2015	2015	Percent change in counts between 2005 and 2015	Percent change in rates between 2005 and 2015
Neonatal sepsis and other neonatal infections	0.0 (0.0 to 0.0)	0.0 (0.0 to 0.0)	0.0 (0.0 to 0.0)	0.0 (0.0 to 0.0)	0.0 (0.0 to 0.0)	0.0 (0.0 to 0.0)
Severe infection due to neonatal sepsis and other neonatal infections	0.0 (0.0 to 0.0)	0.0 (0.0 to 0.0)	0.0 (0.0 to 0.0)	0.0 (0.0 to 0.0)	0.0 (0.0 to 0.0)	0.0 (0.0 to 0.0)
Complications of neonatal sepsis and other neonatal infections aggregate	0.0 (0.0 to 0.0)	0.0 (0.0 to 0.0)	0.0 (0.0 to 0.0)	0.0 (0.0 to 0.0)	0.0 (0.0 to 0.0)	0.0 (0.0 to 0.0)
Hemolytic disease and other neonatal jaundice	221.4 (196.1 to 253.2)	12.9 (11.6 to 14.2)*	4.2 (3.0 to 5.4)*	58.5 (42.1 to 76.3)	10.7 (4.3 to 16.7)*	2.1 (-3.7 to 7.7)
Other neonatal disorders	--	--	--	113.8 (73.5 to 163.7)	14.9 (-9.2 to 42.7)	6.0 (-16.2 to 31.7)
Nutritional deficiencies	169,284.5 (166,179.7 to 172,336.2)	1.7 (-0.7 to 4.1)	-6.2 (-8.3 to -4.0)*	9,037.4 (6,123.3 to 12,741.5)	-6.4 (-10.9 to -1.6)*	-13.6 (-17.7 to -9.2)*
Protein-energy malnutrition	17,180.6 (16,358.3 to 18,048.3)	-4.5 (-10.0 to 1.2)	-11.8 (-16.9 to -6.6)*	2,129.4 (1,378.3 to 3,011.8)	-4.2 (-9.6 to 1.5)	-11.6 (-16.6 to -6.3)*
Iodine deficiency	707.5 (590.5 to 845.7)	-6.8 (-9.2 to -4.8)*	-14.0 (-16.2 to -12.1)*	35.8 (15.3 to 60.9)	-1.3 (-9.6 to 4.7)	-8.9 (-16.6 to -3.4)*
Visible goiter due to iodine deficiency aggregate	566.8 (503.7 to 646.0)	-8.6 (-10.6 to -6.4)*	-15.6 (-17.5 to -13.6)*	10.4 (6.4 to 16.5)	-8.5 (-12.2 to -4.6)*	-15.5 (-18.9 to -12.0)*
Visible goiter with heart failure due to iodine deficiency aggregate	0.0 (0.0 to 0.0)	0.0 (0.0 to 0.0)	0.0 (0.0 to 0.0)	0.0 (0.0 to 0.0)	0.0 (0.0 to 0.0)	0.0 (0.0 to 0.0)
Intellectual disability due to iodine deficiency aggregate	140.7 (38.6 to 239.2)	1.1 (-8.3 to 4.1)	-6.7 (-15.3 to -4.0)*	25.4 (6.6 to 48.2)	2.0 (-10.4 to 9.6)	-5.9 (-17.3 to 1.2)
Vitamin A deficiency	597.8 (369.3 to 858.6)	4.6 (1.4 to 7.4)*	-3.5 (-6.4 to -0.9)*	24.6 (13.0 to 40.9)	4.4 (-1.3 to 10.2)	-3.6 (-8.9 to 1.7)
Iron-deficiency anemia	175,767.8 (170,825.2 to 180,911.0)	1.0 (-2.3 to 4.2)	-6.8 (-9.9 to -3.8)*	6,342.3 (4,202.0 to 9,147.7)	-6.3 (-11.7 to -0.3)*	-13.5 (-18.5 to -8.0)*
Iron-deficiency anemia without heart failure aggregate	175,767.7 (170,825.0 to 180,910.8)	1.0 (-2.3 to 4.2)	-6.8 (-9.9 to -3.8)*	6,342.2 (4,202.0 to 9,147.7)	-6.3 (-11.7 to -0.3)*	-13.5 (-18.5 to -8.0)*
Iron-deficiency anemia with heart failure aggregate	0.2 (0.1 to 0.2)	15.8 (13.3 to 18.4)*	6.9 (4.5 to 9.3)*	0.0 (0.0 to 0.0)	15.8 (13.3 to 18.4)*	6.9 (4.5 to 9.3)*
Other nutritional deficiencies	--	--	--	505.4 (301.6 to 786.5)	-16.5 (-36.6 to 6.2)	-22.9 (-41.5 to -2.0)*
Other communicable, maternal, neonatal, and nutritional diseases	35,691.1 (34,996.6 to 36,296.2)	10.6 (8.5 to 12.7)*	2.1 (0.1 to 4.0)*	428.9 (283.3 to 619.3)	-2.9 (-9.5 to 4.0)	-10.4 (-16.5 to -4.0)*
Sexually transmitted diseases excluding HIV	0.0 (0.0 to 0.0)	0.0 (0.0 to 0.0)	0.0 (0.0 to 0.0)	0.0 (0.0 to 0.0)	0.0 (0.0 to 0.0)	0.0 (0.0 to 0.0)
Syphilis	0.0 (0.0 to 0.0)	0.0 (0.0 to 0.0)	0.0 (0.0 to 0.0)	0.0 (0.0 to 0.0)	0.0 (0.0 to 0.0)	0.0 (0.0 to 0.0)
Early syphilis aggregate	0.0 (0.0 to 0.0)	0.0 (0.0 to 0.0)	0.0 (0.0 to 0.0)	0.0 (0.0 to 0.0)	0.0 (0.0 to 0.0)	0.0 (0.0 to 0.0)
Adult tertiary syphilis	0.0 (0.0 to 0.0)	0.0 (0.0 to 0.0)	0.0 (0.0 to 0.0)	0.0 (0.0 to 0.0)	0.0 (0.0 to 0.0)	0.0 (0.0 to 0.0)
Chlamydial infection	0.0 (0.0 to 0.0)	0.0 (0.0 to 0.0)	0.0 (0.0 to 0.0)	0.0 (0.0 to 0.0)	0.0 (0.0 to 0.0)	0.0 (0.0 to 0.0)
Chlamydial infection episodes aggregate	0.0 (0.0 to 0.0)	0.0 (0.0 to 0.0)	0.0 (0.0 to 0.0)	0.0 (0.0 to 0.0)	0.0 (0.0 to 0.0)	0.0 (0.0 to 0.0)
Pelvic inflammatory diseases due to chlamydial infection aggregate	0.0 (0.0 to 0.0)	0.0 (0.0 to 0.0)	0.0 (0.0 to 0.0)	0.0 (0.0 to 0.0)	0.0 (0.0 to 0.0)	0.0 (0.0 to 0.0)
Infertility due to clamydial infection aggregate	0.0 (0.0 to 0.0)	0.0 (0.0 to 0.0)	0.0 (0.0 to 0.0)	0.0 (0.0 to 0.0)	0.0 (0.0 to 0.0)	0.0 (0.0 to 0.0)
Gonococcal infection	0.0 (0.0 to 0.0)	0.0 (0.0 to 0.0)	0.0 (0.0 to 0.0)	0.0 (0.0 to 0.0)	0.0 (0.0 to 0.0)	0.0 (0.0 to 0.0)
Gonococcal infection episodes aggregate	0.0 (0.0 to 0.0)	0.0 (0.0 to 0.0)	0.0 (0.0 to 0.0)	0.0 (0.0 to 0.0)	0.0 (0.0 to 0.0)	0.0 (0.0 to 0.0)
Pelvic inflammatory diseases due to gonococcal infection aggregate	0.0 (0.0 to 0.0)	0.0 (0.0 to 0.0)	0.0 (0.0 to 0.0)	0.0 (0.0 to 0.0)	0.0 (0.0 to 0.0)	0.0 (0.0 to 0.0)
Trichomoniasis	0.0 (0.0 to 0.0)	0.0 (0.0 to 0.0)	0.0 (0.0 to 0.0)	0.0 (0.0 to 0.0)	0.0 (0.0 to 0.0)	0.0 (0.0 to 0.0)
Genital herpes	0.0 (0.0 to 0.0)	0.0 (0.0 to 0.0)	0.0 (0.0 to 0.0)	0.0 (0.0 to 0.0)	0.0 (0.0 to 0.0)	0.0 (0.0 to 0.0)
Moderate infection due to initial genital herpes episode	0.0 (0.0 to 0.0)	0.0 (0.0 to 0.0)	0.0 (0.0 to 0.0)	0.0 (0.0 to 0.0)	0.0 (0.0 to 0.0)	0.0 (0.0 to 0.0)
Complications of genital herpes aggregate	0.0 (0.0 to 0.0)	0.0 (0.0 to 0.0)	0.0 (0.0 to 0.0)	0.0 (0.0 to 0.0)	0.0 (0.0 to 0.0)	0.0 (0.0 to 0.0)
Other sexually transmitted diseases	0.0 (0.0 to 0.0)	0.0 (0.0 to 0.0)	0.0 (0.0 to 0.0)	0.0 (0.0 to 0.0)	0.0 (0.0 to 0.0)	0.0 (0.0 to 0.0)
Pelvic inflammatory diseases due to other sexually transmitted diseases aggregate	0.0 (0.0 to 0.0)	0.0 (0.0 to 0.0)	0.0 (0.0 to 0.0)	0.0 (0.0 to 0.0)	0.0 (0.0 to 0.0)	0.0 (0.0 to 0.0)
Infertility due to other sexually transmitted diseases aggregate	0.0 (0.0 to 0.0)	0.0 (0.0 to 0.0)	0.0 (0.0 to 0.0)	0.0 (0.0 to 0.0)	0.0 (0.0 to 0.0)	0.0 (0.0 to 0.0)
Other sexually transmitted diseases	--	--	--	0.0 (0.0 to 0.0)	0.0 (0.0 to 0.0)	0.0 (0.0 to 0.0)
Hepatitis	25,286.4 (24,648.7 to 25,853.5)	15.2 (12.8 to 17.8)*	6.4 (4.1 to 8.7)*	16.0 (9.3 to 24.6)	5.1 (-29.5 to 50.4)	-3.0 (-34.9 to 38.8)
Acute hepatitis A	2,703.3 (2,082.8 to 3,226.0)	1.4 (-23.3 to 27.5)	-6.4 (-29.2 to 17.7)	11.3 (6.1 to 19.0)	2.0 (-41.9 to 70.8)	-5.9 (-46.3 to 57.6)
Hepatitis B	17,803.1 (16,226.8 to 19,689.4)	17.2 (12.4 to 21.6)*	8.2 (3.8 to 12.2)*	1.9 (1.2 to 2.9)	9.2 (-11.5 to 35.4)	0.8 (-18.3 to 25.0)
Acute Hepatitis B aggregate	266.1 (225.3 to 314.9)	9.2 (-11.4 to 34.2)	0.8 (-18.3 to 23.9)	1.9 (1.2 to 2.9)	9.2 (-11.5 to 35.4)	0.8 (-18.3 to 25.0)
Chronic hepatitis B	17,537.1 (15,950.5 to 19,420.2)	17.3 (12.5 to 21.8)*	8.3 (3.8 to 12.4)*	0.0 (0.0 to 0.0)	0.0 (0.0 to 0.0)	0.0 (0.0 to 0.0)
Hepatitis C	5,245.2 (4,587.6 to 5,951.1)	17.5 (15.1 to 19.5)*	8.5 (6.3 to 10.2)*	2.5 (1.2 to 4.8)	17.5 (3.4 to 33.5)*	8.5 (-4.6 to 23.3)
Acute Hepatitis C aggregate	182.7 (161.7 to 205.6)	17.5 (15.3 to 19.5)*	8.5 (6.4 to 10.3)*	2.5 (1.2 to 4.8)	17.5 (3.4 to 33.5)*	8.5 (-4.6 to 23.3)
Chronic hepatitis C	5,062.2 (4,414.3 to 5,756.3)	17.5 (15.1 to 19.4)*	8.4 (6.2 to 10.2)*	0.0 (0.0 to 0.0)	0.0 (0.0 to 0.0)	0.0 (0.0 to 0.0)
Acute hepatitis E	104.5 (88.8 to 125.4)	4.6 (2.8 to 6.1)*	-3.4 (-5.1 to -2.1)*	0.3 (0.2 to 0.5)	5.1 (-32.7 to 66.6)	-3.0 (-37.9 to 53.7)
Other infectious diseases	11,065.9 (10,742.5 to 11,408.6)	0.9 (-3.3 to 5.5)	-6.9 (-10.7 to -2.6)*	412.9 (274.7 to 597.4)	-3.2 (-9.8 to 3.6)	-10.6 (-16.8 to -4.4)*
Non-communicable diseases	400,549.1 (393,043.3 to 408,503.2)	8.4 (7.5 to 9.2)*	0.0 (-0.7 to 0.8)	9,293.7 (6,540.4 to 12,635.8)	6.6 (5.2 to 8.1)*	-1.6 (-2.9 to -0.2)*
Neoplasms	744.1 (681.4 to 812.5)	10.8 (0.7 to 22.3)*	2.3 (-7.0 to 12.9)	50.5 (30.9 to 76.4)	14.6 (-3.6 to 35.0)	5.8 (-11.0 to 24.6)
Lip and oral cavity cancer	0.0 (0.0 to 0.0)	0.0 (0.0 to 0.0)	0.0 (0.0 to 0.0)	0.0 (0.0 to 0.0)	0.0 (0.0 to 0.0)	0.0 (0.0 to 0.0)
Nasopharynx cancer	0.0 (0.0 to 0.0)	0.0 (0.0 to 0.0)	0.0 (0.0 to 0.0)	0.0 (0.0 to 0.0)	0.0 (0.0 to 0.0)	0.0 (0.0 to 0.0)
Other pharynx cancer	0.0 (0.0 to 0.0)	0.0 (0.0 to 0.0)	0.0 (0.0 to 0.0)	0.0 (0.0 to 0.0)	0.0 (0.0 to 0.0)	0.0 (0.0 to 0.0)
Esophageal cancer	0.0 (0.0 to 0.0)	0.0 (0.0 to 0.0)	0.0 (0.0 to 0.0)	0.0 (0.0 to 0.0)	0.0 (0.0 to 0.0)	0.0 (0.0 to 0.0)
Stomach cancer	0.0 (0.0 to 0.0)	0.0 (0.0 to 0.0)	0.0 (0.0 to 0.0)	0.0 (0.0 to 0.0)	0.0 (0.0 to 0.0)	0.0 (0.0 to 0.0)
Colon and rectum cancer	0.0 (0.0 to 0.0)	0.0 (0.0 to 0.0)	0.0 (0.0 to 0.0)	0.0 (0.0 to 0.0)	0.0 (0.0 to 0.0)	0.0 (0.0 to 0.0)
Colon and rectum cancer aggregate	0.0 (0.0 to 0.0)	0.0 (0.0 to 0.0)	0.0 (0.0 to 0.0)	0.0 (0.0 to 0.0)	0.0 (0.0 to 0.0)	0.0 (0.0 to 0.0)
Stoma due to colon and rectum cancer	0.0 (0.0 to 0.0)	0.0 (0.0 to 0.0)	0.0 (0.0 to 0.0)	0.0 (0.0 to 0.0)	0.0 (0.0 to 0.0)	0.0 (0.0 to 0.0)
Liver cancer	0.0 (0.0 to 0.0)	0.0 (0.0 to 0.0)	0.0 (0.0 to 0.0)	0.0 (0.0 to 0.0)	0.0 (0.0 to 0.0)	0.0 (0.0 to 0.0)
Liver cancer due to hepatitis B	0.0 (0.0 to 0.0)	0.0 (0.0 to 0.0)	0.0 (0.0 to 0.0)	0.0 (0.0 to 0.0)	0.0 (0.0 to 0.0)	0.0 (0.0 to 0.0)
Liver cancer due to hepatitis C	0.0 (0.0 to 0.0)	0.0 (0.0 to 0.0)	0.0 (0.0 to 0.0)	0.0 (0.0 to 0.0)	0.0 (0.0 to 0.0)	0.0 (0.0 to 0.0)
Liver cancer due to alcohol use	0.0 (0.0 to 0.0)	0.0 (0.0 to 0.0)	0.0 (0.0 to 0.0)	0.0 (0.0 to 0.0)	0.0 (0.0 to 0.0)	0.0 (0.0 to 0.0)
Liver cancer due to other causes	0.0 (0.0 to 0.0)	0.0 (0.0 to 0.0)	0.0 (0.0 to 0.0)	0.0 (0.0 to 0.0)	0.0 (0.0 to 0.0)	0.0 (0.0 to 0.0)
Gallbladder and biliary tract cancer	0.0 (0.0 to 0.0)	0.0 (0.0 to 0.0)	0.0 (0.0 to 0.0)	0.0 (0.0 to 0.0)	0.0 (0.0 to 0.0)	0.0 (0.0 to 0.0)
Pancreatic cancer	0.0 (0.0 to 0.0)	0.0 (0.0 to 0.0)	0.0 (0.0 to 0.0)	0.0 (0.0 to 0.0)	0.0 (0.0 to 0.0)	0.0 (0.0 to 0.0)

Cause	Prevalence in thousands (95% confidence interval)			YLDs in thousands (95% confidence interval)		
	2015	Percent change in counts between 2005 and 2015	Percent change in rates between 2005 and 2015	2015	Percent change in counts between 2005 and 2015	Percent change in rates between 2005 and 2015
Larynx cancer	0.0 (0.0 to 0.0)	0.0 (0.0 to 0.0)	0.0 (0.0 to 0.0)	0.0 (0.0 to 0.0)	0.0 (0.0 to 0.0)	0.0 (0.0 to 0.0)
Larynx cancer aggregate	0.0 (0.0 to 0.0)	0.0 (0.0 to 0.0)	0.0 (0.0 to 0.0)	0.0 (0.0 to 0.0)	0.0 (0.0 to 0.0)	0.0 (0.0 to 0.0)
Laryngectomy due to larynx cancer	0.0 (0.0 to 0.0)	0.0 (0.0 to 0.0)	0.0 (0.0 to 0.0)	0.0 (0.0 to 0.0)	0.0 (0.0 to 0.0)	0.0 (0.0 to 0.0)
Tracheal, bronchus, and lung cancer	0.0 (0.0 to 0.0)	0.0 (0.0 to 0.0)	0.0 (0.0 to 0.0)	0.0 (0.0 to 0.0)	0.0 (0.0 to 0.0)	0.0 (0.0 to 0.0)
Malignant skin melanoma	0.0 (0.0 to 0.0)	0.0 (0.0 to 0.0)	0.0 (0.0 to 0.0)	0.0 (0.0 to 0.0)	0.0 (0.0 to 0.0)	0.0 (0.0 to 0.0)
Non-melanoma skin cancer	0.0 (0.0 to 0.0)	0.0 (0.0 to 0.0)	0.0 (0.0 to 0.0)	0.0 (0.0 to 0.0)	0.0 (0.0 to 0.0)	0.0 (0.0 to 0.0)
Non-melanoma skin cancer(squamous-cell carcinoma)	0.0 (0.0 to 0.0)	0.0 (0.0 to 0.0)	0.0 (0.0 to 0.0)	0.0 (0.0 to 0.0)	0.0 (0.0 to 0.0)	0.0 (0.0 to 0.0)
Non-melanoma skin cancer(basal-cell carcinoma)	0.0 (0.0 to 0.0)	0.0 (0.0 to 0.0)	0.0 (0.0 to 0.0)	0.0 (0.0 to 0.0)	0.0 (0.0 to 0.0)	0.0 (0.0 to 0.0)
Breast cancer	0.0 (0.0 to 0.0)	0.0 (0.0 to 0.0)	0.0 (0.0 to 0.0)	0.0 (0.0 to 0.0)	0.0 (0.0 to 0.0)	0.0 (0.0 to 0.0)
Breast cancer aggregate	0.0 (0.0 to 0.0)	0.0 (0.0 to 0.0)	0.0 (0.0 to 0.0)	0.0 (0.0 to 0.0)	0.0 (0.0 to 0.0)	0.0 (0.0 to 0.0)
Mastectomy due to breast cancer	0.0 (0.0 to 0.0)	0.0 (0.0 to 0.0)	0.0 (0.0 to 0.0)	0.0 (0.0 to 0.0)	0.0 (0.0 to 0.0)	0.0 (0.0 to 0.0)
Cervical cancer	0.0 (0.0 to 0.0)	0.0 (0.0 to 0.0)	0.0 (0.0 to 0.0)	0.0 (0.0 to 0.0)	0.0 (0.0 to 0.0)	0.0 (0.0 to 0.0)
Uterine cancer	0.0 (0.0 to 0.0)	0.0 (0.0 to 0.0)	0.0 (0.0 to 0.0)	0.0 (0.0 to 0.0)	0.0 (0.0 to 0.0)	0.0 (0.0 to 0.0)
Ovarian cancer	0.0 (0.0 to 0.0)	0.0 (0.0 to 0.0)	0.0 (0.0 to 0.0)	0.0 (0.0 to 0.0)	0.0 (0.0 to 0.0)	0.0 (0.0 to 0.0)
Prostate cancer	0.0 (0.0 to 0.0)	0.0 (0.0 to 0.0)	0.0 (0.0 to 0.0)	0.0 (0.0 to 0.0)	0.0 (0.0 to 0.0)	0.0 (0.0 to 0.0)
Prostate cancer aggregate	0.0 (0.0 to 0.0)	0.0 (0.0 to 0.0)	0.0 (0.0 to 0.0)	0.0 (0.0 to 0.0)	0.0 (0.0 to 0.0)	0.0 (0.0 to 0.0)
Impotence and incontinence due to prostate cancer aggregate	0.0 (0.0 to 0.0)	0.0 (0.0 to 0.0)	0.0 (0.0 to 0.0)	0.0 (0.0 to 0.0)	0.0 (0.0 to 0.0)	0.0 (0.0 to 0.0)
Testicular cancer	0.0 (0.0 to 0.0)	0.0 (0.0 to 0.0)	0.0 (0.0 to 0.0)	0.0 (0.0 to 0.0)	0.0 (0.0 to 0.0)	0.0 (0.0 to 0.0)
Kidney cancer	89.4 (57.7 to 147.0)	27.1 (-4.8 to 83.3)	17.3 (-12.1 to 69.1)	5.2 (2.8 to 9.2)	26.9 (-7.9 to 82.6)	17.1 (-15.0 to 68.5)
Bladder cancer	0.0 (0.0 to 0.0)	0.0 (0.0 to 0.0)	0.0 (0.0 to 0.0)	0.0 (0.0 to 0.0)	0.0 (0.0 to 0.0)	0.0 (0.0 to 0.0)
Bladder cancer episodes aggregate	0.0 (0.0 to 0.0)	0.0 (0.0 to 0.0)	0.0 (0.0 to 0.0)	0.0 (0.0 to 0.0)	0.0 (0.0 to 0.0)	0.0 (0.0 to 0.0)
Urinary incontinence due to bladder cancer	0.0 (0.0 to 0.0)	0.0 (0.0 to 0.0)	0.0 (0.0 to 0.0)	0.0 (0.0 to 0.0)	0.0 (0.0 to 0.0)	0.0 (0.0 to 0.0)
Brain and nervous system cancer	132.3 (104.4 to 169.0)	3.1 (-17.1 to 26.3)	-4.8 (-23.5 to 16.5)	7.6 (4.5 to 11.4)	3.1 (-16.3 to 25.9)	-4.8 (-22.7 to 16.2)
Thyroid cancer	0.0 (0.0 to 0.0)	0.0 (0.0 to 0.0)	0.0 (0.0 to 0.0)	0.0 (0.0 to 0.0)	0.0 (0.0 to 0.0)	0.0 (0.0 to 0.0)
Mesothelioma	0.0 (0.0 to 0.0)	0.0 (0.0 to 0.0)	0.0 (0.0 to 0.0)	0.0 (0.0 to 0.0)	0.0 (0.0 to 0.0)	0.0 (0.0 to 0.0)
Hodgkin lymphoma	11.0 (8.6 to 14.2)	-29.5 (-41.3 to -11.3)*	-34.9 (-45.8 to -18.2)*	0.6 (0.4 to 1.0)	-29.3 (-42.3 to -9.8)*	-34.7 (-46.8 to -16.8)*
Non-Hodgkin lymphoma	149.3 (96.1 to 236.2)	22.1 (-8.7 to 62.8)	12.7 (-15.7 to 50.2)	8.2 (4.5 to 14.3)	22.3 (-8.5 to 62.9)	12.9 (-15.6 to 50.3)
Multiple myeloma	0.0 (0.0 to 0.0)	0.0 (0.0 to 0.0)	0.0 (0.0 to 0.0)	0.0 (0.0 to 0.0)	0.0 (0.0 to 0.0)	0.0 (0.0 to 0.0)
Leukemia	214.0 (176.3 to 258.1)	9.8 (-12.0 to 35.2)	1.4 (-18.8 to 24.8)	14.4 (7.4 to 25.3)	16.4 (-17.4 to 66.6)	7.4 (-23.8 to 53.8)
Acute lymphoid leukemia	164.2 (98.6 to 252.5)	14.9 (-17.7 to 62.8)	6.0 (-24.0 to 50.3)	9.5 (4.9 to 16.6)	15.2 (-17.9 to 63.5)	6.3 (-24.2 to 50.9)
Chronic lymphoid leukemia	0.0 (0.0 to 0.0)	0.0 (0.0 to 0.0)	0.0 (0.0 to 0.0)	0.0 (0.0 to 0.0)	0.0 (0.0 to 0.0)	0.0 (0.0 to 0.0)
Acute myeloid leukemia	86.7 (49.7 to 144.1)	18.5 (-19.0 to 74.6)	9.3 (-25.2 to 61.2)	5.0 (2.4 to 9.2)	18.7 (-18.1 to 76.2)	9.6 (-24.4 to 62.6)
Chronic myeloid leukemia	0.0 (0.0 to 0.0)	0.0 (0.0 to 0.0)	0.0 (0.0 to 0.0)	0.0 (0.0 to 0.0)	0.0 (0.0 to 0.0)	0.0 (0.0 to 0.0)
Other neoplasms	278.1 (206.0 to 387.2)	14.6 (-4.8 to 40.2)	5.7 (-12.2 to 29.4)	14.4 (8.2 to 22.8)	14.6 (-5.0 to 40.5)	5.8 (-12.3 to 29.7)
Cardiovascular diseases	3,066.8 (2,548.4 to 3,495.9)	8.9 (-2.9 to 23.7)	0.5 (-10.4 to 14.1)	239.6 (141.6 to 404.4)	9.6 (7.4 to 11.7)*	1.1 (-0.9 to 3.1)
Rheumatic heart disease	236.2 (211.1 to 282.3)	-9.0 (-14.0 to -4.3)*	-16.0 (-20.6 to -11.7)*	13.4 (8.6 to 19.7)	-6.9 (-12.9 to -0.1)*	-14.0 (-19.6 to -7.8)*
Rheumatic heart disease, without heart failure	207.8 (182.3 to 253.7)	-10.6 (-16.1 to -5.6)*	-17.5 (-22.6 to -12.9)*	10.0 (6.2 to 15.3)	-10.4 (-17.2 to -2.7)*	-17.3 (-23.6 to -10.2)*
Heart failure fue to rheumatic heart disease aggregate	28.4 (25.3 to 31.4)	5.3 (3.9 to 6.8)*	-2.8 (-4.1 to -1.4)*	3.4 (2.3 to 5.0)	5.4 (-7.4 to 20.9)	-2.7 (-14.5 to 11.6)
Ischemic heart disease	28.2 (24.7 to 32.0)	8.3 (7.2 to 9.3)*	-0.1 (-1.1 to 0.9)	2.5 (1.7 to 3.6)	8.0 (0.8 to 15.8)*	-0.3 (-7.0 to 6.9)
Myocardial infarction episodes aggregate	0.1 (0.0 to 0.2)	9.8 (2.3 to 17.9)*	1.3 (-5.6 to 8.8)	0.0 (0.0 to 0.0)	9.8 (2.3 to 17.9)*	1.3 (-5.6 to 8.8)
Angina due to ischemic heart disease aggregate	18.2 (15.2 to 21.9)	9.8 (8.4 to 11.2)*	1.4 (0.0 to 2.6)*	1.3 (0.8 to 1.9)	9.8 (8.4 to 11.2)*	1.3 (0.0 to 2.6)*
Heart failure fue to ischemic heart disease aggregate	9.8 (8.3 to 11.4)	5.5 (3.7 to 7.2)*	-2.6 (-4.3 to -1.1)*	1.2 (0.8 to 1.8)	6.1 (-8.1 to 22.3)	-2.0 (-15.2 to 12.9)
Cerebrovascular disease	1.5 (1.2 to 1.7)	27.0 (-0.6 to 61.7)	17.2 (-8.2 to 49.2)	18.8 (12.9 to 25.9)	9.4 (8.0 to 10.8)*	1.0 (-0.3 to 2.3)
Ischemic stroke	47.9 (45.2 to 50.9)	11.1 (9.6 to 12.7)*	2.5 (1.1 to 4.0)*	8.5 (5.8 to 11.6)	11.0 (9.2 to 12.7)*	2.4 (0.8 to 4.0)*
Chronic ischemic stroke aggregate	37.7 (35.5 to 40.1)	11.7 (10.3 to 13.2)*	3.1 (1.8 to 4.5)*	6.3 (4.2 to 8.7)	11.7 (10.1 to 13.2)*	3.1 (1.6 to 4.5)*
Ischemic stroke episodes aggregate	10.2 (8.5 to 12.2)	9.0 (3.5 to 14.1)*	0.6 (-4.4 to 5.3)	2.2 (1.4 to 3.2)	9.1 (3.5 to 14.1)*	0.7 (-4.4 to 5.3)
Hemorrhagic stroke	58.4 (55.0 to 62.5)	8.3 (6.8 to 9.9)*	-0.0 (-1.4 to 1.4)	10.3 (7.0 to 14.2)	8.2 (6.5 to 9.9)*	-0.2 (-1.7 to 1.4)
Chronic hemorrhagic stroke aggregate	46.6 (43.9 to 49.5)	8.9 (7.5 to 10.3)*	0.5 (-0.7 to 1.8)	7.7 (5.3 to 10.8)	8.9 (7.4 to 10.4)*	0.5 (-0.9 to 1.9)
Acute hemorrhagic stroke aggregate	11.8 (9.9 to 14.2)	6.1 (0.8 to 11.8)*	-2.1 (-7.0 to 3.2)	2.6 (1.6 to 3.6)	6.1 (0.8 to 11.8)*	-2.1 (-7.0 to 3.2)
Hypertensive heart disease	1.1 (0.9 to 1.2)	6.5 (5.2 to 7.7)*	-1.7 (-2.9 to -0.6)*	0.1 (0.1 to 0.2)	6.5 (5.2 to 7.7)*	-1.7 (-2.9 to -0.6)*
Cardiomyopathy and myocarditis	156.7 (140.2 to 173.2)	12.0 (11.2 to 12.9)*	3.4 (2.6 to 4.2)*	18.6 (12.4 to 26.3)	12.3 (5.4 to 19.9)*	3.7 (-2.7 to 10.7)
Acute myocarditis	3.9 (2.9 to 5.2)	13.0 (11.2 to 15.0)*	4.3 (2.7 to 6.1)*	0.2 (0.1 to 0.3)	13.0 (11.2 to 15.0)*	4.3 (2.7 to 6.1)*
Heart failure due to cardiomyopathy aggregate	152.7 (136.1 to 169.5)	12.0 (11.1 to 12.9)*	3.4 (2.6 to 4.2)*	18.4 (12.3 to 26.1)	12.3 (5.3 to 20.0)*	3.7 (-2.8 to 10.7)
Atrial fibrillation and flutter	0.0 (0.0 to 0.0)	0.0 (0.0 to 0.0)	0.0 (0.0 to 0.0)	0.0 (0.0 to 0.0)	0.0 (0.0 to 0.0)	0.0 (0.0 to 0.0)
Peripheral vascular disease	0.0 (0.0 to 0.0)	0.0 (0.0 to 0.0)	0.0 (0.0 to 0.0)	0.0 (0.0 to 0.0)	0.0 (0.0 to 0.0)	0.0 (0.0 to 0.0)
Endocarditis	1.0 (0.9 to 1.2)	5.3 (0.6 to 10.3)*	-2.8 (-7.2 to 1.8)	0.1 (0.0 to 0.1)	5.2 (0.5 to 10.2)*	-2.9 (-7.2 to 1.7)
Endocarditis episodes aggregate	1.0 (0.9 to 1.2)	5.3 (0.6 to 10.3)*	-2.8 (-7.2 to 1.8)	0.1 (0.0 to 0.1)	5.2 (0.5 to 10.2)*	-2.9 (-7.2 to 1.7)
Heart failure due to endocarditis aggregate	0.0 (0.0 to 0.0)	0.0 (0.0 to 0.0)	0.0 (0.0 to 0.0)	0.0 (0.0 to 0.0)	0.0 (0.0 to 0.0)	0.0 (0.0 to 0.0)
Other cardiovascular and circulatory diseases	2,624.6 (1,583.0 to 4,500.6)	10.6 (9.0 to 12.2)*	2.0 (0.6 to 3.5)*	186.1 (97.7 to 339.0)	10.7 (8.4 to 13.4)*	2.2 (0.0 to 4.7)*
Heart failure due to other cardiovascular diseases aggregate	5.2 (4.6 to 5.9)	10.7 (9.1 to 12.4)*	2.2 (0.7 to 3.7)*	0.6 (0.4 to 0.9)	10.8 (1.3 to 21.9)*	2.3 (-6.5 to 12.5)
Other cardiovascular diseases episodes aggregate	2,619.4 (1,578.4 to 4,495.2)	10.6 (9.0 to 12.2)*	2.0 (0.6 to 3.5)*	185.5 (97.2 to 338.4)	10.7 (8.4 to 13.4)*	2.2 (0.0 to 4.7)*
Chronic respiratory diseases	20,904.9 (18,482.0 to 23,122.8)	3.4 (-0.5 to 7.3)	-4.6 (-8.1 to -1.0)*	992.0 (619.4 to 1,507.5)	2.6 (-2.4 to 7.8)	-5.3 (-9.9 to -0.5)*
Chronic obstructive pulmonary disease	548.3 (441.2 to 672.2)	-4.0 (-7.7 to -0.7)*	-11.4 (-14.8 to -8.3)*	47.6 (36.1 to 61.7)	-6.3 (-13.1 to 0.7)	-13.5 (-19.8 to -7.0)*

Cause	Prevalence in thousands (95% confidence interval)			YLDs in thousands (95% confidence interval)		
	2015	Percent change in counts between 2005 and 2015	Percent change in rates between 2005 and 2015	2015	Percent change in counts between 2005 and 2015	Percent change in rates between 2005 and 2015
Chronic obstructive pulmonary disease without heart failure aggregate	547.8 (440.3 to 672.2)	-3.9 (-7.6 to -0.5)*	-11.3 (-14.7 to -8.2)*	46.7 (35.3 to 60.7)	-6.4 (-13.4 to 0.7)	-13.6 (-20.0 to -7.0)*
Severe chronic obstructive pulmonary disease with heart failure aggregate	2.0 (1.7 to 2.2)	0.2 (-1.3 to 1.8)	-7.5 (-8.9 to -6.1)*	0.9 (0.7 to 1.2)	0.2 (-1.3 to 1.8)	-7.5 (-8.9 to -6.1)*
Pneumoconiosis	0.0 (0.0 to 0.0)	0.0 (0.0 to 0.0)	0.0 (0.0 to 0.0)	1.3 (0.8 to 1.9)	21.3 (16.4 to 26.2)*	11.9 (7.5 to 16.5)*
Silicosis	0.0 (0.0 to 0.0)	0.0 (0.0 to 0.0)	0.0 (0.0 to 0.0)	0.0 (0.0 to 0.0)	0.0 (0.0 to 0.0)	0.0 (0.0 to 0.0)
Silicosis without heart failure aggregate	0.0 (0.0 to 0.0)	0.0 (0.0 to 0.0)	0.0 (0.0 to 0.0)	0.0 (0.0 to 0.0)	0.0 (0.0 to 0.0)	0.0 (0.0 to 0.0)
Severe silicosis with heart failure aggregate	0.0 (0.0 to 0.0)	0.0 (0.0 to 0.0)	0.0 (0.0 to 0.0)	0.0 (0.0 to 0.0)	0.0 (0.0 to 0.0)	0.0 (0.0 to 0.0)
Asbestosis	0.0 (0.0 to 0.0)	0.0 (0.0 to 0.0)	0.0 (0.0 to 0.0)	0.0 (0.0 to 0.0)	0.0 (0.0 to 0.0)	0.0 (0.0 to 0.0)
Asbestosis without heart failure aggregate	0.0 (0.0 to 0.0)	0.0 (0.0 to 0.0)	0.0 (0.0 to 0.0)	0.0 (0.0 to 0.0)	0.0 (0.0 to 0.0)	0.0 (0.0 to 0.0)
Severe asbestosis with heart failure aggregate	0.0 (0.0 to 0.0)	0.0 (0.0 to 0.0)	0.0 (0.0 to 0.0)	0.0 (0.0 to 0.0)	0.0 (0.0 to 0.0)	0.0 (0.0 to 0.0)
Coal workers pneumoconiosis	0.0 (0.0 to 0.0)	0.0 (0.0 to 0.0)	0.0 (0.0 to 0.0)	0.0 (0.0 to 0.0)	0.0 (0.0 to 0.0)	0.0 (0.0 to 0.0)
Coal workers pneumoconiosis without heart failure aggregate	0.0 (0.0 to 0.0)	0.0 (0.0 to 0.0)	0.0 (0.0 to 0.0)	0.0 (0.0 to 0.0)	0.0 (0.0 to 0.0)	0.0 (0.0 to 0.0)
Severe coal workers pneumoconiosis with heart failure aggregate	0.0 (0.0 to 0.0)	0.0 (0.0 to 0.0)	0.0 (0.0 to 0.0)	0.0 (0.0 to 0.0)	0.0 (0.0 to 0.0)	0.0 (0.0 to 0.0)
Other pneumoconiosis	7.7 (6.5 to 9.2)	21.3 (16.4 to 26.2)*	12.0 (7.4 to 16.5)*	1.3 (0.8 to 1.9)	21.3 (16.4 to 26.2)*	11.9 (7.5 to 16.5)*
Other pneumoconiosis without heart failure aggregate	7.7 (6.5 to 9.2)	21.3 (16.4 to 26.2)*	12.0 (7.4 to 16.5)*	1.3 (0.8 to 1.9)	21.3 (16.4 to 26.2)*	11.9 (7.5 to 16.5)*
Severe other pneumoconiosis with heart failure aggregate	0.0 (0.0 to 0.0)	0.0 (0.0 to 0.0)	0.0 (0.0 to 0.0)	0.0 (0.0 to 0.0)	0.0 (0.0 to 0.0)	0.0 (0.0 to 0.0)
Asthma	20,379.6 (14,978.5 to 26,941.3)	3.2 (-2.1 to 8.5)	-4.8 (-9.6 to 0.2)	928.9 (565.5 to 1,444.4)	3.3 (-1.9 to 9.1)	-4.6 (-9.4 to 0.7)
Asymptomatic asthma	6,171.0 (4,461.0 to 8,190.3)	3.2 (-2.1 to 8.5)	-4.8 (-9.6 to 0.2)	0.0 (0.0 to 0.0)	0.0 (0.0 to 0.0)	0.0 (0.0 to 0.0)
Symptomatic asthma aggregate	14,208.6 (10,418.5 to 18,949.5)	3.2 (-2.1 to 8.5)	-4.8 (-9.6 to 0.2)	928.9 (565.5 to 1,444.4)	3.3 (-1.9 to 9.1)	-4.6 (-9.4 to 0.7)
Interstitial lung disease and pulmonary sarcoidosis	15.3 (12.9 to 18.0)	7.9 (4.1 to 11.2)*	-0.4 (-3.9 to 2.6)	2.0 (1.2 to 3.1)	7.6 (2.9 to 11.9)*	-0.7 (-5.0 to 3.3)
Interstitial lung disease and pulmonary sarcoidosis without heart failure aggregate	13.6 (11.3 to 16.1)	8.4 (4.3 to 12.0)*	0.1 (-3.7 to 3.4)	1.2 (0.7 to 2.0)	10.5 (2.9 to 17.7)*	2.0 (-5.0 to 8.6)
Severe interstitial lung disease and pulmonary sarcoidosis with heart failure aggregate	1.7 (1.3 to 2.0)	3.6 (1.8 to 5.9)*	-4.4 (-6.1 to -2.2)*	0.8 (0.5 to 1.1)	3.6 (1.8 to 5.9)*	-4.4 (-6.1 to -2.2)*
Other chronic respiratory diseases	--	--	--	12.1 (8.8 to 16.1)	-16.5 (-28.1 to -3.3)*	-22.9 (-33.7 to -10.7)*
Cirrhosis and other chronic liver diseases	43.7 (39.7 to 47.7)	12.8 (-1.7 to 28.5)	4.1 (-9.3 to 18.6)	8.5 (5.8 to 12.1)	11.1 (-0.2 to 24.6)	2.6 (-7.9 to 15.0)
Cirrhosis and other chronic liver diseases due to hepatitis B	0.4 (0.3 to 0.4)	11.1 (7.8 to 14.3)*	2.5 (-0.5 to 5.5)	0.1 (0.0 to 0.1)	11.1 (7.8 to 14.3)*	2.5 (-0.5 to 5.5)
Cirrhosis and other chronic liver diseases due to hepatitis C	0.2 (0.2 to 0.3)	12.2 (7.0 to 16.8)*	3.6 (-1.2 to 7.8)	0.0 (0.0 to 0.1)	12.2 (7.0 to 16.8)*	3.6 (-1.2 to 7.8)
Cirrhosis and other chronic liver diseases due to alcohol use	0.0 (0.0 to 0.0)	0.0 (0.0 to 0.0)	0.0 (0.0 to 0.0)	0.0 (0.0 to 0.0)	0.0 (0.0 to 0.0)	0.0 (0.0 to 0.0)
Cirrhosis and other chronic liver diseases due to other causes	48.6 (45.4 to 52.0)	11.0 (10.1 to 11.9)*	2.4 (1.6 to 3.3)*	8.4 (5.8 to 11.9)	11.1 (-0.3 to 24.7)	2.6 (-8.0 to 15.1)
Digestive diseases	2,355.8 (2,241.1 to 2,465.6)	-1.6 (-5.4 to 2.1)	-9.2 (-12.7 to -5.8)*	78.2 (52.7 to 113.7)	2.4 (-4.5 to 10.3)	-5.5 (-11.9 to 1.8)
Peptic ulcer disease	0.0 (0.0 to 0.0)	0.0 (0.0 to 0.0)	0.0 (0.0 to 0.0)	0.0 (0.0 to 0.0)	0.0 (0.0 to 0.0)	0.0 (0.0 to 0.0)
Peptic ulcer disease, symptomatic episodes	0.0 (0.0 to 0.0)	0.0 (0.0 to 0.0)	0.0 (0.0 to 0.0)	0.0 (0.0 to 0.0)	0.0 (0.0 to 0.0)	0.0 (0.0 to 0.0)
Anemia due to peptic ulcer disease aggregate	0.0 (0.0 to 0.0)	0.0 (0.0 to 0.0)	0.0 (0.0 to 0.0)	0.0 (0.0 to 0.0)	0.0 (0.0 to 0.0)	0.0 (0.0 to 0.0)
Gastritis and duodenitis	1,069.0 (1,004.3 to 1,133.5)	-0.4 (-6.9 to 6.5)	-8.1 (-14.1 to -1.7)*	40.8 (27.2 to 59.7)	1.2 (-10.2 to 14.1)	-6.6 (-17.1 to 5.3)
Gastritis and duodenitis, symptomatic episodes	21.6 (15.6 to 28.3)	1.2 (-3.6 to 4.7)	-6.6 (-11.0 to -3.4)*	2.4 (1.4 to 3.7)	1.8 (-9.9 to 13.8)	-6.1 (-16.8 to 5.1)
Anemia due to gastritis and duodenitis aggregate	1,047.4 (983.5 to 1,111.2)	-0.4 (-7.1 to 6.6)	-8.1 (-14.3 to -1.6)*	38.4 (25.4 to 56.6)	1.1 (-10.9 to 15.0)	-6.7 (-17.7 to 6.1)
Appendicitis	17.8 (15.1 to 20.7)	11.8 (10.6 to 13.2)*	3.2 (2.1 to 4.5)*	5.7 (3.7 to 8.0)	11.6 (6.4 to 17.3)*	3.0 (-1.8 to 8.3)
Paralytic ileus and intestinal obstruction	12.8 (9.5 to 16.9)	1.9 (-2.9 to 6.6)	-6.0 (-10.4 to -1.6)*	4.1 (2.5 to 6.2)	1.9 (-2.8 to 6.7)	-6.0 (-10.3 to -1.6)*
Inguinal, femoral, and abdominal hernia	1,315.6 (1,020.9 to 1,670.3)	-2.7 (-5.9 to 0.5)	-10.2 (-13.2 to -7.2)*	14.3 (6.7 to 27.7)	-2.6 (-6.3 to 1.0)	-10.1 (-13.5 to -6.8)*
Inflammatory bowel disease	4.7 (3.4 to 6.4)	3.4 (1.8 to 4.8)*	-4.6 (-6.0 to -3.3)*	1.1 (0.6 to 1.7)	3.4 (-1.0 to 7.7)	-4.6 (-8.6 to -0.6)*
Vascular intestinal disorders	0.5 (0.3 to 0.6)	1.4 (-3.5 to 6.5)	-6.4 (-10.9 to -1.7)*	0.1 (0.1 to 0.2)	1.4 (-3.5 to 6.5)	-6.4 (-10.9 to -1.7)*
Gallbladder and biliary diseases	25.1 (18.2 to 34.2)	2.1 (-1.7 to 5.9)	-5.8 (-9.2 to -2.3)*	2.8 (1.7 to 4.3)	2.6 (-10.7 to 17.9)	-5.3 (-17.6 to 8.8)
Pancreatitis	5.9 (4.4 to 7.6)	13.7 (11.0 to 16.3)*	5.0 (2.5 to 7.4)*	1.9 (1.1 to 2.9)	13.6 (3.6 to 25.1)*	4.9 (-4.3 to 15.5)
Other digestive diseases	--	--	--	7.2 (4.7 to 10.8)	10.8 (-1.6 to 26.4)	2.3 (-9.2 to 16.6)
Neurological disorders	1,702.3 (1,568.0 to 1,837.1)	7.9 (2.8 to 13.1)*	-0.4 (-5.1 to 4.4)	518.7 (342.9 to 714.4)	-6.8 (-13.7 to 0.5)	-14.0 (-20.4 to -7.2)*
Alzheimer disease and other dementias	0.0 (0.0 to 0.0)	0.0 (0.0 to 0.0)	0.0 (0.0 to 0.0)	0.0 (0.0 to 0.0)	0.0 (0.0 to 0.0)	0.0 (0.0 to 0.0)
Parkinson disease	0.0 (0.0 to 0.0)	0.0 (0.0 to 0.0)	0.0 (0.0 to 0.0)	0.0 (0.0 to 0.0)	0.0 (0.0 to 0.0)	0.0 (0.0 to 0.0)
Epilepsy	1,718.6 (1,420.9 to 2,041.7)	8.1 (1.7 to 14.5)*	-0.2 (-6.1 to 5.6)	518.0 (342.3 to 713.5)	-6.8 (-13.7 to 0.5)	-14.0 (-20.4 to -7.2)*
Multiple sclerosis	0.0 (0.0 to 0.0)	0.0 (0.0 to 0.0)	0.0 (0.0 to 0.0)	0.0 (0.0 to 0.0)	0.0 (0.0 to 0.0)	0.0 (0.0 to 0.0)
Motor neuron disease	2.4 (2.1 to 2.6)	4.7 (1.7 to 7.6)*	-3.3 (-6.1 to -0.7)*	0.5 (0.4 to 0.7)	4.7 (1.7 to 7.6)*	-3.3 (-6.1 to -0.7)*
Migraine	0.0 (0.0 to 0.0)	0.0 (0.0 to 0.0)	0.0 (0.0 to 0.0)	0.0 (0.0 to 0.0)	0.0 (0.0 to 0.0)	0.0 (0.0 to 0.0)
Tension-type headache	0.0 (0.0 to 0.0)	0.0 (0.0 to 0.0)	0.0 (0.0 to 0.0)	0.0 (0.0 to 0.0)	0.0 (0.0 to 0.0)	0.0 (0.0 to 0.0)
Medication overuse headache	0.0 (0.0 to 0.0)	0.0 (0.0 to 0.0)	0.0 (0.0 to 0.0)	0.0 (0.0 to 0.0)	0.0 (0.0 to 0.0)	0.0 (0.0 to 0.0)
Other neurological disorders	0.8 (0.5 to 1.2)	9.4 (8.6 to 10.0)*	0.9 (0.2 to 1.6)*	0.2 (0.1 to 0.4)	9.4 (8.6 to 10.0)*	0.9 (0.2 to 1.6)*
Mental and substance use disorders	8,999.5 (8,405.5 to 9,784.7)	7.6 (4.3 to 11.1)*	-0.7 (-3.8 to 2.5)	792.8 (531.4 to 1,128.1)	8.5 (7.3 to 9.7)*	0.2 (-1.0 to 1.2)
Schizophrenia	0.0 (0.0 to 0.0)	0.0 (0.0 to 0.0)	0.0 (0.0 to 0.0)	0.0 (0.0 to 0.0)	0.0 (0.0 to 0.0)	0.0 (0.0 to 0.0)
Alcohol use disorders	70.7 (65.6 to 76.3)	7.8 (6.8 to 8.8)*	-0.5 (-1.4 to 0.4)	3.0 (1.9 to 4.5)	7.0 (-1.9 to 17.1)	-1.2 (-9.5 to 8.1)
Alcohol dependence aggregate	0.0 (0.0 to 0.0)	0.0 (0.0 to 0.0)	0.0 (0.0 to 0.0)	0.0 (0.0 to 0.0)	0.0 (0.0 to 0.0)	0.0 (0.0 to 0.0)
Fetal alcohol syndrome aggregate	70.7 (65.6 to 76.3)	7.8 (6.8 to 8.8)*	-0.5 (-1.4 to 0.4)	3.0 (1.9 to 4.5)	7.0 (-1.9 to 17.1)	-1.2 (-9.5 to 8.1)
Drug use disorders	0.0 (0.0 to 0.0)	0.0 (0.0 to 0.0)	0.0 (0.0 to 0.0)	0.0 (0.0 to 0.0)	0.0 (0.0 to 0.0)	0.0 (0.0 to 0.0)
Opioid use disorders	0.0 (0.0 to 0.0)	0.0 (0.0 to 0.0)	0.0 (0.0 to 0.0)	0.0 (0.0 to 0.0)	0.0 (0.0 to 0.0)	0.0 (0.0 to 0.0)
Asymptomatic opioid dependence	0.0 (0.0 to 0.0)	0.0 (0.0 to 0.0)	0.0 (0.0 to 0.0)	0.0 (0.0 to 0.0)	0.0 (0.0 to 0.0)	0.0 (0.0 to 0.0)
Symptomatic opioid dependence aggregate	0.0 (0.0 to 0.0)	0.0 (0.0 to 0.0)	0.0 (0.0 to 0.0)	0.0 (0.0 to 0.0)	0.0 (0.0 to 0.0)	0.0 (0.0 to 0.0)
Cocaine use disorders	0.0 (0.0 to 0.0)	0.0 (0.0 to 0.0)	0.0 (0.0 to 0.0)	0.0 (0.0 to 0.0)	0.0 (0.0 to 0.0)	0.0 (0.0 to 0.0)
Asymptomatic cocaine dependence	0.0 (0.0 to 0.0)	0.0 (0.0 to 0.0)	0.0 (0.0 to 0.0)	0.0 (0.0 to 0.0)	0.0 (0.0 to 0.0)	0.0 (0.0 to 0.0)

Cause	Prevalence in thousands (95% confidence interval)			YLDs in thousands (95% confidence interval)		
	2015	Percent change in counts between 2005 and 2015	Percent change in rates between 2005 and 2015	2015	Percent change in counts between 2005 and 2015	Percent change in rates between 2005 and 2015
Symptomatic cocaine dependence aggregate	0.0 (0.0 to 0.0)	0.0 (0.0 to 0.0)	0.0 (0.0 to 0.0)	0.0 (0.0 to 0.0)	0.0 (0.0 to 0.0)	0.0 (0.0 to 0.0)
Amphetamine use disorders	0.0 (0.0 to 0.0)	0.0 (0.0 to 0.0)	0.0 (0.0 to 0.0)	0.0 (0.0 to 0.0)	0.0 (0.0 to 0.0)	0.0 (0.0 to 0.0)
Asymptomatic amphetamine dependence	0.0 (0.0 to 0.0)	0.0 (0.0 to 0.0)	0.0 (0.0 to 0.0)	0.0 (0.0 to 0.0)	0.0 (0.0 to 0.0)	0.0 (0.0 to 0.0)
Symptomatic amphetamine dependence aggregate	0.0 (0.0 to 0.0)	0.0 (0.0 to 0.0)	0.0 (0.0 to 0.0)	0.0 (0.0 to 0.0)	0.0 (0.0 to 0.0)	0.0 (0.0 to 0.0)
Cannabis use disorders	0.0 (0.0 to 0.0)	0.0 (0.0 to 0.0)	0.0 (0.0 to 0.0)	0.0 (0.0 to 0.0)	0.0 (0.0 to 0.0)	0.0 (0.0 to 0.0)
Asymptomatic cannabis dependence	0.0 (0.0 to 0.0)	0.0 (0.0 to 0.0)	0.0 (0.0 to 0.0)	0.0 (0.0 to 0.0)	0.0 (0.0 to 0.0)	0.0 (0.0 to 0.0)
Symptomatic cannabis dependence aggregate	0.0 (0.0 to 0.0)	0.0 (0.0 to 0.0)	0.0 (0.0 to 0.0)	0.0 (0.0 to 0.0)	0.0 (0.0 to 0.0)	0.0 (0.0 to 0.0)
Other drug use disorders	--	--	--	0.0 (0.0 to 0.0)	0.0 (0.0 to 0.0)	0.0 (0.0 to 0.0)
Depressive disorders	0.0 (0.0 to 0.0)	0.0 (0.0 to 0.0)	0.0 (0.0 to 0.0)	1.6 (0.7 to 3.1)	9.0 (7.8 to 10.6)*	0.6 (-0.5 to 2.1)
Major depressive disorder	5.8 (2.0 to 12.2)	9.2 (7.7 to 11.0)*	0.7 (-0.6 to 2.5)	1.3 (0.4 to 2.7)	9.2 (7.7 to 11.0)*	0.7 (-0.6 to 2.5)
Dysthymia	3.2 (1.2 to 6.8)	8.5 (5.3 to 11.0)*	0.2 (-2.8 to 2.4)	0.3 (0.1 to 0.8)	8.6 (5.3 to 11.0)*	0.2 (-2.8 to 2.4)
Bipolar disorder	0.0 (0.0 to 0.0)	0.0 (0.0 to 0.0)	0.0 (0.0 to 0.0)	0.0 (0.0 to 0.0)	0.0 (0.0 to 0.0)	0.0 (0.0 to 0.0)
Anxiety disorders	666.4 (508.2 to 866.9)	10.1 (7.4 to 13.3)*	1.6 (-0.9 to 4.5)	63.9 (39.7 to 93.3)	10.1 (3.6 to 16.9)*	1.7 (-4.4 to 7.9)
Eating disorders	0.0 (0.0 to 0.0)	0.0 (0.0 to 0.0)	0.0 (0.0 to 0.0)	0.0 (0.0 to 0.0)	0.0 (0.0 to 0.0)	0.0 (0.0 to 0.0)
Anorexia nervosa	0.0 (0.0 to 0.0)	0.0 (0.0 to 0.0)	0.0 (0.0 to 0.0)	0.0 (0.0 to 0.0)	0.0 (0.0 to 0.0)	0.0 (0.0 to 0.0)
Bulimia nervosa	0.0 (0.0 to 0.0)	0.0 (0.0 to 0.0)	0.0 (0.0 to 0.0)	0.0 (0.0 to 0.0)	0.0 (0.0 to 0.0)	0.0 (0.0 to 0.0)
Autistic spectrum disorders	3,432.8 (3,230.2 to 3,672.6)	8.2 (5.4 to 11.1)*	-0.1 (-2.7 to 2.6)	567.7 (373.9 to 812.6)	8.6 (7.1 to 10.1)*	0.3 (-1.1 to 1.7)
Autism	1,328.4 (1,035.7 to 1,660.3)	8.3 (8.0 to 8.6)*	-0.0 (-0.3 to 0.3)	350.2 (214.7 to 517.1)	8.7 (6.4 to 10.8)*	0.3 (-1.8 to 2.3)
Asperger syndrome and other autistic spectrum disorders	2,134.4 (1,600.5 to 2,777.3)	8.3 (8.1 to 8.6)*	-0.0 (-0.2 to 0.2)	217.4 (133.8 to 332.4)	8.5 (6.8 to 10.3)*	0.2 (-1.4 to 1.8)
Attention-deficit/hyperactivity disorder	928.8 (815.3 to 1,079.9)	8.7 (8.1 to 9.4)*	0.3 (-0.2 to 1.0)	11.3 (6.6 to 17.6)	8.8 (4.1 to 13.8)*	0.4 (-3.9 to 5.0)
Conduct disorder	0.0 (0.0 to 0.0)	0.0 (0.0 to 0.0)	0.0 (0.0 to 0.0)	0.0 (0.0 to 0.0)	0.0 (0.0 to 0.0)	0.0 (0.0 to 0.0)
Idiopathic developmental intellectual disability	3,949.0 (2,024.0 to 5,788.0)	6.5 (4.6 to 7.9)*	-1.7 (-3.4 to -0.4)*	145.3 (56.0 to 265.4)	7.4 (5.1 to 9.3)*	-0.9 (-3.0 to 0.9)
Other mental and substance use disorders	0.0 (0.0 to 0.0)	0.0 (0.0 to 0.0)	0.0 (0.0 to 0.0)	0.0 (0.0 to 0.0)	0.0 (0.0 to 0.0)	0.0 (0.0 to 0.0)
Diabetes, urogenital, blood, and endocrine diseases	133,719.2 (131,610.7 to 136,169.9)	5.2 (3.8 to 6.7)*	-2.9 (-4.2 to -1.5)*	1,077.1 (719.0 to 1,544.0)	-1.3 (-7.2 to 4.9)	-8.9 (-14.3 to -3.2)*
Diabetes mellitus	75.5 (59.5 to 92.4)	2.5 (-4.0 to 8.4)	-5.4 (-11.4 to 0.1)	3.6 (2.2 to 5.6)	2.7 (-7.4 to 13.7)	-5.2 (-14.5 to 4.9)
Uncomplicated diabetes mellitus	75.5 (59.5 to 92.4)	2.5 (-4.0 to 8.4)	-5.4 (-11.4 to 0.1)	3.6 (2.2 to 5.6)	2.7 (-7.4 to 13.7)	-5.2 (-14.5 to 4.9)
Neuropathy and other complications of diabetes mellitus aggregate	0.0 (0.0 to 0.0)	1.9 (-4.8 to 8.9)	-6.0 (-12.1 to 0.5)	0.0 (0.0 to 0.0)	1.9 (-4.8 to 8.9)	-6.0 (-12.1 to 0.5)
Vision loss due to diabetes mellitus aggregate	0.0 (0.0 to 0.0)	0.0 (0.0 to 0.0)	0.0 (0.0 to 0.0)	0.0 (0.0 to 0.0)	0.0 (0.0 to 0.0)	0.0 (0.0 to 0.0)
Acute glomerulonephritis	5.1 (4.0 to 6.3)	-8.9 (-13.3 to -4.7)*	-15.9 (-20.0 to -12.1)*	0.3 (0.2 to 0.4)	-8.8 (-13.5 to -4.7)*	-15.8 (-20.1 to -12.0)*
Chronic kidney disease	167.3 (140.5 to 185.0)	7.0 (-0.6 to 14.8)	-1.3 (-8.2 to 6.0)	13.9 (9.4 to 19.1)	7.5 (6.6 to 8.4)*	-0.8 (-1.6 to 0.1)
Chronic kidney disease due to diabetes mellitus	53.3 (33.0 to 82.0)	7.3 (4.7 to 10.0)*	-1.0 (-3.3 to 1.5)	3.3 (1.9 to 5.0)	7.1 (4.8 to 9.5)*	-1.2 (-3.3 to 1.1)
Stage III chronic kidney disease due to diabetes mellitus aggregate	44.3 (25.9 to 70.3)	7.3 (4.8 to 10.1)*	-0.9 (-3.3 to 1.6)	0.3 (0.2 to 0.4)	9.2 (5.4 to 13.2)*	0.8 (-2.7 to 4.5)
Stage IV chronic kidney disease due to diabetes mellitus aggregate	4.7 (2.4 to 8.0)	7.8 (5.1 to 10.6)*	-0.5 (-3.0 to 2.1)	0.8 (0.4 to 1.3)	7.7 (4.9 to 10.7)*	-0.6 (-3.2 to 2.2)
Stage V chronic kidney disease untreated due to diabetes mellitus	3.6 (2.2 to 5.5)	6.9 (4.3 to 9.6)*	-1.3 (-3.8 to 1.1)	2.0 (1.1 to 3.5)	6.9 (4.3 to 9.6)*	-1.3 (-3.8 to 1.1)
End-stage chronic kidney disease due to diabetes aggregate	0.8 (0.5 to 1.2)	1.4 (-2.5 to 5.3)	-6.4 (-10.0 to -2.8)*	0.2 (0.1 to 0.3)	3.7 (-0.1 to 7.2)	-4.3 (-7.8 to -1.1)*
Chronic kidney disease due to hypertension	48.4 (27.4 to 79.7)	7.2 (4.5 to 10.2)*	-1.0 (-3.6 to 1.7)	0.4 (0.3 to 0.7)	7.9 (3.6 to 12.1)*	-0.5 (-4.4 to 3.4)
Stage III chronic kidney disease due to hypertension aggregate	46.9 (26.1 to 78.6)	7.2 (4.4 to 10.1)*	-1.1 (-3.6 to 1.6)	0.0 (0.0 to 0.0)	8.9 (5.3 to 12.8)*	0.5 (-2.9 to 4.1)
Stage IV chronic kidney disease due to hypertension aggregate	1.0 (0.3 to 2.8)	10.6 (3.0 to 20.6)*	2.1 (-4.9 to 11.3)	0.1 (0.0 to 0.4)	9.9 (3.6 to 17.8)*	1.5 (-4.4 to 8.7)
Stage V chronic kidney disease untreated due to hypertension	0.4 (0.3 to 0.7)	7.1 (1.9 to 11.9)*	-1.2 (-5.9 to 3.3)	0.3 (0.1 to 0.4)	7.1 (1.9 to 11.9)*	-1.2 (-5.9 to 3.3)
End-stage chronic kidney disease due to hypertension aggregate	0.1 (0.1 to 0.1)	1.2 (-6.0 to 8.6)	-6.6 (-13.2 to 0.2)	0.0 (0.0 to 0.0)	3.1 (-3.1 to 10.1)	-4.8 (-10.5 to 1.6)
Chronic kidney disease due to glomerulonephritis	19.4 (15.3 to 23.8)	7.0 (5.1 to 8.9)*	-1.3 (-3.0 to 0.5)	3.7 (2.2 to 5.4)	6.6 (4.6 to 8.5)*	-1.6 (-3.4 to 0.2)
Stage III chronic kidney disease due to glomerulonephritis aggregate	9.4 (7.1 to 12.6)	7.5 (4.4 to 10.6)*	-0.8 (-3.6 to 2.0)	0.3 (0.2 to 0.4)	6.6 (3.5 to 9.9)*	-1.6 (-4.5 to 1.5)
Stage IV chronic kidney disease due to glomerulonephritis aggregate	4.9 (2.8 to 6.5)	7.4 (5.2 to 9.8)*	-0.9 (-2.9 to 1.3)	0.8 (0.4 to 1.3)	7.4 (5.1 to 10.0)*	-0.9 (-3.0 to 1.6)
Stage V chronic kidney disease untreated due to glomerulonephritis	4.1 (2.6 to 6.3)	6.6 (4.0 to 8.8)*	-1.6 (-4.0 to 0.4)	2.3 (1.3 to 3.6)	6.6 (4.0 to 8.8)*	-1.6 (-4.0 to 0.4)
End-stage chronic kidney disease due to glomerulonephritis aggregate	1.0 (0.6 to 1.4)	1.2 (-3.0 to 5.2)	-6.6 (-10.4 to -2.9)*	0.2 (0.1 to 0.4)	3.6 (-2.5 to 8.3)	-4.4 (-10.0 to -0.1)*
Chronic kidney disease due to other causes	107.3 (71.7 to 153.6)	8.4 (7.0 to 9.7)*	0.0 (-1.2 to 1.3)	6.5 (4.1 to 9.4)	8.2 (6.9 to 9.5)*	-0.1 (-1.4 to 1.1)
Stage III chronic kidney disease due to other causes aggregate	89.1 (56.1 to 133.0)	8.5 (7.0 to 9.9)*	0.1 (-1.3 to 1.5)	0.5 (0.3 to 0.7)	9.2 (5.5 to 13.3)*	0.8 (-2.6 to 4.6)
Stage IV chronic kidney disease due to other causes aggregate	9.4 (5.5 to 14.4)	9.0 (7.4 to 10.8)*	0.6 (-0.9 to 2.2)	1.5 (0.8 to 2.6)	8.8 (7.1 to 10.8)*	0.5 (-1.1 to 2.3)
Stage V chronic kidney disease untreated due to other causes	7.2 (4.6 to 10.2)	8.0 (6.5 to 9.5)*	-0.3 (-1.7 to 1.0)	4.1 (2.4 to 6.3)	8.0 (6.5 to 9.5)*	-0.3 (-1.7 to 1.0)
End-stage chronic kidney disease due to other causes aggregate	1.6 (1.1 to 2.2)	3.0 (0.3 to 5.7)*	-5.0 (-7.4 to -2.4)*	0.4 (0.2 to 0.6)	6.3 (1.0 to 10.1)*	-1.9 (-6.7 to 1.6)
Urinary diseases and male infertility	657.5 (630.5 to 686.1)	8.6 (4.6 to 12.6)*	0.2 (-3.5 to 3.9)	12.9 (8.1 to 19.6)	13.5 (7.0 to 20.0)*	4.8 (-1.2 to 10.7)
Interstitial nephritis and urinary tract infections	274.3 (258.6 to 292.2)	14.7 (14.1 to 15.3)*	5.8 (5.3 to 6.4)*	9.4 (5.9 to 14.2)	14.8 (8.9 to 21.1)*	6.0 (0.5 to 11.8)*
Urolithiasis	392.4 (324.4 to 464.4)	4.9 (0.9 to 9.1)*	-3.2 (-6.9 to 0.7)	0.3 (0.2 to 0.5)	7.6 (4.2 to 10.8)*	-0.7 (-3.8 to 2.3)
Benign prostatic hyperplasia	0.0 (0.0 to 0.0)	0.0 (0.0 to 0.0)	0.0 (0.0 to 0.0)	0.0 (0.0 to 0.0)	0.0 (0.0 to 0.0)	0.0 (0.0 to 0.0)
Male infertility	0.0 (0.0 to 0.0)	0.0 (0.0 to 0.0)	0.0 (0.0 to 0.0)	0.0 (0.0 to 0.0)	0.0 (0.0 to 0.0)	0.0 (0.0 to 0.0)
Other urinary diseases	--	--	--	3.2 (2.0 to 5.0)	10.4 (-0.2 to 22.0)	1.9 (-7.9 to 12.6)
Gynecological diseases	0.0 (0.0 to 0.0)	0.0 (0.0 to 0.0)	0.0 (0.0 to 0.0)	0.0 (0.0 to 0.0)	0.0 (0.0 to 0.0)	0.0 (0.0 to 0.0)
Uterine fibroids	0.0 (0.0 to 0.0)	0.0 (0.0 to 0.0)	0.0 (0.0 to 0.0)	0.0 (0.0 to 0.0)	0.0 (0.0 to 0.0)	0.0 (0.0 to 0.0)
Uterine fibroids cases aggregate	0.0 (0.0 to 0.0)	0.0 (0.0 to 0.0)	0.0 (0.0 to 0.0)	0.0 (0.0 to 0.0)	0.0 (0.0 to 0.0)	0.0 (0.0 to 0.0)
Mild abdominal pain with anemia due to uterine fibroids aggregate	0.0 (0.0 to 0.0)	0.0 (0.0 to 0.0)	0.0 (0.0 to 0.0)	0.0 (0.0 to 0.0)	0.0 (0.0 to 0.0)	0.0 (0.0 to 0.0)
Polycystic ovarian syndrome	0.0 (0.0 to 0.0)	0.0 (0.0 to 0.0)	0.0 (0.0 to 0.0)	0.0 (0.0 to 0.0)	0.0 (0.0 to 0.0)	0.0 (0.0 to 0.0)
Polycystic ovarian syndrome cases aggregate	0.0 (0.0 to 0.0)	0.0 (0.0 to 0.0)	0.0 (0.0 to 0.0)	0.0 (0.0 to 0.0)	0.0 (0.0 to 0.0)	0.0 (0.0 to 0.0)
Hirsutism and infertility due to polycystic ovarian syndrome aggregate	0.0 (0.0 to 0.0)	0.0 (0.0 to 0.0)	0.0 (0.0 to 0.0)	0.0 (0.0 to 0.0)	0.0 (0.0 to 0.0)	0.0 (0.0 to 0.0)

Cause	Prevalence in thousands (95% confidence interval)			YLDs in thousands (95% confidence interval)		
	2015	Percent change in counts between 2005 and 2015	Percent change in rates between 2005 and 2015	2015	Percent change in counts between 2005 and 2015	Percent change in rates between 2005 and 2015
Female infertility	0.0 (0.0 to 0.0)	0.0 (0.0 to 0.0)	0.0 (0.0 to 0.0)	0.0 (0.0 to 0.0)	0.0 (0.0 to 0.0)	0.0 (0.0 to 0.0)
Endometriosis	0.0 (0.0 to 0.0)	0.0 (0.0 to 0.0)	0.0 (0.0 to 0.0)	0.0 (0.0 to 0.0)	0.0 (0.0 to 0.0)	0.0 (0.0 to 0.0)
Endometriosis cases aggregate	0.0 (0.0 to 0.0)	0.0 (0.0 to 0.0)	0.0 (0.0 to 0.0)	0.0 (0.0 to 0.0)	0.0 (0.0 to 0.0)	0.0 (0.0 to 0.0)
Abdominal pain and infertility due to endometriosis aggregate	0.0 (0.0 to 0.0)	0.0 (0.0 to 0.0)	0.0 (0.0 to 0.0)	0.0 (0.0 to 0.0)	0.0 (0.0 to 0.0)	0.0 (0.0 to 0.0)
Genital prolapse	0.0 (0.0 to 0.0)	0.0 (0.0 to 0.0)	0.0 (0.0 to 0.0)	0.0 (0.0 to 0.0)	0.0 (0.0 to 0.0)	0.0 (0.0 to 0.0)
Premenstrual syndrome	0.0 (0.0 to 0.0)	0.0 (0.0 to 0.0)	0.0 (0.0 to 0.0)	0.0 (0.0 to 0.0)	0.0 (0.0 to 0.0)	0.0 (0.0 to 0.0)
Other gynecological diseases	0.0 (0.0 to 0.0)	0.0 (0.0 to 0.0)	0.0 (0.0 to 0.0)	0.0 (0.0 to 0.0)	0.0 (0.0 to 0.0)	0.0 (0.0 to 0.0)
Other gynecological diseases cases aggregate	0.0 (0.0 to 0.0)	0.0 (0.0 to 0.0)	0.0 (0.0 to 0.0)	0.0 (0.0 to 0.0)	0.0 (0.0 to 0.0)	0.0 (0.0 to 0.0)
Anemia due to other gynecological diseases aggregate	0.0 (0.0 to 0.0)	0.0 (0.0 to 0.0)	0.0 (0.0 to 0.0)	0.0 (0.0 to 0.0)	0.0 (0.0 to 0.0)	0.0 (0.0 to 0.0)
Hemoglobinopathies and hemolytic anemias	126,811.1 (124,621.5 to 129,308.7)	5.3 (3.8 to 6.9)*	-2.8 (-4.2 to -1.3)*	739.2 (488.2 to 1,071.9)	-2.2 (-8.9 to 4.7)	-9.7 (-15.9 to -3.3)*
Thalassemias	199.9 (183.2 to 229.6)	1.0 (-0.7 to 3.4)	-6.8 (-8.3 to -4.5)*	11.3 (7.6 to 16.5)	-1.9 (-11.8 to 8.2)	-9.5 (-18.6 to -0.1)*
Beta-thalassemia major cases aggregate	105.5 (102.1 to 109.6)	2.7 (1.9 to 3.5)*	-5.2 (-6.0 to -4.5)*	5.9 (3.9 to 8.6)	1.2 (-12.8 to 16.8)	-6.6 (-19.5 to 7.8)
Hemoglobin E/beta-thalassemia cases aggregate	30.8 (27.0 to 34.9)	5.1 (2.2 to 8.1)*	-3.0 (-5.7 to -0.2)*	1.9 (1.2 to 2.9)	-3.1 (-27.9 to 26.0)	-10.6 (-33.5 to 16.3)
Hemoglobin H disease cases aggregate	63.3 (47.6 to 92.8)	-3.6 (-8.8 to 3.5)	-11.0 (-15.9 to -4.5)*	3.5 (2.1 to 5.3)	-6.4 (-19.7 to 11.1)	-13.6 (-25.9 to 2.5)
Heart failure due to thalassemias aggregate	0.3 (0.3 to 0.4)	7.7 (5.2 to 10.1)*	-0.6 (-2.9 to 1.6)	0.0 (0.0 to 0.1)	7.7 (5.2 to 10.1)*	-0.6 (-2.9 to 1.6)
Thalassemias trait	18,368.1 (17,851.7 to 19,001.5)	6.8 (6.2 to 7.3)*	-1.4 (-1.9 to -1.0)*	231.5 (150.3 to 340.9)	2.9 (-6.8 to 12.8)	-5.0 (-14.0 to 4.1)
B-thalassemia trait cases aggregate	14,884.4 (14,403.0 to 15,443.0)	7.4 (6.8 to 7.9)*	-0.9 (-1.4 to -0.4)*	215.0 (139.6 to 316.9)	3.5 (-6.2 to 13.8)	-4.5 (-13.4 to 5.0)
Hemoglobin E trait cases aggregate	3,483.7 (3,318.4 to 3,658.9)	4.2 (3.1 to 5.5)*	-3.8 (-4.9 to -2.6)*	16.5 (10.6 to 24.4)	-3.9 (-18.2 to 10.7)	-11.3 (-24.5 to 2.2)
Sickle cell disorders	1,320.6 (1,197.7 to 1,425.3)	3.9 (-4.4 to 13.6)	-4.1 (-11.8 to 4.9)	80.4 (52.8 to 122.0)	-5.4 (-19.8 to 11.0)	-12.7 (-26.0 to 2.4)
Homozygous sickle cell and severe sickle cell/beta-thalassemia cases aggregate	1,156.9 (1,034.9 to 1,258.9)	1.0 (-8.3 to 11.9)	-6.7 (-15.4 to 3.3)	71.0 (46.2 to 109.0)	-7.6 (-22.3 to 10.1)	-14.8 (-28.3 to 1.7)
Hemoglobin SC disease cases aggregate	156.9 (140.4 to 170.6)	30.0 (12.0 to 53.2)*	19.9 (3.4 to 41.4)*	8.8 (5.6 to 13.2)	15.9 (-9.8 to 37.5)	7.0 (-16.7 to 26.9)
Mild sickle cell/beta-thalassemia cases aggregate	6.8 (6.1 to 7.5)	25.3 (13.7 to 37.6)*	15.6 (5.0 to 27.0)*	0.6 (0.4 to 0.9)	20.9 (5.9 to 36.9)*	11.6 (-2.3 to 26.4)
Sickle cell trait	39,032.7 (37,032.7 to 42,407.5)	10.4 (8.4 to 11.8)*	1.9 (0.0 to 3.2)*	177.9 (120.8 to 257.5)	-1.5 (-11.9 to 8.5)	-9.0 (-18.7 to 0.1)
G6PD deficiency	21,122.7 (17,833.5 to 24,576.0)	0.3 (-5.6 to 5.4)	-7.4 (-12.8 to -2.7)*	2.6 (1.7 to 3.8)	-3.5 (-17.0 to 10.8)	-11.0 (-23.4 to 2.2)
G6PD cases aggregate	21,122.7 (17,833.5 to 24,576.0)	0.3 (-5.6 to 5.4)	-7.4 (-12.8 to -2.7)*	2.6 (1.7 to 3.8)	-3.5 (-17.0 to 10.8)	-11.0 (-23.4 to 2.2)
Heart failure due to G6PD deficiency aggregate	0.0 (0.0 to 0.0)	0.0 (0.0 to 0.0)	0.0 (0.0 to 0.0)	0.0 (0.0 to 0.0)	0.0 (0.0 to 0.0)	0.0 (0.0 to 0.0)
G6PD trait	56,108.1 (52,249.0 to 60,054.9)	3.9 (2.1 to 5.6)*	-4.1 (-5.8 to -2.6)*	2.3 (1.5 to 3.2)	4.2 (-10.7 to 21.9)	-3.9 (-17.6 to 12.5)
Other hemoglobinopathies and hemolytic anemias	6,386.5 (6,182.1 to 6,600.8)	1.0 (-2.9 to 4.8)	-6.8 (-10.4 to -3.3)*	233.1 (155.4 to 334.7)	-6.2 (-12.2 to 0.3)	-13.4 (-18.9 to -7.5)*
Other hemoglobinopathies and hemolytic anemias cases aggregate	6,386.5 (6,182.1 to 6,600.8)	1.0 (-2.9 to 4.8)	-6.8 (-10.4 to -3.3)*	225.8 (150.7 to 325.5)	-6.2 (-12.3 to 0.3)	-13.4 (-19.1 to -7.4)*
Heart failure due to other hemoglobinopathies and hemolytic anemias aggregate	0.0 (0.0 to 0.0)	10.1 (8.0 to 12.1)*	1.6 (-0.3 to 3.4)	0.0 (0.0 to 0.0)	10.1 (8.0 to 12.1)*	1.6 (-0.3 to 3.4)
Endocrine, metabolic, blood, and immune disorders	8,521.0 (8,241.5 to 8,811.6)	1.8 (-3.6 to 7.8)	-6.0 (-11.1 to -0.5)*	307.2 (204.9 to 436.5)	-0.2 (-7.4 to 7.9)	-7.9 (-14.5 to -0.4)*
Endocrine, metabolic, blood, and immune disorders cases aggregate	427.7 (388.4 to 466.1)	17.1 (15.8 to 18.7)*	8.1 (6.9 to 9.6)*	17.3 (11.5 to 24.3)	17.3 (11.2 to 23.1)*	8.3 (2.6 to 13.6)*
Anemia due to endocrine, metabolic, blood, and immune disorders aggregate	8,093.2 (7,822.3 to 8,383.4)	1.1 (-4.5 to 7.3)	-6.7 (-11.9 to -1.0)*	289.9 (191.9 to 414.2)	-1.1 (-8.6 to 7.6)	-8.7 (-15.6 to -0.7)*
Heart failure due to endocrine, metabolic, blood, and immune disorders aggregate	0.1 (0.1 to 0.2)	6.1 (5.1 to 7.1)*	-2.1 (-3.0 to -1.1)*	0.0 (0.0 to 0.0)	6.1 (5.1 to 7.1)*	-2.1 (-3.0 to -1.1)*
Musculoskeletal disorders	0.0 (0.0 to 0.0)	0.0 (0.0 to 0.0)	0.0 (0.0 to 0.0)	0.0 (0.0 to 0.0)	0.0 (0.0 to 0.0)	0.0 (0.0 to 0.0)
Rheumatoid arthritis	0.0 (0.0 to 0.0)	0.0 (0.0 to 0.0)	0.0 (0.0 to 0.0)	0.0 (0.0 to 0.0)	0.0 (0.0 to 0.0)	0.0 (0.0 to 0.0)
Osteoarthritis	0.0 (0.0 to 0.0)	0.0 (0.0 to 0.0)	0.0 (0.0 to 0.0)	0.0 (0.0 to 0.0)	0.0 (0.0 to 0.0)	0.0 (0.0 to 0.0)
Osteoarthritis of the hip cases aggregate	0.0 (0.0 to 0.0)	0.0 (0.0 to 0.0)	0.0 (0.0 to 0.0)	0.0 (0.0 to 0.0)	0.0 (0.0 to 0.0)	0.0 (0.0 to 0.0)
Osteoarthritis of the knee cases aggregate	0.0 (0.0 to 0.0)	0.0 (0.0 to 0.0)	0.0 (0.0 to 0.0)	0.0 (0.0 to 0.0)	0.0 (0.0 to 0.0)	0.0 (0.0 to 0.0)
Low back and neck pain	0.0 (0.0 to 0.0)	0.0 (0.0 to 0.0)	0.0 (0.0 to 0.0)	0.0 (0.0 to 0.0)	0.0 (0.0 to 0.0)	0.0 (0.0 to 0.0)
Low back pain	0.0 (0.0 to 0.0)	0.0 (0.0 to 0.0)	0.0 (0.0 to 0.0)	0.0 (0.0 to 0.0)	0.0 (0.0 to 0.0)	0.0 (0.0 to 0.0)
Neck pain	0.0 (0.0 to 0.0)	0.0 (0.0 to 0.0)	0.0 (0.0 to 0.0)	0.0 (0.0 to 0.0)	0.0 (0.0 to 0.0)	0.0 (0.0 to 0.0)
Gout	0.0 (0.0 to 0.0)	0.0 (0.0 to 0.0)	0.0 (0.0 to 0.0)	0.0 (0.0 to 0.0)	0.0 (0.0 to 0.0)	0.0 (0.0 to 0.0)
Asymptomatic gout	0.0 (0.0 to 0.0)	0.0 (0.0 to 0.0)	0.0 (0.0 to 0.0)	0.0 (0.0 to 0.0)	0.0 (0.0 to 0.0)	0.0 (0.0 to 0.0)
Symptomatic gout cases aggregate	0.0 (0.0 to 0.0)	0.0 (0.0 to 0.0)	0.0 (0.0 to 0.0)	0.0 (0.0 to 0.0)	0.0 (0.0 to 0.0)	0.0 (0.0 to 0.0)
Other musculoskeletal disorders	0.0 (0.0 to 0.0)	0.0 (0.0 to 0.0)	0.0 (0.0 to 0.0)	0.0 (0.0 to 0.0)	0.0 (0.0 to 0.0)	0.0 (0.0 to 0.0)
Other non-communicable diseases	343,574.1 (332,058.3 to 355,231.0)	9.1 (7.6 to 10.5)*	0.7 (-0.7 to 2.0)	5,536.3 (3,855.3 to 7,670.3)	10.2 (8.9 to 12.0)*	1.7 (0.5 to 3.4)*
Congenital anomalies	7,283.3 (6,657.9 to 7,843.4)	17.4 (13.5 to 21.7)*	8.4 (4.7 to 12.3)*	683.6 (429.4 to 1,021.8)	23.5 (14.9 to 32.7)*	14.0 (6.0 to 22.5)*
Neural tube defects	149.5 (100.3 to 218.1)	17.7 (13.4 to 21.3)*	8.7 (4.6 to 12.0)*	52.5 (29.6 to 87.6)	19.4 (11.1 to 27.9)*	10.2 (2.5 to 18.0)*
Congenital heart anomalies	3,421.5 (2,385.0 to 5,174.2)	25.3 (21.9 to 29.1)*	15.7 (12.5 to 19.2)*	122.2 (49.4 to 225.1)	25.6 (21.6 to 29.4)*	15.9 (12.2 to 19.4)*
Less severe heart anomalies cases aggregate	3,118.6 (2,035.2 to 4,883.2)	24.6 (21.1 to 28.8)*	15.0 (11.7 to 18.9)*	102.1 (38.0 to 198.7)	24.7 (20.4 to 29.2)*	15.1 (11.1 to 19.2)*
Severe congenital heart anomalies	256.1 (169.0 to 384.3)	35.2 (28.6 to 41.2)*	24.7 (18.7 to 30.3)*	15.4 (6.0 to 30.6)	35.2 (27.1 to 42.2)*	24.7 (17.4 to 31.3)*
Critical congenital heart anomalies	16.4 (9.4 to 26.9)	52.8 (35.3 to 66.8)*	41.1 (24.9 to 54.0)*	1.0 (0.3 to 2.1)	52.7 (28.7 to 74.4)*	40.9 (18.8 to 60.9)*
Heart failure due to congenital heart anomalies aggregate	30.4 (26.9 to 34.0)	10.0 (8.7 to 11.3)*	1.5 (0.3 to 2.7)*	3.7 (2.5 to 5.4)	10.1 (0.3 to 21.3)*	1.6 (-7.4 to 12.0)
Cleft lip and cleft palate	515.6 (290.2 to 874.9)	15.7 (7.0 to 21.2)*	6.8 (-1.2 to 11.9)	6.7 (3.2 to 12.6)	9.5 (-1.2 to 18.5)	1.1 (-8.8 to 9.4)
Down syndrome	466.8 (294.8 to 721.0)	11.7 (8.9 to 14.5)*	3.1 (0.5 to 5.7)*	39.5 (21.6 to 66.3)	11.9 (7.1 to 16.2)*	3.2 (-1.2 to 7.3)
Turner syndrome	29.4 (13.8 to 53.7)	8.3 (6.3 to 10.4)*	-0.1 (-1.9 to 1.9)	0.4 (0.1 to 0.9)	8.3 (4.1 to 12.6)*	-0.0 (-3.9 to 4.0)
Klinefelter syndrome	17.1 (8.9 to 30.7)	7.4 (5.7 to 8.6)*	-0.9 (-2.4 to 0.3)	0.0 (0.0 to 0.1)	7.4 (5.7 to 8.6)*	-0.9 (-2.4 to 0.3)
Other chromosomal abnormalities	395.2 (217.7 to 704.6)	13.0 (10.1 to 15.5)*	4.3 (1.7 to 6.6)*	33.5 (16.7 to 63.5)	13.1 (8.2 to 17.3)*	4.4 (-0.1 to 8.3)
Other congenital anomalies	2,578.9 (1,421.3 to 4,700.6)	9.4 (7.6 to 11.1)*	0.9 (-0.7 to 2.5)	428.8 (250.8 to 674.4)	25.8 (13.3 to 41.3)*	16.1 (4.6 to 30.4)*
Skin and subcutaneous diseases	135,010.3 (130,733.4 to 138,914.9)	8.9 (7.5 to 10.4)*	0.5 (-0.7 to 1.9)	3,706.7 (2,430.6 to 5,291.0)	8.6 (7.6 to 9.5)*	0.2 (-0.7 to 1.1)
Dermatitis	17,295.7 (14,217.5 to 20,501.7)	11.0 (10.6 to 11.4)*	2.5 (2.1 to 2.8)*	1,012.0 (670.2 to 1,462.3)	11.5 (9.9 to 13.0)*	2.9 (1.5 to 4.3)*
Eczema cases aggregate	15,841.7 (12,758.2 to 19,107.1)	11.4 (11.0 to 11.8)*	2.9 (2.5 to 3.2)*	989.9 (654.6 to 1,438.9)	11.6 (10.0 to 13.2)*	3.0 (1.6 to 4.5)*

Cause	Prevalence in thousands (95% confidence interval)			YLDs in thousands (95% confidence interval)		
	2015	Percent change in counts between 2005 and 2015	Percent change in rates between 2005 and 2015	2015	Percent change in counts between 2005 and 2015	Percent change in rates between 2005 and 2015
Contact dermatitis cases aggregate	0.0<br>(0.0 to 0.0)	0.0<br>(0.0 to 0.0)	0.0<br>(0.0 to 0.0)	0.0<br>(0.0 to 0.0)	0.0<br>(0.0 to 0.0)	0.0<br>(0.0 to 0.0)
Seborrhoeic dermatitis cases aggregate	1,453.9<br>(1,104.1 to 1,854.7)	6.4<br>(6.0 to 6.8)*	-1.8<br>(-2.1 to -1.4)*	22.1<br>(11.9 to 37.2)	6.6<br>(3.6 to 9.3)*	-1.7<br>(-4.4 to 0.9)
Psoriasis	1,818.1<br>(1,659.4 to 1,986.6)	8.0<br>(6.4 to 9.7)*	-0.3<br>(-1.8 to 1.3)	153.6<br>(106.4 to 213.7)	8.3<br>(3.7 to 13.0)*	-0.1<br>(-4.3 to 4.3)
Cellulitis	74.1<br>(55.8 to 98.0)	17.7<br>(14.2 to 21.3)*	8.6<br>(5.4 to 11.9)*	5.5<br>(3.2 to 8.8)	17.6<br>(8.4 to 27.2)*	8.6<br>(0.0 to 17.4)*
Pyoderma	540.5<br>(477.5 to 605.6)	11.8<br>(10.0 to 13.9)*	3.2<br>(1.5 to 5.1)*	3.1<br>(1.2 to 6.6)	11.9<br>(7.3 to 16.3)*	3.2<br>(-1.0 to 7.4)
Scabies	23,753.4<br>(16,179.6 to 32,580.1)	4.4<br>(0.9 to 8.2)*	-3.6<br>(-6.8 to -0.1)*	621.6<br>(317.1 to 1,092.6)	4.6<br>(0.9 to 8.5)*	-3.5<br>(-6.9 to 0.2)
Fungal skin diseases	50,951.0<br>(39,026.5 to 64,393.0)	10.1<br>(9.3 to 10.9)*	1.6<br>(0.9 to 2.3)*	292.0<br>(110.7 to 624.7)	10.2<br>(9.4 to 11.1)*	1.7<br>(0.9 to 2.5)*
Viral skin diseases	15,252.5<br>(13,239.9 to 17,578.4)	7.0<br>(6.6 to 7.5)*	-1.2<br>(-1.7 to -0.8)*	478.7<br>(288.4 to 712.9)	7.2<br>(6.1 to 8.2)*	-1.1<br>(-2.0 to -0.1)*
Molluscum contagiosum cases aggregate	11,294.2<br>(9,348.3 to 13,575.9)	6.9<br>(6.4 to 7.6)*	-1.3<br>(-1.8 to -0.7)*	354.4<br>(212.8 to 530.1)	7.1<br>(5.8 to 8.4)*	-1.1<br>(-2.4 to 0.0)
Viral warts cases aggregate	3,958.2<br>(3,356.6 to 4,715.8)	7.2<br>(6.6 to 7.8)*	-1.1<br>(-1.6 to -0.5)*	124.3<br>(75.9 to 187.9)	7.3<br>(5.3 to 9.3)*	-0.9<br>(-2.8 to 0.9)
Acne vulgaris	0.0<br>(0.0 to 0.0)	0.0<br>(0.0 to 0.0)	0.0<br>(0.0 to 0.0)	0.0<br>(0.0 to 0.0)	0.0<br>(0.0 to 0.0)	0.0<br>(0.0 to 0.0)
Alopecia areata	434.7<br>(373.2 to 499.7)	8.1<br>(7.7 to 8.5)*	-0.2<br>(-0.6 to 0.1)	15.0<br>(9.2 to 22.7)	8.5<br>(3.0 to 14.2)*	0.1<br>(-4.9 to 5.4)
Pruritus	3,515.8<br>(2,622.0 to 4,617.5)	8.6<br>(6.3 to 11.4)*	0.2<br>(-1.9 to 2.8)	38.3<br>(16.8 to 72.7)	8.7<br>(6.0 to 12.0)*	0.4<br>(-2.2 to 3.4)
Urticaria	15,138.3<br>(11,100.0 to 20,518.4)	7.8<br>(7.5 to 8.1)*	-0.5<br>(-0.8 to -0.2)*	936.4<br>(532.9 to 1,459.0)	8.1<br>(7.0 to 9.2)*	-0.3<br>(-1.3 to 0.8)
Decubitus ulcer	8.1<br>(5.8 to 11.1)	17.2<br>(12.9 to 21.1)*	8.2<br>(4.2 to 11.8)*	1.3<br>(0.8 to 2.1)	17.2<br>(12.9 to 21.1)*	8.2<br>(4.2 to 11.8)*
Other skin and subcutaneous diseases	24,721.3<br>(22,531.4 to 27,096.2)	11.4<br>(9.6 to 13.0)*	2.8<br>(1.1 to 4.3)*	149.2<br>(71.1 to 280.4)	11.5<br>(9.3 to 13.2)*	2.9<br>(0.9 to 4.5)*
Sense organ diseases	32,270.8<br>(31,244.0 to 33,397.8)	6.9<br>(5.4 to 8.2)*	-1.4<br>(-2.7 to -0.1)*	1,079.9<br>(690.7 to 1,570.1)	8.4<br>(6.2 to 10.5)*	0.0<br>(-2.0 to 2.0)
Glaucoma	0.0<br>(0.0 to 0.0)	0.0<br>(0.0 to 0.0)	0.0<br>(0.0 to 0.0)	0.0<br>(0.0 to 0.0)	0.0<br>(0.0 to 0.0)	0.0<br>(0.0 to 0.0)
Cataract	0.0<br>(0.0 to 0.0)	0.0<br>(0.0 to 0.0)	0.0<br>(0.0 to 0.0)	0.0<br>(0.0 to 0.0)	0.0<br>(0.0 to 0.0)	0.0<br>(0.0 to 0.0)
Macular degeneration	0.0<br>(0.0 to 0.0)	0.0<br>(0.0 to 0.0)	0.0<br>(0.0 to 0.0)	0.0<br>(0.0 to 0.0)	0.0<br>(0.0 to 0.0)	0.0<br>(0.0 to 0.0)
Refraction and accommodation disorders	21,220.3<br>(19,553.3 to 22,955.4)	4.6<br>(3.4 to 5.7)*	-3.5<br>(-4.6 to -2.4)*	436.7<br>(263.4 to 695.7)	6.1<br>(4.7 to 7.7)*	-2.0<br>(-3.4 to -0.6)*
Age-related and other hearing loss	6,064.2<br>(3,513.3 to 9,030.6)	12.9<br>(9.8 to 17.1)*	4.2<br>(1.4 to 8.1)*	492.2<br>(245.0 to 828.9)	10.0<br>(5.3 to 15.5)*	1.5<br>(-2.8 to 6.6)
Other vision loss	424.5<br>(260.0 to 626.7)	12.3<br>(9.7 to 14.7)*	3.6<br>(1.2 to 5.9)*	16.3<br>(8.2 to 28.3)	12.2<br>(7.5 to 17.0)*	3.6<br>(-0.8 to 8.0)
Other sense organ diseases	5,136.4<br>(4,599.0 to 5,743.8)	9.4<br>(7.8 to 11.0)*	1.0<br>(-0.5 to 2.5)	134.7<br>(81.6 to 197.7)	9.7<br>(6.9 to 12.6)*	1.2<br>(-1.3 to 3.9)
Acute other sense organ diseases	178.3<br>(160.7 to 196.3)	9.3<br>(7.4 to 11.2)*	0.8<br>(-0.8 to 2.7)	4.7<br>(2.8 to 7.0)	9.4<br>(-3.6 to 23.0)	1.0<br>(-11.0 to 13.5)
Chronic other sense organ diseases	4,958.1<br>(4,415.9 to 5,568.9)	9.4<br>(7.7 to 11.1)*	1.0<br>(-0.6 to 2.5)	130.0<br>(78.7 to 190.5)	9.7<br>(6.8 to 12.7)*	1.3<br>(-1.4 to 4.0)
Oral disorders	259,956.9<br>(243,461.2 to 276,856.6)	9.5<br>(6.6 to 12.3)*	1.0<br>(-1.7 to 3.7)	66.2<br>(28.4 to 130.1)	9.4<br>(7.5 to 11.0)*	1.0<br>(-0.8 to 2.4)
Deciduous caries	262,336.2<br>(205,023.6 to 319,275.9)	9.4<br>(7.8 to 10.6)*	1.0<br>(-0.5 to 2.0)	66.2<br>(28.4 to 130.1)	9.4<br>(7.5 to 11.0)*	1.0<br>(-0.8 to 2.4)
Permanent caries	0.0<br>(0.0 to 0.0)	0.0<br>(0.0 to 0.0)	0.0<br>(0.0 to 0.0)	0.0<br>(0.0 to 0.0)	0.0<br>(0.0 to 0.0)	0.0<br>(0.0 to 0.0)
Periodontal diseases	0.0<br>(0.0 to 0.0)	0.0<br>(0.0 to 0.0)	0.0<br>(0.0 to 0.0)	0.0<br>(0.0 to 0.0)	0.0<br>(0.0 to 0.0)	0.0<br>(0.0 to 0.0)
Edentulism and severe tooth loss	0.0<br>(0.0 to 0.0)	0.0<br>(0.0 to 0.0)	0.0<br>(0.0 to 0.0)	0.0<br>(0.0 to 0.0)	0.0<br>(0.0 to 0.0)	0.0<br>(0.0 to 0.0)
Other oral disorders	0.0<br>(0.0 to 0.0)	0.0<br>(0.0 to 0.0)	0.0<br>(0.0 to 0.0)	0.0<br>(0.0 to 0.0)	0.0<br>(0.0 to 0.0)	0.0<br>(0.0 to 0.0)
Injuries	21,733.7<br>(18,878.9 to 25,494.3)	19.6<br>(6.7 to 36.0)*	10.3<br>(-1.5 to 25.5)	1,326.7<br>(890.4 to 1,901.2)	22.4<br>(7.0 to 41.8)*	12.9<br>(-1.2 to 30.9)
Transport injuries	284.3<br>(240.9 to 339.7)	5.2<br>(2.5 to 7.9)*	-2.9<br>(-5.4 to -0.4)*	23.7<br>(15.3 to 34.0)	-0.9<br>(-4.6 to 2.4)	-8.6<br>(-11.9 to -5.5)*
Road injuries	202.6<br>(162.6 to 252.7)	5.1<br>(1.4 to 8.7)*	-3.0<br>(-6.5 to 0.3)	16.6<br>(10.3 to 24.6)	0.3<br>(-4.2 to 4.8)	-7.4<br>(-11.6 to -3.3)*
Pedestrian road injuries	26.1<br>(19.9 to 34.4)	-0.8<br>(-4.8 to 3.5)	-8.4<br>(-12.1 to -4.4)*	2.1<br>(1.3 to 3.1)	-7.4<br>(-12.6 to -2.3)*	-14.5<br>(-19.3 to -9.8)*
Cyclist road injuries	57.6<br>(42.8 to 77.1)	1.4<br>(-3.8 to 6.5)	-6.4<br>(-11.2 to -1.7)*	4.3<br>(2.6 to 6.6)	-3.3<br>(-9.4 to 2.6)	-10.7<br>(-16.3 to -5.3)*
Motorcyclist road injuries	36.2<br>(27.0 to 46.5)	16.5<br>(12.6 to 20.5)*	7.5<br>(3.9 to 11.3)*	2.7<br>(1.6 to 4.1)	10.9<br>(6.0 to 16.0)*	2.4<br>(-2.1 to 7.1)
Motor vehicle road injuries	75.4<br>(56.7 to 99.6)	4.2<br>(-0.2 to 8.4)	-3.8<br>(-7.9 to 0.0)	6.9<br>(4.2 to 10.5)	0.5<br>(-4.6 to 5.5)	-7.2<br>(-12.0 to -2.6)*
Other road injuries	7.3<br>(4.8 to 11.2)	15.9<br>(10.5 to 21.5)*	6.9<br>(2.0 to 12.1)*	0.6<br>(0.3 to 1.0)	12.0<br>(5.9 to 18.1)*	3.4<br>(-2.3 to 9.0)
Other transport injuries	81.7<br>(63.6 to 105.7)	5.4<br>(3.7 to 7.0)*	-2.7<br>(-4.3 to -1.3)*	7.1<br>(4.6 to 10.2)	-3.8<br>(-6.8 to -0.7)*	-11.2<br>(-14.0 to -8.3)*
Unintentional injuries	16,180.0<br>(14,867.0 to 17,597.4)	1.9<br>(0.9 to 2.8)*	-5.9<br>(-6.8 to -5.1)*	939.0<br>(653.7 to 1,290.9)	0.4<br>(-1.1 to 1.9)	-7.3<br>(-8.7 to -6.0)*
Falls	2,485.8<br>(2,004.8 to 3,025.0)	14.5<br>(12.0 to 16.9)*	5.7<br>(3.3 to 7.9)*	184.5<br>(118.4 to 267.7)	9.9<br>(6.4 to 13.2)*	1.4<br>(-1.8 to 4.5)
Drowning	38.0<br>(28.2 to 54.1)	-4.3<br>(-5.8 to -3.2)*	-11.7<br>(-13.1 to -10.6)*	2.8<br>(1.8 to 4.2)	-10.8<br>(-13.9 to -7.9)*	-17.7<br>(-20.5 to -15.0)*
Fire, heat, and hot substances	1,522.2<br>(1,151.9 to 1,954.5)	4.4<br>(3.0 to 5.7)*	-3.7<br>(-5.0 to -2.4)*	82.5<br>(54.7 to 117.2)	1.3<br>(-1.9 to 4.7)	-6.5<br>(-9.4 to -3.4)*
Poisonings	243.2<br>(186.8 to 317.6)	12.7<br>(11.4 to 13.9)*	4.0<br>(2.9 to 5.1)*	29.3<br>(18.7 to 44.5)	11.0<br>(7.1 to 14.6)*	2.4<br>(-1.1 to 5.7)
Exposure to mechanical forces	4,559.8<br>(3,950.9 to 5,284.8)	-2.2<br>(-3.7 to -0.7)*	-9.7<br>(-11.1 to -8.4)*	137.0<br>(93.6 to 190.6)	-5.4<br>(-7.7 to -3.0)*	-12.7<br>(-14.9 to -10.5)*
Unintentional firearm injuries	25.0<br>(19.3 to 32.7)	2.2<br>(-1.1 to 6.2)	-5.7<br>(-8.7 to -2.0)*	2.0<br>(1.2 to 3.1)	-0.3<br>(-5.4 to 5.9)	-8.0<br>(-12.7 to -2.2)*
Unintentional suffocation	436.4<br>(318.0 to 606.3)	0.1<br>(-2.3 to 2.9)	-7.6<br>(-9.8 to -5.0)*	17.3<br>(11.0 to 25.0)	-0.0<br>(-2.7 to 2.9)	-7.7<br>(-10.2 to -5.0)*
Other exposure to mechanical forces	4,098.4<br>(3,519.4 to 4,784.6)	-2.5<br>(-4.1 to -1.0)*	-10.0<br>(-11.5 to -8.6)*	117.7<br>(80.3 to 163.7)	-6.2<br>(-8.7 to -3.7)*	-13.4<br>(-15.7 to -11.2)*
Adverse effects of medical treatment	1,190.6<br>(890.9 to 1,549.3)	-1.3<br>(-4.2 to 1.4)	-8.9<br>(-11.6 to -6.4)*	158.7<br>(96.0 to 241.6)	-1.3<br>(-4.2 to 1.4)	-8.9<br>(-11.6 to -6.4)*
Animal contact	738.6<br>(551.7 to 968.7)	-1.9<br>(-5.7 to 2.0)	-9.4<br>(-13.0 to -5.8)*	32.9<br>(21.4 to 47.5)	-2.0<br>(-6.1 to 2.1)	-9.5<br>(-13.3 to -5.8)*
Venomous animal contact	181.3<br>(142.6 to 231.4)	-1.4<br>(-4.4 to 1.6)	-9.0<br>(-11.8 to -6.2)*	19.7<br>(12.3 to 29.2)	-2.2<br>(-6.5 to 2.3)	-9.8<br>(-13.7 to -5.5)*
Non-venomous animal contact	557.3<br>(394.6 to 752.1)	-2.0<br>(-6.2 to 2.6)	-9.6<br>(-13.5 to -5.3)*	13.3<br>(8.2 to 20.1)	-1.5<br>(-6.4 to 3.7)	-9.1<br>(-13.6 to -4.2)*
Foreign body	1,262.3<br>(1,023.8 to 1,548.4)	4.0<br>(2.2 to 5.6)*	-4.0<br>(-5.6 to -2.5)*	71.3<br>(49.4 to 100.2)	-0.8<br>(-3.4 to 1.7)	-8.5<br>(-10.8 to -6.1)*
Pulmonary aspiration and foreign body in airway	566.1<br>(406.6 to 813.1)	2.0<br>(-0.5 to 4.7)	-5.8<br>(-8.2 to -3.4)*	38.5<br>(25.6 to 55.8)	-4.6<br>(-7.6 to -1.6)*	-12.0<br>(-14.7 to -9.2)*
Foreign body in eyes	68.6<br>(20.8 to 132.9)	8.4<br>(7.7 to 9.0)*	0.1<br>(-0.6 to 0.6)	3.9<br>(1.2 to 8.2)	7.9<br>(6.4 to 8.7)*	-0.4<br>(-1.8 to 0.3)
Foreign body in other body part	627.5<br>(477.5 to 798.7)	5.4<br>(2.9 to 7.7)*	-2.7<br>(-5.0 to -0.6)*	28.9<br>(19.1 to 41.2)	3.4<br>(0.3 to 6.6)*	-4.5<br>(-7.4 to -1.6)*
Environmental heat and cold exposure	830.4<br>(673.9 to 1,011.0)	11.6<br>(9.4 to 13.6)*	3.0<br>(1.0 to 4.9)*	57.8<br>(38.2 to 83.1)	8.7<br>(5.5 to 11.9)*	0.3<br>(-2.6 to 3.3)
Other unintentional injuries	3,309.1<br>(2,711.2 to 3,922.8)	-3.0<br>(-4.8 to -1.2)*	-10.5<br>(-12.1 to -8.8)*	182.3<br>(117.3 to 268.0)	-5.1<br>(-7.9 to -2.3)*	-12.4<br>(-15.0 to -9.8)*
Self-harm and interpersonal violence	95.5<br>(80.6 to 113.4)	1.3<br>(-1.3 to 4.2)	-6.5<br>(-8.9 to -3.8)*	6.9<br>(4.7 to 9.7)	-0.7<br>(-3.9 to 3.0)	-8.4<br>(-11.3 to -4.9)*
Self-harm	14.4<br>(11.1 to 18.7)	8.7<br>(7.4 to 9.8)*	0.3<br>(-0.9 to 1.4)	1.5<br>(0.9 to 2.2)	4.3<br>(1.2 to 7.5)*	-3.7<br>(-6.6 to -0.8)*
Interpersonal violence	81.1<br>(66.5 to 98.6)	0.1<br>(-2.9 to 3.4)	-7.6<br>(-10.4 to -4.5)*	5.4<br>(3.6 to 7.7)	-2.0<br>(-5.8 to 2.6)	-9.6<br>(-13.1 to -5.3)*

Cause	Prevalence in thousands (95% confidence interval)			YLDs in thousands (95% confidence interval)		
	2015	Percent change in counts between 2005 and 2015	Percent change in rates between 2005 and 2015	2015	Percent change in counts between 2005 and 2015	Percent change in rates between 2005 and 2015
Assault by firearm	1.5 (1.1 to 1.9)	4.2 (0.6 to 8.0)*	-3.8 (-7.1 to -0.4)*	0.1 (0.1 to 0.2)	2.5 (-3.2 to 8.5)	-5.4 (-10.6 to 0.2)
Assault by sharp object	11.6 (8.9 to 15.1)	-4.1 (-7.3 to -1.0)*	-11.4 (-14.4 to -8.7)*	0.8 (0.5 to 1.2)	-9.3 (-14.7 to -3.7)*	-16.3 (-21.3 to -11.1)*
Assault by other means	68.0 (54.9 to 83.4)	0.7 (-2.6 to 4.5)	-7.0 (-10.1 to -3.5)*	4.5 (3.0 to 6.4)	-0.7 (-4.6 to 3.9)	-8.4 (-12.0 to -4.1)*
Forces of nature, war, and legal intervention	5,174.0 (2,915.7 to 8,423.7)	166.8 (33.3 to 401.5)*	146.2 (23.0 to 362.9)*	357.1 (174.4 to 636.9)	202.4 (48.2 to 455.8)*	179.1 (36.8 to 413.0)*
Exposure to forces of nature	365.2 (165.7 to 702.2)	-76.3 (-90.5 to -45.2)*	-78.1 (-91.2 to -49.4)*	32.6 (12.2 to 68.8)	-66.5 (-86.7 to -22.7)*	-69.1 (-87.7 to -28.7)*
Collective violence and legal intervention	4,808.8 (2,705.5 to 7,857.3)	1,111.5 (672.9 to 1,718.8)*	1,018.2 (613.3 to 1,578.7)*	324.5 (152.8 to 588.9)	1,455.5 (858.4 to 2,418.6)*	1,335.7 (784.6 to 2,224.6)*
**Anemia**	**244,308.5 (237,541.7 to 251,029.8)**	**1.6 (-1.8 to 4.9)**	**-6.2 (-9.3 to -3.2)***	**8,834.6 (5,885.9 to 12,698.1)**	**-5.4 (-11.0 to 0.7)**	**-12.7 (-17.9 to -7.1)***
**Developmental intellectual disability**	**7,424.6 (5,467.6 to 9,268.5)**	**8.0 (7.5 to 8.5)***	**-0.3 (-0.8 to 0.1)**	**829.7 (571.1 to 1,150.5)**	**9.3 (7.4 to 11.5)***	**0.9 (-0.9 to 2.9)**
**Epilepsy**	**2,601.8 (2,150.2 to 3,062.3)**	**11.6 (7.0 to 16.0)***	**3.0 (-1.2 to 7.0)**	**807.3 (574.9 to 1,057.4)**	**1.0 (-4.6 to 7.0)**	**-6.8 (-12.0 to -1.2)***
**Guillain-Barré syndrome**	**2.4 (1.5 to 3.5)**	**9.4 (8.6 to 10.0)***	**0.9 (0.2 to 1.6)***	**0.7 (0.4 to 1.1)**	**9.4 (8.6 to 10.0)***	**0.9 (0.2 to 1.6)***
**Hearing loss**	**19,361.5 (15,989.4 to 23,970.6)**	**9.2 (8.2 to 10.1)***	**0.7 (-0.1 to 1.6)**	**1,169.1 (772.0 to 1,654.4)**	**7.2 (4.8 to 9.8)***	**-1.1 (-3.3 to 1.3)**
**Heart failure**	**232.0 (207.5 to 256.4)**	**10.4 (9.6 to 11.1)***	**1.9 (1.1 to 2.6)***	**29.3 (20.0 to 41.7)**	**10.2 (5.2 to 15.3)***	**1.7 (-2.9 to 6.4)**
**Infertility**	**0.0 (0.0 to 0.0)**	**0.0 (0.0 to 0.0)**	**0.0 (0.0 to 0.0)**	**0.0 (0.0 to 0.0)**	**0.0 (0.0 to 0.0)**	**0.0 (0.0 to 0.0)**
**Pelvic inflammatory disease**	**0.0 (0.0 to 0.0)**	**0.0 (0.0 to 0.0)**	**0.0 (0.0 to 0.0)**	**0.0 (0.0 to 0.0)**	**0.0 (0.0 to 0.0)**	**0.0 (0.0 to 0.0)**
**Vision loss**	**22,685.3 (20,899.0 to 24,582.8)**	**4.7 (3.6 to 5.9)***	**-3.3 (-4.3 to -2.3)***	**593.7 (394.8 to 876.7)**	**6.8 (5.5 to 8.4)***	**-1.4 (-2.7 to 0.1)**

SR Table 11. Prevalent cases and YLDs for 2015, percent change, and change in age-standardised rates between 2005 and 2015 by cause, global, ages 5-9 years						
Cause	Prevalence in thousands (95% confidence interval)			YLDs in thousands (95% confidence interval)		
	2015	Percent change in counts between 2005 and 2015	Percent change in rates between 2005 and 2015	2015	Percent change in counts between 2005 and 2015	Percent change in rates between 2005 and 2015
**All causes**	--	--	--	**35,042.8 (24,932.1 to 47,378.1)**	**3.5 (2.5 to 5.0)***	**-2.8 (-3.8 to -1.4)***
Communicable, maternal, neonatal, and nutritional diseases	359,941.3 (356,579.2 to 363,142.5)	0.6 (-0.4 to 1.5)	-5.5 (-6.5 to -4.7)*	15,720.1 (10,958.5 to 22,324.1)	0.9 (-1.0 to 3.9)	-5.2 (-7.0 to -2.4)*
HIV/AIDS and tuberculosis	1,088.2 (1,055.3 to 1,121.1)	6.5 (2.1 to 10.7)*	-0.0 (-4.1 to 4.0)	174.0 (122.9 to 228.2)	-3.9 (-10.0 to 2.6)	-9.7 (-15.5 to -3.7)*
Tuberculosis	118.6 (83.0 to 166.6)	9.9 (-0.1 to 19.4)	3.2 (-6.2 to 12.1)	37.9 (22.1 to 59.8)	9.7 (-2.7 to 23.0)	3.1 (-8.6 to 15.5)
HIV/AIDS	976.8 (947.6 to 1,008.6)	6.1 (1.4 to 10.8)*	-0.3 (-4.7 to 4.0)	136.1 (97.4 to 174.7)	-7.1 (-13.7 to 0.2)	-12.8 (-19.0 to -5.9)*
HIV/AIDS - Tuberculosis	31.9 (29.0 to 35.0)	-27.8 (-30.6 to -24.4)*	-32.2 (-34.8 to -29.0)*	12.5 (8.4 to 17.2)	-27.3 (-33.7 to -20.6)*	-31.7 (-37.8 to -25.4)*
HIV/AIDS resulting in other diseases	968.8 (933.2 to 1,003.6)	7.9 (4.2 to 11.1)*	1.3 (-2.1 to 4.3)	123.6 (89.5 to 157.9)	-4.4 (-12.0 to 4.0)	-10.2 (-17.4 to -2.4)*
HIV aggregate	627.0 (596.0 to 655.5)	-16.7 (-19.7 to -13.8)*	-21.8 (-24.6 to -19.1)*	47.5 (33.4 to 62.4)	-17.0 (-22.4 to -11.9)*	-22.0 (-27.2 to -17.3)*
AIDS aggregate	341.8 (326.3 to 359.6)	135.6 (112.8 to 163.0)*	121.3 (99.8 to 147.0)*	76.1 (54.5 to 97.7)	5.6 (-8.3 to 22.6)	-0.9 (-13.9 to 15.2)
Diarrhea, lower respiratory, and other common infectious diseases	49,230.5 (46,117.1 to 51,571.0)	6.1 (4.3 to 8.0)*	-0.3 (-2.0 to 1.4)	1,879.2 (1,260.4 to 2,604.1)	5.8 (4.3 to 7.4)*	-0.7 (-2.1 to 0.8)
Diarrheal diseases	3,932.6 (3,527.1 to 4,328.3)	11.6 (10.0 to 13.2)*	4.8 (3.3 to 6.3)*	639.0 (432.7 to 892.1)	11.7 (9.4 to 14.1)*	4.9 (2.7 to 7.2)*
Diarrhoea episodes aggregate	3,932.3 (3,526.9 to 4,328.1)	11.6 (10.0 to 13.2)*	4.8 (3.3 to 6.3)*	639.0 (432.6 to 892.0)	11.7 (9.4 to 14.1)*	4.9 (2.7 to 7.2)*
Guillain-Barré syndrome due to diarrheal diseases	0.3 (0.2 to 0.5)	7.9 (6.8 to 8.7)*	1.3 (0.3 to 2.1)*	0.1 (0.0 to 0.2)	7.9 (6.8 to 8.7)*	1.3 (0.3 to 2.1)*
Intestinal infectious diseases	405.8 (374.7 to 436.1)	-24.8 (-29.6 to -19.7)*	-29.4 (-33.9 to -24.6)*	48.6 (31.9 to 70.4)	-24.2 (-33.7 to -14.1)*	-28.9 (-37.7 to -19.3)*
Typhoid fever	319.8 (246.4 to 405.8)	-23.8 (-28.8 to -18.9)*	-28.5 (-33.1 to -23.8)*	42.6 (27.3 to 62.2)	-23.4 (-34.0 to -12.0)*	-28.1 (-38.0 to -17.4)*
Typhoid fever episodes aggregate	264.5 (203.6 to 338.9)	-23.9 (-31.5 to -15.6)*	-28.5 (-35.7 to -20.8)*	25.2 (16.3 to 37.2)	-23.7 (-33.4 to -12.1)*	-28.4 (-37.5 to -17.5)*
Complications of typhoid fever aggregate	55.3 (36.6 to 78.1)	-23.5 (-47.9 to 13.5)	-28.2 (-51.1 to 6.6)	17.3 (9.8 to 27.3)	-23.0 (-48.3 to 16.5)	-27.7 (-51.4 to 9.4)
Paratyphoid fever	109.6 (77.0 to 155.2)	-22.8 (-30.5 to -14.4)*	-27.5 (-34.8 to -19.6)*	5.7 (3.3 to 9.4)	-22.5 (-37.2 to -3.7)*	-27.2 (-41.0 to -9.5)*
Paratyphoid fever episodes aggregate	104.1 (72.8 to 147.3)	-22.8 (-31.4 to -14.0)*	-27.5 (-35.6 to -19.2)*	5.1 (2.9 to 8.4)	-22.3 (-38.1 to -2.8)*	-27.1 (-41.9 to -8.7)*
Intestinal perforation due to paratyphoid	5.5 (3.1 to 8.9)	-24.2 (-58.6 to 34.6)	-28.9 (-61.1 to 26.4)	0.6 (0.3 to 1.1)	-24.0 (-59.0 to 33.9)	-28.6 (-61.5 to 25.7)
Other intestinal infectious diseases	--	--	--	0.4 (0.1 to 0.9)	-69.8 (-81.2 to -46.8)*	-71.7 (-82.4 to -50.1)*
Lower respiratory infections	945.0 (797.7 to 1,097.9)	0.9 (-1.6 to 3.3)	-5.2 (-7.6 to -3.0)*	58.1 (37.8 to 85.5)	1.0 (-3.0 to 5.0)	-5.2 (-8.9 to -1.4)*
Lower respiratory infection episodes aggregate	944.7 (797.5 to 1,097.7)	0.9 (-1.6 to 3.3)	-5.2 (-7.6 to -3.0)*	58.0 (37.7 to 85.3)	1.0 (-3.0 to 5.0)	-5.2 (-8.9 to -1.4)*
Guillain-Barré syndrome due to lower respiratory infections	0.3 (0.1 to 0.6)	7.9 (6.8 to 8.7)*	1.3 (0.3 to 2.1)*	0.1 (0.0 to 0.2)	7.9 (6.8 to 8.7)*	1.3 (0.3 to 2.1)*
Upper respiratory infections	24,453.3 (17,721.8 to 32,995.9)	6.5 (4.6 to 8.2)*	0.1 (-1.7 to 1.6)	290.9 (149.6 to 533.6)	6.6 (4.6 to 8.3)*	0.1 (-1.8 to 1.7)
Upper respiratory infection episodes aggregate	24,452.4 (17,720.9 to 32,995.0)	6.5 (4.6 to 8.2)*	0.1 (-1.7 to 1.6)	290.6 (149.4 to 533.4)	6.6 (4.6 to 8.3)*	0.1 (-1.8 to 1.7)
Guillain-Barré syndrome due to upper respiratory infections	0.9 (0.5 to 1.4)	7.9 (6.8 to 8.7)*	1.3 (0.3 to 2.1)*	0.3 (0.1 to 0.5)	7.9 (6.8 to 8.7)*	1.3 (0.3 to 2.1)*
Otitis media	19,730.1 (15,907.2 to 23,955.2)	6.2 (4.5 to 7.8)*	-0.3 (-1.9 to 1.2)	638.2 (382.0 to 991.2)	4.1 (0.8 to 7.1)*	-2.3 (-5.3 to 0.6)
Acute otitis media	5,817.5 (3,749.8 to 8,842.2)	7.5 (4.5 to 10.8)*	0.9 (-1.9 to 4.0)	76.3 (35.1 to 149.9)	7.5 (4.2 to 10.9)*	1.0 (-2.1 to 4.2)
Chronic otitis media aggregate	13,912.6 (11,172.0 to 17,000.7)	5.6 (3.8 to 7.6)*	-0.8 (-2.5 to 1.0)	562.0 (331.8 to 870.5)	3.6 (0.1 to 7.1)*	-2.7 (-6.0 to 0.5)
Meningitis	863.0 (823.4 to 901.7)	10.6 (5.5 to 16.1)*	3.9 (-0.9 to 9.0)	158.9 (111.1 to 209.0)	9.0 (5.3 to 12.4)*	2.3 (-1.1 to 5.5)
Pneumococcal meningitis	656.0 (549.1 to 787.2)	18.6 (15.8 to 22.1)*	11.4 (8.7 to 14.6)*	76.1 (52.6 to 102.6)	12.8 (7.4 to 18.5)*	5.9 (0.8 to 11.3)*
Acute pneumococcal meningitis	3.3 (2.1 to 4.8)	23.3 (5.5 to 41.0)*	15.8 (-0.9 to 32.4)	0.4 (0.2 to 0.7)	23.4 (5.5 to 41.0)*	15.9 (-0.9 to 32.4)
Complications of pneumococcal meningitis aggregate	652.7 (546.7 to 783.8)	18.6 (15.8 to 22.0)*	11.4 (8.7 to 14.6)*	75.7 (52.4 to 102.0)	12.8 (7.3 to 18.5)*	5.9 (0.8 to 11.3)*
H influenzae type B meningitis	291.0 (235.4 to 356.1)	-10.0 (-13.9 to -5.3)*	-15.5 (-19.1 to -11.1)*	41.8 (28.9 to 55.8)	2.4 (-5.2 to 10.6)	-3.8 (-11.0 to 3.8)
Acute H influenzae type B meningitis	1.8 (1.0 to 2.9)	-17.0 (-29.3 to -5.6)*	-22.1 (-33.6 to -11.3)*	0.2 (0.1 to 0.4)	-17.0 (-29.3 to -5.6)*	-22.0 (-33.6 to -11.3)*
Complications of of H influenzae type B meningitis aggregate	289.2 (233.9 to 354.2)	-10.0 (-13.8 to -5.2)*	-15.5 (-19.1 to -11.0)*	41.5 (28.7 to 55.5)	2.6 (-5.1 to 10.8)	-3.7 (-10.9 to 4.0)
Meningococcal meningitis	150.4 (105.8 to 206.1)	19.3 (13.5 to 24.9)*	12.0 (6.6 to 17.3)*	12.2 (8.2 to 17.0)	14.7 (9.4 to 20.5)*	7.7 (2.7 to 13.2)*
Acute meningococcal meningitis	1.8 (1.1 to 2.7)	33.2 (13.2 to 54.8)*	25.0 (6.3 to 45.3)*	0.2 (0.1 to 0.4)	33.3 (13.2 to 54.8)*	25.2 (6.3 to 45.3)*
Complications of meningococcal meningitis aggregate	148.6 (104.2 to 203.7)	19.2 (13.3 to 24.9)*	11.9 (6.4 to 17.3)*	12.0 (8.0 to 16.6)	14.3 (9.0 to 20.4)*	7.4 (2.4 to 13.0)*
Other meningitis	243.0 (189.4 to 303.3)	13.6 (10.3 to 17.1)*	6.7 (3.6 to 10.0)*	28.8 (19.8 to 38.7)	7.0 (0.5 to 13.9)*	0.5 (-5.6 to 7.0)
Other meningitis episodes aggregate	10.4 (7.5 to 14.3)	15.9 (2.4 to 30.2)*	8.8 (-3.8 to 22.3)	1.4 (0.8 to 2.2)	16.0 (2.4 to 30.2)*	8.9 (-3.8 to 22.2)
Complications of other meningitis aggregate	232.6 (179.9 to 293.8)	13.5 (10.3 to 17.1)*	6.6 (3.5 to 10.0)*	27.4 (18.8 to 37.1)	6.6 (-0.3 to 14.0)	0.1 (-6.3 to 7.0)
Encephalitis	233.3 (173.6 to 310.6)	-4.0 (-6.3 to -1.3)*	-9.8 (-12.0 to -7.3)*	28.4 (20.0 to 38.7)	-3.5 (-10.7 to 4.1)	-9.4 (-16.2 to -2.2)*
Acute encephalitis	7.5 (5.7 to 10.1)	1.6 (-3.0 to 6.5)	-4.6 (-8.9 to 0.0)	1.0 (0.6 to 1.6)	1.8 (-5.1 to 8.6)	-4.4 (-10.9 to 2.0)
Complications of encephalitis aggregate	225.8 (165.5 to 302.5)	-4.1 (-6.4 to -1.4)*	-10.0 (-12.1 to -7.4)*	27.4 (19.2 to 37.3)	-3.7 (-11.2 to 4.2)	-9.6 (-16.6 to -2.2)*
Diphtheria	0.1 (0.0 to 0.2)	-54.5 (-86.9 to 64.9)	-57.3 (-87.7 to 54.9)	0.0 (0.0 to 0.0)	-54.6 (-86.9 to 64.9)	-57.3 (-87.7 to 54.9)
Whooping cough	106.2 (82.1 to 133.4)	-27.2 (-29.2 to -25.0)*	-31.6 (-33.5 to -29.5)*	5.2 (3.1 to 8.1)	-27.1 (-33.5 to -19.3)*	-31.5 (-37.5 to -24.2)*
Tetanus	21.1 (20.7 to 21.6)	2.9 (1.4 to 3.9)*	-3.4 (-4.8 to -2.4)*	0.9 (0.6 to 1.2)	-8.9 (-17.1 to 1.0)	-14.4 (-22.2 to -5.2)*
Severe tetanus	0.4 (0.2 to 0.6)	-46.3 (-61.3 to -27.0)*	-49.5 (-63.6 to -31.4)*	0.0 (0.0 to 0.1)	-46.1 (-61.3 to -27.0)*	-49.4 (-63.6 to -31.4)*
Complications of tetanus aggregate	20.7 (20.3 to 21.1)	4.6 (4.1 to 5.1)*	-1.8 (-2.3 to -1.3)*	0.8 (0.6 to 1.1)	-5.0 (-13.9 to 5.3)	-10.8 (-19.1 to -1.1)*
Measles	12.4 (5.5 to 24.5)	-69.6 (-73.0 to -65.5)*	-71.5 (-74.7 to -67.6)*	1.1 (0.4 to 2.5)	-68.9 (-73.7 to -62.6)*	-70.8 (-75.3 to -64.9)*
Varicella and herpes zoster	502.8 (364.2 to 673.6)	5.8 (4.5 to 6.6)*	-0.7 (-1.9 to 0.1)	9.8 (5.4 to 16.5)	5.9 (-0.3 to 11.9)	-0.5 (-6.4 to 5.0)
Neglected tropical diseases and malaria	195,573.5 (190,892.3 to 200,391.6)	-6.3 (-8.9 to -3.8)*	-12.1 (-14.5 to -9.7)*	3,077.7 (1,983.0 to 4,920.9)	0.1 (-8.2 to 13.9)	-6.0 (-13.8 to 7.0)
Malaria	63,082.1 (44,805.1 to 83,016.2)	23.8 (11.9 to 33.3)*	16.3 (5.1 to 25.2)*	986.2 (680.9 to 1,369.2)	18.4 (14.9 to 21.8)*	11.2 (7.9 to 14.4)*
Asymptomatic malaria parasitemia(PfPR)	41,905.6 (24,738.9 to 61,172.0)	27.6 (8.9 to 42.2)*	19.9 (2.3 to 33.5)*	0.0 (0.0 to 0.0)	0.0 (0.0 to 0.0)	0.0 (0.0 to 0.0)
Malaria episodes aggregate	2,205.6 (1,189.0 to 3,844.8)	-17.8 (-33.1 to -1.0)*	-22.9 (-37.2 to -7.0)*	45.8 (21.3 to 88.9)	-17.8 (-33.1 to -1.1)*	-22.9 (-37.2 to -7.2)*
Complications of malaria aggregate	81.3 (70.4 to 94.6)	20.8 (18.2 to 23.5)*	13.5 (11.0 to 16.0)*	26.0 (18.9 to 34.0)	17.9 (5.8 to 31.9)*	10.7 (-0.7 to 23.8)
Anemia due to malaria parasitemia(PfPR) aggregate	18,889.6 (17,761.5 to 19,828.5)	23.0 (20.3 to 25.8)*	15.5 (13.0 to 18.2)*	914.5 (623.9 to 1,294.8)	21.1 (18.0 to 24.3)*	13.7 (10.8 to 16.8)*
Chagas disease	169.9 (144.3 to 198.6)	-15.4 (-19.8 to -11.2)*	-20.6 (-24.7 to -16.6)*	0.1 (0.1 to 0.2)	-16.9 (-43.0 to 17.5)	-22.0 (-46.5 to 10.3)
Chagas disease episodes aggregate	168.0 (142.8 to 196.6)	-15.4 (-19.8 to -11.2)*	-20.6 (-24.7 to -16.6)*	0.0 (0.0 to 0.0)	-43.5 (-50.3 to -36.7)*	-47.0 (-53.3 to -40.5)*
Complications of Chagas disease aggregate	1.8 (1.4 to 2.3)	-15.4 (-19.8 to -11.2)*	-20.6 (-24.7 to -16.6)*	0.1 (0.0 to 0.1)	-16.1 (-44.2 to 22.9)	-21.2 (-47.6 to 15.4)

Cause	Prevalence in thousands (95% confidence interval)			YLDs in thousands (95% confidence interval)		
	2015	Percent change in counts between 2005 and 2015	Percent change in rates between 2005 and 2015	2015	Percent change in counts between 2005 and 2015	Percent change in rates between 2005 and 2015
Heart failure due to Chagas disease aggregate	0.1 (0.0 to 0.1)	-15.3 (-20.0 to -11.0)*	-20.5 (-24.9 to -16.4)*	0.0 (0.0 to 0.0)	-15.3 (-20.0 to -11.0)*	-20.5 (-24.8 to -16.4)*
Leishmaniasis	270.4 (229.7 to 347.9)	17.9 (-6.6 to 53.5)	10.7 (-12.3 to 44.2)	3.6 (1.9 to 6.6)	14.9 (5.9 to 24.0)*	7.9 (-0.5 to 16.4)
Visceral leishmaniasis	10.0 (9.0 to 11.1)	5.2 (3.9 to 6.5)*	-1.2 (-2.4 to -0.0)*	0.7 (0.4 to 1.0)	5.1 (-17.4 to 34.4)	-1.3 (-22.5 to 26.2)
Cutaneous and mucocutaneous leishmaniasis	267.6 (216.9 to 348.5)	17.3 (12.8 to 22.6)*	10.2 (5.9 to 15.1)*	2.9 (1.3 to 5.7)	17.6 (8.7 to 26.0)*	10.4 (2.1 to 18.3)*
African trypanosomiasis	1.0 (0.6 to 1.6)	-68.6 (-70.3 to -67.1)*	-70.5 (-72.1 to -69.1)*	0.3 (0.1 to 0.5)	-67.1 (-79.5 to -44.1)*	-69.1 (-80.7 to -47.5)*
Schistosomiasis	22,142.1 (18,728.7 to 27,480.0)	-24.4 (-32.5 to -9.2)*	-29.0 (-36.6 to -14.7)*	214.5 (117.4 to 384.2)	-21.0 (-27.0 to -10.9)*	-25.8 (-31.5 to -16.3)*
Schistosomiasis episodes aggregate	19,398.3 (16,200.7 to 24,223.7)	-24.9 (-33.6 to -8.4)*	-29.5 (-37.6 to -14.0)*	110.5 (43.3 to 237.2)	-25.5 (-34.0 to -8.4)*	-30.1 (-38.0 to -14.0)*
Complications of schistosomiasis aggregate	771.6 (360.1 to 1,482.3)	-26.9 (-41.8 to 9.9)	-31.4 (-45.4 to 3.2)	8.4 (2.9 to 19.2)	-26.4 (-41.1 to 11.0)	-30.9 (-44.7 to 4.2)
Anemia due to schistosomiasis aggregate	2,005.5 (1,932.0 to 2,084.6)	-12.9 (-16.9 to -8.7)*	-18.2 (-22.0 to -14.3)*	95.7 (64.4 to 137.5)	-14.3 (-19.2 to -9.4)*	-19.5 (-24.1 to -15.0)*
Cysticercosis	0.4 (0.2 to 0.7)	-3.0 (-9.8 to 3.3)	-8.9 (-15.3 to -3.0)*	0.1 (0.0 to 0.1)	-14.0 (-21.6 to -7.4)*	-19.2 (-26.4 to -13.0)*
Cystic echinococcosis	72.7 (60.7 to 86.2)	15.1 (12.1 to 18.1)*	8.1 (5.3 to 10.9)*	6.9 (4.5 to 9.8)	15.1 (3.3 to 28.8)*	8.1 (-3.0 to 21.0)
Lymphatic filariasis	2,448.6 (1,988.7 to 2,991.5)	-52.9 (-56.2 to -48.6)*	-55.7 (-58.9 to -51.7)*	87.3 (44.7 to 145.2)	-34.9 (-46.0 to -25.4)*	-38.9 (-49.3 to -29.9)*
Prevalence of detectable microfiliaria due to lymphatic filariasis	1,696.9 (1,363.9 to 2,081.1)	-57.9 (-61.1 to -54.3)*	-60.5 (-63.5 to -57.1)*	0.0 (0.0 to 0.0)	0.0 (0.0 to 0.0)	0.0 (0.0 to 0.0)
Complications of lymphatic filariasis aggregate	751.7 (465.8 to 1,123.1)	-35.1 (-45.5 to -26.6)*	-39.1 (-48.8 to -31.0)*	87.3 (44.7 to 145.2)	-34.9 (-46.0 to -25.4)*	-38.9 (-49.3 to -29.9)*
Onchocerciasis	1,125.6 (624.4 to 1,726.7)	-23.0 (-44.1 to -6.8)*	-27.7 (-47.5 to -12.5)*	80.9 (30.4 to 152.6)	-26.8 (-47.3 to -10.9)*	-31.3 (-50.5 to -16.3)*
Asymptomatic onchocerciasis	0.7 (0.0 to 0.0)	-99.1 (-100.0 to -98.7)*	-99.1 (-100.0 to -98.8)*	0.0 (0.0 to 0.0)	0.0 (0.0 to 0.0)	0.0 (0.0 to 0.0)
Skin disease due to onchocerciasis aggregate	1,124.5 (624.2 to 1,726.1)	-18.8 (-35.9 to -3.4)*	-23.7 (-39.8 to -9.3)*	80.9 (30.4 to 152.6)	-26.8 (-47.3 to -10.9)*	-31.3 (-50.5 to -16.3)*
Vision loss due to onchocerciasis aggregate	0.0 (0.0 to 0.0)	0.0 (0.0 to 0.0)	0.0 (0.0 to 0.0)	0.0 (0.0 to 0.0)	0.0 (0.0 to 0.0)	0.0 (0.0 to 0.0)
Trachoma	0.0 (0.0 to 0.0)	0.0 (0.0 to 0.0)	0.0 (0.0 to 0.0)	0.0 (0.0 to 0.0)	0.0 (0.0 to 0.0)	0.0 (0.0 to 0.0)
Dengue	536.1 (302.3 to 1,143.4)	122.1 (-7.8 to 494.1)	108.6 (-13.4 to 457.9)	88.7 (40.2 to 199.6)	120.5 (-6.5 to 480.8)	107.1 (-12.2 to 445.4)
Dengue episodes aggregate	159.8 (106.8 to 328.5)	117.2 (-10.1 to 459.5)	103.9 (-15.5 to 425.4)	9.4 (5.2 to 21.2)	121.0 (-7.2 to 511.4)	107.6 (-12.9 to 474.1)
Post-dengue chronic fatigue syndrome	376.8 (180.9 to 817.9)	122.3 (-7.8 to 494.1)	108.7 (-13.4 to 457.9)	79.2 (34.4 to 180.9)	120.4 (-7.2 to 477.4)	107.0 (-12.8 to 442.2)
Yellow fever	0.5 (0.1 to 1.3)	-27.1 (-43.1 to -4.9)*	-31.6 (-46.6 to -10.7)*	0.0 (0.0 to 0.0)	-27.0 (-43.1 to -4.9)*	-31.5 (-46.6 to -10.7)*
Rabies	0.1 (0.1 to 0.1)	-45.4 (-63.9 to -14.4)*	-48.7 (-66.1 to -19.6)*	0.0 (0.0 to 0.0)	-45.5 (-63.9 to -14.4)*	-48.8 (-66.1 to -19.6)*
Intestinal nematode infections	131,438.5 (127,114.9 to 135,970.2)	-13.5 (-17.1 to -9.9)*	-18.8 (-22.1 to -15.4)*	385.4 (234.2 to 598.4)	-27.6 (-33.2 to -21.9)*	-32.0 (-37.3 to -26.6)*
Ascariasis	71,088.7 (62,333.2 to 81,893.4)	-21.4 (-34.7 to -7.6)*	-26.2 (-38.6 to -13.2)*	114.3 (61.7 to 191.2)	-39.6 (-48.2 to -29.6)*	-43.2 (-51.4 to -33.9)*
Complications of ascariasis aggregate	6,144.0 (5,662.1 to 6,747.5)	-38.2 (-45.8 to -29.6)*	-42.0 (-49.1 to -33.9)*	114.3 (61.7 to 191.2)	-39.6 (-48.2 to -29.6)*	-43.2 (-51.4 to -33.9)*
Asymptomatic ascariasis	64,944.6 (55,964.1 to 75,675.1)	-19.4 (-34.5 to -3.5)*	-24.3 (-38.5 to -9.3)*	0.0 (0.0 to 0.0)	0.0 (0.0 to 0.0)	0.0 (0.0 to 0.0)
Trichuriasis	45,933.2 (41,580.6 to 50,896.2)	-14.6 (-25.3 to -2.9)*	-19.8 (-29.9 to -8.8)*	57.8 (32.2 to 97.7)	-35.6 (-48.5 to -17.1)*	-39.5 (-51.6 to -22.1)*
Complications of trichuriasis aggregate	2,945.0 (2,588.6 to 3,434.4)	-33.9 (-45.0 to -19.4)*	-38.0 (-48.3 to -24.3)*	57.8 (32.2 to 97.7)	-35.6 (-48.5 to -17.1)*	-39.5 (-51.6 to -22.1)*
Asymptomatic trichuriasis	43,012.2 (38,739.2 to 47,791.5)	-12.8 (-24.3 to -0.0)*	-18.1 (-28.9 to -6.1)*	0.0 (0.0 to 0.0)	0.0 (0.0 to 0.0)	0.0 (0.0 to 0.0)
Hookworm disease	34,249.3 (31,110.3 to 37,668.6)	-16.3 (-27.0 to -4.9)*	-21.4 (-31.4 to -10.7)*	213.3 (137.8 to 315.8)	-15.9 (-20.6 to -11.5)*	-21.0 (-25.4 to -16.9)*
Complications of hookworm disease aggregate	3,798.3 (3,568.7 to 4,054.2)	-21.5 (-29.3 to -13.4)*	-26.3 (-33.6 to -18.6)*	77.4 (43.1 to 128.6)	-22.6 (-32.4 to -13.0)*	-27.4 (-36.5 to -18.3)*
Anemia due to hookworm disease aggregate	2,924.9 (2,885.1 to 2,960.3)	-9.1 (-11.4 to -6.8)*	-14.7 (-16.8 to -12.5)*	135.9 (91.7 to 193.2)	-11.5 (-15.2 to -8.0)*	-16.9 (-20.4 to -13.6)*
Asymptomatic hookworm disease	27,530.8 (24,437.4 to 31,013.7)	-16.2 (-29.5 to -2.3)*	-21.3 (-33.8 to -8.3)*	0.0 (0.0 to 0.0)	0.0 (0.0 to 0.0)	0.0 (0.0 to 0.0)
Food-borne trematodiases	1,711.4 (1,488.3 to 1,973.1)	-13.6 (-16.5 to -10.3)*	-18.9 (-21.6 to -15.7)*	18.3 (9.2 to 31.3)	-18.4 (-25.3 to -10.2)*	-23.4 (-29.9 to -15.7)*
Asymptomatic food-borne trematodiases aggregate	1,552.0 (1,329.7 to 1,813.9)	-13.2 (-16.1 to -9.8)*	-18.5 (-21.2 to -15.3)*	0.0 (0.0 to 0.0)	0.0 (0.0 to 0.0)	0.0 (0.0 to 0.0)
Symptomatic food-borne trematodiases aggregate	159.4 (94.0 to 249.7)	-17.8 (-23.4 to -11.4)*	-22.8 (-28.1 to -16.8)*	18.3 (9.2 to 31.3)	-18.4 (-25.3 to -10.2)*	-23.4 (-29.9 to -15.7)*
Leprosy	0.8 (0.6 to 0.9)	-38.4 (-40.1 to -36.7)*	-42.1 (-43.7 to -40.5)*	0.0 (0.0 to 0.1)	-39.6 (-41.3 to -37.8)*	-43.2 (-44.9 to -41.6)*
Ebola	0.2 (0.1 to 0.3)	0.0 (0.0 to 0.0)	0.0 (0.0 to 0.0)	0.0 (0.0 to 0.1)	0.0 (0.0 to 0.0)	0.0 (0.0 to 0.0)
Other neglected tropical diseases	15,525.6 (15,382.8 to 15,673.7)	0.5 (-0.6 to 1.6)	-5.6 (-6.7 to -4.6)*	1,205.3 (683.3 to 2,355.6)	7.7 (-10.9 to 39.2)	1.1 (-16.4 to 30.8)
Anemia due to other neglected tropical diseases aggregate	15,525.6 (15,382.8 to 15,673.7)	0.5 (-0.6 to 1.6)	-5.6 (-6.7 to -4.6)*	736.7 (495.5 to 1,050.9)	-1.4 (-3.1 to 0.3)	-7.4 (-9.0 to -5.8)*
Maternal disorders	0.0 (0.0 to 0.0)	0.0 (0.0 to 0.0)	0.0 (0.0 to 0.0)	0.0 (0.0 to 0.0)	0.0 (0.0 to 0.0)	0.0 (0.0 to 0.0)
Maternal hemorrhage	0.0 (0.0 to 0.0)	0.0 (0.0 to 0.0)	0.0 (0.0 to 0.0)	0.0 (0.0 to 0.0)	0.0 (0.0 to 0.0)	0.0 (0.0 to 0.0)
Maternal hemorrhage episodes aggregate	0.0 (0.0 to 0.0)	0.0 (0.0 to 0.0)	0.0 (0.0 to 0.0)	0.0 (0.0 to 0.0)	0.0 (0.0 to 0.0)	0.0 (0.0 to 0.0)
Anemia due to maternal hemorrhage aggregate	0.0 (0.0 to 0.0)	0.0 (0.0 to 0.0)	0.0 (0.0 to 0.0)	0.0 (0.0 to 0.0)	0.0 (0.0 to 0.0)	0.0 (0.0 to 0.0)
Maternal sepsis and other maternal infections	0.0 (0.0 to 0.0)	0.0 (0.0 to 0.0)	0.0 (0.0 to 0.0)	0.0 (0.0 to 0.0)	0.0 (0.0 to 0.0)	0.0 (0.0 to 0.0)
Maternal sepsis and other maternal infections aggregate	0.0 (0.0 to 0.0)	0.0 (0.0 to 0.0)	0.0 (0.0 to 0.0)	0.0 (0.0 to 0.0)	0.0 (0.0 to 0.0)	0.0 (0.0 to 0.0)
Infertility due to puerperal sepsis	0.0 (0.0 to 0.0)	0.0 (0.0 to 0.0)	0.0 (0.0 to 0.0)	0.0 (0.0 to 0.0)	0.0 (0.0 to 0.0)	0.0 (0.0 to 0.0)
Maternal hypertensive disorders	0.0 (0.0 to 0.0)	0.0 (0.0 to 0.0)	0.0 (0.0 to 0.0)	0.0 (0.0 to 0.0)	0.0 (0.0 to 0.0)	0.0 (0.0 to 0.0)
Maternal hypertensive disorders episodes aggregate	0.0 (0.0 to 0.0)	0.0 (0.0 to 0.0)	0.0 (0.0 to 0.0)	0.0 (0.0 to 0.0)	0.0 (0.0 to 0.0)	0.0 (0.0 to 0.0)
Eclampsia	0.0 (0.0 to 0.0)	0.0 (0.0 to 0.0)	0.0 (0.0 to 0.0)	0.0 (0.0 to 0.0)	0.0 (0.0 to 0.0)	0.0 (0.0 to 0.0)
Complications of maternal hypertensive disorders aggregate	0.0 (0.0 to 0.0)	0.0 (0.0 to 0.0)	0.0 (0.0 to 0.0)	0.0 (0.0 to 0.0)	0.0 (0.0 to 0.0)	0.0 (0.0 to 0.0)
Maternal obstructed labor and uterine rupture	0.0 (0.0 to 0.0)	0.0 (0.0 to 0.0)	0.0 (0.0 to 0.0)	0.0 (0.0 to 0.0)	0.0 (0.0 to 0.0)	0.0 (0.0 to 0.0)
Obstructed labor, acute event	0.0 (0.0 to 0.0)	0.0 (0.0 to 0.0)	0.0 (0.0 to 0.0)	0.0 (0.0 to 0.0)	0.0 (0.0 to 0.0)	0.0 (0.0 to 0.0)
Fistula due to maternal obstructed labor and uterine rupture aggregate	0.0 (0.0 to 0.0)	0.0 (0.0 to 0.0)	0.0 (0.0 to 0.0)	0.0 (0.0 to 0.0)	0.0 (0.0 to 0.0)	0.0 (0.0 to 0.0)
Maternal abortion, miscarriage, and ectopic pregnancy	0.0 (0.0 to 0.0)	0.0 (0.0 to 0.0)	0.0 (0.0 to 0.0)	0.0 (0.0 to 0.0)	0.0 (0.0 to 0.0)	0.0 (0.0 to 0.0)
Other maternal disorders	--	--	--	0.0 (0.0 to 0.0)	0.0 (0.0 to 0.0)	0.0 (0.0 to 0.0)
Neonatal disorders	4,783.3 (4,528.0 to 4,950.9)	10.5 (7.1 to 14.0)*	3.8 (0.5 to 7.0)*	1,249.7 (939.6 to 1,613.8)	8.9 (4.6 to 13.4)*	2.3 (-1.8 to 6.4)
Neonatal preterm birth complications	3,408.7 (2,976.6 to 3,885.3)	5.2 (-0.0 to 10.5)	-1.2 (-6.1 to 3.8)	571.7 (431.7 to 739.3)	0.3 (-5.6 to 6.5)	-5.9 (-11.3 to 0.0)
Vision loss due to retinopathy of prematurity aggregate	337.5 (263.6 to 426.6)	3.9 (-0.2 to 8.1)	-2.4 (-6.3 to 1.5)	8.4 (4.6 to 13.7)	4.9 (-5.7 to 16.4)	-1.5 (-11.5 to 9.3)
Complications of preterm birth complications aggregate	3,071.1 (2,658.5 to 3,544.2)	5.4 (-0.5 to 11.3)	-1.1 (-6.6 to 4.5)	563.2 (425.5 to 731.2)	0.2 (-5.6 to 6.5)	-5.9 (-11.4 to 0.0)
Neonatal encephalopathy due to birth asphyxia and trauma	1,561.1 (1,090.0 to 2,323.0)	21.5 (18.5 to 24.5)*	14.1 (11.3 to 17.0)*	471.4 (303.7 to 732.1)	19.2 (15.0 to 23.8)*	11.9 (8.0 to 16.3)*

Cause	Prevalence in thousands (95% confidence interval)			YLDs in thousands (95% confidence interval)		
	2015	Percent change in counts between 2005 and 2015	Percent change in rates between 2005 and 2015	2015	Percent change in counts between 2005 and 2015	Percent change in rates between 2005 and 2015
Neonatal sepsis and other neonatal infections	0.0 (0.0 to 0.0)	0.0 (0.0 to 0.0)	0.0 (0.0 to 0.0)	0.0 (0.0 to 0.0)	0.0 (0.0 to 0.0)	0.0 (0.0 to 0.0)
Severe infection due to neonatal sepsis and other neonatal infections	0.0 (0.0 to 0.0)	0.0 (0.0 to 0.0)	0.0 (0.0 to 0.0)	0.0 (0.0 to 0.0)	0.0 (0.0 to 0.0)	0.0 (0.0 to 0.0)
Complications of neonatal sepsis and other neonatal infections aggregate	0.0 (0.0 to 0.0)	0.0 (0.0 to 0.0)	0.0 (0.0 to 0.0)	0.0 (0.0 to 0.0)	0.0 (0.0 to 0.0)	0.0 (0.0 to 0.0)
Hemolytic disease and other neonatal jaundice	230.8 (205.5 to 262.5)	13.7 (12.4 to 15.0)*	6.7 (5.5 to 8.0)*	70.5 (52.5 to 89.4)	11.3 (5.8 to 16.8)*	4.5 (-0.7 to 9.7)
Other neonatal disorders	--	--	--	136.1 (90.0 to 187.0)	15.1 (-7.1 to 41.3)	8.1 (-12.7 to 32.7)
Nutritional deficiencies	171,019.6 (170,054.9 to 171,982.6)	1.2 (0.4 to 2.0)*	-5.0 (-5.8 to -4.3)*	8,749.0 (5,883.8 to 12,501.9)	-0.9 (-1.8 to -0.0)*	-6.9 (-7.7 to -6.1)*
Protein-energy malnutrition	21.0 (12.5 to 32.5)	-15.9 (-28.0 to -1.8)*	-21.1 (-32.4 to -7.8)*	2.6 (1.3 to 4.7)	-15.9 (-33.1 to 5.6)	-21.0 (-37.1 to -0.8)*
Iodine deficiency	4,149.0 (3,697.1 to 4,730.9)	-5.7 (-7.9 to -3.6)*	-11.4 (-13.5 to -9.5)*	140.1 (80.2 to 207.3)	-0.9 (-4.8 to 2.5)	-6.9 (-10.6 to -3.7)*
Visible goiter due to iodine deficiency aggregate	3,746.4 (3,337.4 to 4,264.1)	-6.7 (-8.8 to -4.5)*	-12.4 (-14.4 to -10.3)*	68.2 (41.8 to 108.0)	-6.5 (-9.7 to -3.6)*	-12.2 (-15.2 to -9.5)*
Visible goiter with heart failure due to iodine deficiency aggregate	0.0 (0.0 to 0.0)	0.0 (0.0 to 0.0)	0.0 (0.0 to 0.0)	0.0 (0.0 to 0.0)	0.0 (0.0 to 0.0)	0.0 (0.0 to 0.0)
Intellectual disability due to iodine deficiency aggregate	402.7 (168.6 to 577.3)	4.6 (1.2 to 6.3)*	-1.8 (-5.0 to -0.2)*	71.9 (27.1 to 121.8)	5.2 (-0.2 to 9.9)	-1.2 (-6.3 to 3.2)
Vitamin A deficiency	738.8 (485.8 to 1,103.4)	4.4 (0.8 to 7.7)*	-1.9 (-5.4 to 1.1)	27.9 (15.0 to 46.8)	4.2 (-1.3 to 9.7)	-2.2 (-7.4 to 3.0)
Iron-deficiency anemia	180,048.2 (179,066.7 to 181,017.5)	0.9 (0.1 to 1.6)*	-5.3 (-6.0 to -4.6)*	8,578.2 (5,751.9 to 12,269.0)	-0.9 (-1.8 to -0.0)*	-6.9 (-7.8 to -6.1)*
Iron-deficiency anemia without heart failure aggregate	180,048.1 (179,066.7 to 181,017.5)	0.9 (0.1 to 1.6)*	-5.3 (-6.0 to -4.6)*	8,578.2 (5,751.9 to 12,268.9)	-0.9 (-1.8 to -0.0)*	-6.9 (-7.8 to -6.1)*
Iron-deficiency anemia with heart failure aggregate	0.0 (0.0 to 0.1)	12.7 (9.6 to 15.6)*	5.8 (2.9 to 8.6)*	0.0 (0.0 to 0.0)	12.7 (9.6 to 15.6)*	5.8 (2.9 to 8.6)*
Other nutritional deficiencies	--	--	--	0.1 (0.1 to 0.2)	-31.2 (-48.1 to -10.0)*	-35.4 (-51.3 to -15.5)*
Other communicable, maternal, neonatal, and nutritional diseases	44,082.8 (43,223.2 to 44,745.8)	9.9 (8.3 to 11.5)*	3.2 (1.7 to 4.7)*	590.5 (396.3 to 841.7)	2.7 (-0.8 to 6.6)	-3.5 (-6.8 to 0.1)
Sexually transmitted diseases excluding HIV	36.4 (30.1 to 42.3)	2.1 (-14.1 to 23.0)	-4.1 (-19.3 to 15.5)	0.0 (0.0 to 0.0)	3.8 (-11.6 to 16.4)	-2.5 (-17.0 to 9.3)
Syphilis	48.2 (28.3 to 78.9)	3.6 (-11.6 to 16.1)	-2.7 (-17.0 to 9.1)	0.0 (0.0 to 0.0)	3.8 (-11.6 to 16.4)	-2.5 (-17.0 to 9.3)
Early syphilis aggregate	48.2 (28.3 to 78.9)	3.6 (-11.6 to 16.1)	-2.7 (-17.0 to 9.1)	0.0 (0.0 to 0.0)	3.8 (-11.6 to 16.4)	-2.5 (-17.0 to 9.3)
Adult tertiary syphilis	0.0 (0.0 to 0.0)	0.0 (0.0 to 0.0)	0.0 (0.0 to 0.0)	0.0 (0.0 to 0.0)	0.0 (0.0 to 0.0)	0.0 (0.0 to 0.0)
Chlamydial infection	0.0 (0.0 to 0.0)	0.0 (0.0 to 0.0)	0.0 (0.0 to 0.0)	0.0 (0.0 to 0.0)	0.0 (0.0 to 0.0)	0.0 (0.0 to 0.0)
Chlamydial infection episodes aggregate	0.0 (0.0 to 0.0)	0.0 (0.0 to 0.0)	0.0 (0.0 to 0.0)	0.0 (0.0 to 0.0)	0.0 (0.0 to 0.0)	0.0 (0.0 to 0.0)
Pelvic inflammatory diseases due to chlamydial infection aggregate	0.0 (0.0 to 0.0)	0.0 (0.0 to 0.0)	0.0 (0.0 to 0.0)	0.0 (0.0 to 0.0)	0.0 (0.0 to 0.0)	0.0 (0.0 to 0.0)
Infertility due to clamydial infection aggregate	0.0 (0.0 to 0.0)	0.0 (0.0 to 0.0)	0.0 (0.0 to 0.0)	0.0 (0.0 to 0.0)	0.0 (0.0 to 0.0)	0.0 (0.0 to 0.0)
Gonococcal infection	0.0 (0.0 to 0.0)	0.0 (0.0 to 0.0)	0.0 (0.0 to 0.0)	0.0 (0.0 to 0.0)	0.0 (0.0 to 0.0)	0.0 (0.0 to 0.0)
Gonococcal infection episodes aggregate	0.0 (0.0 to 0.0)	0.0 (0.0 to 0.0)	0.0 (0.0 to 0.0)	0.0 (0.0 to 0.0)	0.0 (0.0 to 0.0)	0.0 (0.0 to 0.0)
Pelvic inflammatory diseases due to gonococcal infection aggregate	0.0 (0.0 to 0.0)	0.0 (0.0 to 0.0)	0.0 (0.0 to 0.0)	0.0 (0.0 to 0.0)	0.0 (0.0 to 0.0)	0.0 (0.0 to 0.0)
Trichomoniasis	0.0 (0.0 to 0.0)	0.0 (0.0 to 0.0)	0.0 (0.0 to 0.0)	0.0 (0.0 to 0.0)	0.0 (0.0 to 0.0)	0.0 (0.0 to 0.0)
Genital herpes	0.0 (0.0 to 0.0)	0.0 (0.0 to 0.0)	0.0 (0.0 to 0.0)	0.0 (0.0 to 0.0)	0.0 (0.0 to 0.0)	0.0 (0.0 to 0.0)
Moderate infection due to initial genital herpes episode	0.0 (0.0 to 0.0)	0.0 (0.0 to 0.0)	0.0 (0.0 to 0.0)	0.0 (0.0 to 0.0)	0.0 (0.0 to 0.0)	0.0 (0.0 to 0.0)
Complications of genital herpes aggregate	0.0 (0.0 to 0.0)	0.0 (0.0 to 0.0)	0.0 (0.0 to 0.0)	0.0 (0.0 to 0.0)	0.0 (0.0 to 0.0)	0.0 (0.0 to 0.0)
Other sexually transmitted diseases	0.0 (0.0 to 0.0)	0.0 (0.0 to 0.0)	0.0 (0.0 to 0.0)	0.0 (0.0 to 0.0)	0.0 (0.0 to 0.0)	0.0 (0.0 to 0.0)
Pelvic inflammatory diseases due to other sexually transmitted diseases aggregate	0.0 (0.0 to 0.0)	0.0 (0.0 to 0.0)	0.0 (0.0 to 0.0)	0.0 (0.0 to 0.0)	0.0 (0.0 to 0.0)	0.0 (0.0 to 0.0)
Infertility due to other sexually transmitted diseases aggregate	0.0 (0.0 to 0.0)	0.0 (0.0 to 0.0)	0.0 (0.0 to 0.0)	0.0 (0.0 to 0.0)	0.0 (0.0 to 0.0)	0.0 (0.0 to 0.0)
Other sexually transmitted diseases	--	--	--	0.0 (0.0 to 0.0)	0.0 (0.0 to 0.0)	0.0 (0.0 to 0.0)
Hepatitis	33,338.0 (32,516.3 to 33,990.7)	13.3 (11.6 to 15.2)*	6.4 (4.8 to 8.2)*	37.6 (23.6 to 56.5)	5.4 (-20.2 to 35.6)	-1.0 (-25.1 to 27.3)
Acute hepatitis A	1,587.3 (1,378.0 to 1,732.2)	5.9 (-8.7 to 20.7)	-0.6 (-14.2 to 13.3)	29.6 (18.1 to 46.0)	5.6 (-24.8 to 45.2)	-0.8 (-29.4 to 36.4)
Hepatitis B	23,443.1 (21,799.0 to 25,298.2)	14.5 (10.7 to 18.1)*	7.6 (4.0 to 10.9)*	4.1 (2.5 to 6.5)	8.1 (-18.9 to 45.7)	1.5 (-23.9 to 36.8)
Acute Hepatitis B aggregate	580.4 (465.6 to 724.7)	8.0 (-18.3 to 43.3)	1.5 (-23.2 to 34.6)	4.1 (2.5 to 6.5)	8.1 (-18.9 to 45.7)	1.5 (-23.9 to 36.8)
Chronic hepatitis B	22,862.8 (21,251.3 to 24,739.5)	14.7 (10.9 to 18.3)*	7.7 (4.2 to 11.1)*	0.0 (0.0 to 0.0)	0.0 (0.0 to 0.0)	0.0 (0.0 to 0.0)
Hepatitis C	8,769.6 (7,786.0 to 9,846.0)	12.4 (10.8 to 13.9)*	5.6 (4.1 to 6.9)*	0.7 (0.3 to 1.3)	10.2 (-0.9 to 22.0)	3.5 (-7.0 to 14.6)
Acute Hepatitis C aggregate	46.3 (33.2 to 60.6)	10.1 (7.0 to 13.6)*	3.4 (0.5 to 6.7)*	0.7 (0.3 to 1.3)	10.2 (-0.9 to 22.0)	3.5 (-7.0 to 14.6)
Chronic hepatitis C	8,723.3 (7,742.4 to 9,793.8)	12.4 (10.8 to 13.9)*	5.6 (4.1 to 7.0)*	0.0 (0.0 to 0.0)	0.0 (0.0 to 0.0)	0.0 (0.0 to 0.0)
Acute hepatitis E	269.2 (217.7 to 326.9)	1.3 (-0.6 to 3.1)	-4.9 (-6.7 to -3.2)*	3.2 (1.8 to 5.2)	0.1 (-33.7 to 46.9)	-6.0 (-37.8 to 38.0)
Other infectious diseases	11,342.6 (11,196.7 to 11,507.1)	0.7 (-2.6 to 4.3)	-5.5 (-8.5 to -2.1)*	552.9 (373.8 to 792.6)	2.6 (-0.8 to 6.2)	-3.7 (-6.8 to -0.3)*
Non-communicable diseases	518,918.7 (511,088.5 to 526,011.3)	6.2 (5.7 to 6.7)*	-0.3 (-0.7 to 0.2)	18,035.2 (12,913.9 to 24,085.4)	6.3 (5.3 to 7.4)*	-0.2 (-1.1 to 0.9)
Neoplasms	354.2 (327.3 to 383.7)	17.7 (7.1 to 29.4)*	10.6 (0.5 to 21.5)*	32.1 (21.4 to 44.5)	16.1 (-0.1 to 34.8)	9.0 (-6.2 to 26.5)
Lip and oral cavity cancer	0.0 (0.0 to 0.0)	0.0 (0.0 to 0.0)	0.0 (0.0 to 0.0)	0.0 (0.0 to 0.0)	0.0 (0.0 to 0.0)	0.0 (0.0 to 0.0)
Nasopharynx cancer	3.4 (1.4 to 6.0)	7.5 (-43.0 to 95.2)	0.9 (-46.5 to 83.3)	0.2 (0.1 to 0.4)	7.0 (-43.0 to 92.1)	0.5 (-46.5 to 80.4)
Other pharynx cancer	0.0 (0.0 to 0.0)	0.0 (0.0 to 0.0)	0.0 (0.0 to 0.0)	0.0 (0.0 to 0.0)	0.0 (0.0 to 0.0)	0.0 (0.0 to 0.0)
Esophageal cancer	0.0 (0.0 to 0.0)	0.0 (0.0 to 0.0)	0.0 (0.0 to 0.0)	0.0 (0.0 to 0.0)	0.0 (0.0 to 0.0)	0.0 (0.0 to 0.0)
Stomach cancer	0.0 (0.0 to 0.0)	0.0 (0.0 to 0.0)	0.0 (0.0 to 0.0)	0.0 (0.0 to 0.0)	0.0 (0.0 to 0.0)	0.0 (0.0 to 0.0)
Colon and rectum cancer	0.0 (0.0 to 0.0)	0.0 (0.0 to 0.0)	0.0 (0.0 to 0.0)	0.0 (0.0 to 0.0)	0.0 (0.0 to 0.0)	0.0 (0.0 to 0.0)
Colon and rectum cancer aggregate	0.0 (0.0 to 0.0)	0.0 (0.0 to 0.0)	0.0 (0.0 to 0.0)	0.0 (0.0 to 0.0)	0.0 (0.0 to 0.0)	0.0 (0.0 to 0.0)
Stoma due to colon and rectum cancer	0.0 (0.0 to 0.0)	0.0 (0.0 to 0.0)	0.0 (0.0 to 0.0)	0.0 (0.0 to 0.0)	0.0 (0.0 to 0.0)	0.0 (0.0 to 0.0)
Liver cancer	0.0 (0.0 to 0.0)	0.0 (0.0 to 0.0)	0.0 (0.0 to 0.0)	0.2 (0.1 to 0.2)	-9.2 (-26.8 to 14.9)	-14.7 (-31.2 to 7.9)
Liver cancer due to hepatitis B	0.4 (0.3 to 0.5)	-2.4 (-25.9 to 32.4)	-8.3 (-30.4 to 24.3)	0.1 (0.0 to 0.1)	-9.9 (-29.2 to 19.8)	-15.3 (-33.5 to 12.5)
Liver cancer due to hepatitis C	0.0 (0.0 to 0.0)	10.3 (-10.0 to 35.2)	3.6 (-15.5 to 26.9)	0.0 (0.0 to 0.0)	0.5 (-16.1 to 20.4)	-5.6 (-21.2 to 13.1)
Liver cancer due to alcohol use	0.0 (0.0 to 0.0)	0.0 (0.0 to 0.0)	0.0 (0.0 to 0.0)	0.0 (0.0 to 0.0)	0.0 (0.0 to 0.0)	0.0 (0.0 to 0.0)
Liver cancer due to other causes	0.4 (0.3 to 0.5)	-1.4 (-21.9 to 24.6)	-7.4 (-26.6 to 17.0)	0.1 (0.1 to 0.1)	-9.0 (-25.6 to 11.3)	-14.6 (-30.1 to 4.6)
Gallbladder and biliary tract cancer	0.0 (0.0 to 0.0)	0.0 (0.0 to 0.0)	0.0 (0.0 to 0.0)	0.0 (0.0 to 0.0)	0.0 (0.0 to 0.0)	0.0 (0.0 to 0.0)
Pancreatic cancer	0.0 (0.0 to 0.0)	0.0 (0.0 to 0.0)	0.0 (0.0 to 0.0)	0.0 (0.0 to 0.0)	0.0 (0.0 to 0.0)	0.0 (0.0 to 0.0)

Cause	Prevalence in thousands (95% confidence interval)			YLDs in thousands (95% confidence interval)		
	2015	Percent change in counts between 2005 and 2015	Percent change in rates between 2005 and 2015	2015	Percent change in counts between 2005 and 2015	Percent change in rates between 2005 and 2015
Larynx cancer	0.0 (0.0 to 0.0)	0.0 (0.0 to 0.0)	0.0 (0.0 to 0.0)	0.0 (0.0 to 0.0)	0.0 (0.0 to 0.0)	0.0 (0.0 to 0.0)
Larynx cancer aggregate	0.0 (0.0 to 0.0)	0.0 (0.0 to 0.0)	0.0 (0.0 to 0.0)	0.0 (0.0 to 0.0)	0.0 (0.0 to 0.0)	0.0 (0.0 to 0.0)
Laryngectomy due to larynx cancer	0.0 (0.0 to 0.0)	0.0 (0.0 to 0.0)	0.0 (0.0 to 0.0)	0.0 (0.0 to 0.0)	0.0 (0.0 to 0.0)	0.0 (0.0 to 0.0)
Tracheal, bronchus, and lung cancer	0.0 (0.0 to 0.0)	0.0 (0.0 to 0.0)	0.0 (0.0 to 0.0)	0.0 (0.0 to 0.0)	0.0 (0.0 to 0.0)	0.0 (0.0 to 0.0)
Malignant skin melanoma	0.0 (0.0 to 0.0)	0.0 (0.0 to 0.0)	0.0 (0.0 to 0.0)	0.0 (0.0 to 0.0)	0.0 (0.0 to 0.0)	0.0 (0.0 to 0.0)
Non-melanoma skin cancer	0.0 (0.0 to 0.0)	0.0 (0.0 to 0.0)	0.0 (0.0 to 0.0)	0.0 (0.0 to 0.0)	0.0 (0.0 to 0.0)	0.0 (0.0 to 0.0)
Non-melanoma skin cancer(squamous-cell carcinoma)	0.0 (0.0 to 0.0)	0.0 (0.0 to 0.0)	0.0 (0.0 to 0.0)	0.0 (0.0 to 0.0)	0.0 (0.0 to 0.0)	0.0 (0.0 to 0.0)
Non-melanoma skin cancer(basal-cell carcinoma)	0.0 (0.0 to 0.0)	0.0 (0.0 to 0.0)	0.0 (0.0 to 0.0)	0.0 (0.0 to 0.0)	0.0 (0.0 to 0.0)	0.0 (0.0 to 0.0)
Breast cancer	0.0 (0.0 to 0.0)	0.0 (0.0 to 0.0)	0.0 (0.0 to 0.0)	0.0 (0.0 to 0.0)	0.0 (0.0 to 0.0)	0.0 (0.0 to 0.0)
Breast cancer aggregate	0.0 (0.0 to 0.0)	0.0 (0.0 to 0.0)	0.0 (0.0 to 0.0)	0.0 (0.0 to 0.0)	0.0 (0.0 to 0.0)	0.0 (0.0 to 0.0)
Mastectomy due to breast cancer	0.0 (0.0 to 0.0)	0.0 (0.0 to 0.0)	0.0 (0.0 to 0.0)	0.0 (0.0 to 0.0)	0.0 (0.0 to 0.0)	0.0 (0.0 to 0.0)
Cervical cancer	0.0 (0.0 to 0.0)	0.0 (0.0 to 0.0)	0.0 (0.0 to 0.0)	0.0 (0.0 to 0.0)	0.0 (0.0 to 0.0)	0.0 (0.0 to 0.0)
Uterine cancer	0.0 (0.0 to 0.0)	0.0 (0.0 to 0.0)	0.0 (0.0 to 0.0)	0.0 (0.0 to 0.0)	0.0 (0.0 to 0.0)	0.0 (0.0 to 0.0)
Ovarian cancer	0.0 (0.0 to 0.0)	0.0 (0.0 to 0.0)	0.0 (0.0 to 0.0)	0.0 (0.0 to 0.0)	0.0 (0.0 to 0.0)	0.0 (0.0 to 0.0)
Prostate cancer	0.0 (0.0 to 0.0)	0.0 (0.0 to 0.0)	0.0 (0.0 to 0.0)	0.0 (0.0 to 0.0)	0.0 (0.0 to 0.0)	0.0 (0.0 to 0.0)
Prostate cancer aggregate	0.0 (0.0 to 0.0)	0.0 (0.0 to 0.0)	0.0 (0.0 to 0.0)	0.0 (0.0 to 0.0)	0.0 (0.0 to 0.0)	0.0 (0.0 to 0.0)
Impotence and incontinence due to prostate cancer aggregate	0.0 (0.0 to 0.0)	0.0 (0.0 to 0.0)	0.0 (0.0 to 0.0)	0.0 (0.0 to 0.0)	0.0 (0.0 to 0.0)	0.0 (0.0 to 0.0)
Testicular cancer	0.0 (0.0 to 0.0)	0.0 (0.0 to 0.0)	0.0 (0.0 to 0.0)	0.0 (0.0 to 0.0)	0.0 (0.0 to 0.0)	0.0 (0.0 to 0.0)
Kidney cancer	25.4 (21.3 to 30.2)	23.6 (5.6 to 44.7)*	16.1 (-0.9 to 35.8)	1.7 (1.1 to 2.4)	22.3 (4.4 to 44.8)*	14.8 (-2.0 to 36.0)
Bladder cancer	0.0 (0.0 to 0.0)	0.0 (0.0 to 0.0)	0.0 (0.0 to 0.0)	0.0 (0.0 to 0.0)	0.0 (0.0 to 0.0)	0.0 (0.0 to 0.0)
Bladder cancer episodes aggregate	0.0 (0.0 to 0.0)	0.0 (0.0 to 0.0)	0.0 (0.0 to 0.0)	0.0 (0.0 to 0.0)	0.0 (0.0 to 0.0)	0.0 (0.0 to 0.0)
Urinary incontinence due to bladder cancer	0.0 (0.0 to 0.0)	0.0 (0.0 to 0.0)	0.0 (0.0 to 0.0)	0.0 (0.0 to 0.0)	0.0 (0.0 to 0.0)	0.0 (0.0 to 0.0)
Brain and nervous system cancer	55.8 (45.4 to 66.7)	12.7 (-6.1 to 33.7)	5.8 (-11.8 to 25.6)	5.1 (3.5 to 7.1)	11.7 (-7.6 to 32.1)	4.9 (-13.2 to 24.0)
Thyroid cancer	0.0 (0.0 to 0.0)	0.0 (0.0 to 0.0)	0.0 (0.0 to 0.0)	0.0 (0.0 to 0.0)	0.0 (0.0 to 0.0)	0.0 (0.0 to 0.0)
Mesothelioma	0.0 (0.0 to 0.0)	0.0 (0.0 to 0.0)	0.0 (0.0 to 0.0)	0.0 (0.0 to 0.0)	0.0 (0.0 to 0.0)	0.0 (0.0 to 0.0)
Hodgkin lymphoma	10.9 (8.5 to 13.7)	-15.8 (-30.1 to 3.2)	-21.0 (-34.3 to -3.1)*	0.7 (0.4 to 1.0)	-18.7 (-32.6 to -0.2)*	-23.6 (-36.7 to -6.3)*
Non-Hodgkin lymphoma	101.2 (75.0 to 125.2)	37.6 (6.0 to 74.2)*	29.2 (-0.5 to 63.6)	6.4 (3.9 to 9.3)	34.1 (2.8 to 72.1)*	25.9 (-3.4 to 61.6)
Multiple myeloma	0.0 (0.0 to 0.0)	0.0 (0.0 to 0.0)	0.0 (0.0 to 0.0)	0.0 (0.0 to 0.0)	0.0 (0.0 to 0.0)	0.0 (0.0 to 0.0)
Leukemia	106.2 (85.6 to 124.8)	5.8 (-12.1 to 27.0)	-0.7 (-17.4 to 19.2)	10.6 (6.0 to 17.6)	9.3 (-19.7 to 52.4)	2.6 (-24.6 to 43.1)
Acute lymphoid leukemia	102.8 (62.6 to 158.7)	11.8 (-21.4 to 59.3)	5.0 (-26.2 to 49.6)	7.7 (4.3 to 12.8)	9.7 (-21.4 to 53.3)	3.0 (-26.2 to 43.9)
Chronic lymphoid leukemia	0.0 (0.0 to 0.0)	0.0 (0.0 to 0.0)	0.0 (0.0 to 0.0)	0.0 (0.0 to 0.0)	0.0 (0.0 to 0.0)	0.0 (0.0 to 0.0)
Acute myeloid leukemia	40.2 (24.0 to 63.0)	9.9 (-23.1 to 55.4)	3.2 (-27.7 to 45.9)	2.9 (1.5 to 4.8)	8.0 (-22.7 to 51.7)	1.5 (-27.5 to 42.5)
Chronic myeloid leukemia	0.0 (0.0 to 0.0)	0.0 (0.0 to 0.0)	0.0 (0.0 to 0.0)	0.0 (0.0 to 0.0)	0.0 (0.0 to 0.0)	0.0 (0.0 to 0.0)
Other neoplasms	117.1 (92.2 to 143.4)	21.8 (1.8 to 44.6)*	14.3 (-4.4 to 35.8)	7.2 (4.6 to 10.6)	20.7 (0.8 to 43.9)*	13.3 (-5.3 to 35.1)
Cardiovascular diseases	8,113.1 (7,362.4 to 8,768.8)	3.7 (-1.5 to 9.2)	-2.6 (-7.5 to 2.5)	620.5 (399.9 to 929.6)	4.9 (2.6 to 7.0)*	-1.5 (-3.7 to 0.4)
Rheumatic heart disease	1,297.9 (1,159.0 to 1,561.8)	-9.2 (-14.0 to -4.6)*	-14.7 (-19.3 to -10.5)*	63.2 (39.5 to 96.4)	-8.9 (-13.9 to -3.9)*	-14.4 (-19.1 to -9.8)*
Rheumatic heart disease, without heart failure	1,285.6 (1,147.0 to 1,549.2)	-9.3 (-14.2 to -4.7)*	-14.8 (-19.5 to -10.5)*	61.7 (38.5 to 94.5)	-9.2 (-14.4 to -4.2)*	-14.7 (-19.6 to -10.0)*
Heart failure fue to rheumatic heart disease aggregate	12.2 (10.3 to 14.6)	7.0 (5.5 to 8.6)*	0.5 (-0.9 to 1.9)	1.5 (1.0 to 2.1)	7.0 (5.6 to 8.7)*	0.5 (-0.9 to 2.1)
Ischemic heart disease	73.1 (63.3 to 83.9)	8.3 (7.1 to 9.4)*	1.7 (0.6 to 2.8)*	5.5 (3.5 to 8.0)	8.3 (3.6 to 13.0)*	1.7 (-2.7 to 6.1)
Myocardial infarction episodes aggregate	0.1 (0.1 to 0.3)	7.5 (1.9 to 13.8)*	0.9 (-4.3 to 6.9)	0.0 (0.0 to 0.0)	7.4 (1.9 to 13.8)*	0.9 (-4.3 to 6.9)
Angina due to ischemic heart disease aggregate	69.2 (59.4 to 79.9)	8.4 (7.0 to 9.5)*	1.8 (0.5 to 2.9)*	5.0 (3.2 to 7.3)	8.3 (3.1 to 13.5)*	1.7 (-3.2 to 6.6)
Heart failure fue to ischemic heart disease aggregate	3.8 (2.9 to 4.7)	7.9 (5.6 to 10.3)*	1.3 (-0.8 to 3.5)	0.5 (0.3 to 0.7)	7.9 (5.6 to 10.3)*	1.3 (-0.9 to 3.5)
Cerebrovascular disease	464.6 (448.9 to 481.8)	8.9 (4.2 to 13.3)*	2.2 (-2.1 to 6.4)	93.5 (63.9 to 129.3)	8.4 (3.0 to 14.4)*	1.8 (-3.3 to 7.4)
Ischemic stroke	108.7 (101.8 to 116.3)	16.6 (14.4 to 18.4)*	9.5 (7.4 to 11.2)*	18.1 (12.1 to 25.5)	16.5 (13.7 to 19.2)*	9.4 (6.8 to 12.0)*
Chronic ischemic stroke aggregate	106.7 (99.6 to 114.3)	16.6 (14.4 to 18.5)*	9.5 (7.5 to 11.3)*	17.7 (11.7 to 25.0)	16.5 (13.7 to 19.3)*	9.4 (6.8 to 12.1)*
Ischemic stroke episodes aggregate	2.0 (1.3 to 2.9)	14.2 (11.9 to 16.8)*	7.2 (5.1 to 9.7)*	0.4 (0.2 to 0.7)	14.2 (11.9 to 16.8)*	7.2 (5.1 to 9.7)*
Hemorrhagic stroke	467.8 (439.0 to 499.6)	6.6 (4.8 to 8.3)*	0.1 (-1.6 to 1.7)	75.4 (51.7 to 104.3)	6.7 (0.3 to 13.9)*	0.2 (-5.8 to 6.9)
Chronic hemorrhagic stroke aggregate	460.2 (431.3 to 492.2)	6.6 (4.7 to 8.3)*	0.1 (-1.6 to 1.7)	73.7 (50.4 to 102.3)	6.7 (0.2 to 14.1)*	0.2 (-5.9 to 7.1)
Acute hemorrhagic stroke aggregate	7.6 (6.3 to 9.1)	4.8 (0.7 to 9.4)*	-1.6 (-5.5 to 2.7)	1.7 (1.0 to 2.3)	4.8 (0.7 to 9.4)*	-1.6 (-5.5 to 2.7)
Hypertensive heart disease	0.6 (0.5 to 0.7)	6.4 (5.0 to 7.7)*	-0.1 (-1.4 to 1.2)	0.1 (0.0 to 0.1)	6.4 (5.0 to 7.7)*	-0.1 (-1.4 to 1.2)
Cardiomyopathy and myocarditis	54.5 (45.8 to 64.3)	8.9 (7.9 to 9.9)*	2.3 (1.3 to 3.2)*	6.3 (4.1 to 8.9)	8.9 (3.2 to 15.2)*	2.2 (-3.1 to 8.1)
Acute myocarditis	4.9 (3.1 to 7.2)	10.3 (7.9 to 12.8)*	3.6 (1.4 to 5.9)*	0.2 (0.1 to 0.4)	10.3 (7.9 to 12.8)*	3.6 (1.4 to 5.9)*
Heart failure due to cardiomyopathy aggregate	49.6 (41.3 to 59.7)	8.8 (7.7 to 9.8)*	2.1 (1.1 to 3.2)*	6.1 (3.9 to 8.6)	8.8 (2.9 to 15.3)*	2.2 (-3.4 to 8.3)
Atrial fibrillation and flutter	0.0 (0.0 to 0.0)	0.0 (0.0 to 0.0)	0.0 (0.0 to 0.0)	0.0 (0.0 to 0.0)	0.0 (0.0 to 0.0)	0.0 (0.0 to 0.0)
Peripheral vascular disease	0.0 (0.0 to 0.0)	0.0 (0.0 to 0.0)	0.0 (0.0 to 0.0)	0.0 (0.0 to 0.0)	0.0 (0.0 to 0.0)	0.0 (0.0 to 0.0)
Endocarditis	0.7 (0.6 to 0.8)	4.6 (0.4 to 9.4)*	-1.8 (-5.7 to 2.7)	0.0 (0.0 to 0.1)	4.6 (0.3 to 9.3)*	-1.8 (-5.8 to 2.6)
Endocarditis episodes aggregate	0.7 (0.6 to 0.8)	4.6 (0.4 to 9.4)*	-1.8 (-5.7 to 2.7)	0.0 (0.0 to 0.1)	4.6 (0.3 to 9.3)*	-1.8 (-5.8 to 2.6)
Heart failure due to endocarditis aggregate	0.0 (0.0 to 0.0)	0.0 (0.0 to 0.0)	0.0 (0.0 to 0.0)	0.0 (0.0 to 0.0)	0.0 (0.0 to 0.0)	0.0 (0.0 to 0.0)
Other cardiovascular and circulatory diseases	6,418.7 (4,529.3 to 8,733.1)	6.2 (3.9 to 8.2)*	-0.3 (-2.5 to 1.6)	452.0 (269.6 to 715.4)	6.3 (3.6 to 8.7)*	-0.2 (-2.7 to 2.1)
Heart failure due to other cardiovascular diseases aggregate	0.9 (0.8 to 1.1)	5.2 (3.2 to 7.2)*	-1.2 (-3.1 to 0.7)	0.1 (0.1 to 0.2)	5.2 (3.2 to 7.2)*	-1.2 (-3.1 to 0.7)
Other cardiovascular diseases episodes aggregate	6,417.8 (4,528.4 to 8,732.1)	6.2 (3.9 to 8.2)*	-0.3 (-2.5 to 1.6)	451.9 (269.5 to 715.2)	6.3 (3.6 to 8.7)*	-0.2 (-2.7 to 2.1)
Chronic respiratory diseases	59,019.6 (54,776.5 to 63,044.5)	4.2 (1.0 to 7.6)*	-2.1 (-5.2 to 1.1)	2,900.5 (1,798.5 to 4,301.2)	3.4 (-0.7 to 8.0)	-2.9 (-6.8 to 1.4)
Chronic obstructive pulmonary disease	1,682.5 (1,374.5 to 2,075.5)	-3.6 (-6.5 to -0.9)*	-9.5 (-12.2 to -7.0)*	142.7 (110.3 to 183.6)	-5.4 (-10.1 to -0.6)*	-11.2 (-15.6 to -6.6)*

Cause	Prevalence in thousands (95% confidence interval)			YLDs in thousands (95% confidence interval)		
	2015	Percent change in counts between 2005 and 2015	Percent change in rates between 2005 and 2015	2015	Percent change in counts between 2005 and 2015	Percent change in rates between 2005 and 2015
Chronic obstructive pulmonary disease without heart failure aggregate	1,685.1 (1,377.2 to 2,076.7)	-3.5 (-6.3 to -0.8)*	-9.4 (-12.0 to -6.9)*	142.5 (110.1 to 183.4)	-5.4 (-10.1 to -0.6)*	-11.2 (-15.6 to -6.6)*
Severe chronic obstructive pulmonary disease with heart failure aggregate	0.5 (0.4 to 0.6)	2.5 (1.1 to 4.0)*	-3.8 (-5.1 to -2.3)*	0.2 (0.2 to 0.3)	2.5 (1.1 to 4.0)*	-3.8 (-5.1 to -2.3)*
Pneumoconiosis	1.7 (1.3 to 2.2)	41.9 (-1.1 to 101.3)	33.2 (-7.2 to 89.0)	8.6 (5.1 to 12.7)	20.4 (15.3 to 25.8)*	13.1 (8.3 to 18.2)*
Silicosis	0.0 (0.0 to 0.0)	0.0 (0.0 to 0.0)	0.0 (0.0 to 0.0)	0.0 (0.0 to 0.0)	0.0 (0.0 to 0.0)	0.0 (0.0 to 0.0)
Silicosis without heart failure aggregate	0.0 (0.0 to 0.0)	0.0 (0.0 to 0.0)	0.0 (0.0 to 0.0)	0.0 (0.0 to 0.0)	0.0 (0.0 to 0.0)	0.0 (0.0 to 0.0)
Severe silicosis with heart failure aggregate	0.0 (0.0 to 0.0)	0.0 (0.0 to 0.0)	0.0 (0.0 to 0.0)	0.0 (0.0 to 0.0)	0.0 (0.0 to 0.0)	0.0 (0.0 to 0.0)
Asbestosis	0.0 (0.0 to 0.0)	0.0 (0.0 to 0.0)	0.0 (0.0 to 0.0)	0.0 (0.0 to 0.0)	0.0 (0.0 to 0.0)	0.0 (0.0 to 0.0)
Asbestosis without heart failure aggregate	0.0 (0.0 to 0.0)	0.0 (0.0 to 0.0)	0.0 (0.0 to 0.0)	0.0 (0.0 to 0.0)	0.0 (0.0 to 0.0)	0.0 (0.0 to 0.0)
Severe asbestosis with heart failure aggregate	0.0 (0.0 to 0.0)	0.0 (0.0 to 0.0)	0.0 (0.0 to 0.0)	0.0 (0.0 to 0.0)	0.0 (0.0 to 0.0)	0.0 (0.0 to 0.0)
Coal workers pneumoconiosis	0.0 (0.0 to 0.0)	0.0 (0.0 to 0.0)	0.0 (0.0 to 0.0)	0.0 (0.0 to 0.0)	0.0 (0.0 to 0.0)	0.0 (0.0 to 0.0)
Coal workers pneumoconiosis without heart failure aggregate	0.0 (0.0 to 0.0)	0.0 (0.0 to 0.0)	0.0 (0.0 to 0.0)	0.0 (0.0 to 0.0)	0.0 (0.0 to 0.0)	0.0 (0.0 to 0.0)
Severe coal workers pneumoconiosis with heart failure aggregate	0.0 (0.0 to 0.0)	0.0 (0.0 to 0.0)	0.0 (0.0 to 0.0)	0.0 (0.0 to 0.0)	0.0 (0.0 to 0.0)	0.0 (0.0 to 0.0)
Other pneumoconiosis	51.3 (43.3 to 60.9)	20.5 (15.4 to 25.5)*	13.1 (8.4 to 17.8)*	8.6 (5.1 to 12.7)	20.4 (15.3 to 25.8)*	13.1 (8.3 to 18.2)*
Other pneumoconiosis without heart failure aggregate	51.3 (43.3 to 60.9)	20.5 (15.4 to 25.5)*	13.1 (8.4 to 17.8)*	8.6 (5.1 to 12.7)	20.4 (15.3 to 25.8)*	13.1 (8.3 to 18.2)*
Severe other pneumoconiosis with heart failure aggregate	0.0 (0.0 to 0.0)	0.0 (0.0 to 0.0)	0.0 (0.0 to 0.0)	0.0 (0.0 to 0.0)	0.0 (0.0 to 0.0)	0.0 (0.0 to 0.0)
Asthma	59,699.2 (45,150.8 to 75,415.5)	4.2 (-0.3 to 9.3)	-2.2 (-6.4 to 2.6)	2,711.3 (1,624.4 to 4,098.2)	4.2 (-0.4 to 9.2)	-2.1 (-6.5 to 2.5)
Asymptomatic asthma	18,073.9 (13,623.5 to 23,185.9)	4.2 (-0.3 to 9.3)	-2.2 (-6.4 to 2.6)	0.0 (0.0 to 0.0)	0.0 (0.0 to 0.0)	0.0 (0.0 to 0.0)
Symptomatic asthma aggregate	41,625.3 (31,360.0 to 53,358.0)	4.2 (-0.3 to 9.3)	-2.2 (-6.4 to 2.6)	2,711.3 (1,624.4 to 4,098.2)	4.2 (-0.4 to 9.2)	-2.1 (-6.5 to 2.5)
Interstitial lung disease and pulmonary sarcoidosis	25.7 (21.8 to 30.4)	5.8 (2.2 to 8.9)*	-0.6 (-4.1 to 2.3)	3.3 (1.9 to 5.0)	5.8 (1.1 to 10.2)*	-0.6 (-5.1 to 3.5)
Interstitial lung disease and pulmonary sarcoidosis without heart failure aggregate	24.2 (20.4 to 28.9)	6.1 (2.2 to 9.3)*	-0.4 (-4.0 to 2.6)	2.6 (1.5 to 4.2)	6.9 (0.9 to 12.5)*	0.3 (-5.2 to 5.6)
Severe interstitial lung disease and pulmonary sarcoidosis with heart failure aggregate	1.5 (1.2 to 1.7)	2.2 (0.7 to 3.7)*	-4.1 (-5.5 to -2.6)*	0.7 (0.5 to 0.9)	2.2 (0.7 to 3.7)*	-4.1 (-5.5 to -2.6)*
Other chronic respiratory diseases	--	--	--	34.6 (26.3 to 45.2)	-17.0 (-26.0 to -6.1)*	-22.1 (-30.6 to -11.9)*
Cirrhosis and other chronic liver diseases	62.0 (56.6 to 67.1)	15.8 (2.5 to 29.2)*	8.7 (-3.7 to 21.3)	12.1 (8.2 to 17.0)	12.7 (1.7 to 24.1)*	5.9 (-4.5 to 16.5)
Cirrhosis and other chronic liver diseases due to hepatitis B	1.4 (1.1 to 1.7)	12.1 (9.0 to 15.1)*	5.3 (2.4 to 8.1)*	0.3 (0.2 to 0.4)	12.1 (9.0 to 15.1)*	5.3 (2.4 to 8.1)*
Cirrhosis and other chronic liver diseases due to hepatitis C	0.9 (0.7 to 1.1)	14.0 (9.0 to 18.2)*	7.1 (2.4 to 11.0)*	0.2 (0.1 to 0.2)	14.0 (9.0 to 18.2)*	7.0 (2.4 to 11.0)*
Cirrhosis and other chronic liver diseases due to alcohol use	0.0 (0.0 to 0.0)	0.0 (0.0 to 0.0)	0.0 (0.0 to 0.0)	0.0 (0.0 to 0.0)	0.0 (0.0 to 0.0)	0.0 (0.0 to 0.0)
Cirrhosis and other chronic liver diseases due to other causes	67.9 (63.4 to 72.4)	12.6 (11.7 to 13.6)*	5.8 (4.9 to 6.7)*	11.7 (7.9 to 16.4)	12.7 (1.2 to 24.4)*	5.9 (-5.0 to 16.9)
Digestive diseases	4,106.2 (3,987.3 to 4,230.3)	-0.7 (-3.4 to 2.3)	-6.7 (-9.3 to -3.9)*	265.3 (182.7 to 367.3)	6.1 (2.8 to 10.3)*	-0.3 (-3.5 to 3.6)
Peptic ulcer disease	224.9 (216.5 to 234.0)	-20.7 (-24.1 to -17.0)*	-25.5 (-28.7 to -22.1)*	13.2 (8.9 to 18.8)	-17.8 (-24.3 to -10.7)*	-22.8 (-28.9 to -16.2)*
Peptic ulcer disease, symptomatic episodes	11.2 (8.4 to 14.5)	-23.6 (-29.4 to -18.5)*	-28.2 (-33.7 to -23.5)*	1.3 (0.8 to 2.0)	-23.0 (-33.7 to -12.0)*	-27.7 (-37.7 to -17.4)*
Anemia due to peptic ulcer disease aggregate	213.7 (205.7 to 222.3)	-20.5 (-24.2 to -16.6)*	-25.3 (-28.8 to -21.7)*	11.9 (8.0 to 17.0)	-17.2 (-24.5 to -9.5)*	-22.3 (-29.1 to -15.0)*
Gastritis and duodenitis	3,044.4 (2,953.5 to 3,142.7)	3.5 (1.0 to 6.2)*	-2.8 (-5.2 to -0.3)*	189.0 (129.7 to 264.1)	8.3 (4.2 to 13.1)*	1.7 (-2.1 to 6.2)
Gastritis and duodenitis, symptomatic episodes	57.2 (43.5 to 72.1)	0.8 (-2.6 to 3.2)	-5.4 (-8.5 to -3.1)*	6.3 (3.9 to 9.2)	0.7 (-10.1 to 13.4)	-5.4 (-15.6 to 6.4)
Anemia due to gastritis and duodenitis aggregate	2,987.3 (2,898.5 to 3,084.2)	3.5 (0.9 to 6.3)*	-2.8 (-5.2 to -0.2)*	182.7 (124.9 to 256.8)	8.5 (4.3 to 13.6)*	1.9 (-2.0 to 6.6)
Appendicitis	31.9 (26.6 to 38.0)	9.2 (7.4 to 10.9)*	2.5 (0.8 to 4.1)*	10.0 (6.3 to 14.5)	8.8 (-5.6 to 25.2)	2.2 (-11.3 to 17.5)
Paralytic ileus and intestinal obstruction	3.8 (2.8 to 5.0)	1.0 (-5.2 to 6.5)	-5.2 (-11.0 to -0.0)*	1.2 (0.7 to 1.9)	1.0 (-5.2 to 6.5)	-5.2 (-11.0 to -0.0)*
Inguinal, femoral, and abdominal hernia	804.5 (553.5 to 1,102.1)	-8.1 (-12.2 to -4.1)*	-13.7 (-17.5 to -9.9)*	8.7 (3.9 to 17.3)	-8.0 (-13.0 to -3.0)*	-13.6 (-18.3 to -8.9)*
Inflammatory bowel disease	57.1 (45.9 to 70.3)	4.7 (3.9 to 5.6)*	-1.7 (-2.5 to -0.8)*	12.9 (7.9 to 18.9)	5.1 (-2.0 to 12.5)	-1.3 (-8.0 to 5.6)
Vascular intestinal disorders	0.3 (0.2 to 0.4)	-0.8 (-7.0 to 4.4)	-6.9 (-12.6 to -1.9)*	0.1 (0.1 to 0.2)	-0.8 (-7.0 to 4.4)	-6.9 (-12.6 to -1.9)*
Gallbladder and biliary diseases	29.6 (21.8 to 39.4)	-2.3 (-8.0 to 2.6)	-8.2 (-13.6 to -3.6)*	3.3 (2.0 to 5.1)	-2.0 (-15.8 to 12.9)	-8.0 (-20.9 to 6.1)
Pancreatitis	7.1 (5.1 to 9.2)	8.1 (3.6 to 11.9)*	1.5 (-2.7 to 5.1)	2.3 (1.4 to 3.5)	8.0 (-1.9 to 17.8)	1.5 (-7.9 to 10.6)
Other digestive diseases	--	--	--	24.6 (16.4 to 35.2)	14.0 (4.9 to 26.6)*	7.0 (-1.5 to 18.9)
Neurological disorders	40,480.8 (38,116.0 to 43,267.6)	5.2 (2.7 to 7.9)*	-1.2 (-3.6 to 1.4)	1,195.2 (801.2 to 1,679.1)	-1.1 (-5.2 to 3.1)	-7.1 (-11.0 to -3.2)*
Alzheimer disease and other dementias	0.0 (0.0 to 0.0)	0.0 (0.0 to 0.0)	0.0 (0.0 to 0.0)	0.0 (0.0 to 0.0)	0.0 (0.0 to 0.0)	0.0 (0.0 to 0.0)
Parkinson disease	0.0 (0.0 to 0.0)	0.0 (0.0 to 0.0)	0.0 (0.0 to 0.0)	0.0 (0.0 to 0.0)	0.0 (0.0 to 0.0)	0.0 (0.0 to 0.0)
Epilepsy	2,142.2 (1,747.5 to 2,642.2)	8.9 (3.0 to 14.9)*	2.3 (-3.3 to 7.9)	635.8 (411.7 to 895.8)	-6.4 (-13.0 to 0.8)	-12.1 (-18.3 to -5.3)*
Multiple sclerosis	3.9 (2.6 to 5.7)	2.4 (-0.4 to 4.7)	-3.9 (-6.5 to -1.6)*	1.4 (0.8 to 2.2)	2.4 (-0.4 to 4.7)	-3.9 (-6.5 to -1.6)*
Motor neuron disease	3.7 (3.4 to 4.1)	8.6 (5.9 to 11.4)*	2.0 (-0.5 to 4.6)	0.8 (0.6 to 1.1)	8.6 (5.9 to 11.4)*	2.0 (-0.5 to 4.6)
Migraine	13,878.7 (10,671.0 to 17,645.8)	5.6 (2.4 to 8.9)*	-0.8 (-3.8 to 2.3)	495.2 (279.5 to 785.1)	5.8 (1.9 to 10.0)*	-0.7 (-4.3 to 3.3)
Tension-type headache	25,125.1 (16,919.1 to 34,775.7)	4.7 (3.7 to 5.4)*	-1.7 (-2.6 to -1.0)*	38.3 (16.1 to 72.7)	4.8 (2.0 to 7.6)*	-1.6 (-4.3 to 1.1)
Medication overuse headache	142.6 (79.4 to 225.2)	7.5 (-2.5 to 18.2)	1.0 (-8.4 to 11.0)	23.3 (11.6 to 42.2)	7.6 (-5.3 to 21.5)	1.0 (-11.1 to 14.1)
Other neurological disorders	0.8 (0.4 to 1.4)	7.9 (6.8 to 8.7)*	1.3 (0.3 to 2.1)*	0.3 (0.2 to 0.5)	6.1 (4.0 to 7.5)*	-0.4 (-2.4 to 1.0)
Mental and substance use disorders	43,703.1 (41,693.3 to 45,657.0)	6.7 (5.2 to 8.5)*	0.2 (-1.3 to 1.9)	3,599.9 (2,492.3 to 4,876.7)	7.1 (6.1 to 8.2)*	0.6 (-0.3 to 1.6)
Schizophrenia	0.0 (0.0 to 0.0)	0.0 (0.0 to 0.0)	0.0 (0.0 to 0.0)	0.0 (0.0 to 0.0)	0.0 (0.0 to 0.0)	0.0 (0.0 to 0.0)
Alcohol use disorders	92.0 (87.1 to 96.4)	6.7 (6.1 to 7.2)*	0.2 (-0.4 to 0.6)	4.9 (3.2 to 7.0)	6.9 (-0.3 to 14.1)	0.3 (-6.4 to 7.1)
Alcohol dependence aggregate	0.0 (0.0 to 0.0)	0.0 (0.0 to 0.0)	0.0 (0.0 to 0.0)	0.0 (0.0 to 0.0)	0.0 (0.0 to 0.0)	0.0 (0.0 to 0.0)
Fetal alcohol syndrome aggregate	92.0 (87.1 to 96.4)	6.7 (6.1 to 7.2)*	0.2 (-0.4 to 0.6)	4.9 (3.2 to 7.0)	6.9 (-0.3 to 14.1)	0.3 (-6.4 to 7.1)
Drug use disorders	0.0 (0.0 to 0.0)	0.0 (0.0 to 0.0)	0.0 (0.0 to 0.0)	0.0 (0.0 to 0.0)	0.0 (0.0 to 0.0)	0.0 (0.0 to 0.0)
Opioid use disorders	0.0 (0.0 to 0.0)	0.0 (0.0 to 0.0)	0.0 (0.0 to 0.0)	0.0 (0.0 to 0.0)	0.0 (0.0 to 0.0)	0.0 (0.0 to 0.0)
Asymptomatic opioid dependence	0.0 (0.0 to 0.0)	0.0 (0.0 to 0.0)	0.0 (0.0 to 0.0)	0.0 (0.0 to 0.0)	0.0 (0.0 to 0.0)	0.0 (0.0 to 0.0)
Symptomatic opioid dependence aggregate	0.0 (0.0 to 0.0)	0.0 (0.0 to 0.0)	0.0 (0.0 to 0.0)	0.0 (0.0 to 0.0)	0.0 (0.0 to 0.0)	0.0 (0.0 to 0.0)
Cocaine use disorders	0.0 (0.0 to 0.0)	0.0 (0.0 to 0.0)	0.0 (0.0 to 0.0)	0.0 (0.0 to 0.0)	0.0 (0.0 to 0.0)	0.0 (0.0 to 0.0)
Asymptomatic cocaine dependence	0.0 (0.0 to 0.0)	0.0 (0.0 to 0.0)	0.0 (0.0 to 0.0)	0.0 (0.0 to 0.0)	0.0 (0.0 to 0.0)	0.0 (0.0 to 0.0)

Cause	Prevalence in thousands (95% confidence interval)			YLDs in thousands (95% confidence interval)		
	2015	Percent change in counts between 2005 and 2015	Percent change in rates between 2005 and 2015	2015	Percent change in counts between 2005 and 2015	Percent change in rates between 2005 and 2015
Symptomatic cocaine dependence aggregate	0.0 (0.0 to 0.0)	0.0 (0.0 to 0.0)	0.0 (0.0 to 0.0)	0.0 (0.0 to 0.0)	0.0 (0.0 to 0.0)	0.0 (0.0 to 0.0)
Amphetamine use disorders	0.0 (0.0 to 0.0)	0.0 (0.0 to 0.0)	0.0 (0.0 to 0.0)	0.0 (0.0 to 0.0)	0.0 (0.0 to 0.0)	0.0 (0.0 to 0.0)
Asymptomatic amphetamine dependence	0.0 (0.0 to 0.0)	0.0 (0.0 to 0.0)	0.0 (0.0 to 0.0)	0.0 (0.0 to 0.0)	0.0 (0.0 to 0.0)	0.0 (0.0 to 0.0)
Symptomatic amphetamine dependence aggregate	0.0 (0.0 to 0.0)	0.0 (0.0 to 0.0)	0.0 (0.0 to 0.0)	0.0 (0.0 to 0.0)	0.0 (0.0 to 0.0)	0.0 (0.0 to 0.0)
Cannabis use disorders	0.0 (0.0 to 0.0)	0.0 (0.0 to 0.0)	0.0 (0.0 to 0.0)	0.0 (0.0 to 0.0)	0.0 (0.0 to 0.0)	0.0 (0.0 to 0.0)
Asymptomatic cannabis dependence	0.0 (0.0 to 0.0)	0.0 (0.0 to 0.0)	0.0 (0.0 to 0.0)	0.0 (0.0 to 0.0)	0.0 (0.0 to 0.0)	0.0 (0.0 to 0.0)
Symptomatic cannabis dependence aggregate	0.0 (0.0 to 0.0)	0.0 (0.0 to 0.0)	0.0 (0.0 to 0.0)	0.0 (0.0 to 0.0)	0.0 (0.0 to 0.0)	0.0 (0.0 to 0.0)
Other drug use disorders	--	--	--	0.0 (0.0 to 0.0)	0.0 (0.0 to 0.0)	0.0 (0.0 to 0.0)
Depressive disorders	981.4 (864.8 to 1,122.8)	7.9 (2.7 to 13.4)*	1.3 (-3.6 to 6.5)	174.5 (94.2 to 282.8)	8.3 (4.3 to 12.4)*	1.7 (-2.0 to 5.6)
Major depressive disorder	695.1 (383.8 to 1,070.5)	8.4 (7.2 to 9.6)*	1.8 (0.6 to 2.9)*	148.5 (70.4 to 252.1)	8.5 (4.1 to 13.4)*	1.9 (-2.2 to 6.5)
Dysthymia	259.3 (149.3 to 389.7)	6.9 (5.1 to 8.7)*	0.3 (-1.3 to 2.1)	26.0 (12.6 to 45.5)	7.0 (0.6 to 14.3)*	0.5 (-5.5 to 7.3)
Bipolar disorder	0.0 (0.0 to 0.0)	0.0 (0.0 to 0.0)	0.0 (0.0 to 0.0)	0.0 (0.0 to 0.0)	0.0 (0.0 to 0.0)	0.0 (0.0 to 0.0)
Anxiety disorders	9,259.3 (7,149.5 to 11,898.7)	7.8 (5.2 to 11.0)*	1.3 (-1.2 to 4.2)	883.9 (563.5 to 1,278.2)	8.0 (4.9 to 11.6)*	1.4 (-1.5 to 4.8)
Eating disorders	27.9 (23.1 to 32.6)	27.4 (9.6 to 49.9)*	19.6 (2.9 to 40.8)*	9.7 (5.1 to 16.4)	13.9 (3.0 to 24.7)*	7.0 (-3.3 to 17.1)
Anorexia nervosa	39.0 (23.3 to 61.4)	12.5 (8.2 to 16.2)*	5.7 (1.6 to 9.1)*	8.5 (4.1 to 14.9)	12.3 (0.1 to 24.3)*	5.4 (-6.0 to 16.7)
Bulimia nervosa	5.0 (2.8 to 8.3)	28.1 (23.4 to 33.3)*	20.3 (15.9 to 25.2)*	1.1 (0.6 to 1.9)	28.1 (23.4 to 33.3)*	20.3 (15.9 to 25.2)*
Autistic spectrum disorders	5,521.0 (5,224.0 to 5,764.2)	6.6 (4.2 to 8.9)*	0.1 (-2.2 to 2.3)	919.0 (616.9 to 1,284.6)	6.8 (5.5 to 8.2)*	0.3 (-0.9 to 1.6)
Autism	2,234.9 (1,821.5 to 2,670.9)	6.8 (6.6 to 7.0)*	0.3 (0.1 to 0.5)*	585.2 (381.7 to 836.1)	7.0 (5.2 to 8.9)*	0.5 (-1.2 to 2.2)
Asperger syndrome and other autistic spectrum disorders	3,290.4 (2,668.4 to 4,077.6)	6.5 (6.4 to 6.7)*	0.0 (-0.1 to 0.2)	333.8 (212.4 to 500.1)	6.6 (5.0 to 8.1)*	0.1 (-1.4 to 1.5)
Attention-deficit/hyperactivity disorder	9,216.6 (8,110.1 to 10,703.6)	5.7 (5.0 to 6.3)*	-0.8 (-1.4 to -0.2)*	112.2 (66.7 to 174.3)	5.8 (4.2 to 7.4)*	-0.7 (-2.2 to 0.8)
Conduct disorder	9,046.0 (6,627.4 to 12,103.6)	6.8 (6.2 to 7.4)*	0.3 (-0.3 to 0.9)	1,086.3 (633.6 to 1,729.5)	6.9 (5.5 to 8.3)*	0.4 (-0.9 to 1.7)
Idiopathic developmental intellectual disability	11,318.0 (6,805.0 to 15,685.2)	6.7 (5.7 to 7.4)*	0.2 (-0.8 to 0.9)	409.4 (183.7 to 721.1)	6.2 (4.1 to 7.4)*	-0.3 (-2.2 to 0.9)
Other mental and substance use disorders	0.0 (0.0 to 0.0)	0.0 (0.0 to 0.0)	0.0 (0.0 to 0.0)	0.0 (0.0 to 0.0)	0.0 (0.0 to 0.0)	0.0 (0.0 to 0.0)
Diabetes, urogenital, blood, and endocrine diseases	159,091.4 (156,674.3 to 161,936.5)	5.3 (3.8 to 6.8)*	-1.1 (-2.6 to 0.3)	2,062.2 (1,398.2 to 2,913.7)	8.0 (6.6 to 9.4)*	1.5 (0.1 to 2.7)*
Diabetes mellitus	724.3 (572.3 to 885.4)	1.6 (-4.8 to 7.3)	-4.6 (-10.6 to 0.8)	34.8 (21.4 to 54.1)	1.6 (-5.5 to 7.9)	-4.6 (-11.3 to 1.3)
Uncomplicated diabetes mellitus	724.1 (572.1 to 885.1)	1.6 (-4.8 to 7.3)	-4.6 (-10.6 to 0.8)	34.8 (21.4 to 54.1)	1.6 (-5.5 to 7.9)	-4.6 (-11.3 to 1.3)
Neuropathy and other complications of diabetes mellitus aggregate	0.2 (0.1 to 0.6)	1.1 (-5.3 to 7.9)	-5.0 (-11.1 to 1.3)	0.0 (0.0 to 0.1)	1.2 (-5.3 to 7.9)	-5.0 (-11.1 to 1.3)
Vision loss due to diabetes mellitus aggregate	0.0 (0.0 to 0.0)	0.0 (0.0 to 0.0)	0.0 (0.0 to 0.0)	0.0 (0.0 to 0.0)	0.0 (0.0 to 0.0)	0.0 (0.0 to 0.0)
Acute glomerulonephritis	6.1 (4.7 to 7.9)	-11.4 (-15.2 to -7.3)*	-16.8 (-20.4 to -13.0)*	0.3 (0.2 to 0.5)	-11.2 (-15.9 to -6.6)*	-16.6 (-21.0 to -12.3)*
Chronic kidney disease	348.4 (291.6 to 397.6)	6.2 (0.2 to 13.2)*	-0.3 (-5.9 to 6.3)	57.9 (39.8 to 81.1)	7.2 (5.3 to 9.1)*	0.6 (-1.1 to 2.4)
Chronic kidney disease due to diabetes mellitus	168.8 (101.6 to 255.0)	5.3 (3.1 to 7.7)*	-1.1 (-3.2 to 1.1)	16.0 (9.7 to 24.9)	6.2 (2.3 to 10.1)*	-0.3 (-3.9 to 3.4)
Stage III chronic kidney disease due to diabetes mellitus aggregate	99.9 (53.0 to 165.7)	5.5 (3.1 to 7.9)*	-0.9 (-3.2 to 1.4)	1.2 (0.8 to 1.7)	9.0 (5.0 to 13.1)*	2.4 (-1.4 to 6.2)
Stage IV chronic kidney disease due to diabetes mellitus aggregate	58.5 (34.5 to 92.2)	5.1 (2.7 to 7.7)*	-1.3 (-3.5 to 1.1)	9.8 (5.5 to 15.8)	6.5 (0.5 to 12.3)*	-0.0 (-5.6 to 5.5)
Stage V chronic kidney disease untreated due to diabetes mellitus	8.0 (4.8 to 12.1)	5.0 (2.5 to 7.5)*	-1.4 (-3.7 to 1.0)	4.6 (2.4 to 7.4)	5.0 (2.5 to 7.5)*	-1.4 (-3.7 to 1.0)
End-stage chronic kidney disease due to diabetes aggregate	2.4 (1.5 to 3.6)	1.8 (-1.5 to 5.0)	-4.4 (-7.5 to -1.4)*	0.5 (0.3 to 0.8)	3.8 (0.7 to 6.8)*	-2.5 (-5.5 to 0.3)
Chronic kidney disease due to hypertension	99.4 (50.0 to 178.4)	5.1 (1.4 to 8.3)*	-1.3 (-4.8 to 1.7)	3.2 (1.6 to 6.2)	4.7 (-0.1 to 9.7)	-1.7 (-6.2 to 3.0)
Stage III chronic kidney disease due to hypertension aggregate	81.5 (39.5 to 144.6)	5.7 (2.2 to 8.6)*	-0.7 (-4.0 to 2.0)	0.2 (0.1 to 0.2)	8.9 (7.2 to 10.8)*	2.2 (0.7 to 4.1)*
Stage IV chronic kidney disease due to hypertension aggregate	16.4 (6.1 to 41.0)	2.3 (-5.4 to 10.0)	-4.0 (-11.2 to 3.3)	2.3 (0.9 to 5.1)	4.2 (-2.0 to 10.5)	-2.1 (-8.0 to 3.8)
Stage V chronic kidney disease untreated due to hypertension	1.1 (0.7 to 1.7)	5.6 (1.0 to 10.7)*	-0.9 (-5.2 to 3.9)	0.6 (0.4 to 1.0)	5.6 (1.0 to 10.7)*	-0.9 (-5.2 to 3.9)
End-stage chronic kidney disease due to hypertension aggregate	0.3 (0.2 to 0.5)	2.6 (-4.0 to 9.6)	-3.6 (-9.8 to 2.9)	0.1 (0.0 to 0.1)	4.4 (-1.1 to 10.3)	-2.0 (-7.1 to 3.6)
Chronic kidney disease due to glomerulonephritis	84.5 (65.4 to 100.4)	7.0 (5.0 to 8.8)*	0.4 (-1.4 to 2.2)	14.4 (9.2 to 20.1)	7.2 (4.1 to 10.1)*	0.6 (-2.2 to 3.4)
Stage III chronic kidney disease due to glomerulonephritis aggregate	27.4 (20.8 to 36.3)	7.5 (4.7 to 10.5)*	0.9 (-1.7 to 3.7)	1.1 (0.7 to 1.5)	10.0 (6.4 to 13.4)*	3.3 (-0.1 to 6.5)
Stage IV chronic kidney disease due to glomerulonephritis aggregate	47.2 (32.6 to 55.6)	7.2 (4.4 to 9.4)*	0.6 (-2.0 to 2.7)	8.5 (5.2 to 12.3)	8.0 (3.3 to 12.4)*	1.5 (-3.0 to 5.5)
Stage V chronic kidney disease untreated due to glomerulonephritis	7.7 (4.6 to 11.5)	5.4 (2.9 to 7.7)*	-1.0 (-3.4 to 1.1)	4.4 (2.4 to 6.9)	5.4 (2.9 to 7.7)*	-1.0 (-3.4 to 1.1)
End-stage chronic kidney disease due to glomerulonephritis aggregate	2.2 (1.3 to 3.3)	1.7 (-1.7 to 5.0)	-4.5 (-7.7 to -1.4)*	0.5 (0.2 to 0.8)	2.5 (-3.1 to 6.6)	-3.8 (-9.0 to 0.1)
Chronic kidney disease due to other causes	259.0 (168.3 to 375.2)	7.0 (5.5 to 8.5)*	0.5 (-0.9 to 1.9)	24.3 (14.9 to 36.3)	8.2 (4.7 to 11.6)*	1.6 (-1.7 to 4.8)
Stage III chronic kidney disease due to other causes aggregate	153.0 (87.0 to 247.7)	7.2 (5.5 to 8.9)*	0.7 (-0.9 to 2.2)	1.7 (1.2 to 2.4)	13.0 (8.4 to 17.7)*	6.1 (1.8 to 10.6)*
Stage IV chronic kidney disease due to other causes aggregate	90.0 (56.2 to 131.2)	6.7 (4.9 to 8.6)*	0.2 (-1.5 to 2.0)	14.8 (8.4 to 23.8)	8.5 (2.7 to 14.1)*	1.9 (-3.6 to 7.1)
Stage V chronic kidney disease untreated due to other causes	12.5 (8.0 to 18.1)	6.6 (5.0 to 8.3)*	0.1 (-1.4 to 1.7)	7.1 (3.9 to 11.0)	6.6 (5.0 to 8.3)*	0.1 (-1.4 to 1.7)
End-stage chronic kidney disease due to other causes aggregate	3.5 (2.3 to 5.1)	3.8 (1.3 to 6.5)*	-2.5 (-4.9 to -0.0)*	0.8 (0.4 to 1.3)	6.1 (0.8 to 9.8)*	-0.4 (-5.3 to 3.1)
Urinary diseases and male infertility	2,710.1 (2,544.2 to 2,880.6)	4.5 (0.5 to 8.5)*	-1.9 (-5.6 to 1.9)	7.9 (4.9 to 11.8)	9.4 (0.8 to 17.7)*	2.7 (-5.4 to 10.5)
Interstitial nephritis and urinary tract infections	156.3 (141.2 to 172.7)	11.2 (10.4 to 12.1)*	4.4 (3.6 to 5.2)*	5.3 (3.2 to 8.0)	11.3 (2.4 to 20.3)*	4.5 (-3.9 to 13.0)
Urolithiasis	2,584.7 (2,136.2 to 3,060.8)	3.9 (-0.4 to 8.2)	-2.4 (-6.4 to 1.6)	0.5 (0.3 to 0.8)	5.4 (0.4 to 9.9)*	-1.0 (-5.7 to 3.2)
Benign prostatic hyperplasia	0.0 (0.0 to 0.0)	0.0 (0.0 to 0.0)	0.0 (0.0 to 0.0)	0.0 (0.0 to 0.0)	0.0 (0.0 to 0.0)	0.0 (0.0 to 0.0)
Male infertility	0.0 (0.0 to 0.0)	0.0 (0.0 to 0.0)	0.0 (0.0 to 0.0)	0.0 (0.0 to 0.0)	0.0 (0.0 to 0.0)	0.0 (0.0 to 0.0)
Other urinary diseases	--	--	--	2.0 (1.2 to 3.0)	5.6 (-6.1 to 18.4)	-0.8 (-11.8 to 11.2)
Gynecological diseases	0.0 (0.0 to 0.0)	0.0 (0.0 to 0.0)	0.0 (0.0 to 0.0)	0.0 (0.0 to 0.0)	0.0 (0.0 to 0.0)	0.0 (0.0 to 0.0)
Uterine fibroids	0.0 (0.0 to 0.0)	0.0 (0.0 to 0.0)	0.0 (0.0 to 0.0)	0.0 (0.0 to 0.0)	0.0 (0.0 to 0.0)	0.0 (0.0 to 0.0)
Uterine fibroids cases aggregate	0.0 (0.0 to 0.0)	0.0 (0.0 to 0.0)	0.0 (0.0 to 0.0)	0.0 (0.0 to 0.0)	0.0 (0.0 to 0.0)	0.0 (0.0 to 0.0)
Mild abdominal pain with anemia due to uterine fibroids aggregate	0.0 (0.0 to 0.0)	0.0 (0.0 to 0.0)	0.0 (0.0 to 0.0)	0.0 (0.0 to 0.0)	0.0 (0.0 to 0.0)	0.0 (0.0 to 0.0)
Polycystic ovarian syndrome	0.0 (0.0 to 0.0)	0.0 (0.0 to 0.0)	0.0 (0.0 to 0.0)	0.0 (0.0 to 0.0)	0.0 (0.0 to 0.0)	0.0 (0.0 to 0.0)
Polycystic ovarian syndrome cases aggregate	0.0 (0.0 to 0.0)	0.0 (0.0 to 0.0)	0.0 (0.0 to 0.0)	0.0 (0.0 to 0.0)	0.0 (0.0 to 0.0)	0.0 (0.0 to 0.0)
Hirsutism and infertility due to polycystic ovarian syndrome aggregate	0.0 (0.0 to 0.0)	0.0 (0.0 to 0.0)	0.0 (0.0 to 0.0)	0.0 (0.0 to 0.0)	0.0 (0.0 to 0.0)	0.0 (0.0 to 0.0)

Cause	Prevalence in thousands (95% confidence interval)			YLDs in thousands (95% confidence interval)		
	2015	Percent change in counts between 2005 and 2015	Percent change in rates between 2005 and 2015	2015	Percent change in counts between 2005 and 2015	Percent change in rates between 2005 and 2015
Female infertility	0.0 (0.0 to 0.0)	0.0 (0.0 to 0.0)	0.0 (0.0 to 0.0)	0.0 (0.0 to 0.0)	0.0 (0.0 to 0.0)	0.0 (0.0 to 0.0)
Endometriosis	0.0 (0.0 to 0.0)	0.0 (0.0 to 0.0)	0.0 (0.0 to 0.0)	0.0 (0.0 to 0.0)	0.0 (0.0 to 0.0)	0.0 (0.0 to 0.0)
Endometriosis cases aggregate	0.0 (0.0 to 0.0)	0.0 (0.0 to 0.0)	0.0 (0.0 to 0.0)	0.0 (0.0 to 0.0)	0.0 (0.0 to 0.0)	0.0 (0.0 to 0.0)
Abdominal pain and infertility due to endometriosis aggregate	0.0 (0.0 to 0.0)	0.0 (0.0 to 0.0)	0.0 (0.0 to 0.0)	0.0 (0.0 to 0.0)	0.0 (0.0 to 0.0)	0.0 (0.0 to 0.0)
Genital prolapse	0.0 (0.0 to 0.0)	0.0 (0.0 to 0.0)	0.0 (0.0 to 0.0)	0.0 (0.0 to 0.0)	0.0 (0.0 to 0.0)	0.0 (0.0 to 0.0)
Premenstrual syndrome	0.0 (0.0 to 0.0)	0.0 (0.0 to 0.0)	0.0 (0.0 to 0.0)	0.0 (0.0 to 0.0)	0.0 (0.0 to 0.0)	0.0 (0.0 to 0.0)
Other gynecological diseases	0.0 (0.0 to 0.0)	0.0 (0.0 to 0.0)	0.0 (0.0 to 0.0)	0.0 (0.0 to 0.0)	0.0 (0.0 to 0.0)	0.0 (0.0 to 0.0)
Other gynecological diseases cases aggregate	0.0 (0.0 to 0.0)	0.0 (0.0 to 0.0)	0.0 (0.0 to 0.0)	0.0 (0.0 to 0.0)	0.0 (0.0 to 0.0)	0.0 (0.0 to 0.0)
Anemia due to other gynecological diseases aggregate	0.0 (0.0 to 0.0)	0.0 (0.0 to 0.0)	0.0 (0.0 to 0.0)	0.0 (0.0 to 0.0)	0.0 (0.0 to 0.0)	0.0 (0.0 to 0.0)
Hemoglobinopathies and hemolytic anemias	149,681.6 (147,090.6 to 152,610.9)	5.5 (3.8 to 7.1)*	-0.9 (-2.5 to 0.6)	1,555.0 (1,047.9 to 2,210.9)	9.6 (7.9 to 11.0)*	2.9 (1.3 to 4.2)*
Thalassemias	70.1 (63.6 to 80.9)	-3.5 (-5.7 to -0.9)*	-9.4 (-11.5 to -6.9)*	6.2 (4.2 to 8.7)	-0.8 (-7.8 to 7.0)	-6.9 (-13.4 to 0.5)
Beta-thalassemia major cases aggregate	29.8 (28.6 to 30.9)	2.7 (1.8 to 3.5)*	-3.6 (-4.4 to -2.8)*	2.6 (1.8 to 3.7)	2.6 (-8.7 to 15.3)	-3.6 (-14.3 to 8.3)
Hemoglobin E/beta-thalassemia cases aggregate	9.9 (9.0 to 11.0)	3.2 (0.7 to 5.9)*	-3.1 (-5.4 to -0.5)*	0.9 (0.6 to 1.3)	2.1 (-10.9 to 16.8)	-4.1 (-16.4 to 9.7)
Hemoglobin H disease cases aggregate	30.4 (24.3 to 41.4)	-10.7 (-15.4 to -5.1)*	-16.1 (-20.5 to -10.9)*	2.6 (1.7 to 3.8)	-5.0 (-15.2 to 7.3)	-10.8 (-20.4 to 0.7)
Heart failure due to thalassemias aggregate	0.0 (0.0 to 0.0)	0.0 (0.0 to 0.0)	0.0 (0.0 to 0.0)	0.0 (0.0 to 0.0)	0.0 (0.0 to 0.0)	0.0 (0.0 to 0.0)
Thalassemias trait	22,248.6 (21,723.8 to 22,893.7)	3.6 (3.0 to 4.2)*	-2.7 (-3.3 to -2.2)*	757.7 (510.6 to 1,079.8)	10.9 (9.4 to 12.5)*	4.1 (2.8 to 5.6)*
B-thalassemia trait cases aggregate	17,953.5 (17,491.5 to 18,541.5)	3.9 (3.2 to 4.6)*	-2.5 (-3.1 to -1.8)*	699.0 (469.8 to 992.9)	10.6 (9.1 to 12.1)*	3.8 (2.4 to 5.3)*
Hemoglobin E trait cases aggregate	4,295.1 (4,098.5 to 4,502.7)	2.4 (1.3 to 3.6)*	-3.8 (-4.9 to -2.8)*	58.7 (39.7 to 83.8)	15.3 (9.6 to 20.7)*	8.2 (2.9 to 13.4)*
Sickle cell disorders	1,153.6 (1,123.2 to 1,193.2)	8.5 (6.3 to 10.6)*	1.9 (-0.2 to 3.9)	108.6 (74.3 to 151.2)	12.0 (7.5 to 16.8)*	5.2 (0.9 to 9.7)*
Homozygous sickle cell and severe sickle cell/beta-thalassemia cases aggregate	1,079.6 (1,051.4 to 1,115.3)	7.1 (4.9 to 9.3)*	0.5 (-1.5 to 2.6)	102.1 (69.8 to 142.2)	11.0 (6.7 to 15.9)*	4.2 (0.2 to 8.8)*
Hemoglobin SC disease cases aggregate	72.2 (66.3 to 78.4)	34.4 (25.1 to 46.9)*	26.2 (17.5 to 38.0)*	6.2 (4.1 to 9.0)	32.0 (6.3 to 61.5)*	23.9 (-0.2 to 51.6)
Mild sickle cell/beta-thalassemia cases aggregate	1.9 (1.7 to 2.0)	17.6 (12.9 to 22.6)*	10.5 (6.0 to 15.1)*	0.2 (0.2 to 0.3)	17.7 (12.9 to 21.4)*	10.5 (6.1 to 14.0)*
Sickle cell trait	45,915.1 (43,415.8 to 50,289.3)	13.7 (11.6 to 15.2)*	6.8 (4.8 to 8.1)*	356.9 (239.9 to 506.8)	16.5 (11.7 to 19.9)*	9.4 (4.9 to 12.6)*
G6PD deficiency	24,976.9 (21,107.2 to 29,032.7)	1.2 (-4.5 to 6.3)	-4.9 (-10.3 to -0.2)*	4.7 (3.2 to 6.6)	1.2 (-10.2 to 14.6)	-5.0 (-15.7 to 7.6)
G6PD cases aggregate	24,976.9 (21,107.2 to 29,032.7)	1.2 (-4.5 to 6.3)	-4.9 (-10.3 to -0.2)*	4.7 (3.2 to 6.6)	1.2 (-10.2 to 14.6)	-5.0 (-15.7 to 7.6)
Heart failure due to G6PD deficiency aggregate	0.0 (0.0 to 0.0)	0.0 (0.0 to 0.0)	0.0 (0.0 to 0.0)	0.0 (0.0 to 0.0)	0.0 (0.0 to 0.0)	0.0 (0.0 to 0.0)
G6PD trait	66,792.5 (62,226.5 to 71,470.8)	3.7 (2.0 to 5.3)*	-2.6 (-4.3 to -1.1)*	4.1 (2.7 to 5.7)	-0.4 (-12.8 to 13.1)	-6.5 (-18.1 to 6.2)
Other hemoglobinopathies and hemolytic anemias	6,553.0 (6,434.2 to 6,656.3)	0.8 (-2.0 to 3.8)	-5.3 (-7.9 to -2.6)*	316.9 (213.3 to 449.9)	-0.3 (-3.4 to 3.4)	-6.3 (-9.3 to -2.9)*
Other hemoglobinopathies and hemolytic anemias cases aggregate	6,553.0 (6,434.1 to 6,656.2)	0.8 (-2.0 to 3.8)	-5.3 (-7.9 to -2.6)*	307.4 (207.0 to 436.1)	-0.5 (-3.7 to 3.3)	-6.5 (-9.6 to -3.0)*
Heart failure due to other hemoglobinopathies and hemolytic anemias aggregate	0.0 (0.0 to 0.0)	5.7 (3.7 to 7.7)*	-0.8 (-2.6 to 1.2)	0.0 (0.0 to 0.0)	5.7 (3.7 to 7.7)*	-0.8 (-2.6 to 1.2)
Endocrine, metabolic, blood, and immune disorders	8,618.1 (8,427.3 to 8,794.2)	1.1 (-2.9 to 5.1)	-5.1 (-8.8 to -1.3)*	406.4 (275.5 to 574.8)	3.2 (-1.3 to 7.8)	-3.1 (-7.3 to 1.2)
Endocrine, metabolic, blood, and immune disorders cases aggregate	321.5 (290.2 to 354.6)	6.4 (4.6 to 8.3)*	-0.1 (-1.8 to 1.7)	13.0 (8.6 to 18.5)	6.4 (1.5 to 11.3)*	-0.0 (-4.7 to 4.6)
Anemia due to endocrine, metabolic, blood, and immune disorders aggregate	8,296.5 (8,106.1 to 8,469.8)	0.9 (-3.2 to 5.0)	-5.3 (-9.1 to -1.4)*	393.3 (266.0 to 558.6)	3.1 (-1.5 to 7.8)	-3.2 (-7.5 to 1.3)
Heart failure due to endocrine, metabolic, blood, and immune disorders aggregate	0.1 (0.1 to 0.1)	3.0 (1.6 to 4.4)*	-3.3 (-4.6 to -2.0)*	0.0 (0.0 to 0.0)	3.0 (1.6 to 4.4)*	-3.3 (-4.6 to -2.0)*
Musculoskeletal disorders	2,778.0 (2,617.2 to 2,910.3)	5.5 (3.0 to 8.3)*	-0.9 (-3.2 to 1.7)	339.3 (229.5 to 477.5)	5.5 (2.4 to 8.3)*	-1.0 (-3.8 to 1.7)
Rheumatoid arthritis	109.7 (91.5 to 129.4)	4.8 (-0.8 to 10.2)	-1.6 (-6.9 to 3.5)	28.1 (18.5 to 39.5)	5.0 (-1.9 to 12.7)	-1.4 (-7.9 to 5.8)
Osteoarthritis	0.0 (0.0 to 0.0)	0.0 (0.0 to 0.0)	0.0 (0.0 to 0.0)	0.0 (0.0 to 0.0)	0.0 (0.0 to 0.0)	0.0 (0.0 to 0.0)
Osteoarthritis of the hip cases aggregate	0.0 (0.0 to 0.0)	0.0 (0.0 to 0.0)	0.0 (0.0 to 0.0)	0.0 (0.0 to 0.0)	0.0 (0.0 to 0.0)	0.0 (0.0 to 0.0)
Osteoarthritis of the knee cases aggregate	0.0 (0.0 to 0.0)	0.0 (0.0 to 0.0)	0.0 (0.0 to 0.0)	0.0 (0.0 to 0.0)	0.0 (0.0 to 0.0)	0.0 (0.0 to 0.0)
Low back and neck pain	2,697.3 (2,543.0 to 2,825.2)	5.6 (3.0 to 8.3)*	-0.8 (-3.3 to 1.7)	311.3 (207.4 to 441.2)	5.5 (2.3 to 8.5)*	-0.9 (-3.9 to 1.9)
Low back pain	2,304.0 (1,947.1 to 2,722.0)	5.2 (2.9 to 7.5)*	-1.2 (-3.4 to 0.9)	269.0 (176.6 to 385.4)	5.3 (1.6 to 8.9)*	-1.1 (-4.6 to 2.2)
Neck pain	413.9 (239.0 to 656.8)	6.7 (3.8 to 9.0)*	0.2 (-2.5 to 2.4)	42.3 (21.5 to 72.3)	6.7 (1.3 to 12.4)*	0.1 (-4.9 to 5.5)
Gout	0.0 (0.0 to 0.0)	0.0 (0.0 to 0.0)	0.0 (0.0 to 0.0)	0.0 (0.0 to 0.0)	0.0 (0.0 to 0.0)	0.0 (0.0 to 0.0)
Asymptomatic gout	0.0 (0.0 to 0.0)	0.0 (0.0 to 0.0)	0.0 (0.0 to 0.0)	0.0 (0.0 to 0.0)	0.0 (0.0 to 0.0)	0.0 (0.0 to 0.0)
Symptomatic gout cases aggregate	0.0 (0.0 to 0.0)	0.0 (0.0 to 0.0)	0.0 (0.0 to 0.0)	0.0 (0.0 to 0.0)	0.0 (0.0 to 0.0)	0.0 (0.0 to 0.0)
Other musculoskeletal disorders	0.0 (0.0 to 0.0)	0.0 (0.0 to 0.0)	0.0 (0.0 to 0.0)	0.0 (0.0 to 0.0)	0.0 (0.0 to 0.0)	0.0 (0.0 to 0.0)
Other non-communicable diseases	433,235.4 (420,970.8 to 445,838.7)	6.3 (5.2 to 7.4)*	-0.2 (-1.2 to 0.8)	7,008.0 (4,871.3 to 9,814.3)	8.2 (6.9 to 10.0)*	1.6 (0.3 to 3.3)*
Congenital anomalies	8,450.7 (7,691.4 to 9,118.1)	15.0 (10.9 to 19.0)*	8.0 (4.1 to 11.7)*	788.1 (495.5 to 1,183.2)	21.8 (13.3 to 31.0)*	14.4 (6.4 to 23.0)*
Neural tube defects	161.7 (110.0 to 235.7)	15.4 (11.0 to 19.0)*	8.4 (4.3 to 11.7)*	57.1 (32.5 to 95.4)	16.8 (8.8 to 24.2)*	9.6 (2.1 to 16.6)*
Congenital heart anomalies	3,949.6 (2,745.6 to 5,978.8)	21.0 (17.7 to 24.7)*	13.7 (10.5 to 17.1)*	137.8 (52.8 to 258.5)	21.5 (17.7 to 25.2)*	14.1 (10.6 to 17.6)*
Less severe heart anomalies cases aggregate	3,625.7 (2,355.2 to 5,659.4)	20.5 (16.9 to 24.6)*	13.1 (9.8 to 17.0)*	118.2 (44.2 to 230.9)	20.6 (16.5 to 24.8)*	13.2 (9.4 to 17.2)*
Severe congenital heart anomalies	301.7 (198.4 to 453.0)	27.5 (21.4 to 32.9)*	19.7 (14.0 to 24.8)*	18.0 (7.1 to 35.9)	27.5 (20.3 to 34.4)*	19.7 (12.9 to 26.2)*
Critical congenital heart anomalies	17.8 (10.1 to 29.1)	42.4 (25.7 to 55.6)*	33.8 (18.0 to 46.1)*	1.1 (0.4 to 2.2)	42.5 (19.4 to 62.3)*	33.9 (12.2 to 52.4)*
Heart failure due to congenital heart anomalies aggregate	4.5 (3.7 to 5.3)	3.6 (2.0 to 5.2)*	-2.7 (-4.2 to -1.2)*	0.6 (0.3 to 0.8)	3.6 (2.0 to 5.2)*	-2.7 (-4.2 to -1.2)*
Cleft lip and cleft palate	588.2 (334.0 to 998.9)	12.7 (3.4 to 18.2)*	5.8 (-2.9 to 11.0)	7.5 (3.6 to 13.7)	7.3 (-2.3 to 15.5)	0.8 (-8.2 to 8.4)
Down syndrome	540.5 (340.3 to 833.2)	11.5 (8.5 to 14.4)*	4.7 (1.9 to 7.4)*	45.5 (24.8 to 77.1)	11.5 (6.9 to 15.8)*	4.7 (0.4 to 8.7)*
Turner syndrome	34.2 (16.1 to 62.8)	7.0 (5.0 to 9.1)*	0.5 (-1.4 to 2.4)	0.5 (0.1 to 1.1)	7.1 (3.0 to 11.8)*	0.6 (-3.3 to 4.9)
Klinefelter syndrome	20.2 (10.6 to 36.4)	6.5 (4.8 to 7.8)*	-0.0 (-1.6 to 1.2)	0.1 (0.0 to 0.1)	6.5 (4.8 to 7.8)*	-0.0 (-1.6 to 1.2)
Other chromosomal abnormalities	457.3 (251.5 to 816.6)	12.0 (8.9 to 14.5)*	5.2 (2.3 to 7.5)*	38.6 (19.2 to 74.2)	11.8 (6.6 to 15.7)*	5.0 (0.1 to 8.7)*
Other congenital anomalies	3,048.5 (1,680.9 to 5,574.2)	8.5 (6.9 to 10.3)*	1.9 (0.3 to 3.6)*	501.0 (295.0 to 790.0)	24.6 (12.3 to 39.7)*	17.0 (5.4 to 31.2)*
Skin and subcutaneous diseases	162,859.7 (156,003.0 to 167,887.1)	7.0 (5.6 to 8.5)*	0.4 (-0.8 to 1.9)	4,146.0 (2,734.9 to 6,064.1)	6.4 (5.5 to 7.4)*	-0.0 (-0.9 to 0.9)
Dermatitis	21,330.9 (17,801.8 to 25,653.4)	9.2 (8.6 to 9.7)*	2.5 (2.0 to 3.0)*	1,140.6 (743.7 to 1,646.0)	10.0 (8.6 to 11.4)*	3.3 (2.0 to 4.6)*
Eczema cases aggregate	16,863.3 (13,417.7 to 20,931.3)	10.3 (9.8 to 10.7)*	3.5 (3.1 to 4.0)*	1,047.9 (679.6 to 1,524.7)	10.4 (8.9 to 11.8)*	3.6 (2.2 to 5.0)*

Cause	Prevalence in thousands (95% confidence interval)			YLDs in thousands (95% confidence interval)		
	2015	Percent change in counts between 2005 and 2015	Percent change in rates between 2005 and 2015	2015	Percent change in counts between 2005 and 2015	Percent change in rates between 2005 and 2015
Contact dermatitis cases aggregate	1,990.4 (1,296.7 to 3,067.5)	7.1 (6.5 to 7.7)*	0.5 (-0.0 to 1.1)	55.2 (29.2 to 95.4)	7.1 (3.4 to 11.0)*	0.6 (-2.9 to 4.3)
Seborrhoeic dermatitis cases aggregate	2,477.2 (1,704.6 to 3,444.6)	4.0 (3.3 to 4.6)*	-2.4 (-3.0 to -1.8)*	37.6 (18.9 to 66.2)	4.0 (1.7 to 6.3)*	-2.3 (-4.5 to -0.2)*
Psoriasis	3,891.0 (3,542.8 to 4,293.8)	6.8 (5.3 to 8.4)*	0.3 (-1.1 to 1.8)	327.3 (225.6 to 451.7)	7.1 (3.9 to 10.3)*	0.5 (-2.5 to 3.6)
Cellulitis	54.9 (40.4 to 73.9)	15.8 (11.8 to 19.4)*	8.7 (5.0 to 12.1)*	4.1 (2.5 to 6.5)	15.6 (9.1 to 21.7)*	8.6 (2.5 to 14.3)*
Pyoderma	455.1 (393.9 to 518.8)	9.6 (7.5 to 11.6)*	2.9 (1.0 to 4.8)*	2.6 (1.0 to 5.7)	9.7 (4.9 to 15.1)*	3.0 (-1.5 to 8.1)
Scabies	24,175.4 (16,243.7 to 35,308.0)	0.5 (-3.2 to 4.2)	-5.6 (-9.1 to -2.2)*	630.5 (312.7 to 1,162.1)	0.6 (-3.2 to 4.3)	-5.5 (-9.1 to -2.0)*
Fungal skin diseases	66,066.4 (51,045.0 to 86,871.9)	9.5 (8.5 to 10.6)*	2.8 (1.9 to 3.9)*	378.8 (141.4 to 803.7)	9.6 (8.6 to 10.7)*	2.9 (1.9 to 4.0)*
Viral skin diseases	27,065.6 (23,526.2 to 31,501.4)	4.7 (4.2 to 5.2)*	-1.7 (-2.1 to -1.2)*	847.2 (534.2 to 1,295.7)	4.8 (4.0 to 5.6)*	-1.6 (-2.4 to -0.8)*
Molluscum contagiosum cases aggregate	13,897.3 (11,013.6 to 17,441.1)	4.9 (4.3 to 5.6)*	-1.5 (-2.1 to -0.8)*	435.1 (260.7 to 697.9)	5.0 (3.8 to 6.2)*	-1.4 (-2.5 to -0.2)*
Viral warts cases aggregate	13,168.3 (11,025.2 to 15,433.7)	4.5 (3.9 to 5.1)*	-1.9 (-2.4 to -1.3)*	412.1 (252.8 to 647.8)	4.6 (3.4 to 5.7)*	-1.8 (-2.9 to -0.7)*
Acne vulgaris	10,805.2 (8,321.3 to 13,731.7)	6.5 (2.4 to 9.9)*	-0.0 (-3.8 to 3.2)	117.6 (50.5 to 224.8)	6.5 (2.6 to 9.9)*	-0.0 (-3.7 to 3.2)
Alopecia areata	523.3 (442.3 to 611.2)	6.2 (5.8 to 6.5)*	-0.3 (-0.6 to 0.0)	18.0 (10.8 to 27.5)	6.2 (1.5 to 11.2)*	-0.3 (-4.7 to 4.4)
Pruritus	3,563.7 (2,481.2 to 5,124.8)	6.4 (3.5 to 9.2)*	-0.1 (-2.8 to 2.6)	38.7 (17.3 to 76.6)	6.4 (3.2 to 9.8)*	-0.1 (-3.1 to 3.1)
Urticaria	8,499.1 (5,573.8 to 12,848.6)	5.8 (5.6 to 6.1)*	-0.6 (-0.9 to -0.4)*	523.7 (287.5 to 887.3)	5.9 (4.5 to 7.5)*	-0.6 (-1.9 to 0.9)
Decubitus ulcer	10.4 (7.2 to 14.3)	15.1 (9.7 to 19.8)*	8.1 (3.0 to 12.5)*	1.7 (1.0 to 2.6)	15.1 (9.7 to 19.8)*	8.1 (3.0 to 12.5)*
Other skin and subcutaneous diseases	19,084.7 (17,181.9 to 21,149.5)	9.2 (7.3 to 11.0)*	2.5 (0.8 to 4.3)*	115.1 (53.9 to 215.4)	9.3 (7.3 to 11.2)*	2.6 (0.7 to 4.4)*
Sense organ diseases	45,772.6 (44,171.1 to 47,429.5)	6.0 (4.6 to 7.6)*	-0.4 (-1.8 to 1.1)	1,739.2 (1,115.1 to 2,546.2)	7.2 (5.1 to 9.6)*	0.7 (-1.3 to 2.9)
Glaucoma	0.0 (0.0 to 0.0)	0.0 (0.0 to 0.0)	0.0 (0.0 to 0.0)	0.0 (0.0 to 0.0)	0.0 (0.0 to 0.0)	0.0 (0.0 to 0.0)
Cataract	0.0 (0.0 to 0.0)	0.0 (0.0 to 0.0)	0.0 (0.0 to 0.0)	0.0 (0.0 to 0.0)	0.0 (0.0 to 0.0)	0.0 (0.0 to 0.0)
Macular degeneration	0.0 (0.0 to 0.0)	0.0 (0.0 to 0.0)	0.0 (0.0 to 0.0)	0.0 (0.0 to 0.0)	0.0 (0.0 to 0.0)	0.0 (0.0 to 0.0)
Refraction and accommodation disorders	26,901.9 (24,695.9 to 29,612.6)	3.7 (2.7 to 4.8)*	-2.6 (-3.6 to -1.6)*	561.4 (349.6 to 896.4)	5.7 (4.3 to 7.2)*	-0.8 (-2.1 to 0.7)
Age-related and other hearing loss	13,706.7 (8,983.1 to 19,304.1)	9.9 (7.4 to 12.5)*	3.2 (0.8 to 5.6)*	1,006.1 (538.1 to 1,629.0)	8.1 (4.5 to 12.1)*	1.5 (-1.9 to 5.2)
Other vision loss	772.3 (502.8 to 1,143.0)	8.9 (6.4 to 11.4)*	2.3 (-0.1 to 4.6)	28.5 (15.4 to 48.0)	8.8 (5.1 to 12.9)*	2.2 (-1.3 to 6.0)
Other sense organ diseases	5,481.8 (4,766.8 to 6,249.9)	7.3 (5.2 to 9.3)*	0.7 (-1.2 to 2.6)	143.3 (87.1 to 214.8)	7.4 (4.6 to 10.3)*	0.9 (-1.7 to 3.6)
Acute other sense organ diseases	143.0 (127.5 to 160.1)	7.5 (5.3 to 9.7)*	1.0 (-1.1 to 3.0)	3.8 (2.3 to 5.8)	7.5 (5.1 to 9.7)*	1.0 (-1.3 to 3.0)
Chronic other sense organ diseases	5,338.8 (4,627.2 to 6,095.9)	7.3 (5.1 to 9.3)*	0.7 (-1.3 to 2.7)	139.5 (84.6 to 209.1)	7.4 (4.6 to 10.4)*	0.9 (-1.8 to 3.7)
Oral disorders	338,933.3 (321,493.4 to 356,215.5)	5.8 (3.8 to 7.9)*	-0.7 (-2.5 to 1.3)	334.6 (192.4 to 542.7)	6.2 (5.2 to 7.3)*	-0.2 (-1.2 to 0.8)
Deciduous caries	291,198.5 (226,213.8 to 363,896.7)	5.4 (2.9 to 7.6)*	-1.0 (-3.4 to 1.0)	76.1 (32.4 to 152.1)	4.9 (1.9 to 7.4)*	-1.5 (-4.3 to 0.8)
Permanent caries	79,102.9 (57,446.6 to 109,551.5)	7.3 (5.0 to 9.2)*	0.8 (-1.4 to 2.6)	54.4 (22.3 to 108.8)	7.1 (4.2 to 9.4)*	0.6 (-2.1 to 2.8)
Periodontal diseases	0.0 (0.0 to 0.0)	0.0 (0.0 to 0.0)	0.0 (0.0 to 0.0)	0.0 (0.0 to 0.0)	0.0 (0.0 to 0.0)	0.0 (0.0 to 0.0)
Edentulism and severe tooth loss	0.0 (0.0 to 0.0)	0.0 (0.0 to 0.0)	0.0 (0.0 to 0.0)	0.0 (0.0 to 0.0)	0.0 (0.0 to 0.0)	0.0 (0.0 to 0.0)
Other oral disorders	6,812.6 (5,848.6 to 8,088.5)	6.4 (6.1 to 6.7)*	-0.1 (-0.4 to 0.2)	204.1 (123.1 to 317.0)	6.5 (5.2 to 8.0)*	0.0 (-1.2 to 1.4)
Injuries	27,569.5 (25,241.4 to 30,042.7)	3.9 (-0.2 to 8.2)	-2.4 (-6.3 to 1.6)	1,287.5 (916.5 to 1,745.4)	-1.3 (-6.8 to 3.7)	-7.4 (-12.5 to -2.6)*
Transport injuries	785.3 (691.8 to 883.9)	1.6 (-0.4 to 3.5)	-4.6 (-6.5 to -2.8)*	57.6 (39.2 to 79.0)	-5.8 (-9.2 to -2.6)*	-11.5 (-14.7 to -8.5)*
Road injuries	632.4 (549.5 to 725.7)	1.1 (-1.3 to 3.4)	-5.0 (-7.3 to -2.9)*	45.6 (30.3 to 63.4)	-4.9 (-8.7 to -1.4)*	-10.7 (-14.3 to -7.4)*
Pedestrian road injuries	86.7 (69.3 to 108.6)	-5.2 (-7.8 to -2.5)*	-11.0 (-13.4 to -8.4)*	6.0 (3.8 to 8.6)	-14.1 (-18.4 to -9.9)*	-19.3 (-23.3 to -15.4)*
Cyclist road injuries	209.4 (168.0 to 257.1)	-2.6 (-5.8 to 0.5)	-8.5 (-11.6 to -5.6)*	14.1 (9.1 to 20.4)	-8.2 (-13.0 to -3.8)*	-13.8 (-18.3 to -9.6)*
Motorcyclist road injuries	98.5 (80.1 to 124.3)	14.5 (12.1 to 16.8)*	7.5 (5.3 to 9.7)*	6.6 (4.3 to 9.5)	6.2 (2.0 to 10.1)*	-0.3 (-4.2 to 3.4)
Motor vehicle road injuries	204.7 (163.4 to 256.8)	1.0 (-2.5 to 3.8)	-5.2 (-8.5 to -2.6)*	16.5 (10.6 to 23.7)	-3.7 (-7.8 to 0.3)	-9.6 (-13.4 to -5.8)*
Other road injuries	33.1 (24.8 to 45.0)	9.6 (5.3 to 13.7)*	3.0 (-1.1 to 6.8)	2.4 (1.4 to 3.7)	5.3 (-0.1 to 10.1)	-1.1 (-6.2 to 3.4)
Other transport injuries	152.9 (118.2 to 193.9)	3.5 (2.0 to 4.9)*	-2.8 (-4.3 to -1.5)*	12.0 (8.1 to 17.0)	-8.7 (-11.5 to -5.6)*	-14.3 (-16.9 to -11.4)*
Unintentional injuries	23,844.5 (21,863.6 to 25,951.8)	2.8 (2.1 to 3.6)*	-3.4 (-4.2 to -2.7)*	1,102.1 (780.4 to 1,501.1)	-0.4 (-2.3 to 1.2)	-6.5 (-8.2 to -4.9)*
Falls	5,091.1 (4,222.8 to 6,066.7)	11.5 (9.5 to 13.4)*	4.7 (2.8 to 6.5)*	340.2 (224.1 to 483.8)	4.3 (0.8 to 7.5)*	-2.1 (-5.4 to 1.0)
Drowning	84.5 (62.9 to 118.0)	-7.6 (-8.8 to -6.5)*	-13.2 (-14.3 to -12.2)*	5.7 (3.7 to 8.4)	-16.2 (-19.1 to -13.0)*	-21.3 (-24.1 to -18.3)*
Fire, heat, and hot substances	2,140.7 (1,581.1 to 2,764.0)	3.1 (2.0 to 4.1)*	-3.2 (-4.2 to -2.2)*	85.0 (57.8 to 118.6)	-3.2 (-7.3 to 0.2)	-9.1 (-13.0 to -5.9)*
Poisonings	382.5 (295.1 to 494.8)	11.3 (9.8 to 12.8)*	4.5 (3.1 to 5.9)*	45.4 (28.9 to 69.1)	9.1 (5.7 to 12.7)*	2.4 (-0.7 to 5.8)
Exposure to mechanical forces	7,142.4 (6,149.4 to 8,217.2)	0.8 (-0.3 to 2.0)	-5.3 (-6.4 to -4.2)*	159.8 (109.3 to 224.6)	-3.4 (-5.9 to -1.0)*	-9.3 (-11.6 to -7.0)*
Unintentional firearm injuries	36.6 (28.5 to 46.8)	4.0 (1.3 to 6.8)*	-2.3 (-4.8 to 0.3)	2.0 (1.3 to 2.9)	-1.5 (-6.5 to 3.2)	-7.5 (-12.2 to -3.1)*
Unintentional suffocation	431.3 (290.2 to 627.1)	-2.3 (-4.8 to 0.4)	-8.3 (-10.6 to -5.7)*	14.2 (9.4 to 20.5)	-1.9 (-4.2 to 0.6)	-7.8 (-10.0 to -5.5)*
Other exposure to mechanical forces	6,674.4 (5,707.6 to 7,756.3)	1.0 (-0.2 to 2.3)	-5.1 (-6.3 to -4.0)*	143.6 (97.8 to 201.2)	-3.6 (-6.1 to -1.1)*	-9.5 (-11.8 to -7.2)*
Adverse effects of medical treatment	646.6 (472.1 to 851.3)	3.3 (0.1 to 6.4)*	-3.0 (-6.0 to -0.1)*	86.2 (51.3 to 133.9)	3.3 (0.1 to 6.4)*	-3.0 (-6.0 to -0.1)*
Animal contact	1,289.7 (1,037.6 to 1,592.1)	-3.1 (-6.5 to 0.3)	-9.0 (-12.2 to -5.9)*	58.8 (38.9 to 83.8)	-3.6 (-7.2 to -0.1)*	-9.5 (-12.8 to -6.2)*
Venomous animal contact	403.5 (322.0 to 502.2)	-1.4 (-3.9 to 1.3)	-7.4 (-9.8 to -4.9)*	41.0 (26.6 to 59.6)	-2.9 (-6.8 to 1.3)	-8.8 (-12.5 to -4.9)*
Non-venomous animal contact	886.2 (667.0 to 1,150.8)	-3.9 (-7.9 to 0.0)	-9.7 (-13.5 to -6.1)*	17.9 (11.2 to 26.7)	-5.1 (-10.1 to -0.4)*	-10.9 (-15.6 to -6.5)*
Foreign body	1,603.1 (1,292.5 to 1,939.0)	4.2 (2.5 to 5.9)*	-2.1 (-3.8 to -0.5)*	78.2 (54.6 to 109.6)	-3.2 (-6.0 to -0.1)*	-9.1 (-11.8 to -6.2)*
Pulmonary aspiration and foreign body in airway	767.2 (542.3 to 1,085.1)	1.4 (-1.5 to 4.3)	-4.7 (-7.5 to -2.1)*	46.4 (30.4 to 68.4)	-7.3 (-10.5 to -3.8)*	-12.9 (-15.9 to -9.6)*
Foreign body in eyes	75.9 (24.1 to 146.8)	7.9 (7.1 to 8.5)*	1.3 (0.6 to 1.9)*	4.3 (1.4 to 9.1)	7.3 (6.0 to 8.1)*	0.8 (-0.5 to 1.5)
Foreign body in other body part	760.0 (590.0 to 949.3)	6.9 (5.2 to 8.6)*	0.4 (-1.2 to 2.0)	27.5 (18.4 to 38.8)	2.9 (-0.1 to 5.6)	-3.4 (-6.2 to -0.9)*
Environmental heat and cold exposure	1,360.6 (1,106.4 to 1,637.7)	10.5 (8.9 to 11.9)*	3.7 (2.2 to 5.1)*	79.5 (53.5 to 116.2)	5.6 (2.8 to 8.4)*	-0.8 (-3.4 to 1.8)
Other unintentional injuries	4,103.4 (3,372.9 to 4,905.6)	-4.5 (-6.0 to -3.2)*	-10.4 (-11.7 to -9.1)*	163.2 (107.9 to 237.6)	-8.5 (-11.2 to -6.3)*	-14.1 (-16.6 to -12.0)*
Self-harm and interpersonal violence	162.5 (142.1 to 188.2)	3.1 (0.7 to 5.1)*	-3.2 (-5.5 to -1.3)*	11.1 (7.5 to 15.1)	-1.2 (-3.9 to 1.2)	-7.3 (-9.8 to -5.0)*
Self-harm	48.2 (37.3 to 61.3)	8.6 (7.7 to 9.5)*	2.0 (1.1 to 2.8)*	4.8 (3.0 to 7.0)	3.1 (0.4 to 6.1)*	-3.2 (-5.8 to -0.3)*
Interpersonal violence	114.3 (96.6 to 135.4)	0.9 (-2.0 to 3.7)	-5.3 (-8.0 to -2.6)*	6.3 (4.2 to 8.7)	-4.3 (-7.8 to -0.9)*	-10.2 (-13.4 to -6.9)*

Cause	Prevalence in thousands (95% confidence interval)			YLDs in thousands (95% confidence interval)		
	2015	Percent change in counts between 2005 and 2015	Percent change in rates between 2005 and 2015	2015	Percent change in counts between 2005 and 2015	Percent change in rates between 2005 and 2015
Assault by firearm	2.4 (1.9 to 3.1)	2.9 (-0.2 to 6.2)	-3.4 (-6.3 to -0.3)*	0.1 (0.1 to 0.2)	-4.6 (-9.1 to 0.2)	-10.4 (-14.7 to -5.9)*
Assault by sharp object	19.7 (15.8 to 24.8)	-6.6 (-8.8 to -4.6)*	-12.3 (-14.4 to -10.4)*	1.0 (0.6 to 1.4)	-16.4 (-20.8 to -11.4)*	-21.5 (-25.7 to -16.8)*
Assault by other means	92.2 (75.4 to 111.7)	2.6 (-1.0 to 6.0)	-3.7 (-7.1 to -0.4)*	5.2 (3.5 to 7.2)	-1.7 (-5.4 to 1.9)	-7.7 (-11.2 to -4.3)*
Forces of nature, war, and legal intervention	2,777.3 (1,700.1 to 4,044.1)	15.3 (-24.0 to 71.1)	8.3 (-28.6 to 60.7)	116.8 (63.7 to 196.9)	-7.2 (-44.8 to 56.7)	-12.9 (-48.2 to 47.1)
Exposure to forces of nature	902.5 (488.3 to 1,527.6)	-42.7 (-69.4 to 4.9)	-46.2 (-71.3 to -1.5)*	43.2 (23.0 to 76.9)	-58.6 (-76.7 to -23.9)*	-61.1 (-78.1 to -28.6)*
Collective violence and legal intervention	1,874.8 (1,023.8 to 2,906.8)	124.6 (31.4 to 258.9)*	110.9 (23.4 to 237.1)*	73.6 (33.5 to 128.6)	239.7 (78.9 to 452.1)*	219.0 (68.0 to 418.5)*
**Anemia**	**272,528.4 (270,655.7 to 274,278.8)**	**2.8 (2.1 to 3.5)***	**-3.5 (-4.1 to -2.8)***	**13,163.1 (8,839.0 to 18,823.4)**	**1.7 (0.8 to 2.5)***	**-4.5 (-5.3 to -3.7)***
**Developmental intellectual disability**	**17,529.1 (13,062.6 to 21,856.7)**	**7.9 (7.5 to 8.3)***	**1.3 (1.0 to 1.7)***	**1,860.0 (1,316.7 to 2,453.7)**	**10.2 (8.3 to 12.5)***	**3.5 (1.7 to 5.6)***
**Epilepsy**	**3,959.7 (3,301.7 to 4,619.5)**	**13.8 (9.9 to 17.5)***	**6.8 (3.2 to 10.3)***	**1,296.3 (930.1 to 1,689.2)**	**5.3 (-0.0 to 10.6)**	**-1.1 (-6.1 to 3.9)**
**Guillain-Barré syndrome**	**2.5 (1.3 to 4.1)**	**7.9 (6.8 to 8.7)***	**1.3 (0.3 to 2.1)***	**0.7 (0.3 to 1.4)**	**7.9 (6.8 to 8.7)***	**1.3 (0.3 to 2.1)***
**Hearing loss**	**30,792.8 (25,014.1 to 37,795.5)**	**7.8 (7.0 to 8.6)***	**1.2 (0.4 to 2.0)***	**1,813.8 (1,181.8 to 2,631.5)**	**6.4 (4.1 to 8.7)***	**-0.1 (-2.3 to 2.1)**
**Heart failure**	**73.7 (62.1 to 87.7)**	**7.8 (6.8 to 8.8)***	**1.3 (0.3 to 2.2)***	**9.7 (6.3 to 13.7)**	**7.5 (3.7 to 11.5)***	**0.9 (-2.6 to 4.7)**
**Infertility**	**0.0 (0.0 to 0.0)**	**0.0 (0.0 to 0.0)**	**0.0 (0.0 to 0.0)**	**0.0 (0.0 to 0.0)**	**0.0 (0.0 to 0.0)**	**0.0 (0.0 to 0.0)**
**Pelvic inflammatory disease**	**0.0 (0.0 to 0.0)**	**0.0 (0.0 to 0.0)**	**0.0 (0.0 to 0.0)**	**0.0 (0.0 to 0.0)**	**0.0 (0.0 to 0.0)**	**0.0 (0.0 to 0.0)**
**Vision loss**	**29,212.1 (26,664.6 to 32,202.2)**	**4.0 (3.0 to 5.1)***	**-2.3 (-3.3 to -1.4)***	**872.9 (596.3 to 1,261.1)**	**7.6 (6.0 to 9.5)***	**1.0 (-0.5 to 2.8)**

SR Table 11. Prevalent cases and YLDs for 2015, percent change, and change in age-standardised rates between 2005 and 2015 by cause, global, ages 10-14 years

Cause	Prevalence in thousands (95% confidence interval)			YLDs in thousands (95% confidence interval)		
	2015	Percent change in counts between 2005 and 2015	Percent change in rates between 2005 and 2015	2015	Percent change in counts between 2005 and 2015	Percent change in rates between 2005 and 2015
**All causes**	--	--	--	**40,944.1 (29,773.1 to 54,874.9)**	**-2.2 (-3.1 to -0.8)***	**-1.1 (-2.1 to 0.2)**
Communicable, maternal, neonatal, and nutritional diseases	334,735.8 (331,421.0 to 337,538.2)	-5.5 (-6.3 to -4.5)*	-4.5 (-5.4 to -3.5)*	13,153.9 (9,219.1 to 18,913.4)	-4.7 (-7.2 to -0.6)*	-3.7 (-6.2 to 0.4)
HIV/AIDS and tuberculosis	1,024.6 (983.9 to 1,063.2)	59.4 (52.8 to 66.8)*	61.1 (54.3 to 68.6)*	234.9 (162.7 to 314.0)	28.0 (20.6 to 36.0)*	29.4 (21.8 to 37.4)*
Tuberculosis	289.3 (215.2 to 389.7)	9.1 (2.2 to 15.5)*	10.2 (3.3 to 16.7)*	91.6 (54.9 to 138.6)	9.2 (0.2 to 18.0)*	10.4 (1.3 to 19.2)*
HIV/AIDS	740.8 (717.1 to 767.2)	94.4 (84.2 to 105.8)*	96.5 (86.1 to 107.9)*	143.3 (102.6 to 185.1)	43.8 (30.7 to 56.7)*	45.3 (32.0 to 58.4)*
HIV/AIDS - Tuberculosis	24.3 (22.0 to 26.7)	38.2 (33.1 to 43.0)*	39.6 (34.5 to 44.4)*	9.5 (6.3 to 13.1)	38.4 (23.6 to 54.5)*	39.8 (24.9 to 56.1)*
HIV/AIDS resulting in other diseases	738.6 (712.3 to 765.7)	93.9 (84.3 to 103.1)*	95.9 (86.2 to 105.2)*	133.8 (95.8 to 172.6)	44.3 (30.4 to 58.3)*	45.8 (31.8 to 60.0)*
HIV aggregate	381.7 (356.1 to 406.3)	60.4 (51.0 to 71.4)*	62.1 (52.6 to 73.2)*	30.0 (20.8 to 39.9)	58.6 (45.3 to 73.1)*	60.3 (46.8 to 74.9)*
AIDS aggregate	356.9 (333.9 to 384.8)	149.7 (125.3 to 175.2)*	152.3 (127.7 to 178.0)*	103.8 (73.5 to 134.6)	40.6 (24.3 to 58.7)*	42.0 (25.6 to 60.3)*
Diarrhea, lower respiratory, and other common infectious diseases	36,610.5 (34,146.9 to 39,020.7)	-2.5 (-4.3 to -0.7)*	-1.4 (-3.3 to 0.4)	1,271.5 (867.8 to 1,788.2)	-1.8 (-3.4 to -0.2)*	-0.8 (-2.4 to 0.9)
Diarrheal diseases	2,233.9 (2,017.8 to 2,464.6)	1.8 (-0.2 to 3.6)	2.9 (0.8 to 4.7)*	361.0 (240.5 to 509.8)	2.0 (-0.9 to 4.8)	3.0 (0.2 to 5.8)*
Diarrhoea episodes aggregate	2,233.7 (2,017.5 to 2,464.4)	1.8 (-0.2 to 3.6)	2.9 (0.8 to 4.7)*	360.9 (240.5 to 509.7)	2.0 (-0.9 to 4.8)	3.0 (0.2 to 5.8)*
Guillain-Barré syndrome due to diarrheal diseases	0.3 (0.1 to 0.5)	0.5 (-1.1 to 1.5)	1.5 (-0.0 to 2.6)	0.1 (0.0 to 0.2)	0.5 (-1.1 to 1.5)	1.5 (-0.0 to 2.6)
Intestinal infectious diseases	302.0 (274.9 to 332.9)	-20.2 (-26.0 to -13.8)*	-19.4 (-25.2 to -12.9)*	36.4 (23.3 to 53.5)	-20.1 (-30.2 to -8.2)*	-19.3 (-29.5 to -7.2)*
Typhoid fever	240.5 (181.3 to 314.2)	-19.4 (-24.3 to -14.7)*	-18.6 (-23.5 to -13.8)*	31.7 (19.8 to 47.1)	-19.2 (-30.4 to -6.5)*	-18.4 (-29.7 to -5.5)*
Typhoid fever episodes aggregate	199.6 (148.3 to 260.4)	-19.3 (-27.0 to -10.4)*	-18.5 (-26.2 to -9.5)*	19.0 (11.3 to 29.2)	-19.1 (-30.2 to -7.2)*	-18.3 (-29.5 to -6.2)*
Complications of typhoid fever aggregate	41.0 (27.3 to 58.2)	-19.8 (-47.3 to 16.4)	-19.0 (-46.8 to 17.6)	12.7 (7.0 to 20.4)	-19.3 (-47.7 to 21.6)	-18.5 (-47.1 to 22.9)
Paratyphoid fever	85.6 (58.9 to 123.3)	-18.8 (-26.0 to -10.8)*	-18.0 (-25.3 to -9.8)*	4.4 (2.4 to 7.3)	-18.8 (-34.0 to 0.3)	-18.0 (-33.3 to 1.3)
Paratyphoid fever episodes aggregate	81.2 (55.8 to 117.5)	-18.8 (-26.5 to -9.8)*	-18.0 (-25.8 to -8.9)*	3.9 (2.2 to 6.5)	-19.0 (-35.5 to 1.5)	-18.1 (-34.8 to 2.6)
Intestinal perforation due to paratyphoid	4.4 (2.5 to 7.4)	-18.0 (-54.5 to 50.2)	-17.2 (-54.0 to 51.8)	0.5 (0.2 to 0.9)	-17.9 (-54.5 to 50.2)	-17.1 (-54.0 to 51.8)
Other intestinal infectious diseases	--	--	--	0.3 (0.1 to 0.7)	-67.5 (-80.2 to -44.9)*	-67.2 (-80.0 to -44.3)*
Lower respiratory infections	395.8 (342.0 to 452.8)	0.5 (-2.1 to 2.9)	1.5 (-1.1 to 4.0)	24.3 (15.6 to 35.0)	0.4 (-4.8 to 5.3)	1.4 (-3.8 to 6.4)
Lower respiratory infection episodes aggregate	395.5 (341.6 to 452.6)	0.5 (-2.1 to 2.9)	1.5 (-1.1 to 4.0)	24.2 (15.5 to 35.0)	0.4 (-4.8 to 5.3)	1.4 (-3.8 to 6.4)
Guillain-Barré syndrome due to lower respiratory infections	0.3 (0.1 to 0.5)	0.5 (-1.1 to 1.5)	1.6 (-0.0 to 2.6)	0.1 (0.0 to 0.2)	0.5 (-1.1 to 1.5)	1.6 (-0.0 to 2.6)
Upper respiratory infections	21,150.0 (14,750.2 to 28,433.7)	-3.1 (-4.4 to -2.0)*	-2.1 (-3.4 to -1.0)*	250.5 (128.2 to 440.7)	-3.1 (-4.6 to -1.7)*	-2.1 (-3.6 to -0.7)*
Upper respiratory infection episodes aggregate	21,149.2 (14,749.5 to 28,433.3)	-3.1 (-4.4 to -2.0)*	-2.1 (-3.4 to -1.0)*	250.3 (128.0 to 440.5)	-3.1 (-4.6 to -1.7)*	-2.1 (-3.6 to -0.7)*
Guillain-Barré syndrome due to upper respiratory infections	0.7 (0.4 to 1.3)	0.5 (-1.1 to 1.5)	1.5 (-0.0 to 2.6)	0.2 (0.1 to 0.4)	0.5 (-1.1 to 1.5)	1.6 (-0.0 to 2.6)
Otitis media	11,935.0 (9,618.0 to 14,649.4)	-2.1 (-4.3 to -0.1)*	-1.0 (-3.3 to 1.0)	402.5 (241.3 to 622.7)	-4.3 (-8.1 to -0.8)*	-3.3 (-7.1 to 0.2)
Acute otitis media	1,878.2 (1,258.4 to 2,751.2)	2.2 (-1.5 to 5.9)	3.2 (-0.5 to 7.0)	24.6 (10.8 to 45.4)	2.2 (-1.9 to 6.3)	3.3 (-0.9 to 7.4)
Chronic otitis media aggregate	10,056.8 (7,920.1 to 12,625.8)	-2.8 (-5.3 to -0.5)*	-1.8 (-4.3 to 0.5)	377.9 (224.7 to 583.6)	-4.7 (-8.7 to -1.1)*	-3.7 (-7.8 to -0.1)*
Meningitis	833.0 (791.6 to 872.9)	11.8 (6.7 to 16.6)*	13.0 (7.8 to 17.8)*	149.9 (105.2 to 196.2)	5.6 (2.4 to 9.0)*	6.7 (3.4 to 10.1)*
Pneumococcal meningitis	664.9 (552.2 to 807.4)	15.9 (12.8 to 19.4)*	17.1 (14.0 to 20.6)*	73.0 (50.6 to 97.1)	9.2 (3.6 to 14.9)*	10.3 (4.6 to 16.0)*
Acute pneumococcal meningitis	2.3 (1.5 to 3.4)	12.7 (-4.2 to 28.3)	13.8 (-3.2 to 29.7)	0.3 (0.2 to 0.5)	12.6 (-4.2 to 28.3)	13.8 (-3.2 to 29.7)
Complications of pneumococcal meningitis aggregate	662.6 (549.6 to 804.5)	15.9 (12.8 to 19.4)*	17.1 (14.0 to 20.6)*	72.7 (50.5 to 96.6)	9.2 (3.6 to 14.9)*	10.3 (4.6 to 16.1)*
H influenzae type B meningitis	270.3 (216.4 to 336.3)	-11.9 (-15.5 to -7.6)*	-11.0 (-14.6 to -6.7)*	36.6 (25.2 to 48.9)	-0.3 (-7.0 to 6.9)	0.8 (-6.0 to 8.0)
Acute H influenzae type B meningitis	1.0 (0.6 to 1.6)	-22.4 (-34.1 to -11.6)*	-21.6 (-33.4 to -10.7)*	0.1 (0.1 to 0.2)	-22.4 (-34.1 to -11.6)*	-21.6 (-33.4 to -10.7)*
Complications of of H influenzae type B meningitis aggregate	269.3 (215.6 to 335.1)	-11.9 (-15.5 to -7.5)*	-10.9 (-14.6 to -6.6)*	36.5 (25.1 to 48.7)	-0.2 (-6.9 to 7.0)	0.9 (-5.9 to 8.1)
Meningococcal meningitis	159.4 (113.3 to 219.6)	17.0 (11.6 to 22.4)*	18.2 (12.8 to 23.7)*	13.1 (8.7 to 17.7)	10.4 (7.2 to 14.3)*	11.5 (8.3 to 15.5)*
Acute meningococcal meningitis	1.2 (0.7 to 1.8)	20.4 (0.9 to 37.8)*	21.6 (1.9 to 39.2)*	0.2 (0.1 to 0.3)	20.3 (0.9 to 37.8)*	21.6 (1.9 to 39.2)*
Complications of meningococcal meningitis aggregate	158.2 (111.9 to 218.9)	17.0 (11.6 to 22.3)*	18.2 (12.7 to 23.6)*	12.9 (8.6 to 17.5)	10.3 (7.1 to 14.2)*	11.4 (8.2 to 15.4)*
Other meningitis	242.7 (188.7 to 305.0)	10.0 (6.8 to 13.8)*	11.1 (7.9 to 14.9)*	27.1 (18.8 to 36.0)	2.3 (-3.4 to 8.6)	3.4 (-2.4 to 9.7)
Other meningitis episodes aggregate	7.7 (5.3 to 10.2)	7.1 (-7.2 to 18.8)	8.2 (-6.2 to 20.0)	1.0 (0.6 to 1.5)	7.0 (-7.1 to 18.8)	8.1 (-6.2 to 20.0)
Complications of other meningitis aggregate	235.0 (181.3 to 297.1)	10.1 (6.8 to 14.0)*	11.2 (7.9 to 15.1)*	26.1 (18.1 to 34.7)	2.2 (-3.9 to 8.7)	3.2 (-2.9 to 9.9)
Encephalitis	285.5 (208.3 to 384.5)	-5.3 (-7.8 to -2.5)*	-4.4 (-6.8 to -1.5)*	33.5 (23.2 to 45.2)	-4.3 (-10.5 to 2.7)	-3.3 (-9.6 to 3.7)
Acute encephalitis	6.2 (4.8 to 7.8)	-3.7 (-9.2 to 1.6)	-2.7 (-8.3 to 2.6)	0.8 (0.5 to 1.2)	-3.5 (-11.3 to 3.5)	-2.5 (-10.3 to 4.5)
Complications of encephalitis aggregate	279.3 (201.6 to 378.5)	-5.4 (-7.9 to -2.5)*	-4.4 (-6.9 to -1.5)*	32.7 (22.6 to 43.8)	-4.4 (-10.6 to 2.9)	-3.4 (-9.7 to 3.9)
Diphtheria	0.0 (0.0 to 0.1)	-58.8 (-84.7 to 12.8)	-58.4 (-84.5 to 14.0)	0.0 (0.0 to 0.0)	-58.8 (-84.7 to 12.8)	-58.4 (-84.5 to 14.0)
Whooping cough	19.7 (15.2 to 24.8)	-29.1 (-30.9 to -27.2)*	-28.4 (-30.2 to -26.4)*	1.0 (0.6 to 1.5)	-28.6 (-39.5 to -15.0)*	-27.8 (-38.9 to -14.1)*
Tetanus	20.5 (20.0 to 21.0)	1.9 (0.1 to 3.0)*	3.0 (1.1 to 4.0)*	0.8 (0.6 to 1.1)	-13.8 (-22.5 to -4.5)*	-12.9 (-21.7 to -3.5)*
Severe tetanus	0.4 (0.3 to 0.8)	-45.2 (-57.7 to -27.6)*	-44.6 (-57.3 to -26.8)*	0.1 (0.0 to 0.1)	-45.1 (-57.7 to -27.6)*	-44.5 (-57.3 to -26.8)*
Complications of tetanus aggregate	20.1 (19.7 to 20.4)	3.8 (3.3 to 4.3)*	4.8 (4.3 to 5.4)*	0.8 (0.6 to 1.1)	-10.2 (-18.6 to 1.2)	-9.3 (-17.8 to 2.2)
Measles	3.2 (1.4 to 6.4)	-69.7 (-72.9 to -65.8)*	-69.3 (-72.6 to -65.4)*	0.3 (0.1 to 0.7)	-69.7 (-72.9 to -65.7)*	-69.3 (-72.6 to -65.4)*
Varicella and herpes zoster	337.3 (270.0 to 419.2)	-1.4 (-2.3 to -0.6)*	-0.4 (-1.3 to 0.4)	11.4 (6.4 to 18.8)	-2.9 (-8.3 to 2.5)	-1.9 (-7.3 to 3.5)
Neglected tropical diseases and malaria	185,250.7 (180,302.7 to 189,680.8)	-10.2 (-12.6 to -7.6)*	-9.3 (-11.7 to -6.7)*	2,480.1 (1,500.3 to 4,280.4)	-3.5 (-15.3 to 16.0)	-2.5 (-14.4 to 17.2)
Malaria	41,751.4 (32,788.5 to 54,270.1)	40.2 (26.2 to 58.1)*	41.7 (27.5 to 59.7)*	592.6 (413.5 to 820.5)	26.9 (21.2 to 32.1)*	28.2 (22.5 to 33.5)*
Asymptomatic malaria parasitemia(PfPR)	28,548.6 (19,572.3 to 40,921.9)	51.6 (28.8 to 78.1)*	53.1 (30.1 to 79.9)*	0.0 (0.0 to 0.0)	0.0 (0.0 to 0.0)	0.0 (0.0 to 0.0)
Malaria episodes aggregate	2,393.8 (1,266.5 to 4,032.3)	-21.7 (-33.0 to -4.8)*	-20.9 (-32.3 to -3.9)*	49.5 (22.4 to 93.1)	-21.5 (-33.2 to -4.3)*	-20.7 (-32.5 to -3.3)*
Complications of malaria aggregate	130.5 (117.9 to 144.0)	23.6 (21.5 to 25.7)*	24.8 (22.7 to 27.0)*	41.6 (31.5 to 53.2)	20.6 (11.7 to 29.4)*	21.9 (12.8 to 30.8)*
Anemia due to malaria parasitemia(PfPR) aggregate	10,678.4 (10,351.7 to 10,983.3)	37.3 (34.5 to 40.0)*	38.7 (35.9 to 41.5)*	501.5 (337.9 to 714.6)	35.8 (32.1 to 39.8)*	37.2 (33.5 to 41.3)*
Chagas disease	216.7 (183.3 to 253.7)	-17.6 (-21.3 to -13.9)*	-16.8 (-20.5 to -13.0)*	0.5 (0.3 to 0.8)	-16.9 (-30.2 to 0.3)	-16.1 (-29.4 to 1.4)
Chagas disease episodes aggregate	207.1 (174.3 to 242.7)	-17.6 (-21.3 to -13.9)*	-16.8 (-20.5 to -13.0)*	0.0 (0.0 to 0.0)	-26.9 (-32.4 to -21.0)*	-26.1 (-31.7 to -20.2)*
Complications of Chagas disease aggregate	9.2 (6.9 to 11.7)	-17.6 (-21.3 to -13.9)*	-16.8 (-20.5 to -13.0)*	0.5 (0.3 to 0.7)	-16.8 (-30.6 to 1.2)	-15.9 (-29.8 to 2.3)

Cause	Prevalence in thousands (95% confidence interval)			YLDs in thousands (95% confidence interval)		
	2015	Percent change in counts between 2005 and 2015	Percent change in rates between 2005 and 2015	2015	Percent change in counts between 2005 and 2015	Percent change in rates between 2005 and 2015
Heart failure due to Chagas disease aggregate	0.3<br>(0.2 to 0.5)	-18.1<br>(-22.0 to -14.4)*	-17.2<br>(-21.2 to -13.5)*	0.0<br>(0.0 to 0.0)	-17.8<br>(-25.9 to -9.7)*	-17.0<br>(-25.2 to -8.8)*
Leishmaniasis	342.3<br>(293.2 to 426.5)	22.6<br>(-0.9 to 57.8)	23.8<br>(0.1 to 59.4)*	4.3<br>(2.1 to 7.9)	20.5<br>(12.0 to 28.8)*	21.7<br>(13.2 to 30.1)*
Visceral leishmaniasis	7.9<br>(7.1 to 8.8)	7.0<br>(5.3 to 8.7)*	8.1<br>(6.3 to 9.9)*	0.6<br>(0.3 to 0.8)	7.8<br>(-18.5 to 43.4)	8.9<br>(-17.7 to 44.9)
Cutaneous and mucocutaneous leishmaniasis	341.5<br>(278.4 to 438.6)	22.3<br>(17.8 to 27.1)*	23.5<br>(19.0 to 28.4)*	3.7<br>(1.7 to 7.3)	22.6<br>(15.0 to 30.2)*	23.9<br>(16.1 to 31.5)*
African trypanosomiasis	0.8<br>(0.5 to 1.3)	-68.1<br>(-69.8 to -66.6)*	-67.7<br>(-69.5 to -66.2)*	0.2<br>(0.1 to 0.4)	-66.1<br>(-76.0 to -49.5)*	-65.7<br>(-75.7 to -48.9)*
Schistosomiasis	33,896.0<br>(28,613.3 to 42,071.9)	-23.8<br>(-32.2 to -8.3)*	-23.1<br>(-31.5 to -7.3)*	339.4<br>(180.9 to 609.0)	-20.7<br>(-27.6 to -7.6)*	-19.9<br>(-26.9 to -6.6)*
Schistosomiasis episodes aggregate	24,717.3<br>(20,356.6 to 30,268.7)	-24.0<br>(-32.1 to -11.7)*	-23.2<br>(-31.4 to -10.8)*	140.7<br>(55.1 to 305.1)	-24.8<br>(-32.6 to -11.9)*	-24.0<br>(-31.9 to -11.0)*
Complications of schistosomiasis aggregate	6,469.8<br>(3,642.3 to 11,014.1)	-25.8<br>(-40.8 to 11.6)	-25.1<br>(-40.2 to 12.8)	70.4<br>(27.1 to 154.7)	-25.4<br>(-40.3 to 13.2)	-24.6<br>(-39.7 to 14.4)
Anemia due to schistosomiasis aggregate	2,754.4<br>(2,661.7 to 2,861.9)	-11.5<br>(-15.0 to -7.1)*	-10.5<br>(-14.2 to -6.2)*	128.3<br>(86.1 to 181.6)	-12.5<br>(-17.0 to -7.8)*	-11.6<br>(-16.2 to -6.9)*
Cysticercosis	13.8<br>(8.2 to 21.6)	-2.2<br>(-8.3 to 4.2)	-1.2<br>(-7.3 to 5.3)	2.3<br>(1.2 to 3.9)	-11.1<br>(-26.7 to 8.1)	-10.2<br>(-26.0 to 9.2)
Cystic echinococcosis	73.6<br>(58.7 to 90.7)	5.1<br>(1.5 to 8.5)*	6.2<br>(2.6 to 9.7)*	6.9<br>(4.5 to 10.1)	4.9<br>(-4.5 to 15.7)	6.0<br>(-3.5 to 16.9)
Lymphatic filariasis	3,073.8<br>(2,507.4 to 3,695.5)	-49.7<br>(-53.3 to -45.6)*	-49.2<br>(-52.9 to -45.0)*	126.7<br>(68.5 to 203.2)	-25.5<br>(-37.8 to -16.2)*	-24.7<br>(-37.2 to -15.3)*
Prevalence of detectable microfiliaria due to lymphatic filariasis	1,962.7<br>(1,602.2 to 2,359.3)	-57.6<br>(-60.8 to -54.1)*	-57.2<br>(-60.4 to -53.6)*	0.0<br>(0.0 to 0.0)	0.0<br>(0.0 to 0.0)	0.0<br>(0.0 to 0.0)
Complications of lymphatic filariasis aggregate	1,111.1<br>(725.2 to 1,619.8)	-25.0<br>(-36.7 to -16.4)*	-24.2<br>(-36.1 to -15.5)*	126.7<br>(68.5 to 203.2)	-25.5<br>(-37.8 to -16.2)*	-24.7<br>(-37.2 to -15.3)*
Onchocerciasis	1,458.7<br>(1,037.2 to 2,009.4)	-40.4<br>(-51.9 to -28.0)*	-39.8<br>(-51.4 to -27.3)*	100.2<br>(41.9 to 185.7)	-29.9<br>(-46.7 to -16.4)*	-29.2<br>(-46.1 to -15.6)*
Asymptomatic onchocerciasis	78.2<br>(7.2 to 185.1)	-88.5<br>(-98.6 to -79.2)*	-88.3<br>(-98.6 to -79.0)*	0.0<br>(0.0 to 0.0)	0.0<br>(0.0 to 0.0)	0.0<br>(0.0 to 0.0)
Skin disease due to onchocerciasis aggregate	1,379.8<br>(908.0 to 1,951.5)	-22.0<br>(-35.7 to -7.9)*	-21.2<br>(-35.0 to -7.0)*	100.2<br>(41.9 to 185.7)	-29.9<br>(-46.6 to -15.7)*	-29.2<br>(-46.1 to -14.9)*
Vision loss due to onchocerciasis aggregate	0.1<br>(0.0 to 0.1)	-50.0<br>(-60.8 to -38.5)*	-49.5<br>(-60.4 to -37.8)*	0.0<br>(0.0 to 0.0)	-51.8<br>(-68.4 to -25.8)*	-51.2<br>(-68.1 to -25.1)*
Trachoma	0.0<br>(0.0 to 0.0)	0.0<br>(0.0 to 0.0)	0.0<br>(0.0 to 0.0)	0.0<br>(0.0 to 0.0)	0.0<br>(0.0 to 0.0)	0.0<br>(0.0 to 0.0)
Dengue	545.2<br>(305.9 to 1,174.9)	123.7<br>(-7.8 to 502.4)	126.1<br>(-6.9 to 508.7)	89.7<br>(41.3 to 201.4)	121.9<br>(-6.5 to 518.4)	124.2<br>(-5.5 to 524.8)
Dengue episodes aggregate	162.5<br>(108.1 to 337.1)	116.6<br>(-10.4 to 467.1)	118.9<br>(-9.5 to 473.0)	9.6<br>(5.2 to 21.1)	122.7<br>(-8.5 to 495.4)	125.1<br>(-7.5 to 501.6)
Post-dengue chronic fatigue syndrome	383.2<br>(183.1 to 840.6)	123.9<br>(-7.8 to 502.4)	126.2<br>(-6.9 to 508.7)	80.1<br>(35.1 to 185.1)	121.8<br>(-6.7 to 520.2)	124.1<br>(-5.7 to 526.7)
Yellow fever	0.4<br>(0.1 to 1.2)	-25.8<br>(-41.7 to -5.0)*	-25.0<br>(-41.1 to -4.1)*	0.0<br>(0.0 to 0.0)	-25.9<br>(-41.7 to -5.0)*	-25.1<br>(-41.1 to -4.1)*
Rabies	0.1<br>(0.0 to 0.1)	-52.4<br>(-67.9 to -28.8)*	-51.9<br>(-67.6 to -28.0)*	0.0<br>(0.0 to 0.0)	-52.4<br>(-67.9 to -28.8)*	-51.9<br>(-67.6 to -28.0)*
Intestinal nematode infections	136,236.9<br>(132,090.3 to 140,631.7)	-14.9<br>(-17.9 to -11.9)*	-14.1<br>(-17.0 to -11.0)*	375.4<br>(227.8 to 578.4)	-30.6<br>(-36.0 to -24.7)*	-29.9<br>(-35.4 to -23.9)*
Ascariasis	72,436.6<br>(63,475.7 to 82,516.9)	-23.6<br>(-35.1 to -9.5)*	-22.8<br>(-34.4 to -8.6)*	90.5<br>(48.6 to 150.9)	-46.5<br>(-55.1 to -36.8)*	-45.9<br>(-54.6 to -36.2)*
Complications of ascariasis aggregate	4,875.3<br>(4,428.5 to 5,375.8)	-45.5<br>(-52.6 to -37.4)*	-44.9<br>(-52.2 to -36.7)*	90.5<br>(48.6 to 150.9)	-46.5<br>(-55.1 to -36.8)*	-45.9<br>(-54.6 to -36.2)*
Asymptomatic ascariasis	67,560.2<br>(58,484.6 to 77,662.8)	-21.3<br>(-34.1 to -6.0)*	-20.5<br>(-33.5 to -5.0)*	0.0<br>(0.0 to 0.0)	0.0<br>(0.0 to 0.0)	0.0<br>(0.0 to 0.0)
Trichuriasis	46,169.3<br>(41,671.0 to 51,146.4)	-15.9<br>(-26.0 to -3.0)*	-15.0<br>(-25.3 to -2.0)*	57.5<br>(31.3 to 99.8)	-34.0<br>(-47.9 to -13.1)*	-33.3<br>(-47.4 to -12.2)*
Complications of trichuriasis aggregate	2,873.5<br>(2,476.3 to 3,479.5)	-32.5<br>(-44.6 to -15.0)*	-31.8<br>(-44.1 to -14.1)*	57.5<br>(31.3 to 99.8)	-34.0<br>(-47.9 to -13.1)*	-33.3<br>(-47.4 to -12.2)*
Asymptomatic trichuriasis	43,316.1<br>(38,793.9 to 48,245.2)	-14.5<br>(-25.8 to -0.5)*	-13.6<br>(-25.0 to 0.6)	0.0<br>(0.0 to 0.0)	0.0<br>(0.0 to 0.0)	0.0<br>(0.0 to 0.0)
Hookworm disease	38,767.8<br>(35,464.4 to 42,870.8)	-18.1<br>(-28.2 to -7.1)*	-17.3<br>(-27.4 to -6.2)*	227.3<br>(145.1 to 338.6)	-20.1<br>(-25.0 to -15.6)*	-19.3<br>(-24.2 to -14.8)*
Complications of hookworm disease aggregate	4,582.7<br>(4,304.9 to 4,922.3)	-24.2<br>(-31.8 to -16.6)*	-23.4<br>(-31.1 to -15.7)*	94.5<br>(51.9 to 157.2)	-25.3<br>(-34.6 to -15.8)*	-24.5<br>(-33.9 to -14.9)*
Anemia due to hookworm disease aggregate	2,915.9<br>(2,880.0 to 2,955.8)	-13.6<br>(-15.6 to -11.6)*	-12.7<br>(-14.7 to -10.7)*	132.8<br>(89.9 to 190.1)	-16.0<br>(-19.1 to -12.9)*	-15.1<br>(-18.2 to -12.0)*
Asymptomatic hookworm disease	31,274.1<br>(27,879.0 to 35,366.1)	-17.5<br>(-30.1 to -3.7)*	-16.7<br>(-29.4 to -2.7)*	0.0<br>(0.0 to 0.0)	0.0<br>(0.0 to 0.0)	0.0<br>(0.0 to 0.0)
Food-borne trematodiases	2,822.6<br>(2,555.7 to 3,133.5)	-30.7<br>(-33.3 to -28.0)*	-29.9<br>(-32.6 to -27.3)*	45.8<br>(22.8 to 83.2)	-34.4<br>(-39.3 to -27.6)*	-33.7<br>(-38.7 to -26.9)*
Asymptomatic food-borne trematodiases aggregate	2,424.8<br>(2,084.9 to 2,781.0)	-30.1<br>(-32.8 to -27.2)*	-29.4<br>(-32.1 to -26.5)*	0.0<br>(0.0 to 0.0)	0.0<br>(0.0 to 0.0)	0.0<br>(0.0 to 0.0)
Symptomatic food-borne trematodiases aggregate	397.9<br>(229.9 to 663.1)	-33.8<br>(-38.1 to -27.9)*	-33.1<br>(-37.5 to -27.2)*	45.8<br>(22.8 to 83.2)	-34.4<br>(-39.3 to -27.6)*	-33.7<br>(-38.7 to -26.9)*
Leprosy	2.4<br>(2.1 to 2.7)	-32.2<br>(-33.8 to -30.6)*	-31.5<br>(-33.1 to -29.9)*	0.1<br>(0.1 to 0.2)	-32.9<br>(-36.5 to -29.0)*	-32.2<br>(-35.8 to -28.3)*
Ebola	0.2<br>(0.1 to 0.3)	0.0<br>(0.0 to 0.0)	0.0<br>(0.0 to 0.0)	0.0<br>(0.0 to 0.1)	0.0<br>(0.0 to 0.0)	0.0<br>(0.0 to 0.0)
Other neglected tropical diseases	4,425.2<br>(4,361.7 to 4,487.9)	-6.0<br>(-7.4 to -4.7)*	-5.1<br>(-6.5 to -3.7)*	795.9<br>(318.8 to 2,101.8)	14.3<br>(-21.4 to 66.8)	15.5<br>(-20.5 to 68.5)
Anemia due to other neglected tropical diseases aggregate	4,425.2<br>(4,361.7 to 4,487.9)	-6.0<br>(-7.4 to -4.7)*	-5.1<br>(-6.5 to -3.7)*	204.3<br>(138.0 to 288.7)	-8.9<br>(-10.9 to -6.9)*	-8.0<br>(-10.0 to -6.0)*
Maternal disorders	16.9<br>(13.7 to 20.6)	-27.7<br>(-42.7 to -7.4)*	-26.9<br>(-42.1 to -6.5)*	3.4<br>(2.2 to 4.8)	-15.7<br>(-31.5 to 4.1)	-14.8<br>(-30.8 to 5.2)
Maternal hemorrhage	8.9<br>(8.5 to 9.4)	-12.8<br>(-16.7 to -8.4)*	-11.9<br>(-15.8 to -7.5)*	0.7<br>(0.5 to 0.9)	-12.1<br>(-18.9 to -4.3)*	-11.1<br>(-18.1 to -3.3)*
Maternal hemorrhage episodes aggregate	0.8<br>(0.4 to 1.3)	-14.0<br>(-46.7 to 38.9)	-13.1<br>(-46.2 to 40.3)	0.1<br>(0.1 to 0.2)	-13.6<br>(-42.5 to 30.4)	-12.7<br>(-41.9 to 31.7)
Anemia due to maternal hemorrhage aggregate	8.1<br>(8.0 to 8.2)	-12.7<br>(-14.3 to -11.0)*	-11.8<br>(-13.4 to -10.1)*	0.6<br>(0.4 to 0.8)	-11.7<br>(-13.4 to -9.7)*	-10.8<br>(-12.5 to -8.8)*
Maternal sepsis and other maternal infections	7.5<br>(4.5 to 12.3)	-11.6<br>(-47.4 to 51.2)	-10.7<br>(-46.9 to 52.7)	0.3<br>(0.1 to 0.6)	-15.3<br>(-55.9 to 63.9)	-14.4<br>(-55.4 to 65.6)
Maternal sepsis and other maternal infections aggregate	5.8<br>(2.8 to 10.4)	-15.9<br>(-56.3 to 66.4)	-15.1<br>(-55.9 to 68.1)	0.3<br>(0.1 to 0.6)	-15.8<br>(-56.6 to 66.2)	-15.0<br>(-56.2 to 67.9)
Infertility due to puerperal sepsis	1.8<br>(1.2 to 2.5)	6.1<br>(1.3 to 12.9)*	7.2<br>(2.4 to 14.1)*	0.0<br>(0.0 to 0.0)	6.1<br>(1.3 to 12.9)*	7.2<br>(2.4 to 14.1)*
Maternal hypertensive disorders	19.3<br>(10.8 to 30.3)	-20.6<br>(-52.1 to 30.6)	-19.8<br>(-51.6 to 31.9)	0.9<br>(0.4 to 1.7)	-20.8<br>(-51.8 to 28.5)	-19.9<br>(-51.3 to 29.9)
Maternal hypertensive disorders episodes aggregate	19.3<br>(10.8 to 30.3)	-20.6<br>(-52.1 to 30.6)	-19.8<br>(-51.6 to 32.0)	0.9<br>(0.4 to 1.7)	-20.9<br>(-52.6 to 30.8)	-20.1<br>(-52.2 to 32.2)
Eclampsia	0.0<br>(0.0 to 0.1)	-14.8<br>(-72.3 to 158.0)	-13.9<br>(-72.1 to 160.7)	0.0<br>(0.0 to 0.0)	-14.8<br>(-72.3 to 158.0)	-13.9<br>(-72.1 to 160.7)
Complications of maternal hypertensive disorders aggregate	0.0<br>(0.0 to 0.0)	0.0<br>(0.0 to 0.0)	0.0<br>(0.0 to 0.0)	0.0<br>(0.0 to 0.0)	0.0<br>(0.0 to 0.0)	0.0<br>(0.0 to 0.0)
Maternal obstructed labor and uterine rupture	2.2<br>(1.4 to 3.6)	-6.3<br>(-20.1 to 9.5)	-5.3<br>(-19.3 to 10.6)	0.8<br>(0.4 to 1.4)	-5.7<br>(-28.4 to 23.2)	-4.7<br>(-27.7 to 24.5)
Obstructed labor, acute event	0.5<br>(0.2 to 0.8)	-14.9<br>(-52.0 to 53.6)	-14.0<br>(-51.5 to 55.2)	0.2<br>(0.1 to 0.3)	-15.0<br>(-52.0 to 53.6)	-14.2<br>(-51.5 to 55.2)
Fistula due to maternal obstructed labor and uterine rupture aggregate	1.8<br>(1.0 to 3.1)	-3.7<br>(-10.7 to 3.4)	-2.7<br>(-9.7 to 4.5)	0.6<br>(0.3 to 1.2)	-3.1<br>(-30.1 to 32.5)	-2.1<br>(-29.4 to 33.9)
Maternal abortion, miscarriage, and ectopic pregnancy	1.5<br>(0.9 to 2.4)	-21.6<br>(-50.0 to 23.6)	-20.8<br>(-49.5 to 24.9)	0.2<br>(0.1 to 0.3)	-21.7<br>(-50.0 to 23.6)	-20.9<br>(-49.5 to 24.9)
Other maternal disorders	--	--	--	0.5<br>(0.3 to 0.7)	-22.4<br>(-37.3 to -4.3)*	-21.6<br>(-36.7 to -3.3)*
Neonatal disorders	4,387.3<br>(4,164.4 to 4,558.7)	3.8<br>(0.5 to 7.1)*	4.8<br>(1.5 to 8.2)*	1,134.5<br>(854.8 to 1,464.2)	2.2<br>(-1.7 to 6.6)	3.3<br>(-0.7 to 7.7)
Neonatal preterm birth complications	3,183.1<br>(2,779.2 to 3,632.0)	-2.8<br>(-7.5 to 2.2)	-1.8<br>(-6.5 to 3.3)	525.7<br>(393.7 to 675.8)	-8.0<br>(-13.1 to -2.6)*	-7.0<br>(-12.2 to -1.6)*
Vision loss due to retinopathy of prematurity aggregate	325.0<br>(255.5 to 408.7)	-2.9<br>(-6.7 to 1.1)	-1.9<br>(-5.8 to 2.2)	8.4<br>(4.7 to 13.7)	1.1<br>(-9.4 to 11.4)	2.2<br>(-8.5 to 12.6)
Complications of preterm birth complications aggregate	2,858.1<br>(2,475.9 to 3,289.9)	-2.8<br>(-8.0 to 2.7)	-1.7<br>(-7.0 to 3.8)	517.4<br>(386.7 to 671.4)	-8.1<br>(-13.3 to -2.6)*	-7.2<br>(-12.4 to -1.6)*
Neonatal encephalopathy due to birth asphyxia and trauma	1,390.4<br>(966.9 to 2,071.5)	18.4<br>(15.5 to 21.0)*	19.6<br>(16.7 to 22.3)*	421.1<br>(271.8 to 651.4)	16.0<br>(11.7 to 20.4)*	17.2<br>(12.8 to 21.6)*

Cause	Prevalence in thousands (95% confidence interval)			YLDs in thousands (95% confidence interval)		
	2015	Percent change in counts between 2005 and 2015	Percent change in rates between 2005 and 2015	2015	Percent change in counts between 2005 and 2015	Percent change in rates between 2005 and 2015
Neonatal sepsis and other neonatal infections	0.0 (0.0 to 0.0)	0.0 (0.0 to 0.0)	0.0 (0.0 to 0.0)	0.0 (0.0 to 0.0)	0.0 (0.0 to 0.0)	0.0 (0.0 to 0.0)
Severe infection due to neonatal sepsis and other neonatal infections	0.0 (0.0 to 0.0)	0.0 (0.0 to 0.0)	0.0 (0.0 to 0.0)	0.0 (0.0 to 0.0)	0.0 (0.0 to 0.0)	0.0 (0.0 to 0.0)
Complications of neonatal sepsis and other neonatal infections aggregate	0.0 (0.0 to 0.0)	0.0 (0.0 to 0.0)	0.0 (0.0 to 0.0)	0.0 (0.0 to 0.0)	0.0 (0.0 to 0.0)	0.0 (0.0 to 0.0)
Hemolytic disease and other neonatal jaundice	209.3 (186.9 to 237.4)	8.4 (7.2 to 9.7)*	9.6 (8.3 to 10.8)*	64.3 (48.0 to 81.6)	5.8 (1.0 to 11.0)*	6.9 (2.0 to 12.1)*
Other neonatal disorders	--	--	--	123.4 (81.6 to 168.1)	7.4 (-11.0 to 29.8)	8.5 (-10.0 to 31.1)
Nutritional deficiencies	157,220.1 (156,423.5 to 158,128.4)	-5.9 (-6.6 to -5.2)*	-4.9 (-5.6 to -4.2)*	7,734.6 (5,212.6 to 10,997.8)	-7.2 (-7.9 to -6.4)*	-6.2 (-6.9 to -5.5)*
Protein-energy malnutrition	9.3 (5.7 to 14.3)	-18.8 (-28.5 to -7.3)*	-18.0 (-27.7 to -6.4)*	1.2 (0.6 to 2.0)	-18.0 (-32.7 to -0.7)*	-17.1 (-32.0 to 0.3)
Iodine deficiency	8,874.7 (7,960.7 to 10,079.5)	-7.7 (-10.0 to -5.6)*	-6.7 (-9.0 to -4.6)*	221.1 (137.6 to 324.5)	-4.5 (-7.7 to -1.7)*	-3.5 (-6.7 to -0.7)*
Visible goiter due to iodine deficiency aggregate	8,497.9 (7,590.3 to 9,653.0)	-8.2 (-10.4 to -6.0)*	-7.2 (-9.5 to -5.0)*	154.1 (94.7 to 243.1)	-8.1 (-10.6 to -5.6)*	-7.1 (-9.7 to -4.6)*
Visible goiter with heart failure due to iodine deficiency aggregate	0.0 (0.0 to 0.0)	0.0 (0.0 to 0.0)	0.0 (0.0 to 0.0)	0.0 (0.0 to 0.0)	0.0 (0.0 to 0.0)	0.0 (0.0 to 0.0)
Intellectual disability due to iodine deficiency aggregate	376.9 (159.6 to 512.7)	4.4 (1.3 to 6.1)*	5.5 (2.3 to 7.2)*	67.0 (24.9 to 109.0)	4.9 (-0.4 to 9.3)	6.0 (0.6 to 10.4)*
Vitamin A deficiency	599.8 (388.0 to 882.1)	1.8 (-2.2 to 5.2)	2.8 (-1.1 to 6.3)	23.1 (12.6 to 38.3)	1.6 (-3.9 to 7.2)	2.7 (-2.9 to 8.3)
Iron-deficiency anemia	160,714.5 (159,843.3 to 161,545.5)	-6.2 (-6.8 to -5.6)*	-5.2 (-5.9 to -4.6)*	7,489.2 (5,033.6 to 10,632.0)	-7.3 (-8.0 to -6.5)*	-6.3 (-7.0 to -5.6)*
Iron-deficiency anemia without heart failure aggregate	160,714.5 (159,843.3 to 161,545.5)	-6.2 (-6.8 to -5.6)*	-5.2 (-5.9 to -4.6)*	7,489.2 (5,033.6 to 10,632.0)	-7.3 (-8.0 to -6.5)*	-6.3 (-7.0 to -5.6)*
Iron-deficiency anemia with heart failure aggregate	0.0 (0.0 to 0.0)	7.9 (4.1 to 11.3)*	9.0 (5.1 to 12.5)*	0.0 (0.0 to 0.0)	7.9 (4.1 to 11.3)*	9.0 (5.1 to 12.5)*
Other nutritional deficiencies	--	--	--	0.1 (0.0 to 0.1)	-32.6 (-46.0 to -16.6)*	-31.9 (-45.5 to -15.8)*
Other communicable, maternal, neonatal, and nutritional diseases	45,286.1 (44,540.8 to 46,119.2)	1.9 (0.8 to 3.0)*	3.0 (1.8 to 4.1)*	295.0 (196.7 to 418.4)	-5.5 (-8.7 to -2.4)*	-4.5 (-7.8 to -1.4)*
Sexually transmitted diseases excluding HIV	6,426.7 (6,038.4 to 6,824.4)	0.7 (-2.0 to 3.5)	1.8 (-0.9 to 4.5)	8.1 (4.4 to 14.0)	4.3 (-4.9 to 13.4)	5.4 (-3.9 to 14.5)
Syphilis	821.0 (561.6 to 1,225.8)	-5.5 (-18.3 to 5.6)	-4.5 (-17.4 to 6.7)	0.2 (0.1 to 0.5)	-5.4 (-24.8 to 12.4)	-4.4 (-24.1 to 13.6)
Early syphilis aggregate	821.0 (561.6 to 1,225.8)	-5.5 (-18.3 to 5.6)	-4.5 (-17.4 to 6.7)	0.2 (0.1 to 0.5)	-5.4 (-24.8 to 12.4)	-4.4 (-24.1 to 13.6)
Adult tertiary syphilis	0.0 (0.0 to 0.0)	0.0 (0.0 to 0.0)	0.0 (0.0 to 0.0)	0.0 (0.0 to 0.0)	0.0 (0.0 to 0.0)	0.0 (0.0 to 0.0)
Chlamydial infection	358.4 (227.4 to 545.1)	-3.2 (-6.2 to -1.0)*	-2.2 (-5.3 to 0.0)	1.9 (1.0 to 3.2)	-3.5 (-11.2 to 4.1)	-2.5 (-10.3 to 5.2)
Chlamydial infection episodes aggregate	356.1 (225.4 to 542.6)	-3.2 (-6.2 to -1.0)*	-2.1 (-5.2 to 0.1)	1.5 (0.7 to 2.8)	-1.7 (-11.2 to 8.5)	-0.7 (-10.2 to 9.6)
Pelvic inflammatory diseases due to chlamydial infection aggregate	2.3 (1.6 to 3.1)	-11.4 (-14.5 to -8.6)*	-10.5 (-13.6 to -7.6)*	0.3 (0.2 to 0.5)	-11.4 (-14.5 to -8.6)*	-10.5 (-13.6 to -7.6)*
Infertility due to clamydial infection aggregate	0.0 (0.0 to 0.0)	0.0 (0.0 to 0.0)	0.0 (0.0 to 0.0)	0.0 (0.0 to 0.0)	0.0 (0.0 to 0.0)	0.0 (0.0 to 0.0)
Gonococcal infection	328.0 (144.4 to 669.2)	13.5 (-29.3 to 42.9)	14.7 (-28.6 to 44.4)	2.2 (0.9 to 4.6)	12.9 (-22.5 to 38.2)	14.1 (-21.7 to 39.6)
Gonococcal infection episodes aggregate	327.3 (143.9 to 668.7)	13.6 (-29.4 to 43.0)	14.8 (-28.6 to 44.5)	2.1 (0.8 to 4.4)	14.5 (-23.0 to 41.0)	15.7 (-22.2 to 42.4)
Pelvic inflammatory diseases due to gonococcal infection aggregate	0.7 (0.5 to 0.9)	-14.2 (-18.3 to -9.4)*	-13.3 (-17.4 to -8.4)*	0.1 (0.1 to 0.1)	-14.2 (-18.3 to -9.4)*	-13.3 (-17.4 to -8.4)*
Trichomoniasis	867.5 (537.8 to 1,287.9)	6.7 (5.2 to 8.2)*	7.8 (6.3 to 9.4)*	1.1 (0.4 to 2.6)	9.3 (3.0 to 16.1)*	10.4 (4.1 to 17.3)*
Genital herpes	4,163.7 (3,233.2 to 5,227.3)	0.1 (-2.5 to 2.3)	1.1 (-1.5 to 3.4)	2.4 (0.8 to 5.1)	5.0 (-1.8 to 12.5)	6.1 (-0.8 to 13.6)
Moderate infection due to initial genital herpes episode	27.2 (5.9 to 62.5)	9.2 (6.3 to 12.5)*	10.3 (7.4 to 13.6)*	1.4 (0.3 to 3.2)	9.0 (-3.1 to 22.8)	10.1 (-2.1 to 24.1)
Complications of genital herpes aggregate	4,136.5 (3,215.5 to 5,182.7)	0.0 (-2.5 to 2.3)	1.0 (-1.5 to 3.4)	1.0 (0.3 to 2.5)	0.3 (-5.7 to 6.6)	1.3 (-4.7 to 7.7)
Other sexually transmitted diseases	2.9 (2.0 to 4.0)	-9.6 (-11.8 to -7.1)*	-8.6 (-10.9 to -6.1)*	0.4 (0.2 to 0.6)	-9.5 (-11.9 to -7.0)*	-8.6 (-11.0 to -6.1)*
Pelvic inflammatory diseases due to other sexually transmitted diseases aggregate	2.9 (2.0 to 4.0)	-9.6 (-11.8 to -7.1)*	-8.6 (-10.9 to -6.1)*	0.4 (0.2 to 0.6)	-9.5 (-11.9 to -7.0)*	-8.6 (-11.0 to -6.1)*
Infertility due to other sexually transmitted diseases aggregate	0.0 (0.0 to 0.0)	0.0 (0.0 to 0.0)	0.0 (0.0 to 0.0)	0.0 (0.0 to 0.0)	0.0 (0.0 to 0.0)	0.0 (0.0 to 0.0)
Other sexually transmitted diseases	--	--	--	1.6 (0.8 to 2.8)	6.9 (-12.9 to 25.2)	8.0 (-12.0 to 26.5)
Hepatitis	34,693.4 (34,008.0 to 35,371.9)	3.5 (2.0 to 5.0)*	4.6 (3.1 to 6.1)*	42.2 (27.3 to 61.8)	-0.9 (-17.4 to 17.1)	0.1 (-16.5 to 18.3)
Acute hepatitis A	882.0 (749.3 to 988.5)	0.7 (-15.9 to 17.9)	1.7 (-15.0 to 19.2)	29.2 (17.9 to 43.8)	0.3 (-21.4 to 24.1)	1.4 (-20.6 to 25.4)
Hepatitis B	25,326.3 (23,771.3 to 26,914.7)	4.8 (2.0 to 7.8)*	5.9 (3.0 to 8.9)*	6.4 (3.7 to 10.5)	-0.1 (-31.0 to 46.6)	0.9 (-30.3 to 48.1)
Acute Hepatitis B aggregate	908.0 (686.2 to 1,213.2)	-0.2 (-30.9 to 43.7)	0.9 (-30.2 to 45.2)	6.4 (3.7 to 10.5)	-0.1 (-31.0 to 46.6)	0.9 (-30.3 to 48.1)
Chronic hepatitis B	24,418.3 (22,943.8 to 26,003.9)	5.0 (2.4 to 7.7)*	6.1 (3.5 to 8.8)*	0.0 (0.0 to 0.0)	0.0 (0.0 to 0.0)	0.0 (0.0 to 0.0)
Hepatitis C	8,941.7 (7,916.9 to 10,047.4)	1.1 (-0.1 to 2.2)	2.1 (0.9 to 3.3)*	0.3 (0.1 to 0.6)	-2.3 (-4.6 to -0.5)*	-1.3 (-3.6 to 0.5)
Acute Hepatitis C aggregate	20.3 (14.8 to 26.8)	-2.4 (-4.7 to -0.5)*	-1.4 (-3.7 to 0.5)	0.3 (0.1 to 0.6)	-2.3 (-4.6 to -0.5)*	-1.3 (-3.6 to 0.5)
Chronic hepatitis C	8,921.3 (7,898.8 to 10,024.1)	1.1 (-0.1 to 2.2)	2.1 (0.9 to 3.3)*	0.0 (0.0 to 0.0)	0.0 (0.0 to 0.0)	0.0 (0.0 to 0.0)
Acute hepatitis E	286.1 (245.2 to 332.0)	-7.0 (-8.5 to -5.5)*	-6.0 (-7.6 to -4.5)*	6.3 (3.8 to 9.6)	-6.9 (-31.7 to 24.9)	-6.0 (-31.0 to 26.2)
Other infectious diseases	4,930.4 (4,890.0 to 4,967.8)	-6.6 (-7.8 to -5.4)*	-5.6 (-6.9 to -4.4)*	244.7 (166.5 to 347.9)	-6.6 (-9.4 to -4.0)*	-5.6 (-8.4 to -3.0)*
Non-communicable diseases	519,093.8 (515,297.4 to 522,056.4)	-0.8 (-1.1 to -0.5)*	0.2 (-0.1 to 0.5)	26,332.8 (19,193.9 to 34,353.1)	-0.5 (-1.1 to 0.2)	0.5 (-0.1 to 1.2)
Neoplasms	250.0 (232.1 to 269.1)	5.0 (-4.3 to 15.7)	6.1 (-3.3 to 16.9)	35.0 (23.6 to 48.4)	2.7 (-12.6 to 18.5)	3.8 (-11.7 to 19.7)
Lip and oral cavity cancer	0.0 (0.0 to 0.0)	0.0 (0.0 to 0.0)	0.0 (0.0 to 0.0)	0.0 (0.0 to 0.0)	0.0 (0.0 to 0.0)	0.0 (0.0 to 0.0)
Nasopharynx cancer	3.7 (1.5 to 6.4)	-6.2 (-49.7 to 70.9)	-5.2 (-49.2 to 72.7)	0.3 (0.1 to 0.6)	-6.6 (-49.1 to 68.7)	-5.6 (-48.6 to 70.5)
Other pharynx cancer	0.0 (0.0 to 0.0)	0.0 (0.0 to 0.0)	0.0 (0.0 to 0.0)	0.0 (0.0 to 0.0)	0.0 (0.0 to 0.0)	0.0 (0.0 to 0.0)
Esophageal cancer	0.0 (0.0 to 0.0)	0.0 (0.0 to 0.0)	0.0 (0.0 to 0.0)	0.0 (0.0 to 0.0)	0.0 (0.0 to 0.0)	0.0 (0.0 to 0.0)
Stomach cancer	0.0 (0.0 to 0.0)	0.0 (0.0 to 0.0)	0.0 (0.0 to 0.0)	0.0 (0.0 to 0.0)	0.0 (0.0 to 0.0)	0.0 (0.0 to 0.0)
Colon and rectum cancer	0.0 (0.0 to 0.0)	0.0 (0.0 to 0.0)	0.0 (0.0 to 0.0)	0.0 (0.0 to 0.0)	0.0 (0.0 to 0.0)	0.0 (0.0 to 0.0)
Colon and rectum cancer aggregate	0.0 (0.0 to 0.0)	0.0 (0.0 to 0.0)	0.0 (0.0 to 0.0)	0.0 (0.0 to 0.0)	0.0 (0.0 to 0.0)	0.0 (0.0 to 0.0)
Stoma due to colon and rectum cancer	0.0 (0.0 to 0.0)	0.0 (0.0 to 0.0)	0.0 (0.0 to 0.0)	0.0 (0.0 to 0.0)	0.0 (0.0 to 0.0)	0.0 (0.0 to 0.0)
Liver cancer	0.0 (0.0 to 0.0)	0.0 (0.0 to 0.0)	0.0 (0.0 to 0.0)	0.2 (0.1 to 0.3)	-19.3 (-36.2 to 5.2)	-18.4 (-35.5 to 6.3)
Liver cancer due to hepatitis B	0.6 (0.5 to 0.8)	-15.5 (-38.3 to 14.2)	-14.6 (-37.7 to 15.3)	0.1 (0.1 to 0.2)	-19.9 (-38.8 to 8.4)	-19.1 (-38.2 to 9.5)
Liver cancer due to hepatitis C	0.0 (0.0 to 0.0)	3.8 (-14.1 to 25.4)	4.8 (-13.3 to 26.7)	0.0 (0.0 to 0.0)	-3.1 (-17.8 to 14.2)	-2.1 (-17.0 to 15.4)
Liver cancer due to alcohol use	0.0 (0.0 to 0.0)	0.0 (0.0 to 0.0)	0.0 (0.0 to 0.0)	0.0 (0.0 to 0.0)	0.0 (0.0 to 0.0)	0.0 (0.0 to 0.0)
Liver cancer due to other causes	0.3 (0.3 to 0.4)	-14.6 (-34.0 to 9.7)	-13.7 (-33.3 to 10.9)	0.1 (0.1 to 0.1)	-19.3 (-35.1 to 2.2)	-18.4 (-34.4 to 3.3)
Gallbladder and biliary tract cancer	0.0 (0.0 to 0.0)	0.0 (0.0 to 0.0)	0.0 (0.0 to 0.0)	0.0 (0.0 to 0.0)	0.0 (0.0 to 0.0)	0.0 (0.0 to 0.0)
Pancreatic cancer	0.0 (0.0 to 0.0)	0.0 (0.0 to 0.0)	0.0 (0.0 to 0.0)	0.0 (0.0 to 0.0)	0.0 (0.0 to 0.0)	0.0 (0.0 to 0.0)

Cause	Prevalence in thousands (95% confidence interval)			YLDs in thousands (95% confidence interval)		
	2015	Percent change in counts between 2005 and 2015	Percent change in rates between 2005 and 2015	2015	Percent change in counts between 2005 and 2015	Percent change in rates between 2005 and 2015
Larynx cancer	0.0 (0.0 to 0.0)	0.0 (0.0 to 0.0)	0.0 (0.0 to 0.0)	0.0 (0.0 to 0.0)	0.0 (0.0 to 0.0)	0.0 (0.0 to 0.0)
Larynx cancer aggregate	0.0 (0.0 to 0.0)	0.0 (0.0 to 0.0)	0.0 (0.0 to 0.0)	0.0 (0.0 to 0.0)	0.0 (0.0 to 0.0)	0.0 (0.0 to 0.0)
Laryngectomy due to larynx cancer	0.0 (0.0 to 0.0)	0.0 (0.0 to 0.0)	0.0 (0.0 to 0.0)	0.0 (0.0 to 0.0)	0.0 (0.0 to 0.0)	0.0 (0.0 to 0.0)
Tracheal, bronchus, and lung cancer	0.0 (0.0 to 0.0)	0.0 (0.0 to 0.0)	0.0 (0.0 to 0.0)	0.0 (0.0 to 0.0)	0.0 (0.0 to 0.0)	0.0 (0.0 to 0.0)
Malignant skin melanoma	0.0 (0.0 to 0.0)	0.0 (0.0 to 0.0)	0.0 (0.0 to 0.0)	0.0 (0.0 to 0.0)	0.0 (0.0 to 0.0)	0.0 (0.0 to 0.0)
Non-melanoma skin cancer	0.0 (0.0 to 0.0)	0.0 (0.0 to 0.0)	0.0 (0.0 to 0.0)	0.0 (0.0 to 0.0)	0.0 (0.0 to 0.0)	0.0 (0.0 to 0.0)
Non-melanoma skin cancer(squamous-cell carcinoma)	0.0 (0.0 to 0.0)	0.0 (0.0 to 0.0)	0.0 (0.0 to 0.0)	0.0 (0.0 to 0.0)	0.0 (0.0 to 0.0)	0.0 (0.0 to 0.0)
Non-melanoma skin cancer(basal-cell carcinoma)	0.0 (0.0 to 0.0)	0.0 (0.0 to 0.0)	0.0 (0.0 to 0.0)	0.0 (0.0 to 0.0)	0.0 (0.0 to 0.0)	0.0 (0.0 to 0.0)
Breast cancer	0.0 (0.0 to 0.0)	0.0 (0.0 to 0.0)	0.0 (0.0 to 0.0)	0.0 (0.0 to 0.0)	0.0 (0.0 to 0.0)	0.0 (0.0 to 0.0)
Breast cancer aggregate	0.0 (0.0 to 0.0)	0.0 (0.0 to 0.0)	0.0 (0.0 to 0.0)	0.0 (0.0 to 0.0)	0.0 (0.0 to 0.0)	0.0 (0.0 to 0.0)
Mastectomy due to breast cancer	0.0 (0.0 to 0.0)	0.0 (0.0 to 0.0)	0.0 (0.0 to 0.0)	0.0 (0.0 to 0.0)	0.0 (0.0 to 0.0)	0.0 (0.0 to 0.0)
Cervical cancer	0.0 (0.0 to 0.0)	0.0 (0.0 to 0.0)	0.0 (0.0 to 0.0)	0.0 (0.0 to 0.0)	0.0 (0.0 to 0.0)	0.0 (0.0 to 0.0)
Uterine cancer	0.0 (0.0 to 0.0)	0.0 (0.0 to 0.0)	0.0 (0.0 to 0.0)	0.0 (0.0 to 0.0)	0.0 (0.0 to 0.0)	0.0 (0.0 to 0.0)
Ovarian cancer	0.0 (0.0 to 0.0)	0.0 (0.0 to 0.0)	0.0 (0.0 to 0.0)	0.0 (0.0 to 0.0)	0.0 (0.0 to 0.0)	0.0 (0.0 to 0.0)
Prostate cancer	0.0 (0.0 to 0.0)	0.0 (0.0 to 0.0)	0.0 (0.0 to 0.0)	0.0 (0.0 to 0.0)	0.0 (0.0 to 0.0)	0.0 (0.0 to 0.0)
Prostate cancer aggregate	0.0 (0.0 to 0.0)	0.0 (0.0 to 0.0)	0.0 (0.0 to 0.0)	0.0 (0.0 to 0.0)	0.0 (0.0 to 0.0)	0.0 (0.0 to 0.0)
Impotence and incontinence due to prostate cancer aggregate	0.0 (0.0 to 0.0)	0.0 (0.0 to 0.0)	0.0 (0.0 to 0.0)	0.0 (0.0 to 0.0)	0.0 (0.0 to 0.0)	0.0 (0.0 to 0.0)
Testicular cancer	0.0 (0.0 to 0.0)	0.0 (0.0 to 0.0)	0.0 (0.0 to 0.0)	0.0 (0.0 to 0.0)	0.0 (0.0 to 0.0)	0.0 (0.0 to 0.0)
Kidney cancer	10.9 (8.8 to 13.5)	24.9 (4.0 to 51.8)*	26.2 (5.1 to 53.4)*	0.9 (0.6 to 1.2)	23.5 (2.1 to 49.8)*	24.8 (3.2 to 51.4)*
Bladder cancer	0.0 (0.0 to 0.0)	0.0 (0.0 to 0.0)	0.0 (0.0 to 0.0)	0.0 (0.0 to 0.0)	0.0 (0.0 to 0.0)	0.0 (0.0 to 0.0)
Bladder cancer episodes aggregate	0.0 (0.0 to 0.0)	0.0 (0.0 to 0.0)	0.0 (0.0 to 0.0)	0.0 (0.0 to 0.0)	0.0 (0.0 to 0.0)	0.0 (0.0 to 0.0)
Urinary incontinence due to bladder cancer	0.0 (0.0 to 0.0)	0.0 (0.0 to 0.0)	0.0 (0.0 to 0.0)	0.0 (0.0 to 0.0)	0.0 (0.0 to 0.0)	0.0 (0.0 to 0.0)
Brain and nervous system cancer	36.3 (29.4 to 43.0)	-4.0 (-20.1 to 14.9)	-3.0 (-19.3 to 16.0)	4.1 (2.8 to 5.7)	-4.8 (-21.0 to 14.1)	-3.8 (-20.2 to 15.3)
Thyroid cancer	12.9 (11.5 to 14.1)	5.1 (-4.1 to 16.5)	6.1 (-3.1 to 17.7)	0.7 (0.5 to 1.1)	4.1 (-6.9 to 16.2)	5.2 (-5.9 to 17.4)
Mesothelioma	0.0 (0.0 to 0.0)	0.0 (0.0 to 0.0)	0.0 (0.0 to 0.0)	0.0 (0.0 to 0.0)	0.0 (0.0 to 0.0)	0.0 (0.0 to 0.0)
Hodgkin lymphoma	13.5 (10.3 to 16.7)	-16.8 (-29.8 to 0.0)	-15.9 (-29.1 to 1.1)	1.1 (0.7 to 1.7)	-20.5 (-33.1 to -3.7)*	-19.6 (-32.4 to -2.7)*
Non-Hodgkin lymphoma	74.4 (55.1 to 92.2)	29.6 (2.1 to 62.3)*	30.9 (3.1 to 63.9)*	5.7 (3.6 to 8.2)	25.6 (-0.3 to 58.3)	26.9 (0.8 to 59.9)*
Multiple myeloma	0.0 (0.0 to 0.0)	0.0 (0.0 to 0.0)	0.0 (0.0 to 0.0)	0.0 (0.0 to 0.0)	0.0 (0.0 to 0.0)	0.0 (0.0 to 0.0)
Leukemia	64.9 (54.4 to 75.1)	-7.1 (-23.2 to 13.5)	-6.2 (-22.4 to 14.7)	13.7 (7.7 to 21.3)	-3.5 (-29.9 to 33.1)	-2.5 (-29.2 to 34.4)
Acute lymphoid leukemia	72.9 (45.6 to 111.2)	-0.8 (-29.0 to 38.7)	0.2 (-28.3 to 40.2)	9.4 (5.3 to 14.6)	-2.9 (-30.2 to 34.4)	-1.8 (-29.5 to 35.8)
Chronic lymphoid leukemia	0.0 (0.0 to 0.0)	0.0 (0.0 to 0.0)	0.0 (0.0 to 0.0)	0.0 (0.0 to 0.0)	0.0 (0.0 to 0.0)	0.0 (0.0 to 0.0)
Acute myeloid leukemia	35.6 (22.5 to 54.9)	-3.1 (-31.1 to 34.4)	-2.1 (-30.4 to 35.8)	4.4 (2.4 to 7.0)	-4.8 (-32.1 to 31.7)	-3.8 (-31.4 to 33.0)
Chronic myeloid leukemia	0.0 (0.0 to 0.0)	0.0 (0.0 to 0.0)	0.0 (0.0 to 0.0)	0.0 (0.0 to 0.0)	0.0 (0.0 to 0.0)	0.0 (0.0 to 0.0)
Other neoplasms	110.9 (88.5 to 138.3)	9.2 (-9.4 to 29.8)	10.3 (-8.5 to 31.1)	8.2 (5.4 to 11.6)	8.3 (-10.1 to 27.4)	9.5 (-9.2 to 28.7)
Cardiovascular diseases	5,678.2 (5,330.4 to 6,027.9)	-10.0 (-13.7 to -6.0)*	-9.1 (-12.8 to -5.1)*	440.2 (289.3 to 620.0)	-7.7 (-10.1 to -5.4)*	-6.8 (-9.1 to -4.5)*
Rheumatic heart disease	2,297.2 (2,050.5 to 2,771.7)	-13.0 (-17.1 to -8.8)*	-12.1 (-16.2 to -7.9)*	110.5 (68.7 to 169.1)	-12.8 (-17.2 to -8.3)*	-11.9 (-16.3 to -7.3)*
Rheumatic heart disease, without heart failure	2,288.6 (2,042.4 to 2,763.8)	-13.0 (-17.1 to -8.9)*	-12.1 (-16.2 to -7.9)*	109.4 (68.0 to 167.7)	-13.0 (-17.3 to -8.4)*	-12.1 (-16.5 to -7.4)*
Heart failure fue to rheumatic heart disease aggregate	8.6 (7.1 to 10.4)	2.9 (1.1 to 4.7)*	4.0 (2.2 to 5.8)*	1.1 (0.7 to 1.5)	2.9 (1.1 to 4.7)*	4.0 (2.2 to 5.8)*
Ischemic heart disease	140.1 (120.2 to 160.5)	1.2 (0.0 to 2.2)*	2.2 (1.0 to 3.3)*	10.4 (6.8 to 15.0)	1.4 (-6.3 to 10.0)	2.5 (-5.3 to 11.1)
Myocardial infarction episodes aggregate	0.2 (0.1 to 0.4)	-0.0 (-3.9 to 4.7)	1.0 (-2.9 to 5.8)	0.0 (0.0 to 0.0)	-0.0 (-3.9 to 4.7)	1.0 (-2.9 to 5.8)
Angina due to ischemic heart disease aggregate	131.2 (111.9 to 151.3)	1.3 (0.1 to 2.4)*	2.4 (1.1 to 3.5)*	9.3 (6.0 to 13.5)	1.8 (-7.1 to 11.3)	2.8 (-6.2 to 12.5)
Heart failure fue to ischemic heart disease aggregate	8.8 (7.2 to 10.6)	-1.3 (-2.8 to 0.2)	-0.3 (-1.8 to 1.3)	1.1 (0.7 to 1.6)	-1.3 (-2.8 to 0.2)	-0.3 (-1.8 to 1.3)
Cerebrovascular disease	720.9 (696.8 to 744.0)	1.0 (-2.5 to 4.2)	2.0 (-1.4 to 5.3)	125.8 (85.3 to 173.7)	1.2 (-3.0 to 5.7)	2.3 (-2.0 to 6.8)
Ischemic stroke	221.4 (208.7 to 236.1)	0.4 (-0.9 to 1.5)	1.4 (0.2 to 2.5)*	36.1 (24.4 to 50.4)	0.4 (-4.5 to 5.2)	1.5 (-3.6 to 6.3)
Chronic ischemic stroke aggregate	220.8 (208.1 to 235.5)	0.4 (-0.9 to 1.5)	1.4 (0.2 to 2.5)*	36.0 (24.3 to 50.3)	0.4 (-4.6 to 5.2)	1.5 (-3.6 to 6.3)
Ischemic stroke episodes aggregate	0.7 (0.4 to 1.0)	4.0 (2.0 to 5.9)*	5.0 (3.1 to 7.0)*	0.1 (0.1 to 0.2)	4.0 (2.0 to 5.9)*	5.0 (3.1 to 7.0)*
Hemorrhagic stroke	564.0 (532.5 to 600.5)	1.4 (0.2 to 2.6)*	2.4 (1.2 to 3.6)*	89.7 (60.8 to 123.8)	1.5 (-4.0 to 7.6)	2.6 (-3.0 to 8.8)
Chronic hemorrhagic stroke aggregate	562.3 (530.8 to 598.4)	1.4 (0.2 to 2.6)*	2.4 (1.2 to 3.6)*	89.3 (60.4 to 123.3)	1.5 (-4.0 to 7.7)	2.6 (-3.1 to 8.8)
Acute hemorrhagic stroke aggregate	1.7 (1.0 to 2.8)	3.8 (1.3 to 6.5)*	4.9 (2.4 to 7.6)*	0.4 (0.2 to 0.7)	3.8 (1.3 to 6.5)*	4.9 (2.4 to 7.6)*
Hypertensive heart disease	0.4 (0.4 to 0.5)	0.1 (-1.6 to 1.7)	1.1 (-0.6 to 2.8)	0.1 (0.0 to 0.1)	0.1 (-1.6 to 1.7)	1.1 (-0.6 to 2.8)
Cardiomyopathy and myocarditis	40.3 (33.9 to 47.3)	1.7 (0.6 to 2.8)*	2.8 (1.6 to 3.9)*	4.5 (2.9 to 6.4)	1.9 (-1.4 to 5.0)	2.9 (-0.3 to 6.1)
Acute myocarditis	6.0 (4.0 to 8.7)	-0.3 (-2.3 to 1.9)	0.7 (-1.3 to 2.9)	0.3 (0.2 to 0.5)	-0.3 (-2.2 to 1.9)	0.7 (-1.2 to 3.0)
Heart failure due to cardiomyopathy aggregate	34.4 (28.2 to 41.3)	2.1 (0.8 to 3.4)*	3.2 (1.9 to 4.4)*	4.2 (2.7 to 6.0)	2.0 (-1.5 to 5.4)	3.1 (-0.5 to 6.5)
Atrial fibrillation and flutter	0.0 (0.0 to 0.0)	0.0 (0.0 to 0.0)	0.0 (0.0 to 0.0)	0.0 (0.0 to 0.0)	0.0 (0.0 to 0.0)	0.0 (0.0 to 0.0)
Peripheral vascular disease	0.0 (0.0 to 0.0)	0.0 (0.0 to 0.0)	0.0 (0.0 to 0.0)	0.0 (0.0 to 0.0)	0.0 (0.0 to 0.0)	0.0 (0.0 to 0.0)
Endocarditis	0.2 (0.2 to 0.3)	5.1 (-1.1 to 11.9)	6.2 (-0.1 to 13.0)	0.0 (0.0 to 0.0)	5.0 (-1.1 to 11.9)	6.1 (-0.1 to 13.0)
Endocarditis episodes aggregate	0.2 (0.2 to 0.3)	5.1 (-1.1 to 11.9)	6.2 (-0.1 to 13.0)	0.0 (0.0 to 0.0)	5.0 (-1.1 to 11.9)	6.1 (-0.1 to 13.0)
Heart failure due to endocarditis aggregate	0.0 (0.0 to 0.0)	0.0 (0.0 to 0.0)	0.0 (0.0 to 0.0)	0.0 (0.0 to 0.0)	0.0 (0.0 to 0.0)	0.0 (0.0 to 0.0)
Other cardiovascular and circulatory diseases	2,698.0 (1,766.3 to 3,935.5)	-10.6 (-13.1 to -8.2)*	-9.7 (-12.2 to -7.3)*	189.0 (104.5 to 309.8)	-10.6 (-13.5 to -7.9)*	-9.7 (-12.6 to -6.9)*
Heart failure due to other cardiovascular diseases aggregate	0.7 (0.5 to 0.8)	-12.2 (-14.4 to -10.2)*	-11.3 (-13.6 to -9.3)*	0.1 (0.1 to 0.1)	-12.2 (-14.4 to -10.2)*	-11.3 (-13.6 to -9.3)*
Other cardiovascular diseases episodes aggregate	2,697.3 (1,765.7 to 3,934.9)	-10.6 (-13.1 to -8.2)*	-9.7 (-12.2 to -7.3)*	188.9 (104.4 to 309.7)	-10.6 (-13.5 to -7.9)*	-9.7 (-12.6 to -6.9)*
Chronic respiratory diseases	46,822.4 (44,331.9 to 49,847.2)	-0.9 (-3.3 to 1.5)	0.1 (-2.3 to 2.6)	2,386.9 (1,565.4 to 3,418.3)	-1.8 (-5.9 to 1.5)	-0.7 (-4.9 to 2.6)
Chronic obstructive pulmonary disease	3,055.4 (2,524.1 to 3,617.1)	-8.5 (-11.3 to -6.0)*	-7.5 (-10.3 to -5.0)*	254.0 (200.3 to 312.1)	-8.0 (-11.8 to -4.5)*	-7.1 (-10.9 to -3.5)*

Cause	Prevalence in thousands (95% confidence interval)			YLDs in thousands (95% confidence interval)		
	2015	Percent change in counts between 2005 and 2015	Percent change in rates between 2005 and 2015	2015	Percent change in counts between 2005 and 2015	Percent change in rates between 2005 and 2015
Chronic obstructive pulmonary disease without heart failure aggregate	3,057.9 (2,527.0 to 3,618.8)	-8.4 (-11.2 to -5.9)*	-7.5 (-10.3 to -4.9)*	253.9 (200.2 to 312.0)	-8.0 (-11.8 to -4.5)*	-7.1 (-10.9 to -3.5)*
Severe chronic obstructive pulmonary disease with heart failure aggregate	0.3 (0.2 to 0.3)	-3.1 (-4.8 to -1.4)*	-2.1 (-3.8 to -0.4)*	0.1 (0.1 to 0.2)	-3.1 (-4.8 to -1.4)*	-2.1 (-3.8 to -0.4)*
Pneumoconiosis	78.7 (71.4 to 86.3)	19.6 (7.4 to 33.2)*	20.9 (8.5 to 34.6)*	19.5 (11.7 to 29.4)	11.3 (-0.3 to 24.2)	12.4 (0.7 to 25.5)*
Silicosis	0.7 (0.5 to 0.9)	-10.3 (-14.5 to -6.3)*	-9.4 (-13.6 to -5.3)*	0.1 (0.1 to 0.2)	-10.3 (-14.5 to -6.3)*	-9.4 (-13.6 to -5.3)*
Silicosis without heart failure aggregate	0.7 (0.5 to 0.9)	-10.3 (-14.5 to -6.3)*	-9.4 (-13.6 to -5.3)*	0.1 (0.1 to 0.2)	-10.3 (-14.5 to -6.3)*	-9.4 (-13.6 to -5.3)*
Severe silicosis with heart failure aggregate	0.0 (0.0 to 0.0)	0.0 (0.0 to 0.0)	0.0 (0.0 to 0.0)	0.0 (0.0 to 0.0)	0.0 (0.0 to 0.0)	0.0 (0.0 to 0.0)
Asbestosis	0.1 (0.1 to 0.1)	-10.9 (-14.9 to -6.8)*	-10.0 (-14.0 to -5.8)*	0.0 (0.0 to 0.0)	-10.9 (-14.9 to -6.8)*	-10.0 (-14.0 to -5.8)*
Asbestosis without heart failure aggregate	0.1 (0.1 to 0.1)	-10.9 (-14.9 to -6.8)*	-10.0 (-14.0 to -5.8)*	0.0 (0.0 to 0.0)	-10.9 (-14.9 to -6.8)*	-10.0 (-14.0 to -5.8)*
Severe asbestosis with heart failure aggregate	0.0 (0.0 to 0.0)	0.0 (0.0 to 0.0)	0.0 (0.0 to 0.0)	0.0 (0.0 to 0.0)	0.0 (0.0 to 0.0)	0.0 (0.0 to 0.0)
Coal workers pneumoconiosis	0.1 (0.1 to 0.1)	3.0 (-3.1 to 9.1)	4.1 (-2.1 to 10.2)	0.0 (0.0 to 0.0)	3.0 (-3.1 to 9.1)	4.1 (-2.1 to 10.2)
Coal workers pneumoconiosis without heart failure aggregate	0.1 (0.1 to 0.1)	3.0 (-3.1 to 9.1)	4.1 (-2.1 to 10.2)	0.0 (0.0 to 0.0)	3.0 (-3.1 to 9.1)	4.1 (-2.1 to 10.2)
Severe coal workers pneumoconiosis with heart failure aggregate	0.0 (0.0 to 0.0)	0.0 (0.0 to 0.0)	0.0 (0.0 to 0.0)	0.0 (0.0 to 0.0)	0.0 (0.0 to 0.0)	0.0 (0.0 to 0.0)
Other pneumoconiosis	119.4 (101.7 to 140.6)	11.8 (6.9 to 16.6)*	13.0 (8.0 to 17.8)*	19.4 (11.6 to 29.2)	11.5 (-0.2 to 24.5)	12.6 (0.8 to 25.8)*
Other pneumoconiosis without heart failure aggregate	119.4 (101.7 to 140.6)	11.8 (6.9 to 16.6)*	13.0 (8.0 to 17.8)*	19.4 (11.6 to 29.2)	11.5 (-0.2 to 24.5)	12.6 (0.8 to 25.8)*
Severe other pneumoconiosis with heart failure aggregate	0.0 (0.0 to 0.0)	0.0 (0.0 to 0.0)	0.0 (0.0 to 0.0)	0.0 (0.0 to 0.0)	0.0 (0.0 to 0.0)	0.0 (0.0 to 0.0)
Asthma	45,335.1 (35,342.5 to 56,668.8)	-0.6 (-5.0 to 3.2)	0.5 (-4.1 to 4.2)	2,050.9 (1,252.6 to 3,032.2)	-0.5 (-4.9 to 3.1)	0.5 (-3.9 to 4.2)
Asymptomatic asthma	13,724.7 (10,448.5 to 17,444.7)	-0.6 (-5.0 to 3.2)	0.5 (-4.1 to 4.2)	0.0 (0.0 to 0.0)	0.0 (0.0 to 0.0)	0.0 (0.0 to 0.0)
Symptomatic asthma aggregate	31,610.4 (24,463.3 to 40,252.2)	-0.6 (-5.0 to 3.2)	0.5 (-4.1 to 4.2)	2,050.9 (1,252.6 to 3,032.2)	-0.5 (-4.9 to 3.1)	0.5 (-3.9 to 4.2)
Interstitial lung disease and pulmonary sarcoidosis	26.6 (22.8 to 31.1)	-4.2 (-7.4 to -1.4)*	-3.2 (-6.5 to -0.3)*	3.4 (2.0 to 5.2)	-4.1 (-8.4 to 0.0)	-3.1 (-7.4 to 1.1)
Interstitial lung disease and pulmonary sarcoidosis without heart failure aggregate	25.9 (22.2 to 30.4)	-4.2 (-7.6 to -1.4)*	-3.2 (-6.6 to -0.3)*	3.0 (1.7 to 4.8)	-4.3 (-9.1 to 0.3)	-3.3 (-8.2 to 1.3)
Severe interstitial lung disease and pulmonary sarcoidosis with heart failure aggregate	0.7 (0.6 to 0.8)	-2.1 (-3.7 to -0.6)*	-1.1 (-2.7 to 0.4)	0.3 (0.2 to 0.5)	-2.1 (-3.7 to -0.6)*	-1.1 (-2.7 to 0.4)
Other chronic respiratory diseases	--	--	--	59.0 (46.0 to 74.0)	-17.0 (-25.4 to -7.2)*	-16.1 (-24.6 to -6.2)*
Cirrhosis and other chronic liver diseases	58.0 (52.6 to 63.1)	11.5 (-1.6 to 25.8)	12.7 (-0.6 to 27.1)	11.8 (8.1 to 16.4)	7.1 (-3.2 to 18.4)	8.2 (-2.2 to 19.6)
Cirrhosis and other chronic liver diseases due to hepatitis B	2.6 (2.1 to 3.2)	6.7 (3.6 to 9.7)*	7.8 (4.7 to 10.8)*	0.5 (0.3 to 0.7)	6.7 (3.6 to 9.7)*	7.8 (4.7 to 10.8)*
Cirrhosis and other chronic liver diseases due to hepatitis C	1.6 (1.3 to 1.9)	9.9 (4.8 to 13.9)*	11.0 (5.9 to 15.1)*	0.3 (0.2 to 0.4)	9.9 (4.8 to 13.9)*	11.0 (5.9 to 15.1)*
Cirrhosis and other chronic liver diseases due to alcohol use	0.0 (0.0 to 0.0)	0.0 (0.0 to 0.0)	0.0 (0.0 to 0.0)	0.0 (0.0 to 0.0)	0.0 (0.0 to 0.0)	0.0 (0.0 to 0.0)
Cirrhosis and other chronic liver diseases due to other causes	64.5 (59.8 to 69.0)	7.1 (6.2 to 8.1)*	8.3 (7.3 to 9.2)*	11.1 (7.6 to 15.3)	7.1 (-3.9 to 19.1)	8.2 (-2.9 to 20.3)
Digestive diseases	4,888.5 (4,785.8 to 4,988.5)	-7.8 (-10.1 to -5.6)*	-6.9 (-9.2 to -4.6)*	338.7 (234.2 to 463.6)	-5.4 (-8.3 to -2.6)*	-4.4 (-7.3 to -1.6)*
Peptic ulcer disease	644.9 (625.1 to 663.5)	-17.7 (-20.0 to -15.3)*	-16.8 (-19.2 to -14.4)*	32.6 (21.8 to 46.9)	-21.1 (-26.0 to -16.5)*	-20.3 (-25.3 to -15.6)*
Peptic ulcer disease, symptomatic episodes	37.5 (27.9 to 48.3)	-20.7 (-24.7 to -17.3)*	-19.9 (-23.9 to -16.4)*	4.2 (2.5 to 6.1)	-20.9 (-31.6 to -10.2)*	-20.1 (-30.8 to -9.3)*
Anemia due to peptic ulcer disease aggregate	607.5 (590.8 to 622.9)	-17.5 (-19.9 to -14.9)*	-16.6 (-19.1 to -14.0)*	28.4 (19.0 to 41.4)	-21.1 (-26.3 to -16.1)*	-20.3 (-25.5 to -15.2)*
Gastritis and duodenitis	3,408.9 (3,327.1 to 3,498.9)	-6.2 (-8.7 to -3.9)*	-5.3 (-7.7 to -2.9)*	197.1 (133.8 to 275.5)	-4.4 (-8.0 to -0.3)*	-3.4 (-7.0 to 0.7)
Gastritis and duodenitis, symptomatic episodes	71.7 (52.0 to 95.2)	-7.8 (-11.0 to -5.1)*	-6.8 (-10.1 to -4.1)*	7.9 (4.8 to 12.0)	-7.6 (-16.2 to 1.3)	-6.7 (-15.3 to 2.3)
Anemia due to gastritis and duodenitis aggregate	3,337.3 (3,257.7 to 3,424.7)	-6.2 (-8.7 to -3.8)*	-5.2 (-7.8 to -2.8)*	189.2 (128.4 to 266.0)	-4.2 (-8.1 to 0.0)	-3.2 (-7.2 to 1.1)
Appendicitis	46.5 (38.6 to 55.4)	0.9 (-0.8 to 2.6)	2.0 (0.2 to 3.6)*	14.5 (9.3 to 20.7)	1.2 (-9.4 to 13.2)	2.2 (-8.5 to 14.4)
Paralytic ileus and intestinal obstruction	2.8 (2.1 to 3.6)	-0.2 (-5.5 to 4.6)	0.9 (-4.5 to 5.7)	0.9 (0.6 to 1.4)	-0.2 (-5.5 to 4.6)	0.8 (-4.5 to 5.7)
Inguinal, femoral, and abdominal hernia	576.1 (405.3 to 780.4)	-9.5 (-14.0 to -4.8)*	-8.6 (-13.2 to -3.8)*	6.3 (2.8 to 12.4)	-9.4 (-14.9 to -3.9)*	-8.5 (-14.0 to -2.9)*
Inflammatory bowel disease	204.2 (167.8 to 249.0)	-2.5 (-3.2 to -1.7)*	-1.4 (-2.2 to -0.7)*	45.3 (28.6 to 66.7)	-2.3 (-7.2 to 2.9)	-1.3 (-6.2 to 4.0)
Vascular intestinal disorders	0.3 (0.2 to 0.5)	-5.8 (-12.2 to -0.1)*	-4.8 (-11.2 to 0.9)	0.1 (0.1 to 0.2)	-5.8 (-12.2 to -0.1)*	-4.8 (-11.2 to 0.9)
Gallbladder and biliary diseases	65.4 (48.1 to 86.2)	-6.2 (-11.8 to -0.8)*	-5.2 (-10.9 to 0.2)	7.3 (4.3 to 11.0)	-6.1 (-16.9 to 5.8)	-5.1 (-16.0 to 6.9)
Pancreatitis	10.6 (7.7 to 14.1)	-1.3 (-6.0 to 2.3)	-0.3 (-5.0 to 3.4)	3.4 (2.1 to 5.4)	-1.3 (-9.6 to 6.4)	-0.3 (-8.7 to 7.5)
Other digestive diseases	--	--	--	31.3 (21.1 to 44.3)	1.5 (-5.6 to 11.4)	2.6 (-4.6 to 12.6)
Neurological disorders	131,554.4 (120,338.5 to 140,468.3)	0.7 (-1.6 to 2.9)	1.7 (-0.6 to 4.0)	2,995.7 (1,956.9 to 4,266.2)	-2.0 (-4.8 to 0.6)	-1.0 (-3.8 to 1.6)
Alzheimer disease and other dementias	0.0 (0.0 to 0.0)	0.0 (0.0 to 0.0)	0.0 (0.0 to 0.0)	0.0 (0.0 to 0.0)	0.0 (0.0 to 0.0)	0.0 (0.0 to 0.0)
Parkinson disease	0.0 (0.0 to 0.0)	0.0 (0.0 to 0.0)	0.0 (0.0 to 0.0)	0.0 (0.0 to 0.0)	0.0 (0.0 to 0.0)	0.0 (0.0 to 0.0)
Epilepsy	2,121.2 (1,746.8 to 2,585.1)	1.2 (-4.9 to 7.7)	2.2 (-3.9 to 8.8)	621.4 (404.7 to 854.9)	-12.6 (-19.5 to -5.9)*	-11.7 (-18.6 to -4.9)*
Multiple sclerosis	11.6 (8.4 to 15.9)	-4.6 (-7.0 to -2.8)*	-3.6 (-6.0 to -1.7)*	4.2 (2.6 to 6.4)	-4.5 (-7.3 to -2.1)*	-3.6 (-6.4 to -1.1)*
Motor neuron disease	4.4 (4.1 to 4.9)	-2.7 (-5.0 to -0.4)*	-1.7 (-4.0 to 0.7)	0.9 (0.7 to 1.3)	-2.7 (-5.0 to -0.4)*	-1.7 (-4.0 to 0.7)
Migraine	57,772.7 (46,801.9 to 70,515.6)	1.2 (-1.2 to 3.6)	2.2 (-0.2 to 4.6)	2,046.4 (1,183.7 to 3,123.2)	1.2 (-1.4 to 4.2)	2.3 (-0.3 to 5.3)
Tension-type headache	80,335.6 (59,605.4 to 105,541.8)	0.3 (-0.5 to 1.0)	1.4 (0.5 to 2.1)*	122.4 (54.0 to 230.3)	0.4 (-1.2 to 2.0)	1.4 (-0.1 to 3.1)
Medication overuse headache	1,232.6 (827.4 to 1,725.3)	1.6 (-7.4 to 11.8)	2.7 (-6.5 to 13.0)	199.9 (112.3 to 319.7)	1.7 (-7.7 to 12.4)	2.7 (-6.7 to 13.6)
Other neurological disorders	0.7 (0.4 to 1.3)	0.5 (-1.1 to 1.5)	1.6 (-0.0 to 2.6)	0.4 (0.2 to 0.6)	-2.9 (-5.7 to -0.6)*	-1.9 (-4.7 to 0.4)
Mental and substance use disorders	79,068.4 (75,860.2 to 81,400.4)	-0.6 (-1.8 to 0.6)	0.4 (-0.7 to 1.7)	8,303.1 (5,688.4 to 11,347.6)	0.8 (0.0 to 1.7)*	1.9 (1.0 to 2.8)*
Schizophrenia	20.0 (10.7 to 33.2)	-2.2 (-3.6 to -0.7)*	-1.2 (-2.6 to 0.3)	14.2 (6.9 to 24.4)	-2.2 (-3.6 to -0.7)*	-1.2 (-2.6 to 0.3)
Alcohol use disorders	527.5 (381.9 to 698.9)	-6.7 (-10.9 to -2.3)*	-5.7 (-9.9 to -1.3)*	50.5 (29.3 to 79.3)	-7.1 (-12.3 to -1.3)*	-6.2 (-11.3 to -0.3)*
Alcohol dependence aggregate	442.8 (296.3 to 615.5)	-7.7 (-13.0 to -2.6)*	-6.8 (-12.0 to -1.5)*	46.0 (26.0 to 74.6)	-7.7 (-13.6 to -1.2)*	-6.7 (-12.7 to -0.2)*
Fetal alcohol syndrome aggregate	84.7 (79.8 to 88.6)	-1.0 (-1.5 to -0.6)*	0.0 (-0.5 to 0.4)	4.5 (3.0 to 6.6)	-0.9 (-7.1 to 5.2)	0.1 (-6.2 to 6.3)
Drug use disorders	315.6 (286.1 to 345.1)	-10.9 (-16.1 to -5.2)*	-10.0 (-15.2 to -4.3)*	15.3 (9.6 to 21.7)	-5.4 (-10.7 to 0.2)	-4.4 (-9.8 to 1.3)
Opioid use disorders	0.0 (0.0 to 0.0)	0.0 (0.0 to 0.0)	0.0 (0.0 to 0.0)	0.0 (0.0 to 0.0)	0.0 (0.0 to 0.0)	0.0 (0.0 to 0.0)
Asymptomatic opioid dependence	0.0 (0.0 to 0.0)	0.0 (0.0 to 0.0)	0.0 (0.0 to 0.0)	0.0 (0.0 to 0.0)	0.0 (0.0 to 0.0)	0.0 (0.0 to 0.0)
Symptomatic opioid dependence aggregate	0.0 (0.0 to 0.0)	0.0 (0.0 to 0.0)	0.0 (0.0 to 0.0)	0.0 (0.0 to 0.0)	0.0 (0.0 to 0.0)	0.0 (0.0 to 0.0)
Cocaine use disorders	0.0 (0.0 to 0.0)	0.0 (0.0 to 0.0)	0.0 (0.0 to 0.0)	0.0 (0.0 to 0.0)	0.0 (0.0 to 0.0)	0.0 (0.0 to 0.0)
Asymptomatic cocaine dependence	0.0 (0.0 to 0.0)	0.0 (0.0 to 0.0)	0.0 (0.0 to 0.0)	0.0 (0.0 to 0.0)	0.0 (0.0 to 0.0)	0.0 (0.0 to 0.0)

Cause	Prevalence in thousands (95% confidence interval)			YLDs in thousands (95% confidence interval)		
	2015	Percent change in counts between 2005 and 2015	Percent change in rates between 2005 and 2015	2015	Percent change in counts between 2005 and 2015	Percent change in rates between 2005 and 2015
Symptomatic cocaine dependence aggregate	0.0 (0.0 to 0.0)	0.0 (0.0 to 0.0)	0.0 (0.0 to 0.0)	0.0 (0.0 to 0.0)	0.0 (0.0 to 0.0)	0.0 (0.0 to 0.0)
Amphetamine use disorders	24.5 (17.9 to 32.0)	5.8 (2.0 to 9.9)*	6.9 (3.0 to 11.1)*	3.4 (1.9 to 5.3)	5.2 (-3.2 to 14.0)	6.3 (-2.2 to 15.2)
Asymptomatic amphetamine dependence	13.4 (9.2 to 18.1)	5.8 (2.0 to 9.9)*	6.9 (3.0 to 11.1)*	0.0 (0.0 to 0.0)	0.0 (0.0 to 0.0)	0.0 (0.0 to 0.0)
Symptomatic amphetamine dependence aggregate	11.1 (7.5 to 15.2)	5.8 (2.0 to 9.9)*	6.9 (3.0 to 11.1)*	3.4 (1.9 to 5.3)	5.2 (-3.2 to 14.0)	6.3 (-2.2 to 15.2)
Cannabis use disorders	324.8 (231.3 to 431.2)	-10.8 (-14.6 to -8.0)*	-9.8 (-13.7 to -7.1)*	9.7 (5.6 to 14.9)	-10.5 (-17.1 to -3.6)*	-9.6 (-16.2 to -2.6)*
Asymptomatic cannabis dependence	187.9 (131.9 to 251.7)	-10.8 (-14.6 to -8.0)*	-9.8 (-13.7 to -7.1)*	0.0 (0.0 to 0.0)	0.0 (0.0 to 0.0)	0.0 (0.0 to 0.0)
Symptomatic cannabis dependence aggregate	136.9 (96.3 to 185.7)	-10.8 (-14.6 to -8.0)*	-9.8 (-13.7 to -7.1)*	9.7 (5.6 to 14.9)	-10.5 (-17.1 to -3.6)*	-9.6 (-16.2 to -2.6)*
Other drug use disorders	--	--	--	2.2 (1.2 to 3.5)	5.1 (-4.1 to 14.9)	6.2 (-3.1 to 16.1)
Depressive disorders	8,903.0 (7,995.4 to 9,669.2)	3.6 (0.5 to 6.8)*	4.7 (1.5 to 7.9)*	1,678.1 (988.1 to 2,561.1)	4.5 (2.9 to 6.0)*	5.6 (4.0 to 7.1)*
Major depressive disorder	6,939.4 (4,845.7 to 9,748.9)	5.1 (3.9 to 6.2)*	6.2 (5.0 to 7.3)*	1,472.9 (831.4 to 2,292.6)	5.2 (3.5 to 6.9)*	6.3 (4.6 to 8.1)*
Dysthymia	2,058.2 (1,454.2 to 2,862.8)	-0.1 (-1.4 to 1.5)	1.0 (-0.4 to 2.6)	205.2 (115.7 to 338.9)	0.0 (-2.4 to 2.6)	1.1 (-1.4 to 3.6)
Bipolar disorder	984.5 (702.1 to 1,289.4)	0.4 (0.0 to 0.7)*	1.4 (1.0 to 1.7)*	207.2 (120.4 to 332.3)	0.4 (-3.1 to 3.8)	1.5 (-2.1 to 4.9)
Anxiety disorders	22,157.5 (17,406.7 to 28,015.0)	-0.1 (-2.5 to 2.6)	0.9 (-1.5 to 3.7)	2,101.0 (1,340.8 to 3,000.9)	-0.1 (-2.7 to 2.9)	1.0 (-1.7 to 4.0)
Eating disorders	410.5 (376.5 to 439.7)	10.0 (4.9 to 15.6)*	11.2 (6.0 to 16.8)*	90.5 (57.6 to 134.3)	9.9 (4.7 to 15.2)*	11.0 (5.8 to 16.4)*
Anorexia nervosa	265.9 (192.2 to 352.2)	4.0 (0.9 to 7.0)*	5.1 (1.9 to 8.1)*	57.3 (35.7 to 85.5)	4.1 (-1.6 to 10.0)	5.2 (-0.6 to 11.1)
Bulimia nervosa	155.2 (97.6 to 241.5)	21.5 (17.2 to 26.2)*	22.8 (18.5 to 27.5)*	33.2 (18.0 to 57.0)	21.5 (12.2 to 30.8)*	22.8 (13.4 to 32.1)*
Autistic spectrum disorders	6,027.9 (5,699.9 to 6,286.3)	-0.7 (-2.5 to 1.2)	0.4 (-1.5 to 2.3)	990.2 (664.2 to 1,373.3)	-0.5 (-1.5 to 0.6)	0.6 (-0.5 to 1.6)
Autism	2,405.8 (2,013.2 to 2,863.5)	-0.3 (-0.6 to -0.1)*	0.7 (0.5 to 1.0)*	626.6 (405.6 to 895.2)	-0.2 (-1.8 to 1.3)	0.8 (-0.8 to 2.3)
Asperger syndrome and other autistic spectrum disorders	3,598.2 (3,029.9 to 4,329.4)	-1.0 (-1.1 to -0.8)*	0.1 (-0.1 to 0.2)	363.6 (241.4 to 532.6)	-0.9 (-2.2 to 0.5)	0.1 (-1.1 to 1.5)
Attention-deficit/hyperactivity disorder	11,947.4 (10,538.8 to 13,791.2)	-6.4 (-7.3 to -5.6)*	-5.4 (-6.3 to -4.6)*	145.0 (87.1 to 225.4)	-6.4 (-7.8 to -5.1)*	-5.4 (-6.9 to -4.1)*
Conduct disorder	21,652.7 (16,417.0 to 27,059.9)	0.3 (-0.1 to 0.6)	1.3 (1.0 to 1.7)*	2,595.2 (1,516.5 to 4,157.6)	0.4 (-0.5 to 1.2)	1.4 (0.6 to 2.3)*
Idiopathic developmental intellectual disability	11,380.2 (6,807.7 to 15,783.7)	0.4 (-0.5 to 1.0)	1.4 (0.5 to 2.1)*	416.0 (186.4 to 729.8)	-0.6 (-2.9 to 0.7)	0.4 (-1.9 to 1.7)
Other mental and substance use disorders	0.0 (0.0 to 0.0)	0.0 (0.0 to 0.0)	0.0 (0.0 to 0.0)	0.0 (0.0 to 0.0)	0.0 (0.0 to 0.0)	0.0 (0.0 to 0.0)
Diabetes, urogenital, blood, and endocrine diseases	156,976.5 (154,498.9 to 159,716.7)	0.4 (-1.0 to 1.7)	1.4 (0.0 to 2.8)*	1,989.3 (1,349.4 to 2,785.1)	-1.5 (-2.6 to -0.4)*	-0.5 (-1.6 to 0.7)
Diabetes mellitus	2,417.3 (1,981.7 to 2,914.0)	-0.1 (-4.5 to 4.0)	1.0 (-3.5 to 5.1)	122.2 (76.5 to 187.5)	0.1 (-4.8 to 4.5)	1.1 (-3.8 to 5.6)
Uncomplicated diabetes mellitus	2,336.3 (1,905.0 to 2,827.8)	-0.2 (-4.6 to 3.9)	0.9 (-3.6 to 5.0)	111.7 (69.4 to 173.4)	-0.1 (-4.9 to 4.3)	0.9 (-3.9 to 5.4)
Neuropathy and other complications of diabetes mellitus aggregate	81.1 (60.4 to 106.7)	2.1 (-2.8 to 6.7)	3.1 (-1.8 to 7.8)	10.5 (6.5 to 16.1)	2.0 (-7.6 to 11.7)	3.1 (-6.7 to 12.9)
Vision loss due to diabetes mellitus aggregate	0.0 (0.0 to 0.0)	0.0 (0.0 to 0.0)	0.0 (0.0 to 0.0)	0.0 (0.0 to 0.0)	0.0 (0.0 to 0.0)	0.0 (0.0 to 0.0)
Acute glomerulonephritis	5.9 (4.4 to 7.8)	-22.1 (-25.6 to -18.6)*	-21.2 (-24.8 to -17.8)*	0.3 (0.2 to 0.5)	-22.0 (-25.7 to -18.5)*	-21.2 (-24.9 to -17.6)*
Chronic kidney disease	1,218.8 (1,084.8 to 1,325.7)	-1.1 (-4.3 to 2.3)	-0.0 (-3.3 to 3.4)	136.9 (93.3 to 192.8)	-0.8 (-3.1 to 1.7)	0.2 (-2.1 to 2.7)
Chronic kidney disease due to diabetes mellitus	444.5 (285.6 to 653.9)	-2.5 (-5.0 to -0.3)*	-1.5 (-4.0 to 0.7)	41.4 (25.4 to 63.5)	-2.1 (-6.4 to 2.7)	-1.1 (-5.5 to 3.7)
Stage III chronic kidney disease due to diabetes mellitus aggregate	212.4 (115.9 to 335.3)	-2.3 (-4.9 to -0.1)*	-1.3 (-3.9 to 1.0)	2.1 (1.4 to 2.9)	3.0 (-2.6 to 8.9)	4.0 (-1.6 to 10.0)
Stage IV chronic kidney disease due to diabetes mellitus aggregate	214.6 (134.3 to 320.2)	-2.6 (-5.2 to -0.0)*	-1.5 (-4.2 to 1.0)	31.2 (18.1 to 50.2)	-2.3 (-8.1 to 3.7)	-1.3 (-7.1 to 4.8)
Stage V chronic kidney disease untreated due to diabetes mellitus	12.9 (8.0 to 18.7)	-2.4 (-4.7 to -0.1)*	-1.4 (-3.8 to 0.9)	7.3 (4.1 to 11.9)	-2.4 (-4.7 to -0.1)*	-1.4 (-3.8 to 0.9)
End-stage chronic kidney disease due to diabetes aggregate	4.6 (2.9 to 6.9)	-9.4 (-12.4 to -6.8)*	-8.5 (-11.5 to -5.9)*	0.8 (0.4 to 1.4)	-3.9 (-6.8 to -1.3)*	-2.9 (-5.8 to -0.3)*
Chronic kidney disease due to hypertension	230.3 (114.4 to 394.1)	-1.2 (-5.2 to 2.0)	-0.2 (-4.3 to 3.1)	11.1 (5.8 to 20.7)	-3.4 (-9.6 to 2.9)	-2.4 (-8.7 to 3.9)
Stage III chronic kidney disease due to hypertension aggregate	156.8 (75.2 to 263.2)	0.5 (-2.7 to 3.2)	1.5 (-1.7 to 4.3)	0.4 (0.3 to 0.5)	1.3 (-1.1 to 3.5)	2.3 (-0.1 to 4.6)
Stage IV chronic kidney disease due to hypertension aggregate	70.6 (28.0 to 157.9)	-4.6 (-11.9 to 2.1)	-3.6 (-11.0 to 3.1)	9.4 (4.4 to 18.8)	-3.7 (-11.3 to 3.7)	-2.7 (-10.4 to 4.8)
Stage V chronic kidney disease untreated due to hypertension	2.1 (1.3 to 3.2)	-2.5 (-6.5 to 1.6)	-1.5 (-5.6 to 2.7)	1.2 (0.7 to 2.0)	-2.5 (-6.5 to 1.6)	-1.5 (-5.6 to 2.7)
End-stage chronic kidney disease due to hypertension aggregate	0.8 (0.5 to 1.2)	-9.0 (-14.6 to -3.5)*	-8.0 (-13.7 to -2.5)*	0.1 (0.1 to 0.2)	-4.1 (-8.9 to 0.6)	-3.1 (-8.0 to 1.6)
Chronic kidney disease due to glomerulonephritis	218.4 (162.3 to 264.6)	0.9 (-1.5 to 3.1)	1.9 (-0.4 to 4.2)	32.0 (20.0 to 45.1)	1.5 (-3.5 to 6.6)	2.5 (-2.5 to 7.7)
Stage III chronic kidney disease due to glomerulonephritis aggregate	56.4 (40.2 to 79.4)	-0.4 (-3.4 to 2.7)	0.6 (-2.4 to 3.7)	1.7 (1.2 to 2.4)	3.3 (-0.7 to 7.1)	4.3 (0.3 to 8.2)*
Stage IV chronic kidney disease due to glomerulonephritis aggregate	147.4 (101.2 to 179.4)	1.7 (-1.2 to 4.3)	2.8 (-0.2 to 5.4)	23.3 (14.2 to 33.7)	2.0 (-4.6 to 9.2)	3.1 (-3.6 to 10.3)
Stage V chronic kidney disease untreated due to glomerulonephritis	11.1 (6.3 to 17.1)	-0.4 (-2.9 to 1.9)	0.6 (-1.9 to 3.0)	6.3 (3.3 to 10.2)	-0.4 (-2.9 to 1.9)	0.6 (-1.9 to 3.0)
End-stage chronic kidney disease due to glomerulonephritis aggregate	3.5 (2.0 to 5.4)	-6.7 (-9.9 to -3.5)*	-5.7 (-8.9 to -2.5)*	0.7 (0.4 to 1.2)	-4.0 (-7.8 to -0.9)*	-3.0 (-6.9 to 0.1)
Chronic kidney disease due to other causes	562.0 (374.5 to 816.0)	-0.1 (-1.7 to 1.6)	0.9 (-0.7 to 2.6)	52.4 (32.6 to 80.6)	-0.5 (-4.4 to 3.5)	0.5 (-3.4 to 4.6)
Stage III chronic kidney disease due to other causes aggregate	267.5 (151.5 to 424.5)	0.4 (-1.3 to 2.1)	1.4 (-0.3 to 3.2)	2.5 (1.7 to 3.6)	4.4 (0.5 to 8.8)*	5.5 (1.5 to 9.9)*
Stage IV chronic kidney disease due to other causes aggregate	272.5 (175.1 to 399.8)	-0.4 (-2.4 to 1.8)	0.6 (-1.4 to 2.9)	39.4 (23.2 to 62.0)	-0.7 (-6.1 to 4.6)	0.3 (-5.1 to 5.7)
Stage V chronic kidney disease untreated due to other causes	16.6 (10.4 to 24.3)	-0.4 (-2.1 to 1.4)	0.6 (-1.1 to 2.4)	9.4 (5.4 to 14.9)	-0.5 (-2.2 to 1.4)	0.5 (-1.2 to 2.4)
End-stage chronic kidney disease due to other causes aggregate	5.4 (3.4 to 8.0)	-6.6 (-8.7 to -4.0)*	-5.6 (-7.8 to -3.0)*	1.1 (0.6 to 1.8)	-2.6 (-5.6 to 0.2)	-1.6 (-4.6 to 1.2)
Urinary diseases and male infertility	6,414.6 (6,007.1 to 6,787.9)	-0.7 (-3.8 to 2.9)	0.3 (-2.8 to 3.9)	7.9 (4.9 to 11.8)	0.5 (-6.1 to 7.5)	1.5 (-5.1 to 8.6)
Interstitial nephritis and urinary tract infections	142.6 (129.0 to 157.6)	2.3 (1.5 to 3.0)*	3.3 (2.5 to 4.1)*	4.8 (2.9 to 7.4)	2.3 (-5.4 to 10.4)	3.4 (-4.4 to 11.5)
Urolithiasis	6,333.9 (5,327.8 to 7,433.4)	-0.9 (-4.8 to 3.1)	0.2 (-3.8 to 4.2)	1.0 (0.6 to 1.6)	-2.2 (-6.9 to 2.3)	-1.2 (-5.9 to 3.3)
Benign prostatic hyperplasia	0.0 (0.0 to 0.0)	0.0 (0.0 to 0.0)	0.0 (0.0 to 0.0)	0.0 (0.0 to 0.0)	0.0 (0.0 to 0.0)	0.0 (0.0 to 0.0)
Male infertility	0.0 (0.0 to 0.0)	0.0 (0.0 to 0.0)	0.0 (0.0 to 0.0)	0.0 (0.0 to 0.0)	0.0 (0.0 to 0.0)	0.0 (0.0 to 0.0)
Other urinary diseases	--	--	--	2.1 (1.3 to 3.1)	-2.4 (-12.5 to 8.8)	-1.4 (-11.6 to 9.9)
Gynecological diseases	0.0 (0.0 to 0.0)	0.0 (0.0 to 0.0)	0.0 (0.0 to 0.0)	0.0 (0.0 to 0.0)	0.0 (0.0 to 0.0)	0.0 (0.0 to 0.0)
Uterine fibroids	0.0 (0.0 to 0.0)	0.0 (0.0 to 0.0)	0.0 (0.0 to 0.0)	0.0 (0.0 to 0.0)	0.0 (0.0 to 0.0)	0.0 (0.0 to 0.0)
Uterine fibroids cases aggregate	0.0 (0.0 to 0.0)	0.0 (0.0 to 0.0)	0.0 (0.0 to 0.0)	0.0 (0.0 to 0.0)	0.0 (0.0 to 0.0)	0.0 (0.0 to 0.0)
Mild abdominal pain with anemia due to uterine fibroids aggregate	0.0 (0.0 to 0.0)	0.0 (0.0 to 0.0)	0.0 (0.0 to 0.0)	0.0 (0.0 to 0.0)	0.0 (0.0 to 0.0)	0.0 (0.0 to 0.0)
Polycystic ovarian syndrome	0.0 (0.0 to 0.0)	0.0 (0.0 to 0.0)	0.0 (0.0 to 0.0)	0.0 (0.0 to 0.0)	0.0 (0.0 to 0.0)	0.0 (0.0 to 0.0)
Polycystic ovarian syndrome cases aggregate	0.0 (0.0 to 0.0)	0.0 (0.0 to 0.0)	0.0 (0.0 to 0.0)	0.0 (0.0 to 0.0)	0.0 (0.0 to 0.0)	0.0 (0.0 to 0.0)
Hirsutism and infertility due to polycystic ovarian syndrome aggregate	0.0 (0.0 to 0.0)	0.0 (0.0 to 0.0)	0.0 (0.0 to 0.0)	0.0 (0.0 to 0.0)	0.0 (0.0 to 0.0)	0.0 (0.0 to 0.0)

Cause	Prevalence in thousands (95% confidence interval)			YLDs in thousands (95% confidence interval)		
	2015	Percent change in counts between 2005 and 2015	Percent change in rates between 2005 and 2015	2015	Percent change in counts between 2005 and 2015	Percent change in rates between 2005 and 2015
Female infertility	0.0 (0.0 to 0.0)	0.0 (0.0 to 0.0)	0.0 (0.0 to 0.0)	0.0 (0.0 to 0.0)	0.0 (0.0 to 0.0)	0.0 (0.0 to 0.0)
Endometriosis	0.0 (0.0 to 0.0)	0.0 (0.0 to 0.0)	0.0 (0.0 to 0.0)	0.0 (0.0 to 0.0)	0.0 (0.0 to 0.0)	0.0 (0.0 to 0.0)
Endometriosis cases aggregate	0.0 (0.0 to 0.0)	0.0 (0.0 to 0.0)	0.0 (0.0 to 0.0)	0.0 (0.0 to 0.0)	0.0 (0.0 to 0.0)	0.0 (0.0 to 0.0)
Abdominal pain and infertility due to endometriosis aggregate	0.0 (0.0 to 0.0)	0.0 (0.0 to 0.0)	0.0 (0.0 to 0.0)	0.0 (0.0 to 0.0)	0.0 (0.0 to 0.0)	0.0 (0.0 to 0.0)
Genital prolapse	0.0 (0.0 to 0.0)	0.0 (0.0 to 0.0)	0.0 (0.0 to 0.0)	0.0 (0.0 to 0.0)	0.0 (0.0 to 0.0)	0.0 (0.0 to 0.0)
Premenstrual syndrome	0.0 (0.0 to 0.0)	0.0 (0.0 to 0.0)	0.0 (0.0 to 0.0)	0.0 (0.0 to 0.0)	0.0 (0.0 to 0.0)	0.0 (0.0 to 0.0)
Other gynecological diseases	0.0 (0.0 to 0.0)	0.0 (0.0 to 0.0)	0.0 (0.0 to 0.0)	0.0 (0.0 to 0.0)	0.0 (0.0 to 0.0)	0.0 (0.0 to 0.0)
Other gynecological diseases cases aggregate	0.0 (0.0 to 0.0)	0.0 (0.0 to 0.0)	0.0 (0.0 to 0.0)	0.0 (0.0 to 0.0)	0.0 (0.0 to 0.0)	0.0 (0.0 to 0.0)
Anemia due to other gynecological diseases aggregate	0.0 (0.0 to 0.0)	0.0 (0.0 to 0.0)	0.0 (0.0 to 0.0)	0.0 (0.0 to 0.0)	0.0 (0.0 to 0.0)	0.0 (0.0 to 0.0)
Hemoglobinopathies and hemolytic anemias	144,763.7 (142,312.3 to 147,581.1)	0.7 (-0.9 to 2.1)	1.7 (0.1 to 3.2)*	1,439.7 (976.3 to 2,052.4)	-1.2 (-2.5 to 0.1)	-0.2 (-1.5 to 1.2)
Thalassemias	20.0 (18.2 to 23.5)	-12.2 (-14.9 to -8.5)*	-11.3 (-14.0 to -7.5)*	1.9 (1.3 to 2.8)	-13.0 (-20.9 to -4.3)*	-12.1 (-20.1 to -3.3)*
Beta-thalassemia major cases aggregate	7.2 (6.9 to 7.5)	-11.3 (-12.7 to -9.9)*	-10.3 (-11.8 to -8.9)*	0.8 (0.5 to 1.1)	-14.6 (-28.2 to 1.5)	-13.7 (-27.5 to 2.6)
Hemoglobin E/beta-thalassemia cases aggregate	3.2 (3.0 to 3.5)	-5.0 (-6.7 to -2.8)*	-4.1 (-5.7 to -1.8)*	0.3 (0.2 to 0.5)	-6.9 (-16.1 to 2.1)	-5.9 (-15.2 to 3.1)
Hemoglobin H disease cases aggregate	9.5 (7.7 to 13.1)	-15.0 (-20.6 to -7.9)*	-14.2 (-19.8 to -6.9)*	0.8 (0.5 to 1.2)	-13.8 (-26.4 to -0.9)*	-12.9 (-25.7 to 0.2)
Heart failure due to thalassemias aggregate	0.1 (0.1 to 0.1)	-14.5 (-17.4 to -11.5)*	-13.6 (-16.5 to -10.6)*	0.0 (0.0 to 0.0)	-14.5 (-17.4 to -11.5)*	-13.6 (-16.5 to -10.6)*
Thalassemias trait	21,790.0 (21,291.1 to 22,424.4)	-8.2 (-8.7 to -7.8)*	-7.3 (-7.8 to -6.8)*	634.3 (429.4 to 905.6)	-4.3 (-5.7 to -2.9)*	-3.3 (-4.7 to -1.9)*
B-thalassemia trait cases aggregate	17,452.3 (16,994.3 to 18,002.4)	-9.2 (-9.7 to -8.6)*	-8.2 (-8.8 to -7.7)*	584.4 (395.1 to 832.5)	-5.0 (-6.3 to -3.7)*	-4.1 (-5.4 to -2.7)*
Hemoglobin E trait cases aggregate	4,337.7 (4,139.5 to 4,550.3)	-4.4 (-5.4 to -3.3)*	-3.4 (-4.4 to -2.3)*	50.0 (33.4 to 71.1)	4.8 (-0.1 to 9.3)	5.9 (1.0 to 10.5)*
Sickle cell disorders	740.8 (711.8 to 773.5)	8.7 (5.7 to 11.5)*	9.8 (6.8 to 12.6)*	65.2 (44.8 to 92.2)	12.6 (6.6 to 18.6)*	13.8 (7.7 to 19.8)*
Homozygous sickle cell and severe sickle cell/beta-thalassemia cases aggregate	709.3 (681.0 to 740.1)	7.8 (4.6 to 10.6)*	8.9 (5.7 to 11.7)*	62.5 (42.7 to 87.8)	11.9 (5.9 to 17.8)*	13.1 (7.0 to 19.0)*
Hemoglobin SC disease cases aggregate	30.9 (28.1 to 33.8)	34.4 (22.8 to 46.9)*	35.8 (24.1 to 48.4)*	2.6 (1.7 to 3.9)	33.1 (-1.6 to 76.4)	34.5 (-0.6 to 78.2)
Mild sickle cell/beta-thalassemia cases aggregate	0.7 (0.6 to 0.7)	0.7 (-4.0 to 6.2)	1.7 (-3.0 to 7.3)	0.1 (0.1 to 0.1)	3.0 (-2.0 to 8.9)	4.1 (-1.0 to 10.0)
Sickle cell trait	42,672.3 (40,195.0 to 47,243.6)	14.4 (13.0 to 15.6)*	15.6 (14.2 to 16.8)*	280.1 (188.8 to 400.7)	15.0 (9.1 to 18.5)*	16.2 (10.2 to 19.7)*
G6PD deficiency	23,539.0 (19,907.2 to 27,341.8)	-1.8 (-7.1 to 2.9)	-0.8 (-6.1 to 4.0)	3.9 (2.6 to 5.4)	-5.0 (-15.7 to 6.3)	-4.0 (-14.8 to 7.4)
G6PD cases aggregate	23,539.0 (19,907.2 to 27,341.8)	-1.8 (-7.1 to 2.9)	-0.8 (-6.1 to 4.0)	3.9 (2.6 to 5.4)	-5.0 (-15.7 to 6.3)	-4.0 (-14.8 to 7.4)
Heart failure due to G6PD deficiency aggregate	0.0 (0.0 to 0.0)	0.0 (0.0 to 0.0)	0.0 (0.0 to 0.0)	0.0 (0.0 to 0.0)	0.0 (0.0 to 0.0)	0.0 (0.0 to 0.0)
G6PD trait	63,591.8 (59,256.5 to 68,045.4)	-0.9 (-2.5 to 0.5)	0.1 (-1.5 to 1.5)	3.4 (2.3 to 4.8)	-8.0 (-18.0 to 3.0)	-7.0 (-17.2 to 4.1)
Other hemoglobinopathies and hemolytic anemias	9,324.7 (9,224.5 to 9,421.5)	-6.7 (-8.0 to -5.3)*	-5.7 (-7.0 to -4.4)*	450.9 (306.3 to 642.7)	-6.6 (-8.4 to -4.7)*	-5.6 (-7.4 to -3.7)*
Other hemoglobinopathies and hemolytic anemias cases aggregate	9,324.6 (9,224.3 to 9,421.3)	-6.7 (-8.0 to -5.3)*	-5.7 (-7.0 to -4.4)*	445.1 (302.3 to 633.5)	-6.7 (-8.5 to -4.9)*	-5.7 (-7.5 to -3.9)*
Heart failure due to other hemoglobinopathies and hemolytic anemias aggregate	0.2 (0.1 to 0.2)	-8.7 (-11.1 to -6.5)*	-7.7 (-10.2 to -5.5)*	0.0 (0.0 to 0.0)	-8.7 (-11.1 to -6.5)*	-7.8 (-10.2 to -5.5)*
Endocrine, metabolic, blood, and immune disorders	6,087.0 (6,029.3 to 6,149.1)	-6.4 (-9.3 to -3.2)*	-5.4 (-8.4 to -2.2)*	282.3 (190.0 to 402.8)	-3.9 (-7.3 to -0.3)*	-2.9 (-6.4 to 0.7)
Endocrine, metabolic, blood, and immune disorders cases aggregate	261.1 (235.2 to 286.2)	-7.2 (-9.1 to -5.5)*	-6.2 (-8.2 to -4.6)*	10.5 (6.9 to 14.9)	-6.9 (-11.4 to -2.5)*	-5.9 (-10.4 to -1.5)*
Anemia due to endocrine, metabolic, blood, and immune disorders aggregate	5,825.8 (5,777.2 to 5,879.5)	-6.4 (-9.4 to -3.0)*	-5.4 (-8.5 to -2.0)*	271.7 (183.1 to 388.8)	-3.8 (-7.4 to -0.1)*	-2.8 (-6.4 to 1.0)
Heart failure due to endocrine, metabolic, blood, and immune disorders aggregate	0.0 (0.0 to 0.1)	-6.2 (-7.6 to -4.8)*	-5.3 (-6.7 to -3.8)*	0.0 (0.0 to 0.0)	-6.2 (-7.6 to -4.8)*	-5.3 (-6.7 to -3.8)*
Musculoskeletal disorders	17,083.3 (16,650.3 to 17,603.4)	-4.6 (-6.2 to -3.1)*	-3.7 (-5.2 to -2.1)*	1,986.5 (1,370.3 to 2,711.7)	-4.6 (-6.6 to -2.6)*	-3.6 (-5.6 to -1.6)*
Rheumatoid arthritis	557.9 (468.0 to 654.0)	-4.0 (-9.1 to 1.0)	-3.0 (-8.1 to 2.0)	139.4 (92.6 to 195.3)	-3.9 (-10.1 to 2.0)	-2.9 (-9.2 to 3.1)
Osteoarthritis	0.0 (0.0 to 0.0)	0.0 (0.0 to 0.0)	0.0 (0.0 to 0.0)	0.0 (0.0 to 0.0)	0.0 (0.0 to 0.0)	0.0 (0.0 to 0.0)
Osteoarthritis of the hip cases aggregate	0.0 (0.0 to 0.0)	0.0 (0.0 to 0.0)	0.0 (0.0 to 0.0)	0.0 (0.0 to 0.0)	0.0 (0.0 to 0.0)	0.0 (0.0 to 0.0)
Osteoarthritis of the knee cases aggregate	0.0 (0.0 to 0.0)	0.0 (0.0 to 0.0)	0.0 (0.0 to 0.0)	0.0 (0.0 to 0.0)	0.0 (0.0 to 0.0)	0.0 (0.0 to 0.0)
Low back and neck pain	12,899.7 (12,400.5 to 13,400.1)	-2.8 (-4.3 to -1.3)*	-1.8 (-3.3 to -0.3)*	1,478.4 (1,025.3 to 2,101.2)	-2.8 (-4.7 to -0.9)*	-1.8 (-3.8 to 0.1)
Low back pain	10,940.4 (9,752.6 to 12,260.5)	-3.1 (-5.0 to -1.5)*	-2.1 (-4.0 to -0.4)*	1,269.9 (867.1 to 1,794.5)	-3.1 (-5.2 to -0.9)*	-2.0 (-4.2 to 0.2)
Neck pain	2,060.5 (1,454.6 to 2,776.3)	-1.4 (-3.8 to 0.5)	-0.4 (-2.8 to 1.5)	208.5 (121.1 to 322.8)	-1.4 (-4.3 to 1.5)	-0.3 (-3.3 to 2.6)
Gout	0.0 (0.0 to 0.0)	0.0 (0.0 to 0.0)	0.0 (0.0 to 0.0)	0.0 (0.0 to 0.0)	0.0 (0.0 to 0.0)	0.0 (0.0 to 0.0)
Asymptomatic gout	0.0 (0.0 to 0.0)	0.0 (0.0 to 0.0)	0.0 (0.0 to 0.0)	0.0 (0.0 to 0.0)	0.0 (0.0 to 0.0)	0.0 (0.0 to 0.0)
Symptomatic gout cases aggregate	0.0 (0.0 to 0.0)	0.0 (0.0 to 0.0)	0.0 (0.0 to 0.0)	0.0 (0.0 to 0.0)	0.0 (0.0 to 0.0)	0.0 (0.0 to 0.0)
Other musculoskeletal disorders	3,786.4 (2,838.0 to 5,147.6)	-11.3 (-17.4 to -4.8)*	-10.3 (-16.5 to -3.8)*	368.7 (219.6 to 561.5)	-11.2 (-17.7 to -4.5)*	-10.3 (-16.9 to -3.5)*
Other non-communicable diseases	414,491.1 (406,000.3 to 420,893.0)	-0.8 (-1.6 to -0.1)*	0.2 (-0.5 to 0.9)	7,845.4 (5,151.4 to 11,417.8)	1.1 (-0.0 to 2.8)	2.2 (1.0 to 3.8)*
Congenital anomalies	8,109.1 (7,371.5 to 8,770.3)	6.4 (2.8 to 9.9)*	7.5 (3.8 to 11.1)*	751.8 (468.7 to 1,138.3)	13.7 (6.1 to 22.0)*	14.9 (7.2 to 23.3)*
Neural tube defects	150.0 (102.1 to 217.4)	6.6 (2.7 to 10.0)*	7.7 (3.8 to 11.2)*	52.7 (29.9 to 88.1)	7.5 (0.1 to 14.2)*	8.6 (1.2 to 15.4)*
Congenital heart anomalies	3,843.8 (2,684.4 to 5,795.7)	9.8 (6.7 to 13.0)*	10.9 (7.8 to 14.2)*	133.5 (51.8 to 249.2)	9.9 (6.4 to 13.4)*	11.1 (7.5 to 14.6)*
Less severe heart anomalies cases aggregate	3,528.9 (2,300.2 to 5,502.1)	9.6 (5.9 to 13.0)*	10.7 (7.0 to 14.2)*	114.6 (42.9 to 222.7)	9.5 (5.5 to 13.5)*	10.7 (6.6 to 14.6)*
Severe congenital heart anomalies	295.6 (194.7 to 443.6)	11.9 (6.7 to 16.5)*	13.0 (7.8 to 17.7)*	17.5 (6.9 to 35.2)	11.8 (5.3 to 17.9)*	13.0 (6.4 to 19.1)*
Critical congenital heart anomalies	16.7 (9.4 to 27.1)	30.8 (15.9 to 42.1)*	32.2 (17.1 to 43.6)*	1.0 (0.3 to 2.1)	31.1 (11.5 to 48.2)*	32.4 (12.7 to 49.8)*
Heart failure due to congenital heart anomalies aggregate	2.7 (2.2 to 3.2)	-10.3 (-11.9 to -8.6)*	-9.3 (-11.0 to -7.7)*	0.3 (0.2 to 0.5)	-10.3 (-11.9 to -8.6)*	-9.3 (-11.0 to -7.7)*
Cleft lip and cleft palate	563.2 (322.5 to 951.6)	1.4 (-8.0 to 7.1)	2.4 (-7.0 to 8.2)	7.1 (3.5 to 13.3)	-3.0 (-12.9 to 5.1)	-2.0 (-12.0 to 6.2)
Down syndrome	511.8 (322.7 to 788.7)	6.6 (3.5 to 9.6)*	7.7 (4.6 to 10.8)*	43.0 (23.7 to 72.1)	6.5 (1.5 to 10.8)*	7.6 (2.6 to 11.9)*
Turner syndrome	32.6 (15.3 to 59.6)	-1.8 (-3.9 to 0.1)	-0.7 (-2.9 to 1.2)	0.5 (0.1 to 1.0)	-1.7 (-5.8 to 2.4)	-0.7 (-4.8 to 3.5)
Klinefelter syndrome	19.3 (10.1 to 34.6)	0.3 (-1.4 to 1.4)	1.3 (-0.3 to 2.5)	0.1 (0.0 to 0.1)	0.3 (-1.4 to 1.4)	1.3 (-0.3 to 2.5)
Other chromosomal abnormalities	432.9 (238.8 to 770.8)	5.4 (2.4 to 7.8)*	6.5 (3.5 to 8.9)*	36.5 (18.2 to 69.3)	5.3 (0.6 to 9.2)*	6.4 (1.6 to 10.4)*
Other congenital anomalies	2,883.4 (1,587.9 to 5,288.3)	2.5 (0.8 to 4.4)*	3.6 (1.8 to 5.5)*	478.5 (282.3 to 759.3)	17.4 (5.6 to 31.9)*	18.6 (6.7 to 33.3)*
Skin and subcutaneous diseases	242,996.9 (238,959.5 to 246,264.2)	-0.7 (-1.5 to 0.0)	0.3 (-0.5 to 1.1)	4,891.8 (3,057.9 to 7,722.9)	-0.7 (-1.6 to 0.1)	0.3 (-0.6 to 1.2)
Dermatitis	20,949.8 (17,261.0 to 25,248.9)	1.8 (0.9 to 2.7)*	2.8 (1.9 to 3.8)*	906.1 (590.5 to 1,290.8)	3.9 (2.3 to 5.4)*	5.0 (3.4 to 6.5)*
Eczema cases aggregate	10,946.8 (8,737.2 to 13,984.2)	6.1 (5.4 to 6.9)*	7.2 (6.5 to 8.0)*	676.0 (439.2 to 997.4)	6.2 (4.3 to 7.8)*	7.3 (5.4 to 9.0)*

Cause	Prevalence in thousands (95% confidence interval)			YLDs in thousands (95% confidence interval)		
	2015	Percent change in counts between 2005 and 2015	Percent change in rates between 2005 and 2015	2015	Percent change in counts between 2005 and 2015	Percent change in rates between 2005 and 2015
Contact dermatitis cases aggregate	6,330.7 (4,201.8 to 9,395.5)	-1.5 (-2.0 to -1.0)*	-0.5 (-1.0 to 0.0)	174.7 (92.5 to 282.6)	-1.5 (-3.5 to 0.4)	-0.4 (-2.5 to 1.5)
Seborrhoeic dermatitis cases aggregate	3,672.3 (2,544.4 to 5,173.2)	-4.6 (-5.5 to -3.6)*	-3.6 (-4.5 to -2.6)*	55.4 (27.8 to 95.4)	-4.5 (-6.4 to -2.8)*	-3.5 (-5.5 to -1.7)*
Psoriasis	4,431.2 (4,019.7 to 4,875.3)	1.1 (-0.2 to 2.3)	2.2 (0.8 to 3.4)*	370.3 (255.1 to 508.1)	1.2 (-1.5 to 3.8)	2.3 (-0.5 to 4.9)
Cellulitis	52.1 (38.7 to 69.3)	6.9 (2.8 to 11.3)*	8.0 (3.9 to 12.5)*	3.9 (2.3 to 6.1)	6.8 (0.2 to 13.3)*	7.9 (1.2 to 14.5)*
Pyoderma	413.9 (361.1 to 482.8)	0.7 (-1.2 to 2.9)	1.8 (-0.2 to 4.0)	2.4 (1.0 to 4.9)	0.8 (-3.6 to 5.2)	1.9 (-2.6 to 6.3)
Scabies	22,213.6 (14,396.0 to 32,970.1)	-7.7 (-11.1 to -4.4)*	-6.8 (-10.2 to -3.4)*	576.5 (280.1 to 993.1)	-7.7 (-11.0 to -4.3)*	-6.7 (-10.1 to -3.3)*
Fungal skin diseases	52,326.2 (40,919.9 to 66,528.9)	2.3 (1.4 to 3.3)*	3.3 (2.4 to 4.4)*	298.3 (116.1 to 623.9)	2.3 (1.4 to 3.4)*	3.4 (2.5 to 4.5)*
Viral skin diseases	24,159.0 (20,647.6 to 27,893.6)	-2.9 (-3.6 to -2.0)*	-1.9 (-2.6 to -1.0)*	752.7 (476.2 to 1,137.0)	-2.8 (-3.9 to -1.7)*	-1.8 (-2.9 to -0.7)*
Molluscum contagiosum cases aggregate	5,830.7 (4,499.3 to 7,634.3)	-1.5 (-2.4 to -0.3)*	-0.5 (-1.4 to 0.7)	181.8 (107.3 to 297.1)	-1.4 (-3.2 to 0.4)	-0.4 (-2.2 to 1.4)
Viral warts cases aggregate	18,328.4 (15,306.0 to 21,659.9)	-3.3 (-4.2 to -2.3)*	-2.3 (-3.2 to -1.3)*	570.9 (355.1 to 866.3)	-3.2 (-4.5 to -2.0)*	-2.2 (-3.5 to -1.0)*
Acne vulgaris	135,688.4 (122,493.0 to 149,694.5)	-0.6 (-2.2 to 0.8)	0.5 (-1.2 to 1.8)	1,475.7 (705.6 to 2,724.6)	-0.5 (-2.1 to 0.8)	0.5 (-1.1 to 1.9)
Alopecia areata	651.1 (556.3 to 752.3)	-1.2 (-1.6 to -0.7)*	-0.2 (-0.6 to 0.3)	22.3 (13.6 to 34.1)	-1.1 (-5.1 to 2.8)	-0.1 (-4.1 to 3.9)
Pruritus	3,030.5 (2,049.7 to 4,329.5)	-1.3 (-4.7 to 1.3)	-0.3 (-3.7 to 2.4)	32.9 (14.1 to 69.6)	-1.3 (-4.7 to 1.8)	-0.3 (-3.7 to 2.8)
Urticaria	5,119.0 (3,322.7 to 7,488.7)	-1.5 (-2.1 to -1.0)*	-0.5 (-1.0 to -0.0)*	315.0 (165.4 to 523.2)	-1.5 (-3.0 to 0.2)	-0.4 (-2.0 to 1.2)
Decubitus ulcer	13.9 (9.9 to 19.1)	6.0 (1.0 to 10.8)*	7.1 (2.0 to 11.9)*	2.3 (1.4 to 3.5)	6.0 (1.0 to 10.8)*	7.1 (2.1 to 11.9)*
Other skin and subcutaneous diseases	22,210.2 (20,014.7 to 24,539.9)	1.0 (-0.6 to 2.6)	2.1 (0.5 to 3.7)*	133.5 (63.6 to 248.3)	1.0 (-0.7 to 2.9)	2.1 (0.3 to 3.9)*
Sense organ diseases	47,317.1 (45,476.0 to 48,982.7)	0.3 (-1.2 to 1.7)	1.3 (-0.1 to 2.8)	1,844.4 (1,147.1 to 2,705.4)	1.9 (-0.3 to 4.3)	2.9 (0.7 to 5.3)*
Glaucoma	0.0 (0.0 to 0.0)	0.0 (0.0 to 0.0)	0.0 (0.0 to 0.0)	0.0 (0.0 to 0.0)	0.0 (0.0 to 0.0)	0.0 (0.0 to 0.0)
Cataract	0.0 (0.0 to 0.0)	0.0 (0.0 to 0.0)	0.0 (0.0 to 0.0)	0.0 (0.0 to 0.0)	0.0 (0.0 to 0.0)	0.0 (0.0 to 0.0)
Macular degeneration	0.0 (0.0 to 0.0)	0.0 (0.0 to 0.0)	0.0 (0.0 to 0.0)	0.0 (0.0 to 0.0)	0.0 (0.0 to 0.0)	0.0 (0.0 to 0.0)
Refraction and accommodation disorders	26,171.4 (23,775.9 to 29,162.4)	-2.1 (-3.2 to -0.9)*	-1.1 (-2.2 to 0.1)	539.9 (339.3 to 853.0)	0.2 (-1.2 to 1.7)	1.2 (-0.1 to 2.7)
Age-related and other hearing loss	15,986.5 (11,097.3 to 21,777.3)	4.7 (2.4 to 6.9)*	5.7 (3.5 to 8.0)*	1,122.7 (621.9 to 1,750.0)	3.1 (-0.5 to 6.9)	4.1 (0.5 to 8.0)*
Other vision loss	977.3 (608.1 to 1,562.9)	-0.5 (-2.6 to 1.7)	0.6 (-1.6 to 2.8)	37.3 (18.9 to 65.5)	-0.2 (-3.6 to 3.5)	0.9 (-2.6 to 4.6)
Other sense organ diseases	5,548.4 (4,841.9 to 6,324.7)	-0.5 (-2.3 to 1.7)	0.6 (-1.3 to 2.8)	144.4 (85.1 to 212.3)	-0.4 (-3.1 to 2.3)	0.6 (-2.1 to 3.4)
Acute other sense organ diseases	96.5 (85.3 to 107.7)	-0.2 (-2.4 to 1.9)	0.8 (-1.4 to 3.0)	2.6 (1.6 to 3.8)	-0.2 (-2.4 to 1.9)	0.8 (-1.4 to 2.9)
Chronic other sense organ diseases	5,451.9 (4,742.6 to 6,227.7)	-0.5 (-2.3 to 1.7)	0.6 (-1.3 to 2.8)	141.8 (83.5 to 209.0)	-0.4 (-3.2 to 2.4)	0.6 (-2.2 to 3.5)
Oral disorders	255,239.3 (240,483.8 to 267,770.5)	-1.1 (-3.2 to 0.9)	-0.0 (-2.2 to 1.9)	357.4 (196.5 to 594.9)	-0.7 (-1.9 to 0.4)	0.4 (-0.8 to 1.4)
Deciduous caries	19,159.5 (5,233.6 to 46,126.5)	-40.3 (-72.5 to -20.0)*	-39.7 (-72.2 to -19.2)*	4.9 (0.9 to 13.2)	-41.9 (-73.9 to -21.1)*	-41.3 (-73.6 to -20.2)*
Permanent caries	247,767.4 (198,544.6 to 297,711.4)	2.8 (1.1 to 4.3)*	3.9 (2.1 to 5.4)*	165.6 (71.3 to 322.5)	2.0 (0.2 to 3.6)*	3.0 (1.2 to 4.7)*
Periodontal diseases	0.0 (0.0 to 0.0)	0.0 (0.0 to 0.0)	0.0 (0.0 to 0.0)	0.0 (0.0 to 0.0)	0.0 (0.0 to 0.0)	0.0 (0.0 to 0.0)
Edentulism and severe tooth loss	0.0 (0.0 to 0.0)	0.0 (0.0 to 0.0)	0.0 (0.0 to 0.0)	0.0 (0.0 to 0.0)	0.0 (0.0 to 0.0)	0.0 (0.0 to 0.0)
Other oral disorders	6,263.8 (5,342.9 to 7,243.4)	-1.2 (-1.5 to -0.8)*	-0.1 (-0.4 to 0.2)	187.0 (113.8 to 288.1)	-1.1 (-2.4 to 0.1)	-0.1 (-1.3 to 1.1)
Injuries	33,560.4 (31,133.9 to 36,536.6)	-1.8 (-5.2 to 2.1)	-0.7 (-4.2 to 3.2)	1,457.4 (1,043.5 to 1,973.0)	-7.8 (-11.9 to -3.9)*	-6.8 (-11.0 to -2.9)*
Transport injuries	1,358.0 (1,213.3 to 1,498.6)	-8.6 (-10.1 to -7.1)*	-7.7 (-9.2 to -6.2)*	92.7 (64.9 to 128.5)	-15.2 (-18.0 to -12.6)*	-14.4 (-17.2 to -11.7)*
Road injuries	1,156.5 (1,025.9 to 1,293.7)	-9.2 (-10.9 to -7.5)*	-8.2 (-10.0 to -6.5)*	77.8 (53.7 to 108.2)	-14.9 (-17.9 to -12.1)*	-14.1 (-17.0 to -11.2)*
Pedestrian road injuries	154.6 (126.5 to 189.9)	-15.8 (-17.4 to -14.0)*	-14.9 (-16.5 to -13.1)*	10.0 (6.6 to 14.1)	-24.3 (-27.7 to -20.9)*	-23.5 (-26.9 to -20.1)*
Cyclist road injuries	363.5 (295.8 to 438.7)	-14.7 (-17.1 to -12.6)*	-13.8 (-16.2 to -11.7)*	22.2 (14.3 to 32.0)	-20.6 (-24.2 to -17.2)*	-19.8 (-23.4 to -16.3)*
Motorcyclist road injuries	197.8 (162.4 to 240.1)	3.6 (1.2 to 5.7)*	4.6 (2.3 to 6.8)*	13.0 (8.6 to 18.7)	-5.0 (-9.2 to -1.2)*	-4.0 (-8.2 to -0.2)*
Motor vehicle road injuries	366.8 (299.2 to 453.5)	-8.5 (-11.0 to -6.4)*	-7.6 (-10.0 to -5.4)*	27.6 (18.3 to 39.1)	-12.5 (-15.7 to -9.4)*	-11.6 (-14.9 to -8.5)*
Other road injuries	73.7 (55.1 to 93.4)	2.8 (-0.1 to 5.7)	3.9 (0.9 to 6.8)*	5.0 (3.1 to 7.5)	-1.5 (-5.5 to 2.4)	-0.5 (-4.5 to 3.4)
Other transport injuries	201.5 (164.1 to 246.0)	-5.1 (-6.4 to -3.8)*	-4.1 (-5.4 to -2.8)*	14.9 (10.2 to 20.9)	-16.8 (-19.5 to -13.7)*	-16.0 (-18.7 to -12.9)*
Unintentional injuries	28,647.9 (26,607.6 to 30,817.9)	-1.8 (-2.6 to -1.1)*	-0.8 (-1.5 to -0.1)*	1,227.7 (873.2 to 1,670.3)	-5.9 (-7.7 to -4.1)*	-4.9 (-6.8 to -3.2)*
Falls	6,741.4 (5,745.5 to 7,801.9)	6.3 (5.0 to 7.5)*	7.4 (6.1 to 8.6)*	419.9 (288.2 to 591.2)	-1.9 (-5.1 to 1.3)	-0.9 (-4.1 to 2.3)
Drowning	110.8 (87.7 to 141.4)	-16.9 (-18.2 to -15.4)*	-16.1 (-17.4 to -14.6)*	7.3 (4.9 to 10.5)	-24.9 (-27.6 to -22.0)*	-24.1 (-26.8 to -21.2)*
Fire, heat, and hot substances	2,297.8 (1,779.3 to 2,893.9)	-3.7 (-4.8 to -2.8)*	-2.7 (-3.8 to -1.8)*	89.7 (62.3 to 124.4)	-9.9 (-13.6 to -6.6)*	-8.9 (-12.7 to -5.6)*
Poisonings	393.7 (309.8 to 501.5)	4.1 (2.1 to 6.0)*	5.2 (3.2 to 7.1)*	45.9 (29.9 to 68.4)	2.0 (-1.4 to 5.0)	3.0 (-0.4 to 6.1)
Exposure to mechanical forces	8,567.6 (7,458.5 to 9,726.6)	-2.2 (-3.4 to -1.1)*	-1.2 (-2.3 to -0.1)*	171.9 (119.5 to 240.1)	-7.2 (-9.4 to -4.9)*	-6.2 (-8.5 to -4.0)*
Unintentional firearm injuries	47.2 (37.7 to 58.7)	-4.3 (-6.5 to -1.7)*	-3.3 (-5.5 to -0.7)*	2.4 (1.6 to 3.4)	-10.1 (-14.1 to -6.3)*	-9.2 (-13.2 to -5.4)*
Unintentional suffocation	416.7 (286.7 to 597.4)	-14.8 (-17.8 to -11.6)*	-14.0 (-16.9 to -10.7)*	13.5 (9.0 to 19.3)	-12.5 (-14.7 to -10.2)*	-11.5 (-13.8 to -9.3)*
Other exposure to mechanical forces	8,103.7 (7,025.7 to 9,232.9)	-1.5 (-2.6 to -0.4)*	-0.4 (-1.6 to 0.6)	155.9 (108.1 to 218.9)	-6.6 (-9.1 to -4.2)*	-5.6 (-8.1 to -3.2)*
Adverse effects of medical treatment	416.1 (308.8 to 541.8)	-1.7 (-5.0 to 1.5)	-0.7 (-4.1 to 2.5)	55.5 (33.4 to 85.5)	-1.7 (-5.0 to 1.5)	-0.6 (-4.1 to 2.5)
Animal contact	1,745.5 (1,436.3 to 2,088.9)	-4.0 (-6.8 to -1.3)*	-3.0 (-5.8 to -0.3)*	79.0 (52.7 to 111.3)	-4.7 (-7.8 to -1.8)*	-3.7 (-6.9 to -0.7)*
Venomous animal contact	651.1 (534.0 to 799.6)	-2.4 (-4.7 to -0.2)*	-1.4 (-3.7 to 0.9)	60.0 (39.1 to 86.5)	-4.1 (-7.4 to -0.8)*	-3.1 (-6.4 to 0.2)
Non-venomous animal contact	1,094.3 (843.1 to 1,395.6)	-5.0 (-8.5 to -1.5)*	-4.0 (-7.6 to -0.4)*	19.0 (11.8 to 29.7)	-6.7 (-10.9 to -1.8)*	-5.7 (-10.0 to -0.8)*
Foreign body	1,645.8 (1,359.6 to 2,002.1)	-0.1 (-2.0 to 1.6)	1.0 (-1.0 to 2.7)	75.5 (52.3 to 106.2)	-7.9 (-10.7 to -5.2)*	-6.9 (-9.7 to -4.2)*
Pulmonary aspiration and foreign body in airway	765.1 (550.5 to 1,067.7)	-4.3 (-7.3 to -1.5)*	-3.3 (-6.3 to -0.4)*	45.4 (29.8 to 66.6)	-12.3 (-15.2 to -9.3)*	-11.4 (-14.3 to -8.4)*
Foreign body in eyes	60.6 (22.3 to 115.7)	2.9 (2.0 to 3.7)*	4.0 (3.1 to 4.8)*	3.4 (1.2 to 7.3)	2.4 (0.9 to 3.3)*	3.5 (1.9 to 4.3)*
Foreign body in other body part	820.1 (650.1 to 1,025.7)	4.0 (2.7 to 5.4)*	5.1 (3.7 to 6.5)*	26.7 (18.2 to 38.3)	-0.6 (-3.2 to 1.7)	0.4 (-2.1 to 2.8)
Environmental heat and cold exposure	1,865.4 (1,559.3 to 2,200.9)	3.4 (2.2 to 4.7)*	4.5 (3.3 to 5.7)*	100.7 (68.7 to 141.7)	-1.2 (-3.5 to 1.0)	-0.2 (-2.5 to 2.0)
Other unintentional injuries	4,864.0 (4,120.9 to 5,645.8)	-11.3 (-12.5 to -10.2)*	-10.4 (-11.5 to -9.3)*	182.3 (118.3 to 259.4)	-14.9 (-17.1 to -12.8)*	-14.0 (-16.3 to -11.9)*
Self-harm and interpersonal violence	298.2 (266.4 to 333.2)	-6.1 (-7.7 to -4.5)*	-5.1 (-6.7 to -3.5)*	20.8 (14.3 to 28.3)	-11.1 (-13.2 to -8.9)*	-10.2 (-12.3 to -8.0)*
Self-harm	92.8 (73.6 to 113.8)	-1.9 (-3.0 to -0.7)*	-0.9 (-1.9 to 0.3)	8.8 (5.7 to 12.5)	-7.5 (-10.0 to -5.0)*	-6.5 (-9.0 to -4.0)*
Interpersonal violence	205.4 (178.5 to 234.5)	-7.8 (-10.1 to -5.5)*	-6.9 (-9.1 to -4.5)*	12.0 (8.3 to 16.6)	-13.6 (-16.5 to -10.7)*	-12.7 (-15.6 to -9.8)*

Cause	Prevalence in thousands (95% confidence interval)			YLDs in thousands (95% confidence interval)		
	2015	Percent change in counts between 2005 and 2015	Percent change in rates between 2005 and 2015	2015	Percent change in counts between 2005 and 2015	Percent change in rates between 2005 and 2015
Assault by firearm	4.2 (3.5 to 5.0)	-6.6 (-9.0 to -4.0)*	-5.6 (-8.0 to -3.0)*	0.3 (0.2 to 0.4)	-13.0 (-17.5 to -8.2)*	-12.0 (-16.7 to -7.2)*
Assault by sharp object	36.3 (29.6 to 44.5)	-17.3 (-19.5 to -15.3)*	-16.4 (-18.6 to -14.4)*	2.0 (1.3 to 2.8)	-25.3 (-29.6 to -20.7)*	-24.6 (-28.9 to -19.9)*
Assault by other means	165.0 (140.0 to 191.2)	-5.5 (-8.2 to -2.9)*	-4.5 (-7.2 to -1.9)*	9.8 (6.7 to 13.5)	-10.9 (-13.8 to -7.9)*	-9.9 (-12.9 to -6.9)*
Forces of nature, war, and legal intervention	3,256.3 (2,071.1 to 5,002.6)	2.5 (-28.9 to 53.6)	3.5 (-28.2 to 55.2)	116.1 (69.4 to 183.6)	-18.9 (-48.2 to 27.4)	-18.0 (-47.7 to 28.8)
Exposure to forces of nature	1,770.9 (872.0 to 3,265.7)	6.4 (-35.5 to 78.1)	7.5 (-34.9 to 80.0)	56.5 (31.4 to 95.6)	-45.7 (-68.4 to -3.7)*	-45.2 (-68.1 to -2.7)*
Collective violence and legal intervention	1,485.4 (900.3 to 2,276.8)	-1.8 (-43.8 to 89.0)	-0.8 (-43.2 to 90.9)	59.6 (27.8 to 104.5)	52.9 (-25.5 to 228.7)	54.5 (-24.7 to 232.1)
**Anemia**	**226,095.1 (224,794.8 to 227,309.9)**	**-4.4 (-5.0 to -3.8)***	**-3.4 (-4.0 to -2.8)***	**10,700.1 (7,197.1 to 15,187.6)**	**-5.1 (-5.8 to -4.4)***	**-4.1 (-4.9 to -3.4)***
**Developmental intellectual disability**	**17,597.2 (13,090.7 to 22,009.9)**	**1.5 (1.2 to 1.9)***	**2.6 (2.2 to 3.0)***	**1,883.5 (1,345.8 to 2,486.1)**	**3.1 (1.4 to 5.1)***	**4.2 (2.4 to 6.2)***
**Epilepsy**	**3,857.6 (3,239.3 to 4,492.4)**	**5.6 (1.8 to 9.4)***	**6.7 (2.9 to 10.5)***	**1,260.2 (915.9 to 1,635.0)**	**-1.9 (-6.9 to 2.9)**	**-0.9 (-5.9 to 4.0)**
**Guillain-Barré syndrome**	**2.1 (1.1 to 3.9)**	**0.5 (-1.1 to 1.5)**	**1.6 (-0.0 to 2.6)**	**0.6 (0.3 to 1.3)**	**0.5 (-1.1 to 1.5)**	**1.6 (-0.0 to 2.6)**
**Hearing loss**	**29,094.2 (23,806.6 to 35,308.9)**	**1.8 (0.8 to 2.8)***	**2.8 (1.8 to 3.9)***	**1,736.7 (1,083.8 to 2,523.7)**	**1.0 (-1.5 to 3.5)**	**2.0 (-0.5 to 4.6)**
**Heart failure**	**57.2 (47.9 to 68.3)**	**0.5 (-0.6 to 1.7)**	**1.6 (0.4 to 2.7)***	**7.4 (4.8 to 10.5)**	**0.4 (-1.8 to 2.5)**	**1.5 (-0.7 to 3.6)**
**Infertility**	**1.8 (1.2 to 2.5)**	**6.1 (1.3 to 12.9)***	**7.2 (2.4 to 14.1)***	**0.0 (0.0 to 0.0)**	**6.1 (1.3 to 12.9)***	**7.2 (2.4 to 14.1)***
**Pelvic inflammatory disease**	**5.9 (4.2 to 8.0)**	**-10.8 (-12.4 to -9.4)***	**-9.9 (-11.5 to -8.4)***	**0.8 (0.5 to 1.2)**	**-10.8 (-12.4 to -9.2)***	**-9.9 (-11.5 to -8.3)***
**Vision loss**	**28,534.2 (25,760.4 to 31,943.7)**	**-1.9 (-2.8 to -0.7)***	**-0.8 (-1.8 to 0.3)**	**849.9 (580.2 to 1,224.2)**	**1.5 (0.0 to 3.2)***	**2.6 (1.1 to 4.3)***

SR Table 11. Prevalent cases and YLDs for 2015, percent change, and change in age-standardised rates between 2005 and 2015 by cause, global, ages 15-19 years						
Cause	Prevalence in thousands (95% confidence interval)			YLDs in thousands (95% confidence interval)		
	2015	Percent change in counts between 2005 and 2015	Percent change in rates between 2005 and 2015	2015	Percent change in counts between 2005 and 2015	Percent change in rates between 2005 and 2015
**All causes**	--	--	--	**44,429.2 (32,948.2 to 58,028.0)**	**-6.4 (-7.3 to -5.4)***	**-1.2 (-2.1 to -0.1)***
Communicable, maternal, neonatal, and nutritional diseases	328,917.6 (325,756.2 to 331,810.6)	-8.6 (-9.4 to -7.7)*	-3.4 (-4.3 to -2.5)*	8,278.3 (5,847.9 to 11,932.5)	-12.3 (-15.2 to -8.0)*	-7.4 (-10.4 to -2.8)*
HIV/AIDS and tuberculosis	1,326.9 (1,265.9 to 1,391.5)	24.2 (16.9 to 31.2)*	31.2 (23.5 to 38.6)*	246.9 (163.9 to 342.1)	14.3 (8.6 to 20.7)*	20.8 (14.7 to 27.5)*
Tuberculosis	504.1 (382.4 to 664.0)	2.0 (-4.3 to 7.8)	7.7 (1.1 to 13.9)*	159.5 (97.8 to 235.3)	2.1 (-4.7 to 9.0)	7.9 (0.7 to 15.1)*
HIV/AIDS	832.7 (789.0 to 881.7)	43.0 (29.7 to 56.5)*	51.1 (37.1 to 65.3)*	87.4 (62.0 to 114.8)	46.1 (34.4 to 59.1)*	54.4 (42.0 to 68.0)*
HIV/AIDS - Tuberculosis	28.9 (26.1 to 31.8)	-10.6 (-13.8 to -7.1)*	-5.5 (-8.9 to -1.9)*	11.2 (7.5 to 15.4)	-10.3 (-18.3 to -0.8)*	-5.2 (-13.7 to 4.8)
HIV/AIDS resulting in other diseases	833.6 (780.5 to 892.5)	45.8 (36.4 to 56.0)*	54.0 (44.1 to 64.8)*	76.1 (54.2 to 100.5)	61.1 (46.1 to 77.1)*	70.2 (54.3 to 87.1)*
HIV aggregate	531.5 (483.9 to 587.2)	-4.2 (-11.4 to 4.2)	1.1 (-6.4 to 10.1)	39.0 (27.3 to 51.8)	-3.2 (-11.5 to 6.4)	2.2 (-6.5 to 12.4)
AIDS aggregate	302.1 (285.5 to 320.1)	1,717.5 (1,197.9 to 2,559.8)*	1,819.9 (1,270.9 to 2,709.6)*	37.2 (26.5 to 49.9)	431.2 (262.6 to 744.7)*	461.1 (283.0 to 792.3)*
Diarrhea, lower respiratory, and other common infectious diseases	31,995.9 (29,372.2 to 34,071.0)	-6.7 (-9.1 to -4.5)*	-1.5 (-4.0 to 0.9)	1,105.3 (765.5 to 1,554.6)	-3.9 (-5.4 to -2.2)*	1.5 (-0.1 to 3.3)
Diarrheal diseases	2,100.8 (1,996.0 to 2,222.1)	2.9 (1.8 to 4.0)*	8.7 (7.6 to 9.8)*	338.4 (228.6 to 467.4)	3.1 (0.9 to 5.4)*	8.9 (6.6 to 11.3)*
Diarrhoea episodes aggregate	2,100.6 (1,995.8 to 2,221.9)	2.9 (1.8 to 4.0)*	8.7 (7.6 to 9.8)*	338.3 (228.6 to 467.3)	3.1 (0.9 to 5.4)*	8.9 (6.6 to 11.3)*
Guillain-Barré syndrome due to diarrheal diseases	0.2 (0.1 to 0.4)	-3.3 (-4.6 to -2.1)*	2.2 (0.7 to 3.4)*	0.1 (0.0 to 0.1)	-3.3 (-4.6 to -2.1)*	2.2 (0.7 to 3.4)*
Intestinal infectious diseases	175.0 (156.8 to 193.6)	-22.8 (-29.7 to -15.3)*	-18.4 (-25.7 to -10.6)*	22.6 (13.7 to 34.7)	-19.6 (-30.2 to -7.7)*	-15.1 (-26.2 to -2.5)*
Typhoid fever	148.5 (107.2 to 204.0)	-19.2 (-23.8 to -14.7)*	-14.6 (-19.5 to -9.8)*	19.6 (11.7 to 30.3)	-18.6 (-30.5 to -4.9)*	-14.0 (-26.6 to 0.5)
Typhoid fever episodes aggregate	122.9 (87.7 to 170.5)	-19.2 (-27.3 to -11.3)*	-14.7 (-23.2 to -6.3)*	11.7 (6.8 to 18.8)	-18.9 (-29.3 to -6.6)*	-14.3 (-25.3 to -1.4)*
Complications of typhoid fever aggregate	25.6 (16.3 to 37.2)	-19.0 (-44.0 to 18.9)	-14.4 (-40.8 to 25.6)	8.0 (4.2 to 13.4)	-18.2 (-46.0 to 25.6)	-13.5 (-43.0 to 32.7)
Paratyphoid fever	54.3 (34.6 to 81.4)	-20.1 (-27.0 to -12.2)*	-15.6 (-22.9 to -7.3)*	2.8 (1.5 to 4.8)	-19.6 (-34.9 to 0.4)	-15.1 (-31.2 to 6.1)
Paratyphoid fever episodes aggregate	51.5 (33.0 to 77.5)	-20.1 (-27.4 to -11.9)*	-15.6 (-23.3 to -6.9)*	2.5 (1.3 to 4.2)	-19.7 (-35.4 to 0.3)	-15.2 (-31.8 to 6.0)
Intestinal perforation due to paratyphoid	2.8 (1.5 to 4.7)	-19.0 (-54.4 to 49.0)	-14.4 (-51.9 to 57.4)	0.3 (0.2 to 0.6)	-19.0 (-54.4 to 49.0)	-14.4 (-51.9 to 57.4)
Other intestinal infectious diseases	--	--	--	0.2 (0.1 to 0.4)	-66.1 (-78.9 to -43.1)*	-64.2 (-77.7 to -39.9)*
Lower respiratory infections	323.5 (298.2 to 348.8)	-0.6 (-2.5 to 1.2)	5.0 (3.0 to 6.9)*	19.8 (13.1 to 28.6)	-0.6 (-5.4 to 4.5)	5.0 (-0.1 to 10.3)
Lower respiratory infection episodes aggregate	323.2 (297.9 to 348.5)	-0.6 (-2.5 to 1.2)	5.0 (3.0 to 6.9)*	19.8 (13.0 to 28.5)	-0.6 (-5.4 to 4.5)	5.0 (-0.1 to 10.4)
Guillain-Barré syndrome due to lower respiratory infections	0.2 (0.1 to 0.4)	-3.3 (-4.6 to -2.1)*	2.2 (0.7 to 3.4)*	0.1 (0.0 to 0.1)	-3.3 (-4.6 to -2.1)*	2.2 (0.7 to 3.4)*
Upper respiratory infections	19,404.2 (13,411.5 to 27,010.1)	-8.0 (-9.3 to -7.0)*	-2.8 (-4.2 to -1.7)*	229.5 (114.5 to 410.1)	-8.0 (-9.6 to -6.7)*	-2.8 (-4.5 to -1.5)*
Upper respiratory infection episodes aggregate	19,403.5 (13,410.8 to 27,009.4)	-8.0 (-9.3 to -7.0)*	-2.8 (-4.2 to -1.7)*	229.3 (114.3 to 410.0)	-8.0 (-9.6 to -6.7)*	-2.8 (-4.5 to -1.5)*
Guillain-Barré syndrome due to upper respiratory infections	0.7 (0.4 to 1.1)	-3.3 (-4.6 to -2.1)*	2.2 (0.7 to 3.4)*	0.2 (0.1 to 0.4)	-3.3 (-4.6 to -2.1)*	2.2 (0.7 to 3.4)*
Otitis media	9,322.9 (7,562.5 to 11,125.8)	-7.2 (-9.2 to -5.2)*	-1.9 (-4.1 to 0.1)	302.3 (184.2 to 456.6)	-9.2 (-13.0 to -5.6)*	-4.1 (-8.1 to -0.3)*
Acute otitis media	1,311.8 (1,019.8 to 1,688.8)	-0.3 (-3.4 to 3.2)	5.3 (2.0 to 9.0)*	17.2 (8.3 to 31.2)	-0.2 (-3.9 to 3.8)	5.4 (1.5 to 9.6)*
Chronic otitis media aggregate	8,011.1 (6,356.7 to 9,773.7)	-8.2 (-10.5 to -6.0)*	-3.0 (-5.5 to -0.8)*	285.1 (173.1 to 433.0)	-9.7 (-13.6 to -5.9)*	-4.6 (-8.7 to -0.6)*
Meningitis	802.1 (765.5 to 842.6)	10.9 (6.3 to 15.8)*	17.1 (12.3 to 22.3)*	143.9 (99.1 to 188.3)	3.0 (0.1 to 6.1)*	8.8 (5.7 to 12.1)*
Pneumococcal meningitis	661.4 (550.8 to 797.4)	12.6 (9.5 to 16.0)*	18.9 (15.7 to 22.6)*	70.7 (48.4 to 93.8)	6.2 (1.1 to 11.6)*	12.2 (6.7 to 17.9)*
Acute pneumococcal meningitis	2.1 (1.4 to 2.9)	10.4 (-3.5 to 23.1)	16.6 (2.0 to 30.1)*	0.3 (0.2 to 0.4)	10.4 (-3.5 to 23.1)	16.6 (2.0 to 30.1)*
Complications of pneumococcal meningitis aggregate	659.3 (549.1 to 795.4)	12.6 (9.5 to 16.0)*	18.9 (15.7 to 22.6)*	70.4 (48.2 to 93.5)	6.2 (1.0 to 11.6)*	12.2 (6.7 to 17.9)*
H influenzae type B meningitis	250.9 (200.1 to 314.2)	-13.5 (-16.9 to -9.4)*	-8.7 (-12.2 to -4.3)*	32.8 (22.7 to 44.6)	-2.1 (-8.3 to 5.0)	3.5 (-3.2 to 10.9)
Acute H influenzae type B meningitis	0.8 (0.5 to 1.2)	-24.0 (-33.9 to -15.2)*	-19.7 (-30.1 to -10.4)*	0.1 (0.1 to 0.2)	-24.1 (-33.9 to -15.2)*	-19.8 (-30.1 to -10.4)*
Complications of of H influenzae type B meningitis aggregate	250.1 (199.6 to 313.1)	-13.5 (-16.9 to -9.4)*	-8.6 (-12.2 to -4.3)*	32.7 (22.7 to 44.5)	-2.0 (-8.3 to 5.1)	3.6 (-3.1 to 11.1)
Meningococcal meningitis	159.4 (117.5 to 214.3)	13.6 (8.9 to 18.8)*	20.0 (15.0 to 25.5)*	13.7 (9.2 to 18.7)	6.3 (2.4 to 10.5)*	12.3 (8.1 to 16.8)*
Acute meningococcal meningitis	1.1 (0.7 to 1.6)	13.3 (-2.2 to 26.9)	19.7 (3.3 to 34.1)*	0.1 (0.1 to 0.2)	13.3 (-2.2 to 26.9)	19.7 (3.3 to 34.1)*
Complications of meningococcal meningitis aggregate	158.3 (116.3 to 213.0)	13.6 (8.8 to 18.8)*	20.0 (14.9 to 25.5)*	13.5 (9.0 to 18.5)	6.2 (2.3 to 10.6)*	12.2 (8.1 to 16.8)*
Other meningitis	242.8 (188.5 to 301.4)	6.8 (3.8 to 10.3)*	12.8 (9.6 to 16.6)*	26.7 (18.4 to 35.8)	-0.5 (-5.6 to 5.3)	5.1 (-0.3 to 11.2)
Other meningitis episodes aggregate	7.5 (5.8 to 9.3)	5.0 (-6.7 to 14.9)	10.9 (-1.5 to 21.4)	1.0 (0.6 to 1.5)	4.8 (-6.8 to 14.7)	10.7 (-1.6 to 21.2)
Complications of other meningitis aggregate	235.3 (180.3 to 293.4)	6.9 (3.7 to 10.4)*	12.9 (9.6 to 16.7)*	25.7 (17.6 to 34.4)	-0.7 (-6.0 to 5.3)	4.9 (-0.7 to 11.2)
Encephalitis	319.4 (235.1 to 430.9)	-7.0 (-9.3 to -4.4)*	-1.8 (-4.1 to 1.0)	36.9 (25.8 to 49.0)	-5.5 (-11.0 to 0.5)	-0.2 (-6.0 to 6.2)
Acute encephalitis	5.2 (4.3 to 6.2)	-4.3 (-9.6 to -0.2)*	1.1 (-4.5 to 5.4)	0.7 (0.4 to 1.0)	-4.1 (-12.4 to 3.5)	1.2 (-7.4 to 9.3)
Complications of encephalitis aggregate	314.3 (229.7 to 425.7)	-7.1 (-9.3 to -4.4)*	-1.8 (-4.2 to 0.9)	36.2 (25.2 to 48.0)	-5.6 (-11.1 to 0.5)	-0.2 (-6.1 to 6.2)
Diphtheria	0.0 (0.0 to 0.0)	-62.0 (-85.9 to -2.1)*	-59.9 (-85.1 to 3.4)	0.0 (0.0 to 0.0)	-61.7 (-85.9 to -2.1)*	-59.6 (-85.1 to 3.4)
Whooping cough	7.3 (5.6 to 9.2)	-30.6 (-32.2 to -28.7)*	-26.6 (-28.4 to -24.7)*	0.4 (0.2 to 0.6)	-30.0 (-37.5 to -21.7)*	-26.1 (-34.0 to -17.3)*
Tetanus	19.3 (18.9 to 19.8)	0.6 (-0.9 to 1.7)	6.3 (4.7 to 7.4)*	0.8 (0.6 to 1.1)	-16.9 (-24.1 to -7.8)*	-12.2 (-19.9 to -2.6)*
Severe tetanus	0.4 (0.3 to 0.8)	-41.1 (-53.9 to -23.1)*	-37.8 (-51.3 to -18.7)*	0.1 (0.0 to 0.1)	-40.9 (-53.9 to -23.1)*	-37.6 (-51.3 to -18.7)*
Complications of tetanus aggregate	18.9 (18.5 to 19.2)	2.3 (1.8 to 2.9)*	8.1 (7.5 to 8.6)*	0.7 (0.5 to 1.0)	-14.2 (-22.1 to -5.0)*	-9.4 (-17.7 to 0.3)
Measles	1.1 (0.5 to 2.2)	-69.7 (-72.9 to -65.9)*	-68.0 (-71.4 to -64.0)*	0.1 (0.0 to 0.2)	-69.8 (-72.9 to -65.9)*	-68.1 (-71.4 to -64.0)*
Varicella and herpes zoster	261.1 (207.1 to 322.6)	-5.6 (-6.5 to -4.5)*	-0.2 (-1.3 to 0.9)	10.6 (6.3 to 16.9)	-7.2 (-12.3 to -2.4)*	-2.0 (-7.3 to 3.1)
Neglected tropical diseases and malaria	183,126.2 (178,808.5 to 187,380.9)	-11.5 (-13.7 to -9.0)*	-6.5 (-8.8 to -3.9)*	1,836.0 (1,117.1 to 3,227.6)	-13.7 (-23.8 to 4.4)	-8.9 (-19.6 to 10.2)
Malaria	31,254.3 (26,258.0 to 38,476.4)	35.0 (23.9 to 47.3)*	42.6 (30.9 to 55.6)*	278.4 (197.6 to 386.5)	14.8 (9.0 to 20.0)*	21.3 (15.1 to 26.7)*
Asymptomatic malaria parasitemia(PfPR)	22,172.3 (17,231.7 to 29,312.6)	43.8 (28.2 to 61.4)*	51.9 (35.4 to 70.5)*	0.0 (0.0 to 0.0)	0.0 (0.0 to 0.0)	0.0 (0.0 to 0.0)
Malaria episodes aggregate	1,829.0 (1,040.9 to 3,096.7)	-13.1 (-27.6 to 1.8)	-8.2 (-23.5 to 7.5)	37.8 (17.8 to 70.1)	-13.1 (-27.4 to 1.7)	-8.2 (-23.4 to 7.5)
Complications of malaria aggregate	135.7 (122.1 to 148.7)	24.6 (21.9 to 27.8)*	31.6 (28.8 to 35.0)*	42.9 (32.8 to 54.1)	21.4 (13.4 to 29.6)*	28.3 (19.8 to 36.9)*
Anemia due to malaria parasitemia(PfPR) aggregate	7,117.4 (6,944.3 to 7,270.1)	29.1 (26.0 to 32.3)*	36.3 (33.1 to 39.8)*	197.6 (132.0 to 286.6)	20.8 (15.4 to 26.4)*	27.6 (21.9 to 33.6)*
Chagas disease	273.6 (231.1 to 323.2)	-18.2 (-21.4 to -15.2)*	-13.6 (-17.0 to -10.4)*	1.1 (0.7 to 1.6)	-18.2 (-27.9 to -7.3)*	-13.6 (-23.8 to -2.1)*
Chagas disease episodes aggregate	253.0 (214.0 to 299.3)	-18.2 (-21.4 to -15.2)*	-13.6 (-17.0 to -10.4)*	0.0 (0.0 to 0.0)	-20.7 (-24.5 to -16.5)*	-16.3 (-20.3 to -11.8)*
Complications of Chagas disease aggregate	19.5 (14.9 to 24.8)	-18.3 (-21.4 to -15.2)*	-13.6 (-17.0 to -10.4)*	1.0 (0.6 to 1.5)	-18.1 (-28.3 to -6.8)*	-13.5 (-24.3 to -1.6)*

Cause	Prevalence in thousands (95% confidence interval)			YLDs in thousands (95% confidence interval)		
	2015	Percent change in counts between 2005 and 2015	Percent change in rates between 2005 and 2015	2015	Percent change in counts between 2005 and 2015	Percent change in rates between 2005 and 2015
Heart failure due to Chagas disease aggregate	1.1 (0.7 to 1.8)	-19.3 (-22.7 to -16.1)*	-14.7 (-18.4 to -11.4)*	0.1 (0.0 to 0.1)	-19.6 (-33.7 to -3.3)*	-15.1 (-30.0 to 2.1)
Leishmaniasis	368.6 (318.6 to 446.4)	24.2 (-0.2 to 56.9)	31.2 (5.4 to 65.7)*	4.4 (2.2 to 8.5)	22.5 (15.6 to 29.8)*	29.4 (22.1 to 37.1)*
Visceral leishmaniasis	6.2 (5.7 to 6.8)	7.6 (5.9 to 9.4)*	13.6 (11.8 to 15.6)*	0.4 (0.3 to 0.6)	8.0 (-16.2 to 42.3)	14.1 (-11.5 to 50.3)
Cutaneous and mucocutaneous leishmaniasis	369.4 (305.9 to 466.2)	24.0 (19.8 to 28.1)*	31.0 (26.5 to 35.4)*	4.0 (1.8 to 7.9)	24.4 (17.2 to 31.0)*	31.4 (23.8 to 38.4)*
African trypanosomiasis	0.7 (0.4 to 1.1)	-68.1 (-69.8 to -66.7)*	-66.3 (-68.1 to -64.8)*	0.2 (0.1 to 0.4)	-66.0 (-75.8 to -49.3)*	-64.1 (-74.4 to -46.4)*
Schistosomiasis	35,221.9 (29,600.0 to 44,207.1)	-25.6 (-34.1 to -7.5)*	-21.4 (-30.4 to -2.3)*	339.0 (169.4 to 628.8)	-25.1 (-32.9 to -6.0)*	-20.9 (-29.1 to -0.7)*
Schistosomiasis episodes aggregate	16,218.9 (12,279.4 to 20,895.2)	-29.1 (-37.9 to -15.4)*	-25.1 (-34.4 to -10.6)*	92.9 (35.8 to 196.9)	-30.0 (-38.6 to -15.9)*	-26.1 (-35.2 to -11.2)*
Complications of schistosomiasis aggregate	16,738.1 (11,096.5 to 24,967.4)	-21.6 (-34.7 to 11.1)	-17.2 (-31.1 to 17.4)	183.1 (77.6 to 375.0)	-21.3 (-33.9 to 11.7)	-16.8 (-30.2 to 18.0)
Anemia due to schistosomiasis aggregate	2,330.3 (2,244.8 to 2,404.9)	-22.1 (-25.1 to -19.1)*	-17.7 (-20.9 to -14.6)*	63.0 (42.4 to 91.4)	-27.7 (-32.4 to -22.8)*	-23.7 (-28.6 to -18.4)*
Cysticercosis	97.8 (63.7 to 149.3)	-7.8 (-13.6 to -2.1)*	-2.6 (-8.7 to 3.4)	15.6 (8.6 to 25.7)	-16.6 (-25.9 to -8.3)*	-11.9 (-21.7 to -3.2)*
Cystic echinococcosis	82.2 (69.2 to 97.7)	-1.1 (-3.9 to 1.5)	4.4 (1.5 to 7.2)*	7.7 (5.0 to 11.0)	-1.3 (-10.5 to 8.4)	4.2 (-5.5 to 14.5)
Lymphatic filariasis	3,428.8 (2,804.6 to 4,099.7)	-48.8 (-52.9 to -44.4)*	-45.9 (-50.2 to -41.3)*	153.1 (81.7 to 244.8)	-20.3 (-33.6 to -9.8)*	-15.8 (-29.8 to -4.7)*
Prevalence of detectable microfiliaria due to lymphatic filariasis	2,079.9 (1,736.3 to 2,407.4)	-58.6 (-61.9 to -55.2)*	-56.2 (-59.7 to -52.7)*	0.0 (0.0 to 0.0)	0.0 (0.0 to 0.0)	0.0 (0.0 to 0.0)
Complications of lymphatic filariasis aggregate	1,349.0 (881.0 to 1,901.7)	-19.3 (-32.0 to -9.5)*	-14.8 (-28.1 to -4.4)*	153.1 (81.7 to 244.8)	-20.3 (-33.6 to -9.8)*	-15.8 (-29.8 to -4.7)*
Onchocerciasis	1,715.4 (1,327.1 to 2,241.0)	-40.4 (-50.1 to -30.2)*	-37.0 (-47.3 to -26.2)*	108.4 (47.4 to 196.0)	-30.2 (-46.7 to -15.7)*	-26.2 (-43.7 to -11.0)*
Asymptomatic onchocerciasis	281.5 (171.5 to 400.2)	-71.9 (-76.9 to -68.9)*	-70.3 (-75.6 to -67.2)*	0.0 (0.0 to 0.0)	0.0 (0.0 to 0.0)	0.0 (0.0 to 0.0)
Skin disease due to onchocerciasis aggregate	1,435.7 (994.0 to 1,975.7)	-23.5 (-35.9 to -10.4)*	-19.2 (-32.3 to -5.3)*	108.4 (47.3 to 196.0)	-30.2 (-46.7 to -15.1)*	-26.2 (-43.7 to -10.3)*
Vision loss due to onchocerciasis aggregate	1.1 (0.6 to 1.9)	-45.8 (-56.6 to -36.5)*	-42.8 (-54.2 to -32.9)*	0.1 (0.0 to 0.1)	-50.8 (-65.9 to -32.0)*	-48.0 (-64.0 to -28.1)*
Trachoma	22.2 (11.0 to 42.4)	-7.7 (-21.5 to 8.5)	-2.5 (-17.1 to 14.7)	1.1 (0.5 to 2.0)	-11.6 (-27.3 to 6.9)	-6.6 (-23.2 to 12.9)
Dengue	489.3 (275.3 to 1,056.2)	123.1 (-7.7 to 498.6)	135.6 (-2.5 to 532.3)	80.2 (36.0 to 183.0)	120.8 (-6.8 to 490.4)	133.3 (-1.5 to 523.7)
Dengue episodes aggregate	146.3 (97.2 to 303.9)	115.7 (-10.2 to 465.4)	127.9 (-5.1 to 497.3)	8.6 (4.7 to 19.3)	121.8 (-7.7 to 483.6)	134.3 (-2.5 to 516.5)
Post-dengue chronic fatigue syndrome	343.9 (165.4 to 754.4)	123.2 (-7.7 to 498.6)	135.8 (-2.5 to 532.3)	71.7 (31.0 to 165.7)	120.7 (-6.4 to 490.0)	133.2 (-1.1 to 523.2)
Yellow fever	0.3 (0.1 to 0.9)	-25.9 (-41.3 to -1.9)*	-21.7 (-38.0 to 3.6)	0.0 (0.0 to 0.0)	-26.0 (-41.3 to -1.9)*	-21.8 (-38.0 to 3.6)
Rabies	0.0 (0.0 to 0.0)	-47.0 (-64.6 to -20.4)*	-44.0 (-62.6 to -15.9)*	0.0 (0.0 to 0.0)	-47.0 (-64.6 to -20.4)*	-44.0 (-62.6 to -15.9)*
Intestinal nematode infections	138,616.7 (134,505.7 to 142,221.1)	-14.2 (-17.1 to -11.3)*	-9.4 (-12.4 to -6.3)*	306.9 (174.4 to 502.6)	-30.7 (-36.4 to -23.6)*	-26.8 (-32.8 to -19.3)*
Ascariasis	72,547.5 (63,144.6 to 82,774.2)	-21.4 (-34.1 to -7.6)*	-17.0 (-30.4 to -2.4)*	80.2 (43.9 to 134.9)	-44.8 (-54.6 to -33.7)*	-41.7 (-52.0 to -30.0)*
Complications of ascariasis aggregate	4,390.0 (3,965.0 to 4,892.1)	-42.6 (-50.7 to -33.2)*	-39.3 (-47.9 to -29.4)*	80.2 (43.9 to 134.9)	-44.8 (-54.6 to -33.7)*	-41.7 (-52.0 to -30.0)*
Asymptomatic ascariasis	68,165.7 (58,609.1 to 78,517.3)	-19.5 (-33.4 to -4.3)*	-14.9 (-29.6 to 1.0)	0.0 (0.0 to 0.0)	0.0 (0.0 to 0.0)	0.0 (0.0 to 0.0)
Trichuriasis	44,094.4 (39,985.6 to 48,730.2)	-12.8 (-23.9 to 0.2)	-7.9 (-19.6 to 5.8)	55.7 (29.0 to 98.9)	-20.1 (-38.1 to 8.2)	-15.6 (-34.6 to 14.3)
Complications of trichuriasis aggregate	2,744.1 (2,349.9 to 3,414.8)	-19.3 (-35.4 to 5.3)	-14.8 (-31.7 to 11.2)	55.7 (29.0 to 98.9)	-20.1 (-38.1 to 8.2)	-15.6 (-34.6 to 14.3)
Asymptomatic trichuriasis	41,351.6 (37,202.2 to 46,187.4)	-12.3 (-24.2 to 1.6)	-7.4 (-19.9 to 7.4)	0.0 (0.0 to 0.0)	0.0 (0.0 to 0.0)	0.0 (0.0 to 0.0)
Hookworm disease	43,610.1 (39,390.8 to 48,429.7)	-19.1 (-29.5 to -8.1)*	-14.6 (-25.5 to -2.9)*	171.0 (102.2 to 270.7)	-25.0 (-31.2 to -18.2)*	-20.8 (-27.3 to -13.6)*
Complications of hookworm disease aggregate	5,417.0 (5,076.1 to 5,801.1)	-24.4 (-31.9 to -15.9)*	-20.1 (-28.1 to -11.2)*	112.6 (61.8 to 187.9)	-25.5 (-34.2 to -15.3)*	-21.3 (-30.5 to -10.5)*
Anemia due to hookworm disease aggregate	2,428.9 (2,382.2 to 2,474.1)	-18.4 (-20.7 to -16.1)*	-13.9 (-16.3 to -11.3)*	58.4 (38.1 to 85.5)	-24.0 (-28.3 to -19.4)*	-19.7 (-24.2 to -14.8)*
Asymptomatic hookworm disease	35,768.4 (31,434.4 to 40,555.9)	-18.3 (-31.0 to -4.2)*	-13.7 (-27.1 to 1.2)	0.0 (0.0 to 0.0)	0.0 (0.0 to 0.0)	0.0 (0.0 to 0.0)
Food-borne trematodiases	3,906.8 (3,601.6 to 4,246.4)	-43.1 (-44.8 to -41.3)*	-39.9 (-41.7 to -38.0)*	72.0 (30.0 to 142.7)	-45.5 (-48.4 to -40.9)*	-42.4 (-45.5 to -37.6)*
Asymptomatic food-borne trematodiases aggregate	3,288.0 (2,725.1 to 3,753.5)	-42.7 (-44.5 to -40.7)*	-39.5 (-41.4 to -37.4)*	0.0 (0.0 to 0.0)	0.0 (0.0 to 0.0)	0.0 (0.0 to 0.0)
Symptomatic food-borne trematodiases aggregate	618.8 (289.2 to 1,127.7)	-45.1 (-47.7 to -41.0)*	-42.0 (-44.7 to -37.7)*	72.0 (30.0 to 142.7)	-45.5 (-48.4 to -40.9)*	-42.4 (-45.5 to -37.6)*
Leprosy	5.2 (4.6 to 5.8)	-33.8 (-35.2 to -32.3)*	-30.1 (-31.5 to -28.5)*	0.3 (0.2 to 0.4)	-34.2 (-39.0 to -29.2)*	-30.5 (-35.6 to -25.2)*
Ebola	0.2 (0.1 to 0.4)	13,427.2 (9,115.5 to 17,781.9)*	14,188.9 (9,634.4 to 18,788.7)*	0.0 (0.0 to 0.1)	12,644.0 (6,407.9 to 19,272.1)*	13,361.5 (6,774.4 to 20,362.9)*
Other neglected tropical diseases	3,492.3 (3,423.0 to 3,561.3)	-14.9 (-17.0 to -12.8)*	-10.1 (-12.3 to -7.9)*	467.5 (157.0 to 1,279.9)	6.1 (-31.5 to 62.3)	12.1 (-27.6 to 71.4)
Anemia due to other neglected tropical diseases aggregate	3,492.3 (3,423.0 to 3,561.3)	-14.9 (-17.0 to -12.8)*	-10.1 (-12.3 to -7.9)*	73.9 (48.3 to 109.9)	-24.5 (-28.7 to -20.7)*	-20.3 (-24.6 to -16.2)*
Maternal disorders	1,257.5 (1,143.1 to 1,357.6)	-17.1 (-24.2 to -8.7)*	-12.4 (-19.9 to -3.6)*	92.9 (61.6 to 133.0)	-18.4 (-35.9 to 4.4)	-13.8 (-32.3 to 10.3)
Maternal hemorrhage	253.2 (238.9 to 268.8)	-18.3 (-24.1 to -12.3)*	-13.7 (-19.9 to -7.3)*	10.7 (7.2 to 15.5)	-14.1 (-27.6 to 2.2)	-9.3 (-23.5 to 7.9)
Maternal hemorrhage episodes aggregate	24.5 (14.3 to 38.1)	-15.7 (-47.6 to 38.1)	-11.0 (-44.6 to 45.9)	4.0 (2.1 to 6.6)	-15.5 (-43.2 to 29.6)	-10.7 (-40.0 to 36.9)
Anemia due to maternal hemorrhage aggregate	228.8 (219.5 to 238.1)	-18.6 (-23.5 to -12.8)*	-14.0 (-19.1 to -7.9)*	6.7 (4.4 to 9.9)	-13.2 (-22.3 to -2.4)*	-8.3 (-17.9 to 3.1)
Maternal sepsis and other maternal infections	199.1 (117.5 to 312.9)	-17.8 (-52.5 to 42.7)	-13.2 (-49.8 to 50.7)	8.4 (4.0 to 16.0)	-19.1 (-56.5 to 55.3)	-14.5 (-54.1 to 64.1)
Maternal sepsis and other maternal infections aggregate	163.6 (80.7 to 277.1)	-18.9 (-58.3 to 60.9)	-14.3 (-55.9 to 70.0)	8.2 (3.9 to 15.8)	-19.3 (-56.9 to 57.1)	-14.7 (-54.5 to 66.0)
Infertility due to puerperal sepsis	36.0 (29.6 to 43.9)	-11.7 (-14.6 to -8.7)*	-6.7 (-9.8 to -3.6)*	0.2 (0.1 to 0.4)	-11.4 (-21.9 to 0.7)	-6.4 (-17.5 to 6.4)
Maternal hypertensive disorders	708.6 (404.5 to 1,101.1)	-18.3 (-51.3 to 34.9)	-13.7 (-48.5 to 42.5)	34.4 (16.4 to 58.8)	-18.4 (-50.7 to 33.5)	-13.8 (-47.9 to 41.0)
Maternal hypertensive disorders episodes aggregate	710.8 (405.3 to 1,104.8)	-18.0 (-51.2 to 35.6)	-13.4 (-48.5 to 43.2)	33.7 (15.9 to 58.2)	-18.3 (-51.2 to 34.1)	-13.8 (-48.4 to 41.7)
Eclampsia	1.1 (0.4 to 2.2)	-20.5 (-74.5 to 135.8)	-16.0 (-73.0 to 149.1)	0.7 (0.2 to 1.4)	-20.5 (-74.5 to 135.8)	-16.0 (-73.0 to 149.1)
Complications of maternal hypertensive disorders aggregate	0.0 (0.0 to 0.0)	0.0 (0.0 to 0.0)	0.0 (0.0 to 0.0)	0.0 (0.0 to 0.0)	0.0 (0.0 to 0.0)	0.0 (0.0 to 0.0)
Maternal obstructed labor and uterine rupture	62.1 (47.1 to 79.7)	-15.2 (-28.1 to -0.9)*	-10.4 (-24.0 to 4.7)	20.5 (13.0 to 30.2)	-14.8 (-28.2 to 2.3)	-10.0 (-24.2 to 8.1)
Obstructed labor, acute event	15.8 (9.0 to 26.5)	-18.0 (-54.0 to 49.3)	-13.4 (-51.4 to 57.7)	4.9 (2.4 to 8.9)	-17.8 (-55.0 to 52.1)	-13.1 (-52.5 to 60.6)
Fistula due to maternal obstructed labor and uterine rupture aggregate	46.3 (32.6 to 61.9)	-14.2 (-18.8 to -10.1)*	-9.4 (-14.2 to -5.0)*	15.6 (9.4 to 23.6)	-13.8 (-25.3 to -1.2)*	-8.9 (-21.1 to 4.4)
Maternal abortion, miscarriage, and ectopic pregnancy	52.0 (31.3 to 78.8)	-24.9 (-52.2 to 20.9)	-20.6 (-49.5 to 27.7)	5.7 (3.0 to 9.6)	-24.8 (-53.3 to 24.3)	-20.6 (-50.6 to 31.3)
Other maternal disorders	--	--	--	13.1 (8.4 to 19.5)	-23.1 (-40.7 to -1.5)*	-18.8 (-37.3 to 4.0)
Neonatal disorders	4,100.9 (3,869.4 to 4,247.4)	-0.1 (-3.1 to 3.2)	5.6 (2.4 to 9.0)*	1,035.9 (778.7 to 1,333.3)	-1.6 (-5.5 to 2.7)	4.0 (-0.1 to 8.4)
Neonatal preterm birth complications	3,032.5 (2,647.3 to 3,443.9)	-6.5 (-11.0 to -1.8)*	-1.2 (-5.9 to 3.7)	483.2 (364.7 to 625.5)	-11.9 (-16.9 to -6.9)*	-7.0 (-12.2 to -1.7)*
Vision loss due to retinopathy of prematurity aggregate	323.9 (255.4 to 407.2)	-6.3 (-10.0 to -2.6)*	-1.1 (-4.9 to 2.9)	9.0 (5.1 to 14.7)	-2.1 (-10.6 to 7.0)	3.4 (-5.5 to 13.0)
Complications of preterm birth complications aggregate	2,708.3 (2,353.2 to 3,108.4)	-6.5 (-11.4 to -1.2)*	-1.3 (-6.4 to 4.3)	474.2 (356.5 to 615.2)	-12.1 (-17.3 to -7.1)*	-7.2 (-12.7 to -1.8)*
Neonatal encephalopathy due to birth asphyxia and trauma	1,262.6 (880.3 to 1,875.9)	15.6 (12.9 to 18.0)*	22.1 (19.3 to 24.6)*	380.1 (243.6 to 585.8)	13.2 (9.2 to 17.2)*	19.6 (15.3 to 23.8)*

Cause	Prevalence in thousands (95% confidence interval)			YLDs in thousands (95% confidence interval)		
	2015	Percent change in counts between 2005 and 2015	Percent change in rates between 2005 and 2015	2015	Percent change in counts between 2005 and 2015	Percent change in rates between 2005 and 2015
Neonatal sepsis and other neonatal infections	0.0 (0.0 to 0.0)	0.0 (0.0 to 0.0)	0.0 (0.0 to 0.0)	0.0 (0.0 to 0.0)	0.0 (0.0 to 0.0)	0.0 (0.0 to 0.0)
Severe infection due to neonatal sepsis and other neonatal infections	0.0 (0.0 to 0.0)	0.0 (0.0 to 0.0)	0.0 (0.0 to 0.0)	0.0 (0.0 to 0.0)	0.0 (0.0 to 0.0)	0.0 (0.0 to 0.0)
Complications of neonatal sepsis and other neonatal infections aggregate	0.0 (0.0 to 0.0)	0.0 (0.0 to 0.0)	0.0 (0.0 to 0.0)	0.0 (0.0 to 0.0)	0.0 (0.0 to 0.0)	0.0 (0.0 to 0.0)
Hemolytic disease and other neonatal jaundice	191.9 (170.8 to 217.6)	5.4 (4.2 to 6.5)*	11.3 (10.1 to 12.5)*	58.7 (43.6 to 75.3)	2.6 (-1.8 to 7.5)	8.3 (3.7 to 13.6)*
Other neonatal disorders	--	--	--	113.9 (75.9 to 153.8)	3.1 (-12.9 to 24.0)	8.9 (-8.0 to 31.0)
Nutritional deficiencies	130,323.7 (128,835.6 to 131,794.4)	-12.5 (-13.7 to -11.2)*	-7.5 (-8.8 to -6.2)*	3,754.5 (2,488.0 to 5,476.0)	-17.5 (-19.5 to -15.5)*	-12.8 (-15.0 to -10.7)*
Protein-energy malnutrition	5.1 (3.1 to 8.2)	-27.0 (-35.9 to -16.0)*	-22.8 (-32.3 to -11.3)*	0.6 (0.3 to 1.1)	-26.9 (-37.8 to -13.3)*	-22.8 (-34.3 to -8.5)*
Iodine deficiency	11,281.1 (10,112.4 to 12,795.1)	-8.8 (-11.1 to -6.6)*	-3.7 (-6.0 to -1.4)*	257.6 (161.7 to 384.6)	-6.2 (-8.8 to -3.6)*	-1.0 (-3.7 to 1.8)
Visible goiter due to iodine deficiency aggregate	10,945.2 (9,789.2 to 12,412.9)	-9.2 (-11.3 to -6.9)*	-4.0 (-6.4 to -1.7)*	198.1 (121.0 to 311.5)	-9.0 (-11.4 to -6.6)*	-3.9 (-6.4 to -1.3)*
Visible goiter with heart failure due to iodine deficiency aggregate	0.0 (0.0 to 0.0)	0.0 (0.0 to 0.0)	0.0 (0.0 to 0.0)	0.0 (0.0 to 0.0)	0.0 (0.0 to 0.0)	0.0 (0.0 to 0.0)
Intellectual disability due to iodine deficiency aggregate	335.9 (141.4 to 457.9)	3.7 (0.3 to 5.5)*	9.5 (5.9 to 11.5)*	59.5 (22.0 to 98.6)	4.4 (-1.3 to 9.5)	10.3 (4.2 to 15.6)*
Vitamin A deficiency	534.9 (370.1 to 750.5)	-0.9 (-4.6 to 2.8)	4.6 (0.8 to 8.6)*	22.2 (12.8 to 36.2)	-1.3 (-6.7 to 4.6)	4.3 (-1.4 to 10.5)
Iron-deficiency anemia	130,036.2 (128,423.9 to 131,631.9)	-13.8 (-15.0 to -12.6)*	-8.9 (-10.2 to -7.6)*	3,474.1 (2,300.5 to 5,049.0)	-18.3 (-20.5 to -16.2)*	-13.7 (-16.0 to -11.4)*
Iron-deficiency anemia without heart failure aggregate	130,036.1 (128,423.8 to 131,631.7)	-13.8 (-15.0 to -12.6)*	-8.9 (-10.2 to -7.6)*	3,474.1 (2,300.5 to 5,049.0)	-18.3 (-20.5 to -16.2)*	-13.7 (-16.0 to -11.4)*
Iron-deficiency anemia with heart failure aggregate	0.1 (0.1 to 0.2)	8.2 (4.7 to 11.6)*	14.3 (10.6 to 17.9)*	0.0 (0.0 to 0.0)	8.2 (4.7 to 11.6)*	14.3 (10.6 to 17.9)*
Other nutritional deficiencies	--	--	--	0.1 (0.0 to 0.1)	-33.4 (-47.5 to -18.2)*	-29.6 (-44.6 to -13.6)*
Other communicable, maternal, neonatal, and nutritional diseases	83,908.8 (81,292.7 to 85,918.1)	-3.6 (-4.7 to -2.6)*	1.8 (0.6 to 2.8)*	206.8 (130.5 to 316.9)	-10.7 (-15.3 to -5.8)*	-5.6 (-10.5 to -0.5)*
Sexually transmitted diseases excluding HIV	46,890.7 (44,272.5 to 49,423.3)	-2.3 (-4.1 to -0.4)*	3.2 (1.3 to 5.3)*	75.7 (40.8 to 131.0)	0.5 (-5.9 to 7.4)	6.2 (-0.6 to 13.5)
Syphilis	3,022.5 (2,147.0 to 4,316.4)	-7.3 (-17.3 to 1.0)	-2.0 (-12.7 to 6.7)	1.9 (1.2 to 3.0)	-4.9 (-10.5 to -0.1)*	0.4 (-5.4 to 5.5)
Early syphilis aggregate	3,017.1 (2,141.2 to 4,310.5)	-7.3 (-17.3 to 1.0)	-2.0 (-12.7 to 6.7)	0.7 (0.2 to 1.9)	-7.4 (-19.0 to 3.0)	-2.1 (-14.4 to 8.8)
Adult tertiary syphilis	5.5 (4.6 to 6.2)	-3.3 (-7.3 to 0.2)	2.2 (-2.1 to 5.9)	1.1 (0.7 to 1.6)	-3.3 (-7.3 to 0.2)	2.2 (-2.1 to 5.9)
Chlamydial infection	6,845.6 (4,911.4 to 9,151.9)	-4.1 (-6.8 to -0.8)*	1.3 (-1.5 to 4.8)	25.5 (13.5 to 46.3)	-5.9 (-11.4 to 0.8)	-0.6 (-6.4 to 6.5)
Chlamydial infection episodes aggregate	6,824.5 (4,890.1 to 9,131.7)	-4.1 (-6.7 to -0.7)*	1.3 (-1.5 to 4.9)	24.1 (12.6 to 44.1)	-5.4 (-11.2 to 2.0)	-0.0 (-6.2 to 7.8)
Pelvic inflammatory diseases due to chlamydial infection aggregate	9.1 (6.2 to 12.6)	-14.5 (-17.6 to -11.7)*	-9.7 (-13.0 to -6.8)*	1.2 (0.7 to 1.9)	-14.5 (-20.0 to -9.0)*	-9.6 (-15.5 to -3.9)*
Infertility due to clamydial infection aggregate	12.1 (10.6 to 13.6)	-10.5 (-11.8 to -9.3)*	-5.5 (-6.8 to -4.2)*	0.1 (0.0 to 0.2)	-10.9 (-17.7 to -4.2)*	-5.9 (-13.0 to 1.2)
Gonococcal infection	3,598.6 (1,968.7 to 6,305.0)	11.4 (-18.2 to 30.2)	17.6 (-13.6 to 37.5)	23.7 (10.2 to 47.9)	9.5 (-13.7 to 23.5)	15.7 (-8.9 to 30.4)
Gonococcal infection episodes aggregate	3,591.3 (1,961.2 to 6,298.4)	11.4 (-18.2 to 30.3)	17.7 (-13.6 to 37.6)	23.2 (9.9 to 47.4)	10.2 (-13.8 to 24.4)	16.5 (-9.0 to 31.4)
Pelvic inflammatory diseases due to gonococcal infection aggregate	7.3 (6.1 to 8.8)	-13.4 (-15.8 to -11.3)*	-8.6 (-11.1 to -6.3)*	0.5 (0.3 to 0.7)	-16.3 (-19.9 to -12.0)*	-11.6 (-15.4 to -7.0)*
Trichomoniasis	9,530.3 (6,526.7 to 13,404.0)	1.5 (0.4 to 2.6)*	7.2 (6.0 to 8.4)*	12.3 (4.4 to 26.5)	3.4 (1.0 to 5.9)*	9.2 (6.7 to 11.9)*
Genital herpes	26,296.8 (20,546.9 to 32,790.2)	-3.7 (-6.0 to -1.7)*	1.7 (-0.7 to 3.9)	10.4 (3.6 to 22.2)	-0.2 (-5.0 to 5.3)	5.4 (0.4 to 11.2)*
Moderate infection due to initial genital herpes episode	78.4 (17.1 to 180.3)	6.2 (3.4 to 9.8)*	12.2 (9.2 to 16.0)*	3.8 (0.8 to 9.0)	6.2 (-5.4 to 18.2)	12.1 (-0.1 to 24.9)
Complications of genital herpes aggregate	26,218.4 (20,530.2 to 32,656.3)	-3.7 (-6.0 to -1.7)*	1.7 (-0.7 to 3.8)	6.5 (1.8 to 16.2)	-3.7 (-7.0 to -0.5)*	1.8 (-1.8 to 5.1)
Other sexually transmitted diseases	31.9 (26.9 to 37.3)	-13.8 (-15.3 to -12.4)*	-8.9 (-10.5 to -7.5)*	1.9 (1.2 to 2.9)	-15.2 (-20.4 to -10.0)*	-10.4 (-15.9 to -4.9)*
Pelvic inflammatory diseases due to other sexually transmitted diseases aggregate	13.3 (9.2 to 18.1)	-15.3 (-17.5 to -12.9)*	-10.6 (-12.8 to -8.0)*	1.8 (1.1 to 2.7)	-15.4 (-20.8 to -9.9)*	-10.6 (-16.4 to -4.8)*
Infertility due to other sexually transmitted diseases aggregate	18.6 (16.3 to 21.2)	-12.7 (-14.5 to -10.9)*	-7.7 (-9.7 to -5.9)*	0.1 (0.0 to 0.2)	-12.6 (-21.3 to -3.7)*	-7.7 (-16.9 to 1.7)
Other sexually transmitted diseases	--	--	--	20.3 (10.7 to 35.5)	1.5 (-9.4 to 12.7)	7.2 (-4.3 to 19.0)
Hepatitis	36,881.3 (36,193.9 to 37,639.9)	-3.7 (-4.8 to -2.5)*	1.7 (0.5 to 3.0)*	38.3 (24.0 to 55.8)	-6.8 (-22.8 to 12.7)	-1.6 (-18.5 to 19.0)
Acute hepatitis A	570.5 (475.6 to 664.7)	-6.0 (-23.5 to 16.8)	-0.7 (-19.2 to 23.4)	22.8 (13.9 to 34.2)	-6.0 (-26.7 to 17.6)	-0.7 (-22.5 to 24.2)
Hepatitis B	27,808.9 (26,373.3 to 29,413.0)	-3.0 (-5.8 to -0.1)*	2.4 (-0.5 to 5.5)	9.6 (5.5 to 15.6)	-7.2 (-43.3 to 49.5)	-2.0 (-40.2 to 57.9)
Acute Hepatitis B aggregate	1,340.4 (975.9 to 1,888.1)	-7.3 (-43.0 to 49.7)	-2.1 (-39.8 to 58.1)	9.6 (5.5 to 15.6)	-7.2 (-43.3 to 49.5)	-2.0 (-40.2 to 57.9)
Chronic hepatitis B	26,468.5 (25,136.5 to 27,924.4)	-2.8 (-4.6 to -0.8)*	2.7 (0.7 to 4.8)*	0.0 (0.0 to 0.0)	0.0 (0.0 to 0.0)	0.0 (0.0 to 0.0)
Hepatitis C	9,093.0 (8,070.8 to 10,271.3)	-5.0 (-6.0 to -4.1)*	0.3 (-0.7 to 1.3)	0.2 (0.1 to 0.5)	-7.2 (-9.8 to -5.4)*	-2.0 (-4.7 to -0.1)*
Acute Hepatitis C aggregate	16.8 (12.4 to 22.1)	-7.2 (-9.8 to -5.4)*	-2.0 (-4.7 to -0.0)*	0.2 (0.1 to 0.5)	-7.2 (-9.8 to -5.4)*	-2.0 (-4.7 to -0.1)*
Chronic hepatitis C	9,076.2 (8,053.5 to 10,251.9)	-5.0 (-6.0 to -4.0)*	0.3 (-0.7 to 1.4)	0.0 (0.0 to 0.0)	0.0 (0.0 to 0.0)	0.0 (0.0 to 0.0)
Acute hepatitis E	201.3 (168.8 to 244.8)	-9.5 (-11.9 to -7.6)*	-4.4 (-6.9 to -2.4)*	5.6 (3.4 to 8.6)	-9.4 (-29.1 to 17.3)	-4.3 (-25.1 to 23.9)
Other infectious diseases	3,768.5 (3,702.4 to 3,831.0)	-15.1 (-16.9 to -13.3)*	-10.4 (-12.2 to -8.4)*	92.9 (60.1 to 140.9)	-19.4 (-23.9 to -14.4)*	-14.8 (-19.7 to -9.6)*
Non-communicable diseases	536,115.4 (532,669.5 to 538,938.6)	-4.8 (-5.0 to -4.6)*	0.5 (0.3 to 0.7)*	34,360.0 (25,261.1 to 44,343.0)	-4.7 (-5.3 to -4.0)*	0.7 (0.1 to 1.4)*
Neoplasms	320.9 (298.9 to 345.4)	-2.8 (-10.7 to 5.9)	2.7 (-5.7 to 11.9)	50.3 (34.7 to 68.9)	-6.4 (-17.0 to 5.4)	-1.1 (-12.4 to 11.4)
Lip and oral cavity cancer	12.9 (9.6 to 17.7)	3.7 (-16.3 to 30.5)	9.5 (-11.6 to 37.8)	1.1 (0.7 to 1.6)	3.5 (-17.2 to 32.1)	9.3 (-12.5 to 39.6)
Nasopharynx cancer	7.6 (3.1 to 13.1)	-13.6 (-53.8 to 43.1)	-8.7 (-51.2 to 51.1)	0.7 (0.3 to 1.3)	-13.9 (-52.4 to 43.6)	-9.1 (-49.7 to 51.7)
Other pharynx cancer	2.0 (1.7 to 2.3)	-7.7 (-19.6 to 6.9)	-2.6 (-15.1 to 12.9)	0.2 (0.1 to 0.2)	-8.6 (-20.6 to 5.9)	-3.5 (-16.1 to 11.8)
Esophageal cancer	0.6 (0.4 to 0.8)	-26.3 (-46.5 to 2.9)	-22.2 (-43.5 to 8.7)	0.1 (0.1 to 0.2)	-29.6 (-48.0 to -4.1)*	-25.7 (-45.1 to 1.3)
Stomach cancer	2.8 (2.4 to 3.2)	-32.2 (-42.3 to -21.9)*	-28.4 (-39.0 to -17.5)*	0.3 (0.2 to 0.4)	-33.7 (-43.5 to -23.7)*	-30.0 (-40.4 to -19.4)*
Colon and rectum cancer	12.4 (10.7 to 14.3)	0.1 (-13.0 to 17.6)	5.7 (-8.1 to 24.3)	1.1 (0.8 to 1.5)	-2.6 (-16.9 to 15.6)	2.8 (-12.2 to 22.1)
Colon and rectum cancer aggregate	12.4 (10.7 to 14.3)	0.1 (-13.0 to 17.8)	5.8 (-8.1 to 24.4)	1.1 (0.8 to 1.5)	-2.6 (-16.9 to 15.8)	2.9 (-12.2 to 22.3)
Stoma due to colon and rectum cancer	0.0 (0.0 to 0.0)	-15.9 (-17.0 to -14.8)*	-11.1 (-12.3 to -10.0)*	0.0 (0.0 to 0.0)	-15.9 (-17.0 to -14.8)*	-11.1 (-12.3 to -10.0)*
Liver cancer	0.0 (0.0 to 0.0)	0.0 (0.0 to 0.0)	0.0 (0.0 to 0.0)	0.5 (0.3 to 0.7)	-25.2 (-44.6 to -1.3)*	-21.0 (-41.5 to 4.3)
Liver cancer due to hepatitis B	1.4 (1.1 to 1.9)	-22.6 (-46.4 to 7.6)	-18.2 (-43.4 to 13.7)	0.3 (0.2 to 0.5)	-26.0 (-47.0 to 0.6)	-21.8 (-44.0 to 6.3)
Liver cancer due to hepatitis C	0.1 (0.0 to 0.1)	-4.1 (-24.4 to 16.6)	1.3 (-20.1 to 23.2)	0.0 (0.0 to 0.0)	-9.6 (-27.1 to 8.9)	-4.5 (-23.0 to 15.0)
Liver cancer due to alcohol use	0.0 (0.0 to 0.0)	0.4 (-23.2 to 30.3)	6.0 (-18.8 to 37.7)	0.0 (0.0 to 0.0)	-2.4 (-25.3 to 27.4)	3.1 (-21.1 to 34.6)
Liver cancer due to other causes	0.6 (0.5 to 0.7)	-21.2 (-41.8 to 4.3)	-16.8 (-38.5 to 10.2)	0.1 (0.1 to 0.2)	-24.9 (-40.9 to -3.9)*	-20.6 (-37.6 to 1.5)
Gallbladder and biliary tract cancer	0.1 (0.1 to 0.1)	-9.8 (-18.2 to 0.1)	-4.7 (-13.6 to 5.8)	0.0 (0.0 to 0.0)	-10.9 (-19.1 to -1.2)*	-5.9 (-14.5 to 4.3)
Pancreatic cancer	0.2 (0.2 to 0.2)	0.6 (-11.5 to 13.9)	6.3 (-6.6 to 20.3)	0.1 (0.0 to 0.1)	-2.0 (-13.8 to 10.9)	3.6 (-8.9 to 17.1)

Cause	Prevalence in thousands (95% confidence interval)			YLDs in thousands (95% confidence interval)		
	2015	Percent change in counts between 2005 and 2015	Percent change in rates between 2005 and 2015	2015	Percent change in counts between 2005 and 2015	Percent change in rates between 2005 and 2015
Larynx cancer	7.7 (7.3 to 8.3)	-8.2 (-12.7 to -3.5)*	-3.0 (-7.8 to 1.9)	0.9 (0.6 to 1.2)	-9.1 (-14.3 to -3.8)*	-4.0 (-9.5 to 1.6)
Larynx cancer aggregate	0.9 (0.6 to 1.4)	-19.7 (-43.3 to 18.1)	-15.2 (-40.1 to 24.7)	0.2 (0.1 to 0.3)	-16.6 (-32.0 to 4.3)	-11.9 (-28.2 to 10.1)
Laryngectomy due to larynx cancer	6.8 (6.5 to 7.1)	-6.4 (-7.0 to -5.9)*	-1.2 (-1.8 to -0.6)*	0.7 (0.4 to 1.0)	-6.4 (-7.0 to -5.9)*	-1.2 (-1.8 to -0.6)*
Tracheal, bronchus, and lung cancer	2.7 (1.6 to 4.2)	-11.2 (-39.9 to 33.8)	-6.2 (-36.5 to 41.4)	0.5 (0.2 to 0.8)	-13.9 (-42.5 to 33.4)	-9.1 (-39.2 to 40.9)
Malignant skin melanoma	16.6 (14.1 to 20.5)	-3.8 (-13.1 to 7.5)	1.7 (-8.2 to 13.6)	1.0 (0.6 to 1.4)	-5.0 (-15.8 to 7.6)	0.3 (-11.1 to 13.6)
Non-melanoma skin cancer	7.1 (5.3 to 9.3)	46.0 (-2.0 to 129.3)	54.2 (3.5 to 142.2)*	1.1 (0.7 to 1.6)	13.6 (-6.8 to 38.3)	20.0 (-1.5 to 46.1)
Non-melanoma skin cancer(squamous-cell carcinoma)	18.7 (15.6 to 22.1)	16.5 (-2.8 to 41.3)	23.1 (2.6 to 49.2)*	1.1 (0.7 to 1.6)	13.7 (-6.8 to 38.8)	20.1 (-1.5 to 46.6)
Non-melanoma skin cancer(basal-cell carcinoma)	0.5 (0.3 to 0.8)	-9.4 (-13.6 to -6.6)*	-4.3 (-8.7 to -1.4)*	0.0 (0.0 to 0.0)	-9.4 (-13.6 to -6.6)*	-4.3 (-8.7 to -1.4)*
Breast cancer	18.7 (12.4 to 24.7)	11.3 (-14.7 to 48.4)	17.6 (-9.9 to 56.7)	1.4 (0.8 to 2.1)	7.3 (-19.9 to 43.8)	13.4 (-15.4 to 51.9)
Breast cancer aggregate	17.4 (11.1 to 23.3)	13.1 (-15.5 to 53.9)	19.5 (-10.7 to 62.6)	1.3 (0.7 to 1.9)	8.9 (-21.0 to 49.1)	15.0 (-16.5 to 57.5)
Mastectomy due to breast cancer	1.3 (1.2 to 1.4)	-8.2 (-9.6 to -6.8)*	-3.0 (-4.5 to -1.6)*	0.1 (0.1 to 0.2)	-8.2 (-9.6 to -6.8)*	-3.0 (-4.5 to -1.6)*
Cervical cancer	10.4 (5.4 to 14.1)	-25.2 (-53.0 to 4.4)	-21.0 (-50.3 to 10.3)	0.8 (0.4 to 1.3)	-24.2 (-50.4 to 7.3)	-20.0 (-47.6 to 13.3)
Uterine cancer	9.6 (6.9 to 12.3)	-17.1 (-38.8 to 30.3)	-12.4 (-35.4 to 37.7)	0.6 (0.4 to 1.0)	-17.6 (-39.6 to 34.8)	-12.9 (-36.2 to 42.4)
Ovarian cancer	13.3 (8.2 to 17.9)	-4.4 (-33.4 to 38.3)	1.0 (-29.6 to 46.1)	1.8 (1.0 to 2.7)	-5.0 (-33.2 to 35.5)	0.3 (-29.4 to 43.1)
Prostate cancer	4.9 (3.6 to 5.9)	-4.9 (-18.3 to 10.6)	0.5 (-13.7 to 16.9)	0.4 (0.2 to 0.5)	-12.9 (-26.6 to 2.9)	-8.0 (-22.4 to 8.7)
Prostate cancer aggregate	4.9 (3.6 to 5.9)	-4.9 (-18.3 to 10.6)	0.5 (-13.7 to 16.9)	0.4 (0.2 to 0.5)	-12.9 (-26.6 to 2.9)	-8.0 (-22.4 to 8.7)
Impotence and incontinence due to prostate cancer aggregate	0.0 (0.0 to 0.0)	0.0 (0.0 to 0.0)	0.0 (0.0 to 0.0)	0.0 (0.0 to 0.0)	0.0 (0.0 to 0.0)	0.0 (0.0 to 0.0)
Testicular cancer	25.7 (20.8 to 30.3)	3.1 (-17.6 to 28.7)	8.9 (-13.0 to 36.0)	1.6 (1.0 to 2.3)	1.7 (-19.0 to 27.7)	7.4 (-14.4 to 34.9)
Kidney cancer	7.5 (6.1 to 8.7)	13.0 (-5.2 to 33.1)	19.3 (0.1 to 40.6)*	0.6 (0.4 to 0.8)	11.4 (-6.6 to 30.9)	17.7 (-1.4 to 38.3)
Bladder cancer	5.7 (4.6 to 6.6)	-2.0 (-16.2 to 18.2)	3.6 (-11.5 to 24.8)	0.4 (0.3 to 0.6)	-2.6 (-16.3 to 15.9)	2.9 (-11.6 to 22.5)
Bladder cancer episodes aggregate	5.7 (4.6 to 6.6)	-1.9 (-16.2 to 18.4)	3.6 (-11.5 to 25.0)	0.4 (0.3 to 0.6)	-2.5 (-16.4 to 16.4)	3.0 (-11.7 to 22.9)
Urinary incontinence due to bladder cancer	0.0 (0.0 to 0.0)	-11.2 (-12.6 to -9.6)*	-6.2 (-7.7 to -4.6)*	0.0 (0.0 to 0.0)	-11.2 (-12.6 to -9.6)*	-6.2 (-7.7 to -4.6)*
Brain and nervous system cancer	27.5 (22.7 to 32.3)	-14.3 (-28.7 to 4.6)	-9.4 (-24.7 to 10.5)	3.1 (2.1 to 4.3)	-14.9 (-28.6 to 4.7)	-10.1 (-24.6 to 10.6)
Thyroid cancer	13.3 (11.9 to 14.4)	-1.7 (-10.1 to 10.5)	3.8 (-5.1 to 16.8)	0.8 (0.5 to 1.1)	-2.9 (-12.7 to 10.1)	2.6 (-7.8 to 16.3)
Mesothelioma	0.3 (0.2 to 0.3)	-13.2 (-23.6 to -0.4)*	-8.3 (-19.3 to 5.2)	0.0 (0.0 to 0.1)	-13.2 (-24.0 to -0.5)*	-8.3 (-19.8 to 5.1)
Hodgkin lymphoma	22.7 (18.1 to 28.5)	-21.2 (-32.2 to -6.3)*	-16.8 (-28.4 to -1.0)*	1.9 (1.2 to 2.7)	-23.7 (-35.4 to -8.8)*	-19.4 (-31.8 to -3.6)*
Non-Hodgkin lymphoma	77.3 (59.0 to 95.9)	15.8 (-6.7 to 41.5)	22.3 (-1.5 to 49.4)	5.9 (3.8 to 8.3)	12.3 (-9.6 to 39.1)	18.6 (-4.5 to 46.9)
Multiple myeloma	1.7 (1.3 to 2.3)	6.4 (-16.2 to 36.9)	12.4 (-11.4 to 44.6)	0.4 (0.3 to 0.6)	3.2 (-18.0 to 31.6)	9.0 (-13.4 to 39.0)
Leukemia	45.2 (36.4 to 54.4)	-20.6 (-36.7 to -0.7)*	-16.1 (-33.1 to 4.9)	13.3 (7.5 to 21.4)	-13.5 (-37.8 to 18.6)	-8.7 (-34.3 to 25.3)
Acute lymphoid leukemia	42.3 (26.4 to 69.6)	-0.1 (-28.8 to 42.3)	5.5 (-24.8 to 50.3)	5.4 (3.0 to 9.2)	-2.5 (-30.2 to 37.8)	2.9 (-26.3 to 45.5)
Chronic lymphoid leukemia	28.8 (18.0 to 43.8)	-32.2 (-54.2 to -3.7)*	-28.3 (-51.6 to 1.8)	3.6 (2.0 to 5.9)	-32.5 (-54.4 to -4.0)*	-28.8 (-51.8 to 1.4)
Acute myeloid leukemia	27.2 (16.8 to 44.9)	0.8 (-28.2 to 43.2)	6.4 (-24.1 to 51.2)	3.3 (1.8 to 5.8)	-1.1 (-29.9 to 40.3)	4.5 (-26.0 to 48.2)
Chronic myeloid leukemia	7.0 (4.3 to 11.6)	-13.2 (-39.1 to 24.3)	-8.3 (-35.6 to 31.3)	0.9 (0.5 to 1.5)	-16.0 (-41.0 to 20.2)	-11.3 (-37.7 to 27.0)
Other neoplasms	132.4 (106.2 to 162.0)	2.2 (-17.2 to 23.0)	8.0 (-12.5 to 29.9)	9.8 (6.5 to 13.8)	1.3 (-17.9 to 22.2)	7.0 (-13.3 to 29.1)
Cardiovascular diseases	7,166.3 (6,886.3 to 7,535.3)	-12.4 (-15.1 to -9.7)*	-7.4 (-10.3 to -4.6)*	524.3 (362.1 to 714.4)	-10.5 (-12.7 to -8.5)*	-5.5 (-7.8 to -3.4)*
Rheumatic heart disease	3,005.4 (2,683.7 to 3,647.5)	-15.7 (-20.0 to -11.9)*	-11.0 (-15.4 to -6.9)*	144.4 (90.4 to 219.2)	-15.5 (-19.9 to -11.2)*	-10.8 (-15.4 to -6.2)*
Rheumatic heart disease, without heart failure	2,992.6 (2,672.0 to 3,633.9)	-15.8 (-20.0 to -11.9)*	-11.0 (-15.5 to -6.9)*	142.8 (89.1 to 217.5)	-15.7 (-20.1 to -11.3)*	-10.9 (-15.6 to -6.3)*
Heart failure fue to rheumatic heart disease aggregate	12.9 (11.5 to 14.4)	0.7 (-0.5 to 2.0)	6.4 (5.1 to 7.7)*	1.6 (1.1 to 2.2)	0.7 (-0.6 to 2.0)	6.3 (5.0 to 7.8)*
Ischemic heart disease	239.8 (207.2 to 273.4)	-3.5 (-4.6 to -2.5)*	1.9 (0.8 to 3.0)*	18.3 (12.0 to 26.1)	-3.5 (-9.4 to 2.6)	2.0 (-4.3 to 8.4)
Myocardial infarction episodes aggregate	0.3 (0.1 to 0.5)	-3.2 (-5.4 to 1.3)	2.3 (-0.1 to 7.0)	0.0 (0.0 to 0.1)	-3.2 (-5.4 to 1.3)	2.2 (-0.1 to 7.0)
Angina due to ischemic heart disease aggregate	213.9 (181.6 to 247.0)	-3.3 (-4.4 to -2.1)*	2.2 (1.0 to 3.4)*	15.1 (9.7 to 21.8)	-3.0 (-9.8 to 4.9)	2.5 (-4.8 to 10.8)
Heart failure fue to ischemic heart disease aggregate	25.6 (22.4 to 29.1)	-5.8 (-6.9 to -4.8)*	-0.5 (-1.7 to 0.6)	3.1 (2.1 to 4.5)	-5.9 (-9.3 to -2.1)*	-0.6 (-4.2 to 3.4)
Cerebrovascular disease	784.9 (764.9 to 808.1)	-3.6 (-6.1 to -0.6)*	1.9 (-0.8 to 5.0)	133.2 (91.2 to 184.3)	-3.6 (-7.4 to 0.6)	1.8 (-2.2 to 6.3)
Ischemic stroke	269.0 (253.9 to 284.8)	-3.9 (-4.9 to -3.1)*	1.5 (0.5 to 2.4)*	43.4 (29.7 to 60.0)	-4.0 (-9.7 to 1.9)	1.4 (-4.6 to 7.7)
Chronic ischemic stroke aggregate	268.4 (253.4 to 284.3)	-3.9 (-4.9 to -3.0)*	1.5 (0.5 to 2.4)*	43.2 (29.6 to 59.8)	-4.0 (-9.7 to 2.0)	1.4 (-4.6 to 7.7)
Ischemic stroke episodes aggregate	0.6 (0.4 to 0.8)	-4.6 (-7.1 to -1.9)*	0.8 (-1.9 to 3.6)	0.1 (0.1 to 0.2)	-4.6 (-7.1 to -1.9)*	0.8 (-1.9 to 3.6)
Hemorrhagic stroke	567.1 (535.8 to 600.3)	-3.5 (-4.4 to -2.6)*	2.0 (1.0 to 2.9)*	89.9 (61.6 to 124.7)	-3.5 (-8.6 to 2.0)	2.0 (-3.5 to 7.8)
Chronic hemorrhagic stroke aggregate	565.9 (534.8 to 599.2)	-3.5 (-4.4 to -2.6)*	2.0 (1.0 to 2.9)*	89.6 (61.4 to 124.4)	-3.5 (-8.6 to 2.1)	2.0 (-3.5 to 7.8)
Acute hemorrhagic stroke aggregate	1.2 (0.8 to 1.8)	-4.9 (-7.4 to -2.4)*	0.4 (-2.2 to 3.1)	0.3 (0.1 to 0.4)	-4.9 (-7.4 to -2.4)*	0.5 (-2.2 to 3.1)
Hypertensive heart disease	0.7 (0.6 to 0.8)	-2.3 (-3.4 to -1.2)*	3.2 (2.0 to 4.4)*	0.1 (0.1 to 0.1)	-2.3 (-3.4 to -1.2)*	3.2 (2.0 to 4.4)*
Cardiomyopathy and myocarditis	59.2 (53.3 to 65.5)	-1.2 (-2.0 to -0.4)*	4.4 (3.5 to 5.2)*	6.5 (4.4 to 9.2)	-1.1 (-7.5 to 5.8)	4.5 (-2.3 to 11.8)
Acute myocarditis	8.7 (5.9 to 12.7)	-5.1 (-7.2 to -3.1)*	0.2 (-2.0 to 2.4)	0.4 (0.2 to 0.7)	-5.1 (-8.0 to -2.4)*	0.3 (-2.8 to 3.1)
Heart failure due to cardiomyopathy aggregate	50.5 (45.4 to 55.6)	-0.5 (-1.3 to 0.3)	5.2 (4.2 to 6.0)*	6.1 (4.0 to 8.6)	-0.8 (-7.5 to 6.6)	4.8 (-2.3 to 12.6)
Atrial fibrillation and flutter	0.0 (0.0 to 0.0)	0.0 (0.0 to 0.0)	0.0 (0.0 to 0.0)	0.0 (0.0 to 0.0)	0.0 (0.0 to 0.0)	0.0 (0.0 to 0.0)
Peripheral vascular disease	0.0 (0.0 to 0.0)	0.0 (0.0 to 0.0)	0.0 (0.0 to 0.0)	0.0 (0.0 to 0.0)	0.0 (0.0 to 0.0)	0.0 (0.0 to 0.0)
Endocarditis	2.8 (2.1 to 3.7)	4.5 (1.0 to 8.0)*	10.4 (6.7 to 14.0)*	0.3 (0.2 to 0.5)	4.8 (1.1 to 8.4)*	10.7 (6.8 to 14.5)*
Endocarditis episodes aggregate	0.2 (0.2 to 0.3)	-1.1 (-7.5 to 5.7)	4.5 (-2.3 to 11.6)	0.0 (0.0 to 0.0)	-1.0 (-7.5 to 5.7)	4.6 (-2.3 to 11.7)
Heart failure due to endocarditis aggregate	2.5 (1.9 to 3.4)	5.1 (1.2 to 8.8)*	11.0 (6.9 to 14.9)*	0.3 (0.2 to 0.5)	5.1 (1.2 to 8.8)*	11.0 (6.9 to 14.9)*
Other cardiovascular and circulatory diseases	3,164.5 (2,400.8 to 4,129.6)	-11.7 (-13.6 to -9.7)*	-6.7 (-8.7 to -4.6)*	221.4 (135.2 to 342.2)	-11.7 (-13.9 to -9.2)*	-6.7 (-9.0 to -4.1)*
Heart failure due to other cardiovascular diseases aggregate	2.1 (1.8 to 2.4)	-13.8 (-15.3 to -12.1)*	-8.9 (-10.6 to -7.2)*	0.3 (0.2 to 0.4)	-13.8 (-15.6 to -11.9)*	-9.0 (-10.9 to -6.9)*
Other cardiovascular diseases episodes aggregate	3,162.4 (2,398.9 to 4,127.3)	-11.7 (-13.6 to -9.7)*	-6.7 (-8.7 to -4.6)*	221.2 (135.0 to 341.9)	-11.7 (-13.9 to -9.2)*	-6.7 (-9.0 to -4.1)*
Chronic respiratory diseases	36,577.7 (34,674.9 to 38,186.6)	-5.6 (-7.7 to -3.5)*	-0.3 (-2.5 to 1.9)	1,961.3 (1,331.5 to 2,736.3)	-6.4 (-9.8 to -3.6)*	-1.1 (-4.8 to 1.9)
Chronic obstructive pulmonary disease	4,463.5 (3,795.2 to 5,155.6)	-11.5 (-14.1 to -9.3)*	-6.5 (-9.2 to -4.1)*	367.4 (295.4 to 445.6)	-9.3 (-12.5 to -6.2)*	-4.2 (-7.5 to -0.9)*

Cause	Prevalence in thousands (95% confidence interval)			YLDs in thousands (95% confidence interval)		
	2015	Percent change in counts between 2005 and 2015	Percent change in rates between 2005 and 2015	2015	Percent change in counts between 2005 and 2015	Percent change in rates between 2005 and 2015
Chronic obstructive pulmonary disease without heart failure aggregate	4,466.0 (3,796.6 to 5,160.6)	-11.5 (-14.0 to -9.2)*	-6.5 (-9.2 to -4.1)*	367.3 (295.3 to 445.4)	-9.3 (-12.5 to -6.2)*	-4.2 (-7.5 to -0.9)*
Severe chronic obstructive pulmonary disease with heart failure aggregate	0.4 (0.3 to 0.4)	-8.9 (-10.1 to -7.8)*	-3.8 (-5.0 to -2.6)*	0.2 (0.1 to 0.2)	-8.9 (-10.1 to -7.8)*	-3.8 (-5.0 to -2.6)*
Pneumoconiosis	136.3 (127.0 to 145.7)	15.0 (6.4 to 24.5)*	21.4 (12.4 to 31.5)*	27.1 (16.8 to 39.9)	4.8 (-4.2 to 14.6)	10.7 (1.2 to 21.1)*
Silicosis	4.2 (3.4 to 5.2)	-16.1 (-19.3 to -13.1)*	-11.4 (-14.7 to -8.2)*	0.7 (0.4 to 1.1)	-16.1 (-19.5 to -13.1)*	-11.4 (-14.9 to -8.2)*
Silicosis without heart failure aggregate	4.2 (3.4 to 5.2)	-16.1 (-19.3 to -13.1)*	-11.4 (-14.7 to -8.2)*	0.7 (0.4 to 1.1)	-16.1 (-19.5 to -13.1)*	-11.4 (-14.9 to -8.2)*
Severe silicosis with heart failure aggregate	0.0 (0.0 to 0.0)	0.0 (0.0 to 0.0)	0.0 (0.0 to 0.0)	0.0 (0.0 to 0.0)	0.0 (0.0 to 0.0)	0.0 (0.0 to 0.0)
Asbestosis	0.8 (0.7 to 1.0)	-18.1 (-21.9 to -14.2)*	-13.5 (-17.5 to -9.4)*	0.1 (0.1 to 0.2)	-18.1 (-21.9 to -14.2)*	-13.5 (-17.5 to -9.4)*
Asbestosis without heart failure aggregate	0.8 (0.7 to 1.0)	-18.1 (-21.9 to -14.2)*	-13.5 (-17.5 to -9.4)*	0.1 (0.1 to 0.2)	-18.1 (-21.9 to -14.2)*	-13.5 (-17.5 to -9.4)*
Severe asbestosis with heart failure aggregate	0.0 (0.0 to 0.0)	0.0 (0.0 to 0.0)	0.0 (0.0 to 0.0)	0.0 (0.0 to 0.0)	0.0 (0.0 to 0.0)	0.0 (0.0 to 0.0)
Coal workers pneumoconiosis	0.4 (0.3 to 0.5)	0.0 (-6.2 to 5.9)	5.6 (-0.9 to 11.9)	0.1 (0.0 to 0.1)	0.0 (-6.2 to 5.9)	5.7 (-0.9 to 11.9)
Coal workers pneumoconiosis without heart failure aggregate	0.4 (0.3 to 0.5)	0.0 (-6.2 to 5.9)	5.6 (-0.9 to 11.9)	0.1 (0.0 to 0.1)	0.0 (-6.2 to 5.9)	5.7 (-0.9 to 11.9)
Severe coal workers pneumoconiosis with heart failure aggregate	0.0 (0.0 to 0.0)	0.0 (0.0 to 0.0)	0.0 (0.0 to 0.0)	0.0 (0.0 to 0.0)	0.0 (0.0 to 0.0)	0.0 (0.0 to 0.0)
Other pneumoconiosis	163.3 (141.3 to 189.9)	6.1 (1.9 to 10.4)*	12.1 (7.6 to 16.6)*	26.2 (16.3 to 38.5)	5.7 (-3.8 to 16.2)	11.6 (1.7 to 22.7)*
Other pneumoconiosis without heart failure aggregate	163.3 (141.3 to 189.9)	6.1 (1.9 to 10.4)*	12.1 (7.6 to 16.6)*	26.2 (16.3 to 38.5)	5.7 (-3.8 to 16.2)	11.6 (1.7 to 22.7)*
Severe other pneumoconiosis with heart failure aggregate	0.0 (0.0 to 0.0)	-2.4 (-5.7 to 0.9)	3.1 (-0.4 to 6.6)	0.0 (0.0 to 0.0)	-2.4 (-5.7 to 0.9)	3.1 (-0.4 to 6.6)
Asthma	32,737.5 (26,327.9 to 39,419.1)	-5.3 (-9.9 to -1.7)*	0.0 (-4.9 to 3.8)	1,479.8 (904.9 to 2,198.1)	-5.2 (-9.8 to -1.5)*	0.1 (-4.7 to 4.0)
Asymptomatic asthma	9,914.6 (7,737.6 to 12,449.5)	-5.3 (-9.9 to -1.7)*	0.0 (-4.9 to 3.8)	0.0 (0.0 to 0.0)	0.0 (0.0 to 0.0)	0.0 (0.0 to 0.0)
Symptomatic asthma aggregate	22,822.9 (18,309.5 to 27,886.3)	-5.3 (-9.9 to -1.7)*	0.0 (-4.9 to 3.8)	1,479.8 (904.9 to 2,198.1)	-5.2 (-9.8 to -1.5)*	0.1 (-4.7 to 4.0)
Interstitial lung disease and pulmonary sarcoidosis	28.8 (24.8 to 33.3)	-10.5 (-13.6 to -7.7)*	-5.4 (-8.7 to -2.5)*	3.6 (2.2 to 5.5)	-10.2 (-14.7 to -5.6)*	-5.2 (-9.9 to -0.3)*
Interstitial lung disease and pulmonary sarcoidosis without heart failure aggregate	28.4 (24.3 to 32.9)	-10.6 (-13.8 to -7.8)*	-5.6 (-9.0 to -2.6)*	3.4 (2.0 to 5.3)	-10.7 (-15.5 to -5.9)*	-5.7 (-10.8 to -0.6)*
Severe interstitial lung disease and pulmonary sarcoidosis with heart failure aggregate	0.4 (0.4 to 0.5)	-0.1 (-2.3 to 1.9)	5.5 (3.2 to 7.6)*	0.2 (0.1 to 0.3)	-0.1 (-2.3 to 1.9)	5.5 (3.2 to 7.6)*
Other chronic respiratory diseases	--	--	--	83.4 (65.8 to 102.9)	-15.4 (-24.0 to -6.0)*	-10.6 (-19.7 to -0.7)*
Cirrhosis and other chronic liver diseases	57.2 (52.4 to 62.0)	5.9 (-5.6 to 18.9)	11.8 (-0.3 to 25.5)	12.6 (8.7 to 17.5)	3.6 (-4.9 to 13.5)	9.4 (0.4 to 19.9)*
Cirrhosis and other chronic liver diseases due to hepatitis B	4.3 (3.5 to 5.3)	1.8 (-1.4 to 5.0)	7.6 (4.1 to 10.9)*	0.8 (0.5 to 1.1)	1.8 (-1.4 to 5.0)	7.6 (4.1 to 10.9)*
Cirrhosis and other chronic liver diseases due to hepatitis C	2.6 (2.0 to 3.3)	7.0 (2.3 to 10.9)*	13.0 (8.0 to 17.2)*	0.5 (0.3 to 0.7)	7.0 (2.3 to 10.9)*	13.0 (8.0 to 17.2)*
Cirrhosis and other chronic liver diseases due to alcohol use	2.6 (1.6 to 4.0)	11.1 (3.6 to 16.0)*	17.3 (9.5 to 22.5)*	0.5 (0.2 to 0.8)	11.1 (3.6 to 16.0)*	17.3 (9.5 to 22.5)*
Cirrhosis and other chronic liver diseases due to other causes	63.6 (58.8 to 68.5)	3.1 (2.2 to 4.2)*	8.9 (7.9 to 10.1)*	10.9 (7.4 to 15.1)	3.3 (-6.4 to 14.8)	9.1 (-1.2 to 21.2)
Digestive diseases	7,004.4 (6,808.4 to 7,196.3)	-13.0 (-15.6 to -9.9)*	-8.1 (-10.9 to -4.8)*	371.5 (258.7 to 509.9)	-11.1 (-14.6 to -6.6)*	-6.1 (-9.8 to -1.4)*
Peptic ulcer disease	1,383.2 (1,342.8 to 1,422.4)	-23.4 (-25.4 to -21.4)*	-19.1 (-21.2 to -17.0)*	43.9 (29.2 to 63.5)	-28.3 (-32.3 to -23.9)*	-24.3 (-28.5 to -19.6)*
Peptic ulcer disease, symptomatic episodes	86.8 (66.8 to 112.1)	-22.5 (-27.1 to -18.0)*	-18.1 (-23.0 to -13.4)*	9.6 (5.9 to 14.2)	-22.3 (-30.0 to -14.4)*	-18.0 (-26.1 to -9.6)*
Anemia due to peptic ulcer disease aggregate	1,296.4 (1,265.8 to 1,325.2)	-23.5 (-25.5 to -21.4)*	-19.2 (-21.3 to -16.9)*	34.3 (22.4 to 50.7)	-29.8 (-34.4 to -24.7)*	-25.8 (-30.7 to -20.5)*
Gastritis and duodenitis	4,304.3 (4,139.4 to 4,474.4)	-11.1 (-15.1 to -7.2)*	-6.1 (-10.3 to -1.9)*	143.9 (95.1 to 208.0)	-13.0 (-19.6 to -3.7)*	-8.1 (-15.0 to 1.8)
Gastritis and duodenitis, symptomatic episodes	95.7 (67.5 to 130.7)	-10.4 (-13.5 to -7.5)*	-5.4 (-8.6 to -2.3)*	10.5 (6.3 to 16.8)	-10.1 (-17.9 to -2.3)*	-5.1 (-13.3 to 3.2)
Anemia due to gastritis and duodenitis aggregate	4,208.6 (4,043.9 to 4,376.2)	-11.1 (-15.2 to -7.1)*	-6.1 (-10.4 to -1.9)*	133.3 (87.4 to 193.7)	-13.2 (-20.3 to -3.1)*	-8.3 (-15.8 to 2.3)
Appendicitis	54.8 (45.6 to 65.0)	-1.4 (-2.9 to 0.2)	4.1 (2.5 to 5.9)*	17.0 (10.9 to 24.1)	-1.4 (-10.8 to 8.9)	4.1 (-5.8 to 15.0)
Paralytic ileus and intestinal obstruction	3.4 (2.5 to 4.4)	1.3 (-4.5 to 7.0)	7.0 (0.8 to 13.0)*	1.1 (0.6 to 1.6)	1.3 (-4.5 to 7.0)	7.0 (0.8 to 13.0)*
Inguinal, femoral, and abdominal hernia	705.9 (505.9 to 955.0)	-9.3 (-14.7 to -3.7)*	-4.2 (-9.9 to 1.7)	7.6 (3.6 to 14.7)	-9.3 (-14.9 to -3.1)*	-4.2 (-10.2 to 2.4)
Inflammatory bowel disease	447.0 (377.1 to 528.8)	-5.1 (-5.8 to -4.2)*	0.3 (-0.5 to 1.2)	98.8 (64.2 to 142.6)	-4.9 (-8.3 to -1.5)*	0.4 (-3.1 to 4.1)
Vascular intestinal disorders	0.6 (0.4 to 0.8)	-1.1 (-7.0 to 4.7)	4.5 (-1.8 to 10.6)	0.2 (0.1 to 0.3)	-1.1 (-7.0 to 4.7)	4.5 (-1.8 to 10.6)
Gallbladder and biliary diseases	156.3 (119.8 to 206.8)	-4.4 (-9.5 to 1.1)	1.0 (-4.5 to 6.8)	17.2 (10.5 to 26.4)	-4.1 (-12.4 to 4.6)	1.3 (-7.5 to 10.5)
Pancreatitis	23.0 (17.6 to 29.2)	-1.0 (-6.0 to 3.6)	4.6 (-0.7 to 9.4)	7.3 (4.5 to 10.6)	-1.2 (-12.5 to 10.9)	4.4 (-7.6 to 17.2)
Other digestive diseases	--	--	--	34.5 (23.7 to 48.8)	-1.9 (-8.6 to 7.5)	3.6 (-3.4 to 13.5)
Neurological disorders	167,608.2 (154,074.9 to 179,311.5)	-2.5 (-4.9 to -0.2)*	3.0 (0.5 to 5.4)*	4,009.5 (2,545.9 to 5,716.3)	-4.4 (-6.8 to -1.9)*	1.0 (-1.6 to 3.6)
Alzheimer disease and other dementias	0.0 (0.0 to 0.0)	0.0 (0.0 to 0.0)	0.0 (0.0 to 0.0)	0.0 (0.0 to 0.0)	0.0 (0.0 to 0.0)	0.0 (0.0 to 0.0)
Parkinson disease	0.0 (0.0 to 0.0)	0.0 (0.0 to 0.0)	0.0 (0.0 to 0.0)	0.0 (0.0 to 0.0)	0.0 (0.0 to 0.0)	0.0 (0.0 to 0.0)
Epilepsy	2,107.8 (1,769.6 to 2,498.7)	-3.9 (-9.9 to 2.3)	1.5 (-4.8 to 8.1)	610.1 (395.3 to 829.8)	-16.9 (-22.8 to -10.7)*	-12.2 (-18.5 to -5.7)*
Multiple sclerosis	30.9 (23.7 to 40.1)	-6.3 (-9.5 to -3.8)*	-1.0 (-4.4 to 1.6)	11.1 (7.2 to 16.0)	-6.1 (-10.6 to -2.0)*	-0.8 (-5.6 to 3.5)
Motor neuron disease	5.4 (4.9 to 5.9)	-4.4 (-6.6 to -2.3)*	1.0 (-1.4 to 3.2)	1.1 (0.8 to 1.5)	-4.4 (-6.6 to -2.3)*	1.0 (-1.4 to 3.2)
Migraine	76,830.9 (59,578.8 to 97,354.3)	-1.7 (-3.7 to 0.2)	3.9 (1.7 to 5.8)*	2,709.4 (1,563.3 to 4,160.2)	-1.5 (-3.6 to 0.7)	4.0 (1.8 to 6.4)*
Tension-type headache	104,871.6 (74,476.6 to 140,955.7)	-2.7 (-3.7 to -1.8)*	2.7 (1.7 to 3.8)*	159.8 (67.4 to 311.6)	-2.6 (-4.2 to -1.1)*	2.9 (1.2 to 4.5)*
Medication overuse headache	3,202.3 (2,268.6 to 4,430.7)	-2.5 (-11.7 to 6.7)	3.0 (-6.7 to 12.7)	517.3 (305.2 to 792.7)	-2.4 (-11.6 to 6.8)	3.1 (-6.6 to 12.8)
Other neurological disorders	0.6 (0.4 to 1.1)	-3.3 (-4.6 to -2.1)*	2.2 (0.7 to 3.4)*	0.7 (0.5 to 1.1)	-6.9 (-11.3 to -3.2)*	-1.7 (-6.3 to 2.3)
Mental and substance use disorders	96,756.4 (94,113.6 to 99,062.2)	-4.5 (-5.3 to -3.7)*	0.9 (0.0 to 1.8)*	12,247.5 (8,473.7 to 16,235.6)	-2.7 (-3.5 to -1.9)*	2.8 (2.0 to 3.6)*
Schizophrenia	249.1 (176.1 to 343.6)	-9.7 (-11.0 to -8.6)*	-4.6 (-6.0 to -3.4)*	166.5 (105.4 to 239.9)	-9.8 (-14.0 to -5.5)*	-4.7 (-9.2 to -0.1)*
Alcohol use disorders	2,962.8 (2,280.6 to 3,903.6)	-12.0 (-16.3 to -7.8)*	-7.0 (-11.5 to -2.6)*	299.8 (181.8 to 446.0)	-12.0 (-16.5 to -7.0)*	-7.0 (-11.8 to -1.8)*
Alcohol dependence aggregate	2,882.6 (2,198.3 to 3,822.3)	-12.2 (-16.6 to -7.8)*	-7.2 (-11.9 to -2.7)*	295.6 (178.0 to 442.1)	-12.1 (-16.7 to -7.1)*	-7.1 (-12.0 to -1.8)*
Fetal alcohol syndrome aggregate	80.1 (75.3 to 84.1)	-5.1 (-5.7 to -4.6)*	0.2 (-0.4 to 0.7)	4.2 (2.7 to 6.1)	-5.5 (-11.6 to 1.0)	-0.2 (-6.6 to 6.7)
Drug use disorders	5,879.2 (5,600.0 to 6,172.1)	-9.8 (-12.0 to -7.6)*	-4.7 (-7.1 to -2.4)*	668.5 (454.0 to 903.4)	-5.2 (-8.0 to -2.4)*	0.1 (-2.9 to 3.1)
Opioid use disorders	491.3 (385.4 to 621.6)	-7.5 (-11.3 to -4.2)*	-2.3 (-6.3 to 1.2)	210.5 (138.1 to 295.3)	-7.4 (-12.3 to -2.9)*	-2.2 (-7.3 to 2.6)
Asymptomatic opioid dependence	79.7 (54.1 to 114.9)	-7.5 (-11.3 to -4.2)*	-2.3 (-6.3 to 1.2)	0.0 (0.0 to 0.0)	0.0 (0.0 to 0.0)	0.0 (0.0 to 0.0)
Symptomatic opioid dependence aggregate	411.6 (314.0 to 521.9)	-7.5 (-11.3 to -4.2)*	-2.3 (-6.3 to 1.2)	210.5 (138.1 to 295.3)	-7.4 (-12.3 to -2.9)*	-2.2 (-7.3 to 2.6)
Cocaine use disorders	441.5 (342.7 to 548.0)	3.7 (0.2 to 7.4)*	9.5 (5.8 to 13.5)*	61.8 (37.1 to 91.7)	3.7 (-1.8 to 9.9)	9.5 (3.8 to 16.1)*
Asymptomatic cocaine dependence	221.3 (162.9 to 294.0)	3.7 (0.2 to 7.4)*	9.5 (5.8 to 13.5)*	0.0 (0.0 to 0.0)	0.0 (0.0 to 0.0)	0.0 (0.0 to 0.0)

Cause	Prevalence in thousands (95% confidence interval)			YLDs in thousands (95% confidence interval)		
	2015	Percent change in counts between 2005 and 2015	Percent change in rates between 2005 and 2015	2015	Percent change in counts between 2005 and 2015	Percent change in rates between 2005 and 2015
Symptomatic cocaine dependence aggregate	220.2 (161.3 to 291.4)	3.7 (0.2 to 7.4)*	9.5 (5.8 to 13.5)*	61.8 (37.1 to 91.7)	3.7 (-1.8 to 9.9)	9.5 (3.8 to 16.1)*
Amphetamine use disorders	1,093.6 (805.4 to 1,415.9)	-2.7 (-5.9 to 0.9)	2.7 (-0.6 to 6.6)	146.2 (83.1 to 229.0)	-2.7 (-7.8 to 2.4)	2.8 (-2.6 to 8.2)
Asymptomatic amphetamine dependence	598.8 (415.2 to 806.0)	-2.7 (-5.9 to 0.9)	2.7 (-0.6 to 6.6)	0.0 (0.0 to 0.0)	0.0 (0.0 to 0.0)	0.0 (0.0 to 0.0)
Symptomatic amphetamine dependence aggregate	494.8 (341.7 to 679.8)	-2.7 (-5.9 to 0.9)	2.7 (-0.6 to 6.6)	146.2 (83.1 to 229.0)	-2.7 (-7.8 to 2.4)	2.8 (-2.6 to 8.2)
Cannabis use disorders	3,900.1 (3,163.9 to 4,739.8)	-13.1 (-15.1 to -11.0)*	-8.2 (-10.4 to -6.0)*	114.9 (71.9 to 168.1)	-13.0 (-15.9 to -10.1)*	-8.1 (-11.2 to -5.1)*
Asymptomatic cannabis dependence	2,255.8 (1,806.3 to 2,757.4)	-13.1 (-15.1 to -11.0)*	-8.2 (-10.4 to -6.0)*	0.0 (0.0 to 0.0)	0.0 (0.0 to 0.0)	0.0 (0.0 to 0.0)
Symptomatic cannabis dependence aggregate	1,644.2 (1,317.4 to 2,039.0)	-13.1 (-15.1 to -11.0)*	-8.2 (-10.4 to -6.0)*	114.9 (71.9 to 168.1)	-13.0 (-15.9 to -10.1)*	-8.1 (-11.2 to -5.1)*
Other drug use disorders	--	--	--	135.0 (80.3 to 201.7)	-0.8 (-5.6 to 4.5)	4.7 (-0.3 to 10.4)
Depressive disorders	22,185.0 (20,409.5 to 23,817.2)	-0.2 (-2.5 to 2.1)	5.4 (3.0 to 7.9)*	4,199.9 (2,670.7 to 6,057.4)	0.6 (-0.4 to 1.6)	6.3 (5.2 to 7.4)*
Major depressive disorder	17,239.4 (13,099.4 to 22,115.9)	1.1 (0.3 to 1.9)*	6.8 (6.0 to 7.7)*	3,646.3 (2,264.8 to 5,393.4)	1.3 (0.2 to 2.4)*	7.0 (5.9 to 8.1)*
Dysthymia	5,566.8 (3,957.6 to 7,648.7)	-3.5 (-5.2 to -2.0)*	1.9 (0.2 to 3.5)*	553.6 (313.8 to 871.0)	-3.4 (-5.4 to -1.4)*	2.0 (-0.1 to 4.1)
Bipolar disorder	3,921.2 (3,023.0 to 4,852.1)	-3.1 (-3.5 to -2.8)*	2.3 (2.0 to 2.6)*	822.6 (491.0 to 1,274.1)	-3.0 (-4.6 to -1.3)*	2.5 (0.7 to 4.2)*
Anxiety disorders	26,567.4 (21,432.3 to 32,111.3)	-3.8 (-5.8 to -1.6)*	1.6 (-0.5 to 3.9)	2,510.5 (1,617.2 to 3,544.9)	-3.8 (-5.9 to -1.6)*	1.6 (-0.6 to 4.0)
Eating disorders	1,025.7 (932.0 to 1,099.3)	4.2 (-0.1 to 8.6)	10.0 (5.5 to 14.7)*	224.8 (146.4 to 331.0)	4.1 (0.2 to 8.0)*	9.9 (5.8 to 14.1)*
Anorexia nervosa	661.0 (461.1 to 899.9)	-1.0 (-4.9 to 2.8)	4.5 (0.5 to 8.6)*	141.6 (87.1 to 218.8)	-1.0 (-5.8 to 3.7)	4.5 (-0.5 to 9.5)
Bulimia nervosa	390.0 (250.4 to 588.7)	14.0 (10.9 to 17.1)*	20.5 (17.2 to 23.7)*	83.2 (45.1 to 139.4)	14.1 (8.9 to 19.1)*	20.5 (15.1 to 25.8)*
Autistic spectrum disorders	5,964.3 (5,706.1 to 6,236.3)	-4.9 (-6.8 to -3.1)*	0.4 (-1.5 to 2.3)	978.2 (654.1 to 1,359.9)	-4.6 (-5.7 to -3.5)*	0.8 (-0.3 to 1.9)
Autism	2,372.7 (1,991.9 to 2,818.7)	-4.4 (-4.7 to -4.1)*	1.0 (0.7 to 1.3)*	617.8 (395.7 to 890.2)	-4.3 (-5.8 to -2.7)*	1.1 (-0.5 to 2.7)
Asperger syndrome and other autistic spectrum disorders	3,563.7 (3,016.7 to 4,266.7)	-5.1 (-5.3 to -5.0)*	0.2 (0.0 to 0.4)*	360.5 (238.1 to 528.5)	-5.1 (-6.2 to -3.8)*	0.3 (-1.0 to 1.7)
Attention-deficit/hyperactivity disorder	9,897.7 (8,774.1 to 11,189.5)	-12.3 (-13.3 to -11.3)*	-7.3 (-8.4 to -6.3)*	120.2 (70.5 to 184.8)	-12.2 (-13.8 to -10.7)*	-7.3 (-8.9 to -5.7)*
Conduct disorder	15,636.5 (11,370.6 to 20,254.1)	-3.6 (-4.1 to -3.1)*	1.8 (1.3 to 2.4)*	1,874.3 (1,061.5 to 3,018.0)	-3.5 (-4.5 to -2.5)*	2.0 (0.9 to 3.0)*
Idiopathic developmental intellectual disability	10,403.2 (6,122.8 to 14,466.3)	-3.8 (-4.8 to -3.1)*	1.6 (0.6 to 2.4)*	382.2 (170.0 to 667.5)	-4.9 (-7.4 to -3.6)*	0.4 (-2.1 to 1.9)
Other mental and substance use disorders	0.0 (0.0 to 0.0)	0.0 (0.0 to 0.0)	0.0 (0.0 to 0.0)	0.0 (0.0 to 0.0)	0.0 (0.0 to 0.0)	0.0 (0.0 to 0.0)
Diabetes, urogenital, blood, and endocrine diseases	192,649.6 (190,733.2 to 195,714.5)	-4.6 (-5.5 to -3.6)*	0.8 (-0.2 to 1.8)	1,858.2 (1,276.0 to 2,577.7)	-9.2 (-10.7 to -7.7)*	-4.1 (-5.7 to -2.5)*
Diabetes mellitus	6,040.7 (4,870.6 to 7,357.1)	0.2 (-3.1 to 3.9)	5.9 (2.4 to 9.7)*	334.7 (208.1 to 501.2)	0.9 (-2.6 to 4.6)	6.6 (2.9 to 10.4)*
Uncomplicated diabetes mellitus	5,484.4 (4,398.7 to 6,667.1)	-0.2 (-3.5 to 3.4)	5.4 (1.9 to 9.2)*	262.4 (159.3 to 397.5)	-0.1 (-3.6 to 3.5)	5.5 (1.8 to 9.4)*
Neuropathy and other complications of diabetes mellitus aggregate	556.3 (413.0 to 744.7)	4.7 (0.6 to 9.0)*	10.6 (6.3 to 15.2)*	72.4 (43.7 to 111.9)	4.9 (-0.4 to 10.6)	10.8 (5.2 to 16.8)*
Vision loss due to diabetes mellitus aggregate	0.0 (0.0 to 0.0)	0.0 (0.0 to 0.0)	0.0 (0.0 to 0.0)	0.0 (0.0 to 0.0)	0.0 (0.0 to 0.0)	0.0 (0.0 to 0.0)
Acute glomerulonephritis	5.2 (3.9 to 7.0)	-32.6 (-36.7 to -29.2)*	-28.8 (-33.1 to -25.2)*	0.3 (0.2 to 0.4)	-32.5 (-36.8 to -28.8)*	-28.8 (-33.3 to -24.7)*
Chronic kidney disease	2,870.1 (2,635.2 to 3,103.2)	-3.3 (-6.1 to -0.3)*	2.1 (-0.8 to 5.3)	180.1 (123.8 to 254.0)	-5.8 (-7.9 to -3.7)*	-0.5 (-2.7 to 1.7)
Chronic kidney disease due to diabetes mellitus	1,051.8 (683.2 to 1,510.5)	-5.0 (-7.4 to -2.8)*	0.4 (-2.2 to 2.7)	60.3 (36.9 to 90.4)	-6.1 (-9.8 to -2.5)*	-0.8 (-4.8 to 3.0)
Stage III chronic kidney disease due to diabetes mellitus aggregate	695.4 (424.8 to 1,056.5)	-4.7 (-7.2 to -2.3)*	0.7 (-2.0 to 3.1)	4.0 (2.7 to 5.6)	-6.1 (-10.0 to -2.1)*	-0.8 (-5.0 to 3.4)
Stage IV chronic kidney disease due to diabetes mellitus aggregate	328.3 (208.8 to 483.9)	-5.3 (-8.3 to -2.5)*	0.1 (-3.1 to 3.0)	43.7 (25.7 to 68.9)	-5.8 (-10.7 to -1.0)*	-0.5 (-5.7 to 4.6)
Stage V chronic kidney disease untreated due to diabetes mellitus	19.5 (12.9 to 27.9)	-6.6 (-8.7 to -4.4)*	-1.3 (-3.6 to 1.0)	11.1 (6.3 to 17.1)	-6.6 (-9.8 to -3.5)*	-1.4 (-4.7 to 2.0)
End-stage chronic kidney disease due to diabetes aggregate	8.6 (5.7 to 12.1)	-15.1 (-17.7 to -12.2)*	-10.3 (-13.1 to -7.3)*	1.5 (0.9 to 2.4)	-9.6 (-12.3 to -7.0)*	-4.5 (-7.4 to -1.8)*
Chronic kidney disease due to hypertension	632.9 (341.1 to 1,023.7)	-1.8 (-5.1 to 1.5)	3.8 (0.3 to 7.2)*	18.0 (9.1 to 33.0)	-3.2 (-9.2 to 3.0)	2.2 (-4.0 to 8.8)
Stage III chronic kidney disease due to hypertension aggregate	509.0 (277.5 to 816.2)	-1.9 (-4.7 to 0.9)	3.7 (0.6 to 6.6)*	0.8 (0.5 to 1.1)	-7.2 (-9.8 to -4.2)*	-2.0 (-4.7 to 1.1)
Stage IV chronic kidney disease due to hypertension aggregate	118.6 (48.8 to 245.3)	-1.0 (-8.3 to 8.4)	4.6 (-3.2 to 14.5)	14.9 (6.6 to 29.1)	-2.3 (-9.2 to 5.6)	3.2 (-4.1 to 11.6)
Stage V chronic kidney disease untreated due to hypertension	3.7 (2.3 to 5.5)	-7.2 (-11.4 to -3.1)*	-2.0 (-6.5 to 2.3)	2.1 (1.1 to 3.3)	-7.2 (-11.4 to -3.1)*	-2.0 (-6.5 to 2.3)
End-stage chronic kidney disease due to hypertension aggregate	1.6 (1.1 to 2.4)	-15.0 (-20.5 to -9.5)*	-10.2 (-16.0 to -4.4)*	0.3 (0.2 to 0.5)	-10.0 (-14.5 to -5.0)*	-4.9 (-9.7 to 0.3)
Chronic kidney disease due to glomerulonephritis	395.1 (314.5 to 498.7)	-5.3 (-7.6 to -2.9)*	0.0 (-2.4 to 2.5)	41.5 (26.8 to 58.8)	-6.3 (-10.5 to -1.9)*	-1.1 (-5.4 to 3.6)
Stage III chronic kidney disease due to glomerulonephritis aggregate	170.4 (118.9 to 246.2)	-4.8 (-8.2 to -1.3)*	0.6 (-3.0 to 4.3)	2.8 (1.9 to 3.9)	-5.3 (-7.9 to -2.7)*	0.0 (-2.7 to 2.8)
Stage IV chronic kidney disease due to glomerulonephritis aggregate	203.3 (151.4 to 246.0)	-5.5 (-8.3 to -2.6)*	-0.2 (-3.1 to 2.9)	28.8 (18.3 to 41.2)	-6.6 (-12.4 to -0.3)*	-1.4 (-7.5 to 5.3)
Stage V chronic kidney disease untreated due to glomerulonephritis	15.5 (9.0 to 24.2)	-5.1 (-7.5 to -2.7)*	0.3 (-2.3 to 2.8)	8.8 (4.7 to 14.5)	-5.0 (-8.4 to -1.7)*	0.4 (-3.2 to 3.8)
End-stage chronic kidney disease due to glomerulonephritis aggregate	5.9 (3.5 to 8.9)	-12.9 (-15.9 to -10.0)*	-8.0 (-11.1 to -5.0)*	1.1 (0.6 to 1.8)	-11.0 (-14.1 to -8.2)*	-6.0 (-9.3 to -3.0)*
Chronic kidney disease due to other causes	1,087.7 (689.1 to 1,543.0)	-2.8 (-4.6 to -0.8)*	2.7 (0.8 to 4.8)*	60.3 (36.7 to 90.4)	-6.0 (-9.6 to -2.5)*	-0.7 (-4.5 to 3.0)
Stage III chronic kidney disease due to other causes aggregate	718.8 (431.8 to 1,094.0)	-2.3 (-4.2 to -0.2)*	3.2 (1.2 to 5.4)*	3.4 (2.3 to 4.7)	-7.2 (-10.6 to -4.1)*	-2.0 (-5.6 to 1.3)
Stage IV chronic kidney disease due to other causes aggregate	340.6 (212.5 to 500.5)	-3.5 (-6.1 to -0.8)*	2.0 (-0.8 to 4.8)	43.8 (25.3 to 67.6)	-6.1 (-10.7 to -1.6)*	-0.8 (-5.7 to 3.9)
Stage V chronic kidney disease untreated due to other causes	20.4 (12.9 to 29.7)	-4.8 (-6.6 to -3.0)*	0.5 (-1.4 to 2.5)	11.5 (6.2 to 18.0)	-4.8 (-7.8 to -1.5)*	0.6 (-2.6 to 4.0)
End-stage chronic kidney disease due to other causes aggregate	8.0 (5.2 to 11.5)	-12.9 (-15.5 to -10.0)*	-8.0 (-10.7 to -5.0)*	1.5 (0.8 to 2.3)	-9.3 (-11.8 to -6.6)*	-4.2 (-6.9 to -1.4)*
Urinary diseases and male infertility	11,686.4 (10,964.3 to 12,271.7)	-1.8 (-4.3 to 1.0)	3.8 (1.1 to 6.7)*	20.4 (11.8 to 32.7)	2.5 (-3.3 to 8.2)	8.3 (2.2 to 14.3)*
Interstitial nephritis and urinary tract infections	207.1 (194.1 to 221.8)	-0.1 (-0.8 to 0.6)	5.5 (4.8 to 6.2)*	7.0 (4.4 to 10.7)	-0.2 (-6.3 to 6.3)	5.4 (-1.0 to 12.2)
Urolithiasis	10,446.1 (9,009.6 to 12,112.2)	-3.2 (-6.2 to 0.1)	2.3 (-0.9 to 5.8)	2.5 (1.5 to 3.8)	-4.5 (-15.1 to 7.1)	0.9 (-10.3 to 13.2)
Benign prostatic hyperplasia	0.0 (0.0 to 0.0)	0.0 (0.0 to 0.0)	0.0 (0.0 to 0.0)	0.0 (0.0 to 0.0)	0.0 (0.0 to 0.0)	0.0 (0.0 to 0.0)
Male infertility	1,167.1 (745.1 to 1,672.8)	11.7 (2.5 to 20.6)*	18.0 (8.3 to 27.4)*	7.7 (2.8 to 16.5)	11.2 (1.2 to 21.2)*	17.5 (6.9 to 28.0)*
Other urinary diseases	--	--	--	3.3 (2.0 to 4.8)	-4.0 (-13.5 to 6.8)	1.4 (-8.6 to 12.8)
Gynecological diseases	56,609.4 (54,994.9 to 58,012.6)	-6.6 (-8.1 to -5.0)*	-1.3 (-2.9 to 0.3)	639.0 (418.3 to 918.0)	-7.9 (-10.1 to -5.7)*	-2.8 (-5.0 to -0.4)*
Uterine fibroids	550.9 (497.0 to 612.6)	-5.5 (-6.1 to -5.0)*	-0.2 (-0.8 to 0.3)	15.2 (9.4 to 23.4)	-7.9 (-12.2 to -3.2)*	-2.7 (-7.2 to 2.3)
Uterine fibroids cases aggregate	173.1 (145.8 to 201.1)	-5.5 (-6.1 to -5.0)*	-0.2 (-0.8 to 0.3)	0.0 (0.0 to 0.0)	-17.4 (-100.0 to inf)	-12.8 (-100.0 to inf)
Mild abdominal pain with anemia due to uterine fibroids aggregate	377.8 (334.3 to 423.5)	-5.5 (-6.1 to -5.0)*	-0.2 (-0.8 to 0.3)	15.2 (9.4 to 23.4)	-7.9 (-12.2 to -3.2)*	-2.7 (-7.2 to 2.3)
Polycystic ovarian syndrome	1,746.9 (1,149.9 to 2,615.1)	-4.0 (-4.7 to -3.2)*	1.4 (0.7 to 2.3)*	16.0 (6.8 to 34.2)	-4.1 (-6.0 to -2.2)*	1.3 (-0.7 to 3.4)
Polycystic ovarian syndrome cases aggregate	1,432.5 (938.6 to 2,167.6)	-3.8 (-4.5 to -3.0)*	1.6 (0.9 to 2.5)*	12.5 (5.3 to 26.0)	-3.8 (-5.8 to -1.7)*	1.7 (-0.5 to 3.8)
Hirsutism and infertility due to polycystic ovarian syndrome aggregate	314.4 (183.7 to 516.6)	-4.6 (-5.8 to -3.2)*	0.7 (-0.5 to 2.2)	3.6 (1.4 to 8.2)	-5.1 (-10.6 to 0.1)	0.2 (-5.5 to 5.8)

Cause	Prevalence in thousands (95% confidence interval)			YLDs in thousands (95% confidence interval)		
	2015	Percent change in counts between 2005 and 2015	Percent change in rates between 2005 and 2015	2015	Percent change in counts between 2005 and 2015	Percent change in rates between 2005 and 2015
Female infertility	1,873.4 (1,042.4 to 2,835.6)	14.6 (3.0 to 27.3)*	21.0 (8.8 to 34.5)*	11.7 (4.0 to 26.2)	14.3 (2.2 to 28.6)*	20.7 (7.9 to 35.8)*
Endometriosis	168.3 (120.5 to 237.1)	-8.4 (-9.5 to -7.1)*	-3.2 (-4.4 to -1.8)*	16.0 (9.3 to 26.0)	-8.1 (-14.5 to -1.6)*	-3.0 (-9.7 to 3.9)
Endometriosis cases aggregate	159.1 (114.3 to 222.9)	-8.3 (-9.4 to -7.0)*	-3.2 (-4.3 to -1.8)*	15.0 (8.8 to 24.3)	-8.1 (-14.8 to -1.2)*	-2.9 (-10.0 to 4.4)
Abdominal pain and infertility due to endometriosis aggregate	9.1 (5.6 to 13.7)	-9.0 (-10.4 to -7.6)*	-3.9 (-5.4 to -2.4)*	1.0 (0.5 to 1.6)	-9.0 (-10.6 to -7.4)*	-3.9 (-5.6 to -2.2)*
Genital prolapse	367.1 (247.8 to 527.5)	-9.6 (-13.7 to -5.3)*	-4.5 (-8.8 to 0.1)	1.2 (0.5 to 2.3)	-9.4 (-18.4 to 0.8)	-4.3 (-13.8 to 6.5)
Premenstrual syndrome	50,272.4 (45,137.7 to 55,954.3)	-7.0 (-9.9 to -4.1)*	-1.7 (-4.8 to 1.3)	429.1 (261.1 to 666.8)	-6.9 (-9.9 to -3.8)*	-1.7 (-4.8 to 1.6)
Other gynecological diseases	4,218.7 (4,116.2 to 4,347.1)	-10.5 (-11.7 to -9.2)*	-5.5 (-6.8 to -4.1)*	149.7 (104.3 to 209.2)	-12.4 (-15.0 to -9.8)*	-7.5 (-10.3 to -4.8)*
Other gynecological diseases cases aggregate	1,055.1 (962.7 to 1,167.2)	-6.7 (-7.2 to -6.1)*	-1.4 (-2.0 to -0.8)*	42.1 (28.5 to 59.1)	-6.5 (-11.0 to -1.4)*	-1.3 (-6.0 to 4.1)
Anemia due to other gynecological diseases aggregate	3,163.6 (3,115.8 to 3,217.6)	-11.7 (-13.3 to -10.0)*	-6.8 (-8.5 to -5.0)*	107.6 (72.5 to 154.5)	-14.5 (-17.7 to -11.5)*	-9.7 (-13.0 to -6.5)*
Hemoglobinopathies and hemolytic anemias	135,481.2 (133,171.5 to 138,291.8)	-3.6 (-5.1 to -2.1)*	1.8 (0.3 to 3.4)*	565.1 (375.9 to 820.6)	-14.7 (-16.8 to -12.5)*	-9.9 (-12.1 to -7.6)*
Thalassemias	8.0 (7.5 to 9.2)	-16.7 (-18.9 to -13.6)*	-12.0 (-14.4 to -8.7)*	0.7 (0.5 to 1.0)	-20.0 (-26.8 to -12.0)*	-15.5 (-22.7 to -7.1)*
Beta-thalassemia major cases aggregate	3.2 (3.1 to 3.4)	-19.5 (-21.7 to -17.3)*	-14.9 (-17.3 to -12.6)*	0.3 (0.2 to 0.4)	-23.6 (-33.4 to -12.8)*	-19.3 (-29.6 to -7.9)*
Hemoglobin E/beta-thalassemia cases aggregate	1.6 (1.5 to 1.7)	-7.3 (-8.7 to -6.0)*	-2.1 (-3.5 to -0.7)*	0.1 (0.1 to 0.2)	-11.3 (-23.3 to 1.9)	-6.4 (-18.9 to 7.7)
Hemoglobin H disease cases aggregate	3.0 (2.5 to 4.2)	-17.9 (-23.6 to -10.4)*	-13.3 (-19.3 to -5.3)*	0.2 (0.2 to 0.3)	-20.0 (-33.5 to -5.4)*	-15.5 (-29.7 to -0.1)*
Heart failure due to thalassemias aggregate	0.2 (0.2 to 0.2)	-17.4 (-19.3 to -15.7)*	-12.8 (-14.8 to -10.9)*	0.0 (0.0 to 0.0)	-17.5 (-19.3 to -15.7)*	-12.8 (-14.8 to -10.9)*
Thalassemias trait	21,168.4 (20,619.0 to 21,804.3)	-16.1 (-16.6 to -15.7)*	-11.4 (-11.9 to -10.9)*	236.1 (156.0 to 346.0)	-21.4 (-24.0 to -18.7)*	-17.0 (-19.7 to -14.1)*
B-thalassemia trait cases aggregate	16,917.8 (16,435.0 to 17,481.6)	-17.7 (-18.4 to -17.2)*	-13.1 (-13.8 to -12.5)*	219.4 (145.1 to 320.4)	-22.0 (-24.6 to -19.4)*	-17.6 (-20.4 to -14.9)*
Hemoglobin E trait cases aggregate	4,250.6 (4,050.5 to 4,463.2)	-9.0 (-10.0 to -7.9)*	-3.9 (-5.0 to -2.7)*	16.7 (10.8 to 24.9)	-12.2 (-17.9 to -5.7)*	-7.3 (-13.3 to -0.4)*
Sickle cell disorders	399.1 (377.2 to 421.1)	18.2 (14.8 to 21.9)*	24.9 (21.2 to 28.8)*	38.5 (26.9 to 52.6)	15.8 (7.9 to 23.9)*	22.3 (14.0 to 30.9)*
Homozygous sickle cell and severe sickle cell/beta-thalassemia cases aggregate	378.1 (357.5 to 399.3)	17.7 (14.2 to 21.3)*	24.4 (20.6 to 28.2)*	36.3 (25.4 to 49.8)	15.3 (7.2 to 23.3)*	21.8 (13.3 to 30.2)*
Hemoglobin SC disease cases aggregate	20.4 (18.1 to 22.7)	29.1 (12.6 to 64.0)*	36.4 (18.9 to 73.2)*	2.1 (1.3 to 3.0)	25.9 (-8.3 to 73.0)	32.9 (-3.1 to 82.8)
Mild sickle cell/beta-thalassemia cases aggregate	0.6 (0.5 to 0.6)	-3.3 (-6.8 to 0.5)	2.1 (-1.5 to 6.1)	0.1 (0.1 to 0.1)	-0.6 (-3.7 to 2.6)	5.0 (1.7 to 8.4)*
Sickle cell trait	38,956.0 (36,574.6 to 43,412.8)	12.9 (11.9 to 13.7)*	19.2 (18.2 to 20.1)*	132.7 (88.2 to 191.3)	1.9 (-2.3 to 5.9)	7.6 (3.2 to 11.9)*
G6PD deficiency	22,133.4 (18,724.5 to 25,707.8)	-5.0 (-10.0 to -0.5)*	0.4 (-5.0 to 5.1)	2.3 (1.5 to 3.2)	-13.8 (-23.8 to -3.4)*	-8.9 (-19.5 to 2.0)
G6PD cases aggregate	22,133.4 (18,724.5 to 25,707.8)	-5.0 (-10.0 to -0.5)*	0.4 (-5.0 to 5.1)	2.3 (1.5 to 3.2)	-13.8 (-23.8 to -3.4)*	-8.9 (-19.5 to 2.0)
Heart failure due to G6PD deficiency aggregate	0.0 (0.0 to 0.0)	0.0 (0.0 to 0.0)	0.0 (0.0 to 0.0)	0.0 (0.0 to 0.0)	0.0 (0.0 to 0.0)	0.0 (0.0 to 0.0)
G6PD trait	60,977.2 (56,796.3 to 65,281.3)	-4.7 (-6.2 to -3.4)*	0.6 (-0.9 to 2.1)	2.7 (1.8 to 3.7)	-8.2 (-18.5 to 5.2)	-3.0 (-13.9 to 11.1)
Other hemoglobinopathies and hemolytic anemias	7,066.7 (6,934.1 to 7,190.1)	-15.3 (-17.1 to -13.6)*	-10.6 (-12.4 to -8.7)*	152.2 (99.5 to 226.0)	-20.8 (-24.3 to -17.6)*	-16.3 (-20.0 to -13.0)*
Other hemoglobinopathies and hemolytic anemias cases aggregate	7,066.4 (6,933.7 to 7,189.7)	-15.3 (-17.1 to -13.6)*	-10.6 (-12.4 to -8.7)*	148.7 (96.0 to 221.1)	-21.2 (-24.7 to -17.9)*	-16.8 (-20.5 to -13.3)*
Heart failure due to other hemoglobinopathies and hemolytic anemias aggregate	0.4 (0.3 to 0.4)	-12.7 (-14.3 to -11.0)*	-7.8 (-9.5 to -6.0)*	0.0 (0.0 to 0.1)	-12.7 (-14.3 to -11.0)*	-7.8 (-9.5 to -6.0)*
Endocrine, metabolic, blood, and immune disorders	4,800.9 (4,718.1 to 4,887.8)	-14.3 (-16.0 to -12.5)*	-9.5 (-11.3 to -7.6)*	118.7 (78.5 to 170.8)	-18.7 (-21.9 to -15.4)*	-14.1 (-17.5 to -10.6)*
Endocrine, metabolic, blood, and immune disorders cases aggregate	240.8 (218.0 to 265.7)	-11.1 (-12.9 to -9.3)*	-6.1 (-8.0 to -4.2)*	9.7 (6.4 to 13.5)	-10.9 (-15.7 to -5.8)*	-5.9 (-10.9 to -0.5)*
Anemia due to endocrine, metabolic, blood, and immune disorders aggregate	4,559.9 (4,481.8 to 4,638.4)	-14.5 (-16.2 to -12.7)*	-9.7 (-11.5 to -7.7)*	109.0 (72.2 to 158.9)	-19.3 (-22.9 to -15.9)*	-14.8 (-18.5 to -11.1)*
Heart failure due to endocrine, metabolic, blood, and immune disorders aggregate	0.1 (0.1 to 0.2)	-7.4 (-8.6 to -6.3)*	-2.2 (-3.5 to -1.0)*	0.0 (0.0 to 0.0)	-7.5 (-8.6 to -6.3)*	-2.2 (-3.5 to -1.0)*
Musculoskeletal disorders	44,202.1 (42,584.6 to 45,750.1)	-8.3 (-9.7 to -6.7)*	-3.1 (-4.7 to -1.5)*	4,975.6 (3,393.2 to 6,760.3)	-8.3 (-10.3 to -6.2)*	-3.2 (-5.2 to -0.9)*
Rheumatoid arthritis	837.1 (712.3 to 974.8)	-9.0 (-13.5 to -4.5)*	-3.9 (-8.7 to 0.9)	208.3 (139.9 to 290.5)	-9.0 (-14.2 to -3.6)*	-3.9 (-9.4 to 1.8)
Osteoarthritis	0.0 (0.0 to 0.0)	0.0 (0.0 to 0.0)	0.0 (0.0 to 0.0)	0.0 (0.0 to 0.0)	0.0 (0.0 to 0.0)	0.0 (0.0 to 0.0)
Osteoarthritis of the hip cases aggregate	0.0 (0.0 to 0.0)	0.0 (0.0 to 0.0)	0.0 (0.0 to 0.0)	0.0 (0.0 to 0.0)	0.0 (0.0 to 0.0)	0.0 (0.0 to 0.0)
Osteoarthritis of the knee cases aggregate	0.0 (0.0 to 0.0)	0.0 (0.0 to 0.0)	0.0 (0.0 to 0.0)	0.0 (0.0 to 0.0)	0.0 (0.0 to 0.0)	0.0 (0.0 to 0.0)
Low back and neck pain	25,603.4 (24,512.7 to 26,540.2)	-7.1 (-8.3 to -5.9)*	-1.9 (-3.2 to -0.6)*	2,941.5 (2,027.8 to 4,059.2)	-7.1 (-8.6 to -5.4)*	-1.8 (-3.4 to -0.1)*
Low back pain	21,382.2 (19,076.5 to 23,796.4)	-7.3 (-9.0 to -5.6)*	-2.0 (-3.9 to -0.2)*	2,478.3 (1,692.5 to 3,485.7)	-7.2 (-9.0 to -5.3)*	-1.9 (-3.8 to 0.0)
Neck pain	4,584.8 (3,254.6 to 6,217.7)	-6.6 (-8.3 to -4.5)*	-1.4 (-3.2 to 0.9)	463.2 (280.8 to 721.6)	-6.6 (-8.7 to -4.1)*	-1.3 (-3.6 to 1.3)
Gout	86.6 (56.9 to 123.8)	-4.1 (-5.3 to -2.5)*	1.3 (0.0 to 3.0)*	3.1 (1.7 to 5.2)	-4.0 (-6.3 to -1.4)*	1.4 (-1.0 to 4.1)
Asymptomatic gout	77.3 (50.9 to 110.3)	-4.1 (-5.3 to -2.5)*	1.3 (0.0 to 3.0)*	0.0 (0.0 to 0.0)	0.0 (0.0 to 0.0)	0.0 (0.0 to 0.0)
Symptomatic gout cases aggregate	9.2 (5.9 to 13.6)	-4.1 (-5.3 to -2.5)*	1.3 (0.0 to 3.0)*	3.1 (1.7 to 5.2)	-4.0 (-6.3 to -1.4)*	1.4 (-1.0 to 4.1)
Other musculoskeletal disorders	18,790.4 (14,977.2 to 23,541.4)	-10.3 (-15.2 to -5.3)*	-5.3 (-10.4 to 0.0)	1,822.8 (1,141.2 to 2,613.0)	-10.2 (-15.1 to -5.2)*	-5.2 (-10.3 to 0.2)
Other non-communicable diseases	437,920.0 (430,766.0 to 445,373.7)	-4.2 (-4.8 to -3.5)*	1.2 (0.6 to 1.9)*	8,349.1 (5,449.2 to 12,350.0)	-3.2 (-4.3 to -1.6)*	2.3 (1.1 to 3.9)*
Congenital anomalies	7,927.9 (7,233.2 to 8,504.9)	0.1 (-3.3 to 3.6)	5.7 (2.1 to 9.4)*	735.3 (457.8 to 1,103.9)	8.5 (1.7 to 16.7)*	14.6 (7.4 to 23.2)*
Neural tube defects	142.6 (96.9 to 206.6)	1.8 (-1.5 to 4.6)	7.5 (4.0 to 10.5)*	49.9 (28.4 to 82.8)	2.3 (-4.4 to 8.5)	8.0 (1.0 to 14.6)*
Congenital heart anomalies	3,812.6 (2,673.3 to 5,714.0)	0.7 (-2.8 to 4.1)	6.4 (2.7 to 9.9)*	132.8 (51.6 to 246.4)	1.0 (-2.8 to 4.6)	6.7 (2.7 to 10.5)*
Less severe heart anomalies cases aggregate	3,486.6 (2,284.8 to 5,406.1)	0.3 (-3.6 to 4.0)	6.0 (1.8 to 9.9)*	113.2 (42.1 to 218.7)	0.4 (-4.2 to 4.3)	6.0 (1.2 to 10.2)*
Severe congenital heart anomalies	305.4 (201.8 to 457.3)	4.5 (-0.3 to 8.8)	10.4 (5.3 to 14.9)*	18.1 (7.2 to 35.9)	4.5 (-1.3 to 10.2)	10.4 (4.3 to 16.4)*
Critical congenital heart anomalies	16.1 (9.2 to 26.6)	29.7 (15.4 to 41.0)*	37.0 (21.9 to 48.9)*	1.0 (0.3 to 2.0)	29.3 (8.0 to 47.5)*	36.6 (14.1 to 55.8)*
Heart failure due to congenital heart anomalies aggregate	4.5 (4.0 to 5.2)	-15.7 (-16.9 to -14.4)*	-10.9 (-12.2 to -9.6)*	0.6 (0.4 to 0.8)	-15.6 (-17.5 to -13.8)*	-10.9 (-12.9 to -8.9)*
Cleft lip and cleft palate	555.4 (321.0 to 935.6)	-4.6 (-14.2 to 1.6)	0.8 (-9.3 to 7.3)	6.9 (3.4 to 12.7)	-8.7 (-19.1 to -0.3)*	-3.5 (-14.5 to 5.3)
Down syndrome	494.8 (312.4 to 761.5)	3.7 (0.2 to 6.8)*	9.5 (5.9 to 12.8)*	41.5 (22.9 to 69.5)	3.6 (-1.3 to 7.7)	9.5 (4.3 to 13.7)*
Turner syndrome	31.9 (15.0 to 58.3)	-6.3 (-8.5 to -4.6)*	-1.0 (-3.4 to 0.8)	0.7 (0.2 to 1.5)	-6.3 (-11.1 to -1.3)*	-1.0 (-6.1 to 4.2)
Klinefelter syndrome	18.7 (9.8 to 33.5)	-2.9 (-4.5 to -1.8)*	2.5 (0.9 to 3.8)*	0.1 (0.0 to 0.1)	-2.9 (-4.5 to -1.8)*	2.6 (0.9 to 3.8)*
Other chromosomal abnormalities	420.0 (232.3 to 745.9)	1.4 (-1.6 to 3.8)	7.1 (3.9 to 9.7)*	35.3 (17.7 to 68.0)	1.3 (-3.1 to 5.3)	7.0 (2.4 to 11.2)*
Other congenital anomalies	2,764.4 (1,521.1 to 5,086.2)	-1.6 (-3.5 to 0.4)	4.0 (1.9 to 6.1)*	468.2 (279.1 to 746.9)	13.1 (2.0 to 27.3)*	19.4 (7.7 to 34.5)*
Skin and subcutaneous diseases	283,425.8 (275,994.0 to 290,216.1)	-5.2 (-6.0 to -4.4)*	0.2 (-0.7 to 1.0)	5,226.8 (3,098.0 to 8,343.1)	-5.1 (-6.1 to -4.1)*	0.2 (-0.8 to 1.3)
Dermatitis	20,397.6 (16,833.8 to 24,920.4)	-3.3 (-4.2 to -2.2)*	2.2 (1.2 to 3.3)*	770.6 (498.0 to 1,084.4)	-0.5 (-2.2 to 1.3)	5.1 (3.4 to 7.0)*
Eczema cases aggregate	7,717.2 (6,085.4 to 9,881.2)	3.3 (2.5 to 4.2)*	9.1 (8.3 to 10.0)*	475.5 (305.0 to 700.1)	3.4 (1.5 to 5.5)*	9.2 (7.2 to 11.4)*

Cause	Prevalence in thousands (95% confidence interval)			YLDs in thousands (95% confidence interval)		
	2015	Percent change in counts between 2005 and 2015	Percent change in rates between 2005 and 2015	2015	Percent change in counts between 2005 and 2015	Percent change in rates between 2005 and 2015
Contact dermatitis cases aggregate	8,331.8 (5,679.0 to 12,118.8)	-5.4 (-6.0 to -4.8)*	-0.1 (-0.7 to 0.5)	229.4 (123.4 to 387.9)	-5.3 (-6.9 to -3.7)*	0.0 (-1.7 to 1.7)
Seborrhoeic dermatitis cases aggregate	4,348.6 (3,058.9 to 5,987.8)	-9.5 (-10.6 to -8.4)*	-4.4 (-5.6 to -3.3)*	65.7 (33.9 to 112.7)	-9.4 (-11.3 to -7.8)*	-4.3 (-6.4 to -2.6)*
Psoriasis	4,634.7 (4,212.4 to 5,100.9)	-2.1 (-3.3 to -0.9)*	3.4 (2.2 to 4.7)*	385.8 (266.3 to 527.0)	-2.0 (-4.4 to 0.5)	3.5 (1.0 to 6.2)*
Cellulitis	60.7 (45.9 to 78.6)	6.9 (2.6 to 11.5)*	13.0 (8.4 to 17.8)*	4.5 (2.7 to 6.9)	6.9 (-0.9 to 15.1)	12.9 (4.7 to 21.6)*
Pyoderma	463.9 (400.8 to 532.6)	-4.0 (-6.0 to -1.6)*	1.4 (-0.7 to 3.9)	2.6 (1.0 to 5.6)	-3.9 (-7.9 to 0.3)	1.5 (-2.7 to 6.0)
Scabies	23,244.1 (15,666.6 to 34,046.4)	-12.3 (-15.7 to -8.9)*	-7.3 (-11.0 to -3.7)*	601.7 (300.6 to 1,048.7)	-12.3 (-15.9 to -8.8)*	-7.3 (-11.2 to -3.6)*
Fungal skin diseases	39,053.4 (30,750.3 to 49,710.0)	-3.8 (-4.4 to -3.1)*	1.7 (1.0 to 2.4)*	221.9 (85.6 to 461.1)	-3.7 (-4.4 to -2.9)*	1.7 (1.0 to 2.6)*
Viral skin diseases	18,875.4 (16,207.3 to 22,344.3)	-6.9 (-8.1 to -5.6)*	-1.6 (-2.9 to -0.3)*	587.1 (364.2 to 882.2)	-6.8 (-8.2 to -5.4)*	-1.6 (-3.1 to -0.1)*
Molluscum contagiosum cases aggregate	2,764.8 (2,143.6 to 3,639.0)	-4.8 (-5.9 to -3.4)*	0.6 (-0.6 to 2.0)	86.1 (50.9 to 134.6)	-4.7 (-7.0 to -2.4)*	0.7 (-1.8 to 3.1)
Viral warts cases aggregate	16,110.6 (13,539.2 to 19,614.7)	-7.2 (-8.7 to -5.8)*	-2.0 (-3.5 to -0.5)*	501.1 (306.8 to 766.6)	-7.2 (-8.8 to -5.5)*	-2.0 (-3.7 to -0.2)*
Acne vulgaris	197,915.2 (177,591.4 to 219,630.8)	-4.9 (-6.5 to -3.3)*	0.5 (-1.2 to 2.2)	2,151.4 (1,025.8 to 3,990.6)	-4.8 (-6.4 to -3.2)*	0.5 (-1.1 to 2.3)
Alopecia areata	1,158.1 (996.3 to 1,334.1)	-5.5 (-6.1 to -4.9)*	-0.2 (-0.8 to 0.4)	39.6 (24.3 to 60.3)	-5.5 (-8.6 to -2.8)*	-0.2 (-3.4 to 2.6)
Pruritus	3,414.0 (2,426.6 to 4,774.1)	-5.8 (-8.2 to -3.0)*	-0.5 (-3.0 to 2.4)	37.1 (16.4 to 77.0)	-5.8 (-8.4 to -2.7)*	-0.5 (-3.2 to 2.8)
Urticaria	4,199.2 (2,792.5 to 6,168.9)	-6.1 (-6.7 to -5.4)*	-0.8 (-1.5 to -0.1)*	257.4 (141.5 to 432.5)	-6.0 (-7.7 to -4.2)*	-0.7 (-2.5 to 1.1)
Decubitus ulcer	21.8 (15.3 to 29.8)	4.1 (-1.0 to 8.6)	9.9 (4.6 to 14.7)*	3.6 (2.2 to 5.5)	4.1 (-1.1 to 8.7)	9.9 (4.5 to 14.8)*
Other skin and subcutaneous diseases	27,240.6 (24,636.7 to 29,959.2)	-3.2 (-5.1 to -1.4)*	2.2 (0.3 to 4.1)*	163.6 (76.8 to 297.3)	-3.1 (-5.0 to -1.2)*	2.3 (0.3 to 4.3)*
Sense organ diseases	50,207.1 (48,261.5 to 52,155.6)	-3.6 (-4.8 to -2.4)*	1.9 (0.6 to 3.1)*	1,904.1 (1,224.0 to 2,753.9)	-1.5 (-3.5 to 0.8)	4.1 (1.9 to 6.5)*
Glaucoma	0.0 (0.0 to 0.0)	0.0 (0.0 to 0.0)	0.0 (0.0 to 0.0)	0.0 (0.0 to 0.0)	0.0 (0.0 to 0.0)	0.0 (0.0 to 0.0)
Cataract	0.0 (0.0 to 0.0)	0.0 (0.0 to 0.0)	0.0 (0.0 to 0.0)	0.0 (0.0 to 0.0)	0.0 (0.0 to 0.0)	0.0 (0.0 to 0.0)
Macular degeneration	0.0 (0.0 to 0.0)	0.0 (0.0 to 0.0)	0.0 (0.0 to 0.0)	0.0 (0.0 to 0.0)	0.0 (0.0 to 0.0)	0.0 (0.0 to 0.0)
Refraction and accommodation disorders	25,907.6 (23,524.4 to 28,399.2)	-6.1 (-7.3 to -4.7)*	-0.8 (-2.1 to 0.7)	539.2 (345.3 to 830.6)	-3.7 (-5.0 to -2.2)*	1.7 (0.3 to 3.3)*
Age-related and other hearing loss	17,195.0 (12,419.4 to 22,319.8)	1.2 (-0.9 to 3.3)	6.9 (4.7 to 9.1)*	1,123.3 (640.4 to 1,752.1)	0.3 (-3.0 to 3.9)	6.0 (2.5 to 9.7)*
Other vision loss	1,165.9 (799.3 to 1,655.4)	-4.9 (-6.9 to -3.0)*	0.5 (-1.7 to 2.5)	50.0 (28.1 to 79.6)	-4.2 (-7.9 to -0.4)*	1.2 (-2.8 to 5.2)
Other sense organ diseases	7,367.1 (6,418.4 to 8,313.6)	-4.9 (-6.9 to -2.7)*	0.4 (-1.7 to 2.8)	191.6 (115.1 to 286.2)	-4.8 (-7.5 to -2.2)*	0.6 (-2.3 to 3.3)
Acute other sense organ diseases	87.8 (77.7 to 98.3)	-4.9 (-6.7 to -3.0)*	0.5 (-1.5 to 2.5)	2.4 (1.4 to 3.5)	-4.9 (-6.8 to -3.0)*	0.5 (-1.5 to 2.5)
Chronic other sense organ diseases	7,279.3 (6,330.5 to 8,228.0)	-4.9 (-6.9 to -2.6)*	0.4 (-1.7 to 2.8)	189.3 (113.7 to 282.5)	-4.8 (-7.5 to -2.1)*	0.6 (-2.3 to 3.4)
Oral disorders	267,425.5 (251,959.9 to 282,910.4)	-1.3 (-3.4 to 0.9)	4.2 (2.0 to 6.6)*	482.8 (268.5 to 787.7)	-3.8 (-4.8 to -2.9)*	1.6 (0.6 to 2.6)*
Deciduous caries	0.0 (0.0 to 0.0)	0.0 (0.0 to 0.0)	0.0 (0.0 to 0.0)	0.0 (0.0 to 0.0)	0.0 (0.0 to 0.0)	0.0 (0.0 to 0.0)
Permanent caries	269,074.4 (217,556.4 to 322,676.3)	-1.1 (-2.5 to 0.3)	4.5 (2.9 to 6.0)*	177.7 (75.7 to 349.2)	-1.9 (-3.4 to -0.4)*	3.6 (2.1 to 5.2)*
Periodontal diseases	5,062.5 (3,673.8 to 7,010.9)	-1.4 (-2.7 to 0.1)	4.2 (2.7 to 5.8)*	34.0 (12.6 to 73.8)	-1.3 (-3.1 to 0.5)	4.3 (2.4 to 6.2)*
Edentulism and severe tooth loss	0.0 (0.0 to 0.0)	0.0 (0.0 to 0.0)	0.0 (0.0 to 0.0)	0.0 (0.0 to 0.0)	0.0 (0.0 to 0.0)	0.0 (0.0 to 0.0)
Other oral disorders	9,096.3 (7,993.7 to 10,062.3)	-5.5 (-5.9 to -5.0)*	-0.1 (-0.6 to 0.4)	271.1 (161.6 to 409.7)	-5.4 (-6.5 to -4.3)*	-0.1 (-1.2 to 1.1)
Injuries	41,412.1 (38,728.0 to 44,466.9)	-7.7 (-10.9 to -4.3)*	-2.5 (-5.9 to 1.1)	1,790.9 (1,285.3 to 2,444.7)	-10.7 (-14.7 to -6.7)*	-5.7 (-9.9 to -1.4)*
Transport injuries	2,432.0 (2,226.5 to 2,646.1)	-12.1 (-13.4 to -10.7)*	-7.2 (-8.6 to -5.7)*	161.3 (111.7 to 218.3)	-18.7 (-21.2 to -16.3)*	-14.1 (-16.8 to -11.6)*
Road injuries	2,178.3 (1,980.0 to 2,391.8)	-12.4 (-13.8 to -10.9)*	-7.5 (-9.0 to -5.9)*	143.2 (98.2 to 194.8)	-18.3 (-20.9 to -15.7)*	-13.7 (-16.5 to -11.0)*
Pedestrian road injuries	266.3 (219.9 to 317.3)	-18.5 (-20.1 to -16.9)*	-14.0 (-15.6 to -12.2)*	16.8 (11.4 to 23.7)	-26.8 (-29.9 to -23.6)*	-22.7 (-26.0 to -19.3)*
Cyclist road injuries	534.2 (443.7 to 631.2)	-18.1 (-19.7 to -16.4)*	-13.5 (-15.2 to -11.7)*	30.7 (20.5 to 43.4)	-24.8 (-28.0 to -21.6)*	-20.5 (-23.9 to -17.1)*
Motorcyclist road injuries	471.1 (397.0 to 562.1)	-3.0 (-4.9 to -1.0)*	2.5 (0.4 to 4.6)*	30.2 (19.6 to 42.9)	-11.2 (-14.9 to -7.6)*	-6.2 (-10.1 to -2.4)*
Motor vehicle road injuries	783.3 (660.1 to 919.2)	-13.0 (-15.2 to -10.9)*	-8.1 (-10.4 to -5.9)*	57.4 (38.3 to 80.0)	-17.0 (-20.2 to -13.8)*	-12.3 (-15.7 to -8.9)*
Other road injuries	123.4 (95.9 to 154.1)	2.1 (-0.3 to 4.6)	7.8 (5.3 to 10.5)*	8.1 (5.1 to 11.5)	-2.5 (-6.1 to 1.1)	2.9 (-0.8 to 6.8)
Other transport injuries	253.7 (214.6 to 301.2)	-9.7 (-10.9 to -8.6)*	-4.7 (-5.8 to -3.5)*	18.1 (12.7 to 25.3)	-21.7 (-24.5 to -18.8)*	-17.3 (-20.2 to -14.2)*
Unintentional injuries	33,828.1 (31,649.1 to 36,054.1)	-6.0 (-6.6 to -5.4)*	-0.7 (-1.3 to -0.1)*	1,403.7 (1,001.8 to 1,909.8)	-10.8 (-12.7 to -9.0)*	-5.8 (-7.8 to -3.9)*
Falls	8,037.4 (6,892.1 to 9,242.8)	0.1 (-0.8 to 1.1)	5.8 (4.7 to 6.8)*	472.4 (324.5 to 651.3)	-8.7 (-11.8 to -5.5)*	-3.5 (-6.8 to -0.2)*
Drowning	137.8 (112.4 to 170.2)	-22.4 (-23.8 to -20.8)*	-18.1 (-19.5 to -16.4)*	8.8 (6.0 to 12.4)	-30.2 (-32.8 to -27.4)*	-26.3 (-29.0 to -23.4)*
Fire, heat, and hot substances	2,597.6 (2,079.0 to 3,210.0)	-8.1 (-9.1 to -7.1)*	-2.9 (-3.9 to -1.9)*	105.4 (73.0 to 147.7)	-13.9 (-17.1 to -11.0)*	-9.1 (-12.4 to -6.0)*
Poisonings	406.0 (322.8 to 509.0)	-1.7 (-3.6 to 0.1)	3.8 (1.8 to 5.7)*	46.6 (30.6 to 68.9)	-3.5 (-6.4 to -0.7)*	1.9 (-1.1 to 4.9)
Exposure to mechanical forces	9,975.8 (8,765.7 to 11,232.1)	-5.1 (-6.1 to -4.0)*	0.2 (-0.8 to 1.4)	196.1 (136.7 to 272.8)	-10.5 (-12.8 to -8.3)*	-5.4 (-7.9 to -3.2)*
Unintentional firearm injuries	74.5 (62.3 to 89.5)	-9.8 (-11.9 to -7.7)*	-4.7 (-6.9 to -2.5)*	4.0 (2.7 to 5.5)	-15.0 (-18.7 to -11.2)*	-10.3 (-14.2 to -6.2)*
Unintentional suffocation	419.7 (290.1 to 595.5)	-20.9 (-24.2 to -17.4)*	-16.5 (-19.9 to -12.8)*	13.7 (9.0 to 19.5)	-18.0 (-20.4 to -15.7)*	-13.4 (-15.9 to -10.9)*
Other exposure to mechanical forces	9,481.6 (8,331.6 to 10,796.3)	-4.2 (-5.2 to -3.3)*	1.2 (0.2 to 2.2)*	178.5 (123.6 to 249.2)	-9.7 (-12.3 to -7.4)*	-4.7 (-7.3 to -2.2)*
Adverse effects of medical treatment	408.3 (307.1 to 531.5)	-5.0 (-8.6 to -1.7)*	0.3 (-3.5 to 3.8)	54.4 (33.2 to 81.4)	-5.0 (-8.6 to -1.7)*	0.3 (-3.5 to 3.8)
Animal contact	2,201.9 (1,846.6 to 2,588.0)	-4.2 (-6.6 to -1.7)*	1.2 (-1.4 to 3.8)	95.0 (63.1 to 130.3)	-5.2 (-8.1 to -2.4)*	0.1 (-2.9 to 3.1)
Venomous animal contact	908.4 (759.3 to 1,070.0)	-2.2 (-4.2 to -0.2)*	3.3 (1.2 to 5.4)*	74.9 (49.5 to 104.3)	-4.6 (-7.6 to -1.6)*	0.8 (-2.4 to 3.9)
Non-venomous animal contact	1,293.4 (1,024.1 to 1,603.1)	-5.5 (-8.7 to -2.5)*	-0.1 (-3.5 to 3.0)	20.0 (12.8 to 31.3)	-7.6 (-11.9 to -3.5)*	-2.4 (-6.9 to 1.9)
Foreign body	1,751.4 (1,477.7 to 2,081.7)	-3.0 (-5.3 to -1.4)*	2.4 (0.1 to 4.1)*	77.6 (53.9 to 108.9)	-11.0 (-13.8 to -8.2)*	-6.0 (-8.9 to -3.0)*
Pulmonary aspiration and foreign body in airway	762.6 (557.6 to 1,054.7)	-8.0 (-11.1 to -5.2)*	-2.9 (-6.1 to 0.1)	44.6 (29.5 to 65.0)	-16.0 (-18.9 to -12.9)*	-11.3 (-14.4 to -8.0)*
Foreign body in eyes	61.9 (23.6 to 117.5)	-0.6 (-2.1 to 0.5)	5.0 (3.4 to 6.1)*	3.5 (1.3 to 7.3)	-1.2 (-3.1 to 0.0)	4.4 (2.3 to 5.7)*
Foreign body in other body part	926.9 (759.4 to 1,147.1)	1.3 (-0.0 to 2.6)	7.0 (5.6 to 8.4)*	29.5 (20.4 to 42.0)	-3.5 (-5.9 to -1.2)*	1.9 (-0.6 to 4.3)
Environmental heat and cold exposure	2,399.9 (2,055.3 to 2,746.2)	-2.6 (-3.6 to -1.5)*	2.9 (1.8 to 4.0)*	123.6 (85.4 to 168.1)	-7.5 (-9.6 to -5.4)*	-2.3 (-4.5 to -0.1)*
Other unintentional injuries	5,912.0 (5,062.3 to 6,785.5)	-15.9 (-16.9 to -15.0)*	-11.2 (-12.2 to -10.2)*	223.8 (148.9 to 320.0)	-19.0 (-20.8 to -17.1)*	-14.4 (-16.4 to -12.5)*
Self-harm and interpersonal violence	631.4 (563.6 to 705.9)	-12.8 (-14.3 to -11.3)*	-7.9 (-9.5 to -6.4)*	41.9 (28.6 to 57.5)	-18.4 (-20.7 to -16.0)*	-13.8 (-16.3 to -11.2)*
Self-harm	159.2 (131.9 to 189.4)	-9.6 (-10.5 to -8.7)*	-4.5 (-5.5 to -3.5)*	14.2 (9.6 to 19.9)	-15.0 (-17.2 to -12.8)*	-10.2 (-12.5 to -7.9)*
Interpersonal violence	472.3 (414.3 to 537.2)	-13.9 (-15.6 to -12.0)*	-9.0 (-10.9 to -7.0)*	27.7 (18.9 to 38.3)	-20.1 (-23.0 to -16.9)*	-15.6 (-18.6 to -12.2)*

Cause	Prevalence in thousands (95% confidence interval)			YLDs in thousands (95% confidence interval)		
	2015	Percent change in counts between 2005 and 2015	Percent change in rates between 2005 and 2015	2015	Percent change in counts between 2005 and 2015	Percent change in rates between 2005 and 2015
Assault by firearm	16.0 (13.7 to 18.5)	-9.4 (-11.8 to -7.1)*	-4.3 (-6.9 to -1.9)*	1.2 (0.8 to 1.6)	-14.2 (-18.6 to -9.8)*	-9.4 (-14.1 to -4.7)*
Assault by sharp object	95.1 (78.8 to 113.0)	-22.5 (-24.7 to -20.7)*	-18.2 (-20.4 to -16.3)*	5.4 (3.5 to 7.6)	-29.5 (-33.5 to -25.5)*	-25.5 (-29.7 to -21.3)*
Assault by other means	361.2 (309.3 to 419.2)	-11.4 (-13.4 to -9.4)*	-6.5 (-8.5 to -4.3)*	21.1 (14.2 to 29.2)	-17.6 (-20.5 to -14.5)*	-12.9 (-16.0 to -9.7)*
Forces of nature, war, and legal intervention	4,520.6 (3,151.7 to 6,381.8)	-16.1 (-38.3 to 15.0)	-11.3 (-34.8 to 21.4)	184.1 (108.1 to 284.6)	1.1 (-33.9 to 53.0)	6.8 (-30.1 to 61.6)
Exposure to forces of nature	1,746.0 (1,016.8 to 2,758.2)	-10.3 (-39.8 to 31.0)	-5.3 (-36.4 to 38.4)	55.7 (33.8 to 83.7)	-45.2 (-65.8 to -14.1)*	-42.1 (-63.9 to -9.3)*
Collective violence and legal intervention	2,774.6 (1,800.5 to 4,118.4)	-19.3 (-46.8 to 27.3)	-14.8 (-43.8 to 34.5)	128.4 (62.4 to 213.6)	59.6 (-11.2 to 170.7)	68.5 (-6.2 to 186.0)
**Anemia**	**185,827.4 (183,668.9 to 188,003.5)**	**-12.7 (-13.9 to -11.5)***	**-7.8 (-9.0 to -6.5)***	**5,012.4 (3,315.2 to 7,284.5)**	**-16.9 (-19.0 to -15.0)***	**-12.2 (-14.5 to -10.2)***
**Developmental intellectual disability**	**16,350.1 (12,167.2 to 20,385.7)**	**-2.5 (-2.9 to -2.1)***	**3.0 (2.5 to 3.4)***	**1,767.9 (1,259.0 to 2,340.8)**	**-1.1 (-2.7 to 0.8)**	**4.4 (2.8 to 6.5)***
**Epilepsy**	**3,728.2 (3,133.4 to 4,302.9)**	**0.4 (-3.4 to 4.2)**	**6.0 (2.0 to 10.1)***	**1,196.5 (849.0 to 1,576.2)**	**-6.9 (-12.0 to -2.5)***	**-1.7 (-7.0 to 3.0)**
**Guillain-Barré syndrome**	**1.9 (1.1 to 3.1)**	**-3.3 (-4.6 to -2.1)***	**2.2 (0.7 to 3.4)***	**0.6 (0.3 to 1.0)**	**-3.3 (-4.6 to -2.1)***	**2.2 (0.7 to 3.4)***
**Hearing loss**	**28,157.2 (23,004.4 to 33,302.5)**	**-1.9 (-3.1 to -0.6)***	**3.6 (2.4 to 5.0)***	**1,636.7 (1,049.7 to 2,377.8)**	**-2.0 (-4.5 to 0.5)**	**3.5 (0.8 to 6.1)***
**Heart failure**	**101.5 (92.9 to 110.5)**	**-3.1 (-3.8 to -2.4)***	**2.4 (1.7 to 3.1)***	**12.6 (8.7 to 17.7)**	**-3.1 (-6.4 to 0.4)**	**2.3 (-1.1 to 6.0)**
**Infertility**	**3,459.3 (2,196.0 to 4,940.5)**	**10.7 (1.6 to 20.1)***	**16.9 (7.3 to 26.8)***	**24.5 (9.6 to 52.8)**	**8.3 (-0.2 to 16.8)**	**14.4 (5.4 to 23.4)***
**Pelvic inflammatory disease**	**25.8 (17.9 to 34.8)**	**-15.2 (-16.7 to -13.6)***	**-10.4 (-12.0 to -8.7)***	**3.5 (2.1 to 5.2)**	**-15.2 (-18.5 to -11.9)***	**-10.4 (-13.9 to -6.9)***
**Vision loss**	**28,392.8 (25,761.0 to 31,208.9)**	**-5.8 (-6.9 to -4.5)***	**-0.5 (-1.7 to 0.9)**	**846.0 (567.5 to 1,206.5)**	**-2.5 (-4.0 to -1.0)***	**2.9 (1.4 to 4.6)***

SR Table 11. Prevalent cases and YLDs for 2015, percent change, and change in age-standardised rates between 2005 and 2015 by cause, global, ages 20-24 years						
Cause	Prevalence in thousands (95% confidence interval)			YLDs in thousands (95% confidence interval)		
	2015	Percent change in counts between 2005 and 2015	Percent change in rates between 2005 and 2015	2015	Percent change in counts between 2005 and 2015	Percent change in rates between 2005 and 2015
**All causes**	--	--	--	**51,741.4 (38,402.6 to 66,679.1)**	**5.3 (4.5 to 6.1)***	**-2.9 (-3.7 to -2.1)***
Communicable, maternal, neonatal, and nutritional diseases	351,715.1 (348,333.1 to 355,360.4)	3.0 (2.1 to 3.8)*	-5.0 (-5.8 to -4.3)*	8,063.9 (5,777.9 to 11,284.8)	-4.6 (-7.1 to -1.5)*	-12.0 (-14.4 to -9.1)*
HIV/AIDS and tuberculosis	3,605.2 (3,470.4 to 3,758.4)	-3.0 (-7.3 to 1.5)	-10.6 (-14.5 to -6.4)*	547.7 (370.8 to 764.4)	-2.2 (-7.3 to 2.9)	-9.8 (-14.5 to -5.1)*
Tuberculosis	991.1 (742.6 to 1,322.1)	1.8 (-6.2 to 9.1)	-6.1 (-13.5 to 0.6)	312.6 (194.0 to 470.4)	2.1 (-6.2 to 9.7)	-5.8 (-13.5 to 1.2)
HIV/AIDS	2,641.3 (2,522.5 to 2,771.0)	-4.6 (-10.1 to 1.0)	-12.0 (-17.1 to -6.9)*	235.1 (167.8 to 310.0)	-7.4 (-12.5 to -2.2)*	-14.6 (-19.3 to -9.8)*
HIV/AIDS - Tuberculosis	94.5 (85.3 to 104.0)	-37.3 (-39.4 to -34.9)*	-42.1 (-44.2 to -40.0)*	36.3 (24.7 to 49.9)	-36.8 (-40.6 to -32.7)*	-41.7 (-45.2 to -38.0)*
HIV/AIDS resulting in other diseases	2,615.9 (2,462.8 to 2,783.5)	-2.6 (-8.4 to 2.8)	-10.2 (-15.5 to -5.2)*	198.7 (142.3 to 260.7)	1.2 (-5.0 to 7.5)	-6.7 (-12.4 to -0.9)*
HIV aggregate	2,336.2 (2,195.8 to 2,497.7)	-12.9 (-18.1 to -7.6)*	-19.7 (-24.5 to -14.8)*	169.1 (120.5 to 222.5)	-13.4 (-18.6 to -8.0)*	-20.2 (-24.9 to -15.1)*
AIDS aggregate	279.7 (247.6 to 310.7)	9,635.0 (4,394.1 to 15,694.6)*	8,877.6 (4,044.5 to 14,465.7)*	29.6 (21.0 to 39.8)	2,555.6 (985.0 to 4,986.5)*	2,349.0 (900.6 to 4,590.7)*
Diarrhea, lower respiratory, and other common infectious diseases	31,963.7 (29,227.1 to 34,091.8)	4.4 (1.9 to 6.9)*	-3.7 (-6.0 to -1.4)*	1,016.8 (700.9 to 1,387.1)	4.8 (2.9 to 6.6)*	-3.4 (-5.1 to -1.7)*
Diarrheal diseases	1,906.7 (1,779.8 to 2,032.3)	8.5 (7.2 to 9.9)*	0.1 (-1.1 to 1.3)	305.9 (209.7 to 417.2)	8.9 (6.5 to 11.2)*	0.4 (-1.8 to 2.6)
Diarrhoea episodes aggregate	1,906.5 (1,779.5 to 2,032.0)	8.5 (7.2 to 9.9)*	0.1 (-1.1 to 1.3)	305.9 (209.7 to 417.1)	8.9 (6.5 to 11.2)*	0.4 (-1.8 to 2.6)
Guillain-Barré syndrome due to diarrheal diseases	0.2 (0.1 to 0.4)	9.5 (8.0 to 10.6)*	1.0 (-0.4 to 2.0)	0.1 (0.0 to 0.1)	9.5 (8.0 to 10.6)*	1.0 (-0.4 to 2.0)
Intestinal infectious diseases	106.5 (95.1 to 117.7)	-19.9 (-27.2 to -11.8)*	-26.1 (-32.9 to -18.7)*	14.6 (8.8 to 23.0)	-15.3 (-26.9 to -1.7)*	-21.8 (-32.6 to -9.3)*
Typhoid fever	95.9 (66.9 to 131.6)	-14.5 (-19.3 to -9.6)*	-21.2 (-25.6 to -16.6)*	12.6 (7.4 to 20.0)	-14.2 (-27.0 to 0.9)	-20.8 (-32.7 to -7.0)*
Typhoid fever episodes aggregate	79.4 (54.7 to 109.5)	-14.4 (-22.1 to -5.8)*	-21.1 (-28.2 to -13.1)*	7.5 (4.5 to 11.8)	-14.3 (-27.4 to 0.3)	-21.0 (-33.0 to -7.5)*
Complications of typhoid fever aggregate	16.4 (10.5 to 24.3)	-15.1 (-43.1 to 27.7)	-21.7 (-47.6 to 17.7)	5.1 (2.6 to 8.6)	-13.9 (-45.4 to 36.8)	-20.6 (-49.6 to 26.2)
Paratyphoid fever	36.1 (23.3 to 54.3)	-15.6 (-23.0 to -7.9)*	-22.2 (-29.0 to -15.0)*	1.9 (1.0 to 3.2)	-15.4 (-31.4 to 5.5)	-22.0 (-36.7 to -2.7)*
Paratyphoid fever episodes aggregate	34.2 (22.0 to 51.6)	-15.6 (-23.0 to -7.4)*	-22.2 (-29.0 to -14.6)*	1.7 (0.9 to 2.9)	-15.4 (-33.1 to 7.3)	-22.0 (-38.3 to -1.1)*
Intestinal perforation due to paratyphoid	1.8 (0.9 to 3.1)	-15.7 (-52.4 to 44.4)	-22.2 (-56.1 to 33.2)	0.2 (0.1 to 0.4)	-15.7 (-52.4 to 44.4)	-22.3 (-56.1 to 33.2)
Other intestinal infectious diseases	--	--	--	0.1 (0.0 to 0.3)	-63.8 (-77.8 to -40.8)*	-66.6 (-79.5 to -45.4)*
Lower respiratory infections	285.7 (256.3 to 316.6)	8.7 (6.1 to 11.1)*	0.2 (-2.1 to 2.4)	17.5 (11.6 to 25.5)	8.9 (3.5 to 14.7)*	0.4 (-4.5 to 5.8)
Lower respiratory infection episodes aggregate	285.4 (256.0 to 316.3)	8.7 (6.1 to 11.1)*	0.2 (-2.1 to 2.4)	17.4 (11.5 to 25.4)	8.9 (3.5 to 14.7)*	0.4 (-4.5 to 5.8)
Guillain-Barré syndrome due to lower respiratory infections	0.2 (0.1 to 0.5)	9.5 (8.0 to 10.6)*	1.0 (-0.4 to 2.0)	0.1 (0.0 to 0.2)	9.5 (8.0 to 10.6)*	1.0 (-0.4 to 2.0)
Upper respiratory infections	19,535.6 (13,641.8 to 26,601.4)	6.9 (5.9 to 8.0)*	-1.4 (-2.4 to -0.4)*	230.5 (118.3 to 398.7)	7.0 (5.7 to 8.3)*	-1.3 (-2.6 to -0.1)*
Upper respiratory infection episodes aggregate	19,535.0 (13,641.3 to 26,600.7)	6.9 (5.9 to 8.0)*	-1.4 (-2.4 to -0.4)*	230.3 (118.2 to 398.4)	7.0 (5.7 to 8.3)*	-1.3 (-2.6 to -0.1)*
Guillain-Barré syndrome due to upper respiratory infections	0.7 (0.3 to 1.2)	9.5 (8.0 to 10.6)*	1.0 (-0.4 to 2.0)	0.2 (0.1 to 0.4)	9.5 (8.0 to 10.6)*	1.0 (-0.4 to 2.0)
Otitis media	9,185.0 (6,520.6 to 11,998.1)	-2.0 (-5.5 to 1.1)	-9.6 (-12.9 to -6.7)*	255.3 (142.4 to 393.8)	-2.4 (-7.7 to 2.7)	-10.0 (-14.9 to -5.3)*
Acute otitis media	990.8 (741.4 to 1,325.1)	9.7 (5.6 to 14.2)*	1.2 (-2.6 to 5.4)	13.0 (6.4 to 24.2)	9.9 (4.8 to 15.0)*	1.4 (-3.3 to 6.0)
Chronic otitis media aggregate	8,194.2 (5,628.3 to 11,049.9)	-3.2 (-7.0 to 0.2)	-10.7 (-14.3 to -7.6)*	242.3 (134.5 to 374.4)	-3.0 (-8.5 to 2.3)	-10.5 (-15.6 to -5.6)*
Meningitis	804.3 (763.9 to 844.6)	12.3 (7.7 to 17.3)*	3.5 (-0.7 to 8.2)	140.4 (98.3 to 183.1)	10.1 (6.8 to 13.6)*	1.6 (-1.5 to 4.8)
Pneumococcal meningitis	666.7 (556.7 to 795.6)	16.2 (13.4 to 19.7)*	7.2 (4.6 to 10.3)*	68.7 (47.9 to 90.5)	11.9 (6.5 to 17.4)*	3.2 (-1.8 to 8.3)
Acute pneumococcal meningitis	2.0 (1.3 to 2.8)	15.6 (-0.1 to 29.8)	6.6 (-7.9 to 19.7)	0.3 (0.1 to 0.4)	15.6 (-0.1 to 29.8)	6.6 (-7.9 to 19.7)
Complications of pneumococcal meningitis aggregate	664.7 (555.0 to 793.7)	16.2 (13.4 to 19.6)*	7.2 (4.6 to 10.3)*	68.4 (47.8 to 90.2)	11.9 (6.5 to 17.4)*	3.2 (-1.8 to 8.3)
H influenzae type B meningitis	237.8 (190.0 to 295.3)	-11.0 (-14.9 to -6.5)*	-17.9 (-21.5 to -13.7)*	30.0 (20.4 to 40.8)	4.3 (-2.4 to 11.9)	-3.8 (-10.0 to 3.2)
Acute H influenzae type B meningitis	0.6 (0.4 to 1.0)	-21.2 (-31.6 to -10.8)*	-27.3 (-36.9 to -17.8)*	0.1 (0.0 to 0.1)	-21.2 (-31.6 to -10.8)*	-27.4 (-36.9 to -17.8)*
Complications of of H influenzae type B meningitis aggregate	237.2 (189.4 to 294.7)	-11.0 (-14.8 to -6.4)*	-17.9 (-21.5 to -13.7)*	29.9 (20.3 to 40.7)	4.4 (-2.3 to 12.0)	-3.7 (-9.9 to 3.3)
Meningococcal meningitis	161.5 (121.5 to 212.6)	17.6 (12.8 to 22.3)*	8.5 (4.0 to 12.8)*	14.3 (9.8 to 19.6)	13.8 (8.8 to 19.0)*	4.9 (0.4 to 9.7)*
Acute meningococcal meningitis	1.0 (0.6 to 1.4)	15.1 (-4.5 to 32.5)	6.1 (-11.9 to 22.2)	0.1 (0.1 to 0.2)	15.1 (-4.5 to 32.5)	6.1 (-11.9 to 22.2)
Complications of meningococcal meningitis aggregate	160.6 (120.6 to 211.6)	17.6 (12.8 to 22.4)*	8.5 (4.1 to 12.8)*	14.2 (9.6 to 19.4)	13.8 (8.8 to 19.0)*	4.9 (0.4 to 9.7)*
Other meningitis	251.3 (199.8 to 308.2)	15.0 (12.0 to 18.3)*	6.1 (3.3 to 9.1)*	27.4 (18.5 to 36.6)	10.6 (5.2 to 16.0)*	2.0 (-3.0 to 7.0)
Other meningitis episodes aggregate	7.2 (5.3 to 9.2)	11.7 (-3.1 to 23.7)	3.0 (-10.7 to 14.1)	1.0 (0.6 to 1.4)	11.6 (-3.1 to 23.7)	2.9 (-10.7 to 14.1)
Complications of other meningitis aggregate	244.1 (193.1 to 300.6)	15.1 (12.1 to 18.4)*	6.2 (3.4 to 9.2)*	26.4 (17.8 to 35.2)	10.6 (5.1 to 16.4)*	2.0 (-3.1 to 7.3)
Encephalitis	351.3 (258.8 to 476.9)	-1.1 (-3.5 to 1.8)	-8.8 (-11.0 to -6.1)*	40.5 (27.9 to 54.0)	1.6 (-4.5 to 7.8)	-6.3 (-12.0 to -0.5)*
Acute encephalitis	4.7 (3.8 to 5.7)	3.5 (-2.4 to 9.1)	-4.6 (-10.0 to 0.6)	0.6 (0.4 to 1.0)	3.8 (-4.8 to 12.2)	-4.3 (-12.2 to 3.4)
Complications of encephalitis aggregate	346.5 (254.7 to 472.1)	-1.2 (-3.6 to 1.7)	-8.9 (-11.1 to -6.2)*	39.9 (27.5 to 53.3)	1.6 (-4.6 to 7.8)	-6.3 (-12.0 to -0.5)*
Diphtheria	0.0 (0.0 to 0.0)	-59.2 (-84.3 to 4.5)	-62.4 (-85.5 to -3.6)*	0.0 (0.0 to 0.0)	-59.3 (-84.3 to 4.5)	-62.5 (-85.5 to -3.6)*
Whooping cough	5.7 (4.4 to 7.2)	-25.5 (-27.7 to -23.1)*	-31.3 (-33.3 to -29.1)*	0.3 (0.2 to 0.4)	-25.3 (-32.0 to -18.3)*	-31.1 (-37.3 to -24.7)*
Tetanus	17.9 (17.5 to 18.4)	9.0 (7.2 to 10.2)*	0.5 (-1.2 to 1.6)	0.8 (0.6 to 1.0)	-4.7 (-12.5 to 3.5)	-12.1 (-19.3 to -4.6)*
Severe tetanus	0.4 (0.2 to 0.7)	-40.3 (-53.3 to -20.7)*	-44.9 (-56.9 to -26.8)*	0.0 (0.0 to 0.1)	-40.2 (-53.4 to -20.7)*	-44.9 (-57.1 to -26.8)*
Complications of tetanus aggregate	17.5 (17.2 to 17.9)	10.9 (10.4 to 11.4)*	2.3 (1.8 to 2.8)*	0.7 (0.5 to 1.0)	-0.7 (-8.3 to 7.9)	-8.5 (-15.4 to -0.5)*
Measles	0.6 (0.3 to 1.2)	-66.5 (-70.2 to -61.8)*	-69.1 (-72.5 to -64.8)*	0.1 (0.0 to 0.1)	-66.5 (-70.2 to -61.8)*	-69.1 (-72.5 to -64.8)*
Varicella and herpes zoster	256.9 (198.1 to 327.4)	8.9 (8.3 to 9.5)*	0.5 (-0.1 to 1.0)	10.9 (6.0 to 17.3)	8.6 (3.0 to 14.9)*	0.2 (-5.1 to 5.9)
Neglected tropical diseases and malaria	175,095.1 (170,891.4 to 179,272.0)	-3.8 (-6.1 to -1.0)*	-11.3 (-13.5 to -8.7)*	1,653.3 (1,050.0 to 2,672.9)	-11.5 (-19.8 to 1.5)	-18.4 (-26.1 to -6.4)*
Malaria	24,178.2 (20,566.4 to 29,306.1)	33.2 (23.3 to 45.2)*	22.8 (13.7 to 33.9)*	200.0 (141.9 to 276.9)	17.4 (12.2 to 22.9)*	8.3 (3.5 to 13.4)*
Asymptomatic malaria parasitemia(PfPR)	17,935.4 (14,349.4 to 23,082.1)	39.2 (25.4 to 54.8)*	28.3 (15.6 to 42.8)*	0.0 (0.0 to 0.0)	0.0 (0.0 to 0.0)	0.0 (0.0 to 0.0)
Malaria episodes aggregate	1,287.7 (728.6 to 2,221.3)	-6.4 (-18.1 to 6.2)	-13.7 (-24.5 to -2.0)*	26.6 (12.3 to 49.6)	-6.2 (-18.7 to 6.7)	-13.5 (-25.0 to -1.6)*
Complications of malaria aggregate	116.3 (104.5 to 128.1)	25.6 (23.1 to 28.8)*	15.9 (13.5 to 18.8)*	36.6 (27.6 to 46.3)	22.2 (13.2 to 31.2)*	12.7 (4.4 to 21.0)*
Anemia due to malaria parasitemia(PfPR) aggregate	4,838.8 (4,708.7 to 4,965.1)	27.3 (23.5 to 31.3)*	17.4 (13.9 to 21.1)*	136.8 (91.7 to 198.9)	22.1 (15.9 to 28.8)*	12.6 (6.9 to 18.7)*
Chagas disease	343.4 (288.9 to 400.0)	-17.5 (-20.2 to -14.8)*	-24.0 (-26.4 to -21.4)*	1.7 (1.1 to 2.6)	-17.6 (-25.4 to -9.4)*	-24.0 (-31.2 to -16.5)*
Chagas disease episodes aggregate	310.5 (260.4 to 363.0)	-17.5 (-20.2 to -14.8)*	-23.9 (-26.4 to -21.4)*	0.0 (0.0 to 0.0)	-24.4 (-29.7 to -20.0)*	-30.2 (-35.1 to -26.3)*
Complications of Chagas disease aggregate	29.7 (22.6 to 37.7)	-17.6 (-20.2 to -14.8)*	-24.0 (-26.4 to -21.5)*	1.5 (1.0 to 2.3)	-17.4 (-25.8 to -8.4)*	-23.8 (-31.6 to -15.5)*

Cause	Prevalence in thousands (95% confidence interval)			YLDs in thousands (95% confidence interval)		
	2015	Percent change in counts between 2005 and 2015	Percent change in rates between 2005 and 2015	2015	Percent change in counts between 2005 and 2015	Percent change in rates between 2005 and 2015
Heart failure due to Chagas disease aggregate	3.2 (1.9 to 5.1)	-18.7 (-21.5 to -15.8)*	-25.0 (-27.6 to -22.4)*	0.2 (0.1 to 0.3)	-18.7 (-30.3 to -4.5)*	-25.0 (-35.7 to -11.9)*
Leishmaniasis	357.9 (313.7 to 435.2)	24.1 (0.1 to 54.3)*	14.4 (-7.6 to 42.3)	4.3 (2.1 to 8.0)	22.7 (15.9 to 28.6)*	13.2 (6.8 to 18.6)*
Visceral leishmaniasis	5.2 (4.7 to 5.7)	9.5 (8.0 to 11.0)*	1.0 (-0.4 to 2.4)	0.4 (0.2 to 0.5)	9.9 (-15.6 to 42.3)	1.4 (-22.2 to 31.2)
Cutaneous and mucocutaneous leishmaniasis	359.9 (300.5 to 447.7)	23.7 (19.8 to 27.2)*	14.1 (10.5 to 17.3)*	3.9 (1.8 to 7.6)	24.1 (17.5 to 30.3)*	14.4 (8.4 to 20.1)*
African trypanosomiasis	1.5 (0.8 to 2.4)	-68.6 (-70.2 to -67.1)*	-71.0 (-72.5 to -69.7)*	0.4 (0.1 to 0.8)	-67.5 (-83.5 to -46.0)*	-70.1 (-84.8 to -50.2)*
Schistosomiasis	30,458.6 (25,238.8 to 39,611.1)	-24.7 (-34.0 to -3.2)*	-30.6 (-39.1 to -10.7)*	313.2 (156.6 to 604.6)	-24.1 (-32.6 to -1.8)*	-30.0 (-37.8 to -9.5)*
Schistosomiasis episodes aggregate	9,853.5 (6,877.2 to 13,621.0)	-29.6 (-39.8 to -12.2)*	-35.1 (-44.5 to -19.1)*	57.2 (21.7 to 126.0)	-30.3 (-39.9 to -12.1)*	-35.8 (-44.6 to -18.9)*
Complications of schistosomiasis aggregate	18,853.6 (12,678.6 to 28,776.9)	-22.2 (-34.9 to 10.1)	-28.2 (-39.9 to 1.5)	208.1 (91.7 to 428.3)	-21.3 (-33.8 to 11.4)	-27.5 (-38.9 to 2.7)
Anemia due to schistosomiasis aggregate	1,742.3 (1,677.8 to 1,806.0)	-23.3 (-26.4 to -20.1)*	-29.2 (-32.1 to -26.3)*	47.9 (32.0 to 69.3)	-27.1 (-31.6 to -22.1)*	-32.8 (-36.9 to -28.2)*
Cysticercosis	216.9 (143.1 to 314.5)	-9.6 (-16.0 to -3.6)*	-16.6 (-22.6 to -11.1)*	34.2 (19.2 to 55.9)	-18.6 (-26.3 to -11.8)*	-25.0 (-32.0 to -18.6)*
Cystic echinococcosis	100.4 (83.3 to 120.5)	13.2 (9.5 to 16.7)*	4.4 (1.0 to 7.6)*	9.4 (6.1 to 13.5)	13.2 (3.4 to 23.2)*	4.4 (-4.7 to 13.6)
Lymphatic filariasis	3,484.7 (2,836.0 to 4,213.0)	-48.8 (-53.3 to -44.3)*	-52.8 (-56.9 to -48.6)*	169.7 (89.7 to 270.6)	-20.7 (-34.0 to -9.8)*	-26.8 (-39.2 to -16.9)*
Prevalence of detectable microfiliaria due to lymphatic filariasis	1,985.6 (1,677.4 to 2,291.8)	-59.8 (-63.3 to -56.5)*	-63.0 (-66.1 to -59.8)*	0.0 (0.0 to 0.0)	0.0 (0.0 to 0.0)	0.0 (0.0 to 0.0)
Complications of lymphatic filariasis aggregate	1,499.1 (980.2 to 2,147.6)	-19.5 (-32.6 to -9.6)*	-25.8 (-37.9 to -16.6)*	169.7 (89.7 to 270.6)	-20.7 (-34.0 to -9.8)*	-26.8 (-39.2 to -16.9)*
Onchocerciasis	1,904.9 (1,528.0 to 2,427.0)	-33.9 (-42.6 to -24.2)*	-39.0 (-47.0 to -30.1)*	112.9 (49.3 to 199.7)	-24.8 (-42.9 to -8.6)*	-30.7 (-47.4 to -15.7)*
Asymptomatic onchocerciasis	469.8 (349.2 to 606.6)	-57.9 (-60.2 to -56.0)*	-61.1 (-63.3 to -59.4)*	0.0 (0.0 to 0.0)	0.0 (0.0 to 0.0)	0.0 (0.0 to 0.0)
Skin disease due to onchocerciasis aggregate	1,432.4 (1,015.9 to 1,943.4)	-18.4 (-31.5 to -4.1)*	-24.8 (-36.8 to -11.5)*	112.5 (48.9 to 199.4)	-24.7 (-42.5 to -8.0)*	-30.5 (-47.0 to -15.1)*
Vision loss due to onchocerciasis aggregate	4.8 (3.2 to 6.9)	-54.8 (-57.8 to -52.0)*	-58.3 (-61.1 to -55.7)*	0.3 (0.2 to 0.5)	-57.4 (-66.8 to -45.5)*	-60.7 (-69.3 to -49.7)*
Trachoma	57.9 (31.4 to 96.6)	-4.1 (-15.7 to 8.2)	-11.5 (-22.3 to -0.2)*	3.7 (1.6 to 6.8)	-8.7 (-22.5 to 6.7)	-15.8 (-28.5 to -1.6)*
Dengue	435.1 (248.8 to 924.4)	127.7 (-4.6 to 504.2)	110.0 (-12.1 to 457.2)	71.2 (32.5 to 160.7)	126.2 (-7.0 to 507.2)	108.6 (-14.3 to 460.0)
Dengue episodes aggregate	129.8 (87.6 to 265.3)	121.5 (-7.7 to 470.0)	104.3 (-14.9 to 425.7)	7.6 (4.2 to 17.0)	126.7 (-7.0 to 487.3)	109.1 (-14.3 to 441.6)
Post-dengue chronic fatigue syndrome	305.8 (149.5 to 662.4)	127.8 (-4.6 to 504.2)	110.1 (-12.1 to 457.2)	63.6 (27.6 to 146.1)	126.1 (-7.3 to 515.5)	108.5 (-14.5 to 467.6)
Yellow fever	0.3 (0.1 to 0.8)	-26.4 (-41.5 to -4.7)*	-32.1 (-46.1 to -12.1)*	0.0 (0.0 to 0.0)	-26.4 (-41.5 to -4.7)*	-32.1 (-46.1 to -12.1)*
Rabies	0.0 (0.0 to 0.0)	-43.3 (-63.9 to -17.1)*	-47.7 (-66.7 to -23.5)*	0.0 (0.0 to 0.0)	-43.2 (-63.9 to -17.1)*	-47.6 (-66.7 to -23.5)*
Intestinal nematode infections	135,632.0 (131,804.3 to 139,169.7)	-5.3 (-8.7 to -2.1)*	-12.7 (-15.8 to -9.7)*	291.3 (165.2 to 475.9)	-25.5 (-31.8 to -17.8)*	-31.3 (-37.1 to -24.2)*
Ascariasis	71,068.1 (61,599.9 to 81,851.6)	-13.2 (-27.5 to 2.7)	-19.9 (-33.1 to -5.3)*	77.5 (42.9 to 131.4)	-40.3 (-50.9 to -28.5)*	-45.0 (-54.7 to -34.1)*
Complications of ascariasis aggregate	4,240.7 (3,815.4 to 4,716.3)	-38.0 (-46.8 to -27.9)*	-42.8 (-51.0 to -33.5)*	77.5 (42.9 to 131.4)	-40.3 (-50.9 to -28.5)*	-45.0 (-54.7 to -34.1)*
Asymptomatic ascariasis	66,831.9 (57,321.2 to 77,547.1)	-10.9 (-26.2 to 6.8)	-17.8 (-32.0 to -1.5)*	0.0 (0.0 to 0.0)	0.0 (0.0 to 0.0)	0.0 (0.0 to 0.0)
Trichuriasis	43,176.1 (38,942.4 to 47,777.4)	-4.1 (-16.4 to 10.5)	-11.6 (-22.9 to 1.9)	54.5 (28.3 to 96.8)	-14.2 (-33.1 to 15.2)	-20.9 (-38.3 to 6.3)
Complications of trichuriasis aggregate	2,674.3 (2,294.8 to 3,319.0)	-13.8 (-30.7 to 12.0)	-20.5 (-36.1 to 3.2)	54.5 (28.3 to 96.8)	-14.2 (-33.1 to 15.2)	-20.9 (-38.3 to 6.3)
Asymptomatic trichuriasis	40,493.9 (36,308.3 to 45,237.5)	-3.4 (-16.5 to 12.2)	-11.0 (-23.0 to 3.5)	0.0 (0.0 to 0.0)	0.0 (0.0 to 0.0)	0.0 (0.0 to 0.0)
Hookworm disease	42,108.8 (38,072.9 to 46,969.7)	-11.7 (-23.0 to 0.5)	-18.6 (-29.0 to -7.3)*	159.3 (95.8 to 252.7)	-19.4 (-26.1 to -11.7)*	-25.7 (-31.9 to -18.6)*
Complications of hookworm disease aggregate	5,183.5 (4,848.4 to 5,582.7)	-18.5 (-26.8 to -9.3)*	-24.9 (-32.5 to -16.4)*	107.6 (59.0 to 179.5)	-19.7 (-29.5 to -8.4)*	-25.9 (-35.0 to -15.5)*
Anemia due to hookworm disease aggregate	2,068.1 (2,032.0 to 2,109.1)	-12.8 (-15.0 to -10.3)*	-19.6 (-21.6 to -17.3)*	51.7 (34.0 to 76.0)	-18.7 (-23.0 to -14.7)*	-25.1 (-29.0 to -21.3)*
Asymptomatic hookworm disease	34,850.8 (30,682.7 to 39,573.4)	-10.5 (-24.6 to 4.8)	-17.5 (-30.4 to -3.4)*	0.0 (0.0 to 0.0)	0.0 (0.0 to 0.0)	0.0 (0.0 to 0.0)
Food-borne trematodiases	5,937.6 (5,453.0 to 6,481.0)	-6.2 (-9.1 to -3.1)*	-13.5 (-16.2 to -10.6)*	144.1 (69.7 to 269.2)	-8.1 (-12.5 to -1.7)*	-15.2 (-19.3 to -9.3)*
Asymptomatic food-borne trematodiases aggregate	4,687.6 (3,733.9 to 5,452.1)	-5.9 (-9.0 to -2.4)*	-13.2 (-16.1 to -10.0)*	0.0 (0.0 to 0.0)	0.0 (0.0 to 0.0)	0.0 (0.0 to 0.0)
Symptomatic food-borne trematodiases aggregate	1,250.0 (686.2 to 2,136.4)	-7.5 (-11.4 to -1.4)*	-14.7 (-18.3 to -9.1)*	144.1 (69.7 to 269.2)	-8.1 (-12.5 to -1.7)*	-15.2 (-19.3 to -9.3)*
Leprosy	10.2 (9.2 to 11.5)	-31.6 (-32.8 to -30.3)*	-36.9 (-38.0 to -35.7)*	0.6 (0.4 to 0.9)	-31.5 (-41.5 to -19.6)*	-36.8 (-46.1 to -25.9)*
Ebola	0.3 (0.1 to 0.5)	0.0 (0.0 to 0.0)	0.0 (0.0 to 0.0)	0.1 (0.0 to 0.1)	0.0 (0.0 to 0.0)	0.0 (0.0 to 0.0)
Other neglected tropical diseases	2,626.9 (2,568.1 to 2,687.8)	-7.1 (-9.5 to -4.6)*	-14.3 (-16.5 to -12.0)*	296.6 (104.2 to 805.0)	6.0 (-29.6 to 61.5)	-2.2 (-35.1 to 48.9)
Anemia due to other neglected tropical diseases aggregate	2,626.9 (2,568.1 to 2,687.8)	-7.1 (-9.5 to -4.6)*	-14.3 (-16.5 to -12.0)*	50.2 (32.9 to 76.0)	-19.4 (-24.5 to -14.8)*	-25.7 (-30.4 to -21.4)*
Maternal disorders	2,453.3 (2,220.7 to 2,626.8)	-3.4 (-10.7 to 4.8)	-10.9 (-17.6 to -3.4)*	194.2 (132.1 to 275.3)	-7.5 (-23.7 to 13.5)	-14.7 (-29.6 to 4.6)
Maternal hemorrhage	519.3 (497.7 to 546.9)	-7.4 (-12.0 to -2.4)*	-14.6 (-18.9 to -10.0)*	20.0 (13.3 to 29.0)	-6.8 (-20.1 to 8.8)	-14.1 (-26.3 to 0.3)
Maternal hemorrhage episodes aggregate	44.6 (25.8 to 71.5)	-2.9 (-39.9 to 57.8)	-10.5 (-44.6 to 45.5)	7.3 (3.9 to 12.2)	-2.4 (-36.7 to 46.0)	-10.0 (-41.6 to 34.7)
Anemia due to maternal hemorrhage aggregate	474.7 (464.4 to 485.6)	-7.8 (-10.7 to -4.6)*	-14.9 (-17.6 to -12.0)*	12.7 (8.3 to 18.5)	-9.2 (-15.1 to -2.9)*	-16.3 (-21.7 to -10.5)*
Maternal sepsis and other maternal infections	430.1 (299.0 to 616.4)	-7.7 (-37.6 to 36.7)	-14.8 (-42.5 to 26.0)	13.7 (6.4 to 26.3)	-7.3 (-49.7 to 73.3)	-14.5 (-53.7 to 59.8)
Maternal sepsis and other maternal infections aggregate	252.6 (126.7 to 441.7)	-7.0 (-52.3 to 83.5)	-14.2 (-56.0 to 69.2)	12.8 (5.7 to 24.8)	-7.2 (-51.4 to 82.3)	-14.5 (-55.2 to 68.1)
Infertility due to puerperal sepsis	177.4 (147.4 to 208.7)	-8.7 (-11.4 to -5.9)*	-15.8 (-18.3 to -13.2)*	0.9 (0.3 to 1.9)	-8.3 (-14.4 to -2.3)*	-15.4 (-21.1 to -9.9)*
Maternal hypertensive disorders	1,245.0 (721.3 to 1,912.8)	-1.1 (-40.6 to 63.5)	-8.8 (-45.3 to 50.8)	60.1 (29.2 to 101.6)	-1.2 (-39.3 to 61.3)	-8.9 (-44.1 to 48.8)
Maternal hypertensive disorders episodes aggregate	1,246.5 (720.6 to 1,915.3)	-1.0 (-40.6 to 63.7)	-8.7 (-45.2 to 51.0)	59.1 (28.2 to 100.8)	-1.1 (-39.9 to 63.8)	-8.8 (-44.6 to 51.0)
Eclampsia	1.5 (0.6 to 3.2)	-7.7 (-69.8 to 174.1)	-14.9 (-72.2 to 152.7)	0.9 (0.3 to 1.9)	-7.7 (-69.8 to 174.1)	-14.9 (-72.2 to 152.7)
Complications of maternal hypertensive disorders aggregate	0.0 (0.0 to 0.0)	0.0 (0.0 to 0.0)	0.0 (0.0 to 0.0)	0.0 (0.0 to 0.0)	0.0 (0.0 to 0.0)	0.0 (0.0 to 0.0)
Maternal obstructed labor and uterine rupture	181.5 (136.9 to 231.5)	-14.1 (-21.5 to -5.4)*	-20.7 (-27.6 to -12.8)*	59.9 (37.3 to 88.9)	-13.7 (-22.3 to -4.0)*	-20.4 (-28.4 to -11.5)*
Obstructed labor, acute event	25.9 (13.9 to 45.3)	-7.8 (-48.2 to 67.4)	-14.9 (-52.3 to 54.3)	8.0 (3.7 to 15.1)	-7.2 (-49.0 to 70.2)	-14.4 (-53.0 to 57.0)
Fistula due to maternal obstructed labor and uterine rupture aggregate	155.5 (111.1 to 203.2)	-15.0 (-19.3 to -10.7)*	-21.6 (-25.6 to -17.6)*	52.0 (31.8 to 79.1)	-14.6 (-21.6 to -7.7)*	-21.2 (-27.7 to -14.9)*
Maternal abortion, miscarriage, and ectopic pregnancy	104.7 (63.8 to 163.3)	-8.5 (-42.2 to 46.8)	-15.7 (-46.7 to 35.4)	11.5 (6.1 to 19.3)	-8.3 (-41.5 to 50.4)	-15.4 (-46.1 to 38.7)
Other maternal disorders	--	--	--	29.1 (19.1 to 43.5)	-6.5 (-24.7 to 16.7)	-13.7 (-30.5 to 7.6)
Neonatal disorders	4,035.6 (3,825.6 to 4,177.7)	13.4 (9.8 to 17.0)*	4.6 (1.3 to 7.9)*	995.7 (747.0 to 1,283.7)	11.5 (7.4 to 15.6)*	2.8 (-1.0 to 6.6)
Neonatal preterm birth complications	3,073.5 (2,684.9 to 3,493.6)	8.4 (3.1 to 13.8)*	-0.0 (-4.9 to 5.0)	474.0 (358.1 to 615.1)	3.3 (-2.2 to 9.8)	-4.7 (-9.8 to 1.2)
Vision loss due to retinopathy of prematurity aggregate	341.9 (268.5 to 424.8)	5.8 (1.9 to 9.8)*	-2.4 (-6.0 to 1.3)	10.5 (6.1 to 16.8)	6.3 (-1.8 to 15.7)	-2.0 (-9.5 to 6.7)
Complications of preterm birth complications aggregate	2,731.5 (2,371.3 to 3,122.7)	8.7 (2.8 to 15.0)*	0.3 (-5.2 to 6.0)	463.5 (347.6 to 601.7)	3.3 (-2.4 to 9.8)	-4.8 (-10.0 to 1.3)
Neonatal encephalopathy due to birth asphyxia and trauma	1,185.3 (831.5 to 1,748.6)	25.1 (22.2 to 27.9)*	15.4 (12.7 to 18.0)*	354.0 (228.0 to 544.3)	22.4 (18.0 to 26.7)*	12.9 (8.8 to 16.8)*

Cause	Prevalence in thousands (95% confidence interval)			YLDs in thousands (95% confidence interval)		
	2015	Percent change in counts between 2005 and 2015	Percent change in rates between 2005 and 2015	2015	Percent change in counts between 2005 and 2015	Percent change in rates between 2005 and 2015
Neonatal sepsis and other neonatal infections	0.0<br>(0.0 to 0.0)	0.0<br>(0.0 to 0.0)	0.0<br>(0.0 to 0.0)	0.0<br>(0.0 to 0.0)	0.0<br>(0.0 to 0.0)	0.0<br>(0.0 to 0.0)
Severe infection due to neonatal sepsis and other neonatal infections	0.0<br>(0.0 to 0.0)	0.0<br>(0.0 to 0.0)	0.0<br>(0.0 to 0.0)	0.0<br>(0.0 to 0.0)	0.0<br>(0.0 to 0.0)	0.0<br>(0.0 to 0.0)
Complications of neonatal sepsis and other neonatal infections aggregate	0.0<br>(0.0 to 0.0)	0.0<br>(0.0 to 0.0)	0.0<br>(0.0 to 0.0)	0.0<br>(0.0 to 0.0)	0.0<br>(0.0 to 0.0)	0.0<br>(0.0 to 0.0)
Hemolytic disease and other neonatal jaundice	184.2<br>(163.8 to 208.4)	17.9<br>(16.5 to 19.2)*	8.7<br>(7.5 to 9.9)*	56.1<br>(41.9 to 72.1)	15.0<br>(10.1 to 20.3)*	6.0<br>(1.5 to 11.0)*
Other neonatal disorders	--	--	--	111.6<br>(75.5 to 149.3)	15.6<br>(-1.6 to 36.7)	6.6<br>(-9.2 to 26.1)
Nutritional deficiencies	116,082.6<br>(114,760.7 to 117,400.7)	-2.9<br>(-4.4 to -1.4)*	-10.5<br>(-11.8 to -9.1)*	3,329.6<br>(2,218.9 to 4,829.0)	-8.6<br>(-10.6 to -6.7)*	-15.7<br>(-17.5 to -13.9)*
Protein-energy malnutrition	5.9<br>(3.6 to 9.9)	-23.0<br>(-33.1 to -10.5)*	-29.0<br>(-38.3 to -17.5)*	0.7<br>(0.4 to 1.4)	-22.9<br>(-34.4 to -8.3)*	-28.9<br>(-39.5 to -15.5)*
Iodine deficiency	11,603.6<br>(10,407.3 to 13,163.4)	0.0<br>(-2.4 to 2.5)	-7.7<br>(-10.0 to -5.5)*	257.3<br>(161.5 to 387.4)	1.9<br>(-0.6 to 4.5)	-6.1<br>(-8.3 to -3.6)*
Visible goiter due to iodine deficiency aggregate	11,301.6<br>(10,133.8 to 12,810.3)	-0.2<br>(-2.5 to 2.3)	-7.9<br>(-10.1 to -5.7)*	204.1<br>(125.7 to 321.6)	0.0<br>(-2.5 to 2.8)	-7.8<br>(-10.1 to -5.2)*
Visible goiter with heart failure due to iodine deficiency aggregate	0.0<br>(0.0 to 0.0)	0.0<br>(0.0 to 0.0)	0.0<br>(0.0 to 0.0)	0.0<br>(0.0 to 0.0)	0.0<br>(0.0 to 0.0)	0.0<br>(0.0 to 0.0)
Intellectual disability due to iodine deficiency aggregate	302.0<br>(125.2 to 413.1)	8.8<br>(4.7 to 10.9)*	0.3<br>(-3.4 to 2.3)	53.2<br>(19.1 to 87.9)	9.5<br>(2.5 to 15.3)*	1.0<br>(-5.5 to 6.4)
Vitamin A deficiency	481.5<br>(333.0 to 646.6)	7.1<br>(1.9 to 11.8)*	-1.2<br>(-6.0 to 3.1)	22.2<br>(12.8 to 34.8)	6.6<br>(0.2 to 13.1)*	-1.7<br>(-7.6 to 4.3)
Iron-deficiency anemia	113,696.6<br>(112,375.5 to 115,051.9)	-4.6<br>(-5.9 to -3.3)*	-12.0<br>(-13.2 to -10.8)*	3,049.3<br>(2,032.0 to 4,418.3)	-9.5<br>(-11.6 to -7.4)*	-16.5<br>(-18.5 to -14.6)*
Iron-deficiency anemia without heart failure aggregate	113,696.3<br>(112,375.2 to 115,051.7)	-4.6<br>(-5.9 to -3.3)*	-12.0<br>(-13.2 to -10.8)*	3,049.3<br>(2,032.0 to 4,418.2)	-9.5<br>(-11.6 to -7.4)*	-16.5<br>(-18.5 to -14.6)*
Iron-deficiency anemia with heart failure aggregate	0.3<br>(0.2 to 0.3)	16.3<br>(13.5 to 19.0)*	7.3<br>(4.7 to 9.7)*	0.0<br>(0.0 to 0.0)	16.3<br>(13.5 to 19.0)*	7.3<br>(4.7 to 9.7)*
Other nutritional deficiencies	--	--	--	0.1<br>(0.0 to 0.1)	-21.7<br>(-37.8 to -3.5)*	-27.8<br>(-42.6 to -11.0)*
Other communicable, maternal, neonatal, and nutritional diseases	146,677.3<br>(141,951.6 to 151,630.9)	7.8<br>(6.7 to 9.1)*	-0.5<br>(-1.6 to 0.6)	326.6<br>(204.9 to 513.8)	5.9<br>(0.9 to 10.5)*	-2.3<br>(-6.9 to 1.9)
Sexually transmitted diseases excluding HIV	110,610.0<br>(104,688.6 to 116,364.8)	7.2<br>(5.5 to 8.9)*	-1.2<br>(-2.7 to 0.4)	218.6<br>(130.2 to 357.3)	10.7<br>(6.9 to 14.7)*	2.1<br>(-1.4 to 5.8)
Syphilis	5,062.5<br>(3,566.0 to 7,123.1)	0.1<br>(-9.2 to 7.8)	-7.7<br>(-16.2 to -0.6)*	7.6<br>(5.0 to 10.7)	2.3<br>(-8.0 to 13.9)	-5.6<br>(-15.1 to 5.0)
Early syphilis aggregate	5,030.0<br>(3,533.3 to 7,090.9)	0.1<br>(-9.3 to 7.8)	-7.7<br>(-16.3 to -0.6)*	1.2<br>(0.3 to 3.1)	0.3<br>(-9.8 to 9.1)	-7.5<br>(-16.8 to 0.6)
Adult tertiary syphilis	32.5<br>(27.7 to 36.8)	2.3<br>(-1.7 to 5.9)	-5.6<br>(-9.3 to -2.3)*	6.3<br>(4.0 to 9.1)	2.7<br>(-9.8 to 16.3)	-5.3<br>(-16.8 to 7.3)
Chlamydial infection	21,992.9<br>(16,089.4 to 28,740.8)	7.5<br>(4.8 to 10.2)*	-0.8<br>(-3.3 to 1.7)	85.9<br>(47.8 to 147.7)	10.4<br>(6.4 to 14.8)*	1.8<br>(-1.9 to 5.9)
Chlamydial infection episodes aggregate	21,899.5<br>(15,990.8 to 28,639.1)	7.6<br>(4.9 to 10.3)*	-0.8<br>(-3.3 to 1.7)	82.9<br>(45.8 to 143.7)	11.0<br>(6.9 to 16.0)*	2.4<br>(-1.4 to 6.9)
Pelvic inflammatory diseases due to chlamydial infection aggregate	19.3<br>(14.9 to 24.3)	-5.5<br>(-9.4 to -2.1)*	-12.8<br>(-16.4 to -9.7)*	2.6<br>(1.6 to 3.8)	-5.5<br>(-15.2 to 4.8)	-12.8<br>(-21.8 to -3.3)*
Infertility due to clamydial infection aggregate	74.1<br>(66.9 to 81.3)	1.1<br>(-0.3 to 2.3)	-6.8<br>(-8.1 to -5.7)*	0.5<br>(0.2 to 0.9)	0.2<br>(-7.7 to 8.2)	-7.6<br>(-14.9 to -0.2)*
Gonococcal infection	8,706.3<br>(5,324.5 to 13,680.0)	15.3<br>(-1.1 to 26.8)	6.3<br>(-8.8 to 16.9)	68.7<br>(33.6 to 121.0)	14.2<br>(2.0 to 23.3)*	5.3<br>(-5.9 to 13.7)
Gonococcal infection episodes aggregate	8,669.2<br>(5,287.1 to 13,643.0)	15.4<br>(-1.1 to 26.9)	6.4<br>(-8.8 to 17.0)	67.4<br>(32.7 to 119.5)	14.7<br>(2.3 to 24.0)*	5.8<br>(-5.7 to 14.4)
Pelvic inflammatory diseases due to gonococcal infection aggregate	37.1<br>(33.5 to 41.1)	-1.5<br>(-3.4 to 0.1)	-9.2<br>(-10.9 to -7.7)*	1.4<br>(0.9 to 2.0)	-7.1<br>(-12.5 to -1.3)*	-14.3<br>(-19.3 to -8.9)*
Trichomoniasis	24,716.3<br>(17,630.3 to 32,592.6)	10.3<br>(9.4 to 11.3)*	1.7<br>(0.8 to 2.6)*	30.7<br>(11.6 to 67.4)	10.3<br>(8.6 to 12.1)*	1.7<br>(0.2 to 3.3)*
Genital herpes	62,027.1<br>(49,836.3 to 75,870.1)	5.8<br>(3.5 to 8.1)*	-2.4<br>(-4.6 to -0.3)*	20.0<br>(6.8 to 44.2)	9.1<br>(4.9 to 14.7)*	0.6<br>(-3.2 to 5.8)
Moderate infection due to initial genital herpes episode	93.3<br>(21.0 to 213.6)	20.9<br>(18.0 to 24.1)*	11.4<br>(8.8 to 14.4)*	4.6<br>(1.0 to 11.1)	21.0<br>(9.6 to 32.3)*	11.6<br>(1.1 to 22.0)*
Complications of genital herpes aggregate	61,933.8<br>(49,733.4 to 75,774.8)	5.8<br>(3.5 to 8.0)*	-2.4<br>(-4.6 to -0.4)*	15.5<br>(4.3 to 37.9)	6.0<br>(3.3 to 8.5)*	-2.3<br>(-4.8 to 0.1)
Other sexually transmitted diseases	159.1<br>(144.1 to 175.2)	0.3<br>(-1.0 to 1.7)	-7.5<br>(-8.7 to -6.2)*	5.7<br>(3.7 to 8.2)	-3.4<br>(-10.4 to 4.5)	-10.9<br>(-17.4 to -3.6)*
Pelvic inflammatory diseases due to other sexually transmitted diseases aggregate	36.8<br>(29.2 to 45.8)	-4.2<br>(-6.3 to -2.0)*	-11.7<br>(-13.6 to -9.7)*	4.9<br>(3.1 to 7.1)	-4.0<br>(-12.0 to 5.0)	-11.5<br>(-18.9 to -3.1)*
Infertility due to other sexually transmitted diseases aggregate	122.3<br>(109.6 to 135.6)	1.8<br>(0.2 to 3.5)*	-6.1<br>(-7.6 to -4.6)*	0.7<br>(0.3 to 1.5)	0.8<br>(-5.4 to 7.0)	-7.0<br>(-12.8 to -1.3)*
Other sexually transmitted diseases	--	--	--	64.6<br>(36.2 to 106.4)	12.6<br>(6.6 to 18.8)*	3.9<br>(-1.7 to 9.6)
Hepatitis	41,776.8<br>(40,991.0 to 42,544.2)	11.3<br>(10.1 to 12.6)*	2.7<br>(1.5 to 3.8)*	38.3<br>(24.0 to 57.4)	10.9<br>(-14.6 to 42.7)	2.2<br>(-21.2 to 31.6)
Acute hepatitis A	435.1<br>(356.7 to 504.3)	11.7<br>(-11.4 to 38.4)	3.0<br>(-18.3 to 27.6)	18.2<br>(11.4 to 27.6)	11.7<br>(-13.2 to 41.5)	3.0<br>(-19.9 to 30.5)
Hepatitis B	32,259.5<br>(30,726.4 to 33,957.6)	12.0<br>(8.3 to 15.8)*	3.3<br>(-0.1 to 6.8)	15.1<br>(8.0 to 25.3)	10.7<br>(-37.2 to 86.4)	2.1<br>(-42.1 to 71.9)
Acute Hepatitis B aggregate	1,851.5<br>(1,260.1 to 2,757.1)	10.8<br>(-35.8 to 84.8)	2.2<br>(-40.8 to 70.5)	15.1<br>(8.0 to 25.3)	10.7<br>(-37.2 to 86.4)	2.1<br>(-42.1 to 71.9)
Chronic hepatitis B	30,408.0<br>(29,095.7 to 31,899.0)	12.0<br>(10.3 to 13.8)*	3.3<br>(1.7 to 5.0)*	0.0<br>(0.0 to 0.0)	0.0<br>(0.0 to 0.0)	0.0<br>(0.0 to 0.0)
Hepatitis C	9,837.4<br>(8,712.5 to 11,102.6)	10.3<br>(8.9 to 11.5)*	1.7<br>(0.4 to 2.8)*	0.3<br>(0.1 to 0.5)	9.0<br>(5.2 to 11.7)*	0.5<br>(-3.0 to 3.0)
Acute Hepatitis C aggregate	18.5<br>(13.7 to 24.5)	9.0<br>(5.1 to 11.7)*	0.5<br>(-3.1 to 3.0)	0.3<br>(0.1 to 0.5)	9.0<br>(5.2 to 11.7)*	0.5<br>(-3.0 to 3.0)
Chronic hepatitis C	9,818.9<br>(8,696.6 to 11,081.9)	10.3<br>(8.9 to 11.5)*	1.7<br>(0.4 to 2.9)*	0.0<br>(0.0 to 0.0)	0.0<br>(0.0 to 0.0)	0.0<br>(0.0 to 0.0)
Acute hepatitis E	154.5<br>(114.4 to 209.1)	7.1<br>(4.0 to 10.2)*	-1.3<br>(-4.1 to 1.7)	4.7<br>(2.7 to 7.5)	8.0<br>(-13.4 to 34.3)	-0.4<br>(-20.1 to 23.8)
Other infectious diseases	2,864.4<br>(2,813.9 to 2,913.2)	-6.8<br>(-8.9 to -4.6)*	-14.0<br>(-16.0 to -12.0)*	69.7<br>(43.7 to 106.9)	-8.7<br>(-14.6 to -2.2)*	-15.8<br>(-21.2 to -9.8)*
Non-communicable diseases	549,558.1<br>(547,191.5 to 552,645.7)	8.4<br>(8.1 to 8.6)*	-0.1<br>(-0.3 to 0.1)	41,421.3<br>(30,468.1 to 53,204.0)	8.0<br>(7.4 to 8.7)*	-0.4<br>(-1.0 to 0.3)
Neoplasms	505.1<br>(477.5 to 534.6)	22.5<br>(14.8 to 31.5)*	13.0<br>(5.9 to 21.3)*	68.1<br>(47.8 to 91.7)	14.2<br>(4.5 to 24.4)*	5.3<br>(-3.7 to 14.7)
Lip and oral cavity cancer	22.9<br>(17.9 to 31.3)	18.7<br>(-2.7 to 45.6)	9.4<br>(-10.3 to 34.3)	2.0<br>(1.3 to 2.9)	18.2<br>(-4.0 to 46.3)	9.0<br>(-11.4 to 34.9)
Nasopharynx cancer	12.8<br>(5.4 to 21.4)	1.3<br>(-42.9 to 71.6)	-6.6<br>(-47.4 to 58.3)	1.2<br>(0.5 to 2.2)	1.0<br>(-43.7 to 68.9)	-6.8<br>(-48.0 to 55.8)
Other pharynx cancer	2.6<br>(2.2 to 3.1)	2.4<br>(-12.0 to 18.5)	-5.6<br>(-18.9 to 9.3)	0.2<br>(0.1 to 0.3)	1.7<br>(-12.6 to 17.6)	-6.2<br>(-19.4 to 8.4)
Esophageal cancer	1.3<br>(1.0 to 1.8)	-0.6<br>(-26.7 to 34.2)	-8.3<br>(-32.4 to 23.8)	0.3<br>(0.2 to 0.4)	-5.1<br>(-27.5 to 25.9)	-12.5<br>(-33.1 to 16.1)
Stomach cancer	7.4<br>(6.1 to 8.4)	-14.1<br>(-24.2 to -1.3)*	-20.8<br>(-30.1 to -9.0)*	0.9<br>(0.6 to 1.2)	-15.8<br>(-25.9 to -3.2)*	-22.4<br>(-31.7 to -10.7)*
Colon and rectum cancer	32.4<br>(27.3 to 37.6)	21.4<br>(4.5 to 43.5)*	12.0<br>(-3.7 to 32.3)	2.9<br>(2.0 to 3.9)	18.1<br>(0.2 to 39.9)*	9.0<br>(-7.6 to 29.0)
Colon and rectum cancer aggregate	32.3<br>(27.2 to 37.5)	21.5<br>(4.5 to 43.7)*	12.1<br>(-3.7 to 32.5)	2.9<br>(2.0 to 3.8)	18.2<br>(0.2 to 40.1)*	9.0<br>(-7.6 to 29.2)
Stoma due to colon and rectum cancer	0.1<br>(0.1 to 0.1)	3.0<br>(2.0 to 3.9)*	-5.0<br>(-5.9 to -4.1)*	0.0<br>(0.0 to 0.0)	3.0<br>(2.0 to 3.9)*	-5.0<br>(-5.9 to -4.1)*
Liver cancer	0.0<br>(0.0 to 0.0)	82.2<br>(-100.0 to 1,092.1)	68.0<br>(-100.0 to 999.4)	0.9<br>(0.6 to 1.4)	-1.8<br>(-26.9 to 27.8)	-9.5<br>(-32.6 to 17.9)
Liver cancer due to hepatitis B	3.1<br>(2.2 to 4.6)	4.7<br>(-25.6 to 44.6)	-3.4<br>(-31.3 to 33.4)	0.7<br>(0.4 to 1.0)	-1.3<br>(-28.2 to 31.4)	-8.9<br>(-33.8 to 21.2)
Liver cancer due to hepatitis C	0.2<br>(0.1 to 0.2)	14.5<br>(-5.9 to 39.5)	5.6<br>(-13.2 to 28.7)	0.0<br>(0.0 to 0.1)	7.3<br>(-11.6 to 28.7)	-1.1<br>(-18.5 to 18.6)
Liver cancer due to alcohol use	0.1<br>(0.0 to 0.1)	24.5<br>(-6.4 to 62.8)	14.8<br>(-13.7 to 50.2)	0.0<br>(0.0 to 0.0)	18.2<br>(-11.9 to 55.3)	9.0<br>(-18.7 to 43.2)
Liver cancer due to other causes	0.9<br>(0.7 to 1.2)	-0.4<br>(-24.8 to 30.0)	-8.2<br>(-30.7 to 19.9)	0.2<br>(0.1 to 0.3)	-6.2<br>(-27.1 to 18.3)	-13.5<br>(-32.8 to 9.1)
Gallbladder and biliary tract cancer	0.2<br>(0.1 to 0.2)	-4.9<br>(-12.3 to 3.9)	-12.3<br>(-19.1 to -4.2)*	0.0<br>(0.0 to 0.1)	-6.3<br>(-13.4 to 2.5)	-13.6<br>(-20.1 to -5.5)*
Pancreatic cancer	0.4<br>(0.4 to 0.5)	16.9<br>(2.9 to 31.9)*	7.8<br>(-5.1 to 21.7)	0.1<br>(0.1 to 0.2)	14.5<br>(0.9 to 29.6)*	5.5<br>(-7.0 to 19.5)

Cause	Prevalence in thousands (95% confidence interval)			YLDs in thousands (95% confidence interval)		
	2015	Percent change in counts between 2005 and 2015	Percent change in rates between 2005 and 2015	2015	Percent change in counts between 2005 and 2015	Percent change in rates between 2005 and 2015
Larynx cancer	10.5 (10.0 to 11.2)	5.8 (0.6 to 10.3)*	-2.5 (-7.2 to 1.7)	1.2 (0.8 to 1.7)	4.8 (-0.9 to 9.9)	-3.3 (-8.7 to 1.4)
Larynx cancer aggregate	1.1 (0.8 to 1.7)	-2.2 (-32.0 to 41.4)	-9.8 (-37.3 to 30.4)	0.3 (0.2 to 0.4)	-1.0 (-19.0 to 20.5)	-8.7 (-25.3 to 11.1)
Laryngectomy due to larynx cancer	9.4 (9.0 to 9.8)	6.8 (6.1 to 7.4)*	-1.5 (-2.2 to -1.0)*	0.9 (0.6 to 1.4)	6.8 (6.1 to 7.4)*	-1.5 (-2.2 to -1.0)*
Tracheal, bronchus, and lung cancer	4.8 (3.2 to 8.5)	11.2 (-18.5 to 71.0)	2.6 (-24.8 to 57.7)	0.9 (0.5 to 1.7)	7.8 (-22.7 to 69.4)	-0.6 (-28.8 to 56.3)
Malignant skin melanoma	36.3 (30.9 to 47.0)	10.2 (1.1 to 20.3)*	1.6 (-6.7 to 10.9)	2.1 (1.4 to 3.2)	8.8 (-2.2 to 21.6)	0.4 (-9.8 to 12.2)
Non-melanoma skin cancer	13.2 (10.8 to 15.8)	58.6 (15.3 to 117.6)*	46.3 (6.3 to 100.7)*	1.4 (0.9 to 2.1)	28.9 (8.5 to 56.3)*	18.9 (0.0 to 44.1)*
Non-melanoma skin cancer(squamous-cell carcinoma)	24.3 (19.5 to 29.1)	32.8 (13.2 to 61.2)*	22.5 (4.3 to 48.7)*	1.4 (0.9 to 2.1)	29.5 (8.5 to 57.3)*	19.4 (0.1 to 45.0)*
Non-melanoma skin cancer(basal-cell carcinoma)	2.6 (1.9 to 3.7)	4.7 (1.5 to 7.5)*	-3.4 (-6.4 to -0.8)*	0.0 (0.0 to 0.1)	4.8 (1.4 to 8.0)*	-3.4 (-6.5 to -0.4)*
Breast cancer	43.8 (32.5 to 52.8)	18.5 (-4.8 to 47.7)	9.3 (-12.2 to 36.2)	3.5 (2.3 to 5.0)	14.2 (-6.6 to 40.5)	5.3 (-13.9 to 29.6)
Breast cancer aggregate	32.7 (21.3 to 41.7)	24.0 (-8.7 to 68.7)	14.4 (-15.8 to 55.6)	2.6 (1.6 to 3.8)	17.9 (-10.6 to 59.0)	8.7 (-17.6 to 46.7)
Mastectomy due to breast cancer	11.1 (10.5 to 11.7)	4.9 (3.9 to 6.0)*	-3.3 (-4.2 to -2.3)*	0.9 (0.6 to 1.4)	4.8 (-4.3 to 14.3)	-3.3 (-11.7 to 5.4)
Cervical cancer	29.1 (14.2 to 42.0)	-18.1 (-48.0 to 16.9)	-24.4 (-52.1 to 7.8)	2.3 (1.1 to 3.7)	-17.3 (-47.7 to 18.8)	-23.7 (-51.8 to 9.6)
Uterine cancer	17.9 (12.5 to 22.5)	-4.0 (-29.0 to 45.5)	-11.5 (-34.5 to 34.2)	1.2 (0.7 to 1.8)	-4.8 (-30.8 to 47.2)	-12.2 (-36.2 to 35.8)
Ovarian cancer	20.6 (12.1 to 29.8)	14.9 (-25.4 to 64.2)	5.9 (-31.2 to 51.4)	2.8 (1.5 to 4.5)	13.7 (-26.2 to 64.6)	4.9 (-31.9 to 51.8)
Prostate cancer	6.8 (4.8 to 7.9)	13.3 (-0.7 to 28.8)	4.5 (-8.4 to 18.8)	0.5 (0.3 to 0.8)	3.6 (-12.4 to 24.5)	-4.5 (-19.2 to 14.8)
Prostate cancer aggregate	6.8 (4.8 to 7.9)	13.3 (-0.7 to 28.8)	4.5 (-8.4 to 18.8)	0.5 (0.3 to 0.8)	3.6 (-12.4 to 24.5)	-4.5 (-19.2 to 14.8)
Impotence and incontinence due to prostate cancer aggregate	0.0 (0.0 to 0.0)	0.0 (0.0 to 0.0)	0.0 (0.0 to 0.0)	0.0 (0.0 to 0.0)	0.0 (0.0 to 0.0)	0.0 (0.0 to 0.0)
Testicular cancer	79.6 (69.4 to 91.0)	36.7 (18.9 to 56.7)*	26.1 (9.7 to 44.5)*	4.8 (3.2 to 6.9)	34.8 (15.7 to 54.7)*	24.3 (6.7 to 42.7)*
Kidney cancer	11.1 (9.0 to 12.7)	36.7 (15.5 to 56.6)*	26.1 (6.5 to 44.4)*	0.8 (0.6 to 1.2)	34.2 (13.5 to 53.8)*	23.8 (4.7 to 41.9)*
Bladder cancer	7.6 (5.7 to 9.3)	16.3 (-4.8 to 44.0)	7.2 (-12.2 to 32.8)	0.6 (0.4 to 0.9)	13.2 (-6.4 to 37.8)	4.4 (-13.7 to 27.1)
Bladder cancer episodes aggregate	7.4 (5.6 to 9.1)	16.6 (-5.0 to 45.2)	7.5 (-12.4 to 33.9)	0.6 (0.4 to 0.9)	13.8 (-7.0 to 40.2)	4.9 (-14.2 to 29.3)
Urinary incontinence due to bladder cancer	0.2 (0.2 to 0.2)	2.6 (1.0 to 4.3)*	-5.4 (-6.9 to -3.8)*	0.0 (0.0 to 0.0)	2.6 (1.0 to 4.3)*	-5.4 (-6.9 to -3.8)*
Brain and nervous system cancer	32.6 (27.6 to 37.8)	12.1 (-2.5 to 31.0)	3.4 (-10.1 to 20.8)	3.7 (2.5 to 5.0)	10.8 (-3.7 to 29.6)	2.2 (-11.2 to 19.5)
Thyroid cancer	19.4 (16.9 to 21.4)	5.2 (-6.8 to 22.2)	-3.0 (-14.0 to 12.7)	1.1 (0.7 to 1.6)	4.2 (-9.1 to 22.2)	-3.9 (-16.1 to 12.6)
Mesothelioma	0.4 (0.3 to 0.4)	2.5 (-10.1 to 16.6)	-5.4 (-17.1 to 7.5)	0.1 (0.0 to 0.1)	2.5 (-9.5 to 16.4)	-5.5 (-16.6 to 7.3)
Hodgkin lymphoma	43.4 (36.3 to 52.3)	-5.2 (-16.7 to 8.2)	-12.6 (-23.1 to -0.2)*	3.5 (2.3 to 4.8)	-7.9 (-19.6 to 6.4)	-15.1 (-25.9 to -1.9)*
Non-Hodgkin lymphoma	94.2 (71.3 to 118.6)	37.8 (10.9 to 69.5)*	27.1 (2.2 to 56.3)*	7.1 (4.6 to 10.3)	33.6 (7.2 to 65.1)*	23.2 (-1.2 to 52.3)
Multiple myeloma	2.5 (2.0 to 3.0)	43.9 (20.8 to 69.4)*	32.7 (11.4 to 56.2)*	0.6 (0.4 to 0.8)	39.1 (17.0 to 63.3)*	28.2 (7.9 to 50.6)*
Leukemia	33.7 (28.4 to 39.5)	72.5 (41.3 to 111.8)*	59.1 (30.3 to 95.3)*	12.0 (7.7 to 17.6)	20.7 (-4.7 to 58.8)	11.3 (-12.1 to 46.4)
Acute lymphoid leukemia	31.6 (23.6 to 44.2)	36.2 (5.3 to 84.4)*	25.6 (-2.9 to 70.1)	4.1 (2.6 to 6.3)	32.9 (2.4 to 80.2)*	22.5 (-5.5 to 66.2)
Chronic lymphoid leukemia	30.0 (21.9 to 40.8)	10.3 (-18.8 to 51.9)	1.7 (-25.1 to 40.1)	3.7 (2.3 to 5.5)	8.4 (-20.0 to 50.1)	-0.0 (-26.3 to 38.4)
Acute myeloid leukemia	24.7 (18.4 to 34.7)	29.4 (-0.2 to 75.2)	19.3 (-7.9 to 61.6)	3.1 (1.9 to 4.7)	27.2 (-1.4 to 72.1)	17.3 (-9.1 to 58.7)
Chronic myeloid leukemia	8.5 (6.2 to 11.9)	12.5 (-14.2 to 51.9)	3.7 (-20.9 to 40.1)	1.1 (0.7 to 1.7)	9.1 (-16.4 to 46.7)	0.6 (-22.9 to 35.3)
Other neoplasms	125.6 (100.1 to 157.3)	18.5 (-10.0 to 51.0)	9.2 (-17.0 to 39.3)	9.3 (6.1 to 13.8)	17.4 (-10.2 to 49.0)	8.2 (-17.2 to 37.4)
Cardiovascular diseases	6,750.8 (6,423.4 to 7,039.5)	-0.7 (-3.9 to 2.5)	-8.4 (-11.3 to -5.5)*	497.4 (350.2 to 659.6)	2.7 (-0.6 to 5.6)	-5.3 (-8.3 to -2.7)*
Rheumatic heart disease	3,519.0 (3,144.6 to 4,316.7)	-6.9 (-11.5 to -2.5)*	-14.1 (-18.4 to -10.1)*	168.9 (106.0 to 258.4)	-6.6 (-11.3 to -2.0)*	-13.9 (-18.2 to -9.6)*
Rheumatic heart disease, without heart failure	3,500.9 (3,126.5 to 4,298.1)	-7.0 (-11.6 to -2.6)*	-14.2 (-18.5 to -10.2)*	166.7 (104.3 to 255.2)	-6.8 (-11.6 to -2.2)*	-14.1 (-18.5 to -9.8)*
Heart failure fue to rheumatic heart disease aggregate	18.2 (16.6 to 19.9)	14.5 (13.2 to 15.7)*	5.6 (4.4 to 6.7)*	2.2 (1.5 to 3.2)	14.5 (11.6 to 18.4)*	5.5 (2.9 to 9.2)*
Ischemic heart disease	402.1 (349.0 to 457.1)	10.5 (9.4 to 11.6)*	1.9 (0.9 to 3.0)*	31.0 (20.3 to 44.3)	10.9 (5.6 to 16.1)*	2.3 (-2.6 to 7.1)
Myocardial infarction episodes aggregate	0.5 (0.3 to 0.7)	9.3 (7.7 to 11.1)*	0.8 (-0.6 to 2.5)	0.0 (0.0 to 0.1)	9.3 (7.7 to 11.1)*	0.8 (-0.6 to 2.5)
Angina due to ischemic heart disease aggregate	346.8 (293.3 to 401.3)	10.3 (9.0 to 11.6)*	1.7 (0.6 to 2.9)*	24.3 (15.5 to 35.4)	10.6 (4.6 to 16.8)*	2.0 (-3.6 to 7.7)
Heart failure fue to ischemic heart disease aggregate	54.9 (49.7 to 60.6)	12.0 (11.2 to 12.9)*	3.3 (2.5 to 4.1)*	6.6 (4.4 to 9.4)	12.0 (4.0 to 21.7)*	3.3 (-4.0 to 12.3)
Cerebrovascular disease	868.6 (845.7 to 888.3)	12.0 (8.7 to 14.9)*	3.3 (0.2 to 6.0)*	145.6 (100.2 to 200.4)	11.9 (7.0 to 16.2)*	3.2 (-1.3 to 7.2)
Ischemic stroke	329.8 (311.1 to 349.1)	15.4 (14.2 to 16.4)*	6.4 (5.3 to 7.4)*	52.8 (35.7 to 73.0)	15.4 (8.9 to 22.8)*	6.4 (0.4 to 13.3)*
Chronic ischemic stroke aggregate	329.0 (310.4 to 348.4)	15.4 (14.2 to 16.5)*	6.4 (5.3 to 7.4)*	52.7 (35.6 to 72.8)	15.4 (8.9 to 22.9)*	6.4 (0.4 to 13.3)*
Ischemic stroke episodes aggregate	0.8 (0.5 to 1.1)	9.5 (6.7 to 12.3)*	1.0 (-1.6 to 3.6)	0.2 (0.1 to 0.3)	9.5 (6.7 to 12.3)*	1.0 (-1.6 to 3.6)
Hemorrhagic stroke	588.0 (556.0 to 621.8)	9.8 (8.8 to 10.8)*	1.3 (0.3 to 2.2)*	92.8 (64.3 to 127.0)	10.0 (4.2 to 15.9)*	1.4 (-3.9 to 6.8)
Chronic hemorrhagic stroke aggregate	586.6 (554.6 to 620.6)	9.8 (8.8 to 10.8)*	1.3 (0.3 to 2.2)*	92.5 (64.1 to 126.7)	10.0 (4.2 to 15.9)*	1.5 (-3.9 to 6.9)
Acute hemorrhagic stroke aggregate	1.4 (0.9 to 1.8)	5.1 (0.4 to 9.4)*	-3.1 (-7.4 to 0.9)	0.3 (0.2 to 0.5)	5.1 (0.4 to 9.4)*	-3.0 (-7.4 to 0.9)
Hypertensive heart disease	11.8 (10.6 to 13.0)	11.3 (10.3 to 12.5)*	2.7 (1.7 to 3.7)*	1.5 (1.0 to 2.1)	11.3 (10.3 to 12.5)*	2.7 (1.7 to 3.7)*
Cardiomyopathy and myocarditis	70.4 (64.0 to 77.7)	10.6 (9.7 to 11.5)*	2.0 (1.2 to 2.8)*	7.7 (5.3 to 10.7)	10.6 (3.3 to 18.6)*	2.0 (-4.8 to 9.4)
Acute myocarditis	11.3 (7.8 to 15.9)	10.2 (8.0 to 12.4)*	1.6 (-0.4 to 3.7)	0.6 (0.3 to 1.0)	10.1 (3.5 to 17.5)*	1.5 (-4.6 to 8.3)
Heart failure due to cardiomyopathy aggregate	59.1 (53.9 to 64.7)	10.7 (9.7 to 11.7)*	2.0 (1.2 to 3.0)*	7.1 (4.8 to 9.9)	10.7 (2.9 to 19.3)*	2.1 (-5.1 to 10.0)
Atrial fibrillation and flutter	0.0 (0.0 to 0.0)	0.0 (0.0 to 0.0)	0.0 (0.0 to 0.0)	0.0 (0.0 to 0.0)	0.0 (0.0 to 0.0)	0.0 (0.0 to 0.0)
Peripheral vascular disease	0.0 (0.0 to 0.0)	0.0 (0.0 to 0.0)	0.0 (0.0 to 0.0)	0.0 (0.0 to 0.0)	0.0 (0.0 to 0.0)	0.0 (0.0 to 0.0)
Endocarditis	3.7 (2.9 to 4.7)	13.5 (9.9 to 16.8)*	4.6 (1.3 to 7.7)*	0.4 (0.3 to 0.6)	13.9 (10.2 to 17.3)*	5.0 (1.6 to 8.2)*
Endocarditis episodes aggregate	0.4 (0.3 to 0.4)	6.6 (0.0 to 12.8)*	-1.7 (-7.8 to 4.0)	0.0 (0.0 to 0.0)	6.7 (0.2 to 12.8)*	-1.6 (-7.6 to 4.1)
Heart failure due to endocarditis aggregate	3.3 (2.5 to 4.3)	14.3 (10.3 to 17.8)*	5.4 (1.7 to 8.7)*	0.4 (0.3 to 0.6)	14.3 (10.3 to 17.8)*	5.4 (1.7 to 8.7)*
Other cardiovascular and circulatory diseases	2,038.6 (1,510.3 to 2,727.2)	4.0 (2.2 to 5.7)*	-4.1 (-5.8 to -2.5)*	142.3 (88.2 to 213.5)	4.1 (1.4 to 7.0)*	-4.0 (-6.5 to -1.3)*
Heart failure due to other cardiovascular diseases aggregate	3.2 (2.8 to 3.6)	1.3 (-0.3 to 2.9)	-6.6 (-8.0 to -5.1)*	0.4 (0.3 to 0.5)	1.3 (-0.5 to 3.1)	-6.5 (-8.2 to -4.9)*
Other cardiovascular diseases episodes aggregate	2,035.5 (1,507.3 to 2,724.0)	4.0 (2.2 to 5.7)*	-4.1 (-5.8 to -2.5)*	141.9 (87.9 to 213.0)	4.2 (1.4 to 7.0)*	-3.9 (-6.5 to -1.3)*
Chronic respiratory diseases	27,781.8 (26,004.7 to 29,626.6)	4.6 (2.4 to 7.0)*	-3.6 (-5.5 to -1.3)*	1,609.8 (1,167.7 to 2,143.4)	3.1 (0.4 to 5.7)*	-5.0 (-7.4 to -2.5)*
Chronic obstructive pulmonary disease	5,783.3 (4,983.4 to 6,687.9)	0.5 (-1.9 to 2.7)	-7.3 (-9.5 to -5.2)*	461.2 (370.1 to 551.2)	0.9 (-2.4 to 4.4)	-6.9 (-10.0 to -3.7)*

Cause	Prevalence in thousands (95% confidence interval)			YLDs in thousands (95% confidence interval)		
	2015	Percent change in counts between 2005 and 2015	Percent change in rates between 2005 and 2015	2015	Percent change in counts between 2005 and 2015	Percent change in rates between 2005 and 2015
Chronic obstructive pulmonary disease without heart failure aggregate	5,786.3 (4,986.0 to 6,688.6)	0.5 (-1.8 to 2.8)	-7.3 (-9.5 to -5.2)*	460.8 (369.9 to 550.9)	0.9 (-2.4 to 4.4)	-6.9 (-10.0 to -3.7)*
Severe chronic obstructive pulmonary disease with heart failure aggregate	0.8 (0.7 to 0.8)	6.8 (5.7 to 7.9)*	-1.5 (-2.5 to -0.5)*	0.4 (0.3 to 0.5)	6.8 (5.7 to 7.9)*	-1.5 (-2.5 to -0.5)*
Pneumoconiosis	162.5 (151.9 to 172.4)	30.1 (21.4 to 39.1)*	20.0 (11.9 to 28.3)*	32.0 (20.0 to 46.3)	20.1 (10.9 to 30.6)*	10.8 (2.3 to 20.5)*
Silicosis	9.8 (8.1 to 12.0)	5.4 (2.3 to 8.3)*	-2.8 (-5.7 to -0.1)*	1.6 (1.0 to 2.5)	5.4 (2.1 to 8.3)*	-2.8 (-5.9 to -0.1)*
Silicosis without heart failure aggregate	9.8 (8.1 to 12.0)	5.4 (2.3 to 8.3)*	-2.8 (-5.7 to -0.1)*	1.6 (1.0 to 2.5)	5.4 (2.1 to 8.3)*	-2.8 (-5.9 to -0.1)*
Severe silicosis with heart failure aggregate	0.0 (0.0 to 0.0)	0.0 (0.0 to 0.0)	0.0 (0.0 to 0.0)	0.0 (0.0 to 0.0)	0.0 (0.0 to 0.0)	0.0 (0.0 to 0.0)
Asbestosis	2.3 (1.9 to 2.7)	3.9 (-0.0 to 7.8)	-4.2 (-7.8 to -0.6)*	0.4 (0.2 to 0.6)	3.9 (-0.0 to 7.8)	-4.2 (-7.8 to -0.6)*
Asbestosis without heart failure aggregate	2.3 (1.9 to 2.7)	3.9 (-0.0 to 7.8)	-4.2 (-7.8 to -0.6)*	0.4 (0.2 to 0.6)	3.9 (-0.0 to 7.8)	-4.2 (-7.8 to -0.6)*
Severe asbestosis with heart failure aggregate	0.0 (0.0 to 0.0)	0.0 (0.0 to 0.0)	0.0 (0.0 to 0.0)	0.0 (0.0 to 0.0)	0.0 (0.0 to 0.0)	0.0 (0.0 to 0.0)
Coal workers pneumoconiosis	1.1 (0.9 to 1.4)	7.6 (1.8 to 12.9)*	-0.8 (-6.1 to 4.1)	0.2 (0.1 to 0.3)	7.6 (1.8 to 12.9)*	-0.8 (-6.1 to 4.1)
Coal workers pneumoconiosis without heart failure aggregate	1.1 (0.9 to 1.4)	7.6 (1.8 to 12.9)*	-0.8 (-6.1 to 4.1)	0.2 (0.1 to 0.3)	7.6 (1.8 to 12.9)*	-0.8 (-6.1 to 4.1)
Severe coal workers pneumoconiosis with heart failure aggregate	0.0 (0.0 to 0.0)	0.0 (0.0 to 0.0)	0.0 (0.0 to 0.0)	0.0 (0.0 to 0.0)	0.0 (0.0 to 0.0)	0.0 (0.0 to 0.0)
Other pneumoconiosis	186.4 (162.8 to 216.2)	21.5 (17.4 to 25.6)*	12.1 (8.3 to 15.8)*	29.8 (18.6 to 43.1)	21.4 (11.4 to 32.9)*	12.0 (2.7 to 22.6)*
Other pneumoconiosis without heart failure aggregate	186.4 (162.8 to 216.2)	21.5 (17.4 to 25.6)*	12.1 (8.3 to 15.8)*	29.8 (18.6 to 43.1)	21.4 (11.4 to 32.9)*	12.0 (2.7 to 22.6)*
Severe other pneumoconiosis with heart failure aggregate	0.0 (0.0 to 0.0)	0.0 (0.0 to 0.0)	0.0 (0.0 to 0.0)	0.0 (0.0 to 0.0)	0.0 (0.0 to 0.0)	0.0 (0.0 to 0.0)
Asthma	22,493.9 (17,734.1 to 27,325.1)	5.1 (1.9 to 8.8)*	-3.0 (-6.1 to 0.3)	1,013.3 (627.4 to 1,497.6)	5.3 (2.0 to 9.0)*	-2.9 (-6.0 to 0.5)
Asymptomatic asthma	6,811.0 (5,235.4 to 8,605.3)	5.1 (1.9 to 8.8)*	-3.0 (-6.1 to 0.3)	0.0 (0.0 to 0.0)	0.0 (0.0 to 0.0)	0.0 (0.0 to 0.0)
Symptomatic asthma aggregate	15,682.9 (12,420.5 to 19,153.5)	5.1 (1.9 to 8.8)*	-3.0 (-6.1 to 0.3)	1,013.3 (627.4 to 1,497.6)	5.3 (2.0 to 9.0)*	-2.9 (-6.0 to 0.5)
Interstitial lung disease and pulmonary sarcoidosis	36.4 (31.6 to 41.6)	4.0 (1.0 to 6.5)*	-4.1 (-6.8 to -1.8)*	4.6 (2.7 to 6.8)	4.2 (-0.9 to 9.1)	-3.9 (-8.6 to 0.6)
Interstitial lung disease and pulmonary sarcoidosis without heart failure aggregate	35.9 (31.1 to 41.1)	3.9 (0.9 to 6.5)*	-4.2 (-6.9 to -1.8)*	4.3 (2.5 to 6.5)	4.0 (-1.5 to 9.2)	-4.1 (-9.1 to 0.7)
Severe interstitial lung disease and pulmonary sarcoidosis with heart failure aggregate	0.5 (0.5 to 0.6)	9.1 (6.4 to 11.2)*	0.6 (-1.8 to 2.6)	0.3 (0.2 to 0.3)	9.1 (6.4 to 11.2)*	0.6 (-1.8 to 2.6)
Other chronic respiratory diseases	--	--	--	98.8 (77.7 to 121.5)	-11.4 (-19.8 to -1.3)*	-18.3 (-26.1 to -9.0)*
Cirrhosis and other chronic liver diseases	47.6 (43.3 to 52.1)	23.1 (8.7 to 40.0)*	13.5 (0.2 to 29.1)*	14.5 (9.8 to 20.3)	13.1 (5.8 to 20.9)*	4.3 (-2.5 to 11.5)
Cirrhosis and other chronic liver diseases due to hepatitis B	14.1 (11.7 to 17.0)	9.8 (7.3 to 12.3)*	1.3 (-1.0 to 3.6)	2.5 (1.6 to 3.7)	9.6 (-2.3 to 23.0)	1.1 (-9.9 to 13.4)
Cirrhosis and other chronic liver diseases due to hepatitis C	8.6 (6.9 to 10.6)	13.6 (9.4 to 17.5)*	4.7 (0.9 to 8.4)*	1.5 (0.9 to 2.3)	13.5 (1.2 to 27.1)*	4.7 (-6.7 to 17.2)
Cirrhosis and other chronic liver diseases due to alcohol use	17.3 (14.9 to 20.0)	19.7 (17.2 to 22.0)*	10.4 (8.1 to 12.5)*	3.0 (2.0 to 4.4)	18.6 (6.4 to 32.9)*	9.4 (-1.9 to 22.6)
Cirrhosis and other chronic liver diseases due to other causes	43.8 (39.4 to 48.5)	12.0 (10.5 to 13.5)*	3.3 (1.9 to 4.6)*	7.5 (5.1 to 10.7)	12.2 (0.1 to 25.9)*	3.5 (-7.7 to 16.1)
Digestive diseases	8,792.3 (8,583.7 to 9,006.9)	-3.1 (-5.8 to 0.0)	-10.6 (-13.1 to -7.8)*	518.9 (362.4 to 710.8)	-1.6 (-4.1 to 1.2)	-9.2 (-11.6 to -6.7)*
Peptic ulcer disease	2,077.0 (2,026.5 to 2,133.9)	-16.3 (-18.9 to -13.7)*	-22.8 (-25.3 to -20.4)*	70.5 (47.4 to 100.9)	-22.2 (-26.0 to -18.6)*	-28.2 (-31.8 to -25.0)*
Peptic ulcer disease, symptomatic episodes	159.7 (126.9 to 201.3)	-14.2 (-18.8 to -9.8)*	-20.9 (-25.1 to -16.8)*	17.5 (11.1 to 26.2)	-14.1 (-21.0 to -6.8)*	-20.8 (-27.1 to -14.0)*
Anemia due to peptic ulcer disease aggregate	1,917.3 (1,876.3 to 1,958.8)	-16.5 (-19.2 to -13.6)*	-23.0 (-25.5 to -20.3)*	52.9 (35.6 to 76.7)	-24.5 (-28.9 to -20.4)*	-30.4 (-34.4 to -26.6)*
Gastritis and duodenitis	4,720.6 (4,567.9 to 4,869.3)	0.9 (-3.5 to 4.9)	-7.0 (-11.0 to -3.3)*	160.8 (109.3 to 231.5)	-4.0 (-9.5 to 1.5)	-11.5 (-16.5 to -6.4)*
Gastritis and duodenitis, symptomatic episodes	125.4 (91.7 to 167.0)	3.4 (-0.5 to 6.2)	-4.6 (-8.2 to -2.1)*	13.8 (8.3 to 21.1)	3.6 (-4.7 to 11.6)	-4.5 (-12.1 to 2.9)
Anemia due to gastritis and duodenitis aggregate	4,595.1 (4,441.3 to 4,742.4)	0.8 (-3.7 to 4.9)	-7.0 (-11.2 to -3.2)*	147.1 (98.7 to 210.1)	-4.7 (-10.4 to 1.3)	-12.1 (-17.4 to -6.5)*
Appendicitis	55.1 (46.1 to 65.5)	10.4 (8.7 to 12.0)*	1.8 (0.2 to 3.3)*	17.0 (10.9 to 24.4)	10.5 (-0.2 to 22.6)	1.9 (-8.0 to 13.1)
Paralytic ileus and intestinal obstruction	4.2 (3.0 to 5.4)	10.0 (3.3 to 16.0)*	1.5 (-4.7 to 6.9)	1.4 (0.8 to 2.1)	10.0 (3.3 to 16.0)*	1.5 (-4.7 to 6.9)
Inguinal, femoral, and abdominal hernia	973.7 (690.9 to 1,347.2)	0.4 (-5.3 to 6.4)	-7.4 (-12.7 to -1.9)*	10.5 (4.8 to 20.9)	0.6 (-5.8 to 6.8)	-7.2 (-13.1 to -1.5)*
Inflammatory bowel disease	739.1 (641.2 to 844.4)	6.5 (5.8 to 7.0)*	-1.8 (-2.4 to -1.3)*	162.6 (105.2 to 225.7)	6.6 (3.9 to 9.5)*	-1.7 (-4.2 to 1.0)
Vascular intestinal disorders	1.0 (0.8 to 1.4)	9.4 (3.6 to 15.4)*	0.9 (-4.5 to 6.4)	0.3 (0.2 to 0.5)	9.4 (3.6 to 15.4)*	0.9 (-4.5 to 6.4)
Gallbladder and biliary diseases	289.4 (223.4 to 370.0)	4.2 (-1.4 to 9.9)	-3.9 (-9.0 to 1.3)	31.8 (19.6 to 47.6)	4.4 (-3.2 to 12.1)	-3.7 (-10.7 to 3.4)
Pancreatitis	45.1 (35.0 to 56.1)	13.6 (8.9 to 18.5)*	4.8 (0.4 to 9.3)*	14.0 (8.8 to 20.7)	13.8 (1.6 to 27.7)*	5.0 (-6.3 to 17.7)
Other digestive diseases	--	--	--	50.0 (34.4 to 69.5)	7.7 (0.8 to 17.7)*	-0.7 (-7.0 to 8.6)
Neurological disorders	223,686.2 (209,462.3 to 235,619.9)	7.6 (5.7 to 9.7)*	-0.8 (-2.5 to 1.1)	4,810.6 (3,083.1 to 6,777.1)	5.7 (3.0 to 8.2)*	-2.6 (-5.0 to -0.2)*
Alzheimer disease and other dementias	0.0 (0.0 to 0.0)	0.0 (0.0 to 0.0)	0.0 (0.0 to 0.0)	0.0 (0.0 to 0.0)	0.0 (0.0 to 0.0)	0.0 (0.0 to 0.0)
Parkinson disease	5.0 (4.2 to 5.7)	3.2 (-2.1 to 8.5)	-4.9 (-9.7 to 0.1)	0.7 (0.4 to 1.0)	3.2 (-2.1 to 8.5)	-4.8 (-9.7 to 0.1)
Epilepsy	2,102.7 (1,779.0 to 2,440.8)	4.3 (-2.0 to 10.3)	-3.8 (-9.6 to 1.7)	594.9 (392.1 to 816.8)	-10.9 (-17.2 to -4.5)*	-17.8 (-23.7 to -11.9)*
Multiple sclerosis	75.1 (61.3 to 90.7)	5.7 (1.5 to 9.8)*	-2.5 (-6.4 to 1.2)	26.3 (17.4 to 37.1)	5.8 (-0.3 to 11.4)	-2.4 (-8.1 to 2.8)
Motor neuron disease	6.5 (5.9 to 7.2)	10.3 (7.8 to 12.6)*	1.7 (-0.6 to 3.8)	1.4 (1.0 to 1.8)	10.3 (7.8 to 12.6)*	1.7 (-0.6 to 3.8)
Migraine	93,251.3 (73,297.1 to 115,527.9)	8.4 (6.4 to 10.4)*	-0.1 (-1.9 to 1.8)	3,272.4 (1,934.4 to 4,995.5)	8.6 (6.4 to 11.0)*	0.2 (-1.9 to 2.4)
Tension-type headache	153,542.0 (108,918.5 to 203,354.7)	7.2 (6.1 to 8.4)*	-1.1 (-2.1 to -0.0)*	233.3 (104.8 to 455.6)	7.3 (5.8 to 8.9)*	-1.0 (-2.4 to 0.4)
Medication overuse headache	4,227.1 (2,847.1 to 6,179.4)	8.3 (-1.3 to 19.6)	-0.1 (-9.0 to 10.3)	680.0 (385.1 to 1,084.7)	8.4 (-1.1 to 19.3)	-0.0 (-8.8 to 10.1)
Other neurological disorders	0.6 (0.3 to 1.2)	9.5 (8.0 to 10.6)*	1.0 (-0.4 to 2.0)	1.5 (1.0 to 2.2)	3.3 (-3.0 to 9.8)	-4.7 (-10.5 to 1.3)
Mental and substance use disorders	119,602.1 (116,826.2 to 122,665.2)	9.1 (8.1 to 10.0)*	0.6 (-0.3 to 1.5)	16,443.8 (11,729.6 to 21,239.5)	10.0 (9.3 to 10.8)*	1.5 (0.8 to 2.2)*
Schizophrenia	1,352.2 (985.0 to 1,837.6)	8.9 (7.7 to 10.2)*	0.5 (-0.7 to 1.6)	899.7 (586.9 to 1,256.7)	9.2 (6.5 to 11.7)*	0.7 (-1.8 to 3.0)
Alcohol use disorders	6,703.4 (5,172.7 to 8,890.4)	4.6 (-0.3 to 10.1)	-3.6 (-8.1 to 1.5)	680.8 (422.4 to 1,003.6)	4.7 (-0.6 to 10.3)	-3.5 (-8.4 to 1.7)
Alcohol dependence aggregate	6,622.9 (5,093.7 to 8,804.4)	4.5 (-0.5 to 10.1)	-3.6 (-8.2 to 1.5)	676.5 (418.5 to 997.9)	4.7 (-0.7 to 10.3)	-3.5 (-8.4 to 1.7)
Fetal alcohol syndrome aggregate	80.5 (75.4 to 84.7)	8.3 (7.6 to 8.9)*	-0.1 (-0.7 to 0.4)	4.2 (2.7 to 6.1)	8.3 (1.7 to 15.3)*	-0.2 (-6.2 to 6.3)
Drug use disorders	9,590.0 (9,292.3 to 9,882.4)	12.3 (10.4 to 14.1)*	3.5 (1.9 to 5.3)*	1,709.2 (1,189.0 to 2,292.0)	14.8 (11.4 to 18.2)*	5.9 (2.7 to 9.0)*
Opioid use disorders	2,404.6 (1,899.1 to 2,997.6)	9.3 (5.4 to 13.2)*	0.8 (-2.8 to 4.4)	1,024.4 (683.0 to 1,405.8)	9.5 (5.3 to 13.8)*	1.0 (-2.9 to 5.0)
Asymptomatic opioid dependence	390.0 (271.3 to 560.7)	9.3 (5.4 to 13.2)*	0.8 (-2.8 to 4.4)	0.0 (0.0 to 0.0)	0.0 (0.0 to 0.0)	0.0 (0.0 to 0.0)
Symptomatic opioid dependence aggregate	2,014.6 (1,581.4 to 2,521.3)	9.3 (5.4 to 13.2)*	0.8 (-2.8 to 4.4)	1,024.4 (683.0 to 1,405.8)	9.5 (5.3 to 13.8)*	1.0 (-2.9 to 5.0)
Cocaine use disorders	664.5 (535.8 to 796.5)	22.7 (19.1 to 26.2)*	13.2 (9.9 to 16.4)*	92.4 (57.0 to 134.6)	22.8 (16.9 to 29.3)*	13.2 (7.8 to 19.2)*
Asymptomatic cocaine dependence	333.0 (251.9 to 425.3)	22.7 (19.1 to 26.2)*	13.2 (9.9 to 16.4)*	0.0 (0.0 to 0.0)	0.0 (0.0 to 0.0)	0.0 (0.0 to 0.0)

Cause	Prevalence in thousands (95% confidence interval)			YLDs in thousands (95% confidence interval)		
	2015	Percent change in counts between 2005 and 2015	Percent change in rates between 2005 and 2015	2015	Percent change in counts between 2005 and 2015	Percent change in rates between 2005 and 2015
Symptomatic cocaine dependence aggregate	331.5<br>(248.7 to 420.7)	22.7<br>(19.1 to 26.2)*	13.2<br>(9.9 to 16.4)*	92.4<br>(57.0 to 134.6)	22.8<br>(16.9 to 29.3)*	13.2<br>(7.8 to 19.2)*
Amphetamine use disorders	1,790.2<br>(1,353.0 to 2,279.7)	31.1<br>(26.8 to 35.6)*	20.9<br>(16.9 to 25.0)*	238.2<br>(134.7 to 365.7)	31.3<br>(25.8 to 37.8)*	21.0<br>(16.0 to 27.1)*
Asymptomatic amphetamine dependence	980.2<br>(700.3 to 1,290.8)	31.1<br>(26.8 to 35.6)*	20.9<br>(16.9 to 25.0)*	0.0<br>(0.0 to 0.0)	0.0<br>(0.0 to 0.0)	0.0<br>(0.0 to 0.0)
Symptomatic amphetamine dependence aggregate	810.0<br>(571.1 to 1,104.0)	31.1<br>(26.8 to 35.6)*	20.9<br>(16.9 to 25.0)*	238.2<br>(134.7 to 365.7)	31.3<br>(25.8 to 37.8)*	21.0<br>(16.0 to 27.1)*
Cannabis use disorders	4,752.9<br>(4,126.8 to 5,486.3)	6.9<br>(5.5 to 8.3)*	-1.4<br>(-2.7 to -0.2)*	139.5<br>(86.9 to 204.4)	7.1<br>(4.2 to 9.8)*	-1.3<br>(-3.9 to 1.3)
Asymptomatic cannabis dependence	2,749.1<br>(2,360.1 to 3,217.8)	6.9<br>(5.5 to 8.3)*	-1.4<br>(-2.7 to -0.2)*	0.0<br>(0.0 to 0.0)	0.0<br>(0.0 to 0.0)	0.0<br>(0.0 to 0.0)
Symptomatic cannabis dependence aggregate	2,003.8<br>(1,679.8 to 2,403.8)	6.9<br>(5.5 to 8.3)*	-1.4<br>(-2.7 to -0.2)*	139.5<br>(86.9 to 204.4)	7.1<br>(4.2 to 9.8)*	-1.3<br>(-3.9 to 1.3)
Other drug use disorders	--	--	--	214.7<br>(127.3 to 318.7)	28.8<br>(22.7 to 35.5)*	18.8<br>(13.2 to 24.9)*
Depressive disorders	29,970.5<br>(27,414.0 to 31,936.1)	10.1<br>(7.5 to 12.5)*	1.5<br>(-0.8 to 3.8)	5,575.0<br>(3,465.4 to 8,220.0)	10.5<br>(9.4 to 11.5)*	1.9<br>(0.9 to 2.8)*
Major depressive disorder	22,620.8<br>(16,289.7 to 30,741.6)	10.5<br>(9.5 to 11.4)*	1.9<br>(0.9 to 2.7)*	4,757.7<br>(2,903.2 to 7,162.3)	10.7<br>(9.5 to 11.8)*	2.1<br>(1.0 to 3.1)*
Dysthymia	8,250.8<br>(6,231.6 to 10,665.7)	9.0<br>(7.2 to 10.9)*	0.5<br>(-1.2 to 2.3)	817.3<br>(496.5 to 1,240.3)	9.1<br>(7.1 to 11.2)*	0.7<br>(-1.3 to 2.6)
Bipolar disorder	6,113.3<br>(4,976.0 to 7,405.5)	8.4<br>(8.2 to 8.6)*	-0.0<br>(-0.2 to 0.2)	1,276.6<br>(758.2 to 1,965.3)	8.7<br>(7.2 to 10.0)*	0.2<br>(-1.1 to 1.5)
Anxiety disorders	26,752.5<br>(20,958.5 to 33,253.4)	9.0<br>(6.8 to 11.4)*	0.6<br>(-1.5 to 2.7)	2,517.7<br>(1,595.5 to 3,709.5)	9.3<br>(6.8 to 11.8)*	0.8<br>(-1.5 to 3.1)
Eating disorders	1,219.6<br>(1,107.9 to 1,301.5)	20.2<br>(16.0 to 24.9)*	10.9<br>(6.9 to 15.2)*	261.4<br>(166.5 to 387.1)	20.5<br>(17.0 to 24.1)*	11.1<br>(7.9 to 14.5)*
Anorexia nervosa	680.8<br>(502.0 to 913.7)	15.3<br>(12.0 to 18.8)*	6.3<br>(3.2 to 9.6)*	145.3<br>(92.0 to 221.0)	15.4<br>(10.6 to 20.3)*	6.4<br>(2.0 to 10.9)*
Bulimia nervosa	547.2<br>(367.8 to 787.7)	27.5<br>(24.6 to 30.4)*	17.6<br>(14.9 to 20.3)*	116.1<br>(66.1 to 188.5)	27.6<br>(23.3 to 32.3)*	17.7<br>(13.7 to 22.0)*
Autistic spectrum disorders	5,956.9<br>(5,673.5 to 6,241.5)	9.0<br>(7.0 to 11.1)*	0.6<br>(-1.3 to 2.5)	973.8<br>(647.3 to 1,344.2)	9.3<br>(8.1 to 10.4)*	0.8<br>(-0.3 to 1.8)
Autism	2,367.9<br>(1,982.1 to 2,806.7)	9.2<br>(9.0 to 9.4)*	0.7<br>(0.6 to 0.9)*	614.1<br>(398.2 to 876.1)	9.4<br>(7.7 to 11.0)*	0.9<br>(-0.7 to 2.3)
Asperger syndrome and other autistic spectrum disorders	3,564.1<br>(3,006.3 to 4,260.6)	9.0<br>(8.9 to 9.0)*	0.5<br>(0.5 to 0.5)*	359.8<br>(237.3 to 525.5)	9.1<br>(7.7 to 10.6)*	0.6<br>(-0.7 to 2.0)
Attention-deficit/hyperactivity disorder	7,772.3<br>(6,828.3 to 8,767.3)	7.4<br>(6.8 to 8.0)*	-0.9<br>(-1.5 to -0.4)*	94.3<br>(55.2 to 144.5)	7.5<br>(5.6 to 9.3)*	-0.8<br>(-2.6 to 0.8)
Conduct disorder	1,800.3<br>(1,240.5 to 2,456.1)	8.6<br>(8.0 to 9.2)*	0.1<br>(-0.4 to 0.7)	214.6<br>(118.9 to 351.3)	8.8<br>(6.1 to 11.5)*	0.3<br>(-2.2 to 2.9)
Idiopathic developmental intellectual disability	9,913.2<br>(5,771.8 to 13,861.1)	8.7<br>(7.7 to 9.5)*	0.3<br>(-0.7 to 1.0)	366.8<br>(163.9 to 637.9)	8.1<br>(6.0 to 9.4)*	-0.3<br>(-2.3 to 0.9)
Other mental and substance use disorders	24,334.6<br>(23,982.1 to 24,699.4)	8.6<br>(6.4 to 11.0)*	0.2<br>(-1.8 to 2.3)	1,873.9<br>(1,295.4 to 2,528.2)	8.8<br>(6.4 to 11.4)*	0.3<br>(-1.9 to 2.7)
Diabetes, urogenital, blood, and endocrine diseases	217,395.8<br>(214,602.4 to 219,992.5)	7.5<br>(6.5 to 8.4)*	-0.8<br>(-1.8 to -0.0)*	2,548.8<br>(1,740.5 to 3,556.7)	6.5<br>(4.8 to 8.2)*	-1.8<br>(-3.3 to -0.3)*
Diabetes mellitus	11,043.7<br>(9,236.7 to 13,081.8)	17.6<br>(13.4 to 22.2)*	8.5<br>(4.6 to 12.7)*	661.3<br>(417.6 to 966.3)	17.9<br>(13.6 to 22.6)*	8.7<br>(4.8 to 13.1)*
Uncomplicated diabetes mellitus	9,421.9<br>(7,846.6 to 11,154.5)	17.6<br>(13.3 to 22.3)*	8.4<br>(4.5 to 12.8)*	450.1<br>(267.1 to 673.0)	17.7<br>(13.5 to 22.4)*	8.6<br>(4.6 to 12.9)*
Neuropathy and other complications of diabetes mellitus aggregate	1,608.0<br>(1,188.4 to 2,127.6)	18.1<br>(13.5 to 22.9)*	8.9<br>(4.6 to 13.4)*	210.5<br>(125.8 to 316.0)	18.3<br>(13.2 to 23.4)*	9.1<br>(4.4 to 13.8)*
Vision loss due to diabetes mellitus aggregate	13.8<br>(7.9 to 22.3)	7.6<br>(4.8 to 10.5)*	-0.8<br>(-3.4 to 1.9)	0.7<br>(0.3 to 1.1)	7.8<br>(2.4 to 13.1)*	-0.6<br>(-5.6 to 4.3)
Acute glomerulonephritis	5.8<br>(4.2 to 7.8)	-10.2<br>(-15.4 to -4.5)*	-17.2<br>(-22.0 to -12.0)*	0.3<br>(0.2 to 0.5)	-10.1<br>(-16.6 to -3.4)*	-17.1<br>(-23.1 to -10.9)*
Chronic kidney disease	6,712.8<br>(6,119.2 to 7,282.3)	9.4<br>(6.4 to 12.2)*	0.9<br>(-1.9 to 3.5)	247.0<br>(172.9 to 332.2)	7.1<br>(4.4 to 9.7)*	-1.2<br>(-3.7 to 1.2)
Chronic kidney disease due to diabetes mellitus	2,512.2<br>(1,624.6 to 3,601.7)	9.6<br>(7.1 to 12.1)*	1.1<br>(-1.2 to 3.4)	89.5<br>(57.0 to 128.7)	7.7<br>(2.7 to 12.7)*	-0.7<br>(-5.3 to 3.9)
Stage III chronic kidney disease due to diabetes mellitus aggregate	2,090.9<br>(1,317.4 to 3,080.2)	9.8<br>(7.2 to 12.6)*	1.3<br>(-1.1 to 3.8)	8.4<br>(5.8 to 11.7)	4.9<br>(-4.3 to 15.3)	-3.3<br>(-11.7 to 6.3)
Stage IV chronic kidney disease due to diabetes mellitus aggregate	343.7<br>(206.3 to 508.3)	8.8<br>(6.2 to 11.4)*	0.3<br>(-2.1 to 2.7)	45.7<br>(25.8 to 70.3)	7.8<br>(2.7 to 13.1)*	-0.6<br>(-5.3 to 4.3)
Stage V chronic kidney disease untreated due to diabetes mellitus	60.9<br>(38.3 to 89.6)	8.7<br>(6.4 to 11.1)*	0.2<br>(-1.8 to 2.5)	32.5<br>(17.9 to 50.9)	8.7<br>(-2.4 to 20.6)	0.3<br>(-10.0 to 11.2)
End-stage chronic kidney disease due to diabetes aggregate	16.7<br>(11.4 to 22.9)	3.2<br>(0.4 to 6.1)*	-4.8<br>(-7.4 to -2.2)*	2.8<br>(1.7 to 4.4)	3.8<br>(-1.9 to 9.5)	-4.3<br>(-9.5 to 1.0)
Chronic kidney disease due to hypertension	1,632.2<br>(1,011.3 to 2,506.6)	9.8<br>(6.5 to 13.1)*	1.2<br>(-1.8 to 4.3)	26.0<br>(15.2 to 42.8)	9.1<br>(4.1 to 14.7)*	0.6<br>(-4.0 to 5.8)
Stage III chronic kidney disease due to hypertension aggregate	1,485.2<br>(905.4 to 2,304.5)	9.6<br>(6.3 to 13.0)*	1.1<br>(-2.0 to 4.2)	1.9<br>(1.3 to 2.7)	4.4<br>(1.7 to 7.3)*	-3.7<br>(-6.2 to -1.1)*
Stage IV chronic kidney disease due to hypertension aggregate	130.9<br>(56.2 to 258.3)	11.7<br>(4.0 to 21.9)*	3.0<br>(-4.1 to 12.4)	16.3<br>(7.8 to 31.5)	10.5<br>(2.8 to 19.8)*	1.9<br>(-5.2 to 10.5)
Stage V chronic kidney disease untreated due to hypertension	12.6<br>(7.7 to 18.8)	7.7<br>(2.8 to 12.8)*	-0.7<br>(-5.2 to 4.0)	7.1<br>(3.9 to 11.4)	7.7<br>(2.8 to 12.8)*	-0.7<br>(-5.2 to 4.0)
End-stage chronic kidney disease due to hypertension aggregate	3.5<br>(2.4 to 5.0)	3.1<br>(-4.6 to 10.9)	-4.9<br>(-12.0 to 2.3)	0.6<br>(0.4 to 1.0)	3.3<br>(-2.5 to 10.0)	-4.7<br>(-10.1 to 1.5)
Chronic kidney disease due to glomerulonephritis	772.3<br>(589.9 to 1,009.3)	7.3<br>(3.9 to 10.9)*	-1.1<br>(-4.2 to 2.3)	59.5<br>(39.2 to 82.5)	5.8<br>(-0.3 to 11.1)	-2.5<br>(-8.0 to 2.4)
Stage III chronic kidney disease due to glomerulonephritis aggregate	515.3<br>(354.0 to 734.7)	8.0<br>(3.6 to 12.8)*	-0.4<br>(-4.5 to 4.0)	4.6<br>(3.2 to 6.4)	-2.6<br>(-11.9 to 8.0)	-10.1<br>(-18.8 to -0.4)*
Stage IV chronic kidney disease due to glomerulonephritis aggregate	200.7<br>(149.2 to 249.9)	5.6<br>(1.8 to 9.3)*	-2.6<br>(-6.1 to 0.8)	28.3<br>(18.0 to 39.9)	5.6<br>(-0.8 to 12.5)	-2.6<br>(-8.5 to 3.7)
Stage V chronic kidney disease untreated due to glomerulonephritis	46.1<br>(27.5 to 69.0)	7.6<br>(5.0 to 10.2)*	-0.7<br>(-3.2 to 1.6)	24.8<br>(13.8 to 39.3)	8.0<br>(-3.1 to 19.2)	-0.4<br>(-10.7 to 10.0)
End-stage chronic kidney disease due to glomerulonephritis aggregate	10.3<br>(6.6 to 14.9)	2.9<br>(-0.4 to 6.3)	-5.1<br>(-8.1 to -2.0)*	1.9<br>(1.1 to 3.1)	1.9<br>(-1.8 to 5.5)	-6.0<br>(-9.5 to -2.7)*
Chronic kidney disease due to other causes	2,134.6<br>(1,354.5 to 3,161.3)	9.5<br>(7.5 to 11.6)*	1.0<br>(-0.9 to 2.9)	72.0<br>(43.0 to 106.2)	6.9<br>(1.4 to 11.9)*	-1.5<br>(-6.5 to 3.2)
Stage III chronic kidney disease due to other causes aggregate	1,777.2<br>(1,097.3 to 2,702.0)	9.7<br>(7.6 to 12.0)*	1.2<br>(-0.8 to 3.2)	4.4<br>(3.0 to 6.2)	-1.9<br>(-12.7 to 9.7)	-9.6<br>(-19.5 to 1.2)
Stage IV chronic kidney disease due to other causes aggregate	293.4<br>(165.4 to 439.9)	8.5<br>(6.4 to 10.8)*	0.1<br>(-1.9 to 2.2)	37.7<br>(20.9 to 59.0)	6.7<br>(1.3 to 11.7)*	-1.6<br>(-6.6 to 3.0)
Stage V chronic kidney disease untreated due to other causes	51.7<br>(29.7 to 78.5)	8.4<br>(6.5 to 10.2)*	-0.0<br>(-1.8 to 1.7)	27.6<br>(14.1 to 44.9)	8.9<br>(-1.5 to 21.4)	0.5<br>(-9.1 to 12.0)
End-stage chronic kidney disease due to other causes aggregate	12.3<br>(7.8 to 18.0)	3.7<br>(0.8 to 6.6)*	-4.3<br>(-7.0 to -1.7)*	2.3<br>(1.3 to 3.6)	3.2<br>(-2.2 to 8.7)	-4.9<br>(-9.8 to 0.3)
Urinary diseases and male infertility	20,124.1<br>(19,051.2 to 20,948.9)	12.3<br>(9.8 to 15.1)*	3.6<br>(1.2 to 6.2)*	51.5<br>(27.1 to 93.3)	19.6<br>(13.8 to 24.8)*	10.3<br>(5.0 to 15.1)*
Interstitial nephritis and urinary tract infections	246.6<br>(230.9 to 264.2)	11.7<br>(10.9 to 12.5)*	3.0<br>(2.3 to 3.7)*	8.4<br>(5.1 to 12.7)	11.8<br>(5.7 to 18.3)*	3.1<br>(-2.5 to 9.1)
Urolithiasis	15,011.4<br>(12,555.5 to 17,639.7)	8.3<br>(5.1 to 11.3)*	-0.2<br>(-3.1 to 2.6)	5.1<br>(3.2 to 7.7)	8.3<br>(-3.5 to 22.3)	-0.1<br>(-11.0 to 12.8)
Benign prostatic hyperplasia	0.0<br>(0.0 to 0.0)	0.0<br>(0.0 to 0.0)	0.0<br>(0.0 to 0.0)	0.0<br>(0.0 to 0.0)	0.0<br>(0.0 to 0.0)	0.0<br>(0.0 to 0.0)
Male infertility	5,128.8<br>(3,721.3 to 7,003.1)	26.8<br>(20.8 to 32.4)*	17.0<br>(11.4 to 22.1)*	33.4<br>(13.5 to 69.3)	25.5<br>(18.7 to 31.9)*	15.7<br>(9.5 to 21.6)*
Other urinary diseases	--	--	--	4.6<br>(3.0 to 6.9)	9.2<br>(-1.8 to 22.1)	0.7<br>(-9.5 to 12.6)
Gynecological diseases	80,258.7<br>(78,217.3 to 81,919.5)	8.2<br>(6.7 to 9.5)*	-0.3<br>(-1.6 to 1.0)	1,032.0<br>(685.4 to 1,456.6)	6.0<br>(3.8 to 8.1)*	-2.2<br>(-4.2 to -0.3)*
Uterine fibroids	2,666.0<br>(2,337.3 to 3,047.8)	7.4<br>(7.1 to 7.7)*	-0.9<br>(-1.2 to -0.6)*	72.4<br>(45.5 to 109.0)	3.4<br>(0.3 to 6.2)*	-4.7<br>(-7.5 to -2.1)*
Uterine fibroids cases aggregate	863.0<br>(704.2 to 1,021.5)	10.1<br>(8.5 to 11.6)*	1.5<br>(0.0 to 2.9)*	0.3<br>(0.1 to 0.6)	526.5<br>(116.1 to 2,450.0)*	477.8<br>(99.3 to 2,251.6)*
Mild abdominal pain with anemia due to uterine fibroids aggregate	1,803.0<br>(1,563.1 to 2,086.8)	6.2<br>(5.6 to 6.9)*	-2.1<br>(-2.6 to -1.4)*	72.1<br>(45.3 to 108.4)	3.1<br>(-0.0 to 5.9)	-5.0<br>(-7.8 to -2.3)*
Polycystic ovarian syndrome	6,655.0<br>(4,579.3 to 9,155.5)	7.3<br>(6.9 to 7.7)*	-1.1<br>(-1.4 to -0.7)*	61.6<br>(26.5 to 125.2)	6.8<br>(5.5 to 8.0)*	-1.5<br>(-2.7 to -0.4)*
Polycystic ovarian syndrome cases aggregate	5,268.5<br>(3,535.5 to 7,455.6)	7.2<br>(6.8 to 7.6)*	-1.1<br>(-1.5 to -0.8)*	45.9<br>(19.9 to 90.7)	7.3<br>(6.0 to 8.6)*	-1.0<br>(-2.3 to 0.1)
Hirsutism and infertility due to polycystic ovarian syndrome aggregate	1,386.5<br>(838.0 to 2,111.8)	7.5<br>(6.5 to 8.5)*	-0.9<br>(-1.8 to 0.0)	15.7<br>(6.2 to 34.9)	5.3<br>(1.6 to 8.3)*	-2.9<br>(-6.3 to -0.1)*

Cause	Prevalence in thousands (95% confidence interval)			YLDs in thousands (95% confidence interval)		
	2015	Percent change in counts between 2005 and 2015	Percent change in rates between 2005 and 2015	2015	Percent change in counts between 2005 and 2015	Percent change in rates between 2005 and 2015
Female infertility	9,320.0 (6,231.7 to 13,283.4)	32.2 (24.6 to 40.7)*	21.9 (14.9 to 29.8)*	57.2 (22.2 to 122.5)	30.7 (23.0 to 39.0)*	20.5 (13.4 to 28.2)*
Endometriosis	873.8 (620.9 to 1,207.0)	5.2 (4.4 to 5.7)*	-3.0 (-3.7 to -2.5)*	82.2 (49.1 to 127.2)	5.4 (1.9 to 8.9)*	-2.8 (-6.0 to 0.4)
Endometriosis cases aggregate	822.6 (586.0 to 1,149.2)	5.2 (4.4 to 5.7)*	-3.0 (-3.7 to -2.5)*	76.9 (45.8 to 119.7)	5.4 (1.6 to 9.0)*	-2.8 (-6.3 to 0.5)
Abdominal pain and infertility due to endometriosis aggregate	51.2 (32.5 to 77.9)	5.4 (4.5 to 6.1)*	-2.8 (-3.7 to -2.2)*	5.3 (2.9 to 8.9)	5.8 (0.1 to 11.9)*	-2.4 (-7.7 to 3.2)
Genital prolapse	2,372.2 (1,545.6 to 3,484.2)	-3.1 (-8.6 to 2.3)	-10.6 (-15.7 to -5.6)*	7.6 (3.3 to 15.5)	-2.9 (-10.0 to 3.9)	-10.4 (-17.0 to -4.2)*
Premenstrual syndrome	59,863.9 (53,734.1 to 67,081.4)	6.7 (3.2 to 9.9)*	-1.6 (-4.8 to 1.4)	509.2 (315.7 to 775.6)	6.8 (3.1 to 10.5)*	-1.5 (-5.0 to 1.9)
Other gynecological diseases	6,590.4 (6,366.4 to 6,870.1)	2.5 (1.4 to 3.6)*	-5.5 (-6.5 to -4.5)*	241.9 (168.2 to 332.0)	1.0 (-1.5 to 3.4)	-6.8 (-9.2 to -4.6)*
Other gynecological diseases cases aggregate	3,283.5 (3,066.2 to 3,557.6)	7.7 (7.5 to 7.9)*	-0.7 (-0.8 to -0.5)*	130.3 (89.2 to 180.9)	7.8 (4.5 to 11.1)*	-0.6 (-3.6 to 2.5)
Anemia due to other gynecological diseases aggregate	3,306.9 (3,255.0 to 3,364.4)	-2.2 (-4.1 to -0.3)*	-9.8 (-11.6 to -8.0)*	111.5 (74.9 to 160.5)	-5.9 (-9.5 to -2.6)*	-13.3 (-16.5 to -10.2)*
Hemoglobinopathies and hemolytic anemias	131,804.2 (129,514.3 to 134,445.3)	6.2 (4.5 to 7.8)*	-2.1 (-3.6 to -0.6)*	461.1 (308.1 to 670.3)	-4.1 (-6.7 to -1.6)*	-11.5 (-14.0 to -9.2)*
Thalassemias	4.8 (4.6 to 5.1)	-0.5 (-3.0 to 1.9)	-8.2 (-10.5 to -6.0)*	0.4 (0.3 to 0.6)	0.2 (-8.9 to 10.4)	-7.6 (-16.0 to 1.8)
Beta-thalassemia major cases aggregate	2.7 (2.6 to 2.8)	-0.1 (-3.5 to 3.3)	-7.9 (-11.0 to -4.7)*	0.2 (0.2 to 0.3)	1.9 (-12.2 to 17.0)	-6.0 (-19.1 to 7.9)
Hemoglobin E/beta-thalassemia cases aggregate	0.9 (0.9 to 1.0)	5.1 (3.9 to 6.2)*	-3.1 (-4.2 to -2.0)*	0.1 (0.1 to 0.1)	1.0 (-15.4 to 22.6)	-6.8 (-22.0 to 13.1)
Hemoglobin H disease cases aggregate	1.0 (0.9 to 1.3)	-6.4 (-11.9 to -0.3)*	-13.7 (-18.7 to -8.0)*	0.1 (0.1 to 0.1)	-4.9 (-24.4 to 18.5)	-12.3 (-30.3 to 9.3)
Heart failure due to thalassemias aggregate	0.2 (0.1 to 0.2)	2.9 (0.9 to 4.8)*	-5.2 (-7.0 to -3.3)*	0.0 (0.0 to 0.0)	2.8 (0.9 to 4.8)*	-5.2 (-7.0 to -3.3)*
Thalassemias trait	22,481.7 (21,920.0 to 23,130.6)	4.7 (4.2 to 5.2)*	-3.4 (-3.9 to -3.0)*	231.3 (153.2 to 339.2)	-3.6 (-7.4 to -0.1)*	-11.1 (-14.6 to -7.9)*
B-thalassemia trait cases aggregate	18,119.9 (17,611.5 to 18,703.3)	4.4 (3.8 to 4.9)*	-3.7 (-4.3 to -3.3)*	216.0 (142.8 to 316.9)	-3.6 (-7.4 to 0.0)	-11.1 (-14.6 to -7.8)*
Hemoglobin E trait cases aggregate	4,361.8 (4,162.7 to 4,575.7)	6.0 (5.0 to 7.1)*	-2.2 (-3.2 to -1.2)*	15.3 (10.0 to 22.8)	-4.2 (-12.5 to 3.4)	-11.6 (-19.3 to -4.6)*
Sickle cell disorders	137.5 (128.8 to 146.0)	14.7 (9.8 to 19.4)*	5.8 (1.3 to 10.2)*	14.1 (9.7 to 19.2)	14.1 (4.9 to 24.2)*	5.2 (-3.3 to 14.6)
Homozygous sickle cell and severe sickle cell/beta-thalassemia cases aggregate	124.7 (117.0 to 132.4)	13.6 (8.9 to 17.6)*	4.8 (0.4 to 8.4)*	12.7 (8.8 to 17.2)	14.1 (4.3 to 25.1)*	5.2 (-3.9 to 15.4)
Hemoglobin SC disease cases aggregate	12.3 (10.8 to 13.7)	28.6 (5.6 to 68.6)*	18.6 (-2.6 to 55.5)	1.3 (0.9 to 2.0)	14.9 (-11.9 to 50.9)	5.9 (-18.7 to 39.2)
Mild sickle cell/beta-thalassemia cases aggregate	0.5 (0.5 to 0.5)	-1.5 (-5.8 to 3.0)	-9.2 (-13.1 to -5.0)*	0.1 (0.1 to 0.1)	2.0 (-1.7 to 5.7)	-5.9 (-9.4 to -2.6)*
Sickle cell trait	35,694.0 (33,522.5 to 39,812.1)	14.8 (13.8 to 15.6)*	5.8 (4.9 to 6.6)*	107.7 (71.7 to 155.7)	3.1 (-1.6 to 7.3)	-4.9 (-9.2 to -1.1)*
G6PD deficiency	21,429.1 (18,143.0 to 24,868.5)	2.7 (-2.9 to 7.6)	-5.3 (-10.4 to -0.8)*	1.7 (1.2 to 2.4)	-5.4 (-13.5 to 3.9)	-12.8 (-20.2 to -4.2)*
G6PD cases aggregate	21,429.1 (18,143.0 to 24,868.5)	2.7 (-2.9 to 7.6)	-5.3 (-10.4 to -0.8)*	1.7 (1.2 to 2.4)	-5.4 (-13.5 to 3.9)	-12.8 (-20.2 to -4.2)*
Heart failure due to G6PD deficiency aggregate	0.0 (0.0 to 0.0)	0.0 (0.0 to 0.0)	0.0 (0.0 to 0.0)	0.0 (0.0 to 0.0)	0.0 (0.0 to 0.0)	0.0 (0.0 to 0.0)
G6PD trait	60,742.3 (56,539.9 to 65,097.5)	4.8 (3.0 to 6.4)*	-3.3 (-5.0 to -1.9)*	2.6 (1.7 to 3.6)	2.1 (-11.4 to 16.7)	-5.8 (-18.3 to 7.6)
Other hemoglobinopathies and hemolytic anemias	5,234.4 (5,134.3 to 5,335.5)	-7.1 (-9.2 to -5.1)*	-14.4 (-16.3 to -12.4)*	103.3 (68.0 to 153.5)	-13.3 (-17.3 to -9.4)*	-20.0 (-23.7 to -16.4)*
Other hemoglobinopathies and hemolytic anemias cases aggregate	5,233.6 (5,133.6 to 5,334.7)	-7.1 (-9.2 to -5.1)*	-14.4 (-16.3 to -12.4)*	101.5 (66.6 to 151.3)	-13.5 (-17.6 to -9.6)*	-20.2 (-24.0 to -16.6)*
Heart failure due to other hemoglobinopathies and hemolytic anemias aggregate	0.8 (0.7 to 0.9)	2.2 (0.6 to 4.0)*	-5.7 (-7.3 to -4.1)*	0.1 (0.1 to 0.1)	2.2 (0.6 to 4.0)*	-5.7 (-7.3 to -4.1)*
Endocrine, metabolic, blood, and immune disorders	3,954.9 (3,888.3 to 4,017.9)	-4.9 (-6.9 to -3.0)*	-12.3 (-14.1 to -10.5)*	95.6 (63.9 to 137.7)	-8.9 (-12.5 to -5.0)*	-15.9 (-19.3 to -12.4)*
Endocrine, metabolic, blood, and immune disorders cases aggregate	264.1 (239.6 to 291.8)	7.1 (4.9 to 9.0)*	-1.3 (-3.2 to 0.5)	10.6 (7.0 to 14.8)	7.1 (1.5 to 12.9)*	-1.2 (-6.4 to 4.1)
Anemia due to endocrine, metabolic, blood, and immune disorders aggregate	3,690.5 (3,629.3 to 3,750.8)	-5.7 (-7.7 to -3.6)*	-13.0 (-14.9 to -11.1)*	85.0 (56.8 to 123.3)	-10.5 (-14.5 to -6.4)*	-17.5 (-21.2 to -13.7)*
Heart failure due to endocrine, metabolic, blood, and immune disorders aggregate	0.3 (0.3 to 0.4)	5.8 (4.6 to 7.1)*	-2.4 (-3.6 to -1.2)*	0.0 (0.0 to 0.1)	5.8 (4.6 to 7.1)*	-2.4 (-3.6 to -1.2)*
Musculoskeletal disorders	65,899.9 (63,472.9 to 68,866.8)	5.2 (3.7 to 6.8)*	-3.0 (-4.4 to -1.6)*	7,370.8 (5,085.5 to 10,145.5)	5.2 (3.1 to 7.4)*	-3.0 (-5.0 to -0.9)*
Rheumatoid arthritis	944.9 (797.9 to 1,100.9)	6.6 (1.7 to 11.5)*	-1.7 (-6.2 to 2.8)	233.7 (159.4 to 328.1)	6.7 (1.5 to 12.5)*	-1.6 (-6.4 to 3.7)
Osteoarthritis	0.0 (0.0 to 0.0)	0.0 (0.0 to 0.0)	0.0 (0.0 to 0.0)	0.0 (0.0 to 0.0)	0.0 (0.0 to 0.0)	0.0 (0.0 to 0.0)
Osteoarthritis of the hip cases aggregate	0.0 (0.0 to 0.0)	0.0 (0.0 to 0.0)	0.0 (0.0 to 0.0)	0.0 (0.0 to 0.0)	0.0 (0.0 to 0.0)	0.0 (0.0 to 0.0)
Osteoarthritis of the knee cases aggregate	0.0 (0.0 to 0.0)	0.0 (0.0 to 0.0)	0.0 (0.0 to 0.0)	0.0 (0.0 to 0.0)	0.0 (0.0 to 0.0)	0.0 (0.0 to 0.0)
Low back and neck pain	37,920.1 (36,354.4 to 39,446.6)	5.6 (4.2 to 7.1)*	-2.6 (-3.9 to -1.2)*	4,348.6 (2,965.7 to 6,133.3)	5.7 (4.1 to 7.3)*	-2.5 (-4.0 to -1.0)*
Low back pain	29,193.7 (25,950.9 to 32,945.4)	5.4 (3.4 to 7.2)*	-2.8 (-4.6 to -1.2)*	3,370.3 (2,285.2 to 4,780.2)	5.6 (3.5 to 7.5)*	-2.7 (-4.5 to -0.9)*
Neck pain	9,700.5 (6,834.4 to 13,237.9)	6.2 (4.3 to 8.6)*	-2.0 (-3.8 to 0.2)	978.3 (596.2 to 1,560.1)	6.4 (4.2 to 9.0)*	-1.9 (-3.9 to 0.5)
Gout	545.2 (404.7 to 709.7)	9.3 (8.3 to 10.3)*	0.8 (-0.1 to 1.7)	18.7 (11.3 to 28.9)	9.3 (0.9 to 19.6)*	0.8 (-7.0 to 10.3)
Asymptomatic gout	487.1 (361.9 to 634.6)	9.3 (8.3 to 10.3)*	0.8 (-0.1 to 1.7)	0.0 (0.0 to 0.0)	0.0 (0.0 to 0.0)	0.0 (0.0 to 0.0)
Symptomatic gout cases aggregate	58.2 (42.0 to 77.7)	9.3 (8.3 to 10.3)*	0.8 (-0.1 to 1.7)	18.7 (11.3 to 28.9)	9.3 (0.9 to 19.6)*	0.8 (-7.0 to 10.3)
Other musculoskeletal disorders	28,692.3 (23,120.6 to 35,399.0)	4.0 (-0.9 to 8.9)	-4.1 (-8.6 to 0.5)	2,769.9 (1,743.7 to 3,995.4)	4.2 (-0.6 to 9.5)	-3.9 (-8.4 to 0.9)
Other non-communicable diseases	401,092.2 (391,255.8 to 409,509.0)	8.9 (8.1 to 9.7)*	0.4 (-0.3 to 1.2)	7,538.5 (5,040.3 to 10,984.2)	10.9 (9.7 to 12.7)*	2.3 (1.1 to 3.9)*
Congenital anomalies	8,346.7 (7,642.8 to 8,961.7)	17.5 (13.7 to 21.1)*	8.3 (4.9 to 11.7)*	757.8 (470.5 to 1,145.3)	24.5 (15.7 to 33.9)*	14.8 (6.7 to 23.5)*
Neural tube defects	140.2 (95.3 to 203.0)	15.0 (11.8 to 17.8)*	6.0 (3.1 to 8.6)*	48.8 (27.6 to 81.1)	16.0 (8.8 to 23.1)*	7.0 (0.4 to 13.5)*
Congenital heart anomalies	4,210.2 (2,965.3 to 6,278.6)	23.5 (19.8 to 27.5)*	13.9 (10.4 to 17.6)*	146.8 (57.0 to 272.4)	24.0 (20.1 to 28.3)*	14.4 (10.8 to 18.3)*
Less severe heart anomalies cases aggregate	3,833.5 (2,551.3 to 5,885.8)	22.8 (18.4 to 27.2)*	13.2 (9.2 to 17.3)*	124.2 (46.5 to 240.6)	22.8 (17.9 to 27.7)*	13.2 (8.7 to 17.8)*
Severe congenital heart anomalies	354.9 (236.2 to 531.1)	31.4 (25.2 to 36.7)*	21.2 (15.5 to 26.0)*	21.0 (8.3 to 42.0)	31.4 (23.7 to 38.5)*	21.2 (14.1 to 27.7)*
Critical congenital heart anomalies	16.4 (9.3 to 27.3)	53.2 (34.8 to 67.5)*	41.3 (24.3 to 54.5)*	1.0 (0.3 to 2.0)	53.5 (26.0 to 78.1)*	41.6 (16.2 to 64.2)*
Heart failure due to congenital heart anomalies aggregate	5.4 (4.8 to 6.0)	6.1 (4.8 to 7.6)*	-2.1 (-3.4 to -0.8)*	0.7 (0.4 to 0.9)	6.1 (4.0 to 8.4)*	-2.1 (-4.1 to -0.0)*
Cleft lip and cleft palate	585.7 (340.0 to 980.6)	13.5 (2.8 to 20.7)*	4.7 (-5.2 to 11.3)	7.0 (3.5 to 12.9)	7.1 (-3.8 to 16.8)	-1.2 (-11.3 to 7.7)
Down syndrome	493.4 (312.2 to 756.8)	12.6 (8.8 to 16.1)*	3.8 (0.3 to 7.1)*	41.3 (22.8 to 70.0)	12.6 (7.6 to 17.4)*	3.9 (-0.8 to 8.3)
Turner syndrome	32.9 (15.4 to 59.8)	7.9 (6.0 to 9.8)*	-0.5 (-2.3 to 1.2)	0.7 (0.3 to 1.5)	8.0 (1.9 to 14.0)*	-0.4 (-6.0 to 5.1)
Klinefelter syndrome	18.9 (9.9 to 33.9)	9.2 (7.6 to 10.5)*	0.7 (-0.8 to 1.9)	0.2 (0.1 to 0.5)	9.2 (3.2 to 14.5)*	0.7 (-4.8 to 5.6)
Other chromosomal abnormalities	426.0 (236.3 to 754.8)	12.2 (8.5 to 15.1)*	3.4 (0.0 to 6.2)*	35.8 (17.9 to 68.9)	12.2 (7.3 to 16.3)*	3.4 (-1.0 to 7.3)
Other congenital anomalies	2,767.2 (1,520.5 to 5,083.5)	10.9 (8.7 to 13.0)*	2.2 (0.3 to 4.2)*	477.3 (281.1 to 755.5)	28.2 (15.4 to 43.6)*	18.2 (6.4 to 32.5)*
Skin and subcutaneous diseases	203,885.8 (198,738.6 to 207,989.8)	9.3 (8.3 to 10.3)*	0.8 (-0.2 to 1.7)	4,020.0 (2,515.8 to 6,292.7)	9.1 (8.1 to 10.1)*	0.6 (-0.3 to 1.5)
Dermatitis	19,433.1 (15,701.8 to 23,946.7)	8.9 (8.6 to 9.2)*	0.4 (0.1 to 0.7)*	668.0 (422.4 to 970.7)	9.7 (8.5 to 11.2)*	1.2 (0.0 to 2.5)*
Eczema cases aggregate	5,782.1 (4,258.5 to 7,456.5)	11.0 (10.4 to 11.6)*	2.4 (1.8 to 2.9)*	355.2 (219.9 to 516.7)	11.2 (9.1 to 13.6)*	2.6 (0.6 to 4.8)*

Cause	Prevalence in thousands (95% confidence interval)			YLDs in thousands (95% confidence interval)		
	2015	Percent change in counts between 2005 and 2015	Percent change in rates between 2005 and 2015	2015	Percent change in counts between 2005 and 2015	Percent change in rates between 2005 and 2015
Contact dermatitis cases aggregate	8,616.5 (5,602.4 to 12,268.5)	8.0 (7.7 to 8.2)*	-0.4 (-0.6 to -0.2)*	236.8 (124.5 to 395.8)	8.1 (6.6 to 9.8)*	-0.3 (-1.7 to 1.3)
Seborrhoeic dermatitis cases aggregate	5,034.4 (3,629.8 to 6,681.6)	8.0 (7.5 to 8.6)*	-0.4 (-0.8 to 0.2)	76.0 (38.7 to 126.9)	8.2 (6.4 to 9.7)*	-0.3 (-1.8 to 1.2)
Psoriasis	5,238.7 (4,758.0 to 5,753.8)	9.2 (7.8 to 10.5)*	0.7 (-0.6 to 1.9)	434.4 (298.6 to 601.2)	9.4 (6.8 to 12.0)*	0.9 (-1.5 to 3.3)
Cellulitis	65.1 (48.6 to 85.6)	19.1 (14.7 to 24.2)*	9.9 (5.8 to 14.6)*	4.8 (2.9 to 7.3)	19.1 (11.4 to 27.9)*	9.9 (2.8 to 17.9)*
Pyoderma	492.1 (426.4 to 561.6)	9.6 (7.2 to 12.3)*	1.0 (-1.1 to 3.6)	2.8 (1.1 to 5.9)	9.6 (5.2 to 14.3)*	1.1 (-3.0 to 5.4)
Scabies	22,661.7 (14,741.7 to 33,659.2)	7.9 (3.6 to 12.5)*	-0.5 (-4.5 to 3.7)	588.8 (281.5 to 1,069.4)	8.0 (3.6 to 12.5)*	-0.4 (-4.5 to 3.7)
Fungal skin diseases	36,305.5 (30,886.1 to 42,303.7)	9.0 (8.8 to 9.2)*	0.5 (0.4 to 0.7)*	206.7 (81.5 to 435.4)	9.1 (8.7 to 9.6)*	0.6 (0.2 to 1.1)*
Viral skin diseases	15,765.8 (13,356.0 to 19,005.1)	7.6 (7.0 to 8.3)*	-0.8 (-1.3 to -0.1)*	489.6 (302.0 to 761.3)	7.7 (6.6 to 8.7)*	-0.6 (-1.7 to 0.3)
Molluscum contagiosum cases aggregate	2,086.1 (1,601.3 to 2,681.6)	8.3 (7.7 to 9.0)*	-0.1 (-0.7 to 0.5)	64.9 (37.0 to 103.7)	8.5 (6.1 to 10.9)*	0.1 (-2.1 to 2.3)
Viral warts cases aggregate	13,679.7 (11,359.4 to 16,864.5)	7.5 (6.8 to 8.3)*	-0.9 (-1.5 to -0.1)*	424.7 (260.5 to 668.1)	7.6 (6.4 to 8.8)*	-0.7 (-1.9 to 0.3)
Acne vulgaris	97,178.7 (83,829.6 to 112,583.9)	9.7 (7.3 to 12.0)*	1.1 (-1.0 to 3.3)	1,054.7 (493.7 to 1,992.1)	9.7 (7.3 to 12.1)*	1.2 (-1.0 to 3.4)
Alopecia areata	2,114.8 (1,834.5 to 2,408.7)	7.9 (7.8 to 8.1)*	-0.5 (-0.6 to -0.3)*	72.4 (44.3 to 110.4)	8.1 (5.8 to 10.3)*	-0.3 (-2.4 to 1.7)
Pruritus	4,392.3 (3,087.1 to 6,120.2)	8.5 (5.7 to 11.2)*	0.1 (-2.5 to 2.6)	47.6 (21.2 to 99.8)	8.6 (5.4 to 11.4)*	0.1 (-2.8 to 2.8)
Urticaria	4,299.4 (2,989.9 to 5,985.2)	8.1 (8.0 to 8.3)*	-0.3 (-0.4 to -0.1)*	262.4 (144.5 to 426.4)	8.3 (6.3 to 10.2)*	-0.1 (-1.9 to 1.6)
Decubitus ulcer	29.0 (21.2 to 39.3)	16.4 (11.5 to 21.2)*	7.4 (2.8 to 11.8)*	4.8 (2.9 to 7.3)	16.5 (11.4 to 21.8)*	7.4 (2.7 to 12.4)*
Other skin and subcutaneous diseases	30,487.9 (27,889.0 to 33,533.5)	11.2 (9.2 to 13.2)*	2.6 (0.7 to 4.4)*	182.9 (87.7 to 340.2)	11.3 (9.3 to 13.5)*	2.7 (0.8 to 4.6)*
Sense organ diseases	56,722.1 (54,265.3 to 59,529.3)	10.1 (8.4 to 11.7)*	1.5 (-0.0 to 3.0)	2,048.5 (1,301.0 to 3,006.1)	10.9 (8.6 to 13.3)*	2.3 (0.1 to 4.5)*
Glaucoma	0.0 (0.0 to 0.0)	0.0 (0.0 to 0.0)	0.0 (0.0 to 0.0)	0.0 (0.0 to 0.0)	0.0 (0.0 to 0.0)	0.0 (0.0 to 0.0)
Cataract	46.3 (29.0 to 70.7)	4.8 (1.4 to 8.5)*	-3.3 (-6.5 to 0.1)	3.7 (1.9 to 6.8)	3.5 (-5.5 to 12.9)	-4.5 (-12.8 to 4.1)
Macular degeneration	0.0 (0.0 to 0.0)	0.0 (0.0 to 0.0)	0.0 (0.0 to 0.0)	0.0 (0.0 to 0.0)	0.0 (0.0 to 0.0)	0.0 (0.0 to 0.0)
Refraction and accommodation disorders	27,762.5 (25,398.3 to 30,302.8)	5.6 (4.4 to 7.0)*	-2.6 (-3.8 to -1.3)*	583.7 (374.4 to 901.1)	7.3 (5.9 to 8.8)*	-1.1 (-2.3 to 0.3)
Age-related and other hearing loss	19,587.4 (13,132.4 to 27,052.1)	17.9 (14.0 to 25.1)*	8.7 (5.2 to 15.4)*	1,141.6 (640.4 to 1,779.6)	13.4 (9.3 to 17.8)*	4.5 (0.8 to 8.7)*
Other vision loss	1,478.9 (1,059.2 to 1,995.1)	8.6 (6.5 to 10.5)*	0.2 (-1.8 to 1.9)	75.8 (44.6 to 118.9)	8.4 (4.6 to 12.0)*	-0.0 (-3.5 to 3.3)
Other sense organ diseases	9,407.6 (8,291.1 to 10,625.9)	9.4 (7.2 to 11.4)*	0.9 (-1.1 to 2.8)	243.7 (145.6 to 366.6)	9.5 (6.9 to 12.2)*	1.0 (-1.4 to 3.5)
Acute other sense organ diseases	91.8 (81.8 to 102.1)	9.3 (7.1 to 11.6)*	0.8 (-1.2 to 2.9)	2.5 (1.5 to 3.7)	9.3 (7.1 to 11.6)*	0.8 (-1.2 to 2.9)
Chronic other sense organ diseases	9,315.8 (8,204.5 to 10,535.5)	9.4 (7.2 to 11.4)*	0.9 (-1.2 to 2.8)	241.3 (144.1 to 363.2)	9.5 (6.9 to 12.3)*	1.0 (-1.4 to 3.5)
Oral disorders	260,175.1 (246,309.2 to 274,082.2)	8.7 (6.8 to 10.6)*	0.3 (-1.5 to 2.0)	712.2 (411.1 to 1,142.7)	8.6 (7.8 to 9.3)*	0.1 (-0.5 to 0.8)
Deciduous caries	0.0 (0.0 to 0.0)	0.0 (0.0 to 0.0)	0.0 (0.0 to 0.0)	0.0 (0.0 to 0.0)	0.0 (0.0 to 0.0)	0.0 (0.0 to 0.0)
Permanent caries	248,204.6 (203,111.2 to 296,158.2)	8.7 (7.0 to 10.2)*	0.2 (-1.3 to 1.6)	168.2 (72.7 to 321.0)	7.9 (6.4 to 9.5)*	-0.5 (-1.9 to 1.0)
Periodontal diseases	13,226.1 (9,763.8 to 17,387.6)	8.9 (7.6 to 9.9)*	0.4 (-0.7 to 1.4)	88.8 (33.1 to 193.1)	9.0 (7.6 to 10.3)*	0.6 (-0.8 to 1.7)
Edentulism and severe tooth loss	3,725.8 (3,388.7 to 4,106.3)	9.8 (9.1 to 10.3)*	1.2 (0.6 to 1.7)*	108.0 (71.6 to 153.4)	9.9 (8.0 to 11.7)*	1.3 (-0.4 to 3.0)
Other oral disorders	11,664.8 (10,420.0 to 12,780.4)	8.3 (8.0 to 8.6)*	-0.1 (-0.4 to 0.1)	347.2 (210.2 to 527.5)	8.4 (7.5 to 9.5)*	0.0 (-0.9 to 0.9)
Injuries	53,132.6 (49,925.2 to 56,600.3)	-2.0 (-7.6 to 2.2)	-9.6 (-14.8 to -5.8)*	2,256.2 (1,631.6 to 3,035.1)	-3.9 (-9.0 to 0.8)	-11.3 (-16.1 to -7.0)*
Transport injuries	4,250.0 (3,928.3 to 4,614.4)	5.9 (4.6 to 7.2)*	-2.4 (-3.5 to -1.1)*	269.3 (187.6 to 367.3)	-3.2 (-6.6 to -0.1)*	-10.8 (-13.8 to -7.9)*
Road injuries	3,911.7 (3,588.4 to 4,271.8)	6.0 (4.6 to 7.5)*	-2.2 (-3.5 to -0.9)*	246.5 (170.8 to 336.8)	-2.3 (-5.7 to 0.8)	-9.9 (-13.1 to -7.0)*
Pedestrian road injuries	454.2 (378.2 to 543.6)	3.9 (2.3 to 5.4)*	-4.2 (-5.6 to -2.8)*	27.6 (18.9 to 39.0)	-7.7 (-11.6 to -3.7)*	-14.9 (-18.5 to -11.2)*
Cyclist road injuries	769.4 (638.3 to 891.3)	6.0 (4.4 to 7.6)*	-2.2 (-3.7 to -0.8)*	42.9 (29.1 to 60.7)	-3.9 (-8.3 to 0.2)	-11.4 (-15.4 to -7.6)*
Motorcyclist road injuries	945.3 (801.1 to 1,121.5)	12.8 (11.1 to 14.4)*	4.1 (2.5 to 5.5)*	57.1 (37.6 to 79.7)	1.6 (-2.7 to 5.8)	-6.3 (-10.3 to -2.4)*
Motor vehicle road injuries	1,557.1 (1,302.6 to 1,818.1)	1.6 (-0.5 to 3.7)	-6.3 (-8.3 to -4.3)*	107.4 (71.2 to 148.6)	-3.6 (-7.0 to -0.5)*	-11.1 (-14.2 to -8.2)*
Other road injuries	185.7 (147.7 to 235.5)	19.1 (16.7 to 21.0)*	9.8 (7.6 to 11.6)*	11.6 (7.4 to 16.5)	12.8 (8.8 to 16.2)*	4.0 (0.3 to 7.2)*
Other transport injuries	338.3 (291.5 to 392.5)	4.2 (3.4 to 5.1)*	-3.9 (-4.7 to -3.1)*	22.7 (16.0 to 31.4)	-12.2 (-15.8 to -8.1)*	-19.0 (-22.4 to -15.3)*
Unintentional injuries	41,930.5 (39,543.2 to 44,651.0)	3.0 (2.5 to 3.5)*	-5.0 (-5.5 to -4.5)*	1,692.9 (1,209.8 to 2,288.4)	-3.5 (-5.8 to -1.3)*	-11.0 (-13.1 to -9.0)*
Falls	10,030.1 (8,792.7 to 11,397.6)	11.3 (10.5 to 12.1)*	2.6 (1.9 to 3.3)*	570.7 (395.1 to 777.6)	-0.6 (-4.5 to 3.4)	-8.3 (-12.0 to -4.7)*
Drowning	169.6 (143.8 to 205.7)	-8.7 (-9.5 to -7.9)*	-15.8 (-16.6 to -15.0)*	10.4 (7.2 to 14.7)	-20.5 (-23.8 to -16.8)*	-26.7 (-29.7 to -23.3)*
Fire, heat, and hot substances	3,466.7 (2,886.3 to 4,183.8)	0.1 (-0.7 to 1.1)	-7.7 (-8.5 to -6.8)*	142.6 (100.3 to 194.8)	-7.5 (-11.0 to -4.3)*	-14.7 (-17.9 to -11.7)*
Poisonings	437.1 (356.2 to 541.0)	6.6 (5.0 to 8.2)*	-1.7 (-3.1 to -0.2)*	48.5 (32.2 to 70.4)	3.9 (0.6 to 6.7)*	-4.2 (-7.2 to -1.6)*
Exposure to mechanical forces	11,975.2 (10,635.1 to 13,397.0)	4.1 (3.3 to 5.0)*	-4.0 (-4.7 to -3.2)*	230.7 (160.7 to 315.2)	-3.4 (-6.2 to -0.8)*	-11.0 (-13.5 to -8.5)*
Unintentional firearm injuries	121.4 (100.7 to 147.1)	-0.4 (-2.2 to 1.4)	-8.1 (-9.8 to -6.5)*	6.1 (4.1 to 8.7)	-7.7 (-11.4 to -4.1)*	-14.8 (-18.3 to -11.6)*
Unintentional suffocation	473.9 (339.0 to 655.1)	-3.0 (-5.7 to 0.1)	-10.5 (-13.0 to -7.7)*	15.6 (10.4 to 22.0)	-2.9 (-5.5 to -0.5)*	-10.5 (-12.8 to -8.2)*
Other exposure to mechanical forces	11,379.8 (10,053.7 to 12,783.1)	4.5 (3.7 to 5.4)*	-3.6 (-4.4 to -2.8)*	209.0 (143.9 to 288.2)	-3.4 (-6.3 to -0.6)*	-10.9 (-13.5 to -8.3)*
Adverse effects of medical treatment	493.7 (364.1 to 656.0)	4.2 (0.3 to 8.2)*	-3.9 (-7.5 to -0.2)*	65.8 (40.0 to 99.7)	4.2 (0.3 to 8.2)*	-3.9 (-7.5 to -0.2)*
Animal contact	2,547.4 (2,182.0 to 2,971.5)	0.7 (-1.5 to 2.9)	-7.1 (-9.1 to -5.1)*	103.7 (71.3 to 142.2)	-1.2 (-3.8 to 1.7)	-8.9 (-11.3 to -6.2)*
Venomous animal contact	1,109.7 (951.2 to 1,303.0)	2.8 (1.2 to 4.4)*	-5.2 (-6.7 to -3.7)*	82.1 (55.2 to 113.2)	-0.4 (-3.0 to 2.5)	-8.2 (-10.6 to -5.5)*
Non-venomous animal contact	1,437.8 (1,160.4 to 1,777.0)	-0.9 (-3.9 to 2.4)	-8.6 (-11.4 to -5.6)*	21.6 (14.1 to 33.1)	-4.1 (-8.5 to 0.1)	-11.6 (-15.6 to -7.6)*
Foreign body	1,985.1 (1,689.9 to 2,322.6)	5.5 (3.9 to 6.8)*	-2.7 (-4.2 to -1.5)*	85.5 (59.6 to 117.2)	-3.5 (-6.4 to -0.5)*	-11.0 (-13.7 to -8.2)*
Pulmonary aspiration and foreign body in airway	805.9 (604.6 to 1,093.7)	3.8 (1.1 to 6.5)*	-4.2 (-6.8 to -1.8)*	45.9 (30.7 to 66.0)	-7.3 (-10.6 to -3.8)*	-14.5 (-17.6 to -11.3)*
Foreign body in eyes	76.3 (31.0 to 139.1)	11.0 (10.1 to 11.8)*	2.3 (1.5 to 3.1)*	4.3 (1.6 to 8.7)	10.2 (8.4 to 11.3)*	1.6 (-0.0 to 2.6)
Foreign body in other body part	1,102.9 (904.8 to 1,322.6)	6.3 (5.1 to 7.5)*	-2.0 (-3.1 to -0.8)*	35.3 (24.0 to 50.0)	0.3 (-2.2 to 2.6)	-7.5 (-9.8 to -5.3)*
Environmental heat and cold exposure	3,131.4 (2,717.8 to 3,618.9)	6.5 (5.5 to 7.3)*	-1.8 (-2.7 to -1.1)*	153.0 (107.2 to 213.7)	-1.0 (-3.4 to 1.2)	-8.7 (-10.9 to -6.6)*
Other unintentional injuries	7,694.1 (6,700.0 to 8,681.3)	-7.4 (-8.3 to -6.5)*	-14.6 (-15.4 to -13.8)*	282.0 (188.0 to 393.1)	-10.9 (-13.0 to -9.1)*	-17.8 (-19.8 to -16.1)*
Self-harm and interpersonal violence	1,146.1 (1,038.9 to 1,264.4)	2.8 (1.4 to 4.1)*	-5.2 (-6.4 to -4.0)*	67.5 (46.3 to 92.8)	-6.2 (-8.9 to -3.2)*	-13.5 (-15.9 to -10.8)*
Self-harm	265.2 (223.0 to 311.9)	2.7 (1.8 to 3.5)*	-5.3 (-6.1 to -4.6)*	21.2 (14.3 to 29.4)	-5.3 (-7.9 to -2.6)*	-12.6 (-15.1 to -10.2)*
Interpersonal violence	880.9 (777.2 to 982.1)	2.9 (1.2 to 4.4)*	-5.1 (-6.7 to -3.7)*	46.3 (32.0 to 64.4)	-6.6 (-9.7 to -3.0)*	-13.9 (-16.8 to -10.6)*

Cause	Prevalence in thousands (95% confidence interval)			YLDs in thousands (95% confidence interval)		
	2015	Percent change in counts between 2005 and 2015	Percent change in rates between 2005 and 2015	2015	Percent change in counts between 2005 and 2015	Percent change in rates between 2005 and 2015
Assault by firearm	42.9 (35.4 to 52.4)	-3.1 (-4.8 to -1.6)*	-10.6 (-12.2 to -9.2)*	2.6 (1.8 to 3.6)	-11.7 (-15.7 to -7.9)*	-18.5 (-22.3 to -15.1)*
Assault by sharp object	209.1 (170.8 to 252.4)	-2.0 (-4.3 to -0.1)*	-9.6 (-11.8 to -7.9)*	9.7 (6.5 to 13.6)	-14.2 (-18.3 to -9.9)*	-20.9 (-24.7 to -16.9)*
Assault by other means	628.9 (540.7 to 720.2)	5.0 (3.3 to 6.6)*	-3.2 (-4.7 to -1.7)*	34.1 (23.4 to 47.9)	-3.8 (-7.1 to -0.4)*	-11.3 (-14.3 to -8.2)*
Forces of nature, war, and legal intervention	5,806.0 (4,018.9 to 7,821.1)	-30.7 (-52.7 to -4.4)*	-36.1 (-56.4 to -11.8)*	226.6 (132.0 to 341.9)	-6.3 (-41.0 to 37.0)	-13.6 (-45.6 to 26.3)
Exposure to forces of nature	1,853.6 (1,074.9 to 2,894.2)	-7.5 (-36.7 to 35.3)	-14.7 (-41.6 to 24.7)	58.0 (36.6 to 85.8)	-40.6 (-62.0 to -7.5)*	-45.3 (-64.9 to -14.7)*
Collective violence and legal intervention	3,952.5 (2,509.3 to 5,588.2)	-38.0 (-61.8 to -5.0)*	-42.8 (-64.8 to -12.4)*	168.6 (86.3 to 276.6)	17.0 (-39.3 to 105.1)	7.9 (-44.1 to 89.2)
**Anemia**	**163,284.8 (161,384.6 to 165,172.1)**	**-3.6 (-4.9 to -2.4)***	**-11.1 (-12.3 to -10.0)***	**4,444.1 (2,972.7 to 6,439.5)**	**-8.3 (-10.3 to -6.3)***	**-15.4 (-17.3 to -13.6)***
**Developmental intellectual disability**	**15,773.2 (11,728.3 to 19,688.5)**	**9.9 (9.6 to 10.3)***	**1.4 (1.0 to 1.7)***	**1,713.4 (1,214.3 to 2,270.8)**	**12.4 (10.5 to 14.8)***	**3.6 (1.9 to 5.8)***
**Epilepsy**	**3,717.1 (3,127.4 to 4,252.5)**	**10.9 (6.5 to 14.9)***	**2.3 (-1.8 to 6.0)**	**1,163.6 (818.9 to 1,529.0)**	**2.7 (-3.6 to 8.7)**	**-5.3 (-11.1 to 0.3)**
**Guillain-Barré syndrome**	**1.9 (1.0 to 3.4)**	**9.5 (8.0 to 10.6)***	**1.0 (-0.4 to 2.0)**	**0.6 (0.3 to 1.1)**	**9.5 (8.0 to 10.6)***	**1.0 (-0.4 to 2.0)**
**Hearing loss**	**30,764.7 (24,989.6 to 37,149.7)**	**10.7 (9.5 to 12.1)***	**2.1 (1.0 to 3.4)***	**1,610.5 (1,010.3 to 2,327.4)**	**9.9 (6.9 to 12.6)***	**1.3 (-1.4 to 3.9)**
**Heart failure**	**161.8 (151.3 to 173.6)**	**10.4 (9.8 to 11.0)***	**1.8 (1.2 to 2.4)***	**19.9 (13.6 to 27.7)**	**10.8 (6.9 to 15.2)***	**2.2 (-1.5 to 6.2)**
**Infertility**	**16,329.0 (12,063.5 to 21,869.3)**	**26.7 (20.9 to 32.5)***	**16.9 (11.5 to 22.2)***	**114.1 (48.5 to 231.0)**	**22.9 (17.0 to 28.3)***	**13.3 (7.9 to 18.3)***
**Pelvic inflammatory disease**	**64.8 (51.8 to 79.6)**	**-5.1 (-6.3 to -4.0)***	**-12.5 (-13.6 to -11.4)***	**8.7 (5.6 to 12.4)**	**-5.0 (-10.4 to 0.6)**	**-12.4 (-17.4 to -7.2)***
**Vision loss**	**30,633.8 (28,035.7 to 33,447.6)**	**5.9 (4.7 to 7.2)***	**-2.3 (-3.4 to -1.1)***	**923.8 (626.1 to 1,303.3)**	**9.0 (7.5 to 10.7)***	**0.5 (-0.9 to 2.1)**

SR Table 11. Prevalent cases and YLDs for 2015, percent change, and change in age-standardised rates between 2005 and 2015 by cause, global, ages 25-29 years

Cause	Prevalence in thousands (95% confidence interval)			YLDs in thousands (95% confidence interval)		
	2015	Percent change in counts between 2005 and 2015	Percent change in rates between 2005 and 2015	2015	Percent change in counts between 2005 and 2015	Percent change in rates between 2005 and 2015
**All causes**	--	--	--	**56,070.7 (41,502.3 to 72,376.5)**	**16.3 (15.5 to 17.1)***	**-3.2 (-3.9 to -2.6)***
Communicable, maternal, neonatal, and nutritional diseases	363,850.1 (360,788.4 to 367,683.6)	14.1 (13.2 to 15.0)*	-5.1 (-5.8 to -4.3)*	8,000.5 (5,717.4 to 11,105.2)	3.4 (1.0 to 6.5)*	-14.0 (-16.0 to -11.4)*
HIV/AIDS and tuberculosis	5,756.2 (5,570.3 to 5,965.8)	-3.4 (-6.9 to 0.3)	-19.6 (-22.6 to -16.6)*	820.9 (565.1 to 1,128.8)	-3.6 (-8.5 to 1.7)	-19.8 (-23.9 to -15.4)*
Tuberculosis	1,242.1 (898.6 to 1,709.7)	6.6 (-2.3 to 15.6)	-11.3 (-18.8 to -3.8)*	389.5 (239.8 to 599.7)	7.0 (-2.3 to 16.2)	-11.0 (-18.7 to -3.3)*
HIV/AIDS	4,518.8 (4,368.4 to 4,694.6)	-5.9 (-10.0 to -1.7)*	-21.8 (-25.1 to -18.2)*	431.5 (309.8 to 564.8)	-11.5 (-16.0 to -6.8)*	-26.4 (-30.2 to -22.5)*
HIV/AIDS - Tuberculosis	166.4 (150.3 to 183.2)	-37.6 (-39.8 to -35.3)*	-48.1 (-49.9 to -46.2)*	63.5 (43.0 to 86.1)	-37.2 (-40.4 to -34.0)*	-47.7 (-50.4 to -45.1)*
HIV/AIDS resulting in other diseases	4,525.9 (4,337.5 to 4,752.6)	-4.0 (-7.8 to -0.1)*	-20.1 (-23.3 to -16.9)*	368.0 (264.6 to 480.6)	-4.7 (-10.4 to 0.9)	-20.8 (-25.5 to -16.1)*
HIV aggregate	3,685.6 (3,513.6 to 3,896.0)	-20.1 (-23.8 to -16.3)*	-33.5 (-36.6 to -30.4)*	268.4 (191.6 to 352.7)	-21.0 (-24.7 to -17.0)*	-34.3 (-37.4 to -30.9)*
AIDS aggregate	840.3 (780.3 to 902.0)	742.3 (550.1 to 1,000.8)*	600.7 (440.8 to 815.7)*	99.6 (71.6 to 129.8)	114.3 (56.9 to 208.1)*	78.3 (30.5 to 156.3)*
Diarrhea, lower respiratory, and other common infectious diseases	28,735.1 (26,446.9 to 30,500.5)	15.6 (12.9 to 18.2)*	-3.9 (-6.1 to -1.7)*	879.6 (611.7 to 1,240.6)	14.6 (12.8 to 16.3)*	-4.7 (-6.1 to -3.3)*
Diarrheal diseases	1,647.0 (1,564.8 to 1,725.8)	15.6 (14.6 to 16.6)*	-3.8 (-4.7 to -3.0)*	263.4 (181.7 to 360.1)	16.0 (13.4 to 18.5)*	-3.5 (-5.6 to -1.4)*
Diarrhoea episodes aggregate	1,646.8 (1,564.5 to 1,725.5)	15.6 (14.6 to 16.6)*	-3.8 (-4.7 to -3.0)*	263.4 (181.7 to 360.0)	16.0 (13.4 to 18.5)*	-3.5 (-5.6 to -1.4)*
Guillain-Barré syndrome due to diarrheal diseases	0.2 (0.1 to 0.4)	20.9 (19.4 to 21.9)*	0.5 (-0.7 to 1.4)	0.1 (0.0 to 0.1)	20.9 (19.4 to 21.9)*	0.5 (-0.7 to 1.4)
Intestinal infectious diseases	68.9 (62.7 to 75.7)	-21.7 (-29.7 to -13.1)*	-34.9 (-41.5 to -27.7)*	10.8 (6.7 to 15.7)	-8.6 (-20.4 to 5.1)	-24.0 (-33.8 to -12.5)*
Typhoid fever	70.1 (53.8 to 89.2)	-8.0 (-12.3 to -4.0)*	-23.5 (-27.0 to -20.2)*	9.3 (5.6 to 13.7)	-7.4 (-21.5 to 8.3)	-23.0 (-34.7 to -9.9)*
Typhoid fever episodes aggregate	58.0 (44.3 to 74.1)	-8.3 (-16.3 to 0.4)	-23.7 (-30.3 to -16.5)*	5.5 (3.4 to 8.1)	-8.3 (-22.1 to 6.7)	-23.7 (-35.2 to -11.2)*
Complications of typhoid fever aggregate	12.2 (8.0 to 17.2)	-6.9 (-37.7 to 38.1)	-22.5 (-48.2 to 14.9)	3.8 (2.0 to 6.0)	-6.2 (-38.6 to 43.9)	-21.9 (-48.9 to 19.7)
Paratyphoid fever	27.5 (19.3 to 37.6)	-8.7 (-15.4 to -1.1)*	-24.1 (-29.6 to -17.7)*	1.4 (0.8 to 2.3)	-8.5 (-25.3 to 13.9)	-23.9 (-37.8 to -5.3)*
Paratyphoid fever episodes aggregate	26.1 (18.3 to 35.9)	-8.7 (-16.0 to 0.0)	-24.0 (-30.1 to -16.8)*	1.3 (0.7 to 2.1)	-8.3 (-25.8 to 15.9)	-23.8 (-38.2 to -3.6)*
Intestinal perforation due to paratyphoid	1.4 (0.8 to 2.2)	-9.8 (-48.9 to 54.8)	-25.0 (-57.5 to 28.8)	0.2 (0.1 to 0.3)	-9.8 (-48.9 to 54.8)	-24.9 (-57.5 to 28.8)
Other intestinal infectious diseases	--	--	--	0.1 (0.0 to 0.2)	-61.5 (-75.2 to -38.0)*	-68.0 (-79.4 to -48.4)*
Lower respiratory infections	272.6 (254.2 to 292.0)	18.3 (16.3 to 20.2)*	-1.6 (-3.3 to -0.0)*	16.7 (11.0 to 24.2)	18.6 (12.5 to 24.2)*	-1.4 (-6.4 to 3.3)
Lower respiratory infection episodes aggregate	272.3 (254.0 to 291.4)	18.3 (16.3 to 20.2)*	-1.6 (-3.3 to -0.0)*	16.6 (11.0 to 24.1)	18.5 (12.5 to 24.2)*	-1.4 (-6.4 to 3.4)
Guillain-Barré syndrome due to lower respiratory infections	0.3 (0.1 to 0.5)	20.9 (19.4 to 21.9)*	0.5 (-0.7 to 1.4)	0.1 (0.0 to 0.2)	20.9 (19.4 to 21.9)*	0.5 (-0.7 to 1.4)
Upper respiratory infections	19,239.6 (13,201.6 to 25,804.7)	18.6 (17.5 to 19.9)*	-1.3 (-2.2 to -0.3)*	226.6 (118.6 to 405.3)	18.8 (17.3 to 20.4)*	-1.2 (-2.5 to 0.2)
Upper respiratory infection episodes aggregate	19,238.9 (13,200.7 to 25,804.1)	18.6 (17.5 to 19.9)*	-1.3 (-2.2 to -0.3)*	226.4 (118.4 to 405.0)	18.8 (17.3 to 20.4)*	-1.2 (-2.5 to 0.2)
Guillain-Barré syndrome due to upper respiratory infections	0.7 (0.4 to 1.1)	20.9 (19.4 to 21.9)*	0.5 (-0.7 to 1.4)	0.2 (0.1 to 0.4)	20.9 (19.4 to 21.9)*	0.5 (-0.7 to 1.4)
Otitis media	6,660.5 (4,861.6 to 8,551.7)	7.7 (4.4 to 10.7)*	-10.4 (-13.1 to -7.9)*	182.6 (102.6 to 282.0)	7.0 (2.2 to 12.3)*	-11.0 (-15.0 to -6.6)*
Acute otitis media	868.1 (687.4 to 1,103.6)	20.4 (16.1 to 25.1)*	0.2 (-3.4 to 4.0)	11.3 (5.6 to 21.6)	20.5 (15.0 to 26.1)*	0.2 (-4.4 to 4.9)
Chronic otitis media aggregate	5,792.4 (4,027.1 to 7,655.2)	6.0 (2.4 to 9.3)*	-11.8 (-14.8 to -9.1)*	171.2 (95.9 to 265.4)	6.2 (1.0 to 11.9)*	-11.7 (-16.0 to -7.0)*
Meningitis	692.3 (649.0 to 726.5)	19.1 (13.9 to 24.3)*	-0.9 (-5.2 to 3.4)	122.5 (85.8 to 160.8)	19.7 (16.3 to 23.3)*	-0.4 (-3.2 to 2.5)
Pneumococcal meningitis	600.9 (503.1 to 716.5)	24.0 (21.1 to 27.4)*	3.2 (0.7 to 6.0)*	58.1 (40.3 to 77.1)	20.5 (14.7 to 26.4)*	0.2 (-4.6 to 5.2)
Acute pneumococcal meningitis	1.8 (1.2 to 2.5)	24.3 (10.5 to 37.3)*	3.4 (-8.1 to 14.2)	0.2 (0.1 to 0.4)	24.3 (10.5 to 37.3)*	3.4 (-8.1 to 14.2)
Complications of pneumococcal meningitis aggregate	599.2 (501.2 to 714.8)	24.0 (21.1 to 27.4)*	3.2 (0.7 to 6.0)*	57.9 (40.1 to 76.8)	20.5 (14.6 to 26.4)*	0.2 (-4.6 to 5.2)
H influenzae type B meningitis	205.4 (160.8 to 258.2)	-5.9 (-10.4 to -0.5)*	-21.7 (-25.5 to -17.2)*	24.4 (16.4 to 33.2)	13.2 (5.8 to 20.5)*	-5.8 (-12.0 to 0.2)
Acute H influenzae type B meningitis	0.5 (0.3 to 0.8)	-15.3 (-26.0 to -5.6)*	-29.6 (-38.5 to -21.5)*	0.1 (0.0 to 0.1)	-15.4 (-26.0 to -5.6)*	-29.6 (-38.5 to -21.5)*
Complications of of H influenzae type B meningitis aggregate	204.8 (160.2 to 257.6)	-5.9 (-10.4 to -0.4)*	-21.7 (-25.5 to -17.2)*	24.4 (16.4 to 33.1)	13.4 (5.9 to 20.6)*	-5.7 (-11.9 to 0.3)
Meningococcal meningitis	154.1 (116.8 to 198.0)	26.1 (21.3 to 30.8)*	4.9 (0.9 to 8.8)*	14.5 (10.1 to 19.8)	24.0 (18.6 to 29.1)*	3.2 (-1.3 to 7.4)
Acute meningococcal meningitis	0.8 (0.5 to 1.2)	21.8 (5.0 to 37.5)*	1.3 (-12.7 to 14.4)	0.1 (0.1 to 0.2)	21.8 (5.0 to 37.5)*	1.3 (-12.7 to 14.4)
Complications of meningococcal meningitis aggregate	153.3 (115.8 to 197.2)	26.1 (21.3 to 30.8)*	4.9 (0.9 to 8.8)*	14.4 (10.0 to 19.6)	24.1 (18.6 to 29.2)*	3.2 (-1.3 to 7.5)
Other meningitis	239.4 (190.3 to 292.8)	24.3 (21.1 to 27.9)*	3.4 (0.7 to 6.4)*	25.4 (17.4 to 34.5)	22.0 (15.7 to 28.4)*	1.5 (-3.7 to 6.8)
Other meningitis episodes aggregate	6.7 (5.1 to 8.4)	19.9 (7.3 to 31.0)*	-0.3 (-10.8 to 9.0)	0.9 (0.6 to 1.3)	19.8 (7.2 to 30.9)*	-0.3 (-10.8 to 8.9)
Complications of other meningitis aggregate	232.7 (183.7 to 287.0)	24.5 (21.2 to 28.1)*	3.5 (0.8 to 6.6)*	24.5 (16.8 to 33.4)	22.1 (15.5 to 28.7)*	1.6 (-3.9 to 7.1)
Encephalitis	379.6 (281.3 to 506.6)	7.3 (4.7 to 10.6)*	-10.8 (-12.9 to -8.0)*	44.5 (31.1 to 59.7)	10.6 (4.7 to 17.1)*	-8.0 (-12.9 to -2.6)*
Acute encephalitis	4.8 (3.9 to 5.8)	13.4 (8.3 to 18.0)*	-5.6 (-9.9 to -1.8)*	0.6 (0.4 to 0.9)	13.3 (4.9 to 21.4)*	-5.7 (-12.7 to 1.0)
Complications of encephalitis aggregate	374.8 (276.2 to 502.3)	7.2 (4.6 to 10.5)*	-10.8 (-13.0 to -8.1)*	43.8 (30.6 to 58.9)	10.5 (4.6 to 17.0)*	-8.1 (-13.0 to -2.6)*
Diphtheria	0.0 (0.0 to 0.0)	-57.0 (-83.7 to 4.7)	-64.2 (-86.4 to -12.9)*	0.0 (0.0 to 0.0)	-57.0 (-83.7 to 4.7)	-64.3 (-86.4 to -12.9)*
Whooping cough	3.9 (3.0 to 4.8)	-20.1 (-22.7 to -17.3)*	-33.6 (-35.7 to -31.2)*	0.2 (0.1 to 0.3)	-20.2 (-22.7 to -17.3)*	-33.6 (-35.7 to -31.2)*
Tetanus	17.2 (16.8 to 17.6)	19.6 (17.5 to 20.9)*	-0.5 (-2.3 to 0.6)	0.8 (0.6 to 1.0)	7.0 (-1.0 to 15.1)	-11.0 (-17.6 to -4.3)*
Severe tetanus	0.4 (0.2 to 0.6)	-34.1 (-49.2 to -12.9)*	-45.2 (-57.8 to -27.6)*	0.0 (0.0 to 0.1)	-34.0 (-49.4 to -12.9)*	-45.1 (-57.9 to -27.5)*
Complications of tetanus aggregate	16.8 (16.5 to 17.2)	21.7 (21.2 to 22.3)*	1.3 (0.8 to 1.7)*	0.7 (0.5 to 1.0)	11.6 (4.1 to 20.3)*	-7.2 (-13.4 to 0.0)
Measles	0.4 (0.2 to 0.7)	-62.3 (-66.7 to -57.0)*	-68.7 (-72.3 to -64.2)*	0.0 (0.0 to 0.1)	-62.4 (-66.7 to -57.0)*	-68.7 (-72.3 to -64.2)*
Varicella and herpes zoster	266.5 (201.0 to 339.9)	20.7 (19.8 to 21.4)*	0.4 (-0.3 to 1.0)	11.5 (6.2 to 18.5)	21.0 (14.9 to 27.5)*	0.6 (-4.5 to 6.1)
Neglected tropical diseases and malaria	168,074.6 (163,717.4 to 172,152.4)	4.3 (1.6 to 7.5)*	-13.2 (-15.5 to -10.6)*	1,580.3 (1,029.6 to 2,449.5)	-5.2 (-13.9 to 6.8)	-21.1 (-28.4 to -11.1)*
Malaria	19,776.3 (16,969.1 to 23,275.7)	34.6 (25.9 to 45.0)*	12.0 (4.7 to 20.6)*	157.1 (113.0 to 213.3)	20.6 (15.8 to 25.8)*	0.3 (-3.6 to 4.7)
Asymptomatic malaria parasitemia(PfPR)	14,977.9 (12,169.6 to 18,408.4)	38.9 (26.8 to 53.2)*	15.6 (5.5 to 27.4)*	0.0 (0.0 to 0.0)	0.0 (0.0 to 0.0)	0.0 (0.0 to 0.0)
Malaria episodes aggregate	798.9 (450.6 to 1,373.8)	-2.6 (-13.7 to 10.1)	-19.0 (-28.2 to -8.4)*	16.5 (7.7 to 30.9)	-2.4 (-13.7 to 10.9)	-18.8 (-28.2 to -7.8)*
Complications of malaria aggregate	98.1 (88.8 to 107.2)	29.9 (27.5 to 32.9)*	8.1 (6.1 to 10.5)*	30.8 (23.5 to 38.8)	26.2 (17.2 to 36.0)*	4.9 (-2.5 to 13.1)
Anemia due to malaria parasitemia(PfPR) aggregate	3,901.4 (3,804.7 to 4,007.5)	29.5 (25.7 to 34.0)*	7.7 (4.6 to 11.5)*	109.8 (73.8 to 159.1)	23.4 (17.8 to 30.0)*	2.7 (-2.0 to 8.1)
Chagas disease	425.1 (355.3 to 491.8)	-15.3 (-17.9 to -12.6)*	-29.5 (-31.7 to -27.3)*	2.5 (1.6 to 3.7)	-15.3 (-22.1 to -8.6)*	-29.6 (-35.2 to -23.9)*
Chagas disease episodes aggregate	378.4 (314.8 to 439.9)	-15.3 (-17.9 to -12.6)*	-29.5 (-31.7 to -27.3)*	0.0 (0.0 to 0.0)	-26.0 (-32.2 to -20.8)*	-38.4 (-43.6 to -34.1)*
Complications of Chagas disease aggregate	39.6 (30.2 to 49.9)	-15.3 (-17.9 to -12.6)*	-29.5 (-31.7 to -27.3)*	2.1 (1.3 to 3.1)	-15.2 (-22.6 to -7.5)*	-29.5 (-35.7 to -23.1)*

Cause	Prevalence in thousands (95% confidence interval)			YLDs in thousands (95% confidence interval)		
	2015	Percent change in counts between 2005 and 2015	Percent change in rates between 2005 and 2015	2015	Percent change in counts between 2005 and 2015	Percent change in rates between 2005 and 2015
Heart failure due to Chagas disease aggregate	7.1 (4.2 to 11.2)	-16.1 (-19.0 to -13.4)*	-30.3 (-32.6 to -28.0)*	0.4 (0.2 to 0.7)	-16.0 (-28.2 to -2.9)*	-30.1 (-40.3 to -19.2)*
Leishmaniasis	341.5 (302.8 to 414.0)	27.8 (3.5 to 59.0)*	6.3 (-13.9 to 32.2)	4.0 (2.0 to 7.7)	26.8 (20.2 to 33.6)*	5.5 (-0.0 to 11.1)
Visceral leishmaniasis	4.7 (4.3 to 5.1)	16.7 (15.3 to 18.1)*	-2.9 (-4.1 to -1.8)*	0.3 (0.2 to 0.5)	17.3 (-11.8 to 53.4)	-2.5 (-26.6 to 27.6)
Cutaneous and mucocutaneous leishmaniasis	343.0 (286.7 to 427.3)	27.4 (23.5 to 30.8)*	6.0 (2.7 to 8.8)*	3.7 (1.7 to 7.3)	27.7 (21.3 to 34.3)*	6.3 (0.9 to 11.7)*
African trypanosomiasis	1.2 (0.7 to 2.0)	-68.2 (-69.9 to -66.7)*	-73.5 (-74.9 to -72.3)*	0.3 (0.1 to 0.7)	-67.1 (-82.6 to -43.3)*	-72.6 (-85.5 to -52.8)*
Schistosomiasis	26,292.4 (21,658.8 to 35,511.2)	-22.1 (-31.8 to 0.9)	-35.2 (-43.2 to -16.1)*	275.6 (136.3 to 538.2)	-21.1 (-30.1 to 2.4)	-34.4 (-41.8 to -14.8)*
Schistosomiasis episodes aggregate	7,754.1 (5,368.3 to 10,740.0)	-27.1 (-37.5 to -9.6)*	-39.3 (-48.0 to -24.8)*	45.2 (17.8 to 95.9)	-28.4 (-38.5 to -9.6)*	-40.5 (-48.8 to -24.8)*
Complications of schistosomiasis aggregate	17,095.3 (11,808.3 to 26,196.5)	-19.8 (-32.7 to 14.0)	-33.2 (-44.0 to -5.2)*	190.6 (84.5 to 401.2)	-18.4 (-30.7 to 15.1)	-32.2 (-42.4 to -4.3)*
Anemia due to schistosomiasis aggregate	1,438.5 (1,382.6 to 1,498.8)	-19.9 (-23.4 to -16.2)*	-33.4 (-36.3 to -30.3)*	39.8 (26.5 to 57.7)	-24.2 (-29.1 to -19.2)*	-37.0 (-41.0 to -32.8)*
Cysticercosis	253.2 (166.9 to 379.6)	-7.1 (-14.5 to -0.3)*	-22.7 (-28.9 to -17.1)*	39.4 (22.4 to 63.9)	-16.4 (-24.9 to -8.8)*	-30.4 (-37.5 to -24.1)*
Cystic echinococcosis	111.6 (94.7 to 132.6)	26.3 (22.3 to 30.1)*	5.0 (1.7 to 8.2)*	10.4 (6.9 to 15.0)	26.6 (16.7 to 37.9)*	5.3 (-3.0 to 14.7)
Lymphatic filariasis	3,441.8 (2,789.5 to 4,209.5)	-47.6 (-52.4 to -42.9)*	-56.4 (-60.4 to -52.5)*	185.7 (97.8 to 299.6)	-20.1 (-34.7 to -8.4)*	-33.6 (-45.7 to -23.8)*
Prevalence of detectable microfiliaria due to lymphatic filariasis	1,799.0 (1,531.5 to 2,056.9)	-60.4 (-63.6 to -56.9)*	-67.1 (-69.7 to -64.1)*	0.0 (0.0 to 0.0)	0.0 (0.0 to 0.0)	0.0 (0.0 to 0.0)
Complications of lymphatic filariasis aggregate	1,642.7 (1,070.6 to 2,343.6)	-19.0 (-33.1 to -7.9)*	-32.6 (-44.3 to -23.4)*	185.7 (97.8 to 299.6)	-20.1 (-34.7 to -8.4)*	-33.6 (-45.7 to -23.8)*
Onchocerciasis	1,839.7 (1,488.0 to 2,341.0)	-25.9 (-35.0 to -15.3)*	-38.3 (-46.0 to -29.6)*	110.9 (48.4 to 194.8)	-17.3 (-37.5 to 1.4)	-31.2 (-48.0 to -15.6)*
Asymptomatic onchocerciasis	486.6 (367.3 to 625.1)	-48.7 (-51.2 to -46.4)*	-57.3 (-59.4 to -55.4)*	0.0 (0.0 to 0.0)	0.0 (0.0 to 0.0)	0.0 (0.0 to 0.0)
Skin disease due to onchocerciasis aggregate	1,340.2 (959.4 to 1,793.9)	-10.8 (-25.1 to 5.2)	-25.8 (-37.7 to -12.4)*	109.7 (47.0 to 192.9)	-16.7 (-37.0 to 2.6)	-30.7 (-47.6 to -14.4)*
Vision loss due to onchocerciasis aggregate	14.4 (9.6 to 21.5)	-55.8 (-60.0 to -50.3)*	-63.2 (-66.7 to -58.7)*	1.2 (0.7 to 1.9)	-53.6 (-62.8 to -42.8)*	-61.4 (-69.1 to -52.5)*
Trachoma	81.7 (47.5 to 132.6)	4.7 (-5.4 to 16.1)	-12.9 (-21.3 to -3.4)*	6.7 (3.1 to 13.3)	-0.7 (-15.1 to 15.9)	-17.4 (-29.3 to -3.6)*
Dengue	395.2 (225.6 to 836.6)	133.2 (-4.3 to 531.7)	94.0 (-20.4 to 425.5)	64.4 (29.8 to 145.2)	130.7 (-6.7 to 528.0)	91.9 (-22.4 to 422.4)
Dengue episodes aggregate	117.7 (79.9 to 239.2)	125.9 (-8.3 to 489.4)	87.9 (-23.7 to 390.3)	6.9 (3.8 to 14.8)	131.7 (-5.1 to 524.7)	92.7 (-21.1 to 419.6)
Post-dengue chronic fatigue syndrome	277.8 (136.1 to 600.3)	133.4 (-4.3 to 531.7)	94.2 (-20.4 to 425.5)	57.5 (25.3 to 131.8)	130.6 (-7.3 to 530.7)	91.8 (-22.9 to 424.7)
Yellow fever	0.2 (0.1 to 0.6)	-25.8 (-42.3 to -3.3)*	-38.3 (-52.0 to -19.5)*	0.0 (0.0 to 0.0)	-25.9 (-42.3 to -3.3)*	-38.3 (-52.0 to -19.5)*
Rabies	0.0 (0.0 to 0.0)	-43.7 (-64.1 to -14.4)*	-53.2 (-70.2 to -28.8)*	0.0 (0.0 to 0.0)	-43.7 (-64.1 to -14.4)*	-53.2 (-70.2 to -28.8)*
Intestinal nematode infections	131,113.3 (127,235.8 to 134,587.4)	3.0 (-0.7 to 6.5)	-14.3 (-17.4 to -11.4)*	276.0 (157.8 to 452.3)	-19.5 (-26.3 to -11.2)*	-33.0 (-38.7 to -26.2)*
Ascariasis	68,940.6 (59,371.7 to 79,953.1)	-5.2 (-21.0 to 13.0)	-21.2 (-34.3 to -6.0)*	73.3 (40.0 to 124.0)	-36.2 (-47.4 to -23.5)*	-46.9 (-56.2 to -36.3)*
Complications of ascariasis aggregate	4,020.0 (3,615.4 to 4,484.7)	-33.6 (-43.2 to -22.5)*	-44.8 (-52.8 to -35.5)*	73.3 (40.0 to 124.0)	-36.2 (-47.4 to -23.5)*	-46.9 (-56.2 to -36.3)*
Asymptomatic ascariasis	64,924.0 (55,287.2 to 76,075.1)	-2.7 (-19.5 to 17.6)	-19.0 (-33.0 to -2.2)*	0.0 (0.0 to 0.0)	0.0 (0.0 to 0.0)	0.0 (0.0 to 0.0)
Trichuriasis	41,269.2 (37,244.1 to 45,823.3)	2.8 (-10.4 to 19.2)	-14.5 (-25.5 to -0.8)*	50.4 (26.3 to 88.8)	-10.8 (-30.3 to 19.2)	-25.8 (-42.0 to -0.9)*
Complications of trichuriasis aggregate	2,482.9 (2,140.3 to 3,055.3)	-10.0 (-27.6 to 15.1)	-25.1 (-39.8 to -4.3)*	50.4 (26.3 to 88.8)	-10.8 (-30.3 to 19.2)	-25.8 (-42.0 to -0.9)*
Asymptomatic trichuriasis	38,784.8 (34,640.0 to 43,439.9)	3.7 (-10.6 to 21.5)	-13.7 (-25.6 to 1.0)	0.0 (0.0 to 0.0)	0.0 (0.0 to 0.0)	0.0 (0.0 to 0.0)
Hookworm disease	40,335.8 (36,371.2 to 45,131.7)	-3.9 (-16.5 to 10.2)	-20.1 (-30.6 to -8.3)*	152.2 (91.2 to 241.7)	-11.1 (-18.4 to -3.2)*	-26.1 (-32.1 to -19.5)*
Complications of hookworm disease aggregate	4,925.0 (4,595.6 to 5,310.0)	-11.8 (-20.8 to -2.0)*	-26.6 (-34.1 to -18.5)*	101.8 (55.7 to 169.5)	-13.4 (-23.9 to -1.5)*	-27.9 (-36.7 to -18.0)*
Anemia due to hookworm disease aggregate	1,998.4 (1,960.2 to 2,037.0)	-0.1 (-3.0 to 2.9)	-16.9 (-19.3 to -14.4)*	50.4 (33.5 to 74.3)	-6.3 (-11.4 to -0.9)*	-22.1 (-26.3 to -17.6)*
Asymptomatic hookworm disease	33,410.6 (29,395.4 to 38,081.6)	-2.9 (-18.0 to 14.4)	-19.2 (-31.8 to -4.8)*	0.0 (0.0 to 0.0)	0.0 (0.0 to 0.0)	0.0 (0.0 to 0.0)
Food-borne trematodiases	8,577.2 (7,969.1 to 9,236.5)	30.2 (26.0 to 34.6)*	8.3 (4.8 to 12.0)*	208.7 (98.8 to 393.5)	27.3 (20.3 to 38.9)*	5.9 (0.1 to 15.5)*
Asymptomatic food-borne trematodiases aggregate	6,765.2 (5,390.0 to 7,817.8)	30.7 (25.9 to 35.8)*	8.7 (4.7 to 12.9)*	0.0 (0.0 to 0.0)	0.0 (0.0 to 0.0)	0.0 (0.0 to 0.0)
Symptomatic food-borne trematodiases aggregate	1,812.0 (976.0 to 3,129.3)	28.1 (21.7 to 38.8)*	6.6 (1.2 to 15.4)*	208.7 (98.8 to 393.5)	27.3 (20.3 to 38.9)*	5.9 (0.1 to 15.5)*
Leprosy	16.8 (15.2 to 18.7)	-25.4 (-26.7 to -24.0)*	-38.0 (-39.1 to -36.7)*	1.0 (0.7 to 1.5)	-25.1 (-35.2 to -13.7)*	-37.7 (-46.1 to -28.2)*
Ebola	0.3 (0.2 to 0.6)	76,080.9 (44,544.3 to 274,844.9)*	63,269.6 (37,036.5 to 228,607.4)*	0.1 (0.0 to 0.1)	75,947.2 (38,495.8 to 392,059.0)*	63,158.3 (32,005.2 to 326,109.7)*
Other neglected tropical diseases	2,399.9 (2,335.8 to 2,462.0)	3.1 (-0.1 to 6.3)	-14.2 (-16.9 to -11.6)*	237.4 (84.8 to 608.9)	10.3 (-24.9 to 67.9)	-8.3 (-37.6 to 39.6)
Anemia due to other neglected tropical diseases aggregate	2,399.9 (2,335.8 to 2,462.0)	3.1 (-0.1 to 6.3)	-14.2 (-16.9 to -11.6)*	43.7 (28.3 to 65.1)	-12.0 (-17.8 to -6.2)*	-26.8 (-31.6 to -22.0)*
Maternal disorders	2,714.9 (2,530.6 to 2,887.9)	10.1 (2.2 to 16.8)*	-8.4 (-15.0 to -2.8)*	213.7 (147.5 to 300.2)	2.3 (-13.1 to 22.0)	-14.9 (-27.7 to 1.5)
Maternal hemorrhage	661.0 (633.1 to 696.4)	13.0 (7.3 to 19.4)*	-6.0 (-10.7 to -0.7)*	24.1 (16.0 to 34.7)	9.5 (-6.1 to 27.7)	-8.9 (-21.9 to 6.2)
Maternal hemorrhage episodes aggregate	52.5 (31.2 to 82.7)	12.3 (-30.9 to 80.1)	-6.6 (-42.5 to 49.8)	8.5 (4.5 to 14.0)	12.5 (-25.9 to 68.1)	-6.4 (-38.3 to 39.9)
Anemia due to maternal hemorrhage aggregate	608.5 (591.4 to 626.2)	13.1 (8.8 to 17.6)*	-5.9 (-9.5 to -2.2)*	15.6 (10.1 to 23.0)	8.0 (-0.6 to 16.5)	-10.2 (-17.3 to -3.1)*
Maternal sepsis and other maternal infections	576.3 (450.5 to 749.4)	6.2 (-18.0 to 41.5)	-11.6 (-31.8 to 17.7)	13.6 (6.6 to 25.1)	8.7 (-38.7 to 94.1)	-9.6 (-49.0 to 61.5)
Maternal sepsis and other maternal infections aggregate	236.7 (122.0 to 399.0)	9.7 (-44.3 to 117.5)	-8.7 (-53.7 to 80.9)	11.9 (5.6 to 21.9)	9.4 (-43.1 to 111.1)	-9.0 (-52.6 to 75.6)
Infertility due to puerperal sepsis	340.2 (296.5 to 395.5)	4.1 (1.7 to 6.6)*	-13.4 (-15.4 to -11.3)*	1.7 (0.6 to 3.7)	4.3 (-0.6 to 9.1)	-13.3 (-17.3 to -9.3)*
Maternal hypertensive disorders	1,176.9 (702.1 to 1,799.4)	11.6 (-31.9 to 84.8)	-7.1 (-43.4 to 53.7)	56.7 (28.4 to 98.4)	11.8 (-31.8 to 83.1)	-7.0 (-43.3 to 52.3)
Maternal hypertensive disorders episodes aggregate	1,177.5 (702.6 to 1,801.6)	11.8 (-31.8 to 85.0)	-7.0 (-43.3 to 53.9)	55.8 (27.6 to 97.7)	11.8 (-31.7 to 84.1)	-7.0 (-43.2 to 53.2)
Eclampsia	1.4 (0.5 to 2.8)	9.0 (-64.1 to 215.7)	-9.3 (-70.1 to 162.6)	0.8 (0.3 to 1.7)	9.1 (-64.1 to 215.7)	-9.3 (-70.1 to 162.6)
Complications of maternal hypertensive disorders aggregate	0.0 (0.0 to 0.0)	0.0 (0.0 to 0.0)	0.0 (0.0 to 0.0)	0.0 (0.0 to 0.0)	0.0 (0.0 to 0.0)	0.0 (0.0 to 0.0)
Maternal obstructed labor and uterine rupture	219.1 (180.6 to 266.4)	-10.6 (-16.3 to -3.6)*	-25.6 (-30.4 to -19.8)*	72.3 (47.0 to 103.6)	-10.0 (-17.2 to -2.1)*	-25.1 (-31.1 to -18.5)*
Obstructed labor, acute event	24.1 (12.9 to 40.9)	5.6 (-40.6 to 89.6)	-12.2 (-50.6 to 57.7)	7.4 (3.6 to 13.1)	6.3 (-40.2 to 89.5)	-11.6 (-50.2 to 57.6)
Fistula due to maternal obstructed labor and uterine rupture aggregate	195.0 (157.8 to 239.8)	-12.2 (-15.9 to -8.7)*	-27.0 (-30.1 to -24.1)*	64.9 (41.8 to 95.6)	-11.5 (-17.3 to -4.8)*	-26.4 (-31.2 to -20.8)*
Maternal abortion, miscarriage, and ectopic pregnancy	119.2 (68.3 to 186.7)	11.6 (-29.2 to 79.2)	-7.2 (-41.1 to 49.1)	13.1 (6.9 to 22.2)	11.8 (-29.2 to 83.7)	-7.0 (-41.1 to 52.8)
Other maternal disorders	--	--	--	34.0 (22.7 to 48.9)	7.3 (-12.0 to 31.0)	-10.7 (-26.8 to 9.0)
Neonatal disorders	3,952.9 (3,759.0 to 4,093.7)	25.4 (21.3 to 29.3)*	4.3 (0.9 to 7.6)*	960.4 (718.9 to 1,226.4)	23.4 (19.0 to 28.2)*	2.6 (-1.0 to 6.7)
Neonatal preterm birth complications	3,087.1 (2,696.2 to 3,506.5)	20.6 (14.8 to 26.6)*	0.3 (-4.5 to 5.3)	468.5 (352.4 to 606.3)	15.4 (8.9 to 22.3)*	-4.0 (-9.4 to 1.7)
Vision loss due to retinopathy of prematurity aggregate	350.4 (276.9 to 434.2)	16.2 (12.0 to 20.4)*	-3.4 (-6.8 to 0.1)	11.8 (7.1 to 18.3)	15.7 (6.8 to 24.7)*	-3.8 (-11.1 to 3.7)
Complications of preterm birth complications aggregate	2,736.4 (2,374.0 to 3,131.4)	21.2 (14.6 to 27.9)*	0.8 (-4.7 to 6.4)	456.7 (342.9 to 593.7)	15.4 (8.9 to 22.4)*	-4.0 (-9.4 to 1.8)
Neonatal encephalopathy due to birth asphyxia and trauma	1,110.4 (780.0 to 1,635.1)	37.2 (34.0 to 40.4)*	14.1 (11.4 to 16.8)*	329.3 (212.7 to 507.5)	34.1 (29.4 to 39.4)*	11.6 (7.7 to 16.0)*

Cause	Prevalence in thousands (95% confidence interval)			YLDs in thousands (95% confidence interval)		
	2015	Percent change in counts between 2005 and 2015	Percent change in rates between 2005 and 2015	2015	Percent change in counts between 2005 and 2015	Percent change in rates between 2005 and 2015
Neonatal sepsis and other neonatal infections	0.0 (0.0 to 0.0)	0.0 (0.0 to 0.0)	0.0 (0.0 to 0.0)	0.0 (0.0 to 0.0)	0.0 (0.0 to 0.0)	0.0 (0.0 to 0.0)
Severe infection due to neonatal sepsis and other neonatal infections	0.0 (0.0 to 0.0)	0.0 (0.0 to 0.0)	0.0 (0.0 to 0.0)	0.0 (0.0 to 0.0)	0.0 (0.0 to 0.0)	0.0 (0.0 to 0.0)
Complications of neonatal sepsis and other neonatal infections aggregate	0.0 (0.0 to 0.0)	0.0 (0.0 to 0.0)	0.0 (0.0 to 0.0)	0.0 (0.0 to 0.0)	0.0 (0.0 to 0.0)	0.0 (0.0 to 0.0)
Hemolytic disease and other neonatal jaundice	176.7 (157.4 to 199.7)	30.5 (29.1 to 32.0)*	8.6 (7.4 to 9.8)*	53.5 (39.9 to 69.0)	27.3 (21.8 to 33.1)*	5.9 (1.3 to 10.7)*
Other neonatal disorders	--	--	--	109.1 (74.3 to 145.1)	28.4 (9.3 to 50.2)*	6.8 (-9.1 to 24.9)
Nutritional deficiencies	111,000.4 (109,667.5 to 112,424.9)	7.5 (5.8 to 9.2)*	-10.6 (-12.0 to -9.1)*	3,131.1 (2,090.4 to 4,548.4)	0.5 (-2.1 to 3.2)	-16.4 (-18.6 to -14.2)*
Protein-energy malnutrition	7.2 (4.5 to 11.2)	-19.0 (-28.5 to -8.2)*	-32.6 (-40.5 to -23.7)*	0.9 (0.5 to 1.6)	-17.5 (-31.0 to -1.2)*	-31.4 (-42.6 to -17.8)*
Iodine deficiency	11,355.5 (10,214.7 to 12,875.1)	10.1 (7.5 to 12.7)*	-8.4 (-10.5 to -6.2)*	246.5 (155.9 to 370.2)	11.7 (9.1 to 14.6)*	-7.1 (-9.3 to -4.7)*
Visible goiter due to iodine deficiency aggregate	11,090.1 (9,949.3 to 12,546.8)	9.9 (7.5 to 12.5)*	-8.6 (-10.6 to -6.4)*	199.9 (123.4 to 314.3)	10.2 (7.3 to 13.0)*	-8.4 (-10.8 to -6.0)*
Visible goiter with heart failure due to iodine deficiency aggregate	0.0 (0.0 to 0.0)	0.0 (0.0 to 0.0)	0.0 (0.0 to 0.0)	0.0 (0.0 to 0.0)	0.0 (0.0 to 0.0)	0.0 (0.0 to 0.0)
Intellectual disability due to iodine deficiency aggregate	265.4 (109.6 to 364.5)	18.1 (13.1 to 20.7)*	-1.8 (-5.9 to 0.4)	46.7 (16.5 to 76.6)	19.0 (11.5 to 24.7)*	-1.0 (-7.2 to 3.7)
Vitamin A deficiency	425.0 (297.1 to 570.7)	17.9 (12.3 to 23.5)*	-2.0 (-6.6 to 2.7)	21.4 (12.7 to 33.1)	17.1 (10.2 to 24.1)*	-2.6 (-8.3 to 3.2)
Iron-deficiency anemia	107,955.9 (106,511.9 to 109,446.5)	5.6 (4.0 to 7.3)*	-12.1 (-13.5 to -10.7)*	2,862.2 (1,911.6 to 4,149.9)	-0.4 (-3.2 to 2.4)	-17.2 (-19.5 to -14.8)*
Iron-deficiency anemia without heart failure aggregate	107,955.6 (106,511.6 to 109,446.2)	5.6 (4.0 to 7.3)*	-12.1 (-13.5 to -10.7)*	2,862.1 (1,911.6 to 4,149.9)	-0.4 (-3.2 to 2.4)	-17.2 (-19.5 to -14.8)*
Iron-deficiency anemia with heart failure aggregate	0.3 (0.2 to 0.4)	26.7 (23.5 to 30.2)*	5.4 (2.7 to 8.3)*	0.0 (0.0 to 0.1)	26.7 (23.5 to 30.2)*	5.4 (2.7 to 8.3)*
Other nutritional deficiencies	--	--	--	0.1 (0.0 to 0.2)	-10.4 (-31.6 to 12.4)	-25.4 (-43.1 to -6.5)*
Other communicable, maternal, neonatal, and nutritional diseases	180,339.6 (174,516.6 to 185,583.6)	18.5 (17.2 to 19.9)*	-1.4 (-2.5 to -0.3)*	414.5 (255.2 to 652.8)	18.5 (12.1 to 24.3)*	-1.4 (-6.7 to 3.4)
Sexually transmitted diseases excluding HIV	144,190.2 (137,119.4 to 149,707.0)	17.3 (15.5 to 19.1)*	-2.4 (-3.9 to -1.0)*	308.9 (183.3 to 501.7)	21.7 (17.4 to 26.0)*	1.3 (-2.3 to 4.8)
Syphilis	6,013.1 (4,150.4 to 8,648.4)	6.4 (-3.1 to 14.3)	-11.5 (-19.4 to -4.9)*	16.0 (10.8 to 22.2)	10.3 (1.7 to 19.5)*	-8.2 (-15.4 to -0.6)*
Early syphilis aggregate	5,937.8 (4,085.1 to 8,578.1)	6.3 (-3.3 to 14.4)	-11.6 (-19.5 to -4.9)*	1.4 (0.4 to 3.6)	6.3 (-5.0 to 16.0)	-11.6 (-20.9 to -3.5)*
Adult tertiary syphilis	75.2 (64.3 to 85.2)	10.3 (6.0 to 14.0)*	-8.2 (-11.8 to -5.1)*	14.5 (9.5 to 20.8)	10.8 (1.2 to 20.9)*	-7.9 (-15.9 to 0.6)
Chlamydial infection	26,803.6 (18,765.9 to 38,351.8)	16.0 (12.7 to 19.8)*	-3.5 (-6.2 to -0.3)*	115.0 (62.9 to 194.0)	21.4 (16.3 to 27.3)*	1.0 (-3.2 to 5.9)
Chlamydial infection episodes aggregate	26,616.1 (18,587.7 to 38,160.1)	16.1 (12.7 to 19.9)*	-3.5 (-6.2 to -0.3)*	110.3 (58.8 to 189.2)	22.2 (16.7 to 28.0)*	1.6 (-2.9 to 6.5)
Pelvic inflammatory diseases due to chlamydial infection aggregate	28.3 (20.8 to 38.3)	4.3 (0.2 to 8.5)*	-13.2 (-16.7 to -9.8)*	3.8 (2.4 to 5.9)	4.7 (-5.0 to 15.7)	-12.9 (-21.0 to -3.7)*
Infertility due to clamydial infection aggregate	159.2 (145.5 to 173.2)	14.0 (12.7 to 15.2)*	-5.2 (-6.2 to -4.2)*	0.9 (0.4 to 1.9)	13.4 (6.5 to 20.4)*	-5.7 (-11.4 to 0.1)
Gonococcal infection	11,237.6 (6,418.7 to 17,751.4)	26.6 (12.4 to 34.6)*	5.3 (-6.5 to 12.0)	106.6 (55.0 to 188.8)	27.5 (17.0 to 35.5)*	6.0 (-2.7 to 12.7)
Gonococcal infection episodes aggregate	11,142.3 (6,315.0 to 17,649.4)	26.8 (12.4 to 34.8)*	5.5 (-6.5 to 12.1)	103.9 (52.7 to 185.5)	28.2 (17.5 to 36.4)*	6.6 (-2.3 to 13.5)
Pelvic inflammatory diseases due to gonococcal infection aggregate	95.3 (86.9 to 104.0)	11.5 (9.5 to 13.4)*	-7.3 (-8.9 to -5.7)*	2.7 (1.7 to 4.0)	4.5 (-5.0 to 14.9)	-13.1 (-21.0 to -4.4)*
Trichomoniasis	27,878.0 (19,885.2 to 38,001.7)	19.7 (18.5 to 20.9)*	-0.4 (-1.4 to 0.5)	33.0 (12.7 to 72.8)	18.9 (16.9 to 20.8)*	-1.1 (-2.8 to 0.5)
Genital herpes	89,149.3 (75,438.5 to 104,813.7)	16.6 (14.5 to 18.6)*	-3.0 (-4.8 to -1.3)*	25.6 (8.3 to 58.4)	19.8 (16.3 to 26.4)*	-0.3 (-3.2 to 5.2)
Moderate infection due to initial genital herpes episode	69.3 (15.9 to 158.0)	44.9 (37.9 to 52.9)*	20.5 (14.7 to 27.2)*	3.4 (0.7 to 8.1)	45.0 (29.2 to 63.4)*	20.6 (7.4 to 35.9)*
Complications of genital herpes aggregate	89,080.1 (75,395.9 to 104,786.6)	16.6 (14.4 to 18.6)*	-3.0 (-4.8 to -1.4)*	22.2 (6.3 to 54.2)	16.7 (14.2 to 18.9)*	-2.9 (-5.0 to -1.1)*
Other sexually transmitted diseases	406.9 (368.7 to 444.0)	13.6 (12.0 to 15.4)*	-5.5 (-6.8 to -4.0)*	12.7 (8.4 to 18.9)	7.4 (1.0 to 13.5)*	-10.7 (-16.0 to -5.6)*
Pelvic inflammatory diseases due to other sexually transmitted diseases aggregate	81.6 (61.8 to 107.6)	6.1 (4.0 to 8.4)*	-11.8 (-13.5 to -9.8)*	10.8 (7.0 to 16.1)	6.2 (-1.1 to 13.1)	-11.7 (-17.7 to -5.9)*
Infertility due to other sexually transmitted diseases aggregate	325.2 (295.9 to 357.4)	15.6 (13.7 to 17.9)*	-3.8 (-5.4 to -1.9)*	1.9 (0.8 to 3.9)	14.9 (9.5 to 20.0)*	-4.4 (-8.9 to -0.2)*
Other sexually transmitted diseases	--	--	--	92.5 (54.6 to 149.3)	25.2 (19.2 to 31.6)*	4.2 (-0.8 to 9.5)
Hepatitis	45,201.1 (44,503.9 to 45,948.7)	24.2 (22.8 to 25.6)*	3.3 (2.1 to 4.5)*	42.7 (25.5 to 67.8)	25.6 (-12.3 to 77.8)	4.5 (-27.0 to 47.9)
Acute hepatitis A	351.9 (293.0 to 404.6)	27.8 (1.6 to 60.1)*	6.3 (-15.4 to 33.2)	14.9 (9.2 to 22.2)	27.8 (-1.3 to 60.9)	6.3 (-17.9 to 33.9)
Hepatitis B	35,122.5 (33,619.2 to 36,880.9)	24.5 (20.1 to 28.8)*	3.6 (-0.1 to 7.1)	23.5 (12.5 to 41.5)	25.3 (-33.9 to 129.0)	4.2 (-45.0 to 90.5)
Acute Hepatitis B aggregate	1,913.0 (1,249.0 to 2,813.9)	24.6 (-33.8 to 124.3)	3.6 (-45.0 to 86.6)	23.5 (12.5 to 41.5)	25.3 (-33.9 to 129.0)	4.2 (-45.0 to 90.5)
Chronic hepatitis B	33,209.4 (31,799.2 to 34,755.6)	24.5 (22.8 to 26.1)*	3.5 (2.2 to 4.9)*	0.0 (0.0 to 0.0)	0.0 (0.0 to 0.0)	0.0 (0.0 to 0.0)
Hepatitis C	10,520.8 (9,301.7 to 11,810.2)	23.4 (21.9 to 24.8)*	2.6 (1.4 to 3.8)*	0.3 (0.2 to 0.7)	21.1 (16.3 to 24.7)*	0.8 (-3.2 to 3.8)
Acute Hepatitis C aggregate	23.7 (17.4 to 32.3)	21.2 (16.3 to 24.8)*	0.8 (-3.3 to 3.8)	0.3 (0.2 to 0.7)	21.1 (16.3 to 24.7)*	0.8 (-3.2 to 3.8)
Chronic hepatitis C	10,497.1 (9,280.7 to 11,784.3)	23.4 (21.9 to 24.8)*	2.6 (1.4 to 3.9)*	0.0 (0.0 to 0.0)	0.0 (0.0 to 0.0)	0.0 (0.0 to 0.0)
Acute hepatitis E	129.3 (102.2 to 165.0)	19.9 (17.4 to 22.6)*	-0.3 (-2.3 to 2.0)	4.0 (2.4 to 6.2)	20.3 (-2.5 to 48.9)	0.1 (-18.9 to 23.9)
Other infectious diseases	2,638.4 (2,586.0 to 2,689.7)	3.7 (0.9 to 6.6)*	-13.7 (-16.1 to -11.4)*	62.8 (39.5 to 98.9)	1.3 (-6.3 to 9.2)	-15.7 (-22.1 to -9.2)*
Non-communicable diseases	562,951.2 (559,724.0 to 565,719.9)	20.0 (19.8 to 20.2)*	-0.2 (-0.4 to 0.0)	45,404.2 (33,436.7 to 58,796.3)	19.6 (18.9 to 20.3)*	-0.5 (-1.1 to 0.1)
Neoplasms	955.9 (914.3 to 998.8)	33.1 (25.8 to 40.3)*	10.8 (4.6 to 16.7)*	108.9 (77.6 to 143.6)	27.6 (16.8 to 38.8)*	6.1 (-2.8 to 15.5)
Lip and oral cavity cancer	40.2 (29.5 to 57.6)	25.4 (-0.5 to 57.4)	4.3 (-17.2 to 30.9)	3.5 (2.1 to 5.6)	24.8 (-2.0 to 59.0)	3.8 (-18.4 to 32.3)
Nasopharynx cancer	22.8 (10.1 to 38.6)	20.5 (-29.4 to 102.7)	0.2 (-41.3 to 68.6)	2.1 (0.9 to 3.8)	19.9 (-29.8 to 98.3)	-0.3 (-41.6 to 64.9)
Other pharynx cancer	4.7 (3.8 to 5.6)	13.8 (-3.6 to 34.9)	-5.3 (-19.8 to 12.2)	0.4 (0.3 to 0.5)	13.1 (-4.2 to 34.0)	-5.9 (-20.3 to 11.5)
Esophageal cancer	1.9 (1.5 to 2.5)	13.1 (-10.4 to 45.2)	-5.9 (-25.5 to 20.8)	0.4 (0.2 to 0.5)	7.3 (-13.4 to 35.9)	-10.8 (-28.0 to 13.0)
Stomach cancer	16.1 (13.1 to 18.4)	-2.4 (-16.0 to 11.9)	-18.9 (-30.2 to -6.9)*	1.9 (1.3 to 2.6)	-4.1 (-18.1 to 10.8)	-20.2 (-31.8 to -7.8)*
Colon and rectum cancer	61.2 (51.5 to 71.4)	39.2 (16.4 to 64.1)*	15.8 (-3.1 to 36.5)	5.3 (3.6 to 7.3)	35.6 (13.2 to 59.7)*	12.8 (-5.8 to 32.9)
Colon and rectum cancer aggregate	61.0 (51.4 to 71.2)	39.3 (16.5 to 64.3)*	15.9 (-3.1 to 36.6)	5.3 (3.6 to 7.2)	35.8 (13.2 to 60.1)*	12.9 (-5.8 to 33.2)
Stoma due to colon and rectum cancer	0.2 (0.2 to 0.2)	15.9 (14.7 to 17.0)*	-3.6 (-4.6 to -2.7)*	0.0 (0.0 to 0.0)	15.9 (14.7 to 17.0)*	-3.6 (-4.6 to -2.7)*
Liver cancer	0.2 (0.0 to 0.7)	274.7 (-77.4 to 6,100.8)	211.7 (-81.2 to 5,058.0)	2.0 (1.2 to 3.4)	13.9 (-22.8 to 70.0)	-5.3 (-35.8 to 41.4)
Liver cancer due to hepatitis B	6.7 (4.5 to 10.9)	22.2 (-20.8 to 93.9)	1.7 (-34.1 to 61.3)	1.5 (0.9 to 2.4)	14.7 (-24.0 to 76.5)	-4.6 (-36.8 to 46.9)
Liver cancer due to hepatitis C	0.5 (0.3 to 0.7)	28.3 (0.4 to 63.9)*	6.7 (-16.5 to 36.3)	0.1 (0.1 to 0.2)	20.1 (-4.6 to 50.0)	-0.1 (-20.6 to 24.8)
Liver cancer due to alcohol use	0.2 (0.1 to 0.4)	44.4 (1.3 to 105.5)*	20.1 (-15.7 to 70.9)	0.1 (0.0 to 0.1)	36.0 (-4.5 to 91.5)	13.2 (-20.6 to 59.3)
Liver cancer due to other causes	1.8 (1.2 to 2.7)	14.0 (-21.6 to 70.8)	-5.2 (-34.8 to 42.1)	0.4 (0.2 to 0.6)	7.2 (-25.1 to 53.9)	-10.8 (-37.7 to 28.0)
Gallbladder and biliary tract cancer	0.3 (0.3 to 0.3)	3.7 (-6.7 to 16.7)	-13.8 (-22.4 to -2.9)*	0.1 (0.1 to 0.1)	2.2 (-8.0 to 14.5)	-15.0 (-23.5 to -4.8)*
Pancreatic cancer	0.9 (0.8 to 1.0)	31.8 (18.4 to 47.1)*	9.6 (-1.5 to 22.4)	0.2 (0.2 to 0.3)	29.0 (16.0 to 43.9)*	7.3 (-3.5 to 19.7)

Cause	Prevalence in thousands (95% confidence interval)			YLDs in thousands (95% confidence interval)		
	2015	Percent change in counts between 2005 and 2015	Percent change in rates between 2005 and 2015	2015	Percent change in counts between 2005 and 2015	Percent change in rates between 2005 and 2015
Larynx cancer	14.0 (13.3 to 15.3)	18.3 (15.6 to 21.3)*	-1.6 (-3.9 to 0.9)	1.6 (1.1 to 2.3)	17.4 (9.2 to 25.8)*	-2.4 (-9.2 to 4.7)
Larynx cancer aggregate	1.3 (1.0 to 1.7)	11.9 (-10.4 to 42.0)	-6.9 (-25.5 to 18.1)	0.4 (0.3 to 0.5)	12.8 (-1.0 to 28.8)	-6.2 (-17.6 to 7.2)
Laryngectomy due to larynx cancer	12.7 (12.0 to 13.9)	19.0 (18.1 to 19.9)*	-1.0 (-1.7 to -0.3)*	1.2 (0.8 to 1.8)	18.9 (9.7 to 29.9)*	-1.1 (-8.8 to 8.0)
Tracheal, bronchus, and lung cancer	10.4 (7.1 to 16.4)	30.9 (-9.5 to 94.6)	8.8 (-24.7 to 61.9)	1.8 (1.1 to 3.2)	25.8 (-12.6 to 87.5)	4.7 (-27.3 to 55.9)
Malignant skin melanoma	67.2 (57.4 to 86.6)	24.3 (14.7 to 35.0)*	3.4 (-4.6 to 12.3)	3.9 (2.5 to 5.8)	23.1 (11.6 to 34.9)*	2.4 (-7.2 to 12.2)
Non-melanoma skin cancer	21.6 (18.1 to 25.3)	108.4 (61.8 to 167.0)*	73.4 (34.6 to 122.1)*	1.9 (1.2 to 2.9)	59.0 (32.6 to 90.9)*	32.3 (10.3 to 58.8)*
Non-melanoma skin cancer(squamous-cell carcinoma)	31.7 (24.9 to 40.3)	65.2 (39.7 to 97.2)*	37.4 (16.2 to 64.1)*	1.8 (1.2 to 2.8)	60.6 (33.0 to 94.0)*	33.6 (10.6 to 61.4)*
Non-melanoma skin cancer(basal-cell carcinoma)	5.1 (3.7 to 6.7)	19.4 (15.6 to 22.9)*	-0.7 (-3.9 to 2.3)	0.1 (0.0 to 0.1)	19.5 (15.3 to 23.4)*	-0.6 (-4.1 to 2.6)
Breast cancer	159.2 (130.9 to 180.7)	22.9 (5.3 to 43.5)*	2.2 (-12.4 to 19.4)	12.2 (8.4 to 16.9)	19.0 (1.7 to 40.4)*	-1.0 (-15.4 to 16.8)
Breast cancer aggregate	128.3 (100.2 to 149.4)	24.7 (3.0 to 51.3)*	3.7 (-14.3 to 25.9)	9.7 (6.6 to 13.4)	19.8 (-1.6 to 46.8)	-0.4 (-18.2 to 22.1)
Mastectomy due to breast cancer	30.9 (29.2 to 32.7)	16.1 (14.8 to 17.4)*	-3.5 (-4.5 to -2.3)*	2.5 (1.6 to 3.7)	16.0 (1.9 to 32.4)*	-3.5 (-15.2 to 10.2)
Cervical cancer	96.6 (62.8 to 139.1)	-10.4 (-39.2 to 26.5)	-25.5 (-49.4 to 5.2)	7.5 (4.3 to 11.6)	-10.1 (-38.7 to 25.6)	-25.2 (-49.0 to 4.4)
Uterine cancer	40.1 (21.1 to 54.0)	21.6 (-38.3 to 124.9)	1.1 (-48.7 to 87.0)	2.6 (1.3 to 4.2)	20.6 (-38.7 to 123.9)	0.3 (-49.0 to 86.2)
Ovarian cancer	31.6 (23.5 to 40.6)	33.9 (2.6 to 70.5)*	11.4 (-14.6 to 41.8)	4.2 (2.7 to 6.0)	31.5 (-0.2 to 69.5)	9.4 (-17.0 to 41.0)
Prostate cancer	7.9 (5.7 to 9.7)	34.9 (13.2 to 61.5)*	12.2 (-5.8 to 34.3)	0.6 (0.4 to 0.9)	24.2 (2.6 to 50.3)*	3.3 (-14.7 to 25.0)
Prostate cancer aggregate	7.7 (5.5 to 9.5)	35.5 (13.1 to 63.1)*	12.7 (-5.9 to 35.7)	0.6 (0.4 to 0.9)	24.5 (2.1 to 51.4)*	3.5 (-15.0 to 25.9)
Impotence and incontinence due to prostate cancer aggregate	0.2 (0.2 to 0.2)	17.5 (16.9 to 18.1)*	-2.2 (-2.7 to -1.8)*	0.0 (0.0 to 0.0)	17.5 (16.9 to 18.1)*	-2.2 (-2.7 to -1.8)*
Testicular cancer	116.9 (101.2 to 134.4)	42.8 (25.5 to 62.7)*	18.8 (4.4 to 35.4)*	6.9 (4.5 to 10.2)	41.0 (22.3 to 61.5)*	17.3 (1.7 to 34.3)*
Kidney cancer	18.2 (15.1 to 20.7)	52.9 (30.2 to 74.5)*	27.2 (8.3 to 45.1)*	1.4 (0.9 to 1.9)	50.3 (27.3 to 73.3)*	25.0 (5.9 to 44.1)*
Bladder cancer	11.0 (8.4 to 13.4)	30.8 (7.7 to 63.4)*	8.8 (-10.4 to 35.9)	1.0 (0.7 to 1.4)	25.4 (6.6 to 51.0)*	4.3 (-11.3 to 25.6)
Bladder cancer episodes aggregate	10.5 (7.9 to 12.8)	31.7 (7.3 to 66.4)*	9.6 (-10.7 to 38.4)	0.9 (0.6 to 1.3)	26.5 (5.7 to 55.5)*	5.3 (-12.0 to 29.3)
Urinary incontinence due to bladder cancer	0.5 (0.5 to 0.6)	15.2 (13.5 to 17.1)*	-4.2 (-5.6 to -2.6)*	0.1 (0.1 to 0.1)	15.2 (13.5 to 17.1)*	-4.2 (-5.6 to -2.6)*
Brain and nervous system cancer	43.0 (35.2 to 50.9)	32.5 (12.8 to 55.9)*	10.2 (-6.2 to 29.7)	4.8 (3.3 to 6.7)	30.9 (11.8 to 54.0)*	8.9 (-7.0 to 28.1)
Thyroid cancer	36.5 (31.8 to 40.5)	16.0 (2.1 to 32.6)*	-3.5 (-15.1 to 10.3)	2.1 (1.4 to 3.0)	15.4 (-0.1 to 33.3)	-4.0 (-16.9 to 10.9)
Mesothelioma	0.6 (0.6 to 0.7)	19.6 (4.9 to 37.6)*	-0.5 (-12.8 to 14.5)	0.1 (0.1 to 0.2)	19.1 (4.7 to 36.6)*	-0.9 (-12.9 to 13.7)
Hodgkin lymphoma	49.8 (42.6 to 60.6)	11.5 (-1.1 to 27.1)	-7.3 (-17.8 to 5.7)	4.0 (2.7 to 5.6)	8.0 (-5.1 to 24.7)	-10.1 (-21.1 to 3.8)
Non-Hodgkin lymphoma	117.6 (93.8 to 142.5)	57.4 (29.9 to 87.3)*	30.9 (8.0 to 55.8)*	8.9 (5.8 to 12.7)	52.7 (26.5 to 83.2)*	27.0 (5.2 to 52.4)*
Multiple myeloma	3.2 (2.4 to 4.1)	68.6 (37.1 to 107.4)*	40.2 (14.0 to 72.5)*	0.8 (0.5 to 1.1)	62.6 (33.1 to 100.5)*	35.2 (10.7 to 66.8)*
Leukemia	49.8 (41.9 to 57.4)	119.6 (77.2 to 167.5)*	82.7 (47.4 to 122.5)*	15.3 (9.8 to 22.5)	51.3 (14.7 to 102.2)*	25.8 (-4.6 to 68.2)
Acute lymphoid leukemia	32.4 (23.5 to 45.1)	64.3 (22.2 to 123.3)*	36.6 (1.7 to 85.8)*	4.2 (2.6 to 6.5)	61.0 (19.8 to 118.2)*	33.9 (-0.4 to 81.5)
Chronic lymphoid leukemia	41.3 (29.4 to 58.9)	51.3 (9.5 to 115.1)*	25.8 (-8.9 to 79.0)	5.1 (3.2 to 7.9)	48.5 (6.8 to 108.2)*	23.5 (-11.2 to 73.2)
Acute myeloid leukemia	32.9 (24.4 to 44.7)	55.2 (19.2 to 109.2)*	29.1 (-0.8 to 74.0)	4.1 (2.6 to 6.1)	53.4 (18.0 to 108.0)*	27.6 (-1.9 to 73.0)
Chronic myeloid leukemia	13.9 (10.2 to 19.3)	38.5 (3.9 to 85.9)*	15.2 (-13.6 to 54.7)	1.8 (1.1 to 2.7)	35.0 (1.3 to 80.7)*	12.3 (-15.8 to 50.3)
Other neoplasms	152.3 (120.5 to 192.7)	40.8 (20.0 to 64.9)*	17.1 (-0.2 to 37.1)	11.2 (7.2 to 16.3)	38.4 (16.9 to 62.7)*	15.1 (-2.8 to 35.3)
Cardiovascular diseases	7,192.0 (6,874.9 to 7,420.5)	10.5 (6.9 to 13.8)*	-8.1 (-11.1 to -5.3)*	535.0 (373.4 to 723.9)	14.8 (10.5 to 18.3)*	-4.5 (-8.1 to -1.6)*
Rheumatic heart disease	3,810.5 (3,393.7 to 4,758.6)	0.8 (-4.1 to 5.6)	-16.1 (-20.2 to -12.2)*	182.9 (114.5 to 281.7)	1.2 (-3.8 to 6.4)	-15.8 (-20.0 to -11.5)*
Rheumatic heart disease, without heart failure	3,785.8 (3,369.2 to 4,732.5)	0.7 (-4.3 to 5.5)	-16.3 (-20.4 to -12.3)*	179.9 (112.4 to 277.6)	0.8 (-4.2 to 6.0)	-16.1 (-20.3 to -11.8)*
Heart failure fue to rheumatic heart disease aggregate	24.7 (21.7 to 27.9)	30.4 (28.7 to 32.2)*	8.5 (7.1 to 9.9)*	3.0 (2.0 to 4.3)	30.1 (20.5 to 40.3)*	8.2 (0.2 to 16.7)*
Ischemic heart disease	641.9 (559.8 to 728.4)	23.8 (22.5 to 25.1)*	3.0 (1.9 to 4.1)*	49.9 (32.8 to 71.0)	24.3 (19.3 to 29.1)*	3.4 (-0.8 to 7.4)
Myocardial infarction episodes aggregate	0.9 (0.5 to 1.3)	18.8 (17.1 to 20.1)*	-1.2 (-2.6 to -0.1)*	0.1 (0.0 to 0.1)	18.8 (17.1 to 20.1)*	-1.2 (-2.6 to -0.1)*
Angina due to ischemic heart disease aggregate	541.4 (456.7 to 627.2)	23.4 (21.9 to 24.9)*	2.7 (1.4 to 3.9)*	37.9 (24.2 to 55.0)	23.6 (18.0 to 29.8)*	2.8 (-1.8 to 7.9)
Heart failure fue to ischemic heart disease aggregate	99.6 (88.0 to 112.5)	26.2 (24.9 to 27.5)*	4.9 (3.9 to 6.0)*	11.9 (7.9 to 16.9)	26.4 (16.4 to 36.3)*	5.2 (-3.2 to 13.4)
Cerebrovascular disease	987.2 (964.4 to 1,010.8)	24.8 (21.7 to 28.0)*	3.8 (1.3 to 6.4)*	162.0 (110.1 to 222.2)	24.5 (19.6 to 29.3)*	3.6 (-0.5 to 7.6)
Ischemic stroke	412.9 (392.6 to 435.2)	31.0 (29.8 to 32.2)*	9.0 (8.0 to 9.9)*	65.6 (44.5 to 89.4)	31.1 (23.6 to 38.4)*	9.0 (2.8 to 15.1)*
Chronic ischemic stroke aggregate	411.6 (391.4 to 434.0)	31.0 (29.8 to 32.2)*	9.0 (8.0 to 10.0)*	65.3 (44.3 to 89.0)	31.1 (23.6 to 38.5)*	9.1 (2.9 to 15.2)*
Ischemic stroke episodes aggregate	1.3 (0.9 to 1.9)	22.4 (18.2 to 26.1)*	1.8 (-1.7 to 4.9)	0.3 (0.2 to 0.5)	22.4 (18.2 to 26.1)*	1.9 (-1.7 to 4.9)
Hemorrhagic stroke	614.0 (584.4 to 646.5)	20.2 (19.2 to 21.2)*	-0.0 (-0.8 to 0.8)	96.4 (66.0 to 131.5)	20.4 (14.6 to 26.1)*	0.2 (-4.7 to 4.9)
Chronic hemorrhagic stroke aggregate	612.0 (582.6 to 644.1)	20.2 (19.2 to 21.2)*	0.0 (-0.8 to 0.8)	96.0 (65.7 to 131.1)	20.5 (14.5 to 26.2)*	0.2 (-4.7 to 5.0)
Acute hemorrhagic stroke aggregate	2.0 (1.5 to 2.6)	14.4 (6.5 to 21.5)*	-4.8 (-11.4 to 1.1)	0.4 (0.3 to 0.7)	14.4 (6.5 to 21.5)*	-4.8 (-11.4 to 1.1)
Hypertensive heart disease	38.9 (34.3 to 43.8)	24.2 (22.9 to 25.6)*	3.3 (2.3 to 4.5)*	4.7 (3.2 to 6.6)	24.1 (13.9 to 35.2)*	3.2 (-5.2 to 12.5)
Cardiomyopathy and myocarditis	62.2 (55.3 to 70.5)	22.1 (20.9 to 23.3)*	1.6 (0.5 to 2.6)*	6.6 (4.6 to 9.2)	22.2 (14.7 to 29.2)*	1.6 (-4.6 to 7.5)
Acute myocarditis	12.2 (8.4 to 17.0)	22.2 (19.0 to 25.1)*	1.6 (-1.0 to 4.1)	0.6 (0.3 to 1.0)	22.2 (15.7 to 29.2)*	1.6 (-3.8 to 7.5)
Heart failure due to cardiomyopathy aggregate	49.9 (44.0 to 56.4)	22.1 (20.7 to 23.6)*	1.5 (0.4 to 2.8)*	6.0 (4.1 to 8.4)	22.2 (14.1 to 29.9)*	1.6 (-5.1 to 8.1)
Atrial fibrillation and flutter	0.0 (0.0 to 0.0)	0.0 (0.0 to 0.0)	0.0 (0.0 to 0.0)	0.0 (0.0 to 0.0)	0.0 (0.0 to 0.0)	0.0 (0.0 to 0.0)
Peripheral vascular disease	0.0 (0.0 to 0.0)	0.0 (0.0 to 0.0)	0.0 (0.0 to 0.0)	0.0 (0.0 to 0.0)	0.0 (0.0 to 0.0)	0.0 (0.0 to 0.0)
Endocarditis	4.5 (3.4 to 5.8)	22.7 (18.7 to 26.4)*	2.1 (-1.3 to 5.1)	0.5 (0.3 to 0.8)	23.0 (18.7 to 27.0)*	2.3 (-1.3 to 5.6)
Endocarditis episodes aggregate	0.7 (0.5 to 0.8)	19.1 (10.6 to 25.8)*	-0.9 (-8.0 to 4.7)	0.0 (0.0 to 0.1)	19.1 (10.7 to 25.8)*	-0.9 (-7.9 to 4.6)
Heart failure due to endocarditis aggregate	3.8 (2.8 to 5.1)	23.3 (18.6 to 27.4)*	2.6 (-1.3 to 6.0)	0.5 (0.3 to 0.7)	23.3 (18.6 to 27.4)*	2.6 (-1.3 to 6.0)
Other cardiovascular and circulatory diseases	1,841.2 (1,407.4 to 2,377.0)	21.8 (19.6 to 24.1)*	1.3 (-0.5 to 3.2)	128.4 (79.5 to 188.1)	21.9 (18.6 to 25.3)*	1.4 (-1.3 to 4.3)
Heart failure due to other cardiovascular diseases aggregate	3.5 (3.0 to 4.0)	20.3 (18.1 to 22.4)*	0.0 (-1.7 to 1.8)	0.4 (0.3 to 0.6)	20.3 (18.1 to 22.5)*	0.1 (-1.8 to 1.9)
Other cardiovascular diseases episodes aggregate	1,837.7 (1,404.1 to 2,372.6)	21.8 (19.6 to 24.1)*	1.3 (-0.5 to 3.2)	127.9 (79.2 to 187.6)	21.9 (18.6 to 25.3)*	1.4 (-1.3 to 4.3)
Chronic respiratory diseases	25,422.6 (24,085.2 to 26,727.1)	14.5 (12.4 to 16.6)*	-4.7 (-6.5 to -3.0)*	1,549.3 (1,147.5 to 2,014.9)	12.4 (9.7 to 15.1)*	-6.5 (-8.7 to -4.3)*
Chronic obstructive pulmonary disease	7,118.2 (6,179.5 to 8,159.8)	11.4 (9.0 to 13.6)*	-7.4 (-9.3 to -5.5)*	549.9 (447.3 to 656.7)	10.7 (7.0 to 14.1)*	-8.0 (-11.0 to -5.1)*

Cause	Prevalence in thousands (95% confidence interval)			YLDs in thousands (95% confidence interval)		
	2015	Percent change in counts between 2005 and 2015	Percent change in rates between 2005 and 2015	2015	Percent change in counts between 2005 and 2015	Percent change in rates between 2005 and 2015
Chronic obstructive pulmonary disease without heart failure aggregate	7,120.9 (6,181.2 to 8,162.1)	11.4 (9.0 to 13.7)*	-7.3 (-9.3 to -5.4)*	549.2 (446.8 to 656.0)	10.6 (7.0 to 14.1)*	-8.0 (-11.0 to -5.1)*
Severe chronic obstructive pulmonary disease with heart failure aggregate	1.3 (1.1 to 1.5)	21.9 (20.1 to 23.8)*	1.4 (-0.1 to 3.0)	0.6 (0.4 to 0.8)	21.9 (20.1 to 23.8)*	1.4 (-0.1 to 3.0)
Pneumoconiosis	179.0 (168.2 to 189.9)	36.3 (28.0 to 45.5)*	13.4 (6.5 to 21.0)*	35.9 (22.6 to 52.6)	31.8 (22.9 to 42.0)*	9.7 (2.3 to 18.2)*
Silicosis	17.8 (14.8 to 21.1)	18.6 (15.3 to 21.4)*	-1.4 (-4.1 to 1.0)	3.0 (1.8 to 4.5)	18.9 (9.7 to 28.4)*	-1.1 (-8.7 to 6.8)
Silicosis without heart failure aggregate	17.8 (14.8 to 21.1)	18.6 (15.3 to 21.4)*	-1.4 (-4.1 to 1.0)	3.0 (1.8 to 4.5)	18.9 (9.7 to 28.4)*	-1.1 (-8.7 to 6.8)
Severe silicosis with heart failure aggregate	0.0 (0.0 to 0.0)	0.0 (0.0 to 0.0)	0.0 (0.0 to 0.0)	0.0 (0.0 to 0.0)	0.0 (0.0 to 0.0)	0.0 (0.0 to 0.0)
Asbestosis	3.9 (3.4 to 4.5)	21.7 (18.2 to 25.4)*	1.2 (-1.7 to 4.3)	0.7 (0.4 to 1.0)	21.7 (18.1 to 25.4)*	1.2 (-1.7 to 4.4)
Asbestosis without heart failure aggregate	3.9 (3.4 to 4.5)	21.7 (18.2 to 25.4)*	1.2 (-1.7 to 4.3)	0.7 (0.4 to 1.0)	21.7 (18.1 to 25.4)*	1.2 (-1.7 to 4.4)
Severe asbestosis with heart failure aggregate	0.0 (0.0 to 0.0)	0.0 (0.0 to 0.0)	0.0 (0.0 to 0.0)	0.0 (0.0 to 0.0)	0.0 (0.0 to 0.0)	0.0 (0.0 to 0.0)
Coal workers pneumoconiosis	1.8 (1.5 to 2.1)	18.9 (13.9 to 23.6)*	-1.1 (-5.2 to 2.8)	0.3 (0.2 to 0.4)	18.9 (13.9 to 23.6)*	-1.1 (-5.2 to 2.8)
Coal workers pneumoconiosis without heart failure aggregate	1.8 (1.5 to 2.1)	18.9 (13.9 to 23.6)*	-1.1 (-5.2 to 2.8)	0.3 (0.2 to 0.4)	18.9 (13.9 to 23.6)*	-1.1 (-5.2 to 2.8)
Severe coal workers pneumoconiosis with heart failure aggregate	0.0 (0.0 to 0.0)	0.0 (0.0 to 0.0)	0.0 (0.0 to 0.0)	0.0 (0.0 to 0.0)	0.0 (0.0 to 0.0)	0.0 (0.0 to 0.0)
Other pneumoconiosis	200.5 (175.8 to 230.0)	33.6 (29.4 to 37.5)*	11.2 (7.6 to 14.4)*	31.9 (19.9 to 47.3)	33.6 (23.3 to 45.3)*	11.1 (2.6 to 20.9)*
Other pneumoconiosis without heart failure aggregate	200.5 (175.8 to 230.0)	33.6 (29.4 to 37.5)*	11.2 (7.6 to 14.4)*	31.9 (19.9 to 47.3)	33.6 (23.3 to 45.3)*	11.1 (2.6 to 20.9)*
Severe other pneumoconiosis with heart failure aggregate	0.0 (0.0 to 0.0)	0.0 (0.0 to 0.0)	0.0 (0.0 to 0.0)	0.0 (0.0 to 0.0)	0.0 (0.0 to 0.0)	0.0 (0.0 to 0.0)
Asthma	18,845.9 (15,402.3 to 22,808.1)	15.5 (11.9 to 18.9)*	-4.0 (-6.9 to -1.1)*	846.3 (518.9 to 1,243.3)	15.6 (12.1 to 19.1)*	-3.8 (-6.7 to -0.9)*
Asymptomatic asthma	5,705.7 (4,585.6 to 7,162.6)	15.5 (11.9 to 18.9)*	-4.0 (-6.9 to -1.1)*	0.0 (0.0 to 0.0)	0.0 (0.0 to 0.0)	0.0 (0.0 to 0.0)
Symptomatic asthma aggregate	13,140.2 (10,654.4 to 15,928.3)	15.5 (11.9 to 18.9)*	-4.0 (-6.9 to -1.1)*	846.3 (518.9 to 1,243.3)	15.6 (12.1 to 19.1)*	-3.8 (-6.7 to -0.9)*
Interstitial lung disease and pulmonary sarcoidosis	53.4 (45.9 to 62.1)	17.7 (14.8 to 20.6)*	-2.1 (-4.5 to 0.3)	6.7 (4.1 to 9.9)	17.9 (10.1 to 25.5)*	-1.9 (-8.4 to 4.4)
Interstitial lung disease and pulmonary sarcoidosis without heart failure aggregate	52.1 (44.7 to 60.6)	17.7 (14.8 to 20.6)*	-2.1 (-4.5 to 0.3)	6.1 (3.6 to 9.1)	17.8 (9.2 to 26.3)*	-2.0 (-9.2 to 5.1)
Severe interstitial lung disease and pulmonary sarcoidosis with heart failure aggregate	1.3 (1.1 to 1.5)	18.5 (16.5 to 20.6)*	-1.5 (-3.1 to 0.3)	0.6 (0.4 to 0.8)	18.5 (16.5 to 20.6)*	-1.5 (-3.1 to 0.3)
Other chronic respiratory diseases	--	--	--	110.6 (87.4 to 135.2)	-5.0 (-14.1 to 5.7)	-21.0 (-28.5 to -12.1)*
Cirrhosis and other chronic liver diseases	68.1 (62.6 to 73.5)	31.4 (17.3 to 46.0)*	9.3 (-2.4 to 21.5)	18.5 (12.6 to 25.6)	25.8 (17.5 to 33.8)*	4.7 (-2.2 to 11.3)
Cirrhosis and other chronic liver diseases due to hepatitis B	25.2 (20.2 to 30.6)	20.6 (17.0 to 23.6)*	0.3 (-2.6 to 2.8)	4.4 (2.8 to 6.5)	21.3 (5.4 to 39.0)*	0.9 (-12.3 to 15.6)
Cirrhosis and other chronic liver diseases due to hepatitis C	15.3 (11.9 to 19.2)	24.4 (19.3 to 29.0)*	3.5 (-0.8 to 7.3)	2.7 (1.6 to 4.1)	24.3 (6.4 to 45.3)*	3.4 (-11.5 to 20.8)
Cirrhosis and other chronic liver diseases due to alcohol use	33.8 (29.0 to 39.4)	31.3 (28.3 to 34.2)*	9.2 (6.7 to 11.6)*	5.8 (3.9 to 8.4)	31.4 (19.1 to 45.2)*	9.3 (-0.9 to 20.7)
Cirrhosis and other chronic liver diseases due to other causes	32.7 (27.7 to 38.7)	25.0 (22.4 to 27.7)*	3.9 (1.9 to 6.2)*	5.6 (3.7 to 8.2)	24.7 (9.9 to 42.0)*	3.7 (-8.6 to 18.2)
Digestive diseases	10,412.3 (10,131.2 to 10,666.6)	7.2 (4.4 to 10.0)*	-10.8 (-13.2 to -8.5)*	645.5 (449.2 to 871.1)	9.7 (7.1 to 12.4)*	-8.8 (-10.9 to -6.5)*
Peptic ulcer disease	2,552.3 (2,476.4 to 2,619.4)	-6.0 (-8.4 to -3.3)*	-21.8 (-23.8 to -19.6)*	88.2 (59.9 to 125.9)	-10.8 (-14.7 to -6.8)*	-25.8 (-29.0 to -22.4)*
Peptic ulcer disease, symptomatic episodes	206.7 (161.8 to 255.4)	-4.9 (-10.4 to -0.2)*	-20.9 (-25.5 to -17.0)*	22.7 (14.3 to 32.3)	-4.7 (-12.5 to 2.6)	-20.7 (-27.2 to -14.6)*
Anemia due to peptic ulcer disease aggregate	2,345.6 (2,284.7 to 2,398.5)	-6.1 (-8.7 to -3.2)*	-21.9 (-24.1 to -19.5)*	65.6 (43.5 to 95.6)	-12.7 (-16.9 to -8.2)*	-27.4 (-30.9 to -23.7)*
Gastritis and duodenitis	5,343.0 (5,135.6 to 5,524.6)	11.6 (6.7 to 16.3)*	-7.2 (-11.2 to -3.3)*	188.3 (127.6 to 270.0)	8.5 (3.3 to 14.2)*	-9.7 (-14.1 to -5.0)*
Gastritis and duodenitis, symptomatic episodes	154.6 (112.5 to 208.2)	13.5 (8.5 to 16.1)*	-5.6 (-9.8 to -3.4)*	17.0 (10.2 to 26.0)	13.9 (5.4 to 21.9)*	-5.3 (-12.4 to 1.4)
Anemia due to gastritis and duodenitis aggregate	5,188.4 (4,998.8 to 5,366.2)	11.5 (6.5 to 16.4)*	-7.2 (-11.4 to -3.2)*	171.3 (115.9 to 245.7)	8.0 (2.1 to 14.2)*	-10.1 (-15.0 to -5.0)*
Appendicitis	48.3 (40.5 to 56.5)	21.3 (19.4 to 23.1)*	0.9 (-0.7 to 2.4)	14.9 (9.3 to 21.2)	22.1 (9.5 to 35.5)*	1.6 (-8.9 to 12.7)
Paralytic ileus and intestinal obstruction	5.0 (3.6 to 6.5)	18.3 (10.4 to 25.3)*	-1.6 (-8.1 to 4.3)	1.6 (1.0 to 2.4)	18.3 (10.4 to 25.3)*	-1.6 (-8.1 to 4.3)
Inguinal, femoral, and abdominal hernia	1,125.8 (778.3 to 1,494.3)	8.0 (2.0 to 13.8)*	-10.1 (-15.1 to -5.4)*	12.2 (5.6 to 24.4)	8.3 (1.6 to 14.5)*	-9.9 (-15.5 to -4.8)*
Inflammatory bowel disease	963.2 (858.9 to 1,068.3)	16.4 (15.8 to 17.0)*	-3.2 (-3.7 to -2.6)*	211.3 (139.1 to 294.0)	16.6 (13.9 to 19.3)*	-3.0 (-5.2 to -0.7)*
Vascular intestinal disorders	1.4 (1.0 to 1.8)	18.7 (11.6 to 25.4)*	-1.2 (-7.2 to 4.3)	0.4 (0.3 to 0.7)	18.7 (11.6 to 25.4)*	-1.3 (-7.2 to 4.3)
Gallbladder and biliary diseases	415.1 (316.1 to 531.4)	13.1 (6.5 to 19.5)*	-5.9 (-11.4 to -0.6)*	45.5 (27.5 to 69.8)	13.3 (5.4 to 20.9)*	-5.8 (-12.4 to 0.5)
Pancreatitis	66.6 (52.4 to 82.1)	26.3 (20.7 to 31.2)*	5.0 (0.4 to 9.2)*	20.5 (13.1 to 30.1)	26.5 (13.9 to 39.6)*	5.3 (-5.2 to 16.1)
Other digestive diseases	--	--	--	62.6 (43.2 to 86.0)	16.9 (9.4 to 28.1)*	-2.8 (-9.0 to 6.5)
Neurological disorders	268,228.2 (247,157.1 to 285,461.1)	19.1 (16.7 to 21.4)*	-1.0 (-2.9 to 1.0)	5,562.9 (3,570.9 to 8,160.4)	16.4 (13.7 to 18.9)*	-3.2 (-5.4 to -1.1)*
Alzheimer disease and other dementias	0.0 (0.0 to 0.0)	0.0 (0.0 to 0.0)	0.0 (0.0 to 0.0)	0.0 (0.0 to 0.0)	0.0 (0.0 to 0.0)	0.0 (0.0 to 0.0)
Parkinson disease	35.9 (30.6 to 41.3)	16.6 (10.8 to 22.7)*	-3.0 (-7.8 to 2.1)	4.9 (3.2 to 7.0)	16.7 (9.4 to 24.0)*	-3.0 (-9.0 to 3.1)
Epilepsy	1,949.0 (1,670.2 to 2,241.5)	13.5 (7.3 to 19.7)*	-5.6 (-10.7 to -0.5)*	540.6 (359.7 to 747.3)	-3.7 (-10.5 to 3.1)	-19.9 (-25.5 to -14.2)*
Multiple sclerosis	136.8 (117.0 to 157.2)	17.3 (13.7 to 21.1)*	-2.4 (-5.4 to 0.8)	47.3 (31.9 to 64.3)	17.5 (11.2 to 23.8)*	-2.3 (-7.5 to 3.0)
Motor neuron disease	7.5 (6.7 to 8.2)	18.8 (16.3 to 21.3)*	-1.2 (-3.3 to 0.9)	1.6 (1.1 to 2.1)	18.8 (16.3 to 21.3)*	-1.2 (-3.3 to 0.9)
Migraine	110,775.4 (88,725.7 to 136,245.1)	18.4 (16.5 to 20.4)*	-1.5 (-3.1 to 0.1)	3,874.2 (2,260.6 to 6,025.6)	18.7 (16.6 to 20.9)*	-1.2 (-3.0 to 0.5)
Tension-type headache	189,236.7 (135,764.5 to 260,346.7)	19.2 (17.8 to 20.6)*	-0.8 (-2.0 to 0.3)	287.6 (119.3 to 551.4)	19.4 (17.6 to 21.1)*	-0.7 (-2.2 to 0.8)
Medication overuse headache	4,996.5 (3,154.5 to 7,100.7)	20.2 (9.1 to 32.5)*	0.0 (-9.2 to 10.2)	803.9 (436.1 to 1,337.9)	20.5 (9.3 to 33.2)*	0.3 (-9.1 to 10.8)
Other neurological disorders	0.7 (0.4 to 1.1)	20.9 (19.4 to 21.9)*	0.5 (-0.7 to 1.4)	2.8 (1.9 to 3.9)	12.9 (6.0 to 19.9)*	-6.1 (-11.8 to -0.3)*
Mental and substance use disorders	110,698.6 (107,999.8 to 114,161.7)	20.8 (19.7 to 21.9)*	0.5 (-0.4 to 1.4)	16,875.5 (12,170.5 to 21,545.0)	22.0 (21.2 to 22.9)*	1.5 (0.8 to 2.2)*
Schizophrenia	2,567.1 (2,047.3 to 3,204.4)	23.0 (22.0 to 24.0)*	2.3 (1.5 to 3.1)*	1,700.3 (1,172.9 to 2,235.0)	23.3 (21.2 to 25.3)*	2.6 (0.8 to 4.3)*
Alcohol use disorders	8,820.3 (7,434.3 to 10,491.6)	19.2 (15.4 to 23.6)*	-0.8 (-4.0 to 2.8)	894.8 (595.5 to 1,276.6)	19.4 (15.3 to 23.8)*	-0.6 (-4.1 to 3.0)
Alcohol dependence aggregate	8,741.3 (7,355.0 to 10,410.8)	19.2 (15.3 to 23.6)*	-0.8 (-4.1 to 2.8)	890.6 (592.2 to 1,270.2)	19.4 (15.3 to 23.8)*	-0.6 (-4.1 to 3.0)
Fetal alcohol syndrome aggregate	79.0 (73.9 to 83.3)	19.6 (18.8 to 20.5)*	-0.5 (-1.2 to 0.2)	4.1 (2.6 to 6.0)	20.0 (12.6 to 27.6)*	-0.2 (-6.4 to 6.1)
Drug use disorders	8,938.6 (8,645.2 to 9,238.4)	28.0 (25.9 to 30.2)*	6.5 (4.7 to 8.3)*	1,951.3 (1,352.6 to 2,565.4)	31.5 (28.1 to 35.0)*	9.4 (6.5 to 12.3)*
Opioid use disorders	3,205.3 (2,638.5 to 3,876.1)	26.6 (23.1 to 30.4)*	5.3 (2.4 to 8.5)*	1,359.7 (934.9 to 1,809.7)	26.9 (22.9 to 31.1)*	5.5 (2.2 to 9.1)*
Asymptomatic opioid dependence	520.1 (364.6 to 728.1)	26.6 (23.1 to 30.4)*	5.3 (2.4 to 8.5)*	0.0 (0.0 to 0.0)	0.0 (0.0 to 0.0)	0.0 (0.0 to 0.0)
Symptomatic opioid dependence aggregate	2,685.2 (2,184.7 to 3,286.7)	26.6 (23.1 to 30.4)*	5.3 (2.4 to 8.5)*	1,359.7 (934.9 to 1,809.7)	26.9 (22.9 to 31.1)*	5.5 (2.2 to 9.1)*
Cocaine use disorders	596.7 (498.8 to 702.8)	37.8 (34.2 to 41.4)*	14.6 (11.6 to 17.7)*	82.7 (52.5 to 119.1)	37.9 (31.5 to 44.4)*	14.7 (9.4 to 20.1)*
Asymptomatic cocaine dependence	299.0 (231.5 to 378.6)	37.8 (34.2 to 41.4)*	14.6 (11.6 to 17.7)*	0.0 (0.0 to 0.0)	0.0 (0.0 to 0.0)	0.0 (0.0 to 0.0)

Cause	Prevalence in thousands (95% confidence interval)			YLDs in thousands (95% confidence interval)		
	2015	Percent change in counts between 2005 and 2015	Percent change in rates between 2005 and 2015	2015	Percent change in counts between 2005 and 2015	Percent change in rates between 2005 and 2015
Symptomatic cocaine dependence aggregate	297.7 (228.2 to 377.5)	37.8 (34.2 to 41.4)*	14.6 (11.6 to 17.7)*	82.7 (52.5 to 119.1)	37.9 (31.5 to 44.4)*	14.7 (9.4 to 20.1)*
Amphetamine use disorders	1,596.3 (1,249.6 to 1,962.4)	55.3 (50.5 to 60.3)*	29.2 (25.2 to 33.3)*	211.8 (122.7 to 319.2)	55.4 (48.5 to 62.4)*	29.3 (23.5 to 35.1)*
Asymptomatic amphetamine dependence	874.0 (652.1 to 1,128.4)	55.3 (50.5 to 60.3)*	29.2 (25.2 to 33.3)*	0.0 (0.0 to 0.0)	0.0 (0.0 to 0.0)	0.0 (0.0 to 0.0)
Symptomatic amphetamine dependence aggregate	722.3 (512.5 to 955.8)	55.3 (50.5 to 60.3)*	29.2 (25.2 to 33.3)*	211.8 (122.7 to 319.2)	55.4 (48.5 to 62.4)*	29.3 (23.5 to 35.1)*
Cannabis use disorders	3,619.3 (3,062.3 to 4,238.5)	18.8 (17.4 to 20.2)*	-1.1 (-2.4 to 0.0)	105.9 (67.2 to 154.8)	19.1 (15.5 to 22.6)*	-1.0 (-3.9 to 2.0)
Asymptomatic cannabis dependence	2,093.5 (1,739.9 to 2,498.3)	18.8 (17.4 to 20.2)*	-1.1 (-2.4 to 0.0)	0.0 (0.0 to 0.0)	0.0 (0.0 to 0.0)	0.0 (0.0 to 0.0)
Symptomatic cannabis dependence aggregate	1,525.8 (1,252.1 to 1,835.3)	18.8 (17.4 to 20.2)*	-1.1 (-2.4 to 0.0)	105.9 (67.2 to 154.8)	19.1 (15.5 to 22.6)*	-1.0 (-3.9 to 2.0)
Other drug use disorders	--	--	--	191.2 (115.1 to 279.8)	50.1 (42.9 to 57.8)*	24.9 (18.9 to 31.3)*
Depressive disorders	31,483.4 (29,584.5 to 34,107.1)	20.5 (18.3 to 22.7)*	0.3 (-1.6 to 2.1)	5,792.1 (3,726.5 to 8,222.3)	20.9 (19.8 to 22.0)*	0.6 (-0.3 to 1.5)
Major depressive disorder	23,120.9 (17,426.5 to 29,191.2)	20.7 (19.7 to 21.7)*	0.4 (-0.5 to 1.2)	4,843.7 (3,052.3 to 6,929.3)	21.1 (19.8 to 22.2)*	0.7 (-0.4 to 1.7)
Dysthymia	9,605.5 (7,453.0 to 12,475.6)	20.0 (18.0 to 22.0)*	-0.2 (-1.8 to 1.5)	948.5 (593.6 to 1,440.4)	20.2 (18.0 to 22.4)*	-0.0 (-1.8 to 1.8)
Bipolar disorder	6,731.2 (5,472.0 to 8,272.8)	19.1 (18.8 to 19.3)*	-0.9 (-1.2 to -0.7)*	1,400.4 (834.3 to 2,132.9)	19.4 (17.9 to 20.9)*	-0.7 (-1.9 to 0.6)
Anxiety disorders	26,329.8 (20,614.5 to 33,779.2)	19.4 (16.7 to 22.2)*	-0.7 (-2.9 to 1.6)	2,469.8 (1,556.9 to 3,668.9)	19.6 (16.8 to 22.5)*	-0.5 (-2.9 to 1.9)
Eating disorders	1,186.1 (1,111.7 to 1,263.1)	30.3 (26.1 to 34.6)*	8.4 (4.9 to 12.0)*	252.0 (165.7 to 356.9)	30.6 (27.4 to 34.0)*	8.6 (6.0 to 11.4)*
Anorexia nervosa	518.8 (420.1 to 640.5)	25.5 (22.3 to 28.8)*	4.4 (1.7 to 7.1)*	110.5 (72.4 to 162.9)	25.8 (20.8 to 30.5)*	4.6 (0.5 to 8.5)*
Bulimia nervosa	668.6 (495.8 to 917.6)	34.5 (32.1 to 37.1)*	11.9 (9.9 to 14.0)*	141.5 (86.4 to 218.7)	34.6 (30.7 to 38.8)*	12.0 (8.7 to 15.5)*
Autistic spectrum disorders	5,789.1 (5,503.8 to 6,022.2)	20.7 (18.5 to 23.0)*	0.4 (-1.4 to 2.3)	943.4 (627.3 to 1,310.5)	21.0 (19.8 to 22.3)*	0.6 (-0.4 to 1.8)
Autism	2,298.0 (1,926.2 to 2,725.1)	20.8 (20.5 to 21.0)*	0.5 (0.2 to 0.7)*	594.3 (385.6 to 844.0)	21.0 (19.2 to 22.9)*	0.7 (-0.9 to 2.2)
Asperger syndrome and other autistic spectrum disorders	3,464.7 (2,915.9 to 4,157.5)	20.7 (20.6 to 20.8)*	0.4 (0.3 to 0.5)*	349.1 (229.6 to 508.5)	20.9 (19.4 to 22.3)*	0.5 (-0.6 to 1.7)
Attention-deficit/hyperactivity disorder	5,577.7 (4,883.7 to 6,399.6)	21.7 (20.9 to 22.5)*	1.2 (0.6 to 1.9)*	67.6 (39.7 to 103.1)	21.8 (19.6 to 24.1)*	1.4 (-0.5 to 3.2)
Conduct disorder	0.0 (0.0 to 0.0)	0.0 (0.0 to 0.0)	0.0 (0.0 to 0.0)	0.0 (0.0 to 0.0)	0.0 (0.0 to 0.0)	0.0 (0.0 to 0.0)
Idiopathic developmental intellectual disability	9,266.2 (5,376.4 to 12,986.1)	21.2 (20.1 to 22.2)*	0.8 (-0.1 to 1.6)	344.7 (151.9 to 602.9)	20.5 (18.4 to 22.1)*	0.3 (-1.5 to 1.5)
Other mental and substance use disorders	13,772.6 (13,493.1 to 14,076.2)	20.8 (17.4 to 24.3)*	0.4 (-2.3 to 3.4)	1,059.2 (730.2 to 1,428.4)	20.9 (17.2 to 24.8)*	0.6 (-2.5 to 3.8)
Diabetes, urogenital, blood, and endocrine diseases	236,095.6 (233,784.6 to 238,725.7)	19.4 (18.5 to 20.2)*	-0.7 (-1.5 to 0.0)	3,416.3 (2,364.9 to 4,725.6)	20.0 (18.0 to 21.9)*	-0.2 (-1.8 to 1.4)
Diabetes mellitus	16,934.5 (14,386.8 to 19,608.7)	30.1 (25.6 to 34.7)*	8.2 (4.4 to 12.1)*	1,095.6 (704.9 to 1,568.7)	30.1 (25.5 to 34.8)*	8.2 (4.4 to 12.2)*
Uncomplicated diabetes mellitus	13,475.0 (11,256.5 to 15,882.0)	30.3 (25.8 to 35.2)*	8.4 (4.7 to 12.5)*	642.7 (389.9 to 954.8)	30.5 (25.8 to 35.5)*	8.6 (4.6 to 12.7)*
Neuropathy and other complications of diabetes mellitus aggregate	3,380.4 (2,532.8 to 4,385.4)	29.3 (24.2 to 34.4)*	7.6 (3.3 to 11.8)*	448.5 (275.3 to 663.9)	29.6 (24.6 to 35.2)*	7.8 (3.6 to 12.5)*
Vision loss due to diabetes mellitus aggregate	79.1 (49.0 to 125.9)	21.2 (18.2 to 24.6)*	0.8 (-1.7 to 3.6)	4.4 (2.4 to 7.4)	21.6 (15.5 to 27.8)*	1.2 (-3.9 to 6.3)
Acute glomerulonephritis	6.4 (4.6 to 8.8)	7.7 (1.3 to 13.2)*	-10.4 (-15.8 to -5.8)*	0.3 (0.2 to 0.5)	7.8 (-0.3 to 14.7)	-10.3 (-17.1 to -4.6)*
Chronic kidney disease	12,894.2 (11,475.5 to 13,883.8)	21.3 (17.4 to 25.2)*	0.9 (-2.4 to 4.2)	395.7 (280.0 to 522.3)	18.8 (16.0 to 21.4)*	-1.2 (-3.5 to 1.0)
Chronic kidney disease due to diabetes mellitus	4,734.5 (2,979.8 to 6,892.6)	21.5 (18.5 to 24.9)*	1.1 (-1.5 to 3.9)	144.5 (95.6 to 206.3)	19.3 (14.5 to 24.3)*	-0.8 (-4.7 to 3.4)
Stage III chronic kidney disease due to diabetes mellitus aggregate	4,186.7 (2,582.0 to 6,217.7)	21.7 (18.5 to 25.3)*	1.3 (-1.5 to 4.2)	13.2 (9.0 to 18.5)	15.7 (6.5 to 25.5)*	-3.7 (-11.4 to 4.4)
Stage IV chronic kidney disease due to diabetes mellitus aggregate	374.6 (227.2 to 562.2)	20.2 (17.6 to 23.1)*	-0.0 (-2.2 to 2.4)	49.8 (28.5 to 80.0)	19.2 (13.4 to 25.5)*	-0.9 (-5.7 to 4.4)
Stage V chronic kidney disease untreated due to diabetes mellitus	144.3 (96.0 to 210.7)	20.1 (17.4 to 22.9)*	-0.1 (-2.3 to 2.2)	76.7 (45.4 to 118.3)	20.3 (12.7 to 29.2)*	0.0 (-6.3 to 7.5)
End-stage chronic kidney disease due to diabetes aggregate	28.9 (20.1 to 38.3)	16.4 (13.0 to 20.4)*	-3.2 (-6.0 to 0.2)	4.9 (3.1 to 7.3)	14.9 (6.3 to 24.7)*	-4.4 (-11.6 to 3.7)
Chronic kidney disease due to hypertension	3,228.8 (1,989.7 to 4,972.3)	21.1 (17.2 to 25.2)*	0.7 (-2.5 to 4.1)	42.8 (27.2 to 62.9)	19.7 (12.1 to 27.5)*	-0.5 (-6.7 to 6.0)
Stage III chronic kidney disease due to hypertension aggregate	3,039.3 (1,852.7 to 4,729.2)	21.1 (16.9 to 25.1)*	0.7 (-2.7 to 4.1)	3.1 (2.2 to 4.3)	13.0 (5.7 to 20.3)*	-6.0 (-12.1 to 0.1)
Stage IV chronic kidney disease due to hypertension aggregate	145.3 (76.0 to 277.8)	22.5 (13.6 to 34.2)*	1.9 (-5.5 to 11.6)	18.6 (9.3 to 33.9)	21.1 (11.6 to 31.2)*	0.7 (-7.2 to 9.1)
Stage V chronic kidney disease untreated due to hypertension	36.6 (23.6 to 53.8)	19.4 (14.1 to 25.1)*	-0.7 (-5.1 to 4.1)	19.8 (11.5 to 31.5)	19.8 (6.3 to 34.5)*	-0.4 (-11.6 to 11.9)
End-stage chronic kidney disease due to hypertension aggregate	7.6 (5.3 to 10.6)	17.0 (7.9 to 26.1)*	-2.7 (-10.2 to 4.9)	1.3 (0.8 to 2.0)	15.1 (7.6 to 23.0)*	-4.3 (-10.5 to 2.3)
Chronic kidney disease due to glomerulonephritis	1,579.1 (1,155.8 to 2,120.3)	19.9 (15.6 to 24.9)*	-0.3 (-3.8 to 3.9)	100.3 (64.6 to 145.9)	18.0 (12.4 to 23.5)*	-1.8 (-6.5 to 2.7)
Stage III chronic kidney disease due to glomerulonephritis aggregate	1,214.1 (816.5 to 1,740.2)	20.4 (14.9 to 26.5)*	0.1 (-4.4 to 5.2)	5.5 (3.6 to 8.1)	8.6 (-4.6 to 22.9)	-9.7 (-20.7 to 2.2)
Stage IV chronic kidney disease due to glomerulonephritis aggregate	236.0 (162.9 to 302.4)	17.8 (12.6 to 23.4)*	-2.0 (-6.3 to 2.7)	32.2 (19.6 to 45.7)	17.3 (9.4 to 25.7)*	-2.4 (-9.0 to 4.5)
Stage V chronic kidney disease untreated due to glomerulonephritis	111.7 (70.5 to 164.5)	19.5 (16.6 to 22.4)*	-0.6 (-3.0 to 1.8)	59.3 (34.2 to 96.7)	19.7 (11.5 to 28.4)*	-0.5 (-7.3 to 6.8)
End-stage chronic kidney disease due to glomerulonephritis aggregate	17.3 (11.3 to 25.4)	15.5 (11.3 to 20.3)*	-3.9 (-7.5 to 0.1)	3.2 (1.8 to 5.0)	12.9 (6.1 to 20.6)*	-6.0 (-11.7 to 0.3)
Chronic kidney disease due to other causes	3,740.5 (2,266.7 to 5,791.7)	20.9 (18.4 to 23.4)*	0.5 (-1.5 to 2.7)	108.1 (63.5 to 164.7)	18.4 (13.0 to 23.4)*	-1.5 (-6.0 to 2.6)
Stage III chronic kidney disease due to other causes aggregate	3,312.8 (1,958.6 to 5,187.9)	21.1 (18.5 to 23.8)*	0.7 (-1.5 to 3.0)	5.5 (3.6 to 8.1)	8.6 (-4.5 to 23.8)	-9.7 (-20.6 to 2.9)
Stage IV chronic kidney disease due to other causes aggregate	294.2 (167.9 to 459.0)	19.7 (17.7 to 21.6)*	-0.5 (-2.1 to 1.2)	38.4 (21.1 to 62.0)	18.2 (12.2 to 23.9)*	-1.6 (-6.6 to 3.1)
Stage V chronic kidney disease untreated due to other causes	113.9 (68.8 to 169.8)	19.4 (17.3 to 21.3)*	-0.7 (-2.4 to 0.9)	60.6 (32.4 to 100.4)	19.8 (11.0 to 28.3)*	-0.4 (-7.7 to 6.7)
End-stage chronic kidney disease due to other causes aggregate	19.6 (12.3 to 28.1)	16.1 (12.6 to 19.5)*	-3.4 (-6.4 to -0.6)*	3.5 (2.0 to 5.6)	13.7 (4.8 to 23.3)*	-5.5 (-12.8 to 2.5)
Urinary diseases and male infertility	25,710.8 (24,731.2 to 26,660.0)	24.4 (21.8 to 27.0)*	3.5 (1.3 to 5.6)*	57.4 (29.6 to 104.0)	31.3 (24.8 to 36.9)*	9.2 (3.8 to 13.9)*
Interstitial nephritis and urinary tract infections	232.9 (217.2 to 248.0)	22.8 (22.1 to 23.4)*	2.1 (1.6 to 2.7)*	7.9 (4.9 to 12.0)	23.0 (16.1 to 30.2)*	2.3 (-3.4 to 8.3)
Urolithiasis	19,724.8 (16,823.9 to 22,886.4)	20.6 (17.4 to 23.4)*	0.3 (-2.4 to 2.6)	7.3 (4.4 to 10.8)	20.2 (8.7 to 33.1)*	-0.1 (-9.5 to 10.7)
Benign prostatic hyperplasia	0.0 (0.0 to 0.0)	0.0 (0.0 to 0.0)	0.0 (0.0 to 0.0)	0.0 (0.0 to 0.0)	0.0 (0.0 to 0.0)	0.0 (0.0 to 0.0)
Male infertility	5,962.3 (4,155.2 to 8,173.7)	39.1 (33.8 to 44.2)*	15.7 (11.3 to 20.0)*	36.8 (13.7 to 78.8)	38.2 (32.3 to 43.6)*	14.9 (10.0 to 19.5)*
Other urinary diseases	--	--	--	5.5 (3.5 to 8.1)	18.0 (5.5 to 32.3)*	-1.9 (-12.2 to 10.1)
Gynecological diseases	93,280.6 (91,278.8 to 95,126.4)	19.8 (18.4 to 21.1)*	-0.4 (-1.5 to 0.7)	1,329.6 (897.3 to 1,901.8)	17.0 (14.5 to 19.2)*	-2.7 (-4.7 to -0.9)*
Uterine fibroids	8,806.6 (7,793.8 to 9,863.9)	19.2 (18.9 to 19.6)*	-0.8 (-1.1 to -0.5)*	224.7 (141.6 to 349.3)	13.5 (10.9 to 15.8)*	-5.6 (-7.7 to -3.7)*
Uterine fibroids cases aggregate	3,380.6 (2,843.2 to 4,005.4)	24.2 (22.0 to 26.7)*	3.4 (1.5 to 5.4)*	6.6 (2.9 to 13.3)	53.7 (38.4 to 77.6)*	27.9 (15.1 to 47.7)*
Mild abdominal pain with anemia due to uterine fibroids aggregate	5,426.0 (4,890.5 to 5,984.0)	16.3 (15.0 to 17.4)*	-3.3 (-4.4 to -2.3)*	218.1 (138.4 to 336.8)	12.6 (10.0 to 15.0)*	-6.3 (-8.5 to -4.4)*
Polycystic ovarian syndrome	10,898.4 (7,708.4 to 14,616.9)	18.5 (17.8 to 19.1)*	-1.4 (-2.0 to -0.9)*	98.6 (42.3 to 201.7)	18.2 (16.9 to 19.4)*	-1.7 (-2.8 to -0.7)*
Polycystic ovarian syndrome cases aggregate	8,505.9 (6,015.5 to 11,687.0)	18.3 (17.6 to 18.9)*	-1.6 (-2.2 to -1.1)*	74.0 (31.4 to 150.3)	18.5 (17.2 to 19.6)*	-1.5 (-2.5 to -0.5)*
Hirsutism and infertility due to polycystic ovarian syndrome aggregate	2,392.5 (1,511.6 to 3,538.1)	19.1 (18.0 to 20.4)*	-0.9 (-1.9 to 0.2)	24.6 (9.2 to 54.4)	17.4 (13.7 to 20.4)*	-2.4 (-5.4 to 0.1)

Cause	Prevalence in thousands (95% confidence interval)			YLDs in thousands (95% confidence interval)		
	2015	Percent change in counts between 2005 and 2015	Percent change in rates between 2005 and 2015	2015	Percent change in counts between 2005 and 2015	Percent change in rates between 2005 and 2015
Female infertility	11,960.3 (7,327.6 to 17,847.7)	47.4 (38.2 to 58.3)*	22.6 (14.9 to 31.6)*	69.1 (24.1 to 153.5)	46.7 (38.0 to 57.4)*	22.1 (14.8 to 30.9)*
Endometriosis	1,631.2 (1,286.9 to 2,075.0)	17.1 (16.4 to 17.8)*	-2.6 (-3.2 to -2.0)*	152.9 (94.9 to 226.0)	17.3 (14.5 to 20.1)*	-2.4 (-4.7 to -0.1)*
Endometriosis cases aggregate	1,531.1 (1,203.9 to 1,944.8)	17.1 (16.3 to 17.7)*	-2.6 (-3.2 to -2.1)*	142.8 (88.7 to 210.4)	17.3 (14.3 to 20.3)*	-2.4 (-4.9 to 0.1)
Abdominal pain and infertility due to endometriosis aggregate	100.1 (68.5 to 142.1)	17.7 (16.6 to 19.4)*	-2.1 (-3.0 to -0.7)*	10.1 (5.7 to 16.2)	17.9 (10.3 to 26.2)*	-1.9 (-8.2 to 5.0)
Genital prolapse	5,291.1 (4,080.6 to 6,666.2)	8.6 (4.3 to 12.8)*	-9.7 (-13.3 to -6.2)*	16.9 (8.0 to 32.3)	8.9 (3.8 to 14.2)*	-9.4 (-13.6 to -5.0)*
Premenstrual syndrome	60,032.8 (53,842.1 to 65,974.8)	17.7 (12.2 to 21.6)*	-2.1 (-6.7 to 1.1)	509.0 (315.7 to 765.9)	17.9 (12.3 to 22.3)*	-1.9 (-6.6 to 1.7)
Other gynecological diseases	7,079.6 (6,804.2 to 7,373.7)	14.2 (13.1 to 15.3)*	-5.0 (-5.9 to -4.1)*	258.4 (181.9 to 353.4)	12.3 (9.3 to 15.1)*	-6.6 (-9.1 to -4.2)*
Other gynecological diseases cases aggregate	3,836.7 (3,549.3 to 4,118.0)	20.5 (20.3 to 20.8)*	0.3 (0.0 to 0.5)*	151.9 (103.3 to 210.5)	20.8 (17.7 to 24.0)*	0.5 (-2.1 to 3.2)
Anemia due to other gynecological diseases aggregate	3,242.9 (3,187.6 to 3,297.6)	7.6 (5.5 to 9.6)*	-10.5 (-12.3 to -8.9)*	106.5 (71.6 to 153.0)	2.0 (-2.2 to 6.0)	-15.1 (-18.6 to -11.8)*
Hemoglobinopathies and hemolytic anemias	129,623.2 (127,433.2 to 132,238.5)	16.9 (15.1 to 18.6)*	-2.8 (-4.3 to -1.3)*	449.0 (296.8 to 654.0)	11.6 (8.2 to 15.1)*	-7.2 (-10.0 to -4.3)*
Thalassemias	3.6 (3.5 to 3.7)	8.1 (5.0 to 11.2)*	-10.0 (-12.7 to -7.5)*	0.3 (0.2 to 0.5)	14.7 (0.4 to 31.1)*	-4.6 (-16.4 to 9.0)
Beta-thalassemia major cases aggregate	2.5 (2.4 to 2.6)	6.9 (2.6 to 11.2)*	-11.1 (-14.6 to -7.5)*	0.2 (0.1 to 0.3)	16.3 (-5.1 to 41.9)	-3.2 (-21.1 to 18.0)
Hemoglobin E/beta-thalassemia cases aggregate	0.5 (0.5 to 0.5)	9.6 (8.4 to 10.9)*	-8.8 (-9.8 to -7.8)*	0.0 (0.0 to 0.1)	7.9 (-10.9 to 28.8)	-10.2 (-25.9 to 7.1)
Hemoglobin H disease cases aggregate	0.4 (0.3 to 0.5)	8.8 (2.4 to 15.1)*	-9.5 (-14.8 to -4.3)*	0.0 (0.0 to 0.1)	10.8 (4.0 to 17.9)*	-7.9 (-13.5 to -1.9)*
Heart failure due to thalassemias aggregate	0.2 (0.1 to 0.2)	23.2 (20.2 to 26.1)*	2.5 (-0.0 to 4.9)	0.0 (0.0 to 0.0)	23.2 (20.2 to 26.1)*	2.5 (-0.0 to 4.9)
Thalassemias trait	24,104.1 (23,539.2 to 24,767.5)	21.7 (21.1 to 22.3)*	1.3 (0.8 to 1.7)*	249.8 (165.0 to 365.6)	18.4 (14.4 to 23.4)*	-1.5 (-4.9 to 2.7)
B-thalassemia trait cases aggregate	19,632.6 (19,123.1 to 20,247.1)	22.6 (21.9 to 23.2)*	2.0 (1.4 to 2.5)*	234.2 (154.5 to 342.5)	18.8 (14.6 to 23.8)*	-1.2 (-4.6 to 3.0)
Hemoglobin E trait cases aggregate	4,471.5 (4,276.9 to 4,678.9)	18.1 (16.9 to 19.1)*	-1.8 (-2.8 to -0.9)*	15.5 (10.2 to 23.0)	13.6 (4.1 to 23.8)*	-5.5 (-13.4 to 3.0)
Sickle cell disorders	59.4 (55.6 to 63.1)	19.3 (14.5 to 24.2)*	-0.7 (-4.8 to 3.3)	6.7 (4.6 to 9.1)	20.7 (9.6 to 32.7)*	0.4 (-8.8 to 10.4)
Homozygous sickle cell and severe sickle cell/beta-thalassemia cases aggregate	51.6 (48.4 to 54.6)	18.5 (13.7 to 23.3)*	-1.4 (-5.4 to 2.6)	5.6 (3.9 to 7.7)	20.0 (7.8 to 33.2)*	-0.2 (-10.3 to 10.8)
Hemoglobin SC disease cases aggregate	7.4 (6.7 to 8.2)	26.6 (14.0 to 38.3)*	5.3 (-5.2 to 15.0)	1.0 (0.7 to 1.4)	26.0 (7.8 to 47.5)*	4.8 (-10.3 to 22.7)
Mild sickle cell/beta-thalassemia cases aggregate	0.4 (0.4 to 0.5)	2.8 (-1.9 to 7.5)	-14.5 (-18.4 to -10.6)*	0.1 (0.1 to 0.1)	5.5 (1.5 to 9.1)*	-12.3 (-15.5 to -9.2)*
Sickle cell trait	32,992.0 (30,989.6 to 36,825.2)	22.2 (21.0 to 23.1)*	1.6 (0.6 to 2.4)*	96.8 (65.0 to 139.7)	10.8 (5.3 to 15.6)*	-7.8 (-12.4 to -3.8)*
G6PD deficiency	20,784.3 (17,624.4 to 24,084.5)	12.2 (6.3 to 17.5)*	-6.6 (-11.5 to -2.2)*	1.6 (1.1 to 2.2)	3.7 (-4.8 to 13.3)	-13.7 (-20.8 to -5.8)*
G6PD cases aggregate	20,784.3 (17,624.4 to 24,084.5)	12.2 (6.3 to 17.5)*	-6.6 (-11.5 to -2.2)*	1.6 (1.1 to 2.2)	3.7 (-4.8 to 13.3)	-13.7 (-20.8 to -5.8)*
Heart failure due to G6PD deficiency aggregate	0.0 (0.0 to 0.0)	0.0 (0.0 to 0.0)	0.0 (0.0 to 0.0)	0.0 (0.0 to 0.0)	0.0 (0.0 to 0.0)	0.0 (0.0 to 0.0)
G6PD trait	60,145.2 (55,948.9 to 64,465.6)	15.2 (13.1 to 16.9)*	-4.2 (-5.9 to -2.7)*	2.2 (1.5 to 3.0)	8.6 (-6.3 to 26.8)	-9.7 (-22.0 to 5.5)
Other hemoglobinopathies and hemolytic anemias	4,791.3 (4,689.5 to 4,894.3)	3.4 (0.7 to 6.3)*	-14.0 (-16.3 to -11.6)*	91.7 (59.5 to 135.8)	-3.3 (-8.6 to 2.0)	-19.6 (-24.0 to -15.2)*
Other hemoglobinopathies and hemolytic anemias cases aggregate	4,790.1 (4,688.4 to 4,893.2)	3.4 (0.7 to 6.3)*	-14.0 (-16.3 to -11.6)*	90.5 (58.5 to 134.5)	-3.5 (-8.8 to 1.9)	-19.7 (-24.1 to -15.2)*
Heart failure due to other hemoglobinopathies and hemolytic anemias aggregate	1.2 (1.0 to 1.3)	20.4 (18.2 to 22.8)*	0.1 (-1.7 to 2.1)	0.1 (0.1 to 0.2)	20.4 (18.2 to 22.8)*	0.1 (-1.7 to 2.1)
Endocrine, metabolic, blood, and immune disorders	3,750.9 (3,676.6 to 3,821.7)	5.8 (3.2 to 8.2)*	-12.0 (-14.1 to -10.0)*	88.6 (59.3 to 128.1)	0.1 (-4.3 to 4.4)	-16.8 (-20.4 to -13.1)*
Endocrine, metabolic, blood, and immune disorders cases aggregate	300.5 (272.0 to 332.2)	19.5 (17.4 to 22.0)*	-0.6 (-2.4 to 1.4)	12.0 (8.0 to 16.7)	19.7 (13.9 to 26.2)*	-0.4 (-5.2 to 5.0)
Anemia due to endocrine, metabolic, blood, and immune disorders aggregate	3,450.0 (3,386.4 to 3,511.4)	4.8 (2.1 to 7.3)*	-12.9 (-15.1 to -10.7)*	76.5 (50.3 to 112.7)	-2.5 (-7.4 to 2.4)	-18.9 (-23.0 to -14.8)*
Heart failure due to endocrine, metabolic, blood, and immune disorders aggregate	0.4 (0.3 to 0.4)	18.6 (17.1 to 20.3)*	-1.4 (-2.6 to 0.1)	0.0 (0.0 to 0.1)	18.6 (17.1 to 20.3)*	-1.4 (-2.6 to 0.1)
Musculoskeletal disorders	80,625.5 (77,187.7 to 84,329.1)	16.9 (15.3 to 18.5)*	-2.7 (-4.1 to -1.4)*	9,140.5 (6,392.3 to 12,652.0)	16.9 (14.6 to 19.0)*	-2.8 (-4.7 to -1.0)*
Rheumatoid arthritis	1,055.7 (901.6 to 1,224.7)	19.8 (15.2 to 24.2)*	-0.3 (-4.2 to 3.3)	260.3 (178.3 to 361.1)	20.1 (14.4 to 25.8)*	-0.1 (-4.9 to 4.7)
Osteoarthritis	0.0 (0.0 to 0.0)	0.0 (0.0 to 0.0)	0.0 (0.0 to 0.0)	0.0 (0.0 to 0.0)	0.0 (0.0 to 0.0)	0.0 (0.0 to 0.0)
Osteoarthritis of the hip cases aggregate	0.0 (0.0 to 0.0)	0.0 (0.0 to 0.0)	0.0 (0.0 to 0.0)	0.0 (0.0 to 0.0)	0.0 (0.0 to 0.0)	0.0 (0.0 to 0.0)
Osteoarthritis of the knee cases aggregate	0.0 (0.0 to 0.0)	0.0 (0.0 to 0.0)	0.0 (0.0 to 0.0)	0.0 (0.0 to 0.0)	0.0 (0.0 to 0.0)	0.0 (0.0 to 0.0)
Low back and neck pain	53,666.6 (50,868.2 to 56,109.5)	16.4 (14.7 to 18.3)*	-3.2 (-4.6 to -1.6)*	6,159.5 (4,211.7 to 8,789.7)	16.5 (14.7 to 18.2)*	-3.1 (-4.6 to -1.7)*
Low back pain	38,062.4 (33,799.0 to 42,901.4)	15.8 (13.8 to 17.9)*	-3.6 (-5.4 to -1.9)*	4,379.7 (2,999.1 to 6,119.8)	16.1 (13.8 to 18.3)*	-3.5 (-5.3 to -1.6)*
Neck pain	17,689.0 (12,193.6 to 24,948.9)	17.4 (14.6 to 20.1)*	-2.4 (-4.7 to -0.1)*	1,779.8 (1,032.6 to 2,891.7)	17.5 (14.6 to 20.3)*	-2.2 (-4.6 to 0.1)
Gout	1,371.4 (1,070.3 to 1,698.8)	20.7 (19.7 to 21.9)*	0.4 (-0.4 to 1.4)	46.4 (29.6 to 68.2)	20.8 (14.6 to 27.4)*	0.5 (-4.7 to 6.0)
Asymptomatic gout	1,225.1 (957.0 to 1,524.6)	20.7 (19.7 to 21.9)*	0.4 (-0.4 to 1.4)	0.0 (0.0 to 0.0)	0.0 (0.0 to 0.0)	0.0 (0.0 to 0.0)
Symptomatic gout cases aggregate	146.3 (110.2 to 187.3)	20.7 (19.7 to 21.9)*	0.4 (-0.4 to 1.4)	46.4 (29.6 to 68.2)	20.8 (14.6 to 27.4)*	0.5 (-4.7 to 6.0)
Other musculoskeletal disorders	27,812.2 (22,098.2 to 34,629.2)	17.1 (10.7 to 22.6)*	-2.6 (-8.0 to 2.0)	2,674.3 (1,701.1 to 3,981.7)	17.4 (10.7 to 23.0)*	-2.3 (-7.9 to 2.3)
Other non-communicable diseases	403,431.1 (393,827.7 to 411,872.9)	20.2 (19.4 to 21.0)*	-0.0 (-0.7 to 0.7)	7,551.7 (5,176.4 to 10,971.6)	22.6 (21.1 to 24.7)*	2.0 (0.8 to 3.7)*
Congenital anomalies	8,652.6 (7,949.1 to 9,357.7)	34.3 (30.0 to 39.0)*	11.7 (8.1 to 15.6)*	765.0 (474.4 to 1,148.1)	39.9 (29.4 to 50.9)*	16.4 (7.6 to 25.5)*
Neural tube defects	132.8 (90.4 to 191.5)	26.3 (23.1 to 29.1)*	5.1 (2.4 to 7.4)*	46.0 (26.1 to 75.9)	27.4 (20.3 to 34.2)*	6.0 (0.0 to 11.6)*
Congenital heart anomalies	4,567.7 (3,218.4 to 6,711.1)	45.9 (41.8 to 50.6)*	21.4 (18.0 to 25.3)*	159.0 (61.8 to 291.7)	46.3 (41.5 to 50.9)*	21.7 (17.7 to 25.5)*
Less severe heart anomalies cases aggregate	4,162.0 (2,798.9 to 6,349.0)	45.4 (40.9 to 50.6)*	20.9 (17.2 to 25.3)*	134.7 (50.1 to 257.0)	45.6 (40.0 to 50.6)*	21.1 (16.4 to 25.3)*
Severe congenital heart anomalies	385.5 (258.1 to 576.7)	51.1 (44.3 to 56.9)*	25.7 (20.0 to 30.5)*	22.8 (9.0 to 45.5)	51.2 (41.7 to 59.0)*	25.7 (17.8 to 32.3)*
Critical congenital heart anomalies	14.9 (8.5 to 24.3)	66.3 (44.6 to 83.2)*	38.3 (20.3 to 52.4)*	0.9 (0.3 to 1.8)	67.0 (36.4 to 94.4)*	38.9 (13.4 to 61.7)*
Heart failure due to congenital heart anomalies aggregate	5.3 (4.7 to 6.1)	22.6 (21.1 to 24.3)*	2.0 (0.7 to 3.4)*	0.7 (0.4 to 0.9)	22.6 (21.1 to 24.3)*	2.0 (0.7 to 3.4)*
Cleft lip and cleft palate	598.4 (349.2 to 998.2)	28.0 (16.0 to 36.3)*	6.4 (-3.5 to 13.3)	7.0 (3.4 to 12.6)	20.1 (7.1 to 30.7)*	-0.1 (-10.9 to 8.7)
Down syndrome	483.1 (306.5 to 739.5)	23.7 (19.6 to 27.5)*	2.9 (-0.5 to 6.0)	40.4 (22.6 to 67.5)	23.6 (17.8 to 28.3)*	2.8 (-2.0 to 6.7)
Turner syndrome	32.9 (15.4 to 60.1)	19.4 (17.3 to 21.5)*	-0.7 (-2.4 to 1.1)	0.7 (0.3 to 1.5)	19.5 (13.2 to 26.2)*	-0.6 (-5.8 to 4.9)
Klinefelter syndrome	18.6 (9.7 to 33.4)	18.9 (17.0 to 20.3)*	-1.1 (-2.7 to 0.1)	0.2 (0.1 to 0.5)	18.9 (12.8 to 24.9)*	-1.1 (-6.2 to 3.9)
Other chromosomal abnormalities	420.5 (233.5 to 743.6)	22.4 (17.9 to 25.9)*	1.8 (-1.9 to 4.7)	35.3 (17.5 to 66.6)	22.4 (16.5 to 27.0)*	1.8 (-3.1 to 5.6)
Other congenital anomalies	2,728.0 (1,498.3 to 5,005.7)	22.0 (19.5 to 24.4)*	1.5 (-0.6 to 3.5)	476.6 (282.2 to 751.0)	42.7 (28.2 to 60.4)*	18.7 (6.6 to 33.5)*
Skin and subcutaneous diseases	177,662.5 (174,247.0 to 181,549.5)	21.4 (20.4 to 22.3)*	1.0 (0.2 to 1.8)*	3,511.8 (2,209.9 to 5,458.6)	21.0 (19.9 to 22.2)*	0.7 (-0.2 to 1.7)
Dermatitis	19,040.5 (15,565.8 to 23,298.6)	19.6 (19.3 to 19.8)*	-0.6 (-0.7 to -0.4)*	602.6 (385.7 to 876.1)	19.8 (18.4 to 21.1)*	-0.4 (-1.5 to 0.8)
Eczema cases aggregate	4,548.5 (3,401.4 to 5,929.0)	19.8 (19.4 to 20.1)*	-0.4 (-0.7 to -0.1)*	279.0 (169.0 to 417.0)	20.0 (17.5 to 22.6)*	-0.2 (-2.3 to 2.0)

Cause	Prevalence in thousands (95% confidence interval)			YLDs in thousands (95% confidence interval)		
	2015	Percent change in counts between 2005 and 2015	Percent change in rates between 2005 and 2015	2015	Percent change in counts between 2005 and 2015	Percent change in rates between 2005 and 2015
Contact dermatitis cases aggregate	8,544.5 (5,965.0 to 12,403.5)	19.1 (18.7 to 19.4)*	-1.0 (-1.2 to -0.7)*	234.0 (130.2 to 397.6)	19.3 (17.3 to 21.2)*	-0.8 (-2.4 to 0.8)
Seborrhoeic dermatitis cases aggregate	5,947.5 (4,295.5 to 8,035.2)	20.1 (19.9 to 20.5)*	-0.1 (-0.3 to 0.2)	89.6 (46.0 to 150.3)	20.3 (18.7 to 22.0)*	0.1 (-1.3 to 1.4)
Psoriasis	6,083.4 (5,506.5 to 6,679.4)	19.3 (17.8 to 20.8)*	-0.7 (-2.0 to 0.5)	502.8 (344.8 to 695.4)	19.6 (16.8 to 22.3)*	-0.5 (-2.8 to 1.8)
Cellulitis	62.6 (45.6 to 84.1)	28.8 (23.3 to 33.7)*	7.1 (2.6 to 11.2)*	4.6 (2.7 to 7.2)	28.7 (20.7 to 36.7)*	7.0 (0.4 to 13.7)*
Pyoderma	421.4 (364.4 to 483.7)	21.7 (18.6 to 24.7)*	1.2 (-1.3 to 3.7)	2.4 (1.0 to 5.2)	22.0 (16.8 to 27.6)*	1.5 (-2.8 to 6.1)
Scabies	17,305.0 (10,942.4 to 26,008.0)	22.6 (17.1 to 29.1)*	2.0 (-2.6 to 7.4)	450.0 (215.5 to 870.7)	22.8 (17.4 to 29.5)*	2.1 (-2.4 to 7.7)
Fungal skin diseases	36,829.9 (30,656.6 to 43,380.3)	20.7 (20.6 to 20.9)*	0.4 (0.3 to 0.6)*	209.7 (82.0 to 447.5)	20.9 (20.4 to 21.4)*	0.6 (0.1 to 1.0)*
Viral skin diseases	13,076.7 (11,078.1 to 15,433.5)	19.4 (19.1 to 19.8)*	-0.7 (-0.9 to -0.4)*	405.1 (253.0 to 604.4)	19.6 (18.5 to 20.7)*	-0.5 (-1.5 to 0.4)
Molluscum contagiosum cases aggregate	1,441.5 (1,104.3 to 1,789.2)	19.6 (19.2 to 20.1)*	-0.5 (-0.8 to -0.1)*	44.7 (26.5 to 70.8)	19.8 (16.7 to 23.0)*	-0.4 (-3.0 to 2.3)
Viral warts cases aggregate	11,635.1 (9,669.8 to 14,018.0)	19.4 (19.0 to 19.8)*	-0.7 (-1.0 to -0.4)*	360.4 (224.7 to 539.1)	19.6 (18.3 to 20.8)*	-0.5 (-1.6 to 0.5)
Acne vulgaris	64,233.5 (55,355.0 to 75,136.8)	22.4 (19.1 to 25.5)*	1.8 (-0.9 to 4.4)	695.5 (315.1 to 1,294.0)	22.5 (19.3 to 25.8)*	1.9 (-0.8 to 4.6)
Alopecia areata	2,830.7 (2,457.9 to 3,215.1)	19.1 (18.8 to 19.3)*	-1.0 (-1.2 to -0.7)*	96.8 (58.5 to 148.3)	19.3 (17.3 to 21.4)*	-0.8 (-2.4 to 1.0)
Pruritus	5,287.7 (3,579.8 to 7,395.6)	20.9 (17.8 to 24.7)*	0.6 (-2.0 to 3.7)	57.3 (25.4 to 112.7)	21.0 (17.4 to 25.0)*	0.7 (-2.3 to 4.0)
Urticaria	4,529.3 (3,233.1 to 6,256.3)	20.5 (20.2 to 20.7)*	0.2 (-0.0 to 0.4)	275.8 (157.7 to 443.1)	20.7 (18.6 to 22.7)*	0.4 (-1.3 to 2.1)
Decubitus ulcer	31.9 (22.6 to 43.8)	26.6 (20.9 to 32.2)*	5.3 (0.5 to 9.9)*	5.3 (3.1 to 8.3)	26.5 (20.2 to 32.7)*	5.2 (-0.0 to 10.4)
Other skin and subcutaneous diseases	34,012.9 (30,605.3 to 37,456.3)	23.7 (21.6 to 25.9)*	2.9 (1.1 to 4.7)*	203.8 (96.8 to 382.8)	23.8 (21.6 to 26.2)*	3.0 (1.1 to 5.0)*
Sense organ diseases	70,949.6 (68,465.1 to 73,836.1)	21.0 (19.7 to 22.5)*	0.7 (-0.4 to 1.9)	2,350.2 (1,568.8 to 3,303.5)	21.4 (19.3 to 23.6)*	1.0 (-0.8 to 2.8)
Glaucoma	0.0 (0.0 to 0.0)	0.0 (0.0 to 0.0)	0.0 (0.0 to 0.0)	0.0 (0.0 to 0.0)	0.0 (0.0 to 0.0)	0.0 (0.0 to 0.0)
Cataract	284.3 (186.1 to 417.0)	16.4 (13.0 to 20.0)*	-3.2 (-6.0 to -0.2)*	25.2 (13.0 to 44.0)	14.7 (7.4 to 22.4)*	-4.6 (-10.7 to 1.8)
Macular degeneration	0.0 (0.0 to 0.0)	0.0 (0.0 to 0.0)	0.0 (0.0 to 0.0)	0.0 (0.0 to 0.0)	0.0 (0.0 to 0.0)	0.0 (0.0 to 0.0)
Refraction and accommodation disorders	30,142.5 (27,438.6 to 33,529.8)	16.8 (15.0 to 18.6)*	-2.8 (-4.3 to -1.4)*	646.2 (413.9 to 990.7)	18.3 (16.8 to 19.7)*	-1.6 (-2.9 to -0.4)*
Age-related and other hearing loss	29,707.4 (22,756.5 to 37,148.8)	25.8 (23.3 to 28.7)*	4.6 (2.6 to 7.1)*	1,256.5 (750.9 to 1,862.1)	23.2 (19.1 to 27.3)*	2.5 (-0.9 to 5.9)
Other vision loss	1,720.8 (1,291.8 to 2,273.9)	21.9 (19.7 to 24.0)*	1.4 (-0.4 to 3.2)	104.4 (63.6 to 160.8)	22.0 (17.8 to 25.9)*	1.5 (-2.0 to 4.8)
Other sense organ diseases	12,290.6 (10,878.4 to 13,844.1)	21.4 (18.8 to 23.9)*	1.0 (-1.1 to 3.1)	317.9 (190.9 to 481.0)	21.6 (18.5 to 24.7)*	1.1 (-1.4 to 3.7)
Acute other sense organ diseases	92.5 (82.5 to 102.7)	21.3 (18.8 to 24.1)*	0.9 (-1.2 to 3.2)	2.5 (1.5 to 3.7)	21.3 (18.7 to 24.0)*	0.9 (-1.2 to 3.2)
Chronic other sense organ diseases	12,198.0 (10,787.1 to 13,755.8)	21.4 (18.8 to 23.9)*	1.0 (-1.1 to 3.1)	315.4 (189.4 to 477.3)	21.6 (18.5 to 24.7)*	1.1 (-1.4 to 3.8)
Oral disorders	273,698.3 (256,556.7 to 288,002.5)	19.2 (17.3 to 21.0)*	-0.8 (-2.4 to 0.7)	924.7 (542.2 to 1,500.9)	19.4 (18.7 to 20.0)*	-0.7 (-1.3 to -0.1)*
Deciduous caries	0.0 (0.0 to 0.0)	0.0 (0.0 to 0.0)	0.0 (0.0 to 0.0)	0.0 (0.0 to 0.0)	0.0 (0.0 to 0.0)	0.0 (0.0 to 0.0)
Permanent caries	251,073.8 (203,238.3 to 299,096.9)	18.9 (17.1 to 20.5)*	-1.1 (-2.6 to 0.2)	172.9 (73.2 to 332.2)	17.3 (15.3 to 19.0)*	-2.4 (-4.1 to -1.0)*
Periodontal diseases	24,926.0 (18,403.2 to 32,473.9)	19.8 (18.8 to 20.7)*	-0.3 (-1.2 to 0.4)	166.9 (61.9 to 350.3)	20.0 (18.8 to 21.1)*	-0.2 (-1.2 to 0.7)
Edentulism and severe tooth loss	6,550.7 (5,897.4 to 7,285.6)	18.8 (18.2 to 19.3)*	-1.2 (-1.6 to -0.8)*	189.6 (125.8 to 270.9)	18.9 (17.4 to 20.5)*	-1.1 (-2.3 to 0.2)
Other oral disorders	13,301.1 (11,915.6 to 14,880.1)	20.2 (19.9 to 20.6)*	0.0 (-0.3 to 0.3)	395.3 (238.0 to 606.5)	20.4 (19.3 to 21.4)*	0.1 (-0.8 to 1.0)
Injuries	65,585.5 (61,936.1 to 69,619.3)	13.7 (10.6 to 16.6)*	-5.4 (-8.0 to -3.0)*	2,665.9 (1,922.6 to 3,564.6)	6.7 (2.9 to 10.3)*	-11.2 (-14.4 to -8.2)*
Transport injuries	6,187.2 (5,687.1 to 6,699.4)	19.6 (18.6 to 20.6)*	-0.5 (-1.4 to 0.3)	376.0 (264.3 to 515.3)	8.6 (4.9 to 12.3)*	-9.6 (-12.7 to -6.6)*
Road injuries	5,761.2 (5,249.0 to 6,267.7)	19.9 (18.7 to 20.9)*	-0.3 (-1.2 to 0.6)	348.3 (243.7 to 477.3)	9.7 (6.1 to 13.3)*	-8.8 (-11.8 to -5.8)*
Pedestrian road injuries	668.6 (558.0 to 813.9)	20.1 (18.4 to 21.6)*	-0.1 (-1.5 to 1.2)	39.3 (26.6 to 55.7)	5.8 (1.5 to 10.4)*	-12.0 (-15.6 to -8.2)*
Cyclist road injuries	1,037.6 (873.3 to 1,203.3)	22.4 (20.9 to 23.8)*	1.8 (0.5 to 3.0)*	56.5 (37.8 to 79.9)	10.1 (4.8 to 14.9)*	-8.4 (-12.8 to -4.4)*
Motorcyclist road injuries	1,477.2 (1,240.5 to 1,766.1)	25.1 (23.8 to 26.3)*	4.1 (3.0 to 5.1)*	84.4 (56.5 to 118.5)	11.2 (6.4 to 15.7)*	-7.5 (-11.5 to -3.7)*
Motor vehicle road injuries	2,331.0 (1,932.9 to 2,761.0)	14.5 (12.7 to 16.1)*	-4.8 (-6.3 to -3.5)*	153.1 (102.3 to 214.6)	8.4 (5.2 to 11.5)*	-9.8 (-12.5 to -7.2)*
Other road injuries	246.7 (198.6 to 297.8)	33.8 (31.8 to 35.7)*	11.3 (9.7 to 12.9)*	15.0 (10.0 to 21.2)	25.8 (21.7 to 30.0)*	4.6 (1.3 to 8.1)*
Other transport injuries	426.1 (368.6 to 486.2)	16.2 (15.4 to 17.0)*	-3.4 (-4.0 to -2.7)*	27.7 (19.5 to 38.0)	-3.3 (-7.6 to 1.5)	-19.6 (-23.1 to -15.6)*
Unintentional injuries	50,218.9 (47,334.0 to 53,063.2)	13.7 (13.1 to 14.2)*	-5.4 (-5.9 to -5.0)*	1,973.7 (1,393.9 to 2,664.5)	5.5 (2.8 to 8.3)*	-12.2 (-14.5 to -9.9)*
Falls	12,367.2 (10,887.5 to 13,952.6)	23.7 (23.0 to 24.5)*	2.9 (2.3 to 3.6)*	681.3 (476.0 to 949.6)	9.6 (5.3 to 14.0)*	-8.8 (-12.4 to -5.1)*
Drowning	204.3 (173.7 to 240.6)	2.1 (1.3 to 2.8)*	-15.1 (-15.7 to -14.4)*	12.1 (8.3 to 17.1)	-12.8 (-16.6 to -8.4)*	-27.5 (-30.6 to -23.8)*
Fire, heat, and hot substances	4,439.1 (3,723.0 to 5,310.1)	10.9 (10.0 to 11.9)*	-7.7 (-8.5 to -7.0)*	176.9 (122.6 to 245.2)	1.7 (-2.3 to 5.6)	-15.4 (-18.8 to -12.2)*
Poisonings	461.3 (382.2 to 570.4)	17.7 (16.2 to 19.1)*	-2.1 (-3.4 to -0.9)*	48.9 (32.7 to 70.9)	13.8 (10.7 to 16.8)*	-5.3 (-8.0 to -2.8)*
Exposure to mechanical forces	13,944.2 (12,556.5 to 15,355.7)	15.1 (14.3 to 15.9)*	-4.2 (-4.9 to -3.6)*	265.2 (185.1 to 365.8)	6.0 (2.8 to 9.0)*	-11.8 (-14.5 to -9.3)*
Unintentional firearm injuries	171.6 (146.3 to 204.6)	11.0 (9.5 to 12.6)*	-7.6 (-8.9 to -6.4)*	7.9 (5.3 to 11.1)	2.1 (-1.7 to 6.1)	-15.1 (-18.3 to -11.8)*
Unintentional suffocation	543.0 (405.4 to 722.5)	9.4 (6.6 to 12.3)*	-9.0 (-11.3 to -6.6)*	18.5 (12.3 to 25.8)	9.1 (6.4 to 11.5)*	-9.3 (-11.5 to -7.3)*
Other exposure to mechanical forces	13,229.5 (11,835.7 to 14,612.3)	15.4 (14.6 to 16.3)*	-4.0 (-4.6 to -3.3)*	238.8 (165.9 to 331.6)	5.9 (2.5 to 9.1)*	-11.9 (-14.8 to -9.2)*
Adverse effects of medical treatment	581.0 (429.9 to 754.8)	10.7 (6.9 to 14.7)*	-7.9 (-11.1 to -4.6)*	77.4 (46.0 to 118.9)	10.7 (6.9 to 14.7)*	-7.9 (-11.1 to -4.6)*
Animal contact	2,780.1 (2,405.5 to 3,192.0)	8.9 (6.7 to 10.8)*	-9.4 (-11.2 to -7.8)*	104.2 (71.2 to 143.1)	6.4 (3.7 to 9.0)*	-11.5 (-13.8 to -9.3)*
Venomous animal contact	1,241.1 (1,077.6 to 1,416.9)	11.8 (10.2 to 13.4)*	-7.0 (-8.4 to -5.7)*	82.3 (54.9 to 113.8)	7.4 (4.6 to 10.2)*	-10.7 (-13.0 to -8.3)*
Non-venomous animal contact	1,539.0 (1,248.4 to 1,868.6)	6.7 (3.7 to 9.2)*	-11.3 (-13.7 to -9.1)*	21.9 (14.3 to 32.8)	2.9 (-1.3 to 6.9)	-14.4 (-17.9 to -11.0)*
Foreign body	2,286.0 (1,974.4 to 2,606.2)	15.5 (14.2 to 16.8)*	-3.9 (-5.0 to -2.9)*	95.1 (66.8 to 131.1)	5.2 (2.0 to 8.5)*	-12.5 (-15.2 to -9.7)*
Pulmonary aspiration and foreign body in airway	885.9 (682.3 to 1,163.7)	16.3 (13.6 to 18.9)*	-3.2 (-5.5 to -1.1)*	49.2 (33.1 to 70.3)	2.0 (-1.9 to 6.3)	-15.1 (-18.4 to -11.6)*
Foreign body in eyes	90.6 (35.1 to 167.3)	25.0 (23.3 to 27.3)*	4.0 (2.6 to 5.9)*	5.1 (1.9 to 10.1)	24.0 (21.4 to 26.2)*	3.1 (1.0 to 5.0)*
Foreign body in other body part	1,309.5 (1,116.2 to 1,525.9)	14.4 (13.3 to 15.6)*	-4.8 (-5.8 to -3.8)*	40.8 (28.1 to 56.4)	7.2 (4.6 to 9.9)*	-10.8 (-13.0 to -8.6)*
Environmental heat and cold exposure	3,948.6 (3,441.2 to 4,703.9)	16.0 (15.2 to 16.7)*	-3.5 (-4.2 to -2.9)*	183.0 (129.8 to 255.5)	6.4 (3.7 to 9.3)*	-11.5 (-13.8 to -9.1)*
Other unintentional injuries	9,207.3 (7,885.0 to 10,641.5)	2.2 (1.3 to 3.3)*	-15.0 (-15.7 to -14.1)*	329.5 (216.7 to 460.0)	-2.3 (-4.3 to -0.3)*	-18.7 (-20.4 to -17.0)*
Self-harm and interpersonal violence	1,585.5 (1,437.7 to 1,740.2)	15.4 (14.4 to 16.2)*	-4.0 (-4.8 to -3.3)*	84.1 (59.8 to 113.8)	3.4 (0.2 to 6.7)*	-14.0 (-16.7 to -11.2)*
Self-harm	358.7 (302.1 to 421.7)	12.1 (11.2 to 13.0)*	-6.8 (-7.5 to -6.0)*	25.7 (17.8 to 35.3)	1.8 (-1.1 to 4.8)	-15.3 (-17.8 to -12.8)*
Interpersonal violence	1,226.8 (1,087.1 to 1,368.4)	16.4 (15.3 to 17.4)*	-3.2 (-4.1 to -2.3)*	58.4 (40.7 to 79.1)	4.2 (0.6 to 7.9)*	-13.4 (-16.3 to -10.3)*

Cause	Prevalence in thousands (95% confidence interval)			YLDs in thousands (95% confidence interval)		
	2015	Percent change in counts between 2005 and 2015	Percent change in rates between 2005 and 2015	2015	Percent change in counts between 2005 and 2015	Percent change in rates between 2005 and 2015
Assault by firearm	68.0<br>(55.7 to 86.3)	5.6<br>(4.5 to 6.7)*	-12.1<br>(-13.1 to -11.2)*	3.5<br>(2.4 to 4.9)	-4.6<br>(-8.3 to -1.0)*	-20.6<br>(-23.7 to -17.6)*
Assault by sharp object	303.6<br>(243.0 to 361.9)	14.1<br>(12.6 to 15.5)*	-5.1<br>(-6.3 to -3.9)*	11.4<br>(7.7 to 16.1)	-3.1<br>(-7.8 to 2.0)	-19.4<br>(-23.3 to -15.2)*
Assault by other means	855.2<br>(742.5 to 989.6)	18.2<br>(16.9 to 19.3)*	-1.7<br>(-2.8 to -0.8)*	43.5<br>(30.2 to 59.6)	7.1<br>(3.5 to 10.5)*	-10.9<br>(-13.9 to -8.1)*
Forces of nature, war, and legal intervention	7,593.9<br>(5,451.1 to 10,275.1)	9.2<br>(-11.3 to 34.6)	-9.1<br>(-26.2 to 11.9)	232.2<br>(150.0 to 335.6)	15.6<br>(-13.2 to 54.2)	-3.8<br>(-27.8 to 28.3)
Exposure to forces of nature	2,089.4<br>(1,290.9 to 3,223.5)	15.4<br>(-14.0 to 55.1)	-4.0<br>(-28.4 to 29.1)	61.0<br>(39.9 to 87.9)	-24.9<br>(-48.8 to 9.9)	-37.5<br>(-57.4 to -8.5)*
Collective violence and legal intervention	5,504.5<br>(3,734.8 to 7,626.7)	7.1<br>(-19.4 to 42.7)	-10.9<br>(-32.9 to 18.7)	171.2<br>(101.6 to 259.3)	43.2<br>(-6.9 to 110.8)	19.1<br>(-22.5 to 75.4)
**Anemia**	**160,647.4<br>(158,612.8 to 162,728.1)**	**7.3<br>(5.7 to 8.9)***	**-10.8<br>(-12.1 to -9.4)***	**4,393.8<br>(2,942.3 to 6,407.5)**	**1.8<br>(-0.8 to 4.4)**	**-15.3<br>(-17.5 to -13.2)***
**Developmental intellectual disability**	**14,905.4<br>(11,079.4 to 18,611.0)**	**22.0<br>(21.6 to 22.6)***	**1.5<br>(1.2 to 2.0)***	**1,629.1<br>(1,158.6 to 2,158.5)**	**24.7<br>(22.1 to 27.5)***	**3.7<br>(1.6 to 6.1)***
**Epilepsy**	**3,512.9<br>(2,945.8 to 4,041.4)**	**21.4<br>(16.7 to 25.7)***	**1.0<br>(-2.9 to 4.6)**	**1,085.3<br>(757.2 to 1,430.5)**	**12.8<br>(5.5 to 19.7)***	**-6.2<br>(-12.2 to -0.5)***
**Guillain-Barré syndrome**	**2.0<br>(1.2 to 3.2)**	**20.9<br>(19.4 to 21.9)***	**0.5<br>(-0.7 to 1.4)**	**0.6<br>(0.3 to 1.1)**	**20.9<br>(19.4 to 21.9)***	**0.5<br>(-0.7 to 1.4)**
**Hearing loss**	**38,422.1<br>(31,829.0 to 45,626.4)**	**22.0<br>(20.7 to 23.4)***	**1.5<br>(0.4 to 2.6)***	**1,648.9<br>(1,071.5 to 2,358.2)**	**20.6<br>(17.5 to 23.5)***	**0.4<br>(-2.2 to 2.7)**
**Heart failure**	**237.6<br>(219.7 to 259.0)**	**23.2<br>(22.3 to 24.1)***	**2.5<br>(1.7 to 3.2)***	**29.1<br>(19.9 to 40.2)**	**24.1<br>(19.7 to 28.6)***	**3.3<br>(-0.5 to 7.0)**
**Infertility**	**21,359.1<br>(15,021.3 to 29,180.3)**	**39.2<br>(33.4 to 45.0)***	**15.8<br>(10.9 to 20.6)***	**145.9<br>(58.8 to 295.9)**	**35.1<br>(29.5 to 40.1)***	**12.3<br>(7.7 to 16.5)***
**Pelvic inflammatory disease**	**126.1<br>(95.2 to 166.0)**	**5.1<br>(3.4 to 6.8)***	**-12.6<br>(-14.0 to -11.1)***	**16.8<br>(10.8 to 24.9)**	**5.4<br>(-0.1 to 11.0)**	**-12.3<br>(-16.9 to -7.6)***
**Vision loss**	**33,563.4<br>(30,563.7 to 37,042.1)**	**17.1<br>(15.4 to 18.7)***	**-2.6<br>(-4.0 to -1.3)***	**1,044.0<br>(714.4 to 1,456.4)**	**20.0<br>(18.4 to 21.6)***	**-0.2<br>(-1.5 to 1.1)**

SR Table 11. Prevalent cases and YLDs for 2015, percent change, and change in age-standardised rates between 2005 and 2015 by cause, global, ages 30-34 years						
Cause	Prevalence in thousands (95% confidence interval)			YLDs in thousands (95% confidence interval)		
	2015	Percent change in counts between 2005 and 2015	Percent change in rates between 2005 and 2015	2015	Percent change in counts between 2005 and 2015	Percent change in rates between 2005 and 2015
**All causes**	--	--	--	**55,459.8 (41,239.3 to 71,749.7)**	**9.6 (8.9 to 10.3)***	**-1.1 (-1.7 to -0.4)***
Communicable, maternal, neonatal, and nutritional diseases	328,762.1 (325,879.1 to 332,011.5)	7.1 (6.4 to 8.0)*	-3.3 (-3.9 to -2.5)*	7,153.8 (5,115.6 to 9,980.8)	0.2 (-2.2 to 3.2)	-9.5 (-11.7 to -6.8)*
HIV/AIDS and tuberculosis	6,116.3 (5,930.1 to 6,314.9)	2.3 (-1.3 to 6.1)	-7.7 (-10.8 to -4.2)*	783.6 (553.4 to 1,038.4)	-6.3 (-12.7 to -0.1)*	-15.4 (-21.2 to -9.8)*
Tuberculosis	774.5 (565.7 to 1,029.4)	9.8 (0.7 to 19.9)*	-0.9 (-9.1 to 8.2)	241.4 (149.7 to 362.9)	10.0 (0.2 to 20.5)*	-0.6 (-9.5 to 8.8)
HIV/AIDS	5,351.2 (5,190.3 to 5,528.4)	1.3 (-2.7 to 5.4)	-8.5 (-12.1 to -4.8)*	542.2 (390.6 to 707.5)	-12.1 (-19.2 to -4.8)*	-20.6 (-27.0 to -14.1)*
HIV/AIDS - Tuberculosis	192.3 (172.6 to 212.4)	-27.0 (-30.0 to -23.4)*	-34.0 (-36.8 to -30.8)*	72.6 (49.2 to 99.2)	-26.6 (-30.7 to -22.1)*	-33.7 (-37.5 to -29.7)*
HIV/AIDS resulting in other diseases	5,429.8 (5,233.6 to 5,668.4)	3.6 (-0.4 to 7.1)	-6.4 (-10.0 to -3.3)*	469.5 (336.9 to 615.3)	-9.3 (-17.8 to -0.8)*	-18.1 (-25.8 to -10.4)*
HIV aggregate	3,791.7 (3,620.1 to 4,004.7)	-22.5 (-25.8 to -19.0)*	-30.0 (-33.0 to -26.9)*	277.0 (197.7 to 362.0)	-23.7 (-27.1 to -19.9)*	-31.1 (-34.2 to -27.7)*
AIDS aggregate	1,638.1 (1,551.8 to 1,734.9)	368.3 (269.4 to 508.1)*	322.8 (233.5 to 449.0)*	192.5 (139.1 to 252.8)	24.5 (-6.1 to 71.6)	12.4 (-15.2 to 54.9)
Diarrhea, lower respiratory, and other common infectious diseases	23,394.9 (21,113.1 to 25,796.6)	8.3 (5.7 to 10.8)*	-2.2 (-4.6 to 0.0)	704.3 (473.8 to 992.6)	11.4 (9.6 to 13.3)*	0.6 (-1.1 to 2.3)
Diarrheal diseases	1,370.0 (1,312.4 to 1,425.9)	18.7 (17.8 to 19.6)*	7.2 (6.4 to 8.0)*	217.9 (149.7 to 296.9)	19.0 (16.3 to 21.9)*	7.5 (5.0 to 10.1)*
Diarrhoea episodes aggregate	1,369.8 (1,312.0 to 1,425.6)	18.7 (17.8 to 19.6)*	7.2 (6.4 to 8.0)*	217.9 (149.7 to 296.8)	19.0 (16.3 to 21.9)*	7.5 (5.0 to 10.1)*
Guillain-Barré syndrome due to diarrheal diseases	0.2 (0.1 to 0.4)	13.4 (11.6 to 14.9)*	2.4 (0.8 to 3.7)*	0.1 (0.0 to 0.1)	13.4 (11.6 to 14.9)*	2.4 (0.8 to 3.7)*
Intestinal infectious diseases	45.6 (39.8 to 51.9)	-23.6 (-33.1 to -14.4)*	-31.0 (-39.6 to -22.7)*	8.3 (5.0 to 12.4)	-4.6 (-16.2 to 9.2)	-13.9 (-24.4 to -1.4)*
Typhoid fever	53.2 (38.8 to 70.6)	-4.9 (-9.7 to -0.2)*	-14.1 (-18.5 to -9.9)*	7.1 (4.3 to 10.9)	-2.9 (-16.3 to 12.4)	-12.3 (-24.4 to 1.5)
Typhoid fever episodes aggregate	44.0 (31.7 to 58.8)	-5.0 (-12.9 to 3.9)	-14.2 (-21.3 to -6.2)*	4.2 (2.4 to 6.4)	-4.6 (-18.7 to 10.7)	-13.8 (-26.6 to -0.0)*
Complications of typhoid fever aggregate	9.1 (6.0 to 13.3)	-4.5 (-34.7 to 39.9)	-13.8 (-41.0 to 26.3)	2.9 (1.7 to 4.7)	-0.3 (-32.7 to 50.6)	-10.0 (-39.3 to 36.0)
Paratyphoid fever	21.6 (14.8 to 30.9)	-7.0 (-14.2 to 1.7)	-16.0 (-22.5 to -8.2)*	1.1 (0.6 to 1.9)	-7.2 (-25.8 to 15.7)	-16.2 (-33.0 to 4.4)
Paratyphoid fever episodes aggregate	20.5 (13.9 to 29.1)	-7.0 (-14.7 to 2.2)	-16.0 (-23.0 to -7.7)*	1.0 (0.6 to 1.6)	-7.3 (-28.0 to 18.0)	-16.3 (-35.0 to 6.6)
Intestinal perforation due to paratyphoid	1.1 (0.6 to 1.9)	-6.0 (-46.7 to 65.7)	-15.1 (-51.9 to 49.6)	0.1 (0.1 to 0.2)	-6.0 (-46.7 to 65.7)	-15.1 (-51.9 to 49.6)
Other intestinal infectious diseases	--	--	--	0.1 (0.0 to 0.2)	-62.3 (-75.3 to -40.2)*	-66.0 (-77.7 to -46.0)*
Lower respiratory infections	244.9 (230.4 to 260.3)	17.8 (16.0 to 19.6)*	6.4 (4.8 to 7.9)*	14.9 (9.9 to 21.4)	17.9 (12.4 to 23.9)*	6.5 (1.5 to 11.9)*
Lower respiratory infection episodes aggregate	244.6 (230.2 to 260.1)	17.8 (16.0 to 19.6)*	6.4 (4.8 to 7.9)*	14.9 (9.8 to 21.3)	18.0 (12.4 to 24.1)*	6.5 (1.5 to 12.0)*
Guillain-Barré syndrome due to lower respiratory infections	0.3 (0.1 to 0.5)	13.4 (11.6 to 14.9)*	2.4 (0.8 to 3.7)*	0.1 (0.0 to 0.1)	13.4 (11.6 to 14.9)*	2.4 (0.8 to 3.7)*
Upper respiratory infections	16,548.5 (11,590.8 to 22,592.0)	8.9 (7.8 to 10.1)*	-1.6 (-2.7 to -0.6)*	194.5 (100.7 to 350.2)	9.0 (7.5 to 10.4)*	-1.6 (-2.9 to -0.3)*
Upper respiratory infection episodes aggregate	16,547.8 (11,590.2 to 22,591.3)	8.9 (7.8 to 10.1)*	-1.6 (-2.7 to -0.6)*	194.3 (100.5 to 350.0)	9.0 (7.5 to 10.4)*	-1.6 (-2.9 to -0.3)*
Guillain-Barré syndrome due to upper respiratory infections	0.7 (0.4 to 1.1)	13.4 (11.6 to 14.9)*	2.4 (0.8 to 3.7)*	0.2 (0.1 to 0.4)	13.4 (11.6 to 14.9)*	2.4 (0.8 to 3.7)*
Otitis media	4,463.2 (3,356.5 to 5,866.1)	2.0 (-1.1 to 5.4)	-7.9 (-10.7 to -4.8)*	120.2 (65.8 to 199.0)	0.9 (-4.4 to 6.3)	-8.9 (-13.7 to -4.1)*
Acute otitis media	708.5 (554.1 to 920.3)	16.5 (12.1 to 21.3)*	5.2 (1.2 to 9.6)*	9.2 (4.5 to 17.7)	16.6 (11.2 to 22.5)*	5.3 (0.4 to 10.6)*
Chronic otitis media aggregate	3,754.7 (2,666.5 to 5,160.3)	-0.4 (-4.0 to 3.3)	-10.1 (-13.3 to -6.7)*	111.0 (60.5 to 185.6)	-0.3 (-5.7 to 5.4)	-9.9 (-14.9 to -4.9)*
Meningitis	566.3 (536.0 to 595.8)	22.7 (17.2 to 28.6)*	10.8 (5.8 to 16.2)*	93.6 (66.1 to 123.7)	17.6 (14.3 to 20.9)*	6.2 (3.2 to 9.1)*
Pneumococcal meningitis	505.9 (416.9 to 612.9)	25.7 (22.6 to 29.2)*	13.5 (10.7 to 16.6)*	43.5 (30.5 to 57.4)	19.6 (13.5 to 25.0)*	8.0 (2.5 to 12.9)*
Acute pneumococcal meningitis	1.5 (1.0 to 2.2)	27.3 (12.1 to 41.4)*	14.9 (1.2 to 27.7)*	0.2 (0.1 to 0.3)	27.2 (12.1 to 41.4)*	14.9 (1.2 to 27.7)*
Complications of pneumococcal meningitis aggregate	504.4 (415.5 to 611.0)	25.7 (22.6 to 29.2)*	13.5 (10.7 to 16.7)*	43.3 (30.4 to 57.2)	19.6 (13.5 to 25.1)*	8.0 (2.4 to 12.9)*
H influenzae type B meningitis	162.1 (123.2 to 208.0)	-7.1 (-11.5 to -1.8)*	-16.2 (-20.1 to -11.4)*	16.9 (11.4 to 23.4)	11.8 (5.1 to 18.6)*	0.9 (-5.1 to 7.0)
Acute H influenzae type B meningitis	0.5 (0.3 to 0.7)	-12.6 (-24.1 to -1.2)*	-21.0 (-31.5 to -10.8)*	0.1 (0.0 to 0.1)	-12.6 (-24.1 to -1.2)*	-21.1 (-31.5 to -10.8)*
Complications of of H influenzae type B meningitis aggregate	161.6 (122.4 to 207.4)	-7.1 (-11.5 to -1.8)*	-16.1 (-20.1 to -11.3)*	16.8 (11.4 to 23.3)	11.9 (5.2 to 18.7)*	1.0 (-5.0 to 7.2)
Meningococcal meningitis	139.7 (106.9 to 177.1)	27.8 (23.3 to 32.4)*	15.4 (11.4 to 19.5)*	13.6 (9.4 to 18.2)	23.4 (17.9 to 29.2)*	11.5 (6.4 to 16.6)*
Acute meningococcal meningitis	0.7 (0.4 to 1.1)	24.5 (6.4 to 40.3)*	12.4 (-3.9 to 26.7)	0.1 (0.1 to 0.2)	24.5 (6.4 to 40.3)*	12.4 (-3.9 to 26.7)
Complications of meningococcal meningitis aggregate	139.0 (106.3 to 176.6)	27.8 (23.4 to 32.4)*	15.4 (11.4 to 19.6)*	13.5 (9.3 to 18.0)	23.4 (17.9 to 29.2)*	11.5 (6.4 to 16.6)*
Other meningitis	204.0 (160.1 to 251.6)	19.3 (16.0 to 22.9)*	7.7 (4.8 to 11.0)*	19.6 (13.3 to 26.9)	14.8 (9.4 to 20.8)*	3.7 (-1.3 to 9.0)
Other meningitis episodes aggregate	5.8 (4.3 to 7.5)	19.9 (7.0 to 31.3)*	8.2 (-3.4 to 18.5)	0.8 (0.5 to 1.2)	19.8 (7.0 to 31.2)*	8.2 (-3.4 to 18.5)
Complications of other meningitis aggregate	198.2 (153.8 to 244.9)	19.3 (16.0 to 22.8)*	7.7 (4.7 to 10.9)*	18.8 (12.9 to 25.9)	14.6 (9.0 to 20.8)*	3.5 (-1.5 to 9.1)
Encephalitis	372.0 (271.2 to 500.2)	6.4 (3.7 to 9.9)*	-3.9 (-6.4 to -0.8)*	42.7 (29.7 to 57.5)	9.1 (3.4 to 14.9)*	-1.5 (-6.7 to 3.8)
Acute encephalitis	4.7 (3.8 to 5.8)	13.3 (8.0 to 17.8)*	2.3 (-2.5 to 6.4)	0.6 (0.4 to 0.9)	13.3 (5.2 to 20.8)*	2.3 (-5.0 to 9.1)
Complications of encephalitis aggregate	367.3 (266.8 to 495.8)	6.3 (3.6 to 9.9)*	-4.0 (-6.5 to -0.8)*	42.1 (29.3 to 56.5)	9.1 (3.2 to 14.9)*	-1.5 (-6.8 to 3.7)
Diphtheria	0.0 (0.0 to 0.0)	-55.3 (-82.3 to 13.3)	-59.7 (-84.0 to 2.3)	0.0 (0.0 to 0.0)	-55.3 (-82.3 to 13.3)	-59.6 (-84.0 to 2.3)
Whooping cough	3.0 (2.3 to 3.7)	-22.5 (-24.7 to -20.1)*	-30.0 (-32.0 to -27.9)*	0.1 (0.1 to 0.2)	-22.5 (-24.7 to -20.1)*	-30.0 (-32.0 to -27.9)*
Tetanus	15.4 (15.1 to 15.8)	12.3 (10.5 to 13.4)*	1.4 (-0.3 to 2.4)	0.7 (0.5 to 0.9)	-4.7 (-12.0 to 3.4)	-14.0 (-20.5 to -6.6)*
Severe tetanus	0.3 (0.2 to 0.6)	-34.5 (-48.3 to -15.0)*	-40.9 (-53.3 to -23.3)*	0.0 (0.0 to 0.1)	-34.4 (-48.5 to -14.7)*	-40.8 (-53.5 to -22.9)*
Complications of tetanus aggregate	15.1 (14.8 to 15.4)	14.0 (13.5 to 14.6)*	3.0 (2.5 to 3.5)*	0.6 (0.5 to 0.8)	-1.6 (-9.2 to 7.3)	-11.2 (-18.0 to -3.1)*
Measles	0.2 (0.1 to 0.4)	-63.1 (-67.1 to -58.3)*	-66.7 (-70.3 to -62.3)*	0.0 (0.0 to 0.0)	-63.1 (-67.1 to -58.3)*	-66.7 (-70.3 to -62.3)*
Varicella and herpes zoster	260.4 (212.4 to 312.2)	10.2 (9.3 to 11.0)*	-0.5 (-1.3 to 0.2)	11.3 (6.4 to 17.7)	8.8 (3.6 to 14.5)*	-1.8 (-6.4 to 3.3)
Neglected tropical diseases and malaria	147,564.1 (143,984.1 to 151,142.4)	-0.7 (-3.2 to 2.2)	-10.3 (-12.6 to -7.7)*	1,425.1 (932.4 to 2,188.5)	-8.4 (-16.1 to 4.2)	-17.3 (-24.2 to -6.0)*
Malaria	16,171.3 (14,049.3 to 18,669.1)	39.1 (30.7 to 48.6)*	25.6 (18.0 to 34.1)*	131.7 (94.5 to 181.2)	26.0 (20.9 to 31.9)*	13.8 (9.2 to 19.1)*
Asymptomatic malaria parasitemia(PfPR)	12,157.7 (10,048.6 to 14,683.3)	43.1 (31.6 to 56.1)*	29.2 (18.8 to 41.0)*	0.0 (0.0 to 0.0)	0.0 (0.0 to 0.0)	0.0 (0.0 to 0.0)
Malaria episodes aggregate	620.3 (342.6 to 1,039.7)	4.4 (-8.8 to 17.8)	-5.8 (-17.7 to 6.4)	12.7 (6.0 to 23.8)	4.6 (-8.9 to 19.6)	-5.5 (-17.7 to 8.0)
Complications of malaria aggregate	81.6 (73.8 to 89.1)	35.0 (32.7 to 38.0)*	21.9 (19.8 to 24.6)*	25.5 (19.3 to 32.1)	31.0 (21.9 to 39.9)*	18.3 (10.1 to 26.4)*
Anemia due to malaria parasitemia(PfPR) aggregate	3,311.8 (3,231.3 to 3,402.0)	33.7 (29.9 to 37.7)*	20.7 (17.3 to 24.4)*	93.5 (62.9 to 135.2)	28.3 (21.6 to 35.4)*	15.9 (9.8 to 22.3)*
Chagas disease	506.3 (418.6 to 584.1)	-10.3 (-13.1 to -7.2)*	-19.0 (-21.6 to -16.2)*	3.3 (2.0 to 4.9)	-10.4 (-16.6 to -4.1)*	-19.1 (-24.7 to -13.4)*
Chagas disease episodes aggregate	445.2 (369.6 to 517.4)	-10.3 (-13.1 to -7.2)*	-19.0 (-21.5 to -16.2)*	0.0 (0.0 to 0.0)	-22.3 (-29.9 to -14.8)*	-29.9 (-36.7 to -23.1)*
Complications of Chagas disease aggregate	48.7 (36.9 to 62.1)	-10.3 (-13.1 to -7.2)*	-19.0 (-21.6 to -16.2)*	2.6 (1.6 to 3.9)	-10.3 (-17.2 to -2.9)*	-19.0 (-25.2 to -12.3)*

Cause	Prevalence in thousands (95% confidence interval)			YLDs in thousands (95% confidence interval)		
	2015	Percent change in counts between 2005 and 2015	Percent change in rates between 2005 and 2015	2015	Percent change in counts between 2005 and 2015	Percent change in rates between 2005 and 2015
Heart failure due to Chagas disease aggregate	12.4 (7.3 to 19.1)	-10.9 (-13.9 to -7.6)*	-19.5 (-22.2 to -16.6)*	0.7 (0.3 to 1.2)	-10.6 (-20.8 to 1.2)	-19.3 (-28.5 to -8.6)*
Leishmaniasis	322.2 (284.9 to 386.4)	34.1 (7.1 to 65.6)*	21.0 (-3.3 to 49.5)	3.8 (1.9 to 7.2)	32.7 (26.3 to 39.6)*	19.9 (14.1 to 26.1)*
Visceral leishmaniasis	4.4 (4.0 to 4.7)	19.4 (18.1 to 20.9)*	7.8 (6.7 to 9.1)*	0.3 (0.2 to 0.4)	20.1 (-8.6 to 57.9)	8.4 (-17.5 to 42.5)
Cutaneous and mucocutaneous leishmaniasis	325.0 (273.6 to 405.5)	33.7 (29.6 to 37.4)*	20.7 (17.0 to 24.1)*	3.5 (1.6 to 6.8)	34.0 (27.7 to 40.3)*	21.0 (15.3 to 26.7)*
African trypanosomiasis	1.0 (0.6 to 1.6)	-68.3 (-70.1 to -66.7)*	-71.4 (-73.0 to -70.0)*	0.3 (0.1 to 0.5)	-66.9 (-82.9 to -46.4)*	-70.1 (-84.6 to -51.6)*
Schistosomiasis	22,086.9 (18,214.2 to 28,935.5)	-19.5 (-29.5 to 3.1)	-27.4 (-36.3 to -6.9)*	230.7 (114.6 to 449.9)	-18.0 (-26.9 to 4.5)	-25.9 (-34.0 to -5.6)*
Schistosomiasis episodes aggregate	7,084.6 (4,696.9 to 10,022.4)	-25.2 (-36.2 to -6.9)*	-32.5 (-42.4 to -15.9)*	40.9 (15.6 to 86.0)	-27.2 (-37.6 to -8.1)*	-34.2 (-43.7 to -17.0)*
Complications of schistosomiasis aggregate	13,784.3 (9,367.3 to 21,333.8)	-15.8 (-29.5 to 19.7)	-23.9 (-36.4 to 8.0)	155.9 (69.3 to 328.9)	-13.9 (-26.8 to 20.8)	-22.3 (-34.0 to 9.1)
Anemia due to schistosomiasis aggregate	1,220.3 (1,178.7 to 1,261.7)	-19.2 (-22.3 to -15.9)*	-27.1 (-29.9 to -24.1)*	33.9 (22.8 to 49.1)	-22.9 (-27.8 to -17.5)*	-30.4 (-34.8 to -25.5)*
Cysticercosis	220.2 (134.8 to 325.2)	-8.0 (-15.6 to -1.4)*	-16.9 (-23.8 to -11.0)*	33.8 (19.1 to 54.2)	-17.0 (-26.2 to -9.4)*	-25.1 (-33.3 to -18.2)*
Cystic echinococcosis	106.7 (92.9 to 123.3)	20.0 (17.1 to 22.8)*	8.3 (5.7 to 10.8)*	9.9 (6.6 to 14.2)	19.8 (10.4 to 29.3)*	8.2 (-0.4 to 16.8)
Lymphatic filariasis	3,456.6 (2,787.1 to 4,282.6)	-44.1 (-49.3 to -38.9)*	-49.5 (-54.2 to -44.8)*	200.8 (107.5 to 324.5)	-16.4 (-32.3 to -4.1)*	-24.5 (-38.9 to -13.4)*
Prevalence of detectable microfiliaria due to lymphatic filariasis	1,672.3 (1,439.1 to 1,901.3)	-59.0 (-62.3 to -55.2)*	-63.0 (-65.9 to -59.5)*	0.0 (0.0 to 0.0)	0.0 (0.0 to 0.0)	0.0 (0.0 to 0.0)
Complications of lymphatic filariasis aggregate	1,784.3 (1,170.9 to 2,585.2)	-15.2 (-30.2 to -3.2)*	-23.4 (-37.0 to -12.6)*	200.8 (107.5 to 324.5)	-16.4 (-32.3 to -4.1)*	-24.5 (-38.9 to -13.4)*
Onchocerciasis	1,537.1 (1,227.5 to 1,973.3)	-23.2 (-33.4 to -12.2)*	-30.6 (-39.9 to -20.7)*	101.0 (44.9 to 178.9)	-14.5 (-36.1 to 5.2)	-22.8 (-42.3 to -5.0)*
Asymptomatic onchocerciasis	351.2 (256.3 to 459.8)	-49.0 (-51.8 to -46.7)*	-54.0 (-56.5 to -51.9)*	0.0 (0.0 to 0.0)	0.0 (0.0 to 0.0)	0.0 (0.0 to 0.0)
Skin disease due to onchocerciasis aggregate	1,151.0 (820.8 to 1,546.3)	-8.4 (-23.5 to 8.3)	-17.3 (-30.9 to -1.8)*	98.4 (42.3 to 176.1)	-13.5 (-35.5 to 6.5)	-21.9 (-41.8 to -3.4)*
Vision loss due to onchocerciasis aggregate	34.2 (22.5 to 52.7)	-38.2 (-47.3 to -25.7)*	-44.2 (-52.5 to -32.9)*	2.5 (1.5 to 4.0)	-39.9 (-50.4 to -28.5)*	-45.8 (-55.2 to -35.4)*
Trachoma	92.3 (55.1 to 143.4)	8.4 (-2.3 to 19.8)	-2.1 (-11.8 to 8.2)	7.6 (3.9 to 14.4)	3.0 (-10.4 to 17.5)	-7.0 (-19.1 to 6.1)
Dengue	358.7 (199.6 to 790.0)	149.7 (-0.8 to 601.5)	125.4 (-10.4 to 533.4)	58.2 (26.3 to 137.9)	147.6 (-1.0 to 599.7)	123.6 (-10.6 to 531.8)
Dengue episodes aggregate	106.6 (70.2 to 227.4)	137.5 (-5.0 to 554.3)	114.4 (-14.3 to 490.7)	6.3 (3.4 to 14.4)	148.1 (-0.9 to 583.7)	124.0 (-10.5 to 517.4)
Post-dengue chronic fatigue syndrome	252.1 (120.6 to 569.6)	149.8 (-0.8 to 601.5)	125.6 (-10.4 to 533.4)	52.0 (22.1 to 125.9)	147.6 (-0.8 to 602.4)	123.6 (-10.4 to 534.2)
Yellow fever	0.2 (0.0 to 0.5)	-21.5 (-41.1 to 1.1)	-29.1 (-46.8 to -8.7)*	0.0 (0.0 to 0.0)	-21.4 (-41.1 to 1.1)	-29.1 (-46.8 to -8.7)*
Rabies	0.0 (0.0 to 0.0)	-45.1 (-65.8 to -16.2)*	-50.5 (-69.1 to -24.3)*	0.0 (0.0 to 0.0)	-45.0 (-65.8 to -16.2)*	-50.3 (-69.1 to -24.3)*
Intestinal nematode infections	116,249.5 (112,848.5 to 119,261.8)	-1.6 (-5.1 to 1.8)	-11.2 (-14.3 to -8.1)*	247.3 (141.1 to 404.7)	-20.1 (-26.7 to -12.2)*	-27.9 (-33.8 to -20.7)*
Ascariasis	61,451.7 (53,055.7 to 71,010.5)	-9.3 (-24.5 to 7.6)	-18.1 (-31.9 to -2.8)*	66.1 (36.2 to 111.6)	-36.2 (-47.2 to -23.7)*	-42.4 (-52.3 to -31.1)*
Complications of ascariasis aggregate	3,623.8 (3,260.8 to 4,044.2)	-33.9 (-43.1 to -23.4)*	-40.4 (-48.6 to -30.8)*	66.1 (36.2 to 111.6)	-36.2 (-47.2 to -23.7)*	-42.4 (-52.3 to -31.1)*
Asymptomatic ascariasis	57,825.9 (49,334.4 to 67,379.3)	-7.2 (-23.7 to 11.9)	-16.2 (-31.1 to 1.1)	0.0 (0.0 to 0.0)	0.0 (0.0 to 0.0)	0.0 (0.0 to 0.0)
Trichuriasis	36,501.5 (32,908.5 to 40,625.6)	-0.2 (-13.4 to 16.6)	-9.9 (-21.8 to 5.3)	45.0 (23.4 to 78.9)	-8.3 (-28.3 to 22.6)	-17.2 (-35.2 to 10.7)
Complications of trichuriasis aggregate	2,229.7 (1,921.1 to 2,740.5)	-7.4 (-25.3 to 18.5)	-16.4 (-32.6 to 7.0)	45.0 (23.4 to 78.9)	-8.3 (-28.3 to 22.6)	-17.2 (-35.2 to 10.7)
Asymptomatic trichuriasis	34,280.1 (30,654.5 to 38,381.8)	0.3 (-13.9 to 18.3)	-9.4 (-22.3 to 6.8)	0.0 (0.0 to 0.0)	0.0 (0.0 to 0.0)	0.0 (0.0 to 0.0)
Hookworm disease	35,588.3 (32,038.8 to 39,977.6)	-6.9 (-19.4 to 7.1)	-15.9 (-27.3 to -3.3)*	136.2 (81.6 to 216.0)	-13.2 (-20.9 to -5.2)*	-21.6 (-28.6 to -14.4)*
Complications of hookworm disease aggregate	4,361.9 (4,064.8 to 4,706.3)	-13.2 (-22.3 to -3.6)*	-21.7 (-29.9 to -13.0)*	89.8 (48.7 to 149.1)	-14.6 (-25.1 to -2.4)*	-22.9 (-32.4 to -11.9)*
Anemia due to hookworm disease aggregate	1,805.9 (1,767.9 to 1,841.5)	-5.1 (-7.5 to -2.4)*	-14.3 (-16.5 to -11.8)*	46.4 (30.9 to 67.8)	-10.2 (-15.1 to -5.6)*	-18.9 (-23.3 to -14.8)*
Asymptomatic hookworm disease	29,425.7 (25,842.9 to 33,691.0)	-5.9 (-21.2 to 11.7)	-15.1 (-28.9 to 0.9)	0.0 (0.0 to 0.0)	0.0 (0.0 to 0.0)	0.0 (0.0 to 0.0)
Food-borne trematodiases	6,905.1 (6,378.7 to 7,444.1)	-14.3 (-17.2 to -11.3)*	-22.6 (-25.2 to -19.9)*	184.9 (94.5 to 337.5)	-15.7 (-21.1 to -7.5)*	-23.9 (-28.8 to -16.5)*
Asymptomatic food-borne trematodiases aggregate	5,286.5 (4,164.7 to 6,117.4)	-14.1 (-17.4 to -10.3)*	-22.4 (-25.4 to -19.0)*	0.0 (0.0 to 0.0)	0.0 (0.0 to 0.0)	0.0 (0.0 to 0.0)
Symptomatic food-borne trematodiases aggregate	1,618.7 (948.1 to 2,683.4)	-15.0 (-20.1 to -7.2)*	-23.3 (-27.9 to -16.2)*	184.9 (94.5 to 337.5)	-15.7 (-21.1 to -7.5)*	-23.9 (-28.8 to -16.5)*
Leprosy	24.4 (22.3 to 27.0)	-17.0 (-18.2 to -15.7)*	-25.0 (-26.1 to -23.9)*	1.5 (1.0 to 2.1)	-16.8 (-27.6 to -4.8)*	-24.9 (-34.6 to -14.0)*
Ebola	0.3 (0.1 to 0.6)	72,355.7 (42,380.9 to 261,513.3)*	65,321.0 (38,256.5 to 236,113.3)*	0.1 (0.0 to 0.1)	71,886.3 (37,534.9 to 353,475.7)*	64,897.2 (33,880.9 to 319,147.0)*
Other neglected tropical diseases	2,184.2 (2,129.0 to 2,239.6)	-2.5 (-5.3 to 0.2)	-12.0 (-14.5 to -9.5)*	210.4 (74.8 to 548.5)	8.7 (-25.8 to 64.4)	-1.9 (-33.0 to 48.4)
Anemia due to other neglected tropical diseases aggregate	2,184.2 (2,129.0 to 2,239.6)	-2.5 (-5.3 to 0.2)	-12.0 (-14.5 to -9.5)*	40.0 (25.6 to 60.1)	-14.2 (-19.8 to -8.8)*	-22.5 (-27.6 to -17.7)*
Maternal disorders	2,170.2 (2,030.7 to 2,279.2)	10.9 (3.9 to 17.6)*	0.2 (-6.2 to 6.2)	168.8 (115.5 to 234.6)	1.8 (-12.2 to 19.0)	-8.1 (-20.7 to 7.5)
Maternal hemorrhage	495.3 (474.5 to 518.9)	8.1 (2.4 to 14.4)*	-2.4 (-7.6 to 3.3)	16.8 (11.0 to 23.8)	3.7 (-11.5 to 23.4)	-6.4 (-20.1 to 11.4)
Maternal hemorrhage episodes aggregate	38.8 (23.7 to 61.4)	9.8 (-32.0 to 76.6)	-0.8 (-38.6 to 59.4)	6.3 (3.5 to 10.4)	10.5 (-25.3 to 67.2)	-0.2 (-32.6 to 51.0)
Anemia due to maternal hemorrhage aggregate	456.6 (442.1 to 469.7)	7.9 (3.4 to 12.6)*	-2.6 (-6.6 to 1.6)	10.5 (6.8 to 15.4)	-0.0 (-8.3 to 8.5)	-9.7 (-17.2 to -2.1)*
Maternal sepsis and other maternal infections	581.3 (490.7 to 693.3)	9.8 (-7.0 to 31.5)	-0.9 (-16.0 to 18.7)	9.3 (4.8 to 16.9)	8.7 (-33.9 to 83.2)	-1.8 (-40.3 to 65.4)
Maternal sepsis and other maternal infections aggregate	141.3 (70.5 to 247.5)	8.3 (-44.8 to 113.7)	-2.2 (-50.2 to 92.9)	7.1 (3.3 to 13.6)	8.2 (-42.8 to 109.0)	-2.3 (-48.4 to 88.7)
Infertility due to puerperal sepsis	440.2 (391.8 to 492.0)	10.3 (8.2 to 12.8)*	-0.4 (-2.3 to 1.8)	2.2 (0.8 to 4.8)	10.5 (6.2 to 15.2)*	-0.2 (-4.2 to 4.0)
Maternal hypertensive disorders	825.8 (519.1 to 1,261.6)	16.1 (-29.3 to 90.2)	4.8 (-36.2 to 71.7)	39.5 (20.2 to 70.3)	16.0 (-29.2 to 89.4)	4.8 (-36.1 to 71.0)
Maternal hypertensive disorders episodes aggregate	826.6 (519.2 to 1,262.6)	16.2 (-29.3 to 90.3)	4.9 (-36.1 to 71.8)	39.0 (19.9 to 69.7)	16.2 (-29.0 to 90.5)	4.9 (-35.9 to 72.0)
Eclampsia	0.8 (0.3 to 1.6)	2.4 (-65.9 to 203.2)	-7.5 (-69.2 to 173.8)	0.5 (0.2 to 1.0)	2.6 (-65.9 to 203.2)	-7.4 (-69.2 to 173.8)
Complications of maternal hypertensive disorders aggregate	0.0 (0.0 to 0.0)	0.0 (0.0 to 0.0)	0.0 (0.0 to 0.0)	0.0 (0.0 to 0.0)	0.0 (0.0 to 0.0)	0.0 (0.0 to 0.0)
Maternal obstructed labor and uterine rupture	204.2 (163.9 to 250.5)	-8.5 (-13.5 to -3.2)*	-17.4 (-21.9 to -12.6)*	66.9 (42.9 to 97.8)	-8.2 (-14.9 to -1.8)*	-17.1 (-23.2 to -11.3)*
Obstructed labor, acute event	14.8 (7.8 to 24.8)	5.5 (-40.9 to 90.5)	-4.7 (-46.6 to 72.0)	4.6 (2.2 to 8.3)	5.9 (-41.5 to 84.6)	-4.4 (-47.1 to 66.6)
Fistula due to maternal obstructed labor and uterine rupture aggregate	189.5 (149.8 to 234.9)	-9.5 (-13.5 to -5.7)*	-18.3 (-21.9 to -14.8)*	62.4 (39.7 to 91.4)	-9.1 (-15.4 to -3.5)*	-17.9 (-23.6 to -12.9)*
Maternal abortion, miscarriage, and ectopic pregnancy	87.4 (52.0 to 134.1)	8.7 (-30.9 to 74.0)	-1.8 (-37.6 to 57.1)	9.5 (5.0 to 16.2)	8.5 (-30.8 to 74.5)	-2.0 (-37.5 to 57.6)
Other maternal disorders	--	--	--	26.8 (17.5 to 37.5)	5.6 (-12.6 to 26.7)	-4.7 (-21.1 to 14.4)
Neonatal disorders	3,473.4 (3,296.5 to 3,604.1)	17.7 (14.1 to 21.5)*	6.3 (3.0 to 9.7)*	823.8 (622.2 to 1,055.8)	16.4 (12.0 to 20.9)*	5.1 (1.1 to 9.1)*
Neonatal preterm birth complications	2,718.5 (2,378.2 to 3,090.8)	10.9 (5.7 to 16.5)*	0.2 (-4.5 to 5.2)	397.7 (299.2 to 515.4)	4.8 (-1.1 to 11.0)	-5.3 (-10.7 to 0.2)
Vision loss due to retinopathy of prematurity aggregate	320.3 (254.2 to 397.7)	8.9 (5.0 to 13.0)*	-1.7 (-5.2 to 2.0)	11.1 (6.8 to 17.2)	11.5 (2.6 to 20.2)*	0.6 (-7.3 to 8.5)
Complications of preterm birth complications aggregate	2,398.2 (2,086.6 to 2,747.9)	11.2 (5.4 to 17.5)*	0.4 (-4.8 to 6.1)	386.6 (290.9 to 500.6)	4.7 (-1.4 to 11.1)	-5.5 (-11.0 to 0.3)
Neonatal encephalopathy due to birth asphyxia and trauma	969.6 (679.9 to 1,423.5)	36.2 (33.1 to 39.0)*	22.9 (20.2 to 25.5)*	284.4 (185.7 to 436.6)	32.8 (28.3 to 37.7)*	19.9 (15.9 to 24.3)*

Cause	Prevalence in thousands (95% confidence interval)			YLDs in thousands (95% confidence interval)		
	2015	Percent change in counts between 2005 and 2015	Percent change in rates between 2005 and 2015	2015	Percent change in counts between 2005 and 2015	Percent change in rates between 2005 and 2015
Neonatal sepsis and other neonatal infections	0.0 (0.0 to 0.0)	0.0 (0.0 to 0.0)	0.0 (0.0 to 0.0)	0.0 (0.0 to 0.0)	0.0 (0.0 to 0.0)	0.0 (0.0 to 0.0)
Severe infection due to neonatal sepsis and other neonatal infections	0.0 (0.0 to 0.0)	0.0 (0.0 to 0.0)	0.0 (0.0 to 0.0)	0.0 (0.0 to 0.0)	0.0 (0.0 to 0.0)	0.0 (0.0 to 0.0)
Complications of neonatal sepsis and other neonatal infections aggregate	0.0 (0.0 to 0.0)	0.0 (0.0 to 0.0)	0.0 (0.0 to 0.0)	0.0 (0.0 to 0.0)	0.0 (0.0 to 0.0)	0.0 (0.0 to 0.0)
Hemolytic disease and other neonatal jaundice	153.6 (137.0 to 173.6)	25.4 (24.1 to 26.6)*	13.2 (12.1 to 14.3)*	46.1 (34.5 to 59.6)	21.7 (16.1 to 27.7)*	9.9 (4.9 to 15.3)*
Other neonatal disorders	--	--	--	95.6 (65.1 to 126.7)	25.1 (7.3 to 45.7)*	12.9 (-3.1 to 31.6)
Nutritional deficiencies	101,125.0 (100,035.6 to 102,249.8)	3.1 (1.6 to 4.7)*	-6.9 (-8.3 to -5.5)*	2,885.0 (1,924.2 to 4,199.9)	-1.2 (-3.2 to 0.9)	-10.8 (-12.6 to -8.9)*
Protein-energy malnutrition	7.2 (4.5 to 11.7)	-15.9 (-27.0 to -3.8)*	-24.1 (-34.1 to -13.2)*	0.9 (0.5 to 1.7)	-15.4 (-30.8 to 1.8)	-23.6 (-37.5 to -8.1)*
Iodine deficiency	10,203.9 (9,212.2 to 11,524.7)	7.1 (4.6 to 9.5)*	-3.3 (-5.5 to -1.1)*	217.6 (137.5 to 327.1)	9.4 (6.5 to 12.1)*	-1.2 (-3.8 to 1.2)
Visible goiter due to iodine deficiency aggregate	9,984.8 (8,978.8 to 11,282.4)	6.8 (4.3 to 9.4)*	-3.5 (-5.8 to -1.3)*	179.2 (110.3 to 283.5)	7.0 (4.2 to 9.5)*	-3.4 (-5.9 to -1.1)*
Visible goiter with heart failure due to iodine deficiency aggregate	0.0 (0.0 to 0.0)	16.6 (14.0 to 19.4)*	5.3 (2.9 to 7.8)*	0.0 (0.0 to 0.0)	16.6 (14.0 to 19.4)*	5.3 (2.9 to 7.8)*
Intellectual disability due to iodine deficiency aggregate	219.1 (90.0 to 301.0)	21.3 (15.6 to 24.4)*	9.5 (4.4 to 12.3)*	38.3 (13.3 to 62.8)	22.2 (13.3 to 28.5)*	10.4 (2.3 to 16.1)*
Vitamin A deficiency	348.0 (242.7 to 469.8)	17.6 (11.9 to 23.8)*	6.2 (1.0 to 11.8)*	18.4 (11.0 to 27.6)	16.8 (9.1 to 24.2)*	5.5 (-1.5 to 12.1)
Iron-deficiency anemia	98,790.1 (97,690.3 to 99,980.0)	1.8 (0.4 to 3.2)*	-8.1 (-9.4 to -6.8)*	2,648.0 (1,767.4 to 3,840.4)	-2.0 (-4.3 to 0.2)	-11.6 (-13.5 to -9.6)*
Iron-deficiency anemia without heart failure aggregate	98,789.7 (97,689.9 to 99,979.7)	1.8 (0.4 to 3.2)*	-8.1 (-9.4 to -6.8)*	2,647.9 (1,767.3 to 3,840.4)	-2.0 (-4.3 to 0.2)	-11.6 (-13.5 to -9.6)*
Iron-deficiency anemia with heart failure aggregate	0.4 (0.3 to 0.4)	22.6 (20.0 to 25.5)*	10.7 (8.3 to 13.3)*	0.0 (0.0 to 0.1)	22.6 (20.0 to 25.5)*	10.7 (8.3 to 13.3)*
Other nutritional deficiencies	--	--	--	0.1 (0.0 to 0.2)	-13.0 (-37.7 to 15.1)	-21.4 (-43.8 to 4.0)
Other communicable, maternal, neonatal, and nutritional diseases	169,503.2 (165,532.3 to 172,956.6)	10.6 (9.5 to 11.7)*	-0.1 (-1.1 to 0.9)	363.3 (226.8 to 562.5)	12.1 (4.0 to 18.9)*	1.2 (-6.1 to 7.4)
Sexually transmitted diseases excluding HIV	137,353.7 (133,205.5 to 141,188.1)	11.2 (9.8 to 12.7)*	0.4 (-0.9 to 1.7)	266.1 (160.5 to 424.9)	15.9 (10.1 to 21.5)*	4.6 (-0.6 to 9.7)
Syphilis	6,095.0 (4,188.7 to 8,640.1)	1.3 (-8.2 to 9.0)	-8.5 (-17.1 to -1.6)*	23.6 (15.7 to 33.3)	11.0 (3.5 to 18.8)*	0.2 (-6.6 to 7.3)
Early syphilis aggregate	5,979.4 (4,073.6 to 8,542.4)	1.1 (-8.6 to 8.9)	-8.7 (-17.5 to -1.6)*	1.5 (0.4 to 3.7)	1.1 (-9.6 to 10.5)	-8.7 (-18.3 to -0.3)*
Adult tertiary syphilis	115.6 (99.9 to 131.7)	11.3 (7.2 to 15.0)*	0.5 (-3.2 to 3.9)	22.1 (14.4 to 31.8)	11.7 (3.6 to 19.7)*	0.8 (-6.5 to 8.1)
Chlamydial infection	14,977.0 (9,812.6 to 21,233.9)	2.1 (-1.1 to 5.7)	-7.8 (-10.7 to -4.5)*	68.1 (36.9 to 112.7)	1.9 (-3.7 to 7.7)	-8.0 (-13.0 to -2.8)*
Chlamydial infection episodes aggregate	14,712.4 (9,560.1 to 20,970.7)	2.0 (-1.3 to 5.6)	-7.9 (-10.9 to -4.6)*	63.1 (32.7 to 106.5)	1.7 (-4.4 to 8.2)	-8.2 (-13.7 to -2.3)*
Pelvic inflammatory diseases due to chlamydial infection aggregate	27.7 (20.8 to 37.1)	1.2 (-3.2 to 5.7)	-8.6 (-12.6 to -4.5)*	3.7 (2.3 to 5.7)	1.6 (-8.2 to 12.5)	-8.3 (-17.1 to 1.5)
Infertility due to clamydial infection aggregate	236.9 (211.1 to 261.0)	11.2 (10.1 to 12.4)*	0.4 (-0.5 to 1.5)	1.3 (0.5 to 2.7)	11.0 (5.7 to 16.0)*	0.2 (-4.6 to 4.7)
Gonococcal infection	10,293.1 (6,170.7 to 17,206.5)	33.4 (21.7 to 41.5)*	20.5 (9.9 to 27.8)*	105.3 (53.3 to 183.2)	33.8 (23.1 to 42.1)*	20.8 (11.2 to 28.3)*
Gonococcal infection episodes aggregate	10,138.6 (6,011.1 to 17,042.8)	33.8 (21.9 to 42.0)*	20.8 (10.0 to 28.2)*	102.3 (50.9 to 179.3)	34.9 (24.2 to 43.3)*	21.8 (12.1 to 29.4)*
Pelvic inflammatory diseases due to gonococcal infection aggregate	154.6 (140.1 to 170.2)	10.6 (8.4 to 12.6)*	-0.2 (-2.1 to 1.7)	3.0 (1.9 to 4.4)	3.8 (-4.7 to 12.6)	-6.3 (-14.0 to 1.6)
Trichomoniasis	23,038.0 (16,379.7 to 31,672.3)	11.8 (10.9 to 12.6)*	1.0 (0.1 to 1.7)*	26.6 (10.2 to 58.7)	12.5 (10.8 to 14.0)*	1.6 (0.1 to 3.0)*
Genital herpes	96,627.5 (83,315.7 to 110,950.5)	11.7 (10.0 to 13.3)*	0.9 (-0.7 to 2.3)	26.3 (8.4 to 62.0)	14.5 (11.5 to 20.9)*	3.4 (0.7 to 9.2)*
Moderate infection due to initial genital herpes episode	45.8 (10.3 to 105.7)	55.9 (39.4 to 82.2)*	40.8 (25.9 to 64.5)*	2.2 (0.5 to 5.4)	55.5 (34.5 to 91.8)*	40.4 (21.4 to 73.1)*
Complications of genital herpes aggregate	96,581.7 (83,238.1 to 110,893.8)	11.7 (10.0 to 13.3)*	0.9 (-0.7 to 2.3)	24.1 (6.9 to 58.7)	11.8 (9.8 to 13.8)*	1.0 (-0.9 to 2.7)
Other sexually transmitted diseases	672.1 (609.2 to 736.5)	7.1 (5.3 to 8.9)*	-3.3 (-4.9 to -1.6)*	16.2 (10.7 to 23.7)	0.0 (-4.9 to 4.9)	-9.7 (-14.2 to -5.3)*
Pelvic inflammatory diseases due to other sexually transmitted diseases aggregate	98.6 (75.9 to 125.7)	-1.9 (-3.7 to -0.1)*	-11.4 (-13.0 to -9.8)*	13.0 (8.4 to 19.1)	-1.9 (-7.9 to 3.8)	-11.4 (-16.8 to -6.2)*
Infertility due to other sexually transmitted diseases aggregate	573.6 (514.2 to 634.8)	8.8 (6.7 to 10.9)*	-1.7 (-3.7 to 0.2)	3.2 (1.3 to 6.5)	8.6 (4.7 to 12.5)*	-1.9 (-5.5 to 1.5)
Other sexually transmitted diseases	--	--	--	71.4 (40.8 to 114.8)	20.0 (10.2 to 29.3)*	8.3 (-0.5 to 16.7)
Hepatitis	40,852.8 (40,388.0 to 41,402.6)	9.6 (8.6 to 10.6)*	-1.1 (-2.0 to -0.1)*	40.9 (24.3 to 67.2)	12.4 (-28.1 to 71.4)	1.5 (-35.1 to 54.8)
Acute hepatitis A	250.0 (201.6 to 296.1)	12.4 (-13.3 to 44.7)	1.5 (-21.7 to 30.6)	10.5 (6.6 to 15.9)	12.4 (-15.1 to 46.7)	1.5 (-23.3 to 32.4)
Hepatitis B	31,353.3 (29,954.1 to 32,917.0)	8.5 (5.3 to 11.9)*	-2.0 (-4.9 to 1.1)	27.1 (13.4 to 50.5)	12.8 (-43.6 to 105.8)	1.8 (-49.1 to 85.8)
Acute Hepatitis B aggregate	1,425.1 (939.1 to 2,137.3)	12.3 (-39.3 to 100.2)	1.4 (-45.2 to 80.7)	27.1 (13.4 to 50.5)	12.8 (-43.6 to 105.8)	1.8 (-49.1 to 85.8)
Chronic hepatitis B	29,928.3 (28,764.8 to 31,264.4)	8.4 (7.1 to 9.7)*	-2.2 (-3.3 to -0.9)*	0.0 (0.0 to 0.0)	0.0 (0.0 to 0.0)	0.0 (0.0 to 0.0)
Hepatitis C	9,986.1 (8,813.2 to 11,165.7)	12.2 (11.1 to 13.3)*	1.3 (0.3 to 2.3)*	0.4 (0.2 to 0.7)	10.6 (7.6 to 12.7)*	-0.2 (-2.8 to 1.8)
Acute Hepatitis C aggregate	26.0 (20.2 to 33.5)	10.6 (7.6 to 12.7)*	-0.2 (-2.9 to 1.8)	0.4 (0.2 to 0.7)	10.6 (7.6 to 12.7)*	-0.2 (-2.8 to 1.8)
Chronic hepatitis C	9,960.1 (8,788.3 to 11,139.0)	12.2 (11.1 to 13.3)*	1.3 (0.3 to 2.3)*	0.0 (0.0 to 0.0)	0.0 (0.0 to 0.0)	0.0 (0.0 to 0.0)
Acute hepatitis E	92.9 (76.3 to 111.8)	9.3 (6.9 to 11.6)*	-1.3 (-3.5 to 0.8)	2.9 (1.8 to 4.4)	9.4 (-11.1 to 35.9)	-1.2 (-19.7 to 22.7)
Other infectious diseases	2,419.6 (2,375.5 to 2,463.2)	-2.0 (-4.3 to 0.4)	-11.5 (-13.5 to -9.4)*	56.2 (35.3 to 86.2)	-3.2 (-9.8 to 4.7)	-12.6 (-18.6 to -5.5)*
Non-communicable diseases	512,882.2 (510,173.0 to 515,086.7)	11.0 (10.9 to 11.2)*	0.3 (0.1 to 0.4)*	45,455.7 (33,833.9 to 58,920.8)	11.7 (10.9 to 12.4)*	0.8 (0.2 to 1.5)*
Neoplasms	1,547.2 (1,486.5 to 1,618.3)	14.1 (8.6 to 20.4)*	3.0 (-2.0 to 8.7)	158.6 (111.7 to 212.9)	10.7 (2.9 to 19.6)*	-0.0 (-7.1 to 8.0)
Lip and oral cavity cancer	53.6 (42.5 to 71.5)	18.5 (-4.2 to 45.8)	7.0 (-13.5 to 31.6)	4.6 (2.9 to 7.1)	17.8 (-5.0 to 46.5)	6.3 (-14.2 to 32.3)
Nasopharynx cancer	27.6 (12.4 to 47.6)	-5.3 (-46.9 to 54.6)	-14.5 (-52.1 to 39.6)	2.6 (1.1 to 4.7)	-5.9 (-46.7 to 51.1)	-15.0 (-51.9 to 36.4)
Other pharynx cancer	8.4 (7.3 to 9.6)	14.4 (1.8 to 28.4)*	3.3 (-8.1 to 15.9)	0.7 (0.5 to 1.0)	13.5 (0.7 to 27.9)*	2.5 (-9.1 to 15.5)
Esophageal cancer	3.6 (2.9 to 4.4)	1.5 (-18.5 to 24.6)	-8.4 (-26.4 to 12.5)	0.7 (0.5 to 1.0)	-2.9 (-21.2 to 18.3)	-12.3 (-28.9 to 6.8)
Stomach cancer	31.4 (26.4 to 34.8)	-19.6 (-29.7 to -8.1)*	-27.4 (-36.5 to -17.0)*	3.7 (2.4 to 4.9)	-21.4 (-31.7 to -9.9)*	-29.0 (-38.3 to -18.7)*
Colon and rectum cancer	102.4 (89.2 to 116.2)	21.0 (6.7 to 38.2)*	9.3 (-3.7 to 24.8)	8.8 (6.1 to 11.9)	17.3 (1.9 to 35.4)*	5.9 (-8.0 to 22.2)
Colon and rectum cancer aggregate	102.1 (88.9 to 115.9)	21.1 (6.7 to 38.3)*	9.3 (-3.7 to 24.9)	8.7 (6.0 to 11.8)	17.3 (1.9 to 35.6)*	6.0 (-8.0 to 22.4)
Stoma due to colon and rectum cancer	0.3 (0.2 to 0.3)	2.8 (2.0 to 3.8)*	-7.1 (-7.9 to -6.3)*	0.0 (0.0 to 0.1)	2.8 (2.0 to 3.8)*	-7.1 (-7.9 to -6.3)*
Liver cancer	1.8 (0.8 to 3.3)	-3.4 (-62.2 to 144.1)	-12.8 (-65.8 to 120.4)	3.3 (2.0 to 5.7)	-15.8 (-46.4 to 35.7)	-23.9 (-51.6 to 22.5)
Liver cancer due to hepatitis B	10.4 (6.8 to 18.1)	-11.4 (-47.6 to 55.3)	-20.0 (-52.7 to 40.2)	2.2 (1.3 to 4.0)	-17.1 (-48.0 to 37.7)	-25.2 (-53.0 to 24.3)
Liver cancer due to hepatitis C	1.0 (0.7 to 1.4)	9.5 (-20.2 to 48.9)	-1.1 (-28.0 to 34.4)	0.2 (0.1 to 0.3)	2.3 (-23.5 to 36.0)	-7.6 (-30.9 to 22.8)
Liver cancer due to alcohol use	0.9 (0.6 to 1.4)	14.3 (-26.8 to 83.7)	3.2 (-33.9 to 65.9)	0.2 (0.1 to 0.4)	7.2 (-31.5 to 66.4)	-3.2 (-38.1 to 50.2)
Liver cancer due to other causes	2.8 (1.8 to 4.4)	-17.2 (-48.9 to 39.2)	-25.2 (-53.9 to 25.7)	0.6 (0.4 to 1.1)	-22.0 (-49.5 to 23.4)	-29.6 (-54.4 to 11.4)
Gallbladder and biliary tract cancer	0.7 (0.6 to 0.8)	-6.3 (-15.4 to 4.2)	-15.4 (-23.6 to -5.9)*	0.2 (0.1 to 0.3)	-7.6 (-16.5 to 2.4)	-16.6 (-24.6 to -7.6)*
Pancreatic cancer	1.8 (1.6 to 2.1)	18.7 (6.7 to 32.9)*	7.2 (-3.7 to 20.0)	0.5 (0.3 to 0.6)	15.5 (4.3 to 29.1)*	4.3 (-5.9 to 16.6)

Cause	Prevalence in thousands (95% confidence interval)			YLDs in thousands (95% confidence interval)		
	2015	Percent change in counts between 2005 and 2015	Percent change in rates between 2005 and 2015	2015	Percent change in counts between 2005 and 2015	Percent change in rates between 2005 and 2015
Larynx cancer	19.9 (18.6 to 22.1)	10.2 (4.8 to 15.7)*	-0.5 (-5.3 to 4.5)	2.3 (1.6 to 3.2)	8.4 (0.5 to 16.7)*	-2.1 (-9.3 to 5.4)
Larynx cancer aggregate	2.7 (1.9 to 3.9)	2.7 (-24.7 to 43.2)	-7.3 (-32.1 to 29.3)	0.6 (0.4 to 0.9)	1.5 (-14.3 to 20.8)	-8.4 (-22.6 to 9.1)
Laryngectomy due to larynx cancer	17.2 (16.2 to 19.3)	11.5 (10.5 to 13.0)*	0.6 (-0.2 to 2.0)	1.7 (1.1 to 2.4)	11.4 (3.3 to 20.3)*	0.6 (-6.7 to 8.7)
Tracheal, bronchus, and lung cancer	18.7 (14.0 to 25.7)	-1.1 (-36.7 to 45.6)	-10.7 (-42.8 to 31.5)	3.3 (2.0 to 5.0)	-5.3 (-40.5 to 42.1)	-14.5 (-46.3 to 28.3)
Malignant skin melanoma	95.9 (82.2 to 126.4)	17.4 (8.5 to 26.9)*	6.0 (-2.1 to 14.6)	5.6 (3.5 to 8.4)	16.2 (5.7 to 27.3)*	4.9 (-4.5 to 15.0)
Non-melanoma skin cancer	33.1 (28.7 to 38.2)	50.1 (24.2 to 83.0)*	35.5 (12.2 to 65.2)*	2.5 (1.6 to 3.7)	39.3 (14.7 to 67.4)*	25.8 (3.5 to 51.1)*
Non-melanoma skin cancer(squamous-cell carcinoma)	41.8 (34.0 to 51.0)	44.6 (19.8 to 72.7)*	30.5 (8.1 to 55.9)*	2.4 (1.6 to 3.6)	40.4 (14.7 to 69.7)*	26.8 (3.6 to 53.2)*
Non-melanoma skin cancer(basal-cell carcinoma)	7.3 (5.5 to 9.5)	12.3 (9.2 to 14.7)*	1.4 (-1.4 to 3.6)	0.1 (0.0 to 0.2)	12.4 (6.4 to 17.2)*	1.5 (-4.0 to 5.8)
Breast cancer	460.4 (402.1 to 508.1)	17.2 (3.5 to 33.3)*	5.8 (-6.6 to 20.4)	34.0 (23.3 to 46.7)	13.6 (0.3 to 29.0)*	2.6 (-9.4 to 16.5)
Breast cancer aggregate	392.7 (333.8 to 440.2)	19.2 (2.9 to 38.8)*	7.7 (-7.1 to 25.3)	28.6 (19.7 to 38.8)	15.0 (-0.7 to 34.1)	3.8 (-10.4 to 21.1)
Mastectomy due to breast cancer	67.6 (64.9 to 70.5)	6.7 (5.7 to 7.8)*	-3.6 (-4.6 to -2.7)*	5.4 (3.3 to 7.9)	6.6 (-1.5 to 15.5)	-3.7 (-11.0 to 4.3)
Cervical cancer	219.9 (152.0 to 297.4)	-13.9 (-39.2 to 18.1)	-22.2 (-45.1 to 6.6)	16.8 (10.5 to 25.5)	-13.2 (-38.4 to 19.6)	-21.7 (-44.4 to 8.0)
Uterine cancer	55.0 (29.8 to 78.8)	-9.7 (-57.5 to 95.9)	-18.5 (-61.6 to 76.9)	3.6 (1.7 to 6.1)	-10.0 (-56.9 to 89.6)	-18.7 (-61.1 to 71.2)
Ovarian cancer	40.9 (32.1 to 51.1)	25.3 (0.7 to 54.6)*	13.1 (-9.1 to 39.6)	5.4 (3.5 to 7.7)	23.4 (-1.3 to 53.6)	11.4 (-10.9 to 38.6)
Prostate cancer	11.0 (7.9 to 13.6)	25.8 (8.9 to 47.0)*	13.6 (-1.7 to 32.8)	0.9 (0.6 to 1.3)	16.0 (-0.6 to 36.8)	4.8 (-10.2 to 23.6)
Prostate cancer aggregate	9.8 (6.8 to 12.4)	28.3 (9.0 to 52.5)*	15.9 (-1.6 to 37.7)	0.8 (0.5 to 1.1)	17.3 (-1.6 to 41.5)	5.9 (-11.2 to 27.8)
Impotence and incontinence due to prostate cancer aggregate	1.2 (1.1 to 1.2)	8.1 (7.5 to 8.7)*	-2.4 (-3.0 to -1.8)*	0.1 (0.1 to 0.2)	8.1 (7.5 to 8.7)*	-2.4 (-3.0 to -1.8)*
Testicular cancer	113.8 (98.9 to 130.4)	33.5 (17.0 to 51.2)*	20.6 (5.6 to 36.5)*	6.8 (4.4 to 9.6)	32.0 (15.4 to 49.6)*	19.2 (4.2 to 35.1)*
Kidney cancer	24.8 (21.6 to 28.1)	29.3 (13.9 to 47.8)*	16.7 (2.9 to 33.4)*	1.9 (1.2 to 2.5)	27.2 (10.7 to 46.1)*	14.8 (-0.1 to 31.9)
Bladder cancer	21.9 (16.9 to 27.0)	17.1 (-5.4 to 50.3)	5.8 (-14.6 to 35.7)	2.0 (1.3 to 2.8)	13.8 (-4.1 to 38.6)	2.7 (-13.4 to 25.2)
Bladder cancer episodes aggregate	20.0 (15.0 to 25.1)	18.0 (-6.7 to 55.5)	6.5 (-15.7 to 40.4)	1.7 (1.1 to 2.4)	14.8 (-7.1 to 46.2)	3.7 (-16.1 to 32.0)
Urinary incontinence due to bladder cancer	1.9 (1.8 to 2.0)	8.9 (7.8 to 10.0)*	-1.6 (-2.6 to -0.7)*	0.3 (0.2 to 0.5)	8.9 (7.8 to 10.0)*	-1.6 (-2.6 to -0.7)*
Brain and nervous system cancer	46.4 (39.1 to 54.9)	12.4 (-5.0 to 33.7)	1.5 (-14.2 to 20.7)	5.2 (3.6 to 7.2)	11.2 (-6.8 to 32.5)	0.4 (-15.9 to 19.7)
Thyroid cancer	42.7 (35.9 to 48.5)	-1.2 (-15.8 to 13.2)	-10.7 (-24.0 to 2.3)	2.4 (1.6 to 3.5)	-1.8 (-17.5 to 14.6)	-11.4 (-25.5 to 3.5)
Mesothelioma	0.6 (0.6 to 0.7)	7.3 (-5.2 to 21.2)	-3.1 (-14.4 to 9.5)	0.1 (0.1 to 0.2)	7.1 (-5.4 to 21.1)	-3.3 (-14.6 to 9.3)
Hodgkin lymphoma	45.1 (38.7 to 54.3)	8.8 (-3.5 to 24.0)	-1.8 (-12.9 to 12.0)	3.6 (2.5 to 5.0)	5.7 (-7.2 to 22.1)	-4.5 (-16.2 to 10.2)
Non-Hodgkin lymphoma	133.4 (113.0 to 155.4)	47.1 (27.4 to 67.7)*	32.8 (15.0 to 51.4)*	10.0 (6.8 to 14.0)	42.9 (23.2 to 63.2)*	29.0 (11.3 to 47.4)*
Multiple myeloma	4.7 (3.8 to 5.8)	43.2 (21.5 to 67.8)*	29.3 (9.7 to 51.5)*	1.1 (0.7 to 1.6)	40.1 (19.8 to 63.4)*	26.5 (8.2 to 47.5)*
Leukemia	45.8 (39.5 to 52.5)	35.0 (12.4 to 60.6)*	21.9 (1.5 to 45.0)*	13.8 (9.4 to 18.9)	20.9 (1.4 to 48.0)*	9.1 (-8.4 to 33.7)
Acute lymphoid leukemia	26.8 (21.1 to 34.4)	36.3 (8.6 to 70.7)*	23.0 (-1.9 to 54.2)	3.5 (2.3 to 5.0)	32.3 (5.4 to 65.8)*	19.4 (-4.8 to 49.7)
Chronic lymphoid leukemia	34.4 (26.1 to 45.1)	5.9 (-20.2 to 40.9)	-4.3 (-28.0 to 27.2)	4.3 (2.7 to 6.2)	4.4 (-20.9 to 39.1)	-5.8 (-28.6 to 25.6)
Acute myeloid leukemia	33.3 (26.3 to 43.1)	38.0 (13.8 to 69.7)*	24.6 (2.8 to 53.2)*	4.2 (2.7 to 5.9)	35.1 (10.3 to 65.9)*	22.0 (-0.5 to 49.8)
Chronic myeloid leukemia	14.7 (11.6 to 19.1)	20.3 (-2.6 to 49.6)	8.6 (-12.1 to 35.1)	1.9 (1.3 to 2.8)	16.8 (-5.4 to 45.4)	5.4 (-14.6 to 31.3)
Other neoplasms	164.1 (133.1 to 202.9)	29.5 (9.2 to 50.4)*	16.9 (-1.4 to 35.8)	12.2 (8.0 to 17.4)	28.0 (7.5 to 49.5)*	15.5 (-3.0 to 35.0)
Cardiovascular diseases	7,254.6 (6,960.4 to 7,449.1)	2.6 (-0.4 to 5.4)	-7.4 (-10.1 to -4.8)*	552.3 (391.6 to 746.1)	6.1 (1.0 to 9.0)*	-4.2 (-8.9 to -1.6)*
Rheumatic heart disease	3,599.4 (3,207.3 to 4,510.8)	-6.9 (-14.0 to -2.2)*	-15.9 (-22.3 to -11.7)*	172.7 (108.0 to 267.2)	-6.6 (-13.8 to -1.4)*	-15.7 (-22.1 to -11.0)*
Rheumatic heart disease, without heart failure	3,568.2 (3,174.0 to 4,478.3)	-7.0 (-14.1 to -2.3)*	-16.0 (-22.5 to -11.8)*	169.0 (105.2 to 261.9)	-7.0 (-14.0 to -1.7)*	-16.0 (-22.4 to -11.3)*
Heart failure fue to rheumatic heart disease aggregate	31.2 (27.9 to 34.7)	14.2 (12.8 to 15.7)*	3.1 (1.8 to 4.5)*	3.8 (2.5 to 5.3)	13.9 (3.2 to 25.6)*	2.9 (-6.8 to 13.4)
Ischemic heart disease	920.3 (802.2 to 1,041.0)	15.2 (14.1 to 16.3)*	4.0 (3.0 to 5.0)*	71.8 (47.8 to 102.0)	15.2 (11.6 to 18.7)*	4.0 (0.8 to 7.2)*
Myocardial infarction episodes aggregate	1.9 (1.4 to 2.5)	6.1 (4.4 to 7.6)*	-4.2 (-5.7 to -2.9)*	0.2 (0.1 to 0.3)	6.0 (4.4 to 7.6)*	-4.2 (-5.7 to -2.9)*
Angina due to ischemic heart disease aggregate	761.2 (642.5 to 881.3)	15.4 (14.1 to 16.7)*	4.2 (3.0 to 5.4)*	53.0 (34.1 to 76.7)	15.5 (11.0 to 19.6)*	4.3 (0.2 to 8.0)*
Heart failure fue to ischemic heart disease aggregate	157.2 (141.6 to 174.2)	14.3 (13.4 to 15.2)*	3.2 (2.4 to 4.0)*	18.6 (12.6 to 26.2)	14.3 (7.7 to 21.5)*	3.2 (-2.7 to 9.7)
Cerebrovascular disease	1,034.2 (1,010.9 to 1,056.1)	12.2 (9.5 to 15.1)*	1.3 (-1.1 to 3.9)	167.2 (114.4 to 230.1)	11.8 (7.8 to 15.7)*	1.0 (-2.7 to 4.4)
Ischemic stroke	370.6 (353.3 to 388.9)	17.9 (17.0 to 18.6)*	6.4 (5.7 to 7.1)*	58.5 (40.1 to 80.9)	17.6 (11.6 to 23.8)*	6.2 (0.8 to 11.8)*
Chronic ischemic stroke aggregate	368.9 (351.3 to 387.3)	17.9 (17.1 to 18.7)*	6.5 (5.7 to 7.2)*	58.2 (39.9 to 80.3)	17.7 (11.7 to 23.9)*	6.2 (0.8 to 11.9)*
Ischemic stroke episodes aggregate	1.7 (1.2 to 2.3)	9.4 (5.0 to 13.4)*	-1.2 (-5.2 to 2.4)	0.4 (0.2 to 0.6)	9.4 (5.0 to 13.4)*	-1.2 (-5.2 to 2.4)
Hemorrhagic stroke	696.0 (664.0 to 729.8)	8.9 (8.2 to 9.6)*	-1.7 (-2.4 to -1.0)*	108.7 (73.8 to 149.2)	9.0 (4.4 to 13.7)*	-1.6 (-5.8 to 2.6)
Chronic hemorrhagic stroke aggregate	692.8 (660.8 to 726.7)	8.9 (8.2 to 9.6)*	-1.6 (-2.3 to -1.0)*	108.0 (73.4 to 148.4)	9.0 (4.4 to 13.7)*	-1.6 (-5.8 to 2.7)
Acute hemorrhagic stroke aggregate	3.2 (2.6 to 3.8)	3.1 (-2.6 to 8.1)	-6.9 (-12.1 to -2.4)*	0.7 (0.4 to 1.0)	3.1 (-2.6 to 8.1)	-6.9 (-12.1 to -2.4)*
Hypertensive heart disease	62.6 (56.7 to 69.2)	14.6 (13.4 to 15.6)*	3.4 (2.4 to 4.4)*	7.4 (5.1 to 10.4)	14.6 (4.6 to 25.9)*	3.5 (-5.6 to 13.7)
Cardiomyopathy and myocarditis	61.5 (53.9 to 70.5)	16.0 (14.8 to 17.2)*	4.8 (3.7 to 5.8)*	6.6 (4.5 to 9.1)	16.2 (9.6 to 23.5)*	4.9 (-1.1 to 11.5)
Acute myocarditis	11.4 (7.5 to 15.8)	12.6 (9.7 to 15.3)*	1.7 (-1.0 to 4.1)	0.6 (0.3 to 0.9)	12.6 (6.5 to 19.7)*	1.7 (-3.8 to 8.1)
Heart failure due to cardiomyopathy aggregate	50.1 (43.8 to 56.8)	16.8 (15.6 to 18.1)*	5.5 (4.4 to 6.6)*	6.0 (4.1 to 8.4)	16.5 (9.3 to 24.8)*	5.2 (-1.3 to 12.7)
Atrial fibrillation and flutter	342.8 (256.8 to 443.9)	8.0 (6.2 to 10.1)*	-2.5 (-4.1 to -0.6)*	29.1 (18.1 to 45.1)	8.2 (1.8 to 15.0)*	-2.3 (-8.1 to 3.9)
Peripheral vascular disease	0.0 (0.0 to 0.0)	0.0 (0.0 to 0.0)	0.0 (0.0 to 0.0)	0.0 (0.0 to 0.0)	0.0 (0.0 to 0.0)	0.0 (0.0 to 0.0)
Endocarditis	4.1 (3.5 to 5.0)	20.7 (17.6 to 23.6)*	9.0 (6.2 to 11.6)*	0.4 (0.3 to 0.6)	20.7 (17.7 to 23.7)*	9.0 (6.3 to 11.7)*
Endocarditis episodes aggregate	1.0 (0.7 to 1.2)	20.3 (13.0 to 27.2)*	8.6 (2.0 to 14.8)*	0.1 (0.0 to 0.1)	20.2 (12.9 to 27.1)*	8.5 (1.9 to 14.7)*
Heart failure due to endocarditis aggregate	3.2 (2.6 to 4.0)	20.8 (17.4 to 24.1)*	9.0 (6.0 to 12.0)*	0.4 (0.2 to 0.6)	20.8 (17.4 to 24.1)*	9.0 (6.0 to 12.0)*
Other cardiovascular and circulatory diseases	1,398.9 (1,164.8 to 1,682.9)	15.0 (13.0 to 17.0)*	3.8 (2.1 to 5.6)*	97.0 (63.3 to 138.3)	15.0 (11.8 to 18.3)*	3.8 (1.0 to 6.8)*
Heart failure due to other cardiovascular diseases aggregate	5.1 (4.4 to 5.8)	15.2 (13.2 to 17.2)*	4.0 (2.2 to 5.8)*	0.6 (0.4 to 0.9)	15.1 (12.8 to 17.6)*	4.0 (1.8 to 6.1)*
Other cardiovascular diseases episodes aggregate	1,393.8 (1,159.7 to 1,677.3)	15.0 (13.0 to 17.0)*	3.8 (2.1 to 5.6)*	96.3 (62.9 to 137.6)	15.0 (11.8 to 18.3)*	3.8 (1.0 to 6.8)*
Chronic respiratory diseases	24,366.9 (23,064.1 to 25,534.3)	9.2 (7.2 to 11.2)*	-1.4 (-3.2 to 0.4)	1,519.8 (1,149.5 to 1,971.6)	8.1 (5.3 to 10.9)*	-2.4 (-4.9 to 0.2)
Chronic obstructive pulmonary disease	7,743.6 (6,694.8 to 8,924.2)	4.1 (1.9 to 6.3)*	-6.0 (-8.0 to -4.1)*	592.9 (480.8 to 700.2)	5.6 (2.1 to 9.1)*	-4.7 (-7.8 to -1.5)*

Cause	Prevalence in thousands (95% confidence interval)			YLDs in thousands (95% confidence interval)		
	2015	Percent change in counts between 2005 and 2015	Percent change in rates between 2005 and 2015	2015	Percent change in counts between 2005 and 2015	Percent change in rates between 2005 and 2015
Chronic obstructive pulmonary disease without heart failure aggregate	7,745.0 (6,694.1 to 8,923.4)	4.1 (2.0 to 6.3)*	-6.0 (-7.9 to -4.0)*	591.7 (479.8 to 698.5)	5.6 (2.1 to 9.1)*	-4.7 (-7.8 to -1.5)*
Severe chronic obstructive pulmonary disease with heart failure aggregate	2.5 (2.2 to 2.8)	6.8 (5.6 to 8.2)*	-3.6 (-4.7 to -2.3)*	1.2 (0.8 to 1.6)	6.8 (5.6 to 8.2)*	-3.6 (-4.7 to -2.3)*
Pneumoconiosis	176.6 (167.1 to 185.9)	18.8 (12.1 to 26.3)*	7.2 (1.2 to 14.0)*	34.9 (22.1 to 50.7)	15.6 (7.9 to 23.7)*	4.4 (-2.6 to 11.7)
Silicosis	23.9 (20.5 to 27.7)	-9.0 (-12.0 to -6.4)*	-17.9 (-20.6 to -15.5)*	4.0 (2.4 to 5.9)	-8.3 (-17.1 to 1.0)	-17.2 (-25.1 to -8.8)*
Silicosis without heart failure aggregate	23.9 (20.5 to 27.7)	-9.0 (-12.0 to -6.4)*	-17.9 (-20.6 to -15.5)*	4.0 (2.4 to 5.9)	-8.3 (-17.1 to 1.0)	-17.2 (-25.1 to -8.8)*
Severe silicosis with heart failure aggregate	0.0 (0.0 to 0.0)	0.0 (0.0 to 0.0)	0.0 (0.0 to 0.0)	0.0 (0.0 to 0.0)	0.0 (0.0 to 0.0)	0.0 (0.0 to 0.0)
Asbestosis	4.6 (3.8 to 5.3)	4.0 (1.0 to 7.0)*	-6.1 (-8.8 to -3.4)*	0.8 (0.5 to 1.1)	4.0 (1.0 to 7.0)*	-6.1 (-8.8 to -3.4)*
Asbestosis without heart failure aggregate	4.6 (3.8 to 5.3)	4.0 (1.0 to 7.0)*	-6.1 (-8.8 to -3.4)*	0.8 (0.5 to 1.1)	4.0 (1.0 to 7.0)*	-6.1 (-8.8 to -3.4)*
Severe asbestosis with heart failure aggregate	0.0 (0.0 to 0.0)	0.0 (0.0 to 0.0)	0.0 (0.0 to 0.0)	0.0 (0.0 to 0.0)	0.0 (0.0 to 0.0)	0.0 (0.0 to 0.0)
Coal workers pneumoconiosis	2.3 (1.9 to 2.7)	22.0 (17.4 to 26.5)*	10.2 (6.0 to 14.2)*	0.4 (0.2 to 0.6)	22.0 (17.4 to 26.5)*	10.2 (6.0 to 14.2)*
Coal workers pneumoconiosis without heart failure aggregate	2.3 (1.9 to 2.7)	22.0 (17.4 to 26.5)*	10.2 (6.0 to 14.2)*	0.4 (0.2 to 0.6)	22.0 (17.4 to 26.5)*	10.2 (6.0 to 14.2)*
Severe coal workers pneumoconiosis with heart failure aggregate	0.0 (0.0 to 0.0)	0.0 (0.0 to 0.0)	0.0 (0.0 to 0.0)	0.0 (0.0 to 0.0)	0.0 (0.0 to 0.0)	0.0 (0.0 to 0.0)
Other pneumoconiosis	188.8 (166.0 to 214.3)	20.4 (16.6 to 23.9)*	8.7 (5.3 to 11.9)*	29.8 (18.7 to 43.5)	20.0 (10.6 to 30.0)*	8.4 (-0.1 to 17.4)
Other pneumoconiosis without heart failure aggregate	188.8 (166.0 to 214.3)	20.4 (16.6 to 23.9)*	8.7 (5.3 to 11.9)*	29.8 (18.7 to 43.5)	20.0 (10.6 to 30.0)*	8.4 (-0.1 to 17.4)
Severe other pneumoconiosis with heart failure aggregate	0.0 (0.0 to 0.0)	0.0 (0.0 to 0.0)	0.0 (0.0 to 0.0)	0.0 (0.0 to 0.0)	0.0 (0.0 to 0.0)	0.0 (0.0 to 0.0)
Asthma	17,115.2 (13,950.0 to 20,738.9)	11.4 (7.7 to 15.0)*	0.6 (-2.8 to 3.9)	764.7 (480.0 to 1,126.2)	11.5 (7.6 to 15.3)*	0.6 (-2.8 to 4.1)
Asymptomatic asthma	5,182.1 (4,040.1 to 6,479.8)	11.4 (7.7 to 15.0)*	0.6 (-2.8 to 3.9)	0.0 (0.0 to 0.0)	0.0 (0.0 to 0.0)	0.0 (0.0 to 0.0)
Symptomatic asthma aggregate	11,933.1 (9,578.7 to 14,573.1)	11.4 (7.7 to 15.0)*	0.6 (-2.8 to 3.9)	764.7 (480.0 to 1,126.2)	11.5 (7.6 to 15.3)*	0.6 (-2.8 to 4.1)
Interstitial lung disease and pulmonary sarcoidosis	70.9 (61.2 to 82.2)	12.1 (9.3 to 14.7)*	1.2 (-1.3 to 3.6)	8.9 (5.4 to 13.3)	12.2 (5.2 to 19.5)*	1.3 (-5.0 to 7.9)
Interstitial lung disease and pulmonary sarcoidosis without heart failure aggregate	69.2 (59.5 to 80.4)	11.9 (9.1 to 14.6)*	1.1 (-1.5 to 3.5)	8.0 (4.8 to 12.2)	11.7 (3.9 to 19.7)*	0.9 (-6.2 to 8.1)
Severe interstitial lung disease and pulmonary sarcoidosis with heart failure aggregate	1.7 (1.5 to 2.0)	17.0 (15.2 to 18.9)*	5.6 (4.1 to 7.3)*	0.8 (0.6 to 1.1)	17.0 (15.2 to 18.9)*	5.6 (4.1 to 7.3)*
Other chronic respiratory diseases	--	--	--	118.4 (94.0 to 144.0)	-1.0 (-10.5 to 9.7)	-10.6 (-19.2 to -1.0)*
Cirrhosis and other chronic liver diseases	108.3 (102.1 to 114.5)	25.8 (17.0 to 35.8)*	13.6 (5.6 to 22.6)*	23.8 (16.3 to 33.3)	18.6 (12.7 to 25.7)*	7.1 (1.7 to 13.5)*
Cirrhosis and other chronic liver diseases due to hepatitis B	39.0 (33.6 to 44.8)	10.2 (7.1 to 12.8)*	-0.5 (-3.3 to 1.8)	6.7 (4.5 to 9.7)	10.1 (-0.8 to 22.8)	-0.6 (-10.5 to 10.9)
Cirrhosis and other chronic liver diseases due to hepatitis C	26.7 (22.6 to 31.2)	21.4 (18.4 to 24.4)*	9.6 (6.9 to 12.3)*	4.6 (3.0 to 6.6)	21.3 (6.6 to 37.6)*	9.6 (-3.8 to 24.3)
Cirrhosis and other chronic liver diseases due to alcohol use	46.3 (41.1 to 51.8)	24.5 (22.2 to 26.7)*	12.4 (10.3 to 14.4)*	7.9 (5.2 to 11.1)	24.7 (13.7 to 37.5)*	12.6 (2.7 to 24.1)*
Cirrhosis and other chronic liver diseases due to other causes	26.9 (22.6 to 31.9)	19.5 (16.9 to 21.9)*	7.9 (5.6 to 10.1)*	4.7 (3.0 to 6.8)	19.4 (5.2 to 35.5)*	7.8 (-5.0 to 22.4)
Digestive diseases	11,743.5 (11,483.4 to 12,036.9)	2.0 (-0.6 to 4.7)	-7.9 (-10.3 to -5.4)*	705.3 (495.3 to 954.5)	4.3 (1.8 to 6.7)*	-5.8 (-8.1 to -3.6)*
Peptic ulcer disease	2,696.4 (2,628.0 to 2,771.0)	-10.9 (-13.1 to -8.5)*	-19.6 (-21.5 to -17.4)*	93.3 (62.9 to 131.8)	-14.6 (-17.9 to -11.5)*	-22.9 (-25.9 to -20.1)*
Peptic ulcer disease, symptomatic episodes	211.2 (164.4 to 266.4)	-10.4 (-15.6 to -5.3)*	-19.1 (-23.8 to -14.5)*	23.1 (14.5 to 34.2)	-10.2 (-17.0 to -3.6)*	-19.0 (-25.0 to -13.0)*
Anemia due to peptic ulcer disease aggregate	2,485.3 (2,439.5 to 2,535.4)	-11.0 (-13.3 to -8.4)*	-19.6 (-21.7 to -17.3)*	70.2 (46.9 to 102.5)	-16.0 (-19.6 to -12.3)*	-24.1 (-27.5 to -20.8)*
Gastritis and duodenitis	6,406.4 (6,143.4 to 6,642.8)	5.5 (0.9 to 9.8)*	-4.8 (-8.9 to -0.8)*	219.8 (148.3 to 312.3)	4.2 (-1.4 to 10.1)	-5.9 (-11.0 to -0.6)*
Gastritis and duodenitis, symptomatic episodes	180.4 (133.7 to 241.1)	7.3 (2.8 to 10.1)*	-3.1 (-7.2 to -0.6)*	19.7 (11.9 to 30.3)	7.4 (0.6 to 14.0)*	-3.0 (-9.1 to 3.0)
Anemia due to gastritis and duodenitis aggregate	6,226.0 (5,970.0 to 6,456.0)	5.4 (0.8 to 9.9)*	-4.8 (-9.0 to -0.7)*	200.1 (133.7 to 287.4)	3.9 (-2.3 to 10.2)	-6.2 (-11.8 to -0.5)*
Appendicitis	37.8 (31.4 to 45.2)	19.3 (17.4 to 20.9)*	7.7 (6.0 to 9.2)*	11.6 (7.4 to 16.7)	19.5 (6.1 to 32.9)*	7.9 (-4.2 to 20.0)
Paralytic ileus and intestinal obstruction	5.5 (4.0 to 7.4)	18.8 (11.3 to 25.5)*	7.2 (0.5 to 13.3)*	1.8 (1.1 to 2.7)	18.7 (11.3 to 25.5)*	7.2 (0.5 to 13.3)*
Inguinal, femoral, and abdominal hernia	1,186.6 (835.8 to 1,687.5)	5.5 (-0.5 to 11.1)	-4.8 (-10.2 to 0.3)	12.8 (5.7 to 25.7)	5.6 (-1.0 to 11.5)	-4.7 (-10.6 to 0.7)
Inflammatory bowel disease	1,007.7 (907.8 to 1,102.3)	8.7 (8.1 to 9.2)*	-1.9 (-2.4 to -1.4)*	219.7 (149.3 to 303.5)	8.8 (6.7 to 10.9)*	-1.8 (-3.7 to 0.2)
Vascular intestinal disorders	1.5 (1.1 to 1.9)	18.4 (11.5 to 25.2)*	6.9 (0.7 to 13.0)*	0.5 (0.3 to 0.7)	18.4 (11.5 to 25.2)*	6.9 (0.7 to 13.0)*
Gallbladder and biliary diseases	479.1 (358.8 to 618.6)	10.5 (4.4 to 16.7)*	-0.2 (-5.7 to 5.3)	52.3 (31.2 to 78.5)	10.6 (3.6 to 17.7)*	-0.1 (-6.4 to 6.3)
Pancreatitis	80.1 (62.4 to 101.0)	18.4 (12.2 to 23.7)*	6.9 (1.3 to 11.7)*	24.4 (15.4 to 36.0)	18.3 (7.4 to 29.5)*	6.8 (-3.0 to 16.9)
Other digestive diseases	--	--	--	69.1 (47.9 to 95.7)	10.6 (3.7 to 20.4)*	-0.1 (-6.4 to 8.7)
Neurological disorders	259,679.9 (243,539.4 to 273,480.5)	12.2 (10.0 to 14.3)*	1.3 (-0.7 to 3.2)	5,600.0 (3,618.4 to 8,039.1)	12.0 (9.6 to 14.2)*	1.1 (-1.1 to 3.1)
Alzheimer disease and other dementias	0.0 (0.0 to 0.0)	0.0 (0.0 to 0.0)	0.0 (0.0 to 0.0)	0.0 (0.0 to 0.0)	0.0 (0.0 to 0.0)	0.0 (0.0 to 0.0)
Parkinson disease	78.4 (67.3 to 89.6)	1.3 (-3.8 to 6.5)	-8.6 (-13.2 to -3.8)*	10.4 (6.9 to 14.7)	1.4 (-7.4 to 10.4)	-8.4 (-16.4 to -0.4)*
Epilepsy	1,605.9 (1,384.8 to 1,875.3)	9.0 (3.2 to 14.7)*	-1.6 (-6.8 to 3.5)	437.5 (296.4 to 588.3)	-7.3 (-13.3 to -1.2)*	-16.3 (-21.7 to -10.8)*
Multiple sclerosis	184.5 (165.5 to 203.9)	12.9 (10.5 to 15.6)*	2.0 (-0.2 to 4.4)	63.0 (44.4 to 82.2)	12.8 (7.8 to 18.3)*	1.9 (-2.6 to 6.8)
Motor neuron disease	7.8 (7.0 to 8.8)	9.1 (6.9 to 11.6)*	-1.5 (-3.5 to 0.7)	1.7 (1.2 to 2.2)	9.1 (6.9 to 11.6)*	-1.5 (-3.5 to 0.7)
Migraine	111,769.3 (88,559.9 to 140,271.4)	13.5 (11.6 to 15.3)*	2.5 (0.8 to 4.1)*	3,879.2 (2,316.0 to 5,912.2)	13.7 (11.8 to 15.7)*	2.7 (0.9 to 4.4)*
Tension-type headache	190,095.2 (139,044.0 to 254,131.9)	11.8 (10.3 to 13.2)*	0.9 (-0.4 to 2.2)	287.0 (122.6 to 548.3)	11.8 (10.2 to 13.6)*	1.0 (-0.5 to 2.6)
Medication overuse headache	5,729.1 (3,754.2 to 8,402.4)	16.0 (5.1 to 26.7)*	4.8 (-5.1 to 14.4)	917.2 (472.4 to 1,562.6)	16.2 (5.4 to 26.6)*	4.9 (-4.8 to 14.4)
Other neurological disorders	0.7 (0.4 to 1.1)	13.4 (11.6 to 14.9)*	2.4 (0.8 to 3.7)*	3.9 (2.7 to 5.3)	6.1 (0.4 to 11.5)*	-4.2 (-9.3 to 0.7)
Mental and substance use disorders	98,786.4 (96,297.5 to 101,919.2)	12.8 (11.8 to 13.8)*	1.9 (0.9 to 2.8)*	15,425.2 (10,997.4 to 19,879.1)	13.7 (12.9 to 14.5)*	2.7 (1.9 to 3.4)*
Schizophrenia	2,877.1 (2,396.7 to 3,419.6)	7.8 (7.0 to 8.5)*	-2.7 (-3.4 to -2.1)*	1,892.3 (1,340.1 to 2,437.6)	7.8 (6.3 to 9.4)*	-2.6 (-4.1 to -1.3)*
Alcohol use disorders	8,625.5 (6,990.4 to 10,394.9)	9.1 (4.7 to 13.9)*	-1.5 (-5.4 to 2.8)	871.9 (551.7 to 1,297.8)	9.1 (4.5 to 14.2)*	-1.4 (-5.7 to 3.1)
Alcohol dependence aggregate	8,556.6 (6,920.5 to 10,323.4)	9.1 (4.7 to 13.9)*	-1.5 (-5.5 to 2.9)	868.4 (548.7 to 1,293.7)	9.1 (4.5 to 14.3)*	-1.5 (-5.7 to 3.2)
Fetal alcohol syndrome aggregate	68.9 (64.3 to 73.1)	9.9 (9.1 to 10.7)*	-0.8 (-1.5 to -0.0)*	3.6 (2.3 to 5.1)	10.0 (2.9 to 17.4)*	-0.7 (-7.1 to 6.0)
Drug use disorders	6,074.5 (5,867.0 to 6,264.8)	15.6 (13.7 to 17.6)*	4.4 (2.7 to 6.2)*	1,427.8 (982.1 to 1,868.2)	20.6 (17.7 to 23.6)*	8.9 (6.3 to 11.6)*
Opioid use disorders	2,550.7 (2,064.7 to 3,020.5)	20.2 (16.9 to 23.7)*	8.5 (5.6 to 11.6)*	1,074.3 (709.7 to 1,438.0)	20.3 (16.4 to 24.1)*	8.6 (5.1 to 12.1)*
Asymptomatic opioid dependence	414.1 (283.4 to 584.7)	20.2 (16.9 to 23.7)*	8.5 (5.6 to 11.6)*	0.0 (0.0 to 0.0)	0.0 (0.0 to 0.0)	0.0 (0.0 to 0.0)
Symptomatic opioid dependence aggregate	2,136.6 (1,729.3 to 2,562.2)	20.2 (16.9 to 23.7)*	8.5 (5.6 to 11.6)*	1,074.3 (709.7 to 1,438.0)	20.3 (16.4 to 24.1)*	8.6 (5.1 to 12.1)*
Cocaine use disorders	412.1 (341.6 to 486.9)	23.6 (20.3 to 27.2)*	11.6 (8.6 to 14.9)*	56.8 (36.1 to 81.8)	23.4 (16.6 to 30.4)*	11.4 (5.3 to 17.7)*
Asymptomatic cocaine dependence	206.5 (159.7 to 258.4)	23.6 (20.3 to 27.2)*	11.6 (8.6 to 14.9)*	0.0 (0.0 to 0.0)	0.0 (0.0 to 0.0)	0.0 (0.0 to 0.0)

Cause	Prevalence in thousands (95% confidence interval)			YLDs in thousands (95% confidence interval)		
	2015	Percent change in counts between 2005 and 2015	Percent change in rates between 2005 and 2015	2015	Percent change in counts between 2005 and 2015	Percent change in rates between 2005 and 2015
Symptomatic cocaine dependence aggregate	205.6 (159.1 to 257.7)	23.6 (20.3 to 27.2)*	11.6 (8.6 to 14.9)*	56.8 (36.1 to 81.8)	23.4 (16.6 to 30.4)*	11.4 (5.3 to 17.7)*
Amphetamine use disorders	886.6 (708.2 to 1,102.3)	27.5 (23.5 to 31.5)*	15.1 (11.5 to 18.8)*	117.0 (67.7 to 178.6)	27.4 (20.7 to 34.3)*	15.0 (9.0 to 21.3)*
Asymptomatic amphetamine dependence	485.4 (360.3 to 631.1)	27.5 (23.5 to 31.5)*	15.1 (11.5 to 18.8)*	0.0 (0.0 to 0.0)	0.0 (0.0 to 0.0)	0.0 (0.0 to 0.0)
Symptomatic amphetamine dependence aggregate	401.2 (286.4 to 536.4)	27.5 (23.5 to 31.5)*	15.1 (11.5 to 18.8)*	117.0 (67.7 to 178.6)	27.4 (20.7 to 34.3)*	15.0 (9.0 to 21.3)*
Cannabis use disorders	2,293.9 (1,956.7 to 2,678.2)	6.2 (4.8 to 7.8)*	-4.1 (-5.4 to -2.7)*	66.8 (42.5 to 97.4)	6.2 (2.6 to 9.9)*	-4.1 (-7.4 to -0.8)*
Asymptomatic cannabis dependence	1,326.8 (1,113.3 to 1,571.8)	6.2 (4.8 to 7.8)*	-4.1 (-5.4 to -2.7)*	0.0 (0.0 to 0.0)	0.0 (0.0 to 0.0)	0.0 (0.0 to 0.0)
Symptomatic cannabis dependence aggregate	967.1 (801.3 to 1,171.9)	6.2 (4.8 to 7.8)*	-4.1 (-5.4 to -2.7)*	66.8 (42.5 to 97.4)	6.2 (2.6 to 9.9)*	-4.1 (-7.4 to -0.8)*
Other drug use disorders	--	--	--	112.9 (69.5 to 165.3)	26.1 (19.7 to 33.2)*	13.9 (8.1 to 20.2)*
Depressive disorders	29,865.8 (27,938.4 to 32,072.6)	14.7 (12.4 to 17.0)*	3.6 (1.5 to 5.6)*	5,430.2 (3,451.3 to 7,759.5)	15.6 (14.3 to 16.7)*	4.4 (3.2 to 5.4)*
Major depressive disorder	21,686.5 (15,400.6 to 28,340.0)	16.2 (14.9 to 17.2)*	4.9 (3.8 to 5.8)*	4,514.8 (2,724.6 to 6,660.7)	16.4 (15.0 to 17.7)*	5.1 (3.8 to 6.3)*
Dysthymia	9,317.0 (7,502.9 to 11,562.2)	11.7 (10.0 to 13.4)*	0.8 (-0.7 to 2.4)	915.5 (597.0 to 1,335.7)	11.7 (9.9 to 13.7)*	0.9 (-0.7 to 2.7)
Bipolar disorder	5,718.7 (4,673.8 to 6,952.5)	12.7 (12.4 to 13.0)*	1.8 (1.5 to 2.1)*	1,182.1 (702.2 to 1,796.0)	12.8 (11.5 to 14.3)*	1.9 (0.7 to 3.2)*
Anxiety disorders	24,252.1 (19,677.9 to 30,291.6)	14.4 (12.0 to 16.8)*	3.3 (1.1 to 5.5)*	2,261.0 (1,461.5 to 3,311.3)	14.5 (11.9 to 17.2)*	3.4 (1.0 to 5.8)*
Eating disorders	1,085.3 (1,010.4 to 1,155.0)	21.7 (18.3 to 25.3)*	9.9 (6.8 to 13.1)*	230.0 (149.7 to 335.1)	22.0 (19.1 to 25.1)*	10.2 (7.5 to 13.0)*
Anorexia nervosa	330.4 (269.0 to 405.1)	15.9 (13.0 to 19.0)*	4.6 (2.1 to 7.4)*	70.0 (45.1 to 102.3)	16.0 (10.8 to 21.3)*	4.7 (0.1 to 9.5)*
Bulimia nervosa	759.8 (588.7 to 980.4)	24.9 (22.7 to 27.1)*	12.7 (10.8 to 14.8)*	160.0 (99.1 to 244.6)	24.8 (21.5 to 28.4)*	12.7 (9.7 to 15.9)*
Autistic spectrum disorders	4,986.7 (4,729.8 to 5,202.0)	10.9 (8.8 to 12.8)*	0.1 (-1.7 to 1.9)	809.7 (542.4 to 1,106.3)	11.1 (9.9 to 12.3)*	0.3 (-0.8 to 1.4)
Autism	1,984.2 (1,671.0 to 2,357.1)	11.3 (11.0 to 11.6)*	0.5 (0.2 to 0.8)*	510.2 (326.4 to 721.5)	11.4 (9.7 to 13.1)*	0.6 (-0.9 to 2.1)
Asperger syndrome and other autistic spectrum disorders	2,983.0 (2,509.7 to 3,563.5)	10.5 (10.3 to 10.7)*	-0.2 (-0.4 to -0.0)*	299.4 (198.1 to 438.5)	10.6 (9.2 to 12.0)*	-0.2 (-1.4 to 1.1)
Attention-deficit/hyperactivity disorder	3,188.1 (2,720.7 to 3,700.1)	4.7 (3.4 to 6.0)*	-5.5 (-6.7 to -4.3)*	38.5 (22.6 to 59.3)	4.7 (2.1 to 7.4)*	-5.5 (-7.8 to -3.1)*
Conduct disorder	0.0 (0.0 to 0.0)	0.0 (0.0 to 0.0)	0.0 (0.0 to 0.0)	0.0 (0.0 to 0.0)	0.0 (0.0 to 0.0)	0.0 (0.0 to 0.0)
Idiopathic developmental intellectual disability	7,685.3 (4,414.8 to 10,821.2)	13.7 (12.4 to 14.7)*	2.7 (1.5 to 3.6)*	286.2 (124.6 to 502.7)	12.1 (9.3 to 13.7)*	1.2 (-1.4 to 2.7)
Other mental and substance use disorders	13,058.3 (12,830.2 to 13,272.2)	10.8 (8.3 to 13.4)*	-0.0 (-2.2 to 2.4)	995.4 (687.8 to 1,352.0)	10.9 (8.0 to 13.9)*	0.1 (-2.5 to 2.8)
Diabetes, urogenital, blood, and endocrine diseases	231,470.7 (229,378.1 to 233,650.8)	11.4 (10.7 to 12.1)*	0.6 (-0.0 to 1.2)	3,957.0 (2,783.2 to 5,451.0)	11.0 (9.0 to 12.9)*	0.2 (-1.6 to 1.9)
Diabetes mellitus	20,948.0 (17,818.3 to 24,117.6)	15.3 (11.0 to 19.3)*	4.1 (0.2 to 7.7)*	1,463.6 (963.5 to 2,097.9)	17.9 (13.3 to 22.0)*	6.4 (2.3 to 10.2)*
Uncomplicated diabetes mellitus	15,397.6 (12,955.6 to 18,127.2)	12.5 (8.0 to 16.9)*	1.6 (-2.5 to 5.5)	731.7 (449.9 to 1,096.5)	12.5 (8.0 to 16.9)*	1.6 (-2.5 to 5.5)
Neuropathy and other complications of diabetes mellitus aggregate	5,391.7 (4,194.9 to 6,856.6)	23.7 (18.8 to 28.8)*	11.7 (7.3 to 16.3)*	722.8 (459.0 to 1,053.1)	23.8 (18.7 to 29.0)*	11.8 (7.2 to 16.5)*
Vision loss due to diabetes mellitus aggregate	158.6 (104.9 to 233.4)	18.6 (15.9 to 21.5)*	7.1 (4.7 to 9.7)*	9.1 (5.3 to 14.9)	18.5 (12.8 to 24.1)*	7.0 (1.8 to 12.0)*
Acute glomerulonephritis	5.9 (4.3 to 7.7)	-18.5 (-23.7 to -14.4)*	-26.4 (-31.1 to -22.7)*	0.3 (0.2 to 0.5)	-18.0 (-25.6 to -11.0)*	-26.0 (-32.8 to -19.6)*
Chronic kidney disease	17,534.5 (16,036.1 to 18,727.2)	13.9 (10.7 to 17.1)*	2.8 (-0.1 to 5.7)	485.1 (343.9 to 654.9)	10.7 (8.4 to 12.9)*	-0.1 (-2.1 to 1.9)
Chronic kidney disease due to diabetes mellitus	6,233.7 (3,934.1 to 8,802.6)	12.2 (8.6 to 15.6)*	1.3 (-1.9 to 4.4)	172.3 (109.5 to 253.1)	8.9 (4.5 to 13.4)*	-1.7 (-5.6 to 2.4)
Stage III chronic kidney disease due to diabetes mellitus aggregate	5,623.7 (3,488.3 to 8,082.5)	12.6 (8.7 to 16.1)*	1.6 (-1.8 to 4.8)	12.0 (8.2 to 16.9)	2.6 (-9.5 to 15.8)	-7.3 (-18.3 to 4.6)
Stage IV chronic kidney disease due to diabetes mellitus aggregate	373.3 (231.5 to 564.2)	10.5 (7.7 to 13.3)*	-0.2 (-2.8 to 2.3)	48.7 (27.8 to 78.1)	9.4 (4.7 to 14.8)*	-1.2 (-5.5 to 3.6)
Stage V chronic kidney disease untreated due to diabetes mellitus	198.5 (130.1 to 290.6)	10.0 (7.0 to 12.8)*	-0.6 (-3.3 to 1.9)	105.0 (60.8 to 160.4)	10.2 (4.0 to 16.8)*	-0.5 (-6.1 to 5.4)
End-stage chronic kidney disease due to diabetes aggregate	38.2 (26.5 to 51.4)	-5.5 (-8.9 to -2.2)*	-14.7 (-17.8 to -11.7)*	6.6 (4.0 to 10.0)	-1.4 (-8.8 to 5.7)	-11.0 (-17.6 to -4.6)*
Chronic kidney disease due to hypertension	4,450.9 (2,758.6 to 6,473.8)	15.9 (11.9 to 20.2)*	4.6 (1.0 to 8.5)*	57.9 (36.9 to 84.3)	10.0 (3.0 to 17.5)*	-0.6 (-7.0 to 6.1)
Stage III chronic kidney disease due to hypertension aggregate	4,224.6 (2,548.7 to 6,168.0)	16.1 (11.9 to 20.6)*	4.8 (1.1 to 8.9)*	3.5 (2.4 to 4.9)	-2.0 (-14.7 to 12.3)	-11.6 (-23.0 to 1.4)
Stage IV chronic kidney disease due to hypertension aggregate	152.5 (92.1 to 264.2)	15.7 (6.9 to 25.8)*	4.5 (-3.4 to 13.6)	19.6 (10.7 to 33.6)	14.0 (4.4 to 23.6)*	3.0 (-5.8 to 11.6)
Stage V chronic kidney disease untreated due to hypertension	61.5 (39.1 to 88.2)	9.7 (4.6 to 14.6)*	-1.0 (-5.6 to 3.5)	32.6 (18.1 to 50.9)	9.9 (-0.2 to 21.8)	-0.8 (-9.9 to 10.0)
End-stage chronic kidney disease due to hypertension aggregate	12.3 (8.5 to 17.4)	-4.4 (-12.3 to 2.9)	-13.7 (-20.8 to -7.1)*	2.2 (1.3 to 3.3)	0.2 (-8.0 to 8.7)	-9.5 (-16.9 to -1.8)*
Chronic kidney disease due to glomerulonephritis	2,367.1 (1,756.6 to 3,240.3)	11.7 (7.2 to 17.2)*	0.9 (-3.2 to 5.8)	128.1 (80.8 to 187.1)	12.0 (7.1 to 17.1)*	1.2 (-3.3 to 5.7)
Stage III chronic kidney disease due to glomerulonephritis aggregate	1,930.0 (1,355.0 to 2,787.3)	11.8 (6.4 to 18.3)*	1.0 (-3.9 to 6.9)	6.3 (4.1 to 9.6)	10.1 (-3.7 to 26.1)	-0.6 (-13.1 to 13.9)
Stage IV chronic kidney disease due to glomerulonephritis aggregate	255.5 (178.3 to 331.3)	11.4 (6.2 to 16.8)*	0.6 (-4.1 to 5.5)	33.9 (20.6 to 50.4)	10.4 (3.8 to 17.8)*	-0.3 (-6.3 to 6.4)
Stage V chronic kidney disease untreated due to glomerulonephritis	158.8 (97.7 to 231.2)	13.4 (10.3 to 16.4)*	2.4 (-0.4 to 5.1)	83.6 (45.3 to 130.8)	13.5 (6.8 to 20.3)*	2.5 (-3.6 to 8.6)
End-stage chronic kidney disease due to glomerulonephritis aggregate	22.9 (15.0 to 32.8)	-2.5 (-6.7 to 1.9)	-11.9 (-15.7 to -8.0)*	4.2 (2.5 to 6.5)	0.6 (-7.3 to 8.9)	-9.1 (-16.3 to -1.7)*
Chronic kidney disease due to other causes	4,730.0 (2,800.0 to 7,408.6)	15.3 (12.6 to 18.1)*	4.1 (1.6 to 6.7)*	126.8 (74.3 to 194.0)	12.1 (7.5 to 17.0)*	1.2 (-2.9 to 5.6)
Stage III chronic kidney disease due to other causes aggregate	4,272.5 (2,515.7 to 6,767.2)	15.6 (12.8 to 18.6)*	4.4 (1.8 to 7.1)*	5.6 (3.5 to 8.4)	11.4 (-3.3 to 28.9)	0.6 (-12.7 to 16.4)
Stage IV chronic kidney disease due to other causes aggregate	281.2 (167.1 to 440.5)	13.8 (11.6 to 15.8)*	2.7 (0.7 to 4.6)*	36.8 (20.0 to 59.0)	11.9 (6.6 to 18.0)*	1.1 (-3.8 to 6.5)
Stage V chronic kidney disease untreated due to other causes	151.1 (91.3 to 228.4)	12.8 (10.8 to 14.7)*	1.9 (0.0 to 3.6)*	79.8 (43.0 to 128.2)	13.0 (6.3 to 20.0)*	2.0 (-4.1 to 8.4)
End-stage chronic kidney disease due to other causes aggregate	25.1 (15.1 to 37.0)	-2.3 (-5.6 to 1.4)	-11.8 (-14.8 to -8.4)*	4.6 (2.5 to 7.6)	0.9 (-7.7 to 10.7)	-8.9 (-16.7 to -0.0)*
Urinary diseases and male infertility	27,865.7 (26,487.3 to 29,212.9)	17.3 (14.8 to 19.6)*	5.9 (3.7 to 8.0)*	52.3 (27.7 to 95.8)	17.5 (13.0 to 22.2)*	6.1 (2.0 to 10.4)*
Interstitial nephritis and urinary tract infections	196.0 (181.0 to 212.1)	17.0 (16.4 to 17.9)*	5.7 (5.1 to 6.4)*	6.6 (4.1 to 10.0)	17.4 (11.1 to 24.0)*	6.0 (0.3 to 12.0)*
Urolithiasis	22,621.4 (19,563.7 to 26,563.7)	16.4 (13.8 to 19.1)*	5.1 (2.7 to 7.6)*	8.0 (4.9 to 11.8)	12.1 (2.8 to 22.0)*	1.2 (-7.2 to 10.2)
Benign prostatic hyperplasia	0.0 (0.0 to 0.0)	0.0 (0.0 to 0.0)	0.0 (0.0 to 0.0)	0.0 (0.0 to 0.0)	0.0 (0.0 to 0.0)	0.0 (0.0 to 0.0)
Male infertility	5,501.9 (3,858.0 to 7,869.9)	21.0 (16.1 to 26.3)*	9.3 (4.8 to 14.1)*	32.3 (12.3 to 71.5)	20.9 (15.6 to 26.1)*	9.2 (4.4 to 13.8)*
Other urinary diseases	--	--	--	5.4 (3.4 to 7.9)	7.3 (-2.8 to 18.6)	-3.1 (-12.2 to 7.1)
Gynecological diseases	98,258.4 (96,255.5 to 100,416.7)	9.9 (8.7 to 11.2)*	-0.7 (-1.9 to 0.4)	1,466.7 (989.2 to 2,122.3)	8.6 (6.4 to 10.4)*	-2.0 (-3.9 to -0.4)*
Uterine fibroids	15,459.5 (13,778.0 to 17,290.9)	10.8 (10.1 to 11.4)*	0.0 (-0.6 to 0.6)	338.9 (212.1 to 528.0)	8.2 (6.1 to 10.2)*	-2.3 (-4.2 to -0.5)*
Uterine fibroids cases aggregate	8,172.2 (6,895.4 to 9,585.8)	11.4 (9.4 to 13.5)*	0.6 (-1.2 to 2.5)	35.7 (16.4 to 67.9)	12.3 (7.8 to 17.4)*	1.4 (-2.7 to 6.0)
Mild abdominal pain with anemia due to uterine fibroids aggregate	7,287.2 (6,825.8 to 7,752.9)	10.1 (8.4 to 11.8)*	-0.6 (-2.1 to 1.0)	303.3 (192.6 to 460.6)	7.7 (5.4 to 10.2)*	-2.7 (-4.9 to -0.5)*
Polycystic ovarian syndrome	13,287.7 (9,767.3 to 17,330.6)	11.4 (10.8 to 12.0)*	0.6 (0.0 to 1.1)*	117.6 (50.4 to 233.9)	11.4 (10.3 to 12.5)*	0.6 (-0.4 to 1.6)
Polycystic ovarian syndrome cases aggregate	10,363.7 (7,372.1 to 13,837.1)	11.2 (10.7 to 11.8)*	0.4 (-0.1 to 0.9)	89.8 (38.8 to 180.9)	11.2 (10.2 to 12.3)*	0.4 (-0.5 to 1.4)
Hirsutism and infertility due to polycystic ovarian syndrome aggregate	2,924.0 (1,865.4 to 4,325.8)	12.1 (10.9 to 13.3)*	1.2 (0.1 to 2.3)*	27.8 (10.6 to 61.0)	11.9 (8.6 to 14.7)*	1.1 (-1.9 to 3.6)

Cause	Prevalence in thousands (95% confidence interval)			YLDs in thousands (95% confidence interval)		
	2015	Percent change in counts between 2005 and 2015	Percent change in rates between 2005 and 2015	2015	Percent change in counts between 2005 and 2015	Percent change in rates between 2005 and 2015
Female infertility	11,675.4 (6,592.8 to 19,262.0)	24.2 (15.9 to 34.9)*	12.1 (4.7 to 21.8)*	64.4 (22.4 to 151.6)	24.3 (16.0 to 34.7)*	12.3 (4.7 to 21.6)*
Endometriosis	1,971.6 (1,509.9 to 2,548.5)	7.7 (7.0 to 8.5)*	-2.7 (-3.4 to -2.0)*	183.6 (112.9 to 271.6)	7.8 (5.3 to 10.2)*	-2.6 (-5.0 to -0.5)*
Endometriosis cases aggregate	1,850.5 (1,414.6 to 2,406.9)	7.7 (6.9 to 8.5)*	-2.8 (-3.5 to -2.0)*	171.5 (106.0 to 254.3)	7.8 (5.1 to 10.2)*	-2.7 (-5.1 to -0.5)*
Abdominal pain and infertility due to endometriosis aggregate	121.1 (81.5 to 173.2)	8.5 (7.2 to 9.9)*	-2.0 (-3.2 to -0.8)*	12.1 (6.5 to 19.3)	8.6 (2.5 to 14.7)*	-2.0 (-7.5 to 3.5)
Genital prolapse	8,015.7 (6,235.4 to 10,116.6)	7.7 (3.7 to 12.0)*	-2.8 (-6.4 to 1.1)	25.5 (11.9 to 48.3)	7.9 (3.3 to 12.8)*	-2.6 (-6.7 to 1.8)
Premenstrual syndrome	58,792.5 (52,657.9 to 64,599.3)	7.4 (2.2 to 11.3)*	-3.0 (-7.7 to 0.5)	495.2 (301.0 to 727.7)	7.5 (2.4 to 11.5)*	-2.9 (-7.5 to 0.7)
Other gynecological diseases	6,619.9 (6,299.8 to 6,949.9)	8.4 (7.2 to 9.4)*	-2.2 (-3.2 to -1.3)*	241.4 (169.1 to 328.5)	7.1 (4.6 to 9.4)*	-3.3 (-5.6 to -1.2)*
Other gynecological diseases cases aggregate	3,659.1 (3,350.3 to 3,984.7)	10.6 (10.1 to 11.1)*	-0.1 (-0.6 to 0.3)	143.9 (97.5 to 200.4)	10.7 (7.7 to 13.6)*	-0.0 (-2.7 to 2.6)
Anemia due to other gynecological diseases aggregate	2,960.7 (2,911.6 to 3,006.9)	5.7 (3.3 to 7.9)*	-4.6 (-6.8 to -2.6)*	97.5 (65.7 to 140.0)	2.1 (-1.9 to 5.8)	-7.8 (-11.5 to -4.5)*
Hemoglobinopathies and hemolytic anemias	116,670.2 (114,647.2 to 119,226.9)	11.1 (9.5 to 12.8)*	0.3 (-1.2 to 1.8)	407.1 (268.6 to 591.4)	0.5 (-2.2 to 3.4)	-9.3 (-11.7 to -6.7)*
Thalassemias	2.8 (2.7 to 2.8)	-8.6 (-12.0 to -5.3)*	-17.5 (-20.5 to -14.5)*	0.3 (0.2 to 0.4)	-8.4 (-27.0 to 12.5)	-17.3 (-34.1 to 1.6)
Beta-thalassemia major cases aggregate	2.1 (2.0 to 2.2)	-10.9 (-15.0 to -6.7)*	-19.6 (-23.3 to -15.8)*	0.2 (0.1 to 0.3)	-11.4 (-34.9 to 18.0)	-20.0 (-41.3 to 6.6)
Hemoglobin E/beta-thalassemia cases aggregate	0.3 (0.3 to 0.3)	-8.0 (-9.0 to -7.0)*	-17.0 (-17.9 to -16.0)*	0.0 (0.0 to 0.0)	-8.5 (-19.4 to -1.3)*	-17.4 (-27.3 to -10.9)*
Hemoglobin H disease cases aggregate	0.1 (0.1 to 0.2)	-0.6 (-5.7 to 4.4)	-10.3 (-14.9 to -5.7)*	0.0 (0.0 to 0.0)	0.3 (-4.9 to 5.8)	-9.5 (-14.1 to -4.4)*
Heart failure due to thalassemias aggregate	0.2 (0.2 to 0.3)	10.9 (8.6 to 13.1)*	0.1 (-1.9 to 2.1)	0.0 (0.0 to 0.0)	10.9 (8.6 to 13.1)*	0.1 (-1.9 to 2.1)
Thalassemias trait	21,188.3 (20,664.5 to 21,777.3)	2.0 (1.4 to 2.5)*	-7.9 (-8.4 to -7.4)*	223.9 (147.5 to 328.0)	-1.9 (-5.7 to 1.9)	-11.4 (-14.9 to -8.0)*
B-thalassemia trait cases aggregate	17,060.5 (16,614.7 to 17,608.4)	0.8 (0.1 to 1.3)*	-9.0 (-9.6 to -8.5)*	210.1 (138.6 to 308.3)	-2.2 (-6.1 to 1.6)	-11.7 (-15.2 to -8.2)*
Hemoglobin E trait cases aggregate	4,127.8 (3,943.6 to 4,324.6)	7.3 (6.2 to 8.5)*	-3.1 (-4.1 to -2.1)*	13.8 (9.0 to 20.4)	3.3 (-5.5 to 12.9)	-6.7 (-14.7 to 2.0)
Sickle cell disorders	32.4 (30.0 to 34.7)	23.7 (15.1 to 34.3)*	11.7 (3.9 to 21.3)*	3.9 (2.7 to 5.3)	17.6 (5.5 to 30.3)*	6.2 (-4.7 to 17.6)
Homozygous sickle cell and severe sickle cell/beta-thalassemia cases aggregate	28.0 (25.8 to 30.0)	23.2 (13.7 to 35.2)*	11.2 (2.7 to 22.0)*	3.3 (2.2 to 4.4)	16.0 (2.2 to 30.6)*	4.7 (-7.7 to 17.9)
Hemoglobin SC disease cases aggregate	4.1 (3.7 to 4.6)	30.7 (20.7 to 45.1)*	18.0 (9.0 to 31.1)*	0.6 (0.4 to 0.8)	29.2 (13.4 to 46.0)*	16.7 (2.4 to 31.8)*
Mild sickle cell/beta-thalassemia cases aggregate	0.4 (0.4 to 0.4)	1.7 (-2.2 to 6.0)	-8.2 (-11.7 to -4.3)*	0.1 (0.0 to 0.1)	6.2 (2.3 to 10.2)*	-4.1 (-7.6 to -0.5)*
Sickle cell trait	29,827.0 (27,991.0 to 33,354.8)	26.3 (25.4 to 27.1)*	14.0 (13.2 to 14.8)*	90.7 (60.4 to 130.8)	15.9 (10.8 to 20.8)*	4.7 (-0.0 to 9.1)
G6PD deficiency	18,757.3 (15,919.8 to 21,718.7)	9.2 (3.7 to 14.1)*	-1.4 (-6.4 to 3.1)	1.5 (1.0 to 2.1)	0.4 (-7.5 to 9.3)	-9.3 (-16.5 to -1.3)*
G6PD cases aggregate	18,757.3 (15,919.8 to 21,718.7)	9.2 (3.7 to 14.1)*	-1.4 (-6.4 to 3.1)	1.5 (1.0 to 2.1)	0.4 (-7.5 to 9.3)	-9.3 (-16.5 to -1.3)*
Heart failure due to G6PD deficiency aggregate	0.0 (0.0 to 0.0)	0.0 (0.0 to 0.0)	0.0 (0.0 to 0.0)	0.0 (0.0 to 0.0)	0.0 (0.0 to 0.0)	0.0 (0.0 to 0.0)
G6PD trait	54,503.4 (50,684.5 to 58,417.4)	10.4 (8.6 to 12.1)*	-0.3 (-1.9 to 1.2)	1.8 (1.2 to 2.6)	5.8 (-9.1 to 23.9)	-4.5 (-17.9 to 11.9)
Other hemoglobinopathies and hemolytic anemias	4,395.5 (4,309.6 to 4,477.8)	-2.7 (-5.0 to -0.4)*	-12.2 (-14.2 to -10.0)*	84.9 (55.6 to 126.1)	-7.4 (-11.5 to -3.3)*	-16.4 (-20.1 to -12.7)*
Other hemoglobinopathies and hemolytic anemias cases aggregate	4,394.0 (4,308.1 to 4,476.3)	-2.7 (-5.0 to -0.4)*	-12.2 (-14.2 to -10.0)*	83.9 (54.7 to 124.9)	-7.5 (-11.7 to -3.4)*	-16.5 (-20.3 to -12.8)*
Heart failure due to other hemoglobinopathies and hemolytic anemias aggregate	1.5 (1.3 to 1.8)	11.6 (9.6 to 13.7)*	0.7 (-1.0 to 2.7)	0.2 (0.1 to 0.3)	11.6 (9.6 to 13.7)*	0.7 (-1.0 to 2.7)
Endocrine, metabolic, blood, and immune disorders	3,489.3 (3,420.2 to 3,554.8)	0.7 (-1.6 to 3.1)	-9.0 (-11.1 to -6.9)*	81.8 (55.2 to 117.4)	-3.2 (-7.1 to 0.7)	-12.6 (-16.1 to -9.0)*
Endocrine, metabolic, blood, and immune disorders cases aggregate	327.8 (297.0 to 362.2)	10.4 (8.4 to 12.7)*	-0.3 (-2.1 to 1.8)	13.1 (8.7 to 18.4)	10.5 (5.0 to 16.4)*	-0.2 (-5.2 to 5.1)
Anemia due to endocrine, metabolic, blood, and immune disorders aggregate	3,161.1 (3,101.1 to 3,216.8)	-0.2 (-2.7 to 2.4)	-9.9 (-12.1 to -7.5)*	68.7 (45.7 to 100.5)	-5.4 (-9.8 to -0.8)*	-14.6 (-18.6 to -10.4)*
Heart failure due to endocrine, metabolic, blood, and immune disorders aggregate	0.5 (0.4 to 0.5)	10.6 (9.4 to 11.7)*	-0.2 (-1.3 to 0.9)	0.1 (0.0 to 0.1)	10.6 (9.4 to 11.7)*	-0.2 (-1.3 to 0.9)
Musculoskeletal disorders	91,186.5 (87,408.3 to 93,382.6)	8.9 (7.4 to 10.4)*	-1.7 (-3.0 to -0.3)*	10,410.0 (7,232.7 to 14,309.0)	8.9 (6.8 to 11.1)*	-1.7 (-3.6 to 0.3)
Rheumatoid arthritis	1,122.4 (964.4 to 1,302.6)	10.4 (6.9 to 13.9)*	-0.4 (-3.5 to 2.8)	274.8 (189.9 to 379.3)	10.4 (5.9 to 14.5)*	-0.3 (-4.4 to 3.4)
Osteoarthritis	918.5 (853.5 to 991.6)	9.9 (8.7 to 11.3)*	-0.7 (-1.8 to 0.5)	49.8 (34.1 to 69.5)	11.5 (7.9 to 15.1)*	0.7 (-2.6 to 3.9)
Osteoarthritis of the hip cases aggregate	104.6 (70.0 to 147.1)	13.8 (10.5 to 16.9)*	2.7 (-0.2 to 5.5)	5.7 (3.3 to 9.2)	15.9 (9.3 to 22.7)*	4.6 (-1.3 to 10.8)
Osteoarthritis of the knee cases aggregate	813.9 (763.2 to 877.4)	9.5 (8.2 to 10.9)*	-1.2 (-2.3 to 0.1)	44.1 (30.2 to 61.5)	11.0 (7.1 to 15.1)*	0.2 (-3.3 to 3.9)
Low back and neck pain	65,364.3 (62,369.6 to 67,686.3)	7.6 (5.9 to 9.2)*	-2.9 (-4.4 to -1.4)*	7,515.4 (5,095.1 to 10,449.5)	7.6 (5.9 to 9.3)*	-2.8 (-4.4 to -1.3)*
Low back pain	43,320.0 (38,598.3 to 48,749.1)	9.5 (7.5 to 11.6)*	-1.1 (-2.9 to 0.8)	4,959.9 (3,362.1 to 6,924.7)	9.7 (7.6 to 11.8)*	-1.0 (-2.8 to 1.0)
Neck pain	25,539.5 (17,793.6 to 36,375.3)	3.9 (0.9 to 6.7)*	-6.2 (-8.9 to -3.6)*	2,555.5 (1,479.0 to 4,081.8)	3.9 (0.8 to 6.8)*	-6.2 (-9.0 to -3.5)*
Gout	2,171.9 (1,670.0 to 2,742.5)	8.6 (7.6 to 9.8)*	-2.0 (-2.9 to -0.9)*	72.6 (46.4 to 107.0)	8.6 (3.5 to 13.8)*	-1.9 (-6.6 to 2.8)
Asymptomatic gout	1,940.3 (1,487.1 to 2,454.7)	8.6 (7.6 to 9.8)*	-2.0 (-2.9 to -0.9)*	0.0 (0.0 to 0.0)	0.0 (0.0 to 0.0)	0.0 (0.0 to 0.0)
Symptomatic gout cases aggregate	231.6 (172.2 to 304.0)	8.6 (7.6 to 9.8)*	-2.0 (-2.9 to -0.9)*	72.6 (46.4 to 107.0)	8.6 (3.5 to 13.8)*	-1.9 (-6.6 to 2.8)
Other musculoskeletal disorders	26,147.3 (19,436.6 to 33,244.8)	12.5 (4.8 to 19.1)*	1.5 (-5.3 to 7.5)	2,497.4 (1,542.6 to 3,820.5)	12.6 (4.9 to 19.4)*	1.7 (-5.3 to 7.8)
Other non-communicable diseases	371,913.7 (362,208.9 to 378,888.7)	11.6 (10.9 to 12.4)*	0.8 (0.2 to 1.5)*	7,103.7 (4,823.4 to 10,292.2)	13.7 (12.4 to 15.4)*	2.6 (1.4 to 4.2)*
Congenital anomalies	7,530.9 (6,903.0 to 8,095.8)	20.6 (16.6 to 24.6)*	8.9 (5.3 to 12.5)*	667.8 (413.8 to 1,014.4)	28.4 (19.0 to 38.2)*	15.9 (7.5 to 24.8)*
Neural tube defects	111.4 (75.8 to 159.5)	17.3 (14.1 to 20.0)*	5.9 (3.0 to 8.3)*	38.1 (22.1 to 62.8)	17.7 (10.8 to 24.7)*	6.3 (0.0 to 12.6)*
Congenital heart anomalies	3,918.1 (2,752.7 to 5,770.5)	26.3 (22.0 to 30.3)*	14.0 (10.1 to 17.6)*	135.7 (53.2 to 250.6)	26.2 (21.4 to 30.5)*	14.0 (9.6 to 17.9)*
Less severe heart anomalies cases aggregate	3,572.8 (2,386.7 to 5,460.7)	26.3 (21.5 to 30.6)*	14.0 (9.7 to 17.9)*	115.1 (43.3 to 218.8)	26.3 (21.0 to 31.0)*	14.0 (9.2 to 18.3)*
Severe congenital heart anomalies	330.2 (221.4 to 494.3)	26.1 (20.6 to 30.7)*	13.8 (8.9 to 18.0)*	19.4 (7.7 to 38.7)	26.1 (19.3 to 32.8)*	13.8 (7.7 to 19.9)*
Critical congenital heart anomalies	10.8 (6.3 to 17.5)	41.4 (24.4 to 54.7)*	27.7 (12.3 to 39.7)*	0.6 (0.2 to 1.3)	41.9 (17.7 to 66.6)*	28.1 (6.3 to 50.4)*
Heart failure due to congenital heart anomalies aggregate	4.3 (3.7 to 4.9)	5.7 (4.5 to 6.9)*	-4.6 (-5.7 to -3.4)*	0.5 (0.4 to 0.8)	5.7 (4.5 to 6.9)*	-4.6 (-5.7 to -3.4)*
Cleft lip and cleft palate	524.2 (308.0 to 870.8)	13.6 (1.6 to 22.8)*	2.6 (-8.2 to 10.8)	6.0 (3.0 to 10.8)	8.1 (-4.8 to 19.2)	-2.4 (-14.1 to 7.7)
Down syndrome	435.6 (276.4 to 669.5)	22.8 (18.2 to 27.3)*	10.9 (6.7 to 14.9)*	36.2 (20.2 to 61.0)	22.5 (16.4 to 27.8)*	10.6 (5.1 to 15.4)*
Turner syndrome	29.4 (13.7 to 53.1)	9.7 (7.3 to 11.7)*	-0.9 (-3.1 to 0.9)	0.6 (0.2 to 1.3)	9.8 (3.9 to 16.1)*	-0.9 (-6.2 to 4.8)
Klinefelter syndrome	16.7 (8.8 to 30.0)	11.2 (9.2 to 12.6)*	0.4 (-1.4 to 1.7)	0.2 (0.1 to 0.4)	11.1 (5.9 to 16.0)*	0.3 (-4.3 to 4.8)
Other chromosomal abnormalities	378.5 (210.5 to 667.5)	16.7 (12.1 to 20.4)*	5.4 (1.2 to 8.7)*	31.7 (15.9 to 60.2)	16.6 (10.8 to 21.1)*	5.3 (0.0 to 9.4)*
Other congenital anomalies	2,413.9 (1,326.8 to 4,430.5)	13.2 (10.6 to 15.7)*	2.2 (-0.2 to 4.5)	419.2 (248.8 to 660.6)	32.2 (18.3 to 48.9)*	19.3 (6.8 to 34.4)*
Skin and subcutaneous diseases	147,254.4 (144,627.4 to 150,192.6)	11.0 (10.1 to 11.9)*	0.3 (-0.6 to 1.1)	2,926.3 (1,896.6 to 4,441.5)	10.9 (9.8 to 12.0)*	0.2 (-0.8 to 1.1)
Dermatitis	17,310.1 (14,052.4 to 21,657.6)	9.1 (8.5 to 9.7)*	-1.5 (-2.0 to -1.0)*	524.3 (333.4 to 772.5)	11.0 (9.5 to 12.6)*	0.2 (-1.1 to 1.6)
Eczema cases aggregate	3,688.6 (2,828.1 to 4,729.2)	14.6 (13.9 to 15.1)*	3.4 (2.9 to 4.0)*	225.3 (139.5 to 339.3)	14.7 (11.9 to 17.2)*	3.5 (1.1 to 5.8)*

Cause	Prevalence in thousands (95% confidence interval)			YLDs in thousands (95% confidence interval)		
	2015	Percent change in counts between 2005 and 2015	Percent change in rates between 2005 and 2015	2015	Percent change in counts between 2005 and 2015	Percent change in rates between 2005 and 2015
Contact dermatitis cases aggregate	7,740.6 (5,240.5 to 11,383.6)	9.7 (9.1 to 10.2)*	-1.0 (-1.5 to -0.5)*	210.5 (117.0 to 354.4)	9.7 (8.0 to 11.5)*	-0.9 (-2.5 to 0.7)
Seborrhoeic dermatitis cases aggregate	5,880.9 (4,277.1 to 7,869.8)	5.2 (4.5 to 6.0)*	-5.0 (-5.7 to -4.3)*	88.5 (46.1 to 147.9)	5.2 (3.6 to 6.7)*	-5.0 (-6.4 to -3.7)*
Psoriasis	6,401.3 (5,797.6 to 7,056.9)	13.2 (11.9 to 14.6)*	2.2 (1.0 to 3.5)*	526.3 (359.1 to 718.1)	13.3 (11.1 to 15.8)*	2.3 (0.3 to 4.5)*
Cellulitis	57.8 (43.3 to 74.0)	22.9 (17.4 to 28.7)*	11.0 (6.0 to 16.2)*	4.3 (2.5 to 6.5)	22.8 (15.3 to 30.4)*	10.8 (4.1 to 17.8)*
Pyoderma	337.4 (290.0 to 387.3)	12.6 (9.6 to 15.2)*	1.6 (-1.0 to 4.0)	1.9 (0.7 to 4.0)	12.6 (7.1 to 17.8)*	1.6 (-3.3 to 6.4)
Scabies	11,331.5 (7,185.8 to 17,281.0)	4.6 (-0.6 to 10.8)	-5.5 (-10.3 to -0.0)*	292.8 (143.9 to 527.7)	4.7 (-0.4 to 10.9)	-5.5 (-10.1 to 0.1)
Fungal skin diseases	32,590.3 (26,688.7 to 39,459.2)	11.4 (11.0 to 11.8)*	0.6 (0.2 to 1.0)*	185.2 (69.5 to 395.7)	11.4 (10.8 to 12.1)*	0.6 (0.0 to 1.2)*
Viral skin diseases	10,316.6 (8,619.0 to 12,266.8)	10.2 (9.1 to 11.3)*	-0.5 (-1.4 to 0.5)	318.8 (194.5 to 489.3)	10.2 (8.8 to 11.7)*	-0.5 (-1.8 to 0.9)
Molluscum contagiosum cases aggregate	843.1 (657.4 to 1,071.4)	10.7 (9.8 to 11.7)*	-0.0 (-0.9 to 0.8)	26.0 (15.3 to 41.9)	10.7 (7.0 to 14.4)*	-0.0 (-3.4 to 3.3)
Viral warts cases aggregate	9,473.4 (7,814.6 to 11,460.2)	10.1 (9.0 to 11.3)*	-0.6 (-1.6 to 0.5)	292.7 (178.8 to 446.9)	10.2 (8.6 to 11.7)*	-0.5 (-2.0 to 0.9)
Acne vulgaris	42,945.0 (36,600.6 to 50,087.7)	11.0 (8.4 to 14.0)*	0.2 (-2.1 to 2.9)	463.5 (217.0 to 880.1)	11.0 (8.4 to 14.0)*	0.3 (-2.1 to 2.9)
Alopecia areata	2,734.5 (2,363.9 to 3,178.1)	9.5 (8.8 to 10.1)*	-1.1 (-1.7 to -0.6)*	93.1 (55.1 to 142.1)	9.5 (7.6 to 11.5)*	-1.1 (-2.8 to 0.7)
Pruritus	5,434.7 (3,837.8 to 7,473.0)	11.3 (8.5 to 14.7)*	0.5 (-2.1 to 3.5)	58.7 (25.7 to 110.4)	11.4 (8.3 to 14.8)*	0.6 (-2.2 to 3.7)
Urticaria	4,024.4 (2,887.3 to 5,766.9)	11.8 (11.2 to 12.4)*	0.9 (0.4 to 1.5)*	243.8 (141.8 to 405.7)	11.9 (10.0 to 13.9)*	1.0 (-0.7 to 2.8)
Decubitus ulcer	32.9 (23.9 to 44.9)	16.3 (10.9 to 21.0)*	5.0 (0.1 to 9.3)*	5.4 (3.4 to 8.2)	16.2 (9.6 to 22.4)*	4.9 (-1.0 to 10.5)
Other skin and subcutaneous diseases	34,856.8 (31,738.3 to 38,608.8)	13.6 (11.4 to 15.8)*	2.6 (0.6 to 4.6)*	208.2 (98.8 to 379.8)	13.7 (11.4 to 15.8)*	2.6 (0.6 to 4.6)*
Sense organ diseases	81,837.8 (79,058.7 to 83,894.9)	12.6 (11.4 to 13.8)*	1.6 (0.6 to 2.8)*	2,497.5 (1,652.1 to 3,574.7)	14.0 (11.8 to 16.1)*	2.9 (1.0 to 4.8)*
Glaucoma	0.0 (0.0 to 0.0)	0.0 (0.0 to 0.0)	0.0 (0.0 to 0.0)	0.0 (0.0 to 0.0)	0.0 (0.0 to 0.0)	0.0 (0.0 to 0.0)
Cataract	588.2 (421.2 to 815.8)	13.8 (10.7 to 17.2)*	2.7 (-0.0 to 5.9)	47.1 (27.5 to 75.2)	11.3 (6.2 to 16.7)*	0.5 (-4.1 to 5.4)
Macular degeneration	0.0 (0.0 to 0.0)	0.0 (0.0 to 0.0)	0.0 (0.0 to 0.0)	0.0 (0.0 to 0.0)	0.0 (0.0 to 0.0)	0.0 (0.0 to 0.0)
Refraction and accommodation disorders	32,459.2 (29,696.3 to 35,576.6)	11.5 (9.8 to 13.2)*	0.7 (-0.9 to 2.2)	654.9 (418.8 to 978.0)	13.5 (12.1 to 15.2)*	2.5 (1.2 to 4.0)*
Age-related and other hearing loss	39,383.8 (31,144.4 to 48,547.5)	13.9 (12.0 to 15.9)*	2.8 (1.2 to 4.6)*	1,351.2 (826.3 to 2,071.4)	14.6 (10.9 to 18.3)*	3.5 (0.1 to 6.9)*
Other vision loss	1,615.2 (1,216.9 to 2,085.1)	17.3 (15.1 to 19.6)*	5.9 (3.9 to 8.0)*	101.5 (64.4 to 155.8)	18.4 (14.7 to 22.5)*	6.9 (3.5 to 10.6)*
Other sense organ diseases	13,295.7 (11,848.4 to 14,866.2)	11.4 (8.9 to 13.8)*	0.6 (-1.6 to 2.7)	342.6 (204.0 to 517.1)	11.5 (8.7 to 14.1)*	0.6 (-1.8 to 3.0)
Acute other sense organ diseases	80.8 (72.5 to 90.2)	11.8 (9.5 to 14.2)*	0.9 (-1.2 to 3.1)	2.2 (1.3 to 3.3)	11.8 (9.5 to 14.2)*	0.9 (-1.2 to 3.1)
Chronic other sense organ diseases	13,214.9 (11,766.1 to 14,778.5)	11.4 (8.9 to 13.8)*	0.6 (-1.7 to 2.8)	340.5 (202.8 to 514.0)	11.5 (8.7 to 14.1)*	0.6 (-1.9 to 3.0)
Oral disorders	259,802.7 (244,935.1 to 271,133.8)	12.2 (10.6 to 13.8)*	1.3 (-0.1 to 2.8)	1,012.1 (591.6 to 1,646.8)	12.6 (11.9 to 13.2)*	1.7 (1.0 to 2.2)*
Deciduous caries	0.0 (0.0 to 0.0)	0.0 (0.0 to 0.0)	0.0 (0.0 to 0.0)	0.0 (0.0 to 0.0)	0.0 (0.0 to 0.0)	0.0 (0.0 to 0.0)
Permanent caries	234,088.7 (193,616.1 to 279,270.1)	12.0 (10.4 to 13.6)*	1.2 (-0.3 to 2.6)	162.4 (69.9 to 319.3)	10.8 (9.1 to 12.4)*	0.1 (-1.5 to 1.5)
Periodontal diseases	35,965.2 (27,943.5 to 45,284.4)	14.2 (13.1 to 15.3)*	3.1 (2.2 to 4.1)*	240.3 (89.6 to 510.0)	14.3 (13.1 to 15.4)*	3.2 (2.1 to 4.2)*
Edentulism and severe tooth loss	7,998.5 (7,310.1 to 8,878.9)	14.9 (14.3 to 15.5)*	3.7 (3.2 to 4.3)*	230.6 (153.3 to 324.9)	15.0 (13.5 to 16.4)*	3.8 (2.5 to 5.1)*
Other oral disorders	12,790.2 (11,436.5 to 14,173.3)	10.8 (10.3 to 11.3)*	0.1 (-0.4 to 0.5)	378.9 (229.9 to 578.6)	10.9 (10.0 to 11.9)*	0.1 (-0.7 to 1.1)
Injuries	72,757.5 (68,345.1 to 77,732.1)	14.5 (11.3 to 19.1)*	3.4 (0.5 to 7.6)*	2,850.3 (2,037.9 to 3,820.8)	3.0 (-0.5 to 6.8)	-7.0 (-10.2 to -3.5)*
Transport injuries	7,138.9 (6,651.0 to 7,715.4)	6.0 (5.2 to 6.7)*	-4.3 (-5.0 to -3.7)*	423.2 (292.5 to 572.3)	-3.6 (-6.8 to -0.6)*	-12.9 (-15.8 to -10.2)*
Road injuries	6,661.8 (6,167.9 to 7,261.0)	5.8 (5.0 to 6.6)*	-4.4 (-5.2 to -3.8)*	392.4 (270.4 to 533.0)	-3.1 (-6.2 to -0.2)*	-12.5 (-15.3 to -9.9)*
Pedestrian road injuries	759.1 (636.9 to 932.2)	0.9 (-0.5 to 2.1)	-8.9 (-10.1 to -7.9)*	44.0 (29.9 to 62.8)	-10.9 (-14.4 to -7.6)*	-19.6 (-22.7 to -16.5)*
Cyclist road injuries	1,104.8 (938.8 to 1,298.6)	-0.5 (-1.8 to 0.7)	-10.2 (-11.3 to -9.1)*	59.0 (39.8 to 82.1)	-10.4 (-14.3 to -6.9)*	-19.1 (-22.6 to -15.9)*
Motorcyclist road injuries	1,730.4 (1,465.1 to 2,064.3)	10.6 (9.6 to 11.5)*	-0.1 (-1.0 to 0.6)	97.2 (65.2 to 137.3)	-1.7 (-6.0 to 2.1)	-11.2 (-15.1 to -7.8)*
Motor vehicle road injuries	2,788.7 (2,368.6 to 3,244.8)	5.5 (4.4 to 6.6)*	-4.7 (-5.8 to -3.7)*	175.6 (119.5 to 242.7)	-0.7 (-3.5 to 2.0)	-10.3 (-12.8 to -7.9)*
Other road injuries	278.8 (224.1 to 335.4)	24.5 (22.6 to 26.0)*	12.4 (10.7 to 13.8)*	16.6 (11.0 to 22.8)	17.3 (13.5 to 20.6)*	5.9 (2.5 to 8.9)*
Other transport injuries	477.1 (416.3 to 548.7)	7.7 (6.9 to 8.5)*	-2.8 (-3.5 to -2.0)*	30.8 (21.8 to 41.7)	-9.1 (-12.8 to -5.0)*	-17.9 (-21.3 to -14.2)*
Unintentional injuries	53,766.0 (50,761.0 to 56,832.1)	8.9 (8.4 to 9.4)*	-1.7 (-2.2 to -1.2)*	2,073.6 (1,459.8 to 2,801.6)	0.8 (-1.8 to 3.4)	-9.0 (-11.3 to -6.6)*
Falls	13,007.3 (11,566.5 to 14,816.6)	15.7 (15.0 to 16.4)*	4.5 (3.9 to 5.1)*	710.5 (493.1 to 992.4)	2.8 (-1.7 to 6.9)	-7.2 (-11.2 to -3.5)*
Drowning	212.1 (184.1 to 248.0)	-7.4 (-8.5 to -6.3)*	-16.3 (-17.4 to -15.4)*	12.6 (8.6 to 17.6)	-19.5 (-22.5 to -15.8)*	-27.3 (-30.0 to -24.0)*
Fire, heat, and hot substances	4,981.6 (4,111.2 to 5,828.2)	9.1 (8.4 to 9.9)*	-1.5 (-2.2 to -0.8)*	194.8 (136.0 to 264.2)	0.7 (-3.0 to 4.4)	-9.0 (-12.4 to -5.7)*
Poisonings	433.9 (360.9 to 526.4)	13.7 (11.8 to 15.6)*	2.7 (1.0 to 4.4)*	44.4 (29.6 to 64.2)	10.4 (7.1 to 13.5)*	-0.3 (-3.3 to 2.5)
Exposure to mechanical forces	14,413.9 (13,013.4 to 15,760.5)	11.2 (10.3 to 12.1)*	0.4 (-0.4 to 1.2)	272.7 (189.4 to 380.2)	2.9 (-0.0 to 5.7)	-7.1 (-9.7 to -4.5)*
Unintentional firearm injuries	193.1 (165.3 to 225.9)	10.6 (9.3 to 11.7)*	-0.1 (-1.3 to 0.9)	8.3 (5.6 to 11.7)	1.9 (-1.7 to 5.4)	-8.0 (-11.2 to -4.8)*
Unintentional suffocation	548.4 (433.9 to 706.2)	-2.4 (-6.5 to 2.5)	-11.9 (-15.6 to -7.5)*	19.6 (13.4 to 27.0)	1.9 (-1.1 to 5.3)	-8.0 (-10.7 to -4.9)*
Other exposure to mechanical forces	13,672.4 (12,320.8 to 15,000.1)	11.8 (11.0 to 12.6)*	1.0 (0.2 to 1.7)*	244.7 (169.2 to 342.6)	3.0 (-0.1 to 5.9)	-7.0 (-9.8 to -4.4)*
Adverse effects of medical treatment	620.7 (467.3 to 793.6)	6.6 (2.7 to 10.5)*	-3.7 (-7.3 to -0.2)*	82.7 (50.7 to 124.4)	6.6 (2.7 to 10.5)*	-3.7 (-7.3 to -0.2)*
Animal contact	2,882.9 (2,524.1 to 3,271.2)	10.7 (8.9 to 12.3)*	-0.1 (-1.7 to 1.4)	99.2 (67.6 to 136.1)	7.7 (5.0 to 10.2)*	-2.8 (-5.2 to -0.5)*
Venomous animal contact	1,322.9 (1,149.7 to 1,522.8)	13.9 (12.5 to 15.4)*	2.9 (1.6 to 4.2)*	78.3 (52.6 to 108.1)	8.6 (5.7 to 11.5)*	-2.0 (-4.5 to 0.6)
Non-venomous animal contact	1,560.0 (1,289.8 to 1,877.3)	8.0 (5.8 to 10.1)*	-2.5 (-4.5 to -0.6)*	20.9 (13.3 to 31.5)	4.4 (0.3 to 7.8)*	-5.7 (-9.4 to -2.6)*
Foreign body	2,426.6 (2,117.2 to 2,749.2)	14.0 (12.1 to 15.3)*	2.9 (1.3 to 4.2)*	96.0 (67.6 to 131.7)	2.1 (-1.3 to 5.8)	-7.8 (-10.8 to -4.5)*
Pulmonary aspiration and foreign body in airway	892.8 (706.0 to 1,152.7)	9.0 (6.1 to 11.8)*	-1.5 (-4.2 to 0.9)	48.0 (32.5 to 68.2)	-4.9 (-8.8 to -0.6)*	-14.2 (-17.6 to -10.2)*
Foreign body in eyes	87.7 (34.1 to 155.5)	16.1 (14.8 to 17.1)*	4.8 (3.7 to 5.7)*	4.9 (1.9 to 9.5)	15.1 (13.0 to 16.4)*	4.0 (2.0 to 5.1)*
Foreign body in other body part	1,446.1 (1,239.9 to 1,694.7)	17.1 (16.0 to 18.2)*	5.7 (4.7 to 6.8)*	43.1 (30.0 to 59.3)	9.7 (7.0 to 12.5)*	-0.9 (-3.4 to 1.6)
Environmental heat and cold exposure	4,331.6 (3,790.6 to 5,179.4)	8.4 (7.7 to 9.0)*	-2.1 (-2.8 to -1.6)*	192.9 (134.5 to 272.9)	-0.5 (-3.1 to 2.1)	-10.2 (-12.5 to -7.8)*
Other unintentional injuries	10,455.4 (9,087.8 to 12,008.9)	-2.2 (-3.0 to -1.4)*	-11.7 (-12.4 to -11.0)*	367.7 (245.3 to 508.4)	-6.5 (-8.4 to -4.8)*	-15.6 (-17.3 to -14.0)*
Self-harm and interpersonal violence	1,748.3 (1,579.1 to 1,919.4)	4.2 (3.6 to 4.8)*	-5.9 (-6.5 to -5.4)*	87.0 (61.6 to 117.2)	-6.5 (-9.4 to -3.3)*	-15.6 (-18.2 to -12.7)*
Self-harm	419.8 (359.8 to 486.2)	4.2 (3.5 to 4.9)*	-5.9 (-6.6 to -5.3)*	27.0 (18.5 to 36.5)	-5.8 (-8.4 to -3.3)*	-15.0 (-17.3 to -12.6)*
Interpersonal violence	1,328.4 (1,183.2 to 1,484.0)	4.2 (3.4 to 4.9)*	-5.9 (-6.6 to -5.3)*	60.0 (42.1 to 81.5)	-6.9 (-10.3 to -3.1)*	-15.9 (-19.0 to -12.5)*

Cause	Prevalence in thousands (95% confidence interval)			YLDs in thousands (95% confidence interval)		
	**2015**	**Percent change in counts between 2005 and 2015**	**Percent change in rates between 2005 and 2015**	**2015**	**Percent change in counts between 2005 and 2015**	**Percent change in rates between 2005 and 2015**
Assault by firearm	83.0 (68.7 to 100.5)	6.9 (5.9 to 7.9)*	-3.4 (-4.4 to -2.5)*	4.1 (2.8 to 5.6)	-3.0 (-6.5 to 0.8)	-12.5 (-15.6 to -9.0)*
Assault by sharp object	331.0 (266.4 to 397.3)	0.2 (-0.8 to 1.3)	-9.5 (-10.4 to -8.6)*	11.0 (7.5 to 15.4)	-14.9 (-19.2 to -10.2)*	-23.1 (-27.1 to -19.0)*
Assault by other means	914.4 (799.2 to 1,042.6)	5.5 (4.6 to 6.3)*	-4.8 (-5.5 to -4.0)*	44.9 (31.6 to 61.7)	-5.0 (-8.2 to -1.6)*	-14.2 (-17.2 to -11.1)*
Forces of nature, war, and legal intervention	10,104.3 (7,072.8 to 13,962.8)	76.0 (39.2 to 126.8)*	59.0 (25.7 to 104.8)*	266.5 (182.2 to 375.3)	49.1 (12.2 to 105.8)*	34.6 (1.3 to 85.8)*
Exposure to forces of nature	2,191.1 (1,363.1 to 3,332.8)	23.9 (-7.2 to 61.7)	11.9 (-16.2 to 46.0)	63.5 (43.0 to 88.1)	-19.9 (-46.0 to 16.1)	-27.6 (-51.2 to 4.8)
Collective violence and legal intervention	7,913.2 (5,341.1 to 11,486.1)	99.3 (48.0 to 172.8)*	79.9 (33.7 to 146.3)*	203.0 (129.8 to 299.9)	104.0 (42.5 to 191.8)*	84.2 (28.6 to 163.5)*
**Anemia**	**151,137.5 (149,394.5 to 152,846.7)**	**2.5 (1.2 to 3.8)***	**-7.5 (-8.6 to -6.2)***	**4,207.5 (2,814.9 to 6,143.3)**	**-0.8 (-2.9 to 1.2)**	**-10.4 (-12.3 to -8.6)***
**Developmental intellectual disability**	**12,559.3 (9,347.2 to 15,662.7)**	**14.6 (14.1 to 15.2)***	**3.5 (3.1 to 4.0)***	**1,373.2 (978.4 to 1,817.3)**	**15.9 (13.7 to 18.2)***	**4.6 (2.7 to 6.8)***
**Epilepsy**	**2,921.3 (2,477.0 to 3,371.5)**	**14.9 (10.8 to 18.9)***	**3.7 (0.1 to 7.3)***	**892.9 (631.8 to 1,170.7)**	**6.9 (0.5 to 12.9)***	**-3.5 (-9.3 to 2.0)**
**Guillain-Barré syndrome**	**2.0 (1.2 to 3.2)**	**13.4 (11.6 to 14.9)***	**2.4 (0.8 to 3.7)***	**0.6 (0.3 to 1.1)**	**13.4 (11.6 to 14.9)***	**2.4 (0.8 to 3.7)***
**Hearing loss**	**45,722.1 (37,600.9 to 54,995.8)**	**12.5 (11.1 to 14.1)***	**1.6 (0.3 to 3.0)***	**1,657.0 (1,071.2 to 2,441.3)**	**13.0 (9.9 to 15.8)***	**2.0 (-0.8 to 4.6)**
**Heart failure**	**332.9 (310.9 to 357.5)**	**13.4 (12.6 to 14.2)***	**2.4 (1.7 to 3.1)***	**40.3 (27.9 to 56.0)**	**13.9 (10.0 to 17.7)***	**2.8 (-0.7 to 6.3)**
**Infertility**	**21,647.0 (15,020.4 to 31,828.6)**	**20.5 (15.1 to 26.5)***	**8.8 (3.9 to 14.2)***	**144.4 (61.4 to 299.7)**	**18.8 (14.2 to 23.5)***	**7.2 (3.1 to 11.5)***
**Pelvic inflammatory disease**	**143.0 (110.5 to 182.0)**	**-1.1 (-2.5 to 0.4)**	**-10.7 (-12.0 to -9.4)***	**18.9 (12.3 to 27.9)**	**-0.9 (-5.6 to 4.2)**	**-10.5 (-14.8 to -6.0)***
**Vision loss**	**36,010.4 (32,837.7 to 39,585.9)**	**11.9 (10.2 to 13.5)***	**1.0 (-0.5 to 2.5)**	**1,040.2 (711.6 to 1,455.1)**	**14.5 (13.2 to 15.9)***	**3.4 (2.2 to 4.7)***

SR Table 11. Prevalent cases and YLDs for 2015, percent change, and change in age-standardised rates between 2005 and 2015 by cause, global, ages 35-39 years						
Cause	Prevalence in thousands (95% confidence interval)			YLDs in thousands (95% confidence interval)		
	2015	Percent change in counts between 2005 and 2015	Percent change in rates between 2005 and 2015	2015	Percent change in counts between 2005 and 2015	Percent change in rates between 2005 and 2015
**All causes**	--	--	--	**55,373.8 (41,286.3 to 71,225.1)**	**5.6 (5.0 to 6.3)***	**-0.9 (-1.5 to -0.2)***
Communicable, maternal, neonatal, and nutritional diseases	296,203.9 (293,468.8 to 298,744.8)	3.3 (2.6 to 4.1)*	-3.0 (-3.7 to -2.3)*	6,301.2 (4,542.7 to 8,676.5)	-2.5 (-5.0 to 0.5)	-8.5 (-10.9 to -5.7)*
HIV/AIDS and tuberculosis	6,072.7 (5,906.7 to 6,250.8)	15.7 (11.2 to 20.1)*	8.6 (4.4 to 12.7)*	790.5 (570.6 to 1,063.2)	-4.9 (-13.2 to 3.3)	-10.8 (-18.5 to -3.1)*
Tuberculosis	621.7 (450.6 to 844.0)	5.2 (-4.0 to 14.6)	-1.3 (-9.9 to 7.5)	192.8 (117.6 to 302.8)	5.5 (-3.8 to 15.0)	-1.0 (-9.7 to 8.0)
HIV/AIDS	5,453.8 (5,300.5 to 5,628.4)	17.1 (12.1 to 22.0)*	9.9 (5.2 to 14.5)*	597.8 (435.0 to 774.3)	-7.9 (-17.0 to 1.8)	-13.5 (-22.1 to -4.5)*
HIV/AIDS - Tuberculosis	186.9 (166.8 to 208.7)	-15.1 (-18.5 to -11.2)*	-20.3 (-23.5 to -16.7)*	70.1 (46.9 to 96.5)	-14.7 (-19.5 to -9.7)*	-19.9 (-24.4 to -15.2)*
HIV/AIDS resulting in other diseases	5,562.4 (5,385.2 to 5,790.6)	19.9 (15.6 to 23.9)*	12.5 (8.4 to 16.3)*	527.6 (382.4 to 686.5)	-6.9 (-17.4 to 4.2)	-12.6 (-22.5 to -2.2)*
HIV aggregate	3,161.8 (3,014.4 to 3,338.5)	-20.9 (-24.3 to -17.5)*	-25.8 (-29.0 to -22.6)*	232.8 (167.4 to 308.9)	-22.2 (-25.6 to -18.6)*	-27.0 (-30.1 to -23.6)*
AIDS aggregate	2,400.7 (2,290.9 to 2,518.0)	274.1 (214.1 to 353.1)*	251.1 (194.8 to 325.2)*	294.8 (212.3 to 383.6)	10.3 (-12.7 to 42.8)	3.5 (-18.1 to 34.0)
Diarrhea, lower respiratory, and other common infectious diseases	21,094.9 (19,280.8 to 23,206.8)	4.4 (1.4 to 7.3)*	-2.1 (-4.8 to 0.7)	604.2 (414.7 to 851.5)	8.2 (6.4 to 10.1)*	1.6 (-0.1 to 3.3)
Diarrheal diseases	1,090.4 (1,030.6 to 1,151.8)	17.7 (16.6 to 18.7)*	10.5 (9.4 to 11.4)*	172.4 (117.9 to 238.4)	18.0 (15.0 to 20.9)*	10.8 (7.9 to 13.4)*
Diarrhoea episodes aggregate	1,090.1 (1,030.2 to 1,151.6)	17.7 (16.6 to 18.7)*	10.5 (9.4 to 11.4)*	172.3 (117.9 to 238.3)	18.0 (15.0 to 20.9)*	10.8 (7.9 to 13.4)*
Guillain-Barré syndrome due to diarrheal diseases	0.2 (0.1 to 0.4)	8.1 (6.0 to 9.8)*	1.4 (-0.5 to 3.0)	0.1 (0.0 to 0.1)	8.1 (6.0 to 9.8)*	1.4 (-0.5 to 3.0)
Intestinal infectious diseases	37.1 (32.7 to 41.5)	-22.2 (-31.8 to -12.6)*	-27.0 (-36.0 to -17.9)*	6.8 (4.2 to 9.9)	-6.3 (-18.7 to 8.6)	-12.1 (-23.7 to 1.9)
Typhoid fever	43.1 (33.3 to 55.2)	-6.8 (-11.6 to -1.8)*	-12.5 (-17.0 to -7.8)*	5.8 (3.6 to 8.6)	-4.5 (-18.9 to 13.0)	-10.4 (-23.9 to 6.1)
Typhoid fever episodes aggregate	35.7 (27.2 to 45.8)	-7.0 (-15.8 to 2.4)	-12.7 (-21.0 to -3.9)*	3.3 (2.1 to 5.2)	-6.5 (-21.2 to 11.6)	-12.3 (-26.0 to 4.7)
Complications of typhoid fever aggregate	7.5 (4.9 to 10.8)	-5.9 (-37.0 to 42.7)	-11.6 (-40.8 to 33.9)	2.4 (1.3 to 3.8)	-1.5 (-37.0 to 52.8)	-7.6 (-40.9 to 43.4)
Paratyphoid fever	18.2 (13.2 to 24.9)	-9.4 (-15.8 to -1.8)*	-15.0 (-21.0 to -7.8)*	1.0 (0.5 to 1.5)	-9.2 (-26.9 to 11.0)	-14.8 (-31.4 to 4.1)
Paratyphoid fever episodes aggregate	17.3 (12.6 to 23.7)	-9.5 (-16.6 to -1.3)*	-15.0 (-21.8 to -7.3)*	0.8 (0.5 to 1.3)	-9.3 (-27.7 to 12.6)	-14.9 (-32.1 to 5.6)
Intestinal perforation due to paratyphoid	0.9 (0.5 to 1.5)	-8.6 (-47.0 to 60.2)	-14.2 (-50.3 to 50.4)	0.1 (0.1 to 0.2)	-8.6 (-47.0 to 60.2)	-14.2 (-50.3 to 50.4)
Other intestinal infectious diseases	--	--	--	0.0 (0.0 to 0.1)	-64.7 (-77.4 to -43.6)*	-66.9 (-78.8 to -47.1)*
Lower respiratory infections	214.3 (198.0 to 231.9)	15.6 (13.4 to 17.9)*	8.5 (6.4 to 10.6)*	13.0 (8.8 to 18.6)	15.9 (10.0 to 22.1)*	8.8 (3.2 to 14.6)*
Lower respiratory infection episodes aggregate	214.1 (197.8 to 231.7)	15.7 (13.4 to 17.9)*	8.5 (6.4 to 10.6)*	13.0 (8.7 to 18.5)	16.0 (10.0 to 22.2)*	8.8 (3.2 to 14.6)*
Guillain-Barré syndrome due to lower respiratory infections	0.3 (0.1 to 0.5)	8.1 (6.0 to 9.8)*	1.4 (-0.5 to 3.0)	0.1 (0.0 to 0.1)	8.1 (6.0 to 9.8)*	1.4 (-0.5 to 3.0)
Upper respiratory infections	15,161.1 (10,110.4 to 20,726.4)	4.6 (3.7 to 5.7)*	-1.8 (-2.7 to -0.8)*	177.3 (88.8 to 310.3)	4.7 (3.3 to 6.1)*	-1.7 (-3.0 to -0.4)*
Upper respiratory infection episodes aggregate	15,160.4 (10,109.9 to 20,725.7)	4.6 (3.7 to 5.7)*	-1.8 (-2.7 to -0.8)*	177.1 (88.6 to 310.2)	4.7 (3.3 to 6.1)*	-1.7 (-3.0 to -0.4)*
Guillain-Barré syndrome due to upper respiratory infections	0.7 (0.4 to 1.1)	8.1 (6.0 to 9.8)*	1.4 (-0.5 to 3.0)	0.2 (0.1 to 0.4)	8.1 (6.0 to 9.8)*	1.5 (-0.5 to 3.0)
Otitis media	3,818.4 (2,929.3 to 4,861.9)	-1.7 (-4.3 to 0.8)	-7.8 (-10.2 to -5.4)*	102.5 (60.8 to 167.6)	-2.7 (-7.5 to 1.9)	-8.7 (-13.2 to -4.4)*
Acute otitis media	589.7 (463.6 to 740.6)	12.6 (8.7 to 16.8)*	5.7 (2.0 to 9.6)*	7.6 (3.8 to 14.8)	12.6 (7.8 to 17.8)*	5.7 (1.1 to 10.5)*
Chronic otitis media aggregate	3,228.8 (2,391.0 to 4,278.2)	-4.0 (-6.7 to -1.1)*	-9.9 (-12.5 to -7.2)*	94.8 (55.5 to 154.8)	-3.8 (-8.9 to 1.0)	-9.7 (-14.5 to -5.2)*
Meningitis	492.3 (466.2 to 521.2)	22.2 (16.5 to 28.1)*	14.7 (9.4 to 20.2)*	80.1 (56.7 to 106.4)	14.9 (11.7 to 18.1)*	7.9 (4.8 to 10.9)*
Pneumococcal meningitis	443.9 (366.5 to 536.1)	23.4 (20.4 to 26.8)*	15.8 (13.0 to 19.0)*	37.0 (26.1 to 49.0)	17.1 (11.5 to 22.6)*	9.9 (4.6 to 15.0)*
Acute pneumococcal meningitis	1.3 (0.9 to 1.9)	24.9 (10.9 to 39.3)*	17.2 (4.1 to 30.8)*	0.2 (0.1 to 0.3)	24.9 (10.9 to 39.3)*	17.2 (4.1 to 30.8)*
Complications of pneumococcal meningitis aggregate	442.6 (365.1 to 534.7)	23.4 (20.4 to 26.8)*	15.8 (13.0 to 19.0)*	36.8 (26.0 to 48.8)	17.0 (11.5 to 22.6)*	9.8 (4.6 to 15.0)*
H influenzae type B meningitis	135.3 (101.7 to 174.0)	-9.1 (-13.3 to -3.8)*	-14.7 (-18.6 to -9.7)*	13.5 (9.2 to 18.6)	9.8 (3.9 to 16.5)*	3.0 (-2.5 to 9.3)
Acute H influenzae type B meningitis	0.4 (0.3 to 0.6)	-12.3 (-23.6 to -1.2)*	-17.7 (-28.3 to -7.2)*	0.1 (0.0 to 0.1)	-12.3 (-23.6 to -1.2)*	-17.7 (-28.3 to -7.2)*
Complications of of H influenzae type B meningitis aggregate	134.9 (101.1 to 173.7)	-9.1 (-13.3 to -3.8)*	-14.6 (-18.6 to -9.7)*	13.5 (9.1 to 18.5)	9.9 (4.0 to 16.6)*	3.1 (-2.4 to 9.4)
Meningococcal meningitis	122.6 (94.0 to 156.3)	25.7 (21.7 to 29.9)*	18.0 (14.3 to 21.9)*	12.4 (8.6 to 16.8)	20.7 (15.5 to 26.3)*	13.3 (8.4 to 18.6)*
Acute meningococcal meningitis	0.6 (0.4 to 0.9)	24.7 (8.3 to 41.1)*	17.0 (1.7 to 32.5)*	0.1 (0.0 to 0.1)	24.7 (8.3 to 41.1)*	17.0 (1.7 to 32.5)*
Complications of meningococcal meningitis aggregate	122.1 (93.6 to 155.6)	25.7 (21.7 to 29.9)*	18.0 (14.2 to 21.9)*	12.3 (8.6 to 16.7)	20.7 (15.5 to 26.4)*	13.3 (8.4 to 18.6)*
Other meningitis	182.8 (142.3 to 223.1)	14.9 (12.2 to 18.2)*	7.9 (5.3 to 10.9)*	17.2 (11.8 to 23.0)	10.9 (5.0 to 16.6)*	4.1 (-1.5 to 9.4)
Other meningitis episodes aggregate	4.8 (3.5 to 6.3)	18.8 (6.6 to 30.4)*	11.5 (0.1 to 22.4)*	0.6 (0.4 to 1.0)	18.6 (6.5 to 30.2)*	11.3 (-0.1 to 22.2)
Complications of other meningitis aggregate	178.0 (138.1 to 218.3)	14.8 (11.9 to 18.2)*	7.8 (5.0 to 11.0)*	16.6 (11.3 to 22.2)	10.7 (4.5 to 16.3)*	3.9 (-1.9 to 9.2)
Encephalitis	357.5 (260.8 to 481.5)	3.8 (1.1 to 7.0)*	-2.6 (-5.1 to 0.5)	40.2 (28.4 to 53.2)	6.6 (1.5 to 11.9)*	0.0 (-4.8 to 5.1)
Acute encephalitis	4.6 (3.8 to 5.6)	10.2 (4.6 to 15.4)*	3.4 (-1.8 to 8.3)	0.6 (0.4 to 0.9)	10.2 (2.8 to 17.8)*	3.4 (-3.6 to 10.5)
Complications of encephalitis aggregate	352.9 (256.1 to 477.2)	3.7 (1.1 to 7.0)*	-2.7 (-5.1 to 0.4)	39.5 (28.0 to 52.5)	6.5 (1.4 to 12.0)*	-0.0 (-4.9 to 5.1)
Diphtheria	0.0 (0.0 to 0.0)	-53.7 (-82.2 to 24.6)	-56.6 (-83.3 to 17.0)	0.0 (0.0 to 0.0)	-53.6 (-82.2 to 24.6)	-56.5 (-83.3 to 17.0)
Whooping cough	2.2 (1.7 to 2.7)	-24.2 (-26.3 to -22.0)*	-28.9 (-30.8 to -26.8)*	0.1 (0.1 to 0.2)	-24.2 (-26.3 to -22.0)*	-28.9 (-30.8 to -26.8)*
Tetanus	13.8 (13.4 to 14.2)	9.1 (6.8 to 10.4)*	2.4 (0.3 to 3.6)*	0.6 (0.4 to 0.8)	-9.6 (-16.7 to -1.5)*	-15.1 (-21.8 to -7.6)*
Severe tetanus	0.4 (0.2 to 0.7)	-34.9 (-48.5 to -17.3)*	-38.9 (-51.7 to -22.3)*	0.0 (0.0 to 0.1)	-34.9 (-48.4 to -17.2)*	-38.9 (-51.6 to -22.3)*
Complications of tetanus aggregate	13.4 (13.1 to 13.6)	11.2 (10.7 to 11.8)*	4.4 (3.9 to 4.9)*	0.5 (0.4 to 0.7)	-6.2 (-13.3 to 2.0)	-12.0 (-18.6 to -4.2)*
Measles	0.1 (0.1 to 0.3)	-63.4 (-67.3 to -58.8)*	-65.7 (-69.3 to -61.3)*	0.0 (0.0 to 0.0)	-63.5 (-67.3 to -58.8)*	-65.7 (-69.3 to -61.3)*
Varicella and herpes zoster	254.0 (206.9 to 305.4)	5.7 (4.7 to 6.7)*	-0.8 (-1.7 to 0.1)	11.2 (6.7 to 18.0)	4.0 (-0.8 to 8.9)	-2.4 (-6.9 to 2.2)
Neglected tropical diseases and malaria	129,501.1 (126,435.5 to 132,814.8)	-3.9 (-6.4 to -1.0)*	-9.8 (-12.2 to -7.1)*	1,270.2 (835.8 to 1,949.5)	-10.2 (-17.9 to 2.3)	-15.7 (-23.0 to -4.0)*
Malaria	12,893.5 (11,212.8 to 15,037.3)	41.2 (33.3 to 51.3)*	32.5 (25.1 to 42.0)*	105.2 (75.8 to 144.6)	24.6 (19.1 to 30.2)*	17.0 (11.8 to 22.2)*
Asymptomatic malaria parasitemia(PfPR)	9,599.0 (7,962.2 to 11,631.0)	46.5 (34.9 to 60.8)*	37.5 (26.6 to 50.9)*	0.0 (0.0 to 0.0)	0.0 (0.0 to 0.0)	0.0 (0.0 to 0.0)
Malaria episodes aggregate	534.9 (293.0 to 908.4)	7.2 (-12.3 to 33.1)	0.6 (-17.7 to 24.9)	10.9 (5.1 to 21.0)	7.7 (-11.9 to 34.2)	1.1 (-17.3 to 26.0)
Complications of malaria aggregate	63.3 (56.6 to 69.5)	36.3 (32.9 to 40.5)*	28.0 (24.8 to 31.9)*	19.6 (14.7 to 24.7)	31.9 (22.2 to 42.1)*	23.8 (14.7 to 33.4)*
Anemia due to malaria parasitemia(PfPR) aggregate	2,696.4 (2,628.4 to 2,761.9)	32.6 (29.2 to 36.3)*	24.4 (21.3 to 27.9)*	74.6 (50.3 to 106.7)	25.7 (19.2 to 31.7)*	18.0 (11.9 to 23.6)*
Chagas disease	572.8 (469.5 to 664.8)	-11.3 (-14.4 to -7.8)*	-16.8 (-19.6 to -13.5)*	4.0 (2.5 to 6.0)	-11.2 (-17.5 to -5.1)*	-16.6 (-22.6 to -10.9)*
Chagas disease episodes aggregate	498.7 (408.1 to 580.2)	-11.3 (-14.4 to -7.8)*	-16.7 (-19.6 to -13.5)*	0.0 (0.0 to 0.0)	-20.6 (-28.3 to -13.8)*	-25.5 (-32.7 to -19.1)*
Complications of Chagas disease aggregate	56.1 (42.4 to 71.0)	-11.3 (-14.4 to -7.8)*	-16.8 (-19.7 to -13.5)*	3.0 (1.9 to 4.5)	-11.1 (-18.4 to -3.7)*	-16.6 (-23.4 to -9.6)*

Cause	Prevalence in thousands (95% confidence interval)			YLDs in thousands (95% confidence interval)		
	2015	Percent change in counts between 2005 and 2015	Percent change in rates between 2005 and 2015	2015	Percent change in counts between 2005 and 2015	Percent change in rates between 2005 and 2015
Heart failure due to Chagas disease aggregate	18.1 (10.7 to 27.6)	-11.8 (-15.2 to -8.2)*	-17.3 (-20.4 to -13.8)*	1.0 (0.5 to 1.7)	-11.3 (-20.8 to -1.6)*	-16.8 (-25.7 to -7.6)*
Leishmaniasis	291.7 (257.6 to 347.9)	35.5 (10.3 to 67.0)*	27.2 (3.5 to 56.8)*	3.4 (1.7 to 6.5)	34.0 (26.9 to 40.5)*	25.7 (19.1 to 31.8)*
Visceral leishmaniasis	3.9 (3.7 to 4.1)	18.9 (17.8 to 20.0)*	11.6 (10.5 to 12.6)*	0.3 (0.2 to 0.4)	19.2 (-9.6 to 59.4)	11.8 (-15.2 to 49.6)
Cutaneous and mucocutaneous leishmaniasis	294.4 (247.5 to 366.1)	34.7 (30.9 to 38.4)*	26.5 (22.9 to 29.9)*	3.2 (1.5 to 6.1)	35.4 (28.9 to 41.3)*	27.1 (21.0 to 32.6)*
African trypanosomiasis	0.8 (0.5 to 1.3)	-68.6 (-70.5 to -67.0)*	-70.5 (-72.3 to -69.0)*	0.2 (0.1 to 0.4)	-67.4 (-83.8 to -44.8)*	-69.4 (-84.8 to -48.2)*
Schistosomiasis	17,788.3 (14,856.5 to 22,734.6)	-19.4 (-29.0 to 1.0)	-24.3 (-33.3 to -5.3)*	179.6 (91.0 to 342.9)	-17.1 (-25.9 to 4.0)	-22.2 (-30.5 to -2.4)*
Schistosomiasis episodes aggregate	7,231.9 (5,389.5 to 9,235.4)	-24.7 (-34.7 to -8.2)*	-29.3 (-38.7 to -13.9)*	41.3 (15.9 to 87.0)	-26.6 (-36.1 to -9.7)*	-31.1 (-40.0 to -15.2)*
Complications of schistosomiasis aggregate	9,548.3 (6,739.9 to 14,455.5)	-13.5 (-27.4 to 22.6)	-18.9 (-31.9 to 15.1)	110.3 (51.0 to 222.0)	-11.2 (-24.2 to 24.1)	-16.7 (-28.8 to 16.5)
Anemia due to schistosomiasis aggregate	1,025.7 (986.0 to 1,061.6)	-18.5 (-21.9 to -15.3)*	-23.5 (-26.7 to -20.5)*	28.0 (18.5 to 40.8)	-22.5 (-28.0 to -17.5)*	-27.2 (-32.5 to -22.6)*
Cysticercosis	182.8 (116.2 to 282.0)	-11.5 (-19.6 to -4.7)*	-17.0 (-24.6 to -10.6)*	27.5 (15.6 to 45.0)	-20.3 (-29.3 to -12.3)*	-25.2 (-33.6 to -17.7)*
Cystic echinococcosis	102.6 (88.0 to 119.1)	18.5 (15.5 to 21.5)*	11.3 (8.4 to 14.0)*	9.5 (6.4 to 13.3)	18.5 (9.9 to 27.7)*	11.2 (3.1 to 19.8)*
Lymphatic filariasis	3,277.9 (2,623.6 to 4,142.1)	-42.4 (-48.1 to -36.6)*	-45.9 (-51.2 to -40.5)*	201.1 (108.2 to 328.0)	-15.5 (-31.8 to -2.3)*	-20.7 (-36.0 to -8.3)*
Prevalence of detectable microfiliaria due to lymphatic filariasis	1,478.3 (1,269.5 to 1,700.8)	-58.8 (-62.2 to -54.8)*	-61.3 (-64.6 to -57.6)*	0.0 (0.0 to 0.0)	0.0 (0.0 to 0.0)	0.0 (0.0 to 0.0)
Complications of lymphatic filariasis aggregate	1,799.6 (1,195.9 to 2,615.3)	-14.2 (-29.9 to -2.1)*	-19.4 (-34.2 to -8.1)*	201.1 (108.2 to 328.0)	-15.5 (-31.8 to -2.3)*	-20.7 (-36.0 to -8.3)*
Onchocerciasis	1,214.5 (953.2 to 1,557.1)	-25.5 (-36.2 to -14.1)*	-30.1 (-40.2 to -19.4)*	86.6 (39.1 to 155.4)	-17.5 (-38.0 to 2.6)	-22.6 (-41.8 to -3.7)*
Asymptomatic onchocerciasis	232.9 (162.0 to 314.9)	-53.4 (-56.5 to -51.0)*	-56.3 (-59.2 to -54.0)*	0.0 (0.0 to 0.0)	0.0 (0.0 to 0.0)	0.0 (0.0 to 0.0)
Skin disease due to onchocerciasis aggregate	925.3 (647.5 to 1,276.4)	-12.5 (-27.6 to 4.0)	-17.9 (-32.1 to -2.4)*	82.2 (34.7 to 149.8)	-17.1 (-39.3 to 4.0)	-22.2 (-43.1 to -1.9)*
Vision loss due to onchocerciasis aggregate	58.0 (38.4 to 83.5)	-20.7 (-32.7 to -7.0)*	-25.6 (-36.9 to -12.7)*	4.3 (2.6 to 6.8)	-24.3 (-36.2 to -10.2)*	-29.0 (-40.1 to -15.7)*
Trachoma	112.2 (71.9 to 166.0)	4.8 (-4.1 to 14.9)	-1.7 (-10.0 to 7.9)	9.5 (5.0 to 16.7)	0.3 (-11.0 to 14.2)	-5.9 (-16.5 to 7.2)
Dengue	319.1 (175.9 to 712.3)	149.4 (-1.5 to 605.5)	134.1 (-7.6 to 562.1)	51.4 (23.1 to 121.8)	147.2 (-2.2 to 628.9)	132.0 (-8.2 to 584.1)
Dengue episodes aggregate	95.1 (61.7 to 206.3)	135.8 (-5.7 to 559.1)	121.3 (-11.5 to 518.6)	5.5 (3.0 to 12.7)	147.4 (-5.3 to 595.7)	132.2 (-11.2 to 552.9)
Post-dengue chronic fatigue syndrome	224.3 (106.6 to 512.4)	149.6 (-1.5 to 605.5)	134.2 (-7.6 to 562.2)	45.9 (19.3 to 112.7)	147.2 (-2.1 to 626.9)	132.0 (-8.1 to 582.2)
Yellow fever	0.1 (0.0 to 0.4)	-20.5 (-37.6 to 5.9)	-25.4 (-41.5 to -0.6)*	0.0 (0.0 to 0.0)	-20.4 (-37.6 to 5.9)	-25.3 (-41.5 to -0.6)*
Rabies	0.0 (0.0 to 0.1)	-45.9 (-68.5 to -10.7)*	-49.3 (-70.4 to -16.2)*	0.0 (0.0 to 0.0)	-45.9 (-68.5 to -10.7)*	-49.2 (-70.4 to -16.2)*
Intestinal nematode infections	102,880.2 (99,916.7 to 105,675.5)	-4.8 (-8.2 to -1.4)*	-10.6 (-13.8 to -7.5)*	215.0 (122.7 to 350.1)	-22.8 (-29.4 to -14.6)*	-27.5 (-33.7 to -19.9)*
Ascariasis	54,326.7 (46,787.0 to 63,074.1)	-12.6 (-27.3 to 4.4)	-18.0 (-31.8 to -2.0)*	57.6 (31.7 to 97.4)	-38.4 (-49.0 to -26.2)*	-42.2 (-52.2 to -30.7)*
Complications of ascariasis aggregate	3,181.6 (2,854.0 to 3,557.2)	-36.1 (-45.0 to -25.4)*	-40.0 (-48.4 to -30.0)*	57.6 (31.7 to 97.4)	-38.4 (-49.0 to -26.2)*	-42.2 (-52.2 to -30.7)*
Asymptomatic ascariasis	51,150.4 (43,558.1 to 59,933.2)	-10.6 (-26.4 to 8.1)	-16.1 (-31.0 to 1.5)	0.0 (0.0 to 0.0)	0.0 (0.0 to 0.0)	0.0 (0.0 to 0.0)
Trichuriasis	32,295.1 (29,063.6 to 35,987.2)	-2.8 (-16.1 to 13.9)	-8.8 (-21.3 to 6.9)	38.9 (20.2 to 68.6)	-11.4 (-31.2 to 18.4)	-16.9 (-35.4 to 11.1)
Complications of trichuriasis aggregate	1,932.9 (1,659.2 to 2,389.1)	-10.4 (-28.0 to 15.6)	-15.9 (-32.4 to 8.5)	38.9 (20.2 to 68.6)	-11.4 (-31.2 to 18.4)	-16.9 (-35.4 to 11.1)
Asymptomatic trichuriasis	30,365.7 (27,116.9 to 34,053.0)	-2.3 (-16.3 to 15.5)	-8.3 (-21.4 to 8.4)	0.0 (0.0 to 0.0)	0.0 (0.0 to 0.0)	0.0 (0.0 to 0.0)
Hookworm disease	31,297.6 (28,113.1 to 35,158.9)	-9.3 (-21.5 to 4.7)	-14.9 (-26.3 to -1.8)*	118.5 (71.0 to 187.0)	-15.9 (-23.2 to -8.2)*	-21.1 (-27.9 to -13.9)*
Complications of hookworm disease aggregate	3,823.2 (3,554.0 to 4,146.8)	-15.2 (-24.1 to -5.5)*	-20.4 (-28.8 to -11.3)*	78.3 (42.7 to 129.5)	-16.6 (-27.5 to -4.9)*	-21.7 (-32.0 to -10.8)*
Anemia due to hookworm disease aggregate	1,604.9 (1,569.4 to 1,639.9)	-9.6 (-12.2 to -7.0)*	-15.2 (-17.6 to -12.7)*	40.2 (26.6 to 58.3)	-14.6 (-19.4 to -10.2)*	-19.9 (-24.3 to -15.7)*
Asymptomatic hookworm disease	25,874.2 (22,640.8 to 29,663.1)	-8.3 (-23.5 to 9.7)	-13.9 (-28.2 to 3.0)	0.0 (0.0 to 0.0)	0.0 (0.0 to 0.0)	0.0 (0.0 to 0.0)
Food-borne trematodiases	6,470.0 (5,970.6 to 7,023.1)	-21.2 (-23.7 to -18.5)*	-26.0 (-28.4 to -23.5)*	171.9 (88.9 to 311.9)	-22.4 (-27.2 to -15.7)*	-27.2 (-31.7 to -20.9)*
Asymptomatic food-borne trematodiases aggregate	4,957.3 (3,947.2 to 5,743.0)	-20.9 (-23.9 to -17.7)*	-25.8 (-28.5 to -22.7)*	0.0 (0.0 to 0.0)	0.0 (0.0 to 0.0)	0.0 (0.0 to 0.0)
Symptomatic food-borne trematodiases aggregate	1,512.6 (871.2 to 2,503.5)	-21.9 (-26.2 to -15.4)*	-26.7 (-30.7 to -20.6)*	171.9 (88.9 to 311.9)	-22.4 (-27.2 to -15.7)*	-27.2 (-31.7 to -20.9)*
Leprosy	31.3 (28.7 to 34.4)	-11.8 (-13.2 to -10.5)*	-17.3 (-18.5 to -16.0)*	1.9 (1.3 to 2.8)	-11.1 (-20.8 to -0.9)*	-16.5 (-25.6 to -7.0)*
Ebola	0.3 (0.2 to 0.6)	75,215.9 (44,065.4 to 271,839.5)*	70,585.5 (41,350.0 to 255,120.5)*	0.1 (0.0 to 0.1)	73,889.7 (39,240.2 to 358,000.4)*	69,340.8 (36,821.6 to 335,984.1)*
Other neglected tropical diseases	2,004.3 (1,953.8 to 2,051.3)	-5.7 (-8.6 to -3.1)*	-11.5 (-14.2 to -9.0)*	203.3 (72.2 to 559.7)	8.0 (-27.5 to 64.1)	1.4 (-32.0 to 54.0)
Anemia due to other neglected tropical diseases aggregate	2,004.3 (1,953.8 to 2,051.3)	-5.7 (-8.6 to -3.1)*	-11.5 (-14.2 to -9.0)*	36.9 (23.7 to 54.8)	-16.2 (-21.0 to -10.8)*	-21.3 (-25.9 to -16.3)*
Maternal disorders	1,391.7 (1,325.3 to 1,455.7)	8.9 (2.8 to 14.4)*	2.2 (-3.5 to 7.4)	107.2 (73.4 to 147.3)	-1.0 (-12.1 to 13.2)	-7.0 (-17.5 to 6.2)
Maternal hemorrhage	254.9 (241.1 to 270.7)	6.9 (0.3 to 13.3)*	0.3 (-5.8 to 6.3)	8.4 (5.4 to 11.9)	5.7 (-10.9 to 27.0)	-0.8 (-16.4 to 19.2)
Maternal hemorrhage episodes aggregate	20.5 (12.0 to 32.6)	9.7 (-32.0 to 75.8)	2.9 (-36.2 to 65.0)	3.4 (1.8 to 5.5)	10.5 (-26.7 to 69.2)	3.7 (-31.2 to 58.8)
Anemia due to maternal hemorrhage aggregate	234.3 (224.8 to 243.8)	6.6 (1.1 to 12.5)*	0.1 (-5.2 to 5.6)	5.1 (3.3 to 7.4)	2.7 (-8.5 to 14.9)	-3.6 (-14.1 to 7.8)
Maternal sepsis and other maternal infections	513.2 (458.4 to 577.8)	9.7 (0.5 to 21.1)*	3.0 (-5.7 to 13.6)	5.5 (2.8 to 9.7)	8.6 (-27.6 to 64.1)	1.9 (-32.1 to 54.0)
Maternal sepsis and other maternal infections aggregate	64.4 (31.6 to 119.0)	7.7 (-45.3 to 111.8)	1.1 (-48.7 to 98.8)	3.2 (1.5 to 6.5)	7.3 (-44.8 to 107.1)	0.7 (-48.2 to 94.4)
Infertility due to puerperal sepsis	448.9 (408.1 to 495.0)	10.0 (8.1 to 12.4)*	3.2 (1.5 to 5.5)*	2.2 (0.9 to 4.9)	10.4 (6.3 to 14.5)*	3.6 (-0.2 to 7.4)
Maternal hypertensive disorders	450.8 (275.4 to 682.7)	13.9 (-31.2 to 86.9)	6.9 (-35.4 to 75.4)	21.4 (11.2 to 36.3)	13.7 (-30.7 to 84.5)	6.7 (-35.0 to 73.2)
Maternal hypertensive disorders episodes aggregate	450.6 (275.0 to 682.7)	13.8 (-31.2 to 86.9)	6.8 (-35.5 to 75.4)	21.2 (11.0 to 36.0)	13.9 (-31.0 to 84.7)	6.9 (-35.3 to 73.3)
Eclampsia	0.4 (0.1 to 0.7)	-4.1 (-68.1 to 184.9)	-10.0 (-70.1 to 167.4)	0.2 (0.1 to 0.4)	-4.0 (-68.1 to 184.9)	-9.9 (-70.1 to 167.4)
Complications of maternal hypertensive disorders aggregate	0.0 (0.0 to 0.0)	0.0 (0.0 to 0.0)	0.0 (0.0 to 0.0)	0.0 (0.0 to 0.0)	0.0 (0.0 to 0.0)	0.0 (0.0 to 0.0)
Maternal obstructed labor and uterine rupture	153.6 (124.5 to 189.6)	-10.5 (-14.3 to -6.5)*	-16.0 (-19.6 to -12.2)*	50.1 (32.7 to 72.8)	-10.1 (-16.4 to -4.2)*	-15.6 (-21.5 to -10.1)*
Obstructed labor, acute event	6.1 (3.3 to 10.4)	6.1 (-40.3 to 90.2)	-0.4 (-43.9 to 78.5)	1.9 (0.9 to 3.5)	6.4 (-40.4 to 89.2)	-0.2 (-44.0 to 77.6)
Fistula due to maternal obstructed labor and uterine rupture aggregate	147.5 (118.4 to 183.3)	-11.1 (-14.6 to -7.8)*	-16.6 (-19.9 to -13.5)*	48.2 (31.2 to 70.4)	-10.6 (-17.0 to -5.0)*	-16.1 (-22.1 to -10.8)*
Maternal abortion, miscarriage, and ectopic pregnancy	51.2 (29.8 to 82.7)	11.0 (-29.3 to 76.9)	4.2 (-33.7 to 66.0)	5.5 (2.8 to 9.6)	11.2 (-30.7 to 79.1)	4.4 (-34.9 to 68.1)
Other maternal disorders	--	--	--	16.3 (10.6 to 22.8)	3.6 (-13.4 to 22.8)	-2.8 (-18.7 to 15.2)
Neonatal disorders	3,051.0 (2,902.6 to 3,180.3)	13.1 (9.4 to 16.9)*	6.1 (2.7 to 9.8)*	702.0 (529.7 to 898.3)	11.8 (7.5 to 16.1)*	4.9 (0.9 to 8.9)*
Neonatal preterm birth complications	2,423.9 (2,125.2 to 2,758.3)	6.1 (1.1 to 11.3)*	-0.5 (-5.1 to 4.4)	341.6 (258.1 to 440.7)	-0.3 (-5.9 to 5.6)	-6.4 (-11.6 to -0.9)*
Vision loss due to retinopathy of prematurity aggregate	294.4 (235.2 to 363.5)	4.8 (1.1 to 8.6)*	-1.6 (-5.2 to 1.9)	10.4 (6.3 to 16.0)	9.0 (0.6 to 17.7)*	2.3 (-5.6 to 10.4)
Complications of preterm birth complications aggregate	2,129.2 (1,854.8 to 2,438.0)	6.2 (0.7 to 12.1)*	-0.3 (-5.5 to 5.2)	331.2 (249.2 to 427.7)	-0.6 (-6.3 to 5.5)	-6.7 (-12.1 to -1.0)*
Neonatal encephalopathy due to birth asphyxia and trauma	822.5 (580.8 to 1,207.2)	33.0 (30.3 to 35.7)*	24.9 (22.2 to 27.3)*	238.2 (154.0 to 364.7)	29.5 (24.8 to 34.3)*	21.6 (17.1 to 26.1)*

Cause	Prevalence in thousands (95% confidence interval)			YLDs in thousands (95% confidence interval)		
	2015	Percent change in counts between 2005 and 2015	Percent change in rates between 2005 and 2015	2015	Percent change in counts between 2005 and 2015	Percent change in rates between 2005 and 2015
Neonatal sepsis and other neonatal infections	0.0 (0.0 to 0.0)	0.0 (0.0 to 0.0)	0.0 (0.0 to 0.0)	0.0 (0.0 to 0.0)	0.0 (0.0 to 0.0)	0.0 (0.0 to 0.0)
Severe infection due to neonatal sepsis and other neonatal infections	0.0 (0.0 to 0.0)	0.0 (0.0 to 0.0)	0.0 (0.0 to 0.0)	0.0 (0.0 to 0.0)	0.0 (0.0 to 0.0)	0.0 (0.0 to 0.0)
Complications of neonatal sepsis and other neonatal infections aggregate	0.0 (0.0 to 0.0)	0.0 (0.0 to 0.0)	0.0 (0.0 to 0.0)	0.0 (0.0 to 0.0)	0.0 (0.0 to 0.0)	0.0 (0.0 to 0.0)
Hemolytic disease and other neonatal jaundice	132.2 (118.3 to 149.3)	22.0 (20.8 to 23.3)*	14.5 (13.4 to 15.8)*	39.3 (29.3 to 50.4)	17.9 (12.7 to 23.7)*	10.7 (5.8 to 16.1)*
Other neonatal disorders	--	--	--	82.9 (57.4 to 109.4)	21.6 (5.5 to 39.8)*	14.1 (-1.0 to 31.2)
Nutritional deficiencies	91,144.2 (90,110.5 to 92,290.2)	-0.3 (-1.9 to 1.3)	-6.5 (-7.9 to -4.9)*	2,547.8 (1,698.1 to 3,682.0)	-4.5 (-6.7 to -2.3)*	-10.3 (-12.4 to -8.3)*
Protein-energy malnutrition	8.3 (5.2 to 13.3)	-16.3 (-26.2 to -4.5)*	-21.5 (-30.7 to -10.3)*	1.0 (0.5 to 1.8)	-15.0 (-29.1 to 1.7)	-20.3 (-33.5 to -4.6)*
Iodine deficiency	9,136.0 (8,276.1 to 10,297.7)	4.8 (2.5 to 7.2)*	-1.7 (-3.8 to 0.6)	190.1 (121.0 to 285.1)	6.9 (4.1 to 9.7)*	0.3 (-2.3 to 2.9)
Visible goiter due to iodine deficiency aggregate	8,964.1 (8,080.4 to 10,119.4)	4.5 (2.2 to 7.0)*	-1.9 (-4.1 to 0.4)	160.2 (98.9 to 252.2)	4.7 (2.2 to 7.2)*	-1.7 (-4.1 to 0.6)
Visible goiter with heart failure due to iodine deficiency aggregate	0.0 (0.0 to 0.0)	0.0 (0.0 to 0.0)	0.0 (0.0 to 0.0)	0.0 (0.0 to 0.0)	0.0 (0.0 to 0.0)	0.0 (0.0 to 0.0)
Intellectual disability due to iodine deficiency aggregate	172.0 (69.8 to 234.9)	19.5 (13.1 to 22.8)*	12.1 (6.2 to 15.3)*	29.9 (10.0 to 48.9)	20.5 (10.5 to 27.2)*	13.1 (3.7 to 19.4)*
Vitamin A deficiency	281.4 (195.2 to 372.1)	13.5 (7.7 to 19.8)*	6.5 (1.1 to 12.5)*	15.6 (9.4 to 23.4)	13.7 (6.6 to 20.8)*	6.8 (0.0 to 13.3)*
Iron-deficiency anemia	88,785.2 (87,558.8 to 89,914.2)	-1.7 (-3.2 to -0.1)*	-7.7 (-9.2 to -6.2)*	2,340.9 (1,556.5 to 3,372.7)	-5.4 (-7.7 to -3.1)*	-11.2 (-13.4 to -9.0)*
Iron-deficiency anemia without heart failure aggregate	88,784.7 (87,558.1 to 89,913.7)	-1.7 (-3.2 to -0.1)*	-7.7 (-9.2 to -6.2)*	2,340.9 (1,556.5 to 3,372.6)	-5.4 (-7.7 to -3.1)*	-11.2 (-13.4 to -9.0)*
Iron-deficiency anemia with heart failure aggregate	0.5 (0.4 to 0.6)	19.2 (16.3 to 22.1)*	11.8 (9.2 to 14.6)*	0.1 (0.0 to 0.1)	19.2 (16.3 to 22.1)*	11.8 (9.2 to 14.6)*
Other nutritional deficiencies	--	--	--	0.1 (0.1 to 0.2)	-14.0 (-35.5 to 12.9)	-19.3 (-39.4 to 6.0)
Other communicable, maternal, neonatal, and nutritional diseases	154,059.9 (150,437.3 to 157,433.8)	6.6 (5.7 to 7.6)*	0.1 (-0.8 to 1.0)	279.3 (174.9 to 433.1)	8.0 (0.5 to 15.4)*	1.3 (-5.7 to 8.3)
Sexually transmitted diseases excluding HIV	124,450.6 (120,463.8 to 128,255.2)	7.5 (6.3 to 8.9)*	0.9 (-0.3 to 2.2)	194.2 (118.7 to 311.0)	12.8 (7.5 to 18.3)*	5.9 (0.9 to 11.1)*
Syphilis	5,624.1 (3,846.4 to 8,097.4)	-0.3 (-9.8 to 7.3)	-6.4 (-15.3 to 0.7)	27.0 (18.1 to 38.1)	9.1 (2.9 to 15.5)*	2.4 (-3.4 to 8.4)
Early syphilis aggregate	5,489.1 (3,710.6 to 7,952.8)	-0.5 (-10.3 to 7.2)	-6.7 (-15.8 to 0.6)	1.3 (0.4 to 3.4)	-0.5 (-11.1 to 8.0)	-6.6 (-16.6 to 1.4)
Adult tertiary syphilis	135.0 (116.9 to 154.3)	9.4 (5.9 to 12.7)*	2.7 (-0.6 to 5.7)	25.7 (16.9 to 36.3)	9.6 (3.1 to 16.3)*	2.9 (-3.2 to 9.2)
Chlamydial infection	6,644.5 (4,462.8 to 9,295.2)	1.7 (-1.9 to 5.2)	-4.6 (-7.9 to -1.3)*	33.7 (19.2 to 55.2)	-0.9 (-7.4 to 6.0)	-7.0 (-13.1 to -0.5)*
Chlamydial infection episodes aggregate	6,274.5 (4,100.2 to 8,931.8)	1.4 (-2.4 to 5.0)	-4.9 (-8.4 to -1.4)*	28.5 (15.6 to 47.8)	-1.5 (-9.0 to 6.4)	-7.6 (-14.6 to -0.1)*
Pelvic inflammatory diseases due to chlamydial infection aggregate	25.2 (19.1 to 33.3)	-1.2 (-5.5 to 2.9)	-7.3 (-11.3 to -3.4)*	3.3 (2.1 to 5.0)	-0.6 (-11.3 to 10.5)	-6.7 (-16.7 to 3.7)
Infertility due to clamydial infection aggregate	344.7 (312.3 to 372.6)	8.7 (7.8 to 9.7)*	2.0 (1.2 to 2.9)*	1.9 (0.8 to 4.0)	8.6 (4.7 to 12.7)*	2.0 (-1.7 to 5.8)
Gonococcal infection	6,532.9 (3,768.6 to 10,469.4)	30.7 (20.3 to 38.8)*	22.7 (12.9 to 30.3)*	69.9 (35.4 to 122.8)	31.1 (19.6 to 40.0)*	23.0 (12.3 to 31.4)*
Gonococcal infection episodes aggregate	6,327.4 (3,568.7 to 10,261.2)	31.6 (21.1 to 39.7)*	23.5 (13.7 to 31.1)*	67.2 (33.2 to 119.6)	32.5 (20.8 to 41.6)*	24.3 (13.3 to 32.9)*
Pelvic inflammatory diseases due to gonococcal infection aggregate	205.5 (184.5 to 228.7)	8.9 (6.5 to 10.6)*	2.2 (-0.0 to 3.8)	2.7 (1.7 to 4.2)	3.6 (-3.8 to 10.9)	-2.8 (-9.7 to 4.1)
Trichomoniasis	19,525.5 (13,904.0 to 26,139.3)	7.8 (6.9 to 8.6)*	1.2 (0.3 to 1.9)*	22.1 (8.2 to 49.0)	8.3 (6.8 to 9.8)*	1.7 (0.2 to 3.1)*
Genital herpes	95,676.3 (82,965.5 to 109,451.9)	7.4 (5.9 to 9.0)*	0.8 (-0.6 to 2.3)	25.2 (7.7 to 59.1)	10.1 (7.3 to 16.9)*	3.3 (0.7 to 9.7)*
Moderate infection due to initial genital herpes episode	29.4 (6.8 to 69.7)	82.1 (62.6 to 111.3)*	70.9 (52.6 to 98.3)*	1.5 (0.3 to 3.5)	79.5 (56.6 to 113.5)*	68.4 (47.0 to 100.3)*
Complications of genital herpes aggregate	95,646.9 (82,948.7 to 109,421.7)	7.4 (5.9 to 9.0)*	0.8 (-0.6 to 2.3)	23.7 (6.8 to 58.2)	7.5 (5.6 to 9.5)*	0.9 (-0.9 to 2.8)
Other sexually transmitted diseases	1,028.9 (944.7 to 1,102.4)	3.4 (2.0 to 4.6)*	-3.0 (-4.3 to -1.8)*	16.4 (10.4 to 23.8)	-2.7 (-7.3 to 1.4)	-8.7 (-13.0 to -4.8)*
Pelvic inflammatory diseases due to other sexually transmitted diseases aggregate	85.5 (66.5 to 108.2)	-5.8 (-7.4 to -4.1)*	-11.6 (-13.1 to -10.0)*	11.2 (7.2 to 16.6)	-5.7 (-11.2 to -0.0)*	-11.5 (-16.6 to -6.2)*
Infertility due to other sexually transmitted diseases aggregate	943.4 (860.8 to 1,013.2)	4.3 (2.9 to 5.6)*	-2.1 (-3.5 to -0.8)*	5.2 (2.1 to 10.7)	4.4 (1.6 to 7.2)*	-2.0 (-4.6 to 0.6)
Other sexually transmitted diseases	--	--	--	41.4 (22.7 to 67.9)	20.3 (8.7 to 29.9)*	12.9 (2.0 to 22.0)*
Hepatitis	37,397.9 (36,878.7 to 37,930.5)	4.4 (3.6 to 5.3)*	-2.0 (-2.8 to -1.1)*	33.6 (19.8 to 53.2)	6.2 (-30.7 to 62.0)	-0.4 (-35.0 to 52.0)
Acute hepatitis A	188.5 (149.8 to 225.4)	5.1 (-18.5 to 37.4)	-1.3 (-23.5 to 28.9)	7.9 (4.9 to 11.9)	5.1 (-18.5 to 40.1)	-1.4 (-23.5 to 31.5)
Hepatitis B	28,117.1 (26,936.8 to 29,413.6)	3.5 (0.9 to 6.2)*	-2.8 (-5.3 to -0.4)*	23.2 (12.6 to 40.8)	6.5 (-41.7 to 97.2)	-0.1 (-45.3 to 85.1)
Acute Hepatitis B aggregate	1,039.1 (682.9 to 1,585.9)	6.8 (-40.4 to 94.6)	0.2 (-44.1 to 82.6)	23.2 (12.6 to 40.8)	6.5 (-41.7 to 97.2)	-0.1 (-45.3 to 85.1)
Chronic hepatitis B	27,078.0 (26,022.6 to 28,227.7)	3.4 (2.3 to 4.5)*	-3.0 (-4.0 to -1.9)*	0.0 (0.0 to 0.0)	0.0 (0.0 to 0.0)	0.0 (0.0 to 0.0)
Hepatitis C	9,787.1 (8,664.4 to 10,931.5)	7.0 (6.1 to 8.0)*	0.4 (-0.5 to 1.3)	0.4 (0.2 to 0.8)	6.7 (3.4 to 9.3)*	0.1 (-2.9 to 2.6)
Acute Hepatitis C aggregate	28.7 (20.9 to 37.5)	6.7 (3.4 to 9.4)*	0.2 (-2.9 to 2.6)	0.4 (0.2 to 0.8)	6.7 (3.4 to 9.3)*	0.1 (-2.9 to 2.6)
Chronic hepatitis C	9,758.4 (8,632.1 to 10,898.2)	7.0 (6.1 to 8.0)*	0.4 (-0.5 to 1.3)	0.0 (0.0 to 0.0)	0.0 (0.0 to 0.0)	0.0 (0.0 to 0.0)
Acute hepatitis E	65.5 (49.3 to 91.0)	6.2 (2.4 to 10.0)*	-0.3 (-3.9 to 3.2)	2.0 (1.2 to 3.3)	6.5 (-14.3 to 31.1)	-0.1 (-19.6 to 23.0)
Other infectious diseases	2,253.5 (2,207.3 to 2,296.6)	-4.9 (-7.3 to -2.6)*	-10.7 (-13.0 to -8.6)*	51.4 (32.3 to 79.4)	-6.2 (-12.2 to 0.7)	-12.0 (-17.6 to -5.5)*
Non-communicable diseases	471,161.2 (469,216.8 to 473,109.1)	6.9 (6.7 to 7.0)*	0.3 (0.2 to 0.5)*	46,169.0 (34,544.0 to 59,308.5)	7.4 (6.8 to 8.1)*	0.8 (0.2 to 1.5)*
Neoplasms	2,554.1 (2,450.5 to 2,665.3)	8.3 (2.8 to 14.0)*	1.7 (-3.5 to 7.0)	241.4 (170.9 to 317.4)	3.9 (-3.9 to 12.1)	-2.5 (-9.8 to 5.2)
Lip and oral cavity cancer	96.2 (73.7 to 134.1)	15.1 (-5.8 to 42.1)	8.0 (-11.6 to 33.4)	8.2 (5.3 to 12.7)	14.2 (-7.2 to 41.6)	7.2 (-12.9 to 32.9)
Nasopharynx cancer	41.7 (21.8 to 67.5)	-13.7 (-48.1 to 32.1)	-19.0 (-51.3 to 23.9)	3.9 (1.9 to 6.7)	-14.0 (-47.3 to 31.2)	-19.3 (-50.6 to 23.2)
Other pharynx cancer	17.6 (15.0 to 20.3)	10.3 (-3.9 to 25.3)	3.5 (-9.8 to 17.6)	1.5 (1.0 to 2.1)	9.6 (-5.9 to 26.8)	2.9 (-11.7 to 19.0)
Esophageal cancer	7.0 (5.8 to 8.8)	-8.6 (-29.7 to 12.1)	-14.2 (-34.1 to 5.2)	1.4 (0.9 to 1.9)	-12.6 (-32.2 to 6.1)	-18.0 (-36.4 to -0.4)*
Stomach cancer	53.4 (46.9 to 58.5)	-25.9 (-34.1 to -17.8)*	-30.5 (-38.1 to -22.8)*	6.2 (4.3 to 8.2)	-28.3 (-36.3 to -19.9)*	-32.7 (-40.2 to -24.8)*
Colon and rectum cancer	161.2 (143.9 to 178.8)	16.8 (3.5 to 33.7)*	9.6 (-2.8 to 25.4)	13.6 (9.5 to 18.4)	12.6 (-1.5 to 30.1)	5.7 (-7.6 to 22.1)
Colon and rectum cancer aggregate	160.7 (143.5 to 178.3)	16.8 (3.5 to 33.8)*	9.6 (-2.8 to 25.6)	13.5 (9.5 to 18.3)	12.7 (-1.5 to 30.3)	5.8 (-7.6 to 22.3)
Stoma due to colon and rectum cancer	0.4 (0.4 to 0.5)	-0.6 (-1.6 to 0.4)	-6.7 (-7.6 to -5.8)*	0.1 (0.0 to 0.1)	-0.6 (-1.6 to 0.4)	-6.8 (-7.6 to -5.8)*
Liver cancer	4.8 (2.5 to 7.7)	-52.7 (-75.5 to -13.2)*	-55.6 (-77.0 to -18.5)*	5.1 (3.1 to 9.2)	-29.0 (-58.4 to 23.1)	-33.4 (-61.0 to 15.5)
Liver cancer due to hepatitis B	15.0 (9.8 to 28.4)	-27.0 (-62.3 to 44.5)	-31.5 (-64.6 to 35.6)	3.1 (1.8 to 5.9)	-31.4 (-60.6 to 23.9)	-35.7 (-63.0 to 16.3)
Liver cancer due to hepatitis C	1.9 (1.3 to 2.6)	1.0 (-31.6 to 39.5)	-5.2 (-35.8 to 30.9)	0.4 (0.3 to 0.6)	-8.2 (-35.7 to 23.7)	-13.9 (-39.6 to 16.1)
Liver cancer due to alcohol use	2.5 (1.7 to 4.4)	-4.3 (-44.5 to 64.2)	-10.2 (-48.0 to 54.1)	0.6 (0.4 to 1.1)	-11.5 (-48.9 to 49.1)	-17.0 (-52.1 to 39.9)
Liver cancer due to other causes	4.1 (2.7 to 7.1)	-32.1 (-61.3 to 25.3)	-36.3 (-63.6 to 17.6)	0.9 (0.6 to 1.6)	-36.2 (-61.2 to 6.9)	-40.2 (-63.6 to 0.3)
Gallbladder and biliary tract cancer	1.5 (1.3 to 1.7)	-8.8 (-17.9 to 2.6)	-14.4 (-22.9 to -3.7)*	0.4 (0.3 to 0.6)	-10.0 (-19.0 to 1.6)	-15.6 (-23.9 to -4.6)*
Pancreatic cancer	3.5 (3.1 to 4.0)	11.9 (2.0 to 23.7)*	5.0 (-4.3 to 16.1)	0.9 (0.6 to 1.2)	7.7 (-1.8 to 18.7)	1.0 (-7.8 to 11.4)

Cause	Prevalence in thousands (95% confidence interval)			YLDs in thousands (95% confidence interval)		
	2015	Percent change in counts between 2005 and 2015	Percent change in rates between 2005 and 2015	2015	Percent change in counts between 2005 and 2015	Percent change in rates between 2005 and 2015
Larynx cancer	36.1 (33.9 to 40.3)	7.0 (-0.4 to 14.9)	0.4 (-6.6 to 7.9)	4.1 (2.8 to 5.7)	4.9 (-5.5 to 16.1)	-1.5 (-11.3 to 9.0)
Larynx cancer aggregate	5.1 (3.4 to 9.1)	-6.2 (-36.7 to 43.0)	-12.0 (-40.5 to 34.2)	1.2 (0.7 to 1.9)	-5.2 (-23.6 to 21.0)	-11.0 (-28.3 to 13.5)
Laryngectomy due to larynx cancer	31.0 (29.8 to 33.1)	9.5 (8.9 to 10.2)*	2.8 (2.2 to 3.4)*	2.9 (1.9 to 4.2)	9.7 (-1.9 to 23.0)	2.9 (-7.9 to 15.4)
Tracheal, bronchus, and lung cancer	32.7 (25.3 to 44.1)	-11.7 (-34.2 to 15.4)	-17.1 (-38.2 to 8.3)	5.7 (3.6 to 8.5)	-14.8 (-36.6 to 12.4)	-20.1 (-40.5 to 5.5)
Malignant skin melanoma	138.6 (120.1 to 181.2)	14.3 (5.5 to 23.6)*	7.3 (-1.0 to 16.0)	8.0 (5.2 to 11.8)	13.3 (3.2 to 23.2)*	6.3 (-3.1 to 15.6)
Non-melanoma skin cancer	46.3 (41.4 to 51.8)	33.5 (14.8 to 54.3)*	25.3 (7.7 to 44.8)*	3.1 (2.0 to 4.5)	30.5 (9.2 to 51.6)*	22.5 (2.4 to 42.3)*
Non-melanoma skin cancer(squamous-cell carcinoma)	50.9 (42.6 to 61.4)	35.8 (14.7 to 57.6)*	27.5 (7.7 to 47.9)*	3.0 (1.9 to 4.3)	31.7 (9.5 to 54.2)*	23.6 (2.7 to 44.7)*
Non-melanoma skin cancer(basal-cell carcinoma)	10.5 (8.1 to 13.0)	5.5 (2.2 to 8.3)*	-0.9 (-4.1 to 1.7)	0.1 (0.1 to 0.2)	5.5 (-3.4 to 15.0)	-1.0 (-9.4 to 8.0)
Breast cancer	958.0 (876.2 to 1,046.4)	12.5 (0.3 to 26.5)*	5.6 (-5.9 to 18.7)	70.8 (49.4 to 95.4)	8.7 (-2.5 to 21.4)	2.0 (-8.5 to 13.9)
Breast cancer aggregate	789.6 (706.9 to 878.4)	15.3 (0.3 to 33.1)*	8.2 (-5.9 to 24.9)	57.5 (40.1 to 76.1)	10.6 (-3.5 to 27.2)	3.8 (-9.5 to 19.3)
Mastectomy due to breast cancer	168.4 (160.5 to 175.2)	0.9 (-0.5 to 2.1)	-5.3 (-6.6 to -4.2)*	13.3 (8.3 to 19.6)	1.1 (-3.7 to 6.1)	-5.1 (-9.6 to -0.4)*
Cervical cancer	345.6 (287.2 to 413.1)	-13.3 (-27.0 to 6.0)	-18.7 (-31.5 to -0.5)*	26.2 (17.7 to 36.2)	-13.0 (-27.1 to 6.8)	-18.3 (-31.6 to 0.2)
Uterine cancer	108.0 (72.1 to 145.5)	-9.2 (-53.2 to 88.5)	-14.8 (-56.1 to 76.9)	7.1 (4.1 to 10.9)	-9.6 (-53.0 to 86.3)	-15.1 (-55.9 to 74.8)
Ovarian cancer	56.5 (45.0 to 72.6)	17.9 (-6.8 to 45.8)	10.6 (-12.5 to 36.8)	7.3 (4.9 to 10.4)	16.2 (-9.9 to 44.7)	9.0 (-15.4 to 35.8)
Prostate cancer	18.0 (13.9 to 22.0)	29.3 (12.7 to 49.6)*	21.3 (5.8 to 40.4)*	1.5 (0.9 to 2.1)	18.0 (1.6 to 36.8)*	10.8 (-4.7 to 28.3)
Prostate cancer aggregate	15.1 (11.0 to 19.1)	35.9 (15.0 to 64.5)*	27.6 (7.9 to 54.4)*	1.2 (0.7 to 1.7)	22.1 (1.3 to 46.7)*	14.6 (-4.9 to 37.7)
Impotence and incontinence due to prostate cancer aggregate	2.9 (2.8 to 3.0)	3.1 (2.5 to 3.7)*	-3.3 (-3.8 to -2.7)*	0.3 (0.2 to 0.4)	3.1 (2.5 to 3.7)*	-3.3 (-3.8 to -2.7)*
Testicular cancer	93.2 (80.6 to 107.3)	25.5 (9.1 to 42.5)*	17.7 (2.4 to 33.8)*	5.5 (3.6 to 7.9)	24.2 (7.4 to 42.4)*	16.6 (0.8 to 33.6)*
Kidney cancer	44.7 (40.2 to 49.5)	19.2 (7.0 to 33.2)*	11.8 (0.4 to 25.0)*	3.3 (2.3 to 4.5)	17.5 (4.2 to 33.1)*	10.3 (-2.2 to 25.0)
Bladder cancer	39.7 (29.5 to 50.8)	5.9 (-19.7 to 37.2)	-0.7 (-24.6 to 28.8)	3.8 (2.6 to 5.4)	4.9 (-15.3 to 28.3)	-1.6 (-20.5 to 20.4)
Bladder cancer episodes aggregate	34.8 (24.7 to 45.9)	5.9 (-23.1 to 42.6)	-0.6 (-27.8 to 33.8)	2.9 (1.9 to 4.3)	4.7 (-20.8 to 35.9)	-1.7 (-25.7 to 27.6)
Urinary incontinence due to bladder cancer	4.9 (4.6 to 5.2)	5.4 (4.1 to 6.4)*	-1.1 (-2.3 to -0.1)*	0.9 (0.6 to 1.2)	5.4 (4.1 to 6.4)*	-1.1 (-2.3 to -0.1)*
Brain and nervous system cancer	53.7 (45.1 to 63.8)	5.4 (-12.4 to 26.8)	-1.1 (-17.8 to 19.0)	6.0 (4.1 to 8.1)	4.1 (-14.0 to 25.2)	-2.3 (-19.3 to 17.5)
Thyroid cancer	68.4 (60.3 to 76.9)	-3.7 (-15.0 to 8.2)	-9.6 (-20.2 to 1.5)	3.9 (2.5 to 5.5)	-4.5 (-17.0 to 8.9)	-10.4 (-22.1 to 2.2)
Mesothelioma	1.0 (0.9 to 1.2)	1.3 (-10.7 to 14.9)	-4.9 (-16.2 to 7.8)	0.2 (0.1 to 0.3)	1.3 (-10.6 to 14.9)	-5.0 (-16.1 to 7.9)
Hodgkin lymphoma	42.4 (35.7 to 51.7)	10.6 (-4.2 to 28.7)	3.8 (-10.0 to 20.8)	3.4 (2.3 to 4.8)	6.9 (-8.1 to 25.4)	0.3 (-13.7 to 17.7)
Non-Hodgkin lymphoma	151.1 (128.7 to 175.3)	39.0 (20.6 to 56.8)*	30.4 (13.2 to 47.2)*	11.3 (7.8 to 15.6)	35.1 (16.5 to 53.8)*	26.8 (9.3 to 44.4)*
Multiple myeloma	7.7 (6.1 to 9.4)	30.0 (8.3 to 56.7)*	22.0 (1.6 to 47.1)*	1.8 (1.2 to 2.5)	27.5 (6.8 to 52.9)*	19.7 (0.3 to 43.5)*
Leukemia	56.7 (49.7 to 64.3)	23.1 (4.0 to 44.5)*	15.5 (-2.4 to 35.6)	14.7 (10.2 to 19.8)	9.4 (-4.7 to 25.1)	2.6 (-10.6 to 17.4)
Acute lymphoid leukemia	27.2 (22.0 to 34.0)	19.9 (-0.2 to 43.3)	12.6 (-6.3 to 34.5)	3.6 (2.4 to 5.0)	15.8 (-3.5 to 38.2)	8.7 (-9.5 to 29.7)
Chronic lymphoid leukemia	34.5 (26.4 to 44.4)	-3.9 (-24.6 to 24.9)	-9.8 (-29.3 to 17.2)	4.3 (2.7 to 6.0)	-5.5 (-25.5 to 23.4)	-11.4 (-30.1 to 15.8)
Acute myeloid leukemia	38.2 (31.7 to 46.1)	27.3 (9.2 to 47.1)*	19.4 (2.5 to 38.0)*	4.8 (3.2 to 6.6)	24.5 (7.3 to 44.0)*	16.8 (0.7 to 35.1)*
Chronic myeloid leukemia	16.1 (13.1 to 19.6)	7.6 (-9.7 to 28.9)	1.0 (-15.3 to 20.9)	2.1 (1.4 to 2.9)	4.2 (-12.5 to 25.0)	-2.2 (-17.9 to 17.3)
Other neoplasms	174.0 (144.8 to 210.1)	18.6 (-0.6 to 37.9)	11.3 (-6.7 to 29.5)	12.7 (8.6 to 17.8)	17.6 (-1.4 to 36.5)	10.4 (-7.5 to 28.1)
Cardiovascular diseases	8,423.7 (8,128.7 to 8,637.4)	-0.5 (-3.0 to 1.9)	-6.6 (-9.0 to -4.4)*	658.9 (462.2 to 875.0)	2.2 (-2.8 to 4.5)	-4.1 (-8.8 to -1.9)*
Rheumatic heart disease	3,302.5 (2,945.5 to 4,174.0)	-10.6 (-19.5 to -5.7)*	-16.1 (-24.4 to -11.5)*	158.8 (99.6 to 244.9)	-10.2 (-19.2 to -5.3)*	-15.8 (-24.2 to -11.1)*
Rheumatic heart disease, without heart failure	3,260.3 (2,902.7 to 4,131.9)	-10.8 (-19.7 to -5.9)*	-16.2 (-24.6 to -11.6)*	153.8 (96.4 to 237.8)	-10.7 (-19.8 to -5.8)*	-16.2 (-24.7 to -11.6)*
Heart failure fue to rheumatic heart disease aggregate	42.1 (37.2 to 47.9)	8.1 (6.6 to 9.6)*	1.4 (0.0 to 2.9)*	5.0 (3.3 to 7.2)	8.1 (-2.4 to 21.1)	1.4 (-8.4 to 13.7)
Ischemic heart disease	1,452.6 (1,278.4 to 1,639.7)	9.4 (8.3 to 10.4)*	2.7 (1.7 to 3.6)*	109.1 (72.4 to 155.5)	10.3 (7.5 to 13.1)*	3.5 (0.9 to 6.2)*
Myocardial infarction episodes aggregate	58.7 (43.0 to 79.9)	-11.7 (-19.4 to -4.9)*	-17.2 (-24.3 to -10.7)*	0.4 (0.2 to 0.6)	-0.2 (-2.8 to 1.8)	-6.3 (-8.8 to -4.5)*
Angina due to ischemic heart disease aggregate	1,140.8 (965.1 to 1,323.7)	11.1 (9.8 to 12.3)*	4.3 (3.0 to 5.4)*	79.0 (51.0 to 114.2)	11.2 (7.6 to 14.8)*	4.4 (1.0 to 7.7)*
Heart failure fue to ischemic heart disease aggregate	253.2 (225.7 to 281.7)	8.2 (7.4 to 9.1)*	1.6 (0.8 to 2.4)*	29.6 (20.0 to 41.4)	8.2 (3.1 to 13.1)*	1.5 (-3.3 to 6.1)
Cerebrovascular disease	1,231.4 (1,208.3 to 1,255.6)	5.9 (3.7 to 8.1)*	-0.6 (-2.7 to 1.5)	196.0 (134.0 to 266.9)	5.6 (2.1 to 8.9)*	-0.9 (-4.1 to 2.2)
Ischemic stroke	371.2 (356.4 to 386.6)	13.5 (12.7 to 14.2)*	6.6 (5.8 to 7.2)*	58.2 (39.5 to 79.6)	13.5 (8.0 to 19.6)*	6.5 (1.4 to 12.2)*
Chronic ischemic stroke aggregate	369.2 (353.8 to 384.5)	13.6 (12.8 to 14.2)*	6.6 (5.8 to 7.2)*	57.8 (39.1 to 79.1)	13.6 (8.1 to 19.7)*	6.6 (1.4 to 12.4)*
Ischemic stroke episodes aggregate	2.1 (1.4 to 2.8)	3.9 (-2.2 to 10.0)	-2.5 (-8.2 to 3.3)	0.4 (0.3 to 0.7)	3.9 (-2.2 to 10.0)	-2.5 (-8.2 to 3.3)
Hemorrhagic stroke	889.8 (855.3 to 925.5)	2.7 (1.9 to 3.3)*	-3.6 (-4.3 to -3.0)*	137.8 (94.8 to 187.8)	2.6 (-1.1 to 6.4)	-3.7 (-7.2 to -0.1)*
Chronic hemorrhagic stroke aggregate	884.5 (850.1 to 920.5)	2.7 (2.0 to 3.4)*	-3.6 (-4.3 to -3.0)*	136.7 (93.7 to 186.4)	2.7 (-1.1 to 6.5)	-3.6 (-7.1 to -0.0)*
Acute hemorrhagic stroke aggregate	5.3 (4.2 to 6.3)	-3.7 (-10.7 to 2.6)	-9.6 (-16.2 to -3.7)*	1.1 (0.7 to 1.6)	-3.8 (-10.7 to 2.6)	-9.7 (-16.2 to -3.7)*
Hypertensive heart disease	81.6 (71.6 to 93.0)	9.9 (8.7 to 11.1)*	3.1 (2.0 to 4.3)*	9.6 (6.3 to 13.6)	9.7 (1.7 to 19.0)*	2.9 (-4.6 to 11.6)
Cardiomyopathy and myocarditis	85.6 (74.7 to 97.5)	10.0 (8.8 to 11.2)*	3.2 (2.1 to 4.4)*	9.4 (6.2 to 13.5)	10.1 (2.9 to 18.2)*	3.3 (-3.4 to 10.9)
Acute myocarditis	10.3 (7.0 to 14.1)	8.6 (6.1 to 11.3)*	1.9 (-0.4 to 4.5)	0.5 (0.3 to 0.8)	8.6 (2.2 to 14.7)*	1.9 (-4.1 to 7.7)
Heart failure due to cardiomyopathy aggregate	75.3 (65.6 to 86.5)	10.2 (8.9 to 11.6)*	3.4 (2.2 to 4.7)*	8.9 (5.9 to 12.8)	10.1 (2.7 to 18.8)*	3.4 (-3.6 to 11.5)
Atrial fibrillation and flutter	500.1 (405.5 to 614.1)	1.8 (0.3 to 3.5)*	-4.5 (-5.9 to -2.9)*	42.2 (27.4 to 62.0)	1.9 (-2.7 to 6.9)	-4.3 (-8.7 to 0.3)
Peripheral vascular disease	0.0 (0.0 to 0.0)	0.0 (0.0 to 0.0)	0.0 (0.0 to 0.0)	0.0 (0.0 to 0.0)	0.0 (0.0 to 0.0)	0.0 (0.0 to 0.0)
Endocarditis	5.4 (4.4 to 6.5)	16.5 (13.0 to 20.1)*	9.4 (6.1 to 12.8)*	0.6 (0.4 to 0.9)	16.5 (13.0 to 20.3)*	9.3 (6.0 to 12.9)*
Endocarditis episodes aggregate	1.1 (0.8 to 1.3)	16.6 (9.3 to 23.6)*	9.4 (2.6 to 16.0)*	0.1 (0.0 to 0.1)	16.3 (9.0 to 23.4)*	9.2 (2.3 to 15.8)*
Heart failure due to endocarditis aggregate	4.3 (3.3 to 5.4)	16.5 (12.6 to 20.8)*	9.3 (5.7 to 13.4)*	0.5 (0.3 to 0.8)	16.5 (12.6 to 20.8)*	9.3 (5.7 to 13.4)*
Other cardiovascular and circulatory diseases	1,928.4 (1,621.4 to 2,297.3)	7.3 (5.4 to 9.0)*	0.7 (-1.1 to 2.3)	133.3 (85.5 to 191.5)	7.2 (4.6 to 9.9)*	0.6 (-1.9 to 3.1)
Heart failure due to other cardiovascular diseases aggregate	7.8 (6.7 to 9.0)	7.6 (5.8 to 9.4)*	1.0 (-0.7 to 2.7)	1.0 (0.6 to 1.4)	7.4 (-0.6 to 16.5)	0.8 (-6.7 to 9.4)
Other cardiovascular diseases episodes aggregate	1,920.6 (1,614.6 to 2,289.1)	7.3 (5.4 to 9.0)*	0.7 (-1.1 to 2.3)	132.3 (84.9 to 190.2)	7.2 (4.5 to 9.9)*	0.6 (-1.9 to 3.2)
Chronic respiratory diseases	24,523.9 (23,368.7 to 25,602.4)	5.3 (3.5 to 6.9)*	-1.2 (-2.8 to 0.4)	1,516.9 (1,169.2 to 1,929.8)	4.4 (1.9 to 6.9)*	-2.0 (-4.4 to 0.4)
Chronic obstructive pulmonary disease	8,341.8 (7,214.4 to 9,475.9)	-0.4 (-2.4 to 1.5)	-6.5 (-8.4 to -4.7)*	622.7 (503.7 to 744.6)	1.4 (-2.2 to 4.7)	-4.9 (-8.2 to -1.7)*

Cause	Prevalence in thousands (95% confidence interval)			YLDs in thousands (95% confidence interval)		
	2015	Percent change in counts between 2005 and 2015	Percent change in rates between 2005 and 2015	2015	Percent change in counts between 2005 and 2015	Percent change in rates between 2005 and 2015
Chronic obstructive pulmonary disease without heart failure aggregate	8,341.0 (7,210.6 to 9,473.6)	-0.4 (-2.4 to 1.6)	-6.5 (-8.4 to -4.7)*	620.2 (501.9 to 741.1)	1.4 (-2.3 to 4.7)	-4.9 (-8.3 to -1.7)*
Severe chronic obstructive pulmonary disease with heart failure aggregate	5.2 (4.5 to 6.1)	2.7 (1.2 to 4.2)*	-3.6 (-5.0 to -2.2)*	2.5 (1.7 to 3.4)	2.7 (1.2 to 4.2)*	-3.6 (-5.0 to -2.2)*
Pneumoconiosis	176.3 (167.6 to 185.0)	10.4 (4.5 to 16.6)*	3.6 (-1.9 to 9.4)	34.6 (22.2 to 50.5)	9.7 (2.9 to 17.0)*	3.0 (-3.4 to 9.8)
Silicosis	29.8 (25.8 to 34.4)	-14.0 (-16.5 to -11.4)*	-19.3 (-21.7 to -16.8)*	4.9 (2.9 to 7.2)	-13.8 (-25.1 to -1.9)*	-19.1 (-29.7 to -7.9)*
Silicosis without heart failure aggregate	29.8 (25.8 to 34.4)	-14.0 (-16.5 to -11.4)*	-19.3 (-21.7 to -16.8)*	4.9 (2.9 to 7.2)	-13.8 (-25.1 to -1.9)*	-19.1 (-29.7 to -7.9)*
Severe silicosis with heart failure aggregate	0.0 (0.0 to 0.0)	0.0 (0.0 to 0.0)	0.0 (0.0 to 0.0)	0.0 (0.0 to 0.0)	0.0 (0.0 to 0.0)	0.0 (0.0 to 0.0)
Asbestosis	5.4 (4.6 to 6.3)	0.2 (-2.5 to 2.8)	-6.0 (-8.5 to -3.6)*	0.9 (0.5 to 1.4)	0.2 (-2.5 to 2.8)	-6.0 (-8.5 to -3.6)*
Asbestosis without heart failure aggregate	5.4 (4.6 to 6.3)	0.2 (-2.5 to 2.8)	-6.0 (-8.5 to -3.6)*	0.9 (0.5 to 1.4)	0.2 (-2.5 to 2.8)	-6.0 (-8.5 to -3.6)*
Severe asbestosis with heart failure aggregate	0.0 (0.0 to 0.0)	0.0 (0.0 to 0.0)	0.0 (0.0 to 0.0)	0.0 (0.0 to 0.0)	0.0 (0.0 to 0.0)	0.0 (0.0 to 0.0)
Coal workers pneumoconiosis	2.7 (2.3 to 3.2)	23.6 (19.6 to 27.3)*	16.0 (12.3 to 19.5)*	0.5 (0.3 to 0.7)	23.6 (19.6 to 27.2)*	16.0 (12.3 to 19.4)*
Coal workers pneumoconiosis without heart failure aggregate	2.7 (2.3 to 3.2)	23.7 (19.8 to 27.6)*	16.1 (12.4 to 19.7)*	0.4 (0.3 to 0.7)	23.9 (20.0 to 27.8)*	16.3 (12.6 to 20.0)*
Severe coal workers pneumoconiosis with heart failure aggregate	0.0 (0.0 to 0.0)	14.9 (6.0 to 25.0)*	7.8 (-0.5 to 17.3)	0.0 (0.0 to 0.0)	14.9 (6.0 to 25.0)*	7.8 (-0.5 to 17.3)
Other pneumoconiosis	180.3 (158.7 to 203.4)	15.2 (11.7 to 18.5)*	8.1 (4.9 to 11.2)*	28.3 (18.1 to 41.7)	15.2 (6.8 to 23.8)*	8.2 (0.2 to 16.2)*
Other pneumoconiosis without heart failure aggregate	180.1 (158.6 to 203.3)	15.2 (11.7 to 18.5)*	8.1 (4.9 to 11.2)*	28.3 (18.0 to 41.6)	15.2 (6.7 to 23.9)*	8.2 (0.2 to 16.2)*
Severe other pneumoconiosis with heart failure aggregate	0.1 (0.1 to 0.1)	17.1 (13.2 to 21.7)*	9.9 (6.2 to 14.2)*	0.1 (0.0 to 0.1)	17.1 (13.2 to 21.7)*	9.9 (6.2 to 14.2)*
Asthma	16,427.6 (13,675.0 to 19,489.0)	8.0 (5.1 to 11.3)*	1.4 (-1.4 to 4.5)	730.1 (464.3 to 1,064.2)	8.1 (5.1 to 11.5)*	1.5 (-1.3 to 4.6)
Asymptomatic asthma	4,974.5 (3,978.3 to 6,067.4)	8.0 (5.1 to 11.3)*	1.4 (-1.4 to 4.5)	0.0 (0.0 to 0.0)	0.0 (0.0 to 0.0)	0.0 (0.0 to 0.0)
Symptomatic asthma aggregate	11,453.1 (9,407.7 to 13,802.2)	8.0 (5.1 to 11.3)*	1.4 (-1.4 to 4.5)	730.1 (464.3 to 1,064.2)	8.1 (5.1 to 11.5)*	1.5 (-1.3 to 4.6)
Interstitial lung disease and pulmonary sarcoidosis	91.4 (78.0 to 105.4)	10.4 (7.6 to 13.1)*	3.6 (1.0 to 6.1)*	11.3 (6.8 to 16.8)	10.5 (3.6 to 17.6)*	3.8 (-2.7 to 10.4)
Interstitial lung disease and pulmonary sarcoidosis without heart failure aggregate	89.1 (75.8 to 103.1)	10.4 (7.5 to 13.1)*	3.6 (0.9 to 6.2)*	10.2 (6.1 to 15.5)	10.4 (2.7 to 18.3)*	3.6 (-3.7 to 11.0)
Severe interstitial lung disease and pulmonary sarcoidosis with heart failure aggregate	2.3 (1.9 to 2.7)	11.8 (9.9 to 13.7)*	4.9 (3.1 to 6.7)*	1.1 (0.7 to 1.5)	11.8 (9.9 to 13.7)*	4.9 (3.1 to 6.7)*
Other chronic respiratory diseases	--	--	--	118.2 (94.0 to 143.6)	-2.4 (-11.9 to 8.2)	-8.4 (-17.4 to 1.5)
Cirrhosis and other chronic liver diseases	154.7 (147.4 to 162.1)	15.8 (9.1 to 23.1)*	8.7 (2.4 to 15.6)*	30.6 (21.0 to 42.4)	14.6 (9.0 to 20.2)*	7.6 (2.3 to 12.8)*
Cirrhosis and other chronic liver diseases due to hepatitis B	55.7 (46.9 to 64.8)	6.5 (2.7 to 9.9)*	-0.1 (-3.6 to 3.2)	9.4 (6.2 to 13.7)	6.7 (-3.7 to 17.2)	0.1 (-9.6 to 10.0)
Cirrhosis and other chronic liver diseases due to hepatitis C	41.9 (34.3 to 50.7)	18.6 (15.1 to 22.0)*	11.3 (8.0 to 14.5)*	7.1 (4.6 to 10.3)	18.9 (5.9 to 31.1)*	11.5 (-0.6 to 23.1)
Cirrhosis and other chronic liver diseases due to alcohol use	58.0 (50.2 to 66.7)	19.4 (16.5 to 22.1)*	12.0 (9.3 to 14.6)*	9.8 (6.5 to 14.0)	19.5 (9.4 to 30.6)*	12.1 (2.7 to 22.6)*
Cirrhosis and other chronic liver diseases due to other causes	24.2 (19.8 to 29.6)	16.3 (13.2 to 19.5)*	9.1 (6.2 to 12.2)*	4.2 (2.7 to 6.2)	15.8 (3.7 to 29.5)*	8.7 (-2.7 to 21.6)
Digestive diseases	13,325.7 (13,014.2 to 13,641.9)	-1.3 (-3.9 to 1.4)	-7.4 (-9.8 to -4.9)*	738.2 (524.7 to 983.7)	-0.1 (-2.6 to 2.6)	-6.2 (-8.6 to -3.7)*
Peptic ulcer disease	2,831.7 (2,748.8 to 2,923.2)	-13.3 (-15.9 to -10.7)*	-18.7 (-21.1 to -16.2)*	95.7 (63.9 to 134.8)	-17.0 (-20.1 to -13.9)*	-22.1 (-25.0 to -19.2)*
Peptic ulcer disease, symptomatic episodes	213.3 (161.1 to 275.5)	-12.6 (-17.5 to -7.3)*	-17.9 (-22.6 to -13.0)*	23.2 (14.5 to 34.2)	-12.5 (-18.5 to -6.1)*	-17.9 (-23.5 to -11.9)*
Anemia due to peptic ulcer disease aggregate	2,618.4 (2,551.9 to 2,684.9)	-13.4 (-16.2 to -10.6)*	-18.7 (-21.4 to -16.1)*	72.5 (48.0 to 104.8)	-18.3 (-21.7 to -14.7)*	-23.4 (-26.6 to -20.0)*
Gastritis and duodenitis	7,689.3 (7,373.7 to 8,008.0)	1.7 (-2.5 to 6.1)	-4.5 (-8.5 to -0.4)*	246.1 (167.6 to 351.2)	0.2 (-5.0 to 6.5)	-5.9 (-10.8 to -0.0)*
Gastritis and duodenitis, symptomatic episodes	201.1 (149.4 to 263.4)	2.5 (-2.6 to 5.3)	-3.8 (-8.6 to -1.2)*	21.9 (13.3 to 33.6)	2.6 (-3.7 to 8.4)	-3.7 (-9.7 to 1.7)
Anemia due to gastritis and duodenitis aggregate	7,488.1 (7,185.3 to 7,799.7)	1.7 (-2.6 to 6.2)	-4.5 (-8.6 to -0.3)*	224.2 (149.7 to 321.2)	-0.0 (-5.7 to 6.8)	-6.2 (-11.5 to 0.3)
Appendicitis	28.4 (23.7 to 34.0)	16.7 (15.0 to 18.4)*	9.5 (7.9 to 11.1)*	8.7 (5.6 to 12.4)	16.7 (4.4 to 29.8)*	9.5 (-2.0 to 21.8)
Paralytic ileus and intestinal obstruction	6.0 (4.4 to 7.6)	16.0 (9.5 to 22.2)*	8.9 (2.7 to 14.7)*	1.9 (1.2 to 2.9)	16.1 (9.0 to 22.6)*	9.0 (2.3 to 15.1)*
Inguinal, femoral, and abdominal hernia	1,342.2 (961.8 to 1,830.5)	1.9 (-3.9 to 8.0)	-4.3 (-9.8 to 1.3)	14.4 (6.5 to 29.1)	2.1 (-4.4 to 8.2)	-4.2 (-10.3 to 1.5)
Inflammatory bowel disease	999.5 (913.9 to 1,084.5)	2.7 (2.2 to 3.2)*	-3.6 (-4.1 to -3.2)*	216.5 (148.6 to 298.7)	2.8 (0.9 to 4.9)*	-3.5 (-5.3 to -1.5)*
Vascular intestinal disorders	1.6 (1.2 to 2.2)	13.2 (6.0 to 20.4)*	6.2 (-0.5 to 13.0)	0.5 (0.3 to 0.8)	13.2 (6.0 to 20.4)*	6.2 (-0.5 to 13.0)
Gallbladder and biliary diseases	507.8 (385.2 to 654.9)	7.5 (1.5 to 13.6)*	0.9 (-4.8 to 6.6)	55.1 (34.4 to 82.4)	7.6 (0.6 to 15.1)*	1.0 (-5.6 to 8.0)
Pancreatitis	88.1 (71.0 to 109.0)	14.4 (8.9 to 19.3)*	7.4 (2.2 to 11.9)*	26.7 (17.2 to 39.4)	14.4 (4.6 to 24.2)*	7.4 (-1.9 to 16.6)
Other digestive diseases	--	--	--	72.6 (50.4 to 99.9)	5.3 (-1.7 to 14.6)	-1.2 (-7.7 to 7.5)
Neurological disorders	235,470.4 (218,512.2 to 246,945.1)	8.6 (6.6 to 10.7)*	1.9 (0.0 to 3.9)*	5,339.5 (3,566.8 to 7,490.4)	8.7 (6.0 to 11.2)*	2.0 (-0.5 to 4.4)
Alzheimer disease and other dementias	0.0 (0.0 to 0.0)	0.0 (0.0 to 0.0)	0.0 (0.0 to 0.0)	0.0 (0.0 to 0.0)	0.0 (0.0 to 0.0)	0.0 (0.0 to 0.0)
Parkinson disease	106.7 (92.4 to 121.2)	-0.9 (-5.3 to 3.4)	-7.0 (-11.2 to -2.9)*	14.0 (9.4 to 19.7)	-0.7 (-8.6 to 7.6)	-6.8 (-14.2 to 1.0)
Epilepsy	1,360.5 (1,171.2 to 1,578.3)	3.8 (-1.5 to 9.1)	-2.6 (-7.6 to 2.3)	361.0 (245.1 to 480.8)	-12.1 (-18.1 to -6.2)*	-17.5 (-23.1 to -12.0)*
Multiple sclerosis	206.2 (187.6 to 225.9)	4.4 (2.3 to 6.6)*	-2.0 (-4.0 to 0.0)	69.7 (50.2 to 90.3)	4.3 (-0.1 to 9.0)	-2.1 (-6.2 to 2.3)
Motor neuron disease	8.3 (7.4 to 9.5)	3.8 (1.2 to 6.1)*	-2.6 (-5.0 to -0.4)*	1.8 (1.3 to 2.4)	3.8 (1.2 to 6.1)*	-2.6 (-5.0 to -0.4)*
Migraine	105,034.3 (84,179.1 to 126,110.8)	10.2 (8.3 to 12.4)*	3.4 (1.6 to 5.5)*	3,615.3 (2,204.7 to 5,586.6)	10.4 (8.3 to 12.6)*	3.6 (1.6 to 5.7)*
Tension-type headache	166,241.2 (119,245.1 to 222,450.2)	8.2 (6.5 to 9.7)*	1.5 (-0.0 to 3.0)	250.2 (110.3 to 480.3)	8.3 (6.4 to 10.0)*	1.6 (-0.2 to 3.3)
Medication overuse headache	6,443.1 (4,383.7 to 9,370.0)	12.3 (1.6 to 23.0)*	5.4 (-4.6 to 15.4)	1,023.0 (582.7 to 1,687.4)	12.4 (1.7 to 23.0)*	5.5 (-4.6 to 15.5)
Other neurological disorders	0.7 (0.4 to 1.1)	8.1 (6.0 to 9.8)*	1.4 (-0.5 to 3.0)	4.5 (3.1 to 6.1)	-1.2 (-6.2 to 3.8)	-7.3 (-12.0 to -2.6)*
Mental and substance use disorders	89,517.7 (87,049.4 to 91,864.5)	7.9 (6.9 to 8.8)*	1.3 (0.4 to 2.1)*	14,010.8 (10,104.3 to 17,959.4)	8.6 (7.9 to 9.4)*	1.9 (1.2 to 2.7)*
Schizophrenia	2,927.8 (2,482.4 to 3,454.6)	3.6 (2.9 to 4.3)*	-2.8 (-3.5 to -2.2)*	1,910.2 (1,376.3 to 2,422.6)	3.6 (2.2 to 5.0)*	-2.7 (-4.0 to -1.4)*
Alcohol use disorders	7,690.8 (6,547.4 to 9,002.1)	1.4 (-2.1 to 4.8)	-4.8 (-8.2 to -1.7)*	773.7 (496.1 to 1,144.7)	1.4 (-2.4 to 5.0)	-4.8 (-8.4 to -1.4)*
Alcohol dependence aggregate	7,629.8 (6,486.9 to 8,939.1)	1.4 (-2.2 to 4.8)	-4.9 (-8.2 to -1.7)*	770.6 (493.5 to 1,139.5)	1.4 (-2.4 to 5.0)	-4.8 (-8.4 to -1.4)*
Fetal alcohol syndrome aggregate	60.9 (56.4 to 65.1)	5.8 (4.9 to 6.6)*	-0.7 (-1.5 to 0.0)	3.1 (2.0 to 4.5)	5.4 (-1.4 to 12.3)	-1.0 (-7.5 to 5.4)
Drug use disorders	4,188.0 (4,021.9 to 4,326.9)	10.8 (8.8 to 12.9)*	4.0 (2.1 to 5.9)*	1,025.1 (704.9 to 1,339.9)	16.1 (13.1 to 19.4)*	9.0 (6.2 to 12.0)*
Opioid use disorders	1,948.7 (1,575.6 to 2,340.3)	15.8 (12.4 to 19.1)*	8.6 (5.5 to 11.8)*	814.2 (551.3 to 1,094.0)	15.9 (12.0 to 19.7)*	8.8 (5.1 to 12.3)*
Asymptomatic opioid dependence	316.3 (216.9 to 448.1)	15.8 (12.4 to 19.1)*	8.6 (5.5 to 11.8)*	0.0 (0.0 to 0.0)	0.0 (0.0 to 0.0)	0.0 (0.0 to 0.0)
Symptomatic opioid dependence aggregate	1,632.4 (1,321.5 to 1,984.9)	15.8 (12.4 to 19.1)*	8.6 (5.5 to 11.8)*	814.2 (551.3 to 1,094.0)	15.9 (12.0 to 19.7)*	8.8 (5.1 to 12.3)*
Cocaine use disorders	269.7 (222.4 to 319.0)	16.9 (13.8 to 20.5)*	9.7 (6.8 to 13.1)*	37.1 (23.1 to 54.3)	16.8 (10.5 to 23.6)*	9.6 (3.7 to 16.0)*
Asymptomatic cocaine dependence	135.2 (104.0 to 170.6)	16.9 (13.8 to 20.5)*	9.7 (6.8 to 13.1)*	0.0 (0.0 to 0.0)	0.0 (0.0 to 0.0)	0.0 (0.0 to 0.0)

Cause	Prevalence in thousands (95% confidence interval)			YLDs in thousands (95% confidence interval)		
	2015	Percent change in counts between 2005 and 2015	Percent change in rates between 2005 and 2015	2015	Percent change in counts between 2005 and 2015	Percent change in rates between 2005 and 2015
Symptomatic cocaine dependence aggregate	134.5 (102.7 to 171.5)	16.9 (13.8 to 20.5)*	9.7 (6.8 to 13.1)*	37.1 (23.1 to 54.3)	16.8 (10.5 to 23.6)*	9.6 (3.7 to 16.0)*
Amphetamine use disorders	487.0 (386.7 to 610.5)	25.1 (21.2 to 29.6)*	17.4 (13.7 to 21.6)*	64.0 (37.0 to 97.6)	25.3 (17.7 to 33.4)*	17.6 (10.4 to 25.2)*
Asymptomatic amphetamine dependence	266.6 (196.0 to 348.2)	25.1 (21.2 to 29.6)*	17.5 (13.7 to 21.6)*	0.0 (0.0 to 0.0)	0.0 (0.0 to 0.0)	0.0 (0.0 to 0.0)
Symptomatic amphetamine dependence aggregate	220.4 (159.8 to 294.8)	25.1 (21.2 to 29.6)*	17.4 (13.7 to 21.6)*	64.0 (37.0 to 97.6)	25.3 (17.7 to 33.4)*	17.6 (10.4 to 25.2)*
Cannabis use disorders	1,523.4 (1,293.7 to 1,807.6)	0.8 (-0.5 to 2.3)	-5.4 (-6.6 to -4.0)*	44.2 (27.8 to 64.4)	1.0 (-2.9 to 5.0)	-5.2 (-8.9 to -1.4)*
Asymptomatic cannabis dependence	881.1 (733.5 to 1,067.8)	0.8 (-0.5 to 2.3)	-5.4 (-6.6 to -4.0)*	0.0 (0.0 to 0.0)	0.0 (0.0 to 0.0)	0.0 (0.0 to 0.0)
Symptomatic cannabis dependence aggregate	642.3 (525.1 to 783.4)	0.8 (-0.5 to 2.3)	-5.4 (-6.6 to -4.0)*	44.2 (27.8 to 64.4)	1.0 (-2.9 to 5.0)	-5.2 (-8.9 to -1.4)*
Other drug use disorders	--	--	--	65.6 (40.5 to 95.2)	22.1 (15.5 to 28.8)*	14.6 (8.4 to 20.9)*
Depressive disorders	28,649.4 (26,818.8 to 30,303.5)	9.4 (7.4 to 11.2)*	2.7 (0.8 to 4.4)*	5,094.0 (3,295.4 to 7,152.3)	10.3 (9.2 to 11.4)*	3.5 (2.5 to 4.5)*
Major depressive disorder	20,441.8 (15,819.9 to 25,595.0)	11.0 (9.9 to 12.0)*	4.1 (3.1 to 5.1)*	4,229.1 (2,599.1 to 6,025.9)	11.1 (9.9 to 12.3)*	4.3 (3.2 to 5.4)*
Dysthymia	8,849.3 (7,144.8 to 10,829.0)	6.4 (4.7 to 8.0)*	-0.2 (-1.7 to 1.4)	864.9 (552.1 to 1,273.4)	6.4 (4.7 to 8.2)*	-0.1 (-1.8 to 1.6)
Bipolar disorder	4,636.4 (3,809.2 to 5,569.3)	8.6 (8.3 to 8.9)*	1.9 (1.6 to 2.2)*	951.6 (569.9 to 1,438.9)	8.7 (7.2 to 10.2)*	2.0 (0.6 to 3.4)*
Anxiety disorders	22,473.6 (18,676.8 to 27,278.3)	9.7 (7.4 to 12.1)*	3.0 (0.8 to 5.2)*	2,082.2 (1,362.5 to 2,975.4)	9.8 (7.3 to 12.1)*	3.1 (0.7 to 5.2)*
Eating disorders	841.2 (789.2 to 893.0)	17.8 (13.7 to 21.8)*	10.6 (6.7 to 14.3)*	177.2 (113.6 to 261.7)	18.2 (15.1 to 21.3)*	10.9 (8.0 to 13.8)*
Anorexia nervosa	217.6 (175.4 to 268.3)	13.2 (10.0 to 16.3)*	6.3 (3.2 to 9.1)*	45.8 (29.0 to 67.1)	13.4 (7.6 to 19.2)*	6.4 (1.0 to 11.8)*
Bulimia nervosa	626.7 (475.7 to 814.7)	19.9 (17.7 to 22.0)*	12.5 (10.5 to 14.5)*	131.3 (81.4 to 198.4)	20.0 (16.5 to 23.5)*	12.6 (9.3 to 15.9)*
Autistic spectrum disorders	4,373.2 (4,138.8 to 4,554.1)	6.8 (4.9 to 8.7)*	0.2 (-1.5 to 2.0)	706.2 (474.5 to 972.3)	7.1 (5.8 to 8.4)*	0.5 (-0.7 to 1.7)
Autism	1,742.0 (1,466.7 to 2,066.9)	7.3 (6.9 to 7.7)*	0.7 (0.4 to 1.0)*	445.1 (284.7 to 637.1)	7.4 (5.6 to 9.3)*	0.8 (-0.9 to 2.5)
Asperger syndrome and other autistic spectrum disorders	2,614.9 (2,195.4 to 3,122.4)	6.4 (6.2 to 6.7)*	-0.1 (-0.3 to 0.1)	261.1 (172.9 to 382.3)	6.5 (5.1 to 8.1)*	-0.0 (-1.4 to 1.4)
Attention-deficit/hyperactivity disorder	1,646.8 (1,378.7 to 1,943.1)	-1.0 (-2.6 to 0.7)	-7.1 (-8.6 to -5.5)*	19.8 (11.5 to 30.5)	-1.0 (-4.4 to 2.3)	-7.1 (-10.3 to -4.0)*
Conduct disorder	0.0 (0.0 to 0.0)	0.0 (0.0 to 0.0)	0.0 (0.0 to 0.0)	0.0 (0.0 to 0.0)	0.0 (0.0 to 0.0)	0.0 (0.0 to 0.0)
Idiopathic developmental intellectual disability	6,351.2 (3,596.8 to 9,000.7)	9.2 (7.8 to 10.3)*	2.5 (1.2 to 3.5)*	237.7 (102.4 to 411.7)	7.5 (4.4 to 9.2)*	0.9 (-2.0 to 2.5)
Other mental and substance use disorders	13,620.0 (13,421.7 to 13,835.0)	6.6 (4.2 to 8.9)*	0.0 (-2.2 to 2.2)	1,033.0 (717.0 to 1,397.5)	6.6 (4.1 to 9.3)*	0.1 (-2.3 to 2.6)
Diabetes, urogenital, blood, and endocrine diseases	225,393.4 (223,302.2 to 227,913.1)	7.1 (6.5 to 7.8)*	0.5 (-0.1 to 1.2)	4,487.0 (3,140.7 to 6,172.6)	7.4 (5.6 to 9.3)*	0.8 (-0.9 to 2.6)
Diabetes mellitus	26,623.4 (23,199.3 to 30,360.4)	10.4 (6.9 to 13.9)*	3.6 (0.3 to 6.9)*	1,951.8 (1,292.8 to 2,724.4)	13.6 (9.5 to 17.7)*	6.6 (2.8 to 10.5)*
Uncomplicated diabetes mellitus	18,377.6 (15,545.0 to 21,574.8)	6.6 (3.1 to 10.3)*	0.1 (-3.3 to 3.5)	869.7 (544.1 to 1,297.4)	6.6 (3.1 to 10.4)*	0.1 (-3.2 to 3.7)
Neuropathy and other complications of diabetes mellitus aggregate	7,990.8 (6,422.0 to 9,815.3)	20.0 (15.7 to 24.5)*	12.6 (8.6 to 16.9)*	1,067.1 (694.3 to 1,529.0)	20.0 (15.4 to 24.5)*	12.6 (8.4 to 16.9)*
Vision loss due to diabetes mellitus aggregate	254.9 (180.1 to 359.3)	16.6 (14.2 to 19.2)*	9.4 (7.2 to 11.8)*	15.0 (9.1 to 23.0)	16.3 (11.0 to 21.7)*	9.1 (4.1 to 14.2)*
Acute glomerulonephritis	6.0 (4.3 to 8.2)	-24.9 (-30.0 to -20.7)*	-29.5 (-34.3 to -25.5)*	0.3 (0.2 to 0.5)	-24.4 (-32.0 to -17.1)*	-29.1 (-36.2 to -22.2)*
Chronic kidney disease	20,034.2 (18,259.9 to 21,512.0)	11.6 (8.3 to 15.1)*	4.7 (1.6 to 8.0)*	560.3 (399.2 to 750.6)	6.9 (4.9 to 9.0)*	0.3 (-1.6 to 2.3)
Chronic kidney disease due to diabetes mellitus	6,852.4 (4,319.2 to 9,822.8)	10.1 (6.3 to 13.7)*	3.4 (-0.2 to 6.7)	192.3 (119.6 to 277.6)	4.5 (0.2 to 8.6)*	-1.9 (-5.9 to 1.9)
Stage III chronic kidney disease due to diabetes mellitus aggregate	6,146.6 (3,805.9 to 8,938.6)	10.8 (6.9 to 14.6)*	4.0 (0.3 to 7.5)*	10.0 (6.6 to 14.5)	-6.8 (-18.9 to 6.4)	-12.5 (-23.9 to -0.1)*
Stage IV chronic kidney disease due to diabetes mellitus aggregate	429.2 (258.8 to 626.1)	6.0 (3.0 to 8.9)*	-0.5 (-3.4 to 2.2)	54.5 (29.3 to 85.8)	5.1 (0.3 to 9.4)*	-1.4 (-5.8 to 2.7)
Stage V chronic kidney disease untreated due to diabetes mellitus	226.7 (148.9 to 324.0)	6.0 (3.1 to 8.9)*	-0.5 (-3.3 to 2.3)	118.9 (67.7 to 184.3)	6.1 (0.5 to 11.7)*	-0.5 (-5.7 to 4.9)
End-stage chronic kidney disease due to diabetes aggregate	50.0 (34.5 to 66.1)	-11.3 (-14.8 to -8.0)*	-16.8 (-20.1 to -13.7)*	9.0 (5.4 to 13.7)	-5.0 (-11.7 to 1.8)	-10.8 (-17.1 to -4.5)*
Chronic kidney disease due to hypertension	5,359.3 (3,156.7 to 8,128.0)	13.6 (9.6 to 17.6)*	6.6 (2.9 to 10.4)*	73.1 (46.6 to 105.1)	5.1 (-1.6 to 12.0)	-1.4 (-7.6 to 5.1)
Stage III chronic kidney disease due to hypertension aggregate	5,068.0 (2,874.4 to 7,802.5)	14.1 (10.0 to 18.5)*	7.1 (3.2 to 11.2)*	2.8 (1.8 to 4.3)	-2.5 (-15.8 to 12.3)	-8.5 (-21.0 to 5.4)
Stage IV chronic kidney disease due to hypertension aggregate	191.3 (113.3 to 329.9)	6.5 (-2.0 to 15.2)	-0.1 (-8.1 to 8.1)	24.1 (12.7 to 41.4)	6.5 (-2.7 to 15.9)	-0.1 (-8.6 to 8.7)
Stage V chronic kidney disease untreated due to hypertension	81.4 (54.7 to 115.2)	5.4 (0.0 to 10.6)*	-1.0 (-6.1 to 3.8)	42.8 (24.8 to 64.7)	5.8 (-3.2 to 15.1)	-0.7 (-9.2 to 8.0)
End-stage chronic kidney disease due to hypertension aggregate	18.6 (13.3 to 25.6)	-11.0 (-17.5 to -3.9)*	-16.4 (-22.6 to -9.8)*	3.3 (2.1 to 5.1)	-4.9 (-13.6 to 4.9)	-10.7 (-18.9 to -1.5)*
Chronic kidney disease due to glomerulonephritis	2,977.1 (2,196.4 to 3,965.5)	9.7 (4.8 to 14.8)*	3.0 (-1.6 to 7.8)	156.4 (97.8 to 225.8)	9.5 (5.3 to 14.2)*	2.8 (-1.1 to 7.2)
Stage III chronic kidney disease due to glomerulonephritis aggregate	2,429.4 (1,642.0 to 3,413.8)	9.9 (4.0 to 15.8)*	3.2 (-2.4 to 8.7)	7.6 (4.9 to 11.5)	12.1 (0.3 to 25.3)*	5.2 (-5.9 to 17.6)
Stage IV chronic kidney disease due to glomerulonephritis aggregate	322.5 (215.6 to 430.9)	10.0 (4.9 to 15.2)*	3.3 (-1.6 to 8.1)	41.6 (23.9 to 61.9)	10.2 (3.9 to 17.1)*	3.4 (-2.5 to 9.9)
Stage V chronic kidney disease untreated due to glomerulonephritis	193.7 (124.6 to 284.9)	9.7 (7.0 to 12.7)*	3.0 (0.4 to 5.8)*	101.3 (58.1 to 159.2)	9.9 (4.3 to 16.2)*	3.1 (-2.1 to 9.1)
End-stage chronic kidney disease due to glomerulonephritis aggregate	31.5 (20.5 to 46.0)	-8.2 (-11.9 to -4.1)*	-13.9 (-17.3 to -10.0)*	5.8 (3.4 to 9.1)	-3.3 (-11.7 to 6.3)	-9.2 (-17.1 to -0.3)*
Chronic kidney disease due to other causes	5,077.3 (3,021.4 to 7,777.4)	13.2 (10.3 to 16.1)*	6.2 (3.5 to 9.0)*	138.5 (82.8 to 211.8)	8.5 (4.4 to 12.8)*	1.9 (-2.0 to 5.9)
Stage III chronic kidney disease due to other causes aggregate	4,567.0 (2,669.7 to 7,070.7)	13.8 (10.6 to 16.9)*	6.8 (3.8 to 9.7)*	5.8 (3.6 to 8.9)	11.5 (-1.0 to 26.7)	4.6 (-7.1 to 18.9)
Stage IV chronic kidney disease due to other causes aggregate	311.9 (179.4 to 479.8)	9.8 (7.8 to 11.9)*	3.0 (1.1 to 5.0)*	39.6 (20.6 to 64.1)	7.7 (2.9 to 12.5)*	1.1 (-3.5 to 5.6)
Stage V chronic kidney disease untreated due to other causes	166.4 (100.3 to 246.3)	9.3 (7.0 to 11.6)*	2.5 (0.4 to 4.7)*	87.1 (47.0 to 136.4)	9.6 (3.5 to 15.7)*	2.8 (-2.8 to 8.6)
End-stage chronic kidney disease due to other causes aggregate	32.0 (20.6 to 47.2)	-7.3 (-10.3 to -4.0)*	-13.0 (-15.8 to -9.9)*	6.0 (3.5 to 9.7)	-2.0 (-10.3 to 7.9)	-8.0 (-15.8 to 1.2)
Urinary diseases and male infertility	30,055.6 (28,676.1 to 31,296.1)	11.4 (9.4 to 13.6)*	4.6 (2.6 to 6.6)*	49.5 (26.9 to 94.8)	10.5 (5.7 to 14.8)*	3.7 (-0.8 to 7.7)
Interstitial nephritis and urinary tract infections	160.6 (149.6 to 171.9)	13.1 (12.5 to 13.8)*	6.2 (5.6 to 6.8)*	5.4 (3.4 to 8.1)	13.3 (7.1 to 20.7)*	6.4 (0.5 to 13.3)*
Urolithiasis	24,977.5 (21,796.5 to 28,863.2)	11.5 (9.1 to 14.1)*	4.6 (2.4 to 7.0)*	8.4 (5.2 to 12.6)	8.4 (-0.5 to 17.8)	1.7 (-6.6 to 10.6)
Benign prostatic hyperplasia	0.0 (0.0 to 0.0)	0.0 (0.0 to 0.0)	0.0 (0.0 to 0.0)	0.0 (0.0 to 0.0)	0.0 (0.0 to 0.0)	0.0 (0.0 to 0.0)
Male infertility	5,375.1 (3,631.0 to 7,963.3)	11.6 (5.1 to 16.9)*	4.7 (-1.3 to 9.7)	30.6 (11.6 to 70.6)	12.0 (6.0 to 17.1)*	5.1 (-0.5 to 9.9)
Other urinary diseases	--	--	--	5.1 (3.3 to 7.5)	2.7 (-8.2 to 14.7)	-3.7 (-13.9 to 7.6)
Gynecological diseases	99,738.3 (97,874.6 to 101,795.5)	5.4 (4.3 to 6.5)*	-1.1 (-2.2 to -0.0)*	1,479.1 (1,005.9 to 2,162.9)	4.0 (2.4 to 5.4)*	-2.4 (-3.9 to -1.1)*
Uterine fibroids	22,426.2 (20,582.3 to 24,561.0)	6.2 (5.6 to 6.8)*	-0.3 (-0.9 to 0.2)	395.1 (244.7 to 627.7)	2.3 (-0.2 to 4.5)	-4.0 (-6.4 to -1.9)*
Uterine fibroids cases aggregate	15,104.4 (13,318.1 to 17,194.4)	7.7 (6.2 to 9.3)*	1.1 (-0.4 to 2.6)	86.3 (41.2 to 161.9)	9.1 (6.1 to 11.9)*	2.4 (-0.4 to 5.0)
Mild abdominal pain with anemia due to uterine fibroids aggregate	7,321.8 (7,179.3 to 7,444.6)	3.3 (0.8 to 5.8)*	-3.1 (-5.4 to -0.7)*	308.8 (201.2 to 459.9)	0.6 (-2.6 to 3.9)	-5.6 (-8.6 to -2.5)*
Polycystic ovarian syndrome	16,238.3 (12,486.1 to 20,871.3)	6.7 (6.1 to 7.4)*	0.1 (-0.5 to 0.8)	141.4 (63.9 to 275.4)	6.8 (5.9 to 7.8)*	0.3 (-0.6 to 1.2)
Polycystic ovarian syndrome cases aggregate	12,721.0 (9,629.7 to 16,538.2)	6.2 (5.7 to 7.0)*	-0.3 (-0.8 to 0.4)	109.9 (49.0 to 214.1)	6.3 (5.3 to 7.3)*	-0.2 (-1.1 to 0.7)
Hirsutism and infertility due to polycystic ovarian syndrome aggregate	3,517.3 (2,325.8 to 5,201.1)	8.2 (7.0 to 9.3)*	1.6 (0.4 to 2.6)*	31.4 (11.4 to 69.8)	8.8 (6.1 to 11.5)*	2.1 (-0.4 to 4.6)

Cause	Prevalence in thousands (95% confidence interval)			YLDs in thousands (95% confidence interval)		
	2015	Percent change in counts between 2005 and 2015	Percent change in rates between 2005 and 2015	2015	Percent change in counts between 2005 and 2015	Percent change in rates between 2005 and 2015
Female infertility	12,015.7 (6,071.9 to 21,051.2)	9.5 (-0.9 to 18.8)	2.7 (-7.0 to 11.5)	64.7 (20.9 to 159.7)	10.0 (0.1 to 19.1)*	3.3 (-6.0 to 11.8)
Endometriosis	2,007.7 (1,607.3 to 2,506.6)	4.5 (3.8 to 5.2)*	-1.9 (-2.6 to -1.3)*	185.9 (116.1 to 269.0)	4.7 (2.5 to 6.8)*	-1.8 (-3.8 to 0.3)
Endometriosis cases aggregate	1,886.1 (1,510.4 to 2,365.4)	4.4 (3.6 to 5.1)*	-2.0 (-2.7 to -1.4)*	173.8 (109.8 to 251.4)	4.6 (2.3 to 6.9)*	-1.9 (-4.0 to 0.3)
Abdominal pain and infertility due to endometriosis aggregate	121.5 (81.5 to 169.6)	6.1 (4.8 to 7.2)*	-0.4 (-1.6 to 0.6)	12.1 (6.7 to 18.7)	6.1 (-0.3 to 11.8)	-0.5 (-6.4 to 4.9)
Genital prolapse	10,638.1 (8,722.3 to 12,743.8)	3.5 (0.5 to 6.8)*	-2.9 (-5.7 to 0.2)	33.8 (15.9 to 62.7)	3.6 (0.1 to 7.3)*	-2.8 (-6.1 to 0.7)
Premenstrual syndrome	52,860.0 (48,978.9 to 57,323.6)	3.7 (0.9 to 6.3)*	-2.6 (-5.3 to -0.3)*	443.1 (271.0 to 668.7)	3.8 (0.6 to 6.6)*	-2.5 (-5.6 to 0.1)
Other gynecological diseases	5,941.0 (5,687.9 to 6,183.8)	4.3 (3.2 to 5.4)*	-2.1 (-3.1 to -1.1)*	215.1 (151.3 to 295.0)	3.2 (1.0 to 5.5)*	-3.1 (-5.2 to -0.9)*
Other gynecological diseases cases aggregate	3,379.7 (3,138.4 to 3,626.5)	6.1 (5.7 to 6.5)*	-0.4 (-0.8 to -0.0)*	132.3 (88.7 to 181.7)	6.2 (3.5 to 9.0)*	-0.4 (-2.8 to 2.3)
Anemia due to other gynecological diseases aggregate	2,561.3 (2,512.9 to 2,606.5)	2.0 (-0.4 to 4.4)	-4.3 (-6.5 to -2.0)*	82.8 (55.9 to 118.0)	-1.2 (-5.1 to 2.6)	-7.2 (-10.9 to -3.7)*
Hemoglobinopathies and hemolytic anemias	104,194.5 (102,416.3 to 106,436.5)	7.0 (5.5 to 8.6)*	0.4 (-1.0 to 1.9)	371.1 (246.0 to 541.9)	-4.5 (-8.0 to -1.3)*	-10.4 (-13.7 to -7.3)*
Thalassemias	2.4 (2.3 to 2.5)	-5.3 (-9.3 to -1.0)*	-11.1 (-14.9 to -7.1)*	0.2 (0.1 to 0.3)	-8.1 (-34.2 to 26.0)	-13.8 (-38.2 to 18.2)
Beta-thalassemia major cases aggregate	2.0 (1.9 to 2.1)	-4.7 (-9.6 to 0.5)	-10.6 (-15.2 to -5.7)*	0.2 (0.1 to 0.3)	-8.3 (-39.9 to 37.8)	-13.9 (-43.6 to 29.4)
Hemoglobin E/beta-thalassemia cases aggregate	0.2 (0.2 to 0.2)	-16.6 (-17.3 to -15.9)*	-21.7 (-22.4 to -21.0)*	0.0 (0.0 to 0.0)	-15.9 (-19.6 to -11.2)*	-21.1 (-24.6 to -16.6)*
Hemoglobin H disease cases aggregate	0.1 (0.0 to 0.1)	3.0 (-2.0 to 7.9)	-3.3 (-8.0 to 1.3)	0.0 (0.0 to 0.0)	0.2 (-7.1 to 6.3)	-5.9 (-12.8 to -0.2)*
Heart failure due to thalassemias aggregate	0.2 (0.1 to 0.2)	0.3 (-1.7 to 2.2)	-5.9 (-7.8 to -4.0)*	0.0 (0.0 to 0.0)	0.3 (-1.7 to 2.2)	-5.9 (-7.8 to -4.0)*
Thalassemias trait	19,748.5 (19,291.5 to 20,267.3)	-2.5 (-3.0 to -1.9)*	-8.5 (-9.0 to -8.0)*	207.0 (135.5 to 299.8)	-8.0 (-13.0 to -3.3)*	-13.6 (-18.4 to -9.3)*
B-thalassemia trait cases aggregate	15,936.3 (15,531.3 to 16,416.9)	-3.6 (-4.2 to -3.0)*	-9.5 (-10.1 to -9.0)*	194.8 (128.1 to 282.4)	-8.3 (-13.3 to -3.6)*	-13.9 (-18.7 to -9.6)*
Hemoglobin E trait cases aggregate	3,812.2 (3,647.2 to 3,987.9)	2.5 (1.3 to 3.7)*	-3.8 (-4.9 to -2.7)*	12.2 (7.8 to 18.3)	-2.8 (-12.7 to 7.2)	-8.7 (-18.1 to 0.6)
Sickle cell disorders	19.4 (18.0 to 20.6)	30.8 (24.4 to 36.7)*	22.7 (16.8 to 28.3)*	2.4 (1.7 to 3.2)	26.1 (14.9 to 39.5)*	18.3 (7.9 to 30.9)*
Homozygous sickle cell and severe sickle cell/beta-thalassemia cases aggregate	17.0 (15.8 to 18.2)	32.9 (25.6 to 39.6)*	24.7 (17.9 to 31.1)*	2.0 (1.4 to 2.8)	27.2 (13.7 to 43.1)*	19.3 (6.7 to 34.3)*
Hemoglobin SC disease cases aggregate	2.0 (1.8 to 2.2)	23.4 (15.0 to 30.5)*	15.8 (8.0 to 22.5)*	0.3 (0.2 to 0.4)	26.4 (18.2 to 34.7)*	18.6 (10.9 to 26.4)*
Mild sickle cell/beta-thalassemia cases aggregate	0.3 (0.3 to 0.4)	-9.9 (-12.9 to -6.0)*	-15.4 (-18.3 to -11.8)*	0.1 (0.0 to 0.1)	-4.4 (-7.6 to -0.7)*	-10.2 (-13.3 to -6.8)*
Sickle cell trait	25,764.2 (24,177.1 to 28,858.5)	25.6 (24.7 to 26.5)*	17.9 (17.0 to 18.7)*	79.0 (53.3 to 113.1)	12.9 (7.5 to 18.2)*	5.9 (0.9 to 10.9)*
G6PD deficiency	16,418.1 (13,929.9 to 19,017.8)	5.2 (0.0 to 9.8)*	-1.3 (-6.1 to 3.1)	1.4 (0.9 to 1.9)	-3.3 (-10.8 to 4.9)	-9.2 (-16.3 to -1.5)*
G6PD cases aggregate	16,418.1 (13,929.9 to 19,017.8)	5.2 (0.0 to 9.8)*	-1.3 (-6.1 to 3.1)	1.4 (0.9 to 1.9)	-3.3 (-10.8 to 4.9)	-9.2 (-16.3 to -1.5)*
Heart failure due to G6PD deficiency aggregate	0.0 (0.0 to 0.0)	0.0 (0.0 to 0.0)	0.0 (0.0 to 0.0)	0.0 (0.0 to 0.0)	0.0 (0.0 to 0.0)	0.0 (0.0 to 0.0)
G6PD trait	48,723.3 (45,262.0 to 52,247.1)	5.7 (4.0 to 7.3)*	-0.8 (-2.4 to 0.7)	1.5 (1.0 to 2.1)	2.8 (-12.6 to 21.7)	-3.6 (-18.0 to 14.2)
Other hemoglobinopathies and hemolytic anemias	4,127.4 (4,041.1 to 4,206.3)	-5.4 (-7.6 to -3.2)*	-11.2 (-13.3 to -9.1)*	79.6 (51.6 to 117.2)	-10.3 (-14.3 to -6.3)*	-15.8 (-19.6 to -12.1)*
Other hemoglobinopathies and hemolytic anemias cases aggregate	4,125.0 (4,038.7 to 4,204.2)	-5.4 (-7.6 to -3.2)*	-11.2 (-13.3 to -9.1)*	78.5 (50.8 to 115.8)	-10.4 (-14.5 to -6.3)*	-15.9 (-19.7 to -12.1)*
Heart failure due to other hemoglobinopathies and hemolytic anemias aggregate	2.4 (2.0 to 2.7)	6.8 (4.7 to 8.8)*	0.3 (-1.7 to 2.1)	0.3 (0.2 to 0.4)	6.8 (4.7 to 8.8)*	0.3 (-1.7 to 2.1)
Endocrine, metabolic, blood, and immune disorders	3,247.2 (3,176.6 to 3,310.1)	-2.4 (-4.7 to -0.2)*	-8.4 (-10.6 to -6.3)*	75.0 (50.2 to 107.6)	-6.1 (-9.9 to -2.1)*	-11.9 (-15.5 to -8.1)*
Endocrine, metabolic, blood, and immune disorders cases aggregate	350.6 (317.3 to 389.1)	5.9 (3.6 to 8.1)*	-0.6 (-2.7 to 1.5)	13.9 (9.2 to 19.5)	6.0 (0.6 to 11.6)*	-0.5 (-5.6 to 4.8)
Anemia due to endocrine, metabolic, blood, and immune disorders aggregate	2,895.8 (2,838.2 to 2,947.2)	-3.3 (-5.8 to -0.8)*	-9.3 (-11.6 to -6.9)*	61.0 (40.2 to 89.2)	-8.5 (-13.0 to -3.8)*	-14.1 (-18.3 to -9.7)*
Heart failure due to endocrine, metabolic, blood, and immune disorders aggregate	0.8 (0.7 to 0.9)	4.3 (3.2 to 5.5)*	-2.1 (-3.2 to -1.0)*	0.1 (0.1 to 0.1)	4.3 (3.2 to 5.5)*	-2.1 (-3.2 to -1.0)*
Musculoskeletal disorders	105,471.6 (101,699.8 to 108,873.9)	5.3 (3.9 to 6.6)*	-1.2 (-2.5 to 0.1)	12,077.2 (8,377.8 to 16,351.4)	5.4 (3.6 to 7.2)*	-1.1 (-2.7 to 0.6)
Rheumatoid arthritis	1,374.6 (1,207.9 to 1,579.1)	7.0 (4.0 to 9.7)*	0.4 (-2.4 to 3.0)	334.3 (227.7 to 463.1)	7.1 (3.4 to 10.7)*	0.5 (-2.9 to 3.9)
Osteoarthritis	5,823.5 (5,519.9 to 6,120.7)	7.0 (5.5 to 8.6)*	0.4 (-1.0 to 1.9)	341.1 (235.2 to 473.3)	8.0 (5.9 to 10.2)*	1.3 (-0.6 to 3.5)
Osteoarthritis of the hip cases aggregate	463.6 (373.0 to 564.6)	11.0 (9.0 to 13.3)*	4.2 (2.3 to 6.3)*	25.6 (16.3 to 37.2)	13.6 (8.3 to 19.0)*	6.6 (1.6 to 11.7)*
Osteoarthritis of the knee cases aggregate	5,359.8 (5,073.0 to 5,661.9)	6.6 (5.1 to 8.4)*	0.1 (-1.4 to 1.7)	315.4 (218.3 to 439.2)	7.5 (5.3 to 9.9)*	0.9 (-1.2 to 3.1)
Low back and neck pain	73,106.5 (69,110.8 to 77,026.6)	3.9 (2.1 to 5.6)*	-2.5 (-4.1 to -0.9)*	8,504.4 (5,824.6 to 11,811.4)	4.1 (2.4 to 5.7)*	-2.3 (-3.9 to -0.8)*
Low back pain	45,869.9 (40,676.3 to 51,607.3)	5.5 (3.3 to 7.5)*	-0.9 (-3.0 to 0.9)	5,218.5 (3,589.3 to 7,401.1)	5.7 (3.4 to 7.6)*	-0.8 (-3.0 to 0.9)
Neck pain	33,004.7 (22,879.1 to 46,573.6)	1.6 (-1.0 to 3.9)	-4.6 (-7.1 to -2.5)*	3,285.9 (1,929.4 to 5,164.2)	1.6 (-1.1 to 4.0)	-4.6 (-7.2 to -2.4)*
Gout	2,906.5 (2,345.0 to 3,556.8)	3.8 (3.0 to 4.7)*	-2.6 (-3.4 to -1.8)*	96.4 (63.3 to 137.3)	3.9 (0.1 to 7.8)*	-2.5 (-6.1 to 1.1)
Asymptomatic gout	2,596.6 (2,096.2 to 3,170.6)	3.8 (3.0 to 4.7)*	-2.6 (-3.4 to -1.8)*	0.0 (0.0 to 0.0)	0.0 (0.0 to 0.0)	0.0 (0.0 to 0.0)
Symptomatic gout cases aggregate	309.9 (242.5 to 388.4)	3.8 (3.0 to 4.7)*	-2.6 (-3.4 to -1.8)*	96.4 (63.3 to 137.3)	3.9 (0.1 to 7.8)*	-2.5 (-6.1 to 1.1)
Other musculoskeletal disorders	29,517.0 (22,679.9 to 37,051.5)	9.1 (3.4 to 15.0)*	2.4 (-3.0 to 7.9)	2,801.0 (1,761.7 to 4,119.7)	9.2 (3.2 to 15.2)*	2.5 (-3.1 to 8.1)
Other non-communicable diseases	345,576.9 (339,486.9 to 352,048.6)	7.9 (7.2 to 8.5)*	1.2 (0.6 to 1.8)*	7,068.6 (4,711.6 to 10,168.9)	9.8 (8.7 to 11.5)*	3.1 (2.0 to 4.7)*
Congenital anomalies	6,753.5 (6,185.4 to 7,264.8)	15.4 (11.6 to 19.2)*	8.3 (4.8 to 11.9)*	592.3 (365.0 to 900.0)	23.0 (14.3 to 32.3)*	15.4 (7.2 to 24.2)*
Neural tube defects	95.0 (64.6 to 135.6)	13.5 (10.7 to 16.1)*	6.6 (3.9 to 8.9)*	32.3 (18.5 to 52.8)	14.3 (6.9 to 21.3)*	7.2 (0.4 to 13.8)*
Congenital heart anomalies	3,517.1 (2,469.3 to 5,194.2)	19.1 (14.4 to 23.3)*	11.8 (7.4 to 15.7)*	121.7 (47.9 to 224.5)	19.2 (14.5 to 23.5)*	11.9 (7.5 to 15.9)*
Less severe heart anomalies cases aggregate	3,197.3 (2,131.8 to 4,890.7)	18.9 (13.4 to 23.3)*	11.6 (6.5 to 15.7)*	102.7 (38.2 to 195.9)	18.9 (13.3 to 23.8)*	11.6 (6.4 to 16.1)*
Severe congenital heart anomalies	306.6 (204.9 to 460.9)	21.5 (15.7 to 26.5)*	14.0 (8.6 to 18.7)*	18.0 (7.2 to 36.0)	21.5 (14.6 to 28.3)*	14.0 (7.6 to 20.4)*
Critical congenital heart anomalies	8.3 (4.8 to 13.4)	38.9 (22.1 to 52.2)*	30.4 (14.6 to 42.8)*	0.5 (0.2 to 1.0)	39.2 (13.1 to 64.5)*	30.7 (6.2 to 54.4)*
Heart failure due to congenital heart anomalies aggregate	4.9 (4.2 to 5.6)	1.6 (0.5 to 2.8)*	-4.6 (-5.7 to -3.5)*	0.6 (0.4 to 0.9)	1.6 (0.5 to 2.8)*	-4.6 (-5.7 to -3.5)*
Cleft lip and cleft palate	477.9 (283.2 to 793.6)	9.6 (-2.6 to 19.3)	2.9 (-8.6 to 11.9)	5.4 (2.7 to 9.5)	3.9 (-9.4 to 15.5)	-2.5 (-15.0 to 8.4)
Down syndrome	387.5 (245.8 to 593.7)	20.6 (15.4 to 25.4)*	13.2 (8.3 to 17.7)*	32.1 (18.1 to 54.0)	20.3 (14.2 to 25.7)*	12.9 (7.1 to 17.9)*
Turner syndrome	26.3 (12.2 to 47.6)	4.3 (1.8 to 6.2)*	-2.1 (-4.4 to -0.3)*	0.6 (0.2 to 1.2)	4.2 (-2.1 to 9.9)	-2.2 (-8.1 to 3.1)
Klinefelter syndrome	15.2 (8.0 to 27.2)	7.7 (5.6 to 9.1)*	1.1 (-0.9 to 2.4)	0.2 (0.1 to 0.4)	7.8 (2.6 to 12.6)*	1.1 (-3.7 to 5.7)
Other chromosomal abnormalities	344.3 (191.9 to 606.7)	15.0 (9.9 to 19.1)*	7.9 (3.2 to 11.8)*	28.7 (14.4 to 54.3)	14.8 (8.1 to 19.8)*	7.7 (1.5 to 12.4)*
Other congenital anomalies	2,157.5 (1,184.8 to 3,957.0)	9.1 (6.3 to 11.8)*	2.3 (-0.2 to 4.9)	371.3 (221.0 to 593.0)	26.5 (13.2 to 42.4)*	18.7 (6.2 to 33.7)*
Skin and subcutaneous diseases	127,860.6 (125,215.7 to 129,716.5)	7.0 (6.2 to 7.7)*	0.4 (-0.3 to 1.1)	2,562.1 (1,664.0 to 3,835.2)	6.9 (5.9 to 7.9)*	0.3 (-0.6 to 1.3)
Dermatitis	16,273.7 (12,938.0 to 20,054.9)	4.8 (4.2 to 5.5)*	-1.6 (-2.3 to -1.0)*	484.1 (307.5 to 700.4)	6.9 (5.5 to 8.6)*	0.4 (-1.0 to 1.9)
Eczema cases aggregate	3,257.2 (2,499.3 to 4,124.4)	11.6 (10.9 to 12.4)*	4.8 (4.0 to 5.4)*	197.6 (122.5 to 297.6)	11.8 (9.0 to 14.3)*	4.9 (2.3 to 7.3)*

Cause	Prevalence in thousands (95% confidence interval)			YLDs in thousands (95% confidence interval)		
	2015	Percent change in counts between 2005 and 2015	Percent change in rates between 2005 and 2015	2015	Percent change in counts between 2005 and 2015	Percent change in rates between 2005 and 2015
Contact dermatitis cases aggregate	7,556.6 (5,030.1 to 10,673.7)	5.1 (4.5 to 5.8)*	-1.3 (-2.0 to -0.7)*	204.7 (111.7 to 333.0)	5.2 (3.4 to 6.9)*	-1.3 (-2.9 to 0.3)
Seborrhoeic dermatitis cases aggregate	5,459.9 (3,886.0 to 7,409.9)	0.7 (-0.0 to 1.5)	-5.5 (-6.2 to -4.8)*	81.8 (42.1 to 140.8)	0.7 (-0.8 to 2.1)	-5.5 (-6.9 to -4.2)*
Psoriasis	6,306.2 (5,682.8 to 6,922.0)	9.6 (8.3 to 10.8)*	2.8 (1.6 to 4.0)*	515.4 (353.9 to 711.4)	9.6 (7.4 to 11.8)*	2.9 (0.8 to 5.0)*
Cellulitis	51.2 (38.4 to 66.6)	18.3 (12.7 to 24.6)*	11.0 (5.8 to 16.9)*	3.8 (2.2 to 6.0)	18.4 (9.9 to 27.2)*	11.1 (3.1 to 19.4)*
Pyoderma	306.3 (264.8 to 352.4)	9.1 (6.2 to 12.1)*	2.4 (-0.3 to 5.2)	1.7 (0.7 to 3.7)	9.2 (4.1 to 14.4)*	2.5 (-2.3 to 7.4)
Scabies	9,270.2 (6,025.3 to 13,483.1)	-1.0 (-5.5 to 4.2)	-7.1 (-11.3 to -2.2)*	238.5 (115.9 to 431.2)	-1.0 (-5.6 to 4.7)	-7.1 (-11.4 to -1.8)*
Fungal skin diseases	28,377.8 (24,342.7 to 32,833.2)	7.5 (7.2 to 7.9)*	0.9 (0.6 to 1.2)*	160.6 (63.6 to 338.4)	7.6 (7.0 to 8.1)*	0.9 (0.4 to 1.5)*
Viral skin diseases	8,812.4 (7,391.4 to 10,550.4)	5.9 (4.9 to 6.9)*	-0.6 (-1.6 to 0.3)	270.7 (167.7 to 414.9)	5.9 (4.4 to 7.4)*	-0.6 (-2.0 to 0.8)
Molluscum contagiosum cases aggregate	618.8 (487.8 to 777.6)	6.5 (5.6 to 7.4)*	-0.1 (-0.9 to 0.8)	19.0 (11.4 to 29.7)	6.6 (2.9 to 10.5)*	0.0 (-3.4 to 3.7)
Viral warts cases aggregate	8,193.5 (6,800.9 to 9,874.9)	5.8 (4.7 to 6.9)*	-0.7 (-1.7 to 0.4)	251.7 (155.7 to 385.8)	5.9 (4.3 to 7.3)*	-0.6 (-2.1 to 0.7)
Acne vulgaris	28,796.2 (23,955.5 to 34,684.8)	7.5 (4.9 to 10.5)*	0.9 (-1.5 to 3.7)	309.9 (140.8 to 588.9)	7.5 (4.9 to 10.6)*	0.9 (-1.6 to 3.8)
Alopecia areata	2,279.2 (1,948.3 to 2,645.4)	5.2 (4.4 to 6.0)*	-1.3 (-2.0 to -0.6)*	77.2 (47.0 to 117.0)	5.2 (3.2 to 7.2)*	-1.3 (-3.1 to 0.6)
Pruritus	5,501.5 (3,798.9 to 7,688.0)	7.0 (3.8 to 10.4)*	0.4 (-2.6 to 3.6)	59.2 (25.4 to 120.7)	7.0 (3.8 to 10.4)*	0.4 (-2.6 to 3.6)
Urticaria	3,629.4 (2,518.5 to 5,167.4)	7.6 (6.9 to 8.3)*	1.0 (0.3 to 1.6)*	219.1 (118.5 to 356.8)	7.7 (5.9 to 9.6)*	1.1 (-0.6 to 2.8)
Decubitus ulcer	35.1 (25.3 to 48.2)	9.8 (4.1 to 14.8)*	3.1 (-2.3 to 7.7)	5.8 (3.5 to 9.0)	9.8 (2.1 to 16.6)*	3.0 (-4.2 to 9.5)
Other skin and subcutaneous diseases	36,290.8 (32,931.0 to 40,445.8)	8.9 (7.0 to 11.0)*	2.2 (0.5 to 4.2)*	216.1 (102.0 to 403.7)	9.0 (7.1 to 11.1)*	2.3 (0.5 to 4.3)*
Sense organ diseases	97,980.3 (94,674.4 to 101,255.9)	8.7 (7.6 to 9.8)*	2.0 (1.0 to 3.0)*	2,843.3 (1,867.5 to 4,074.5)	10.4 (8.7 to 12.5)*	3.6 (2.0 to 5.6)*
Glaucoma	0.0 (0.0 to 0.0)	0.0 (0.0 to 0.0)	0.0 (0.0 to 0.0)	0.0 (0.0 to 0.0)	0.0 (0.0 to 0.0)	0.0 (0.0 to 0.0)
Cataract	1,041.1 (759.8 to 1,375.5)	11.3 (8.1 to 14.6)*	4.5 (1.5 to 7.6)*	76.0 (46.4 to 113.3)	8.8 (4.5 to 12.9)*	2.1 (-1.9 to 5.9)
Macular degeneration	0.0 (0.0 to 0.0)	0.0 (0.0 to 0.0)	0.0 (0.0 to 0.0)	0.0 (0.0 to 0.0)	0.0 (0.0 to 0.0)	0.0 (0.0 to 0.0)
Refraction and accommodation disorders	37,282.4 (33,553.7 to 41,523.2)	9.3 (7.0 to 11.7)*	2.6 (0.4 to 4.8)*	705.2 (441.0 to 1,086.1)	10.9 (9.3 to 12.5)*	4.1 (2.6 to 5.6)*
Age-related and other hearing loss	52,646.2 (42,788.8 to 62,263.5)	8.8 (7.2 to 10.5)*	2.1 (0.7 to 3.7)*	1,590.8 (978.8 to 2,378.2)	10.6 (7.6 to 13.9)*	3.8 (1.0 to 6.9)*
Other vision loss	1,561.8 (1,210.8 to 1,994.5)	14.7 (12.2 to 17.4)*	7.7 (5.3 to 10.2)*	103.6 (65.2 to 153.3)	16.1 (12.6 to 19.8)*	9.0 (5.7 to 12.5)*
Other sense organ diseases	14,351.9 (12,654.7 to 16,236.1)	7.3 (4.8 to 9.9)*	0.7 (-1.6 to 3.2)	367.8 (221.5 to 542.2)	7.4 (4.6 to 10.2)*	0.8 (-1.8 to 3.4)
Acute other sense organ diseases	71.0 (63.2 to 79.8)	7.5 (5.0 to 9.8)*	0.9 (-1.4 to 3.1)	1.9 (1.2 to 2.9)	7.5 (5.0 to 9.8)*	0.9 (-1.4 to 3.1)
Chronic other sense organ diseases	14,281.0 (12,583.4 to 16,161.2)	7.3 (4.8 to 9.9)*	0.7 (-1.7 to 3.2)	365.9 (220.2 to 539.4)	7.4 (4.6 to 10.2)*	0.8 (-1.8 to 3.4)
Oral disorders	238,025.8 (226,852.1 to 248,094.9)	8.9 (7.3 to 10.4)*	2.2 (0.7 to 3.6)*	1,070.9 (612.2 to 1,748.7)	9.2 (8.6 to 9.8)*	2.5 (1.9 to 3.1)*
Deciduous caries	0.0 (0.0 to 0.0)	0.0 (0.0 to 0.0)	0.0 (0.0 to 0.0)	0.0 (0.0 to 0.0)	0.0 (0.0 to 0.0)	0.0 (0.0 to 0.0)
Permanent caries	208,241.3 (169,690.1 to 248,422.2)	9.2 (7.4 to 10.9)*	2.4 (0.8 to 4.1)*	145.7 (62.4 to 284.4)	8.5 (6.7 to 10.2)*	1.8 (0.2 to 3.4)*
Periodontal diseases	46,027.0 (35,931.2 to 57,560.9)	9.9 (8.5 to 11.1)*	3.2 (1.9 to 4.2)*	307.0 (112.0 to 638.6)	10.0 (8.6 to 11.2)*	3.3 (1.9 to 4.4)*
Edentulism and severe tooth loss	9,230.8 (8,453.4 to 10,290.6)	12.0 (11.5 to 12.6)*	5.2 (4.6 to 5.7)*	264.9 (174.3 to 371.2)	12.1 (11.0 to 13.4)*	5.2 (4.1 to 6.4)*
Other oral disorders	11,968.3 (10,552.3 to 13,421.8)	6.6 (6.0 to 7.2)*	0.0 (-0.6 to 0.6)	353.3 (214.0 to 538.0)	6.6 (5.6 to 7.6)*	0.1 (-0.9 to 1.0)
Injuries	74,431.2 (70,379.7 to 79,023.1)	7.0 (4.4 to 10.1)*	0.4 (-2.0 to 3.4)	2,903.6 (2,078.3 to 3,872.1)	-2.8 (-6.1 to 0.6)	-8.7 (-11.9 to -5.6)*
Transport injuries	8,074.9 (7,528.6 to 8,647.9)	1.5 (0.9 to 2.1)*	-4.8 (-5.3 to -4.2)*	469.5 (329.5 to 651.9)	-7.8 (-10.8 to -4.8)*	-13.5 (-16.3 to -10.7)*
Road injuries	7,551.7 (6,993.0 to 8,130.7)	1.3 (0.7 to 2.0)*	-4.9 (-5.5 to -4.2)*	436.3 (304.4 to 606.8)	-7.4 (-10.4 to -4.5)*	-13.1 (-15.9 to -10.4)*
Pedestrian road injuries	882.8 (756.4 to 1,085.9)	-3.5 (-4.6 to -2.5)*	-9.5 (-10.4 to -8.5)*	50.3 (34.7 to 71.2)	-14.9 (-18.4 to -11.6)*	-20.2 (-23.4 to -17.0)*
Cyclist road injuries	1,213.2 (1,039.8 to 1,448.0)	-6.0 (-7.1 to -4.9)*	-11.8 (-12.8 to -10.7)*	64.5 (43.3 to 90.3)	-15.7 (-19.3 to -12.2)*	-20.9 (-24.2 to -17.6)*
Motorcyclist road injuries	1,988.0 (1,674.9 to 2,316.9)	5.9 (5.1 to 6.8)*	-0.6 (-1.3 to 0.2)	110.0 (74.0 to 153.0)	-6.1 (-10.4 to -2.2)*	-11.9 (-15.9 to -8.2)*
Motor vehicle road injuries	3,154.7 (2,740.0 to 3,621.6)	1.5 (0.6 to 2.4)*	-4.8 (-5.6 to -3.9)*	193.2 (132.0 to 264.4)	-4.6 (-7.2 to -2.1)*	-10.4 (-12.9 to -8.1)*
Other road injuries	313.0 (252.3 to 370.4)	20.4 (19.0 to 21.6)*	13.0 (11.7 to 14.1)*	18.3 (12.4 to 25.6)	13.3 (9.7 to 16.3)*	6.3 (3.0 to 9.1)*
Other transport injuries	523.2 (461.6 to 590.2)	3.5 (2.8 to 4.2)*	-2.9 (-3.5 to -2.2)*	33.3 (23.4 to 45.1)	-12.4 (-16.1 to -8.3)*	-17.8 (-21.3 to -14.0)*
Unintentional injuries	55,898.6 (52,935.7 to 59,214.2)	5.4 (4.9 to 5.9)*	-1.1 (-1.5 to -0.6)*	2,113.5 (1,484.0 to 2,877.5)	-2.7 (-5.3 to -0.2)*	-8.7 (-11.2 to -6.3)*
Falls	14,022.0 (12,467.7 to 15,800.5)	12.0 (11.4 to 12.5)*	5.1 (4.5 to 5.6)*	749.1 (524.3 to 1,038.9)	-0.8 (-5.1 to 3.1)	-6.9 (-10.9 to -3.3)*
Drowning	228.7 (198.6 to 263.5)	-10.1 (-11.2 to -9.0)*	-15.6 (-16.7 to -14.5)*	13.3 (9.1 to 18.3)	-22.0 (-25.0 to -18.2)*	-26.8 (-29.6 to -23.3)*
Fire, heat, and hot substances	5,289.5 (4,500.6 to 6,085.6)	5.7 (4.9 to 6.4)*	-0.8 (-1.6 to -0.2)*	196.7 (136.5 to 266.5)	-2.2 (-5.9 to 1.0)	-8.2 (-11.6 to -5.2)*
Poisonings	407.7 (345.4 to 487.7)	9.5 (7.6 to 11.4)*	2.8 (1.0 to 4.6)*	39.9 (26.9 to 57.1)	6.2 (2.6 to 9.4)*	-0.3 (-3.7 to 2.7)
Exposure to mechanical forces	14,511.3 (13,130.5 to 15,809.1)	7.8 (7.0 to 8.7)*	1.2 (0.4 to 2.0)*	272.9 (188.7 to 379.5)	-0.1 (-2.9 to 2.8)	-6.2 (-8.9 to -3.5)*
Unintentional firearm injuries	205.9 (177.5 to 238.2)	7.4 (6.3 to 8.4)*	0.8 (-0.2 to 1.7)	8.5 (5.8 to 11.9)	-1.0 (-4.6 to 2.6)	-7.1 (-10.5 to -3.7)*
Unintentional suffocation	561.1 (457.9 to 698.3)	-5.2 (-10.0 to 0.5)	-11.0 (-15.6 to -5.7)*	20.4 (14.1 to 27.9)	-0.0 (-3.3 to 3.4)	-6.2 (-9.2 to -3.0)*
Other exposure to mechanical forces	13,744.3 (12,379.4 to 15,051.9)	8.4 (7.6 to 9.1)*	1.8 (1.0 to 2.4)*	244.1 (167.3 to 342.0)	-0.1 (-3.1 to 2.9)	-6.2 (-9.1 to -3.5)*
Adverse effects of medical treatment	621.6 (465.4 to 789.3)	1.8 (-2.1 to 5.6)	-4.5 (-8.1 to -0.9)*	82.8 (51.3 to 123.8)	1.8 (-2.1 to 5.6)	-4.5 (-8.1 to -0.9)*
Animal contact	2,816.2 (2,494.7 to 3,156.7)	9.0 (7.5 to 10.3)*	2.3 (0.9 to 3.5)*	90.2 (61.9 to 123.9)	5.6 (3.4 to 7.9)*	-0.9 (-2.9 to 1.2)
Venomous animal contact	1,325.4 (1,160.4 to 1,517.4)	12.3 (11.1 to 13.6)*	5.4 (4.3 to 6.6)*	70.4 (48.2 to 97.1)	6.5 (4.2 to 8.9)*	-0.0 (-2.2 to 2.2)
Non-venomous animal contact	1,490.8 (1,242.0 to 1,765.5)	6.2 (4.2 to 7.9)*	-0.3 (-2.2 to 1.3)	19.8 (12.8 to 29.7)	2.5 (-1.0 to 5.7)	-3.8 (-7.1 to -0.8)*
Foreign body	2,426.0 (2,118.3 to 2,731.1)	11.9 (10.0 to 13.3)*	5.0 (3.2 to 6.3)*	91.2 (64.0 to 126.5)	-0.9 (-4.5 to 3.0)	-7.0 (-10.4 to -3.3)*
Pulmonary aspiration and foreign body in airway	868.2 (701.1 to 1,107.1)	5.5 (2.7 to 8.2)*	-1.0 (-3.6 to 1.5)	45.0 (31.0 to 63.6)	-9.0 (-12.9 to -4.6)*	-14.6 (-18.3 to -10.4)*
Foreign body in eyes	79.2 (33.4 to 144.5)	10.9 (9.1 to 12.3)*	4.1 (2.4 to 5.4)*	4.4 (1.8 to 8.7)	9.9 (7.5 to 11.4)*	3.2 (0.9 to 4.6)*
Foreign body in other body part	1,478.6 (1,276.7 to 1,723.7)	16.0 (15.1 to 17.0)*	8.9 (8.0 to 9.8)*	41.8 (29.0 to 58.7)	8.3 (5.5 to 11.2)*	1.7 (-1.0 to 4.4)
Environmental heat and cold exposure	4,572.7 (3,968.3 to 5,469.7)	5.3 (4.7 to 6.0)*	-1.2 (-1.8 to -0.5)*	195.8 (135.2 to 277.0)	-3.8 (-6.4 to -1.1)*	-9.7 (-12.2 to -7.2)*
Other unintentional injuries	11,002.9 (9,660.8 to 12,649.1)	-6.0 (-6.6 to -5.2)*	-11.7 (-12.3 to -11.1)*	381.6 (252.8 to 539.7)	-10.2 (-12.1 to -8.6)*	-15.7 (-17.5 to -14.2)*
Self-harm and interpersonal violence	1,897.7 (1,722.9 to 2,085.6)	-1.2 (-1.7 to -0.7)*	-7.2 (-7.8 to -6.8)*	90.8 (63.0 to 122.5)	-11.7 (-14.5 to -8.5)*	-17.1 (-19.8 to -14.2)*
Self-harm	462.5 (399.5 to 535.9)	1.8 (1.1 to 2.4)*	-4.5 (-5.1 to -3.9)*	27.6 (19.1 to 37.2)	-9.3 (-12.1 to -6.3)*	-14.8 (-17.5 to -12.0)*
Interpersonal violence	1,435.2 (1,270.1 to 1,616.6)	-2.1 (-2.8 to -1.5)*	-8.1 (-8.7 to -7.6)*	63.3 (43.7 to 86.2)	-12.7 (-15.9 to -9.4)*	-18.0 (-21.1 to -14.9)*

Cause	Prevalence in thousands (95% confidence interval)			YLDs in thousands (95% confidence interval)		
	2015	Percent change in counts between 2005 and 2015	Percent change in rates between 2005 and 2015	2015	Percent change in counts between 2005 and 2015	Percent change in rates between 2005 and 2015
Assault by firearm	95.7 (80.5 to 115.6)	2.5 (1.4 to 3.4)*	-3.8 (-4.9 to -3.0)*	4.5 (3.1 to 6.3)	-7.2 (-10.8 to -3.6)*	-12.9 (-16.3 to -9.5)*
Assault by sharp object	356.4 (288.7 to 429.3)	-5.4 (-6.4 to -4.3)*	-11.2 (-12.1 to -10.2)*	11.4 (7.7 to 15.9)	-19.7 (-23.8 to -15.4)*	-24.7 (-28.5 to -20.6)*
Assault by other means	983.1 (859.2 to 1,124.3)	-1.3 (-2.0 to -0.6)*	-7.3 (-8.1 to -6.7)*	47.4 (32.6 to 64.8)	-11.3 (-14.4 to -8.1)*	-16.8 (-19.7 to -13.7)*
Forces of nature, war, and legal intervention	8,560.1 (6,081.5 to 11,732.7)	28.4 (2.2 to 63.1)*	20.5 (-4.0 to 53.1)	229.7 (156.5 to 318.5)	13.4 (-15.9 to 51.2)	6.4 (-21.0 to 41.9)
Exposure to forces of nature	2,129.8 (1,323.3 to 3,313.3)	30.5 (-1.1 to 76.1)	22.5 (-7.2 to 65.3)	62.1 (41.7 to 89.2)	-13.9 (-42.9 to 23.1)	-19.2 (-46.4 to 15.5)
Collective violence and legal intervention	6,430.2 (4,410.6 to 9,090.0)	27.7 (-3.6 to 69.9)	19.8 (-9.5 to 59.4)	167.6 (106.5 to 244.8)	28.4 (-12.0 to 86.5)	20.5 (-17.4 to 75.1)
**Anemia**	**139,594.5 (137,768.5 to 141,245.7)**	**-1.2 (-2.7 to 0.4)**	**-7.2 (-8.6 to -5.8)***	**3,849.7 (2,556.4 to 5,545.8)**	**-4.3 (-6.5 to -2.2)***	**-10.2 (-12.3 to -8.2)***
**Developmental intellectual disability**	**10,592.9 (7,888.1 to 13,219.5)**	**10.3 (9.8 to 10.9)***	**3.5 (3.1 to 4.0)***	**1,163.7 (827.4 to 1,536.9)**	**11.3 (9.1 to 13.7)***	**4.4 (2.4 to 6.7)***
**Epilepsy**	**2,460.3 (2,078.3 to 2,851.3)**	**9.4 (5.5 to 13.1)***	**2.6 (-0.9 to 6.2)**	**740.3 (527.8 to 970.4)**	**1.6 (-4.4 to 7.5)**	**-4.6 (-10.3 to 0.9)**
**Guillain-Barré syndrome**	**2.0 (1.2 to 3.2)**	**8.1 (6.0 to 9.8)***	**1.4 (-0.5 to 3.0)**	**0.6 (0.3 to 1.0)**	**8.1 (6.0 to 9.8)***	**1.4 (-0.5 to 3.0)**
**Hearing loss**	**58,178.2 (48,722.5 to 67,942.8)**	**8.0 (6.6 to 9.5)***	**1.4 (0.1 to 2.7)***	**1,858.8 (1,174.9 to 2,725.2)**	**9.4 (6.8 to 12.2)***	**2.6 (0.3 to 5.3)***
**Heart failure**	**498.7 (462.3 to 538.4)**	**7.8 (7.1 to 8.6)***	**1.2 (0.5 to 1.9)***	**60.3 (41.6 to 85.1)**	**8.1 (4.9 to 11.4)***	**1.5 (-1.5 to 4.5)**
**Infertility**	**22,992.5 (15,282.8 to 34,649.6)**	**9.5 (2.9 to 15.4)***	**2.8 (-3.4 to 8.3)**	**149.4 (64.2 to 322.7)**	**9.6 (4.2 to 14.2)***	**2.9 (-2.2 to 7.2)**
**Pelvic inflammatory disease**	**122.9 (95.8 to 154.9)**	**-4.4 (-5.7 to -3.2)***	**-10.3 (-11.5 to -9.2)***	**16.2 (10.5 to 23.7)**	**-4.1 (-8.7 to 0.3)**	**-10.0 (-14.3 to -5.8)***
**Vision loss**	**41,219.6 (37,292.0 to 45,582.9)**	**9.6 (7.5 to 11.7)***	**2.9 (0.9 to 4.8)***	**1,098.2 (746.5 to 1,543.2)**	**11.4 (10.0 to 12.8)***	**4.5 (3.3 to 5.8)***

SR Table 11. Prevalent cases and YLDs for 2015, percent change, and change in age-standardised rates between 2005 and 2015 by cause, global, ages 40-44 years						
Cause	Prevalence in thousands (95% confidence interval)			YLDs in thousands (95% confidence interval)		
	2015	Percent change in counts between 2005 and 2015	Percent change in rates between 2005 and 2015	2015	Percent change in counts between 2005 and 2015	Percent change in rates between 2005 and 2015
**All causes**	--	--	--	**57,909.9 (43,128.0 to 74,427.3)**	**12.5 (11.8 to 13.2)***	**-2.7 (-3.3 to -2.1)***
Communicable, maternal, neonatal, and nutritional diseases	281,100.5 (278,095.2 to 283,205.8)	11.5 (10.6 to 12.4)*	-3.6 (-4.4 to -2.8)*	5,712.2 (4,149.8 to 7,810.3)	2.1 (-0.5 to 5.6)	-11.7 (-13.9 to -8.7)*
HIV/AIDS and tuberculosis	5,413.9 (5,258.6 to 5,598.6)	28.5 (22.8 to 34.0)*	11.2 (6.2 to 15.8)*	737.7 (531.2 to 993.4)	-0.3 (-9.4 to 8.5)	-13.8 (-21.7 to -6.2)*
Tuberculosis	672.5 (505.7 to 898.9)	5.5 (-3.8 to 14.6)	-8.8 (-16.8 to -0.9)*	207.0 (129.3 to 315.4)	5.9 (-4.2 to 15.6)	-8.4 (-17.1 to 0.0)
HIV/AIDS	4,753.4 (4,612.7 to 4,919.6)	32.7 (25.8 to 39.3)*	14.7 (8.8 to 20.5)*	530.7 (385.7 to 690.5)	-2.6 (-13.8 to 9.2)	-15.7 (-25.5 to -5.6)*
HIV/AIDS - Tuberculosis	162.7 (146.9 to 182.3)	-6.1 (-9.5 to -2.6)*	-18.8 (-21.7 to -15.8)*	60.5 (41.4 to 84.0)	-5.5 (-10.5 to -0.1)*	-18.3 (-22.6 to -13.6)*
HIV/AIDS resulting in other diseases	4,828.9 (4,655.4 to 5,043.4)	35.7 (30.8 to 40.1)*	17.3 (13.1 to 21.2)*	470.2 (340.5 to 615.8)	-2.2 (-14.9 to 11.2)	-15.4 (-26.4 to -3.8)*
HIV aggregate	2,235.1 (2,117.6 to 2,382.0)	-21.8 (-25.0 to -18.1)*	-32.4 (-35.1 to -29.1)*	164.1 (116.9 to 215.1)	-23.0 (-26.5 to -19.2)*	-33.4 (-36.4 to -30.1)*
AIDS aggregate	2,593.8 (2,465.6 to 2,742.9)	270.3 (216.3 to 340.0)*	220.3 (173.5 to 280.5)*	306.1 (221.5 to 400.1)	14.4 (-9.5 to 47.2)	-1.1 (-21.7 to 27.3)
Diarrhea, lower respiratory, and other common infectious diseases	20,124.2 (18,590.1 to 21,429.4)	10.8 (8.3 to 13.4)*	-4.2 (-6.4 to -1.9)*	574.6 (395.4 to 800.5)	12.8 (11.1 to 14.6)*	-2.4 (-3.9 to -0.9)*
Diarrheal diseases	940.0 (884.4 to 995.1)	18.2 (16.9 to 19.3)*	2.2 (1.1 to 3.2)*	147.7 (100.8 to 204.3)	18.6 (15.6 to 21.3)*	2.6 (-0.0 to 4.9)
Diarrhoea episodes aggregate	939.8 (884.2 to 994.9)	18.2 (16.9 to 19.3)*	2.2 (1.1 to 3.2)*	147.6 (100.8 to 204.2)	18.6 (15.6 to 21.3)*	2.6 (-0.0 to 4.9)
Guillain-Barré syndrome due to diarrheal diseases	0.3 (0.1 to 0.4)	13.4 (11.2 to 14.9)*	-1.9 (-3.9 to -0.6)*	0.1 (0.0 to 0.1)	13.4 (11.2 to 14.9)*	-1.9 (-3.9 to -0.6)*
Intestinal infectious diseases	31.3 (27.8 to 35.4)	-27.5 (-36.0 to -17.7)*	-37.3 (-44.6 to -28.8)*	5.8 (3.7 to 8.6)	-4.8 (-17.5 to 10.1)	-17.6 (-28.7 to -4.7)*
Typhoid fever	37.1 (28.6 to 47.7)	-5.3 (-10.1 to 0.6)	-18.1 (-22.3 to -13.0)*	4.9 (3.1 to 7.5)	-2.9 (-17.2 to 13.9)	-16.0 (-28.4 to -1.5)*
Typhoid fever episodes aggregate	30.8 (23.4 to 39.9)	-5.3 (-14.1 to 4.2)	-18.1 (-25.7 to -9.9)*	2.9 (1.8 to 4.3)	-4.9 (-20.0 to 12.2)	-17.8 (-30.8 to -2.9)*
Complications of typhoid fever aggregate	6.4 (4.4 to 9.1)	-5.2 (-35.5 to 39.8)	-18.0 (-44.2 to 20.9)	2.1 (1.2 to 3.3)	0.0 (-32.9 to 51.8)	-13.5 (-42.0 to 31.2)
Paratyphoid fever	16.3 (11.7 to 22.3)	-8.2 (-15.2 to -1.1)*	-20.6 (-26.6 to -14.5)*	0.8 (0.5 to 1.4)	-7.6 (-25.2 to 12.5)	-20.1 (-35.3 to -2.7)*
Paratyphoid fever episodes aggregate	15.5 (11.2 to 21.2)	-8.2 (-15.4 to -0.4)*	-20.6 (-26.9 to -13.9)*	0.8 (0.4 to 1.2)	-7.4 (-26.4 to 16.1)	-19.9 (-36.3 to 0.4)
Intestinal perforation due to paratyphoid	0.8 (0.5 to 1.3)	-9.1 (-47.2 to 57.3)	-21.4 (-54.4 to 36.0)	0.1 (0.0 to 0.2)	-9.1 (-47.2 to 57.3)	-21.3 (-54.4 to 36.0)
Other intestinal infectious diseases	--	--	--	0.0 (0.0 to 0.1)	-64.8 (-77.3 to -44.1)*	-69.6 (-80.4 to -51.7)*
Lower respiratory infections	216.7 (200.9 to 232.8)	16.5 (14.5 to 18.6)*	0.8 (-1.0 to 2.5)	13.1 (8.8 to 18.8)	16.6 (11.0 to 22.5)*	0.9 (-4.0 to 6.0)
Lower respiratory infection episodes aggregate	216.4 (200.6 to 232.6)	16.5 (14.5 to 18.6)*	0.8 (-1.0 to 2.5)	13.0 (8.7 to 18.7)	16.6 (11.0 to 22.6)*	0.9 (-4.1 to 6.0)
Guillain-Barré syndrome due to lower respiratory infections	0.3 (0.1 to 0.5)	13.4 (11.2 to 14.9)*	-1.9 (-3.9 to -0.6)*	0.1 (0.0 to 0.2)	13.4 (11.2 to 14.9)*	-1.9 (-3.9 to -0.6)*
Upper respiratory infections	14,300.6 (10,119.5 to 19,056.6)	12.1 (11.0 to 13.2)*	-3.1 (-4.0 to -2.1)*	167.1 (87.9 to 308.4)	12.2 (10.7 to 13.7)*	-3.0 (-4.3 to -1.7)*
Upper respiratory infection episodes aggregate	14,299.8 (10,117.9 to 19,055.9)	12.1 (11.0 to 13.2)*	-3.1 (-4.0 to -2.1)*	166.9 (87.7 to 308.2)	12.2 (10.7 to 13.7)*	-3.0 (-4.3 to -1.7)*
Guillain-Barré syndrome due to upper respiratory infections	0.8 (0.4 to 1.2)	13.4 (11.2 to 14.9)*	-1.9 (-3.9 to -0.6)*	0.2 (0.1 to 0.4)	13.4 (11.2 to 14.9)*	-1.9 (-3.9 to -0.6)*
Otitis media	4,011.1 (2,872.7 to 5,447.6)	5.1 (1.7 to 8.0)*	-9.1 (-12.0 to -6.6)*	108.7 (61.3 to 173.6)	4.5 (-0.7 to 9.9)	-9.6 (-14.1 to -4.9)*
Acute otitis media	507.0 (393.7 to 663.4)	16.5 (12.4 to 21.1)*	0.7 (-2.8 to 4.7)	6.5 (3.2 to 12.4)	16.5 (11.0 to 22.5)*	0.8 (-4.0 to 5.9)
Chronic otitis media aggregate	3,504.1 (2,355.8 to 4,880.5)	3.6 (-0.1 to 6.9)	-10.4 (-13.6 to -7.5)*	102.2 (57.1 to 162.9)	3.8 (-1.7 to 9.5)	-10.2 (-15.0 to -5.3)*
Meningitis	461.4 (438.2 to 486.1)	19.3 (14.4 to 24.9)*	3.1 (-1.1 to 8.0)	79.6 (56.2 to 105.7)	18.9 (15.8 to 22.1)*	2.8 (0.1 to 5.6)*
Pneumococcal meningitis	417.3 (348.5 to 498.6)	22.9 (20.3 to 25.9)*	6.3 (4.0 to 8.8)*	36.8 (25.9 to 49.2)	19.6 (14.2 to 25.1)*	3.4 (-1.2 to 8.2)
Acute pneumococcal meningitis	1.2 (0.8 to 1.7)	22.1 (7.6 to 37.0)*	5.6 (-7.0 to 18.5)	0.2 (0.1 to 0.3)	22.1 (7.6 to 37.0)*	5.6 (-7.0 to 18.5)
Complications of pneumococcal meningitis aggregate	416.1 (347.6 to 497.3)	22.9 (20.3 to 25.8)*	6.3 (4.0 to 8.8)*	36.6 (25.8 to 48.9)	19.6 (14.2 to 25.2)*	3.4 (-1.3 to 8.2)
H influenzae type B meningitis	124.7 (95.4 to 159.1)	-7.3 (-11.7 to -1.9)*	-19.9 (-23.6 to -15.2)*	13.1 (8.9 to 18.0)	14.1 (7.3 to 21.2)*	-1.3 (-7.2 to 4.8)
Acute H influenzae type B meningitis	0.4 (0.2 to 0.6)	-13.1 (-24.7 to -1.8)*	-24.8 (-34.9 to -15.1)*	0.1 (0.0 to 0.1)	-13.1 (-24.7 to -1.8)*	-24.8 (-34.9 to -15.1)*
Complications of of H influenzae type B meningitis aggregate	124.3 (95.1 to 158.7)	-7.3 (-11.6 to -1.9)*	-19.8 (-23.6 to -15.1)*	13.0 (8.9 to 18.0)	14.3 (7.4 to 21.4)*	-1.2 (-7.1 to 5.0)
Meningococcal meningitis	107.7 (83.8 to 133.5)	23.1 (19.5 to 26.7)*	6.5 (3.3 to 9.6)*	11.5 (7.9 to 15.4)	21.5 (16.7 to 26.7)*	5.1 (0.9 to 9.6)*
Acute meningococcal meningitis	0.5 (0.3 to 0.8)	20.8 (4.2 to 36.2)*	4.5 (-9.9 to 17.8)	0.1 (0.0 to 0.1)	20.9 (4.2 to 36.2)*	4.5 (-9.9 to 17.8)
Complications of meningococcal meningitis aggregate	107.2 (83.3 to 133.1)	23.1 (19.5 to 26.8)*	6.5 (3.3 to 9.7)*	11.5 (7.8 to 15.3)	21.5 (16.7 to 26.8)*	5.1 (0.9 to 9.6)*
Other meningitis	180.8 (143.1 to 218.5)	20.3 (17.6 to 23.4)*	4.1 (1.7 to 6.7)*	18.2 (12.4 to 24.7)	19.5 (14.1 to 25.4)*	3.4 (-1.3 to 8.5)
Other meningitis episodes aggregate	4.2 (3.1 to 5.6)	19.5 (6.4 to 31.4)*	3.4 (-8.0 to 13.6)	0.6 (0.3 to 0.9)	19.5 (6.5 to 31.3)*	3.4 (-7.9 to 13.6)
Complications of other meningitis aggregate	176.6 (139.5 to 214.1)	20.3 (17.5 to 23.4)*	4.1 (1.6 to 6.8)*	17.6 (12.0 to 23.9)	19.5 (14.0 to 25.6)*	3.4 (-1.4 to 8.6)
Encephalitis	356.1 (258.3 to 484.7)	5.1 (2.6 to 8.3)*	-9.1 (-11.2 to -6.3)*	39.9 (27.8 to 54.1)	9.1 (3.4 to 14.5)*	-5.6 (-10.6 to -1.0)*
Acute encephalitis	4.5 (3.7 to 5.5)	9.7 (4.0 to 15.0)*	-5.1 (-10.1 to -0.5)*	0.6 (0.4 to 0.9)	11.2 (-0.3 to 23.7)	-3.9 (-13.8 to 7.0)
Complications of encephalitis aggregate	351.6 (253.9 to 479.4)	5.1 (2.5 to 8.3)*	-9.1 (-11.3 to -6.4)*	39.3 (27.5 to 53.3)	9.1 (3.3 to 14.6)*	-5.6 (-10.6 to -0.9)*
Diphtheria	0.0 (0.0 to 0.0)	-53.6 (-81.7 to 24.2)	-59.9 (-84.2 to 7.4)	0.0 (0.0 to 0.0)	-53.6 (-81.7 to 24.2)	-59.9 (-84.2 to 7.4)
Whooping cough	2.5 (1.9 to 3.1)	-21.0 (-23.5 to -18.4)*	-31.7 (-33.9 to -29.4)*	0.1 (0.1 to 0.2)	-21.1 (-23.5 to -18.4)*	-31.7 (-33.9 to -29.4)*
Tetanus	12.9 (12.6 to 13.3)	17.8 (14.2 to 19.7)*	1.9 (-1.2 to 3.5)	0.6 (0.4 to 0.8)	1.3 (-7.2 to 10.4)	-12.4 (-19.8 to -4.5)*
Severe tetanus	0.4 (0.3 to 0.8)	-34.3 (-48.0 to -13.9)*	-43.2 (-55.0 to -25.6)*	0.1 (0.0 to 0.1)	-34.4 (-48.0 to -14.0)*	-43.2 (-55.1 to -25.7)*
Complications of tetanus aggregate	12.5 (12.2 to 12.7)	21.2 (20.6 to 21.7)*	4.8 (4.3 to 5.3)*	0.5 (0.4 to 0.7)	7.8 (-0.0 to 16.1)	-6.8 (-13.5 to 0.4)
Measles	0.1 (0.0 to 0.2)	-59.6 (-64.2 to -53.8)*	-65.0 (-69.1 to -60.1)*	0.0 (0.0 to 0.0)	-59.6 (-64.2 to -53.8)*	-65.1 (-69.1 to -60.1)*
Varicella and herpes zoster	261.5 (199.9 to 335.1)	16.1 (15.2 to 16.9)*	0.4 (-0.4 to 1.1)	12.0 (7.0 to 19.7)	16.0 (10.8 to 21.7)*	0.4 (-4.2 to 5.2)
Neglected tropical diseases and malaria	119,881.0 (117,042.7 to 122,825.9)	4.3 (1.3 to 7.3)*	-9.8 (-12.4 to -7.2)*	1,174.8 (776.1 to 1,787.2)	-3.9 (-12.9 to 8.6)	-16.9 (-24.7 to -6.1)*
Malaria	10,061.2 (8,760.9 to 11,671.7)	38.4 (30.3 to 47.9)*	19.7 (12.7 to 27.9)*	84.4 (60.2 to 115.4)	25.0 (18.5 to 31.1)*	8.1 (2.5 to 13.3)*
Asymptomatic malaria parasitemia(PfPR)	7,387.2 (6,102.5 to 8,956.4)	43.0 (31.5 to 56.9)*	23.7 (13.7 to 35.7)*	0.0 (0.0 to 0.0)	0.0 (0.0 to 0.0)	0.0 (0.0 to 0.0)
Malaria episodes aggregate	405.0 (224.1 to 700.2)	2.3 (-16.4 to 26.8)	-11.5 (-27.7 to 9.6)	8.2 (3.8 to 15.7)	2.8 (-16.2 to 27.4)	-11.1 (-27.5 to 10.2)
Complications of malaria aggregate	48.5 (43.3 to 53.5)	36.6 (33.1 to 41.1)*	18.1 (15.1 to 22.1)*	14.9 (11.3 to 18.6)	31.6 (21.5 to 42.8)*	13.8 (5.0 to 23.5)*
Anemia due to malaria parasitemia(PfPR) aggregate	2,220.5 (2,160.8 to 2,282.8)	32.8 (29.3 to 37.1)*	14.8 (11.8 to 18.6)*	61.3 (41.2 to 88.4)	27.1 (19.8 to 34.6)*	9.9 (3.6 to 16.4)*
Chagas disease	589.1 (484.7 to 685.8)	-16.6 (-19.8 to -13.2)*	-27.9 (-30.7 to -24.9)*	4.5 (2.9 to 6.6)	-16.5 (-21.6 to -11.0)*	-27.8 (-32.2 to -23.0)*
Chagas disease episodes aggregate	507.7 (417.8 to 594.7)	-16.6 (-19.8 to -13.1)*	-27.9 (-30.7 to -24.9)*	0.0 (0.0 to 0.0)	-18.9 (-25.2 to -13.6)*	-29.8 (-35.3 to -25.2)*
Complications of Chagas disease aggregate	58.5 (44.6 to 73.7)	-16.7 (-19.8 to -13.2)*	-27.9 (-30.7 to -24.9)*	3.3 (2.1 to 4.9)	-16.4 (-22.6 to -10.1)*	-27.7 (-33.0 to -22.3)*

Cause	Prevalence in thousands (95% confidence interval)			YLDs in thousands (95% confidence interval)		
	2015	Percent change in counts between 2005 and 2015	Percent change in rates between 2005 and 2015	2015	Percent change in counts between 2005 and 2015	Percent change in rates between 2005 and 2015
Heart failure due to Chagas disease aggregate	22.9 (13.7 to 34.5)	-17.1 (-20.4 to -13.6)*	-28.3 (-31.2 to -25.3)*	1.2 (0.6 to 2.1)	-16.7 (-24.8 to -8.4)*	-27.9 (-35.0 to -20.8)*
Leishmaniasis	258.1 (229.2 to 306.3)	32.3 (7.6 to 61.6)*	14.4 (-7.0 to 39.8)	3.0 (1.5 to 5.7)	31.3 (24.9 to 37.4)*	13.5 (8.0 to 18.8)*
Visceral leishmaniasis	3.1 (2.9 to 3.3)	17.1 (15.7 to 18.5)*	1.3 (0.1 to 2.5)*	0.2 (0.1 to 0.3)	17.8 (-13.4 to 59.9)	1.9 (-25.1 to 38.3)
Cutaneous and mucocutaneous leishmaniasis	261.5 (220.1 to 322.1)	31.8 (28.3 to 34.9)*	14.0 (10.9 to 16.6)*	2.8 (1.3 to 5.4)	32.5 (26.6 to 38.2)*	14.6 (9.5 to 19.5)*
African trypanosomiasis	0.7 (0.4 to 1.0)	-68.5 (-70.4 to -67.0)*	-72.8 (-74.4 to -71.5)*	0.2 (0.1 to 0.3)	-67.4 (-83.0 to -44.3)*	-71.8 (-85.3 to -51.8)*
Schistosomiasis	14,283.8 (11,939.6 to 18,011.2)	-20.9 (-30.4 to -1.8)*	-31.6 (-39.8 to -15.1)*	140.4 (72.3 to 259.3)	-18.3 (-26.7 to 0.9)	-29.4 (-36.6 to -12.8)*
Schistosomiasis episodes aggregate	6,735.3 (4,887.0 to 8,709.2)	-25.1 (-35.0 to -10.5)*	-35.2 (-43.8 to -22.6)*	38.2 (15.0 to 81.9)	-26.5 (-35.8 to -11.6)*	-36.4 (-44.5 to -23.5)*
Complications of schistosomiasis aggregate	6,659.2 (4,504.9 to 10,375.8)	-15.6 (-28.8 to 19.1)	-27.0 (-38.4 to 3.0)	78.3 (37.0 to 155.0)	-12.5 (-24.8 to 21.1)	-24.3 (-35.0 to 4.8)
Anemia due to schistosomiasis aggregate	863.1 (830.2 to 894.8)	-19.0 (-22.7 to -15.8)*	-29.9 (-33.1 to -27.1)*	23.9 (16.1 to 34.4)	-21.7 (-27.0 to -15.8)*	-32.3 (-36.8 to -27.1)*
Cysticercosis	165.3 (107.0 to 241.9)	-11.0 (-19.0 to -4.3)*	-23.0 (-29.9 to -17.2)*	24.4 (13.8 to 38.5)	-20.4 (-29.2 to -12.7)*	-31.2 (-38.8 to -24.5)*
Cystic echinococcosis	101.8 (85.2 to 120.7)	21.9 (18.4 to 25.8)*	5.4 (2.4 to 8.8)*	9.4 (6.1 to 13.3)	21.8 (12.8 to 32.3)*	5.3 (-2.5 to 14.4)
Lymphatic filariasis	3,022.7 (2,398.0 to 3,830.7)	-41.0 (-47.1 to -35.1)*	-49.0 (-54.2 to -43.9)*	191.6 (104.0 to 312.5)	-14.3 (-31.3 to -0.3)*	-25.9 (-40.6 to -13.8)*
Prevalence of detectable microfiliaria due to lymphatic filariasis	1,293.8 (1,116.5 to 1,493.1)	-58.8 (-62.2 to -54.8)*	-64.4 (-67.3 to -60.9)*	0.0 (0.0 to 0.0)	0.0 (0.0 to 0.0)	0.0 (0.0 to 0.0)
Complications of lymphatic filariasis aggregate	1,728.9 (1,141.2 to 2,487.8)	-12.8 (-28.9 to -0.2)*	-24.6 (-38.5 to -13.7)*	191.6 (104.0 to 312.5)	-14.3 (-31.3 to -0.3)*	-25.9 (-40.6 to -13.8)*
Onchocerciasis	990.7 (770.1 to 1,271.8)	-26.3 (-37.3 to -14.0)*	-36.2 (-45.8 to -25.6)*	76.3 (36.1 to 138.2)	-18.9 (-38.3 to 1.3)	-29.8 (-46.6 to -12.4)*
Asymptomatic onchocerciasis	159.3 (102.8 to 223.6)	-56.3 (-60.1 to -53.7)*	-62.2 (-65.5 to -60.0)*	0.0 (0.0 to 0.0)	0.0 (0.0 to 0.0)	0.0 (0.0 to 0.0)
Skin disease due to onchocerciasis aggregate	750.0 (519.1 to 1,039.5)	-15.7 (-31.1 to 1.0)	-27.1 (-40.4 to -12.5)*	69.7 (29.7 to 131.2)	-19.3 (-41.9 to 2.6)	-30.2 (-49.8 to -11.0)*
Vision loss due to onchocerciasis aggregate	81.7 (55.8 to 117.2)	-8.9 (-21.8 to 4.9)	-21.2 (-32.4 to -9.3)*	6.6 (4.1 to 10.6)	-13.8 (-26.5 to -0.4)*	-25.4 (-36.5 to -13.9)*
Trachoma	165.8 (105.6 to 249.7)	3.8 (-5.3 to 13.5)	-10.2 (-18.1 to -1.9)*	16.1 (8.2 to 29.0)	1.4 (-9.8 to 13.4)	-12.3 (-22.0 to -1.9)*
Dengue	271.7 (148.3 to 613.9)	158.9 (1.5 to 635.1)*	123.9 (-12.3 to 535.7)	43.4 (19.1 to 103.3)	156.4 (0.5 to 660.8)*	121.8 (-13.1 to 558.0)
Dengue episodes aggregate	81.1 (52.1 to 178.0)	146.1 (-2.4 to 590.2)	112.9 (-15.6 to 496.9)	4.7 (2.5 to 11.2)	156.6 (1.0 to 656.4)*	121.9 (-12.7 to 554.1)
Post-dengue chronic fatigue syndrome	190.9 (90.2 to 441.0)	159.1 (1.5 to 635.1)*	124.0 (-12.2 to 535.7)	38.8 (16.1 to 92.7)	156.4 (0.8 to 659.0)*	121.8 (-12.8 to 556.4)
Yellow fever	0.1 (0.0 to 0.3)	-20.6 (-38.3 to 3.1)	-31.3 (-46.7 to -10.8)*	0.0 (0.0 to 0.0)	-20.7 (-38.3 to 3.1)	-31.4 (-46.7 to -10.8)*
Rabies	0.0 (0.0 to 0.0)	-46.1 (-62.2 to -23.6)*	-53.4 (-67.3 to -33.9)*	0.0 (0.0 to 0.0)	-46.0 (-62.2 to -23.6)*	-53.3 (-67.3 to -33.9)*
Intestinal nematode infections	96,050.9 (93,142.9 to 98,834.8)	3.7 (0.1 to 7.5)*	-10.3 (-13.5 to -7.1)*	193.3 (110.4 to 315.2)	-19.1 (-25.9 to -10.8)*	-30.0 (-35.9 to -22.9)*
Ascariasis	50,582.6 (43,255.5 to 60,060.3)	-5.3 (-21.8 to 15.6)	-18.1 (-32.4 to 0.0)	51.4 (28.2 to 87.1)	-36.2 (-47.2 to -23.1)*	-44.9 (-54.4 to -33.5)*
Complications of ascariasis aggregate	2,849.8 (2,548.0 to 3,202.4)	-33.8 (-43.3 to -22.5)*	-42.7 (-51.0 to -33.0)*	51.4 (28.2 to 87.1)	-36.2 (-47.2 to -23.1)*	-44.9 (-54.4 to -33.5)*
Asymptomatic ascariasis	47,733.1 (40,432.1 to 57,217.1)	-2.8 (-20.6 to 20.4)	-15.9 (-31.3 to 4.1)	0.0 (0.0 to 0.0)	0.0 (0.0 to 0.0)	0.0 (0.0 to 0.0)
Trichuriasis	29,937.6 (26,761.8 to 33,513.8)	5.2 (-9.5 to 23.1)	-9.1 (-21.7 to 6.5)	34.3 (17.9 to 60.3)	-9.6 (-29.7 to 21.8)	-21.8 (-39.2 to 5.4)
Complications of trichuriasis aggregate	1,709.7 (1,464.7 to 2,125.2)	-8.9 (-26.9 to 17.4)	-21.2 (-36.8 to 1.5)	34.3 (17.9 to 60.3)	-9.6 (-29.7 to 21.8)	-21.8 (-39.2 to 5.4)
Asymptomatic trichuriasis	28,224.7 (25,063.3 to 31,745.0)	6.1 (-9.4 to 25.8)	-8.2 (-21.6 to 8.8)	0.0 (0.0 to 0.0)	0.0 (0.0 to 0.0)	0.0 (0.0 to 0.0)
Hookworm disease	28,988.6 (25,857.7 to 32,600.1)	-2.0 (-15.6 to 13.7)	-15.3 (-27.0 to -1.6)*	107.6 (65.2 to 169.2)	-10.6 (-18.4 to -2.2)*	-22.7 (-29.4 to -15.4)*
Complications of hookworm disease aggregate	3,488.7 (3,223.9 to 3,779.9)	-10.1 (-19.8 to 0.6)	-22.3 (-30.7 to -13.0)*	71.1 (39.2 to 118.6)	-11.6 (-23.4 to 1.4)	-23.5 (-33.7 to -12.3)*
Anemia due to hookworm disease aggregate	1,528.0 (1,493.4 to 1,562.0)	-1.2 (-4.3 to 2.0)	-14.6 (-17.2 to -11.8)*	36.5 (24.1 to 53.0)	-8.6 (-13.9 to -3.2)*	-20.9 (-25.5 to -16.3)*
Asymptomatic hookworm disease	23,967.4 (20,867.1 to 27,602.9)	-0.8 (-17.1 to 19.2)	-14.2 (-28.3 to 3.1)	0.0 (0.0 to 0.0)	0.0 (0.0 to 0.0)	0.0 (0.0 to 0.0)
Food-borne trematodiases	7,542.0 (6,922.1 to 8,285.6)	14.9 (11.3 to 18.4)*	-0.7 (-3.7 to 2.4)	197.9 (97.1 to 366.5)	13.5 (8.1 to 20.1)*	-1.8 (-6.5 to 3.8)
Asymptomatic food-borne trematodiases aggregate	5,790.3 (4,567.2 to 6,808.0)	15.1 (11.3 to 19.0)*	-0.5 (-3.7 to 2.9)	0.0 (0.0 to 0.0)	0.0 (0.0 to 0.0)	0.0 (0.0 to 0.0)
Symptomatic food-borne trematodiases aggregate	1,751.8 (958.4 to 2,974.5)	14.1 (9.4 to 20.2)*	-1.3 (-5.4 to 4.0)	197.9 (97.1 to 366.5)	13.5 (8.1 to 20.1)*	-1.8 (-6.5 to 3.8)
Leprosy	39.7 (36.7 to 43.1)	-7.3 (-9.0 to -5.6)*	-19.9 (-21.3 to -18.3)*	2.4 (1.6 to 3.4)	-6.4 (-15.4 to 3.4)	-19.1 (-26.9 to -10.6)*
Ebola	0.3 (0.1 to 0.5)	14,579.0 (13,412.8 to 17,613.5)*	12,594.3 (11,585.8 to 15,218.6)*	0.1 (0.0 to 0.1)	13,522.0 (10,221.1 to 17,794.3)*	11,680.2 (8,825.7 to 15,374.9)*
Other neglected tropical diseases	1,942.4 (1,897.3 to 1,986.7)	4.2 (1.6 to 7.0)*	-9.9 (-12.2 to -7.5)*	187.4 (68.5 to 516.1)	10.3 (-26.4 to 64.5)	-4.6 (-36.3 to 42.2)
Anemia due to other neglected tropical diseases aggregate	1,942.4 (1,897.3 to 1,986.7)	4.2 (1.6 to 7.0)*	-9.9 (-12.2 to -7.5)*	35.8 (23.2 to 53.4)	-8.9 (-14.3 to -3.3)*	-21.2 (-25.9 to -16.4)*
Maternal disorders	777.7 (741.0 to 806.9)	3.2 (-0.8 to 7.4)	-10.7 (-14.2 to -7.2)*	56.6 (38.8 to 76.1)	-7.2 (-15.7 to 2.2)	-19.8 (-27.1 to -11.6)*
Maternal hemorrhage	89.3 (83.6 to 95.6)	7.1 (-1.6 to 17.6)	-7.4 (-14.9 to 1.7)	3.1 (2.0 to 4.4)	6.8 (-12.6 to 30.8)	-7.6 (-24.4 to 13.1)
Maternal hemorrhage episodes aggregate	7.7 (4.7 to 11.8)	5.4 (-34.3 to 68.9)	-8.9 (-43.2 to 46.1)	1.3 (0.7 to 2.1)	6.1 (-30.7 to 63.1)	-8.2 (-40.0 to 41.0)
Anemia due to maternal hemorrhage aggregate	81.6 (77.0 to 86.5)	7.3 (-1.1 to 17.6)	-7.2 (-14.5 to 1.7)	1.8 (1.2 to 2.6)	7.3 (-9.7 to 26.5)	-7.2 (-21.9 to 9.4)
Maternal sepsis and other maternal infections	443.1 (402.2 to 485.8)	6.4 (1.6 to 11.7)*	-7.9 (-12.1 to -3.4)*	3.4 (1.7 to 6.4)	4.6 (-21.6 to 40.7)	-9.6 (-32.2 to 21.6)
Maternal sepsis and other maternal infections aggregate	26.3 (13.5 to 46.8)	1.0 (-48.7 to 99.1)	-12.7 (-55.7 to 72.2)	1.3 (0.6 to 2.5)	0.8 (-48.0 to 98.8)	-12.9 (-55.0 to 71.9)
Infertility due to puerperal sepsis	416.7 (380.2 to 456.4)	6.8 (5.1 to 9.2)*	-7.6 (-9.1 to -5.6)*	2.1 (0.8 to 4.5)	7.2 (3.3 to 10.7)*	-7.3 (-10.6 to -4.2)*
Maternal hypertensive disorders	157.7 (95.8 to 237.3)	5.5 (-36.4 to 70.2)	-8.8 (-45.0 to 47.2)	7.4 (3.8 to 12.7)	5.3 (-36.6 to 71.7)	-8.9 (-45.2 to 48.5)
Maternal hypertensive disorders episodes aggregate	157.6 (95.7 to 237.0)	5.4 (-36.5 to 70.0)	-8.9 (-45.1 to 47.1)	7.4 (3.8 to 12.6)	5.5 (-36.8 to 72.0)	-8.7 (-45.3 to 48.7)
Eclampsia	0.1 (0.0 to 0.3)	-11.2 (-71.2 to 168.6)	-23.2 (-75.1 to 132.3)	0.1 (0.0 to 0.2)	-11.2 (-71.2 to 168.6)	-23.2 (-75.1 to 132.3)
Complications of maternal hypertensive disorders aggregate	0.0 (0.0 to 0.0)	0.0 (0.0 to 0.0)	0.0 (0.0 to 0.0)	0.0 (0.0 to 0.0)	0.0 (0.0 to 0.0)	0.0 (0.0 to 0.0)
Maternal obstructed labor and uterine rupture	100.8 (84.3 to 120.3)	-14.0 (-17.6 to -10.3)*	-25.6 (-28.7 to -22.4)*	32.7 (21.3 to 46.8)	-13.4 (-19.5 to -7.5)*	-25.1 (-30.4 to -20.0)*
Obstructed labor, acute event	1.9 (1.0 to 3.2)	1.5 (-42.6 to 81.0)	-12.2 (-50.4 to 56.5)	0.6 (0.3 to 1.1)	2.5 (-43.3 to 85.9)	-11.4 (-51.0 to 60.8)
Fistula due to maternal obstructed labor and uterine rupture aggregate	99.0 (82.2 to 118.4)	-14.2 (-18.0 to -10.8)*	-25.8 (-29.1 to -22.9)*	32.1 (20.8 to 45.9)	-13.7 (-19.9 to -7.7)*	-25.3 (-30.8 to -20.2)*
Maternal abortion, miscarriage, and ectopic pregnancy	19.7 (11.7 to 30.1)	5.8 (-32.3 to 68.3)	-8.5 (-41.5 to 45.5)	2.1 (1.1 to 3.6)	6.2 (-34.4 to 72.9)	-8.2 (-43.3 to 49.5)
Other maternal disorders	--	--	--	7.8 (5.2 to 10.9)	-2.3 (-16.9 to 12.8)	-15.5 (-28.1 to -2.4)*
Neonatal disorders	2,807.6 (2,688.3 to 2,931.7)	20.5 (16.6 to 24.5)*	4.2 (0.8 to 7.7)*	622.4 (472.2 to 791.2)	19.5 (15.3 to 24.1)*	3.4 (-0.3 to 7.3)
Neonatal preterm birth complications	2,300.6 (2,014.7 to 2,617.1)	15.3 (9.9 to 21.1)*	-0.3 (-5.0 to 4.7)	312.7 (235.7 to 400.8)	10.9 (5.2 to 17.2)*	-4.1 (-9.0 to 1.3)
Vision loss due to retinopathy of prematurity aggregate	287.1 (229.8 to 355.2)	11.7 (7.8 to 15.7)*	-3.4 (-6.8 to 0.1)	10.3 (6.4 to 15.6)	14.7 (6.3 to 24.0)*	-0.8 (-8.1 to 7.2)
Complications of preterm birth complications aggregate	2,013.3 (1,754.7 to 2,309.7)	15.9 (9.7 to 22.7)*	0.2 (-5.1 to 6.1)	302.4 (227.6 to 389.0)	10.7 (4.8 to 17.3)*	-4.2 (-9.4 to 1.5)
Neonatal encephalopathy due to birth asphyxia and trauma	704.7 (499.5 to 1,041.8)	35.6 (32.7 to 38.3)*	17.3 (14.8 to 19.6)*	201.9 (130.5 to 306.8)	32.5 (27.5 to 37.4)*	14.6 (10.2 to 18.8)*

Cause	Prevalence in thousands (95% confidence interval)			YLDs in thousands (95% confidence interval)		
	2015	Percent change in counts between 2005 and 2015	Percent change in rates between 2005 and 2015	2015	Percent change in counts between 2005 and 2015	Percent change in rates between 2005 and 2015
Neonatal sepsis and other neonatal infections	0.0 (0.0 to 0.0)	0.0 (0.0 to 0.0)	0.0 (0.0 to 0.0)	0.0 (0.0 to 0.0)	0.0 (0.0 to 0.0)	0.0 (0.0 to 0.0)
Severe infection due to neonatal sepsis and other neonatal infections	0.0 (0.0 to 0.0)	0.0 (0.0 to 0.0)	0.0 (0.0 to 0.0)	0.0 (0.0 to 0.0)	0.0 (0.0 to 0.0)	0.0 (0.0 to 0.0)
Complications of neonatal sepsis and other neonatal infections aggregate	0.0 (0.0 to 0.0)	0.0 (0.0 to 0.0)	0.0 (0.0 to 0.0)	0.0 (0.0 to 0.0)	0.0 (0.0 to 0.0)	0.0 (0.0 to 0.0)
Hemolytic disease and other neonatal jaundice	117.8 (105.4 to 132.3)	29.6 (27.9 to 31.2)*	12.1 (10.6 to 13.5)*	34.8 (26.1 to 44.8)	26.1 (20.5 to 31.9)*	9.0 (4.2 to 14.1)*
Other neonatal disorders	--	--	--	73.0 (51.0 to 95.2)	24.6 (9.3 to 42.1)*	7.7 (-5.4 to 22.9)
Nutritional deficiencies	85,463.1 (84,437.1 to 86,540.5)	5.9 (4.2 to 7.7)*	-8.4 (-9.9 to -6.9)*	2,326.4 (1,547.9 to 3,354.9)	-0.6 (-3.2 to 1.9)	-14.1 (-16.3 to -11.9)*
Protein-energy malnutrition	9.2 (5.7 to 14.6)	-14.0 (-23.6 to -2.0)*	-25.7 (-33.9 to -15.3)*	1.1 (0.6 to 2.0)	-13.1 (-27.3 to 2.5)	-24.8 (-37.1 to -11.3)*
Iodine deficiency	8,508.9 (7,712.5 to 9,572.2)	10.3 (7.9 to 12.7)*	-4.6 (-6.7 to -2.5)*	171.8 (109.4 to 259.2)	11.2 (8.8 to 13.8)*	-3.8 (-5.9 to -1.6)*
Visible goiter due to iodine deficiency aggregate	8,377.0 (7,578.0 to 9,424.6)	10.2 (7.9 to 12.7)*	-4.7 (-6.7 to -2.5)*	149.1 (92.2 to 234.7)	10.5 (7.9 to 13.2)*	-4.4 (-6.7 to -2.1)*
Visible goiter with heart failure due to iodine deficiency aggregate	0.0 (0.0 to 0.0)	18.3 (15.9 to 20.8)*	2.3 (0.2 to 4.5)*	0.0 (0.0 to 0.0)	18.3 (15.9 to 20.8)*	2.3 (0.2 to 4.5)*
Intellectual disability due to iodine deficiency aggregate	131.9 (53.0 to 177.8)	14.9 (8.9 to 18.4)*	-0.6 (-5.9 to 2.4)	22.8 (7.9 to 37.0)	16.0 (7.2 to 23.2)*	0.3 (-7.3 to 6.5)
Vitamin A deficiency	226.6 (150.4 to 312.4)	16.3 (9.4 to 23.9)*	0.6 (-5.4 to 7.1)	13.5 (8.0 to 21.0)	18.0 (10.3 to 26.3)*	2.0 (-4.6 to 9.2)
Iron-deficiency anemia	83,223.0 (82,091.1 to 84,406.7)	4.3 (2.8 to 5.9)*	-9.8 (-11.1 to -8.4)*	2,139.8 (1,431.2 to 3,091.6)	-1.6 (-4.3 to 1.2)	-14.9 (-17.3 to -12.5)*
Iron-deficiency anemia without heart failure aggregate	83,222.3 (82,090.3 to 84,405.9)	4.3 (2.8 to 5.9)*	-9.8 (-11.1 to -8.4)*	2,139.7 (1,431.1 to 3,091.5)	-1.6 (-4.3 to 1.2)	-14.9 (-17.3 to -12.5)*
Iron-deficiency anemia with heart failure aggregate	0.7 (0.6 to 0.9)	26.2 (23.5 to 28.9)*	9.2 (6.8 to 11.5)*	0.1 (0.1 to 0.1)	26.2 (23.5 to 28.9)*	9.2 (6.8 to 11.5)*
Other nutritional deficiencies	--	--	--	0.1 (0.1 to 0.2)	-12.8 (-32.5 to 10.5)	-24.6 (-41.6 to -4.4)*
Other communicable, maternal, neonatal, and nutritional diseases	146,664.9 (142,421.0 to 151,320.1)	14.5 (13.5 to 15.6)*	-1.0 (-1.8 to -0.1)*	219.6 (140.1 to 337.2)	10.9 (4.0 to 18.3)*	-4.1 (-10.1 to 2.3)
Sexually transmitted diseases excluding HIV	117,197.4 (112,410.2 to 122,114.7)	13.8 (12.4 to 15.1)*	-1.6 (-2.8 to -0.4)*	143.6 (87.7 to 231.2)	15.0 (11.1 to 19.3)*	-0.5 (-3.9 to 3.2)
Syphilis	4,870.2 (3,368.8 to 6,953.5)	2.3 (-6.8 to 10.0)	-11.5 (-19.4 to -4.9)*	27.4 (18.4 to 38.1)	6.7 (0.1 to 12.8)*	-7.7 (-13.4 to -2.5)*
Early syphilis aggregate	4,731.1 (3,241.2 to 6,812.4)	2.2 (-7.3 to 10.1)	-11.6 (-19.8 to -4.8)*	1.1 (0.3 to 2.9)	2.0 (-8.7 to 11.0)	-11.8 (-21.1 to -4.0)*
Adult tertiary syphilis	139.2 (121.6 to 158.5)	6.6 (3.4 to 9.4)*	-7.8 (-10.6 to -5.4)*	26.3 (17.2 to 36.9)	6.9 (0.1 to 13.1)*	-7.5 (-13.4 to -2.2)*
Chlamydial infection	3,096.9 (2,218.1 to 4,516.2)	17.7 (14.4 to 21.6)*	1.8 (-1.1 to 5.1)	18.4 (10.5 to 30.6)	14.5 (7.7 to 21.2)*	-1.0 (-6.9 to 4.8)
Chlamydial infection episodes aggregate	2,610.2 (1,726.0 to 4,053.9)	20.1 (16.0 to 25.1)*	3.8 (0.3 to 8.2)*	12.9 (6.7 to 23.3)	19.6 (10.8 to 28.9)*	3.4 (-4.2 to 11.5)
Pelvic inflammatory diseases due to chlamydial infection aggregate	22.3 (16.1 to 30.9)	0.7 (-3.2 to 4.4)	-12.9 (-16.3 to -9.7)*	3.0 (1.8 to 4.5)	1.6 (-8.3 to 12.8)	-12.1 (-20.7 to -2.4)*
Infertility due to clamydial infection aggregate	464.4 (416.3 to 509.7)	6.7 (5.3 to 8.1)*	-7.7 (-8.9 to -6.5)*	2.5 (1.0 to 5.3)	7.2 (3.7 to 10.8)*	-7.3 (-10.3 to -4.2)*
Gonococcal infection	3,514.1 (1,969.7 to 6,082.5)	26.9 (15.1 to 36.8)*	9.8 (-0.4 to 18.3)	36.8 (17.9 to 71.2)	28.5 (15.7 to 38.1)*	11.1 (0.1 to 19.4)*
Gonococcal infection episodes aggregate	3,266.2 (1,725.8 to 5,820.5)	28.9 (16.2 to 39.1)*	11.4 (0.5 to 20.3)*	34.5 (16.2 to 68.8)	30.5 (17.2 to 40.4)*	12.9 (1.3 to 21.4)*
Pelvic inflammatory diseases due to gonococcal infection aggregate	247.9 (218.8 to 281.4)	6.1 (3.6 to 8.0)*	-8.2 (-10.4 to -6.6)*	2.3 (1.4 to 4.1)	4.2 (-1.5 to 10.2)	-9.9 (-14.8 to -4.7)*
Trichomoniasis	17,955.8 (12,600.1 to 24,988.8)	15.6 (14.6 to 16.3)*	-0.1 (-0.9 to 0.6)	19.9 (7.3 to 43.7)	15.2 (13.4 to 16.7)*	-0.4 (-1.9 to 0.9)
Genital herpes	94,704.8 (82,806.1 to 107,354.0)	13.5 (11.9 to 15.1)*	-1.8 (-3.2 to -0.4)*	24.4 (7.4 to 58.9)	16.4 (13.5 to 24.4)*	0.6 (-1.9 to 7.5)
Moderate infection due to initial genital herpes episode	20.3 (4.6 to 48.3)	170.3 (109.9 to 277.5)*	133.7 (81.6 to 226.5)*	1.0 (0.2 to 2.4)	164.6 (103.0 to 278.1)*	128.8 (75.6 to 227.0)*
Complications of genital herpes aggregate	94,684.4 (82,789.9 to 107,329.2)	13.5 (11.9 to 15.1)*	-1.8 (-3.2 to -0.4)*	23.4 (6.7 to 57.5)	13.7 (11.7 to 15.6)*	-1.7 (-3.4 to 0.0)
Other sexually transmitted diseases	1,467.4 (1,322.6 to 1,604.9)	6.2 (4.5 to 7.9)*	-8.2 (-9.6 to -6.7)*	16.7 (10.0 to 26.3)	3.0 (-1.2 to 6.9)	-10.9 (-14.5 to -7.6)*
Pelvic inflammatory diseases due to other sexually transmitted diseases aggregate	69.7 (51.7 to 92.7)	-0.2 (-2.1 to 2.2)	-13.7 (-15.3 to -11.6)*	9.1 (5.5 to 13.8)	-0.1 (-6.2 to 6.1)	-13.6 (-18.9 to -8.2)*
Infertility due to other sexually transmitted diseases aggregate	1,397.8 (1,259.8 to 1,531.2)	6.5 (4.7 to 8.3)*	-7.9 (-9.4 to -6.3)*	7.6 (3.0 to 15.5)	7.0 (4.4 to 9.6)*	-7.4 (-9.7 to -5.2)*
Other sexually transmitted diseases	--	--	--	20.6 (11.0 to 36.7)	27.4 (16.5 to 36.7)*	10.2 (0.8 to 18.2)*
Hepatitis	36,766.6 (36,213.4 to 37,284.0)	18.3 (17.4 to 19.3)*	2.3 (1.5 to 3.1)*	28.0 (16.8 to 44.5)	17.8 (-20.4 to 73.2)	1.9 (-31.1 to 49.8)
Acute hepatitis A	165.2 (134.7 to 192.3)	22.3 (-4.3 to 54.5)	5.8 (-17.2 to 33.6)	6.9 (4.3 to 10.5)	22.0 (-7.2 to 56.8)	5.5 (-19.7 to 35.6)
Hepatitis B	27,001.1 (25,993.6 to 28,076.0)	19.1 (16.8 to 21.5)*	3.0 (1.0 to 5.1)*	19.1 (10.4 to 32.6)	16.4 (-34.2 to 104.8)	0.7 (-43.1 to 77.1)
Acute Hepatitis B aggregate	828.3 (541.5 to 1,231.2)	16.1 (-34.6 to 105.7)	0.4 (-43.4 to 77.9)	19.1 (10.4 to 32.6)	16.4 (-34.2 to 104.8)	0.7 (-43.1 to 77.1)
Chronic hepatitis B	26,172.8 (25,161.3 to 27,242.9)	19.2 (18.1 to 20.4)*	3.1 (2.1 to 4.1)*	0.0 (0.0 to 0.0)	0.0 (0.0 to 0.0)	0.0 (0.0 to 0.0)
Hepatitis C	10,349.0 (9,146.4 to 11,577.6)	16.1 (14.7 to 17.4)*	0.4 (-0.8 to 1.6)	0.5 (0.2 to 0.9)	15.0 (10.8 to 18.3)*	-0.5 (-4.2 to 2.3)
Acute Hepatitis C aggregate	32.5 (25.1 to 41.5)	15.1 (10.8 to 18.3)*	-0.5 (-4.2 to 2.3)	0.5 (0.2 to 0.9)	15.0 (10.8 to 18.3)*	-0.5 (-4.2 to 2.3)
Chronic hepatitis C	10,316.4 (9,119.0 to 11,545.5)	16.1 (14.7 to 17.4)*	0.4 (-0.8 to 1.6)	0.0 (0.0 to 0.0)	0.0 (0.0 to 0.0)	0.0 (0.0 to 0.0)
Acute hepatitis E	49.0 (32.2 to 72.4)	17.5 (14.7 to 21.4)*	1.6 (-0.8 to 5.0)	1.5 (0.8 to 2.6)	17.4 (-6.8 to 49.4)	1.5 (-19.4 to 29.2)
Other infectious diseases	2,195.6 (2,154.8 to 2,237.4)	3.3 (1.1 to 5.8)*	-10.7 (-12.6 to -8.5)*	48.0 (30.4 to 73.0)	-2.8 (-9.0 to 3.9)	-15.9 (-21.3 to -10.1)*
Non-communicable diseases	459,575.8 (458,131.6 to 461,101.4)	15.6 (15.5 to 15.8)*	-0.0 (-0.1 to 0.1)	49,150.5 (36,423.6 to 62,939.1)	14.9 (14.2 to 15.6)*	-0.7 (-1.3 to -0.0)*
Neoplasms	4,061.9 (3,932.3 to 4,214.0)	15.9 (11.4 to 20.6)*	0.2 (-3.7 to 4.3)	372.5 (263.8 to 492.3)	11.2 (3.8 to 18.9)*	-3.8 (-10.2 to 2.8)
Lip and oral cavity cancer	130.4 (106.0 to 163.8)	19.1 (0.2 to 41.5)*	3.0 (-13.3 to 22.4)	11.1 (7.3 to 15.7)	18.2 (-0.7 to 39.9)	2.2 (-14.2 to 21.0)
Nasopharynx cancer	62.3 (33.3 to 104.2)	-5.8 (-45.3 to 56.3)	-18.5 (-52.7 to 35.2)	5.7 (2.9 to 10.2)	-6.4 (-44.7 to 53.3)	-19.1 (-52.2 to 32.5)
Other pharynx cancer	35.3 (31.2 to 39.7)	7.9 (-3.1 to 19.7)	-6.6 (-16.2 to 3.5)	3.0 (2.1 to 4.0)	7.1 (-5.0 to 19.8)	-7.4 (-17.9 to 3.6)
Esophageal cancer	16.7 (13.1 to 23.2)	-7.8 (-29.5 to 20.0)	-20.3 (-39.1 to 3.8)	3.2 (2.1 to 4.8)	-12.5 (-32.1 to 13.2)	-24.4 (-41.3 to -2.1)*
Stomach cancer	96.9 (88.8 to 105.5)	-16.8 (-23.9 to -9.2)*	-28.0 (-34.2 to -21.5)*	11.2 (8.0 to 14.5)	-18.6 (-26.8 to -9.8)*	-29.6 (-36.7 to -22.0)*
Colon and rectum cancer	267.4 (235.9 to 298.5)	23.3 (10.2 to 38.4)*	6.6 (-4.7 to 19.7)	22.2 (15.6 to 29.9)	20.3 (7.2 to 36.1)*	4.0 (-7.3 to 17.7)
Colon and rectum cancer aggregate	266.8 (235.3 to 297.8)	23.3 (10.2 to 38.5)*	6.6 (-4.7 to 19.7)	22.1 (15.6 to 29.8)	20.4 (7.2 to 36.2)*	4.1 (-7.3 to 17.8)
Stoma due to colon and rectum cancer	0.6 (0.5 to 0.7)	6.5 (5.0 to 7.8)*	-7.9 (-9.2 to -6.7)*	0.1 (0.1 to 0.1)	6.5 (5.0 to 7.8)*	-7.9 (-9.2 to -6.7)*
Liver cancer	11.9 (7.0 to 17.5)	-15.7 (-49.5 to 38.5)	-27.1 (-56.3 to 19.8)	8.9 (5.4 to 16.6)	-6.3 (-33.6 to 60.2)	-19.0 (-42.6 to 38.5)
Liver cancer due to hepatitis B	24.6 (15.7 to 49.9)	-2.4 (-39.0 to 96.2)	-15.6 (-47.2 to 69.7)	5.0 (3.0 to 9.7)	-8.6 (-36.7 to 63.3)	-21.0 (-45.3 to 41.2)
Liver cancer due to hepatitis C	3.7 (2.8 to 5.2)	15.7 (-16.6 to 55.4)	0.1 (-27.8 to 34.4)	0.8 (0.5 to 1.1)	4.7 (-19.8 to 37.4)	-9.5 (-30.6 to 18.8)
Liver cancer due to alcohol use	6.9 (4.7 to 12.8)	16.8 (-24.5 to 108.9)	1.0 (-34.7 to 80.6)	1.6 (1.0 to 3.0)	8.4 (-26.6 to 84.1)	-6.2 (-36.5 to 59.2)
Liver cancer due to other causes	6.9 (4.6 to 12.6)	-10.3 (-43.3 to 60.7)	-22.4 (-51.0 to 38.9)	1.5 (0.9 to 2.6)	-16.2 (-41.0 to 36.6)	-27.5 (-49.0 to 18.1)
Gallbladder and biliary tract cancer	2.7 (2.4 to 3.0)	-4.3 (-12.5 to 4.7)	-17.2 (-24.3 to -9.4)*	0.8 (0.5 to 1.0)	-5.4 (-13.3 to 3.7)	-18.2 (-25.0 to -10.3)*
Pancreatic cancer	7.2 (6.5 to 8.0)	15.2 (4.5 to 26.1)*	-0.4 (-9.6 to 9.1)	1.8 (1.3 to 2.4)	11.7 (1.7 to 22.3)*	-3.4 (-12.0 to 5.8)

Cause	Prevalence in thousands (95% confidence interval)			YLDs in thousands (95% confidence interval)		
	2015	Percent change in counts between 2005 and 2015	Percent change in rates between 2005 and 2015	2015	Percent change in counts between 2005 and 2015	Percent change in rates between 2005 and 2015
Larynx cancer	64.8 (60.2 to 72.8)	10.7 (3.4 to 20.0)*	-4.2 (-10.6 to 3.7)	7.3 (5.1 to 10.0)	8.7 (-0.9 to 19.6)	-6.0 (-14.3 to 3.5)
Larynx cancer aggregate	13.2 (9.0 to 21.1)	-8.8 (-33.6 to 28.1)	-21.1 (-42.6 to 10.8)	2.5 (1.6 to 3.7)	-4.5 (-20.2 to 17.0)	-17.5 (-30.9 to 1.2)
Laryngectomy due to larynx cancer	51.6 (49.9 to 54.0)	17.2 (15.8 to 18.1)*	1.3 (0.1 to 2.2)*	4.8 (3.1 to 6.9)	17.2 (6.8 to 29.0)*	1.4 (-7.6 to 11.5)
Tracheal, bronchus, and lung cancer	71.4 (56.0 to 98.5)	4.2 (-15.8 to 32.4)	-9.9 (-27.2 to 14.5)	12.2 (7.9 to 18.3)	1.3 (-19.4 to 29.9)	-12.4 (-30.3 to 12.3)
Malignant skin melanoma	204.2 (172.1 to 267.8)	21.1 (11.8 to 31.6)*	4.7 (-3.4 to 13.9)	11.7 (7.6 to 17.4)	19.8 (10.0 to 31.0)*	3.6 (-4.9 to 13.3)
Non-melanoma skin cancer	74.8 (67.5 to 82.1)	39.7 (24.2 to 57.5)*	20.9 (7.4 to 36.2)*	4.4 (2.9 to 6.3)	43.9 (24.7 to 66.1)*	24.4 (7.9 to 43.6)*
Non-melanoma skin cancer(squamous-cell carcinoma)	72.3 (61.6 to 87.9)	50.7 (31.6 to 74.1)*	30.4 (13.8 to 50.5)*	4.2 (2.8 to 6.1)	46.3 (26.4 to 70.6)*	26.5 (9.3 to 47.5)*
Non-melanoma skin cancer(basal-cell carcinoma)	15.9 (12.7 to 19.6)	3.1 (-0.2 to 6.0)	-10.8 (-13.7 to -8.3)*	0.2 (0.1 to 0.3)	3.0 (-7.3 to 13.8)	-10.9 (-19.8 to -1.6)*
Breast cancer	1,677.2 (1,534.9 to 1,801.2)	20.8 (9.5 to 33.1)*	4.4 (-5.3 to 15.1)	125.0 (86.4 to 168.8)	16.1 (6.1 to 27.3)*	0.4 (-8.3 to 10.1)
Breast cancer aggregate	1,327.6 (1,183.8 to 1,453.7)	25.0 (10.3 to 41.6)*	8.1 (-4.6 to 22.5)	97.5 (68.4 to 130.0)	18.8 (5.9 to 34.3)*	2.8 (-8.4 to 16.1)
Mastectomy due to breast cancer	349.6 (332.7 to 365.5)	7.0 (5.4 to 8.2)*	-7.5 (-8.8 to -6.4)*	27.5 (17.3 to 39.8)	7.2 (3.4 to 11.0)*	-7.3 (-10.5 to -4.0)*
Cervical cancer	427.0 (355.0 to 514.4)	-10.2 (-25.9 to 9.9)	-22.3 (-35.9 to -5.0)*	32.2 (21.7 to 43.7)	-9.7 (-25.7 to 10.5)	-21.9 (-35.8 to -4.4)*
Uterine cancer	197.2 (128.8 to 264.1)	12.1 (-22.1 to 64.6)	-3.1 (-32.6 to 42.3)	12.8 (7.2 to 19.7)	11.2 (-23.4 to 63.9)	-3.8 (-33.8 to 41.7)
Ovarian cancer	81.6 (66.8 to 103.3)	19.5 (1.4 to 40.7)*	3.3 (-12.3 to 21.7)	10.4 (7.0 to 14.5)	18.5 (-2.5 to 42.4)	2.4 (-15.7 to 23.2)
Prostate cancer	44.4 (35.3 to 55.1)	20.1 (3.5 to 38.9)*	3.9 (-10.5 to 20.1)	3.6 (2.4 to 5.0)	14.5 (-0.1 to 30.0)	-1.0 (-13.6 to 12.4)
Prostate cancer aggregate	36.0 (26.9 to 46.8)	24.0 (3.0 to 48.3)*	7.3 (-10.9 to 28.2)	2.8 (1.8 to 4.0)	17.1 (-1.7 to 38.3)	1.3 (-15.0 to 19.6)
Impotence and incontinence due to prostate cancer aggregate	8.4 (8.2 to 8.6)	5.8 (5.4 to 6.2)*	-8.5 (-8.9 to -8.1)*	0.8 (0.5 to 1.1)	6.0 (0.7 to 11.0)*	-8.3 (-12.9 to -4.0)*
Testicular cancer	73.0 (63.0 to 83.7)	31.9 (13.5 to 49.4)*	14.0 (-1.9 to 29.2)	4.3 (2.9 to 6.3)	29.7 (11.3 to 49.5)*	12.2 (-3.8 to 29.3)
Kidney cancer	86.9 (79.0 to 95.6)	21.3 (9.8 to 33.1)*	4.9 (-5.0 to 15.1)	6.3 (4.3 to 8.6)	19.7 (7.4 to 32.8)*	3.6 (-7.1 to 14.9)
Bladder cancer	72.7 (57.7 to 89.0)	15.8 (-3.7 to 38.8)	0.1 (-16.7 to 20.0)	6.8 (4.6 to 9.4)	13.2 (-1.8 to 31.1)	-2.1 (-15.1 to 13.4)
Bladder cancer episodes aggregate	64.1 (49.0 to 80.5)	16.5 (-5.6 to 43.2)	0.7 (-18.4 to 23.8)	5.3 (3.4 to 7.4)	13.9 (-4.9 to 36.8)	-1.5 (-17.8 to 18.3)
Urinary incontinence due to bladder cancer	8.6 (8.1 to 9.2)	10.9 (9.3 to 12.2)*	-4.1 (-5.5 to -3.0)*	1.6 (1.1 to 2.2)	11.0 (4.3 to 17.8)*	-4.0 (-9.8 to 1.9)
Brain and nervous system cancer	67.1 (55.3 to 83.4)	16.4 (-2.6 to 38.2)	0.7 (-15.8 to 19.5)	7.5 (5.1 to 10.6)	14.8 (-3.2 to 36.4)	-0.7 (-16.3 to 18.0)
Thyroid cancer	125.0 (109.3 to 141.2)	7.3 (-5.9 to 21.1)	-7.2 (-18.6 to 4.7)	7.0 (4.6 to 10.1)	6.4 (-7.3 to 20.6)	-8.0 (-19.9 to 4.3)
Mesothelioma	1.7 (1.5 to 2.0)	9.1 (-3.8 to 24.3)	-5.7 (-16.8 to 7.5)	0.3 (0.2 to 0.5)	8.7 (-4.3 to 24.2)	-6.0 (-17.2 to 7.4)
Hodgkin lymphoma	38.0 (31.8 to 46.7)	8.9 (-6.0 to 26.6)	-5.8 (-18.7 to 9.5)	3.1 (2.0 to 4.4)	5.1 (-10.2 to 23.6)	-9.1 (-22.3 to 6.9)
Non-Hodgkin lymphoma	203.8 (173.5 to 234.3)	44.5 (26.2 to 63.7)*	25.0 (9.1 to 41.6)*	15.1 (10.2 to 20.7)	40.4 (21.7 to 60.4)*	21.4 (5.2 to 38.7)*
Multiple myeloma	14.6 (12.1 to 17.5)	33.5 (15.6 to 57.4)*	15.4 (-0.0 to 36.1)	3.4 (2.3 to 4.5)	31.0 (14.2 to 53.4)*	13.3 (-1.2 to 32.7)
Leukemia	68.3 (61.5 to 76.5)	28.2 (11.7 to 46.6)*	10.9 (-3.4 to 26.8)	16.1 (11.3 to 21.5)	17.6 (5.3 to 32.1)*	1.7 (-8.9 to 14.2)
Acute lymphoid leukemia	27.0 (22.1 to 33.2)	26.4 (7.8 to 47.6)*	9.3 (-6.8 to 27.7)	3.5 (2.4 to 4.9)	22.8 (4.1 to 43.7)*	6.2 (-10.0 to 24.2)
Chronic lymphoid leukemia	44.4 (36.1 to 54.3)	18.7 (-2.3 to 45.4)	2.7 (-15.5 to 25.8)	5.5 (3.8 to 7.6)	15.9 (-5.2 to 42.4)	0.3 (-18.0 to 23.1)
Acute myeloid leukemia	39.3 (33.2 to 46.9)	27.1 (12.9 to 42.6)*	9.9 (-2.4 to 23.3)	4.9 (3.4 to 6.7)	24.5 (10.7 to 40.8)*	7.7 (-4.3 to 21.7)
Chronic myeloid leukemia	16.5 (13.7 to 19.8)	4.7 (-8.6 to 20.8)	-9.5 (-21.0 to 4.4)	2.1 (1.5 to 3.0)	1.5 (-11.7 to 17.3)	-12.3 (-23.6 to 1.4)
Other neoplasms	208.3 (174.5 to 250.3)	22.5 (8.0 to 40.4)*	5.9 (-6.6 to 21.4)	15.1 (10.2 to 21.3)	21.1 (5.9 to 39.3)*	4.7 (-8.4 to 20.4)
Cardiovascular diseases	15,291.6 (14,785.6 to 15,700.8)	11.8 (9.8 to 13.5)*	-3.3 (-5.0 to -1.8)*	960.0 (682.3 to 1,283.9)	12.7 (9.0 to 14.6)*	-2.5 (-5.7 to -0.9)*
Rheumatic heart disease	3,102.7 (2,754.7 to 4,036.1)	-0.9 (-10.2 to 4.7)	-14.3 (-22.3 to -9.4)*	150.1 (94.1 to 230.4)	-0.2 (-9.6 to 5.5)	-13.7 (-21.8 to -8.8)*
Rheumatic heart disease, without heart failure	3,039.7 (2,688.2 to 3,975.8)	-1.3 (-10.6 to 4.4)	-14.6 (-22.7 to -9.7)*	142.7 (89.2 to 221.8)	-1.2 (-10.6 to 4.5)	-14.5 (-22.7 to -9.6)*
Heart failure fue to rheumatic heart disease aggregate	63.1 (54.7 to 71.8)	24.0 (22.2 to 25.8)*	7.3 (5.6 to 8.8)*	7.4 (5.0 to 10.6)	23.9 (13.6 to 35.5)*	7.1 (-1.8 to 17.2)
Ischemic heart disease	2,902.5 (2,577.1 to 3,241.2)	14.0 (12.9 to 15.0)*	-1.4 (-2.3 to -0.6)*	208.5 (138.4 to 294.4)	17.4 (15.3 to 19.6)*	1.5 (-0.3 to 3.4)
Myocardial infarction episodes aggregate	182.2 (138.3 to 233.8)	-18.8 (-25.5 to -12.5)*	-29.7 (-35.6 to -24.3)*	0.8 (0.5 to 1.2)	0.9 (-7.0 to 8.2)	-12.7 (-19.5 to -6.4)*
Angina due to ischemic heart disease aggregate	2,293.8 (1,973.0 to 2,639.4)	17.0 (15.9 to 18.1)*	1.2 (0.3 to 2.2)*	158.0 (101.5 to 224.3)	17.3 (14.7 to 19.8)*	1.4 (-0.8 to 3.6)
Heart failure fue to ischemic heart disease aggregate	426.6 (383.5 to 470.7)	18.0 (17.1 to 18.9)*	2.0 (1.3 to 2.8)*	49.6 (33.4 to 69.6)	18.1 (14.3 to 22.5)*	2.1 (-1.2 to 5.9)
Cerebrovascular disease	1,720.3 (1,691.4 to 1,749.0)	14.1 (12.0 to 16.2)*	-1.4 (-3.1 to 0.5)	268.2 (184.8 to 366.1)	14.2 (11.2 to 17.2)*	-1.3 (-3.9 to 1.3)
Ischemic stroke	646.5 (620.4 to 671.8)	16.1 (15.2 to 16.9)*	0.4 (-0.4 to 1.1)	99.7 (68.6 to 135.8)	16.4 (12.1 to 21.4)*	0.6 (-3.1 to 5.0)
Chronic ischemic stroke aggregate	642.0 (616.2 to 667.9)	16.2 (15.3 to 16.9)*	0.5 (-0.3 to 1.1)	98.8 (67.7 to 134.8)	16.5 (12.2 to 21.5)*	0.7 (-3.0 to 5.1)
Ischemic stroke episodes aggregate	4.4 (3.6 to 5.5)	6.1 (0.8 to 11.7)*	-8.2 (-12.9 to -3.4)*	1.0 (0.6 to 1.4)	6.1 (0.8 to 11.7)*	-8.2 (-12.9 to -3.4)*
Hemorrhagic stroke	1,093.7 (1,050.2 to 1,137.2)	12.6 (11.7 to 13.3)*	-2.6 (-3.4 to -2.0)*	168.5 (116.1 to 228.0)	12.9 (9.0 to 16.8)*	-2.4 (-5.7 to 1.0)
Chronic hemorrhagic stroke aggregate	1,085.4 (1,042.5 to 1,128.7)	12.7 (11.7 to 13.4)*	-2.6 (-3.4 to -1.9)*	166.6 (114.9 to 225.5)	13.0 (9.1 to 16.9)*	-2.3 (-5.6 to 1.1)
Acute hemorrhagic stroke aggregate	8.3 (7.1 to 9.7)	4.0 (-1.6 to 9.4)	-10.1 (-14.9 to -5.4)*	1.8 (1.2 to 2.5)	4.0 (-1.6 to 9.4)	-10.1 (-14.9 to -5.4)*
Hypertensive heart disease	126.1 (111.2 to 142.3)	21.5 (20.4 to 22.7)*	5.1 (4.1 to 6.1)*	14.7 (9.9 to 20.7)	21.5 (13.8 to 30.1)*	5.0 (-1.6 to 12.5)
Cardiomyopathy and myocarditis	111.4 (96.5 to 127.0)	11.7 (10.4 to 13.0)*	-3.4 (-4.5 to -2.3)*	12.4 (8.2 to 17.7)	11.5 (4.8 to 17.9)*	-3.6 (-9.4 to 2.0)
Acute myocarditis	10.3 (7.0 to 14.8)	17.7 (14.4 to 20.9)*	1.8 (-1.1 to 4.5)	0.5 (0.3 to 0.8)	17.6 (10.5 to 25.0)*	1.7 (-4.4 to 8.1)
Heart failure due to cardiomyopathy aggregate	101.1 (87.5 to 116.3)	11.1 (9.8 to 12.5)*	-3.9 (-5.1 to -2.7)*	11.8 (7.9 to 16.8)	11.2 (4.2 to 18.0)*	-3.8 (-9.9 to 2.0)
Atrial fibrillation and flutter	832.5 (680.0 to 1,006.6)	10.5 (8.9 to 11.9)*	-4.5 (-5.8 to -3.3)*	69.9 (45.2 to 100.5)	10.7 (6.9 to 14.4)*	-4.2 (-7.6 to -1.0)*
Peripheral vascular disease	3,368.6 (2,637.8 to 4,280.3)	17.1 (16.2 to 18.3)*	1.3 (0.5 to 2.3)*	0.2 (0.1 to 0.4)	10.3 (1.3 to 20.3)*	-4.6 (-12.4 to 4.1)
Endocarditis	6.0 (5.1 to 7.1)	17.3 (14.5 to 20.0)*	1.4 (-1.0 to 3.8)	0.7 (0.4 to 1.0)	17.2 (14.4 to 20.1)*	1.4 (-1.1 to 3.8)
Endocarditis episodes aggregate	1.1 (0.8 to 1.3)	17.2 (9.9 to 23.7)*	1.4 (-4.9 to 7.0)	0.1 (0.0 to 0.1)	16.8 (9.5 to 23.2)*	1.0 (-5.3 to 6.6)
Heart failure due to endocarditis aggregate	5.0 (4.1 to 6.1)	17.3 (14.3 to 20.3)*	1.4 (-1.2 to 4.0)	0.6 (0.4 to 0.9)	17.3 (14.3 to 20.3)*	1.4 (-1.2 to 4.0)
Other cardiovascular and circulatory diseases	3,426.7 (2,920.9 to 4,003.4)	16.7 (15.0 to 18.6)*	0.9 (-0.6 to 2.5)	235.4 (156.5 to 337.4)	16.8 (14.4 to 19.3)*	1.0 (-1.1 to 3.1)
Heart failure due to other cardiovascular diseases aggregate	12.9 (11.1 to 14.8)	16.3 (14.6 to 18.1)*	0.6 (-0.9 to 2.1)	1.6 (1.0 to 2.3)	16.4 (8.5 to 24.5)*	0.6 (-6.1 to 7.7)
Other cardiovascular diseases episodes aggregate	3,413.8 (2,909.7 to 3,988.5)	16.7 (15.0 to 18.6)*	0.9 (-0.6 to 2.5)	233.9 (155.4 to 335.0)	16.8 (14.4 to 19.3)*	1.0 (-1.1 to 3.2)
Chronic respiratory diseases	26,184.5 (25,017.3 to 27,264.2)	10.3 (8.4 to 12.3)*	-4.6 (-6.3 to -2.9)*	1,611.5 (1,237.2 to 2,027.8)	9.5 (7.0 to 12.3)*	-5.3 (-7.5 to -2.9)*
Chronic obstructive pulmonary disease	9,730.1 (8,426.6 to 11,279.6)	7.2 (4.8 to 9.4)*	-7.3 (-9.4 to -5.4)*	705.2 (574.5 to 847.5)	8.9 (5.2 to 12.5)*	-5.8 (-9.0 to -2.7)*

Cause	Prevalence in thousands (95% confidence interval)			YLDs in thousands (95% confidence interval)		
	2015	Percent change in counts between 2005 and 2015	Percent change in rates between 2005 and 2015	2015	Percent change in counts between 2005 and 2015	Percent change in rates between 2005 and 2015
Chronic obstructive pulmonary disease without heart failure aggregate	9,718.7 (8,413.8 to 11,267.9)	7.2 (4.8 to 9.4)*	-7.3 (-9.4 to -5.4)*	697.1 (567.3 to 839.6)	8.7 (5.0 to 12.4)*	-6.0 (-9.2 to -2.8)*
Severe chronic obstructive pulmonary disease with heart failure aggregate	17.1 (14.9 to 19.5)	23.7 (22.2 to 25.1)*	6.9 (5.7 to 8.2)*	8.1 (5.7 to 11.0)	23.3 (17.7 to 29.9)*	6.6 (1.8 to 12.3)*
Pneumoconiosis	188.2 (179.0 to 198.1)	26.1 (19.8 to 33.1)*	9.0 (3.6 to 15.1)*	36.5 (23.3 to 52.5)	24.0 (16.7 to 32.0)*	7.3 (0.9 to 14.2)*
Silicosis	38.4 (33.2 to 43.7)	12.6 (9.8 to 15.6)*	-2.6 (-5.0 to -0.0)*	6.2 (3.8 to 9.3)	13.2 (0.4 to 27.2)*	-2.1 (-13.2 to 10.0)
Silicosis without heart failure aggregate	38.4 (33.2 to 43.7)	12.6 (9.8 to 15.6)*	-2.6 (-5.0 to -0.0)*	6.2 (3.8 to 9.3)	13.2 (0.4 to 27.2)*	-2.1 (-13.2 to 10.0)
Severe silicosis with heart failure aggregate	0.0 (0.0 to 0.0)	0.0 (0.0 to 0.0)	0.0 (0.0 to 0.0)	0.0 (0.0 to 0.0)	0.0 (0.0 to 0.0)	0.0 (0.0 to 0.0)
Asbestosis	6.9 (5.8 to 8.1)	12.8 (10.1 to 15.6)*	-2.5 (-4.8 to 0.0)	1.1 (0.7 to 1.7)	12.8 (10.0 to 15.7)*	-2.5 (-4.9 to 0.1)
Asbestosis without heart failure aggregate	6.9 (5.8 to 8.1)	12.8 (10.1 to 15.6)*	-2.5 (-4.8 to 0.0)	1.1 (0.7 to 1.7)	12.8 (10.0 to 15.7)*	-2.5 (-4.9 to 0.1)
Severe asbestosis with heart failure aggregate	0.0 (0.0 to 0.0)	0.0 (0.0 to 0.0)	0.0 (0.0 to 0.0)	0.0 (0.0 to 0.0)	0.0 (0.0 to 0.0)	0.0 (0.0 to 0.0)
Coal workers pneumoconiosis	3.2 (2.7 to 3.9)	17.8 (14.3 to 21.1)*	1.9 (-1.1 to 4.7)	0.5 (0.3 to 0.8)	17.8 (14.4 to 21.2)*	1.9 (-1.0 to 4.8)
Coal workers pneumoconiosis without heart failure aggregate	3.2 (2.6 to 3.8)	17.7 (14.2 to 21.1)*	1.8 (-1.3 to 4.7)	0.5 (0.3 to 0.8)	17.6 (14.0 to 21.0)*	1.7 (-1.5 to 4.7)
Severe coal workers pneumoconiosis with heart failure aggregate	0.1 (0.0 to 0.1)	22.3 (13.5 to 31.5)*	5.8 (-1.8 to 13.7)	0.0 (0.0 to 0.0)	22.3 (13.5 to 31.5)*	5.8 (-1.8 to 13.7)
Other pneumoconiosis	183.1 (162.1 to 207.3)	27.2 (24.0 to 30.6)*	10.0 (7.2 to 12.9)*	28.5 (18.1 to 41.5)	27.3 (18.1 to 37.4)*	10.1 (2.1 to 18.8)*
Other pneumoconiosis without heart failure aggregate	182.9 (161.9 to 207.1)	27.2 (23.9 to 30.6)*	10.0 (7.2 to 12.9)*	28.4 (18.0 to 41.3)	27.3 (18.1 to 37.4)*	10.1 (2.1 to 18.8)*
Severe other pneumoconiosis with heart failure aggregate	0.2 (0.2 to 0.2)	25.1 (21.3 to 29.4)*	8.2 (4.9 to 11.9)*	0.1 (0.1 to 0.1)	25.1 (21.3 to 29.4)*	8.2 (4.9 to 11.9)*
Asthma	16,616.3 (13,317.5 to 20,561.4)	11.5 (8.2 to 15.6)*	-3.6 (-6.5 to 0.0)	734.2 (458.9 to 1,094.1)	11.6 (8.1 to 15.8)*	-3.5 (-6.5 to 0.2)
Asymptomatic asthma	5,032.1 (3,903.7 to 6,354.5)	11.5 (8.2 to 15.6)*	-3.6 (-6.5 to 0.0)	0.0 (0.0 to 0.0)	0.0 (0.0 to 0.0)	0.0 (0.0 to 0.0)
Symptomatic asthma aggregate	11,584.3 (9,306.9 to 14,452.2)	11.5 (8.2 to 15.6)*	-3.6 (-6.5 to 0.0)	734.2 (458.9 to 1,094.1)	11.6 (8.1 to 15.8)*	-3.5 (-6.5 to 0.2)
Interstitial lung disease and pulmonary sarcoidosis	112.1 (96.3 to 129.2)	12.5 (9.7 to 15.2)*	-2.7 (-5.1 to -0.4)*	13.9 (8.5 to 20.5)	12.9 (6.2 to 19.7)*	-2.4 (-8.1 to 3.5)
Interstitial lung disease and pulmonary sarcoidosis without heart failure aggregate	108.1 (92.8 to 124.8)	12.5 (9.6 to 15.3)*	-2.7 (-5.2 to -0.3)*	12.0 (7.0 to 18.2)	12.9 (5.3 to 20.9)*	-2.3 (-8.9 to 4.6)
Severe interstitial lung disease and pulmonary sarcoidosis with heart failure aggregate	4.0 (3.4 to 4.6)	12.4 (10.8 to 14.0)*	-2.8 (-4.2 to -1.4)*	1.9 (1.3 to 2.6)	12.4 (10.6 to 14.2)*	-2.8 (-4.3 to -1.2)*
Other chronic respiratory diseases	--	--	--	121.7 (96.8 to 148.8)	-2.0 (-11.1 to 8.4)	-15.2 (-23.1 to -6.2)*
Cirrhosis and other chronic liver diseases	227.0 (218.7 to 236.1)	21.2 (15.7 to 26.9)*	4.8 (0.1 to 9.7)*	41.0 (28.4 to 56.8)	21.0 (15.8 to 25.9)*	4.6 (0.2 to 8.9)*
Cirrhosis and other chronic liver diseases due to hepatitis B	76.9 (66.8 to 88.9)	19.5 (16.3 to 22.4)*	3.3 (0.6 to 5.8)*	12.9 (8.7 to 18.1)	19.6 (10.5 to 29.4)*	3.5 (-4.4 to 11.9)
Cirrhosis and other chronic liver diseases due to hepatitis C	61.3 (52.1 to 72.3)	21.1 (17.8 to 24.2)*	4.8 (1.9 to 7.4)*	10.3 (6.9 to 14.8)	21.3 (10.8 to 31.7)*	4.9 (-4.2 to 13.9)
Cirrhosis and other chronic liver diseases due to alcohol use	77.3 (67.7 to 87.4)	22.7 (19.8 to 25.3)*	6.1 (3.6 to 8.4)*	13.0 (8.8 to 18.4)	22.9 (14.0 to 32.4)*	6.3 (-1.4 to 14.5)
Cirrhosis and other chronic liver diseases due to other causes	28.4 (23.3 to 34.0)	18.4 (15.3 to 21.3)*	2.4 (-0.3 to 4.9)	4.8 (3.1 to 7.0)	18.7 (5.6 to 32.5)*	2.7 (-8.7 to 14.6)
Digestive diseases	15,661.3 (15,265.1 to 16,049.7)	3.3 (0.6 to 6.3)*	-10.6 (-13.0 to -8.1)*	816.3 (574.0 to 1,103.4)	4.8 (2.3 to 7.4)*	-9.4 (-11.5 to -7.1)*
Peptic ulcer disease	3,183.5 (3,088.7 to 3,276.9)	-12.4 (-14.8 to -9.9)*	-24.2 (-26.3 to -22.1)*	106.0 (71.4 to 150.8)	-14.3 (-17.2 to -11.4)*	-25.9 (-28.4 to -23.4)*
Peptic ulcer disease, symptomatic episodes	232.2 (180.7 to 296.4)	-11.1 (-16.5 to -6.0)*	-23.1 (-27.8 to -18.7)*	25.1 (15.5 to 36.8)	-11.0 (-17.4 to -4.7)*	-23.0 (-28.6 to -17.6)*
Anemia due to peptic ulcer disease aggregate	2,951.3 (2,879.8 to 3,022.0)	-12.5 (-15.0 to -9.9)*	-24.3 (-26.5 to -22.1)*	80.9 (54.0 to 117.4)	-15.3 (-18.4 to -12.0)*	-26.7 (-29.5 to -23.9)*
Gastritis and duodenitis	9,611.4 (9,251.6 to 9,992.9)	8.4 (4.2 to 12.5)*	-6.3 (-9.9 to -2.7)*	299.4 (202.9 to 427.7)	6.7 (1.6 to 12.1)*	-7.8 (-12.1 to -3.1)*
Gastritis and duodenitis, symptomatic episodes	241.9 (175.9 to 320.8)	9.5 (2.8 to 12.8)*	-5.3 (-11.1 to -2.5)*	26.1 (15.9 to 40.1)	9.8 (2.0 to 15.2)*	-5.1 (-11.8 to -0.3)*
Anemia due to gastritis and duodenitis aggregate	9,369.5 (9,017.7 to 9,726.9)	8.3 (4.1 to 12.5)*	-6.3 (-10.0 to -2.7)*	273.2 (181.9 to 390.7)	6.4 (1.0 to 12.4)*	-8.0 (-12.7 to -2.8)*
Appendicitis	21.7 (18.0 to 26.2)	20.1 (18.0 to 22.1)*	3.9 (2.1 to 5.6)*	6.7 (4.3 to 9.6)	20.1 (6.6 to 34.4)*	3.8 (-7.8 to 16.2)
Paralytic ileus and intestinal obstruction	6.3 (4.8 to 7.9)	16.0 (9.1 to 22.0)*	0.3 (-5.6 to 5.5)	2.0 (1.2 to 3.0)	16.1 (9.2 to 22.1)*	0.4 (-5.6 to 5.6)
Inguinal, femoral, and abdominal hernia	1,420.8 (989.3 to 1,922.6)	2.2 (-3.4 to 8.1)	-11.6 (-16.5 to -6.5)*	15.1 (6.4 to 28.7)	2.3 (-3.6 to 8.4)	-11.5 (-16.6 to -6.2)*
Inflammatory bowel disease	1,040.0 (958.0 to 1,123.7)	8.7 (8.2 to 9.3)*	-6.0 (-6.5 to -5.5)*	223.9 (153.2 to 307.3)	8.9 (6.6 to 11.0)*	-5.9 (-7.8 to -4.0)*
Vascular intestinal disorders	1.8 (1.3 to 2.4)	11.5 (4.3 to 18.0)*	-3.6 (-9.8 to 2.0)	0.6 (0.3 to 0.8)	11.5 (4.3 to 18.0)*	-3.6 (-9.8 to 2.0)
Gallbladder and biliary diseases	499.1 (385.4 to 640.3)	10.2 (4.3 to 15.8)*	-4.7 (-9.8 to 0.1)	53.9 (34.3 to 79.6)	10.4 (3.8 to 16.8)*	-4.6 (-10.2 to 1.0)
Pancreatitis	90.3 (69.4 to 110.6)	9.3 (3.4 to 14.2)*	-5.5 (-10.6 to -1.2)*	27.2 (17.1 to 39.0)	9.7 (0.3 to 18.7)*	-5.2 (-13.2 to 2.6)
Other digestive diseases	--	--	--	81.6 (56.2 to 112.4)	12.0 (5.1 to 21.5)*	-3.2 (-9.1 to 5.1)
Neurological disorders	207,275.4 (193,627.8 to 220,194.2)	15.3 (13.2 to 17.5)*	-0.3 (-2.1 to 1.6)	4,946.7 (3,273.1 to 7,163.5)	12.9 (10.1 to 15.8)*	-2.3 (-4.8 to 0.2)
Alzheimer disease and other dementias	39.7 (23.1 to 66.0)	8.5 (4.5 to 11.4)*	-6.2 (-9.6 to -3.7)*	5.3 (2.8 to 9.3)	8.8 (2.3 to 13.5)*	-5.9 (-11.5 to -1.8)*
Parkinson disease	133.7 (115.6 to 154.1)	15.4 (10.9 to 20.1)*	-0.2 (-4.1 to 3.9)	17.4 (11.7 to 24.2)	15.4 (6.6 to 25.0)*	-0.2 (-7.8 to 8.1)
Epilepsy	1,260.0 (1,081.0 to 1,435.3)	8.8 (3.1 to 14.6)*	-5.9 (-10.8 to -0.9)*	323.6 (221.3 to 440.0)	-8.5 (-14.9 to -1.9)*	-20.9 (-26.4 to -15.1)*
Multiple sclerosis	225.3 (208.3 to 243.7)	6.2 (4.2 to 8.1)*	-8.2 (-9.9 to -6.5)*	75.6 (53.7 to 96.9)	6.3 (2.2 to 9.9)*	-8.1 (-11.6 to -4.9)*
Motor neuron disease	10.0 (8.6 to 11.4)	8.3 (4.8 to 11.3)*	-6.4 (-9.3 to -3.8)*	2.1 (1.5 to 2.8)	8.3 (4.8 to 11.3)*	-6.4 (-9.3 to -3.8)*
Migraine	94,333.7 (76,239.7 to 115,403.3)	14.9 (12.9 to 17.0)*	-0.6 (-2.4 to 1.1)	3,223.5 (1,919.9 to 4,827.6)	15.2 (13.1 to 17.3)*	-0.3 (-2.2 to 1.4)
Tension-type headache	139,847.9 (101,795.4 to 192,609.9)	15.6 (13.4 to 17.5)*	0.0 (-1.9 to 1.6)	210.3 (91.2 to 407.0)	15.8 (13.3 to 18.1)*	0.1 (-2.0 to 2.1)
Medication overuse headache	6,869.2 (4,435.6 to 9,643.8)	14.0 (3.2 to 25.2)*	-1.4 (-10.8 to 8.3)	1,083.5 (601.6 to 1,768.2)	14.1 (3.0 to 25.4)*	-1.3 (-10.9 to 8.5)
Other neurological disorders	0.7 (0.4 to 1.2)	13.4 (11.2 to 14.9)*	-1.9 (-3.9 to -0.6)*	5.3 (3.7 to 7.1)	3.0 (-1.6 to 7.3)	-11.0 (-14.9 to -7.2)*
Mental and substance use disorders	82,794.4 (80,225.8 to 85,371.6)	14.7 (13.6 to 15.8)*	-0.8 (-1.8 to 0.1)	13,032.3 (9,269.1 to 16,904.7)	15.3 (14.5 to 16.1)*	-0.3 (-1.0 to 0.4)
Schizophrenia	2,990.4 (2,590.7 to 3,443.0)	16.1 (15.4 to 16.8)*	0.4 (-0.2 to 1.0)	1,936.0 (1,385.7 to 2,431.9)	16.4 (14.9 to 18.0)*	0.7 (-0.7 to 2.1)
Alcohol use disorders	7,128.1 (5,862.7 to 8,494.5)	4.8 (-0.2 to 9.0)	-9.4 (-13.7 to -5.8)*	713.7 (446.8 to 1,049.9)	4.9 (-0.4 to 9.4)	-9.3 (-13.8 to -5.4)*
Alcohol dependence aggregate	7,071.1 (5,804.5 to 8,437.6)	4.7 (-0.3 to 9.0)	-9.5 (-13.8 to -5.8)*	710.9 (444.4 to 1,046.5)	4.9 (-0.4 to 9.4)	-9.3 (-13.9 to -5.4)*
Fetal alcohol syndrome aggregate	56.9 (52.5 to 61.1)	14.2 (13.1 to 15.3)*	-1.2 (-2.2 to -0.3)*	2.9 (1.8 to 4.1)	14.6 (7.1 to 22.9)*	-0.9 (-7.4 to 6.2)
Drug use disorders	3,212.3 (3,076.0 to 3,310.4)	18.8 (16.5 to 21.2)*	2.7 (0.8 to 4.8)*	798.3 (548.9 to 1,069.9)	22.3 (18.9 to 26.0)*	5.8 (2.9 to 8.9)*
Opioid use disorders	1,596.8 (1,289.1 to 1,955.8)	20.5 (17.0 to 24.4)*	4.2 (1.2 to 7.5)*	661.8 (448.1 to 899.6)	20.8 (16.8 to 24.8)*	4.5 (1.0 to 8.0)*
Asymptomatic opioid dependence	258.9 (182.0 to 367.2)	20.5 (17.0 to 24.4)*	4.2 (1.2 to 7.5)*	0.0 (0.0 to 0.0)	0.0 (0.0 to 0.0)	0.0 (0.0 to 0.0)
Symptomatic opioid dependence aggregate	1,337.9 (1,063.8 to 1,656.9)	20.5 (17.0 to 24.4)*	4.2 (1.2 to 7.5)*	661.8 (448.1 to 899.6)	20.8 (16.8 to 24.8)*	4.5 (1.0 to 8.0)*
Cocaine use disorders	183.7 (148.7 to 220.2)	25.9 (22.1 to 30.2)*	8.9 (5.6 to 12.6)*	25.3 (15.8 to 36.7)	26.0 (18.2 to 34.6)*	9.0 (2.2 to 16.4)*
Asymptomatic cocaine dependence	92.1 (70.3 to 118.0)	25.9 (22.1 to 30.2)*	8.9 (5.6 to 12.6)*	0.0 (0.0 to 0.0)	0.0 (0.0 to 0.0)	0.0 (0.0 to 0.0)

Cause	Prevalence in thousands (95% confidence interval)			YLDs in thousands (95% confidence interval)		
	2015	Percent change in counts between 2005 and 2015	Percent change in rates between 2005 and 2015	2015	Percent change in counts between 2005 and 2015	Percent change in rates between 2005 and 2015
Symptomatic cocaine dependence aggregate	91.6 (70.3 to 116.8)	25.9 (22.1 to 30.2)*	8.9 (5.6 to 12.6)*	25.3 (15.8 to 36.7)	26.0 (18.2 to 34.6)*	9.0 (2.2 to 16.4)*
Amphetamine use disorders	281.4 (219.3 to 351.2)	46.4 (40.7 to 52.9)*	26.6 (21.7 to 32.2)*	36.7 (20.9 to 56.5)	46.6 (35.9 to 59.0)*	26.8 (17.5 to 37.5)*
Asymptomatic amphetamine dependence	154.1 (112.7 to 202.1)	46.4 (40.7 to 52.9)*	26.6 (21.7 to 32.2)*	0.0 (0.0 to 0.0)	0.0 (0.0 to 0.0)	0.0 (0.0 to 0.0)
Symptomatic amphetamine dependence aggregate	127.3 (92.5 to 170.1)	46.4 (40.7 to 52.9)*	26.6 (21.7 to 32.2)*	36.7 (20.9 to 56.5)	46.6 (35.9 to 59.0)*	26.8 (17.5 to 37.5)*
Cannabis use disorders	1,187.4 (1,005.7 to 1,399.4)	11.4 (10.1 to 12.8)*	-3.7 (-4.8 to -2.4)*	34.2 (21.7 to 51.0)	11.5 (6.4 to 16.3)*	-3.5 (-8.0 to 0.6)
Asymptomatic cannabis dependence	686.9 (571.8 to 822.2)	11.4 (10.1 to 12.8)*	-3.7 (-4.8 to -2.4)*	0.0 (0.0 to 0.0)	0.0 (0.0 to 0.0)	0.0 (0.0 to 0.0)
Symptomatic cannabis dependence aggregate	500.5 (406.4 to 604.2)	11.4 (10.1 to 12.8)*	-3.7 (-4.8 to -2.4)*	34.2 (21.7 to 51.0)	11.5 (6.4 to 16.3)*	-3.5 (-8.0 to 0.6)
Other drug use disorders	--	--	--	40.3 (24.6 to 58.1)	37.6 (29.3 to 46.4)*	19.0 (11.8 to 26.6)*
Depressive disorders	28,361.1 (26,364.8 to 30,028.9)	14.9 (12.8 to 17.2)*	-0.6 (-2.5 to 1.4)	4,936.1 (3,198.7 to 7,043.3)	15.2 (13.9 to 16.5)*	-0.4 (-1.5 to 0.7)
Major depressive disorder	19,733.3 (14,559.1 to 25,908.6)	14.7 (13.4 to 16.1)*	-0.8 (-1.9 to 0.4)	4,056.4 (2,416.3 to 5,959.4)	15.0 (13.5 to 16.5)*	-0.5 (-1.9 to 0.8)
Dysthymia	9,049.2 (6,967.9 to 11,447.6)	15.7 (14.0 to 17.6)*	0.1 (-1.4 to 1.7)	879.7 (549.1 to 1,354.3)	15.9 (13.9 to 17.9)*	0.2 (-1.5 to 1.9)
Bipolar disorder	3,885.2 (3,251.1 to 4,629.1)	14.8 (14.5 to 15.1)*	-0.7 (-1.0 to -0.5)*	791.8 (476.5 to 1,194.5)	15.1 (13.5 to 16.8)*	-0.5 (-1.8 to 1.0)
Anxiety disorders	21,857.1 (17,687.0 to 26,953.7)	14.7 (11.8 to 17.6)*	-0.8 (-3.3 to 1.7)	2,011.9 (1,298.3 to 2,910.4)	15.0 (12.0 to 17.9)*	-0.6 (-3.1 to 2.0)
Eating disorders	495.8 (463.8 to 531.0)	24.5 (19.6 to 29.8)*	7.7 (3.4 to 12.2)*	103.7 (65.7 to 155.1)	24.9 (21.1 to 28.9)*	8.0 (4.7 to 11.5)*
Anorexia nervosa	135.7 (107.8 to 166.7)	20.7 (17.1 to 24.0)*	4.4 (1.2 to 7.2)*	28.4 (17.8 to 41.6)	20.8 (13.7 to 28.5)*	4.5 (-1.7 to 11.1)
Bulimia nervosa	362.0 (268.1 to 477.5)	26.4 (24.1 to 28.6)*	9.3 (7.3 to 11.3)*	75.4 (46.0 to 116.0)	26.5 (22.0 to 31.0)*	9.4 (5.5 to 13.3)*
Autistic spectrum disorders	4,083.9 (3,868.2 to 4,266.1)	16.0 (13.8 to 18.2)*	0.3 (-1.6 to 2.2)	654.8 (437.8 to 896.3)	16.3 (15.0 to 17.6)*	0.5 (-0.5 to 1.7)
Autism	1,624.6 (1,365.4 to 1,927.4)	16.1 (15.8 to 16.3)*	0.4 (0.1 to 0.6)*	412.2 (262.1 to 590.9)	16.3 (14.5 to 18.2)*	0.6 (-1.0 to 2.2)
Asperger syndrome and other autistic spectrum disorders	2,441.5 (2,068.9 to 2,925.5)	16.0 (15.9 to 16.1)*	0.3 (0.2 to 0.4)*	242.6 (161.7 to 355.3)	16.2 (14.6 to 17.7)*	0.5 (-0.9 to 1.8)
Attention-deficit/hyperactivity disorder	732.3 (587.0 to 896.3)	13.7 (12.4 to 14.9)*	-1.7 (-2.8 to -0.7)*	8.8 (5.0 to 13.7)	13.8 (9.3 to 18.8)*	-1.6 (-5.5 to 2.7)
Conduct disorder	0.0 (0.0 to 0.0)	0.0 (0.0 to 0.0)	0.0 (0.0 to 0.0)	0.0 (0.0 to 0.0)	0.0 (0.0 to 0.0)	0.0 (0.0 to 0.0)
Idiopathic developmental intellectual disability	5,542.6 (3,085.0 to 7,935.5)	17.7 (16.4 to 19.0)*	1.8 (0.6 to 2.9)*	209.9 (90.4 to 364.7)	16.7 (14.4 to 18.5)*	1.0 (-1.1 to 2.5)
Other mental and substance use disorders	11,496.0 (11,293.2 to 11,688.7)	15.7 (12.8 to 18.7)*	0.1 (-2.4 to 2.6)	867.4 (604.8 to 1,164.2)	16.0 (13.0 to 19.1)*	0.3 (-2.3 to 3.0)
Diabetes, urogenital, blood, and endocrine diseases	232,820.3 (230,510.8 to 235,668.2)	16.0 (15.4 to 16.6)*	0.3 (-0.2 to 0.9)	5,318.0 (3,735.0 to 7,302.0)	16.7 (14.5 to 19.1)*	0.9 (-1.0 to 3.0)
Diabetes mellitus	35,495.2 (31,072.9 to 40,455.6)	21.0 (16.8 to 25.1)*	4.6 (1.0 to 8.2)*	2,676.2 (1,788.0 to 3,756.4)	22.4 (18.0 to 26.8)*	5.9 (2.1 to 9.7)*
Uncomplicated diabetes mellitus	23,407.5 (19,174.8 to 27,763.0)	19.2 (15.2 to 23.5)*	3.1 (-0.4 to 6.8)	1,103.5 (684.5 to 1,643.7)	19.4 (15.3 to 23.6)*	3.2 (-0.3 to 6.9)
Neuropathy and other complications of diabetes mellitus aggregate	11,727.3 (8,951.9 to 14,959.5)	24.6 (19.3 to 29.5)*	7.7 (3.2 to 12.0)*	1,549.7 (988.0 to 2,285.0)	24.7 (19.4 to 29.8)*	7.9 (3.3 to 12.2)*
Vision loss due to diabetes mellitus aggregate	360.4 (246.1 to 514.3)	21.5 (18.8 to 24.6)*	5.1 (2.7 to 7.7)*	23.1 (14.1 to 35.4)	22.8 (16.7 to 29.3)*	6.2 (0.9 to 11.9)*
Acute glomerulonephritis	7.2 (5.3 to 9.5)	-1.2 (-6.9 to 3.3)	-14.6 (-19.5 to -10.6)*	0.4 (0.2 to 0.6)	-0.4 (-10.6 to 9.8)	-13.8 (-22.7 to -5.0)*
Chronic kidney disease	22,223.8 (19,533.7 to 24,453.1)	19.1 (15.5 to 22.6)*	3.0 (-0.1 to 6.0)	634.7 (450.4 to 831.9)	14.2 (12.1 to 16.1)*	-1.3 (-3.1 to 0.4)
Chronic kidney disease due to diabetes mellitus	7,714.4 (4,817.3 to 10,834.2)	20.2 (16.5 to 24.1)*	4.0 (0.7 to 7.3)*	217.6 (134.7 to 318.9)	14.3 (10.1 to 18.5)*	-1.1 (-4.8 to 2.5)
Stage III chronic kidney disease due to diabetes mellitus aggregate	6,870.7 (4,218.4 to 9,847.2)	20.9 (16.9 to 25.0)*	4.6 (1.1 to 8.1)*	9.2 (5.8 to 13.5)	-6.9 (-21.9 to 9.1)	-19.5 (-32.4 to -5.7)*
Stage IV chronic kidney disease due to diabetes mellitus aggregate	521.3 (305.4 to 778.9)	15.9 (13.2 to 18.9)*	0.2 (-2.1 to 2.8)	65.1 (35.4 to 104.1)	15.3 (10.7 to 19.9)*	-0.3 (-4.2 to 3.7)
Stage V chronic kidney disease untreated due to diabetes mellitus	251.0 (162.6 to 363.1)	15.8 (13.0 to 18.8)*	0.1 (-2.2 to 2.8)	130.5 (74.7 to 200.6)	16.2 (10.5 to 22.5)*	0.4 (-4.4 to 6.0)
End-stage chronic kidney disease due to diabetes aggregate	71.4 (48.6 to 95.3)	6.6 (2.6 to 10.6)*	-7.8 (-11.2 to -4.4)*	12.9 (7.6 to 19.4)	9.6 (-0.8 to 19.8)	-5.2 (-14.2 to 3.6)
Chronic kidney disease due to hypertension	6,068.2 (3,871.5 to 9,034.4)	17.6 (13.4 to 21.5)*	1.7 (-1.9 to 5.1)	91.7 (60.0 to 130.8)	15.3 (8.3 to 22.2)*	-0.3 (-6.3 to 5.7)
Stage III chronic kidney disease due to hypertension aggregate	5,686.4 (3,555.9 to 8,616.5)	17.8 (13.6 to 21.9)*	1.9 (-1.8 to 5.4)	2.7 (1.7 to 4.2)	6.5 (-9.8 to 25.2)	-7.9 (-22.0 to 8.2)
Stage IV chronic kidney disease due to hypertension aggregate	251.0 (148.4 to 425.8)	14.6 (5.2 to 23.4)*	-0.9 (-9.1 to 6.7)	31.0 (16.8 to 52.9)	14.3 (4.7 to 24.2)*	-1.1 (-9.4 to 7.4)
Stage V chronic kidney disease untreated due to hypertension	101.2 (69.2 to 146.7)	16.3 (10.8 to 22.0)*	0.6 (-4.2 to 5.5)	52.7 (31.5 to 78.8)	16.7 (7.8 to 26.5)*	0.9 (-6.8 to 9.4)
End-stage chronic kidney disease due to hypertension aggregate	29.6 (21.0 to 40.0)	8.5 (0.4 to 17.0)*	-6.2 (-13.2 to 1.2)	5.3 (3.3 to 8.2)	11.5 (0.6 to 22.6)*	-3.6 (-13.0 to 6.0)
Chronic kidney disease due to glomerulonephritis	3,627.5 (2,740.3 to 4,840.2)	18.6 (13.5 to 23.7)*	2.6 (-1.8 to 7.0)	175.7 (111.0 to 252.8)	13.3 (9.2 to 17.8)*	-2.1 (-5.6 to 1.8)
Stage III chronic kidney disease due to glomerulonephritis aggregate	2,983.9 (2,090.4 to 4,167.0)	19.9 (13.9 to 26.2)*	3.7 (-1.5 to 9.1)	8.6 (5.5 to 13.0)	17.8 (6.2 to 32.3)*	1.8 (-8.2 to 14.4)
Stage IV chronic kidney disease due to glomerulonephritis aggregate	387.0 (252.6 to 517.6)	13.7 (7.9 to 19.8)*	-1.7 (-6.7 to 3.6)	49.1 (29.0 to 72.5)	14.7 (8.2 to 22.3)*	-0.8 (-6.4 to 5.8)
Stage V chronic kidney disease untreated due to glomerulonephritis	212.0 (137.5 to 316.8)	12.6 (9.5 to 15.4)*	-2.6 (-5.3 to -0.2)*	109.8 (61.4 to 170.7)	12.6 (6.9 to 18.0)*	-2.6 (-7.6 to 2.1)
End-stage chronic kidney disease due to glomerulonephritis aggregate	44.5 (29.2 to 64.3)	6.8 (2.4 to 11.4)*	-7.7 (-11.4 to -3.7)*	8.2 (4.7 to 12.9)	8.5 (-1.8 to 18.6)	-6.2 (-15.1 to 2.5)
Chronic kidney disease due to other causes	5,439.5 (3,403.8 to 8,141.8)	19.3 (16.3 to 22.5)*	3.2 (0.6 to 5.9)*	149.7 (89.5 to 223.2)	14.4 (10.5 to 18.7)*	-1.0 (-4.5 to 2.7)
Stage III chronic kidney disease due to other causes aggregate	4,866.6 (3,008.2 to 7,356.4)	19.9 (16.7 to 23.3)*	3.7 (0.9 to 6.6)*	6.3 (4.0 to 9.6)	10.7 (-0.5 to 22.6)	-4.3 (-14.0 to 6.0)
Stage IV chronic kidney disease due to other causes aggregate	355.0 (209.1 to 546.5)	15.6 (13.5 to 17.7)*	-0.1 (-1.9 to 1.8)	44.5 (23.9 to 71.9)	13.4 (8.7 to 18.0)*	-1.9 (-6.0 to 2.0)
Stage V chronic kidney disease untreated due to other causes	174.7 (109.9 to 260.7)	15.2 (13.0 to 17.6)*	-0.3 (-2.3 to 1.7)	90.7 (51.0 to 145.0)	15.6 (10.0 to 21.7)*	-0.0 (-4.9 to 5.3)
End-stage chronic kidney disease due to other causes aggregate	43.2 (28.0 to 62.2)	8.1 (4.6 to 11.8)*	-6.5 (-9.5 to -3.4)*	8.2 (4.6 to 13.1)	10.4 (1.8 to 20.6)*	-4.5 (-11.9 to 4.3)
Urinary diseases and male infertility	34,009.9 (32,260.0 to 35,180.7)	17.0 (14.9 to 19.0)*	1.1 (-0.6 to 2.9)	72.2 (42.2 to 121.4)	15.0 (10.8 to 18.6)*	-0.5 (-4.2 to 2.6)
Interstitial nephritis and urinary tract infections	143.6 (133.0 to 155.1)	17.7 (16.9 to 18.6)*	1.8 (1.1 to 2.5)*	4.8 (3.0 to 7.1)	17.9 (10.4 to 25.0)*	1.9 (-4.6 to 8.1)
Urolithiasis	28,373.8 (24,753.5 to 32,316.7)	16.9 (14.4 to 19.6)*	1.1 (-1.0 to 3.5)	8.9 (5.6 to 13.1)	13.9 (5.6 to 24.1)*	-1.5 (-8.7 to 7.3)
Benign prostatic hyperplasia	616.4 (446.5 to 837.5)	10.2 (6.1 to 13.9)*	-4.7 (-8.2 to -1.5)*	23.3 (13.4 to 36.7)	10.3 (5.2 to 15.3)*	-4.6 (-9.0 to -0.3)*
Male infertility	5,323.8 (3,707.7 to 7,407.9)	18.7 (13.0 to 23.7)*	2.6 (-2.3 to 7.0)	29.9 (11.2 to 67.8)	19.3 (14.0 to 24.3)*	3.2 (-1.4 to 7.5)
Other urinary diseases	--	--	--	5.3 (3.4 to 7.7)	12.3 (0.9 to 25.0)*	-2.9 (-12.7 to 8.1)
Gynecological diseases	106,343.0 (104,311.7 to 108,239.2)	14.2 (13.1 to 15.3)*	-1.3 (-2.2 to -0.3)*	1,491.4 (1,001.0 to 2,161.5)	11.1 (9.5 to 12.7)*	-4.0 (-5.3 to -2.5)*
Uterine fibroids	29,533.7 (27,286.5 to 31,684.6)	15.1 (14.8 to 15.3)*	-0.5 (-0.7 to -0.3)*	422.5 (253.6 to 687.2)	7.1 (3.6 to 9.8)*	-7.4 (-10.4 to -5.1)*
Uterine fibroids cases aggregate	22,870.6 (20,655.8 to 25,000.7)	18.1 (17.3 to 19.0)*	2.1 (1.4 to 2.9)*	145.4 (70.7 to 273.0)	20.4 (18.9 to 22.0)*	4.1 (2.8 to 5.5)*
Mild abdominal pain with anemia due to uterine fibroids aggregate	6,663.1 (6,544.5 to 6,791.0)	5.7 (3.3 to 8.0)*	-8.6 (-10.7 to -6.6)*	277.1 (177.8 to 416.1)	1.2 (-2.2 to 4.6)	-12.5 (-15.4 to -9.5)*
Polycystic ovarian syndrome	10,898.3 (8,527.1 to 14,413.0)	13.6 (12.6 to 14.4)*	-1.8 (-2.6 to -1.1)*	94.2 (41.8 to 189.5)	14.0 (12.7 to 15.3)*	-1.4 (-2.5 to -0.3)*
Polycystic ovarian syndrome cases aggregate	8,509.7 (6,550.8 to 11,177.0)	13.1 (12.3 to 13.9)*	-2.2 (-2.9 to -1.5)*	73.3 (32.5 to 143.4)	13.2 (12.0 to 14.5)*	-2.1 (-3.1 to -1.0)*
Hirsutism and infertility due to polycystic ovarian syndrome aggregate	2,388.6 (1,566.6 to 3,563.7)	15.2 (12.9 to 16.9)*	-0.4 (-2.3 to 1.1)	20.9 (7.4 to 48.1)	16.7 (13.3 to 20.2)*	1.0 (-2.0 to 4.0)

Cause	Prevalence in thousands (95% confidence interval)			YLDs in thousands (95% confidence interval)		
	2015	Percent change in counts between 2005 and 2015	Percent change in rates between 2005 and 2015	2015	Percent change in counts between 2005 and 2015	Percent change in rates between 2005 and 2015
Female infertility	13,947.0<br>(8,034.5 to 21,803.1)	19.9<br>(10.8 to 27.4)*	3.7<br>(-4.2 to 10.1)	74.7<br>(26.0 to 171.4)	20.6<br>(11.9 to 27.7)*	4.3<br>(-3.3 to 10.5)
Endometriosis	1,920.6<br>(1,490.9 to 2,417.2)	11.3<br>(10.1 to 12.2)*	-3.7<br>(-4.8 to -3.0)*	176.7<br>(112.5 to 259.8)	11.5<br>(9.0 to 14.0)*	-3.6<br>(-5.8 to -1.4)*
Endometriosis cases aggregate	1,802.4<br>(1,394.5 to 2,250.8)	11.2<br>(10.1 to 12.1)*	-3.8<br>(-4.8 to -3.0)*	165.0<br>(104.9 to 245.2)	11.4<br>(8.8 to 14.0)*	-3.7<br>(-5.9 to -1.4)*
Abdominal pain and infertility due to endometriosis aggregate	118.2<br>(80.0 to 166.1)	12.7<br>(10.8 to 14.4)*	-2.6<br>(-4.2 to -1.1)*	11.7<br>(6.7 to 17.9)	12.9<br>(6.6 to 19.3)*	-2.3<br>(-7.8 to 3.1)
Genital prolapse	13,953.8<br>(11,019.0 to 17,273.9)	8.8<br>(5.2 to 11.8)*	-5.9<br>(-9.0 to -3.3)*	44.1<br>(20.9 to 82.4)	8.9<br>(5.0 to 12.6)*	-5.8<br>(-9.2 to -2.6)*
Premenstrual syndrome	57,605.2<br>(52,929.6 to 62,779.3)	13.3<br>(10.7 to 16.2)*	-2.0<br>(-4.3 to 0.5)	480.5<br>(298.4 to 726.0)	13.5<br>(10.6 to 16.6)*	-1.8<br>(-4.3 to 0.8)
Other gynecological diseases	5,552.7<br>(5,275.2 to 5,873.3)	11.1<br>(9.4 to 12.4)*	-4.0<br>(-5.4 to -2.8)*	198.7<br>(138.1 to 272.0)	9.4<br>(6.5 to 12.1)*	-5.4<br>(-7.9 to -3.1)*
Other gynecological diseases cases aggregate	3,249.4<br>(2,979.6 to 3,579.8)	15.4<br>(15.0 to 15.7)*	-0.2<br>(-0.5 to 0.1)	126.4<br>(84.9 to 176.2)	15.5<br>(12.8 to 18.3)*	-0.1<br>(-2.5 to 2.3)
Anemia due to other gynecological diseases aggregate	2,303.3<br>(2,253.6 to 2,361.6)	5.5<br>(2.0 to 8.8)*	-8.8<br>(-11.8 to -6.0)*	72.3<br>(48.9 to 103.3)	0.1<br>(-5.0 to 4.8)	-13.4<br>(-17.9 to -9.4)*
Hemoglobinopathies and hemolytic anemias	98,388.0<br>(96,699.8 to 100,550.9)	15.7<br>(14.1 to 17.3)*	0.0<br>(-1.3 to 1.4)	371.5<br>(246.2 to 542.4)	10.9<br>(7.4 to 14.3)*	-4.1<br>(-7.2 to -1.1)*
Thalassemias	2.0<br>(1.9 to 2.1)	16.0<br>(10.3 to 22.3)*	0.3<br>(-4.6 to 5.8)	0.2<br>(0.1 to 0.3)	19.7<br>(-13.4 to 67.9)	3.5<br>(-25.1 to 45.2)
Beta-thalassemia major cases aggregate	1.8<br>(1.7 to 1.9)	16.2<br>(9.7 to 23.3)*	0.5<br>(-5.1 to 6.6)	0.2<br>(0.1 to 0.2)	21.2<br>(-17.0 to 80.2)	4.8<br>(-28.2 to 55.9)
Hemoglobin E/beta-thalassemia cases aggregate	0.1<br>(0.1 to 0.1)	11.7<br>(10.9 to 12.6)*	-3.4<br>(-4.1 to -2.6)*	0.0<br>(0.0 to 0.0)	6.6<br>(1.0 to 13.0)*	-7.8<br>(-12.7 to -2.3)*
Hemoglobin H disease cases aggregate	0.0<br>(0.0 to 0.0)	18.9<br>(14.0 to 24.2)*	2.8<br>(-1.4 to 7.4)	0.0<br>(0.0 to 0.0)	17.1<br>(10.3 to 24.7)*	1.2<br>(-4.6 to 7.9)
Heart failure due to thalassemias aggregate	0.0<br>(0.0 to 0.0)	20.9<br>(18.5 to 23.4)*	4.5<br>(2.5 to 6.7)*	0.0<br>(0.0 to 0.0)	20.9<br>(18.5 to 23.4)*	4.5<br>(2.5 to 6.7)*
Thalassemias trait	20,531.9<br>(20,085.2 to 21,062.9)	21.2<br>(20.6 to 21.9)*	4.8<br>(4.3 to 5.4)*	217.8<br>(143.2 to 319.1)	16.0<br>(11.0 to 21.3)*	0.3<br>(-4.0 to 4.9)
B-thalassemia trait cases aggregate	16,660.6<br>(16,260.6 to 17,145.1)	21.0<br>(20.3 to 21.7)*	4.6<br>(4.0 to 5.3)*	205.7<br>(135.3 to 301.3)	16.1<br>(11.0 to 21.6)*	0.4<br>(-4.0 to 5.1)
Hemoglobin E trait cases aggregate	3,871.2<br>(3,711.0 to 4,042.5)	22.2<br>(21.1 to 23.4)*	5.7<br>(4.7 to 6.7)*	12.1<br>(7.8 to 17.9)	14.9<br>(5.9 to 25.6)*	-0.6<br>(-8.5 to 8.7)
Sickle cell disorders	12.3<br>(11.3 to 13.2)	28.3<br>(16.7 to 41.0)*	10.9<br>(0.9 to 21.9)*	1.6<br>(1.1 to 2.1)	28.8<br>(15.5 to 43.8)*	11.4<br>(-0.1 to 24.4)
Homozygous sickle cell and severe sickle cell/beta-thalassemia cases aggregate	11.1<br>(10.1 to 12.0)	31.8<br>(18.6 to 46.5)*	14.0<br>(2.5 to 26.7)*	1.4<br>(1.0 to 1.9)	32.5<br>(17.1 to 50.6)*	14.6<br>(1.3 to 30.3)*
Hemoglobin SC disease cases aggregate	0.9<br>(0.9 to 1.0)	11.0<br>(7.4 to 14.5)*	-4.0<br>(-7.2 to -1.0)*	0.1<br>(0.1 to 0.2)	14.5<br>(9.7 to 19.8)*	-1.0<br>(-5.1 to 3.6)
Mild sickle cell/beta-thalassemia cases aggregate	0.3<br>(0.3 to 0.3)	-14.0<br>(-17.9 to -9.4)*	-25.6<br>(-29.0 to -21.7)*	0.1<br>(0.0 to 0.1)	-8.1<br>(-11.7 to -4.3)*	-20.5<br>(-23.6 to -17.3)*
Sickle cell trait	22,299.5<br>(20,982.2 to 24,912.9)	24.3<br>(23.5 to 25.1)*	7.5<br>(6.8 to 8.2)*	70.0<br>(46.7 to 101.4)	11.6<br>(6.2 to 16.9)*	-3.5<br>(-8.1 to 1.1)
G6PD deficiency	15,177.7<br>(12,884.6 to 17,570.1)	11.9<br>(6.5 to 16.6)*	-3.3<br>(-7.9 to 0.9)	1.3<br>(0.9 to 1.9)	3.4<br>(-5.0 to 12.0)	-10.6<br>(-17.9 to -3.2)*
G6PD cases aggregate	15,177.7<br>(12,884.6 to 17,570.1)	11.9<br>(6.5 to 16.6)*	-3.3<br>(-7.9 to 0.9)	1.3<br>(0.9 to 1.9)	3.4<br>(-5.0 to 12.0)	-10.6<br>(-17.9 to -3.2)*
Heart failure due to G6PD deficiency aggregate	0.0<br>(0.0 to 0.0)	0.0<br>(0.0 to 0.0)	0.0<br>(0.0 to 0.0)	0.0<br>(0.0 to 0.0)	0.0<br>(0.0 to 0.0)	0.0<br>(0.0 to 0.0)
G6PD trait	46,046.4<br>(42,658.7 to 49,415.9)	12.9<br>(11.1 to 14.5)*	-2.4<br>(-3.9 to -1.0)*	1.4<br>(0.9 to 1.9)	12.9<br>(-1.9 to 31.0)	-2.3<br>(-15.2 to 13.3)
Other hemoglobinopathies and hemolytic anemias	4,058.5<br>(3,980.6 to 4,137.9)	3.3<br>(1.3 to 5.5)*	-10.7<br>(-12.4 to -8.8)*	79.2<br>(52.3 to 116.7)	-1.5<br>(-5.6 to 2.6)	-14.8<br>(-18.4 to -11.3)*
Other hemoglobinopathies and hemolytic anemias cases aggregate	4,054.8<br>(3,977.5 to 4,134.2)	3.3<br>(1.3 to 5.5)*	-10.7<br>(-12.4 to -8.8)*	78.0<br>(51.6 to 115.2)	-1.6<br>(-5.8 to 2.5)	-14.9<br>(-18.5 to -11.3)*
Heart failure due to other hemoglobinopathies and hemolytic anemias aggregate	3.7<br>(3.2 to 4.3)	19.1<br>(17.4 to 20.9)*	3.0<br>(1.6 to 4.5)*	0.5<br>(0.3 to 0.7)	19.1<br>(17.4 to 20.9)*	3.0<br>(1.6 to 4.5)*
Endocrine, metabolic, blood, and immune disorders	3,177.0<br>(3,108.3 to 3,247.2)	4.6<br>(2.2 to 7.1)*	-9.5<br>(-11.6 to -7.4)*	71.7<br>(48.8 to 102.7)	-2.6<br>(-6.7 to 1.8)	-15.8<br>(-19.3 to -12.0)*
Endocrine, metabolic, blood, and immune disorders cases aggregate	400.7<br>(366.1 to 438.8)	9.9<br>(7.8 to 12.0)*	-5.0<br>(-6.8 to -3.2)*	15.8<br>(10.5 to 22.2)	10.0<br>(4.5 to 15.4)*	-4.9<br>(-9.7 to -0.2)*
Anemia due to endocrine, metabolic, blood, and immune disorders aggregate	2,775.0<br>(2,716.9 to 2,826.0)	3.9<br>(1.1 to 6.8)*	-10.2<br>(-12.5 to -7.7)*	55.8<br>(36.9 to 81.5)	-5.7<br>(-10.9 to -0.1)*	-18.4<br>(-22.9 to -13.6)*
Heart failure due to endocrine, metabolic, blood, and immune disorders aggregate	1.3<br>(1.1 to 1.5)	11.2<br>(10.0 to 12.4)*	-3.8<br>(-4.9 to -2.8)*	0.2<br>(0.1 to 0.2)	11.2<br>(10.0 to 12.4)*	-3.8<br>(-4.9 to -2.8)*
Musculoskeletal disorders	125,438.7<br>(121,489.4 to 128,883.2)	14.5<br>(13.2 to 15.9)*	-1.0<br>(-2.1 to 0.2)	14,327.7<br>(10,211.3 to 19,191.6)	14.4<br>(12.6 to 16.2)*	-1.1<br>(-2.7 to 0.5)
Rheumatoid arthritis	1,836.4<br>(1,560.2 to 2,158.2)	16.1<br>(12.0 to 20.2)*	0.4<br>(-3.1 to 4.0)	443.6<br>(297.6 to 610.3)	16.3<br>(11.7 to 20.7)*	0.6<br>(-3.4 to 4.4)
Osteoarthritis	14,621.1<br>(13,851.5 to 15,407.0)	19.3<br>(17.8 to 21.3)*	3.2<br>(1.9 to 4.9)*	871.2<br>(606.0 to 1,202.0)	21.1<br>(19.2 to 23.3)*	4.7<br>(3.1 to 6.6)*
Osteoarthritis of the hip cases aggregate	998.2<br>(804.4 to 1,186.4)	16.9<br>(14.8 to 19.3)*	1.1<br>(-0.7 to 3.2)	55.6<br>(34.9 to 79.5)	20.0<br>(16.3 to 24.1)*	3.7<br>(0.6 to 7.3)*
Osteoarthritis of the knee cases aggregate	13,622.9<br>(12,888.0 to 14,389.7)	19.5<br>(17.8 to 21.5)*	3.4<br>(1.9 to 5.1)*	815.6<br>(565.8 to 1,128.4)	21.1<br>(19.2 to 23.5)*	4.8<br>(3.1 to 6.8)*
Low back and neck pain	84,490.4<br>(80,380.2 to 88,235.2)	13.0<br>(11.2 to 14.9)*	-2.3<br>(-3.8 to -0.6)*	9,848.1<br>(6,905.8 to 13,639.4)	12.9<br>(11.1 to 14.7)*	-2.4<br>(-3.9 to -0.8)*
Low back pain	49,896.7<br>(44,168.9 to 55,848.3)	11.5<br>(9.4 to 13.6)*	-3.6<br>(-5.4 to -1.8)*	5,636.8<br>(3,836.4 to 7,974.6)	11.7<br>(9.5 to 13.8)*	-3.4<br>(-5.3 to -1.6)*
Neck pain	42,543.7<br>(30,329.3 to 58,947.2)	14.4<br>(11.6 to 17.0)*	-1.1<br>(-3.5 to 1.2)	4,211.3<br>(2,550.7 to 6,542.4)	14.6<br>(11.7 to 17.2)*	-0.9<br>(-3.4 to 1.4)
Gout	3,778.8<br>(2,935.2 to 4,791.4)	15.5<br>(14.3 to 16.7)*	-0.1<br>(-1.1 to 1.0)	124.1<br>(80.2 to 182.7)	15.7<br>(11.4 to 19.8)*	0.1<br>(-3.6 to 3.6)
Asymptomatic gout	3,376.0<br>(2,620.0 to 4,276.9)	15.5<br>(14.3 to 16.7)*	-0.1<br>(-1.1 to 1.0)	0.0<br>(0.0 to 0.0)	0.0<br>(0.0 to 0.0)	0.0<br>(0.0 to 0.0)
Symptomatic gout cases aggregate	402.8<br>(305.7 to 515.5)	15.5<br>(14.3 to 16.7)*	-0.1<br>(-1.1 to 1.0)	124.1<br>(80.2 to 182.7)	15.7<br>(11.4 to 19.8)*	0.1<br>(-3.6 to 3.6)
Other musculoskeletal disorders	32,277.1<br>(24,530.3 to 41,521.7)	16.9<br>(10.0 to 23.8)*	1.1<br>(-4.8 to 7.0)	3,040.8<br>(1,861.8 to 4,524.2)	17.1<br>(10.4 to 23.9)*	1.3<br>(-4.6 to 7.1)
Other non-communicable diseases	348,043.7<br>(342,870.4 to 352,809.2)	16.0<br>(15.4 to 16.6)*	0.3<br>(-0.2 to 0.8)	7,724.4<br>(5,144.8 to 11,180.4)	17.8<br>(16.6 to 19.2)*	1.8<br>(0.8 to 3.1)*
Congenital anomalies	6,519.8<br>(5,999.2 to 7,027.2)	26.8<br>(22.7 to 31.1)*	9.7<br>(6.1 to 13.4)*	556.2<br>(340.6 to 843.8)	31.9<br>(23.2 to 41.7)*	14.1<br>(6.5 to 22.5)*
Neural tube defects	84.6<br>(57.8 to 120.5)	21.9<br>(19.0 to 24.5)*	5.4<br>(2.9 to 7.7)*	28.5<br>(16.1 to 46.3)	23.1<br>(16.1 to 29.9)*	6.5<br>(0.4 to 12.3)*
Congenital heart anomalies	3,476.8<br>(2,448.8 to 5,140.7)	34.2<br>(28.7 to 39.0)*	16.1<br>(11.3 to 20.2)*	120.1<br>(47.0 to 221.5)	34.5<br>(28.9 to 39.4)*	16.4<br>(11.5 to 20.6)*
Less severe heart anomalies cases aggregate	3,153.5<br>(2,115.9 to 4,832.5)	33.9<br>(27.6 to 39.1)*	15.8<br>(10.4 to 20.3)*	100.8<br>(37.7 to 195.0)	34.0<br>(27.1 to 39.7)*	15.9<br>(9.9 to 20.8)*
Severe congenital heart anomalies	311.5<br>(208.5 to 472.3)	38.0<br>(30.8 to 44.6)*	19.3<br>(13.2 to 25.1)*	18.2<br>(7.2 to 36.5)	38.2<br>(29.7 to 46.4)*	19.5<br>(12.1 to 26.6)*
Critical congenital heart anomalies	6.3<br>(3.6 to 10.3)	52.2<br>(32.4 to 68.0)*	31.6<br>(14.5 to 45.3)*	0.4<br>(0.1 to 0.8)	52.1<br>(22.0 to 85.1)*	31.5<br>(5.5 to 60.1)*
Heart failure due to congenital heart anomalies aggregate	5.6<br>(4.8 to 6.4)	14.2<br>(12.7 to 15.5)*	-1.3<br>(-2.5 to -0.1)*	0.7<br>(0.5 to 1.0)	14.2<br>(12.7 to 15.5)*	-1.3<br>(-2.6 to -0.1)*
Cleft lip and cleft palate	467.9<br>(276.4 to 775.4)	22.4<br>(9.1 to 32.8)*	5.9<br>(-5.7 to 14.8)	5.1<br>(2.5 to 9.0)	15.8<br>(0.5 to 28.2)*	0.1<br>(-13.1 to 10.8)
Down syndrome	346.1<br>(219.6 to 531.0)	20.4<br>(14.7 to 25.6)*	4.2<br>(-0.8 to 8.6)	28.6<br>(16.2 to 48.1)	20.4<br>(13.3 to 26.6)*	4.1<br>(-2.1 to 9.5)
Turner syndrome	25.2<br>(11.6 to 45.3)	13.5<br>(11.2 to 15.8)*	-1.9<br>(-3.8 to 0.1)	0.5<br>(0.2 to 1.1)	13.6<br>(7.0 to 19.7)*	-1.8<br>(-7.5 to 3.5)
Klinefelter syndrome	14.4<br>(7.6 to 25.8)	15.1<br>(12.8 to 16.7)*	-0.5<br>(-2.5 to 0.9)	0.1<br>(0.1 to 0.3)	15.1<br>(8.3 to 20.2)*	-0.5<br>(-6.3 to 4.0)
Other chromosomal abnormalities	318.1<br>(176.5 to 560.4)	18.7<br>(12.9 to 23.7)*	2.7<br>(-2.4 to 6.9)	26.5<br>(13.3 to 49.8)	18.6<br>(11.5 to 24.1)*	2.6<br>(-3.6 to 7.3)
Other congenital anomalies	2,045.0<br>(1,114.9 to 3,752.5)	17.9<br>(15.0 to 20.9)*	2.0<br>(-0.6 to 4.6)	346.8<br>(206.2 to 556.3)	34.3<br>(21.5 to 50.7)*	16.2<br>(5.1 to 30.3)*
Skin and subcutaneous diseases	120,958.4<br>(118,164.4 to 123,224.7)	16.8<br>(16.0 to 17.7)*	1.0<br>(0.3 to 1.7)*	2,404.1<br>(1,561.4 to 3,559.3)	16.2<br>(15.2 to 17.2)*	0.5<br>(-0.4 to 1.3)
Dermatitis	15,911.9<br>(12,778.9 to 20,095.3)	14.5<br>(14.1 to 15.0)*	-1.0<br>(-1.3 to -0.6)*	466.6<br>(297.0 to 676.7)	14.7<br>(13.5 to 16.1)*	-0.8<br>(-1.8 to 0.4)
Eczema cases aggregate	2,972.8<br>(2,279.0 to 3,766.8)	15.9<br>(15.3 to 16.5)*	0.2<br>(-0.3 to 0.7)	179.2<br>(113.3 to 265.5)	16.1<br>(13.5 to 18.8)*	0.4<br>(-1.9 to 2.8)

Cause	Prevalence in thousands (95% confidence interval)			YLDs in thousands (95% confidence interval)		
	2015	Percent change in counts between 2005 and 2015	Percent change in rates between 2005 and 2015	2015	Percent change in counts between 2005 and 2015	Percent change in rates between 2005 and 2015
Contact dermatitis cases aggregate	7,807.7 (5,229.8 to 11,380.0)	12.8 (12.3 to 13.3)*	-2.4 (-2.9 to -2.1)*	210.8 (115.2 to 353.4)	12.9 (11.4 to 14.6)*	-2.3 (-3.6 to -0.9)*
Seborrhoeic dermatitis cases aggregate	5,131.4 (3,752.3 to 6,952.3)	16.4 (16.0 to 16.8)*	0.7 (0.3 to 1.0)*	76.5 (39.8 to 130.0)	16.5 (14.8 to 18.0)*	0.7 (-0.7 to 2.0)
Psoriasis	6,381.9 (5,789.7 to 6,982.5)	15.2 (13.9 to 16.6)*	-0.3 (-1.5 to 0.8)	518.3 (359.7 to 719.5)	15.5 (13.2 to 17.6)*	-0.1 (-2.1 to 1.7)
Cellulitis	51.8 (38.6 to 68.3)	16.2 (10.0 to 22.2)*	0.5 (-4.9 to 5.7)	3.8 (2.2 to 5.9)	16.4 (7.0 to 25.9)*	0.6 (-7.4 to 8.9)
Pyoderma	314.2 (274.7 to 358.4)	16.7 (13.4 to 20.2)*	0.9 (-1.9 to 3.9)	1.8 (0.7 to 3.7)	16.8 (11.8 to 22.3)*	1.0 (-3.3 to 5.8)
Scabies	9,464.1 (6,046.9 to 13,961.6)	18.1 (12.4 to 25.0)*	2.1 (-2.8 to 8.1)	243.1 (114.0 to 430.3)	18.2 (12.4 to 24.5)*	2.2 (-2.8 to 7.7)
Fungal skin diseases	26,170.0 (22,646.1 to 30,528.5)	17.5 (17.3 to 17.7)*	1.6 (1.5 to 1.7)*	147.5 (58.4 to 315.2)	17.6 (17.1 to 18.1)*	1.7 (1.3 to 2.1)*
Viral skin diseases	8,128.7 (6,803.1 to 9,704.9)	14.8 (14.4 to 15.2)*	-0.7 (-1.1 to -0.4)*	249.0 (152.1 to 386.2)	14.9 (13.8 to 16.1)*	-0.6 (-1.6 to 0.4)
Molluscum contagiosum cases aggregate	509.0 (405.0 to 648.4)	15.0 (14.4 to 15.4)*	-0.6 (-1.0 to -0.2)*	15.6 (9.4 to 24.4)	15.2 (10.8 to 20.3)*	-0.4 (-4.2 to 4.0)
Viral warts cases aggregate	7,619.8 (6,299.1 to 9,224.4)	14.8 (14.4 to 15.2)*	-0.8 (-1.1 to -0.4)*	233.4 (142.7 to 363.9)	14.9 (13.8 to 16.1)*	-0.6 (-1.6 to 0.4)
Acne vulgaris	20,281.8 (17,252.2 to 24,520.4)	17.4 (14.3 to 20.4)*	1.5 (-1.2 to 4.1)	217.2 (103.5 to 415.4)	17.5 (14.6 to 20.6)*	1.6 (-0.9 to 4.3)
Alopecia areata	1,834.3 (1,554.9 to 2,182.0)	14.0 (13.6 to 14.4)*	-1.4 (-1.7 to -1.1)*	61.9 (38.3 to 94.4)	14.2 (11.8 to 16.4)*	-1.3 (-3.3 to 0.6)
Pruritus	5,388.7 (3,688.0 to 7,609.2)	16.4 (12.5 to 20.4)*	0.7 (-2.7 to 4.2)	57.7 (26.1 to 118.8)	16.5 (12.5 to 20.5)*	0.7 (-2.7 to 4.2)
Urticaria	3,212.0 (2,184.1 to 4,537.2)	15.9 (15.3 to 16.3)*	0.2 (-0.3 to 0.6)	193.0 (105.7 to 332.7)	16.1 (14.1 to 18.0)*	0.4 (-1.3 to 2.0)
Decubitus ulcer	40.8 (28.4 to 54.6)	17.1 (10.6 to 22.5)*	1.3 (-4.3 to 5.9)	6.6 (4.0 to 10.2)	17.4 (7.7 to 26.1)*	1.5 (-6.8 to 9.1)
Other skin and subcutaneous diseases	40,023.2 (36,169.1 to 44,171.3)	18.1 (16.0 to 20.2)*	2.1 (0.3 to 4.0)*	237.7 (112.8 to 440.9)	18.2 (16.2 to 20.3)*	2.2 (0.5 to 4.1)*
Sense organ diseases	125,618.0 (122,245.7 to 129,743.1)	16.6 (15.5 to 17.7)*	0.8 (-0.1 to 1.8)	3,569.4 (2,360.0 to 5,184.4)	17.7 (15.7 to 19.7)*	1.8 (0.1 to 3.5)*
Glaucoma	0.0 (0.0 to 0.0)	0.0 (0.0 to 0.0)	0.0 (0.0 to 0.0)	0.0 (0.0 to 0.0)	0.0 (0.0 to 0.0)	0.0 (0.0 to 0.0)
Cataract	1,727.2 (1,302.4 to 2,272.0)	14.9 (11.8 to 17.9)*	-0.7 (-3.3 to 2.0)	127.7 (79.7 to 188.5)	14.2 (10.3 to 17.7)*	-1.3 (-4.6 to 1.8)
Macular degeneration	0.0 (0.0 to 0.0)	0.0 (0.0 to 0.0)	0.0 (0.0 to 0.0)	0.0 (0.0 to 0.0)	0.0 (0.0 to 0.0)	0.0 (0.0 to 0.0)
Refraction and accommodation disorders	49,616.5 (45,777.6 to 53,807.7)	14.0 (12.2 to 16.0)*	-1.4 (-3.0 to 0.4)	868.9 (541.7 to 1,387.1)	15.6 (14.0 to 17.3)*	-0.1 (-1.4 to 1.4)
Age-related and other hearing loss	72,695.1 (59,305.6 to 86,464.3)	18.4 (16.8 to 20.3)*	2.4 (1.0 to 4.0)*	2,029.8 (1,250.4 to 3,055.1)	18.7 (15.4 to 22.1)*	2.6 (-0.2 to 5.6)
Other vision loss	1,695.9 (1,289.4 to 2,195.1)	22.5 (19.7 to 25.6)*	6.0 (3.5 to 8.6)*	127.2 (82.6 to 191.2)	23.7 (20.0 to 27.5)*	7.0 (3.8 to 10.3)*
Other sense organ diseases	16,301.9 (14,410.8 to 18,469.3)	16.5 (13.8 to 19.3)*	0.7 (-1.6 to 3.2)	415.7 (252.0 to 612.6)	16.6 (13.8 to 19.9)*	0.9 (-1.6 to 3.7)
Acute other sense organ diseases	68.1 (61.1 to 76.4)	16.7 (14.3 to 19.5)*	0.9 (-1.2 to 3.4)	1.8 (1.1 to 2.7)	16.7 (14.3 to 19.5)*	0.9 (-1.2 to 3.4)
Chronic other sense organ diseases	16,233.8 (14,347.3 to 18,404.0)	16.5 (13.8 to 19.3)*	0.7 (-1.6 to 3.2)	413.9 (250.9 to 609.8)	16.6 (13.8 to 19.9)*	0.9 (-1.6 to 3.7)
Oral disorders	236,016.1 (226,216.6 to 246,371.3)	15.6 (14.1 to 17.2)*	-0.0 (-1.3 to 1.4)	1,194.8 (682.1 to 1,968.1)	15.4 (14.6 to 16.1)*	-0.2 (-0.9 to 0.4)
Deciduous caries	0.0 (0.0 to 0.0)	0.0 (0.0 to 0.0)	0.0 (0.0 to 0.0)	0.0 (0.0 to 0.0)	0.0 (0.0 to 0.0)	0.0 (0.0 to 0.0)
Permanent caries	193,945.1 (162,981.7 to 232,505.0)	15.4 (13.5 to 17.3)*	-0.2 (-1.9 to 1.4)	134.6 (58.8 to 258.6)	13.0 (11.0 to 15.2)*	-2.2 (-4.0 to -0.4)*
Periodontal diseases	58,298.1 (45,669.4 to 74,072.4)	16.9 (15.4 to 18.0)*	1.1 (-0.2 to 2.1)	387.5 (139.8 to 834.0)	17.1 (15.5 to 18.2)*	1.2 (-0.1 to 2.2)
Edentulism and severe tooth loss	11,465.1 (10,472.5 to 12,558.0)	13.9 (13.4 to 14.3)*	-1.5 (-1.9 to -1.1)*	327.7 (215.1 to 460.7)	14.0 (13.0 to 15.0)*	-1.4 (-2.2 to -0.5)*
Other oral disorders	11,740.9 (10,544.5 to 13,150.2)	15.6 (15.2 to 16.0)*	0.0 (-0.3 to 0.3)	345.0 (208.1 to 523.4)	15.8 (14.9 to 16.7)*	0.1 (-0.7 to 0.9)
Injuries	78,078.8 (74,128.4 to 81,864.9)	8.0 (5.6 to 10.1)*	-6.6 (-8.7 to -4.8)*	3,047.2 (2,178.8 to 4,134.0)	-1.0 (-4.5 to 2.4)	-14.4 (-17.4 to -11.5)*
Transport injuries	9,879.7 (9,283.8 to 10,579.4)	13.1 (12.4 to 13.8)*	-2.2 (-2.8 to -1.6)*	564.4 (393.2 to 775.2)	2.5 (-1.0 to 6.0)	-11.3 (-14.4 to -8.4)*
Road injuries	9,265.5 (8,684.0 to 9,996.8)	13.3 (12.5 to 14.0)*	-2.1 (-2.7 to -1.5)*	526.3 (365.2 to 721.4)	3.3 (-0.2 to 6.7)	-10.7 (-13.7 to -7.7)*
Pedestrian road injuries	1,132.3 (976.2 to 1,339.3)	13.9 (12.8 to 14.9)*	-1.5 (-2.5 to -0.6)*	63.5 (43.1 to 89.2)	0.4 (-3.9 to 4.5)	-13.2 (-16.9 to -9.6)*
Cyclist road injuries	1,568.5 (1,350.8 to 1,808.8)	13.8 (12.6 to 14.9)*	-1.6 (-2.6 to -0.6)*	82.5 (55.5 to 116.0)	2.2 (-2.3 to 6.3)	-11.6 (-15.5 to -8.1)*
Motorcyclist road injuries	2,476.8 (2,130.3 to 2,858.9)	20.6 (19.7 to 21.6)*	4.3 (3.6 to 5.1)*	135.0 (91.5 to 185.8)	6.6 (1.7 to 11.2)*	-7.8 (-12.1 to -3.9)*
Motor vehicle road injuries	3,726.0 (3,309.6 to 4,300.3)	7.0 (5.8 to 8.1)*	-7.5 (-8.5 to -6.5)*	224.3 (152.2 to 308.1)	1.0 (-1.9 to 3.6)	-12.6 (-15.1 to -10.4)*
Other road injuries	361.8 (301.1 to 425.2)	33.0 (31.6 to 34.2)*	15.0 (13.8 to 16.0)*	20.9 (13.9 to 29.4)	25.2 (21.0 to 28.6)*	8.2 (4.6 to 11.2)*
Other transport injuries	614.2 (541.9 to 704.2)	10.4 (9.8 to 11.1)*	-4.5 (-5.0 to -3.9)*	38.1 (26.9 to 51.5)	-7.0 (-11.3 to -2.5)*	-19.6 (-23.3 to -15.7)*
Unintentional injuries	59,144.6 (56,134.6 to 62,221.6)	8.6 (8.1 to 9.0)*	-6.1 (-6.5 to -5.7)*	2,197.1 (1,538.7 to 2,986.3)	-0.2 (-3.3 to 2.6)	-13.7 (-16.4 to -11.2)*
Falls	15,332.5 (13,660.4 to 17,197.8)	16.2 (15.7 to 16.8)*	0.5 (0.0 to 1.0)*	804.8 (557.3 to 1,115.4)	2.1 (-2.6 to 6.5)	-11.7 (-15.8 to -7.9)*
Drowning	261.3 (222.9 to 300.0)	-1.1 (-1.7 to -0.5)*	-14.5 (-14.9 to -13.9)*	14.8 (10.2 to 20.5)	-15.6 (-19.4 to -11.3)*	-27.0 (-30.3 to -23.3)*
Fire, heat, and hot substances	5,478.5 (4,640.0 to 6,292.5)	4.2 (3.4 to 4.8)*	-9.9 (-10.6 to -9.4)*	196.4 (137.0 to 267.5)	-3.5 (-7.3 to -0.2)*	-16.6 (-19.9 to -13.7)*
Poisonings	393.9 (334.4 to 464.6)	10.6 (9.3 to 12.1)*	-4.3 (-5.5 to -3.1)*	36.5 (24.7 to 52.2)	6.1 (2.9 to 9.3)*	-8.2 (-11.0 to -5.4)*
Exposure to mechanical forces	14,808.9 (13,528.5 to 16,096.7)	10.9 (10.2 to 11.6)*	-4.1 (-4.7 to -3.5)*	276.4 (190.1 to 385.9)	2.4 (-0.7 to 5.3)	-11.5 (-14.1 to -8.9)*
Unintentional firearm injuries	212.9 (185.9 to 245.5)	7.9 (7.0 to 8.7)*	-6.7 (-7.4 to -6.0)*	8.4 (5.8 to 11.7)	-0.9 (-4.6 to 2.6)	-14.3 (-17.5 to -11.3)*
Unintentional suffocation	614.4 (492.5 to 770.2)	6.3 (2.7 to 9.8)*	-8.1 (-11.2 to -5.0)*	22.1 (15.2 to 30.4)	8.3 (5.6 to 10.8)*	-6.4 (-8.7 to -4.2)*
Other exposure to mechanical forces	13,981.6 (12,674.5 to 15,291.1)	11.2 (10.6 to 11.8)*	-3.8 (-4.4 to -3.3)*	245.9 (168.7 to 345.7)	2.0 (-1.4 to 5.1)	-11.8 (-14.7 to -9.1)*
Adverse effects of medical treatment	638.7 (488.0 to 822.5)	1.3 (-2.9 to 5.4)	-12.4 (-16.1 to -8.9)*	85.2 (51.8 to 127.6)	1.3 (-2.9 to 5.4)	-12.4 (-16.1 to -8.9)*
Animal contact	2,739.5 (2,455.4 to 3,077.5)	8.0 (6.6 to 9.2)*	-6.6 (-7.8 to -5.5)*	81.9 (56.5 to 111.7)	4.4 (1.9 to 6.7)*	-9.8 (-11.9 to -7.7)*
Venomous animal contact	1,284.8 (1,135.6 to 1,471.5)	11.6 (10.5 to 12.7)*	-3.5 (-4.4 to -2.5)*	62.6 (42.6 to 85.1)	5.4 (2.8 to 8.2)*	-8.8 (-11.1 to -6.4)*
Non-venomous animal contact	1,454.8 (1,238.6 to 1,706.2)	5.0 (2.9 to 6.7)*	-9.2 (-11.0 to -7.7)*	19.3 (12.5 to 29.5)	1.0 (-2.5 to 4.2)	-12.6 (-15.7 to -9.9)*
Foreign body	2,369.3 (2,081.6 to 2,664.2)	14.5 (13.4 to 15.5)*	-1.0 (-1.9 to -0.1)*	86.3 (61.1 to 119.8)	1.9 (-2.0 to 5.8)	-11.9 (-15.2 to -8.5)*
Pulmonary aspiration and foreign body in airway	903.0 (736.2 to 1,131.4)	15.7 (13.1 to 18.3)*	0.1 (-2.2 to 2.3)	45.0 (31.1 to 63.1)	-2.0 (-6.6 to 3.0)	-15.3 (-19.3 to -10.9)*
Foreign body in eyes	72.8 (30.0 to 131.5)	16.4 (15.2 to 17.6)*	0.7 (-0.3 to 1.7)	4.0 (1.7 to 7.8)	15.2 (12.7 to 16.5)*	-0.4 (-2.5 to 0.8)
Foreign body in other body part	1,393.4 (1,210.0 to 1,611.6)	13.7 (13.0 to 14.5)*	-1.7 (-2.3 to -1.0)*	37.3 (25.9 to 53.3)	5.7 (2.8 to 8.5)*	-8.6 (-11.1 to -6.2)*
Environmental heat and cold exposure	4,808.8 (4,212.0 to 5,635.4)	9.9 (9.3 to 10.5)*	-5.0 (-5.5 to -4.5)*	199.1 (136.6 to 278.1)	-0.8 (-4.1 to 2.5)	-14.2 (-17.1 to -11.4)*
Other unintentional injuries	12,313.2 (10,910.8 to 14,040.2)	-1.0 (-1.7 to -0.2)*	-14.4 (-15.0 to -13.7)*	415.7 (275.3 to 583.0)	-5.4 (-7.4 to -3.6)*	-18.2 (-19.9 to -16.7)*
Self-harm and interpersonal violence	2,188.8 (1,984.1 to 2,372.8)	5.0 (4.5 to 5.4)*	-9.2 (-9.7 to -8.8)*	100.5 (70.6 to 135.4)	-6.8 (-10.1 to -3.2)*	-19.4 (-22.3 to -16.3)*
Self-harm	524.2 (450.6 to 599.1)	4.7 (4.0 to 5.3)*	-9.5 (-10.1 to -9.0)*	28.8 (20.0 to 39.1)	-6.9 (-10.2 to -3.6)*	-19.5 (-22.3 to -16.7)*
Interpersonal violence	1,664.6 (1,486.5 to 1,832.7)	5.1 (4.5 to 5.6)*	-9.1 (-9.7 to -8.6)*	71.8 (50.2 to 97.9)	-6.7 (-10.3 to -2.9)*	-19.4 (-22.4 to -16.1)*

Cause	Prevalence in thousands (95% confidence interval)			YLDs in thousands (95% confidence interval)		
	2015	Percent change in counts between 2005 and 2015	Percent change in rates between 2005 and 2015	2015	Percent change in counts between 2005 and 2015	Percent change in rates between 2005 and 2015
Assault by firearm	105.1 (88.7 to 122.9)	4.2 (2.8 to 5.3)*	-9.9 (-11.1 to -8.9)*	4.8 (3.3 to 6.7)	-5.9 (-9.6 to -2.5)*	-18.6 (-21.8 to -15.6)*
Assault by sharp object	409.4 (329.9 to 491.6)	4.6 (3.7 to 5.3)*	-9.6 (-10.3 to -8.9)*	12.5 (8.5 to 17.4)	-12.0 (-17.0 to -6.7)*	-23.9 (-28.2 to -19.3)*
Assault by other means	1,150.1 (1,011.5 to 1,290.8)	5.3 (4.6 to 6.0)*	-8.9 (-9.5 to -8.3)*	54.5 (37.8 to 73.8)	-5.5 (-9.0 to -2.0)*	-18.3 (-21.3 to -15.2)*
Forces of nature, war, and legal intervention	6,865.7 (4,932.2 to 9,265.3)	-2.2 (-21.7 to 22.0)	-15.4 (-32.3 to 5.5)	185.2 (129.5 to 250.0)	-15.2 (-35.3 to 10.9)	-26.7 (-44.1 to -4.1)*
Exposure to forces of nature	2,056.4 (1,275.2 to 3,117.8)	38.0 (1.8 to 88.4)*	19.4 (-11.9 to 63.0)	60.4 (40.2 to 85.6)	-11.3 (-42.8 to 33.7)	-23.3 (-50.5 to 15.6)
Collective violence and legal intervention	4,809.2 (3,379.4 to 6,545.5)	-13.0 (-33.3 to 14.6)	-24.8 (-42.3 to -0.9)*	124.8 (80.8 to 178.1)	-17.0 (-41.4 to 24.9)	-28.2 (-49.4 to 8.0)
**Anemia**	**134,885.3 (133,105.2 to 136,602.8)**	**5.7 (4.3 to 7.2)***	**-8.6 (-9.8 to -7.3)***	**3,656.9 (2,441.4 to 5,324.6)**	**0.6 (-1.8 to 3.1)**	**-13.0 (-15.1 to -10.8)***
**Developmental intellectual disability**	**9,425.8 (7,010.2 to 11,787.1)**	**18.3 (17.8 to 18.8)***	**2.3 (1.9 to 2.8)***	**1,046.0 (745.0 to 1,389.9)**	**20.5 (17.8 to 23.5)***	**4.2 (1.9 to 6.8)***
**Epilepsy**	**2,238.0 (1,882.9 to 2,564.3)**	**16.5 (12.1 to 20.7)***	**0.7 (-3.1 to 4.4)**	**659.8 (470.3 to 867.0)**	**8.4 (0.5 to 15.5)***	**-6.3 (-13.0 to -0.2)***
**Guillain-Barré syndrome**	**2.2 (1.2 to 3.5)**	**13.4 (11.2 to 14.9)***	**-1.9 (-3.9 to -0.6)***	**0.6 (0.3 to 1.1)**	**13.4 (11.2 to 14.9)***	**-1.9 (-3.9 to -0.6)***
**Hearing loss**	**78,372.5 (65,043.0 to 92,295.4)**	**17.7 (16.2 to 19.3)***	**1.8 (0.5 to 3.2)***	**2,295.1 (1,437.4 to 3,383.6)**	**17.7 (14.8 to 20.5)***	**1.8 (-0.7 to 4.2)**
**Heart failure**	**790.2 (732.2 to 849.4)**	**16.7 (15.8 to 17.5)***	**0.9 (0.2 to 1.6)***	**98.6 (67.9 to 136.8)**	**17.8 (15.0 to 20.5)***	**1.8 (-0.5 to 4.2)**
**Infertility**	**24,327.8 (16,623.2 to 34,121.0)**	**17.6 (11.4 to 23.2)***	**1.7 (-3.7 to 6.6)**	**150.9 (65.1 to 328.5)**	**17.8 (13.0 to 22.6)***	**1.9 (-2.2 to 6.1)**
**Pelvic inflammatory disease**	**99.6 (74.0 to 132.8)**	**0.1 (-1.5 to 1.7)**	**-13.4 (-14.8 to -12.0)***	**13.1 (8.0 to 19.9)**	**0.4 (-4.8 to 5.5)**	**-13.2 (-17.6 to -8.8)***
**Vision loss**	**54,471.9 (50,260.4 to 59,211.8)**	**14.3 (12.5 to 16.2)***	**-1.2 (-2.7 to 0.5)**	**1,338.8 (909.6 to 1,941.2)**	**16.7 (15.2 to 18.3)***	**1.0 (-0.3 to 2.3)**

SR Table 11. Prevalent cases and YLDs for 2015, percent change, and change in age-standardised rates between 2005 and 2015 by cause, global, ages 45-49 years						
Cause	Prevalence in thousands (95% confidence interval)			YLDs in thousands (95% confidence interval)		
	2015	Percent change in counts between 2005 and 2015	Percent change in rates between 2005 and 2015	2015	Percent change in counts between 2005 and 2015	Percent change in rates between 2005 and 2015
**All causes**	--	--	--	**59,885.5 (45,061.4 to 76,882.9)**	**23.2 (22.4 to 23.9)***	**-3.1 (-3.7 to -2.5)***
Communicable, maternal, neonatal, and nutritional diseases	263,040.5 (260,505.7 to 265,095.7)	22.1 (21.1 to 23.0)*	-3.9 (-4.7 to -3.2)*	5,128.1 (3,711.6 to 6,984.0)	8.4 (5.6 to 11.8)*	-14.7 (-16.9 to -12.0)*
HIV/AIDS and tuberculosis	4,409.8 (4,269.7 to 4,564.1)	42.8 (36.5 to 49.2)*	12.4 (7.4 to 17.4)*	643.4 (462.8 to 863.9)	2.9 (-6.1 to 12.2)	-19.0 (-26.1 to -11.7)*
Tuberculosis	719.2 (537.4 to 979.9)	10.3 (1.3 to 21.3)*	-13.1 (-20.3 to -4.5)*	219.2 (135.7 to 332.1)	10.8 (1.6 to 22.0)*	-12.8 (-20.0 to -4.0)*
HIV/AIDS	3,693.7 (3,569.4 to 3,836.4)	51.7 (43.1 to 60.0)*	19.4 (12.6 to 25.9)*	424.2 (306.7 to 549.0)	-0.8 (-12.5 to 11.8)	-21.9 (-31.2 to -12.0)*
HIV/AIDS - Tuberculosis	124.1 (110.4 to 138.1)	5.4 (0.8 to 11.3)*	-17.0 (-20.7 to -12.4)*	45.8 (31.0 to 62.3)	6.1 (-0.2 to 13.4)	-16.5 (-21.4 to -10.7)*
HIV/AIDS resulting in other diseases	3,723.8 (3,571.2 to 3,914.3)	54.8 (49.3 to 59.6)*	21.8 (17.5 to 25.6)*	378.4 (273.8 to 490.3)	-1.6 (-15.1 to 12.7)	-22.5 (-33.1 to -11.3)*
HIV aggregate	1,363.6 (1,287.8 to 1,457.4)	-19.9 (-23.1 to -16.1)*	-37.0 (-39.5 to -34.0)*	99.6 (71.2 to 130.3)	-21.1 (-24.6 to -17.1)*	-37.9 (-40.6 to -34.8)*
AIDS aggregate	2,360.2 (2,241.3 to 2,508.7)	235.9 (200.2 to 275.0)*	164.4 (136.3 to 195.2)*	278.7 (202.0 to 363.3)	8.0 (-12.1 to 33.3)	-15.0 (-30.8 to 4.9)
Diarrhea, lower respiratory, and other common infectious diseases	19,552.6 (17,940.2 to 20,728.7)	21.3 (18.3 to 24.6)*	-4.5 (-6.9 to -1.9)*	567.8 (393.0 to 786.5)	20.7 (18.8 to 22.5)*	-5.0 (-6.5 to -3.6)*
Diarrheal diseases	902.0 (863.3 to 940.2)	21.7 (20.8 to 22.6)*	-4.2 (-4.9 to -3.5)*	140.6 (96.7 to 193.4)	22.2 (19.5 to 25.0)*	-3.8 (-6.0 to -1.6)*
Diarrhoea episodes aggregate	901.7 (863.0 to 939.9)	21.7 (20.8 to 22.6)*	-4.2 (-4.9 to -3.5)*	140.5 (96.6 to 193.3)	22.2 (19.5 to 25.0)*	-3.8 (-6.0 to -1.6)*
Guillain-Barré syndrome due to diarrheal diseases	0.3 (0.2 to 0.5)	24.0 (21.8 to 25.5)*	-2.4 (-4.1 to -1.2)*	0.1 (0.0 to 0.1)	24.0 (21.8 to 25.5)*	-2.4 (-4.1 to -1.2)*
Intestinal infectious diseases	29.4 (26.2 to 32.9)	-23.5 (-32.2 to -13.6)*	-39.7 (-46.6 to -32.0)*	5.2 (3.3 to 7.6)	0.3 (-13.3 to 16.5)	-21.1 (-31.8 to -8.3)*
Typhoid fever	33.3 (25.5 to 43.1)	-0.3 (-5.8 to 5.7)	-21.5 (-25.9 to -16.8)*	4.4 (2.7 to 6.5)	2.6 (-13.9 to 23.4)	-19.2 (-32.2 to -2.9)*
Typhoid fever episodes aggregate	27.6 (20.8 to 36.0)	-0.2 (-9.2 to 9.1)	-21.5 (-28.5 to -14.2)*	2.6 (1.6 to 3.8)	0.6 (-15.1 to 19.4)	-20.8 (-33.2 to -6.0)*
Complications of typhoid fever aggregate	5.7 (3.9 to 8.0)	-0.5 (-32.4 to 49.8)	-21.7 (-46.8 to 17.9)	1.8 (1.0 to 2.9)	5.5 (-28.9 to 62.4)	-16.9 (-44.0 to 27.9)
Paratyphoid fever	15.1 (10.3 to 21.2)	-5.0 (-12.9 to 3.1)	-25.2 (-31.5 to -18.8)*	0.8 (0.4 to 1.2)	-4.4 (-21.6 to 17.5)	-24.7 (-38.3 to -7.5)*
Paratyphoid fever episodes aggregate	14.3 (9.8 to 20.2)	-5.0 (-13.4 to 3.4)	-25.3 (-31.8 to -18.6)*	0.7 (0.4 to 1.1)	-4.5 (-23.3 to 20.5)	-24.8 (-39.7 to -5.2)*
Intestinal perforation due to paratyphoid	0.8 (0.4 to 1.3)	-3.4 (-43.0 to 73.3)	-24.0 (-55.2 to 36.4)	0.1 (0.0 to 0.2)	-3.5 (-43.0 to 73.3)	-24.0 (-55.2 to 36.4)
Other intestinal infectious diseases	--	--	--	0.0 (0.0 to 0.1)	-62.1 (-75.2 to -38.7)*	-70.2 (-80.5 to -51.7)*
Lower respiratory infections	248.7 (235.1 to 262.4)	21.3 (19.6 to 23.0)*	-4.5 (-5.9 to -3.2)*	14.9 (10.1 to 21.1)	21.6 (16.8 to 26.7)*	-4.3 (-8.1 to -0.3)*
Lower respiratory infection episodes aggregate	248.4 (234.7 to 262.0)	21.3 (19.6 to 23.0)*	-4.6 (-5.9 to -3.2)*	14.9 (10.0 to 21.0)	21.6 (16.7 to 26.7)*	-4.3 (-8.1 to -0.2)*
Guillain-Barré syndrome due to lower respiratory infections	0.3 (0.1 to 0.6)	24.0 (21.8 to 25.5)*	-2.4 (-4.1 to -1.2)*	0.1 (0.0 to 0.2)	24.0 (21.8 to 25.5)*	-2.4 (-4.1 to -1.2)*
Upper respiratory infections	12,935.1 (8,914.5 to 18,188.0)	24.1 (22.8 to 25.6)*	-2.3 (-3.4 to -1.1)*	150.5 (76.1 to 267.7)	24.3 (22.6 to 26.1)*	-2.2 (-3.5 to -0.7)*
Upper respiratory infection episodes aggregate	12,934.3 (8,913.6 to 18,187.1)	24.1 (22.8 to 25.6)*	-2.3 (-3.4 to -1.1)*	150.3 (75.7 to 267.6)	24.3 (22.6 to 26.1)*	-2.2 (-3.5 to -0.7)*
Guillain-Barré syndrome due to upper respiratory infections	0.8 (0.5 to 1.3)	24.0 (21.8 to 25.5)*	-2.4 (-4.1 to -1.2)*	0.2 (0.1 to 0.4)	24.0 (21.8 to 25.5)*	-2.4 (-4.1 to -1.2)*
Otitis media	4,633.2 (3,592.5 to 5,845.4)	15.4 (12.2 to 18.6)*	-9.2 (-11.7 to -6.6)*	127.3 (75.1 to 196.4)	15.2 (10.0 to 21.0)*	-9.3 (-13.4 to -4.8)*
Acute otitis media	433.1 (347.3 to 544.1)	25.1 (21.1 to 29.6)*	-1.6 (-4.7 to 2.0)	5.6 (2.7 to 10.2)	25.3 (20.1 to 31.3)*	-1.4 (-5.5 to 3.3)
Chronic otitis media aggregate	4,200.1 (3,157.2 to 5,415.0)	14.5 (11.0 to 18.0)*	-9.9 (-12.7 to -7.1)*	121.8 (71.6 to 187.9)	14.7 (9.3 to 20.7)*	-9.7 (-14.0 to -5.0)*
Meningitis	437.5 (414.8 to 461.0)	18.7 (14.1 to 23.6)*	-6.6 (-10.2 to -2.7)*	78.1 (55.3 to 102.4)	24.6 (21.3 to 27.8)*	-2.0 (-4.5 to 0.6)
Pneumococcal meningitis	396.6 (335.5 to 469.2)	25.9 (23.5 to 28.7)*	-0.9 (-2.8 to 1.3)	36.6 (25.8 to 48.7)	25.1 (19.7 to 30.9)*	-1.6 (-5.8 to 3.0)
Acute pneumococcal meningitis	1.2 (0.8 to 1.7)	24.2 (10.3 to 37.6)*	-2.2 (-13.2 to 8.3)	0.2 (0.1 to 0.3)	24.3 (10.3 to 37.6)*	-2.2 (-13.2 to 8.3)
Complications of pneumococcal meningitis aggregate	395.5 (334.1 to 468.2)	26.0 (23.5 to 28.7)*	-0.9 (-2.8 to 1.3)	36.5 (25.8 to 48.5)	25.1 (19.7 to 30.9)*	-1.5 (-5.8 to 3.1)
H influenzae type B meningitis	113.0 (88.1 to 142.6)	-4.9 (-9.3 to 0.6)	-25.1 (-28.6 to -20.8)*	12.3 (8.5 to 16.9)	19.1 (12.0 to 26.3)*	-6.2 (-11.9 to -0.6)*
Acute H influenzae type B meningitis	0.4 (0.2 to 0.6)	-13.2 (-23.5 to -3.1)*	-31.7 (-39.8 to -23.7)*	0.1 (0.0 to 0.1)	-13.2 (-23.5 to -3.1)*	-31.6 (-39.8 to -23.7)*
Complications of of H influenzae type B meningitis aggregate	112.6 (87.7 to 142.2)	-4.9 (-9.3 to 0.6)	-25.1 (-28.6 to -20.8)*	12.2 (8.4 to 16.8)	19.3 (12.1 to 26.6)*	-6.1 (-11.8 to -0.4)*
Meningococcal meningitis	96.2 (75.8 to 119.4)	24.5 (21.2 to 28.1)*	-2.0 (-4.6 to 0.8)	10.7 (7.4 to 14.3)	25.4 (20.8 to 30.8)*	-1.3 (-4.9 to 3.0)
Acute meningococcal meningitis	0.5 (0.3 to 0.7)	21.7 (7.0 to 36.9)*	-4.2 (-15.8 to 7.7)	0.1 (0.0 to 0.1)	21.8 (7.0 to 36.9)*	-4.1 (-15.8 to 7.7)
Complications of meningococcal meningitis aggregate	95.7 (75.2 to 119.0)	24.5 (21.2 to 28.1)*	-2.0 (-4.6 to 0.8)	10.6 (7.4 to 14.2)	25.5 (20.8 to 30.9)*	-1.3 (-4.9 to 3.0)
Other meningitis	175.6 (140.2 to 211.0)	25.8 (23.0 to 28.6)*	-0.9 (-3.2 to 1.2)	18.5 (12.6 to 24.7)	26.9 (21.6 to 32.6)*	-0.2 (-4.3 to 4.4)
Other meningitis episodes aggregate	3.9 (3.0 to 5.1)	22.9 (10.5 to 34.6)*	-3.3 (-13.0 to 5.9)	0.5 (0.3 to 0.8)	22.9 (10.5 to 34.5)*	-3.2 (-13.0 to 5.9)
Complications of other meningitis aggregate	171.7 (136.6 to 207.3)	25.9 (23.1 to 28.7)*	-0.9 (-3.1 to 1.3)	18.0 (12.3 to 24.0)	27.0 (21.6 to 32.9)*	-0.1 (-4.3 to 4.6)
Encephalitis	335.3 (241.9 to 453.9)	8.3 (5.6 to 11.7)*	-14.7 (-16.9 to -12.1)*	36.2 (25.4 to 48.7)	13.2 (7.5 to 18.8)*	-10.9 (-15.4 to -6.5)*
Acute encephalitis	4.3 (3.6 to 5.2)	12.7 (6.9 to 18.1)*	-11.3 (-15.8 to -7.0)*	0.6 (0.4 to 0.8)	14.0 (0.2 to 28.9)*	-10.2 (-21.1 to 1.4)
Complications of encephalitis aggregate	331.1 (237.5 to 449.8)	8.3 (5.5 to 11.7)*	-14.8 (-16.9 to -12.0)*	35.7 (24.9 to 47.9)	13.2 (7.3 to 18.9)*	-10.9 (-15.5 to -6.4)*
Diphtheria	0.0 (0.0 to 0.0)	-53.9 (-80.2 to 12.1)	-63.7 (-84.4 to -11.7)*	0.0 (0.0 to 0.0)	-53.9 (-80.2 to 12.1)	-63.7 (-84.4 to -11.7)*
Whooping cough	2.3 (1.7 to 2.8)	-16.2 (-19.1 to -13.0)*	-34.0 (-36.3 to -31.5)*	0.1 (0.1 to 0.2)	-16.2 (-19.1 to -13.0)*	-34.0 (-36.3 to -31.5)*
Tetanus	11.6 (11.3 to 12.0)	25.8 (21.0 to 28.4)*	-0.9 (-4.8 to 1.1)	0.5 (0.4 to 0.7)	11.2 (-0.4 to 21.0)	-12.5 (-21.6 to -4.7)*
Severe tetanus	0.5 (0.3 to 0.8)	-30.0 (-46.6 to -4.9)*	-44.9 (-58.0 to -25.2)*	0.1 (0.0 to 0.1)	-29.9 (-46.6 to -4.9)*	-44.8 (-58.0 to -25.2)*
Complications of tetanus aggregate	11.1 (10.9 to 11.4)	30.2 (29.6 to 30.8)*	2.5 (2.0 to 2.9)*	0.5 (0.3 to 0.6)	20.5 (12.7 to 29.7)*	-5.1 (-11.3 to 2.1)
Measles	0.1 (0.0 to 0.2)	-56.3 (-61.6 to -49.7)*	-65.6 (-69.7 to -60.4)*	0.0 (0.0 to 0.0)	-56.4 (-61.6 to -49.7)*	-65.6 (-69.7 to -60.4)*
Varicella and herpes zoster	300.7 (234.0 to 377.3)	28.2 (27.1 to 29.2)*	0.9 (0.0 to 1.7)*	14.1 (8.3 to 23.0)	29.1 (24.0 to 34.2)*	1.6 (-2.4 to 5.7)
Neglected tropical diseases and malaria	108,668.5 (106,003.4 to 111,392.1)	13.9 (10.5 to 17.3)*	-10.3 (-13.1 to -7.6)*	1,053.2 (698.3 to 1,611.1)	2.3 (-7.9 to 14.8)	-19.5 (-27.5 to -9.6)*
Malaria	7,811.2 (6,840.4 to 8,894.5)	35.0 (28.1 to 43.4)*	6.3 (0.8 to 12.8)*	65.9 (46.7 to 90.1)	22.8 (16.9 to 28.8)*	-3.3 (-8.0 to 1.3)
Asymptomatic malaria parasitemia(PfPR)	5,693.0 (4,740.7 to 6,776.4)	38.5 (28.2 to 50.4)*	9.0 (0.9 to 18.3)*	0.0 (0.0 to 0.0)	0.0 (0.0 to 0.0)	0.0 (0.0 to 0.0)
Malaria episodes aggregate	227.9 (124.7 to 393.9)	-4.1 (-22.7 to 22.0)	-24.5 (-39.1 to -4.0)*	4.6 (2.2 to 8.9)	-3.7 (-23.3 to 22.8)	-24.2 (-39.7 to -3.3)*
Complications of malaria aggregate	36.6 (33.2 to 40.3)	36.1 (33.2 to 40.4)*	7.2 (4.9 to 10.5)*	11.3 (8.5 to 14.1)	31.3 (21.0 to 42.1)*	3.3 (-4.8 to 11.9)
Anemia due to malaria parasitemia(PfPR) aggregate	1,853.7 (1,806.1 to 1,901.5)	31.6 (27.4 to 35.5)*	3.6 (0.3 to 6.7)*	50.0 (34.0 to 71.7)	24.1 (17.2 to 31.7)*	-2.3 (-7.8 to 3.7)
Chagas disease	608.7 (502.4 to 715.7)	-11.5 (-14.9 to -8.1)*	-30.4 (-33.0 to -27.6)*	5.1 (3.3 to 7.6)	-11.4 (-17.1 to -5.9)*	-30.3 (-34.7 to -26.0)*
Chagas disease episodes aggregate	518.1 (427.7 to 612.8)	-11.5 (-14.9 to -8.0)*	-30.4 (-33.0 to -27.6)*	0.0 (0.0 to 0.0)	-4.8 (-10.1 to 0.4)	-25.1 (-29.2 to -20.9)*
Complications of Chagas disease aggregate	61.7 (47.0 to 77.8)	-11.5 (-14.9 to -8.1)*	-30.4 (-33.0 to -27.6)*	3.6 (2.3 to 5.3)	-11.4 (-18.0 to -5.3)*	-30.3 (-35.5 to -25.4)*

Cause	Prevalence in thousands (95% confidence interval)			YLDs in thousands (95% confidence interval)		
	2015	Percent change in counts between 2005 and 2015	Percent change in rates between 2005 and 2015	2015	Percent change in counts between 2005 and 2015	Percent change in rates between 2005 and 2015
Heart failure due to Chagas disease aggregate	28.9 (17.3 to 43.1)	-11.7 (-15.2 to -8.1)*	-30.5 (-33.2 to -27.6)*	1.5 (0.8 to 2.6)	-11.5 (-18.8 to -3.1)*	-30.3 (-36.1 to -23.7)*
Leishmaniasis	231.0 (205.6 to 271.5)	29.9 (6.4 to 57.3)*	2.2 (-16.3 to 23.8)	2.6 (1.3 to 5.0)	29.8 (24.0 to 35.8)*	2.2 (-2.4 to 6.9)
Visceral leishmaniasis	2.1 (2.0 to 2.2)	19.8 (18.5 to 21.1)*	-5.7 (-6.8 to -4.7)*	0.1 (0.1 to 0.2)	19.2 (-13.8 to 68.1)	-6.1 (-32.2 to 32.3)
Cutaneous and mucocutaneous leishmaniasis	235.4 (200.4 to 286.6)	30.0 (26.7 to 32.7)*	2.3 (-0.3 to 4.5)	2.5 (1.2 to 4.8)	30.5 (25.0 to 35.8)*	2.7 (-1.6 to 6.9)
African trypanosomiasis	0.5 (0.3 to 0.8)	-68.9 (-70.6 to -67.5)*	-75.5 (-76.9 to -74.4)*	0.1 (0.0 to 0.3)	-67.9 (-83.8 to -44.8)*	-74.7 (-87.2 to -56.5)*
Schistosomiasis	11,440.5 (9,550.1 to 14,490.9)	-23.6 (-32.6 to -5.0)*	-39.8 (-46.9 to -25.3)*	110.5 (57.0 to 205.0)	-21.2 (-29.1 to -2.5)*	-38.0 (-44.2 to -23.3)*
Schistosomiasis episodes aggregate	5,728.8 (4,463.4 to 7,235.2)	-27.1 (-36.3 to -13.7)*	-42.6 (-49.9 to -32.0)*	32.3 (12.8 to 68.8)	-28.4 (-37.2 to -14.7)*	-43.6 (-50.6 to -32.9)*
Complications of schistosomiasis aggregate	4,979.6 (3,433.6 to 7,779.0)	-18.2 (-31.0 to 14.8)	-35.6 (-45.7 to -9.6)*	58.3 (27.9 to 115.3)	-15.3 (-27.5 to 16.3)	-33.3 (-42.9 to -8.5)*
Anemia due to schistosomiasis aggregate	717.4 (696.0 to 740.6)	-22.3 (-24.9 to -19.6)*	-38.8 (-40.9 to -36.7)*	19.9 (13.3 to 28.7)	-24.5 (-29.2 to -20.0)*	-40.6 (-44.3 to -37.0)*
Cysticercosis	151.3 (97.1 to 232.2)	-8.3 (-16.2 to -1.4)*	-27.8 (-34.0 to -22.4)*	21.9 (12.3 to 35.6)	-18.8 (-26.8 to -11.7)*	-36.1 (-42.4 to -30.5)*
Cystic echinococcosis	107.0 (92.0 to 124.2)	30.1 (26.5 to 33.8)*	2.4 (-0.5 to 5.3)	9.8 (6.6 to 13.7)	29.9 (20.4 to 40.5)*	2.2 (-5.2 to 10.6)
Lymphatic filariasis	2,673.4 (2,111.9 to 3,363.0)	-39.5 (-46.1 to -33.3)*	-52.4 (-57.6 to -47.5)*	172.8 (94.7 to 277.2)	-11.8 (-29.5 to 2.3)	-30.6 (-44.5 to -19.5)*
Prevalence of detectable microfiliaria due to lymphatic filariasis	1,100.9 (954.5 to 1,273.2)	-58.8 (-62.1 to -54.7)*	-67.5 (-70.2 to -64.4)*	0.0 (0.0 to 0.0)	0.0 (0.0 to 0.0)	0.0 (0.0 to 0.0)
Complications of lymphatic filariasis aggregate	1,572.4 (1,038.5 to 2,235.0)	-10.2 (-27.7 to 3.5)	-29.3 (-43.1 to -18.6)*	172.8 (94.7 to 277.2)	-11.8 (-29.5 to 2.3)	-30.6 (-44.5 to -19.5)*
Onchocerciasis	835.7 (642.8 to 1,088.8)	-24.6 (-36.7 to -11.5)*	-40.6 (-50.2 to -30.3)*	69.3 (33.5 to 127.2)	-17.8 (-37.5 to 2.6)	-35.3 (-50.8 to -19.3)*
Asymptomatic onchocerciasis	110.2 (63.4 to 162.4)	-57.7 (-64.0 to -54.8)*	-66.7 (-71.6 to -64.4)*	0.0 (0.0 to 0.0)	0.0 (0.0 to 0.0)	0.0 (0.0 to 0.0)
Skin disease due to onchocerciasis aggregate	628.3 (429.6 to 895.3)	-16.0 (-32.0 to 1.4)	-33.9 (-46.5 to -20.1)*	61.0 (26.2 to 115.3)	-19.2 (-42.9 to 3.6)	-36.4 (-55.1 to -18.2)*
Vision loss due to onchocerciasis aggregate	98.8 (69.0 to 135.3)	-0.7 (-13.8 to 12.0)	-21.8 (-32.2 to -11.8)*	8.2 (5.0 to 12.7)	-5.3 (-18.1 to 7.3)	-25.4 (-35.6 to -15.6)*
Trachoma	197.1 (127.3 to 290.9)	1.4 (-7.0 to 10.6)	-20.2 (-26.8 to -13.0)*	19.0 (10.4 to 33.3)	-0.8 (-11.4 to 10.9)	-21.9 (-30.3 to -12.7)*
Dengue	233.7 (128.0 to 526.5)	180.9 (11.8 to 680.5)*	121.1 (-12.0 to 514.3)	37.1 (16.8 to 90.4)	178.2 (9.4 to 686.3)*	119.0 (-13.9 to 518.9)
Dengue episodes aggregate	69.9 (45.0 to 152.7)	167.9 (7.9 to 638.1)*	110.9 (-15.0 to 480.9)	4.0 (2.2 to 9.0)	178.3 (12.0 to 652.9)*	119.0 (-11.9 to 492.6)
Post-dengue chronic fatigue syndrome	164.3 (77.8 to 378.1)	181.1 (11.8 to 680.5)*	121.2 (-12.0 to 514.3)	33.1 (14.3 to 81.5)	178.2 (9.6 to 678.5)*	118.9 (-13.7 to 512.8)
Yellow fever	0.1 (0.0 to 0.2)	-23.5 (-39.7 to 2.5)	-39.8 (-52.5 to -19.3)*	0.0 (0.0 to 0.0)	-23.5 (-39.7 to 2.5)	-39.7 (-52.5 to -19.3)*
Rabies	0.1 (0.0 to 0.1)	-39.2 (-62.4 to -12.6)*	-52.2 (-70.4 to -31.2)*	0.0 (0.0 to 0.0)	-39.1 (-62.4 to -12.6)*	-52.1 (-70.4 to -31.2)*
Intestinal nematode infections	87,779.1 (85,106.4 to 90,407.7)	14.0 (10.0 to 18.3)*	-10.3 (-13.4 to -6.9)*	173.1 (98.9 to 280.9)	-12.1 (-19.6 to -3.0)*	-30.8 (-36.7 to -23.7)*
Ascariasis	46,161.0 (39,112.9 to 55,854.9)	4.0 (-14.7 to 29.1)	-18.1 (-32.9 to 1.6)	45.8 (25.2 to 78.2)	-30.7 (-42.8 to -16.2)*	-45.5 (-55.0 to -34.1)*
Complications of ascariasis aggregate	2,560.7 (2,283.6 to 2,883.4)	-27.8 (-38.3 to -15.4)*	-43.2 (-51.4 to -33.4)*	45.8 (25.2 to 78.2)	-30.7 (-42.8 to -16.2)*	-45.5 (-55.0 to -34.1)*
Asymptomatic ascariasis	43,605.4 (36,476.8 to 53,323.7)	6.8 (-13.5 to 33.9)	-15.9 (-31.9 to 5.4)	0.0 (0.0 to 0.0)	0.0 (0.0 to 0.0)	0.0 (0.0 to 0.0)
Trichuriasis	27,398.4 (24,453.1 to 30,746.1)	16.1 (-0.3 to 36.0)	-8.6 (-21.5 to 7.0)	30.9 (16.0 to 54.5)	-2.6 (-24.4 to 31.0)	-23.3 (-40.5 to 3.1)
Complications of trichuriasis aggregate	1,542.7 (1,319.8 to 1,921.2)	-1.8 (-21.5 to 26.6)	-22.7 (-38.2 to -0.4)*	30.9 (16.0 to 54.5)	-2.6 (-24.4 to 31.0)	-23.3 (-40.5 to 3.1)
Asymptomatic trichuriasis	25,851.0 (22,864.7 to 29,242.9)	17.3 (0.1 to 39.2)*	-7.6 (-21.2 to 9.5)	0.0 (0.0 to 0.0)	0.0 (0.0 to 0.0)	0.0 (0.0 to 0.0)
Hookworm disease	26,349.0 (23,420.4 to 29,793.1)	7.3 (-7.9 to 24.9)	-15.5 (-27.5 to -1.7)*	96.4 (58.8 to 151.9)	-2.9 (-11.7 to 6.7)	-23.5 (-30.5 to -16.0)*
Complications of hookworm disease aggregate	3,150.0 (2,903.1 to 3,420.5)	-1.9 (-12.7 to 10.3)	-22.8 (-31.3 to -13.2)*	63.7 (35.1 to 106.7)	-3.6 (-16.6 to 10.9)	-24.2 (-34.3 to -12.7)*
Anemia due to hookworm disease aggregate	1,454.6 (1,413.7 to 1,497.0)	8.2 (4.1 to 12.6)*	-14.8 (-18.1 to -11.4)*	32.7 (21.5 to 48.0)	-1.3 (-7.4 to 4.8)	-22.3 (-27.1 to -17.5)*
Asymptomatic hookworm disease	21,741.6 (18,807.9 to 25,116.1)	8.7 (-10.3 to 30.8)	-14.4 (-29.4 to 2.9)	0.0 (0.0 to 0.0)	0.0 (0.0 to 0.0)	0.0 (0.0 to 0.0)
Food-borne trematodiases	7,664.3 (7,037.2 to 8,392.5)	52.2 (47.5 to 57.1)*	19.8 (16.1 to 23.7)*	199.9 (97.6 to 370.7)	50.9 (44.7 to 57.6)*	18.8 (13.9 to 24.1)*
Asymptomatic food-borne trematodiases aggregate	5,881.7 (4,669.9 to 6,903.0)	52.4 (47.7 to 57.3)*	19.9 (16.3 to 23.8)*	0.0 (0.0 to 0.0)	0.0 (0.0 to 0.0)	0.0 (0.0 to 0.0)
Symptomatic food-borne trematodiases aggregate	1,782.6 (981.3 to 3,027.4)	51.6 (46.1 to 57.3)*	19.3 (15.0 to 23.8)*	199.9 (97.6 to 370.7)	50.9 (44.7 to 57.6)*	18.8 (13.9 to 24.1)*
Leprosy	47.6 (44.4 to 51.4)	-3.9 (-5.5 to -2.1)*	-24.3 (-25.6 to -22.9)*	2.9 (1.9 to 4.1)	-2.9 (-10.8 to 5.3)	-23.6 (-29.8 to -17.1)*
Ebola	0.1 (0.1 to 0.3)	66,217.2 (43,239.7 to 208,792.9)*	52,098.7 (34,012.9 to 164,320.8)*	0.0 (0.0 to 0.1)	63,392.1 (36,152.6 to 279,026.3)*	49,875.0 (28,434.7 to 219,601.8)*
Other neglected tropical diseases	1,869.3 (1,825.2 to 1,913.4)	15.2 (12.4 to 18.1)*	-9.3 (-11.6 to -7.0)*	163.2 (60.7 to 434.5)	10.4 (-26.1 to 65.8)	-13.1 (-41.8 to 30.5)
Anemia due to other neglected tropical diseases aggregate	1,869.3 (1,825.2 to 1,913.4)	15.2 (12.4 to 18.1)*	-9.3 (-11.6 to -7.0)*	34.5 (22.5 to 50.7)	-1.8 (-7.8 to 3.6)	-22.7 (-27.4 to -18.4)*
Maternal disorders	500.5 (484.0 to 516.6)	3.7 (0.4 to 7.4)*	-18.3 (-21.0 to -15.5)*	29.8 (20.7 to 40.4)	-11.8 (-18.4 to -4.7)*	-30.6 (-35.7 to -25.0)*
Maternal hemorrhage	22.9 (20.3 to 25.4)	3.7 (-9.8 to 20.8)	-18.4 (-29.0 to -4.9)*	1.0 (0.6 to 1.5)	2.6 (-15.8 to 27.8)	-19.3 (-33.8 to 0.6)
Maternal hemorrhage episodes aggregate	2.2 (1.2 to 3.7)	-0.3 (-38.3 to 61.6)	-21.5 (-51.5 to 27.2)	0.4 (0.2 to 0.7)	0.5 (-33.5 to 55.3)	-20.9 (-47.7 to 22.3)
Anemia due to maternal hemorrhage aggregate	20.7 (18.4 to 22.8)	4.2 (-10.2 to 21.4)	-18.0 (-29.3 to -4.5)*	0.6 (0.4 to 0.9)	3.9 (-16.5 to 29.5)	-18.2 (-34.3 to 2.0)
Maternal sepsis and other maternal infections	390.2 (356.2 to 426.4)	9.5 (6.7 to 12.6)*	-13.8 (-16.0 to -11.4)*	2.4 (1.0 to 4.6)	6.6 (-11.8 to 25.3)	-16.1 (-30.5 to -1.4)*
Maternal sepsis and other maternal infections aggregate	9.4 (4.4 to 16.7)	-5.8 (-51.6 to 84.5)	-25.9 (-61.9 to 45.2)	0.5 (0.2 to 0.9)	-6.1 (-52.7 to 87.0)	-26.1 (-62.8 to 47.2)
Infertility due to puerperal sepsis	380.9 (347.8 to 417.3)	10.0 (8.1 to 12.4)*	-13.4 (-14.9 to -11.5)*	1.9 (0.7 to 4.1)	10.3 (6.4 to 14.6)*	-13.2 (-16.2 to -9.8)*
Maternal hypertensive disorders	40.2 (24.0 to 60.4)	-1.6 (-42.6 to 60.3)	-22.5 (-54.8 to 26.2)	1.9 (1.0 to 3.2)	-1.4 (-39.5 to 58.2)	-22.4 (-52.4 to 24.5)
Maternal hypertensive disorders episodes aggregate	40.3 (24.0 to 60.5)	-1.3 (-42.5 to 60.9)	-22.3 (-54.7 to 26.7)	1.9 (0.9 to 3.1)	-1.3 (-40.6 to 59.6)	-22.3 (-53.3 to 25.7)
Eclampsia	0.0 (0.0 to 0.1)	-13.1 (-73.0 to 159.4)	-31.6 (-78.8 to 104.1)	0.0 (0.0 to 0.1)	-13.0 (-73.0 to 159.4)	-31.5 (-78.8 to 104.1)
Complications of maternal hypertensive disorders aggregate	0.0 (0.0 to 0.0)	0.0 (0.0 to 0.0)	0.0 (0.0 to 0.0)	0.0 (0.0 to 0.0)	0.0 (0.0 to 0.0)	0.0 (0.0 to 0.0)
Maternal obstructed labor and uterine rupture	62.4 (51.9 to 74.7)	-16.7 (-20.3 to -13.2)*	-34.4 (-37.2 to -31.7)*	20.1 (13.1 to 28.7)	-16.3 (-22.5 to -9.6)*	-34.1 (-39.0 to -28.8)*
Obstructed labor, acute event	0.5 (0.3 to 0.8)	-3.5 (-45.5 to 71.4)	-24.1 (-57.1 to 34.9)	0.2 (0.1 to 0.3)	-3.6 (-45.6 to 71.4)	-24.1 (-57.2 to 34.9)
Fistula due to maternal obstructed labor and uterine rupture aggregate	61.9 (51.4 to 74.1)	-16.8 (-20.4 to -13.3)*	-34.5 (-37.3 to -31.8)*	19.9 (13.1 to 28.5)	-16.4 (-22.7 to -9.7)*	-34.2 (-39.2 to -28.9)*
Maternal abortion, miscarriage, and ectopic pregnancy	5.4 (3.1 to 8.6)	-1.5 (-37.3 to 57.9)	-22.5 (-50.7 to 24.3)	0.6 (0.3 to 1.1)	-0.7 (-38.2 to 67.4)	-21.8 (-51.4 to 31.7)
Other maternal disorders	--	--	--	3.8 (2.5 to 5.5)	-5.6 (-18.3 to 8.7)	-25.7 (-35.7 to -14.5)*
Neonatal disorders	2,540.7 (2,425.6 to 2,639.6)	32.2 (28.0 to 36.6)*	4.1 (0.7 to 7.5)*	538.9 (409.3 to 689.0)	31.2 (26.5 to 35.9)*	3.2 (-0.5 to 7.0)
Neonatal preterm birth complications	2,129.0 (1,865.7 to 2,408.2)	28.1 (21.8 to 34.4)*	0.8 (-4.2 to 5.8)	274.2 (208.9 to 352.1)	24.8 (18.1 to 32.0)*	-1.8 (-7.0 to 3.9)
Vision loss due to retinopathy of prematurity aggregate	275.4 (219.2 to 341.2)	22.1 (17.7 to 26.4)*	-3.9 (-7.4 to -0.5)*	9.8 (6.0 to 15.0)	23.5 (14.2 to 33.6)*	-2.8 (-10.1 to 5.2)
Complications of preterm birth complications aggregate	1,853.3 (1,616.2 to 2,118.2)	29.0 (21.8 to 36.3)*	1.6 (-4.1 to 7.3)	264.4 (200.3 to 341.2)	24.9 (17.8 to 32.1)*	-1.7 (-7.3 to 4.0)
Neonatal encephalopathy due to birth asphyxia and trauma	598.7 (424.6 to 887.5)	43.8 (40.7 to 46.8)*	13.2 (10.7 to 15.6)*	169.9 (111.0 to 259.3)	40.5 (35.7 to 45.9)*	10.6 (6.8 to 14.8)*

Cause	Prevalence in thousands (95% confidence interval)			YLDs in thousands (95% confidence interval)		
	2015	Percent change in counts between 2005 and 2015	Percent change in rates between 2005 and 2015	2015	Percent change in counts between 2005 and 2015	Percent change in rates between 2005 and 2015
Neonatal sepsis and other neonatal infections	0.0 (0.0 to 0.0)	0.0 (0.0 to 0.0)	0.0 (0.0 to 0.0)	0.0 (0.0 to 0.0)	0.0 (0.0 to 0.0)	0.0 (0.0 to 0.0)
Severe infection due to neonatal sepsis and other neonatal infections	0.0 (0.0 to 0.0)	0.0 (0.0 to 0.0)	0.0 (0.0 to 0.0)	0.0 (0.0 to 0.0)	0.0 (0.0 to 0.0)	0.0 (0.0 to 0.0)
Complications of neonatal sepsis and other neonatal infections aggregate	0.0 (0.0 to 0.0)	0.0 (0.0 to 0.0)	0.0 (0.0 to 0.0)	0.0 (0.0 to 0.0)	0.0 (0.0 to 0.0)	0.0 (0.0 to 0.0)
Hemolytic disease and other neonatal jaundice	102.1 (91.1 to 114.5)	39.8 (38.0 to 41.6)*	10.1 (8.6 to 11.5)*	30.0 (22.3 to 38.4)	35.6 (29.2 to 42.6)*	6.7 (1.7 to 12.2)*
Other neonatal disorders	--	--	--	64.7 (45.9 to 84.2)	34.5 (18.3 to 51.8)*	5.9 (-6.9 to 19.5)
Nutritional deficiencies	80,463.6 (79,421.0 to 81,597.6)	15.1 (13.1 to 17.2)*	-9.4 (-10.9 to -7.8)*	2,116.8 (1,401.6 to 3,070.4)	5.4 (2.7 to 8.2)*	-17.0 (-19.2 to -14.9)*
Protein-energy malnutrition	9.6 (6.0 to 15.5)	-8.5 (-18.7 to 3.4)	-27.9 (-36.0 to -18.6)*	1.2 (0.6 to 2.1)	-7.1 (-20.6 to 9.1)	-26.9 (-37.5 to -14.1)*
Iodine deficiency	7,849.5 (7,123.0 to 8,821.5)	17.8 (15.4 to 20.4)*	-7.2 (-9.1 to -5.2)*	154.3 (98.2 to 235.8)	18.1 (15.6 to 20.5)*	-7.0 (-9.0 to -5.1)*
Visible goiter due to iodine deficiency aggregate	7,748.7 (7,010.1 to 8,706.2)	17.9 (15.4 to 20.5)*	-7.2 (-9.2 to -5.2)*	137.1 (85.3 to 215.0)	18.2 (15.6 to 20.9)*	-7.0 (-9.0 to -4.8)*
Visible goiter with heart failure due to iodine deficiency aggregate	0.0 (0.0 to 0.0)	28.7 (26.8 to 30.7)*	1.3 (-0.2 to 2.9)	0.0 (0.0 to 0.0)	28.7 (26.8 to 30.7)*	1.3 (-0.2 to 2.9)
Intellectual disability due to iodine deficiency aggregate	100.7 (40.8 to 133.7)	16.5 (9.9 to 20.2)*	-8.3 (-13.5 to -5.4)*	17.2 (6.0 to 27.6)	17.3 (7.6 to 25.3)*	-7.7 (-15.3 to -1.4)*
Vitamin A deficiency	180.6 (117.1 to 254.4)	21.4 (13.8 to 29.7)*	-4.4 (-10.5 to 2.1)	11.4 (6.7 to 17.3)	24.0 (15.1 to 34.0)*	-2.4 (-9.4 to 5.5)
Iron-deficiency anemia	78,276.5 (77,136.4 to 79,493.3)	13.2 (11.4 to 15.1)*	-10.9 (-12.3 to -9.4)*	1,949.7 (1,286.6 to 2,824.3)	4.5 (1.5 to 7.5)*	-17.8 (-20.1 to -15.4)*
Iron-deficiency anemia without heart failure aggregate	78,275.3 (77,134.9 to 79,491.9)	13.2 (11.4 to 15.1)*	-10.9 (-12.3 to -9.4)*	1,949.6 (1,286.5 to 2,824.1)	4.5 (1.5 to 7.5)*	-17.8 (-20.1 to -15.4)*
Iron-deficiency anemia with heart failure aggregate	1.3 (1.1 to 1.5)	32.6 (28.4 to 36.6)*	4.4 (1.1 to 7.5)*	0.2 (0.1 to 0.2)	32.6 (28.4 to 36.6)*	4.4 (1.1 to 7.5)*
Other nutritional deficiencies	--	--	--	0.1 (0.1 to 0.2)	-4.2 (-22.8 to 18.2)	-24.6 (-39.2 to -6.9)*
Other communicable, maternal, neonatal, and nutritional diseases	137,220.0 (133,136.8 to 139,769.0)	25.0 (23.9 to 26.2)*	-1.6 (-2.5 to -0.7)*	178.3 (111.9 to 274.6)	17.7 (10.3 to 25.3)*	-7.3 (-13.2 to -1.4)*
Sexually transmitted diseases excluding HIV	108,806.5 (104,775.8 to 112,239.3)	23.5 (22.0 to 25.0)*	-2.8 (-4.0 to -1.6)*	108.5 (66.1 to 176.1)	21.7 (17.4 to 25.6)*	-4.2 (-7.6 to -1.2)*
Syphilis	4,360.5 (3,017.4 to 6,233.1)	7.2 (-4.3 to 15.5)	-15.6 (-24.7 to -9.1)*	27.1 (18.2 to 37.6)	10.9 (5.5 to 16.9)*	-12.7 (-17.0 to -8.0)*
Early syphilis aggregate	4,221.7 (2,881.1 to 6,101.9)	7.1 (-4.9 to 15.6)	-15.7 (-25.1 to -9.0)*	1.0 (0.3 to 2.7)	7.2 (-5.7 to 16.8)	-15.6 (-25.8 to -8.1)*
Adult tertiary syphilis	138.8 (121.4 to 158.2)	10.7 (7.5 to 13.8)*	-12.8 (-15.4 to -10.5)*	26.0 (17.0 to 36.4)	11.0 (5.3 to 17.3)*	-12.6 (-17.1 to -7.6)*
Chlamydial infection	1,400.2 (1,010.5 to 1,896.0)	33.7 (30.0 to 37.5)*	5.2 (2.3 to 8.2)*	9.5 (5.6 to 15.1)	27.7 (18.8 to 35.8)*	0.5 (-6.5 to 6.9)
Chlamydial infection episodes aggregate	1,098.2 (722.1 to 1,602.6)	36.4 (32.1 to 41.2)*	7.4 (3.9 to 11.1)*	5.7 (3.0 to 9.7)	37.6 (24.2 to 51.0)*	8.3 (-2.2 to 18.9)
Pelvic inflammatory diseases due to chlamydial infection aggregate	16.8 (12.6 to 21.6)	8.3 (4.6 to 12.2)*	-14.7 (-17.7 to -11.7)*	2.2 (1.4 to 3.3)	8.7 (-2.1 to 21.0)	-14.4 (-23.0 to -4.8)*
Infertility due to clamydial infection aggregate	285.2 (205.2 to 352.1)	25.7 (21.2 to 31.3)*	-1.1 (-4.6 to 3.4)	1.6 (0.6 to 3.3)	26.3 (20.1 to 33.5)*	-0.6 (-5.4 to 5.1)
Gonococcal infection	2,034.9 (1,143.5 to 3,504.7)	30.1 (14.4 to 41.1)*	2.4 (-9.9 to 11.1)	20.3 (9.7 to 37.6)	29.9 (15.2 to 40.6)*	2.2 (-9.3 to 10.7)
Gonococcal infection episodes aggregate	1,880.6 (1,004.2 to 3,344.3)	30.8 (13.5 to 42.3)*	3.0 (-10.6 to 12.0)	18.9 (8.6 to 36.4)	30.8 (14.8 to 42.4)*	3.0 (-9.6 to 12.1)
Pelvic inflammatory diseases due to gonococcal infection aggregate	154.3 (112.6 to 194.4)	22.5 (16.7 to 27.5)*	-3.6 (-8.2 to 0.3)	1.4 (0.8 to 2.3)	17.7 (11.8 to 24.2)*	-7.4 (-12.0 to -2.3)*
Trichomoniasis	15,833.8 (10,658.3 to 22,507.0)	26.1 (25.1 to 26.9)*	-0.8 (-1.6 to -0.1)*	17.3 (6.4 to 37.6)	25.5 (23.7 to 27.4)*	-1.2 (-2.7 to 0.3)
Genital herpes	91,097.3 (79,833.0 to 103,177.4)	23.4 (21.6 to 25.1)*	-2.9 (-4.3 to -1.5)*	23.1 (6.9 to 56.2)	26.3 (23.2 to 33.8)*	-0.6 (-3.0 to 5.3)
Moderate infection due to initial genital herpes episode	14.4 (3.4 to 33.3)	325.8 (240.5 to 429.4)*	235.2 (168.0 to 316.7)*	0.7 (0.1 to 1.7)	314.6 (224.5 to 461.2)*	226.3 (155.4 to 341.7)*
Complications of genital herpes aggregate	91,082.9 (79,827.3 to 103,160.6)	23.4 (21.6 to 25.1)*	-2.9 (-4.3 to -1.6)*	22.5 (6.4 to 55.2)	23.6 (21.5 to 25.8)*	-2.7 (-4.4 to -1.0)*
Other sexually transmitted diseases	979.9 (731.0 to 1,169.5)	26.5 (22.3 to 32.1)*	-0.4 (-3.8 to 4.0)	11.2 (6.8 to 17.7)	17.0 (10.2 to 23.1)*	-7.9 (-13.3 to -3.1)*
Pelvic inflammatory diseases due to other sexually transmitted diseases aggregate	46.2 (34.8 to 58.7)	8.8 (6.6 to 10.7)*	-14.4 (-16.1 to -12.9)*	6.0 (3.7 to 8.9)	9.1 (1.3 to 16.9)*	-14.2 (-20.2 to -8.0)*
Infertility due to other sexually transmitted diseases aggregate	933.7 (681.4 to 1,125.6)	27.6 (23.0 to 33.5)*	0.4 (-3.2 to 5.1)	5.1 (2.0 to 10.8)	28.0 (23.3 to 34.7)*	0.8 (-2.9 to 6.0)
Other sexually transmitted diseases	--	--	--	10.7 (5.6 to 19.7)	32.1 (18.9 to 42.3)*	3.9 (-6.4 to 12.0)
Hepatitis	35,093.4 (34,649.6 to 35,713.5)	31.4 (30.3 to 32.6)*	3.4 (2.6 to 4.3)*	23.7 (14.2 to 37.3)	30.2 (-14.8 to 96.2)	2.4 (-32.9 to 54.4)
Acute hepatitis A	142.1 (115.9 to 166.5)	41.1 (9.4 to 77.7)*	11.1 (-13.9 to 39.9)	5.9 (3.6 to 8.9)	41.5 (6.9 to 81.7)*	11.3 (-15.9 to 43.0)
Hepatitis B	24,902.1 (23,970.9 to 25,887.3)	33.2 (30.4 to 35.9)*	4.9 (2.6 to 7.0)*	16.1 (8.6 to 28.0)	26.7 (-29.6 to 131.3)	-0.3 (-44.6 to 82.0)
Acute Hepatitis B aggregate	700.0 (455.6 to 1,098.1)	26.3 (-30.1 to 132.2)	-0.6 (-45.0 to 82.8)	16.1 (8.6 to 28.0)	26.7 (-29.6 to 131.3)	-0.3 (-44.6 to 82.0)
Chronic hepatitis B	24,202.1 (23,280.3 to 25,121.6)	33.4 (32.3 to 34.7)*	5.0 (4.1 to 6.0)*	0.0 (0.0 to 0.0)	0.0 (0.0 to 0.0)	0.0 (0.0 to 0.0)
Hepatitis C	10,829.2 (9,601.3 to 12,104.0)	27.4 (25.5 to 29.1)*	0.2 (-1.2 to 1.6)	0.5 (0.2 to 1.0)	25.6 (16.4 to 32.7)*	-1.2 (-8.4 to 4.4)
Acute Hepatitis C aggregate	35.6 (25.2 to 46.8)	25.7 (17.5 to 31.3)*	-1.1 (-7.5 to 3.3)	0.5 (0.2 to 1.0)	25.6 (16.4 to 32.7)*	-1.2 (-8.4 to 4.4)
Chronic hepatitis C	10,793.6 (9,561.5 to 12,067.5)	27.4 (25.5 to 29.1)*	0.3 (-1.2 to 1.6)	0.0 (0.0 to 0.0)	0.0 (0.0 to 0.0)	0.0 (0.0 to 0.0)
Acute hepatitis E	37.6 (26.1 to 52.4)	28.4 (25.3 to 32.0)*	1.1 (-1.4 to 3.9)	1.2 (0.6 to 2.0)	28.7 (2.7 to 57.6)*	1.3 (-19.2 to 24.1)
Other infectious diseases	2,169.4 (2,125.2 to 2,213.3)	13.8 (11.0 to 16.8)*	-10.4 (-12.6 to -8.1)*	46.1 (29.4 to 69.4)	4.6 (-2.0 to 10.8)	-17.7 (-22.9 to -12.8)*
Non-communicable diseases	441,093.0 (439,936.1 to 442,389.5)	26.9 (26.8 to 27.0)*	-0.1 (-0.2 to -0.0)*	51,527.9 (38,793.7 to 66,137.1)	25.9 (25.1 to 26.7)*	-0.9 (-1.5 to -0.3)*
Neoplasms	6,405.0 (6,213.0 to 6,603.4)	33.9 (29.2 to 38.8)*	5.4 (1.7 to 9.3)*	576.8 (410.7 to 757.5)	27.8 (20.5 to 35.7)*	0.6 (-5.1 to 6.8)
Lip and oral cavity cancer	213.1 (184.7 to 251.4)	31.0 (13.3 to 52.2)*	3.1 (-10.8 to 19.8)	18.0 (12.3 to 24.5)	30.3 (11.9 to 53.0)*	2.5 (-11.9 to 20.4)
Nasopharynx cancer	94.0 (58.6 to 153.2)	31.2 (-16.9 to 104.4)	3.2 (-34.6 to 60.9)	8.7 (4.7 to 14.9)	29.8 (-16.6 to 99.2)	2.1 (-34.4 to 56.8)
Other pharynx cancer	81.8 (72.1 to 92.0)	14.7 (2.5 to 27.6)*	-9.7 (-19.3 to 0.4)	6.8 (4.7 to 9.1)	13.4 (0.8 to 27.0)*	-10.7 (-20.6 to -0.0)*
Esophageal cancer	35.8 (27.7 to 51.4)	5.9 (-19.5 to 43.2)	-16.6 (-36.7 to 12.7)	6.7 (4.3 to 10.0)	0.2 (-22.8 to 32.5)	-21.1 (-39.2 to 4.3)
Stomach cancer	172.2 (158.2 to 189.2)	0.6 (-8.3 to 10.9)	-20.8 (-27.8 to -12.7)*	19.8 (14.2 to 25.8)	-1.3 (-11.5 to 10.5)	-22.3 (-30.4 to -13.0)*
Colon and rectum cancer	475.0 (422.0 to 534.2)	40.9 (27.5 to 55.8)*	10.9 (0.4 to 22.6)*	38.6 (27.2 to 52.5)	38.5 (25.0 to 54.2)*	9.0 (-1.6 to 21.3)
Colon and rectum cancer aggregate	474.0 (421.1 to 533.2)	41.0 (27.5 to 55.9)*	11.0 (0.4 to 22.7)*	38.5 (27.1 to 52.3)	38.6 (25.0 to 54.3)*	9.1 (-1.6 to 21.4)
Stoma due to colon and rectum cancer	1.0 (0.9 to 1.1)	21.8 (19.9 to 23.7)*	-4.1 (-5.6 to -2.6)*	0.1 (0.1 to 0.2)	21.8 (19.9 to 23.7)*	-4.1 (-5.6 to -2.6)*
Liver cancer	25.0 (18.0 to 32.9)	38.8 (-1.8 to 94.2)	9.2 (-22.7 to 52.9)	13.9 (8.9 to 24.5)	22.3 (-5.3 to 84.3)	-3.7 (-25.5 to 45.1)
Liver cancer due to hepatitis B	33.6 (23.0 to 69.8)	26.6 (-12.7 to 122.8)	-0.3 (-31.3 to 75.4)	6.7 (4.1 to 12.7)	17.6 (-13.7 to 87.3)	-7.4 (-32.1 to 47.5)
Liver cancer due to hepatitis C	7.6 (5.8 to 10.4)	50.6 (18.0 to 93.5)*	18.5 (-7.1 to 52.3)	1.5 (1.0 to 2.2)	32.6 (11.3 to 66.4)*	4.4 (-12.4 to 30.9)
Liver cancer due to alcohol use	16.0 (11.3 to 31.1)	50.5 (8.7 to 145.1)*	18.4 (-14.5 to 92.9)	3.5 (2.2 to 6.6)	37.5 (5.5 to 114.9)*	8.2 (-16.9 to 69.1)
Liver cancer due to other causes	10.2 (7.2 to 18.2)	19.7 (-20.3 to 91.9)	-5.8 (-37.3 to 51.1)	2.2 (1.3 to 3.7)	10.2 (-17.9 to 61.9)	-13.3 (-35.4 to 27.4)
Gallbladder and biliary tract cancer	4.9 (4.3 to 5.4)	6.2 (-2.9 to 17.1)	-16.4 (-23.6 to -7.8)*	1.4 (1.0 to 1.9)	4.8 (-3.9 to 15.6)	-17.5 (-24.3 to -9.0)*
Pancreatic cancer	14.3 (12.9 to 15.8)	30.2 (20.0 to 42.1)*	2.4 (-5.5 to 11.8)	3.5 (2.4 to 4.7)	26.3 (16.8 to 36.7)*	-0.6 (-8.0 to 7.6)

Cause	Prevalence in thousands (95% confidence interval)			YLDs in thousands (95% confidence interval)		
	2015	Percent change in counts between 2005 and 2015	Percent change in rates between 2005 and 2015	2015	Percent change in counts between 2005 and 2015	Percent change in rates between 2005 and 2015
Larynx cancer	112.4 (101.3 to 128.4)	18.4 (8.5 to 33.0)*	-6.8 (-14.6 to 4.7)	12.5 (9.0 to 16.8)	17.2 (7.2 to 30.4)*	-7.8 (-15.6 to 2.6)
Larynx cancer aggregate	39.3 (28.5 to 55.0)	6.0 (-18.6 to 43.6)	-16.6 (-35.9 to 13.1)	5.8 (3.9 to 8.2)	7.9 (-9.5 to 33.7)	-15.1 (-28.8 to 5.3)
Laryngectomy due to larynx cancer	73.1 (70.6 to 76.1)	26.4 (24.6 to 27.6)*	-0.5 (-1.9 to 0.5)	6.7 (4.4 to 9.7)	26.6 (18.3 to 36.5)*	-0.3 (-6.9 to 7.5)
Tracheal, bronchus, and lung cancer	148.3 (123.8 to 194.2)	25.5 (9.3 to 51.5)*	-1.2 (-13.9 to 19.2)	24.5 (17.2 to 35.0)	21.8 (5.2 to 45.8)*	-4.1 (-17.2 to 14.8)
Malignant skin melanoma	254.3 (214.0 to 339.8)	37.7 (26.4 to 49.2)*	8.4 (-0.5 to 17.5)	14.5 (9.4 to 21.7)	35.9 (24.0 to 48.6)*	7.0 (-2.4 to 17.0)
Non-melanoma skin cancer	110.3 (102.4 to 119.5)	52.3 (37.9 to 67.5)*	19.9 (8.5 to 31.9)*	6.0 (3.9 to 8.6)	61.4 (42.8 to 82.9)*	27.0 (12.4 to 44.0)*
Non-melanoma skin cancer(squamous-cell carcinoma)	97.5 (83.5 to 115.8)	70.7 (52.2 to 93.6)*	34.4 (19.8 to 52.4)*	5.7 (3.7 to 8.2)	65.5 (44.7 to 88.5)*	30.2 (13.9 to 48.3)*
Non-melanoma skin cancer(basal-cell carcinoma)	26.3 (21.0 to 33.1)	7.8 (3.5 to 11.6)*	-15.2 (-18.5 to -12.1)*	0.3 (0.1 to 0.5)	8.1 (-1.3 to 17.1)	-14.9 (-22.3 to -7.9)*
Breast cancer	2,543.0 (2,360.0 to 2,724.6)	37.2 (24.4 to 49.5)*	8.0 (-2.1 to 17.7)	193.6 (135.0 to 259.3)	31.1 (20.3 to 42.1)*	3.2 (-5.3 to 11.8)
Breast cancer aggregate	1,874.1 (1,683.8 to 2,052.1)	45.2 (26.6 to 63.6)*	14.3 (-0.3 to 28.8)	141.3 (100.5 to 188.4)	36.3 (20.4 to 52.4)*	7.3 (-5.2 to 20.0)
Mastectomy due to breast cancer	668.9 (645.0 to 690.7)	18.7 (17.5 to 19.9)*	-6.5 (-7.5 to -5.6)*	52.3 (33.4 to 75.8)	18.9 (15.9 to 22.0)*	-6.4 (-8.8 to -4.0)*
Cervical cancer	484.7 (412.3 to 577.1)	3.2 (-11.7 to 21.9)	-18.8 (-30.5 to -4.1)*	36.4 (24.5 to 49.4)	3.5 (-11.4 to 22.9)	-18.5 (-30.3 to -3.3)*
Uterine cancer	365.9 (248.0 to 513.8)	37.2 (0.8 to 86.3)*	8.0 (-20.7 to 46.7)	23.5 (13.5 to 37.4)	36.0 (0.7 to 86.8)*	7.1 (-20.7 to 47.0)
Ovarian cancer	117.5 (101.4 to 145.2)	26.8 (12.3 to 46.0)*	-0.2 (-11.6 to 14.9)	14.8 (10.4 to 19.8)	24.9 (7.3 to 46.2)*	-1.7 (-15.6 to 15.1)
Prostate cancer	187.7 (150.0 to 239.5)	40.7 (29.4 to 53.6)*	10.7 (1.9 to 20.9)*	13.7 (9.2 to 19.4)	33.5 (22.2 to 46.6)*	5.1 (-3.8 to 15.4)
Prostate cancer aggregate	162.1 (124.7 to 214.1)	46.6 (32.8 to 62.6)*	15.4 (4.5 to 28.0)*	11.4 (7.5 to 16.5)	38.9 (24.6 to 55.7)*	9.3 (-1.9 to 22.5)
Impotence and incontinence due to prostate cancer aggregate	25.6 (24.9 to 26.3)	11.9 (11.3 to 12.5)*	-11.9 (-12.4 to -11.5)*	2.3 (1.5 to 3.3)	12.4 (4.8 to 21.0)*	-11.5 (-17.5 to -4.7)*
Testicular cancer	58.0 (47.6 to 68.8)	57.3 (30.4 to 87.5)*	23.8 (2.6 to 47.6)*	3.5 (2.3 to 5.0)	53.8 (25.7 to 83.0)*	21.1 (-1.1 to 44.1)
Kidney cancer	170.0 (152.3 to 191.1)	36.3 (23.5 to 51.9)*	7.3 (-2.8 to 19.5)	12.2 (8.4 to 16.2)	34.6 (20.5 to 51.1)*	5.9 (-5.2 to 19.0)
Bladder cancer	133.0 (110.5 to 162.2)	30.8 (12.4 to 54.6)*	2.9 (-11.6 to 21.7)	12.0 (8.2 to 16.4)	26.6 (11.9 to 45.5)*	-0.4 (-11.9 to 14.5)
Bladder cancer episodes aggregate	118.9 (96.3 to 147.9)	32.1 (11.4 to 59.1)*	4.0 (-12.3 to 25.2)	9.4 (6.4 to 13.1)	28.5 (10.3 to 53.0)*	1.1 (-13.2 to 20.4)
Urinary incontinence due to bladder cancer	14.1 (13.3 to 15.0)	20.2 (18.9 to 21.4)*	-5.4 (-6.4 to -4.5)*	2.5 (1.7 to 3.5)	20.1 (12.2 to 29.3)*	-5.5 (-11.7 to 1.8)
Brain and nervous system cancer	83.7 (69.8 to 104.5)	34.5 (15.4 to 62.2)*	5.9 (-9.2 to 27.6)	9.3 (6.2 to 13.0)	32.4 (13.4 to 60.5)*	4.2 (-10.7 to 26.3)
Thyroid cancer	223.9 (196.1 to 251.2)	28.9 (13.4 to 45.5)*	1.5 (-10.8 to 14.5)	12.6 (8.4 to 17.9)	28.1 (12.4 to 46.1)*	0.8 (-11.6 to 15.0)
Mesothelioma	2.4 (2.2 to 2.8)	21.1 (8.6 to 34.9)*	-4.7 (-14.5 to 6.2)	0.5 (0.3 to 0.6)	20.3 (7.7 to 34.5)*	-5.3 (-15.2 to 5.9)
Hodgkin lymphoma	42.5 (35.4 to 52.8)	28.2 (8.8 to 50.9)*	0.9 (-14.4 to 18.8)	3.5 (2.4 to 4.9)	22.8 (3.2 to 45.6)*	-3.4 (-18.8 to 14.6)
Non-Hodgkin lymphoma	278.1 (238.1 to 322.5)	64.8 (44.1 to 87.2)*	29.7 (13.4 to 47.4)*	20.5 (13.9 to 27.6)	60.2 (38.9 to 83.1)*	26.1 (9.3 to 44.1)*
Multiple myeloma	24.7 (20.4 to 29.8)	47.7 (24.7 to 73.7)*	16.2 (-1.9 to 36.7)	5.5 (3.7 to 7.5)	43.4 (21.0 to 68.2)*	12.8 (-4.7 to 32.4)
Leukemia	107.3 (98.1 to 117.4)	50.5 (34.8 to 68.2)*	18.5 (6.1 to 32.4)*	21.2 (15.0 to 27.8)	37.5 (23.9 to 54.2)*	8.2 (-2.5 to 21.4)
Acute lymphoid leukemia	37.1 (30.1 to 45.4)	44.5 (23.9 to 70.2)*	13.7 (-2.5 to 34.0)	4.9 (3.3 to 6.8)	41.0 (19.6 to 67.7)*	11.0 (-5.9 to 32.0)
Chronic lymphoid leukemia	52.9 (42.9 to 66.5)	50.7 (25.5 to 86.0)*	18.6 (-1.2 to 46.4)	6.7 (4.5 to 9.2)	45.8 (21.2 to 79.1)*	14.8 (-4.6 to 41.0)
Acute myeloid leukemia	53.1 (45.4 to 62.2)	42.7 (27.6 to 61.3)*	12.3 (0.5 to 27.0)*	6.7 (4.6 to 9.0)	39.2 (24.1 to 57.8)*	9.5 (-2.3 to 24.2)
Chronic myeloid leukemia	22.5 (18.5 to 27.8)	18.7 (4.0 to 37.0)*	-6.6 (-18.1 to 7.8)	2.9 (2.0 to 4.0)	14.8 (0.2 to 31.5)*	-9.7 (-21.1 to 3.5)
Other neoplasms	267.8 (220.8 to 340.3)	36.1 (20.7 to 57.6)*	7.2 (-5.0 to 24.1)	19.2 (12.7 to 28.0)	34.4 (18.9 to 55.6)*	5.8 (-6.4 to 22.5)
Cardiovascular diseases	23,589.2 (22,801.0 to 24,317.2)	22.4 (20.6 to 23.8)*	-3.7 (-5.1 to -2.6)*	1,399.1 (994.7 to 1,873.2)	23.5 (20.9 to 25.1)*	-2.8 (-4.8 to -1.5)*
Rheumatic heart disease	2,700.4 (2,379.4 to 3,620.3)	5.5 (-3.5 to 11.6)	-16.9 (-24.0 to -12.2)*	133.0 (83.5 to 206.6)	6.9 (-2.3 to 13.0)	-15.9 (-23.1 to -11.1)*
Rheumatic heart disease, without heart failure	2,599.7 (2,273.4 to 3,519.4)	4.7 (-4.3 to 10.9)	-17.6 (-24.6 to -12.7)*	121.4 (74.8 to 192.9)	5.0 (-4.2 to 11.4)	-17.4 (-24.6 to -12.3)*
Heart failure fue to rheumatic heart disease aggregate	100.8 (89.7 to 112.4)	32.4 (30.6 to 34.1)*	4.2 (2.8 to 5.5)*	11.7 (8.0 to 16.2)	32.6 (23.7 to 41.8)*	4.4 (-2.6 to 11.6)
Ischemic heart disease	6,468.3 (5,352.9 to 7,804.6)	20.1 (18.2 to 21.9)*	-5.4 (-7.0 to -4.0)*	444.3 (291.4 to 650.7)	25.1 (23.1 to 27.0)*	-1.5 (-3.1 to -0.0)*
Myocardial infarction episodes aggregate	525.6 (424.2 to 647.4)	-14.5 (-22.4 to -7.1)*	-32.7 (-38.9 to -26.9)*	1.6 (1.0 to 2.4)	7.9 (-2.3 to 17.8)	-15.0 (-23.1 to -7.3)*
Angina due to ischemic heart disease aggregate	5,179.4 (4,059.3 to 6,505.8)	24.0 (22.1 to 25.7)*	-2.4 (-3.9 to -1.0)*	354.5 (222.5 to 529.1)	24.3 (21.8 to 26.6)*	-2.1 (-4.1 to -0.3)*
Heart failure fue to ischemic heart disease aggregate	763.3 (703.4 to 827.5)	28.7 (27.9 to 29.5)*	1.3 (0.7 to 2.0)*	88.3 (60.4 to 123.2)	28.8 (25.7 to 32.2)*	1.4 (-1.0 to 4.0)
Cerebrovascular disease	2,557.5 (2,523.9 to 2,596.7)	23.6 (21.8 to 25.4)*	-2.7 (-4.1 to -1.3)*	395.4 (273.6 to 535.7)	23.8 (21.0 to 26.5)*	-2.6 (-4.8 to -0.4)*
Ischemic stroke	1,099.7 (1,063.7 to 1,134.6)	24.2 (23.2 to 25.0)*	-2.2 (-3.0 to -1.6)*	168.3 (116.6 to 230.1)	24.6 (20.6 to 29.1)*	-2.0 (-5.1 to 1.6)
Chronic ischemic stroke aggregate	1,091.1 (1,055.0 to 1,125.3)	24.3 (23.3 to 25.2)*	-2.2 (-3.0 to -1.5)*	166.4 (115.3 to 227.6)	24.7 (20.7 to 29.3)*	-1.8 (-5.0 to 1.8)
Ischemic stroke episodes aggregate	8.6 (6.9 to 10.7)	13.7 (6.2 to 21.0)*	-10.5 (-16.4 to -4.7)*	1.9 (1.2 to 2.7)	13.7 (6.2 to 21.0)*	-10.5 (-16.4 to -4.7)*
Hemorrhagic stroke	1,486.7 (1,438.6 to 1,533.6)	23.0 (22.0 to 23.8)*	-3.2 (-4.0 to -2.5)*	227.1 (157.8 to 309.5)	23.2 (19.6 to 26.4)*	-3.0 (-5.9 to -0.5)*
Chronic hemorrhagic stroke aggregate	1,474.5 (1,426.2 to 1,521.2)	23.0 (22.0 to 23.9)*	-3.2 (-3.9 to -2.4)*	224.5 (155.9 to 306.6)	23.3 (19.7 to 26.6)*	-2.9 (-5.8 to -0.3)*
Acute hemorrhagic stroke aggregate	12.2 (10.0 to 14.6)	13.8 (6.3 to 20.8)*	-10.5 (-16.3 to -4.9)*	2.7 (1.7 to 3.7)	13.8 (6.3 to 20.8)*	-10.4 (-16.3 to -4.9)*
Hypertensive heart disease	237.4 (212.5 to 263.9)	34.5 (33.2 to 35.7)*	5.8 (4.9 to 6.8)*	27.4 (18.5 to 38.0)	34.9 (29.3 to 41.3)*	6.2 (1.8 to 11.2)*
Cardiomyopathy and myocarditis	134.1 (119.4 to 150.8)	20.1 (18.7 to 21.4)*	-5.5 (-6.6 to -4.4)*	14.9 (10.1 to 21.0)	19.8 (13.9 to 26.4)*	-5.7 (-10.4 to -0.5)*
Acute myocarditis	10.6 (7.3 to 14.7)	30.4 (26.6 to 34.2)*	2.6 (-0.3 to 5.7)	0.5 (0.3 to 0.9)	30.4 (26.6 to 34.5)*	2.6 (-0.4 to 5.9)
Heart failure due to cardiomyopathy aggregate	123.4 (109.3 to 139.4)	19.3 (17.8 to 20.7)*	-6.1 (-7.3 to -5.0)*	14.3 (9.7 to 20.3)	19.4 (13.3 to 26.2)*	-6.0 (-10.8 to -0.6)*
Atrial fibrillation and flutter	1,291.5 (1,031.7 to 1,599.4)	22.6 (20.8 to 24.2)*	-3.5 (-4.9 to -2.2)*	107.6 (69.0 to 156.2)	22.8 (19.0 to 26.7)*	-3.3 (-6.3 to -0.2)*
Peripheral vascular disease	6,706.7 (5,437.1 to 8,317.6)	27.3 (26.5 to 28.2)*	0.2 (-0.5 to 0.9)	1.2 (0.5 to 2.6)	21.7 (13.5 to 29.8)*	-4.2 (-10.7 to 2.2)
Endocarditis	2.6 (2.2 to 2.9)	24.3 (20.4 to 27.8)*	-2.2 (-5.2 to 0.6)	0.3 (0.2 to 0.4)	24.2 (20.5 to 27.6)*	-2.2 (-5.2 to 0.5)
Endocarditis episodes aggregate	1.0 (0.8 to 1.2)	24.2 (17.0 to 31.0)*	-2.3 (-7.9 to 3.1)	0.1 (0.0 to 0.1)	23.7 (16.6 to 30.4)*	-2.7 (-8.3 to 2.6)
Heart failure due to endocarditis aggregate	1.6 (1.3 to 1.9)	24.4 (20.1 to 28.7)*	-2.1 (-5.5 to 1.3)	0.2 (0.1 to 0.3)	24.4 (20.0 to 28.7)*	-2.1 (-5.5 to 1.3)
Other cardiovascular and circulatory diseases	4,019.0 (3,517.3 to 4,637.0)	29.3 (27.7 to 30.9)*	1.8 (0.5 to 3.0)*	274.8 (183.9 to 382.3)	29.6 (27.3 to 32.0)*	2.0 (0.2 to 3.9)*
Heart failure due to other cardiovascular diseases aggregate	25.1 (21.8 to 28.9)	29.1 (27.5 to 30.8)*	1.6 (0.4 to 3.0)*	3.0 (2.0 to 4.3)	29.5 (20.9 to 39.1)*	1.9 (-4.9 to 9.5)
Other cardiovascular diseases episodes aggregate	3,993.9 (3,495.6 to 4,607.9)	29.3 (27.7 to 30.9)*	1.8 (0.5 to 3.0)*	271.8 (181.9 to 378.2)	29.6 (27.2 to 32.0)*	2.0 (0.1 to 3.9)*
Chronic respiratory diseases	28,221.7 (27,164.0 to 29,331.4)	19.7 (17.8 to 21.5)*	-5.8 (-7.3 to -4.3)*	1,749.1 (1,348.5 to 2,162.2)	17.8 (15.1 to 20.4)*	-7.3 (-9.4 to -5.2)*
Chronic obstructive pulmonary disease	11,690.7 (10,019.3 to 13,556.3)	17.8 (15.3 to 20.3)*	-7.3 (-9.3 to -5.3)*	817.6 (659.9 to 993.2)	17.7 (13.5 to 21.8)*	-7.3 (-10.7 to -4.1)*

Cause	Prevalence in thousands (95% confidence interval)			YLDs in thousands (95% confidence interval)		
	2015	Percent change in counts between 2005 and 2015	Percent change in rates between 2005 and 2015	2015	Percent change in counts between 2005 and 2015	Percent change in rates between 2005 and 2015
Chronic obstructive pulmonary disease without heart failure aggregate	11,637.8<br>(9,968.5 to 13,508.2)	17.7<br>(15.2 to 20.2)*	-7.3<br>(-9.3 to -5.4)*	790.9<br>(638.4 to 963.9)	17.1<br>(12.9 to 21.3)*	-7.8<br>(-11.2 to -4.5)*
Severe chronic obstructive pulmonary disease with heart failure aggregate	59.2<br>(52.1 to 66.8)	38.7<br>(37.2 to 40.2)*	9.1<br>(8.0 to 10.4)*	26.7<br>(18.8 to 35.2)	38.5<br>(29.5 to 48.7)*	9.0<br>(1.9 to 17.0)*
Pneumoconiosis	192.1<br>(182.7 to 201.5)	39.7<br>(32.9 to 47.0)*	10.0<br>(4.6 to 15.7)*	37.2<br>(23.7 to 53.6)	35.8<br>(28.3 to 43.4)*	6.9<br>(1.0 to 12.9)*
Silicosis	42.4<br>(36.7 to 48.1)	31.1<br>(28.2 to 34.1)*	3.2<br>(0.9 to 5.5)*	6.8<br>(4.3 to 10.0)	30.9<br>(17.2 to 45.3)*	3.0<br>(-7.7 to 14.4)
Silicosis without heart failure aggregate	42.4<br>(36.7 to 48.1)	31.1<br>(28.2 to 34.1)*	3.2<br>(0.9 to 5.5)*	6.8<br>(4.3 to 10.0)	30.9<br>(17.2 to 45.3)*	3.0<br>(-7.7 to 14.4)
Severe silicosis with heart failure aggregate	0.0<br>(0.0 to 0.0)	0.0<br>(0.0 to 0.0)	0.0<br>(0.0 to 0.0)	0.0<br>(0.0 to 0.0)	0.0<br>(0.0 to 0.0)	0.0<br>(0.0 to 0.0)
Asbestosis	8.9<br>(7.7 to 10.4)	22.7<br>(19.7 to 25.6)*	-3.5<br>(-5.8 to -1.2)*	1.5<br>(0.9 to 2.2)	22.9<br>(19.5 to 26.4)*	-3.3<br>(-5.9 to -0.5)*
Asbestosis without heart failure aggregate	8.9<br>(7.6 to 10.4)	22.6<br>(19.7 to 25.5)*	-3.5<br>(-5.8 to -1.2)*	1.5<br>(0.9 to 2.2)	22.8<br>(19.4 to 26.3)*	-3.3<br>(-6.0 to -0.6)*
Severe asbestosis with heart failure aggregate	0.0<br>(0.0 to 0.0)	36.7<br>(32.1 to 41.7)*	7.6<br>(4.0 to 11.6)*	0.0<br>(0.0 to 0.0)	36.7<br>(32.1 to 41.7)*	7.6<br>(4.0 to 11.6)*
Coal workers pneumoconiosis	4.3<br>(3.6 to 5.1)	21.8<br>(18.9 to 25.2)*	-4.1<br>(-6.4 to -1.5)*	0.7<br>(0.4 to 1.1)	21.8<br>(18.4 to 25.4)*	-4.1<br>(-6.8 to -1.3)*
Coal workers pneumoconiosis without heart failure aggregate	4.2<br>(3.5 to 5.0)	21.6<br>(18.7 to 25.0)*	-4.3<br>(-6.6 to -1.6)*	0.7<br>(0.4 to 1.0)	21.3<br>(17.8 to 25.2)*	-4.5<br>(-7.3 to -1.5)*
Severe coal workers pneumoconiosis with heart failure aggregate	0.1<br>(0.1 to 0.1)	33.0<br>(23.4 to 44.7)*	4.7<br>(-2.9 to 13.9)	0.0<br>(0.0 to 0.0)	33.0<br>(23.4 to 44.7)*	4.7<br>(-2.9 to 13.9)
Other pneumoconiosis	181.9<br>(161.5 to 204.6)	38.2<br>(35.0 to 41.5)*	8.8<br>(6.3 to 11.3)*	28.1<br>(17.8 to 40.9)	38.2<br>(28.6 to 47.9)*	8.8<br>(1.2 to 16.4)*
Other pneumoconiosis without heart failure aggregate	181.9<br>(161.5 to 204.6)	38.2<br>(35.0 to 41.5)*	8.8<br>(6.3 to 11.3)*	28.1<br>(17.8 to 40.9)	38.2<br>(28.6 to 47.9)*	8.8<br>(1.2 to 16.4)*
Severe other pneumoconiosis with heart failure aggregate	0.0<br>(0.0 to 0.0)	0.0<br>(0.0 to 0.0)	0.0<br>(0.0 to 0.0)	0.0<br>(0.0 to 0.0)	0.0<br>(0.0 to 0.0)	0.0<br>(0.0 to 0.0)
Asthma	17,041.0<br>(14,252.4 to 20,462.7)	20.1<br>(17.2 to 23.3)*	-5.4<br>(-7.7 to -2.9)*	748.4<br>(471.2 to 1,063.3)	20.3<br>(17.2 to 23.6)*	-5.3<br>(-7.7 to -2.7)*
Asymptomatic asthma	5,160.2<br>(4,183.9 to 6,332.5)	20.1<br>(17.2 to 23.3)*	-5.4<br>(-7.7 to -2.9)*	0.0<br>(0.0 to 0.0)	0.0<br>(0.0 to 0.0)	0.0<br>(0.0 to 0.0)
Symptomatic asthma aggregate	11,880.8<br>(9,850.3 to 14,377.9)	20.1<br>(17.2 to 23.3)*	-5.4<br>(-7.7 to -2.9)*	748.4<br>(471.2 to 1,063.3)	20.3<br>(17.2 to 23.6)*	-5.3<br>(-7.7 to -2.7)*
Interstitial lung disease and pulmonary sarcoidosis	139.5<br>(119.5 to 161.7)	19.1<br>(16.0 to 22.0)*	-6.3<br>(-8.7 to -3.9)*	17.2<br>(10.4 to 25.3)	19.5<br>(13.3 to 26.6)*	-5.9<br>(-10.8 to -0.4)*
Interstitial lung disease and pulmonary sarcoidosis without heart failure aggregate	133.0<br>(113.8 to 154.7)	19.0<br>(15.8 to 22.1)*	-6.4<br>(-8.8 to -3.9)*	14.1<br>(8.2 to 21.3)	19.2<br>(11.4 to 27.4)*	-6.2<br>(-12.3 to 0.3)
Severe interstitial lung disease and pulmonary sarcoidosis with heart failure aggregate	6.4<br>(5.5 to 7.4)	21.0<br>(19.3 to 23.0)*	-4.8<br>(-6.1 to -3.2)*	3.1<br>(2.1 to 4.1)	21.2<br>(16.2 to 26.3)*	-4.6<br>(-8.6 to -0.6)*
Other chronic respiratory diseases	--	--	--	128.7<br>(102.0 to 159.5)	1.6<br>(-8.3 to 11.8)	-20.1<br>(-27.8 to -12.0)*
Cirrhosis and other chronic liver diseases	302.8<br>(292.4 to 313.3)	32.0<br>(27.2 to 37.2)*	3.9<br>(0.1 to 8.0)*	52.1<br>(35.5 to 71.3)	32.1<br>(27.3 to 36.8)*	4.0<br>(0.2 to 7.6)*
Cirrhosis and other chronic liver diseases due to hepatitis B	91.8<br>(78.8 to 107.9)	33.3<br>(29.1 to 36.9)*	4.9<br>(1.6 to 7.8)*	15.3<br>(10.2 to 21.7)	33.4<br>(23.5 to 43.9)*	5.0<br>(-2.8 to 13.3)
Cirrhosis and other chronic liver diseases due to hepatitis C	83.8<br>(70.3 to 98.5)	30.5<br>(26.5 to 34.4)*	2.7<br>(-0.4 to 5.8)	14.0<br>(9.1 to 20.1)	30.7<br>(21.3 to 40.7)*	2.9<br>(-4.6 to 10.7)
Cirrhosis and other chronic liver diseases due to alcohol use	99.0<br>(84.4 to 112.7)	33.3<br>(29.6 to 36.9)*	4.9<br>(2.0 to 7.8)*	16.5<br>(11.0 to 23.4)	33.9<br>(25.5 to 42.7)*	5.4<br>(-1.2 to 12.3)
Cirrhosis and other chronic liver diseases due to other causes	37.9<br>(31.4 to 44.9)	27.9<br>(24.5 to 30.9)*	0.7<br>(-2.0 to 3.0)	6.3<br>(4.0 to 9.2)	27.8<br>(15.3 to 41.5)*	0.6<br>(-9.2 to 11.4)
Digestive diseases	19,349.8<br>(18,828.2 to 19,853.6)	12.7<br>(9.5 to 16.1)*	-11.3<br>(-13.8 to -8.6)*	945.8<br>(665.2 to 1,290.9)	14.3<br>(11.0 to 17.9)*	-10.1<br>(-12.6 to -7.2)*
Peptic ulcer disease	4,017.5<br>(3,905.5 to 4,137.8)	-5.1<br>(-7.7 to -2.3)*	-25.3<br>(-27.4 to -23.1)*	133.6<br>(89.6 to 192.1)	-6.2<br>(-9.4 to -2.9)*	-26.1<br>(-28.7 to -23.6)*
Peptic ulcer disease, symptomatic episodes	275.5<br>(217.1 to 334.8)	-4.0<br>(-9.7 to 1.4)	-24.5<br>(-28.9 to -20.2)*	29.6<br>(18.6 to 43.3)	-3.8<br>(-11.1 to 2.8)	-24.3<br>(-30.0 to -19.1)*
Anemia due to peptic ulcer disease aggregate	3,742.0<br>(3,636.0 to 3,842.9)	-5.2<br>(-7.9 to -2.1)*	-25.4<br>(-27.5 to -23.0)*	104.0<br>(69.4 to 150.5)	-6.8<br>(-10.3 to -3.0)*	-26.7<br>(-29.4 to -23.6)*
Gastritis and duodenitis	12,752.4<br>(12,199.3 to 13,325.7)	19.1<br>(13.8 to 24.4)*	-6.3<br>(-10.4 to -2.1)*	386.5<br>(256.5 to 555.4)	17.1<br>(10.6 to 24.3)*	-7.8<br>(-12.9 to -2.2)*
Gastritis and duodenitis, symptomatic episodes	295.9<br>(217.8 to 391.6)	19.0<br>(13.8 to 22.4)*	-6.3<br>(-10.4 to -3.6)*	31.8<br>(19.0 to 48.0)	19.3<br>(12.9 to 24.6)*	-6.1<br>(-11.1 to -1.9)*
Anemia due to gastritis and duodenitis aggregate	12,456.5<br>(11,896.0 to 13,012.8)	19.1<br>(13.8 to 24.5)*	-6.3<br>(-10.4 to -2.0)*	354.6<br>(234.3 to 509.8)	16.9<br>(10.0 to 24.7)*	-8.0<br>(-13.4 to -1.9)*
Appendicitis	17.9<br>(14.7 to 21.3)	27.4<br>(25.3 to 29.5)*	0.2<br>(-1.3 to 1.9)	5.5<br>(3.5 to 7.9)	27.7<br>(15.4 to 41.1)*	0.5<br>(-9.2 to 11.0)
Paralytic ileus and intestinal obstruction	6.7<br>(5.1 to 8.5)	22.2<br>(15.6 to 28.3)*	-3.8<br>(-9.0 to 1.0)	2.2<br>(1.4 to 3.1)	22.2<br>(15.2 to 28.8)*	-3.8<br>(-9.3 to 1.4)
Inguinal, femoral, and abdominal hernia	1,354.2<br>(950.6 to 1,824.8)	8.7<br>(2.8 to 14.7)*	-14.4<br>(-19.1 to -9.7)*	14.4<br>(6.4 to 27.9)	8.9<br>(2.4 to 15.1)*	-14.3<br>(-19.4 to -9.4)*
Inflammatory bowel disease	1,068.0<br>(985.0 to 1,150.5)	19.5<br>(18.8 to 20.1)*	-6.0<br>(-6.5 to -5.5)*	228.3<br>(157.6 to 312.2)	19.7<br>(17.6 to 21.8)*	-5.8<br>(-7.4 to -4.1)*
Vascular intestinal disorders	2.0<br>(1.5 to 2.6)	16.7<br>(9.0 to 23.7)*	-8.1<br>(-14.2 to -2.6)*	0.7<br>(0.4 to 1.0)	16.7<br>(9.0 to 23.7)*	-8.1<br>(-14.2 to -2.6)*
Gallbladder and biliary diseases	470.7<br>(363.2 to 610.6)	17.7<br>(10.8 to 23.7)*	-7.4<br>(-12.8 to -2.6)*	50.4<br>(31.3 to 74.9)	17.9<br>(10.4 to 25.5)*	-7.2<br>(-13.1 to -1.2)*
Pancreatitis	96.2<br>(76.0 to 118.6)	16.6<br>(9.7 to 22.8)*	-8.3<br>(-13.6 to -3.4)*	28.6<br>(18.1 to 41.5)	16.8<br>(6.3 to 26.9)*	-8.1<br>(-16.3 to -0.1)*
Other digestive diseases	--	--	--	95.6<br>(66.8 to 132.6)	23.5<br>(15.3 to 34.4)*	-2.8<br>(-9.2 to 5.8)
Neurological disorders	187,362.0<br>(175,126.7 to 196,027.1)	26.0<br>(23.7 to 28.2)*	-0.9<br>(-2.7 to 0.9)	4,363.7<br>(2,814.2 to 6,182.0)	22.1<br>(19.0 to 24.9)*	-3.9<br>(-6.3 to -1.7)*
Alzheimer disease and other dementias	265.9<br>(188.7 to 388.7)	22.7<br>(20.2 to 24.7)*	-3.5<br>(-5.4 to -1.8)*	34.0<br>(20.4 to 54.1)	23.0<br>(16.3 to 28.6)*	-3.2<br>(-8.5 to 1.2)
Parkinson disease	185.2<br>(160.1 to 212.6)	30.0<br>(25.3 to 34.9)*	2.3<br>(-1.4 to 6.2)	23.9<br>(16.0 to 33.8)	30.3<br>(21.4 to 38.7)*	2.6<br>(-4.4 to 9.2)
Epilepsy	1,194.3<br>(1,024.8 to 1,363.7)	19.7<br>(13.5 to 26.0)*	-5.8<br>(-10.7 to -0.9)*	297.4<br>(205.7 to 408.3)	-0.4<br>(-7.1 to 6.7)	-21.6<br>(-26.9 to -16.0)*
Multiple sclerosis	238.9<br>(221.3 to 256.9)	18.6<br>(16.7 to 20.7)*	-6.6<br>(-8.2 to -5.0)*	79.5<br>(56.4 to 102.6)	18.8<br>(14.4 to 23.2)*	-6.5<br>(-10.0 to -3.1)*
Motor neuron disease	13.0<br>(10.9 to 14.9)	19.9<br>(15.3 to 24.4)*	-5.6<br>(-9.3 to -2.1)*	2.8<br>(1.9 to 3.7)	19.9<br>(15.3 to 24.3)*	-5.6<br>(-9.2 to -2.1)*
Migraine	83,923.5<br>(68,060.1 to 103,320.8)	24.0<br>(21.8 to 26.2)*	-2.4<br>(-4.1 to -0.7)*	2,841.5<br>(1,690.4 to 4,353.2)	24.4<br>(22.1 to 26.6)*	-2.1<br>(-3.9 to -0.3)*
Tension-type headache	129,505.0<br>(94,546.7 to 170,148.3)	27.0<br>(24.7 to 29.2)*	-0.1<br>(-1.9 to 1.7)	193.5<br>(82.8 to 365.8)	27.1<br>(24.7 to 29.6)*	0.1<br>(-1.9 to 2.0)
Medication overuse headache	5,639.2<br>(3,710.0 to 7,989.2)	22.6<br>(11.8 to 35.1)*	-3.5<br>(-12.0 to 6.4)	883.8<br>(480.5 to 1,456.1)	22.9<br>(12.2 to 35.7)*	-3.2<br>(-11.7 to 6.8)
Other neurological disorders	0.8<br>(0.5 to 1.3)	24.0<br>(21.8 to 25.5)*	-2.4<br>(-4.1 to -1.2)*	7.2<br>(5.1 to 9.8)	17.0<br>(11.6 to 22.1)*	-7.9<br>(-12.1 to -3.9)*
Mental and substance use disorders	77,701.3<br>(75,208.9 to 80,262.4)	25.5<br>(24.4 to 26.6)*	-1.2<br>(-2.1 to -0.4)*	12,094.9<br>(8,741.8 to 15,629.2)	26.1<br>(25.3 to 26.9)*	-0.8<br>(-1.4 to -0.1)*
Schizophrenia	2,840.1<br>(2,468.1 to 3,224.2)	29.0<br>(28.1 to 29.9)*	1.5<br>(0.9 to 2.2)*	1,821.0<br>(1,322.0 to 2,292.2)	29.3<br>(27.5 to 31.1)*	1.8<br>(0.4 to 3.2)*
Alcohol use disorders	6,297.5<br>(5,441.7 to 7,212.6)	14.6<br>(10.6 to 18.2)*	-9.8<br>(-13.0 to -7.0)*	625.7<br>(402.0 to 913.1)	14.9<br>(10.5 to 18.6)*	-9.6<br>(-13.0 to -6.7)*
Alcohol dependence aggregate	6,245.4<br>(5,388.1 to 7,159.7)	14.5<br>(10.4 to 18.1)*	-9.9<br>(-13.1 to -7.0)*	623.1<br>(400.2 to 909.6)	14.8<br>(10.4 to 18.5)*	-9.6<br>(-13.1 to -6.7)*
Fetal alcohol syndrome aggregate	52.1<br>(48.0 to 56.2)	25.5<br>(24.1 to 27.0)*	-1.2<br>(-2.3 to -0.0)*	2.6<br>(1.7 to 3.8)	26.2<br>(17.1 to 35.3)*	-0.7<br>(-7.8 to 6.5)
Drug use disorders	2,433.8<br>(2,323.3 to 2,511.3)	32.2<br>(29.4 to 35.1)*	4.1<br>(1.9 to 6.4)*	651.5<br>(453.0 to 871.7)	34.9<br>(31.0 to 38.8)*	6.2<br>(3.1 to 9.2)*
Opioid use disorders	1,366.5<br>(1,122.2 to 1,655.7)	32.8<br>(29.3 to 36.5)*	4.6<br>(1.8 to 7.4)*	560.7<br>(380.6 to 758.1)	33.2<br>(29.0 to 37.3)*	4.8<br>(1.5 to 8.1)*
Asymptomatic opioid dependence	221.5<br>(154.2 to 308.1)	32.8<br>(29.3 to 36.5)*	4.6<br>(1.8 to 7.4)*	0.0<br>(0.0 to 0.0)	0.0<br>(0.0 to 0.0)	0.0<br>(0.0 to 0.0)
Symptomatic opioid dependence aggregate	1,145.0<br>(926.1 to 1,400.7)	32.8<br>(29.3 to 36.5)*	4.6<br>(1.8 to 7.4)*	560.7<br>(380.6 to 758.1)	33.2<br>(29.0 to 37.3)*	4.8<br>(1.5 to 8.1)*
Cocaine use disorders	138.4<br>(113.5 to 166.1)	44.0<br>(39.0 to 50.0)*	13.4<br>(9.4 to 18.1)*	19.0<br>(11.7 to 27.8)	44.0<br>(34.0 to 54.8)*	13.4<br>(5.5 to 21.9)*
Asymptomatic cocaine dependence	69.4<br>(53.0 to 88.6)	44.0<br>(39.0 to 50.0)*	13.4<br>(9.4 to 18.1)*	0.0<br>(0.0 to 0.0)	0.0<br>(0.0 to 0.0)	0.0<br>(0.0 to 0.0)

Cause	Prevalence in thousands (95% confidence interval)			YLDs in thousands (95% confidence interval)		
	2015	Percent change in counts between 2005 and 2015	Percent change in rates between 2005 and 2015	2015	Percent change in counts between 2005 and 2015	Percent change in rates between 2005 and 2015
Symptomatic cocaine dependence aggregate	69.0 (53.2 to 88.5)	44.0 (39.0 to 50.0)*	13.4 (9.4 to 18.1)*	19.0 (11.7 to 27.8)	44.0 (34.0 to 54.8)*	13.4 (5.5 to 21.9)*
Amphetamine use disorders	168.4 (132.0 to 210.9)	68.6 (60.7 to 77.2)*	32.7 (26.5 to 39.5)*	21.9 (12.3 to 33.6)	68.9 (53.9 to 86.0)*	32.9 (21.2 to 46.4)*
Asymptomatic amphetamine dependence	92.3 (66.6 to 121.4)	68.6 (60.7 to 77.2)*	32.7 (26.5 to 39.5)*	0.0 (0.0 to 0.0)	0.0 (0.0 to 0.0)	0.0 (0.0 to 0.0)
Symptomatic amphetamine dependence aggregate	76.1 (54.4 to 100.9)	68.6 (60.7 to 77.2)*	32.7 (26.5 to 39.5)*	21.9 (12.3 to 33.6)	68.9 (53.9 to 86.0)*	32.9 (21.2 to 46.4)*
Cannabis use disorders	810.1 (672.3 to 961.7)	24.0 (22.5 to 25.6)*	-2.4 (-3.6 to -1.1)*	23.2 (14.6 to 34.7)	24.1 (17.9 to 31.0)*	-2.3 (-7.2 to 3.1)
Asymptomatic cannabis dependence	468.6 (385.1 to 564.7)	24.0 (22.5 to 25.6)*	-2.4 (-3.6 to -1.1)*	0.0 (0.0 to 0.0)	0.0 (0.0 to 0.0)	0.0 (0.0 to 0.0)
Symptomatic cannabis dependence aggregate	341.5 (277.1 to 419.3)	24.0 (22.5 to 25.6)*	-2.4 (-3.6 to -1.1)*	23.2 (14.6 to 34.7)	24.1 (17.9 to 31.0)*	-2.3 (-7.2 to 3.1)
Other drug use disorders	--	--	--	26.6 (16.1 to 39.0)	56.5 (46.9 to 67.9)*	23.2 (15.6 to 32.2)*
Depressive disorders	27,455.8 (26,311.2 to 29,063.8)	25.2 (23.3 to 27.2)*	-1.5 (-2.9 to 0.1)	4,694.9 (3,130.6 to 6,602.2)	24.9 (23.7 to 26.0)*	-1.7 (-2.6 to -0.8)*
Major depressive disorder	18,555.7 (14,928.1 to 22,585.4)	23.7 (22.5 to 24.8)*	-2.7 (-3.6 to -1.8)*	3,781.9 (2,414.1 to 5,394.6)	24.0 (22.6 to 25.3)*	-2.4 (-3.5 to -1.4)*
Dysthymia	9,448.1 (7,444.1 to 11,771.3)	28.4 (26.5 to 30.4)*	1.1 (-0.5 to 2.7)	913.0 (569.9 to 1,376.2)	28.7 (26.8 to 30.8)*	1.3 (-0.2 to 3.0)
Bipolar disorder	3,281.9 (2,735.7 to 3,905.6)	25.3 (25.0 to 25.7)*	-1.3 (-1.6 to -1.1)*	663.4 (400.1 to 988.8)	25.7 (24.0 to 27.6)*	-1.1 (-2.4 to 0.4)
Anxiety disorders	20,701.7 (16,393.4 to 25,365.9)	25.6 (22.4 to 29.0)*	-1.1 (-3.6 to 1.5)	1,890.7 (1,187.3 to 2,744.7)	25.9 (22.5 to 29.3)*	-0.9 (-3.6 to 1.8)
Eating disorders	176.4 (160.4 to 192.4)	42.5 (33.2 to 51.6)*	12.2 (4.9 to 19.3)*	36.9 (22.4 to 54.6)	39.4 (32.1 to 46.3)*	9.7 (3.9 to 15.1)*
Anorexia nervosa	63.0 (48.6 to 81.5)	32.0 (27.3 to 36.3)*	3.9 (0.2 to 7.3)*	13.1 (8.1 to 19.6)	31.8 (22.0 to 42.7)*	3.8 (-4.0 to 12.4)
Bulimia nervosa	114.9 (77.2 to 163.7)	44.0 (40.5 to 48.1)*	13.4 (10.6 to 16.6)*	23.8 (13.2 to 37.8)	43.9 (34.5 to 53.4)*	13.3 (5.9 to 20.8)*
Autistic spectrum disorders	3,733.0 (3,543.4 to 3,898.4)	27.3 (25.0 to 29.5)*	0.2 (-1.6 to 2.0)	594.6 (397.6 to 814.3)	27.5 (26.1 to 28.9)*	0.3 (-0.7 to 1.4)
Autism	1,488.1 (1,247.3 to 1,764.9)	27.1 (26.7 to 27.4)*	0.0 (-0.3 to 0.3)	374.7 (242.1 to 532.2)	27.4 (25.5 to 29.4)*	0.3 (-1.2 to 1.8)
Asperger syndrome and other autistic spectrum disorders	2,227.5 (1,894.5 to 2,677.8)	27.3 (27.2 to 27.5)*	0.2 (0.1 to 0.3)*	219.9 (146.0 to 321.3)	27.6 (25.9 to 29.1)*	0.4 (-0.9 to 1.6)
Attention-deficit/hyperactivity disorder	166.6 (120.5 to 223.8)	29.9 (28.0 to 32.0)*	2.3 (0.8 to 3.9)*	2.0 (1.1 to 3.3)	29.7 (19.6 to 41.3)*	2.1 (-5.8 to 11.2)
Conduct disorder	0.0 (0.0 to 0.0)	0.0 (0.0 to 0.0)	0.0 (0.0 to 0.0)	0.0 (0.0 to 0.0)	0.0 (0.0 to 0.0)	0.0 (0.0 to 0.0)
Idiopathic developmental intellectual disability	4,736.2 (2,568.7 to 6,823.1)	28.0 (26.5 to 29.6)*	0.8 (-0.4 to 2.0)	181.6 (78.9 to 313.7)	27.7 (25.1 to 29.9)*	0.5 (-1.6 to 2.2)
Other mental and substance use disorders	12,454.8 (12,243.5 to 12,657.0)	27.1 (24.1 to 30.2)*	0.0 (-2.4 to 2.5)	932.7 (649.3 to 1,251.3)	27.4 (24.0 to 30.6)*	0.3 (-2.4 to 2.8)
Diabetes, urogenital, blood, and endocrine diseases	228,546.5 (226,702.7 to 230,419.6)	26.5 (26.0 to 27.1)*	-0.4 (-0.8 to 0.0)	6,054.8 (4,260.2 to 8,215.2)	27.3 (24.8 to 29.9)*	0.2 (-1.8 to 2.2)
Diabetes mellitus	45,056.1 (40,109.4 to 50,509.4)	31.3 (27.4 to 35.1)*	3.3 (0.2 to 6.4)*	3,492.7 (2,336.8 to 4,955.5)	31.8 (27.6 to 35.9)*	3.8 (0.4 to 7.0)*
Uncomplicated diabetes mellitus	28,265.4 (22,516.7 to 33,692.9)	30.7 (26.5 to 34.6)*	2.9 (-0.4 to 6.0)	1,326.1 (798.4 to 2,029.5)	30.9 (26.7 to 34.8)*	3.0 (-0.3 to 6.1)
Neuropathy and other complications of diabetes mellitus aggregate	16,362.3 (11,890.4 to 21,649.8)	32.2 (27.0 to 37.3)*	4.1 (0.0 to 8.0)*	2,138.4 (1,289.0 to 3,208.7)	32.4 (27.1 to 37.5)*	4.2 (0.1 to 8.2)*
Vision loss due to diabetes mellitus aggregate	428.4 (305.3 to 589.7)	32.3 (29.1 to 35.5)*	4.2 (1.6 to 6.7)*	28.3 (17.5 to 41.6)	33.8 (27.4 to 40.1)*	5.3 (0.3 to 10.3)*
Acute glomerulonephritis	7.8 (5.7 to 10.2)	20.8 (14.7 to 25.3)*	-4.9 (-9.7 to -1.3)*	0.4 (0.2 to 0.7)	21.8 (9.6 to 34.2)*	-4.1 (-13.8 to 5.6)
Chronic kidney disease	25,154.6 (23,257.4 to 27,047.2)	28.4 (25.6 to 31.4)*	1.1 (-1.2 to 3.4)	684.1 (492.0 to 884.7)	24.2 (22.3 to 26.3)*	-2.2 (-3.8 to -0.6)*
Chronic kidney disease due to diabetes mellitus	8,484.7 (5,222.0 to 12,123.7)	30.6 (26.7 to 35.4)*	2.8 (-0.3 to 6.6)	224.5 (137.3 to 323.1)	25.6 (21.4 to 30.0)*	-1.1 (-4.5 to 2.3)
Stage III chronic kidney disease due to diabetes mellitus aggregate	7,611.7 (4,612.4 to 11,026.8)	31.2 (27.0 to 36.3)*	3.2 (-0.0 to 7.3)	8.3 (5.3 to 12.8)	6.7 (-11.4 to 28.4)	-16.0 (-30.2 to 1.1)
Stage IV chronic kidney disease due to diabetes mellitus aggregate	522.2 (324.5 to 797.6)	26.7 (23.4 to 30.4)*	-0.3 (-2.9 to 2.6)	64.1 (35.2 to 103.8)	25.4 (20.4 to 30.2)*	-1.3 (-5.2 to 2.5)
Stage V chronic kidney disease untreated due to diabetes mellitus	263.3 (168.0 to 363.5)	27.2 (24.0 to 30.8)*	0.1 (-2.4 to 3.0)	135.7 (78.3 to 200.8)	27.6 (21.9 to 33.6)*	0.4 (-4.0 to 5.1)
End-stage chronic kidney disease due to diabetes aggregate	87.6 (59.1 to 116.7)	22.0 (17.4 to 26.8)*	-4.0 (-7.6 to -0.2)*	16.4 (9.6 to 23.9)	21.7 (10.8 to 33.2)*	-4.2 (-12.8 to 4.9)
Chronic kidney disease due to hypertension	6,656.0 (4,297.4 to 9,912.9)	25.7 (21.1 to 29.9)*	-1.1 (-4.7 to 2.2)	107.3 (71.8 to 155.7)	27.7 (20.9 to 35.2)*	0.5 (-4.8 to 6.4)
Stage III chronic kidney disease due to hypertension aggregate	6,226.3 (3,907.4 to 9,356.7)	25.6 (20.7 to 30.2)*	-1.1 (-5.0 to 2.4)	4.0 (2.5 to 6.3)	29.3 (13.6 to 45.2)*	1.7 (-10.6 to 14.3)
Stage IV chronic kidney disease due to hypertension aggregate	268.1 (167.6 to 441.2)	26.1 (16.8 to 35.8)*	-0.8 (-8.1 to 6.9)	33.3 (18.5 to 59.6)	25.1 (15.1 to 34.8)*	-1.5 (-9.4 to 6.1)
Stage V chronic kidney disease untreated due to hypertension	120.8 (86.2 to 172.1)	28.5 (22.9 to 34.4)*	1.2 (-3.2 to 5.8)	62.3 (39.0 to 95.6)	29.1 (20.1 to 39.4)*	1.6 (-5.5 to 9.7)
End-stage chronic kidney disease due to hypertension aggregate	40.8 (30.2 to 53.7)	26.4 (17.8 to 35.5)*	-0.5 (-7.3 to 6.7)	7.7 (4.9 to 11.2)	27.1 (15.7 to 38.3)*	0.0 (-8.9 to 8.9)
Chronic kidney disease due to glomerulonephritis	4,391.8 (3,237.7 to 5,904.0)	28.9 (23.5 to 35.1)*	1.5 (-2.8 to 6.3)	184.0 (122.1 to 264.9)	21.4 (16.8 to 25.9)*	-4.5 (-8.1 to -0.9)*
Stage III chronic kidney disease due to glomerulonephritis aggregate	3,715.9 (2,575.1 to 5,163.9)	30.4 (24.1 to 37.2)*	2.7 (-2.3 to 8.0)	9.6 (6.1 to 14.8)	19.6 (9.4 to 30.2)*	-5.8 (-13.9 to 2.5)
Stage IV chronic kidney disease due to glomerulonephritis aggregate	399.8 (266.2 to 539.4)	21.1 (15.9 to 27.6)*	-4.6 (-8.8 to 0.4)	49.8 (29.8 to 76.7)	20.7 (14.2 to 27.7)*	-5.0 (-10.1 to 0.6)
Stage V chronic kidney disease untreated due to glomerulonephritis	222.1 (143.0 to 317.9)	21.2 (17.9 to 24.4)*	-4.6 (-7.2 to -2.1)*	114.2 (67.2 to 172.8)	21.7 (15.5 to 28.0)*	-4.2 (-9.1 to 0.7)
End-stage chronic kidney disease due to glomerulonephritis aggregate	54.1 (34.9 to 78.4)	23.1 (18.1 to 28.3)*	-3.1 (-7.0 to 1.0)	10.4 (6.2 to 16.2)	23.0 (10.9 to 35.1)*	-3.2 (-12.7 to 6.3)
Chronic kidney disease due to other causes	6,396.6 (4,001.5 to 9,404.4)	27.8 (24.6 to 31.1)*	0.6 (-1.9 to 3.2)	168.2 (106.0 to 252.6)	23.5 (19.5 to 27.8)*	-2.8 (-5.9 to 0.6)
Stage III chronic kidney disease due to other causes aggregate	5,760.2 (3,567.5 to 8,597.8)	28.2 (24.8 to 31.7)*	0.9 (-1.8 to 3.7)	8.1 (5.1 to 12.5)	17.3 (5.9 to 29.6)*	-7.7 (-16.6 to 2.0)
Stage IV chronic kidney disease due to other causes aggregate	382.6 (225.8 to 597.5)	24.0 (21.6 to 26.4)*	-2.4 (-4.3 to -0.5)*	47.5 (25.9 to 80.1)	21.7 (16.8 to 26.2)*	-4.2 (-8.1 to -0.7)*
Stage V chronic kidney disease untreated due to other causes	197.0 (125.4 to 295.7)	24.6 (22.1 to 27.2)*	-2.0 (-3.9 to 0.1)	101.5 (58.7 to 160.9)	24.9 (19.4 to 31.0)*	-1.7 (-6.0 to 3.1)
End-stage chronic kidney disease due to other causes aggregate	56.7 (37.0 to 80.9)	23.5 (19.5 to 28.0)*	-2.8 (-6.0 to 0.7)	11.2 (6.4 to 17.3)	22.9 (13.5 to 32.9)*	-3.3 (-10.7 to 4.6)
Urinary diseases and male infertility	34,833.8 (33,499.0 to 36,016.5)	25.8 (23.8 to 27.8)*	-1.0 (-2.5 to 0.6)	138.0 (84.8 to 207.8)	24.9 (20.4 to 29.3)*	-1.7 (-5.2 to 1.8)
Interstitial nephritis and urinary tract infections	138.8 (129.7 to 148.3)	26.8 (26.0 to 27.6)*	-0.2 (-0.8 to 0.4)	4.6 (2.9 to 7.0)	26.9 (20.0 to 34.8)*	-0.1 (-5.5 to 6.1)
Urolithiasis	31,396.2 (27,717.8 to 35,717.2)	25.9 (23.4 to 28.4)*	-0.9 (-2.9 to 1.1)	9.6 (5.9 to 14.4)	24.2 (14.8 to 34.4)*	-2.2 (-9.6 to 5.8)
Benign prostatic hyperplasia	3,061.0 (2,294.4 to 4,078.1)	24.7 (19.8 to 29.7)*	-1.9 (-5.7 to 2.1)	115.0 (68.0 to 177.4)	24.9 (19.4 to 30.5)*	-1.7 (-6.0 to 2.7)
Male infertility	574.1 (357.9 to 885.6)	28.0 (19.6 to 36.0)*	0.7 (-5.9 to 7.1)	3.3 (1.2 to 7.4)	28.7 (19.9 to 36.5)*	1.3 (-5.6 to 7.4)
Other urinary diseases	--	--	--	5.5 (3.5 to 7.9)	23.9 (11.3 to 37.8)*	-2.5 (-12.4 to 8.4)
Gynecological diseases	100,136.3 (98,739.6 to 101,377.1)	24.7 (23.7 to 25.7)*	-1.9 (-2.6 to -1.1)*	1,312.7 (891.3 to 1,833.8)	20.8 (19.2 to 22.6)*	-4.9 (-6.2 to -3.5)*
Uterine fibroids	28,457.3 (26,710.3 to 30,201.7)	26.9 (26.6 to 27.2)*	-0.1 (-0.4 to 0.1)	380.6 (225.9 to 621.7)	15.5 (11.4 to 18.7)*	-9.1 (-12.4 to -6.6)*
Uterine fibroids cases aggregate	22,577.9 (20,827.9 to 24,312.3)	31.3 (30.5 to 32.2)*	3.3 (2.7 to 4.1)*	145.1 (72.2 to 267.0)	34.5 (32.9 to 36.2)*	5.8 (4.6 to 7.2)*
Mild abdominal pain with anemia due to uterine fibroids aggregate	5,879.5 (5,766.1 to 5,989.4)	12.4 (9.9 to 14.7)*	-11.5 (-13.5 to -9.7)*	235.5 (152.8 to 352.3)	6.3 (2.9 to 9.6)*	-16.3 (-19.0 to -13.7)*
Polycystic ovarian syndrome	379.4 (288.9 to 514.4)	25.4 (24.2 to 26.6)*	-1.3 (-2.2 to -0.3)*	3.3 (1.4 to 6.8)	25.7 (21.2 to 30.1)*	-1.0 (-4.6 to 2.4)
Polycystic ovarian syndrome cases aggregate	313.1 (234.8 to 422.0)	23.7 (22.7 to 24.6)*	-2.6 (-3.4 to -1.9)*	2.7 (1.2 to 5.3)	23.8 (18.8 to 29.0)*	-2.5 (-6.5 to 1.5)
Hirsutism and infertility due to polycystic ovarian syndrome aggregate	66.3 (39.4 to 104.3)	34.0 (29.6 to 39.5)*	5.5 (2.0 to 9.8)*	0.6 (0.2 to 1.4)	35.0 (24.0 to 46.0)*	6.3 (-2.4 to 14.9)

Cause	Prevalence in thousands (95% confidence interval)			YLDs in thousands (95% confidence interval)		
	2015	Percent change in counts between 2005 and 2015	Percent change in rates between 2005 and 2015	2015	Percent change in counts between 2005 and 2015	Percent change in rates between 2005 and 2015
Female infertility	508.4 (106.9 to 1,435.5)	37.1 (-14.2 to 65.5)	7.9 (-32.4 to 30.3)	2.7 (0.4 to 8.8)	38.0 (-12.4 to 66.3)	8.6 (-31.0 to 30.9)
Endometriosis	1,515.8 (1,255.9 to 1,810.2)	24.1 (23.1 to 24.9)*	-2.3 (-3.1 to -1.7)*	138.6 (89.1 to 197.7)	24.3 (21.5 to 27.1)*	-2.1 (-4.4 to 0.0)
Endometriosis cases aggregate	1,446.4 (1,195.4 to 1,720.9)	23.6 (22.6 to 24.5)*	-2.7 (-3.5 to -2.0)*	131.7 (84.8 to 188.4)	23.8 (20.8 to 26.7)*	-2.5 (-4.9 to -0.3)*
Abdominal pain and infertility due to endometriosis aggregate	69.4 (41.8 to 101.0)	35.1 (30.8 to 41.0)*	6.3 (3.0 to 11.0)*	6.9 (3.6 to 11.0)	34.8 (26.5 to 45.0)*	6.1 (-0.5 to 14.1)
Genital prolapse	18,665.3 (15,575.2 to 22,036.0)	17.8 (14.6 to 20.7)*	-7.3 (-9.8 to -5.0)*	58.7 (28.2 to 106.9)	18.0 (14.7 to 21.2)*	-7.1 (-9.7 to -4.6)*
Premenstrual syndrome	66,684.5 (62,385.9 to 71,419.8)	24.8 (21.6 to 28.5)*	-1.8 (-4.3 to 1.1)	552.9 (346.0 to 836.4)	25.0 (21.7 to 28.8)*	-1.6 (-4.2 to 1.4)
Other gynecological diseases	5,015.2 (4,809.9 to 5,231.4)	20.4 (19.1 to 21.7)*	-5.3 (-6.3 to -4.2)*	175.9 (122.7 to 237.8)	18.0 (15.2 to 20.9)*	-7.1 (-9.3 to -4.9)*
Other gynecological diseases cases aggregate	2,985.4 (2,790.1 to 3,190.7)	26.5 (26.1 to 26.9)*	-0.5 (-0.8 to -0.1)*	115.4 (77.5 to 158.2)	26.8 (23.5 to 30.0)*	-0.2 (-2.8 to 2.4)
Anemia due to other gynecological diseases aggregate	2,029.9 (1,984.3 to 2,080.3)	12.4 (9.7 to 15.4)*	-11.5 (-13.7 to -9.1)*	60.5 (40.4 to 86.7)	4.2 (-0.1 to 9.3)	-17.9 (-21.4 to -14.0)*
Hemoglobinopathies and hemolytic anemias	90,919.5 (89,321.1 to 92,865.3)	25.2 (23.6 to 26.8)*	-1.5 (-2.7 to -0.2)*	355.6 (235.3 to 518.4)	22.7 (18.8 to 27.1)*	-3.4 (-6.5 to 0.0)
Thalassemias	1.5 (1.4 to 1.6)	33.1 (25.7 to 41.0)*	4.7 (-1.1 to 11.0)	0.2 (0.1 to 0.3)	27.1 (-28.6 to 113.2)	0.0 (-43.8 to 67.8)
Beta-thalassemia major cases aggregate	1.3 (1.3 to 1.4)	33.4 (25.2 to 42.2)*	5.0 (-1.5 to 12.0)	0.1 (0.1 to 0.3)	26.8 (-35.4 to 128.3)	-0.2 (-49.1 to 79.7)
Hemoglobin E/beta-thalassemia cases aggregate	0.1 (0.1 to 0.1)	26.1 (25.2 to 26.9)*	-0.8 (-1.5 to -0.1)*	0.0 (0.0 to 0.0)	25.1 (16.3 to 34.3)*	-1.5 (-8.4 to 5.7)
Hemoglobin H disease cases aggregate	0.0 (0.0 to 0.0)	39.4 (32.9 to 45.5)*	9.7 (4.6 to 14.6)*	0.0 (0.0 to 0.0)	35.6 (26.5 to 44.6)*	6.8 (-0.5 to 13.8)
Heart failure due to thalassemias aggregate	0.0 (0.0 to 0.0)	40.2 (37.0 to 43.4)*	10.3 (7.8 to 12.8)*	0.0 (0.0 to 0.0)	40.2 (37.0 to 43.4)*	10.3 (7.8 to 12.8)*
Thalassemias trait	19,536.2 (19,091.4 to 20,064.6)	38.1 (37.3 to 38.8)*	8.7 (8.1 to 9.3)*	210.2 (139.0 to 307.5)	33.7 (27.9 to 39.8)*	5.2 (0.7 to 10.1)*
B-thalassemia trait cases aggregate	15,873.9 (15,491.9 to 16,383.6)	38.3 (37.4 to 39.1)*	8.9 (8.2 to 9.5)*	199.1 (131.5 to 292.0)	33.9 (28.1 to 39.9)*	5.4 (0.8 to 10.2)*
Hemoglobin E trait cases aggregate	3,662.2 (3,509.7 to 3,825.2)	37.2 (36.0 to 38.5)*	8.0 (7.1 to 9.0)*	11.1 (7.3 to 16.6)	30.1 (18.3 to 43.3)*	2.4 (-6.9 to 12.8)
Sickle cell disorders	6.7 (6.2 to 7.1)	22.4 (14.2 to 31.2)*	-3.7 (-10.1 to 3.3)	0.9 (0.6 to 1.2)	19.0 (10.4 to 27.1)*	-6.3 (-13.1 to 0.0)
Homozygous sickle cell and severe sickle cell/beta-thalassemia cases aggregate	5.9 (5.4 to 6.3)	25.6 (15.9 to 36.1)*	-1.1 (-8.8 to 7.1)	0.8 (0.5 to 1.0)	21.5 (11.2 to 31.5)*	-4.4 (-12.4 to 3.5)
Hemoglobin SC disease cases aggregate	0.5 (0.5 to 0.5)	7.6 (4.3 to 10.8)*	-15.3 (-17.9 to -12.8)*	0.1 (0.1 to 0.1)	13.1 (9.6 to 16.8)*	-11.0 (-13.7 to -8.0)*
Mild sickle cell/beta-thalassemia cases aggregate	0.3 (0.3 to 0.3)	-5.1 (-9.7 to 0.6)	-25.3 (-28.9 to -20.8)*	0.0 (0.0 to 0.1)	-3.1 (-7.1 to 1.6)	-23.7 (-26.9 to -20.0)*
Sickle cell trait	19,255.1 (18,097.9 to 21,598.4)	26.5 (25.2 to 28.7)*	-0.4 (-1.5 to 1.3)	62.8 (42.0 to 90.1)	14.0 (9.2 to 18.7)*	-10.3 (-14.0 to -6.6)*
G6PD deficiency	13,713.7 (11,641.5 to 15,882.0)	19.4 (13.8 to 24.4)*	-6.0 (-10.4 to -2.1)*	1.4 (1.0 to 2.0)	15.2 (5.5 to 25.5)*	-9.3 (-17.0 to -1.3)*
G6PD cases aggregate	13,713.3 (11,641.1 to 15,881.6)	19.4 (13.8 to 24.4)*	-6.0 (-10.4 to -2.1)*	1.3 (0.9 to 1.9)	14.7 (4.7 to 25.2)*	-9.7 (-17.6 to -1.5)*
Heart failure due to G6PD deficiency aggregate	0.4 (0.3 to 0.5)	31.5 (28.4 to 34.7)*	3.5 (1.0 to 6.0)*	0.0 (0.0 to 0.1)	31.5 (28.4 to 34.7)*	3.5 (1.0 to 6.0)*
G6PD trait	43,076.4 (39,828.4 to 46,274.8)	22.7 (20.7 to 24.6)*	-3.4 (-5.0 to -2.0)*	1.2 (0.8 to 1.7)	18.3 (0.9 to 38.7)*	-6.9 (-20.6 to 9.1)
Other hemoglobinopathies and hemolytic anemias	4,054.2 (3,973.0 to 4,144.3)	14.3 (11.7 to 17.0)*	-10.1 (-12.1 to -7.9)*	78.9 (51.6 to 114.7)	6.2 (1.3 to 11.0)*	-16.4 (-20.2 to -12.6)*
Other hemoglobinopathies and hemolytic anemias cases aggregate	4,046.7 (3,966.0 to 4,137.0)	14.2 (11.7 to 17.0)*	-10.1 (-12.1 to -7.9)*	77.2 (50.2 to 112.7)	5.9 (0.9 to 10.7)*	-16.7 (-20.6 to -12.8)*
Heart failure due to other hemoglobinopathies and hemolytic anemias aggregate	7.6 (6.6 to 8.7)	37.0 (35.2 to 39.0)*	7.9 (6.4 to 9.4)*	0.9 (0.6 to 1.3)	37.2 (31.7 to 43.0)*	8.0 (3.7 to 12.5)*
Endocrine, metabolic, blood, and immune disorders	3,165.7 (3,099.2 to 3,233.3)	14.6 (12.1 to 17.2)*	-9.8 (-11.8 to -7.7)*	71.4 (49.1 to 101.2)	5.7 (1.2 to 10.1)*	-16.8 (-20.4 to -13.4)*
Endocrine, metabolic, blood, and immune disorders cases aggregate	482.4 (438.0 to 532.1)	20.3 (17.5 to 22.9)*	-5.3 (-7.5 to -3.2)*	18.8 (12.8 to 26.9)	20.4 (14.4 to 26.6)*	-5.2 (-10.0 to -0.4)*
Anemia due to endocrine, metabolic, blood, and immune disorders aggregate	2,681.0 (2,626.1 to 2,733.5)	13.6 (10.8 to 16.7)*	-10.6 (-12.8 to -8.1)*	52.3 (34.6 to 76.4)	1.1 (-4.3 to 6.9)	-20.4 (-24.7 to -15.9)*
Heart failure due to endocrine, metabolic, blood, and immune disorders aggregate	2.3 (2.0 to 2.7)	21.5 (20.2 to 22.8)*	-4.4 (-5.4 to -3.3)*	0.3 (0.2 to 0.4)	21.5 (19.7 to 23.2)*	-4.4 (-5.8 to -3.0)*
Musculoskeletal disorders	137,394.5 (132,008.3 to 141,913.3)	27.2 (25.8 to 28.5)*	0.1 (-1.0 to 1.2)	15,651.5 (11,212.2 to 20,943.6)	27.0 (25.0 to 29.1)*	-0.0 (-1.6 to 1.6)
Rheumatoid arthritis	2,298.0 (1,985.9 to 2,663.1)	28.0 (23.8 to 32.6)*	0.8 (-2.5 to 4.3)	550.9 (372.0 to 744.8)	28.3 (23.6 to 33.1)*	1.0 (-2.7 to 4.7)
Osteoarthritis	23,624.3 (22,648.5 to 24,577.1)	32.2 (30.8 to 33.7)*	4.0 (2.9 to 5.2)*	1,387.8 (959.7 to 1,905.2)	34.3 (32.4 to 36.3)*	5.7 (4.2 to 7.3)*
Osteoarthritis of the hip cases aggregate	1,608.8 (1,354.8 to 1,896.6)	27.4 (25.7 to 29.2)*	0.2 (-1.1 to 1.7)	88.2 (57.6 to 125.1)	30.0 (26.8 to 33.5)*	2.3 (-0.2 to 5.1)
Osteoarthritis of the knee cases aggregate	22,015.5 (21,104.1 to 22,920.6)	32.6 (31.1 to 34.2)*	4.3 (3.2 to 5.6)*	1,299.6 (901.9 to 1,795.5)	34.6 (32.6 to 36.8)*	5.9 (4.4 to 7.7)*
Low back and neck pain	91,061.6 (86,170.1 to 94,596.1)	25.3 (23.4 to 27.2)*	-1.4 (-2.9 to 0.1)	10,648.3 (7,408.6 to 14,731.4)	25.1 (22.8 to 27.4)*	-1.5 (-3.3 to 0.3)
Low back pain	53,048.7 (47,458.3 to 59,648.2)	21.8 (19.4 to 24.2)*	-4.2 (-6.0 to -2.3)*	5,951.0 (4,113.5 to 8,339.9)	22.1 (19.8 to 24.5)*	-3.9 (-5.7 to -2.0)*
Neck pain	47,773.4 (33,934.9 to 65,532.4)	29.0 (25.7 to 32.2)*	1.5 (-1.1 to 4.1)	4,697.3 (2,890.1 to 7,107.3)	29.2 (26.0 to 32.5)*	1.7 (-0.9 to 4.3)
Gout	4,520.8 (3,606.3 to 5,536.7)	28.8 (27.6 to 29.9)*	1.4 (0.4 to 2.2)*	147.1 (97.1 to 214.4)	29.0 (25.1 to 33.2)*	1.5 (-1.6 to 4.8)
Asymptomatic gout	4,038.9 (3,221.0 to 4,944.7)	28.8 (27.6 to 29.9)*	1.4 (0.4 to 2.2)*	0.0 (0.0 to 0.0)	0.0 (0.0 to 0.0)	0.0 (0.0 to 0.0)
Symptomatic gout cases aggregate	481.9 (372.0 to 600.2)	28.8 (27.6 to 29.9)*	1.4 (0.4 to 2.2)*	147.1 (97.1 to 214.4)	29.0 (25.1 to 33.2)*	1.5 (-1.6 to 4.8)
Other musculoskeletal disorders	31,225.8 (23,777.5 to 40,041.0)	30.3 (22.9 to 36.7)*	2.6 (-3.3 to 7.6)	2,917.4 (1,874.1 to 4,203.8)	30.7 (23.1 to 37.0)*	2.9 (-3.1 to 7.9)
Other non-communicable diseases	346,412.8 (341,815.6 to 349,764.2)	26.9 (26.4 to 27.4)*	-0.1 (-0.5 to 0.3)	8,640.1 (5,743.8 to 12,628.5)	28.0 (26.7 to 29.5)*	0.7 (-0.3 to 1.9)
Congenital anomalies	6,108.8 (5,628.7 to 6,602.3)	42.2 (37.2 to 47.0)*	11.9 (8.0 to 15.7)*	517.7 (318.2 to 788.3)	45.6 (34.9 to 57.0)*	14.6 (6.2 to 23.6)*
Neural tube defects	73.3 (50.8 to 104.1)	36.4 (33.3 to 39.4)*	7.4 (4.9 to 9.8)*	24.4 (13.8 to 39.6)	38.1 (30.2 to 45.5)*	8.7 (2.5 to 14.5)*
Congenital heart anomalies	3,344.3 (2,361.4 to 4,927.5)	54.0 (48.1 to 59.4)*	21.2 (16.5 to 25.4)*	114.7 (45.1 to 210.8)	54.4 (48.7 to 59.7)*	21.5 (17.0 to 25.7)*
Less severe heart anomalies cases aggregate	3,038.5 (2,042.3 to 4,648.3)	53.5 (47.0 to 59.3)*	20.9 (15.7 to 25.4)*	96.6 (36.1 to 185.3)	53.7 (47.0 to 60.1)*	21.0 (15.7 to 26.0)*
Severe congenital heart anomalies	297.4 (198.9 to 448.2)	58.8 (50.2 to 66.5)*	25.0 (18.2 to 31.1)*	17.3 (6.9 to 34.4)	59.1 (48.8 to 69.0)*	25.2 (17.1 to 33.0)*
Critical congenital heart anomalies	3.9 (2.3 to 6.5)	80.0 (54.0 to 101.3)*	41.7 (21.2 to 58.4)*	0.2 (0.1 to 0.5)	79.3 (37.0 to 118.9)*	41.1 (7.9 to 72.3)*
Heart failure due to congenital heart anomalies aggregate	4.4 (3.8 to 5.1)	30.3 (28.8 to 31.9)*	2.6 (1.4 to 3.8)*	0.5 (0.4 to 0.8)	30.3 (28.8 to 31.9)*	2.6 (1.4 to 3.8)*
Cleft lip and cleft palate	444.6 (262.1 to 728.8)	38.6 (23.8 to 50.2)*	9.1 (-2.6 to 18.3)	4.7 (2.4 to 8.3)	30.8 (14.4 to 44.4)*	2.9 (-9.9 to 13.7)
Down syndrome	308.9 (197.8 to 474.0)	29.4 (23.0 to 35.0)*	1.9 (-3.2 to 6.3)	27.7 (15.7 to 46.0)	29.4 (22.7 to 35.1)*	1.9 (-3.4 to 6.4)
Turner syndrome	23.6 (10.9 to 42.7)	27.2 (24.6 to 30.0)*	0.1 (-1.9 to 2.3)	0.5 (0.2 to 1.1)	27.3 (19.4 to 35.3)*	0.2 (-6.0 to 6.5)
Klinefelter syndrome	13.5 (7.1 to 24.1)	25.7 (23.1 to 27.6)*	-1.1 (-3.1 to 0.4)	0.1 (0.0 to 0.3)	25.7 (19.0 to 32.2)*	-1.0 (-6.4 to 4.1)
Other chromosomal abnormalities	287.8 (159.4 to 505.6)	28.1 (21.4 to 33.8)*	0.8 (-4.4 to 5.3)	26.0 (13.1 to 48.0)	28.3 (20.0 to 34.3)*	1.0 (-5.5 to 5.7)
Other congenital anomalies	1,900.5 (1,033.9 to 3,495.6)	28.1 (24.9 to 31.4)*	0.8 (-1.7 to 3.5)	319.7 (191.1 to 513.4)	46.8 (32.5 to 64.4)*	15.5 (4.3 to 29.4)*
Skin and subcutaneous diseases	113,126.2 (110,371.5 to 115,182.6)	28.8 (27.8 to 29.8)*	1.4 (0.6 to 2.1)*	2,241.8 (1,489.2 to 3,299.6)	27.9 (26.7 to 29.1)*	0.6 (-0.3 to 1.6)
Dermatitis	15,191.1 (12,467.0 to 18,812.4)	26.4 (25.6 to 27.2)*	-0.5 (-1.1 to 0.1)	441.1 (284.4 to 634.4)	25.7 (24.2 to 27.0)*	-1.1 (-2.2 to -0.0)*
Eczema cases aggregate	2,774.9 (2,128.3 to 3,543.4)	25.1 (24.5 to 25.7)*	-1.5 (-2.0 to -1.1)*	166.2 (102.6 to 242.0)	25.4 (22.5 to 28.3)*	-1.3 (-3.6 to 1.0)

Cause	Prevalence in thousands (95% confidence interval)			YLDs in thousands (95% confidence interval)		
	2015	Percent change in counts between 2005 and 2015	Percent change in rates between 2005 and 2015	2015	Percent change in counts between 2005 and 2015	Percent change in rates between 2005 and 2015
Contact dermatitis cases aggregate	7,542.7 (5,232.9 to 10,667.1)	23.6 (22.9 to 24.3)*	-2.7 (-3.3 to -2.2)*	202.7 (113.2 to 326.6)	23.8 (21.9 to 25.5)*	-2.5 (-4.0 to -1.2)*
Seborrhoeic dermatitis cases aggregate	4,873.5 (3,481.6 to 6,708.3)	31.8 (31.4 to 32.1)*	3.7 (3.4 to 4.0)*	72.2 (38.4 to 121.6)	32.0 (30.3 to 33.6)*	3.9 (2.5 to 5.1)*
Psoriasis	6,471.5 (5,924.8 to 7,082.9)	25.1 (23.7 to 26.6)*	-1.5 (-2.6 to -0.4)*	521.8 (364.0 to 724.3)	25.4 (23.2 to 27.8)*	-1.3 (-3.0 to 0.6)
Cellulitis	56.1 (42.2 to 73.6)	22.9 (17.2 to 28.7)*	-3.2 (-7.7 to 1.3)	4.0 (2.5 to 6.2)	23.1 (13.2 to 33.0)*	-3.1 (-10.9 to 4.7)
Pyoderma	320.7 (280.6 to 367.4)	27.3 (24.1 to 31.0)*	0.2 (-2.3 to 3.1)	1.8 (0.7 to 3.8)	27.5 (22.0 to 33.1)*	0.3 (-4.0 to 4.8)
Scabies	8,969.7 (5,856.2 to 13,269.2)	35.8 (29.3 to 43.3)*	6.9 (1.8 to 12.8)*	229.6 (113.9 to 422.3)	36.0 (29.5 to 43.3)*	7.1 (1.9 to 12.8)*
Fungal skin diseases	23,514.8 (19,085.4 to 29,085.0)	29.2 (29.0 to 29.4)*	1.7 (1.5 to 1.9)*	131.9 (50.7 to 287.2)	29.4 (28.8 to 29.9)*	1.8 (1.4 to 2.3)*
Viral skin diseases	7,331.9 (6,217.6 to 8,639.2)	26.8 (26.3 to 27.2)*	-0.2 (-0.6 to 0.2)	223.5 (134.5 to 337.7)	27.0 (25.6 to 28.3)*	-0.1 (-1.2 to 1.0)
Molluscum contagiosum cases aggregate	379.8 (298.8 to 481.6)	26.2 (25.7 to 26.8)*	-0.6 (-1.1 to -0.2)*	11.6 (6.9 to 17.5)	26.5 (20.9 to 31.7)*	-0.4 (-4.8 to 3.7)
Viral warts cases aggregate	6,952.1 (5,829.2 to 8,264.6)	26.8 (26.3 to 27.3)*	-0.2 (-0.6 to 0.2)	211.9 (126.9 to 323.4)	27.0 (25.6 to 28.3)*	-0.0 (-1.2 to 1.0)
Acne vulgaris	13,186.5 (11,281.3 to 15,328.8)	28.5 (25.2 to 31.6)*	1.1 (-1.4 to 3.6)	140.6 (65.8 to 265.8)	28.7 (25.4 to 31.9)*	1.3 (-1.3 to 3.8)
Alopecia areata	1,522.4 (1,290.3 to 1,793.4)	25.4 (24.8 to 25.9)*	-1.3 (-1.8 to -0.9)*	51.2 (31.3 to 80.4)	25.6 (23.0 to 28.1)*	-1.2 (-3.2 to 0.8)
Pruritus	5,127.8 (3,467.9 to 7,268.1)	27.7 (23.5 to 33.0)*	0.5 (-2.8 to 4.7)	55.0 (23.6 to 114.7)	27.9 (23.8 to 33.4)*	0.7 (-2.6 to 5.0)
Urticaria	2,959.2 (2,093.1 to 4,229.6)	26.9 (26.2 to 27.5)*	-0.1 (-0.7 to 0.4)	176.2 (101.2 to 288.5)	27.2 (25.0 to 29.2)*	0.1 (-1.6 to 1.7)
Decubitus ulcer	49.2 (36.2 to 64.7)	27.2 (20.3 to 32.7)*	0.1 (-5.3 to 4.5)	7.9 (4.9 to 11.9)	27.5 (15.1 to 38.9)*	0.4 (-9.4 to 9.3)
Other skin and subcutaneous diseases	43,491.7 (39,544.9 to 47,677.0)	30.2 (28.0 to 32.5)*	2.5 (0.7 to 4.3)*	257.3 (122.1 to 486.9)	30.4 (28.0 to 32.8)*	2.6 (0.7 to 4.6)*
Sense organ diseases	158,994.5 (154,808.5 to 163,234.9)	26.6 (25.6 to 27.6)*	-0.4 (-1.2 to 0.5)	4,530.8 (2,978.2 to 6,641.3)	27.1 (25.1 to 29.1)*	0.1 (-1.5 to 1.6)
Glaucoma	151.5 (102.3 to 214.8)	31.3 (28.3 to 34.6)*	3.4 (1.0 to 6.0)*	12.1 (7.1 to 19.1)	32.4 (23.7 to 41.1)*	4.2 (-2.7 to 11.1)
Cataract	2,517.6 (1,914.8 to 3,236.4)	21.1 (18.0 to 24.1)*	-4.7 (-7.1 to -2.3)*	175.2 (112.9 to 255.8)	20.9 (17.7 to 23.8)*	-4.8 (-7.3 to -2.6)*
Macular degeneration	123.0 (77.9 to 181.5)	40.9 (37.8 to 44.1)*	10.9 (8.5 to 13.5)*	11.3 (6.2 to 18.7)	40.7 (30.9 to 50.9)*	10.8 (3.0 to 18.8)*
Refraction and accommodation disorders	66,316.7 (60,178.3 to 72,617.7)	21.4 (18.6 to 24.8)*	-4.5 (-6.7 to -1.8)*	1,070.9 (650.7 to 1,706.7)	22.9 (20.9 to 25.3)*	-3.2 (-4.9 to -1.4)*
Age-related and other hearing loss	98,166.3 (84,094.7 to 113,828.4)	29.5 (28.1 to 31.1)*	1.9 (0.8 to 3.2)*	2,622.4 (1,653.6 to 3,937.5)	28.6 (25.1 to 32.0)*	1.3 (-1.5 to 3.9)
Other vision loss	1,709.2 (1,303.0 to 2,185.8)	34.3 (31.0 to 38.2)*	5.7 (3.1 to 8.8)*	133.4 (87.9 to 195.4)	34.2 (30.1 to 38.2)*	5.6 (2.4 to 8.8)*
Other sense organ diseases	19,937.0 (17,745.2 to 22,394.2)	28.4 (25.5 to 31.2)*	1.1 (-1.2 to 3.3)	505.5 (308.9 to 748.6)	28.6 (25.6 to 31.7)*	1.2 (-1.1 to 3.6)
Acute other sense organ diseases	65.8 (58.6 to 73.2)	28.3 (25.4 to 31.3)*	1.0 (-1.3 to 3.3)	1.8 (1.1 to 2.6)	28.3 (25.4 to 31.3)*	1.0 (-1.3 to 3.3)
Chronic other sense organ diseases	19,871.2 (17,678.3 to 22,327.3)	28.4 (25.5 to 31.2)*	1.1 (-1.3 to 3.3)	503.7 (307.8 to 746.2)	28.6 (25.6 to 31.7)*	1.2 (-1.1 to 3.6)
Oral disorders	227,240.1 (219,729.4 to 234,119.6)	26.1 (24.6 to 27.5)*	-0.8 (-1.9 to 0.4)	1,349.7 (788.7 to 2,169.3)	25.3 (24.4 to 26.2)*	-1.4 (-2.0 to -0.7)*
Deciduous caries	0.0 (0.0 to 0.0)	0.0 (0.0 to 0.0)	0.0 (0.0 to 0.0)	0.0 (0.0 to 0.0)	0.0 (0.0 to 0.0)	0.0 (0.0 to 0.0)
Permanent caries	175,586.6 (144,519.7 to 207,471.6)	25.3 (23.2 to 27.7)*	-1.4 (-3.1 to 0.5)	123.2 (53.3 to 242.7)	22.4 (20.1 to 25.0)*	-3.6 (-5.5 to -1.6)*
Periodontal diseases	68,070.2 (53,995.7 to 86,285.4)	27.7 (26.5 to 28.6)*	0.5 (-0.4 to 1.2)	449.9 (165.2 to 953.1)	27.9 (26.7 to 28.9)*	0.6 (-0.3 to 1.4)
Edentulism and severe tooth loss	15,813.0 (14,583.6 to 17,485.9)	22.1 (21.7 to 22.5)*	-3.9 (-4.2 to -3.6)*	449.9 (297.4 to 629.8)	22.3 (21.4 to 23.2)*	-3.7 (-4.5 to -3.1)*
Other oral disorders	11,183.6 (9,883.3 to 12,366.1)	27.1 (26.5 to 27.6)*	0.0 (-0.4 to 0.5)	326.7 (201.6 to 502.6)	27.3 (26.3 to 28.2)*	0.2 (-0.6 to 0.9)
Injuries	83,491.7 (79,353.5 to 87,525.0)	19.2 (17.5 to 21.0)*	-6.2 (-7.5 to -4.8)*	3,229.5 (2,293.0 to 4,370.8)	8.8 (5.1 to 12.5)*	-14.3 (-17.2 to -11.5)*
Transport injuries	11,462.0 (10,775.7 to 12,325.3)	27.6 (26.7 to 28.4)*	0.4 (-0.2 to 1.0)	643.8 (444.2 to 878.3)	15.7 (11.5 to 19.5)*	-9.0 (-12.3 to -5.9)*
Road injuries	10,766.3 (10,094.2 to 11,608.2)	28.1 (27.2 to 28.9)*	0.8 (0.1 to 1.5)*	602.0 (413.7 to 822.6)	16.9 (12.8 to 20.7)*	-8.0 (-11.2 to -5.0)*
Pedestrian road injuries	1,368.2 (1,176.3 to 1,609.3)	30.8 (29.6 to 31.9)*	2.9 (2.0 to 3.8)*	75.9 (51.4 to 104.5)	15.4 (10.4 to 19.9)*	-9.2 (-13.1 to -5.6)*
Cyclist road injuries	1,805.2 (1,567.2 to 2,062.0)	32.4 (31.2 to 33.7)*	4.2 (3.3 to 5.2)*	94.4 (63.5 to 130.9)	19.0 (13.5 to 23.9)*	-6.3 (-10.6 to -2.5)*
Motorcyclist road injuries	2,846.2 (2,444.1 to 3,263.7)	38.5 (37.4 to 39.8)*	9.0 (8.1 to 10.1)*	152.5 (103.8 to 209.5)	22.4 (16.5 to 27.6)*	-3.6 (-8.3 to 0.4)
Motor vehicle road injuries	4,336.8 (3,820.5 to 5,057.1)	18.3 (17.1 to 19.5)*	-6.9 (-7.8 to -5.9)*	255.8 (173.2 to 354.3)	11.9 (8.6 to 14.8)*	-11.9 (-14.5 to -9.6)*
Other road injuries	409.8 (344.7 to 472.4)	49.0 (47.7 to 50.2)*	17.3 (16.3 to 18.2)*	23.3 (15.7 to 32.7)	40.4 (35.8 to 44.1)*	10.5 (6.9 to 13.4)*
Other transport injuries	695.8 (613.9 to 790.5)	19.9 (19.3 to 20.4)*	-5.6 (-6.1 to -5.2)*	41.8 (29.7 to 57.1)	0.3 (-4.6 to 5.9)	-21.1 (-24.9 to -16.6)*
Unintentional injuries	62,169.4 (59,175.7 to 65,533.8)	16.3 (15.8 to 16.7)*	-8.5 (-8.9 to -8.2)*	2,286.1 (1,593.3 to 3,115.3)	6.8 (3.3 to 10.2)*	-15.9 (-18.7 to -13.3)*
Falls	17,038.9 (15,238.7 to 18,962.4)	26.9 (26.4 to 27.4)*	-0.1 (-0.5 to 0.3)	877.9 (600.5 to 1,208.2)	11.2 (5.9 to 16.1)*	-12.5 (-16.6 to -8.6)*
Drowning	300.5 (254.2 to 347.5)	8.6 (8.2 to 9.3)*	-14.5 (-14.9 to -14.0)*	16.8 (11.5 to 22.9)	-7.5 (-11.8 to -2.7)*	-27.2 (-30.6 to -23.4)*
Fire, heat, and hot substances	5,785.1 (5,002.4 to 6,545.7)	8.8 (8.1 to 9.4)*	-14.3 (-14.9 to -13.9)*	192.5 (135.6 to 263.2)	0.4 (-3.8 to 4.3)	-20.9 (-24.3 to -17.9)*
Poisonings	387.5 (331.2 to 455.1)	16.6 (15.6 to 17.8)*	-8.2 (-9.0 to -7.3)*	34.0 (23.1 to 48.5)	10.8 (7.6 to 13.9)*	-12.8 (-15.3 to -10.4)*
Exposure to mechanical forces	15,056.4 (13,826.9 to 16,295.9)	18.5 (17.8 to 19.1)*	-6.7 (-7.3 to -6.3)*	280.2 (195.2 to 390.9)	9.1 (5.5 to 12.4)*	-14.1 (-17.0 to -11.5)*
Unintentional firearm injuries	218.5 (190.1 to 250.8)	15.9 (15.1 to 16.8)*	-8.7 (-9.4 to -8.1)*	8.3 (5.7 to 11.6)	5.9 (1.7 to 9.8)*	-16.6 (-20.0 to -13.6)*
Unintentional suffocation	657.4 (532.7 to 808.3)	16.0 (13.3 to 18.8)*	-8.7 (-10.9 to -6.5)*	23.7 (16.1 to 32.4)	15.5 (13.1 to 17.5)*	-9.1 (-11.0 to -7.5)*
Other exposure to mechanical forces	14,180.6 (12,964.2 to 15,419.3)	18.6 (18.0 to 19.2)*	-6.6 (-7.1 to -6.2)*	248.2 (172.2 to 346.5)	8.6 (4.7 to 12.2)*	-14.5 (-17.6 to -11.7)*
Adverse effects of medical treatment	700.5 (521.5 to 896.6)	6.7 (2.0 to 11.2)*	-16.0 (-19.7 to -12.5)*	93.5 (57.2 to 140.5)	6.7 (2.0 to 11.2)*	-16.0 (-19.7 to -12.5)*
Animal contact	2,630.9 (2,362.4 to 2,925.9)	10.9 (9.6 to 12.1)*	-12.7 (-13.7 to -11.8)*	73.5 (50.2 to 99.6)	6.7 (4.2 to 9.2)*	-16.0 (-18.0 to -14.1)*
Venomous animal contact	1,218.5 (1,078.1 to 1,380.9)	14.5 (13.6 to 15.5)*	-9.8 (-10.6 to -9.1)*	54.9 (37.3 to 74.4)	7.9 (5.1 to 10.6)*	-15.1 (-17.3 to -13.0)*
Non-venomous animal contact	1,412.4 (1,212.8 to 1,635.5)	7.9 (6.2 to 9.6)*	-15.0 (-16.4 to -13.8)*	18.7 (12.1 to 28.3)	3.5 (-0.1 to 6.6)	-18.5 (-21.4 to -16.1)*
Foreign body	2,302.1 (2,035.8 to 2,592.0)	20.3 (19.3 to 21.3)*	-5.3 (-6.1 to -4.5)*	81.8 (57.7 to 112.8)	7.2 (2.7 to 11.7)*	-15.6 (-19.1 to -12.1)*
Pulmonary aspiration and foreign body in airway	918.1 (768.8 to 1,139.6)	25.9 (23.4 to 28.4)*	-0.9 (-2.9 to 1.1)	44.6 (31.0 to 62.1)	5.5 (0.3 to 11.6)*	-16.9 (-21.0 to -12.1)*
Foreign body in eyes	68.9 (31.3 to 115.6)	26.1 (24.5 to 27.9)*	-0.7 (-2.0 to 0.7)	3.8 (1.6 to 7.1)	24.6 (21.9 to 26.6)*	-1.9 (-4.1 to -0.4)*
Foreign body in other body part	1,315.2 (1,144.1 to 1,526.9)	16.3 (15.7 to 17.0)*	-8.4 (-8.9 to -7.9)*	33.4 (22.9 to 47.1)	7.7 (4.6 to 10.7)*	-15.2 (-17.7 to -12.8)*
Environmental heat and cold exposure	4,999.4 (4,373.7 to 5,820.0)	15.1 (14.5 to 15.7)*	-9.4 (-9.9 to -8.9)*	202.2 (139.8 to 278.2)	3.2 (-0.5 to 6.9)	-18.8 (-21.7 to -15.9)*
Other unintentional injuries	12,968.2 (11,601.2 to 14,604.2)	6.9 (6.2 to 7.7)*	-15.8 (-16.4 to -15.2)*	433.7 (287.8 to 613.3)	2.3 (0.2 to 4.2)*	-19.5 (-21.2 to -18.0)*
Self-harm and interpersonal violence	2,467.1 (2,252.2 to 2,655.8)	17.9 (17.3 to 18.4)*	-7.2 (-7.6 to -6.8)*	108.2 (76.3 to 145.7)	3.9 (-0.4 to 8.3)	-18.3 (-21.6 to -14.8)*
Self-harm	585.2 (507.6 to 676.0)	12.9 (12.2 to 13.5)*	-11.2 (-11.7 to -10.6)*	29.9 (20.9 to 40.6)	-0.4 (-4.0 to 3.6)	-21.6 (-24.5 to -18.5)*
Interpersonal violence	1,881.9 (1,702.0 to 2,058.5)	19.5 (18.9 to 20.1)*	-5.9 (-6.4 to -5.5)*	78.3 (54.0 to 106.9)	5.6 (1.1 to 10.1)*	-16.9 (-20.4 to -13.3)*

Cause	Prevalence in thousands (95% confidence interval)			YLDs in thousands (95% confidence interval)		
	2015	Percent change in counts between 2005 and 2015	Percent change in rates between 2005 and 2015	2015	Percent change in counts between 2005 and 2015	Percent change in rates between 2005 and 2015
Assault by firearm	116.5<br>(99.4 to 136.8)	17.0<br>(15.7 to 18.2)*	-7.9<br>(-9.0 to -7.0)*	5.1<br>(3.5 to 7.2)	5.6<br>(1.0 to 9.5)*	-16.9<br>(-20.5 to -13.8)*
Assault by sharp object	445.9<br>(363.8 to 527.5)	20.4<br>(19.6 to 21.2)*	-5.2<br>(-5.9 to -4.6)*	12.7<br>(8.5 to 17.6)	0.3<br>(-6.1 to 6.5)	-21.1<br>(-26.1 to -16.2)*
Assault by other means	1,319.5<br>(1,180.2 to 1,480.2)	19.5<br>(18.7 to 20.2)*	-6.0<br>(-6.6 to -5.4)*	60.5<br>(41.9 to 82.7)	6.8<br>(2.5 to 11.0)*	-16.0<br>(-19.3 to -12.6)*
Forces of nature, war, and legal intervention	7,393.1<br>(5,365.3 to 9,810.8)	34.9<br>(13.4 to 59.2)*	6.2<br>(-10.7 to 25.3)	191.4<br>(138.4 to 251.6)	14.7<br>(-4.8 to 39.8)	-9.7<br>(-25.1 to 10.0)
Exposure to forces of nature	1,904.3<br>(1,198.1 to 2,872.8)	66.4<br>(24.0 to 113.6)*	31.0<br>(-2.4 to 68.1)	55.2<br>(36.5 to 76.8)	13.4<br>(-24.9 to 62.0)	-10.7<br>(-40.9 to 27.5)
Collective violence and legal intervention	5,488.9<br>(3,829.1 to 7,321.8)	26.5<br>(3.4 to 54.6)*	-0.4<br>(-18.6 to 21.7)	136.2<br>(92.5 to 189.7)	15.2<br>(-10.5 to 49.2)	-9.3<br>(-29.6 to 17.4)
**Anemia**	**132,059.0<br>(130,369.9 to 133,893.8)**	**14.9<br>(13.2 to 16.5)***	**-9.5<br>(-10.9 to -8.3)***	**3,487.5<br>(2,309.4 to 5,044.3)**	**7.7<br>(5.1 to 10.4)***	**-15.3<br>(-17.3 to -13.1)***
**Developmental intellectual disability**	**8,241.0<br>(6,130.1 to 10,308.4)**	**28.5<br>(28.0 to 29.2)***	**1.2<br>(0.8 to 1.7)***	**926.7<br>(664.8 to 1,229.9)**	**31.7<br>(28.9 to 35.0)***	**3.7<br>(1.4 to 6.3)***
**Epilepsy**	**2,046.2<br>(1,728.4 to 2,355.5)**	**27.7<br>(23.0 to 32.1)***	**0.5<br>(-3.2 to 4.0)**	**588.3<br>(416.9 to 772.6)**	**18.2<br>(9.3 to 25.9)***	**-7.0<br>(-14.0 to -0.9)***
**Guillain-Barré syndrome**	**2.4<br>(1.5 to 3.7)**	**24.0<br>(21.8 to 25.5)***	**-2.4<br>(-4.1 to -1.2)***	**0.7<br>(0.4 to 1.2)**	**24.0<br>(21.8 to 25.5)***	**-2.4<br>(-4.1 to -1.2)***
**Hearing loss**	**104,382.1<br>(90,030.1 to 119,659.5)**	**28.8<br>(27.4 to 30.3)***	**1.4<br>(0.3 to 2.6)***	**2,895.2<br>(1,856.2 to 4,269.9)**	**27.8<br>(24.5 to 30.7)***	**0.6<br>(-2.0 to 2.9)**
**Heart failure**	**1,362.2<br>(1,296.1 to 1,431.0)**	**28.2<br>(27.5 to 28.8)***	**0.9<br>(0.3 to 1.4)***	**178.3<br>(125.1 to 243.6)**	**29.9<br>(27.6 to 32.3)***	**2.3<br>(0.5 to 4.1)***
**Infertility**	**2,997.3<br>(1,977.5 to 4,515.8)**	**26.4<br>(17.9 to 34.7)***	**-0.5<br>(-7.2 to 6.0)**	**23.1<br>(11.5 to 43.7)**	**29.3<br>(22.8 to 35.8)***	**1.8<br>(-3.3 to 6.9)**
**Pelvic inflammatory disease**	**66.9<br>(50.7 to 84.4)**	**8.7<br>(7.1 to 10.1)***	**-14.4<br>(-15.7 to -13.3)***	**8.8<br>(5.4 to 12.9)**	**9.0<br>(3.2 to 15.3)***	**-14.2<br>(-18.8 to -9.3)***
**Vision loss**	**72,259.2<br>(65,704.4 to 78,626.2)**	**21.7<br>(19.1 to 24.7)***	**-4.2<br>(-6.3 to -1.8)***	**1,605.4<br>(1,078.1 to 2,369.6)**	**24.3<br>(22.5 to 26.1)***	**-2.2<br>(-3.6 to -0.8)***

SR Table 11. Prevalent cases and YLDs for 2015, percent change, and change in age-standardised rates between 2005 and 2015 by cause, global, ages 50-54 years						
Cause	Prevalence in thousands (95% confidence interval)			YLDs in thousands (95% confidence interval)		
	2015	Percent change in counts between 2005 and 2015	Percent change in rates between 2005 and 2015	2015	Percent change in counts between 2005 and 2015	Percent change in rates between 2005 and 2015
**All causes**	--	--	--	**57,408.0 (42,880.3 to 73,630.8)**	**22.9 (22.2 to 23.6)***	**-1.6 (-2.2 to -1.0)***
Communicable, maternal, neonatal, and nutritional diseases	223,390.6 (220,881.1 to 225,872.0)	20.5 (19.5 to 21.5)*	-3.5 (-4.3 to -2.7)*	4,040.4 (2,936.5 to 5,543.1)	9.0 (6.1 to 12.4)*	-12.7 (-15.0 to -10.0)*
HIV/AIDS and tuberculosis	3,237.2 (3,119.1 to 3,363.3)	60.1 (52.4 to 67.9)*	28.2 (22.0 to 34.4)*	464.6 (324.3 to 632.9)	10.0 (-1.2 to 21.1)	-11.9 (-20.9 to -3.1)*
Tuberculosis	585.4 (431.9 to 776.5)	9.1 (0.5 to 18.3)*	-12.7 (-19.5 to -5.3)*	176.7 (107.6 to 257.5)	9.4 (-0.1 to 19.1)	-12.4 (-20.0 to -4.7)*
HIV/AIDS	2,665.6 (2,561.5 to 2,781.5)	78.4 (67.5 to 89.2)*	42.8 (34.1 to 51.5)*	287.9 (208.8 to 375.1)	10.3 (-4.8 to 27.4)	-11.7 (-23.8 to 2.0)
HIV/AIDS - Tuberculosis	83.0 (74.2 to 92.0)	17.9 (12.6 to 24.8)*	-5.6 (-9.9 to -0.1)*	30.4 (20.7 to 41.5)	18.5 (10.8 to 27.5)*	-5.1 (-11.3 to 2.1)
HIV/AIDS resulting in other diseases	2,670.5 (2,545.6 to 2,822.9)	81.8 (75.5 to 88.2)*	45.5 (40.5 to 50.7)*	257.5 (186.7 to 336.5)	9.4 (-7.4 to 27.9)	-12.4 (-25.8 to 2.4)
HIV aggregate	837.0 (783.7 to 898.7)	-15.1 (-18.6 to -11.1)*	-32.0 (-34.8 to -28.9)*	60.3 (43.3 to 79.1)	-16.4 (-20.4 to -11.8)*	-33.0 (-36.3 to -29.4)*
AIDS aggregate	1,833.5 (1,733.2 to 1,959.4)	279.6 (239.7 to 326.9)*	203.9 (172.0 to 241.8)*	197.2 (142.7 to 261.4)	20.8 (-4.3 to 52.0)	-3.3 (-23.4 to 21.7)
Diarrhea, lower respiratory, and other common infectious diseases	17,828.2 (16,525.3 to 19,291.7)	20.8 (17.8 to 23.6)*	-3.3 (-5.7 to -1.1)*	532.2 (362.2 to 740.8)	21.0 (19.1 to 22.9)*	-3.1 (-4.6 to -1.6)*
Diarrheal diseases	847.6 (811.2 to 882.3)	24.8 (23.9 to 25.7)*	-0.1 (-0.8 to 0.7)	131.1 (89.8 to 179.9)	25.2 (22.7 to 27.9)*	0.3 (-1.8 to 2.4)
Diarrhoea episodes aggregate	847.3 (810.8 to 881.9)	24.8 (23.9 to 25.7)*	-0.1 (-0.8 to 0.7)	131.0 (89.7 to 179.8)	25.2 (22.7 to 27.9)*	0.3 (-1.8 to 2.4)
Guillain-Barré syndrome due to diarrheal diseases	0.3 (0.2 to 0.5)	24.4 (22.6 to 25.9)*	-0.4 (-1.8 to 0.8)	0.1 (0.0 to 0.2)	24.4 (22.6 to 25.9)*	-0.4 (-1.8 to 0.8)
Intestinal infectious diseases	25.6 (22.7 to 28.6)	-19.6 (-29.5 to -9.3)*	-35.6 (-43.5 to -27.4)*	4.5 (2.8 to 6.5)	3.5 (-9.3 to 18.8)	-17.1 (-27.4 to -4.9)*
Typhoid fever	28.5 (21.6 to 37.0)	2.9 (-2.9 to 9.3)	-17.6 (-22.2 to -12.5)*	3.8 (2.3 to 5.5)	6.2 (-9.7 to 24.1)	-14.9 (-27.7 to -0.6)*
Typhoid fever episodes aggregate	23.6 (17.8 to 30.6)	2.9 (-6.5 to 13.9)	-17.6 (-25.1 to -8.8)*	2.2 (1.3 to 3.3)	3.6 (-12.7 to 23.7)	-17.0 (-30.1 to -1.0)*
Complications of typhoid fever aggregate	4.9 (3.3 to 6.9)	2.9 (-29.8 to 50.3)	-17.6 (-43.8 to 20.3)	1.6 (0.9 to 2.5)	10.1 (-26.9 to 66.4)	-11.9 (-41.5 to 33.2)
Paratyphoid fever	12.9 (8.6 to 17.9)	-2.8 (-11.5 to 6.2)	-22.2 (-29.1 to -14.9)*	0.7 (0.4 to 1.1)	-2.4 (-20.1 to 19.8)	-21.8 (-36.0 to -4.1)*
Paratyphoid fever episodes aggregate	12.2 (8.0 to 17.1)	-2.8 (-11.6 to 6.4)	-22.2 (-29.2 to -14.8)*	0.6 (0.3 to 1.0)	-2.4 (-21.2 to 23.1)	-21.8 (-36.9 to -1.4)*
Intestinal perforation due to paratyphoid	0.7 (0.4 to 1.1)	-2.0 (-46.5 to 82.6)	-21.6 (-57.2 to 46.2)	0.1 (0.0 to 0.1)	-2.3 (-46.5 to 82.6)	-21.8 (-57.2 to 46.2)
Other intestinal infectious diseases	--	--	--	0.0 (0.0 to 0.1)	-61.1 (-74.5 to -38.7)*	-68.9 (-79.6 to -50.9)*
Lower respiratory infections	266.1 (249.6 to 284.2)	24.2 (22.2 to 26.4)*	-0.5 (-2.1 to 1.2)	15.9 (10.6 to 22.4)	24.3 (19.1 to 29.6)*	-0.5 (-4.7 to 3.7)
Lower respiratory infection episodes aggregate	265.8 (249.3 to 283.8)	24.2 (22.2 to 26.4)*	-0.5 (-2.1 to 1.2)	15.8 (10.6 to 22.2)	24.3 (19.0 to 29.6)*	-0.5 (-4.7 to 3.8)
Guillain-Barré syndrome due to lower respiratory infections	0.3 (0.2 to 0.6)	24.4 (22.6 to 25.9)*	-0.4 (-1.8 to 0.8)	0.1 (0.0 to 0.2)	24.5 (22.6 to 25.9)*	-0.4 (-1.8 to 0.8)
Upper respiratory infections	11,012.3 (7,802.0 to 15,246.7)	23.6 (22.2 to 24.9)*	-1.1 (-2.2 to 0.0)	127.3 (64.7 to 231.1)	23.7 (21.9 to 25.6)*	-0.9 (-2.4 to 0.5)
Upper respiratory infection episodes aggregate	11,011.4 (7,801.2 to 15,245.6)	23.6 (22.2 to 24.9)*	-1.1 (-2.2 to 0.0)	127.0 (64.4 to 230.8)	23.7 (21.9 to 25.6)*	-0.9 (-2.4 to 0.5)
Guillain-Barré syndrome due to upper respiratory infections	0.9 (0.5 to 1.4)	24.4 (22.6 to 25.9)*	-0.4 (-1.8 to 0.8)	0.3 (0.1 to 0.5)	24.4 (22.6 to 25.9)*	-0.4 (-1.8 to 0.8)
Otitis media	4,769.5 (3,508.4 to 6,387.0)	14.6 (10.8 to 18.6)*	-8.3 (-11.3 to -5.1)*	131.9 (74.1 to 206.8)	14.3 (9.0 to 20.5)*	-8.5 (-12.7 to -3.5)*
Acute otitis media	351.5 (277.4 to 446.4)	26.6 (22.1 to 31.1)*	1.4 (-2.2 to 5.0)	4.5 (2.2 to 8.3)	26.7 (21.1 to 32.4)*	1.4 (-3.0 to 6.0)
Chronic otitis media aggregate	4,418.0 (3,184.0 to 6,011.0)	13.7 (9.6 to 18.0)*	-8.9 (-12.2 to -5.5)*	127.4 (70.9 to 201.4)	13.9 (8.4 to 20.2)*	-8.8 (-13.2 to -3.7)*
Meningitis	423.4 (404.8 to 443.0)	22.1 (17.5 to 26.4)*	-2.3 (-5.9 to 1.2)	74.1 (52.7 to 97.6)	24.7 (21.4 to 27.8)*	-0.2 (-2.8 to 2.3)
Pneumococcal meningitis	373.3 (317.9 to 436.7)	28.2 (25.9 to 30.9)*	2.7 (0.8 to 4.8)*	35.4 (24.8 to 47.3)	26.2 (21.1 to 31.2)*	1.1 (-3.0 to 5.0)
Acute pneumococcal meningitis	1.3 (0.9 to 1.8)	28.6 (16.0 to 41.6)*	3.0 (-7.1 to 13.4)	0.2 (0.1 to 0.3)	28.7 (16.0 to 41.6)*	3.0 (-7.1 to 13.4)
Complications of pneumococcal meningitis aggregate	372.0 (316.6 to 435.1)	28.2 (25.9 to 30.9)*	2.7 (0.8 to 4.8)*	35.3 (24.7 to 47.0)	26.2 (21.0 to 31.2)*	1.0 (-3.1 to 5.0)
H influenzae type B meningitis	100.6 (77.6 to 127.0)	-3.2 (-7.6 to 2.3)	-22.5 (-26.0 to -18.1)*	11.2 (7.8 to 15.2)	19.5 (12.6 to 26.6)*	-4.3 (-9.9 to 1.4)
Acute H influenzae type B meningitis	0.4 (0.3 to 0.6)	-9.9 (-20.3 to 0.6)	-27.9 (-36.2 to -19.5)*	0.1 (0.0 to 0.1)	-9.9 (-20.3 to 0.6)	-27.9 (-36.2 to -19.5)*
Complications of of H influenzae type B meningitis aggregate	100.2 (77.2 to 126.5)	-3.2 (-7.6 to 2.3)	-22.5 (-26.0 to -18.1)*	11.1 (7.7 to 15.1)	19.7 (12.7 to 26.9)*	-4.2 (-9.8 to 1.6)
Meningococcal meningitis	86.3 (68.8 to 107.0)	26.3 (23.3 to 29.7)*	1.1 (-1.3 to 3.8)	9.8 (6.7 to 13.1)	26.7 (21.7 to 32.0)*	1.5 (-2.6 to 5.7)
Acute meningococcal meningitis	0.5 (0.3 to 0.8)	27.0 (12.7 to 41.6)*	1.7 (-9.8 to 13.3)	0.1 (0.0 to 0.1)	27.0 (12.7 to 41.6)*	1.7 (-9.8 to 13.3)
Complications of meningococcal meningitis aggregate	85.8 (68.3 to 106.4)	26.3 (23.3 to 29.7)*	1.1 (-1.3 to 3.8)	9.7 (6.7 to 13.0)	26.7 (21.6 to 32.0)*	1.5 (-2.6 to 5.7)
Other meningitis	164.6 (132.0 to 196.5)	23.4 (20.9 to 26.0)*	-1.2 (-3.2 to 0.9)	17.8 (12.4 to 24.0)	23.9 (18.6 to 29.2)*	-0.8 (-5.0 to 3.4)
Other meningitis episodes aggregate	3.7 (2.8 to 4.7)	26.3 (14.9 to 37.3)*	1.1 (-8.0 to 9.9)	0.5 (0.3 to 0.8)	26.4 (14.9 to 37.3)*	1.2 (-8.0 to 9.9)
Complications of other meningitis aggregate	160.9 (128.5 to 193.1)	23.3 (20.9 to 25.9)*	-1.3 (-3.2 to 0.8)	17.3 (12.1 to 23.3)	23.8 (18.4 to 29.3)*	-0.9 (-5.2 to 3.5)
Encephalitis	299.6 (210.0 to 411.8)	9.1 (6.3 to 12.5)*	-12.7 (-14.9 to -9.9)*	30.4 (21.3 to 41.3)	14.0 (8.5 to 19.8)*	-8.7 (-13.1 to -4.1)*
Acute encephalitis	3.8 (3.0 to 4.6)	17.3 (10.9 to 23.5)*	-6.1 (-11.2 to -1.1)*	0.5 (0.3 to 0.7)	18.7 (5.3 to 34.3)*	-5.0 (-15.7 to 7.6)
Complications of encephalitis aggregate	295.8 (206.1 to 407.5)	9.0 (6.2 to 12.5)*	-12.7 (-15.0 to -9.9)*	29.9 (21.0 to 40.4)	14.0 (8.3 to 19.9)*	-8.7 (-13.3 to -4.0)*
Diphtheria	0.0 (0.0 to 0.0)	-50.2 (-78.5 to 19.6)	-60.1 (-82.8 to -4.2)*	0.0 (0.0 to 0.0)	-49.8 (-78.5 to 19.6)	-59.8 (-82.8 to -4.2)*
Whooping cough	2.0 (1.6 to 2.5)	-15.8 (-18.7 to -12.8)*	-32.6 (-34.9 to -30.2)*	0.1 (0.1 to 0.2)	-15.9 (-18.7 to -12.8)*	-32.6 (-34.9 to -30.2)*
Tetanus	9.6 (9.4 to 10.0)	18.8 (14.7 to 21.3)*	-4.9 (-8.2 to -2.8)*	0.4 (0.3 to 0.6)	3.5 (-6.6 to 12.7)	-17.1 (-25.2 to -9.8)*
Severe tetanus	0.4 (0.2 to 0.8)	-32.4 (-48.0 to -6.3)*	-45.9 (-58.3 to -25.0)*	0.1 (0.0 to 0.1)	-32.4 (-47.7 to -6.4)*	-45.9 (-58.1 to -25.0)*
Complications of tetanus aggregate	9.2 (9.0 to 9.4)	23.1 (22.5 to 23.6)*	-1.5 (-2.0 to -1.0)*	0.4 (0.3 to 0.5)	12.3 (4.8 to 20.5)*	-10.1 (-16.1 to -3.5)*
Measles	0.1 (0.0 to 0.1)	-57.2 (-62.1 to -50.9)*	-65.7 (-69.7 to -60.7)*	0.0 (0.0 to 0.0)	-57.2 (-62.1 to -50.9)*	-65.7 (-69.7 to -60.7)*
Varicella and herpes zoster	341.3 (283.3 to 405.8)	24.6 (23.7 to 25.5)*	-0.3 (-0.9 to 0.5)	16.6 (9.7 to 25.8)	24.4 (20.3 to 28.6)*	-0.4 (-3.7 to 3.0)
Neglected tropical diseases and malaria	91,169.5 (88,953.4 to 93,508.8)	11.5 (8.2 to 15.0)*	-10.7 (-13.4 to -7.9)*	859.1 (574.5 to 1,312.6)	-1.1 (-10.6 to 12.1)	-20.8 (-28.4 to -10.2)*
Malaria	6,116.4 (5,381.4 to 6,925.0)	33.9 (27.2 to 41.1)*	7.2 (1.8 to 13.0)*	47.4 (33.9 to 64.9)	21.1 (14.4 to 28.3)*	-3.1 (-8.4 to 2.8)
Asymptomatic malaria parasitemia(PfPR)	4,533.6 (3,773.3 to 5,353.5)	37.0 (27.9 to 47.5)*	9.7 (2.4 to 18.1)*	0.0 (0.0 to 0.0)	0.0 (0.0 to 0.0)	0.0 (0.0 to 0.0)
Malaria episodes aggregate	173.8 (95.6 to 302.2)	-2.8 (-22.2 to 25.4)	-22.2 (-37.7 to 0.4)	3.5 (1.6 to 6.8)	-2.4 (-23.1 to 26.0)	-21.8 (-38.4 to 0.9)
Complications of malaria aggregate	26.8 (24.1 to 29.6)	37.7 (34.6 to 42.0)*	10.2 (7.8 to 13.7)*	8.3 (6.3 to 10.4)	32.4 (22.3 to 44.4)*	6.0 (-2.1 to 15.6)
Anemia due to malaria parasitemia(PfPR) aggregate	1,382.1 (1,346.2 to 1,422.0)	30.2 (26.0 to 35.3)*	4.2 (0.9 to 8.3)*	35.6 (23.8 to 51.2)	21.5 (13.6 to 29.6)*	-2.7 (-9.1 to 3.8)
Chagas disease	610.4 (504.8 to 717.3)	-4.6 (-8.1 to -1.1)*	-23.6 (-26.5 to -20.8)*	5.8 (3.7 to 8.4)	-4.4 (-9.7 to 1.1)	-23.4 (-27.7 to -19.1)*
Chagas disease episodes aggregate	511.7 (420.8 to 600.1)	-4.6 (-8.1 to -1.1)*	-23.6 (-26.4 to -20.8)*	0.0 (0.0 to 0.0)	10.3 (5.0 to 16.0)*	-11.7 (-15.9 to -7.1)*
Complications of Chagas disease aggregate	63.5 (48.4 to 80.1)	-4.6 (-8.1 to -1.1)*	-23.6 (-26.5 to -20.8)*	3.9 (2.5 to 5.7)	-4.5 (-10.9 to 2.1)	-23.6 (-28.7 to -18.2)*

Cause	Prevalence in thousands (95% confidence interval)			YLDs in thousands (95% confidence interval)		
	2015	Percent change in counts between 2005 and 2015	Percent change in rates between 2005 and 2015	2015	Percent change in counts between 2005 and 2015	Percent change in rates between 2005 and 2015
Heart failure due to Chagas disease aggregate	35.2 (21.3 to 52.3)	-4.6 (-8.2 to -0.9)*	-23.6 (-26.5 to -20.7)*	1.9 (0.9 to 3.1)	-4.1 (-11.4 to 3.3)	-23.2 (-29.1 to -17.3)*
Leishmaniasis	208.4 (185.8 to 242.6)	29.1 (6.7 to 54.5)*	3.3 (-14.6 to 23.7)	2.3 (1.1 to 4.5)	28.6 (23.0 to 34.0)*	3.0 (-1.5 to 7.3)
Visceral leishmaniasis	1.3 (1.2 to 1.4)	21.4 (20.2 to 22.8)*	-2.8 (-3.8 to -1.7)*	0.1 (0.1 to 0.1)	22.0 (-15.3 to 71.7)	-2.3 (-32.2 to 37.4)
Cutaneous and mucocutaneous leishmaniasis	213.1 (183.0 to 258.0)	28.8 (25.8 to 31.4)*	3.1 (0.7 to 5.2)*	2.2 (1.1 to 4.4)	28.9 (23.8 to 33.8)*	3.2 (-0.9 to 7.2)
African trypanosomiasis	0.4 (0.2 to 0.7)	-69.4 (-70.9 to -68.1)*	-75.5 (-76.7 to -74.5)*	0.1 (0.0 to 0.2)	-69.2 (-84.4 to -47.7)*	-75.3 (-87.5 to -58.1)*
Schistosomiasis	9,213.7 (7,693.1 to 11,634.6)	-25.1 (-34.0 to -7.4)*	-40.0 (-47.1 to -25.8)*	85.1 (43.5 to 159.9)	-23.0 (-31.0 to -4.5)*	-38.3 (-44.8 to -23.6)*
Schistosomiasis episodes aggregate	4,999.8 (4,038.3 to 6,152.0)	-28.1 (-36.6 to -15.3)*	-42.4 (-49.3 to -32.2)*	28.0 (11.0 to 59.3)	-29.5 (-37.9 to -15.9)*	-43.6 (-50.2 to -32.7)*
Complications of schistosomiasis aggregate	3,662.9 (2,518.5 to 5,603.5)	-19.0 (-31.9 to 13.9)	-35.1 (-45.5 to -8.8)*	43.0 (20.4 to 84.5)	-15.9 (-27.9 to 15.6)	-32.7 (-42.3 to -7.4)*
Anemia due to schistosomiasis aggregate	534.7 (512.0 to 556.1)	-26.0 (-29.3 to -22.5)*	-40.8 (-43.4 to -37.9)*	14.2 (9.6 to 20.6)	-28.2 (-34.1 to -21.7)*	-42.5 (-47.2 to -37.3)*
Cysticercosis	139.7 (87.4 to 218.5)	-9.5 (-17.9 to -2.4)*	-27.6 (-34.2 to -21.9)*	20.0 (11.2 to 34.4)	-20.1 (-28.3 to -12.4)*	-36.0 (-42.6 to -29.8)*
Cystic echinococcosis	110.2 (97.0 to 124.7)	34.2 (31.0 to 37.7)*	7.5 (4.9 to 10.2)*	10.0 (6.7 to 13.9)	33.9 (25.0 to 43.9)*	7.2 (0.0 to 15.3)*
Lymphatic filariasis	2,328.5 (1,826.2 to 2,926.8)	-38.4 (-45.6 to -31.8)*	-50.6 (-56.4 to -45.4)*	152.1 (84.4 to 242.7)	-10.0 (-28.3 to 4.6)	-28.0 (-42.6 to -16.2)*
Prevalence of detectable microfiliaria due to lymphatic filariasis	932.8 (804.5 to 1,074.0)	-58.6 (-62.0 to -54.6)*	-66.9 (-69.5 to -63.6)*	0.0 (0.0 to 0.0)	0.0 (0.0 to 0.0)	0.0 (0.0 to 0.0)
Complications of lymphatic filariasis aggregate	1,395.7 (915.5 to 1,981.7)	-8.4 (-26.4 to 5.8)	-26.6 (-41.1 to -15.3)*	152.1 (84.4 to 242.7)	-10.0 (-28.3 to 4.6)	-28.0 (-42.6 to -16.2)*
Onchocerciasis	695.0 (525.4 to 927.9)	-24.4 (-36.8 to -10.3)*	-39.5 (-49.4 to -28.2)*	62.8 (31.4 to 113.5)	-17.9 (-37.2 to 3.1)	-34.2 (-49.7 to -17.5)*
Asymptomatic onchocerciasis	60.7 (22.8 to 101.3)	-65.1 (-80.0 to -59.5)*	-72.0 (-84.0 to -67.5)*	0.0 (0.0 to 0.0)	0.0 (0.0 to 0.0)	0.0 (0.0 to 0.0)
Skin disease due to onchocerciasis aggregate	521.9 (347.9 to 761.0)	-18.0 (-34.6 to 0.4)	-34.3 (-47.7 to -19.5)*	53.4 (22.5 to 101.6)	-20.3 (-44.6 to 3.7)	-36.2 (-55.6 to -16.8)*
Vision loss due to onchocerciasis aggregate	112.6 (77.1 to 157.5)	3.3 (-10.4 to 16.2)	-17.3 (-28.2 to -7.0)*	9.5 (5.7 to 15.0)	-0.8 (-14.2 to 12.4)	-20.6 (-31.3 to -10.0)*
Trachoma	225.0 (143.9 to 335.3)	-2.3 (-10.0 to 6.4)	-21.8 (-27.9 to -14.8)*	19.9 (10.8 to 34.6)	-5.6 (-14.2 to 4.9)	-24.4 (-31.3 to -16.0)*
Dengue	211.9 (117.4 to 470.9)	198.8 (21.6 to 713.2)*	139.3 (-2.7 to 551.1)	33.4 (14.9 to 79.3)	196.6 (19.9 to 732.8)*	137.5 (-4.0 to 566.8)
Dengue episodes aggregate	63.2 (41.2 to 135.8)	179.8 (16.3 to 655.2)*	124.0 (-6.9 to 504.7)	3.6 (2.0 to 8.3)	195.8 (21.2 to 696.2)*	136.9 (-3.0 to 537.4)
Post-dengue chronic fatigue syndrome	148.9 (70.5 to 339.0)	199.0 (21.6 to 713.2)*	139.4 (-2.7 to 551.1)	29.8 (12.6 to 70.7)	196.7 (19.5 to 732.9)*	137.6 (-4.3 to 566.8)
Yellow fever	0.1 (0.0 to 0.2)	-23.7 (-41.8 to -1.0)*	-38.9 (-53.4 to -20.7)*	0.0 (0.0 to 0.0)	-23.5 (-41.8 to -1.0)*	-38.8 (-53.4 to -20.7)*
Rabies	0.0 (0.0 to 0.0)	-47.5 (-67.6 to -19.0)*	-57.9 (-74.1 to -35.2)*	0.0 (0.0 to 0.0)	-47.5 (-67.6 to -19.0)*	-58.0 (-74.1 to -35.2)*
Intestinal nematode infections	74,465.3 (72,131.0 to 76,641.5)	12.7 (8.8 to 17.1)*	-9.8 (-12.9 to -6.2)*	143.2 (81.1 to 232.8)	-9.8 (-17.7 to -0.3)*	-27.8 (-34.1 to -20.2)*
Ascariasis	39,275.7 (33,372.6 to 47,139.8)	3.7 (-15.0 to 28.1)	-17.0 (-31.9 to 2.6)	39.1 (21.4 to 66.1)	-27.1 (-39.8 to -11.3)*	-41.6 (-51.8 to -29.0)*
Complications of ascariasis aggregate	2,190.5 (1,955.3 to 2,461.8)	-24.6 (-35.6 to -11.2)*	-39.6 (-48.4 to -28.9)*	39.1 (21.4 to 66.1)	-27.1 (-39.8 to -11.3)*	-41.6 (-51.8 to -29.0)*
Asymptomatic ascariasis	37,085.1 (31,107.2 to 44,922.4)	6.1 (-14.1 to 32.8)	-15.1 (-31.2 to 6.3)	0.0 (0.0 to 0.0)	0.0 (0.0 to 0.0)	0.0 (0.0 to 0.0)
Trichuriasis	23,201.3 (20,701.1 to 26,019.6)	15.7 (-1.1 to 36.2)	-7.4 (-20.8 to 9.0)	26.4 (13.8 to 46.6)	3.0 (-20.3 to 37.2)	-17.5 (-36.2 to 9.9)
Complications of trichuriasis aggregate	1,326.7 (1,138.3 to 1,643.3)	3.6 (-16.8 to 33.1)	-17.0 (-33.4 to 6.6)	26.4 (13.8 to 46.6)	3.0 (-20.3 to 37.2)	-17.5 (-36.2 to 9.9)
Asymptomatic trichuriasis	21,870.6 (19,356.6 to 24,684.0)	16.5 (-1.6 to 38.6)	-6.7 (-21.2 to 11.0)	0.0 (0.0 to 0.0)	0.0 (0.0 to 0.0)	0.0 (0.0 to 0.0)
Hookworm disease	22,314.9 (19,818.3 to 25,252.3)	6.9 (-8.6 to 24.8)	-14.4 (-26.8 to -0.1)*	77.7 (46.6 to 123.0)	-2.2 (-11.5 to 8.0)	-21.7 (-29.1 to -13.5)*
Complications of hookworm disease aggregate	2,670.4 (2,460.8 to 2,899.6)	-0.5 (-11.5 to 11.8)	-20.3 (-29.2 to -10.5)*	53.8 (29.5 to 89.2)	-1.8 (-14.7 to 12.9)	-21.4 (-31.7 to -9.6)*
Anemia due to hookworm disease aggregate	1,131.2 (1,102.6 to 1,160.1)	7.5 (3.9 to 11.1)*	-14.0 (-16.8 to -11.0)*	23.8 (15.8 to 34.9)	-3.2 (-9.5 to 2.4)	-22.5 (-27.5 to -18.0)*
Asymptomatic hookworm disease	18,508.0 (16,041.2 to 21,436.8)	8.0 (-11.1 to 30.1)	-13.5 (-28.8 to 4.2)	0.0 (0.0 to 0.0)	0.0 (0.0 to 0.0)	0.0 (0.0 to 0.0)
Food-borne trematodiases	5,749.0 (5,309.1 to 6,269.4)	22.6 (18.9 to 26.5)*	-1.9 (-4.8 to 1.2)	136.2 (62.4 to 257.8)	21.0 (15.6 to 26.7)*	-3.1 (-7.4 to 1.5)
Asymptomatic food-borne trematodiases aggregate	4,527.6 (3,666.4 to 5,226.3)	22.8 (19.1 to 26.7)*	-1.7 (-4.6 to 1.5)	0.0 (0.0 to 0.0)	0.0 (0.0 to 0.0)	0.0 (0.0 to 0.0)
Symptomatic food-borne trematodiases aggregate	1,221.4 (662.0 to 2,108.2)	21.7 (17.2 to 26.7)*	-2.6 (-6.2 to 1.5)	136.2 (62.4 to 257.8)	21.0 (15.6 to 26.7)*	-3.1 (-7.4 to 1.5)
Leprosy	53.7 (50.5 to 57.5)	-0.8 (-2.0 to 0.7)	-20.5 (-21.6 to -19.4)*	3.3 (2.2 to 4.6)	-0.1 (-6.9 to 8.1)	-20.0 (-25.4 to -13.5)*
Ebola	0.1 (0.0 to 0.2)	0.0 (0.0 to 0.0)	0.0 (0.0 to 0.0)	0.0 (0.0 to 0.0)	0.0 (0.0 to 0.0)	0.0 (0.0 to 0.0)
Other neglected tropical diseases	1,430.9 (1,400.0 to 1,464.3)	11.4 (8.6 to 14.2)*	-10.8 (-13.1 to -8.6)*	137.6 (50.1 to 369.8)	9.3 (-28.2 to 65.9)	-12.5 (-42.5 to 32.8)
Anemia due to other neglected tropical diseases aggregate	1,430.9 (1,400.0 to 1,464.3)	11.4 (8.6 to 14.2)*	-10.8 (-13.1 to -8.6)*	25.1 (16.4 to 37.4)	-4.5 (-11.2 to 1.7)	-23.5 (-28.9 to -18.6)*
Maternal disorders	36.5 (33.3 to 39.9)	-20.1 (-28.2 to -11.8)*	-36.1 (-42.5 to -29.4)*	13.3 (8.6 to 19.0)	-18.4 (-25.5 to -11.0)*	-34.6 (-40.4 to -28.8)*
Maternal hemorrhage	0.0 (0.0 to 0.0)	0.0 (0.0 to 0.0)	0.0 (0.0 to 0.0)	0.0 (0.0 to 0.0)	0.0 (0.0 to 0.0)	0.0 (0.0 to 0.0)
Maternal hemorrhage episodes aggregate	0.0 (0.0 to 0.0)	0.0 (0.0 to 0.0)	0.0 (0.0 to 0.0)	0.0 (0.0 to 0.0)	0.0 (0.0 to 0.0)	0.0 (0.0 to 0.0)
Anemia due to maternal hemorrhage aggregate	0.0 (0.0 to 0.0)	0.0 (0.0 to 0.0)	0.0 (0.0 to 0.0)	0.0 (0.0 to 0.0)	0.0 (0.0 to 0.0)	0.0 (0.0 to 0.0)
Maternal sepsis and other maternal infections	0.0 (0.0 to 0.0)	0.0 (0.0 to 0.0)	0.0 (0.0 to 0.0)	0.0 (0.0 to 0.0)	0.0 (0.0 to 0.0)	0.0 (0.0 to 0.0)
Maternal sepsis and other maternal infections aggregate	0.0 (0.0 to 0.0)	0.0 (0.0 to 0.0)	0.0 (0.0 to 0.0)	0.0 (0.0 to 0.0)	0.0 (0.0 to 0.0)	0.0 (0.0 to 0.0)
Infertility due to puerperal sepsis	0.0 (0.0 to 0.0)	0.0 (0.0 to 0.0)	0.0 (0.0 to 0.0)	0.0 (0.0 to 0.0)	0.0 (0.0 to 0.0)	0.0 (0.0 to 0.0)
Maternal hypertensive disorders	0.0 (0.0 to 0.0)	0.0 (0.0 to 0.0)	0.0 (0.0 to 0.0)	0.0 (0.0 to 0.0)	0.0 (0.0 to 0.0)	0.0 (0.0 to 0.0)
Maternal hypertensive disorders episodes aggregate	0.0 (0.0 to 0.0)	0.0 (0.0 to 0.0)	0.0 (0.0 to 0.0)	0.0 (0.0 to 0.0)	0.0 (0.0 to 0.0)	0.0 (0.0 to 0.0)
Eclampsia	0.0 (0.0 to 0.0)	0.0 (0.0 to 0.0)	0.0 (0.0 to 0.0)	0.0 (0.0 to 0.0)	0.0 (0.0 to 0.0)	0.0 (0.0 to 0.0)
Complications of maternal hypertensive disorders aggregate	0.0 (0.0 to 0.0)	0.0 (0.0 to 0.0)	0.0 (0.0 to 0.0)	0.0 (0.0 to 0.0)	0.0 (0.0 to 0.0)	0.0 (0.0 to 0.0)
Maternal obstructed labor and uterine rupture	36.7 (30.5 to 43.9)	-19.6 (-22.9 to -16.2)*	-35.6 (-38.3 to -32.9)*	11.7 (7.6 to 16.7)	-19.2 (-26.0 to -12.1)*	-35.3 (-40.7 to -29.7)*
Obstructed labor, acute event	0.0 (0.0 to 0.1)	1.4 (-43.4 to 80.8)	-18.8 (-54.6 to 44.7)	0.0 (0.0 to 0.0)	1.3 (-43.4 to 80.8)	-18.9 (-54.6 to 44.7)
Fistula due to maternal obstructed labor and uterine rupture aggregate	36.6 (30.5 to 43.9)	-19.6 (-22.9 to -16.2)*	-35.6 (-38.3 to -32.9)*	11.7 (7.6 to 16.6)	-19.2 (-26.0 to -12.2)*	-35.3 (-40.7 to -29.7)*
Maternal abortion, miscarriage, and ectopic pregnancy	0.0 (0.0 to 0.0)	0.0 (0.0 to 0.0)	0.0 (0.0 to 0.0)	0.0 (0.0 to 0.0)	0.0 (0.0 to 0.0)	0.0 (0.0 to 0.0)
Other maternal disorders	--	--	--	1.5 (0.9 to 2.4)	-11.6 (-26.8 to 5.8)	-29.2 (-41.4 to -15.3)*
Neonatal disorders	2,108.4 (2,014.4 to 2,181.6)	34.1 (29.6 to 38.9)*	7.3 (3.8 to 11.2)*	429.2 (327.5 to 546.7)	31.8 (26.9 to 36.9)*	5.5 (1.6 to 9.6)*
Neonatal preterm birth complications	1,796.0 (1,571.7 to 2,034.2)	28.5 (22.0 to 34.8)*	2.9 (-2.3 to 8.0)	214.3 (163.5 to 272.7)	23.4 (16.4 to 30.6)*	-1.2 (-6.8 to 4.6)
Vision loss due to retinopathy of prematurity aggregate	246.9 (197.3 to 305.4)	23.0 (18.7 to 27.2)*	-1.6 (-5.0 to 1.9)	8.7 (5.4 to 13.5)	23.4 (14.0 to 32.8)*	-1.2 (-8.7 to 6.4)
Complications of preterm birth complications aggregate	1,549.0 (1,344.4 to 1,775.5)	29.4 (22.1 to 36.8)*	3.6 (-2.3 to 9.6)	205.6 (155.8 to 262.1)	23.4 (16.2 to 30.9)*	-1.2 (-7.0 to 4.8)
Neonatal encephalopathy due to birth asphyxia and trauma	483.2 (341.8 to 718.9)	48.1 (44.9 to 51.2)*	18.6 (16.0 to 21.1)*	137.0 (88.7 to 208.4)	43.9 (38.6 to 49.3)*	15.2 (11.0 to 19.5)*

Cause	Prevalence in thousands (95% confidence interval)			YLDs in thousands (95% confidence interval)		
	2015	Percent change in counts between 2005 and 2015	Percent change in rates between 2005 and 2015	2015	Percent change in counts between 2005 and 2015	Percent change in rates between 2005 and 2015
Neonatal sepsis and other neonatal infections	0.0 (0.0 to 0.0)	0.0 (0.0 to 0.0)	0.0 (0.0 to 0.0)	0.0 (0.0 to 0.0)	0.0 (0.0 to 0.0)	0.0 (0.0 to 0.0)
Severe infection due to neonatal sepsis and other neonatal infections	0.0 (0.0 to 0.0)	0.0 (0.0 to 0.0)	0.0 (0.0 to 0.0)	0.0 (0.0 to 0.0)	0.0 (0.0 to 0.0)	0.0 (0.0 to 0.0)
Complications of neonatal sepsis and other neonatal infections aggregate	0.0 (0.0 to 0.0)	0.0 (0.0 to 0.0)	0.0 (0.0 to 0.0)	0.0 (0.0 to 0.0)	0.0 (0.0 to 0.0)	0.0 (0.0 to 0.0)
Hemolytic disease and other neonatal jaundice	80.9 (72.1 to 91.4)	39.2 (37.6 to 40.7)*	11.4 (10.2 to 12.7)*	24.0 (18.0 to 30.8)	33.7 (27.8 to 40.3)*	7.1 (2.3 to 12.3)*
Other neonatal disorders	--	--	--	53.9 (38.5 to 70.3)	38.6 (22.8 to 55.6)*	11.0 (-1.7 to 24.6)
Nutritional deficiencies	63,597.4 (62,683.8 to 64,507.2)	14.6 (12.8 to 16.8)*	-8.2 (-9.7 to -6.5)*	1,612.8 (1,081.3 to 2,327.0)	5.8 (2.5 to 8.9)*	-15.3 (-17.9 to -12.8)*
Protein-energy malnutrition	12.7 (7.9 to 20.1)	-9.3 (-18.8 to 2.8)	-27.4 (-35.0 to -17.7)*	1.5 (0.8 to 2.7)	-8.3 (-21.8 to 7.2)	-26.6 (-37.4 to -14.2)*
Iodine deficiency	6,856.1 (6,215.0 to 7,711.8)	17.0 (14.6 to 19.4)*	-6.3 (-8.2 to -4.4)*	132.1 (83.8 to 202.2)	17.9 (15.4 to 20.5)*	-5.6 (-7.6 to -3.6)*
Visible goiter due to iodine deficiency aggregate	6,780.2 (6,132.0 to 7,618.1)	16.9 (14.5 to 19.3)*	-6.4 (-8.3 to -4.5)*	119.3 (74.0 to 187.3)	17.2 (14.7 to 20.0)*	-6.2 (-8.2 to -3.9)*
Visible goiter with heart failure due to iodine deficiency aggregate	0.0 (0.0 to 0.0)	29.2 (27.1 to 31.5)*	3.5 (1.7 to 5.3)*	0.0 (0.0 to 0.0)	29.2 (27.1 to 31.5)*	3.5 (1.7 to 5.3)*
Intellectual disability due to iodine deficiency aggregate	75.9 (33.6 to 98.5)	23.6 (17.2 to 27.8)*	-1.1 (-6.2 to 2.4)	12.8 (4.8 to 20.1)	24.7 (14.7 to 33.6)*	-0.2 (-8.2 to 6.9)
Vitamin A deficiency	141.9 (97.5 to 199.3)	19.8 (12.5 to 27.4)*	-4.1 (-9.9 to 2.0)	9.2 (5.4 to 13.9)	22.1 (13.6 to 31.2)*	-2.2 (-9.0 to 5.1)
Iron-deficiency anemia	60,903.7 (59,946.8 to 61,928.0)	13.1 (11.0 to 15.2)*	-9.5 (-11.1 to -7.8)*	1,469.8 (984.9 to 2,129.0)	4.8 (1.3 to 8.0)*	-16.1 (-18.9 to -13.5)*
Iron-deficiency anemia without heart failure aggregate	60,901.9 (59,945.3 to 61,926.1)	13.1 (11.0 to 15.2)*	-9.5 (-11.1 to -7.8)*	1,469.6 (984.8 to 2,128.7)	4.8 (1.3 to 8.0)*	-16.1 (-18.9 to -13.5)*
Iron-deficiency anemia with heart failure aggregate	1.7 (1.5 to 2.1)	31.5 (27.7 to 35.3)*	5.3 (2.3 to 8.3)*	0.2 (0.1 to 0.3)	31.9 (26.9 to 36.9)*	5.6 (1.6 to 9.6)*
Other nutritional deficiencies	--	--	--	0.2 (0.1 to 0.3)	-6.8 (-27.7 to 16.7)	-25.4 (-42.1 to -6.5)*
Other communicable, maternal, neonatal, and nutritional diseases	117,665.5 (114,663.1 to 120,628.1)	23.9 (22.7 to 24.9)*	-0.8 (-1.7 to 0.0)	129.2 (81.6 to 198.4)	18.2 (10.2 to 26.1)*	-5.4 (-11.8 to 0.9)
Sexually transmitted diseases excluding HIV	92,671.5 (88,906.2 to 95,897.6)	23.5 (22.0 to 25.0)*	-1.1 (-2.3 to 0.0)	74.7 (45.5 to 121.4)	22.5 (18.3 to 26.2)*	-2.0 (-5.3 to 1.0)
Syphilis	4,003.9 (2,835.7 to 5,619.7)	11.1 (1.0 to 19.0)*	-11.0 (-19.2 to -4.7)*	25.7 (17.1 to 35.6)	17.3 (11.0 to 23.6)*	-6.0 (-11.1 to -1.0)*
Early syphilis aggregate	3,871.0 (2,688.9 to 5,487.8)	10.9 (0.4 to 19.3)*	-11.2 (-19.6 to -4.5)*	0.9 (0.3 to 2.4)	11.3 (0.6 to 20.8)*	-10.9 (-19.5 to -3.3)*
Adult tertiary syphilis	132.8 (116.2 to 153.6)	17.0 (13.7 to 20.4)*	-6.3 (-9.0 to -3.6)*	24.7 (16.2 to 34.4)	17.6 (11.1 to 24.2)*	-5.9 (-11.1 to -0.6)*
Chlamydial infection	446.7 (291.8 to 638.7)	30.6 (26.0 to 34.9)*	4.6 (0.9 to 8.0)*	3.6 (2.1 to 5.6)	22.1 (11.5 to 33.3)*	-2.2 (-10.7 to 6.7)
Chlamydial infection episodes aggregate	436.8 (283.2 to 627.6)	31.2 (26.5 to 35.6)*	5.0 (1.2 to 8.6)*	2.3 (1.2 to 4.0)	29.3 (14.3 to 46.2)*	3.5 (-8.4 to 17.0)
Pelvic inflammatory diseases due to chlamydial infection aggregate	9.8 (6.5 to 13.8)	9.8 (5.9 to 13.9)*	-12.1 (-15.2 to -8.8)*	1.3 (0.7 to 2.1)	11.3 (-1.1 to 23.9)	-10.9 (-20.8 to -0.8)*
Infertility due to clamydial infection aggregate	0.0 (0.0 to 0.0)	0.0 (0.0 to 0.0)	0.0 (0.0 to 0.0)	0.0 (0.0 to 0.0)	0.0 (0.0 to 0.0)	0.0 (0.0 to 0.0)
Gonococcal infection	913.7 (445.9 to 1,654.9)	35.0 (7.4 to 52.1)*	8.1 (-14.0 to 21.8)	8.8 (3.9 to 17.6)	33.5 (10.4 to 48.0)*	6.9 (-11.6 to 18.5)
Gonococcal infection episodes aggregate	911.9 (443.6 to 1,653.0)	35.1 (7.4 to 52.2)*	8.2 (-14.0 to 21.8)	8.5 (3.7 to 17.2)	34.3 (10.2 to 49.6)*	7.5 (-11.8 to 19.8)
Pelvic inflammatory diseases due to gonococcal infection aggregate	1.9 (1.2 to 2.7)	12.5 (6.8 to 18.6)*	-9.9 (-14.5 to -5.0)*	0.3 (0.1 to 0.4)	12.5 (6.8 to 18.6)*	-9.9 (-14.5 to -5.0)*
Trichomoniasis	11,555.9 (8,081.6 to 15,891.7)	25.2 (24.1 to 26.2)*	0.2 (-0.6 to 1.0)	12.6 (4.8 to 27.4)	25.4 (23.5 to 27.5)*	0.4 (-1.1 to 2.1)
Genital herpes	81,160.7 (71,251.1 to 91,763.9)	23.7 (22.0 to 25.3)*	-1.0 (-2.3 to 0.3)	20.3 (6.0 to 49.5)	26.0 (23.4 to 32.4)*	0.8 (-1.2 to 6.0)
Moderate infection due to initial genital herpes episode	8.6 (2.0 to 20.8)	616.8 (471.4 to 825.6)*	473.9 (357.5 to 641.1)*	0.4 (0.1 to 1.0)	602.7 (390.8 to 1,042.3)*	462.6 (293.0 to 814.6)*
Complications of genital herpes aggregate	81,152.0 (71,236.9 to 91,753.2)	23.7 (22.0 to 25.3)*	-1.0 (-2.3 to 0.3)	19.9 (5.7 to 48.9)	23.9 (21.7 to 26.0)*	-0.8 (-2.5 to 0.9)
Other sexually transmitted diseases	27.7 (18.6 to 38.7)	8.8 (6.3 to 11.0)*	-12.9 (-14.9 to -11.2)*	3.6 (1.9 to 5.7)	8.5 (-0.6 to 18.0)	-13.1 (-20.4 to -5.5)*
Pelvic inflammatory diseases due to other sexually transmitted diseases aggregate	27.7 (18.6 to 38.7)	8.8 (6.3 to 11.0)*	-12.9 (-14.9 to -11.2)*	3.6 (1.9 to 5.7)	8.5 (-0.6 to 18.0)	-13.1 (-20.4 to -5.5)*
Infertility due to other sexually transmitted diseases aggregate	0.0 (0.0 to 0.0)	0.0 (0.0 to 0.0)	0.0 (0.0 to 0.0)	0.0 (0.0 to 0.0)	0.0 (0.0 to 0.0)	0.0 (0.0 to 0.0)
Other sexually transmitted diseases	--	--	--	4.7 (2.4 to 8.7)	32.4 (13.7 to 45.9)*	6.0 (-8.9 to 16.9)
Hepatitis	30,749.0 (30,261.1 to 31,394.1)	25.7 (24.7 to 26.7)*	0.7 (-0.2 to 1.5)	18.6 (11.4 to 30.0)	28.4 (-16.2 to 90.5)	2.8 (-32.9 to 52.5)
Acute hepatitis A	110.6 (89.7 to 129.1)	37.3 (7.8 to 77.8)*	9.9 (-13.7 to 42.4)	4.6 (2.8 to 6.8)	37.9 (5.9 to 81.9)*	10.4 (-15.2 to 45.6)
Hepatitis B	20,850.1 (20,106.8 to 21,608.9)	25.5 (23.1 to 27.8)*	0.4 (-1.4 to 2.3)	12.6 (6.9 to 21.9)	25.6 (-30.1 to 122.9)	0.6 (-44.0 to 78.4)
Acute Hepatitis B aggregate	550.9 (366.7 to 828.8)	26.0 (-30.6 to 121.6)	0.9 (-44.4 to 77.4)	12.6 (6.9 to 21.9)	25.6 (-30.1 to 122.9)	0.6 (-44.0 to 78.4)
Chronic hepatitis B	20,299.2 (19,559.4 to 21,011.8)	25.4 (24.4 to 26.5)*	0.4 (-0.4 to 1.3)	0.0 (0.0 to 0.0)	0.0 (0.0 to 0.0)	0.0 (0.0 to 0.0)
Hepatitis C	10,529.4 (9,371.5 to 11,732.7)	25.8 (24.2 to 27.3)*	0.7 (-0.5 to 1.9)	0.5 (0.2 to 0.9)	23.9 (15.7 to 29.9)*	-0.8 (-7.3 to 4.0)
Acute Hepatitis C aggregate	33.2 (24.6 to 43.2)	24.0 (16.7 to 29.1)*	-0.7 (-6.5 to 3.4)	0.5 (0.2 to 0.9)	23.9 (15.7 to 29.9)*	-0.8 (-7.3 to 4.0)
Chronic hepatitis C	10,496.3 (9,341.0 to 11,699.2)	25.8 (24.3 to 27.3)*	0.8 (-0.5 to 1.9)	0.0 (0.0 to 0.0)	0.0 (0.0 to 0.0)	0.0 (0.0 to 0.0)
Acute hepatitis E	27.3 (18.8 to 36.5)	24.7 (21.8 to 27.4)*	-0.2 (-2.5 to 2.0)	0.9 (0.5 to 1.4)	24.3 (1.3 to 51.7)*	-0.5 (-18.9 to 21.5)
Other infectious diseases	1,711.2 (1,677.8 to 1,745.1)	12.2 (9.3 to 15.3)*	-10.1 (-12.5 to -7.7)*	35.9 (22.6 to 55.1)	6.1 (-1.0 to 13.7)	-15.0 (-20.7 to -8.9)*
Non-communicable diseases	391,077.8 (390,296.7 to 391,804.7)	24.9 (24.8 to 25.0)*	0.0 (-0.0 to 0.1)	50,127.1 (37,764.0 to 64,338.1)	25.0 (24.3 to 25.8)*	0.1 (-0.5 to 0.7)
Neoplasms	8,596.1 (8,386.5 to 8,821.6)	37.3 (33.5 to 41.5)*	9.9 (6.9 to 13.3)*	770.5 (554.8 to 1,013.4)	29.2 (22.7 to 35.7)*	3.4 (-1.8 to 8.7)
Lip and oral cavity cancer	283.5 (251.1 to 322.8)	33.5 (18.0 to 52.5)*	6.8 (-5.5 to 22.1)	23.8 (16.8 to 31.9)	31.5 (15.5 to 51.1)*	5.3 (-7.5 to 21.0)
Nasopharynx cancer	102.2 (67.0 to 152.2)	12.5 (-20.3 to 62.5)	-9.9 (-36.2 to 30.1)	9.5 (5.6 to 15.2)	11.3 (-20.2 to 58.6)	-10.9 (-36.1 to 27.0)
Other pharynx cancer	128.9 (116.7 to 141.8)	21.2 (10.7 to 33.3)*	-3.0 (-11.3 to 6.7)	10.7 (7.6 to 14.4)	19.9 (8.3 to 33.4)*	-4.0 (-13.3 to 6.8)
Esophageal cancer	61.1 (48.9 to 89.4)	-1.0 (-23.4 to 29.2)	-20.7 (-38.7 to 3.4)	11.0 (7.3 to 17.0)	-7.1 (-26.4 to 18.2)	-25.6 (-41.1 to -5.4)*
Stomach cancer	260.1 (239.5 to 284.7)	-7.1 (-15.1 to 2.3)	-25.6 (-32.1 to -18.1)*	29.6 (21.2 to 38.6)	-9.0 (-17.8 to 0.9)	-27.2 (-34.1 to -19.2)*
Colon and rectum cancer	730.6 (663.3 to 805.8)	40.3 (30.1 to 51.1)*	12.3 (4.2 to 21.0)*	58.2 (41.2 to 77.2)	36.8 (25.7 to 48.2)*	9.6 (0.7 to 18.6)*
Colon and rectum cancer aggregate	729.2 (662.0 to 804.6)	40.3 (30.2 to 51.1)*	12.4 (4.2 to 21.0)*	58.1 (41.1 to 77.0)	36.9 (25.7 to 48.3)*	9.6 (0.7 to 18.7)*
Stoma due to colon and rectum cancer	1.3 (1.2 to 1.5)	22.0 (19.8 to 24.1)*	-2.3 (-4.1 to -0.6)*	0.2 (0.1 to 0.3)	22.0 (19.8 to 24.1)*	-2.3 (-4.1 to -0.6)*
Liver cancer	42.4 (34.5 to 52.0)	23.8 (-7.5 to 60.0)	-0.9 (-26.0 to 28.1)	17.6 (11.6 to 25.0)	8.8 (-10.7 to 35.8)	-12.9 (-28.5 to 8.7)
Liver cancer due to hepatitis B	36.4 (26.9 to 55.5)	9.7 (-19.7 to 52.0)	-12.2 (-35.7 to 21.7)	7.1 (4.7 to 10.4)	-0.7 (-21.7 to 31.6)	-20.5 (-37.3 to 5.4)
Liver cancer due to hepatitis C	13.2 (10.7 to 16.4)	46.3 (20.2 to 79.4)*	17.1 (-3.8 to 43.7)	2.5 (1.7 to 3.5)	27.4 (10.2 to 48.9)*	2.0 (-11.7 to 19.2)
Liver cancer due to alcohol use	25.6 (19.3 to 37.8)	39.4 (7.0 to 90.3)*	11.6 (-14.4 to 52.3)	5.3 (3.5 to 7.7)	23.1 (-1.5 to 59.6)	-1.5 (-21.2 to 27.8)
Liver cancer due to other causes	12.9 (9.5 to 19.1)	9.3 (-23.8 to 53.3)	-12.5 (-39.0 to 22.7)	2.7 (1.7 to 3.9)	-2.2 (-24.1 to 24.5)	-21.7 (-39.2 to -0.3)*
Gallbladder and biliary tract cancer	8.0 (7.1 to 8.9)	6.8 (-2.2 to 16.6)	-14.5 (-21.7 to -6.6)*	2.3 (1.6 to 3.0)	5.0 (-4.2 to 14.7)	-15.9 (-23.3 to -8.2)*
Pancreatic cancer	24.6 (22.6 to 27.0)	31.6 (21.6 to 42.7)*	5.4 (-2.6 to 14.2)	6.0 (4.2 to 8.0)	26.1 (17.6 to 35.3)*	0.9 (-5.8 to 8.4)

Cause	Prevalence in thousands (95% confidence interval)			YLDs in thousands (95% confidence interval)		
	2015	Percent change in counts between 2005 and 2015	Percent change in rates between 2005 and 2015	2015	Percent change in counts between 2005 and 2015	Percent change in rates between 2005 and 2015
Larynx cancer	168.5 (151.7 to 193.0)	17.4 (7.0 to 30.7)*	-6.0 (-14.3 to 4.6)	18.1 (12.7 to 24.3)	16.5 (5.8 to 29.3)*	-6.7 (-15.3 to 3.5)
Larynx cancer aggregate	81.9 (64.8 to 105.9)	9.6 (-10.1 to 35.1)	-12.3 (-28.0 to 8.2)	10.2 (6.8 to 14.0)	10.1 (-6.1 to 30.9)	-11.9 (-24.8 to 4.8)
Laryngectomy due to larynx cancer	86.5 (83.7 to 89.2)	26.0 (25.2 to 26.7)*	0.9 (0.2 to 1.5)*	7.9 (5.3 to 11.5)	26.0 (17.6 to 34.8)*	0.9 (-5.9 to 7.9)
Tracheal, bronchus, and lung cancer	249.8 (219.2 to 314.5)	21.6 (8.6 to 39.5)*	-2.6 (-13.0 to 11.7)	40.3 (28.3 to 54.3)	16.8 (3.4 to 35.7)*	-6.5 (-17.2 to 8.6)
Malignant skin melanoma	321.2 (262.6 to 432.2)	54.4 (41.6 to 68.9)*	23.6 (13.4 to 35.2)*	18.4 (11.9 to 27.3)	52.2 (38.3 to 66.5)*	21.8 (10.7 to 33.3)*
Non-melanoma skin cancer	156.8 (146.9 to 167.1)	51.7 (39.9 to 64.1)*	21.5 (12.0 to 31.3)*	8.2 (5.4 to 11.7)	59.6 (43.5 to 77.8)*	27.8 (14.9 to 42.4)*
Non-melanoma skin cancer(squamous-cell carcinoma)	132.1 (114.8 to 154.3)	68.3 (50.8 to 88.5)*	34.8 (20.7 to 50.9)*	7.8 (5.2 to 11.0)	62.7 (45.7 to 81.4)*	30.2 (16.7 to 45.2)*
Non-melanoma skin cancer(basal-cell carcinoma)	39.6 (31.6 to 49.2)	18.3 (14.2 to 22.4)*	-5.3 (-8.6 to -2.0)*	0.4 (0.2 to 0.8)	18.4 (9.5 to 27.2)*	-5.2 (-12.3 to 1.9)
Breast cancer	2,860.0 (2,655.5 to 3,060.6)	44.4 (32.9 to 57.6)*	15.6 (6.4 to 26.2)*	226.8 (157.6 to 303.5)	36.5 (26.6 to 46.6)*	9.3 (1.4 to 17.3)*
Breast cancer aggregate	1,842.0 (1,625.1 to 2,051.9)	63.3 (42.7 to 88.4)*	30.7 (14.2 to 50.9)*	147.7 (105.1 to 193.5)	47.8 (31.2 to 66.6)*	18.3 (5.1 to 33.3)*
Mastectomy due to breast cancer	1,017.9 (977.6 to 1,060.2)	19.4 (18.0 to 20.9)*	-4.4 (-5.6 to -3.2)*	79.1 (50.3 to 116.0)	19.4 (16.8 to 21.9)*	-4.4 (-6.5 to -2.4)*
Cervical cancer	453.2 (379.5 to 552.9)	-0.2 (-16.9 to 20.9)	-20.1 (-33.4 to -3.2)*	34.1 (23.4 to 47.0)	0.0 (-16.5 to 20.9)	-19.9 (-33.1 to -3.2)*
Uterine cancer	508.5 (370.2 to 708.8)	32.0 (-1.1 to 74.4)	5.7 (-20.8 to 39.6)	32.5 (19.0 to 51.9)	30.6 (-2.8 to 72.4)	4.6 (-22.2 to 38.1)
Ovarian cancer	143.9 (126.5 to 171.0)	27.6 (13.3 to 43.8)*	2.2 (-9.2 to 15.2)	18.0 (12.6 to 24.1)	25.7 (9.0 to 44.8)*	0.6 (-12.7 to 15.9)
Prostate cancer	600.8 (476.5 to 818.1)	47.9 (37.0 to 58.6)*	18.4 (9.7 to 27.0)*	42.2 (28.2 to 61.4)	41.0 (30.2 to 52.5)*	12.9 (4.2 to 22.1)*
Prostate cancer aggregate	549.3 (424.4 to 766.2)	51.2 (39.0 to 63.9)*	21.1 (11.3 to 31.2)*	37.7 (24.9 to 55.1)	44.0 (31.9 to 58.0)*	15.3 (5.6 to 26.5)*
Impotence and incontinence due to prostate cancer aggregate	51.5 (50.0 to 53.0)	19.9 (19.2 to 20.6)*	-4.0 (-4.6 to -3.5)*	4.6 (3.1 to 6.5)	19.9 (13.1 to 27.6)*	-4.0 (-9.4 to 2.1)
Testicular cancer	39.5 (31.3 to 48.3)	73.0 (39.5 to 111.7)*	38.5 (11.7 to 69.5)*	2.4 (1.6 to 3.6)	68.0 (35.0 to 105.5)*	34.5 (8.1 to 64.6)*
Kidney cancer	270.3 (243.8 to 305.6)	48.0 (34.0 to 65.5)*	18.5 (7.3 to 32.5)*	19.1 (13.4 to 25.6)	45.3 (30.2 to 63.6)*	16.3 (4.2 to 31.0)*
Bladder cancer	232.1 (202.0 to 269.2)	27.7 (11.8 to 45.3)*	2.2 (-10.5 to 16.3)	19.8 (13.6 to 27.0)	25.0 (11.8 to 39.7)*	0.1 (-10.5 to 11.9)
Bladder cancer episodes aggregate	210.5 (180.2 to 247.1)	28.6 (11.0 to 48.2)*	2.9 (-11.1 to 18.6)	16.1 (11.1 to 22.0)	26.3 (9.7 to 44.2)*	1.1 (-12.2 to 15.4)
Urinary incontinence due to bladder cancer	21.6 (20.5 to 22.7)	19.4 (18.3 to 20.5)*	-4.4 (-5.2 to -3.5)*	3.7 (2.5 to 5.2)	19.5 (7.7 to 32.0)*	-4.3 (-13.8 to 5.7)
Brain and nervous system cancer	95.7 (82.2 to 115.4)	25.9 (7.5 to 47.5)*	0.8 (-13.9 to 18.1)	10.6 (7.3 to 14.8)	23.7 (6.3 to 45.3)*	-0.9 (-14.9 to 16.3)
Thyroid cancer	387.7 (352.4 to 418.5)	77.9 (60.9 to 94.8)*	42.4 (28.8 to 56.0)*	22.0 (14.8 to 31.0)	74.5 (57.4 to 92.2)*	39.7 (26.0 to 53.9)*
Mesothelioma	3.9 (3.6 to 4.3)	25.5 (14.8 to 37.6)*	0.5 (-8.1 to 10.2)	0.8 (0.5 to 1.0)	24.6 (14.1 to 36.5)*	-0.3 (-8.7 to 9.3)
Hodgkin lymphoma	45.1 (37.0 to 56.2)	35.4 (16.0 to 57.5)*	8.4 (-7.1 to 26.1)	3.7 (2.5 to 5.4)	28.8 (9.4 to 51.7)*	3.1 (-12.4 to 21.4)
Non-Hodgkin lymphoma	363.6 (311.3 to 419.5)	69.6 (49.2 to 90.3)*	35.8 (19.4 to 52.4)*	26.5 (18.1 to 35.7)	63.8 (43.8 to 84.1)*	31.2 (15.2 to 47.4)*
Multiple myeloma	40.9 (34.1 to 49.7)	49.7 (28.4 to 75.8)*	19.9 (2.8 to 40.7)*	8.9 (6.1 to 12.2)	45.5 (23.3 to 71.7)*	16.5 (-1.3 to 37.5)
Leukemia	149.9 (140.1 to 162.2)	47.8 (34.2 to 62.9)*	18.3 (7.5 to 30.4)*	26.0 (18.5 to 33.9)	34.6 (22.0 to 48.4)*	7.8 (-2.3 to 18.8)
Acute lymphoid leukemia	40.8 (33.4 to 49.7)	40.0 (22.0 to 62.3)*	12.1 (-2.4 to 30.0)	5.4 (3.6 to 7.5)	36.0 (17.0 to 57.9)*	8.9 (-6.3 to 26.4)
Chronic lymphoid leukemia	68.2 (56.1 to 83.1)	38.9 (19.9 to 63.2)*	11.2 (-4.0 to 30.6)	8.6 (5.9 to 11.8)	34.4 (14.9 to 58.5)*	7.6 (-8.0 to 26.9)
Acute myeloid leukemia	67.9 (57.3 to 79.8)	48.2 (31.9 to 67.7)*	18.6 (5.6 to 34.3)*	8.6 (5.9 to 11.4)	43.1 (27.2 to 61.0)*	14.6 (1.9 to 28.9)*
Chronic myeloid leukemia	25.4 (21.4 to 29.8)	20.3 (5.1 to 37.5)*	-3.7 (-15.9 to 10.1)	3.3 (2.3 to 4.5)	15.5 (1.1 to 32.4)*	-7.5 (-19.0 to 6.0)
Other neoplasms	329.5 (275.5 to 399.4)	35.9 (20.1 to 54.0)*	8.8 (-3.9 to 23.3)	23.5 (16.0 to 32.4)	33.6 (16.9 to 51.1)*	6.9 (-6.4 to 21.0)
Cardiovascular diseases	34,296.1 (33,188.5 to 35,413.8)	22.5 (20.9 to 24.0)*	-1.9 (-3.2 to -0.8)*	1,994.1 (1,409.9 to 2,679.1)	22.5 (20.1 to 23.8)*	-1.9 (-3.8 to -0.9)*
Rheumatic heart disease	2,122.1 (1,846.7 to 2,887.9)	-1.5 (-12.3 to 4.3)	-21.2 (-29.8 to -16.5)*	106.9 (66.9 to 166.8)	-0.0 (-10.9 to 5.7)	-20.0 (-28.6 to -15.3)*
Rheumatic heart disease, without heart failure	1,997.8 (1,726.0 to 2,762.1)	-2.6 (-13.3 to 3.4)	-22.0 (-30.6 to -17.2)*	92.7 (56.8 to 150.6)	-2.5 (-13.4 to 3.9)	-22.0 (-30.7 to -16.8)*
Heart failure fue to rheumatic heart disease aggregate	124.3 (110.1 to 139.3)	19.7 (17.9 to 21.4)*	-4.2 (-5.6 to -2.8)*	14.3 (9.8 to 19.7)	19.8 (12.7 to 27.4)*	-4.1 (-9.8 to 2.0)
Ischemic heart disease	10,881.7 (8,814.8 to 13,252.4)	21.7 (19.6 to 23.6)*	-2.6 (-4.2 to -1.1)*	713.3 (466.2 to 1,057.3)	25.6 (23.6 to 27.4)*	0.5 (-1.0 to 2.0)
Myocardial infarction episodes aggregate	1,218.3 (999.7 to 1,494.4)	-0.5 (-9.5 to 7.8)	-20.4 (-27.5 to -13.7)*	2.4 (1.6 to 3.4)	11.9 (3.8 to 20.0)*	-10.4 (-16.9 to -3.9)*
Angina due to ischemic heart disease aggregate	8,501.2 (6,441.3 to 10,859.8)	24.8 (22.7 to 26.6)*	-0.1 (-1.8 to 1.4)	577.5 (357.4 to 876.7)	25.0 (22.7 to 27.1)*	0.1 (-1.7 to 1.7)
Heart failure fue to ischemic heart disease aggregate	1,162.2 (1,070.3 to 1,251.3)	28.2 (27.3 to 29.1)*	2.6 (2.0 to 3.3)*	133.4 (91.4 to 185.2)	28.3 (26.0 to 30.6)*	2.7 (0.9 to 4.5)*
Cerebrovascular disease	3,494.2 (3,438.8 to 3,536.9)	19.1 (17.7 to 20.6)*	-4.6 (-5.7 to -3.5)*	536.7 (372.0 to 725.9)	19.1 (16.9 to 21.3)*	-4.6 (-6.4 to -2.9)*
Ischemic stroke	1,799.8 (1,738.3 to 1,860.6)	19.3 (18.1 to 20.4)*	-4.5 (-5.4 to -3.6)*	272.9 (189.8 to 368.5)	19.5 (16.3 to 22.2)*	-4.3 (-6.9 to -2.1)*
Chronic ischemic stroke aggregate	1,785.5 (1,724.1 to 1,845.5)	19.4 (18.2 to 20.4)*	-4.4 (-5.4 to -3.6)*	269.8 (187.5 to 364.8)	19.6 (16.4 to 22.4)*	-4.3 (-6.8 to -2.0)*
Ischemic stroke episodes aggregate	14.3 (12.3 to 16.5)	10.6 (6.0 to 15.4)*	-11.5 (-15.2 to -7.6)*	3.1 (2.0 to 4.3)	10.6 (6.0 to 15.4)*	-11.5 (-15.2 to -7.6)*
Hemorrhagic stroke	1,743.4 (1,685.3 to 1,802.0)	18.7 (17.6 to 19.8)*	-5.0 (-5.9 to -4.1)*	263.8 (182.9 to 356.6)	18.8 (16.0 to 21.5)*	-4.9 (-7.1 to -2.7)*
Chronic hemorrhagic stroke aggregate	1,728.8 (1,669.9 to 1,786.8)	18.8 (17.6 to 19.8)*	-4.9 (-5.8 to -4.0)*	260.6 (180.6 to 352.6)	18.9 (16.1 to 21.7)*	-4.8 (-7.1 to -2.6)*
Acute hemorrhagic stroke aggregate	14.6 (12.4 to 17.1)	10.6 (4.5 to 16.1)*	-11.4 (-16.3 to -7.0)*	3.2 (2.0 to 4.4)	10.7 (4.5 to 16.2)*	-11.4 (-16.3 to -7.0)*
Hypertensive heart disease	347.7 (310.4 to 387.8)	30.3 (29.1 to 31.4)*	4.3 (3.3 to 5.2)*	39.8 (27.1 to 55.3)	30.5 (26.1 to 35.5)*	4.4 (1.0 to 8.5)*
Cardiomyopathy and myocarditis	146.4 (127.1 to 166.6)	25.2 (23.4 to 26.8)*	0.2 (-1.2 to 1.5)	16.1 (11.0 to 22.8)	25.0 (19.5 to 31.4)*	0.1 (-4.3 to 5.2)
Acute myocarditis	10.7 (7.3 to 14.7)	28.6 (25.2 to 32.5)*	3.0 (0.2 to 6.1)*	0.5 (0.3 to 0.8)	28.6 (24.9 to 32.5)*	3.0 (0.0 to 6.1)*
Heart failure due to cardiomyopathy aggregate	135.7 (116.4 to 154.7)	24.9 (23.1 to 26.7)*	-0.0 (-1.5 to 1.5)	15.6 (10.6 to 22.1)	24.9 (19.4 to 31.5)*	-0.0 (-4.4 to 5.3)
Atrial fibrillation and flutter	1,836.3 (1,477.1 to 2,244.4)	22.5 (21.1 to 23.7)*	-1.9 (-3.1 to -0.9)*	151.7 (99.0 to 220.8)	22.7 (19.4 to 25.6)*	-1.8 (-4.4 to 0.6)
Peripheral vascular disease	10,627.0 (8,407.2 to 13,192.8)	26.9 (26.1 to 27.7)*	1.6 (0.9 to 2.3)*	11.2 (5.1 to 21.8)	24.5 (21.7 to 27.3)*	-0.3 (-2.6 to 1.9)
Endocarditis	3.1 (2.8 to 3.5)	27.7 (24.5 to 30.7)*	2.2 (-0.3 to 4.6)	0.3 (0.2 to 0.5)	27.6 (24.5 to 30.8)*	2.2 (-0.3 to 4.8)
Endocarditis episodes aggregate	1.0 (0.8 to 1.3)	27.6 (19.7 to 34.1)*	2.1 (-4.1 to 7.3)	0.1 (0.0 to 0.1)	27.3 (19.5 to 33.9)*	2.0 (-4.3 to 7.2)
Heart failure due to endocarditis aggregate	2.1 (1.9 to 2.4)	27.8 (24.3 to 31.1)*	2.3 (-0.5 to 5.0)	0.3 (0.2 to 0.4)	27.7 (24.1 to 31.6)*	2.3 (-0.7 to 5.3)
Other cardiovascular and circulatory diseases	6,156.2 (5,391.7 to 7,024.3)	28.0 (26.5 to 29.5)*	2.5 (1.3 to 3.7)*	418.0 (275.0 to 583.7)	28.1 (26.1 to 30.0)*	2.6 (1.0 to 4.1)*
Heart failure due to other cardiovascular diseases aggregate	42.2 (36.8 to 48.5)	27.9 (26.3 to 29.6)*	2.4 (1.2 to 3.7)*	4.9 (3.2 to 6.9)	28.0 (17.4 to 39.4)*	2.5 (-6.0 to 11.6)
Other cardiovascular diseases episodes aggregate	6,114.0 (5,354.8 to 6,976.1)	28.0 (26.5 to 29.5)*	2.5 (1.3 to 3.7)*	413.1 (271.6 to 577.0)	28.1 (26.1 to 30.1)*	2.6 (1.0 to 4.1)*
Chronic respiratory diseases	29,449.6 (28,000.7 to 30,658.6)	19.5 (17.6 to 21.5)*	-4.3 (-5.8 to -2.7)*	1,866.2 (1,445.0 to 2,305.8)	17.5 (14.4 to 20.4)*	-5.9 (-8.4 to -3.6)*
Chronic obstructive pulmonary disease	13,672.1 (11,891.6 to 16,135.4)	15.2 (12.5 to 18.1)*	-7.8 (-9.9 to -5.4)*	934.4 (756.0 to 1,123.9)	15.1 (10.9 to 19.6)*	-7.9 (-11.2 to -4.3)*

Cause	Prevalence in thousands (95% confidence interval)			YLDs in thousands (95% confidence interval)		
	2015	Percent change in counts between 2005 and 2015	Percent change in rates between 2005 and 2015	2015	Percent change in counts between 2005 and 2015	Percent change in rates between 2005 and 2015
Chronic obstructive pulmonary disease without heart failure aggregate	13,538.7<br>(11,753.5 to 15,980.0)	15.1<br>(12.4 to 18.0)*	-7.8<br>(-10.0 to -5.5)*	873.4<br>(705.8 to 1,056.5)	14.3<br>(9.7 to 18.9)*	-8.5<br>(-12.1 to -4.8)*
Severe chronic obstructive pulmonary disease with heart failure aggregate	140.3<br>(124.8 to 157.8)	27.9<br>(26.7 to 29.1)*	2.4<br>(1.4 to 3.3)*	61.0<br>(43.6 to 78.7)	28.0<br>(22.5 to 34.5)*	2.5<br>(-1.9 to 7.7)
Pneumoconiosis	179.5<br>(170.2 to 188.2)	30.5<br>(24.4 to 37.2)*	4.5<br>(-0.4 to 9.8)	35.5<br>(22.8 to 51.7)	28.4<br>(21.3 to 35.5)*	2.8<br>(-2.9 to 8.5)
Silicosis	40.3<br>(34.9 to 45.5)	16.8<br>(14.2 to 19.4)*	-6.5<br>(-8.6 to -4.4)*	6.5<br>(4.0 to 9.6)	17.3<br>(6.5 to 28.9)*	-6.1<br>(-14.8 to 3.2)
Silicosis without heart failure aggregate	39.9<br>(34.5 to 45.2)	16.7<br>(14.0 to 19.3)*	-6.6<br>(-8.8 to -4.5)*	6.3<br>(3.9 to 9.4)	16.9<br>(5.8 to 28.7)*	-6.4<br>(-15.3 to 3.0)
Severe silicosis with heart failure aggregate	0.4<br>(0.3 to 0.4)	32.7<br>(30.2 to 35.3)*	6.3<br>(4.2 to 8.3)*	0.2<br>(0.1 to 0.2)	32.7<br>(30.2 to 35.3)*	6.3<br>(4.2 to 8.3)*
Asbestosis	11.5<br>(9.8 to 13.6)	19.3<br>(16.7 to 21.9)*	-4.4<br>(-6.5 to -2.4)*	1.9<br>(1.2 to 2.9)	19.8<br>(15.7 to 23.5)*	-4.0<br>(-7.4 to -1.1)*
Asbestosis without heart failure aggregate	11.5<br>(9.8 to 13.6)	19.3<br>(16.7 to 21.9)*	-4.5<br>(-6.6 to -2.4)*	1.9<br>(1.2 to 2.9)	19.8<br>(15.6 to 23.5)*	-4.1<br>(-7.4 to -1.1)*
Severe asbestosis with heart failure aggregate	0.0<br>(0.0 to 0.0)	23.9<br>(22.5 to 25.5)*	-0.8<br>(-1.9 to 0.5)	0.0<br>(0.0 to 0.0)	23.9<br>(22.5 to 25.5)*	-0.8<br>(-1.9 to 0.5)
Coal workers pneumoconiosis	6.0<br>(5.0 to 7.1)	32.7<br>(29.4 to 36.0)*	6.3<br>(3.6 to 8.9)*	1.0<br>(0.6 to 1.5)	32.9<br>(29.1 to 36.9)*	6.4<br>(3.4 to 9.6)*
Coal workers pneumoconiosis without heart failure aggregate	5.8<br>(4.8 to 6.9)	32.4<br>(28.9 to 35.8)*	6.0<br>(3.2 to 8.7)*	0.9<br>(0.6 to 1.4)	32.0<br>(27.9 to 36.0)*	5.7<br>(2.4 to 8.9)*
Severe coal workers pneumoconiosis with heart failure aggregate	0.1<br>(0.1 to 0.2)	48.0<br>(37.6 to 58.8)*	18.5<br>(10.1 to 27.1)*	0.1<br>(0.0 to 0.1)	47.9<br>(37.6 to 58.8)*	18.4<br>(10.1 to 27.1)*
Other pneumoconiosis	170.1<br>(149.4 to 192.8)	32.1<br>(29.1 to 35.1)*	5.8<br>(3.3 to 8.2)*	26.1<br>(16.4 to 37.9)	32.1<br>(22.6 to 41.1)*	5.7<br>(-1.8 to 13.0)
Other pneumoconiosis without heart failure aggregate	170.0<br>(149.4 to 192.7)	32.1<br>(29.1 to 35.1)*	5.8<br>(3.3 to 8.2)*	26.0<br>(16.3 to 37.9)	32.1<br>(22.6 to 41.1)*	5.7<br>(-1.8 to 13.0)
Severe other pneumoconiosis with heart failure aggregate	0.1<br>(0.1 to 0.1)	31.9<br>(28.8 to 35.1)*	5.6<br>(3.1 to 8.1)*	0.0<br>(0.0 to 0.1)	31.9<br>(28.8 to 35.1)*	5.6<br>(3.1 to 8.1)*
Asthma	16,839.3<br>(13,261.1 to 20,819.2)	22.5<br>(19.0 to 25.8)*	-2.0<br>(-4.8 to 0.7)	734.9<br>(460.5 to 1,062.3)	22.6<br>(19.0 to 26.0)*	-1.8<br>(-4.7 to 0.9)
Asymptomatic asthma	5,098.6<br>(3,967.7 to 6,471.2)	22.5<br>(19.0 to 25.8)*	-2.0<br>(-4.8 to 0.7)	0.0<br>(0.0 to 0.0)	0.0<br>(0.0 to 0.0)	0.0<br>(0.0 to 0.0)
Symptomatic asthma aggregate	11,740.7<br>(9,197.5 to 14,607.4)	22.5<br>(19.0 to 25.8)*	-2.0<br>(-4.8 to 0.7)	734.9<br>(460.5 to 1,062.3)	22.6<br>(19.0 to 26.0)*	-1.8<br>(-4.7 to 0.9)
Interstitial lung disease and pulmonary sarcoidosis	163.3<br>(141.2 to 192.5)	23.1<br>(20.2 to 25.9)*	-1.4<br>(-3.7 to 0.8)	20.2<br>(12.5 to 29.2)	23.5<br>(16.5 to 30.0)*	-1.1<br>(-6.7 to 4.1)
Interstitial lung disease and pulmonary sarcoidosis without heart failure aggregate	152.0<br>(130.0 to 180.7)	22.9<br>(19.8 to 25.9)*	-1.6<br>(-4.1 to 0.8)	14.8<br>(8.6 to 22.6)	22.5<br>(13.2 to 31.4)*	-1.9<br>(-9.3 to 5.2)
Severe interstitial lung disease and pulmonary sarcoidosis with heart failure aggregate	11.3<br>(9.7 to 13.0)	26.1<br>(24.5 to 27.9)*	0.9<br>(-0.3 to 2.4)	5.4<br>(3.7 to 7.2)	26.4<br>(22.6 to 30.3)*	1.2<br>(-1.8 to 4.3)
Other chronic respiratory diseases	--	--	--	141.1<br>(113.2 to 173.5)	6.8<br>(-3.0 to 18.2)	-14.5<br>(-22.3 to -5.4)*
Cirrhosis and other chronic liver diseases	350.4<br>(340.0 to 360.6)	34.1<br>(29.7 to 39.0)*	7.4<br>(3.8 to 11.3)*	58.4<br>(40.0 to 79.1)	34.3<br>(29.8 to 38.6)*	7.5<br>(3.9 to 11.0)*
Cirrhosis and other chronic liver diseases due to hepatitis B	97.7<br>(82.7 to 113.4)	29.4<br>(24.7 to 33.9)*	3.6<br>(-0.2 to 7.2)	16.2<br>(10.7 to 23.1)	30.1<br>(21.3 to 38.3)*	4.2<br>(-2.9 to 10.7)
Cirrhosis and other chronic liver diseases due to hepatitis C	95.4<br>(79.7 to 113.1)	35.3<br>(31.1 to 39.0)*	8.3<br>(5.0 to 11.3)*	15.8<br>(10.4 to 22.2)	35.6<br>(27.2 to 44.6)*	8.5<br>(1.8 to 15.8)*
Cirrhosis and other chronic liver diseases due to alcohol use	111.7<br>(96.5 to 128.3)	37.1<br>(33.5 to 40.7)*	9.8<br>(6.9 to 12.7)*	18.4<br>(12.2 to 25.9)	37.4<br>(29.2 to 45.9)*	10.0<br>(3.4 to 16.8)*
Cirrhosis and other chronic liver diseases due to other causes	48.9<br>(41.2 to 57.8)	33.6<br>(30.1 to 36.9)*	7.0<br>(4.2 to 9.6)*	8.1<br>(5.3 to 11.4)	33.7<br>(21.6 to 46.7)*	7.0<br>(-2.6 to 17.4)
Digestive diseases	20,925.6<br>(20,437.4 to 21,452.0)	10.8<br>(7.6 to 13.9)*	-11.3<br>(-13.8 to -8.8)*	952.6<br>(662.1 to 1,304.8)	13.3<br>(10.3 to 16.9)*	-9.3<br>(-11.7 to -6.4)*
Peptic ulcer disease	4,571.6<br>(4,436.6 to 4,700.3)	-1.0<br>(-3.4 to 1.8)	-20.7<br>(-22.7 to -18.5)*	145.2<br>(98.0 to 207.7)	-4.1<br>(-7.7 to -0.7)*	-23.2<br>(-26.1 to -20.5)*
Peptic ulcer disease, symptomatic episodes	322.1<br>(254.6 to 392.9)	-0.1<br>(-5.2 to 4.4)	-20.0<br>(-24.1 to -16.4)*	34.3<br>(21.9 to 50.1)	0.1<br>(-5.9 to 5.9)	-19.8<br>(-24.7 to -15.2)*
Anemia due to peptic ulcer disease aggregate	4,249.5<br>(4,132.6 to 4,356.0)	-1.0<br>(-3.7 to 2.0)	-20.8<br>(-22.9 to -18.4)*	110.9<br>(74.1 to 160.7)	-5.3<br>(-9.7 to -1.3)*	-24.2<br>(-27.7 to -21.0)*
Gastritis and duodenitis	13,887.2<br>(13,363.3 to 14,477.7)	13.4<br>(8.7 to 18.3)*	-9.2<br>(-13.0 to -5.3)*	392.0<br>(263.5 to 559.0)	12.2<br>(6.5 to 19.3)*	-10.1<br>(-14.7 to -4.5)*
Gastritis and duodenitis, symptomatic episodes	331.4<br>(243.3 to 430.3)	14.2<br>(8.8 to 17.0)*	-8.6<br>(-12.9 to -6.3)*	35.4<br>(21.3 to 54.5)	14.4<br>(8.5 to 19.1)*	-8.4<br>(-13.1 to -4.6)*
Anemia due to gastritis and duodenitis aggregate	13,555.8<br>(13,017.5 to 14,153.3)	13.4<br>(8.7 to 18.4)*	-9.2<br>(-13.0 to -5.2)*	356.6<br>(238.4 to 513.0)	12.0<br>(5.9 to 19.5)*	-10.3<br>(-15.2 to -4.3)*
Appendicitis	16.1<br>(13.3 to 19.3)	29.3<br>(27.3 to 31.2)*	3.6<br>(2.0 to 5.0)*	4.9<br>(3.2 to 7.0)	29.8<br>(18.1 to 41.9)*	3.9<br>(-5.4 to 13.6)
Paralytic ileus and intestinal obstruction	7.0<br>(5.4 to 8.9)	25.7<br>(19.4 to 32.2)*	0.7<br>(-4.4 to 5.9)	2.3<br>(1.4 to 3.2)	25.7<br>(18.7 to 32.3)*	0.7<br>(-4.9 to 5.9)
Inguinal, femoral, and abdominal hernia	1,445.4<br>(1,054.5 to 1,934.1)	11.2<br>(5.2 to 16.9)*	-11.0<br>(-15.8 to -6.4)*	15.3<br>(7.1 to 29.8)	11.3<br>(4.9 to 17.2)*	-10.9<br>(-16.0 to -6.2)*
Inflammatory bowel disease	1,020.6<br>(948.3 to 1,097.2)	21.4<br>(20.9 to 21.9)*	-2.8<br>(-3.2 to -2.4)*	216.5<br>(148.9 to 292.9)	21.5<br>(19.5 to 23.5)*	-2.7<br>(-4.3 to -1.1)*
Vascular intestinal disorders	2.5<br>(1.9 to 3.2)	23.6<br>(16.8 to 30.6)*	-1.1<br>(-6.4 to 4.6)	0.8<br>(0.5 to 1.2)	23.7<br>(16.7 to 30.8)*	-1.0<br>(-6.6 to 4.7)
Gallbladder and biliary diseases	463.3<br>(357.0 to 598.4)	18.6<br>(12.4 to 24.8)*	-5.1<br>(-10.0 to -0.0)*	49.4<br>(30.8 to 75.1)	18.7<br>(11.9 to 25.7)*	-5.0<br>(-10.4 to 0.7)
Pancreatitis	95.4<br>(76.1 to 116.2)	26.6<br>(20.0 to 32.6)*	1.4<br>(-4.0 to 6.2)	28.2<br>(18.1 to 40.6)	27.0<br>(16.9 to 38.0)*	1.7<br>(-6.4 to 10.5)
Other digestive diseases	--	--	--	98.0<br>(67.1 to 136.5)	25.6<br>(17.1 to 37.0)*	0.6<br>(-6.2 to 9.7)
Neurological disorders	152,432.9<br>(142,791.3 to 160,469.5)	25.7<br>(23.5 to 28.1)*	0.7<br>(-1.1 to 2.6)	3,727.3<br>(2,468.1 to 5,262.2)	23.4<br>(20.4 to 26.6)*	-1.2<br>(-3.6 to 1.4)
Alzheimer disease and other dementias	675.1<br>(517.6 to 883.6)	23.4<br>(22.2 to 24.7)*	-1.2<br>(-2.2 to -0.2)*	85.1<br>(55.5 to 124.8)	23.6<br>(19.7 to 27.5)*	-1.1<br>(-4.1 to 2.1)
Parkinson disease	260.6<br>(224.2 to 303.3)	24.0<br>(19.6 to 28.7)*	-0.7<br>(-4.2 to 3.0)	33.1<br>(22.2 to 46.8)	24.2<br>(16.4 to 31.6)*	-0.6<br>(-6.8 to 5.3)
Epilepsy	1,077.5<br>(907.2 to 1,266.6)	21.1<br>(14.8 to 27.4)*	-3.0<br>(-8.1 to 2.0)	261.5<br>(177.5 to 361.1)	-0.2<br>(-7.2 to 6.9)	-20.1<br>(-25.7 to -14.4)*
Multiple sclerosis	225.1<br>(209.7 to 241.8)	26.3<br>(24.1 to 28.4)*	1.1<br>(-0.6 to 2.8)	74.3<br>(52.8 to 95.6)	26.2<br>(21.1 to 31.6)*	1.1<br>(-3.1 to 5.4)
Motor neuron disease	15.9<br>(13.5 to 18.7)	23.2<br>(17.8 to 28.6)*	-1.4<br>(-5.6 to 2.9)	3.4<br>(2.3 to 4.6)	23.2<br>(18.0 to 28.7)*	-1.3<br>(-5.5 to 3.0)
Migraine	69,143.2<br>(55,524.8 to 83,622.6)	25.5<br>(23.5 to 27.8)*	0.5<br>(-1.2 to 2.3)	2,320.8<br>(1,384.7 to 3,463.6)	25.8<br>(23.4 to 28.2)*	0.7<br>(-1.2 to 2.6)
Tension-type headache	102,023.8<br>(73,251.9 to 138,138.8)	26.3<br>(23.8 to 28.4)*	1.1<br>(-0.9 to 2.8)	151.4<br>(67.7 to 289.2)	26.4<br>(23.6 to 29.0)*	1.2<br>(-1.1 to 3.3)
Medication overuse headache	5,073.2<br>(3,242.1 to 7,333.6)	25.4<br>(13.9 to 37.6)*	0.4<br>(-8.8 to 10.2)	788.0<br>(438.1 to 1,251.9)	25.5<br>(14.0 to 38.0)*	0.5<br>(-8.7 to 10.5)
Other neurological disorders	0.9<br>(0.5 to 1.4)	24.4<br>(22.6 to 25.9)*	-0.4<br>(-1.8 to 0.8)	9.7<br>(6.8 to 13.3)	20.6<br>(15.8 to 25.5)*	-3.4<br>(-7.3 to 0.4)
Mental and substance use disorders	68,030.6<br>(66,245.1 to 69,819.8)	24.9<br>(23.8 to 25.8)*	-0.0<br>(-0.9 to 0.8)	10,423.5<br>(7,572.2 to 13,455.1)	25.3<br>(24.5 to 26.2)*	0.3<br>(-0.3 to 1.0)
Schizophrenia	2,390.9<br>(2,077.5 to 2,714.1)	24.9<br>(24.2 to 25.6)*	-0.0<br>(-0.6 to 0.6)	1,516.8<br>(1,110.4 to 1,911.2)	25.1<br>(23.4 to 26.8)*	0.2<br>(-1.2 to 1.5)
Alcohol use disorders	5,031.1<br>(4,202.0 to 5,945.3)	17.0<br>(13.0 to 21.2)*	-6.3<br>(-9.6 to -3.0)*	495.1<br>(315.5 to 716.4)	17.2<br>(13.0 to 21.9)*	-6.2<br>(-9.5 to -2.4)*
Alcohol dependence aggregate	4,986.5<br>(4,156.1 to 5,901.8)	16.9<br>(12.9 to 21.2)*	-6.4<br>(-9.6 to -3.0)*	492.8<br>(314.0 to 714.1)	17.1<br>(12.9 to 21.9)*	-6.2<br>(-9.6 to -2.4)*
Fetal alcohol syndrome aggregate	44.6<br>(41.0 to 48.1)	23.9<br>(22.7 to 25.1)*	-0.8<br>(-1.7 to 0.2)	2.2<br>(1.4 to 3.2)	23.4<br>(15.8 to 31.6)*	-1.2<br>(-7.3 to 5.4)
Drug use disorders	1,863.2<br>(1,771.4 to 1,949.0)	36.7<br>(33.6 to 39.9)*	9.5<br>(7.0 to 12.0)*	519.0<br>(357.2 to 687.8)	40.7<br>(36.4 to 44.9)*	12.7<br>(9.2 to 16.0)*
Opioid use disorders	1,117.2<br>(900.7 to 1,338.6)	39.1<br>(35.1 to 43.1)*	11.3<br>(8.1 to 14.6)*	453.2<br>(304.8 to 608.4)	39.3<br>(34.4 to 43.9)*	11.5<br>(7.6 to 15.2)*
Asymptomatic opioid dependence	181.2<br>(125.8 to 250.9)	39.0<br>(35.1 to 43.1)*	11.3<br>(8.1 to 14.6)*	0.0<br>(0.0 to 0.0)	0.0<br>(0.0 to 0.0)	0.0<br>(0.0 to 0.0)
Symptomatic opioid dependence aggregate	936.0<br>(751.2 to 1,134.5)	39.1<br>(35.1 to 43.1)*	11.3<br>(8.1 to 14.6)*	453.2<br>(304.8 to 608.4)	39.3<br>(34.4 to 43.9)*	11.5<br>(7.6 to 15.2)*
Cocaine use disorders	125.0<br>(99.1 to 154.9)	57.7<br>(52.7 to 63.6)*	26.3<br>(22.3 to 31.0)*	17.0<br>(10.3 to 25.5)	56.8<br>(46.3 to 68.4)*	25.5<br>(17.1 to 34.8)*
Asymptomatic cocaine dependence	62.7<br>(46.4 to 81.6)	57.7<br>(52.7 to 63.6)*	26.3<br>(22.3 to 31.0)*	0.0<br>(0.0 to 0.0)	0.0<br>(0.0 to 0.0)	0.0<br>(0.0 to 0.0)

Cause	Prevalence in thousands (95% confidence interval)			YLDs in thousands (95% confidence interval)		
	2015	Percent change in counts between 2005 and 2015	Percent change in rates between 2005 and 2015	2015	Percent change in counts between 2005 and 2015	Percent change in rates between 2005 and 2015
Symptomatic cocaine dependence aggregate	62.3 (47.4 to 80.7)	57.7 (52.7 to 63.6)*	26.3 (22.3 to 31.0)*	17.0 (10.3 to 25.5)	56.8 (46.3 to 68.4)*	25.5 (17.1 to 34.8)*
Amphetamine use disorders	99.7 (75.9 to 129.2)	72.9 (64.8 to 81.1)*	38.5 (31.9 to 45.0)*	13.0 (7.5 to 20.0)	71.7 (53.7 to 92.7)*	37.5 (23.1 to 54.3)*
Asymptomatic amphetamine dependence	54.7 (39.1 to 73.4)	73.0 (64.8 to 81.1)*	38.5 (31.9 to 45.0)*	0.0 (0.0 to 0.0)	0.0 (0.0 to 0.0)	0.0 (0.0 to 0.0)
Symptomatic amphetamine dependence aggregate	45.1 (32.1 to 61.7)	72.9 (64.8 to 81.1)*	38.5 (31.9 to 45.0)*	13.0 (7.5 to 20.0)	71.7 (53.7 to 92.7)*	37.5 (23.1 to 54.3)*
Cannabis use disorders	572.8 (501.0 to 650.3)	23.4 (22.0 to 24.8)*	-1.2 (-2.4 to -0.0)*	16.4 (10.5 to 23.5)	23.5 (17.1 to 30.2)*	-1.1 (-6.3 to 4.2)
Asymptomatic cannabis dependence	331.3 (285.8 to 384.7)	23.4 (22.0 to 24.8)*	-1.2 (-2.4 to -0.0)*	0.0 (0.0 to 0.0)	0.0 (0.0 to 0.0)	0.0 (0.0 to 0.0)
Symptomatic cannabis dependence aggregate	241.5 (203.9 to 285.3)	23.4 (22.0 to 24.8)*	-1.2 (-2.4 to -0.0)*	16.4 (10.5 to 23.5)	23.5 (17.1 to 30.2)*	-1.1 (-6.3 to 4.2)
Other drug use disorders	--	--	--	19.5 (11.9 to 28.5)	63.1 (52.5 to 75.2)*	30.6 (22.1 to 40.3)*
Depressive disorders	24,942.7 (23,785.9 to 26,099.5)	24.1 (22.3 to 25.8)*	-0.7 (-2.1 to 0.7)	4,200.8 (2,851.6 to 5,817.1)	24.0 (22.8 to 25.1)*	-0.7 (-1.6 to 0.2)
Major depressive disorder	16,433.6 (13,733.5 to 19,879.6)	23.5 (22.2 to 24.5)*	-1.2 (-2.1 to -0.3)*	3,320.3 (2,146.6 to 4,682.1)	23.7 (22.2 to 24.9)*	-1.0 (-2.2 to 0.0)
Dysthymia	9,175.4 (7,495.1 to 11,085.1)	25.3 (23.6 to 26.9)*	0.3 (-1.0 to 1.6)	880.5 (569.0 to 1,300.2)	25.4 (23.5 to 27.1)*	0.4 (-1.1 to 1.8)
Bipolar disorder	2,629.1 (2,186.8 to 3,140.3)	25.2 (24.9 to 25.5)*	0.2 (-0.0 to 0.5)	526.7 (317.2 to 781.2)	25.3 (23.4 to 27.2)*	0.3 (-1.2 to 1.9)
Anxiety disorders	17,909.2 (14,569.2 to 21,695.3)	27.2 (24.3 to 30.2)*	1.9 (-0.5 to 4.2)	1,623.4 (1,047.3 to 2,310.4)	27.4 (24.4 to 30.6)*	2.0 (-0.4 to 4.6)
Eating disorders	0.0 (0.0 to 0.0)	0.0 (0.0 to 0.0)	0.0 (0.0 to 0.0)	0.0 (0.0 to 0.0)	0.0 (0.0 to 0.0)	0.0 (0.0 to 0.0)
Anorexia nervosa	0.0 (0.0 to 0.0)	0.0 (0.0 to 0.0)	0.0 (0.0 to 0.0)	0.0 (0.0 to 0.0)	0.0 (0.0 to 0.0)	0.0 (0.0 to 0.0)
Bulimia nervosa	0.0 (0.0 to 0.0)	0.0 (0.0 to 0.0)	0.0 (0.0 to 0.0)	0.0 (0.0 to 0.0)	0.0 (0.0 to 0.0)	0.0 (0.0 to 0.0)
Autistic spectrum disorders	3,155.2 (3,000.7 to 3,307.9)	25.0 (22.7 to 27.4)*	0.1 (-1.8 to 2.0)	499.5 (335.0 to 678.2)	25.3 (23.9 to 26.6)*	0.3 (-0.8 to 1.4)
Autism	1,264.3 (1,058.4 to 1,508.0)	25.2 (24.9 to 25.6)*	0.3 (-0.0 to 0.5)	315.4 (204.7 to 448.1)	25.4 (23.4 to 27.3)*	0.4 (-1.2 to 1.9)
Asperger syndrome and other autistic spectrum disorders	1,877.6 (1,597.8 to 2,257.9)	24.9 (24.8 to 25.0)*	-0.0 (-0.1 to 0.1)	184.1 (123.4 to 268.8)	25.0 (23.4 to 26.6)*	0.1 (-1.2 to 1.4)
Attention-deficit/hyperactivity disorder	17.6 (9.7 to 28.6)	22.7 (19.3 to 26.4)*	-1.8 (-4.5 to 1.2)	0.2 (0.1 to 0.4)	22.8 (3.8 to 45.6)*	-1.7 (-16.9 to 16.6)
Conduct disorder	0.0 (0.0 to 0.0)	0.0 (0.0 to 0.0)	0.0 (0.0 to 0.0)	0.0 (0.0 to 0.0)	0.0 (0.0 to 0.0)	0.0 (0.0 to 0.0)
Idiopathic developmental intellectual disability	3,721.2 (1,954.4 to 5,389.3)	25.6 (23.8 to 27.1)*	0.5 (-0.9 to 1.8)	144.2 (62.4 to 250.4)	24.8 (21.4 to 27.1)*	-0.1 (-2.8 to 1.8)
Other mental and substance use disorders	12,097.9 (11,901.9 to 12,303.8)	24.9 (22.0 to 27.9)*	-0.0 (-2.3 to 2.4)	897.9 (627.0 to 1,211.3)	25.1 (21.9 to 28.4)*	0.1 (-2.4 to 2.8)
Diabetes, urogenital, blood, and endocrine diseases	197,666.0 (195,697.4 to 199,766.1)	24.4 (23.9 to 25.0)*	-0.4 (-0.8 to 0.1)	6,087.6 (4,214.3 to 8,266.4)	26.8 (23.8 to 29.5)*	1.5 (-0.9 to 3.7)
Diabetes mellitus	51,284.7 (45,680.0 to 57,237.7)	28.8 (24.6 to 32.3)*	3.1 (-0.2 to 5.9)	4,050.6 (2,704.9 to 5,687.6)	30.6 (26.1 to 34.5)*	4.5 (0.9 to 7.7)*
Uncomplicated diabetes mellitus	30,958.6 (24,683.2 to 37,028.5)	26.2 (21.6 to 30.2)*	1.0 (-2.7 to 4.2)	1,444.4 (857.8 to 2,225.8)	26.3 (21.6 to 30.3)*	1.2 (-2.6 to 4.3)
Neuropathy and other complications of diabetes mellitus aggregate	19,889.0 (14,654.4 to 25,757.9)	33.0 (27.8 to 37.7)*	6.5 (2.3 to 10.3)*	2,577.4 (1,592.1 to 3,837.5)	33.1 (27.9 to 37.8)*	6.5 (2.4 to 10.3)*
Vision loss due to diabetes mellitus aggregate	437.1 (303.6 to 602.5)	30.7 (27.4 to 33.7)*	4.6 (2.0 to 7.0)*	28.7 (17.9 to 42.4)	30.2 (24.6 to 36.3)*	4.3 (-0.2 to 9.2)
Acute glomerulonephritis	7.4 (5.4 to 9.5)	8.1 (2.2 to 13.0)*	-13.4 (-18.2 to -9.5)*	0.4 (0.2 to 0.6)	9.3 (-2.5 to 22.3)	-12.5 (-22.0 to -2.1)*
Chronic kidney disease	28,052.2 (25,884.1 to 30,309.8)	27.1 (24.3 to 30.2)*	1.8 (-0.5 to 4.2)	701.8 (500.4 to 923.2)	24.3 (22.4 to 26.4)*	-0.4 (-2.0 to 1.2)
Chronic kidney disease due to diabetes mellitus	8,516.5 (5,564.2 to 12,302.9)	27.7 (23.8 to 31.2)*	2.2 (-0.9 to 5.0)	207.8 (126.2 to 307.7)	23.9 (19.5 to 28.1)*	-0.8 (-4.3 to 2.5)
Stage III chronic kidney disease due to diabetes mellitus aggregate	7,731.7 (4,989.4 to 11,424.2)	28.1 (23.9 to 31.8)*	2.5 (-0.8 to 5.5)	6.3 (3.8 to 10.2)	8.4 (-9.3 to 29.2)	-13.2 (-27.4 to 3.5)
Stage IV chronic kidney disease due to diabetes mellitus aggregate	443.9 (257.7 to 679.2)	24.6 (21.6 to 27.7)*	-0.2 (-2.6 to 2.2)	53.6 (28.7 to 89.0)	23.7 (19.0 to 28.7)*	-1.0 (-4.7 to 3.1)
Stage V chronic kidney disease untreated due to diabetes mellitus	252.7 (163.2 to 349.7)	25.9 (22.7 to 29.1)*	0.8 (-1.8 to 3.3)	129.0 (73.2 to 198.3)	26.0 (20.6 to 31.5)*	0.9 (-3.5 to 5.3)
End-stage chronic kidney disease due to diabetes aggregate	88.1 (59.9 to 119.6)	15.5 (11.3 to 19.7)*	-7.5 (-10.9 to -4.1)*	18.8 (11.2 to 28.8)	16.8 (7.2 to 27.1)*	-6.5 (-14.1 to 1.8)
Chronic kidney disease due to hypertension	7,412.9 (4,661.5 to 11,007.8)	27.0 (23.4 to 30.6)*	1.7 (-1.2 to 4.5)	120.6 (79.8 to 169.6)	26.1 (20.2 to 33.6)*	1.0 (-3.8 to 6.9)
Stage III chronic kidney disease due to hypertension aggregate	6,951.9 (4,202.2 to 10,398.6)	27.2 (23.2 to 31.0)*	1.8 (-1.4 to 4.9)	5.1 (3.1 to 8.0)	23.2 (11.3 to 35.2)*	-1.4 (-10.9 to 8.3)
Stage IV chronic kidney disease due to hypertension aggregate	270.5 (174.4 to 421.8)	25.4 (18.2 to 32.5)*	0.4 (-5.3 to 6.1)	33.0 (19.8 to 53.5)	24.7 (16.0 to 33.8)*	-0.2 (-7.1 to 7.1)
Stage V chronic kidney disease untreated due to hypertension	141.2 (99.0 to 194.9)	27.1 (21.5 to 33.5)*	1.8 (-2.7 to 6.9)	72.0 (43.1 to 106.8)	27.4 (18.8 to 37.0)*	2.0 (-4.9 to 9.7)
End-stage chronic kidney disease due to hypertension aggregate	49.3 (35.8 to 65.8)	20.7 (13.1 to 29.4)*	-3.4 (-9.4 to 3.6)	10.4 (6.6 to 15.4)	23.2 (9.9 to 38.4)*	-1.3 (-12.0 to 10.8)
Chronic kidney disease due to glomerulonephritis	5,206.7 (3,715.5 to 7,069.9)	27.7 (22.6 to 34.0)*	2.3 (-1.8 to 7.3)	189.8 (120.4 to 271.7)	24.3 (20.1 to 28.9)*	-0.5 (-3.8 to 3.2)
Stage III chronic kidney disease due to glomerulonephritis aggregate	4,525.1 (3,067.2 to 6,376.7)	28.4 (22.6 to 35.6)*	2.8 (-1.8 to 8.6)	10.2 (6.5 to 15.2)	18.7 (9.5 to 27.7)*	-4.9 (-12.3 to 2.3)
Stage IV chronic kidney disease due to glomerulonephritis aggregate	387.6 (254.5 to 527.5)	23.5 (18.8 to 28.9)*	-1.1 (-4.9 to 3.2)	47.1 (27.3 to 72.4)	23.3 (17.6 to 29.7)*	-1.3 (-5.9 to 3.9)
Stage V chronic kidney disease untreated due to glomerulonephritis	235.1 (149.5 to 339.9)	25.1 (21.8 to 28.4)*	0.2 (-2.5 to 2.8)	119.7 (66.7 to 183.7)	25.5 (19.2 to 31.5)*	0.5 (-4.6 to 5.2)
End-stage chronic kidney disease due to glomerulonephritis aggregate	58.9 (37.6 to 83.5)	19.7 (14.9 to 24.6)*	-4.2 (-8.0 to -0.2)*	12.8 (7.0 to 19.8)	21.6 (10.0 to 34.8)*	-2.6 (-11.9 to 7.9)
Chronic kidney disease due to other causes	7,510.9 (4,776.3 to 11,170.3)	26.9 (24.0 to 30.0)*	1.6 (-0.7 to 4.0)	183.6 (115.0 to 272.7)	23.7 (20.0 to 27.4)*	-0.9 (-3.9 to 2.0)
Stage III chronic kidney disease due to other causes aggregate	6,835.6 (4,238.4 to 10,341.8)	27.3 (24.1 to 30.5)*	1.9 (-0.6 to 4.5)	9.6 (6.1 to 14.6)	19.7 (10.5 to 29.0)*	-4.2 (-11.5 to 3.3)
Stage IV chronic kidney disease due to other causes aggregate	387.8 (236.5 to 612.0)	23.8 (21.5 to 26.1)*	-0.9 (-2.8 to 0.9)	46.7 (25.4 to 75.3)	22.3 (17.8 to 26.6)*	-2.1 (-5.7 to 1.3)
Stage V chronic kidney disease untreated due to other causes	221.3 (144.3 to 321.5)	25.1 (22.9 to 27.6)*	0.2 (-1.6 to 2.1)	112.7 (65.0 to 176.2)	25.4 (20.0 to 30.1)*	0.4 (-3.9 to 4.2)
End-stage chronic kidney disease due to other causes aggregate	66.2 (44.6 to 94.0)	18.4 (15.0 to 22.3)*	-5.2 (-8.0 to -2.1)*	14.6 (8.9 to 22.1)	18.6 (8.5 to 29.3)*	-5.1 (-13.1 to 3.6)
Urinary diseases and male infertility	38,337.0 (36,777.0 to 39,647.0)	24.3 (22.3 to 26.2)*	-0.5 (-2.1 to 1.0)	279.7 (170.8 to 414.7)	21.9 (17.5 to 26.7)*	-2.4 (-5.9 to 1.4)
Interstitial nephritis and urinary tract infections	137.0 (127.6 to 146.2)	29.0 (28.2 to 29.9)*	3.3 (2.7 to 4.0)*	4.5 (2.8 to 6.8)	29.1 (21.7 to 36.7)*	3.4 (-2.6 to 9.5)
Urolithiasis	32,064.4 (28,416.0 to 36,408.9)	24.9 (22.6 to 27.3)*	0.0 (-1.9 to 1.9)	9.0 (5.6 to 13.2)	27.1 (17.3 to 37.7)*	1.8 (-6.1 to 10.2)
Benign prostatic hyperplasia	6,992.2 (5,301.4 to 9,048.4)	21.5 (16.8 to 26.4)*	-2.7 (-6.5 to 1.2)	261.0 (159.1 to 389.8)	21.6 (17.1 to 26.6)*	-2.6 (-6.3 to 1.4)
Male infertility	0.0 (0.0 to 0.0)	0.0 (0.0 to 0.0)	0.0 (0.0 to 0.0)	0.0 (0.0 to 0.0)	0.0 (0.0 to 0.0)	0.0 (0.0 to 0.0)
Other urinary diseases	--	--	--	5.1 (3.3 to 7.5)	23.4 (11.6 to 37.8)*	-1.2 (-10.7 to 10.3)
Gynecological diseases	61,149.5 (60,452.4 to 61,922.8)	21.8 (20.9 to 22.8)*	-2.5 (-3.2 to -1.7)*	731.3 (494.9 to 1,032.3)	18.1 (16.4 to 20.0)*	-5.4 (-6.8 to -3.9)*
Uterine fibroids	18,528.1 (17,346.6 to 19,776.5)	24.6 (24.3 to 24.9)*	-0.2 (-0.5 to 0.0)	258.3 (154.3 to 421.1)	14.7 (10.7 to 18.1)*	-8.2 (-11.4 to -5.4)*
Uterine fibroids cases aggregate	14,107.7 (12,965.9 to 15,336.0)	28.4 (27.1 to 29.7)*	2.8 (1.8 to 3.9)*	87.9 (42.7 to 161.4)	31.2 (29.0 to 33.6)*	5.1 (3.3 to 7.0)*
Mild abdominal pain with anemia due to uterine fibroids aggregate	4,420.5 (4,309.3 to 4,529.7)	13.8 (10.6 to 17.0)*	-8.9 (-11.5 to -6.3)*	170.4 (110.9 to 256.8)	7.7 (3.1 to 12.3)*	-13.7 (-17.5 to -10.1)*
Polycystic ovarian syndrome	2.4 (1.9 to 3.3)	24.1 (23.7 to 24.6)*	-0.6 (-1.0 to -0.3)*	0.0 (0.0 to 0.0)	24.1 (23.7 to 24.6)*	-0.6 (-1.0 to -0.3)*
Polycystic ovarian syndrome cases aggregate	2.4 (1.9 to 3.3)	24.1 (23.7 to 24.6)*	-0.6 (-1.0 to -0.3)*	0.0 (0.0 to 0.0)	24.1 (23.7 to 24.6)*	-0.6 (-1.0 to -0.3)*
Hirsutism and infertility due to polycystic ovarian syndrome aggregate	0.0 (0.0 to 0.0)	0.0 (0.0 to 0.0)	0.0 (0.0 to 0.0)	0.0 (0.0 to 0.0)	0.0 (0.0 to 0.0)	0.0 (0.0 to 0.0)

Cause	Prevalence in thousands (95% confidence interval)			YLDs in thousands (95% confidence interval)		
	2015	Percent change in counts between 2005 and 2015	Percent change in rates between 2005 and 2015	2015	Percent change in counts between 2005 and 2015	Percent change in rates between 2005 and 2015
Female infertility	0.0 (0.0 to 0.0)	0.0 (0.0 to 0.0)	0.0 (0.0 to 0.0)	0.0 (0.0 to 0.0)	0.0 (0.0 to 0.0)	0.0 (0.0 to 0.0)
Endometriosis	669.3 (553.4 to 799.8)	24.4 (23.9 to 24.8)*	-0.4 (-0.8 to -0.1)*	60.6 (38.2 to 87.5)	24.6 (20.8 to 28.5)*	-0.3 (-3.3 to 2.9)
Endometriosis cases aggregate	669.3 (553.4 to 799.8)	24.4 (23.9 to 24.8)*	-0.4 (-0.8 to -0.1)*	60.6 (38.2 to 87.5)	24.6 (20.8 to 28.5)*	-0.3 (-3.3 to 2.9)
Abdominal pain and infertility due to endometriosis aggregate	0.0 (0.0 to 0.0)	0.0 (0.0 to 0.0)	0.0 (0.0 to 0.0)	0.0 (0.0 to 0.0)	0.0 (0.0 to 0.0)	0.0 (0.0 to 0.0)
Genital prolapse	22,738.1 (19,738.0 to 26,026.2)	17.6 (14.6 to 20.4)*	-5.9 (-8.2 to -3.6)*	71.2 (34.3 to 128.7)	17.7 (14.7 to 20.8)*	-5.7 (-8.2 to -3.3)*
Premenstrual syndrome	24,585.5 (22,833.1 to 26,456.9)	22.4 (18.7 to 26.3)*	-2.0 (-4.9 to 1.2)	202.6 (126.7 to 303.0)	22.6 (18.4 to 27.6)*	-1.9 (-5.2 to 2.1)
Other gynecological diseases	4,001.3 (3,786.8 to 4,226.8)	18.7 (16.9 to 20.5)*	-5.0 (-6.4 to -3.5)*	138.6 (96.1 to 187.1)	15.9 (12.6 to 19.3)*	-7.2 (-9.8 to -4.5)*
Other gynecological diseases cases aggregate	2,459.1 (2,246.3 to 2,675.9)	23.9 (23.5 to 24.3)*	-0.8 (-1.1 to -0.5)*	94.5 (64.0 to 130.3)	24.0 (20.6 to 27.4)*	-0.7 (-3.4 to 2.0)
Anemia due to other gynecological diseases aggregate	1,542.3 (1,503.8 to 1,584.1)	11.3 (7.2 to 15.5)*	-10.9 (-14.2 to -7.5)*	44.2 (29.9 to 62.5)	1.7 (-4.6 to 8.2)	-18.6 (-23.6 to -13.4)*
Hemoglobinopathies and hemolytic anemias	77,684.0 (76,300.3 to 79,345.6)	22.3 (21.0 to 23.8)*	-2.1 (-3.1 to -0.9)*	261.8 (174.0 to 377.8)	14.1 (10.3 to 18.0)*	-8.7 (-11.7 to -5.5)*
Thalassemias	0.8 (0.8 to 0.9)	19.4 (12.6 to 27.0)*	-4.4 (-9.9 to 1.7)	0.1 (0.1 to 0.2)	22.6 (-32.4 to 118.5)	-1.8 (-45.9 to 74.9)
Beta-thalassemia major cases aggregate	0.7 (0.7 to 0.7)	21.0 (12.8 to 30.4)*	-3.1 (-9.7 to 4.4)	0.1 (0.0 to 0.2)	24.6 (-38.2 to 147.1)	-0.2 (-50.5 to 97.8)
Hemoglobin E/beta-thalassemia cases aggregate	0.1 (0.1 to 0.1)	-5.1 (-5.7 to -4.4)*	-24.0 (-24.5 to -23.5)*	0.0 (0.0 to 0.0)	-7.3 (-14.6 to 2.8)	-25.8 (-31.6 to -17.7)*
Hemoglobin H disease cases aggregate	0.0 (0.0 to 0.0)	28.1 (22.7 to 34.2)*	2.6 (-1.8 to 7.5)	0.0 (0.0 to 0.0)	24.8 (16.6 to 32.8)*	-0.1 (-6.7 to 6.4)
Heart failure due to thalassemias aggregate	0.1 (0.0 to 0.1)	34.7 (31.9 to 37.4)*	7.9 (5.6 to 10.0)*	0.0 (0.0 to 0.0)	34.7 (31.9 to 37.4)*	7.9 (5.6 to 10.0)*
Thalassemias trait	15,926.8 (15,549.9 to 16,369.8)	23.5 (22.9 to 24.1)*	-1.1 (-1.6 to -0.6)*	150.8 (99.8 to 216.6)	19.1 (14.3 to 24.6)*	-4.6 (-8.5 to -0.2)*
B-thalassemia trait cases aggregate	12,867.2 (12,533.3 to 13,282.0)	22.0 (21.3 to 22.7)*	-2.3 (-2.9 to -1.8)*	142.8 (94.8 to 205.1)	18.9 (13.9 to 24.6)*	-4.8 (-8.8 to -0.2)*
Hemoglobin E trait cases aggregate	3,059.6 (2,925.7 to 3,201.7)	30.2 (28.9 to 31.6)*	4.3 (3.2 to 5.4)*	8.0 (5.2 to 11.9)	23.8 (12.2 to 36.2)*	-0.9 (-10.2 to 9.1)
Sickle cell disorders	4.0 (3.7 to 4.2)	30.0 (23.1 to 37.5)*	4.1 (-1.5 to 10.1)	0.6 (0.4 to 0.8)	26.6 (17.9 to 36.0)*	1.4 (-5.6 to 8.9)
Homozygous sickle cell and severe sickle cell/beta-thalassemia cases aggregate	3.4 (3.2 to 3.6)	32.7 (24.4 to 41.8)*	6.2 (-0.4 to 13.6)	0.5 (0.3 to 0.6)	29.0 (18.4 to 40.8)*	3.3 (-5.2 to 12.8)
Hemoglobin SC disease cases aggregate	0.3 (0.3 to 0.3)	21.5 (17.5 to 26.3)*	-2.7 (-5.9 to 1.1)	0.1 (0.0 to 0.1)	25.3 (20.7 to 29.8)*	0.3 (-3.3 to 3.9)
Mild sickle cell/beta-thalassemia cases aggregate	0.3 (0.2 to 0.3)	9.2 (5.7 to 13.7)*	-12.6 (-15.3 to -9.0)*	0.0 (0.0 to 0.1)	7.1 (3.6 to 10.7)*	-14.3 (-17.1 to -11.3)*
Sickle cell trait	16,555.5 (15,593.3 to 18,484.9)	29.7 (27.9 to 32.9)*	3.8 (2.4 to 6.4)*	48.4 (32.7 to 69.2)	15.0 (9.6 to 20.7)*	-7.9 (-12.3 to -3.4)*
G6PD deficiency	11,685.4 (9,913.7 to 13,545.1)	17.9 (12.6 to 22.7)*	-5.6 (-9.9 to -1.8)*	1.2 (0.8 to 1.7)	14.7 (5.4 to 24.0)*	-8.1 (-15.6 to -0.8)*
G6PD cases aggregate	11,684.9 (9,913.0 to 13,544.6)	17.9 (12.6 to 22.7)*	-5.6 (-9.9 to -1.8)*	1.1 (0.8 to 1.5)	13.7 (3.9 to 23.3)*	-9.0 (-16.8 to -1.2)*
Heart failure due to G6PD deficiency aggregate	0.6 (0.5 to 0.7)	33.8 (31.1 to 36.8)*	7.1 (4.9 to 9.5)*	0.1 (0.0 to 0.1)	33.8 (31.1 to 36.8)*	7.1 (4.9 to 9.5)*
G6PD trait	37,528.7 (34,680.9 to 40,341.3)	21.3 (19.4 to 23.1)*	-2.9 (-4.4 to -1.5)*	1.0 (0.7 to 1.4)	18.4 (2.8 to 38.1)*	-5.2 (-17.7 to 10.6)
Other hemoglobinopathies and hemolytic anemias	3,211.2 (3,145.0 to 3,290.8)	12.5 (9.5 to 15.5)*	-9.9 (-12.3 to -7.5)*	59.7 (39.6 to 87.1)	2.3 (-3.3 to 8.3)	-18.1 (-22.6 to -13.3)*
Other hemoglobinopathies and hemolytic anemias cases aggregate	3,202.2 (3,135.7 to 3,282.1)	12.4 (9.5 to 15.5)*	-10.0 (-12.4 to -7.5)*	58.0 (38.4 to 84.6)	1.8 (-4.0 to 8.0)	-18.5 (-23.2 to -13.5)*
Heart failure due to other hemoglobinopathies and hemolytic anemias aggregate	9.0 (7.8 to 10.3)	29.6 (28.0 to 31.2)*	3.8 (2.5 to 5.1)*	1.1 (0.7 to 1.6)	29.7 (23.1 to 37.2)*	3.8 (-1.5 to 9.8)
Endocrine, metabolic, blood, and immune disorders	2,669.5 (2,602.5 to 2,740.7)	15.3 (12.8 to 17.7)*	-7.7 (-9.7 to -5.8)*	62.2 (42.7 to 87.8)	9.0 (4.9 to 13.3)*	-12.7 (-16.0 to -9.3)*
Endocrine, metabolic, blood, and immune disorders cases aggregate	565.8 (516.9 to 623.0)	26.6 (23.7 to 29.4)*	1.3 (-1.0 to 3.6)	21.9 (14.9 to 30.5)	26.7 (20.8 to 33.2)*	1.5 (-3.3 to 6.6)
Anemia due to endocrine, metabolic, blood, and immune disorders aggregate	2,099.8 (2,058.2 to 2,142.0)	12.6 (9.7 to 15.5)*	-9.9 (-12.1 to -7.5)*	39.8 (26.7 to 58.2)	1.1 (-5.2 to 7.1)	-19.0 (-24.1 to -14.2)*
Heart failure due to endocrine, metabolic, blood, and immune disorders aggregate	3.8 (3.3 to 4.4)	24.3 (22.8 to 25.8)*	-0.5 (-1.6 to 0.7)	0.5 (0.3 to 0.7)	24.2 (22.7 to 25.9)*	-0.5 (-1.8 to 0.8)
Musculoskeletal disorders	132,900.0 (128,934.5 to 137,681.6)	25.4 (24.2 to 26.7)*	0.4 (-0.6 to 1.5)	15,078.4 (10,840.5 to 20,098.7)	25.5 (23.7 to 27.5)*	0.5 (-1.0 to 2.1)
Rheumatoid arthritis	2,537.1 (2,208.6 to 2,929.9)	26.7 (23.0 to 30.8)*	1.5 (-1.5 to 4.7)	603.1 (408.5 to 819.8)	26.9 (22.6 to 31.0)*	1.6 (-1.8 to 4.9)
Osteoarthritis	29,526.3 (28,326.7 to 30,688.9)	27.8 (26.5 to 29.2)*	2.3 (1.3 to 3.4)*	1,696.5 (1,184.5 to 2,325.2)	29.4 (27.8 to 31.1)*	3.6 (2.3 to 4.9)*
Osteoarthritis of the hip cases aggregate	2,323.1 (1,963.7 to 2,754.0)	28.5 (27.0 to 30.2)*	2.9 (1.7 to 4.2)*	125.4 (84.9 to 177.0)	30.8 (28.1 to 33.7)*	4.7 (2.5 to 7.0)*
Osteoarthritis of the knee cases aggregate	27,203.2 (26,087.7 to 28,265.6)	27.7 (26.4 to 29.2)*	2.3 (1.2 to 3.5)*	1,571.1 (1,089.9 to 2,144.8)	29.3 (27.7 to 31.1)*	3.5 (2.2 to 4.9)*
Low back and neck pain	84,160.2 (80,272.7 to 87,879.4)	23.4 (21.6 to 25.3)*	-1.2 (-2.7 to 0.3)	9,870.6 (6,866.1 to 13,531.8)	23.3 (21.4 to 25.2)*	-1.3 (-2.8 to 0.3)
Low back pain	51,144.0 (45,845.0 to 57,217.1)	21.9 (19.7 to 24.0)*	-2.4 (-4.2 to -0.7)*	5,688.0 (3,952.8 to 7,859.0)	22.1 (19.7 to 24.3)*	-2.3 (-4.1 to -0.5)*
Neck pain	42,831.1 (31,319.4 to 57,622.8)	25.0 (21.8 to 28.1)*	0.1 (-2.5 to 2.6)	4,182.6 (2,647.8 to 6,249.9)	25.1 (21.9 to 28.3)*	0.2 (-2.4 to 2.7)
Gout	4,783.7 (3,645.4 to 6,076.5)	26.4 (25.1 to 27.6)*	1.2 (0.2 to 2.1)*	154.2 (97.3 to 225.8)	26.5 (23.0 to 30.0)*	1.3 (-1.6 to 4.0)
Asymptomatic gout	4,273.7 (3,245.2 to 5,447.1)	26.4 (25.1 to 27.6)*	1.2 (0.2 to 2.1)*	0.0 (0.0 to 0.0)	0.0 (0.0 to 0.0)	0.0 (0.0 to 0.0)
Symptomatic gout cases aggregate	510.0 (381.5 to 667.0)	26.4 (25.1 to 27.6)*	1.2 (0.2 to 2.1)*	154.2 (97.3 to 225.8)	26.5 (23.0 to 30.0)*	1.3 (-1.6 to 4.0)
Other musculoskeletal disorders	29,736.7 (21,698.3 to 38,889.4)	30.8 (23.2 to 37.8)*	4.7 (-1.4 to 10.3)	2,754.0 (1,736.9 to 4,073.6)	31.0 (23.1 to 38.0)*	4.9 (-1.5 to 10.5)
Other non-communicable diseases	322,387.0 (318,515.3 to 325,902.0)	25.3 (24.8 to 25.7)*	0.3 (-0.0 to 0.7)	9,168.5 (6,128.4 to 13,420.6)	26.5 (25.3 to 28.0)*	1.3 (0.4 to 2.5)*
Congenital anomalies	4,996.2 (4,584.1 to 5,378.2)	39.0 (34.0 to 43.8)*	11.3 (7.3 to 15.2)*	451.6 (281.9 to 685.5)	43.9 (33.8 to 55.3)*	15.2 (7.1 to 24.3)*
Neural tube defects	56.4 (39.2 to 80.4)	37.7 (34.5 to 40.6)*	10.2 (7.7 to 12.6)*	18.5 (10.5 to 29.8)	38.7 (30.4 to 47.4)*	11.1 (4.4 to 18.0)*
Congenital heart anomalies	2,805.8 (1,978.1 to 4,155.1)	49.1 (43.0 to 54.4)*	19.4 (14.5 to 23.6)*	95.4 (37.4 to 174.8)	49.4 (43.4 to 55.1)*	19.6 (14.8 to 24.2)*
Less severe heart anomalies cases aggregate	2,559.9 (1,723.2 to 3,905.2)	48.6 (41.8 to 54.4)*	19.0 (13.5 to 23.6)*	80.9 (30.7 to 155.5)	48.7 (41.6 to 55.0)*	19.1 (13.4 to 24.1)*
Severe congenital heart anomalies	240.9 (160.0 to 362.0)	54.4 (46.4 to 61.7)*	23.6 (17.2 to 29.5)*	13.9 (5.4 to 27.7)	54.3 (44.4 to 63.2)*	23.5 (15.6 to 30.7)*
Critical congenital heart anomalies	1.8 (1.0 to 3.0)	79.1 (49.1 to 103.2)*	43.4 (19.3 to 62.7)*	0.1 (0.0 to 0.2)	79.7 (45.0 to 109.8)*	43.9 (16.1 to 68.0)*
Heart failure due to congenital heart anomalies aggregate	3.3 (2.9 to 3.8)	26.0 (24.6 to 27.5)*	0.9 (-0.3 to 2.1)	0.4 (0.3 to 0.6)	26.0 (24.6 to 27.5)*	0.9 (-0.3 to 2.1)
Cleft lip and cleft palate	379.3 (222.3 to 622.0)	36.5 (21.1 to 49.0)*	9.3 (-3.0 to 19.3)	3.9 (2.0 to 6.9)	28.7 (11.0 to 44.0)*	3.1 (-11.1 to 15.3)
Down syndrome	238.4 (154.7 to 359.6)	35.4 (28.5 to 41.2)*	8.4 (2.9 to 13.1)*	31.8 (18.6 to 51.4)	35.4 (28.6 to 41.0)*	8.4 (3.0 to 12.9)*
Turner syndrome	20.1 (9.3 to 36.2)	26.7 (24.2 to 29.6)*	1.4 (-0.5 to 3.7)	0.3 (0.1 to 0.6)	26.6 (19.3 to 33.5)*	1.4 (-4.4 to 6.9)
Klinefelter syndrome	11.7 (6.1 to 21.0)	24.9 (22.3 to 26.8)*	0.0 (-2.1 to 1.5)	0.0 (0.0 to 0.1)	25.0 (22.3 to 26.8)*	0.0 (-2.1 to 1.5)
Other chromosomal abnormalities	221.5 (123.5 to 386.4)	28.9 (21.7 to 35.3)*	3.2 (-2.5 to 8.3)	29.8 (15.8 to 54.8)	29.6 (22.2 to 36.0)*	3.7 (-2.2 to 8.9)
Other congenital anomalies	1,649.2 (897.5 to 3,034.9)	25.9 (22.6 to 29.3)*	0.8 (-1.9 to 3.5)	271.8 (161.0 to 435.2)	45.5 (31.7 to 63.5)*	16.5 (5.4 to 30.9)*
Skin and subcutaneous diseases	104,499.4 (102,472.5 to 106,451.5)	26.5 (25.6 to 27.3)*	1.3 (0.6 to 1.9)*	2,017.1 (1,353.3 to 2,955.5)	25.8 (24.8 to 26.8)*	0.7 (-0.1 to 1.6)
Dermatitis	13,714.0 (11,039.2 to 17,039.5)	24.1 (23.9 to 24.4)*	-0.6 (-0.8 to -0.4)*	395.5 (257.9 to 574.3)	24.5 (23.1 to 25.8)*	-0.3 (-1.5 to 0.7)
Eczema cases aggregate	2,470.7 (1,882.0 to 3,202.1)	25.4 (24.8 to 25.9)*	0.4 (-0.1 to 0.8)	147.0 (89.3 to 218.4)	25.5 (22.5 to 28.4)*	0.5 (-1.9 to 2.8)

Cause	Prevalence in thousands (95% confidence interval)			YLDs in thousands (95% confidence interval)		
	2015	Percent change in counts between 2005 and 2015	Percent change in rates between 2005 and 2015	2015	Percent change in counts between 2005 and 2015	Percent change in rates between 2005 and 2015
Contact dermatitis cases aggregate	6,946.5<br>(4,774.4 to 9,610.1)	23.6<br>(23.2 to 23.9)*	-1.1<br>(-1.4 to -0.8)*	185.2<br>(102.0 to 295.2)	23.7<br>(22.0 to 25.3)*	-1.0<br>(-2.3 to 0.3)
Seborrhoeic dermatitis cases aggregate	4,296.9<br>(3,079.8 to 5,749.7)	24.4<br>(24.1 to 24.6)*	-0.4<br>(-0.7 to -0.2)*	63.2<br>(33.3 to 105.0)	24.5<br>(23.1 to 25.9)*	-0.3<br>(-1.5 to 0.8)
Psoriasis	6,330.8<br>(5,761.1 to 6,946.9)	25.5<br>(24.1 to 26.8)*	0.5<br>(-0.6 to 1.5)	506.4<br>(353.0 to 704.3)	25.7<br>(23.5 to 27.8)*	0.6<br>(-1.1 to 2.3)
Cellulitis	59.4<br>(43.9 to 77.5)	31.5<br>(26.1 to 38.1)*	5.2<br>(0.9 to 10.6)*	4.3<br>(2.5 to 6.6)	31.2<br>(19.7 to 43.5)*	5.1<br>(-4.2 to 14.9)
Pyoderma	305.6<br>(266.9 to 347.4)	25.8<br>(22.5 to 29.4)*	0.7<br>(-1.9 to 3.6)	1.7<br>(0.7 to 3.5)	25.9<br>(20.4 to 31.6)*	0.8<br>(-3.6 to 5.4)
Scabies	7,085.0<br>(4,463.1 to 10,844.2)	25.8<br>(19.9 to 32.2)*	0.7<br>(-4.0 to 5.9)	180.1<br>(89.4 to 335.8)	25.9<br>(19.9 to 32.4)*	0.8<br>(-4.0 to 6.0)
Fungal skin diseases	20,372.3<br>(16,366.7 to 25,150.2)	26.3<br>(26.1 to 26.5)*	1.1<br>(0.9 to 1.3)*	113.7<br>(44.8 to 249.2)	26.4<br>(25.8 to 27.0)*	1.2<br>(0.8 to 1.7)*
Viral skin diseases	6,453.3<br>(5,393.4 to 7,582.6)	25.1<br>(24.4 to 25.8)*	0.1<br>(-0.4 to 0.7)	195.5<br>(119.8 to 295.0)	25.2<br>(23.5 to 26.6)*	0.2<br>(-1.1 to 1.4)
Molluscum contagiosum cases aggregate	257.6<br>(202.6 to 330.9)	24.4<br>(24.0 to 24.9)*	-0.4<br>(-0.7 to -0.0)*	7.8<br>(4.6 to 12.3)	24.5<br>(18.6 to 31.0)*	-0.3<br>(-5.1 to 4.9)
Viral warts cases aggregate	6,195.7<br>(5,105.1 to 7,346.5)	25.1<br>(24.4 to 25.8)*	0.1<br>(-0.4 to 0.7)	187.7<br>(114.9 to 283.8)	25.2<br>(23.5 to 26.7)*	0.3<br>(-1.1 to 1.4)
Acne vulgaris	8,224.4<br>(6,983.9 to 9,666.5)	27.2<br>(23.7 to 30.3)*	1.8<br>(-1.0 to 4.3)	87.4<br>(40.1 to 164.8)	27.3<br>(23.6 to 30.5)*	1.9<br>(-1.0 to 4.5)
Alopecia areata	1,243.7<br>(1,046.6 to 1,464.1)	23.7<br>(23.3 to 24.1)*	-0.9<br>(-1.3 to -0.6)*	41.5<br>(25.6 to 62.8)	23.8<br>(21.4 to 26.2)*	-0.9<br>(-2.8 to 1.1)
Pruritus	4,941.4<br>(3,444.2 to 6,933.6)	24.8<br>(20.7 to 29.2)*	-0.1<br>(-3.4 to 3.4)	52.5<br>(23.8 to 103.9)	25.0<br>(20.6 to 29.5)*	0.1<br>(-3.4 to 3.7)
Urticaria	2,589.5<br>(1,812.2 to 3,640.0)	25.3<br>(25.1 to 25.6)*	0.3<br>(0.2 to 0.5)*	153.1<br>(85.9 to 239.0)	25.5<br>(23.4 to 27.6)*	0.5<br>(-1.2 to 2.2)
Decubitus ulcer	56.2<br>(42.0 to 72.7)	29.3<br>(23.2 to 35.0)*	3.6<br>(-1.3 to 8.1)	8.8<br>(5.2 to 13.3)	29.1<br>(16.8 to 42.8)*	3.4<br>(-6.5 to 14.4)
Other skin and subcutaneous diseases	46,998.0<br>(43,206.4 to 51,108.6)	28.3<br>(26.2 to 30.7)*	2.7<br>(1.0 to 4.6)*	276.6<br>(133.1 to 517.5)	28.3<br>(26.2 to 30.7)*	2.8<br>(1.0 to 4.7)*
Sense organ diseases	177,653.2<br>(174,277.4 to 181,642.9)	25.5<br>(24.7 to 26.4)*	0.5<br>(-0.2 to 1.2)	5,235.0<br>(3,425.0 to 7,836.2)	25.9<br>(23.9 to 27.8)*	0.8<br>(-0.8 to 2.3)
Glaucoma	584.7<br>(398.2 to 833.8)	30.3<br>(26.7 to 33.6)*	4.3<br>(1.4 to 7.0)*	45.1<br>(26.7 to 70.7)	30.0<br>(24.4 to 35.8)*	4.1<br>(-0.4 to 8.7)
Cataract	3,166.5<br>(2,426.0 to 4,096.5)	20.6<br>(17.1 to 24.0)*	-3.5<br>(-6.2 to -0.7)*	201.6<br>(133.5 to 287.9)	18.6<br>(15.4 to 21.8)*	-5.1<br>(-7.6 to -2.4)*
Macular degeneration	419.5<br>(265.3 to 610.4)	38.7<br>(35.0 to 42.2)*	11.0<br>(8.1 to 13.8)*	31.1<br>(18.5 to 48.1)	37.2<br>(30.8 to 44.1)*	9.8<br>(4.7 to 15.3)*
Refraction and accommodation disorders	77,842.6<br>(72,266.7 to 83,881.4)	23.8<br>(21.4 to 26.5)*	-0.9<br>(-2.8 to 1.2)	1,211.7<br>(729.2 to 1,971.2)	23.9<br>(22.1 to 25.8)*	-0.8<br>(-2.3 to 0.7)
Age-related and other hearing loss	118,071.8<br>(100,392.1 to 135,155.8)	26.5<br>(25.2 to 28.0)*	1.3<br>(0.2 to 2.5)*	3,085.5<br>(1,924.3 to 4,672.1)	26.7<br>(23.4 to 29.9)*	1.4<br>(-1.2 to 4.0)
Other vision loss	1,615.1<br>(1,216.1 to 2,066.1)	31.5<br>(27.7 to 35.3)*	5.3<br>(2.2 to 8.3)*	127.2<br>(82.8 to 188.2)	30.8<br>(26.8 to 34.9)*	4.7<br>(1.5 to 8.0)*
Other sense organ diseases	21,146.3<br>(18,753.6 to 23,852.0)	26.4<br>(23.7 to 29.2)*	1.2<br>(-1.0 to 3.5)	532.8<br>(319.7 to 796.4)	26.6<br>(23.7 to 29.5)*	1.3<br>(-0.9 to 3.7)
Acute other sense organ diseases	62.3<br>(55.7 to 69.8)	26.2<br>(23.3 to 29.0)*	1.0<br>(-1.2 to 3.3)	1.7<br>(1.0 to 2.5)	26.2<br>(23.3 to 29.0)*	1.0<br>(-1.2 to 3.3)
Chronic other sense organ diseases	21,084.0<br>(18,686.4 to 23,787.8)	26.4<br>(23.7 to 29.2)*	1.2<br>(-1.0 to 3.5)	531.1<br>(318.7 to 794.0)	26.6<br>(23.7 to 29.5)*	1.3<br>(-0.9 to 3.7)
Oral disorders	205,730.2<br>(197,336.5 to 212,567.7)	25.1<br>(23.6 to 26.6)*	0.1<br>(-1.0 to 1.3)	1,464.9<br>(866.9 to 2,326.6)	25.3<br>(24.7 to 25.9)*	0.3<br>(-0.2 to 0.8)
Deciduous caries	0.0<br>(0.0 to 0.0)	0.0<br>(0.0 to 0.0)	0.0<br>(0.0 to 0.0)	0.0<br>(0.0 to 0.0)	0.0<br>(0.0 to 0.0)	0.0<br>(0.0 to 0.0)
Permanent caries	150,256.4<br>(122,481.2 to 182,776.1)	24.7<br>(22.4 to 26.9)*	-0.1<br>(-2.0 to 1.6)	106.5<br>(45.8 to 209.0)	23.1<br>(20.8 to 25.4)*	-1.4<br>(-3.3 to 0.4)
Periodontal diseases	68,537.1<br>(54,689.0 to 85,667.1)	26.3<br>(25.4 to 27.1)*	1.1<br>(0.4 to 1.8)*	450.5<br>(168.8 to 934.4)	26.5<br>(25.5 to 27.3)*	1.2<br>(0.5 to 1.9)*
Edentulism and severe tooth loss	21,897.0<br>(20,099.7 to 23,788.7)	24.9<br>(24.4 to 25.4)*	0.0<br>(-0.4 to 0.4)	619.6<br>(410.1 to 860.1)	25.0<br>(24.2 to 25.9)*	0.1<br>(-0.6 to 0.8)
Other oral disorders	9,926.7<br>(8,813.9 to 11,017.6)	24.8<br>(24.2 to 25.4)*	-0.1<br>(-0.6 to 0.4)	288.3<br>(176.5 to 436.9)	24.9<br>(23.8 to 26.0)*	0.0<br>(-0.9 to 0.8)
Injuries	84,071.7<br>(80,192.5 to 88,171.2)	22.3<br>(20.6 to 24.1)*	-2.1<br>(-3.4 to -0.6)*	3,240.5<br>(2,314.1 to 4,384.2)	11.3<br>(7.6 to 14.9)*	-10.9<br>(-13.8 to -8.0)*
Transport injuries	11,855.1<br>(11,177.2 to 12,787.1)	26.6<br>(26.0 to 27.1)*	1.3<br>(0.9 to 1.8)*	657.1<br>(459.4 to 894.1)	15.0<br>(11.1 to 18.9)*	-7.9<br>(-11.0 to -4.8)*
Road injuries	11,117.3<br>(10,428.9 to 12,045.6)	27.0<br>(26.4 to 27.6)*	1.7<br>(1.2 to 2.2)*	613.5<br>(428.2 to 837.0)	16.2<br>(12.3 to 19.9)*	-7.0<br>(-10.1 to -4.0)*
Pedestrian road injuries	1,413.6<br>(1,238.6 to 1,629.2)	25.1<br>(24.2 to 26.0)*	0.2<br>(-0.6 to 0.9)	77.2<br>(52.4 to 105.9)	10.8<br>(6.3 to 15.2)*	-11.3<br>(-14.9 to -7.8)*
Cyclist road injuries	1,831.2<br>(1,566.6 to 2,073.2)	25.0<br>(23.7 to 26.2)*	0.1<br>(-0.9 to 1.1)	94.7<br>(63.3 to 132.7)	12.7<br>(7.6 to 17.1)*	-9.8<br>(-13.9 to -6.2)*
Motorcyclist road injuries	2,835.7<br>(2,484.9 to 3,224.2)	34.6<br>(33.5 to 35.8)*	7.7<br>(6.9 to 8.7)*	149.8<br>(102.1 to 207.1)	19.6<br>(14.1 to 24.7)*	-4.3<br>(-8.6 to -0.2)*
Motor vehicle road injuries	4,622.6<br>(4,100.5 to 5,347.0)	22.5<br>(21.6 to 23.7)*	-1.9<br>(-2.7 to -1.0)*	268.5<br>(182.8 to 377.2)	15.6<br>(12.3 to 18.5)*	-7.5<br>(-10.1 to -5.1)*
Other road injuries	414.2<br>(347.2 to 485.7)	48.0<br>(46.8 to 49.2)*	18.5<br>(17.5 to 19.4)*	23.2<br>(15.9 to 32.6)	39.7<br>(35.6 to 43.2)*	11.8<br>(8.6 to 14.7)*
Other transport injuries	737.7<br>(647.9 to 831.2)	20.3<br>(19.7 to 20.9)*	-3.6<br>(-4.2 to -3.2)*	43.6<br>(30.6 to 58.6)	0.9<br>(-4.0 to 6.5)	-19.2<br>(-23.1 to -14.8)*
Unintentional injuries	62,357.9<br>(59,372.3 to 65,299.6)	17.6<br>(17.2 to 18.1)*	-5.8<br>(-6.2 to -5.4)*	2,283.6<br>(1,592.6 to 3,138.7)	8.3<br>(4.7 to 11.7)*	-13.3<br>(-16.2 to -10.6)*
Falls	17,682.2<br>(15,913.3 to 19,340.4)	28.6<br>(28.1 to 29.2)*	3.0<br>(2.5 to 3.5)*	904.6<br>(626.0 to 1,246.0)	12.8<br>(7.4 to 17.9)*	-9.7<br>(-14.0 to -5.6)*
Drowning	330.8<br>(284.5 to 379.9)	9.3<br>(8.6 to 10.0)*	-12.5<br>(-13.1 to -12.0)*	18.5<br>(12.7 to 25.5)	-6.1<br>(-10.5 to -1.4)*	-24.8<br>(-28.3 to -21.0)*
Fire, heat, and hot substances	5,941.4<br>(5,300.0 to 6,687.5)	13.4<br>(12.8 to 14.0)*	-9.2<br>(-9.7 to -8.7)*	182.8<br>(129.1 to 254.0)	5.0<br>(0.6 to 9.0)*	-16.0<br>(-19.5 to -12.8)*
Poisonings	364.2<br>(314.8 to 425.2)	18.2<br>(17.1 to 19.4)*	-5.4<br>(-6.2 to -4.4)*	30.6<br>(20.9 to 43.0)	12.4<br>(8.7 to 15.5)*	-10.0<br>(-13.0 to -7.5)*
Exposure to mechanical forces	14,458.3<br>(13,273.8 to 15,570.6)	20.2<br>(19.5 to 20.9)*	-3.8<br>(-4.3 to -3.2)*	269.3<br>(186.5 to 377.9)	11.1<br>(7.5 to 14.5)*	-11.1<br>(-13.9 to -8.3)*
Unintentional firearm injuries	214.1<br>(186.5 to 245.3)	20.2<br>(19.3 to 21.1)*	-3.7<br>(-4.5 to -3.1)*	7.9<br>(5.5 to 11.0)	10.2<br>(5.8 to 14.0)*	-11.7<br>(-15.3 to -8.7)*
Unintentional suffocation	657.8<br>(537.3 to 796.9)	13.0<br>(9.5 to 16.3)*	-9.5<br>(-12.3 to -6.9)*	24.2<br>(16.7 to 32.8)	13.9<br>(11.2 to 16.4)*	-8.8<br>(-10.9 to -6.8)*
Other exposure to mechanical forces	13,586.4<br>(12,401.0 to 14,746.8)	20.6<br>(19.9 to 21.2)*	-3.5<br>(-4.0 to -3.0)*	237.2<br>(163.3 to 336.1)	10.8<br>(6.9 to 14.5)*	-11.3<br>(-14.4 to -8.3)*
Adverse effects of medical treatment	731.7<br>(541.0 to 953.4)	8.0<br>(3.2 to 13.3)*	-13.5<br>(-17.4 to -9.3)*	97.5<br>(59.3 to 147.6)	8.0<br>(3.2 to 13.3)*	-13.5<br>(-17.4 to -9.3)*
Animal contact	2,526.7<br>(2,283.0 to 2,802.6)	13.8<br>(12.6 to 14.9)*	-8.9<br>(-9.9 to -8.0)*	67.1<br>(45.5 to 90.8)	9.0<br>(6.5 to 11.5)*	-12.7<br>(-14.7 to -10.7)*
Venomous animal contact	1,145.1<br>(1,009.4 to 1,282.2)	17.6<br>(16.7 to 18.7)*	-5.8<br>(-6.6 to -5.0)*	48.9<br>(33.1 to 66.6)	10.0<br>(7.4 to 12.9)*	-11.9<br>(-14.0 to -9.6)*
Non-venomous animal contact	1,381.6<br>(1,197.3 to 1,585.8)	10.8<br>(9.1 to 12.4)*	-11.3<br>(-12.6 to -10.0)*	18.1<br>(11.5 to 27.2)	6.5<br>(2.9 to 9.6)*	-14.7<br>(-17.6 to -12.3)*
Foreign body	2,173.7<br>(1,932.9 to 2,428.7)	22.6<br>(21.5 to 23.6)*	-1.9<br>(-2.7 to -1.0)*	75.4<br>(52.9 to 104.9)	8.5<br>(3.9 to 13.5)*	-13.1<br>(-16.8 to -9.2)*
Pulmonary aspiration and foreign body in airway	881.8<br>(751.9 to 1,069.3)	24.4<br>(22.1 to 26.8)*	-0.4<br>(-2.2 to 1.5)	41.7<br>(29.0 to 57.1)	5.0<br>(-0.2 to 10.9)	-15.9<br>(-20.1 to -11.2)*
Foreign body in eyes	60.6<br>(29.7 to 103.4)	26.1<br>(24.6 to 27.9)*	0.9<br>(-0.2 to 2.4)	3.3<br>(1.5 to 6.2)	24.5<br>(22.3 to 26.5)*	-0.3<br>(-2.1 to 1.3)
Foreign body in other body part	1,231.3<br>(1,063.1 to 1,412.5)	21.1<br>(20.4 to 21.9)*	-3.0<br>(-3.6 to -2.4)*	30.4<br>(21.1 to 42.9)	12.0<br>(8.6 to 15.4)*	-10.3<br>(-13.0 to -7.6)*
Environmental heat and cold exposure	5,016.0<br>(4,448.9 to 5,732.0)	14.5<br>(14.1 to 15.0)*	-8.3<br>(-8.7 to -7.9)*	200.3<br>(138.4 to 275.2)	3.4<br>(-0.2 to 7.0)	-17.2<br>(-20.1 to -14.3)*
Other unintentional injuries	13,133.0<br>(11,822.7 to 14,800.3)	6.5<br>(5.9 to 7.1)*	-14.7<br>(-15.2 to -14.2)*	437.5<br>(293.0 to 626.2)	2.0<br>(0.0 to 3.8)*	-18.3<br>(-19.9 to -16.9)*
Self-harm and interpersonal violence	2,477.1<br>(2,264.7 to 2,672.7)	20.0<br>(19.5 to 20.4)*	-3.9<br>(-4.3 to -3.6)*	104.3<br>(73.1 to 140.9)	5.4<br>(0.9 to 10.2)*	-15.6<br>(-19.2 to -11.8)*
Self-harm	614.7<br>(536.7 to 703.7)	15.3<br>(14.7 to 15.8)*	-7.7<br>(-8.1 to -7.3)*	29.6<br>(20.7 to 40.1)	0.9<br>(-3.3 to 5.2)	-19.2<br>(-22.6 to -15.8)*
Interpersonal violence	1,862.4<br>(1,675.1 to 2,062.9)	21.6<br>(21.1 to 22.1)*	-2.6<br>(-3.1 to -2.3)*	74.8<br>(51.8 to 101.6)	7.3<br>(2.5 to 12.2)*	-14.1<br>(-17.9 to -10.2)*

Cause	Prevalence in thousands (95% confidence interval)			YLDs in thousands (95% confidence interval)		
	**2015**	**Percent change in counts between 2005 and 2015**	**Percent change in rates between 2005 and 2015**	**2015**	**Percent change in counts between 2005 and 2015**	**Percent change in rates between 2005 and 2015**
Assault by firearm	114.1<br>(98.5 to 132.8)	22.8<br>(21.7 to 23.7)*	-1.7<br>(-2.6 to -0.9)*	4.9<br>(3.3 to 6.8)	10.5<br>(5.7 to 14.7)*	-11.5<br>(-15.4 to -8.1)*
Assault by sharp object	426.0<br>(349.8 to 502.4)	20.3<br>(19.5 to 21.1)*	-3.7<br>(-4.3 to -3.1)*	11.4<br>(7.7 to 16.0)	-0.1<br>(-6.8 to 6.5)	-20.0<br>(-25.4 to -14.8)*
Assault by other means	1,322.3<br>(1,173.2 to 1,497.2)	21.9<br>(21.3 to 22.5)*	-2.4<br>(-2.8 to -1.9)*	58.5<br>(40.4 to 79.8)	8.6<br>(4.0 to 13.3)*	-13.1<br>(-16.7 to -9.3)*
Forces of nature, war, and legal intervention	7,381.6<br>(5,170.6 to 10,022.5)	71.3<br>(47.1 to 100.9)*	37.2<br>(17.8 to 60.9)*	195.5<br>(137.3 to 261.8)	47.2<br>(20.2 to 77.5)*	17.9<br>(-3.8 to 42.1)
Exposure to forces of nature	1,694.7<br>(1,046.8 to 2,597.3)	58.1<br>(14.7 to 108.8)*	26.6<br>(-8.2 to 67.2)	51.2<br>(33.5 to 71.7)	8.8<br>(-29.8 to 55.9)	-12.9<br>(-43.8 to 24.8)
Collective violence and legal intervention	5,686.9<br>(3,874.4 to 7,825.2)	75.7<br>(45.8 to 112.3)*	40.7<br>(16.8 to 70.0)*	144.3<br>(95.0 to 205.6)	68.2<br>(35.5 to 106.7)*	34.7<br>(8.5 to 65.5)*
**Anemia**	**108,319.3<br>(106,855.5 to 109,848.0)**	**13.3<br>(11.5 to 15.1)***	**-9.3<br>(-10.7 to -7.9)***	**2,770.0<br>(1,843.9 to 3,978.6)**	**6.9<br>(3.9 to 9.7)***	**-14.4<br>(-16.8 to -12.1)***
**Developmental intellectual disability**	**6,660.5<br>(4,953.8 to 8,345.5)**	**26.9<br>(26.3 to 27.5)***	**1.6<br>(1.1 to 2.1)***	**782.7<br>(563.2 to 1,030.4)**	**29.6<br>(27.1 to 32.5)***	**3.8<br>(1.8 to 6.1)***
**Epilepsy**	**1,777.5<br>(1,509.0 to 2,068.6)**	**27.1<br>(22.3 to 31.6)***	**1.7<br>(-2.1 to 5.3)**	**499.8<br>(356.0 to 658.1)**	**16.4<br>(8.3 to 24.0)***	**-6.8<br>(-13.3 to -0.7)***
**Guillain-Barré syndrome**	**2.6<br>(1.5 to 4.1)**	**24.4<br>(22.6 to 25.9)***	**-0.4<br>(-1.8 to 0.8)**	**0.8<br>(0.4 to 1.3)**	**24.4<br>(22.6 to 25.9)***	**-0.4<br>(-1.8 to 0.8)**
**Hearing loss**	**124,242.6<br>(107,122.7 to 141,390.2)**	**26.0<br>(24.8 to 27.4)***	**0.9<br>(-0.1 to 2.0)**	**3,343.4<br>(2,115.4 to 5,040.3)**	**26.0<br>(23.0 to 28.9)***	**0.9<br>(-1.5 to 3.2)**
**Heart failure**	**2,020.2<br>(1,918.8 to 2,125.7)**	**27.0<br>(26.2 to 27.6)***	**1.6<br>(1.1 to 2.1)***	**279.2<br>(196.0 to 376.9)**	**27.5<br>(25.6 to 29.5)***	**2.1<br>(0.6 to 3.7)***
**Infertility**	**0.0<br>(0.0 to 0.0)**	**0.0<br>(0.0 to 0.0)**	**0.0<br>(0.0 to 0.0)**	**0.0<br>(0.0 to 0.0)**	**0.0<br>(0.0 to 0.0)**	**0.0<br>(0.0 to 0.0)**
**Pelvic inflammatory disease**	**39.4<br>(26.4 to 55.0)**	**9.2<br>(7.1 to 10.9)***	**-12.5<br>(-14.2 to -11.2)***	**5.2<br>(2.8 to 8.1)**	**9.4<br>(1.9 to 16.7)***	**-12.4<br>(-18.4 to -6.6)***
**Vision loss**	**84,996.5<br>(78,900.8 to 91,540.7)**	**23.9<br>(21.6 to 26.3)***	**-0.8<br>(-2.7 to 1.1)**	**1,794.6<br>(1,169.0 to 2,680.7)**	**24.1<br>(22.5 to 25.6)***	**-0.7<br>(-1.9 to 0.6)**

SR Table 11. Prevalent cases and YLDs for 2015, percent change, and change in age-standardised rates between 2005 and 2015 by cause, global, ages 55-59 years

Cause	Prevalence in thousands (95% confidence interval)			YLDs in thousands (95% confidence interval)		
	2015	Percent change in counts between 2005 and 2015	Percent change in rates between 2005 and 2015	2015	Percent change in counts between 2005 and 2015	Percent change in rates between 2005 and 2015
**All causes**	--	--	--	**53,681.6 (40,138.3 to 68,926.1)**	**32.2 (31.5 to 32.9)***	**-1.3 (-1.9 to -0.8)***
Communicable, maternal, neonatal, and nutritional diseases	182,113.6 (180,019.7 to 183,953.1)	29.9 (28.8 to 30.9)*	-3.1 (-3.8 to -2.3)*	3,193.7 (2,308.0 to 4,372.4)	17.0 (13.7 to 20.7)*	-12.7 (-15.1 to -9.9)*
HIV/AIDS and tuberculosis	2,319.7 (2,230.1 to 2,414.8)	70.3 (61.0 to 79.9)*	27.1 (20.2 to 34.3)*	339.2 (240.1 to 450.2)	19.8 (9.1 to 31.1)*	-10.6 (-18.6 to -2.1)*
Tuberculosis	511.9 (384.6 to 660.4)	14.9 (4.2 to 25.3)*	-14.2 (-22.2 to -6.5)*	152.6 (94.3 to 220.4)	15.4 (4.3 to 26.1)*	-13.9 (-22.1 to -5.8)*
HIV/AIDS	1,818.7 (1,740.1 to 1,902.5)	96.2 (82.4 to 110.9)*	46.5 (36.1 to 57.4)*	186.6 (136.3 to 244.0)	23.6 (6.0 to 42.1)*	-7.7 (-20.9 to 6.0)
HIV/AIDS - Tuberculosis	52.3 (47.4 to 57.8)	25.1 (18.9 to 32.5)*	-6.6 (-11.3 to -1.1)*	19.0 (12.8 to 25.6)	25.7 (16.8 to 34.4)*	-6.1 (-12.8 to 0.3)
HIV/AIDS resulting in other diseases	1,819.4 (1,722.5 to 1,939.8)	100.4 (93.1 to 107.7)*	49.6 (44.2 to 55.0)*	167.6 (121.0 to 219.7)	23.4 (4.0 to 44.1)*	-7.9 (-22.3 to 7.6)
HIV aggregate	564.9 (523.1 to 611.5)	-8.9 (-12.8 to -4.2)*	-32.0 (-34.9 to -28.5)*	40.2 (28.6 to 52.5)	-10.2 (-14.7 to -4.8)*	-33.0 (-36.3 to -28.9)*
AIDS aggregate	1,254.5 (1,179.3 to 1,350.6)	335.9 (286.6 to 392.6)*	225.4 (188.6 to 267.7)*	127.4 (91.9 to 168.9)	39.8 (9.4 to 79.2)*	4.4 (-18.3 to 33.8)
Diarrhea, lower respiratory, and other common infectious diseases	14,643.8 (13,601.4 to 15,336.5)	28.5 (25.5 to 31.5)*	-4.1 (-6.3 to -1.8)*	454.1 (314.1 to 627.9)	29.4 (27.6 to 31.5)*	-3.4 (-4.8 to -1.9)*
Diarrheal diseases	761.3 (721.0 to 803.0)	32.8 (31.5 to 34.1)*	-0.9 (-1.8 to 0.1)	116.4 (80.4 to 160.6)	33.2 (30.2 to 36.0)*	-0.6 (-2.8 to 1.5)
Diarrhoea episodes aggregate	761.0 (720.7 to 802.7)	32.8 (31.5 to 34.1)*	-0.9 (-1.8 to 0.1)	116.3 (80.3 to 160.5)	33.2 (30.2 to 36.0)*	-0.6 (-2.8 to 1.5)
Guillain-Barré syndrome due to diarrheal diseases	0.3 (0.2 to 0.5)	31.0 (28.8 to 32.8)*	-2.2 (-3.9 to -0.8)*	0.1 (0.0 to 0.2)	31.0 (28.8 to 32.8)*	-2.2 (-3.9 to -0.8)*
Intestinal infectious diseases	20.2 (17.9 to 22.4)	-7.9 (-18.6 to 3.6)	-31.2 (-39.2 to -22.6)*	3.6 (2.2 to 5.3)	12.5 (-3.8 to 31.0)	-16.0 (-28.2 to -2.2)*
Typhoid fever	23.0 (17.7 to 29.2)	11.6 (4.3 to 18.4)*	-16.7 (-22.1 to -11.6)*	3.0 (1.9 to 4.5)	15.4 (-3.1 to 36.6)	-13.8 (-27.6 to 2.0)
Typhoid fever episodes aggregate	19.1 (14.4 to 24.4)	11.7 (1.3 to 23.1)*	-16.6 (-24.4 to -8.1)*	1.8 (1.1 to 2.7)	12.8 (-5.6 to 31.5)	-15.8 (-29.5 to -1.8)*
Complications of typhoid fever aggregate	3.9 (2.6 to 5.6)	10.9 (-26.1 to 64.4)	-17.2 (-44.8 to 22.7)	1.3 (0.7 to 2.0)	19.3 (-22.5 to 85.5)	-10.9 (-42.2 to 38.5)
Paratyphoid fever	10.1 (7.1 to 13.8)	7.3 (-1.4 to 16.1)	-19.9 (-26.4 to -13.3)*	0.5 (0.3 to 0.8)	7.5 (-14.5 to 33.5)	-19.8 (-36.2 to -0.3)*
Paratyphoid fever episodes aggregate	9.6 (6.7 to 13.1)	7.3 (-1.8 to 17.1)	-19.9 (-26.7 to -12.6)*	0.5 (0.3 to 0.7)	7.4 (-16.1 to 35.1)	-19.8 (-37.3 to 0.9)
Intestinal perforation due to paratyphoid	0.5 (0.3 to 0.8)	8.0 (-38.3 to 89.6)	-19.4 (-53.9 to 41.5)	0.1 (0.0 to 0.1)	7.8 (-38.3 to 89.6)	-19.6 (-53.9 to 41.5)
Other intestinal infectious diseases	--	--	--	0.0 (0.0 to 0.1)	-58.0 (-72.0 to -34.0)*	-68.6 (-79.1 to -50.7)*
Lower respiratory infections	262.9 (242.5 to 284.9)	33.6 (31.0 to 36.5)*	-0.2 (-2.2 to 1.9)	15.6 (10.4 to 21.9)	33.9 (28.7 to 38.7)*	-0.1 (-4.0 to 3.5)
Lower respiratory infection episodes aggregate	262.6 (242.2 to 284.6)	33.6 (31.0 to 36.5)*	-0.2 (-2.2 to 1.9)	15.5 (10.4 to 21.8)	33.9 (28.6 to 38.7)*	-0.1 (-4.0 to 3.6)
Guillain-Barré syndrome due to lower respiratory infections	0.3 (0.2 to 0.6)	31.0 (28.8 to 32.8)*	-2.2 (-3.9 to -0.8)*	0.1 (0.0 to 0.2)	31.0 (28.8 to 32.8)*	-2.2 (-3.9 to -0.8)*
Upper respiratory infections	9,170.3 (6,494.4 to 12,235.6)	30.8 (29.5 to 32.4)*	-2.3 (-3.3 to -1.2)*	105.5 (53.6 to 189.2)	30.9 (29.1 to 33.0)*	-2.3 (-3.6 to -0.7)*
Upper respiratory infection episodes aggregate	9,169.5 (6,493.6 to 12,234.7)	30.8 (29.5 to 32.4)*	-2.3 (-3.3 to -1.2)*	105.2 (53.3 to 189.0)	30.9 (29.1 to 33.0)*	-2.3 (-3.6 to -0.7)*
Guillain-Barré syndrome due to upper respiratory infections	0.9 (0.5 to 1.4)	31.0 (28.8 to 32.8)*	-2.2 (-3.9 to -0.8)*	0.3 (0.1 to 0.4)	31.0 (28.8 to 32.8)*	-2.2 (-3.9 to -0.8)*
Otitis media	3,777.4 (2,964.8 to 4,839.3)	22.2 (18.9 to 25.6)*	-8.8 (-11.2 to -6.2)*	103.8 (60.3 to 160.9)	21.9 (16.2 to 28.1)*	-9.0 (-13.3 to -4.4)*
Acute otitis media	270.1 (219.0 to 341.0)	34.1 (29.5 to 38.7)*	0.1 (-3.3 to 3.5)	3.4 (1.7 to 6.2)	34.2 (27.8 to 40.4)*	0.1 (-4.6 to 4.8)
Chronic otitis media aggregate	3,507.3 (2,707.2 to 4,593.9)	21.4 (17.9 to 25.1)*	-9.4 (-12.0 to -6.6)*	100.4 (58.3 to 156.0)	21.5 (15.5 to 28.0)*	-9.3 (-13.8 to -4.4)*
Meningitis	401.1 (382.4 to 420.1)	28.7 (24.2 to 32.9)*	-3.9 (-7.3 to -0.8)*	68.0 (48.8 to 89.2)	33.7 (30.2 to 37.0)*	-0.2 (-2.8 to 2.2)
Pneumococcal meningitis	341.6 (294.0 to 399.4)	35.8 (33.6 to 38.4)*	1.4 (-0.3 to 3.3)	33.0 (23.7 to 44.0)	35.1 (29.7 to 40.8)*	0.8 (-3.2 to 5.1)
Acute pneumococcal meningitis	1.4 (0.9 to 1.9)	36.3 (22.1 to 49.8)*	1.8 (-8.8 to 11.8)	0.2 (0.1 to 0.3)	36.4 (22.1 to 49.8)*	1.8 (-8.8 to 11.8)
Complications of pneumococcal meningitis aggregate	340.2 (292.4 to 397.4)	35.8 (33.6 to 38.4)*	1.4 (-0.3 to 3.3)	32.8 (23.5 to 43.8)	35.1 (29.6 to 40.8)*	0.8 (-3.2 to 5.1)
H influenzae type B meningitis	87.5 (68.3 to 107.8)	2.9 (-1.6 to 8.5)	-23.2 (-26.6 to -19.0)*	9.8 (6.8 to 13.3)	27.9 (20.7 to 35.4)*	-4.5 (-9.9 to 1.0)
Acute H influenzae type B meningitis	0.4 (0.2 to 0.7)	-0.5 (-12.5 to 12.8)	-25.7 (-34.7 to -15.8)*	0.1 (0.0 to 0.1)	-0.5 (-12.5 to 12.8)	-25.7 (-34.7 to -15.8)*
Complications of of H influenzae type B meningitis aggregate	87.0 (68.0 to 107.5)	2.9 (-1.6 to 8.5)	-23.2 (-26.6 to -19.0)*	9.8 (6.8 to 13.2)	28.1 (20.8 to 35.6)*	-4.4 (-9.8 to 1.2)
Meningococcal meningitis	76.3 (61.5 to 93.4)	31.9 (28.5 to 35.4)*	-1.5 (-4.1 to 1.1)	8.5 (5.9 to 11.5)	34.7 (29.3 to 40.2)*	0.6 (-3.4 to 4.7)
Acute meningococcal meningitis	0.5 (0.3 to 0.8)	33.7 (17.5 to 49.0)*	-0.2 (-12.3 to 11.2)	0.1 (0.0 to 0.1)	33.7 (17.5 to 49.0)*	-0.2 (-12.3 to 11.2)
Complications of meningococcal meningitis aggregate	75.8 (60.9 to 93.0)	31.9 (28.5 to 35.4)*	-1.5 (-4.1 to 1.1)	8.4 (5.9 to 11.4)	34.8 (29.3 to 40.3)*	0.6 (-3.5 to 4.7)
Other meningitis	152.2 (123.7 to 180.2)	32.5 (30.0 to 35.2)*	-1.1 (-3.0 to 0.9)	16.6 (11.6 to 22.1)	33.9 (27.8 to 39.9)*	-0.1 (-4.6 to 4.5)
Other meningitis episodes aggregate	3.4 (2.6 to 4.4)	37.6 (24.5 to 49.5)*	2.7 (-7.0 to 11.6)	0.5 (0.3 to 0.7)	37.3 (24.4 to 49.2)*	2.5 (-7.2 to 11.3)
Complications of other meningitis aggregate	148.8 (120.4 to 176.8)	32.4 (29.9 to 35.1)*	-1.2 (-3.1 to 0.9)	16.2 (11.3 to 21.5)	33.8 (27.6 to 40.0)*	-0.1 (-4.7 to 4.5)
Encephalitis	255.6 (174.6 to 357.8)	20.7 (17.8 to 24.4)*	-9.9 (-12.1 to -7.2)*	23.4 (16.4 to 31.4)	26.5 (20.6 to 33.5)*	-5.6 (-10.0 to -0.4)*
Acute encephalitis	2.9 (2.3 to 3.6)	34.0 (27.6 to 39.4)*	0.0 (-4.7 to 4.1)	0.4 (0.2 to 0.6)	34.4 (25.6 to 42.7)*	0.3 (-6.3 to 6.5)
Complications of encephalitis aggregate	252.8 (171.6 to 354.2)	20.6 (17.7 to 24.2)*	-10.0 (-12.1 to -7.3)*	23.0 (16.2 to 30.9)	26.3 (20.3 to 33.4)*	-5.7 (-10.2 to -0.4)*
Diphtheria	0.0 (0.0 to 0.0)	-48.5 (-77.9 to 23.8)	-61.6 (-83.5 to -7.6)*	0.0 (0.0 to 0.0)	-48.6 (-77.9 to 23.8)	-61.6 (-83.5 to -7.6)*
Whooping cough	2.4 (1.9 to 3.0)	-8.9 (-12.0 to -5.6)*	-32.0 (-34.3 to -29.5)*	0.1 (0.1 to 0.2)	-8.1 (-14.9 to -1.6)*	-31.4 (-36.5 to -26.5)*
Tetanus	8.0 (7.8 to 8.4)	31.0 (25.5 to 34.4)*	-2.2 (-6.3 to 0.3)	0.3 (0.3 to 0.5)	13.3 (0.6 to 25.2)*	-15.4 (-24.9 to -6.6)*
Severe tetanus	0.4 (0.3 to 0.7)	-28.8 (-44.0 to -4.0)*	-46.8 (-58.2 to -28.4)*	0.1 (0.0 to 0.1)	-28.7 (-43.9 to -4.1)*	-46.8 (-58.1 to -28.4)*
Complications of tetanus aggregate	7.6 (7.5 to 7.8)	37.3 (36.7 to 37.9)*	2.5 (2.0 to 3.0)*	0.3 (0.2 to 0.4)	27.6 (18.0 to 38.5)*	-4.7 (-11.9 to 3.4)
Measles	0.1 (0.0 to 0.1)	-53.2 (-58.6 to -46.5)*	-65.1 (-69.1 to -60.0)*	0.0 (0.0 to 0.0)	-53.3 (-58.6 to -46.5)*	-65.1 (-69.1 to -60.0)*
Varicella and herpes zoster	345.5 (285.7 to 419.4)	33.5 (32.2 to 34.7)*	-0.3 (-1.3 to 0.5)	17.4 (10.1 to 27.7)	33.6 (29.2 to 38.0)*	-0.2 (-3.5 to 3.0)
Neglected tropical diseases and malaria	73,814.7 (72,032.5 to 75,682.5)	20.3 (16.8 to 24.2)*	-10.2 (-12.8 to -7.3)*	707.7 (470.8 to 1,081.5)	3.7 (-6.4 to 16.7)	-22.6 (-30.2 to -12.9)*
Malaria	4,741.4 (4,089.5 to 5,437.5)	34.8 (27.9 to 42.0)*	0.6 (-4.5 to 6.0)	36.5 (26.1 to 49.7)	23.6 (17.2 to 30.9)*	-7.7 (-12.5 to -2.3)*
Asymptomatic malaria parasitemia(PfPR)	3,538.4 (2,892.7 to 4,262.5)	37.6 (28.2 to 47.7)*	2.7 (-4.3 to 10.3)	0.0 (0.0 to 0.0)	0.0 (0.0 to 0.0)	0.0 (0.0 to 0.0)
Malaria episodes aggregate	104.1 (57.9 to 184.7)	-3.5 (-24.5 to 25.7)	-28.0 (-43.6 to -6.2)*	2.1 (1.0 to 4.1)	-3.1 (-25.2 to 26.9)	-27.7 (-44.2 to -5.3)*
Complications of malaria aggregate	18.1 (16.3 to 19.9)	44.4 (41.4 to 48.6)*	7.8 (5.5 to 10.9)*	5.6 (4.3 to 7.2)	38.8 (28.6 to 50.2)*	3.6 (-4.0 to 12.1)
Anemia due to malaria parasitemia(PfPR) aggregate	1,080.8 (1,049.8 to 1,117.9)	30.9 (26.3 to 35.3)*	-2.3 (-5.7 to 1.0)	28.8 (19.7 to 41.0)	23.5 (15.6 to 32.1)*	-7.8 (-13.7 to -1.4)*
Chagas disease	561.4 (466.8 to 655.9)	2.6 (-1.1 to 6.2)	-23.4 (-26.2 to -20.7)*	6.0 (3.9 to 8.9)	2.8 (-3.0 to 9.1)	-23.3 (-27.6 to -18.6)*
Chagas disease episodes aggregate	461.8 (383.0 to 545.2)	2.6 (-1.1 to 6.3)	-23.4 (-26.2 to -20.7)*	0.0 (0.0 to 0.0)	23.0 (17.7 to 28.3)*	-8.2 (-12.1 to -4.2)*
Complications of Chagas disease aggregate	60.6 (46.9 to 77.2)	2.6 (-1.1 to 6.3)	-23.4 (-26.2 to -20.7)*	4.0 (2.5 to 5.8)	2.7 (-4.0 to 10.3)	-23.3 (-28.3 to -17.7)*

Cause	Prevalence in thousands (95% confidence interval)			YLDs in thousands (95% confidence interval)		
	2015	Percent change in counts between 2005 and 2015	Percent change in rates between 2005 and 2015	2015	Percent change in counts between 2005 and 2015	Percent change in rates between 2005 and 2015
Heart failure due to Chagas disease aggregate	39.0 (23.8 to 57.4)	2.5 (-1.3 to 6.3)	-23.5 (-26.3 to -20.6)*	2.0 (1.1 to 3.4)	2.8 (-4.7 to 9.6)	-23.3 (-28.9 to -18.1)*
Leishmaniasis	186.1 (167.1 to 211.9)	30.7 (9.4 to 54.7)*	-2.5 (-18.3 to 15.5)	2.0 (1.0 to 3.8)	30.2 (25.5 to 35.1)*	-2.8 (-6.3 to 0.8)
Visceral leishmaniasis	0.7 (0.6 to 0.8)	31.6 (30.4 to 32.9)*	-1.7 (-2.7 to -0.8)*	0.0 (0.0 to 0.1)	31.5 (6.3 to 65.4)*	-1.8 (-20.7 to 23.5)
Cutaneous and mucocutaneous leishmaniasis	190.1 (163.8 to 228.2)	30.0 (27.1 to 32.7)*	-2.9 (-5.1 to -1.0)*	2.0 (0.9 to 3.8)	30.2 (25.4 to 35.0)*	-2.8 (-6.4 to 0.8)
African trypanosomiasis	0.3 (0.2 to 0.5)	-70.3 (-71.9 to -69.0)*	-77.9 (-79.0 to -76.9)*	0.1 (0.0 to 0.2)	-68.8 (-84.6 to -49.7)*	-76.7 (-88.5 to -62.5)*
Schistosomiasis	7,453.5 (6,217.4 to 9,403.6)	-24.8 (-33.8 to -7.0)*	-43.9 (-50.5 to -30.6)*	67.1 (34.3 to 125.6)	-23.3 (-31.1 to -5.3)*	-42.7 (-48.6 to -29.3)*
Schistosomiasis episodes aggregate	4,353.3 (3,595.8 to 5,243.2)	-26.5 (-35.2 to -13.0)*	-45.1 (-51.7 to -35.0)*	24.0 (9.3 to 51.5)	-28.2 (-36.6 to -14.2)*	-46.4 (-52.7 to -35.9)*
Complications of schistosomiasis aggregate	2,690.9 (1,862.1 to 4,188.7)	-19.0 (-31.7 to 13.4)	-39.6 (-49.0 to -15.3)*	31.3 (14.8 to 61.4)	-16.1 (-28.0 to 15.3)	-37.4 (-46.2 to -13.9)*
Anemia due to schistosomiasis aggregate	430.3 (412.3 to 460.0)	-27.3 (-30.3 to -22.5)*	-45.7 (-48.0 to -42.1)*	11.8 (8.0 to 16.7)	-29.5 (-34.7 to -21.8)*	-47.3 (-51.2 to -41.6)*
Cysticercosis	125.3 (81.9 to 187.0)	1.2 (-6.4 to 7.9)	-24.5 (-30.2 to -19.5)*	17.8 (10.3 to 28.9)	-10.8 (-19.4 to -3.6)*	-33.4 (-39.8 to -28.1)*
Cystic echinococcosis	105.8 (93.0 to 120.0)	47.1 (42.7 to 51.6)*	9.8 (6.5 to 13.2)*	9.5 (6.4 to 13.2)	46.7 (36.9 to 58.1)*	9.5 (2.2 to 18.0)*
Lymphatic filariasis	1,984.1 (1,548.5 to 2,492.8)	-35.8 (-43.5 to -28.8)*	-52.1 (-57.8 to -46.8)*	129.5 (71.9 to 204.6)	-5.0 (-24.4 to 11.1)	-29.1 (-43.6 to -17.1)*
Prevalence of detectable microfiliaria due to lymphatic filariasis	782.9 (675.8 to 909.6)	-57.7 (-61.2 to -53.3)*	-68.4 (-71.1 to -65.1)*	0.0 (0.0 to 0.0)	0.0 (0.0 to 0.0)	0.0 (0.0 to 0.0)
Complications of lymphatic filariasis aggregate	1,201.2 (790.6 to 1,702.2)	-3.1 (-22.1 to 11.8)	-27.7 (-41.8 to -16.5)*	129.5 (71.9 to 204.6)	-5.0 (-24.4 to 11.1)	-29.1 (-43.6 to -17.1)*
Onchocerciasis	599.3 (448.8 to 815.9)	-23.1 (-35.5 to -8.4)*	-42.6 (-51.8 to -31.6)*	57.3 (29.4 to 102.4)	-17.4 (-35.9 to 2.8)	-38.3 (-52.1 to -23.2)*
Asymptomatic onchocerciasis	29.8 (2.8 to 63.9)	-73.7 (-96.2 to -62.3)*	-80.4 (-97.2 to -71.8)*	0.0 (0.0 to 0.0)	0.0 (0.0 to 0.0)	0.0 (0.0 to 0.0)
Skin disease due to onchocerciasis aggregate	434.7 (277.6 to 656.0)	-18.7 (-36.4 to 0.6)	-39.3 (-52.5 to -24.7)*	46.5 (19.9 to 90.2)	-20.6 (-44.8 to 3.7)	-40.8 (-58.8 to -22.4)*
Vision loss due to onchocerciasis aggregate	136.3 (94.1 to 193.1)	3.9 (-9.4 to 17.7)	-22.4 (-32.4 to -12.2)*	10.9 (6.7 to 16.3)	-0.1 (-12.7 to 12.9)	-25.4 (-34.8 to -15.7)*
Trachoma	309.2 (201.5 to 452.1)	3.9 (-5.0 to 13.3)	-22.5 (-29.1 to -15.4)*	24.5 (14.2 to 38.5)	-1.0 (-10.2 to 9.3)	-26.1 (-33.0 to -18.4)*
Dengue	179.0 (100.1 to 393.7)	221.4 (28.8 to 793.0)*	139.9 (-3.8 to 566.6)	27.8 (12.6 to 63.9)	218.2 (27.3 to 803.0)*	137.5 (-5.0 to 574.1)
Dengue episodes aggregate	53.6 (35.3 to 113.6)	203.4 (23.7 to 723.1)*	126.5 (-7.6 to 514.5)	3.0 (1.6 to 6.7)	217.0 (32.5 to 772.3)*	136.6 (-1.1 to 551.1)
Post-dengue chronic fatigue syndrome	125.8 (59.8 to 283.3)	221.6 (28.8 to 793.1)*	140.1 (-3.8 to 566.6)	24.8 (10.7 to 58.5)	218.3 (26.6 to 800.5)*	137.6 (-5.5 to 572.2)
Yellow fever	0.1 (0.0 to 0.1)	-24.7 (-43.0 to -1.1)*	-43.8 (-57.4 to -26.2)*	0.0 (0.0 to 0.0)	-24.5 (-43.0 to -1.1)*	-43.7 (-57.4 to -26.2)*
Rabies	0.0 (0.0 to 0.1)	-40.9 (-60.8 to -14.0)*	-55.9 (-70.7 to -35.8)*	0.0 (0.0 to 0.0)	-40.7 (-60.8 to -14.0)*	-55.7 (-70.7 to -35.8)*
Intestinal nematode infections	60,679.8 (58,750.7 to 62,420.5)	23.0 (18.7 to 27.8)*	-8.2 (-11.4 to -4.6)*	116.3 (66.2 to 189.9)	-1.3 (-9.6 to 8.9)	-26.3 (-32.5 to -18.7)*
Ascariasis	32,003.0 (27,203.4 to 38,341.8)	13.1 (-7.4 to 39.3)	-15.5 (-30.9 to 4.0)	31.4 (17.3 to 53.2)	-20.3 (-34.1 to -4.1)*	-40.5 (-50.8 to -28.4)*
Complications of ascariasis aggregate	1,780.6 (1,587.4 to 2,003.1)	-17.5 (-29.3 to -3.3)*	-38.4 (-47.2 to -27.8)*	31.4 (17.3 to 53.2)	-20.3 (-34.1 to -4.1)*	-40.5 (-50.8 to -28.4)*
Asymptomatic ascariasis	30,228.4 (25,352.9 to 36,518.1)	15.7 (-6.3 to 45.0)	-13.6 (-30.1 to 8.2)	0.0 (0.0 to 0.0)	0.0 (0.0 to 0.0)	0.0 (0.0 to 0.0)
Trichuriasis	18,845.3 (16,821.9 to 21,122.9)	26.6 (8.2 to 48.8)*	-5.5 (-19.2 to 11.1)	21.4 (11.2 to 37.3)	16.2 (-9.4 to 55.0)	-13.3 (-32.4 to 15.7)
Complications of trichuriasis aggregate	1,088.7 (934.2 to 1,347.7)	17.0 (-5.7 to 50.3)	-12.6 (-29.6 to 12.2)	21.4 (11.2 to 37.3)	16.2 (-9.4 to 55.0)	-13.3 (-32.4 to 15.7)
Asymptomatic trichuriasis	17,759.3 (15,728.2 to 20,067.9)	27.3 (7.8 to 51.4)*	-5.0 (-19.5 to 13.0)	0.0 (0.0 to 0.0)	0.0 (0.0 to 0.0)	0.0 (0.0 to 0.0)
Hookworm disease	18,188.3 (16,170.1 to 20,585.9)	16.3 (-0.3 to 35.9)	-13.2 (-25.5 to 1.5)	63.4 (38.4 to 99.6)	5.9 (-4.1 to 16.6)	-21.0 (-28.4 to -12.9)*
Complications of hookworm disease aggregate	2,182.8 (2,012.2 to 2,371.6)	8.4 (-3.3 to 21.7)	-19.1 (-27.8 to -9.1)*	43.5 (24.0 to 71.7)	6.5 (-7.2 to 22.9)	-20.5 (-30.7 to -8.2)*
Anemia due to hookworm disease aggregate	921.9 (898.2 to 947.6)	16.2 (12.1 to 20.5)*	-13.2 (-16.3 to -10.1)*	20.0 (13.5 to 29.1)	4.7 (-2.4 to 11.7)	-21.9 (-27.1 to -16.6)*
Asymptomatic hookworm disease	15,088.8 (13,066.7 to 17,470.2)	17.6 (-3.0 to 41.5)	-12.2 (-27.6 to 5.6)	0.0 (0.0 to 0.0)	0.0 (0.0 to 0.0)	0.0 (0.0 to 0.0)
Food-borne trematodiases	4,098.7 (3,767.0 to 4,467.1)	35.4 (31.6 to 39.8)*	1.1 (-1.7 to 4.3)	96.9 (45.4 to 178.2)	34.3 (28.5 to 40.8)*	0.2 (-4.1 to 5.1)
Asymptomatic food-borne trematodiases aggregate	3,220.6 (2,609.7 to 3,735.3)	35.6 (31.7 to 40.0)*	1.2 (-1.7 to 4.5)	0.0 (0.0 to 0.0)	0.0 (0.0 to 0.0)	0.0 (0.0 to 0.0)
Symptomatic food-borne trematodiases aggregate	878.1 (476.8 to 1,466.8)	34.7 (29.9 to 40.5)*	0.5 (-3.0 to 4.9)	96.9 (45.4 to 178.2)	34.3 (28.5 to 40.8)*	0.2 (-4.1 to 5.1)
Leprosy	57.4 (54.1 to 60.9)	9.6 (8.1 to 11.1)*	-18.2 (-19.3 to -17.1)*	3.5 (2.3 to 4.9)	10.8 (3.7 to 17.6)*	-17.3 (-22.6 to -12.2)*
Ebola	0.1 (0.0 to 0.1)	33,126.8 (21,603.0 to 104,607.0)*	24,703.1 (16,100.9 to 78,061.6)*	0.0 (0.0 to 0.0)	31,443.2 (16,986.0 to 146,319.4)*	23,446.4 (12,654.4 to 109,199.0)*
Other neglected tropical diseases	980.0 (957.3 to 1,002.7)	20.6 (17.1 to 24.3)*	-10.0 (-12.6 to -7.2)*	112.7 (39.1 to 321.1)	13.6 (-26.7 to 72.6)	-15.2 (-45.3 to 28.8)
Anemia due to other neglected tropical diseases aggregate	980.0 (957.3 to 1,002.7)	20.6 (17.1 to 24.3)*	-10.0 (-12.6 to -7.2)*	16.1 (10.4 to 23.9)	0.2 (-7.6 to 7.7)	-25.2 (-31.0 to -19.6)*
Maternal disorders	22.6 (20.6 to 24.8)	-23.7 (-31.1 to -15.0)*	-43.0 (-48.6 to -36.5)*	8.0 (5.3 to 11.6)	-22.6 (-30.1 to -14.7)*	-42.2 (-47.8 to -36.3)*
Maternal hemorrhage	0.0 (0.0 to 0.0)	0.0 (0.0 to 0.0)	0.0 (0.0 to 0.0)	0.0 (0.0 to 0.0)	0.0 (0.0 to 0.0)	0.0 (0.0 to 0.0)
Maternal hemorrhage episodes aggregate	0.0 (0.0 to 0.0)	0.0 (0.0 to 0.0)	0.0 (0.0 to 0.0)	0.0 (0.0 to 0.0)	0.0 (0.0 to 0.0)	0.0 (0.0 to 0.0)
Anemia due to maternal hemorrhage aggregate	0.0 (0.0 to 0.0)	0.0 (0.0 to 0.0)	0.0 (0.0 to 0.0)	0.0 (0.0 to 0.0)	0.0 (0.0 to 0.0)	0.0 (0.0 to 0.0)
Maternal sepsis and other maternal infections	0.0 (0.0 to 0.0)	0.0 (0.0 to 0.0)	0.0 (0.0 to 0.0)	0.0 (0.0 to 0.0)	0.0 (0.0 to 0.0)	0.0 (0.0 to 0.0)
Maternal sepsis and other maternal infections aggregate	0.0 (0.0 to 0.0)	0.0 (0.0 to 0.0)	0.0 (0.0 to 0.0)	0.0 (0.0 to 0.0)	0.0 (0.0 to 0.0)	0.0 (0.0 to 0.0)
Infertility due to puerperal sepsis	0.0 (0.0 to 0.0)	0.0 (0.0 to 0.0)	0.0 (0.0 to 0.0)	0.0 (0.0 to 0.0)	0.0 (0.0 to 0.0)	0.0 (0.0 to 0.0)
Maternal hypertensive disorders	0.0 (0.0 to 0.0)	0.0 (0.0 to 0.0)	0.0 (0.0 to 0.0)	0.0 (0.0 to 0.0)	0.0 (0.0 to 0.0)	0.0 (0.0 to 0.0)
Maternal hypertensive disorders episodes aggregate	0.0 (0.0 to 0.0)	0.0 (0.0 to 0.0)	0.0 (0.0 to 0.0)	0.0 (0.0 to 0.0)	0.0 (0.0 to 0.0)	0.0 (0.0 to 0.0)
Eclampsia	0.0 (0.0 to 0.0)	0.0 (0.0 to 0.0)	0.0 (0.0 to 0.0)	0.0 (0.0 to 0.0)	0.0 (0.0 to 0.0)	0.0 (0.0 to 0.0)
Complications of maternal hypertensive disorders aggregate	0.0 (0.0 to 0.0)	0.0 (0.0 to 0.0)	0.0 (0.0 to 0.0)	0.0 (0.0 to 0.0)	0.0 (0.0 to 0.0)	0.0 (0.0 to 0.0)
Maternal obstructed labor and uterine rupture	22.6 (18.7 to 27.1)	-23.2 (-26.8 to -19.9)*	-42.7 (-45.3 to -40.2)*	7.1 (4.6 to 10.2)	-23.2 (-30.5 to -15.7)*	-42.6 (-48.1 to -37.1)*
Obstructed labor, acute event	0.0 (0.0 to 0.0)	0.0 (0.0 to 0.0)	0.0 (0.0 to 0.0)	0.0 (0.0 to 0.0)	0.0 (0.0 to 0.0)	0.0 (0.0 to 0.0)
Fistula due to maternal obstructed labor and uterine rupture aggregate	22.6 (18.7 to 27.1)	-23.2 (-26.8 to -19.9)*	-42.7 (-45.3 to -40.2)*	7.1 (4.6 to 10.2)	-23.2 (-30.5 to -15.7)*	-42.6 (-48.1 to -37.1)*
Maternal abortion, miscarriage, and ectopic pregnancy	0.0 (0.0 to 0.0)	0.0 (0.0 to 0.0)	0.0 (0.0 to 0.0)	0.0 (0.0 to 0.0)	0.0 (0.0 to 0.0)	0.0 (0.0 to 0.0)
Other maternal disorders	--	--	--	0.9 (0.5 to 1.4)	-17.7 (-33.4 to -1.7)*	-38.6 (-50.3 to -26.6)*
Neonatal disorders	1,613.1 (1,544.5 to 1,670.6)	44.5 (39.2 to 50.1)*	7.8 (3.9 to 12.0)*	309.8 (236.8 to 395.9)	43.7 (38.4 to 49.0)*	7.2 (3.3 to 11.2)*
Neonatal preterm birth complications	1,406.3 (1,230.2 to 1,593.5)	38.3 (31.1 to 45.5)*	3.2 (-2.1 to 8.6)	149.4 (114.3 to 189.4)	34.6 (27.0 to 42.5)*	0.5 (-5.2 to 6.3)
Vision loss due to retinopathy of prematurity aggregate	209.6 (168.0 to 258.8)	31.6 (26.9 to 36.3)*	-1.8 (-5.3 to 1.8)	7.4 (4.6 to 11.1)	32.4 (22.7 to 42.4)*	-1.2 (-8.4 to 6.3)
Complications of preterm birth complications aggregate	1,196.6 (1,035.8 to 1,373.7)	39.6 (31.0 to 48.1)*	4.2 (-2.2 to 10.5)	142.0 (107.4 to 181.0)	34.7 (26.8 to 42.9)*	0.6 (-5.4 to 6.6)
Neonatal encephalopathy due to birth asphyxia and trauma	361.5 (256.8 to 541.3)	62.0 (58.3 to 65.7)*	21.0 (18.2 to 23.7)*	102.2 (66.1 to 157.2)	57.0 (51.1 to 63.3)*	17.2 (12.8 to 21.9)*

Cause	Prevalence in thousands (95% confidence interval)			YLDs in thousands (95% confidence interval)		
	2015	Percent change in counts between 2005 and 2015	Percent change in rates between 2005 and 2015	2015	Percent change in counts between 2005 and 2015	Percent change in rates between 2005 and 2015
Neonatal sepsis and other neonatal infections	0.0 (0.0 to 0.0)	0.0 (0.0 to 0.0)	0.0 (0.0 to 0.0)	0.0 (0.0 to 0.0)	0.0 (0.0 to 0.0)	0.0 (0.0 to 0.0)
Severe infection due to neonatal sepsis and other neonatal infections	0.0 (0.0 to 0.0)	0.0 (0.0 to 0.0)	0.0 (0.0 to 0.0)	0.0 (0.0 to 0.0)	0.0 (0.0 to 0.0)	0.0 (0.0 to 0.0)
Complications of neonatal sepsis and other neonatal infections aggregate	0.0 (0.0 to 0.0)	0.0 (0.0 to 0.0)	0.0 (0.0 to 0.0)	0.0 (0.0 to 0.0)	0.0 (0.0 to 0.0)	0.0 (0.0 to 0.0)
Hemolytic disease and other neonatal jaundice	59.5 (53.1 to 67.4)	52.5 (50.8 to 54.3)*	13.9 (12.5 to 15.2)*	17.8 (13.4 to 23.1)	46.9 (41.0 to 53.1)*	9.7 (5.3 to 14.3)*
Other neonatal disorders	--	--	--	40.4 (29.1 to 52.6)	47.0 (30.8 to 64.4)*	9.8 (-2.3 to 22.7)
Nutritional deficiencies	49,461.6 (48,800.5 to 50,161.5)	23.7 (21.5 to 25.9)*	-7.7 (-9.3 to -6.0)*	1,282.7 (863.3 to 1,817.8)	15.1 (11.5 to 18.9)*	-14.1 (-16.8 to -11.2)*
Protein-energy malnutrition	16.2 (10.3 to 26.1)	-4.0 (-14.2 to 7.6)	-28.4 (-36.0 to -19.7)*	1.9 (1.0 to 3.4)	-3.2 (-16.1 to 12.1)	-27.7 (-37.4 to -16.4)*
Iodine deficiency	5,765.3 (5,221.9 to 6,484.8)	26.3 (23.8 to 28.8)*	-5.7 (-7.6 to -3.8)*	107.8 (69.2 to 164.4)	27.1 (24.3 to 29.9)*	-5.1 (-7.2 to -3.0)*
Visible goiter due to iodine deficiency aggregate	5,717.0 (5,170.7 to 6,426.5)	26.2 (23.7 to 28.8)*	-5.8 (-7.7 to -3.8)*	99.8 (62.0 to 155.4)	26.5 (23.4 to 29.4)*	-5.6 (-7.9 to -3.4)*
Visible goiter with heart failure due to iodine deficiency aggregate	0.0 (0.0 to 0.0)	38.6 (36.1 to 41.2)*	3.5 (1.6 to 5.4)*	0.0 (0.0 to 0.0)	38.6 (36.1 to 41.2)*	3.5 (1.6 to 5.4)*
Intellectual disability due to iodine deficiency aggregate	48.2 (22.3 to 61.7)	34.5 (28.6 to 39.2)*	0.4 (-4.0 to 3.9)	8.0 (3.0 to 12.3)	35.8 (23.7 to 47.2)*	1.4 (-7.7 to 9.9)
Vitamin A deficiency	116.5 (80.7 to 161.7)	32.0 (24.3 to 39.8)*	-1.4 (-7.2 to 4.3)	7.4 (4.4 to 11.2)	33.7 (23.4 to 43.8)*	-0.2 (-7.8 to 7.3)
Iron-deficiency anemia	46,755.7 (45,960.2 to 47,573.0)	21.9 (19.7 to 24.7)*	-9.0 (-10.7 to -6.9)*	1,165.3 (785.6 to 1,652.5)	14.0 (10.1 to 18.2)*	-14.9 (-17.8 to -11.8)*
Iron-deficiency anemia without heart failure aggregate	46,752.5 (45,957.6 to 47,569.4)	21.9 (19.7 to 24.7)*	-9.0 (-10.7 to -6.9)*	1,164.9 (785.3 to 1,652.0)	14.0 (10.1 to 18.2)*	-14.9 (-17.8 to -11.8)*
Iron-deficiency anemia with heart failure aggregate	3.3 (2.6 to 4.1)	37.8 (33.6 to 42.4)*	2.9 (-0.2 to 6.3)	0.4 (0.2 to 0.6)	38.4 (16.5 to 65.7)*	3.3 (-13.0 to 23.7)
Other nutritional deficiencies	--	--	--	0.2 (0.1 to 0.4)	2.9 (-20.8 to 29.3)	-23.2 (-40.9 to -3.5)*
Other communicable, maternal, neonatal, and nutritional diseases	96,260.8 (93,681.6 to 98,800.5)	33.3 (32.1 to 34.5)*	-0.5 (-1.4 to 0.4)	92.1 (58.9 to 141.5)	26.4 (17.2 to 35.0)*	-5.6 (-12.5 to 0.8)
Sexually transmitted diseases excluding HIV	75,011.9 (71,938.0 to 77,996.0)	32.6 (30.9 to 34.2)*	-1.0 (-2.3 to 0.2)	53.0 (32.3 to 85.6)	29.9 (25.3 to 33.4)*	-3.0 (-6.5 to -0.4)*
Syphilis	2,452.9 (1,796.2 to 3,268.2)	19.0 (8.2 to 28.2)*	-11.2 (-19.2 to -4.3)*	22.5 (14.9 to 31.4)	26.0 (19.7 to 32.6)*	-6.0 (-10.7 to -1.0)*
Early syphilis aggregate	2,333.6 (1,687.5 to 3,146.0)	18.7 (7.5 to 28.2)*	-11.4 (-19.7 to -4.3)*	0.6 (0.2 to 1.4)	18.9 (6.4 to 29.4)*	-11.3 (-20.6 to -3.4)*
Adult tertiary syphilis	119.3 (103.6 to 137.7)	25.9 (22.4 to 29.5)*	-6.0 (-8.7 to -3.3)*	21.9 (14.4 to 30.8)	26.2 (19.6 to 33.2)*	-5.8 (-10.7 to -0.6)*
Chlamydial infection	170.7 (112.4 to 248.9)	42.7 (38.4 to 48.5)*	6.5 (3.3 to 10.8)*	1.6 (1.0 to 2.4)	30.5 (23.8 to 38.0)*	-2.6 (-7.6 to 3.0)
Chlamydial infection episodes aggregate	165.8 (107.9 to 244.5)	43.7 (39.2 to 49.7)*	7.2 (3.9 to 11.7)*	0.9 (0.5 to 1.6)	43.2 (32.9 to 55.9)*	6.9 (-0.8 to 16.4)
Pelvic inflammatory diseases due to chlamydial infection aggregate	4.9 (3.7 to 6.4)	16.8 (13.3 to 20.4)*	-12.8 (-15.4 to -10.2)*	0.7 (0.4 to 1.0)	16.7 (11.0 to 22.9)*	-12.9 (-17.2 to -8.2)*
Infertility due to clamydial infection aggregate	0.0 (0.0 to 0.0)	0.0 (0.0 to 0.0)	0.0 (0.0 to 0.0)	0.0 (0.0 to 0.0)	0.0 (0.0 to 0.0)	0.0 (0.0 to 0.0)
Gonococcal infection	248.3 (112.9 to 506.3)	41.0 (-4.2 to 74.0)	5.3 (-28.5 to 29.9)	2.1 (0.9 to 4.3)	39.6 (5.1 to 65.0)*	4.2 (-21.5 to 23.2)
Gonococcal infection episodes aggregate	247.3 (111.8 to 505.4)	41.1 (-4.3 to 74.1)	5.3 (-28.6 to 29.9)	2.0 (0.8 to 4.2)	41.2 (4.3 to 68.7)*	5.4 (-22.2 to 25.9)
Pelvic inflammatory diseases due to gonococcal infection aggregate	0.9 (0.7 to 1.2)	18.7 (13.9 to 23.6)*	-11.4 (-15.0 to -7.7)*	0.1 (0.1 to 0.2)	18.7 (13.9 to 23.6)*	-11.4 (-15.0 to -7.7)*
Trichomoniasis	7,104.4 (4,851.0 to 10,005.5)	34.5 (33.3 to 35.5)*	0.4 (-0.5 to 1.1)	7.8 (2.9 to 17.0)	35.1 (32.9 to 37.2)*	0.8 (-0.8 to 2.4)
Genital herpes	68,354.2 (60,009.5 to 77,262.5)	32.8 (31.0 to 34.5)*	-0.9 (-2.2 to 0.4)	16.8 (4.9 to 40.9)	33.6 (31.4 to 36.5)*	-0.3 (-1.9 to 1.9)
Moderate infection due to initial genital herpes episode	2.3 (0.5 to 5.5)	620.1 (476.9 to 821.8)*	437.5 (330.6 to 588.1)*	0.1 (0.0 to 0.3)	609.2 (332.4 to 1,211.5)*	429.4 (222.8 to 879.0)*
Complications of genital herpes aggregate	68,351.9 (60,006.5 to 77,260.7)	32.8 (31.0 to 34.5)*	-0.9 (-2.2 to 0.4)	16.6 (4.8 to 40.7)	32.9 (30.7 to 35.1)*	-0.8 (-2.4 to 0.9)
Other sexually transmitted diseases	17.0 (12.7 to 22.3)	18.1 (16.2 to 20.0)*	-11.8 (-13.3 to -10.4)*	2.2 (1.3 to 3.3)	18.2 (6.6 to 29.8)*	-11.8 (-20.4 to -3.1)*
Pelvic inflammatory diseases due to other sexually transmitted diseases aggregate	17.0 (12.7 to 22.3)	18.1 (16.2 to 20.0)*	-11.8 (-13.3 to -10.4)*	2.2 (1.3 to 3.3)	18.2 (6.6 to 29.8)*	-11.8 (-20.4 to -3.1)*
Infertility due to other sexually transmitted diseases aggregate	0.0 (0.0 to 0.0)	0.0 (0.0 to 0.0)	0.0 (0.0 to 0.0)	0.0 (0.0 to 0.0)	0.0 (0.0 to 0.0)	0.0 (0.0 to 0.0)
Other sexually transmitted diseases	--	--	--	1.2 (0.6 to 2.2)	40.3 (14.4 to 60.9)*	4.7 (-14.6 to 20.1)
Hepatitis	26,085.9 (25,591.6 to 26,558.0)	36.4 (35.4 to 37.4)*	1.8 (1.1 to 2.6)*	14.0 (8.1 to 22.1)	39.5 (-6.4 to 106.3)	4.1 (-30.1 to 54.0)
Acute hepatitis A	82.4 (65.9 to 97.2)	47.0 (12.6 to 89.4)*	9.8 (-15.9 to 41.4)	3.4 (2.1 to 5.2)	47.4 (11.4 to 95.2)*	10.0 (-16.8 to 45.7)
Hepatitis B	16,985.7 (16,371.4 to 17,635.1)	37.2 (35.0 to 39.6)*	2.4 (0.8 to 4.2)*	9.6 (5.1 to 16.7)	37.4 (-23.8 to 136.9)	2.6 (-43.1 to 76.8)
Acute Hepatitis B aggregate	421.7 (274.0 to 635.1)	36.7 (-22.7 to 137.6)	2.1 (-42.3 to 77.3)	9.6 (5.1 to 16.7)	37.4 (-23.8 to 136.9)	2.6 (-43.1 to 76.8)
Chronic hepatitis B	16,564.1 (15,992.9 to 17,153.2)	37.2 (36.1 to 38.4)*	2.4 (1.6 to 3.3)*	0.0 (0.0 to 0.0)	0.0 (0.0 to 0.0)	0.0 (0.0 to 0.0)
Hepatitis C	9,678.0 (8,630.0 to 10,757.0)	34.8 (32.8 to 36.6)*	0.6 (-0.9 to 2.0)	0.4 (0.2 to 0.8)	33.0 (26.4 to 38.8)*	-0.7 (-5.7 to 3.6)
Acute Hepatitis C aggregate	27.3 (20.9 to 34.8)	32.7 (26.2 to 38.6)*	-0.9 (-5.8 to 3.5)	0.4 (0.2 to 0.8)	33.0 (26.4 to 38.8)*	-0.7 (-5.7 to 3.6)
Chronic hepatitis C	9,650.6 (8,599.8 to 10,726.7)	34.8 (32.8 to 36.6)*	0.6 (-0.9 to 2.0)	0.0 (0.0 to 0.0)	0.0 (0.0 to 0.0)	0.0 (0.0 to 0.0)
Acute hepatitis E	18.6 (12.3 to 27.9)	35.8 (33.2 to 38.4)*	1.4 (-0.5 to 3.3)	0.6 (0.3 to 1.0)	35.6 (11.1 to 68.6)*	1.2 (-17.1 to 25.8)
Other infectious diseases	1,196.9 (1,173.0 to 1,221.2)	20.1 (16.7 to 23.7)*	-10.3 (-12.9 to -7.7)*	25.2 (15.9 to 40.1)	14.1 (5.1 to 23.5)*	-14.8 (-21.6 to -7.8)*
Non-communicable diseases	332,233.8 (331,828.6 to 332,644.5)	34.0 (34.0 to 34.1)*	0.0 (0.0 to 0.1)*	47,453.0 (35,474.0 to 60,915.3)	34.3 (33.5 to 35.0)*	0.2 (-0.3 to 0.8)
Neoplasms	10,074.8 (9,868.6 to 10,297.5)	45.6 (42.2 to 49.2)*	8.7 (6.1 to 11.4)*	907.2 (656.7 to 1,191.5)	37.3 (31.3 to 43.1)*	2.5 (-2.0 to 6.8)
Lip and oral cavity cancer	326.9 (291.1 to 370.3)	43.9 (29.5 to 61.3)*	7.4 (-3.4 to 20.4)	27.5 (19.4 to 37.0)	42.2 (27.4 to 59.6)*	6.2 (-4.9 to 19.2)
Nasopharynx cancer	95.0 (70.9 to 127.2)	17.9 (-5.2 to 53.6)	-12.0 (-29.2 to 14.6)	8.8 (5.5 to 12.9)	16.7 (-6.5 to 52.0)	-12.9 (-30.2 to 13.5)
Other pharynx cancer	155.6 (141.0 to 170.8)	33.8 (21.7 to 46.5)*	-0.2 (-9.2 to 9.3)	12.9 (9.0 to 17.1)	32.4 (19.2 to 46.6)*	-1.1 (-11.0 to 9.5)
Esophageal cancer	82.2 (67.5 to 116.8)	10.0 (-11.2 to 40.7)	-17.9 (-33.7 to 5.1)	14.4 (9.7 to 21.0)	2.9 (-15.8 to 27.8)	-23.2 (-37.2 to -4.6)*
Stomach cancer	347.5 (320.3 to 378.0)	2.6 (-6.4 to 12.1)	-23.4 (-30.1 to -16.3)*	39.0 (28.3 to 50.5)	0.1 (-9.2 to 10.2)	-25.2 (-32.2 to -17.7)*
Colon and rectum cancer	909.0 (846.3 to 979.6)	38.7 (30.5 to 47.9)*	3.6 (-2.6 to 10.4)	71.9 (51.8 to 93.4)	36.1 (27.5 to 45.9)*	1.6 (-4.9 to 8.9)
Colon and rectum cancer aggregate	907.4 (844.8 to 978.2)	38.8 (30.5 to 48.0)*	3.6 (-2.6 to 10.5)	71.6 (51.7 to 93.1)	36.1 (27.5 to 46.0)*	1.6 (-4.8 to 9.0)
Stoma due to colon and rectum cancer	1.6 (1.4 to 1.8)	21.6 (18.6 to 24.6)*	-9.2 (-11.5 to -7.0)*	0.2 (0.1 to 0.3)	21.7 (18.6 to 24.6)*	-9.2 (-11.5 to -7.0)*
Liver cancer	57.9 (50.8 to 66.0)	41.9 (13.0 to 71.3)*	5.9 (-15.6 to 27.9)	20.5 (14.0 to 28.1)	22.7 (4.9 to 44.1)*	-8.4 (-21.7 to 7.6)
Liver cancer due to hepatitis B	35.9 (27.8 to 48.9)	22.7 (-5.0 to 62.3)	-8.4 (-29.1 to 21.2)	6.9 (4.6 to 9.6)	10.9 (-8.1 to 36.2)	-17.2 (-31.4 to 1.7)
Liver cancer due to hepatitis C	21.0 (17.0 to 25.7)	56.2 (28.1 to 89.4)*	16.6 (-4.4 to 41.4)	3.8 (2.5 to 5.2)	35.3 (16.8 to 56.0)*	1.0 (-12.8 to 16.5)
Liver cancer due to alcohol use	33.8 (27.0 to 43.7)	56.2 (25.3 to 98.9)*	16.6 (-6.5 to 48.5)	6.7 (4.5 to 9.3)	37.4 (16.1 to 65.7)*	2.6 (-13.3 to 23.7)
Liver cancer due to other causes	14.8 (11.4 to 21.2)	23.5 (-7.4 to 64.1)	-7.8 (-30.9 to 22.5)	3.0 (2.0 to 4.3)	10.2 (-8.2 to 35.6)	-17.7 (-31.5 to 1.2)
Gallbladder and biliary tract cancer	11.6 (10.4 to 12.7)	14.8 (6.1 to 24.1)*	-14.3 (-20.8 to -7.4)*	3.3 (2.3 to 4.4)	13.0 (4.3 to 22.7)*	-15.6 (-22.2 to -8.4)*
Pancreatic cancer	35.8 (33.2 to 39.6)	40.9 (31.1 to 52.9)*	5.2 (-2.1 to 14.1)	8.4 (5.8 to 11.1)	35.2 (25.3 to 46.5)*	0.9 (-6.5 to 9.3)

Cause	Prevalence in thousands (95% confidence interval)			YLDs in thousands (95% confidence interval)		
	2015	Percent change in counts between 2005 and 2015	Percent change in rates between 2005 and 2015	2015	Percent change in counts between 2005 and 2015	Percent change in rates between 2005 and 2015
Larynx cancer	211.7 (190.0 to 243.0)	29.5 (16.7 to 46.7)*	-3.4 (-12.9 to 9.5)	21.9 (15.6 to 29.5)	28.4 (16.7 to 43.4)*	-4.2 (-12.9 to 7.1)
Larynx cancer aggregate	123.5 (102.0 to 155.4)	24.3 (4.0 to 52.4)*	-7.2 (-22.3 to 13.8)	14.0 (9.6 to 19.0)	23.6 (6.6 to 46.0)*	-7.7 (-20.4 to 9.0)
Laryngectomy due to larynx cancer	88.1 (85.0 to 90.9)	37.4 (36.4 to 38.5)*	2.6 (1.9 to 3.4)*	8.0 (5.2 to 11.4)	37.7 (29.7 to 45.8)*	2.8 (-3.2 to 8.8)
Tracheal, bronchus, and lung cancer	347.1 (314.0 to 406.1)	31.0 (20.1 to 44.0)*	-2.2 (-10.3 to 7.5)	54.9 (38.4 to 72.0)	26.4 (14.7 to 40.5)*	-5.7 (-14.4 to 4.9)
Malignant skin melanoma	347.0 (280.3 to 448.8)	62.1 (49.1 to 77.1)*	21.0 (11.3 to 32.2)*	19.9 (12.8 to 29.3)	59.9 (46.4 to 75.9)*	19.3 (9.3 to 31.3)*
Non-melanoma skin cancer	199.2 (186.3 to 214.3)	59.0 (47.8 to 71.7)*	18.7 (10.3 to 28.2)*	10.1 (6.8 to 14.3)	67.6 (50.9 to 86.1)*	25.1 (12.7 to 38.9)*
Non-melanoma skin cancer(squamous-cell carcinoma)	158.7 (139.3 to 184.5)	76.3 (58.6 to 96.1)*	31.6 (18.4 to 46.4)*	9.5 (6.4 to 13.3)	70.9 (53.2 to 91.2)*	27.6 (14.4 to 42.8)*
Non-melanoma skin cancer(basal-cell carcinoma)	53.4 (42.3 to 66.2)	26.1 (21.5 to 31.4)*	-5.8 (-9.3 to -1.9)*	0.6 (0.3 to 1.1)	26.3 (17.8 to 34.8)*	-5.8 (-12.1 to 0.6)
Breast cancer	2,726.3 (2,524.0 to 2,929.0)	54.0 (41.7 to 66.6)*	14.9 (5.8 to 24.4)*	226.1 (159.6 to 302.9)	44.6 (35.1 to 54.3)*	7.9 (0.8 to 15.2)*
Breast cancer aggregate	1,459.7 (1,244.7 to 1,671.8)	91.5 (61.0 to 124.3)*	43.0 (20.2 to 67.4)*	128.4 (90.3 to 169.1)	63.4 (43.7 to 86.0)*	22.0 (7.2 to 38.9)*
Mastectomy due to breast cancer	1,266.5 (1,227.7 to 1,314.5)	25.6 (24.2 to 26.9)*	-6.3 (-7.3 to -5.3)*	97.7 (62.6 to 142.1)	25.5 (23.2 to 27.8)*	-6.3 (-8.0 to -4.6)*
Cervical cancer	353.0 (289.9 to 451.2)	-6.9 (-25.5 to 20.6)	-30.5 (-44.4 to -10.0)*	26.8 (17.7 to 38.0)	-6.5 (-25.3 to 22.0)	-30.2 (-44.2 to -8.9)*
Uterine cancer	570.5 (466.6 to 734.4)	43.3 (21.4 to 72.7)*	7.0 (-9.4 to 28.9)	36.3 (24.6 to 53.6)	41.5 (18.9 to 69.9)*	5.6 (-11.2 to 26.8)
Ovarian cancer	137.2 (123.8 to 154.6)	30.1 (18.3 to 43.0)*	-2.9 (-11.7 to 6.8)	17.1 (12.4 to 22.4)	28.2 (14.5 to 42.9)*	-4.3 (-14.5 to 6.7)
Prostate cancer	1,357.5 (1,056.2 to 1,872.1)	60.6 (44.6 to 74.1)*	19.9 (8.0 to 30.0)*	94.9 (63.7 to 138.0)	53.6 (39.4 to 66.2)*	14.7 (4.1 to 24.1)*
Prostate cancer aggregate	1,264.0 (963.4 to 1,779.3)	64.7 (47.1 to 79.6)*	22.9 (9.8 to 34.1)*	86.7 (57.1 to 127.1)	57.7 (41.9 to 72.6)*	17.8 (5.9 to 28.8)*
Impotence and incontinence due to prostate cancer aggregate	93.5 (91.4 to 95.6)	20.3 (19.8 to 21.0)*	-10.2 (-10.6 to -9.7)*	8.2 (5.6 to 11.6)	20.4 (15.4 to 25.9)*	-10.1 (-13.8 to -6.0)*
Testicular cancer	24.6 (19.3 to 31.2)	83.7 (48.7 to 130.8)*	37.1 (11.0 to 72.3)*	1.6 (1.0 to 2.3)	78.3 (40.8 to 124.3)*	33.1 (5.1 to 67.4)*
Kidney cancer	341.1 (308.8 to 378.5)	54.1 (40.8 to 68.6)*	15.0 (5.1 to 25.8)*	24.0 (16.9 to 32.2)	51.6 (37.4 to 67.2)*	13.1 (2.6 to 24.8)*
Bladder cancer	324.3 (287.6 to 367.6)	31.0 (17.0 to 46.4)*	-2.2 (-12.6 to 9.3)	26.5 (18.6 to 36.0)	29.0 (16.0 to 42.3)*	-3.7 (-13.4 to 6.2)
Bladder cancer episodes aggregate	299.1 (262.5 to 341.7)	31.5 (16.2 to 48.3)*	-1.8 (-13.3 to 10.7)	22.3 (15.7 to 30.5)	29.6 (14.1 to 46.1)*	-3.2 (-14.8 to 9.1)
Urinary incontinence due to bladder cancer	25.2 (23.5 to 27.0)	25.8 (24.3 to 27.5)*	-6.1 (-7.2 to -4.8)*	4.2 (2.9 to 5.8)	26.0 (14.7 to 38.8)*	-5.9 (-14.4 to 3.6)
Brain and nervous system cancer	102.8 (89.5 to 123.0)	33.8 (17.3 to 53.5)*	-0.1 (-12.4 to 14.6)	11.4 (7.8 to 15.6)	31.8 (15.3 to 52.8)*	-1.6 (-14.0 to 14.0)
Thyroid cancer	493.3 (449.4 to 534.0)	106.8 (88.3 to 126.8)*	54.4 (40.6 to 69.3)*	28.4 (19.2 to 39.6)	102.7 (83.0 to 122.9)*	51.3 (36.6 to 66.4)*
Mesothelioma	5.5 (5.1 to 6.1)	24.7 (14.5 to 35.9)*	-6.9 (-14.5 to 1.4)	1.1 (0.8 to 1.5)	24.1 (13.8 to 36.3)*	-7.3 (-15.0 to 1.7)
Hodgkin lymphoma	45.8 (37.0 to 58.6)	46.2 (25.5 to 70.6)*	9.1 (-6.3 to 27.4)	3.9 (2.5 to 5.7)	39.7 (20.1 to 65.9)*	4.2 (-10.4 to 23.9)
Non-Hodgkin lymphoma	418.0 (365.0 to 472.5)	76.4 (59.2 to 92.2)*	31.7 (18.9 to 43.5)*	30.2 (20.8 to 40.8)	70.2 (53.5 to 87.0)*	27.0 (14.6 to 39.6)*
Multiple myeloma	51.8 (43.7 to 60.7)	53.1 (33.6 to 76.0)*	14.3 (-0.3 to 31.4)	11.0 (7.4 to 14.9)	49.1 (27.8 to 71.2)*	11.3 (-4.6 to 27.8)
Leukemia	178.5 (166.4 to 191.7)	54.6 (42.4 to 68.9)*	15.4 (6.3 to 26.1)*	29.1 (20.7 to 37.5)	43.0 (31.5 to 56.4)*	6.8 (-1.9 to 16.7)
Acute lymphoid leukemia	44.0 (36.8 to 52.6)	52.2 (32.6 to 74.0)*	13.6 (-1.0 to 29.9)	5.9 (4.1 to 8.0)	47.0 (27.8 to 72.0)*	9.7 (-4.6 to 28.4)
Chronic lymphoid leukemia	79.4 (67.1 to 92.7)	46.2 (27.9 to 68.1)*	9.2 (-4.5 to 25.5)	10.1 (7.1 to 13.6)	41.3 (22.4 to 64.0)*	5.5 (-8.7 to 22.5)
Acute myeloid leukemia	74.6 (63.9 to 87.0)	56.8 (39.2 to 74.1)*	17.0 (3.9 to 29.9)*	9.5 (6.5 to 12.3)	50.6 (33.8 to 68.0)*	12.4 (-0.1 to 25.4)
Chronic myeloid leukemia	26.5 (22.7 to 30.8)	30.1 (15.9 to 48.0)*	-2.9 (-13.4 to 10.5)	3.5 (2.4 to 4.7)	25.1 (10.5 to 42.0)*	-6.6 (-17.5 to 6.0)
Other neoplasms	359.9 (307.7 to 440.7)	39.7 (24.5 to 58.2)*	4.3 (-7.0 to 18.1)	25.5 (17.3 to 35.5)	37.7 (22.2 to 56.2)*	2.8 (-8.7 to 16.6)
Cardiovascular diseases	43,984.1 (42,976.4 to 44,839.9)	33.1 (31.7 to 34.4)*	-0.7 (-1.7 to 0.3)	2,546.7 (1,803.3 to 3,411.2)	33.0 (31.5 to 34.3)*	-0.7 (-1.8 to 0.2)
Rheumatic heart disease	1,623.3 (1,400.4 to 2,282.7)	7.0 (-3.1 to 12.8)	-20.1 (-27.7 to -15.8)*	83.7 (52.9 to 132.3)	9.6 (-1.1 to 15.7)	-18.2 (-26.1 to -13.7)*
Rheumatic heart disease, without heart failure	1,491.1 (1,268.3 to 2,156.0)	5.1 (-4.8 to 11.1)	-21.6 (-28.9 to -17.0)*	68.6 (41.5 to 114.8)	5.2 (-5.0 to 11.5)	-21.5 (-29.1 to -16.8)*
Heart failure fue to rheumatic heart disease aggregate	132.1 (118.8 to 146.1)	35.2 (33.4 to 37.0)*	1.0 (-0.4 to 2.3)	15.0 (10.1 to 20.9)	35.4 (28.8 to 41.9)*	1.0 (-3.8 to 6.0)
Ischemic heart disease	13,845.9 (11,767.3 to 16,220.4)	32.6 (30.6 to 34.4)*	-1.0 (-2.5 to 0.3)	874.1 (571.4 to 1,256.0)	37.4 (35.6 to 39.0)*	2.6 (1.2 to 3.8)*
Myocardial infarction episodes aggregate	1,990.0 (1,678.8 to 2,294.5)	9.9 (2.9 to 16.6)*	-18.0 (-23.2 to -13.0)*	3.0 (1.9 to 4.2)	18.1 (10.0 to 25.9)*	-11.8 (-17.9 to -6.0)*
Angina due to ischemic heart disease aggregate	10,272.0 (8,182.2 to 12,746.3)	37.5 (35.8 to 39.1)*	2.7 (1.3 to 3.8)*	691.3 (435.6 to 1,021.5)	37.8 (35.7 to 39.8)*	2.9 (1.3 to 4.3)*
Heart failure fue to ischemic heart disease aggregate	1,583.9 (1,453.1 to 1,711.5)	36.2 (35.1 to 37.2)*	1.7 (0.9 to 2.4)*	179.8 (123.7 to 250.2)	36.3 (34.3 to 38.4)*	1.7 (0.2 to 3.3)*
Cerebrovascular disease	4,424.8 (4,376.9 to 4,471.6)	28.3 (27.0 to 29.6)*	-4.2 (-5.2 to -3.3)*	672.8 (466.0 to 908.3)	28.4 (26.4 to 30.3)*	-4.2 (-5.6 to -2.7)*
Ischemic stroke	2,603.5 (2,519.4 to 2,687.8)	27.8 (26.7 to 28.8)*	-4.6 (-5.4 to -3.8)*	389.8 (269.4 to 526.9)	27.9 (25.6 to 30.4)*	-4.5 (-6.3 to -2.7)*
Chronic ischemic stroke aggregate	2,582.4 (2,500.2 to 2,667.8)	27.8 (26.7 to 28.8)*	-4.6 (-5.4 to -3.8)*	385.3 (266.3 to 520.7)	28.0 (25.6 to 30.4)*	-4.4 (-6.2 to -2.6)*
Ischemic stroke episodes aggregate	21.0 (17.2 to 25.3)	20.5 (13.2 to 27.3)*	-10.1 (-15.5 to -5.0)*	4.6 (2.9 to 6.4)	20.5 (13.2 to 27.3)*	-10.0 (-15.5 to -5.0)*
Hemorrhagic stroke	1,891.6 (1,831.6 to 1,953.7)	28.9 (27.8 to 30.0)*	-3.8 (-4.6 to -3.0)*	282.9 (196.7 to 381.2)	29.0 (26.2 to 31.8)*	-3.7 (-5.8 to -1.6)*
Chronic hemorrhagic stroke aggregate	1,875.9 (1,816.2 to 1,938.4)	29.0 (27.8 to 30.0)*	-3.7 (-4.6 to -2.9)*	279.5 (194.2 to 376.0)	29.1 (26.2 to 31.9)*	-3.6 (-5.8 to -1.6)*
Acute hemorrhagic stroke aggregate	15.7 (13.0 to 18.6)	21.4 (14.5 to 28.0)*	-9.4 (-14.5 to -4.5)*	3.4 (2.2 to 4.8)	21.5 (14.6 to 28.0)*	-9.3 (-14.5 to -4.5)*
Hypertensive heart disease	434.9 (386.9 to 485.1)	40.5 (39.2 to 41.8)*	4.9 (3.9 to 5.9)*	49.3 (33.7 to 68.6)	40.7 (36.8 to 45.0)*	5.0 (2.1 to 8.2)*
Cardiomyopathy and myocarditis	159.6 (140.4 to 180.3)	29.5 (27.7 to 31.2)*	-3.3 (-4.7 to -2.1)*	17.5 (11.7 to 24.5)	29.2 (23.6 to 35.1)*	-3.5 (-7.8 to 0.8)
Acute myocarditis	10.8 (7.5 to 15.3)	37.3 (33.2 to 41.3)*	2.5 (-0.5 to 5.5)	0.5 (0.3 to 0.9)	37.4 (32.0 to 42.2)*	2.6 (-1.4 to 6.2)
Heart failure due to cardiomyopathy aggregate	148.8 (130.1 to 169.3)	29.0 (27.1 to 30.8)*	-3.7 (-5.1 to -2.4)*	16.9 (11.4 to 23.6)	29.0 (23.1 to 35.1)*	-3.7 (-8.1 to 0.8)
Atrial fibrillation and flutter	2,707.0 (2,256.0 to 3,213.4)	29.9 (28.4 to 31.1)*	-3.1 (-4.1 to -2.1)*	221.2 (144.8 to 311.3)	30.0 (27.5 to 32.4)*	-2.9 (-4.8 to -1.1)*
Peripheral vascular disease	14,957.7 (12,069.9 to 18,118.8)	36.4 (35.6 to 37.2)*	1.8 (1.2 to 2.4)*	39.6 (17.5 to 77.8)	33.3 (31.6 to 34.9)*	-0.5 (-1.8 to 0.7)
Endocarditis	3.9 (3.3 to 4.4)	31.9 (27.5 to 36.0)*	-1.5 (-4.8 to 1.5)	0.4 (0.3 to 0.6)	31.3 (12.8 to 50.2)*	-2.0 (-15.8 to 12.1)
Endocarditis episodes aggregate	1.1 (0.9 to 1.4)	32.9 (24.2 to 40.9)*	-0.8 (-7.3 to 5.2)	0.1 (0.0 to 0.1)	32.7 (24.0 to 40.6)*	-1.0 (-7.4 to 5.0)
Heart failure due to endocarditis aggregate	2.7 (2.3 to 3.3)	31.5 (26.3 to 36.7)*	-1.8 (-5.7 to 2.0)	0.3 (0.2 to 0.5)	31.0 (9.8 to 53.8)*	-2.2 (-18.1 to 14.8)
Other cardiovascular and circulatory diseases	8,741.2 (7,593.5 to 10,052.3)	36.9 (35.2 to 38.8)*	2.2 (0.9 to 3.6)*	588.1 (390.7 to 827.1)	37.1 (34.9 to 39.2)*	2.3 (0.7 to 3.9)*
Heart failure due to other cardiovascular diseases aggregate	66.6 (57.4 to 76.9)	37.0 (35.0 to 39.2)*	2.3 (0.8 to 3.9)*	7.6 (5.1 to 10.7)	37.1 (28.4 to 47.0)*	2.3 (-4.2 to 9.7)
Other cardiovascular diseases episodes aggregate	8,674.6 (7,535.8 to 9,975.4)	36.9 (35.2 to 38.8)*	2.2 (0.9 to 3.6)*	580.5 (386.1 to 816.1)	37.1 (34.9 to 39.2)*	2.3 (0.7 to 3.9)*
Chronic respiratory diseases	30,822.7 (29,608.1 to 32,070.6)	27.0 (25.2 to 28.9)*	-5.2 (-6.6 to -3.8)*	1,988.1 (1,551.2 to 2,431.7)	26.4 (23.0 to 29.6)*	-5.7 (-8.1 to -3.2)*
Chronic obstructive pulmonary disease	15,695.6 (13,452.6 to 18,300.3)	24.5 (20.9 to 28.1)*	-7.1 (-9.8 to -4.3)*	1,066.8 (854.8 to 1,300.4)	25.8 (20.6 to 30.9)*	-6.1 (-10.0 to -2.3)*

Cause	Prevalence in thousands (95% confidence interval)			YLDs in thousands (95% confidence interval)		
	2015	Percent change in counts between 2005 and 2015	Percent change in rates between 2005 and 2015	2015	Percent change in counts between 2005 and 2015	Percent change in rates between 2005 and 2015
Chronic obstructive pulmonary disease without heart failure aggregate	15,413.1<br>(13,172.4 to 18,038.4)	24.3<br>(20.6 to 28.0)*	-7.2<br>(-10.0 to -4.5)*	943.0<br>(749.8 to 1,153.0)	24.2<br>(18.5 to 30.0)*	-7.3<br>(-11.6 to -3.0)*
Severe chronic obstructive pulmonary disease with heart failure aggregate	289.7<br>(254.8 to 331.4)	39.1<br>(37.6 to 40.5)*	3.8<br>(2.7 to 4.9)*	123.9<br>(88.5 to 160.6)	39.4<br>(35.3 to 43.7)*	4.0<br>(1.0 to 7.3)*
Pneumoconiosis	176.1<br>(167.4 to 184.5)	40.2<br>(34.4 to 46.3)*	4.6<br>(0.3 to 9.2)*	34.3<br>(21.6 to 49.9)	39.0<br>(32.3 to 45.8)*	3.8<br>(-1.2 to 8.8)
Silicosis	38.8<br>(33.7 to 44.2)	28.7<br>(25.4 to 31.7)*	-4.0<br>(-6.4 to -1.7)*	6.2<br>(3.8 to 9.1)	29.1<br>(18.7 to 41.1)*	-3.6<br>(-11.4 to 5.3)
Silicosis without heart failure aggregate	38.8<br>(33.7 to 44.2)	28.7<br>(25.4 to 31.7)*	-4.0<br>(-6.4 to -1.7)*	6.2<br>(3.8 to 9.1)	29.1<br>(18.7 to 41.1)*	-3.6<br>(-11.4 to 5.3)
Severe silicosis with heart failure aggregate	0.0<br>(0.0 to 0.0)	0.0<br>(0.0 to 0.0)	0.0<br>(0.0 to 0.0)	0.0<br>(0.0 to 0.0)	0.0<br>(0.0 to 0.0)	0.0<br>(0.0 to 0.0)
Asbestosis	14.8<br>(12.9 to 16.9)	29.8<br>(26.9 to 32.7)*	-3.1<br>(-5.3 to -0.9)*	2.4<br>(1.5 to 3.6)	30.8<br>(20.3 to 42.3)*	-2.3<br>(-10.2 to 6.2)
Asbestosis without heart failure aggregate	14.7<br>(12.8 to 16.9)	29.8<br>(26.9 to 32.7)*	-3.1<br>(-5.3 to -0.9)*	2.4<br>(1.5 to 3.5)	30.8<br>(20.1 to 42.5)*	-2.3<br>(-10.4 to 6.4)
Severe asbestosis with heart failure aggregate	0.1<br>(0.1 to 0.1)	31.0<br>(29.0 to 33.2)*	-2.2<br>(-3.7 to -0.6)*	0.0<br>(0.0 to 0.0)	31.0<br>(29.0 to 33.2)*	-2.2<br>(-3.7 to -0.6)*
Coal workers pneumoconiosis	8.0<br>(6.8 to 9.2)	42.0<br>(37.6 to 45.7)*	6.0<br>(2.7 to 8.8)*	1.3<br>(0.8 to 2.0)	41.1<br>(26.9 to 58.6)*	5.3<br>(-5.3 to 18.4)
Coal workers pneumoconiosis without heart failure aggregate	7.8<br>(6.6 to 9.1)	41.9<br>(37.5 to 45.7)*	5.9<br>(2.6 to 8.8)*	1.2<br>(0.7 to 1.9)	40.8<br>(25.9 to 59.4)*	5.1<br>(-6.0 to 19.0)
Severe coal workers pneumoconiosis with heart failure aggregate	0.2<br>(0.1 to 0.2)	46.4<br>(35.1 to 57.6)*	9.3<br>(0.8 to 17.6)*	0.1<br>(0.1 to 0.1)	46.3<br>(35.1 to 57.6)*	9.2<br>(0.8 to 17.6)*
Other pneumoconiosis	160.7<br>(139.8 to 183.4)	42.5<br>(39.6 to 45.5)*	6.4<br>(4.2 to 8.6)*	24.4<br>(15.5 to 35.6)	42.6<br>(34.2 to 51.6)*	6.4<br>(0.2 to 13.2)*
Other pneumoconiosis without heart failure aggregate	160.5<br>(139.6 to 183.3)	42.5<br>(39.6 to 45.6)*	6.4<br>(4.2 to 8.7)*	24.3<br>(15.4 to 35.5)	42.6<br>(34.3 to 51.7)*	6.5<br>(0.2 to 13.2)*
Severe other pneumoconiosis with heart failure aggregate	0.2<br>(0.1 to 0.2)	29.4<br>(25.6 to 33.2)*	-3.4<br>(-6.2 to -0.5)*	0.1<br>(0.1 to 0.1)	29.4<br>(25.6 to 33.2)*	-3.4<br>(-6.2 to -0.5)*
Asthma	16,469.7<br>(13,858.1 to 19,630.5)	28.7<br>(25.8 to 31.8)*	-3.9<br>(-6.1 to -1.6)*	712.5<br>(456.3 to 1,017.1)	28.7<br>(25.8 to 31.9)*	-3.9<br>(-6.1 to -1.6)*
Asymptomatic asthma	4,987.3<br>(4,024.0 to 6,159.4)	28.7<br>(25.8 to 31.8)*	-3.9<br>(-6.1 to -1.6)*	0.0<br>(0.0 to 0.0)	0.0<br>(0.0 to 0.0)	0.0<br>(0.0 to 0.0)
Symptomatic asthma aggregate	11,482.5<br>(9,539.4 to 13,702.1)	28.7<br>(25.8 to 31.8)*	-3.9<br>(-6.1 to -1.6)*	712.5<br>(456.3 to 1,017.1)	28.7<br>(25.8 to 31.9)*	-3.9<br>(-6.1 to -1.6)*
Interstitial lung disease and pulmonary sarcoidosis	181.7<br>(154.8 to 213.8)	32.3<br>(28.6 to 35.4)*	-1.3<br>(-4.0 to 1.1)	22.5<br>(14.1 to 32.9)	32.2<br>(26.1 to 38.7)*	-1.3<br>(-5.8 to 3.5)
Interstitial lung disease and pulmonary sarcoidosis without heart failure aggregate	165.0<br>(139.0 to 196.3)	32.7<br>(28.9 to 36.0)*	-1.0<br>(-3.7 to 1.5)	14.7<br>(8.4 to 22.7)	34.0<br>(25.1 to 43.5)*	0.1<br>(-6.6 to 7.1)
Severe interstitial lung disease and pulmonary sarcoidosis with heart failure aggregate	16.7<br>(13.3 to 19.6)	28.5<br>(26.6 to 31.8)*	-4.0<br>(-5.5 to -1.6)*	7.8<br>(5.3 to 10.6)	28.9<br>(23.6 to 34.6)*	-3.8<br>(-7.8 to 0.5)
Other chronic respiratory diseases	--	--	--	152.0<br>(119.9 to 186.7)	16.7<br>(5.1 to 28.8)*	-12.9<br>(-21.5 to -3.9)*
Cirrhosis and other chronic liver diseases	348.6<br>(339.6 to 357.8)	45.5<br>(41.3 to 50.0)*	8.6<br>(5.5 to 12.0)*	57.2<br>(38.8 to 78.4)	45.4<br>(40.9 to 49.9)*	8.5<br>(5.2 to 11.9)*
Cirrhosis and other chronic liver diseases due to hepatitis B	96.2<br>(80.2 to 113.9)	42.5<br>(37.5 to 47.4)*	6.4<br>(2.6 to 10.1)*	15.7<br>(10.4 to 22.8)	42.7<br>(32.8 to 52.5)*	6.5<br>(-0.9 to 13.8)
Cirrhosis and other chronic liver diseases due to hepatitis C	87.1<br>(73.9 to 100.9)	42.5<br>(37.6 to 46.9)*	6.4<br>(2.8 to 9.7)*	14.2<br>(9.5 to 19.8)	42.5<br>(34.1 to 50.9)*	6.4<br>(0.1 to 12.6)*
Cirrhosis and other chronic liver diseases due to alcohol use	109.8<br>(93.9 to 125.9)	48.1<br>(44.0 to 52.0)*	10.6<br>(7.5 to 13.5)*	17.9<br>(12.1 to 25.3)	48.4<br>(40.0 to 56.8)*	10.8<br>(4.5 to 17.1)*
Cirrhosis and other chronic liver diseases due to other causes	57.5<br>(48.1 to 66.9)	48.6<br>(44.6 to 52.6)*	10.9<br>(8.0 to 13.9)*	9.3<br>(6.3 to 13.2)	48.9<br>(37.6 to 61.1)*	11.1<br>(2.7 to 20.2)*
Digestive diseases	22,333.5<br>(21,857.1 to 22,767.1)	18.9<br>(15.9 to 21.9)*	-11.3<br>(-13.4 to -9.0)*	978.7<br>(684.0 to 1,325.6)	19.2<br>(16.3 to 22.2)*	-11.0<br>(-13.1 to -8.8)*
Peptic ulcer disease	5,128.7<br>(4,973.9 to 5,276.2)	8.1<br>(5.0 to 11.4)*	-19.3<br>(-21.6 to -16.9)*	164.9<br>(111.5 to 232.1)	5.4<br>(1.9 to 8.8)*	-21.4<br>(-23.9 to -18.8)*
Peptic ulcer disease, symptomatic episodes	367.8<br>(288.4 to 455.7)	10.5<br>(4.7 to 16.2)*	-17.5<br>(-21.8 to -13.3)*	38.8<br>(25.3 to 56.6)	10.8<br>(4.2 to 16.9)*	-17.3<br>(-22.2 to -12.8)*
Anemia due to peptic ulcer disease aggregate	4,760.9<br>(4,634.0 to 4,889.1)	7.9<br>(4.7 to 11.4)*	-19.4<br>(-21.8 to -16.9)*	126.1<br>(84.5 to 179.1)	3.8<br>(-0.2 to 8.0)	-22.5<br>(-25.5 to -19.4)*
Gastritis and duodenitis	14,958.0<br>(14,554.7 to 15,407.7)	21.2<br>(17.3 to 25.2)*	-9.6<br>(-12.4 to -6.6)*	425.6<br>(289.7 to 608.0)	17.6<br>(12.8 to 22.8)*	-12.2<br>(-15.8 to -8.4)*
Gastritis and duodenitis, symptomatic episodes	367.7<br>(274.2 to 477.0)	24.2<br>(17.5 to 28.1)*	-7.3<br>(-12.3 to -4.4)*	38.9<br>(24.1 to 58.0)	24.4<br>(17.3 to 29.4)*	-7.1<br>(-12.4 to -3.4)*
Anemia due to gastritis and duodenitis aggregate	14,590.3<br>(14,182.3 to 15,013.5)	21.1<br>(17.1 to 25.2)*	-9.6<br>(-12.6 to -6.5)*	386.7<br>(262.2 to 552.0)	16.9<br>(11.8 to 22.6)*	-12.7<br>(-16.5 to -8.5)*
Appendicitis	14.8<br>(12.4 to 17.5)	38.3<br>(35.9 to 40.3)*	3.2<br>(1.4 to 4.8)*	4.5<br>(2.9 to 6.3)	38.6<br>(24.9 to 53.3)*	3.4<br>(-6.7 to 14.5)
Paralytic ileus and intestinal obstruction	7.0<br>(5.5 to 8.7)	31.3<br>(24.8 to 38.0)*	-2.0<br>(-6.8 to 3.0)	2.3<br>(1.4 to 3.3)	31.5<br>(24.5 to 38.7)*	-1.8<br>(-7.0 to 3.5)
Inguinal, femoral, and abdominal hernia	1,489.9<br>(1,085.0 to 2,018.8)	18.2<br>(11.6 to 24.9)*	-11.8<br>(-16.7 to -6.8)*	15.7<br>(7.1 to 30.5)	18.3<br>(11.4 to 25.2)*	-11.7<br>(-16.8 to -6.6)*
Inflammatory bowel disease	915.4<br>(854.4 to 979.8)	26.9<br>(26.3 to 27.5)*	-5.3<br>(-5.7 to -4.8)*	192.0<br>(132.6 to 261.8)	26.9<br>(24.9 to 29.1)*	-5.2<br>(-6.8 to -3.7)*
Vascular intestinal disorders	2.9<br>(2.3 to 3.7)	30.6<br>(23.2 to 38.5)*	-2.5<br>(-8.1 to 3.4)	0.9<br>(0.6 to 1.3)	31.9<br>(18.8 to 44.6)*	-1.5<br>(-11.3 to 7.9)
Gallbladder and biliary diseases	443.9<br>(339.3 to 573.8)	27.9<br>(21.4 to 34.7)*	-4.5<br>(-9.4 to 0.5)	47.0<br>(29.2 to 69.7)	28.0<br>(20.9 to 35.6)*	-4.4<br>(-9.8 to 1.2)
Pancreatitis	88.5<br>(69.8 to 108.8)	35.4<br>(28.1 to 42.2)*	1.1<br>(-4.4 to 6.1)	25.8<br>(16.5 to 36.8)	35.5<br>(24.5 to 46.1)*	1.1<br>(-7.1 to 9.0)
Other digestive diseases	--	--	--	100.1<br>(69.7 to 137.4)	30.3<br>(21.6 to 42.2)*	-2.7<br>(-9.2 to 6.2)
Neurological disorders	124,007.6<br>(117,292.7 to 130,935.5)	35.6<br>(33.4 to 37.9)*	1.2<br>(-0.4 to 2.9)	3,213.6<br>(2,187.4 to 4,432.8)	32.2<br>(28.6 to 35.8)*	-1.3<br>(-4.0 to 1.3)
Alzheimer disease and other dementias	1,357.0<br>(1,018.2 to 1,731.0)	34.9<br>(33.7 to 35.9)*	0.7<br>(-0.2 to 1.4)	168.5<br>(111.2 to 244.6)	35.0<br>(32.0 to 37.8)*	0.7<br>(-1.5 to 2.9)
Parkinson disease	391.9<br>(342.3 to 450.8)	34.7<br>(29.6 to 40.1)*	0.6<br>(-3.3 to 4.6)	48.5<br>(32.8 to 67.0)	34.8<br>(27.5 to 42.5)*	0.6<br>(-4.8 to 6.4)
Epilepsy	951.4<br>(815.8 to 1,118.5)	31.4<br>(25.0 to 38.1)*	-1.9<br>(-6.7 to 3.1)	225.5<br>(155.8 to 315.1)	8.8<br>(1.1 to 16.4)*	-18.8<br>(-24.5 to -13.1)*
Multiple sclerosis	190.2<br>(176.6 to 204.8)	29.1<br>(26.5 to 31.5)*	-3.6<br>(-5.5 to -1.8)*	62.1<br>(44.4 to 79.2)	29.0<br>(24.1 to 34.1)*	-3.7<br>(-7.4 to 0.1)
Motor neuron disease	18.2<br>(15.5 to 21.3)	20.4<br>(14.7 to 25.7)*	-10.1<br>(-14.4 to -6.2)*	3.9<br>(2.7 to 5.3)	20.4<br>(14.6 to 25.9)*	-10.1<br>(-14.4 to -6.0)*
Migraine	55,252.6<br>(44,720.3 to 67,386.4)	34.5<br>(32.1 to 36.8)*	0.4<br>(-1.4 to 2.1)	1,832.4<br>(1,094.4 to 2,818.8)	34.7<br>(32.1 to 37.4)*	0.6<br>(-1.4 to 2.6)
Tension-type headache	80,392.0<br>(57,989.4 to 106,358.2)	36.7<br>(34.7 to 38.9)*	2.1<br>(0.6 to 3.7)*	118.3<br>(50.9 to 218.7)	36.9<br>(34.4 to 39.4)*	2.2<br>(0.4 to 4.0)*
Medication overuse headache	4,830.4<br>(3,101.4 to 6,951.2)	33.6<br>(20.7 to 48.1)*	-0.3<br>(-9.9 to 10.5)	740.9<br>(413.7 to 1,171.4)	33.7<br>(20.6 to 48.3)*	-0.2<br>(-10.0 to 10.7)
Other neurological disorders	0.8<br>(0.5 to 1.3)	31.0<br>(28.8 to 32.8)*	-2.2<br>(-3.9 to -0.8)*	13.4<br>(9.3 to 18.9)	27.2<br>(22.0 to 31.6)*	-5.0<br>(-8.9 to -1.8)*
Mental and substance use disorders	54,547.2<br>(53,418.7 to 56,058.1)	34.1<br>(33.0 to 35.2)*	0.1<br>(-0.7 to 0.9)	8,307.9<br>(6,059.0 to 10,811.4)	34.6<br>(33.6 to 35.5)*	0.4<br>(-0.3 to 1.2)
Schizophrenia	1,878.4<br>(1,645.4 to 2,113.0)	34.4<br>(33.6 to 35.3)*	0.4<br>(-0.2 to 1.0)	1,175.3<br>(863.2 to 1,475.3)	34.7<br>(32.8 to 36.5)*	0.5<br>(-0.8 to 1.9)
Alcohol use disorders	3,578.1<br>(3,058.9 to 4,151.3)	29.7<br>(25.3 to 33.8)*	-3.2<br>(-6.4 to -0.1)*	347.9<br>(223.5 to 499.5)	29.8<br>(25.2 to 34.2)*	-3.1<br>(-6.6 to 0.2)
Alcohol dependence aggregate	3,542.5<br>(3,024.7 to 4,115.0)	29.6<br>(25.3 to 33.8)*	-3.2<br>(-6.5 to -0.1)*	346.2<br>(222.3 to 497.2)	29.8<br>(25.2 to 34.2)*	-3.1<br>(-6.6 to 0.2)
Fetal alcohol syndrome aggregate	35.6<br>(32.6 to 38.6)	32.1<br>(30.6 to 33.7)*	-1.4<br>(-2.5 to -0.2)*	1.8<br>(1.2 to 2.6)	32.0<br>(23.9 to 40.4)*	-1.5<br>(-7.5 to 4.8)
Drug use disorders	1,347.0<br>(1,284.0 to 1,426.4)	48.3<br>(44.9 to 51.8)*	10.7<br>(8.2 to 13.3)*	382.1<br>(262.7 to 500.9)	52.4<br>(47.6 to 57.3)*	13.8<br>(10.2 to 17.4)*
Opioid use disorders	811.1<br>(662.5 to 972.8)	51.0<br>(46.7 to 55.1)*	12.7<br>(9.5 to 15.8)*	324.3<br>(217.5 to 433.3)	51.2<br>(45.6 to 56.9)*	12.9<br>(8.7 to 17.1)*
Asymptomatic opioid dependence	131.5<br>(92.0 to 182.9)	50.9<br>(46.7 to 55.1)*	12.7<br>(9.5 to 15.8)*	0.0<br>(0.0 to 0.0)	0.0<br>(0.0 to 0.0)	0.0<br>(0.0 to 0.0)
Symptomatic opioid dependence aggregate	679.5<br>(545.0 to 822.2)	51.0<br>(46.7 to 55.1)*	12.7<br>(9.5 to 15.8)*	324.3<br>(217.5 to 433.3)	51.2<br>(45.6 to 56.9)*	12.9<br>(8.7 to 17.1)*
Cocaine use disorders	158.1<br>(130.0 to 193.9)	61.3<br>(57.7 to 65.4)*	20.4<br>(17.7 to 23.4)*	21.0<br>(13.0 to 31.3)	60.1<br>(50.4 to 70.7)*	19.5<br>(12.2 to 27.4)*
Asymptomatic cocaine dependence	79.3<br>(60.5 to 102.2)	61.3<br>(57.7 to 65.4)*	20.4<br>(17.7 to 23.4)*	0.0<br>(0.0 to 0.0)	0.0<br>(0.0 to 0.0)	0.0<br>(0.0 to 0.0)

Cause	Prevalence in thousands (95% confidence interval)			YLDs in thousands (95% confidence interval)		
	2015	Percent change in counts between 2005 and 2015	Percent change in rates between 2005 and 2015	2015	Percent change in counts between 2005 and 2015	Percent change in rates between 2005 and 2015
Symptomatic cocaine dependence aggregate	78.9 (60.4 to 102.5)	61.3 (57.7 to 65.4)*	20.4 (17.7 to 23.4)*	21.0 (13.0 to 31.3)	60.1 (50.4 to 70.7)*	19.5 (12.2 to 27.4)*
Amphetamine use disorders	60.6 (45.8 to 78.0)	90.2 (81.5 to 99.3)*	42.0 (35.5 to 48.8)*	7.9 (4.6 to 12.1)	88.0 (68.4 to 111.8)*	40.3 (25.7 to 58.1)*
Asymptomatic amphetamine dependence	33.2 (23.8 to 45.7)	90.2 (81.5 to 99.3)*	42.0 (35.5 to 48.8)*	0.0 (0.0 to 0.0)	0.0 (0.0 to 0.0)	0.0 (0.0 to 0.0)
Symptomatic amphetamine dependence aggregate	27.4 (19.5 to 37.4)	90.2 (81.5 to 99.3)*	42.0 (35.5 to 48.8)*	7.9 (4.6 to 12.1)	88.0 (68.4 to 111.8)*	40.3 (25.7 to 58.1)*
Cannabis use disorders	356.4 (316.6 to 396.7)	30.8 (29.3 to 32.2)*	-2.4 (-3.5 to -1.3)*	10.2 (6.5 to 14.5)	30.9 (23.6 to 38.9)*	-2.3 (-7.8 to 3.7)
Asymptomatic cannabis dependence	206.1 (179.4 to 233.3)	30.8 (29.3 to 32.2)*	-2.4 (-3.5 to -1.3)*	0.0 (0.0 to 0.0)	0.0 (0.0 to 0.0)	0.0 (0.0 to 0.0)
Symptomatic cannabis dependence aggregate	150.2 (126.7 to 176.0)	30.8 (29.3 to 32.2)*	-2.4 (-3.5 to -1.3)*	10.2 (6.5 to 14.5)	30.9 (23.6 to 38.9)*	-2.3 (-7.8 to 3.7)
Other drug use disorders	--	--	--	18.7 (11.6 to 27.4)	67.0 (56.3 to 78.0)*	24.7 (16.7 to 32.9)*
Depressive disorders	21,701.2 (20,426.1 to 23,067.2)	33.6 (31.5 to 35.7)*	-0.3 (-1.9 to 1.3)	3,577.3 (2,398.2 to 5,042.2)	33.4 (32.0 to 34.6)*	-0.4 (-1.5 to 0.5)
Major depressive disorder	13,833.9 (10,954.0 to 17,308.2)	32.6 (30.9 to 33.8)*	-1.1 (-2.3 to -0.1)*	2,761.3 (1,813.7 to 3,951.2)	32.8 (30.9 to 34.3)*	-0.9 (-2.3 to 0.2)
Dysthymia	8,579.7 (6,974.5 to 10,268.5)	35.3 (33.5 to 37.2)*	1.0 (-0.3 to 2.4)	816.0 (520.0 to 1,185.9)	35.5 (33.4 to 37.6)*	1.1 (-0.4 to 2.7)
Bipolar disorder	2,013.0 (1,664.3 to 2,404.4)	33.1 (32.6 to 33.6)*	-0.6 (-1.0 to -0.3)*	398.4 (240.5 to 600.3)	33.2 (31.0 to 35.3)*	-0.6 (-2.2 to 1.0)
Anxiety disorders	14,433.7 (11,887.6 to 17,335.9)	35.0 (32.0 to 37.8)*	0.8 (-1.4 to 2.9)	1,293.7 (854.2 to 1,820.8)	35.1 (32.1 to 38.0)*	0.8 (-1.4 to 3.0)
Eating disorders	0.0 (0.0 to 0.0)	0.0 (0.0 to 0.0)	0.0 (0.0 to 0.0)	0.0 (0.0 to 0.0)	0.0 (0.0 to 0.0)	0.0 (0.0 to 0.0)
Anorexia nervosa	0.0 (0.0 to 0.0)	0.0 (0.0 to 0.0)	0.0 (0.0 to 0.0)	0.0 (0.0 to 0.0)	0.0 (0.0 to 0.0)	0.0 (0.0 to 0.0)
Bulimia nervosa	0.0 (0.0 to 0.0)	0.0 (0.0 to 0.0)	0.0 (0.0 to 0.0)	0.0 (0.0 to 0.0)	0.0 (0.0 to 0.0)	0.0 (0.0 to 0.0)
Autistic spectrum disorders	2,548.7 (2,423.6 to 2,677.9)	34.1 (31.7 to 36.7)*	0.1 (-1.7 to 2.0)	399.6 (268.8 to 543.5)	34.4 (33.0 to 35.7)*	0.3 (-0.7 to 1.3)
Autism	1,025.5 (858.1 to 1,225.4)	34.4 (33.8 to 34.9)*	0.3 (-0.1 to 0.7)	252.8 (164.3 to 360.3)	34.5 (32.8 to 36.3)*	0.4 (-0.9 to 1.8)
Asperger syndrome and other autistic spectrum disorders	1,511.8 (1,282.6 to 1,815.5)	34.1 (33.9 to 34.2)*	0.1 (-0.1 to 0.2)	146.7 (98.6 to 212.8)	34.2 (32.4 to 36.0)*	0.1 (-1.2 to 1.5)
Attention-deficit/hyperactivity disorder	1.7 (1.3 to 2.2)	44.8 (33.5 to 60.7)*	8.1 (-0.4 to 20.0)	0.0 (0.0 to 0.0)	43.5 (-25.2 to 162.0)	7.1 (-44.2 to 95.5)
Conduct disorder	0.0 (0.0 to 0.0)	0.0 (0.0 to 0.0)	0.0 (0.0 to 0.0)	0.0 (0.0 to 0.0)	0.0 (0.0 to 0.0)	0.0 (0.0 to 0.0)
Idiopathic developmental intellectual disability	2,740.3 (1,389.9 to 4,037.4)	36.7 (34.8 to 38.8)*	2.1 (0.6 to 3.6)*	107.9 (46.1 to 188.1)	35.3 (31.7 to 38.1)*	1.0 (-1.7 to 3.1)
Other mental and substance use disorders	8,522.0 (8,359.8 to 8,686.3)	34.0 (30.5 to 37.6)*	0.0 (-2.6 to 2.7)	625.7 (436.1 to 834.9)	34.1 (30.5 to 37.9)*	0.1 (-2.6 to 3.0)
Diabetes, urogenital, blood, and endocrine diseases	168,931.5 (166,581.8 to 171,318.2)	33.9 (33.2 to 34.5)*	-0.1 (-0.6 to 0.4)	5,899.3 (4,163.2 to 7,972.7)	37.9 (34.7 to 40.8)*	2.9 (0.6 to 5.1)*
Diabetes mellitus	52,530.5 (47,297.2 to 58,166.8)	40.1 (35.8 to 43.7)*	4.6 (1.4 to 7.3)*	4,175.5 (2,841.3 to 5,760.6)	41.6 (37.0 to 45.6)*	5.7 (2.3 to 8.7)*
Uncomplicated diabetes mellitus	31,006.1 (25,803.2 to 35,970.6)	37.8 (33.2 to 41.7)*	2.9 (-0.6 to 5.8)	1,435.1 (889.3 to 2,174.6)	37.9 (33.2 to 41.8)*	3.0 (-0.6 to 5.9)
Neuropathy and other complications of diabetes mellitus aggregate	21,056.5 (16,583.1 to 25,787.1)	43.6 (38.8 to 48.2)*	7.2 (3.6 to 10.6)*	2,710.1 (1,746.6 to 3,922.7)	43.7 (38.7 to 48.2)*	7.3 (3.5 to 10.6)*
Vision loss due to diabetes mellitus aggregate	467.9 (328.7 to 647.8)	41.4 (37.9 to 45.0)*	5.6 (3.0 to 8.2)*	30.3 (19.0 to 45.6)	40.8 (35.2 to 46.6)*	5.1 (1.0 to 9.4)*
Acute glomerulonephritis	7.3 (5.5 to 9.6)	21.3 (15.0 to 27.3)*	-9.5 (-14.1 to -5.0)*	0.4 (0.2 to 0.6)	23.0 (6.0 to 41.4)*	-8.2 (-20.9 to 5.6)
Chronic kidney disease	30,436.4 (27,688.4 to 32,796.0)	35.8 (32.9 to 38.7)*	1.4 (-0.8 to 3.5)	711.1 (516.3 to 936.7)	33.4 (31.4 to 35.4)*	-0.4 (-1.9 to 1.1)
Chronic kidney disease due to diabetes mellitus	8,941.1 (5,842.4 to 12,389.7)	36.9 (33.0 to 41.0)*	2.2 (-0.7 to 5.2)	200.7 (125.6 to 287.6)	33.3 (28.8 to 37.8)*	-0.5 (-3.8 to 2.9)
Stage III chronic kidney disease due to diabetes mellitus aggregate	8,188.2 (5,262.0 to 11,536.6)	37.1 (33.0 to 41.5)*	2.3 (-0.7 to 5.6)	6.0 (3.6 to 10.0)	14.0 (-2.1 to 32.5)	-14.9 (-26.9 to -1.1)*
Stage IV chronic kidney disease due to diabetes mellitus aggregate	422.2 (257.4 to 663.3)	36.0 (32.5 to 39.3)*	1.5 (-1.1 to 4.0)	50.0 (27.4 to 82.1)	35.6 (30.9 to 40.8)*	1.2 (-2.3 to 5.1)
Stage V chronic kidney disease untreated due to diabetes mellitus	241.8 (163.6 to 349.4)	35.2 (31.7 to 38.7)*	0.9 (-1.7 to 3.5)	121.5 (72.9 to 178.8)	35.3 (29.7 to 41.0)*	1.0 (-3.2 to 5.2)
End-stage chronic kidney disease due to diabetes aggregate	89.0 (62.4 to 117.6)	24.4 (19.9 to 28.6)*	-7.1 (-10.5 to -4.0)*	23.1 (13.9 to 34.0)	24.8 (15.3 to 34.5)*	-6.8 (-13.9 to 0.4)
Chronic kidney disease due to hypertension	8,012.5 (5,027.6 to 11,310.0)	36.3 (32.1 to 41.0)*	1.7 (-1.4 to 5.2)	135.4 (90.9 to 188.4)	35.5 (29.9 to 42.3)*	1.2 (-3.0 to 6.2)
Stage III chronic kidney disease due to hypertension aggregate	7,503.4 (4,548.1 to 10,750.5)	36.3 (31.8 to 41.5)*	1.8 (-1.6 to 5.6)	6.4 (4.1 to 9.8)	28.1 (16.9 to 41.3)*	-4.3 (-12.8 to 5.5)
Stage IV chronic kidney disease due to hypertension aggregate	294.2 (186.4 to 471.1)	35.9 (28.4 to 43.8)*	1.4 (-4.2 to 7.4)	35.1 (20.1 to 58.9)	34.6 (26.4 to 43.0)*	0.5 (-5.7 to 6.7)
Stage V chronic kidney disease untreated due to hypertension	157.5 (109.7 to 222.2)	36.8 (31.1 to 43.2)*	2.2 (-2.1 to 6.9)	79.3 (50.4 to 122.6)	37.1 (29.3 to 46.3)*	2.4 (-3.5 to 9.2)
End-stage chronic kidney disease due to hypertension aggregate	57.4 (41.9 to 75.7)	30.5 (22.7 to 39.5)*	-2.6 (-8.4 to 4.1)	14.7 (9.1 to 21.7)	32.6 (19.1 to 47.5)*	-1.0 (-11.1 to 10.1)
Chronic kidney disease due to glomerulonephritis	6,179.5 (4,478.3 to 8,272.7)	37.6 (32.3 to 44.2)*	2.7 (-1.2 to 7.6)	186.8 (116.3 to 267.0)	34.0 (29.7 to 39.0)*	0.1 (-3.2 to 3.7)
Stage III chronic kidney disease due to glomerulonephritis aggregate	5,504.9 (3,839.2 to 7,491.3)	38.0 (32.0 to 45.3)*	3.0 (-1.5 to 8.5)	10.7 (6.9 to 16.2)	25.8 (16.0 to 35.4)*	-6.1 (-13.4 to 1.0)
Stage IV chronic kidney disease due to glomerulonephritis aggregate	386.6 (240.8 to 541.2)	35.3 (29.7 to 41.6)*	1.0 (-3.2 to 5.7)	46.1 (27.1 to 71.1)	35.0 (28.4 to 42.0)*	0.8 (-4.1 to 6.0)
Stage V chronic kidney disease untreated due to glomerulonephritis	228.1 (141.5 to 323.6)	34.6 (31.0 to 38.3)*	0.4 (-2.2 to 3.3)	114.4 (65.4 to 175.9)	34.9 (28.8 to 41.0)*	0.7 (-3.9 to 5.2)
End-stage chronic kidney disease due to glomerulonephritis aggregate	59.8 (38.0 to 87.7)	28.9 (23.8 to 34.2)*	-3.8 (-7.6 to 0.2)	15.6 (8.6 to 24.7)	30.8 (18.9 to 44.4)*	-2.3 (-11.2 to 7.8)
Chronic kidney disease due to other causes	8,331.0 (5,277.2 to 11,979.5)	34.0 (30.6 to 37.6)*	0.0 (-2.5 to 2.7)	188.2 (118.4 to 277.7)	31.4 (27.5 to 35.1)*	-1.9 (-4.8 to 0.9)
Stage III chronic kidney disease due to other causes aggregate	7,642.6 (4,745.9 to 11,120.5)	34.2 (30.6 to 38.0)*	0.2 (-2.5 to 3.0)	10.6 (6.9 to 16.1)	21.4 (12.3 to 30.8)*	-9.4 (-16.2 to -2.4)*
Stage IV chronic kidney disease due to other causes aggregate	395.4 (234.4 to 611.2)	33.1 (30.3 to 35.8)*	-0.6 (-2.7 to 1.4)	47.0 (26.1 to 75.5)	32.8 (28.3 to 37.2)*	-0.9 (-4.2 to 2.4)
Stage V chronic kidney disease untreated due to other causes	223.0 (142.8 to 317.6)	32.9 (30.3 to 35.6)*	-0.8 (-2.8 to 1.2)	112.2 (65.0 to 176.3)	33.0 (27.6 to 38.7)*	-0.7 (-4.7 to 3.5)
End-stage chronic kidney disease due to other causes aggregate	70.0 (46.2 to 97.3)	24.8 (20.6 to 29.0)*	-6.8 (-10.0 to -3.7)*	18.4 (10.9 to 28.0)	24.6 (15.6 to 34.7)*	-7.0 (-13.7 to 0.5)
Urinary diseases and male infertility	41,862.3 (40,592.7 to 43,063.7)	34.2 (32.5 to 36.1)*	0.2 (-1.1 to 1.6)	499.8 (311.4 to 730.3)	30.2 (26.9 to 33.8)*	-2.8 (-5.3 to -0.1)*
Interstitial nephritis and urinary tract infections	138.0 (128.9 to 148.3)	36.2 (35.2 to 37.1)*	1.7 (1.0 to 2.4)*	4.5 (2.8 to 6.8)	36.2 (29.3 to 43.7)*	1.7 (-3.5 to 7.2)
Urolithiasis	30,532.9 (27,362.5 to 34,609.4)	36.1 (33.5 to 38.5)*	1.6 (-0.3 to 3.4)	7.8 (5.0 to 11.5)	39.8 (29.2 to 51.5)*	4.4 (-3.6 to 13.1)
Benign prostatic hyperplasia	13,027.9 (10,462.8 to 16,036.5)	29.9 (26.6 to 33.5)*	-3.0 (-5.5 to -0.4)*	482.8 (298.7 to 706.9)	30.0 (26.6 to 33.6)*	-3.0 (-5.5 to -0.3)*
Male infertility	0.0 (0.0 to 0.0)	0.0 (0.0 to 0.0)	0.0 (0.0 to 0.0)	0.0 (0.0 to 0.0)	0.0 (0.0 to 0.0)	0.0 (0.0 to 0.0)
Other urinary diseases	--	--	--	4.7 (2.9 to 6.7)	33.3 (20.8 to 47.7)*	-0.5 (-9.8 to 10.3)
Gynecological diseases	30,580.2 (29,786.8 to 31,433.9)	27.4 (25.7 to 29.3)*	-4.9 (-6.2 to -3.4)*	245.3 (146.4 to 397.9)	25.7 (22.8 to 27.9)*	-6.1 (-8.3 to -4.5)*
Uterine fibroids	10,676.4 (9,977.5 to 11,375.6)	32.8 (32.4 to 33.1)*	-0.9 (-1.2 to -0.6)*	176.3 (108.1 to 280.2)	26.2 (22.4 to 29.2)*	-5.8 (-8.6 to -3.5)*
Uterine fibroids cases aggregate	7,075.3 (6,439.5 to 7,740.7)	35.2 (33.2 to 37.1)*	0.9 (-0.6 to 2.4)	39.2 (18.5 to 73.2)	37.4 (33.4 to 41.2)*	2.6 (-0.5 to 5.4)
Mild abdominal pain with anemia due to uterine fibroids aggregate	3,601.1 (3,465.5 to 3,719.8)	28.2 (24.7 to 31.8)*	-4.3 (-6.9 to -1.6)*	137.0 (88.5 to 206.1)	23.3 (19.0 to 27.4)*	-7.9 (-11.2 to -4.9)*
Polycystic ovarian syndrome	0.0 (0.0 to 0.0)	0.0 (0.0 to 0.0)	0.0 (0.0 to 0.0)	0.0 (0.0 to 0.0)	0.0 (0.0 to 0.0)	0.0 (0.0 to 0.0)
Polycystic ovarian syndrome cases aggregate	0.0 (0.0 to 0.0)	0.0 (0.0 to 0.0)	0.0 (0.0 to 0.0)	0.0 (0.0 to 0.0)	0.0 (0.0 to 0.0)	0.0 (0.0 to 0.0)
Hirsutism and infertility due to polycystic ovarian syndrome aggregate	0.0 (0.0 to 0.0)	0.0 (0.0 to 0.0)	0.0 (0.0 to 0.0)	0.0 (0.0 to 0.0)	0.0 (0.0 to 0.0)	0.0 (0.0 to 0.0)

Cause	Prevalence in thousands (95% confidence interval)			YLDs in thousands (95% confidence interval)		
	2015	Percent change in counts between 2005 and 2015	Percent change in rates between 2005 and 2015	2015	Percent change in counts between 2005 and 2015	Percent change in rates between 2005 and 2015
Female infertility	0.0<br>(0.0 to 0.0)	0.0<br>(0.0 to 0.0)	0.0<br>(0.0 to 0.0)	0.0<br>(0.0 to 0.0)	0.0<br>(0.0 to 0.0)	0.0<br>(0.0 to 0.0)
Endometriosis	0.0<br>(0.0 to 0.0)	0.0<br>(0.0 to 0.0)	0.0<br>(0.0 to 0.0)	0.0<br>(0.0 to 0.0)	0.0<br>(0.0 to 0.0)	0.0<br>(0.0 to 0.0)
Endometriosis cases aggregate	0.0<br>(0.0 to 0.0)	0.0<br>(0.0 to 0.0)	0.0<br>(0.0 to 0.0)	0.0<br>(0.0 to 0.0)	0.0<br>(0.0 to 0.0)	0.0<br>(0.0 to 0.0)
Abdominal pain and infertility due to endometriosis aggregate	0.0<br>(0.0 to 0.0)	0.0<br>(0.0 to 0.0)	0.0<br>(0.0 to 0.0)	0.0<br>(0.0 to 0.0)	0.0<br>(0.0 to 0.0)	0.0<br>(0.0 to 0.0)
Genital prolapse	22,177.2<br>(19,399.4 to 25,538.2)	24.3<br>(20.9 to 27.3)*	-7.2<br>(-9.8 to -5.0)*	69.0<br>(33.2 to 123.5)	24.5<br>(21.0 to 27.8)*	-7.1<br>(-9.7 to -4.6)*
Premenstrual syndrome	0.0<br>(0.0 to 0.0)	0.0<br>(0.0 to 0.0)	0.0<br>(0.0 to 0.0)	0.0<br>(0.0 to 0.0)	0.0<br>(0.0 to 0.0)	0.0<br>(0.0 to 0.0)
Other gynecological diseases	0.0<br>(0.0 to 0.0)	0.0<br>(0.0 to 0.0)	0.0<br>(0.0 to 0.0)	0.0<br>(0.0 to 0.0)	0.0<br>(0.0 to 0.0)	0.0<br>(0.0 to 0.0)
Other gynecological diseases cases aggregate	0.0<br>(0.0 to 0.0)	0.0<br>(0.0 to 0.0)	0.0<br>(0.0 to 0.0)	0.0<br>(0.0 to 0.0)	0.0<br>(0.0 to 0.0)	0.0<br>(0.0 to 0.0)
Anemia due to other gynecological diseases aggregate	0.0<br>(0.0 to 0.0)	0.0<br>(0.0 to 0.0)	0.0<br>(0.0 to 0.0)	0.0<br>(0.0 to 0.0)	0.0<br>(0.0 to 0.0)	0.0<br>(0.0 to 0.0)
Hemoglobinopathies and hemolytic anemias	64,182.9<br>(63,073.1 to 65,346.7)	31.2<br>(29.7 to 32.8)*	-2.1<br>(-3.2 to -0.9)*	214.1<br>(145.1 to 309.0)	23.5<br>(18.7 to 28.1)*	-7.8<br>(-11.4 to -4.4)*
Thalassemias	0.4<br>(0.4 to 0.4)	6.5<br>(-0.8 to 13.6)	-20.5<br>(-25.9 to -15.2)*	0.1<br>(0.0 to 0.1)	2.8<br>(-42.4 to 86.4)	-23.3<br>(-57.0 to 39.1)
Beta-thalassemia major cases aggregate	0.3<br>(0.3 to 0.3)	1.3<br>(-7.5 to 10.2)	-24.4<br>(-30.9 to -17.8)*	0.0<br>(0.0 to 0.1)	-2.3<br>(-53.7 to 104.7)	-27.1<br>(-65.4 to 52.8)
Hemoglobin E/beta-thalassemia cases aggregate	0.0<br>(0.0 to 0.0)	5.8<br>(5.1 to 6.6)*	-21.0<br>(-21.5 to -20.5)*	0.0<br>(0.0 to 0.0)	4.8<br>(-1.6 to 12.2)	-21.8<br>(-26.6 to -16.2)*
Hemoglobin H disease cases aggregate	0.0<br>(0.0 to 0.0)	24.9<br>(19.0 to 30.8)*	-6.8<br>(-11.2 to -2.4)*	0.0<br>(0.0 to 0.0)	22.7<br>(15.2 to 30.1)*	-8.4<br>(-14.0 to -2.8)*
Heart failure due to thalassemias aggregate	0.1<br>(0.0 to 0.1)	47.7<br>(43.8 to 51.2)*	10.2<br>(7.3 to 12.9)*	0.0<br>(0.0 to 0.0)	47.6<br>(43.8 to 51.2)*	10.2<br>(7.3 to 12.9)*
Thalassemias trait	13,148.1<br>(12,835.0 to 13,540.4)	34.7<br>(33.9 to 35.4)*	0.6<br>(-0.0 to 1.1)	130.8<br>(87.9 to 188.9)	29.2<br>(22.3 to 36.1)*	-3.6<br>(-8.7 to 1.6)
B-thalassemia trait cases aggregate	10,608.1<br>(10,331.5 to 10,969.8)	33.1<br>(32.1 to 33.9)*	-0.7<br>(-1.4 to -0.1)*	123.9<br>(83.3 to 179.5)	28.7<br>(21.7 to 35.3)*	-4.0<br>(-9.2 to 1.0)
Hemoglobin E trait cases aggregate	2,540.0<br>(2,432.6 to 2,654.5)	42.1<br>(40.8 to 43.5)*	6.1<br>(5.1 to 7.1)*	6.9<br>(4.4 to 10.3)	39.4<br>(24.0 to 57.6)*	4.1<br>(-7.4 to 17.7)
Sickle cell disorders	2.2<br>(2.1 to 2.3)	27.9<br>(23.6 to 32.2)*	-4.6<br>(-7.7 to -1.3)*	0.3<br>(0.2 to 0.5)	27.9<br>(22.0 to 34.2)*	-4.5<br>(-9.0 to 0.2)
Homozygous sickle cell and severe sickle cell/beta-thalassemia cases aggregate	1.8<br>(1.7 to 1.9)	30.6<br>(25.5 to 36.1)*	-2.5<br>(-6.3 to 1.6)	0.3<br>(0.2 to 0.4)	31.2<br>(23.9 to 39.4)*	-2.0<br>(-7.5 to 4.1)
Hemoglobin SC disease cases aggregate	0.2<br>(0.2 to 0.2)	21.7<br>(18.1 to 25.6)*	-9.1<br>(-11.8 to -6.3)*	0.0<br>(0.0 to 0.0)	22.8<br>(19.5 to 25.9)*	-8.3<br>(-10.8 to -6.0)*
Mild sickle cell/beta-thalassemia cases aggregate	0.2<br>(0.2 to 0.2)	11.2<br>(8.3 to 14.4)*	-17.0<br>(-19.2 to -14.6)*	0.0<br>(0.0 to 0.0)	8.4<br>(5.3 to 11.7)*	-19.0<br>(-21.4 to -16.6)*
Sickle cell trait	13,675.0<br>(12,969.3 to 15,064.8)	36.8<br>(35.8 to 37.8)*	2.1<br>(1.4 to 2.9)*	40.7<br>(27.7 to 58.0)	21.6<br>(15.4 to 27.8)*	-9.2<br>(-13.9 to -4.6)*
G6PD deficiency	9,568.6<br>(8,111.8 to 11,099.0)	26.9<br>(20.9 to 32.2)*	-5.3<br>(-9.7 to -1.4)*	1.0<br>(0.7 to 1.5)	24.8<br>(16.8 to 32.8)*	-6.8<br>(-12.8 to -0.9)*
G6PD cases aggregate	9,567.3<br>(8,110.5 to 11,097.7)	26.9<br>(20.9 to 32.1)*	-5.3<br>(-9.7 to -1.4)*	0.9<br>(0.6 to 1.2)	21.4<br>(12.2 to 30.4)*	-9.4<br>(-16.2 to -2.7)*
Heart failure due to G6PD deficiency aggregate	1.3<br>(1.1 to 1.5)	48.1<br>(44.9 to 51.7)*	10.5<br>(8.2 to 13.2)*	0.2<br>(0.1 to 0.2)	48.0<br>(42.5 to 53.8)*	10.5<br>(6.3 to 14.8)*
G6PD trait	31,473.3<br>(29,064.1 to 33,860.7)	29.6<br>(27.4 to 31.5)*	-3.3<br>(-4.9 to -1.8)*	0.9<br>(0.6 to 1.2)	31.5<br>(14.3 to 52.1)*	-1.8<br>(-14.7 to 13.6)
Other hemoglobinopathies and hemolytic anemias	2,211.4<br>(2,168.3 to 2,255.3)	20.0<br>(17.0 to 23.3)*	-10.5<br>(-12.6 to -8.0)*	40.2<br>(27.0 to 59.0)	9.2<br>(3.4 to 14.4)*	-18.5<br>(-22.8 to -14.6)*
Other hemoglobinopathies and hemolytic anemias cases aggregate	2,198.6<br>(2,154.7 to 2,241.2)	19.9<br>(16.9 to 23.2)*	-10.5<br>(-12.7 to -8.0)*	38.2<br>(25.5 to 56.4)	8.2<br>(2.2 to 13.8)*	-19.3<br>(-23.7 to -15.1)*
Heart failure due to other hemoglobinopathies and hemolytic anemias aggregate	12.9<br>(11.1 to 14.9)	38.2<br>(36.3 to 40.0)*	3.1<br>(1.7 to 4.5)*	1.6<br>(1.0 to 2.2)	38.5<br>(29.8 to 47.7)*	3.4<br>(-3.1 to 10.2)
Endocrine, metabolic, blood, and immune disorders	2,144.9<br>(2,080.1 to 2,211.7)	23.8<br>(20.9 to 26.8)*	-7.6<br>(-9.7 to -5.4)*	53.2<br>(36.8 to 73.6)	19.7<br>(14.5 to 25.2)*	-10.6<br>(-14.6 to -6.5)*
Endocrine, metabolic, blood, and immune disorders cases aggregate	606.5<br>(553.0 to 664.2)	31.3<br>(28.5 to 34.2)*	-2.0<br>(-4.1 to 0.1)	23.2<br>(15.8 to 32.1)	31.4<br>(26.0 to 37.5)*	-1.9<br>(-6.0 to 2.7)
Anemia due to endocrine, metabolic, blood, and immune disorders aggregate	1,532.5<br>(1,503.1 to 1,563.7)	21.0<br>(17.2 to 24.8)*	-9.7<br>(-12.5 to -6.8)*	29.2<br>(19.7 to 42.6)	11.6<br>(3.9 to 19.3)*	-16.7<br>(-22.4 to -10.9)*
Heart failure due to endocrine, metabolic, blood, and immune disorders aggregate	5.9<br>(5.1 to 6.8)	28.8<br>(27.1 to 30.4)*	-3.8<br>(-5.1 to -2.6)*	0.7<br>(0.5 to 1.0)	28.8<br>(26.9 to 30.9)*	-3.8<br>(-5.3 to -2.3)*
Musculoskeletal disorders	123,249.7<br>(120,372.3 to 126,026.9)	34.1<br>(32.9 to 35.1)*	0.1<br>(-0.8 to 0.9)	13,978.8<br>(9,912.1 to 18,480.1)	34.3<br>(32.6 to 36.1)*	0.3<br>(-1.0 to 1.6)
Rheumatoid arthritis	2,586.8<br>(2,266.9 to 2,959.9)	34.2<br>(30.3 to 38.1)*	0.2<br>(-2.7 to 3.1)	608.2<br>(416.8 to 829.7)	34.3<br>(30.1 to 38.5)*	0.2<br>(-2.8 to 3.4)
Osteoarthritis	32,229.1<br>(31,057.3 to 33,445.3)	36.3<br>(35.0 to 37.5)*	1.7<br>(0.8 to 2.6)*	1,810.1<br>(1,267.1 to 2,470.1)	40.1<br>(38.2 to 41.9)*	4.6<br>(3.2 to 6.0)*
Osteoarthritis of the hip cases aggregate	3,655.3<br>(3,190.2 to 4,155.5)	35.6<br>(34.2 to 37.0)*	1.2<br>(0.2 to 2.3)*	194.3<br>(135.2 to 273.4)	40.4<br>(37.8 to 43.4)*	4.8<br>(2.9 to 7.0)*
Osteoarthritis of the knee cases aggregate	28,573.8<br>(27,479.7 to 29,694.7)	36.3<br>(34.9 to 37.7)*	1.8<br>(0.7 to 2.8)*	1,615.9<br>(1,122.7 to 2,193.4)	40.0<br>(38.1 to 42.0)*	4.5<br>(3.1 to 6.0)*
Low back and neck pain	74,200.0<br>(71,572.3 to 76,650.7)	31.7<br>(30.0 to 33.4)*	-1.7<br>(-3.0 to -0.4)*	8,687.8<br>(6,100.6 to 11,898.4)	31.5<br>(29.5 to 33.3)*	-1.8<br>(-3.3 to -0.5)*
Low back pain	47,564.7<br>(42,528.6 to 53,282.4)	30.3<br>(28.0 to 32.5)*	-2.7<br>(-4.5 to -1.1)*	5,233.5<br>(3,674.1 to 7,169.9)	30.5<br>(28.1 to 32.6)*	-2.6<br>(-4.3 to -1.0)*
Neck pain	35,723.1<br>(25,860.3 to 47,205.3)	33.0<br>(30.0 to 35.8)*	-0.7<br>(-2.9 to 1.4)	3,454.2<br>(2,206.1 to 5,177.3)	33.1<br>(30.1 to 36.0)*	-0.7<br>(-2.9 to 1.5)
Gout	4,854.2<br>(3,929.8 to 5,954.5)	34.1<br>(33.0 to 35.3)*	0.1<br>(-0.7 to 1.0)	154.5<br>(102.2 to 220.1)	34.3<br>(30.8 to 37.7)*	0.2<br>(-2.3 to 2.8)
Asymptomatic gout	4,336.6<br>(3,495.7 to 5,319.6)	34.1<br>(33.0 to 35.3)*	0.1<br>(-0.7 to 1.0)	0.0<br>(0.0 to 0.0)	0.0<br>(0.0 to 0.0)	0.0<br>(0.0 to 0.0)
Symptomatic gout cases aggregate	517.6<br>(404.0 to 651.1)	34.1<br>(33.0 to 35.3)*	0.1<br>(-0.7 to 1.0)	154.5<br>(102.2 to 220.1)	34.3<br>(30.8 to 37.7)*	0.2<br>(-2.3 to 2.8)
Other musculoskeletal disorders	29,682.4<br>(22,618.5 to 37,052.5)	39.9<br>(33.0 to 46.1)*	4.5<br>(-0.7 to 9.0)	2,718.1<br>(1,734.1 to 3,946.9)	40.2<br>(33.1 to 46.3)*	4.7<br>(-0.6 to 9.2)
Other non-communicable diseases	287,972.6<br>(285,457.2 to 290,071.0)	34.5<br>(34.2 to 34.9)*	0.4<br>(0.2 to 0.7)*	9,575.6<br>(6,480.6 to 13,866.1)	35.8<br>(34.4 to 37.2)*	1.4<br>(0.4 to 2.4)*
Congenital anomalies	3,957.7<br>(3,623.9 to 4,209.3)	49.5<br>(44.2 to 54.7)*	11.6<br>(7.6 to 15.5)*	361.7<br>(227.6 to 545.6)	51.8<br>(42.5 to 62.6)*	13.3<br>(6.4 to 21.4)*
Neural tube defects	38.4<br>(26.9 to 54.8)	46.3<br>(42.6 to 50.2)*	9.2<br>(6.4 to 12.1)*	12.3<br>(7.0 to 20.1)	47.7<br>(38.8 to 56.2)*	10.2<br>(3.6 to 16.6)*
Congenital heart anomalies	2,195.3<br>(1,535.1 to 3,254.7)	59.2<br>(52.2 to 65.1)*	18.8<br>(13.6 to 23.3)*	73.8<br>(28.6 to 135.7)	58.8<br>(52.0 to 65.1)*	18.5<br>(13.4 to 23.3)*
Less severe heart anomalies cases aggregate	2,014.1<br>(1,348.4 to 3,069.9)	59.5<br>(51.8 to 65.9)*	19.1<br>(13.3 to 23.8)*	63.2<br>(23.9 to 121.1)	59.5<br>(51.7 to 66.3)*	19.1<br>(13.2 to 24.2)*
Severe congenital heart anomalies	177.6<br>(117.5 to 266.6)	55.4<br>(46.8 to 63.8)*	16.0<br>(9.6 to 22.3)*	10.2<br>(3.9 to 20.1)	55.3<br>(44.9 to 65.0)*	15.9<br>(8.2 to 23.1)*
Critical congenital heart anomalies	0.5<br>(0.3 to 0.9)	64.6<br>(37.8 to 90.5)*	22.9<br>(2.9 to 42.2)*	0.0<br>(0.0 to 0.1)	64.7<br>(37.8 to 90.6)*	23.0<br>(2.9 to 42.3)*
Heart failure due to congenital heart anomalies aggregate	3.1<br>(2.7 to 3.6)	30.3<br>(28.7 to 31.8)*	-2.8<br>(-3.9 to -1.6)*	0.4<br>(0.3 to 0.6)	30.2<br>(28.7 to 31.8)*	-2.8<br>(-3.9 to -1.6)*
Cleft lip and cleft palate	305.5<br>(177.9 to 502.8)	45.6<br>(28.9 to 58.5)*	8.7<br>(-3.8 to 18.3)	3.1<br>(1.5 to 5.4)	38.7<br>(20.2 to 53.2)*	3.5<br>(-10.3 to 14.3)
Down syndrome	176.4<br>(114.5 to 262.6)	43.3<br>(35.6 to 50.1)*	7.0<br>(1.2 to 12.1)*	29.1<br>(16.9 to 46.6)	43.3<br>(35.4 to 50.1)*	6.9<br>(1.1 to 12.0)*
Turner syndrome	15.8<br>(7.4 to 28.5)	36.5<br>(33.5 to 39.9)*	1.9<br>(-0.3 to 4.4)	0.2<br>(0.1 to 0.5)	36.2<br>(29.2 to 43.6)*	1.7<br>(-3.6 to 7.2)
Klinefelter syndrome	9.6<br>(5.0 to 17.2)	32.3<br>(29.2 to 34.6)*	-1.2<br>(-3.6 to 0.5)	0.0<br>(0.0 to 0.1)	32.3<br>(29.2 to 34.6)*	-1.2<br>(-3.6 to 0.5)
Other chromosomal abnormalities	166.1<br>(94.3 to 287.4)	33.6<br>(24.8 to 41.7)*	-0.3<br>(-6.8 to 5.8)	27.5<br>(14.7 to 50.6)	34.3<br>(25.3 to 42.5)*	0.2<br>(-6.4 to 6.4)
Other congenital anomalies	1,370.8<br>(745.3 to 2,522.3)	37.2<br>(33.7 to 40.8)*	2.4<br>(-0.2 to 5.1)	215.7<br>(125.8 to 343.7)	53.8<br>(40.9 to 70.6)*	14.8<br>(5.2 to 27.4)*
Skin and subcutaneous diseases	94,717.1<br>(92,967.2 to 96,243.0)	35.6<br>(34.8 to 36.5)*	1.3<br>(0.6 to 1.9)*	1,771.2<br>(1,191.9 to 2,541.4)	34.4<br>(33.3 to 35.5)*	0.3<br>(-0.5 to 1.1)
Dermatitis	11,896.9<br>(9,413.9 to 14,763.3)	32.0<br>(31.6 to 32.3)*	-1.5<br>(-1.7 to -1.2)*	345.7<br>(217.0 to 497.8)	31.9<br>(30.4 to 33.3)*	-1.5<br>(-2.7 to -0.5)*
Eczema cases aggregate	2,145.2<br>(1,610.1 to 2,767.2)	32.1<br>(31.3 to 32.9)*	-1.4<br>(-2.0 to -0.8)*	126.2<br>(75.9 to 185.6)	32.2<br>(29.2 to 35.2)*	-1.3<br>(-3.6 to 0.9)

Cause	Prevalence in thousands (95% confidence interval)			YLDs in thousands (95% confidence interval)		
	2015	Percent change in counts between 2005 and 2015	Percent change in rates between 2005 and 2015	2015	Percent change in counts between 2005 and 2015	Percent change in rates between 2005 and 2015
Contact dermatitis cases aggregate	6,500.8 (4,318.9 to 9,249.9)	31.2 (30.7 to 31.7)*	-2.1 (-2.5 to -1.7)*	171.8 (94.2 to 281.3)	31.3 (29.6 to 33.0)*	-2.0 (-3.3 to -0.7)*
Seborrhoeic dermatitis cases aggregate	3,250.8 (2,247.1 to 4,553.4)	33.4 (33.0 to 33.8)*	-0.4 (-0.7 to -0.1)*	47.6 (24.6 to 81.4)	33.5 (31.8 to 35.2)*	-0.3 (-1.6 to 1.0)
Psoriasis	5,902.5 (5,407.5 to 6,423.4)	32.6 (31.1 to 34.0)*	-1.0 (-2.1 to 0.0)	466.8 (324.8 to 634.5)	32.7 (30.5 to 35.1)*	-0.9 (-2.6 to 0.8)
Cellulitis	58.0 (44.2 to 74.8)	36.7 (30.4 to 43.6)*	2.1 (-2.7 to 7.2)	4.1 (2.4 to 6.2)	36.5 (25.9 to 47.8)*	1.9 (-6.1 to 10.4)
Pyoderma	288.2 (250.4 to 326.5)	35.9 (32.3 to 39.5)*	1.5 (-1.2 to 4.2)	1.6 (0.6 to 3.4)	36.2 (30.4 to 42.1)*	1.7 (-2.6 to 6.1)
Scabies	5,370.2 (3,634.9 to 7,914.8)	39.9 (33.0 to 46.5)*	4.4 (-0.7 to 9.3)	135.4 (66.0 to 233.6)	40.0 (33.0 to 47.0)*	4.5 (-0.7 to 9.7)
Fungal skin diseases	17,970.2 (15,295.1 to 21,127.1)	36.2 (35.9 to 36.5)*	1.7 (1.4 to 1.9)*	99.7 (40.9 to 210.2)	36.3 (35.6 to 36.9)*	1.8 (1.3 to 2.2)*
Viral skin diseases	5,523.0 (4,684.7 to 6,613.5)	33.7 (33.0 to 34.4)*	-0.2 (-0.7 to 0.3)	166.0 (102.7 to 255.4)	33.9 (32.4 to 35.4)*	-0.1 (-1.2 to 1.0)
Molluscum contagiosum cases aggregate	177.4 (137.7 to 222.8)	32.7 (32.2 to 33.2)*	-0.9 (-1.3 to -0.5)*	5.3 (3.2 to 8.2)	32.8 (25.9 to 40.6)*	-0.9 (-6.0 to 4.9)
Viral warts cases aggregate	5,345.6 (4,502.4 to 6,442.1)	33.8 (33.0 to 34.5)*	-0.1 (-0.7 to 0.4)	160.7 (99.0 to 247.5)	33.9 (32.4 to 35.4)*	-0.0 (-1.2 to 1.1)
Acne vulgaris	4,955.3 (4,259.8 to 5,764.7)	35.8 (32.5 to 39.1)*	1.4 (-1.1 to 3.8)	52.3 (23.9 to 97.1)	35.8 (32.5 to 39.4)*	1.4 (-1.1 to 4.0)
Alopecia areata	1,054.7 (895.2 to 1,224.5)	30.8 (30.1 to 31.4)*	-2.4 (-2.9 to -1.9)*	34.9 (21.2 to 53.4)	30.8 (28.0 to 33.5)*	-2.4 (-4.5 to -0.3)*
Pruritus	4,546.7 (3,156.8 to 6,345.0)	34.5 (30.4 to 39.0)*	0.4 (-2.7 to 3.8)	47.8 (21.5 to 93.9)	34.6 (30.4 to 39.4)*	0.5 (-2.6 to 4.0)
Urticaria	2,118.3 (1,489.3 to 2,974.9)	35.2 (34.9 to 35.5)*	0.9 (0.7 to 1.2)*	124.1 (71.0 to 203.9)	35.3 (33.0 to 37.7)*	1.0 (-0.7 to 2.8)
Decubitus ulcer	61.4 (45.9 to 79.8)	37.5 (31.2 to 43.2)*	2.6 (-2.1 to 6.9)	9.4 (5.9 to 13.9)	37.7 (26.0 to 50.5)*	2.8 (-6.0 to 12.3)
Other skin and subcutaneous diseases	48,494.4 (44,097.3 to 52,716.1)	37.1 (34.8 to 39.5)*	2.4 (0.6 to 4.1)*	283.2 (133.1 to 518.3)	37.2 (34.9 to 39.6)*	2.4 (0.7 to 4.2)*
Sense organ diseases	184,656.4 (181,558.7 to 188,131.9)	35.9 (35.2 to 36.6)*	1.5 (0.9 to 2.0)*	5,893.8 (3,960.6 to 8,654.5)	35.9 (33.9 to 37.8)*	1.5 (-0.1 to 2.9)
Glaucoma	830.1 (597.4 to 1,097.6)	40.6 (37.2 to 44.0)*	5.0 (2.4 to 7.5)*	66.7 (41.1 to 99.1)	39.5 (34.8 to 44.2)*	4.1 (0.6 to 7.7)*
Cataract	4,777.5 (3,737.1 to 6,030.1)	30.3 (26.4 to 33.8)*	-2.7 (-5.6 to -0.1)*	287.9 (193.5 to 416.6)	27.8 (24.7 to 30.8)*	-4.6 (-6.9 to -2.3)*
Macular degeneration	605.2 (429.4 to 838.3)	47.0 (43.6 to 50.6)*	9.8 (7.2 to 12.4)*	40.7 (25.5 to 60.5)	43.6 (38.1 to 49.3)*	7.2 (3.1 to 11.5)*
Refraction and accommodation disorders	83,468.8 (76,609.3 to 90,871.1)	36.4 (32.8 to 39.8)*	1.8 (-0.9 to 4.4)	1,322.6 (810.9 to 2,153.6)	35.1 (32.5 to 37.7)*	0.9 (-1.1 to 2.8)
Age-related and other hearing loss	133,312.5 (116,780.9 to 147,837.3)	36.6 (35.6 to 37.8)*	2.0 (1.2 to 2.9)*	3,441.1 (2,185.1 to 5,144.0)	36.8 (33.6 to 39.9)*	2.1 (-0.3 to 4.4)
Other vision loss	1,688.4 (1,275.3 to 2,181.9)	45.3 (40.7 to 49.9)*	8.5 (5.0 to 11.9)*	130.6 (83.4 to 189.7)	43.1 (38.9 to 47.7)*	6.8 (3.7 to 10.2)*
Other sense organ diseases	24,193.2 (21,728.2 to 27,039.7)	34.7 (32.0 to 37.3)*	0.5 (-1.4 to 2.5)	604.2 (367.4 to 881.0)	34.8 (31.9 to 37.6)*	0.6 (-1.5 to 2.7)
Acute other sense organ diseases	56.9 (50.7 to 63.7)	35.1 (32.4 to 38.0)*	0.8 (-1.2 to 3.0)	1.5 (0.9 to 2.3)	35.1 (32.4 to 38.0)*	0.8 (-1.2 to 3.0)
Chronic other sense organ diseases	24,136.3 (21,672.9 to 26,981.6)	34.7 (32.0 to 37.3)*	0.5 (-1.5 to 2.5)	602.7 (366.5 to 878.7)	34.8 (31.9 to 37.6)*	0.6 (-1.5 to 2.7)
Oral disorders	181,483.8 (175,247.7 to 186,669.3)	34.0 (32.5 to 35.5)*	-0.0 (-1.1 to 1.1)	1,548.9 (950.4 to 2,359.4)	33.7 (33.2 to 34.3)*	-0.2 (-0.5 to 0.2)
Deciduous caries	0.0 (0.0 to 0.0)	0.0 (0.0 to 0.0)	0.0 (0.0 to 0.0)	0.0 (0.0 to 0.0)	0.0 (0.0 to 0.0)	0.0 (0.0 to 0.0)
Permanent caries	127,343.8 (105,782.8 to 151,892.7)	33.6 (31.6 to 35.7)*	-0.3 (-1.8 to 1.3)	90.2 (39.2 to 174.0)	30.7 (28.3 to 33.0)*	-2.5 (-4.2 to -0.7)*
Periodontal diseases	62,508.0 (50,382.2 to 74,874.4)	35.1 (34.2 to 35.8)*	0.8 (0.2 to 1.4)*	407.9 (158.1 to 840.5)	35.2 (34.2 to 35.9)*	0.9 (0.2 to 1.5)*
Edentulism and severe tooth loss	28,692.9 (26,469.6 to 30,847.2)	33.3 (32.7 to 33.8)*	-0.5 (-0.9 to -0.1)*	805.6 (529.9 to 1,135.5)	33.3 (32.6 to 34.1)*	-0.5 (-1.1 to 0.1)
Other oral disorders	8,504.5 (7,610.8 to 9,479.2)	33.8 (33.0 to 34.5)*	-0.1 (-0.7 to 0.4)	245.2 (148.5 to 363.7)	33.9 (32.7 to 35.1)*	-0.0 (-0.9 to 0.8)
Injuries	78,542.8 (75,373.5 to 82,155.3)	30.8 (29.2 to 32.5)*	-2.4 (-3.6 to -1.1)*	3,034.9 (2,164.6 to 4,125.9)	19.6 (16.0 to 23.1)*	-10.7 (-13.4 to -8.1)*
Transport injuries	11,439.5 (10,798.6 to 12,293.9)	37.4 (36.7 to 38.0)*	2.5 (2.1 to 3.0)*	624.2 (435.6 to 852.4)	26.1 (21.9 to 30.0)*	-5.9 (-9.0 to -2.9)*
Road injuries	10,709.4 (10,051.6 to 11,551.5)	37.8 (37.1 to 38.5)*	2.8 (2.3 to 3.4)*	581.9 (403.6 to 800.3)	27.2 (23.2 to 31.0)*	-5.1 (-8.0 to -2.2)*
Pedestrian road injuries	1,389.6 (1,226.8 to 1,600.3)	36.2 (35.1 to 37.5)*	1.7 (0.9 to 2.6)*	75.2 (51.8 to 104.6)	21.9 (16.9 to 26.6)*	-9.0 (-12.7 to -5.5)*
Cyclist road injuries	1,770.5 (1,520.9 to 1,999.7)	36.6 (34.9 to 38.0)*	2.0 (0.7 to 3.0)*	90.4 (60.4 to 126.5)	23.9 (18.3 to 28.8)*	-7.5 (-11.7 to -3.9)*
Motorcyclist road injuries	2,706.6 (2,371.5 to 3,039.1)	46.2 (44.7 to 47.7)*	9.1 (8.0 to 10.3)*	140.9 (96.3 to 197.4)	31.0 (25.5 to 36.2)*	-2.2 (-6.3 to 1.7)
Motor vehicle road injuries	4,447.4 (3,898.6 to 5,080.9)	32.3 (31.0 to 33.5)*	-1.2 (-2.2 to -0.3)*	253.6 (171.5 to 352.5)	26.1 (22.9 to 29.2)*	-5.8 (-8.3 to -3.6)*
Other road injuries	395.3 (329.0 to 463.4)	61.6 (60.1 to 63.1)*	20.6 (19.5 to 21.8)*	21.9 (14.9 to 30.6)	53.1 (48.6 to 57.3)*	14.3 (11.0 to 17.4)*
Other transport injuries	730.1 (647.0 to 821.4)	31.8 (31.1 to 32.4)*	-1.6 (-2.1 to -1.1)*	42.2 (29.6 to 57.3)	12.4 (7.3 to 18.0)*	-16.1 (-19.9 to -11.9)*
Unintentional injuries	59,062.2 (56,600.7 to 61,832.6)	26.3 (25.9 to 26.8)*	-5.7 (-6.0 to -5.4)*	2,162.7 (1,503.9 to 2,998.5)	16.3 (12.5 to 19.7)*	-13.2 (-16.0 to -10.7)*
Falls	17,639.2 (16,019.8 to 19,278.8)	35.9 (35.2 to 36.6)*	1.5 (1.0 to 1.9)*	889.7 (620.5 to 1,214.3)	20.0 (14.5 to 25.4)*	-10.4 (-14.5 to -6.4)*
Drowning	352.4 (301.8 to 403.6)	20.1 (19.3 to 20.8)*	-10.4 (-11.0 to -9.9)*	19.7 (13.3 to 26.8)	3.3 (-1.5 to 8.2)	-22.9 (-26.5 to -19.2)*
Fire, heat, and hot substances	5,564.4 (4,945.4 to 6,252.8)	21.6 (21.1 to 22.3)*	-9.2 (-9.6 to -8.7)*	158.4 (109.5 to 224.0)	15.0 (10.7 to 18.5)*	-14.1 (-17.4 to -11.6)*
Poisonings	327.7 (285.4 to 381.4)	27.6 (26.5 to 28.7)*	-4.8 (-5.5 to -3.9)*	26.1 (18.0 to 36.6)	21.2 (17.7 to 24.4)*	-9.5 (-12.1 to -7.1)*
Exposure to mechanical forces	13,004.7 (11,975.5 to 14,022.1)	29.4 (28.8 to 30.0)*	-3.4 (-3.9 to -2.9)*	242.9 (168.2 to 342.3)	20.3 (16.9 to 23.3)*	-10.2 (-12.8 to -7.9)*
Unintentional firearm injuries	189.6 (165.6 to 217.1)	26.1 (25.2 to 27.1)*	-5.9 (-6.5 to -5.1)*	6.8 (4.7 to 9.5)	17.8 (13.9 to 21.1)*	-12.1 (-15.0 to -9.6)*
Unintentional suffocation	650.3 (547.6 to 779.1)	26.0 (22.7 to 29.2)*	-6.0 (-8.4 to -3.6)*	24.5 (17.0 to 33.2)	27.6 (25.0 to 30.0)*	-4.8 (-6.7 to -3.0)*
Other exposure to mechanical forces	12,164.7 (11,127.2 to 13,191.4)	29.7 (29.0 to 30.2)*	-3.2 (-3.7 to -2.8)*	211.6 (145.4 to 301.9)	19.6 (15.8 to 22.9)*	-10.7 (-13.6 to -8.3)*
Adverse effects of medical treatment	696.8 (517.2 to 909.0)	6.3 (0.9 to 10.8)*	-20.7 (-24.7 to -17.3)*	92.8 (57.1 to 142.0)	6.3 (0.9 to 10.8)*	-20.7 (-24.7 to -17.3)*
Animal contact	2,296.1 (2,084.2 to 2,524.2)	23.9 (22.7 to 25.0)*	-7.5 (-8.4 to -6.7)*	58.1 (40.5 to 79.2)	19.0 (16.5 to 21.6)*	-11.2 (-13.0 to -9.2)*
Venomous animal contact	1,036.6 (923.7 to 1,150.8)	30.4 (29.5 to 31.4)*	-2.6 (-3.3 to -1.9)*	41.9 (29.0 to 56.6)	20.9 (18.2 to 24.1)*	-9.7 (-11.8 to -7.4)*
Non-venomous animal contact	1,259.6 (1,103.8 to 1,436.8)	19.0 (17.4 to 20.5)*	-11.2 (-12.4 to -10.1)*	16.3 (10.4 to 24.6)	14.2 (10.7 to 16.9)*	-14.8 (-17.4 to -12.7)*
Foreign body	1,956.8 (1,751.7 to 2,159.4)	32.3 (31.3 to 33.3)*	-1.2 (-2.0 to -0.5)*	66.6 (47.1 to 92.1)	18.2 (13.2 to 23.2)*	-11.8 (-15.5 to -8.1)*
Pulmonary aspiration and foreign body in airway	814.3 (707.9 to 973.6)	37.7 (35.4 to 40.0)*	2.8 (1.1 to 4.5)*	37.7 (26.3 to 51.6)	16.7 (10.8 to 23.2)*	-12.9 (-17.3 to -8.1)*
Foreign body in eyes	48.9 (23.8 to 82.2)	33.6 (32.2 to 35.3)*	-0.3 (-1.3 to 1.0)	2.7 (1.2 to 5.0)	31.6 (29.8 to 33.2)*	-1.8 (-3.1 to -0.6)*
Foreign body in other body part	1,093.6 (951.5 to 1,244.8)	28.5 (27.9 to 29.2)*	-4.1 (-4.6 to -3.5)*	26.2 (18.0 to 37.1)	19.1 (15.4 to 22.6)*	-11.1 (-13.8 to -8.5)*
Environmental heat and cold exposure	4,799.2 (4,304.8 to 5,456.2)	26.3 (25.8 to 26.7)*	-5.7 (-6.1 to -5.4)*	191.1 (131.5 to 263.8)	15.0 (11.2 to 18.6)*	-14.2 (-17.0 to -11.5)*
Other unintentional injuries	12,424.9 (11,233.7 to 13,780.4)	14.9 (14.2 to 15.5)*	-14.2 (-14.7 to -13.7)*	417.3 (282.5 to 590.0)	9.9 (7.9 to 11.6)*	-18.0 (-19.5 to -16.7)*
Self-harm and interpersonal violence	2,222.7 (2,052.1 to 2,390.0)	26.5 (26.0 to 27.1)*	-5.5 (-5.9 to -5.2)*	90.8 (63.7 to 122.1)	13.0 (8.7 to 17.5)*	-15.7 (-18.9 to -12.3)*
Self-harm	590.4 (523.4 to 668.7)	21.5 (20.8 to 22.2)*	-9.3 (-9.8 to -8.8)*	27.0 (19.0 to 36.6)	8.6 (4.6 to 12.7)*	-18.9 (-21.9 to -15.9)*
Interpersonal violence	1,632.2 (1,480.9 to 1,801.6)	28.5 (27.9 to 29.0)*	-4.1 (-4.5 to -3.7)*	63.7 (43.8 to 87.0)	14.9 (10.3 to 19.7)*	-14.2 (-17.7 to -10.6)*

Cause	Prevalence in thousands (95% confidence interval)			YLDs in thousands (95% confidence interval)		
	2015	Percent change in counts between 2005 and 2015	Percent change in rates between 2005 and 2015	2015	Percent change in counts between 2005 and 2015	Percent change in rates between 2005 and 2015
Assault by firearm	100.7 (86.7 to 116.4)	28.3 (27.0 to 29.4)*	-4.3 (-5.2 to -3.4)*	4.2 (2.8 to 5.8)	17.2 (13.0 to 21.1)*	-12.5 (-15.6 to -9.6)*
Assault by sharp object	362.2 (299.9 to 423.4)	28.7 (27.9 to 29.5)*	-3.9 (-4.5 to -3.3)*	9.4 (6.3 to 13.1)	8.6 (1.6 to 15.1)*	-18.9 (-24.2 to -14.1)*
Assault by other means	1,169.3 (1,042.6 to 1,317.8)	28.4 (27.7 to 29.0)*	-4.2 (-4.7 to -3.7)*	50.1 (34.4 to 68.6)	16.0 (11.7 to 20.5)*	-13.4 (-16.6 to -10.0)*
Forces of nature, war, and legal intervention	5,818.4 (4,098.4 to 7,940.2)	80.2 (51.5 to 109.5)*	34.5 (13.1 to 56.4)*	157.2 (111.3 to 210.7)	54.4 (20.0 to 89.5)*	15.2 (-10.5 to 41.5)
Exposure to forces of nature	1,411.6 (841.4 to 2,245.3)	44.5 (4.0 to 94.8)*	7.9 (-22.4 to 45.5)	43.2 (28.4 to 61.1)	-0.5 (-39.2 to 45.8)	-25.7 (-54.6 to 8.8)
Collective violence and legal intervention	4,406.7 (2,976.5 to 6,042.5)	95.6 (63.5 to 132.4)*	46.0 (22.1 to 73.5)*	114.0 (74.9 to 161.3)	95.2 (58.9 to 136.1)*	45.7 (18.6 to 76.2)*
**Anemia**	**88,958.1 (87,787.5 to 90,106.4)**	**21.8 (19.9 to 24.0)***	**-9.1 (-10.5 to -7.5)***	**2,342.2 (1,587.3 to 3,339.4)**	**15.9 (12.9 to 19.2)***	**-13.5 (-15.7 to -11.0)***
**Developmental intellectual disability**	**5,062.9 (3,760.5 to 6,328.0)**	**37.3 (36.7 to 38.0)***	**2.5 (2.0 to 3.0)***	**611.4 (441.1 to 802.9)**	**39.9 (36.8 to 43.4)***	**4.4 (2.1 to 7.0)***
**Epilepsy**	**1,476.1 (1,268.9 to 1,713.1)**	**37.9 (32.6 to 43.2)***	**2.9 (-1.0 to 6.9)**	**402.2 (287.0 to 527.1)**	**25.7 (16.8 to 33.9)***	**-6.2 (-12.8 to -0.0)***
**Guillain-Barré syndrome**	**2.5 (1.6 to 3.8)**	**31.0 (28.8 to 32.8)***	**-2.2 (-3.9 to -0.8)***	**0.7 (0.4 to 1.3)**	**31.0 (28.8 to 32.8)***	**-2.2 (-3.9 to -0.8)***
**Hearing loss**	**138,282.4 (121,955.0 to 152,829.9)**	**36.2 (35.2 to 37.3)***	**1.7 (0.9 to 2.5)***	**3,649.4 (2,352.0 to 5,405.1)**	**36.2 (33.2 to 39.1)***	**1.7 (-0.6 to 3.9)**
**Heart failure**	**2,741.5 (2,587.8 to 2,886.6)**	**36.0 (35.2 to 36.8)***	**1.5 (0.9 to 2.1)***	**406.3 (285.4 to 543.9)**	**37.0 (35.2 to 38.7)***	**2.3 (0.9 to 3.6)***
**Infertility**	**0.0 (0.0 to 0.0)**	**0.0 (0.0 to 0.0)**	**0.0 (0.0 to 0.0)**	**0.0 (0.0 to 0.0)**	**0.0 (0.0 to 0.0)**	**0.0 (0.0 to 0.0)**
**Pelvic inflammatory disease**	**22.9 (17.2 to 30.0)**	**17.9 (16.2 to 19.4)***	**-12.0 (-13.2 to -10.9)***	**3.0 (1.8 to 4.6)**	**17.9 (8.9 to 26.5)***	**-12.0 (-18.7 to -5.5)***
**Vision loss**	**92,757.0 (84,944.8 to 100,761.6)**	**36.1 (32.8 to 39.2)***	**1.6 (-0.8 to 3.9)**	**2,003.9 (1,321.7 to 3,017.4)**	**34.4 (32.3 to 36.5)***	**0.3 (-1.2 to 1.9)**

SR Table 11. Prevalent cases and YLDs for 2015, percent change, and change in age-standardised rates between 2005 and 2015 by cause, global, ages 60-64 years

Cause	Prevalence in thousands (95% confidence interval)			YLDs in thousands (95% confidence interval)		
	2015	Percent change in counts between 2005 and 2015	Percent change in rates between 2005 and 2015	2015	Percent change in counts between 2005 and 2015	Percent change in rates between 2005 and 2015
**All causes**	--	--	--	**53,923.3 (40,245.7 to 69,969.8)**	**52.1 (51.2 to 52.9)***	**-2.3 (-2.8 to -1.7)***
Communicable, maternal, neonatal, and nutritional diseases	155,583.1 (153,766.3 to 157,326.7)	46.3 (44.9 to 47.6)*	-6.0 (-6.9 to -5.2)*	2,570.6 (1,862.4 to 3,535.8)	26.7 (23.1 to 31.1)*	-18.6 (-20.9 to -15.7)*
HIV/AIDS and tuberculosis	1,759.8 (1,684.3 to 1,843.5)	65.1 (56.1 to 74.5)*	6.1 (0.3 to 12.1)*	281.0 (196.9 to 380.2)	25.6 (15.1 to 36.2)*	-19.3 (-26.0 to -12.5)*
Tuberculosis	535.8 (407.4 to 678.8)	30.2 (19.8 to 41.1)*	-16.3 (-23.0 to -9.3)*	157.6 (99.0 to 226.6)	30.8 (20.1 to 41.9)*	-15.9 (-22.8 to -8.8)*
HIV/AIDS	1,229.8 (1,167.3 to 1,301.5)	86.3 (72.0 to 101.6)*	19.7 (10.5 to 29.6)*	123.4 (89.7 to 160.5)	19.5 (2.3 to 39.1)*	-23.2 (-34.3 to -10.6)*
HIV/AIDS - Tuberculosis	34.3 (30.8 to 37.8)	11.8 (6.8 to 17.0)*	-28.2 (-31.4 to -24.8)*	12.3 (8.4 to 16.5)	12.8 (4.7 to 20.9)*	-27.5 (-32.7 to -22.3)*
HIV/AIDS resulting in other diseases	1,228.2 (1,153.6 to 1,317.0)	89.6 (82.0 to 98.3)*	21.8 (17.0 to 27.4)*	111.1 (80.7 to 145.1)	20.3 (1.1 to 42.1)*	-22.7 (-35.0 to -8.7)*
HIV aggregate	405.4 (375.3 to 441.3)	-12.4 (-16.4 to -6.8)*	-43.7 (-46.3 to -40.1)*	28.4 (20.6 to 37.1)	-13.4 (-18.4 to -7.3)*	-44.4 (-47.5 to -40.4)*
AIDS aggregate	822.8 (763.9 to 893.1)	345.1 (297.1 to 402.5)*	186.0 (155.2 to 222.9)*	82.7 (59.6 to 109.8)	38.8 (8.8 to 79.7)*	-10.8 (-30.1 to 15.4)
Diarrhea, lower respiratory, and other common infectious diseases	12,615.2 (11,608.7 to 13,174.4)	48.5 (44.6 to 52.1)*	-4.6 (-7.1 to -2.3)*	400.8 (279.1 to 555.9)	42.0 (39.4 to 44.6)*	-8.8 (-10.4 to -7.1)*
Diarrheal diseases	743.2 (707.8 to 776.4)	34.8 (33.6 to 36.0)*	-13.4 (-14.1 to -12.6)*	112.1 (77.1 to 153.6)	35.4 (32.7 to 38.1)*	-13.0 (-14.7 to -11.3)*
Diarrhoea episodes aggregate	742.9 (707.5 to 776.3)	34.8 (33.6 to 36.0)*	-13.4 (-14.1 to -12.6)*	112.0 (77.1 to 153.5)	35.4 (32.7 to 38.1)*	-13.0 (-14.7 to -11.3)*
Guillain-Barré syndrome due to diarrheal diseases	0.3 (0.2 to 0.5)	53.0 (49.5 to 55.4)*	-1.7 (-3.9 to -0.1)*	0.1 (0.0 to 0.2)	53.0 (49.5 to 55.4)*	-1.7 (-3.9 to -0.1)*
Intestinal infectious diseases	18.5 (16.6 to 20.6)	-5.5 (-15.9 to 6.3)	-39.3 (-46.0 to -31.7)*	3.1 (2.0 to 4.5)	12.8 (-1.7 to 29.2)	-27.5 (-36.8 to -17.0)*
Typhoid fever	20.2 (15.5 to 25.7)	12.5 (5.5 to 18.7)*	-27.7 (-32.2 to -23.7)*	2.6 (1.7 to 3.8)	15.2 (-1.9 to 33.8)	-26.0 (-37.0 to -14.0)*
Typhoid fever episodes aggregate	16.7 (12.7 to 21.3)	12.5 (1.5 to 23.4)*	-27.7 (-34.8 to -20.7)*	1.5 (0.9 to 2.3)	13.2 (-3.7 to 33.3)	-27.3 (-38.1 to -14.4)*
Complications of typhoid fever aggregate	3.5 (2.4 to 4.9)	12.8 (-24.2 to 61.9)	-27.5 (-51.3 to 4.0)	1.1 (0.6 to 1.7)	18.1 (-22.4 to 78.2)	-24.1 (-50.1 to 14.5)
Paratyphoid fever	8.6 (6.1 to 11.4)	9.6 (1.3 to 18.9)*	-29.6 (-34.9 to -23.6)*	0.4 (0.3 to 0.7)	10.5 (-9.2 to 36.9)	-29.0 (-41.7 to -12.1)*
Paratyphoid fever episodes aggregate	8.1 (5.8 to 10.9)	9.5 (0.5 to 19.4)*	-29.6 (-35.4 to -23.3)*	0.4 (0.2 to 0.6)	10.6 (-12.0 to 39.0)	-28.9 (-43.4 to -10.7)*
Intestinal perforation due to paratyphoid	0.4 (0.3 to 0.7)	10.0 (-37.8 to 91.1)	-29.3 (-60.0 to 22.8)	0.1 (0.0 to 0.1)	9.9 (-37.8 to 91.1)	-29.4 (-60.0 to 22.8)
Other intestinal infectious diseases	--	--	--	0.0 (0.0 to 0.1)	-53.8 (-70.2 to -24.7)*	-70.3 (-80.9 to -51.6)*
Lower respiratory infections	302.2 (285.3 to 321.3)	45.4 (43.2 to 47.7)*	-6.6 (-8.0 to -5.1)*	17.6 (12.0 to 24.8)	45.7 (41.1 to 50.6)*	-6.3 (-9.3 to -3.2)*
Lower respiratory infection episodes aggregate	301.9 (284.9 to 321.0)	45.4 (43.2 to 47.6)*	-6.6 (-8.0 to -5.1)*	17.5 (11.9 to 24.7)	45.7 (41.0 to 50.6)*	-6.4 (-9.4 to -3.2)*
Guillain-Barré syndrome due to lower respiratory infections	0.3 (0.2 to 0.6)	53.0 (49.5 to 55.4)*	-1.7 (-3.9 to -0.1)*	0.1 (0.0 to 0.2)	53.0 (49.5 to 55.4)*	-1.7 (-3.9 to -0.1)*
Upper respiratory infections	7,967.4 (5,570.7 to 11,063.7)	55.9 (54.2 to 57.7)*	0.2 (-0.9 to 1.4)	90.8 (46.6 to 166.2)	56.1 (53.8 to 58.4)*	0.3 (-1.1 to 1.8)
Upper respiratory infection episodes aggregate	7,966.5 (5,570.0 to 11,062.5)	55.9 (54.2 to 57.7)*	0.2 (-0.9 to 1.4)	90.5 (46.3 to 165.9)	56.1 (53.8 to 58.4)*	0.3 (-1.1 to 1.8)
Guillain-Barré syndrome due to upper respiratory infections	0.9 (0.6 to 1.5)	53.0 (49.5 to 55.4)*	-1.7 (-3.9 to -0.1)*	0.3 (0.1 to 0.5)	53.0 (49.5 to 55.4)*	-1.7 (-3.9 to -0.1)*
Otitis media	2,875.3 (2,091.7 to 3,953.4)	38.0 (33.7 to 43.2)*	-11.3 (-14.1 to -8.0)*	78.1 (44.7 to 129.1)	37.8 (31.1 to 45.5)*	-11.5 (-15.8 to -6.5)*
Acute otitis media	218.8 (171.7 to 283.8)	51.0 (45.2 to 56.4)*	-3.0 (-6.7 to 0.5)	2.8 (1.4 to 4.9)	51.3 (43.8 to 59.1)*	-2.8 (-7.6 to 2.3)
Chronic otitis media aggregate	2,656.5 (1,879.0 to 3,714.8)	37.1 (32.3 to 42.6)*	-11.9 (-15.0 to -8.4)*	75.3 (42.9 to 125.4)	37.3 (30.5 to 45.3)*	-11.8 (-16.1 to -6.6)*
Meningitis	361.6 (347.4 to 377.1)	27.8 (23.7 to 31.9)*	-17.9 (-20.5 to -15.2)*	62.3 (43.7 to 80.5)	40.6 (36.9 to 44.2)*	-9.7 (-12.0 to -7.3)*
Pneumococcal meningitis	309.9 (269.7 to 361.4)	40.0 (37.7 to 42.5)*	-10.1 (-11.5 to -8.4)*	30.5 (21.7 to 40.2)	41.4 (35.5 to 47.1)*	-9.2 (-12.9 to -5.5)*
Acute pneumococcal meningitis	1.4 (0.9 to 1.9)	43.1 (28.7 to 56.9)*	-8.1 (-17.3 to 0.8)	0.2 (0.1 to 0.3)	43.1 (28.7 to 56.9)*	-8.0 (-17.3 to 0.8)
Complications of pneumococcal meningitis aggregate	308.6 (268.5 to 359.8)	39.9 (37.7 to 42.5)*	-10.1 (-11.5 to -8.5)*	30.4 (21.5 to 40.1)	41.3 (35.5 to 47.1)*	-9.2 (-12.9 to -5.5)*
H influenzae type B meningitis	76.4 (60.7 to 93.9)	6.3 (1.4 to 12.7)*	-31.7 (-34.9 to -27.6)*	8.8 (6.1 to 11.9)	32.8 (25.2 to 40.5)*	-14.7 (-19.5 to -9.7)*
Acute H influenzae type B meningitis	0.4 (0.2 to 0.6)	6.8 (-6.1 to 19.4)	-31.4 (-39.7 to -23.3)*	0.1 (0.0 to 0.1)	6.8 (-6.1 to 19.4)	-31.4 (-39.7 to -23.3)*
Complications of of H influenzae type B meningitis aggregate	76.0 (60.3 to 93.3)	6.3 (1.4 to 12.7)*	-31.7 (-34.9 to -27.6)*	8.8 (6.0 to 11.8)	33.0 (25.3 to 40.8)*	-14.5 (-19.5 to -9.5)*
Meningococcal meningitis	65.5 (52.6 to 80.7)	37.1 (33.7 to 41.0)*	-11.9 (-14.1 to -9.4)*	7.2 (5.0 to 9.7)	42.9 (37.1 to 48.4)*	-8.2 (-11.9 to -4.6)*
Acute meningococcal meningitis	0.5 (0.3 to 0.8)	41.7 (24.8 to 56.9)*	-8.9 (-19.8 to 0.8)	0.1 (0.0 to 0.1)	41.7 (24.8 to 56.9)*	-8.9 (-19.8 to 0.8)
Complications of meningococcal meningitis aggregate	65.0 (52.0 to 80.1)	37.1 (33.7 to 40.9)*	-11.9 (-14.1 to -9.5)*	7.2 (4.9 to 9.6)	42.9 (37.1 to 48.5)*	-8.2 (-11.9 to -4.5)*
Other meningitis	141.7 (116.0 to 168.3)	40.1 (37.4 to 42.8)*	-10.0 (-11.7 to -8.2)*	15.7 (10.9 to 20.8)	42.7 (35.8 to 48.9)*	-8.3 (-12.7 to -4.3)*
Other meningitis episodes aggregate	3.0 (2.3 to 3.8)	45.3 (31.6 to 57.8)*	-6.6 (-15.4 to 1.4)	0.4 (0.2 to 0.6)	46.9 (33.1 to 59.6)*	-5.6 (-14.4 to 2.6)
Complications of other meningitis aggregate	138.6 (113.1 to 165.2)	39.9 (37.2 to 42.8)*	-10.1 (-11.9 to -8.3)*	15.3 (10.6 to 20.3)	42.6 (35.6 to 48.9)*	-8.3 (-12.9 to -4.3)*
Encephalitis	212.4 (140.4 to 303.9)	27.3 (24.3 to 31.4)*	-18.2 (-20.1 to -15.6)*	17.7 (12.1 to 23.3)	34.9 (28.5 to 41.5)*	-13.3 (-17.4 to -9.1)*
Acute encephalitis	2.4 (1.9 to 3.0)	46.4 (39.4 to 52.1)*	-5.9 (-10.4 to -2.3)*	0.3 (0.2 to 0.5)	46.7 (38.3 to 54.7)*	-5.7 (-11.1 to -0.6)*
Complications of encephalitis aggregate	209.6 (137.6 to 300.7)	26.9 (23.8 to 31.1)*	-18.4 (-20.4 to -15.7)*	17.4 (11.8 to 23.0)	34.7 (28.1 to 41.5)*	-13.5 (-17.7 to -9.1)*
Diphtheria	0.0 (0.0 to 0.0)	0.0 (0.0 to 0.0)	0.0 (0.0 to 0.0)	0.0 (0.0 to 0.0)	0.0 (0.0 to 0.0)	0.0 (0.0 to 0.0)
Whooping cough	0.0 (0.0 to 0.0)	0.0 (0.0 to 0.0)	0.0 (0.0 to 0.0)	0.0 (0.0 to 0.0)	0.0 (0.0 to 0.0)	0.0 (0.0 to 0.0)
Tetanus	6.9 (6.7 to 7.3)	41.8 (33.2 to 46.4)*	-8.9 (-14.4 to -5.9)*	0.3 (0.2 to 0.4)	22.9 (5.3 to 38.6)*	-21.0 (-32.4 to -11.0)*
Severe tetanus	0.4 (0.2 to 0.8)	-31.2 (-46.9 to -9.1)*	-55.8 (-65.9 to -41.6)*	0.0 (0.0 to 0.1)	-30.8 (-47.4 to -5.3)*	-55.5 (-66.2 to -39.1)*
Complications of tetanus aggregate	6.6 (6.4 to 6.7)	50.9 (50.2 to 51.6)*	-3.0 (-3.5 to -2.6)*	0.2 (0.2 to 0.3)	46.5 (34.6 to 60.4)*	-5.8 (-13.5 to 3.1)
Measles	0.0 (0.0 to 0.0)	0.0 (0.0 to 0.0)	0.0 (0.0 to 0.0)	0.0 (0.0 to 0.0)	0.0 (0.0 to 0.0)	0.0 (0.0 to 0.0)
Varicella and herpes zoster	364.7 (285.2 to 464.3)	55.2 (52.9 to 57.1)*	-0.3 (-1.8 to 1.0)	18.8 (10.9 to 29.5)	55.5 (50.9 to 59.9)*	-0.1 (-3.1 to 2.7)
Neglected tropical diseases and malaria	60,689.1 (59,167.2 to 62,263.4)	27.3 (23.5 to 31.4)*	-18.2 (-20.6 to -15.6)*	594.8 (398.4 to 888.3)	7.1 (-4.2 to 20.6)	-31.2 (-38.4 to -22.5)*
Malaria	3,514.6 (3,006.0 to 4,071.5)	32.4 (25.7 to 39.5)*	-14.9 (-19.2 to -10.4)*	26.3 (18.7 to 36.8)	20.3 (13.9 to 26.5)*	-22.7 (-26.8 to -18.7)*
Asymptomatic malaria parasitemia(PfPR)	2,606.5 (2,101.3 to 3,154.9)	34.6 (25.5 to 44.0)*	-13.5 (-19.4 to -7.5)*	0.0 (0.0 to 0.0)	0.0 (0.0 to 0.0)	0.0 (0.0 to 0.0)
Malaria episodes aggregate	66.4 (36.1 to 115.5)	-0.3 (-20.0 to 26.5)	-35.9 (-48.6 to -18.7)*	1.3 (0.6 to 2.6)	0.4 (-20.8 to 29.1)	-35.5 (-49.1 to -17.0)*
Complications of malaria aggregate	10.8 (9.6 to 12.0)	42.4 (39.4 to 45.7)*	-8.5 (-10.4 to -6.4)*	3.4 (2.5 to 4.3)	37.2 (26.5 to 48.9)*	-11.9 (-18.7 to -4.3)*
Anemia due to malaria parasitemia(PfPR) aggregate	831.0 (810.8 to 851.0)	29.2 (25.0 to 33.5)*	-17.0 (-19.7 to -14.2)*	21.6 (14.6 to 31.0)	19.4 (11.8 to 26.4)*	-23.3 (-28.1 to -18.8)*
Chagas disease	494.5 (413.4 to 580.6)	8.2 (4.5 to 11.8)*	-30.5 (-32.8 to -28.1)*	6.2 (4.0 to 9.0)	8.4 (2.9 to 14.1)*	-30.4 (-33.9 to -26.7)*
Chagas disease episodes aggregate	397.4 (331.0 to 468.5)	8.2 (4.5 to 11.8)*	-30.5 (-32.9 to -28.2)*	0.0 (0.0 to 0.0)	32.7 (26.7 to 38.6)*	-14.7 (-18.6 to -11.0)*
Complications of Chagas disease aggregate	56.1 (43.3 to 71.0)	8.2 (4.5 to 11.8)*	-30.5 (-32.8 to -28.1)*	4.0 (2.6 to 5.9)	8.3 (1.6 to 15.3)*	-30.4 (-34.7 to -25.9)*

Cause	Prevalence in thousands (95% confidence interval)			YLDs in thousands (95% confidence interval)		
	2015	Percent change in counts between 2005 and 2015	Percent change in rates between 2005 and 2015	2015	Percent change in counts between 2005 and 2015	Percent change in rates between 2005 and 2015
Heart failure due to Chagas disease aggregate	41.1 (25.4 to 59.2)	8.3 (4.5 to 12.1)*	-30.4 (-32.8 to -28.0)*	2.1 (1.1 to 3.5)	8.4 (1.7 to 15.1)*	-30.3 (-34.7 to -26.0)*
Leishmaniasis	162.9 (146.6 to 184.5)	31.8 (11.9 to 54.2)*	-15.3 (-28.1 to -0.9)*	1.8 (0.8 to 3.3)	32.0 (27.0 to 36.7)*	-15.2 (-18.4 to -12.1)*
Visceral leishmaniasis	0.4 (0.4 to 0.5)	38.9 (36.7 to 41.3)*	-10.7 (-12.2 to -9.2)*	0.0 (0.0 to 0.0)	39.4 (6.6 to 82.7)*	-10.4 (-31.5 to 17.4)
Cutaneous and mucocutaneous leishmaniasis	166.9 (144.8 to 197.6)	31.7 (28.7 to 34.4)*	-15.3 (-17.3 to -13.6)*	1.7 (0.8 to 3.3)	31.9 (26.8 to 36.6)*	-15.3 (-18.5 to -12.2)*
African trypanosomiasis	0.3 (0.2 to 0.4)	-70.8 (-72.3 to -69.5)*	-81.2 (-82.2 to -80.4)*	0.1 (0.0 to 0.1)	-69.1 (-84.3 to -50.4)*	-80.1 (-89.9 to -68.1)*
Schistosomiasis	5,826.3 (4,869.2 to 7,431.1)	-25.0 (-33.9 to -6.4)*	-51.8 (-57.5 to -39.9)*	50.7 (26.1 to 95.3)	-23.9 (-31.4 to -5.8)*	-51.1 (-55.9 to -39.5)*
Schistosomiasis episodes aggregate	3,525.7 (2,908.3 to 4,312.9)	-27.4 (-35.9 to -12.2)*	-53.3 (-58.8 to -43.6)*	19.7 (7.9 to 42.4)	-27.1 (-35.7 to -11.0)*	-53.2 (-58.7 to -42.8)*
Complications of schistosomiasis aggregate	1,817.4 (1,246.3 to 2,880.5)	-22.8 (-35.0 to 8.2)	-50.4 (-58.3 to -30.5)*	21.8 (10.3 to 42.6)	-16.8 (-28.7 to 13.7)	-46.5 (-54.2 to -27.0)*
Anemia due to schistosomiasis aggregate	340.7 (330.3 to 351.4)	-29.4 (-32.0 to -26.4)*	-54.6 (-56.3 to -52.7)*	9.2 (6.3 to 13.0)	-31.3 (-35.6 to -26.7)*	-55.8 (-58.6 to -52.9)*
Cysticercosis	110.8 (73.0 to 174.1)	10.1 (3.3 to 16.4)*	-29.3 (-33.6 to -25.2)*	15.5 (9.0 to 26.9)	-3.5 (-11.3 to 3.8)	-38.0 (-43.0 to -33.3)*
Cystic echinococcosis	103.8 (88.9 to 120.7)	76.6 (69.9 to 83.2)*	13.5 (9.2 to 17.7)*	9.2 (6.0 to 12.9)	76.3 (64.3 to 88.8)*	13.3 (5.6 to 21.3)*
Lymphatic filariasis	1,614.5 (1,258.1 to 2,043.5)	-36.7 (-44.7 to -29.7)*	-59.3 (-64.4 to -54.8)*	105.5 (59.3 to 167.5)	-5.8 (-25.6 to 10.5)	-39.5 (-52.2 to -29.0)*
Prevalence of detectable microfiliaria due to lymphatic filariasis	622.1 (536.7 to 724.1)	-59.0 (-62.5 to -54.8)*	-73.7 (-75.9 to -70.9)*	0.0 (0.0 to 0.0)	0.0 (0.0 to 0.0)	0.0 (0.0 to 0.0)
Complications of lymphatic filariasis aggregate	992.3 (656.2 to 1,411.4)	-3.9 (-23.1 to 11.2)	-38.3 (-50.6 to -28.5)*	105.5 (59.3 to 167.5)	-5.8 (-25.6 to 10.5)	-39.5 (-52.2 to -29.0)*
Onchocerciasis	520.3 (379.3 to 726.3)	-21.9 (-34.4 to -5.8)*	-49.8 (-57.8 to -39.5)*	51.9 (27.4 to 93.2)	-17.4 (-34.6 to 2.8)	-46.9 (-58.0 to -33.9)*
Asymptomatic onchocerciasis	13.6 (0.4 to 38.8)	-81.5 (-98.8 to -66.3)*	-88.1 (-99.2 to -78.4)*	0.0 (0.0 to 0.0)	0.0 (0.0 to 0.0)	0.0 (0.0 to 0.0)
Skin disease due to onchocerciasis aggregate	351.8 (217.7 to 552.4)	-22.1 (-39.8 to -1.9)*	-49.9 (-61.3 to -37.0)*	40.4 (17.9 to 78.1)	-21.2 (-45.2 to 3.8)	-49.4 (-64.8 to -33.2)*
Vision loss due to onchocerciasis aggregate	146.2 (97.7 to 211.2)	3.5 (-10.1 to 17.8)	-33.5 (-42.2 to -24.3)*	11.5 (7.0 to 17.9)	-0.6 (-13.1 to 12.6)	-36.1 (-44.2 to -27.7)*
Trachoma	414.3 (265.3 to 626.1)	4.9 (-4.5 to 15.5)	-32.6 (-38.6 to -25.8)*	34.1 (19.0 to 56.7)	-1.0 (-10.0 to 8.4)	-36.4 (-42.2 to -30.4)*
Dengue	145.4 (83.3 to 308.7)	211.8 (27.5 to 746.9)*	100.4 (-18.0 to 444.2)	22.3 (10.5 to 49.7)	208.7 (30.0 to 747.7)*	98.3 (-16.5 to 444.8)
Dengue episodes aggregate	42.4 (28.7 to 86.1)	187.3 (20.3 to 647.2)*	84.6 (-22.7 to 380.2)	2.4 (1.4 to 5.4)	207.7 (21.7 to 748.2)*	97.7 (-21.8 to 445.0)
Post-dengue chronic fatigue syndrome	102.2 (49.8 to 221.2)	212.0 (27.5 to 746.9)*	100.5 (-18.0 to 444.2)	19.9 (8.7 to 45.3)	208.8 (29.7 to 761.9)*	98.4 (-16.7 to 453.8)
Yellow fever	0.0 (0.0 to 0.1)	-24.8 (-42.3 to -2.9)*	-51.7 (-62.9 to -37.6)*	0.0 (0.0 to 0.0)	-24.7 (-42.3 to -2.9)*	-51.6 (-62.9 to -37.6)*
Rabies	0.0 (0.0 to 0.0)	-41.3 (-65.1 to -17.1)*	-62.3 (-77.6 to -46.7)*	0.0 (0.0 to 0.0)	-41.2 (-65.1 to -17.1)*	-62.2 (-77.6 to -46.7)*
Intestinal nematode infections	49,950.0 (48,416.1 to 51,370.0)	29.9 (25.3 to 35.1)*	-16.5 (-19.5 to -13.2)*	91.1 (52.1 to 147.7)	-0.6 (-9.2 to 9.7)	-36.1 (-41.7 to -29.5)*
Ascariasis	26,205.6 (21,905.3 to 32,171.2)	18.7 (-4.2 to 49.3)	-23.7 (-38.4 to -4.1)*	24.2 (13.5 to 41.1)	-21.3 (-35.1 to -5.1)*	-49.4 (-58.3 to -39.0)*
Complications of ascariasis aggregate	1,353.2 (1,199.0 to 1,534.8)	-20.6 (-32.4 to -6.8)*	-49.0 (-56.6 to -40.1)*	24.2 (13.5 to 41.1)	-21.3 (-35.1 to -5.1)*	-49.4 (-58.3 to -39.0)*
Asymptomatic ascariasis	24,816.9 (20,503.1 to 30,826.1)	21.8 (-3.3 to 56.2)	-21.7 (-37.9 to 0.4)	0.0 (0.0 to 0.0)	0.0 (0.0 to 0.0)	0.0 (0.0 to 0.0)
Trichuriasis	15,372.4 (13,672.2 to 17,311.8)	32.0 (12.7 to 55.1)*	-15.2 (-27.6 to -0.3)*	16.3 (8.6 to 28.4)	12.8 (-12.4 to 49.1)	-27.5 (-43.7 to -4.2)*
Complications of trichuriasis aggregate	815.7 (702.5 to 1,003.4)	11.2 (-9.7 to 42.0)	-28.5 (-41.9 to -8.7)*	16.3 (8.6 to 28.4)	12.8 (-12.4 to 49.1)	-27.5 (-43.7 to -4.2)*
Asymptomatic trichuriasis	14,536.2 (12,807.5 to 16,439.7)	33.3 (13.2 to 58.1)*	-14.4 (-27.3 to 1.6)	0.0 (0.0 to 0.0)	0.0 (0.0 to 0.0)	0.0 (0.0 to 0.0)
Hookworm disease	14,970.7 (13,252.1 to 17,057.6)	22.4 (4.9 to 43.5)*	-21.3 (-32.6 to -7.8)*	50.6 (30.6 to 79.7)	9.1 (-1.5 to 20.6)	-29.9 (-36.7 to -22.5)*
Complications of hookworm disease aggregate	1,729.3 (1,587.1 to 1,873.3)	9.4 (-2.5 to 23.3)	-29.7 (-37.3 to -20.8)*	34.9 (19.5 to 57.7)	9.8 (-4.6 to 26.8)	-29.4 (-38.7 to -18.5)*
Anemia due to hookworm disease aggregate	799.9 (775.7 to 825.5)	24.7 (20.0 to 29.3)*	-19.9 (-22.9 to -16.9)*	15.7 (10.2 to 22.9)	7.4 (-0.2 to 15.0)	-31.0 (-35.9 to -26.1)*
Asymptomatic hookworm disease	12,399.5 (10,694.8 to 14,475.4)	24.0 (2.3 to 49.7)*	-20.3 (-34.3 to -3.8)*	0.0 (0.0 to 0.0)	0.0 (0.0 to 0.0)	0.0 (0.0 to 0.0)
Food-borne trematodiases	3,636.6 (3,348.0 to 3,956.5)	76.3 (71.4 to 82.3)*	13.3 (10.2 to 17.1)*	83.1 (39.6 to 153.5)	75.9 (68.5 to 84.1)*	13.1 (8.3 to 18.3)*
Asymptomatic food-borne trematodiases aggregate	2,874.6 (2,301.1 to 3,333.9)	76.2 (71.3 to 82.2)*	13.3 (10.1 to 17.1)*	0.0 (0.0 to 0.0)	0.0 (0.0 to 0.0)	0.0 (0.0 to 0.0)
Symptomatic food-borne trematodiases aggregate	762.0 (402.8 to 1,315.4)	76.4 (70.9 to 83.2)*	13.3 (9.8 to 17.7)*	83.1 (39.6 to 153.5)	75.9 (68.5 to 84.1)*	13.1 (8.3 to 18.3)*
Leprosy	58.4 (55.3 to 61.7)	13.5 (11.8 to 15.3)*	-27.0 (-28.2 to -25.9)*	3.5 (2.4 to 4.9)	14.8 (8.7 to 21.4)*	-26.3 (-30.2 to -22.0)*
Ebola	0.1 (0.0 to 0.1)	0.0 (0.0 to 0.0)	0.0 (0.0 to 0.0)	0.0 (0.0 to 0.0)	0.0 (0.0 to 0.0)	0.0 (0.0 to 0.0)
Other neglected tropical diseases	653.5 (623.9 to 682.1)	32.2 (26.0 to 38.6)*	-15.0 (-19.1 to -10.9)*	93.6 (32.9 to 262.5)	17.3 (-24.1 to 78.9)	-24.6 (-51.2 to 15.0)
Anemia due to other neglected tropical diseases aggregate	653.5 (623.9 to 682.1)	32.2 (26.0 to 38.6)*	-15.0 (-19.1 to -10.9)*	13.6 (9.1 to 19.6)	6.4 (-2.2 to 15.5)	-31.6 (-37.1 to -25.8)*
Maternal disorders	14.0 (12.6 to 15.5)	-29.5 (-38.0 to -20.2)*	-54.7 (-60.2 to -48.7)*	4.9 (3.2 to 7.1)	-27.9 (-36.0 to -20.4)*	-53.7 (-58.9 to -48.8)*
Maternal hemorrhage	0.0 (0.0 to 0.0)	0.0 (0.0 to 0.0)	0.0 (0.0 to 0.0)	0.0 (0.0 to 0.0)	0.0 (0.0 to 0.0)	0.0 (0.0 to 0.0)
Maternal hemorrhage episodes aggregate	0.0 (0.0 to 0.0)	0.0 (0.0 to 0.0)	0.0 (0.0 to 0.0)	0.0 (0.0 to 0.0)	0.0 (0.0 to 0.0)	0.0 (0.0 to 0.0)
Anemia due to maternal hemorrhage aggregate	0.0 (0.0 to 0.0)	0.0 (0.0 to 0.0)	0.0 (0.0 to 0.0)	0.0 (0.0 to 0.0)	0.0 (0.0 to 0.0)	0.0 (0.0 to 0.0)
Maternal sepsis and other maternal infections	0.0 (0.0 to 0.0)	0.0 (0.0 to 0.0)	0.0 (0.0 to 0.0)	0.0 (0.0 to 0.0)	0.0 (0.0 to 0.0)	0.0 (0.0 to 0.0)
Maternal sepsis and other maternal infections aggregate	0.0 (0.0 to 0.0)	0.0 (0.0 to 0.0)	0.0 (0.0 to 0.0)	0.0 (0.0 to 0.0)	0.0 (0.0 to 0.0)	0.0 (0.0 to 0.0)
Infertility due to puerperal sepsis	0.0 (0.0 to 0.0)	0.0 (0.0 to 0.0)	0.0 (0.0 to 0.0)	0.0 (0.0 to 0.0)	0.0 (0.0 to 0.0)	0.0 (0.0 to 0.0)
Maternal hypertensive disorders	0.0 (0.0 to 0.0)	0.0 (0.0 to 0.0)	0.0 (0.0 to 0.0)	0.0 (0.0 to 0.0)	0.0 (0.0 to 0.0)	0.0 (0.0 to 0.0)
Maternal hypertensive disorders episodes aggregate	0.0 (0.0 to 0.0)	0.0 (0.0 to 0.0)	0.0 (0.0 to 0.0)	0.0 (0.0 to 0.0)	0.0 (0.0 to 0.0)	0.0 (0.0 to 0.0)
Eclampsia	0.0 (0.0 to 0.0)	0.0 (0.0 to 0.0)	0.0 (0.0 to 0.0)	0.0 (0.0 to 0.0)	0.0 (0.0 to 0.0)	0.0 (0.0 to 0.0)
Complications of maternal hypertensive disorders aggregate	0.0 (0.0 to 0.0)	0.0 (0.0 to 0.0)	0.0 (0.0 to 0.0)	0.0 (0.0 to 0.0)	0.0 (0.0 to 0.0)	0.0 (0.0 to 0.0)
Maternal obstructed labor and uterine rupture	14.2 (11.7 to 17.0)	-28.3 (-31.4 to -25.4)*	-54.0 (-55.9 to -52.1)*	4.4 (2.9 to 6.3)	-28.3 (-36.5 to -20.8)*	-53.9 (-59.2 to -49.1)*
Obstructed labor, acute event	0.0 (0.0 to 0.0)	0.0 (0.0 to 0.0)	0.0 (0.0 to 0.0)	0.0 (0.0 to 0.0)	0.0 (0.0 to 0.0)	0.0 (0.0 to 0.0)
Fistula due to maternal obstructed labor and uterine rupture aggregate	14.2 (11.7 to 17.0)	-28.3 (-31.4 to -25.4)*	-54.0 (-55.9 to -52.1)*	4.4 (2.9 to 6.3)	-28.3 (-36.5 to -20.8)*	-53.9 (-59.2 to -49.1)*
Maternal abortion, miscarriage, and ectopic pregnancy	0.0 (0.0 to 0.0)	0.0 (0.0 to 0.0)	0.0 (0.0 to 0.0)	0.0 (0.0 to 0.0)	0.0 (0.0 to 0.0)	0.0 (0.0 to 0.0)
Other maternal disorders	--	--	--	0.5 (0.3 to 0.8)	-25.0 (-39.6 to -10.6)*	-51.8 (-61.2 to -42.6)*
Neonatal disorders	1,284.7 (1,223.1 to 1,331.2)	68.1 (61.6 to 75.1)*	8.0 (3.9 to 12.5)*	222.5 (169.5 to 282.4)	68.7 (62.7 to 75.1)*	8.4 (4.6 to 12.5)*
Neonatal preterm birth complications	1,166.8 (1,018.1 to 1,332.7)	63.3 (54.8 to 72.2)*	4.9 (-0.6 to 10.6)	105.7 (79.4 to 134.0)	62.3 (53.7 to 71.6)*	4.3 (-1.2 to 10.3)
Vision loss due to retinopathy of prematurity aggregate	189.0 (151.4 to 232.9)	53.1 (48.0 to 58.6)*	-1.6 (-4.9 to 1.9)	6.6 (4.1 to 10.0)	52.2 (41.1 to 65.3)*	-2.2 (-9.3 to 6.2)
Complications of preterm birth complications aggregate	977.8 (842.3 to 1,125.6)	65.5 (54.8 to 76.3)*	6.3 (-0.5 to 13.3)	99.1 (74.2 to 126.8)	63.0 (53.8 to 72.9)*	4.7 (-1.2 to 11.1)
Neonatal encephalopathy due to birth asphyxia and trauma	261.3 (184.8 to 393.7)	81.3 (76.6 to 86.1)*	16.5 (13.5 to 19.6)*	73.2 (48.1 to 112.2)	75.4 (68.2 to 83.3)*	12.7 (8.1 to 17.8)*

Cause	Prevalence in thousands (95% confidence interval)			YLDs in thousands (95% confidence interval)		
	2015	Percent change in counts between 2005 and 2015	Percent change in rates between 2005 and 2015	2015	Percent change in counts between 2005 and 2015	Percent change in rates between 2005 and 2015
Neonatal sepsis and other neonatal infections	0.0 (0.0 to 0.0)	0.0 (0.0 to 0.0)	0.0 (0.0 to 0.0)	0.0 (0.0 to 0.0)	0.0 (0.0 to 0.0)	0.0 (0.0 to 0.0)
Severe infection due to neonatal sepsis and other neonatal infections	0.0 (0.0 to 0.0)	0.0 (0.0 to 0.0)	0.0 (0.0 to 0.0)	0.0 (0.0 to 0.0)	0.0 (0.0 to 0.0)	0.0 (0.0 to 0.0)
Complications of neonatal sepsis and other neonatal infections aggregate	0.0 (0.0 to 0.0)	0.0 (0.0 to 0.0)	0.0 (0.0 to 0.0)	0.0 (0.0 to 0.0)	0.0 (0.0 to 0.0)	0.0 (0.0 to 0.0)
Hemolytic disease and other neonatal jaundice	43.4 (38.5 to 49.3)	72.9 (70.7 to 75.2)*	11.1 (9.7 to 12.6)*	13.1 (9.6 to 16.8)	65.6 (59.4 to 72.1)*	6.4 (2.4 to 10.6)*
Other neonatal disorders	--	--	--	30.6 (22.2 to 39.5)	78.6 (58.4 to 97.8)*	14.8 (1.8 to 27.1)*
Nutritional deficiencies	39,886.5 (39,207.6 to 40,575.6)	41.1 (38.3 to 44.0)*	-9.3 (-11.1 to -7.5)*	989.4 (663.0 to 1,413.0)	27.7 (23.6 to 32.1)*	-17.9 (-20.6 to -15.1)*
Protein-energy malnutrition	21.0 (13.2 to 33.2)	-1.5 (-11.4 to 10.9)	-36.7 (-43.1 to -28.7)*	2.5 (1.3 to 4.3)	-0.6 (-14.2 to 14.0)	-36.1 (-44.9 to -26.7)*
Iodine deficiency	5,068.5 (4,594.9 to 5,694.0)	40.9 (38.3 to 43.8)*	-9.4 (-11.1 to -7.6)*	91.4 (58.0 to 140.2)	41.5 (38.6 to 44.7)*	-9.1 (-10.9 to -7.0)*
Visible goiter due to iodine deficiency aggregate	5,042.3 (4,566.2 to 5,667.0)	40.9 (38.3 to 43.8)*	-9.4 (-11.2 to -7.6)*	87.1 (54.2 to 135.5)	41.4 (38.4 to 44.9)*	-9.1 (-11.1 to -6.9)*
Visible goiter with heart failure due to iodine deficiency aggregate	0.0 (0.0 to 0.0)	49.8 (47.2 to 52.5)*	-3.8 (-5.4 to -2.0)*	0.0 (0.0 to 0.0)	49.8 (47.2 to 52.5)*	-3.8 (-5.4 to -2.0)*
Intellectual disability due to iodine deficiency aggregate	26.2 (12.5 to 34.0)	40.9 (33.8 to 46.8)*	-9.4 (-14.0 to -5.7)*	4.3 (1.8 to 6.7)	42.4 (28.7 to 59.4)*	-8.5 (-17.3 to 2.4)
Vitamin A deficiency	86.4 (59.3 to 127.5)	40.9 (32.1 to 49.7)*	-9.5 (-15.1 to -3.8)*	5.8 (3.4 to 8.8)	42.4 (30.3 to 53.5)*	-8.5 (-16.3 to -1.4)*
Iron-deficiency anemia	37,112.7 (36,410.8 to 37,834.9)	39.5 (36.6 to 42.5)*	-10.3 (-12.2 to -8.4)*	889.5 (595.2 to 1,269.9)	26.5 (22.0 to 30.9)*	-18.7 (-21.6 to -15.9)*
Iron-deficiency anemia without heart failure aggregate	37,107.1 (36,405.6 to 37,829.3)	39.5 (36.6 to 42.5)*	-10.3 (-12.2 to -8.4)*	888.8 (594.7 to 1,269.0)	26.5 (22.0 to 30.9)*	-18.7 (-21.6 to -15.9)*
Iron-deficiency anemia with heart failure aggregate	5.7 (4.6 to 7.1)	41.5 (37.7 to 45.9)*	-9.1 (-11.5 to -6.3)*	0.7 (0.4 to 1.0)	42.7 (24.6 to 64.4)*	-8.3 (-19.9 to 5.7)
Other nutritional deficiencies	--	--	--	0.3 (0.1 to 0.5)	9.6 (-15.4 to 36.7)	-29.6 (-45.6 to -12.2)*
Other communicable, maternal, neonatal, and nutritional diseases	83,026.8 (80,708.6 to 85,060.0)	53.1 (51.7 to 54.5)*	-1.6 (-2.5 to -0.7)*	77.1 (50.2 to 118.1)	43.5 (33.3 to 54.0)*	-7.8 (-14.4 to -1.1)*
Sexually transmitted diseases excluding HIV	63,594.4 (60,932.5 to 66,104.5)	51.4 (49.5 to 53.4)*	-2.7 (-3.9 to -1.4)*	41.6 (25.6 to 66.8)	44.1 (38.1 to 48.6)*	-7.4 (-11.3 to -4.5)*
Syphilis	863.7 (639.2 to 1,165.2)	21.1 (9.0 to 31.8)*	-22.2 (-30.0 to -15.3)*	19.8 (13.0 to 27.8)	37.0 (30.1 to 43.9)*	-12.0 (-16.4 to -7.5)*
Early syphilis aggregate	755.5 (533.3 to 1,055.4)	19.2 (5.0 to 31.2)*	-23.4 (-32.5 to -15.7)*	0.2 (0.0 to 0.4)	19.5 (3.7 to 35.1)*	-23.2 (-33.3 to -13.2)*
Adult tertiary syphilis	108.2 (93.0 to 124.6)	36.6 (33.1 to 40.0)*	-12.2 (-14.5 to -10.0)*	19.6 (13.0 to 27.6)	37.1 (30.2 to 44.2)*	-11.9 (-16.3 to -7.3)*
Chlamydial infection	63.7 (44.9 to 87.1)	62.3 (56.3 to 68.3)*	4.3 (0.4 to 8.2)*	0.7 (0.5 to 1.1)	46.0 (39.5 to 54.0)*	-6.2 (-10.4 to -1.1)*
Chlamydial infection episodes aggregate	60.9 (42.4 to 84.1)	64.0 (57.6 to 70.4)*	5.4 (1.2 to 9.5)*	0.3 (0.2 to 0.6)	65.1 (53.9 to 77.0)*	6.1 (-1.1 to 13.7)
Pelvic inflammatory diseases due to chlamydial infection aggregate	2.8 (1.9 to 3.9)	33.0 (27.8 to 38.3)*	-14.5 (-17.9 to -11.1)*	0.4 (0.2 to 0.6)	33.0 (27.8 to 38.3)*	-14.5 (-17.9 to -11.1)*
Infertility due to clamydial infection aggregate	0.0 (0.0 to 0.0)	0.0 (0.0 to 0.0)	0.0 (0.0 to 0.0)	0.0 (0.0 to 0.0)	0.0 (0.0 to 0.0)	0.0 (0.0 to 0.0)
Gonococcal infection	47.7 (25.2 to 108.1)	61.4 (29.5 to 79.5)*	3.7 (-16.8 to 15.4)	0.4 (0.2 to 0.6)	50.7 (32.8 to 64.5)*	-3.2 (-14.6 to 5.7)
Gonococcal infection episodes aggregate	47.2 (24.8 to 107.7)	61.7 (29.4 to 80.1)*	3.9 (-16.9 to 15.7)	0.3 (0.1 to 0.6)	54.9 (31.7 to 70.4)*	-0.5 (-15.4 to 9.5)
Pelvic inflammatory diseases due to gonococcal infection aggregate	0.5 (0.4 to 0.7)	36.4 (29.7 to 43.4)*	-12.3 (-16.6 to -7.9)*	0.1 (0.0 to 0.1)	36.4 (29.7 to 43.4)*	-12.3 (-16.6 to -7.9)*
Trichomoniasis	4,247.9 (3,566.2 to 5,014.0)	54.9 (53.9 to 56.1)*	-0.5 (-1.1 to 0.3)	4.7 (1.8 to 10.0)	55.0 (52.3 to 57.9)*	-0.4 (-2.1 to 1.4)
Genital herpes	60,439.1 (52,997.0 to 68,344.4)	51.6 (49.5 to 53.5)*	-2.6 (-4.0 to -1.3)*	14.6 (4.2 to 35.5)	51.7 (49.0 to 54.3)*	-2.5 (-4.2 to -0.8)*
Moderate infection due to initial genital herpes episode	0.0 (0.0 to 0.0)	0.0 (0.0 to 0.0)	0.0 (0.0 to 0.0)	0.0 (0.0 to 0.0)	0.0 (0.0 to 0.0)	0.0 (0.0 to 0.0)
Complications of genital herpes aggregate	60,439.1 (52,997.0 to 68,344.4)	51.6 (49.5 to 53.5)*	-2.6 (-4.0 to -1.3)*	14.6 (4.2 to 35.5)	51.7 (49.0 to 54.3)*	-2.5 (-4.2 to -0.8)*
Other sexually transmitted diseases	11.0 (7.5 to 15.1)	37.9 (34.7 to 40.9)*	-11.4 (-13.5 to -9.4)*	1.4 (0.8 to 2.3)	38.5 (23.5 to 55.3)*	-11.0 (-20.6 to -0.2)*
Pelvic inflammatory diseases due to other sexually transmitted diseases aggregate	11.0 (7.5 to 15.1)	37.9 (34.7 to 40.9)*	-11.4 (-13.5 to -9.4)*	1.4 (0.8 to 2.3)	38.5 (23.5 to 55.3)*	-11.0 (-20.6 to -0.2)*
Infertility due to other sexually transmitted diseases aggregate	0.0 (0.0 to 0.0)	0.0 (0.0 to 0.0)	0.0 (0.0 to 0.0)	0.0 (0.0 to 0.0)	0.0 (0.0 to 0.0)	0.0 (0.0 to 0.0)
Other sexually transmitted diseases	--	--	--	0.3 (0.1 to 0.4)	58.8 (47.4 to 68.6)*	2.1 (-5.3 to 8.3)
Hepatitis	23,667.0 (23,213.8 to 24,105.7)	58.2 (57.1 to 59.5)*	1.7 (0.9 to 2.5)*	11.4 (7.0 to 17.7)	61.7 (8.7 to 149.6)*	3.9 (-30.2 to 60.4)
Acute hepatitis A	69.3 (56.3 to 80.6)	84.0 (44.7 to 130.7)*	18.2 (-7.0 to 48.2)	2.8 (1.7 to 4.2)	84.0 (40.7 to 135.0)*	18.3 (-9.6 to 51.0)
Hepatitis B	14,907.2 (14,379.6 to 15,454.6)	59.5 (56.8 to 62.2)*	2.5 (0.7 to 4.2)*	7.8 (4.3 to 12.9)	55.7 (-13.3 to 188.9)	0.1 (-44.3 to 85.7)
Acute Hepatitis B aggregate	345.6 (227.1 to 521.3)	56.0 (-13.5 to 186.4)	0.2 (-44.4 to 84.0)	7.8 (4.3 to 12.9)	55.7 (-13.3 to 188.9)	0.1 (-44.3 to 85.7)
Chronic hepatitis B	14,561.6 (14,053.9 to 15,105.8)	59.5 (58.2 to 61.0)*	2.5 (1.7 to 3.4)*	0.0 (0.0 to 0.0)	0.0 (0.0 to 0.0)	0.0 (0.0 to 0.0)
Hepatitis C	9,298.6 (8,283.2 to 10,356.6)	55.6 (53.1 to 57.9)*	0.0 (-1.6 to 1.5)	0.3 (0.1 to 0.6)	56.1 (46.5 to 63.6)*	0.3 (-5.9 to 5.1)
Acute Hepatitis C aggregate	21.8 (15.6 to 28.8)	56.9 (47.2 to 64.8)*	0.8 (-5.4 to 5.9)	0.3 (0.1 to 0.6)	56.1 (46.5 to 63.6)*	0.3 (-5.9 to 5.1)
Chronic hepatitis C	9,277.0 (8,264.3 to 10,334.7)	55.6 (53.2 to 57.9)*	0.0 (-1.6 to 1.5)	0.0 (0.0 to 0.0)	0.0 (0.0 to 0.0)	0.0 (0.0 to 0.0)
Acute hepatitis E	15.1 (9.9 to 23.7)	51.6 (48.3 to 55.5)*	-2.6 (-4.7 to -0.1)*	0.5 (0.2 to 0.8)	51.3 (23.4 to 87.7)*	-2.8 (-20.7 to 20.6)
Other infectious diseases	904.5 (878.3 to 930.4)	40.0 (33.2 to 47.1)*	-10.0 (-14.4 to -5.5)*	24.2 (15.7 to 35.6)	35.3 (23.1 to 48.1)*	-13.1 (-20.9 to -4.8)*
Non-communicable diseases	300,880.3 (300,653.3 to 301,085.2)	55.6 (55.5 to 55.6)*	-0.0 (-0.1 to 0.0)	48,344.6 (36,075.9 to 62,488.5)	54.5 (53.6 to 55.4)*	-0.7 (-1.3 to -0.2)*
Neoplasms	13,129.0 (12,861.4 to 13,387.9)	86.6 (82.3 to 90.8)*	19.9 (17.1 to 22.6)*	1,186.3 (862.7 to 1,550.0)	74.6 (67.2 to 81.7)*	12.2 (7.5 to 16.8)*
Lip and oral cavity cancer	353.8 (314.3 to 399.2)	63.8 (46.7 to 83.8)*	5.3 (-5.8 to 18.1)	29.8 (21.3 to 39.9)	61.8 (45.0 to 81.8)*	3.9 (-6.9 to 16.8)
Nasopharynx cancer	95.8 (71.9 to 127.3)	60.0 (26.6 to 105.8)*	2.8 (-18.6 to 32.3)	9.0 (5.6 to 13.2)	56.7 (23.6 to 100.9)*	0.7 (-20.6 to 29.1)
Other pharynx cancer	176.2 (158.8 to 196.2)	59.1 (42.5 to 75.5)*	2.3 (-8.5 to 12.8)	14.5 (10.6 to 18.9)	56.9 (39.8 to 74.7)*	0.8 (-10.2 to 12.3)
Esophageal cancer	128.0 (102.7 to 193.7)	52.0 (23.7 to 98.0)*	-2.3 (-20.5 to 27.2)	21.6 (14.5 to 32.1)	39.1 (15.0 to 78.4)*	-10.6 (-26.1 to 14.6)
Stomach cancer	551.1 (507.7 to 603.6)	44.8 (32.0 to 59.8)*	-6.9 (-15.2 to 2.7)	61.1 (44.3 to 78.8)	40.9 (27.7 to 56.3)*	-9.4 (-17.9 to 0.5)
Colon and rectum cancer	1,289.3 (1,207.2 to 1,382.2)	73.8 (64.4 to 83.5)*	11.7 (5.6 to 17.9)*	101.2 (73.0 to 131.4)	70.2 (60.8 to 80.6)*	9.4 (3.3 to 16.0)*
Colon and rectum cancer aggregate	1,287.0 (1,205.0 to 1,379.7)	73.8 (64.4 to 83.6)*	11.7 (5.6 to 18.0)*	100.9 (72.8 to 131.0)	70.3 (60.8 to 80.7)*	9.4 (3.3 to 16.1)*
Stoma due to colon and rectum cancer	2.4 (2.1 to 2.6)	52.4 (48.6 to 55.9)*	-2.1 (-4.5 to 0.2)	0.3 (0.2 to 0.5)	52.4 (48.6 to 55.9)*	-2.1 (-4.5 to 0.2)
Liver cancer	93.4 (80.5 to 106.5)	114.1 (82.8 to 152.2)*	37.6 (17.4 to 62.0)*	27.0 (18.8 to 36.6)	62.5 (38.9 to 94.2)*	4.4 (-10.7 to 24.8)
Liver cancer due to hepatitis B	40.2 (30.8 to 56.8)	64.4 (27.3 to 118.1)*	5.6 (-18.2 to 40.1)	7.7 (5.1 to 11.1)	49.1 (23.3 to 86.7)*	-4.2 (-20.8 to 20.0)
Liver cancer due to hepatitis C	34.8 (27.5 to 44.6)	102.6 (65.4 to 157.2)*	30.2 (6.3 to 65.2)*	5.9 (4.0 to 7.9)	68.7 (44.6 to 100.1)*	8.4 (-7.1 to 28.6)
Liver cancer due to alcohol use	48.7 (38.9 to 63.1)	107.9 (68.9 to 161.7)*	33.6 (8.5 to 68.1)*	9.4 (6.3 to 12.9)	80.8 (52.7 to 116.0)*	16.2 (-1.9 to 38.8)
Liver cancer due to other causes	19.4 (14.8 to 29.9)	63.5 (23.8 to 119.3)*	5.1 (-20.5 to 40.9)	4.0 (2.6 to 5.7)	45.5 (20.8 to 83.6)*	-6.5 (-22.4 to 18.0)
Gallbladder and biliary tract cancer	18.2 (16.4 to 20.1)	41.5 (30.5 to 53.7)*	-9.1 (-16.1 to -1.2)*	5.1 (3.6 to 6.7)	38.9 (27.0 to 51.9)*	-10.8 (-18.4 to -2.4)*
Pancreatic cancer	55.6 (51.0 to 61.0)	80.9 (68.9 to 95.3)*	16.2 (8.5 to 25.5)*	12.6 (8.9 to 16.4)	71.4 (59.4 to 85.5)*	10.1 (2.4 to 19.2)*

Cause	Prevalence in thousands (95% confidence interval)			YLDs in thousands (95% confidence interval)		
	2015	Percent change in counts between 2005 and 2015	Percent change in rates between 2005 and 2015	2015	Percent change in counts between 2005 and 2015	Percent change in rates between 2005 and 2015
Larynx cancer	242.9 (220.0 to 271.1)	52.5 (39.6 to 69.5)*	-2.0 (-10.3 to 9.0)	24.5 (17.3 to 32.3)	50.8 (37.9 to 65.6)*	-3.1 (-11.4 to 6.4)
Larynx cancer aggregate	159.2 (136.8 to 186.4)	50.3 (31.0 to 75.9)*	-3.4 (-15.8 to 13.0)	17.0 (12.0 to 22.7)	48.1 (30.0 to 69.8)*	-4.8 (-16.4 to 9.1)
Laryngectomy due to larynx cancer	83.7 (80.2 to 86.8)	56.7 (54.7 to 59.4)*	0.7 (-0.6 to 2.4)	7.5 (5.0 to 10.8)	57.2 (48.2 to 66.7)*	1.0 (-4.8 to 7.1)
Tracheal, bronchus, and lung cancer	531.0 (486.6 to 598.3)	74.4 (60.6 to 92.1)*	12.1 (3.2 to 23.4)*	82.6 (59.8 to 106.3)	67.4 (53.6 to 85.7)*	7.6 (-1.3 to 19.3)
Malignant skin melanoma	410.1 (317.6 to 529.2)	117.5 (98.7 to 137.0)*	39.8 (27.7 to 52.3)*	23.4 (15.1 to 34.2)	113.6 (94.6 to 133.0)*	37.2 (25.1 to 49.7)*
Non-melanoma skin cancer	306.4 (288.1 to 325.6)	108.7 (96.4 to 123.1)*	34.1 (26.2 to 43.4)*	15.9 (10.7 to 22.1)	115.1 (93.5 to 139.5)*	38.2 (24.3 to 53.9)*
Non-melanoma skin cancer(squamous-cell carcinoma)	248.3 (218.2 to 288.3)	127.8 (105.7 to 153.9)*	46.4 (32.2 to 63.2)*	15.2 (10.3 to 20.8)	119.0 (95.4 to 145.2)*	40.7 (25.6 to 57.6)*
Non-melanoma skin cancer(basal-cell carcinoma)	73.5 (58.2 to 93.0)	59.1 (53.1 to 65.5)*	2.2 (-1.6 to 6.4)	0.8 (0.4 to 1.4)	59.1 (48.9 to 69.6)*	2.2 (-4.3 to 9.0)
Breast cancer	2,837.0 (2,627.0 to 3,048.0)	92.3 (78.6 to 106.6)*	23.6 (14.8 to 32.8)*	243.9 (172.2 to 329.0)	78.6 (67.8 to 89.6)*	14.8 (7.8 to 21.8)*
Breast cancer aggregate	1,242.8 (1,025.5 to 1,467.8)	186.7 (134.9 to 244.2)*	84.2 (50.9 to 121.2)*	122.0 (88.9 to 158.3)	114.1 (85.5 to 147.3)*	37.6 (19.2 to 58.9)*
Mastectomy due to breast cancer	1,593.9 (1,530.0 to 1,657.2)	53.1 (51.3 to 54.8)*	-1.6 (-2.8 to -0.5)*	121.9 (78.2 to 175.8)	53.2 (50.6 to 55.8)*	-1.5 (-3.2 to 0.1)
Cervical cancer	348.3 (295.1 to 413.0)	22.8 (4.2 to 45.2)*	-21.1 (-33.1 to -6.7)*	26.5 (18.3 to 36.2)	22.3 (3.2 to 44.8)*	-21.4 (-33.7 to -7.0)*
Uterine cancer	665.2 (547.9 to 822.6)	96.4 (68.0 to 134.0)*	26.2 (8.0 to 50.4)*	42.5 (28.4 to 61.6)	92.9 (64.8 to 130.6)*	23.9 (5.9 to 48.2)*
Ovarian cancer	152.8 (138.3 to 173.2)	63.7 (49.1 to 81.2)*	5.2 (-4.2 to 16.5)	19.0 (13.8 to 24.8)	60.3 (43.9 to 78.4)*	3.0 (-7.5 to 14.6)
Prostate cancer	2,544.4 (2,001.8 to 3,529.1)	98.8 (82.8 to 117.1)*	27.7 (17.4 to 39.5)*	182.7 (120.7 to 264.5)	89.8 (74.4 to 106.3)*	22.0 (12.1 to 32.6)*
Prostate cancer aggregate	2,327.8 (1,784.7 to 3,312.0)	104.6 (86.7 to 125.0)*	31.5 (20.0 to 44.6)*	164.0 (107.5 to 240.8)	95.3 (77.7 to 114.0)*	25.5 (14.2 to 37.5)*
Impotence and incontinence due to prostate cancer aggregate	216.6 (213.1 to 220.2)	52.2 (51.7 to 52.7)*	-2.2 (-2.5 to -1.9)*	18.7 (12.7 to 25.8)	51.9 (47.2 to 56.4)*	-2.4 (-5.4 to 0.5)
Testicular cancer	20.1 (15.9 to 25.5)	132.5 (91.0 to 185.3)*	49.4 (22.7 to 83.3)*	1.3 (0.9 to 1.9)	122.3 (80.8 to 175.9)*	42.9 (16.2 to 77.3)*
Kidney cancer	443.7 (399.7 to 493.2)	108.7 (89.5 to 129.9)*	34.1 (21.8 to 47.7)*	30.9 (21.6 to 41.9)	104.0 (84.1 to 125.6)*	31.1 (18.3 to 45.0)*
Bladder cancer	497.2 (443.6 to 563.5)	68.7 (51.7 to 89.1)*	8.4 (-2.6 to 21.5)	38.5 (27.4 to 51.7)	63.9 (48.2 to 82.3)*	5.3 (-4.7 to 17.1)
Bladder cancer episodes aggregate	472.4 (418.5 to 539.0)	70.0 (51.9 to 91.9)*	9.2 (-2.4 to 23.3)	34.4 (24.6 to 46.2)	66.0 (48.7 to 86.5)*	6.7 (-4.4 to 19.9)
Urinary incontinence due to bladder cancer	24.9 (23.4 to 26.4)	47.5 (46.1 to 48.9)*	-5.2 (-6.1 to -4.3)*	4.1 (2.8 to 5.6)	48.3 (35.5 to 62.4)*	-4.7 (-13.0 to 4.4)
Brain and nervous system cancer	123.7 (108.1 to 154.0)	76.5 (55.7 to 101.7)*	13.4 (0.0 to 29.6)*	13.5 (9.4 to 18.5)	71.7 (52.2 to 97.7)*	10.4 (-2.2 to 27.1)
Thyroid cancer	549.5 (501.1 to 601.7)	195.3 (163.0 to 226.9)*	89.8 (69.0 to 110.1)*	31.8 (21.9 to 44.4)	183.3 (151.2 to 214.4)*	82.0 (61.4 to 102.0)*
Mesothelioma	7.3 (6.7 to 8.1)	57.2 (43.8 to 72.3)*	1.0 (-7.6 to 10.7)	1.5 (1.0 to 1.9)	55.1 (39.2 to 72.2)*	-0.3 (-10.5 to 10.6)
Hodgkin lymphoma	47.6 (38.6 to 62.5)	83.1 (59.4 to 115.2)*	17.6 (2.4 to 38.3)*	4.1 (2.7 to 5.9)	72.5 (48.7 to 104.3)*	10.9 (-4.4 to 31.3)
Non-Hodgkin lymphoma	529.2 (462.0 to 595.1)	118.8 (96.5 to 140.1)*	40.6 (26.3 to 54.3)*	38.0 (26.8 to 51.4)	109.4 (87.4 to 130.8)*	34.5 (20.4 to 48.3)*
Multiple myeloma	70.0 (60.5 to 82.6)	92.3 (70.3 to 120.0)*	23.6 (9.4 to 41.4)*	14.6 (10.2 to 19.7)	84.8 (62.2 to 110.3)*	18.8 (4.2 to 35.1)*
Leukemia	242.5 (227.1 to 260.0)	82.5 (68.8 to 96.8)*	17.2 (8.5 to 26.5)*	37.0 (26.5 to 47.1)	70.0 (56.9 to 85.4)*	9.3 (0.8 to 19.1)*
Acute lymphoid leukemia	51.9 (43.9 to 60.6)	76.3 (54.0 to 99.9)*	13.3 (-1.0 to 28.5)	7.2 (4.9 to 9.6)	71.1 (48.3 to 97.8)*	10.0 (-4.7 to 27.1)
Chronic lymphoid leukemia	111.0 (96.3 to 129.3)	80.4 (59.4 to 103.8)*	16.0 (2.5 to 31.0)*	14.2 (9.8 to 18.7)	73.1 (51.5 to 95.3)*	11.2 (-2.7 to 25.5)
Acute myeloid leukemia	93.4 (81.1 to 107.0)	82.6 (65.4 to 101.8)*	17.4 (6.3 to 29.7)*	11.9 (8.3 to 15.4)	76.6 (58.7 to 96.9)*	13.5 (2.0 to 26.5)*
Chronic myeloid leukemia	27.1 (23.5 to 31.3)	47.7 (33.1 to 65.9)*	-5.1 (-14.5 to 6.6)	3.7 (2.6 to 5.0)	41.9 (26.7 to 59.5)*	-8.8 (-18.6 to 2.5)
Other neoplasms	455.3 (384.4 to 544.5)	78.3 (59.6 to 104.1)*	14.6 (2.5 to 31.2)*	32.1 (21.9 to 44.5)	74.5 (56.0 to 99.0)*	12.1 (0.2 to 27.9)*
Cardiovascular diseases	55,405.6 (53,823.6 to 56,590.4)	54.4 (53.0 to 56.0)*	-0.8 (-1.7 to 0.2)	3,227.6 (2,279.9 to 4,256.6)	54.2 (52.9 to 55.3)*	-0.9 (-1.7 to -0.2)*
Rheumatic heart disease	1,245.5 (1,050.8 to 1,874.9)	15.2 (7.7 to 20.6)*	-26.0 (-30.8 to -22.5)*	66.8 (42.5 to 110.1)	21.0 (11.7 to 27.6)*	-22.3 (-28.2 to -18.0)*
Rheumatic heart disease, without heart failure	1,095.2 (899.1 to 1,721.2)	10.6 (4.4 to 16.2)*	-28.9 (-32.9 to -25.4)*	49.9 (29.5 to 90.4)	10.9 (4.5 to 16.7)*	-28.8 (-32.8 to -25.0)*
Heart failure fue to rheumatic heart disease aggregate	150.3 (133.2 to 169.8)	65.0 (62.5 to 67.4)*	6.0 (4.5 to 7.6)*	16.9 (11.4 to 24.0)	65.5 (57.5 to 73.1)*	6.4 (1.2 to 11.3)*
Ischemic heart disease	16,753.8 (13,926.9 to 19,974.5)	56.0 (54.0 to 58.1)*	0.2 (-1.0 to 1.6)	1,029.1 (662.5 to 1,456.9)	58.7 (56.9 to 60.3)*	2.0 (0.8 to 3.0)*
Myocardial infarction episodes aggregate	2,910.7 (2,466.5 to 3,389.8)	44.1 (35.3 to 53.0)*	-7.4 (-13.0 to -1.7)*	4.0 (2.6 to 5.6)	35.3 (27.1 to 43.1)*	-13.1 (-18.3 to -8.1)*
Angina due to ischemic heart disease aggregate	11,545.6 (8,815.1 to 14,833.2)	59.3 (57.4 to 61.2)*	2.3 (1.2 to 3.6)*	767.5 (463.7 to 1,127.6)	59.7 (57.4 to 62.0)*	2.6 (1.1 to 4.1)*
Heart failure fue to ischemic heart disease aggregate	2,297.6 (2,104.1 to 2,493.9)	56.0 (54.8 to 57.1)*	0.2 (-0.5 to 1.0)	257.6 (176.2 to 356.3)	56.2 (54.0 to 58.1)*	0.4 (-1.1 to 1.6)
Cerebrovascular disease	5,723.9 (5,666.6 to 5,786.6)	48.8 (47.4 to 50.3)*	-4.4 (-5.3 to -3.4)*	858.8 (591.5 to 1,155.5)	49.1 (47.0 to 51.1)*	-4.2 (-5.5 to -2.9)*
Ischemic stroke	3,681.5 (3,542.6 to 3,819.8)	47.6 (46.3 to 49.0)*	-5.2 (-6.0 to -4.3)*	543.2 (375.6 to 726.2)	48.0 (45.8 to 50.4)*	-4.9 (-6.3 to -3.4)*
Chronic ischemic stroke aggregate	3,650.0 (3,510.8 to 3,785.3)	47.7 (46.4 to 49.0)*	-5.1 (-6.0 to -4.2)*	536.4 (369.6 to 718.4)	48.1 (45.8 to 50.6)*	-4.8 (-6.3 to -3.2)*
Ischemic stroke episodes aggregate	31.5 (27.2 to 36.1)	38.8 (32.8 to 44.6)*	-10.8 (-14.7 to -7.1)*	6.8 (4.4 to 9.5)	39.1 (32.4 to 45.5)*	-10.6 (-14.9 to -6.5)*
Hemorrhagic stroke	2,141.1 (2,059.1 to 2,220.7)	50.6 (49.2 to 52.0)*	-3.2 (-4.1 to -2.3)*	315.6 (217.1 to 425.3)	50.9 (48.0 to 54.2)*	-3.0 (-4.9 to -0.9)*
Chronic hemorrhagic stroke aggregate	2,122.3 (2,041.3 to 2,201.7)	50.7 (49.3 to 52.1)*	-3.2 (-4.0 to -2.3)*	311.5 (214.0 to 419.6)	51.1 (48.2 to 54.4)*	-2.9 (-4.8 to -0.8)*
Acute hemorrhagic stroke aggregate	18.8 (16.0 to 21.9)	41.2 (34.3 to 47.6)*	-9.2 (-13.7 to -5.2)*	4.1 (2.6 to 5.7)	41.3 (34.3 to 47.7)*	-9.2 (-13.7 to -5.1)*
Hypertensive heart disease	603.7 (536.6 to 679.0)	58.7 (57.1 to 60.4)*	2.0 (1.0 to 3.1)*	67.7 (46.2 to 94.6)	59.2 (55.2 to 63.3)*	2.3 (-0.3 to 5.0)
Cardiomyopathy and myocarditis	180.7 (156.6 to 208.2)	48.5 (46.3 to 50.7)*	-4.6 (-6.0 to -3.1)*	19.6 (13.1 to 27.6)	48.2 (42.4 to 53.8)*	-4.8 (-8.5 to -1.2)*
Acute myocarditis	11.5 (7.8 to 16.1)	59.1 (54.5 to 63.9)*	2.2 (-0.7 to 5.3)	0.6 (0.3 to 1.0)	59.0 (50.7 to 67.3)*	2.2 (-3.2 to 7.5)
Heart failure due to cardiomyopathy aggregate	169.2 (145.1 to 195.9)	47.9 (45.5 to 50.2)*	-5.0 (-6.5 to -3.5)*	19.0 (12.6 to 26.8)	47.9 (41.9 to 53.6)*	-5.0 (-8.8 to -1.3)*
Atrial fibrillation and flutter	4,225.8 (3,521.5 to 5,112.1)	57.5 (56.2 to 58.9)*	1.2 (0.4 to 2.1)*	340.9 (223.1 to 485.1)	57.7 (55.3 to 60.2)*	1.4 (-0.2 to 3.0)
Peripheral vascular disease	20,750.1 (16,726.9 to 25,260.5)	56.4 (55.4 to 57.5)*	0.5 (-0.2 to 1.2)	80.2 (37.3 to 149.5)	57.3 (55.3 to 59.4)*	1.1 (-0.2 to 2.4)
Endocarditis	7.4 (6.4 to 8.5)	46.4 (42.9 to 49.9)*	-5.9 (-8.2 to -3.7)*	0.8 (0.5 to 1.2)	47.0 (32.5 to 63.4)*	-5.5 (-14.8 to 5.0)
Endocarditis episodes aggregate	1.3 (1.0 to 1.6)	49.9 (42.0 to 57.2)*	-3.7 (-8.8 to 1.0)	0.1 (0.0 to 0.1)	50.0 (42.1 to 57.2)*	-3.6 (-8.7 to 1.0)
Heart failure due to endocarditis aggregate	6.1 (5.2 to 7.2)	45.7 (41.6 to 49.5)*	-6.4 (-9.0 to -3.9)*	0.7 (0.5 to 1.1)	46.7 (31.0 to 64.7)*	-5.7 (-15.8 to 5.9)
Other cardiovascular and circulatory diseases	11,463.2 (9,933.2 to 13,164.0)	55.5 (53.6 to 57.5)*	-0.1 (-1.3 to 1.2)	763.7 (507.4 to 1,059.5)	55.8 (53.7 to 58.0)*	0.1 (-1.2 to 1.5)
Heart failure due to other cardiovascular diseases aggregate	110.7 (95.5 to 127.5)	54.7 (52.4 to 57.1)*	-0.6 (-2.1 to 0.9)	12.4 (8.3 to 17.5)	55.1 (47.6 to 62.8)*	-0.3 (-5.2 to 4.6)
Other cardiovascular diseases episodes aggregate	11,352.5 (9,838.9 to 13,037.1)	55.5 (53.6 to 57.5)*	-0.1 (-1.3 to 1.2)	751.2 (499.4 to 1,043.4)	55.8 (53.7 to 58.0)*	0.1 (-1.2 to 1.5)
Chronic respiratory diseases	34,793.4 (33,311.0 to 36,237.3)	45.3 (43.2 to 47.4)*	-6.6 (-8.0 to -5.3)*	2,232.2 (1,763.9 to 2,711.6)	40.9 (37.1 to 44.5)*	-9.5 (-11.9 to -7.2)*
Chronic obstructive pulmonary disease	19,563.4 (16,800.8 to 22,820.1)	46.0 (41.9 to 49.8)*	-6.2 (-8.8 to -3.7)*	1,289.9 (1,040.8 to 1,559.1)	42.3 (36.5 to 47.9)*	-8.5 (-12.3 to -5.0)*

Cause	Prevalence in thousands (95% confidence interval)			YLDs in thousands (95% confidence interval)		
	2015	Percent change in counts between 2005 and 2015	Percent change in rates between 2005 and 2015	2015	Percent change in counts between 2005 and 2015	Percent change in rates between 2005 and 2015
Chronic obstructive pulmonary disease without heart failure aggregate	18,962.7 (16,234.2 to 22,182.9)	45.6 (41.4 to 49.5)*	-6.4 (-9.1 to -3.9)*	1,033.2 (813.8 to 1,278.3)	37.9 (30.6 to 44.4)*	-11.4 (-16.1 to -7.2)*
Severe chronic obstructive pulmonary disease with heart failure aggregate	610.7 (545.0 to 684.2)	62.5 (60.8 to 64.1)*	4.4 (3.3 to 5.5)*	256.7 (185.0 to 326.0)	63.3 (59.5 to 67.0)*	4.9 (2.5 to 7.3)*
Pneumoconiosis	192.0 (180.8 to 201.2)	56.1 (50.1 to 62.9)*	0.3 (-3.6 to 4.7)	36.5 (23.0 to 53.3)	57.8 (50.7 to 65.5)*	1.4 (-3.2 to 6.3)
Silicosis	43.3 (37.3 to 49.5)	54.7 (50.8 to 58.4)*	-0.6 (-3.1 to 1.8)	7.0 (4.3 to 10.3)	55.7 (43.1 to 68.8)*	0.0 (-8.1 to 8.4)
Silicosis without heart failure aggregate	42.2 (36.1 to 48.4)	54.7 (50.8 to 58.6)*	-0.6 (-3.1 to 1.9)	6.4 (3.8 to 9.6)	55.8 (42.0 to 70.2)*	0.1 (-8.7 to 9.4)
Severe silicosis with heart failure aggregate	1.2 (1.0 to 1.3)	54.4 (51.0 to 57.9)*	-0.8 (-2.9 to 1.5)	0.6 (0.4 to 0.7)	54.4 (51.1 to 58.1)*	-0.8 (-2.9 to 1.6)
Asbestosis	20.8 (17.9 to 23.9)	62.4 (58.9 to 66.5)*	4.4 (2.1 to 7.0)*	3.3 (2.1 to 4.8)	62.9 (48.4 to 78.1)*	4.7 (-4.6 to 14.4)
Asbestosis without heart failure aggregate	20.6 (17.7 to 23.7)	62.5 (58.8 to 66.6)*	4.4 (2.1 to 7.0)*	3.2 (2.0 to 4.7)	63.0 (48.1 to 78.7)*	4.7 (-4.9 to 14.8)
Severe asbestosis with heart failure aggregate	0.2 (0.2 to 0.3)	61.1 (58.7 to 63.4)*	3.5 (2.0 to 5.0)*	0.1 (0.1 to 0.1)	61.1 (58.7 to 63.4)*	3.5 (2.0 to 5.0)*
Coal workers pneumoconiosis	10.9 (9.1 to 12.8)	57.7 (51.6 to 62.9)*	1.3 (-2.6 to 4.7)	1.8 (1.1 to 2.6)	57.8 (33.2 to 89.2)*	1.4 (-14.4 to 21.6)
Coal workers pneumoconiosis without heart failure aggregate	10.6 (8.8 to 12.6)	57.9 (51.6 to 63.2)*	1.5 (-2.6 to 4.8)	1.6 (1.0 to 2.4)	58.4 (32.4 to 92.4)*	1.8 (-14.9 to 23.6)
Severe coal workers pneumoconiosis with heart failure aggregate	0.2 (0.2 to 0.3)	49.5 (37.9 to 57.6)*	-3.9 (-11.4 to 1.2)	0.1 (0.1 to 0.1)	49.5 (37.9 to 57.6)*	-3.9 (-11.4 to 1.2)
Other pneumoconiosis	163.5 (139.8 to 190.4)	57.4 (54.4 to 60.7)*	1.1 (-0.8 to 3.3)	24.4 (15.1 to 35.9)	57.7 (47.8 to 68.3)*	1.4 (-5.0 to 8.2)
Other pneumoconiosis without heart failure aggregate	162.9 (139.1 to 189.7)	57.5 (54.5 to 60.8)*	1.2 (-0.7 to 3.3)	24.1 (14.8 to 35.6)	58.0 (47.9 to 68.9)*	1.6 (-4.9 to 8.6)
Severe other pneumoconiosis with heart failure aggregate	0.6 (0.6 to 0.7)	38.1 (35.4 to 40.9)*	-11.2 (-13.0 to -9.4)*	0.3 (0.2 to 0.4)	38.1 (35.4 to 40.9)*	-11.2 (-13.0 to -9.4)*
Asthma	16,838.8 (13,498.7 to 20,610.1)	43.0 (40.1 to 46.4)*	-8.1 (-10.0 to -5.9)*	720.9 (458.8 to 1,068.4)	43.3 (40.1 to 46.7)*	-7.9 (-10.0 to -5.7)*
Asymptomatic asthma	5,099.7 (3,947.8 to 6,431.6)	43.1 (40.1 to 46.4)*	-8.1 (-10.0 to -5.9)*	0.0 (0.0 to 0.0)	0.0 (0.0 to 0.0)	0.0 (0.0 to 0.0)
Symptomatic asthma aggregate	11,739.1 (9,310.4 to 14,461.2)	43.0 (40.1 to 46.4)*	-8.1 (-10.0 to -5.9)*	720.9 (458.8 to 1,068.4)	43.3 (40.1 to 46.7)*	-7.9 (-10.0 to -5.7)*
Interstitial lung disease and pulmonary sarcoidosis	217.4 (185.8 to 255.5)	59.8 (55.7 to 63.5)*	2.7 (0.1 to 5.1)*	26.8 (16.9 to 38.9)	59.4 (52.9 to 66.6)*	2.4 (-1.7 to 7.0)
Interstitial lung disease and pulmonary sarcoidosis without heart failure aggregate	192.9 (162.5 to 229.9)	60.7 (56.4 to 64.7)*	3.3 (0.5 to 5.8)*	15.6 (9.1 to 24.1)	64.0 (52.9 to 75.5)*	5.4 (-1.8 to 12.8)
Severe interstitial lung disease and pulmonary sarcoidosis with heart failure aggregate	24.5 (18.2 to 29.3)	52.9 (49.3 to 57.3)*	-1.7 (-4.0 to 1.1)	11.2 (7.2 to 15.4)	53.3 (46.1 to 61.2)*	-1.5 (-6.1 to 3.6)
Other chronic respiratory diseases	--	--	--	158.2 (124.8 to 194.5)	17.2 (6.0 to 29.8)*	-24.7 (-31.9 to -16.6)*
Cirrhosis and other chronic liver diseases	343.7 (334.3 to 353.6)	68.1 (63.3 to 72.9)*	8.0 (5.0 to 11.1)*	55.6 (39.0 to 75.0)	68.4 (63.0 to 73.7)*	8.2 (4.8 to 11.6)*
Cirrhosis and other chronic liver diseases due to hepatitis B	95.7 (79.7 to 113.8)	62.4 (56.7 to 67.2)*	4.3 (0.7 to 7.4)*	15.4 (10.1 to 21.8)	62.8 (52.0 to 73.0)*	4.6 (-2.3 to 11.2)
Cirrhosis and other chronic liver diseases due to hepatitis C	82.9 (69.1 to 97.1)	65.5 (59.9 to 71.2)*	6.4 (2.8 to 10.0)*	13.3 (8.8 to 18.6)	65.8 (55.6 to 75.6)*	6.5 (0.0 to 12.8)*
Cirrhosis and other chronic liver diseases due to alcohol use	104.2 (87.4 to 121.5)	73.3 (68.5 to 77.9)*	11.4 (8.3 to 14.3)*	16.7 (11.4 to 23.6)	73.5 (63.9 to 83.2)*	11.5 (5.3 to 17.7)*
Cirrhosis and other chronic liver diseases due to other causes	62.7 (52.3 to 74.0)	71.8 (67.5 to 75.9)*	10.4 (7.6 to 13.1)*	10.1 (6.8 to 14.1)	72.3 (59.8 to 85.2)*	10.7 (2.7 to 19.0)*
Digestive diseases	29,446.0 (28,909.7 to 29,969.6)	37.3 (34.2 to 40.3)*	-11.8 (-13.8 to -9.8)*	1,209.8 (842.0 to 1,650.3)	35.9 (32.7 to 39.5)*	-12.6 (-14.7 to -10.3)*
Peptic ulcer disease	7,213.8 (6,997.8 to 7,433.7)	25.0 (21.7 to 28.2)*	-19.7 (-21.8 to -17.6)*	227.8 (152.9 to 318.1)	19.7 (15.6 to 23.6)*	-23.1 (-25.7 to -20.6)*
Peptic ulcer disease, symptomatic episodes	469.1 (358.3 to 606.9)	25.5 (19.1 to 31.3)*	-19.4 (-23.5 to -15.6)*	48.8 (31.3 to 71.0)	25.8 (18.7 to 32.3)*	-19.2 (-23.7 to -15.0)*
Anemia due to peptic ulcer disease aggregate	6,744.7 (6,555.8 to 6,907.8)	24.9 (21.5 to 28.4)*	-19.7 (-21.9 to -17.5)*	178.9 (119.9 to 257.4)	18.1 (12.8 to 22.7)*	-24.1 (-27.5 to -21.2)*
Gastritis and duodenitis	20,731.4 (20,251.4 to 21,249.0)	39.3 (35.6 to 43.1)*	-10.5 (-12.9 to -8.1)*	578.0 (391.1 to 819.0)	33.3 (28.4 to 38.1)*	-14.3 (-17.5 to -11.3)*
Gastritis and duodenitis, symptomatic episodes	466.6 (355.0 to 599.3)	40.4 (34.9 to 44.0)*	-9.8 (-13.3 to -7.5)*	48.8 (29.7 to 71.5)	40.8 (34.8 to 45.8)*	-9.5 (-13.4 to -6.3)*
Anemia due to gastritis and duodenitis aggregate	20,264.8 (19,791.4 to 20,740.5)	39.2 (35.5 to 43.1)*	-10.5 (-12.9 to -8.0)*	529.2 (354.1 to 758.1)	32.7 (27.2 to 37.8)*	-14.7 (-18.3 to -11.4)*
Appendicitis	14.5 (11.8 to 17.5)	46.8 (44.1 to 49.3)*	-5.7 (-7.4 to -4.1)*	4.3 (2.8 to 6.1)	48.2 (33.2 to 64.0)*	-4.8 (-14.4 to 5.4)
Paralytic ileus and intestinal obstruction	7.7 (6.1 to 9.6)	47.7 (40.9 to 54.6)*	-5.1 (-9.5 to -0.7)*	2.4 (1.5 to 3.5)	48.1 (38.1 to 58.5)*	-4.8 (-11.3 to 1.9)
Inguinal, femoral, and abdominal hernia	1,470.5 (1,089.6 to 1,925.9)	33.5 (27.1 to 40.2)*	-14.2 (-18.4 to -9.9)*	15.3 (6.8 to 28.9)	33.8 (27.1 to 40.8)*	-14.0 (-18.4 to -9.5)*
Inflammatory bowel disease	887.8 (828.7 to 955.1)	51.5 (50.7 to 52.4)*	-2.7 (-3.2 to -2.1)*	183.9 (126.6 to 248.9)	51.6 (48.7 to 54.2)*	-2.6 (-4.4 to -0.9)*
Vascular intestinal disorders	3.7 (2.9 to 4.7)	56.5 (48.1 to 64.1)*	0.6 (-4.8 to 5.5)	1.1 (0.7 to 1.7)	56.3 (42.0 to 71.8)*	0.4 (-8.7 to 10.4)
Gallbladder and biliary diseases	469.9 (361.4 to 609.9)	49.5 (41.7 to 57.9)*	-4.0 (-8.9 to 1.5)	49.1 (30.5 to 70.6)	49.7 (41.3 to 58.4)*	-3.8 (-9.2 to 1.8)
Pancreatitis	90.0 (72.8 to 110.5)	69.1 (61.7 to 76.1)*	8.6 (3.9 to 13.2)*	25.8 (17.0 to 35.8)	69.1 (56.8 to 81.5)*	8.7 (0.8 to 16.6)*
Other digestive diseases	--	--	--	122.1 (82.4 to 168.1)	52.5 (41.3 to 68.4)*	-2.0 (-9.2 to 8.2)
Neurological disorders	96,323.7 (89,368.6 to 102,487.8)	55.3 (52.0 to 58.8)*	-0.2 (-2.3 to 2.0)	2,831.1 (1,920.9 to 3,982.5)	52.7 (48.6 to 56.9)*	-1.9 (-4.5 to 0.8)
Alzheimer disease and other dementias	2,737.7 (2,048.5 to 3,377.9)	58.5 (57.1 to 59.5)*	1.8 (1.0 to 2.5)*	335.8 (222.5 to 489.3)	58.8 (56.3 to 61.2)*	2.1 (0.4 to 3.6)*
Parkinson disease	635.5 (546.6 to 735.3)	56.3 (49.7 to 62.7)*	0.4 (-3.8 to 4.6)	77.3 (52.5 to 106.7)	56.7 (48.3 to 64.4)*	0.7 (-4.7 to 5.6)
Epilepsy	928.0 (794.9 to 1,066.7)	53.1 (45.9 to 60.4)*	-1.6 (-6.2 to 3.0)	211.1 (143.9 to 289.5)	23.8 (14.9 to 32.5)*	-20.5 (-26.2 to -14.9)*
Multiple sclerosis	174.3 (161.5 to 187.7)	60.4 (57.3 to 63.4)*	3.1 (1.1 to 5.0)*	56.2 (39.8 to 72.4)	60.2 (54.0 to 67.2)*	2.9 (-1.1 to 7.5)
Motor neuron disease	25.0 (20.6 to 29.9)	54.8 (46.3 to 62.5)*	-0.5 (-6.0 to 4.4)	5.2 (3.5 to 7.2)	54.5 (45.0 to 63.8)*	-0.7 (-6.8 to 5.3)
Migraine	41,481.2 (33,247.2 to 50,550.9)	53.9 (51.0 to 56.7)*	-1.1 (-3.0 to 0.7)	1,354.2 (808.5 to 2,062.7)	54.4 (51.3 to 57.4)*	-0.8 (-2.8 to 1.2)
Tension-type headache	59,887.6 (42,058.1 to 83,550.2)	55.7 (53.0 to 58.8)*	0.1 (-1.7 to 2.0)	87.2 (36.4 to 171.3)	56.1 (52.7 to 59.3)*	0.3 (-1.9 to 2.3)
Medication overuse headache	4,491.6 (2,964.8 to 6,575.9)	56.0 (40.4 to 72.6)*	0.2 (-9.8 to 10.9)	681.8 (376.6 to 1,116.5)	56.2 (40.4 to 72.7)*	0.4 (-9.7 to 10.9)
Other neurological disorders	0.9 (0.5 to 1.4)	53.0 (49.5 to 55.4)*	-1.7 (-3.9 to -0.1)*	22.1 (15.5 to 30.8)	52.1 (45.7 to 57.7)*	-2.3 (-6.4 to 1.3)
Mental and substance use disorders	46,304.5 (44,985.6 to 47,522.9)	55.7 (54.3 to 57.1)*	0.1 (-0.9 to 1.0)	6,978.2 (5,064.2 to 9,174.1)	56.5 (55.3 to 57.7)*	0.6 (-0.2 to 1.3)
Schizophrenia	1,539.2 (1,352.7 to 1,730.1)	58.7 (57.6 to 59.9)*	2.0 (1.3 to 2.7)*	947.0 (703.0 to 1,181.5)	59.2 (56.8 to 61.7)*	2.3 (0.8 to 3.9)*
Alcohol use disorders	2,623.8 (2,191.4 to 3,122.3)	58.7 (53.3 to 64.1)*	2.0 (-1.5 to 5.5)	251.7 (167.4 to 370.1)	59.1 (52.8 to 65.3)*	2.3 (-1.8 to 6.2)
Alcohol dependence aggregate	2,594.1 (2,162.5 to 3,091.0)	58.8 (53.3 to 64.2)*	2.0 (-1.5 to 5.5)	250.2 (166.0 to 368.5)	59.2 (52.8 to 65.4)*	2.3 (-1.8 to 6.3)
Fetal alcohol syndrome aggregate	29.7 (26.9 to 32.7)	55.2 (53.5 to 56.9)*	-0.3 (-1.4 to 0.8)	1.5 (0.9 to 2.1)	53.9 (44.4 to 62.9)*	-1.1 (-7.2 to 4.7)
Drug use disorders	1,075.1 (1,034.5 to 1,115.1)	81.1 (77.1 to 85.1)*	16.4 (13.8 to 18.9)*	307.8 (211.9 to 397.5)	84.2 (78.8 to 89.8)*	18.4 (14.9 to 22.0)*
Opioid use disorders	614.6 (518.8 to 731.6)	81.0 (76.7 to 85.5)*	16.3 (13.6 to 19.2)*	242.0 (163.3 to 321.6)	81.6 (75.4 to 88.2)*	16.7 (12.7 to 20.9)*
Asymptomatic opioid dependence	99.7 (71.6 to 138.0)	81.0 (76.7 to 85.5)*	16.3 (13.6 to 19.2)*	0.0 (0.0 to 0.0)	0.0 (0.0 to 0.0)	0.0 (0.0 to 0.0)
Symptomatic opioid dependence aggregate	514.9 (429.8 to 617.1)	81.0 (76.7 to 85.5)*	16.3 (13.6 to 19.2)*	242.0 (163.3 to 321.6)	81.6 (75.4 to 88.2)*	16.7 (12.7 to 20.9)*
Cocaine use disorders	232.6 (182.6 to 291.7)	92.4 (88.1 to 97.0)*	23.7 (20.9 to 26.6)*	30.2 (18.9 to 45.4)	92.1 (81.5 to 104.7)*	23.4 (16.6 to 31.5)*
Asymptomatic cocaine dependence	116.6 (85.8 to 153.8)	92.4 (88.1 to 97.0)*	23.7 (20.9 to 26.6)*	0.0 (0.0 to 0.0)	0.0 (0.0 to 0.0)	0.0 (0.0 to 0.0)

Cause	Prevalence in thousands (95% confidence interval)			YLDs in thousands (95% confidence interval)		
	2015	Percent change in counts between 2005 and 2015	Percent change in rates between 2005 and 2015	2015	Percent change in counts between 2005 and 2015	Percent change in rates between 2005 and 2015
Symptomatic cocaine dependence aggregate	116.0 (85.9 to 151.7)	92.4 (88.1 to 97.0)*	23.7 (20.9 to 26.6)*	30.2 (18.9 to 45.4)	92.1 (81.5 to 104.7)*	23.4 (16.6 to 31.5)*
Amphetamine use disorders	46.2 (34.8 to 59.3)	149.1 (138.0 to 162.6)*	60.1 (52.9 to 68.8)*	6.0 (3.5 to 9.0)	142.6 (114.3 to 178.3)*	55.9 (37.7 to 78.8)*
Asymptomatic amphetamine dependence	25.3 (18.0 to 34.4)	149.1 (138.0 to 162.6)*	60.1 (52.9 to 68.8)*	0.0 (0.0 to 0.0)	0.0 (0.0 to 0.0)	0.0 (0.0 to 0.0)
Symptomatic amphetamine dependence aggregate	20.9 (14.9 to 28.1)	149.1 (138.0 to 162.6)*	60.1 (52.9 to 68.8)*	6.0 (3.5 to 9.0)	142.6 (114.3 to 178.3)*	55.9 (37.7 to 78.8)*
Cannabis use disorders	215.5 (196.9 to 236.1)	58.0 (56.3 to 59.8)*	1.5 (0.4 to 2.7)*	6.2 (4.1 to 8.8)	57.8 (48.9 to 67.7)*	1.4 (-4.3 to 7.8)
Asymptomatic cannabis dependence	124.7 (111.1 to 138.8)	58.0 (56.3 to 59.8)*	1.5 (0.4 to 2.7)*	0.0 (0.0 to 0.0)	0.0 (0.0 to 0.0)	0.0 (0.0 to 0.0)
Symptomatic cannabis dependence aggregate	90.9 (78.5 to 104.7)	58.0 (56.3 to 59.8)*	1.5 (0.4 to 2.7)*	6.2 (4.1 to 8.8)	57.8 (48.9 to 67.7)*	1.4 (-4.3 to 7.8)
Other drug use disorders	--	--	--	23.5 (14.7 to 34.9)	99.1 (88.2 to 112.1)*	27.9 (20.9 to 36.3)*
Depressive disorders	19,915.4 (18,839.5 to 21,158.0)	54.3 (51.6 to 56.8)*	-0.9 (-2.6 to 0.8)	3,195.0 (2,153.9 to 4,507.3)	53.7 (52.0 to 55.2)*	-1.2 (-2.3 to -0.2)*
Major depressive disorder	12,203.1 (9,426.2 to 15,460.5)	52.0 (50.0 to 53.8)*	-2.3 (-3.6 to -1.2)*	2,401.3 (1,566.1 to 3,429.0)	52.4 (50.3 to 54.3)*	-2.0 (-3.4 to -0.9)*
Dysthymia	8,441.1 (6,522.8 to 10,457.8)	57.3 (54.9 to 60.1)*	1.1 (-0.5 to 2.9)	793.7 (500.5 to 1,206.0)	57.7 (54.9 to 60.5)*	1.3 (-0.4 to 3.1)
Bipolar disorder	1,643.5 (1,366.3 to 1,965.5)	54.6 (54.2 to 55.0)*	-0.6 (-0.9 to -0.4)*	320.4 (192.6 to 483.9)	55.0 (52.5 to 57.8)*	-0.4 (-2.0 to 1.4)
Anxiety disorders	12,250.2 (9,860.2 to 15,127.3)	56.0 (51.9 to 59.7)*	0.2 (-2.4 to 2.6)	1,083.5 (714.9 to 1,548.4)	56.2 (52.1 to 60.1)*	0.4 (-2.2 to 2.9)
Eating disorders	0.0 (0.0 to 0.0)	0.0 (0.0 to 0.0)	0.0 (0.0 to 0.0)	0.0 (0.0 to 0.0)	0.0 (0.0 to 0.0)	0.0 (0.0 to 0.0)
Anorexia nervosa	0.0 (0.0 to 0.0)	0.0 (0.0 to 0.0)	0.0 (0.0 to 0.0)	0.0 (0.0 to 0.0)	0.0 (0.0 to 0.0)	0.0 (0.0 to 0.0)
Bulimia nervosa	0.0 (0.0 to 0.0)	0.0 (0.0 to 0.0)	0.0 (0.0 to 0.0)	0.0 (0.0 to 0.0)	0.0 (0.0 to 0.0)	0.0 (0.0 to 0.0)
Autistic spectrum disorders	2,202.9 (2,103.9 to 2,326.8)	55.9 (53.0 to 58.8)*	0.2 (-1.7 to 2.1)	341.0 (229.2 to 463.4)	56.1 (53.9 to 58.2)*	0.3 (-1.1 to 1.7)
Autism	888.4 (744.7 to 1,064.8)	55.8 (55.1 to 56.4)*	0.1 (-0.3 to 0.5)	215.9 (140.6 to 306.0)	56.1 (52.9 to 59.3)*	0.3 (-1.8 to 2.4)
Asperger syndrome and other autistic spectrum disorders	1,303.5 (1,104.8 to 1,558.3)	55.8 (55.6 to 55.9)*	0.1 (-0.0 to 0.2)	125.1 (84.0 to 181.2)	56.0 (53.9 to 58.2)*	0.3 (-1.1 to 1.6)
Attention-deficit/hyperactivity disorder	0.5 (0.4 to 0.5)	79.1 (61.2 to 98.5)*	15.1 (3.6 to 27.6)*	0.0 (0.0 to 0.0)	72.1 (-53.2 to 551.1)	10.6 (-69.9 to 318.4)
Conduct disorder	0.0 (0.0 to 0.0)	0.0 (0.0 to 0.0)	0.0 (0.0 to 0.0)	0.0 (0.0 to 0.0)	0.0 (0.0 to 0.0)	0.0 (0.0 to 0.0)
Idiopathic developmental intellectual disability	2,070.9 (1,007.2 to 3,092.6)	54.7 (51.6 to 57.5)*	-0.6 (-2.6 to 1.2)	83.6 (35.6 to 145.4)	53.7 (48.8 to 57.6)*	-1.2 (-4.4 to 1.2)
Other mental and substance use disorders	6,189.4 (6,019.7 to 6,356.8)	55.4 (49.9 to 61.2)*	-0.1 (-3.7 to 3.6)	448.3 (314.8 to 603.5)	55.8 (49.8 to 61.8)*	0.1 (-3.8 to 4.0)
Diabetes, urogenital, blood, and endocrine diseases	164,949.7 (162,869.5 to 167,228.9)	52.6 (51.8 to 53.3)*	-2.0 (-2.4 to -1.5)*	6,227.1 (4,378.6 to 8,347.3)	56.7 (53.9 to 59.3)*	0.7 (-1.1 to 2.4)
Diabetes mellitus	53,051.8 (48,247.1 to 58,408.9)	58.1 (54.1 to 61.6)*	1.6 (-1.0 to 3.8)	4,220.9 (2,897.5 to 5,840.8)	59.3 (55.3 to 63.0)*	2.3 (-0.2 to 4.7)
Uncomplicated diabetes mellitus	30,830.5 (25,965.4 to 35,954.9)	56.2 (51.8 to 60.2)*	0.4 (-2.4 to 3.0)	1,412.9 (894.2 to 2,104.0)	56.5 (52.1 to 60.4)*	0.5 (-2.3 to 3.1)
Neuropathy and other complications of diabetes mellitus aggregate	21,708.4 (17,354.4 to 26,460.1)	60.6 (56.0 to 65.1)*	3.2 (0.2 to 6.1)*	2,773.3 (1,828.4 to 4,022.3)	60.7 (56.0 to 64.9)*	3.2 (0.2 to 5.9)*
Vision loss due to diabetes mellitus aggregate	512.9 (353.5 to 716.5)	65.1 (60.6 to 70.3)*	6.1 (3.2 to 9.4)*	34.7 (21.9 to 51.0)	65.3 (59.0 to 72.3)*	6.2 (2.2 to 10.7)*
Acute glomerulonephritis	7.8 (5.6 to 10.4)	48.0 (40.6 to 54.1)*	-4.9 (-9.6 to -0.9)*	0.4 (0.2 to 0.6)	48.9 (26.3 to 72.6)*	-4.3 (-18.9 to 10.9)
Chronic kidney disease	35,621.1 (32,598.6 to 38,193.2)	53.1 (50.0 to 56.3)*	-1.6 (-3.6 to 0.5)	772.1 (553.7 to 992.4)	54.8 (52.7 to 57.0)*	-0.5 (-1.9 to 0.9)
Chronic kidney disease due to diabetes mellitus	10,815.3 (7,075.4 to 15,640.7)	53.9 (49.2 to 58.8)*	-1.1 (-4.1 to 2.0)	220.9 (143.0 to 324.5)	56.2 (51.2 to 61.2)*	0.4 (-2.8 to 3.6)
Stage III chronic kidney disease due to diabetes mellitus aggregate	9,986.8 (6,495.4 to 14,643.7)	53.8 (48.8 to 58.8)*	-1.2 (-4.4 to 2.1)	8.6 (5.2 to 14.0)	51.8 (37.0 to 69.1)*	-2.4 (-12.0 to 8.7)
Stage IV chronic kidney disease due to diabetes mellitus aggregate	472.5 (286.4 to 729.2)	54.4 (50.3 to 58.8)*	-0.8 (-3.4 to 2.0)	55.1 (29.9 to 91.2)	54.4 (49.1 to 59.8)*	-0.8 (-4.2 to 2.7)
Stage V chronic kidney disease untreated due to diabetes mellitus	256.3 (166.2 to 372.1)	55.2 (50.9 to 59.6)*	-0.2 (-3.0 to 2.6)	127.0 (75.6 to 189.5)	55.7 (49.0 to 62.5)*	0.0 (-4.3 to 4.4)
End-stage chronic kidney disease due to diabetes aggregate	99.6 (67.7 to 134.7)	61.2 (55.4 to 67.1)*	3.6 (-0.1 to 7.4)	30.1 (17.6 to 45.0)	63.6 (52.4 to 74.9)*	5.1 (-2.1 to 12.4)
Chronic kidney disease due to hypertension	8,969.9 (5,838.7 to 12,910.2)	53.6 (48.5 to 58.4)*	-1.3 (-4.6 to 1.8)	160.9 (109.9 to 225.7)	55.7 (49.4 to 62.2)*	0.1 (-4.0 to 4.2)
Stage III chronic kidney disease due to hypertension aggregate	8,371.4 (5,193.5 to 12,235.4)	53.5 (47.9 to 58.7)*	-1.4 (-4.9 to 2.0)	9.3 (5.9 to 14.4)	50.5 (39.0 to 62.2)*	-3.3 (-10.7 to 4.2)
Stage IV chronic kidney disease due to hypertension aggregate	347.0 (228.1 to 523.3)	52.6 (44.5 to 60.9)*	-1.9 (-7.1 to 3.4)	40.9 (23.6 to 66.7)	52.2 (44.0 to 60.3)*	-2.2 (-7.4 to 3.0)
Stage V chronic kidney disease untreated due to hypertension	182.2 (130.0 to 248.1)	55.1 (48.9 to 61.5)*	-0.3 (-4.3 to 3.8)	90.3 (56.2 to 136.7)	55.6 (47.2 to 64.3)*	-0.0 (-5.4 to 5.6)
End-stage chronic kidney disease due to hypertension aggregate	69.3 (50.5 to 91.7)	64.3 (55.5 to 73.9)*	5.5 (-0.1 to 11.7)	20.5 (12.9 to 31.4)	66.6 (53.0 to 81.1)*	7.1 (-1.7 to 16.4)
Chronic kidney disease due to glomerulonephritis	7,888.1 (5,692.5 to 10,921.5)	53.8 (47.6 to 60.3)*	-1.2 (-5.2 to 3.0)	185.9 (119.0 to 261.2)	55.3 (50.4 to 60.6)*	-0.2 (-3.4 to 3.2)
Stage III chronic kidney disease due to glomerulonephritis aggregate	7,204.8 (4,989.5 to 10,181.0)	53.6 (46.7 to 60.6)*	-1.3 (-5.7 to 3.2)	12.4 (8.0 to 18.4)	43.3 (32.4 to 54.3)*	-7.9 (-14.9 to -0.8)*
Stage IV chronic kidney disease due to glomerulonephritis aggregate	405.0 (259.3 to 559.9)	54.5 (47.7 to 61.9)*	-0.7 (-5.1 to 4.0)	47.6 (27.4 to 72.5)	54.8 (47.3 to 62.9)*	-0.5 (-5.3 to 4.7)
Stage V chronic kidney disease untreated due to glomerulonephritis	218.9 (138.9 to 305.6)	54.9 (50.7 to 59.3)*	-0.5 (-3.2 to 2.4)	107.9 (61.4 to 163.7)	55.3 (48.4 to 62.6)*	-0.2 (-4.6 to 4.5)
End-stage chronic kidney disease due to glomerulonephritis aggregate	59.4 (37.7 to 84.7)	63.6 (56.8 to 70.2)*	5.1 (0.8 to 9.3)*	17.8 (10.0 to 27.8)	66.1 (52.2 to 80.7)*	6.8 (-2.2 to 16.1)
Chronic kidney disease due to other causes	10,047.2 (6,197.4 to 14,596.8)	51.0 (46.9 to 55.5)*	-3.0 (-5.6 to -0.1)*	204.5 (124.2 to 294.5)	52.2 (47.7 to 56.6)*	-2.2 (-5.1 to 0.6)
Stage III chronic kidney disease due to other causes aggregate	9,297.7 (5,698.5 to 13,533.9)	50.9 (46.6 to 55.6)*	-3.0 (-5.8 to -0.0)*	14.2 (9.1 to 21.6)	39.7 (28.6 to 50.9)*	-10.2 (-17.4 to -3.1)*
Stage IV chronic kidney disease due to other causes aggregate	439.4 (267.3 to 682.4)	51.3 (48.1 to 54.5)*	-2.8 (-4.8 to -0.7)*	51.6 (27.8 to 84.8)	50.7 (45.6 to 56.1)*	-3.1 (-6.4 to 0.3)
Stage V chronic kidney disease untreated due to other causes	233.5 (147.7 to 336.3)	52.5 (49.3 to 55.8)*	-2.0 (-4.1 to 0.1)	115.5 (66.0 to 179.1)	52.9 (47.0 to 58.9)*	-1.8 (-5.5 to 2.1)
End-stage chronic kidney disease due to other causes aggregate	76.6 (50.7 to 108.7)	59.9 (54.1 to 65.8)*	2.7 (-1.0 to 6.5)	23.1 (13.0 to 35.2)	60.7 (48.4 to 73.3)*	3.3 (-4.6 to 11.3)
Urinary diseases and male infertility	47,021.1 (45,205.4 to 48,427.0)	53.6 (51.6 to 55.8)*	-1.3 (-2.6 to 0.1)	769.4 (472.5 to 1,134.9)	56.0 (51.9 to 60.4)*	0.3 (-2.4 to 3.1)
Interstitial nephritis and urinary tract infections	142.6 (133.3 to 152.9)	58.6 (57.6 to 59.6)*	1.9 (1.3 to 2.6)*	4.6 (2.9 to 7.0)	58.7 (50.5 to 66.7)*	2.0 (-3.3 to 7.1)
Urolithiasis	29,625.7 (26,601.2 to 32,850.6)	52.3 (49.9 to 54.7)*	-2.2 (-3.7 to -0.6)*	7.8 (4.9 to 11.4)	73.1 (61.5 to 86.3)*	11.2 (3.8 to 19.7)*
Benign prostatic hyperplasia	20,502.8 (16,235.6 to 25,473.3)	55.7 (51.6 to 60.0)*	0.0 (-2.6 to 2.8)	752.3 (459.7 to 1,113.6)	55.8 (51.6 to 60.3)*	0.1 (-2.6 to 3.0)
Male infertility	0.0 (0.0 to 0.0)	0.0 (0.0 to 0.0)	0.0 (0.0 to 0.0)	0.0 (0.0 to 0.0)	0.0 (0.0 to 0.0)	0.0 (0.0 to 0.0)
Other urinary diseases	--	--	--	4.7 (3.0 to 6.7)	59.1 (45.2 to 74.9)*	2.2 (-6.7 to 12.4)
Gynecological diseases	25,378.8 (24,612.3 to 26,109.1)	44.3 (42.1 to 46.5)*	-7.3 (-8.7 to -5.8)*	111.3 (56.5 to 194.7)	46.5 (43.8 to 49.0)*	-5.8 (-7.6 to -4.3)*
Uterine fibroids	6,925.4 (6,543.9 to 7,352.4)	54.6 (54.1 to 55.0)*	-0.7 (-1.0 to -0.4)*	49.5 (24.7 to 93.0)	54.8 (53.6 to 56.1)*	-0.5 (-1.3 to 0.3)
Uterine fibroids cases aggregate	6,925.4 (6,543.9 to 7,352.4)	54.6 (54.1 to 55.0)*	-0.7 (-1.0 to -0.4)*	49.5 (24.7 to 93.0)	54.8 (53.6 to 56.1)*	-0.5 (-1.3 to 0.3)
Mild abdominal pain with anemia due to uterine fibroids aggregate	0.0 (0.0 to 0.0)	0.0 (0.0 to 0.0)	0.0 (0.0 to 0.0)	0.0 (0.0 to 0.0)	0.0 (0.0 to 0.0)	0.0 (0.0 to 0.0)
Polycystic ovarian syndrome	0.0 (0.0 to 0.0)	0.0 (0.0 to 0.0)	0.0 (0.0 to 0.0)	0.0 (0.0 to 0.0)	0.0 (0.0 to 0.0)	0.0 (0.0 to 0.0)
Polycystic ovarian syndrome cases aggregate	0.0 (0.0 to 0.0)	0.0 (0.0 to 0.0)	0.0 (0.0 to 0.0)	0.0 (0.0 to 0.0)	0.0 (0.0 to 0.0)	0.0 (0.0 to 0.0)
Hirsutism and infertility due to polycystic ovarian syndrome aggregate	0.0 (0.0 to 0.0)	0.0 (0.0 to 0.0)	0.0 (0.0 to 0.0)	0.0 (0.0 to 0.0)	0.0 (0.0 to 0.0)	0.0 (0.0 to 0.0)

Cause	Prevalence in thousands (95% confidence interval)			YLDs in thousands (95% confidence interval)		
	2015	Percent change in counts between 2005 and 2015	Percent change in rates between 2005 and 2015	2015	Percent change in counts between 2005 and 2015	Percent change in rates between 2005 and 2015
Female infertility	0.0 (0.0 to 0.0)	0.0 (0.0 to 0.0)	0.0 (0.0 to 0.0)	0.0 (0.0 to 0.0)	0.0 (0.0 to 0.0)	0.0 (0.0 to 0.0)
Endometriosis	0.0 (0.0 to 0.0)	0.0 (0.0 to 0.0)	0.0 (0.0 to 0.0)	0.0 (0.0 to 0.0)	0.0 (0.0 to 0.0)	0.0 (0.0 to 0.0)
Endometriosis cases aggregate	0.0 (0.0 to 0.0)	0.0 (0.0 to 0.0)	0.0 (0.0 to 0.0)	0.0 (0.0 to 0.0)	0.0 (0.0 to 0.0)	0.0 (0.0 to 0.0)
Abdominal pain and infertility due to endometriosis aggregate	0.0 (0.0 to 0.0)	0.0 (0.0 to 0.0)	0.0 (0.0 to 0.0)	0.0 (0.0 to 0.0)	0.0 (0.0 to 0.0)	0.0 (0.0 to 0.0)
Genital prolapse	20,014.4 (17,522.1 to 23,082.3)	40.1 (36.4 to 43.6)*	-10.0 (-12.3 to -7.7)*	61.8 (29.8 to 110.6)	40.5 (36.6 to 44.3)*	-9.7 (-12.2 to -7.3)*
Premenstrual syndrome	0.0 (0.0 to 0.0)	0.0 (0.0 to 0.0)	0.0 (0.0 to 0.0)	0.0 (0.0 to 0.0)	0.0 (0.0 to 0.0)	0.0 (0.0 to 0.0)
Other gynecological diseases	0.0 (0.0 to 0.0)	0.0 (0.0 to 0.0)	0.0 (0.0 to 0.0)	0.0 (0.0 to 0.0)	0.0 (0.0 to 0.0)	0.0 (0.0 to 0.0)
Other gynecological diseases cases aggregate	0.0 (0.0 to 0.0)	0.0 (0.0 to 0.0)	0.0 (0.0 to 0.0)	0.0 (0.0 to 0.0)	0.0 (0.0 to 0.0)	0.0 (0.0 to 0.0)
Anemia due to other gynecological diseases aggregate	0.0 (0.0 to 0.0)	0.0 (0.0 to 0.0)	0.0 (0.0 to 0.0)	0.0 (0.0 to 0.0)	0.0 (0.0 to 0.0)	0.0 (0.0 to 0.0)
Hemoglobinopathies and hemolytic anemias	57,632.1 (56,688.1 to 58,738.1)	46.4 (44.6 to 47.9)*	-6.0 (-7.1 to -4.9)*	266.1 (178.4 to 379.5)	38.0 (33.5 to 42.8)*	-11.3 (-14.2 to -8.3)*
Thalassemias	0.3 (0.2 to 0.3)	45.3 (35.1 to 56.0)*	-6.6 (-13.2 to 0.2)	0.0 (0.0 to 0.1)	42.5 (34.6 to 51.4)*	-8.4 (-13.5 to -2.7)*
Beta-thalassemia major cases aggregate	0.2 (0.2 to 0.2)	41.3 (27.7 to 55.8)*	-9.2 (-17.9 to 0.1)	0.0 (0.0 to 0.0)	39.0 (29.5 to 49.7)*	-10.7 (-16.8 to -3.8)*
Hemoglobin E/beta-thalassemia cases aggregate	0.0 (0.0 to 0.0)	50.4 (49.4 to 51.5)*	-3.4 (-4.0 to -2.7)*	0.0 (0.0 to 0.0)	48.6 (33.4 to 64.6)*	-4.5 (-14.3 to 5.7)
Hemoglobin H disease cases aggregate	0.0 (0.0 to 0.0)	58.9 (52.7 to 65.8)*	2.1 (-1.9 to 6.6)	0.0 (0.0 to 0.0)	54.5 (46.8 to 62.8)*	-0.8 (-5.7 to 4.6)
Heart failure due to thalassemias aggregate	0.0 (0.0 to 0.0)	65.2 (60.6 to 69.5)*	6.2 (3.2 to 8.9)*	0.0 (0.0 to 0.0)	65.2 (60.6 to 69.5)*	6.1 (3.2 to 8.9)*
Thalassemias trait	11,987.1 (11,716.4 to 12,329.2)	61.3 (60.3 to 62.2)*	3.7 (3.0 to 4.2)*	122.6 (80.7 to 177.6)	55.5 (48.8 to 62.4)*	-0.1 (-4.4 to 4.3)
B-thalassemia trait cases aggregate	9,797.1 (9,543.9 to 10,123.9)	61.7 (60.4 to 62.7)*	3.9 (3.1 to 4.5)*	116.9 (77.2 to 168.9)	55.9 (49.0 to 62.6)*	0.2 (-4.3 to 4.5)
Hemoglobin E trait cases aggregate	2,190.0 (2,104.2 to 2,278.3)	59.6 (58.2 to 61.0)*	2.6 (1.7 to 3.5)*	5.7 (3.6 to 8.7)	48.3 (31.8 to 64.5)*	-4.7 (-15.3 to 5.7)
Sickle cell disorders	1.3 (1.3 to 1.4)	51.6 (45.1 to 58.8)*	-2.6 (-6.8 to 2.0)	0.2 (0.2 to 0.3)	54.7 (48.1 to 61.3)*	-0.6 (-4.8 to 3.6)
Homozygous sickle cell and severe sickle cell/beta-thalassemia cases aggregate	1.1 (1.0 to 1.1)	53.4 (45.1 to 62.3)*	-1.4 (-6.7 to 4.3)	0.2 (0.1 to 0.3)	58.6 (50.1 to 67.1)*	1.9 (-3.6 to 7.4)
Hemoglobin SC disease cases aggregate	0.1 (0.1 to 0.1)	56.7 (52.2 to 61.5)*	0.7 (-2.2 to 3.8)	0.0 (0.0 to 0.0)	52.6 (49.3 to 55.8)*	-1.9 (-4.0 to 0.1)
Mild sickle cell/beta-thalassemia cases aggregate	0.1 (0.1 to 0.1)	35.4 (31.6 to 39.2)*	-13.0 (-15.4 to -10.5)*	0.0 (0.0 to 0.0)	31.5 (27.5 to 35.5)*	-15.5 (-18.1 to -12.9)*
Sickle cell trait	11,194.0 (10,681.5 to 12,183.5)	42.0 (37.9 to 44.3)*	-8.8 (-11.4 to -7.3)*	34.1 (23.0 to 48.2)	24.4 (17.9 to 31.0)*	-20.1 (-24.2 to -15.8)*
G6PD deficiency	7,987.8 (6,768.2 to 9,275.8)	37.4 (30.8 to 43.2)*	-11.7 (-16.0 to -8.0)*	0.9 (0.6 to 1.2)	39.8 (27.3 to 52.6)*	-10.1 (-18.2 to -1.9)*
G6PD cases aggregate	7,987.5 (6,767.9 to 9,275.5)	37.4 (30.8 to 43.2)*	-11.7 (-16.0 to -8.0)*	0.8 (0.6 to 1.2)	39.2 (26.3 to 52.6)*	-10.5 (-18.8 to -1.9)*
Heart failure due to G6PD deficiency aggregate	0.3 (0.3 to 0.4)	54.4 (51.2 to 58.0)*	-0.8 (-2.8 to 1.5)	0.0 (0.0 to 0.1)	54.4 (51.2 to 58.0)*	-0.8 (-2.8 to 1.5)
G6PD trait	27,530.7 (25,406.6 to 29,658.3)	44.6 (42.0 to 47.0)*	-7.1 (-8.7 to -5.6)*	0.7 (0.5 to 1.0)	39.2 (20.6 to 59.7)*	-10.6 (-22.5 to 2.6)
Other hemoglobinopathies and hemolytic anemias	4,546.2 (4,454.9 to 4,640.1)	40.3 (37.1 to 43.9)*	-9.8 (-11.9 to -7.6)*	107.5 (73.1 to 151.5)	26.1 (21.0 to 31.0)*	-19.0 (-22.3 to -15.8)*
Other hemoglobinopathies and hemolytic anemias cases aggregate	4,525.5 (4,434.3 to 4,619.7)	40.3 (37.0 to 43.8)*	-9.9 (-12.0 to -7.6)*	104.6 (70.9 to 148.1)	25.5 (20.2 to 30.5)*	-19.4 (-22.8 to -16.2)*
Heart failure due to other hemoglobinopathies and hemolytic anemias aggregate	20.7 (17.8 to 23.9)	55.3 (53.3 to 57.3)*	-0.2 (-1.5 to 1.1)	2.5 (1.6 to 3.5)	55.7 (44.4 to 68.7)*	0.0 (-7.2 to 8.4)
Endocrine, metabolic, blood, and immune disorders	3,413.1 (3,324.8 to 3,511.4)	43.9 (38.7 to 48.4)*	-7.5 (-10.9 to -4.6)*	86.9 (60.5 to 120.4)	37.2 (31.3 to 43.7)*	-11.8 (-15.6 to -7.6)*
Endocrine, metabolic, blood, and immune disorders cases aggregate	682.7 (618.0 to 750.3)	61.7 (58.2 to 65.1)*	3.9 (1.7 to 6.1)*	25.8 (17.4 to 35.0)	61.7 (54.8 to 68.7)*	3.9 (-0.5 to 8.4)
Anemia due to endocrine, metabolic, blood, and immune disorders aggregate	2,720.7 (2,656.5 to 2,786.1)	40.1 (33.8 to 45.5)*	-10.0 (-14.0 to -6.5)*	60.0 (40.4 to 84.7)	28.6 (20.8 to 37.2)*	-17.4 (-22.4 to -11.8)*
Heart failure due to endocrine, metabolic, blood, and immune disorders aggregate	9.7 (8.4 to 11.2)	47.1 (45.2 to 49.1)*	-5.5 (-6.7 to -4.2)*	1.2 (0.8 to 1.7)	48.1 (39.4 to 57.0)*	-4.9 (-10.4 to 0.9)
Musculoskeletal disorders	120,561.2 (117,888.1 to 123,738.9)	55.5 (54.2 to 56.7)*	-0.1 (-0.9 to 0.7)	13,578.2 (9,667.7 to 17,951.6)	55.4 (53.3 to 57.6)*	-0.1 (-1.5 to 1.3)
Rheumatoid arthritis	2,783.0 (2,453.0 to 3,133.7)	59.7 (55.5 to 64.0)*	2.6 (-0.1 to 5.4)	646.3 (448.1 to 879.1)	60.0 (55.2 to 64.7)*	2.8 (-0.3 to 5.8)
Osteoarthritis	36,018.2 (34,653.5 to 37,451.2)	60.5 (59.3 to 61.7)*	3.2 (2.4 to 3.9)*	1,955.4 (1,365.4 to 2,676.7)	63.3 (61.6 to 65.1)*	4.9 (3.8 to 6.1)*
Osteoarthritis of the hip cases aggregate	5,707.7 (4,870.7 to 6,547.9)	60.9 (58.8 to 63.1)*	3.4 (2.0 to 4.8)*	293.0 (197.0 to 418.0)	64.0 (61.4 to 67.3)*	5.4 (3.7 to 7.5)*
Osteoarthritis of the knee cases aggregate	30,310.5 (29,226.2 to 31,452.4)	60.5 (59.1 to 61.8)*	3.1 (2.2 to 4.0)*	1,662.4 (1,155.9 to 2,244.1)	63.2 (61.3 to 65.0)*	4.9 (3.6 to 6.0)*
Low back and neck pain	70,510.3 (67,870.5 to 73,094.5)	53.2 (51.3 to 55.1)*	-1.6 (-2.7 to -0.3)*	8,186.6 (5,741.6 to 11,193.1)	53.0 (51.0 to 55.2)*	-1.7 (-3.0 to -0.2)*
Low back pain	46,488.5 (41,479.3 to 52,214.6)	50.5 (47.8 to 53.2)*	-3.3 (-5.0 to -1.5)*	5,048.7 (3,499.8 to 6,969.6)	50.8 (48.2 to 53.6)*	-3.1 (-4.8 to -1.3)*
Neck pain	32,869.1 (24,086.4 to 43,361.7)	56.5 (53.2 to 60.3)*	0.5 (-1.5 to 3.0)	3,137.9 (1,975.7 to 4,734.9)	56.7 (53.4 to 60.7)*	0.7 (-1.4 to 3.3)
Gout	5,209.9 (4,168.6 to 6,571.0)	57.6 (56.2 to 59.2)*	1.3 (0.4 to 2.3)*	163.3 (107.4 to 231.0)	57.9 (54.1 to 62.0)*	1.5 (-1.0 to 4.1)
Asymptomatic gout	4,654.3 (3,712.6 to 5,851.2)	57.6 (56.2 to 59.2)*	1.3 (0.4 to 2.3)*	0.0 (0.0 to 0.0)	0.0 (0.0 to 0.0)	0.0 (0.0 to 0.0)
Symptomatic gout cases aggregate	555.6 (429.2 to 704.8)	57.6 (56.2 to 59.2)*	1.3 (0.4 to 2.3)*	163.3 (107.4 to 231.0)	57.9 (54.1 to 62.0)*	1.5 (-1.0 to 4.1)
Other musculoskeletal disorders	29,065.0 (21,654.4 to 37,515.1)	55.6 (47.1 to 63.2)*	-0.0 (-5.5 to 4.9)	2,626.5 (1,641.2 to 3,861.6)	56.1 (47.5 to 63.6)*	0.3 (-5.2 to 5.1)
Other non-communicable diseases	271,175.3 (270,051.1 to 272,574.7)	55.8 (55.5 to 56.1)*	0.1 (-0.1 to 0.3)	10,818.6 (7,405.3 to 15,455.8)	55.1 (53.3 to 56.7)*	-0.4 (-1.5 to 0.7)
Congenital anomalies	3,431.3 (3,135.6 to 3,632.0)	78.9 (72.2 to 85.7)*	15.0 (10.6 to 19.3)*	304.0 (190.7 to 462.9)	80.4 (67.3 to 95.5)*	15.9 (7.5 to 25.6)*
Neural tube defects	27.3 (19.3 to 38.9)	82.3 (76.9 to 88.0)*	17.1 (13.7 to 20.8)*	8.6 (4.8 to 13.9)	84.1 (71.5 to 95.5)*	18.3 (10.2 to 25.6)*
Congenital heart anomalies	1,900.4 (1,321.7 to 2,831.2)	96.2 (86.6 to 104.7)*	26.1 (19.9 to 31.6)*	63.1 (24.6 to 115.7)	96.2 (86.6 to 104.7)*	26.1 (19.9 to 31.5)*
Less severe heart anomalies cases aggregate	1,751.2 (1,180.7 to 2,657.1)	95.9 (85.2 to 105.0)*	25.9 (19.0 to 31.7)*	54.4 (20.6 to 103.9)	96.0 (84.9 to 105.6)*	25.9 (18.8 to 32.1)*
Severe congenital heart anomalies	145.9 (95.9 to 221.5)	100.7 (88.6 to 112.7)*	29.0 (21.2 to 36.7)*	8.3 (3.2 to 16.2)	100.6 (85.9 to 114.4)*	28.9 (19.4 to 37.8)*
Critical congenital heart anomalies	0.2 (0.1 to 0.3)	128.9 (89.9 to 166.6)*	47.1 (22.0 to 71.3)*	0.0 (0.0 to 0.0)	129.1 (89.9 to 166.6)*	47.2 (22.0 to 71.3)*
Heart failure due to congenital heart anomalies aggregate	3.1 (2.7 to 3.6)	51.5 (49.9 to 53.2)*	-2.6 (-3.7 to -1.5)*	0.4 (0.2 to 0.5)	51.5 (49.5 to 53.6)*	-2.6 (-3.9 to -1.3)*
Cleft lip and cleft palate	276.8 (162.2 to 459.4)	88.3 (67.8 to 104.6)*	21.0 (7.8 to 31.5)*	2.7 (1.4 to 4.7)	79.3 (56.0 to 98.1)*	15.2 (0.3 to 27.3)*
Down syndrome	144.8 (94.4 to 215.0)	70.1 (60.9 to 78.5)*	9.3 (3.4 to 14.7)*	23.8 (13.9 to 38.2)	69.9 (60.1 to 78.5)*	9.2 (2.9 to 14.7)*
Turner syndrome	13.0 (6.1 to 23.8)	65.3 (61.2 to 70.2)*	6.2 (3.6 to 9.3)*	0.2 (0.1 to 0.4)	65.3 (58.0 to 72.3)*	6.2 (1.5 to 10.7)*
Klinefelter syndrome	8.4 (4.4 to 15.0)	54.6 (50.9 to 57.5)*	-0.6 (-3.0 to 1.2)	0.0 (0.0 to 0.1)	54.7 (50.9 to 57.5)*	-0.6 (-3.0 to 1.2)
Other chromosomal abnormalities	142.8 (82.2 to 246.0)	63.8 (53.0 to 73.8)*	5.3 (-1.7 to 11.7)	23.5 (12.6 to 42.8)	64.4 (53.4 to 74.5)*	5.6 (-1.4 to 12.1)
Other congenital anomalies	1,189.9 (641.3 to 2,194.9)	55.0 (51.0 to 59.2)*	-0.4 (-3.0 to 2.3)	182.2 (106.7 to 293.7)	79.0 (62.3 to 100.7)*	15.0 (4.3 to 29.0)*
Skin and subcutaneous diseases	93,697.4 (91,528.4 to 95,277.4)	57.7 (56.7 to 58.7)*	1.3 (0.7 to 2.0)*	1,692.8 (1,138.3 to 2,487.5)	56.6 (55.1 to 57.7)*	0.6 (-0.3 to 1.3)
Dermatitis	10,794.0 (8,450.7 to 13,686.1)	55.0 (54.5 to 55.4)*	-0.4 (-0.7 to -0.1)*	322.1 (197.8 to 470.7)	54.6 (52.7 to 56.3)*	-0.7 (-1.9 to 0.4)
Eczema cases aggregate	2,053.4 (1,545.6 to 2,649.0)	52.3 (51.3 to 53.4)*	-2.1 (-2.8 to -1.4)*	119.5 (74.0 to 177.5)	52.5 (49.1 to 56.2)*	-2.0 (-4.2 to 0.4)

Cause	Prevalence in thousands (95% confidence interval)			YLDs in thousands (95% confidence interval)		
	2015	Percent change in counts between 2005 and 2015	Percent change in rates between 2005 and 2015	2015	Percent change in counts between 2005 and 2015	Percent change in rates between 2005 and 2015
Contact dermatitis cases aggregate	6,486.7 (4,407.7 to 9,499.5)	55.6 (55.1 to 56.2)*	0.0 (-0.3 to 0.4)	169.8 (88.0 to 272.9)	55.9 (53.9 to 58.1)*	0.2 (-1.1 to 1.6)
Seborrhoeic dermatitis cases aggregate	2,253.8 (1,726.2 to 2,854.2)	55.4 (54.9 to 55.9)*	-0.1 (-0.4 to 0.2)	32.8 (17.8 to 54.6)	55.7 (53.2 to 58.0)*	0.0 (-1.5 to 1.5)
Psoriasis	5,747.7 (5,278.0 to 6,297.1)	54.0 (52.3 to 55.7)*	-1.1 (-2.1 to 0.0)	448.4 (310.4 to 615.0)	54.2 (51.6 to 56.7)*	-0.9 (-2.6 to 0.7)
Cellulitis	59.0 (45.5 to 76.1)	63.4 (57.1 to 70.2)*	5.0 (0.9 to 9.4)*	4.1 (2.5 to 6.1)	63.3 (50.8 to 77.1)*	4.9 (-3.1 to 13.8)
Pyoderma	284.4 (248.7 to 322.6)	58.4 (54.2 to 62.5)*	1.8 (-0.9 to 4.4)	1.6 (0.6 to 3.2)	58.7 (52.5 to 65.5)*	2.0 (-2.0 to 6.4)
Scabies	4,731.3 (3,238.0 to 6,742.1)	62.2 (54.0 to 71.1)*	4.3 (-1.0 to 9.9)	118.2 (59.6 to 205.6)	62.5 (54.4 to 71.2)*	4.4 (-0.8 to 10.0)
Fungal skin diseases	16,735.6 (13,634.2 to 20,402.7)	55.4 (54.9 to 55.9)*	-0.1 (-0.5 to 0.2)	92.1 (35.9 to 193.3)	55.7 (54.8 to 56.5)*	0.0 (-0.5 to 0.6)
Viral skin diseases	5,202.3 (4,431.6 to 6,202.4)	55.8 (53.5 to 57.8)*	0.1 (-1.4 to 1.4)	154.8 (95.0 to 232.9)	56.1 (53.3 to 58.7)*	0.3 (-1.5 to 2.0)
Molluscum contagiosum cases aggregate	151.6 (118.5 to 191.3)	54.5 (53.5 to 55.4)*	-0.7 (-1.3 to -0.1)*	4.5 (2.7 to 7.1)	54.9 (46.6 to 63.6)*	-0.5 (-5.8 to 5.1)
Viral warts cases aggregate	5,050.7 (4,281.6 to 6,058.8)	55.9 (53.5 to 57.9)*	0.2 (-1.4 to 1.5)	150.3 (91.6 to 226.4)	56.1 (53.2 to 58.8)*	0.3 (-1.5 to 2.0)
Acne vulgaris	4,032.7 (3,535.1 to 4,602.3)	59.1 (55.0 to 63.5)*	2.2 (-0.4 to 5.1)	42.1 (20.4 to 78.5)	59.3 (55.0 to 64.1)*	2.4 (-0.4 to 5.5)
Alopecia areata	958.5 (802.1 to 1,116.3)	53.2 (52.6 to 53.8)*	-1.5 (-1.9 to -1.2)*	31.4 (19.5 to 47.0)	53.4 (50.0 to 56.8)*	-1.4 (-3.6 to 0.8)
Pruritus	4,421.2 (3,091.9 to 6,050.6)	56.1 (51.2 to 60.5)*	0.3 (-2.8 to 3.1)	46.2 (20.5 to 91.1)	56.3 (51.3 to 60.8)*	0.5 (-2.7 to 3.3)
Urticaria	1,892.5 (1,335.8 to 2,561.7)	56.1 (55.6 to 56.6)*	0.3 (-0.0 to 0.6)	109.4 (63.0 to 171.2)	56.4 (53.6 to 59.1)*	0.5 (-1.3 to 2.2)
Decubitus ulcer	76.4 (58.0 to 98.9)	58.8 (52.6 to 64.2)*	2.0 (-2.0 to 5.5)	11.3 (7.3 to 17.0)	58.6 (45.8 to 72.4)*	1.9 (-6.3 to 10.8)
Other skin and subcutaneous diseases	53,696.3 (49,202.2 to 58,590.9)	60.1 (57.4 to 62.5)*	2.8 (1.1 to 4.4)*	311.1 (147.7 to 578.1)	60.3 (57.5 to 62.7)*	3.0 (1.2 to 4.5)*
Sense organ diseases	192,705.7 (189,868.2 to 195,560.3)	56.1 (55.3 to 56.8)*	0.3 (-0.2 to 0.8)	7,073.7 (4,781.4 to 10,101.7)	53.8 (51.1 to 56.2)*	-1.2 (-2.9 to 0.4)
Glaucoma	974.9 (695.2 to 1,311.5)	60.6 (56.3 to 65.0)*	3.2 (0.5 to 6.0)*	87.6 (52.8 to 132.5)	59.9 (54.9 to 65.5)*	2.7 (-0.5 to 6.3)
Cataract	7,339.3 (5,731.7 to 9,247.2)	39.1 (34.6 to 43.1)*	-10.6 (-13.5 to -8.0)*	456.8 (302.0 to 650.6)	36.2 (32.5 to 39.5)*	-12.5 (-14.8 to -10.3)*
Macular degeneration	738.0 (511.0 to 1,060.5)	73.7 (68.9 to 78.7)*	11.6 (8.6 to 14.8)*	49.0 (30.2 to 74.9)	70.7 (64.4 to 76.7)*	9.7 (5.6 to 13.6)*
Refraction and accommodation disorders	87,810.3 (80,741.4 to 94,903.9)	55.2 (51.8 to 59.0)*	-0.2 (-2.5 to 2.2)	1,446.9 (894.9 to 2,321.5)	51.1 (48.0 to 54.2)*	-2.9 (-4.9 to -0.9)*
Age-related and other hearing loss	154,166.2 (136,767.1 to 170,286.0)	56.8 (55.5 to 58.1)*	0.8 (-0.1 to 1.6)	4,152.5 (2,715.2 to 6,036.9)	55.6 (51.6 to 59.6)*	0.0 (-2.6 to 2.5)
Other vision loss	1,912.6 (1,444.7 to 2,481.2)	70.9 (65.3 to 77.0)*	9.8 (6.2 to 13.7)*	152.7 (95.5 to 224.8)	65.8 (60.5 to 71.5)*	6.5 (3.1 to 10.2)*
Other sense organ diseases	29,474.8 (26,121.9 to 32,867.2)	56.8 (53.7 to 60.1)*	0.8 (-1.2 to 2.9)	728.2 (442.2 to 1,072.7)	57.0 (53.8 to 60.4)*	0.9 (-1.2 to 3.1)
Acute other sense organ diseases	60.0 (53.7 to 67.1)	57.5 (54.4 to 61.2)*	1.2 (-0.8 to 3.6)	1.6 (1.0 to 2.4)	57.6 (54.2 to 61.5)*	1.3 (-0.9 to 3.8)
Chronic other sense organ diseases	29,414.8 (26,060.5 to 32,808.2)	56.8 (53.7 to 60.1)*	0.8 (-1.3 to 2.9)	726.6 (441.3 to 1,070.2)	57.0 (53.8 to 60.4)*	0.9 (-1.2 to 3.1)
Oral disorders	171,238.5 (166,862.7 to 174,979.3)	55.1 (53.9 to 56.4)*	-0.3 (-1.1 to 0.5)	1,748.1 (1,095.3 to 2,563.7)	55.1 (54.5 to 55.8)*	-0.3 (-0.7 to 0.1)
Deciduous caries	0.0 (0.0 to 0.0)	0.0 (0.0 to 0.0)	0.0 (0.0 to 0.0)	0.0 (0.0 to 0.0)	0.0 (0.0 to 0.0)	0.0 (0.0 to 0.0)
Permanent caries	118,256.7 (97,808.8 to 138,424.0)	55.3 (52.4 to 58.2)*	-0.2 (-2.0 to 1.6)	84.7 (36.9 to 164.7)	55.3 (52.5 to 57.8)*	-0.2 (-2.0 to 1.4)
Periodontal diseases	56,884.9 (46,659.2 to 67,961.6)	53.9 (52.8 to 54.9)*	-1.1 (-1.8 to -0.5)*	368.1 (142.9 to 759.3)	54.2 (53.0 to 55.2)*	-0.9 (-1.7 to -0.3)*
Edentulism and severe tooth loss	38,803.3 (35,750.3 to 42,069.9)	55.2 (54.7 to 55.7)*	-0.3 (-0.6 to 0.1)	1,079.4 (718.0 to 1,525.7)	55.4 (54.5 to 56.2)*	-0.2 (-0.7 to 0.4)
Other oral disorders	7,561.0 (6,725.1 to 8,421.7)	55.4 (54.6 to 56.3)*	-0.1 (-0.7 to 0.4)	216.0 (131.3 to 323.1)	55.7 (54.3 to 57.0)*	0.0 (-0.8 to 0.9)
Injuries	78,216.9 (75,040.9 to 81,747.6)	51.9 (50.4 to 53.6)*	-2.4 (-3.3 to -1.3)*	3,008.1 (2,122.8 to 4,104.0)	40.2 (35.6 to 44.5)*	-9.9 (-12.9 to -7.1)*
Transport injuries	11,863.7 (11,233.3 to 12,707.7)	68.4 (67.5 to 69.2)*	8.2 (7.6 to 8.7)*	632.0 (439.4 to 867.6)	54.9 (49.8 to 59.7)*	-0.5 (-3.7 to 2.6)
Road injuries	11,115.1 (10,474.3 to 11,961.1)	69.5 (68.6 to 70.3)*	8.9 (8.4 to 9.5)*	590.7 (408.9 to 816.2)	57.1 (52.2 to 61.5)*	1.0 (-2.2 to 3.8)
Pedestrian road injuries	1,518.5 (1,336.4 to 1,722.5)	70.9 (69.5 to 72.3)*	9.8 (8.9 to 10.7)*	80.5 (55.1 to 111.8)	53.8 (47.8 to 59.6)*	-1.2 (-5.0 to 2.6)
Cyclist road injuries	1,896.6 (1,611.2 to 2,130.4)	73.2 (71.1 to 75.1)*	11.3 (9.9 to 12.5)*	95.3 (64.1 to 135.2)	57.9 (51.1 to 63.8)*	1.5 (-2.9 to 5.3)
Motorcyclist road injuries	2,688.2 (2,340.6 to 3,002.9)	75.5 (73.7 to 77.1)*	12.8 (11.6 to 13.8)*	136.5 (94.5 to 191.0)	58.2 (51.4 to 64.0)*	1.6 (-2.7 to 5.4)
Motor vehicle road injuries	4,624.2 (4,138.0 to 5,229.9)	63.2 (61.9 to 64.5)*	4.9 (4.0 to 5.7)*	257.5 (176.9 to 354.2)	55.9 (52.2 to 59.2)*	0.2 (-2.2 to 2.3)
Other road injuries	387.6 (328.6 to 449.1)	85.5 (83.8 to 87.2)*	19.2 (18.1 to 20.3)*	20.9 (14.1 to 29.2)	76.3 (71.3 to 80.7)*	13.3 (10.1 to 16.1)*
Other transport injuries	748.6 (659.9 to 850.4)	53.0 (52.2 to 53.6)*	-1.7 (-2.2 to -1.3)*	41.3 (28.8 to 56.2)	28.6 (22.0 to 35.5)*	-17.3 (-21.6 to -12.9)*
Unintentional injuries	59,599.9 (56,961.2 to 62,327.7)	46.8 (46.2 to 47.4)*	-5.6 (-6.0 to -5.3)*	2,171.1 (1,509.9 to 2,992.7)	35.8 (31.1 to 40.0)*	-12.7 (-15.7 to -10.0)*
Falls	18,675.8 (17,028.5 to 20,331.4)	63.0 (62.3 to 63.7)*	4.7 (4.3 to 5.2)*	921.2 (640.6 to 1,257.1)	43.4 (36.2 to 49.7)*	-7.9 (-12.5 to -3.8)*
Drowning	410.3 (355.6 to 481.0)	38.7 (37.9 to 39.4)*	-10.9 (-11.4 to -10.4)*	22.2 (15.1 to 30.8)	18.4 (12.5 to 24.5)*	-23.9 (-27.7 to -20.0)*
Fire, heat, and hot substances	5,350.6 (4,713.9 to 6,020.6)	40.2 (39.3 to 41.0)*	-9.9 (-10.5 to -9.4)*	135.8 (93.0 to 197.2)	31.0 (25.1 to 35.5)*	-15.8 (-19.6 to -12.9)*
Poisonings	311.7 (272.5 to 360.6)	48.5 (47.7 to 49.2)*	-4.6 (-5.1 to -4.1)*	23.1 (16.1 to 32.2)	38.5 (34.7 to 42.5)*	-11.0 (-13.4 to -8.4)*
Exposure to mechanical forces	12,289.4 (11,377.7 to 13,254.9)	49.4 (48.6 to 50.2)*	-4.0 (-4.5 to -3.5)*	228.4 (157.2 to 321.3)	39.1 (34.9 to 43.2)*	-10.6 (-13.3 to -8.0)*
Unintentional firearm injuries	178.0 (156.0 to 202.9)	43.7 (42.7 to 44.6)*	-7.6 (-8.3 to -7.1)*	6.1 (4.3 to 8.6)	33.4 (28.8 to 37.7)*	-14.3 (-17.2 to -11.5)*
Unintentional suffocation	700.8 (598.9 to 833.3)	48.0 (44.5 to 51.4)*	-4.9 (-7.1 to -2.7)*	26.3 (18.3 to 36.0)	48.4 (45.4 to 51.1)*	-4.6 (-6.5 to -2.9)*
Other exposure to mechanical forces	11,410.6 (10,476.9 to 12,390.2)	49.6 (48.8 to 50.3)*	-3.9 (-4.4 to -3.4)*	196.0 (134.3 to 280.5)	38.2 (33.3 to 42.7)*	-11.2 (-14.4 to -8.3)*
Adverse effects of medical treatment	723.9 (534.9 to 942.1)	26.0 (19.8 to 31.3)*	-19.1 (-23.0 to -15.6)*	96.5 (59.0 to 146.8)	26.0 (19.8 to 31.3)*	-19.1 (-23.0 to -15.6)*
Animal contact	2,065.0 (1,862.6 to 2,269.4)	30.0 (28.7 to 31.1)*	-16.5 (-17.3 to -15.8)*	49.4 (34.8 to 67.4)	23.4 (20.6 to 26.1)*	-20.7 (-22.5 to -18.9)*
Venomous animal contact	916.0 (823.0 to 1,016.1)	33.6 (32.6 to 34.7)*	-14.2 (-14.8 to -13.5)*	34.8 (24.1 to 47.0)	23.7 (20.6 to 26.8)*	-20.5 (-22.5 to -18.5)*
Non-venomous animal contact	1,149.0 (1,002.3 to 1,310.0)	27.2 (25.6 to 28.6)*	-18.2 (-19.3 to -17.4)*	14.6 (9.3 to 22.4)	22.7 (19.1 to 25.8)*	-21.1 (-23.5 to -19.2)*
Foreign body	1,810.9 (1,638.1 to 1,996.5)	45.8 (44.6 to 47.0)*	-6.3 (-7.1 to -5.5)*	60.2 (42.9 to 83.7)	31.1 (25.2 to 37.2)*	-15.8 (-19.5 to -11.9)*
Pulmonary aspiration and foreign body in airway	800.8 (698.6 to 937.7)	59.6 (56.8 to 62.5)*	2.6 (0.8 to 4.4)*	35.5 (24.8 to 48.5)	34.2 (26.9 to 42.1)*	-13.8 (-18.5 to -8.7)*
Foreign body in eyes	43.7 (22.4 to 70.8)	52.0 (50.0 to 54.4)*	-2.4 (-3.6 to -0.8)*	2.4 (1.1 to 4.2)	49.8 (47.9 to 51.8)*	-3.7 (-5.0 to -2.5)*
Foreign body in other body part	966.4 (845.2 to 1,099.2)	35.7 (35.1 to 36.5)*	-12.8 (-13.2 to -12.3)*	22.3 (15.3 to 31.5)	24.8 (20.5 to 29.2)*	-19.8 (-22.6 to -17.0)*
Environmental heat and cold exposure	4,951.5 (4,425.5 to 5,526.0)	49.1 (48.3 to 49.8)*	-4.2 (-4.7 to -3.8)*	195.0 (135.3 to 270.4)	35.9 (31.3 to 40.3)*	-12.7 (-15.6 to -9.8)*
Other unintentional injuries	13,010.9 (11,741.3 to 14,417.7)	32.0 (31.1 to 32.9)*	-15.1 (-15.7 to -14.6)*	439.3 (296.9 to 620.7)	26.8 (24.1 to 28.8)*	-18.5 (-20.3 to -17.2)*
Self-harm and interpersonal violence	2,166.9 (2,004.2 to 2,314.8)	52.7 (52.2 to 53.2)*	-1.9 (-2.2 to -1.6)*	84.7 (59.4 to 113.6)	35.9 (30.5 to 41.6)*	-12.7 (-16.2 to -9.0)*
Self-harm	616.9 (546.4 to 693.9)	50.9 (50.1 to 51.5)*	-3.0 (-3.5 to -2.7)*	26.3 (18.3 to 35.6)	31.6 (25.9 to 37.4)*	-15.5 (-19.1 to -11.7)*
Interpersonal violence	1,549.9 (1,410.0 to 1,701.5)	53.4 (52.8 to 54.0)*	-1.4 (-1.8 to -1.1)*	58.4 (40.2 to 79.7)	37.9 (32.5 to 43.4)*	-11.4 (-14.9 to -7.8)*

Cause	Prevalence in thousands (95% confidence interval)			YLDs in thousands (95% confidence interval)		
	2015	Percent change in counts between 2005 and 2015	Percent change in rates between 2005 and 2015	2015	Percent change in counts between 2005 and 2015	Percent change in rates between 2005 and 2015
Assault by firearm	91.1<br>(78.9 to 104.4)	46.5<br>(45.2 to 47.6)*	-5.9<br>(-6.7 to -5.1)*	3.6<br>(2.5 to 5.1)	33.6<br>(28.6 to 37.8)*	-14.2<br>(-17.3 to -11.4)*
Assault by sharp object	332.3<br>(275.0 to 387.4)	51.9<br>(50.9 to 53.0)*	-2.4<br>(-3.0 to -1.7)*	8.4<br>(5.6 to 11.7)	28.4<br>(20.2 to 36.4)*	-17.5<br>(-22.8 to -12.3)*
Assault by other means	1,126.4<br>(1,004.5 to 1,258.3)	54.4<br>(53.7 to 55.1)*	-0.8<br>(-1.2 to -0.3)*	46.4<br>(31.9 to 63.6)	40.1<br>(35.0 to 45.2)*	-9.9<br>(-13.3 to -6.7)*
Forces of nature, war, and legal intervention	4,586.5<br>(3,282.3 to 6,251.1)	87.9<br>(57.0 to 121.4)*	20.7<br>(0.9 to 42.3)*	120.4<br>(87.4 to 159.5)	57.2<br>(19.5 to 94.4)*	1.0<br>(-23.2 to 24.9)
Exposure to forces of nature	1,346.7<br>(791.0 to 2,208.5)	60.3<br>(9.8 to 127.3)*	3.0<br>(-29.5 to 46.0)	40.5<br>(26.1 to 57.7)	13.4<br>(-32.9 to 71.1)	-27.1<br>(-56.9 to 9.9)
Collective violence and legal intervention	3,239.8<br>(2,269.9 to 4,396.9)	102.3<br>(66.7 to 144.4)*	30.0<br>(7.2 to 57.0)*	79.9<br>(54.9 to 111.2)	95.5<br>(56.1 to 139.7)*	25.6<br>(0.3 to 54.0)*
**Anemia**	**86,444.2<br>(85,276.4 to 87,536.6)**	**39.2<br>(37.3 to 41.2)***	**-10.6<br>(-11.8 to -9.3)***	**2,219.1<br>(1,502.4 to 3,147.2)**	**29.8<br>(26.4 to 33.2)***	**-16.6<br>(-18.8 to -14.4)***
**Developmental intellectual disability**	**3,982.5<br>(2,950.2 to 5,005.4)**	**55.9<br>(55.3 to 56.6)***	**0.2<br>(-0.2 to 0.6)**	**485.2<br>(351.1 to 635.5)**	**60.8<br>(57.4 to 64.7)***	**3.3<br>(1.1 to 5.8)***
**Epilepsy**	**1,314.2<br>(1,121.4 to 1,504.5)**	**58.5<br>(52.4 to 64.4)***	**1.9<br>(-2.1 to 5.7)**	**338.8<br>(243.3 to 440.9)**	**40.9<br>(30.0 to 50.6)***	**-9.5<br>(-16.4 to -3.2)***
**Guillain-Barré syndrome**	**2.6<br>(1.7 to 4.1)**	**53.0<br>(49.5 to 55.4)***	**-1.7<br>(-3.9 to -0.1)***	**0.8<br>(0.4 to 1.3)**	**53.0<br>(49.5 to 55.4)***	**-1.7<br>(-3.9 to -0.1)***
**Hearing loss**	**158,096.5<br>(141,020.9 to 174,440.7)**	**56.4<br>(55.1 to 57.6)***	**0.5<br>(-0.3 to 1.3)**	**4,320.9<br>(2,841.8 to 6,233.7)**	**55.2<br>(51.3 to 58.9)***	**-0.3<br>(-2.7 to 2.1)**
**Heart failure**	**4,055.7<br>(3,835.9 to 4,271.1)**	**56.5<br>(55.6 to 57.3)***	**0.6<br>(-0.0 to 1.1)**	**650.1<br>(466.8 to 851.7)**	**58.8<br>(57.1 to 60.6)***	**2.1<br>(0.9 to 3.2)***
**Infertility**	**0.0<br>(0.0 to 0.0)**	**0.0<br>(0.0 to 0.0)**	**0.0<br>(0.0 to 0.0)**	**0.0<br>(0.0 to 0.0)**	**0.0<br>(0.0 to 0.0)**	**0.0<br>(0.0 to 0.0)**
**Pelvic inflammatory disease**	**14.3<br>(9.7 to 19.8)**	**36.8<br>(33.7 to 39.3)***	**-12.1<br>(-14.1 to -10.5)***	**1.9<br>(1.1 to 3.0)**	**37.2<br>(25.7 to 49.8)***	**-11.8<br>(-19.2 to -3.8)***
**Vision loss**	**100,228.7<br>(91,820.8 to 108,633.0)**	**54.0<br>(50.8 to 57.3)***	**-1.1<br>(-3.1 to 1.0)**	**2,340.8<br>(1,560.8 to 3,461.5)**	**48.4<br>(45.5 to 51.1)***	**-4.6<br>(-6.5 to -2.9)***

SR Table 11. Prevalent cases and YLDs for 2015, percent change, and change in age-standardised rates between 2005 and 2015 by cause, global, ages 65-69 years						
Cause	Prevalence in thousands (95% confidence interval)			YLDs in thousands (95% confidence interval)		
	2015	Percent change in counts between 2005 and 2015	Percent change in rates between 2005 and 2015	2015	Percent change in counts between 2005 and 2015	Percent change in rates between 2005 and 2015
**All causes**	--	--	--	**40,557.3 (30,437.8 to 52,142.3)**	**20.0 (19.3 to 20.6)***	**-1.9 (-2.5 to -1.3)***
Communicable, maternal, neonatal, and nutritional diseases	101,485.8 (100,064.8 to 102,594.2)	17.9 (16.7 to 18.9)*	-3.6 (-4.5 to -2.7)*	1,546.9 (1,126.6 to 2,108.3)	5.8 (2.1 to 10.2)*	-13.5 (-16.5 to -9.8)*
HIV/AIDS and tuberculosis	1,143.8 (1,079.4 to 1,204.4)	43.3 (36.1 to 51.3)*	17.2 (11.3 to 23.8)*	195.8 (133.9 to 271.6)	12.8 (3.8 to 21.5)*	-7.7 (-15.1 to -0.6)*
Tuberculosis	429.7 (318.9 to 556.9)	16.1 (6.9 to 24.7)*	-5.1 (-12.5 to 2.0)	124.7 (79.6 to 187.1)	16.6 (7.2 to 25.5)*	-4.6 (-12.3 to 2.7)
HIV/AIDS	718.0 (681.5 to 757.6)	65.9 (52.8 to 79.4)*	35.7 (25.0 to 46.8)*	71.2 (52.2 to 92.4)	6.7 (-8.1 to 23.8)	-12.7 (-24.8 to 1.3)
HIV/AIDS - Tuberculosis	20.9 (18.7 to 23.2)	4.3 (0.1 to 8.3)*	-14.7 (-18.2 to -11.4)*	7.4 (5.1 to 10.1)	5.2 (-2.8 to 13.0)	-13.9 (-20.5 to -7.6)*
HIV/AIDS resulting in other diseases	714.5 (669.8 to 762.2)	68.2 (61.1 to 75.8)*	37.6 (31.7 to 43.8)*	63.8 (46.5 to 83.6)	6.9 (-9.7 to 26.2)	-12.6 (-26.1 to 3.2)
HIV aggregate	247.8 (229.2 to 267.3)	-18.6 (-22.3 to -14.4)*	-33.4 (-36.4 to -30.0)*	17.1 (12.3 to 22.3)	-19.7 (-24.0 to -14.9)*	-34.3 (-37.8 to -30.4)*
AIDS aggregate	466.7 (431.8 to 506.6)	287.8 (246.4 to 335.8)*	217.2 (183.3 to 256.4)*	46.7 (33.9 to 61.6)	21.7 (-4.7 to 56.5)	-0.4 (-22.1 to 28.0)
Diarrhea, lower respiratory, and other common infectious diseases	8,109.2 (7,547.9 to 8,762.1)	15.9 (12.9 to 19.0)*	-5.2 (-7.6 to -2.6)*	295.5 (210.7 to 402.3)	18.6 (17.0 to 20.2)*	-3.0 (-4.3 to -1.7)*
Diarrheal diseases	707.2 (684.8 to 729.0)	24.1 (23.4 to 24.8)*	1.5 (0.9 to 2.1)*	105.2 (73.4 to 143.4)	24.4 (22.3 to 26.5)*	1.7 (0.0 to 3.4)*
Diarrhoea episodes aggregate	707.0 (684.5 to 728.7)	24.1 (23.4 to 24.8)*	1.5 (0.9 to 2.1)*	105.1 (73.3 to 143.2)	24.4 (22.3 to 26.5)*	1.7 (0.0 to 3.4)*
Guillain-Barré syndrome due to diarrheal diseases	0.2 (0.1 to 0.3)	19.6 (15.4 to 22.3)*	-2.2 (-5.6 to 0.1)	0.1 (0.0 to 0.1)	19.6 (15.4 to 22.3)*	-2.2 (-5.6 to 0.1)
Intestinal infectious diseases	16.6 (14.8 to 18.4)	-11.2 (-20.2 to -1.1)*	-27.4 (-34.7 to -19.1)*	2.5 (1.6 to 3.6)	0.6 (-12.7 to 15.0)	-17.8 (-28.6 to -5.9)*
Typhoid fever	17.1 (13.0 to 21.9)	2.3 (-4.7 to 8.8)	-16.3 (-22.0 to -11.0)*	2.1 (1.3 to 3.1)	3.0 (-12.6 to 20.2)	-15.8 (-28.5 to -1.7)*
Typhoid fever episodes aggregate	14.2 (10.6 to 18.3)	2.3 (-7.4 to 13.6)	-16.3 (-24.2 to -7.1)*	1.3 (0.8 to 1.9)	3.0 (-12.2 to 21.9)	-15.8 (-28.2 to -0.3)*
Complications of typhoid fever aggregate	3.0 (2.0 to 4.1)	2.0 (-30.2 to 47.0)	-16.6 (-42.9 to 20.3)	0.9 (0.5 to 1.4)	2.9 (-32.2 to 57.5)	-15.8 (-44.5 to 28.8)
Paratyphoid fever	6.9 (4.7 to 9.3)	-2.0 (-11.4 to 8.0)	-19.8 (-27.5 to -11.7)*	0.4 (0.2 to 0.6)	-1.8 (-19.9 to 21.6)	-19.7 (-34.5 to -0.5)*
Paratyphoid fever episodes aggregate	6.5 (4.5 to 8.9)	-1.9 (-11.6 to 8.5)	-19.7 (-27.7 to -11.2)*	0.3 (0.2 to 0.5)	-1.5 (-20.7 to 24.6)	-19.4 (-35.1 to 1.9)
Intestinal perforation due to paratyphoid	0.3 (0.2 to 0.6)	-4.0 (-47.7 to 76.4)	-21.5 (-57.3 to 44.3)	0.0 (0.0 to 0.1)	-4.2 (-47.7 to 76.4)	-21.6 (-57.3 to 44.3)
Other intestinal infectious diseases	--	--	--	0.0 (0.0 to 0.1)	-61.3 (-74.6 to -39.2)*	-68.4 (-79.2 to -50.3)*
Lower respiratory infections	307.2 (292.2 to 323.3)	20.5 (18.9 to 22.1)*	-1.4 (-2.8 to -0.2)*	17.7 (12.0 to 24.7)	20.7 (17.3 to 24.0)*	-1.3 (-4.1 to 1.5)
Lower respiratory infection episodes aggregate	307.0 (292.0 to 323.1)	20.5 (18.9 to 22.1)*	-1.4 (-2.8 to -0.2)*	17.6 (12.0 to 24.6)	20.7 (17.3 to 24.1)*	-1.3 (-4.1 to 1.5)
Guillain-Barré syndrome due to lower respiratory infections	0.2 (0.1 to 0.4)	19.6 (15.4 to 22.3)*	-2.2 (-5.6 to 0.1)	0.1 (0.0 to 0.1)	19.6 (15.4 to 22.3)*	-2.2 (-5.6 to 0.1)
Upper respiratory infections	4,837.1 (3,353.7 to 6,614.5)	16.5 (14.7 to 18.2)*	-4.7 (-6.2 to -3.3)*	54.7 (28.2 to 101.0)	16.7 (14.4 to 18.9)*	-4.6 (-6.4 to -2.8)*
Upper respiratory infection episodes aggregate	4,836.4 (3,352.5 to 6,614.1)	16.5 (14.7 to 18.2)*	-4.7 (-6.2 to -3.3)*	54.5 (28.0 to 100.9)	16.6 (14.4 to 18.9)*	-4.6 (-6.4 to -2.8)*
Guillain-Barré syndrome due to upper respiratory infections	0.7 (0.4 to 1.0)	19.6 (15.4 to 22.3)*	-2.2 (-5.6 to 0.1)	0.2 (0.1 to 0.3)	19.6 (15.4 to 22.3)*	-2.2 (-5.6 to 0.1)
Otitis media	1,624.5 (1,252.2 to 2,109.0)	10.5 (7.4 to 13.9)*	-9.6 (-12.2 to -6.8)*	43.4 (25.9 to 70.0)	10.3 (4.7 to 16.1)*	-9.8 (-14.4 to -5.1)*
Acute otitis media	137.5 (106.9 to 174.6)	19.9 (16.1 to 23.6)*	-1.9 (-5.0 to 1.1)	1.7 (0.8 to 3.1)	20.1 (14.3 to 25.8)*	-1.8 (-6.5 to 2.9)
Chronic otitis media aggregate	1,486.9 (1,107.7 to 1,957.7)	9.7 (6.3 to 13.4)*	-10.3 (-13.1 to -7.2)*	41.7 (24.9 to 67.4)	9.9 (4.2 to 15.9)*	-10.1 (-14.8 to -5.2)*
Meningitis	292.4 (282.4 to 303.5)	14.8 (11.2 to 18.2)*	-6.1 (-9.1 to -3.3)*	46.9 (33.2 to 61.0)	18.0 (14.9 to 21.1)*	-3.5 (-6.0 to -1.0)*
Pneumococcal meningitis	241.2 (208.5 to 280.4)	20.0 (18.2 to 22.2)*	-1.8 (-3.3 to -0.1)*	23.6 (16.7 to 30.6)	19.0 (14.4 to 23.7)*	-2.7 (-6.5 to 1.1)
Acute pneumococcal meningitis	1.0 (0.7 to 1.4)	25.0 (13.6 to 35.9)*	2.2 (-7.1 to 11.2)	0.1 (0.1 to 0.2)	25.0 (13.6 to 35.9)*	2.2 (-7.1 to 11.2)
Complications of pneumococcal meningitis aggregate	240.2 (207.5 to 279.4)	20.0 (18.2 to 22.2)*	-1.8 (-3.3 to -0.1)*	23.4 (16.6 to 30.5)	19.0 (14.3 to 23.6)*	-2.7 (-6.5 to 1.1)
H influenzae type B meningitis	57.4 (46.5 to 70.7)	-6.9 (-10.9 to -1.9)*	-23.8 (-27.1 to -19.8)*	6.6 (4.6 to 9.0)	13.5 (6.6 to 19.4)*	-7.2 (-12.8 to -2.4)*
Acute H influenzae type B meningitis	0.3 (0.2 to 0.5)	-7.0 (-17.1 to 3.3)	-24.0 (-32.2 to -15.5)*	0.0 (0.0 to 0.1)	-7.0 (-17.1 to 3.3)	-24.0 (-32.2 to -15.5)*
Complications of of H influenzae type B meningitis aggregate	57.1 (46.1 to 70.4)	-6.9 (-10.9 to -1.9)*	-23.8 (-27.2 to -19.8)*	6.6 (4.6 to 8.9)	13.6 (6.7 to 19.5)*	-7.1 (-12.7 to -2.2)*
Meningococcal meningitis	46.6 (36.9 to 58.1)	16.1 (13.1 to 19.3)*	-5.1 (-7.5 to -2.4)*	4.7 (3.2 to 6.3)	16.7 (12.4 to 21.1)*	-4.5 (-8.0 to -1.0)*
Acute meningococcal meningitis	0.4 (0.3 to 0.6)	18.8 (6.0 to 30.5)*	-2.8 (-13.3 to 6.7)	0.1 (0.0 to 0.1)	18.8 (6.0 to 30.5)*	-2.8 (-13.3 to 6.7)
Complications of meningococcal meningitis aggregate	46.1 (36.4 to 57.7)	16.1 (13.0 to 19.4)*	-5.1 (-7.6 to -2.4)*	4.6 (3.2 to 6.2)	16.7 (12.3 to 21.1)*	-4.5 (-8.1 to -1.0)*
Other meningitis	110.9 (91.3 to 131.3)	20.3 (18.1 to 22.6)*	-1.6 (-3.4 to 0.2)	12.0 (8.4 to 16.0)	19.3 (13.9 to 24.4)*	-2.4 (-6.9 to 1.7)
Other meningitis episodes aggregate	2.1 (1.6 to 2.7)	25.9 (15.4 to 35.5)*	3.0 (-5.7 to 10.8)	0.3 (0.2 to 0.4)	25.9 (15.3 to 35.3)*	2.9 (-5.7 to 10.7)
Complications of other meningitis aggregate	108.8 (89.3 to 129.3)	20.2 (18.0 to 22.4)*	-1.7 (-3.5 to 0.1)	11.8 (8.2 to 15.6)	19.1 (13.6 to 24.4)*	-2.6 (-7.1 to 1.7)
Encephalitis	144.1 (94.2 to 210.0)	4.6 (2.2 to 7.7)*	-14.5 (-16.4 to -11.9)*	10.5 (7.2 to 14.1)	9.3 (3.9 to 14.9)*	-10.6 (-15.0 to -6.0)*
Acute encephalitis	1.7 (1.3 to 2.1)	12.2 (5.9 to 18.0)*	-8.3 (-13.4 to -3.5)*	0.2 (0.1 to 0.3)	12.2 (5.3 to 19.4)*	-8.3 (-13.9 to -2.3)*
Complications of encephalitis aggregate	142.4 (92.5 to 208.3)	4.5 (2.1 to 7.6)*	-14.6 (-16.5 to -12.0)*	10.3 (7.0 to 13.8)	9.2 (3.8 to 14.9)*	-10.7 (-15.1 to -6.0)*
Diphtheria	0.0 (0.0 to 0.0)	0.0 (0.0 to 0.0)	0.0 (0.0 to 0.0)	0.0 (0.0 to 0.0)	0.0 (0.0 to 0.0)	0.0 (0.0 to 0.0)
Whooping cough	0.0 (0.0 to 0.0)	0.0 (0.0 to 0.0)	0.0 (0.0 to 0.0)	0.0 (0.0 to 0.0)	0.0 (0.0 to 0.0)	0.0 (0.0 to 0.0)
Tetanus	4.8 (4.6 to 5.1)	22.2 (14.8 to 26.5)*	-0.1 (-6.1 to 3.5)	0.2 (0.1 to 0.3)	1.5 (-12.9 to 14.6)	-16.9 (-28.8 to -6.2)*
Severe tetanus	0.3 (0.2 to 0.6)	-37.2 (-51.0 to -19.2)*	-48.6 (-59.9 to -33.9)*	0.0 (0.0 to 0.1)	-36.6 (-51.0 to -18.6)*	-48.2 (-59.9 to -33.4)*
Complications of tetanus aggregate	4.4 (4.4 to 4.5)	31.6 (31.0 to 32.2)*	7.6 (7.1 to 8.1)*	0.1 (0.1 to 0.2)	25.0 (13.9 to 36.6)*	2.3 (-6.9 to 11.7)
Measles	0.0 (0.0 to 0.0)	0.0 (0.0 to 0.0)	0.0 (0.0 to 0.0)	0.0 (0.0 to 0.0)	0.0 (0.0 to 0.0)	0.0 (0.0 to 0.0)
Varicella and herpes zoster	276.1 (218.0 to 351.2)	23.8 (21.8 to 25.5)*	1.3 (-0.4 to 2.7)	14.4 (8.5 to 22.3)	24.1 (20.2 to 28.0)*	1.5 (-1.7 to 4.7)
Neglected tropical diseases and malaria	42,178.3 (41,191.4 to 43,314.4)	11.4 (8.0 to 15.0)*	-8.9 (-11.7 to -6.0)*	431.1 (290.7 to 651.4)	-3.4 (-13.1 to 9.7)	-21.0 (-29.0 to -10.3)*
Malaria	2,443.5 (2,112.7 to 2,785.5)	32.5 (26.1 to 39.0)*	8.4 (3.2 to 13.7)*	14.6 (10.3 to 20.5)	16.8 (9.8 to 24.1)*	-4.5 (-10.2 to 1.5)
Asymptomatic malaria parasitemia(PfPR)	1,909.8 (1,577.3 to 2,252.6)	35.6 (27.5 to 44.3)*	10.9 (4.3 to 18.0)*	0.0 (0.0 to 0.0)	0.0 (0.0 to 0.0)	0.0 (0.0 to 0.0)
Malaria episodes aggregate	34.2 (19.0 to 59.6)	-10.2 (-28.6 to 15.8)	-26.5 (-41.6 to -5.3)*	0.7 (0.3 to 1.3)	-9.6 (-29.7 to 18.0)	-26.0 (-42.5 to -3.5)*
Complications of malaria aggregate	5.3 (4.6 to 5.9)	35.1 (32.7 to 38.0)*	10.5 (8.5 to 12.8)*	1.7 (1.2 to 2.2)	30.0 (20.4 to 41.3)*	6.4 (-1.5 to 15.6)
Anemia due to malaria parasitemia(PfPR) aggregate	494.2 (480.0 to 510.3)	25.7 (20.8 to 30.4)*	2.8 (-1.2 to 6.7)	12.2 (8.3 to 17.4)	17.0 (8.9 to 25.3)*	-4.3 (-10.9 to 2.5)
Chagas disease	393.3 (327.6 to 462.3)	5.2 (1.8 to 8.4)*	-14.0 (-16.7 to -11.3)*	5.8 (3.8 to 8.4)	5.6 (0.4 to 11.1)*	-13.6 (-17.8 to -9.1)*
Chagas disease episodes aggregate	306.8 (254.7 to 361.9)	5.1 (1.7 to 8.3)*	-14.1 (-16.8 to -11.4)*	0.0 (0.0 to 0.0)	25.9 (20.2 to 31.9)*	3.0 (-1.7 to 7.9)
Complications of Chagas disease aggregate	47.9 (37.3 to 60.8)	5.2 (1.9 to 8.5)*	-13.9 (-16.7 to -11.2)*	3.8 (2.5 to 5.6)	5.5 (-0.7 to 11.9)	-13.7 (-18.8 to -8.4)*

Cause	Prevalence in thousands (95% confidence interval)			YLDs in thousands (95% confidence interval)		
	2015	Percent change in counts between 2005 and 2015	Percent change in rates between 2005 and 2015	2015	Percent change in counts between 2005 and 2015	Percent change in rates between 2005 and 2015
Heart failure due to Chagas disease aggregate	38.7 (24.4 to 54.4)	5.6 (2.1 to 9.1)*	-13.6 (-16.5 to -10.8)*	2.0 (1.1 to 3.2)	5.7 (-0.7 to 11.9)	-13.6 (-18.7 to -8.4)*
Leishmaniasis	134.8 (121.4 to 151.7)	31.7 (13.9 to 52.0)*	7.7 (-6.8 to 24.3)	1.4 (0.7 to 2.7)	31.5 (26.8 to 36.0)*	7.5 (3.7 to 11.2)*
Visceral leishmaniasis	0.3 (0.2 to 0.3)	27.1 (25.4 to 28.9)*	3.9 (2.6 to 5.4)*	0.0 (0.0 to 0.0)	27.7 (19.9 to 35.7)*	4.5 (-2.0 to 11.0)
Cutaneous and mucocutaneous leishmaniasis	138.2 (120.3 to 163.2)	31.4 (28.5 to 34.3)*	7.4 (5.1 to 9.8)*	1.4 (0.7 to 2.7)	31.5 (26.9 to 36.1)*	7.6 (3.8 to 11.3)*
African trypanosomiasis	0.2 (0.1 to 0.3)	-69.4 (-71.0 to -68.1)*	-75.0 (-76.3 to -73.9)*	0.1 (0.0 to 0.1)	-68.0 (-83.4 to -45.8)*	-73.8 (-86.4 to -55.7)*
Schistosomiasis	4,291.4 (3,591.8 to 5,426.2)	-25.1 (-34.4 to -6.7)*	-38.7 (-46.3 to -23.7)*	35.1 (17.5 to 66.5)	-24.0 (-32.0 to -5.5)*	-37.9 (-44.4 to -22.7)*
Schistosomiasis episodes aggregate	2,849.5 (2,358.2 to 3,493.0)	-25.5 (-34.4 to -10.4)*	-39.1 (-46.4 to -26.7)*	15.3 (6.1 to 33.1)	-27.4 (-36.1 to -12.0)*	-40.6 (-47.8 to -28.0)*
Complications of schistosomiasis aggregate	1,239.6 (818.4 to 1,988.9)	-19.2 (-32.3 to 14.4)	-33.9 (-44.6 to -6.4)*	14.3 (6.7 to 28.0)	-15.7 (-28.0 to 16.1)	-31.1 (-41.1 to -5.0)*
Anemia due to schistosomiasis aggregate	216.5 (206.7 to 226.5)	-30.3 (-33.7 to -26.5)*	-43.0 (-45.8 to -39.9)*	5.5 (3.7 to 7.8)	-32.5 (-38.1 to -26.8)*	-44.8 (-49.4 to -40.1)*
Cysticercosis	90.5 (58.3 to 133.0)	0.1 (-5.8 to 6.5)	-18.1 (-22.9 to -12.9)*	12.6 (7.0 to 20.1)	-11.3 (-18.2 to -3.5)*	-27.4 (-33.1 to -21.1)*
Cystic echinococcosis	62.3 (54.2 to 71.9)	14.3 (10.7 to 18.3)*	-6.5 (-9.5 to -3.3)*	5.5 (3.6 to 7.5)	14.1 (6.5 to 21.8)*	-6.7 (-12.9 to -0.4)*
Lymphatic filariasis	1,204.2 (944.4 to 1,517.8)	-40.1 (-47.4 to -33.7)*	-51.0 (-57.0 to -45.8)*	77.0 (42.9 to 121.5)	-12.7 (-31.0 to 1.7)	-28.6 (-43.6 to -16.8)*
Prevalence of detectable microfiliaria due to lymphatic filariasis	469.4 (405.6 to 539.0)	-60.4 (-63.5 to -56.5)*	-67.6 (-70.2 to -64.5)*	0.0 (0.0 to 0.0)	0.0 (0.0 to 0.0)	0.0 (0.0 to 0.0)
Complications of lymphatic filariasis aggregate	734.8 (484.3 to 1,039.5)	-11.0 (-28.4 to 2.7)	-27.2 (-41.4 to -16.0)*	77.0 (42.9 to 121.5)	-12.7 (-31.0 to 1.7)	-28.6 (-43.6 to -16.8)*
Onchocerciasis	428.7 (307.0 to 616.3)	-20.5 (-34.9 to -3.8)*	-35.0 (-46.7 to -21.3)*	44.2 (23.3 to 79.0)	-17.5 (-34.6 to 2.8)	-32.6 (-46.5 to -15.9)*
Asymptomatic onchocerciasis	3.9 (0.0 to 18.8)	-91.2 (-100.0 to -75.0)*	-92.8 (-100.0 to -79.6)*	0.0 (0.0 to 0.0)	0.0 (0.0 to 0.0)	0.0 (0.0 to 0.0)
Skin disease due to onchocerciasis aggregate	284.1 (168.6 to 460.9)	-21.8 (-40.1 to -1.2)*	-36.1 (-51.0 to -19.2)*	33.2 (14.9 to 64.7)	-22.5 (-45.6 to 2.3)	-36.6 (-55.5 to -16.2)*
Vision loss due to onchocerciasis aggregate	141.0 (94.6 to 210.4)	6.7 (-7.5 to 22.6)	-12.7 (-24.4 to 0.3)	11.0 (6.6 to 17.1)	2.3 (-10.6 to 15.3)	-16.4 (-26.9 to -5.7)*
Trachoma	476.4 (319.3 to 678.7)	-4.4 (-13.0 to 4.2)	-21.8 (-28.9 to -14.8)*	39.6 (22.1 to 62.7)	-9.4 (-18.6 to -0.2)*	-25.9 (-33.4 to -18.4)*
Dengue	104.0 (59.9 to 220.0)	175.7 (14.1 to 635.7)*	125.5 (-6.7 to 501.7)	15.7 (7.3 to 34.7)	172.7 (14.3 to 622.3)*	123.0 (-6.5 to 490.8)
Dengue episodes aggregate	31.2 (21.2 to 63.2)	160.3 (9.4 to 567.2)*	112.9 (-10.5 to 445.7)	1.7 (1.0 to 3.8)	171.7 (10.6 to 629.9)*	122.2 (-9.5 to 497.0)
Post-dengue chronic fatigue syndrome	73.1 (35.9 to 157.3)	175.9 (14.1 to 635.7)*	125.7 (-6.7 to 501.7)	14.0 (6.1 to 31.9)	172.8 (14.5 to 621.7)*	123.1 (-6.3 to 490.3)
Yellow fever	0.0 (0.0 to 0.1)	-23.8 (-42.6 to 1.0)	-37.7 (-53.1 to -17.4)*	0.0 (0.0 to 0.0)	-23.7 (-42.6 to 1.0)	-37.6 (-53.1 to -17.4)*
Rabies	0.0 (0.0 to 0.0)	-42.3 (-60.5 to -18.6)*	-52.8 (-67.7 to -33.4)*	0.0 (0.0 to 0.0)	-42.3 (-60.5 to -18.6)*	-52.8 (-67.7 to -33.4)*
Intestinal nematode infections	34,783.2 (33,738.5 to 35,785.2)	13.5 (9.4 to 17.8)*	-7.2 (-10.5 to -3.7)*	60.5 (34.1 to 99.3)	-11.8 (-19.5 to -2.3)*	-27.8 (-34.2 to -20.1)*
Ascariasis	18,251.1 (15,340.4 to 22,246.1)	3.5 (-15.6 to 29.5)	-15.3 (-31.0 to 5.9)	16.9 (9.3 to 28.4)	-29.9 (-42.0 to -15.8)*	-42.7 (-52.5 to -31.1)*
Complications of ascariasis aggregate	980.0 (869.2 to 1,106.9)	-27.2 (-38.2 to -14.7)*	-40.5 (-49.4 to -30.3)*	16.9 (9.3 to 28.4)	-29.9 (-42.0 to -15.8)*	-42.7 (-52.5 to -31.1)*
Asymptomatic ascariasis	17,271.9 (14,369.2 to 21,227.6)	6.1 (-14.8 to 34.9)	-13.2 (-30.3 to 10.3)	0.0 (0.0 to 0.0)	0.0 (0.0 to 0.0)	0.0 (0.0 to 0.0)
Trichuriasis	10,761.4 (9,608.1 to 12,067.9)	17.4 (0.2 to 38.1)*	-4.0 (-18.0 to 13.0)	11.5 (6.1 to 20.0)	5.6 (-17.9 to 41.1)	-13.6 (-32.9 to 15.4)
Complications of trichuriasis aggregate	601.2 (517.0 to 740.0)	6.7 (-13.5 to 35.9)	-12.7 (-29.2 to 11.2)	11.5 (6.1 to 20.0)	5.6 (-17.9 to 41.1)	-13.6 (-32.9 to 15.4)
Asymptomatic trichuriasis	10,164.1 (8,985.7 to 11,493.5)	18.1 (0.2 to 40.2)*	-3.4 (-18.0 to 14.7)	0.0 (0.0 to 0.0)	0.0 (0.0 to 0.0)	0.0 (0.0 to 0.0)
Hookworm disease	10,473.6 (9,281.6 to 11,856.1)	8.2 (-7.3 to 26.5)	-11.5 (-24.2 to 3.5)	32.0 (18.8 to 51.2)	-4.3 (-14.6 to 6.5)	-21.8 (-30.1 to -12.9)*
Complications of hookworm disease aggregate	1,248.4 (1,147.4 to 1,355.7)	-0.2 (-10.8 to 12.3)	-18.4 (-27.1 to -8.2)*	24.3 (13.5 to 40.2)	-2.1 (-14.7 to 12.5)	-19.9 (-30.2 to -8.0)*
Anemia due to hookworm disease aggregate	456.4 (442.0 to 470.7)	6.6 (2.4 to 11.1)*	-12.8 (-16.2 to -9.2)*	7.7 (5.0 to 11.3)	-10.8 (-19.3 to -2.5)*	-27.0 (-34.0 to -20.2)*
Asymptomatic hookworm disease	8,771.2 (7,601.8 to 10,154.7)	9.6 (-9.3 to 32.2)	-10.3 (-25.8 to 8.2)	0.0 (0.0 to 0.0)	0.0 (0.0 to 0.0)	0.0 (0.0 to 0.0)
Food-borne trematodiases	2,088.4 (1,922.1 to 2,260.8)	22.1 (18.4 to 25.8)*	-0.2 (-3.1 to 2.9)	47.3 (22.4 to 87.6)	20.9 (9.0 to 30.6)*	-1.2 (-10.9 to 6.8)
Asymptomatic food-borne trematodiases aggregate	1,649.3 (1,319.6 to 1,915.9)	22.4 (18.2 to 26.9)*	0.1 (-3.3 to 3.8)	0.0 (0.0 to 0.0)	0.0 (0.0 to 0.0)	0.0 (0.0 to 0.0)
Symptomatic food-borne trematodiases aggregate	439.1 (233.7 to 751.9)	20.7 (9.1 to 29.9)*	-1.3 (-10.7 to 6.3)	47.3 (22.4 to 87.6)	20.9 (9.0 to 30.6)*	-1.2 (-10.9 to 6.8)
Leprosy	51.3 (48.6 to 54.0)	4.2 (2.8 to 5.7)*	-14.8 (-16.0 to -13.5)*	3.1 (2.1 to 4.3)	5.1 (-0.3 to 10.7)	-14.0 (-18.4 to -9.4)*
Ebola	0.0 (0.0 to 0.1)	0.0 (0.0 to 0.0)	0.0 (0.0 to 0.0)	0.0 (0.0 to 0.0)	0.0 (0.0 to 0.0)	0.0 (0.0 to 0.0)
Other neglected tropical diseases	307.9 (293.1 to 322.2)	11.3 (5.8 to 17.1)*	-9.0 (-13.4 to -4.2)*	68.7 (21.6 to 209.1)	15.0 (-29.4 to 81.7)	-5.9 (-42.3 to 48.6)
Anemia due to other neglected tropical diseases aggregate	307.9 (293.1 to 322.2)	11.3 (5.8 to 17.1)*	-9.0 (-13.4 to -4.2)*	6.1 (4.0 to 8.8)	-10.9 (-19.8 to -2.1)*	-27.1 (-34.4 to -20.0)*
Maternal disorders	8.7 (7.8 to 9.6)	-30.6 (-38.8 to -20.8)*	-43.2 (-49.9 to -35.2)*	3.0 (2.0 to 4.3)	-29.3 (-37.2 to -20.9)*	-42.2 (-48.6 to -35.3)*
Maternal hemorrhage	0.0 (0.0 to 0.0)	0.0 (0.0 to 0.0)	0.0 (0.0 to 0.0)	0.0 (0.0 to 0.0)	0.0 (0.0 to 0.0)	0.0 (0.0 to 0.0)
Maternal hemorrhage episodes aggregate	0.0 (0.0 to 0.0)	0.0 (0.0 to 0.0)	0.0 (0.0 to 0.0)	0.0 (0.0 to 0.0)	0.0 (0.0 to 0.0)	0.0 (0.0 to 0.0)
Anemia due to maternal hemorrhage aggregate	0.0 (0.0 to 0.0)	0.0 (0.0 to 0.0)	0.0 (0.0 to 0.0)	0.0 (0.0 to 0.0)	0.0 (0.0 to 0.0)	0.0 (0.0 to 0.0)
Maternal sepsis and other maternal infections	0.0 (0.0 to 0.0)	0.0 (0.0 to 0.0)	0.0 (0.0 to 0.0)	0.0 (0.0 to 0.0)	0.0 (0.0 to 0.0)	0.0 (0.0 to 0.0)
Maternal sepsis and other maternal infections aggregate	0.0 (0.0 to 0.0)	0.0 (0.0 to 0.0)	0.0 (0.0 to 0.0)	0.0 (0.0 to 0.0)	0.0 (0.0 to 0.0)	0.0 (0.0 to 0.0)
Infertility due to puerperal sepsis	0.0 (0.0 to 0.0)	0.0 (0.0 to 0.0)	0.0 (0.0 to 0.0)	0.0 (0.0 to 0.0)	0.0 (0.0 to 0.0)	0.0 (0.0 to 0.0)
Maternal hypertensive disorders	0.0 (0.0 to 0.0)	0.0 (0.0 to 0.0)	0.0 (0.0 to 0.0)	0.0 (0.0 to 0.0)	0.0 (0.0 to 0.0)	0.0 (0.0 to 0.0)
Maternal hypertensive disorders episodes aggregate	0.0 (0.0 to 0.0)	0.0 (0.0 to 0.0)	0.0 (0.0 to 0.0)	0.0 (0.0 to 0.0)	0.0 (0.0 to 0.0)	0.0 (0.0 to 0.0)
Eclampsia	0.0 (0.0 to 0.0)	0.0 (0.0 to 0.0)	0.0 (0.0 to 0.0)	0.0 (0.0 to 0.0)	0.0 (0.0 to 0.0)	0.0 (0.0 to 0.0)
Complications of maternal hypertensive disorders aggregate	0.0 (0.0 to 0.0)	0.0 (0.0 to 0.0)	0.0 (0.0 to 0.0)	0.0 (0.0 to 0.0)	0.0 (0.0 to 0.0)	0.0 (0.0 to 0.0)
Maternal obstructed labor and uterine rupture	8.8 (7.3 to 10.5)	-29.9 (-33.1 to -27.1)*	-42.7 (-45.2 to -40.3)*	2.7 (1.8 to 3.8)	-29.7 (-37.3 to -21.3)*	-42.5 (-48.7 to -35.6)*
Obstructed labor, acute event	0.0 (0.0 to 0.0)	0.0 (0.0 to 0.0)	0.0 (0.0 to 0.0)	0.0 (0.0 to 0.0)	0.0 (0.0 to 0.0)	0.0 (0.0 to 0.0)
Fistula due to maternal obstructed labor and uterine rupture aggregate	8.8 (7.3 to 10.5)	-29.9 (-33.1 to -27.1)*	-42.7 (-45.2 to -40.3)*	2.7 (1.8 to 3.8)	-29.7 (-37.3 to -21.3)*	-42.5 (-48.7 to -35.6)*
Maternal abortion, miscarriage, and ectopic pregnancy	0.0 (0.0 to 0.0)	0.0 (0.0 to 0.0)	0.0 (0.0 to 0.0)	0.0 (0.0 to 0.0)	0.0 (0.0 to 0.0)	0.0 (0.0 to 0.0)
Other maternal disorders	--	--	--	0.3 (0.2 to 0.5)	-25.9 (-40.5 to -9.2)*	-39.4 (-51.4 to -25.7)*
Neonatal disorders	748.1 (712.7 to 779.8)	29.8 (24.4 to 36.1)*	6.2 (1.8 to 11.4)*	109.6 (82.0 to 141.0)	34.1 (29.1 to 39.0)*	9.6 (5.6 to 13.6)*
Neonatal preterm birth complications	713.8 (620.5 to 823.7)	26.8 (19.3 to 34.0)*	3.7 (-2.4 to 9.6)	50.8 (38.5 to 65.8)	28.4 (21.7 to 35.5)*	5.0 (-0.5 to 10.8)
Vision loss due to retinopathy of prematurity aggregate	127.7 (102.3 to 157.3)	19.1 (15.0 to 23.4)*	-2.6 (-5.9 to 1.0)	4.4 (2.7 to 6.8)	18.6 (9.7 to 28.8)*	-3.0 (-10.2 to 5.3)
Complications of preterm birth complications aggregate	586.0 (502.9 to 682.1)	28.6 (19.3 to 37.8)*	5.2 (-2.4 to 12.7)	46.3 (34.8 to 60.1)	29.4 (22.2 to 37.3)*	5.9 (-0.0 to 12.3)
Neonatal encephalopathy due to birth asphyxia and trauma	129.0 (89.9 to 193.5)	45.8 (42.2 to 49.5)*	19.3 (16.3 to 22.3)*	36.4 (23.5 to 55.9)	41.6 (35.1 to 48.6)*	15.8 (10.5 to 21.5)*

Cause	Prevalence in thousands (95% confidence interval)			YLDs in thousands (95% confidence interval)		
	2015	Percent change in counts between 2005 and 2015	Percent change in rates between 2005 and 2015	2015	Percent change in counts between 2005 and 2015	Percent change in rates between 2005 and 2015
Neonatal sepsis and other neonatal infections	0.0 (0.0 to 0.0)	0.0 (0.0 to 0.0)	0.0 (0.0 to 0.0)	0.0 (0.0 to 0.0)	0.0 (0.0 to 0.0)	0.0 (0.0 to 0.0)
Severe infection due to neonatal sepsis and other neonatal infections	0.0 (0.0 to 0.0)	0.0 (0.0 to 0.0)	0.0 (0.0 to 0.0)	0.0 (0.0 to 0.0)	0.0 (0.0 to 0.0)	0.0 (0.0 to 0.0)
Complications of neonatal sepsis and other neonatal infections aggregate	0.0 (0.0 to 0.0)	0.0 (0.0 to 0.0)	0.0 (0.0 to 0.0)	0.0 (0.0 to 0.0)	0.0 (0.0 to 0.0)	0.0 (0.0 to 0.0)
Hemolytic disease and other neonatal jaundice	22.4 (19.7 to 25.6)	35.4 (33.4 to 37.6)*	10.8 (9.1 to 12.6)*	6.9 (5.0 to 8.8)	31.1 (26.4 to 35.9)*	7.2 (3.4 to 11.2)*
Other neonatal disorders	--	--	--	15.5 (11.3 to 19.8)	38.1 (23.3 to 52.5)*	13.0 (0.8 to 24.8)*
Nutritional deficiencies	20,140.8 (19,816.9 to 20,482.4)	9.9 (7.5 to 12.3)*	-10.1 (-12.1 to -8.2)*	465.7 (312.8 to 670.0)	-0.1 (-3.8 to 3.5)	-18.3 (-21.3 to -15.3)*
Protein-energy malnutrition	27.3 (17.1 to 42.8)	-7.6 (-17.6 to 4.4)	-24.4 (-32.6 to -14.6)*	3.1 (1.7 to 5.4)	-7.1 (-18.8 to 7.5)	-24.0 (-33.6 to -12.1)*
Iodine deficiency	3,471.0 (3,154.7 to 3,895.1)	14.3 (12.1 to 16.6)*	-6.5 (-8.3 to -4.6)*	60.7 (38.4 to 93.5)	14.4 (11.8 to 17.2)*	-6.4 (-8.6 to -4.1)*
Visible goiter due to iodine deficiency aggregate	3,461.3 (3,145.7 to 3,884.2)	14.3 (12.2 to 16.6)*	-6.5 (-8.3 to -4.6)*	59.2 (37.1 to 91.6)	14.7 (11.9 to 17.5)*	-6.2 (-8.5 to -3.9)*
Visible goiter with heart failure due to iodine deficiency aggregate	0.0 (0.0 to 0.1)	26.3 (24.1 to 28.6)*	3.3 (1.5 to 5.2)*	0.0 (0.0 to 0.0)	26.3 (24.1 to 28.6)*	3.3 (1.5 to 5.2)*
Intellectual disability due to iodine deficiency aggregate	9.6 (4.7 to 12.4)	6.1 (3.9 to 9.3)*	-13.2 (-15.0 to -10.6)*	1.5 (0.6 to 2.4)	6.5 (-7.1 to 24.7)	-12.9 (-24.0 to 2.0)
Vitamin A deficiency	57.1 (38.9 to 82.3)	23.6 (16.2 to 30.9)*	1.1 (-4.9 to 7.1)	3.9 (2.3 to 6.0)	25.3 (14.8 to 36.6)*	2.5 (-6.1 to 11.7)
Iron-deficiency anemia	17,568.6 (17,194.5 to 17,936.7)	7.9 (5.1 to 10.7)*	-11.8 (-14.1 to -9.5)*	397.5 (271.7 to 567.7)	-2.1 (-6.3 to 1.9)	-19.9 (-23.4 to -16.7)*
Iron-deficiency anemia without heart failure aggregate	17,560.8 (17,187.1 to 17,929.9)	7.9 (5.1 to 10.7)*	-11.8 (-14.1 to -9.5)*	396.6 (271.1 to 566.4)	-2.2 (-6.4 to 1.8)	-20.0 (-23.4 to -16.7)*
Iron-deficiency anemia with heart failure aggregate	7.8 (6.0 to 9.8)	34.8 (31.5 to 37.9)*	10.2 (7.5 to 12.8)*	0.9 (0.6 to 1.3)	34.6 (21.6 to 48.1)*	10.1 (-0.5 to 21.1)
Other nutritional deficiencies	--	--	--	0.3 (0.2 to 0.6)	-3.2 (-25.0 to 18.7)	-20.8 (-38.7 to -2.9)*
Other communicable, maternal, neonatal, and nutritional diseases	55,658.7 (54,077.0 to 57,092.2)	21.0 (19.8 to 22.1)*	-1.1 (-2.0 to -0.1)*	46.2 (30.0 to 69.5)	11.0 (2.9 to 19.2)*	-9.2 (-15.9 to -2.5)*
Sexually transmitted diseases excluding HIV	42,488.5 (40,646.2 to 44,161.3)	20.1 (18.5 to 21.7)*	-1.8 (-3.1 to -0.5)*	28.1 (17.8 to 44.6)	14.0 (8.8 to 17.8)*	-6.8 (-11.0 to -3.6)*
Syphilis	188.0 (146.7 to 246.8)	7.5 (-0.8 to 15.3)	-12.1 (-18.9 to -5.7)*	14.2 (9.5 to 19.9)	8.6 (3.2 to 14.1)*	-11.1 (-15.6 to -6.7)*
Early syphilis aggregate	108.6 (69.8 to 166.2)	6.9 (-8.8 to 18.5)	-12.6 (-25.4 to -3.1)*	0.0 (0.0 to 0.1)	7.4 (-13.5 to 29.0)	-12.2 (-29.2 to 5.5)
Adult tertiary syphilis	79.4 (68.0 to 91.5)	8.4 (5.1 to 11.5)*	-11.4 (-14.0 to -8.8)*	14.2 (9.5 to 19.9)	8.6 (3.2 to 14.1)*	-11.1 (-15.6 to -6.7)*
Chlamydial infection	18.0 (12.8 to 24.2)	26.7 (22.4 to 31.4)*	3.6 (0.1 to 7.4)*	0.3 (0.2 to 0.5)	9.3 (5.2 to 13.8)*	-10.6 (-13.9 to -6.9)*
Chlamydial infection episodes aggregate	16.2 (11.0 to 22.6)	30.0 (25.3 to 35.3)*	6.3 (2.5 to 10.6)*	0.1 (0.0 to 0.2)	32.2 (22.5 to 42.5)*	8.1 (0.2 to 16.6)*
Pelvic inflammatory diseases due to chlamydial infection aggregate	1.8 (1.3 to 2.4)	3.0 (-0.4 to 6.4)	-15.8 (-18.5 to -12.9)*	0.2 (0.2 to 0.4)	3.0 (-0.4 to 6.4)	-15.8 (-18.5 to -12.9)*
Infertility due to clamydial infection aggregate	0.0 (0.0 to 0.0)	0.0 (0.0 to 0.0)	0.0 (0.0 to 0.0)	0.0 (0.0 to 0.0)	0.0 (0.0 to 0.0)	0.0 (0.0 to 0.0)
Gonococcal infection	12.6 (6.7 to 29.6)	40.2 (12.8 to 60.4)*	14.6 (-7.7 to 31.2)	0.1 (0.1 to 0.2)	23.3 (10.9 to 36.8)*	0.8 (-9.3 to 11.9)
Gonococcal infection episodes aggregate	12.3 (6.3 to 29.2)	41.4 (12.9 to 61.4)*	15.6 (-7.7 to 32.0)	0.1 (0.0 to 0.1)	36.7 (15.8 to 53.4)*	11.8 (-5.3 to 25.4)
Pelvic inflammatory diseases due to gonococcal infection aggregate	0.3 (0.3 to 0.4)	7.3 (2.9 to 12.2)*	-12.2 (-15.8 to -8.2)*	0.0 (0.0 to 0.1)	7.3 (2.9 to 12.2)*	-12.2 (-15.8 to -8.2)*
Trichomoniasis	2,447.0 (2,049.9 to 2,905.4)	24.2 (23.3 to 25.2)*	1.6 (0.8 to 2.4)*	2.7 (1.1 to 5.9)	25.2 (22.4 to 27.7)*	2.4 (0.1 to 4.5)*
Genital herpes	41,030.3 (35,953.6 to 46,392.2)	20.0 (18.4 to 21.5)*	-1.9 (-3.2 to -0.7)*	9.8 (2.8 to 23.9)	20.1 (18.1 to 22.2)*	-1.7 (-3.4 to -0.1)*
Moderate infection due to initial genital herpes episode	0.0 (0.0 to 0.0)	0.0 (0.0 to 0.0)	0.0 (0.0 to 0.0)	0.0 (0.0 to 0.0)	0.0 (0.0 to 0.0)	0.0 (0.0 to 0.0)
Complications of genital herpes aggregate	41,030.3 (35,953.6 to 46,392.2)	20.0 (18.4 to 21.5)*	-1.9 (-3.2 to -0.7)*	9.8 (2.8 to 23.9)	20.1 (18.1 to 22.2)*	-1.7 (-3.4 to -0.1)*
Other sexually transmitted diseases	6.9 (5.2 to 9.0)	7.5 (5.9 to 9.3)*	-12.1 (-13.4 to -10.6)*	0.9 (0.6 to 1.4)	8.9 (-2.4 to 21.8)	-10.9 (-20.2 to -0.4)*
Pelvic inflammatory diseases due to other sexually transmitted diseases aggregate	6.9 (5.2 to 9.0)	7.5 (5.9 to 9.3)*	-12.1 (-13.4 to -10.6)*	0.9 (0.6 to 1.4)	8.9 (-2.4 to 21.8)	-10.9 (-20.2 to -0.4)*
Infertility due to other sexually transmitted diseases aggregate	0.0 (0.0 to 0.0)	0.0 (0.0 to 0.0)	0.0 (0.0 to 0.0)	0.0 (0.0 to 0.0)	0.0 (0.0 to 0.0)	0.0 (0.0 to 0.0)
Other sexually transmitted diseases	--	--	--	0.1 (0.0 to 0.1)	33.1 (22.9 to 43.4)*	8.9 (0.5 to 17.3)*
Hepatitis	16,169.7 (15,821.4 to 16,451.4)	23.7 (22.8 to 24.7)*	1.2 (0.4 to 2.0)*	7.0 (4.2 to 11.2)	24.4 (-17.7 to 89.6)	1.7 (-32.7 to 55.1)
Acute hepatitis A	40.7 (32.7 to 48.1)	33.4 (2.6 to 69.3)*	9.1 (-16.1 to 38.5)	1.6 (1.0 to 2.4)	33.8 (1.3 to 76.4)*	9.4 (-17.2 to 44.3)
Hepatitis B	9,897.2 (9,556.8 to 10,241.0)	26.2 (24.2 to 28.1)*	3.2 (1.6 to 4.8)*	4.9 (2.7 to 8.2)	21.9 (-30.7 to 124.7)	-0.3 (-43.3 to 83.8)
Acute Hepatitis B aggregate	219.0 (146.7 to 334.7)	21.9 (-29.9 to 119.9)	-0.3 (-42.7 to 79.9)	4.9 (2.7 to 8.2)	21.9 (-30.7 to 124.7)	-0.3 (-43.3 to 83.8)
Chronic hepatitis B	9,678.3 (9,350.1 to 10,017.9)	26.3 (25.3 to 27.3)*	3.3 (2.5 to 4.1)*	0.0 (0.0 to 0.0)	0.0 (0.0 to 0.0)	0.0 (0.0 to 0.0)
Hepatitis C	6,665.3 (5,923.9 to 7,406.6)	20.2 (18.2 to 22.2)*	-1.7 (-3.3 to -0.1)*	0.2 (0.1 to 0.3)	20.2 (13.4 to 24.9)*	-1.7 (-7.2 to 2.2)
Acute Hepatitis C aggregate	10.8 (8.0 to 13.8)	20.1 (13.3 to 24.8)*	-1.8 (-7.3 to 2.1)	0.2 (0.1 to 0.3)	20.2 (13.4 to 24.9)*	-1.7 (-7.2 to 2.2)
Chronic hepatitis C	6,654.5 (5,912.7 to 7,395.7)	20.2 (18.2 to 22.2)*	-1.7 (-3.3 to -0.1)*	0.0 (0.0 to 0.0)	0.0 (0.0 to 0.0)	0.0 (0.0 to 0.0)
Acute hepatitis E	11.3 (7.7 to 16.2)	21.0 (18.1 to 24.6)*	-1.1 (-3.4 to 1.9)	0.4 (0.2 to 0.6)	20.5 (-2.2 to 47.5)	-1.4 (-20.0 to 20.6)
Other infectious diseases	423.3 (411.1 to 435.7)	8.2 (1.6 to 15.4)*	-11.5 (-16.9 to -5.6)*	11.1 (7.2 to 16.7)	-2.2 (-11.5 to 8.9)	-20.0 (-27.7 to -11.0)*
Non-communicable diseases	204,628.8 (204,577.7 to 204,699.9)	22.2 (22.2 to 22.3)*	-0.0 (-0.0 to -0.0)*	36,793.4 (27,380.3 to 47,334.2)	21.5 (20.8 to 22.2)*	-0.6 (-1.2 to -0.1)*
Neoplasms	11,391.1 (11,200.5 to 11,584.3)	44.7 (41.9 to 47.4)*	18.4 (16.0 to 20.6)*	1,050.0 (762.7 to 1,348.3)	35.0 (29.6 to 39.9)*	10.4 (6.0 to 14.5)*
Lip and oral cavity cancer	285.3 (254.2 to 321.2)	35.0 (21.2 to 50.5)*	10.4 (-0.9 to 23.1)	24.3 (17.1 to 32.4)	32.1 (18.2 to 47.9)*	8.1 (-3.3 to 21.0)
Nasopharynx cancer	62.5 (46.9 to 83.5)	23.4 (-0.3 to 59.5)	0.9 (-18.5 to 30.5)	6.1 (3.9 to 8.8)	20.6 (-4.3 to 54.0)	-1.3 (-21.8 to 25.9)
Other pharynx cancer	127.5 (113.7 to 142.8)	30.6 (16.5 to 46.2)*	6.8 (-4.7 to 19.6)	10.6 (7.5 to 14.0)	27.3 (13.2 to 43.3)*	4.1 (-7.4 to 17.2)
Esophageal cancer	115.2 (95.1 to 162.3)	15.4 (-6.4 to 47.5)	-5.6 (-23.4 to 20.6)	19.5 (13.3 to 27.8)	5.8 (-12.4 to 31.6)	-13.5 (-28.3 to 7.7)
Stomach cancer	518.0 (477.7 to 564.5)	11.4 (1.8 to 22.0)*	-8.9 (-16.8 to -0.2)*	57.1 (41.9 to 72.7)	7.2 (-3.1 to 18.5)	-12.3 (-20.7 to -3.1)*
Colon and rectum cancer	1,196.6 (1,137.3 to 1,260.7)	29.4 (23.2 to 35.8)*	5.8 (0.8 to 11.1)*	93.8 (68.5 to 121.9)	25.1 (18.6 to 31.7)*	2.3 (-3.0 to 7.7)
Colon and rectum cancer aggregate	1,194.3 (1,135.0 to 1,258.6)	29.4 (23.3 to 35.9)*	5.9 (0.8 to 11.1)*	93.5 (68.2 to 121.5)	25.1 (18.6 to 31.7)*	2.4 (-3.0 to 7.7)
Stoma due to colon and rectum cancer	2.3 (2.0 to 2.5)	14.9 (12.4 to 17.7)*	-6.0 (-8.0 to -3.7)*	0.3 (0.2 to 0.4)	14.9 (12.4 to 17.7)*	-6.0 (-8.0 to -3.7)*
Liver cancer	83.1 (74.1 to 93.1)	45.2 (25.7 to 66.3)*	18.7 (2.8 to 36.0)*	22.9 (16.0 to 30.7)	22.3 (3.9 to 42.8)*	0.1 (-15.0 to 16.8)
Liver cancer due to hepatitis B	29.2 (22.6 to 40.9)	24.2 (-3.2 to 65.2)	1.6 (-20.8 to 35.1)	5.6 (3.7 to 7.8)	14.1 (-6.4 to 40.3)	-6.7 (-23.5 to 14.8)
Liver cancer due to hepatitis C	34.1 (27.4 to 44.2)	54.4 (24.7 to 99.4)*	26.2 (2.0 to 63.1)*	5.7 (3.9 to 7.7)	29.5 (10.7 to 56.5)*	5.9 (-9.5 to 28.0)
Liver cancer due to alcohol use	41.4 (34.0 to 53.3)	44.9 (18.4 to 77.8)*	18.5 (-3.2 to 45.4)	8.0 (5.5 to 10.7)	29.4 (10.6 to 51.2)*	5.9 (-9.5 to 23.6)
Liver cancer due to other causes	16.9 (13.0 to 26.1)	19.9 (-13.4 to 69.8)	-1.9 (-29.2 to 38.9)	3.5 (2.3 to 5.1)	11.2 (-10.0 to 42.4)	-9.1 (-26.4 to 16.5)
Gallbladder and biliary tract cancer	18.6 (16.7 to 20.3)	10.0 (0.4 to 19.9)*	-10.1 (-17.9 to -1.9)*	5.1 (3.6 to 6.8)	8.0 (-1.5 to 17.8)	-11.7 (-19.5 to -3.7)*
Pancreatic cancer	52.4 (49.0 to 56.6)	33.2 (26.1 to 42.8)*	9.0 (3.1 to 16.8)*	11.7 (8.2 to 15.2)	26.7 (18.1 to 35.9)*	3.6 (-3.4 to 11.2)

Cause	Prevalence in thousands (95% confidence interval)			YLDs in thousands (95% confidence interval)		
	2015	Percent change in counts between 2005 and 2015	Percent change in rates between 2005 and 2015	2015	Percent change in counts between 2005 and 2015	Percent change in rates between 2005 and 2015
Larynx cancer	190.6 (174.7 to 211.1)	19.5 (9.8 to 30.9)*	-2.3 (-10.2 to 7.1)	18.8 (13.6 to 24.5)	16.9 (7.0 to 28.0)*	-4.4 (-12.5 to 4.7)
Larynx cancer aggregate	137.3 (121.3 to 157.9)	18.5 (5.4 to 34.1)*	-3.0 (-13.8 to 9.7)	14.1 (10.1 to 18.3)	15.3 (2.7 to 29.7)*	-5.7 (-16.0 to 6.1)
Laryngectomy due to larynx cancer	53.2 (50.7 to 55.2)	21.9 (20.8 to 23.3)*	-0.3 (-1.2 to 0.9)	4.7 (3.2 to 6.7)	22.2 (15.5 to 29.3)*	-0.0 (-5.5 to 5.7)
Tracheal, bronchus, and lung cancer	492.9 (458.3 to 544.3)	30.0 (21.3 to 41.3)*	6.4 (-0.8 to 15.6)	75.5 (55.5 to 96.2)	23.0 (13.8 to 35.3)*	0.6 (-6.9 to 10.7)
Malignant skin melanoma	315.7 (239.7 to 402.2)	69.3 (53.3 to 86.9)*	38.5 (25.4 to 52.9)*	18.2 (11.6 to 26.4)	64.0 (48.5 to 81.2)*	34.1 (21.5 to 48.2)*
Non-melanoma skin cancer	297.2 (281.7 to 314.5)	64.1 (55.2 to 74.2)*	34.2 (26.9 to 42.5)*	15.6 (10.7 to 21.7)	69.0 (51.9 to 90.1)*	38.2 (24.2 to 55.5)*
Non-melanoma skin cancer(squamous-cell carcinoma)	236.7 (205.1 to 281.3)	81.3 (62.5 to 105.1)*	48.2 (32.9 to 67.7)*	14.9 (10.1 to 20.6)	72.1 (53.7 to 94.5)*	40.7 (25.7 to 59.1)*
Non-melanoma skin cancer(basal-cell carcinoma)	70.5 (59.4 to 84.3)	23.8 (19.6 to 27.6)*	1.2 (-2.2 to 4.4)	0.7 (0.3 to 1.3)	23.8 (17.7 to 30.8)*	1.3 (-3.8 to 7.0)
Breast cancer	2,070.3 (1,943.7 to 2,185.9)	45.7 (38.2 to 53.9)*	19.1 (13.0 to 25.8)*	186.1 (131.6 to 250.7)	37.0 (30.6 to 44.1)*	12.0 (6.8 to 17.9)*
Breast cancer aggregate	666.0 (541.7 to 789.9)	137.7 (95.3 to 186.6)*	94.4 (59.7 to 134.4)*	79.7 (57.7 to 102.2)	61.2 (42.3 to 85.0)*	31.9 (16.4 to 51.3)*
Mastectomy due to breast cancer	1,403.1 (1,349.2 to 1,455.6)	23.1 (21.7 to 24.5)*	0.6 (-0.5 to 1.8)	106.4 (68.0 to 153.4)	23.1 (21.2 to 25.0)*	0.7 (-0.9 to 2.2)
Cervical cancer	232.5 (199.5 to 282.6)	-1.3 (-17.5 to 21.1)	-19.3 (-32.5 to -0.9)*	18.0 (12.3 to 24.6)	-2.1 (-18.3 to 18.9)	-19.9 (-33.2 to -2.8)*
Uterine cancer	424.6 (364.3 to 497.0)	28.2 (12.3 to 46.7)*	4.9 (-8.2 to 20.0)	27.5 (18.7 to 37.5)	25.8 (9.9 to 44.9)*	2.9 (-10.1 to 18.5)
Ovarian cancer	108.8 (100.7 to 120.7)	15.6 (7.3 to 26.1)*	-5.4 (-12.2 to 3.1)	13.7 (10.0 to 17.8)	13.4 (3.8 to 25.1)*	-7.3 (-15.1 to 2.3)
Prostate cancer	2,932.2 (2,358.8 to 4,106.8)	69.0 (57.0 to 82.8)*	38.2 (28.4 to 49.5)*	216.2 (147.4 to 311.8)	58.6 (47.6 to 70.9)*	29.7 (20.7 to 39.8)*
Prostate cancer aggregate	2,652.3 (2,080.2 to 3,828.7)	74.8 (60.9 to 90.9)*	43.0 (31.6 to 56.1)*	192.5 (130.1 to 286.1)	63.4 (50.6 to 78.0)*	33.6 (23.2 to 45.6)*
Impotence and incontinence due to prostate cancer aggregate	279.9 (274.3 to 285.2)	28.5 (28.1 to 28.9)*	5.1 (4.8 to 5.5)*	23.8 (16.3 to 32.7)	28.3 (25.3 to 31.4)*	4.9 (2.4 to 7.5)*
Testicular cancer	12.2 (9.5 to 15.3)	75.5 (44.2 to 118.8)*	43.5 (17.9 to 78.9)*	0.8 (0.6 to 1.2)	64.3 (33.5 to 107.4)*	34.4 (9.2 to 69.6)*
Kidney cancer	339.3 (313.6 to 367.7)	45.2 (33.4 to 56.8)*	18.7 (9.1 to 28.2)*	23.6 (17.0 to 31.4)	41.0 (28.9 to 53.0)*	15.4 (5.5 to 25.2)*
Bladder cancer	490.6 (439.3 to 548.8)	31.4 (17.8 to 46.2)*	7.5 (-3.6 to 19.6)	36.5 (25.9 to 48.8)	27.2 (15.0 to 40.5)*	4.1 (-5.9 to 14.9)
Bladder cancer episodes aggregate	477.3 (425.7 to 535.4)	31.9 (17.8 to 47.2)*	7.9 (-3.6 to 20.4)	34.3 (24.1 to 45.8)	28.0 (14.7 to 42.5)*	4.7 (-6.2 to 16.5)
Urinary incontinence due to bladder cancer	13.3 (12.3 to 14.4)	15.8 (14.4 to 17.2)*	-5.3 (-6.4 to -4.1)*	2.2 (1.5 to 3.1)	16.4 (5.2 to 27.4)*	-4.8 (-13.9 to 4.2)
Brain and nervous system cancer	93.9 (83.1 to 113.0)	31.5 (18.8 to 48.9)*	7.5 (-2.8 to 21.8)	10.3 (7.3 to 13.8)	28.3 (14.3 to 46.4)*	4.9 (-6.5 to 19.7)
Thyroid cancer	447.8 (394.9 to 505.7)	168.2 (134.0 to 204.4)*	119.3 (91.4 to 149.0)*	26.8 (18.4 to 36.4)	152.9 (118.9 to 188.3)*	106.8 (79.1 to 135.8)*
Mesothelioma	7.8 (7.0 to 8.8)	33.1 (20.2 to 49.2)*	8.9 (-1.7 to 22.0)	1.6 (1.1 to 2.1)	31.1 (14.3 to 52.1)*	7.3 (-6.5 to 24.4)
Hodgkin lymphoma	35.5 (28.9 to 47.3)	32.4 (13.8 to 56.7)*	8.3 (-6.9 to 28.1)	3.2 (2.1 to 4.6)	23.4 (5.6 to 47.0)*	1.0 (-13.6 to 20.2)
Non-Hodgkin lymphoma	457.5 (389.9 to 508.7)	69.9 (53.4 to 85.2)*	39.0 (25.5 to 51.5)*	32.9 (23.5 to 43.3)	62.8 (46.7 to 77.6)*	33.1 (20.0 to 45.2)*
Multiple myeloma	64.7 (56.8 to 75.1)	47.0 (31.2 to 67.5)*	20.2 (7.3 to 37.0)*	13.5 (9.4 to 17.8)	41.7 (25.9 to 62.2)*	15.9 (3.0 to 32.7)*
Leukemia	217.3 (206.5 to 229.3)	36.9 (27.6 to 46.1)*	12.0 (4.4 to 19.5)*	32.6 (23.5 to 41.7)	28.4 (19.2 to 37.8)*	5.0 (-2.5 to 12.7)
Acute lymphoid leukemia	44.0 (37.5 to 51.8)	43.6 (26.8 to 60.8)*	17.4 (3.7 to 31.5)*	6.2 (4.2 to 8.3)	37.4 (20.1 to 55.6)*	12.4 (-1.7 to 27.3)
Chronic lymphoid leukemia	92.3 (82.8 to 102.6)	29.0 (17.8 to 41.7)*	5.5 (-3.7 to 15.9)	12.1 (8.7 to 15.5)	23.6 (10.7 to 38.2)*	1.1 (-9.5 to 13.1)
Acute myeloid leukemia	83.6 (74.2 to 94.2)	45.5 (33.1 to 59.7)*	19.0 (8.9 to 30.6)*	10.9 (7.7 to 14.1)	38.9 (24.2 to 54.7)*	13.6 (1.6 to 26.5)*
Chronic myeloid leukemia	24.8 (22.0 to 28.1)	9.6 (-1.3 to 22.0)	-10.4 (-19.3 to -0.2)*	3.5 (2.5 to 4.6)	5.3 (-7.4 to 19.5)	-13.9 (-24.2 to -2.2)*
Other neoplasms	389.2 (339.2 to 457.5)	32.4 (18.3 to 48.5)*	8.3 (-3.2 to 21.4)	27.6 (19.4 to 37.4)	29.9 (15.2 to 46.9)*	6.2 (-5.8 to 20.1)
Cardiovascular diseases	50,179.2 (48,926.3 to 51,067.7)	18.3 (17.3 to 19.3)*	-3.2 (-4.0 to -2.4)*	2,906.9 (2,069.3 to 3,819.8)	18.1 (17.0 to 19.0)*	-3.4 (-4.3 to -2.7)*
Rheumatic heart disease	760.0 (628.7 to 1,192.6)	-2.5 (-9.5 to 2.3)	-20.3 (-26.0 to -16.3)*	43.0 (27.5 to 71.0)	3.3 (-5.3 to 9.5)	-15.5 (-22.5 to -10.5)*
Rheumatic heart disease, without heart failure	627.3 (494.2 to 1,057.8)	-7.7 (-13.2 to -2.1)*	-24.5 (-29.0 to -19.9)*	28.3 (16.6 to 55.0)	-7.6 (-13.3 to -1.6)*	-24.4 (-29.1 to -19.5)*
Heart failure fue to rheumatic heart disease aggregate	132.7 (118.2 to 146.0)	32.9 (31.0 to 34.6)*	8.7 (7.1 to 10.1)*	14.7 (9.9 to 20.6)	33.3 (27.4 to 38.9)*	9.0 (4.2 to 13.6)*
Ischemic heart disease	14,849.6 (12,741.6 to 17,253.9)	7.6 (5.8 to 9.4)*	-12.0 (-13.5 to -10.5)*	916.1 (607.2 to 1,257.1)	16.6 (15.5 to 17.8)*	-4.6 (-5.5 to -3.6)*
Myocardial infarction episodes aggregate	2,681.0 (2,288.5 to 3,106.6)	-18.6 (-24.5 to -12.5)*	-33.4 (-38.3 to -28.4)*	3.9 (2.6 to 5.5)	3.2 (-3.1 to 9.3)	-15.6 (-20.7 to -10.6)*
Angina due to ischemic heart disease aggregate	9,643.5 (7,598.0 to 12,071.4)	14.5 (13.1 to 15.9)*	-6.3 (-7.5 to -5.2)*	632.6 (391.0 to 908.2)	14.9 (13.3 to 16.3)*	-6.1 (-7.3 to -4.9)*
Heart failure fue to ischemic heart disease aggregate	2,525.1 (2,319.6 to 2,743.8)	21.0 (19.9 to 22.3)*	-1.0 (-2.0 to 0.0)	279.6 (194.8 to 381.1)	21.1 (19.5 to 22.8)*	-0.9 (-2.3 to 0.4)
Cerebrovascular disease	5,359.9 (5,284.6 to 5,409.7)	10.6 (9.7 to 11.5)*	-9.5 (-10.2 to -8.8)*	794.0 (550.5 to 1,054.6)	10.8 (9.2 to 12.3)*	-9.4 (-10.7 to -8.2)*
Ischemic stroke	3,604.8 (3,476.2 to 3,738.2)	8.6 (7.5 to 9.7)*	-11.2 (-12.1 to -10.3)*	524.0 (363.8 to 697.5)	9.0 (7.4 to 10.6)*	-10.9 (-12.2 to -9.6)*
Chronic ischemic stroke aggregate	3,572.2 (3,444.5 to 3,704.6)	8.7 (7.6 to 9.8)*	-11.1 (-12.0 to -10.2)*	517.1 (358.7 to 688.3)	9.1 (7.5 to 10.7)*	-10.8 (-12.1 to -9.5)*
Ischemic stroke episodes aggregate	32.6 (27.2 to 38.8)	1.3 (-4.6 to 7.8)	-17.2 (-22.0 to -11.8)*	6.9 (4.4 to 9.8)	2.5 (-4.8 to 10.4)	-16.2 (-22.2 to -9.7)*
Hemorrhagic stroke	1,858.7 (1,792.1 to 1,926.5)	14.2 (13.1 to 15.4)*	-6.6 (-7.5 to -5.6)*	270.0 (187.7 to 359.1)	14.5 (12.3 to 16.7)*	-6.3 (-8.1 to -4.6)*
Chronic hemorrhagic stroke aggregate	1,841.5 (1,775.7 to 1,909.6)	14.3 (13.1 to 15.5)*	-6.5 (-7.5 to -5.6)*	266.2 (184.7 to 355.4)	14.6 (12.4 to 16.8)*	-6.2 (-8.0 to -4.5)*
Acute hemorrhagic stroke aggregate	17.3 (14.4 to 20.7)	6.8 (-0.2 to 13.2)	-12.7 (-18.4 to -7.5)*	3.8 (2.4 to 5.3)	7.0 (-0.4 to 13.4)	-12.5 (-18.5 to -7.3)*
Hypertensive heart disease	693.4 (621.7 to 775.6)	32.2 (31.1 to 33.3)*	8.1 (7.2 to 9.1)*	76.8 (52.3 to 106.7)	32.5 (30.0 to 35.3)*	8.3 (6.3 to 10.6)*
Cardiomyopathy and myocarditis	157.3 (138.4 to 177.8)	22.0 (20.2 to 23.7)*	-0.2 (-1.7 to 1.2)	16.8 (11.5 to 23.1)	21.8 (17.5 to 26.2)*	-0.4 (-3.9 to 3.2)
Acute myocarditis	9.2 (6.2 to 12.9)	26.9 (23.3 to 30.4)*	3.8 (0.8 to 6.6)*	0.5 (0.2 to 0.7)	25.7 (17.4 to 34.6)*	2.8 (-4.0 to 10.1)
Heart failure due to cardiomyopathy aggregate	148.2 (130.2 to 168.4)	21.7 (19.8 to 23.5)*	-0.4 (-2.0 to 1.0)	16.4 (11.1 to 22.5)	21.7 (17.3 to 26.2)*	-0.5 (-4.1 to 3.2)
Atrial fibrillation and flutter	4,422.8 (3,777.2 to 5,164.0)	19.2 (18.3 to 20.2)*	-2.5 (-3.3 to -1.7)*	351.9 (233.6 to 493.9)	19.4 (17.9 to 21.0)*	-2.4 (-3.5 to -1.1)*
Peripheral vascular disease	21,606.6 (18,040.7 to 25,822.1)	22.6 (21.9 to 23.4)*	0.3 (-0.3 to 0.9)	98.7 (46.2 to 182.5)	24.5 (22.1 to 27.3)*	1.8 (-0.2 to 4.1)
Endocarditis	8.3 (7.0 to 9.8)	17.3 (13.2 to 21.5)*	-4.0 (-7.4 to -0.6)*	0.9 (0.6 to 1.3)	17.3 (3.5 to 32.2)*	-4.0 (-15.4 to 8.1)
Endocarditis episodes aggregate	1.2 (0.9 to 1.4)	21.0 (14.0 to 27.0)*	-1.1 (-6.8 to 3.9)	0.1 (0.0 to 0.1)	21.2 (14.4 to 27.2)*	-0.9 (-6.4 to 4.0)
Heart failure due to endocarditis aggregate	7.1 (5.9 to 8.7)	16.8 (12.0 to 21.1)*	-4.5 (-8.4 to -1.0)*	0.8 (0.5 to 1.2)	17.0 (2.3 to 33.2)*	-4.3 (-16.3 to 9.0)
Other cardiovascular and circulatory diseases	9,225.3 (8,037.6 to 10,495.0)	28.8 (27.3 to 30.3)*	5.4 (4.1 to 6.6)*	608.7 (413.0 to 852.2)	29.0 (27.3 to 30.7)*	5.5 (4.1 to 6.9)*
Heart failure due to other cardiovascular diseases aggregate	129.3 (110.9 to 148.9)	29.3 (27.6 to 31.1)*	5.7 (4.3 to 7.2)*	14.3 (9.7 to 20.4)	29.5 (24.2 to 34.8)*	5.9 (1.6 to 10.2)*
Other cardiovascular diseases episodes aggregate	9,096.0 (7,926.2 to 10,343.0)	28.8 (27.3 to 30.3)*	5.4 (4.1 to 6.6)*	594.3 (403.1 to 833.0)	29.0 (27.3 to 30.7)*	5.5 (4.1 to 6.9)*
Chronic respiratory diseases	29,633.4 (28,555.5 to 30,752.2)	15.9 (14.3 to 17.4)*	-5.2 (-6.5 to -4.0)*	1,977.6 (1,593.8 to 2,408.5)	16.3 (13.2 to 19.2)*	-4.9 (-7.4 to -2.5)*
Chronic obstructive pulmonary disease	17,791.3 (15,410.8 to 20,516.2)	15.2 (11.9 to 18.6)*	-5.8 (-8.5 to -3.0)*	1,200.4 (987.8 to 1,439.5)	17.9 (13.2 to 22.4)*	-3.6 (-7.4 to 0.1)

Cause	Prevalence in thousands (95% confidence interval)			YLDs in thousands (95% confidence interval)		
	2015	Percent change in counts between 2005 and 2015	Percent change in rates between 2005 and 2015	2015	Percent change in counts between 2005 and 2015	Percent change in rates between 2005 and 2015
Chronic obstructive pulmonary disease without heart failure aggregate	16,865.7 (14,529.6 to 19,576.0)	14.4 (10.8 to 17.8)*	-6.5 (-9.4 to -3.6)*	815.6 (638.9 to 1,027.6)	11.5 (5.1 to 17.5)*	-8.8 (-14.1 to -3.9)*
Severe chronic obstructive pulmonary disease with heart failure aggregate	932.2 (832.1 to 1,035.3)	33.9 (31.1 to 35.6)*	9.5 (7.2 to 10.9)*	384.8 (287.0 to 473.1)	34.2 (31.2 to 37.0)*	9.8 (7.3 to 12.1)*
Pneumoconiosis	169.2 (160.4 to 176.7)	27.5 (22.5 to 32.2)*	4.2 (0.2 to 8.1)*	30.6 (19.5 to 44.7)	27.0 (21.5 to 32.3)*	3.8 (-0.6 to 8.2)
Silicosis	35.4 (30.4 to 41.0)	17.9 (14.5 to 21.1)*	-3.6 (-6.3 to -0.9)*	5.8 (3.6 to 8.6)	18.7 (9.8 to 28.5)*	-2.9 (-10.2 to 5.1)
Silicosis without heart failure aggregate	32.4 (27.5 to 37.7)	17.3 (13.7 to 20.8)*	-4.0 (-7.0 to -1.2)*	4.4 (2.5 to 6.7)	16.9 (5.2 to 29.7)*	-4.4 (-14.0 to 6.1)
Severe silicosis with heart failure aggregate	3.0 (2.7 to 3.4)	24.0 (21.1 to 26.6)*	1.4 (-1.0 to 3.5)	1.4 (1.0 to 1.9)	24.7 (13.4 to 36.6)*	2.0 (-7.3 to 11.7)
Asbestosis	20.8 (18.4 to 23.3)	34.3 (32.0 to 37.0)*	9.9 (7.9 to 12.1)*	3.2 (2.1 to 4.6)	34.2 (24.8 to 44.5)*	9.7 (2.1 to 18.2)*
Asbestosis without heart failure aggregate	20.5 (18.1 to 23.0)	34.3 (31.9 to 37.0)*	9.8 (7.9 to 12.1)*	3.1 (1.9 to 4.5)	34.0 (24.1 to 44.9)*	9.6 (1.5 to 18.5)*
Severe asbestosis with heart failure aggregate	0.3 (0.3 to 0.4)	37.2 (34.9 to 39.4)*	12.2 (10.3 to 14.0)*	0.2 (0.1 to 0.2)	37.2 (34.9 to 39.5)*	12.2 (10.4 to 14.1)*
Coal workers pneumoconiosis	10.6 (9.1 to 12.3)	16.3 (12.1 to 19.9)*	-4.9 (-8.3 to -1.9)*	1.6 (1.0 to 2.4)	15.6 (-2.6 to 37.1)	-5.4 (-20.3 to 12.1)
Coal workers pneumoconiosis without heart failure aggregate	10.3 (8.9 to 12.0)	15.9 (11.6 to 19.6)*	-5.2 (-8.7 to -2.2)*	1.5 (0.9 to 2.2)	14.3 (-4.9 to 37.6)	-6.5 (-22.2 to 12.5)
Severe coal workers pneumoconiosis with heart failure aggregate	0.3 (0.2 to 0.3)	33.1 (27.3 to 39.0)*	8.8 (4.1 to 13.7)*	0.1 (0.1 to 0.2)	33.1 (27.3 to 39.0)*	8.8 (4.1 to 13.7)*
Other pneumoconiosis	134.6 (115.8 to 155.8)	29.1 (26.6 to 31.4)*	5.6 (3.5 to 7.5)*	19.9 (12.6 to 29.6)	29.5 (22.2 to 37.3)*	5.9 (-0.0 to 12.3)
Other pneumoconiosis without heart failure aggregate	133.1 (114.2 to 154.3)	29.1 (26.6 to 31.5)*	5.6 (3.6 to 7.6)*	19.2 (12.0 to 28.8)	29.7 (22.0 to 37.9)*	6.1 (-0.2 to 12.8)
Severe other pneumoconiosis with heart failure aggregate	1.5 (1.3 to 1.7)	25.0 (22.7 to 27.5)*	2.2 (0.3 to 4.2)*	0.7 (0.5 to 1.0)	25.0 (21.7 to 28.5)*	2.2 (-0.5 to 5.1)
Asthma	13,672.8 (11,361.8 to 16,059.7)	15.6 (13.3 to 18.2)*	-5.5 (-7.3 to -3.3)*	578.5 (369.9 to 838.9)	15.7 (13.4 to 18.5)*	-5.4 (-7.3 to -3.1)*
Asymptomatic asthma	4,140.9 (3,339.7 to 5,065.5)	15.6 (13.3 to 18.2)*	-5.5 (-7.3 to -3.3)*	0.0 (0.0 to 0.0)	0.0 (0.0 to 0.0)	0.0 (0.0 to 0.0)
Symptomatic asthma aggregate	9,531.9 (7,979.6 to 11,334.0)	15.6 (13.3 to 18.2)*	-5.5 (-7.3 to -3.3)*	578.5 (369.9 to 838.9)	15.7 (13.4 to 18.5)*	-5.4 (-7.3 to -3.1)*
Interstitial lung disease and pulmonary sarcoidosis	193.4 (164.0 to 229.3)	23.7 (20.4 to 27.2)*	1.2 (-1.6 to 4.0)	23.7 (14.4 to 34.9)	24.3 (18.5 to 30.0)*	1.7 (-3.0 to 6.4)
Interstitial lung disease and pulmonary sarcoidosis without heart failure aggregate	165.6 (137.6 to 199.5)	22.7 (19.1 to 26.4)*	0.3 (-2.6 to 3.4)	11.5 (6.7 to 18.1)	18.9 (10.9 to 26.9)*	-2.8 (-9.3 to 3.8)
Severe interstitial lung disease and pulmonary sarcoidosis with heart failure aggregate	27.8 (19.0 to 35.2)	30.2 (26.9 to 32.7)*	6.5 (3.8 to 8.6)*	12.2 (7.5 to 17.3)	29.9 (22.6 to 37.0)*	6.3 (0.3 to 12.1)*
Other chronic respiratory diseases	--	--	--	144.3 (115.6 to 177.1)	4.0 (-6.3 to 16.1)	-15.0 (-23.3 to -5.0)*
Cirrhosis and other chronic liver diseases	243.5 (236.3 to 250.6)	29.4 (25.8 to 33.5)*	5.8 (2.9 to 9.2)*	39.0 (26.9 to 53.7)	29.5 (25.6 to 33.1)*	5.9 (2.7 to 8.8)*
Cirrhosis and other chronic liver diseases due to hepatitis B	71.4 (59.7 to 85.0)	28.6 (24.5 to 32.3)*	5.2 (1.8 to 8.2)*	11.4 (7.4 to 16.0)	28.9 (20.9 to 36.5)*	5.4 (-1.1 to 11.7)
Cirrhosis and other chronic liver diseases due to hepatitis C	61.6 (53.8 to 69.9)	31.6 (27.9 to 35.0)*	7.6 (4.6 to 10.4)*	9.8 (6.7 to 13.6)	31.9 (24.6 to 40.3)*	7.9 (1.9 to 14.7)*
Cirrhosis and other chronic liver diseases due to alcohol use	65.8 (55.9 to 76.3)	29.9 (26.2 to 33.5)*	6.3 (3.2 to 9.2)*	10.4 (7.1 to 14.7)	30.3 (22.4 to 38.5)*	6.5 (0.1 to 13.3)*
Cirrhosis and other chronic liver diseases due to other causes	46.9 (39.5 to 54.9)	25.8 (22.7 to 28.7)*	2.9 (0.3 to 5.3)*	7.4 (5.1 to 10.6)	26.2 (18.5 to 34.3)*	3.2 (-3.1 to 9.8)
Digestive diseases	24,153.3 (23,788.1 to 24,531.9)	10.1 (8.0 to 12.3)*	-10.0 (-11.7 to -8.1)*	931.2 (654.3 to 1,274.3)	8.2 (5.8 to 10.7)*	-11.5 (-13.4 to -9.5)*
Peptic ulcer disease	6,224.2 (6,063.2 to 6,395.6)	-5.7 (-7.9 to -3.3)*	-22.9 (-24.7 to -20.9)*	191.6 (130.7 to 269.0)	-7.3 (-10.1 to -4.6)*	-24.2 (-26.4 to -22.0)*
Peptic ulcer disease, symptomatic episodes	453.7 (354.3 to 584.1)	-3.7 (-9.8 to 1.8)	-21.2 (-26.2 to -16.7)*	46.7 (29.8 to 67.4)	-3.4 (-9.6 to 2.2)	-21.0 (-26.1 to -16.4)*
Anemia due to peptic ulcer disease aggregate	5,770.4 (5,652.0 to 5,890.1)	-5.8 (-8.1 to -3.2)*	-23.0 (-24.9 to -20.9)*	144.9 (98.7 to 207.8)	-8.5 (-11.4 to -5.3)*	-25.1 (-27.6 to -22.5)*
Gastritis and duodenitis	17,315.1 (16,997.7 to 17,653.8)	15.7 (13.3 to 18.4)*	-5.4 (-7.3 to -3.2)*	445.7 (303.3 to 631.5)	9.8 (6.6 to 13.0)*	-10.2 (-12.8 to -7.6)*
Gastritis and duodenitis, symptomatic episodes	444.5 (340.9 to 562.2)	16.2 (12.4 to 19.2)*	-4.9 (-8.1 to -2.5)*	45.9 (29.8 to 66.0)	16.5 (12.1 to 20.5)*	-4.7 (-8.3 to -1.5)*
Anemia due to gastritis and duodenitis aggregate	16,870.7 (16,562.9 to 17,183.1)	15.6 (13.2 to 18.4)*	-5.4 (-7.4 to -3.1)*	399.8 (271.2 to 572.0)	9.1 (5.5 to 12.5)*	-10.8 (-13.7 to -8.0)*
Appendicitis	12.3 (10.2 to 14.8)	23.1 (21.2 to 25.0)*	0.7 (-0.9 to 2.2)	3.6 (2.3 to 5.2)	23.7 (12.7 to 35.8)*	1.2 (-7.8 to 11.1)
Paralytic ileus and intestinal obstruction	6.7 (5.3 to 8.3)	20.7 (15.2 to 26.0)*	-1.3 (-5.7 to 3.1)	2.1 (1.3 to 3.0)	20.9 (10.7 to 31.4)*	-1.1 (-9.5 to 7.5)
Inguinal, femoral, and abdominal hernia	1,099.1 (794.9 to 1,468.4)	1.8 (-3.5 to 6.8)	-16.8 (-21.0 to -12.7)*	11.3 (5.1 to 22.0)	2.0 (-3.4 to 7.3)	-16.5 (-21.0 to -12.3)*
Inflammatory bowel disease	628.0 (583.0 to 685.9)	19.8 (19.0 to 20.5)*	-2.0 (-2.7 to -1.4)*	128.3 (89.1 to 173.9)	19.9 (17.9 to 22.0)*	-1.9 (-3.6 to -0.2)*
Vascular intestinal disorders	3.3 (2.6 to 4.1)	21.3 (14.7 to 27.3)*	-0.8 (-6.2 to 4.1)	1.0 (0.7 to 1.5)	20.6 (9.1 to 32.5)*	-1.3 (-10.7 to 8.4)
Gallbladder and biliary diseases	347.4 (268.1 to 439.6)	8.6 (1.0 to 15.3)*	-11.2 (-17.4 to -5.7)*	35.9 (23.4 to 54.4)	8.7 (0.8 to 15.8)*	-11.1 (-17.6 to -5.3)*
Pancreatitis	62.8 (48.9 to 78.1)	15.2 (8.4 to 21.6)*	-5.8 (-11.3 to -0.6)*	17.8 (11.6 to 25.6)	15.3 (6.3 to 24.4)*	-5.7 (-13.1 to 1.8)
Other digestive diseases	--	--	--	94.0 (65.3 to 129.0)	23.7 (14.3 to 35.8)*	1.2 (-6.5 to 11.1)
Neurological disorders	57,708.2 (54,017.3 to 60,948.7)	20.7 (18.4 to 23.1)*	-1.3 (-3.2 to 0.7)	1,913.5 (1,318.0 to 2,625.5)	19.0 (16.2 to 21.6)*	-2.7 (-5.0 to -0.5)*
Alzheimer disease and other dementias	4,006.5 (3,017.1 to 5,040.8)	26.9 (26.0 to 27.8)*	3.8 (3.0 to 4.5)*	484.7 (312.5 to 710.8)	27.1 (25.5 to 28.5)*	3.9 (2.7 to 5.1)*
Parkinson disease	809.2 (715.7 to 918.9)	24.9 (20.1 to 30.0)*	2.1 (-1.8 to 6.3)	96.9 (66.1 to 133.3)	25.1 (19.5 to 30.8)*	2.3 (-2.3 to 7.0)
Epilepsy	718.4 (619.3 to 823.6)	24.5 (18.7 to 30.4)*	1.9 (-2.9 to 6.7)	161.1 (110.3 to 218.8)	1.9 (-4.7 to 8.5)	-16.7 (-22.1 to -11.2)*
Multiple sclerosis	112.4 (103.8 to 121.6)	27.6 (24.8 to 30.2)*	4.3 (2.0 to 6.5)*	35.8 (25.4 to 46.2)	27.2 (21.7 to 32.9)*	4.0 (-0.5 to 8.7)
Motor neuron disease	22.5 (19.1 to 26.8)	28.2 (20.7 to 35.4)*	4.9 (-1.2 to 10.7)	4.6 (3.2 to 6.4)	28.0 (18.8 to 36.6)*	4.7 (-2.8 to 11.7)
Migraine	21,457.1 (17,254.7 to 26,415.5)	18.7 (16.3 to 21.0)*	-2.9 (-4.8 to -1.0)*	689.7 (418.5 to 1,084.4)	19.0 (16.5 to 21.8)*	-2.7 (-4.7 to -0.4)*
Tension-type headache	34,031.6 (23,810.0 to 46,772.5)	21.4 (18.5 to 24.2)*	-0.7 (-3.0 to 1.6)	49.2 (21.3 to 93.8)	21.6 (18.5 to 24.7)*	-0.5 (-3.1 to 2.0)
Medication overuse headache	2,423.0 (1,503.6 to 3,693.1)	14.8 (3.9 to 27.4)*	-6.1 (-15.0 to 4.2)	362.7 (198.8 to 583.4)	15.0 (3.8 to 27.9)*	-6.0 (-15.1 to 4.6)
Other neurological disorders	0.6 (0.4 to 0.9)	19.6 (15.4 to 22.3)*	-2.2 (-5.6 to 0.1)	28.9 (19.6 to 40.7)	19.3 (15.0 to 23.3)*	-2.5 (-6.0 to 0.8)
Mental and substance use disorders	28,926.4 (28,212.3 to 29,645.6)	21.3 (20.1 to 22.5)*	-0.8 (-1.8 to 0.2)	4,305.6 (3,137.6 to 5,596.6)	21.6 (20.6 to 22.6)*	-0.6 (-1.4 to 0.2)
Schizophrenia	879.1 (777.9 to 991.9)	26.5 (25.6 to 27.4)*	3.5 (2.8 to 4.2)*	531.4 (394.0 to 658.9)	26.8 (24.7 to 28.9)*	3.7 (2.0 to 5.4)*
Alcohol use disorders	1,306.3 (1,108.0 to 1,552.5)	9.5 (5.6 to 13.5)*	-10.4 (-13.7 to -7.1)*	123.6 (82.9 to 177.5)	9.9 (5.3 to 14.4)*	-10.1 (-13.9 to -6.4)*
Alcohol dependence aggregate	1,288.3 (1,089.9 to 1,534.1)	9.4 (5.3 to 13.4)*	-10.5 (-13.8 to -7.2)*	122.7 (82.2 to 176.4)	9.8 (5.1 to 14.4)*	-10.2 (-14.0 to -6.5)*
Fetal alcohol syndrome aggregate	18.0 (16.2 to 20.1)	22.3 (20.9 to 23.8)*	0.1 (-1.1 to 1.2)	0.9 (0.6 to 1.3)	20.7 (14.4 to 26.8)*	-1.3 (-6.4 to 3.7)
Drug use disorders	657.4 (636.9 to 682.3)	37.2 (34.0 to 40.6)*	12.3 (9.6 to 15.0)*	181.6 (126.3 to 236.1)	36.6 (32.0 to 41.2)*	11.7 (8.0 to 15.5)*
Opioid use disorders	326.3 (271.2 to 387.7)	31.0 (27.9 to 34.7)*	7.1 (4.6 to 10.2)*	126.4 (86.2 to 168.9)	31.5 (26.2 to 36.5)*	7.5 (3.2 to 11.7)*
Asymptomatic opioid dependence	52.9 (38.0 to 73.1)	31.0 (27.9 to 34.7)*	7.1 (4.6 to 10.2)*	0.0 (0.0 to 0.0)	0.0 (0.0 to 0.0)	0.0 (0.0 to 0.0)
Symptomatic opioid dependence aggregate	273.4 (225.1 to 329.0)	31.0 (27.9 to 34.7)*	7.1 (4.6 to 10.2)*	126.4 (86.2 to 168.9)	31.5 (26.2 to 36.5)*	7.5 (3.2 to 11.7)*
Cocaine use disorders	222.4 (183.7 to 267.9)	49.3 (46.7 to 51.9)*	22.1 (20.0 to 24.3)*	28.4 (17.7 to 42.1)	49.6 (42.1 to 57.2)*	22.3 (16.2 to 28.6)*
Asymptomatic cocaine dependence	111.4 (86.1 to 141.7)	49.3 (46.7 to 51.9)*	22.1 (20.0 to 24.3)*	0.0 (0.0 to 0.0)	0.0 (0.0 to 0.0)	0.0 (0.0 to 0.0)

Cause	Prevalence in thousands (95% confidence interval)			YLDs in thousands (95% confidence interval)		
	2015	Percent change in counts between 2005 and 2015	Percent change in rates between 2005 and 2015	2015	Percent change in counts between 2005 and 2015	Percent change in rates between 2005 and 2015
Symptomatic cocaine dependence aggregate	110.9 (85.1 to 140.3)	49.3 (46.7 to 51.9)*	22.1 (20.0 to 24.3)*	28.4 (17.7 to 42.1)	49.6 (42.1 to 57.2)*	22.3 (16.2 to 28.6)*
Amphetamine use disorders	25.1 (18.8 to 32.5)	79.7 (73.0 to 87.8)*	47.0 (41.5 to 53.6)*	3.3 (1.9 to 5.1)	73.3 (54.7 to 94.9)*	41.8 (26.5 to 59.4)*
Asymptomatic amphetamine dependence	13.8 (9.7 to 18.9)	79.7 (73.0 to 87.8)*	47.0 (41.5 to 53.6)*	0.0 (0.0 to 0.0)	0.0 (0.0 to 0.0)	0.0 (0.0 to 0.0)
Symptomatic amphetamine dependence aggregate	11.4 (7.9 to 15.4)	79.7 (73.0 to 87.8)*	47.0 (41.5 to 53.6)*	3.3 (1.9 to 5.1)	73.3 (54.7 to 94.9)*	41.8 (26.5 to 59.4)*
Cannabis use disorders	103.9 (94.0 to 115.1)	23.8 (22.3 to 25.3)*	1.2 (0.0 to 2.5)*	3.0 (1.9 to 4.3)	23.6 (18.8 to 29.0)*	1.1 (-2.8 to 5.5)
Asymptomatic cannabis dependence	60.1 (53.0 to 67.2)	23.8 (22.3 to 25.3)*	1.2 (0.0 to 2.5)*	0.0 (0.0 to 0.0)	0.0 (0.0 to 0.0)	0.0 (0.0 to 0.0)
Symptomatic cannabis dependence aggregate	43.8 (37.4 to 50.5)	23.8 (22.3 to 25.3)*	1.2 (0.0 to 2.5)*	3.0 (1.9 to 4.3)	23.6 (18.8 to 29.0)*	1.1 (-2.8 to 5.5)
Other drug use disorders	--	--	--	20.6 (13.0 to 30.6)	51.8 (43.8 to 60.0)*	24.2 (17.6 to 30.9)*
Depressive disorders	13,406.4 (12,803.9 to 14,087.4)	19.6 (17.7 to 21.3)*	-2.2 (-3.7 to -0.8)*	2,105.4 (1,460.2 to 2,889.5)	18.8 (17.5 to 20.0)*	-2.8 (-3.9 to -1.8)*
Major depressive disorder	8,052.2 (6,622.3 to 9,639.5)	16.9 (15.6 to 18.1)*	-4.4 (-5.5 to -3.4)*	1,562.5 (1,056.0 to 2,176.2)	17.2 (15.8 to 18.6)*	-4.1 (-5.3 to -3.0)*
Dysthymia	5,845.6 (4,533.3 to 7,262.9)	23.3 (21.3 to 25.4)*	0.8 (-0.8 to 2.6)	542.9 (343.6 to 824.1)	23.5 (21.5 to 25.8)*	1.0 (-0.6 to 2.9)
Bipolar disorder	1,003.6 (825.6 to 1,204.9)	22.0 (21.6 to 22.5)*	-0.2 (-0.6 to 0.2)	192.8 (116.5 to 286.3)	22.3 (20.0 to 24.6)*	0.0 (-1.8 to 1.9)
Anxiety disorders	8,067.1 (6,449.1 to 10,063.4)	24.0 (20.8 to 27.1)*	1.4 (-1.2 to 3.9)	703.9 (454.6 to 1,013.1)	24.2 (20.8 to 27.5)*	1.6 (-1.2 to 4.3)
Eating disorders	0.0 (0.0 to 0.0)	0.0 (0.0 to 0.0)	0.0 (0.0 to 0.0)	0.0 (0.0 to 0.0)	0.0 (0.0 to 0.0)	0.0 (0.0 to 0.0)
Anorexia nervosa	0.0 (0.0 to 0.0)	0.0 (0.0 to 0.0)	0.0 (0.0 to 0.0)	0.0 (0.0 to 0.0)	0.0 (0.0 to 0.0)	0.0 (0.0 to 0.0)
Bulimia nervosa	0.0 (0.0 to 0.0)	0.0 (0.0 to 0.0)	0.0 (0.0 to 0.0)	0.0 (0.0 to 0.0)	0.0 (0.0 to 0.0)	0.0 (0.0 to 0.0)
Autistic spectrum disorders	1,429.5 (1,360.4 to 1,506.3)	23.0 (20.6 to 25.4)*	0.6 (-1.4 to 2.6)	218.6 (149.0 to 294.5)	23.2 (21.6 to 24.8)*	0.8 (-0.5 to 2.1)
Autism	579.0 (485.2 to 697.3)	22.9 (22.4 to 23.5)*	0.5 (0.1 to 1.0)*	138.7 (90.2 to 195.9)	23.2 (20.8 to 25.7)*	0.7 (-1.2 to 2.8)
Asperger syndrome and other autistic spectrum disorders	842.9 (714.3 to 1,009.4)	23.0 (22.8 to 23.3)*	0.6 (0.4 to 0.8)*	79.9 (54.2 to 115.6)	23.3 (21.6 to 25.0)*	0.8 (-0.6 to 2.2)
Attention-deficit/hyperactivity disorder	0.2 (0.1 to 0.2)	45.3 (24.3 to 71.1)*	18.8 (1.6 to 39.9)*	0.0 (0.0 to 0.0)	42.2 (-99.8 to 115,068.7)	16.3 (-99.8 to 94,094.6)
Conduct disorder	0.0 (0.0 to 0.0)	0.0 (0.0 to 0.0)	0.0 (0.0 to 0.0)	0.0 (0.0 to 0.0)	0.0 (0.0 to 0.0)	0.0 (0.0 to 0.0)
Idiopathic developmental intellectual disability	1,180.6 (544.1 to 1,787.2)	23.0 (21.0 to 25.2)*	0.6 (-1.0 to 2.4)	49.1 (21.1 to 87.1)	22.0 (18.4 to 25.0)*	-0.2 (-3.2 to 2.2)
Other mental and substance use disorders	2,791.7 (2,710.0 to 2,872.4)	22.4 (17.4 to 27.8)*	0.1 (-4.0 to 4.5)	199.3 (138.7 to 268.6)	22.6 (17.2 to 28.7)*	0.3 (-4.2 to 5.2)
Diabetes, urogenital, blood, and endocrine diseases	120,867.7 (119,125.2 to 122,331.5)	22.0 (21.4 to 22.6)*	-0.2 (-0.7 to 0.3)	4,773.2 (3,343.5 to 6,424.8)	25.3 (23.0 to 27.5)*	2.5 (0.6 to 4.3)*
Diabetes mellitus	40,052.4 (36,643.3 to 43,873.9)	29.4 (26.3 to 32.2)*	5.8 (3.3 to 8.1)*	3,192.7 (2,187.9 to 4,439.1)	29.6 (26.3 to 32.9)*	6.0 (3.3 to 8.7)*
Uncomplicated diabetes mellitus	22,898.7 (18,495.6 to 27,539.4)	29.6 (25.4 to 33.1)*	6.0 (2.6 to 8.9)*	1,038.4 (646.4 to 1,562.0)	29.7 (25.6 to 33.3)*	6.1 (2.7 to 9.1)*
Neuropathy and other complications of diabetes mellitus aggregate	16,700.7 (12,849.3 to 21,048.3)	29.2 (24.4 to 33.5)*	5.7 (1.7 to 9.2)*	2,122.6 (1,378.5 to 3,119.9)	29.6 (24.9 to 33.7)*	6.0 (2.2 to 9.4)*
Vision loss due to diabetes mellitus aggregate	453.0 (326.6 to 611.7)	28.9 (25.8 to 32.2)*	5.4 (2.9 to 8.1)*	31.7 (20.3 to 46.5)	31.0 (26.5 to 35.9)*	7.2 (3.5 to 11.2)*
Acute glomerulonephritis	5.7 (4.2 to 7.4)	17.1 (11.3 to 21.9)*	-4.2 (-9.0 to -0.3)*	0.3 (0.2 to 0.4)	18.0 (1.0 to 37.3)*	-3.5 (-17.4 to 12.3)
Chronic kidney disease	32,148.0 (28,439.3 to 35,461.1)	23.0 (19.8 to 26.2)*	0.6 (-2.0 to 3.2)	632.7 (470.6 to 816.7)	23.5 (21.8 to 25.2)*	1.0 (-0.4 to 2.4)
Chronic kidney disease due to diabetes mellitus	9,965.6 (6,302.2 to 14,726.1)	28.2 (24.4 to 32.3)*	4.9 (1.8 to 8.2)*	180.4 (114.4 to 263.1)	29.5 (25.4 to 34.0)*	5.9 (2.6 to 9.6)*
Stage III chronic kidney disease due to diabetes mellitus aggregate	9,250.1 (5,799.0 to 13,769.6)	28.1 (24.2 to 32.4)*	4.8 (1.6 to 8.3)*	6.9 (4.2 to 11.2)	26.7 (15.1 to 38.5)*	3.6 (-5.8 to 13.3)
Stage IV chronic kidney disease due to diabetes mellitus aggregate	441.1 (275.9 to 650.2)	28.6 (25.1 to 32.2)*	5.1 (2.3 to 8.1)*	49.7 (27.0 to 79.2)	27.6 (23.5 to 32.0)*	4.4 (1.0 to 7.9)*
Stage V chronic kidney disease untreated due to diabetes mellitus	205.5 (135.3 to 294.4)	28.1 (24.6 to 31.7)*	4.7 (1.9 to 7.7)*	100.1 (57.6 to 150.7)	28.4 (23.2 to 33.9)*	5.0 (0.8 to 9.5)*
End-stage chronic kidney disease due to diabetes aggregate	69.0 (47.2 to 93.6)	38.0 (33.4 to 43.2)*	12.9 (9.1 to 17.2)*	23.6 (13.6 to 34.3)	40.0 (31.4 to 49.8)*	14.5 (7.5 to 22.5)*
Chronic kidney disease due to hypertension	7,198.1 (4,557.5 to 10,619.0)	15.9 (11.7 to 20.2)*	-5.2 (-8.6 to -1.7)*	146.6 (98.9 to 200.6)	28.0 (23.1 to 33.2)*	4.7 (0.7 to 9.0)*
Stage III chronic kidney disease due to hypertension aggregate	6,626.4 (4,038.0 to 9,986.0)	15.0 (10.3 to 19.6)*	-5.9 (-9.8 to -2.2)*	7.9 (5.1 to 12.3)	17.9 (8.9 to 26.7)*	-3.6 (-10.9 to 3.6)
Stage IV chronic kidney disease due to hypertension aggregate	353.2 (246.8 to 522.5)	25.8 (19.7 to 31.8)*	2.9 (-2.1 to 7.8)	40.3 (24.4 to 64.2)	25.7 (19.8 to 32.2)*	2.8 (-2.0 to 8.2)
Stage V chronic kidney disease untreated due to hypertension	165.2 (118.9 to 230.5)	27.7 (22.7 to 32.8)*	4.4 (0.4 to 8.6)*	80.5 (49.5 to 117.9)	27.9 (21.9 to 34.5)*	4.6 (-0.3 to 10.0)
End-stage chronic kidney disease due to hypertension aggregate	53.3 (39.2 to 70.7)	37.0 (29.8 to 44.6)*	12.1 (6.2 to 18.3)*	17.9 (11.1 to 27.0)	38.9 (27.4 to 52.1)*	13.6 (4.2 to 24.4)*
Chronic kidney disease due to glomerulonephritis	7,849.1 (5,439.6 to 10,948.6)	26.0 (20.9 to 31.0)*	3.0 (-1.1 to 7.1)	127.8 (79.9 to 187.3)	14.7 (10.2 to 18.8)*	-6.2 (-9.8 to -2.8)*
Stage III chronic kidney disease due to glomerulonephritis aggregate	7,339.3 (4,955.0 to 10,474.5)	26.7 (21.3 to 32.0)*	3.6 (-0.8 to 8.0)	7.5 (4.8 to 11.2)	0.2 (-8.2 to 8.7)	-18.0 (-24.9 to -11.1)*
Stage IV chronic kidney disease due to glomerulonephritis aggregate	327.9 (217.3 to 463.5)	17.1 (10.9 to 24.2)*	-4.2 (-9.3 to 1.6)	37.0 (21.1 to 57.5)	16.7 (10.3 to 23.6)*	-4.6 (-9.8 to 1.1)
Stage V chronic kidney disease untreated due to glomerulonephritis	146.8 (91.0 to 220.8)	12.5 (8.1 to 16.6)*	-8.0 (-11.6 to -4.7)*	71.3 (41.2 to 115.4)	13.0 (7.1 to 18.6)*	-7.6 (-12.4 to -3.0)*
End-stage chronic kidney disease due to glomerulonephritis aggregate	35.2 (22.6 to 52.8)	30.5 (24.7 to 36.1)*	6.8 (2.0 to 11.3)*	12.0 (6.9 to 19.0)	31.7 (20.2 to 44.0)*	7.8 (-1.7 to 17.8)
Chronic kidney disease due to other causes	9,898.1 (6,324.9 to 14,476.4)	20.8 (17.6 to 24.2)*	-1.2 (-3.8 to 1.6)	178.0 (113.4 to 255.0)	20.9 (17.6 to 24.3)*	-1.1 (-3.8 to 1.7)
Stage III chronic kidney disease due to other causes aggregate	9,203.6 (5,830.0 to 13,598.5)	20.7 (17.3 to 24.2)*	-1.2 (-4.1 to 1.6)	11.4 (7.4 to 17.2)	7.3 (-0.4 to 14.4)	-12.3 (-18.5 to -6.5)*
Stage IV chronic kidney disease due to other causes aggregate	437.2 (265.0 to 668.0)	21.4 (18.6 to 23.9)*	-0.7 (-3.0 to 1.3)	49.8 (27.5 to 77.8)	20.6 (17.2 to 24.2)*	-1.3 (-4.1 to 1.6)
Stage V chronic kidney disease untreated due to other causes	200.5 (129.3 to 284.4)	20.4 (17.6 to 23.3)*	-1.6 (-3.8 to 0.9)	97.5 (55.5 to 146.0)	20.7 (16.4 to 24.9)*	-1.3 (-4.8 to 2.2)
End-stage chronic kidney disease due to other causes aggregate	56.8 (38.3 to 78.7)	32.3 (28.0 to 36.6)*	8.2 (4.7 to 11.8)*	19.3 (11.3 to 29.3)	33.0 (23.3 to 44.0)*	8.8 (0.8 to 17.8)*
Urinary diseases and male infertility	37,653.9 (36,613.8 to 38,657.1)	18.8 (17.2 to 20.5)*	-2.8 (-4.2 to -1.5)*	699.2 (442.9 to 1,020.1)	14.5 (12.0 to 17.3)*	-6.4 (-8.4 to -4.1)*
Interstitial nephritis and urinary tract infections	105.9 (99.1 to 113.6)	21.3 (20.4 to 22.2)*	-0.8 (-1.5 to -0.1)*	3.4 (2.2 to 5.0)	21.6 (15.7 to 27.4)*	-0.6 (-5.4 to 4.2)
Urolithiasis	21,961.7 (19,768.6 to 24,171.1)	21.4 (19.3 to 23.6)*	-0.7 (-2.4 to 1.1)	5.1 (3.3 to 7.4)	27.3 (17.9 to 38.1)*	4.1 (-3.6 to 13.0)
Benign prostatic hyperplasia	18,941.9 (15,749.5 to 22,291.4)	14.1 (11.5 to 16.9)*	-6.7 (-8.8 to -4.4)*	687.4 (434.6 to 1,005.2)	14.3 (11.8 to 17.1)*	-6.5 (-8.6 to -4.2)*
Male infertility	0.0 (0.0 to 0.0)	0.0 (0.0 to 0.0)	0.0 (0.0 to 0.0)	0.0 (0.0 to 0.0)	0.0 (0.0 to 0.0)	0.0 (0.0 to 0.0)
Other urinary diseases	--	--	--	3.3 (2.2 to 4.7)	32.6 (21.5 to 46.6)*	8.5 (-0.6 to 19.9)
Gynecological diseases	16,499.5 (15,945.3 to 17,006.5)	16.7 (14.9 to 18.8)*	-4.5 (-6.1 to -2.9)*	67.4 (34.2 to 118.2)	17.7 (15.5 to 19.8)*	-3.7 (-5.5 to -2.0)*
Uterine fibroids	3,512.0 (3,332.2 to 3,709.6)	21.7 (21.4 to 22.1)*	-0.4 (-0.7 to -0.1)*	24.9 (12.5 to 46.7)	21.9 (20.8 to 23.0)*	-0.3 (-1.2 to 0.6)
Uterine fibroids cases aggregate	3,512.0 (3,332.2 to 3,709.6)	21.7 (21.4 to 22.1)*	-0.4 (-0.7 to -0.1)*	24.9 (12.5 to 46.7)	21.9 (20.8 to 23.0)*	-0.3 (-1.2 to 0.6)
Mild abdominal pain with anemia due to uterine fibroids aggregate	0.0 (0.0 to 0.0)	0.0 (0.0 to 0.0)	0.0 (0.0 to 0.0)	0.0 (0.0 to 0.0)	0.0 (0.0 to 0.0)	0.0 (0.0 to 0.0)
Polycystic ovarian syndrome	0.0 (0.0 to 0.0)	0.0 (0.0 to 0.0)	0.0 (0.0 to 0.0)	0.0 (0.0 to 0.0)	0.0 (0.0 to 0.0)	0.0 (0.0 to 0.0)
Polycystic ovarian syndrome cases aggregate	0.0 (0.0 to 0.0)	0.0 (0.0 to 0.0)	0.0 (0.0 to 0.0)	0.0 (0.0 to 0.0)	0.0 (0.0 to 0.0)	0.0 (0.0 to 0.0)
Hirsutism and infertility due to polycystic ovarian syndrome aggregate	0.0 (0.0 to 0.0)	0.0 (0.0 to 0.0)	0.0 (0.0 to 0.0)	0.0 (0.0 to 0.0)	0.0 (0.0 to 0.0)	0.0 (0.0 to 0.0)

Cause	Prevalence in thousands (95% confidence interval)			YLDs in thousands (95% confidence interval)		
	2015	Percent change in counts between 2005 and 2015	Percent change in rates between 2005 and 2015	2015	Percent change in counts between 2005 and 2015	Percent change in rates between 2005 and 2015
Female infertility	0.0 (0.0 to 0.0)	0.0 (0.0 to 0.0)	0.0 (0.0 to 0.0)	0.0 (0.0 to 0.0)	0.0 (0.0 to 0.0)	0.0 (0.0 to 0.0)
Endometriosis	0.0 (0.0 to 0.0)	0.0 (0.0 to 0.0)	0.0 (0.0 to 0.0)	0.0 (0.0 to 0.0)	0.0 (0.0 to 0.0)	0.0 (0.0 to 0.0)
Endometriosis cases aggregate	0.0 (0.0 to 0.0)	0.0 (0.0 to 0.0)	0.0 (0.0 to 0.0)	0.0 (0.0 to 0.0)	0.0 (0.0 to 0.0)	0.0 (0.0 to 0.0)
Abdominal pain and infertility due to endometriosis aggregate	0.0 (0.0 to 0.0)	0.0 (0.0 to 0.0)	0.0 (0.0 to 0.0)	0.0 (0.0 to 0.0)	0.0 (0.0 to 0.0)	0.0 (0.0 to 0.0)
Genital prolapse	13,895.4 (12,168.0 to 16,061.0)	15.2 (12.0 to 17.9)*	-5.8 (-8.4 to -3.6)*	42.5 (20.6 to 76.3)	15.4 (12.2 to 18.5)*	-5.6 (-8.2 to -3.1)*
Premenstrual syndrome	0.0 (0.0 to 0.0)	0.0 (0.0 to 0.0)	0.0 (0.0 to 0.0)	0.0 (0.0 to 0.0)	0.0 (0.0 to 0.0)	0.0 (0.0 to 0.0)
Other gynecological diseases	0.0 (0.0 to 0.0)	0.0 (0.0 to 0.0)	0.0 (0.0 to 0.0)	0.0 (0.0 to 0.0)	0.0 (0.0 to 0.0)	0.0 (0.0 to 0.0)
Other gynecological diseases cases aggregate	0.0 (0.0 to 0.0)	0.0 (0.0 to 0.0)	0.0 (0.0 to 0.0)	0.0 (0.0 to 0.0)	0.0 (0.0 to 0.0)	0.0 (0.0 to 0.0)
Anemia due to other gynecological diseases aggregate	0.0 (0.0 to 0.0)	0.0 (0.0 to 0.0)	0.0 (0.0 to 0.0)	0.0 (0.0 to 0.0)	0.0 (0.0 to 0.0)	0.0 (0.0 to 0.0)
Hemoglobinopathies and hemolytic anemias	39,285.3 (38,591.0 to 39,999.5)	21.9 (20.5 to 23.3)*	-0.3 (-1.5 to 0.8)	132.2 (89.5 to 188.0)	10.3 (6.4 to 14.0)*	-9.8 (-12.9 to -6.8)*
Thalassemias	0.1 (0.1 to 0.1)	14.7 (7.1 to 23.3)*	-6.1 (-12.4 to 0.9)	0.0 (0.0 to 0.0)	8.7 (-4.0 to 18.3)	-11.1 (-21.5 to -3.2)*
Beta-thalassemia major cases aggregate	0.1 (0.1 to 0.1)	5.0 (-5.7 to 17.6)	-14.1 (-22.9 to -3.8)*	0.0 (0.0 to 0.0)	0.3 (-16.8 to 13.0)	-18.0 (-32.0 to -7.6)*
Hemoglobin E/beta-thalassemia cases aggregate	0.0 (0.0 to 0.0)	37.0 (36.1 to 38.0)*	12.0 (11.3 to 12.9)*	0.0 (0.0 to 0.0)	36.2 (23.2 to 48.6)*	11.4 (0.8 to 21.6)*
Hemoglobin H disease cases aggregate	0.0 (0.0 to 0.0)	33.5 (28.5 to 39.4)*	9.2 (5.1 to 14.0)*	0.0 (0.0 to 0.0)	29.2 (24.2 to 34.4)*	5.7 (1.6 to 9.9)*
Heart failure due to thalassemias aggregate	0.0 (0.0 to 0.0)	28.9 (25.5 to 32.1)*	5.5 (2.7 to 8.0)*	0.0 (0.0 to 0.0)	28.9 (25.5 to 32.1)*	5.4 (2.7 to 8.0)*
Thalassemias trait	8,276.8 (8,087.7 to 8,527.0)	30.5 (29.9 to 31.2)*	6.8 (6.2 to 7.3)*	61.7 (41.0 to 88.8)	23.5 (18.3 to 29.2)*	1.0 (-3.2 to 5.6)
B-thalassemia trait cases aggregate	6,747.6 (6,581.0 to 6,988.3)	29.5 (28.8 to 30.2)*	5.9 (5.3 to 6.5)*	58.7 (39.0 to 84.3)	23.5 (18.1 to 29.5)*	1.0 (-3.4 to 5.9)
Hemoglobin E trait cases aggregate	1,529.2 (1,471.8 to 1,588.6)	35.2 (34.1 to 36.4)*	10.6 (9.7 to 11.6)*	3.0 (1.9 to 4.5)	22.4 (10.2 to 36.8)*	0.1 (-9.8 to 11.9)
Sickle cell disorders	0.6 (0.6 to 0.6)	33.1 (30.5 to 35.7)*	8.9 (6.7 to 11.0)*	0.1 (0.1 to 0.1)	32.3 (28.5 to 36.4)*	8.2 (5.1 to 11.6)*
Homozygous sickle cell and severe sickle cell/beta-thalassemia cases aggregate	0.4 (0.4 to 0.5)	34.1 (30.9 to 37.1)*	9.7 (7.1 to 12.1)*	0.1 (0.1 to 0.1)	34.2 (29.5 to 39.1)*	9.7 (5.9 to 13.8)*
Hemoglobin SC disease cases aggregate	0.1 (0.0 to 0.1)	39.0 (35.7 to 42.6)*	13.7 (11.0 to 16.6)*	0.0 (0.0 to 0.0)	35.1 (31.7 to 38.4)*	10.5 (7.7 to 13.2)*
Mild sickle cell/beta-thalassemia cases aggregate	0.1 (0.1 to 0.1)	23.3 (19.7 to 26.9)*	0.9 (-2.1 to 3.8)	0.0 (0.0 to 0.0)	19.5 (15.5 to 23.7)*	-2.3 (-5.5 to 1.2)
Sickle cell trait	7,873.9 (7,453.7 to 8,698.5)	28.0 (26.9 to 28.9)*	4.7 (3.8 to 5.4)*	20.2 (13.7 to 28.9)	10.6 (4.5 to 16.9)*	-9.5 (-14.5 to -4.4)*
G6PD deficiency	5,449.0 (4,612.6 to 6,330.3)	17.3 (11.7 to 22.2)*	-4.0 (-8.6 to -0.1)*	0.7 (0.5 to 1.0)	22.7 (14.3 to 32.0)*	0.4 (-6.5 to 8.0)
G6PD cases aggregate	5,447.6 (4,611.2 to 6,328.9)	17.3 (11.7 to 22.2)*	-4.1 (-8.7 to -0.1)*	0.5 (0.4 to 0.7)	19.6 (9.1 to 30.9)*	-2.1 (-10.8 to 7.0)
Heart failure due to G6PD deficiency aggregate	1.4 (1.2 to 1.7)	33.4 (31.2 to 35.6)*	9.1 (7.3 to 10.9)*	0.2 (0.1 to 0.3)	33.4 (25.4 to 42.0)*	9.1 (2.6 to 16.1)*
G6PD trait	19,276.9 (17,792.6 to 20,768.6)	19.2 (17.3 to 21.0)*	-2.5 (-4.0 to -1.0)*	0.4 (0.3 to 0.6)	15.5 (2.4 to 28.0)*	-5.6 (-16.3 to 4.7)
Other hemoglobinopathies and hemolytic anemias	2,158.4 (2,108.1 to 2,211.4)	8.7 (5.8 to 11.7)*	-11.1 (-13.4 to -8.7)*	49.0 (33.7 to 69.0)	-3.0 (-7.7 to 1.7)	-20.7 (-24.5 to -16.8)*
Other hemoglobinopathies and hemolytic anemias cases aggregate	2,138.7 (2,087.9 to 2,190.9)	8.5 (5.6 to 11.6)*	-11.2 (-13.6 to -8.8)*	46.4 (31.6 to 65.3)	-4.2 (-9.2 to 0.9)	-21.6 (-25.7 to -17.5)*
Heart failure due to other hemoglobinopathies and hemolytic anemias aggregate	19.7 (17.0 to 22.5)	28.2 (26.3 to 30.0)*	4.9 (3.3 to 6.3)*	2.3 (1.5 to 3.2)	28.1 (17.7 to 39.1)*	4.8 (-3.7 to 13.8)
Endocrine, metabolic, blood, and immune disorders	1,830.6 (1,767.6 to 1,896.2)	11.2 (7.1 to 15.5)*	-9.1 (-12.4 to -5.5)*	48.8 (33.7 to 66.6)	7.0 (1.6 to 13.8)*	-12.5 (-16.9 to -6.9)*
Endocrine, metabolic, blood, and immune disorders cases aggregate	539.7 (488.8 to 591.5)	17.8 (15.3 to 20.7)*	-3.6 (-5.7 to -1.3)*	20.1 (13.7 to 27.4)	18.0 (13.3 to 23.3)*	-3.5 (-7.3 to 0.8)
Anemia due to endocrine, metabolic, blood, and immune disorders aggregate	1,277.7 (1,241.8 to 1,313.6)	8.5 (3.3 to 14.4)*	-11.3 (-15.5 to -6.5)*	27.1 (18.5 to 38.3)	-0.8 (-8.5 to 9.8)	-18.9 (-25.2 to -10.2)*
Heart failure due to endocrine, metabolic, blood, and immune disorders aggregate	13.3 (11.5 to 15.3)	27.0 (25.3 to 28.8)*	3.9 (2.5 to 5.3)*	1.5 (1.0 to 2.2)	26.8 (18.1 to 34.8)*	3.7 (-3.4 to 10.3)
Musculoskeletal disorders	86,530.8 (84,812.9 to 88,309.4)	22.2 (21.3 to 23.1)*	-0.1 (-0.8 to 0.7)	9,632.3 (6,964.5 to 12,627.7)	21.9 (20.3 to 23.6)*	-0.3 (-1.6 to 1.1)
Rheumatoid arthritis	2,161.3 (1,915.3 to 2,415.1)	26.1 (22.9 to 29.3)*	3.2 (0.5 to 5.8)*	495.2 (350.0 to 671.0)	26.4 (22.8 to 29.9)*	3.4 (0.4 to 6.3)*
Osteoarthritis	28,632.5 (27,588.0 to 29,713.7)	25.1 (24.3 to 26.0)*	2.3 (1.6 to 3.0)*	1,513.8 (1,061.4 to 2,057.2)	25.9 (24.7 to 27.1)*	2.9 (2.0 to 3.9)*
Osteoarthritis of the hip cases aggregate	5,548.3 (4,907.1 to 6,268.8)	24.4 (22.7 to 26.3)*	1.8 (0.3 to 3.3)*	277.0 (190.1 to 391.0)	24.7 (22.6 to 26.9)*	2.0 (0.3 to 3.8)*
Osteoarthritis of the knee cases aggregate	23,084.2 (22,286.6 to 23,947.2)	25.3 (24.4 to 26.2)*	2.5 (1.8 to 3.2)*	1,236.7 (864.3 to 1,676.7)	26.1 (24.8 to 27.4)*	3.2 (2.1 to 4.2)*
Low back and neck pain	49,505.6 (47,338.4 to 51,638.2)	18.4 (16.8 to 20.2)*	-3.1 (-4.5 to -1.7)*	5,679.5 (4,003.3 to 7,743.4)	17.9 (16.2 to 19.5)*	-3.6 (-4.9 to -2.3)*
Low back pain	32,842.5 (29,299.1 to 36,588.1)	15.5 (13.4 to 17.6)*	-5.5 (-7.3 to -3.8)*	3,515.8 (2,474.1 to 4,844.7)	15.8 (13.7 to 18.0)*	-5.3 (-7.0 to -3.5)*
Neck pain	22,941.8 (16,637.2 to 30,196.3)	21.4 (18.9 to 24.1)*	-0.7 (-2.8 to 1.5)	2,163.7 (1,348.1 to 3,235.5)	21.5 (19.0 to 24.4)*	-0.6 (-2.6 to 1.7)
Gout	3,982.2 (3,262.5 to 4,803.3)	25.6 (24.6 to 26.8)*	2.7 (1.9 to 3.7)*	123.0 (81.8 to 170.7)	25.9 (23.0 to 29.0)*	3.0 (0.6 to 5.5)*
Asymptomatic gout	3,557.6 (2,919.8 to 4,297.3)	25.6 (24.6 to 26.8)*	2.7 (1.9 to 3.7)*	0.0 (0.0 to 0.0)	0.0 (0.0 to 0.0)	0.0 (0.0 to 0.0)
Symptomatic gout cases aggregate	424.7 (337.6 to 525.1)	25.6 (24.6 to 26.8)*	2.7 (1.9 to 3.7)*	123.0 (81.8 to 170.7)	25.9 (23.0 to 29.0)*	3.0 (0.6 to 5.5)*
Other musculoskeletal disorders	20,428.2 (15,912.6 to 25,591.6)	30.3 (24.5 to 35.9)*	6.6 (1.8 to 11.1)*	1,820.8 (1,194.0 to 2,583.1)	30.6 (24.7 to 36.2)*	6.8 (2.0 to 11.4)*
Other non-communicable diseases	189,863.2 (189,280.8 to 190,424.6)	22.3 (22.1 to 22.4)*	0.0 (-0.1 to 0.1)	9,264.0 (6,362.1 to 13,090.4)	22.1 (20.6 to 23.6)*	-0.1 (-1.4 to 1.1)
Congenital anomalies	2,134.0 (1,929.0 to 2,287.1)	38.8 (33.3 to 43.9)*	13.5 (9.0 to 17.7)*	183.7 (114.9 to 283.2)	40.5 (30.9 to 53.2)*	14.9 (7.0 to 25.3)*
Neural tube defects	12.7 (8.9 to 18.0)	50.9 (45.9 to 56.2)*	23.4 (19.3 to 27.8)*	3.8 (2.1 to 6.6)	53.8 (39.7 to 66.1)*	25.8 (14.2 to 35.8)*
Congenital heart anomalies	1,132.9 (781.8 to 1,723.7)	48.5 (39.1 to 57.0)*	21.5 (13.7 to 28.4)*	37.0 (14.1 to 67.9)	49.0 (39.9 to 56.8)*	21.8 (14.5 to 28.2)*
Less severe heart anomalies cases aggregate	1,055.6 (703.9 to 1,649.7)	47.9 (37.7 to 56.9)*	21.0 (12.6 to 28.3)*	32.5 (12.1 to 62.8)	48.0 (37.7 to 56.7)*	21.1 (12.6 to 28.2)*
Severe congenital heart anomalies	74.8 (48.8 to 113.8)	58.9 (48.5 to 69.6)*	30.0 (21.5 to 38.7)*	4.2 (1.7 to 8.2)	58.7 (46.4 to 71.7)*	29.8 (19.7 to 40.4)*
Critical congenital heart anomalies	0.0 (0.0 to 0.0)	113.2 (79.7 to 144.8)*	74.3 (47.0 to 100.2)*	0.0 (0.0 to 0.0)	113.3 (79.7 to 144.8)*	74.4 (47.0 to 100.2)*
Heart failure due to congenital heart anomalies aggregate	2.5 (2.1 to 2.9)	27.5 (26.1 to 29.1)*	4.3 (3.1 to 5.6)*	0.3 (0.2 to 0.4)	27.5 (25.1 to 29.8)*	4.3 (2.3 to 6.2)*
Cleft lip and cleft palate	173.1 (101.3 to 291.1)	53.4 (35.8 to 67.7)*	25.5 (11.0 to 37.2)*	1.7 (0.8 to 3.0)	47.8 (27.5 to 65.2)*	20.9 (4.3 to 35.1)*
Down syndrome	84.9 (55.2 to 126.6)	32.1 (23.9 to 38.9)*	8.0 (1.3 to 13.6)*	13.9 (8.1 to 21.9)	31.4 (23.1 to 38.5)*	7.5 (0.7 to 13.3)*
Turner syndrome	7.6 (3.6 to 13.8)	36.1 (32.4 to 40.4)*	11.3 (8.3 to 14.8)*	0.1 (0.0 to 0.2)	36.1 (31.1 to 40.9)*	11.3 (7.3 to 15.2)*
Klinefelter syndrome	5.5 (2.8 to 9.9)	26.9 (23.2 to 29.8)*	3.8 (0.8 to 6.1)*	0.0 (0.0 to 0.0)	26.9 (23.2 to 29.8)*	3.8 (0.8 to 6.1)*
Other chromosomal abnormalities	85.0 (49.2 to 146.2)	25.8 (16.3 to 34.7)*	2.9 (-4.9 to 10.2)	13.9 (7.5 to 25.2)	25.9 (16.4 to 34.6)*	2.9 (-4.8 to 10.1)
Other congenital anomalies	797.9 (429.9 to 1,470.5)	23.9 (20.7 to 27.4)*	1.4 (-1.3 to 4.2)	113.3 (65.3 to 186.4)	40.5 (28.2 to 59.7)*	14.9 (4.8 to 30.6)*
Skin and subcutaneous diseases	69,771.6 (68,535.8 to 70,885.2)	24.0 (23.2 to 24.9)*	1.4 (0.8 to 2.1)*	1,197.7 (798.5 to 1,761.0)	23.7 (22.8 to 24.7)*	1.2 (0.4 to 2.0)*
Dermatitis	8,258.3 (6,561.0 to 10,347.3)	24.2 (23.7 to 24.6)*	1.5 (1.2 to 1.9)*	234.3 (147.5 to 342.0)	24.1 (22.8 to 25.4)*	1.5 (0.4 to 2.6)*
Eczema cases aggregate	1,480.9 (1,120.7 to 1,906.5)	22.9 (22.0 to 23.9)*	0.5 (-0.2 to 1.4)	85.1 (51.8 to 129.1)	23.2 (20.3 to 26.1)*	0.8 (-1.6 to 3.1)

Cause	Prevalence in thousands (95% confidence interval)			YLDs in thousands (95% confidence interval)		
	2015	Percent change in counts between 2005 and 2015	Percent change in rates between 2005 and 2015	2015	Percent change in counts between 2005 and 2015	Percent change in rates between 2005 and 2015
Contact dermatitis cases aggregate	4,505.5<br>(3,036.7 to 6,480.1)	24.6<br>(23.9 to 25.3)*	1.9<br>(1.3 to 2.5)*	116.5<br>(64.9 to 193.1)	24.7<br>(23.1 to 26.5)*	2.0<br>(0.7 to 3.5)*
Seborrhoeic dermatitis cases aggregate	2,271.9<br>(1,716.2 to 2,893.4)	24.1<br>(23.5 to 24.6)*	1.5<br>(1.0 to 1.9)*	32.7<br>(17.6 to 55.2)	24.2<br>(22.7 to 25.8)*	1.6<br>(0.4 to 2.9)*
Psoriasis	3,974.7<br>(3,640.0 to 4,329.9)	21.6<br>(20.4 to 23.0)*	-0.5<br>(-1.5 to 0.6)	305.8<br>(214.2 to 416.2)	21.9<br>(19.9 to 24.0)*	-0.3<br>(-2.0 to 1.4)
Cellulitis	42.4<br>(32.5 to 56.0)	28.9<br>(23.9 to 34.8)*	5.4<br>(1.3 to 10.3)*	2.9<br>(1.8 to 4.5)	28.6<br>(19.0 to 39.4)*	5.1<br>(-2.6 to 14.0)
Pyoderma	205.5<br>(181.3 to 233.3)	15.3<br>(12.6 to 18.1)*	-5.7<br>(-7.9 to -3.4)*	1.1<br>(0.4 to 2.4)	15.6<br>(10.8 to 20.6)*	-5.4<br>(-9.4 to -1.4)*
Scabies	3,324.7<br>(2,199.1 to 4,739.2)	28.9<br>(21.9 to 36.1)*	5.4<br>(-0.3 to 11.3)	82.4<br>(42.0 to 145.7)	29.2<br>(22.3 to 36.7)*	5.7<br>(-0.0 to 11.8)
Fungal skin diseases	12,061.7<br>(9,753.2 to 14,776.6)	22.2<br>(21.9 to 22.5)*	-0.0<br>(-0.3 to 0.2)	65.8<br>(25.3 to 139.5)	22.4<br>(21.9 to 23.1)*	0.1<br>(-0.3 to 0.7)
Viral skin diseases	3,510.1<br>(3,024.1 to 4,168.4)	21.8<br>(20.7 to 22.9)*	-0.3<br>(-1.3 to 0.5)	103.5<br>(63.1 to 156.1)	22.0<br>(20.4 to 23.5)*	-0.2<br>(-1.5 to 1.0)
Molluscum contagiosum cases aggregate	97.3<br>(76.2 to 123.9)	22.5<br>(21.9 to 23.1)*	0.2<br>(-0.3 to 0.7)	2.9<br>(1.7 to 4.5)	22.9<br>(15.5 to 30.2)*	0.5<br>(-5.5 to 6.5)
Viral warts cases aggregate	3,412.8<br>(2,929.6 to 4,064.4)	21.8<br>(20.6 to 22.9)*	-0.4<br>(-1.3 to 0.5)	100.6<br>(61.2 to 152.1)	22.0<br>(20.4 to 23.6)*	-0.2<br>(-1.6 to 1.1)
Acne vulgaris	2,047.9<br>(1,837.4 to 2,298.1)	23.6<br>(20.6 to 26.8)*	1.1<br>(-1.4 to 3.7)	21.2<br>(10.2 to 39.1)	23.8<br>(20.5 to 27.1)*	1.2<br>(-1.5 to 4.0)
Alopecia areata	544.5<br>(470.4 to 625.2)	21.5<br>(20.6 to 22.4)*	-0.6<br>(-1.4 to 0.1)	17.7<br>(10.8 to 26.2)	21.6<br>(18.7 to 25.0)*	-0.5<br>(-2.9 to 2.2)
Pruritus	3,284.2<br>(2,324.6 to 4,468.7)	22.7<br>(18.7 to 26.4)*	0.4<br>(-2.9 to 3.4)	34.0<br>(14.5 to 69.0)	22.9<br>(18.9 to 26.7)*	0.5<br>(-2.8 to 3.6)
Urticaria	1,213.8<br>(858.0 to 1,694.1)	22.0<br>(21.4 to 22.7)*	-0.2<br>(-0.7 to 0.4)	69.4<br>(39.8 to 108.6)	22.2<br>(19.9 to 24.6)*	-0.1<br>(-1.9 to 1.9)
Decubitus ulcer	78.7<br>(60.5 to 100.8)	32.2<br>(27.4 to 36.6)*	8.1<br>(4.2 to 11.7)*	11.4<br>(7.2 to 16.5)	32.0<br>(21.5 to 42.7)*	8.0<br>(-0.7 to 16.7)
Other skin and subcutaneous diseases	43,252.4<br>(39,290.7 to 47,219.8)	25.3<br>(23.4 to 27.5)*	2.5<br>(0.9 to 4.3)*	248.1<br>(120.4 to 451.5)	25.5<br>(23.5 to 27.7)*	2.6<br>(1.0 to 4.4)*
Sense organ diseases	146,520.8<br>(145,308.3 to 148,030.8)	22.6<br>(22.2 to 23.1)*	0.3<br>(-0.1 to 0.7)	6,420.5<br>(4,378.4 to 8,989.7)	22.0<br>(19.7 to 24.1)*	-0.3<br>(-2.1 to 1.5)
Glaucoma	899.0<br>(660.8 to 1,155.6)	27.4<br>(24.7 to 30.2)*	4.2<br>(2.0 to 6.5)*	85.1<br>(51.9 to 125.3)	27.5<br>(23.7 to 31.3)*	4.3<br>(1.1 to 7.4)*
Cataract	8,262.8<br>(6,782.2 to 10,113.9)	16.4<br>(13.6 to 18.9)*	-4.8<br>(-7.1 to -2.7)*	539.0<br>(357.3 to 761.6)	16.3<br>(13.8 to 18.5)*	-4.9<br>(-6.9 to -3.1)*
Macular degeneration	650.9<br>(471.2 to 905.6)	36.9<br>(33.5 to 40.3)*	11.9<br>(9.2 to 14.7)*	43.0<br>(27.8 to 64.2)	35.4<br>(31.0 to 40.2)*	10.7<br>(7.2 to 14.7)*
Refraction and accommodation disorders	68,581.3<br>(62,789.5 to 74,274.5)	22.9<br>(20.1 to 26.0)*	0.5<br>(-1.8 to 3.1)	1,205.3<br>(779.8 to 1,894.2)	21.5<br>(19.6 to 23.4)*	-0.6<br>(-2.2 to 1.0)
Age-related and other hearing loss	128,944.0<br>(118,644.1 to 139,284.4)	22.8<br>(22.0 to 23.6)*	0.4<br>(-0.2 to 1.1)	3,790.2<br>(2,553.3 to 5,360.4)	22.2<br>(18.6 to 25.6)*	-0.1<br>(-3.0 to 2.7)
Other vision loss	1,683.2<br>(1,257.3 to 2,161.8)	33.1<br>(29.1 to 37.1)*	8.9<br>(5.6 to 12.1)*	136.8<br>(85.4 to 200.3)	30.0<br>(26.2 to 33.8)*	6.3<br>(3.2 to 9.4)*
Other sense organ diseases	25,417.7<br>(22,652.9 to 28,315.2)	23.3<br>(21.0 to 25.6)*	0.9<br>(-1.1 to 2.8)	621.0<br>(376.5 to 904.5)	23.5<br>(21.0 to 26.0)*	1.0<br>(-1.0 to 3.1)
Acute other sense organ diseases	50.9<br>(45.7 to 56.3)	23.4<br>(21.1 to 26.1)*	1.0<br>(-0.9 to 3.1)	1.3<br>(0.7 to 1.9)	24.0<br>(10.5 to 40.8)*	1.4<br>(-9.6 to 15.1)
Chronic other sense organ diseases	25,366.9<br>(22,598.5 to 28,264.0)	23.3<br>(20.9 to 25.6)*	0.9<br>(-1.1 to 2.8)	619.8<br>(375.8 to 902.8)	23.5<br>(21.0 to 26.0)*	1.0<br>(-1.0 to 3.1)
Oral disorders	119,942.0<br>(117,855.2 to 122,055.2)	21.0<br>(20.2 to 21.8)*	-1.0<br>(-1.7 to -0.4)*	1,462.1<br>(934.7 to 2,127.2)	19.7<br>(19.0 to 20.5)*	-2.1<br>(-2.6 to -1.5)*
Deciduous caries	0.0<br>(0.0 to 0.0)	0.0<br>(0.0 to 0.0)	0.0<br>(0.0 to 0.0)	0.0<br>(0.0 to 0.0)	0.0<br>(0.0 to 0.0)	0.0<br>(0.0 to 0.0)
Permanent caries	78,416.6<br>(68,524.6 to 88,824.3)	19.6<br>(17.8 to 21.4)*	-2.2<br>(-3.7 to -0.7)*	55.1<br>(24.1 to 108.3)	16.6<br>(14.4 to 18.8)*	-4.7<br>(-6.4 to -2.8)*
Periodontal diseases	37,920.0<br>(31,350.0 to 44,535.9)	24.6<br>(23.4 to 25.5)*	1.9<br>(1.0 to 2.7)*	242.9<br>(96.4 to 501.6)	24.8<br>(23.6 to 25.8)*	2.1<br>(1.1 to 2.9)*
Edentulism and severe tooth loss	37,199.7<br>(34,700.7 to 39,925.5)	18.3<br>(17.8 to 18.7)*	-3.3<br>(-3.6 to -2.9)*	1,025.1<br>(680.9 to 1,418.8)	18.4<br>(17.9 to 19.0)*	-3.1<br>(-3.6 to -2.7)*
Other oral disorders	4,915.8<br>(4,438.4 to 5,458.3)	22.2<br>(21.4 to 22.9)*	-0.1<br>(-0.7 to 0.5)	139.0<br>(86.4 to 207.6)	22.4<br>(21.2 to 23.5)*	0.1<br>(-0.8 to 1.0)
Injuries	57,769.0<br>(55,583.9 to 60,297.9)	14.9<br>(13.7 to 16.1)*	-6.1<br>(-7.0 to -5.0)*	2,216.9<br>(1,570.2 to 3,022.9)	7.3<br>(4.3 to 9.9)*	-12.3<br>(-14.7 to -10.1)*
Transport injuries	8,497.6<br>(8,044.6 to 9,059.1)	22.2<br>(21.4 to 22.9)*	-0.1<br>(-0.7 to 0.5)	443.1<br>(309.6 to 602.2)	14.1<br>(11.0 to 17.0)*	-6.7<br>(-9.2 to -4.3)*
Road injuries	7,928.5<br>(7,447.6 to 8,489.1)	22.3<br>(21.5 to 23.0)*	0.0<br>(-0.6 to 0.6)	412.4<br>(286.0 to 562.8)	15.0<br>(12.1 to 17.7)*	-5.9<br>(-8.3 to -3.8)*
Pedestrian road injuries	1,138.2<br>(997.6 to 1,282.1)	23.5<br>(22.4 to 24.5)*	1.0<br>(0.1 to 1.8)*	59.4<br>(40.6 to 82.7)	13.4<br>(9.5 to 17.2)*	-7.2<br>(-10.4 to -4.2)*
Cyclist road injuries	1,357.1<br>(1,176.8 to 1,532.8)	24.4<br>(23.1 to 25.5)*	1.7<br>(0.7 to 2.6)*	67.0<br>(45.4 to 93.7)	15.6<br>(11.5 to 19.2)*	-5.4<br>(-8.8 to -2.5)*
Motorcyclist road injuries	1,923.5<br>(1,692.0 to 2,144.7)	35.0<br>(33.4 to 36.4)*	10.4<br>(9.1 to 11.6)*	95.6<br>(66.4 to 133.6)	24.6<br>(20.3 to 28.5)*	1.9<br>(-1.6 to 5.1)
Motor vehicle road injuries	3,229.6<br>(2,892.9 to 3,612.0)	13.5<br>(12.2 to 14.6)*	-7.2<br>(-8.2 to -6.3)*	175.6<br>(122.6 to 241.0)	9.4<br>(7.2 to 11.4)*	-10.5<br>(-12.3 to -8.9)*
Other road injuries	280.1<br>(238.2 to 324.0)	39.6<br>(38.6 to 40.8)*	14.2<br>(13.3 to 15.2)*	14.8<br>(10.1 to 20.8)	34.6<br>(31.6 to 37.1)*	10.1<br>(7.6 to 12.2)*
Other transport injuries	569.1<br>(505.7 to 641.0)	20.3<br>(19.7 to 21.1)*	-1.6<br>(-2.1 to -1.0)*	30.7<br>(21.7 to 41.9)	3.1<br>(-1.7 to 8.0)	-15.7<br>(-19.6 to -11.6)*
Unintentional injuries	44,585.4<br>(42,781.5 to 46,672.2)	11.4<br>(10.9 to 11.9)*	-8.9<br>(-9.3 to -8.5)*	1,634.1<br>(1,138.6 to 2,243.4)	4.8<br>(1.9 to 7.6)*	-14.3<br>(-16.7 to -12.0)*
Falls	14,725.2<br>(13,398.2 to 16,212.7)	23.2<br>(22.3 to 23.9)*	0.7<br>(0.1 to 1.3)*	718.6<br>(493.8 to 985.5)	11.1<br>(6.3 to 15.5)*	-9.2<br>(-13.1 to -5.5)*
Drowning	351.5<br>(307.8 to 403.9)	-3.1<br>(-3.6 to -2.6)*	-20.8<br>(-21.2 to -20.4)*	19.0<br>(13.3 to 26.5)	-12.7<br>(-16.0 to -9.6)*	-28.6<br>(-31.3 to -26.0)*
Fire, heat, and hot substances	3,797.6<br>(3,318.6 to 4,277.8)	4.6<br>(4.1 to 5.2)*	-14.4<br>(-14.8 to -13.9)*	86.9<br>(57.4 to 130.6)	0.1<br>(-3.4 to 2.7)	-18.2<br>(-21.0 to -16.0)*
Poisonings	216.9<br>(190.3 to 248.8)	6.8<br>(6.0 to 7.6)*	-12.6<br>(-13.3 to -12.0)*	15.3<br>(10.6 to 21.2)	1.1<br>(-2.0 to 3.9)	-17.3<br>(-19.9 to -15.1)*
Exposure to mechanical forces	8,612.5<br>(7,998.8 to 9,276.6)	14.8<br>(14.2 to 15.3)*	-6.1<br>(-6.6 to -5.7)*	161.3<br>(111.7 to 226.7)	8.7<br>(5.9 to 11.4)*	-11.1<br>(-13.4 to -8.9)*
Unintentional firearm injuries	127.7<br>(111.7 to 145.1)	14.4<br>(13.7 to 15.0)*	-6.5<br>(-7.0 to -5.9)*	4.2<br>(3.0 to 6.0)	7.2<br>(3.9 to 10.0)*	-12.3<br>(-15.0 to -10.0)*
Unintentional suffocation	573.9<br>(495.2 to 675.9)	19.4<br>(16.0 to 22.8)*	-2.3<br>(-5.1 to 0.4)	21.5<br>(14.8 to 29.4)	19.3<br>(16.6 to 21.7)*	-2.4<br>(-4.6 to -0.4)*
Other exposure to mechanical forces	7,910.9<br>(7,285.2 to 8,570.7)	14.5<br>(14.0 to 14.9)*	-6.4<br>(-6.8 to -6.0)*	135.5<br>(93.4 to 190.9)	7.3<br>(4.1 to 10.2)*	-12.3<br>(-14.8 to -9.9)*
Adverse effects of medical treatment	577.5<br>(424.3 to 756.1)	1.6<br>(-3.4 to 5.8)	-16.9<br>(-21.0 to -13.4)*	77.0<br>(47.3 to 117.7)	1.6<br>(-3.4 to 5.8)	-16.9<br>(-21.0 to -13.4)*
Animal contact	1,540.9<br>(1,406.0 to 1,688.6)	12.8<br>(11.9 to 13.7)*	-7.7<br>(-8.5 to -7.0)*	35.6<br>(24.8 to 48.7)	8.0<br>(5.6 to 10.3)*	-11.7<br>(-13.6 to -9.8)*
Venomous animal contact	679.1<br>(612.6 to 755.1)	17.0<br>(16.1 to 17.9)*	-4.3<br>(-5.0 to -3.6)*	24.7<br>(17.4 to 32.9)	8.7<br>(6.1 to 11.4)*	-11.1<br>(-13.2 to -8.9)*
Non-venomous animal contact	861.8<br>(758.6 to 968.1)	9.8<br>(8.6 to 10.9)*	-10.2<br>(-11.2 to -9.3)*	10.9<br>(7.0 to 16.6)	6.4<br>(3.4 to 8.9)*	-13.0<br>(-15.4 to -10.9)*
Foreign body	1,319.9<br>(1,210.3 to 1,444.9)	12.0<br>(11.3 to 12.7)*	-8.4<br>(-9.0 to -7.9)*	43.4<br>(31.0 to 59.7)	1.6<br>(-2.2 to 5.4)	-16.9<br>(-20.0 to -13.8)*
Pulmonary aspiration and foreign body in airway	575.9<br>(509.2 to 675.2)	14.5<br>(12.8 to 16.0)*	-6.3<br>(-7.7 to -5.2)*	25.3<br>(17.8 to 34.4)	-0.1<br>(-4.4 to 4.5)	-18.3<br>(-21.8 to -14.5)*
Foreign body in eyes	31.2<br>(16.2 to 50.5)	17.9<br>(15.9 to 20.0)*	-3.5<br>(-5.2 to -1.9)*	1.7<br>(0.8 to 3.1)	16.7<br>(14.6 to 18.7)*	-4.5<br>(-6.2 to -2.9)*
Foreign body in other body part	712.9<br>(632.5 to 801.7)	9.7<br>(9.2 to 10.2)*	-10.3<br>(-10.7 to -9.8)*	16.4<br>(11.2 to 23.2)	2.9<br>(0.1 to 5.6)*	-15.8<br>(-18.1 to -13.6)*
Environmental heat and cold exposure	3,695.8<br>(3,256.8 to 4,121.9)	2.4<br>(1.6 to 3.2)*	-16.2<br>(-16.9 to -15.6)*	146.9<br>(100.7 to 203.9)	-3.7<br>(-6.1 to -1.4)*	-21.2<br>(-23.2 to -19.4)*
Other unintentional injuries	9,747.6<br>(8,819.5 to 10,837.2)	1.1<br>(0.4 to 1.7)*	-17.3<br>(-17.9 to -16.8)*	330.0<br>(223.2 to 464.1)	-1.6<br>(-3.1 to -0.4)*	-19.5<br>(-20.8 to -18.5)*
Self-harm and interpersonal violence	1,498.3<br>(1,388.4 to 1,602.2)	14.8<br>(14.3 to 15.2)*	-6.1<br>(-6.5 to -5.8)*	56.7<br>(39.8 to 76.2)	3.5<br>(-0.3 to 7.4)	-15.4<br>(-18.5 to -12.2)*
Self-harm	448.3<br>(398.2 to 502.8)	14.5<br>(13.9 to 15.2)*	-6.3<br>(-6.9 to -5.8)*	18.4<br>(13.0 to 24.9)	0.8<br>(-3.3 to 5.2)	-17.6<br>(-20.9 to -14.0)*
Interpersonal violence	1,050.0<br>(958.2 to 1,150.8)	14.9<br>(14.3 to 15.4)*	-6.0<br>(-6.5 to -5.6)*	38.3<br>(26.7 to 52.2)	4.8<br>(1.2 to 8.5)*	-14.3<br>(-17.3 to -11.3)*

Cause	Prevalence in thousands (95% confidence interval)			YLDs in thousands (95% confidence interval)		
	2015	Percent change in counts between 2005 and 2015	Percent change in rates between 2005 and 2015	2015	Percent change in counts between 2005 and 2015	Percent change in rates between 2005 and 2015
Assault by firearm	66.5<br>(57.4 to 76.2)	25.9<br>(25.0 to 26.8)*	3.0<br>(2.3 to 3.7)*	2.6<br>(1.8 to 3.6)	17.5<br>(13.9 to 20.4)*	-3.9<br>(-6.9 to -1.5)*
Assault by sharp object	221.7<br>(184.4 to 257.5)	12.8<br>(11.6 to 13.8)*	-7.8<br>(-8.7 to -6.9)*	5.5<br>(3.7 to 7.6)	-3.5<br>(-9.2 to 2.3)	-21.0<br>(-25.8 to -16.3)*
Assault by other means	761.7<br>(684.5 to 844.6)	14.6<br>(14.0 to 15.3)*	-6.2<br>(-6.8 to -5.7)*	30.3<br>(21.1 to 41.2)	5.5<br>(2.0 to 8.9)*	-13.8<br>(-16.6 to -10.9)*
Forces of nature, war, and legal intervention	3,187.7<br>(2,256.4 to 4,351.5)	58.0<br>(32.9 to 87.6)*	29.2<br>(8.7 to 53.4)*	83.0<br>(60.0 to 106.5)	28.6<br>(-2.8 to 60.7)	5.1<br>(-20.5 to 31.4)
Exposure to forces of nature	1,080.8<br>(628.7 to 1,803.5)	28.8<br>(-9.2 to 80.5)	5.3<br>(-25.7 to 47.6)	32.8<br>(20.8 to 47.8)	-6.4<br>(-42.4 to 39.1)	-23.4<br>(-52.9 to 13.8)
Collective violence and legal intervention	2,106.9<br>(1,498.8 to 2,888.9)	78.9<br>(48.6 to 114.4)*	46.3<br>(21.5 to 75.3)*	50.2<br>(34.7 to 68.3)	70.0<br>(38.0 to 107.5)*	39.0<br>(12.9 to 69.7)*
**Anemia**	**53,114.1<br>(52,354.8 to 53,802.7)**	**10.4<br>(8.7 to 12.1)***	**-9.7<br>(-11.1 to -8.3)***	**1,320.0<br>(905.8 to 1,855.8)**	**3.9<br>(1.2 to 6.4)***	**-15.0<br>(-17.2 to -13.0)***
**Developmental intellectual disability**	**2,362.2<br>(1,735.7 to 2,950.0)**	**23.4<br>(23.0 to 24.0)***	**1.0<br>(0.6 to 1.4)***	**286.2<br>(207.7 to 375.2)**	**26.7<br>(24.1 to 29.6)***	**3.6<br>(1.5 to 6.0)***
**Epilepsy**	**928.4<br>(801.1 to 1,060.4)**	**27.6<br>(22.6 to 32.5)***	**4.4<br>(0.3 to 8.4)***	**226.3<br>(163.6 to 294.4)**	**11.6<br>(4.0 to 18.9)***	**-8.7<br>(-14.9 to -2.7)***
**Guillain-Barré syndrome**	**1.9<br>(1.2 to 2.8)**	**19.6<br>(15.4 to 22.3)***	**-2.2<br>(-5.6 to 0.1)**	**0.6<br>(0.3 to 0.9)**	**19.6<br>(15.4 to 22.3)***	**-2.2<br>(-5.6 to 0.1)**
**Hearing loss**	**131,297.3<br>(120,939.0 to 141,687.3)**	**22.6<br>(21.9 to 23.4)***	**0.3<br>(-0.3 to 0.9)**	**3,894.6<br>(2,624.9 to 5,496.2)**	**22.0<br>(18.5 to 25.3)***	**-0.2<br>(-3.1 to 2.5)**
**Heart failure**	**4,684.4<br>(4,487.4 to 4,884.3)**	**25.5<br>(24.6 to 26.3)***	**2.7<br>(1.9 to 3.3)***	**809.3<br>(598.1 to 1,039.3)**	**28.7<br>(27.0 to 30.2)***	**5.2<br>(3.9 to 6.5)***
**Infertility**	**0.0<br>(0.0 to 0.0)**	**0.0<br>(0.0 to 0.0)**	**0.0<br>(0.0 to 0.0)**	**0.0<br>(0.0 to 0.0)**	**0.0<br>(0.0 to 0.0)**	**0.0<br>(0.0 to 0.0)**
**Pelvic inflammatory disease**	**9.0<br>(6.8 to 11.8)**	**6.6<br>(5.2 to 8.1)***	**-12.8<br>(-14.0 to -11.6)***	**1.2<br>(0.8 to 1.8)**	**7.6<br>(-0.8 to 17.1)**	**-12.0<br>(-18.8 to -4.2)***
**Vision loss**	**81,392.3<br>(74,829.9 to 88,282.3)**	**22.4<br>(19.9 to 25.0)***	**0.1<br>(-1.9 to 2.2)**	**2,128.7<br>(1,452.1 to 3,021.2)**	**20.5<br>(18.8 to 22.0)***	**-1.5<br>(-2.8 to -0.2)***

SR Table 11. Prevalent cases and YLDs for 2015, percent change, and change in age-standardised rates between 2005 and 2015 by cause, global, ages 70-74 years

Cause	Prevalence in thousands (95% confidence interval)			YLDs in thousands (95% confidence interval)		
	2015	Percent change in counts between 2005 and 2015	Percent change in rates between 2005 and 2015	2015	Percent change in counts between 2005 and 2015	Percent change in rates between 2005 and 2015
**All causes**	--	--	--	**35,402.6 (26,536.6 to 45,512.2)**	**21.7 (21.0 to 22.3)***	**-0.9 (-1.4 to -0.4)***
Communicable, maternal, neonatal, and nutritional diseases	75,540.6 (74,510.7 to 76,590.1)	18.4 (17.2 to 19.5)*	-3.6 (-4.5 to -2.6)*	1,129.8 (823.4 to 1,537.6)	9.8 (5.8 to 14.1)*	-10.6 (-13.8 to -7.0)*
HIV/AIDS and tuberculosis	858.5 (818.9 to 898.5)	35.0 (27.6 to 42.5)*	10.0 (4.0 to 16.1)*	154.3 (105.3 to 217.8)	9.0 (1.4 to 16.4)*	-11.2 (-17.4 to -5.2)*
Tuberculosis	371.6 (279.4 to 497.8)	11.3 (3.2 to 18.5)*	-9.3 (-15.9 to -3.5)*	105.9 (66.3 to 158.6)	11.7 (3.1 to 19.3)*	-9.0 (-16.0 to -2.8)*
HIV/AIDS	496.7 (468.1 to 528.6)	60.5 (47.5 to 76.8)*	30.8 (20.2 to 44.1)*	48.4 (35.4 to 62.7)	3.7 (-9.9 to 20.9)	-15.5 (-26.6 to -1.5)*
HIV/AIDS - Tuberculosis	13.9 (11.9 to 15.9)	0.8 (-3.6 to 5.5)	-17.9 (-21.4 to -14.0)*	4.8 (3.2 to 6.5)	1.4 (-5.3 to 9.6)	-17.4 (-22.8 to -10.7)*
HIV/AIDS resulting in other diseases	496.9 (463.3 to 538.0)	62.5 (54.3 to 70.8)*	32.4 (25.7 to 39.1)*	43.6 (31.7 to 56.7)	4.0 (-11.2 to 23.1)	-15.3 (-27.7 to 0.3)
HIV aggregate	177.4 (162.8 to 194.3)	-18.3 (-23.2 to -12.4)*	-33.4 (-37.4 to -28.6)*	12.0 (8.8 to 15.6)	-19.5 (-25.2 to -12.8)*	-34.4 (-39.1 to -28.9)*
AIDS aggregate	319.5 (295.1 to 349.1)	260.9 (223.6 to 308.1)*	194.1 (163.7 to 232.5)*	31.6 (22.9 to 41.3)	16.9 (-7.7 to 50.4)	-4.8 (-24.8 to 22.5)
Diarrhea, lower respiratory, and other common infectious diseases	6,171.7 (5,744.0 to 6,512.5)	20.6 (17.6 to 23.9)*	-1.7 (-4.2 to 0.9)	233.6 (167.1 to 311.4)	20.9 (19.4 to 22.3)*	-1.5 (-2.7 to -0.3)*
Diarrheal diseases	630.3 (611.1 to 649.8)	24.0 (23.5 to 24.6)*	1.0 (0.6 to 1.5)*	92.2 (64.4 to 124.7)	24.3 (22.4 to 26.1)*	1.2 (-0.2 to 2.8)
Diarrhoea episodes aggregate	630.1 (611.0 to 649.7)	24.0 (23.5 to 24.6)*	1.0 (0.6 to 1.5)*	92.2 (64.3 to 124.6)	24.3 (22.4 to 26.1)*	1.2 (-0.2 to 2.8)
Guillain-Barré syndrome due to diarrheal diseases	0.2 (0.1 to 0.3)	21.5 (17.4 to 24.4)*	-1.0 (-4.4 to 1.4)	0.1 (0.0 to 0.1)	21.4 (17.4 to 24.4)*	-1.0 (-4.4 to 1.4)
Intestinal infectious diseases	14.8 (13.5 to 16.2)	-1.9 (-11.4 to 7.9)	-20.1 (-27.8 to -12.0)*	2.0 (1.3 to 2.8)	2.4 (-11.2 to 18.1)	-16.6 (-27.6 to -3.8)*
Typhoid fever	13.7 (10.8 to 17.0)	3.4 (-2.7 to 9.8)	-15.7 (-20.8 to -10.5)*	1.7 (1.1 to 2.4)	4.8 (-10.6 to 24.2)	-14.6 (-27.1 to 1.2)
Typhoid fever episodes aggregate	11.4 (8.8 to 14.2)	3.3 (-6.9 to 14.8)	-15.8 (-24.1 to -6.5)*	1.0 (0.6 to 1.5)	3.8 (-11.2 to 22.8)	-15.5 (-27.7 to 0.0)
Complications of typhoid fever aggregate	2.4 (1.6 to 3.3)	3.9 (-32.1 to 56.4)	-15.3 (-44.6 to 27.4)	0.7 (0.4 to 1.0)	6.4 (-33.8 to 64.2)	-13.3 (-46.0 to 33.8)
Paratyphoid fever	5.4 (3.7 to 7.3)	-0.8 (-9.8 to 8.9)	-19.1 (-26.5 to -11.2)*	0.3 (0.2 to 0.4)	-0.7 (-20.6 to 23.7)	-19.1 (-35.3 to 0.8)
Paratyphoid fever episodes aggregate	5.1 (3.5 to 6.9)	-0.7 (-9.9 to 9.2)	-19.1 (-26.6 to -11.0)*	0.2 (0.1 to 0.4)	-0.6 (-22.0 to 25.7)	-19.0 (-36.4 to 2.4)
Intestinal perforation due to paratyphoid	0.3 (0.2 to 0.5)	-1.3 (-44.0 to 73.9)	-19.6 (-54.4 to 41.7)	0.0 (0.0 to 0.1)	-1.4 (-44.0 to 73.9)	-19.7 (-54.4 to 41.7)
Other intestinal infectious diseases	--	--	--	0.0 (0.0 to 0.1)	-54.7 (-70.5 to -27.3)*	-63.1 (-76.0 to -40.7)*
Lower respiratory infections	304.9 (287.6 to 324.3)	20.1 (18.1 to 21.9)*	-2.1 (-3.8 to -0.7)*	17.3 (11.8 to 23.9)	20.2 (16.7 to 23.7)*	-2.1 (-4.9 to 0.8)
Lower respiratory infection episodes aggregate	304.7 (287.4 to 324.1)	20.1 (18.1 to 21.9)*	-2.1 (-3.8 to -0.7)*	17.3 (11.8 to 23.8)	20.2 (16.7 to 23.7)*	-2.1 (-4.9 to 0.8)
Guillain-Barré syndrome due to lower respiratory infections	0.2 (0.1 to 0.4)	21.4 (17.4 to 24.4)*	-1.1 (-4.4 to 1.4)	0.1 (0.0 to 0.1)	21.4 (17.4 to 24.4)*	-1.1 (-4.4 to 1.4)
Upper respiratory infections	3,628.5 (2,531.8 to 5,000.5)	23.4 (21.6 to 25.1)*	0.5 (-0.9 to 1.9)	40.5 (20.8 to 72.4)	23.4 (21.2 to 25.8)*	0.6 (-1.2 to 2.5)
Upper respiratory infection episodes aggregate	3,627.9 (2,531.4 to 4,999.9)	23.4 (21.6 to 25.1)*	0.5 (-0.9 to 1.9)	40.3 (20.6 to 72.3)	23.4 (21.2 to 25.8)*	0.6 (-1.2 to 2.5)
Guillain-Barré syndrome due to upper respiratory infections	0.6 (0.4 to 0.8)	21.5 (17.4 to 24.4)*	-1.0 (-4.4 to 1.4)	0.2 (0.1 to 0.3)	21.4 (17.4 to 24.4)*	-1.0 (-4.4 to 1.4)
Otitis media	1,071.5 (748.8 to 1,470.3)	12.2 (8.2 to 16.7)*	-8.6 (-11.8 to -4.9)*	28.1 (15.7 to 46.3)	11.9 (5.8 to 18.5)*	-8.8 (-13.8 to -3.4)*
Acute otitis media	98.1 (73.9 to 128.8)	23.3 (19.7 to 27.0)*	0.4 (-2.5 to 3.4)	1.2 (0.6 to 2.2)	23.3 (16.6 to 30.2)*	0.5 (-5.0 to 6.1)
Chronic otitis media aggregate	973.4 (641.8 to 1,372.5)	11.2 (7.1 to 16.0)*	-9.4 (-12.7 to -5.4)*	26.9 (14.9 to 44.9)	11.5 (5.1 to 18.3)*	-9.2 (-14.4 to -3.6)*
Meningitis	233.3 (224.0 to 241.8)	18.1 (14.7 to 21.6)*	-3.8 (-6.5 to -0.9)*	35.6 (25.4 to 46.0)	20.4 (17.4 to 23.6)*	-1.9 (-4.4 to 0.7)
Pneumococcal meningitis	196.6 (169.9 to 227.3)	23.1 (21.4 to 25.2)*	0.3 (-1.1 to 2.0)	19.0 (13.6 to 24.7)	22.0 (17.4 to 26.5)*	-0.6 (-4.3 to 3.0)
Acute pneumococcal meningitis	0.8 (0.5 to 1.1)	26.1 (14.6 to 38.3)*	2.7 (-6.6 to 12.7)	0.1 (0.1 to 0.2)	26.1 (14.6 to 38.3)*	2.7 (-6.6 to 12.7)
Complications of pneumococcal meningitis aggregate	195.8 (169.1 to 226.7)	23.1 (21.4 to 25.1)*	0.3 (-1.1 to 2.0)	18.9 (13.5 to 24.6)	22.0 (17.4 to 26.4)*	-0.6 (-4.3 to 3.0)
H influenzae type B meningitis	43.1 (34.4 to 52.4)	-6.1 (-9.9 to -1.1)*	-23.5 (-26.6 to -19.4)*	4.9 (3.4 to 6.5)	15.6 (8.4 to 23.2)*	-5.8 (-11.7 to 0.4)
Acute H influenzae type B meningitis	0.2 (0.1 to 0.3)	-7.8 (-18.4 to 3.2)	-24.9 (-33.5 to -15.9)*	0.0 (0.0 to 0.1)	-7.8 (-18.4 to 3.2)	-24.8 (-33.5 to -15.9)*
Complications of of H influenzae type B meningitis aggregate	42.8 (34.2 to 52.2)	-6.1 (-10.0 to -1.1)*	-23.5 (-26.6 to -19.4)*	4.9 (3.4 to 6.5)	15.8 (8.5 to 23.4)*	-5.7 (-11.6 to 0.6)
Meningococcal meningitis	31.1 (23.0 to 40.4)	21.9 (18.2 to 25.9)*	-0.7 (-3.7 to 2.5)	2.2 (1.5 to 2.9)	22.2 (19.0 to 25.5)*	-0.5 (-3.1 to 2.3)
Acute meningococcal meningitis	0.3 (0.2 to 0.5)	23.8 (10.9 to 35.9)*	0.8 (-9.6 to 10.8)	0.0 (0.0 to 0.1)	24.0 (11.0 to 36.1)*	1.0 (-9.5 to 10.9)
Complications of meningococcal meningitis aggregate	30.8 (22.7 to 39.9)	21.9 (18.2 to 25.9)*	-0.7 (-3.7 to 2.6)	2.1 (1.5 to 2.9)	22.1 (18.9 to 25.6)*	-0.5 (-3.1 to 2.3)
Other meningitis	88.8 (72.5 to 105.0)	20.6 (18.6 to 22.8)*	-1.8 (-3.4 to 0.0)	9.5 (6.7 to 12.3)	19.4 (14.5 to 24.8)*	-2.7 (-6.7 to 1.7)
Other meningitis episodes aggregate	1.6 (1.2 to 2.0)	26.4 (15.7 to 36.3)*	3.0 (-5.7 to 11.1)	0.2 (0.1 to 0.3)	27.2 (16.5 to 37.2)*	3.7 (-5.1 to 11.8)
Complications of other meningitis aggregate	87.2 (70.9 to 103.4)	20.5 (18.4 to 22.7)*	-1.9 (-3.5 to -0.0)*	9.2 (6.5 to 12.1)	19.2 (14.2 to 24.8)*	-2.9 (-6.9 to 1.7)
Encephalitis	105.6 (65.1 to 158.4)	8.7 (6.2 to 12.4)*	-11.4 (-13.4 to -8.4)*	6.5 (4.6 to 8.6)	13.8 (9.2 to 18.9)*	-7.3 (-11.0 to -3.1)*
Acute encephalitis	1.5 (1.2 to 1.8)	22.5 (16.8 to 27.9)*	-0.2 (-4.8 to 4.2)	0.2 (0.1 to 0.3)	27.0 (13.2 to 42.3)*	3.4 (-7.7 to 16.0)
Complications of encephalitis aggregate	104.1 (63.8 to 156.8)	8.5 (6.1 to 12.1)*	-11.6 (-13.6 to -8.6)*	6.3 (4.4 to 8.4)	13.4 (8.7 to 18.6)*	-7.6 (-11.4 to -3.4)*
Diphtheria	0.0 (0.0 to 0.0)	0.0 (0.0 to 0.0)	0.0 (0.0 to 0.0)	0.0 (0.0 to 0.0)	0.0 (0.0 to 0.0)	0.0 (0.0 to 0.0)
Whooping cough	0.0 (0.0 to 0.0)	0.0 (0.0 to 0.0)	0.0 (0.0 to 0.0)	0.0 (0.0 to 0.0)	0.0 (0.0 to 0.0)	0.0 (0.0 to 0.0)
Tetanus	3.3 (3.2 to 3.5)	16.9 (8.9 to 21.8)*	-4.7 (-11.3 to -0.8)*	0.1 (0.1 to 0.2)	-4.7 (-20.4 to 10.2)	-22.4 (-35.1 to -10.2)*
Severe tetanus	0.3 (0.2 to 0.5)	-38.4 (-52.7 to -18.9)*	-49.8 (-61.5 to -33.9)*	0.0 (0.0 to 0.1)	-37.4 (-52.9 to -17.6)*	-49.0 (-61.6 to -32.9)*
Complications of tetanus aggregate	3.0 (3.0 to 3.1)	26.7 (26.2 to 27.2)*	3.3 (2.8 to 3.7)*	0.1 (0.1 to 0.1)	20.6 (9.1 to 33.8)*	-1.8 (-11.1 to 9.0)
Measles	0.0 (0.0 to 0.0)	0.0 (0.0 to 0.0)	0.0 (0.0 to 0.0)	0.0 (0.0 to 0.0)	0.0 (0.0 to 0.0)	0.0 (0.0 to 0.0)
Varicella and herpes zoster	218.3 (181.7 to 259.3)	21.1 (19.5 to 22.4)*	-1.3 (-2.6 to -0.2)*	11.4 (6.9 to 17.2)	21.1 (17.5 to 24.9)*	-1.3 (-4.3 to 1.8)
Neglected tropical diseases and malaria	29,316.9 (28,636.2 to 30,078.2)	6.1 (3.0 to 9.4)*	-13.6 (-16.1 to -10.8)*	308.5 (207.5 to 460.4)	-4.1 (-14.0 to 9.0)	-21.8 (-29.9 to -11.2)*
Malaria	1,567.7 (1,368.7 to 1,774.3)	33.6 (27.5 to 39.8)*	8.9 (3.9 to 13.9)*	9.5 (6.7 to 13.2)	20.3 (11.9 to 28.4)*	-2.0 (-8.9 to 4.6)
Asymptomatic malaria parasitemia(PfPR)	1,220.0 (1,018.7 to 1,425.3)	36.1 (28.1 to 44.6)*	10.9 (4.4 to 17.8)*	0.0 (0.0 to 0.0)	0.0 (0.0 to 0.0)	0.0 (0.0 to 0.0)
Malaria episodes aggregate	20.2 (11.2 to 35.1)	-8.2 (-27.3 to 17.7)	-25.2 (-40.8 to -4.1)*	0.4 (0.2 to 0.8)	-7.5 (-28.5 to 20.3)	-24.6 (-41.7 to -1.9)*
Complications of malaria aggregate	2.0 (1.7 to 2.4)	36.0 (33.1 to 39.1)*	10.8 (8.4 to 13.4)*	0.7 (0.5 to 0.9)	31.5 (24.8 to 38.9)*	7.1 (1.7 to 13.1)*
Anemia due to malaria parasitemia(PfPR) aggregate	325.5 (315.0 to 337.3)	28.6 (23.0 to 34.4)*	4.8 (0.2 to 9.5)*	8.4 (5.8 to 11.9)	21.2 (11.8 to 29.9)*	-1.3 (-8.9 to 5.8)
Chagas disease	303.2 (250.6 to 355.8)	3.9 (0.7 to 6.9)*	-15.4 (-18.0 to -12.9)*	5.3 (3.4 to 7.7)	4.3 (-0.8 to 9.2)	-15.0 (-19.1 to -11.0)*
Chagas disease episodes aggregate	227.9 (188.1 to 268.7)	3.8 (0.5 to 6.8)*	-15.5 (-18.1 to -13.0)*	0.0 (0.0 to 0.0)	25.2 (20.2 to 32.0)*	2.0 (-2.0 to 7.6)
Complications of Chagas disease aggregate	40.5 (31.5 to 51.3)	4.1 (0.8 to 7.1)*	-15.2 (-17.9 to -12.8)*	3.6 (2.3 to 5.4)	4.2 (-1.6 to 10.1)	-15.1 (-19.8 to -10.3)*

Cause	Prevalence in thousands (95% confidence interval)			YLDs in thousands (95% confidence interval)		
	2015	Percent change in counts between 2005 and 2015	Percent change in rates between 2005 and 2015	2015	Percent change in counts between 2005 and 2015	Percent change in rates between 2005 and 2015
Heart failure due to Chagas disease aggregate	34.8 (22.7 to 47.7)	4.5 (1.2 to 7.7)*	-14.8 (-17.5 to -12.2)*	1.7 (0.9 to 2.7)	4.5 (-1.2 to 10.1)	-14.9 (-19.5 to -10.3)*
Leishmaniasis	102.5 (93.2 to 114.6)	30.0 (13.4 to 48.9)*	6.0 (-7.6 to 21.3)	1.1 (0.5 to 2.0)	29.9 (25.3 to 34.1)*	5.8 (2.1 to 9.2)*
Visceral leishmaniasis	0.2 (0.1 to 0.2)	27.7 (26.0 to 29.5)*	4.1 (2.7 to 5.6)*	0.0 (0.0 to 0.0)	28.2 (19.9 to 36.6)*	4.5 (-2.3 to 11.3)
Cutaneous and mucocutaneous leishmaniasis	105.3 (92.6 to 122.5)	29.7 (27.3 to 32.2)*	5.7 (3.7 to 7.7)*	1.1 (0.5 to 2.0)	29.9 (25.2 to 34.1)*	5.8 (2.0 to 9.2)*
African trypanosomiasis	0.1 (0.1 to 0.2)	-69.0 (-70.5 to -67.8)*	-74.8 (-75.9 to -73.7)*	0.0 (0.0 to 0.1)	-67.4 (-83.8 to -45.7)*	-73.5 (-86.8 to -55.8)*
Schistosomiasis	2,933.5 (2,452.8 to 3,691.9)	-26.7 (-35.8 to -9.2)*	-40.3 (-47.7 to -26.0)*	23.5 (11.8 to 43.8)	-25.4 (-33.3 to -8.2)*	-39.2 (-45.6 to -25.2)*
Schistosomiasis episodes aggregate	2,007.9 (1,667.5 to 2,479.9)	-28.3 (-36.9 to -13.8)*	-41.6 (-48.6 to -29.8)*	10.8 (4.3 to 22.9)	-29.2 (-37.9 to -13.9)*	-42.3 (-49.4 to -29.9)*
Complications of schistosomiasis aggregate	738.4 (474.1 to 1,215.5)	-20.9 (-34.4 to 11.4)	-35.6 (-46.5 to -9.2)*	8.5 (4.0 to 16.8)	-16.6 (-28.9 to 14.8)	-32.0 (-42.1 to -6.5)*
Anemia due to schistosomiasis aggregate	155.1 (147.6 to 163.3)	-28.9 (-32.4 to -24.8)*	-42.0 (-44.9 to -38.7)*	4.2 (2.8 to 5.9)	-30.5 (-36.8 to -23.9)*	-43.4 (-48.5 to -38.0)*
Cysticercosis	74.5 (45.9 to 115.0)	-2.3 (-8.8 to 4.0)	-20.4 (-25.7 to -15.3)*	10.2 (5.8 to 16.7)	-13.2 (-21.0 to -6.0)*	-29.3 (-35.6 to -23.5)*
Cystic echinococcosis	52.9 (46.7 to 59.8)	41.1 (36.9 to 45.4)*	15.0 (11.5 to 18.5)*	4.6 (3.1 to 6.3)	40.4 (29.3 to 52.8)*	14.4 (5.4 to 24.5)*
Lymphatic filariasis	871.0 (682.0 to 1,108.7)	-39.8 (-46.9 to -33.2)*	-50.9 (-56.7 to -45.6)*	55.1 (30.6 to 86.8)	-12.5 (-30.6 to 1.9)	-28.7 (-43.5 to -17.0)*
Prevalence of detectable microfiliaria due to lymphatic filariasis	336.3 (288.8 to 386.7)	-60.3 (-63.5 to -56.3)*	-67.7 (-70.3 to -64.4)*	0.0 (0.0 to 0.0)	0.0 (0.0 to 0.0)	0.0 (0.0 to 0.0)
Complications of lymphatic filariasis aggregate	534.7 (351.3 to 762.7)	-10.7 (-28.1 to 2.9)	-27.3 (-41.4 to -16.2)*	55.1 (30.6 to 86.8)	-12.5 (-30.6 to 1.9)	-28.7 (-43.5 to -17.0)*
Onchocerciasis	319.1 (218.5 to 469.8)	-16.9 (-32.9 to 1.6)	-32.3 (-45.3 to -17.3)*	33.6 (18.0 to 59.5)	-15.7 (-33.2 to 5.6)	-31.3 (-45.5 to -14.0)*
Asymptomatic onchocerciasis	1.4 (0.0 to 7.6)	-93.6 (-100.0 to -82.2)*	-94.8 (-100.0 to -85.5)*	0.0 (0.0 to 0.0)	0.0 (0.0 to 0.0)	0.0 (0.0 to 0.0)
Skin disease due to onchocerciasis aggregate	212.8 (122.0 to 354.9)	-20.9 (-39.6 to 0.0)	-35.5 (-50.8 to -18.4)*	26.1 (12.0 to 50.5)	-20.5 (-43.3 to 3.9)	-35.2 (-53.8 to -14.6)*
Vision loss due to onchocerciasis aggregate	102.8 (66.9 to 158.0)	11.4 (-3.4 to 29.4)	-9.2 (-21.3 to 5.4)	7.5 (4.5 to 11.5)	6.5 (-6.2 to 20.4)	-13.2 (-23.6 to -1.9)*
Trachoma	417.1 (269.2 to 615.2)	-3.0 (-13.3 to 7.4)	-20.9 (-29.4 to -12.5)*	29.9 (18.1 to 45.3)	-7.9 (-17.1 to 2.5)	-24.9 (-32.4 to -16.5)*
Dengue	75.4 (43.3 to 160.5)	166.1 (10.4 to 605.5)*	116.8 (-10.1 to 474.8)	11.2 (5.3 to 25.4)	163.5 (7.1 to 614.6)*	114.7 (-12.7 to 482.3)
Dengue episodes aggregate	22.4 (15.1 to 45.4)	146.8 (4.5 to 537.6)*	101.1 (-14.9 to 419.5)	1.2 (0.7 to 2.7)	162.4 (10.0 to 594.9)*	113.8 (-10.4 to 466.2)
Post-dengue chronic fatigue syndrome	53.0 (25.9 to 115.3)	166.3 (10.4 to 605.5)*	116.9 (-10.1 to 474.8)	10.0 (4.5 to 23.2)	163.6 (7.2 to 619.0)*	114.8 (-12.7 to 485.8)
Yellow fever	0.0 (0.0 to 0.1)	-24.4 (-44.5 to 1.9)	-38.4 (-54.8 to -16.9)*	0.0 (0.0 to 0.0)	-24.4 (-44.5 to 1.9)	-38.4 (-54.8 to -16.9)*
Rabies	0.0 (0.0 to 0.0)	-44.2 (-64.4 to -12.5)*	-54.6 (-71.0 to -28.7)*	0.0 (0.0 to 0.0)	-44.1 (-64.4 to -12.5)*	-54.5 (-71.0 to -28.7)*
Intestinal nematode infections	24,112.8 (23,399.9 to 24,828.7)	7.0 (3.2 to 10.9)*	-12.8 (-15.9 to -9.6)*	41.1 (23.4 to 66.8)	-17.7 (-24.6 to -9.3)*	-32.9 (-38.5 to -26.1)*
Ascariasis	12,667.4 (10,701.5 to 15,294.9)	-2.0 (-19.9 to 21.5)	-20.1 (-34.7 to -1.0)*	11.7 (6.5 to 19.6)	-34.3 (-45.3 to -21.4)*	-46.5 (-55.5 to -36.0)*
Complications of ascariasis aggregate	676.9 (601.7 to 762.1)	-32.3 (-42.0 to -20.6)*	-44.9 (-52.8 to -35.3)*	11.7 (6.5 to 19.6)	-34.3 (-45.3 to -21.4)*	-46.5 (-55.5 to -36.0)*
Asymptomatic ascariasis	11,983.8 (10,036.8 to 14,576.6)	0.5 (-19.0 to 26.3)	-18.1 (-34.0 to 2.9)	0.0 (0.0 to 0.0)	0.0 (0.0 to 0.0)	0.0 (0.0 to 0.0)
Trichuriasis	7,449.1 (6,652.9 to 8,345.4)	9.4 (-6.5 to 28.5)	-10.9 (-23.8 to 4.7)	7.7 (4.1 to 13.2)	-5.1 (-25.5 to 24.0)	-22.7 (-39.3 to 1.0)
Complications of trichuriasis aggregate	400.4 (347.2 to 487.9)	-5.3 (-22.6 to 19.9)	-22.9 (-37.0 to -2.3)*	7.7 (4.1 to 13.2)	-5.1 (-25.5 to 24.0)	-22.7 (-39.3 to 1.0)
Asymptomatic trichuriasis	7,043.5 (6,230.0 to 7,964.5)	10.3 (-6.6 to 31.1)	-10.2 (-23.9 to 6.8)	0.0 (0.0 to 0.0)	0.0 (0.0 to 0.0)	0.0 (0.0 to 0.0)
Hookworm disease	7,256.1 (6,443.6 to 8,206.7)	2.0 (-12.5 to 18.8)	-16.9 (-28.7 to -3.2)*	21.7 (12.8 to 34.6)	-9.7 (-18.8 to 0.8)	-26.4 (-33.8 to -17.8)*
Complications of hookworm disease aggregate	856.7 (789.0 to 928.5)	-7.2 (-17.0 to 3.8)	-24.4 (-32.4 to -15.4)*	16.7 (9.3 to 27.1)	-7.9 (-20.0 to 5.9)	-25.0 (-34.8 to -13.7)*
Anemia due to hookworm disease aggregate	317.4 (307.0 to 328.7)	2.2 (-2.5 to 6.9)	-16.8 (-20.5 to -12.9)*	5.0 (3.3 to 7.5)	-15.0 (-23.7 to -6.1)*	-30.8 (-37.9 to -23.5)*
Asymptomatic hookworm disease	6,072.1 (5,253.6 to 7,023.9)	3.2 (-13.9 to 24.0)	-15.9 (-29.8 to 1.0)	0.0 (0.0 to 0.0)	0.0 (0.0 to 0.0)	0.0 (0.0 to 0.0)
Food-borne trematodiases	1,472.8 (1,358.0 to 1,601.5)	22.8 (16.5 to 27.8)*	0.0 (-5.1 to 4.2)	33.3 (15.2 to 60.4)	22.2 (13.3 to 32.4)*	-0.5 (-7.7 to 7.8)
Asymptomatic food-borne trematodiases aggregate	1,156.5 (926.5 to 1,359.8)	22.8 (16.8 to 28.4)*	0.1 (-4.9 to 4.6)	0.0 (0.0 to 0.0)	0.0 (0.0 to 0.0)	0.0 (0.0 to 0.0)
Symptomatic food-borne trematodiases aggregate	316.3 (161.2 to 527.1)	22.8 (14.7 to 32.4)*	0.0 (-6.5 to 7.9)	33.3 (15.2 to 60.4)	22.2 (13.3 to 32.4)*	-0.5 (-7.7 to 7.8)
Leprosy	43.7 (41.5 to 45.9)	4.3 (3.1 to 5.6)*	-15.0 (-16.0 to -14.0)*	2.6 (1.8 to 3.6)	5.0 (0.2 to 10.0)*	-14.4 (-18.4 to -10.4)*
Ebola	0.0 (0.0 to 0.1)	0.0 (0.0 to 0.0)	0.0 (0.0 to 0.0)	0.0 (0.0 to 0.0)	0.0 (0.0 to 0.0)	0.0 (0.0 to 0.0)
Other neglected tropical diseases	179.1 (169.3 to 189.5)	11.8 (4.2 to 20.3)*	-8.9 (-15.1 to -2.0)*	47.5 (14.4 to 146.2)	13.2 (-33.6 to 83.1)	-7.8 (-45.9 to 49.2)
Anemia due to other neglected tropical diseases aggregate	179.1 (169.3 to 189.5)	11.8 (4.2 to 20.3)*	-8.9 (-15.1 to -2.0)*	3.0 (2.0 to 4.3)	-9.9 (-23.7 to 7.0)	-26.6 (-37.8 to -12.8)*
Maternal disorders	5.1 (4.5 to 5.8)	-32.6 (-41.0 to -22.9)*	-45.1 (-51.9 to -37.2)*	1.8 (1.1 to 2.5)	-31.1 (-39.8 to -22.3)*	-43.8 (-51.0 to -36.7)*
Maternal hemorrhage	0.0 (0.0 to 0.0)	0.0 (0.0 to 0.0)	0.0 (0.0 to 0.0)	0.0 (0.0 to 0.0)	0.0 (0.0 to 0.0)	0.0 (0.0 to 0.0)
Maternal hemorrhage episodes aggregate	0.0 (0.0 to 0.0)	0.0 (0.0 to 0.0)	0.0 (0.0 to 0.0)	0.0 (0.0 to 0.0)	0.0 (0.0 to 0.0)	0.0 (0.0 to 0.0)
Anemia due to maternal hemorrhage aggregate	0.0 (0.0 to 0.0)	0.0 (0.0 to 0.0)	0.0 (0.0 to 0.0)	0.0 (0.0 to 0.0)	0.0 (0.0 to 0.0)	0.0 (0.0 to 0.0)
Maternal sepsis and other maternal infections	0.0 (0.0 to 0.0)	0.0 (0.0 to 0.0)	0.0 (0.0 to 0.0)	0.0 (0.0 to 0.0)	0.0 (0.0 to 0.0)	0.0 (0.0 to 0.0)
Maternal sepsis and other maternal infections aggregate	0.0 (0.0 to 0.0)	0.0 (0.0 to 0.0)	0.0 (0.0 to 0.0)	0.0 (0.0 to 0.0)	0.0 (0.0 to 0.0)	0.0 (0.0 to 0.0)
Infertility due to puerperal sepsis	0.0 (0.0 to 0.0)	0.0 (0.0 to 0.0)	0.0 (0.0 to 0.0)	0.0 (0.0 to 0.0)	0.0 (0.0 to 0.0)	0.0 (0.0 to 0.0)
Maternal hypertensive disorders	0.0 (0.0 to 0.0)	0.0 (0.0 to 0.0)	0.0 (0.0 to 0.0)	0.0 (0.0 to 0.0)	0.0 (0.0 to 0.0)	0.0 (0.0 to 0.0)
Maternal hypertensive disorders episodes aggregate	0.0 (0.0 to 0.0)	0.0 (0.0 to 0.0)	0.0 (0.0 to 0.0)	0.0 (0.0 to 0.0)	0.0 (0.0 to 0.0)	0.0 (0.0 to 0.0)
Eclampsia	0.0 (0.0 to 0.0)	0.0 (0.0 to 0.0)	0.0 (0.0 to 0.0)	0.0 (0.0 to 0.0)	0.0 (0.0 to 0.0)	0.0 (0.0 to 0.0)
Complications of maternal hypertensive disorders aggregate	0.0 (0.0 to 0.0)	0.0 (0.0 to 0.0)	0.0 (0.0 to 0.0)	0.0 (0.0 to 0.0)	0.0 (0.0 to 0.0)	0.0 (0.0 to 0.0)
Maternal obstructed labor and uterine rupture	5.2 (4.3 to 6.3)	-32.0 (-35.0 to -29.0)*	-44.6 (-47.1 to -42.2)*	1.6 (1.0 to 2.2)	-31.6 (-40.1 to -23.4)*	-44.2 (-51.2 to -37.6)*
Obstructed labor, acute event	0.0 (0.0 to 0.0)	0.0 (0.0 to 0.0)	0.0 (0.0 to 0.0)	0.0 (0.0 to 0.0)	0.0 (0.0 to 0.0)	0.0 (0.0 to 0.0)
Fistula due to maternal obstructed labor and uterine rupture aggregate	5.2 (4.3 to 6.3)	-32.0 (-35.0 to -29.0)*	-44.6 (-47.1 to -42.2)*	1.6 (1.0 to 2.2)	-31.6 (-40.1 to -23.4)*	-44.2 (-51.2 to -37.6)*
Maternal abortion, miscarriage, and ectopic pregnancy	0.0 (0.0 to 0.0)	0.0 (0.0 to 0.0)	0.0 (0.0 to 0.0)	0.0 (0.0 to 0.0)	0.0 (0.0 to 0.0)	0.0 (0.0 to 0.0)
Other maternal disorders	--	--	--	0.2 (0.1 to 0.3)	-26.7 (-41.8 to -8.9)*	-40.3 (-52.6 to -25.8)*
Neonatal disorders	508.0 (476.7 to 532.3)	30.1 (23.2 to 37.4)*	6.0 (0.4 to 11.9)*	55.5 (41.1 to 71.9)	35.0 (29.9 to 40.4)*	10.0 (5.8 to 14.4)*
Neonatal preterm birth complications	507.9 (438.6 to 586.6)	27.1 (19.2 to 35.2)*	3.5 (-2.9 to 10.2)	25.9 (19.3 to 33.4)	28.4 (22.2 to 36.0)*	4.6 (-0.4 to 10.8)
Vision loss due to retinopathy of prematurity aggregate	99.8 (79.7 to 122.7)	21.2 (17.1 to 25.5)*	-1.3 (-4.6 to 2.3)	3.5 (2.1 to 5.3)	22.9 (13.4 to 33.2)*	0.1 (-7.6 to 8.5)
Complications of preterm birth complications aggregate	408.1 (347.9 to 482.4)	28.6 (18.5 to 38.7)*	4.8 (-3.4 to 13.0)	22.4 (16.8 to 29.2)	29.3 (22.1 to 37.7)*	5.3 (-0.5 to 12.2)
Neonatal encephalopathy due to birth asphyxia and trauma	61.6 (41.9 to 92.5)	50.1 (45.6 to 54.3)*	22.3 (18.6 to 25.7)*	17.5 (11.0 to 27.2)	44.1 (36.6 to 51.3)*	17.4 (11.3 to 23.3)*

Cause	Prevalence in thousands (95% confidence interval)			YLDs in thousands (95% confidence interval)		
	2015	Percent change in counts between 2005 and 2015	Percent change in rates between 2005 and 2015	2015	Percent change in counts between 2005 and 2015	Percent change in rates between 2005 and 2015
Neonatal sepsis and other neonatal infections	0.0 (0.0 to 0.0)	0.0 (0.0 to 0.0)	0.0 (0.0 to 0.0)	0.0 (0.0 to 0.0)	0.0 (0.0 to 0.0)	0.0 (0.0 to 0.0)
Severe infection due to neonatal sepsis and other neonatal infections	0.0 (0.0 to 0.0)	0.0 (0.0 to 0.0)	0.0 (0.0 to 0.0)	0.0 (0.0 to 0.0)	0.0 (0.0 to 0.0)	0.0 (0.0 to 0.0)
Complications of neonatal sepsis and other neonatal infections aggregate	0.0 (0.0 to 0.0)	0.0 (0.0 to 0.0)	0.0 (0.0 to 0.0)	0.0 (0.0 to 0.0)	0.0 (0.0 to 0.0)	0.0 (0.0 to 0.0)
Hemolytic disease and other neonatal jaundice	11.7 (9.9 to 13.6)	39.2 (37.2 to 41.4)*	13.4 (11.8 to 15.2)*	3.6 (2.6 to 4.7)	34.1 (30.4 to 37.6)*	9.2 (6.3 to 12.1)*
Other neonatal disorders	--	--	--	8.5 (6.2 to 11.1)	39.3 (24.4 to 53.3)*	13.5 (1.4 to 24.9)*
Nutritional deficiencies	14,526.0 (14,224.5 to 14,844.5)	19.6 (16.4 to 23.1)*	-2.6 (-5.2 to 0.3)	340.1 (229.5 to 486.4)	13.3 (8.7 to 17.6)*	-7.7 (-11.4 to -4.2)*
Protein-energy malnutrition	37.4 (23.5 to 57.1)	-9.5 (-19.0 to 2.2)	-26.3 (-34.0 to -16.8)*	4.2 (2.2 to 7.2)	-9.3 (-20.1 to 4.0)	-26.1 (-34.9 to -15.3)*
Iodine deficiency	2,675.1 (2,414.6 to 3,006.0)	17.6 (15.6 to 19.8)*	-4.2 (-5.8 to -2.4)*	45.7 (28.8 to 70.4)	18.0 (15.7 to 20.6)*	-3.9 (-5.7 to -1.7)*
Visible goiter due to iodine deficiency aggregate	2,671.5 (2,412.0 to 3,001.9)	17.6 (15.6 to 19.8)*	-4.2 (-5.8 to -2.4)*	45.1 (28.4 to 69.5)	17.8 (15.6 to 20.5)*	-4.0 (-5.8 to -1.8)*
Visible goiter with heart failure due to iodine deficiency aggregate	0.1 (0.0 to 0.1)	30.1 (28.4 to 31.9)*	6.0 (4.6 to 7.5)*	0.0 (0.0 to 0.0)	30.1 (28.4 to 31.9)*	6.0 (4.6 to 7.5)*
Intellectual disability due to iodine deficiency aggregate	3.6 (1.5 to 4.7)	26.9 (21.1 to 31.4)*	3.4 (-1.3 to 7.1)	0.6 (0.3 to 1.0)	27.9 (19.4 to 35.7)*	4.2 (-2.7 to 10.5)
Vitamin A deficiency	35.7 (24.0 to 49.7)	18.5 (12.8 to 25.4)*	-3.4 (-8.1 to 2.2)	2.4 (1.4 to 3.7)	19.7 (10.1 to 30.0)*	-2.5 (-10.3 to 5.9)
Iron-deficiency anemia	12,459.0 (12,137.5 to 12,798.1)	19.9 (16.6 to 23.3)*	-2.3 (-5.0 to 0.4)	287.3 (195.0 to 407.7)	13.0 (7.8 to 17.7)*	-8.0 (-12.2 to -4.1)*
Iron-deficiency anemia without heart failure aggregate	12,445.8 (12,124.1 to 12,784.4)	19.9 (16.6 to 23.2)*	-2.3 (-5.0 to 0.4)	285.8 (193.7 to 405.6)	12.9 (7.7 to 17.7)*	-8.0 (-12.2 to -4.1)*
Iron-deficiency anemia with heart failure aggregate	13.3 (10.8 to 15.8)	34.6 (31.9 to 37.0)*	9.6 (7.5 to 11.6)*	1.5 (1.0 to 2.1)	34.4 (24.4 to 44.5)*	9.5 (1.4 to 17.7)*
Other nutritional deficiencies	--	--	--	0.4 (0.2 to 0.8)	-4.6 (-23.1 to 15.8)	-22.3 (-37.3 to -5.7)*
Other communicable, maternal, neonatal, and nutritional diseases	42,628.7 (41,429.6 to 43,746.0)	22.5 (21.2 to 23.8)*	-0.2 (-1.3 to 0.8)	36.1 (24.2 to 54.6)	23.2 (15.0 to 32.5)*	0.3 (-6.3 to 7.9)
Sexually transmitted diseases excluding HIV	32,720.4 (31,328.7 to 34,022.4)	22.5 (20.7 to 24.4)*	-0.2 (-1.7 to 1.3)	22.9 (14.7 to 35.1)	23.6 (19.6 to 27.7)*	0.7 (-2.5 to 4.1)
Syphilis	78.1 (67.2 to 91.5)	23.8 (20.1 to 27.0)*	0.8 (-2.2 to 3.5)	12.7 (8.4 to 17.6)	25.4 (19.0 to 32.0)*	2.2 (-3.1 to 7.6)
Early syphilis aggregate	5.9 (2.7 to 12.5)	8.9 (-20.7 to 28.6)	-11.2 (-35.4 to 4.8)	0.0 (0.0 to 0.0)	8.8 (-28.5 to 34.8)	-11.4 (-41.8 to 9.8)
Adult tertiary syphilis	72.1 (62.1 to 83.1)	25.2 (21.8 to 28.3)*	2.0 (-0.8 to 4.5)	12.7 (8.4 to 17.6)	25.4 (19.0 to 32.0)*	2.2 (-3.1 to 7.6)
Chlamydial infection	2.6 (1.9 to 3.6)	15.4 (10.5 to 20.4)*	-6.0 (-10.0 to -1.9)*	0.2 (0.1 to 0.3)	8.7 (5.2 to 12.3)*	-11.5 (-14.3 to -8.5)*
Chlamydial infection episodes aggregate	1.3 (0.7 to 2.1)	23.9 (16.0 to 32.4)*	0.9 (-5.5 to 7.9)	0.0 (0.0 to 0.0)	25.1 (13.0 to 38.1)*	1.9 (-8.0 to 12.5)
Pelvic inflammatory diseases due to chlamydial infection aggregate	1.3 (0.9 to 1.9)	8.2 (4.7 to 11.8)*	-11.9 (-14.7 to -8.9)*	0.2 (0.1 to 0.3)	8.2 (4.7 to 11.8)*	-11.9 (-14.7 to -8.9)*
Infertility due to clamydial infection aggregate	0.0 (0.0 to 0.0)	0.0 (0.0 to 0.0)	0.0 (0.0 to 0.0)	0.0 (0.0 to 0.0)	0.0 (0.0 to 0.0)	0.0 (0.0 to 0.0)
Gonococcal infection	0.4 (0.3 to 0.6)	19.6 (7.7 to 33.2)*	-2.6 (-12.2 to 8.5)	0.0 (0.0 to 0.1)	14.7 (9.5 to 20.2)*	-6.6 (-10.8 to -2.0)*
Gonococcal infection episodes aggregate	0.1 (0.1 to 0.3)	30.4 (-5.1 to 54.3)	6.2 (-22.6 to 25.7)	0.0 (0.0 to 0.0)	27.9 (-0.3 to 45.2)	4.2 (-18.8 to 18.3)
Pelvic inflammatory diseases due to gonococcal infection aggregate	0.3 (0.2 to 0.4)	14.4 (9.1 to 20.1)*	-6.8 (-11.1 to -2.1)*	0.0 (0.0 to 0.1)	14.4 (9.1 to 20.1)*	-6.8 (-11.1 to -2.1)*
Trichomoniasis	1,584.7 (1,325.5 to 1,872.3)	22.8 (21.9 to 23.8)*	0.1 (-0.7 to 0.9)	1.8 (0.7 to 3.8)	22.2 (19.4 to 25.2)*	-0.4 (-2.7 to 2.0)
Genital herpes	31,900.2 (28,035.1 to 36,070.4)	22.5 (21.0 to 24.0)*	-0.2 (-1.4 to 1.1)	7.5 (2.2 to 18.3)	22.6 (20.6 to 24.7)*	-0.1 (-1.8 to 1.6)
Moderate infection due to initial genital herpes episode	0.0 (0.0 to 0.0)	0.0 (0.0 to 0.0)	0.0 (0.0 to 0.0)	0.0 (0.0 to 0.0)	0.0 (0.0 to 0.0)	0.0 (0.0 to 0.0)
Complications of genital herpes aggregate	31,900.2 (28,035.1 to 36,070.4)	22.5 (21.0 to 24.0)*	-0.2 (-1.4 to 1.1)	7.5 (2.2 to 18.3)	22.6 (20.6 to 24.7)*	-0.1 (-1.8 to 1.6)
Other sexually transmitted diseases	5.0 (3.5 to 6.8)	9.4 (7.4 to 11.4)*	-10.9 (-12.5 to -9.3)*	0.6 (0.4 to 1.0)	11.3 (0.3 to 23.3)*	-9.3 (-18.3 to 0.5)
Pelvic inflammatory diseases due to other sexually transmitted diseases aggregate	5.0 (3.5 to 6.8)	9.4 (7.4 to 11.4)*	-10.9 (-12.5 to -9.3)*	0.6 (0.4 to 1.0)	11.3 (0.3 to 23.3)*	-9.3 (-18.3 to 0.5)
Infertility due to other sexually transmitted diseases aggregate	0.0 (0.0 to 0.0)	0.0 (0.0 to 0.0)	0.0 (0.0 to 0.0)	0.0 (0.0 to 0.0)	0.0 (0.0 to 0.0)	0.0 (0.0 to 0.0)
Other sexually transmitted diseases	--	--	--	0.0 (0.0 to 0.0)	24.7 (13.2 to 37.2)*	1.6 (-7.7 to 11.8)
Hepatitis	12,197.8 (11,929.2 to 12,419.8)	22.4 (21.5 to 23.4)*	-0.3 (-1.0 to 0.5)	5.0 (3.1 to 7.9)	27.1 (-16.0 to 93.1)	3.6 (-31.5 to 57.3)
Acute hepatitis A	29.9 (24.1 to 34.7)	37.3 (6.7 to 73.6)*	11.9 (-13.1 to 41.5)	1.2 (0.7 to 1.8)	37.4 (4.1 to 79.7)*	11.9 (-15.1 to 46.5)
Hepatitis B	7,133.1 (6,888.9 to 7,392.6)	22.8 (20.8 to 24.8)*	0.1 (-1.5 to 1.7)	3.4 (1.9 to 6.0)	24.6 (-30.7 to 122.2)	1.5 (-43.5 to 81.0)
Acute Hepatitis B aggregate	157.9 (103.8 to 239.6)	24.4 (-30.8 to 118.9)	1.3 (-43.6 to 78.4)	3.4 (1.9 to 6.0)	24.6 (-30.7 to 122.2)	1.5 (-43.5 to 81.0)
Chronic hepatitis B	6,975.2 (6,740.6 to 7,218.0)	22.8 (21.8 to 23.8)*	0.0 (-0.7 to 0.9)	0.0 (0.0 to 0.0)	0.0 (0.0 to 0.0)	0.0 (0.0 to 0.0)
Hepatitis C	5,361.9 (4,785.2 to 5,943.4)	21.9 (20.0 to 23.7)*	-0.7 (-2.2 to 0.8)	0.1 (0.0 to 0.2)	22.9 (14.5 to 28.5)*	0.2 (-6.7 to 4.7)
Acute Hepatitis C aggregate	6.2 (4.2 to 8.4)	23.1 (14.6 to 28.8)*	0.3 (-6.6 to 4.9)	0.1 (0.0 to 0.2)	22.9 (14.5 to 28.5)*	0.2 (-6.7 to 4.7)
Chronic hepatitis C	5,355.7 (4,779.0 to 5,937.9)	21.9 (20.0 to 23.7)*	-0.7 (-2.2 to 0.8)	0.0 (0.0 to 0.0)	0.0 (0.0 to 0.0)	0.0 (0.0 to 0.0)
Acute hepatitis E	9.5 (5.7 to 15.9)	20.8 (18.8 to 22.5)*	-1.6 (-3.2 to -0.2)*	0.3 (0.1 to 0.6)	20.7 (-0.3 to 46.9)	-1.7 (-18.8 to 19.7)
Other infectious diseases	297.4 (283.8 to 313.0)	19.6 (10.6 to 28.7)*	-2.6 (-9.9 to 4.8)	8.2 (5.2 to 12.5)	19.7 (3.4 to 36.9)*	-2.5 (-15.7 to 11.6)
Non-communicable diseases	158,012.2 (157,984.6 to 158,038.8)	22.7 (22.7 to 22.7)*	0.0 (-0.0 to 0.0)	32,312.5 (24,372.1 to 41,357.0)	22.7 (22.1 to 23.4)*	0.0 (-0.5 to 0.5)
Neoplasms	10,349.9 (10,178.2 to 10,528.7)	41.9 (39.2 to 44.6)*	15.6 (13.4 to 17.8)*	981.5 (725.2 to 1,252.6)	32.5 (28.2 to 37.1)*	7.9 (4.4 to 11.7)*
Lip and oral cavity cancer	210.9 (191.1 to 234.0)	31.7 (20.8 to 44.6)*	7.3 (-1.6 to 17.8)	18.4 (13.1 to 23.9)	29.0 (17.1 to 42.6)*	5.1 (-4.6 to 16.2)
Nasopharynx cancer	43.6 (31.7 to 58.9)	18.8 (-6.4 to 59.8)	-3.2 (-23.8 to 30.2)	4.4 (2.9 to 6.5)	15.3 (-10.1 to 53.0)	-6.1 (-26.7 to 24.7)
Other pharynx cancer	91.8 (81.4 to 102.1)	30.3 (15.6 to 45.7)*	6.1 (-5.8 to 18.7)	7.8 (5.6 to 10.3)	27.3 (12.3 to 43.3)*	3.7 (-8.5 to 16.8)
Esophageal cancer	101.2 (86.2 to 137.1)	4.9 (-11.3 to 28.3)	-14.5 (-27.7 to 4.5)	16.9 (12.2 to 23.1)	-5.1 (-19.0 to 13.1)	-22.7 (-34.0 to -7.8)*
Stomach cancer	493.1 (458.7 to 530.7)	9.9 (1.4 to 19.5)*	-10.4 (-17.4 to -2.6)*	54.4 (40.2 to 69.3)	5.1 (-3.6 to 14.8)	-14.3 (-21.4 to -6.4)*
Colon and rectum cancer	1,235.4 (1,180.2 to 1,293.5)	31.0 (25.5 to 36.3)*	6.7 (2.2 to 11.0)*	98.2 (73.0 to 127.6)	26.5 (20.6 to 32.0)*	3.1 (-1.7 to 7.6)
Colon and rectum cancer aggregate	1,232.8 (1,177.5 to 1,290.9)	31.0 (25.5 to 36.3)*	6.8 (2.3 to 11.1)*	97.8 (72.8 to 127.1)	26.5 (20.6 to 32.1)*	3.1 (-1.7 to 7.6)
Stoma due to colon and rectum cancer	2.6 (2.3 to 2.9)	17.8 (14.9 to 20.7)*	-4.0 (-6.4 to -1.7)*	0.4 (0.2 to 0.5)	18.0 (13.9 to 21.9)*	-3.9 (-7.2 to -0.6)*
Liver cancer	84.8 (75.2 to 98.2)	41.2 (22.6 to 64.0)*	15.1 (-0.1 to 33.6)	21.0 (14.6 to 28.0)	17.7 (-0.1 to 40.2)	-4.1 (-18.6 to 14.2)
Liver cancer due to hepatitis B	22.4 (17.3 to 30.9)	14.8 (-11.0 to 49.2)	-6.4 (-27.5 to 21.5)	4.2 (2.8 to 5.9)	3.7 (-15.3 to 27.0)	-15.5 (-31.0 to 3.5)
Liver cancer due to hepatitis C	40.0 (31.2 to 53.3)	60.6 (25.6 to 112.8)*	30.9 (2.3 to 73.4)*	6.4 (4.5 to 9.0)	31.5 (9.1 to 61.7)*	7.2 (-11.1 to 31.8)
Liver cancer due to alcohol use	38.4 (31.6 to 48.8)	42.6 (17.2 to 75.7)*	16.2 (-4.5 to 43.2)	7.1 (4.9 to 9.4)	23.9 (6.8 to 45.7)*	1.0 (-13.0 to 18.7)
Liver cancer due to other causes	16.2 (12.4 to 25.3)	12.3 (-20.2 to 56.4)	-8.5 (-35.0 to 27.4)	3.2 (2.1 to 4.8)	2.9 (-19.9 to 30.6)	-16.1 (-34.7 to 6.4)
Gallbladder and biliary tract cancer	20.9 (18.9 to 22.9)	10.8 (2.0 to 20.4)*	-9.7 (-16.9 to -1.9)*	5.5 (3.8 to 7.3)	8.6 (-2.0 to 19.8)	-11.5 (-20.2 to -2.4)*
Pancreatic cancer	57.3 (53.4 to 61.7)	38.0 (30.4 to 46.6)*	12.4 (6.2 to 19.5)*	12.2 (8.6 to 15.8)	29.0 (19.5 to 39.2)*	5.1 (-2.6 to 13.4)

Cause	Prevalence in thousands (95% confidence interval)			YLDs in thousands (95% confidence interval)		
	2015	Percent change in counts between 2005 and 2015	Percent change in rates between 2005 and 2015	2015	Percent change in counts between 2005 and 2015	Percent change in rates between 2005 and 2015
Larynx cancer	142.6 (132.3 to 155.4)	17.4 (8.8 to 27.3)*	-4.4 (-11.3 to 3.7)	14.2 (10.3 to 18.4)	14.9 (6.4 to 24.8)*	-6.3 (-13.3 to 1.7)
Larynx cancer aggregate	103.9 (93.8 to 116.2)	14.1 (2.8 to 27.4)*	-7.0 (-16.2 to 3.8)	10.9 (7.9 to 14.1)	11.6 (1.2 to 24.0)*	-9.1 (-17.5 to 1.0)
Laryngectomy due to larynx cancer	38.6 (37.0 to 40.0)	27.0 (25.7 to 29.1)*	3.5 (2.4 to 5.2)*	3.4 (2.3 to 4.8)	27.1 (18.3 to 35.9)*	3.6 (-3.6 to 10.8)
Tracheal, bronchus, and lung cancer	470.5 (441.4 to 501.9)	24.0 (17.2 to 32.2)*	1.1 (-4.5 to 7.7)	71.6 (52.3 to 90.0)	17.3 (10.0 to 25.6)*	-4.4 (-10.4 to 2.4)
Malignant skin melanoma	298.0 (219.9 to 366.9)	77.8 (58.9 to 97.0)*	44.8 (29.5 to 60.5)*	17.5 (11.2 to 25.4)	72.8 (54.5 to 92.7)*	40.8 (25.9 to 57.0)*
Non-melanoma skin cancer	290.9 (276.7 to 306.2)	61.2 (52.8 to 70.6)*	31.4 (24.5 to 39.0)*	15.5 (10.8 to 21.1)	64.3 (48.3 to 80.8)*	33.9 (20.8 to 47.3)*
Non-melanoma skin cancer(squamous-cell carcinoma)	225.2 (201.1 to 255.4)	77.0 (60.6 to 95.9)*	44.2 (30.8 to 59.6)*	14.7 (10.3 to 19.9)	66.7 (49.6 to 85.0)*	35.9 (21.9 to 50.7)*
Non-melanoma skin cancer(basal-cell carcinoma)	73.5 (61.6 to 86.7)	28.0 (23.9 to 32.1)*	4.3 (0.9 to 7.6)*	0.8 (0.4 to 1.4)	28.0 (20.8 to 34.7)*	4.3 (-1.6 to 9.8)
Breast cancer	1,725.2 (1,649.8 to 1,800.2)	32.3 (27.8 to 36.9)*	7.8 (4.1 to 11.5)*	160.4 (113.8 to 214.1)	29.4 (24.6 to 34.3)*	5.4 (1.5 to 9.4)*
Breast cancer aggregate	369.3 (301.4 to 440.8)	106.8 (73.1 to 141.1)*	68.5 (41.1 to 96.5)*	59.1 (41.9 to 76.2)	48.5 (33.7 to 65.3)*	21.0 (8.9 to 34.6)*
Mastectomy due to breast cancer	1,354.7 (1,306.2 to 1,403.3)	20.4 (19.1 to 21.6)*	-1.9 (-3.0 to -0.9)*	101.3 (65.4 to 146.8)	20.4 (18.5 to 22.3)*	-1.9 (-3.4 to -0.4)*
Cervical cancer	176.1 (156.5 to 203.2)	1.4 (-11.0 to 15.7)	-17.4 (-27.5 to -5.7)*	14.0 (10.0 to 18.5)	-0.1 (-13.1 to 14.2)	-18.6 (-29.2 to -7.0)*
Uterine cancer	364.9 (309.2 to 434.0)	45.3 (25.4 to 68.5)*	18.4 (2.2 to 37.3)*	24.1 (16.7 to 32.6)	41.3 (21.9 to 65.8)*	15.2 (-0.7 to 35.1)
Ovarian cancer	99.9 (93.8 to 107.4)	25.2 (17.3 to 33.8)*	2.0 (-4.4 to 9.0)	12.8 (9.5 to 16.2)	22.1 (10.8 to 34.2)*	-0.6 (-9.7 to 9.3)
Prostate cancer	2,684.6 (2,111.8 to 3,785.9)	62.1 (49.8 to 75.5)*	32.1 (22.1 to 43.0)*	211.4 (143.2 to 307.7)	52.8 (41.9 to 64.6)*	24.5 (15.6 to 34.1)*
Prostate cancer aggregate	2,477.6 (1,908.2 to 3,579.3)	66.6 (53.0 to 81.2)*	35.8 (24.6 to 47.7)*	194.1 (132.0 to 286.8)	56.3 (44.1 to 69.6)*	27.4 (17.4 to 38.2)*
Impotence and incontinence due to prostate cancer aggregate	207.0 (202.2 to 211.7)	22.0 (21.5 to 22.6)*	-0.6 (-1.0 to -0.1)*	17.3 (11.8 to 23.7)	21.8 (18.6 to 25.0)*	-0.8 (-3.3 to 1.8)
Testicular cancer	9.3 (7.2 to 12.2)	71.8 (37.9 to 116.9)*	40.0 (12.3 to 76.7)*	0.7 (0.5 to 1.0)	59.3 (26.7 to 105.5)*	29.8 (3.3 to 67.5)*
Kidney cancer	355.3 (324.3 to 393.6)	58.9 (45.2 to 75.3)*	29.5 (18.3 to 42.8)*	24.9 (17.8 to 33.6)	54.2 (40.1 to 70.3)*	25.6 (14.2 to 38.7)*
Bladder cancer	487.0 (446.3 to 532.8)	20.7 (10.4 to 32.6)*	-1.7 (-10.1 to 8.1)	36.1 (25.9 to 47.6)	18.1 (8.0 to 29.8)*	-3.8 (-12.0 to 5.8)
Bladder cancer episodes aggregate	478.5 (437.3 to 524.3)	20.9 (10.3 to 33.1)*	-1.5 (-10.1 to 8.5)	34.7 (24.9 to 45.7)	18.4 (7.9 to 30.6)*	-3.5 (-12.1 to 6.4)
Urinary incontinence due to bladder cancer	8.5 (8.0 to 9.1)	10.4 (8.9 to 11.9)*	-10.1 (-11.3 to -8.8)*	1.4 (1.0 to 1.9)	11.1 (-0.2 to 23.7)	-9.5 (-18.6 to 0.8)
Brain and nervous system cancer	78.6 (70.0 to 92.2)	29.7 (16.8 to 45.5)*	5.7 (-4.8 to 18.6)	8.7 (6.2 to 11.4)	26.0 (12.4 to 42.6)*	2.7 (-8.4 to 16.2)
Thyroid cancer	363.2 (312.7 to 406.4)	188.8 (148.1 to 227.2)*	135.3 (102.1 to 166.6)*	22.8 (15.8 to 30.8)	169.7 (130.8 to 205.8)*	119.8 (88.1 to 149.1)*
Mesothelioma	8.8 (8.0 to 9.8)	43.8 (29.8 to 59.6)*	17.2 (5.8 to 30.0)*	1.8 (1.2 to 2.4)	41.7 (20.8 to 65.5)*	15.4 (-1.5 to 34.8)
Hodgkin lymphoma	32.6 (26.8 to 41.7)	37.6 (17.9 to 61.2)*	12.1 (-3.9 to 31.4)	3.1 (2.1 to 4.5)	26.1 (7.8 to 50.4)*	2.8 (-12.2 to 22.5)
Non-Hodgkin lymphoma	408.7 (339.4 to 458.8)	62.2 (48.8 to 76.3)*	32.2 (21.3 to 43.6)*	29.6 (21.0 to 39.4)	54.8 (41.3 to 68.4)*	26.1 (15.1 to 37.2)*
Multiple myeloma	65.6 (58.0 to 74.2)	49.6 (34.6 to 67.7)*	21.9 (9.7 to 36.6)*	13.5 (9.6 to 17.7)	42.9 (27.7 to 61.5)*	16.4 (4.1 to 31.6)*
Leukemia	222.0 (208.6 to 237.7)	38.6 (28.9 to 49.3)*	12.9 (5.0 to 21.7)*	33.4 (24.0 to 42.2)	30.2 (19.5 to 40.8)*	6.1 (-2.6 to 14.7)
Acute lymphoid leukemia	38.5 (33.1 to 44.4)	39.9 (25.2 to 57.4)*	14.0 (2.0 to 28.3)*	5.7 (4.0 to 7.5)	34.0 (17.9 to 53.5)*	9.2 (-4.0 to 25.0)
Chronic lymphoid leukemia	92.0 (82.1 to 103.9)	32.1 (20.4 to 45.7)*	7.7 (-1.9 to 18.8)	12.4 (8.9 to 16.2)	26.3 (11.8 to 42.4)*	2.9 (-8.9 to 16.0)
Acute myeloid leukemia	86.0 (75.7 to 96.8)	45.2 (33.9 to 57.9)*	18.3 (9.1 to 28.6)*	11.6 (8.3 to 15.0)	38.6 (25.4 to 52.5)*	12.9 (2.1 to 24.3)*
Chronic myeloid leukemia	25.3 (22.1 to 29.0)	20.4 (8.2 to 35.1)*	-1.9 (-11.8 to 10.0)	3.7 (2.6 to 4.8)	15.2 (1.0 to 32.6)*	-6.1 (-17.7 to 8.0)
Other neoplasms	371.5 (322.4 to 434.0)	37.6 (24.5 to 53.0)*	12.1 (1.4 to 24.7)*	26.6 (18.9 to 36.0)	33.6 (20.4 to 48.2)*	8.8 (-1.9 to 20.7)
Cardiovascular diseases	51,478.3 (49,839.1 to 52,656.5)	23.3 (22.2 to 24.5)*	0.5 (-0.4 to 1.4)	3,029.0 (2,204.4 to 3,967.0)	21.1 (20.2 to 21.9)*	-1.4 (-2.0 to -0.7)*
Rheumatic heart disease	448.8 (362.5 to 723.8)	-8.6 (-16.4 to -2.8)*	-25.6 (-31.9 to -20.8)*	27.5 (17.9 to 45.5)	-0.8 (-11.1 to 6.1)	-19.1 (-27.5 to -13.6)*
Rheumatic heart disease, without heart failure	331.9 (245.4 to 606.3)	-17.0 (-22.3 to -9.5)*	-32.4 (-36.7 to -26.2)*	14.8 (8.3 to 31.1)	-16.9 (-22.4 to -9.5)*	-32.3 (-36.7 to -26.2)*
Heart failure fue to rheumatic heart disease aggregate	116.9 (103.6 to 130.0)	28.0 (25.8 to 30.0)*	4.3 (2.5 to 5.9)*	12.7 (8.9 to 17.8)	28.1 (22.8 to 33.4)*	4.4 (0.0 to 8.7)*
Ischemic heart disease	16,084.8 (13,664.7 to 18,843.9)	23.9 (22.3 to 25.5)*	1.0 (-0.4 to 2.3)	961.2 (645.1 to 1,332.4)	24.5 (23.1 to 25.8)*	1.4 (0.3 to 2.5)*
Myocardial infarction episodes aggregate	3,393.1 (2,896.0 to 3,917.7)	21.0 (15.6 to 26.9)*	-1.4 (-5.8 to 3.4)	4.2 (2.9 to 5.8)	6.0 (0.2 to 11.6)*	-13.6 (-18.4 to -9.1)*
Angina due to ischemic heart disease aggregate	9,585.7 (7,244.9 to 12,242.6)	25.3 (23.6 to 26.9)*	2.1 (0.7 to 3.4)*	618.6 (383.6 to 902.5)	25.5 (23.5 to 27.4)*	2.3 (0.7 to 3.8)*
Heart failure fue to ischemic heart disease aggregate	3,105.9 (2,835.1 to 3,398.5)	23.0 (22.0 to 24.1)*	0.3 (-0.6 to 1.1)	338.3 (234.3 to 457.6)	23.0 (21.7 to 24.3)*	0.2 (-0.9 to 1.3)
Cerebrovascular disease	5,661.1 (5,560.1 to 5,723.1)	16.4 (15.3 to 17.5)*	-5.1 (-6.1 to -4.3)*	825.8 (573.1 to 1,090.6)	16.5 (15.0 to 18.0)*	-5.1 (-6.3 to -3.9)*
Ischemic stroke	3,944.5 (3,779.2 to 4,134.1)	15.2 (14.0 to 16.5)*	-6.1 (-7.1 to -5.1)*	561.9 (390.7 to 741.0)	15.4 (13.7 to 17.0)*	-6.0 (-7.3 to -4.6)*
Chronic ischemic stroke aggregate	3,906.6 (3,739.0 to 4,097.8)	15.3 (14.1 to 16.6)*	-6.0 (-7.0 to -5.0)*	554.2 (385.4 to 731.4)	15.5 (13.9 to 17.1)*	-5.9 (-7.2 to -4.6)*
Ischemic stroke episodes aggregate	37.9 (32.9 to 43.8)	6.8 (2.3 to 11.8)*	-13.0 (-16.6 to -8.9)*	7.7 (5.1 to 10.7)	8.4 (0.6 to 16.7)*	-11.7 (-18.0 to -4.9)*
Hemorrhagic stroke	1,852.7 (1,772.8 to 1,944.5)	18.9 (17.6 to 20.2)*	-3.2 (-4.2 to -2.1)*	263.9 (183.7 to 347.1)	18.9 (16.6 to 21.1)*	-3.1 (-5.0 to -1.4)*
Chronic hemorrhagic stroke aggregate	1,834.5 (1,754.4 to 1,925.7)	18.9 (17.7 to 20.3)*	-3.1 (-4.1 to -2.0)*	260.0 (181.0 to 341.7)	19.0 (16.8 to 21.2)*	-3.0 (-4.8 to -1.2)*
Acute hemorrhagic stroke aggregate	18.3 (15.7 to 21.7)	10.5 (5.1 to 15.3)*	-9.9 (-14.4 to -6.1)*	3.9 (2.5 to 5.5)	10.9 (4.7 to 16.6)*	-9.6 (-14.7 to -5.0)*
Hypertensive heart disease	803.5 (718.1 to 900.9)	24.5 (23.6 to 25.6)*	1.5 (0.7 to 2.3)*	87.6 (60.2 to 120.2)	24.6 (22.7 to 26.6)*	1.5 (-0.0 to 3.1)
Cardiomyopathy and myocarditis	136.3 (117.1 to 157.7)	20.4 (18.4 to 22.4)*	-1.9 (-3.5 to -0.3)*	14.3 (9.7 to 19.8)	20.0 (15.0 to 24.8)*	-2.3 (-6.3 to 1.7)
Acute myocarditis	8.4 (5.7 to 11.7)	25.6 (22.1 to 28.1)*	2.3 (-0.5 to 4.4)	0.4 (0.2 to 0.6)	25.6 (13.1 to 37.7)*	2.3 (-7.9 to 12.2)
Heart failure due to cardiomyopathy aggregate	128.0 (109.4 to 148.9)	20.1 (17.9 to 22.2)*	-2.2 (-3.9 to -0.5)*	13.9 (9.4 to 19.4)	19.8 (15.0 to 24.7)*	-2.4 (-6.3 to 1.6)
Atrial fibrillation and flutter	5,131.3 (4,383.1 to 6,072.1)	22.0 (21.1 to 23.0)*	-0.6 (-1.4 to 0.2)	401.1 (266.2 to 563.1)	22.1 (20.7 to 23.5)*	-0.5 (-1.7 to 0.6)
Peripheral vascular disease	24,064.9 (19,478.0 to 29,288.2)	26.4 (25.3 to 27.4)*	3.0 (2.1 to 3.8)*	115.9 (53.5 to 215.8)	25.3 (24.0 to 26.6)*	2.1 (1.1 to 3.1)*
Endocarditis	10.2 (9.1 to 11.6)	15.9 (12.8 to 18.9)*	-5.6 (-8.1 to -3.1)*	1.1 (0.7 to 1.6)	16.0 (1.9 to 30.7)*	-5.5 (-17.0 to 6.5)
Endocarditis episodes aggregate	1.2 (1.0 to 1.4)	20.0 (14.7 to 24.9)*	-2.2 (-6.5 to 1.8)	0.1 (0.0 to 0.1)	19.8 (14.6 to 24.7)*	-2.4 (-6.7 to 1.6)
Heart failure due to endocarditis aggregate	9.0 (7.9 to 10.4)	15.4 (11.9 to 18.8)*	-6.0 (-8.8 to -3.2)*	1.0 (0.7 to 1.5)	15.7 (0.9 to 31.2)*	-5.7 (-17.8 to 6.9)
Other cardiovascular and circulatory diseases	9,137.7 (8,067.7 to 10,397.0)	21.5 (20.1 to 23.0)*	-1.0 (-2.2 to 0.2)	594.5 (412.9 to 832.4)	21.6 (20.1 to 23.2)*	-0.9 (-2.2 to 0.4)
Heart failure due to other cardiovascular diseases aggregate	164.1 (142.1 to 189.0)	22.8 (21.1 to 24.5)*	0.1 (-1.3 to 1.4)	17.9 (12.3 to 25.1)	22.8 (18.7 to 27.0)*	0.1 (-3.3 to 3.5)
Other cardiovascular diseases episodes aggregate	8,973.6 (7,920.6 to 10,204.7)	21.5 (20.0 to 22.9)*	-1.0 (-2.2 to 0.2)	576.6 (399.9 to 803.7)	21.6 (20.0 to 23.2)*	-1.0 (-2.2 to 0.4)
Chronic respiratory diseases	27,188.1 (26,140.1 to 28,277.1)	16.4 (14.8 to 18.0)*	-5.2 (-6.5 to -3.9)*	1,799.2 (1,447.5 to 2,193.8)	13.9 (10.8 to 16.9)*	-7.2 (-9.8 to -4.7)*
Chronic obstructive pulmonary disease	17,250.0 (15,025.4 to 19,781.8)	14.5 (11.4 to 17.7)*	-6.7 (-9.2 to -4.1)*	1,117.3 (923.4 to 1,340.1)	13.3 (8.9 to 17.8)*	-7.7 (-11.2 to -4.0)*

Cause	Prevalence in thousands (95% confidence interval)			YLDs in thousands (95% confidence interval)		
	2015	Percent change in counts between 2005 and 2015	Percent change in rates between 2005 and 2015	2015	Percent change in counts between 2005 and 2015	Percent change in rates between 2005 and 2015
Chronic obstructive pulmonary disease without heart failure aggregate	16,082.8 (13,892.1 to 18,610.4)	14.0 (10.8 to 17.4)*	-7.1 (-9.8 to -4.4)*	643.9 (488.6 to 823.0)	7.0 (0.2 to 13.9)*	-12.8 (-18.4 to -7.2)*
Severe chronic obstructive pulmonary disease with heart failure aggregate	1,172.4 (1,018.9 to 1,303.7)	23.0 (18.1 to 25.4)*	0.3 (-3.8 to 2.2)	473.4 (362.9 to 566.6)	23.1 (17.8 to 26.0)*	0.3 (-4.0 to 2.7)
Pneumoconiosis	158.5 (152.9 to 165.3)	27.1 (23.1 to 31.3)*	3.6 (0.3 to 7.0)*	27.6 (17.8 to 40.2)	26.5 (21.9 to 31.3)*	3.0 (-0.7 to 7.0)
Silicosis	32.5 (27.5 to 37.3)	15.9 (12.6 to 19.1)*	-5.5 (-8.3 to -3.0)*	5.3 (3.3 to 7.8)	17.0 (8.6 to 25.1)*	-4.7 (-11.5 to 1.9)
Silicosis without heart failure aggregate	28.7 (23.9 to 33.5)	16.0 (12.4 to 19.4)*	-5.5 (-8.4 to -2.7)*	3.6 (2.1 to 5.6)	16.9 (6.4 to 28.4)*	-4.7 (-13.3 to 4.6)
Severe silicosis with heart failure aggregate	3.8 (3.2 to 4.3)	15.4 (11.4 to 18.6)*	-6.0 (-9.3 to -3.3)*	1.7 (1.2 to 2.3)	17.0 (4.2 to 30.5)*	-4.6 (-15.1 to 6.3)
Asbestosis	21.2 (18.9 to 23.5)	32.0 (29.8 to 34.3)*	7.6 (5.8 to 9.4)*	3.2 (2.1 to 4.6)	31.2 (22.3 to 41.4)*	6.9 (-0.4 to 15.2)
Asbestosis without heart failure aggregate	20.8 (18.4 to 23.1)	32.2 (30.0 to 34.6)*	7.7 (5.9 to 9.7)*	3.0 (1.9 to 4.3)	31.8 (22.3 to 43.1)*	7.4 (-0.3 to 16.6)
Severe asbestosis with heart failure aggregate	0.4 (0.4 to 0.5)	22.4 (20.7 to 24.1)*	-0.3 (-1.6 to 1.1)	0.2 (0.1 to 0.3)	22.4 (20.5 to 24.4)*	-0.3 (-1.8 to 1.3)
Coal workers pneumoconiosis	13.1 (11.3 to 14.9)	43.8 (39.9 to 48.2)*	17.2 (14.0 to 20.7)*	2.0 (1.2 to 2.9)	43.4 (21.3 to 68.0)*	16.8 (-1.2 to 36.9)
Coal workers pneumoconiosis without heart failure aggregate	12.8 (11.0 to 14.6)	44.0 (40.0 to 48.6)*	17.3 (14.1 to 21.1)*	1.8 (1.1 to 2.7)	44.2 (20.4 to 70.7)*	17.5 (-1.9 to 39.1)
Severe coal workers pneumoconiosis with heart failure aggregate	0.3 (0.2 to 0.3)	33.8 (27.8 to 40.1)*	9.1 (4.1 to 14.2)*	0.1 (0.1 to 0.2)	33.8 (27.8 to 40.1)*	9.0 (4.1 to 14.2)*
Other pneumoconiosis	117.4 (98.7 to 139.7)	27.2 (24.8 to 29.6)*	3.7 (1.7 to 5.6)*	17.1 (10.9 to 25.6)	27.1 (20.4 to 33.8)*	3.6 (-1.9 to 9.0)
Other pneumoconiosis without heart failure aggregate	115.3 (96.8 to 137.6)	27.4 (24.9 to 29.8)*	3.8 (1.7 to 5.7)*	16.1 (10.1 to 24.4)	27.5 (20.6 to 34.5)*	3.9 (-1.7 to 9.6)
Severe other pneumoconiosis with heart failure aggregate	2.2 (1.9 to 2.5)	20.5 (18.7 to 22.3)*	-1.8 (-3.3 to -0.3)*	1.0 (0.7 to 1.3)	20.6 (11.3 to 31.4)*	-1.7 (-9.3 to 7.1)
Asthma	12,000.5 (9,968.4 to 14,219.1)	17.3 (14.9 to 20.1)*	-4.4 (-6.4 to -2.1)*	500.2 (319.8 to 712.5)	17.3 (14.8 to 20.2)*	-4.4 (-6.5 to -2.0)*
Asymptomatic asthma	3,634.4 (2,863.4 to 4,427.8)	17.3 (14.9 to 20.1)*	-4.4 (-6.4 to -2.1)*	0.0 (0.0 to 0.0)	0.0 (0.0 to 0.0)	0.0 (0.0 to 0.0)
Symptomatic asthma aggregate	8,366.1 (6,840.6 to 9,915.6)	17.3 (14.9 to 20.1)*	-4.4 (-6.4 to -2.1)*	500.2 (319.8 to 712.5)	17.3 (14.8 to 20.2)*	-4.4 (-6.5 to -2.0)*
Interstitial lung disease and pulmonary sarcoidosis	198.0 (165.0 to 238.0)	25.7 (21.9 to 29.1)*	2.4 (-0.6 to 5.2)	23.8 (14.4 to 35.7)	25.4 (19.8 to 31.5)*	2.2 (-2.4 to 7.1)
Interstitial lung disease and pulmonary sarcoidosis without heart failure aggregate	167.6 (138.1 to 204.6)	26.2 (22.2 to 30.0)*	2.9 (-0.5 to 5.9)	10.9 (6.2 to 17.2)	28.5 (19.5 to 37.5)*	4.7 (-2.6 to 12.0)
Severe interstitial lung disease and pulmonary sarcoidosis with heart failure aggregate	30.4 (20.8 to 39.7)	23.0 (19.8 to 26.5)*	0.2 (-2.4 to 3.1)	12.9 (7.8 to 18.5)	23.0 (16.1 to 30.8)*	0.2 (-5.4 to 6.5)
Other chronic respiratory diseases	--	--	--	130.3 (104.4 to 159.8)	3.2 (-7.3 to 15.0)	-15.9 (-24.5 to -6.3)*
Cirrhosis and other chronic liver diseases	193.7 (187.5 to 199.4)	33.9 (29.8 to 37.8)*	9.1 (5.8 to 12.3)*	30.5 (21.1 to 41.6)	33.6 (29.7 to 38.2)*	8.9 (5.7 to 12.6)*
Cirrhosis and other chronic liver diseases due to hepatitis B	57.0 (47.6 to 67.7)	27.7 (23.4 to 31.5)*	4.1 (0.6 to 7.2)*	8.9 (5.9 to 12.7)	27.8 (19.9 to 35.2)*	4.2 (-2.3 to 10.2)
Cirrhosis and other chronic liver diseases due to hepatitis C	50.0 (43.5 to 57.4)	34.2 (30.2 to 37.9)*	9.3 (6.1 to 12.4)*	7.8 (5.3 to 10.8)	34.2 (25.3 to 42.7)*	9.4 (2.1 to 16.3)*
Cirrhosis and other chronic liver diseases due to alcohol use	48.4 (40.3 to 57.7)	41.4 (36.3 to 45.9)*	15.2 (11.0 to 18.9)*	7.5 (5.1 to 10.4)	41.4 (31.1 to 50.5)*	15.2 (6.8 to 22.6)*
Cirrhosis and other chronic liver diseases due to other causes	40.1 (33.6 to 46.5)	33.0 (29.6 to 36.2)*	8.4 (5.6 to 11.0)*	6.2 (4.3 to 8.9)	32.8 (24.0 to 42.0)*	8.2 (1.0 to 15.7)*
Digestive diseases	22,631.4 (22,293.5 to 22,977.1)	10.9 (8.8 to 12.9)*	-9.7 (-11.4 to -8.0)*	891.2 (617.2 to 1,235.7)	8.5 (6.2 to 10.5)*	-11.6 (-13.4 to -9.9)*
Peptic ulcer disease	7,545.7 (7,304.3 to 7,759.5)	4.2 (1.8 to 6.5)*	-15.1 (-17.0 to -13.2)*	234.7 (159.1 to 326.3)	1.5 (-1.7 to 4.3)	-17.3 (-19.9 to -15.0)*
Peptic ulcer disease, symptomatic episodes	533.7 (419.5 to 671.8)	4.5 (-1.5 to 9.8)	-14.9 (-19.8 to -10.5)*	54.1 (33.8 to 77.1)	4.6 (-1.6 to 9.9)	-14.8 (-19.8 to -10.4)*
Anemia due to peptic ulcer disease aggregate	7,012.0 (6,799.2 to 7,173.4)	4.2 (1.6 to 6.5)*	-15.1 (-17.2 to -13.2)*	180.6 (122.4 to 257.3)	0.6 (-3.1 to 3.7)	-18.0 (-21.1 to -15.5)*
Gastritis and duodenitis	15,129.2 (14,859.1 to 15,403.1)	11.9 (9.6 to 14.2)*	-8.9 (-10.7 to -6.9)*	402.4 (271.9 to 566.1)	6.3 (3.2 to 9.4)*	-13.4 (-15.9 to -10.8)*
Gastritis and duodenitis, symptomatic episodes	393.0 (306.1 to 485.6)	9.1 (4.5 to 11.9)*	-11.1 (-14.8 to -8.8)*	40.0 (26.4 to 56.4)	9.3 (4.3 to 12.8)*	-10.9 (-15.0 to -8.1)*
Anemia due to gastritis and duodenitis aggregate	14,736.2 (14,478.0 to 14,988.8)	11.9 (9.6 to 14.4)*	-8.8 (-10.7 to -6.8)*	362.4 (244.0 to 516.4)	6.0 (2.5 to 9.4)*	-13.7 (-16.5 to -10.8)*
Appendicitis	11.0 (9.2 to 12.8)	21.7 (19.8 to 23.3)*	-0.8 (-2.4 to 0.5)	3.2 (2.1 to 4.4)	21.7 (12.1 to 31.7)*	-0.9 (-8.7 to 7.3)
Paralytic ileus and intestinal obstruction	6.7 (5.3 to 8.5)	21.4 (15.8 to 27.0)*	-1.1 (-5.6 to 3.5)	2.0 (1.2 to 2.9)	22.0 (8.2 to 37.1)*	-0.6 (-11.8 to 11.7)
Inguinal, femoral, and abdominal hernia	916.7 (641.9 to 1,247.5)	8.9 (3.5 to 14.5)*	-11.2 (-15.7 to -6.7)*	9.3 (4.2 to 17.9)	9.1 (3.6 to 14.7)*	-11.1 (-15.6 to -6.5)*
Inflammatory bowel disease	502.5 (466.0 to 549.3)	19.2 (18.1 to 19.9)*	-2.9 (-3.7 to -2.3)*	100.8 (70.2 to 136.9)	19.2 (17.0 to 21.6)*	-2.9 (-4.7 to -0.9)*
Vascular intestinal disorders	3.5 (2.8 to 4.4)	21.8 (14.6 to 27.6)*	-0.8 (-6.6 to 4.0)	1.1 (0.7 to 1.6)	22.0 (11.5 to 32.0)*	-0.6 (-9.1 to 7.5)
Gallbladder and biliary diseases	306.9 (235.5 to 390.3)	18.1 (11.6 to 25.5)*	-3.8 (-9.0 to 2.3)	31.2 (19.6 to 46.1)	18.2 (11.2 to 25.6)*	-3.7 (-9.4 to 2.4)
Pancreatitis	64.8 (51.7 to 83.3)	36.9 (29.8 to 43.7)*	11.5 (5.8 to 17.1)*	18.0 (11.6 to 26.2)	37.2 (26.6 to 47.6)*	11.8 (3.2 to 20.3)*
Other digestive diseases	--	--	--	88.5 (60.9 to 122.0)	20.0 (11.6 to 30.8)*	-2.2 (-9.0 to 6.6)
Neurological disorders	42,575.4 (39,685.7 to 44,441.2)	23.6 (21.0 to 26.2)*	0.7 (-1.4 to 2.9)	1,881.3 (1,321.7 to 2,557.1)	22.5 (20.4 to 24.8)*	-0.2 (-1.9 to 1.7)
Alzheimer disease and other dementias	6,138.0 (4,785.5 to 7,587.8)	24.9 (23.8 to 25.9)*	1.8 (0.9 to 2.5)*	865.1 (576.0 to 1,230.1)	25.0 (23.6 to 26.2)*	1.8 (0.7 to 2.8)*
Parkinson disease	1,008.8 (887.3 to 1,139.7)	20.5 (15.4 to 25.4)*	-1.8 (-5.9 to 2.2)	118.6 (81.1 to 161.4)	20.5 (15.3 to 25.9)*	-1.8 (-6.1 to 2.6)
Epilepsy	625.8 (542.2 to 729.5)	26.2 (19.4 to 32.5)*	2.8 (-2.7 to 8.0)	134.7 (92.8 to 181.5)	2.4 (-4.6 to 9.7)	-16.6 (-22.3 to -10.6)*
Multiple sclerosis	84.6 (77.4 to 92.3)	27.2 (24.1 to 30.0)*	3.7 (1.1 to 5.9)*	26.5 (18.9 to 33.9)	27.0 (20.3 to 34.3)*	3.5 (-2.0 to 9.5)
Motor neuron disease	20.7 (17.3 to 24.5)	24.8 (17.0 to 32.2)*	1.7 (-4.7 to 7.7)	4.2 (2.9 to 5.7)	24.6 (15.8 to 34.7)*	1.5 (-5.7 to 9.7)
Migraine	13,329.6 (10,950.0 to 16,073.1)	23.8 (21.3 to 26.2)*	0.9 (-1.2 to 2.8)	419.5 (249.1 to 645.1)	23.9 (21.0 to 26.7)*	1.0 (-1.4 to 3.2)
Tension-type headache	24,942.0 (17,303.7 to 34,643.5)	23.5 (20.7 to 26.3)*	0.6 (-1.7 to 2.9)	35.5 (15.5 to 69.5)	23.6 (20.1 to 26.8)*	0.7 (-2.2 to 3.3)
Medication overuse headache	1,544.6 (971.5 to 2,282.8)	25.3 (13.2 to 40.0)*	2.1 (-7.8 to 14.0)	227.2 (127.3 to 373.9)	25.5 (13.2 to 40.3)*	2.2 (-7.7 to 14.3)
Other neurological disorders	0.5 (0.3 to 0.8)	21.5 (17.4 to 24.4)*	-1.0 (-4.4 to 1.4)	49.8 (34.7 to 67.7)	23.3 (18.7 to 27.4)*	0.4 (-3.3 to 3.8)
Mental and substance use disorders	22,312.9 (21,710.6 to 22,937.0)	23.0 (21.7 to 24.2)*	0.2 (-0.8 to 1.2)	3,154.3 (2,299.1 to 4,084.7)	23.3 (22.3 to 24.2)*	0.4 (-0.3 to 1.2)
Schizophrenia	514.2 (453.9 to 582.5)	23.8 (23.0 to 24.7)*	0.9 (0.2 to 1.6)*	303.8 (226.8 to 378.4)	23.9 (21.8 to 26.2)*	1.0 (-0.8 to 2.8)
Alcohol use disorders	879.6 (722.1 to 1,066.3)	25.3 (20.9 to 29.5)*	2.1 (-1.5 to 5.6)	81.7 (53.0 to 119.1)	25.4 (20.2 to 30.3)*	2.2 (-2.0 to 6.1)
Alcohol dependence aggregate	867.6 (710.1 to 1,054.0)	25.4 (20.9 to 29.7)*	2.2 (-1.5 to 5.6)	81.2 (52.5 to 118.4)	25.5 (20.2 to 30.3)*	2.2 (-2.0 to 6.2)
Fetal alcohol syndrome aggregate	12.0 (10.6 to 13.6)	21.2 (19.6 to 22.8)*	-1.2 (-2.5 to 0.0)	0.6 (0.4 to 0.9)	19.5 (16.0 to 22.4)*	-2.6 (-5.5 to -0.3)*
Drug use disorders	466.5 (447.7 to 490.6)	38.9 (35.7 to 42.2)*	13.2 (10.5 to 15.9)*	122.6 (85.4 to 161.4)	39.8 (35.1 to 44.5)*	13.9 (10.1 to 17.7)*
Opioid use disorders	197.1 (159.2 to 237.0)	36.9 (33.4 to 40.6)*	11.5 (8.7 to 14.6)*	74.6 (51.0 to 100.2)	37.2 (31.3 to 43.1)*	11.8 (7.0 to 16.6)*
Asymptomatic opioid dependence	32.0 (22.6 to 44.2)	36.8 (33.4 to 40.6)*	11.5 (8.7 to 14.6)*	0.0 (0.0 to 0.0)	0.0 (0.0 to 0.0)	0.0 (0.0 to 0.0)
Symptomatic opioid dependence aggregate	165.2 (131.9 to 201.8)	36.9 (33.4 to 40.6)*	11.5 (8.7 to 14.6)*	74.6 (51.0 to 100.2)	37.2 (31.3 to 43.1)*	11.8 (7.0 to 16.6)*
Cocaine use disorders	205.4 (163.0 to 253.0)	42.7 (40.0 to 45.5)*	16.3 (14.1 to 18.6)*	25.7 (16.0 to 37.6)	42.6 (36.1 to 50.4)*	16.2 (10.9 to 22.6)*
Asymptomatic cocaine dependence	102.9 (77.9 to 133.3)	42.7 (40.0 to 45.5)*	16.3 (14.1 to 18.6)*	0.0 (0.0 to 0.0)	0.0 (0.0 to 0.0)	0.0 (0.0 to 0.0)

Cause	Prevalence in thousands (95% confidence interval)			YLDs in thousands (95% confidence interval)		
	2015	Percent change in counts between 2005 and 2015	Percent change in rates between 2005 and 2015	2015	Percent change in counts between 2005 and 2015	Percent change in rates between 2005 and 2015
Symptomatic cocaine dependence aggregate	102.5 (76.4 to 132.6)	42.7 (40.0 to 45.5)*	16.3 (14.1 to 18.6)*	25.7 (16.0 to 37.6)	42.6 (36.1 to 50.4)*	16.2 (10.9 to 22.6)*
Amphetamine use disorders	17.7 (13.9 to 22.2)	83.8 (76.2 to 92.6)*	49.7 (43.6 to 56.9)*	2.3 (1.4 to 3.6)	77.4 (61.2 to 95.6)*	44.5 (31.3 to 59.3)*
Asymptomatic amphetamine dependence	9.7 (7.2 to 12.9)	83.8 (76.2 to 92.6)*	49.8 (43.6 to 56.9)*	0.0 (0.0 to 0.0)	0.0 (0.0 to 0.0)	0.0 (0.0 to 0.0)
Symptomatic amphetamine dependence aggregate	8.0 (5.8 to 10.7)	83.8 (76.2 to 92.6)*	49.7 (43.6 to 56.9)*	2.3 (1.4 to 3.6)	77.4 (61.2 to 95.6)*	44.5 (31.3 to 59.3)*
Cannabis use disorders	58.7 (52.6 to 65.6)	21.4 (20.0 to 23.0)*	-1.1 (-2.3 to 0.2)	1.7 (1.1 to 2.4)	21.4 (15.6 to 26.9)*	-1.1 (-5.8 to 3.4)
Asymptomatic cannabis dependence	34.0 (29.7 to 38.6)	21.4 (20.0 to 23.0)*	-1.1 (-2.3 to 0.2)	0.0 (0.0 to 0.0)	0.0 (0.0 to 0.0)	0.0 (0.0 to 0.0)
Symptomatic cannabis dependence aggregate	24.7 (21.3 to 28.7)	21.4 (20.0 to 23.0)*	-1.1 (-2.3 to 0.2)	1.7 (1.1 to 2.4)	21.4 (15.6 to 26.9)*	-1.1 (-5.8 to 3.4)
Other drug use disorders	--	--	--	18.2 (11.3 to 26.7)	45.1 (37.6 to 53.5)*	18.2 (12.1 to 25.1)*
Depressive disorders	10,274.4 (9,764.9 to 10,756.5)	22.1 (20.1 to 24.1)*	-0.5 (-2.1 to 1.1)	1,592.7 (1,078.8 to 2,174.4)	21.9 (20.9 to 23.1)*	-0.7 (-1.5 to 0.3)
Major depressive disorder	6,256.8 (5,217.9 to 7,349.5)	21.3 (20.2 to 22.4)*	-1.2 (-2.0 to -0.3)*	1,191.5 (815.6 to 1,679.1)	21.4 (20.1 to 22.8)*	-1.1 (-2.1 to 0.0)
Dysthymia	4,390.5 (3,579.6 to 5,322.1)	23.3 (21.7 to 24.9)*	0.5 (-0.8 to 1.7)	401.2 (260.1 to 592.8)	23.4 (21.5 to 25.1)*	0.5 (-1.0 to 2.0)
Bipolar disorder	692.3 (568.4 to 836.8)	22.3 (21.6 to 23.0)*	-0.3 (-0.9 to 0.2)	130.3 (79.5 to 192.2)	22.4 (19.6 to 25.0)*	-0.3 (-2.5 to 1.9)
Anxiety disorders	6,078.0 (5,033.2 to 7,397.8)	23.8 (21.0 to 26.5)*	0.9 (-1.4 to 3.1)	520.7 (342.3 to 742.0)	23.8 (20.7 to 26.7)*	0.9 (-1.7 to 3.2)
Eating disorders	0.0 (0.0 to 0.0)	0.0 (0.0 to 0.0)	0.0 (0.0 to 0.0)	0.0 (0.0 to 0.0)	0.0 (0.0 to 0.0)	0.0 (0.0 to 0.0)
Anorexia nervosa	0.0 (0.0 to 0.0)	0.0 (0.0 to 0.0)	0.0 (0.0 to 0.0)	0.0 (0.0 to 0.0)	0.0 (0.0 to 0.0)	0.0 (0.0 to 0.0)
Bulimia nervosa	0.0 (0.0 to 0.0)	0.0 (0.0 to 0.0)	0.0 (0.0 to 0.0)	0.0 (0.0 to 0.0)	0.0 (0.0 to 0.0)	0.0 (0.0 to 0.0)
Autistic spectrum disorders	1,044.4 (994.3 to 1,096.6)	23.3 (20.9 to 25.8)*	0.5 (-1.5 to 2.5)	157.3 (107.6 to 212.0)	23.4 (21.7 to 25.1)*	0.5 (-0.9 to 1.9)
Autism	426.2 (356.7 to 514.9)	23.5 (23.0 to 23.9)*	0.6 (0.3 to 1.0)*	100.2 (65.1 to 141.3)	23.6 (21.1 to 26.0)*	0.7 (-1.3 to 2.7)
Asperger syndrome and other autistic spectrum disorders	612.0 (517.7 to 733.6)	23.0 (23.0 to 23.0)*	0.2 (0.2 to 0.3)*	57.1 (38.6 to 82.1)	23.1 (21.2 to 25.0)*	0.3 (-1.3 to 1.8)
Attention-deficit/hyperactivity disorder	0.1 (0.0 to 0.1)	55.5 (22.5 to 95.2)*	26.7 (-0.2 to 59.1)	0.0 (0.0 to 0.0)	55.5 (22.5 to 95.2)*	26.7 (-0.2 to 59.1)
Conduct disorder	0.0 (0.0 to 0.0)	0.0 (0.0 to 0.0)	0.0 (0.0 to 0.0)	0.0 (0.0 to 0.0)	0.0 (0.0 to 0.0)	0.0 (0.0 to 0.0)
Idiopathic developmental intellectual disability	730.2 (318.2 to 1,143.6)	21.5 (18.4 to 23.6)*	-1.0 (-3.6 to 0.7)	31.4 (13.3 to 55.1)	19.9 (14.8 to 23.1)*	-2.3 (-6.5 to 0.3)
Other mental and substance use disorders	3,048.5 (2,958.2 to 3,137.1)	22.8 (18.1 to 27.7)*	0.1 (-3.8 to 4.0)	213.7 (151.5 to 282.9)	22.9 (18.1 to 27.9)*	0.1 (-3.7 to 4.2)
Diabetes, urogenital, blood, and endocrine diseases	99,023.8 (97,872.1 to 100,453.4)	22.9 (22.2 to 23.5)*	0.1 (-0.5 to 0.6)	4,074.9 (2,872.1 to 5,414.7)	27.3 (25.2 to 29.1)*	3.7 (2.0 to 5.2)*
Diabetes mellitus	31,950.4 (29,336.0 to 34,875.0)	28.3 (25.5 to 30.9)*	4.6 (2.3 to 6.7)*	2,588.0 (1,789.2 to 3,557.2)	29.7 (26.5 to 32.6)*	5.7 (3.1 to 8.0)*
Uncomplicated diabetes mellitus	17,319.7 (13,976.4 to 20,846.7)	26.0 (22.3 to 29.1)*	2.7 (-0.3 to 5.2)	774.9 (482.9 to 1,137.1)	26.0 (22.4 to 29.0)*	2.6 (-0.3 to 5.1)
Neuropathy and other complications of diabetes mellitus aggregate	14,227.7 (11,194.2 to 17,700.9)	31.3 (27.4 to 35.1)*	7.0 (3.8 to 10.1)*	1,786.0 (1,166.9 to 2,590.4)	31.4 (27.6 to 35.0)*	7.0 (3.9 to 10.0)*
Vision loss due to diabetes mellitus aggregate	403.0 (271.9 to 581.6)	28.3 (24.9 to 31.3)*	4.5 (1.8 to 7.0)*	27.2 (17.0 to 39.8)	29.4 (24.9 to 33.8)*	5.4 (1.8 to 9.0)*
Acute glomerulonephritis	4.3 (3.0 to 5.6)	10.0 (4.2 to 14.8)*	-10.4 (-15.1 to -6.5)*	0.2 (0.1 to 0.3)	11.1 (-4.5 to 28.2)	-9.4 (-22.2 to 4.4)
Chronic kidney disease	33,022.4 (30,162.0 to 35,664.8)	25.3 (22.5 to 28.1)*	2.1 (-0.2 to 4.4)	627.5 (459.9 to 823.0)	24.8 (23.4 to 26.4)*	1.7 (0.5 to 3.0)*
Chronic kidney disease due to diabetes mellitus	9,680.4 (6,382.5 to 13,787.3)	26.3 (22.4 to 30.3)*	2.9 (-0.3 to 6.2)	166.4 (106.0 to 239.9)	26.0 (22.4 to 29.7)*	2.7 (-0.2 to 5.7)
Stage III chronic kidney disease due to diabetes mellitus aggregate	8,943.1 (5,865.5 to 12,834.5)	26.4 (22.4 to 30.4)*	3.0 (-0.3 to 6.3)	8.1 (5.2 to 12.5)	26.4 (18.1 to 35.2)*	3.0 (-3.8 to 10.2)
Stage IV chronic kidney disease due to diabetes mellitus aggregate	515.6 (318.8 to 760.1)	25.6 (22.1 to 28.9)*	2.3 (-0.5 to 5.0)	57.1 (30.9 to 91.6)	25.4 (21.6 to 29.1)*	2.1 (-0.9 to 5.2)
Stage V chronic kidney disease untreated due to diabetes mellitus	175.5 (117.2 to 257.0)	25.9 (22.5 to 29.3)*	2.6 (-0.1 to 5.3)	83.7 (48.6 to 130.7)	25.9 (21.3 to 31.1)*	2.6 (-1.2 to 6.8)
End-stage chronic kidney disease due to diabetes aggregate	46.2 (31.6 to 63.1)	26.5 (21.9 to 31.5)*	3.0 (-0.7 to 7.2)	17.5 (10.0 to 26.6)	28.5 (20.5 to 37.5)*	4.7 (-1.8 to 12.0)
Chronic kidney disease due to hypertension	7,345.4 (4,623.5 to 10,884.7)	27.6 (23.3 to 31.7)*	4.0 (0.5 to 7.3)*	149.6 (103.8 to 203.7)	23.7 (19.8 to 28.1)*	0.8 (-2.4 to 4.4)
Stage III chronic kidney disease due to hypertension aggregate	6,698.3 (4,056.0 to 10,077.2)	27.9 (23.2 to 32.4)*	4.2 (0.4 to 7.9)*	9.0 (5.9 to 13.7)	14.7 (6.8 to 23.0)*	-6.6 (-13.0 to 0.2)
Stage IV chronic kidney disease due to hypertension aggregate	449.4 (307.2 to 648.5)	24.4 (19.1 to 29.9)*	1.4 (-3.0 to 5.9)	50.6 (30.1 to 80.1)	24.1 (18.4 to 30.0)*	1.1 (-3.6 to 5.9)
Stage V chronic kidney disease untreated due to hypertension	158.0 (114.8 to 219.0)	23.9 (19.4 to 28.4)*	0.9 (-2.7 to 4.7)	75.2 (48.3 to 109.9)	24.1 (18.5 to 30.4)*	1.1 (-3.4 to 6.3)
End-stage chronic kidney disease due to hypertension aggregate	39.6 (28.6 to 52.6)	24.4 (18.4 to 31.0)*	1.3 (-3.5 to 6.7)	14.8 (9.1 to 21.7)	26.1 (17.2 to 35.9)*	2.8 (-4.5 to 10.7)
Chronic kidney disease due to glomerulonephritis	8,484.6 (5,998.1 to 11,522.4)	24.8 (20.1 to 29.8)*	1.7 (-2.1 to 5.7)	124.4 (78.2 to 182.8)	26.8 (22.4 to 31.2)*	3.3 (-0.2 to 6.9)
Stage III chronic kidney disease due to glomerulonephritis aggregate	7,925.0 (5,459.5 to 10,882.3)	24.6 (19.7 to 29.7)*	1.6 (-2.5 to 5.7)	7.8 (5.1 to 11.6)	17.3 (5.3 to 28.8)*	-4.5 (-14.2 to 5.0)
Stage IV chronic kidney disease due to glomerulonephritis aggregate	402.6 (249.9 to 604.4)	26.8 (20.0 to 33.9)*	3.3 (-2.3 to 9.1)	44.4 (24.0 to 71.6)	27.0 (20.3 to 34.4)*	3.5 (-2.0 to 9.5)
Stage V chronic kidney disease untreated due to glomerulonephritis	133.3 (81.0 to 200.0)	27.9 (24.1 to 32.2)*	4.2 (1.1 to 7.7)*	63.2 (35.4 to 102.9)	27.9 (21.8 to 34.8)*	4.2 (-0.7 to 9.8)
End-stage chronic kidney disease due to glomerulonephritis aggregate	23.6 (14.5 to 35.3)	25.4 (20.1 to 30.9)*	2.2 (-2.1 to 6.7)	9.0 (5.0 to 14.5)	27.3 (15.8 to 41.4)*	3.7 (-5.7 to 15.2)
Chronic kidney disease due to other causes	10,852.5 (7,111.5 to 15,420.3)	24.5 (21.3 to 27.8)*	1.4 (-1.2 to 4.1)	187.0 (122.9 to 265.9)	23.4 (20.6 to 26.3)*	0.6 (-1.8 to 2.9)
Stage III chronic kidney disease due to other causes aggregate	10,044.0 (6,504.0 to 14,469.1)	24.5 (21.1 to 28.0)*	1.4 (-1.3 to 4.3)	13.6 (8.9 to 20.2)	14.7 (6.1 to 23.6)*	-6.5 (-13.6 to 0.7)
Stage IV chronic kidney disease due to other causes aggregate	570.2 (354.2 to 860.9)	24.1 (21.7 to 26.6)*	1.1 (-0.9 to 3.1)	64.1 (36.5 to 106.9)	23.5 (20.0 to 26.7)*	0.6 (-2.2 to 3.2)
Stage V chronic kidney disease untreated due to other causes	195.8 (132.2 to 278.7)	24.3 (21.7 to 27.1)*	1.3 (-0.8 to 3.6)	93.2 (56.7 to 141.4)	24.6 (20.5 to 28.9)*	1.6 (-1.8 to 5.1)
End-stage chronic kidney disease due to other causes aggregate	42.5 (28.5 to 59.4)	23.0 (18.8 to 27.4)*	0.2 (-3.2 to 3.8)	16.1 (9.7 to 25.1)	24.5 (16.4 to 33.6)*	1.4 (-5.2 to 8.9)
Urinary diseases and male infertility	32,795.9 (31,754.9 to 33,917.0)	23.2 (21.3 to 25.1)*	0.4 (-1.2 to 1.9)	673.8 (427.1 to 985.2)	23.8 (21.4 to 26.2)*	0.9 (-1.1 to 2.8)
Interstitial nephritis and urinary tract infections	106.0 (99.4 to 113.4)	29.4 (28.4 to 30.4)*	5.4 (4.6 to 6.2)*	3.4 (2.1 to 5.0)	29.5 (22.9 to 36.3)*	5.5 (0.2 to 11.0)*
Urolithiasis	17,937.0 (16,096.2 to 19,800.6)	23.1 (21.3 to 25.1)*	0.3 (-1.2 to 2.0)	3.8 (2.5 to 5.7)	39.2 (28.5 to 50.5)*	13.4 (4.7 to 22.6)*
Benign prostatic hyperplasia	18,568.6 (15,098.4 to 22,073.9)	23.6 (21.4 to 26.0)*	0.7 (-1.1 to 2.6)	664.1 (420.4 to 972.8)	23.7 (21.3 to 26.1)*	0.8 (-1.2 to 2.7)
Male infertility	0.0 (0.0 to 0.0)	0.0 (0.0 to 0.0)	0.0 (0.0 to 0.0)	0.0 (0.0 to 0.0)	0.0 (0.0 to 0.0)	0.0 (0.0 to 0.0)
Other urinary diseases	--	--	--	2.5 (1.6 to 3.6)	24.5 (14.3 to 37.1)*	1.4 (-6.8 to 11.7)
Gynecological diseases	12,188.5 (11,791.1 to 12,551.7)	16.9 (14.8 to 19.1)*	-4.8 (-6.4 to -3.0)*	46.7 (23.5 to 81.6)	17.6 (15.3 to 19.6)*	-4.2 (-6.1 to -2.6)*
Uterine fibroids	2,040.9 (1,943.1 to 2,149.0)	21.6 (21.2 to 22.0)*	-0.9 (-1.2 to -0.6)*	14.3 (7.2 to 26.7)	21.7 (20.2 to 23.0)*	-0.9 (-2.1 to 0.2)
Uterine fibroids cases aggregate	2,040.9 (1,943.1 to 2,149.0)	21.6 (21.2 to 22.0)*	-0.9 (-1.2 to -0.6)*	14.3 (7.2 to 26.7)	21.7 (20.2 to 23.0)*	-0.9 (-2.1 to 0.2)
Mild abdominal pain with anemia due to uterine fibroids aggregate	0.0 (0.0 to 0.0)	0.0 (0.0 to 0.0)	0.0 (0.0 to 0.0)	0.0 (0.0 to 0.0)	0.0 (0.0 to 0.0)	0.0 (0.0 to 0.0)
Polycystic ovarian syndrome	0.0 (0.0 to 0.0)	0.0 (0.0 to 0.0)	0.0 (0.0 to 0.0)	0.0 (0.0 to 0.0)	0.0 (0.0 to 0.0)	0.0 (0.0 to 0.0)
Polycystic ovarian syndrome cases aggregate	0.0 (0.0 to 0.0)	0.0 (0.0 to 0.0)	0.0 (0.0 to 0.0)	0.0 (0.0 to 0.0)	0.0 (0.0 to 0.0)	0.0 (0.0 to 0.0)
Hirsutism and infertility due to polycystic ovarian syndrome aggregate	0.0 (0.0 to 0.0)	0.0 (0.0 to 0.0)	0.0 (0.0 to 0.0)	0.0 (0.0 to 0.0)	0.0 (0.0 to 0.0)	0.0 (0.0 to 0.0)

Cause	Prevalence in thousands (95% confidence interval)			YLDs in thousands (95% confidence interval)		
	2015	Percent change in counts between 2005 and 2015	Percent change in rates between 2005 and 2015	2015	Percent change in counts between 2005 and 2015	Percent change in rates between 2005 and 2015
Female infertility	0.0 (0.0 to 0.0)	0.0 (0.0 to 0.0)	0.0 (0.0 to 0.0)	0.0 (0.0 to 0.0)	0.0 (0.0 to 0.0)	0.0 (0.0 to 0.0)
Endometriosis	0.0 (0.0 to 0.0)	0.0 (0.0 to 0.0)	0.0 (0.0 to 0.0)	0.0 (0.0 to 0.0)	0.0 (0.0 to 0.0)	0.0 (0.0 to 0.0)
Endometriosis cases aggregate	0.0 (0.0 to 0.0)	0.0 (0.0 to 0.0)	0.0 (0.0 to 0.0)	0.0 (0.0 to 0.0)	0.0 (0.0 to 0.0)	0.0 (0.0 to 0.0)
Abdominal pain and infertility due to endometriosis aggregate	0.0 (0.0 to 0.0)	0.0 (0.0 to 0.0)	0.0 (0.0 to 0.0)	0.0 (0.0 to 0.0)	0.0 (0.0 to 0.0)	0.0 (0.0 to 0.0)
Genital prolapse	10,734.0 (9,383.2 to 12,386.1)	15.7 (12.7 to 18.2)*	-5.7 (-8.2 to -3.7)*	32.4 (15.6 to 57.9)	15.9 (12.8 to 18.6)*	-5.6 (-8.1 to -3.3)*
Premenstrual syndrome	0.0 (0.0 to 0.0)	0.0 (0.0 to 0.0)	0.0 (0.0 to 0.0)	0.0 (0.0 to 0.0)	0.0 (0.0 to 0.0)	0.0 (0.0 to 0.0)
Other gynecological diseases	0.0 (0.0 to 0.0)	0.0 (0.0 to 0.0)	0.0 (0.0 to 0.0)	0.0 (0.0 to 0.0)	0.0 (0.0 to 0.0)	0.0 (0.0 to 0.0)
Other gynecological diseases cases aggregate	0.0 (0.0 to 0.0)	0.0 (0.0 to 0.0)	0.0 (0.0 to 0.0)	0.0 (0.0 to 0.0)	0.0 (0.0 to 0.0)	0.0 (0.0 to 0.0)
Anemia due to other gynecological diseases aggregate	0.0 (0.0 to 0.0)	0.0 (0.0 to 0.0)	0.0 (0.0 to 0.0)	0.0 (0.0 to 0.0)	0.0 (0.0 to 0.0)	0.0 (0.0 to 0.0)
Hemoglobinopathies and hemolytic anemias	28,835.7 (28,336.2 to 29,440.4)	18.6 (17.2 to 20.1)*	-3.3 (-4.5 to -2.2)*	96.2 (65.1 to 137.2)	14.8 (10.1 to 19.3)*	-6.5 (-10.3 to -2.8)*
Thalassemias	0.1 (0.1 to 0.1)	14.4 (7.0 to 22.0)*	-6.8 (-12.8 to -0.6)*	0.0 (0.0 to 0.0)	17.2 (5.1 to 30.1)*	-4.5 (-14.4 to 6.0)
Beta-thalassemia major cases aggregate	0.0 (0.0 to 0.0)	15.7 (2.7 to 30.2)*	-5.7 (-16.3 to 6.1)	0.0 (0.0 to 0.0)	18.4 (0.1 to 40.2)*	-3.6 (-18.4 to 14.3)
Hemoglobin E/beta-thalassemia cases aggregate	0.0 (0.0 to 0.0)	10.0 (9.3 to 10.8)*	-10.4 (-11.0 to -9.7)*	0.0 (0.0 to 0.0)	13.7 (3.3 to 23.9)*	-7.4 (-15.8 to 0.9)
Hemoglobin H disease cases aggregate	0.0 (0.0 to 0.0)	14.0 (9.6 to 18.7)*	-7.1 (-10.7 to -3.3)*	0.0 (0.0 to 0.0)	10.7 (5.8 to 15.6)*	-9.8 (-13.8 to -5.8)*
Heart failure due to thalassemias aggregate	0.0 (0.0 to 0.0)	16.8 (13.3 to 20.2)*	-4.9 (-7.7 to -2.0)*	0.0 (0.0 to 0.0)	16.7 (13.3 to 20.2)*	-4.9 (-7.7 to -2.0)*
Thalassemias trait	5,883.5 (5,743.8 to 6,068.2)	20.9 (20.3 to 21.5)*	-1.5 (-2.0 to -1.0)*	46.2 (30.8 to 66.8)	18.5 (13.0 to 24.4)*	-3.5 (-8.0 to 1.3)
B-thalassemia trait cases aggregate	4,823.6 (4,692.3 to 5,005.4)	20.3 (19.7 to 21.0)*	-1.9 (-2.5 to -1.4)*	44.0 (29.3 to 63.4)	18.6 (12.8 to 24.6)*	-3.4 (-8.1 to 1.6)
Hemoglobin E trait cases aggregate	1,060.0 (1,019.8 to 1,100.8)	23.4 (22.3 to 24.5)*	0.5 (-0.3 to 1.4)	2.2 (1.4 to 3.2)	16.9 (6.1 to 28.6)*	-4.7 (-13.6 to 4.8)
Sickle cell disorders	0.2 (0.2 to 0.2)	17.8 (15.6 to 19.9)*	-4.0 (-5.8 to -2.3)*	0.0 (0.0 to 0.1)	15.2 (12.7 to 17.7)*	-6.2 (-8.2 to -4.1)*
Homozygous sickle cell and severe sickle cell/beta-thalassemia cases aggregate	0.2 (0.2 to 0.2)	19.4 (17.0 to 21.9)*	-2.7 (-4.7 to -0.6)*	0.0 (0.0 to 0.0)	17.1 (14.2 to 20.1)*	-4.6 (-7.0 to -2.2)*
Hemoglobin SC disease cases aggregate	0.0 (0.0 to 0.0)	15.9 (12.7 to 19.2)*	-5.5 (-8.2 to -2.8)*	0.0 (0.0 to 0.0)	12.0 (9.1 to 14.9)*	-8.8 (-11.1 to -6.4)*
Mild sickle cell/beta-thalassemia cases aggregate	0.0 (0.0 to 0.0)	9.7 (6.2 to 13.3)*	-10.6 (-13.5 to -7.7)*	0.0 (0.0 to 0.0)	6.1 (1.9 to 10.4)*	-13.5 (-17.0 to -10.0)*
Sickle cell trait	5,545.9 (5,236.6 to 6,162.3)	26.0 (25.3 to 26.7)*	2.7 (2.1 to 3.3)*	15.3 (10.3 to 21.3)	9.2 (2.1 to 16.4)*	-11.0 (-16.8 to -5.1)*
G6PD deficiency	3,848.9 (3,249.9 to 4,479.8)	13.4 (7.9 to 18.3)*	-7.6 (-12.1 to -3.6)*	0.4 (0.3 to 0.6)	17.4 (7.7 to 28.0)*	-4.3 (-12.3 to 4.3)
G6PD cases aggregate	3,848.6 (3,249.7 to 4,479.6)	13.4 (7.9 to 18.3)*	-7.6 (-12.1 to -3.6)*	0.4 (0.3 to 0.5)	16.5 (6.4 to 27.9)*	-5.0 (-13.3 to 4.2)
Heart failure due to G6PD deficiency aggregate	0.2 (0.2 to 0.3)	30.3 (27.5 to 33.2)*	6.2 (3.9 to 8.6)*	0.0 (0.0 to 0.0)	30.3 (27.5 to 33.2)*	6.2 (3.9 to 8.6)*
G6PD trait	14,670.0 (13,479.0 to 15,852.9)	16.5 (14.3 to 18.7)*	-5.0 (-6.9 to -3.3)*	0.4 (0.2 to 0.5)	16.3 (4.5 to 29.6)*	-5.2 (-14.9 to 5.6)
Other hemoglobinopathies and hemolytic anemias	1,515.1 (1,464.9 to 1,566.1)	20.5 (15.7 to 25.1)*	-1.8 (-5.7 to 1.9)	34.0 (23.2 to 47.7)	12.5 (4.8 to 19.7)*	-8.3 (-14.6 to -2.5)*
Other hemoglobinopathies and hemolytic anemias cases aggregate	1,507.0 (1,457.0 to 1,558.0)	20.5 (15.7 to 25.1)*	-1.8 (-5.8 to 1.9)	32.8 (22.4 to 46.2)	12.2 (4.3 to 19.6)*	-8.5 (-15.0 to -2.5)*
Heart failure due to other hemoglobinopathies and hemolytic anemias aggregate	8.0 (7.0 to 9.2)	21.6 (19.6 to 23.7)*	-0.9 (-2.5 to 0.8)	0.9 (0.6 to 1.3)	22.0 (11.3 to 33.7)*	-0.6 (-9.3 to 8.9)
Endocrine, metabolic, blood, and immune disorders	1,489.6 (1,430.1 to 1,553.6)	22.1 (16.6 to 26.8)*	-0.5 (-5.0 to 3.3)	42.4 (29.8 to 57.5)	20.6 (14.7 to 27.4)*	-1.8 (-6.5 to 3.8)
Endocrine, metabolic, blood, and immune disorders cases aggregate	569.1 (521.3 to 621.2)	25.1 (22.4 to 28.0)*	2.0 (-0.2 to 4.3)	20.9 (14.4 to 28.7)	25.1 (20.5 to 30.2)*	2.0 (-1.8 to 6.1)
Anemia due to endocrine, metabolic, blood, and immune disorders aggregate	902.0 (870.1 to 935.0)	20.2 (11.4 to 28.0)*	-2.0 (-9.3 to 4.3)	19.4 (13.3 to 27.5)	15.9 (5.8 to 29.9)*	-5.6 (-13.8 to 5.8)
Heart failure due to endocrine, metabolic, blood, and immune disorders aggregate	18.5 (16.2 to 21.3)	20.9 (19.2 to 22.6)*	-1.5 (-2.9 to -0.1)*	2.1 (1.4 to 2.9)	20.9 (13.1 to 30.3)*	-1.5 (-7.9 to 6.1)
Musculoskeletal disorders	70,369.8 (68,677.8 to 71,985.3)	23.3 (22.3 to 24.2)*	0.5 (-0.3 to 1.2)	7,782.6 (5,732.3 to 10,345.5)	23.4 (21.9 to 24.9)*	0.6 (-0.7 to 1.7)
Rheumatoid arthritis	1,848.9 (1,667.1 to 2,048.6)	25.1 (22.0 to 28.2)*	2.0 (-0.6 to 4.4)	415.7 (293.7 to 558.7)	25.2 (21.8 to 28.6)*	2.0 (-0.8 to 4.8)
Osteoarthritis	25,300.4 (24,355.2 to 26,233.9)	26.0 (25.3 to 26.7)*	2.6 (2.1 to 3.2)*	1,302.0 (918.7 to 1,769.3)	27.5 (26.4 to 28.5)*	3.9 (3.0 to 4.7)*
Osteoarthritis of the hip cases aggregate	5,694.3 (5,114.8 to 6,294.3)	28.1 (26.8 to 29.3)*	4.4 (3.3 to 5.4)*	277.9 (192.3 to 387.8)	30.0 (28.3 to 31.9)*	5.9 (4.6 to 7.4)*
Osteoarthritis of the knee cases aggregate	19,606.1 (18,950.9 to 20,306.1)	25.4 (24.6 to 26.2)*	2.2 (1.5 to 2.8)*	1,024.1 (719.0 to 1,393.7)	26.8 (25.6 to 27.9)*	3.3 (2.4 to 4.2)*
Low back and neck pain	40,664.5 (38,751.5 to 42,523.6)	22.2 (20.5 to 23.9)*	-0.4 (-1.9 to 1.0)	4,643.1 (3,279.7 to 6,385.7)	22.1 (20.7 to 23.6)*	-0.5 (-1.7 to 0.7)
Low back pain	28,402.3 (25,120.1 to 31,665.6)	21.3 (19.5 to 23.1)*	-1.2 (-2.7 to 0.3)	2,984.8 (2,092.0 to 4,165.7)	21.4 (19.5 to 23.2)*	-1.1 (-2.7 to 0.4)
Neck pain	17,865.9 (13,145.9 to 23,148.6)	23.3 (20.8 to 26.1)*	0.5 (-1.6 to 2.7)	1,658.4 (1,054.4 to 2,433.4)	23.4 (20.9 to 26.1)*	0.5 (-1.5 to 2.8)
Gout	3,292.1 (2,621.3 to 4,142.7)	23.0 (21.8 to 24.3)*	0.2 (-0.7 to 1.3)	99.7 (64.4 to 143.5)	23.1 (20.0 to 26.0)*	0.3 (-2.2 to 2.7)
Asymptomatic gout	2,941.1 (2,345.8 to 3,704.7)	23.0 (21.8 to 24.3)*	0.2 (-0.7 to 1.3)	0.0 (0.0 to 0.0)	0.0 (0.0 to 0.0)	0.0 (0.0 to 0.0)
Symptomatic gout cases aggregate	351.1 (270.9 to 448.1)	23.0 (21.8 to 24.3)*	0.2 (-0.7 to 1.3)	99.7 (64.4 to 143.5)	23.1 (20.0 to 26.0)*	0.3 (-2.2 to 2.7)
Other musculoskeletal disorders	15,096.5 (11,343.3 to 19,748.8)	23.7 (16.4 to 29.9)*	0.8 (-5.1 to 5.9)	1,322.1 (826.0 to 1,933.4)	23.8 (16.5 to 29.9)*	0.9 (-5.1 to 5.8)
Other non-communicable diseases	149,544.4 (149,246.2 to 149,848.1)	22.8 (22.7 to 22.9)*	0.1 (-0.0 to 0.2)	8,688.1 (6,091.6 to 12,035.3)	23.1 (21.5 to 24.6)*	0.3 (-1.0 to 1.5)
Congenital anomalies	1,519.5 (1,370.9 to 1,609.2)	41.4 (35.1 to 47.2)*	15.2 (10.1 to 19.9)*	130.2 (81.7 to 198.9)	41.7 (31.7 to 54.3)*	15.5 (7.3 to 25.8)*
Neural tube defects	5.9 (4.0 to 8.4)	49.3 (43.6 to 54.9)*	21.6 (17.0 to 26.2)*	1.7 (0.9 to 2.9)	50.6 (36.9 to 61.7)*	22.7 (11.5 to 31.7)*
Congenital heart anomalies	798.3 (552.3 to 1,203.7)	54.6 (45.6 to 62.8)*	26.0 (18.6 to 32.6)*	25.5 (9.8 to 48.3)	53.9 (44.8 to 61.9)*	25.4 (18.0 to 31.9)*
Less severe heart anomalies cases aggregate	756.1 (499.2 to 1,152.6)	55.0 (45.3 to 63.5)*	26.3 (18.4 to 33.2)*	23.0 (8.5 to 44.1)	54.9 (44.5 to 63.8)*	26.2 (17.8 to 33.5)*
Severe congenital heart anomalies	39.6 (24.4 to 60.1)	50.4 (41.0 to 60.4)*	22.5 (14.9 to 30.7)*	2.2 (0.8 to 4.3)	50.0 (36.9 to 62.9)*	22.2 (11.5 to 32.7)*
Critical congenital heart anomalies	0.0 (0.0 to 0.0)	126.5 (92.0 to 161.2)*	84.5 (56.4 to 112.8)*	0.0 (0.0 to 0.0)	126.6 (92.0 to 161.2)*	84.6 (56.4 to 112.8)*
Heart failure due to congenital heart anomalies aggregate	2.6 (2.3 to 3.0)	20.9 (19.6 to 22.3)*	-1.5 (-2.6 to -0.3)*	0.3 (0.2 to 0.5)	20.8 (18.3 to 23.6)*	-1.6 (-3.6 to 0.7)
Cleft lip and cleft palate	122.7 (71.5 to 205.6)	54.1 (36.7 to 67.5)*	25.5 (11.4 to 36.5)*	1.2 (0.6 to 2.1)	52.2 (31.0 to 71.6)*	24.0 (6.8 to 39.8)*
Down syndrome	59.2 (38.0 to 90.0)	40.0 (30.6 to 48.6)*	14.1 (6.4 to 21.1)*	10.9 (6.4 to 17.4)	39.5 (30.5 to 47.7)*	13.7 (6.4 to 20.3)*
Turner syndrome	4.5 (2.1 to 8.2)	33.6 (29.2 to 38.2)*	8.8 (5.3 to 12.6)*	0.1 (0.0 to 0.1)	33.4 (28.2 to 38.9)*	8.7 (4.5 to 13.2)*
Klinefelter syndrome	3.9 (2.0 to 6.9)	27.9 (23.7 to 31.1)*	4.2 (0.8 to 6.8)*	0.0 (0.0 to 0.0)	27.9 (23.7 to 31.1)*	4.2 (0.8 to 6.8)*
Other chromosomal abnormalities	60.5 (34.7 to 102.6)	31.5 (21.1 to 41.3)*	7.1 (-1.3 to 15.1)	11.1 (6.1 to 20.1)	31.9 (21.3 to 42.1)*	7.5 (-1.2 to 15.8)
Other congenital anomalies	592.0 (318.1 to 1,091.2)	24.2 (21.0 to 27.3)*	1.2 (-1.4 to 3.7)	79.7 (46.0 to 130.2)	39.6 (27.3 to 57.8)*	13.7 (3.7 to 28.6)*
Skin and subcutaneous diseases	57,763.6 (56,764.3 to 59,071.5)	24.1 (23.3 to 25.0)*	1.1 (0.4 to 1.9)*	948.8 (633.9 to 1,385.1)	23.6 (22.7 to 24.6)*	0.7 (-0.0 to 1.5)
Dermatitis	7,088.7 (5,733.4 to 8,692.7)	21.9 (21.6 to 22.2)*	-0.7 (-1.0 to -0.5)*	193.4 (125.7 to 282.8)	22.1 (20.8 to 23.4)*	-0.5 (-1.6 to 0.5)
Eczema cases aggregate	1,203.4 (894.9 to 1,572.0)	22.8 (21.9 to 23.7)*	0.1 (-0.7 to 0.8)	67.9 (42.0 to 104.1)	22.8 (20.0 to 25.7)*	0.1 (-2.3 to 2.4)

Cause	Prevalence in thousands (95% confidence interval)			YLDs in thousands (95% confidence interval)		
	2015	Percent change in counts between 2005 and 2015	Percent change in rates between 2005 and 2015	2015	Percent change in counts between 2005 and 2015	Percent change in rates between 2005 and 2015
Contact dermatitis cases aggregate	3,723.7 (2,602.0 to 5,250.6)	21.6 (21.2 to 22.0)*	-0.9 (-1.3 to -0.6)*	94.9 (52.3 to 157.3)	21.6 (20.0 to 23.2)*	-0.9 (-2.2 to 0.4)
Seborrhoeic dermatitis cases aggregate	2,161.6 (1,611.9 to 2,733.8)	21.8 (21.5 to 22.1)*	-0.8 (-1.0 to -0.5)*	30.7 (16.8 to 50.9)	21.9 (20.5 to 23.3)*	-0.7 (-1.9 to 0.4)
Psoriasis	3,006.5 (2,750.3 to 3,269.4)	24.0 (22.9 to 25.2)*	1.0 (0.1 to 2.0)*	227.1 (158.0 to 312.7)	24.0 (21.9 to 26.2)*	1.1 (-0.6 to 2.8)
Cellulitis	39.1 (30.0 to 49.3)	33.2 (27.5 to 39.0)*	8.5 (3.9 to 13.3)*	2.6 (1.6 to 3.9)	32.1 (20.5 to 44.7)*	7.6 (-1.8 to 17.9)
Pyoderma	194.9 (171.4 to 219.9)	25.7 (22.5 to 28.6)*	2.4 (-0.2 to 4.8)	1.0 (0.4 to 2.2)	25.8 (20.9 to 31.0)*	2.5 (-1.5 to 6.8)
Scabies	2,776.1 (1,824.3 to 4,071.2)	20.6 (14.5 to 26.7)*	-1.7 (-6.7 to 3.2)	67.9 (33.9 to 121.5)	20.7 (14.3 to 26.9)*	-1.6 (-6.9 to 3.4)
Fungal skin diseases	10,006.3 (8,502.5 to 11,611.9)	23.8 (23.5 to 24.2)*	0.9 (0.6 to 1.2)*	53.9 (21.5 to 113.5)	23.9 (23.3 to 24.5)*	1.0 (0.5 to 1.4)*
Viral skin diseases	2,571.7 (2,160.0 to 3,028.4)	22.5 (22.2 to 22.9)*	-0.2 (-0.4 to 0.1)	74.7 (46.7 to 111.9)	22.6 (21.2 to 24.1)*	-0.1 (-1.2 to 1.1)
Molluscum contagiosum cases aggregate	58.6 (46.0 to 75.2)	22.4 (22.0 to 22.7)*	-0.3 (-0.6 to -0.0)*	1.7 (1.0 to 2.6)	22.4 (13.9 to 32.0)*	-0.2 (-7.2 to 7.6)
Viral warts cases aggregate	2,513.1 (2,103.7 to 2,967.6)	22.5 (22.2 to 22.9)*	-0.1 (-0.4 to 0.1)	73.0 (45.6 to 109.3)	22.6 (21.2 to 24.1)*	-0.1 (-1.2 to 1.1)
Acne vulgaris	1,288.2 (1,164.7 to 1,436.2)	25.2 (21.9 to 28.1)*	2.0 (-0.6 to 4.4)	13.2 (6.3 to 24.4)	25.2 (21.8 to 28.5)*	2.0 (-0.7 to 4.7)
Alopecia areata	383.2 (323.8 to 449.4)	20.2 (19.9 to 20.6)*	-2.0 (-2.3 to -1.7)*	12.3 (7.4 to 18.6)	20.3 (16.9 to 23.6)*	-2.0 (-4.8 to 0.7)
Pruritus	2,834.1 (1,996.8 to 3,911.7)	23.9 (20.1 to 27.2)*	0.9 (-2.1 to 3.7)	28.9 (12.5 to 56.0)	24.0 (20.1 to 27.5)*	1.0 (-2.1 to 3.9)
Urticaria	861.1 (569.4 to 1,249.7)	22.9 (22.6 to 23.2)*	0.2 (-0.1 to 0.4)	48.6 (27.3 to 77.4)	23.0 (20.6 to 25.7)*	0.2 (-1.8 to 2.5)
Decubitus ulcer	87.4 (67.4 to 111.8)	28.9 (23.8 to 33.5)*	5.0 (0.9 to 8.8)*	12.4 (8.2 to 17.9)	28.7 (20.0 to 37.8)*	4.9 (-2.2 to 12.3)
Other skin and subcutaneous diseases	37,627.4 (34,386.9 to 41,182.9)	25.6 (23.7 to 27.4)*	2.3 (0.8 to 3.8)*	212.8 (104.8 to 385.8)	25.7 (23.7 to 27.4)*	2.4 (0.8 to 3.8)*
Sense organ diseases	122,073.4 (121,229.6 to 123,042.5)	22.9 (22.5 to 23.2)*	0.1 (-0.2 to 0.4)	6,266.4 (4,387.9 to 8,649.5)	22.6 (20.4 to 24.7)*	-0.1 (-1.9 to 1.6)
Glaucoma	781.5 (572.3 to 1,026.4)	29.8 (27.0 to 32.6)*	5.7 (3.5 to 8.0)*	72.8 (45.6 to 105.7)	30.5 (26.8 to 34.6)*	6.3 (3.4 to 9.7)*
Cataract	9,011.5 (7,189.6 to 11,029.9)	19.7 (16.8 to 22.4)*	-2.4 (-4.8 to -0.3)*	563.1 (391.3 to 772.3)	18.2 (15.9 to 20.4)*	-3.7 (-5.6 to -1.9)*
Macular degeneration	598.8 (401.7 to 876.9)	36.9 (33.5 to 40.1)*	11.5 (8.8 to 14.2)*	39.0 (24.9 to 58.9)	37.1 (32.9 to 41.6)*	11.7 (8.3 to 15.4)*
Refraction and accommodation disorders	58,515.5 (54,239.6 to 63,302.7)	22.8 (20.3 to 25.4)*	0.1 (-2.0 to 2.2)	1,042.7 (674.4 to 1,582.7)	22.3 (20.5 to 23.9)*	-0.4 (-1.8 to 1.0)
Age-related and other hearing loss	116,806.2 (109,534.6 to 123,686.0)	22.9 (22.3 to 23.5)*	0.1 (-0.4 to 0.6)	3,842.0 (2,628.2 to 5,457.8)	22.7 (19.3 to 26.0)*	-0.0 (-2.8 to 2.6)
Other vision loss	1,500.7 (1,110.6 to 2,006.0)	31.7 (28.1 to 35.6)*	7.3 (4.3 to 10.5)*	117.3 (73.9 to 168.7)	31.6 (28.0 to 35.6)*	7.2 (4.3 to 10.5)*
Other sense organ diseases	24,488.6 (21,871.0 to 27,104.1)	23.6 (21.4 to 25.7)*	0.7 (-1.1 to 2.4)	589.5 (371.7 to 851.4)	23.6 (21.3 to 25.9)*	0.7 (-1.2 to 2.5)
Acute other sense organ diseases	48.4 (43.3 to 54.3)	24.0 (21.6 to 26.2)*	1.0 (-0.9 to 2.8)	1.2 (0.7 to 1.8)	23.9 (10.3 to 38.4)*	0.9 (-10.1 to 12.8)
Chronic other sense organ diseases	24,440.2 (21,822.6 to 27,052.8)	23.6 (21.4 to 25.7)*	0.7 (-1.1 to 2.4)	588.4 (371.0 to 849.8)	23.6 (21.3 to 25.9)*	0.7 (-1.2 to 2.6)
Oral disorders	95,521.0 (93,349.0 to 96,946.4)	22.9 (21.9 to 23.8)*	0.1 (-0.7 to 0.8)	1,342.7 (871.6 to 1,936.5)	23.3 (22.8 to 23.7)*	0.5 (0.1 to 0.8)*
Deciduous caries	0.0 (0.0 to 0.0)	0.0 (0.0 to 0.0)	0.0 (0.0 to 0.0)	0.0 (0.0 to 0.0)	0.0 (0.0 to 0.0)	0.0 (0.0 to 0.0)
Permanent caries	60,626.4 (52,256.3 to 69,656.4)	22.3 (20.2 to 24.4)*	-0.3 (-2.1 to 1.3)	43.8 (19.6 to 83.1)	22.7 (20.7 to 24.7)*	-0.0 (-1.7 to 1.6)
Periodontal diseases	26,859.1 (22,609.0 to 31,515.8)	23.9 (22.8 to 24.6)*	0.9 (0.1 to 1.6)*	169.9 (66.3 to 344.3)	24.0 (22.9 to 24.8)*	1.0 (0.2 to 1.7)*
Edentulism and severe tooth loss	37,808.4 (35,387.7 to 40,504.6)	23.2 (22.8 to 23.7)*	0.4 (0.0 to 0.8)*	1,028.4 (682.3 to 1,424.9)	23.2 (22.7 to 23.8)*	0.4 (-0.1 to 0.9)
Other oral disorders	3,609.0 (3,215.4 to 4,007.2)	22.8 (22.2 to 23.4)*	0.1 (-0.4 to 0.5)	100.6 (62.6 to 151.0)	22.9 (21.8 to 23.9)*	0.1 (-0.8 to 1.0)
Injuries	51,373.1 (49,408.3 to 53,771.5)	20.1 (19.1 to 21.3)*	-2.1 (-3.0 to -1.1)*	1,960.3 (1,368.3 to 2,687.6)	12.5 (9.3 to 15.3)*	-8.4 (-11.0 to -6.1)*
Transport injuries	7,325.0 (6,940.1 to 7,768.3)	25.3 (24.8 to 25.8)*	2.1 (1.7 to 2.5)*	371.6 (259.3 to 510.1)	18.2 (15.5 to 20.8)*	-3.7 (-5.9 to -1.6)*
Road injuries	6,831.9 (6,445.5 to 7,292.1)	25.7 (25.1 to 26.2)*	2.4 (2.0 to 2.9)*	345.9 (239.4 to 472.9)	19.4 (16.7 to 21.8)*	-2.7 (-4.9 to -0.8)*
Pedestrian road injuries	994.7 (879.3 to 1,132.0)	23.4 (22.4 to 24.4)*	0.5 (-0.3 to 1.4)	50.5 (34.1 to 69.6)	14.1 (10.5 to 17.3)*	-7.1 (-10.0 to -4.5)*
Cyclist road injuries	1,151.5 (1,004.9 to 1,298.8)	24.3 (23.1 to 25.4)*	1.3 (0.3 to 2.1)*	55.4 (37.7 to 78.3)	16.5 (12.9 to 19.6)*	-5.0 (-8.0 to -2.6)*
Motorcyclist road injuries	1,537.0 (1,362.2 to 1,715.0)	32.9 (31.6 to 34.2)*	8.3 (7.2 to 9.4)*	74.0 (51.4 to 102.6)	24.6 (20.9 to 28.0)*	1.5 (-1.5 to 4.3)
Motor vehicle road injuries	2,912.8 (2,645.7 to 3,240.1)	22.4 (21.5 to 23.2)*	-0.3 (-1.0 to 0.4)	153.9 (106.2 to 211.8)	18.6 (16.5 to 20.5)*	-3.4 (-5.1 to -1.8)*
Other road injuries	236.0 (204.3 to 268.7)	41.9 (40.9 to 43.1)*	15.7 (14.8 to 16.6)*	12.1 (8.3 to 16.8)	37.8 (35.3 to 40.0)*	12.3 (10.2 to 14.1)*
Other transport injuries	493.0 (441.0 to 554.2)	20.2 (19.6 to 20.8)*	-2.0 (-2.5 to -1.6)*	25.7 (18.0 to 35.0)	4.7 (0.3 to 9.2)*	-14.7 (-18.2 to -11.0)*
Unintentional injuries	40,501.3 (38,713.6 to 42,716.6)	18.2 (17.7 to 18.8)*	-3.7 (-4.1 to -3.2)*	1,483.3 (1,026.2 to 2,049.2)	11.2 (8.0 to 13.9)*	-9.4 (-12.0 to -7.2)*
Falls	13,935.5 (12,666.4 to 15,493.3)	28.2 (27.5 to 28.8)*	4.4 (3.9 to 5.0)*	667.0 (452.2 to 920.4)	16.0 (10.9 to 20.2)*	-5.5 (-9.6 to -2.0)*
Drowning	390.3 (342.2 to 450.7)	12.3 (11.7 to 12.8)*	-8.5 (-9.0 to -8.1)*	20.2 (14.1 to 27.9)	0.9 (-3.0 to 4.7)	-17.8 (-21.0 to -14.7)*
Fire, heat, and hot substances	3,319.8 (2,893.6 to 3,735.8)	14.3 (13.7 to 15.0)*	-6.8 (-7.3 to -6.3)*	68.9 (44.5 to 105.5)	11.0 (7.6 to 13.1)*	-9.6 (-12.4 to -7.8)*
Poisonings	187.9 (166.4 to 213.8)	17.1 (16.3 to 18.0)*	-4.6 (-5.2 to -3.8)*	12.4 (8.7 to 17.2)	11.9 (8.5 to 15.2)*	-8.8 (-11.6 to -6.1)*
Exposure to mechanical forces	7,080.1 (6,592.8 to 7,602.8)	18.9 (18.3 to 19.4)*	-3.1 (-3.6 to -2.7)*	132.4 (90.7 to 185.2)	13.1 (10.5 to 15.3)*	-7.9 (-10.0 to -6.0)*
Unintentional firearm injuries	104.9 (92.8 to 118.4)	11.9 (11.0 to 12.8)*	-8.8 (-9.5 to -8.1)*	3.4 (2.3 to 4.7)	6.7 (4.0 to 8.9)*	-13.0 (-15.3 to -11.2)*
Unintentional suffocation	541.9 (463.3 to 661.5)	21.7 (18.3 to 24.9)*	-0.8 (-3.6 to 1.8)	20.2 (13.8 to 27.9)	22.6 (20.1 to 24.8)*	-0.1 (-2.1 to 1.7)
Other exposure to mechanical forces	6,433.3 (5,953.4 to 6,939.2)	18.8 (18.3 to 19.2)*	-3.2 (-3.6 to -2.9)*	108.8 (74.5 to 153.8)	11.7 (8.7 to 14.2)*	-9.0 (-11.4 to -6.9)*
Adverse effects of medical treatment	526.4 (388.0 to 686.4)	-4.7 (-9.5 to -0.4)*	-22.4 (-26.3 to -18.8)*	70.2 (42.9 to 107.6)	-4.7 (-9.5 to -0.4)*	-22.4 (-26.3 to -18.8)*
Animal contact	1,206.5 (1,111.8 to 1,317.5)	13.8 (12.9 to 14.7)*	-7.3 (-8.0 to -6.6)*	26.7 (18.6 to 36.4)	8.6 (6.4 to 10.9)*	-11.5 (-13.3 to -9.6)*
Venomous animal contact	519.2 (468.5 to 580.2)	18.2 (17.3 to 19.1)*	-3.7 (-4.4 to -3.0)*	18.1 (12.7 to 24.1)	9.4 (6.7 to 12.2)*	-10.9 (-13.1 to -8.6)*
Non-venomous animal contact	687.3 (610.9 to 767.9)	10.7 (9.6 to 11.8)*	-9.8 (-10.7 to -8.9)*	8.7 (5.5 to 13.2)	7.0 (4.2 to 9.4)*	-12.8 (-15.1 to -10.9)*
Foreign body	1,224.2 (1,116.7 to 1,332.4)	20.3 (19.4 to 21.2)*	-2.0 (-2.7 to -1.2)*	40.0 (28.2 to 55.6)	10.1 (6.2 to 14.2)*	-10.3 (-13.5 to -7.0)*
Pulmonary aspiration and foreign body in airway	549.3 (493.9 to 633.4)	27.9 (25.8 to 29.7)*	4.2 (2.5 to 5.7)*	23.0 (16.2 to 31.5)	11.4 (6.4 to 16.9)*	-9.2 (-13.3 to -4.7)*
Foreign body in eyes	25.4 (13.4 to 43.2)	18.0 (15.7 to 20.0)*	-3.9 (-5.7 to -2.2)*	1.4 (0.7 to 2.6)	16.6 (14.2 to 18.5)*	-5.0 (-6.9 to -3.4)*
Foreign body in other body part	649.5 (575.3 to 723.8)	14.7 (14.0 to 15.4)*	-6.5 (-7.1 to -6.0)*	15.7 (10.7 to 22.1)	7.8 (4.9 to 10.7)*	-12.2 (-14.5 to -9.8)*
Environmental heat and cold exposure	3,639.0 (3,243.7 to 4,125.9)	20.5 (19.8 to 21.0)*	-1.8 (-2.4 to -1.4)*	140.5 (96.5 to 193.1)	13.4 (10.5 to 16.0)*	-7.6 (-10.0 to -5.5)*
Other unintentional injuries	8,991.5 (8,137.3 to 9,965.2)	7.4 (6.8 to 7.9)*	-12.5 (-13.0 to -12.1)*	304.9 (205.6 to 434.6)	5.0 (3.5 to 6.1)*	-14.5 (-15.6 to -13.5)*
Self-harm and interpersonal violence	1,254.3 (1,163.9 to 1,332.9)	19.1 (18.7 to 19.4)*	-3.0 (-3.2 to -2.7)*	45.6 (31.7 to 61.2)	9.0 (5.5 to 12.4)*	-11.2 (-14.1 to -8.4)*
Self-harm	393.5 (351.8 to 440.7)	18.4 (17.8 to 18.9)*	-3.5 (-4.0 to -3.2)*	15.3 (10.7 to 20.5)	5.1 (1.0 to 9.3)*	-14.3 (-17.7 to -11.0)*
Interpersonal violence	860.8 (786.5 to 941.1)	19.4 (19.0 to 19.8)*	-2.7 (-3.0 to -2.4)*	30.3 (21.0 to 41.2)	11.0 (7.7 to 14.1)*	-9.5 (-12.2 to -7.0)*

Cause	Prevalence in thousands (95% confidence interval)			YLDs in thousands (95% confidence interval)		
	**2015**	**Percent change in counts between 2005 and 2015**	**Percent change in rates between 2005 and 2015**	**2015**	**Percent change in counts between 2005 and 2015**	**Percent change in rates between 2005 and 2015**
Assault by firearm	49.9 (43.3 to 57.3)	20.5 (19.5 to 21.3)*	-1.8 (-2.6 to -1.2)*	1.9 (1.3 to 2.6)	14.4 (11.7 to 16.8)*	-6.8 (-9.0 to -4.9)*
Assault by sharp object	177.4 (149.0 to 205.4)	17.3 (16.6 to 18.1)*	-4.4 (-5.0 to -3.7)*	4.3 (2.8 to 5.9)	2.0 (-3.7 to 7.4)	-16.9 (-21.5 to -12.5)*
Assault by other means	633.4 (569.0 to 702.8)	20.0 (19.5 to 20.4)*	-2.3 (-2.7 to -1.9)*	24.1 (16.7 to 32.9)	12.5 (9.6 to 15.3)*	-8.3 (-10.7 to -6.1)*
Forces of nature, war, and legal intervention	2,292.5 (1,608.6 to 3,212.2)	43.3 (17.0 to 75.1)*	16.8 (-4.7 to 42.7)	59.8 (44.0 to 78.0)	14.4 (-17.5 to 46.5)	-6.8 (-32.8 to 19.4)
Exposure to forces of nature	899.3 (536.2 to 1,499.1)	22.8 (-15.1 to 76.9)	0.1 (-30.8 to 44.1)	27.1 (17.7 to 39.5)	-12.9 (-48.0 to 32.4)	-29.0 (-57.6 to 7.9)
Collective violence and legal intervention	1,393.2 (1,001.5 to 1,864.3)	60.6 (32.8 to 94.8)*	30.8 (8.2 to 58.7)*	32.7 (23.4 to 43.9)	54.4 (27.1 to 89.5)*	25.8 (3.5 to 54.4)*
**Anemia**	**44,908.3 (44,268.7 to 45,504.0)**	**14.7 (13.0 to 16.4)***	**-6.6 (-8.0 to -5.1)***	**1,185.5 (806.8 to 1,659.3)**	**10.1 (7.5 to 12.4)***	**-10.3 (-12.4 to -8.4)***
**Developmental intellectual disability**	**1,552.1 (1,148.8 to 1,948.5)**	**23.4 (22.9 to 23.9)***	**0.5 (0.1 to 0.9)***	**189.3 (139.9 to 249.6)**	**26.6 (24.1 to 29.3)***	**3.1 (1.1 to 5.4)***
**Epilepsy**	**745.6 (648.0 to 867.1)**	**27.5 (21.2 to 33.1)***	**3.9 (-1.2 to 8.5)**	**168.2 (121.2 to 223.7)**	**8.7 (1.4 to 16.0)***	**-11.5 (-17.4 to -5.5)***
**Guillain-Barré syndrome**	**1.6 (1.0 to 2.4)**	**21.5 (17.4 to 24.4)***	**-1.0 (-4.4 to 1.4)**	**0.5 (0.3 to 0.8)**	**21.4 (17.4 to 24.4)***	**-1.0 (-4.4 to 1.4)**
**Hearing loss**	**118,430.1 (111,169.1 to 125,204.1)**	**22.8 (22.2 to 23.4)***	**0.0 (-0.5 to 0.5)**	**3,915.0 (2,678.2 to 5,529.7)**	**22.6 (19.3 to 25.8)***	**-0.1 (-2.8 to 2.5)**
**Heart failure**	**5,614.5 (5,331.8 to 5,874.7)**	**23.1 (22.0 to 24.0)***	**0.3 (-0.6 to 1.0)**	**967.4 (727.2 to 1,202.6)**	**23.1 (20.5 to 24.7)***	**0.3 (-1.8 to 1.6)**
**Infertility**	**0.0 (0.0 to 0.0)**	**0.0 (0.0 to 0.0)**	**0.0 (0.0 to 0.0)**	**0.0 (0.0 to 0.0)**	**0.0 (0.0 to 0.0)**	**0.0 (0.0 to 0.0)**
**Pelvic inflammatory disease**	**6.6 (4.6 to 9.0)**	**9.3 (7.4 to 11.1)***	**-10.9 (-12.5 to -9.5)***	**0.9 (0.5 to 1.3)**	**10.7 (1.9 to 20.0)***	**-9.8 (-17.0 to -2.3)***
**Vision loss**	**71,492.5 (65,408.0 to 77,588.6)**	**22.6 (20.3 to 24.8)***	**-0.1 (-2.0 to 1.7)**	**1,919.5 (1,330.6 to 2,671.5)**	**21.6 (19.9 to 23.0)***	**-0.9 (-2.3 to 0.3)**

SR Table 11. Prevalent cases and YLDs for 2015, percent change, and change in age-standardised rates between 2005 and 2015 by cause, global, ages 75-79 years						
Cause	Prevalence in thousands (95% confidence interval)			YLDs in thousands (95% confidence interval)		
	2015	Percent change in counts between 2005 and 2015	Percent change in rates between 2005 and 2015	2015	Percent change in counts between 2005 and 2015	Percent change in rates between 2005 and 2015
**All causes**	**--**	**--**	**--**	**27,535.3 (20,868.1 to 35,290.0)**	**20.1 (19.4 to 20.7)***	**-1.3 (-1.8 to -0.8)***
Communicable, maternal, neonatal, and nutritional diseases	51,519.2 (50,847.3 to 52,268.1)	18.8 (17.6 to 20.1)*	-2.3 (-3.3 to -1.3)*	722.3 (519.5 to 983.8)	11.7 (7.7 to 16.1)*	-8.1 (-11.4 to -4.5)*
HIV/AIDS and tuberculosis	484.3 (460.6 to 510.1)	40.3 (33.3 to 47.8)*	15.3 (9.6 to 21.6)*	89.1 (62.2 to 121.4)	17.6 (9.8 to 25.5)*	-3.3 (-9.7 to 3.2)
Tuberculosis	229.5 (182.5 to 285.9)	21.3 (13.0 to 29.3)*	-0.3 (-7.1 to 6.3)	64.5 (42.3 to 92.7)	21.7 (13.3 to 30.5)*	0.1 (-6.8 to 7.3)
HIV/AIDS	255.2 (240.2 to 272.4)	63.0 (48.6 to 80.0)*	34.0 (22.2 to 48.0)*	24.6 (18.1 to 31.6)	8.1 (-6.4 to 24.0)	-11.1 (-23.0 to 1.9)
HIV/AIDS - Tuberculosis	7.5 (6.7 to 8.3)	13.3 (8.1 to 18.5)*	-6.8 (-11.1 to -2.6)*	2.6 (1.7 to 3.4)	14.0 (4.9 to 23.3)*	-6.2 (-13.7 to 1.4)
HIV/AIDS resulting in other diseases	254.5 (236.9 to 274.2)	64.2 (56.5 to 72.2)*	35.0 (28.7 to 41.6)*	22.0 (16.2 to 28.5)	7.5 (-8.3 to 25.3)	-11.6 (-24.6 to 3.1)
HIV aggregate	88.8 (82.0 to 96.2)	-15.5 (-19.4 to -11.2)*	-30.5 (-33.7 to -27.0)*	5.9 (4.3 to 7.7)	-16.5 (-21.4 to -11.5)*	-31.4 (-35.3 to -27.2)*
AIDS aggregate	165.7 (152.5 to 180.6)	232.0 (195.8 to 273.6)*	173.0 (143.3 to 207.2)*	16.1 (11.8 to 21.2)	20.2 (-4.6 to 51.5)	-1.2 (-21.5 to 24.6)
Diarrhea, lower respiratory, and other common infectious diseases	4,120.5 (3,783.8 to 4,400.4)	18.3 (14.7 to 21.8)*	-2.7 (-5.6 to 0.2)	168.3 (121.8 to 221.0)	24.2 (22.3 to 25.9)*	2.1 (0.6 to 3.6)*
Diarrheal diseases	499.1 (481.9 to 516.8)	31.9 (31.2 to 32.5)*	8.4 (7.9 to 9.0)*	71.8 (50.6 to 96.5)	32.1 (30.3 to 33.8)*	8.6 (7.2 to 10.1)*
Diarrhoea episodes aggregate	498.9 (481.6 to 516.7)	31.9 (31.2 to 32.5)*	8.4 (7.9 to 9.0)*	71.8 (50.6 to 96.5)	32.1 (30.3 to 33.8)*	8.6 (7.2 to 10.1)*
Guillain-Barré syndrome due to diarrheal diseases	0.1 (0.1 to 0.2)	18.8 (15.1 to 21.4)*	-2.3 (-5.4 to -0.2)*	0.0 (0.0 to 0.1)	18.8 (15.1 to 21.4)*	-2.3 (-5.4 to -0.2)*
Intestinal infectious diseases	11.0 (10.1 to 11.8)	9.4 (0.2 to 20.3)*	-10.0 (-17.6 to -1.1)*	1.4 (1.0 to 2.0)	12.3 (-1.1 to 29.0)	-7.6 (-18.7 to 6.1)
Typhoid fever	9.9 (8.2 to 11.8)	13.5 (8.1 to 20.0)*	-6.7 (-11.1 to -1.3)*	1.2 (0.8 to 1.7)	15.3 (-0.1 to 35.6)	-5.2 (-17.8 to 11.5)
Typhoid fever episodes aggregate	8.2 (6.8 to 9.9)	13.3 (2.8 to 24.1)*	-6.8 (-15.4 to 2.0)	0.7 (0.5 to 1.0)	13.8 (-1.8 to 32.7)	-6.4 (-19.3 to 9.1)
Complications of typhoid fever aggregate	1.7 (1.2 to 2.3)	14.5 (-18.9 to 64.8)	-5.9 (-33.3 to 35.5)	0.5 (0.3 to 0.7)	17.5 (-21.2 to 75.8)	-3.4 (-35.2 to 44.5)
Paratyphoid fever	4.0 (3.0 to 5.2)	8.6 (0.0 to 17.8)*	-10.7 (-17.7 to -3.1)*	0.2 (0.1 to 0.3)	9.1 (-12.2 to 36.1)	-10.3 (-27.8 to 11.9)
Paratyphoid fever episodes aggregate	3.7 (2.8 to 4.9)	8.5 (-0.1 to 18.4)	-10.8 (-17.8 to -2.7)*	0.2 (0.1 to 0.3)	9.0 (-14.0 to 37.7)	-10.3 (-29.3 to 13.3)
Intestinal perforation due to paratyphoid	0.2 (0.1 to 0.3)	9.4 (-38.3 to 95.5)	-10.0 (-49.3 to 60.7)	0.0 (0.0 to 0.0)	9.4 (-38.3 to 95.5)	-10.1 (-49.3 to 60.7)
Other intestinal infectious diseases	--	--	--	0.0 (0.0 to 0.0)	-53.8 (-68.8 to -28.0)*	-62.0 (-74.3 to -40.8)*
Lower respiratory infections	265.0 (247.8 to 284.3)	23.1 (20.7 to 25.5)*	1.2 (-0.8 to 3.2)	14.8 (10.1 to 20.6)	23.2 (19.9 to 26.7)*	1.3 (-1.4 to 4.2)
Lower respiratory infection episodes aggregate	264.8 (247.6 to 284.2)	23.1 (20.7 to 25.5)*	1.2 (-0.8 to 3.2)	14.8 (10.1 to 20.5)	23.2 (19.9 to 26.7)*	1.3 (-1.4 to 4.2)
Guillain-Barré syndrome due to lower respiratory infections	0.1 (0.1 to 0.3)	18.8 (15.1 to 21.4)*	-2.3 (-5.4 to -0.2)*	0.0 (0.0 to 0.1)	18.8 (15.1 to 21.4)*	-2.3 (-5.4 to -0.2)*
Upper respiratory infections	2,267.0 (1,543.9 to 3,156.8)	16.7 (14.9 to 18.4)*	-4.0 (-5.5 to -2.6)*	24.9 (12.9 to 44.2)	16.9 (14.6 to 19.2)*	-3.9 (-5.7 to -2.0)*
Upper respiratory infection episodes aggregate	2,266.6 (1,543.4 to 3,156.5)	16.7 (14.9 to 18.4)*	-4.0 (-5.5 to -2.6)*	24.8 (12.8 to 44.1)	16.8 (14.6 to 19.2)*	-3.9 (-5.8 to -2.0)*
Guillain-Barré syndrome due to upper respiratory infections	0.4 (0.2 to 0.6)	18.8 (15.1 to 21.4)*	-2.3 (-5.4 to -0.2)*	0.1 (0.1 to 0.2)	18.8 (15.1 to 21.4)*	-2.3 (-5.4 to -0.2)*
Otitis media	715.8 (541.3 to 926.2)	13.4 (10.6 to 16.6)*	-6.7 (-9.1 to -4.1)*	18.6 (11.0 to 29.0)	13.3 (7.6 to 18.9)*	-6.9 (-11.5 to -2.2)*
Acute otitis media	60.5 (46.0 to 77.6)	19.9 (16.8 to 22.7)*	-1.4 (-3.9 to 0.9)	0.7 (0.3 to 1.4)	19.9 (14.4 to 26.1)*	-1.4 (-5.9 to 3.7)
Chronic otitis media aggregate	655.3 (477.5 to 866.4)	12.8 (9.7 to 16.4)*	-7.2 (-9.8 to -4.3)*	17.8 (10.5 to 28.1)	13.0 (7.4 to 18.8)*	-7.1 (-11.7 to -2.3)*
Meningitis	164.8 (158.8 to 170.4)	22.7 (19.3 to 26.2)*	0.9 (-1.9 to 3.8)	24.4 (17.7 to 31.2)	23.2 (20.2 to 26.2)*	1.3 (-1.1 to 3.8)
Pneumococcal meningitis	140.4 (121.5 to 161.5)	24.3 (22.6 to 26.3)*	2.2 (0.8 to 3.8)*	13.6 (9.8 to 17.5)	24.3 (19.8 to 28.9)*	2.3 (-1.5 to 6.0)
Acute pneumococcal meningitis	0.5 (0.4 to 0.7)	26.7 (14.9 to 38.4)*	4.2 (-5.5 to 13.8)	0.1 (0.0 to 0.1)	26.7 (14.9 to 38.4)*	4.2 (-5.5 to 13.8)
Complications of pneumococcal meningitis aggregate	139.9 (121.0 to 161.0)	24.3 (22.6 to 26.3)*	2.2 (0.8 to 3.8)*	13.5 (9.8 to 17.4)	24.3 (19.7 to 28.9)*	2.2 (-1.5 to 6.0)
H influenzae type B meningitis	29.4 (23.6 to 35.7)	-2.9 (-6.9 to 2.2)	-20.1 (-23.4 to -15.9)*	3.3 (2.3 to 4.4)	17.8 (11.0 to 24.8)*	-3.2 (-8.8 to 2.6)
Acute H influenzae type B meningitis	0.1 (0.1 to 0.2)	-6.1 (-16.6 to 5.2)	-22.8 (-31.4 to -13.5)*	0.0 (0.0 to 0.0)	-6.0 (-16.6 to 5.2)	-22.7 (-31.4 to -13.5)*
Complications of of H influenzae type B meningitis aggregate	29.3 (23.5 to 35.5)	-2.8 (-6.9 to 2.3)	-20.1 (-23.4 to -15.9)*	3.3 (2.3 to 4.4)	17.9 (11.1 to 25.0)*	-3.0 (-8.6 to 2.8)
Meningococcal meningitis	18.0 (12.6 to 24.0)	18.1 (14.5 to 22.0)*	-2.8 (-5.8 to 0.3)	0.7 (0.5 to 1.1)	18.4 (13.0 to 24.0)*	-2.7 (-7.1 to 2.0)
Acute meningococcal meningitis	0.2 (0.2 to 0.4)	24.4 (11.7 to 36.4)*	2.3 (-8.1 to 12.2)	0.0 (0.0 to 0.1)	24.5 (11.7 to 37.1)*	2.4 (-8.2 to 12.7)
Complications of meningococcal meningitis aggregate	17.8 (12.3 to 23.7)	18.1 (14.4 to 22.0)*	-2.9 (-5.9 to 0.3)	0.7 (0.4 to 1.0)	18.1 (12.5 to 24.0)*	-2.9 (-7.5 to 2.0)
Other meningitis	64.0 (52.2 to 75.7)	24.8 (22.9 to 26.9)*	2.6 (1.0 to 4.4)*	6.8 (4.8 to 8.9)	24.4 (19.2 to 29.0)*	2.3 (-2.0 to 6.1)
Other meningitis episodes aggregate	1.0 (0.8 to 1.4)	31.6 (20.4 to 41.6)*	8.2 (-1.0 to 16.4)	0.1 (0.1 to 0.2)	31.7 (20.5 to 41.6)*	8.3 (-0.9 to 16.5)
Complications of other meningitis aggregate	62.9 (51.2 to 74.3)	24.7 (22.7 to 26.8)*	2.5 (0.9 to 4.2)*	6.6 (4.7 to 8.7)	24.3 (18.9 to 29.0)*	2.2 (-2.2 to 6.1)
Encephalitis	70.8 (42.9 to 108.1)	10.6 (8.3 to 13.8)*	-9.1 (-11.0 to -6.4)*	3.8 (2.7 to 5.2)	14.0 (8.9 to 19.2)*	-6.3 (-10.5 to -2.0)*
Acute encephalitis	1.2 (0.9 to 1.5)	24.3 (18.3 to 29.7)*	2.2 (-2.7 to 6.7)	0.1 (0.1 to 0.2)	25.6 (9.4 to 45.8)*	3.3 (-10.1 to 19.9)
Complications of encephalitis aggregate	69.6 (41.9 to 106.9)	10.3 (8.0 to 13.6)*	-9.3 (-11.2 to -6.6)*	3.7 (2.6 to 5.0)	13.6 (8.2 to 19.0)*	-6.6 (-11.0 to -2.1)*
Diphtheria	0.0 (0.0 to 0.0)	0.0 (0.0 to 0.0)	0.0 (0.0 to 0.0)	0.0 (0.0 to 0.0)	0.0 (0.0 to 0.0)	0.0 (0.0 to 0.0)
Whooping cough	0.0 (0.0 to 0.0)	0.0 (0.0 to 0.0)	0.0 (0.0 to 0.0)	0.0 (0.0 to 0.0)	0.0 (0.0 to 0.0)	0.0 (0.0 to 0.0)
Tetanus	2.1 (2.0 to 2.2)	27.0 (18.0 to 32.4)*	4.5 (-3.0 to 8.9)	0.1 (0.0 to 0.1)	3.9 (-15.6 to 22.5)	-14.6 (-30.6 to 0.7)
Severe tetanus	0.2 (0.1 to 0.3)	-30.2 (-47.9 to -5.9)*	-42.6 (-57.2 to -22.6)*	0.0 (0.0 to 0.0)	-29.1 (-47.1 to -1.5)*	-41.7 (-56.5 to -19.0)*
Complications of tetanus aggregate	1.9 (1.9 to 1.9)	37.5 (36.9 to 38.1)*	13.1 (12.6 to 13.5)*	0.0 (0.0 to 0.1)	35.2 (21.9 to 48.1)*	11.2 (0.3 to 21.8)*
Measles	0.0 (0.0 to 0.0)	0.0 (0.0 to 0.0)	0.0 (0.0 to 0.0)	0.0 (0.0 to 0.0)	0.0 (0.0 to 0.0)	0.0 (0.0 to 0.0)
Varicella and herpes zoster	162.5 (132.0 to 195.0)	21.9 (19.9 to 23.6)*	0.2 (-1.4 to 1.6)	8.4 (5.1 to 12.9)	22.1 (18.6 to 25.6)*	0.4 (-2.5 to 3.3)
Neglected tropical diseases and malaria	19,670.8 (19,187.5 to 20,197.5)	14.1 (10.7 to 17.6)*	-6.2 (-9.0 to -3.3)*	205.0 (140.6 to 300.4)	1.1 (-9.2 to 14.5)	-16.9 (-25.3 to -5.8)*
Malaria	852.8 (730.6 to 974.1)	37.5 (31.6 to 44.3)*	13.1 (8.2 to 18.7)*	5.2 (3.6 to 7.1)	27.3 (19.2 to 37.6)*	4.7 (-2.0 to 13.1)
Asymptomatic malaria parasitemia(PfPR)	663.0 (540.9 to 785.6)	39.2 (31.1 to 48.6)*	14.5 (7.8 to 22.2)*	0.0 (0.0 to 0.0)	0.0 (0.0 to 0.0)	0.0 (0.0 to 0.0)
Malaria episodes aggregate	9.3 (5.2 to 17.0)	-10.6 (-33.4 to 18.9)	-26.5 (-45.2 to -2.2)*	0.2 (0.1 to 0.4)	-10.0 (-33.7 to 22.3)	-26.0 (-45.5 to 0.6)
Complications of malaria aggregate	0.5 (0.4 to 0.6)	44.7 (41.0 to 48.8)*	19.0 (16.0 to 22.3)*	0.2 (0.1 to 0.2)	41.1 (36.9 to 45.7)*	16.1 (12.6 to 19.8)*
Anemia due to malaria parasitemia(PfPR) aggregate	179.9 (175.2 to 184.7)	35.2 (28.2 to 42.8)*	11.2 (5.4 to 17.4)*	4.8 (3.3 to 6.7)	28.8 (20.0 to 40.1)*	6.0 (-1.3 to 15.2)
Chagas disease	219.7 (182.2 to 259.5)	3.1 (-0.3 to 6.1)	-15.3 (-18.0 to -12.7)*	4.7 (3.0 to 6.9)	3.6 (-1.2 to 8.2)	-14.8 (-18.8 to -11.0)*
Chagas disease episodes aggregate	157.6 (129.7 to 188.0)	2.9 (-0.5 to 5.9)	-15.4 (-18.1 to -12.9)*	0.0 (0.0 to 0.0)	26.4 (21.1 to 32.5)*	3.9 (-0.4 to 8.9)
Complications of Chagas disease aggregate	33.1 (25.0 to 42.3)	3.3 (0.1 to 6.4)*	-15.0 (-17.6 to -12.5)*	3.3 (1.9 to 5.1)	3.5 (-2.2 to 9.1)	-14.9 (-19.6 to -10.3)*

Cause	Prevalence in thousands (95% confidence interval)			YLDs in thousands (95% confidence interval)		
	2015	Percent change in counts between 2005 and 2015	Percent change in rates between 2005 and 2015	2015	Percent change in counts between 2005 and 2015	Percent change in rates between 2005 and 2015
Heart failure due to Chagas disease aggregate	29.1 (19.3 to 39.1)	3.8 (0.5 to 7.0)*	-14.6 (-17.4 to -12.0)*	1.4 (0.8 to 2.2)	3.9 (-2.1 to 9.7)	-14.6 (-19.5 to -9.8)*
Leishmaniasis	69.5 (64.0 to 76.7)	31.7 (17.8 to 46.8)*	8.3 (-3.1 to 20.8)	0.7 (0.3 to 1.3)	31.5 (27.3 to 35.7)*	8.1 (4.7 to 11.6)*
Visceral leishmaniasis	0.1 (0.1 to 0.1)	34.5 (31.8 to 37.1)*	10.6 (8.4 to 12.7)*	0.0 (0.0 to 0.0)	34.3 (29.0 to 39.8)*	10.4 (6.1 to 14.9)*
Cutaneous and mucocutaneous leishmaniasis	71.3 (63.5 to 82.0)	31.3 (29.2 to 33.4)*	8.0 (6.3 to 9.7)*	0.7 (0.3 to 1.3)	31.5 (27.3 to 35.6)*	8.1 (4.6 to 11.5)*
African trypanosomiasis	0.1 (0.0 to 0.1)	-68.5 (-69.9 to -67.3)*	-74.1 (-75.2 to -73.1)*	0.0 (0.0 to 0.0)	-66.6 (-82.8 to -42.8)*	-72.5 (-85.9 to -53.0)*
Schistosomiasis	1,734.9 (1,457.6 to 2,196.6)	-24.6 (-34.3 to -5.7)*	-38.0 (-46.0 to -22.5)*	13.5 (6.9 to 25.0)	-23.5 (-31.6 to -5.7)*	-37.1 (-43.8 to -22.5)*
Schistosomiasis episodes aggregate	1,248.5 (1,049.0 to 1,534.2)	-25.0 (-34.5 to -8.8)*	-38.4 (-46.1 to -25.0)*	6.5 (2.6 to 14.1)	-26.8 (-36.1 to -9.3)*	-39.8 (-47.4 to -25.4)*
Complications of schistosomiasis aggregate	390.9 (256.2 to 634.5)	-18.4 (-32.2 to 15.8)	-32.9 (-44.2 to -4.7)*	4.4 (2.1 to 8.6)	-14.3 (-27.0 to 18.0)	-29.5 (-40.0 to -3.0)*
Anemia due to schistosomiasis aggregate	94.2 (90.6 to 97.8)	-26.4 (-31.3 to -20.4)*	-39.5 (-43.5 to -34.6)*	2.6 (1.8 to 3.6)	-28.3 (-33.6 to -19.5)*	-41.0 (-45.4 to -33.8)*
Cysticercosis	52.8 (32.7 to 83.5)	2.2 (-4.0 to 8.4)	-15.9 (-21.1 to -10.9)*	7.0 (4.0 to 11.7)	-9.5 (-16.8 to -2.7)*	-25.6 (-31.6 to -20.0)*
Cystic echinococcosis	31.0 (27.4 to 35.0)	26.1 (23.0 to 29.0)*	3.7 (1.1 to 6.1)*	2.7 (1.8 to 3.7)	25.6 (16.1 to 36.0)*	3.3 (-4.5 to 11.9)
Lymphatic filariasis	557.5 (433.4 to 716.9)	-36.2 (-43.8 to -29.0)*	-47.5 (-53.8 to -41.6)*	35.1 (19.7 to 55.1)	-7.7 (-27.7 to 8.4)	-24.1 (-40.6 to -10.8)*
Prevalence of detectable microfiliaria due to lymphatic filariasis	210.9 (179.6 to 245.7)	-58.3 (-61.8 to -53.8)*	-65.7 (-68.5 to -62.0)*	0.0 (0.0 to 0.0)	0.0 (0.0 to 0.0)	0.0 (0.0 to 0.0)
Complications of lymphatic filariasis aggregate	346.6 (226.8 to 497.1)	-5.8 (-25.0 to 9.1)	-22.5 (-38.3 to -10.3)*	35.1 (19.7 to 55.1)	-7.7 (-27.7 to 8.4)	-24.1 (-40.6 to -10.8)*
Onchocerciasis	199.2 (133.9 to 302.0)	-14.0 (-31.3 to 5.4)	-29.3 (-43.5 to -13.3)*	21.9 (11.6 to 39.0)	-13.9 (-31.4 to 7.4)	-29.2 (-43.5 to -11.7)*
Asymptomatic onchocerciasis	0.4 (0.0 to 1.6)	-95.9 (-100.0 to 0.0)	-96.7 (-100.0 to 0.0)	0.0 (0.0 to 0.0)	0.0 (0.0 to 0.0)	0.0 (0.0 to 0.0)
Skin disease due to onchocerciasis aggregate	141.4 (80.6 to 236.8)	-18.1 (-37.6 to 3.7)	-32.6 (-48.7 to -14.5)*	17.8 (8.4 to 34.3)	-18.1 (-40.1 to 7.1)	-32.6 (-50.7 to -11.6)*
Vision loss due to onchocerciasis aggregate	57.6 (36.8 to 89.2)	14.1 (-1.3 to 33.2)	-6.2 (-18.8 to 9.5)	4.1 (2.6 to 6.3)	10.4 (-2.9 to 25.6)	-9.2 (-20.1 to 3.3)
Trachoma	357.3 (241.4 to 506.2)	7.5 (-4.7 to 19.8)	-11.6 (-21.7 to -1.5)*	24.8 (15.7 to 36.4)	1.1 (-10.2 to 13.3)	-16.9 (-26.2 to -6.8)*
Dengue	55.0 (31.9 to 115.7)	195.3 (26.3 to 649.7)*	142.8 (3.9 to 516.5)*	8.1 (3.8 to 17.7)	192.2 (27.3 to 676.2)*	140.3 (4.6 to 538.3)*
Dengue episodes aggregate	16.5 (11.3 to 33.1)	175.9 (21.1 to 593.3)*	126.9 (-0.4 to 470.2)	0.9 (0.5 to 1.9)	190.4 (28.5 to 663.7)*	138.8 (5.6 to 528.0)*
Post-dengue chronic fatigue syndrome	38.7 (19.0 to 82.9)	195.5 (26.3 to 649.7)*	143.0 (3.9 to 516.5)*	7.2 (3.2 to 16.0)	192.4 (27.0 to 682.7)*	140.4 (4.5 to 543.6)*
Yellow fever	0.0 (0.0 to 0.0)	-24.6 (-44.7 to 6.9)	-38.0 (-54.5 to -12.1)*	0.0 (0.0 to 0.0)	-24.7 (-44.7 to 6.9)	-38.1 (-54.5 to -12.1)*
Rabies	0.0 (0.0 to 0.0)	-33.3 (-56.5 to -1.3)*	-45.1 (-64.2 to -18.8)*	0.0 (0.0 to 0.0)	-33.4 (-56.5 to -1.3)*	-45.2 (-64.2 to -18.8)*
Intestinal nematode infections	16,374.7 (15,887.8 to 16,861.1)	16.0 (11.8 to 20.2)*	-4.6 (-8.1 to -1.2)*	27.8 (16.0 to 44.6)	-11.1 (-18.5 to -2.4)*	-26.9 (-33.0 to -19.7)*
Ascariasis	8,592.3 (7,225.4 to 10,465.4)	6.2 (-13.2 to 31.6)	-12.7 (-28.6 to 8.3)	7.8 (4.3 to 13.1)	-29.5 (-41.0 to -16.2)*	-42.0 (-51.5 to -31.1)*
Complications of ascariasis aggregate	461.7 (410.2 to 520.5)	-26.7 (-37.2 to -14.4)*	-39.7 (-48.3 to -29.6)*	7.8 (4.3 to 13.1)	-29.5 (-41.0 to -16.2)*	-42.0 (-51.5 to -31.1)*
Asymptomatic ascariasis	8,130.8 (6,778.6 to 10,015.2)	9.0 (-12.1 to 37.7)	-10.4 (-27.7 to 13.2)	0.0 (0.0 to 0.0)	0.0 (0.0 to 0.0)	0.0 (0.0 to 0.0)
Trichuriasis	5,067.2 (4,519.7 to 5,697.5)	18.2 (1.0 to 39.0)*	-2.8 (-17.0 to 14.3)	5.2 (2.7 to 8.9)	2.8 (-19.3 to 34.5)	-15.5 (-33.6 to 10.6)
Complications of trichuriasis aggregate	276.7 (239.3 to 338.0)	3.1 (-16.1 to 30.7)	-15.2 (-31.0 to 7.5)	5.2 (2.7 to 8.9)	2.8 (-19.3 to 34.5)	-15.5 (-33.6 to 10.6)
Asymptomatic trichuriasis	4,790.1 (4,227.7 to 5,421.8)	19.2 (1.1 to 41.7)*	-2.0 (-16.9 to 16.5)	0.0 (0.0 to 0.0)	0.0 (0.0 to 0.0)	0.0 (0.0 to 0.0)
[truncated: 450,519 more chars]
