# Supplementary material for: Global, regional, and national incidence, prevalence, and years lived with disability for 310 diseases and injuries, 1990–2015: a systematic analysis for the Global Burden of Disease Study 2015
Source: Lancet. 2016 Oct 8;388(10053):1545–602. doi: 10.1016/S0140-6736(16)31678-6 (PMC5055577; doi:10.1016/S0140-6736(16)31678-6)
Supplement: Supplementary appendix [file mmc1.pdf]

THE LANCET

Supplementary appendix

This appendix formed part of the original submission and has been peer reviewed. We post it as supplied by the authors.

Supplement to: GBD 2015 Disease and Injury Incidence and Prevalence Collaborators. Global, regional, and national incidence, prevalence, and years lived with disability for 310 diseases and injuries, 1990–2015: a systematic analysis for the Global Burden of Disease Study 2015. *Lancet* 2016; **388:** 1545–602.

Methods Appendix to Global, regional, and national incidence, prevalence, and YLDs for 310 diseases and injuries for 188 countries, 1990-2015: a systematic analysis for the Global Burden of Disease Study 2015

This appendix provides further methodological detail and supplemental figures and tables The appendix is organized into broad sections following the structure of the main paper.

Supplementary results for incidence, prevalence, and years of life lived with disability are presented in a separate on-line file.

Table of Contents

Methods Appendix: Tables & Figures vii
Preamble 1
Section 1 GBD overview 2
1.1. GATHER statement 2
1.2. Geographic units of the analysis 2
1.3. GBD Cause List 2
1.4. Time period of analysis 3
Section 2 Nonfatal outcome estimation 4
2.1. Data sources, identification and extraction 4
2.1.1. Systematic reviews 4
2.1.2. Search terms 4
2.1.3. Survey data preparation 5
2.1.4. Disease Registers 5
2.1.5. Hospital and claims data 5
2.1.6. Case notifications 6
2.2. Data adjustment 6
2.3. DisMod-MR 2.1 Estimation 7
2.4. Alternative Disease Modeling Strategies 10
2.5. Injury Modeling Strategy 10
2.6. Impairment and Underlying Cause Estimation 12
2.6.1. Impairment squeeze 12
2.7. Severity Distribution 13
2.8. Disability weights 15
2.9. Comorbidity Correction (COMO) 18
2.10. YLD Computation, Uncertainty & Residual YLDs 20
2.11. Decomposition 20
2.12. Socio-demographic Index (SDI) analysis & Epidemiological Transition 22
Section 3. Nonfatal cause-specific estimation process 26
Tuberculosis 27
HIV/AIDS 31
Diarrheal diseases 40
Typhoid and paratyphoid fever 46
Lower respiratory infections (LRI) 49
Upper respiratory infections (URI) 55

Otitis media....58
Meningitis....61
Encephalitis....68
Diptheria....72
Whooping cough....74
Tetanus....76
Measles....79
Varicella and herpes zoster....81
Malaria....84
Chagas disease....88
Visceral leishmaniasis....91
Cutaneous and mucocutaneous leishmaniasis....93
African trypanosomiasis....95
Shistosomiasis....99
Cysticercosis....103
Cystic echinococcosis....106
Lymphatic filariasis....109
Onchocerciasis....111
Dengue....115
Yellow fever....117
Rabies....119
Ascariasis....121
Trichuriasis....125
Hookworm disease....128
Foodborne trematodiases....133
Leprosy....140
Ebola....142
Other neglected tropical diseases....148
Maternal disorders....149
Obstetric fistula....156
Neonatal preerm birth complications....159
Neonatal encephalopathy due to birth asphyxia and birth trauma....163
Neonatal sepsis and other neonatal infections....167
Hemolytic disease and other neonatal jaundice....170
Protein-energy malnutrition....174
Iodine deficiency....176
Vitamin A deficiency....179
Iron deficiency....182

Sexually transmitted diseases excluding HIV....183
Acute hepatitis A....191
Acute hepatitis B....194
Acute hepatitis C....196
Acute hepatitis E....198
Cancer....200
Rheumatic heart disease....215
Ischemic heart disease....219
Cerebrovascular disease....225
Acute myocarditis....231
Atrial fibrillation and flutter....233
Aortic aneurysm....236
Peripheral vascular disease....237
Acute endocarditis....241
Chronic obstructive pulmonary disease (COPD)....245
Pneumoconiosis....249
Asthma....253
Interstitial lung disease and pulmonary sarcoidosis (ILD)....257
Cirrhosis....260
Peptic ulcer disease....263
Gastritis and duodenitis....266
Appendicitis....269
Paralytic ileus and intestinal obstruction....271
Inguinal, femoral, and abdominal hernia....273
Inflammatory bowel disease....276
Vascular intestinal disorders....280
Gallbladder and biliary diseases....282
Pancreatitis....286
Alzheimer disease and other dementias....289
Parkinson disease....293
Multiple sclerosis (MS)....300
Motor neuron diseases....306
Migraine....313
Tension-type headache....316
Medication overuse headache....319
Schizophrenia....322
Alcohol dependence....325

Fetal alcohol syndrome....329
Opioid dependence....332
Cocaine dependence....335
Amphetamine dependence....339
Cannabis dependence....343
Major depressive disorder....347
Dysthymia....352
Bipolar disorder....356
Anxiety disorders....360
Anorexia nervosa....364
Bulimia nervosa....367
Autism....370
Asperger syndrome and other autistic spectrum disorders....374
Attention-deficit/hyperactivity disorder (ADHD) 377
Conduct disorder....381
Diabetes mellitus....385
Acute glomerulonephritis....391
Chronic kidney disease....394
Interstitial nephritis and urinary tract infections....398
Urolithiasis....400
Benign prostatic hyperplasia (BPH)....403
Uterine fibroids....405
Polycystic ovarian syndrome....408
Endometriosis....411
Genital prolapse....414
Premenstrual syndrome (PMS) 417
Other gynecological conditions....420
Hemoglobinopathies and hemolytic anemias....422
Rheumatoid arthritis....428
Osteoarthritis....432
Low back pain (LBP) 436
Neck pain (NP) 441
Gout....444
Other musculoskeletal disorders (MSK) 447
Neural tube defects....451
Congenital heart anomalies....457
Cleft lip and cleft palate....464
Down syndrome....468

Turner syndrome........472
Klinefelter syndrome........476
Other chromosomal anomalies........480
Dermatitis........485
Psoriasis........492
Cellulitis........495
Pyoderma........498
Scabies........502
Fungal skin diseases........505
Viral skin diseases........509
Acne vulgaris........513
Alopecia areata........516
Pruritus........519
Urticaria........522
Decubitus ulcer........525
Other skin and subcutaneous diseases........528
Other sense organ diseases........531
Deciduous caries........533
Permanent caries........538
Chronic periodontal diseases........543
Edentulism and severe tooth loss........547
Other oral disorders........550
Injuries........551
Anemia impairment envelope........558
Epilepsy impairment envelope........562
Infertility impairment envelope........567
Development intellectual disability impairment envelope........570
Guillain-Barré syndrome (GBS) impairment envelope........577
Pelvic inflammatory disease impairment envelope........580
Heart failure impairment envelope........583
Vision impairment envelope........588
Hearing impairment envelope........598

Section 4. Methods Appendix: Tables and Figures........602
Section 5. References........712

Methods Appendix: Tables & Figures

Appendix Figure 1a. Analytical flowchart for the estimation of cause-specific YLDs by location, age, sex, and year for GBD 2015

Appendix Figure 1b. Analytical flowchart for modeling strategies other than DisMod MR 2.1 and injuries for select nonfatal cause groups

Appendix Figure 2. DisMod MR 2.1 Analytical Cascade

Appendix Figure 3. Map of percent of causes with any data available between 1990 and 2015 for 195 countries

Appendix Figure 4. Global decomposition of changes in leading 30 level 3 causes of years lived with disability (YLDs) due to population growth, population ageing, and changes in age-specific YLD rates, 2005 to 2015

Appendix Table 1. Guidelines for Accurate and Transparent Health Estimates Reporting (GATHER) 18-item checklist with description of compliance and location of information for this GBD 2015 nonfatal health outcomes publication

Appendix Table 2. GBD 2015 geography hierarchy with levels

Appendix Table 3. Appendix Table 3. GBD 2015 cause and sequela hierarchy with four levels of causes (1-4) and two levels of sequelae (5-6)

Appendix Table 4. ICD-9 and ICD-10 codes used in the extraction of hospital and claims data, mapped to GBD 2015 nonfatal causes, impairments, and nature of injury categories

Appendix Table 5a. Data Representativeness Index (DRI), the percentage of GBD 2015 geographies with any data, by cause, pertaining to period before 2005, 2005-2015, and all years of data

Appendix Table 5b. Data Representativeness Index (DRI), the percentage of GBD 2015 geographies with any data, by impairment, pertaining to period before 2005, 2005-2015, and all years of data

Appendix Table 6. GBD 2015 sequelae, health states, health state lay descriptions, and disability weights

Appendix Table 7. GBD 2015 methods of estimating years lived with disability (YLDs) for 35 residual categories

Appendix Table 8. Socio-demographic Index (SDI) values for all estimated GBD geographies, 1980-2015

Preamble

This methods appendix provides further methodological detail, supplemental figures, and more detailed results for incidence, prevalence, and years of life lived with disability. The appendix is organized into broad sections following the structure of the main paper. This study complies with the Guidelines for Accurate and Transparent Health Estimates Reporting (GATHER) recommendations, and this appendix is more comprehensive and encyclopedic than previous Global Burden of Disease appendices. It includes detailed tables, figures, cause modeling write-ups and flowcharts, and information on data in an effort to maximize transparency in our estimation processes and provide a comprehensive description of analytical steps. Components of this document are the same as described in the appendix to our GBD 2013 nonfatal paper; substantial components of this appendix are new text. We intend this to be a living document, to be updated with each annual iteration of the Global Burden of Disease.

Section 1. GBD overview

a. GATHER statement

This study complies with the Guidelines for Accurate and Transparent Health Estimates Reporting (GATHER) recommendations. We have documented the steps involved in our analytical procedures and detailed the data sources used in compliance with the Guidelines for Accurate and Transparent Health Estimates Reporting (GATHER). For additional GATHER reporting, please refer to Appendix Table 1 in Section 4.

b. Geographic units of the analysis

The geographies included in GBD 2015 have been arranged into a set of hierarchical categories composed of seven super-regions and a further nested set of 21 regions containing 195 countries and territories. The geographies for which GBD estimated prevalence and incidence of disease, injury incidence, and YLDs, expanded following GBD 2013. The availability of high-quality vital registration data supported the addition of American Samoa, Bermuda, Greenland, Guam, Northern Mariana Islands, Puerto Rico, and US Virgin Islands. These territories had not previously been included in national totals of the United States, United Kingdom, and Denmark but had been included in GBD regional totals. Since GBD 2013, which included subnational assessments for the United Kingdom, Mexico, and China, an additional eight subnational assessments were undertaken. Subnational breakdowns for countries in GBD 2015 included: 26 states and one district for Brazil, 34 provinces and municipalities for China, 31 states and union territories of India that include 62 rural and urban units, 47 prefectures for Japan, 47 counties for Kenya, 32 states and districts for Mexico, 13 regions of Saudi Arabia, nine provinces of South Africa, two regions for Sweden, 13 regions for the United Kingdom, and 51 states and districts for the United States. Combined, there are a total of 256 geographic units at the first subnational unit level. Included in subnational Level 1 geographies are countries that have been subdivided into the first subnational level, such as states or provinces, for the GBD analysis; subnational Level 2 only applies to India and England. For this paper we present data at the national and territory level.

c. GBD Cause List

The GBD cause and sequelae list is organized hierarchically (see Appendix Table 2) to accommodate different purposes and needs of various users.

The first two levels aggregate causes into general grouping. At Level 1 there are three cause groups: communicable, maternal, neonatal, and nutritional diseases (Group 1 diseases); non-communicable diseases (Group 2); and injuries (Group 3). These Level 1 aggregates are subdivided at Level 2 of the hierarchy into 21 cause groupings (e.g., neonatal disorders, neurological disorders, and transport injuries). The disaggregation into Levels 3 and 4 contains the finest level of detail for causes captured in GBD 2015. The greatest detail available for some causes, such as anxiety disorders or rheumatoid arthritis, is at Level 3 of the hierarchy while other specific causes are at Level 4 of the hierarchy with an aggregate category at Level 3 (for example, depressive disorders at Level 3 which encompasses major depressive disorders and dysthymia at Level 4). Sequelae of diseases and injuries are organized at Levels 5 and 6 of the hierarchy. In GBD, sequelae are defined as distinct, mutually exclusive, categories of health

consequences that can be directly attributed to a cause. For example, both neuropathy and blindness due to diabetic retinopathy are sequelae of diabetes; stroke and ischaemic heart disease are not, as these consequences cannot be categorically ascribed to diabetes in an individual despite good evidence for increased risk of these outcomes. The finest detail for all sequelae estimated in GBD is at Level 6 and is aggregated into summary sequelae categories (Level 5) for causes with large numbers of sequelae. Examples include the grouping of the infectious disease episodes and long-term sequelae of meningitis, and the grouping of 47 injury sequelae into seven summary categories (for example all fractures, spinal cord lesions, and head injuries). Sequelae in GBD are mutually exclusive and collectively exhaustive and thus our YLD estimates at each level of the hierarchy sum to the total of the level above. Prevalence and incidence aggregation is estimated at the level of individuals who may have more than one sequela or disease, and therefore are not additive.

The GBD cause list continues to evolve to reflect the policy relevance, public health, and medical care importance of the causes of major losses of health. The cause and sequelae list expanded following feedback from GBD 2013 and input from GBD 2015 collaborators. Eight causes for which nonfatal outcomes are estimated were added to the list for GBD 2015 (nonfatal outcomes for bulimia were included in GBD 2013, for GBD 2015, bulimia was also included as a cause of death). Changes to the cause list derived from policy interest for Ebola; the goal of reducing the size of residual categories, as for the inclusion of motor-neuron disease or environmental heat and cold exposure; or recognition of substantial epidemiological variation within a disease category, such as the addition of four subtypes of leukemia and two subtypes of non-melanoma skin cancer that have distinct epidemiology and outcomes (Appendix Table 3). The incorporation of these changes expanded the cause list from the 301 causes with nonfatal estimates examined in GBD 2013, to 310 causes with nonfatal estimates and from 2,337 to 2,498 unique sequelae at Level 6 of the hierarchy. At the newly created Level 5 of the hierarchy there 154 summary sequela categories. As in GBD 2013, we made no estimates of YLDs for just five causes, either because no disability is possible, as is the case with sudden infant death syndrome; because disability may occur rarely but at levels too low for accurate estimation given the data, as for aortic aneurysm; or because the disability is captured by the injuries or complicating causes that led to that cause of death, as for indirect maternal deaths, late maternal deaths, and maternal deaths aggravated by HIV/AIDS. Appendix Table 4 provides a list of International Classification of Diseases version 9 (ICD-9) and International Classification of Diseases version 10 (ICD-10) codes used in the extraction of hospital and claims data.

d. Time period of the analysis

A complete set of age-, sex-, cause-, and geography-specific incidence and prevalence numbers and rates were computed explicitly for the following years: 1990, 1995, 2000, 2005, 2010, and 2015. For years in between we made estimates by simple log-linear interpolation. Online data visualizations at http://vizhub.healthdata.org/gbd-compare[1] provide access to results for all GBD metrics, including YLDs.

Section 2. Nonfatal outcome estimation

The GBD 2015 nonfatal estimation process is visually represented in Appendix Figures 1a and 1b (section 4). Appendix Figure 1a provides a summary illustration of the steps necessary to estimate incidence, prevalence, and YLDs for disease and injury sequelae in GBD 2015. Appendix Section 3 contains additional detail specific to each disease, impairment and injury and their sequelae. Nonfatal modeling strategies vary significantly between causes. Appendix Figure 1a outlines the general process of nonfatal outcome estimation from data inputs to finalization of YLD burden results; steps 3b and 3c of that process identifies alternative modeling approaches employed for certain causes. These approaches are illustrated in greater detail in Appendix Figure 1b. Conceptually, the estimation effort is divided into eight major components: (1) compiling data sources through data identification and extraction; (2) data adjustment; (3) estimation of prevalence and incidence by cause and sequelae using DisMod-MR 2.1 or alternative modeling strategies for selected cause groups; (4) estimation by impairment; (5) severity distributions; (6) incorporation of disability weights; (7) comorbidity adjustment; and (8) the estimation of YLDs by sequelae and causes.

2.1. Data sources, identification and extraction

2.1.1. Systematic reviews

GBD 2010 collaborators undertook initial systematic reviews for the majority of causes and sequelae; for GBD2013, updates to these reviews were conducted for all causes and sequelae at IHME using data available through August of 2013. For the GBD 2015 study, we conducted systematic literature searches for 101 causes. Additionally, household surveys including the Demographic and Health Surveys (DHS), Multiple Indicator Cluster Surveys, Living Standards Measurement Surveys, Reproductive Health Surveys, and various national health surveys included in the Global Health Data Exchange (ghdx.healthdata.org) were systematically screened for data relevant to sequelae. For some diseases, case notifications reported to the World Health Organization (WHO) have been used as inputs and have been updated through 2015. For other disease sequelae, only a small fraction of the existing data appear in the published literature and other sources predominate such as survey data and vital registration. The new source tool in GHDx offers a comprehensive view of data sources used in GBD 2015.

2.1.2. Search terms

For this iteration of the study, we updated data searches through systematic data and literature reviews for 101 causes through October 2015, including the 9 new causes (Ebola, motor-neuron disease, environmental heat and cold exposure, 4 subtypes of leukemia and squamous and basal cell cancer) that were added in GBD2015. Data were systematically screened from household surveys archived in the Global Health Data Exchange (ghdx.healthdata.org), including Demographic and Health Surveys, Multiple Indicator Cluster Surveys, Living Standards Measurement Surveys, and Reproductive Health Surveys. Other national health surveys were identified based on survey series that had yielded usable data for past rounds of GBD, sources suggested to us by in-country experts, and surveys identified in major multinational survey data catalogs, such as the International Household Survey Network and the World

Health Organization (WHO) Central Data Catalog, as well as through country Ministry of Health and Central Statistical Office websites. Case notifications reported to the WHO were updated through 2015. Citations for all data sources used for nonfatal estimation in GBD 2015 are provided in searchable form through a new web-tool (http://ghdx.healthdata.org/). A description of the search terms employed for cause-specific systematic reviews are detailed by cause in Appendix Section 3.

2.1.3. Survey data preparation

For GBD 2015, survey data for which we have access to the unit record data constitute a substantial part of the underlying data used in the estimation process. During extraction, we concentrate on demographic variables (such as location, gender, age), survey design variables (such as sampling strategy and sampling weights), and the variables used to define the population estimate (such as prevalence or a proportion) and a measure of uncertainty (standard error, confidence interval or sample size and number of cases).

2.1.4. Disease Registers

For GBD 2015 nonfatal estimation, disease registries were an important source for a select number of conditions such as cancers, end-stage renal disease, and congenital disorders.

Registry data is particularly key in the estimation of neoplasms given the increasing attention to non-communicable diseases, particularly cancers, in low and middle-income areas of the world. The GHDx source tool (http://ghdx.healthdata.org/data-type/disease-registry)[2] provides a comprehensive list of registry data used in GBD estimation processes.

2.1.5. Hospital and claims data

For GBD 2015, hospital data – both inpatient and outpatient – played a key role in the nonfatal estimation process of many GBD causes.

For GBD 2015, we accessed aggregate data derived from claims information in a database of US private health insurance and public insurance schemes of Medicaid and Medicare, for the years 2000, 2010, and 2012. The population covered in each year was 3.3 million in 2000, 40.4 million in 2010, and 40.8 million in 2012. For each of these individuals, information on every health service encounter was collected and all episodes of care were linked to individuals by unique identifiers. Outpatient claims could have up to 10 diagnoses (five for inpatient claims). We mapped all ICD-9 four-digit-coded diagnoses to GBD causes see Appendix Table 4). GBD conditions were categorized as "prevalent" for diseases that we estimate to have a duration greater than one year or "incident" if modelled as having a duration less than one year. In a given year, for each individual in the claims data, a prevalent case was defined as any mention in any diagnostic field associated with any claim, including inpatient and outpatient encounters. An incident case was defined the same way, but assumed that claims within a condition-specific duration were the same case. In this way, an individual could have multiple incident cases in a given year, while avoiding double-counting cases with multiple claims from a single illness episode. From the linked claims data we generated several correction factors to account for bias in health service encounter data from elsewhere which were largely available to us aggregated by ICD code and by primary diagnosis only. First, we estimated the ratio between prevalence calculated with only the primary diagnosis and prevalence

calculated using all diagnoses associated with a claim. Second, we used the claims data to generate the average number of outpatient visits per prevalent condition assuming that claims within three days of each other concerned the same visit. Similarly, we generated per person discharge rates from hospital inpatient data in the US and New Zealand, the only sources with unique patient identifiers available for GBD 2015. We computed correction factors for multiple discharges in a year for the same condition from these two sources and applied them to tabulated hospital admission data sources.

Hospital inpatient data were extracted from 284 country-year and 976 subnational-year combinations from 27 countries in North America, Latin America, Europe, and New Zealand. Outpatient encounter data were available from the US, Norway, Sweden, and Canada for 48 country-years. ICD coding was standardized across sources, and versions of ICD (Appendix Table 4). For longer-term conditions and a subset of short-term conditions (maternal disorders, Clostridium difficile diarrhea, myocardial infarction, myocarditis, urinary tract infections, cellulitis, impetigo, decubitus ulcer, pelvic inflammatory disease, and acute episodes of peptic ulcer disease, gastritis and duodenitis, pancreatitis) we used the ratio from US claims data to correct for having primary diagnoses only. We used the average number of outpatient visits per longer term disease from claims data to correct the outpatient data from other sources.

2.1.6. Case notifications

Case notifications, active screening, intervention coverage studies, and surveillance contributed to estimates of infectious diseases. If available, we extracted data from survey and administrative microdata; otherwise, data were extracted from published literature and reports. For many infectious diseases and NTDs, we make use of cases notified by countries to the World Health Organization (WHO) and other global monitoring entities. The causes for which we use WHO case notification data include TB, measles, yellow fever, rabies, dengue, cholera, whooping cough, gonococcal infection, HAT, and other infectious and NTDs, such as Ebola.

2.2 Data adjustment

In addition to the corrections applied to claims and hospital data, a number of other adjustments were applied to extracted nonfatal sources in order to make the data more consistent and suitable for modeling. In this second step of nonfatal estimation, commonly applied adjustments included age-sex splitting, adding study-level covariates, bias correction, adjustments for underreporting of notification data, and computing expected values of excess mortality. Age-sex splitting was commonly applied to literature data reported by age or sex but not by age and sex. We relied on the meta-regression component of our main modeling tool, DisMod-MR 2.1 (see Step 3a below) for most of the bias correction of data for variations in study attributes such as case definitions and measurement method. DisMod-MR 2.1 calculates a single adjustment that is applied regardless of age, sex, or location. If enough data were available to differentiate these adjustments by age, sex, or location, or if detailed survey data were available to make more precise adjustments between different thresholds on a biochemical measure, we applied bias corrections to the data before entry into DisMod-MR 2.1. For instance, we crosswalked between 20 different case definitions with different thresholds of fasting plasma glucose or

glycated hemoglobin levels for diabetes mellitus based on available survey data with individual records of the actual measurements. In another example, we corrected data reporting on one-year prevalence instead of point prevalence of alcohol dependence by age using studies reporting on both measures, as the average duration of alcohol dependence is greater in middle-aged and older individuals compared to young adults. The correction of notification data for underreporting relied on studies that had examined the gap between true incidence and notified cases.

In GBD 2013, we estimated expected values of excess mortality from prevalence or incidence and cause-specific mortality rate (CSMR) data for a few causes only, including tuberculosis and COPD. In order to achieve greater consistency between our cause of death and nonfatal data we adopted this strategy systematically for GBD 2015. We included CSMR in DisMod-MR 2.1 models for every cause for which deaths were estimated with the exception of a few causes with very low mortality rates: uterine fibroids and schizophrenia. We matched every prevalence data point (or incidence data for short-duration conditions) with the CSMR value corresponding to the age range, sex, year, and location of the data point. We restricted this to data points reporting age-groups spanning 20 years or less. The ratio of CSMR to prevalence (or incidence times a short duration) is conceptually equivalent to an excess mortality rate. To reflect a gradient in excess mortality, we added in all relevant models the log of lag distributed income (LDI) as a country covariate for excess mortality, with a strong prior that as LDI increases, excess mortality declines.

2.3. DisMod-MR 2.1 Estimation

a. Estimation of sequelae and causes

The most extensively used estimation method is the Bayesian meta-regression method DisMod-MR 2.1. For some causes such as HIV/AIDS or hepatitis B and C, disease-specific natural history models have been used where the underlying three state model in DisMod-MR 2.1 (susceptible, cases, dead) is insufficient to capture the complexity of a disease process. For some diseases with a range of sequelae differentiated by severity, such as chronic obstructive pulmonary disease (COPD) or diabetes mellitus, DisMod-MR 2.1 is used to meta-analyze the data on overall prevalence. Separate DisMod-MR 2.1 models are then used to analyze data on the proportion of cases with different severity levels or sequelae. Likewise, DisMod-MR 2.1 is used to meta-analyze data on the proportions of liver cancer and cirrhosis due to underlying etiologies such as hepatitis B, hepatitis C, and alcohol use.

b. DisMod-MR 2.1 description

Until GBD 2010, nonfatal estimates in burden of disease assessments were based on a single data source on prevalence, incidence, remission or a mortality risk selected by the researcher as most relevant to a particular geography and time. For GBD 2010, we set a more ambitious goal: to evaluate all available information on a disease that passes a minimum quality standard. That required a different analytical tool that would be able to pool disparate information presented in varying age groupings and from data sources using different methods. The DisMod-MR 1.0 tool used in GBD 2010 evaluated and pooled all available data, adjusted data for systematic bias associated with methods that varied from the reference

and produced estimates by world regions with uncertainty intervals using Bayesian statistical methods. For GBD 2013, the improved DisMod-MR 2.0 had increased computational speed allowing computations that were consistent between all disease parameters at the country rather than region level. The hundred-fold increase in speed of DisMod-MR 2.0 was partly due to a more efficient rewrite of the code in C++ but also by changing to a model specification using log rates rather than a negative binomial model used in DisMod-MR 1.0. In cross-validation tests, the log rates specification worked as well or better than the negative binomial specification.[3] For GBD2015, the computational engine (DisMod-MR 2.1) remained substantively unchanged but we rewrote the ‘wrapper’ code that organizes the flow of data and settings at each level of the analytical cascade. The sequence of estimation occurs at five levels: global, super-region, region, country and, where applicable, subnational geographical unit. The super-region priors are generated at the global level with mixed-effects, nonlinear regression using all available data; the super-region fit, in turn, informs the region fit, and so on down the cascade. The wrapper gives analysts the choice to branch the cascade in terms of time and sex at different levels depending on data density. The default used in most models is to branch by sex after the global fit but to retain all years of data until the lowest level in the cascade. For GBD 2015, we generated fits for the years 1990, 1995, 2000, 2005, 2010, and 2015, see Appendix Figure 2 below.

Appendix Figure 2. GBD 2015 DisMod-MR 2.1 Analytical Cascade

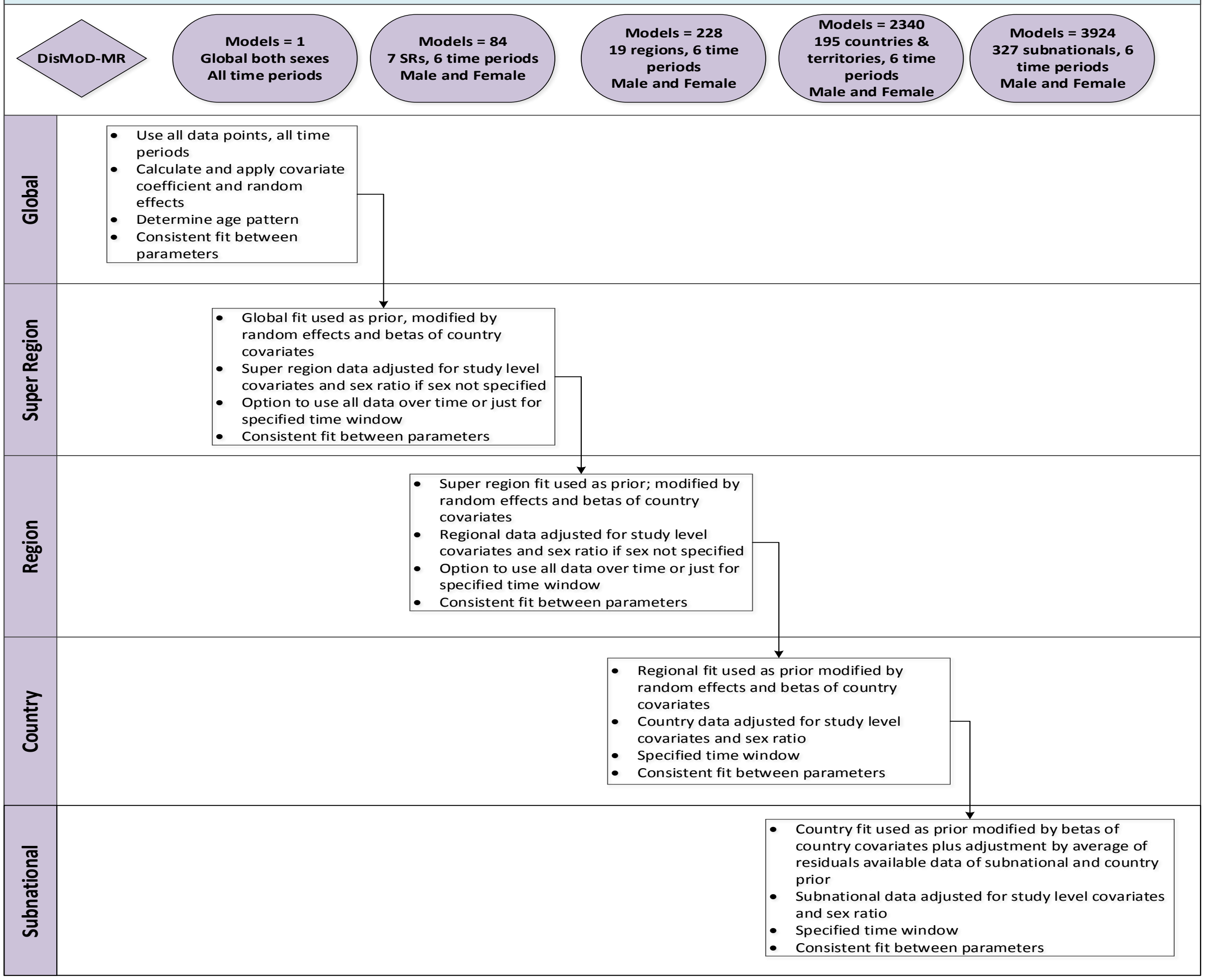

In updating the 'wrapper,' we consolidated the code base into a single language, Python, to make the code more transparent and efficient and to better deal with subnational estimation. The computational engine is limited to three levels of random effects; we differentiate estimates at the super-region, region and country level. In GBD 2013, the subnational units of China, the UK and Mexico were treated as 'countries' such that a random effect was estimated for every geography with contributing data. However, the lack of a hierarchy between country and subnational units meant that the fit to country data contributed as much to the estimation of a subnational unit as the fits for all other countries in the region. We found inconsistency between the country fit and the aggregation of subnational estimates when the country's epidemiology varied from the average of the region. Adding an additional level of random effects required a prohibitively comprehensive rewrite of the underlying DisMod-MR engine. Instead, we added a fifth layer to the cascade, with subnational estimation informed by the country fit and country covariates, plus an adjustment based on the average of the residuals between the subnational unit's available data and its prior. This mimicked the impact of a random effect on estimates between sub nationals.

For GBD 2015 we improved how country covariates differentiate nonfatal estimates for diseases with sparse data. The coefficients for country covariates are re-estimated at each level of the cascade. For a given geography, country coefficients are calculated using both data and prior information available for that geography. In the absence of data, the coefficient of its parent geography is used, in order to utilize the predictive power of our covariates in data sparse situations.

c. DisMod-MR 2.1 likelihood estimation

Analysts have the choice of using a Gaussian, log-Gaussian, Laplace or Log-Laplace likelihood function in DisMod-MR 2.0. The default log-Gaussian equation for the data likelihood is:

$$-log\left[p\left(y_j|\Phi\right)\right] = \log\left(\sqrt{2\pi}\right) + \log\left(\delta_j + s_j\right) + \frac{1}{2}\left(\frac{\log\left(a_j + \eta_j\right) - \log\left(m_j + \eta_j\right)}{\delta_j + s_j}\right)^2$$

where, y_j is a 'measurement value' (i.e., data point); Φ denotes all model random variables; η_j is the offset value, eta, for a particular 'integrand' (prevalence, incidence, remission, excess mortality rate, with-condition mortality rate, cause-specific mortality rate, relative risk or standardized mortality ratio) and a_j is the adjusted measurement for data point j, defined by:

$$a_j = e^{(-u_j - c_j)} y_j$$

where u_j is the total 'area effect' (i.e., the sum of the random effects at three levels of the cascade: super-region, region and country) and c_j is the total covariate effect (i.e., the mean combined fixed effects for sex, study level and country level covariates), defined by:

$$c_j = \sum_{k=0}^{K[I(j)]-1} \beta_{I(j),k} \hat{X}_{k,j}$$

with standard deviation

$$s_j = \sum_{l=0}^{L[I(j)]-1} \zeta_{I(j),l} \hat{Z}_{k,j}$$

where k denotes the mean value of each data point in relation to a covariate (also called x-covariate); I(j) denotes a data point for a particular integrand, j; $\beta_{I(j),k}$ is the multiplier of the kth x-covariate for the ith integrand; $\hat{X}_{k,j}$ is the covariate value corresponding to the data point j for covariate k; l denotes the standard deviation of each data point in relation to a covariate (also called z-covariate); $\zeta_{I(j),k}$ is the multiplier of the lth z-covariate for the ith integrand; and δ_j is the standard deviation for adjusted measurement j, defined by:

$$\delta_j = log\left[y_j + e^{(-u_j - c_j)}\eta_j + c_j\right] - log\left[y_j + e^{(-u_j - c_j)}\eta_j\right]$$

Where m_j denotes the model for the jth measurement, not counting effects or measurement noise and defined by:

$$m_j = \frac{1}{B(j) - A(j)} \int_{A(j)}^{B(j)} I_j(\mathrm{a})\, \mathrm{da}$$

where A(j) is the lower bound of the age range for a data point; B(j) is the upper bound of the age range for a data point; and I_j denotes the function of age corresponding to the integrand for data point j.

2.4. Alternative Disease Modeling Strategies

Most diseases and impairments were modelled by pooling data on incidence and prevalence in DisMod-MR 2.1 followed by a split into disease sequelae and or underlying etiologies. As in previous iterations of GBD, custom models were created for a short list of causes for which the compartment model underpinning DisMod (susceptible, diseased, and dead) was insufficient to capture the complexity of the disease or for which incidence and prevalence needed to be derived from other data. Step 3b of Figure 1a lists the alternative modeling strategies with greater detail shown in Figure 1b for HIV/AIDS, TB, malaria, cancer, neonatal disorders, infectious diseases for which we derived incidence from seroprevalence data, and infectious diseases for which we derived incidence from cause of death rates and pooled estimates of the case fatality proportion.

The full details of alternative disease modeling strategies, disease by disease, and the methods of nonfatal estimation for injuries are provided in Section 3 of this appendix.

2.5. Injury Modeling Strategy

For GBD 2015, we estimated the prevalence of short-term disability for all injury cases by multiplying incidence with an average duration. For treated cases of injuries, short-term durations were determined from two Dutch Injury Surveillance System follow-up studies of 2007–2010 and 2001–2004.[4,5] We used expert opinion to estimate a multiplier for the duration of short-term disability from untreated injuries and scaled all geographies between a minimum of 20% access to treatment and full treatment using GBD's covariate of health system access.

To enable comparison across the six datasets, we mapped all patient-reported outcome measures first to SF-12 and then to a GBD disability weight value. Several years of the US Medical Expenditure Panel Surveys (MEPS) contained individual question responses for EQ5D and SF12. We regressed the log of the SF-12 summary scores on individual EQ5D question responses in MEPS[6] and predicted SF-12 summary scores from the EQ5D responses in the Dutch follow-up studies. Survey participants from a variety of

IHME-conducted surveys were instructed to fill out SF-12 for a selection of 60 health states from GBD based on lay definitions.[7] We first discarded outliers with a SF-12 composite score of two standard deviations or more from the mean. Then we estimated the mean SF-12 score for each health state through a random effects regression of SF-12 on the GBD disability weight for each health state with a constant term and with disability weight included as a random effect. We fitted a Loess curve through these means to obtain a function between the SF-12 score and corresponding GBD disability weight value. For each nature of injury category, the average disability weight as implied by an individual's answers to the SF-12, SF-36, or EQ5D at one year of follow-up, was compared to the disability weight for the long-term disability from that nature of injury and the ratio taken as the average probability of having long-term disability. Fifth, after meta-analyzing standardized mortality ratios for the more severe post-injury sequelae (spinal cord lesions, head trauma, hip fracture, and severe burn injuries).

For some injuries, such as amputations and spinal cord lesion, treatment, e.g., prosthesis or wheelchair, modified the disability weight. In these cases, we approximated the fraction of injuries receiving treatment by the health system access covariate as we did for the short-term duration of treated and untreated injuries.[8]

Comorbidity-corrected YLDs from long-term injuries were then split back to cause of injury categories using the nature by cause of injury distributions from our prevalence estimates. Long-term YLDs were added to short-term YLDs to produce total YLDs by cause of injury. For pragmatic reasons, we excluded short-term injury YLDs from the comorbidity microsimulation because of its multiplier effect on use of limited large computing resources.

Step 3c. of Figure 1a outlines the separate modeling framework used for the estimation of the burden of injuries. We followed a similar strategy as in GBD 2013 to estimate injuries for GBD 2015. Injuries were categorized into 28 mutually exclusive and collectively exhaustive external cause of injury categories and 47 nature of injury categories. For GBD 2015 the external cause list was expanded with one additional cause of injury (environmental heat and cold exposure). For each cause of injury, we modelled injury incidence data from outpatient, inpatient, and survey data in DisMod-MR 2.1 to produce cause of injury incidence by geography, year, age, and sex. Dually coded inpatient and outpatient data were used to create matrices of the distributions of nature of injury in each of the cause of injury categories. We applied these matrices to our cause of injury incidence and produced incidence of injuries by cause and nature of injury combinations. In a person with multiple injuries, we selected the nature of injury category that was likely to be responsible for the largest burden using an empirical severity hierarchy derived from long-term follow-up studies.[9] We estimated the proportion of cases that resulted in permanent disability for each nature of injury category based on seven studies that provided at least one year of follow-up for various nature of injury categories and measured long-term disability with various generic health-related quality of life measures.[5,9–13] We used DisMod-MR 2.1 to estimate prevalence of long-term disability from the incident cases of injury for each cause and nature of injury combination while accounting for excess mortality associated with the more severe post-injury sequelae (spinal cord lesions, head trauma, hip fracture, and severe burn injuries. YLDs for short-term disability were calculated as prevalence multiplied by the appropriate disability weight. To account for comorbidities in YLDs from long-term disability, we first aggregated long-term prevalence for nature of injury categories across all causes of injury; these

estimates were used in the comorbidity simulation together with all other GBD causes.

2.6. Impairment and Underlying Cause Estimation

For GBD 2015, as in GBD 2013, we estimated the country-age-sex-year prevalence of nine impairments – step 4 of Figure 1a. Impairments in GBD are conditions or specific domains of functional health loss which are spread across many GBD causes as sequelae and for which there are better data to estimate the occurrence of the overall impairment than for each sequela based on the underlying cause. These impairments included: anaemia, epilepsy, hearing loss, heart failure, idiopathic developmental intellectual disability, infertility, vision loss, Guillain-Barré syndrome, and pelvic inflammatory disease. Overall impairment prevalence was estimated using DisMod-MR 2.1 except for anaemia, for which spatial-temporal Gaussian process regression (ST- GPR) methods were applied. We constrained cause-specific estimates of impairments, as in the 19 causes of blindness, to sum to the total prevalence estimated for that impairment. Anaemia, epilepsy, hearing loss, heart failure, and idiopathic developmental intellectual disability were estimated at different levels of severity. Estimates were made separately for primary infertility (those unable to conceive), secondary infertility (those having trouble conceiving again), and whether the impairment affected men and/or women. In the case of epilepsy, we determined the proportions with idiopathic and secondary epilepsy as well as for the proportions with severe and less severe epilepsy using mixed effects regressions. The sparse data for the proportion of seizure-free, treated epilepsy were pooled in a random effects meta-analysis. DisMod-MR 2.1 models produced country-, age-, sex-, and year-specific severity levels of hearing loss and vision loss. Due to limited information on the severity levels of intellectual disability, we assumed a similar distribution of severity globally, based on random effects meta-analysis of IQ-specific data for the overall impairment. This was supplemented by cause-specific severity distributions for chromosomal causes and iodine deficiency; the severity of intellectual disability included in the long-term sequelae of causes such as meningitis, neonatal tetanus, and malaria was estimated in combined health states of multiple impairments such as motor impairment, blindness, and/or seizures.[14] We changed the name of the intellectual disability impairment to specify that estimates reflect cases arising during the developmental period which we have defined as ages below 20. The severity of heart failure was derived from our Medical Expenditure Panel Surveys (MEPS) analysis and therefore was not specific for country, year, age, or sex.

A detailed description of the methods of each impairment can be found at the end of Section 3 of this appendix.

2.6.1. Impairment squeeze

For impairments like epilepsy, idiopathic developmental intellectual disability, and blindness, mentioned in Step 4, we often have better information regarding the total prevalence rather than the prevalence of said impairment due to its various causes. For example, we have more data and a better idea of the total number of blind individuals (which we refer to herein as the blindness "envelope") in the world than we do the number of individuals who are blind due to a specific cause like retinopathy of prematurity or

cataract. We achieve this consistency by either "squeezing" or inflating the individual sequela prevalence values so that their sums fit into each appropriate envelope. Blindness, epilepsy and/or intellectual disability appear in various combinations with motor impairment levels as sequelae for a number of neonatal disorders and infectious diseases like malaria and neonatal tetanus ("Moderate motor impairment with blindness and epilepsy due to neonatal tetanus," for example). This present an extra challenge as any squeeze or inflation of one of the impairments making up a sequela will affect the others. We set some rules on how to do these adjustments sequentially. First, when the envelope of an impairment is smaller than the sum of all contributing causes, we redistribute the 'excess' prevalent cases of combined impairment sequelae onto the sequelae that only have motor impairment (at mild, moderate or severe level) within the same cause grouping. Second, we apply the adjustments in a particular order such that we always fit at least one of the envelopes exactly where the other one or two envelopes may be exceeded by some amount. We first enforce a fit to the epilepsy impairment envelope, then intellectual disability and, lastly, blindness. Thus, the epilepsy envelope will always match exactly, whereas the intellectual disability and blindness envelopes may occasionally be exceeded on a draw-by-draw basis.

2.7. Severity Distribution

This section of the appendix outlines the methods used to determine the distribution of severity for conditions which have more than one level of severity and for which there was inadequate information in surveys or published studies on severity distribution.

For Step 5, sequelae were further defined in terms of severity for 194 causes at Level 4 of the hierarchy (Figure 1a). We generally followed the same approach for estimating the distribution of severity as in GBD 2013. For Ebola, we created a health state for the infectious disease episode with duration derived from average hospital admission times, and a health state for ongoing post-infection malaise and joint problems based on four follow-up studies[15–18] from which we derived an average duration. The health states for the subtypes of leukemia and non-melanoma skin cancer were the same as the general cancer health states. For motor-neuron disease we accessed the Pooled Resource Open-Access ALS Clinical Trials (PROACT) database containing detailed information on symptoms and impairments for over 8,500 patients who took part in trials.[19] We grouped the detailed information into three severity levels of motor impairment and respiratory problems, and speech problems all possible combinations between these. In cases in which severity was related to a particular impairment, such as mild, moderate, and severe heart failure due to ischemic heart disease, the analysis was driven by impairment estimation methods. Severity levels for conditions such as chronic kidney disease and COPD were modelled using DisMod-MR 2.1, while we performed meta-analyses to estimate the allocation of severity for causes such as rheumatoid arthritis, dementia, and multiple sclerosis.

For many causes we had inadequate data on severity from surveys or the epidemiological literature. For those diseases, we made use of three population surveys: the US Medical Expenditure Panel Survey (MEPS) 2000–2011, the [US] National Epidemiological Survey on Alcohol and Related Conditions (NESARC) 2000–2001 and 2004–2005, and the Australian National Survey of Mental Health and Wellbeing of Adults

(NSMHWB) 1997.[20–22] Each dataset contained individual-level measurements of functional health status using the SF-12 Health Survey as well as diagnostic information on the conditions affecting each individual.

In order to use the data collected using SF-12 for measuring the distribution of severity, the individual SF-12 summary scores were mapped to an equivalent disability weight. A convenience sample of respondents was asked to complete SF-12 for the hypothetical individual living in a health state described using a selection of 60 of the 235 health states with their lay descriptions from the GBD Disability Weights (DW) surveys, reflecting the full range of severity. Each of these health states has a measured disability weight associated with it on a zero to one scale. In total, we collected 2,202 usable responses. To deal with heterogeneity in responses, we excluded from the statistical analysis responses that were more than two standard deviations from the median for each health state. After correcting for outliers, the simple rank order correlation between SF-12 scores for the hypothetical individuals in each health state characterized by the lay description with the measured disability weight was -0.724. The health states served as random effect groups, such that the composite score would be equal to the intercept plus the random effect estimated for that health state, or

$$DW_i = \alpha + U_{health\ state}.$$

To generate a smooth mapping from SF-12 combined scores to the GBD disability weight space, we used LOESS regression on the random effects for each health state. Because disability weights are defined in the range from 0 to 1, we truncated the function at a combined SF-12 score of 118.64 (any combined score above this level was set to 0) and truncated the function at 44.3 so that any combined score less than that value was set to 1. All SF-12 survey data were thus transformed to disability weight space.

The second stage of the analysis was to build models predicting the transformed SF-12 scores as a function of the number of conditions suffered by each individual. Transformed SF-12 scores into the disability weight scale were modeled, for each measure *m* of each individual *i* over *n* total conditions in the survey, as follows:

$$logit(DW)_{im} = \beta_0 + \beta_1 Condition1_{im} + \cdots + \beta_n Condition\ n_{im} + U_i$$

where the U_i term is a random intercept on individual, to control for individual variations over multiple individual measures. This equation effectively assumes that comorbid conditions act to change SF-12 scores in a multiplicative fashion rather than an additive fashion. The random effect across individuals which is possible in the surveys with multiple measurements (MEPS) allows for individual variation in how they respond to the SF-12 instrument that is not explained by comorbid conditions.

To estimate the comorbidity-corrected effect of each condition (i.e., in isolation) on total disability, we compared the predicted disability weight without the condition of interest ("counterfactual DW") with the predicted disability weight including the condition of interest. Following the multiplicative

comorbidity equation, the joint effect can be written:

$$Condition\ specificDW = 1 - \frac{1 - predictedDW_m}{1 - counterfactualDW_m}$$

The mean of this condition-specific effect over all observations is the population marginal effect of a condition.

Using the model above, we estimate a counterfactual disability weight – the total individual disability weight excluding the effect of the condition of interest. We compared the observed distribution of functional health status with this counterfactual distribution to determine the marginal effect of the condition of interest. In other words, we estimate the health state for each individual and for each condition as the cumulative individual weight minus the effects of all comorbid conditions.

$$Health\ state\ DW = 1 - \frac{1 - individual\ cumulativeDW_m}{1 - counterfactualDW_m}$$

The estimation strategy for health state-specific severity distributions where there are multiple severity categories involved binning individuals' weights into severity cutoffs (e.g., mild, moderate, and severe) for which disability weights were derived. These bins were defined using results from the GBD Disability Weights Studies[23] for conditions which had multiple health states defined. Cutoffs were taken as the midpoints between levels of health state and cases distributed into severity bins accordingly. Cases were considered asymptomatic if the counterfactual weight exceeded the individual cumulative weight.

Severity proportions for most causes are available in the Section 3 of the Appendix.

2.8. Disability weights

To compute years lived with disability for a particular health outcome in a given population, the number of people living with that outcome is multiplied by a disability weight that represents the magnitude of health loss associated with the outcome. Disability weights are measured on a scale from 0 to 1, with 0 implying a state that is equivalent to full health and 1 a state equivalent to death.

Disability weights used in GBD studies prior to GBD 2010 have been criticized for the method used (person trade-off), the small elite panel of international public health experts who determined the weights and the lack of consistency over time as the GBD cause list expanded and additional disability weights from a study in the Netherlands[24] were added or others derived by ad-hoc methods.

GBD 2010 Disability Weights Measurement Study

For GBD 2010 a primary data collection effort focused on measuring health loss rather than welfare loss

using a standardized approach of simple comparison questions directed to the general public across diverse communities.

Multicountry household surveys were conducted between Oct 28, 2009 and June 23, 2010 in five countries (Bangladesh, Indonesia, Peru, Tanzania and the USA) selected to provide diversity across culture, language and socioeconomic status.

Personal face-to-face computer-assisted interviews were conducted for all household surveys with the exception of the survey in the USA which was conducted as computer-assisted telephone interviews. Households were randomly selected using a multistage stratified sampling design where the probability of selection was proportional to the population size. In all cases, samples were designed to be representative for a given geographical area with national representation in the case of the USA.

For every contacted household, an adult respondent aged 18 years or older was randomly selected by the survey program using the Kish approach. For face to face interviews, up to three visits were made to selected households to establish contact. When a respondent was identified, up to three return visits were made in order to do the survey at a time when the respondent was available. For the US telephone surveys, repeat calls were made up to seven times.

A web based survey was posted at a dedicated URL between July 26, 2010 and May 16, 2011. The survey was initially available in English with subsequent availability in Spanish and Mandarin. Recruitment of respondents occurred through several channels, such as news items and editorials in scientific journals, announcements at scientific meetings, postings on websites of institutions participating in the GBD, social networking and communication mobilization channels as well as direct contact with individuals and groups with known global health interests by tapping into the professional networks of the study investigators and their colleagues. Participants in the web based survey were required to be aged 18 or older. Household surveys obtained oral informed consent from all participants; written informed consent was obtained from participants in the web survey. Ethical review board approval was obtained from each household survey site and at the University of Washington, Seattle, WA.

Standardized survey instruments were developed to obtain comparative assessments of the full array of disease and injury sequelae, parsimoniously captured in 220 unique health states. Lay descriptions of health states formed the basis for all comparisons. These descriptions used simple, non-clinical vocabulary that emphasized the major functional consequences and symptoms associated with each health state. Development of these descriptions involved an iterative process of detailed consultation with experts participating in the GBD 2010 study with the goal of both capturing the most relevant details of each health state while avoiding ambiguity and ensuring consistency. Where possible, health states were grounded in standard clinical classifications systems, for example, the Canadian Cardiovascular Society grading scale was referenced for descriptions of stages of angina,[25] while the New York Heart Association functional classification was referenced for severity of heart failure.[26] Pilot testing indicated that the lay descriptions in face-to-face interviews should not exceed 30 words.

A paired comparison question formed the basis of all surveys. The questions in the survey were framed with the following statement, “A person’s health may limit how well parts of his body or mind work. As a result, some people are not able to do all of the things in life that others may do, and some people are

more severely limited than others. I am going to ask you a series of questions about different health problems. In each question, I will describe two different people…" Descriptions of two hypothetical people, each with a particular health state, were presented to respondents who were then asked which person they regarded as the healthier. Health pairs in all surveys were selected by a randomizing computer algorithm. In the five household surveys, paired comparisons were presented for a subset of 108 health states pertaining to chronic conditions. The framing of chronic and acute conditions is different as they were presented as causing life-long or temporary health loss. We chose to only field health states that could be framed as lasting a lifetime in the household surveys as we hypothesized that presenting differently framed comparisons would be difficult to convey in face-to-face interviews. In the web survey we considered this more feasible as respondents could read and refer to the framing of the question for each pair-wise comparison. All 220 health states were thus evaluated in the web survey.

In addition, the web survey included questions relating to population health and health programs specifically – such as "Imagine two different health programs. The first program prevented 1,000 people from getting an illness that causes rapid death. The second program prevented 2,000 people from getting an illness that is not fatal but causes lifelong health problems resulting in moderate to severe disability. Which program would you say produced the greater overall health benefits?" This information was used to anchor the results from the pair-wise comparisons on the 0–1 disability weight scale.

GBD 2013 European Disability Weights Measurement Study

The GBD 2010 disability weights were critically dependent on the ways that outcomes were described to survey respondents. Descriptions for health states were designed to balance validity and parsimony and this necessarily meant that some details of different health states had to be omitted. As lay descriptions were developed collaboratively through individual expert groups organized around a particular set of health issues – some amount of variability in language and detail inevitably occurred. Criticisms and suggestions for improvement came from a number of commentators on the GBD 2010 disability weights measurement study.[27–29]

The GBD 2013 Study expanded the list of disease and injury causes to 306, giving rise to 2337 sequelae, themselves mapped to 235 unique health states. Additional data for the European Disability Weights Measurement Study were collected between September 23, 2013 and November 11, 2013 in Hungary, Italy, the Netherlands and Sweden. This initiation of these surveys was connected to a project sponsored by the European Centre for Disease Prevention and Control (the Burden of Communicable Diseases in Europe project.[30] The four selected countries were chosen to be representative of the four regions of Europe (east, south, middle, and north) in terms of age, sex and education of the respondents. Respondents were recruited from standing internet panels in each country on the basis of quota sampling with reference to age, sex and education in such a way as to maintain population representativeness of these characteristics. Eligible participants were aged 18–65 and were preselected in the case of the Netherlands, where age, sex and education of respondents were already known, or in the case of the other three countries, invited to participate via a web-link and then selected on the basis of their individual characteristics.

The protocol for the European disability weights measurement study followed the protocol that was developed and implemented in the GBD 2010 disability weights measurement study. Lay descriptions for some health states that lacked mention of an important symptom or for which consistency of wording across different levels of severity had been noted were reworded. The European disability weights measurement study included 255 health states, of which 183 were used in the analyses of GBD 2013. Those 183 consisted of 135 of the 220 health states that were included in the European disability weights measurement study with unmodified lay descriptions; 30 from GBD 2010 for which alternative lay descriptions were included. Disability weights were estimated for additional sequelae that were incorporated into GBD 2013 but had not been included in GBD 2010.

Finding high correlation in resulting disability weight values between the country surveys and the web survey, we analyzed the results of all surveys together. We ran probit regression analyses on the answers to the pair-wise comparison questions, with dummies for each health state with a value of 1 for the first state in a pair, –1 for the second of a pair being chosen, and 0 for all states other than the pair being considered. This method formalizes the intuition that if two health states in a pair produce similar health loss, the answers are likely to be evenly split; a pair of health states with very different health loss, will get many more responses favoring one over the other. The statistical methods infer the distances between values attached to different health states based on the frequencies of responses to the paired comparisons. A second analytic step is needed to anchor the resulting estimates onto the 0–1 disability weights scale. We anchored results from the probit regression analysis onto the 0–1 scale using population health equivalence data from the GBD 2010 web survey using a linear regression of the probit coefficients from the analysis of paired comparisons on the logit-transformed disability weight estimates derived from interval regression of the population health equivalence responses. Using numerical integration we then estimated mean values for disability weights on the natural 0–1 scale. Uncertainty was estimated by bootstrapping with 1000 samples.

A complete listing of the GBD sequelae, health states, lay descriptions and DW values for the 235 health states used in GBD 2015 is provided in Appendix Table 6.

2.9. Comorbidity correction (COMO)

The final stage in the estimation of YLDs is a micro-simulation, which adjusts for comorbidity. We refer to this micro-simulation process as “COMO”. For GBD 2015, we estimated the co-occurrence of different diseases by simulating 40,000 individuals in each geography-age-sex-year combination as exposed to the independent probability of having any of the sequelae included in GBD 2015 based on disease prevalence. We tested the contribution of dependent and independent comorbidity in the US MEPS data, and found that independent comorbidity was the dominant factor even though there are well-known examples of dependent comorbidity, i.e., clustering of conditions such as diabetes and stroke or anxiety and alcohol use disorders. Age was the main predictor of comorbidity such that age-specific microsimulations accommodated most of the required comorbidity correction.[31]

The two components necessary for the computation of YLDs, prevalence of each disease sequelae and disability weights, are the two inputs into COMO. The prevalence values are primarily produced using DisMod-MR2.1. The disability weights have been described above.

The micro-simulation, as performed for each age-sex-geography-year, can best be represented as a four-step process. First, simulants are exposed to independent probabilities of having each sequela, where the probability is equal to the prevalence estimate. For each simulant, the probability of having a disease sequela is equal to the estimated prevalence from that draw from the uncertainty distribution. Each simulant is determined to have or not have the disease sequelae based on a draw from a binomial distribution. From this simulation, simulants end up having from none to multiple disease sequelae. Second, the disability weight for each simulant is estimated based on the disease sequelae that they have acquired. The formula for the cumulative disability weight for a simulant is one minus the multiplicative sum of one minus each disability weight present:

$$Simulant\ DW_l = 1 - \prod_{k=i}^{j}(1 - DW_k)$$

where the DW_k is the disability weight for the kth disease sequela that the simulant *l* has acquired. Once the simulant disability weight is computed, the disability weight attributable to each sequela for the simulant is calculated using the following formula:

$$ADW_{lk} = \frac{DW_k}{\sum_{k=i}^{k=j} DW_k} * Simulant\ DW_l$$

where ADW_{lk} is the attributable DW for disease sequela *k* in simulant *l*; DW_k is the disability weight for disease sequela *k*, and simulant DW_l is the disability weight for simulant *l* from the combination of all sequelae that they have acquired. This formula apportions the overall simulant disability weight to each condition in proportion to the disability weight of each condition in isolation.

Finally, YLDs per capita in an age-sex-country-year are computed by taking the sum of the attributable disability weights for a disease sequela across simulants.

$$YLD\ Rate_k = \frac{\sum_{l=1}^{n} ADW_{lk}}{n}$$

The actual number of YLDs from disease sequela *k* in an age-sex-geography-year is then computed as the YLD rate *k* times the appropriate age-sex- geography-year population.

By repeating the simulation process for each age-sex-country-year 1,000 times, the uncertainty in the prevalence of each disease sequelae and the disability weights is propagated into the final comorbidity corrected YLD results. We selected 40,000 simulants for each age-sex- geography-year group on the basis of simulation testing, which has shown that results are stable for YLDs at this number of simulants even in the younger age groups when prevalence is relatively low. Mean results for YLDs which reflect 40 million simulants (40,000 simulants multiplied by 1,000 iterations to capture uncertainty) are very stable in each age-sex-geography-year. For any given location-year-age-sex group, sequelae with prevalence of less than one in 20,000 were excluded from the microsimulation.

One of the outputs from this comorbidity simulation was counts of the number of sequelae for each simulant in the population; these were subsequently scaled to the estimated population in each geography-age-sex-year, which produced the estimated distribution of individuals with comorbidities in each country-year. We created descriptive population pyramids (see Figure 5a-b. from main text) showing individuals by categories of severity of sequelae: asymptomatic, very mild disability (disability weight less than or equal to 0.01), mild disability (from 0.01 to 0.05 inclusive), moderate (from 0.05 to 0.1 inclusive), severe (from 0.1 to 0.3 inclusive), and profound (greater than 0.3). The pyramids classify individuals in our microsimulation to the multiplicatively combined DW of all their comorbid sequelae.

2.10. YLD Computation, Uncertainty & Residual YLDs

For GBD 2015, we computed YLDs by sequela as prevalence multiplied by the disability weight for the health state associated with that sequela. The uncertainty ranges reported around YLDs incorporates uncertainty in prevalence and uncertainty in the disability weight. To do this, we take the 1,000 samples of comorbidity-corrected YLDs and 1,000 samples of the disability weight to generate 1,000 samples of the YLD distribution. We assume no correlation in the uncertainty in YLDs and disability weights. The 95% uncertainty interval is reported as the 25th and 975th values of the distribution. Uncertainty intervals for YLDs at different points in time (1990, 1995, 2000, 2005, 2010, and 2013) for a given disease or sequelae are correlated because of the shared uncertainty in the disability weight. For this reason, changes in YLDs over time can be significant even if the uncertainty intervals of the two estimates of YLDs largely overlap as significance is determined by the uncertainty around the prevalence estimates.

Residual YLDs

Despite expanding our list of causes and sequelae in successive GBD iterations, many diseases remain for which we do not explicitly estimate disease prevalence and YLDs. Less common diseases and their sequelae were included in 35 residual categories (Appendix Table 7). For 22 of these residual categories, epidemiological data on incidence or prevalence were available and so these were modelled accordingly. For 13 residual categories, epidemiological data on incidence and prevalence were not available but sufficient cause of death data allowed for cause of death estimates. For these residual categories, we estimated YLDs by multiplying the residual YLL estimates by the ratio of YLDs to YLL from the estimates for explicitly modelled, Level 3 causes in the same disease category. This scaling was undertaken for each country-sex-year. This approach made the simplifying assumption that the residual diseases caused disability proportionate to the ratio of disability to mortality in explicitly modelled diseases. We did not include causes with large disability but no or little mortality in estimating these ratios, and thus estimated the YLDs from other neurological disorders from the YLD to YLL ratios for dementia, multiple sclerosis, and Parkinson's disease, but did not include the YLDs from headaches and epilepsy in the ratio.

2.11. Decomposition

The numbers of YLDs by cause and geography between 2005 and 2015 were decomposed into three explanatory components: change attributable to growth of the total population, change in the population

structure by age or sex, and change in age-, sex-, and cause-specific rates. All changes in age-, sex-, and cause-specific YLD rates not explained by population growth and aging (demographic change) are referred to as epidemiologic change. The net change of these three components equals the observed change in the total number of YLDs. For reporting purposes, we illustrate the approach for decomposition using the last decade of data (2005-2015). We illustrate the approach for decomposition using the 2005 to 2015 period. We calculated two different sets of numbers for YLDs, using counterfactual scenarios. In the first scenario, for population growth, the number of YLDs in 2015 was the number expected if the total population increased from 2005 as observed, but the age-sex specific population structure and rates of YLDs were the same in 2015 as in 2005. In the second scenario, for population growth and ageing, the number of deaths in 2015 was the number expected using the 2015 age-sex specific population structure, but with the age-sex specific rates of death held constant to 2005. The difference between the number of deaths observed in 2005 and those estimated for 2015 with the population growth scenario is the change in the number of deaths exclusively from population growth. The difference between the population growth scenario and the scenario for population growth and aging is the change exclusively from population aging.[32]

To guarantee consistency between this paper and other GBD papers with decomposition analysis, we changed the decomposition method used to the Das Gupta method which was developed and published more than 30 years ago. There is no detectable differences in the results. The advantage is that the Das Gupta method can be scaled to decompose changes into up to 5 factors. A four factor decomposition was used in the risk factor paper.

Decomposition analysis of changes in global YLDs

The primary factors driving changes in global rates of disability – population growth, aging, and changes to age-standardised rates of cause-specific YLDs– varied by cause from 2005 to 2015 (SR Figure 2). For the leading causes of disability worldwide, changes in total levels of disability ranged from a decrease of 0.93% (1.43–0.38%) for Iron-deficiency anemia to an increase of 39.2% (36.2–42.6%) for diabetes. Population growth accounted for increases in disability across all 30 causes, but spanned from 9.21% (-8.97-9.46%) for cerebrovascular disease to 15.1% (15.0-15.2) for iron-deficiency anemia. In fact, for all Group 1 conditions among the leading 30 causes of disability, population growth was the only factor that hindered further reductions in disability. Population ageing led to rising disability for most causes, but its contribution ranged from 0.32% (-0.40-1.04) to more than 20% for cerebrovascular disease and other dementias. Shifts in population age structures also accounted for more than 8.67% (8.09-9.29%) of increased YLDs due to low back and neck pain, and 19.34% (19.0-19.7%) due to osteoarthritis. On the other hand, for some causes, such as iron-deficiency anaemia and haemoglobinopathies, population aging contributed to decreasing levels of disability. Population aging also accounted for 1.25% (1.5-0.96) of declines in YLDs due to autistic spectrum disorders, diverging from trends observed for other mental health disorders (e.g. population aging was associated with 4.84% (3.76-5.86%) of rising disability due to depressive disorders). Changes in age- and cause-specific YLD rates markedly differed, with various types of injuries experiencing reductions exceeding 15% (e.g falls 16.31% [22.4-10.0%]) and others recording much smaller declines (e.g. sense organ disease 1.14% [1.75-0.47%]).

For more information, see Appendix Figure 4.

2.12. Socio-Demographic Index (SDI) analysis & Epidemiological Transition

a. Development of revised SDI indicator

We began exploring the relationship between a composite indicator of sociodemographic development in GBD 2013. We used lag distributed income per capita (LDI), average educational attainment over the age 15 years, total fertility rate (TFR), and mean population age and called it SDS, sociodemographic status. In response to feedback, we excluded mean population age due its strong relationship to mortality rates. We renamed the indicator Socio-demographic Index (SDI). SDI has an interpretable scale: zero represents the lowest income per capita, lowest educational attainment, and highest TFR observed across all GBD geographies from 1980 to 2015 and one represents the highest income per capita, highest educational attainment, and lowest TFR.

SDI was calculated using the Human Development Index (HDI) methodology, wherein an index value was determined for each covariate input (log LDI, average educational attainment in the population over age 15, and TFR):

$$I_{Cly} = \frac{C_{ly} - \min(C)}{\max(C) - \min(C)}$$

Where I_{cly} – the index for covariate *C*, location *l*, and year *y* – is equal to the difference between the value of that covariate in that location-year and the minimum observed value of the covariate (min(C)) in any location over the 1980–2015 time interval divided by the observed range (max(C)-min(C)). An additional innovation for GBD 2015 was to incorporate subnational locations (resulting in 519 unique administrative units) for the entire estimation period of 1980–2015. The Socio-demographic Index is then the geometric mean of these three indices:

$$SDI = \sqrt[3]{I_{lnLDI} I_{educ} I_{TFR}}$$

In our mortality analyses, for LDI and TFR, we noted depreciating gains in life expectancy at birth and ${}_5q_0$ at the higher and lower terminals, respectively. Due to the significance of these values in indexing, we aimed to identify the point at which increasing income or reducing fertility no longer resulted in improved child mortality or life expectancy. We tested various restrictions, and found that capping LDI at $60,000 and setting a TFR floor at 1 resulted in improved correlations with the resultant health indicators.

We further aimed to validate the use of SDI by regressing it in a variety of forms against life expectancy at birth, ${}_5q_0$, ${}_{35}q_{15}$, and ${}_{20}q_{50}$. We found that SDI generally is as capable of predicting these demographic indicators as the previous SDS, and also as the inputs. We also found that in incorporating year, we did not substantially reduce the coefficients for SDI. Additionally, in testing lags of 2–10 years, we found the version with no lag to be the most predictive. SDI values by GBD geography over time are illustrated in the Supplementary Results.

b. Age-sex-specific relationships between SDI and YLD rates

In order to evaluate the relationship between SDI and morbidity, we fit a simple least-squares regression using a smoothing spline on SDI for every cause in Levels 1, 2, and 3 of the GBD cause hierarchy:

$$\ln(y_{l,y,a,s,c}) = \sum_{i=0}^{d+k} \beta_i B_{i,d}(SDI) + \gamma_U + \gamma_E + \gamma_C + \gamma_O + \varepsilon_{l,y,a,s,c}$$

where:

- $\ln(y_{l,y,a,s,c})$ is the log YLD rate in location *l* and year *y*, and for age *a*, sex *s*, and cause *c*
- $\sum_{i=0}^{d+k} \beta_i B_{i,d}(SDI)$ is resultant parametric curve, of degree *d* and interior knots *k*, of a linear combination of basis splines $B_{i,d}(SDI)$
- γ_U is a dummy variable for the United States
- γ_E is a dummy variable for the GBD region Eastern Europe
- γ_C is a dummy variable for the GBD region Central Asia
- γ_O is a dummy variable for the GBD region Oceania
- $\varepsilon_{l,y,a,s,c}$ is the error term for location *l,* year *y*, age *a*, sex *s*, and cause *c*

Regressions were run separately by age, sex, and cause, using all location-years. Dummy variables were included for locations that were identified in modeling to skew fit due to significant deviation from levels of morbidity observed elsewhere at similar levels of SDI. In the case of the United States because of the inclusion of 50 states, the US collectively had an undue influence on the shape of the relationship which is why a separate dummy variable was included for the US. Because of the mortality crisis in Eastern Europe and Central Asia after the collapse of the Soviet Union, we included a dummy variable to adjust for the mean difference in these regions.

Due to the more reliable estimation at more aggregate levels of cause specificity, we imposed a top-down hierarchical scaling scheme, in which the Level 1 causes were scaled to the predictions for all-cause morbidity, then Level 2 causes were scaled to their scaled Level 1 parents, and once more for Level 3 to Level 2. Having a complete set of age specific YLD rates, we were then able to produce a full set of age-standardized rates for every SDI level. We evaluated this relationship at each centile value of SDI (i.e., by increments of 0.01). The SDI ranged from 0.060 in Mozambique in 1987 to 0.978 in the District of Columbia, United States in 2015.

We used the same modeling set up but used the logit of the share of population in each age-group as the dependent variable to estimate a smoothed relationship between population age-structure and SDI. Predictions for each age-group at each level of SDI were rescaled to sum to 100%.

References

2 Disease registry | GHDx. http://ghdx.healthdata.org/data-type/disease-registry (accessed June 24, 2016).

3 An Integrative Metaregression Framework for Descriptive Epidemiology (Publications on Global Health, Institute for Health Metrics and Evaluation): Abraham D. Flaxman, Theo Vos, Christopher J.L. Murray, Patricia Kiyono: 9780295991849: Amazon.com: Books. https://www.amazon.com/Integrative-Metaregression-Descriptive-Epidemiology-Publications/dp/0295991844 (accessed June 24, 2016).

4 A Haagsma J. Posttraumatic Stress Disorder Following Injury: Trajectories and Impact on Health-Related Quality of Life. *J Depress Anxiety* 2013. DOI:10.4172/2167-1044.S4-002.

5 Polinder S, van Beeck EF, Essink-Bot ML, *et al.* Functional outcome at 2.5, 5, 9, and 24 months after injury in the Netherlands. *J Trauma* 2007; 62: 133–41.

6 Sullivan PW, Ghushchyan V. Mapping the EQ-5D Index from the SF-12. *Med Decis Mak Int J Soc Med Decis Mak* 2006; 26: 401–9.

7 Burstein R, Fleming T, Haagsma J, Salomon JA, Vos T, Murray CJ. Estimating distributions of health state severity for the global burden of disease study. *Popul Health Metr* 2015; 13: 31.

8 Global, regional, and national age-sex specific all-cause and cause-specific mortality for 240 causes of death, 1990-2013: a systematic analysis for the Global Burden of Disease Study 2013. *Lancet* 2015; 385: 117–71.

9 Haagsma JA, Graetz N, Bolliger I, *et al.* The global burden of injury: incidence, mortality, disability-adjusted life years and time trends from the Global Burden of Disease study 2013. *Inj Prev* 2015; : injuryprev-2015-041616.

10 Pickelsimer EE, Selassie AW, Gu JK, Langlois JA. A population-based outcomes study of persons hospitalized with traumatic brain injury: operations of the South Carolina Traumatic Brain Injury Follow-up Registry. *J Head Trauma Rehabil* 2006; 21: 491–504.

11 Mackenzie EJ, Rivara FP, Jurkovich GJ, *et al.* The National Study on Costs and Outcomes of Trauma. *J Trauma* 2007; 63: S54-67-86.

12 van Loey NE, van Beeck EF, Faber BW, van de Schoot R, Bremer M. Health-Related Quality of Life After Burns: A Prospective Multicentre Cohort Study With 18 Months Follow-Up. *J Trauma* 2011; published online Oct 24. DOI:10.1097/TA.0b013e3182199072.

13 Medical Expenditure Panel Survey Home. https://meps.ahrq.gov/mepsweb/ (accessed June 25, 2016).

14 Burstein R, Fleming T, Haagsma J, Salomon JA, Vos T, Murray CJ. Estimating distributions of health state severity for the global burden of disease study. *Popul Health Metr* 2015; 13: 31.

15 Clark DV, Kibuuka H, Millard M, *et al.* Long-term sequelae after Ebola virus disease in Bundibugyo, Uganda: a retrospective cohort study. *Lancet Infect Dis* 2015; 15: 905–12.

16 Qureshi AI, Chughtai M, Loua TO, *et al.* Study of Ebola Virus Disease Survivors in Guinea. *Clin Infect Dis* 2015; 61: 1035–42.

17 Rowe AK, Bertolli J, Khan AS, *et al.* Clinical, Virologic, and Immunologic Follow-Up of Convalescent Ebola Hemorrhagic Fever Patients and Their Household Contacts, Kikwit, Democratic Republic of the Congo. *J Infect Dis* 1999; 179: S28–35.

18 Bwaka MA, Bonnet M-J, Calain P, *et al.* Ebola Hemorrhagic Fever in Kikwit, Democratic Republic of the Congo: Clinical Observations in 103 Patients. *J Infect Dis* 1999; 179: S1–7.

19 PRO-ACT - HOME. https://nctu.partners.org/ProACT/ (accessed May 26, 2016).

20 Medical Expenditure Panel Survey Home. https://meps.ahrq.gov/mepsweb/ (accessed June 25, 2016).

21 NIAAA Publications. http://pubs.niaaa.nih.gov/publications/arh29-2/74-78.htm (accessed June 25, 2016).

22 Statistics c=AU; o=Commonwealth of A ou=Australian B of. Main Features - Main Features. 1998; published online March 12. http://www.abs.gov.au/ausstats/abs@.nsf/ProductsbyReleaseDate/D5A0AC778746378FCA2574EA00122887?OpenDocument (accessed June 25, 2016).

23 Salomon JA, Haagsma JA, Davis A, *et al.* Disability weights for the Global Burden of Disease 2013 study. *Lancet Glob Health* 2015; 3: e712–23.

24 Stouthard M, Essink-Bot ML, Bonsel G, Barendregt J, Kramers P. Disability weights for diseases in the Netherlands. *Eramus Univ Rotterdam* 1997.

25 Campeau L. The Canadian Cardiovascular Society grading of angina pectoris revisited 30 years later. *Can J Cardiol* 2002; 18: 371–9.

26 Nomenclature and criteria for diagnosis of diseases of the heart and great vessels / the Criteria Committee of the New York Heart Association. - Version details. Trove. http://trove.nla.gov.au/version/13288061 (accessed June 25, 2016).

27 Nord E. Disability weights in the Global Burden of Disease 2010: Unclear meaning and overstatement of international agreement. *Health Policy* 2013; 111: 99–104.

28 Taylor HR, Jonas JB, Keeffe J, *et al.* Disability weights for vision disorders in Global Burden of Disease study. *The Lancet* 2013; 381: 23.

29 Voigt K, King NB. Disability weights in the global burden of disease 2010 study: two steps forward, one step back? *Bull World Health Organ* 2014; 92: 226–8.

30 Kretzschmar M, Mangen M-JJ, Pinheiro P, *et al.* New methodology for estimating the burden of infectious diseases in Europe. *PLoS Med* 2012; 9: e1001205.

31 Vos T, Flaxman AD, Naghavi M, *et al.* Years lived with disability (YLDs) for 1160 sequelae of 289 diseases and injuries 1990–2010: a systematic analysis for the Global Burden of Disease Study 2010. *The Lancet* 2012; 380: 2163–96.

32 Gupta PD. A general method of decomposing a difference between two rates into several components. *Demography* 1978; 15: 99–112.

Section 3. Nonfatal cause-specific estimation process

The cause specific modeling write-ups follow the order of the cause hierarchy for GBD 2015. In some cases, multiple cause ids are addressed in a single write-up, for example neoplasms are all included in a single detailed write-up. Similarly, for certain causes with multiple modelable entities, multiple flowcharts are given to indicate differences in modeling approaches, for example – fungal skin diseases and viral skin diseases. Appendix Table 3 illustrates the GBD 2015 cause and sequela hierarchy with levels, Appendix Table 6b matches each sequela with one or more of the 235 GBD health states, and Appendix Table 7 illustrates the methods for estimating 35 residual categories.

Tuberculosis

Flowchart

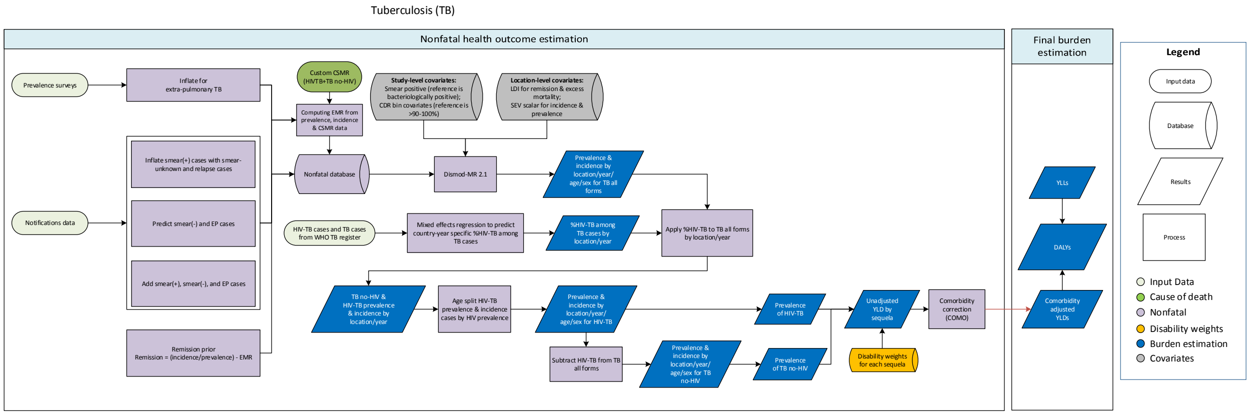

Input data and methodological summary

Case Definition

Tuberculosis (TB) is an infectious disease caused by Mycobacterium tuberculosis. The case definition includes all forms of TB including pulmonary TB and extrapulmonary TB which are bacteriologically confirmed or clinically diagnosed. For TB, the ICD 10 codes are A10-A19.9, B90-B90.9, K67.3, K93.0, M49.0, P37.0, and ICD 9 codes are 010-019.9, 137-137.9, 138.0, 138.9, 139.9, 320.4, 730.4-730.6. For HIV-TB, the ICD 10 code is B20.0.

Input data

Model Inputs

Input data include annual case notifications, data from prevalence surveys, and estimated cause-specific mortality (CSMR) of TB among HIV-positive and HIV-negative individuals. From these inputs, we calculated 'priors' (expected values) on excess mortality and remission to give greater guidance to the model.

A systematic review was done for GBD 2015 using the following PubMed search terms: (((tuberculosis[Title/Abstract]) OR TB[Title/Abstract]) OR Mycobacterium tuberculosis[Title/Abstract]) AND prevalence[Title/Abstract] Filters: Publication date from 2013/01/01 to 2015/12/31; Humans.

The exclusion criteria were:

1. Studies that were not population-based, e.g., hospital or clinic-based studies
2. Studies that did not provide primary data on epidemiological parameters, e.g. commentaries
3. Studies with a sample size of less than 150
4. Reviews

Modeling Strategy

We used DisMod-MR 2.1, the GBD Bayesian meta-regression tool that adjusts for differences in methods between data sources and imposes consistency between data for different parameters.

Modeling TB incidence

We used the age and sex-specific notifications in our analysis. There were missing age data especially for younger age-groups in some countries. We imputed the missing age-groups for three forms of TB notifications (pulmonary smear-positive, pulmonary smear-negative, and extra-pulmonary). Smear-positive age-specific notifications were inflated with the proportion smear-unknown and relapsed cases only reported at the country-year level. Some countries reported only pulmonary smear-positive cases for selected years. Missing smear-negative and extra-pulmonary cases were predicted from the adjusted smear-positive cases using a seemingly unrelated regression. All three types of notifications were added together. We binned countries into case detection rate (CDR) groupings based on the WHO's estimates of country-year specific CDR. We created CDR bins [10-percent CDR groups (0-10%, >10-20%, etc.) up to the group of >90-100%] based on a 5-year moving average of WHO CDR estimates. We assumed all high income countries to be in the group of >90-100%. We added 9 study-level covariates (the reference category was the 90-100% category) as an initial guide by how much notifications need to be increased to reflect the incidence of all TB. We set bounds on the coefficients (in log space) that correspond to the WHO CDR estimates (e.g., -1.9 (-2.3 to -1.6) for the >10-20% category). We then relied on DisMod-MR 2.1 to find an estimate that is consistent with available prevalence and cause-specific mortality rates. We included a country-level covariate, namely, the log-transformed age-standardized Summary Exposure Variables (SEV) scalar covariate to help inform variation over year and geography, especially in location-years with no or sparse data. The SEV scalar covariate reflects the average exposure to all of the risk factors related to TB. It was calculated using the following formula: 1 / (1-PAF), where PAF is the population attributable fraction. We set bounds of 0.75 to 1.25 on the SEV scalar covariate where a value in log space of 1 would reflect perfect agreement with our risk factor estimates.

Modeling TB prevalence

Data from prevalence surveys reporting on pulmonary smear-positive TB and bacteriologically positive TB were included. We included a study covariate indicating whether it was bacteriologically positive TB (reference category) or smear-positive TB. We found no systematic bias between studies that used both symptoms and chest X-ray as screening methods and studies that used only one of the methods. We therefore did not adjust them for systematic bias but added more uncertainty to data points from studies that used only one of the screening methods (by using it as a z-cov in Dismod). We also added more uncertainty to data points from sub-national surveys. Because incidence data are for all forms of TB, we adjusted prevalence surveys to account for extra-pulmonary cases. We predicted location-year-age-sex specific proportions of extra-pulmonary TB among all TB cases using data on the three forms of TB from the incidence data above and the lagged distributed income covariate from the IHME covariate database. We then computed the extra-pulmonary inflation factor as *1+(proportion of extrapulmonary TB /(1-proportion of extrapulmonary TB))*, and applied it to data from prevalence surveys. We included the SEV scalar country-level covariate with priors that as the SEV scalar increases, prevalence increases.

Modeling remission and excess mortality

We matched each prevalence data point and TB CSMR (TB and HIV-TB combined) by location, year, age, and sex to calculate excess mortality rate (EMR) as *EMR=CSMR/prevalence*. We also matched each incidence data point and TB CSMR by location, year, age, and sex to calculate EMR for data-rich countries (i.e., countries with vital registration more than 95% complete for more than 25 years). We calculated remission using data from countries where both incidence and prevalence data were available. We matched incidence and prevalence data by location, year, age, and sex, and calculated remission as *remission=(incidence/prevalence)-EMR.* For data-rich countries, we assumed a remission of 2.0 (1.8-2.2). We ran two DisMod models: one where we used the calculated remission for low- and middle-income countries, and another where we applied the remission assumption for data-rich countries. To reflect a gradient in EMR and remission, we added the log lag distributed income (LDI) as a country covariate, with priors that as LDI increases, EMR decreases and remission increases. For final results we combined results from data rich and data poor countries from their respective models.

HIV-TB incidence and prevalence

The output from the DisMod model described above is for all forms of TB in HIV-negative and HIV-positive individuals. To separate out HIV-TB from all forms of TB, we first estimated the proportions of HIV-TB cases among all TB cases for all locations and years, using the adult HIV death rate as a covariate in a mixed effects regression. The input data for this regression (i.e., proportions of HIV-TB cases among all TB cases) were based on the number of TB cases recorded as HIV-positive and the number of TB cases with an HIV test result recorded in the WHO TB notifications register. We applied the predicted location-year specific proportions to TB incident and prevalent cases from DisMod, to generate HIV-TB incident and prevalent cases by location and year, which were then age-sex splitted based on the age-sex pattern of estimated HIV prevalence by country-year to generate location-year-age-sex specific HIV-TB incident and prevalent cases.

Betas and exponentiated values (which can be interpreted as an odds ratio) from the two DisMod models are shown in the tables below:

Betas and exponentiated values from the data-poor model (using remission calculated based on incidence and prevalence data)

Covariate	Parameter	Beta (95% CI)	Exponentiated beta (95% CI)
Smear positive TB	Prevalence	-0.75	0.47 (0.47 — 0.47)
Sex (male)	Prevalence	0.57	1.77 (1.63 — 1.92)
Sex (male)	Incidence	0.40	1.49 (1.47 — 1.51)
CDR 0 to 10%	Incidence	-2.31	0.099 (0.097 — 0.10)
CDR >10 to 20%	Incidence	-1.62	0.20 (0.19 — 0.20)
CDR >20 to 30%	Incidence	-1.2	0.30 (0.30 — 0.30)
CDR >30 to 40%	Incidence	-0.9	0.41 (0.40 — 0.41)
CDR >40 to 50%	Incidence	-0.7	0.50 (0.49 — 0.50)
CDR >50 to 60%	Incidence	-0.5	0.60 (0.60 — 0.61)
CDR >60 to 70%	Incidence	-0.42	0.66 (0.64 — 0.67)

CDR >70 to 80%	Incidence	-0.28	0.76 (0.73 — 0.79)
CDR >80 to 90%	Incidence	-0.2	0.82 (0.82 — 0.82)
Age-standardized SEV scalar (log-transformed)	Prevalence	0.77	2.16 (2.12 — 2.27)
Age-standardized SEV scalar (log-transformed)	Incidence	0.76	2.13 (2.12 — 2.16)
LDI (log-transformed)	Remission	0.11	1.12 (1.06 — 1.22)
LDI (log-transformed)	Excess mortality	-0.50	0.61 (0.61 — 0.61)

Betas and exponentiated values from the model applying the remission assumption for data-rich countries

Covariate	Parameter	Beta (95% CI)	Exponentiated beta (95% CI)
Smear positive TB	Prevalence	-0.75	0.47 (0.47 — 0.47)
Sex (male)	Prevalence	0.57	1.77 (1.62 — 1.96)
Sex (male)	Incidence	0.39	1.48 (1.47 — 1.49)
CDR 0 to 10%	Incidence	-2.30	0.10 (0.10 — 0.10)
CDR >10 to 20%	Incidence	-1.61	0.20 (0.20 — 0.20)
CDR >20 to 30%	Incidence	-1.20	0.30 (0.30 — 0.30)
CDR >30 to 40%	Incidence	-0.90	0.41 (0.41 — 0.41)
CDR >40 to 50%	Incidence	-0.70	0.50 (0.50 — 0.50)
CDR >50 to 60%	Incidence	-0.50	0.61 (0.60 — 0.61)
CDR >60 to 70%	Incidence	-0.43	0.65 (0.64 — 0.66)
CDR >70 to 80%	Incidence	-0.28	0.75 (0.74 — 0.76)
CDR >80 to 90%	Incidence	-0.20	0.82 (0.82 — 0.82)
Age-standardized SEV scalar (log-transformed)	Prevalence	0.77	2.17 (2.12 — 2.30)
Age-standardized SEV scalar (log-transformed)	Incidence	0.75	2.12 (2.12 — 2.12)
LDI (log-transformed)	Remission	0.11	1.12 (1.07 — 1.20)
LDI (log-transformed)	Excess mortality	-0.50	0.61 (0.61 — 0.61)

The lay descriptions and disability weights for severity levels derived from the GBD Disability Weights study are shown below.

Health state Name	Lay description	DW (95% CI)
Tuberculosis, not HIV infected	has a persistent cough and fever, is short of breath, feels weak, and has lost a lot of weight	0.333 (0.224-0.454)
Tuberculosis, HIV infected	has a persistent cough and fever, shortness of breath, night sweats, weakness and fatigue and severe weight loss	0.408 (0.274-0.549)

HIV/AIDS

Flowchart

HIV/AIDS

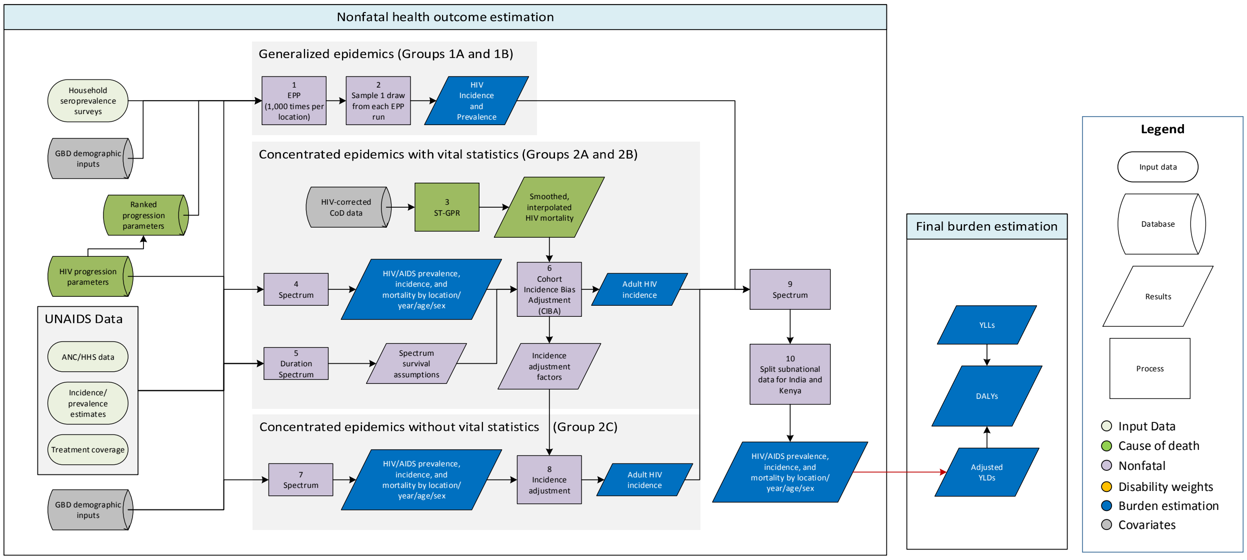

Case definition

Infection with the human immunodeficiency virus (HIV) causes influenza-like symptoms during the acute period following infection and can lead to acquired immunodeficiency syndrome (AIDS) if untreated. HIV attacks the immune system of its host, leaving infected individuals more susceptible to opportunistic infections like tuberculosis. Although there are two different subtypes of HIV, HIV-1 and HIV-2, no distinction is made in our estimation process or presentation of results. For HIV, ICD 10 codes are B20-B24, C46-C469, D84.9; ICD 9 codes are 042-044, 112-118 (after 1980), 130 (after 1980), 136.3-136.8 (after 1980), 176.0-176.9 (after 1980), 279 (after 1980); and ICD9 BTL codes are B184-B185.

Input data

Model inputs

Household seroprevalence surveys

Geographically representative HIV seroprevalence survey results were used as inputs to the model for countries with generalized HIV epidemics where available.

GBD demographic inputs

Location-specific population, fertility, and HIV-free survival rates from GBD 2015 (see Part 1 for details on the generation of these data) and migration data from UNAIDS were used as inputs in modeling all locations.

UNAIDS data

Antenatal care, incidence, prevalence, and treatment coverage data from UNAIDS were used in modeling for all locations.

On-ART literature data

Data were identified by using search terms "HIV," "mortality," and "antiretroviral therapy" in PubMed searches across the literature. To be included, studies must include only HIV-positive people who receive antiretroviral therapy (ART) but who were ART-naïve prior to the study. In addition, studies must report either a duration-specific mortality proportion or a hazard ratio across age or sex, and must not include children.

For duration-specific survival data, studies must report uncertainty on mortality estimates or provide stratum-specific sample sizes and must include duration-specific data to allow for calculation of 0-6, 7-12, or 13-24 month conditional mortality. In addition, studies must either report separate mortality and loss-to-follow-up (LTFU) curves, be corrected for LTFU using vital registration data, or be conducted in a high-income setting. Finally, studies must report the percent of participants who are male, the median age of participants, and either specific data on the number of CD4 T lymphocytes (CD4 counts) or the median CD4 count used for the data.

Hazard ratio data for ages or sexes can only be used if the hazard ratios are controlled for other variables of interest (age, sex, and CD4 category).

Changes for GBD 2015

In GBD 2013, we identified 102 papers for extraction. For GBD 2015, we included 13 additional studies informing the duration-specific mortality estimation process and 26 studies informing the age and sex hazard ratio estimation process (some studies were used and counted in both). We also added one study to our LTFU analysis. In addition, we updated our data from the Antiretroviral Therapy Cohort Collaboration (ART-CC) with country-specific data pre- and post-2001 for enhanced use in estimating time trends for high-income countries. We excluded nine hazard ratio and four duration-specific mortality studies used in GBD 2013 which reported results on populations already present in other extracted studies. The inclusion of new ART-CC data necessitated the exclusion of four additional studies used in GBD 2013.

We also included on-ART cohort mortality data from 10 high-income nations with collaboration from ART-CC. These countries include Austria, Denmark, France, Germany, Italy, the Netherlands, Spain, Switzerland, the United Kingdom, and the United States. We excluded the US data because they were not fully representative of the complete with-HIV on-ART population at the time.

Off-ART literature data

In GBD 2013, to characterize uncertainty in the progression and death rates, we systematically reviewed the literature on mortality without ART. We searched terms related to pre-ART or ART-naive survival since seroconversion.[1] After screening, we identified 13 cohort studies that included the cohorts used by UNAIDS from which we extracted survival at each one-year point after infection. Screening for additional, recently published studies for GBD 2015 identified no new cohort studies for inclusion in this analysis.

Severity splits & disability weights

The basis of the GBD disability weight survey assessments are lay descriptions of sequelae highlighting major functional consequences and symptoms. The lay descriptions and disability weights for HIV/AIDS severity levels are shown below.

Severity level	Lay description	DW (95% CI)
Symptomatic HIV	has weight loss, fatigue, and frequent infections.	0.274 (0.184-0.377)
AIDS with antiretroviral treatment	has occasional fevers and infections. The person takes daily medication that sometimes causes diarrhea.	0.078 (0.052-0.111)
AIDS without antiretroviral treatment	has severe weight loss, weakness, fatigue, cough and fever, and frequent infections, skin rashes, and diarrhea.	0.582 (0.406-0.743)

The proportion of people living with HIV/AIDS who are being treated with anti-retroviral therapy is an output of Spectrum, the compartmental model used to make consistent incidence, prevalence, and mortality estimates described below.

Modeling strategy

In GBD 2015, our general modeling strategy for estimating HIV incidence, prevalence, and mortality is similar in many ways to the strategy used in GBD 2013. In GBD 2015, we continue to use the Spectrum program rewritten in Python for GBD 2013 to facilitate faster and more flexible execution necessary for our more intensive computational needs. We made several changes to Spectrum's assumptions comparing to the Spectrum software used by UNAIDS. A key change in GBD 2015 is the application of EPP using an open-source computer program in R written by Jeffrey Eaton.[4] We ran EPP for all group 1 countries in order to produce incidence curves that were consistent with the demographic and epidemiological assumptions used in GBD 2015. This differed from GBD 2013, where we used the incidence curves provided by UNAIDS.

On-ART

First, we corrected reported probabilities of death for loss to follow-up using an update of the approach developed by Verguet and colleagues.[5] Verguet and colleagues used tracing and follow-up studies to empirically estimate the relationship between death in LTFU and the rate of LTFU.

After extracting the survival data into duration-specific conditional mortality, we used DisMod-MR 2.0 to synthesize the data into estimates of conditional probability of death over initial CD4 count.[1] We modeled the data separately by duration and added a fixed effect on whether the study was conducted prior to 2002. Each analysis was conducted separately for high-income countries, GBD low-income countries outside of sub-Saharan Africa, and sub-Saharan Africa.

To create estimates of age-specific hazard ratios, we synthesized hazard ratio data in five broad age groups: 15-25, 25-35, 35-45, 45-55, and 55-100, and modeled the data using DisMod-MR 2.0.

To create estimates of sex-specific hazard ratios, we use the *metan* function in Stata to create estimates of relative risks separately by region, using female age groups as the reference group.

The age and sex hazard ratios were applied to the CD4-specific mortality rates, accounting for the distribution of ages and sexes in the mortality data. We then subtracted HIV-free mortality from the model life table process to calculate HIV-specific mortality, and used 1,000 draws from the posterior distribution for each age, sex, and CD4 category for conditional probabilities of death for 0-6 months, 7-12 months, and 13-24 months after initiation of ART as inputs into Spectrum.

Changes for GBD 2015

In GBD 2015, our primary methodological change was the analysis of on-ART mortality using a fixed effect on studies before/after 2002, only in the high-income region, to estimate conditional probability of death in DisMod-MR 2.0. By doing so, we incorporated changes over time in the quality of on-ART care, which may improve on-ART mortality. This change was also complemented by the inclusion of time-split data from ART-CC, which allowed us to incorporate this time trend across the large cohort. We then used the estimated post-2002 on-ART mortality through the rest of the on-ART estimation process.

Off-ART

Following UNAIDS assumptions, no-ART mortality is modeled as shown in the figure below.[1]

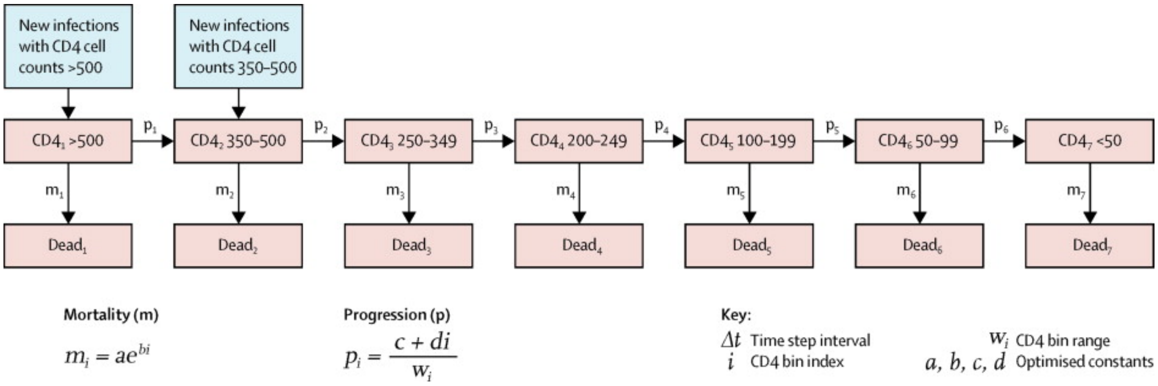

The death and progression rates between CD4 categories vary by age according to four age groups: 15–24 years, 25–34 years, 35–44 years, and 45 years or older. We modeled the logit of the conditional probability of death between years in these studies using the following formula:

$$logit\ (m_{ijk}) = \beta_0 + \sum_{i=1}^{4} \beta_{1i}\, a_i + \sum_{j=1}^{12} \beta_{2j} t_j + u_k + \varepsilon_{ijk}$$

In the formula, *m* is conditional probability of death from year t_j to t_{j+1}, a_i is an indicator variable for age group at seroconversion (15–24 years, 25–34 years, 35–44 years, and 45 years or older), t_j is an indicator variable of year since seroconversion, and u_K is a study-level random effect.

By sampling the variance-covariance matrix of the regression coefficients and the study-level random effect, we generated 1,000 survival curves for each age group that capture the systematic variation in survival across the available studies. For each of the 1,000 survival curves, we used a framework modeled after the UNAIDS optimization framework in which we find a set of progression and death rates that minimizes the sum of the squared errors for the fit to the survival curve.[6,7]

Burden estimation overview

UNAIDS uses two key analytical components in their epidemiological estimation. EPP is used to estimate incidence trajectories that are consistent with prevalence surveys and other prevalence measurements such as antenatal clinic serosurveillance. Spectrum is a compartmental HIV progression model used to generate age-specific incidence, prevalence, and death rates from the EPP incidence curves and assumptions about intervention scale-up and local variation in epidemiology.

For GBD 2013, we created an exact replica of Spectrum in Python. This enabled us to run thousands of iterations of the model at once on our computing cluster and allowed for more flexible input data structures. Additionally, in order to generate estimates with more realistic ranges of uncertainty than those in UNAIDS 2012, we adjusted all input data by uniformly sampled factors between 0.9 and 1.1. These changes, along with our new estimation of with- and without-ART mortality and CD4 progression parameters, persist into GBD 2015.

We have made several substantial improvements elsewhere in the process for GBD 2015. Of particular note, we have integrated EPP into the modeling process when feasible, enabling more robust and internally consistent incorporation of parameter uncertainty in generalized epidemics, and we have vastly improved the accuracy of the incidence adjustment used to fit Spectrum to high-quality vital registration data. Details of the impacts are included in the descriptions of the appropriate country strategies.

Due to the substantial differences in the quality and types of data available across different countries, we used three different methodologies to produce year-, age-, and sex-specific estimates of HIV incidence, prevalence, and mortality.

Countries with seroprevalence surveys and antenatal clinic data (Groups 1A and 1B)

We identified 43 countries – as well as 48 subnational locations from India, Kenya, Mozambique, and South Africa – with at least one geographically representative HIV seroprevalence survey. In order to ensure that our estimates of incidence and prevalence in these places were consistent with our estimates of HIV progression, we used a version of EPP written in R and C++ by Jeffrey Eaton to create new fits to the prevalence data in the UNAIDS files. By substituting in our own assumptions about HIV progression, we were able to ensure that the implied relationship between incidence and mortality/prevalence in EPP is similar to that in Spectrum.

In these locations, most of which experience generalized HIV epidemics, we expect estimates of HIV burden to exhibit substantial uncertainty. To reflect this, we induced a perfect correlation between the previously independent draws of HIV mortality with and without ART and CD4 progression. We paired the draws of the three parameter sets internally and with each other in the following way: we sorted without-ART mortality and CD4 progression internally by age (not CD4), meaning the highest draw of HIV mortality without ART for age a_i and CD4 category c_l will be paired with the highest draw of HIV mortality without ART for age a_k and CD4 category c_l. In the same way, we sorted with-ART mortality internally by age, sex, CD4 count at treatment initiation, and duration on treatment. After this sorting process, the lowest indexed draw of each parameter has the highest values and vice versa. This means that we will use the most extreme possible parameter sets in EPP and Spectrum and should see a commensurate expansion in the range of the uncertainty.

To ensure that this expanded uncertainty is replicated in EPP, we fit the model once for every set of paired draws of the progression parameters for every location. This means that the first iteration of EPP

for Uganda sees the highest draws of all three sets of progression parameters. Such a procedure is necessary because EPP currently has no mechanism for incorporating uncertainty in any inputs except prevalence data. This process (Process 1 in the HIV/AIDS Estimation Flowchart), produced 1,000 sets of EPP output for each of the locations that make up the 47 countries in the group. Every set of EPP outputs contains 500 consistent draws of HIV incidence and prevalence in adults aged 15-49. In many cases, the algorithm used to fit EPP, incremental mixture importance sampling, failed, resulting in fewer than 1,000 sets of EPP results.

For every location in the group, we sampled one of the 500 incidence/prevalence draws from each of the sets of EPP results (Process 2 in the HIV/AIDS Estimation Flowchart). By sampling one draw from each set, we ensured that the distribution of progression parameters dictating the relationship between incidence and prevalence was exactly the same as the distribution of the sorted parameters generated in the previous step. In locations where not all 1,000 iterations of EPP fit successfully, we sampled one draw from every iteration that did succeed and then resampled with replacement from that set of draws. To maintain the link between the input progression draws and the resulting incidence and prevalence draws from EPP, we replaced any parameter draw associated with a failed run of EPP with the parameter draw that that failed draw was replaced with. At the end of this process, for every location in the set of 47 countries, we were left with 1,000 linked draws of adult incidence and prevalence and the exact progression parameters that generated those draws.

We then ran these results, along with the previously described demographic and HIV-specific inputs, through Spectrum to produce location-, year-, age-, and sex-specific estimates of HIV incidence, prevalence, and mortality (Process 9 in the HIV/AIDS Estimation Flowchart).

Countries with vital registration data (Group 2A and 2B)

Vital registration is one of the highest-quality sources of data on HIV burden in many countries, so generating estimates that are consistent with these data, with necessary adjustment to account for any potential underreporting, is critical. We identified 116 countries – as well as 208 subnational locations from Brazil, China, Japan, Mexico, Saudi Arabia, Sweden, the United Kingdom, and the United States – with vital registration data or sample registration systems such as DSP in China.

We imputed missing years of data to generate a complete time series for HIV from the estimated start year of the epidemic using ST-GPR. We analyzed mortality trends using ST-GPR starting in 1981, the year that HIV was first identified in the United States.[8] For ST-GPR, we adjusted the lambda (time weight) and GPR scale according to the completeness of vital registration data, based on whether a country had 10 or more years of complete VR data as analyzed by the Death Distribution Methods (DDM) model described in Part 1. We produced separate splines by country/age group, up to the peak year of death rate. We then ran a linear regression with random effects on region, age, and sex. Following this, we ran space-time residual smoothing, in which time, age, and space weights are used to inform smoothing of the residuals between data points and the linear regression estimate. From this process, we generated space-time estimates with the applied weights, along with the median absolute deviation (MAD) of the space-time estimates from the data. The MAD was calculated at various levels of the geographic hierarchy (e.g., subnational and national), and was added into the data variance term. The data variance and space-time estimates were then analyzed using Gaussian Process Regression to return a final estimate of mortality along with uncertainty.

Although Spectrum produces HIV mortality estimates that are within the realm of possibility in most countries using the incidence curves provided in the UNAIDS 2012/2015 country files, it is a deterministic model that has not yet been integrated into an optimizable framework. Therefore, in order to “fit” it to vital registration data, we need to adjust input incidence. For GBD 2013, we used a process that assumed several different durations between HIV infection and HIV death and adjusted incidence based on death some number of years in the future. Although that method worked relatively well and substantially reduced the disconnect between Spectrum and the VR data, it required very rigid and unrealistic assumptions about these survival durations. For GBD 2015, we have improved the performance of this method, allowing Spectrum to fit to the VR data more closely.

To improve the fit of this process, we restructured Spectrum to add compartments that identify groups of people living with HIV by year of infection (Process 5 in the HIV/AIDS Estimation Flowchart). With this version of Spectrum we can output, among many other metrics, HIV deaths by year, age, sex, and infection cohort. This enables us to adjust incidence to fit to death much more precisely and without making any rigid assumptions about the time from HIV infection to HIV death.

We have incorporated these improvements into a cohort incidence bias adjustment (CIBA) process. First, we ran Spectrum normally to produce 1,000 draws of incidence, prevalence and mortality (Process 4 in the HIV/AIDS Estimation Flowchart). Then, by year, age, and sex, we took the ratio of VR deaths to Spectrum deaths to quantify the amount of bias in Spectrum. Using the mean duration data from the new version of Spectrum, for every year-, age-, and sex-specific infection cohort, we calculated the share of all HIV deaths observed over the course of the projection period in that cohort that would occur in each year after the year of infection. For example, projecting from 1970 through 2015, we identified the cohort of men infected in 1992 at the age of 16, calculated the total number of HIV deaths in that cohort in all subsequent years through the end of 2015, and divided the annual number of deaths by that total. This showed us the distribution of deaths among that cohort over the projection period. In the most extreme case (infections in 2014), we could only produce one point of that distribution (2015), so that single value is exactly 1.0; 100% of the deaths observed in that cohort occurred in 2015.

We then used these distributions of death to weigh the ratio of VR deaths to Spectrum deaths, meaning that ratios in the years where we expect the largest share of deaths were weighed most heavily. We then multiplied the initial size of that cohort from the normal run of Spectrum by the sum of the combined ratios to get a new estimate of new cases in that year/age/sex combination.

We can write this method mathematically in the following way:

$$r_t = \frac{VR_t}{D_t}$$

$$\rho_t^{t-i} = \frac{d_t^{t-i}}{\sum_{k=t-i+1}^{n} d_k^{t-i}}$$

$$\alpha^{t-i} = \sum_{k=t-i+1}^{n} r_k * \rho_t^{t-i}$$

$$n_{\text{adjusted}}^{t-i} = \alpha^{t-i} * n^{t-i}$$

VR_t is the number of HIV/AIDS deaths in year t from ST-GPR, and D_t is the number of HIV/AIDS deaths from the first run of Spectrum. In the second equation, d_t^{t-i} is the number of HIV/AIDS deaths among members of infection cohort $t-i$ in year t, with $i \geq 1$, from the new, duration-tracking version of Spectrum, and n is final year of the projection. Therefore, ρ_t^{t-i} is the share of observed deaths in cohort $t-i$ that we expect to occur in year t. It follows that α^{t-i} is the weighted adjustment ratio described above, which we multiply by the estimated initial size of infection cohort $t-i$ as calculated in the first-stage Spectrum run to get the adjusted number of new cases, $n_{\text{adjusted}}^{t-i}$. This process is run separately for every sex and single-age pair.

CIBA (Process 6 in the HIV/AIDS Estimation Flowchart) allows ratios in each year after a given infection year to influence the final adjustment to incidence. The size of that influence is determined by the relative importance of that year in the cohort-year's distribution of deaths over time. The result is a new set of 1,000 draws of incidence and a set of 1,000 ratios of post-adjustment incidence to pre-adjustment incidence. We perform this adjustment using mean durations from the new version of Spectrum in order to try to shift the mean of the regular distribution of deaths.

Finally, to produce location-, year-, age-, and sex-specific estimates of HIV incidence, prevalence, and mortality, we ran the new estimates of incidence and all previously input data through Spectrum (Process 9 in the HIV/AIDS Estimation Flowchart).

Countries without survey data and vital registration data (Group 2C)

The remaining 24 countries – as well as nine subnational locations from China and Saudi Arabia – had neither geographically representative seroprevalence surveys nor reliable vital registration systems. To produce estimates of HIV burden in these countries, we assumed that Spectrum is similarly biased as in other Group 2 countries. This involved running Spectrum (Process 7 in the HIV/AIDS Estimation Flowchart), adjusting incidence using 1,000 adjustment ratios randomly sampled from the entire set of CIBA results (Process 8), and rerunning Spectrum using the new draws of adjusted incidence (Process 9). As above, the estimates of incidence, prevalence, and mortality were incorporated into the rest of the machinery via the reckoning process.

Originally, Cambodia, which does have a prevalence survey, was included in this group because we have not yet coded the machinery necessary to reproduce the Asian Epidemic Model used by UNAIDS to model prevalence and incidence in Southeast Asian countries.[9] The 2005 DHS survey in Cambodia made clear that we were underestimating the burden due to HIV there by not using survey data during the modeling process. In order to more accurately represent the epidemic, we used the mortality profile from Thailand and scaled it by 80%, the ratio of estimated prevalence rate in Thailand in 2005 and the prevalence rate from the DHS survey in Cambodia. We then treated the scaled death series as VR data and added Cambodia to the 2B group that is run through CIBA.

Subnational splitting for India and Kenya

Spectrum results for India and Kenya subnational locations are modeled at higher levels of geography than our GBD locations. For example, Spectrum results for India are produced at the state level, while GBD 2015 estimates were produced at the state urban-rural level. Similarly, Spectrum is modeled at the province level, while we compute Kenyan subnational estimates for the 47 counties. To split the Spectrum results into more granular results for processing, we assign each GBD subnational unit to a

Spectrum modeling unit. From this, we generate age/sex/year-specific proportions for population, HIV-specific death, and HIV-free mortality.

After this subnational splitting, results were incorporated into the all-cause mortality estimation machinery via the reckoning process described in Part 1.

HIV/AIDS resulting in other diseases

There are two Level 4 causes under the HIV/AIDS Level 3 cause in the GBD 2015 cause hierarchy. The modeling process for HIV/AIDS-tuberculosis is detailed in the capstone paper. We computed the number of people living with HIV resulting in other diseases by subtracting the number of people living with HIV/AIDS-tuberculosis from all people living with HIV/AIDS at the 1,000 draw level.

Diarrheal diseases

Flowchart

Diarrheal diseases

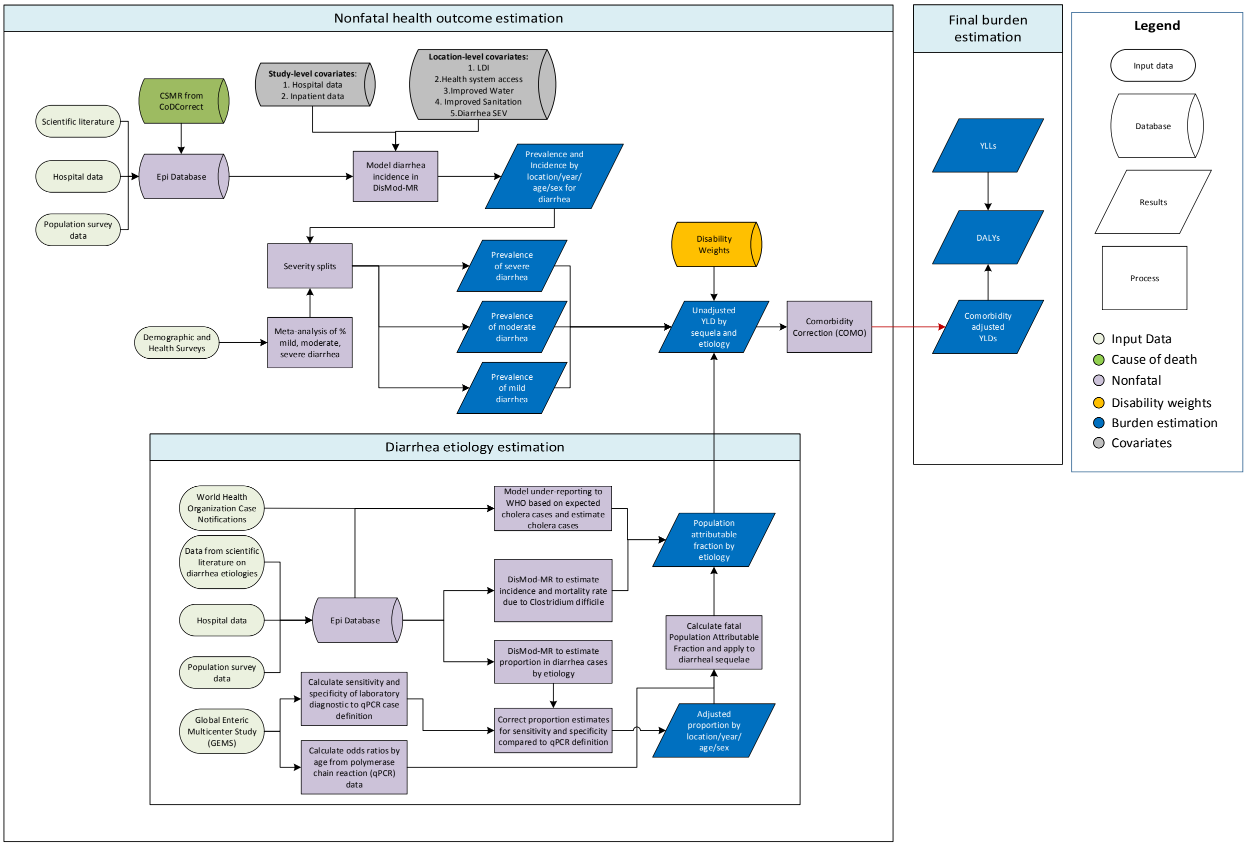

Case definition

We defined diarrheal disease episodes as three or more loose stools in a 24-hour period. Hospital input data use ICD9 codes 001-009.9 and ICD10 codes A00-A09. We also split diarrhea episodes into three severity levels: mild, moderate, and severe.

Input data

Model inputs

We used two main types of data in the diarrhea non-fatal burden estimation and the attribution of diarrheal etiologies. Moreover, we included all data sources used in GBD 2013 and conducted new reviews of scientific literature, surveys, and hospitalization data. We presented a summary of the data sources in Table Data.

The first type of data is the incidence and prevalence of diarrhea in community and hospital settings. We used data from population-representative surveys, such as the Demographic and Health Surveys and the

Multiple Indicator Cluster Surveys. We converted the prevalence of maternal-reported two-week period from surveys to point prevalence in one-year age increments using Equation 1.

1) $$Point\ Prevalence = Period\ Prevalence * \frac{Duration}{(Recall\ Period + Duration - 1)}$$

Hospital data and MarketScan data were identified using the ICD9 codes 001-009.9 and ICD10 codes A00-A09. To be consistent with the survey data, we transformed the hospital and MarketScan data from incidence to prevalence. A summary of the data sources is found in the table below.

Table 1. Summary of the distribution of input data for diarrhea DisMod-MR models

	Prevalence	Incidence
Data dources	616	33
Data points	24,147	316
Countries/subnationals	425	29
GBD world regions	21	13

The second type of data describes diarrhea etiologies. We extracted data on all etiologies except *C. difficile* from scientific literature that reported the proportion of diarrhea cases that tested positive for each pathogen.

We completed a systematic literature review covering the years 2012-2015 for diarrhea prevalence, incidence, and all diarrhea etiologies except *V. cholerae* and *C. difficile*. We excluded studies smaller than 100 samples, on diarrhea outbreaks, on travel-acquired infections, and those based on a subpopulation only, such as HIV-positive individuals or people living in urban slums. We pulled all articles using a PubMed search term that combined non-specific and etiology-specific diarrhea on May 6, 2015 (Search string: *(diarrhoea[title] OR diarrhoea[MeSH Terms] OR diarrhea[title] OR diarrhea[MeSH Terms] OR gastroenteritis[title] OR gastroenteritis[MeSH Terms] OR gastro-enteritis[title] OR salmonella[title/abstract] OR shigell*[title/abstract] OR "enteropathogenic e. coli" [title/abstract] OR enterotoxigenic e. coli[title/abstract] OR campylobacter[title/abstract] OR amoebiasis[title/abstract] OR entamoeb*[title/abstract] OR amoebiasis[title/abstract] OR amebiasis[title/abstract] OR cryptosporidi*[title/abstract] OR rotavirus[title/abstract] OR norovirus[title/abstract] OR adenovirus[title/abstract]) AND ((etiolog*[title/abstract] OR etiology[MeSH Terms] OR cause[title/abstract] OR pathogen[title/abstract]))* ***NOT*** *((colitis[title/abstract] OR enterocolitis[title/abstract] OR inflammatory bowel[title/abstract] OR irritable[title/abstract]OR Crohn*[title/abstract] OR HIV[title] OR treatment[title] OR therapy[title])) NOT ((appendicitis[title/abstract] OR esophag*[title/abstract] OR surger*[title/abstract] OR gastritis[title/abstract] OR liver[title/abstract] OR case report[title] OR case-report[title] OR therapy[title] OR treatment[title])) AND (("2012/01/01"[PDat] : "2015/12/31"[PDat]) AND Humans[Mesh])).*

We identified 2,847 studies, of which 152 met our criteria of inclusion and were included. We extracted data points for location, sex, year, and age. For the data that describe proportion of episodes positive for a given pathogen, we assigned an age range based on the prevalence-weighted mean age of diarrhea in the appropriate year/sex/location if the age of the study participants was not reported.

Our literature review extracted the proportion of any enteropathogenic *Escherichia coli* (EPEC) without differentiating between typical (tEPEC) and atypical (aEPEC). In order to be consistent with the odds ratios that we obtained, we adjusted our proportion estimates of any EPEC to typical EPEC only. This adjustment was informed by a subset of our literature review that reported both atypical and typical EPEC. We estimated a ratio by super-region of tEPEC to any EPEC and adjusted our proportion estimates accordingly. We found that the majority of EPEC diarrhea cases were positive for atypical EPEC, consistent with other published work.[1]

We used the Global Enteric Multicenter Study (GEMS), a seven-site, case-control study of moderate-to-severe diarrhea in children under 5 years,[2] to calculate odds ratios for the diarrheal pathogens. We analyzed raw data for a systematic reanalysis, representative of the distribution of cases and controls by age and site, of roughly half of the 22,000 original GEMS samples that were tested for the presence of pathogen using quantitative polymerase chain reaction (qPCR).[3]

We modeled *Vibrio cholerae* independently from the other etiologies because of its epidemic tendency. We conducted a systematic review of literature for studies published between January 1980 and June 2015 that reported the proportion of diarrhea cases that tested positive for cholera or the case fatality of cholera. We excluded studies specifically about outbreaks and with less than one year of follow-up. From 557 initial results, 43 met our criteria and were extracted. The search was conducted on May 8, 2015 (Search string: *(((diarrhoea[title] OR diarrhoea[MeSH Terms] OR diarrhea[title] OR diarrhea[MeSH Terms] OR gastroenteritis[title] OR gastroenteritis[MeSH Terms] OR gastro-enteritis[title] AND cholera[title/abstract] OR cholera[MeSH Terms]) AND ((etiolog*[title/abstract] OR etiology[MeSH Terms] OR cause[title/abstract] OR pathogen[title/abstract])) NOT ((colitis[title/abstract] OR enterocolitis[title/abstract] OR inflammatory bowel[title/abstract] OR irritable[title/abstract]OR Crohn*[title/abstract] OR HIV[title] OR treatment[title] OR therapy[title])) NOT ((appendicitis[title/abstract] OR esophag*[title/abstract] OR surger*[title/abstract] OR gastritis[title/abstract] OR liver[title/abstract] OR case report[title] OR case-report[title] OR therapy[title] OR treatment[title])) AND (("1990/01/01"[PDat] : "2015/12/31"[PDat]) AND Humans[Mesh]))) NOT ((diarrhoea[title] OR diarrhoea[MeSH Terms] OR diarrhea[title] OR diarrhea[MeSH Terms] OR gastroenteritis[title] OR gastroenteritis[MeSH Terms] OR gastro-enteritis[title]) AND ((etiolog*[title/abstract] OR etiology[MeSH Terms] OR cause[title/abstract] OR pathogen[title/abstract])) NOT ((colitis[title/abstract] OR enterocolitis[title/abstract] OR inflammatory bowel[title/abstract] OR irritable[title/abstract]OR Crohn*[title/abstract] OR HIV[title] OR treatment[title] OR therapy[title])) NOT ((appendicitis[title/abstract] OR esophag*[title/abstract] OR surger*[title/abstract] OR gastritis[title/abstract] OR liver[title/abstract] OR case report[title] OR case-report[title] OR therapy[title] OR treatment[title])) AND (("2012/01/01"[PDat] : "2015/12/31"[PDat]) AND Humans[Mesh]))).*

We also modeled *Clostridium difficile* independently from the etiologies because it was not included as a pathogen in GEMS. We conducted a systematic literature review for the prevalence and incidence of *C. difficile* between January 1990 and May 2015. We used inpatient and outpatient hospital visits coded (ICD9: 008.45, ICD10: A04.7) for *C. difficile* as our incidence data. Of 866 initial results, 50 met our criteria and were extracted. However, nearly all of the hospital data came from Western countries. The search was conducted on May 8, 2015 (Search string: *(((diarrhoea[title] OR diarrhoea[MeSH Terms] OR diarrhea[title] OR diarrhea[MeSH Terms] OR gastroenteritis[title] OR gastroenteritis[MeSH Terms] OR gastro-enteritis[title] AND clostridium difficile[title/abstract] OR c. difficile[title/abstract] OR c. difficile[Mesh Terms] OR clostridium difficile[Mesh Terms]) AND ((etiolog*[title/abstract] OR*

etiology[MeSH Terms] OR cause[title/abstract] OR pathogen[title/abstract])) NOT ((colitis[title/abstract] OR enterocolitis[title/abstract] OR inflammatory bowel[title/abstract] OR irritable[title/abstract]OR Crohn[title/abstract] OR HIV[title] OR treatment[title] OR therapy[title])) NOT ((appendicitis[title/abstract] OR esophag*[title/abstract] OR surger*[title/abstract] OR gastritis[title/abstract] OR liver[title/abstract] OR case report[title] OR case-report[title] OR therapy[title] OR treatment[title])) AND (("1990/01/01"[PDat] : "2015/12/31"[PDat]) AND Humans[Mesh]))) NOT ((diarrhoea[title] OR diarrhoea[MeSH Terms] OR diarrhea[title] OR diarrhea[MeSH Terms] OR gastroenteritis[title] OR gastroenteritis[MeSH Terms] OR gastro-enteritis[title]) AND ((etiolog*[title/abstract] OR etiology[MeSH Terms] OR cause[title/abstract] OR pathogen[title/abstract])) NOT ((colitis[title/abstract] OR enterocolitis[title/abstract] OR inflammatory bowel[title/abstract] OR irritable[title/abstract]OR Crohn*[title/abstract] OR HIV[title] OR treatment[title] OR therapy[title])) NOT ((appendicitis[title/abstract] OR esophag*[title/abstract] OR surger*[title/abstract] OR gastritis[title/abstract] OR liver[title/abstract] OR case report[title] OR case-report[title] OR therapy[title] OR treatment[title])) AND (("2012/01/01"[PDat] : "2015/12/31"[PDat]) AND Humans[Mesh]))).*

Table 2. Data inputs, summary of the distribution of input data for diarrhea DisMod-MR models

	Prevalence	Incidence
Data Sources	616	33
Data points	24,147	316
Countries/subnationals	425	29
GBD world regions	21	13

Severity split inputs

Diarrheal diseases have three severity levels: mild, moderate, and severe. The proportion of diarrhea cases that are assigned to each comes from an analysis of Demographic and Health Surveys. Mild cases are the proportion of diarrhea cases that did not seek medical care; moderate cases are the proportion that sought medical care but did not have severe dehydration or seizures; and severe cases are the proportion that sought medical care with severe dehydration or seizures.

Table 3. Severity splits, details on the severity levels for diarrhea in GBD 2015 and the associated disability weight (DW) with that severity.

Severity level	Lay description	DW (95% CI)
Mild	Has diarrhea defined as 3 or more loose stools in a 24-hour period with no dehydration	0.074 (0.049-0.104)
Moderate	Has diarrhea defined as 3 or more loose stools in a 24-hour period with painful cramps and feeling thirsty and any dehydration	0.188 (0.125-0.264)
Severe	Has diarrhea defined as 3 or more loose stools in a 24-hour period with painful cramps and is very thirsty or feels nauseated	0.247 (0.164-0.348)

	or tired and/or severely dehydrated	

Modeling strategy

The non-fatal diarrheal disease burden is modeled in DisMod-MR, a Bayesian meta-regression modeling framework. DisMod-MR produces estimates of the incidence, prevalence, and remission of diarrhea for each age, sex, geographic location, and year. We defined remission, or the time to recovery, as five days average. The non-fatal burden of diarrheal diseases is informed by survey, hospital, MarketScan, and literature data. Input data are adjusted by study-level covariates that describe if the source is a hospital and/or if the source is from an inpatient sample. Country-level covariates also inform the model. These include the proportion of the population that have access to improved sanitation, access to improved water sources, health system access, income per capita, and the SEV for diarrhea.

Table 4. Covariates. Summary of covariates used in the diarrhea DisMod-MR meta-regression model

Study covariate	Parameter	beta	Exponentiated beta
Improved sanitation	Prevalence	0.25	1.29
Improved water source	Prevalence	-3.03	0.048
Diarrhea SEV	Prevalence	0.015	1.02
Sex	Prevalence	-0.068	0.93
Diarrhea SEV	Incidence	1.2	3.31
Sex	Incidence	-0.17	0.84
Health system access	Excess mortality	-0.32	0.72
Income per capita	Excess mortality	-0.009	0.99

We estimated diarrheal disease etiologies separately from overall diarrhea prevalence and incidence using a counterfactual strategy for enteric adenovirus, *Aeromonas*, *Entamoeba histolytica* (amoebiasis), *Campylobacter enteritis*, cryptosporidiosis, typical enteropathogenic *Escherichia coli* (t-EPEC), enterotoxigenic *Escherichia coli* (ETEC), norovirus, non-typhoidal salmonella infections, rotavirus, and *Shigella*. *Vibrio cholerae* and *Clostridium difficile* were modeled separately.

We dichotomized the continuous qPCR test result from a reanalysis of the Global Enteric Multicenter Study (GEMS) using the value of the cycle threshold (Ct) that most accurately discriminated between cases and controls. We used the lower Ct value that represented the smallest false positive samples (positive in non-diarrhea samples) when we had multiple Ct values for the cutpoint. The Ct values range from 0 to 35 cycles representing the relative concentration of the target gene in the stool sample. A low value indicates a higher concentration of the pathogen while a value of 35 indicates the absence of the target in the sample.

The case definition for each pathogen is a Ct value that is below the established cutoff point. We used a mixed effects conditional logistic regression model to calculate the odds ratio by age for each of our pathogens.

We modeled the proportion data using the meta-regression tool DisMod-MR to estimate the proportion of positive diarrhea cases for each separate etiology by location/year/age/sex and to adjust for testing for

multiple pathogens and sampling from an inpatient population. The population attributable fraction (PAF) was calculated from the proportion of diarrhea cases that are positive for each etiology. This is a counterfactual approach, meaning that the PAF represents the relative reduction in diarrhea morbidity if there was no exposure to a given etiology. As diarrhea can be caused by multiple pathogens and the pathogens may co-infect, PAFs can overlap and add up to more than 100%. We used the following formula to estimate PAF:[4]

$$2)\ PAF = Proportion * (1 - \frac{1}{OR})$$

Where *Proportion* is the proportion of diarrhea cases positive for an etiology and *OR* is the odds ratio of diarrhea given the presence of the pathogen. We used the estimated sensitivity and specificity of the laboratory diagnostic technique used in the GEMS study compared to the qPCR case definition to adjust our proportion before we computed the PAF:[5]

$$3)\ Proportion_{True} = \frac{(Proportion_{Observed} * Specificity - 1)}{(Sensitivity + Specificity - 1)}$$

We used this correction for the "proportion" to account for the fact that the proportions we used are based on a new test that is not consistent with the case definition from GEMS (qPCR versus GEMS conventional laboratory testing for pathogens).

For cholera specifically, we used the literature review to estimate expected number of cholera cases for each country-year using the incidence of diarrhea, estimated using DisMod-MR, and the proportion of diarrhea cases that are positive for cholera. We assigned cholera PAF using odds ratios from the qPCR results. We compared this expected number of cholera cases to the number reported to the World Health Organization.[6] We modeled the underreporting fraction to correct the cholera case notification data for all countries. We used the age-specific proportion of positive cholera samples in DisMod and our incidence estimates to predict the number of cholera cases for each sex/year/location. For *C. difficile*, we modeled incidence in DisMod-MR for each age, sex, year, location. We set remission in our model to one month. All uncertainty in the estimates and the input parameters come from 1,000 draws from a normal or log-normal distribution.

There are several key updates to the approach to diarrhea etiology estimation in GBD 2015 compared to GBD 2013. There are new data used in the diarrhea DisMod-MR models and in the diarrhea proportion DisMod-MR models. We also introduced qPCR diagnostic results from a retest of a systematic sample by age and site from the original GEMS stool samples. The qPCR diagnostic is more sensitive in detecting some pathogens, particularly bacterial ones, than the conventional laboratory test diagnostics used in the primary GEMS analysis and in GBD 2013. These qPCR results impact the odds ratios of diarrhea given pathogen detection and also the exposure which is the proportion of diarrhea cases positive for each pathogen. We adjusted the proportion estimates from the literature review by the sensitivity and specificity of the laboratory diagnostics to the new qPCR case definition to be consistent with the odds ratios. In general, this increased the attributable fractions for each pathogen.

Typhoid and paratyphoid fever

Flowchart

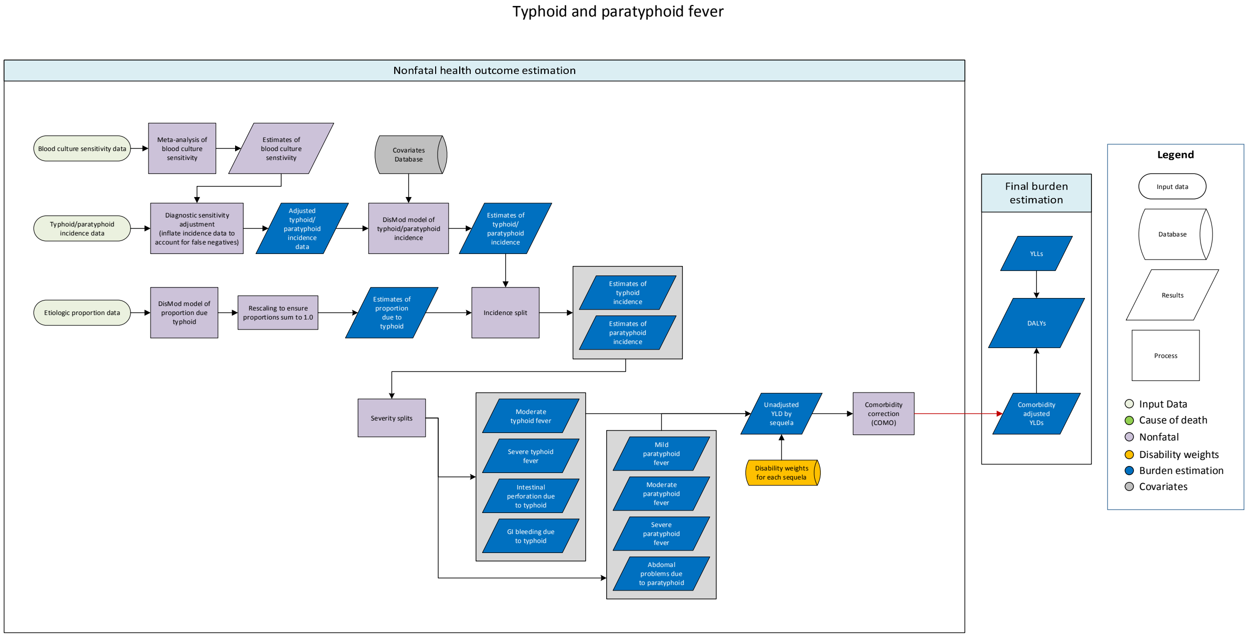

Case definition

Typhoid and paratyphoid are acute bacterial infections that most commonly cause febrile illness and gastrointestinal symptoms. Severe cases are associated with intestinal bleeding and perforation, altered mental state and, in some cases, death. We define a confirmed case as one for which there has been a positive blood culture test for either *Salmonella enterica typhi* or *paratyphi*. Diagnostic criteria do not typically accompany national surveillance reports; however, with blood culture being the standard diagnostic, we treat reported cases as confirmed. Given the poor sensitivity of blood culture, however, we estimated case definition as simply febrile illness resulting from an infection with *Salmonella enterica typhi* or *paratyphi*. This is effectively a counterfactual definition in which we attempt to estimate the number of true infections regardless of test result. These causes include all ICD-10 codes under the heading A01 (Typhoid and paratyphoid fevers).

Input data

Model inputs

Our incidence dataset included a combination of data from prospective cohort studies and national surveillance systems. Similarly, data on proportions due to typhoid and paratyphoid included a combination of prospective cohort studies and national surveillance systems.

Level	Incidence	Proportion
Data points	1,555	414
Studies	80	56
Locations	56	54

Regions	14	11

Updates to systematic reviews are performed on an ongoing schedule across all GBD causes, an update for typhoid and paratyphoid fevers will be performed in the next 1-2 iterations. While no systematic update was conducted, we did incorporate new data that were provided by collaborators.

Severity splits

For GBD 2015, we derived severity splits based on a published review of enteric fever outcomes from (Azmatullah A, Qamar FN, Thaver D, et al. 2005).

Paratyphoid is split into four sequelae: mild (28.5% [15.6 - 44.2]), moderate (52.25% [27.2 - 77.7]), severe (14.25% [8.2 - 21.8]), and abdominal pain and distention (5.0% [2.8 - 7.6]):

Sequela	Description	Disability weight
Mild	Has a low fever and mild discomfort , but no difficulty with daily activities.	0.006 (0.002-0.012)
Moderate	Has a fever and aches, and feels weak, which causes some difficulty with daily activities.	0.051 (0.032-0.074)
Severe	Has a high fever and pain, and feels very weak, which causes great difficulty with daily activities.	0.133 (0.088-0.19)
Abdominal pain & distention due to paratyphoid	Has pain in the belly and feels nauseateds. The person has difficulties with daily activities.	0.114 (0.078-0.159)

Similarly, typhoid is split into four sequelae: moderate (35.0% [26.0 - 44.3]), severe (47.75% [38.0 - 57.4]), severe abdominal pain and distention (17.0% [10.0 - 25.7]), and intestinal bleeding (0.25% [0 - 2.0]):

Sequela	Description	Disability weight
Moderate	Has a fever and aches, and feels weak, which causes some difficulty with daily activities.	0.051 (0.032-0.074)
Severe	Has a high fever and pain, and feels very weak, which causes great difficulty with daily activities.	0.133 (0.088-0.19)
Gastrointestinal bleeding	Vomits blood and feels nauseated.	0.325 (0.209-0.462)
Abdominal pain and distention (includes intestinal perforation)	Has severe pain in the belly and feels nauseated. The person is anxious and unable to carry out daily activities.	0.324 (0.22-0.442)

Modeling strategy

We first model total incidence of typhoid and paratyphoid combined. Second, we model the proportion of this total due to typhoid and the proportion due to paratyphoid. Finally, we split the case estimates into sequelae representing different major symptoms and levels of severity.

Total incidence was modeled using DisMod-MR, using the proportion of the population with access to clean water, and the proportion of the population living in the Indian Ocean monsoon belt as covariates. We performed a crosswalk using a study-level covariate indicating sources that were based on passive versus active surveillance, with active surveillance as the reference. This adjusts for incomplete case capture by passive surveillance. Incidence data were inflated to account for poor diagnostic sensitivity, based on an internal meta-analysis of the sensitivity of blood culture, the most common diagnostic used for typhoid. Similarly, we used two DisMod models to estimate etiologic proportions: one for the proportion of total incidence due to typhoid, and one for the proportion due to paratyphoid.

Typhoid cases are split between four sequelae: moderate typhoid fever, severe typhoid fever, severe typhoid fever with intestinal bleeding, and typhoid fever with abdominal complications. Paratyphoid cases are split between four sequelae: mild paratyphoid fever, moderate paratyphoid fever, severe paratyphoid fever, and paratyphoid fever with abdominal complications.

Changes from GBD 2013 to GBD 2015

We made no major changes in our methods between GBD 2013 and 2015.

Lower respiratory infections (LRI)

Flowchart

Lower respiratory infections

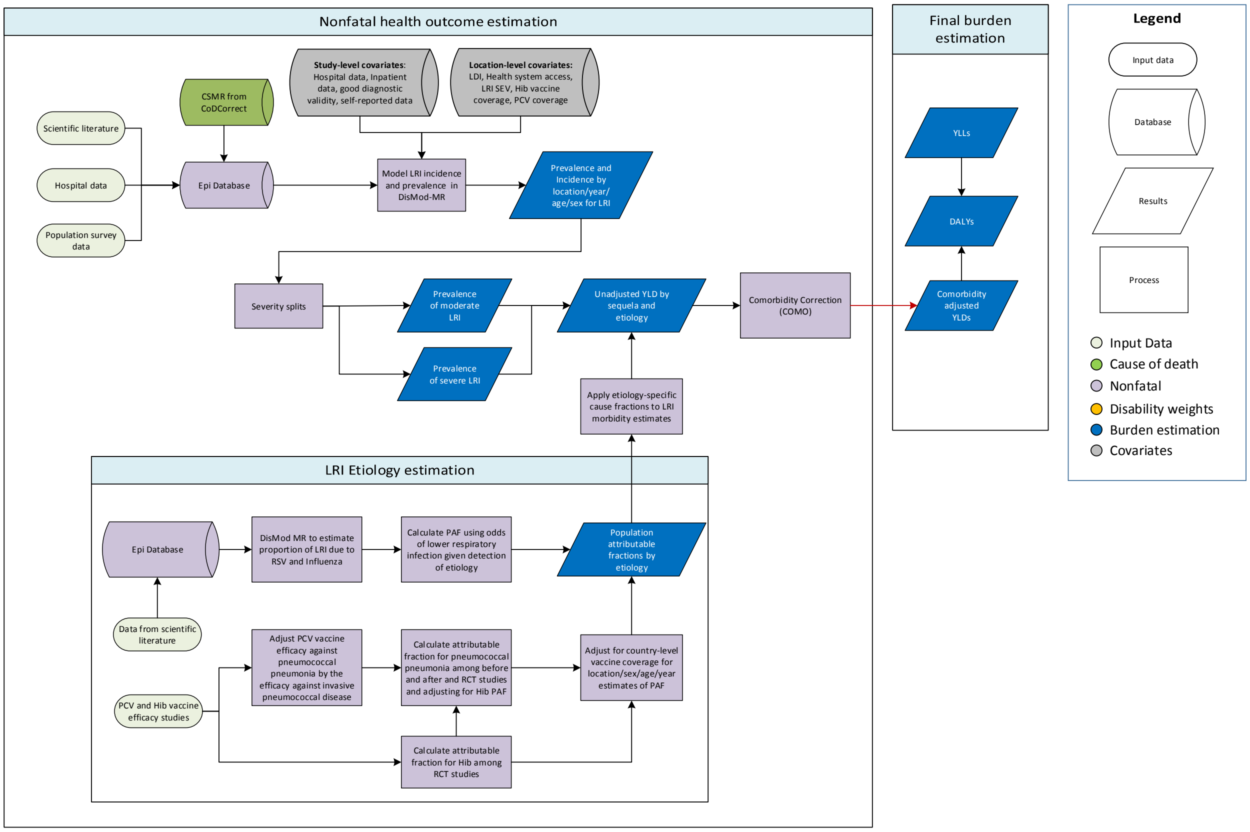

Case definition

We used clinician-diagnosed pneumonia or bronchiolitis as our case definition for lower respiratory infections (LRI). We included ICD9 codes 073.0-073.6, 079.82, 466-469, 480-489, 513.0, and 770.0 and ICD10 codes A48.1, J09-J22, J85.1, P23-P23.9, and U04. LRI etiologies are modeled separately from overall LRI incidence and prevalence. The etiologies include influenza, respiratory syncytial virus, *Streptococcus pneumoniae,* and *Haemophilus influenzae* type b and are episodes of LRI where the etiology is the causal pathogen in the infection.

Input data

Model inputs

We used two primary types of input data for lower respiratory infections. The first is lower respiratory infection incidence and prevalence data. These data come from a systematic literature review, hospital inpatient and outpatient data, claims data from the US, and population-representative surveys (see Table 1: Data Inputs for summary on input data). The second type of data is on the etiologies of LRI. Influenza and respiratory syncytial virus (RSV) population attributable fractions were informed by a systematic

literature review of the proportion of LRI cases that are positive for each pathogen. *Haemophilus influenzae* type B (Hib) and *Streptococcus pneumoniae* (pneumococcal pneumonia) are informed by a systematic review of vaccine efficacy and effectiveness studies.

To estimate the non-fatal burden of lower respiratory infections (LRI), we conducted a systematic review of scientific literature for LRI incidence and prevalence (Search String: ('lower respiratory' [title/abstract] OR pneumonia[title/abstract] AND ('2012/01/01'[PDat] : '2015/12/31'[PDat])AND Humans[MeSH Terms] NOT(autoimmune[title/abstract] OR COPD [title/abstract] OR 'cystic fibrosis'[title/abstract]). Our inclusion criteria were studies that were published between January 1990 and June 2015, had a sample size of at least 100, were at least one year in duration, and included lower respiratory infections, pneumonia, or bronchiolitis in the case definition. Our additional literature review for GBD 2015 from January 2012 through June 2015 identified 1,164 studies, of which 137 met our inclusion criteria and were included.

We also used self-reported prevalence of LRI symptoms from population-representative surveys, such as the Demographic and Health Survey and the Multiple Indicator Cluster Survey. We converted these data from two-week period prevalence to point prevalence using a mean duration of illness of 10 days. The equation for this adjustment is

1) $$Point\ Prevalence = Period\ Prevalence * \frac{Duration}{(Recall\ Period + Duration - 1)}$$

We included a study-level covariate to account for self-reported survey data and a covariate representing the specificity of the symptom prevalence from the surveys. For example, where possible we extracted the prevalence of children under 5 years old with fever, cough, and breathing difficulty located in the chest and/or chest and nose as the most specific symptom definition, but we also extracted prevalence estimates for cough and difficulty breathing as a less specific definition. This is consistent with the DHS and MICS definitions of acute respiratory infection. Some surveys did not include the prevalence of LRI symptoms *and* fever so we adjusted survey prevalence estimates that did not include fever by studies that did using the coefficient from a logistic regression.

In addition to survey data, hospital inpatient, outpatient data, and US claims data were included in the LRI modeling. These data sources described incidence, reflecting the number of new episodes. To make the data more consistent in the modeling process, we converted all incidence data to prevalence data using an average duration of illness of 10 days.

To estimate the attributable fraction for influenza and RSV, we conducted a systematic literature review of the proportion of LRI cases that tested positive for influenza and RSV ((“lower respiratory” [title/abstract] OR pneumonia[title/abstract] AND (influenza[title/abstract] OR influenza[MeSH Terms] OR “respiratory syncytial”[title/abstract] OR etiolog*[title/abstract]) AND ("2012/01/01"[PDat] : "2015/12/31"[PDat])AND Humans[MeSH Terms]). Our inclusion criteria were studies that were published between January 1990 and June 2015, a sample size of at least 100, at least one year in duration, and with lower respiratory infections, pneumonia, or bronchiolitis as the case definition. We excluded studies that described pandemic H1N1 influenza solely and studies that used influenza-like illness as the case definition. If the ages of the study participants were not reported, we assigned an age range based on the prevalence-weighted mean age of LRI, from the LRI prevalence DisMod model, for the appropriate year/sex/location. We extracted study-level covariates such as if the study used diagnostic PCR, if the

sample was from hospitalized cases, and if the study investigated RSV or influenza exclusively from other etiologies.

To estimate the attributable fraction for Hib and pneumococcal pneumonia, we conducted a systematic literature review of studies on the Hib vaccine and PCV effectiveness studies against X-ray-confirmed pneumonia and against pneumococcal and Hib disease up to January 2016 (Search Strings: (“haemophilus influenzae type b” OR “haemophilus influenzae b”) AND vaccine AND (efficacy OR effectiveness) AND ("2012/01/01"[PDat] : "2015/12/31"[PDat])AND Humans[MeSH Terms] NOT (autoimmune[title/abstract] OR COPD [title/abstract] OR "cystic fibrosis"[title/abstract]) ('streptococcus pneumoniae' OR pneumococcus OR pneumococcal) AND ('conjugate vaccine' OR 'polysaccharide vaccine') AND (efficacy OR effectiveness) AND ('2012/01/01'[PDat] : '2015/12/31'[PDat])AND Humans[MeSH Terms]. For PCV studies, we also extracted, if available, the distribution of pneumococcal pneumonia serotypes and the serotypes included in the PCV used in the study. In addition to the studies used in GBD 2013, we included three additional ones for pneumococcal pneumonia but none for Hib. We excluded observational and case-control studies due to implausibly high vaccine efficacy estimates. Since Hib trial data were exclusively for children aged 5 years or younger, we did not include the effect of Hib on ages over 5 years. Moreover, PCV trials are also limited to younger populations. Therefore, to account for the contribution of pneumococcal pneumonia in older populations, we included PCV efficacy studies with before-after approaches to capture the impact of introduction of PCV at the population level.

Table 1: Data Inputs

	Prevalence/Incidence
Sources	585
Countries/subnationals	377
GBD world regions	21

Severity splits

The distribution of moderate (85%) and severe (15%) lower respiratory infections is determined by a meta-analysis of the ratio of severe to all LRI from studies that report the incidence of moderate and severe lower respiratory infections.

We used the health states of acute infectious disease episode, moderate and severe, with the lay descriptions and disability weight values shown in table below:

Table 2: Severity Splits

Severity level	Lay description	DW (95% CI)
Moderate	Cough or difficulty breathing with rapid breathing.<br>Has a fever and aches and feels weak which causes some difficulty with daily activities.	0.051 (0.032-0.074)
Severe	Cough or difficulty breathing with lower chest wall indrawing,	0.133 (0.088-0.19)

	central cyanosis, or the inability to drink.<br>Has a high fever and pain and feels very weak, which causes great difficulty with daily activities.	

Modeling strategy

The non-fatal lower respiratory infection burden is modeled in DisMod-MR, a Bayesian meta-regression modeling framework. DisMod-MR produces estimates of the incidence, prevalence, and remission of LRI for each age, sex, geographic location, and year. We defined the time to recovery as an average of 10 days (9-12 days) which corresponds with a remission 36.5. The models are informed by study-level covariates and by country-level covariates. Hospital data from high-income countries, hospital data from middle-income countries (Mexico, Brazil, and Ecuador), inpatient data, self-reported data, and survey data with poor diagnostic sensitivity were identified and are adjusted in the modeling process.

Table 3: Model covariates

Study covariate	Parameter	Beta	Exponentiated beta
Hospital inpatient population	Prevalence	-0.73	0.48
Hospital data from middle- or low-income country	Prevalence		
Self-reported	Prevalence	0.67	1.96
Severe cases	Prevalence	-1.85	0.16
Poor diagnostic specificity	Prevalence	0.8	2.22
Hib vaccine coverage	Prevalence	-0.33	0.72
PCV vaccine coverage	Prevalence	-0.14	0.87
LRI SEV	Prevalence	-0.21	0.81
Health system access	Excess mortality		
Income per capita	Excess mortality	-0.18	0.84

In addition, the model is informed by cause-specific mortality rate (CSMR) estimates from GBD 2015 with priors on excess mortality rates calculated for each prevalence data point as CSMR/prevalence.

We estimated LRI etiologies separately from overall LRI mortality using two distinct counterfactual modeling strategies to estimate population attributable fractions (PAFs), described in detail below. We did not attribute etiologies to neonatal pneumonia due to a dearth of reliable data in this age group. We used the meta-regression tool DisMod-MR to estimate the proportion of LRI cases that are positive for influenza and RSV, separately, by location/year/age/sex.

We calculated the population attributable fraction (PAF) from the proportion of LRI cases positive for influenza and RSV. We used the following formula to estimate PAF [1]:

$$2)\quad PAF = Proportion * (1 - \frac{1}{OR})$$

Where *Proportion* is the proportion of LRI cases that test positive for influenza or RSV and *OR* is the odds ratio of LRI given the presence of the pathogen. We used an odds ratio of 5.1 (3.19 – 8.14) for influenza and 9.79 (4.98 – 19.27) for RSV from a recently published meta-analysis[2]. These odds ratios are marginally different from those used in GBD 2013. We calculated uncertainty of our PAF estimates from 1,000 draws of each parameter using normal distributions in log space.

For *Streptococcus pneumoniae* (pneumococcal pneumonia) and *Haemophilus influenzae* type B (Hib), we calculated the population attributable fraction using a vaccine probe design[3 4]. The ratio of vaccine effectiveness against nonspecific pneumonia to pathogen-specific disease represents the fraction of pneumonia cases attributable to each pathogen (Equations 3 and 5). A meta-analysis of the study-level PAF estimates from Hib (Equation 3) are used as the base PAF estimate for ages younger than 5 years. Pneumococcal pneumonia study-level PAF estimates (Equation 5) are used as inputs in a global-level DisMod model to determine an age distribution. We then adjusted these Base PAF estimates by vaccine coverage and expected vaccine performance to estimate final country- and year-specific PAF values (Equations 4 and 6). For pneumococcal pneumonia, we further adjusted the PAF using the final Hib PAF estimate and by vaccine serotype coverage (Equation 6).

$$3)\quad PAF_{Study-level} = 1 - \frac{VE}{VE_i}$$

$$4)\quad PAF_{Hib} = PAF_{Base} * \frac{(1 - Cov_{Hib} * VE_{Optimal})}{(1 - PAF_{Base} * Cov_{Hib} * VE_{Optimal})}$$

Where PAF_{Base} is the effect size from a random-effects meta-analysis of Hib study-level PAF values, VE is the vaccine efficacy against nonspecific pneumonia, $VE_i$is the vaccine efficacy against invasive Hib disease, Cov_{Hib} is the country-level coverage of Hib vaccine and $VE_{Optimal}$ is the optimal vaccine effectiveness (0.8, assumed). Optimal vaccine effectiveness is an adjustment for imperfect vaccine performance. We only used randomized-controlled trials (RCTs) in the Hib PAF estimation, which were incorporated using a random effects meta-analysis.

$$5)\quad PAF_{Study-level} = 1 - \frac{VE_{Pneumonia} * (1 - PAF_{Hib} * VE_{Hib\ Optimal})}{VE_{Streptococcus} * Cov_{Serotype}}$$

$$6)\quad PAF_{Pneumo} = \frac{PAF_{Base} * \left(1 - Cov_{PCV} * VE_{PCV\ Optimal}\right)}{\left(1 - PAF_{Hib} * Cov_{Hib} * VE_{Hib\ Optimal}\right) * \left(1 - \frac{PAF_{Base} * Cov_{PCV} * VE_{PCV\ Optimal}}{\left(1 - PAF_{Hib} * Cov_{Hib} * VE_{Hib\ Optimal}\right)}\right)}$$

Where PAF_{Base} is the age-specific PAF estimate from the DisMod model of study-level PAF values, $VE_{Pneumonia}$ is the vaccine efficacy against nonspecific pneumonia, PAF_{Hib} is the final PAF for Hib, $VE_{Hib\ Optimal}$ is the optimal Hib effectiveness (0.8, assumed), $VE_{Streptococcus}$ is the vaccine efficacy against serotype-specific pneumococcal pneumonia, $Cov_{Serotype}$ is the serotype-specific coverage, Cov_{PCV} is the vaccine coverage for region specific serotypes, $VE_{PCV\ Optimal}$ is the optimal vaccine effectiveness (0.8, assumed), and Cov_{Hib} is the Hib coverage by country.

For Hib, we assumed that the vaccine efficacy against invasive Hib disease is the same against Hib pneumonia. For pneumococcal pneumonia, a recent study in adults[5] found that the vaccine efficacy against invasive pneumococcal disease may be significantly higher than against pneumococcal pneumonia. We used this ratio to adjust estimates of vaccine efficacy against invasive pneumococcal disease from other studies. However, recognizing that the study is unique in that it uses a urine antigen test among adults, we added uncertainty around our adjustment using a wide uniform distribution (0.3-1.0). This is an important change from our etiology estimation in GBD 2013.

There are several important differences from the approach to LRI etiology estimation in GBD 2015 compared to GBD 2013. We added new data sources in the LRI DisMod model and in the etiology proportion DisMod models. The most significant change is the above-mentioned adjustment of pneumococcal conjugate vaccine efficacy for the fact that most studies report the vaccine efficacy against invasive, serotype-specific pneumococcal disease rather than overall serotype-specific pneumococcal pneumonia. This has increased the estimates of pneumococcal pneumonia morbidity in a substantial way.

Upper respiratory infections (URI)

Flowchart

Upper respiratory infections (URI)

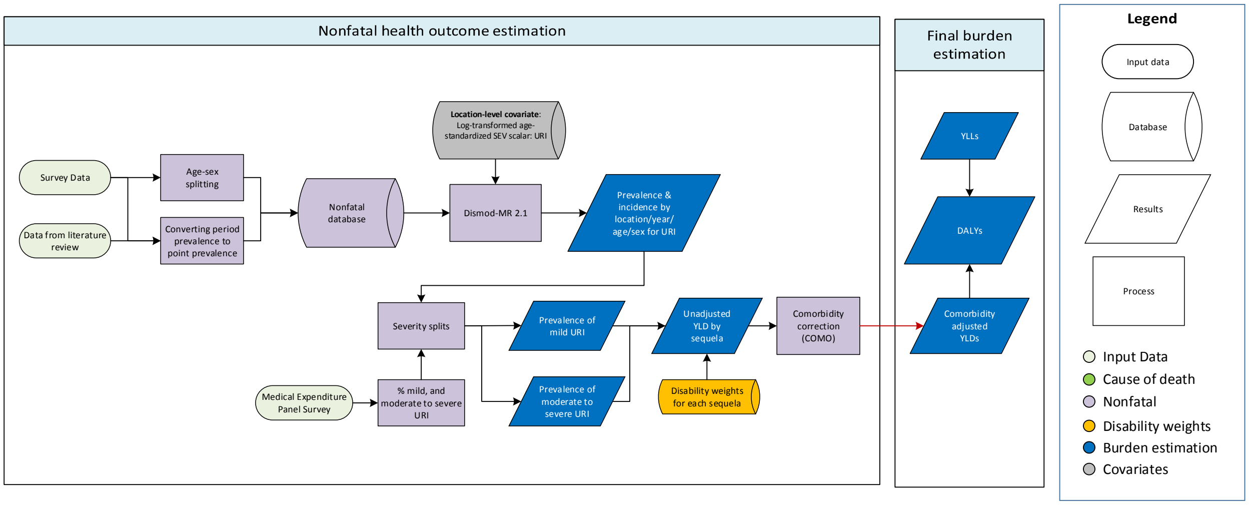

Case definition

Upper respiratory infections (URI) include acute nasopharyngitis, sinusitis, pharyngitis, tonsillitis, laryngitis/tracheitis and epiglottitis. For URI, ICD 10 codes are J00-J02, J02.8-J03, J03.8-J06.9, J36, J36.0, and ICD 9 codes are 460-465.9, 475-475.9, 476.9.

Input data

Model inputs

For GBD 2013, a systematic review of the prevalence of URI was conducted. The PubMed search terms were: ((upper respiratory infection[Title/Abstract] AND prevalence[Title/Abstract]) AND (“2009”[Date – Publication] : “2013”[Date – Publication])).

The exclusion criteria were:

1. Studies that were not population-based, e.g., hospital or clinic-based studies
2. Studies that did not provide primary data on epidemiological parameters, e.g., a commentary piece
3. Studies with a sample size of less than 150
4. Reviews

Updates to systematic reviews are performed on an ongoing schedule across all GBD causes; an update for upper respiratory infections will be performed in the next one to two iterations. The table below shows the number of literature studies included in GBD 2015, as well as the number of countries or subnational units and GBD world regions represented.

Table 1.

	Prevalence	Incidence	Mortality risk
Studies	11	-	-
Countries/subnationals	10	-	-
GBD world regions	8	-	-

In addition, data from nationally representative surveys including United States National Health Interview Surveys, Demographic and Health surveys, and Russia Longitudinal Monitoring Surveys were included.

Severity splits

The table below shows the severity distributions based on the data from Medical Expenditure Panel Surveys where we categorized "acute nasopharyngitis or acute uri multi sites/nos" as mild URI and "acute sinusitis, acute pharyngitis, acute tonsillitis, and acute laryngitis/tracheitis and epiglottitis" as moderate URI.

Table 2.

Age	Mild URI (proportion)	Moderate URI (proportion)	Standard error
0-4	0.8475367	0.1524633	0.0235054
5-9	0.7957522	0.2042478	0.0214788
10-14	0.7510127	0.2489873	0.0219692
15-19	0.7800903	0.2199097	0.0254533
20-24	0.8277971	0.1722029	0.0340625
25-29	0.866052	0.133948	0.0372739
30-34	0.8716526	0.1283474	0.0385933
35-39	0.8711911	0.1288089	0.0404143
40-44	0.8723335	0.1276665	0.0413539
45-49	0.8835218	0.1164782	0.0450838
50-54	0.8862297	0.1137703	0.0483388
55-59	0.8882668	0.1117332	0.0532085
60-64	0.8837538	0.1162462	0.0618551
65-69	0.8844019	0.1155981	0.0741072
70-74	0.8912954	0.1087046	0.0867568
75-79	0.8846442	0.1153558	0.0937314
80-84	0.9130687	0.0869313	0.104148

The lay descriptions and disability weights for severity levels derived from the GBD Disability Weights study are shown below.

Table 3.

Severity level	Lay description	DW (95% CI)
Mild upper respiratory infections	Has a low fever and mild discomfort , but no difficulty with daily activities.	0.006 (0.002-0.012)

Moderate/severe upper respiratory infections	Has a fever and aches, and feels weak, which causes some difficulty with daily activities.	0.051 (0.032-0.074)

Modeling strategy

For GBD 2015, URI was modeled using a standard DisMod-MR 2.1 model. We used the log-transformed age-standardized SEV scalar for URI as a location-level covariate.

Betas and exponentiated values (which can be interpreted as an odds ratio) are shown in the table below.

Table 4.

Covariate	Parameter	beta	Exponentiated beta
Log-transformed age-standardized SEV scalar for URI	Prevalence	4.61 (3.64 — 4.99)	100.23 (38.05 — 147.53)
Sex	Prevalence	-0.034 (-0.056 — -0.014)	0.97 (0.95 — 0.99)

No additional significant changes were made to the modeling process for GBD 2015.

Otitis media

Flowchart

Otitis media (OM)

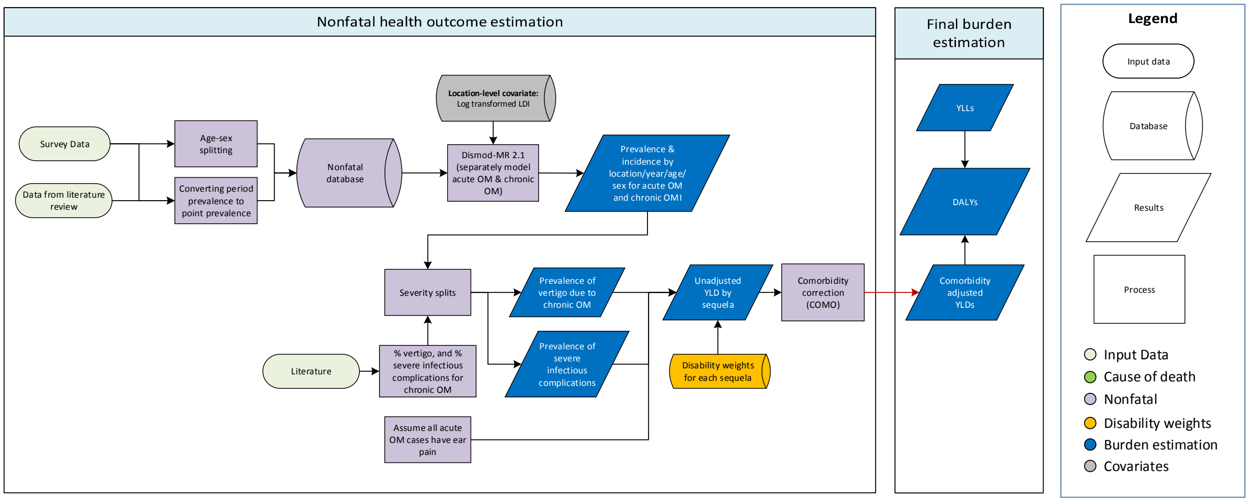

Case definition

Otitis media is an infection of the middle ear space. We included acute otitis media, chronic otitis media, and hearing loss due to otitis media in the GBD non-fatal outcome modeling. (The hearing loss estimation is included in the hearing loss report provided separately.) The ICD 10 codes are H65-H75.83, and ICD 9 codes are 381-384.9.

Input data

Model inputs

A systematic review of the prevalence of otitis media was conducted for GBD 2013. The PubMed search terms were: (((otitis media[Title/Abstract] AND (incidence[Title/Abstract] OR prevalence[Title/Abstract])) AND ("2009"[Date – Publication] : "2013"[Date – Publication])).

The exclusion criteria were:

1. Studies that were not population-based, e.g., hospital or clinic-based studies
2. Studies that did not provide primary data on epidemiological parameters, e.g., commentaries
3. Studies with a sample size of less than 150
4. Reviews
5. Case series

Updates to systematic reviews are performed on an ongoing schedule across all GBD causes, and an update for otitis media will be performed in the next one to two iterations. The table below shows the number of literature studies included in GBD 2015, as well as the number of countries or subnational units and GBD world regions represented.

Table 1. Geographies

	Prevalence	Incidence	Remission
Studies	29	12	5
Countries/subnationals	20	8	4
GBD world regions	11	5	4

In addition, data from the United States Medical Expenditure Panel Surveys and Australia National Health Surveys were included.

Severity splits & disability weights

We assume that all acute otitis media cases would experience ear pain. The severity distributions for chronic otitis media based on the study by Lin and colleagues (2009) were as follows: (i) vertigo (2.9%, 95% CI: 2.4 to 3.6%), and (ii) severe infectious complications (0.05%, 95% CI: 0.01 to 0.2%). We considered the remaining 97% of chronic otitis media cases as asymptomatic. The lay descriptions and disability weights for severity levels derived from the GBD Disability Weights study are shown below.

Table 2. Severity proportions

Severity level	Lay description	DW (95% CI)
Ear pain	Has an earache that causes some difficulty with daily activities.	0.013 (0.007-0.024)
Vertigo due to chronic otitis media*		
Severe infectious complications due to chronic otitis media	Has an earache that causes some difficulty with daily activities.	0.013 (0.007-0.024)

* See the hearing loss report for the lay descriptions and disability weights for different severity levels.

Modeling strategy

For GBD 2015, we modeled acute and chronic otitis media as separate non-fatal health outcomes using DisMod-MR 2.0. We assumed that the incidence of acute otitis media decreases after the age of 5 years. Log transformed LDI covariate was used as a location-level covariate to model chronic otitis media.

For study covariates, the table below illustrates beta and exponentiated beta values (which can be interpreted as an odds ratio):

Table 3. Acute otitis media DisMod model

Covariate	Parameter	Beta (95% CI)	Exponentiated beta (95% CI)
Sex	Prevalence	0.16 (0.00096 — 0.32)	1.18 (1.00 — 1.37)
Sex	Incidence	-0.2 (-0.27 — -0.13)	0.82 (0.76 — 0.88)

Table 4. Chronic otitis media DisMod model

Covariate	Parameter	Beta (95% CI)	Exponentiated beta (95% CI)
Log LDI	Prevalence	-0.45 (-0.5 — -0.35)	0.64 (0.61 — 0.71)
Sex	Prevalence	0.071 (-0.25 — 0.40)	1.07 (0.78 — 1.49)

No other significant changes were made to the modeling strategy in GBD 2015.

Meningitis

Flowchart

Meningitis

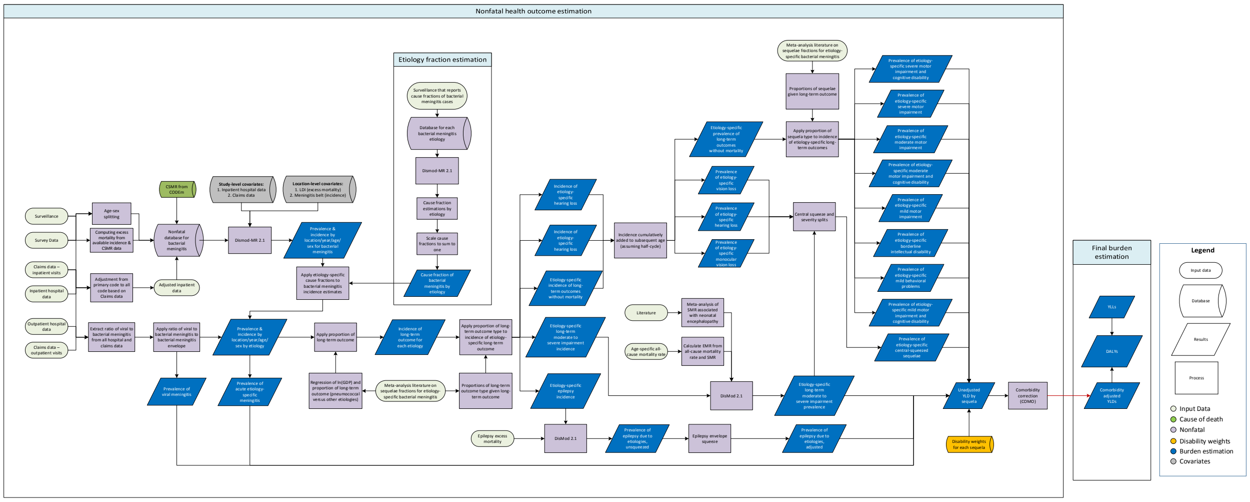

Case definition

Meningitis is a disease caused by inflammation of the meninges, the protective membrane surrounding the brain and spinal cord, and is typically caused by an infection in the cerebrospinal fluid. Symptoms include headache, fever, stiff neck, and sometimes seizures. Included in the GBD modeling were cases meeting ICD-10 diagnostic criteria for meningitis due to bacteria, viruses, or other causes (A39-A39.9, A87-A87.9, D86.81, G00.0-G00.8, G03-G03.8, Z20.811, and Z22.31) (1). In GBD 2015, meningitis encompasses viral meningitis and four bacterial etiologies: pneumococcal, haemophilus influenza type B (HiB), meningococcal, and other.

Input data

Model inputs

In the GBD 2010 study, a systematic review of literature was conducted to capture studies of incidence and excess mortality rate for all bacterial meningitis cases. For each of the four etiologies, literature included excess mortality rate, incidence, proportion, remission, and standardized mortality ratio. The inclusion criteria stipulated that: (1) the publication year must be between 1980 and 2010; (2) "caseness" was based on diagnoses by antigen test, blood test, cerebrospinal fluid test, polymerase chain reaction test, or latex agglutination test; (3) sufficient information must be provided on study method and sample characteristics to assess the quality of the study; and (4) study samples must be representative of the general population. No limitation was set on the language of publication. For GBD 2013, the search strategy was replicated to capture epidemiological studies published between 2010 and 2013. The search strategy was repeated in 2015 only to capture excess mortality – updates to systematic reviews are

performed on an ongoing schedule across all GBD causes, and a complete update for meningitis will be performed in the next one to two iterations.

Additional sources we included in the acute bacterial meningitis model were inpatient-only hospital data and US claims data from 2000, 2010, and 2012, primary diagnosis and inpatient only. Sequelae and severity splits were informed by a meta-analysis, Edmond et al (2), while an internal meta-analysis informed mortality estimates for long-term moderate to severe impairments (3).

Data were outliered or excluded if we found them unreasonable when compared to regional, super-regional, and global rates.

The tables below show the number studies included in GBD 2015, as well as the number of countries or subnational units and GBD world regions represented for the bacterial meningitis model and each model that informs the etiology split.

Table 1a. Acute bacterial meningitis

	Prevalence	Incidence	Mortality risk
Studies	0	112	77
Countries/subnationals	0	189	182
GBD world regions	0	20	17

Tablw 1b. Pneumococcal meningitis proportion

	Proportion
Studies	67
Countries/subnationals	49
GBD world regions	18

Table 1c. Meningococcal meningitis proportion

	Proportion
Studies	62
Countries/subnationals	46
GBD world regions	17

Table 1d. H influenza type B meningitis proportion

	Proportion
Studies	68
Countries/subnationals	49
GBD world regions	18

Table 1e. Other bacterial meningitis proportion

	Proportion
Studies	60
Countries/subnationals	43
GBD world regions	16

Modeling strategy

Non-fatal outcomes were modeled using a combination of custom models and DisMod-MR 2.1, with minor changes from the GBD 2013 modeling process. First, the overall incidence and prevalence of bacterial meningitis was modeled to estimate the short-term morbidity due to acute infection. This DisMod model had a set duration (1/remission) of 4 weeks with a range ±2 weeks. We also imposed caps on excess mortality for neonates and elders based on the highest excess mortality estimates from GBD 2013. Hospital data were flagged with a covariate for inpatient hospital data, as were US claims data with year-specific covariates to be crosswalked to the reference data, which we extracted from literature. We used the function in DisMod-MR 2.1 to pull in cause-specific mortality rate (CSMR) data from our CODEm and CODcorrect analyses and match with prevalence data points for the same geography. We calculated excess mortality rate to estimate priors by dividing CSMR by prevalence, calculated from remission and incidence. To help inform trends where we lack data, we applied a country-level covariate for proportion of the population at the subnational and country levels that lives within the meningitis belt in sub-Saharan Africa. We forced a positive relationship, with a lower bound of 0 and an upper bound of 2. We also applied a lag-distributed income covariate to excess mortality, log transformed and forced negative with an upper bound of -0.1 and a lower bound of -1. Betas and exponentiated values (which can be interpreted as an odd ratio) are shown in the tables below for study-level covariates and country-level covariates.

Table 2. Study covariates

Study covariate	Parameter	beta	Exponentiated beta
Inpatient hHospital	Incidence		
Claims data – 2000	Incidence		
Claims data – 2010	Incidence		
Claims data – 2012	Incidence		

Table 3. Country-level covariates

Country-level covariate	Parameter	beta	Exponentiated beta
Meningitis belt (proportion of population)	Incidence		
LDI (log transformed)	Excess mortality		

Incidence and prevalence of bacterial meningitis were split into four etiologies (pneumococcal, meningococcal, *H. influenzae* type B, and other bacterial meningitis) using four proportion models run in DisMod-MR 2.1. Results from these models were squeezed to sum to 1 at the draw level for each location, year, age, and sex. We applied a Hib3 vaccine coverage covariate to the *H. influenzae* type B proportion model, the proportion of the population living in the meningitis belt covariate and the proportion of the population living in areas covered by the MenAfrivac initiative (meningitis meningococcal type A) to the meningococcal meningitis proportion model, and a PCV3 coverage covariate to the pneumococcal meningitis model. Betas and exponentiated values are shown in the table below.

Table 4. Beta and exponentiated beta values

Country-level covariate	Etiology	Parameter	beta	Exponentiated beta
Hib3 vaccine coverage	Hib	Incidence		
Meningitis belt (proportion of population)	Meningococcal	Incidence		
MenAfrivac coverage	Meningococcal	Incidence		
PCV3 vaccine coverage	Pneumococcal	Incidence		

Data for viral meningitis were only available from hospitals or US claims data, and not from population studies, so incidence and prevalence of viral meningitis were extrapolated from bacterial meningitis incidence by applying age- and sex-specific ratios between bacterial and viral cases from a combination of hospital data and US claims data. In addition to short-term sequelae as a result of acute bacterial and viral meningitis, we also modeled the long-term outcomes from bacterial meningitis infection.

Sequelae splits

We first split the long-term sequelae among survivors of acute infection. We calculated the acute-phase survivors by applying the excess mortality (calculated by the acute meningitis DisMod model) to the incidence of each etiology (excess mortality was converted to case fatality rate by $e^{(\text{-excess mortality x 1/(excess mortality + remission))}}$. The survivors were then subject for long-term sequelae by applying the post-discharge proportions of health consequences calculated by a meta-analysis by Edmond et al (2). We calculated the ratio of acute meningitis survivors that experience major long-term impairments for all etiologies, and the ratio of minor impairments to major impairments for pneumococcal meningitis versus all other etiologies (because pneumococcal meningitis showed significantly higher risk of morbidity than other etiologies). This ratio was based off a regression of log-transformed GDP and ratio values from Edmonds et al. – this was different from last year, which used GNI. The regression is shown below:

$$y = -0.33590 \ln(GDP) + 1.15230$$

We used these two ratios to calculate the proportions of survivors who contract a long-term minor impairment and those who contract a long-term major impairment. The proportion with major impairments were further split (again using pooled proportions from Edmond et al) into specific major impairments, which were grouped into vision loss, hearing loss, moderate-to-severe cognitive impairments, and epilepsy.

The calculated incidence of long-term sequelae was converted to prevalence by two approaches. For the sequelae not associated with excess mortality, which were vision loss, hearing loss, idiopathic developmental intellectual disability, motor impairment, and behavioral problems, the incidence of each age was cumulatively added up to the subsequent age (assuming half-cycle) to construct prevalence at each age. If the sequela is associated with excess mortality (epilepsy and moderate-to-severe cognitive impairments), the calculated incidence was uploaded into DisMod together with the corresponding mortality parameters (excess mortality data from the epilepsy envelope DisMod model, and standardized mortality ratio data from a neonatal encephalopathy meta-analysis, converted to excess mortality using

all-cause mortality estimates) to estimate the prevalence. Vision loss, hearing loss, and epilepsy estimates were squeezed and severity split centrally.

Disability weights

The basis of the GBD disability weight survey assessments are lay descriptions of sequelae highlighting major functional consequences and symptoms. The lay descriptions and disability weights for sequelae associated with each etiology are shown below.

Table 5. Severity splits, lay descriptions, DWs

Severity split	Lay description	DW (95% CI)
Mild behavior problems	This person is hyperactive and has difficulty concentrating, remembering things, and completing tasks.	0.045 (0.028-0.066)
Mild hearing loss	This person has great difficulty hearing and understanding another person talking in a noisy place (for example, on an urban street).	0.01 (0.004-0.019)
Mild hearing loss with ringing	This person is unable to hear and understand another person talking, even in a quiet place, is unable to take part in a phone conversation. Difficulties with communicating and relating to others cause emotional impact at times (for example worry or depression).	0.021 (0.012-0.036)
Moderate hearing loss	This person has great difficulty hearing and understanding another person talking in a noisy place (for example, on an urban street), and sometimes has annoying ringing in the ears.	0.027 (0.015-0.042)
Moderate hearing loss with ringing	This person is unable to hear and understand another person talking, even in a quiet place, is unable to take part in a phone conversation. Difficulties with communicating and relating to others often cause worry, depression, or loneliness.	0.074 (0.048-0.107)
Moderately severe hearing loss	Custom DW from hearing loss impairment envelope	
Severe hearing loss	This person is unable to hear and understand another person talking, even in a quiet place, and unable to take part in a phone conversation. Difficulties with communicating and relating to others cause emotional impact at times (for example worry or depression).	0.158 (0.105-0.227)
Profound hearing loss	This person is unable to hear and understand another person talking, even in a quiet place, is unable to take part in a phone conversation, and has great difficulty hearing anything in any other situation. Difficulties with communicating and	0.204 (0.134-0.288)

	relating to others often cause worry, depression, or loneliness.	
Complete hearing loss	This person cannot hear at all in any situation, including even the loudest sounds, and cannot communicate verbally or use a phone. Difficulties with communicating and relating to others often cause worry, depression, or loneliness.	0.215 (0.144-0.307)
Severe hearing loss with ringing	This person is unable to hear and understand another person talking, even in a quiet place, is unable to take part in a phone conversation, and has annoying ringing in the ears for more than 5 minutes at a time, almost every day. Difficulties with communicating and relating to others cause emotional impact at times (for example worry or depression),	0.261 (0.175-0.36)
Profound hearing loss with ringing	This person is unable to hear and understand another person, even in a quiet place, is unable to take part in a phone conversation, has great difficulty hearing anything in any other situation, and has annoying ringing in the ears for more than 5 minutes at a time, several times a day. Difficulties with communicating and relating to others often cause worry, depression, or loneliness,	0.277 (0.182-0.387)
Complete hearing loss with ringing	This person cannot hear at all in any situation, including even the loudest sounds, and cannot communicate verbally or use a phone, and has very annoying ringing in the ears for more than half of the day. Difficulties with communicating and relating to others often cause worry, depression, or loneliness,	0.316 (0.212-0.435)
Moderate motor impairment	This person has some difficulty in moving around, and difficulty in lifting and holding objects, dressing and sitting upright, but is able to walk without help.	0.061 (0.04-0.089)
Moderate motor plus cognitive impairments	This person has some difficulty in moving around, holding objects, dressing and sitting upright, but can walk without help. This person has low intelligence and is slow in learning to speak and to do simple tasks.	0.203 (0.134-0.29)
Long-term mild motor impairment	This person has some difficulty in moving around but is able to walk without help.	0.01 (0.005-0.02)
Borderline idiopathic developmental intellectual disability	This person is slow in learning at school. As an adult, the person has some difficulty doing complex or unfamiliar tasks but otherwise functions independently.	0.011 (0.005-0.02)
Severe motor impairment	This person is unable to move around without help, and is not able to lift or hold objects, get dressed or sit upright.	0.402 (0.268-0.545)
Epilepsy	(combined DW)	NA

Blindness	Is completely blind, which causes great difficulty in some daily activities, worry and anxiety, and great difficulty going outside the home without assistance.	0.187 (0.124-0.26)
Severe acute episode of infectious disease	This person has a high fever and pain, and feels very weak, which causes great difficulty with daily activities.	0.133 (0.088-0.19)
Mild idiopathic developmental intellectual disability	This person has low intelligence and is slow in learning at school. As an adult, the person can live independently, but often needs help to raise children and can only work at simple supervised jobs.	0.043 (0.026-0.065)
Monocular distance vision loss	This person is blind in one eye and has difficulty judging distances	0.017 (0.009-0.029)
Mild motor plus cognitive impairments	This person has some difficulty in moving around but is able to walk without help. The person is slow in learning at school. As an adult, the person has some difficulty doing complex or unfamiliar tasks but otherwise functions independently.	0.031 (0.018-0.05)
Severe motor plus cognitive impairments	This person cannot move around without help, and cannot lift or hold objects, get dressed or sit upright. The person also has very low intelligence, speaks few words, and needs constant supervision and help with all daily activities.	0.542 (0.37-0.702)

Encephalitis

Flowchart

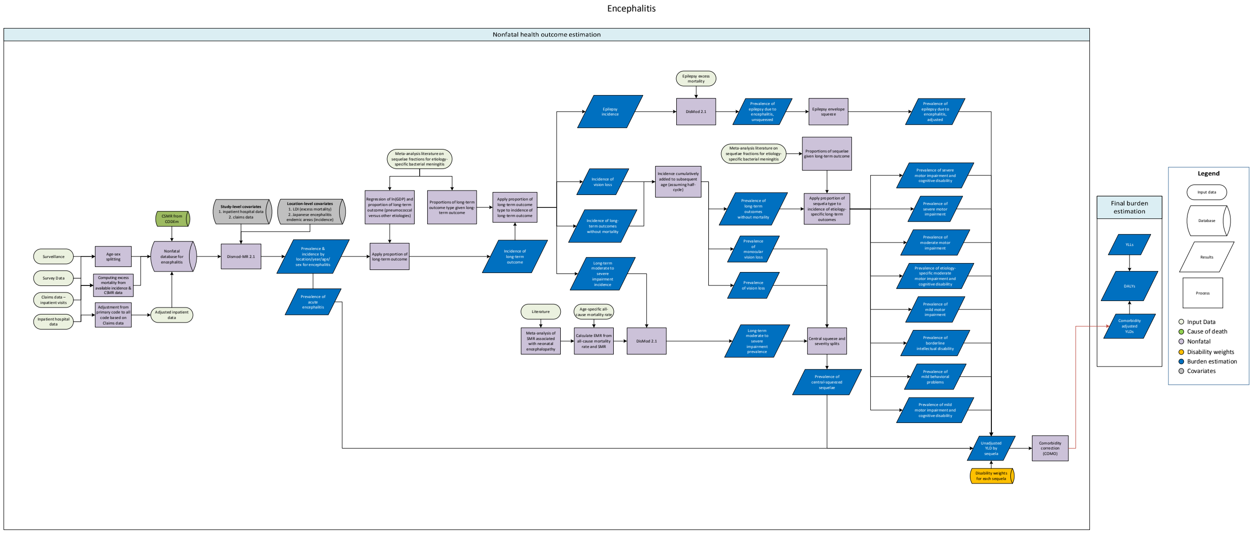

Case definition

Encephalitis is a disease caused by an acute inflammation of the brain. Symptoms of encephalitis can include flu-like symptoms like headache, fever, drowsiness, and fatigue, and at times, seizures, hallucinations, or stroke. Included in the GBD modeling were cases meeting ICD-10 diagnostic criteria for encephalitis (A83-A86.4, B94.1, F07.1, G04-G05.8) (1).

Input data

Model inputs

In the GBD 2010 study, a systematic review of literature was conducted to capture studies of incidence, excess mortality rate, remission, and standardized mortality ratio for encephalitis. These data sources included hospital data and literature. The inclusion criteria stipulated that: (1) the publication year must be between 1980 and 2010; (2) sufficient information must be provided on study method and sample characteristics to assess the quality of the study; and (3) study samples must be representative of the general population. No limitation was set on the language of publication. For GBD 2015, the GBD 2010 search strategy was replicated to capture epidemiological studies published between 2010 and 2013.

Additional sources we included in the acute bacterial meningitis model were inpatient-only hospital data and US claims data from 2000, 2010, and 2012, primary diagnosis and inpatient only. Sequelae and severity splits were informed by a meta-analysis, Edmond et al (2), while an internal meta-analysis informed mortality estimates for long-term moderate-to-severe impairments (3).

Data were outliered or excluded if we found they differed significantly when compared to regional, super-regional, and global rates.

The tables below show the number studies included in GBD 2015, as well as the number of countries or subnational units and GBD world regions represented for the encephalitis.

Table 1. Acute encephalitis

	Prevalence	Incidence	Mortality risk
Studies	0	73	73
Countries/subnationals	0	154	154
GBD world regions	0	12	12

Modeling strategy

Non-fatal outcomes were modeled using a combination of custom models and DisMod-MR 2.1, with minor changes from the GBD 2013 modeling process. First, the overall incidence and prevalence of encephalitis was modeled to estimate the short-term morbidity due to acute infection. This DisMod model had a set duration (1/remission) of three weeks. We also imposed caps on excess mortality for ages 10-50. US claims data were flagged with year-specific covariates to be crosswalked to the reference data, which we extracted from literature and inpatient hospital data. We used the function in DisMod-MR 2.1 to pull in cause-specific mortality rate (CSMR) data from our CODEm and CODcorrect analyses and match with incidence data points for the same geography. We calculated excess mortality rate to estimate priors by dividing CSMR by prevalence, calculated from remission and incidence. To help inform trends where we lack data, we applied a binary country-level covariate at the subnational and country level that indicates if the location is in a Japanese Encephalitis endemic area (4). We forced a positive relationship, with a lower bound of 0 and an upper bound of 2. We also applied a lag-distributed income covariate to excess mortality, log transformed and forced negative with an upper bound of -0.1 and a lower bound of -1. Betas and exponentiated values (which can be interpreted as an odds ratio) are shown in the tables below for study-level covariates and country-level covariates.

Table 2. Study covariates

Study covariate	Parameter	beta	Exponentiated beta
Claims data – 2000	Incidence	-0.84 (-1.02 — -0.65)	0.43 (0.36 — 0.52)
Claims data – 2010	Incidence	-0.51 (-0.58 — -0.44)	0.60 (0.56 — 0.65)
Claims data – 2012	Incidence	-0.4 (-0.48 — -0.33)	0.67 (0.62 — 0.72)

Table 3. Country-level covariates

Country-level covariate	Parameter	beta	Exponentiated beta
Japanese Encephalitis endemic area	Incidence	0.92 (0.75 — 1.04)	2.51 (2.12 — 2.84)
LDI (log transformed)	Excess mortality	-0.18 (-0.22 — -0.088)	0.84 (0.80 — 0.92)

In addition to short-term sequelae as a result of acute encephalitis, we also modeled the long-term outcomes from encephalitis.

Sequelae Splits

We first split the long-term sequelae among survivors of acute infection. We calculated the acute phase survivors by applying the excess mortality (calculated by the acute meningitis DisMod model) to the incidence of each etiology (excess mortality was converted to case fatality rate by $e^{(\text{-excess mortality x 1/(excess mortality + remission))}}$. The survivors were then subject to long-term sequelae by applying the post-discharge proportions of health consequences calculated by a meta-analysis by Edmond et al (2). We calculated the ratio of acute encephalitis survivors that result in a major long-term impairment, and the ratio of minor impairments to major impairments, based off a regression of log-transformed GDP and ratio values from Edmonds et al. This regression was done differently from last year, which previously used GNI. The regression is shown below:

$$y = -0.33590\ln(GDP) + 1.15230$$

We assumed a similar pattern of health outcomes for encephalitis infection survivors as with other bacterial meningitis survivors (except hearing loss, as we could not find evidence of hearing loss as a consequence of encephalitis infection). We used these two ratios to calculate the proportions of survivors who contract a long-term minor impairment and those who contract a long-term major impairment. The proportion with major impairments were further split (again using pooled proportions from Edmond et al) into specific major impairments, which were grouped into vision loss, moderate to severe cognitive impairments, and epilepsy.

The calculated incidence of long-term sequelae was then converted to prevalence by two different approaches. For the sequelae not associated with excess mortality, which were vision loss, intellectual disability, motor impairment, and behavioral problems, the incidence of each age was cumulatively added up to the subsequent age (assuming half-cycle) to construct prevalence at each age. If the sequela is associated with excess mortality (epilepsy and moderate-to-severe cognitive impairments), the calculated incidence was uploaded into DisMod together with the corresponding mortality parameters (excess mortality data from the epilepsy envelope DisMod model, and standardized mortality ratio data from a neonatal encephalopathy meta-analysis, converted to excess mortality using all-cause mortality estimates) to estimate the prevalence. Vision loss and epilepsy estimates were squeezed and severity split centrally.

Disability weights

The basis of the GBD disability weight survey assessments is lay descriptions of sequelae highlighting major functional consequences and symptoms. The lay descriptions and disability weights for sequelae associated with encephalitis are shown below.

Table 4. Severity splits, lay descriptions, and DWs

Severity split	Lay description	DW (95% CI)
Mild behavior problems	This person is hyperactive and has difficulty concentrating, remembering things, and completing tasks.	0.045 (0.028-0.066)
Moderate motor impairment	This person has some difficulty in moving around, and difficulty in lifting and holding objects, dressing and sitting upright, but is able to walk without help.	0.061 (0.04-0.089)

Moderate motor plus cognitive impairments	This person has some difficulty in moving around, holding objects, dressing and sitting upright, but can walk without help. This person has low intelligence and is slow in learning to speak and to do simple tasks.	0.203 (0.134-0.29)
Long- term mild motor impairment	This person has some difficulty in moving around but is able to walk without help.	0.01 (0.005-0.02)
Borderline intellectual disability	This person is slow in learning at school. As an adult, the person has some difficulty doing complex or unfamiliar tasks but otherwise functions independently.	0.011 (0.005-0.02)
Severe motor impairment	This person is unable to move around without help, and is not able to lift or hold objects, get dressed, or sit upright.	0.402 (0.268-0.545)
Epilepsy	(combined DW)	NA
Blindness	Is completely blind, which causes great difficulty in some daily activities, worry and anxiety, and great difficulty going outside the home without assistance.	0.187 (0.124-0.26)
Acute encephalitis	This person has a high fever and pain, and feels very weak, which causes great difficulty with daily activities.	0.133 (0.088-0.19)
Mild intellectual disability	This person has low intelligence and is slow in learning at school. As an adult, the person can live independently but often needs help to raise children and can only work at simple supervised jobs.	0.043 (0.026-0.065)
Monocular distance vision loss	This person is blind in one eye and has difficulty judging distances	0.017 (0.009-0.029)
Mild motor plus cognitive impairments	This person has some difficulty in moving around but is able to walk without help. The person is slow in learning at school. As an adult, the person has some difficulty doing complex or unfamiliar tasks but otherwise functions independently.	0.031 (0.018-0.05)
Severe motor plus cognitive impairments	This person cannot move around without help, and cannot lift or hold objects, get dressed or sit upright. The person also has very low intelligence, speaks few words, and needs constant supervision and help with all daily activities.	0.542 (0.37-0.702)

No other significant changes were made to the modeling process for GBD 2015.

Diphtheria

Flowchart

Diphtheria

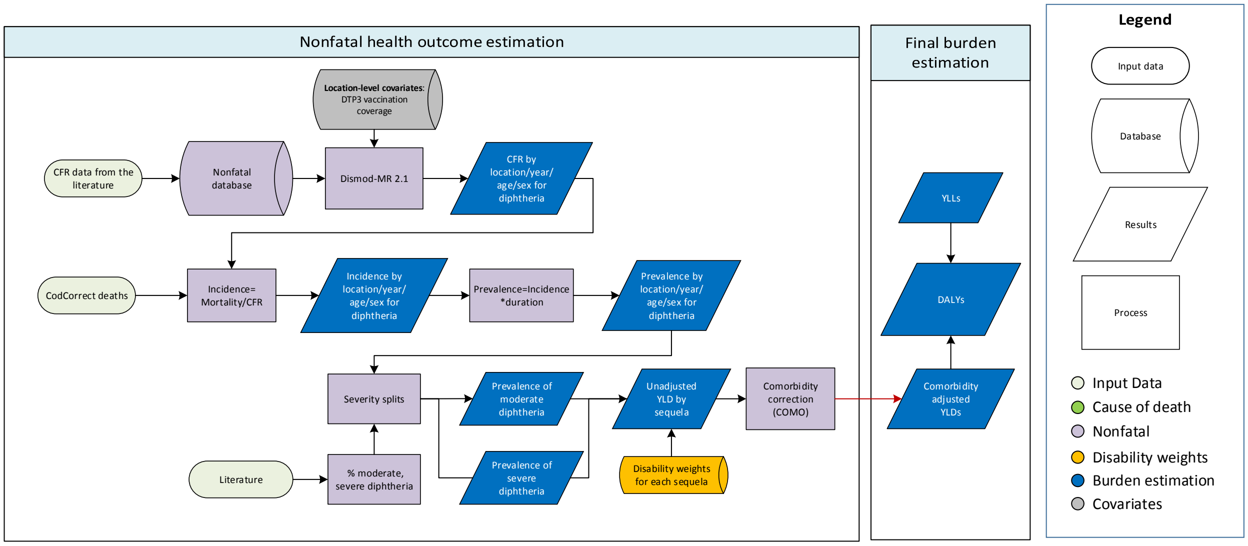

Case definition

Diphtheria is a bacterial infection caused by *Corynebacterium diphtheriae*. For diphtheria, ICD 10 codes are A36-A36.9, Z22.2, Z23.6, and ICD9 codes are 032-032.9, V02.4, V03.5, and V74.3.

Input data

Model inputs

For GBD 2015, we used the following inputs for the estimation process for diphtheria. Input data included case fatality data from the literature and IHME diphtheria mortality estimates created using CODEm.

A systematic review of diphtheria case fatality was conducted for GBD 2013. The PubMed search terms were: ((diphtheria[Title/Abstract] AND case fatality[Title/Abstract])) AND ("2009"[Date - Publication] : "2013"[Date - Publication]).

Updates to systematic reviews are performed on an ongoing schedule across all GBD causes; an update for diphtheria will be performed in the next one to two iterations. The table below shows the number of literature studies included in GBD 2015, as well as the number of countries or subnational units and GBD world regions represented.

Table 1. Geographies represented in systematic review

	Case fatality rate

Studies	19
Countries/subnationals	13
GBD world regions	7

Severity split & disability weights

We draw primarily on the literature, as well as patterns in data observed in South Asia and Central Asia, to assign the following severity distributions for diphtheria: 70% (95% CI:66.5-73.5%) moderate and 30% (95% CI: 26.5-33.5%) severe cases. The lay descriptions and disability weights for diphtheria severity levels derived from the GBD Disability Weights study are shown below.

Table 2. Severity splits, lay descriptions, and DWs

Severity level	Lay description	DW (95% CI)
Moderate diphtheria	Has a fever and aches, and feels weak, which causes some difficulty with daily activities.	0.051 (0.032-0.074)
Severe diphtheria	Has a high fever and pain, and feels very weak, which causes great difficulty with daily activities.	0.133 (0.088-0.19)

Modeling strategy

We used DisMod-MR 2.1 as a meta-regression tool to pool the case fatality data and generate location-year-age-sex-specific case fatality rate estimates. We used DTP3 coverage as a location-level covariate. Diphtheria mortality was modeled using a negative binomial regression and data from the cause of death database with the DTP3 coverage covariate and age dummy variables. Incidence was then calculated as mortality rate divided by case fatality rate. Prevalence was calculated by multiplying incidence and average duration, which was estimated to be 27.5 days, based on a meta-analysis of duration data from the literature.

The table below shows betas and exponentiated values (from the DisMod case-fatality model), which can be interpreted as an odds ratio.

Table 3. Beta and exponentiated beta values

Covariate	Parameter	Beta (95% CI)	Exponentiated beta (95% CI)
DTP3 coverage (proportion)	Case fatality	-0.18 (-0.66, -0.01)	0.84 (0.52, 0.99)
Sex	Case fatality	0.15 (-0.58, 0.90)	1.16 (0.56, 2.46)

No other significant changes were made to the modeling strategy for GBD 2015.

Whooping cough

Flowchart

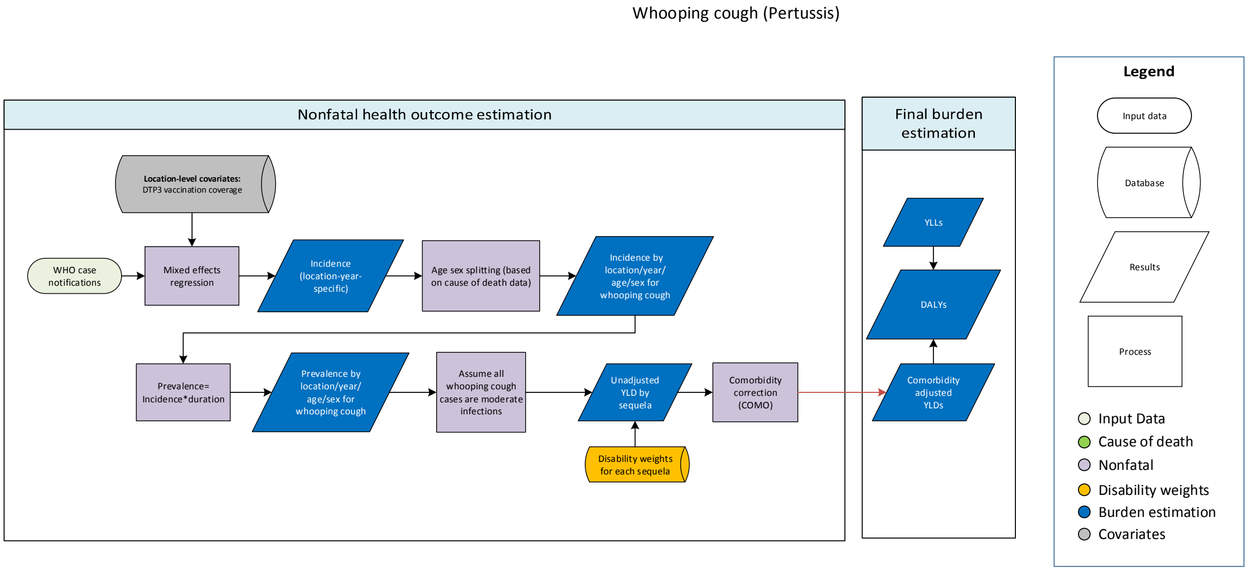

Case definition

For GBD 2015, whooping cough (pertussis), is a contagious respiratory disease caused by the bacterium *Bordetella pertussis*. For pertussis, ICD 10 codes are A37-A37.91, Z23.7, and ICD 9 codes are 033-033.9, 484.3, V03.6.

Input data

Model inputs

For the GBD 2015 nonfatal estimation process, the primary source of input data was pertussis case notifications from the World Health Organization (WHO). We also used historical case notifications for the UK back to 1940 as inputs for the whooping cough model

Severity splits

For GBD 2015, we assumed all pertussis cases were moderate episodes of acute infectious disease because of associated symptoms and MEPS data. The lay description and disability weight derived from the GBD Disability Weights study are shown below.

Table 1. Severity splits, lay descriptions, and DWs

Severity level	Lay description	DW (95% CI)
Moderate	Has a fever and aches and feels weak, which causes some difficulty with daily activities.	0.051 (0.032-0.074)

Modeling strategy

We modeled pertussis incidence using case notifications from the WHO with the DPT3 vaccination coverage covariate. Historical data of cases and DTP3 coverage rates for the UK were also used in the incidence model. More precisely, log-transformed reported incidence rates were regressed on the log of the fraction unvaccinated with a random effect on country. The random effect by country allowed for registration completeness to vary by country. The results of this model were then used to predict incidence as a function of vaccine coverage. We used a value of the random effect that matched the highest random effect in a high income region, which was Switzerland (which has a pertussis monitoring system which should capture cases well). Uncertainty was estimated by taking 1,000 iterations of the predictions based on the variance covariance matrix. Prevalence was calculated by multiplying incidence by an average duration assumed to be 50 days.

The output of the pertussis incidence model is shown in the table below.

Table 2. Beta and random effects

	Beta (95% CI)	Random effects estimate (95% CI)
Fixed effects<br>Proportion unvaccinated (log transformed)	0.931 (0.853, 1.01)	-
Random effects<br>Country	-	1.60 (1.438, 1.787)

There were no additional significant changes to the GBD 2015 modeling process for whooping cough.

Tetanus

Flowchart

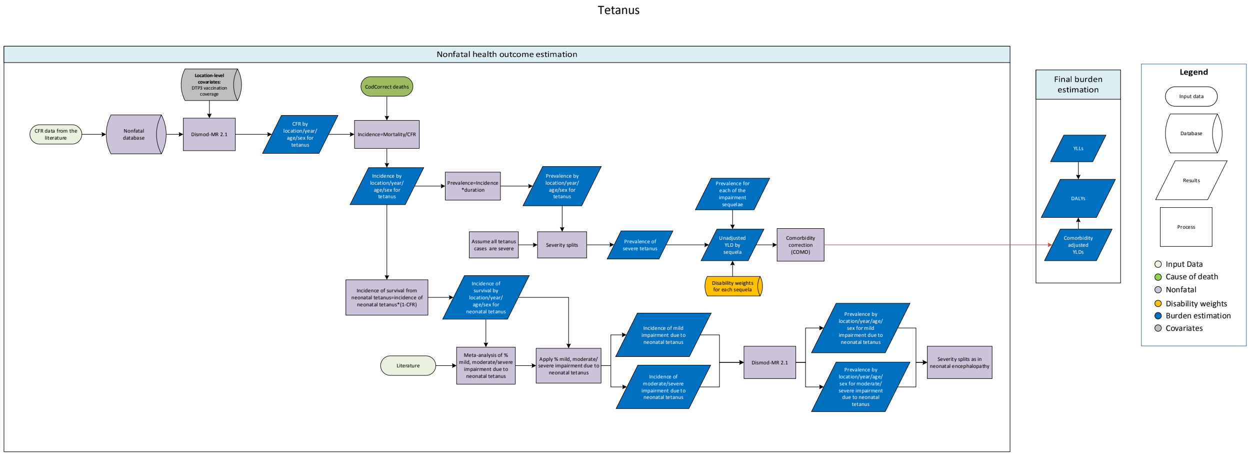

Case definition

Tetanus is a serious bacterial disease caused by the bacterium *Clostridium tetani.* For tetanus, the ICD 10 codes are A33-A35.0, Z23.5, and ICD 9 codes are 037-037.9,771.3,V03.7.

Input data

Model inputs

For GBD 2015, input data for the estimation of tetanus included case fatality data from the literature and IHME tetanus mortality estimates calculated with CODEm.

A systematic review was conducted for GBD 2013. The PubMed search terms were: (tetanus[Title/Abstract]) AND (case fatality[Title/Abstract]) AND ("2009"[Date - Publication] : "2013"[Date - Publication]).

Updates to systematic reviews are performed on an ongoing schedule across all GBD causes, and an update for tetanus will be performed in the next one to two iterations. The table below shows the number of literature studies included in GBD 2015 as well as the number of countries or subnational units and GBD world regions represented.

Table 1. Geographies

	Case fatality rate
Studies	49
Countries/subnationals	19
GBD world regions	12

Severity split & disability weight

We assume that all tetanus cases are severe episodes of acute infectious diseases. The lay descriptions and disability weights for tetanus derived from the GBD Disability Weights study are shown below.

Table 2. Severity splits, lay descriptions, and DWs

Severity level	Lay description	DW (95% CI)
Severe	Has a high fever and pain, and feels very weak, which causes great difficulty with daily activities.	0.133 (0.088-0.19)

Regarding the severity level of impairment due to neonatal tetanus, we assume the same distribution as in neonatal encephalopathy.

Modeling strategy

We used DisMod-MR 2.0 as a meta-regression tool to pool the case fatality data and generate location-year-age-sex-specific case fatality rate estimates. We used DTP3 coverage as a location-level covariate. Mortality was modeled using the standard CODEm tool on neonatal tetanus (ages 0-0.1) and non-neonatal tetanus (ages 1-80) separately for males and females. Incidence was then calculated as:

$$\text{incidence}=\text{mortality rate}/\text{case fatality rate}$$

Prevalence was then computed based on the estimated incidence and duration draws derived from literature review.

To estimate mild and moderate impairment due to neonatal tetanus, we first computed the incidence of survival from neonatal tetanus as:

$$\text{incidence of survival}= \text{incidence}*(1-\text{CFR})$$

We then conducted a meta-analysis of published studies to estimate the proportion of mild impairment due to neonatal tetanus and moderate-to-severe impairment due to neonatal tetanus. We applied these proportions to the estimated incidence of survival, to generate incidence of mild impairment due to neonatal tetanus and moderate-to-severe impairment due to neonatal tetanus, which were used as input data in DisMod 2.0. We ran two separate DisMod models (one for mild impairment due to neonatal tetanus, and one for moderate-to-severe impairment due to neonatal tetanus) to generate age-sex-year-country-specific estimates.

The table below shows betas and exponentiated values for the covariates used in the estimation process (from the DisMod case-fatality model), which can be interpreted as an odds ratio.

Table 3. Beta and exponentiated beta values

Covariate	Parameter	Beta (95% CI)	Exponentiated beta (95% CI)
DTP3 coverage (proportion)	Case fatality	0.52 (-0.061 — 1.32)	1.68 (0.94 — 3.76)
Sex	Case fatality	-0.12 (-0.35 — 0.10)	0.89 (0.71 — 1.11)

No other significant changes were made to the modeling strategy for GBD 2015.

Measles

Flowchart

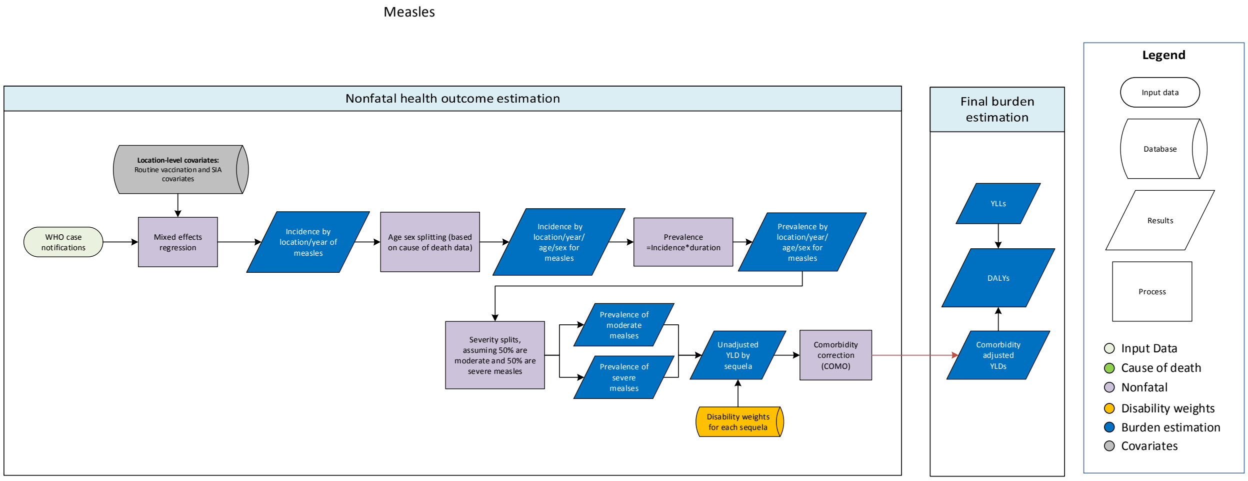

Case definition

Measles is a contagious infection caused by the measles virus. Symptoms include cough, runny nose, fever, conjunctivitis, and red, blotchy skin. For measles, ICD 10 codes are B05-B05.9, Z24.4, and ICD 9 codes are 055-055.9, 484.0, V04.2, V73.2.

Input data

Model inputs

For GBD 2015, the primary source of input data was measles case notifications from the World Health Organization (WHO). This is due to the fact that cases of measles are often geographically specific in distribution.

Severity splits

For GBD 2015, we assume 50% of measles cases were acute episode of moderate infectious diseases and 50% were acute episode of severe infectious diseases. The lay descriptions and disability weights for measles severity levels derived from the GBD Disability Weights study are shown below.

Table 1. Severity splits, lay descriptions, and DWs

Severity level	Lay description	DW (95% CI)
Moderate	Has a fever and aches, and feels weak, which causes some difficulty with daily activities.	0.051 (0.032-0.074)
Severe	Has a high fever and pain, and feels very weak, which causes great difficulty with daily activities.	0.133 (0.088-0.19)

Modeling strategy

For GBD 2015, measles incidence was modeled using case notifications from WHO, routine vaccination rates and supplementary immunization activities (SIAs) data. More precisely, log-transformed incidence rates were regressed on the log of the proportion unvaccinated, SIA variables lagged by one, two, three, four, and five years, with super-region, region and country random effects. The results of this model were then used to predict location-year-specific incidence as a function of vaccine coverage and SIAs. Uncertainty was estimated by taking 1,000 iterations of the predictions based on the variance covariance matrix. We first estimated overall incidence cases which were then assigned an age-sex distribution based on the age-sex-specific patterns found in the cause of death data. For locations in the following three super-regions, namely, high-income, Central Europe/Eastern Europe/Central Asia and Latin America and Caribbean, we directly used reported measles cases as incidence cases. Prevalence was calculated by multiplying incidence by an average duration assumed to be 10 days.

The output of the measles incidence model is shown in the table below.

Table 2. Beta and random effects

	Beta (95% CI)	Random effects estimate (95% CI)
Fixed effects<br>Proportion unvaccinated (log transformed)	1.064 (0.911, 1.217)	
SIA lagged by 1 year	-1.528 (-1.826, -1.230)	
SIA lagged by 2 years	-1.781 (-2.087, -1.476)	
SIA lagged by 3 years	-1.621 (-1.930, -1.313)	
SIA lagged by 4 years	-1.237 (-1.556, -0.918)	
SIA lagged by 5 years	-0.508 (-0.852, -0.164)	
Random effects<br>Super-region		0.854 (0.312, 2.339)
Region		0.589 (0.160, 2.167)
Country		0.936 (0.805, 1.088)

No other significant changes were made to the modeling process for GBD 2015.

Varicella and herpes zoster

Flowchart

Varicella and herpes zoster

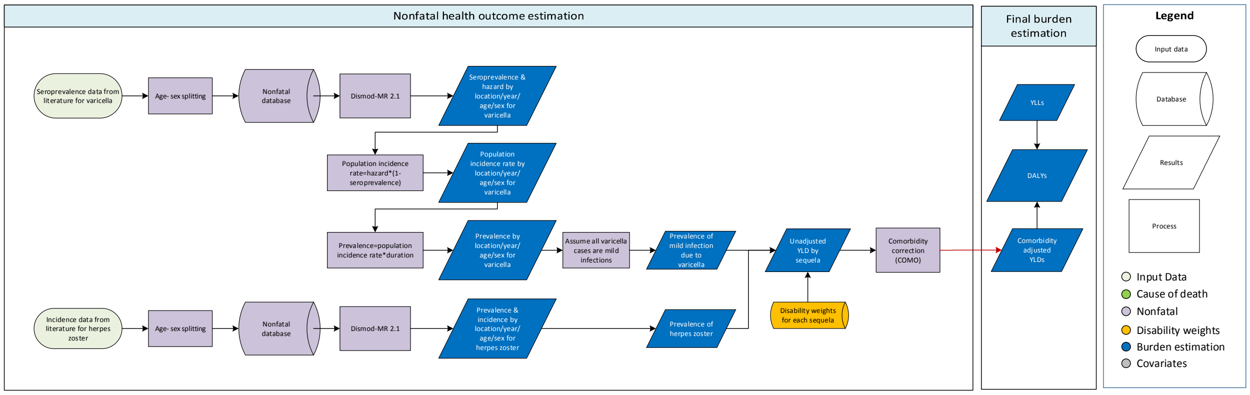

Case definition

Varicella (also known as chicken pox) is an acute infectious disease caused by varicella zoster virus. Herpes zoster (also known as shingles), is caused by the reactivation of the same virus that causes varicella. For varicella and herpes zoster, the ICD 10 codes are B01-B02.9, P35.8, Z20.820, and ICD 9 codes are 052-053.9, V01.71, V01.79, V05.4.

Input data

Model inputs

Input data for varicella were from published seroprevalence studies, and that for herpes zoster were from published incidence studies.

A systematic review was done for both varicella and herpes zoster for GBD 2013. The PubMed search query for varicella was as follows: (varicella[Title/Abstract] AND seroprevalence[Title/Abstract]) AND (incidence[Title/Abstract] OR prevalence[Title/Abstract]) NOT (herpes zoster[Title/Abstract] OR shingles[Title/Abstract]) AND ("2009"[Date - Publication] : "2013"[Date - Publication]).

The PubMed search query for herpes zoster was as follows: ((herpes zoster[Title/Abstract] OR shingles[Title/Abstract]) AND (incidence[Title/Abstract)) NOT (varicella[Title/Abstract] OR chicken pox[Title/Abstract]) AND ("2009"[Date - Publication] : "2013"[Date - Publication])

The exclusion criteria were:

1. Studies that were not population-based, e.g., hospital or clinic-based studies
2. Studies that did not provide primary data on epidemiological parameters, e.g., commentaries
3. Review articles
4. Case series

5. Self-reported cases

Updates to systematic reviews are performed on an ongoing schedule across all GBD causes. An update for varicella and herpes zoster will be performed in the next one to two iterations. The table below shows the number of literature studies included in GBD 2015, as well as the number of countries or subnational units and GBD world regions represented.

Table 1. Geographies

	Seroprevalence	Incidence
Studies	62	38
Countries/subnationals	35	20
GBD world regions	14	7

Severity splits & disability weights

We assume all varicella cases are mild episodes of acute infectious disease. Herpes zoster was a sequela studied in the GBD disability weight study. The lay descriptions and corresponding disability weights are presented in the table below.

Table 2. Severity level, lay description, and DW

Severity level	Lay description	DW (95% CI)
Mild acute infectious disease	Has a low fever and mild discomfort but no difficulty with daily activities.	0.006 (0.002-0.012)
Herpes zoster	Has a blistering skin rash that causes pain, with some burning and itching.	0.058 (0.035-0.09)

Modeling strategy

The modeling strategies for varicella and herpes zoster are outlined below:

I. Varicella seroprevalence data were first run through DisMod-MR 2.0. Detail steps in the estimation of incidence and prevalence are shown below:

(1) Model varicella seroprevalence data as prevalence in DisMod, after specifying zero remission and no excess mortality

(2) Pick up incidence draws (which are actually hazards) from DisMod

(3) Calculate population incidence rate = hazard *(1-prevalence) at the draw level

(4) Calculate prevalence as prevalence= population incidence rate*duration (assumed to be 7 days)

II. Herpes Zoster morbidity was modeled using a standard DisMod model. We assume no excess mortality associated with herpes zoster.

Betas and exponentiated values (which can be interpreted as an odds ratio) are shown in the tables below.

Table 3a. Varicella DisMod model

Covariate	Parameter	Beta (95% CI)	Exponentiated beta (95% CI)
Sex (male)	Seroprevalence	-0.075 (-0.85 — 0.75)	0.93 (0.43 — 2.12)

Table 3b. Herpes zoster DisMod model

Covariate	Parameter	Beta (95% CI)	Exponentiated beta (95% CI)
Sex	incidence	-0.14 (-0.28 — -0.0049)	0.87 (0.76 — 1.00)

No other significant changes were made to the GBD 2015 modeling strategy.

Malaria

Flowchart

Malaria

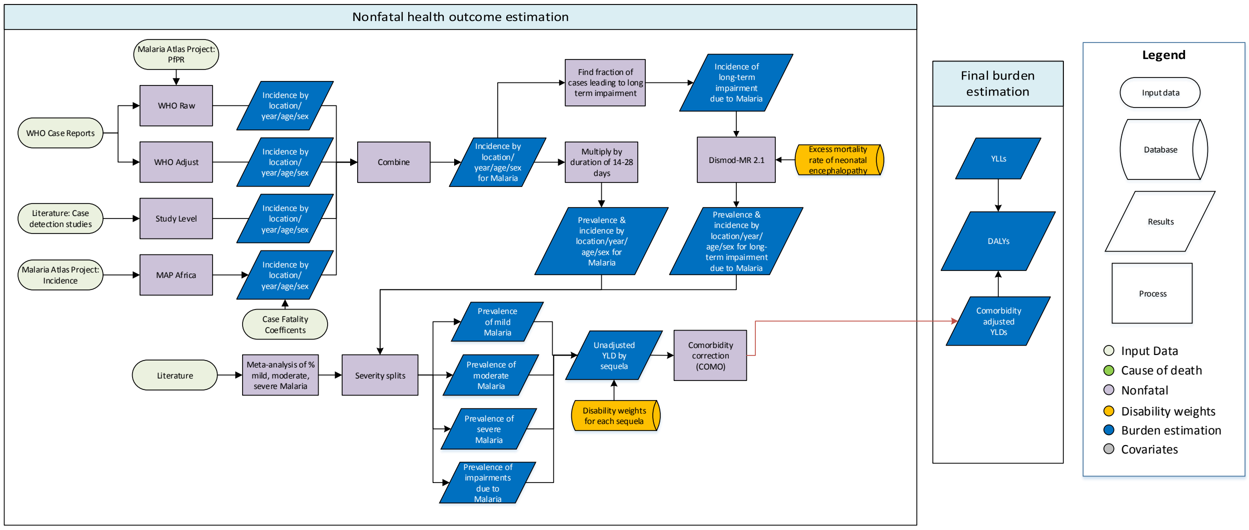

Case definition

Malaria is an acute parasitic mosquito-borne disease. An individual with uncomplicated malaria experiences one to two weeks of persistent fever, chills/shivering, sweating, joint pains and headache. The individual will likely be lethargic and feverish, causing loss of daily function during the attack. Individuals with an untreated *P. falciparum* infection may develop severe malaria, which includes the symptoms of uncomplicated malaria plus potentially swelling, difficulty breathing, unconsciousness, and death. Rapid diagnostic test or microscopy are considered the gold-standard diagnostic approaches for the purposes of GBD. The relevant ICD-10 codes are B50-B54.

Input data

Model inputs

For GBD 2015 a systematic review of malaria was not conducted. Updates to systematic reviews are performed on an ongoing schedule across all GBD causes and an update for will be performed in the next one to two iterations. However, as described below, GBD 2015 does feature a substantial estimation change relative to GBD 2013 through the integration of work by the Malaria Atlas Project (MAP).

Data for the malaria modeling process come from three main sources. For endemic countries in continental Africa, we use estimates from MAP (1). Specifically, we use spatio-temporal cubes of incidence in three broad age-bins (0-5, 5-14 and 15+). It should be noted that these incidence estimates differ slightly from those published previously. First, the cube was re-estimated using a newer antimalarial coverage covariate that includes all types of antimalarial drugs rather than just ACT. This change improves estimates occurring before ACT rollout in the early 2000s and otherwise allows for the expansion of the initial period of interest (from 2000-2015 to 1990-2015). Second, we combined the incidence estimates

with a measure of drug efficacy to produce estimates of untreated incidence that then serve as our quantity of interest for burden assignment. Ultimately, we use MAP data for the following countries: Angola, Benin, Botswana, Burkina Faso, Burundi, Cameroon, Central African Republic, Chad, Congo, Cote d'Ivoire, Democratic Republic of the Congo, Djibouti, Equatorial Guinea, Eritrea, Ethiopia, Gabon, Ghana, Guinea, Guinea-Bissau, Kenya (subnational), Liberia, Madagascar, Malawi, Mali, Mauritania, Mozambique, Namibia, Niger, Nigeria, Rwanda, Senegal, Sierra Leone, Somalia, South Sudan, Sudan, Swaziland, Tanzania, The Gambia, Togo, Uganda, Zambia, and Zimbabwe.

For most other countries with malaria transmission, we use confirmed cases reported in the 2013 World Malaria Report. These include: Afghanistan, Algeria, Argentina, Armenia, Azerbaijan, Bangladesh, Belize, Bhutan, Bolivia, Brazil (subnational), Cambodia, Cape Verde, China (subnational), Colombia, Comoros, Costa Rica, Dominican Republic, Ecuador, Egypt, El Salvador, Georgia, Guatemala, Guyana, Haiti, Honduras, Iran, Iraq, Kyrgyzstan, Laos, Malaysia, Mauritius, Mexico (subnational), Morocco, Nepal, Nicaragua, North Korea, Oman, Pakistan, Panama, Paraguay, Peru, Philippines, Sao Tome and Principe, Saudi Arabia (subnational), Solomon Islands, South Africa (subnational), South Korea, Sri Lanka, Suriname, Syria, Tajikistan, Thailand, Timor-Leste, Turkey, Turkmenistan, United Arab Emirates, Uzbekistan, Vanuatu, Venezuela, and Vietnam.

For the final data type, we used the GBD 2013 systematic review of studies with data on the clinical incidence of malaria. These studies were georeferenced so that the sites could be matched with spatially explicit covariates such as PfPR. Data are standardized into four age categories: 0-4, 5-14, 15+ and all age. Countries whose results are estimated in this group include: India (subnational), Yemen, Indonesia, Myanmar and Papua New Guinea.

Severity splits

As in GBD 2013, we use a two-step process for determining malaria severity. For acute cases, severity splits for mild, moderate, and severe malaria were produced by analysis of MEPS data. These sequelae and their associated disability weights are presented below.

Table 1. Severity level, lay description, and DW

Severity level	Lay description	DW (95% CI)
Mild	Has a low fever and mild discomfort but no difficulty with daily activities.	0.006 (0.002-0.012)
Moderate	Has a fever and aches and feels weak, which causes some difficulty with daily activities.	0.051 (0.032-0.074)
Severe	Has a high fever and pain and feels very weak, which causes great difficulty with daily activities.	0.133 (0.088-0.19)

To determine long-term neurological burden due to malaria, we use the work by Roca-Felter et al. (2008) that examined the number of uncomplicated cases that led to longer-term impairment. Analytically, this means multiplying incidence estimates (described in the section below) for persons under 20 by 0.00029 (0.000077-0.00057). This subset is then combined with excess mortality rates derived from all-cause mortality and standardized mortality ratios for neonatal encephalopathy (NE) in a DisMod model to produce prevalence estimates for all estimation years. Implicit in this process is an assumption that the

disability and trend of impairment due to severe malaria follow NE. The subsequent severity splitting follows NE as well.

Modeling strategy

We stratify analysis of malaria morbidity into four different location sets: MAP Africa, WHO Raw, WHO Adjusted and Study Level.

MAP Africa:

For the subset of countries using data from the Malaria Atlas Project, we combine the broad age and sex untreated incidence estimates from the space-time cube with mortality estimates to generate incidence estimates by GBD standard age and sex groupings. As part of the mortality estimation process, we developed a regression model by age-bin where case fatality is a function of all-cause mortality and sex. More information can be found in the GBD 2015 Mort/CoD paper. Using these models, we generated implied cases estimates where cases = death/case fatality for all age groups and both sexes. Subsequently, we scale the implied cases by age-bin so that they match the corresponding estimates from MAP. Although we carry forward the uncertainty from the MAP incidence estimates, we do not include the uncertainty of the age pattern.

WHO Raw:

Kyrgyzstan, Tajikistan, Belize, Panama, Iran, South Africa, China and Saudi Arabia, Tajikistan, Turkey, Azerbaijan, Uzbekistan, Georgia, South Korea, Argentina, Costa Rica, Armenia, Malaysia, Sri Lanka, Bhutan, Iraq, North Korea, Paraguay, Mexico, El Salvador, Ecuador, Cape Verde and Algeria all feature low case rates and are considered to have complete case reporting—particularly since the turn of the century. As such, our case rates are taken directly from those reported in the World Malaria Report (WMR). Because of systematic review cycles as well as the publication cycle of the WMR, we assume a flat trend of cases forward for the unmeasured years (e.g., 2014 and 2015). For some countries, data from the early 1990s were not reported. For these countries, cases were extrapolated via a mixed effects regression with reported cases as the outcome variable, year as the main predictor and random effects on location. The WMR does not provide information on the demographic characteristics of the malarial case, we apply the 5th percentile age pattern derived from the incidence estimates for MAP Africa to generate age- and sex-specific incidence estimates. In this case, the uncertainty in the broad age-bin pattern is taken into account.

In China, South Africa, and Saudi Arabia, we provide estimates at the subnational level, but the WMR only reports at the national level. Therefore, Chinese subnational estimates are derived from case reports from their malaria control program, while for South Africa and Saudi Arabia, we proportionally assign cases into the subnational geographies by MAP's 2010 world map of prevalence of *Plasmodium Falciparum* (PfPR) (2). This procedure is the assumption that the distribution of PfPR is substantially similar to other malaria strains.

WHO Adjust:

For the remaining countries in the broader WHO grouping (see above) that feature either incomplete reporting systems or higher malaria burden, we adjust the WMR confirmed incidence rate upward via a proxy variable for health systems access. For Mexico, we replace the WHO estimates with subnational estimates from the national malaria control program.

First, we regress the confirmed incidence rate as a function of PfPR and the interaction of health system access and malaria death rate with random effects on location. The regression is fit using all countries

with complete or semi-complete case reports (e.g. not countries in MAP Africa or the study level approach) to derive stable relationships between PfPR and the interaction term. Using the coefficients and associated uncertainty (fixed and random effects), we predict incidence rate with health system access set to the 95th percentile – thereby serving as a proxy for a more complete reporting system.

We apply the 5th percentile MAP Africa pattern to generate age- and sex-specific estimates. Although the uncertainty of the regression is captured, we do not capture uncertainty in the age and sex pattern.

Study Level:

For countries that feature a high malaria burden and poor case reporting infrastructure that are outside of Africa, we use a OLS regression with the natural log of incidence as the outcome. Predictors include the natural log of the malaria death rate, an indicator variable for Africa, the ratio between country and site level PfPR, interaction terms between each age bin and the associated log malaria death rate and an indicator variable on whether the data point relies on passive case detection. We use the 5th percentile age pattern derived from the MAP Africa estimates to distribute cases by age and sex.

Converting to prevalence

Once the separate incidence estimation procedures are complete, the results are combined and converted to prevalence by matching each draw with a draw of duration. Consistent with GBD 2013, we use a uniform distribution between 14 and 28 days for duration.

Comparison to GBD 2013

We have re-evaluated our location groupings for GBD 2013, moving several countries from the WHO adjustment category into the WHO Raw categories. Through the inclusion of the back-casting step, we account for the potential under-reporting that may have occurred pre-2000 in these reassigned countries.

GBD 2013 included an interpolation step to correct for jagged age-patterns as a correction for all-cause mortality during the age- and sex-splitting process. This step created overly smooth time and age trends and has since been removed through the inclusion of high- quality patterns from MAP. As such, we expect more pronounced age patterns (higher in youth, less so in adults) and time trends (sharper declines since 2000) relative to GBD 2013.

The inclusion of MAP incidence estimates in general marks a major step forward in better understanding malarial burden in Africa. In general, we expect a slightly higher amount of disability relative to GBD 2013. Future GBD iterations will expand the geostatistical model currently used in Africa to the rest of the world.

In conjunction with changes in the mortality estimation, GBD 2015 burden due to malaria now features a fully consistent set of results via the integration of MAP incidence data into the modeling framework.

Chagas disease

Flowchart

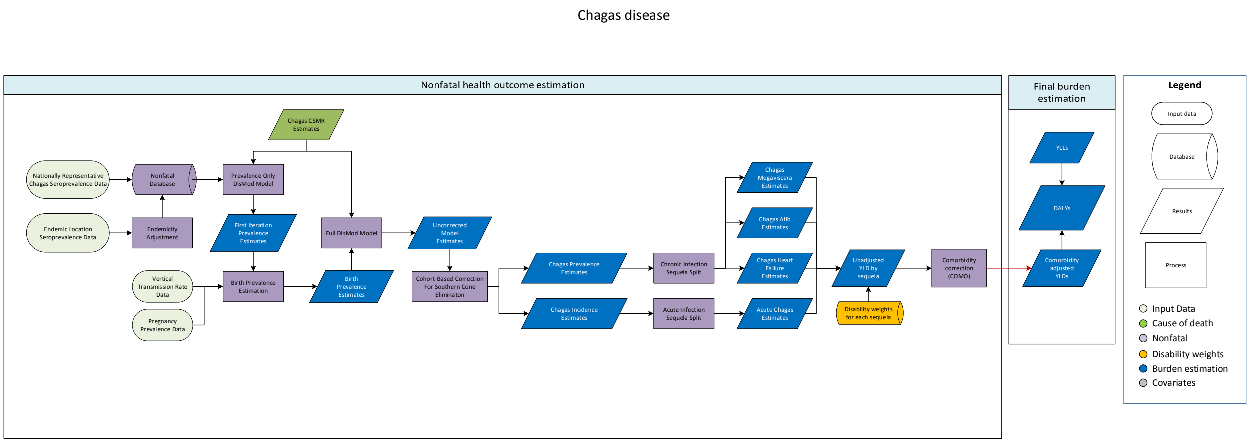

Case definition

Chagas disease is defined by infection with the protozoa *Trypanosoma cruzi,* which is transmitted by *Triatominae* insect vectors (most common), blood transfusion, organ transplant, and congenital transmission. It includes an acute phase corresponding with the time of infection, and is typically asymptomatic. Chronic infection may be latent (i.e., asymptomatic), or result in cardiovascular or digestive sequelae. It includes all ICD-10 codes under the heading B57 (Chagas disease), with codes B57.0-B75.1 corresponding to the acute phase, B57.2 corresponding to chronic cardiovascular sequelae, and B57.3 corresponding to chronic digestive sequelae.

Input data

Model inputs

For GBD 2015 estimation, we used seroprevalence data to model Chagas. The table below illustrates the geographic distribution of model input data for the estimation process.

Table 1. Geographies

Level	Prevalence
Data points	407
Studies	56
Locations	20
Regions	4

We also use CSMR estimates in the modeling process, which will be addressed in further detail below.

Modeling strategy

We modeled Chagas disease using a full DisMod-MR 2.1 Bayesian meta-regression model incorporating seroprevalence data, as above, and CSMR estimates. We assume no remission. We eliminate all new infections, except those via vertical transmission, in Chile and Uruguay for years after the interruption of vector-based transmission. For non-endemic countries, we estimate the prevalence of imported chronic infections based on migration. For each non-endemic country, we estimate the total number of people infected with Chagas as the sum of the number of immigrants from each endemic country multiplied by the corresponding prevalence of Chagas in that endemic country.

We estimate five sequelae: symptomatic acute infection from incidence; and megaviscera, heart failure, atrial fibrillation, and chronic asymptomatic infection from prevalence. We assume that 5% of acute infections will be symptomatic. The proportion of chronic infections resulting in a given sequela varies by sex and age: the prevalence of megaviscera among those infected with Chagas ranges from 0% in children to nearly 10% among older adults; the prevalence of atrial fibrillation attributable to Chagas ranges from 0% among children to approximately 10% in men over 80 years of age; and the prevalence of heart failure attributable to Chagas among those who are infected ranges from 0% among young children to a maximum of 23% among men over 80 years of age.

Severity splits and disability weights

The table below illustrates the sequelae, lay description and DWs for Chagas disease.

Table 2. Sequelae, lay description and DWs

Sequelae	Description	Disability Weight
Atrial fibrillation and flutter due to Chagas disease	Has periods of rapid and irregular heartbeats and occasional fainting.	0.224 (0.151-0.312)
Mild heart failure due to Chagas disease	Is short of breath and easily tires with moderate physical activity, such as walking uphill or more than a quarter-mile on level ground. The person feels comfortable at rest or during activities requiring less effort.	0.041 (0.026-0.062)
Moderate heart failure due to Chagas disease	Is short of breath and easily tires with minimal physical activity, such as walking only a short distance. The person feels comfortable at rest but avoids moderate activity.	0.072 (0.047-0.103)
Severe heart failure due to Chagas disease	Is short of breath and feels tired when at rest. The person avoids any physical activity, for fear of worsening the breathing problems.	0.179 (0.122-0.251)

Mild chronic digestive disease due to Chagas disease	Has some pain in the belly that causes nausea but does not interfere with daily activities.	0.011 (0.005-0.021)
Moderate chronic digestive disease due to Chagas disease	Has pain in the belly and feels nauseous. The person has difficulties with daily activities.	0.114 (0.078-0.159)
Acute Chagas disease	Has a fever and aches, and feels weak, which causes some difficulty with daily activities.	0.051 (0.032-0.074)
Asymptomatic Chagas disease	Latent Chagas infection (i.e., chronic infection with no apparent symptoms)	NA

Changes from GBD 2013 to GBD 2015

We have made no substantive changes in the modeling strategy for endemic countries from GBD 2013 for Chagas endemic countries. One notable improvement, however, is the estimation of Chagas disease among immigrants living in non-endemic countries which offers a more complete picture of Chagas' burden.

Visceral leishmaniasis

Flowchart

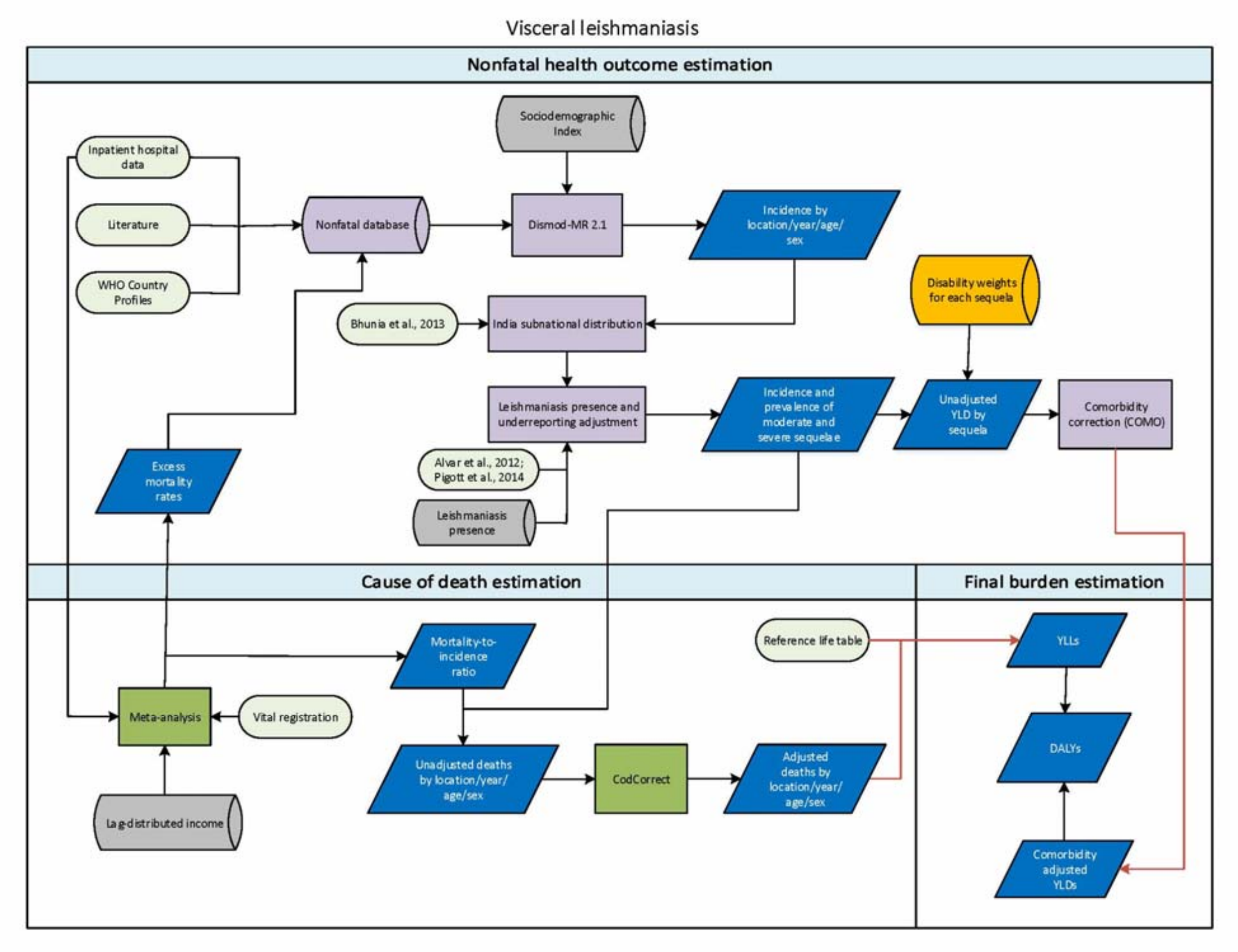

Input Data and Methodological Summary

Case Definition

Visceral leishmaniasis (VL) is the most serious manifestation of disease caused by the *Leishmania* parasite, transmitted through the bite of phlebotomine sand flies. Those infected typically present with fever, weight loss, anemia, leukopenia, thrombocytopenia, and enlargement of the spleen and liver. If left untreated, it can be fatal. Transmission varies by geographic region, as approximately 70 animal species have been identified as potential reservoir hosts of the parasite. The ICD9 code related to visceral leishmaniasis is 085.0, and the ICD10 code is B55.0.

Input data

A systematic review of literature in the PubMed database was done on 17 July 2015 for prevalence and incidence data using the search term:

(leishmaniasis[Title/Abstract] OR "visceral leishmaniasis"[Title/Abstract] OR kala-azar[Title/Abstract] OR "black fever"[Title/Abstract] OR "dumdum fever"[Title/Abstract] OR "cutaneous leishmaniasis"[Title/Abstract] OR "mucosal leishmaniasis"[Title/Abstract] OR "mucocutanaeous leishmaniasis"[Title/Abstract] OR "oriental sore"[Title/Abstract] OR "tropical sore"[Title/Abstract] OR "chiclero ulcer"[Title/Abstract] OR "chiclero's ulcer"[Title/Abstract]) AND ("1990"[Date – Publication] : "2015"[Date – Publication]) AND (epidemiology OR prevalence OR incidence OR mortality OR fatality).

This search returned 3790 results, 274 of which passed title and abstract screening for VL. Upon full text review, 24 studies were selected – five reporting prevalence, 18 reporting exclusively incidence, and one reporting both.

Additionally, incidence data from WHO country profile reports were included for 50 countries, and inpatient hospital data from 75 unique locations was used in incidence estimation. In GBD 2013, we estimated country-year-specific MI ratios by running a linear regression of the logit of the MI ratio on the log of income per capita using vital registration and inpatient hospital data from Brazil and Spain, two countries in which we had both reliable mortality and incidence data at the national level. Those were used here. Assuming a duration of 3 months, excess mortality rates were calculated and used as well.

Modeling strategy

The minimal amount of prevalence data available conflicted with the relationship between incidence and excess mortality data, as well as the remission prior (set to 4 based on duration assumptions), and thus was excluded from the model. No study level covariates were used. The sociodemographic index (SDI) was used as a country level covariate on the incidence data, with a floor of exp(-1) – as to allow a degree of regional and subnational variation while constraining the predictive power such that predictions in hypo-endemic countries with low SDI values and no data would not be unduly high.

In order to best represent the documented distribution of VL in India, we used the national fit from the DisMod model and redistributed it amongst the Indian states based on data from Bhunia, et al. Further, in order to control for DisMod fitting values to locations known to be devoid of VL, we replace estimates in these locations with zeros. Then for locations with confirmed VL presence, we apply an underreporting factor reported in Alvar et al. Resultant incidence draws are then assumed to have a duration of three months, from which prevalence is calculated. Of those three months, three weeks are assumed to be spend with severe infection, and nine with moderate infection.

Cutaneous and mucocutaneous leishmaniasis

Flowchart

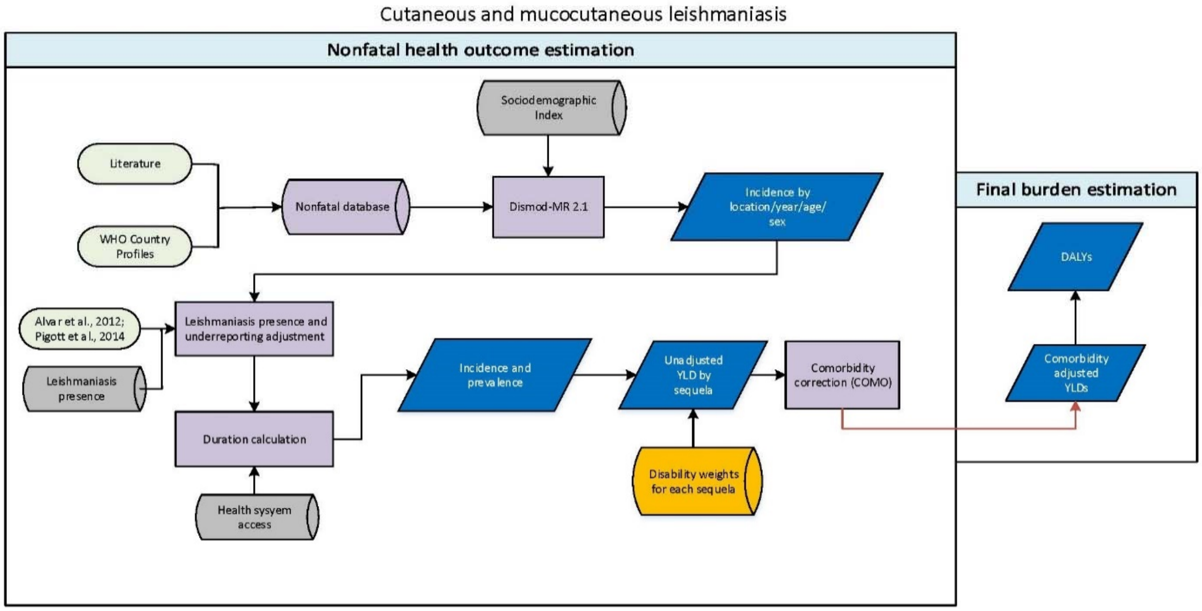

Input Data and Methodological Summary

Case Definition

Cutaneous leishmaniasis (CL) is the most common manifestation of disease caused by the *Leishmania* parasite, transmitted through the bite of phlebotomine sand flies. It causes the appearance of skin lesions, often beginning as papules or nodules and developing in/to ulcers, on parts of the body exposed to the bite of the sand fly. Mucocutaneous leishmaniasis (MCL) is a much more exceptional – and severe – presentation. Primarily isolated to Latin America, MCL infections can result in degradation of the mucous membranes, typically following an ulcerative sore from CL infection. Transmission varies by geographic region, as approximately 70 animal species have been identified as potential reservoir hosts of the parasite.

Input data

A systematic review of literature in the PubMed database was done on 17 July 2015 for prevalence and incidence data using the search term:

> (leishmaniasis[Title/Abstract] OR "visceral leishmaniasis"[Title/Abstract] OR kala-azar[Title/Abstract] OR "black fever"[Title/Abstract] OR "dumdum fever"[Title/Abstract] OR "cutaneous leishmaniasis"[Title/Abstract] OR "mucosal leishmaniasis"[Title/Abstract] OR "mucocutanaeous leishmaniasis"[Title/Abstract] OR "oriental sore"[Title/Abstract] OR "tropical sore"[Title/Abstract] OR "chiclero ulcer"[Title/Abstract] OR "chiclero's ulcer"[Title/Abstract]) AND (“1990”[Date – Publication] : “2015”[Date – Publication]) AND (epidemiology OR prevalence OR incidence OR mortality OR fatality).

This search returned 3790 results, 258 of which passed title and abstract screening for CL and/or MCL. Upon full text review, 35 studies were selected – four reporting prevalence and 31 reporting incidence. For 66 countries, incidence from WHO country profiles were available.

Modeling strategy

The minimal amount of prevalence data conflicted with incidence where available, and thus was excluded from the model. No study level covariates were used. The sociodemographic index (SDI) was used as a country level covariate on the incidence data, with a floor of exp(-1) – as to allow a degree of regional and subnational variation while constraining the predictive power such that predictions in hypo-endemic countries with low SDI values and no data would not be unduly high.

In order to control for DisMod fitting values to locations known to be devoid of CL, we replace estimates in these locations with zeros. Then for locations with confirmed CL presence, we apply an underreporting factor reported in Alvar et al. In order to distinguish prevalence of acute cases and those that endure lifelong disability, we used a normalized version of the health system access (HSA) covariate such that 47.6% of cases with poor access to healthcare – defined as (cases * (1 - norm(HSA)]) – would progress to the lifelong stage. All acute cases were assumed a six month duration.

African trypanosomiasis

Flowchart

African trypanosomiasis

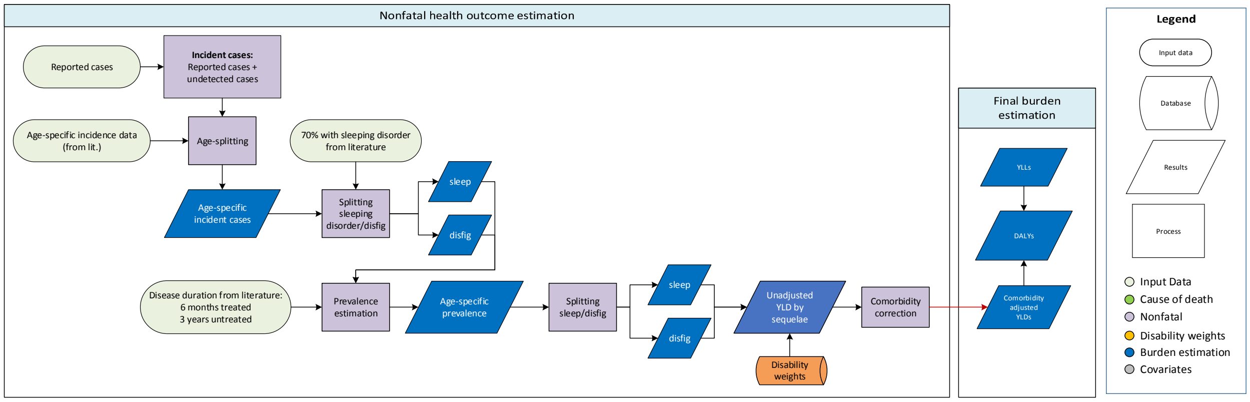

Case Definition

Human African Trypanosomiasis (HAT), also known as sleeping sickness, is a vector-borne disease which is transmitted by the bite of the tsetse fly and considered a neglected tropical disease (NTD). It is caused by the parasite *Trypanosoma brucei* with two subspecies, namely *T.b. rhodesience* (makes up less than 5% of total HAT cases) and *T.b. gambiense*. Cases are diagnosed through laboratory methods which rest on finding the parasite in body fluid or tissue by microscopy. In highly endemic or epidemic areas where the likelihood of false positives in serological tests is deemed lower, a seropositive individual is considered affected even in the absence of parasitological confirmation. The ICD-10 code for HAT are B56.0, B56.1 and B56.9.

Input data

Model inputs

The input data for GBD 2015 included a) population at risk estimates from GBD 2010 ArcGIS analysis using geocoded case notifications for 2000 to 2009 [1] and population Count Grid estimates from Gridded Population of the World 3 [2, 3], b) population screened from 1997 to 2004 [4], c) historical data from GBD 2010 on total number of HAT cases reported [1, 4, 5], and d) cases reported annually to the WHO [6] – for Kenya, a study on cases reported subnationally [7] was used to split the national cases into five counties (HomaBay, Migori, Busia, Bungoma, Kakamega). A systematic review of literature was conducted in PubMed on 8/10/2016 using the following search string: ((African trypanosomiasis[Title/Abstract] AND incidence[Title/Abstract]) AND (“2009”[Date – Publication] : “2013”[Date – Publication])). This yielded 72 studies of which only four met the inclusion criteria and were extracted. The inclusion criteria were:

1. Studies representative of the national population
2. Population-based studies
3. Studies with primary data on incidence

4. Studies of human African trypanosomiasis only (excluded studies on animal African trypanosimiasis)

The four studies extracted had national incidence data similar to the ones extracted from the WHO [6]. Therefore, only two studies with age-specific incidence data from active screening undertaken in the Democratic Republic of Congo [8] and Uganda [9] were used to inform age pattern for incidence and prevalence. Location-years with missing reported cases were excluded and five subnational locations for Kenya were added. The table below shows the number of studies included, and the number of countries or subnational units and GBD world regions represented.

Table 1. Geographies

	incidence
Studies	2
Countries/subnationals	34
GBD world regions	4

Severity splits/Sequelae

The basis of the GBD disability weight (DW) survey assessments are lay descriptions of sequelae highlighting major functional consequences and symptoms. The lay descriptions and disability weights for HAT sequelae due to HAT are shown below.

Table 2. Sequela, ley descriptions, and DWs

Sequela	Lay description	DW (95% CI)
Skin disfigurement, level 2	has a visible physical deformity that causes others to stare and comment. As a result, the person is worried and has trouble sleeping and concentrating	0.067 (0.044-0.096)
Motor plus cognitive impairments, severe	cannot move around without help, and cannot lift or hold objects, get dressed or sit upright. The person also has very low intelligence, speaks few words, and needs constant supervision and help with all daily activities	0.542 (0.37 – 0.702)

Modeling strategy

The non-fatal model for HAT involved estimating prevalence from incidence. First, a multi-level mixed-effects linear regression of natural log-transformed incidence rate (ratio of HAT cases reported to population at risk) on natural log-transformed screening coverage (ratio of number screened for HAT to population at risk), with country random effects, was performed. Gaps were then filled using exponential interpolation between years and extrapolation from 2014 to 2015 for reported cases; for screening coverage only extrapolation from 2014 to 2015 was done. Then 1,000 draws of mortality among treated cases were generated, assuming that 0.7% - 6.0% of all treated (reported) cases die [10, 11, 12].

Using the mean and variance-covariance matrix from the regression as parameters, a multivariate normal distribution was used to generate 1,000 draws of case detection rate (CDR), given the expected screening

coverage. Undetected deaths were then estimated as the difference between the ratio of reported cases to CDR and reported cases (reported cases/CDR – reported cases). Estimates of incidence were obtained by adding the reported cases to the undetected cases. Without information on sex-specific incidence, equal incidence rates between both sexes was assumed. Finally, an age-pattern was applied to the incidence estimates using the incidence studies from DRC and Uganda [8, 9]. Assuming the same proportion in treated and untreated cases, the incidence estimates were then split into the two sequelae, skin disfigurement and sleeping disorder. This was done by generating 1,000 draws of the splitting proportion for the sequelae (70%-74% with sleeping disorder) based on a study that reported presence of symptoms at admission of patients in treatment centers [13] – draws were generated from a beta distribution with alpha parameter = 1884 and beta parameter = 649.

To compute prevalence of HAT, 1,000 draws of total duration of symptoms in untreated cases was generated from a normal distribution with mean = {ln(3) – 0.5 * sigma^2}, and standard deviation = sigma, where sigma = {ln(4.39)-ln(1.92))/(invnormal(0.975)*2)} – these parameters were based on a study of *T.b. gambiense* [14] which estimated an average duration of three years to untreated cases. An estimated duration of six months was applied to cases that received treatment, based on findings from a paper about *T.b. rhodesiense* in Uganda [11]. Prevalence was then estimated from the incident cases before applying age pattern. Prevalence of treated and untreated cases were summed up, assuming that untreated cases have been prevalent up to their death for a certain duration. For untreated cases, it was assumed that half the duration is spent with sleeping disorder (severe motor and cognitive impairment) and disfigurement [14]. Treated (i.e., reported) cases are assumed to have been prevalent for 0.5 years, and for the fraction of treated cases that present with sleeping disorder, it was assumed that this is present for half the total duration and that the rest of the duration is spent suffering from disfiguring skin disease. Treated cases that don't present with sleeping disorder were assigned disfigurement for the entire duration. Lastly, an age-pattern was applied to the prevalence estimates using the incidence studies from DRC and Uganda [8, 9].

Results from the model were assessed by visualizing time trends of incident and prevalent cases across locations and age (similar trends were applied in both sexes). Maps of the global distribution of HAT and the two sequelae were also generated. In addition, the estimated incident cases were compared with the cases reported to the WHO across time – as expected, the estimates from GBD 2015 were higher than the WHO numbers because we accounted for undetected cases.

Changes from GBD 2013 included: a) inclusion of new data on reported cases from WHO [6] (years 2013 and 2014 for 23 locations), b) inclusion of the following country (years) based on available historical data post-1980: Botswana (1983), Ethiopia (1980-1983), Guinea-Bissau (1980-1983, 1985-1987), Rwanda (1980, 1982-1988), and Sierra Leone (1981-1982), c) adding five subnational locations (out of 49) for Kenya, thusd) correcting the age-split proportion such that a 0.32/0.68 proportion was used for adults/kids –in GBD 2013, this proportion was 0.25/0.75 for adults/kids.

References

1. Simarro P, Cecchi G, Paone M, Franco J, Diarra A, Ruiz J, Fevre E, Courtin F, Mattioli R, Jannin J. The Atlas of human African Trypanosomiasis: a contribution to global mapping of neglected tropical diseases. International Journal of Health Geographics, 2010. 9:57
2. Center for International Earth Science Information Network (CIESIN), Columbia University; and Centro Internacional de Agricultura Tropical (CIAT). 2005. Gridded Population of the World, Version 3 (GPWv3): Population Count Grid. Palisades, NY: Socioeconomic Data and Applications Center (SEDAC), Columbia University. Available at http://sedac.ciesin.columbia.edu/gpw
3. Center for International Earth Science Information Network (CIESIN), Columbia University; United Nations Food and Agriculture Programme (FAO); and Centro Internacional de Agricultura Tropical (CIAT). 2005. Gridded Population of the World, Version 3 (GPWv3): Population Count Grid, Future Estimates. Palisades, NY: Socioeconomic Data and Applications Center (SEDAC), Columbia University. Available at http://sedac.ciesin.columbia.edu/gpw
4. WHO. Weekly epidemiological record. 2006, February 24. No. 8, 2006, 81. 69-80
5. WHO Department of Communicable Disease Surveillance and Response (CDS). WHO Report on Global Surveillance of Epidemic-prone Infectious Diseases. 2000. WHO/CDS/CSR/ISR/2000.1.
6. WHO Global Health Observatory Data Repository (http://apps.who.int/gho/data/node.main.A1635?lang=en). Accessed Sept. 2015
7. Ruto JJ, Karuga JW. Temporal and spatial epidemiology of sleeping sickness and use of geographical information system (GIS) in Kenya. J Vector Borne Dis 2009; 1. 18-25
8. Lutumba P, Makieya E, Shaw A, Meheus F, Boelaert M. Human African Trypanosomiasis in a Rural Community, Democratic Republic of Congo. Emerging Infectious Diseases, 2007. Vol 13: No.2, 248-54
9. Fevre E, Odiit M, Coleman P, Woolhouse M, and Welburn S. Estimating the burden of rhodesiense sleeping sickness during an outbreak in Serere, eastern Uganda. BMC Public Health 2008, 8:96
10. Balasegaram M Harris S, Checchi F, Ghorashian S, Hamel C, Karunakara U. Melarsoprol versus eflornithine for treating late-stage Gambian trypanosomiasis in the Republic of Congo. Bulletin of the World Health Organization 2006;84:783-791
11. Odiit M, Kansiime F, Enyaru JCK. Duration of symptoms and case fatality of sleeping sickness caused by Trypanosoma brucei rhodesiense in Tororo, Uganda. East African Medical Journal. December 1997. 792-5
12. Priotto G, Kasparian S, Mutombo W, Ngouama D, Ghorashian S, Arnold U, Ghabri S, Baudin E, Buard V, Kazadi-Kyanza S, Ilunga M, Mutangala W, Pohlig G, Schmid C, Karunakara U, Torreele E, Kande V. Nifurtimox-eflornithine combination therapy for second-stage African Trypanosoma brucei gambiense trypanosomiasis: a multicentre, randomised, phase III, non-inferiority trial. Lancet 2009; 374. 56-64
13. Blum J. Scmid C, Burri C. Clinical aspects of 2541 patients with second stage human African trypanosomiasis. Acta Tropica 97. 2006. 55-64
14. Checchi F, Filipe J, Haydon D, Chandramohan D, and Chappuis F. Estimates of the duration of early and late stage of gambiense sleeping sickness. BMC Infectious Diseases. 2008. 8:16

Schistosomiasis

Flowchart

Schistosomiasis

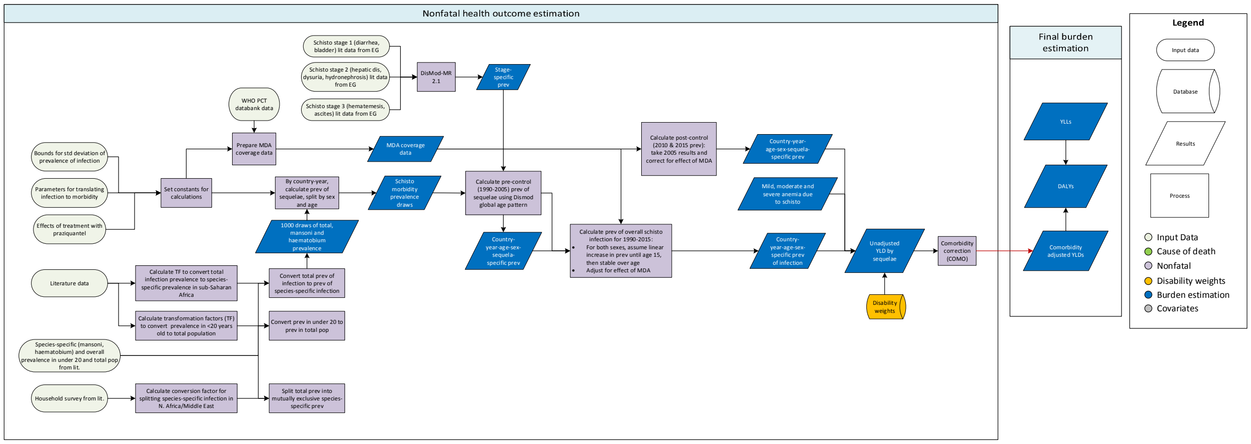

Case definition

Schistosomiasis, also known as bilharzia or “snail fever,” is a helminth disease caused by infection with five species of the parasite *Schistosoma* namely, *S. mansoni, S. japonicum, S. haematobium, S. mekongi, and S. intercalatuma*. It is considered a neglected tropical disease (NTD). The first three species cause the most infection and the last two rarely cause disease. Diagnosis is made by microscopic exam of stool or urine for parasite eggs. For less advanced infections, serologic techniques are used. The ICD-10 codes for schistosomiasis are B565-B65.9.

Input data

Model inputs

To model nonfatal outcomes due to schistosomiasis, data prepared by the expert group (EG) during GBD 2010 was used. To prepare the data, they compiled most recently published estimates of species-specific infection prevalence calculated using Bayesian geo-statistical models based on environmental predictors. Details of materials and methods used by the EG are referenced elsewhere [1-6]. Using citations compiled by the EG, a list of eight possible clinical sequelae and anemia sequelae were defined (mild infection, mild diarrhea, hematemesis (vomiting blood), hepatomegaly, ascites (build-up of fluid in the peritoneal cavity), dysuria (painful urination), bladder pathology, hydronephrosis (swelling of kidney due to build-up of urine in the kidney), mild anemia, moderate anemia, severe anemia).

Stage-specific prevalence data produced by the EG was also used as inputs for three separate single-parameter models in DisMod (stage 1: acute symptoms - diarrhea, active/mild schisto; stage 2: semi-acute/chronic symptoms - hepatomegaly, dysuria, and hydronephrosis; stage 3: long term chronic disease - haematemesis, ascites, and bladder pathology). The table below shows (by stage) the number of literature studies included in GBD 2015, as well as the number of countries or subnational units and GBD world regions represented.

Table 1a. Geographies

	Stage 1	Stage 2	Stage 3
Studies	76	40	10
Countries/subnationals	34	20	7
GBD world regions	8	8	5

Literature data on prevalence of infection by species (mansoni and haematobium) in under 20 and in the total population was also used [3-7] – for Brazil, this data was split into subnational using data from the Schistosomiasis Control Program (PCE) [8]. The table below shows the number of sources included, and the number of countries or subnational units and GBD world regions represented.

Table 1b. Geographies

	prevalence
Sources	5
Countries/subnationals	94
GBD world regions	10

Additional literature sources were used to inform morbidity prevalence estimation. These are highlighted in modeling strategy section of this document.

We did not update the literature review for these data in GBD 2015. Updates to systematic reviews are performed on an ongoing schedule across all GBD causes, an update for schistosomiasis will be performed in the next 1-2 iterations.

Severity splits/Sequelae

The table below shows the list of clinical sequelae [8] (including mild, moderate, and severe anemia) due to schistosomiasis, their lay descriptions, and the associated disease stage and disability weights.

Table 2. Clinical sequela, lay descriptions, disease stage, and DWs

Clinical sequela	Lay description	Disease stage	Disability weights (DWs)
Mild infection	has a low fever and mild discomfort , but no difficulty with daily activities	1	0.006 (0.002-0.012)
Mild diarrhea		1	0.056
Hepatomegaly	has some pain in the belly that causes nausea but does not interfere with daily activities	2	0.011 (0.005-0.021)
Dysuria	has some pain in the belly that causes nausea but does not interfere with daily activities	2	0.011 (0.005-0.021)
Hydronephrosis	has some pain in the belly that causes nausea but does not interfere with daily activities	2	0.011 (0.005-0.021)
Haematemesis	vomits blood and feels nauseous	3	0.325 (0.209-0.463)

Ascites	has pain in the belly and feels nauseous. The person has difficulties with daily activities	3	0.114 (0.078-0.159)
Bladder pathology	has some pain in the belly that causes nausea but does not interfere with daily activities	3	0.011 (0.005-0.021)
Mild anemia	feels slightly tired and weak at times, but this does not interfere with normal daily activities	NA	0.004 (0.001-0.008)
Moderate anemia	feels moderate fatigue, weakness, and shortness of breath after exercise, making daily activities more difficult	NA	0.052 (0.034-0.076)
Severe anemia	feels very weak, tired and short of breath, and has problems with activities that require physical effort or deep concentration	NA	0.149 (0.101-0.210)

Modeling strategy

The morbidity model for schistosomiasis involved six main steps. First, constants were set for calculations on 1) bounds for standard deviation of dispersion of logit prevalence of infection within countries (low=0.6, high=0.8), 2) parameters (a, b, c) for translating infection (x) to morbidity (y): y = (a + bx^c)/(1 + bx^c) – a [9-11], and 3) effects (mean, low, high) of treatment with praziquantel (PZQ) [12, 13]. Next, the conversion factor for splitting species-specific (mansoni, haematobium) infection in North Africa and Middle East was calculated, informed by literature data [14]. In the third step, literature data [4, 5] was used to calculate transformation factors for converting 1) prevalence in individuals under 20 years of age to total population, and 2) total infection prevalence to species-specific prevalence in sub-Saharan Africa.

Next, infection data was prepared by 1) splitting total prevalence in North Africa/Middle East into mutually exclusive species-specific prevalence, 2) converting prevalence in under 20 to prevalence in the total population, and 3) converting total prevalence of infection in sub-Saharan Africa to prevalence of species-specific infection. To generate 1,000 draws of prevalence, the prevalence estimates were scaled using values drawn from a standard normal distribution. Resulting estimates were split by age and sex, based on general pattern (infection: linear increase until age 15, then stable) or DisMod global age pattern for the three different stages of disease, each with a different pattern. All stage-specific DisMod models were run as proportion models and included year as a country-level covariate.

The final step was the prediction of post-control prevalence of infection and morbidity, adjusted for the impact of treatment, given cumulative number of PCT treatments per person at risk (draws of expected reductions in overall infection prevalence were obtained using data from the WHO PCT Databank [15]) and estimated species-specific efficacy, as reported in literature. For reversible sequelae, it was assumed that treatment effects are the same as for infection. For irreversible symptoms (advanced hepatic disease, ascites, hematemesis), it was assumed that incidence among the treated fraction of the population is zero and 10% of individuals with these conditions die each year due their schisto-related sequelae. The burden of anemia due to schistosomiasis was estimated (see anemia documentation for details).

Model evaluation was done by separately assessing the fit of the three stage-specific DisMod models and checking the final estimates produced after age-sex splits. Plots of time trends of prevalence across locations, and age were used to evaluate the results. In addition, maps of the global distribution of total

schistosomiasis prevalence and prevalence of sequelae due to schistosomiasis were also assessed across time.

The main change made from GBD 2013 was splitting Brazil national data into subnational using data from the Schistosomiasis Control program [8]. In addition, newly updated data from the WHO PCT databank was downloaded and used in the model.

References

1. Clements AC, Garba A, Sacko M, et al. Mapping the probability of schistosomiasis and associated uncertainty, West Africa. Emerg Infect Dis. 2008;14(10):1629-32
2. Center Clements AC, Moyeed R, Brooker S. Bayesian geostatistical prediction of the intensityof infection with Schistosoma mansoni in East Africa. Parasitology. 2006;133(Pt 6):711-9
3. Chitsulo L, Engels D, Montresor A, Savioli L. The global status of schistosomiasis and its control. Acta Trop. 2000;77(1):41-51
4. Schur N, Hurlimann E, Stensgaard AS, Chimfwembe K, Mushinge G, Simoonga C, Kabatereine NB, Kristensen TK, Utzinger J, Vounatsou P. Spatially explicit Schistosoma infection risk in eastern Africa using Bayesian geostatistical modelling. Acta Trop. (2011)
5. Schur N, Hurlimann E, Garba A, Traore MS, Ndir O, Ratard RC, Tchuente L-AT, Kristensen TK, Utzinger J, Vounatsou P. Geostatistical Model-Based Estimates of schistosomiasis Prevalence among Individuals Aged <20 Years in West Africa. PLoS Negl Trop Dis 2011;5(6): e1194
6. Steinmann P, Keiser J, Bos R, Tanner M, Utzinger J. schistosomiasis and water resources development: systematic review, meta-analysis, and estimates of people at risk. Lancet Infect Dis. 2006;6(7):411-25
7. Ministry of Health (China). China Schistosomiasis Prevalence National Sample Survey 2004
8. Secretariat of Health Surveillance, Ministry of Health (Brazil). Brazil DATASUS TABNET - Schistosomiasis Control Program (PCE)
9. van der Werf MJ, de Vlas SJ, Brooker S, et al. Quantification of clinical morbidity associated with schistosome infection in sub-Saharan Africa. Acta Trop. 2003;86(2-3):125-39
10. van der Werf MJ, de Vlas SJ, Looman CW, Nagelkerke NJ, Habbema JD, Engels D. Associating community prevalence of Schistosoma mansoni infection with prevalence of signs and symptoms. Acta Trop. 2002;82(2):127-37
11. van der Werf MJ, de Vlas SJ. Diagnosis of urinary schistosomiasis: A novel approach to compare bladder pathology measured by ultrasound and three methods for hematuria detection. Am. J. Trop. Med. Hyg. 2004;82:98-106
12. Danso-Appiah A, Utzinger J, Liu J, Olliaro P. Drugs for treating urinaty schistosomiasis (Review). The Cochrane Library. 2008(3); 1-72
13. Danso-Appiah A, Utzinger J, Liu J, Olliaro P. Drugs for treating urinaty schistosomiasis (Review). The Cochrane Library. 2013(2); 1-177
14. El-Khoby T, Galal N, Fenwick A, et al. The epidemiology of schistosomiasis in Egypt: summary findings in nine governorates. Am. J. Trop. Med. Hyg. 2000; 62: 88–99
15. WHO PCT Databank. 2015; http://www.who.int/neglected_diseases/preventive_chemotherapy/sch/en/

Cysticercosis

Flowchart

Cysticercosis

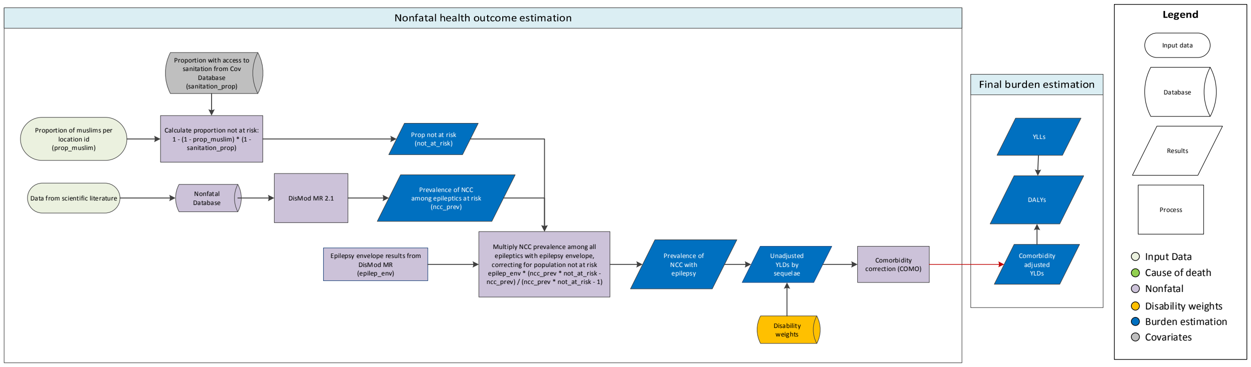

Case Definition

Cysticercosis is a helminth disease caused by the pig tapeworm, *Taenia solium*, transmitted through the fecal oral route or by consumption of pork containing *T. solium* eggs. It is considered an NTD. Diagnosis is made by Magnetic resonance imaging or CT brain scans for neurocysticercosis. The ICD-10 codes for cysticercosis are B69-B69.9.

Input data

Model inputs

The nonfatal estimation for cysticercosis focused on estimating prevalence of neurocysticercosis among epileptics at risk as well as the prevalence of neurocysticercosis with epilepsy. A systematic review of literature was conducted in PubMed using the following search string: ("cysticercosis"[Title/Abstract] OR "neurocysticercosis"[Title/Abstract] OR "cysticerciasis"[Title/Abstract] OR "Taenia solium"[Title/Abstract]) AND (“1990”[Date – Publication] : “2015”[Date – Publication]) AND (epidemiology OR prevalence)). This yielded 1,038 studies of which 166 were included during the title/abstract screening. Following the full-text screening, 17 studies were included and extracted – studies were excluded because of one or more of the following reasons:

1. study not in epileptics
2. study not population-based
3. study does not have primary data on prevalence of neurocysticercosis among epileptics at risk
4. study not in humans (some studies were on cysticercosis in pigs)
5. study on comorbidities with neurocysticercosis (other than epilepsy)
6. study on sub-population, e.g., patients with neurological disorders
7. review study

We combined the newly extracted studies with studies extracted during GBD 2013. The table below shows the number of studies finally included, and the number of countries or subnational units and GBD world regions represented.

Table 1. Geographies

	prevalence
Studies	32
Countries/subnationals	23
GBD world regions	8

A study-level covariate was also created to indicate the type of diagnosis for each study, i.e., definitive or probable. Of the 77 rows of country-year-age-sex data, there were 15 rows with definitive diagnosis and 62 rows with probable diagnosis.

Three additional data sources that were used included 1) epilepsy envelope prevalence (from the epilepsy DisMod MR model), 2) proportion of the population with access to sanitation (from the GBD covariates database), and 3) proportion of the population that is Muslim (from the PEW Research Center [1].)

Modeling strategy

DisMod MR was used to model the prevalence (ONLY) of neurocysticercosis among epileptics at risk. In the model, pigs per capita and religion (binary, >50% Muslim) were used as country-level covariates. In addition, the prevalence of "definitive diagnosis" was crosswalked to that of "probable and definitive diagnosis" so as to not underestimate overall prevalence.

After running DisMod, we adjusted the fraction of people with epilepsy attributable to cysticercosis in endemic countries for the population at risk (based on the proportion of the population without access to sanitation and the proportion of the population that is Muslim). Predicted neurocysticercosis (NCC) prevalence among epileptics at risk such that Prevalence=P×(NM-N)/(NM-1), where P = prevalence of all-cause epilepsy in total population, N = proportion of NCC among epileptics at risk (non-Muslims without access to sanitation), and M = proportion of population not at risk of contracting NCC. It was assumed that the prevalence of epilepsy due to causes other than NCC is the same regardless of whether a population is at risk or not. It was also assumed that Muslims and non-Muslims have equal access to sanitation.

Model evaluation was done by separately assessing the fit of the DisMod MR model and checking the estimates produced after estimating prevalence of NCC with epilepsy. Plots of time trends of prevalence across locations and age were used to evaluate the results. In addition, maps of the global distribution of prevalence of NCC among epileptics at risk and prevalence of NCC with epilepsy were also assessed across time.

Other than using additional data extracted from literature, we updated the proportion of population with Muslim data by filling in subnational locations with national proportions – this was done due to lack of data on this covariate at the subnational level.

No other significant modeling changes were made for GBD 2015.

References

1. "Table: Muslim Population by Country Pew Research Center, Washington, D.C." (March 26, 2014). http://www.pewforum.org/2011/01/27/table-muslim-population-by-country/

Cystic echinococcosis

Flowchart

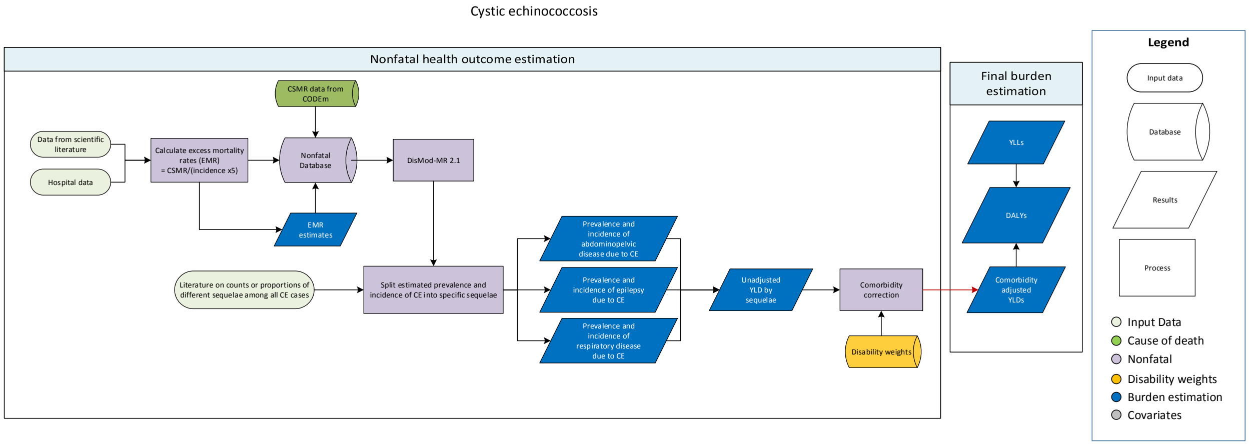

Case definition

Ehinococcosis is a vector-borne disease caused by two species of tapeworm, *Echinococcus granulosis* (most common in sheep) which causes cystic echinococcosis (CE) and *E. multilocularis* (most common in foxes and wild dogs) which causes alveolar echinococcosis. It is considered an NTD. Diagnosis is made by clinical findings, imaging, and serology. The ICD-10 codes for echinococcosis are B67-B67.9.

Input data

Model inputs

The nonfatal estimation for cystic echinococcosis (CE) focused on estimating incidence and prevalence of CE and its sequelae. A systematic review of literature was conducted in PubMed using the following search string: ("echinococcosis"[Title/Abstract] OR "hydatid disease"[Title/Abstract] OR "hydatidosis"[Title/Abstract] OR "echinococcal disease"[Title/Abstract] OR "Echinococcus granulosus infection"[Title/Abstract]) AND (“1990”[Date – Publication] : “2015”[Date – Publication]) AND (epidemiology OR incidence OR prevalence). This yielded 1,619 studies of which 279 were included during the title/abstract screening. Following the full-text screening, 77 studies (32 incidence, 43 prevalence and 2 both) were included and extracted – studies were excluded because of one or more of the following reasons:

1. study not population-based
2. study does not have primary data on prevalence and/or incidence
3. study not in humans
4. study on sub-populations
5. review study

We combined the newly extracted studies with studies extracted during GBD 2013. The table below shows the number of studies finally included, and the number of countries or subnational units and GBD world regions represented.

Table 1a. Geographies, studies

	incidence	prevalence
Studies	47	58
Countries/subnationals	41	24
GBD world regions	12	8

Hospital data on incidence prepared by the GBD team was also used in the CE model. The table below shows the number of studies included in the hospital data, and the number of countries or subnational units and GBD world regions represented.

Table 1b. Geographies, hospital data

	incidence
Studies	38
Countries/subnationals	100
GBD world regions	8

Since we were interested in modeling symptomatic CE cases, we only used data on incidence of patients diagnosed by imaging techniques (mainly ultrasonography). Therefore we excluded prevalence data which were mostly from serological studies.

Two additional data sources that were used, including 1) data on echinococcosis endemicity (0=no cases/no data, 1=sporadic/mostly imported, 2=endemic/limited data, 3=highly endemic) provided by one of our echinococcosis collaborators, and 2) literature data on observed cases of abdominal, respiratory, and epileptic symptoms among echinococcosis cases [1].

Sequelae due to cystic echinoccocosis

The table below shows the sequelae due to echinococcosis and their associated disability weights.

Table 2. Sequela, lay descriptions, and DWs

Sequela	Lay description	DW (95% CI)
Chronic respiratory disease	has cough and shortness of breath after heavy physical activity, but is able to walk long distances and climb stairs.	0.019 (0.011-0.033)
Abdominal problems	has pain in the belly and feels nauseous. The person has difficulties with daily activities	0.114 (0.078 – 0.159)
Epilepsy	(Combined DW)	NA

Modeling strategy

DisMod MR was used to model the nonfatal burden of symptomatic cystic echinococcosis (CE) using only incidence data. Mortality estimates from the custom mortality model were used to inform the excess mortality parameter (CODEm estimates used as cause-specific mortality rate data). Estimates of excess mortality rate were obtained and used to estimate prevalence (CSMR/EMR). A remission of 0.15-0.25 per case per year (duration 2 – 6.7 years, average 5 years) was assumed. The following steps were followed to

estimate excess mortality rate: 1) create custom age groups for CE deaths at the 1,000 draw level; 2) calculate CSMR as CSMR=deaths/population at the 1,000 draw level – calculate mean CSMR, uncertainty interval, and standard error; and 3) calculate EMR as EMR=CSMR/(prevalence), where prevalence = (incidence*5) – standard error of EMR was calculated taking into consideration the standard errors of both prevalence and CSMR.

After running DisMod, a thousand draws of proportions for abdominal, respiratory and epileptic symptoms among echinococcosis cases, that add up to 1, were generated. Uncertainty in the splitting proportions was captured by drawing them from a Dirichlet distribution, informed by published data on cysts localization [1]. On average, the proportions of abdominal, respiratory, and epileptic symptoms due to echinococcosis were 0.8, 0.19, and 0.01, respectively. These proportions were used to split the prevalence and incidence from DisMod into the three sequelae.

Model evaluation was done by separately assessing the fit of the DisMod MR model and checking the estimates produced after estimating incidence and prevalence of sequelae due to cystic echinococcosis. Plots of time trends of incidence and prevalence across locations and age were used to evaluate the results. In addition, maps of the global distribution of incidence and prevalence were assessed across time.

Changes from GBD 2013 included: a) estimation of excess mortality rate using literature/hospital incidence and CSMR data, b) inclusion of echinococcosis endemicity in as a country-level covariate in DisMod, and c) assuming that remission is 0.15-0.25 instead of 0.1 to 1.0 based on discussions with GBD 2015 CE model reviewers (an average remission of five years was used to calculate prevalence of CE).

References

1. Eckert J, Deplazes P. Biological, Epidemiological, and Clinical Aspects of Echinococcosis, a Zoonosis of Increasing Concern. Clin Microbiol Rev, 2004; 17(1): 107-35

Lymphatic flariasis

Flowchart

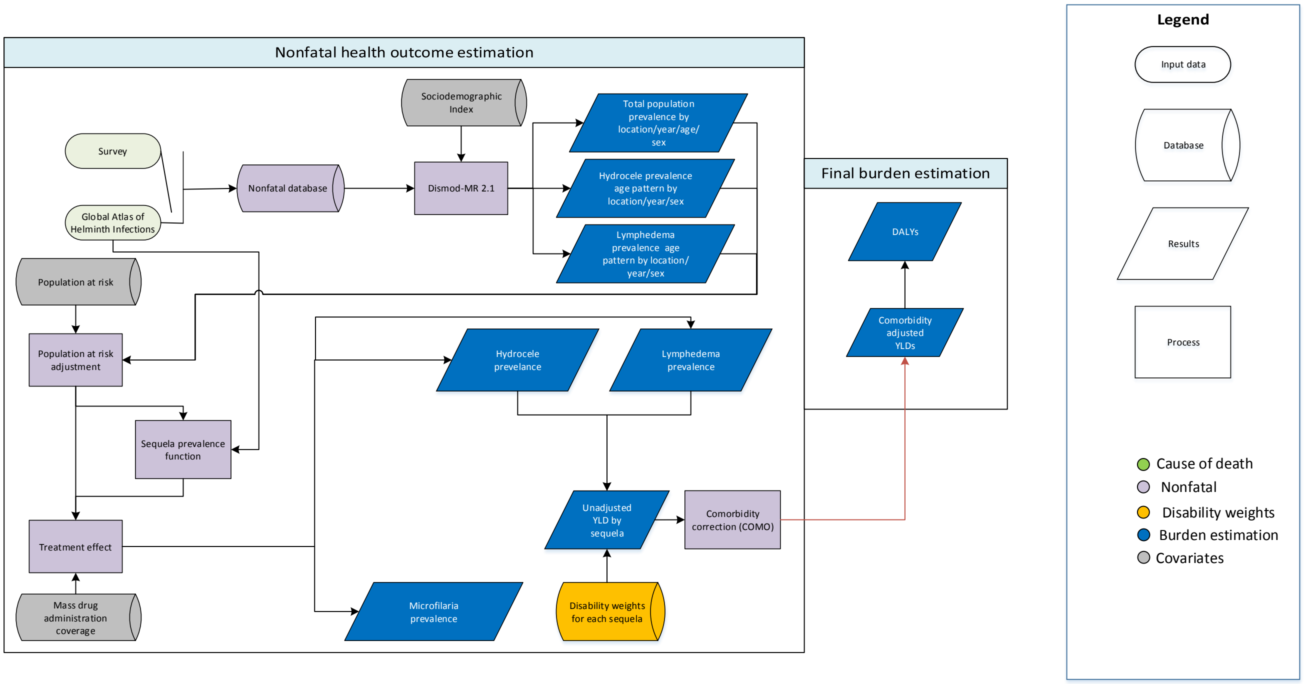

Input Data and Methodological Summary

Case Definition

Lymphatic filariasis (LF) is a neglected tropical disease spread in which threadlike nematodes invade the lymphatic system. The worms responsible – *Wuchereria bancrofti*, *Brugia malayi* and *Brugia timori* – are spread from human to human via mosquitoes. The most prominent clinical manifestations of LF are lymphedema (a swelling of the legs, also known in its more extreme manifestation as elephantiasis) and hydrocele (a collection of fluid in the sac around the testicles).

Input data

A systematic review of literature for GBD 2015 in the PubMed database was done on 10 August 2015 for prevalence and incidence data using the search ("lymphatic filariasis"[Title/Abstract] OR "filariasis"[Title/Abstract] OR "wuchereria"[Title/Abstract] OR "brugia"[Title/Abstract]) AND (“2013”[Date – Publication] : “2015”[Date – Publication]) AND (epidemiology OR incidence OR prevalence). This search returned 185 results, 121 of which passed title and abstract screening. Upon full text review, 37 studies were extracted for inclusion. Below is a summary of the geographic distribution of the data used.

Modeling strategy

Data on prevalence of microfilaria is modeled using Dismod-MR 2.1. Due to the fact that data is collected in endemic locations, we then scaled according to at-risk population in order to attain nationally representative values. We then use non-linear regression to estimate the reduction of microfilaria as a function of treatments per person. Using mass drug administration (MDA) coverage, we are then able to reduce the total estimated prevalence by exposing modeled total prevalence according to treatment efficacy.

For lymphedema and hydrocele, we incorporate survey data from the Global LF Atlas in a non-linear error-in-variables regression that determines the prevalence of lymphedema and hydrocele as functions of microfilaria prevalence, which is then applied to the total microfilaria Dismod model in order to attain an envelope of cases by location-year. Separately, all available prevalence data for these conditions is modeled in Dismod in order to determine an age-sex pattern.

In the estimation of lymphedema and hydrocele prevalence, we perform the same population at-risk correction that is done on microfilaria prevalence. For hydrocele prevalence after treatment, we take the value before MDA rollout in 2000 and reduce that by the same treatment efficacy function described for microfilaria prevalence, using dosage-reduction data specific to hydrocele along with the location-year specific MDA coverage. For lymphedema, we assume no new cases appear among treated individuals. As such, we reduce lymphedema prevalence in post-treatment years in accordance with MDA coverage.

Sequela	Data points	Regions	Countries	Subnational units
Prevalence of detectable microfilaria	1,552	10	40	28
Lymphedema due to lymphatic filariasis	511	10	25	15
Hydrocele due to lymphatic filariasis	265	8	22	12

Onchocerciasis

Flowchart

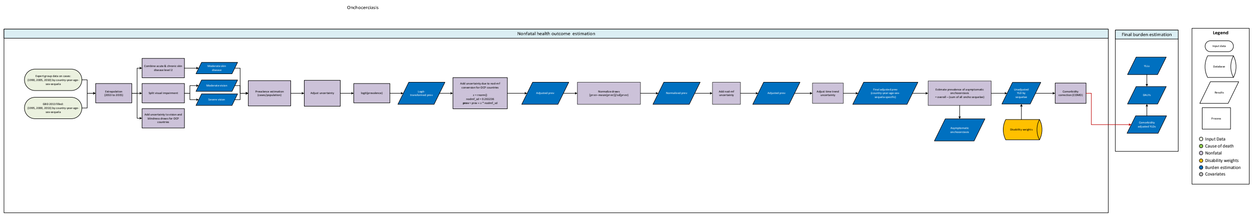

Case definition

Onchocerciasis, also known as river blindness, is a helminth disease caused by a parasitic worm, *Onchocerca volvulus*, transmitted by repeated bites by *Similium* blackflies. Diagnosis is made by different methods including skin snip biopsy to identify larvae, surgical removal of nodules and exam for adult worms, slit lamp exam of anterior part of the eye where larvae or lesions caused by them are visible, and antibody tests (mostly useful to visitors to areas with parasites). The ICD-10 code for onchocerciasis is B73.

Input data

Model inputs

To model nonfatal outcomes due to onchocerciasis, prevalence data prepared by the expert group (EG) during GBD 2013 was used. These included 1,000 draws of infection and morbidity (visual impairment, blindness, and skin conditions) cases with confidence intervals for years 1990, 1995, 2000, 2005, 2010, and 2013 categorized by country, age, and sex. Details of materials and methods used by the EG to generate draws can be found elsewhere [1-5]. The data only represented African countries included in the African Programme for Onchocerciasis Control (APOC) – Angola, Burundi, Cameroon, Central African Republic, Chad, Congo, Democratic Republic of Congo, Ethiopia, Equatorial Guinea, Liberia, Malawi, Nigeria, Sudan, Tanzania, and Uganda – and the Onchocerciasis Control Programme (OCP) – Benin, Burkina Faso, Côte d'Ivoire, Ghana, Guinea Bissau, Guinea, Mali, Niger, Senegal, Sierra Leone, and Togo. The table below shows (by program) the number of countries and GBD world regions represented.

Table 1. Geographies

	APOC	OCP
Countries/subnationals	15	11
GBD world regions	3	1

We did not update the literature review for these data in GBD 2015. Updates to systematic reviews are performed on an ongoing schedule across all GBD causes, and an update for onchocerciasis will be performed in the next 1-2 iterations.

Severity splits/Sequelae

The table below shows the list of sequelae due to onchocerciasis, their lay descriptions, and the associated disability weights (DW).

Table 2. Sequela, lay description, and DWs

Sequela	Lay description	Disability weights (DWs)
Moderate vision impairment	has vision problems that make it difficult to recognize faces or objects across a room	0.031 (0.019-0.049)
Severe vision impairment	has severe vision loss, which causes difficulty in daily activities, some emotional impact (for example worry), and some difficulty going outside the home without assistance	0.184 (0.125-0.258)
Blindness	is completely blind, which causes great difficulty in some daily activities, worry and anxiety, and great difficulty going outside the home without assistance	0.187 (0.124-0.260)
Mild skin disease	has a slight, visible physical deformity that is sometimes sore or itchy. Others notice the deformity, which causes some worry and discomfort	0.027 (0.015-0.042)
Mild skin disease without itch	has a slight, visible physical deformity that others notice, which causes some worry and discomfort	0.011 (0.005-0.021)
Moderate skin disease	has a visible physical deformity that is sore and itchy. Other people stare and comment, which causes the person to worry. The person has trouble sleeping and concentrating	0.188 (0.124-0.267)
Severe skin disease	has a visible physical deformity that is sore and itchy. Other people stare and comment, which causes the person to worry. The person has trouble sleeping and concentrating	0.188 (0.124-0.267)
Severe skin disease without itch	has an obvious physical deformity that makes others uncomfortable, which causes the person to avoid social contact, feel worried, sleep poorly, and think about suicide	0.405 (0.275-0.546)
Asymptomatic onchocerciasis	NA	NA

Modeling strategy

The nonfatal modeling for onchocerciasis included two major steps. In the first step, GBD 2013 prevalence was extrapolated to obtain GBD 2015 estimates. Acute skin disease level 2 and chronic skin disease level 2 were then summed up to create the "moderate skin disease" sequela. Within each of the OCP draws the number of cases with visual impairment and blindness was multiplied by a random value (the exponent of a normally distributed variable with mean zero and standard deviation 0.1) – this was done to add some uncertainty to these estimates. Within each draw, the same randomly drawn value was applied to all country-year-age-sex. The other sequelae already had uncertainty quantified and were used as provided by the EG. Visual impairment was split into moderate and severe vision impairment by first

multiplying the visual impairment estimates by a random value (from a normal distribution with mean 0.84 and standard deviation 0.0031) to generate moderate vision impairment, and then subtracting the resulting estimates from visual impairment to obtain estimates of severe vision impairment. Prevalence of sequelae was calculated by dividing the cases by the population.

The second step in modeling morbidity due to onchocerciasis was the adjustment of uncertainty in 1) conversion of nodule prevalence to microfilaria prevalence and 2) effects of mass treatment. To adjust for uncertainty in nodule to mf prevalence, the final draws from the first step were logit transformed, and then for OCP countries, we added uncertainty from a random value drawn from a normal distribution to the transformed estimates. The resulting estimates were then normalized and scaled using estimates published elsewhere [1]. To adjust for uncertainty due to MDA, the year when MDA with ivermectin started was set to 1990 and adjusted for some countries (1997 for Malawi; 1998 for Chad, Niger, and Tanzania; 1999 for Cameroon, Central African Republic, Equitorial Guinea, Liberia, Nigeria, and Uganda; 2001 for Congo, Ethiopia, and DRC; 2005 for Angola, Burundi, and South Sudan). Time trend uncertainty was then multiplied by the normalized prevalence estimates and then final prevalence was obtained by re-expanding the scaled normalized draws and adjusting the scale back from logit scale.

To estimate the prevalence of asymptomatic onchocerciasis, prevalence of morbidity (vision loss, blindness and skin conditions) was subtracted from the overall onchocerciasis prevalence – moderate vision impairment, severe vision impairment and blindness estimates were each multiplied by a factor of 8/33 before subtraction to account for cases that have concurring symptoms.

Model evaluation was done by separately assessing plots of time trends of prevalence across locations and age for each sequela. In addition, maps of the global distribution of total onchocerciasis prevalence and prevalence of sequelae due to onchocerciasis were also assessed across time. Since the modeling strategy was the same as that applied in GBD 2013, we compared our estimates with those from GBD 2013, and the correlation between the two was about 1.

We have made no substantive changes in the modeling strategy from GBD 2013.

References

1. Zouré HG, Noma M, Tekle AH, Amazigo UV, Diggle PJ, Giorgi E, Remme JH. The geographic distribution of onchocerciasis in the 20 participating countries of the African Programme for Onchocerciasis Control: (2) pre-control endemicity levels and estimated number infected. Parasites & Vectors. 2014. 7-326
2. Coffeng L, Stolk W, Hoerauf A, Habbema D, Bakker R, Hopkins A, de Vlas S. Elimination of African onchocerciasis: modeling the impact of increasing the frequency of ivermectin mass treatment. PLoS One. 2014. 9(12):e115886
3. Coffeng LE, Stolk WA, Zouré HG, Veerman JL, Agblewonu KB, Murdoch ME, Noma M, Fobi G, Richardus JH, Bundy DA, Habbema D, de Vlas SJ, Amazigo UV. African Programme For Onchocerciasis Control 1995-2015: model-estimated health impact and cost. PLoS Negl Trop Dis. 2013; 7(1): e2032
4. Murdoch ME, Asuzu MC, Hagan M, Makunde WH, Ngoumou P, Ogbuagu KF, Okello D, Ozoh G, Remme J. Onchocerciasis: the clinical and epidemiological burden of skin disease in Africa. Ann Trop Med Parasitol. 2002; 96(3): 283-96

5. Brieger WR, Awedoba AK, Eneanya CI, Hagan M, Ogbuagu KF, Okello DO, Ososanya OO, Ovuga EB, Noma M, Kale OO, Burnham GM, Remme JH. The effects of ivermectin on onchocercal skin disease and severe itching: results of a multicentre trial. Trop Med Int Health. 1998; 3(12): 951-61

Dengue

Flowchart

Dengue

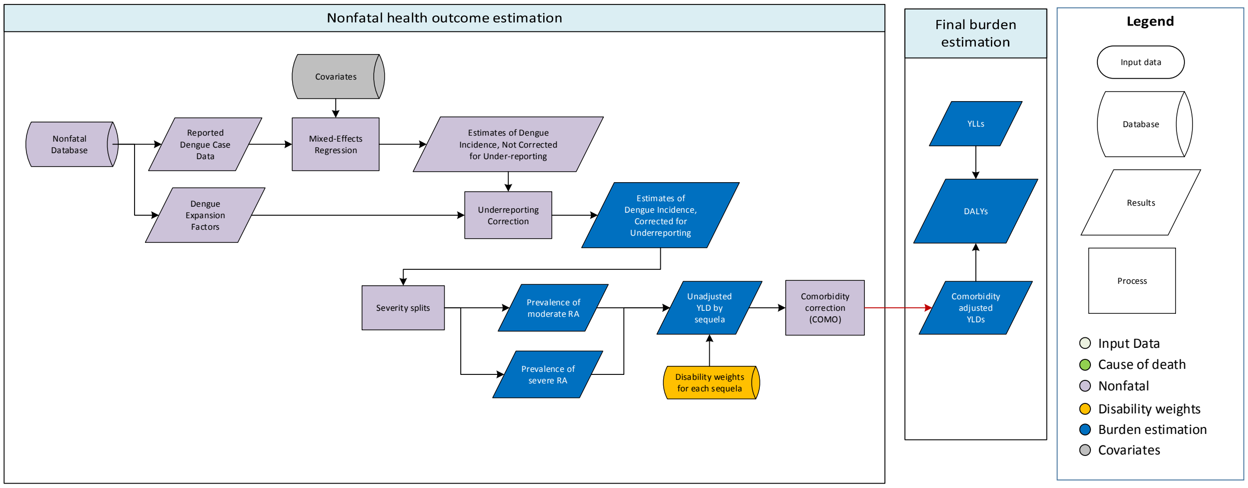

Case definition

Dengue is mosquito-borne viral infection that causes febrile illness and, in severe cases, jaundice, hemorrhage, and death. It includes all ICD-10 codes under the heading A90 (Dengue fever [classical dengue]) and A91 (Dengue hemorrhagic fever).

Input data

Model inputs

For GBD 2015, we modeled dengue incidence based on officially reported cases. The table below illustrates the geographic distribution of data points used in our analysis.

Table 1. Geographies

Level	Incidence
Data points	2515
Studies	70
Locations	115
Regions	14

Updates to systematic reviews are performed on an ongoing schedule across all GBD causes, and an update for dengue fever will be performed in the next one to two iterations. While no systematic update was conducted, we did incorporate new expansion factor data that were provided by collaborators and have updated to the latest available case reports for GBD 2015.

Modeling strategy

We modeled dengue incidence using an improved variant of the methods used for GBD 2013, described by Stanaway et al (Briefly, we derive two dengue-specific covariates: first a variable to define the expected spatial distribution of the disease based on principal components analysis of dengue CSMR estimates and dengue transmission probability and, second, a variable to define the country-specific trends, based on a mixed effects model of reported cases. We then estimate a mixed effects negative binomial model with number of reported cases as the dependent variable, fixed effects on the aforementioned spatial and temporal covariates, and random effects on location. These random effects are assumed to correspond to deviations in reporting completeness and, calibrating against published expansion factor data (i.e., estimates of the degree of underreporting), they are inflated to adjust for underreporting estimates. The resulting incidence estimates are split into moderate (94.5%) and severe (5.5%) sequelae, based on the proportion of reported cases that were severe. We assume that 8.4% of symptomatic infections will produce post-acute chronic fatigue lasting an average of six months.

Severity splits and disability weights

For GBD 2015, we determined the severity proportions for Dengue using survey data.

Table 2. Sequela, lay description and DWs

Sequela	Lay description	Disability Weight (DW)
Moderate	Has a fever and aches, and feels weak, which causes some difficulty with daily activities.	0.051 (0.032-0.074)
Severe	Has a high fever and pain, and feels very weak, which causes great difficulty with daily activities.	0.133 (0.088-0.19)
Asymptomatic	Infection with no apparent illness.	NA

Changes from GBD 2013 to GBD 2015

The approach is largely the same as that used for GBD 2013. One notable change is the addition of the dengue trend covariate described above, which allows for dramatically improved trend estimates.

References

1. Stanaway JD, Shepard DS, Undurraga EA, Halasa YA, Coffeng LE, Brady OJ, et al. The global burden of dengue: an analysis from the Global Burden of Disease Study 2013. The Lancet Infectious Diseases [Internet]. 2016 Feb [cited 2016 May 23].

2. Bhatt S, Gething PW, Brady OJ, Messina JP, Farlow AW, Moyes CL, et al. The global distribution and burden of dengue. Nature. 2013 Apr 25;496(7446):504–7.

Yellow fever

Flowchart

Yellow fever

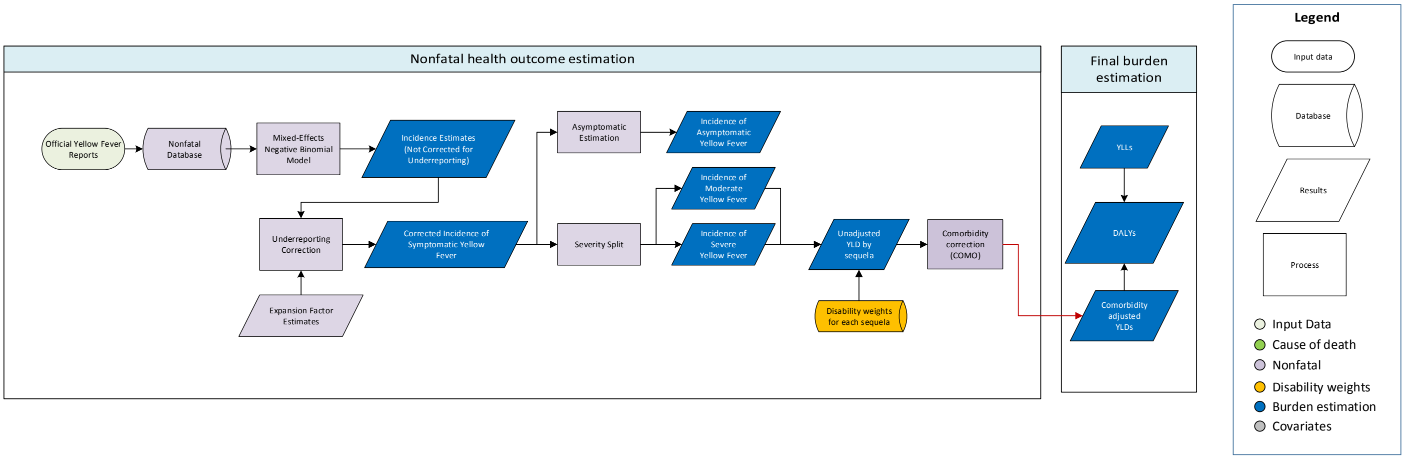

Case definition

Yellow fever is mosquito-borne viral infection that causes febrile illness and, in severe cases, jaundice, hemorrhage, and death. It is considered a neglected tropical disease (NTD). It includes all ICD-10 codes under the heading A95 (Yellow fever).

Input data

Model inputs

Case data for the yellow fever estimate process comes from official case reports filed with the World Health Organization. The table below shows the distribution of said data geographically for the GBD 2015 estimation process.

Table 1. Data Spread

Level	Incidence
Data points	4003
Studies	44
Locations	184
Regions	21

We have updated to the latest available case reports for GBD 2015.

Severity splits

Yellow fever is split into three levels of severity: moderate (33% [13 - 52]), severe (12% [5 - 26]), and asymptomatic (55% [37 - 74]). The table below illustrates this breakdown.

Table 2. Sequela, description and disability weight (DW)

Sequela	Description	Disability weight (DW)
Moderate	Has a fever and aches, and feels weak, which causes some difficulty with daily activities.	0.051 (0.032-0.074)
Severe	Has a high fever and pain, and feels very weak, which causes great difficulty with daily activities.	0.133 (0.088-0.19)
Asymptomatic	Infection with no apparent illness.	NA

Modeling strategy

For GBD 2015, we modeled reported cases of yellow fever using a mixed-effects negative binomial model, with fixed effects for year and random effects for super region, region, and country. We assume that yellow fever cases are underreported, and that this underreporting mirrors that for dengue (a disease for which we have better data on underreporting). With that, we estimate symptomatic cases as the product of our base case estimates and dengue expansion factors (i.e., the factor by which you must multiply reported cases to derive true cases). Based on published estimates from Johansson et al (2014), we assume that 27% of symptomatic cases will be severe.

We have made no major changes in our methods for estimating non-fatal yellow fever between GBD 2013 and 2015.

References

Johansson et al. 2014.

Rabies

Flowchart

Rabies

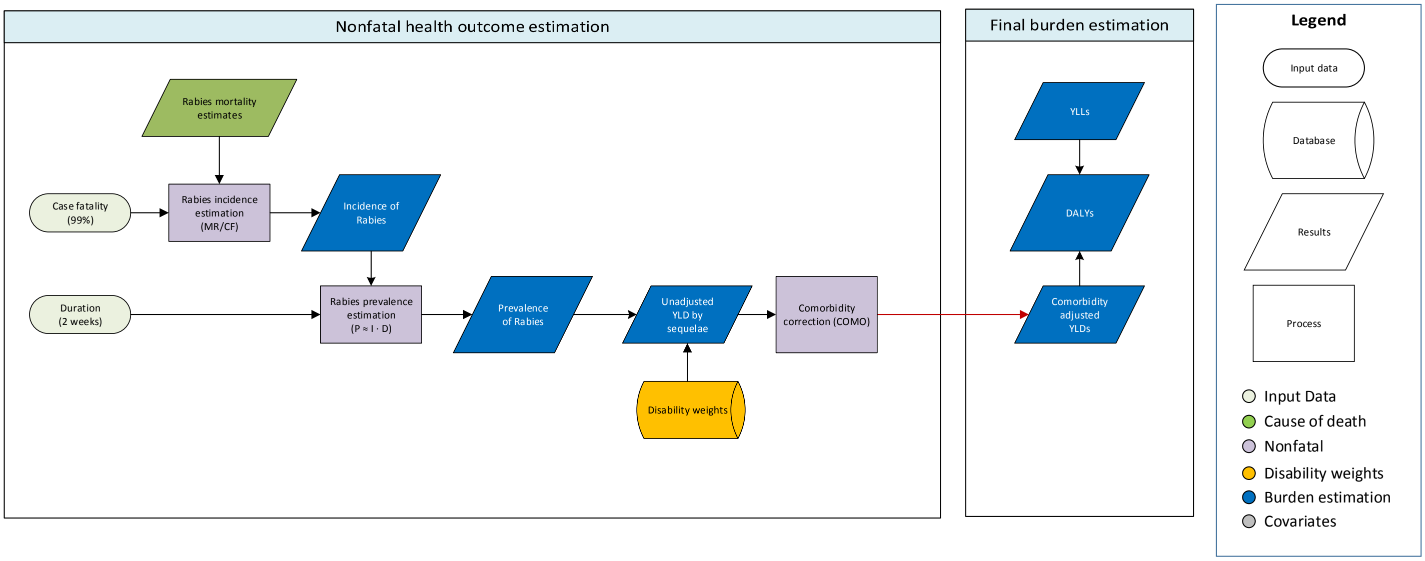

Input data and methodological summary

Case definition

Rabies is a fatal viral infection, transmitted by animal bites. Without prophylactic vaccination the disease is almost universally fatal. The disease has a long incubation period (1-3 months), and early intervention with prophylactic vaccination is nearly 100% effective in preventing symptomatic disease. It is considered a neglected tropical disease (NTD). We model symptomatic infections, not including those infections in which intervention prevented the onset of symptomatic disease, corresponding to the ICD10 code A82.

Input data

Model inputs

As we derive our estimate of cases from our estimate of deaths, there are no incidence data used in the model. For GBD 2015, we modeled rabies mortality using all available data in the cause of death database. Data points were outliered if they reported an improbable number of rabies deaths (e.g., zero rabies deaths in a hyperendemic country) or if their inclusion in the model yielded distorted trends. In some cases multiple data sources for the same location differed dramatically both in their quality and reported rabies mortality (e.g., a verbal autopsy and vital registration source). In these cases the lower-quality data source was outliered.

Modeling strategy

We derive estimates of the number of symptomatic rabies infections (i.e., those not averted through prophylactic vaccination) based on rabies mortality estimates, assuming 99% case fatality. All cases are assumed to be severe.

We modeled rabies mortality using a two-model hybrid approach 1) a global CODEm model of all locations, using all data in the CoD database; and 2) a CODEm model restricted to data-rich countries. We have made two substantive changes in the modeling strategy from GBD 2013. First, we have changed from a single global model to the hybrid global/data rich model approach. Second, we conducted an exploratory analysis to determine the most predictive covariates for rabies and have updated the covariates used in the CODEm model accordingly.

Sequela description and DW

There is only one sequela and associated disability weight for rabies, which is severe. The lay description is included in the table below.

Table 2. Sequela, description and DW

Sequela	Description	Disability Weight (95% CI)
Severe	Has a high fever and pain, and feels very weak, which causes great difficulty with daily activities.	0.133 (0.088-0.19)

Changes from GBD 2013 to GBD 2015

We have made no substantive changes in the modeling strategy for rabies from GBD 2013.

Ascariasis

Flowchart

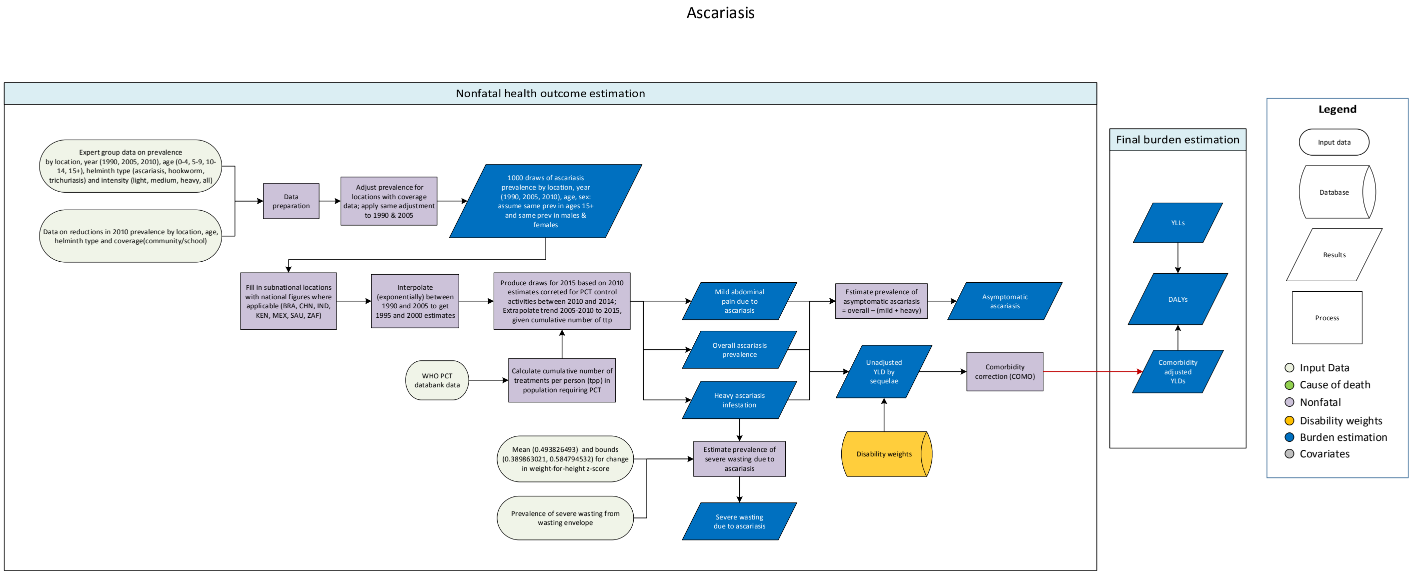

Input data and methodological summary

Case definition

Ascariasis is a helminth diseases caused by the parasitic roundworm, *Ascaris lumbricoides*. It is one of the three intestinal nematode infections (INI)/soil transmitted helminthiasis (STH) that we model in GBD. Diagnosis is made by microscopic exam of stool or by concentration procedures (recommended as eggs may be difficult to see). The ICD-10 codes for ascariasis are B77-B77.9.

Input data

Model inputs

Four different input data were used in the ascariasis nonfatal model. The first was prevalence data prepared by the expert group (EG) during GBD 2010 [1, 2]. They provided the data (mean, upper, lower) by location, year (1990, 2005, 2010), age (0-4, 5-9, 10-14, 15+ years), helminth type (ascariasis, hookworm disease, trichuriasis) and intensity of infection (light, medium, heavy, all). For the model, light infestation was not attributed any disability. The second data, also from the EG, was on reductions in prevalence in 2010, provided by location, age, helminth type, and coverage (community/school). The table below shows the number of countries or subnational units and GBD world regions represented in the data.

Table 1a. Geographic spread of data

	prevalence
Countries/subnationals	163
GBD world regions	16

The third input data was from the WHO PCT Databank [3]. This data was downloaded from the source website and represented 121 locations and six GBD world regions. The last input data was 1,000 draws of wasting envelope prevalence among children under 5 years – the methods used to generate estimates of wasting prevalence are detailed elsewhere (part of risk factors documentation). The table below shows the number of countries or subnational units and GBD world regions represented in the data.

Table 1b. Geographic spread of data

	prevalence
Countries/subnationals	561
GBD world regions	21

Severity splits/Sequelae

The table below shows the list of sequelae due to ascariasis and the associated disability weights (DW). The sequelae were based on prevalence of medium and heavy infestation – medium infestation was assigned mild abdominopelvic problems; heavy infestation was assigned symptomatic worm infection; and light infestation was not attributed any disability.

Table 2. Sequela, lay description and disability weights (DWs)

Sequela	Lay description	DW
Mild abdominopelvic problems	has some pain in the belly that causes nausea but does not interfere with daily activities	0.011 (0.005-0.021)
Heavy infestation	has cramping pain and a bloated feeling in the belly	0.027 (0.015-0.043)
Severe wasting	is extremely skinny and has no energy	0.128 (0.082-0.183)
Asymptomatic ascariasis	N/A	N/A

Modeling strategy

In the estimation of morbidity due to ascariasis, the EG data was first prepared by formatting the location names to be consistent with the GBD 2015 location names and applying the 2010 prevalence to 1990 and 2005 for sub-Saharan Africa countries – estimates for these two years were missing. This was followed by using the data on reductions in 2010 prevalence to adjust the prevalence for locations with coverage data. After this adjustment, only data for medium infection, heavy infection, and all infection was retained.

Using the mean prevalence and the upper and lower bounds of the mean provided by the EG, 1,000 draws of prevalence were generated. This was done by multiplying the mean estimates by the exponent of random draws from a normal distribution with mean = 0 and standard deviation = sd, where sd = abs(abs(ln(upper)-ln(lower))/(invnormal(0.975)*2). These draws were created for all GBD age-groups, assuming the same prevalence in ages 15+ and same prevalence in males and females. Since the draws were only at the national level, subnational locations were filled with national figures where applicable (Brazil, China, India, Kenya, Mexico, Saudi Arabia, and South Africa).

To get 1995 and 2000 estimates, exponential interpolation of estimates between 1990 and 2005 was performed. The draws for 2015 were produced based on 2010 estimates corrected for PCT control activities between 2010 and 2014 – this was done by extrapolating the 2005-2010 trend to 2015, given cumulative number of treatments per person calculated using data from the WHO PCT Databank [3]. The 2005-2010 trend was applied to all intensities of infection. Prevalence was assumed to be zero for the countries with missing input data and also in children younger than 28 days. The resulting estimates were 1,000 draws of ascariasis prevalence by GBD location, year, age, sex, and intensity level (mild, heavy, overall infection). To estimate the prevalence of asymptomatic ascariasis, prevalence of mild and heavy infestation was subtracted from the overall ascariasis prevalence.

The final step in the modeling process was to estimate the prevalence of severe wasting due to ascariasis in age groups 28-364 days and 1-4 years. This was done separately using 1,000 draws of prevalence of heavy infestation due to ascariasis and the wasting envelope prevalence. The initial step in determining prevalence of severe wasting due to ascariasis was generating 1,000 draws of change in weight-for-height z-score per heavy prevalent case from a random normal distribution with mean = 0.493826493 and standard deviation = 0.04972834 (calculated from upper and lower bounds of the mean estimate). The mean, upper and lower bounds were provided by a GBD collaborator who calculated them based on a published article [4]. The prevalence of severe wasting due to ascariasis was then obtained as a function of change in weight-for-height z-score (z_change) such that prevalence = p_wasting_env – Phi(Phi_inv(p_wasting_env) – z_change*p), where p_wasting_env = wasting envelope prevalence, Phi_inv is the inverse standard normal cumulative distribution function (cdf), and p = prevalence of heavy ascariasis infestation.

Model evaluation was done by plotting prevalence of overall ascariasis and that of each sequelae against year for each location and age group. Maps of the global distribution of total ascariasis prevalence and prevalence of sequelae due to ascariasis were also assessed across time and age. Since we used the same data and model as that used in GBD 2013, we compared GBD 2015 estimates for each sequela with those from GBD 2013 for each country and age. As expected, our estimates were very similar to those from GBD 2013.

The only change made from GBD 2013 modeling strategy was the incorporation of updated data from the WHO PCT databank [3] in the correction of estimates for MDA activities.

References

1. Brooker S, Pullan R, Smith J, and Hotez P. Chapter: Intestinal nematodes. Cluster D: Communicable Diseases, Neglected Tropical Diseases Group. Global Burden of Diseases, Injuries, and Risk Factors Study. 2011 (4 July). 1-24
2. Brooker S & Smith JL. Impact of hookworm infection and deworming on anaemia in non-pregnant populations: a systematic review. Tropical Medicine and International Health. 2010. 15,7,776-795
3. WHO PCT Databank. 2015; http://www.who.int/neglected_diseases/preventive_chemotherapy/sth/en/
4. Hall A, Hewitt G, Tuffrey V, de Silva N. A review and meta-analysis of the impact of intestinal worms on child growth and nutrition. Maternal and Child Nutrition. 2008. 4. 118-236.

Trichuriasis

Flowchart

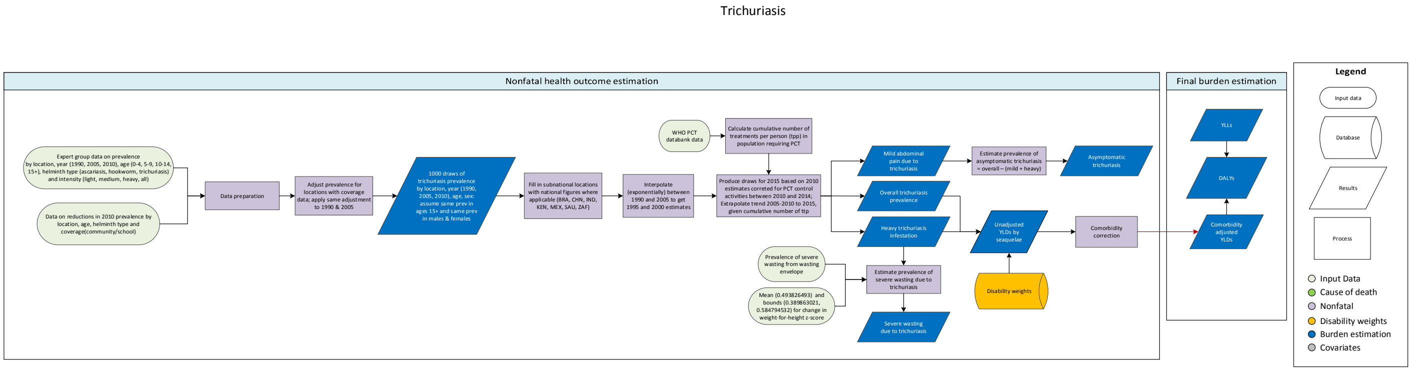

Input data and methodological summary

Case Definition

Trichuriasis is a helminth diseases caused by the parasitic roundworm *Trichuris trichiura*. It is one of the three intestinal nematode infections (INI)/soil transmitted helminthiasis (STH) that we model in GBD. Diagnosis is made by microscopic exam of stool or by concentration procedures (recommended as eggs may be difficult to see). The ICD-10 code for trichuriasis are B79.

Input data

Model inputs

Four different input data were used in the trichuriasis nonfatal model. The first was prevalence data prepared by the expert group (EG) during GBD 2010 [1, 2]. They provided the data (mean, upper, lower) by location, year (1990, 2005, 2010), age (0-4, 5-9, 10-14, 15+ years), helminth type (ascariasis, hookworm disease, trichuriasis) and intensity of infection (light, medium, heavy, all). For the model, light infestation was not attributed any disability. The second data, also from the EG, was on reductions in prevalence in 2010, provided by location, age, helminth type, and coverage (community/school). The table below shows the number of countries or subnational units and GBD world regions represented in the data.

Table 1a. Geographies

	prevalence
Countries/subnationals	163
GBD world regions	16

The third input data was from the WHO PCT Databank [3]. This data was downloaded from the source website and represented 121 locations and 6 GBD world regions. The last input data was 1,000 draws of

wasting envelope prevalence among children under 5 years – the methods used to generate estimates of wasting prevalence are detailed elsewhere (part of risk factors documentation). The table below shows the number of countries or subnational units and GBD world regions represented in the data.

Table 1b. Geographies

	prevalence
Countries/subnationals	561
GBD world regions	21

Severity splits/Sequelae

The table below shows the list of sequelae due to trichuriasis and the associated disability weights (DW). The sequelae were based on prevalence of medium and heavy infestation – medium infestation was assigned mild abdominopelvic problems; heavy infestation was assigned symptomatic worm infection; light infestation was not attributed any disability.

Table 2. Sequela, lay description, and disability weights (DWs)

Sequela	Lay description	DW (95% CI)
Mild abdominopelvic problems	has some pain in the belly that causes nausea but does not interfere with daily activities	0.011 (0.005-0.021)
Heavy infestation	has cramping pain and a bloated feeling in the belly	0.027 (0.015-0.044)
Severe wasting	is extremely skinny and has no energy	0.128 (0.082-0.183)
Asymptomatic trichuriasis	N/A	N/A

Modeling strategy

In the estimation of morbidity due to trichuriasis, the EG data was first prepared by formatting the location names to be consistent with the GBD 2015 location names and applying the 2010 prevalence to 1990 and 2005 for sub-Saharan Africa countries – estimates for these two years were missing. This was followed by using the data on reductions in 2010 prevalence to adjust the prevalence for locations with coverage data. After this adjustment, only data for medium infection, heavy infection, and all infection was retained.

Using the mean prevalence and the upper and lower bounds of the mean provided by the EG, 1,000 draws of prevalence were generated. This was done by multiplying the mean estimates by the exponent of random draws from a normal distribution with mean = 0 and standard deviation = sd, where sd = abs(abs(ln(upper)-ln(lower))/(invnormal(0.975)*2). These draws were created for all GBD age-groups, assuming the same prevalence in ages 15+ and same prevalence in males and females. Since the draws were only at the national level, subnational locations were filled with national figures where applicable (Brazil, China, India, Kenya, Mexico, Saudi Arabia, and South Africa).

To get 1995 and 2000 estimates, exponential interpolation of estimates between 1990 and 2005 was performed. The draws for 2015 were produced based on 2010 estimates corrected for PCT control activities between 2010 and 2014 – this was done by extrapolating the 2005-2010 trend to 2015, given the cumulative number of treatments per person calculated using data from the WHO PCT Databank [3]. The 2005-2010 trend was applied to all intensities of infection. Prevalence was assumed to be zero for

the countries with missing input data and also in children younger than 28 days. The resulting estimates were 1,000 draws of trichuriasis prevalence by GBD location, year, age, sex, and intensity level (mild, heavy, overall infection). To estimate the prevalence of asymptomatic trichuriasis, prevalence of mild and heavy infestation was subtracted from the overall trichuriasis prevalence.

The final step in the modeling process was to estimate the prevalence of severe wasting due to trichuriasis in age groups 28-364 days and 1-4 years. This was done separately using 1,000 draws of prevalence of heavy infestation due to trichuriasis and the wasting envelope prevalence. The initial step in determining prevalence of severe wasting due to trichuriasis was generating 1,000 draws of change in weight-for-height z-score per heavy prevalent case from a random normal distribution with mean = 0. 493826493 and standard deviation = 0.04972834 (calculated from upper and lower bounds of the mean estimate). The mean, upper and lower bounds were provided by a GBD collaborator who calculated them based on a published article [4]. The prevalence of severe wasting due to trichuriasis was then obtained as a function of change in weight-for-height z-score (z_change) such that prevalence = p_wasting_env – Phi(Phi_inv(p_wasting_env) – z_change*p), where p_wasting_env = wasting envelope prevalence, Phi_inv is the inverse standard normal cumulative distribution function (cdf), and p = prevalence of heavy trichuriasis infestation.

Model evaluation was done by plotting prevalence of overall trichuriasis and that of each sequelae against year for each location and age group. Maps of the global distribution of total trichuriasis prevalence and prevalence of sequelae due to trichuriasis were also assessed across time and age. Since we used the same data and model as that used in GBD 2013, we compared GBD 2015 estimates for each sequela with those from GBD 2013 for each country and age. As expected, our estimates were very similar to those from GBD 2013.

The only change made from GBD 2013 modeling strategy was the incorporation of updated data from the WHO PCT databank [3] in the correction of estimates for MDA activities.

References

1. Brooker S, Pullan R, Smith J, and Hotez P. Chapter: Intestinal nematodes. Cluster D: Communicable Diseases, Neglected Tropical Diseases Group. Global Burden of Diseases, Injuries, and Risk Factors Study. 2011 (4 July). 1-24
2. Brooker S & Smith JL. Impact of hookworm infection and deworming on anaemia in non-pregnant populations: a systematic review. Tropical Medicine and International Health. 2010. 15,7,776-795
3. WHO PCT Databank. 2015; http://www.who.int/neglected_diseases/preventive_chemotherapy/sth/en/
4. Hall A, Hewitt G, Tuffrey V, de Silva N. A review and meta-analysis of the impact of intestinal worms on child growth and nutrition. Maternal and Child Nutrition. 2008. 4. 118-236.

Hookworm disease

Flowchart

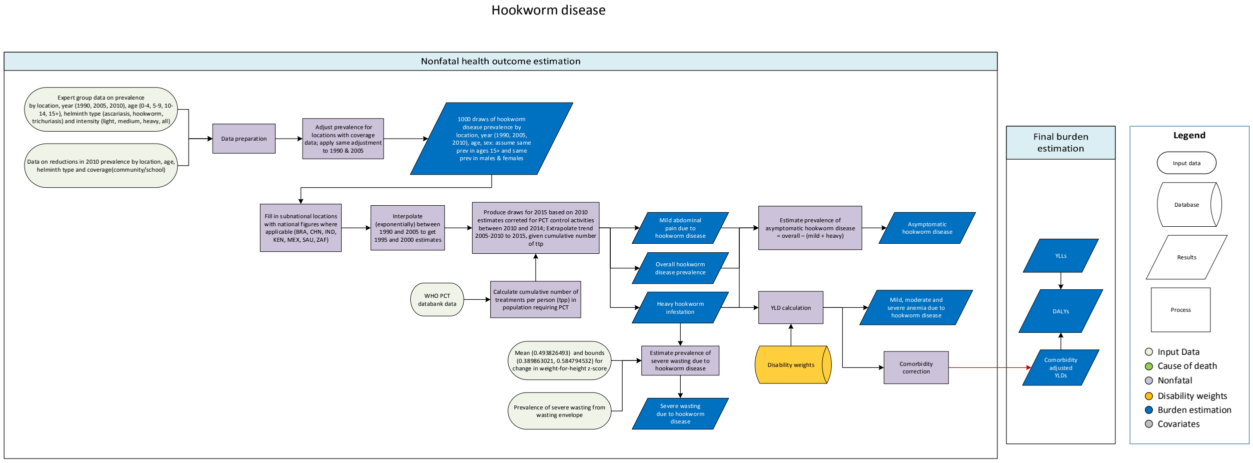

Input data and methodological summary

Case Definition

Hookworm disease is a helminth disease caused by the parasitic roundworms, *Ancylostoma duodenale* and *Necator americanus*. It is one of the three intestinal nematode infections (INI)/soil transmitted helminthiasis (STH) that we model in GBD. Diagnosis is made by a microscopic exam of stool or by concentration procedures (recommended as eggs may be difficult to see). The ICD-10 codes for hookworm disease are B76-B76.9.

Input data

Model inputs

Four different input data were used in the hookworm disease nonfatal model. The first was prevalence data prepared by the expert group (EG) during GBD 2010 [1, 2]. They provided the data (mean, upper, lower) by location, year (1990, 2005, 2010), age (0-4, 5-9, 10-14, 15+ years), helminth type (ascariasis, hookworm disease, trichuriasis) and intensity of infection (light, medium, heavy, all). For the model, light infestation was not attributed any disability. The second data, also from the EG, was on reductions in prevalence in 2010, provided by location, age, helminth type, and coverage (community/school). The table below shows the number of countries or subnational units and GBD world regions represented in the data.

Table 1a. Geographies

	prevalence
Countries/subnationals	163
GBD world regions	16

The third input data was from the WHO PCT Databank [3]. This data was downloaded from the source website and represented 121 locations and 6 GBD world regions. The last input data was 1,000 draws of wasting envelope prevalence among children under 5 years – the methods used to generate estimates of wasting prevalence are detailed elsewhere (part of risk factors documentation). The table below shows the number of countries or subnational units and GBD world regions represented in the data.

Table 1b. Geographies

	prevalence
Countries/subnationals	561
GBD world regions	21

Severity splits/Sequelae

The table below shows the list of sequelae due to hookworm disease and the associated disability weights (DW). The sequelae were based on prevalence of medium and heavy infestation – medium infestation was assigned mild abdominopelvic problems; heavy infestation was assigned symptomatic worm infection; light infestation was not attributed any disability.

Table 2. Sequela, lay description and disability weights (DWs)

Sequela	Lay description	DW
Mild abdominopelvic problems	has some pain in the belly that causes nausea but does not interfere with daily activities	0.011 (0.005-0.021)
Heavy infestation	has cramping pain and a bloated feeling in the belly	0.027 (0.015-0.044)
Severe wasting	is extremely skinny and has no energy	0.128 (0.082-0.183)
Asymptomatic hookworm disease	NA	NA
Mild anemia	feels slightly tired and weak at times, but this does not interfere with normal daily activities	0.004 (0.001-0.008)
Moderate anemia	feels moderate fatigue, weakness, and shortness of breath after exercise, making daily activities more difficult	0.052 (0.034-0.076)
Severe anemia	feels very weak, tired and short of breath, and has problems with activities that require physical effort or deep concentration	0.149 (0.101-0.210)

Modeling strategy

In the estimation of morbidity due to hookworm disease, the EG data was first prepared by formatting the location names to be consistent with the GBD 2015 location names and applying the 2010 prevalence to 1990 and 2005 for sub-Saharan Africa countries – estimates for these two years were missing. This was followed by using the data on reductions in 2010 prevalence to adjust the prevalence for locations with coverage data. After this adjustment, only data for medium infection, heavy infection, and all infection was retained.

Using the mean prevalence and the upper and lower bounds of the mean provided by the EG, 1,000 draws of prevalence were generated. This was done by multiplying the mean estimates by the exponent of random draws from a normal distribution with mean = 0 and standard deviation = sd, where sd = abs(abs(ln(upper)-ln(lower))/(invnormal(0.975)*2). These draws were created for all GBD age-groups, assuming the same prevalence in ages 15+ and same prevalence in males and females. Since the draws were only at the national level, subnational locations were filled with national figures where applicable (Brazil, China, India, Kenya, Mexico, Saudi Arabia, and South Africa).

To get 1995 and 2000 estimates, exponential interpolation of estimates between 1990 and 2005 was performed. The draws for 2015 were produced based on 2010 estimates corrected for PCT control activities between 2010 and 2014 – this was done by extrapolating the 2005-2010 trend to 2015, given cumulative number of treatments per person calculated using data from the WHO PCT Databank [3]. The 2005-2010 trend was applied to all intensities of infection. Prevalence was assumed to be zero for the countries with missing input data and also in children younger than 28 days. The resulting estimates were 1,000 draws of hookworm disease prevalence by GBD location, year, age, sex, and intensity level (mild, heavy, overall infection). To estimate the prevalence of asymptomatic hookworm disease, prevalence of mild and heavy infestation was subtracted from the overall hookworm disease prevalence.

The final step in the modeling process was to estimate the prevalence of severe wasting due to hookworm disease in age groups 28-364 days and 1-4years. This was done separately using 1,000 draws of prevalence of heavy infestation due to hookworm disease and the wasting envelope prevalence. The initial step in determining prevalence of severe wasting due to hookworm disease was generating 1,000 draws of change in weight-for-height z-score per heavy prevalent case from a random normal distribution with mean = 0.493826493 and standard deviation = 0.04972834 (calculated from upper and lower bounds of the mean estimate). The mean, upper and lower bounds were provided by a GBD collaborator who calculated them based on a published article [4]. The prevalence of severe wasting due to hookworm disease was then obtained as a function of change in weight-for-height z-score (z_change) such that prevalence = p_wasting_env – Phi(Phi_inv(p_wasting_env) – z_change*p), where p_wasting_env = wasting envelope prevalence, Phi_inv is the inverse standard normal cumulative distribution function (cdf) and p = prevalence of heavy hookworm infestation. The burden of anemia due to hookworm disease was estimated (see anemia documentation for details).

Model evaluation was done by plotting prevalence of overall hookworm disease and that of each sequelae against year for each location and age group. Maps of the global distribution of total hookworm disease prevalence and prevalence of sequelae due to hookworm disease were also assessed across time and age. Since we used the same data and model as that used in GBD 2013, we compared GBD 2015 estimates for each sequela with those from GBD 2013 for each country and age. As expected, our estimates were very similar to those from GBD 2013.

The only change made from GBD 2013 modeling strategy was the incorporation of updated data from the WHO PCT databank [3] in the correction of estimates for MDA activities.

References

1. Brooker S, Pullan R, Smith J, and Hotez P. Chapter: Intestinal nematodes. Cluster D: Communicable Diseases, Neglected Tropical Diseases Group. Global Burden of Diseases, Injuries, and Risk Factors Study. 2011 (4 July). 1-24

2. Brooker S & Smith JL. Impact of hookworm infection and deworming on anaemia in non-pregnant populations: a systematic review. Tropical Medicine and International Health. 2010. 15,7,776-795
3. WHO PCT Databank. 2015; http://www.who.int/neglected_diseases/preventive_chemotherapy/sth/en/
4. Hall A, Hewitt G, Tuffrey V, de Silva N. A review and meta-analysis of the impact of intestinal worms on child growth and nutrition. Maternal and Child Nutrition. 2008. 4. 118-236.

Intestinal fluke flowchart

Intestinal fluke

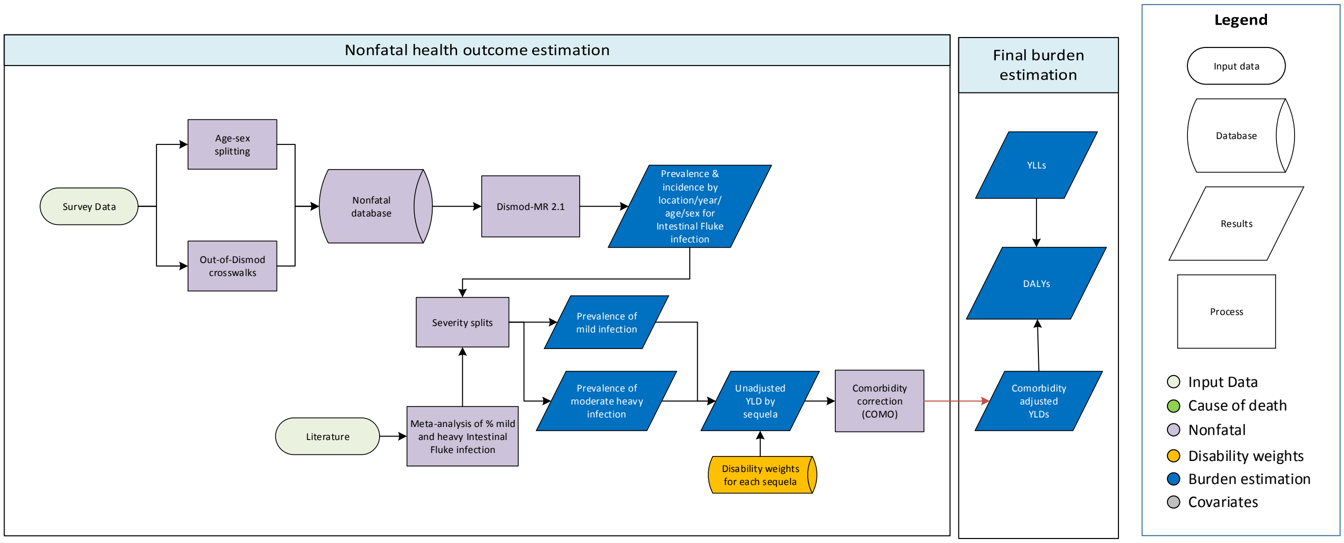

Opisthorchiasis flowchart

Opisthorchiasis

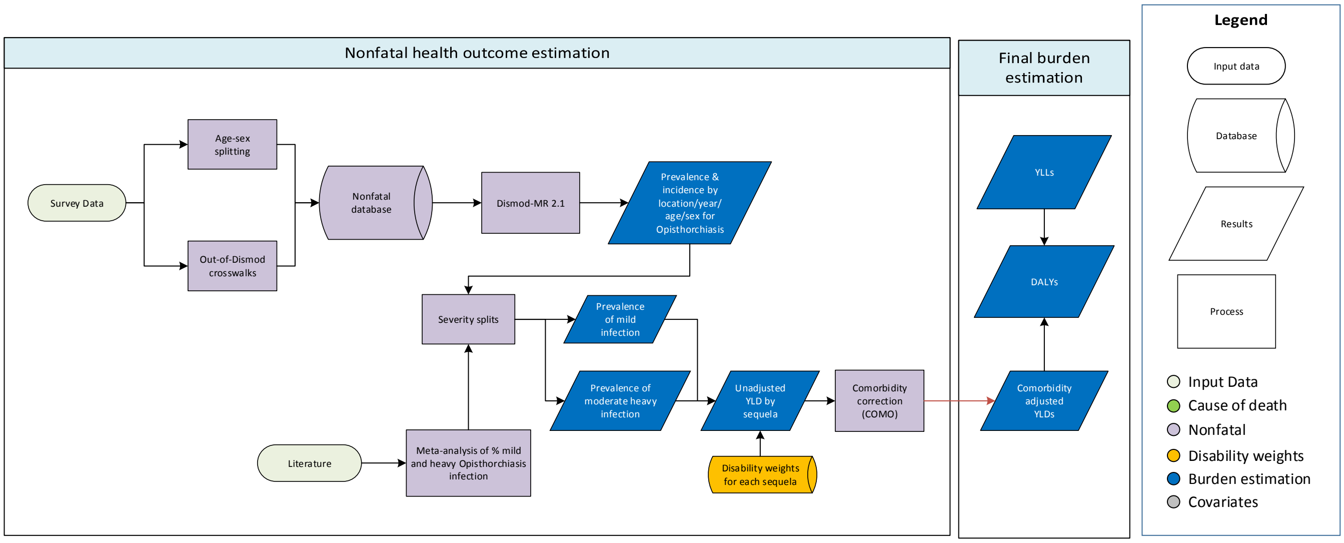

Foodborne trematodiases

Clonorchiasis flowchart

Clonorchiasis

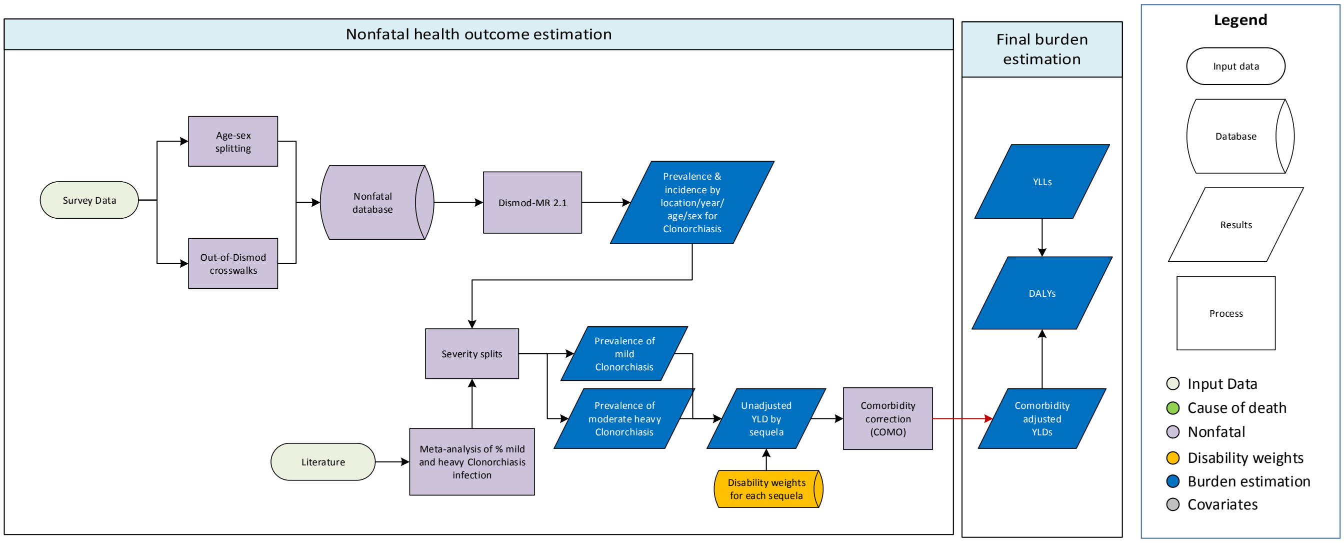

Fascioliasis flowchart

Fascioliasis

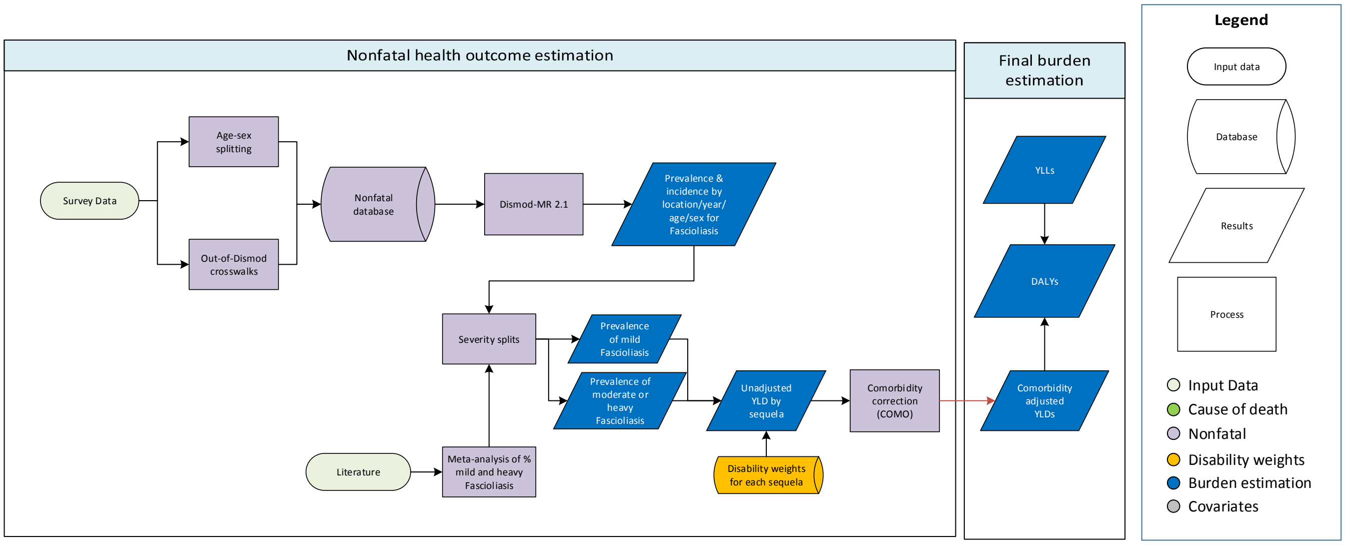

Paragonimiasis flowchart

Paragonimiasis

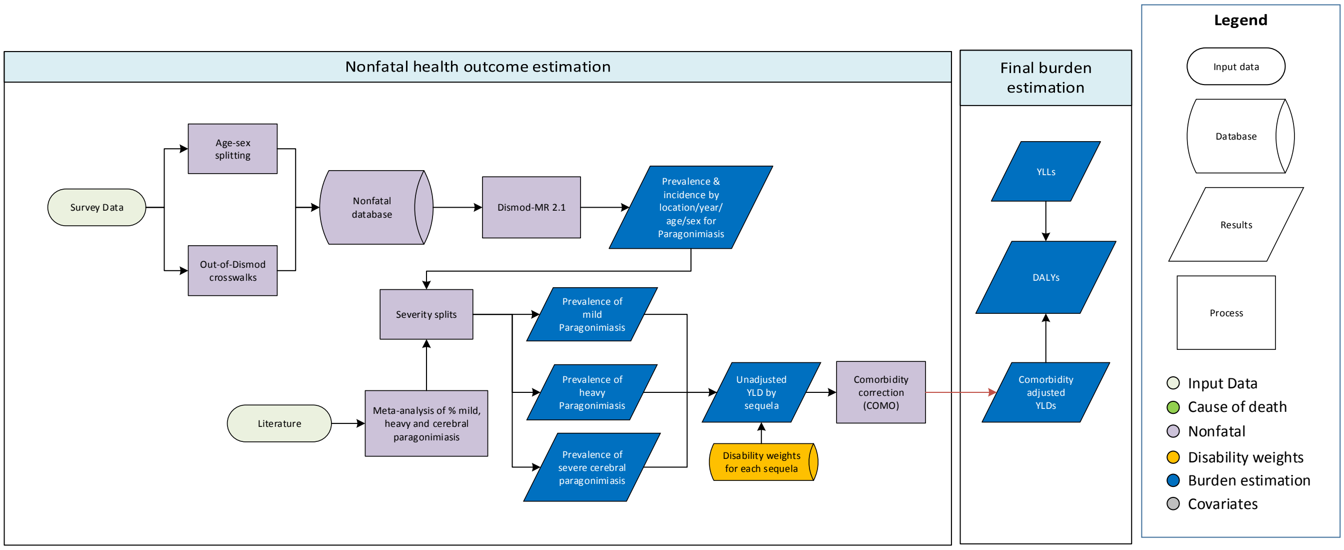

Input data and methodological summary

Case definition

Human foodborne trematodiases (FBT) is defined as the infection with parasitic worms of the class trematoda, which are also known as flukes. Trematodes are transmitted via contaminated food and infection is highly related to food habits. Definitive hosts, including humans, become infected when ingesting viable metacercariae by consuming contaminated aquatic products (e.g. watercrest etc.). In the ICD-10, FBT are listed under code B66 [1].

FBT is subdivided into six types of FBT (*see* Table 1):

- Clonorchiasis
- Fascioliasis
- Intestinal fluke
- Opisthorchiasis
- Paragonimiasis (normal and cerebral infections)

Table 1. Subtypes of FBT

	Species of FBT	Also known as:	Carcinogen
1	Chlonorchiasis	(Chinese) Liver fluke	Associated with choliangiocarcinoma
2	Opisthorchiasis<br><br>(*O viverrini & O felineus*)	Liver fluke	Associated with choliangiocarcinoma<br><br>(*O viverrini*)
3	Fascioliasis	Liver fluke	No available evidence

4	Intenstinal fluke	Liver fluke	No available evidence
5	Paragonimiasis	Lung fluke	

Thresholds for heavy infection and duration by species of FBT

The majority of people infected with FBTs are asymptomatic. When symptoms do occur they are often non-specific. Among the clinical symptomatic group, severity is associated with worm burden, typically measured by fecal egg counts, and the duration of infection. The thresholds for heavy infection and duration by species of FBT are shown in Table 2. The clinical presentation of FBT depends on the target organs (liver, lung, or intestines). Clonorchiasis and opisthorchiasis patients may suffer from loss of appetite, fullness, indigestion, diarrhoea, pain in the right upper quadrant, lassitude, weight loss, ascites, and oedema.[2, 3] Cholangitis, obstructive jaundice, intra-abdominal mass, cholecystitis, and gallbladder or intrahepatic stones may occur as complications.[3, 4]

Table 2. Thresholds for heavy infection and duration by species of FBT

	Species of FBT	Case thresholds for heavy infection	Duration
1	Chlonorchiasis	10,000 eggs per g of feces	lifelong
2	Opisthorchiasis	10,000 eggs per g of feces	lifelong
3	Fascioliasis	1,000 eggs per g of faces	lifelong
4	Intenstinal fluke	1,000 eggs per g of faces	lifelong
5	Paragonimiasis	100 eggs per 5 ml sputum	lifelong
6	Cerebral paragonimiasis	Any infection of the brain with flukes and/or eggs of *Paragonimus* spp.	lifelong

Input data

Model inputs

For GBD 2010, the data came from the expert group and is the result of their analysis. The expert group analysis used the results of a systematic literature review performed by Furst et al. as a starting point for the analysis.[5] Furst et al. searched PubMed, WHOLIS, FAOBIB, Embase, CAB Abstracts, Literatura Latino Americana e do Caribe em Ciências de Saùde (LILACS), ISI Web of Science, BIOSIS preview, Science Direct, African Journals OnLine (AJOL), and the System for Information on Grey Literature in Europe (SIGLE), period Jan 1, 1980 to

Dec 31, 2008. The initial number of studies identified through the literature review was ~34,000 references. The literature review included extracted data from 181 studies. For GBD 2013 and GBD 2015 the search strategy was replicated to capture epidemiological studies published between 2008 and 2015.

Input data for the assessment of the total national number of infected people

Only studies that used countrywide surveys to estimate the national prevalence rates were included (or for China Province-wide surveys). Reason for choosing only national studies is that FBT shows a highly focal spatial distribution and local cross-sectional surveys would profoundly under- or overestimate true national prevalences. We decided not to model national and subnational together and get a coefficient on subnational, because there is not a one fits all relationship across the world. Infection is highly related to food habits and there are highly varying differences between national and sub-national prevalence rates. The final GBD 2015 dataset contained 29 prevalence studies from 17 countries. We used raw data from the selected studies as input for DisMod.

Prevalence intestinal fluke infection

Intestinal fluke is different from the other types of FBT, because there are several pathogens that fall under intestinal fluke infection. It can be caused by pathogens, such as Metagonimus spp., Echinostoma spp., Neodiplostomatidae.[6] When assessing the prevalence of intestinal fluke infection, we added the identified prevalence for each parasite species in order to obtain the overall prevalence of intestinal fluke infections. This approach may lead to a certain overestimation of the true prevalence, because people may be co-infected with more than one intestinal fluke species. There is no sufficient evidence about the proportion of co-infections, but the resulting overestimation of the true prevalence may be more than offset by the assumptions made in our previous modeling approach and the many challenges in generating the underlying epidemiological parameters (e.g., diagnostic inaccuracy in the detection of infections with the more than 50 intestinal fluke species). Also of note: the transmission source of intestinal fluke infections are species-specific and therefore vary. For instance, *Fasciolopsis buski* is usually transmitted by eating raw water plants with the infective parasite stage attached to the water plants, whereas Neodiplostomatidae are transmitted by eating undercooked and infested frogs, snakes, and tadpoles. Because of these different transmission pathways, the rate of co-infection might in fact be smaller than expected.

Input data to differentiate between asymptomatic and heavy infections

We estimated the proportion of heavily-infected among all infected in all available national and regional cross-sectional surveys. It is expected that heavy infection increases with age and there is data available on heavy infection by age group. We therefore decided to include age-dependent rates of heavy infection for clonorchiasis, opisthorchiasis, and intenstinal fluke infection. For (cerebral) paragonimiasis and fascioliasis there was not sufficient age-dependent data on high intensity FBT infection.

Modeling strategy

The GBD 2013 epidemiological modeling strategy for FBT made use DisMod-MR 2.0, a Bayesian meta-regression tool which built on GBD 2010's DisMod-MR. Updated characteristics of DisMod-MR 2.0 included the application of an offset lognormal rather than a negative binomial distribution. Dismod-MR 2.0 also executed its calculations in a cascade from global to country and (where applicable) from country to the subnational geographical unit, thereby

ensuring that estimates were consistent at all levels of the cascade. DisMod-MR 2.0 was used to estimate prevalence, by age, sex, year, and country for FBT.

We used a three-step process for the disease modeling of FBT. In the first step we used DisMod-MR to estimate assess the prevalence of FBT by age, sex, year, and country. In the second we differentiated between asymptomatic and heavy infections. MetaXL (a meta-analysis add in for Microsoft Excel) was used to estimate the proportion of heavy infected among all infected by age group for clonorchiasis, opisthorchiasis, and intenstinal fluke infection (see Table 3 and 4). These proportions were used to estimate the prevalence of heavy FBT infection.
The third step consisted of deselecting countries that have no autochtonous case reports of FBT (input 34,000 references from literature review).

Table 3. Percentage of high intensity infection by age group and type of FBT (based on 8 FBT prevalence studies)

Age category	Clonorchiasis			Opisthorchiasis			Intestinal fluke infection		
	Mean	Low	High	Mean	Low	High	Mean	Low	High
0-9	30%	17%	44%	10%	0%	29%	8%	3%	14%
10-19	15%	0%	43%	15%	0%	69%	11%	8%	14%
20-29	18%	10%	29%	16%	0%	52%	18%	15%	21%
30-39	17%	5%	34%	21%	0%	56%	22%	17%	28%
40-49	22%	13%	32%	28%	1%	68%	22%	13%	32%
50-59	18%	0%	49%	29%	0%	75%	17%	9%	28%
60+	32%	18%	47%	25%	0%	64%	15%	8%	23%

Table 4. Percentage of high intensity infection by type of FBT (based on 4 FBT prevalence studies)

Type of FBT	Mean	Low	High
Paragonimiasis	23%	0%	59%
Fascioliasis	19%	3%	41%

Cerebral paragonimiasis

It was assumed that there was 0.8% of cerebral involvement in paragonimiasis. This proportion was used to estimate the prevalence of cerebral paragonimiasis. This proportion is based on one study. The study was performed in Paju, South Korea. This is an area with 6,738 inhabitants and according to the survey, it was estimated that 29.6% of all individuals would react to intradermal test (= an immunological reaction indicating previous or current contact to the parasite). 25% of all “positive reactors” may have eggs in their sputum (= active infection with the parasite currently present in the human host). If these rates are applied to the community as a whole, the number of patients with active paragonimiasis would be at least 498 (=6,738*0.296*0.250). Furthermore, four cases of cerebral paragonimiasis were found in this community. Therefore, four out of 498 individuals with active paragonimus infection suffered from cerebral infection (=0.80%; 95% confidence interval 0.019%-1.587%).

Severity splits and disability weights

For GBD 2015, FBT was not split into health states with different severities. The table below shows the GBD 2015 disability weights that were used to calculate the burden of FBT in YLDs.

Table 5. Disability weights that were used to calculate FBT YLDs

Sequelae	Severity description	Health state name	Disability weight
Asymptomatic clonorchiasis	Clonorchiasis, currently without symptoms	N/A	0.000 (0.000-0.000)
Hheavy clonorchiasis	Abdominal pain and nausea reported as moderate	Abdominopelvic problem, moderate	0.114 (0.078-0.159)
Asymptomatic opisthorchiasis	Opisthorchiasis, currently without symptoms	N/A	0.000 (0.000-0.000)
Heavy opisthorchiasis	Abdominal pain and nausea reported as moderate	Abdominopelvic problem, moderate	0.114 (0.078-0.159)
Asymptomatic fascioliasis	Fascioliasis, currently without symptoms	N/A	0.000 (0.000-0.000)
Heavy fascioliasis	Abdominal pain and nausea reported as moderate	Abdominopelvic problem, moderate	0.114 (0.078-0.159)
Asymptomatic intestinal fluke infection	Intestinal fluke infection, currently without symptoms	N/A	0.000 (0.000-0.000)
Heavy intestinal fluke infection	Abdominal pain and nausea reported as moderate	Abdominopelvic problem, moderate	0.114 (0.078-0.159)
Asymptomatic paragonimiasis	Paragonimiasis, currently without symptoms	N/A	0.000 (0.000-0.000)
Heavy paragonimiasis	Cough, fever and weight loss	COPD three levels, mild, moderate & severe	0.333 (0.224-0.454)
Cerebral paragonimiasis	Epilepsy due to cerebral paragonimiasis	Epilepsy, less severe (seizures < once per month)	0.263 (0.173-0.367)
		Epilepsy, severe (seizures >= once per month	0.552 (0.375-0.710)

Note. N/A: not applicable

References

1. WHO. *International Statistical Classification of Diseases and Related Health Problems. 10th Revision. Version for 2007.* 2007 [cited 2009 October 14, 2009]; Available from: http://apps.who.int/classifications/apps/icd/icd10online/.
2. Rim, H.J., *Clonorchiasis: an update.* J Helminthol, 2005. **79**(3): p. 269-81.
3. Pungpak, S., et al., *Clinical features in severe opisthorchiasis viverrini.* Southeast Asian J Trop Med Public Health, 1985. **16**(3): p. 405-9.
4. Rim, H.J., *The current pathobiology and chemotherapy of clonorchiasis.* Korean J Parasitol, 1986. **24**(Suppl.): p. 1-141.
5. Furst, T., J. Keiser, and J. Utzinger, *Global burden of human food-borne trematodiasis: a systematic review and meta-analysis.* Lancet Infect Dis, 2012. **12**(3): p. 210-21.
6. Furst, T., et al., *Manifestation, diagnosis, and management of foodborne trematodiasis.* BMJ, 2012. **344**: p. e4093.

Leprosy

Flowchart

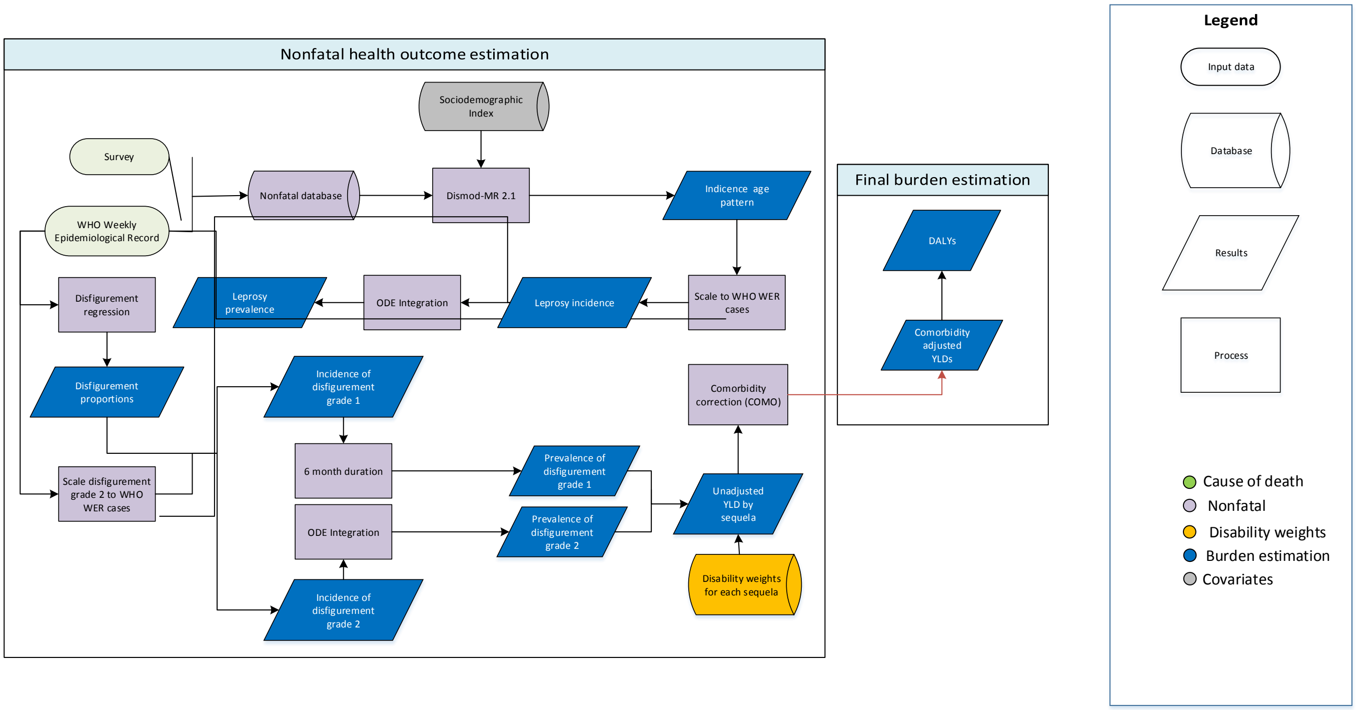

Input Data and Methodological Summary

Case Definition

Leprosy is a chronic bacterial infection caused by *Mycobacterium leprae*, primarily affecting the nervous system, skin, respiratory tract, and eyes. Transmission is facilitated through contact with fluid from the nose and mouth of an infected individual. Disability is associated with cases that develop to disfigurement, which is further subdivided into grade 1 and 2.

Input data

Due to the cyclical nature of systematic review for GBD causes, no data collection was scheduled for GBD 2015. As such, leprosy will be a priority for the next iteration of the study.

Modeling strategy

All available leprosy incidence data was modeled using Dismod-MR 2.1. Following this, the age-sex specific incidence rates are scaled to match the reported incidence in the WHO Weekly Epidemiological Record (WER). Where multiple years are available for a particular country, missing years are interpolated/extrapolated in order to maintain a complete time series. Countries for which only one year

is present, other years are assumed to be zero. We them stream out prevalence of leprosy as a function of time by integrating the ordinary differential equation

$$^{dp}/_{dt} = inc * (1 - prev) - prev * \ EMR$$

Where EMR = excess mortality rate, and is presumed to be zero for this condition.

In order to define the progression of leprosy incidence into disfigurement, we perform a generalized ordered logistic regression on WER data from Brazil, resulting in age-sex-specific probability of grade 2 disfigurement among incident cases of leprosy and grade 1 among incident cases of leprosy without grade 2 disfigurement. Then we use WER data to generate an envelope of grade 2, as was done for total leprosy. We are then able to take leprosy incidence, impose the age-sex-specific disfigurement proportions, and scale to the grade 2 envelope. From there, we apply the grade 1 proportions to the remaining leprosy cases that do not have grade 2 disability. For grade 1 prevalence, a duration of 6 months is applied. In deriving grade 2 prevalence, we use the same ODE integration function as described for total leprosy.

Sequela	Healthstate name	Healthstate description	Disability Weight (95% CI)
Disfigurement level 1 due to leprosy	Disfigurement, level 1	has a slight, visible physical deformity that others notice, which causes some worry and discomfort.	0.011 (0.005-0.021)
Disfigurement level 2 due to leprosy	Disfigurement, level 2	has a visible physical deformity that causes others to stare and comment. As a result, the person is worried and has trouble sleeping and concentrating.	0.067 (0.044-0.096)

Ebola

Flowchart

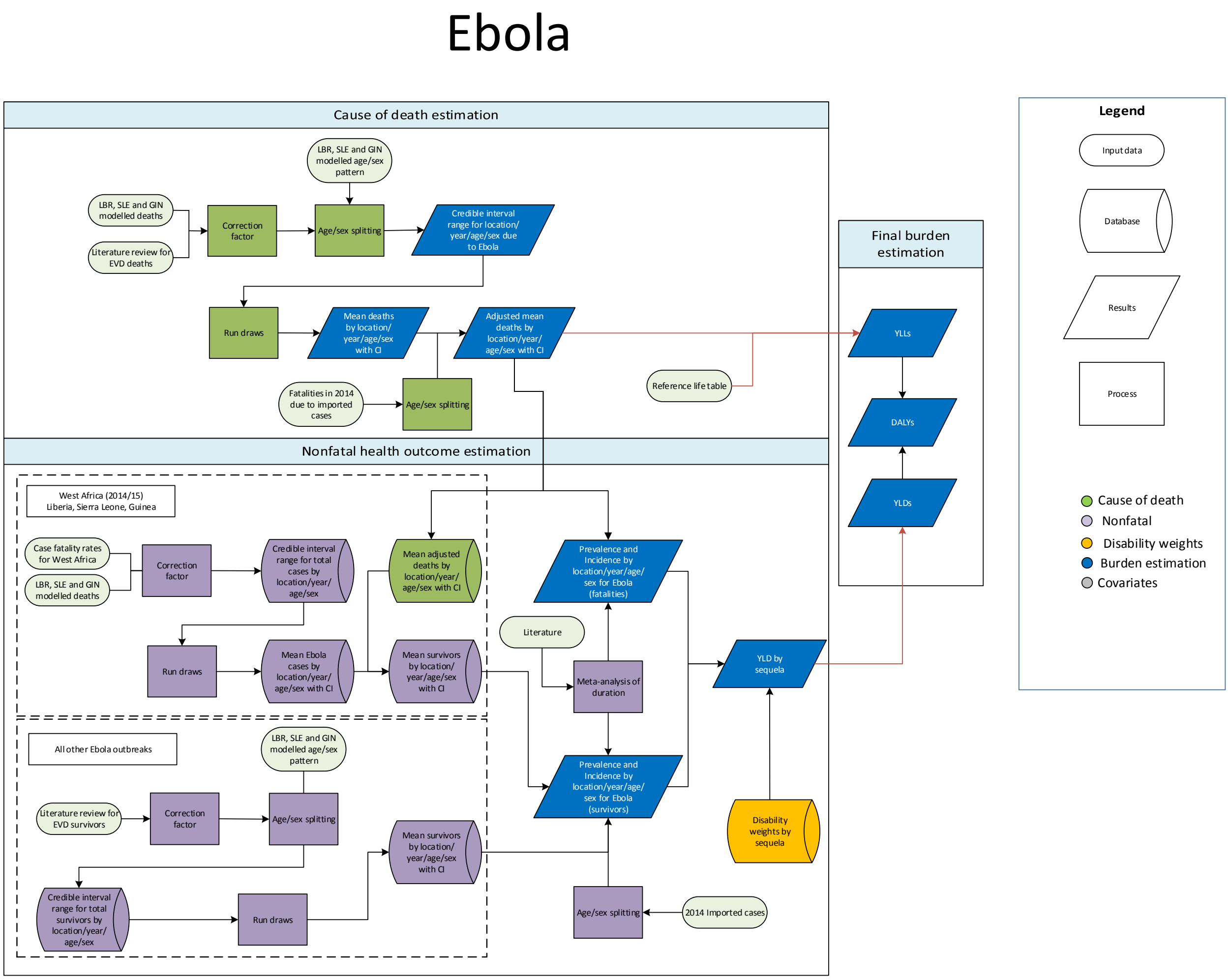

Input data and methodological summary

Case definition

Ebola virus disease is a relatively rare viral pathogen linked with high case fatality rates in both humans and non-human primates. The disease is zoonotic, and whilst bats have been implicated as reservoirs, definitive host species are yet to be identified. Once a human becomes infected after viral transmission from animal sources either directly or indirectly, secondary human-to-human transmission is possible, primarily through exchange of infectious bodily fluids and secretions. Clinical cases typically present initially as a febrile illness, similar to a number of different pathogens, subsequently followed by hemorrhagic complications, and often death. Historically there have been a number of outbreaks, usually

no more than a few hundred cases and typically constrained to one country, focused in Central Africa. The West African outbreak, however, which started in Guinea in 2013, has claimed more lives than all previous outbreaks combined, and spread across the region seeding additional outbreaks. There is an ICD code for Ebola, A98.4, but no data used in the modeling reference that coding (i.e., all the data are from literature extractions).

Input data

Model inputs

Two distinct sequelae were assigned to Ebola virus disease (EVD) to be incorporated into the YLD estimation process: (i) sequela associated with the initial symptomatic phase of the infection (associated with all cases of Ebola virus disease) and (ii) sequela characterizing the long term post-EVD consequences of infection. As such, data were required both to ascertain the number of deaths as well as those surviving from each outbreak.

Data for Ebola virus disease were only included if the case was identified as either "probable" or "confirmed" as per WHO definitions [http://www.who.int/csr/resources/publications/ebola/ebola-case-definition-contact-en.pdf]. A confirmed case is any suspected or probable case with a positive laboratory result through either detection of virus RNA via reverse transcriptase-polymerase chain reaction, or by detection of IgM antibodies directed against Ebola. A probable case is any suspected case evaluated by a clinician or any deceased suspected case with an epidemiological link to a confirmed case.

Data on fatal cases were inherited from the GBD 2015 mortality estimation process and were converted into incidence of cases of Ebola (with fatal outcomes) by cross-referencing locational annualized population estimates.

In order to calculate the numbers of survivors from each outbreak, two data sources were referenced, one based upon modeled estimates of the main three countries in the West African Ebola outbreak (namely Sierra Leone, Liberia, and Guinea) and literature references covering all other subsequent outbreaks.

Researchers from Imperial College, London (UK), as part of the WHO Ebola response team, provided modeled estimates for the number of fatalities that result from a given number of reported cases (provided by line lists from WHO). This method was used in a variety of papers to generate baseline estimates of case fatality rates and other key epidemiological measures whilst correcting for the lag period between initially reporting a case and the final outcome of that case (whether it be death or survival). The full data cleaning and methodology are reported elsewhere (1,2). Bespoke estimates were provided for GBD for Liberia, Sierra Leone, and Guinea and were stratified by age, sex, and year. Death data from Guinea ranged from February 28, 2014, until September 27, 2015, with data from Liberia ranging from March 20, 2014, to May 4, 2015, and data from Sierra Leone ranging from May 21 until September 28, 2015. To these estimates, calculated case fatality ratios (1,2) were applied in order to generate an estimate of the total number of cases stratified by age, sex, and country.

For all other outbreaks, numbers of survivors were directly evaluated based upon numbers published in a previous review (3,4) and consulting original documents describing these outbreaks. This initial review was also updated to include the outbreak that occurred in the Democratic Republic of the Congo in 2014

(5). This resulted in datasets describing each outbreak with variable degrees of detail: some fully describing the age and sex breakdown of all survivors [e.g., (6)] and others simply providing the final total. Only confirmed or probable cases were included. Outbreaks that spanned multiple years, in the absence of sufficient data providing an accurate breakdown, were apportioned between the years by evenly assigning a uniform number of survivors to each month of the outbreak's duration. An additional search was conducted to identify imported cases from the West African outbreak during 2014 and 2015.

Table 1. Sequelae & disability weights (DWs) associated with Ebola

Sequelae	Description	Disability weight
Infectious disease, acute episode, severe	Has a high fever and pain and feels very weak, which causes great difficulty with daily activities	0.133 (0.088-0.19)
Infectious disease, post-acute consequences (fatigue, emotional lability, insomnia)	Is always tired and easily upset. The person feels pain all over the body and is depressed	0.219 (0.148-0.308)

It was not possible to create bespoke disability weights for the more specific sequelae often associated with Ebola virus disease (e.g., hemorrhaging or ocular complications in survivors), so existing disability weights were co-opted. General high fevers and weakness characterize the majority of presenting cases (7) with long-term complications, generally related to weakness and arthralgia (8).

Modeling strategy

Data on cases (both survivors and fatalities) resulting from imported cases from 2014 and 2015 were used as specific count data as it was assumed to be an accurate representation of the cases and outbreaks in these countries, all of which were on high alert for importation of cases (9,10).

The other input data were processed prior to inclusion in GBD to account for any potential underreporting of deaths. The United Nations Mission for Ebola Emergency Response surveys suggested that such underreporting of cases ranged from 1.67 – 2x the reported number of deaths and 1.89 – 2.22x the number of cases (11). In order to capture this potential variation, all input data were multiplied by the lower and upper limit of this estimated correction factor; these numbers then provided the lower and upper bounds from which draw values were taken. For outbreaks where no data were supplied for age and/or sex, the pattern observed in the West African outbreak (for which there were the most comprehensive data) was used to apportion these total values.

One thousand draws were taken from a normal distribution fitted between these lower and upper bound values, which generated mean estimates stratified by age, sex, location, and year along with credible intervals for these numbers. For the West African outbreak, this generated total case numbers, from which the estimated number of deaths was subtracted in order to provide an estimate for the total number of survivors. For all other outbreaks, this data processing directly estimated the total number of

survivors from each outbreak. These count data were converted into prevalence estimates by cross-referencing estimates of population size.

In order to estimate the duration of the sequelae categories, previous modeled assessments of the West African outbreak were consulted (1,2). The duration of initial infection for patients was calculated as the total time period between onset of symptoms to death or to discharge from hospital (8.2 days [7.9–8.4] and 15.1 [14.6–15.6], respectively). These time periods were assumed to be appropriate for characterizing all other outbreaks. This time period was then assigned a disability weight corresponding to "infectious disease, acute episode, severe."

For long-term sequelae estimation, the proportion of survivors still suffering post-acute consequences was modeled using an exponential function with proportions of survivors still reporting poor health states (derived from a small number of survivor studies (12–15)) reported over different time periods. The average duration of post-Ebola sequelae was then calculated as 0.75 years (0.417–1.135).

The final combination of YLDs associated with prevalent initial onset of disease and prevalent post-EVD consequences was then calculated to provide an overall YLD estimate stratified by age, sex, location, and year. Estimates were provided for the years 1990, 1995, 2000, 2005, 2010, and 2015 as per non-fatal GBD estimation protocols.

Potential limitations

Data on Ebola outbreaks prior to 2014 are sparse, and as a result many values derived from the West African outbreak were assumed to be valid for historic outbreaks as well. This may mask significant differences that exist between these outbreaks, some of which were caused by different species of Ebola virus. In order to minimize this problem we chose to implement a data-driven approach – for those outbreaks where sufficiently detailed historical data could be obtained, this was used in preference to any assumed age/sex breakdown.

Similarly, we have only one estimate for potential underreporting, taken from December 2014 in Sierra Leone. It could well be that this rate varies considerably both over time within an outbreak as well as between different outbreaks. As more studies are undertaken in the years following the peak of the outbreak, it is likely that this correction factor will become better parameterized in future GBD iterations.

Hemorrhagic manifestations are currently not considered as an explicit health state for disability weighting, and as a result, the current classification (of infectious disease, acute episode, severe) may be an underestimate. In contrast, the post-Ebola disease sequelae disability weighting may overestimate this burden, particularly when applied over a long period of time. In both instances, however, these disability weightings represent the most relevant linkages in the absence of bespoke values being generated.

Due to so few historical survivors of Ebola virus disease, few studies have tracked the long-term sequelae among cohorts of survivors, and those that have did so only for a relatively limited period. Given the large number of survivors from the West African outbreak, it is likely that future of parameterization of this component will become much better data-driven.

References

1. Agua-Agum J, Ariyarajah A, Aylward B, Blake IM, Brennan R, Cori A, et al. West African Ebola Epidemic after One Year — Slowing but Not Yet under Control. N Engl J Med [Internet]. 2015 Feb 5 [cited 2015 Sep 20];372(6):584–7. Available from: http://www.pubmedcentral.nih.gov/articlerender.fcgi?artid=4368109&tool=pmcentrez&rendertype=abstract

2. Ebola Virus Disease in West Africa - The First 9 Months of the Epidemic and Forward Projections. N Engl J Med [Internet]. 2014 Sep 22 [cited 2014 Sep 23];371(16):1481–95. Available from: http://www.pubmedcentral.nih.gov/articlerender.fcgi?artid=4235004&tool=pmcentrez&rendertype=abstract

3. Pigott DM, Golding N, Mylne A, Huang Z, Henry AJ, Weiss DJ, et al. Mapping the zoonotic niche of Ebola virus disease in Africa. Elife. 2014;3:e04395.

4. Mylne A, Brady OJ, Huang Z, Pigott DM, Golding N, Kraemer MUG, et al. A comprehensive database of the geographic spread of past human Ebola outbreaks. Sci Data. 2014;1:140042.

5. Maganga GD, Kapetshi J, Berthet N, Kebela Ilunga B, Kabange F, Mbala Kingebeni P, et al. Ebola virus disease in the Democratic Republic of Congo. N Engl J Med. 2014;371(22):2083–91.

6. Rosello A, Mossoko M, Flasche S, Van Hoek AJ, Mbala P, Camacho A, et al. Ebola virus disease in the Democratic Republic of the Congo, 1976-2014. Elife. 2015;4.

7. Schieffelin JS, Shaffer JG, Goba A, Gbakie M, Gire SK, Colubri A, et al. Clinical Illness and Outcomes in Patients with Ebola in Sierra Leone. N Engl J Med [Internet]. 2014 Nov 27 [cited 2016 Jun 10];371(22):2092–100. Available from: http://www.nejm.org/doi/abs/10.1056/NEJMoa1411680

8. Tiffany A, Vetter P, Mattia J, Dayer J-A, Bartsch M, Kasztura M, et al. Ebola Virus Disease Complications as Experienced by Survivors in Sierra Leone. Clin Infect Dis [Internet]. 2016 Jun 1 [cited 2016 Jun 10];62(11):1360–6. Available from: http://www.ncbi.nlm.nih.gov/pubmed/27001797

9. Fasina FO, Shittu A, Lazarus D, Tomori O, Simonsen L, Viboud C, et al. Transmission dynamics and control of Ebola virus disease outbreak in Nigeria, July to September 2014. Euro Surveill [Internet]. 2014 Jan [cited 2015 Sep 16];19(40):20920. Available from: http://www.ncbi.nlm.nih.gov/pubmed/25323076

10. Althaus CL, Low N, Musa EO, Shuaib F, Gsteiger S. Ebola virus disease outbreak in Nigeria: Transmission dynamics and rapid control. Epidemics [Internet]. 2015 Jun [cited 2016 Jan 6];11:80–4. Available from: http://www.sciencedirect.com/science/article/pii/S1755436515000341

11. UNMEER. Sierra Leone: Ebola emergency Weekly Situation Report No. 7 [Internet]. 2014. Available from: https://www.humanitarianresponse.info/system/files/documents/files/UNMEER_NERC_SitRep_07Dec.pdf

12. Clark D V, Kibuuka H, Millard M, Wakabi S, Lukwago L, Taylor A, et al. Long-term sequelae after Ebola virus disease in Bundibugyo, Uganda: a retrospective cohort study. Lancet Infect Dis [Internet]. 2015 Aug [cited 2015 Dec 20];15(8):905–12. Available from: http://www.ncbi.nlm.nih.gov/pubmed/25910637

13. Qureshi AI, Chughtai M, Loua TO, Pe Kolie J, Camara HFS, Ishfaq MF, et al. Study of Ebola Virus Disease Survivors in Guinea. Clin Infect Dis [Internet]. 2015 Oct 1 [cited 2016 Mar 3];61(7):1035–42. Available from: http://cid.oxfordjournals.org/content/61/7/1035

14. Rowe AK, Bertolli J, Khan AS, Mukunu R, Muyembe-Tamfum JJ, Bressler D, et al. Clinical, virologic, and immunologic follow-up of convalescent Ebola hemorrhagic fever patients and their household contacts, Kikwit, Democratic Republic of the Congo. Commission de Lutte contre les Epidémies à Kikwit. J Infect Dis [Internet]. 1999 Feb 1 [cited 2015 Dec 20];179 Suppl (Supplement_1):S28–35. Available from: http://jid.oxfordjournals.org/content/179/Supplement_1/S28.full

15. Bwaka MA, Bonnet MJ, Calain P, Colebunders R, De Roo A, Guimard Y, et al. Ebola hemorrhagic fever in Kikwit, Democratic Republic of the Congo: clinical observations in 103 patients. J Infect Dis [Internet]. 1999 Feb 1 [cited 2016 Feb 12];179 Suppl (Supplement_1):S1–7. Available from: http://jid.oxfordjournals.org/content/179/Supplement_1/S1.long

Other neglected tropical diseases

In addition to the neglected tropical diseases described above, there are many diverse types of neglected tropical diseases, which are encompassed by the following ICD 10 codes: A68-A68.9, A69.2-A69.5, A75-A75.9, A77-A79.9, A92-A94.0, A96-A96.9, A98-A98.8, B58, B59-B60.8, B68-B68.9, B70-B72.0, B74.3-B75, B78-B78.9, B80-B81.8, B83-B83.8, P37.1.

Because these neglected tropical diseases are diverse in their underlying causes and risk factors as well as in their associated health outcomes, modeling them together in a DisMod-MR model would not produce reliable estimates of prevalence or excess mortality. Instead, we calculated the YLDs caused by neglected tropical diseases directly using a YLD/YLL ratio.

We calculated the ratio YLDs to YLLs across the specified neglected tropical diseases for which nonfatal outcomes were modeled, using YLL estimates from the GBD 2015 cause of death (CoD) analysis. We then multiplied this YLD/YLL ratio by the YLL estimates for other neglected tropical diseases from the GBD 2015 CoD analysis, providing us with an estimate of the YLDs association with other neglected tropical diseases.

Maternal disorders

1) Abortion, ectopic pregnancy, and miscarriage; 2) Obstructed labor and uterine rupture; 3) Maternal hemorrhage; 4) Maternal sepsis and other maternal infections; 5) Maternal hypertensive disorders; 6) Other maternal disorders

Flowchart

Maternal disorders: 1) Abortion, ectopic pregnancy, and miscarriage; 2) Obstructed labor and uterine rupture; 3) Maternal hemorrhage; 4) Maternal sepsis and other maternal infections; 5) Maternal hypertensive disorders; 6) Other maternal disorders

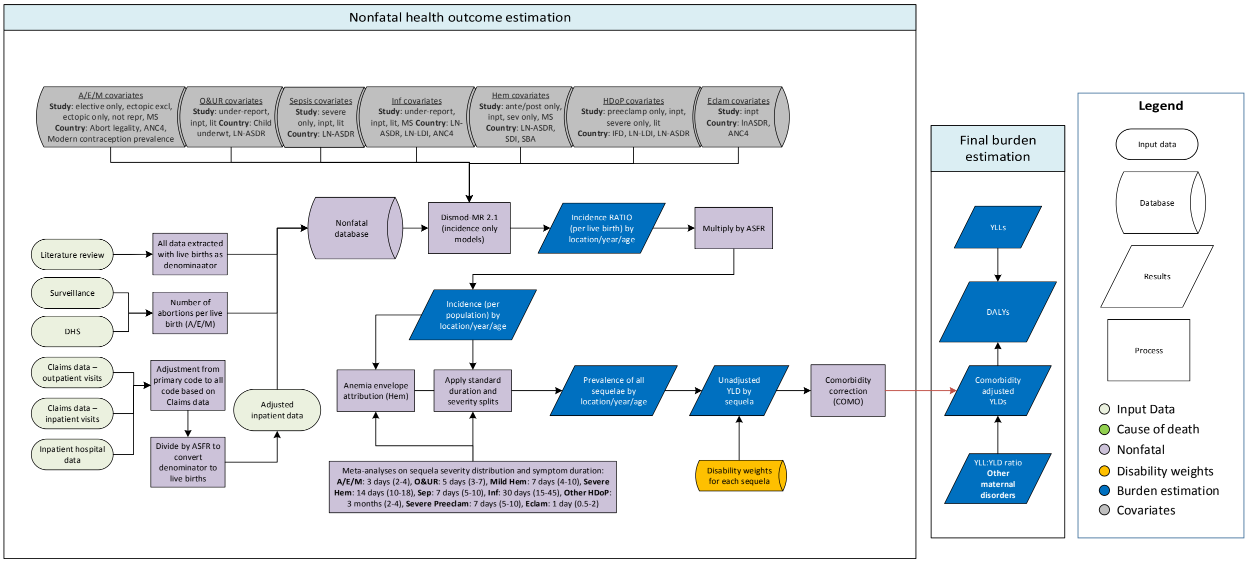

Abbreviations
A/E/M: Abortion, ectopic pregnancy, and miscarriage; O&UR: Obstructed labor and uterine rupture; Hem: Maternal hemorrhage; Sep: Maternal sepsis; Inf: Other maternal infections; HDoP: Maternal hypertensive disorders; Eclam: Eclampsia; ASFR: Age-specific fertility rate; MS: Marketscan; ANC4: coverage of 4 visits of antenatal care; SDI: Socio-Demographic Index; lit: Literature data; inpt: Inpatient data; not repr: Not representative; LN: Natural log; ASDR: Age-standardized death rate (GBD mortality output); LDI: Lag-distributed income per capita

Input data and methodological summary

Case definition

Maternal disorders are those complications occurring during pregnancy, childbirth, and the postpartum period. Seven different statistical models were completed for GBD 2015. These included 1) abortion, ectopic pregnancy, miscarriage, 2) obstructed labor and uterine rupture, 3) maternal hemorrhage (including placental disorders), 4) maternal sepsis, 5) other maternal infections, 6) hypertensive disorders of pregnancy, 7) eclampsia. Other direct maternal disorders was estimated using a YLD-to-YLL ratio approach used for multiple different GBD causes. Late maternal death and indirect maternal disorders, including HIV-related maternal death, were not assigned any disability as the disability associated with them was assumed to be included in estimates for the underlying conditions. ICD-9 and ICD-10 codes for each of the statistical models are contained in the table below.

Table 1. ICD codes for maternal disorders

International classification of diseases codes for maternal disorders in GBD 2015 non-fatal analysis

Model	ICD10 code	ICD9 code
Abortion, ectopic pregnancy, miscarriage	O00-O08, O36.4	631, 633-639
Maternal hemorrhage	O20, O43.2, O44-O46, O62.2, O67, O72	640-641, 661.0, 666
Eclampsia	O15	642.6
Hypertensive disorders of pregnancy	O11-O16	642.3, 642.4, 642.5, 642.6, 642.7, 642.9
Obstructed labor and uterine rupture	O64-O66, O71, O83	659-660, 662, 665, 669.5, 669.6
Other maternal infections	O23, O41, O75.2-3, O86, O91	646.5, 646.6, 659.2, 672.0, 674.1, 674.2, 674.3, 675
Maternal sepsis	O85	659.3, 670

Input data

Systematic literature reviews were completed for GBD 2010 and GBD 2013. These were updated on May 18, 2015 using the search strings below. In addition, we searched ministry of health websites for pregnancy complication data and used from Confidential Enquiry and other sources used in our maternal mortality analyses when they presented data on pregnancy complications. We also performed snowball searches for abortion reporting and surveillance data systems, finding multiple such systems throughout high income countries and several geographies in Central and Eeastern Europe. Inpatient and outpatient data were used, as was claims data from MarketScan in the United States. This data was extracted and processed as described in the section on hospital data, including use of primary-to-any inpatient ratio to correct for under-reporting of pregnancy complications in hospital datasets that rely only on primary discharge codes. All data was extracted in standard fashion, uploaded and stored on a centralized SQL database. The final dataset contents for each of the models is shown below as well.

Abortion, ectopic pregnancy and miscarriage

(((('Abortion, Induced'[Mesh] OR 'Abortion, Therapeutic'[Mesh]) OR 'Abortion, Legal'[Mesh]) OR 'Pregnancy, Ectopic'[Mesh]) NOT ('case report'[Title/Abstract] OR 'birth defect'[Title/Abstract] OR 'congenital'[Title/Abstract])) AND ('2014'[PDAT] : '2015'[PDAT]) AND 'humans'[MeSH Terms] 555 initial hits, 4 new sources extracted

	Incidence
Studies	51
Countries/subnationals	21/50
GBD world regions	12

Maternal hemorrhage

((('Postpartum Hemorrhage'[Mesh] OR 'Uterine Hemorrhage'[Mesh]) OR ('maternal'[Title/Abstract] OR 'pregnant'[Title/Abstract] OR 'pregnancy'[Title/Abstract] OR 'mothers'[MeSH]) AND ('haemorrhage'[Title/Abstract] OR 'hemorrhage'[Title/Abstract]) NOT 'case report'[All fields]) AND ('2014'[PDAT] : '2015'[PDAT])) AND 'humans'[MeSH Terms]. **1029 initial hits, 3 new sources extracted.**

	Incidence
Studies	100
Countries/subnationals	47/19
GBD world regions	16

Hypertensive disorders of pregnancy and Eclampsia

('Pre-Eclampsia'[Mesh] OR 'Eclampsia'[Mesh] OR 'Hypertension, Pregnancy-Induced'[Mesh] OR 'Hypertensive disorders of pregnancy'[Title/Abstract]) NOT ('case report'[All fields] OR kidney don*[Title/Abstract] OR polymorphism*[Title/Abstract] OR endotheli*[Title/Abstract]) AND ('2014'[PDAT] : '2015'[PDAT]) AND 'humans'[MeSH Terms]. **3717 initial hits, 14 new sources extracted.**

Hypertensive disorders of pregnancy	Incidence
Studies	113
Countries/subnationals	66/20
GBD world regions	19

Eclampsia	Incidence
Studies	71
Countries/subnationals	53/11
GBD world regions	17

Obstructed labor and uterine rupture

(((('obstructed labour'[All Fields] OR 'obstructed labor'[All Fields]) OR 'labour dystocia'[All Fields]) OR 'labor dystocia'[All Fields]) OR 'dystocia'[All Fields]) AND ('2013'[PDAT] : '2015'[PDAT]) AND 'humans'[MeSH Terms]. **176 initial hits, 10 new sources extracted**

	Incidence
Studies	31
Countries/subnationals	23/4
GBD world regions	8

Maternal sepsis and other maternal infections:

('Puerperal Infection'[Mesh] OR (('maternal'[Title/Abstract] OR 'pregnant'[Title/Abstract] OR 'pregnancy'[Title/Abstract]) AND ('Sepsis'[Mesh] OR 'infection'[Title/Abstract]))) NOT 'case report'[All

fields] AND ('2014'[PDAT] : '2015'[PDAT]) AND 'humans'[MeSH Terms] AND 'humans'[MeSH Terms]. **197 initial hits, 3 new sources extracted**

Maternal sepsis	Incidence
Studies	33
Countries/subnationals	23/9
GBD world regions	12

Other maternal infections	Incidence
Studies	12
Countries/subnationals	11/5
GBD world regions	8

Modeling strategy

We estimated the incidence ratio of each category of pregnancy complications using DisMod-MR 2.1, with the exception of other maternal disorders which we estimated using a YLD-to-YLL ratio approach used in multiple causes across the GBD 2015. The reason is that most literature and surveillance data is expressed in terms of number of events per live birth rather than per population. Hospital and claims data, which was centrally processed for all GBD 2015 causes to have population as the denominator, was transformed to have live births as the denominator by dividing by age-specific fertility rate (ASFR; live births per population).

We used the datasets described above to estimate incidence ratio for each age-sex-location-year in the GBD 2015 location hierarchy using DisMod-MR 2.1. A number of study-level covariates were used to crosswalk from non-standard sub-populations or case definitions. For most conditions, Marketscan claims data was considered to be the closest approximation of the true incidence of complications so was identified as the reference category. A series of country covariates were then chosen to help drive the magnitude of estimates in areas of sparse or absent data. As mortality is likely at least partially related to the number of cases of a given complication, the natural log of the age-standardized death rate (ASDR) from our cause-specific mortality analysis was used in most of the models. No specific age or slope priors were used. All models were run with a time window of five years. The quantitative results of study-level and country-level covariates for each condition are shown below.

Abortion, ectopic pregnancy and miscarriage

Study-level covariate	Parameter	Geography level	beta	Exponentiated beta
All MarketScan, year 2000	Incidence	Global	-.0215 (-.0526 - -3.8e-04)	.9787 (.9488 - .9996)
All MarketScan, year 2010	Incidence	Global	-.1922 (-.229 - -.161)	.8252 (.7953 - .8513)
Ectopic pregnancies excluded	Incidence	Global	-.6488 (-.8244 - -.4019)	.5227 (.4385 - .669)

Maternal hemorrhage

Study-level covariate	Parameter	Geography level	beta	Exponentiated beta
postpartum hemorrhage only	Incidence	Global	-.0209<br>(-.0761 - -2.4e-04)	.9793<br>(.9267 - .9998)
All MarketScan, year 2012	Incidence	Global	1.068<br>(.6891 - 1.224)	2.909<br>(1.992 - 3.401)
Skilled Birth Attendance (proportion)	Incidence	Global	-.0105<br>(-.048 - -5.6e-05)	.9895<br>(.9531 - .9999)
Sociodemographic Status	Incidence	Global	-.2051<br>(-.2174 - -.2003)	.8146<br>(.8046 - .8185)
All MarketScan, year 2010	Incidence	Global	.9853<br>(.5841 - 1.14)	2.679<br>(1.793 - 3.127)
All MarketScan, year 2000	Incidence	Global	.937<br>(.5328 - 1.113)	2.552<br>(1.704 - 3.044)
Antepartum hemorrhage only	Incidence	Global	-1.459<br>(-1.648 - -1.275)	.2325<br>(.1924 - .2794)
Hospital Inpatient	Incidence	Global	1.228<br>(.8746 - 1.3)	3.416<br>(2.398 - 3.669)
Severe hemorrhage excluded	Incidence	Global	-.0978<br>(-.3343 - -.0037)	.9068<br>(.7158 - .9963)
Severe hemorrhage only	Incidence	Global	-.9551<br>(-1.171 - -.7601)	.3848<br>(.3101 - .4676)

Hypertensive disorders of pregnancy

Study-level covariate	Parameter	Geography level	beta	Exponentiated beta
Literature	Incidence	Global	-.2998<br>(-.3 - -.2998)	.7409<br>(.7408 - .741)
Diagnostic criteria for severe cases	Incidence	Global	-1.575<br>(-1.586 - -1.562)	.2069<br>(.2047 - .2097)
In-Facility Delivery (proportion)	Incidence	Global	-4.5e-05<br>(-2.7e-04 - -2.9e-05)	1<br>(.9997 - 1)
Hospital Inpatient	Incidence	Global	.9999<br>(.9997 - 1)	2.718<br>(2.717 - 2.718)

Eclampsia

Study-level covariate	Parameter	Geography level	beta	Exponentiated beta
Hospital Inpatient	Incidence	Global	1<br>(1 - 1)	2.718<br>(2.718 - 2.718)
Antenatal Care (4 visits) Coverage (proportion)	Incidence	Global	-2.1e-04<br>(-2.2e-04 - -1.4e-04)	.9998<br>(.9998 - .9999)

Obstructed labor and uterine rupture

Study-level covariate	Parameter	Geography level	beta	Exponentiated beta
Under Reported	Incidence	Global	-.0117 (-.0437 - -9.2e-04)	.9883 (.9573 - .9991)
Literature	Incidence	Global	-.0025 (-.0108 - -6.1e-05)	.9975 (.9893 - .9999)
Malnutrition (childhood underweight, proportion <2SD weight for age)	Incidence	Global	.1048 (.1 - .1178)	1.111 (1.105 - 1.125)

Maternal sepsis

Study-level covariate	Parameter	Geography level	beta	Exponentiated beta
All MarketScan, year 2010	Incidence	Global	.1295 (-.1445 - .5427)	1.138 (.8655 - 1.721)
LDI (I$ per capita)	Incidence	Global	-.0018 (-.0072 - -3.0e-05)	.9982 (.9928 - 1)
All MarketScan, year 2000	Incidence	Global	.0857 (-.1859 - .497)	1.089 (.8304 - 1.644)
Literature	Incidence	Global	-.0332 (-.1463 - -.0019)	.9673 (.8639 - .9981)
Antenatal Care (4 visits) Coverage (proportion)	Incidence	Global	-.009 (-.037 - -5.2e-04)	.9911 (.9637 - .9995)

Other maternal infections:

Study-level covariate	Parameter	Geography level	beta	Exponentiated beta
All MarketScan, year 2010	Incidence	Global	.1295 (-.1445 - .5427)	1.138 (.8655 - 1.721)
LDI (I$ per capita)	Incidence	Global	-.0018 (-.0072 - -3.0e-05)	.9982 (.9928 - 1)
All MarketScan, year 2000	Incidence	Global	.0857 (-.1859 - .497)	1.089 (.8304 - 1.644)
Literature	Incidence	Global	-.0332 (-.1463 - -.0019)	.9673 (.8639 - .9981)
Antenatal Care (4 visits) Coverage (proportion)	Incidence	Global	-.009 (-.037 - -5.2e-04)	.9911 (.9637 - .9995)
Hospital Inpatient	Incidence	Global	1.061 (.4314 - 1.999)	2.889 (1.539 - 7.382)
Under Reported	Incidence	Global	-.6689 (-1.463 - -.0209)	.5123 (.2315 - .9793)

Severity splits

After completion of DisMod-MR 2.1 models, all age-specific ratios were then converted to population rates by multiplying by ASFR. Maternal hemorrhage was split between moderate (500-<1000ml blood loss) and severe (>1000 blood loss) on the basis of a meta-analysis of 19 studies[1]. Data on the average duration of acute symptoms were not available so, after consultation with clinician collaborators, we assigned a duration of 7 days (+/-3) for moderate hemorrhage and 14 days (+/- 4) for severe hemorrhage. The total maternal hemorrhage incidence served as input to the causal attribution process of the overall anemia envelope, which is described separately. This is a change from GBD 2013, when only the prevalence of maternal hemorrhage was considered in anemia causal attribution which was inappropriate because the disability from hemorrhage-induced anemia is longer-lasting than the acute hemorrhagic event, a situation that was not previously reflected. Acute disability was calculated assuming incident cases of abortion, ectopic pregnancy, and miscarriage persist for an average of 3 days (+/-1) and obstructed labor was assigned a duration of 5 days (+/-2). Again, these determinations were based on clinical expert determination as we could not identify any data to inform this.

Hypertensive disorders of pregnancy (HDoP) and maternal sepsis and other maternal infections were estimated in two models each. HDoP YLD estimates included severe preeclampsia (proportion = 0.020, 0.0054-0.034) and other HDoP (proportion = 0.98, 0.944-0.9946) which were both derived from a single model of total HDoP. The proportions of each were based on analysis of GBD 2010 hospital data from Austria, Belgium, Ecuador, Spain, Israel, Italy, the Netherlands and USA. The duration of severe preeclampsia was assigned to be 7 days (+/-2) and other HDoP was assigned a duration of 3 months (2-4). Eclampsia was a separate model, assigned a duration of 1 day (+/-1). A large number of those with severe preeclampsia go on to have long term sequelae of the condition[2], as do those with eclampsia[3,4], both of which were included in the estimates of HDoP and were a bulk of the YLD for those conditions. Further disaggregation and characterization of the subtypes of HDoP, especially given their prominent role in our GBD 2015 maternal mortality estimates, will be a goal of future revisions of the GBD estimates. Maternal sepsis was assigned a duration of 5 days (+/-2) and, based on the same data identified in our review of pelvic inflammatory disease (PID; described separately), a proportion of incident cases were estimated to continue on to have secondary infertility due to maternal sepsis. Other maternal infections were assigned a wide potential duration of 15 to 45 days (mean 30).

Uncertainty and model selection

For all maternal disorders, uncertainty bounds include uncertainty due to input data, crosswalks from non-reference definitions, uncertainty in numerical solutions (posteriors) of each DisMod-MR 2.1 model, duration of symptoms, and proportion of all persons with each type of symptom.

In consultation with GBD researchers and collaborators, final models were selected on a combination of qualitative and quantitative goodness of fit to input data, plausibility of geographic and temporal trends, consistency of age pattern, and, when available, comparison with other published studies on the epidemiology of pregnancy complications. Directionality, magnitude, and plausibility of study-level and country-level covariates was also considered in the process of model development. Of note, due to the nature of statistical modeling, final results do not always cover the values reported in input data.

Obstetric fistula

Flowchart

Obstetric fistula

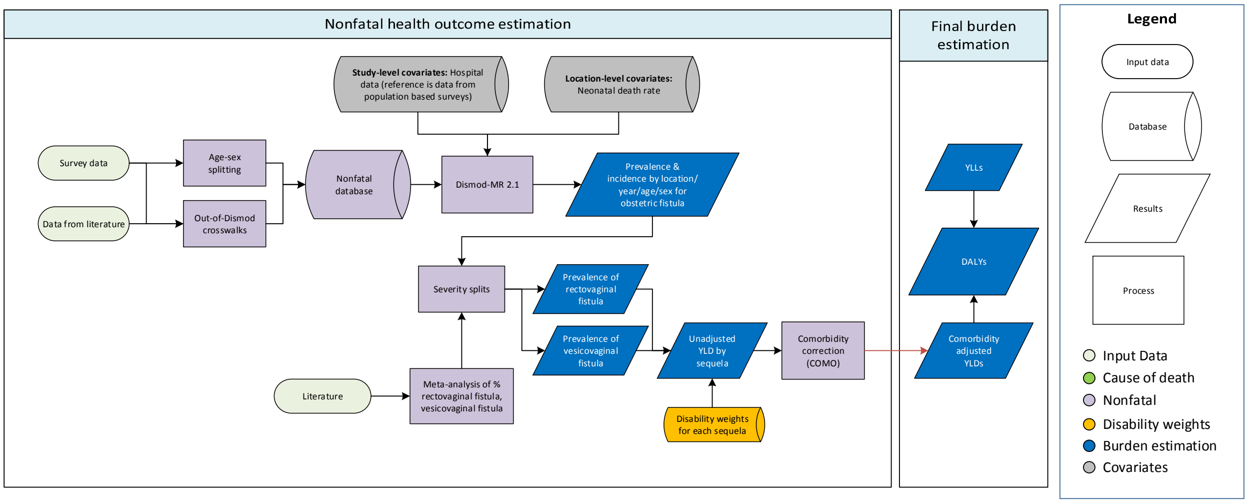

Input data and methodological summary

Case definition

Obstetric fistula is a severe long-term complication of prolonged obstructed labor in which a fistula (hole) develops between the birth canal and the bladder and/or rectum. This is often a consequence of obstructed labor. Please refer to Appendix Table 4 for information regarding ICD codes.

Input data

Model inputs

A systematic review was conducted for GBD 2015. The PubMed search terms were: (('obstetric fistula'[All Fields] OR 'vesicovaginal fistula'[All Fields]) OR 'rectovaginal fistula'[All Fields]) AND ('2013'[PDAT] : '2015'[PDAT]) AND 'humans'[MeSH Terms].

The exclusion criteria were:

1. Studies that did not provide primary data on epidemiological parameters, e.g., commentaries
2. Case series
3. Reviews

The table below shows the number of literature studies included in GBD 2015, as well as the number of countries or subnational units and GBD world regions represented.

Table 1. Literature and geographic representation

	Prevalence	Incidence	Mortality risk
Studies	8	3	-
Countries/subnationals	8	3	-
GBD world regions	3	1	-

In addition to using data from published studies, we also included data from UNFPA reports and nationally representative Demographic and Health Surveys and Multiple Indicator Cluster Surveys.

Severity split & disability weight

The following severity distributions were assigned based a meta-analysis of published studies[1-4] and Pakistan Demographic and Health survey (2006-2007): vesicovaginal fistula (90.8%, 95% CI: 85.0 to 95.4%); rectovaginal fistula (9.2%, 95% CI: 4.6 to 15.0%). The lay descriptions and disability weights for severity levels derived from the GBD Disability Weights study are shown below.

Table 2. Severity level, lay description, and DWs

Severity level	Lay description	DW (95% CI)
Vesicovaginal fistula	Has an abnormal opening between the bladder and the vagina, which makes her unable to control urinating. The woman is anxious and depressed.	0.342 (0.227-0.478)
Rectovaginal fistula	Has an abnormal opening between her vagina and rectum causing flatulence and feces to escape through the vagina. The person gets infections in her vagina, and has pain when urinating.	0.501 (0.339-0.657)

Modeling strategy

Obstetric fistula was modeled using DisMod-MR 2.1. We used neonatal mortality rate as a country-level covariate. We also included a study-level covariate indicating whether it was a hospital-based or community-based study. Remission was calculated, using the cure data from 11 Demographic and Health surveys, by dividing the number of cured obstetric fistula cases by total person-years of follow-up of all cases (cured, uncured, and untreated). The person-year of follow-up for uncured or untreated fistula cases was calculated as the time interval (in years) between the last birth and the date of interview. For cured cases, we assumed that the person-year of follow-up was half the time interval (in years) between the last birth and the date of interview.

Betas and exponentiated values (which can be interpreted as an odds ratio) are shown in the table below.

Table 3. Beta and exponentiated values

Covariate	Parameter	Beta (95% CI)	Exponentiated beta (95% CI)

Neonatal mortality rate	Prevalence	1.93 (1.71 — 2.00)	6.88 (5.55 — 7.37)
Neonatal mortality rate	Incidence	0.38 (0.021 — 1.01)	1.46 (1.02 — 2.73)
Neonatal mortality rate	Remission	-0.67 (-0.98 — -0.18)	0.51 (0.37 — 0.83)
Hospital data	Prevalence	-1.76 (-1.99 — -1.33)	0.17 (0.14 — 0.26)
Hospital data	Incidence	-0.89 (-1.78 — 0.057)	0.41 (0.17 — 1.06)

No other significant changes were made to the modeling strategy in GBD 2015.

Neonatal preterm birth complications

Flowchart

Neonatal preterm birth

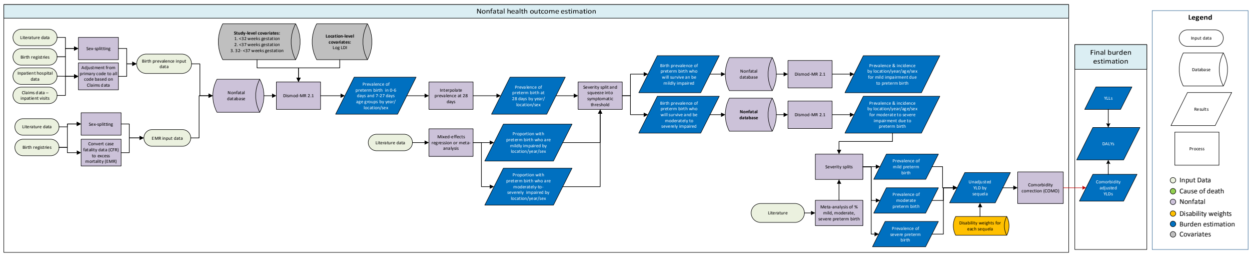

Input data and methodological summary

Case definition

Preterm birth is defined as live birth before 37 completed weeks of gestation. In our analysis, we further break down this cause into three sub-categories of preterm birth, based on gestational age: extremely preterm (<28 weeks), very preterm (28 to <32 weeks), and moderate to late preterm (32 to <37 weeks). These categories are based on the World Health Organization (WHO) definition of preterm birth.[1] Please refer to Appendix Table 4 for information about the ICD codes.

Input data

Model inputs

A systematic review was completed for GBD 2010, and for each following GBD iteration a review was conducted on literature published since the previous addition. Thus, for GBD 2015, a literature review was conducted for the period 2012 – 2015. The PubMed database was searched using the following search string: ((("Infant, Premature"[Mesh] OR ("infant"[All Fields] AND "premature"[All Fields]) OR "premature infant"[All Fields] OR ("preterm"[All Fields] AND "infant"[All Fields]) OR "preterm infant"[All Fields] OR ("infant, newborn"[MeSH Terms] OR ("infant"[All Fields] AND "newborn"[All Fields]) OR "newborn infant"[All Fields] OR ("newborn"[All Fields] AND "infant"[All Fields])) AND (premature[All Fields] OR preterm[All Fields]) OR "premature birth"[MeSH Terms] OR ("premature"[All Fields] AND "birth"[All Fields]) OR "premature birth"[All Fields] OR ("preterm"[All Fields] AND "birth"[All Fields]) OR "preterm birth"[All Fields]) ((("Infant, Premature"[Mesh] OR ("infant"[All Fields] AND "premature"[All Fields]) OR "premature infant"[All Fields] OR ("preterm"[All Fields] AND "infant"[All Fields]) OR "preterm infant"[All Fields] OR ("infant, newborn"[MeSH Terms] OR ("infant"[All Fields] AND "newborn"[All Fields]) OR "newborn infant"[All Fields] OR ("newborn"[All Fields] AND "infant"[All Fields])) AND (premature[All Fields] OR preterm[All Fields]) OR "premature birth"[MeSH Terms] OR ("premature"[All Fields] AND "birth"[All Fields]) OR "premature birth"[All Fields] OR ("preterm"[All Fields] AND "birth"[All Fields]) OR "preterm birth"[All Fields]) AND ("2012"[PDAT] : "3000"[PDAT]) AND "humans"[MeSH Terms].

[1] http://www.who.int/mediacentre/factsheets/fs363/en/

The exclusion criteria were:

1. Studies that did not provide primary data on epidemiological parameters, e.g., a commentary piece
2. Non-representative studies (e.g., only high-risk pregnancies)
3. Reviews

The table below shows the number of literature studies included in GBD 2015, as well as the number of countries or subnational units and GBD world regions represented.

Table 1a-c. Data geographies

1557

	Birth prevalence	Case fatality
Studies	218	105
Countries/subnationals	125	45
GBD world regions	15	16

1558

	Birth prevalence	Case fatality
Studies	225	99
Countries/subnationals	131	44
GBD world regions	17	15

1559

	Birth prevalence	Case fatality
Studies	85	95
Countries/subnationals	83	84
GBD world regions	14	10

In addition to literature data, data from US claims data for 2000, 2010, and 2012 by US state were included. Hospital data from 84 additional locations were used to inform estimates for extremely preterm infants, while hospital data was available for 14 additional locations for the next two oldest gestational age categories. Only inpatient data was included from these datasets, because we believed it would be more representative of the true prevalence of preterm birth than outpatient data: preterm infants in the countries from which hospital data was available are almost sure to be admitted to the hospital, whereas outpatient data is more likely to capture repeated visits by the same child as they grow.

Severity splits and disability weights

The basis of the GBD disability weight (DW) survey assessments are lay descriptions of sequelae highlighting major functional consequences of symptoms. The lay descriptions and disability weights for preterm birth are shown below. Further severity levels are calculated by combining several of these disability weights, e.g., moderate motor plus cognitive impairment with blindness.

Table 2. Severity, lay description, and DWs

Severity level	Lay description	DW (95% CI)
Motor plus cognitive impairments, mild	has some difficulty in moving around but is able to walk without help. The person is slow in learning at school. As an adult, the person has some difficulty doing complex or unfamiliar tasks but otherwise functions independently.	0.031 (0.018-0.05)
Motor impairment, mild	has some difficulty in moving around but is able to walk without help.	0.01 (0.005-0.019)
Motor impairment, moderate	has some difficulty in moving around, and difficulty in lifting and holding objects, dressing and sitting upright, but is able to walk without help.	0.061 (0.04-0.089)
Motor impairment, severe	is unable to move around without help, and is not able to lift or hold objects, get dressed or sit upright.	0.402 (0.268-0.545)
Distance vision, mild impairment	has some difficulty with distance vision, for example reading signs, but no other problems with eyesight.	0.003 (0.001-0.007)
Distance vision, moderate impairment	has vision problems that make it difficult to recognize faces or objects across a room.	0.031 (0.019-0.049)
Distance vision, severe impairment	has severe vision loss, which causes difficulty in daily activities, some emotional impact (for example worry), and some difficulty going outside the home without assistance.	0.184 (0.125-0.259)
Distance vision blindness	is completely blind, which causes great difficulty in some daily activities, worry and anxiety, and great difficulty going outside the home without assistance.	0.187 (0.124-0.26)

To determine the proportion of people within each of these severity levels, one study informed moderate-to-severe impairment splits, and for mild impairments cases were divided equally into both categories.[2]

Modeling strategy

Burden from each of the gestational age categories of preterm birth (extreme, very, and moderate to late) is modeled separately, using similar methods.

For a given gestational age group, an initial DisMod MR 2.1 model is run using prevalence and case fatality data. Prior to input into DisMod, case fatality ratio (CFR) data is transformed into excess mortality rate (EMR) space using the formula:

$$EMR = -\frac{\ln(1-CFR)}{\frac{28}{365.25}}$$

which is analogous to the transformation of cumulative incidence to incidence rate. The denominator in this equation is derived from the definition of our CFR parameter, which is death in the first 28 days of life. The output from this first-step DisMod model is prevalence in the first two neonatal age groups. For this model, remission and incidence are both set to zero, as no one can be born prematurely after birth, and no one can cease to have been born premature after the fact. Study covariates were created for hospital data, US claims data, and literature sources that use non-standard gestational age categories.

Using prevalence in the first two neonatal age groups, prevalence at 28 days is calculated via interpolation. Functionally, this is birth prevalence minus all those who have died in the first 28 days, our case fatality parameter.

[2] Hagberg et al. Acta Paediatrica 1996, 85:954-60

Next, using mild impairment proportion and moderate-to-severe impairment proportion data, we ran either mixed-effect hierarchical regressions or meta-analyses to generate country-year-sex-specific estimates of both parameters (we used a meta-analysis when there was not sufficient data to support a regression model). We then calculated the birth prevalence of each severity level. As mild impairment and moderate-severe impairment were calculated separately, it was possible that they could sum to a value greater than one. To address this, we checked the sum of the two values in any of the 1,000 iterations of the uncertainty analysis, and if greater than 0.9, proportionately rescaled both estimates to sum to 0.9 in any of the 1,000 iterations of the uncertainty analysis (we picked 0.9 rather than 1 to allow at least some probability of a child having no impairment). We then proceeded with the calculation of the birth prevalence of impairment:

Mild impairment birth prevalence = prevalence at 28 days * proportion mild impairment

Moderate/severe impairment birth prevalence = prevalence at 28 days * proportion mod-severe impairment

For moderate/severe impairment, results of these latter calculations were then combined with excess mortality estimates derived from the standard mortality ratios (SMR) of cerebral palsy and used as inputs into a second DisMod MR 2.1 model. For this model, remission and incidence were also set to zero. For mild impairment, we assumed no excess mortality and no remission, and as such simply applied the birth prevalence of mild impairment to every age group.

No other significant changes were made to the modeling strategy for GBD 2015.

Neonatal encephalopathy due to birth asphyxia and trauma

Flowchart

Neonatal encephalopathy due to birth asphyxia and trauma

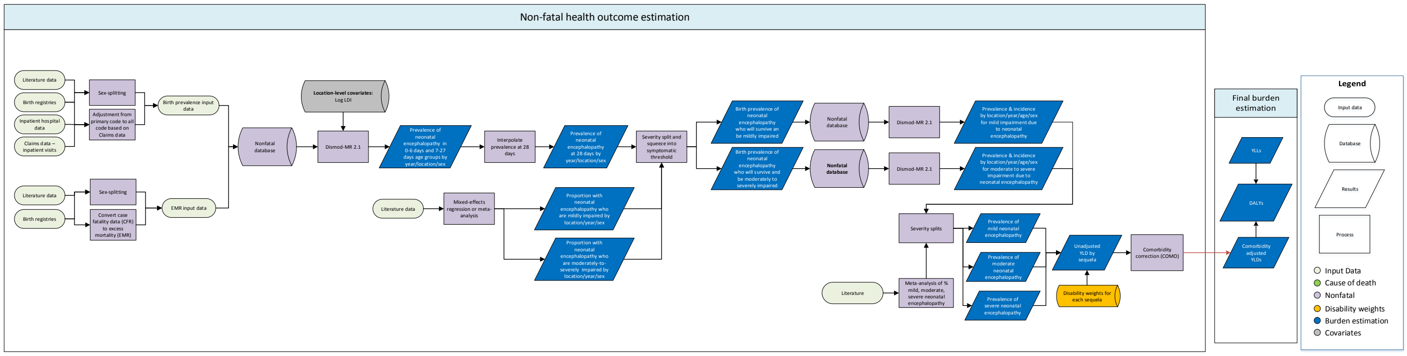

Input data and methodological summary

Case definition

Neonatal encephalopathy (NE) due to birth asphyxia and birth trauma, also called hypoxic-ischemic encephalopathy, is defined as injury to the brain in the first few moments or days of life in an infant born at term. NE has multiple etiologies, and is defined more by its symptoms – abnormal neurological function, including reduced level of consciousness, seizures, depression of tone and reflexes, or difficulty maintaining respiration – than its origin. NE can occur when an infant is deprived of oxygen during delivery or sustains physical trauma to the head, among other causes. ICD codes are listed in Appendix Table 4.

Input data

Model inputs

A systematic review was completed for GBD 2010, and for each following GBD iteration a review was conducted on literature published since the previous addition. Thus, for GBD 2015, a literature review was conducted for the period 2012 – 2015. The PubMed database was searched using the following search string: ((("infant"[Title/Abstract] OR "newborn"[Title/Abstract] OR "newborn infant"[Title/Abstract]) AND ("encephalopathy"[Title/Abstract] OR "neonatal encephalopathy"[Title/Abstract] OR "perinatal asphyxia"[Title/Abstract] OR "asphyxia neonatorum"[Title/Abstract] OR "newborn encephalopathy"[Title/Abstract] OR "hypoxic ischaemic encephalopathy"[Title/Abstract] OR ("birth trauma"[Title/Abstract] AND "birth asphyxia"[Title/Abstract]))) AND ("2012"[PDAT] : "3000"[PDAT]) AND "humans"[MeSH Terms])

The exclusion criteria were:

1. Studies that did not provide primary data on epidemiological parameters, e.g., a commentary piece
2. Non-representative studies (e.g., only high-risk pregnancies)
3. Reviews

The table below shows the number of literature studies included in GBD 2015, as well as the number of countries or subnational units and GBD world regions represented.

Table 1. Geographic representation

	Birth prevalence	Case fatality
Studies	38	35
Countries/subnationals	27	25
GBD world regions	13	12

In addition to literature data, data from US claims data for 2000, 2010, and 2012 by US state were included. Hospital data from 84 additional locations were used to inform estimates for extremely preterm infants, while hospital data was available for 14 additional locations for the next two oldest gestational age categories. Only inpatient data was included from these datasets, because we believed it would be more representative of the true prevalence of preterm birth than outpatient data: preterm infants in the countries from which hospital data was available are almost sure to be admitted to the hospital, whereas outpatient data is more likely to capture repeated visits by the same child as they grow.

Severity splits

The basis of the GBD disability weight (DW) survey assessments are lay descriptions of sequelae highlighting major functional consequences of symptoms. The lay descriptions and disability weights for preterm birth are shown below.

Table 2. Severity level, lay description and DWs

Severity level	Lay description	DW (95% CI)
Motor plus cognitive impairments, mild	has some difficulty in moving around but is able to walk without help. The person is slow in learning at school. As an adult, the person has some difficulty doing complex or unfamiliar tasks but otherwise functions independently.	0.031 (0.018-0.05)
Motor impairment, mild	has some difficulty in moving around but is able to walk without help.	0.01 (0.005-0.019)
Motor impairment, moderate	has some difficulty in moving around, and difficulty in lifting and holding objects, dressing and sitting upright, but is able to walk without help.	0.061 (0.04-0.089)
Motor impairment, severe	is unable to move around without help, and is not able to lift or hold objects, get dressed or sit upright.	0.402 (0.268-0.545)
Distance vision, mild impairment	has some difficulty with distance vision, for example reading signs, but no other problems with eyesight.	0.003 (0.001-0.007)
Distance vision, moderate impairment	has vision problems that make it difficult to recognize faces or objects across a room.	0.031 (0.019-0.049)
Distance vision, severe impairment	has severe vision loss, which causes difficulty in daily activities, some emotional impact (for example worry), and some difficulty going outside the home without assistance.	0.184 (0.125-0.259)
Distance vision blindness	is completely blind, which causes great difficulty in some daily activities, worry and anxiety, and great difficulty going outside the home without assistance.	0.187 (0.124-0.26)

To determine the proportion of people within each of these severity levels, one study informed moderate-to-severe impairment splits, and for mild impairments cases were divided equally into both categories.[1]

Modeling strategy

An initial DisMod MR 2.1 model is run using prevalence and case fatality data. Prior to input into DisMod, case fatality ratio (CFR) data is transformed into excess mortality rate (EMR) space using the formula $EMR = -\frac{\ln(1-CFR)}{\frac{28}{365.25}}$, which is analogous to the transformation of cumulative incidence to incidence rate. The denominator in this equation is derived from the definition of our CFR parameter, which is death in the first 28 days of life. The output from this first-step DisMod model is prevalence in the first two neonatal age groups. For this model, remission and incidence are both set to zero, as no one can be born prematurely after birth, and no one can cease to have been born premature after the fact. Study covariates were created for hospital data, US claims data, and literature sources that use non-standard gestational age categories.

Using prevalence in the first two neonatal age groups, prevalence at 28 days is calculated via interpolation. Functionally, this is birth prevalence minus all those who have died in the first 28 days, our case fatality parameter.

Next, using mild impairment proportion and moderate-to-severe impairment proportion data, we ran either mixed-effect hierarchical regressions or meta-analyses to generate country-year-sex-specific estimates of both parameters (we used a meta-analysis when there was not sufficient data to support a regression model). We then calculated the birth prevalence of each severity level. As mild impairment and moderate-severe impairment were calculated separately, it was possible that they could sum to a value greater than one. To address this, we checked the sum of the two values in any of the 1,000 iterations of the uncertainty analysis, and if greater than 0.9, proportionately rescaled both estimates to sum to 0.9 in any of the 1,000 iterations of the uncertainty analysis (we picked 0.9 rather than 1 to allow at least some probability of a child having no impairment). We then proceeded with the calculation of the birth prevalence of impairment:

Mild impairment birth prevalence = prevalence at 28 days * proportion mild impairment

Moderate/severe impairment birth prevalence = prevalence at 28 days * proportion mod-severe impairment

For moderate/severe impairment, results of these latter calculations were then combined with excess mortality estimates derived from the standard mortality ratios (SMR) of cerebral palsy and used as inputs into a second DIsMod MR 2.1 model. For this model, remission and incidence were also set to zero. For mild impairment, we assumed no excess mortality and no remission, and as such simply applied the birth prevalence of mild impairment to every age group.

[1] Badawi et al Developmental Medicine & Child Neurology, 2005, 47:293-8

No other significant changes were made to the GBD 2015 modeling process.

Neonatal sepsis and other neonatal infections

Flowchart

Neonatal sepsis and other neonatal infections

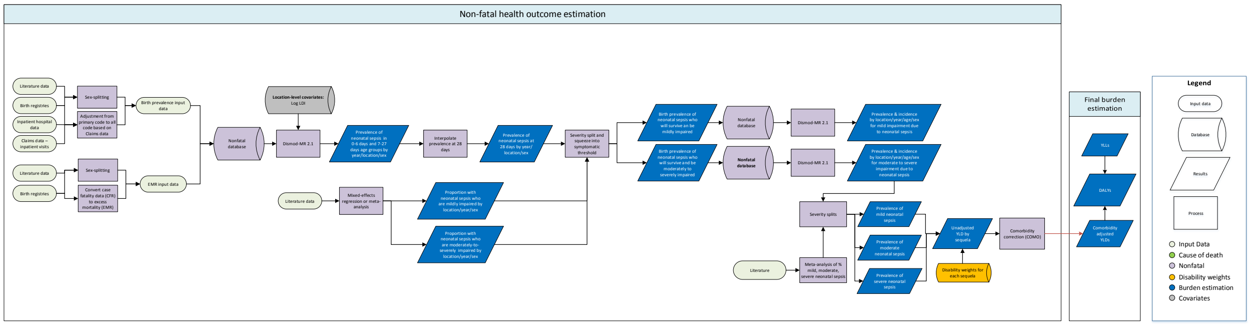

Input data and methodological summary

Case definition

Neonatal sepsis and other neonatal infections are infections during the neonatal period that advance to a systemic blood stream infection, the underlying cause of which can be meningitis, gastroenteritis, or other etiologies. Neonatal pneumonia, however, is not included – it is captured in our modeling of pneumonia as a separate entity. ICD codes associated with these causes include:

Input data

Model inputs

A systematic review was completed for GBD 2010, and for each following GBD iteration a review was conducted on literature published since the previous addition. Thus, for GBD 2015, a literature review was conducted for the period 2012 – 2015. The PubMed database was searched using the following search string: (("infant"[Title/Abstract] OR "newborn"[Title/Abstract] OR "newborn infant"[Title/Abstract]) AND ("neonatal sepsis"[All Fields] OR "neonatal septicaemia"[All Fields] OR "neonatal meningitis"[All Fields] OR "early sepsis"[All Fields] OR "early septicaemia"[All Fields] OR "tetanus"[All Fields] OR "meningitis"[All Fields] OR "sepsis"[All Fields])) AND ("2012"[PDAT] : "3000"[PDAT]) AND "humans"[MeSH Terms]

The exclusion criteria were:

1. Studies that did not provide primary data on epidemiological parameters, e.g., a commentary piece
2. Non-representative studies (e.g., only high-risk pregnancies)
3. Reviews

The table below shows the number of literature studies included in GBD 2015, as well as the number of countries or subnational units and GBD world regions represented.

Table 1. Geographic representation

	Birth prevalence	Case fatality
Studies	2	15
Countries/subnationals	2	15
GBD world regions	2	10

In addition to literature data, data from US claims data for 2000, 2010, and 2012 by US state were included. Hospital data from 84 additional locations were used to inform estimates for extremely preterm infants, while hospital data was available for 14 additional locations for the next two oldest gestational age categories. Only inpatient data was included from these datasets, because we believed it would be more representative of the true prevalence of preterm birth than outpatient data: preterm infants in the countries from which hospital data was available are almost sure to be admitted to the hospital, whereas outpatient data is more likely to capture repeated visits by the same child as they grow.

Severity splits

The basis of the GBD disability weight (DW) survey assessments are lay descriptions of sequelae highlighting major functional consequences of symptoms. The lay descriptions and disability weights for preterm birth are shown below.

Table 2. Severity level, lay description and DWs

Severity level	Lay description	DW (95% CI)
Motor plus cognitive impairments, mild	has some difficulty in moving around but is able to walk without help. The person is slow in learning at school. As an adult, the person has some difficulty doing complex or unfamiliar tasks but otherwise functions independently.	0.031 (0.018-0.05)
Motor impairment, mild	has some difficulty in moving around but is able to walk without help.	0.01 (0.005-0.019)
Motor impairment, moderate	has some difficulty in moving around, and difficulty in lifting and holding objects, dressing and sitting upright, but is able to walk without help.	0.061 (0.04-0.089)
Motor impairment, severe	is unable to move around without help, and is not able to lift or hold objects, get dressed or sit upright.	0.402 (0.268-0.545)
Distance vision, mild impairment	has some difficulty with distance vision, for example reading signs, but no other problems with eyesight.	0.003 (0.001-0.007)
Distance vision, moderate impairment	has vision problems that make it difficult to recognize faces or objects across a room.	0.031 (0.019-0.049)
Distance vision, severe impairment	has severe vision loss, which causes difficulty in daily activities, some emotional impact (for example worry), and some difficulty going outside the home without assistance.	0.184 (0.125-0.259)
Distance vision blindness	is completely blind, which causes great difficulty in some daily activities, worry and anxiety, and great difficulty going outside the home without assistance.	0.187 (0.124-0.26)

To determine the proportion of people within each of these severity levels, one study informed moderate-to-severe impairment splits, and for mild impairments cases were divided equally into both categories.[1]

Modeling strategy

The methodology for sepsis changed for GBD 2015 compared to what had been done in previous

[1] Badawi et al Developmental Medicine & Child Neurology, 2005, 47:293-8

iterations. It has been incorporated into the modeling framework for preterm birth and neonatal encephalopathy. This change was facilitated by the addition of hospital and US claims data and is advantageous because it allows us to model long-term disability from neonatal sepsis, whereas before we only described the disability associated with an acute infectious episode. The new methodology is outlined below.

An initial DisMod MR 2.1 model is run using prevalence and case fatality data. Prior to input into DisMod, case fatality ratio (CFR) data is transformed into excess mortality rate (EMR) space using the formula:

$$EMR = -\frac{\ln(1 - CFR)}{\frac{28}{365.25}}$$

which is analogous to the transformation of cumulative incidence to incidence rate. The denominator in this equation is derived from the definition of our CFR parameter, which is death in the first 28 days of life. The output from this first-step DisMod model is prevalence in the first two neonatal age groups. For this model, remission and incidence are both set to zero, as no one can be born prematurely after birth, and no one can cease to have been born premature after the fact. Study covariates were created for hospital data, US claims data, and literature sources that use non-standard gestational age categories.

Using prevalence in the first two neonatal age groups, prevalence at 28 days is calculated via interpolation. Functionally, this is birth prevalence minus all those who have died in the first 28 days, our case fatality parameter.

Next, using mild impairment proportion and moderate-to-severe impairment proportion data, we ran either mixed-effect hierarchical regressions or meta-analyses to generate country-year-sex-specific estimates of both parameters (we used a meta-analysis when there was not sufficient data to support a regression model). We then calculated the birth prevalence of each severity level. As mild impairment and moderate-severe impairment were calculated separately, it was possible that they could sum to a value greater than one. To address this, we checked the sum of the two values in any of the 1,000 iterations of the uncertainty analysis, and if greater than 0.9, proportionately rescaled both estimates to sum to 0.9 in any of the 1,000 iterations of the uncertainty analysis (we picked 0.9 rather than 1 to allow at least some probability of a child having no impairment). We then proceeded with the calculation of the birth prevalence of impairment:

Mild impairment birth prevalence = prevalence at 28 days * proportion mild impairment

Moderate/severe impairment birth prevalence = prevalence at 28 days * proportion mod-severe impairment

For moderate/severe impairment, results of these latter calculations were then combined with excess mortality estimates derived from the standard mortality ratios (SMR) of cerebral palsy and used as inputs into a second DisMod MR 2.1 model. For this model, remission and incidence were also set to zero. For mild impairment, we assumed no excess mortality and no remission, and as such simply applied the birth prevalence of mild impairment to every age group.

Hemolytic disease and other neonatal jaundice

Flowchart

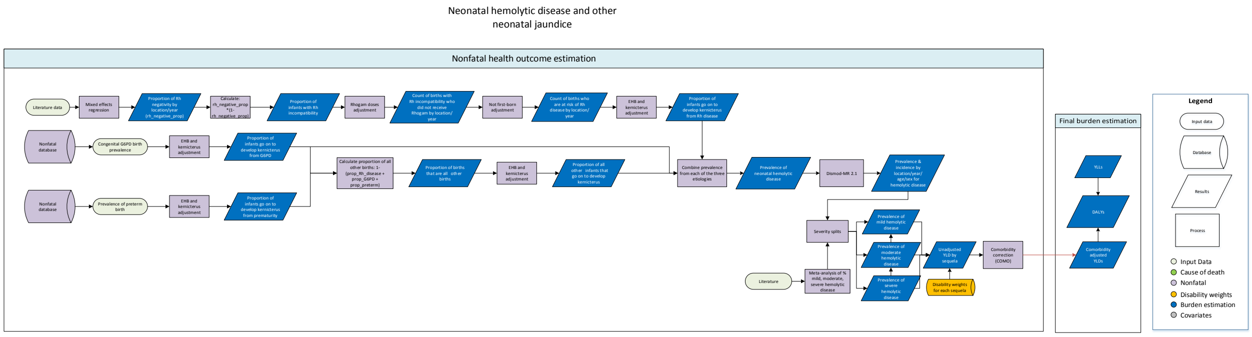

Case definition

Hemolytic disease of the newborn and other neonatal jaundice refers to several etiologies by which an infant develops extreme hyperbilirunemia (EHB) and can then go on to develop kernicterus. The etiologies that we model for GBD are EHB from Rhesus (Rh) disease, preterm birth, glucose-6-phosphate dehydrogenase deficiency (G6PD), and other causes.

Input data

Model inputs

A systematic review was completed for GBD 2010, and for each following GBD iteration a review was conducted on literature published since the previous addition. Thus, for GBD 2015, a literature review was conducted for the period 2012 – 2015. The PubMed database was searched using the following search string: (("infant"[Title/Abstract] OR "newborn"[Title/Abstract] OR "newborn infant"[Title/Abstract]) AND ("haemolytic"[All Fields] OR "hyperbilirubinemia"[All Fields] OR "jaundice"[All Fields] OR "glucose-6-phosphate dehydrogenase deficiency"[All Fields] OR "G6PD deficiency"[All Fields] OR "hyperbilirubinemia"[All Fields] OR "EHB"[All Fields] OR "phototherapy"[All Fields] OR "ABO incompatibility"[All Fields] OR "RH incompatibility"[All Fields] OR "rh blood group system"[All Fields] OR "Rhesus"[All Fields] OR "Rhesus disease"[All Fields] OR "erythroblastosis fetalis"[All Fields] OR "kernicterus"[All Fields])) AND ("2012"[PDAT] : "3000"[PDAT]) Sort by: PublicationDate

The exclusion criteria were:

1. Studies that did not provide primary data on epidemiological parameters, e.g., a commentary piece
2. Non-representative studies (e.g., only high-risk pregnancies)
3. Reviews

For hemolytic disease, much of the input data comes from other GBD models, which are described in detail in the “modeling strategy” section. However, the modeling process for EHB from Rh disease involves literature data for several parameters, the breadth of which are described in the tables below:

Table 1a. Rh negativity

	Prevalence
Studies	54
Countries/subnationals	46
GBD world regions	13

Table 1b. Children who are not first-born

	Prevalence
Studies	82
Countries/subnationals	81
GBD world regions	14

**Please note that US claims data and hospital data were not included in the hemolytic disease modeling process because they are not coded separately by etiology. They could not be slotted into the existing modeling framework.

Severity splits & disability weights

The basis of the GBD disability weight (DW) survey assessments are lay descriptions of sequelae highlighting major functional consequences and symptoms. The lay descriptions and disability weights for hemolytic disease and other neonatal jaundice levels are shown below.

Table 2. Severity, lay description and DWs

Severity level	Lay description	DW (95% CI)
Motor impairment, moderate	has some difficulty in moving around, and difficulty in lifting and holding objects, dressing and sitting upright, but is able to walk without help.	0.061 (0.04-0.089)
Motor impairment, severe	is unable to move around without help, and is not able to lift or hold objects, get dressed or sit upright.	0.402 (0.268-0.545)
Distance vision, mild impairment	has some difficulty with distance vision, for example reading signs, but no other problems with eyesight.	0.003 (0.001-0.007)
Distance vision, moderate impairment	has vision problems that make it difficult to recognize faces or objects across a room.	0.031 (0.019-0.049)
Distance vision, severe impairment	has severe vision loss, which causes difficulty in daily activities, some emotional impact (for example, worry), and some difficulty going outside the home without assistance.	0.184 (0.125-0.259)
Distance vision blindness	is completely blind, which causes great difficulty in some daily activities, worry, and anxiety, and great difficulty going outside the home without assistance.	0.187 (0.124-0.26)

To determine the proportion of people within each of these severity levels, one study informed moderate-to-severe impairment splits, and for mild impairments cases were divided equally into both categories.[1]

[1] Badawi et al. Developmental Medicine & Child Neurology, 2005, 47:293-8

Modeling strategy

The modeling strategy can be described as two main steps. For each of the four etiologies (Rh disease, G6PD deficiency, preterm birth, and other causes) we estimated the prevalence of extreme hyperbilirubinemia (EHB or bilirubin>25 or exchange transfusion). Then, we multiplied this prevalence by an estimated proportion of EHB cases who go on to develop kernicterus. We used development of kernicterus as our criterion for incidence of long-term moderate/severe impairment.

Rh Disease

We began with data on the prevalence of Rh negativity in the population, the number of Rhogam (Rh_0 Immune Globulin) doses distributed to countries in 2010, and the proportion of children who are not firstborn. Mixed effect regressions were run on Rh negativity and birth order greater than one to generate estimates of these values for every country-year. We made the assumptions that the proportion of Rhogam doses to Rh-incompatible children stayed constant over time, and that countries with NMR<5 had complete Rhogam coverage. This yields the following equation for the prevalence of EHB in countries with NMR>5:

EHB Prevalence = Rh negative prevalence * (1 – Rh negative prevalence) * (2010 Rhogam doses / 2010 Rh incompatible babies) * (not-firstborn prevalence) * 0.15

The 0.15 multiplier represents results of previous calculations[5] showing the proportion of women developing Rh isoimmunization with a risk for anti-Rh antibodies complicating subsequent pregnancies.

Finally, to generate estimates of kernicterus prevalence, we multiplied the prevalence of EHB by 0.0072 (0.0038, 0.112) -- the proportion of children with EHB who develop kernicterus (extracted from previously published values).[6,7]

G6PD, Preterm, and Other

The other three pathways of kernicterus modeling simply involve multiplication by scalars. For each of these, given a complete set of prevalence estimates from other GBD models, we multiplied by a scalar representing the proportion of children who are expected to develop EHB (see the table below for the values of these scalars). We then adjusted that estimate upward by a factor of 2.45 (1.44, 4.16) for countries in which the NMR is greater than 15, a value utilized in previous publications[4] to reflect heightened risk in those countries where access to phototherapy for prevention of EHB is not standard. Finally, these EHB prevalence values were multiplied by a set of NMR-dependent scalars representing the proportion of EHB cases that go on to develop kernicterus. See table below, for those values.

Table 3. EHB proportion and CIs

Cause	Source of birth prevalence estimates	EHB proportion	95% CI
G6PD	Congenital DisMod model, GBD 2013.	0.0013	(0.00085, 0.002)
Preterm birth complications	Custom modeling for preterm conditions,	0.00045	(0.00029, 0.0007)

Other	discussed elsewhere in this document (summed over all gestational ages). Global births – (Rh disease birth prevalence + preterm complications birth prevalence + G6PD birth prevalence)	0.00038	(0.00033, 0.00163)

Table 4. EHB proportions used in G6PD, preterm, and other estimates.

NMR	Kernicterus proportion	95% CI
<5	0.23	(0.099, 0.361)
5-15	0.35	(0.12, 0.58)
>=15	0.438	(0.255, 0.621)

Table 5: NMR-dependent kernicterus proportions applied to G6PD, preterm, and other EHB estimates.

Final Kernicterus Prevalence

We estimated preterm kernicterus in order to arrive at a proper value for the number of “other” children at risk of kernicterus but we do not actually include our preterm estimates in our measures of kernicterus since we assume that its disability is captured in our preterm models. Thus, we generate our final birth prevalence of kernicterus by:

Kernicterus birth prevalence = (kernicterus prevalence from Rh disease) + (kernicterus prevalence from G6PD) + (kernicterus prevalence from other disorders).

These estimates, along with estimates of excess mortality associated with kernicterus, are then used as inputs into our disease modeling tool (DisMod) to generate estimates of moderate to severe impairment for all ages.

No other significant changes were made to the GBD 2015 estimation process.

Protein-energy malnutrition

Flowchart

Protein energy malnutrition (PEM)

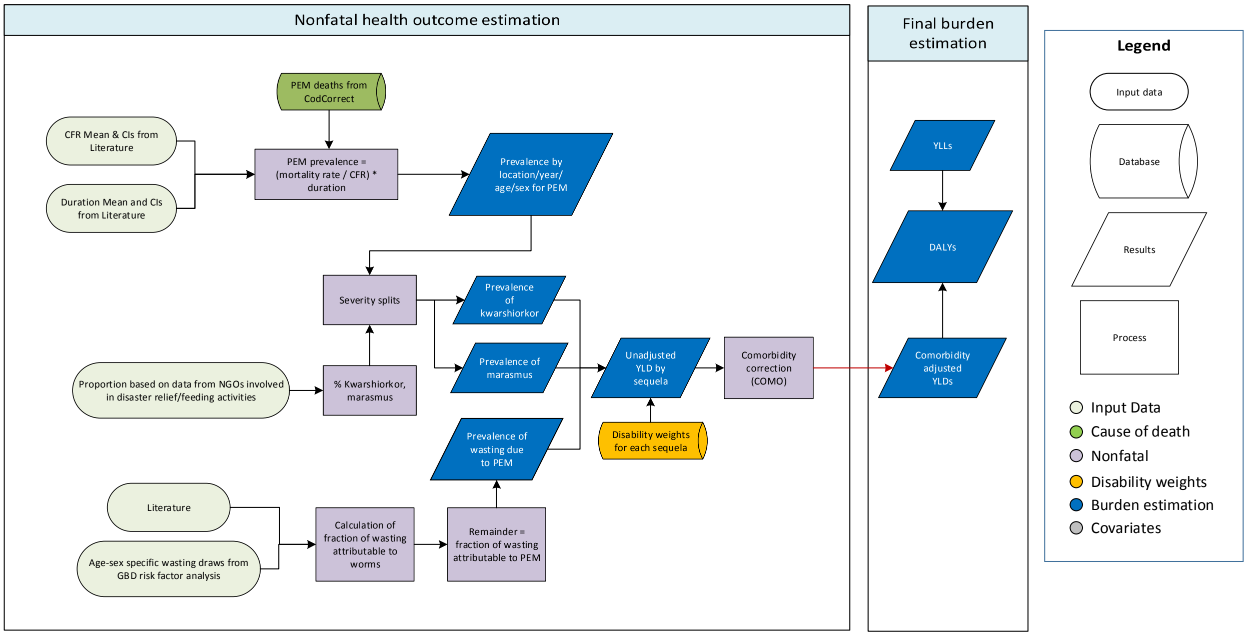

Input data and methodological summary

Case Definition

Our assessment of non-fatal protein energy malnutrition (PEM) includes the quantification of morbidity specific to the following sequelae of disease: kwashiorkor, marasmus, and wasting attributable to PEM. For PEM, ICD 10 codes are E40-E46.9, E64.0, and ICD 9 codes are 260-263.9. These are further detailed in Appendix Table 4.

Input data

Model inputs

For GBD 2015, the data for this analysis come from IHME cause-specific mortality estimates for PEM. The case-fatality rate (CFR) data come from the study by Lapidus et al (2006).[1] The duration estimate comes from the study by Isanaka et al (2011).[2]

Severity splits

We applied disability weights from the GBD disability weight survey to the following distribution for children under age 1 year: 96.6% for marasmus and 3.4% kwashiorkor. For age 1-4 years, we applied the

following distributions: 92.6% for marasmus and 7.4% kwashiorkor. We determined these distributions by calculating the prevalence of marasmus versus kwashiorkor using individual record data on nearly 400,000 children from 34 countries, bundling data from various NGOs involved in disaster relief/feeding activities. This database was provided to us by Valid Nutrition for GBD 2010.

The lay descriptions and disability weights for severity levels derived from the GBD Disability Weights study are shown below.

Table 1. Severity, lay description, and DWs

Severity level	Lay description	DW (95% CI)
Kwashiorkor	Is very tired and irritable and has diarrhea.	0.051 (0.031-0.079)
Marasmus	Is extremely skinny and has no energy.	0.128 (0.082-0.183)
Severe wasting	Is extremely skinny and has no energy.	0.128 (0.082-0.183)

Modeling strategy

We used PEM deaths and estimates of duration and CFR to back-calculate prevalence of kwashiorkor/marasmus. We assumed that all deaths from PEM were due to kwashiorkor/marasmus and not to wasting.

The first steps are to generate draws of the CFR and duration by creating simulations based on the mean and confidence intervals from the two papers described above. The next step is to calculate the death rate (divide deaths by population), and then employ the following formula in order to back-calculate prevalence from death rates:

PEM prevalence = (mortality rate / CFR) * duration

Severe wasting due to PEM was modeled using Gaussian process regression of anthropometric data of prevalence 3 z scores below mean on weight for height. Because both worms and PEM can cause wasting, we needed to divide out the wasting envelope to attribute wasting to both PEM and worms. We determined the amount of wasting attributable to worms by referencing Hall et al. 2008,[3] to determine the mean and confidence interval estimates of the z-score shift. We then calculated the counterfactual malnutrition prevalence given no worms, according to the z-score shift. From this, we calculated the fraction of wasting that is attributable to worms, and assigned the remainder of wasting to PEM.

No other significant changes were made to the GBD 2015 modeling strategy.

Iron-deficiency Anemia

Please refer to Anemia Impairment for details on the modeling strategy for iron-deficiency anemia.

Iodine deficiency

Flowchart

Iodine deficiency

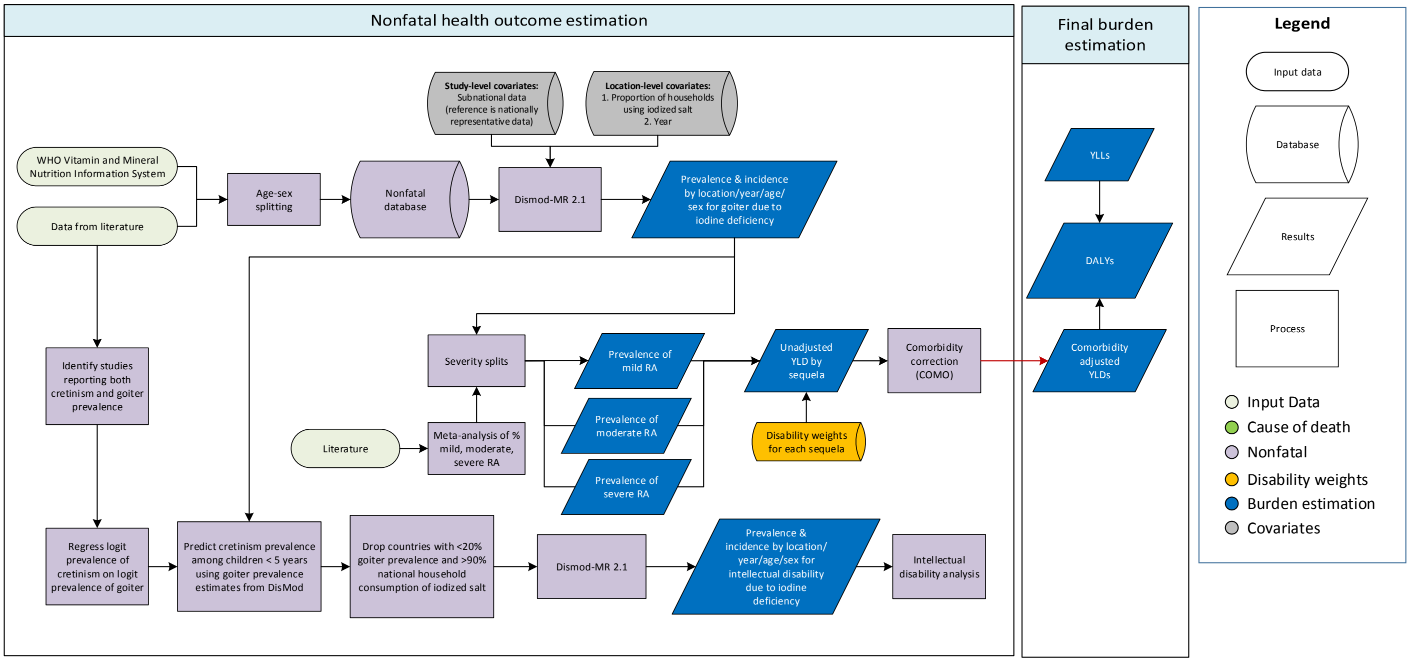

Input data and methodological summary

Case definition

The assessment of non-fatal iodine deficiency involves the quantification of morbidity specific to the following sequelae of disease: grade 2 goiter, intellectual disability due to iodine deficiency, and heart failure attributable to iodine deficiency. The ICD10 codes are E00-E02.

Input data

Model inputs

Data from the WHO Vitamin and Mineral Nutrition Information System and published studies were used. A systematic review was conducted for GBD 2013. The PubMed search terms were: ((iodine deficiency[Title/Abstract] AND prevalence[Title/Abstract]) AND (“2009”[Date – Publication] : “2013”[Date – Publication]))

The exclusion criteria were:

1. Studies that were not population-based, e.g., hospital or clinic-based studies

2. Studies that did not provide primary data on epidemiological parameters, e.g., commentaries
3. Review articles
4. Case series
5. Self-reported cases

Updates to systematic reviews are performed on an ongoing schedule across all GBD causes; an update for iodine deficiency will be performed in the next one to two iterations. The table below shows the number of literature studies included in GBD 2015, as well as the number of countries or subnational units and GBD world regions represented.

Table 1. Geographic representation

	Prevalence	Incidence	Mortality risk
Studies	25	-	-
Countries/subnationals	8	-	-
GBD world regions	5	-	-

Severity splits & disability weights

We applied disability weights from the GBD Disability Weight Survey to the following severity proportions: visible goiter without symptoms of iodine deficiency (proportion=0.90, 95% confidence interval (CI): 0.89-0.91); large mass of goiter without symptoms of iodine deficiency (proportion=0.085, 95% confidence interval (CI): 0.084-0.086); visible goiter with symptoms of iodine deficiency (proportion=0.015, CI: 0.014-0.016).

The lay descriptions and disability weights for severity levels derived from the GBD Disability Weights study are shown below.

Table 2. Severity, lay description, and DWs

Severity level	Lay description	DW (95% CI)
Visible goiter without symptoms	Has a slight, visible physical deformity that others notice, which causes some worry and discomfort.	0.011 (0.005-0.021)
Large mass of goiter without symptoms	Has a visible physical deformity that causes others to stare and comment. As a result, the person is worried and has trouble sleeping and concentrating.	0.067 (0.044-0.096)
Visible goiter with symptoms of iodine deficiency	Has a large mass in the front of the neck. The person sometimes has weakness and fatigue, constipation and weight gain.	0.199 (0.133-0.276)

Modeling strategy

We modeled the prevalence of grade 2 goiter in DisMod-MR 2.1. We chose to model grade 2 goiter over grade 1 goiter because of the greater reliability and consistency of the clinical diagnosis of grade 2 goiter

worldwide. We used a study-level covariate to indicate national and subnational observations, where nationally representative studies were set as the reference category. We used household iodized salt consumption proportion as a country-level covariate.

We estimated the prevalence of intellectual disability due to iodine deficiency (cretinism) by regressing data points from studies reporting both cretinism and goiter prevalence in the same population. To do so, we first transformed cretinism prevalence and goiter prevalence into logit space, regressed the logit prevalence of cretinism on the logit prevalence of goiter, and predicted for all locations using the goiter estimates from the DisMod model above. We dropped locations with total goiter prevalence less than 20% and locations with household iodized salt consumption greater than 90%. We kept observations in children younger than 5 years and use these data as incidence input in DisMod to generate location-year-age-sex-specific estimates. We assumed zero remission as the disease is a lifelong condition, and zero incidence after age 5. We repeated the drop-out criteria of total goiter prevalence and iodized salt consumption on the DisMod output. The severity split for intellectual disability due to iodine deficiency is presented separately in the section for intellectual disability.

Heart failure attributable to iodine deficiency was modeled separately, and the methods for this outcome are presented separately in the section for heart failure and its etiologies.

Betas and exponentiated values (which can be interpreted as an odds ratio) for the grade 2 goiter DisMod model are shown in the table below.

Table 3. Beta and exponentiated beta values

Covariate	Parameter	beta	Exponentiated beta
Proportions of households using iodized salts	Prevalence	-0.55 (-0.60, -0.41)	0.58 (0.55, 0.66)
Subnational	Prevalence	0.50 (0.48, 0.50)	1.64 (1.62, 1.65)
Sex	Prevalence	-0.63 (-0.85, -0.42)	0.53 (0.43, 0.66)

No other significant changes were made to the modeling strategy for GBD 2015.

Vitamin A deficiency

Flowchart

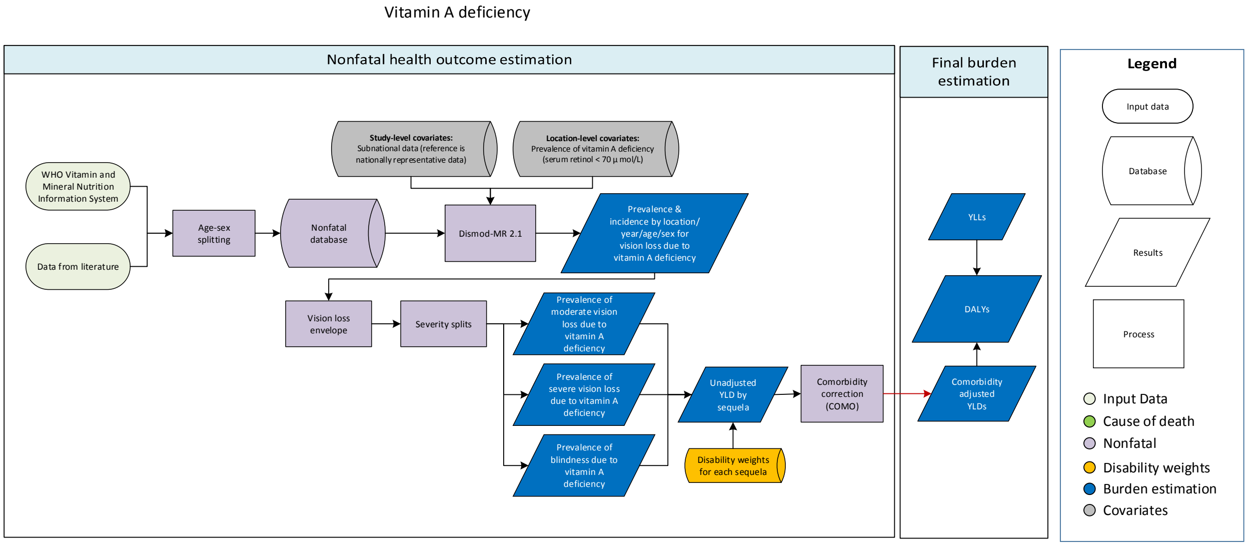

Input data and methodological summary

Case definition

Our assessment of vitamin A deficiency involves the quantification of blindness and vision loss due to corneal ulcerations and corneal scars. ICD 10 codes are E50-E50.9, E64.1, and ICD 9 codes are 264-264.9. These are further detailed in Appendix Table 4.

Input data

Model inputs

For GBD 2015, we used data from the WHO Vitamin and Mineral Nutrition Information System and studies identified through literature review. A systematic review was conducted for GBD 2013. The PubMed search terms were: ((vitamin A deficiency[Title/Abstract] AND prevalence[Title/Abstract]) AND (“2009”[Date – Publication] : “2013”[Date – Publication]))

The exclusion criteria were:

1. Studies that were not population-based, e.g., hospital or clinic-based studies
2. Studies that did not provide primary data on epidemiological parameters, e.g., commentaries
3. Review articles
4. Case series
5. Self-reported cases

Updates to systematic reviews are performed on an ongoing schedule across all GBD causes, and an update for vitamin A deficiency will be performed in the next one to two iterations. The table below shows the number of literature studies included in GBD 2015, as well as the number of countries or subnational units and GBD world regions represented.

Table 1. Geographic representation

	Prevalence	Incidence	Mortality risk
Studies	2	-	-
Countries/subnationals	2	-	-
GBD world regions	2	-	-

Severity splits & disability weights

Dismod-MR 2.0 estimates of prevalence of all vision loss in countries that are known to have vitamin A deficiency are split between moderate and severe vision loss and blindness due to vitamin A deficiency from the vision loss envelope. The lay descriptions and disability weights for severity levels derived from the GBD Disability Weights study are shown below.

Table 2. Severity, lay description and DWs

Severity level	Lay description	DW (95% CI)
Moderate vision loss due to vitamin A deficiency	Has vision problems that make it difficult to recognize faces or objects across a room.	0.031 (0.019-0.049)
Severe vision loss due to vitamin A deficiency	Has severe vision loss, which causes difficulty in daily activities, some emotional impact (for example worry), and some difficulty going outside the home without assistance.	0.184 (0.125-0.259)
Blindness due to vitamin A deficiency	Is completely blind, which causes great difficulty in some daily activities, worry and anxiety, and great difficulty going outside the home without assistance.	0.187 (0.124-0.26)

Modeling strategy

For GBD 2015, we used DisMod MR-2.0 to model prevalence of blindness and vision loss due to vitamin A deficiency. We assumed zero remission since blindness and vision loss due to vitamin A deficiency is permanent. We used a study-level covariate to indicate national and subnational observations, where nationally representative studies were set as the reference category. We used vitamin A deficiency prevalence (serum retinol < 70 micromol/L) as a location-level covariate to inform variation over year and geography, especially in location-years with no or sparse data. We also included a year covariate.

Study covariates, parameters, betas and exponentiated values (which can be interpreted as an odds ratio) are shown in the table below.

Table 3. Beta and exponentiated beta values

Study covariate	Parameter	beta	Exponentiated beta
Vitamin A deficiency prevalence	Prevalence	1.09 (0.13 — 1.96)	2.97 (1.14 — 7.11)
Year	Prevalence	-0.018 (-0.02 — -0.01)	0.98 (0.98 — 0.99)
Sex	Prevalence	0.025 (-1.42 — 1.21)	1.03 (0.24 — 3.34)

No other significant changes were made from GBD 2013 to GBD 2015.

Sexually-transmitted diseases excluding HIV:

Syphilis, chlamydial infection, gonococcal infection, trichomoniasis, genital herpes, syphilis, and other sexually transmitted diseases

Flowchart

Sexually-transmitted diseases excluding HIV:
Syphilis (early and adult tertiary), Chlamydial infection, Gonococcal infection,, Trichomoniasis, Genital herpes due to HSV-2, and Other sexually transmitted diseases

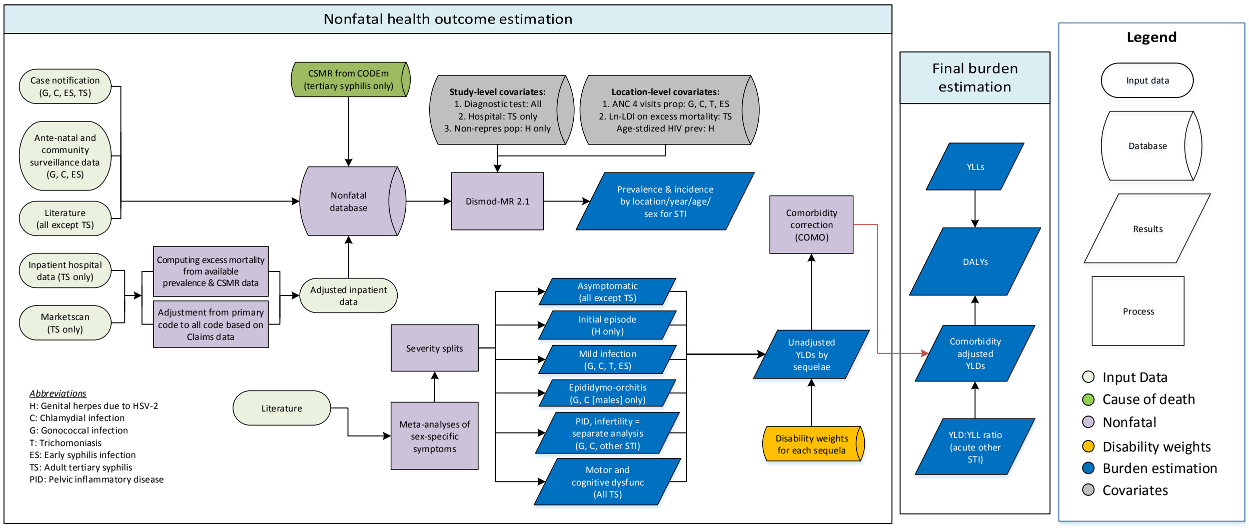

Input data and methodological summary

Case definition

We estimated the prevalence and incidence of genital and reproductive tract infection with several common sexually-transmitted infections, including Chlamydia trachomatis, Neisseria gonorrhea, Trichomonas vaginalis, Treponema pallidum (syphilis), and genital herpes associated with seroprevalent HSV-2. Separate estimates were completed for early syphilis and adult tertiary syphilis. ICD-9 and ICD-10 codes associated with each cause in the GBD 2015 cause-specific mortality analyses are listed below. Of note, mortality was assumed to be zero in trichomoniasis and genital herpes infection and YLD due to congenital syphilis was not estimated. **Appendix Table 4 provides additional information.**

Table 1. International classification of diseases codes for sexually-transmitted infections in GBD 2015 cause of death analysis

Condition	ICD10 code	ICD9 code
Sexually transmitted infections (STI) excl HIV	A50-A60, A63-A64, I98.0, K67.0-K67.2, M73.0-M73.1	090-099,131,614
<u>Syphilis</u>	A50-A53, I98.0, K67.0-K67.2, M73.0-M73.1	090-097
Congenital syphilis	A50	090
Early syphilis	A51	091
Adult tertiary syphilis	A52, I98.0	093-095
Chlamydial infection	A55-A56,K67.0	099.41,099.5
Gonococcal infection	A54,K67.1	098

Trichomoniasis	A59,K67.0	131
Genital herpes	A60	054.1
Other STI	A57-A58, A63-A64, M73.0	099 (except 099.41, 099.5)

Input data

Model inputs

Systematic literature reviews were completed on April 17, 2015 for chlamydia, gonorrhea, trichomonas, genital herpes, and early syphilis. Three related search strings were used as many studies report on multiple infections. With the exception of the early syphilis literature review, which was completed for the first time in GBD 2015, these were the same search strings and strategies as were employed in GBD 2013.

462 initial hits; 54 sources selected for full text review and data extraction: (((chlamydia[Title/Abstract] OR chlamydia tracomatis[Title/Abstract] OR trachoma[Title/Abstract]) AND prevalence[Title/Abstract]) AND ('2013'[Date - Publication] : '2015'[Date - Publication])) /// ((gonorrhea[Title/Abstract] OR Neisseria[Title/Abstract] OR gonococcal[Title/Abstract]) AND prevalence[Title/Abstract]) AND ("2013"[PDAT] : "2015"[PDAT]) /// ((trichomonal[Title/Abstract] OR trichomonas[Title/Abstract]) AND prevalence[Title/Abstract]) AND ('2013'[PDAT] : '2015'[PDAT])

1265 initial hits; 178 sources selected for full text review and data extraction: ("syphilis"[MeSH] OR "Treponema pallidum"[MeSH]) NOT "Yaws"[MeSH] AND "prevalence"[MeSH] AND "1990"[PDAT] : "2015"[PDAT] AND "humans"[MeSH] **///** ("syphilis"[MeSH] OR "Treponema pallidum"[Mesh]) NOT “Yaws”[MeSH] AND ("incidence"[MeSH]) AND ("1990"[PDAT] : "2015"[PDAT]) AND "humans"[MeSH])

13 initial hits; 1 selected for full text review and data extraction: herpes"[Title/Abstract] OR "Herpesvirus 2, Human"[Mesh]) AND ("Prevalence"[Title/Abstract] OR "Incidence"[Title/Abstract] AND ("2015"[PDAT] : "2015"[PDAT])

The genital herpes dataset was supplemented by those sources contained in recent published estimates completed by Looker and colleagues.[1,2] For all other STI, most notably early syphilis, we supplemented our datasets with manual search of national ministry of health websites, antenatal clinic surveillance reports, and case-notification data from locations where centralized reporting is mandatory. To be included, a study must report on laboratory-confirmed diagnosis of an STI. For each STI, the reference category for diagnostic modality was DNA-based test (e.g., PCR or other nucleic acid amplification test) and data using any other diagnostic test was quantitatively crosswalked to the reference category using binary study-level covariates in DisMod-MR 2.1. For genital herpes, any sources that did not use nucleic acid amplification were excluded. For early syphilis, crosswalks were performed in a country-specific manner because non-treponemal false-positive rate is dependent on several epidemiological factors, including age, sex, and endemicity of other infections. Given the potential variability in immunological reasons for false positives in each of the other STI categories, we also did not perform any adjustment for published sensitivity and specificity of tests, as these results are often drawn from particular populations that may not be representative of the global experience for each infection. For all STI except herpes, sources were excluded if the sample population was drawn exclusively from a high-risk group (e.g., HIV positive, men who have sex with men [MSM], or sex workers). Herpes data from such groups was utilized, with quantitative crosswalks completed using binary study-level covariates in DisMod-MR 2.1, because

unlike many STI, genital herpes due to HSV-2 has not been shown to have isolated epidemics in high-risk groups. Composition of final datasets are shown below for each of the different STI models.

Gonococcal infection

	Prevalence	Incidence	Mortality risk
Studies	160	5	1
Countries/subnationals	104/106	3/1	66/66
GBD world regions	21	3	17

Chlamydial infection

	Prevalence	Incidence
Studies	199	3
Countries/subnationals	109/87	2/2
GBD world regions	20	2

Early syphilis

	Prevalence	Incidence
Studies	229	47
Countries/subnationals	93/36	56/140
GBD world regions	7	5

Adult tertiary syphilis

	Prevalence	Mortality
Studies	11	1
Countries/subnationals	34/78	42/116
GBD world regions	6	9

Trichomoniasis infection

	Prevalence	Incidence
Studies	115	2
Countries/subnationals	57/20	1/1
GBD world regions	20	1

Genital herpes

	Prevalence	Incidence	Mortality risk
Studies	284	47	1
Countries/subnationals	133/215	56/140	42/116
GBD world regions	21	12	9

Modeling strategy

We have made no substantive changes to the estimation strategy since 2013. Several details have changed as a result of broader improvements in GBD, including use of CSMR results, subsequent calculation of EMR, and inclusion of large volumes of new data. We estimated the nonfatal burden of STI in three parts. For the first part we estimated the prevalence, incidence, remission, and case fatality from acute infection associated with gonococcus, chlamydia, trichomoniasis, genital herpes (from herpes simplex virus 2), early syphilis, and adult tertiary syphilis using the data above and DisMod-MR 2.1. Not all cases of STI are symptomatic, so we used literature review to guide splitting prevalent cases into asymptomatic and symptomatic health states.[3,4] Specific modeling considerations in DisMod-MR 2.1 for each STI are described below. The second part, which is estimation of prevalence, incidence, remission and case fatality from pelvic inflammatory disease (PID) and PID-induced primary and secondary infertility, is described in a separate section on those conditions. Briefly, for PID we used ICD-9 and ICD-10 coded discharge datasets, Marketscan claims database, and systematic literature review to develop a dataset that was subsequently modeled for all geographies using DisMod-MR 2.1. Processing steps for discharge and claims data are described separately. PID was then split into underlying ideologies (chlamydia, gonorrhea, and other) using results of supplemental literature review and DisMod-MR 2.1 models of the proportion due to each etiology. PID-induced primary and secondary infertility assuming only a fixed subset of incident PID cases develop infertility and that there is no remission in these cases. The third and final part involved finding the ratio of YLD to YLL ratio for all STI (excluding other STI) and then applying that same ratio to other STI YLLs.

Gonococcal infection

Prevalence data was the primary input from literature, case notification systems, and surveillance. Incidence was restricted to only occur between ages 10 and 69. EMR was set to have a maximum value of 0.001. Remission rate bounds were the same as we used in GBD 2013 and GBD 2010.[3,4] Study covariates included crosswalks for data from surveillance systems as they are suspected to underreport population prevalence as well as those data where case diagnosis was based on culture or other non-nucleic acid amplification tests (PCR is gold standard diagnostic method). Female-to-male ratio of prevalence and incidence was restricted to not exceed 1.35:1, an approach that was used to account for relatively sparse data from males. Study covariates used in GBD 2013 to crosswalk data from pregnant women and sexually-active teens were removed from the model in GBD 2015 because, with addition of new data, there was no longer any consistent difference between these groups and general population prevalence. Logit-transformed coverage proportion of four visits of antenatal care coverage (ANC4) was the only country covariate and was assigned to prevalence.

We assigned the proportion of persons who developed symptoms of infection and/or epididymoorchitis. Both were unchanged from the GBD 2010 and are the same as used in WHO analyses. Males were assigned a fixed proportion of each of the following health states.Epididymo-orchitis differed for geographies with long time series of high-quality vital registration data (“data rich”) compared to geographies with poor data (“all others”). This split was performed for developed versus developing countries in GBD 2013 and GBD 2010. Data rich proportion was 0.03 (0.015 – 0.045) and all others was 0.0975 (0.0483 – 0.143). A proportion of the remainder, 0.5875 (0.5288 - 0.6463), were assigned a health state of mild, acute infectious disease. The remainder were assumed to be asymptomatic. Females were estimated to have proportion with mild, acute infectious disease of 0.34 (0.306 - 0.374) and the

remainder asymptomatic.[3,4] Females have fewer symptoms of gonorrhea than males, which results in higher YLDs amongst males, despite a lower number of cases.

Gonococcal infection

Study-level covariate	Parameter	Geography level	beta	Exponentiated beta
Surveillance/notification data	prevalence	Global	-2.6e-04 (-6.0e-04 - -1.7e-05)	.9997 (.9994 - 1)
Culture positive	prevalence	Global	-1.3e-04 (-1.6e-04 - -5.1e-05)	.9999 (.9998 - .9999)
Diagnosis other	prevalence	Global	-.003 (-.0089 - -5.2e-05)	.997 (.9912 - .9999)

Chlamydial infection

Chlamydia estimation methods were the same as used for gonococcal infection described above. The approach to estimating sequelae was the same as for gonorrhea, although the proportions in each state were different. Data rich proportion was 0.02 (0.01 – 0.03) and all others was 0.0625 (0.0325 – 0.0975). A proportion of the remainder, 0.505 (0.4545 - 0.5555), were assigned a health state of mild, acute infectious disease. The remainder were assumed to be asymptomatic. Females were estimated to have proportion with mild, acute infectious disease of 0.17 (0.153 - 0.187) and the remainder asymptomatic.[3,4] Again, despite a higher number of cases amongst females, the YLD-per case amongst males was higher.

Chlamydial infection

Study-level covariate	Parameter	Geography level	beta	Exponentiated beta
Diagnosis other	prevalence	Global	-.0338 (-.2018 - -9.9e-05)	.9667 (.8173 - .9999)
Surveillance/notification data	prevalence	Global	-.5012 (-.9998 - -3.6e-05)	.6058 (.368 - 1)
Culture positive	prevalence	Global	-.0177 (-.0781 - -5.4e-04)	.9825 (.9249 - .9995)

Trichomoniasis

Trichomoniasis estimation methods were the same as used for gonococcal and chlamydia infection with the exception that female-to-male ratio was allowed to rise as high as 2.73:1. Surveillance and case-notification data for trichomonas was considered to be unreliable and not used. We also changed the reference definition for diagnostic test from wet mount to instead be PCR after consultation with STI

experts and GBD collaborators. This change was responsible for the increase compared to GBD 2013. Males are assumed to be 100% asymptomatic with trichomoniasis, while 0.34 (0.306 - 0.374) of prevalent females were assigned a health state of mild, acute infectious disease.[3,4]

Trichomoniasis infection

Study-level covariate	Parameter	Geography level	beta	Exponentiated beta
Diagnostic wet mount	prevalence	Global	-.4129 (-.4965 - -.2513)	.6617 (.6087 - .7778)
Culture positive	prevalence	Global	-.1606 (-.3731 - -.0069)	.8516 (.6886 - .9932)
Antenatal Care (4 visits) Coverage (proportion)	prevalence	Global	-.0516 (-.0974 - -.0032)	.9497 (.9072 - .9968)

Genital herpes due to HSV-2

Genital herpes estimation assumed remission and mortality are both zero. Incidence estimates were thus based on mathematical integration of age-specific prevalence data. Multiple crosswalks were used for known and suspected non-representative populations. This approach was chosen rather than exclusion of high risk groups altogether because, unlike other STI, genital herpes has not been shown to exist as isolated epidemics within high-risk groups. The prevalence amongst these groups is legitimately higher than the general population, however, so crosswalking these data to the reference of general population is the preferred approach. High risk groups in the dataset included MSM, HIV positive, prisoners, sex workers, drug users, and STI clinic samples. Blood donor samples were also crosswalked to the general population assuming the prevalence is lower than the general population. All crosswalks were performed at the region level to allow for variation in the quantitative relationship. A single country covariate, age-standardized HIV prevalence, was used to guide estimates in geographies with sparse data in recognition of the strong relationship between HSV-2 and HIV transmission. Many with initial genital herpes infection have a painful rash that, while not as severe as that accompanying zoster reactivation, is more severe than that associated with recurrent genital herpes episodes. A systematic literature review revealed a few studies that informed our estimation that 0.175 (0.10-0.25) of initial herpes cases have symptoms of moderate, acute infectious disease lasting 3 (2-4) weeks and 0.189 have prevalent persons have 6 (5-7) recurrent episodes per year each lasting 2 (1-3) weeks.[5–7]

Genital herpes

Study-level covariate	Parameter	Geography level	beta	Exponentiated beta
Study Population Sex Workers	prevalence	Region	.6192 (.455 - .7875)	1.858 (1.576 - 2.198)
Study Population institutionalized prisoners	prevalence	Region	.3377 (.0141 - .8743)	1.402 (1.014 - 2.397)
Study Population HIV positive	prevalence	Region	.6767 (.4897 - .8459)	1.967 (1.632 - 2.33)
Study Population Drug Users	prevalence	Region	.0632 (.0018 - .2)	1.065 (1.002 - 1.221)

Study Population STI Clinic	prevalence	Region	.4855 (.3677 - .6121)	1.625 (1.444 - 1.844)
Study Population Blood Donors	prevalence	Region	-.3565 (-.5479 - -.1755)	.7001 (.5782 - .839)
Study Population MSM	prevalence	Region	.1725 (.0093 - .4096)	1.188 (1.009 - 1.506)
HIV age-standardized prevalence	prevalence	Region	.0556 (.0031 - .0982)	1.057 (1.003 - 1.103)

Early syphilis

Early syphilis estimation methods were similar to those used for gonorrhea, chlamydia, and trichomonas infection with a few notable differences. Age range was restricted to be from 10-64 years. Additional crosswalks were performed from blood donor and pregnant study populations. Blood donor samples consistently had prevalence values that were lower than the general population while the difference in prevalence between pregnant and general population samples was near unity. Data from studies using only treponemal tests, or only non-treponemal tests, were additionally crosswalked to the reference definition of both treponemal and non-treponemal testing. Crosswalks were performed in a country-specific manner to reflect the variability in false positive rate in non-treponemal tests as a function of age, sex, and endemicity of other non-STI infectious conditions. Our review of literature on proportion of early syphilis cases with symptoms led to assignment of a 0.686 (0.627-0.745) of prevalent cases to have a combined duration of primary and secondary symptoms lasting 23 (13-44) days.[8–11] The remainder were considered asymptomatic.

Early syphilis infection

Study-level covariate	Parameter	Geography level	beta	Exponentiated beta
Study Population Pregnant	prevalence	Country	.0586 (-.0411 - .1559)	1.06 (.9597 - 1.169)
Antenatal Care (4 visits) Coverage (proportion)	prevalence	Country	-.1951 (-.1996 - -.1804)	.8228 (.8191 - .8349)
Study Population Blood Donors	prevalence	Country	-.1967 (-.1999 - -.1899)	.8214 (.8188 - .827)
Non-trep only	prevalence	Country	.4793 (.4332 - .4984)	1.615 (1.542 - 1.646)

Adult tertiary syphilis

Prevalence data was the primary input. Incidence was assumed to not occur until age 15. Excess mortality rate and remission were both capped at 0.1, which equates to minimum duration of five years. Cause-specific mortality rate (CSMR) results from GBD 2015 mortality and causes of death analysis were included in the model, which was also used to back-calculate excess mortality rate (EMR) data to match each input prevalence datum.[12] CSMR priors were not passed on through the cascade so as to restrict the utility of this data to back calculating prevalence at the country level. Study-level covariates were used to crosswalk hospital and surveillance data to the reference source of Marketscan. Natural log of lag-

distributed income (LN-LDI) was used as a country-level covariate on EMR. All cases of adult tertiary syphilis were assumed to be symptomatic and were assigned the same disability that describes a health state of moderate motor and cognitive dysfunction. Three notable differences exist between GBD 2015 and previous estimates of adult tertiary syphilis. First was inclusion of surveillance data from all available sources. Second was universal application of adjustment factors for the likelihood of tertiary syphilis to appear as primary discharge diagnosis (described in section on hospital data processing). Third was the use of CSMR to back-calculate EMR and addition of a country-covariate on EMR. The net effect of these changes was to elucidate a higher rate of adult tertiary syphilis than previously estimated. The primary limitation of this process is that it has approximated all tertiary syphilis to be of equivalent disability, despite clinical evidence that the manifestations of disease can be variable.

Adult tertiary syphilis

Study-level covariate	Parameter	Geography level	beta	Exponentiated beta
LDI (I$ per capita)	excess mortality rate	Global	-.3 (-.3 - -.2999)	.7408 (.7408 - .7409)
Surveillance/ notification data	prevalence	Global	-1.996 (-2 - -1.987)	.1359 (.1353 - .1371)
Hospital inpatient	prevalence	Global	-1.632 (-1.69 - -1.574)	.1955 (.1845 - .2072)

Other sexually-transmitted infections

To calculate YLD due to acute infection with other STI, we calculated ratio of YLD to YLL ratio for all STI (excluding other STI) and then applied that same ratio to other STI YLLs. YLD were also estimated to other STI as a result of the proportion of PID and PID-induced infertility that was not due to gonorrhea or chlamydia.

Uncertainty and model selection

For all STI estimates, uncertainty bounds include uncertainty due to input data, including CSMR from GBD 2015 mortality and causes of death analysis, crosswalks from non-reference definitions, uncertainty in numerical solutions (posteriors) of each DisMod-MR 2.1 model, and proportion of all infections with each type of symptom.

In consultation with GBD researchers and collaborators, final models were selected on a combination of qualitative and quantitative goodness of fit to input data, plausibility of geographic and temporal trends, consistency of age pattern, and, when available, comparison with other published studies on STI epidemiology. Directionality, magnitude, and plausibility of study-level and country-level covariates was also considered in the process of model development. Of note, due to the nature of statistical modeling, final results do not always cover the values reported in input data.

Acute hepatitis A

Flowchart

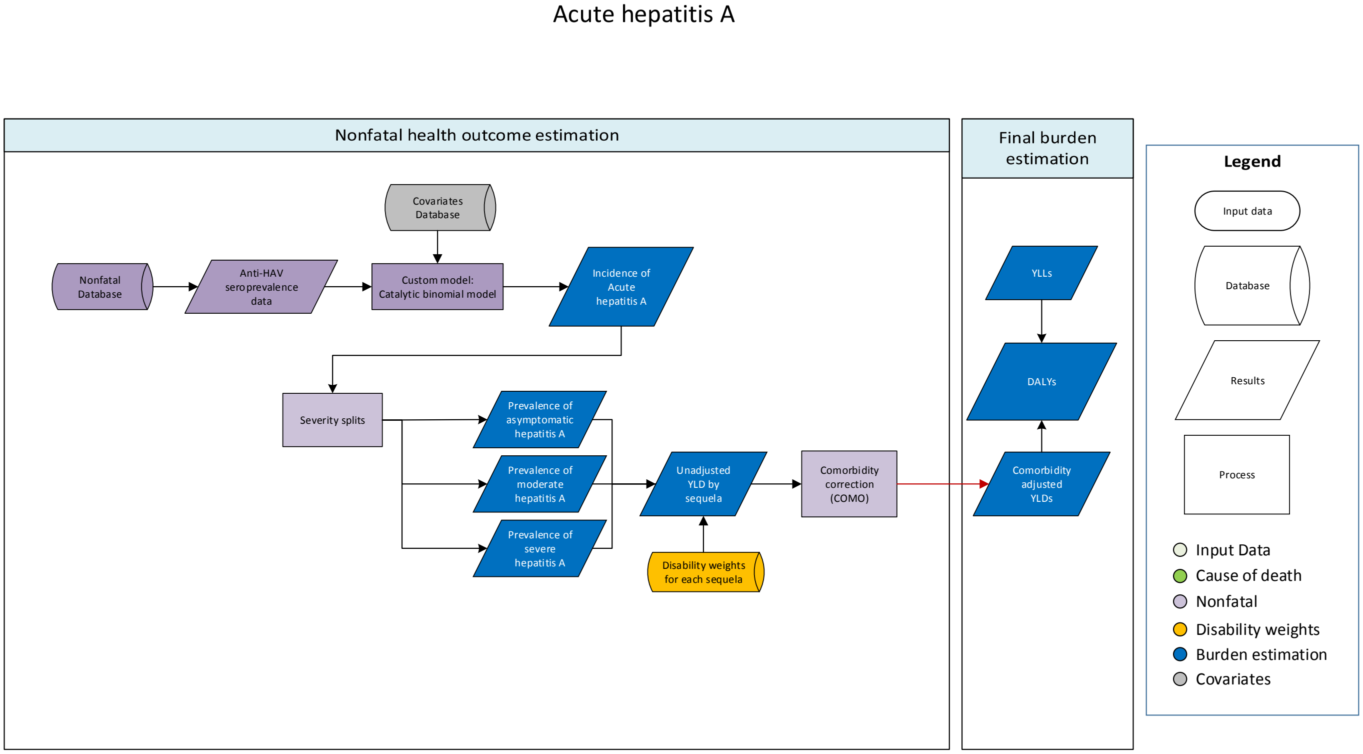

Input data and methodological summary

Case definition

We define acute hepatitis A as an infection with the hepatitis A virus resulting in anti-HAV IgG seroconversion, regardless of symptoms. It includes all ICD-10 codes under the heading B15 (Acute hepatitis A). Appendix Table 4 offers additional information about ICD codes.

Input data

Model inputs

We use anti-HAV seroprevalence data from population-based studies and surveys for the incidence model. See the table below for greater detail.

Level	Prevalence
Data points	3668
Studies	469
Locations	175
Regions	21

Updates to systematic reviews are performed on an ongoing schedule across all GBD causes, and an update for hepatitis A will be performed in the next one to two iterations.

Severity splits & disability weights

Based on information published by Armstrong et al, (2002), we assume that the probability of symptomatic infection increases with age from ~1% in the first year of life to ~85% in adulthood.

The table below illustrates the sequelae associated with acute hepatitis A, as well as the lay descriptions and associated disability weights.

Sequela	Description	Disability weight
Moderate	Has a fever and aches, and feels weak, which causes some difficulty with daily activities.	0.051 (0.032-0.074)
Severe	Has a high fever and pain, and feels very weak, which causes great difficulty with daily activities.	0.133 (0.088-0.19)
Asymptomatic	Infection with no apparent illness.	NA

Modeling strategy

Given its reasonably stable force of infection among susceptible people across age groups, we used a catalytic binomial model to estimate incidence of acute hepatitis A based on anti-HAV seroprevalence. The catalytic binomial model is a binomial generalized linear model with a complementary log-log link, and an offset term for log-age. Since anti-HAV is a lifetime marker of past infection, and a given individual can only be infected once, seroprevalence at age *t* is equal to the cumulative incidence (CI) over *t* years. Assuming constant force of infection, we can estimate the incidence rate (IR) as,

$$CI = 1 - e^{-IR \cdot t}$$

We can rearrange this equation to solve for the log-IR:

$$\ln(IR) = \frac{\ln(-\ln(1 - CI))}{\ln(t)}$$

Thus, by using the complimentary log-log link for CI (i.e., ln(-ln(1-CI)) with an offset for log-age, we are able to model the incidence rate of infection from seroprevalence data. To inform the model in the absence of data we use a predictive covariate derived from principal components analysis of lag-distributed income (LDI) and the proportion of the population with access to improved water. We use a mixed effects model with fixed effects on the aforementioned PCA-derived covariate and nested hierarchical random effects on super-region, region, country, and subnational geographies.

The table below illustrates the study covariates used in the GBD 2015 model, as well as the beta and exponentiated beta values.

Covariate	Coefficient	Exponentiated coefficient
Component of water and LDI	-0.594	0.552
Female sex	-0.124	0.884

Changes from GBD 2013 to GBD 2015

Our overall approach has not changed from that used in GBD 2013. However, whereas we previously used a fixed-effects-only catalytic binominal model for GBD 2013, we have incorporated random effects into the model for GBD 2015, improving the spatial structure of the model and allowing the model to better follow data.

Acute hepatitis B

Flowchart

Acute hepatitis B

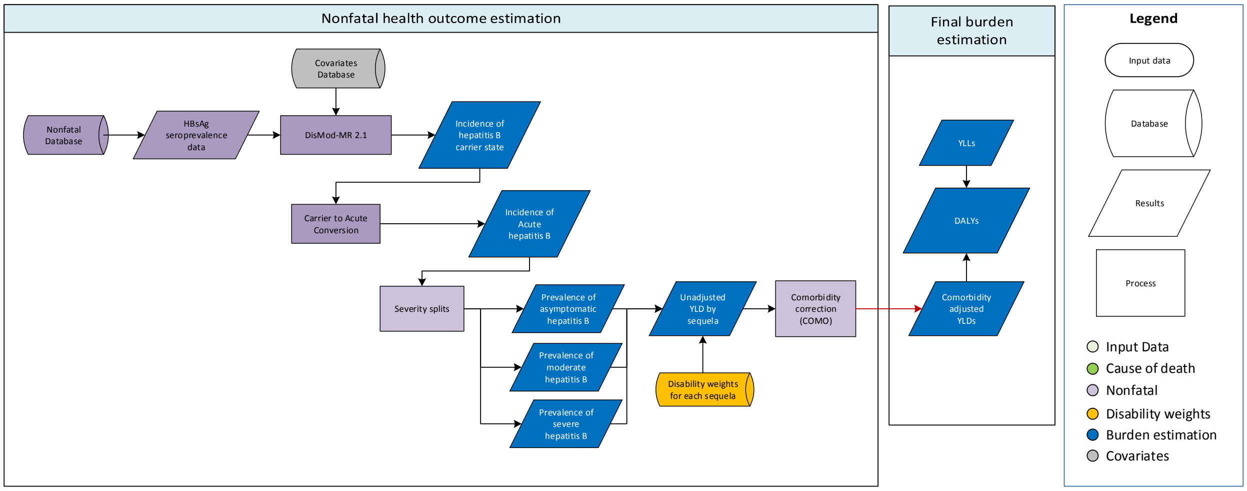

Input data and methodological appendix

Case definition

We define acute hepatitis B as the period corresponding to initial infection with the hepatitis B virus, regardless of symptoms. It includes all ICD-10 codes under the heading B16 (Acute hepatitis B). Appendix Table 4 offers additional information on ICD codes.

Input data

Model inputs

We use hepatitis B surface antigen (HBsAg) seroprevalence data from population-based studies and surveys for the incidence model. See the table below for additional information.

Level	Prevalence
Data points	2,987
Studies	312
Locations	145
Regions	19

Updates to systematic reviews are performed on an ongoing schedule across all GBD causes, and an update for hepatitis A will be performed in the next one to two iterations.

Modeling strategy

We model the incidence of chronic HBsAg carriage using a full DisMod model of HBsAg seroprevalence. We then convert incidence of chronic carriage to total incidence of hepatitis B infection by dividing age-specific estimates of the incidence of chronic carriage by age-specific estimates of the probability of infection resulting in carriage based on Edmunds et al. (1993). The equation is detailed below:

$$P(carrier \mid age \leq 6\ months) = 0.885$$

$$P(carrier \mid 6\ months \leq age < 25\ years) = e^{-0.645 \times age^{0.455}}$$

$$P(carrier \mid age \geq 25\ years) = e^{-0.645 \times 25^{0.455}} = 0.061$$

We then split symptomatic cases into moderate (73%) and severe (27%) severities based on data from McMahon et al. (1985). See references and GHDx source tool for additional information.

Sequela, lay descriptions and disability weights

The below below illustrates the sequela, lay description and DWs associated with acute Hep B.

Sequela	Description	DWs (95% CI)
Moderate	Has a fever and aches, and feels weak, which causes some difficulty with daily activities.	0.051 (0.032-0.074)
Severe	Has a high fever and pain, and feels very weak, which causes great difficulty with daily activities.	0.133 (0.088-0.19)
Asymptomatic	Infection with no apparent illness.	NA

Changes from GBD 2013 to GBD 2015

We have updated the severity splits, but the modeling strategy remains otherwise unchanged from GBD 2013.

Acute hepatitis C

Flowchart

Acute hepatitis C

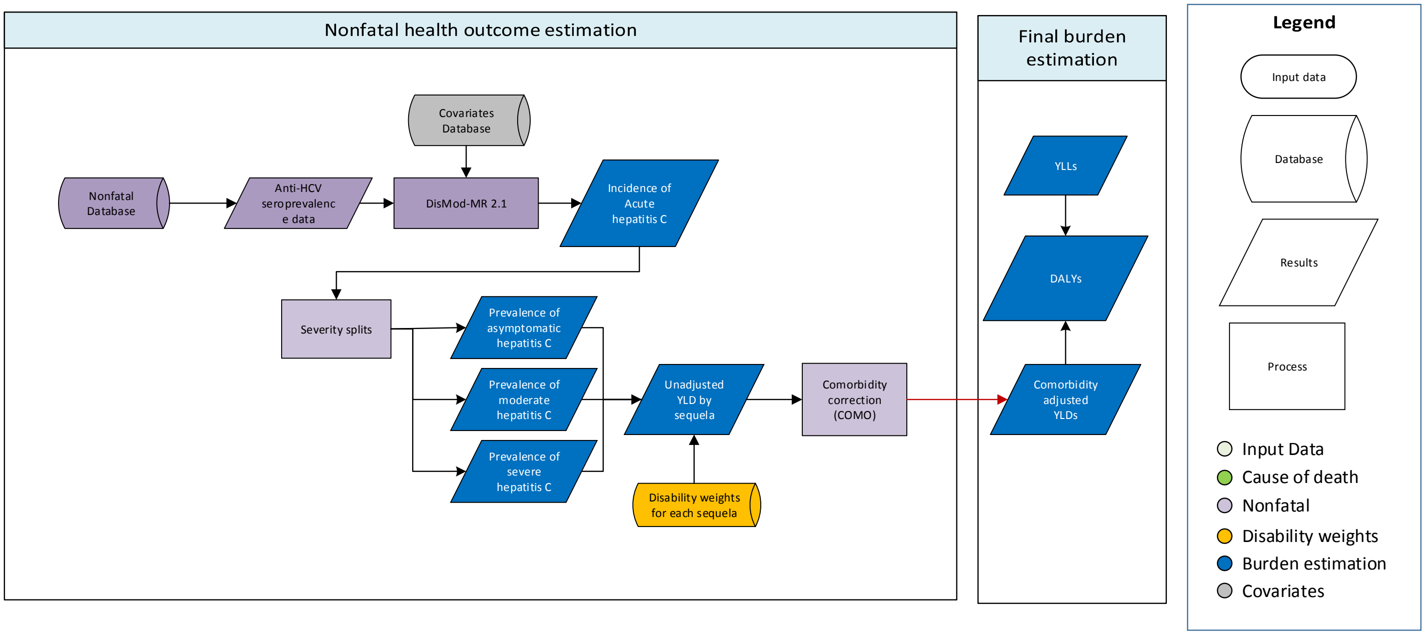

Input data and methodological summary

Case definition

We define acute hepatitis C as the period corresponding to initial infection with the hepatitis C virus, resulting in anti-HCV IgG seroconversion, regardless of symptoms. It includes all ICD-10 codes under the heading B17.1 (Acute hepatitis C). See Appendix Table 4 for additional information on ICD codes.

Input data

Model inputs

To estimate morbidity for hepatitis C, we use anti-HCV seroprevalence data from population-based studies and surveys to estimate incidence and prevalence of hepatitis C infection. The table below illustrates the geographic spread of prevalence data.

Level	Prevalence

Data points	5,242
Studies	239
Locations	521
Regions	21

Modelling strategy

For GBD 2015, we modelled the incidence and prevalence of hepatitis C infection using a full DisMod model of anti-HCV seroprevalence data. We divide incident infections into asymptomatic (75%), moderate (24%), and severe (1%) states. Based on a meta-analysis, we estimate that 75% of anti-HCV positive people are chronically infected.

Severity splits and DWs

The table below illustrates the associated sequela, lay descriptions and disability weights associated the acute Hep C.

Sequela	Description	DWs (95% CI)
Moderate	Has a fever and aches, and feels weak, which causes some difficulty with daily activities.	0.051 (0.032-0.074)
Severe	Has a high fever and pain, and feels very weak, which causes great difficulty with daily activities.	0.133 (0.088-0.19)
Asymptomatic	Infection with no apparent illness.	NA

Changes from GBD 2013 to GBD 2015

We have made no substantive changes in the modeling strategy from GBD 2013.

Acute hepatitis E

Flowchart

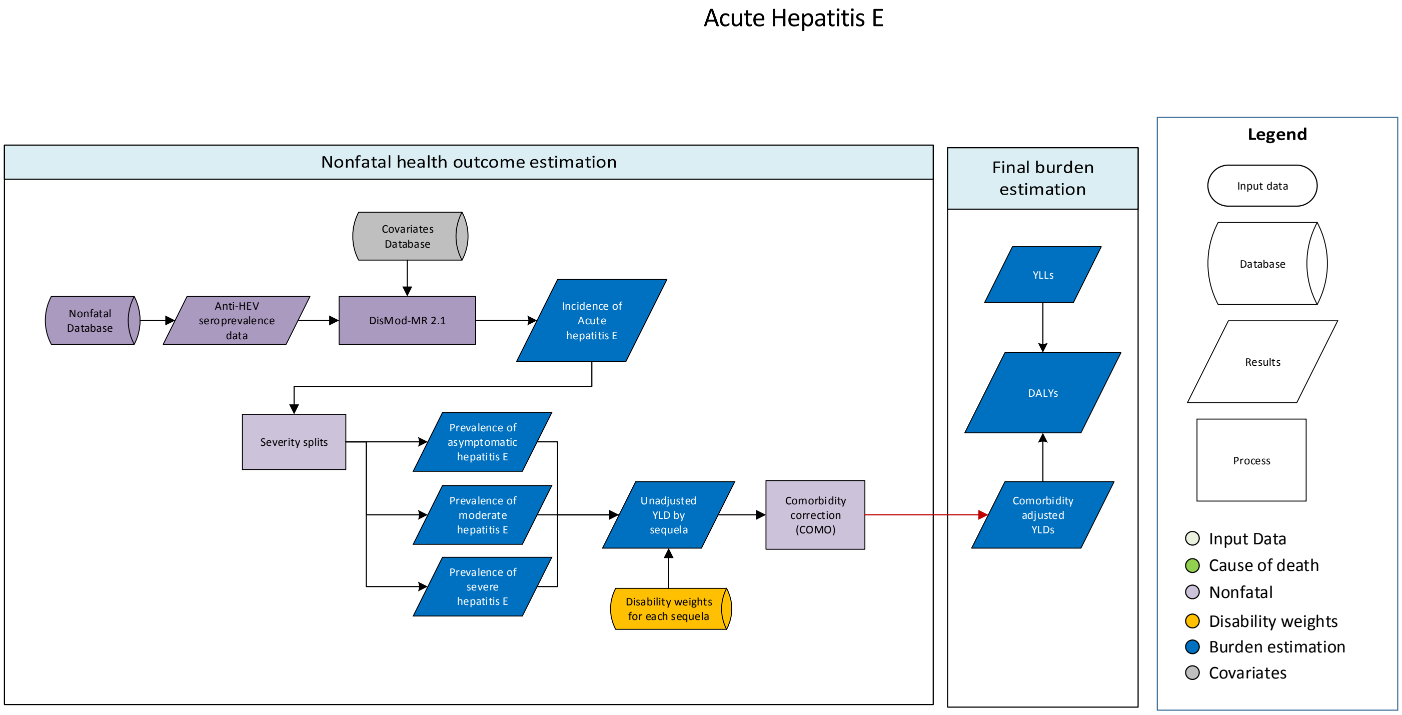

Input data and methodological summary

Case definition

For GBD 2015, we define acute hepatitis E as an infection with the hepatitis E virus resulting in anti-HEV IgG seroconversion, regardless of symptoms. It includes all ICD-10 codes under the heading B17.2 (Acute hepatitis E).

Input data

Model inputs

For GBD 2015 estimation, we used anti-HEV seroprevalence data from population-based studies and surveys to estimate incidence of infection. The table below indicates the number of data points included in terms of prevalence by location hierarchy:

Level	Prevalence
Data points	433
Studies	85
Locations	56

Regions	19

Modeling strategy

The GBD 2015 estimation process for hepatitis E uses a DisMod-MR 2.1 model, which is a Bayesian meta-regression. We model the incidence of Hepatitis E using a full DisMod-MR 2.1 model of anti-HEV seroprevalence, assuming no remission. Based on information published by Rein et al[1] we assume that the probability of symptomatic infection increases with age from ~1% in the first year of life to ~60% in adulthood.

Severity splits

The table below illustrates the sequelae associated with acute hepatitis E, along with their descriptions and disability weights.

Sequela	Description	Disability weight
Moderate	Has a fever and aches, and feels weak, which causes some difficulty with daily activities.	0.051 (0.032-0.074)
Severe	Has a high fever and pain, and feels very weak, which causes great difficulty with daily activities.	0.133 (0.088-0.19)
Asymptomatic	Infection with no apparent illness.	NA

Changes from GBD 2013 to GBD 2015

We have made no substantive changes in the modeling strategy from GBD 2013.

References

1. Rein DB, Stevens GA, Theaker J, Wittenborn JS, Wiersma ST. The global burden of hepatitis E virus genotypes 1 and 2 in 2005. Hepatology. 2012 Apr 1;55(4):988–97.

Cancer

All cancers except for non-melanoma skin cancer (basal cell carcinoma and squamous cell carcinoma)

Flowchart

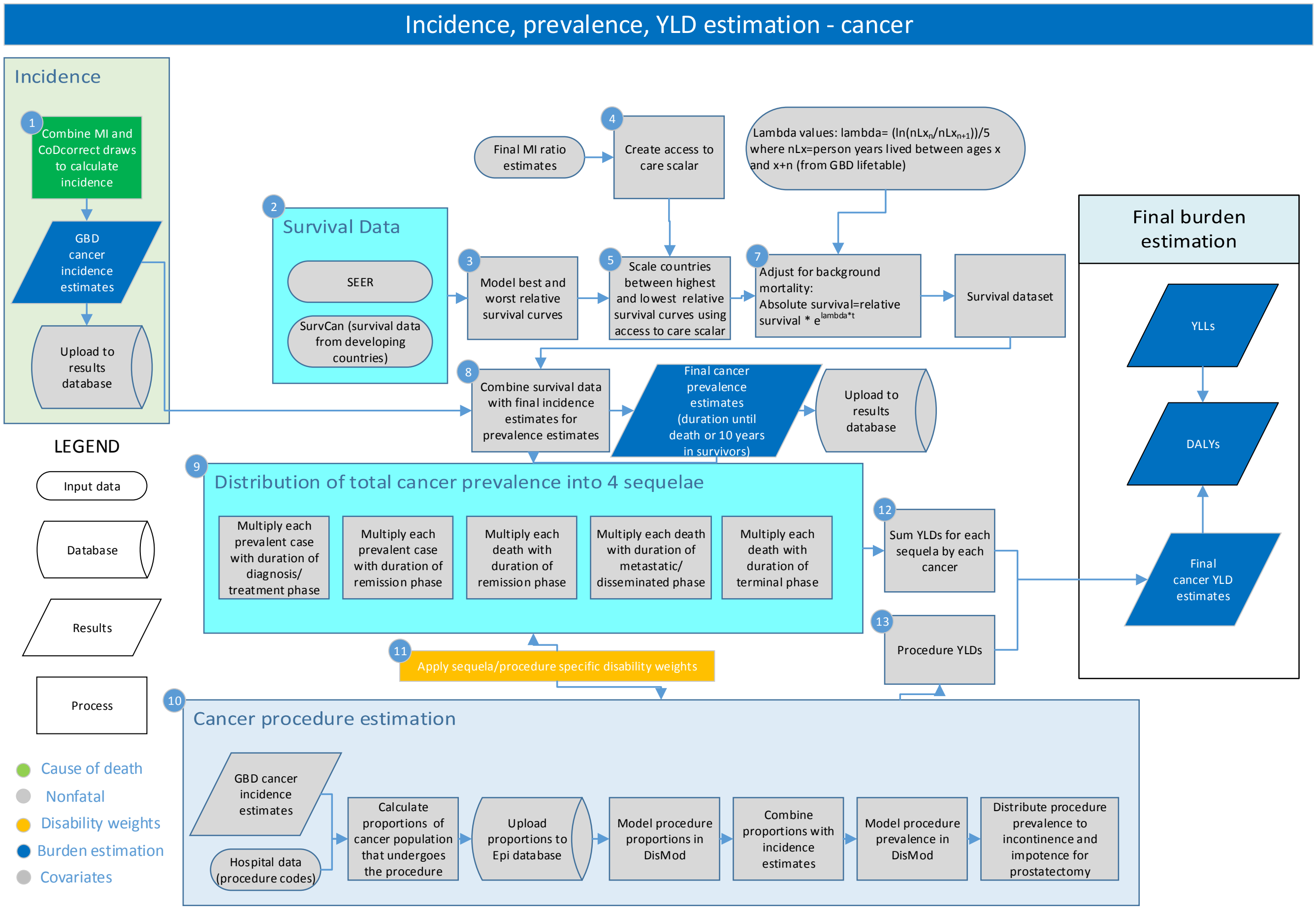

Input data and methodological appendix

Case definition

For GBD 2015, the incidence, prevalence, and disability are estimated for all cancers as defined in ICD-10 (C00-C96). Prevalence for all cancers is estimated for a maximum of 10 years after incidence as in GBD 2013. Prevalence extending beyond the 10 year period is only estimated for permanent sequelae from procedures.

To estimate disability for each cancer, total prevalence is split into four sequelae: 1. diagnosis and primary therapy; 2. controlled phase; 3. metastatic phase; and 4. terminal phase. Diagnosis and primary therapy are defined as the time from symptoms onset to end of treatment. Controlled phase is defined as the time after finishing primary treatment and either cure (defined as survival after 10 years) or metastatic phase. Metastatic phase is defined as the time period of intensive treatment for metastatic disease, terminal phase is defined as the one month period prior to death. Each of these sequelae has a separate disability weight (**Error! Reference source not found.**). Additional disability is estimated for breast cancer (disability due to mastectomy), larynx cancer (disability due to laryngectomy), colon and rectum cancer (disability due to stoma), bladder cancer (disability due to incontinence), and prostatectomy (disability due to incontinence and impotence). The associated ICD codes for neoplasms estimated for GBD 2015 are listed below, as well as in Appendix Table 4.

Table 1. GBD cancer causes with respective ICD codes

GBD cause	ICD9	ICD10
Bladder cancer	188.0-188.9	C67-C67.9
Brain and nervous system cancer	191.0-192.9	C70-C72.9
Breast cancer	174.0-175.9	C50-C50.929
Cervical cancer	180.0-180.9	C53-C53.9, D26.0
Colon and rectum cancer	153.0-154.9, 209.1-209.17	C18-C21.9
Esophageal cancer	150.0-150.9	C15-C15.9
Gallbladder and biliary tract cancer	156.0-156.9, 209.25-209.27	C23-C24.9
Hodgkin disease	201.0-201.98	C81-C81.99
Kidney cancer	189.0-189.1, 209.24	C64-C65.9
Larynx cancer	161.0-161.9	C32-C32.9
Leukemia	208.0-208.92	C94.1, C94.7-C95.92
Acute lymphoid leukemia ALL	204.0-204.02	C91.0-C91.02
Acute myeloid leukemia AML	205.0-205.02, 205.3-205.32, 206.0-206.02, 207.0	C92.0-C92.02, C92.3-C92.62, C93.0-C93.02, C94.0-C94.02, C94.2-C94.22, C94.4-C94.5
Chronic lymphoid leukemia CLL	204.1-204.12	C91.1-C91.12
Chronic myeloid leukemia CML	205.1-205.12, 206.1-206.12, 207.1	C92.1-C92.12
lymphoid leukemia	204.0, 204.2-204.92	C91, C91.2-C91.92
myeloid leukemia	205.0, 205.2-205.22, 205.8-206.0, 206.2-207.9	C92, C92.2-C92.22, C92.7-C93, C93.1-C94, C94.3-C94.32, C94.6
Liver cancer	155.0-155.9	C22-C22.9
Lung, bronchus, and trachea cancer	162.0-162.9, 209.21	C33-C34.92
Non-Hodgkin lymphoma	200.0-200.9, 202.0-202.98	C82-C86.6, C96-C96.9
Malignant skin melanoma	172.0-172.9	C43-C43.9
Mesothelioma	158.9, 163.0-163.9	C45-C45.9
Oral and Lip Cancer	140.0-145.9	C0-C08.9
Multiple myeloma and immunoproliferative diseases	203.0-203.9	C88-C90.9
Nasopharynx cancer	147.0-147.9	C11-C11.9
Non-melanoma skin cancer Basal cell carcinoma	173.0-173.01, 173.09-173.11, 173.19-173.21, 173.29-173.31, 173.39-173.41, 173.49-173.51, 173.59-173.61, 173.69-173.71, 173.79-173.81,	C44.0-C44.01, C44.09-C44.119, C44.19-C44.219, C44.29-C44.319, C44.39-C44.41, C44.49-C44.519, C44.59-C44.619, C44.69-C44.719, C44.79-

	173.89-173.91, 173.99, 216.0-216.9, 232.0-232.9, 238.2	C44.80, C44.82-C44.91, C44.99
Non-melanoma skin cancer Squamous-cell carcinoma	173.02, 173.12, 173.22, 173.32, 173.42, 173.52, 173.62, 173.72, 173.82, 173.92	C44.02, C44.12-C44.129, C44.22-C44.229, C44.32-C44.329, C44.42, C44.52-C44.529, C44.62-C44.629, C44.72-C44.729, C44.81, C44.92, D04-D04.9, D49.2
Other neoplasms	158.0-158.8, 209.4-209.57, 209.61, 209.63-209.67, 210.0-211.8, 212.0-212.8, 213.0-215.9, 217.0-221.8, 222.0-222.8, 223.0-223.89, 224.0-229.0, 229.8, 230.1-230.8, 231.0-231.2, 233.0-233.2, 233.31-233.32, 233.4-233.5, 233.7, 234.0-234.8, 235.0, 235.4, 235.6-235.8, 236.1-236.2, 236.4-236.5, 236.7, 236.91-237.3, 237.5-238.1, 238.3-238.5, 239.2-239.4, 239.6	D00.00-D00.2, D01.0-D01.3, D02.0-D02.3, D03-D03.9, D05-D06.9, D07.0-D07.2, D07.4-D07.5, D09.0, D09.2-D09.3, D09.8, D10.0-D10.7, D11-D12.9, D13.0-D13.7, D14.0-D14.32, D15-D24.9, D27-D27.9, D28.0-D28.7, D29.0-D29.8, D30.0-D30.8, D31-D36.7, D37.01-D37.5, D38.0-D38.5, D39.1-D39.2, D39.8, D40.0-D40.8, D41.0-D41.8, D42-D43.9, D44.0-D44.8, D45-D45.9, D47-D47.0, D47.2-D47.9, D48.0-D48.7, D49.3-D49.4, D49.6
Other cancers	152.0-152.9, 160.0-160.9, 164.0-164.9, 170.0-171.9, 181.0-181.9, 182.9, 183.2-183.8, 184.0-184.4, 184.8, 187.1-187.8, 189.2-189.8, 190.0-190.9, 194.0-194.8, 209.0-209.03, 209.22, 209.31-209.36	C17-C17.9, C3-C31.9, C37-C38.8, C4-C41.9, C47-C5, C51-C52.9, C57-C57.8, C58-C58.0, C60-C60.9, C63-C63.8, C66-C66.9, C68.0-C68.8, C69-C7, C74-C75.8, D49.81
Other pharynx cancer	146.0-146.9, 148.0-148.9	C09-C10.9, C12-C13.9

Ovarian cancer	183.0	C56-C56.9
Pancreatic cancer	157.0-157.9	C25-C25.9
Prostate cancer	185.0-185.9	C61-C61.9
Stomach cancer	151.0-151.9, 209.23	C16-C16.9
Testicular cancer	186.0-186.9	C62-C62.92
Thyroid cancer	193.0-193.9	C73-C73.9
Uterine cancer	182.0-182.8	C54-C54.9
Garbage code	149.0-149.9, 159.0-159.9, 165.0-165.9, 169.0, 173.0, 176.0-179.9, 183.9-184.0, 184.5, 184.9, 187.0, 187.9, 189.0, 189.9, 194.9-199.9, 209.0, 209.2, 209.29-209.3, 209.6, 209.62, 209.69-210.0, 211.0, 211.9-212.0, 212.9, 221.0, 221.9-222.0, 222.9-223.0, 223.9, 229.0-229.1, 229.9-230.0, 230.9-231.0, 231.8-231.9, 233.0, 233.3, 233.39, 233.6, 233.9-234.0, 234.9-235.3, 235.5, 235.9-236.0, 236.3, 236.6, 236.9, 237.4, 238.0, 238.6-239.1, 239.5, 239.7-239.9	C14-C14.9, C26-C29, C35-C36, C39-C39.9, C42, C44, C46-C46.9, C55-C55.9, C57.9, C59-C6, C63.9, C68, C68.9, C75.9-C80.9, C87, C97-D00.0, D01, D01.4-D02, D02.4-D02.9, D07, D07.3-D07.39, D07.6-D09, D09.1-D09.19, D09.7, D09.9-D10, D10.9, D13, D13.9-D14, D14.4, D28, D28.9-D29, D29.9-D30, D30.9, D36.9-D37.0, D37.6-D38, D38.6-D39.0, D39.7, D39.9-D40, D40.9-D41, D41.9, D44, D44.9, D46-D46.9, D47.1, D48, D48.9-D49.1, D49.5, D49.7-D49.8, D49.89-D49.9

Input data

Cancer incidence is directly estimated from cancer mortality using mortality to incidence ratios. Data sources for cancer mortality are described in detail elsewhere.[1] Data sources to scale countries between a hypothetical best- and a hypothetical worst case survival remained the same as in GBD 2013 where we used SEER 2010 data for the “best case” survival and a combination of the 1950 US Mortality Files with “Cancer Survival in Africa, Asia, the Caribbean and Central America” (SurvCan) data for the worst case survival.[2–4] For mesothelioma, gallbladder cancer, and the leukemia subtypes SEER 1973 survival data for the lower boundary was used since these cancers are not included in the US Mortality Files from 1950. To estimate the proportion of cancer patients undergoing procedures we used SEER data form 1983 to 2008[2], Canada Hospital Data from 1994 to 2009[5], and Mexico Hospital Data from 2001 to 2009[6]. Data sources used to adjust procedure sequelae will be listed below.

Modeling strategy

Estimation of cancer mortality and mortality to incidence ratio estimation has been described in the GBD 2015 Mortality and Causes of Death capstone paper.[1] The final GBD cancer mortality estimates are being transformed to incidence estimate by using the separately estimated MI ratios. To summarize the mortality to incidence ratio estimation process, processed incidence and mortality data from cancer registries were matched by cancer, age, sex, year, and location to generate MI ratios. Multiple MI models with different sets of covariates were created. All models were run separately by cancer, and the best model was selected from the following list:

1. $\text{logit}\left(MI\ ratio_{c,a,s,t}\right) = \alpha + \beta_1 SDI_{c,t} + \sum_a^A \beta_2 I_a + \beta_3 I_s + \theta_c + \epsilon_{c,a,s,t}$
2. $\text{logit}\left(MI\ ratio_{c,a,s,t}\right) = \alpha + \beta_1 SDI_{c,t} + \sum_a^A \beta_2 I_a + \beta_3 I_s + \beta_4 t + \theta_c + \epsilon_{c,a,s,t}$
3. $\text{logit}\left(MI\ ratio_{c,a,s,t}\right) = \alpha + \beta_1 SDI_{c,t} + \sum_a^A \beta_2 I_a + \beta_3 I_s + \beta_4 DS + \theta_c + \epsilon_{c,a,s,t}$
4. $\text{logit}\left(MI\ ratio_{c,a,s,t}\right) = \alpha + \beta_1 SDI_{c,t} + \sum_a^A \beta_2 I_a + \beta_3 I_s + \beta_4 DS + \beta_5 t + \theta_c + \epsilon_{c,a,s,t}$
5. $\text{logit}\left(MI\ ratio_{c,a,s,t}\right) = \alpha + \beta_1 SDI_{c,t} + \sum_a^A \beta_2 I_a + \beta_3 I_s + \theta_c + \lambda_{SR}(SDS_{c,t}) + \beta_4 t + \epsilon_{c,a,s,t}$
6. $\text{logit}\left(MI\ ratio_{c,a,s,t}\right) = \alpha + \beta_1 SDI_{c,t} + \sum_a^A \beta_2 I_a + \beta_3 I_s + \theta_c + \lambda_{SR}(SDS_{c,t}) + \epsilon_{c,a,s,t}$
7. $\text{logit}\left(MI\ ratio_{c,a,s,t}\right) = \alpha + \beta_1 SDI_{c,t} + \sum_a^A \beta_2 I_a + \beta_3 I_s + \theta_c + \lambda_{SR}(SDS_{c,t}) + \beta_4 DS + \epsilon_{c,a,s,t}$
8. $\text{logit}\left(MI\ ratio_{c,a,s,t}\right) = \alpha + \beta_1 SDI_{c,t} + \sum_a^A \beta_2 I_a + \beta_3 I_s + \theta_c + \lambda_{SR}(SDS_{c,t}) +$
   $\beta_4 t + \beta_5 DS + \epsilon_{c,a,s,t}$

c: country, a: age group, t: time (years); s: sex
SDI: Socio-demographic index (index using log lag dependent income per capita (LDI), average educational attainment in the population over age 15, and total fertility rate (TFR))
I: indicator variable
DS: binary variable for development status
θ_c: random effect by country (intercept)
$\lambda_{SR}(SDIc, t)$: random effect modifier between SDI and superregion (slope)
$\epsilon_{c,a,s,t}$: error term
All models were tested at multiple stages before generating final predictions. Models were initiated with SDI (Socio-Demographic Index) as covariate and first tested using the complete input dataset. If after that initial test the SDI covariate's coefficient was negative (as expected), the next step was to outlier any data point for which the residual from the prediction was greater than three times the MAD from the mean residual. Next, data were marked as outliers due to a random effect criterion: if the country-level random effect for a developing country was lower than the random effect for the USA, all data points for that country were marked as outliers. This process was run iteratively until all developing countries had country-level random effects greater than that of the USA. All data points marked outliers were dropped from the final dataset, and that dataset was used to create the final model predictions.

If the SDI coefficient was found to be positive (unexpected) after the initial SDI test, it was assumed to indicate an excess of unrealistic data in the input dataset. To remove these unrealistic data, SDI was temporarily removed from the model formula. The model proceeded as above without SDI until all unrealistic data points were removed and the SDI coefficient was found to be negative. Unrealistic data were marked as outliers using the same residual MAD and random effect methodology described above. Once SDI was established as negative (expected) the model proceeded as usual.

To select the best model formula, the initial model results were tested by comparing mean MI predictions and the mean root-mean-squared error (RMSE) values of ten random samples of 80%/20% splits from the input dataset. Mean MI predictions were compared between developing and developed countries. Models were eliminated if the mean MI for developed countries was higher than the mean MI ratio for developing countries. For RMSE testing, the dataset was split into an 80% dataset for model development and a 20% dataset for model testing. The process was repeated ten times. The best model for each cancer was selected based on the lowest mean out-of sample RMSE from those models remaining after checking the mean MI. Table 2 contains the final models selected for each cancer.

Table 2. Final MI ratio model selection

Cancer	Final model number (for model numbers, see text)
Ovarian cancer	1
Uterine cancer	1
Gallbladder cancer	1
Kidney cancer	1
Larynx cancer	1
Acute lymphoid leukemia	1
Chronic myeloid leukemia	1
Lip and oral cavity cancer	1
Pancreatic cancer	1
Hodgkin lymphoma	2
Acute myeloid leukemia	2
Chronic lymphoid leukemia	2
Malignant skin melanoma	2
Bladder cancer	3
Brain and nervous system cancer	3
Esophageal cancer	3
Tracheal, bronchus and lung cancer	3
Mesothelioma	3
Multiple myeloma	3
Other cancer	3
Prostate cancer	4
Testicular cancer	4
Breast cancer	4
Colorectal cancer	4
Leukemia	4
Liver cancer	4
Non-Hodgkin lymphoma	4
Non-melanoma skin cancer (squamous cell carcinoma)	4

Stomach cancer	4
Nasopharynx cancer	6
Cervical cancer	7
Other pharynx cancer	8
Thyroid cancer	8

Once the best models were selected, data points were manually outliered based on the results of the first run of the model algorithm. Data points were outliered if they clearly influenced the model in an unrealistic way. For example, a data point was marked as an outlier if it created a single-year, single age group spike in model predictions. This was mainly the case in countries with a small number of cases or deaths, or in age groups with small numbers of cases or deaths. Manual outliers were removed from the input dataset prior to initiating the second run of the model algorithm.

After best models were selected, all final outliers were dropped from the data input, and final linear predictions were created, the final linear predictions and residuals were used as input for space-time smoothing. The weighted residuals were added to the linear model predictions and used as priors for the third stage, a Gaussian process regression (GPR) implementing a Matern covariance function. Final MI ratio predictions with 95% uncertainty intervals were obtained by back-transforming 1000 draws from the posterior distribution. The MI ratio estimation has undergone a revision compared to the GBD 2010 and GBD 2013. Whereas in GBD 2010 and GBD 2013 only one model was used to predict all MI ratios, for GBD 2015 we generated multiple models and chose a best model based on out-of sample validation. Another major difference is that LDI (lagged distributed income) was used as a covariate, which was replaced by SDI (Socio-demographic index) for GBD 2015. Final MI ratio estimates at the 1000 draw level were combined with final mortality estimates (as well at the 1000 draw level) to generate incidence estimates. It was assumed that uncertainty in the MI ratio is independent of uncertainty in the estimated mortality.

After transforming the final GBD cancer mortality estimates to incidence estimates using the 1,000 mortality draws and the 1,000 MI ratio draws (step 1 in the flowchart), incidence was combined with the relative yearly survival estimates up to 10 years (step 8 in the flowchart). To estimate cancer prevalence, relative cancer survival was estimated by scaling cancer specific survival between the “best case” and “worst case” survival, using the survival data sources listed above (step 2, 3, and 5 in the flowchart). To transform relative to absolute survival (adjusting for background mortality), GBD 2015 lifetables were used (step 6 and 7 in the flowchart) to calculate lambda values: lambda= (ln(nLxn/nLxn+1))/5 where nLx=person years lived between ages x and x+n (from GBD lifetable). Absolute survival was then calculated using an exponential survival function (absolute survival = relative survival * $e^{lambda*t}$).

The access to cancer care variable to scale countries between the best and worst case survival was estimated using the same method as for GBD 2013 (step 4 in the flowchart)[7]:

$$Access\ to\ care = 1 - \frac{Age\ standardized\ MI\ ratio_{cys} - Age\ standardized\ MI\ ratio_{min}}{Age\ standardized\ MI\ ratio_{max} - Age\ standardized\ MI\ ratio_{min}}$$

c=country; y=year; s=sex; Age-standardized MI $ratio_{min}$=lowest MI ratio for all countries and years; Age standardized MI $ratio_{max}$=highest MI ratio for all countries and years

Survivors beyond 10 years were considered cured. The survivor population prevalence was divided into two sequelae (1. diagnosis and primary therapy; 2. controlled phase). The yearly prevalence of the population that did not survive beyond 10 years was then divided into the four sequelae by assigning the fixed durations for the diagnosis and primary therapy phase, metastatic phase, and terminal phase and assigning the remaining prevalence to the controlled phase (step 9 in the flowchart). Duration of the treatment sequelae (1. diagnosis and primary therapy; 2. controlled phases; 3. metastatic phase; 4. terminal phase) remained the same as for GBD 2013.[7] Table 3 lists the duration including sources used.

Table 3. Duration of four prevalence sequelae by cancer

	Diagnosis/ Treatment (months)	Remission	Disseminated/metastatic (months)	Note	Terminal (months)
Esophageal cancer	5[8]	Calculated based on remainder of time after attributing other sequelae.	4.6[9]	SEER Summary Stage 1997 (Distant site/node involved) 1995-2000	1 months
Stomach cancer	5.2[8]		3.88[9]	SEER Summary Stage 1997 (Distant site/node involved) 1995-2000	
Liver cancer	4		2.51[9]	SEER Summary Stage 1997 (Distant site/node involved) 1995-2000	
Larynx cancer	5.3[8]		8.84[9]	SEER Stage IVc	
Lung cancer	3.3[10]		4.51[9]	SEER Summary Stage 1997 (Distant site/node involved) 1995-2000	
Breast cancer	3[10]		17.7[9]	SEER Summary Stage 1997 (Distant site/node involved) 1995-2000	
Cervical cancer	4.8[8]		9.21[9]	SEER Summary Stage 1997 (Distant site/node involved) 1995-2000	
Uterine cancer	4.6[8]		11.6[9]	SEER Summary Stage 1997 (Distant site/node involved) 1995-2000	
Prostate cancer	4[10]		30.35[9]	SEER Summary Stage 1997 (Distant site/node involved) 1995-2000	
Colorectal cancer	4[10]		9.69[9]	SEER Summary Stage 1997 (Distant site/node involved) 1995-2000	
Oral cancer	5.3[8]		9.33[9]	SEER Stage IVc	
Nasopharyngeal cancer	5.3[8]		13.19[9]	SEER Stage IVc	
Cancer of other part of pharynx	5.3[8]		7.91[9]	SEER Stage IVc	

Gallbladder cancer	4		3.47[9]	SEER Summary Stage 1997 (Distant site/node involved) 1995-2000
Pancreas cancer	4.1[8]		2.54[9]	SEER Summary Stage 1997 (Distant site/node involved) 1995-2000
Melanoma	2.9[11]		7.18[9]	SEER Summary Stage 1997 (Distant site/node involved) 1995-2000
NMSC (squamous cell carcinoma)	2.9[11]		17[12]	
Ovarian cancer	3.2[10]		25.6[9]	SEER Summary Stage 1997 (Distant site/node involved) 1995-2000
Testicular cancer	3.7[8]		19.47[9]	SEER Stage III
Kidney cancer	5.3[8]		5.38[9]	SEER Summary Stage 1997 (Distant site/node involved) 1995-2000
Bladder cancer	5.1[8]		5.8[9]	SEER Summary Stage 1997 (Distant site/node involved) 1995-2000
Brain cancer	5		6.93[9]	SEER Median age standardized survival all patients, all years
Thyroid cancer	3		19.39[9]	SEER Stage IVc
Mesothelioma	4		7.75[9]	SEER Summary Stage 1997 (Distant site/node involved) 1995-2000
Hodgkin lymphoma	3.7[10]		26[13]	
Non Hodgkin lymphoma	3.7[10]		7.7[13]	
Multiple myeloma	7[8]		36.82[9]	SEER Median age standardized survival all patients, all years
Leukemia[8]	5		43.67[9]	SEER Median age standardized survival all patients, all years
ALL	12		7.02[9]	SEER Median age standardized survival all patients, all years
AML	6		4.6[9]	SEER Median age standardized survival all patients, all years

CLL	6		48[14]		
CML	6		4.6[9]	SEER Median age standardized survival for AML (patients with CML die in blast crisis, which is treated like AML) all patients, all years	
Other	4.4 (mean of other cancer durations)		15.81[9]	SEER Median age standardized survival all patients, all years	

For cancer specific procedure sequelae hospital data were used to estimate the number of cancer patients undergoing mastectomy, laryngectomy, stoma, prostatectomy, and cystectomy (step 10 in the flowchart). These proportions remained the same as in GBD 2013.[7] Proportions were generated by dividing the rate of procedures generated from the diagnostic codes in the hospital dataset and the coverage population by the GBD age-, and sex-specific disease incidence rates for that country. Diagnostic codes used are listed in table 4:

Table 4. Procedure codes used to estimate cancer procedure proportions		
Procedure	Cancer	Procedure code (ICD-9_CM)
Mastectomy	Breast cancer	854, 8541, 8542, 8543, 8544, 8545,<br>8546, 8547, 8548
Laryngectomy	Larynx cancer	301, 303, 304, 3029
Stoma	Colon and rectum cancer	461, 4610, 4611, 4613, 4862
Cystectomy	Bladder cancer	5771, 5779
Prostatectomy	Prostate	603, 604, 605, 606, 6062

To estimate procedure-related disability for certain cancers, the procedure proportions (proportion of cancer population that undergoes procedures) from hospital data was used as input for a proportion model in Dismod-MR 2.1 in order to estimate the proportions for all locations, by age, and by sex. Since colostomy or ileostomy procedures are done for reasons other than cancer a literature review was done to determine the proportion of ostomies due to colorectal cancer. The “all cause” colostomy proportions were multiplied by 0.58 based on the results of the literature review showing that on average 58% of ostomies are done for colorectal cancer.[15–17] The final procedure proportions were applied to the incidence cases of the respective cancers to determine the incident cases of the cancer population that underwent procedures. These incident cases were used again as an input for DisMod-MR 2.1 with a remission specification of zero and the cause-specific mortality of the specific cancer to obtain prevalence of the sequela. By using cause-specific mortality, the simplifying assumption was made that survival for cancer patients undergoing procedures is the same as for cancer patients who do not need a procedure. Since disability associated with prostatectomy comes from impotence and incontinence and not from the prostatectomy itself 18% of the prostatectomy prevalence was assumed to be incontinent and 55% was assumed to be impotent based on a literature review done for GBD 2013.[18–25]

Since all sequelae for a cause need to be mutually exclusive, the controlled phase for the cancers with additional procedure related disability was adjusted to only include the population without procedure related disability (= controlled phases prevalence of the total population – controlled phase prevalence of the proportion that experienced procedure related disability) (step 11 in the flowchart). The disability weight for the prevalence of the population that experiences additional disability was adjusted to reflect the combined disability of the controlled phase as well as the procedure.

Lastly, the procedure sequelae prevalence and general sequelae prevalence were multiplied with disability weights (Table 5) for the procedures to obtain the number of YLDs (steps 11, 12, 13 in the flowchart). The sum of these YLDs is the final YLD estimate associated with each cancer.

Table 5. Lay description and disability weights

Health state	Lay description	Estimate	Uncertainty interval	
Cancer, diagnosis and primary therapy (cancer_diagnosis)	This person has pain, nausea, fatigue, weight loss and high anxiety.	0.288	0.193	0.399
Cancer, controlled phase (generic_medication)	This person has a chronic disease that requires medication every day and causes some worry but minimal interference with daily activities.	0.049	0.031	0.072
Cancer, metastatic (cancer_metastatic)	This person has severe pain, extreme fatigue, weight loss and high anxiety.	0.451	0.307	0.600
Terminal phase, with medication (cancer_terminal_treat)	This person has lost a lot of weight and regularly uses strong medication to avoid constant pain. The person has no appetite, feels nauseous, and needs to spend most of the day in bed.	0.540	0.377	0.687
Mastectomy (cancer_mastectomy)	This person had one of her breasts removed and sometimes has pain or swelling in the arms.	0.036	0.020	0.057
Stoma (cancer_stoma)	This person has a pouch attached to an opening in the belly to collect and empty stools.	0.095	0.063	0.131
Laryngectomy (speech_problems)	This person has difficulty speaking, and others find it difficult to understand.	0.051	0.032	0.078
Urinary incontinence (incontinence)	This person cannot control urinating.	0.139	0.094	0.198

Impotence (impotence)	This person has difficulty in obtaining or maintaining an erection.	0.017	0.009	0.030

Estimating non-melanoma skin cancer (squamous and basal cell carcinoma)

Mortality due to squamous cell skin cancer was estimated in the same way that all other cancers were estimated using the same methods as in GBD 2013. Cancer registry data was used as input data into the COD database. CODEm models were run to generate estimates for all countries, years, and age groups by sex.

We estimated squamous cell skin cancer incidence by using cancer registry as well as primary literature data for incidence. Only cancer registries that were listed in CI5 VIII as registering squamous cell carcinoma were included in the analysis.[26] For cancer registry data reported at the three digit level (C44: Other and unspecified malignant neoplasm of skin), proportions from Karagas et al were used to split C44 into squamous cell carcinoma and basal cell carcinoma.[27] A systematic literature review was done to update any newly published articles between 2013 and 2015. The search was done on 8/15/2015 and yielded 619 initial results of which five were included as sources for incidence. DisMod-MR 2.1 was used to model incidence. To estimate prevalence the incidence estimates were combined with the yearly survival estimates up to 10 years. Relative survival from squamous cell skin cancer was estimated by scaling cancer specific survival between a "best case" survival of 100% and a "worst case" survival. For the "worst case" survival data for melanoma from the 1950 US Mortality Files was compared to Cancer Survival in Africa, Asia, the Caribbean, and Central America (SurvCan) data and whichever survival was the lowest was used.

As in GBD 2013 for basal cell carcinoma of the skin (BCC) we did not estimate any mortality given that this is a very rare event. Incidence estimates for BCC were generated using the same methods as for GBD 2013. Cancer registry data as well as literature was used for incidence data. A systematic literature review was done to update any newly published articles between 2013 and 2015. The search was done on 8/15/2015 and yielded 619 initial results, of which 14 were included as sources for incidence. DisMod-MR 2.1 was used to model incidence and prevalence. Since prevalence and duration of basal cell skin cancer is not generally reported, we calculated the prevalence of BCC as function of two extreme scenarios (duration 1 versus 5 years). Country, age, sex and year specific duration was estimated using a country-age-sex-year specific relative access-to-care-score.

The access to care score was based on the melanoma mortality to incidence ratio:

$$Access\ to\ cancer\ care = 1 - \frac{Age\ standardized\ MI\ ratio_{cys} - Age\ standardized\ MI\ ratio_{min}}{Age\ standardized\ MI\ ratio_{max} - Age\ standardized\ MI\ ratio_{min}}$$

c=country; y=year; s=sex; Age-standardized MI ratio$_{min}$=lowest MI ratio for all countries and years; Age-standardized MI ratio$_{max}$=highest MI ratio for all countries and years

Remission was calculated as the inverse of the duration estimates and used as additional input for DisMod-MR 2.1.

There are no other significant changes to the GBD 2015 neoplasms modeling process.

References

1 GBD Mortality and Causes of Death Collaborators. Global, regional, and national life expectancy, all-cause and cause specific mortality for 249 causes of death, 1980–2015: a systematic analysis for the Global Burden of Disease Study 2015. *Lancet* 2016; published online Aug.

2 National Cancer Institute (United States). United States SEER Cancer Data 1973-2010. Bethesda, United States: National Cancer Institute (United States). .

3 Sankaranarayanan R, Swaminathan R, World Health Organization, International Agency for Research on Cancer, editors. Cancer survival in Africa, Asia, the Caribbean and Central America. Lyon, France: International Agency for Research on Cancer, World Health Organization, 2011.

4 National Center for Health Statistics, Centers for Disease Control and, Prevention. US Mortality Files. 61-Year Trends in U.S. Cancer Death Rates. http://seer.cancer.gov/archive/csr/1975_2010/results_merged/topic_historical_mort_trends.pdf.

5 Canadian Institute for Health Information (CIHI). Canada Discharge Abstract Database 1994-2009. Ottawa, Canada: Canadian Institute for Health Information (CIHI). .

6 Ministry of Health (Mexico). Mexico Ministry of Health Hospital Discharges 2000-2012. Mexico City, México: Ministry of Health (Mexico). .

7 Fitzmaurice C, Dicker D, Pain A, *et al.* The Global Burden of Cancer 2013. *JAMA Oncol* 2015; published online May 28. DOI:10.1001/jamaoncol.2015.0735.

8 Neal RD, Din NU, Hamilton W, *et al.* Comparison of cancer diagnostic intervals before and after implementation of NICE guidelines: analysis of data from the UK General Practice Research Database. *Br J Cancer* 2014; **110**: 584–92.

9 Surveillance, Epidemiology, and End Results (SEER) Program (www.seer.cancer.gov) SEER*Stat Database: Incidence - SEER 18 Regs Research Data + Hurricane Katrina Impacted Louisiana Cases, Nov 2012 Sub (1973-2010 varying) - Linked To County Attributes - Total U.S., 1969-2011 Counties, National Cancer Institute, DCCPS, Surveillance Research Program, Surveillance Systems Branch, released April 2013, based on the November 2012 submission. .

10 Allgar VL, Neal RD. Delays in the diagnosis of six cancers: analysis of data from the National Survey of NHS Patients: Cancer. *Br J Cancer* 2005; **92**: 1959–70.

11 Neal RD, Cannings-John R, Hood K, *et al.* Excision of malignant melanomas in North Wales: effect of location and surgeon on time to diagnosis and quality of excision. *Fam Pract* 2008; **25**: 221–7.

12 Nolan RC, Chan MT-L, Heenan PJ. A clinicopathologic review of lethal nonmelanoma skin cancers in Western Australia. *J Am Acad Dermatol* 2005; **52**: 101–8.

13 Kewalramani T, Nimer SD, Zelenetz AD, *et al.* Progressive disease following autologous transplantation in patients with chemosensitive relapsed or primary refractory Hodgkin's disease or aggressive non-Hodgkin's lymphoma. *Bone Marrow Transplant* 2003; **32**: 673–9.

14 Esteban D, Tovar N, Jiménez R, *et al.* Patients with relapsed/refractory chronic lymphocytic leukaemia may benefit from inclusion in clinical trials irrespective of the therapy received: a case-control retrospective analsysis. *Blood Cancer J* 2015; **5**: e356.

15 Canova C, Giorato E, Roveron G, Turrini P, Zanotti R. Validation of a stoma-specific quality of life questionnaire in a sample of patients with colostomy or ileostomy. *Colorectal Dis Off J Assoc Coloproctology G B Irel* 2013; **15**: e692-698.

16 Caricato M, Ausania F, Ripetti V, Bartolozzi F, Campoli G, Coppola R. Retrospective analysis of long-term defunctioning stoma complications after colorectal surgery. *Colorectal Dis Off J Assoc Coloproctology G B Irel* 2007; **9**: 559–61.

17 Erwin-Toth P, Thompson SJ, Davis JS. Factors impacting the quality of life of people with an ostomy in North America: results from the Dialogue Study. *J Wound Ostomy Cont Nurs Off Publ Wound Ostomy Cont Nurses Soc WOCN* 2012; **39**: 417-422-424.

18 Catalona WJ, Carvalhal GF, Mager DE, Smith DS. Potency, continence and complication rates in 1,870 consecutive radical retropubic prostatectomies. *J Urol* 1999; **162**: 433–8.

19 Donnellan SM, Duncan HJ, MacGregor RJ, Russell JM. Prospective assessment of incontinence after radical retropubic prostatectomy: objective and subjective analysis. *Urology* 1997; **49**: 225–30.

20 Eastham JA, Kattan MW, Rogers E, *et al.* Risk factors for urinary incontinence after radical prostatectomy. *J Urol* 1996; **156**: 1707–13.

21 Kundu SD, Roehl KA, Eggener SE, Antenor JAV, Han M, Catalona WJ. Potency, continence and complications in 3,477 consecutive radical retropubic prostatectomies. *J Urol* 2004; **172**: 2227–31.

22 Potosky AL, Davis WW, Hoffman RM, *et al.* Five-Year Outcomes After Prostatectomy or Radiotherapy for Prostate Cancer: The Prostate Cancer Outcomes Study. *JNCI J Natl Cancer Inst* 2004; **96**: 1358–67.

23 Sacco E, Prayer-Galetti T, Pinto F, *et al.* Urinary incontinence after radical prostatectomy: incidence by definition, risk factors and temporal trend in a large series with a long-term follow-up. *BJU Int* 2006; **97**: 1234–41.

24 Stanford JL, Feng Z, Hamilton AS, *et al.* Urinary and sexual function after radical prostatectomy for clinically localized prostate cancer: the Prostate Cancer Outcomes Study. *JAMA* 2000; **283**: 354–60.

25 Walsh PC, Marschke P, Ricker D, Burnett AL. Patient-reported urinary continence and sexual function after anatomic radical prostatectomy. *Urology* 2000; **55**: 58–61.

26 Parkin D, Whelan S, Ferlay J, Teppo L, Thomas D. Cancer Incidence in Five Continents VIII. Lyon: IARC, 2002.

27 Karagas MR, Greenberg ER, Spencer SK, Stukel TA, Mott LA. Increase in incidence rates of basal cell and squamous cell skin cancer in New Hampshire, USA. New Hampshire Skin Cancer Study Group. *Int J Cancer J Int Cancer* 1999; **81**: 555–9.

Rheumatic heart disease

Flowchart

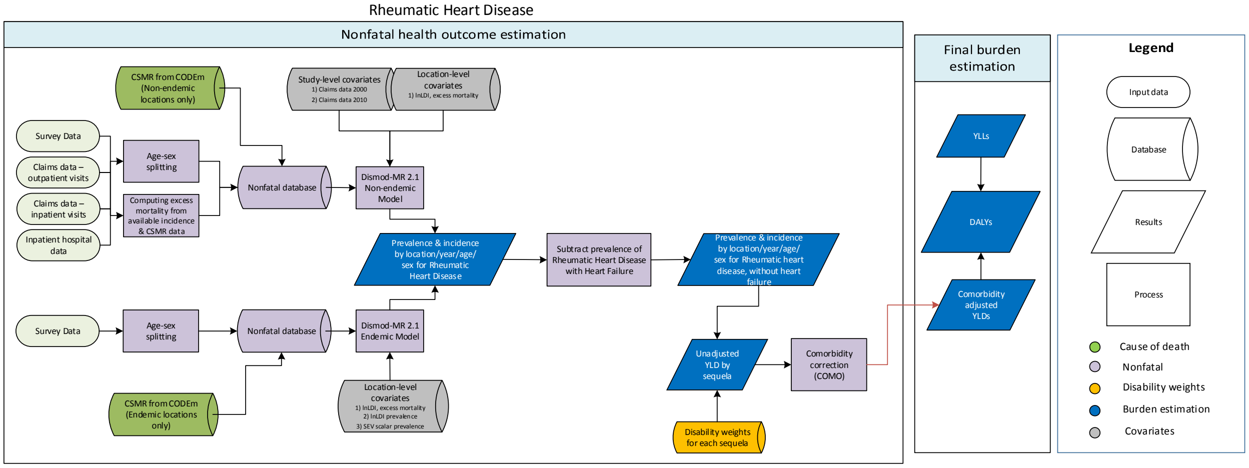

Input data and methodological appendix

Case definition

Rheumatic heart disease (RHD) was defined as a clinical diagnosis by a physician with or without confirmation using echocardiography. This case definition for echocardiographic confirmation of RHD follows the World Heart Federation criteria for echocardiographic diagnosis of rheumatic heart disease (Reményi, B. et al. Nat. Rev. Cardiol. 9, 297–309 (2012); published online 28 February 2012)

ICD codes for data included from hospital records are provided in Appendix Table 4. Please see the table below for additional information on criterion and definition.

Criterion	Definition
1. Echocardiography	Prevalent rheumatic heart disease based on echocardiographic assessment and clinical confirmation
2. Clinical diagnosis	Prevalent rheumatic heart disease based on physician diagnosis

Input data

Model inputs

A systematic review was performed for GBD 2013 and updated for GBD 2015. The GBD 2015 search information encompassed the following:

- Search terms: ('rheumatic heart disease' AND epidemiology[MeSH Subheading]) OR ('acute rheumatic fever' AND epidemiology[MeSH Subheading]) OR ('rheumatic fever' AND epidemiology[MeSH Subheading]) OR (RHD AND epidemiology[MeSH Subheading]) OR ('valvular heart disease' AND epidemiology[MeSH Subheading]) OR (((streptococcus OR streptococci) AND heart) AND epidemiology[MeSH Subheading]) OR (heart AND valve AND disease AND epidemiology[MeSH Subheading]) OR ('mitral valve stenosis' AND epidemiology[MeSH Subheading]) OR (('rheumatic heart disease' OR 'rheumatic fever') AND prevalence) OR (('rheumatic heart disease' OR 'rheumatic fever') AND incidence) OR (('rheumatic heart disease' OR 'rheumatic fever') AND ('standardized mortality ratio' OR SMR)) OR ('rheumatic heart disease' OR 'rheumatic fever' AND 'case fatality')
- Dates included in search: 1/1/2013 – 3/16/2015
- Number of initial hits: 2,045
- Number of sources included: 17

These differed from the GBD 2013 search terms:

- (hasabstract[text] AND Humans[Mesh] AND middle age[MeSH])) OR 21) AND ((rheumatic heart disease/epidemiology[Mesh] OR rheumatic heart disease/mortality[Mesh]) AND (prevalence[Title/Abstract] OR incidence[Title/Abstract]) AND ("2010"[Date - Publication] : "3000"[Date - Publication]) AND (hasabstract[text] AND Humans[Mesh] AND middle age[MeSH]))

The table below illustrates the number of literature studies included in GBD 2015, as well as the number of countries or subnational units and GBD world regions represented.

Low-income model			
	Prevalence	Incidence	Mortality risk
Studies	77	0	0
Countries/subnationals	45	0	0
GBD world regions	12	0	0

High-income model			
	Prevalence	Incidence	Mortality risk
Studies	8	0	0
Countries/subnationals	7	0	0
GBD world regions	4	0	0

We did not include any non-literature-based data types other than the hospital and claims data described elsewhere. Hospital and claims data were available only for the non-endemic country model. We excluded all outpatient data, as they were implausibly low when compared with inpatient data from the same locations and claims data. We also excluded inpatient hospital data from Canada as these were implausibly low when compared with other locations in the region or super-region.

For the endemic country model, we included study-level covariates to crosswalk studies that did not include electrocardiographic confirmation of the disease diagnosis. For the non-endemic country model, we included study-level covariates for inpatient hospital data and claims data from 2000 and 2010 to adjust these data points, using as reference the data obtained from literature and claims data from 2012.

Severity splits and disability weights

Severity level	Lay description	DW (95% CI)
Rheumatic heart disease, not including heart failure	Has a chronic disease that requires medication every day and causes some worry but minimal interference with daily activities.	0.049 (0.031-0.072)

Modeling strategy

For GBD 2015 estimation, we ran two models – one for non-endemic countries and one for endemic countries. We defined endemicity based on GBD 2015 RHD mortality estimates for the year 2015. We used a threshold of 0.15/100,000 deaths in children aged 5-9; countries with less than this number of deaths in 2015 were categorized as high-income, while countries with at least this number of deaths in 2015 were categorized as low-income.

Non-endemic country model: We included hospital data, claims data, and limited literature data on prevalence. We also included CSMR from our mortality estimates of RHD. A prior of no remission was set for all ages, and excess mortality was capped at 0.1 for all ages. We included study-level covariates for inpatient data and claims data from 2000 and 2010, cross-walking them to data from the literature and claims data in 2012. We also included the natural log of lagged distributed income (lnLDI, I$ per capita) as a country-level covariate for excess mortality, with bounds of -0.5 to -0.1.

Endemic country model: We included prevalence data from surveys published in the literature. As with the high-income model, we included CSMR from our mortality estimates of RHD. A prior of no remission was set for all ages, and excess mortality was capped at 0.07, the highest observed mean excess mortality rate data point observed in this model. We also set priors of 0 on incidence for ages 0 to 1, and 50 to 100 to account for patterns of incidence in endemic countries. We used lnLDI as fixed-effect country-level covariates on prevalence and excess mortality, enforcing an inverse relationship for both. The log-transformed, age-standardized SEV scalar was also used as a fixed-effect country-level covariate on prevalence.

We then combined estimates from the endemic and non-endemic models, selecting estimates for the countries identified as non-endemic from the non-endemic model and estimates for the countries identified as endemic from the endemic model. Estimates of heart failure due to RHD were then subtracted from the estimates for RHD, giving the overall prevalence of RHD without heart failure. A description of heart failure due to RHD can be found in the Heart Failure section of the appendix. We evaluated models based on comparing estimates with input data as well as estimates from previous rounds of GBD.

The table below shows the country covariates, parameters, betas, and exponentiated betas:

Covariate	Parameter	beta	Exponentiated beta
Endemic model			
LDI (I$ per capita)	prevalence	-.0815(-.2114 - -.0037)	.9217(.8094 - .9963)
Log-transformed age-standardized SEV scalar: RHD	prevalence	1.086(.5452 - 1.483)	2.962(1.725 - 4.406)
LDI (I$ per capita)	excess mortality rate	-.3748(-.4892 - -.2336)	.6874(.6131 - .7917)

Non-endemic model			
US Claims 2000	prevalence	.3339(.2491 - .4236)	1.396(1.283 - 1.527)
US Claims 2010	prevalence	.6061(.5419 - .6678)	1.833(1.719 - 1.95)
LDI (I$ per capita)	excess mortality rate	-.4849(-.4999 - -.465)	.6158(.6066 - .6281)

We changed the process of selecting countries for the endemic and non-endemic country models. In previous rounds, this decision had been based on country development status and income level, and the models were referred to as low-income and high-income models. However, because our models are trying to capture differences between countries where RHD is endemic and developed countries where the disease is extremely rare, we are now using death data to identify which model should be used for estimates for each country. We set a threshold of 0.15 deaths per 100,000 in children ages 5-9 based on expert opinion. Countries with at least this number of deaths in 2015 were considered endemic for the purposes of RHD modeling, while countries with fewer than this number of deaths were considered non-endemic. Exceptions were applied when data existed to support high childhood endemicity for RHD; because of this, Kenya and Nicaragua were moved to the endemic country category despite relatively low estimates of RHD mortality in this age range. Lists of countries as categorized to endemic or non-endemic are below.

Endemic Locations: North Korea, Cambodia, Indonesia, Laos, Maldives, Myanmar, Philippines, Timor-Leste, Fiji, Kiribati, Marshall Islands, Federated States of Micronesia, Papua New Guinea, Samoa, Solomon Islands, Tonga, Vanuatu, Azerbaijan, Georgia, Kyrgyzstan, Turkmenistan, Uzbekistan, Albania, Belize, Dominica, Dominican Republic, Grenada, Guyana, Haiti, Jamaica, Saint Lucia, Saint Vincent and the Grenadines, Suriname, Bolivia, Nicaragua, Algeria, Egypt, Iraq, Libya, Morocco, Syria, Tunisia, Yemen, Afghanistan, Bangladesh, Bhutan, Nepal, Pakistan, Angola, Central African Republic, Congo, Democratic Republic of the Congo, Equatorial Guinea, Gabon, Burundi, Djibouti, Eritrea, Ethiopia, Madagascar, Malawi, Mauritius, Mozambique, Rwanda, Somalia, Tanzania, Uganda, Zambia, Botswana, Lesotho, Namibia, Swaziland, Zimbabwe, Benin, Burkina Faso, Cameroon, Chad, Cote d'Ivoire, The Gambia, Ghana, Guinea, Guinea-Bissau, Liberia, Mali, Mauritania, Niger, Nigeria, Sao Tome and Principe, Senegal, Sierra Leone, Togo, South Sudan, Sudan, China, India, Kenya, South Africa

Non-endemic Locations: Taiwan, Malaysia, Sri Lanka, Thailand, Vietnam, Armenia, Kazakhstan, Mongolia, Tajikistan, Bosnia and Herzegovina, Bulgaria, Croatia, Czech Republic, Hungary, Macedonia, Montenegro, Poland, Romania, Serbia, Slovakia, Slovenia, Belarus, Estonia, Latvia, Lithuania, Moldova, Russia, Ukraine, Brunei, South Korea, Singapore, Australia, New Zealand, Andorra, Austria, Belgium, Cyprus, Denmark, Finland, France, Germany, Greece, Iceland, Ireland, Israel, Italy, Luxembourg, Malta, Netherlands, Norway, Portugal, Spain, Switzerland, Argentina, Chile, Uruguay, Canada, Antigua and Barbuda, The Bahamas, Barbados, Cuba, Trinidad and Tobago, Ecuador, Peru, Colombia, Costa Rica, El Salvador, Guatemala, Honduras, Panama, Venezuela, Paraguay, Bahrain, Iran, Jordan, Kuwait, Lebanon, Palestine, Oman, Qatar, Turkey, United Arab Emirates, Comoros, Seychelles, Cape Verde, Japan, Sweden, United Kingdom, United States, Mexico, Brazil, Saudi Arabia, Greenland

Other than this difference, there were no substantive changes in the modeling strategy from GBD 2013.

Ischemic heart disease

Flowchart

Ischemic Heart Disease

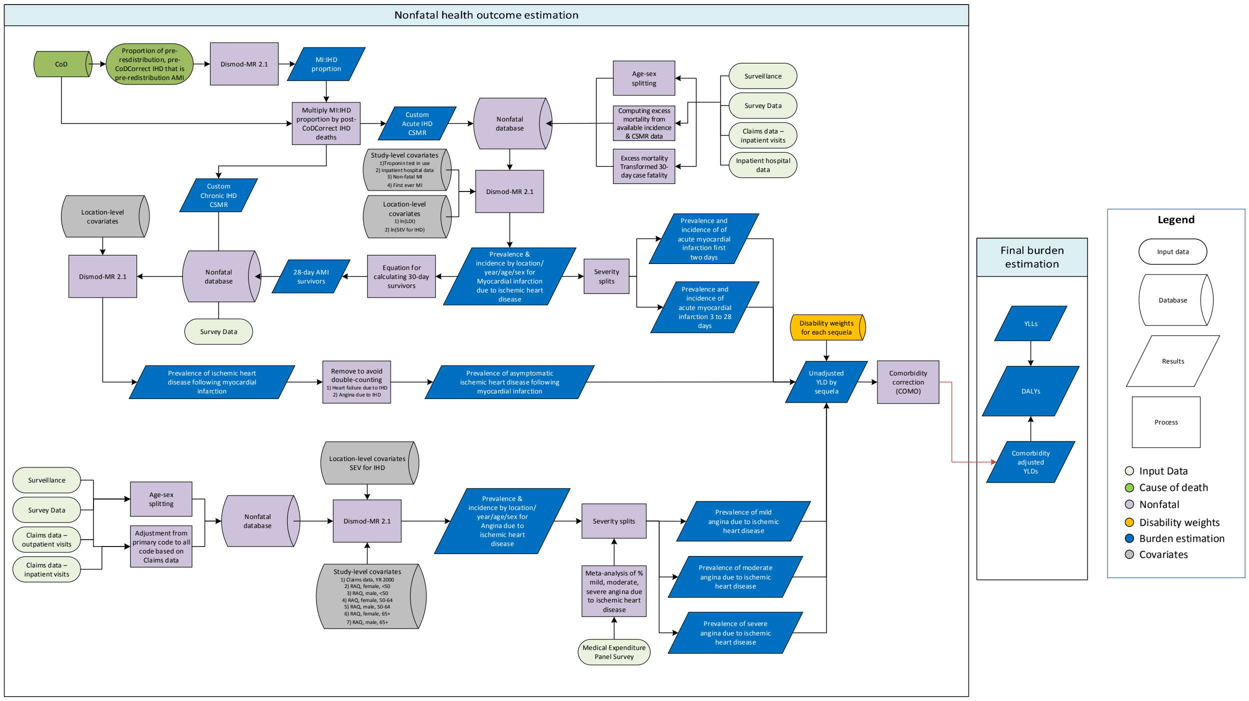

Input data and methodological summary

Case definition

<u>Case definitions:</u>

1) Acute myocardial infarction (AMI): Definite and possible AMI according to the third universal definition of myocardial infarction:
    a. When there is clinical evidence of myocardial necrosis in a clinical setting consistent with myocardial ischemia or
    b. Detection of a rise and/or fall of cardiac biomarker values and with at least one of the following: i) symptoms of ischemia, ii) new or presumed new ST-segment-T wave changes or new left bundle branch block, iii) development of pathological Q waves in the ECG, iv) imaging evidence of new loss of viable myocardium or new regional wall motion abnormality, or v) identification of an intracoronary thrombus by angiography or autopsy.
    c. Sudden (abrupt) unexplained cardiac death, involving cardiac arrest or no evidence of a noncoronary cause of death
    d. Prevalent AMI is condiered to last from the onset of the event to 28 days after the event and is divided into an acute phase (0-2 days) and subacute (3-28 days).
2) Chronic IHD

a. Angina; clinically diagnosed stable exertional angina pectoris or definite angina pectoris according to the Rose Angina Questionnaire, physician diagnosis, or taking nitrate medication for the relief of chest pain.
b. Asymptomatic ischemic heart disease following myocardial infarction; survival to 28 days following incident AMI. The GBD study does not use estimates based on ECG evidence for prior MI, due to its limited specificity and sensitivity (1).

ICD codes used for inclusion of hospital and claims data for AMI, asymptomatic ischemic heart disease after myocardial infarction, and angina are included in Appendix Table 4.

Input data

Model inputs

Myocardial infarction

A systematic review was done for myocardial infarction for GBD 2015.

The search strings used were extensive; a full list can be found here: J:\WORK\04_epi\01_database\02_data\cvd_ihd\00_documentation\IHD_Search_Strings_GBD2015\IHD_Search_Split.docx.

The dates of the search were 1/1/2009 – 2/3/2015. 38,522 studies were returned; 194 were extracted (this number includes extractions that were done for STEMI/NSTEMI models and revascularization models that are not currently part of the MI modeling process but may be in the future).

A systematic review for myocardial infarction was also done for GBD 2013. The extensive search terms for that review can be found here: https://hub.ihme.washington.edu/pages/viewpage.action?spaceKey=SR&title=GBD+2013+Literature+Review+Documentation

Literature data included: Myocardial infarction

	Prevalence	Incidence	Mortality risk
Studies	0	93	61
Countries/subnationals	0	39	32
GBD world regions	0	8	10

Apart from inpatient hospital and inpatient claims data, we did not include any data from sources other than the literature for myocardial infarction. We excluded data with broad age ranges where it was impossible to obtain more granular data, as these data caused the known age pattern for increased risk of myocardial infarction to be masked in the estimates generated from DisMod.

We corrected inpatient hospital data and claims data to account for the fact that these data sources do not capture the out-of-hospital cardiac arrest deaths which are part of the universal definition of AMI. We also included a covariate to correct for the change in diagnostic criteria to include troponin measurements. This adjustment was applied to data collected before 2000. We also adjusted data points that were not specific about whether it was the first AMI for included subjects, using studies where only first events were included as the reference. We also adjusted estimates from studies that only included non-fatal cases using study-level covariates.

Angina

A systematic review was not performed for GBD 2015. Updates to systematic reviews are performed on an ongoing schedule across all GBD causes; an update for angina will be performed in the next one to two iterations.

A systematic review for angina was done for GBD 2013. The search terms for that are here: (Angina Pectoris/epidemiology[Mesh] OR Angina Pectoris/mortality[Mesh]) AND (prevalence[Title/Abstract] OR incidence[Title/Abstract]) AND ("2010"[Date - Publication] : "3000"[Date - Publication])

Literature data included: Angina

	Prevalence	Incidence	Mortality risk
Studies	72	0	7
Countries/subnationals	73	0	7
GBD world regions	20	0	5

We included survey data (including NHANES and World Health Study questionnaires) which included the RAQ items. Prevalence of angina was calculated using the standard algorithm to determine whether the RAQ was positive or negative.

We excluded data with broad age ranges where it was impossible to obtain more granular data, as these data caused the known age pattern for increased risk of angina to be masked in the estimates generated from DisMod.

We included sex- and age group-specific covariates to adjust prevalence data points obtained from the RAQ using the claims data as the reference since the RAQ has been shown to be neither sensitive nor specific.

Severity split inputs

Acute myocardial infarction was split into two severity levels by length of time since the event – days 1 and 2 versus days 3 through 28. Disability weights were established for these two severities using the standard approach for GBD 2015.

Angina was split into mild, moderate, and severe groups using information from MEPS. Disability weights were established for these severities using the standard approach for GBD 2015.

<u>Acute myocardial infarction</u>

Severity level	Lay description	DW (95% CI)
Acute myocardial infarction, days 1-2	Has severe chest pain that becomes worse with any physical activity. The person feels nauseated, short of breath, and very anxious.	0.432 (0.288-0.579)
Acute myocardial infarction, days 3-28	Gets short of breath after heavy physical activity, and tires easily, but has no problems when at rest. The person has to take medication every day and has some anxiety.	0.074 (0.049-0.105)

<u>Angina pectoris</u>

Severity level	Lay description	DW (95% CI)
Mild angina	Has chest pain that occurs with strenuous physical activity, such as running or lifting heavy objects. After a brief rest, the pain goes away.	0.033 (0.02-0.052)
Moderate angina	Has chest pain that occurs with moderate physical activity, such as walking uphill or more than half a kilometer (around a quarter-mile) on level ground. After a brief rest, the pain goes away.	0.08 (0.052-0.113)
Severe angina	Has chest pain that occurs with minimal physical activity, such as walking only a short distance. After a brief rest, the pain goes away. The person avoids most physical activities because of the pain.	0.167 (0.11-0.24)

Modeling strategy

<u>Myocardial infarction</u>

- We first calculated custom cause-specific mortality estimates using data from cause of death data prior to garbage code redistribution, generating age-sex-country specific proportions of IHD deaths that were due to AMI (acute IHD) vs those due to other causes

of IHD (chronic IHD). Estimates of this proportion for all locations were then generated using a DisMod proportion-only model. This proportion was multiplied by post-CodCorrect (final GBD estimtes) IHD deaths to generate CSMR estimates for AMI, even though GBD reports only deaths for all IHD taken together. These data were then used, along with incidence and excess mortality data, in a DisMod model to estimate the prevalence and incidence of myocardial infarction due to ischemic heart disease.

- These estimates were split into prevalence and incidence estimates for days 1-2 and days 3-28 post event. Disability weights were assigned to each of these two groupings.
- We set a value prior of one month for remission (11/13) from the AMI health state. We also set a value prior for the maximum excess mortality rate of 10 for all ages. We included lnLDI as a fixed-effect country-level covariate on excess mortality, forcing an inverse relationship.

Study covariate	Parameter	Beta	Exponentiated beta
Diagnostic blood sample (troponin)	incidence	-.4432(-.4543 to -.44)	.6419 (.6349 to .644)
Hospital data	incidence	-1.5e-04(-4.4e-04 to -4.1e-05)	.9999(.9996 to 1)
First ever MI	incidence	-.002(-.009 to -5.1e-05)	.998(.9911 to .9999)
Non fatal MI	incidence	-8.9e-04(-.0024 to -2.3e-04)	.9991(.9976 to .9998)
LDI (I$ per capita)	excess mortality rate	-.1005(-.1027 to -.1)	.9044(.9024 to .9048)

Asymptomatic ischemic heart disease

- Excess mortality estimates from the myocardial infarction model were used to generate data of the incidence of surviving 28 days post-event.
- We used these data, along with the estimates of CSMR due to chronic IHD (the other part of the proportion described in step 1) and excess mortality data in a DisMod model to estimate the prevalence of persons with IHD following myocardial infarction. This estimate included subjects with angina and heart failure; a proportion of this prevalence was removed in order to avoid double counting based on evidence from the literature(2). The result of this step generates estimates of asymptomatic ischemic heart disease following myocardial infarction.
- We set a value prior of 0 for remission for all ages.

Study covariate	Parameter	Beta	Exponentiated beta
Log-transformed age-standardized SEV scalar: IHD	incidence	.9319(.9187 to .9452)	2.539#(2.506 to 2.573)

Angina

- We used prevalence data from the literature and USA claims databases, along with data on mortality risk to estimate the prevalence and incidence of angina for all locations.
- The proportion of mild, moderate, and severe angina was determined by the standard approach for severity splitting for GBD 2015.

- We included a value prior of 0 for remission for all ages. We also included a value prior of 1 for excess mortality for all ages.
- We included age- and sex-specific study-level covariates to adjust data points based on RAQ, using data points from the claims database as the reference.
- We also included the log-transformed, age-standardized SEV scalar for IHD as a fixed effect country-level covariate.

Describe any cause-specific limitations and modeling assumptions that affect the interpretation of estimates. Study covariate	Parameter	Beta	Exponentiated beta
RAQ, female, less than 50	prevalence	2.435(2.326 - 2.497)	11.42(10.24 - 12.15)
RAQ, male, less than 50	prevalence	.9454(.9349 - .9499)	2.574(2.547 - 2.585)
RAQ, female, 50 to 64	prevalence	1.484(1.447 - 1.5)	4.411(4.25 - 4.482)
RAQ, male, 50 to 64	prevalence	.9897(.9606 - .9997)	2.69(2.613 - 2.717)
RAQ, female, 65 plus	prevalence	.2929(.2719 - .2998)	1.34(1.312 - 1.35)
RAQ, male, 65 plus	prevalence	.2891(.2582 - .2997)	1.335(1.295 - 1.349)
Log-transformed age-standardized SEV scalar: IHD	prevalence	1.238(1.209 - 1.249)	3.449(3.35 - 3.487)

Apart from inclusion of hospital data and claims data, there have been no substantive changes in the modeling strategy for myocardial infarction, asymptomatic ischemic heart disease following myocardial infarction, and angina from GBD 2013.

Cerebrovascular disease

Flowchart

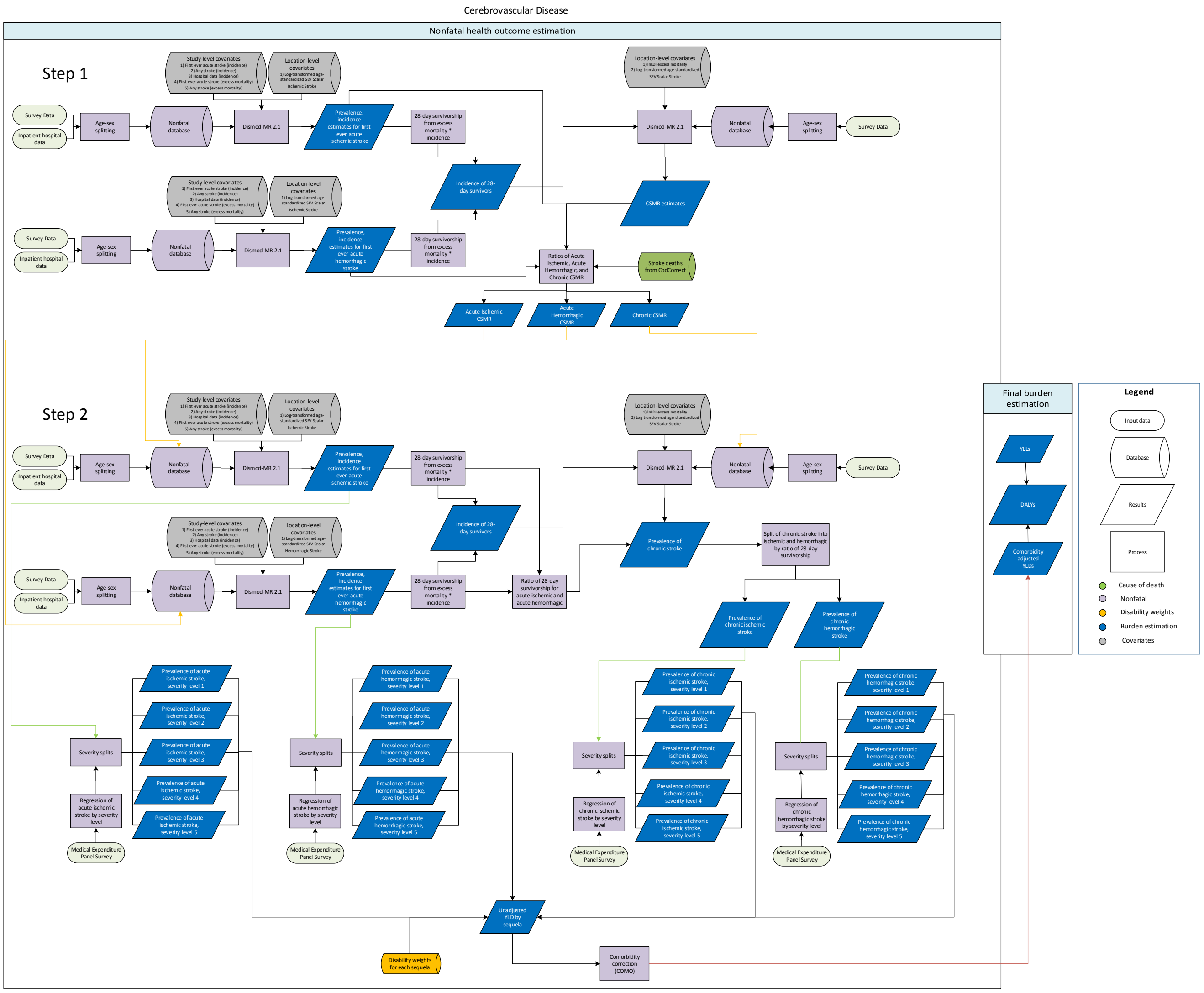

Input data and methodological summary

Case definition

Stroke was defined according to WHO criteria – rapidly developing clinical signs of focal (at times global) disturbance of cerebral function lasting more than 24 hours or leading to death with no apparent cause other than that of vascular origin. Data on transient ischemic attack (TIA) were not included.

Acute stroke: Stroke cases are considered acute from the data of incidence of a first ever stroke through day 28 following the event.

Chronic stroke: Stroke cases are considered chronic beginning 28 days following the occurrence of an event. Chronic stroke includes the sequelae of an acute stroke AND all recurrent stroke events. GBD 2015 adopts this broader definition of chronic stroke than prior iterations in order to model acute strokes using only first-ever incident events.

Ischemic stroke: Incident ischemic stroke is defined as the occurrence of first-ever ischemic stroke, based on clinical diagnosis by a physician using diagnostic imaging. Ischemic strokes are considered to include all vascular events leading to limited blood flow to brain tissue, with resulting infarction, including atherosclerotic and thromboembolic strokes but excluding strokes in which the underlying cause is intracranial hemorrhage.

Hemorrhagic or other strokes: This cause includes all non-ischemic strokes of a vascular cause including subarachnoid and stroke due to intracranial hemorrhage.

ICD codes used for stroke are included in Appendix Table 4.

Input data

Model inputs

A systematic review was not performed for GBD 2015. Updates to systematic reviews are performed on an ongoing schedule across all GBD causes; an update for cerebrovascular disease will be performed in the next iteration.

A systematic review of the literature was performed in GBD 2013

- Search terms:
  - (stroke[Mesh]) AND (prevalence[Title/Abstract] OR incidence[Title/Abstract]) AND ("2010"[Date - Publication] : "3000"[Date - Publication]) AND (hasabstract[text] AND Humans[Mesh] AND middle age[MeSH]))
  - (hasabstract[text] AND Humans[Mesh] AND middle age[MeSH])) OR 21) AND ((hemorrhagic stroke/epidemiology[Mesh] OR hemorrhagic stroke/mortality[Mesh]) AND (prevalence[Title/Abstract] OR incidence[Title/Abstract]) AND ("2010"[Date - Publication] : "3000"[Date - Publication]) AND (hasabstract[text] AND Humans[Mesh] AND middle age[MeSH]))

The tables below indicates the number of literature studies included in GBD 2015, as well as the number of countries or subnational units and GBD world regions represented.

Cerebrovascular disease

	Prevalence	Incidence	Mortality risk
Studies	53	0	8
Countries/subnationals	50	0	4
GBD world regions	14	0	2

Ischemic stroke

	Prevalence	Incidence	Mortality risk
Studies	0	71	45
Countries/subnationals	0	59	48
GBD world regions	0	12	17

Hemorrhagic or other stroke

	Prevalence	Incidence	Mortality risk
Studies	0	71	34
Countries/subnationals	0	59	43
GBD world regions	0	12	11

In addition to inpatient hospital data, we included unpublished stroke registry data for acute ischemic and acute hemorrhagic strokes. We include survey data for chronic cerebrovascular disease. These surveys were identified based on expert opinion and review of major survey series focused on world health that included questions regarding self-reported history of stroke.

We included crosswalks to adjust data for first and recurrent strokes combined, using data for first strokes only as reference. We also included crosswalks for ischemic and hemorrhagic strokes combined (all stroke), using as reference studies with subtype-specific information.

Severity split inputs

The standard GBD approach using MEPS data was used to determine severity splits for stroke. The table below illustrates the severity level, lay description, and disability weights for GBD 2015.

Severity level	Lay description	DW (95% CI)
Stroke, long-term consequences, mild	has some difficulty in moving around and some weakness in one hand, but is able to walk without help.	0.019 (0.01-0.032)
Stroke, long-term consequences, moderate	has some difficulty in moving around, and in using the hands for lifting and holding things, dressing, and grooming.	0.07 (0.046-0.099)
Stroke, long-term consequences, moderate plus cognition problems	has some difficulty in moving around, in using the hands for lifting and holding things, dressing and grooming, and in speaking. The person is often forgetful and confused.	0.316 (0.206-0.437)

Stroke, long-term consequences, severe	is confined to bed or a wheelchair, has difficulty speaking and depends on others for feeding, toileting, and dressing.	0.552 (0.377-0.707)
Stroke, long-term consequences, severe plus cognition problems	is confined to bed or a wheelchair, depends on others for feeding, toileting, and dressing, and has difficulty speaking, thinking clearly, and remembering things.	0.588 (0.411-0.744)

Modeling strategy

Three general approaches were employed for all of the components of the stroke modeling process, detailed in the table below.

- Data were crosswalked from nonstandard to standard case definitions using DisMod for all models. Coefficients for these crosswalks can be found in the tables for fixed effects located below.
- A GBD Standardized Exposure Variable for stroke and a covariate for country income were used as country-level covariates for all models. Coefficients for these covariates can be found in the tables for fixed effects located below.
- DisMod MR-2.1 was set with priors related to the coefficients of variation and heterogeneity for each model. Information for these parameters can be found in the tables of model parameters located below.

Step 1

- We generated estimates for first-ever acute ischemic and first-ever acute hemorrhagic stroke using data collected on stroke incidence and excess mortality. We set value priors of 11 to 13 on remission for all ages to establish a one-month duration for these acute sequelae.
- We then calculated the incidence of surviving 28 days after an acute event for both ischemic and hemorrhagic stroke using the modeled estimates of excess mortality and incidence.
- These survivor data were then uploaded into the chronic stroke, any type model as incidence.
- We then ran the chronic stroke model , using the survivor incidence data, prevalence data, and excess mortality data. We set a value prior of 0 on remission for all ages.
- Implausible or extreme outliers were dropped from these estimation results.
- From these three models, we generated the proportions of deaths for acute ischemic, acute hemorrhagic, and chronic stroke, and split the post-CodCorrect stroke deaths generated from the GBD mortality estimates into these three parts. Thus, the proportion of deaths due to acute ischemic, acute hemorrhagic, and chronic stroke are driven by all available data on incidence, prevalence, and excess mortality data for stroke. These CSMR estimates were then uploaded into the nonfatal database and used to estimates models for Step 2.

Step 2

- We re-ran the first-ever acute ischemic and first-ever acute hemorrhagic models with CSMR as derived from CodCorrect and epidemiologic data as described above. Twenty-eight-day survivorship was recalculated from these models and uploaded into the chronic stroke, any type model with CSMR. As for acute models, this chronic model usises CSMR as derived from CodCorrect and epidemiologic data as described above.

- Implausible or extreme outliers were dropped from these estimation results.
- We then split the overall chronic stroke model into chronic hemorrhagic stroke and chronic ischemic stroke based on the ratio of 28-day survivorship in the acute ischemic and acute hemorrhagic models. The assumption built into this step is that the ratio of prevalent cases of chronic stroke matchs that ratio of chronic stroke survivor cases at 29 days following an incident stroke.

Models were evaluated based on expert opinion, comparison with previous iterations, and model fit.

As described above, in GBD 2015 we are no longer directly estimating first and recurrent stroke combined. This decision was made in consultation with GBD Stroke experts and reflects the fact that standard data reporting for stroke registries is for first-ever stroke. The majority of stroke incidence data available to GBD is for first-ever stroke.

The table below indicates the covariates used by cause in the estimation process, as well as the beta and exponentiated beta values.

Step 1:

Cause	Variable name	Measure	beta	Exponentiated beta
Chronic stroke; any type	Log-transformed age-standardized SEV scalar: Stroke	prevalence	.7833(.7512 to .8785)	2.189(2.12 to-2.407)
Chronic stroke; any type	LDI (I$ per capita)	excess mortality rate	-.1792(-.1819 to -.1769)	.836(.8337 to .8379)
First ever acute hemorrhagic stroke	Hospital data	incidence	.5278(.5223 to .5298)	1.695(1.686 to 1.699)
First ever acute hemorrhagic stroke	Any stroke	incidence	1.359(1.313 to 1.388)	3.892(3.717 to 4.007)
First ever acute hemorrhagic stroke	First-ever acute stroke, ischemic or hemorrhagic	incidence	.4925(.4163 to .5291)	1.636(1.516 to 1.697)
First ever acute hemorrhagic stroke	Log-transformed age-standardized SEV scalar: hemorrhagic stroke	incidence	1.243(1.227 to 1.25)	3.468(3.411 to 3.49)
First ever acute hemorrhagic stroke	Any stroke	excess mortality rate	-.4216(-.5741 to -.2617)	.656(.5632 to .7698)
First ever acute hemorrhagic stroke	First-ever acute stroke, ischemic or hemorrhagic	excess mortality rate	-.1409(-.3484 to .0613)	.8685(.7058 to 1.063)
First ever acute ischemic stroke	Hospital data	incidence	.002(4.3e-05 to .0067)	1.002(1 to 1.007)
First ever acute ischemic stroke	Any stroke	incidence	.4687(.4653 to .47)	1.598(1.592 to 1.6)
First ever acute ischemic stroke	First-ever acute stroke, ischemic or hemorrhagic	incidence	.5142(.4772 to .5296)	1.672(1.612 to 1.698)
First ever acute ischemic stroke	Log-transformed age-standardized SEV scalar: ischemic stroke	incidence	1.106(1.025 to 1.186)	3.021(2.787 to 3.274)

Step 2:

Cause	Variable name	Measure	beta	Exponentiated beta
Chronic stroke, any type with CSMR	Log-transformed age-standardized SEV scalar: Stroke	prevalence	.8185 (.7518 - .9986)	2.267 (2.121 - 2.714)
Chronic stroke, any type with CSMR	LDI (I$ per capita)	excess mortality rate	-.1879 (-.1917 - -.1845)	.8287 (.8256 - .8315)
First-ever acute hemorrhagic stroke with CSMR	Any stroke	incidence	1.401 (1.4 - 1.407)	4.06 (4.055 - 4.084)
First-ever acute hemorrhagic stroke with CSMR	First-ever acute stroke, ischemic or hemorrhagic	incidence	9.8e-04 (2.2e-04 - .0049)	1.001 (1 - 1.005)
First-ever acute hemorrhagic stroke with CSMR	Log-transformed SEV scalar: Hem stroke	incidence	1.152 (1.031 - 1.243)	3.164 (2.804 - 3.466)
First-ever acute hemorrhagic stroke with CSMR	Any stroke	excess mortality rate	-.5999 (-.7527 - -.4538)	.5489 (.4711 - .6352)
First-ever acute hemorrhagic stroke with CSMR	First-ever acute stroke, ischemic or hemorrhagic	excess mortality rate	-.2336 (-.512 - .0366)	.7917 (.5993 - 1.037)
First-ever acute ischemic stroke with CSMR	Any stroke	incidence	.3452 (.3401 - .3575)	1.412 (1.405 - 1.43)
First-ever acute ischemic stroke with CSMR	First-ever acute stroke, ischemic or hemorrhagic	incidence	3.4e-04 (7.3e-05 - 9.8e-04)	1 (1 - 1.001)
First-ever acute ischemic stroke with CSMR	Log-transformed age-standardized SEV scalar: Ischemic stroke	incidence	1.248 (1.24 - 1.25)	3.483 (3.456 - 3.49)
First-ever acute ischemic stroke with CSMR	Any stroke	excess mortality rate	-.6897 (-.8029 - -.5741)	.5017 (.448 - .5632)
First-ever acute ischemic stroke with CSMR	First-ever acute stroke, ischemic or hemorrhagic	excess mortality rate	-.869 (-.9992 - -.7466)	.4194 (.3682 - .474)

Acute myocarditis

Flowchart

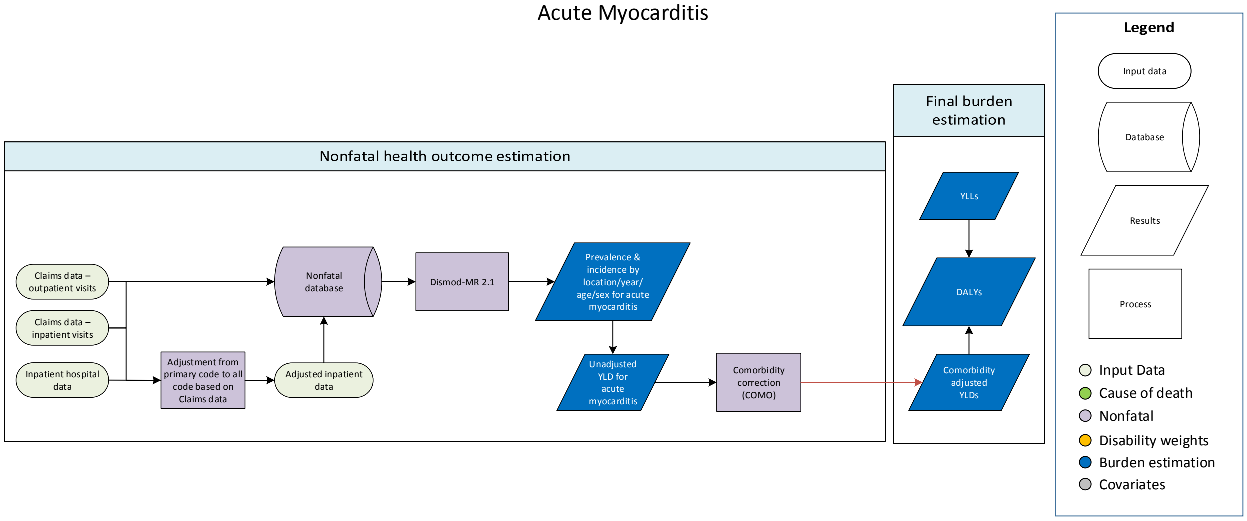

Input data and methodological summary

Case definition

Myocarditis refers to a heterogenous group of diseases with variable clinical and pathological features. Acute myocarditis was defined for GBD as the acute and time-limited symptoms of myocarditis separate from its chronic heart failure-related sequelae. Heart failure due to cardiomyopathy is estimated separately in GBD (see methods for heart failure). Symptoms of acute myocarditis are nonspecific and include a flu-like or gastrointestinal syndrome, followed by anginal-type chest pain, arrhythmias, syncope, or heart failure.

The ICD codes for acute myocarditis are included in Appendix Table 4.

Input data

Model inputs

The preferred data sources for acute myocarditis was hospital admission data and other health facility data identifying cases of acute myocarditis.

A systematic review was performed for GBD 2013 and updated for GBD 2015. The search terms included:

(cardiomyopathy AND epidemiology[MeSH Subheading]) OR (myocarditis AND epidemiology[MeSH Subheading]) OR (cardiomyopathy AND (incidence OR prevalence OR “case fatality”)) OR (myocarditis AND (incidence OR prevalence OR “case fatality”))

- Dates included in search: 1/1/2013 – 3/16/2015
- Number of initial hits: 3,598
- Number of sources included: 0
-

The GBD 2013 search terms included:

(hasabstract[text] AND Humans[Mesh] AND middle age[MeSH])) OR 21) AND ((cardiomyopathy/epidemiology[Mesh] OR cardiomyopathy/mortality[Mesh]) AND (prevalence[Title/Abstract] OR incidence[Title/Abstract]) AND ("2010"[Date - Publication] : "3000"[Date - Publication]) AND (hasabstract[text] AND Humans[Mesh] AND middle age[MeSH]))

We did not include any non-literature-based data, apart from the hospital and claims data described elsewhere. We excluded all outpatient data, as they were implausibly low when compared with inpatient data from the same locations and with claims data. We also excluded inpatient hospital data from countries (Canada, Mexico, Brazil) where the data were implausibly low when compared with other data in the region or super-region.

We included study-level covariates for inpatient hospital data and claims data from 2000 and 2010 to adjust these data points, using as reference the claims data from 2012.

Severity splits and disability weights

Severity level	Lay description	DW (95% CI)
Acute myocarditis	Has a fever and aches, and feels weak, which causes some difficulty with daily activities.	0.051 (0.032-0.074)

Modeling strategy

We used a DisMod MR-2.1 model, which included the following prior settings:

- 1) Setting a minimum of 3 and maximum of 5 on remission to establish an average duration of 3 months; and, 2) Setting excess mortality to 0 for all ages.

For GBD 2015 estimation, we included study-level covariates for inpatient hospital data and claims data from 2000 and 2010 to adjust these data points, using as reference the claims data from 2012. Other than these shifts, we have made no substantive changes in the modeling strategy from GBD 2013.

Study covariate	Parameter	beta	Exponentiated beta
Hospital data	incidence	-1.716(-1.797 to -1.644)	.1798(.1658 to .1932)
All MarketScan, year 2000	incidence	.2966(.2528 to .3362)	1.345(1.288 to 1.4)
All MarketScan, year 2010	incidence	.1523(.1139 to .1933)	1.164(1.121 to 1.213)
Log-transformed age-standardized SEV scalar: CMP	incidence	.695(.5119 to 1.045)	2.004(1.668 to 2.843)

No other significant changes were made to the modeling approach for GBD 2015.

Atrial fibrillation and flutter

Flowchart

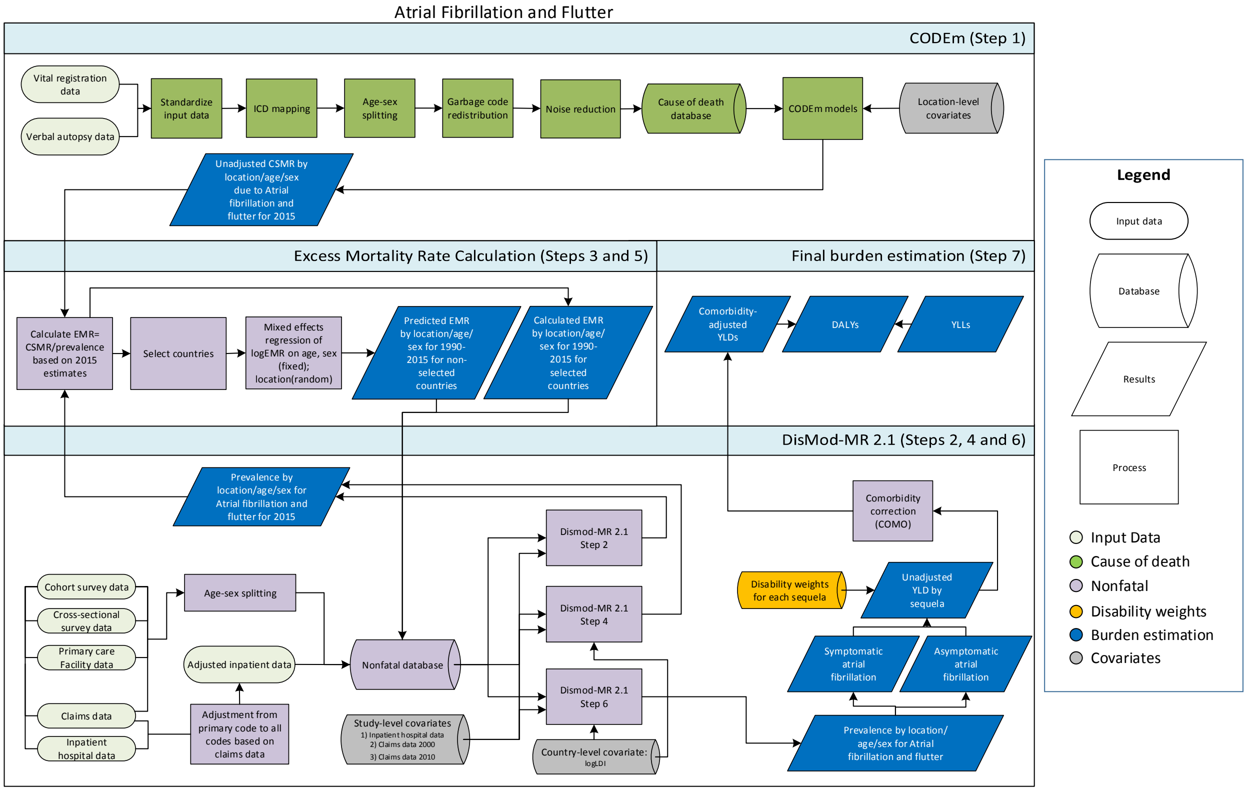

Input data and methodological summary

Case definition

Atrial fibrillation was defined as a diagnosis with atrial fibrillation or atrial flutter by ECG findings. ICD codes used for inclusion of hospital and Marketscan data for atrial fibrillation and flutter are included in Appendix Table 4.

Input data

Model inputs

A systematic review was performed for GBD 2015 with the following search terms:

("atrial fibrillation" AND epidemiology[MeSH Subheading]) OR ("atrial flutter" AND epidemiology[MeSH Subheading]) OR ("atrial fibrillation" AND (prevalence OR incidence OR "case fatality")) OR ("atrial flutter" AND (prevalence OR incidence OR "case fatality")) OR ("heart atrium fibrillation" AND epidemiology[MeSH Subheading]) OR ("heart atrium fibrillation" AND (prevalence OR incidence OR "case fatality"))

The dates of the search were 1/1/2013 – 3/15/2016. There were 5,630 studies returned and, of those, 27 were extracted.

A systematic review was also performed for GBD 2013 and the search terms were:

Search terms: (hasabstract[text] AND Humans[Mesh] AND middle age[MeSH])) OR 21) AND ((atrial fibrillation/epidemiology[Mesh] OR atrial fibrillation/mortality[Mesh]) AND (prevalence[Title/Abstract] OR incidence[Title/Abstract]) AND ("2010"[Date - Publication] : "3000"[Date - Publication]) AND (hasabstract[text] AND Humans[Mesh] AND middle age[MeSH]))

The table below shows the data inputs:

	Prevalence	Incidence	Mortality risk
Studies	71	24	15
Countries/subnationals	42	17	12
GBD world regions	8	3	6

Apart from hospital and claims data points on prevalence, no non-literature-based data were included. We excluded hospital data in certain geographies where the data were implausibly low for all years in both sexes (e.g., Canada, Mexico). We included study-level covariates to crosswalk the inpatient hospital data and the claims data from 2000 and 2010, using as reference literature data and the claims data from 2012.

Severity splits & disability weights

Atrial fibrillation is split into symptomatic and asymptomatic based on standard GBD proportion information. The table below includes lay descriptions and disability weights for the severity levels of atrial fibrillation:

Severity level	Lay description	DW (95% CI)
Asymptomatic	No symptoms	N/A
Symptomatic	Has periods of rapid and irregular heartbeats and occasional fainting	0.224 (0.151-0.312)

Modeling strategy

In order to address changes in coding practices for atrial fibrillation, we used an integrated approach that combined DisMod-MR and CODEm models to generate estimates for atrial fibrillation and flutter. This new approach, a major change from GBD 2013, allowed us to adjust estimates to more accurately reflect the number of deaths for which atrial fibrillation was the true underlying cause of death, thus generating better estimates of prevalence.

- In Step 1, we estimated deaths for atrial fibrillation using a standard CODEm approach.
- In Step 2, we estimated prevalence rates in DisMod-MR using data from published reports of cross-sectional and cohort surveys and primary care facility data. We also used claims data

covering inpatient and outpatient visits for the United States along with inpatient hospital data from 22 countries. As the inpatient hospital data only included information from the primary code for each visit, prevalence rates for these data were adjusted based on the age- and sex-specific proportions of atrial fibrillation in the primary codes vs. secondary codes in the US claims data.

- In Step 3, we calculated the excess mortality rate (EMR) for 2015 (defined as the cause-specific mortality rate (CSMR) estimated from CODEm divided by the prevalence rate from DisMod-MR). We then selected 27 countries based on four conditions: 1) availability of VR data; 2) prevalence rate ≥ 0.005; 3) CSMR ≥ 0.00002; and, 4) EMR ≥ 0.001. Using information from these countries as input data, we ran a linear mixed-effects regression of logEMR on sex, age, and location. Sex and age were treated as fixed effects for the regression, while location was considered a random effect. We then predicted age- and sex-specific EMR using the results of this regression for all non-selected countries. Countries included in the regression were assigned their directly calculated values. These EMR data points were assigned to the time period 1990-2015 and uploaded into the Nonfatal database.
- In Step 4, we re-ran DisMod-MR including the EMR estimated in Step 3 and using log-transformed lagged distributed income (LDI) as a country-level covariate. Based on information from other regressions, we set the bounds at -1.5 to -0.25. We also included study-level covariates to cross-walk the inpatient hospital and claims data from 2000 and 2010 to the reference data, which included literature data and claims data from 2012. We included a value prior of 0 for remission for all ages. We also set a value prior of 0 for excess mortality for ages 0-30.
- In Steps 5 and 6, we repeated the process in Steps 3 and 4. In this iteration, we selected 31 countries that were included in the mixed-effects regression. The criteria were: 1) availability of VR data; 2) prevalence rate ≥ 0.004; 3) CSMR ≥ 0.00002; and, 4) EMR ≥ 0.002.

The prevalence from the DisMod-MR model in Step 6 was used as the finalized output for upload to COMO and further processing into YLDs and DALYs. Models were evaluated based on expert opinion, comparison with results from previous rounds of GBD, and model fit.

The table below includes the study covariates, parameters, betas, and exponentiated betas.

Study covariate	Parameter	Beta	Exponentiated beta
Hospital data	prevalence	-.9192(-.9199 to -.9175)	.3988(.3986 to .3995)
All MarketScan, year 2000	prevalence	-.2752(-.3101 to -.2468)	.7594(.7334 to .7813)
All MarketScan, year 2010	prevalence	.14(.1105 to .1658)	1.15(1.117 to 1.18)
LDI (I$ per capita)	excess mortality rate	-.25(-.2501 to -.25)	.7788(.7787 to .7788)

No other significant changes were made to modeling strategy for GBD 2015.

Peripheral vascular disease

Flowchart

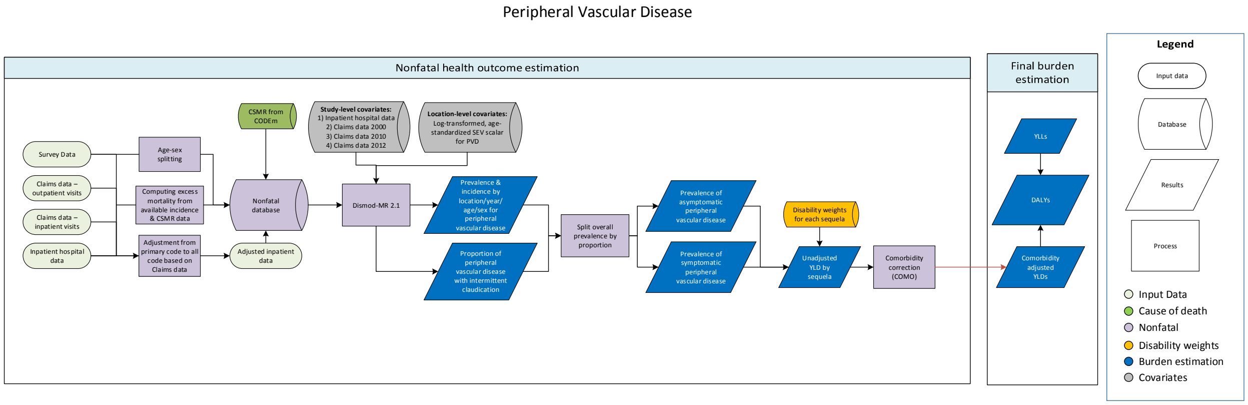

Input data and methodological appendix

Case definition

For GBD 2015, PVD was defined as having an ankle-brachial index (ABI) <0.9. Intermittent claudication was defined clinically.

The ICD codes for peripheral vascular disease are listed in Appendix Table 4.

I70.799, I70.29, I70.538, 443.9, 440.31, 443.24, I70.322, I70.421, 443.0, I70.743, I70.702, I70.639, I70.518, 440.4, I70.391, I70.211, 440.24, I70.329, I70.212, I70.419, I70.744, I70.569, I70.235, I70.638, I70.791, 443.23, 440.22, I70.692, I70.709, I70.409, I70.644, I70.233, I70.401, I70.71, I70.91, I70.539, I70.641, I70.493, I70.745, I70.521, I70.338, I70.542, I70.231, I70.593, I70.731, I70.60, I73.01, I70.62, I70.629, I70.70, 440.20, I70.703, I70.531, I70.301, I70.699, I70.339, I70.5, I70.793, I70.563, I70.313, I70.492, I70.712, I70.602, I73, I70.323, I70.768, I70.508, I70.432, I70.469, I70.345, I70.512, I70.24, I70.461, 443.29, I70.342, I70.613, I70.591, I70.762, I70.541, and I70.312.

Input data

Model inputs

A systematic review was performed for peripheral vascular disease and intermittent claudication for GBD 2015. The search terms used are presented below:

('peripheral vascular disease'[TIAB] AND 'epidemiology'[Subheading]) OR ('peripheral arterial disease'[TIAB] AND 'epidemiology'[Subheading]) OR ('peripheral artery disease'[TIAB] AND 'epidemiology'[Subheading]) OR ('intermittent claudication'[TIAB] AND 'epidemiology'[Subheading]) OR ('ankle-brachial index'[TIAB] AND 'epidemiology'[Subheading]) OR ('ankle brachial index'[TIAB] AND 'epidemiology'[Subheading]) OR ('peripheral artery occlusive disease'[TIAB] AND 'epidemiology'[Subheading]) OR ('peripheral obliterative arteriopathy'[TIAB] AND 'epidemiology'[Subheading]) OR ('peripheral vascular disease'[TIAB] AND 'prevalence'[MeSH Terms]) OR ('peripheral vascular disease'[TIAB] AND 'incidence'[MeSH Terms]) OR ('peripheral vascular disease'[TIAB] AND 'case fatality'[All Fields]) OR ('symptomatic claudication'[TIAB] AND (proportion[All Fields] OR percent[All Fields]))

The search was conducted from 1/1/13 – 3/16/2015, and the number of studies returned was 1,658, of which six were extracted.

A systematic review was also performed for peripheral vascular disease and intermittent claudication for GBD 2013.

Search terms can be found here in the Word document located here: https://hub.ihme.washington.edu/pages/viewpage.action?spaceKey=SR&title=GBD+2013+Literature+Review+Documentation.

The table below shows the number of literature studies included in GBD 2015, as well as the number of countries or subnational units and GBD world regions represented.

Peripheral vascular disease

	Prevalence	Incidence	Mortality risk
Studies	23	3	1
Countries/subnationals	18	2	1
GBD world regions	11	2	1

Proportion with intermittent claudication

	Proportion

Studies	10
Countries/subnationals	6
GBD world regions	4

Apart from the hospital and claims data, we did not include any non-literature-based data types. We excluded hospital data that were implausibly low for all years and both sexes in certain geographies (e.g., Canada, Mexico, Brazil). We included study-level covariates for inpatient hospital data and claims data, using literature data as the reference.

Severity splits and disability weights

We used the proportion of intermittent claudication to split the overall prevalence of peripheral vascular disease into symptomatic and asymptomatic peripheral vascular disease. The table below illustrates these values:

Severity level	Lay description	DW (95% CI)
Asymptomatic	No symptoms	No DW assigned
Symptomatic	Has cramping pains in the legs after walking a medium distance. The pain goes away after a short rest.	0.014 (0.007-0.025)

Modeling strategy

For GBD 2015, we used DisMod to model the overall prevalence of peripheral vascular disease using prevalence data from literature studies, inpatient hospital data, and claims data.

For this model, we included study-level covariates on hospital data and claims data to adjust the data points, using the literature as the reference data. We also included the log-transformed, age-standardized SEV scalar for PVD as a fixed-effect, country-level covariate. We set value priors of 0 for incidence from ages 0 to 30. We also set a value prior of 0 for remission for all ages. Finally, we set a value prior of a maximum value of 0.25 on excess mortality for all ages.

The table below illustrates the study covariates, parameters, beta, and exponentiated beta values for the overall peripheral vascular disease model.

Study covariate	Parameter	beta	Exponentiated beta
Hospital data	prevalence	-1.998#(-2 - -1.993)	.1356#(.1353 - .1363)
All MarketScan, year 2000	prevalence	-.7449#(-.7498 - -.733)	.4748#(.4725 - .4805)
All MarketScan, year 2010	prevalence	-.371#(-.3739 - -.37)	.69#(.688 - .6907)
All MarketScan, year 2012	prevalence	-.3016#(-.3065 - -.3)	.7396#(.736 - .7408)
Log-transformed age-standardized SEV scalar: PVD	prevalence	.7985#(.7506 - .9187)	2.222#(2.118 - 2.506)

We used DisMod MR-2.1 to model the proportion of peripheral vascular disease with intermittent claudication. We set a value prior of 0 for proportion for ages 0 to 40.

To obtain final estimates for asymptomatic and symptomatic peripheral vascular disease, we multiplied the prevalence model by the proportion model at the draw level to generate the prevalence of symptomatic and asymptomatic peripheral vascular disease.

Models were evaluated based on expert review, comparisons with estimates from prior rounds of GBD, and assessing model fit.

Apart from using hospital and claims data in the overall prevalence model, there have been no substantive changes from GBD 2013 in terms of modeling strategy.

Acute endocarditis

Flowchart

Endocarditis

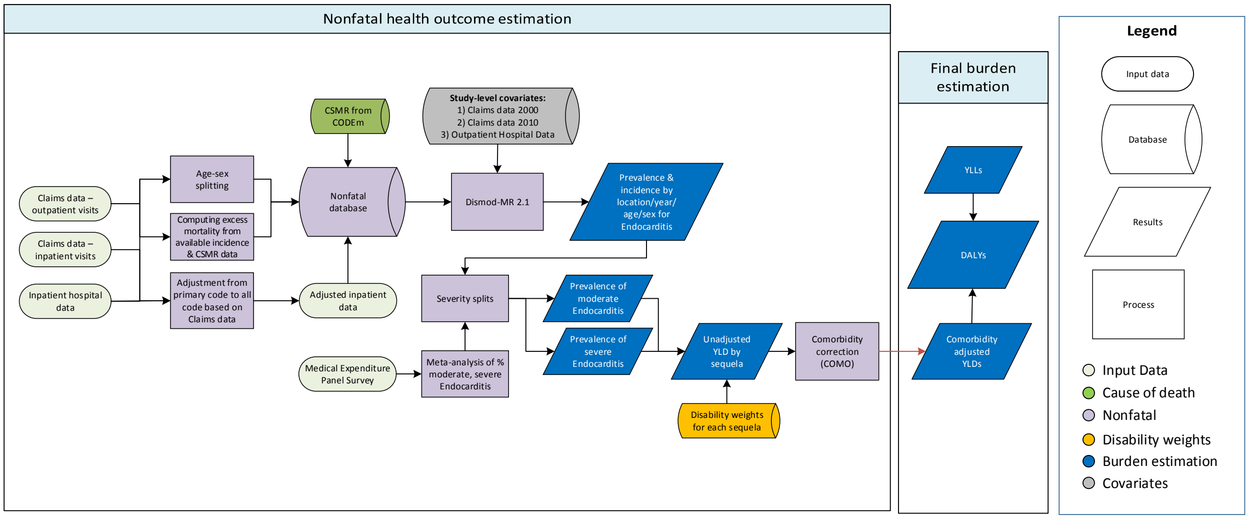

Input data and methodological appendix

Case definition

Our case definition for acute endocarditis was a clinical diagnosis of infective endocarditis. The ICD codes for acute endocarditis are available in Appendix Table 4.

Input data

Model inputs

Prior to the GBD 2015 estimation process, a systematic review was performed for GBD 2013 and subsequently updated in anticipation of GBD 2015. The following search terms were used:

Search terms: ((‘endocarditis’[MeSH Terms] OR ‘endocarditis’[All Fields]) AND ‘epidemiology’[Subheading]) OR ((‘endocarditis’[MeSH Terms] OR ‘endocarditis’[All Fields]) AND ((‘epidemiology’[Subheading] OR ‘epidemiology’[All Fields] OR ‘incidence’[All Fields] OR ‘incidence’[MeSH Terms]) OR (‘epidemiology’[Subheading] OR ‘epidemiology’[All Fields] OR ‘prevalence’[All Fields] OR ‘prevalence’[MeSH Terms]) OR ‘case fatality’[All Fields])) OR ((‘endocardium’[MeSH Terms] OR ‘endocardium’[All Fields]) AND inflammation[TIAB] AND ‘epidemiology’[Subheading]) OR ((‘endocardium’[MeSH Terms] OR ‘endocardium’[All Fields]) AND inflammation[TIAB] AND ((‘epidemiology’[Subheading] OR ‘epidemiology’[All Fields] OR ‘incidence’[All Fields] OR ‘incidence’[MeSH Terms]) OR (‘epidemiology’[Subheading] OR ‘epidemiology’[All Fields] OR ‘prevalence’[All Fields] OR ‘prevalence’[MeSH Terms]) OR ‘case fatality’[All Fields]))

- Dates included in search: 1/1/2013 – 3/16/2015
- Number of initial hits: 1,246
- Number of sources included: 6

The table below shows the number of literature studies included in GBD 2015, as well as the number of countries or subnational units and GBD world regions represented.

	Prevalence	Incidence	Mortality risk
Studies	0	14	1
Countries/subnationals	0	7	1
GBD world regions	0	3	1

We did not include any non-literature-based data types, apart from the hospital and claims data described elsewhere. We excluded all outpatient data, as they were implausibly low when compared with inpatient data from the same locations and claims data. We also excluded inpatient hospital data from countries (Canada, Mexico, Brazil) where the data were implausibly low when compared with other data in the region or super-region.

We included study-level covariates for inpatient hospital data and claims data from 2000 and 2010 to adjust these data points, using as reference the data obtained from literature and claims data from 2012.

Severity split inputs

We used the standard GBD approach, which utilizes MEPS data to split overall estimates of endocarditis into moderate and severe categories. The table below includes the severity level, lay descriptions and DWs associated with acute endocarditis.

Severity level	Lay description	DW (95% CI)
Moderate	Has a fever and aches, and feels weak, which causes some difficulty with daily activities.	0.051 (0.032-0.074)
Severe	Has a high fever and pain, and feels very weak, which causes great difficulty with daily activities.	0.133 (0.088-0.19)

Modeling strategy

For GBD 2015, we estimated acute endocarditis using a DisMod MR-2.1 Bayesian meta-regression model. We included a value prior of 0-20 for ages 0-100 to override the internal defaults of DisMod and allow the model to fit using the input remission data. We included study-level covariates on incidence for inpatient hospital data and claims data from 2000 and 2010.

For GBD 2010, we calculated data for remission from a retrospective cohort study conducted by Landman et al in the Netherlands. The recommended minimum treatment period for IEC is two weeks, which is not always met according to this study. Duration for treated patients, therefore, is more likely about three weeks instead of two because of inconsistent compliance. We then calculated remission with uncertainty,

taking into account the mortality rate of patients in the study and an average duration for survivors of three weeks.

All means and uncertainty intervals were calculated by DisMod-MR 2.1. We evaluated models by comparing model fits with the data and with results from previous GBD estimation cycles. Apart from correcting an error in the specification of the remission parameter, we have made no substantive changes in the modeling strategy from GBD 2013.

The table below illustrates the applicable study covariates, parameters, betas, and exponentiated betas.

Study covariate	Parameter	Beta	Exponentiated beta
Inpatient hospital data	incidence	.4484(.1224 to .5861)	1.566(1.13 to 1.797)
Claims data 2000	incidence	-.0127(-1.913 to 2)	.9874(.1476 to 7.389)
Claims data 2010	incidence	.002(-1.964 to 1.952)	1.002(.1403 to 7.041)
Log LDI	incidence	.5018(.5 to .5073)	1.652(1.649 to 1.661)
Log-transformed age-standardized SEV scalar endocarditis	excess mortality rate	-.2074(-.3416 to -.1001)	.8127(.7106 to .9047)

No other significant changes were made to the modeling strategy in 2015.

Chronic obstructive pulmonary disease (COPD)

Flowchart

Chronic Obstructive Pulmonary Disease (COPD)

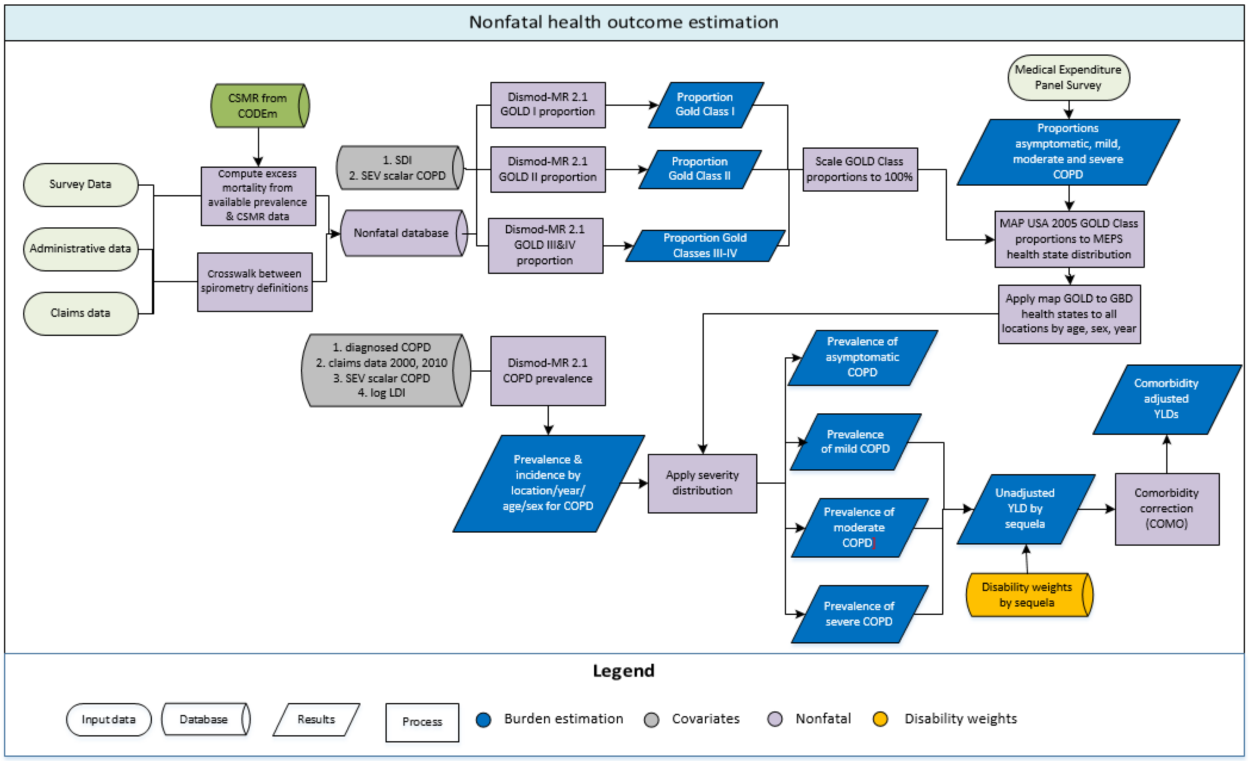

Input data and methodological summary

Case definition

COPD is defined as in the GOLD classification: a measurement of <0.7 FEV1/FVC (1 second of forceful exhalation/total forced expiration) on spirometry after bronchodilation. It should be noted that this a change from GBD 2013 where the 'Lower Limit of Normal (LLN)', i.e. relative to an age and sex-specific norm for the FEV1/FVC ratio, was the reference. We made this decision because the severity grading of COPD follows the GOLD Class definition rather than the LLN concept. The definitions of the severity classes in the GOLD classification are provided below.

GOLD CLASS	FEV1 Score
I: Mild	>=80% of normal
II: Moderate	50-79% of normal
IV: Severe	<50% of normal

ICD-10 codes associated with COPD include J40, J41, J42, J43, J44, and J47. The corresponding ICD-9 codes are 490-492, 494 and 496.

Input data

For GBD 2015, we updated the systematic review from previous iterations. The full search term was:

(chronic obstructive pulmonary disease[Title/Abstract] AND (prevalence[Title/Abstract] or incidence [Title/Abstract] or mortality [Title/Abstract] or death [Title/Abstract]) Filters: Publication date from 2013/01/01 to 2015/12/31; Humans

The search period was between 1/1/2013 and 5/13/2015. Twenty-one new sources were extracted. Studies excluding smokers were excluded from the review.

In addition, we searched for survey data with spirometry – measurements in GHDx, GBD's health data repository. We systematically extracted all spirometry data from the National Health and Nutrition Examination Study series in the United States for which we had only used some published studies in previous GBD studies. The Study of Aging and Global Health (SAGE) series was also examined but ultimately excluded as the spirometry data had implausible FEV1/FVC values (e.g., over 1

Furthermore, claims data for the United States were included. Additional information on the claims data collection and pre-corrections are provided elsewhere. Briefly, we determined US national and state level estimates of COPD prevalence from a database of individual level ICD coded health service encounters. Persons with any claim associated with COPD were marked as a prevalent case for that year.

Studies that provided non-standard cut-offs of COPD prevalence (e.g not .7) were crosswalked before the main analysis step to match the .7 FEV1/FVC case definition.

A table describing the density and distribution of the available data informing the COPD estimation process is provided below.

	Proportion by GOLD Class	Prevalence	Incidence
Studies	15	73	5
Countries or subnational locations	28	116	5
Regions	15	15	4

Modeling strategy

As described above, the estimation of COPD burden occurs in three main steps. The first is the estimation of prevalence and incidence using a DisMoD-MR 2.1 model. The second is the separate estimation of the proportions by three GOLD class groupings in DisMoD-MR 2.1. The third is the combination of these two processes to derive prevalence by severity.

Step 1: Main COPD model

Prior settings include remission of 0 and an incidence ceiling of 0.001 before age 20. The latter was necessary to avoid a kick-up of estimates in childhood at an age range with little or no primary data.

Claims data for 2000 and 2010 were adjusted via study covariates to account for systematically low estimates relative to the 2012 claims data. Implicit in this adjustment is the assumption that variation between years of claims data is a function of data collection inconsistencies.

Similar to other causes, we include estimates of cause-specific mortality rate (CSMR) and derived estimates of excess mortality rate (EMR) by dividing every prevalence data point by the CSMR value for the corresponding location, age, sex year. We did not estimate EMR for data points with an age range greater than 20 years.

To assist estimation, each model includes a series of country-level covariates that describe spatio-temporal patterns. Where available, we use the COPD standardized exposure variables (SEV), which aggregates multiple risk factors into a single variable. We also use the log of LDI on EMR to capture country-level variation of EMR, assuming a negative coefficient (i.e. lower mortality with rising GDP).

Step 2: GOLD class models

The GOLD class models use data from surveys that specified prevalence by GOLD class after expressing the values as a proportion of all COPD cases. We use fixed effects from the SEV scalar and the log of LDI per capita to assist estimation.

Table of model coefficients for COPD and GOLD class models

Cause	Variable_name	Measure	Beta	Exponentiated
COPD	LDI (I$ per capita)	excess mortality rate	-0.5	0.61<br>(0.61 -0 .61)
COPD	Log age-stand SEV scalar: COPD	prevalence	0.76	2.13<br>(2.12 - 2.16)
COPD	Claims data 2010	prevalence	-0.071	0.93<br>(0.90 - 0.96)
COPD	Claims data 2000	prevalence	-0.12	0.89<br>(.86 - .92)
COPD	Diagnosed COPD	prevalence	0.13	1.14<br>(1.02 - 1.31)
GOLD I proportion	Sociodemographic Index	proportion	0.89	2.44<br>(.35 – 7.00)
GOLD I proportion	Log age-stand SEV scalar: COPD	proportion	-0.18	0.8349<br>(0.53 - 1.29)
GOLD II proportion	Sociodemographic Index	proportion	-0.50	0.6062<br>(0.16 - 2.38)
GOLD II proportion	Log age-stand SEV scalar: COPD	proportion	-0.09	0.91<br>(0.59 - 1.6)
GOLD III+IV proportion	Sociodemographic Index	proportion	-0.65	0.5247<br>(0.15 - 3.9)
GOLD III+IV proportion	Log age-stand SEV scalar: COPD	proportion	0.001	1.001<br>(-0.45 - 2.16)

Severity

The three GOLD class groupings reflect a grading based on a physiological measurement rather than a direct measurement of disease severity. In order to map the epidemiological findings by GOLD Class into the three COPD health states for which we have disability weights (DW), we used the 2001-2011 Medical Expenditure Panel Survey (MEPS) data from the United States. Specifically, we convert the GOLD class

designations estimated for the USA in 2005 (the midpoint of MEPS years of analyses) into GBD classifications of asymptomatic, mild, moderate, and severe COPD.

The table below shows the three health states of COPD and the corresponding lay descriptions and disability weights. The graph shows the average proportion by GOLD Class (after scaling to 100%) across all ages for USA in 2005. We also show the proportion of MEPS respondents reporting any health service contact in the past year for COPD with a DW value attributable to COPD of 0, mild range (0 to midpoint between DWs for mild and moderate), moderate range (midpoint of DW values mild and moderate to midpoint of DW values for moderate and severe) and severe range (midpoint between DW values moderate and severe or higher). The DW value for COPD was derived from a regression with indicator variables for all health states reported by MEPS respondents and their reported overall level of disability derived from a conversion of SF-12 answers to GBD DW values. This analysis gave the severity distribution for each GBD cause reported in MEPS after correcting for any comorbid causes individual respondents reported during a year.

Health State	Lay description	DW (95% CI)
Mild COPD	This person has cough and shortness of breath after heavy physical activity, but is able to walk long distances and climb stairs.	0.019 (0.011-0.033)
Moderate COPD	This person has cough, wheezing and shortness of breath, even after light physical activity. The person feels tired and can walk only short distances or climb only a few stairs.	0.225 (0.153-0.31)
Severe COPD	This person has cough, wheezing and shortness of breath all the time. The person has great difficulty walking even short distances or climbing any stairs, feels tired when at rest, and is anxious.	0.408 (0.273-0.556)

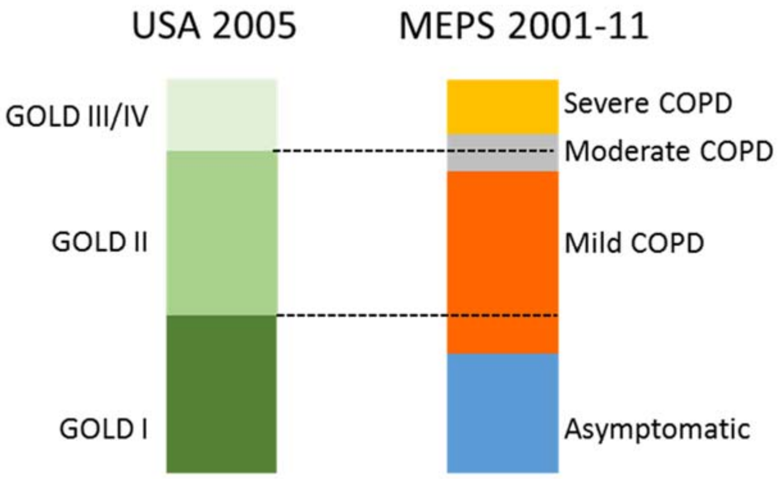

The algorithm to translate GOLD Class to COPD DW categories first assigns GOLD III&IV to severe COPD and what remains to moderate. Next, GOLD class I is assigned to the asymptomatic category first and

what remains goes to mild COPD. This algorithm is repeated for each age and sex category and for all 1,000 draws from the DisMod models of GOLD classes and the MEPS analyses. We end up with proportions of each of the GOLD class categories that map onto GBD COPD health states with uncertainty bounds determined by the 25th and 975th values of the 1,000 draws. These values are then applied to the estimates of the proportion of cases by GOLD class category, after scaling to 100%, by location, year, age and sex. This assumes that the relationship between GOLD class and GBD COPD health states in the United States applies everywhere.

Relative to GBD 2013, the biggest changes to COPD prevalence and incidence derive from changing the basis of the case definition from LLN to GOLD class. This led to decreases in prevalence in young and middle aged adults but greater prevalence in the elderly.

Pneumoconiosis

Coal Worker's Pneumoconiosis, Asbestosis, Silicosis and Other Pneumoconiosis

Flowchart

Pneumoconiosis:
Coal Worker's, Asbestosis, Silicosis, Other

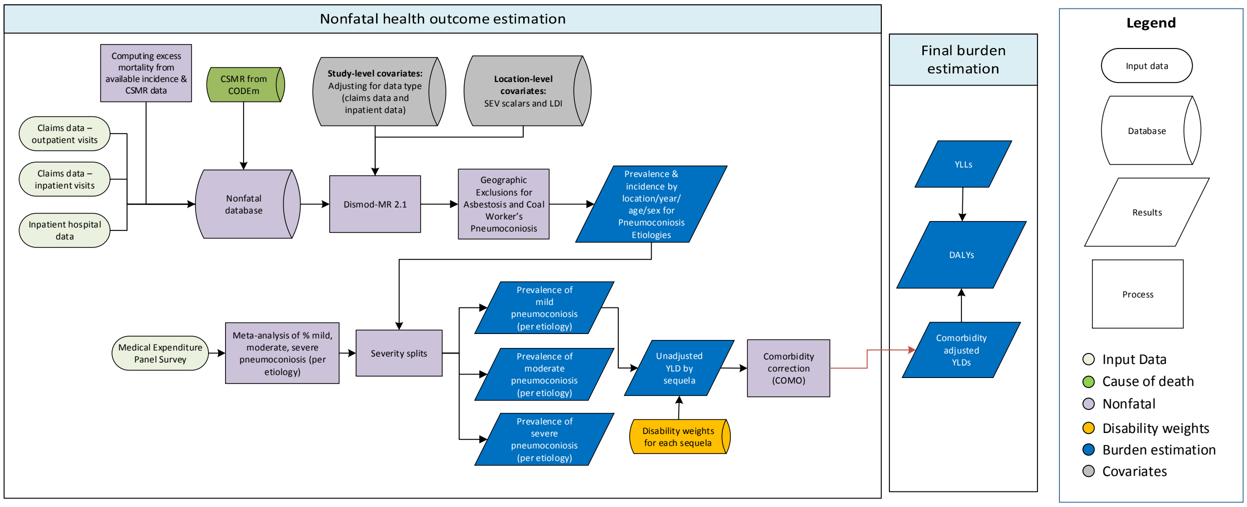

Input data and methodological appendix

Case definition

Pneumoconiosis is a chronic lung disease typified by lung scarring and other interstitial damage caused by exposure to dust and other containments—usually through occupational exposure. For GBD, we model pneumoconiosis by exposure type: coal, asbestosis, silica and other.

Input data

No systematic review of the literature was conducted for any of the pneumoconiosis variants for this iteration of the Global Burden of Disease. These reviews done on a rotating basis and updates will be made in a future iteration.

Data used to make estimates of pneumoconiosis are predominantly from four main sources. The first is literature data from previous systematic reviews—usually from smaller scale studies or surveys of prevalence. The second are occupational exposure reports and registries produced by governmental agencies. The third data format we use are collated hospital inpatient reports. The fourth main category of data are claims data—particularly for the United States. Greater detail on the preparation of the collated inpatient data and claims data is provided elsewhere. An overview of the data density and distribution by measure and location is present below for the four pneumoconiosis variants.

Silicosis	Prevalence	Incidence	Mortality risk
Studies	42	3	1
Subnational Units	99	1	99
Countries	25	3	24
Regions	8	3	8

Coal Worker's	Prevalence	Incidence	Mortality risk
Studies	41	4	1
Subnational Units	83	1	83
Countries	24	4	23
Regions	9	3	8

Asbestosis	Prevalence	Incidence	Mortality risk
Studies	39	3	1
Subnational Units	81	1	81
Countries	26	3	26
Regions	8	3	8

Other	Prevalence	Incidence	Mortality risk
Studies	37	0	1
Subnational Units	111	0	111
Countries	26	0	26
Regions	8	0	8

For all etiologies, we use a sex specific correction factor of the hospital inpatient data where numbers are adjusted upwards by the ratio of primary diagnosis to secondary diagnosis present in the claims data. For sex-cause pairs where the ratio cannot be calculated (e.g. no secondary diagnoses) the data is left unadjusted (ratio of 1). The ratios are presented below.

Asbestosis	37.06452	1
Coal Worker's	36.57143	22.6
Other	12.1	42
Silicosis	9.727273	1

Severity Split Inputs

Data to inform estimates of the severity gradient due to pneumoconiosis etiologies are derived from previous analyses of the Medical Expenditure Panel Survey (MEPS). The disability weights are also shared.

Severity level	Lay description	DW (95% CI)
Mild	has cough and shortness of breath after heavy physical activity, but is able to walk long distances and climb stairs.	0.019 (0.011-0.033)

Moderate	has cough, wheezing and shortness of breath, even after light physical activity. The person feels tired and can walk only short distances or climb only a few stairs.	0.225<br>(0.153-0.312)
Severe	has cough, wheezing and shortness of breath all the time. The person has great difficulty walking even short distances or climbing any stairs, feels tired when at rest, and is anxious.	0.408<br>(0.273-0.556)

Modeling strategy

Estimates for the pneumoconiosis etiologies are produced using a standard DisMod MR 2.1 approach, a Bayesian meta-regression.

For all etiologies, we use prior settings of zero remission. Additionally, we assume no incidence and prevalence before the age of 10.

Claims data for 2000 and 2010 are adjusted via study covariates to account for systematically low estimates relative to the 2012 claims data. Implicit in this adjustment is the assumption that variation between years of claims data is a function of data collection inconsistencies and noise. As pneumoconiosis is a chronic disease largely caused by long-term occupational exposure and there is no evidence of rapid occupational shifts during the period of study, we ascribe observed differences to collection noise/error.

Similar to other causes, we include estimates of cause-specific mortality rate (CSMR) and Excess Mortality Rate (EMR) in this. The source and estimation of these rates are discussedelsewhere. This is a change from GBD2013 where EMR values derived from Chronic Obstructive Pulmonary Disease were used.

To assist estimation, each model includes a series of country-level covariates that describe spatio-temporal patterns. Where available, we use standardized exposure variables (SEV) which aggregates multiple risk factors into a single variable. A full accounting is below:

Cause	Measure	Variable_Name	Beta	Exponentiated
Asbestosis	excess mortality rate	LDI (I$ per capita)	-.5909<br>(-.6625 - -.5183)	.5538<br>(.5156 - .5955)
Asbestosis	prevalence	All MarketScan, year 2010	-.062<br>(-.1164 - -.01)	.9399<br>(.8901 - .9901)
Asbestosis	prevalence	All MarketScan, year 2000	-.0048<br>(-.0167 - -5.3e-04)	.9952<br>(.9835 - .9995)
Coal Worker's	prevalence	All MarketScan, year 2000	-.0016<br>(-.0043 - -8.3e-05)	.9984<br>(.9957 - .9999)
Coal Worker's	prevalence	Log-transformed age-standardized SEV scalar: Coal W	1.166<br>(.9643 - 1.247)	3.209<br>(2.623 - 3.48)
Coal Worker's	prevalence	Coal Production (per capita)	1.036<br>(.0624 - 1.957)	2.819<br>(1.064 - 7.078)

Coal Worker's	prevalence	All MarketScan, year 2010	-.0037 (-.0129 - -1.4e-04)	.9963 (.9872 - .9999)
Coal Worker's	excess mortality rate	LDI (I$ per capita)	-.9896 (-.9997 - -.966)	.3717 (.368 - .3806)
Other pneumoconiosis	prevalence	All MarketScan, year 2010	-1.097 (-1.161 - -1.032)	.3339 (.3132 - .3563)
Other pneumoconiosis	prevalence	Outdoor Air Pollution (PM2.5)	.0495 (.0485 - .05)	1.051 (1.05 - 1.051)
Other pneumoconiosis	prevalence	Log-transformed SEV scalar: Oth Pneum	.7674 (.7515 - .8186)	2.154 (2.12 - 2.267)
Other pneumoconiosis	excess mortality rate	LDI (I$ per capita)	-.0128 (-.019 - -.0102)	.9873 (.9811 - .9899)
Other pneumoconiosis	prevalence	All MarketScan, year 2000	-.9263 (-.9869 - -.8689)	.396 (.3727 - .4194)
Silicosis	prevalence	All MarketScan, year 2000	-.4381 (-.5315 - -.3456)	.6452 (.5877 - .7078)
Silicosis	excess mortality rate	LDI (I$ per capita)	-.4969 (-.4999 - -.4905)	.6084 (.6066 - .6123)
Silicosis	prevalence	All MarketScan, year 2010	-.0527 (-.1285 - -.0019)	.9486 (.8794 - .9981)
Silicosis	prevalence	Log-transformed age-standardized SEV scalar: Silicosis	.9029 (.7532 - 1.179)	2.467 (2.124 - 3.251)

To account for country level differences in excess mortality (perhaps as a function of available medical care) we use ln(lag distributed income) as a proxy measure.

For prevalence and incidence of Coal Worker's Pneumoconiosis and Asbestosis, geographies without a history of coal mining or asbestosis production are set to zero given the causal and necessary relationship between respective occupational exposure and disease. These exclusions are derived from Survey of Energy Resources 2013 and The United States Geological Survey Minerals Yearbook respectively. As the other etiologies do not have such strong single occupational causes, no geographic exclusions are conducted.

Although the general modeling strategy has remained mostly unchanged since GBD 2013, we do expect an across the board increase in prevalence estimates. Driving this shift is the secondary diagnosis correction of the hospital inpatient data.

Asthma

Flowchart

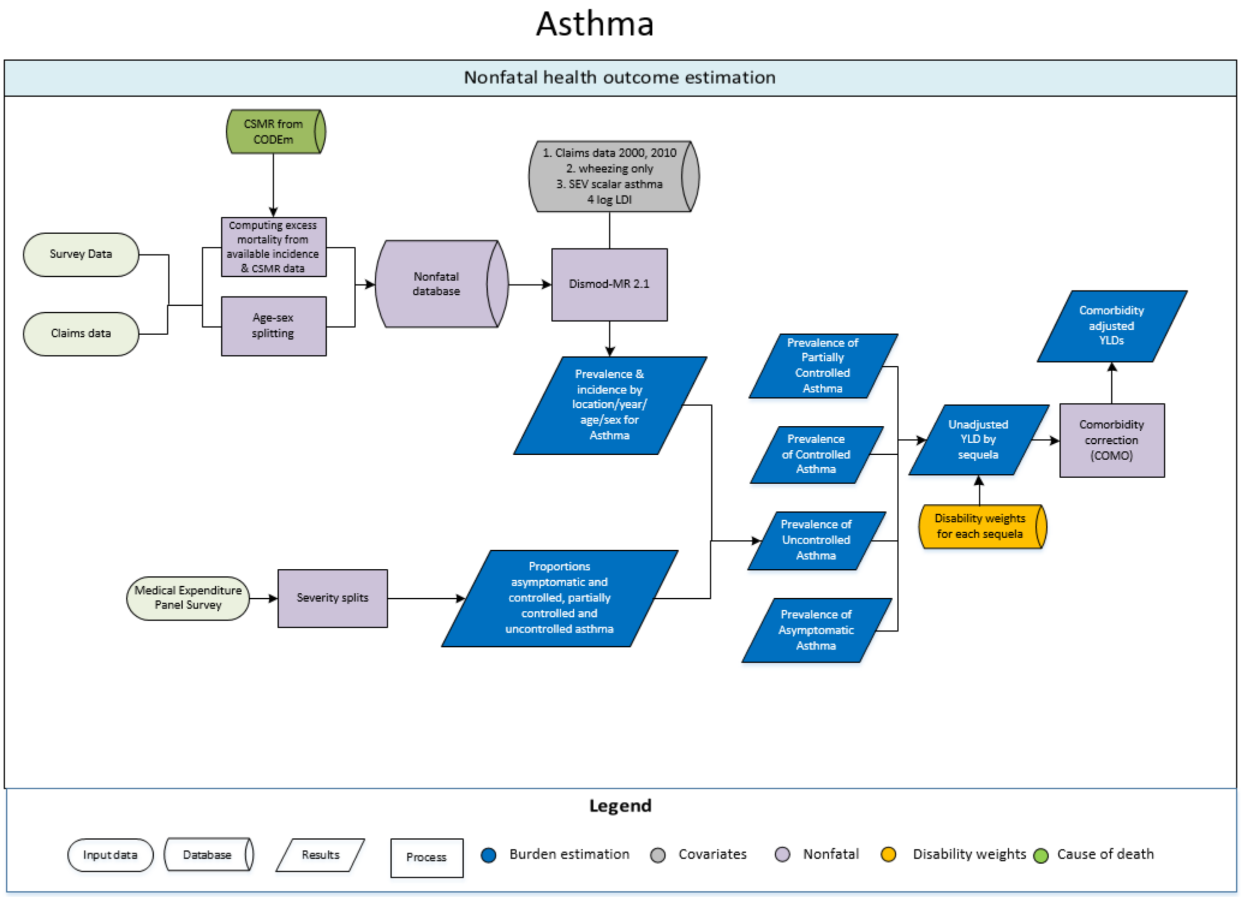

Case definition

Asthma is a chronic lung disease marked by spasms in the bronchi usually resulting from an allergic reaction or hypersensitivity and causing difficulty in breathing. We define asthma as a doctor's diagnosis and wheezing in the past year.   The relevant ICD-10 codes are J45 and J46. ICD-9 code is 493.

Input data

Model inputs

For GBD 2015, we did not undertake a full systematic review of the literature on asthma. Instead, certain studies were re-extracted to ensure accuracy and several survey series for which we have individual records in our GHDx repository were added to the dataset. Data additions and re-analysis include the WHO Study on Global Aging and Adult Health series, the WHO World Health Survey series, and the

Belgian Health Interview Survey. Surveys carried out as part of the International Study of Asthma and Allergies in Childhood (ISAAC) collaboration are the most important source of prevalence data in children.

The following table provides a description of the data density and distribution by geography and epidemiological measure (including the claims data discussed below).

	Prevalence	Incidence	Mortality risk
Studies	267	7	7
Countries/subnational locations	248	5	3
Regions	21	1	1

In addition to literature and survey data, we use claims data from the United States from 2000, 2010, and 2012. Information on the source and preparation of these data are provided in detail elsewhere. Briefly, we determined US national and state level estimates of asthma prevalence from a database of individual level ICD coded health service encounters for three years. Persons with any claim associated with asthma were marked as a prevalent case for that year. Aggregated estimates were then adjusted using a noise reduction algorithm. These corrected data were then used in the modeling process.

Modeling strategy

We use DisMod MR 2.1 as the main modeling tool for asthma. Prior settings include a maximum remission of 0.3 (reflecting the upper bound of the highest observed data) and no incidence between the ages of 0 and 0.5 year as a diagnosis cannot be made in young infants.

Data points from the ISAAC studies were reported for both sexes combined. We sex split before modeling using the ratios derived from the 2012 US claims data (1).

Data that describe wheezing, but do not report presence/absence of an accompanying diagnosis in the last year were crosswalked to the reference category. As the table below shows, studies that only report wheezing are systematically higher than reference data points and are adjusted down-- dividing by the exponentiated coefficient).

To account for country-level differences in excess mortality as a function of available medical care we use log LDI as a covariate and assume a negative coefficient. The effect size is shown below.

Claims data for 2000 and 2010 are adjusted via study covariates to account for systematically lower estimates relative to the 2012 claims data. Implicit in this adjustment is the assumption that variation between years of claims data is a function of data collection inconsistencies.

Similar to other causes, we include estimates of cause-specific mortality rate (CSMR) and excess mortality rate (EMR) derived as a matched value for each prevalence data point dividing CSMR by prevalence. We restrict these EMR calculations to data points of 20 year age span or less.

To assist estimation, the model includes a series of country-level covariates that describe spatio-temporal patterns. Specifically, we use log LDI and the asthma standardized exposure variable (SEV), a scalar that

combines exposure of all GBD risks that influence asthma. A full covariate list, including the study level covariates, described above are presented in the following table with their associated effects:

Variable_name	Measure	Beta	Exponentiated
Wheezing	prevalence	0.13	1.14<br>(1.08 - 1.22)
Claims data 2000	prevalence	-0.51	0.60<br>(.59 - 0.61)
Claims data 2010	prevalence	-0.08	0.92<br>(0.91 - 0.94)
Log LDI (I$ per capita)	prevalence	-0.004	.9938<br>(.9737 - 1.007)
Log SEV scalar: asthma	prevalence	1.202	3.33<br>(2.97 - 3.49)
Log LDI (I$ per capita)	excess mortality rate	-0.42	0.65<br>(0.64 -0 .67)

Severity split inputs

Lay descriptions and disability weights for the asthma health states are shown in table below. The distribution between the three health states is derived from an analysis of the US Medical Expenditure Panel Surveys (MEPS). The methods are described in full in a separate section of this appendix. Briefly, MEPS is an ongoing survey of health service encounters with as its main objective to collect data on health expenditure. Panels are recruited every year and followed up for a period of two years. Diagnostic information provided by respondents on the reasons for any health care contact are coded into three-digit ICD-9 codes by professional coders.

Twice over the two-year follow-up period respondents are asked to fill in SF-12. From convenience samples asking respondents to fill in SF-12 for 60 of the GBD health states, IHME has created a mapping from SF-12 scores to GBD Disability Weights (DW). We perform a regression with indicator variables for all GBD causes that we can identify from the ICD codes in MEPS to derive for each individual with a diagnosis the amount of disability that can be attributed to that condition after controlling for any comorbid conditions. Anyone with a diagnosis of asthma in whom the disability assigned to asthma is negative or zero we assume is asymptomatic (at the time of asking SF-12 question relating to their health status in the past 4 weeks). Non-zero values we bin into the three health states assuming a split between these at the midpoint between DW values. The table below gives the proportions in MEPS in each of the health states and an asymptomatic state.

Severity level	Lay description	DW (95% CI)	Severity distribution
Asymptomatic			30.2%<br>(29.2-31.3%)
Controlled	This person has wheezing and cough once a month, which does not cause difficulty with daily activities.	0.015<br>(0.007-0.026)	20.5%<br>(13.7-28.6%)

Partially controlled	This person has wheezing and cough once a week, which causes some difficulty with daily activities.	0.036 (0.022-0.055)	22.3% (16.6-28.1%)
Uncontrolled	This person has wheezing, cough, and shortness of breath more than twice a week, which causes difficulty with daily activities and sometimes wakes the person at night.	0.133 (0.086-0.192)	26.9% (21.4-35.1%)

Interstitial lung disease and pulmonary sarcoidosis (ILD)

Flowchart

Interstitial Lung Disease and Pulmonary Sarcoidosis (ILD)

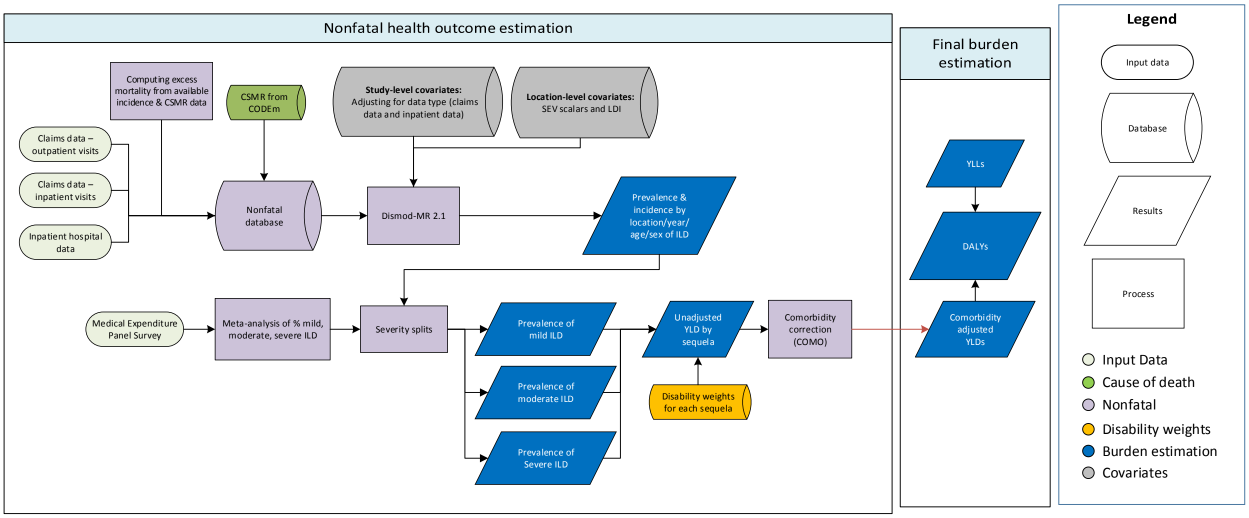

Case definition

Interstitial Lung Diseases and Pulmonary Sarcoidosis are a collection of chronic respiratory diseases that impair lung function and oxygen uptake through scarring and/or inflammation. The relevant ICD codes are D86 and J84. For Interstitial Lung Disease, we use the American Thoracic Society as the gold standard definition.

Input data

Model Inputs

No systematic review of the literature was conducted for ILD for this iteration of the Global Burden of Disease. These reviews done on a rotating basis and updates will be made for a future iteration.

Data used to make estimates of ILD are predominantly from three main sources. The first is literature data from previous systematic reviews—usually from smaller scale studies of prevalence. The second main data type are claims data for the United States. The source and preparation of these data is describe elsewhere. The third main data type is adjusted hospital inpatient records. Because these records only report primary diagnosis, we a priori adjust the numbers by a sex-specific factor based on patterns observed in the US claims data—that is, the ratio of primary to secondary diagnoses. These factors are 6.63 for males and 6.30 for females.

The following table provides a picture of the number of available studies along with their distribution globally and by epidemiological profile. In short, the ILD data landscape is rather sparse. The available

data is largely skewed towards high-income countries like the United States or the member countries of the European Union. The relatively high number of subnational units with data is largely a function of claims data in the United States and hospital data from Mexico and Brazil.

	Prevalence	Incidence	Mortality risk
Studies	47	19	3
Subnational Units	83	3	80
Countries	28	13	28
Regions	8	6	3

Severity Splits

Data to inform estimates of the severity gradient due to ILD are derived from previously analyses of the Medical Expenditure Panel Survey (MEPS). The table below illustrates the lay descriptions and disability weights associated with different levels of severity of interstitial lung disease.

Severity level	Lay description	DW (95% CI)
Mild	has cough and shortness of breath after heavy physical activity, but is able to walk long distances and climb stairs.	0.019 (0.011-0.033)
Moderate	has cough, wheezing and shortness of breath, even after light physical activity. The person feels tired and can walk only short distances or climb only a few stairs.	0.225 (0.153-0.312)
Severe	has cough, wheezing and shortness of breath all the time. The person has great difficulty walking even short distances or climbing any stairs, feels tired when at rest, and is anxious.	0.408 (0.273-0.556)

Modeling strategy

Estimates for ILD are produced using a standard DisMod MR 2.1 approach, a Bayesian meta-regression epidemiological model. We use prior settings of zero remission and we constrain the super region random effects to -.5 - .5 to ensure model stability.

As described above, we use an a priori adjustment of hospital inpatient data to correct for secondary diagnoses available in the claims data. These is a change from GBD2013 where hospital inpatient data was left unadjusted.

Similar to other causes, we include estimates of cause-specific mortality rate (CSMR) and Excess Mortality Rate (EMR) in this. The source and estimation of these rates are discussedelsewhere. This is a change from GBD2013 where EMR values derived from Chronic Obstructive Pulmonary Disease were used.

The GBD ethic is to use all available data sources where reasonable. Because ILD consists of many smaller etiologies not broken out here, we make crosswalks to account for measurement, case definition and study design differences. The full list is below:

Variable_Name	Measure	Beta	Exponentiated
Hospital data	prevalence	.0025 (-1.957 - 2)	1.002 (.1413 - 7.389)
Only idiopathic pulmonary fibrosis and sarcoidosis	prevalence	-1.329 (-1.929 - -.6597)	.2647 (.1453 - .517)
All MarketScan, year 2000	prevalence	-1.006 (-2 - -.0122)	.3656 (.1353 - .9878)
All MarketScan, year 2010	prevalence	-1.001 (-2 - -.0243)	.3675 (.1353 - .976)
Hospital data	incidence	.5903 (-.0277 - 1.208)	1.805 (.9727 - 3.347)
Only idiopathic pulmonary fibrosis	incidence	.0051 (-1.995 - 2)	1.005 (.1361 - 7.389)
Only idiopathic pulmonary fibrosis and sarcoidosis	incidence	-.983 (-1.631 - -.2809)	.3742 (.1957 - .7551)
LDI (I$ per capita)	excess mortality rate	-.1972 (-.2177 - -.1756)	.821 (.8044 - .839)

Claims data for 2000 and 2010 are adjusted via study covariates to account for systematically low estimates relative to the 2012 claims data. Implicit in this adjustment is the assumption that variation between years of claims data is a function of data collection inconsistencies and noise. Additional adjustments are made to literature based hospital data that may not capture all ILD subcauses. This is also the rationale for the remaining study covariates which examine only subsets of the ILD cause.

To account for country level differences in excess mortality (perhaps as a function of available medical care) we use ln(lag distributed income) as a proxy measure. The effect size is shown above.

Although the general modeling strategy has remained mostly unchanged since GBD 2013, we do expect an across the board increase in prevalence estimates. Driving this shift is the secondary diagnosis correction of the hospital inpatient data.

Cirrhosis

Flowchart

Cirrhosis

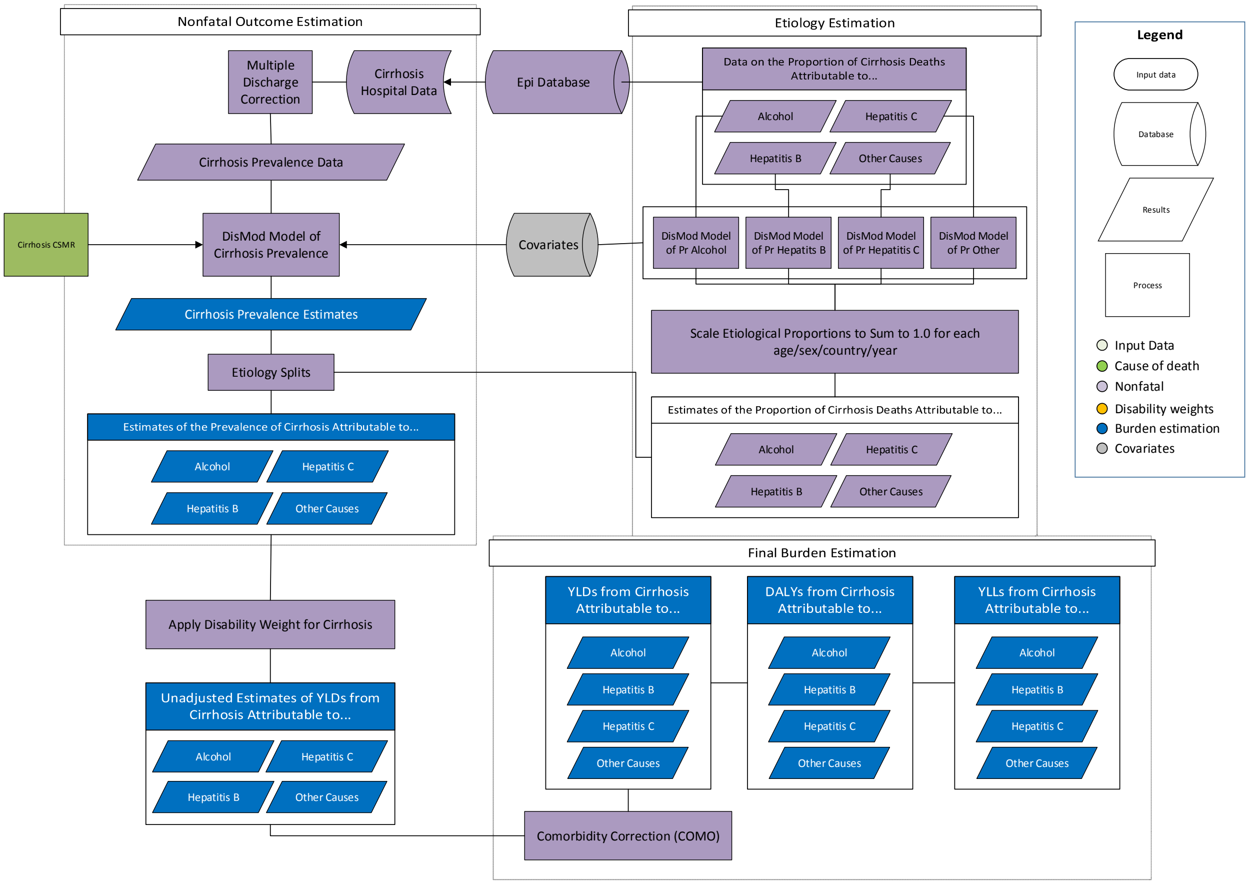

Input data and methodological summary

Case definition

Cirrhosis is a chronic liver disease most often caused by alcohol use, or chronic infection with hepatitis B or C. Early disease is typically asymptomatic as the liver's resilience compensates for cirrhotic damage.Decompensated cirrhosis occurs when the disease progresses beyond the capacity of the liver to compensate for the damage, and is marked by profound symptoms, health loss and, often, death. We model decompensated cirrhosis, defined by cirrhosis (or a closely related diagnosis code) as the primary diagnosis in hospital data. This includes ICD1-0 codes K70-K77, I85, P78.81.

Input data

Model inputs

For GBD 2015, we modeled cirrhosis prevalence based on hospital data, which have been updated to reflect the new estimation cycle. The table below indicates the number of data points, as well as the location and regional breakdown of data.

Level	Prevalence
Data points	7516
Studies	35
Locations	71
Regions	7

We model etiologic proportions based on published estimates of the proportion of cirrhosis due to alcohol use, hepatitis B, hepatitis C, and other causes:

Level	Alcohol	Hepatitis B	Hepatitis C	Other
Data points	73	86	86	55
Studies	47	74	74	33
Locations	23	39	39	16
Regions	13	16	16	10

A systematic review of the literature was conducted to capture studies of proportion of cirrhosis attributable to alcohol, hepatitis B, hepatitis C, and other cause. We searched the peer-reviewed literature via PubMed and solicited sources from GBD collaborators.

The inclusion criteria stipulated that: 1) the publication year must be from 1980 onwards; 2) the sample had to be a representative sample of those with decompensated cirrhosis (e.g., studies of patients with both HCC and cirrhosis were excluded); 3) sufficient information must be provided on study method and sample characteristics to assess the quality of the study; 4) hepatitis B and C were confirmed via HBsAg, in the case of hepatitis B, and anti-HCV IgG, in the case of hepatitis C.

Modeling strategy

We modeled cirrhosis prevalence using hospital data and CSMR estimates, assuming no remission. To estimate the prevalence of cirrhosis due to alcohol, cirrhosis due to hepatitis B, cirrhosis due to hepatitis C, and cirrhosis due to other causes, we develop etiological proportion models using DisMod, and use the results of these models to split the parent cirrhosis prevalence estimates.

Given the similar etiologies for liver cancer and cirrhosis we integrated the etiology models for these two causes. We have more data for liver cancer etiologies than we do for cirrhosis. Therefore, we first developed four single-parameter DisMod models, each to estimate the proportion of liver cancer due to a given cause (i.e., alcohol, hepatitis B, hepatitis C, and other). These models included as covariates alcohol consumption (liters per capita), hepatitis B surface antigen (HBsAg) seroprevalence, and hepatitis C (anti-

HCV IgG) seroprevalence. Moreover, the model for the proportion due alcohol included a binary covariate indicating countries with a predominantly Muslim population (thought to be associated with very low alcohol consumption). Estimates from these liver cancer models were then used as covariates (along with alcohol, HBsAg, and anti-HCV) in the four corresponding cirrhosis etiology models. Estimates from these cirrhosis models were then similarly used as covariates in the corresponding liver cancer models. Proportions from the four etiology models were then rescaled to sum to one at the draw level, and used to split the parent cirrhosis estimates.

Sequelae definitions and associated DWs

Sequela name	Healthstate description	Disability weight (95% CI)
Cirrhosis of the liver due to alcohol	Has a swollen belly and swollen legs. The person feels weakness, fatigue and loss of appetite.	0.178 (0.122-0.25)
Cirrhosis of the liver due to hepatitis B	Has a swollen belly and swollen legs. The person feels weakness, fatigue and loss of appetite.	0.178 (0.123-0.25)
Cirrhosis of the liver due to hepatitis C	Has a swollen belly and swollen legs. The person feels weakness, fatigue and loss of appetite.	0.178 (0.122-0.25)
Cirrhosis of the liver due to other cause	Has a swollen belly and swollen legs. The person feels weakness, fatigue and loss of appetite.	0.178 (0.122-0.25)

Changes from GBD 2013 to GBD 2015

To better integrate our liver cancer and cirrhosis models, we implemented the aforementioned covariate cycle approach as an improvement for GBD 2015.

Peptic ulcer disease

Flowchart

Peptic Ulcer Disease (PUD)

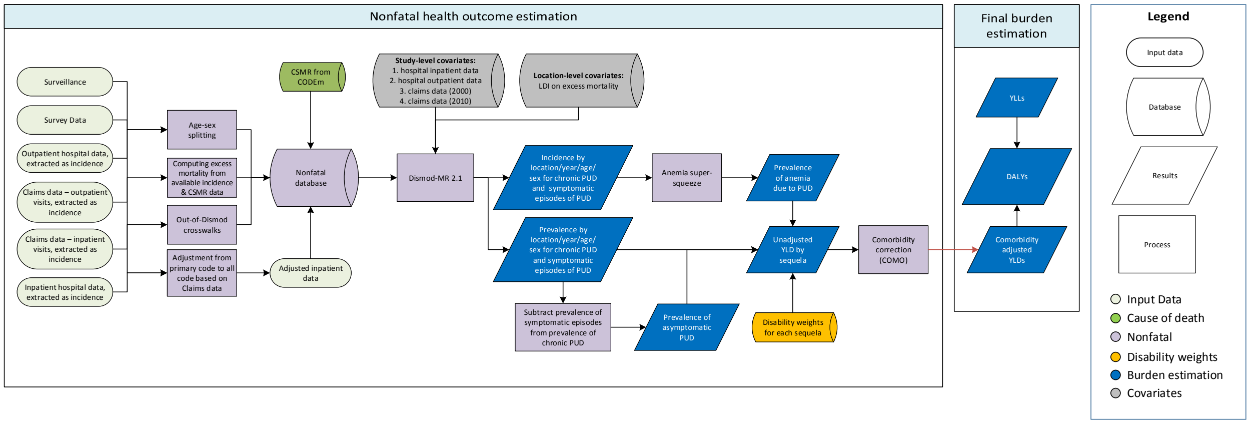

Case definition

Peptic ulcer disease is a digestive disorder involving ulcers in the lining of the stomach (gastric ulcers) or the duodenum (duodenal ulcers), diagnosed by an endoscopy. Peptic ulcer disease is often asymptomatic with periodic symptomatic episodes of heartburn, bloating, nausea or vomiting, and in severe cases, bleeding.[1] ICD codes included are K25, K26, K27, K28, and K31.

Input data

Model inputs

Data inputs were separate for the chronic and symptomatic episode models. For the chronic dataset, a systematic review of literature was conducted to capture studies of prevalence, incidence, case fatality rate, and standardized mortality rate associated with peptic ulcer disease. In summary, the search was conducted in three stages involving searches of the peer-reviewed literature (via Medline, Embase, and PubMed), the grey literature, and expert consultation. The search string used was: (("stomach"[MeSH Terms] OR "stomach"[All Fields] OR "gastric"[All Fields]) AND ("peptic ulcer"[MeSH Terms] OR ("peptic"[All Fields] AND "ulcer"[All Fields]) OR "peptic ulcer"[All Fields]) AND ("duodenal diseases"[MeSH Terms] OR ("duodenal"[All Fields] AND "diseases"[All Fields]) OR "duodenal diseases"[All Fields] OR ("duodenal"[All Fields] AND "disease"[All Fields]) OR "duodenal disease"[All Fields])) AND ("epidemiology"[Subheading] OR "epidemiology"[All Fields] OR "epidemiology"[MeSH Terms]) AND ("2008/01/01"[PDAT] : "2015/12/31"[PDAT]).

The inclusion criteria stipulated that (1) "caseness" must be based on clinical threshold as established by the ICD; (2) sufficient information must be provided on study methods and sample characteristics to assess the quality of the study; and (3) study samples must be representative of the general population (i.e., samples of patients prescribed gastroscopies due to gastric pain or populations with *H. pylori* bacteria were excluded). In addition to literature data, we included hospital inpatient and outpatient data, and US claims data from 2000, 2010, and 2012 at the US state level, extracted as prevalence. The

claims data were extracted using all diagnoses, and the hospital data were adjusted for only recording primary diagnoses, using a correction factor from claims data.

For the symptomatic episode dataset, we used extracted hospital inpatient and outpatient data and US claims data from 2000, 2010, and 2012 at the US state level, extracted as incidence to capture individual episodes. These data were similarly extracted and adjusted for to estimate all diagnoses.

Data were outliered or excluded if we found them unreasonable when compared to regional, super-regional, and global rates.

The tables below show the number studies included in GBD 2015, as well as the number of countries or subnational units and GBD world regions represented.

Chronic PUD

	Prevalence	Incidence	Mortality risk
Studies	7	11	2
Countries/subnationals	9	11	3
GBD world regions	4	3	1

Symptomatic episodes of PUD

	Prevalence	Incidence	Mortality risk
Studies	0	49	1
Countries/subnationals	0	143	143
GBD world regions	0	8	8

Severity split & disability weight

The basis of the GBD disability weight survey assessments are lay descriptions of sequelae highlighting major functional consequences and symptoms. The lay descriptions and disability weights for peptic ulcer disease are shown below:

Severity level	Lay description	DW (95% CI)
Peptic ulcer disease, symptomatic episodes	This person has pain in the belly and feels nauseated. The person has difficulty with daily activities.	0.114 (0.078-0.159)

Modeling strategy

We changed the modeling process for PUD from GBD 2013 to show both prevalence of chronic peptic ulcer disease and symptomatic episodes, rather than just symptomatic episodes. We believe that this more accurately reflects the epidemiology of peptic ulcer disease and is more valuable when considering health policy.

The DisMod model for chronic peptic ulcer disease included bounding remission from 0 to 0.5 (a minimum duration of two years), zero incidence from 0 to 5 years of age, and excess mortality capped at 0.1 for all ages. Reference data were from literature, and we marked inpatient hospital data and outpatient hospital data for study-level covariates, and US claims data with separate year-specific study-level covariates. We used the function in DisMod-MR 2.1 to pull in cause-specific mortality rate (CSMR)

data from our CODEm and CODcorrect analyses and match with prevalence data points for the same geography. We calculated excess mortality rate to estimate priors by dividing CSMR by prevalence. We also applied a lag-distributed income covariate to excess mortality, log transformed and forced negative with an upper bound of -0.1 and a lower bound of -1. Betas and exponentiated values (which can be interpreted as an odds ratio) are shown in the tables below for study-level covariates and country-level covariates.

Study covariate	Parameter	beta	Exponentiated beta
Outpatient	Prevalence	-1.53 (-1.61 — -1.45)	0.22 (0.20 — 0.23)
Inpatient hospital	Prevalence	-1.46 (-1.54 — -1.4)	0.23 (0.22 — 0.25)
Claims data – 2000	Prevalence	-0.29 (-0.35 — -0.24)	0.75 (0.70 — 0.79)
Claims data – 2010	Prevalence	-0.023 (-0.077 — -0.001)	0.98 (0.93 — 1.00)
Claims data – 2012	Prevalence	-0.016 (-0.063 — -0.00027)	0.98 (0.94 — 1.00)

Country-level covariate	Parameter	beta	Exponentiated beta
LDI (log transformed)	Excess mortality	-0.86 (-0.88 — -0.8)	0.42 (0.42 — 0.45)

The symptomatic episodes of peptic ulcer disease DisMod model bounded remission from 16.5-17.5 (a duration of about three weeks). We also assumed no incidence from 0 to 5 years old, and excess mortality capped at 0.1 for all ages. The reference data were US claims data from 2012, and we marked US claims data from 2000 and 2010 with year-specific study-level covariate, and inpatient hospital data with an inpatient study-level covariate. We used the function in DisMod-MR 2.1 to pull in cause-specific mortality rate (CSMR) data from our CODEm and CODcorrect analyses and match with prevalence data points for the same geography. We calculated excess mortality rate to estimate priors by dividing CSMR by prevalence. We also applied a lag-distributed income covariate to excess mortality, log transformed and forced negative with an upper bound of -0.1 and a lower bound of -1, and a log-transformed age-standardized death rate for stomach cancer to incidence. Betas and exponentiated values (which can be interpreted as an odds ratio) are shown in the tables below for study-level covariates and country-level covariates.

Study covariate	Parameter	beta	Exponentiated beta
Outpatient	Incidence	-2.99 (-3 — -2.97)	0.050 (0.050 — 0.051)
Claims data – 2000	Incidence	-0.73 (-0.82 — -0.65)	0.48 (0.44 — 0.52)
Claims data – 2010	Incidence	-0.86 (-0.93 — -0.78)	0.42 (0.39 — 0.46)
Inpatient hospital	Incidence	-2.76 (-2.84 — -2.7)	0.063 (0.058 — 0.067)

Country-level covariate	Parameter	beta	Exponentiated beta
LDI (log transformed)	Excess mortality	-0.000085 (-0.00009 — -0.0000081)	1.00 (1.00 — 1.00)
ln-ASDR (stomach cancer)	Incidence	0.94 (0.86 — 1.00)	2.55 (2.36 — 2.71)

To calculate asymptomatic peptic ulcer disease, we took the estimated prevalence of symptomatic episodes, and subtracted it from our estimated prevalence of chronic peptic ulcer disease.

Gastritis and duodenitis

Flowchart

Gastritis and duodenitis

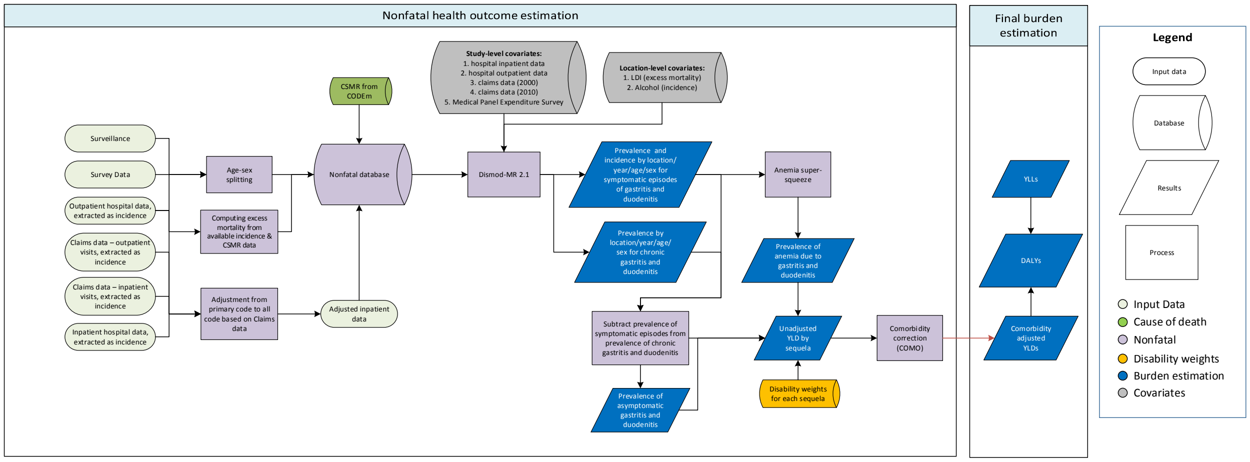

Case definition

Gastritis and duodenitis are digestive disorders involving inflammation of the stomach lining (gastritis) or the duodenum (duodenitis). Gastritis and duodenitis can often be asymptomatic with periodic symptomatic episodes of nausea, vomiting, indigestion, stomach pain, and in severe cases, internal bleeding. ICD codes included are K29.

Input data

Model inputs

Data inputs were separate for the chronic and symptomatic episode models. For the chronic dataset, a systematic review of literature was conducted to capture studies of prevalence, incidence, case fatality rate, and standardized mortality rate associated with gastritis and duodenitis. For GBD 2010, PubMed, Medline, and Embase were searched using the following search terms: (gastritis OR duodenitis) AND epidemiology AND humans). This search was repeated in PubMed for GBD 2015, restricting dates searched from January 1, 2009, to December 31, 2015. The inclusion criteria were:

1. Studies must be representative of the national population, i.e., excluded populations of patients with *H. pylori*, or patients prescribed endoscopies due to stomach pain
2. Sufficient information must be provided on study methods and sample characteristics to assess the quality of the study
3. Excluded reviews

In addition to literature data, we included hospital inpatient and outpatient data, and US claims data from 2000, 2010, and 2012 at the US state level, extracted as prevalence. The claims data were extracted using all diagnoses, and the hospital data were adjusted for only recording primary diagnoses, using a correction factor from claims data.

For the symptomatic episode dataset, we used extracted hospital inpatient and outpatient data and US claims data from 2000, 2010, and 2012 at the US state level, extracted as incidence to capture individual episodes. These data were similarly extracted and adjusted to estimate all diagnoses.

Data were outliered or excluded if we found them unreasonable when compared to regional, super-regional, and global rates.

The tables below show the number studies included in GBD 2015, as well as the number of countries or subnational units and GBD world regions represented.

Chronic gastritis and duodenitis

	Prevalence	Incidence	Mortality risk
Studies	82	1	1
Countries/subnationals	164	1	59
GBD world regions	16	1	5

Symptomatic Episodes of gastritis and duodenitis

	Prevalence	Incidence	Mortality risk
Studies	0	49	1
Countries/subnationals	0	143	143
GBD world regions	0	8	8

Disability weights

The basis of the GBD disability weight survey assessments are lay descriptions of sequelae highlighting major functional consequences and symptoms. The lay descriptions and disability weights for gastritis and duodenitis are shown below:

Severity split	Lay description	DW (95% CI)
Gastritis and duodenitis, symptomatic episodes	This person has pain in the belly and feels nauseated. The person has difficulties with daily activities.	0.114 (0.078 — 0.159)

Modeling strategy

We changed the modeling process from GBD 2013 to show both prevalence of chronic gastritis and duodenitis and symptomatic episodes, rather than just symptomatic episodes. We believe that this more accurately reflects the epidemiology of gastritis and duodenitis, and is more valuable when considering health policy.

The DisMod model for chronic gastritis and duodenitis included bounding remission from 0 to 1 (a minimum duration of one year). Reference data were from literature, and we marked inpatient hospital data and outpatient hospital data for study-level covariates, and US claims data with separate year-specific study-level covariates. We used the function in DisMod-MR 2.1 to pull in cause-specific mortality rate (CSMR) data from our CODEm and CODcorrect analyses and match with prevalence data points for the same geography. We calculated excess mortality rate to estimate priors by dividing CSMR by prevalence. We also applied a country-level covariate for alcohol consumption to prevalence, which we forced positive with a lower bound of 0 and an upper bound of 2. Additionally, we applied a lag-

distributed income covariate to excess mortality, log transformed and forced negative with an upper bound of 0 and a lower bound of -1. Betas and exponentiated values (which can be interpreted as an odds ratio) are shown in the tables below for study-level covariates and country-level covariates.

Study covariate	Parameter	beta	Exponentiated beta
Outpatient	Prevalence	-1.65 (-1.86 — -1.09)	0.19 (0.16 — 0.33)
Inpatient hospital	Prevalence	-1.84 (-2 — -1.24)	0.16 (0.14 — 0.29)
Claims data – 2000	Prevalence	-0.017 (-0.22 — 0.58)	0.98 (0.81 — 1.79)
Claims data – 2010	Prevalence	0.16 (-0.035 — 0.75)	1.17 (0.97 — 2.12)
Claims data – 2012	Prevalence	0.17 (-0.038 — 0.77)	1.18 (0.96 — 2.16)

Country-level covariate	Parameter	beta	Exponentiated beta
Alcohol	Prevalence	0.00047 (0.000039 — 0.0017)	1.00 (1.00 — 1.00)
LDI (log transformed)	Excess mortality	-0.8 (-0.81 — -0.78)	0.45 (0.44 — 0.46)

The symptomatic episodes of gastritis and duodenitis DisMod model bounded remission from 52 to 54 (a duration of about one week). We also assumed no incidence from 0 to 2 years old, and excess mortality capped at 0.01 for all ages. The reference data were US claims data from 2012, and we marked US claims data from 2000 and 2010 with year-specific study-level covariate, and inpatient and outpatient hospital data with specific study-level covariates. We used the function in DisMod-MR 2.1 to pull in cause-specific mortality rate (CSMR) data from our CODEm and CODcorrect analyses and match with incidence data points for the same geography. We calculated excess mortality rate to estimate priors by dividing CSMR by incidence/remission. We also applied a country-level covariate for alcohol consumption to incidence, which we forced positive with a lower bound of 0 and an upper bound of 2. Additionally, we applied a lag-distributed income covariate to excess mortality, log transformed and forced negative with an upper bound of 0 and a lower bound of -1. Betas and exponentiated values (which can be interpreted as an odds ratio) are shown in the tables below for study-level covariates and country-level covariates.

Study covariate	Parameter	beta	Exponentiated beta
Outpatient	Incidence	-0.96 (-1 — -0.9)	0.38 (0.37 — 0.41)
Claims data – 2000	Incidence	-0.085 (-0.13 — -0.045)	0.92 (0.88 — 0.96)
Claims data – 2010	Incidence	0.12 (0.089 — 0.16)	1.13 (1.09 — 1.17)
Inpatient hospital	Incidence	-1 (-1 — -1)	0.37 (0.37 — 0.37)

Country-level covariate	Parameter	beta	Exponentiated beta
Alcohol	Incidence	0 (0 — 0)	1.00 (1.00 — 1.00)
LDI (log transformed)	Excess mortality	-0.00023 (-0.00065 — -0.0000014)	1.00 (1.00 — 1.00)

To calculate asymptomatic gastritis and duodenitis, we took the estimated prevalence of symptomatic episodes, and subtracted it from our estimated prevalence of chronic gastritis and duodenitis.

Appendicitis

Flowchart

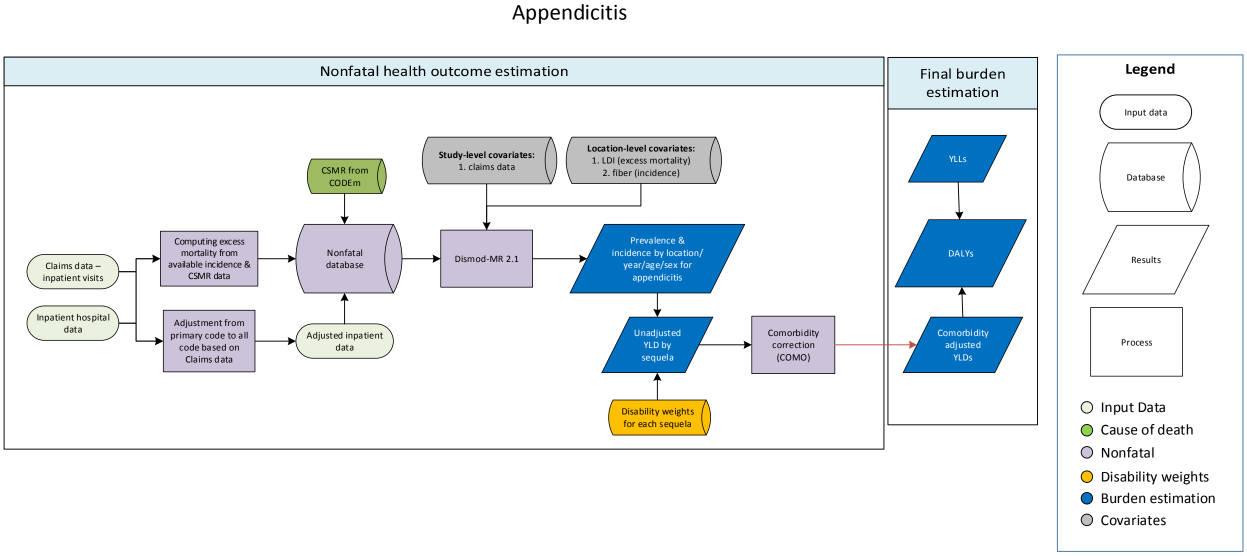

Case definition

Appendicitis is an inflammation of the appendix that causes nausea, vomiting, and sharp pain in the right lower abdomen. Appendicitis requires surgery, or septic shock may set in and the patient will be at risk for severe complications, including sepsis and death. ICD codes included are K35-K35.3, K35.8, K35.80, K35.89, K35.9, K36, K36.0, K37, K37.0, K37.9, and K38.3.

Input data

Model inputs

For GBD 2010, 2013, and 2015, the data used for appendicitis are hospital inpatient data across 103 separate locations and US claims data for 2000, 2010, and 2012 by US state, primary diagnoses only. Descriptions of search strategies for hospital and claims data are included elsewhere in the appendix. The agreed-upon approach for appendicitis was to only use these data sources, and not conduct a literature review, with the exception of one surgical study in Ontario, Canada.[1] Data were outliered or excluded if we found them unreasonable when compared to regional, super-regional, and global rates.

See the table below that shows the number literature studies included in GBD 2015, as well as the number of countries or subnational units and GBD world regions represented.

	Prevalence	Incidence	Mortality risk
Studies	0	1	0
Countries/subnationals	0	140	140
GBD world regions	0	8	8

Disability weights

The basis of the GBD disability weight survey assessments are lay descriptions of sequelae highlighting major functional consequences and symptoms. The lay descriptions and disability weights for appendicitis are shown below:

Severity split	Lay description	DW (95% CI)
Appendicitis, severe	This person has severe pain in the belly and feels nauseated. The person is anxious and unable to carry out daily activities.	0.324 (0.219-0.442)

Modeling strategy

Appendicitis is modeled using DisMoD MR 2.1, a Bayesian meta-regression epidemiological modeling tool. Prior settings in the DisMod model included bounding remission from 25 to 27 (a duration of about two weeks) for all age groups and capping excess mortality at 0.31. The reference data were hospital inpatient data, and we marked US claims data with a separate year-specific study-level covariate.
We used the function in DisMod-MR 2.1 to pull in cause-specific mortality rate (CSMR) data from our CODEm and CODcorrect analyses and match with prevalence data points for the same geography. We calculated excess mortality rate to estimate priors by dividing CSMR by prevalence. We also applied a fiber (g per day) country-level covariate to incidence, forcing a positive relationship with a lower bound of 0, and a lag-distributed income covariate to excess mortality, log transformed and forced negative with an upper bound of -0.1 and a lower bound of -1.

Betas and exponentiated values (which can be interpreted as an odds ratio) are shown in the tables below for study-level covariates and country-level covariates:

Study covariate	Parameter	beta	Exponentiated beta
Inpatient claims data – 2000	Incidence	-0.19 (-0.26 — -0.12)	0.83 (0.77 — 0.88)
Inpatient claims data – 2010	Incidence	-0.13 (-0.17 — -0.084)	0.88 (0.84 — 0.92)
Inpatient claims data – 2012	Incidence	-0.26 (-0.3 — -0.22)	0.77 (0.74 — 0.81)

Country-level covariate	Parameter	beta	Exponentiated beta
Fiber (g per day)	Incidence	-0.00016 (-0.00075 — -0.000016)	1.00 (1.00 — 1.00)
LDI (log transformed)	Excess mortality	-0.34 (-0.35 — -0.33)	0.71 (0.70 — 0.72)

We have made no substantive changes in the modeling strategy from GBD 2013. Significant challenges we faced included lack of data in locations without sufficient hospital data.

There have been no other significant changes to the GBD 2015 modeling strategy.

Paralytic ileus and intestinal obstruction

Flowchart

Paralytic ileus and intestinal obstruction (ileus)

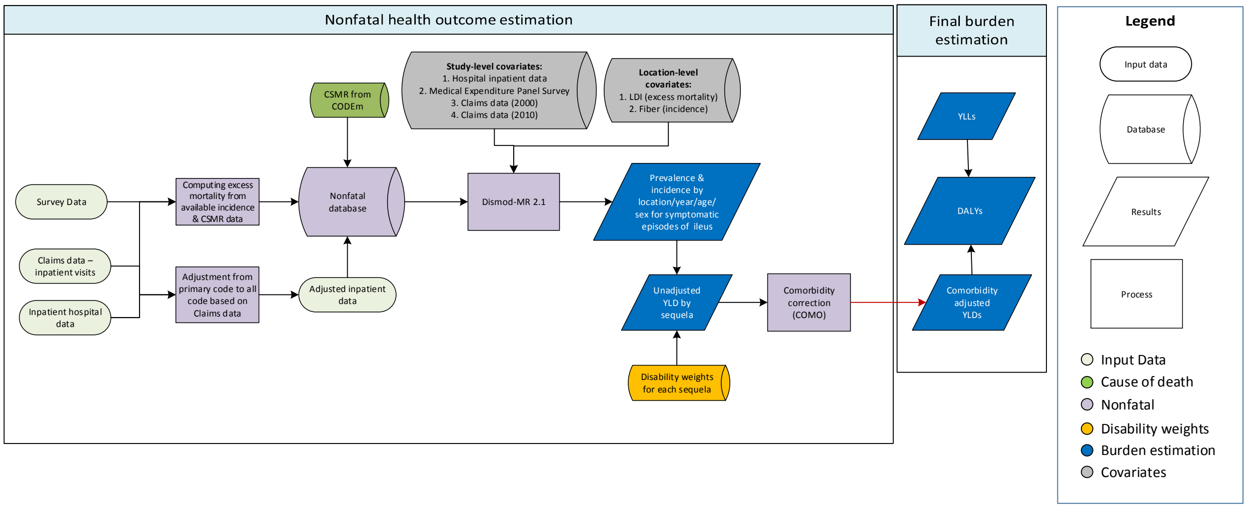

Case definition

Paralytic ileus and intestinal obstruction is a lack of digestive propulsion caused by failed peristalsis, typically requiring surgery. ICD codes for paralytic ileus and intestinal obstruction are K56.

Input data

Model inputs

For GBD 2010, 2013, and 2015, the data used for paralytic ileus and intestinal obstruction are hospital inpatient data and US claims data for 2000, 2010, and 2012 by US state, primary diagnoses only. Descriptions for hospital and claims data are included elsewhere in the appendix. The agreed-upon approach for paralytic ileus and intestinal obstruction was to only use these data sources, and not conduct a literature review. Data were outliered or excluded if we found them unreasonable when compared to regional, super-regional, and global rates.

See the table below that shows the number literature studies included in GBD 2015, as well as the number of countries or subnational units and GBD world regions represented.

	Prevalence	Incidence	Mortality risk
Studies	0	45	1
Countries/subnationals	0	146	142
GBD world regions	0	9	8

Severity split & disability weight

The basis of the GBD disability weight survey assessments is lay descriptions of sequelae highlighting major functional consequences and symptoms. The lay descriptions and disability weights for paralytic ileus and intestinal obstruction are shown below:

Severity level	Lay description	DW (95% CI)
Paralytic ileus and intestinal obstruction, severe	This person has severe pain in the belly and feels nauseated. The person is anxious and unable to carry out daily activities.	0.324 (0.219-0.442)

Modeling strategy

Prior settings in the DisMod model included bounding remission from 25 to 26 (a duration from about two weeks) for all age groups and capping incidence at 0.002 for ages 0 to 5. The reference data were hospital inpatient data, primary diagnosis only, and we marked inpatient US claims data with a separate year-specific study-level covariate.

We used the function in DisMod-MR 2.1 to pull in cause-specific mortality rate (CSMR) data from our CODEm and CODcorrect analyses and match with incidence data points for the same geography. We calculated excess mortality rate to estimate priors by dividing CSMR by incidence/remission. We also applied a country-level fiber consumption covariate to incidence which we forced negative with an upper bound of 0 and a lower bound of -2, and a lag-distributed income covariate to excess mortality, log-transformed and forced negative with an upper bound of 0 and a lower bound of -1.

Betas and exponentiated values (which can be interpreted as an odds ratio) are shown in the tables below for study-level covariates and country-level covariates:

Study covariate	Parameter	beta	Exponentiated beta
Inpatient claims data – 2000	Incidence	-0.28 (-0.34 — -0.21)	0.76 (0.71 — 0.81)
Inpatient claims data – 2010	Incidence	-0.029 (-0.099 — -0.02)	0.97 (0.94 — 1.01)
Inpatient claims data – 2012	Incidence	-0.061 (-0.0035 — -0.000051)	0.94 (0.91 — 0.98)
Medical Expenditure Panel Survey	Incidence	0.26 (0.00048 — 0.55)	1.30 (1.00 — 1.73)

Country-level covariate	Parameter	beta	Exponentiated beta
Fiber	Incidence	-0.0011 (-0.0035 — -0.000051)	1.00 (1.00 — 1.00)
LDI (log transformed)	Excess mortality	-0.84 (-0.85 — -0.83)	0.43 (0.43 — 0.44)

We have made no substantive changes in the modeling strategy from GBD 2013.

Inguinal, femoral, and abdominal hernia

Flowchart

Inguinal, femoral, and abdominal hernia

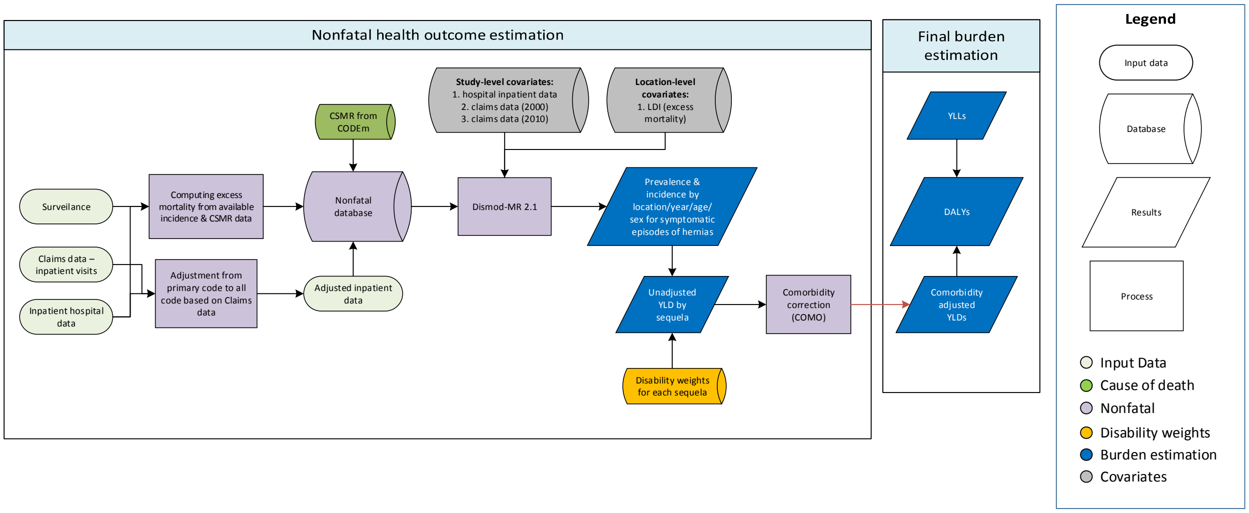

Case definition

A hernia is a digestive disorder that occurs when an internal organ protrudes through an opening in the tissue that holds it in place. Hernias most commonly occur in the inner and outer groin, and in the abdomen, and require surgical intervention. However, it can take several months before surgery occurs, resulting in a chronic condition. ICD codes used are K40, K41, K42, K44, K45, and K46.

Input data

Model inputs

For GBD 2010 and 2013, the data used for hernias are hospital inpatient data and remission data calculated based off a regression from a paper describing mean wait times for elective surgery in OECD countries.[1] For GBD 2015, we use the same data, but only included regression results for OECD countries. We also included US claims data for 2000, 2010, and 2012 by US state. The agreed-upon approach for hernias was to use only these data sources, and not conduct a literature review.

Data were outliered or excluded if we found them unreasonable when compared to regional, super-regional, and global rates.

The tables below show the number studies included in GBD 2015, as well as the number of countries or subnational units and GBD world regions represented.

<u>Chronic inguinal, femoral, and abdominal hernias</u>

	Prevalence	Incidence	Mortality risk	Remission
Studies	0	49	1	1
Countries/subnationals	0	143	143	34
GBD world regions	0	8	8	9

Disability weights

The basis of the GBD disability weight survey assessments are lay descriptions of sequelae highlighting major functional consequences and symptoms. The lay descriptions and disability weights for inguinal, abdominal, and femoral hernias are shown below:

Severity split	Lay description	DW (95% CI)
Inguinal, abdominal, and femoral hernia	This person has some pain in the belly that causes nausea but does not interfere with daily activities.	0.011 (0.005 — 0.021)

Modeling strategy

We changed the modeling process from GBD 2013 to show both prevalence of chronic hernias and symptomatic hernias, rather than just incidence of surgical interventions for hernias. We believe that this more accurately reflects the epidemiology of hernias and is more valuable when considering health policy.

In order to calculate both asymptomatic and symptomatic hernias, we modeled all hernias using a DisMod model, informed by incidence data from hospital and US claims data, paired with the calculated remission from the regression described in Siciliani et al.[1] This DisMod model included bounds on excess mortality for all ages of 0.01, a maximum incidence of 0.02 for ages 0 to 4, and bounds on remission from 0 to 10 (a minimum duration of about five weeks) – we also assumed prevalence at birth due to umbilical hernias. Reference data were US claims data from 2012, and we marked inpatient hospital data and outpatient hospital data with study-level covariates, and the remaining years of US claims data with separate year-specific study-level covariates. We used the function in DisMod-MR 2.1 to pull in cause-specific mortality rate (CSMR) data from our CODEm and CODcorrect analyses and match with incidence data points for the same geography. We calculated excess mortality rate to estimate priors by dividing CSMR by incidence/remission. We also applied a lag-distributed income covariate to both remission and excess mortality, log transformed. We forced this covariate negative for excess mortality (bounds from -1 to 0), and positive for remission (bounds from 0 to 1). Betas and exponentiated values (which can be interpreted as an odds ratio) are shown in the tables below for study-level covariates and country-level covariates.

Study covariate	Parameter	beta	Exponentiated beta
Outpatient	Incidence		
Inpatient hospital	Incidence	-1 (-1 — -1)	0.37 (0.37 — 0.37)
Claims data – 2000	Incidence		
Claims data – 2010	Incidence		

Country-level covariate	Parameter	beta	Exponentiated beta
LDI (log transformed)	Excess mortality	-0.32 (-0.33 — -0.3)	0.73 (0.72 — 0.74)
LDI (log transformed)	Remission	-0.0013 (-0.0052 — -0.00015)	1.00 (0.99 — 1.00)

To calculate symptomatic hernias from prevalence of all hernias, we divided prevalence of all hernias by the primary diagnosis correction factor. The primary diagnosis correction factor is applied to inpatient

hospital data to correct for the bias in how likely codes are to be the primary diagnosis. This is calculated from US claims data, which have data on all diagnoses for an event, and represent all inpatient diagnoses divided by inpatient primary diagnoses. By dividing the prevalence of all hernias by this primary diagnosis correction factor, we calculate symptomatic hernias. We calculated asymptomatic hernias by subtracting the estimated prevalence of symptomatic hernias from our estimated prevalence of all hernias.

Inflammatory bowel disease

Flowchart

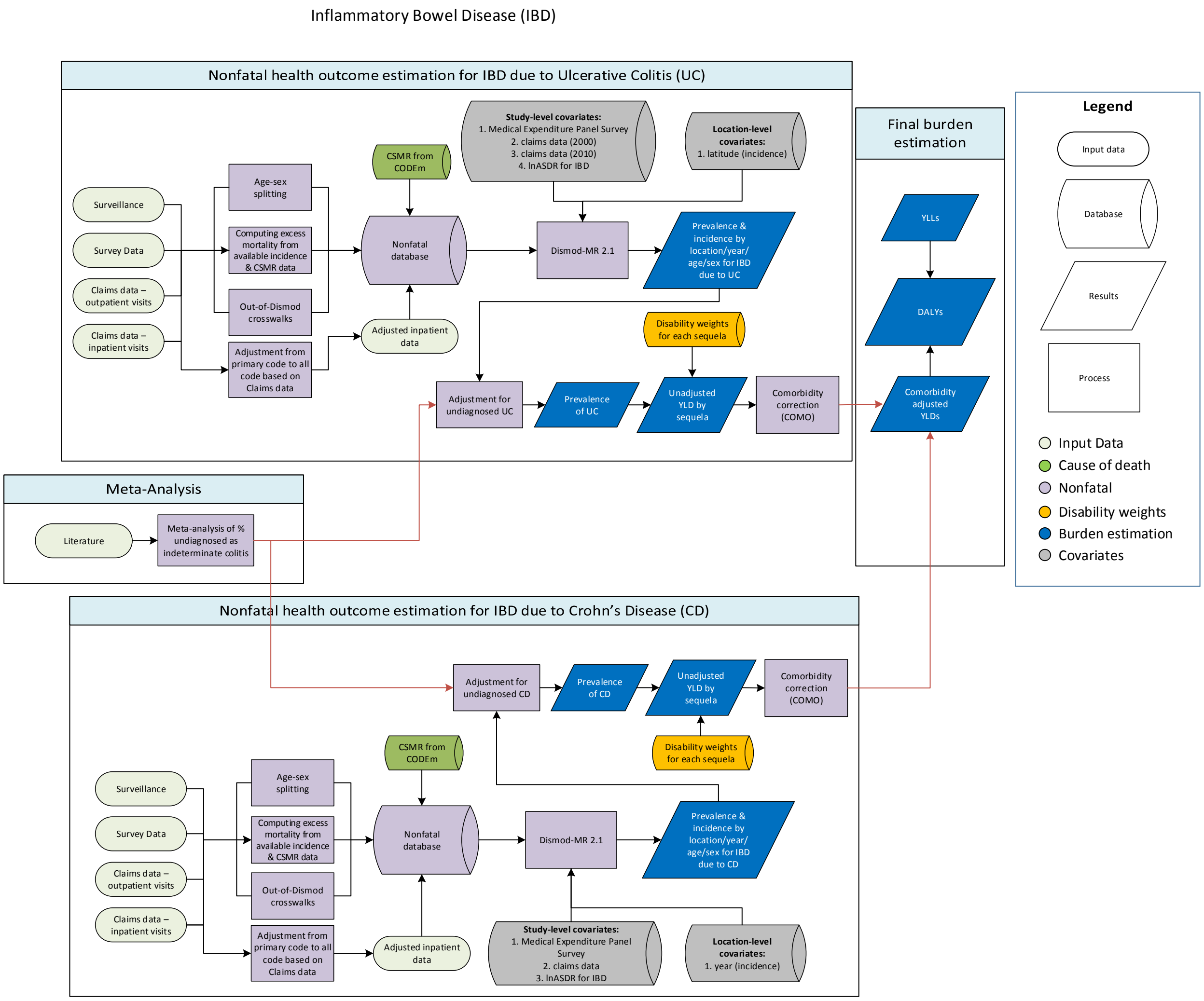

Case definition

Inflammatory bowel disease is a type of digestive disorder involving inflammation of the colon and gastrointestinal tract, most commonly classified as Crohn's disease (inflammation of the small and large intestine) and ulcerative colitis (inflammation of the colon and rectum). In a significant proportion of cases of inflammatory bowel disease, neither Crohn's disease nor ulcerative colitis is definitively the diagnosis, and a diagnosis of indeterminate colitis is applied. ICD codes are K50 for Crohn's disease, K51 for ulcerative colitis, and K52 for indeterminate colitis.

Input data

Model inputs

Data inputs were separate for ulcerative colitis and Crohn's disease, but a single systematic review of literature was conducted to capture studies of prevalence, incidence, case fatality rate, and standardized mortality rate for all inflammatory bowel disease for GBD 2010.

This was repeated for GBD 2015, using the search string: (("crohn disease"[MeSH Terms] OR ("crohn"[All Fields] AND "disease"[All Fields]) OR "crohn disease"[All Fields] OR ("crohn's"[All Fields] AND "disease"[All Fields]) OR "crohn's disease"[All Fields]) OR ("colitis, ulcerative"[MeSH Terms] OR ("colitis"[All Fields] AND "ulcerative"[All Fields]) OR "ulcerative colitis"[All Fields] OR ("ulcerative"[All Fields] AND "colitis"[All Fields])) OR (Inflammatory[All Fields] AND Bowl[All Fields]) OR (("irritable bowel syndrome"[MeSH Terms] OR ("irritable"[All Fields] AND "bowel"[All Fields] AND "syndrome"[All Fields]) OR "irritable bowel syndrome"[All Fields]) AND ("diarrhoea"[All Fields] OR "diarrhea"[MeSH Terms] OR "diarrhea"[All Fields]))) AND ("epidemiology"[Subheading] OR "epidemiology"[All Fields] OR "epidemiology"[MeSH Terms]) AND ("humans [MeSH Terms]). The search was limited to January 1, 2008 to December 31, 2015.

The inclusion criteria were:

1. Studies must be representative of the national population, i.e., prison studies are not considered representative
2. Sufficient information must be provided on study methods and sample characteristics to assess the quality of the study
3. Excluded reviews

In addition to literature data, we included US claims data from 2000, 2010, and 2012 at the US state level, extracted as prevalence. We did not include hospital data because of the large variation across countries and small amount of outpatient data. Data were outliered or excluded if we found them unreasonable when compared to regional, super-regional, and global rates.

The tables below show the number studies included in GBD 2015, as well as the number of countries or subnational units and GBD world regions represented.

Noninfective inflammatory bowel disease due to ulcerative colitis

	Prevalence	Incidence	Mortality risk
Studies	29	79	7
Countries/subnationals	75	70	8
GBD world regions	11	13	3

Noninfective inflammatory bowel disease due to Crohn's disease

	Prevalence	Incidence	Mortality risk
Studies	25	73	7
Countries/subnationals	71	67	8
GBD world regions	9	12	3

Disability weights

The basis of the GBD disability weight survey assessments are lay descriptions of sequelae highlighting major functional consequences and symptoms. The lay descriptions and disability weights for sequelae associated with inflammatory bowel disease are shown below:

Severity split	Lay description	DW (95% CI)
Crohn's disease	This person has cramping abdominal pain, has diarrhea several times a day, and feels very tired for two months every year. When the person does not have symptoms, there is anxiety about them returning.	0.231 (0.156-0.32)
Ulcerative colitis	This person has cramping abdominal pain, has diarrhea several times a day, and feels very tired for two months every year. When the person does not have symptoms, there is anxiety about them returning.	0.231 (0.156-0.32)

Modeling strategy

The modeling strategy for all inflammatory bowel disease encompasses separate DisMod models for ulcerative colitis and Crohn's disease, which are then adjusted to account for inflammatory bowel disease due to indeterminate colitis.

The DisMod model for ulcerative colitis included setting remission to 0 for all age groups, bounding excess mortality from 0 to 0.1 for all ages, and setting incidence to 0 for ages 0 to 1. Reference data were US claims data from 2012, and we marked the remaining years of US claims data with separate year-specific study-level covariates. We also marked Medical Expenditure Panel Survey data and literature with separate covariates. We also applied a country-level covariate of absolute value of average latitude to prevalence, and a ln-ASDR (age standardized death rate) fixed effect on prevalence. The ASDR data are taken from our CODEm and COD correct analyses for all inflammatory bowel disease. Betas and exponentiated values (which can be interpreted as an odds ratio) are shown in the tables below for study-level covariates and country-level covariates.

Study covariate	Parameter	beta	Exponentiated beta
Medical Expenditure Panel Survey	Prevalence	-0.0031 (-0.0086 — -0.000099)	1.00 (0.99 — 1.00)
Literature	Prevalence	-0.0016 (-0.004 — -0.00015)	1.00 (1.00 — 1.00)
Claims data – 2000	Prevalence	-0.33 (-0.36 — -0.3)	0.72 (0.70 — 0.74)
Claims data – 2010	Prevalence	-0.033 (-0.056 — -0.013)	0.97 (0.95 — 0.99)

Country-level covariate	Parameter	beta	Exponentiated beta
ln-ASDR	Prevalence	0.11 (0.051 — 0.17)	1.11 (1.05 — 1.19)
Average latitude	Prevalence	0.014 (0.012 — 0.016)	1.01 (1.05 — 1.19)

The DisMod model for Crohn’s disease included setting remission to 0 for all age groups, bounding excess mortality from 0 to 0.1 for all ages, and setting incidence to 0 for ages 0 to 2. Reference data were US claims data from 2012, and we marked the remaining years of US claims data with separate year-specific study-level covariates. We also marked Medical Expenditure Panel Survey data and literature with separate covariates. We applied a country-level ln-ASDR (age standardized death rate) fixed effect on prevalence. The ASDR data are taken from our CODEm and COD correct analyses for all inflammatory bowel disease. Betas and exponentiated values (which can be interpreted as an odds ratio) are shown in the tables below for study-level covariates and country-level covariates.

Study covariate	Parameter	beta	Exponentiated beta
Medical Expenditure Panel Survey	Prevalence	-0.0025 (-0.012 — -0.00012)	1.00 (0.99 — 1.00)
Literature	Prevalence	-0.0022 (-0.0054 — -0.00041)	1.00 (0.99 — 1.00)
Claims data – 2000	Prevalence	-0.31 (-0.34 — -0.28)	0.74 (0.71 — 0.76)
Claims data – 2010	Prevalence	-0.047 (-0.072 — -0.024)	0.95 (0.93 — 0.98)

Country-level covariate	Parameter	beta	Exponentiated beta
ln-ASDR	Prevalence	0.66 (0.60 — 0.72)	1.94 (1.82 — 2.06)

We conducted a meta-analysis to calculate the ratio of inflammatory bowel disease cases that are categorized as indeterminate colitis to cases that are characterized as either Crohn’s disease or ulcerative colitis. The results of this meta-analysis were a mean ratio of 0.070 (0.054-0.086). We then took the prevalence results of the DisMod models for ulcerative colitis and Crohn’s disease and adjusted them by multiplying by a normal distribution of draws from 1.070 (1.054-1.086) to encompass all inflammatory bowel disease cases.

No additional significant changes were made to the GBD 2015 estimation process.

Vascular intestinal disorders

Flowchart

Vascular Intestinal Disorders

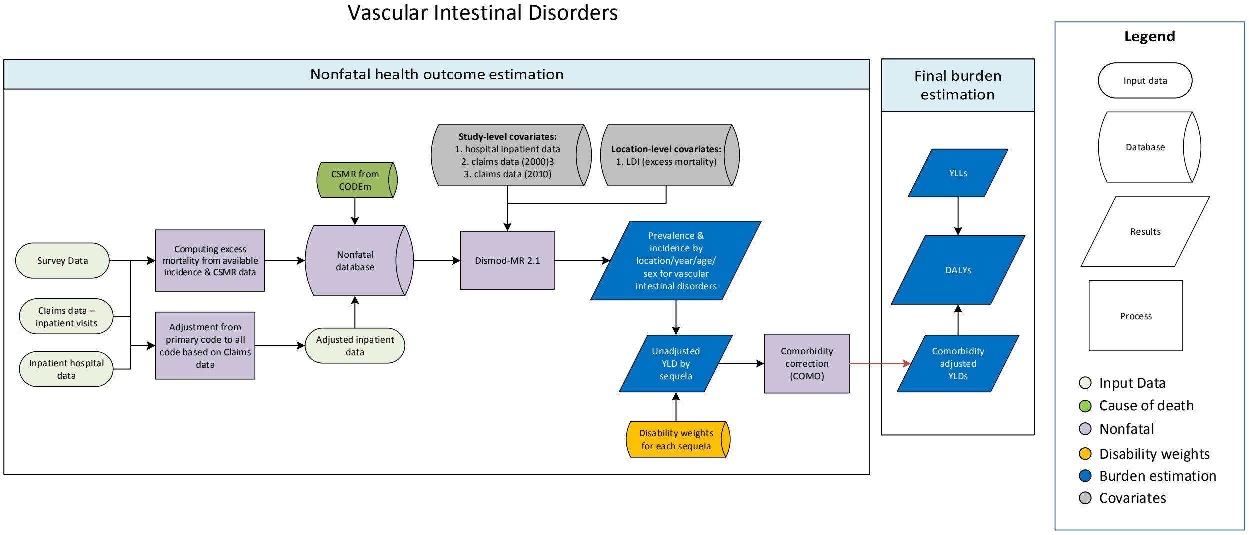

Input data and methodological summary

Case definition

Vascular intestinal disorder, also known as intestinal ischemia, occurs when there is a decreased blood supply to the gastrointestinal tract causing injury to the bowel. Vascular intestinal disorders typically require surgery. ICD codes for vascular intestinal disorders are K55.

Input data

Model inputs

For GBD 2010, 2013, and 2015, the data used for vascular intestinal disorders are hospital inpatient data and US claims data for 2000, 2010, and 2012 by US state, primary diagnoses only. Descriptions of hospital and claims data are included elsewhere in the appendix. The agreed-upon approach for vascular intestinal disorders was to use only these data sources and not conduct a literature review. Data were outliered or excluded if we found them unreasonable when compared to regional, super-regional, and global rates.

See the table below that shows the number literature studies included in GBD 2015, as well as the number of countries or subnational units and GBD world regions represented.

	Prevalence	Incidence	Mortality risk
Studies	0	41	1
Countries/subnationals	0	127	126
GBD world regions	0	6	6

Disability weights

The basis of the GBD disability weight survey assessments are lay descriptions of sequelae highlighting major functional consequences and symptoms. The lay descriptions and disability weights for vascular intestinal disorders are shown below:

Severity split	Lay description	DW (95% CI)
Vascular intestinal disorders, severe	This person has severe pain in the belly and feels nauseated. The person is anxious and unable to carry out daily activities.	0.324 (0.219-0.442)

Modeling strategy

Prior settings in the DisMod model included bounding remission from 2 to 12 (a duration from about four weeks to half a year) for all age groups and capping excess mortality at 10. The reference data were hospital inpatient data, primary diagnosis only, and we marked inpatient US claims data with a separate year-specific study-level covariate.

We used the function in DisMod-MR 2.1 to pull in cause-specific mortality rate (CSMR) data from our CODEm and CODcorrect analyses and match with incidence data points for the same geography. We calculated excess mortality rate to estimate priors by dividing CSMR by incidence/remission. We also applied a lag-distributed income covariate to excess mortality, log-transformed and forced negative with an upper bound of 0 and a lower bound of -1.

Betas and exponentiated values (which can be interpreted as an odds ratio) are shown in the tables below for study-level covariates and country-level covariates:

Study covariate	Parameter	Beta	Exponentiated beta
Inpatient claims data – 2000	Incidence	-0.31 (-0.066 — 0.0024)	0.97 (0.94 — 1.00)
Inpatient claims data – 2010	Incidence	0.55 (0.52 — 0.59)	1.74 (1.68 — 1.80)
Inpatient claims data – 2012	Incidence	0.49 (0.45 — 0.52)	1.63 (1.58 —1.68)
Medical Expenditure Panel Survey	Incidence	-0.52 (-1.13 — 0.042)	0.59 (0.32 — 1.04)

Country-level covariate	Parameter	beta	Exponentiated beta
LDI (log transformed)	Excess mortality	-0.66 (-0.69 — -0.64)	0.51 (0.50 — 0.53)

We have made no substantive changes in the modeling strategy from GBD 2013.

Gallbladder and biliary diseases

Flowchart

Gallbladder and biliary diseases

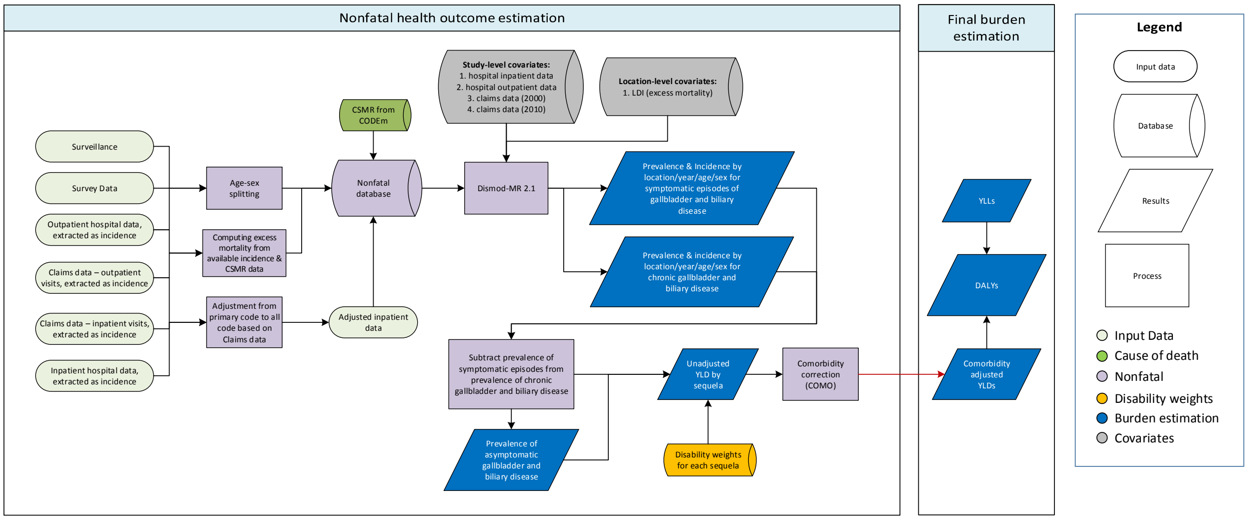

Input data and methodological summary

Case definition

Gallbladder and biliary diseases are digestive disorders including gallstones, cholecystitis, cholangitis, and other diseases of the gallbladder and biliary tract. Cholecystitis is an inflammation of the gallbladder, and cholangitis is an infection or inflammation of the bile duct, the result of a bacterial infection – both are often the result of gallstones. Gallbladder and biliary diseases, especially the presence of gallstones, can often be asymptomatic with periodic symptomatic episodes of severe abdominal pain, nausea, vomiting, and at times fever. ICD codes included are K80, K81, K82, and K83.

Input data

Model inputs

Data inputs were separate for the chronic and symptomatic episode models. For the chronic dataset, a systematic review of literature was conducted to capture studies of prevalence, incidence, case fatality rate, and standardized mortality rate associated with gallbladder and biliary diseases. For GBD 2010, PubMed, Medline, and Embase were searched using the following search terms: gallstones, calculus of gallbladder, gallbladder disease, cholecystitis, biliary tract disease. This search was repeated in PubMed for GBD 2015, restricting dates searched from January 1, 2010, to December 31, 2015. The inclusion criteria were:

1. Studies must be representative of the national population, i.e., excluded populations of patients with *H. pylori*, or patients prescribed an ultrasonography due to stomach pain

2. Sufficient information must be provided on study methods and sample characteristics to assess the quality of the study
3. Excluded reviews

In addition to literature data, we included hospital inpatient and outpatient data and US claims data from 2000, 2010, and 2012 at the US state level, extracted as prevalence. The claims data were extracted using all diagnoses, and the hospital data were adjusted for only recording primary diagnoses, using a correction factor from claims data.

For the symptomatic episode dataset, we used extracted hospital inpatient and outpatient data and US claims data from 2000, 2010, and 2012 at the US state level, extracted as incidence to capture individual episodes. These data were similarly extracted and adjusted to estimate all diagnoses. Data were outliered or excluded if we found them unreasonable when compared to regional, super-regional, and global rates.

The tables below show the number studies included in GBD 2015, as well as the number of countries or subnational units and GBD world regions represented.

<u>Chronic gallbladder and biliary diseases</u>

	Prevalence	Incidence	Mortality risk
Studies	80	4	2
Countries/subnationals	161	5	70
GBD world regions	14	2	9

<u>Symptomatic episodes of gastritis and duodenitis</u>

	Prevalence	Incidence	Mortality risk
Studies	0	52	2
Countries/subnationals	0	145	144
GBD world regions	0	8	8

Disability weights

The basis of the GBD disability weight survey assessments are lay descriptions of sequelae highlighting major functional consequences and symptoms. The lay descriptions and disability weights for gallbladder and biliary disease are shown below:

Severity split	Lay description	DW (95% CI)
Gallbladder and biliary disease, symptomatic episodes	This person has pain in the belly and feels nauseated. The person has difficulties with daily activities.	0.114 (0.078 — 0.159)

Modeling strategy

We changed the modeling process from GBD 2013 to show both prevalence of chronic gallbladder and biliary diseases and symptomatic episodes, rather than just symptomatic episodes. We believe that this

more accurately reflects the epidemiology of gallbladder and biliary diseases, and is more valuable when considering health policy.

The DisMod model for chronic gallbladder and biliary diseases included bounding remission from 0 to 1 (a minimum duration of one year). Reference data were from literature, and we marked inpatient hospital data and outpatient hospital data for study-level covariates, and US claims data with separate year-specific study-level covariates. We used the function in DisMod-MR 2.1 to pull in cause-specific mortality rate (CSMR) data from our CODEm and CODcorrect analyses and match with prevalence data points for the same geography. We calculated excess mortality rate to estimate priors by dividing CSMR by prevalence. We also applied a lag-distributed income covariate to excess mortality, log transformed and forced negative with an upper bound of 0 and a lower bound of -1. Betas and exponentiated values (which can be interpreted as an odds ratio) are shown in the tables below for study-level covariates and country-level covariates.

Study covariate	Parameter	beta	Exponentiated beta
Outpatient	Prevalence	-1.99 (-2 — -1.97)	0.14 (0.14 — 0.14)
Inpatient hospital	Prevalence	-1.97 (-2 — -1.94)	0.14 (0.14 — 0.14)
Claims data – 2000	Prevalence	-0.41 (-0.47 — -0.36)	0.66 (0.63 — 0.70)
Claims data – 2010	Prevalence	-0.59 (-0.65 — -0.54)	0.55 (0.52 — 0.58)
Claims data – 2012	Prevalence	-0.61 (-0.66 — -0.56)	0.54 (0.52 — 0.57)

Country-level covariate	Parameter	beta	Exponentiated beta
LDI (log transformed)	Excess mortality	-0.91 (-0.92 — -0.89)	0.40 (0.40 — 0.41)

The symptomatic episodes of gallbladder and biliary diseases DisMod model bounded remission from 9 to 26 (a duration of about two to six weeks). We also capped excess mortality at 0.1 for all ages. The reference data were US claims data from 2012, and we marked US claims data from 2000 and 2010 with year-specific study-level covariate, and inpatient and outpatient hospital data with specific study-level covariates. We used the function in DisMod-MR 2.1 to pull in cause-specific mortality rate (CSMR) data from our CODEm and CODcorrect analyses and match with incidence data points for the same geography. We calculated excess mortality rate to estimate priors by dividing CSMR by incidence/remission. We also applied a lag-distributed income covariate to excess mortality, log transformed and forced negative with an upper bound of 0 and a lower bound of -1. Betas and exponentiated values (which can be interpreted as an odds ratio) are shown in the tables below for study-level covariates and country-level covariates.

Study covariate	Parameter	beta	Exponentiated beta
Outpatient	Incidence	-1.61 (-1.83 — -1.47)	0.20 (0.16 — 0.23)
Claims data – 2000	Incidence	-0.54 (-0.66 — -0.46)	0.58 (0.52 — 0.63)
Claims data – 2010	Incidence	-0.2 (-0.32 — -0.13)	0.82 (0.73 — 0.88)
Inpatient hospital	Incidence	-1.55 (-1.82 — -1.4)	0.21 (0.16 — 0.25)

Country-level covariate	Parameter	beta	Exponentiated beta
LDI (log transformed)	Excess mortality	-0.25 (-0.26 — -0.23)	0.78 (0.77 — 0.79)

To calculate asymptomatic gallbladder and biliary diseases, we took the estimated prevalence of symptomatic episodes and subtracted it from our estimated prevalence of chronic gallbladder and biliary diseases.

Pancreatitis

Flowchart

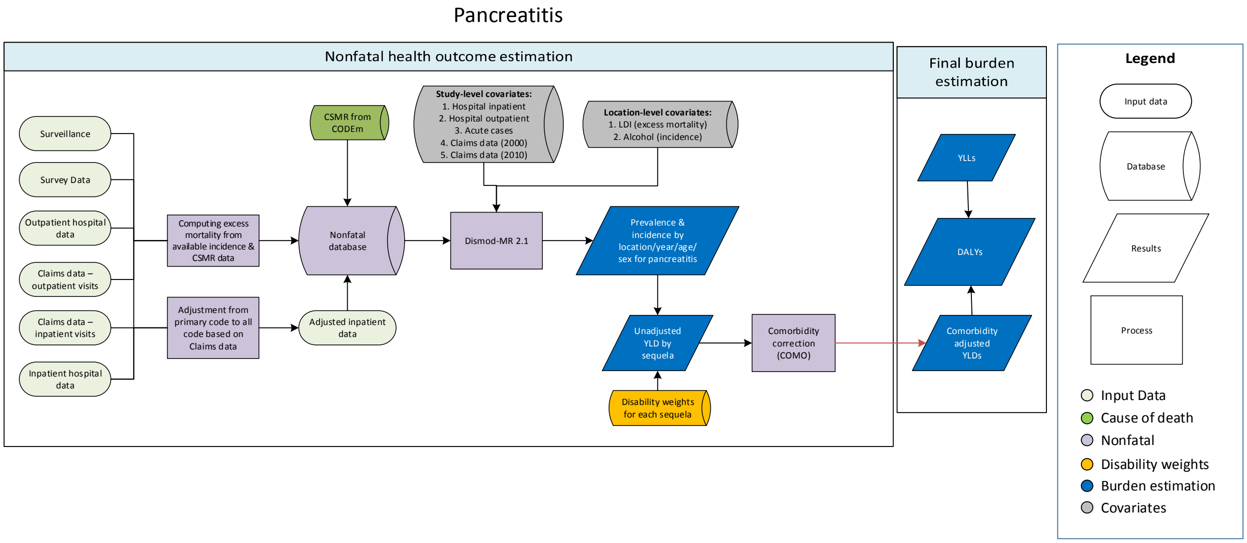

Input data and methodological summary

Case definition

Pancreatitis is the inflammation of the pancreas, and often results in nausea, stomach pain, and vomiting. We model both acute and chronic pancreatitis together, using ICD codes K85 and K86.

Input data

Model inputs

For GBD 2010, a systematic review of the incidence of pancreatitis throughout the world was conducted. PubMed was searched using the following search terms: ([pancreatitis OR pancreatic diseases OR (pancreatic AND diseases) OR pancreatic diseases OR (diseases AND pancreas) OR diseases of pancreas] AND [epidemiology OR epidemiology OR epidemiology] AND humans). The exclusion criteria were:

1. Studies clearly not representative of the national population, i.e., alcoholics or smokers
2. Self-reported data
3. Reviews

A PubMed literature search was conducted this year for GBD 2015, using the above search terms with date specification between January 1, 2005, and December 31, 2015. The most recent literature source dates from 2015. In addition to literature, we included hospital inpatient and outpatient data and US claims data for 2000, 2010, and 2012 by US state. Data were outliered or excluded if we found them unreasonable when compared to regional, super-regional, and global rates.

See the table below, which shows the number of literature studies included in GBD 2015, as well as the number of countries or subnational units and GBD world regions represented.

	Prevalence	Incidence	Mortality risk
Studies	2	52	2
Countries/subnationals	3	147	147
GBD world regions	2	9	9

Disability weights

The basis of the GBD disability weight survey assessments are lay descriptions of sequelae highlighting major functional consequences and symptoms. The lay descriptions and disability weights for pancreatitis are shown below:

Severity split	Lay description	DW (95% CI)
Pancreatitis cases, severe	This person has severe pain in the belly and feels nauseated. The person is anxious and unable to carry out daily activities.	0.324 (0.219-0.442)

Modeling strategy

Prior settings in the DisMod model included bounding remission from 8 to 9 (a duration from about six weeks) for all ages. The reference data were US claims data from 2012, all diagnoses, and we marked hospital inpatient and outpatient with study-level covariates, and 2000 and 2010 US claims data with a separate year-specific study-level covariate. Additionally, we marked literature, which reports acute pancreatitis only, with an acute study-level covariate.

We used the function in DisMod-MR 2.1 to pull in cause-specific mortality rate (CSMR) data from our CODEm and CODcorrect analyses and match with incidence and prevalence data points for the same geography. We calculated excess mortality rate to estimate priors by dividing CSMR by incidence/remission (or prevalence, if applicable). We also applied a country-level alcohol consumption covariate to incidence which we forced positive with a lower bound of 0.25 and an upper bound of 0.75, a log-transformed age-standardized SEV scalar covariate for pancreatitis to incidence with bounds of 0.75 to 1.25, and a lag-distributed income covariate to excess mortality, log-transformed and forced negative with an upper bound of 0 and a lower bound of -1.

Betas and exponentiated values (which can be interpreted as an odds ratio) are shown in the tables below for study-level covariates and country-level covariates:

Study covariate	Parameter	beta	Exponentiated beta
Inpatient claims data – 2000	Incidence	-0.62 (-0.66 — -0.58)	0.54 (0.51 — -0.56)
Inpatient claims data – 2010	Incidence	-0.057 (-0.097 — -0.024)	0.94 (0.91 — 0.98)
Hospital inpatient data	Incidence	-0.44 (-0.47 — -0.4)	0.65 (0.62 — 0.67)

Hospital outpatient data	Incidence	-1.03 (-1.1 — -0.96)	0.36 (0.33 — 0.38)
Acute literature data	Incidence	-1.55 (-1.76 — -1.34)	0.21 (0.17 — 0.26)

Country-level covariate	Parameter	beta	Exponentiated beta
SEV scalar: pancreatitis	Incidence	1.23 (1.20 — 1.25)	3.44 (3.32 — 3.49)
Alcohol	Incidence		
LDI (log transformed)	Excess mortality	-1 (-1 — -1)	0.37 (0.37 — 0.37)

We have made no substantive changes in the modeling strategy from GBD 2013.

Alzheimer disease and other dementias

Flowchart

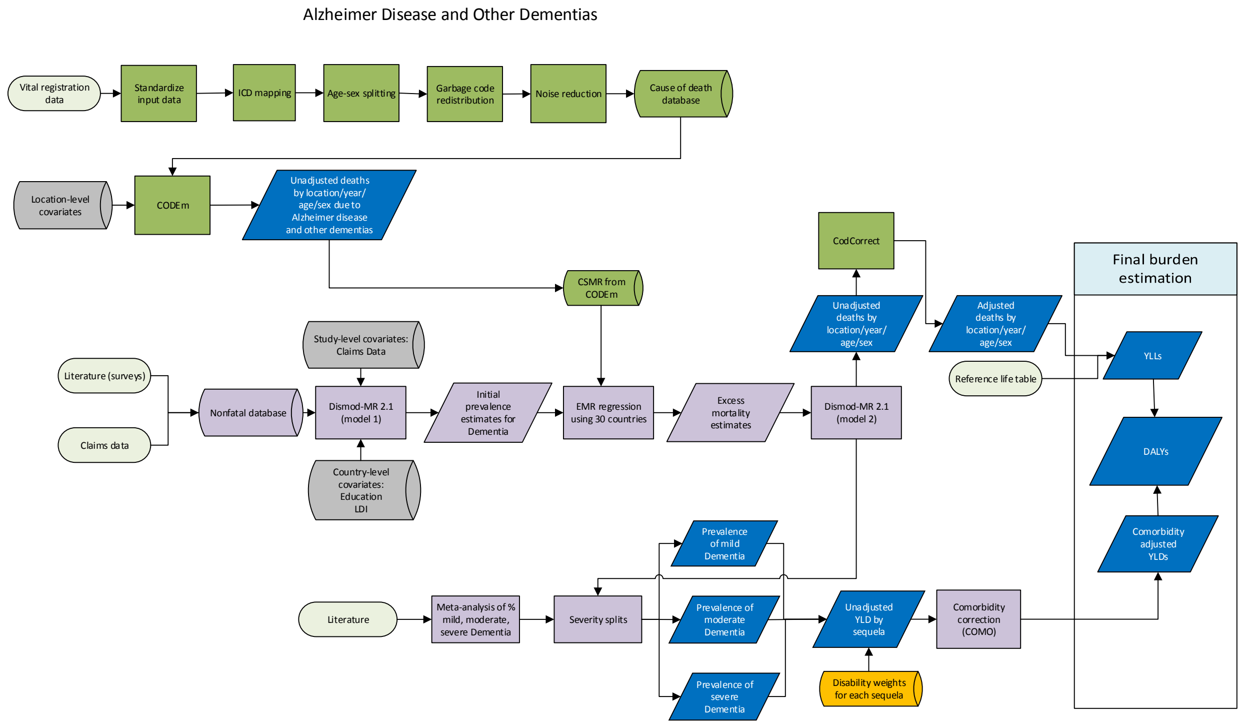

Input data and methodological summary

Case definition

Dementia is a progressive, degenerative, and chronic neurological disorder disease typified by memory impairment and other neurological dysfunctions. For the purposes of GBD 2015, we use the Diagnostic and Statistical Manual of Mental Disorders III or IV, or ICD case definitions as the reference. A wide array of diagnostic and screening instruments exists, including Clinical Dementia Rating scale (CDR), Mini Mental State Examination (MMSE), and the Geriatric Mental State (GMS). For severity rating purposes we use the CDR as the reference. The relevant ICD-10 codes for dementia are F00, F01, F02, F03, G30, and G31. The ICD-9 codes are 290, 291.2, 291.8, 294 and 331.

Unlike most causes in the Global Burden of Disease project, dementia mortality and morbidity estimates are modeled jointly. This is because of marked discrepancies between prevalence data and cause of death data. Specifically, prevalence data suggest little to no variation over time (e.g., 1990-2015) whereas age-standardized mortality rates in vital registrations in high-income countries have increased multiple times over this same period. Additionally, prevalence variation between countries is much smaller than the variation in death rates assigned to dementia in vital registration. We attribute these discrepancies to changing coding practices rather than epidemiological change.

Because of this joint procedure, descriptions of the mortality estimation process are included where relevant.

Input data

Model inputs

To inform our estimates of burden due to dementia, we use mortality data from the vital registration systems, as well as prevalence data from surveys, and administrative data such as claims sources.

An update to earlier GBD systematic reviews was conducted from January 2013 to October 2015 with 1,399 initial hits and 27 marked for extraction. Inclusion criteria identified studies that reported prevalence, incidence, remission rate, excess mortality rate, relative risk of mortality, standardized mortality ratio, or with-condition mortality rate. Studies with no clearly defined sample, or on clinical samples were excluded.

A substantial new source used for GBD 2015 was medical claims data for the years 2000, 2010, and 2012 in all US states.

A table describing the density and distribution of the epidemiological data available for GBD 2015 is presented below:

	Prevalence	Incidence	Mortality risk	Severity
Studies	146	39	16	17
Countries/subnationals	96	13	19	15
GBD world regions	17	10	10	8

Severity splits

In GBD 2013 (and used in GBD 2015), we extracted data from studies reporting on mild, moderate, and severe dementia. As the data indicate an age pattern with greater proportions with more severe disease in the very old we restricted our analyses to studies reporting on severity <70, 70-79, and 80+ ages. Most of these studies reported severity based on the Clinical Dementia Rating scale (CDR): CDR=1 as mild, CDR=2 as moderate, and CDR=3 as severe dementia. Other studies report staging of dementia according to the Mini Mental State Examination (MMSE); DSM III criteria; the Functional capacity scale; the Cambridge Mental Disorders of the Elderly Examination (CAMDEX); the scale of Hughes and the Geriatric Mental State (GMS). We used a random effects meta-analysis to pool the data by severity level.

We multiplied estimations of prevalence (country-year-sex-age specific) by the fractions of mild, moderate, and severe dementia and estimated 95% uncertainty intervals at the 1,000 draw level.

Severity level	Lay description	DW (95% CI)	Severity distribution
Mild	The person has some trouble remembering recent events and finds it hard to concentrate and make decisions and plans.	0.069 (0.046-0.099)	<70: 79% (71-86%)<br>70-79: 71% (63-78%)<br>80+: 61% (53-68%)
Moderate	The person has memory problems and confusion, feels disoriented, at times hears voices that are not real, and needs help with some daily activities.	0.377 (0.252-0.508)	<70: 17% (11-23%)<br>70-79: 19% (14-24%)<br>80+: 26% (22-30%)

Severe	The person has complete memory loss, no longer recognizes close family members, and requires help with all daily activities.	0.449 (0.304-0.595)	<70: 4% (2-7%)<br>70-79: 9% (5-13%)<br>80+: 12% (7-17%)

Modeling strategy

As mentioned above, the estimation of morbidity due to dementia occurs in conjunction with the mortality estimation.

First, we ran a CODEm model for dementia and extracted the cause-specific mortality rates by age, sex, and geography for 2015.

Second, we ran a DisMod-MR 2.1 model with all data on incidence, prevalence, and mortality risk (RR, SMR, or with-condition mortality rates) and a setting of zero remission and extracted 2015 prevalence by age, sex, and geography. To account for potential systematic differences between claims and survey data, we crosswalked for each year of claims data.

Third, we selected 30 countries with high quality vital registration systems with a cause-specific mortality rate to prevalence ratio greater than 0.02 in 2015. These ratios are subsequently used in a regression to estimate general excess mortality—that is, to allow us to correct for the discrepancy between prevalence data and cause of death data described above.

Fourth, we used a mixed effects regression with dummies on age group and sex to predict excess mortality by age and sex, the results of which are found in the table below.

Table: Fixed effect coefficients of EMR regression. Outcome: ln(EMR)					
Independent variables	Coef	Std. error	P value	95% confidence interval	
Female	-0.235	0.020	0.000	-0.274	-0.196
Age 60-64	0.707	0.034	0.000	0.640	0.774
Age 65-69	0.817	0.034	0.000	0.750	0.884
Age 70-74	1.120	0.034	0.000	1.053	1.188
Age 75- 80	1.471	0.034	0.000	1.404	1.539
Age 80+	2.198	0.034	0.000	2.131	2.266
Constant	-4.978	0.074	0.000	-5.122	-4.834

Random effect parameters					
Variance (constant)	0.143	0.038		0.085	0.240
Variance (residual)	0.034	0.003		0.029	0.040

We also fit a variation of the main EMR regression including the natural log of lagged distributed income (lnldi) as an additional covariate. The coefficient estimate and the corresponding confidence interval were then used to set a prior on the relationship between lnldi and EMR in DisMod-MR 2.1. This helped to capture location-specific variation in EMR for locations not included in the regression.

Fifth, these estimates were added to a second DisMod-MR 2.1 model as pertaining to the full 1990–2015 estimation period. For the 30 countries included in the regression, we retained their age- and sex-specific ratios and entered those also as pertaining to the full 1990–2015 estimation period. Thus, the model reflects the cause-specific mortality rate if all countries over time would have had the average propensity to code to dementia as an underlying cause of death similar to the selected 30 countries in 2015.

In this model, we assumed 0 remission as well as 0 excess mortality and incidence until age 40. Because of lack of consistency between prevalence and incidence data in locations where the underlying data , we excluded incidence data from the final model. In a few locations we found good consistency between prevalence and incidence and these were locations where incidence and prevalence were collected as part of the same study. In other locations (Beijing, Germany, Australia, Italy, North West England, Canada, various states in the US, Mexico and Nigeria) we noted that DisMod-MR 2.1 was pushing the fit above the available prevalence data and below incidence – 'averaging the difference'. In all cases the incidence and prevalence data were collected by different studies. We decided to drop the incidence estimates as measuring incidence of dementia when symptoms are still mild is more prone to measurement bias than measuring prevalence when the diagnosis has become more obvious over time.

The table below provides additional information on the country covariates used in this model, as well as beta and exponentiated beta values.

Variable	Measure	Beta	Exponentiated Beta Value (CI)
LDI (I$ per capita)	excess mortality rate	-0.10	0.90 (0.90 -0 .90)
Mean years of education, age-standardized	prevalence	-0.04	0.96 (0.86 -1.00)
US claims data 2000	prevalence	-0.69	0.50 (0.48 -0 .53)
US claims data 2010	prevalence	-0.26	0.77 (0.74 - 0.82)
US claims data 2012	prevalence	-0.20	0.82 (0.79 - 0.86)

As described above, we used crosswalks to standardize the claims data relative to existing literature data and ln-LDI on EMR to capture location specific variation. Age-standardized education was used as a proxy for general brain health/use that may be protective of dementia – specifically Alzheimer's disease.

Parkinson disease

Flowchart

Parkinson Disease

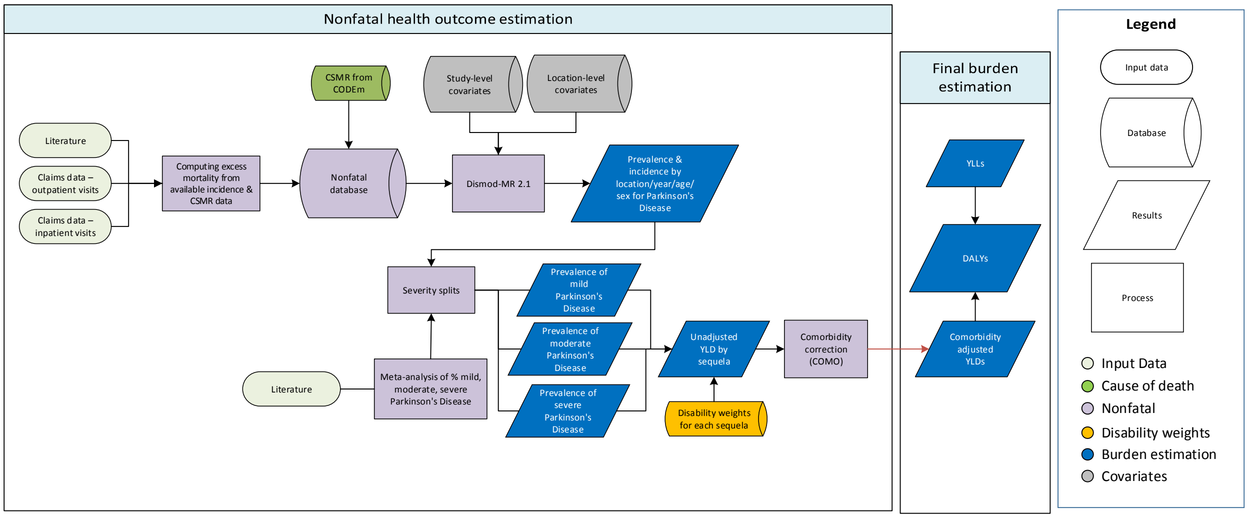

Case definition

Parkinson's disease is a chronic, degenerative, and progressive neurological condition typified by the loss of motor mobility and control – most notably tremors. The corresponding ICD-10 codes are G20, G21, and G22. Our case definition for GBD is the presence of at least two of the four primary symptoms: (1) tremors/trembling, (2) bradykinesia, (3) stiffness of limbs and torso, and (4) posture instability.

Input data

Model inputs

For this iteration of GBD, we updated the systematic review for Parkinson's disease using the following search terms:

(((((Parkinson disease AND epidemiology) AND ("2011/01/01"[PDat] : "2015/12/31"[PDat]))) AND ((Parkinson disease AND epidemiology))))

This search term resulted in 1,433 initial hits with 17 sources marked for extraction. Studies with no clearly defined sample or that drew from specific clinic/patient organizations were excluded.

The data underpinning burden estimates due to Parkinson's are generally of two types. The first type, population-based studies, are part of the literature extraction and consist of cohort studies, surveys, and the like. The second are claims data from the United States for 2000, 2010, and 2012. Additional information on the source and preparation of these data is provided elsewhere.

The following table provides a description of the density and distribution of literature data informing the Parkinson's estimates:

	Prevalence	Incidence	Mortality risk
Studies	120	38	9
Subnational units	70	10	65
Countries	45	21	21
Regions	18	10	10

Beyond the exclusion of studies using non-representative populations, there are no substantial adjustment or outliering criteria for the Parksinson's model. Certain studies have been outliered on a case-by-case basis due to subsequent review and exclusion due to inappropriateness of the study design, and overly broad age and sex groups that conflict with existing gold-standard age-sex-specific data – where possible.

Severity splits

As in GBD 2013, we use Hoehn and Yahr stages to determine severity using the following cut points:

Severity	Stage
Mild	≤2.0
Moderate	2.5-4.0
Severe	>4

We continue to use the severity proportions generated for GBD 2013. In short, we conducted a meta-analysis of studies that reported prevalence of Parkinson's by Hoehn and Yahr stage. The analysis was stratified by high-income and low-middle-income status. The following figures show the results of this analysis:

Figure 1. Percentage of mild cases of Parkinson's disease in population-based studies

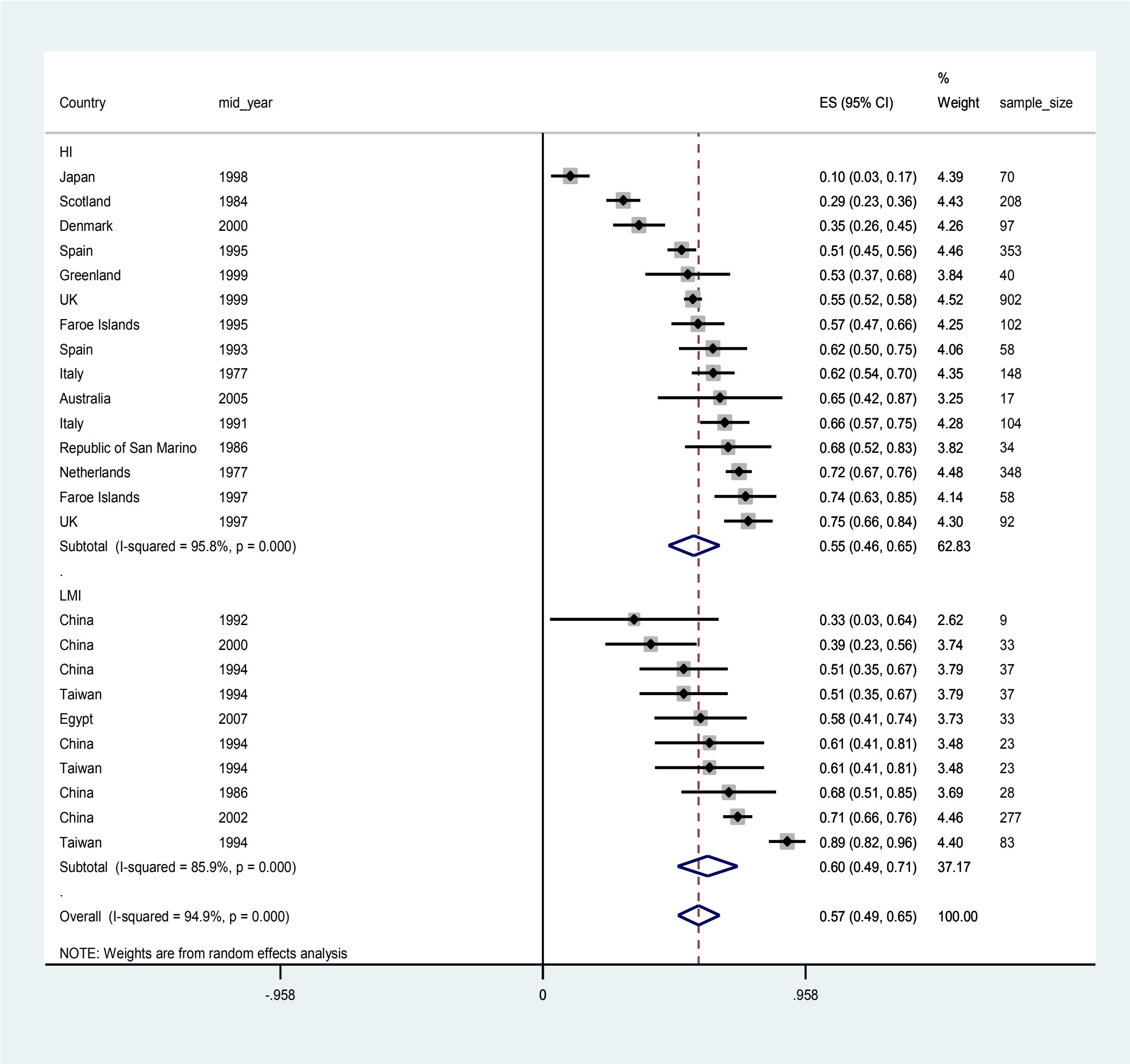

Figure 2. Percentage of moderate cases of Parkinson's disease in population-based studies

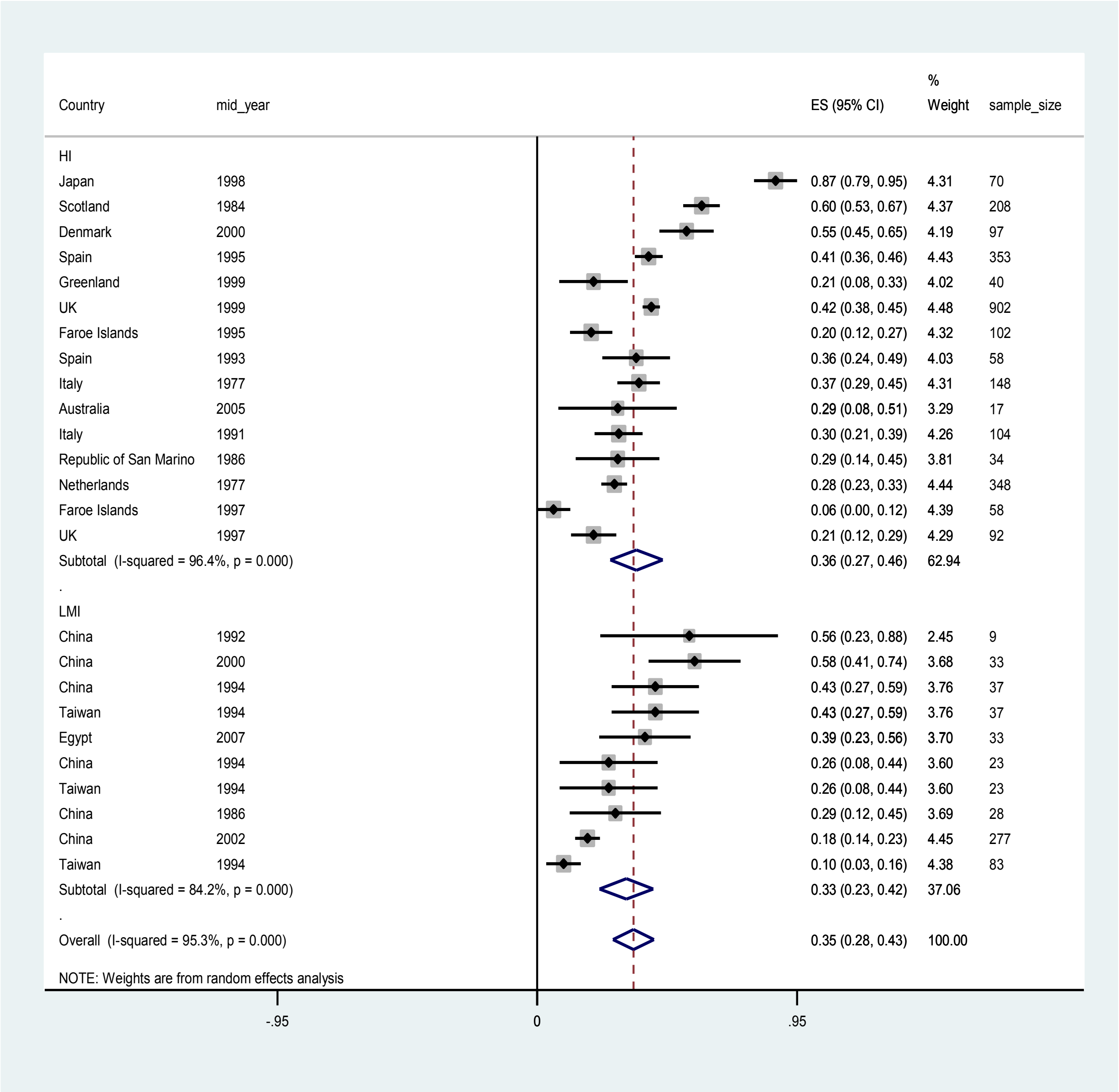

Figure 3. Percentage of severe cases of Parkinson's disease in population-based studies

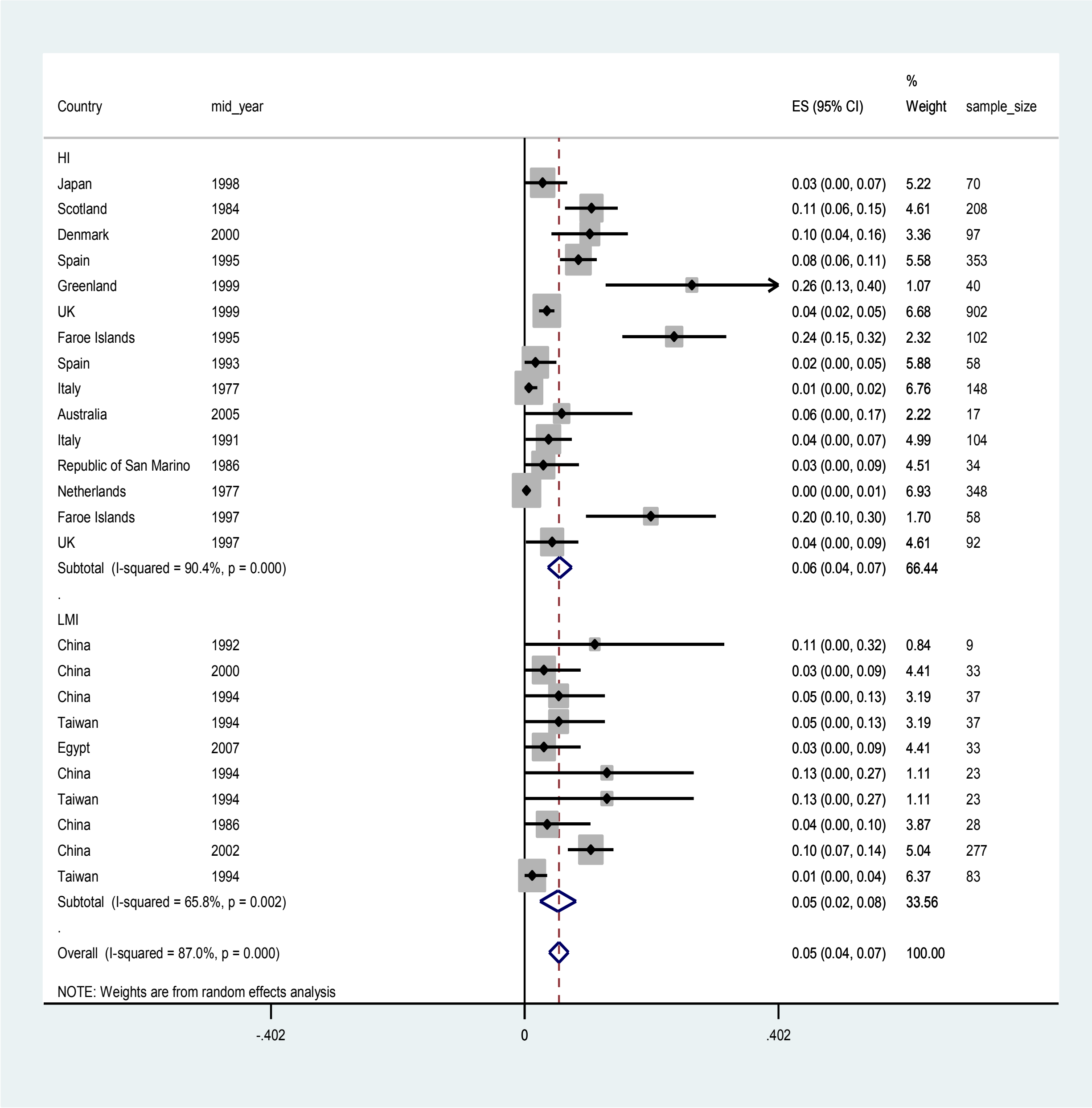

Severity estimates were generated by multiplying estimates of prevalence (country-year-sex-age-specific) by the fractions of mild, moderate, and severe PD and estimated 95% confidence intervals by taking 1,000 draws.

The following table provides the lay description and disability weights associated with Parkinson's disease.

Severity level	Lay description	DW (95% CI)
Mild	Has mild tremors and moves a little slowly, but is able to walk and do daily activities without assistance.	0.01 (0.005-0.019)
Moderate	Has moderate tremors and moves slowly, which causes some difficulty in walking and daily activities. The person has some trouble swallowing, talking, sleeping, and remembering things.	0.267 (0.181-0.372)
Severe	Has severe tremors and moves very slowly, which causes great difficulty in walking and daily activities. The person falls easily and has a lot of difficulty talking, swallowing, sleeping, and remembering things.	0.575 (0.396-0.73)

Modeling strategy

We use DisMod 2.1 as the main analytical tool for the Parkinson's disease estimation process. Prior settings are 0 remission among all ages, with no incidence or excess mortality for ages 0 to 20 years old. We ignore data on incidence, relative risk, standardized mortality ratio, and with-condition mortality as these were shown to be inconsistent with prevalence estimates. We also constrain the super-region random effects for prevalence and incidence to -0.5 and 0.5 to account for spurious inflation of regional differences. Similar to other causes, we use GBD estimates of cause-specific mortality rate (CSMR) and Excess Mortality Rate (EMR) in this model.

We make two study-level crosswalks: Diagnostic Criteria and Ascertainment. Studies that ascertain cases on clinical record review rather than live diagnostic process are crosswalked to match the latter study design. Studies that do not use the gold-standard case definition of presence of at least two of the four main symptoms are crosswalked to meet this gold standard definition. The table below shows the effect of these crosswalks. Both result in an upward adjustment of non-standard data points.

Additionally, claims data for 2000 and 2010 are adjusted via study covariates to account for systematically low estimates relative to the 2012 claims data. Implicit in this adjustment is the assumption that variation between years of claims data is a function of data collection inconsistencies and noise.

For GBD 2015, we added a country-level crosswalk to assist DisMod in estimating global patterns. We use Sociodemographic Index as a proxy to capture possible social and cultural risk factors or modifiers of Parkinson's prevalence.

The following table provides an overview of the study-level and country covariates used in the Parkinson's model.

Covariate	Measure	Beta	Exponentiated
Sociodemographic Index	prevalence	.2302<br>(.0783 - .3789)	1.259<br>(1.081 - 1.461)
(Un)Filled diagnostic criteria	prevalence	-.2797<br>(-.4297 - -.1336)	.756<br>(.6507 - .8749)
All MarketScan, year 2010	prevalence	-.0237<br>(-.0514 - -.0024)	.9765<br>(.9499 - .9976)
All MarketScan, year 2000	prevalence	-.0929<br>(-.1321 - -.0561)	.9113<br>(.8762 - .9454)
Suboptimal Case Ascertainment	prevalence	-.3139<br>(-.4473 - -.1862)	.7306<br>(.6394 - .8301)

Although the foundation of the Parkinson's modeling strategy remains broadly similar, we do expect a few changes. First, the inclusion of SDI as a country covariate may slightly alter country and regional patterns. Second, unlike GBD 2013 we include Parkinson's cause-specific mortality estimates from earlier steps in GBD. We did this to ensure consistency between sets of estimates. However, there is evidence of the non-reliable pattern of death registries for this disease. It is likely that in the next iteration of GBD, we will move toward a single-step natural history model to estimate mortality and morbidity due to Parkinson's and therefore remove this limitation.

Multiple sclerosis (MS)

Flowchart

Multiple Sclerosis

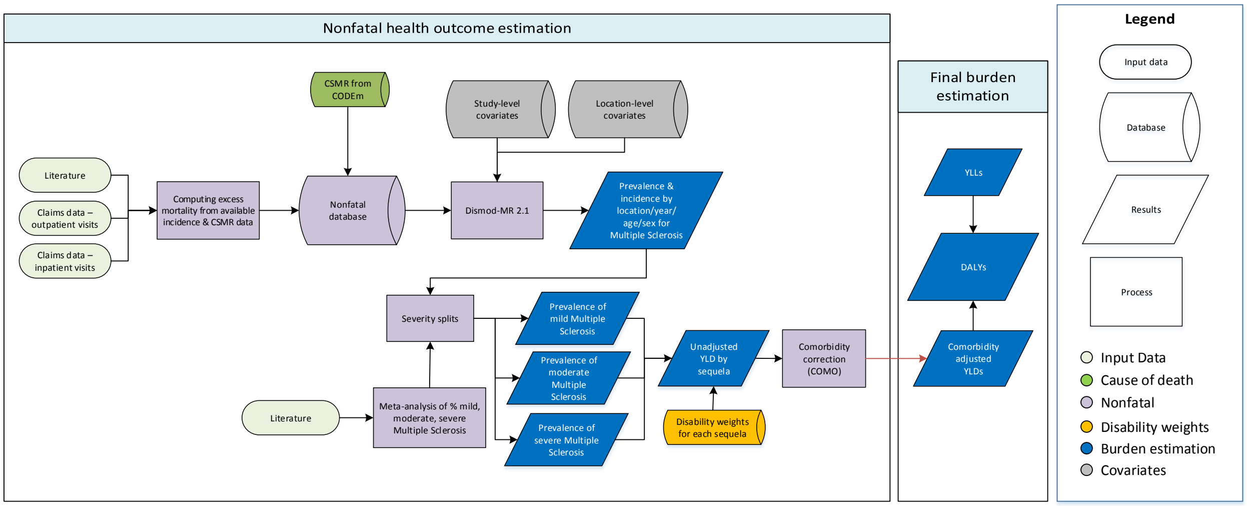

Input data and methodological summary

Case definition

Multiple Sclerosis is a chronic, degenerative and progressive neurological condition typified by the damaging of the myelin sheaths. For GBD, the McDonald's criteria for diagnosis is considered the gold standard, but other definitions such as Poser Committee's criteria and self-report of a doctor's diagnosis are also included. The ICD-10 code for MS is G35.

Input data

A systematic review was conducted for MS for this iteration of GBD. A review was not done for GBD 2013. The search using (multiple sclerosis AND epidemiology AND ("2011/01/01"[PDat] : "2015/12/31"[PDat])) from 1/1/2011-7/15/15 yielded 1756 hits with 28 sources marked for extraction.

The data underpinning estimates of burden due to MS are generally of two types. The first, are representative, population-based surveys. This includes retrospective case/hospital report analysis, nationally representative health studies and the like. Studies with no clearly defined sample or that draw from specific clinic/patient organizations were excluded during the systematic review phase. The second type are claims data from the United States from 2000, 2010 and 2012. Additional information on the source and preparation of these data is provided elsewhere.

The following table provides a description of the density and distribution of literature data informing the MS estimates:

	Prevalence	Incidence	Mortality risk
Studies	147	59	15
Countries/subnationals	44/69	25/6	20/58
GBD world regions	12	8	8

Beyond the exclusion of studies using non-representative populations, there are no substantial adjustment or outliering criteria for the MS model. Certain studies have been outliered on a case by case basis due to: (1) subsequent review and exclusion due to inappropriate of the study design, and overly broad age and sex groups that conflict with existing gold standard age-sex specific data—where possible.

Severity splits

As in GBD 2013, we use Kurtzke's Expanded Disability Status Scale (EDSS) to determine severity splits for MS. They broke down to:

Mild: EDSS ≤ 3.5
Moderate: 3.5 < EDSS ≤ 6.5
Severe: 6.5 < EDSS ≤ 9.5

The table below illustrates the severity level, lay description and DW.

Severity level	Lay description	DW (95% CI)
Mild	has mild loss of feeling in one hand, is a little unsteady while walking, has slight loss of vision in one eye, and often needs to urinate urgently.	0.183 (0.124-0.253)
Moderate	needs help walking, has difficulty with writing and arm coordination, has loss of vision in one eye and cannot control urinating.	0.463 (0.313-0.613)
Severe	has slurred speech and difficulty swallowing. The person has weak arms and hands, very limited and stiff leg movement, has loss of vision in both eyes and cannot control urinating.	0.719 (0.534-0.858)

To generate fractions for population level assignment, we re-use the meta-analysis conducted as part of GBD 2013. In short, we conducted a meta-analysis of all eligible studies that reported both prevalence and EDSS with separate results for High-Income and Low and Middle Income countries. The following figures provide the result of the meta-analysis.

Figure 1. Mild cases of MS

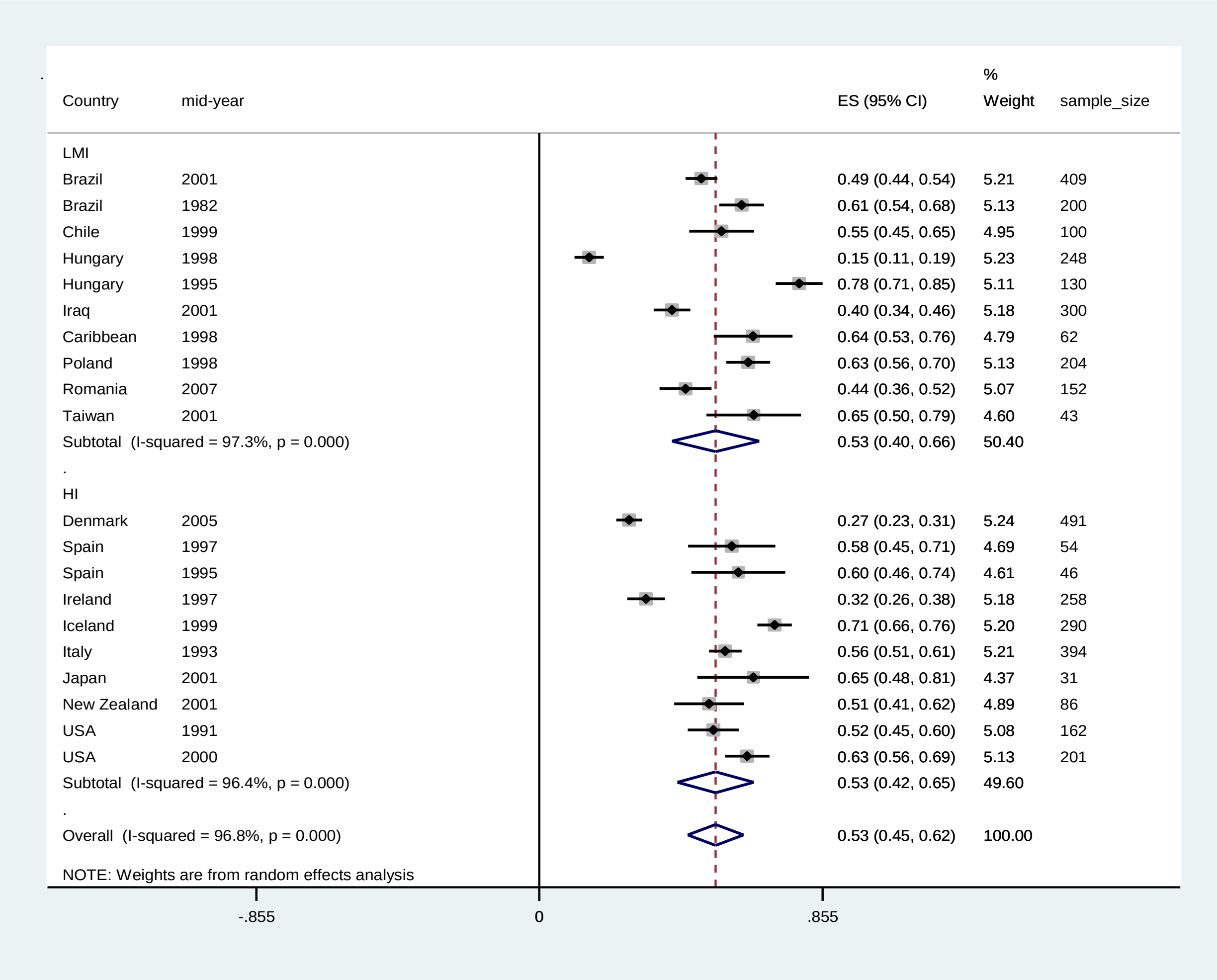

Figure 2. Moderate Cases of MS

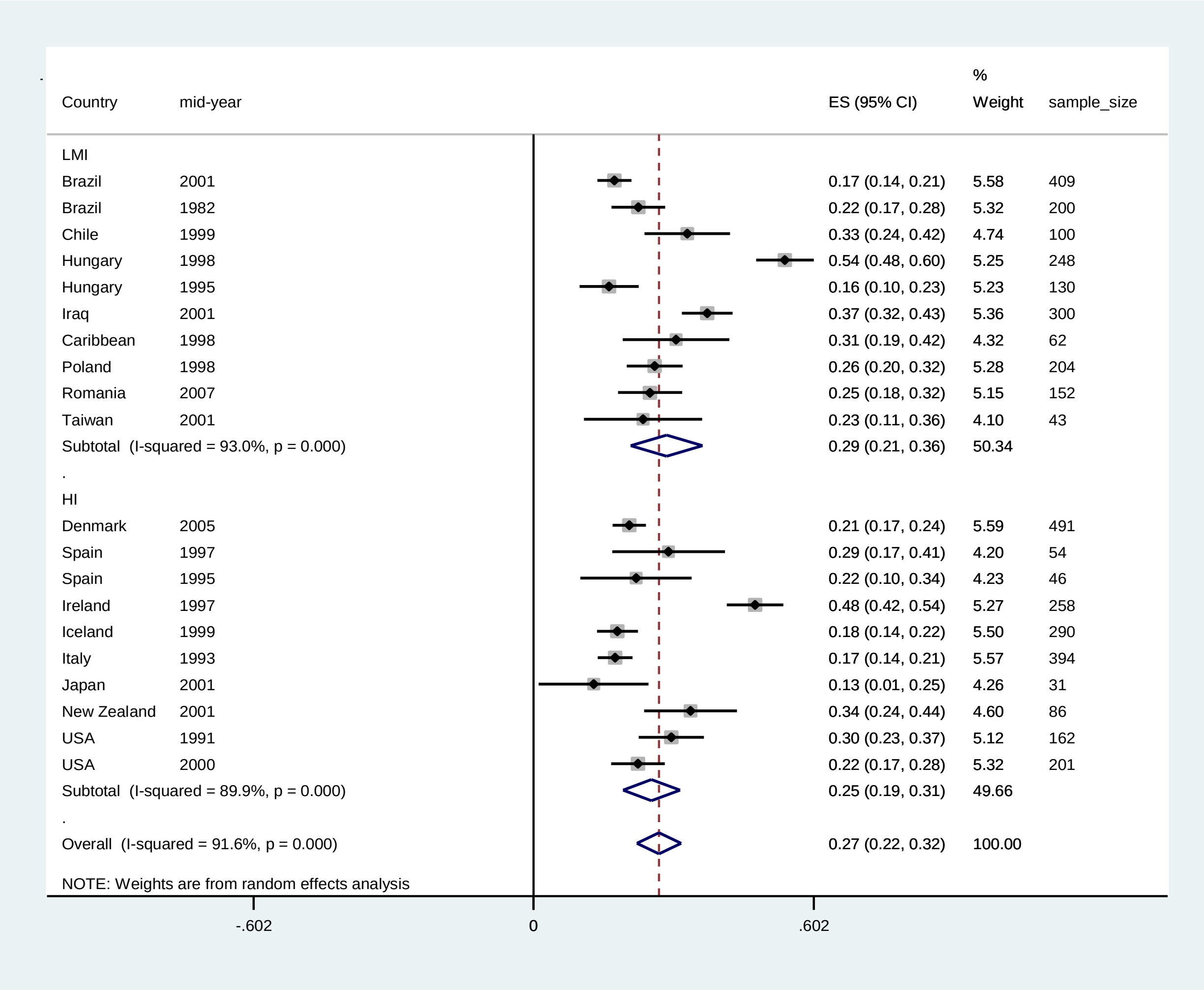

Figure 3. Severe Cases of MS

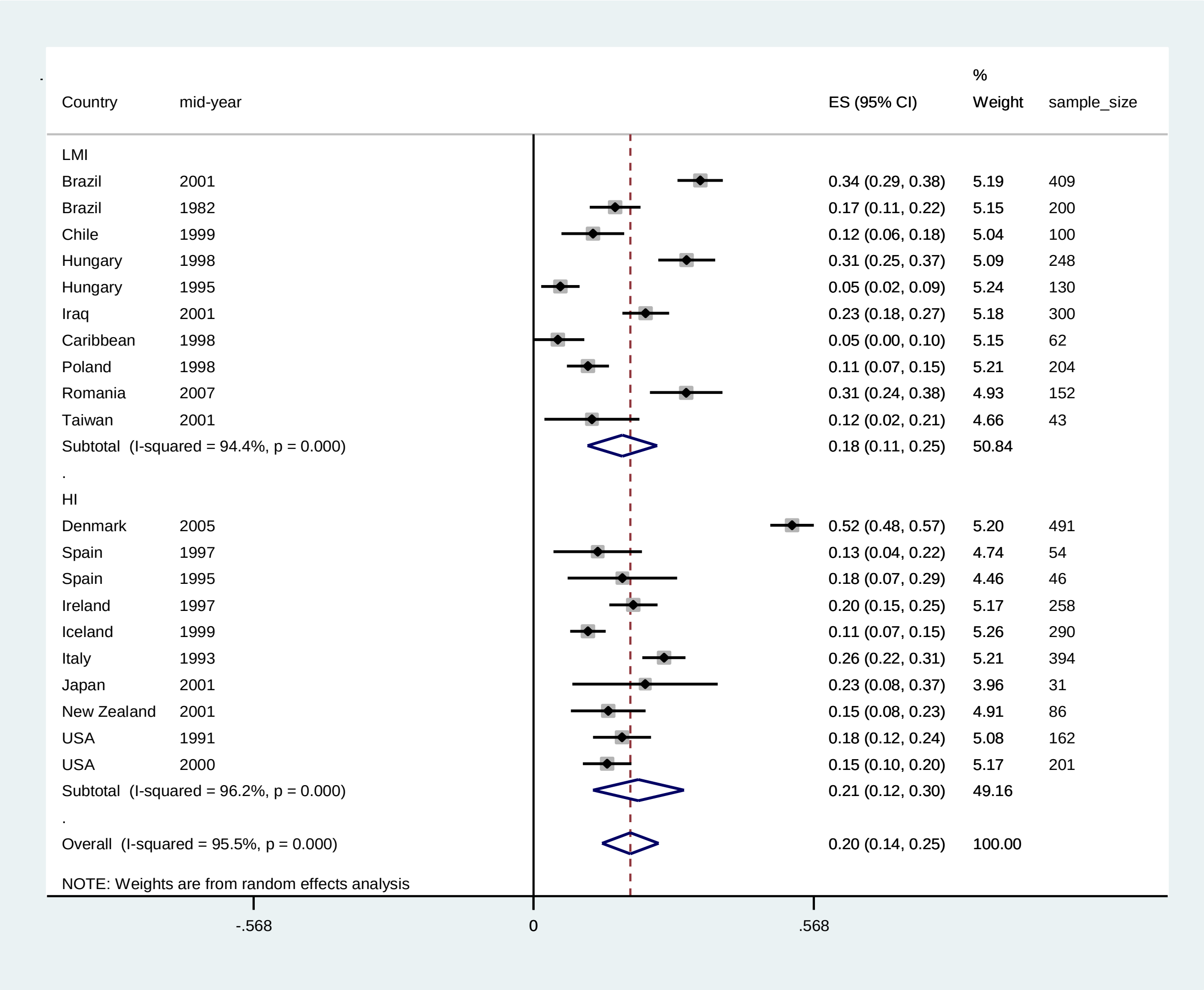

Modeling strategy

We use DisMod 2.1 as the main analytical tool for the MS estimation process. Prior settings include 0 remission for all ages, and no incidence or excess mortality for persons under 4 years old. We also constrain the super region random effects for prevalence and incidence to -.5 and .5 to account for spurious inflation of regional differences.

Unlike GBD 2013, we do not use study covariates to inflate the variance of data points with non-optimal case ascertainment and diagnostic criteria for model parsimony, as they did not meaningfully contribute the modelling process in this cycle.

Claims data for 2000 and 2010 are adjusted via study covariates to account for systematically low estimates relative to the 2012 claims data. Implicit in this adjustment is the assumption that variation between years of claims data is a function of data collection inconsistencies and noise.

Similar to other cases we use GBD estimates of cause-specific mortality rate (CSMR) and Excess Mortality Rate (EMR) in this model.

To assist the estimation process, we use a several country level covariates. These effects plus those of the study covariates are presented below.

Covariate	Measure	Beta	Exponentiated	Parameter Type
Absolute value of average latitude	prevalence	.0377 (.0362 - .0393)	1.038 (1.037 - 1.04)	Country-level
Absolute value of average latitude	incidence	.0229 (.0165 - .0294)	1.023 (1.017 - 1.03)	Country-level
All MarketScan, year 2000	prevalence	-.3526 (-.3799 - -.325)	.7029 (.6839 - .7225)	x-cov
All MarketScan, year 2010	prevalence	-.0111 (-.0281 - -.0016)	.989 (.9723 - .9984)	x-cov
LDI (I$ per capita)	excess mortality rate	-.5187 (-.5358 - -.5013)	.5953 (.5852 - .6057)	Country-level
Sociodemographic Status	prevalence	-2 (-2 - -2)	.1353 (.1353 - .1353)	Country-level

As described in the literature, extreme latitude is associated with higher prevalence and incidence of MS. While the pathway affects MS is not fully understood, our results suggest a sizable relationship. Our operationalization of latitude is created by a population-weighted average of latitude by country and taking the absolute value. The underlying population distribution rasters are part of the Gridded Population of the World dataset.

Although there are no known cures for MS, we expect disease management to differ globally—largely as a function of available resources. To capture this, we use the natural log of lagged distributed income per capita as a proxy to capture this relationship in the estimation of excess mortality.

To capture possible social and cultural risk factors or modifiers of MS prevalence, we include socio-demographic status as a covariate.

In general, we expect little change in the overall patterns of MS relative to GBD 2013. The main data types are consistent with previous iterations of GBD and new data is generally within the bounds of the existing dataset. We also used a substantially similar modelling strategy to GBD 2013.

Motor neuron diseases

Flowchart

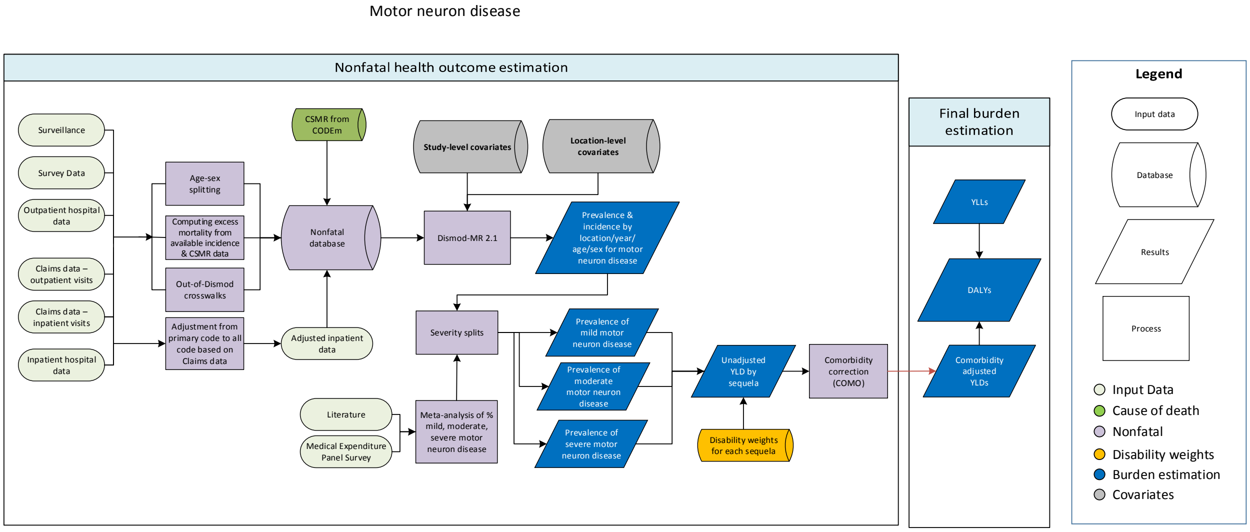

Case definition

Motor neuron diseases (MND) are a set of chronic, degenerative, and progressive neurological conditions typified by the destruction of motor neurons and the subsequent deterioration of voluntary muscle activity. The most common MND is Amyotrophic Lateral Sclerosis. This is a new cause for GBD 2015, with the corresponding ICD-10 code of G12. Our gold standard diagnostic criteria are the El Escorial Criteria, with other similar criteria (e.g., the original set from World Federation of Neurology) if necessary.

Input data

As MND is a new cause in the Global Burden of Disease project, we conducted a full systematic review. The following search string guided our search, which resulted in 3,146 hits with 58 sources meeting extraction criteria: (1) the study is a representative population-based study, (2) reports on prevalence, incidence, remission, excess mortality, relative risk of mortality, standardized mortality ratio, or with-condition mortality rate. Studies with no clearly defined sample were excluded.

(('motor neuron disease'[MeSH Terms] OR ('motor'[All Fields] AND 'neuron'[All Fields] AND 'disease'[All Fields]) OR 'motor neuron disease'[All Fields] OR ('motor'[All Fields] AND 'neuron'[All Fields] AND 'diseases'[All Fields]) OR 'motor neuron diseases'[All Fields]) OR ('amyotrophic lateral sclerosis'[MeSH Terms] OR ('amyotrophic'[All Fields] AND 'lateral'[All Fields] AND 'sclerosis'[All Fields]) OR 'amyotrophic lateral sclerosis'[All Fields]) OR ALS[All Fields] OR ('motor neuron disease'[MeSH Terms] OR ('motor'[All Fields] AND 'neuron'[All Fields] AND 'disease'[All Fields]) OR 'motor neuron disease'[All Fields] OR ('primary'[All Fields] AND 'lateral'[All Fields] AND 'sclerosis'[All Fields]) OR 'primary lateral sclerosis'[All Fields]) OR ('Politics Life Sci'[Journal] OR 'pls'[All Fields]) OR ('muscular atrophy, spinal'[MeSH Terms] OR ('muscular'[All Fields] AND 'atrophy'[All Fields] AND 'spinal'[All Fields]) OR 'spinal muscular atrophy'[All Fields] OR ('progressive'[All Fields] AND 'muscular'[All Fields] AND 'atrophy'[All Fields]) OR 'progressive muscular atrophy'[All Fields]) OR PBP[All Fields] OR ('pseudobulbar palsy'[MeSH Terms] OR

('pseudobulbar'[All Fields] AND 'palsy'[All Fields]) OR 'pseudobulbar palsy'[All Fields])) AND (('epidemiology'[Subheading] OR 'epidemiology'[All Fields] OR 'epidemiology'[MeSH Terms]) OR population-based[All Fields])

The following table provides an overview of the density and distribution of the data extracted from the literature.

	Prevalence	Incidence	Mortality risk
Studies	11	47	5
Subnational units	53	9	53
Countries	6	17	7
Regions	5	7	5

Beyond the literature data, we also make use of claims data from the United States for 2000, 2010, and 2012. Descriptions of the source and preparation of this data are provided elsewhere.

Except for excluding studies using non-representative populations, there are no substantial adjustments or outliering criteria for the MND model. Certain studies have been outliered on a case-by-case basis due to (1) subsequent review and exclusion due to inappropriateness of the study design, and (2) overly broad age and sex groups that conflict with existing gold standard age-sex-specific data – where possible.

Severity splits

To calculate severity and disability due to MND we analyzed a dataset from Pooled Resource Open-access ALS Clinical Trials (PRO-ACT). This dataset contains the largest ALS clinical trials dataset, with a total of 8,635 ALS patient records from multiple completed clinical trials. Among these, we conducted the final analysis with n=4838 (56%) of the patients with complete ALS Function Rating Score (ALSFRS) with average follow-up time of 184 days (min: -22, max: 648), in which 2,999 (62%) received experimental (medication) treatments and 1,301 (27%) received placebo (in these trials, the medications tested were found to be no better than placebo with respect to their effects on ALS progressions).

The ALSFRS is an instrument for evaluating the functional status of patients with Amyotrophic Lateral Sclerosis. It can be used to monitor functional changes in a patient over time. It measures (1) speech, (2) salivation, (3) swallowing, (4) handwriting, (5) cutting food and handling utensils (with or without gastrostomy), (6) dressing and hygiene, (7) turning in bed and adjusting bed clothes, (8) walking, (9) climbing stairs, and (10) breathing. Each task is rated on a 5-point scale from 0 = can't do, to 4 = normal ability. Individual item scores are summed to produce a reported total score of between 0 and 40 (worst to best). ALSFRS has been revised to ALSFRS-R, which includes 12 questions (ALSFRS Q10 changes to (10) Dyspnea, (11) Orthopnea, and (12) Respiratory insufficiency), with individual item scores summed to a score between 0 and 48.

In order to eliminate any bias from the treatment effects on the ALSFRS, only the first observation at the time of trial is selected. If the first observation is missing at the time of trial (or prior), the next non-missing observation is selected to be included in the final analysis.

We subsequently mapped ALSFRS scores into GBD severities, and sequelae into different combinations of speech problems, chronic obstructive pulmonary disease, and motor impairment using the following logic:

Motor Impairment

The ALSFRS assess motor function of the legs through questions on walking (Q8) and stair climbing(Q9).

Combined score	Severity level
8	None
5-7	Mild
2-4	Moderate
0-1	Severe

The ALSFRS also assesses motor impairment through questions on handwriting (Q4), cutting food and handling utensils (Q5), and dressing and hygiene (Q6).

Combined score	Severity level
12	None
9-11	Mild
3-8	Moderate
0-2	Severe

After determining case severity on these two separate metrics, we aggregate by taking the most severe ranking (e.g., severe + mild = a severe case).

Respiratory problems:

Question 10 of the ALSFRS describes breathing difficulty as a function of MND.

ALSFRS score	Description	Severity level
4	Normal	None
3	Shortness of breath with minimal exertion	Mild
2	Shortness of breath at rest	Moderate
0-1	Intermittent ventilator assistance required/ventilator-dependent	Severe

Speech problems

Speech impairment due to MND is derived from ALSFRS question 1, which describes speech impediments. A score of 4 on this question denotes no impairment, while all other values suggest some impairment.

Creating sequelae

After determining the severity status of each case for the three symptom umbrellas, we subsequently estimated the relative proportion of each combination of symptom class and their respective severities.

Those without any symptoms (e.g., no severity) were categorized as having worry about the diagnosis for disability estimation. The following table displays the various sequelae and their associated proportions.

Sequelae	Proportion (Mean)	Proportion (Lower)	Proportion (Upper)
Mild motor impairment, mild respiratory problems and speech problems due to motor neuron disease	0.01779	0.01658	0.01909
Mild motor impairment, moderate respiratory problems and speech problems due to motor neuron disease	0.00270	0.00225	0.00324
Mild motor impairment, severe respiratory problems and speech problems due to motor neuron disease	0.00082	0.00059	0.00113
Mild motor impairment, and speech problems due to motor neuron disease	0.02052	0.01922	0.02190
Moderate motor impairment, mild respiratory problems and speech problems due to motor neuron disease	0.03377	0.03210	0.03552
Moderate motor impairment, moderate respiratory problems and speech problems due to motor neuron disease	0.00715	0.00640	0.00799
Moderate motor impairment, severe respiratory problems and speech problems due to motor neuron disease	0.00286	0.00240	0.00342
Moderate motor impairment, and speech problems due to motor neuron disease	0.03041	0.02883	0.03208
Severe motor impairment, mild respiratory problems and speech problems due to motor neuron disease	0.05242	0.05035	0.05457
Severe motor impairment, moderate respiratory problems and speech problems due to motor neuron disease	0.02247	0.02111	0.02392
Severe motor impairment, severe respiratory problems and speech problems due to motor neuron disease	0.01365	0.01259	0.01479
Severe motor impairment and speech problems due to motor neuron disease	0.04765	0.04567	0.04970
Mild respiratory problems and speech problems due to motor neuron disease	0.01157	0.01060	0.01263
Moderate respiratory problems and speech problems due to motor neuron disease	0.00142	0.00111	0.00182
Severe respiratory problems and speech problems due to motor neuron disease	0.00023	0.00013	0.00043
Speech problems due to motor neuron disease	0.02457	0.02315	0.02608
Mild motor impairment and mild respiratory problems due to motor neuron disease	0.02245	0.02109	0.02389
Mild motor impairment and moderate respiratory problems due to motor neuron disease	0.00275	0.00230	0.00329
Mild motor impairment and severe respiratory problems due to motor neuron disease	0.00068	0.00047	0.00097
Mild motor impairment due to motor neuron disease	0.10388	0.10103	0.10681
Moderate motor impairment and mild respiratory problems due to motor neuron disease	0.06744	0.06511	0.06985
Moderate motor impairment and moderate respiratory problems due to motor neuron disease	0.01302	0.01199	0.01413

Moderate motor impairment and severe respiratory problems due to motor neuron disease	0.00412	0.00356	0.00477
Moderate motor impairment due to motor neuron disease	0.20136	0.19760	0.20518
Severe motor impairment and mild respiratory problems due to motor neuron disease	0.06902	0.06666	0.07146
Severe motor impairment and moderate respiratory problems due to motor neuron disease	0.02000	0.01872	0.02137
Severe motor impairment and severe respiratory problems due to motor neuron disease	0.01062	0.00969	0.01163
Severe motor impairment due to motor neuron disease	0.15037	0.14702	0.15378
Mild respiratory problems due to motor neuron disease	0.00643	0.00571	0.00723
Moderate respiratory problems due to motor neuron disease	0.00044	0.00028	0.00069
Severe respiratory problems due to motor neuron disease	0.00005	0.00001	0.00017
Asymptomatic, but worry about diagnosis due to motor neuron disease	0.03738	0.03562	0.03921

To determine disability due to these sequelae, we use the standard multiplicative aggregation formula as described in the main text. The following table provides description and disability weight assigned to the sequelae as appropriate.

Symptom group	Severity level	Lay description	DW (95%)
Respiratory problems	Asymptomatic		
Respiratory problems	Mild	Has cough and shortness of breath after heavy physical activity, but is able to walk long distances and climb stairs.	0.019<br>(0.011-0.033)
Respiratory problems	Moderate	Has cough, wheezing, and shortness of breath, even after light physical activity. The person feels tired and can walk only short distances or climb only a few stairs.	0.225<br>(0.153-0.31)
Respiratory problems	Severe	Has cough, wheezing, and shortness of breath all the time. The person has great difficulty walking even short distances or climbing any stairs, feels tired when at rest, and is anxious.	0.408<br>(0.273-0.556)
Motor impairment	Asymptomatic		
Motor impairment	Mild	Has some difficulty in moving around but is able to walk without help.	0.01<br>(0.005-0.019)
Motor impairment	Moderate	Has some difficulty in moving around and difficulty in lifting and holding objects, dressing, and sitting upright, but is able to walk without help.	0.061<br>(0.04-0.089)

Motor impairment	Severe	Is unable to move around without help, and is not able to lift or hold objects, get dressed, or sit upright.	0.402 (0.268-0.545)
Speech problems	No		
Speech problems	Yes	Has difficulty speaking, and others find it difficult to understand.	0.051 (0.032-0.078)
Asymptomatic, but worry	Yes	Has a disease diagnosis that causes some worry but minimal interference with daily activities.	0.012 (0.006-0.023)

Modeling strategy

We use DisMod 2.1 as the main analytical tool for MND estimation. Prior settings are limited to 0 remission at all ages. We also constrain the super-region random effects for prevalence and incidence to -0.5 and 0.5 to account for spurious inflation of regional differences.

Claims data for 2000 and 2010 are adjusted via study covariates to account for systematically low estimates relative to the 2012 claims data. Implicit in this adjustment is the assumption that variation between years of claims data is a function of data collection inconsistencies and noise.

Similar to other cases we use GBD estimates of cause-specific mortality rate (CSMR) and Excess Mortality Rate (EMR) in this model. The source and estimation of these rates are discussed elsewhere.

To assist the estimation process we use several country-level covariates.

Covariate	Measure	Beta	Exponentiated
Sociodemographic Index	prevalence	1.626 (1.148 - 1.971)	5.086 (3.152 - 7.178)
All MarketScan, year 2010	prevalence	-.1082 (-.1511 - -.0627)	.8974 (.8598 - .9392)
Absolute value of average latitude	prevalence	.0016 (7.5e-05 - .0041)	1.002 (1 - 1.004)
LDI (I$ per capita)	excess mortality rate	-.4999 (-.5 - -.4998)	.6066 (.6065 - .6067)
Mean BMI	prevalence	-.0922 (-.1336 - -.0581)	.912 (.8749 - .9435)
All MarketScan, year 2000	prevalence	-.1146 (-.1612 - -.0621)	.8918 (.8511 - .9398)

As described in the literature, BMI may be protective of MND. Accordingly, we have included mean BMI as a covariate to assist the estimation of prevalence within the disease model. As expected, the coefficient of BMI on MND prevalence is negative.

Although there are no known cures for MND, we expect disease management to differ globally – largely as a function of available resources. To capture this, we use the natural log of lagged distributed income per capita as a proxy to capture this relationship in the estimation of excess mortality.

To capture possible social and cultural risk factors or modifiers of MS prevalence, we include Sociodemographic Index as a covariate.

Because MND is a new cause for GBD 2015, we have no reference point relative to other GBD iterations.

Migraine

Flowchart

Migraine

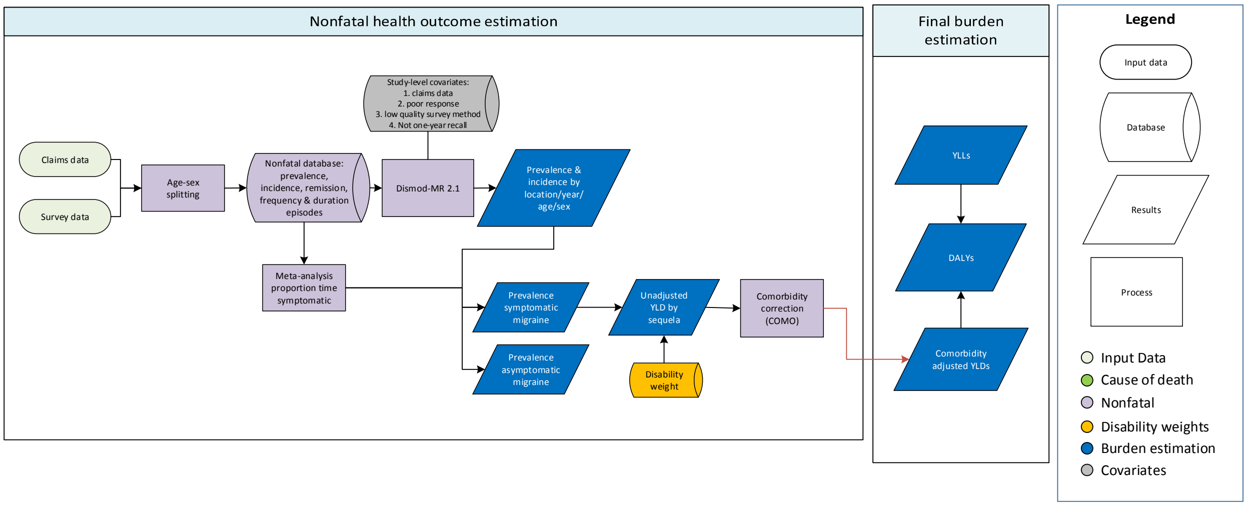

Input data and methodological summary

Case definition

Migraine is a class of disabling primary headache disorders, characterized by recurrent unilateral pulsatile headaches. The two major subtypes are common migraine (without aura) and classic migraine (with aura or neurological symptoms). In GBD we do not distinguish subtypes as most epidemiological studies report on overall migraine only. The ICD-10 code for migraine is G43 and ICD-9 code is 346.

Input data

Model inputs

A systematic review was conducted for GBD 2010 and updated for GBD 2013. For GBD 2015, three new representative surveys conducted by GBD collaborators in Norway, Karnataka, India, and Nepal were added. Inclusion criteria of the systematic reviews were:

- Representative, population-based surveys
- Reporting of prevalence of migraine headache

The table below illustrates the geographic distribution of data points.

	Prevalence	Incidence	Remission	Frequency and duration episodes
Studies	116	3	1	16
Countries/subnational locations	113	3	1	16
GBD world regions	16	2	1	9

In addition, data from US claims data for 2000, 2010, and 2012 by US state were included.

Severity splits

The basis of the GBD disability weight (DW) survey assessments are lay descriptions of sequelae highlighting major functional consequences and symptoms. The lay description and disability weight for migraine are shown below.

Severity level	Lay description	DW (95% CI)
Mild	This person has severe, throbbing head pain and nausea that cause great difficulty in daily activities and sometimes confine the person to bed. Moving around, light, and noise make it worse.	0.434 (0.285 – 0.603)

To determine the proportion of time spent over a year spent in an episode of migraine headache, 16 studies providing data on the frequency of episodes and the average duration of episodes were meta-analyzed. As these studies reported frequency and duration of episodes by disparate categories, an assumption was made that the mean represented each category. For each study the estimated proportion of time symptomatic is 0.085 (0.058-0.112).

Modeling strategy

We used a list of binary covariates which are modified version of quality indicators of epidemiological studies on headache and shown in the table below.

Study covariate	Notation	
	Less desirable (1)	Reference (zero)
Other than one year recall period	Point prevalence	One year prevalence
Not representative	Selected population	General population or community-based sample from whole country OR general population or community-based sample from defined region within a country, or school-based (for children)
Low quality sampling method	Not stated OR no (or failed) attempt to secure representativeness	Total defined population, or random sample corrected for population demographics OR random sample uncorrected for population demographics
Poor response	Not stated, or <70%	70-100%

Low quality survey method and type of interviewer	Not stated OR self-administered (unsupervised) questionnaire OR telephone or face-to-face interview by untrained or unspecified interviewer(s)	Face-to-face interview with headache expert
Low quality validation of diagnostic instrument	Instrument not specified or not validated OR validated, but sensitivity and/or specificity <70% OR validated only in screen-positive sub-sample, or in clinic or unspecified sample, but sensitivity and specificity ≥70%	Validated in target population or similar, and sensitivity and specificity ≥70%, or all diagnoses made in face-to-face or telephone interviews by headache expert
Low quality diagnostic criteria	Not stated OR stated, other than ICHD OR ICHD (or reasonable modification), but uncertain or inappropriate analysis of "probable" diagnoses	ICHD (or reasonable modification) with clear exposition regarding "probable" diagnoses

We added separate covariates for the three years of claims data from Marketscan (2000, 2010, and 2012).

Prior settings in the DisMod model included setting incidence to 0 before age 5 based on expert advice. We also assume no excess mortality due to migraine.

All study covariates were initially evaluated as x-cov (which means that data points are adjusted to the reference value if a systematic bias is detected); those that did not have a significant coefficient, were entered as z-cov (which means that a multiplier is applied to the standard error of such data points to indicate they are less certain values because they did not meet the reference criteria for study quality). The table below shows the fixed effect values of the x-covs which are in log space (as DisMod uses an offset lognormal model) as well as the exponentiated values which for a x-cov can be interpreted as an odds ratio.

The covariates for low quality sampling method, low quality diagnostic criteria, and low quality validation of diagnostic instrument and not representative studies had non-significant coefficients as a x-cov and were subsequently used as z-covs.

Study covariate	Parameter	beta	Exponentiated beta
Low quality survey method and type of interviewer	Prevalence	0.18	1.20 (1.07-1.35)
Other than one year recall period	Prevalence	-0.38	0.68 (0.61-0.76)
Poor response	Prevalence	-0.13	0.88 (0.81-0.95)
Claims data US 2000	Prevalence	-2.35	0.096 (0.071 — 0.12)
Claims data US 2010	Prevalence	-1.96	0.14 (0.11 — 0.17)
Claims data US 2012	Prevalence	-1.86	0.16 (0.12 — 0.19)

No other significant changes were made to modeling strategy from GBD 2013.

Tension-type headache

Flowchart

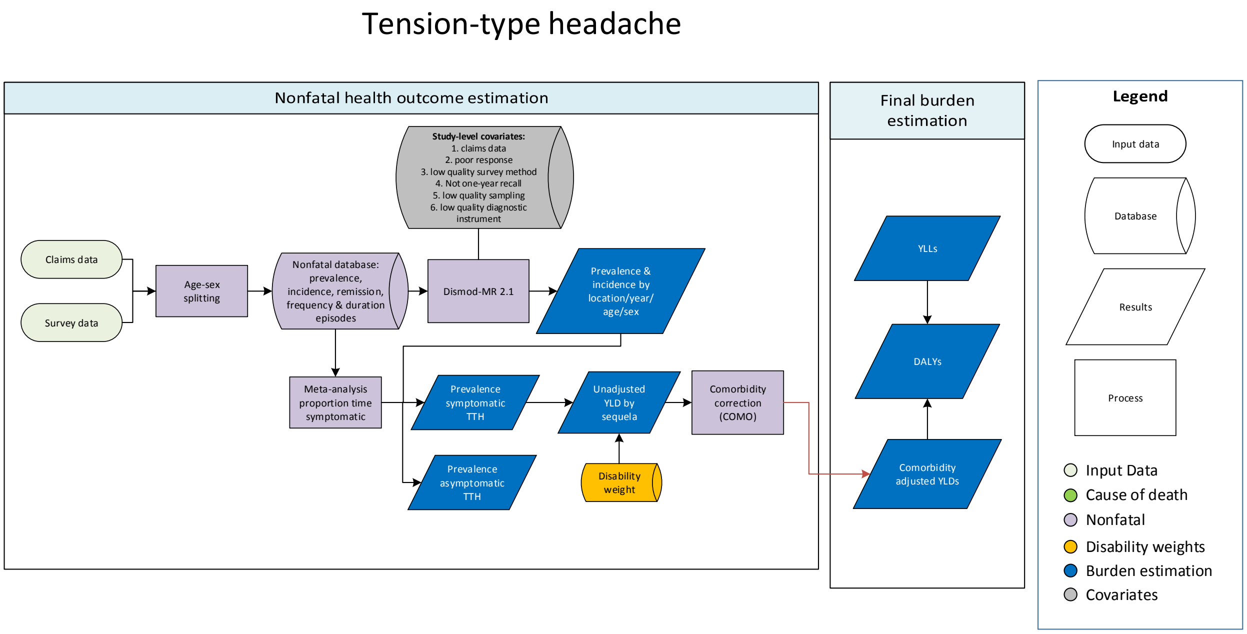

Case Definition

Tension-type headache is characterized by a dull, non-pulsatile, diffuse, band-like (or vice-like) pain of mild to moderate intensity in the head; scalp; or neck. The ICD-10 code for migraine is G44.2 and ICD-9 code is 339.1.

Input data

Model Inputs

A systematic review was conducted for GBD2010 and updated for GBD2013. For GBD2015 three new representative surveys conducted by GBD collaborators in Norway, Karnataka, india and Nepal were added. Inclusion criteria of the systematic reviews were:

- Representative, population-based surveys
- Reporting of prevalence of TTH headache

	Prevalence	Incidence	Remission	Frequency and duration episodes
Studies	84	1	0	9
Countries/subnational locations	103	1	0	7
GBD world regions	15	1	0	6

In addition, data from US claims data for 2000, 2010 and 2012 by US state were included.

Severity Splits

The basis of the GBD disability weight survey assessments are lay descriptions of sequelae highlighting major functional consequences and symptoms. The lay description and disability weight for migraine are shown below.

Severity level	Lay description	DW (95% CI)
Mild	This person has a moderate headache that also affects the neck, which causes difficulty in daily activities	0.036 (0.023 – 0.053)

To determine the proportion of time spent over a year spent in an episode of TTH headache 9 studies providing data on the frequency of episodes and the average duration of episodes were meta-analysed. As these studies reported frequency and duration of episodes by disparate categories, an assumption was made that the mean represented each category. The estimated proportion of time symptomatic is 0.058 (0.023-0.092).

Modeling Strategy

We used a list of binary covariates which are modified version of quality indicators of epidemiological studies on headache (add ref: Steiner TJ, Stovner LJ et al (2013). Improving quality in population surveys of headache prevalence, burden and cost: key methodological considerations. J Headache Pain, 14: 87) and shown in the table below.

Study covariate	Notation	
	Less desirable (1)	Reference (zero)
Other than one-year recall period	Point prevalence	One-year prevalence
Not representative	selected population	general population or community-based sample from whole country OR general population or community-based sample from defined region within a country, or school-based (for children)
Low quality sampling method	not stated OR no (or failed) attempt to secure representativeness	total defined population, or random sample corrected for population demographics OR random sample uncorrected for population demographics
Poor response	not stated, or <70%	70-100%
Low quality survey method and type of interviewer	not stated OR self-administered (unsupervised) questionnaire OR telephone or face-to-face interview by untrained or unspecified interviewer(s)	face-to-face interview with headache expert
Low quality validation of diagnostic instrument	instrument not specified or not validated OR validated, but sensitivity and/or specificity <70% OR validated only in screen-positive sub-sample, or in clinic or unspecified sample, but sensitivity and specificity ≥70%	validated in target population or similar, and sensitivity and specificity ≥70%, or all diagnoses made in face-to-face or telephone interviews by headache expert

Low quality diagnostic criteria	not stated OR stated, other than ICHD OR ICHD (or reasonable modification), but uncertain or inappropriate analysis of “probable” diagnoses	ICHD (or reasonable modification) with clear exposition regarding “probable” diagnoses

We added separate covariates for the three years of claims data from Marketscan (2000, 2010 and 2012).

Prior settings in the DisMod model included setting incidence to 0 before age 5 based on expert advice. We also assume no excess mortality due to TTH. In the absence of any data on remission we set bounds between 0 and 0.5, i.e. ensuring an average duration of at least 2 years.

All study covariates were initially evaluated as x-cov (which means that data points are adjusted to the reference value if a systematic bias is detected); those that did not have a significant coefficient, were entered as z-cov (which means that a multiplier is applied to the standard error of such data points to indicate they are less certain values because they did not meet the reference criteria for study quality). The table below shows the fixed effect values of the x-covs which are in log space (as dismod uses an offset lognormal model) as well as the exponentiated values which for a x-cov can be interpreted as an odds ratio.

The covariate for low quality diagnostic criteria s had non-significant coefficients as a x-cov and were subsequently used as a z-cov.

Study covariate	Parameter	beta	Exponentiated beta
Low quality survey method and type of interviewer	Prevalence	-0.43	0.65 (0.53 — 0.79)
low quality sampling method	Prevalence	1.00	2.72 (2.17 — 3.40)
Low quality validation diagnostic instrument	Prevalence	0.63	1.88 (1.72 — 2.07)
Other than one year recall period	Prevalence	-0.20	0.82 (0.70 — 0.98)
Poor response	Prevalence	-0.38	0.69 (0.60 — 0.79)
Claims data US 2000	Prevalence	-4.39	0.012 (0.012 — 0.013)
Claims data US 2010	Prevalence	-3.99	0.018 (0.018 — 0.019)
Claims data US 2012	Prevalence	-3.89	0.020 (0.020 — 0.021)

The very low coefficients in claims data mean that few cases of TTH are included in claims data. Data points were crosswalked up by a factor 50 or more. We decided to include the data with such large crosswalks as we had no other data for the states of the US and the crosswalks estimated by dismod were within range of the data from three US studies in Massachusetts, Maryland and Kentucky

Medication overuse headache

Flowchart

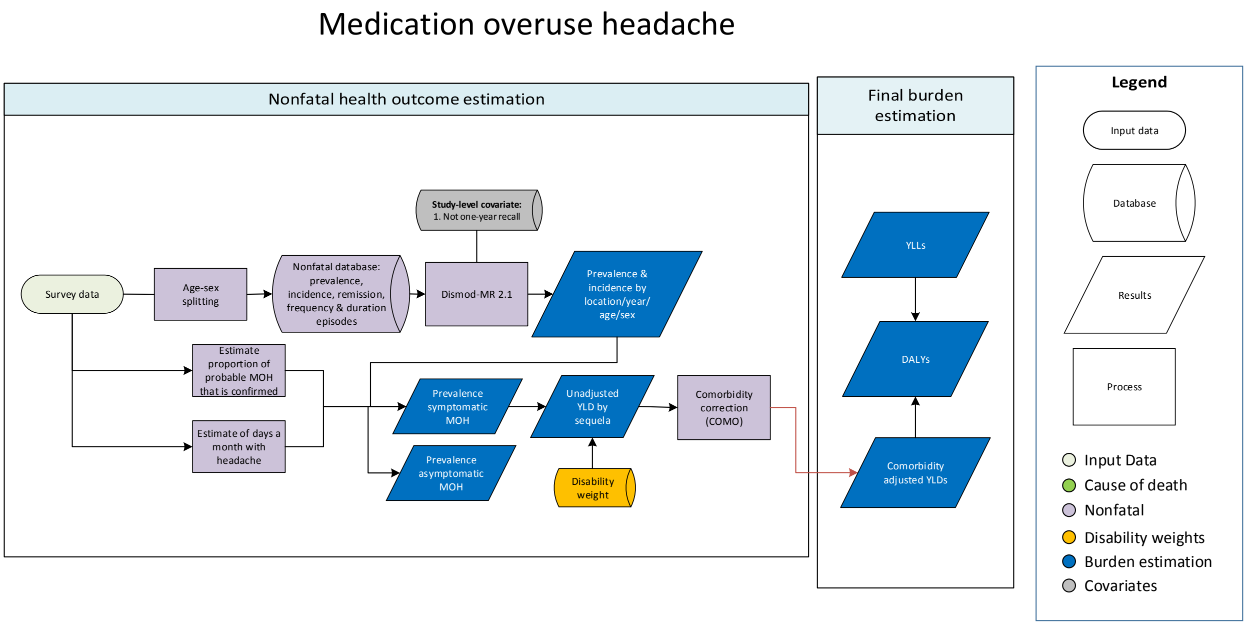

Case Definition

The diagnostic criteria (The International Classification of Headache Disorders, 3rd edition - beta version) for MOH are:

A. Headache present on ≥15 days/month fulfilling criteria C and D

B. Regular overuse (i.e. >2 days per week) for ≥3 months of one or more drugs that can be taken for acute and/or symptomatic treatment of headache

C. Headache has developed or markedly worsened during medication overuse

D. Headache resolves or reverts to its previous pattern within 2 months after discontinuation of overused medication

It also explicitly states that if a person qualifies for chronic migraine or chronic TTH as well as MOH, both diagnoses should be given. However, our headache GBD collaborators, Steiner and Stovner, say that in survey practice, a screening question on chronic headache is used first, followed by questions to determine if medication overuse headache is probable (i.e. fitting all criteria but criterion D).

Only one study was able to meet criterion D making a final diagnosis after a trial of detoxification. Of 25 cases with probably MOH, 15 were confirmed as MOH.

The headache survey in Russia reports an average frequency of 23.1 (SD 6.7; calculated SE 0.46) days with headache per month in people with chronic headache and report that over 2/3 of these are MOH.

Input data

Model Inputs

A systematic review was conducted for GBD2010 and updated for GBD2013. For GBD2015 three new representative surveys conducted by GBD collaborators in Norway, Karnataka, india and Nepal were added. Inclusion criteria of the systematic reviews were:

- Representative, population-based surveys
- Reporting of prevalence of medication overuse headache

	Prevalence	Incidence	Remission
Studies	23	0	0
Countries/subnational locations	19	0	0
GBD world regions	7	0	0

Sequelae Splits

The headache survey in Russia (Ayzenberg 2012) reports an average frequency of 23.1 (SD 6.7; calculated SE 0.46) days with headache per month in people with chronic headache and report that over 2/3 of these are MOH.

	Lay description	DW (95% CI)
Medication overuse headache	This person has daily headaches, felt as dull pain and often lasting all day, with poor sleep, nausea and fatigue. The person takes medicine for the headaches, which provides little relief but is needed to avoid having worse symptoms.	0.217 (0.138 – 0.311)

Modeling Strategy

We used a list of binary covariates which are modified version of quality indicators of epidemiological studies on headache (add ref: Steiner TJ, Stovner LJ et al (2013). Improving quality in population surveys of headache prevalence, burden and cost: key methodological considerations. J Headache Pain, 14: 87) and shown in the table below.

Study covariate	Notation	
	Less desirable (1)	Reference (zero)
Other than one-year recall period	Point prevalence	One-year prevalence
Not representative	selected population	general population or community-based sample from whole country OR general population or community-based sample from defined region within a country, or school-based (for children)

Low quality sampling method	not stated OR no (or failed) attempt to secure representativeness	total defined population, or random sample corrected for population demographics OR random sample uncorrected for population demographics
Poor response	not stated, or <70%	70-100%
Low quality survey method and type of interviewer	not stated OR self-administered (unsupervised) questionnaire OR telephone or face-to-face interview by untrained or unspecified interviewer(s)	face-to-face interview with headache expert
Low quality validation of diagnostic instrument	instrument not specified or not validated OR validated, but sensitivity and/or specificity <70% OR validated only in screen-positive sub-sample, or in clinic or unspecified sample, but sensitivity and specificity ≥70%	validated in target population or similar, and sensitivity and specificity ≥70%, or all diagnoses made in face-to-face or telephone interviews by headache expert
Low quality diagnostic criteria	not stated OR stated, other than ICHD OR ICHD (or reasonable modification), but uncertain or inappropriate analysis of "probable" diagnoses	ICHD (or reasonable modification) with clear exposition regarding "probable" diagnoses

Prior settings in the DisMod model included setting incidence to 0 before age 5 based on expert advice. We also assume no excess mortality due to TTH. In the absence of data on remission we set bounds between 0 and 1, thus ensuring that the average duration is at least one year.

All study covariates were initially evaluated as x-cov (which means that data points are adjusted to the reference value if a systematic bias is detected); those that did not have a significant coefficient, were entered as z-cov (which means that a multiplier is applied to the standard error of such data points to indicate they are less certain values because they did not meet the reference criteria for study quality). The table below shows the fixed effect values of the x-covs which are in log space (as dismod uses an offset lognormal model) as well as the exponentiated values which for a x-cov can be interpreted as an odds ratio.

The covariate for recall period was the only one with a significant coefficient x-cov. The others were subsequently used as a z-cov.

Study covariate	Parameter	beta	Exponentiated beta
Other than one year recall period	Prevalence	-0.16	0.85 (0.62 — 1.23)

To the prevalence output from dismod we first apply the finding from da Silva (2010) that 60% (40.8-79.2%) of 'probable' cases were confirmed cases of MOH. Second, we estimate the proportion of time 'symptomatic', i.e. with headache from the Ayzenberg (2012) estimate of 23.1 days a month with headache and multiply estimates by another 75.9% (72.9-78.8%).

Schizophrenia

Flowchart

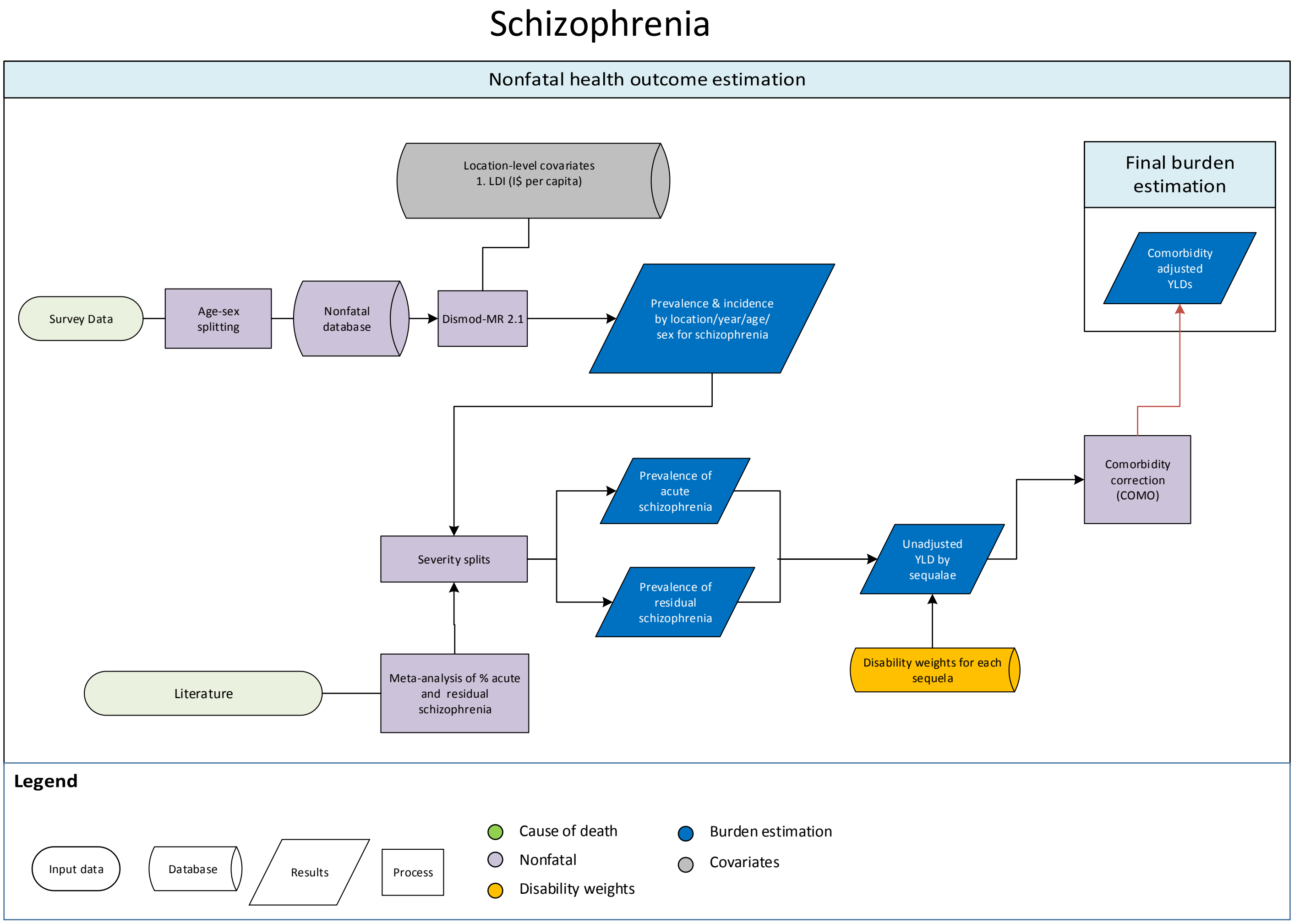

Input data and methodological summary

Case Definition

Schizophrenia is a chronic psychotic disorder which involves the experience of positive symptoms (e.g. delusions, hallucinations, thought disorder) and negative symptoms (e.g. flat affect, loss of interest and emotional withdrawal). Included in the GBD disease modeling, were cases meeting the Diagnostic and Statistical Manual of Mental Disorders (DSM-IV-TR) or the International Classification of Diseases (ICD-10) diagnostic criteria for schizophrenia (DSM: 295.10-295.30, 295.60, 295.90; ICD: F20.0-F20.3 F20.5-F20.9), [1, 2]. Diagnostic criteria include:

A. Two (or more) of the following, each present for a significant portion of time during a 1-month period (or less if successfully treated):

    1. Delusions
    2. Hallucinations
    3. Disorganized speech
    4. Grossly disorganized or catatonic behavior
    5. Negative symptoms

B. Social/occupational dysfunction

C. Continuous signs of the disturbance persist for at least 6 months.

D. Exclusions must be met for schizoaffective and mood disorders, substance and general medical conditions, and a relationship to a pervasive development disorder.

Input data

Model Inputs

For GBD 2015 a systematic review of literature was conducted to capture studies of prevalence, incidence, remission, duration and excess mortality associated with schizophrenia. In summary, the search was conducted in 3 stages involving searches of the peer-reviewed literature (via Medline, Embase and PubMed), the grey literature and, expert consultation. The inclusion criteria stipulated that: (1) the publication year must be from 1980 onwards; (2) 'caseness' must be based on clinical threshold as established by the DSM or ICD; (3) sufficient information must be provided on study method and sample characteristics to assess the quality of the study; and (4) study samples must be representative of the general population (i.e. inpatient or pharmacological treatment samples, case studies, veterans or refugee samples were excluded). No limitation was set on the language of publication.

The final GBD 2015 epidemiological dataset contained 429 prevalence, 19 remission, 93 mortality and 187 incidence data points from 38 countries.

Severity splits

The basis of the GBD disability weight survey assessments are lay descriptions of sequelae highlighting major functional consequences and symptoms. The lay descriptions and disability weights for schizophrenia severity levels are shown below.

Severity level	Lay description	DW (95% CI)
acute state	hears and sees things that are not real and is afraid, confused, and sometimes violent. The person has great difficulty with communication and daily activities, and sometimes wants to harm or kill himself (or herself).	0.778 (0.606-0.9)

residual state	hears and sees things that are not real and has trouble communicating. The person can be forgetful, has difficulty with daily activities, and thinks about hurting himself (or herself).	0.588 (0.411-0.754)

Severity splits used in GBD 2015 were consistent to those used in GBD 2013 for schizophrenia. Information on the distribution of acute and residual states of schizophrenia was obtained from a separate systematic review of the literature [3]. Meta-XL (a Microsoft Excel add in for meta-analysis) was used to pool estimates across all studies to calculate the overall proportion of schizophrenia cases in each health state – acute 63% (29%-91%) and residual state 37% (9%-71%).

Modeling strategy

The GBD 2015 epidemiological modelling strategy for schizophrenia made use DisMod-MR 2.1 to estimate prevalence by age, sex, year, and country. Data across all epidemiological parameters were included in the modelling process. We assumed no incidence and prevalence before age 10 and after age 80. This minimum age of onset was corroborated with expert feedback and existing literature on schizophrenia. Remission was also restricted to a maximum of 0.04 as guided by data available in the dataset.

Study-level covariates can be used in DisMod-MR 2.1 to accommodate for between-study variability in the raw prevalence data. In GBD 2015 tested covariates failed to demonstrate significance resulting in a model without the inclusion of any covariates.

A country-level covariate, LDI, was also included. This covariate represents a moving average of gross domestic product (GDP) over time. LDI was also applied to excess mortality data with a negative relationship assumed.

References

1. American Psychiatric Association, *Diagnostic and Statistical Manual of Mental Disorders (DSM-IV-TR)*. 4th, Text Revision ed. 2000, Washington DC: American Psychiatric Association.

2. World Health Organization, *The ICD-10 Classification of Mental and Behavioural Disorders. Clinical descriptions and diagnostic guidelines.* 1992, World Health Organization: Geneva.

3. Ferrari, A.J., et al., *Health states for schizophrenia and bipolar disorder within the Global Burden of Disease 2010 Study.* Population Health Metrics, 2012. **10**(1): p. 16.

Alcohol dependence

Flowchart

Alcohol dependence

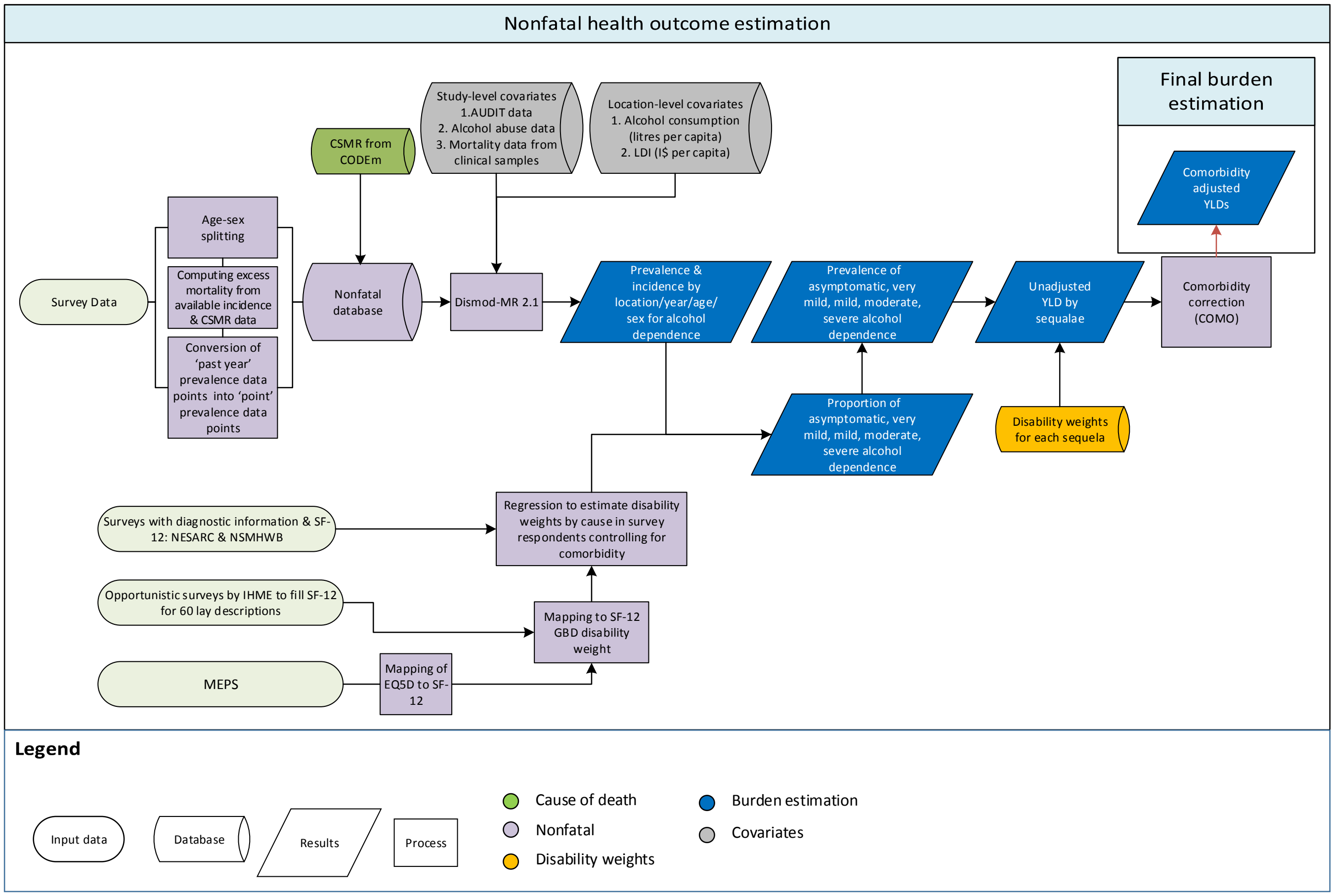

Input data and methodological summary

Case Definition

Alcohol dependence is a substance-related disorder involving a dysfunctional pattern of alcohol use. According to the Diagnostic and Statistical Manual of Mental Disorders (DSM-IV) criteria for alcohol dependence, at least three out of seven of the following criteria must be manifested during a 12 month period:

- Tolerance
- Withdrawal symptoms or clinically defined alcohol withdrawal syndrome
- Use in larger amounts or for longer periods than intended

- Persistent desire or unsuccessful efforts to cut down on alcohol use
- Time is spent obtaining alcohol or recovering from effects
- Social, occupational and recreational pursuits are given up or reduced because of alcohol use
- Use is continued despite knowledge of alcohol-related harm (physical or psychological)

The DSM-IV codes for alcohol dependence is 303.90 and the corresponding International Classification of Diseases (ICD-10) codes are F10.1 and F10.2 [1,2].

Input data

Model Inputs

In GBD2013 a systematic review of literature was conducted to capture studies of prevalence, incidence, remission, duration and excess mortality associated with alcohol dependence. In summary, the search was conducted in 3 stages involving searches of the peer-reviewed literature (via Medline, Embase and PubMed), the grey literature and, expert consultation. For GBD 2015, only stages two and three of the literature review were conducted, with another electronic database search due for alcohol dependence in the next iteration of GBD studies. The inclusion criteria stipulated that: (1) 'caseness' must be based on clinical threshold as established by the DSM and ICD; (2) sufficient information must be provided on study method and sample characteristics to assess the quality of the study; and (3) study samples must be representative of the general population (i.e. inpatient or pharmacological treatment samples (accepted for estimates of mortality), case studies, veterans or refugee samples were excluded).

The final GBD 2015 epidemiological dataset contained 809 prevalence, 13 remission, 96 mortality and 4 incidence data points from 50 countries.

In addition, United States Marketscan claims data for 2000, 2010 and 2012 by state, age, and sex were tested in the model. We decided not to include these data in the final model as we considered it would not be representative of true prevalence of mental and substance use disorders in the general population.

Severity splits

The basis of the GBD disability weight survey assessments are lay descriptions of sequelae highlighting major functional consequences and symptoms. The lay descriptions and disability weights for alcohol dependence severity levels are shown below.

Severity level	Lay description	DW (95% CI)
Very mild	drinks alcohol daily and has difficulty controlling the urge to drink. When sober, the person functions normally.	0.123<br>(0.082-0.177)

Mild	drinks a lot of alcohol and sometimes has difficulty controlling the urge to drink. While intoxicated, the person has difficulty performing daily activities.	0.235 (0.16-0.327)
Moderate	drinks a lot, gets drunk almost every week and has great difficulty controlling the urge to drink. Drinking and recovering cause great difficulty in daily activities, sleep loss, and fatigue.	0.373 (0.248-0.508)
Severe	gets drunk almost every day and is unable to control the urge to drink. Drinking and recovering replace most daily activities. The person has difficulty thinking, remembering and communicating, and feels constant pain and fatigue.	0.57 (0.396-0.732)

**asymptomatic cases carried no disability weight*

Severity splits used in GBD 2015 were consistent to those used in GBD 2013. The US National Epidemiological Survey on Alcohol and Related Conditions (NESARC, conducted in two waved from 2001-2002 and 2004-2005)[3] and the Australian National Survey of Mental Health and Wellbeing of Adults (NSMHWB, conducted in 1997) [4] were used to estimate the proportion of alcohol dependence cases in the asymptomatic 32.0% (25.3% to 40.0%); very mild 58.6% (51.4% to 65.3%), mild 3.8% (1.5% to 6.6%), moderate 3.3% (1.0% to 6.0%) and severe 2.4% (0.5% to 5.8%) disease categories.

Modeling strategy

The GBD 2015 epidemiological modelling strategy for alcohol dependence made use DisMod-MR 2.1 to estimate prevalence by age, sex, year, and country. Data across all epidemiological parameters were included in the modelling process. We assumed no incidence and mortality before age 10. An upper limit of 0.6 was placed on remission (in line with data from the US National Epidemiological Survey on Alcohol and Related Conditions (NESARC) as well as a declining trend with age to restrict DisMod-MR 2.1 from straying too far from the data inputs.

An adjustment was made outside of DisMod MR 2.1 to adjust past year prevalence estimates of alcohol dependence towards the level they would have been had the study measured point prevalence as the latter is less susceptible to recall bias. Given that remission from alcohol dependence (and hence, average disease duration) vary considerably with age, we also applied an age pattern to this adjustment that cannot be replicated within DisMod-MR 2.1 by use of covariates. The first step was to estimate the average duration by taking the inverse of remission. Next, we applied an adjustment factor from one-year to point prevalence using the following formula where average duration is expressed in years:

$$\text{adjustment factor} = \frac{\text{average duration}}{\text{average duration} + 1}$$

Age-specific adjustment factors were applied to all one-year prevalence estimates propagating sampling uncertainty around the prevalence and remission input data using the Ersatz software add-in to Excel (www.epigear.com).

Within DisMod Mr 2.1, study-level covariates were used to accommodate for other sources of between-study variability in the raw prevalence data. Combined abuse and dependence prevalence estimates were crosswalked down towards dependence only estimates. Similarly, prevalence estimates using AUDIT were crosswalked down towards prevalence estimates from diagnostic (non-AUDIT) measures. A final covariate was applied to mortality data derived from clinical samples and resulted in an adjustment down towards mortality estimates derived from non-clinical samples.

Country-level covariates were also included. The LDI covariate represents a moving average of gross domestic product (GDP) over time. LDI was also applied to excess mortality data with a negative relationship assumed. Alcohol consumption was also represented by a covariate representing this in terms of litres of alcohol per capita.

We used the function in DisMod-MR 2.1 to pull in cause-specific mortality rate (CSMR) data from our CODEm and CODcorrect analyses and match it with prevalence data points for the same geography and study year to estimate priors on excess mortality rates (by dividing CSMR by prevalence).

Study/Country covariate	Parameter	beta	Exponentiated beta
cv_abuse and dependence	Prevalence	1.07 (0.45-1.60)	2.92 (1.56-4.96)
cv_AUDIT	Prevalence	1.56 (1.09-1.97)	4.76 (2.97-7.15)
cv_clinical samples	Standardised mortality ratio	0.86 (0.66-1.06)	2.35 (1.93-2.89)
cv_Alcohol (litres per capita)	Prevalence	0.36 (0.04-0.75)	1.43 (1.04-2.12)
cv_LDI (I$ per capita)	Excess mortality	-0.13 (-0.2- -0.1)	0.88 (0.82-0.90)

Changes from GBD2013

YLD estimates have decreased from GBD2013 as a result of changes in data and covariate effects. Changes included:

- GBD2013 employed lnASDR and CSMR data, in GBD2015 only CSMR data was incorporated.
- WMHS survey data was updated with adjusted data to account for ‘skip errors’ in some country surveys. The WMHS covariate became redundant and was excluded
- Alcohol (LPC) and LDI country covariates were introduced in GBD2015

References

1. American Psychiatric Association. Diagnostic and Statistical Manual of Mental Disorders (DSM-IV-TR). 4th, Text Revision ed. Washington DC: American Psychiatric Association; 2000.
2. World Health Organization. The ICD-10 Classification of Mental and Behavioural Disorders. Clinical descriptions and diagnostic guidelines. Geneva: World Health Organization; 1992.
3. Introduction to the National Epidemiologic Survey on Alcohol and Related Conditions [http://pubs.niaaa.nih.gov/publications/arh29-2/74-78.htm]. Access date 1 December 2014.
4. Australian Bureau of Statistics. National Survey of Mental Health and Wellbeing of Adults 1997. Canberra: Australian Bureau of Statistics.

Fetal alcohol syndrome

Flowchart

Fetal alcohol syndrome (FAS)

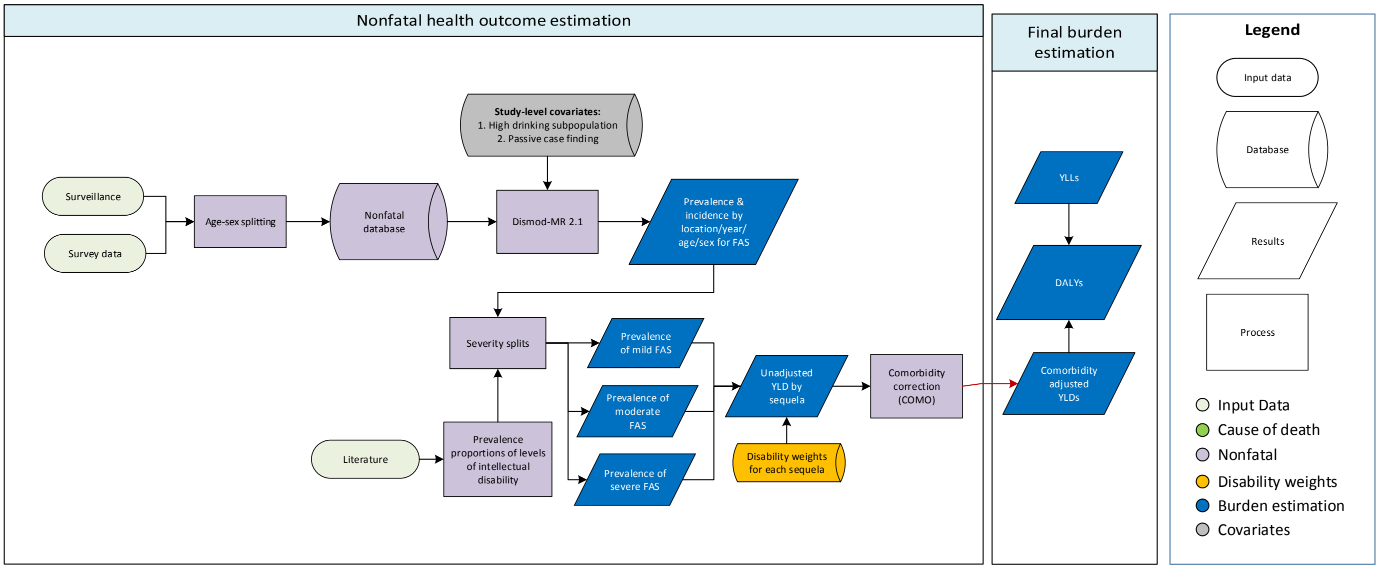

Input data and methodological summary

Case definition

Fetal alcohol syndrome (FAS; ICD-10: Q86.0) is a disorder caused by maternal drinking during pregnancy and is the most severe form of fetal alcohol spectrum disorder (FASD). In GBD, only FAS cases were included in the model. Other manifestations of FASD including partial fetal alcohol syndrome, alcohol-related neurodevelopmental disorder, and alcohol-related birth defects were not included. FAS is characterized by maternal alcohol exposure which results in certain patterns of facial anomalies such as short palpebral fissures and abnormalities in the premaxillary zone (e.g., flat upper lip, flattened philtrum, and flat midface), growth retardation (e.g., decelerating weight over time not due to nutrition), and central nervous system neurodevelopmental abnormalities (e.g., decreased cranial size at birth) in the offspring.[1] Cases were defined according to diagnostic guidelines set by the US institute of Medicine, the British Pediatric Association, and other recognized bodies in the area.

Input data

Model inputs

A series of systematic literature reviews were conducted to capture studies reporting the prevalence, incidence, remission, and excess mortality of FAS. The reviews incorporated searches of peer-reviewed literature via electronic databases and consultation with experts. In order for a study to be included, it must use recognized classifications of FAS (e.g., the US Institute of Medicine) and provide sufficient

details on study methodology and sample characteristics to determine study quality. While studies representative of the general population were preferred, studies of FAS in indigenous and minority communities were also accepted based on expert advice. No limitation was set on the language of publication. Data from the European Surveillance of Congenital Anomalies (EUROCAT) were also included and updated where relevant for GBD 2015. The final dataset for GBD 2015 included 177 prevalence estimates and 13 excess mortality estimates (from studies of individuals with intellectual disability).

	Prevalence	Mortality
Studies	77	5
Countries/subnationals	55	4
GBD world regions	9	3

Severity split inputs

There were no data available which gave prevalence of FAS by severity. As such, severity splits for FAS were calculated by matching FAS severity to categories of IQ in children for which prevalence data is available. Severe FAS was matched to an IQ of less than 50, moderate FAS to an IQ of 50 to 69, mild FAS to an IQ of 74 to 84, and asymptomatic FAS to an IQ of 85 or higher. Prevalence data for these IQ levels were then used to calculate severity splits for FAS.

Severity level	Lay description	DW (95% CI)
Mild	Is a little slow in developing physically and mentally, which causes some difficulty in learning but no other difficulties in daily activities.	0.016 (0.008-0.03)
Moderate	Is slow in developing physically and mentally, which causes some difficulty in daily activities.	0.056 (0.035-0.083)
Severe	Is very slow in developing physically and mentally, which causes great difficulty in daily activities.	0.179 (0.119-0.257)

Modeling strategy

Prevalence was set to begin from birth. Incidence was set to zero given cases cannot manifest after birth (despite the fact they may not be diagnosed immediately at birth). Remission was also set to zero. A covariate was included in the model which addressed the heterogeneity introduced by different case-finding methods, i.e., active versus passive case-finding. In contrast to GBD 2013, all estimates from known high-drinking populations (e.g., indigenous populations) were excluded instead of applying a covariate to adjust such estimates towards non-high-drinking populations. These samples were not considered representative of the general population and, furthermore, the crosswalk on the relevant covariate was extremely large and was not considered a satisfactory approach to dealing with these estimates.

The table below illustrates the covariate, parameter, beta and exponentiated beta values for the model.

Study covariate	Parameter	beta	Exponentiated beta
Passive case finding	Prevalence	0.31 (0.011-1.23)	1.36 (1.01-3.41)

References

1. Stratton K, Howe C, Battaglia F, editors. Fetal alcohol syndrome. Diagnosis, epidemiology, prevention, and treatment. Washington DC: National Academy Press; 1996.

Opioid dependence

Flowchart

Opioid use disorders

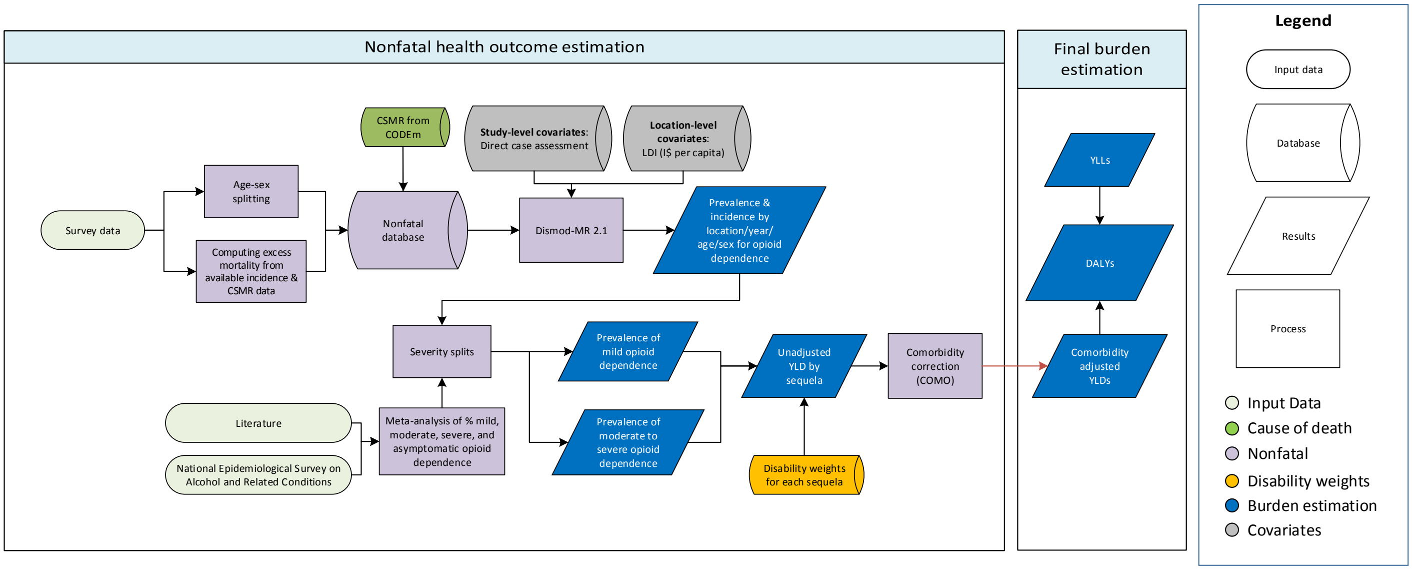

Case definition

Opioid dependence is a substance-related disorder involving a dysfunctional pattern of opioid use. Included in the GBD disease modeling were cases meeting the Diagnostic and Statistical Manual of Mental Disorders (DSM-IV-TR) or the International Classification of Diseases (ICD-10) diagnostic criteria for opioid dependence (DSM:304.00; ICD:F11.2), excluding those cases due to a general medical condition (American Psychiatric Association, 2000; World Health Organization, 1992).

Input data

Model inputs

A systematic review of the literature was conducted to capture studies of prevalence, incidence, remission, and excess mortality associated with opioid dependence. In summary, the search was conducted in 3 stages involving searches of the peer-reviewed literature (via Medline, Embase, and Pubmed), the grey literature, and expert consultation. The agreed-upon approach for mental and substance use disorders was to conduct electronic database searches on a rolling basis. All three stages of GBD 2010's literature review were repeated for GBD 2013 to capture additional data published up to 2013. For GBD 2015, only stages 2 and 3 of the literature review were conducted, with another electronic database search due for opioid dependence in the next iteration of GBD studies.

The inclusion criteria stipulated that 1) the publication year must be from 1980 onward; 2) "caseness" must be based on clinical threshold as established by the DSM or ICD; 3) sufficient information must be provided on study method and sample characteristics to assess the quality of the study; and (4) study

samples must be representative of the general population (i.e., inpatient or pharmacological treatment samples, case studies, veterans, or refugee samples were excluded). No limitation was set on the language of publication. Methods used for this systematic review have been reported in greater detail elsewhere (Degenhardt, et al., 2011; Calabria, et al., 2010).

The table below shows the number of studies included in GBD 2015, as well as the number of countries or subnational units and GBD world regions represented.

	Prevalence	Remission	Mortality
Studies	58	8	42
Countries/subnational geographies	32	9	20
GBD world regions	10	5	6

In addition, United States MarketScan claims data for 2000, 2010, and 2012 by state, age, and sex were tested in the model. We decided not to include these data in the final model as we considered it would not be representative of true prevalence of mental and substance use disorders in the general population. For opioid dependence, prevalence estimated from MarketScan data was extremely low and not comparable with community-representative estimates.

Severity splits

The basis of the GBD disability weight survey assessments is lay descriptions of sequelae highlighting major functional consequences and symptoms. The lay descriptions and disability weights for opioid dependence severity levels are shown below.

Severity level	Lay description	DW (95% CI)
Mild	Uses heroin (or methadone) daily and has difficulty controlling the habit. When not using, the person functions normally.	0.335(0.221-0.473)
Moderate to severe	Uses heroin daily and has difficulty controlling the habit. When the effects wear off, the person feels severe nausea, agitation, vomiting, and fever. The person has a lot of difficulty in daily activities.	0.697 (0.510-0.843)

Based on available data (Shand, Slade, Degenhardt, Baillie, & Nelson, 2011; Shand, Degenhardt, Slade, & Nelson, 2011), the estimated distribution of opioid dependent cases by severity were asymptomatic (16%, 13%–19%), mild (37%, 20%–55%), and moderate/severe (47%, 29%–64%).

Modeling strategy

We ran a DisMod-MR 2.1 model to produce estimates by age, sex, year, and country. We assumed no incidence and excess mortality before age 15. This minimum age of onset was corroborated with expert feedback and existing literature on opioid dependence. We also assumed no incidence after age 64 as supported by data from various sources including the European Monitoring Centre for Drugs and Drug Addiction (European Montioring Centre for Drugs and Drug Addiction, 2014). An upper limit of 0.2 was placed on remission consistent with limits in the dataset. The inclusion of CSMR was a first in GBD 2015 as

improvements to the CODEm modeling strategy allowed the data to better inform the DisMod-MR 2.1 model.

The opioid dependence dataset included data points using "direct" or "indirect" survey methods. "Direct" methods of measuring opioid dependence predominantly involve surveys of the general population that ask if respondents use or are dependent on opioid. Surveys tend to underestimate the prevalence of the most harmful and stigmatized forms of illicit drug use in ways that probably vary between countries and cultures (Reuter & Trautmann, 2009). "Indirect" methods are considered superior; they use different sources of data to indirectly estimate the total number of drug users (methods include "multiplier methods," back projection and capture-recapture methods). Due to insufficient data on dependence from indirect survey methods (considered to be the gold standard for GBD purposes), estimates derived from direct survey methods were included in the modeling. The cv_direct covariate was then used to adjust for whether a direct or indirect survey method was used. A crosswalk was estimated to convert all dependence estimates obtained via direct methods in the dataset, into its equivalent value if the study had measured dependence estimates obtained via indirect methods. A direct: indirect dependence ratio of 0.39 (0.22-0.78) was calculated by DisMod-MR 2.1 based on comparable direct and indirect dependence estimates in the dataset.

Betas and exponentiated values (which can be interpreted as an odds ratio) for each study level covariate are shown in the table below:

Study covariate	Parameter	beta	Exponentiated beta
cv_direct	Prevalence	-0.95 (-1.53 — -0.25)	0.39 (0.22 — 0.78)

References

American Psychiatric Association. (2000). Diagnostic and Statistical Manual of Mental Disorders (DSM-IV-TR). 4th, Text Revision ed. Washington DC: American Psychiatric Association.

Calabria, B., Degenhardt, L., Briegleb, C., Vos, T., Hall, W., Lynskey, M., & et al. (2010). Systematic review of prospective studies investigating "remission" from amphetamine, cannabis, cocaine or opioid dependence. *Addictive Behaviors*.

Degenhardt, L., Bucello, C., Calabria, B., Nelson, P., Roberts, A., Hall, W., & et al. (2011). What data are available on the extent of illicit drug use and dependence globally? Results of four systematic reviews. *Drug and alcohol dependence*.

European Montioring Centre for Drugs and Drug Addiction. (2014). Lisbon, Portugal: EMCDDA.

Reuter, P., & Trautmann, F. (2009). *A Report on Global Illicit Drugs Markets 1998-2007.* Utrecht: Trimbos Institute.

Shand, F. L., Degenhardt, L., Slade, T., & Nelson, E. C. (2011). Sex differences amongst dependent heroin users: Histories, clinical characteristics and predictors of other substance dependence. *Addictive behaviors, 36*(1), 27-36.

Shand, F. L., Slade, T., Degenhardt, L., Baillie, A., & Nelson, E. C. (2011). Opioid dependence latent structure: two classes with differing severity? *Addiction, 106*(3), 590-8.

World Health Organization. (1992). *The ICD-10 Classification of Mental and Behavioural Disorders. Clinical descriptions and diagnostic guidelines.* Geneva: World Health Organization.

Cocaine dependence

Flowchart

Cocaine use disorders

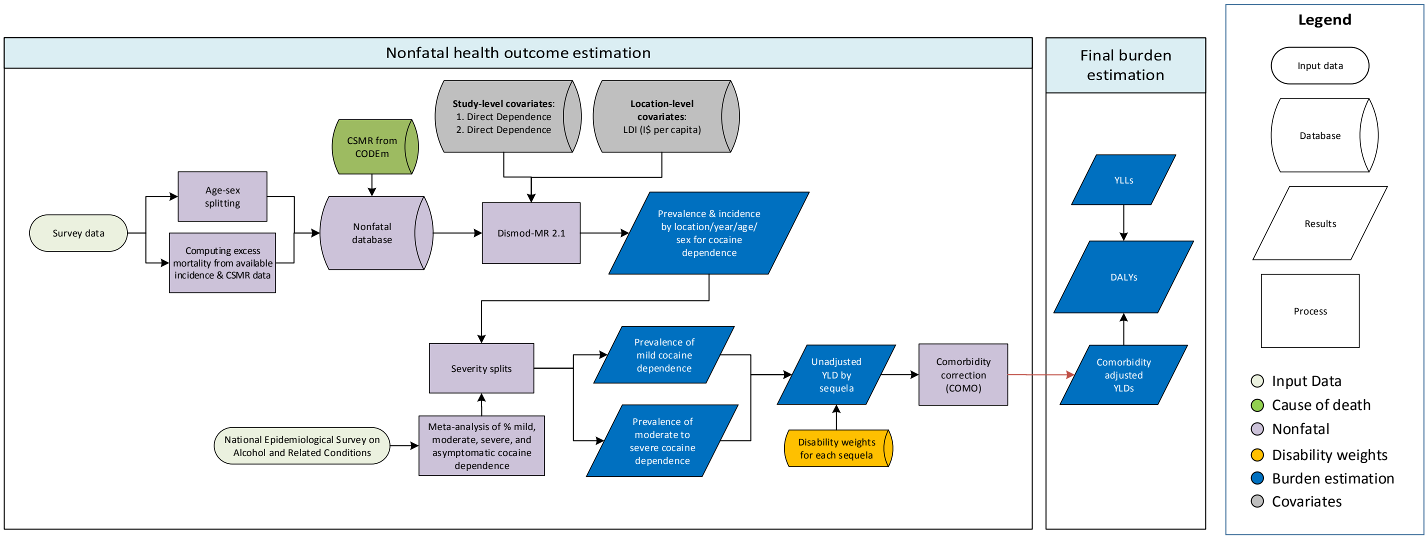

Case definition

Cocaine dependence is a substance-related disorder involving a dysfunctional pattern of cocaine use. Included in the GBD disease modeling were cases meeting the Diagnostic and Statistical Manual of Mental Disorders (DSM-IV-TR) or the International Classification of Diseases (ICD-10) diagnostic criteria for cocaine dependence (DSM:304.20; ICD:F14.2), excluding those cases due to a general medical condition (American Psychiatric Association, 2000; World Health Organization, 1992).

Input data

Model inputs

A systematic review of the literature was conducted to capture studies of prevalence, incidence, remission, and excess mortality associated with cocaine dependence. In summary, the search was conducted in three stages involving searches of the peer-reviewed literature (via Medline, Embase, and Pubmed), the grey literature, and expert consultation. The agreed-upon approach for mental and substance use disorders was to conduct electronic database searches on a rolling basis. All three stages of GBD 2010's literature review were repeated for GBD 2013 to capture additional data published up to 2013. For GBD 2015, only stages 2 and 3 of the literature review were conducted, with another electronic database search due for cocaine dependence in the next iteration of GBD studies.

The inclusion criteria stipulated that 1) the publication year must be from 1980 onward; 2) "caseness" must be based on clinical threshold as established by the DSM or ICD; 3) sufficient information must be provided on study method and sample characteristics to assess the quality of the study; and (4) study

samples must be representative of the general population (i.e., inpatient or pharmacological treatment samples, case studies, veterans or refugee samples were excluded). No limitation was set on the language of publication. Methods used for this systematic review have been reported in greater detail elsewhere (Degenhardt, et al., 2011; Calabria, et al., 2010).

The table below shows the number of studies included in GBD 2015, as well as the number of countries or subnational units and GBD world regions represented.

	Prevalence	Remission	Mortality
Studies	91	3	7
Countries/subnational geographies	43	3	7
GBD world regions	9	2	3

In addition, United States MarketScan claims data for 2000, 2010, and 2012 by state, age, and sex were tested in the model. We decided not to include these data in the final model as we considered it would not be representative of true prevalence of mental and substance use disorders in the general population. For cocaine dependence, prevalence estimated from MarketScan data was extremely low and not comparable with community-representative estimates.

Severity splits

The basis of the GBD disability weight survey assessments are lay descriptions of sequelae highlighting major functional consequences and symptoms. The lay descriptions and disability weights for cocaine dependence severity levels are shown below.

Severity level	Lay description	DW (95% CI)
Mild	Uses cocaine at least once a week and has some difficulty controlling the habit. When not using, the person functions normally.	0.116 (0.074-0.165)
Moderate to severe	Uses cocaine and has difficulty controlling the habit. The person sometimes has mood swings, anxiety, paranoia, hallucinations, and sleep problems, and has some difficulty in daily activities.	0.479 (0.324-0.634)

Based on available data, the estimated distribution of cocaine dependent cases by severity were asymptomatic (50%, 37%–64%), mild (25%, 18%–33%), and moderate/severe (25%, 17%–33%).

Modeling strategy

We ran a DisMod-MR 2.1 model to produce estimates by age, sex, year, and country. We assumed no incidence and excess mortality before age 15. This minimum age of onset was corroborated with expert feedback and existing literature on cocaine dependence. We also assumed no incidence after age 64 as supported by data from various sources including the European Monitoring Centre for Drugs and Drug Addiction (European Montioring Centre for Drugs and Drug Addiction, 2014). An upper limit of 0.06 was placed on remission consistent with limits in the dataset. The inclusion of CSMR was a first in GBD 2015 as improvements to the CODEm modeling strategy allowed the data to better inform the DisMod-MR 2.1 model.

The cocaine dependence dataset included data points of both use and dependence estimated using "direct" or "indirect" survey methods. "Direct" methods of measuring amphetamine dependence predominantly involve surveys of the general population that ask if respondents use or are dependent on amphetamine. Surveys tend to underestimate the prevalence of the most harmful and stigmatized forms of illicit drug use in ways that probably vary between countries and cultures (Reuter & Trautmann, 2009). "Indirect" methods are considered superior; they use different sources of data to indirectly estimate the total number of drug users (methods include "multiplier methods," back projection and capture-recapture methods). Due to the lack of data available on cocaine dependence from indirect survey methods (considered to be the gold standard for GBD purposes), estimates of use and/or estimates from direct survey methods were included in the modeling.

Study-level covariates were then used to accommodate for between-study variability in the raw prevalence data. The cv_direct use covariate was used to adjust for whether direct or indirect survey methods were used. This converted all use estimates obtained via direct methods in the dataset into their equivalent value if the study had measured dependence estimates obtained via indirect methods. A ratio of direct use: indirect dependence was calculated by comparing similar direct use and indirect dependence estimates in the dataset. To allow for meaningful comparisons, paired direct use and indirect dependence estimates needed to be similar in terms of the country they were from, year, age group, sex, and prevalence type. To maximize the number of data points available for this ratio, paired estimates for psychostimulants (i.e., both cocaine and amphetamine) were used.

Once a dataset was set up with paired direct use and indirect dependence estimates, MetaXL (a meta-analysis add-in for Microsoft Excel) was utilized to estimate a ratio of direct use: indirect dependence, whereby direct use estimates were found to be 3.6 (2.6–5.2) times higher than indirect dependence estimates. This ratio was used in DisMod-MR 2.1 to adjust all use estimates in the dataset downward, toward the level they would have been had the study reported indirect dependence. A similar method was used to adjust prevalence estimates of cocaine dependence obtained via direct methods toward the level they would have been had the study measured cocaine dependence using indirect methods. The estimated ratio of direct dependence: indirect dependence was 0.5 (0.2–1.1).

Betas and exponentiated values (which can be interpreted as an odds ratio) for each study level covariate are shown in the table below:

Study covariate	Parameter	beta	Exponentiated beta
cv_direct use	Prevalence	1.29 (0.95—1.64)	3.64 (2.57—5.16)
cv_direct dep	Prevalence	-0.68 (-1.42—.06)	0.50 (0.24—1.06)

References

American Psychiatric Association. (2000). Diagnostic and Statistical Manual of Mental Disorders (DSM-IV-TR). 4th, Text Revision ed. Washington DC: American Psychiatric Association.

Calabria, B., Degenhardt, L., Briegleb, C., Vos, T., Hall, W., Lynskey, M., & et al. (2010). Systematic review of prospective studies investigating "remission" from amphetamine, cannabis, cocaine or opioid dependence. *Addictive Behaviors*.

Degenhardt, L., Bucello, C., Calabria, B., Nelson, P., Roberts, A., Hall, W., & et al. (2011). What data are available on the extent of illicit drug use and dependence globally? Results of four systematic reviews. *Drug and alcohol dependence*.

European Montioring Centre for Drugs and Drug Addiction. (2014). Lisbon, Portugal: EMCDDA.

Reuter, P., & Trautmann, F. (2009). *A Report on Global Illicit Drugs Markets 1998-2007.* Utrecht: Trimbos Institute.

World Health Organization. (1992). *The ICD-10 Classification of Mental and Behavioural Disorders. Clinical descriptions and diagnostic guidelines.* Geneva: World Health Organization.

Amphetamine dependence

Flowchart

Amphetamine use disorders

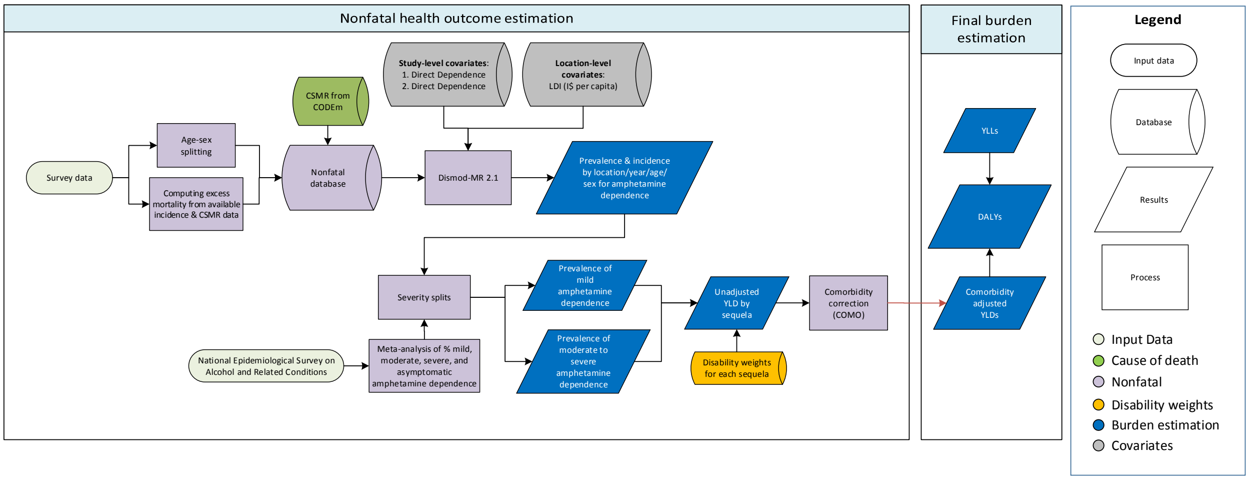

Case definition

Amphetamine dependence is a substance-related disorder involving a dysfunctional pattern of amphetamine use. Included in the GBD 2015 disease modeling were cases meeting the Diagnostic and Statistical Manual of Mental Disorders (DSM-IV-TR) or the International Classification of Diseases (ICD-10) diagnostic criteria for amphetamine dependence (DSM:304.40; ICD:F15.2), excluding those cases due to a general medical condition (American Psychiatric Association, 2000; World Health Organization, 1992).

Input data

Model inputs

A systematic review of the literature was conducted to capture studies of prevalence, incidence, remission, and excess mortality associated with amphetamine dependence. In summary, the search was conducted in three stages involving searches of the peer-reviewed literature (via Medline, Embase, and Pubmed), the grey literature, and expert consultation. The agreed-upon approach for mental and substance use disorders was to conduct electronic database searches on a rolling basis. All three stages of GBD 2010's literature review were repeated for GBD 2013 to capture additional data published up to 2013. For GBD 2015, only stages 2 and 3 of the literature review were conducted, with another electronic database search due for amphetamine dependence in the next iteration of GBD studies.

The inclusion criteria stipulated that 1) the publication year must be from 1980 onward; 2) "caseness" must be based on clinical threshold as established by the DSM or ICD; 3) sufficient information must be provided on study method and sample characteristics to assess the quality of the study; and (4) study samples must be representative of the general population (i.e., inpatient or pharmacological treatment samples, case studies, veterans or refugee samples were excluded). No limitation was set on the language

of publication. The methods used for this systematic review have been reported in greater detail elsewhere (Degenhardt, et al., 2011; Calabria, et al., 2010).

The table below shows the number of studies included in GBD 2015, as well as the number of countries or subnational units and GBD world regions represented.

	Prevalence	Remission	Mortality
Studies	75	1	6
Countries/subnational geographies	55	1	6
GBD world regions	13	1	3

In addition, United States MarketScan claims data for 2000, 2010, and 2012 by state, age, and sex were tested in the model. We decided not to include these data in the final model as we considered it would not be representative of true prevalence of mental and substance use disorders in the general population. For amphetamine dependence, prevalence estimates from MarketScan data were extremely low and not comparable with community-representative estimates.

Severity splits

The basis of the GBD disability weight survey assessments are lay descriptions of sequelae highlighting major functional consequences and symptoms. The lay descriptions and disability weights for amphetamine dependence severity levels are shown below.

Severity level	Lay description	DW (95% CI)
Mild	Uses stimulants (drugs) at least once a week and has some difficulty controlling the habit. When not using, the person functions normally.	0.079 (0.051-0.114)
Moderate to severe	Uses stimulants (drugs) and has difficulty controlling the habit. The person sometimes has depression, hallucinations and mood swings, and has difficulty in daily activities.	0.486 (0.329-0.637)

Based on available data, the estimated distribution of amphetamine dependent cases by severity were asymptomatic (55%, 40%–71%), mild (19%, 12%–27%), and moderate/severe (26%, 16%–35%).

Modeling strategy

We ran a DisMod-MR 2.1 model to produce estimates by age, sex, year, and country. We assumed no incidence and excess mortality before age 15. This minimum age of onset was corroborated with expert feedback and existing literature on amphetamine dependence. We also assumed no incidence after age 64 as supported by data from various sources including the European Monitoring Centre for Drugs and Drug Addiction (European Montioring Centre for Drugs and Drug Addiction, 2014). An upper limit of 0.1 was placed on remission consistent with limits in the dataset. The inclusion of CSMR was a first in GBD 2015 as improvements to the CODEm modelling strategy allowed the data to better inform the DisMod-MR 2.1 model.

The amphetamine dependence dataset included data points of both use and dependence estimated using "direct" or "indirect" survey methods. "Direct" methods of measuring amphetamine dependence predominantly involve surveys of the general population that ask if respondents use or are dependent on amphetamine. Surveys tend to underestimate the prevalence of the most harmful and stigmatized forms of illicit drug use in ways that probably vary between countries and cultures (Reuter & Trautmann, 2009). "Indirect" methods are considered superior; they use different sources of data to indirectly estimate the total number of drug users (methods include "multiplier methods," back projection and capture-recapture methods). Due to the lack of data available on amphetamine dependence from indirect survey methods (considered to be the gold standard for GBD purposes), estimates of use and/or estimates from direct survey methods were included in the modeling.

Study-level covariates were then used to accommodate for between-study variability in the raw prevalence data. The cv_direct use covariate was used to adjust for whether direct or indirect survey methods were used. This converted all use estimates obtained via direct methods in the dataset into their equivalent value if the study had measured dependence estimates obtained via indirect methods. A ratio of direct use: indirect dependence was calculated by comparing similar direct use and indirect dependence estimates in the dataset. To allow for meaningful comparisons, paired direct use and indirect dependence estimates needed to be similar in terms of the country they were from, year, age group, sex, and prevalence type. To maximize the number of data points available for this ratio, paired estimates for psychostimulants (i.e., both cocaine and amphetamine) were used.

Once a dataset was set up with paired direct use and indirect dependence estimates, MetaXL (a meta-analysis add-in for Microsoft Excel) was utilized to estimate a ratio of direct use: indirect dependence, whereby direct use estimates were found to be 3.6 (2.6–5.2) times higher than indirect dependence estimates. This ratio was used in DisMod-MR 2.1 to adjust all use estimates in the dataset downward, toward the level they would have been had the study reported indirect dependence. A similar method was used to adjust prevalence estimates of amphetamine dependence obtained via direct methods toward the level they would have been had the study measured amphetamine dependence using indirect methods. The estimated ratio of direct dependence: indirect dependence was 0.5 (0.2–1.1).

Betas and exponentiated values (which can be interpreted as an odds ratio) for each study level covariate are shown in the table below:

Study covariate	Parameter	beta	Exponentiated beta
cv_direct use	Prevalence	1.29 (0.95—1.64)	3.64 (2.57—5.16)
cv_direct dep	Prevalence	-0.68 (-1.42—.06)	0.50 (0.24—1.06)

References

American Psychiatric Association. (2000). Diagnostic and Statistical Manual of Mental Disorders (DSM-IV-TR). 4th, Text Revision ed. Washington DC: American Psychiatric Association.

Calabria, B., Degenhardt, L., Briegleb, C., Vos, T., Hall, W., Lynskey, M., & et al. (2010). Systematic review of prospective studies investigating "remission" from amphetamine, cannabis, cocaine or opioid dependence. *Addictive Behaviors*.

Degenhardt, L., Bucello, C., Calabria, B., Nelson, P., Roberts, A., Hall, W., & et al. (2011). What data are available on the extent of illicit drug use and dependence globally? Results of four systematic reviews. *Drug and alcohol dependence*.

European Montioring Centre for Drugs and Drug Addiction. (2014). Lisbon, Portugal: EMCDDA.

Reuter, P., & Trautmann, F. (2009). *A Report on Global Illicit Drugs Markets 1998-2007.* Utrecht: Trimbos Institute.

World Health Organization. (1992). *The ICD-10 Classification of Mental and Behavioural Disorders. Clinical descriptions and diagnostic guidelines.* Geneva: World Health Organization.

Cannabis dependence

Flowchart

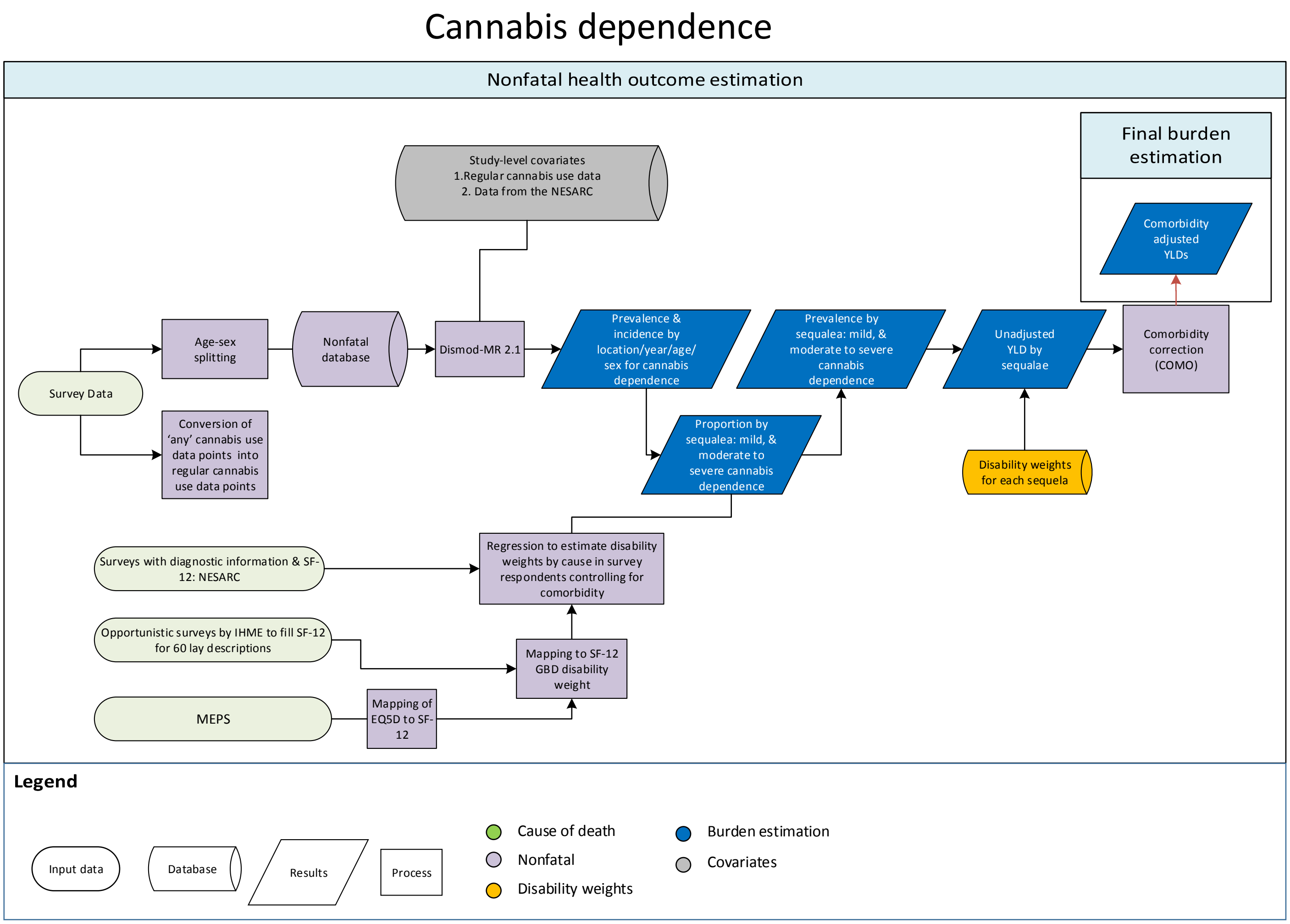

Case Definition

Cannabis dependence is a substance-related disorder involving a dysfunctional pattern of cannabis use. Included in GBD disease modelling were cases meeting diagnostic criteria for cannabis dependence according to the Diagnostic and Statistical Manual of Mental Disorders (DSM), or the equivalent diagnosis in the International Classification of Diseases (ICD). These were identified by the following codes: DSM:304.30, ICD:F12.2; excluding those cases due to a general medical condition [1,2].

According to DSM-IV-TR criteria, cannabis dependence involves a maladaptive pattern of cannabis use, leading to clinically significant impairment or distress. At least three of the following symptoms must be experienced within the same 12-month period:

- Tolerance, characterized by either;
    - a need for increased amounts of the substance to achieve intoxication; or
    - markedly diminished effect with continued use of the same amount of the substance;
- Withdrawal, characterized by either;
    - Withdrawal symptoms characteristic to cannabis dependence; or
    - the same (or similar)substance is taken to avoid withdrawal symptoms;
- substance taken in progressively larger amounts or for longer period;
- persistent desire or unsuccessful efforts to reduce substance use;
- disproportionate time dedicated to obtaining the substance;
- other important activities are given up because of the substance use; and
- substance use is continued despite knowledge of physical or psychological problems occurring as a result of the substance

Input data

Model Inputs

For GBD 2010, a systematic review of the literature was conducted to capture studies of prevalence, incidence, remission, and excess mortality associated with cannabis dependence. In summary, the search was conducted in three stages involving electronic searches of the peer-reviewed literature (via Medline, Embase and PubMed), the grey literature and, expert consultation. The agreed approach for mental and substance use disorders was to conduct electronic database searches on a rolling basis. All three stages of GBD 2010's literature review were repeated for GBD 2013 to capture additional data published up to 2013. For GBD 2015, only stages two and three of the literature review were conducted, with another electronic database search due for cannabis dependence in the next iteration of GBD studies.

The inclusion criteria stipulated that: (1) the publication year must be from 1980 onwards; (2) 'caseness' must be based on clinical threshold as established by the DSM or ICD; (3) sufficient information must be provided on study method and sample characteristics to assess the quality of the study; and (4) study samples must be representative of the general population (i.e. inpatient or pharmacological treatment samples, case studies, veterans or refugee samples were excluded). No limitation was set on the language of publication. Methods used for this systematic review have been reported in greater detail elsewhere [3-6]. The table below shows the number of studies included in GBD 2015, as well as the number of countries or subnational units and GBD world regions represented.

	Prevalence	Remission	Excess-Mortality
Studies	176	3	-
Countries/subnational geographies	151	3	-
GBD world regions	18	3	-

In addition, United States Marketscan claims data for 2000, 2010 and 2012 by state, age, and sex were tested in the model. We decided not to include these data in the final model as we considered it would not be representative of the true prevalence of mental and substance use disorders in the general population.

Severity Splits

The basis of the GBD disability weight survey assessments are lay descriptions of sequelae highlighting major functional consequences and symptoms. The lay descriptions and disability weights for cannabis dependence severity levels are shown below.

Severity level	Lay description	DW (95% CI)
Mild	Uses marijuana at least once a week and has some difficulty controlling the habit. When not using, the person functions normally.	0.039 (0.024-0.06)
Moderate to severe	Uses marijuana daily and has difficulty controlling the habit. The person sometimes has mood swings, anxiety and hallucinations, and has some difficulty in daily activities.	0.266 (0.178-0.364)

The US National Epidemiological Survey on Alcohol and Related Conditions (NESARC, conducted in two waves from 2001-2002 and 2004-2005) [7] was used to estimate the proportion of cannabis dependence cases asymptomatic (58%, 51%-63%), mild (36%, 31%-42%) and moderate to severe (6%, 4%-8%).

Modeling Strategy

The GBD 2015 epidemiological modelling strategy for cannabis dependence made use of DisMod-MR 2.1. Due to insufficient data, estimates of any cannabis use and regular (i.e. weekly) cannabis use were included in the disease modeling of cannabis dependence in a 2-step process. At step 1, a crosswalk was estimated to convert estimates of any use in the dataset into its equivalent value if the study had measured regular use. To do this a ratio of use: regular use was calculated by comparing similar regular use and use estimates in the dataset. To allow for meaningful comparisons, paired regular use and use estimates needed to be similar in terms of the country they were from, year, age group, sex and, prevalence type. Once a dataset was set up with paired regular use and use estimates, MetaXL (a meta-analysis add in for Microsoft Excel) was used to estimate a ratio of use: regular use whereby use estimates were found to be 2.9 (2.5-3.3) times higher than regular use estimates. This ratio was used to adjust all use estimates in the dataset downwards, towards the level they would have been had the study reported regular cannabis use. Step 2 involved the DisMod-MR 2.1 modeling of the regular cannabis use (from step 1) and cannabis dependence data. This cannabis regular use/dependence dataset was modeled using a study-level covariate which adjusted estimates of regular cannabis use towards the desirable which were estimates of cannabis dependence.

Study-level covariates were used to accommodate for between-study variability in the raw prevalence data. As mentioned previously, a cv_regular use covariate adjusted all regular use estimates towards the level they would have been if the study had measured cannabis dependence. This covariate was informed by a cannabis regular use: dependence ratio (4.1, 3.9-4.6) estimated outside of DisMod-MR 2.1 using the same methodology outlined above for the use: regular use ratio. For GBD 2015, the pooled cannabis regular use: dependence ratio changed from 6.8 (5.6-8.2) to 4.1 (3.9-4.6) with the addition of new regular use and dependence data from the United States, France and Canada. This lower ratio in GBD 2015 led to an increase in the prevalence (and therefore burden) of cannabis dependence compared to GBD 2013. Based on expert advice, a cv_nesarc covariate adjusted all estimates derived from the NESARC towards the level they would have been if they had been derived by other surveys. Drug use disorders are not well-captured in household surveys. This is especially an issue in NESARC as the sampling strategy used was biased

towards less severe cases of drug use disorders. Betas and exponentiated values (which can be interpreted as an odds ratio) for each study level covariate are shown in the table below.

Study covariate	Parameter	beta	Exponentiated beta
cv_regular use	Prevalence	1.4 (1.4 — 1.4)	4.1 (4.1 — 4.1)
cv_nesarc	Prevalence	-0.8 (-1.2 — -0.6)	0.4 (0.3 — 0.6)

References

1. American Psychiatric Association. Diagnostic and Statistical Manual of Mental Disorders (DSM-IV-TR). 4th, Text Revision ed. Washington DC: American Psychiatric Association; 2000.
2. World Health Organization. The ICD-10 Classification of Mental and Behavioural Disorders. Clinical descriptions and diagnostic guidelines. Geneva: World Health Organization; 1992.
3. Calabria B, Degenhardt L, Briegleb C, et al. Systematic review of prospective studies investigating "remission" from amphetamine, cannabis, cocaine or opioid dependence. *Addictive Behaviors* 2010; **35**(8): 741-9.
4. Calabria B, Degenhardt L, Hall W, Lynskey M. Does cannabis use increase the risk of death? Systematic review of epidemiological evidence on adverse effects of cannabis use. *Drug Alcohol Rev* 2010; **29**(3): 318-30.
5. Calabria B, Degenhardt L, Nelson P, et al. What do we know about the extent of cannabis use and dependence? Results of a global systematic review. Sydney: National Drug and Alcohol Research Centre, University of NSW, 2010.
6. Degenhardt L, Ferrari AJ, Calabria B, et al. The global epidemiology and contribution of cannabis use and dependence to the global burden of disease: results from the GBD 2010 study. *PloS one* 2013; **8**(10): e76635.
7. Introduction to the National Epidemiologic Survey on Alcohol and Related Conditions [http://pubs.niaaa.nih.gov/publications/arh29-2/74-78.htm]. Access date 1 December 2014.

Major depressive disorder

Flowchart

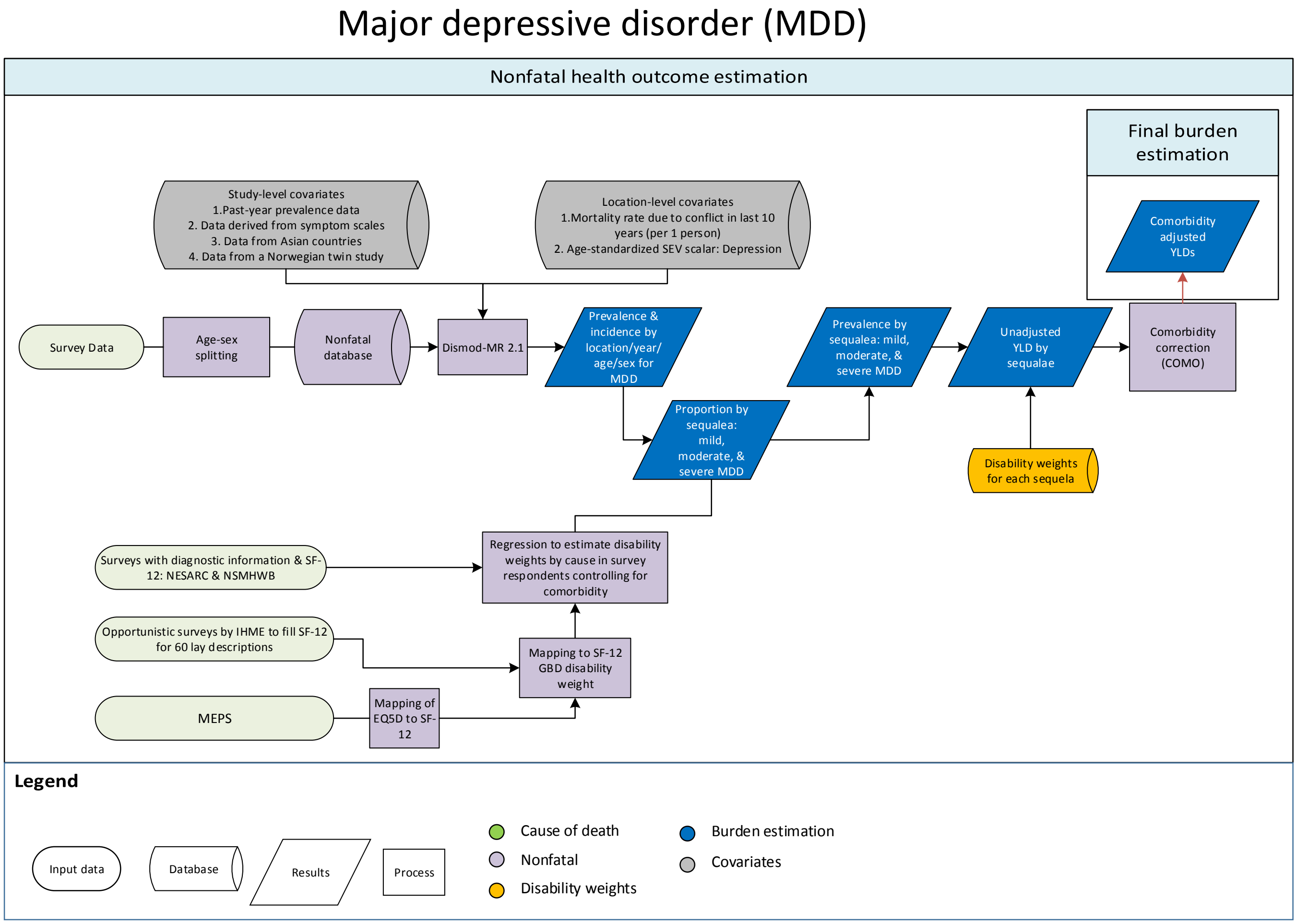

Case Definition

Major depressive disorder (MDD) is an episodic mood disorder involving the experience of one or more major depressive episode(s). Included in GBD disease modelling were cases meeting diagnostic criteria for MDD according to the Diagnostic and Statistical Manual of Mental Disorders (DSM) or the equivalent diagnosis of recurrent depression in the International Classification of Diseases (ICD). These were identified by the following codes: DSM-IV-TR: 296.21–24, 296.31–34; ICD-10: F32.0–9, F33.0–9; excluding those cases due to a general medical condition or substance induced cases [1,2].

According to DSM-IV-TR criteria, MDD involves the presence of at least one major depressive episode, which is the experience of depressed mood almost all day, every day, for at least two weeks. A total of five out nine criteria must be met to make a diagnosis and at least one of the five criteria should either be:

- 'depressed mood' for most of every day; or
- 'loss of interest in nearly all activities' for most of every day.

The other seven criteria are:

- change in eating, appetite or weight;
- excessive sleeping or insomnia;
- agitated or slow motor activity;
- fatigue;
- feeling worthless or inappropriately guilty;
- trouble concentrating; and
- repeated thoughts about death

MDD was modelled as an episodic disorder with the average length of a major depressive episode (i.e. duration) specified. This was consistent with previously proposed methodology for the modelling of MDD for burden of disease purposes [3-5].

Input data

Model Inputs

For GBD 2010, a systematic review of the literature was conducted to capture studies of prevalence, incidence, duration, and excess mortality associated with MDD. In summary, the search was conducted in three stages involving electronic searches of the peer-reviewed literature (via Medline, Embase and PubMed), the grey literature and, expert consultation. The agreed approach for mental and substance use disorders was to conduct electronic database searches on a rolling basis. All three stages of GBD 2010's literature review were repeated for GBD 2013 to capture additional data published up to 2013. For GBD 2015, only stages two and three of the literature review were conducted, with another electronic database search due for MDD in the next iteration of GBD studies.

The inclusion criteria stipulated that: (1) the publication year must be from 1980 onwards; (2) 'caseness' must be based on clinical threshold as established by the DSM or ICD; (3) sufficient information must be provided on study method and sample characteristics to assess the quality of the study; and (4) study samples must be representative of the general population (i.e. inpatient or pharmacological treatment samples, case studies, veterans or refugee samples were excluded). No limitation was set on the language of publication. Methods used for this systematic review have been reported in greater detail elsewhere [6-8]. The table below shows the number of studies included in GBD 2015, as well as the number of countries or subnational units and GBD world regions represented.

	Prevalence	Incidence	Duration	Excess-Mortality
Studies	214	4	5	19
Countries/subnational geographies	114	4	2	13
GBD world regions	16	2	2	5

In addition, United States Marketscan claims data for 2000, 2010 and 2012 by state, age, and sex were tested in the model. We decided not to include these data in the final model as we considered it would not be representative of the true prevalence of mental and substance use disorders in the general population.

Severity Splits

The basis of the GBD disability weight survey assessments are lay descriptions of sequelae highlighting major functional consequences and symptoms. The lay descriptions and disability weights for MDD severity levels are shown below.

Severity level	Lay description	DW (95% CI)
Mild	Feels persistent sadness and has lost interest in usual activities. The person sometimes sleeps badly, feels tired, or has trouble concentrating but still manages to function in daily life with extra effort.	0.145 (0.099-0.209)
Moderate	Has constant sadness and has lost interest in usual activities. The person has some difficulty in daily life, sleeps badly, has trouble concentrating, and sometimes thinks about harming himself (or herself).	0.396 (0.267-0.531)
Severe	Has overwhelming, constant sadness and cannot function in daily life. The person sometimes loses touch with reality and wants to harm or kill himself (or herself).	0.658 (0.477-0.807)

To determine the proportion of people with MDD within each of the severity levels, the US National Epidemiological Survey on Alcohol and Related Conditions (NESARC, conducted in two waves from 2001-2002 and 2004-2005) [9] and the Australian National Survey of Mental Health and Wellbeing of Adults (NSMHWB, conducted in 1997) [10] were used to estimate the proportion of MDD cases asymptomatic (13%, 10%-17%), mild (59%, 49%-69%), moderate (17%, 13%-22%), and severe (10%, 3%-20%).

Modeling Strategy

The GBD 2015 epidemiological modelling strategy for MDD made use of DisMod-MR 2.1. Data across all epidemiological parameters were initially included in the modelling process. However, given that the few incidence data points available typically excluded cases of MDD at baseline, new major depressive episodes in people with previous episodes were not counted and incidence was underestimated. For this reason, we chose to exclude all raw incidence data in the final model and instead, allowed Dismod-MR 2.1 to calculate incidence based on data from other parameters. We assumed no incidence and prevalence before age 3. This minimum age of onset was corroborated with expert feedback and existing MDD literature [6]. An average remission rate for a major depressive episode of 1.45 (1.3-1.6) was used. This was derived from the 4 longitudinal studies [11-14] fitting a lognormal curve with least squared differences to data on the proportion of incident cases still fulfilling the case definition for major depression at intervals over a one year period. As data were only available for a follow-up of one year, a decision had to be made about the maximum allowable duration of an episode. Setting this at 40 years, the average duration implied by the lognormal fit was 0.65 (0.59-0.70) of a year. [15].

Study-level covariates were used to accommodate between-study variability in the raw prevalence data. A cv_past year recall covariate adjusted all data points derived from past year prevalence towards the level they would have been if the study had captured point/past-month prevalence. The latter prevalence period is less affected by recall bias. A cv_symptom scale covariate adjusted all data points derived using a symptom scale towards the level they would have been if the scale had strictly adhered to DSM or ICD

thresholds for MDD. A cv_asian data points covariate was used to adjust all estimates from Asia using a ratio based on a study in China. Phillips and collaborators [16] reported that the prevalence of MDD in China was 2.07% while the prevalence of mood disorders not otherwise specified (NOS) was 2.06%. Of the 808 individuals diagnosed with mood disorders NOS, 467 (58%) met criteria for minor depression (defined by DSM-IV-TR as two to four of nine symptoms of depression lasting for ≥2 weeks). There is evidence to suggest that these reported cases of minor depression are likely misdiagnosed cases MDD as DSM/ICD diagnostic criteria are not sensitive to cross-cultural presentations of MDD in Asia [16-19]. Based on this, a ratio of MDD + minor depression: MDD only (1.53, 1.45-1.63) was derived from data presented by Phillips and collaborators and used to adjust all prevalence estimates from Asia in the model. The aim of this adjustment was not to capture sub-syndromal depression but instead, to pick up on diagnoses of MDD where there is evidence to suggest that the use of Western-based criteria have underestimated prevalence. Finally, a cv_NOR_twin_study covariate was used to flag estimates from a specific study conducted in Norway. Prevalence estimates generated from this study were derived from a twin sample rather than a sample representative of the general population. They were adjusted towards the level of other prevalence data points from Norway in the dataset.

Country-level covariates were also included in the MDD model. Mortality Rates Due to Conflict in Last 10 Years (per 1 person) informed the estimation of prevalence given existing evidence to show a positive association between conflict status and the prevalence of MDD [20,21]. An age-standardized SEV scalar was also included. This made use of the fraction of MDD burden caused by its relevant risk factors combined to inform the estimation of prevalence. Intimate partner violence and childhood sexual violence are the two established risk factors of MDD for which attributable burden is estimated in GBD 2015. Betas and exponentiated values (which can be interpreted as an odds ratio) for each study- and country-level covariate are shown in the table below:

Study/Country covariate	Parameter	beta	Exponentiated beta
cv_asian datapoints	Prevalence	-0.4 (-0.5 — -0.4)	0.7 (0.6 — 0.7)
cv_past year recall	Prevalence	0.3 (0.3 — 0.4)	1.4 (1.3 — 1.5)
cv_symptom scale	Prevalence	0.9 (0.8 — 1.1)	2.5 (2.1 — 2.9)
cv_NOR_twin_study	Prevalence	-1.1 (-1.6 — -0.5)	0.4 (0.2 — 0.6)
Mortality Rate Due to Conflict in Last 10 Years (per 1 person)	Prevalence	0.5 (0.03 — 0.98)	1.7 (1.03 — 2.7)
Age-standardized SEV scalar: Depression	Prevalence	0.9 (0.8 — 1.1)	2.4 (2.1 — 3.04)

References

1. American Psychiatric Association. Diagnostic and Statistical Manual of Mental Disorders (DSM-IV-TR). 4th, Text Revision ed. Washington DC: American Psychiatric Association; 2000.
2. World Health Organization. The ICD-10 Classification of Mental and Behavioural Disorders. Clinical descriptions and diagnostic guidelines. Geneva: World Health Organization; 1992.
3. Vos T, Mathers C, Herrman H, et al. The burden of mental disorders in Victoria, 1996. *Soc Psychiatry Psychiatr Epidemiol* 2001; **36**(2): 53-62.
4. Vos T, Mathers CD. The burden of mental disorders: a comparison of methods between the Australian burden of disease studies and the Global Burden of Disease study. *Bull World Health Organ* 2000; **78**(4): 427-38.

5. Ustun TB, Kessler RC. Global burden of depressive disorders: the issue of duration. *Br J Psychiatry* 2002; **181**: 181-3.
6. Ferrari AJ, Charlson FJ, Norman RE, et al. The epidemiological modelling of major depressive disorder: application for the global burden of disease study 2010. *PloS one* 2013; **8**(7): e69637.
7. Ferrari AJ, Somerville AJ, Baxter AJ, et al. Global variation in the prevalence and incidence of major depressive disorder: a systematic review of the epidemiological literature. *Psychol Med* 2013; **43**(3): 471-81.
8. Ferrari AJ, Charlson FJ, Norman RE, et al. Burden of depressive disorders by country, sex, age, and year: findings from the global burden of disease study 2010. *PLoS Med* 2013; **10**(11): e1001547.
9. Introduction to the National Epidemiologic Survey on Alcohol and Related Conditions [http://pubs.niaaa.nih.gov/publications/arh29-2/74-78.htm]. Access date 1 December 2014.
10. Australian Bureau of Statistics. National Survey of Mental Health and Wellbeing of Adults 1997. Canberra: Australian Bureau of Statistics.
11. Kendler KS, Walters EE, Kessler RC. The prediction of length of major depressive episodes: results from an epidemiological sample of female twins. *Psychol Med* 1997; **27**(1): 107-17.
12. Lewinsohn PM, Clarke GN, Seeley JR, Rohde P. Major depression in community adolescents: age at onset, episode duration, and time to recurrence. *J Am Acad Child Adolesc Psychiatry* 1994; **33**(6): 809-18.
13. Spijker J, de Graaf R, Bijl RV, Beekman AT, Ormel J, Nolen WA. Duration of major depressive episodes in the general population: results from The Netherlands Mental Health Survey and Incidence Study (NEMESIS). *Br J Psychiatry* 2002; **181**: 208-13.
14. McLeod JD, Kessler RC, Landis KR. Speed of recovery from major depressive episodes in a community sample of married men and women. *J Abnorm Psychol* 1992; **101**(2): 277-86.
15. Vos T, Haby MM, Barendregt JJ, Kruijshaar M, Corry J, Andrews G. The burden of major depression avoidable by longer-term treatment strategies. *Archives of General Psychiatry* 2004; **61**(11): 1097-103.
16. Phillips MR, Zhang J, Shi Q, et al. Prevalence, treatment, and associated disability of mental disorders in four provinces in China during 2001–05: an epidemiological survey. *The Lancet* 2009; **373**: 2041–53.
17. Bromet E, Andrade LH, Hwang I, et al. Cross-national epidemiology of DSM-IV major depressive episode. *BMC medicine* 2011; **9**: 90.
18. Wang PS, Aguilar-Gaxiola S, Alonso J, et al. Use of mental health services for anxiety, mood, and substance disorders in 17 countries in the WHO world mental health surveys. *The Lancet* 2007; **370**(9590): 841-50.
19. Simon GE, Goldberg D, Von Korff M, Ustun T. Understanding cross-national differences in depression prevalence. *Psychological Medicine* 2002; **32**(4): 585–94.
20. Karam E, Bou GM. Psychosocial consequences of war among civilian populations. *Current Opinion in Psychiatry* 2013; **16**(413–419).
21. Steel Z, Chey T, Silove D, Marnane C, Bryant RA, van Ommeren M. Association of torture and other potentially traumatic events with mental health outcomes among populations exposed to mass conflict and displacement: a systematic review and meta-analysis. *JAMA* 2009; **302**(5): 537-49.

Dysthymia

Flowchart

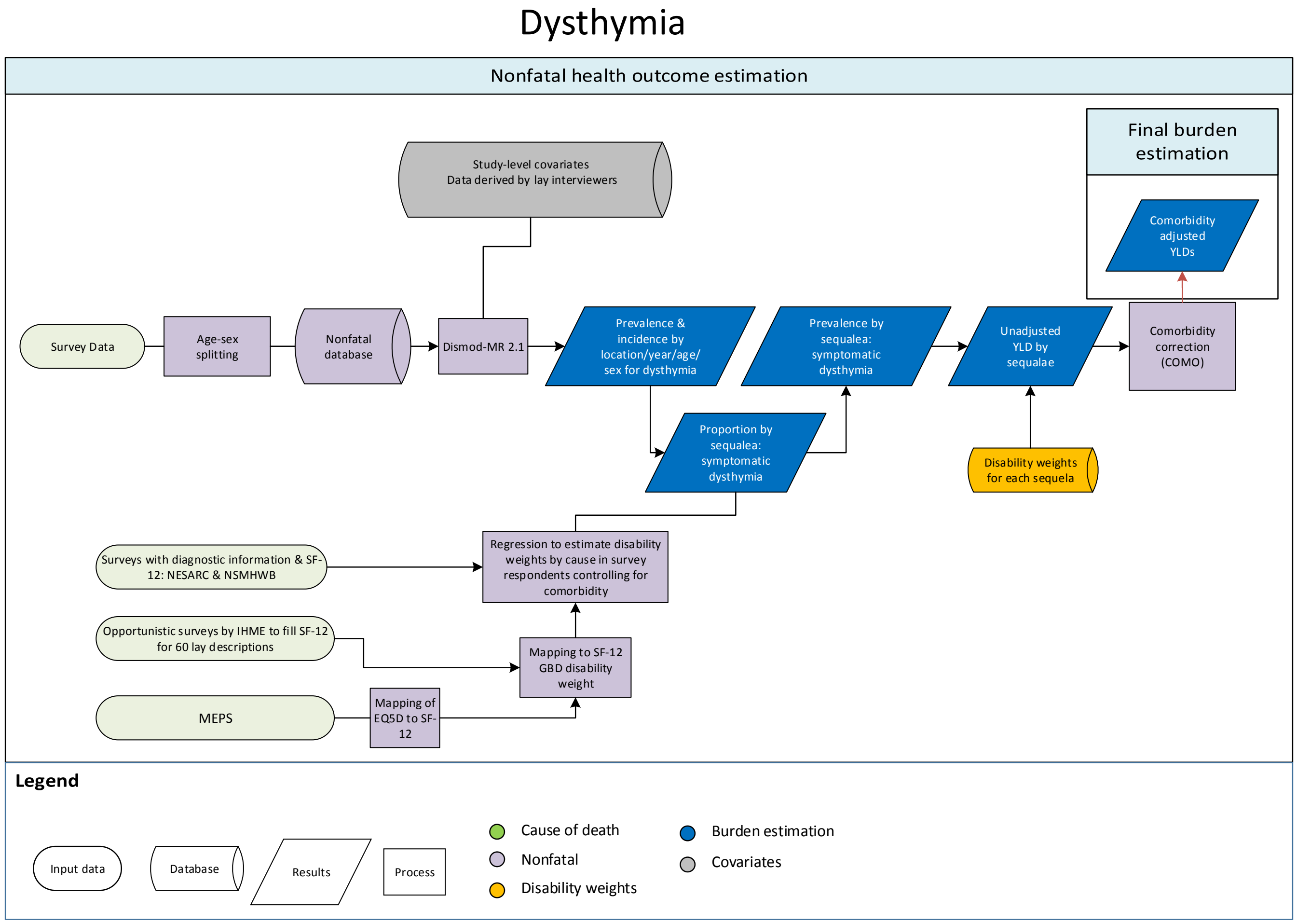

Case Definition

Dysthymia is a mood disorder consisting of chronic depression, demonstrating less severe but longer lasting symptoms than major depressive disorder. Included in GBD disease modelling were cases meeting diagnostic criteria for dysthymia according to the Diagnostic and Statistical Manual of Mental Disorders (DSM), or the equivalent diagnosis in the International Classification of Diseases (ICD). These were identified by the following codes: DSM-IV-TR: 300.4, ICD-10: F34.1; excluding those cases due to a general medical condition or substance induced cases [1,2].

According to DSM-IV TR criteria, dysthymia involves the experience of chronically depressed mood for most of the day, most days that not, for at least two year (or at least one year in children and adolescents). During this period, at least two of the following symptoms must also be experienced;

- poor appetite or overeating;
- insomnia or hypersomnia;

- low energy or fatigue;
- low self-esteem;
- poor concentration or indecisiveness; and
- feelings of hopelessness

Input data

Model Inputs

For GBD 2010, a systematic review of the literature was conducted to capture studies of prevalence, incidence, remission, and excess mortality associated dysthymia. In summary, the search was conducted in three stages involving electronic searches of the peer-reviewed literature (via Medline, Embase and PubMed), the grey literature and, expert consultation. The agreed approach for mental and substance use disorders was to conduct electronic database searches on a rolling basis. All three stages of GBD 2010's literature review were repeated for GBD 2013 to capture additional data published up to 2013. For GBD 2015, only stages two and three of the literature review were conducted, with another electronic database search due for dysthymia in the next iteration of GBD studies.

The inclusion criteria stipulated that: (1) the publication year must be from 1980 onwards; (2) 'caseness' must be based on clinical threshold as established by the DSM or ICD; (3) sufficient information must be provided on study method and sample characteristics to assess the quality of the study; and (4) study samples must be representative of the general population (i.e. inpatient or pharmacological treatment samples, case studies, veterans or refugee samples were excluded). No limitation was set on the language of publication. Methods used for this systematic review have been reported in greater detail elsewhere [3,4]. The table below shows the number of studies included in GBD 2015, as well as the number of countries or subnational units and GBD world regions represented.

	Prevalence	Incidence	Remission	Excess-Mortality
Studies	73	2	2	-
Countries/subnational geographies	57	2	2	-
GBD world regions	10	1	2	-

In addition, United States Marketscan claims data for 2000, 2010 and 2012 by state, age, and sex were tested in the model. We decided not to include these data in the final model as we considered it would not be representative of the true prevalence of mental and substance use disorders in the general population.

Severity Splits

The basis of the GBD disability weight survey assessments are lay descriptions of sequelae highlighting major functional consequences and symptoms. The lay description and disability weight for a symptomatic state of dysthymia are shown below. Given the milder and more stable presentation of dysthymia, it was assigned the same disability weight as that for mild major depressive disorder.

Severity level	Lay description	DW (95% CI)
Symptomatic dysthymia	Feels persistent sadness and has lost interest in usual activities. The person sometimes sleeps badly, feels tired,	0.145 (0.099-0.209)

	or has trouble concentrating but still manages to function in daily life with extra effort.	

To determine the proportion of people with symptomatic and asymptomatic dysthymia, the US National Epidemiological Survey on Alcohol and Related Conditions (NESARC, conducted in two waves from 2001-2002 and 2004-2005) [5] and the Australian National Survey of Mental Health and Wellbeing of Adults (NSMHWB, conducted in 1997)[6] were used to estimate the proportion of dysthymia cases asymptomatic (29%, 23%-36%)and symptomatic (71%, 64%-77%).

Modeling Strategy

The GBD 2015 epidemiological modelling strategy for dysthymia made use of DisMod-MR 2.1. Data across all epidemiological parameters were initially included in the modelling process. The incidence studies reported estimates which were very low, relative to the prevalence data. As prevalence studies contributed much greater world coverage than incidence studies, we excluded the incidence data, relying instead on data from the other parameters. We assumed no incidence and prevalence before age 3. This minimum age of onset was corroborated with expert feedback and was consistent with the available data. Excess-mortality was set to 0 as there is no epidemiological evidence to suggest that dysthymia is associated with a statistically significant risk of mortality [3,4].

Study-level covariates were used to accommodate between-study variability in the raw prevalence data. A cv_lay interviewer covariate created a crosswalk between prevalence derived from clinically trained interviewers (desirable) and prevalence derived from lay-interviewers. A cv_past year prevalence covariate was originally included to adjust all data points derived from past year prevalence towards the level they would have been if the study had captured point/past-month prevalence. As the effect of this covariate was not statistically significant, it was excluded from the final model. Given that dysthymia is being modelled as a chronic disorder with a long duration of between 6 to 10 years, it was not surprising that we did not detect significant variation between point and past year prevalence.

Betas and exponentiated values (which can be interpreted as an odds ratio) for each study level covariate are shown in the table below:

Study covariate	Parameter	beta	Exponentiated beta
cv_lay interviewer	Prevalence	-0.6 (-0.95— -0.2)	0.6 (0.4 — 0.8)

Given that there was an overall paucity in epidemiological data available for dysthymia, and the data available were very heterogeneous given differences in the data collection methodology used between studies, we applied a restriction on location random-effects of -0.3 to 0.3 to further guide the estimation of prevalence.

References

1. American Psychiatric Association. Diagnostic and Statistical Manual of Mental Disorders (DSM-IV-TR). 4th, Text Revision ed. Washington DC: American Psychiatric Association; 2000.

2. World Health Organization. The ICD-10 Classification of Mental and Behavioural Disorders. Clinical descriptions and diagnostic guidelines. Geneva: World Health Organization; 1992.
3. Charlson FJ, Ferrari AJ, Flaxman AD, Whiteford HA. The epidemiological modelling of dysthymia: application for the Global Burden of Disease Study 2010. *J Affect Disord* 2013; **151**(1): 111-20.
4. Ferrari AJ, Charlson FJ, Norman RE, et al. Burden of depressive disorders by country, sex, age, and year: findings from the global burden of disease study 2010. *PLoS Med* 2013; **10**(11): e1001547.
5. Introduction to the National Epidemiologic Survey on Alcohol and Related Conditions [http://pubs.niaaa.nih.gov/publications/arh29-2/74-78.htm]. Access date 1 December 2014.
6. Australian Bureau of Statistics. National Survey of Mental Health and Wellbeing of Adults 1997. Canberra: Australian Bureau of Statistics.

Bipolar disorder

Flowchart

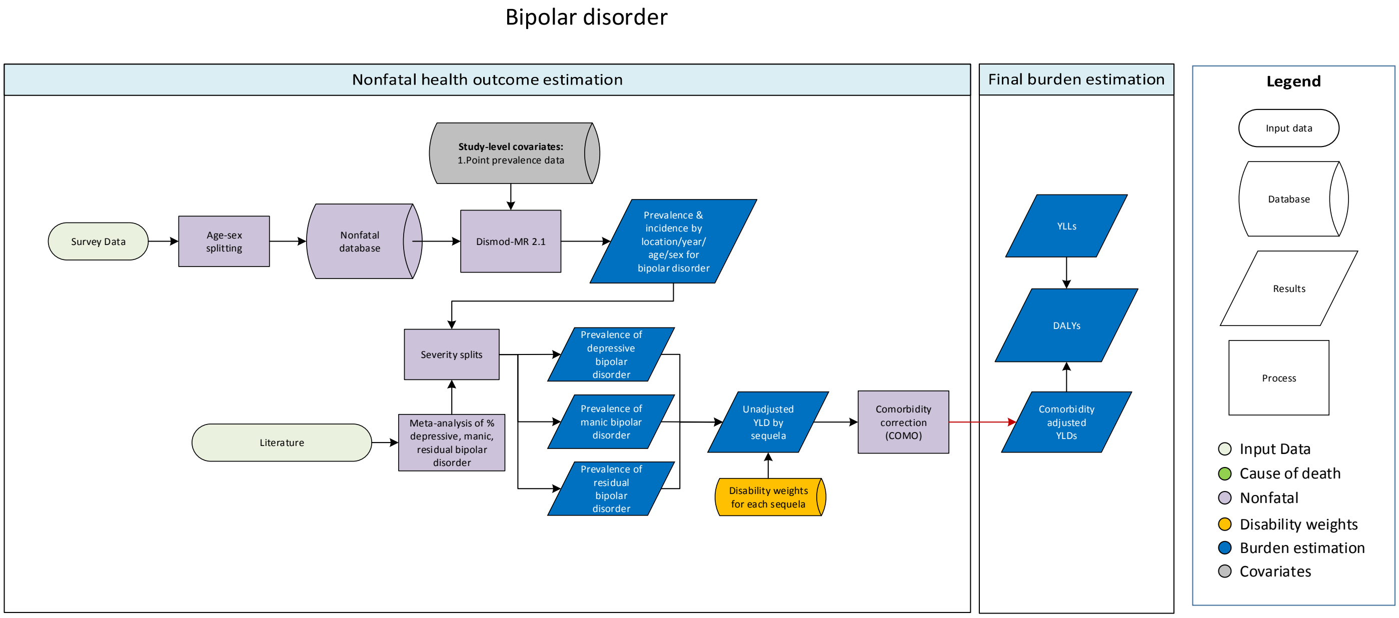

Case Definition

Bipolar disorder is a chronic mood disorder with little or no complete remission. Included in GBD disease modeling were cases meeting diagnostic criteria for bipolar disorder according to the Diagnostic and Statistical Manual of Mental Disorders (DSM), or the equivalent diagnosis in the International Classification of Diseases (ICD)[1,2]. These were identified by the following codes: DSM-IV-TR: 296.0–296.8, 296.89, 301.13; ICD-10: F31.0–F31.6, F31.8–F31.9, F34.0–F34.1, excluding those cases due to a general medical condition or substance-induced cases. A diagnosis of bipolar disorder involves the experience of one or more manic or hypomanic episode(s), which can be accompanied by a major depressive episode.

According to DSM-IV-TR a manic episode involves the experience of elevated, expansive, or irritable mood lasting for at least one week. During this period, at least three (or four is mood is only irritable) of the following symptoms must also be experienced:

- inflated self-esteem or grandiosity;
- decreased need for sleep;
- more talkative;
- flight of ideas or experience that thoughts are racing;
- distractibility;
- increase in goal-directed activity; and
- excessive involvement in pleasurable activities with high potential for painful consequences

A hypomanic episode involves the experience of elevated, expansive, or irritable mood lasting for at least four days. During this period, at least three (or four is mood is only irritable) of the symptoms previously listed for a manic episode must also be experienced.

A major depressive episode involves the experience of depressed mood almost all day, every day, for at least two weeks. A total of five out of nine criteria must be met to make a diagnosis and at least one of the five criteria should either be:

- "depressed mood" for most of every day; or
- "loss of interest in nearly all activities" for most of every day.

The other seven criteria are:

- change in eating, appetite, or weight;
- excessive sleeping or insomnia;
- agitated or slow motor activity;
- fatigue;
- feeling worthless or inappropriately guilty;
- trouble concentrating; and
- repeated thoughts about death

Different subtypes of bipolar disorder can be diagnosed depending on the combination of symptoms experienced. Bipolar I is characterized by at least one manic episode, which can also alternate with a major depressive episode. Bipolar II is characterized by hypomanic episodes, alternating with major depressive episodes. Cyclothymia is characterized by subsyndromal hypomanic and major depressive episodes. Bipolar disorder not otherwise specified is characterized by clinically significant symptoms of bipolar disorder which do not meet criteria for the other diagnoses[1,2]. In GBD 2015 we estimated burden for the entire spectrum of bipolar disorder simultaneously, rather than individually for each subtype of the disorder. At a minimum, epidemiological studies needed to report on bipolar I and bipolar II combined to be included in analyses.

Input data

Model inputs

For GBD 2010, a systematic review of the literature was conducted to capture studies of prevalence, incidence, remission, and excess mortality associated with bipolar disorder. In summary, the search was conducted in three stages involving electronic searches of the peer-reviewed literature (via Medline, Embase, and PubMed), the grey literature, and expert consultation. The agreed approach for mental and substance use disorders was to conduct electronic database searches on a rolling basis. All three stages of GBD 2010's literature review were repeated for GBD 2013 to capture additional data published up to 2013. For GBD 2015, only stages two and three of the literature review were conducted, with another electronic database search due for bipolar disorder in the next iteration of GBD studies.

The inclusion criteria stipulated that (1) the publication year must be from 1980 onward; (2) "caseness" must be based on clinical threshold as established by the DSM or ICD; (3) sufficient information must be provided on study method and sample characteristics to assess the quality of the study; and (4) study

samples must be representative of the general population (i.e., inpatient or pharmacological treatment samples, case studies, veterans or refugee samples were excluded). No limitation was set on the language of publication. Methods used for this systematic review have been reported in greater detail elsewhere[3]. As previously explained, burden was estimated for the entire spectrum of bipolar disorder simultaneously. Combined estimates of all subtypes of bipolar disorders were required. Studies reporting separate estimates for bipolar I, bipolar II, cyclothymia, and/or bipolar not otherwise specified were accepted if sufficient information was available to sum the disorder-specific estimates. At a minimum, studies needed to report on bipolar I and bipolar II. The table below shows the number of studies included in GBD 2015, as well as the number of countries or subnational units and GBD world regions represented.

	Prevalence	Incidence	Remission	Excess mortality
Studies	44	3	-	12
Countries/subnational geographies	41	2	-	8
GBD world regions	11	2	-	3

In addition, United States Marketscan claims data for 2000, 2010, and 2012 by state, age, and sex were tested in the model. We decided not to include these data in the final model as we considered it would not be representative of the true prevalence of mental and substance use disorders in the general population.

Severity splits

The basis of the GBD disability weight survey assessments are lay descriptions of sequelae highlighting major functional consequences and symptoms. The lay descriptions and disability weights for bipolar disorder severity levels are shown below.

Severity level	Lay description	DW (95% CI)
Manic	Is hyperactive, hears and believes things that are not real, and engages in impulsive and aggressive behavior that endanger the person and others.	0.492 (0.341-0.646)
Depressive*	Has constant sadness and has lost interest in usual activities. The person has some difficulty in daily life, sleeps badly, has trouble concentrating, and sometimes thinks about harming himself (or herself).	0.396 (0.267-0.531)
Residual	Has mild mood swings, irritability, and some difficulty with daily activities.	0.032 (0.018-0.051)

**Equivalent to the disability weight estimated for moderate major depressive disorder*

Information on the distribution of manic, depressive, and residual states of bipolar disorder was obtained from a separate systematic review of the literature[4]. Meta-XL (a Microsoft Excel add-in for meta-analysis) was used to pool estimates across all studies to calculate the overall proportion of bipolar cases in each health state. Six studies provided information on the proportion of bipolar disorder cases in a manic (21%, 12%-33%), depressive (23%, 10%-39%), or residual state (52%, 28%-77%).

Modeling strategy

The GBD 2015 epidemiological modeling strategy for bipolar disorder made use of DisMod-MR 2.1. Data across all epidemiological parameters were initially included in the modelling process. The two studies on incidence reported 0% and 0.1% incidence of bipolar disorder and were low relative to the prevalence data. They were excluded from the final model where incidence was estimated using data from other parameters. We assumed no incidence and prevalence before age 10. Remission was set to a maximum of 0.05 in agreement with literature and expert advice suggesting no or very little complete remission from bipolar disorder[5,6].

Study-level covariates were used to accommodate for between-study variability in the raw prevalence data. A cv_point recall covariate adjusted all data points derived from point/past-month prevalence toward the level they would have been if the study had captured 12-month prevalence. We set 12-month prevalence as the desirable level due to the episodic nature of bipolar disorder. Estimates of point prevalence surveying symptoms experienced in the past 30 days or less may fail to diagnose cases of bipolar disorder in a residual state, thereby underestimating prevalence.

The corresponding beta and exponentiated value (which can be interpreted as an odds ratio) is shown in the table below:

Study covariate	Parameter	beta	Exponentiated beta
cv_point recall	Prevalence	-0.5 (-0.8 — -0.2)	0.6 (0.5 — 0.8)

Given that there was an overall paucity in epidemiological data available for bipolar disorder, and the data available were very heterogeneous given differences in the data collection methodology used between studies, we applied a restriction on location random-effects of -0.3 to 0.3 to further guide the estimation of prevalence.

References

1. American Psychiatric Association. Diagnostic and Statistical Manual of Mental Disorders (DSM-IV-TR). Fourth Edition, Text Revision ed. Washington DC: American Psychiatric Association; 2000.
2. World Health Organization. The ICD-10 Classification of Mental and Behavioural Disorders. Clinical descriptions and diagnostic guidelines. Geneva: World Health Organization; 1992.
3. Ferrari AJ, Baxter AJ, Whiteford HA. A systematic review of the global distribution and availability of prevalence data for bipolar disorder. *J Affect Disord* 2011; **134**(1-3): 1-13.
4. Ferrari AJ, Saha S, McGrath JJ, et al. Health states for schizophrenia and bipolar disorder within the Global Burden of Disease 2010 Study. *Population health metrics* 2012; **10**(1): 16.
5. Angst J, Sellaro R. Historical perspectives and natural history of bipolar disorder. *Biological Psychiatry* 2000; **48**(6): 445–57.
6. Colom F, Vieta E. The road to DSM-V. Bipolar disorder episode and course specifiers. *Psychopathology* 2009; **42**(4): 209–18.

Anxiety disorders

Flowchart

Anxiety disorders

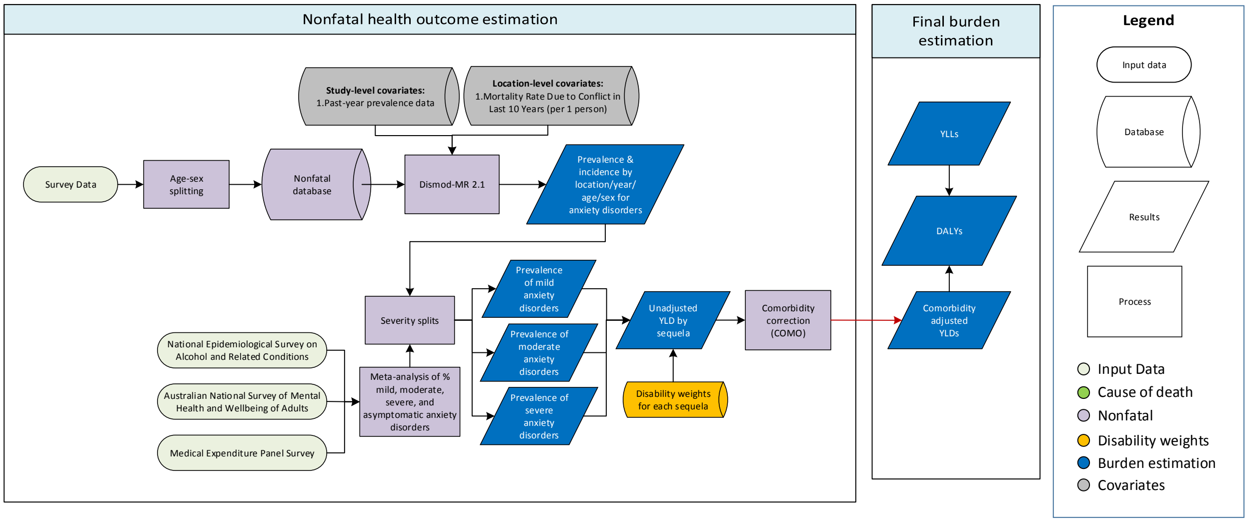

Case Definition

Anxiety disorders are characterized by experiences of intense of fear and distress, typically in combination with other physiological symptoms. We aimed to capture all cases of anxiety disorders reaching diagnostic threshold defined by the Diagnostic and Statistical Manual of Mental Disorders (DSM) or the WHO International Classification of Diseases (ICD-10)[1,2]. Included disorders are listed below and can be identified by the DSM-IV-TR and ICD-10 coding systems as: DSM IV TR: 299.8, 300.0-300.3, 309.21, 309.81 and ICD-10: F40-42, F43.1, F93.0-93.2, F93.8. Excluded were anxiety disorders due to a general medical condition and substance-induced anxiety disorder.

- panic disorder;
- agoraphobia;
- specific phobia;
- social phobia;
- obsessive-compulsive disorder;
- posttraumatic stress disorder;
- acute stress disorder;
- generalized anxiety disorder;
- separation anxiety disorder; and
- anxiety disorder not otherwise specified

As specific anxiety disorders frequently co-occur, anxiety disorders were modeled as a single cause for "any" anxiety disorder in GBD 2015 to avoid the double-counting of individuals meeting criteria for more

than one anxiety disorder. Epidemiological estimates reporting an outcome for "any" or "total" anxiety disorders were included in analyses.

Input data

Model Inputs

For GBD 2010, a systematic review of the literature was conducted to capture studies of prevalence, incidence, remission, and excess mortality associated with anxiety disorders. In summary, the search was conducted in three stages involving electronic searches of the peer-reviewed literature (via Medline, Embase, and PubMed), the grey literature, and expert consultation. The agreed approach for mental and substance use disorders was to conduct electronic database searches on a rolling basis. All three stages of GBD 2010's literature review were repeated for GBD 2013 to capture additional data published up to 2013. For GBD 2015, only stages two and three of the literature review were conducted, with another electronic database search due for anxiety disorders in the next iteration of GBD studies.

The inclusion criteria stipulated that (1) the publication year must be from 1980 onward; (2) "caseness" must be based on clinical threshold as established by the DSM or ICD; (3) sufficient information must be provided on study method and sample characteristics to assess the quality of the study; and (4) study samples must be representative of the general population (i.e., inpatient or pharmacological treatment samples, case studies, veterans or refugee samples were excluded). No limitation was set on the language of publication. Methods used for this systematic review have been reported in greater detail elsewhere[3-5]. The table below shows the number of studies included in GBD 2015, as well as the number of countries or subnational units and GBD world regions represented.

	Prevalence	Incidence	Remission	Excess mortality
Studies	96	2	3	-
Countries/subnational geographies	70	2	3	-
GBD world regions	16	2	2	-

In addition, United States Marketscan claims data for 2000, 2010, and 2012 by state, age, and sex were tested in the model. We decided not to include these data in the final model as we considered it would not be representative of the true prevalence of mental and substance use disorders in the general population.

Severity splits & disability weights

The basis of the GBD disability weight survey assessments are lay descriptions of sequelae highlighting major functional consequences and symptoms. The lay descriptions and disability weights for anxiety disorder severity levels are shown below.

Severity level	Lay description	DW (95% CI)
Mild	Feels mildly anxious and worried, which makes it slightly difficult to concentrate, remember things, and sleep. The person tires easily but is able to perform daily activities.	0.03 (0.018-0.046)

Moderate	Feels anxious and worried, which makes it difficult to concentrate, remember things, and sleep. The person tires easily and finds it difficult to perform daily activities.	0.133 (0.091-0.186)
Severe	Constantly feels very anxious and worried, which makes it difficult to concentrate, remember things, and sleep. The person has lost pleasure in life and thinks about suicide.	0.523 (0.362-0.677)

To determine the proportion of people with anxiety disorders within each of the severity levels, the US National Epidemiological Survey on Alcohol and Related Conditions (NESARC, conducted in two waves, 2001-2002 and 2004-2005)[6] and the Australian National Survey of Mental Health and Wellbeing of Adults (NSMHWB, conducted in 1997)[7] were used to estimate the proportion of anxiety disorder cases that were asymptomatic (20%, 17%-23%), mild (50%, 42%-56%), moderate (19%, 14%-25%), and severe (11%, 7%-16%).

Modeling strategy

The GBD 2015 epidemiological modeling strategy for anxiety disorders made use of DisMod-MR 2.1. Data across all epidemiological parameters were initially included in the modeling process. The incidence studies reported estimates which were very low relative to the prevalence data. As prevalence studies contributed much greater world coverage than incidence studies, we excluded the incidence data, relying instead on data from the other parameters. We assumed no incidence and prevalence before age 2 and after age 95. This minimum age of onset was corroborated with expert feedback and existing literature on anxiety disorders. Remission was set to a maximum of 0.2, consistent with the data points available. Excess mortality was set to 0 as the available epidemiological data showed no statistically significant risk of excess all-cause mortality for anxiety disorders[4,5].

Study-level covariates were used to accommodate for between-study variability in the raw prevalence data. A cv_past year recall covariate adjusted all data points derived from past year prevalence toward the level they would have been if the study had captured point/past-month prevalence. The latter prevalence period is less affected by recall bias. A country-level covariate was also included in the anxiety disorders model. Mortality Rates Due to Conflict in Last 10 Years (per one person) informed the estimation of prevalence given existing evidence to show a positive association between conflict status and prevalence of anxiety disorders[8,9] .

Betas and exponentiated values (which can be interpreted as an odds ratio) for each study level covariate are shown in the table below:

Study/country covariate	Parameter	beta	Exponentiated beta
cv_past year recall	Prevalence	0.4 (0.3 — 0.5)	1.4 (1.3 — 1.6)
Mortality Rate Due to Conflict in Last 10 Years (per 1 person)	Prevalence	0.5 (0.03 — 0.97)	1.6 (1.03 — 2.7)

References

1. American Psychiatric Association. Diagnostic and Statistical Manual of Mental Disorders (DSM-IV-TR). Fourth Edition, Text Revision ed. Washington DC: American Psychiatric Association; 2000.
2. World Health Organization. The ICD-10 Classification of Mental and Behavioural Disorders. Clinical descriptions and diagnostic guidelines. Geneva: World Health Organization; 1992.
3. Baxter AJ, Scott KM, Vos T, Whiteford HA. Global prevalence of anxiety disorders: a systematic review and meta-regression. *Psychological Medicine* 2013; **43**(05): 897-910.
4. Baxter AJ, Vos T, Scott KM, Ferrari AJ, Whiteford HA. The global burden of anxiety disorders in 2010. *Psychological Medicine* 2014; **44**(11): 2363-74.
5. Baxter AJ, Vos T, Scott KM, et al. The regional distribution of anxiety disorders: implications for the Global Burden of Disease Study, 2010. *International Journal of Methods in Psychiatric Research* 2014; **23**(4): 422-38.
6. Introduction to the National Epidemiologic Survey on Alcohol and Related Conditions [http://pubs.niaaa.nih.gov/publications/arh29-2/74-78.htm]. Access date 1 December 2014.
7. Australian Bureau of Statistics. National Survey of Mental Health and Wellbeing of Adults 1997. Canberra: Australian Bureau of Statistics.
8. Karam E, Bou GM. Psychosocial consequences of war among civilian populations. *Current Opinion in Psychiatry* 2013; **16**(413–419).
9. Steel Z, Chey T, Silove D, Marnane C, Bryant RA, van Ommeren M. Association of torture and other potentially traumatic events with mental health outcomes among populations exposed to mass conflict and displacement: a systematic review and meta-analysis. *JAMA* 2009; **302**(5): 537-49.

Anorexia nervosa

Flowchart

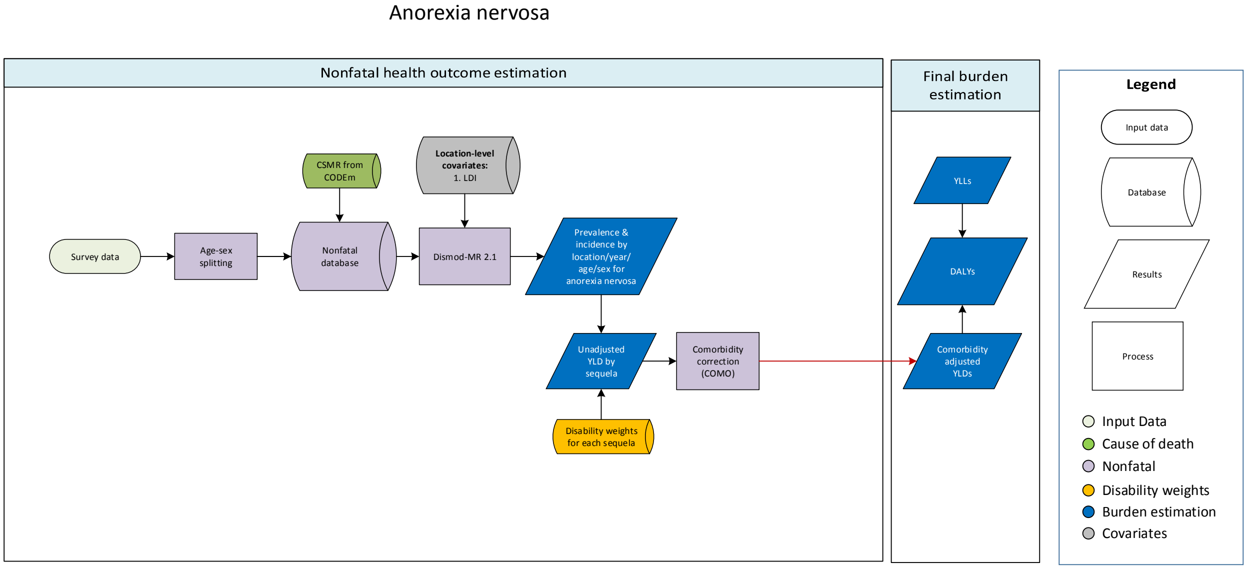

Case definition

According to the Diagnostic and Statistical Manual of Mental Disorders fourth edition, text revision (DSM-IV-TR),[1] anorexia nervosa (AN) is an eating disorder characterized by:

a) Refusal to maintain body weight at or above a minimally normal weight for age and height (e.g., weight loss leading to maintenance of body weight less than 85% of that expected; or failure to make expected weight gain during period of growth, leading to body weight less than 85% of that expected).
b) Intense fear of gaining weight or becoming fat, even though underweight (expanded to include any behavior that interferes with weight gain in DSM-5[2]).
c) Disturbance in the way in which one's body weight or shape is experienced, undue influence of body weight or shape on self-evaluation, or denial of the seriousness of the current low body weight.
d) In postmenarcheal females, amenorrhea, i.e., the absence of at least three consecutive menstrual cycles (this criterion was removed in DSM-5[2]).

Included in GBD were cases meeting diagnostic criteria according to DSM[1] or the International Classification of Diseases (ICD).[3] These were identified by the following codes: 307.1 (DSM-IV-TR) and F50.0 (ICD-10). Different versions of DSM (DSM-III, DSM-III-R, DSM-IV, DSM-IV-TR, and DSM-5) and ICD (ICD-9 and ICD-10) were accepted.

Input data

Model inputs

A series of systematic literature reviews were conducted to capture studies reporting the prevalence, incidence, remission, and excess mortality of AN. The reviews incorporated searches of peer-reviewed literature via electronic databases, investigations of grey literature, and consultation with experts. In order for a study to be included, it must have been published during or after 1980, use DSM or ICD criteria to define cases, provide sufficient details on study methodology and sample characteristics to determine study quality, and be representative of the general population rather than a special population, e.g., prison inmates. No limitation was set on the language of publication. Detailed descriptions of this methodology have been published elsewhere.[4] This methodology was utilized in GBD 2010, GBD 2013 and GBD 2015.

The final dataset for GBD 2015 included 110 prevalence estimates, 35 incidence estimates, 20 remission estimates, and 28 excess mortality estimates. The table below shows the number of studies for each parameter as well as the number of countries/subnationals and GBD world regions covered by the available data.

	Prevalence	Incidence	Remission	Mortality
Studies	49	6	19	22
Countries/subnationals	35	6	15	14
GBD world regions	10	2	4	3

Disability weight

No severity splits were applied to AN. The lay description and disability weight for AN is shown in the table below.

Lay description	DW (95% CI)
Feels an overwhelming need to starve and exercises excessively to lose weight. The person is very thin, weak and anxious.	0.224 (0.150-0.312)

Modeling strategy

Data across all epidemiological parameters were initially included in the modeling process. We assumed no incidence prior to age 5 or from 50 years onward. These settings are in line with those placed on the corresponding cause of death model for eating disorders. A cap of 0.6 was placed on remission in order to obtain a more plausible fit of the model. We used the function in DisMod-MR 2.1 to pull in cause-specific mortality rate (CSMR) data from our CODEm and CODcorrect analyses. The inclusion of CSMR was a first in GBD 2015 as improvements to the CODEm modeling strategy allowed the CSMR data to better inform the DisMod-MR 2.1 model. We also tested using CSMR data to estimate priors on excess mortality rates (EMR) by matching them with prevalence data points for the same geography and study year and dividing CSMR by prevalence. However, the model with CSMR but without EMR was considered more representative of the global distribution of AN. A country-level covariate, lagged distributed income (LDI), was also included. This covariate represents a moving average of gross domestic product (GDP) over time. The limits placed on this covariate meant that prevalence was assumed to increase with rising GDP. LDI was also applied to excess mortality data in order to better inform regional distribution.

Study covariate	Parameter	beta	Exponentiated beta
LDI ($ per capita)	Prevalence	0.61 (0.26-1.16)	1.83 (1.30-3.19)
LDI ($ per capita)	Excess mortality	-0.3 (-0.5- -0.1)	0.74 (0.61-0.90)

References

1. American Psychiatric Association. Diagnostic and Statistical Manual of Mental Disorders (DSM-IV-TR). 4th, Text Revision ed. Washington DC: American Psychiatric Association; 2000.
2. American Psychiatric Association. Diagnostic and Statistical Manual of Mental Disorders, Fifth Edition. Arlington, VA: American Psychiatric Association; 2013.
3. World Health Organisation. ICD-10 Classification of Mental and Behavioural Disorders: Clinical descriptions and diagnostic guidelines. Geneva: World Health Organisation; 1992.
4. Whiteford HA, Degenhardt L, Rehm J, et al. Global burden of disease attributable to mental and substance use disorders: findings from the Global Burden of Disease Study 2010. *The Lancet* 2013; **382**(9904): 1575-86.

Bulimia nervosa

Flowchart

Bulimia nervosa

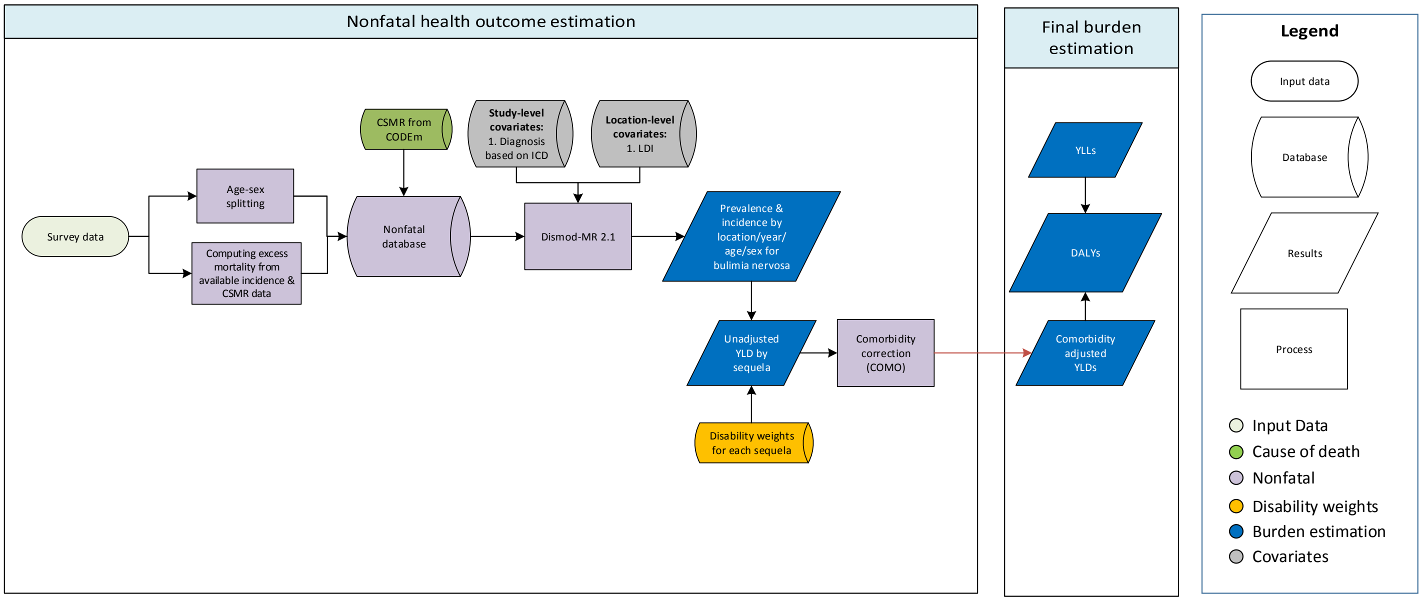

Case definition

According to the Diagnostic and Statistical Manual of Mental Disorders fourth edition, text revision (DSM-IV-TR),[1] bulimia nervosa (BN) is an eating disorder characterized by:

a) Recurrent episodes of binge eating. An episode of binge eating is characterized by both of the following:
   1) eating, in a discrete period of time (e.g., within any two-hour period), an amount of food that is definitely larger than most people would eat during a similar period of time and under similar circumstances
   2) a sense of lack of control over eating during the episode (e.g., a feeling that one cannot stop eating or control what or how much one is eating)

b) Recurrent inappropriate compensatory behavior in order to prevent weight gain, such as self-induced vomiting; misuse of laxatives, diuretics, enemas, or other medications; fasting; or excessive exercise.

c) The binge eating and inappropriate compensatory behaviors both occur, on average, at least twice a week for three months (changed to once a week for three months in DSM-5[2]).

d) Self-evaluation is unduly influenced by body shape and weight.

e) The disturbance does not occur exclusively during episodes of anorexia nervosa.

Included in GBD were cases meeting diagnostic criteria according to DSM[1] or the International Classification of Diseases (ICD).[3] These were identified by the following codes: 307.51 (DSM-IV-TR) and

F50.1 (ICD-10). Different versions of DSM (DSM-III, DSM-III-R, DSM-IV, DSM-IV-TR, and DSM-5) and ICD (ICD-9 and ICD-10) were accepted.

Input data

Model inputs

A series of systematic literature reviews were conducted to capture studies reporting the prevalence, incidence, remission, and excess mortality of BN. The reviews incorporated searches of peer-reviewed literature via electronic databases, investigations of grey literature, and consultation with experts. In order for a study to be included, it must have been published during or after 1980, use DSM or ICD criteria to define cases, provide sufficient details on study methodology and sample characteristics to determine study quality, and be representative of the general population rather than a special population, e.g., prison inmates. No limitation was set on the language of publication. Detailed descriptions of this methodology have been published elsewhere.[4] This methodology was utilized in GBD 2010, GBD 2013 and GBD 2015.

The final dataset for GBD 2015 included 120 prevalence estimates, 14 incidence estimates, 14 remission estimates, and 11 excess mortality estimates. The table below shows the number of studies for each parameter as well as the number of countries/subnationals and GBD world regions covered by the available data.

	Prevalence	Incidence	Remission	Mortality
Studies	49	4	14	11
Countries/subnationals	36	4	11	9
GBD world regions	11	1	3	2

Disability weight

No severity splits were applied to BN. The lay description and disability weight for BN is shown in the table below.

Lay description	DW (95% CI)
Has uncontrolled overeating followed by guilt, starving, and vomiting to lose weight.	0.223 (0.149-0.311)

Modeling strategy

We assumed no incidence prior to 10 years of age or onward from 40 years of age. We used the function in DisMod-MR 2.1 to pull in cause-specific mortality rate (CSMR) data from our CODEm and CODcorrect analyses. The inclusion of CSMR was a first in GBD 2015 as a CODEm model was not run for BN in GBD 2013. We also used CSMR data to estimate priors on excess mortality rates (EMR) by matching them with prevalence data points for the same geography and study year and dividing CSMR by prevalence. A study-level covariate was applied which adjusted estimates based on ICD criteria toward those based on DSM criteria. A country-level covariate, lagged distributed income (LDI), was also included. This covariate represents a moving average of gross domestic product (GDP) over time. The limits placed on this

covariate meant that prevalence was assumed to increase with rising GDP. LDI was also applied to excess mortality data in order to better inform regional distribution.

The table below illustrates the study covariates, parameters, beta and exponentiated beta values for BN.

Study covariate	Parameter	beta	Exponentiated beta
ICD classification	Prevalence	0.90 (-0.11-1.88)	2.45 (0.90-6.57)
LDI	Prevalence	0.47 (0.39-0.50)	1.60 (1.47-1.65)
LDI	Excess mortality	-0.69 (-0.99- -0.2)	0.50 (0.37-0.82)

References

1. American Psychiatric Association. Diagnostic and Statistical Manual of Mental Disorders (DSM-IV-TR). 4th, Text Revision ed. Washington DC: American Psychiatric Association; 2000.
2. American Psychiatric Association. Diagnostic and Statistical Manual of Mental Disorders, Fifth Edition. Arlington, VA: American Psychiatric Association; 2013.
3. World Health Organisation. ICD-10 Classification of Mental and Behavioural Disorders: Clinical descriptions and diagnostic guidelines. Geneva: World Health Organisation; 1992.
4. Whiteford HA, Degenhardt L, Rehm J, et al. Global burden of disease attributable to mental and substance use disorders: findings from the Global Burden of Disease Study 2010. *The Lancet* 2013; 382(9904): 1575-86.

Autism

Flowchart

Autism

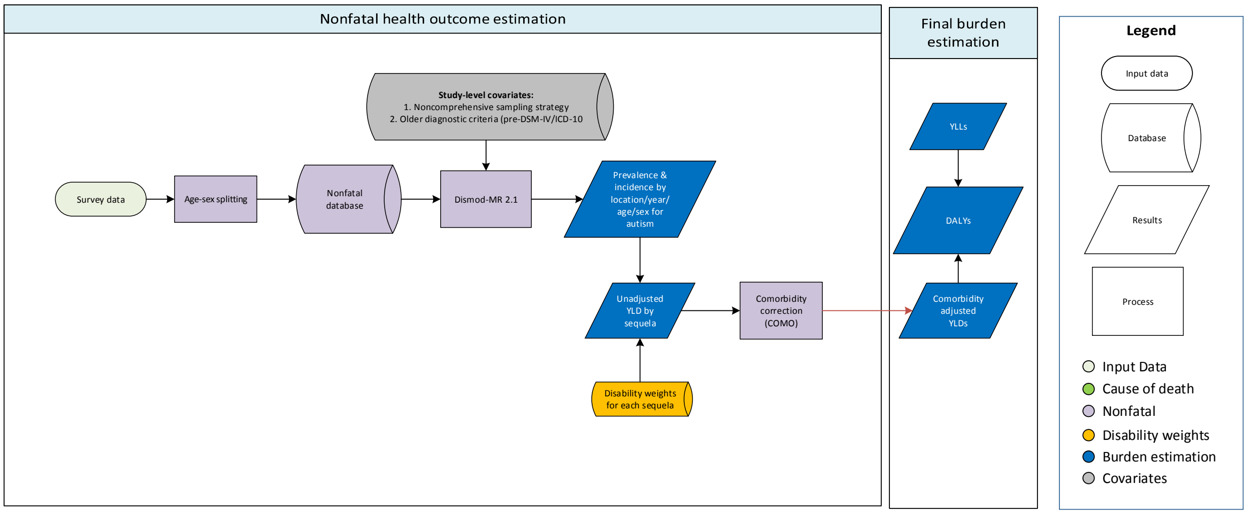

Case definition

Autism (also known as autistic disorder or childhood autism) is an autistic spectrum disorder (ASD) with onset occurring in early childhood. It is characterized by severe and pervasive impairment in several areas of development, including social interaction and communication skills, along with restricted and repetitive patterns of behaviors and/or interests. As per criteria set by the Diagnostic and Statistical Manual of Mental Disorders fourth edition, text revision (DSM-IV-TR),[1] diagnosis requires a total of six (or more) symptoms, with at least two symptoms of qualitative impairment in social interaction and at least one symptom of both qualitative impairment in communication and restricted, repetitive, stereotyped behavior. The recognized symptoms include:

Qualitative impairment in social interaction

a) marked impairment in the use of multiple nonverbal behaviors such as eye-to-eye gaze, facial expression, body postures, and gestures to regulate social interaction
b) failure to develop peer relationships appropriate to developmental level
c) a lack of spontaneous seeking to share enjoyment, interests, or achievements with other people
d) lack of social or emotional reciprocity

Qualitative impairments in communication

a) delay in, or total lack of, the development of spoken language (not accompanied by an attempt to compensate through alternative modes of communication such as gesture)
b) in individuals with adequate speech, marked impairment in the ability to initiate or sustain a conversation with others
c) stereotyped and repetitive use of language or idiosyncratic language
d) lack of varied, spontaneous make-believe play or social imitative play appropriate to developmental level

Restricted repetitive and stereotyped patterns of behavior, interests, and activities

a) encompassing preoccupation with one or more stereotyped and restricted patterns of interest that is abnormal either in intensity or focus
b) apparently inflexible adherence to specific, nonfunctional routines or rituals
c) stereotyped and repetitive motor mannerisms
d) persistent preoccupation with parts of objects

Delays or abnormal functioning with onset prior to three years of age in at social interaction, language interaction, or symbolic or imaginate play is also required. Included in GBD were cases meeting diagnostic criteria according to DSM[1] or the International Classification of Diseases (ICD).[2] These were identified by the following codes: 299.00 (DSM-IV-TR) and F84 (ICD-10). Different versions of DSM (DSM-III, DSM-III-R, DSM-IV, DSM-IV-TR, and DSM-5) and ICD (ICD-9 and ICD-10) were accepted.

Input data

Model inputs

A series of systematic literature reviews were conducted to capture studies reporting the prevalence, incidence, remission, and excess mortality of autism. The reviews incorporated searches of peer-reviewed literature via electronic databases, investigations of grey literature, and consultation with experts. In order for a study to be included, it must have been published during or after 1980, use DSM or ICD criteria to define cases, provide sufficient details on study methodology and sample characteristics to determine study quality, and be representative of the general population rather than a special population e.g. prison inmates. No limitation was set on the language of publication. Detailed descriptions of this methodology have been published elsewhere.[3] This methodology was utilized in GBD 2010 and GBD 2013. GBD 2015 included additional sources identified by GBD experts. Updates to systematic reviews are performed on an ongoing schedule across all GBD causes, an update for autism will be performed in the next 1-2 iterations

The final dataset for GBD 2015 included 102 prevalence estimates, 15 incidence estimates, 4 remission estimates, and 11 excess mortality estimates. The table below shows the number of studies for each parameter as well as the number of countries/subnationals and GBD world regions covered by the available data.

	Prevalence	Incidence	Remission	Mortality
Studies	41	4	4	3
Countries/subnationals	27	4	3	3
GBD world regions	9	3	2	2

Severity split inputs

Autism is one of the causes that contributes to the idiopathic developmental intellectual disability (ID) envelope. As such, a gradation of autism by level of severity was needed. Meta-analyses were conducted using data from six studies reporting information on the IQ level in those with autism in order to calculate the severity splits by six sequelae: autism with 1) no ID, 2) borderline ID, 3) mild ID, 4) moderate ID, 5) severe ID, and 6) profound ID. The lay descriptions and disability weights for autism and each level of intellectual disability are shown in the table below.

Health state	Lay description	DW (95% CI)

Autism	has severe problems interacting with others and difficulty understanding simple questions or directions. The person has great difficulty with basic daily activities and becomes distressed by any change in routine.	0.262 (0.176-0.365)
ID, borderline	is slow in learning at school. As an adult, the person has some difficulty doing complex or unfamiliar tasks but otherwise functions independently.	0.011 (0.005-0.024)
ID, mild	has low intelligence and is slow in learning at school. As an adult, the person can live independently, but often needs help to raise children and can only work at simple supervised jobs.	0.043 (0.028-0.067)
ID, moderate	has low intelligence, and is slow in learning to speak and to do even simple tasks. As an adult, the person requires a lot of support to live independently and raise children. The person can only work at the simplest supervised jobs.	0.098 (0.064-0.142)
ID, severe	has very low intelligence and cannot speak more than a few words, needs constant supervision and help with most daily activities, and can do only the simplest tasks.	0.157 (0.104-0.219)
ID, profound	has very low intelligence, has almost no language, and does not understand even the most basic requests or instructions. The person requires constant supervision and help for all activities.	0.196 (0.126-0.272)

Modeling strategy

We assumed no incidence from 20 years of age onwards. A small setting was placed on remission whereby only minimal remission was allowed over the lifespan. Excess mortality was set to 0 given the limited data demonstrating an association between autism and an increased risk of death with the available excess mortality data too sparse to inform plausible output. Two covariates were applied which: 1) adjusted estimates using a limited sampling strategy towards those using comprehensive sampling strategies (e.g. those including private households and mainstream schools as well as healthcare and remedial therapy facilities), and 2) adjusted estimates based on older diagnostic criteria (prior to DSM-IV and ICD-10) towards estimates made using more current criteria. This approach was largely consistent with that of GBD 2013 with the exception of the addition of the latter covariate.

Study covariate	Parameter	beta	Exponentiated beta
Non-comprehensive sampling strategy	Prevalence	-0.3 (-0.68-0.07)	0.74 (0.51-1.07)
identifies data classified by older criteria (i.e. before DSM-IV and ICD-10)	Prevalence	-0.99 (-1.35- -0.62)	0.37 (0.26-0.54)

References

1. American Psychiatric Association. Diagnostic and Statistical Manual of Mental Disorders (DSM-IV-TR). 4th, Text Revision ed. Washington DC: American Psychiatric Association; 2000.
2. World Health Organisation. ICD-10 Classification of Mental and Behavioural Disorders: Clinical descriptions and diagnostic guidelines. Geneva: World Health Organisation; 1992.
3. Baxter AJ, Brugha TS, Erskine HE, Scheurer RW, Vos T, Scott JG. The epidemiology and global burden of autism spectrum disorders. *Psychological Medicine* 2014; **45**(3): 601-13.

Asperger syndrome and other autistic spectrum disorders

Flowchart

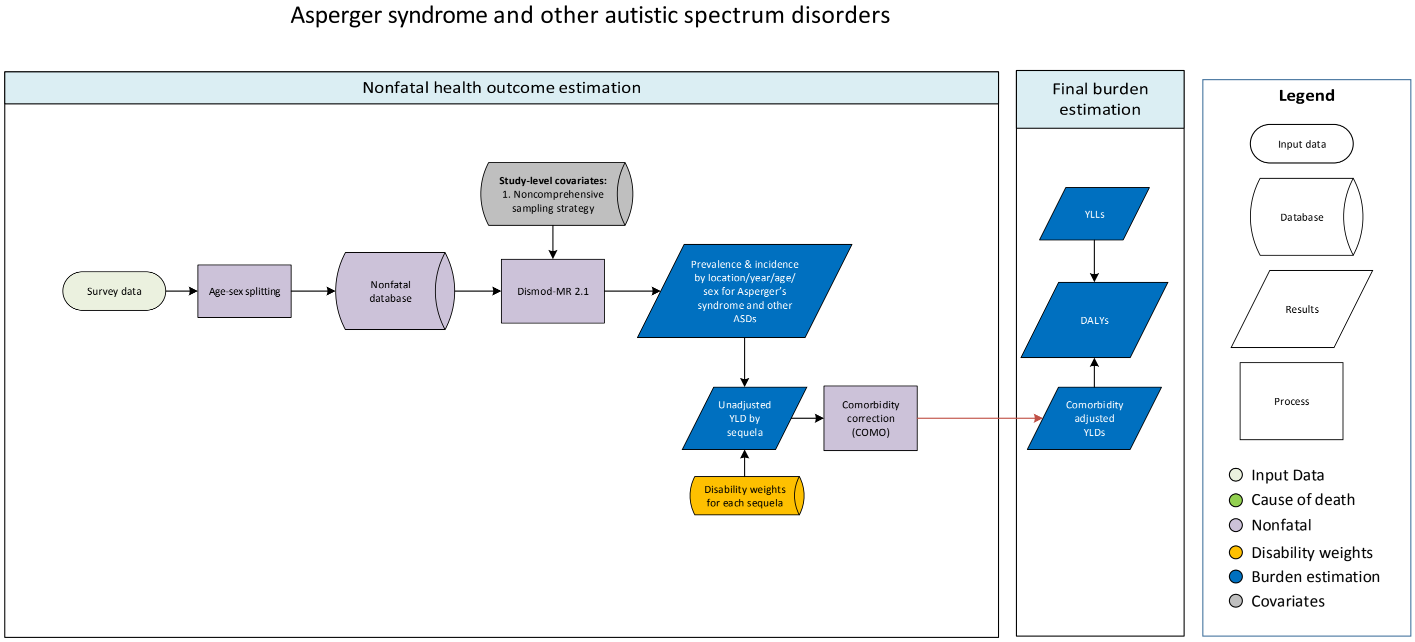

Case definition

Asperger's syndrome is an autistic spectrum disorder (ASD) characterized by severe and sustained impairment in social interaction skills along with restricted and repetitive patterns of behavior or interests. As per criteria set by the Diagnostic and Statistical Manual of Mental Disorders fourth edition, text revision (DSM-IV-TR),[1] diagnosis requires at least two symptoms of qualitative impairment in social interaction and at least one symptom of restricted, repetitive, stereotyped behavior. The recognized symptoms include:

Qualitative impairment in social interaction

a) marked impairment in the use of multiple nonverbal behaviors such as eye-to-eye gaze, facial expression, body postures, and gestures to regulate social interaction
b) failure to develop peer relationships appropriate to developmental level
c) a lack of spontaneous seeking to share enjoyment, interests, or achievements with other people
d) lack of social or emotional reciprocity

Restricted repetitive and stereotyped patterns of behavior, interests, and activities

a) encompassing preoccupation with one or more stereotyped and restricted patterns of interest that is abnormal either in intensity or focus
b) apparently inflexible adherence to specific, nonfunctional routines or rituals

c) stereotyped and repetitive motor mannerisms
d) persistent preoccupation with parts of objects

Unlike autism, there is no clinically significant delay in language acquisition or cognitive development. Included in GBD were cases meeting diagnostic criteria according to DSM[1] or the International Classification of Diseases (ICD).[2] These were identified by the following codes: 299.8 (DSM-IV-TR) and F84.5 (ICD-10). Different versions of DSM (DSM-III, DSM-III-R, DSM-IV, DSM-IV-TR, and DSM-5) and ICD (ICD-9 and ICD-10) were accepted. Estimates of other ASDs were also included such as Rett's disorder (DSM-IV-TR: 299.8, ICD-10: F84.2), childhood disintegrative disorder (DSM-IV-TR: 299.1, ICD-10: F84.3), atypical autism (ICD-10: F84.1), overactive disorder associated with mental retardation and stereotyped movements (ICD-10: F84.4), and pervasive disorder not otherwise specified (DSM-IV-TR: 299.8, ICD-10: F84.8-F84.9).

Input data

Model inputs

A series of systematic literature reviews were conducted to capture studies reporting the prevalence, incidence, remission, and excess mortality of Asperger's syndrome and other ASDs. The reviews incorporated searches of peer-reviewed literature via electronic databases, investigations of grey literature, and consultation with experts. In order for a study to be included, it must have been published during or after 1980, use DSM or ICD criteria to define cases, provide sufficient details on study methodology and sample characteristics to determine study quality, and be representative of the general population rather than a special population, e.g., prison inmates. No limitation was set on the language of publication. Detailed descriptions of this methodology have been published elsewhere.[3] This methodology was utilized in GBD 2010 and GBD 2013. GBD 2015 included additional sources identified by GBD experts. Updates to systematic reviews are performed on an ongoing schedule across all GBD causes, and an update for Asperger's syndrome and other ASDs will be performed in the next one to two iterations.

The final dataset for GBD 2015 included 28 prevalence estimates, 20 incidence estimates, one remission estimate, and one excess mortality estimate. The table below shows the number of studies for each parameter as well as the number of countries/subnationals and GBD world regions covered by the available data.

	Prevalence	Incidence	Remission	Mortality
Studies	18	3	1	1
Countries/subnationals	11	3	1	1
GBD world regions	4	2	1	1

Disability weight

No severity splits were applied to autism. The lay description and disability weight for autism are shown in the table below.

Lay description	DW (95% CI)
Has difficulty interacting with other people and is slow to understand or respond to questions. The person is often	0.104 (0.071-0.147)

preoccupied with one thing and has some difficulty with basic daily activities.	

Modeling strategy

We assumed no incidence from 20 years of age onward. A small setting was placed on remission whereby only minimal remission was allowed over the lifespan. Excess mortality was set to 0 given the limited data demonstrating an association between Asperger's syndrome and other ASDs and an increased risk of death. Only one study reported the excess mortality associated with Asperger's syndrome and other ASDs and found no significantly increased risk of death. A covariate was applied whereby estimates of Asperger's syndrome only were adjusted toward those including both Asperger's syndrome and other ASDs. This approach was largely consistent with that of GBD 2013 with the exception using only one covariate as opposed to multiple as this resulted in a better fit of the model.

Study covariate	Parameter	beta	Exponentiated beta
Asperger's syndrome only	Prevalence	-0.098 (-0.76-0.57)	0.91 (0.47-1.76)

References

1. American Psychiatric Association. Diagnostic and Statistical Manual of Mental Disorders (DSM-IV-TR). 4th, Text Revision ed. Washington DC: American Psychiatric Association; 2000.
2. World Health Organisation. ICD-10 Classification of Mental and Behavioural Disorders: Clinical descriptions and diagnostic guidelines. Geneva: World Health Organisation; 1992.
3. Baxter AJ, Brugha TS, Erskine HE, Scheurer RW, Vos T, Scott JG. The epidemiology and global burden of autism spectrum disorders. *Psychological Medicine* 2014; **45**(3): 601-13.

Attention-deficit/hyperactivity disorder (ADHD)

Flowchart

Attention-deficit/hyperactivity disorder

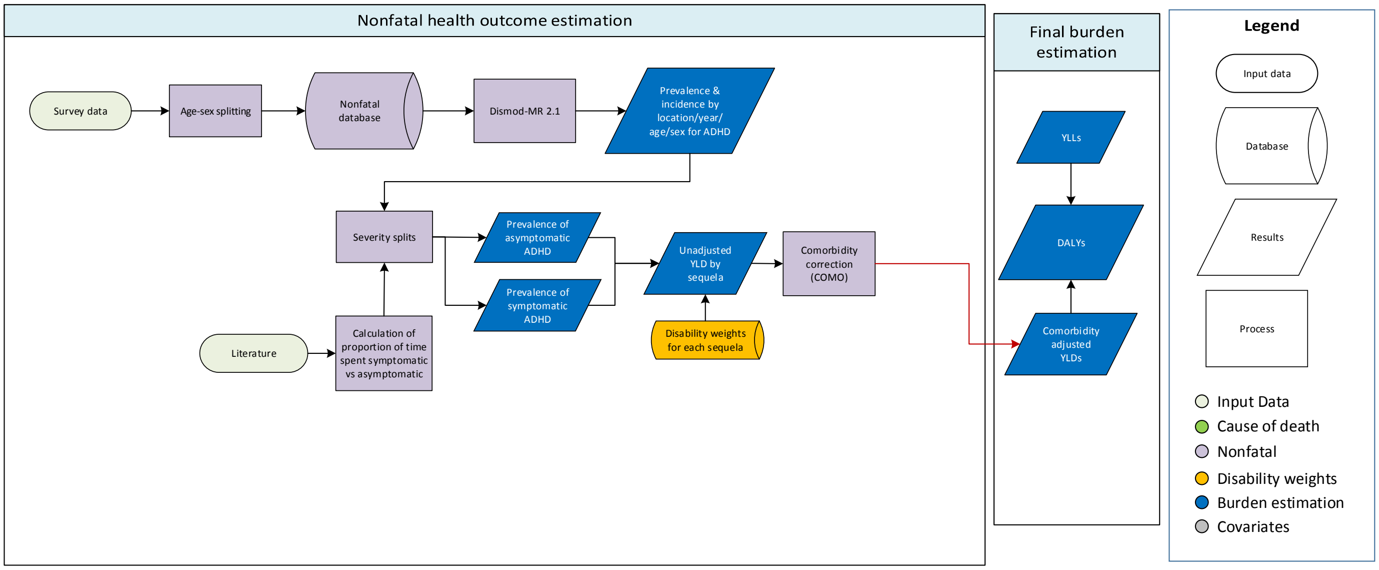

Case definition

Attention-deficit/hyperactivity disorder (ADHD) is an externalizing behavior disorder characterized by persistent inattention and/or hyperactivity-impulsivity. As per criteria set by the Diagnostic and Statistical Manual of Mental Disorders fourth edition, text revision (DSM-IV-TR)[1], diagnosis requires six or more symptoms of inattention or hyperactivity-impulsivity to have persisted for at least six months in two or more settings causing significant impairment to functioning, with at least some impairing symptoms being present prior to 7 years of age (12 years of age in DSM-5[2]). Recognized symptoms include:

Inattention:

- often fails to give close attention to details or makes careless mistakes in schoolwork, work, or other activities
- often has difficulty sustaining attention in tasks or play activities
- often does not seem to listen when spoken to directly
- often does not follow through on instructions and fails to finish school work, chores, or duties in the workplace (not due to oppositional behavior or failure to understand instructions)
- often has difficulty organizing tasks and activities
- often avoids, dislikes, or is reluctant to engage in tasks that require sustained mental effort (such as schoolwork or homework)
- often loses things necessary for tasks or activities (e.g., toys, school assignments, pencils, books, or tools)
- is often easily distracted by extraneous stimuli

- is often forgetful in daily activities

Hyperactivity

- often fidgets with hands or feet or squirms in seat
- often leaves seat in classroom or in other situations in which remaining seated is expected
- often runs about or climbs excessively in situations in which it is inappropriate (in adolescents or adults, may be limited to subjective feelings of restlessness)
- often has difficulty playing or engaging in leisure activities quietly
- is often "on the go" or often acts as if "driven by a motor"
- often talks excessively

Impulsivity

- often blurts out answers before questions have been completed
- often has difficulty awaiting turn
- often interrupts or intrudes on others (e.g., butts into conversations or games)

Included in GBD were cases meeting diagnostic criteria according to DSM[1] or the International Classification of Diseases (ICD)[3] (called "hyperkinetic disorder" in ICD). These were identified by the following codes: 314.0, 314.01 (DSM-IV-TR) and F90 (ICD-10). Different versions of DSM (DSM-III, DSM-III-R, DSM-IV, DSM-IV-TR, and DSM-5) and ICD (ICD-9 and ICD-10) were accepted.

Input data

Model inputs

A series of systematic literature reviews were conducted to capture studies reporting the prevalence, incidence, remission, and excess mortality of ADHD. The reviews incorporated searches of peer-reviewed literature via electronic databases, investigations of grey literature, and consultation with experts. In order for a study to be included, it must have been published during or after 1980, use DSM or ICD criteria to define cases, provide sufficient details on study methodology and sample characteristics to determine study quality, and be representative of the general population rather than a special population, e.g., prison inmates. No limitation was set on the language of publication. Detailed descriptions of this methodology have been published elsewhere.[4] This methodology was utilized in GBD 2010 and GBD 2013. GBD 2015 included additional sources identified by GBD experts and microdata where available. Updates to systematic reviews are performed on an ongoing schedule across all GBD causes, and an update for ADHD will be performed in the next one to two iterations

The final dataset for GBD 2015 included 175 prevalence estimates, five incidence estimates, 14 remission estimates, and three excess mortality estimates. The table below shows the number of studies for each parameter as well as the number of countries/subnationals and GBD world regions covered by the available data.

	Prevalence	Incidence	Remission	Mortality
Studies	96	2	11	2
Countries/subnationals	61	2	8	2
GBD world regions	13	1	2	2

Severity split inputs

A severity split for the proportion of time spent symptomatic versus asymptomatic was based on data from the Great Smoky Mountains Study which assessed the levels of disability found in children and adolescents with mental disorders.[5] Of those with ADHD, 48% reported disability while 20% of individuals with no diagnosis reported disability at the time of survey. Using these as estimates of the proportion of time with disability in the "average case," the proportion of disability in children without a diagnosis was subtracted from the proportion with disability for ADHD, giving an adjusted proportion of 28%. Detailed descriptions of this methodology have been published elsewhere.[6] The lay description and disability weight for ADHD is shown in the table below.

Lay description	DW (95% CI)
Iis hyperactive and has difficulty concentrating, remembering things, and completing tasks	0.045 (0.028-0.066)

Modeling strategy

Data across all epidemiological parameters were initially included in the modeling process. We assumed no incidence prior to 3 years of age or onward from 12 years of age. The minimum age of onset was set in consultation with experts and based on current literature, while the upper age limit on incidence was set in line with the latest DSM-5 criteria. Remission was set to 0 prior to 12 years, in line with the restriction on incidence, while a cap was placed on excess mortality to aid the model in making mortality estimates from extremely limited data. Two covariates were included in the model. The first covariate was an informant covariate which adjusted estimates not requiring agreement between informants (e.g., diagnosis made if either a teacher or parent indicates ADHD) toward estimates which required informant agreement. The second covariate adjusted estimates not requiring impairment (or those not specifying whether impairment was required) for diagnosis toward those which required impairment. Bounds for both covariates were calculated from the epidemiological data and applied in DisMod-MR 2.1.

Study covariate	Parameter	beta	Exponentiated beta
No informant agreement	Prevalence	0.51 (0.27-0.74)	1.66 (1.31-2.09)
No impairment	Prevalence	0.21 (0.12-0.43)	1.23 (1.13-1.53)

References

1. American Psychiatric Association. Diagnostic and Statistical Manual of Mental Disorders (DSM-IV-TR). 4th, Text Revision ed. Washington DC: American Psychiatric Association; 2000.
2. American Psychiatric Association. Diagnostic and Statistical Manual of Mental Disorders, Fifth Edition. Arlington, VA: American Psychiatric Association; 2013.
3. World Health Organisation. ICD-10 Classification of Mental and Behavioural Disorders: Clinical descriptions and diagnostic guidelines. Geneva: World Health Organisation; 1992.
4. Erskine HE, Ferrari AJ, Nelson P, et al. Research Review: Epidemiological modelling of attention-deficit/hyperactivity disorder and conduct disorder for the Global Burden of Disease Study 2010. *Journal of Child Psychology and Psychiatry* 2013; **54**(12): 1263-74.
5. Ezpeleta L, Keeler G, Erkanli A, Costello EJ, Angold A. Epidemiology of Psychiatric Disability in Childhood and Adolescence. *J Child Psychol Psychiatry* 2001; **42**(7): 901-14.

6. Erskine HE, Ferrari AJ, Polanczyk GV, et al. The global burden of conduct disorder and attention-deficit/hyperactivity disorder in 2010. *J Child Psychol Psychiatry* 2014; **55**(4): 328-36.

Conduct disorder

Flowchart

Conduct disorder

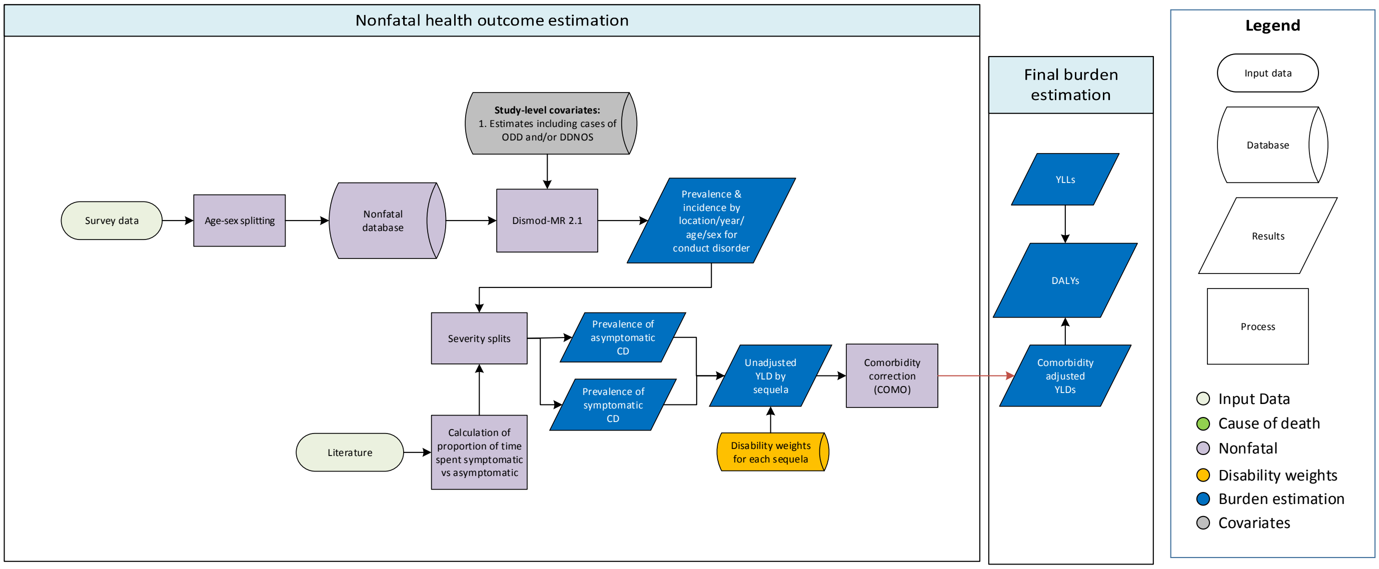

Case definition

Conduct disorder (CD) is an externalizing behavior disorder characterized by a pattern of antisocial behavior that violates the basic rights of others or major age-appropriate societal norms. As per criteria set by the Diagnostic and Statistical Manual of Mental Disorders fourth edition, text revision (DSM-IV-TR),[1] diagnosis requires three or more of the following symptoms to be present in the past 12 months (with at least one present in the last six months) and cause significant impairment in functioning. Symptoms include:

Aggression to people and animals

- often bullies, threatens, or intimidates others
- often initiates physical fights
- has used a weapon that can cause serious physical harm to others (e.g., a bat, brick, broken bottle, knife, gun)
- has been physically cruel to people
- has been physically cruel to animals
- has stolen while confronting a victim (e.g., mugging, purse snatching, extortion, armed robbery)
- has forced someone into sexual activity

Destruction of property

- has deliberately engaged in fire setting with the intention of causing serious damage
- has deliberately destroyed others' property (other than by fire setting)

Deceitfulness or theft

- has broken into someone else's house, building, or car
- often lies to obtain goods or favors or to avoid obligations (i.e., "cons" others)
- has stolen items of nontrivial value without confronting a victim (e.g., shoplifting, but without breaking and entering; forgery)

Serious violations of rules

- often stays out at night despite parental prohibitions, beginning before age 13 years
- has run away from home overnight at least twice while living in parental or parental surrogate home (or once without returning for a lengthy period)
- is often truant from school, beginning before age 13 years

CD is considered a disorder of childhood but can be diagnosed in adults who display such behaviors yet do not meet the criteria for antisocial personality disorder. However, there are almost no studies measuring adult CD as existing studies in this area tend to measure adult antisocial behavior rather than adult CD.[2] As such, only childhood CD (i.e., cases prior to 18 years of age) was modeled in GBD.

Included in GBD were cases meeting diagnostic criteria according to DSM[1] or the International Classification of Diseases (ICD).[3] These were identified by the following codes: 312 (DSM-IV-TR) and F91 (ICD-10). Different versions of DSM (DSM-III, DSM-III-R, DSM-IV, DSM-IV-TR, and DSM-5) and ICD (ICD-9 and ICD-10) were accepted. Estimates also including oppositional defiant disorder (ODD; DSM-IV-TR: 313.81, ICD-10: F91.3) or disruptive behavior disorder not otherwise specified (DDNOS, DSM-IV-TR: 312.9, ICD-10: 91.9) were accepted and adjusted with a covariate during the modeling process.

Input data

Model inputs

A series of systematic literature reviews were conducted to capture studies reporting the prevalence, incidence, remission, and excess mortality of CD. The reviews incorporated searches of peer-reviewed literature via electronic databases, investigations of grey literature, and consultation with experts. In order for a study to be included, it must have been published during or after 1980, use DSM or ICD criteria to define cases, provide sufficient details on study methodology and sample characteristics to determine study quality, and be representative of the general population rather than a special population, e.g., prison inmates. No limitation was set on the language of publication. Detailed descriptions of this methodology have been published elsewhere.[2] This methodology was utilized in GBD 2010 and GBD 2013. GBD 2015 included additional sources identified by GBD experts and microdata where available. Updates to systematic reviews are performed on an ongoing schedule across all GBD causes, and an update for CD will be performed in the next one to two iterations.

The final dataset for GBD 2015 included 108 prevalence estimates, eight incidence estimates, and six remission estimates. No estimates of excess mortality were found for CD. The table below shows the number of studies for each parameter as well as the number of countries/subnationals and GBD world regions covered by the available data.

	Prevalence	Incidence	Remission	Mortality
Studies	48	4	5	0
Countries/subnationals	38	4	4	0
GBD world regions	13	2	2	0

Severity split inputs

A severity split for the proportion of time spent symptomatic versus asymptomatic was based on data from the Great Smoky Mountains Study which assessed the levels of disability found in children and adolescents with mental disorders.[4] Of those with CD, 72% reported disability while 20% of individuals with no diagnosis reported disability at the time of survey. Using these as estimates of the proportion of time with disability in the “average case,” the proportion of disability in children without a diagnosis was subtracted from the proportion with disability for CD, giving an adjusted proportion of 52%. Detailed descriptions of this methodology have been published elsewhere.[5] The lay description and disability weight for CD is shown in the table below.

Lay description	DW (95% CI)
Has frequent behavior problems, which are sometimes violent. The person often has difficulty interacting with other people and feels irritable	0.241 (0.159-0.341)

Modeling strategy

Data across all epidemiological parameters were initially included in the modeling process. We assumed no incidence or prevalence prior to 5 years of age or after 18 years of age. The minimum age of onset was set in consultation with experts while the upper age limit was set in line with DSM criteria. Excess mortality was set to zero given the absence of data demonstrating an association between CD and an increased risk of death. Remission and incidence were capped between ages 4-17 years in order to gain more plausible output. A covariate was used to adjust any prevalence estimates which also included cases of oppositional defiant disorder and/or disruptive behavior disorder not otherwise specified towards those including CD only. Bounds for this covariate was calculated from the epidemiological data and applied in DisMod-MR 2.1. We have made no substantive changes in the modeling strategy from GBD 2013.

Covariate	Parameter	beta	Exponentiated beta
Identifies estimates also containing ODD &/or DDNOS cases	Prevalence	0.51 (0.41-0.76)	1.67 (1.51-2.14)

References

1. American Psychiatric Association. Diagnostic and Statistical Manual of Mental Disorders (DSM-IV-TR). 4th, Text Revision ed. Washington DC: American Psychiatric Association; 2000.
2. Erskine HE, Ferrari AJ, Nelson P, et al. Research Review: Epidemiological modelling of attention-deficit/hyperactivity disorder and conduct disorder for the Global Burden of Disease Study 2010. *Journal of Child Psychology and Psychiatry* 2013; **54**(12): 1263-74.
3. World Health Organisation. ICD-10 Classification of Mental and Behavioural Disorders: Clinical descriptions and diagnostic guidelines. Geneva: World Health Organisation; 1992.
4. Ezpeleta L, Keeler G, Erkanli A, Costello EJ, Angold A. Epidemiology of Psychiatric Disability in Childhood and Adolescence. *J Child Psychol Psychiatry* 2001; **42**(7): 901-14.
5. Erskine HE, Ferrari AJ, Polanczyk GV, et al. The global burden of conduct disorder and attention-deficit/hyperactivity disorder in 2010. *Journal of Child Psychology and Psychiatry* 2014; **55**(4): 328-36.

Diabetes mellitus

Flowchart

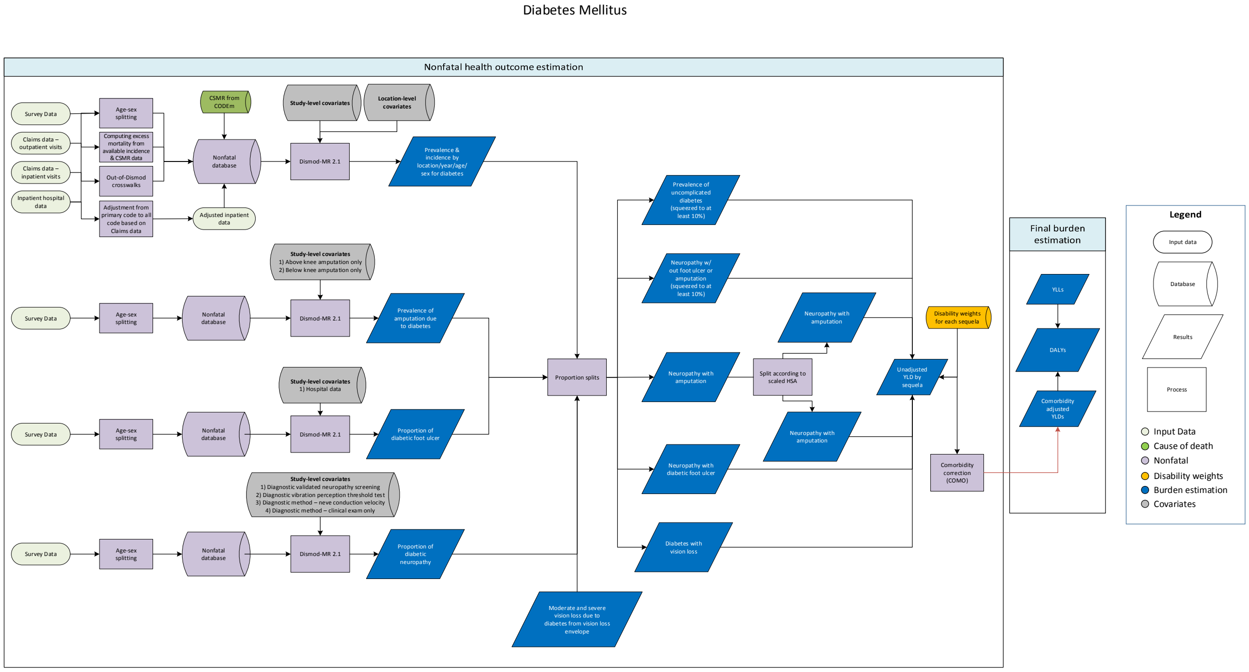

Case definition

The case definitions and diagnostic criteria are presented in the table below. The associated ICD codes include:

Criterion	Definition
1. Diabetes mellitus parent	Chronic cases of diabetes mellitus (DM) defined as all cases meeting either criteria: (1) threshold of fasting plasma glucose (FPG), postprandial plasma glucose (PPG), or hemoglobin A1c, and (2) physician-diagnosed or on current treatment. The goal is to capture all cases of diabetes, regardless of previous diagnosis.
2. Uncomplicated diabetes mellitus	Cases of DM that do not have any of the following complications: neuropathy, foot ulcer, leg amputation, or vision loss
3. Diabetic neuropathy	Cases of DM that experience diagnosable neuropathy
4. Diabetic foot due to neuropathy	Cases of DM that currently have a foot ulcer
5. Diabetic neuropathy and amputation with treatment	Cases of DM that have had a leg amputation above or below the knee, with treatment consisting of a prosthetic limb
6. Diabetic neuropathy and amputation without treatment	Cases of DM that have had a leg amputation above or below the knee, with no prosthetic limb
7. Moderate vision impairment due to diabetes mellitus	Cases of DM that have moderate vision loss due to diabetic retinopathy

8. Severe vision impairment due to diabetes mellitus	Cases of DM that have severe vision loss due to diabetic retinopathy
9. Blindness due to diabetes mellitus	Cases of DM that have blindness due to diabetic retinopathy

Input data

Model inputs

Diabetes mellitus parent:

A systematic review of the literature was done for GBD 2015 with the following search terms:

(diabetes[TI] AND (prevalence[TIAB] OR incidence[TIAB])) OR ('diabetes mellitus'[MeSH Terms] AND 'epidemiology'[MeSH Terms]) OR (diabetes[TI] AND 'epidemiology'[MeSH Terms]) NOT gestational[All Fields] NOT ('neoplasms'[MeSH Terms] OR 'neoplasms'[All Fields] OR 'cancer'[All Fields]) NOT ('mice'[MeSH Terms] OR 'mice'[All Fields]) NOT ('schizophrenia'[MeSH Terms] OR 'schizophrenia'[All Fields]) NOT ('emigrants and immigrants'[MeSH Terms] OR ('emigrants'[All Fields] AND 'immigrants'[All Fields]) OR 'emigrants and immigrants'[All Fields] OR 'immigrants'[All Fields]) NOT ('pregnancy'[MeSH Terms] OR 'pregnancy'[All Fields] OR 'gestation'[All Fields]) NOT ('rats'[MeSH Terms] OR 'rats'[All Fields] OR 'rat'[All Fields]) NOT ('kidney'[MeSH Terms] OR 'kidney'[All Fields]) NOT renal[All Fields] NOT ('vitamins'[Pharmacological Action] OR 'vitamins'[MeSH Terms] OR 'vitamins'[All Fields] OR 'vitamin'[All Fields]) AND ('1990/01/01'[PDAT] : '3000/12/31'[PDAT])

The search took place for the following dates: 1/1/1990 – 9/9/2015. The number of studies returned was 14,938, and the number of studies extracted was 291.

A systematic review of the literature was also done for GBD 2013 with the following search string:

prevalence[Title/Abstract] AND diabetes[Title] AND (“2010”[Date – Publication] : “2013”[Date – Publication])

The table below illustrates the data inputs for the GBD 2015 estimation of diabetes mellitus parent:

	Prevalence	Incidence	Mortality risk
Studies	578	126	8
Countries/subnationals	300	66	8
GBD world regions	21	15	3

We identified survey data with data on diabetes based on information from collaborators and evaluation of available information. Data were crosswalked from alternate definitions to the reference definition using data from NHANES.

Amputation due to diabetes mellitus

A systematic review of the literature was performed for GBD 2015 with the following search terms:

(‘diabetes mellitus’[MeSH Terms] OR (‘diabetes’[All Fields] AND ‘mellitus’[All Fields]) OR ‘diabetes mellitus’[All Fields]) AND ‘amputation’[All Fields] AND (proportion OR prevalence OR incidence) NOT gestational NOT cancer NOT mice NOT schizophrenia NOT immigrants NOT gestation NOT rat NOT kidney NOT renal NOT vitamin

- Dates of search: 1/1/1990 – 8/25/2015
- Number of studies returned: 1,277
- Number of studies extracted: 35

The table below indicates the data inputs for the GBD 2015 estimation of amputation due to diabetes mellitus.

	Prevalence	Incidence	Mortality risk
Studies	12	32	5
Countries/subnationals	10	24	5
GBD world regions	6	7	4

Diabetic neuropathy

A systematic review of the literature was performed for GBD 2015 with the following search terms: (“diabetes mellitus”[MeSH Terms] OR (“diabetes”[All Fields] AND “mellitus”[All Fields]) OR “diabetes mellitus”[All Fields] OR “diabetes”[All Fields] OR “diabetes insipidus”[MeSH Terms] OR (“diabetes”[All Fields] AND “insipidus”[All Fields]) OR “diabetes insipidus”[All Fields]) AND neuropathy[All Fields] AND (proportion OR prevalence OR incidence) NOT gestational NOT cancer NOT mice NOT schizophrenia NOT immigrants NOT gestation NOT rat NOT kidney NOT renal NOT vitamin

- Dates: 1/1/1900 – 8/19/2015
- Number of studies returned: 2,636
- Number of studies extracted: 50

The table below illustrates the model inputs for the GBD 2015 estimation process:

	Proportion
Studies	89
Countries/subnationals	66
GBD world regions	13

Diabetic foot ulcer

A systematic review of the literature was performed for GBD 2015 with the following search terms:

(‘diabetes mellitus’[MeSH Terms] OR (‘diabetes’[All Fields] AND ‘mellitus’[All Fields]) OR ‘diabetes mellitus’[All Fields] OR ‘diabetes’[All Fields] OR ‘diabetes insipidus’[MeSH Terms] OR (‘diabetes’[All Fields] AND ‘insipidus’[All Fields]) OR ‘diabetes insipidus’[All Fields]) AND ulcer[All Fields]AND (proportion OR prevalence OR incidence) NOT gestational NOT cancer NOT mice NOT schizophrenia NOT immigrants NOT gestation NOT rat NOT kidney NOT renal NOT vitamin

- Dates: 1/1/1990 – 7/8/2015
- Number of studies returned: 1,059
- Number of studies extracted: 16

A systematic review of the literature was not performed for GBD 2013. The table below illustrates the data inputs used for the GBD 2015 modeling process.

	Proportion
Studies	43
Countries/subnationals	37
GBD world regions	9

Severity split inputs

Severity splits and disability weights were determined centrally for diabetes mellitus. The table below illustrates the severity levels, lay descriptions, and associated disability weights:

Severity level	Lay description	DW (95% CI)
Uncomplicated diabetes mellitus	Has a chronic disease that requires medication every day and causes some worry, but minimal interference with daily activities	0.049 (0.031 – 0.072)
Diabetic neuropathy	Has pain, tingling, and numbness in the arms, legs, hands, and feet. The person sometimes gets cramps and muscle weakness.	0.133 (0.089 – 0.187)
Diabetic neuropathy with diabetic foot	Has a sore on the foot that is swollen and causes some difficulty in walking.	0.02 (0.01-0.034)
Diabetic neuropathy with treated amputation	Has lost part of one leg, leaving pain and tingling in the stump. The person has an artificial leg that helps in moving around.	0.039 (0.023 – 0.059)
Diabetic neuropathy with untreated amputation	Has lost part of one leg, leaving pain and tingling in the stump. The person does not have an artificial leg, has frequent sores, and uses crutches.	0.173 (0.118 – 0.24)
Moderate vision loss due to diabetes mellitus	Has vision problems that make it difficult to recognize faces or objects across a room.	0.031 (0.019 – 0.049)
Severe vision loss due to diabetes mellitus	Has severe vision loss, which causes difficulty in daily	0.184 (0.125 – 0.259)

	activities, some emotional impact (for example worry), and some difficulty going outside the home without assistance.	
Blindness due to diabetes mellitus	Is completely blind, which causes great difficulty in some daily activities, worry and anxiety, and great difficulty going outside the home without assistance.	0.187 (0.124 – 0.26)

Modeling strategy

For GBD 2015, we estimated the overall prevalence of diabetes using DisMod MR-2.1, a Bayesian meta-regression. We also estimated amputation due to diabetes mellitus, diabetic neuropathy, and diabetic foot using DisMod. We then multiply all proportion draws from neuropathy/foot/amputation models by parent diabetes model so that all estimates are in the same population-space.

Next, we squeeze (neuropathy + moderate vision loss + severe vision loss) to (90% of parent diabetes) prevalence if sum exceeds that 90%. This is to ensure that at least 10% of diabetes cases are uncomplicated for all draws. We then squeeze (amputation + foot ulcer) to (90% of neuropathy) prevalence if sum exceeds 90%. This is to ensure that at least 10% of diabetic neuropathy cases do not have foot ulcer or amputation for all draws. This treats foot ulcer and amputation as mutually exclusive categories by assuming a patient won't have both simultaneously.

From here, we calculate uncomplicated diabetes as the remainder of diabetes cases exclusive of neuropathy and vision loss. In addition, we estimate the prevalence of amputation due to diabetes is split into with and without treatment using scaled HSA values. This split is done centrally.

Diabetes Mellitus

- We set a value prior of 0 for remission for ages 0 to 14
- We set a value prior of a maximum value of 0.01 for remission for ages 15 to 100
- We set a value prior of a maximum value of 0.15 for excess mortality for all ages
- We set a value prior of 0 for incidence for ages 0 to 1
- We set a value prior of a maximum value of 0.1 for incidence for ages 1 to 100
- We crosswalked the prevalence of diabetes in the claims data from 2000, 2010, and 2012 using as reference the prevalence of diabetes in the literature

Study covariate	Parameter	beta	Exponentiated beta
Year	incidence	.0477(.0435 - .05)	1.049#(1.044 - 1.051)
LDI (I$ per capita)	excess mortality rate	-.252(-.2819 - -.2333)	.7772(.7543 - .7919)
All MarketScan, year 2012	prevalence	-.2948(-.3574 - -.2043)	.7447(.6995 - .8152)

Diabetes Fasting Plasma Glucose (mmol/L)	prevalence	.4384(.3794 - .4911)	1.55(1.461 - 1.634)
Year	prevalence	.0103(.008 - .0128)	1.01(1.008 - 1.013)
Diabetes Fasting Plasma Glucose (mmol/L)	incidence	.5846(.2553 - .9064)	1.794(1.291 - 2.475)
All MarketScan, year 2000	prevalence	-.6384(-.7002 - -.5582)	.5281(.4965 - .5722)

Amputation due to diabetes

- We set a value prior of 0 for incidence for ages 0 to 15
- We set a value prior of 0 for remission for all ages
- We crosswalked the incidence of either above or below knee amputation only to the incidence of all amputations

Study covariate	Parameter	beta	Exponentiated beta
Above knee amputation only	incidence	-.2951(-.5573 - -.0495)	.7445(.5728 - .9517)
Below knee amputation only	incidence	-.4264(-.6721 - -.1742)	.6528(.5107 - .8401)

Diabetic neuropathy

- We set a value prior on the proportion of 0 from ages 0 to 1
- We crosswalked data from studies using alternate diagnostic criteria using as reference studies which used the monofilament test as their diagnostic criteria

Study covariate	Parameter	Beta	Exponentiated beta
Diagnostic vibration perception threshold test	proportion	-.1879(-.3693 - -.0015)	.8287 (.6912 - .9985)
Diagnostic method - nerve conduction velocity	proportion	-.2712(-.5187 - -.0419)	.7624(.5953 - .959)
Diagnostic method - clinical exam only	proportion	-.1188(-.3844 - .129)	.888(.6809 - 1.138)
Diagnostic validated neuropathy scoring	proportion	-.0912(-.303 - .1213)	.9129(.7386 - 1.129)

Diabetic foot ulcer

- We set a value prior on the proportion of 0 from ages 0 to 10.
- We crosswalked data from studies investigating hospitalized patients only using as reference studies which captured all diabetic foot ulcers.

Study covariate	Parameter	beta	Exponentiated beta
Hospital data	proportion	.4153(.0641 - .759)	1.515(1.066 - 2.136)

We have made no substantive changes in the modeling strategy from GBD 2013

Acute glomerulonephritis

Flowchart

Acute Glomerulonephritis

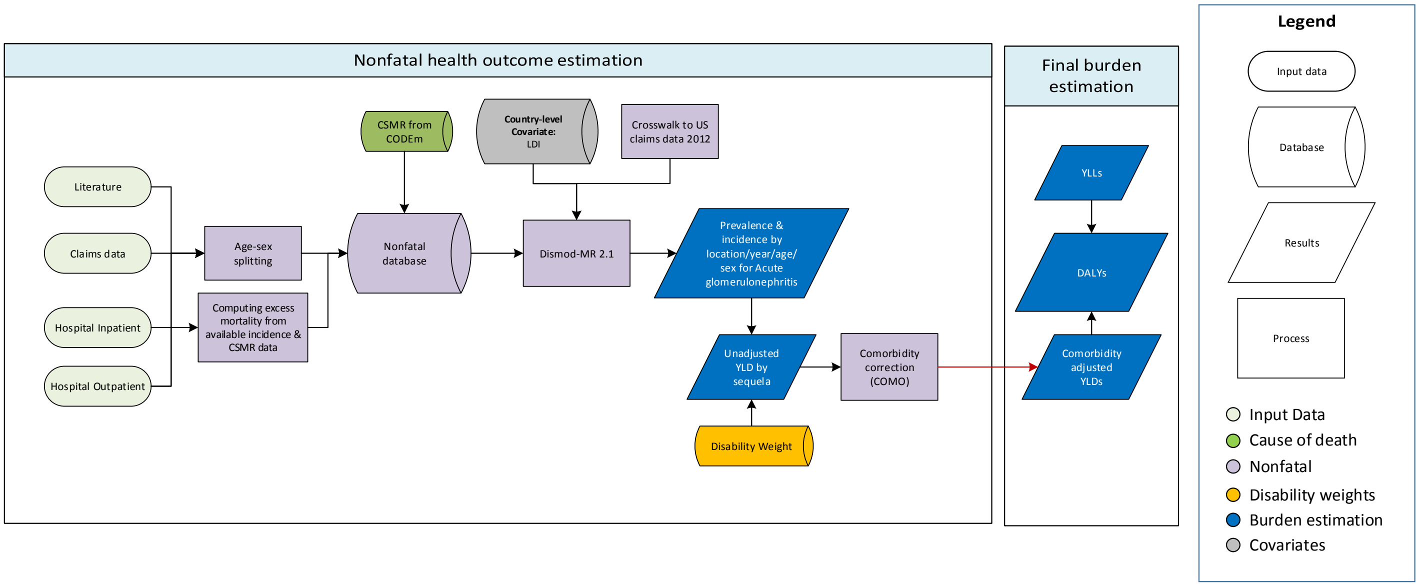

Case Definition

Acute glomerulonephritis (AG) (or post-infectious glomerulonephritis) is an acute episode of hematuria, edema, hypertension, and acute kidney injury that typically follows infection with specific strains of group A beta-hemolytic streptococcus. As used in the GBD study, this term is synonymous with post-streptococcal or post-staphylococcal glomerulonephritis. This disease is typically seen in children but can demonstrate a bimodal distribution as early life immunity wanes within older years. ICD codes include N00, N00.0, N00.1, N00.2, N00.3, N00.4, N00.5, N00.6, N00.7, N00.8, N00.9, N01, N01.1, N01.2, N01.3, N01.4, N01.5, N01.6, N01.7, N01.8, and N01.9.

Input data

Model inputs

For GBD 2010, a systematic review of the prevalence of AG throughout the world was conducted. This search was updated for GBD 2013 and GBD 2015. For GBD 2015 a PubMed search was conducted using the following search terms: ((Acute glomerulonephritis[Title/Abstract] OR GN[Title/Abstract]) AND (incidence[Title/Abstract] OR prevalence[Title/Abstract])) AND ('2013'[PDAT] : '3000'[PDAT]).

The exclusion criteria were:

1. Studies clearly not representative of the national population
2. Studies that did not provide primary data on epidemiological parameters, e.g., a commentary piece
3. Studies that describe non-infectious glomerulonephritis epidemiology

The next planned PubMed search will be conducted for GBD 2016. Opportunistically, additional studies encountered during data review were added. The most recent literature source dates from 2013. Twenty-one articles have been included in total, two of which were added in the last literature review.

The table below shows the number of studies included in GBD 2015, as well as the number of countries and GBD world regions represented by either studies or hospital data.

	Incidence	Mortality
Studies	28	2
Countries	25	2
Regions	9	--

Data from US claims data for 2000, 2010, and 2012 by US state were included. Hospital inpatient and outpatient data was also included. Comparing rates of outpatient data, we detected implausible differences in Canada's rates when compared to outpatient data from all other countries, so data from this country were excluded.

Severity split & disability weight

The basis of the GBD disability weight assessment is lay descriptions of sequelae highlighting major functional consequences and symptoms. Disability weighting (DW) for AG associates with systemic symptoms of fever, aches, weakness, and some difficulty with daily activities (below). The lay description and disability weight for acute glomerulonephritis are shown below.

Cause	Lay description	DW (95% CI)
Acute glomerulonephritis	Has a fever and aches, and feels weak, which causes some difficulty with daily activities.	0.051 (0.032, 0.074)

Modeling strategy

We ran a DisMod-MR 2.1 model to produce estimates by age, sex, year, and country. Prior settings in the DisMod model included setting remission of four to six weeks. It was assumed that no one was born with AG.

We applied a crosswalk to all US claims data and hospital inpatient and outpatient data to adjust to 2012 US claims data. We used the function in DisMod-MR 2.1 to pull in cause-specific mortality rate (CSMR) data from our CODEm and CODcorrect analyses and match them with incidence data points for the same geography and study year to estimate priors on excess mortality rates (by dividing CSMR by incidence).

We included the covariate Lagged Distributed Income (LDI) as a country-level covariate to inform excess mortality, with bounds of -1, -0.1. Incidence was informed by the sociodemographic status covariate, with bounds of (-1, 1).

Betas and exponentiated values (which can be interpreted as an odds ratio) are shown in the table below:

Study covariate	Parameter	beta	Exponentiated beta
Claims data - 2000	Incidence	-0.12<br>(-0.15, -0.088)	0.89<br>(0.86, 0.92)
Claims data - 2010	Incidence	-0.005<br>(-0.015, -0.00027)	1.00<br>(0.98, 1.00)
Hospital, Outpatient	Incidence	-0.32<br>(-0.45, -0.18)	0.73<br>(0.64, 0.83)
Hospital, Inpatient	Incidence	-0.053<br>(-0.076, -0.032)	0.95<br>(0.93, 0.97)

Major changes to our modeling strategy from GBD 2013 include use of Marketscan data.

Chronic kidney disease

Flowchart

Chronic Kidney Disease (CKD)

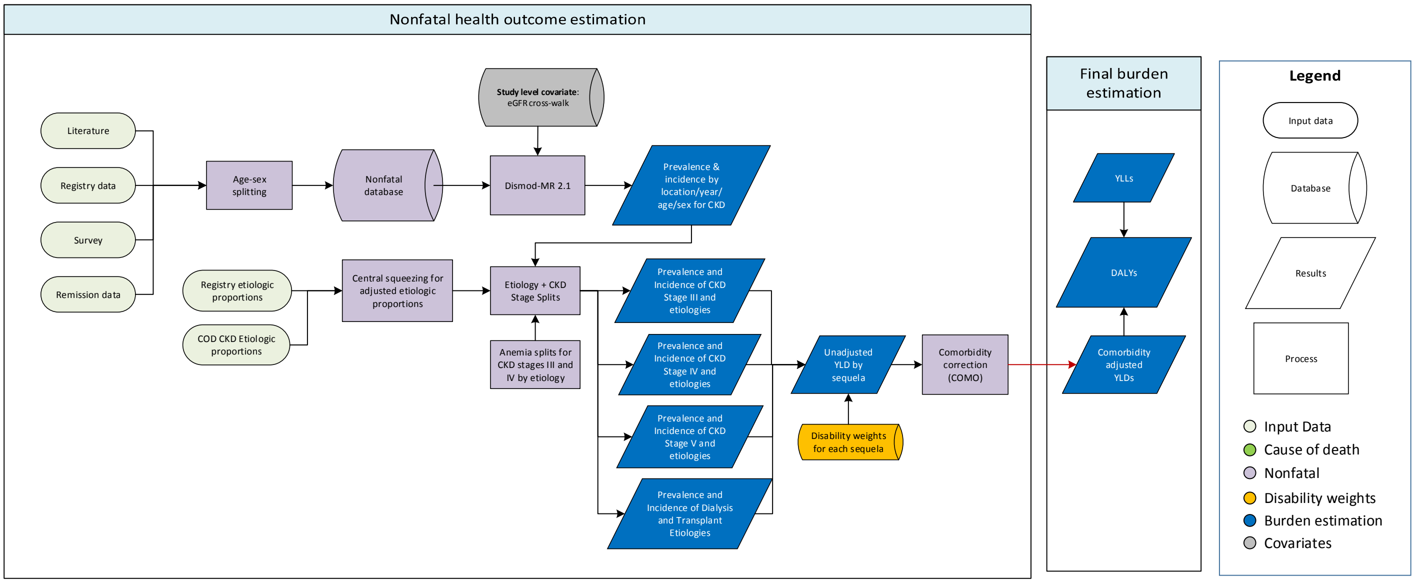

Case definition

Chronic kidney disease (CKD) is defined as a permanent loss of renal function as indicated by estimated glomerular filtration rate (eGFR). The GBD study considers five stages of CKD as defined by degree of loss of renal function or receipt of renal replacement therapy: CKD Stage III (eGFR 30-60ml/min/1.73m^2) CKD Stage IV (eGFR 15-30ml/min/1.73m^2), CKD Stage V (eGFR <15ml/min/1.73m^2, not on renal replacement therapy), maintenance dialysis, and renal transplantation.[1] The ICD-10 codes associated with CKD include N18.1-N18.9.

Input data

Model inputs

For GBD 2010, a systematic review of the prevalence of CKD throughout the world was conducted. This search was updated for GBD 2013. For GBD 2015, this literature search was repeated using PubMed search terms: ((chronic kidney disease[Title/Abstract]) AND prevalence[Title/Abstract]) AND ('2012/01/01'[Date - Publication] : '3000'[Date - Publication]) (humans).

The exclusion criteria were:

1. Studies clearly not representative of the national population
2. Studies that did not provide primary data on epidemiological parameters, e.g., a commentary piece
3. Studies of a specific etiology of CKD

This literature search was augmented by identification of population-based surveys that measured renal function. For maintenance dialysis and renal transplantation, data were largely obtained from registry reports.

The next planned PubMed search will be conducted for GBD 2016.

Disease	Number of sources	Super-regions with data
CKD Stage III	64	All seven super-regions
CKD Stage IV	49	
CKD Stage V	43	
Maintenance dialysis	83	
Renal transplantation	134	

Severity splits & disability weights

CKD stages III-V prevalence estimates are split using CKD etiology proportion models, resulting in CKD estimates by stage and etiology. Then a portion of each etiology split for CKD stages III and IV is attributed disability weighting associated with mild, moderate, and severe anemia.[2] CKD Stage V DW is described as weight loss, constant pain, primarily bedbound (see table below).

Severity level	Lay description	Disability weight (95% CI)
CKD stage III without anemia	Asymptomatic	--
CKD stage III with mild anemia	Feels slightly tired and weak at times, but this does not interfere with normal daily activities.	0.004 (0.001-0.008)
CKD stage III with moderate anemia	Feels moderate fatigue, weakness, and shortness of breath after exercise, making daily activities more difficult.	0.052 (0.034, 0.076)
CKD stage III with severe anemia	Feels very weak, tired, and short of breath, and has problems with activities that require physical effort or deep concentration.	0.149 (0.101, 0.21)
CKD stage IV without anemia	Tires easily, has nausea, reduced appetite, and difficulty sleeping.	0.104 (0.07-0.147)
CKD stage IV with mild anemia		0.108 (0.072, 0.151)
CKD stage IV with moderate anemia		0.15 (0.103, 0.207)
CKD stage IV with severe anemia		0.237 (0.165-0.324)

CKD stage V	Has lost a lot of weight and has constant pain. The person has no appetite, feels nauseated, and needs to spend most of the day in bed.	0.569 (0.389, 0.727)
End-stage renal disease, on dialysis	Is tired and has itching, cramps, headache, joint pains, and shortness of breath. The person needs intensive medical care every other day lasting about half a day.	0.571 (0.397-0.725)
End-stage renal disease, with kidney transplant	Sometimes feels tired and down, and has some difficulty with daily activities.	0.024 (0.014-0.039)

Etiology proportion models are informed by renal registry etiology proportion data, combined with etiology proportions from the GBD Cause of Death database. These two data sources are combined, and the etiology-specific proportions are squeezed to equal 1. These adjusted proportions are applied to the CKD stages as described above, as well as the parent CKD model from the GBD cause of death analysis.

Etiologies included in the GBD study include diabetes mellitus, hypertension, glomerulonephritis, and "other." "Other" excludes cystic diseases, urologic diseases, and toxins/poisons.

Modeling strategy

	Priors (bounds)	Study-level covariates	Country-level covariate
CKD stage III	Remission (0,0.75)<br>Excess mortality (0, 0.05)	Prevalence adjusted for estimating equation used	---
CKD stage IV	Remission (0,0.75)<br>Excess mortality (0, 0.05)	Prevalence adjusted for estimating equation used	---
CKD stage V	Remission (0.5, 2)<br>Incidence (0, 0.001)	Prevalence adjusted for estimating equation used	Age-standardized prevalence of CKD stage IV

As the majority of published data for CKD stages describe prevalence, we informed remission for stages III and IV in order to allow the compartmental model to solve for all parameters. Remission data for CKD stage IV involved the ratio of the incidence of CKD stage V and prevalence of stage IV at the gender, age, and country-matched level. This resulted in a full parameter stage IV model, and enabled us to take the ratio of resulting CKD stage IV incidence and CKD stage III prevalence to determine remission data to convert CKD stage III to a full parameter model.

The maintenance dialysis and renal transplant models included bounds on location random effects for East Asia. As Taiwan has rates of renal replacement therapy far out of proportion to surrounding East Asian countries, these bounds prevent renal replacement therapy estimates for surrounding East Asian countries lacking data to overinflate.

We crosswalked data reporting glomerular filtration rate (GFR) estimated with the CKD-Epi equation to data reported using the MDRD equation as our baseline. We chose the MDRD equation as our baseline as this equation has been validated across multiple populations. GFR reported for children was estimated using the Schwartz equation as the gold standard among the pediatric population. Betas and exponentiated values (which can be interpreted as an odds ratio) are shown in the table below:

Study covariate	Parameter	beta	Exponentiated beta
eGFR calculated with CKD-Epi Equation	Prevalence	-0.099<br>(-0.56, 0.41)	0.91<br>(0.57, 1.51)

Interstitial nephritis and urinary tract infections

Flowchart

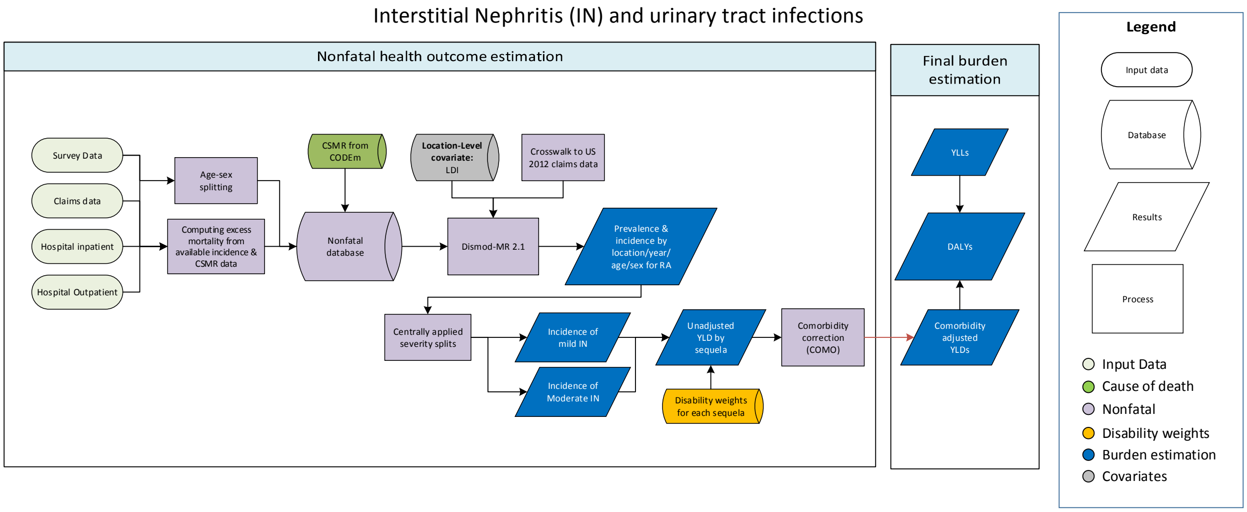

Case definition

Interstitial nephritis (IN) is defined as a kidney infection that can lead to systemic symptoms such as fever and weakness and can cause discomfort and difficulty with daily activities.[1] ICD codes include N10, N10.0, N10.9, N11, N11.0, N11.1, N11.8, N11.9, N12, N12.0, and N12.9.

Input data

Model inputs

The interstitial nephritis model is informed by survey data, US state-level claims data, and global hospital inpatient and outpatient data. US Claims data from years 2000, 2010, and 2012 were included. The table below shows the number of countries and GBD world regions represented.

	Incidence
Countries/subnationals	21
GBD world regions	8

Severity splits & disability weights

The basis of the GBD disability weight survey assessments is lay descriptions of sequelae highlighting major functional consequences and symptoms. Interstitial nephritis is split into mild and moderate severity. Mild severity is associated with a disability weight that correlates with low fever, mild discomfort, but no difficulty with daily activities. Moderate discomfort is associated with a disability

weight that correlates with systemic symptoms of fever, aches, weakness, and some difficulty with daily activities. The lay descriptions and disability weights for IN are shown below.

Severity level	Lay description	DW (95% CI)
Interstitial nephritis and urinary tract infection, mild	Has a low fever and mild discomfort, but no difficulty with daily activities.	0.006 (0.002, 0.012)
Interstitial nephritis and urinary tract infection, moderate	Has a fever and aches, and feels weak, which causes some difficulty with daily activities.	0.051 (0.032, 0.074)

Modeling strategy

We ran a DisMod MR-2.1 model to produce estimates by age, sex, year, and location. Prior settings in the IN DisMod 2.1 model included remission after about one week between ages 0 and 100. We applied a crosswalk to all US claims data and hospital inpatient and outpatient data to adjust to 2012 US claims data. We used the function in DisMod-MR 2.1 to pull in cause-specific mortality rate (CSMR) data from our CODEm and CODcorrect analyses and match them with incidence data points for the same geography and study year to estimate priors on excess mortality rates (by dividing CSMR by incidence).

We included the covariate Lagged Distributed Income (LDI) as a country-level covariate to inform excess mortality, with bounds of -1, -0.1. We used this covariate based on the assumption of a higher likelihood of mortality based on the developmental status of a country.

Betas and exponentiated values (which can be interpreted as an odds ratio) are shown in the table below:

Study covariate	Parameter	beta	Exponentiated beta
Claims data – 2000	Incidence	-0.99 (-1.95, 0)	0.37 (0.14, 1.00)
Claims data –2010	Incidence	-1 (-1.98, 0)	0.37 (0.14, 1.00)
Hospital, outpatient	Incidence	-0.7 (-0.8, -0.6)	0.50 (0.45, 0.55)
Hospital, inpatient	Incidence	-1.8 (-2.05, -1.54)	0.17 (0.13, 0.21)

Major changes from the GBD 2013 analysis for IN include removal of the sex-specific covariate of contraception, as men and women were estimated within the same model.

Urolithiasis

Flowchart

Acute Urolithiasis

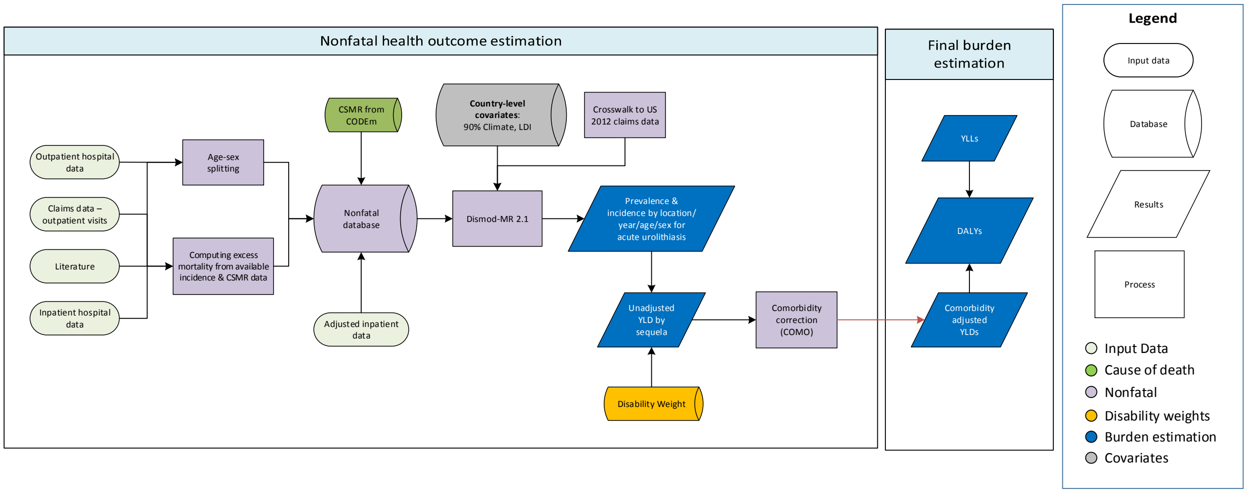

Case definition

Acute urolithiasis (AU) is an acute and usually symptomatic episode of urolithiasis, defined as stone formation located anywhere along the genitourinary tract.[1] Associated ICD codes include N20, N20.0, N20.1, N20.2, N20.9, N21, N21.1, N21.8, N21.9, N22, N22.0, N22.8, N23, and N23.0.

Input data

Model inputs

For GBD 2010, a systematic review of the prevalence of AU throughout the world was conducted. This search was updated for GBD 2013. A PubMed search was conducted using the following search terms: (urolithiasis[Title/Abstract] AND prevalence[Title/Abstract] AND ("2008"[PDAT] : "2013"[PDAT])) AND "humans"[MeSH Terms].

The exclusion criteria were:

1. Studies clearly not representative of the national population
2. Studies that did not provide primary data on epidemiological parameters, e.g., a commentary piece
3. Studies of a specific type of urolithiasis

The next planned PubMed search will be conducted for GBD 2016. Opportunistically, additional studies encountered during data review were added. The most recent literature source dates from 2013. The below table indicates the number of studies included and number of countries/regions represented by either study or hospital data:

	Incidence
Studies	18
Countries/subnationals	26
GBD world regions	9

Data from US claims data for 2000, 2010, and 2012 by US state were included. Hospital inpatient and outpatient data were also included. Comparing rates of outpatient data, we detected implausible differences in Canada and Acra rates when compared to other countries with outpatient data, so data from these locations were excluded.

The table below illustrates the DW associated with AU.

cause	Lay description	DW (95% CI)
Acute urolithiasis	Has pain in the belly and feels nauseated. The person has difficulties with daily activities.	0.114 (0.078, 0.159)

Modeling strategy

For GBD 2015, we modeled AU using DisMod MR 2.1, a Bayesian meta-regression modeling program.

Prior settings in the DisMod model included setting remission of two weeks and an upper bound limit of 0.1 for excess mortality.

We applied a crosswalk to all US claims data and hospital inpatient and outpatient data to adjust to 2012 US claims data.

We used the function in DisMod-MR 2.1 to pull in cause-specific mortality rate (CSMR) data from our CODEm and CODcorrect analyses and match them with incidence data points for the same geography and study year to estimate priors on excess mortality rates (by dividing CSMR by incidence).

We set bounds on location random effects for incidence for five super-regions for which data were sparse: high-income; Central Europe, Eastern Europe, and Central Asia; Southeast Asia, East Asia, and Oceania; Latin America and Caribbean; and North Africa and Middle East.

Betas and exponentiated values (which can be interpreted as an odds ratio) are shown in the table below:

Study covariate	Parameter	beta	Exponentiated beta
Claims data - 2000	Incidence	-0.13 (-0.16, -0.095)	0.88 (0.86, -0.91)
Claims data - 2010	Incidence	-0.00027 (-0.0012, -0.000046)	1.00 (1.00, 1.00)
Hospital, outpatient	Incidence	-0.029 (-0.084, -0.0023)	0.97 (0.92, 1.00)
Hospital, inpatient	Incidence	-0.27 (-0.28, -0.25)	0.77 (0.75, 0.78)

For GBD 2015 we only modeled acute urolithiasis. Chronic urolithiasis estimates were directly derived from acute urolithiasis data in previous GBD iterations. Applying this technique for this GBD iteration

would have required being able to identify acute urolithiasis episodes that repeated within an individual, which is a level of detail we currently do not estimate.

Benign prostatic hyperplasia (BPH)

Flowchart

Benign Prostatic Hyperplasia (BPH)

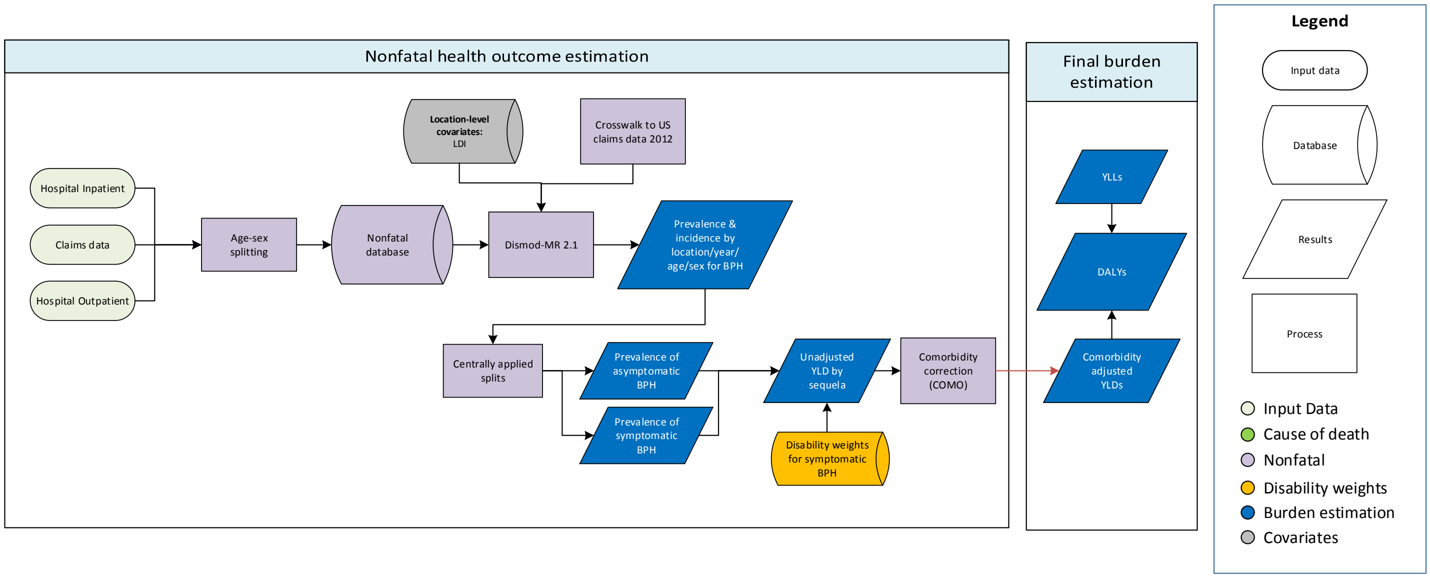

Case definition

Benign prostatic hyperplasia (BPH) is defined as a benign proliferation of prostatic tissue, often leading to symptoms such as urinary retention, bladder outlet obstruction, or urinary tract infection.[1,2] The ICD codes for BPH include N40, N40.0, N40.1, N40.2, N40.3, and N40.9.

Input data

Model inputs

The BPH model is informed by US state-level claims data and global hospital inpatient and outpatient data. US claims data from years 2000, 2010, and 2012 were included. Comparing rates of hospital outpatient BPH prevalence, we detected implausible values for all countries with outpatient data except for Norway, so these countries' data were excluded from the GBD 2015 analysis.

	Prevalence
Studies	33
Countries/subnationals	36
GBD world regions	11

Severity splits

The basis of the GBD disability weight survey assessments are lay descriptions of sequelae highlighting major functional consequences and symptoms of a given cause. BPH is split into symptomatic and asymptomatic types. There is no disability weight (DW) assigned to asymptomatic cases of BPH. The DW associated with symptomatic BPH regards urinary frequency that is sometimes associated with pain – as seen in the table below, which offers further information.

Severity level	Lay description	DW (95% CI)
Asymptomatic	N/A	0
Symptomatic	feels the urge to urinate frequently, but when passing urine it comes out slowly and sometimes is painful.	0.067 (0.043, 0.097)

Modeling strategy

We ran a DisMod 2.1 model to produce estimates by age, sex, year, and location. Prior settings in the BPH DisMod 2.1 model include setting incidence and remission prior to age 40 years to 0. We set an upper limit bound on remission after age 40 to 0.1. We also determined that there was no excess mortality related to BPH.

We applied a crosswalk to all US claims data and hospital inpatient and outpatient data to adjust to 2012 US claims data. We set bounds on location random effects for incidence for five super-regions for which data were sparse: High-income; Central Europe, Eastern Europe, and Central Asia; Southeast Asia, East Asia, and Oceania; Latin America and Caribbean; and North Africa and Middle East.

Betas and exponentiated values (which can be interpreted as an odds ratio) are shown in the table below:

Study covariate	Parameter	beta	Exponentiated beta
Claims data – 2000	Prevalence	-0.022 (-0.077 — -0.00035)	0.98 (0.93 — 1.00)
Claims data – 2010	Prevalence	-0.012 (-0.041 — -0.0002)	0.99 (0.96 — 1.00)
Hospital, outpatient	Prevalence	-1.81 (-1.93 — -1.76)	0.16 (0.14 — 0.17)
Hospital, inpatient	Prevalence	-2.1 (-2.18 — -1.99)	0.12 (0.11 — 0.14)

We have maintained a similar modeling approach for BPH as was performed for the GBD 2013 analysis.

Uterine fibroids

Flowchart

Uterine fibroids

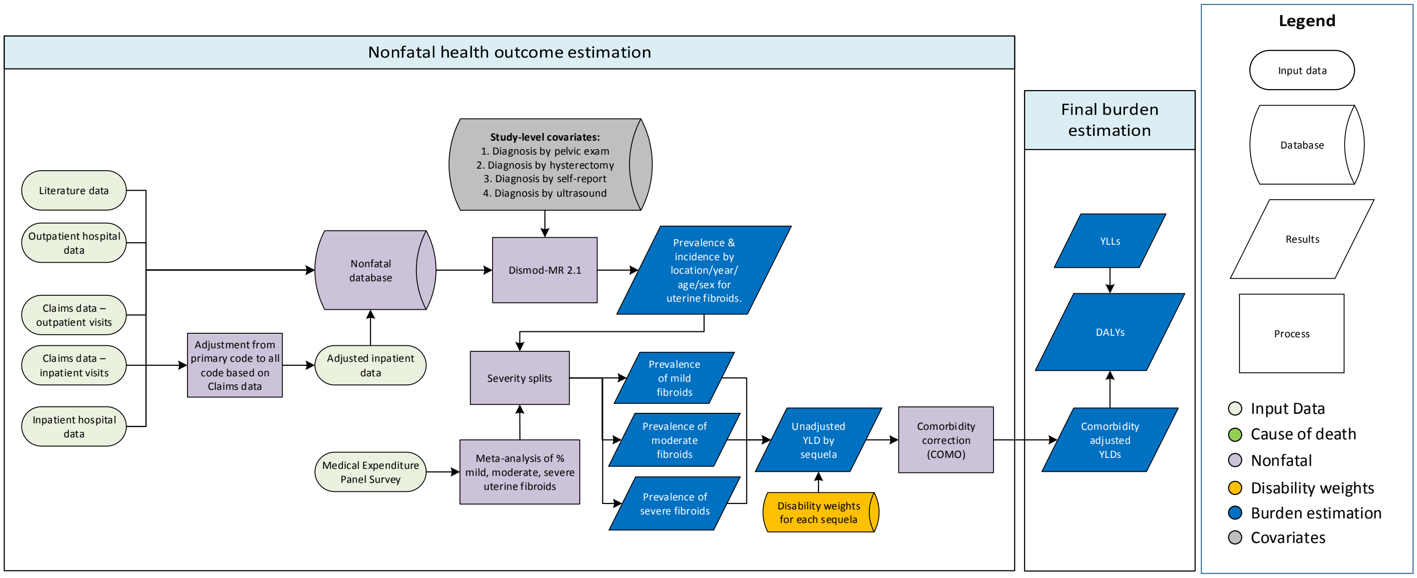

Case definition

Uterine fibroids, also called uterine myomas or leiomyomas, are non-cancerous, compact tumors that occur in the uterus. Fibroids can be diagnosed in a number of ways, including pelvic exam, ultrasound, and hysterectomy. Our reference definition is diagnosis by pelvic exam or ultrasound because it is the most common. However, we incorporate studies that include diagnosis by self-report, pelvic exam only, ultrasound only, hysterectomy only, and all combinations of the three. Refer to Appendix Table 4 for ICD codes.

Input data

Model inputs

For GBD 2010, a systematic review of endometriosis throughout the world was conducted. Ovid MEDLINE, EMBASE, CINAHL, CAB abstracts, WHOLIS, and ISGLE database were searched. The agreed approach for endometriosis was to conduct a PubMed literature search every three years. A PubMed search was conducted as part of the initial review in 2010 and is next due for GBD 2016. Exclusion criteria for the initial systematic review were:

1. Studies that did not provide primary data on epidemiological parameters, e.g., a commentary piece
2. Reviews
3. Clearly non-representative studies (e.g., only high-risk pregnant women)

The table below shows the number of literature studies included in GBD 2015, as well as the number of countries or subnational units and GBD world regions represented.

	Prevalence	Incidence
Studies	12	4
Countries/subnationals	16	17
GBD world regions	7	3

In addition, US claims data for 2000, 2012, and 2012 by US state were included. Inpatient and outpatient hospital data were also included.

Severity splits

The basis of the GBD disability weight (DW) survey assessment are lay descriptions of sequelae highlighting major functional consequences and symptoms. The lay descriptions and disability weights for endometriosis are shown below. Further severity levels are calculated by combining several of these disability weights, e.g., mild abdominal pain with moderate anemia. It should be noted that anemia alone is not ascribed to fibroids, but only in conjunction with mild abdominal pain. The disability weights are listed for reference.

Severity	Lay description	DW (95% CI)
Abdominopelvic problem, mild	has some pain in the belly that causes nausea but does not interfere with daily activities.	0.011<br>(0.005-0.021)
Anemia, mild	feels slightly tired and weak at times, but this does not interfere with normal daily activities.	0.004<br>(0.001-0.008)
Anemia, moderate	feels moderate fatigue, weakness, and shortness of breath after exercise, making daily activities more difficult.	0.052<br>(0.034-0.076)
Anemia, severe	feels very weak, tired and short of breath, and has problems with activities that require physical effort or deep concentration.	0.149<br>(0.101-0.21)

To determine the proportion of women with endometriosis who fall into each severity level, data from the Medical Expenditure Panel Survey (MEPS) is used.

Modeling strategy

The amount of data included in our model increased significantly with GBD 2015 due to the addition of US claims data and hospital data. Many of our DisMod MR 2.1 settings remained the same, but new study-level covariates were added to accommodate the new data.

As in previous GBD iterations, incidence was set to zero prior to 15 years of age and after 51. We assume no excess mortality from endometriosis.

Diagnosis by either ultrasound or pelvic exam was set as the reference category. Study-level covariates for diagnosis by hysterectomy only, pelvic exam only, self report, and pelvic exam, ultrasound or hysterectomy were included. In addition, study-level covariates were added for each year of US claims data, as well as inpatient and outpatient hospital data.

Study covariate	Measure	Parameter	beta	Exponentiated beta
Hysterectomy only	incidence	x-cov	-1.542<br>(-1.683 - -1.402)	.214<br>(.1858 - .2461)

All MarketScan, year 2000	Prevalence	x-cov	-1.03<br>(-1.06 - -1.011)	.3572<br>(.3465 - .3639)
All MarketScan, year 2012	Prevalence	x-cov	-.9597<br>(-.9789 - -.9259)	.383<br>(.3757 - .3962)
All MarketScan, year 2010	Prevalence	x-cov	-.9337<br>(-.9697 - -.8996)	.3931<br>(.3792 - .4067)
Self-reported	prevalence	x-cov	-.2471<br>(-.3161 - -.2217)	.7811<br>(.729 - .8012)

No other significant changes were made to the GBD 2015 modeling strategy.

Polycystic ovarian syndrome

Flowchart

Polycystic ovarian syndrome

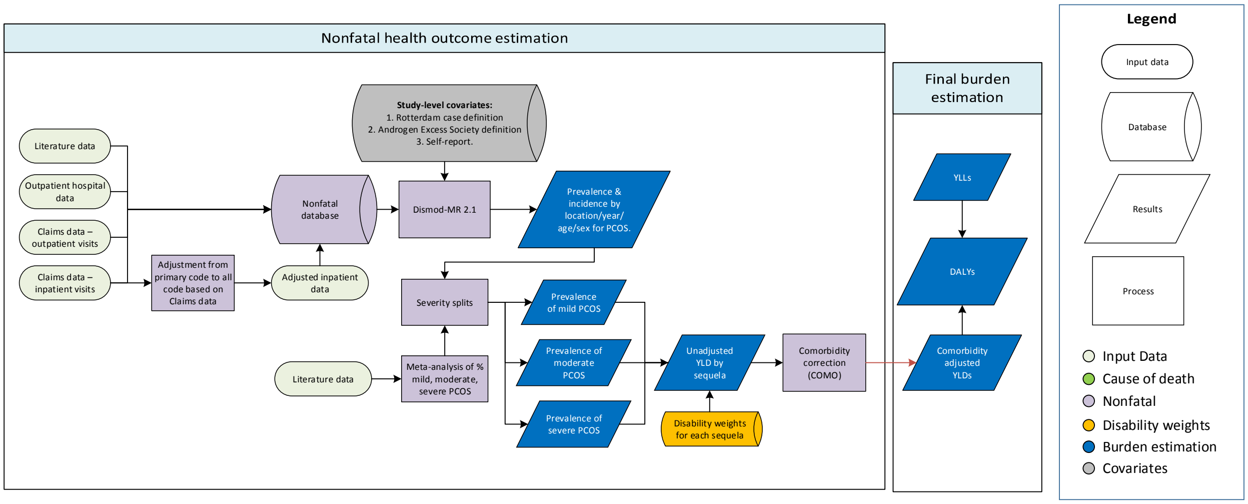

Case definition

Polycystic ovarian syndrome (PCOS) is a condition that affects women's ovaries and can lead to a variety of symptoms. Women with PCOS often have enlarged ovaries that contain pockets of fluid, and symptoms include infrequent menstruation, excess hair growth, acne, and obesity. For ICD codes refer to Appendix Table 4.

Input data

Model inputs

For GBD 2010, a systematic review of endometriosis throughout the world was conducted. Ovid MEDLINE, EMBASE, CINAHL, CAB abstracts, WHOLIS, and ISGLE database were searched. The agreed approach for endometriosis was to conduct a PubMed literature search every three years. A PubMed search was conducted as part of the initial review in 2010 and is next due for GBD 2016. Exclusion criteria for the initial systematic review were:

1. Studies that did not provide primary data on epidemiological parameters, e.g., a commentary piece
2. Reviews
3. Clearly non-representative studies (e.g., only high-risk pregnant women)

The table below shows the number of literature studies included in GBD 2015, as well as the number of countries or subnational units and GBD world regions represented.

	Prevalence
Studies	21
Countries/subnationals	17
GBD world regions	10

In addition, US claims data for 2000, 2012 and 2012 by US state were included. Outpatient hospital data were also included. Inpatient hospital data was not incorporated, as we believed that inpatient data on PCOS would fluctuate wildly with across geographies and different coding practices, and would not represent the true prevalence of PCOS in the population.

Severity splits

The basis of the GBD disability weight (DW) survey assessment are lay descriptions of sequelae highlighting major functional consequences and symptoms. The lay descriptions and disability weights for premenstrual syndrome are shown below. Further severity levels are calculated by combining several of these disability weights, e.g., mild disfigurement and primary infertility.

Severity	Lay description	DW (95% CI)
Disfigurement, level 1	has a slight, visible physical deformity that others notice, which causes some worry and discomfort.	0.011 (0.005-0.021)
Infertility, primary	wants to have a child and has a fertile partner, but the couple cannot conceive.	0.008 (0.003-0.015)
Infertility, secondary	has at least one child, and wants to have more children. The person has a fertile partner, but the couple cannot conceive.	0.005 (0.002-0.011)

To determine the proportion of people within each of these severity levels, one study was consulted.[1] Tehrani et al. included information on the proportion of women who experience primary infertility and hyperandrogenism. Percentages were combined to calculate the proportion of women who fall into both hyperandrogenism and infertility categories.

Modeling strategy

The amount of data included in our model increased significantly with GBD 2015 due to the addition of US claims data. Many of our DisMod MR 2.1 settings remained the same, but new study-level covariates were added to accommodate the new data.

The table below illustrates the study covariates, measures, parameters, beta, and exponentiated beta values.

Study covariate	Measure	parameter	beta	Exponentiated beta
All MarketScan, year 2000	Prevalence	x-cov	-2.518 (-2.559 - -2.5)	.0806 (.0774 - .0821)
All MarketScan, year 2010	Prevalence	x-cov	-1.414 (-1.451 - -1.4)	.2431 (.2343 - .2466)
All MarketScan, year 2012	Prevalence	x-cov	-1.41 (-1.436 - -1.401)	.2441 (.2379 - .2464)

[1] Tehrani FR, Simbar M, Tohidi M, Hosseinpanah F, Azizi F. The prevalence of polycystic ovary syndrome in a community sample of Iranian population: Iranian PCOS prevalence study. <i>Reprod Biol Endocrinol</i>. 2011; 9: 39.

Androgen Excess Society case definition	Prevalence	z-cov	.7526<br>(.0757 - 1.934)	2.122<br>(1.079 - 6.917)
Rotterdam case definition	Prevalence	x-cov	.3154<br>(.208 - .397)	1.371<br>(1.231 - 1.487)
Self-report	prevalence	z-cov	.8094<br>(.0616 - 1.918)	2.246<br>(1.064 - 6.809)

As in previous GBD iterations, incidence was set to zero prior to 15 years of age and after 50. This is because a woman must enter puberty before she can get PCOS, and the condition spontaneously goes into remission with the onset of menopause.

Case definitions for PCOS vary widely, including varying rosters of symptoms over various time periods. We use as our reference definition the NIH/NICHD criteria, for which three signs must be present: clinical or biochemical evidence of hyperandrogenism, oligomenorrhea, and the exclusion of other disorders. We include study-level covariates for other common case definitions, including the Rotterdam and Androgen Excess Society (AES) definitions, as well as self-report. Finally, this year we have added covariates for each year of the US claims data as well as the outpatient hospital data.

No other significant changes took place for GBD 2015.

Endometriosis

Flowchart

Endometriosis

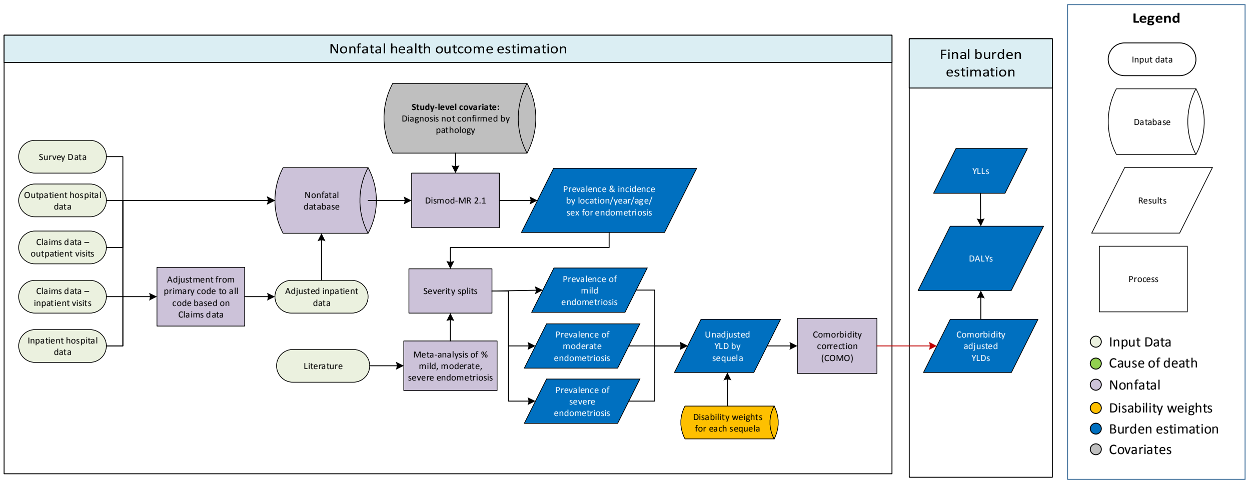

Case definition

Endometriosis is defined as growth of tissue that usually lies inside the uterus grows outside of it. Common symptoms include chronic pain and infertility. Our reference case definition of endometriosis is diagnosis accompanied by pathological confirmation.

Input data

Model inputs

For GBD 2010, a systematic review of endometriosis throughout the world was conducted. Ovid MEDLINE, EMBASE, CINAHL, CAB abstracts, WHOLIS, and ISGLE database were searched. The agreed approach for endometriosis was to conduct a PubMed literature search every three years. A PubMed search was conducted as part of the initial review in 2010 and is next due for GBD 2016. Exclusion criteria for the initial systematic review were:

1. Studies that did not provide primary data on epidemiological parameters, e.g., a commentary piece
2. Reviews
3. Clearly non-representative studies (e.g., only high-risk pregnant women)

The table below shows the number of literature studies included in GBD 2015, as well as the number of countries or subnational units and GBD world regions represented.

	Prevalence	Incidence
Studies	7	10
Countries/subnationals	7	8
GBD world regions	4	2

In addition, US claims data for 2000, 2012 and 2012 by US state were included. Inpatient and outpatient hospital data were also included.

Severity splits & disability weights

The basis of the GBD disability weight (DW) survey assessment are lay descriptions of sequelae highlighting major functional consequences and symptoms. The lay descriptions and disability weights for endometriosis are shown below. Further severity levels are calculated by combining several of these disability weights, e.g., moderate abdominal pain and secondary infertility.

Severity	Lay description	DW (95% CI)
Abdominopelvic problem, mild	has some pain in the belly that causes nausea but does not interfere with daily activities.	0.011 (0.005-0.021)
Abdominopelvic problem, moderate	has pain in the belly and feels nauseous. The person has difficulties with daily activities.	0.114 (0.078-0.159)
Abdominopelvic problem, severe	has severe pain in the belly and feels nauseous. The person is anxious and unable to carry out daily activities.	0.324 (0.219-0.442)
Infertility, primary	wants to have a child and has a fertile partner, but the couple cannot conceive.	0.008 (0.003-0.015)
Infertility, secondary	has at least one child, and wants to have more children. The person has a fertile partner, but the couple cannot conceive.	0.005 (0.002-0.011)

To determine the proportion of people within each of these severity levels, three studies were consulted (ALWHS YEAR; Sinaii et al. 2002 & 2008). One addressed the proportion of women who become infertile, one addressed the proportion with chronic abdominal pain, and the last one severity of abdominal pain. Estimates were combined across studies to calculate the proportion of women who fall into both abdominal pain and infertility categories.

Modeling strategy

We use DisMod-MR 2.1, a Bayesian meta-regression epidemiological model, to generate estimates for Endometriosis by age, sex, year, and country. The amount of data included in our model increased significantly with GBD 2015 due to the addition of US claims data and hospital data. Many of our DisMod MR 2.1 settings remained the same, but new study-level covariates were added to accommodate the new data.

Diagnosis confirmed by pathology was set as the reference definition. In addition to study-level covariate on diagnosis not confirmed by pathology, study-level covariates were added for each year of US claims data as well as inpatient and outpatient hospital data. The table below illustrates covariates, measures, parameters, beta, and exponentiated beta values.

Study covariate	Measure	Parameter	beta	Exponentiated beta
Endometriosis not confirmed by pathology	incidence	x-cov	.4431 (.2076 - .6851)	1.557 (1.231 - 1.984)
All MarketScan, year 2000	prevalence	x-cov	.1311 (.0437 - .2188)	1.14 (1.045 - 1.245)
All MarketScan, year 2010	prevalence	x-cov	.0493 (-.0298 - .1275)	1.051 (.9707 - 1.136)

All MarketScan, year 2012	prevalence	x-cov	.0093<br>(-1.932 - 2)	1.009<br>(.1448 - 7.389)
Endometriosis not confirmed by pathology	prevalence	x-cov	.0995<br>(-.4366 - .6568)	1.105<br>(.6462 - 1.929)
Hospital inpatient	prevalence	x-cov	-1.697<br>(-1.775 - -1.631)	.1832<br>(.1695 - .1957)
Hospital outpatient	prevalence	x-cov	-2.009<br>(-2.173 - -1.842)	.1341<br>(.1138 - .1585)

As in previous GBD iterations, incidence was set to zero prior to 15 years of age and after 51. This is because a woman must enter puberty before she can get endometriosis, and the condition spontaneously goes into remission with the onset of menopause. We assume no excess mortality from endometriosis.

There have been no additional significant changes to the modeling strategy for GBD 2015.

Genital prolapse

Flowchart

Genital prolapse

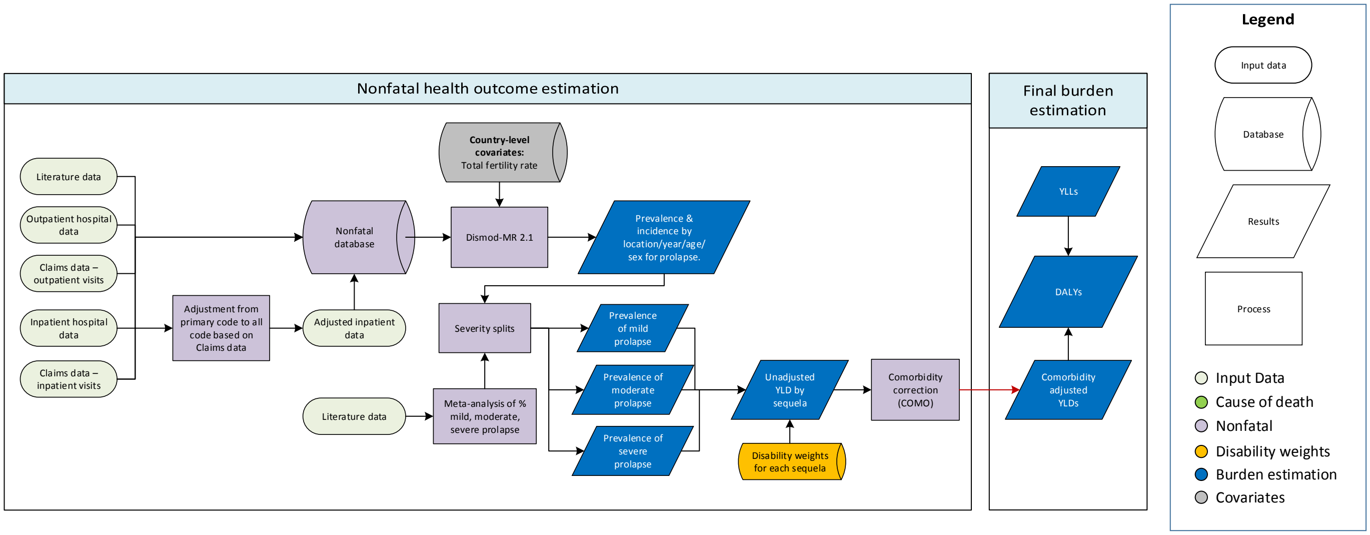

Case definition

Genital prolapse, also called female pelvic organ prolapse, is the clinically relevant descent of one of more of the pelvic structures, including the uterus, bladder, rectum, small or large bowl, or vagina. Risk of prolapse increases with age, and can be exacerbated by vaginal childbirth or physical strain. ICD codes associated with genital prolapse include: N81.

Input data

Model inputs

For GBD 2010, a systematic review of endometriosis throughout the world was conducted. Ovid MEDLINE, EMBASE, CINAHL, CAB abstracts, WHOLIS, and ISGLE database were searched. The agreed approach for endometriosis was to conduct a PubMed literature search every three years. A PubMed search was conducted as part of the initial review in 2010 and is next due for GBD 2016. Exclusion criteria for the initial systematic review were:

1. Studies that did not provide primary data on epidemiological parameters, e.g., a commentary piece
2. Reviews
3. Clearly non-representative studies (e.g., only high-risk pregnant women)

The table below shows the number of literature studies included in GBD 2015, as well as the number of countries or subnational units and GBD world regions represented.

	Prevalence
Studies	13
Countries/subnationals	12
GBD world regions	8

In addition, US claims data for 2000, 2012 and 2012 by US state were included. Outpatient and inpatient hospital data were also included.

Severity splits

The basis of the GBD disability weight (DW) survey assessment are lay descriptions of sequelae highlighting major functional consequences and symptoms. The lay descriptions and disability weights for genital prolapse are shown below. Further severity levels are calculated by combining several of these disability weights, e.g., mild abdominal pain and stress incontinence.

Severity	Lay description	DW (95% CI)
Stress incontinence	loses small amounts of urine without meaning to when coughing, sneezing, laughing or during physical exercise.	0.02 (0.011-0.035)
Abdominopelvic problem, mild	has some pain in the belly that causes nausea but does not interfere with daily activities.	0.011 (0.005-0.021)

To determine the proportion of people within each of these severity levels, two studies were consulted.[1],[2] Scherf and Slieker-Ten Hove included information on the proportion of women with prolapse who experience a bulging sensation as well as stress incontinence. Percentages were combined to calculate the proportion of women who fall into both stress incontinence and bulging sensation categories.

Modeling strategy

The amount of data included in our model increased significantly with GBD 2015 due to the addition of US claims data. Many of our DisMod MR 2.1 settings remained the same, but new study-level covariates were added to accommodate the new data.

As in previous GBD iterations, incidence was set to zero prior to 15 years of age. This is because it is highly unlikely a woman would experience genital prolapse before entering her childbearing years.

This year we have added covariates for each year of the US claims data as well as the outpatient and inpatient hospital data. The table below illustrates covariates, measures, parameters, beta, and exponentiated beta values.

Study covariate	Measure	Parameter	beta	Exponentiated beta
All MarketScan, year 2000	prevalence	x-cov	-1.667 (-1.754 - -1.586)	.1888 (.1731 - .2047)
All MarketScan, year 2010	prevalence	x-cov	-1.676 (-1.757 - -1.593)	.1872 (.1726 - .2033)

[1] Epidemiology of pelvic organ prolapse in rural Gambia, West Africa. Scherf, 2002.
[2] Symptomatic pelvic organ prolapse and possible risk factors in a general population. Slieker-Ten Howe, 2009.

All MarketScan, year 2012	prevalence	x-cov	-1.691<br>(-1.768 - -1.619)	.1843<br>(.1707 - .1981)
Hospital inpatient	prevalence	x-cov	-3.971<br>(-3.998 - -3.93)	.0188<br>(.0184 - .0196)
Hospital outpatient	prevalence	x-cov	-1.001<br>(-4 - 1.904)	.3677<br>(.0183 - 6.711)
Total fertility rate	prevalence	Country-level covariate	.4848<br>(.4419 - .5336)	1.624<br>(1.556 - 1.705)

No other significant changes took place from GBD 2013 to GBD 2015.

Premenstrual syndrome (PMS)

Flowchart

Premenstrual syndrome

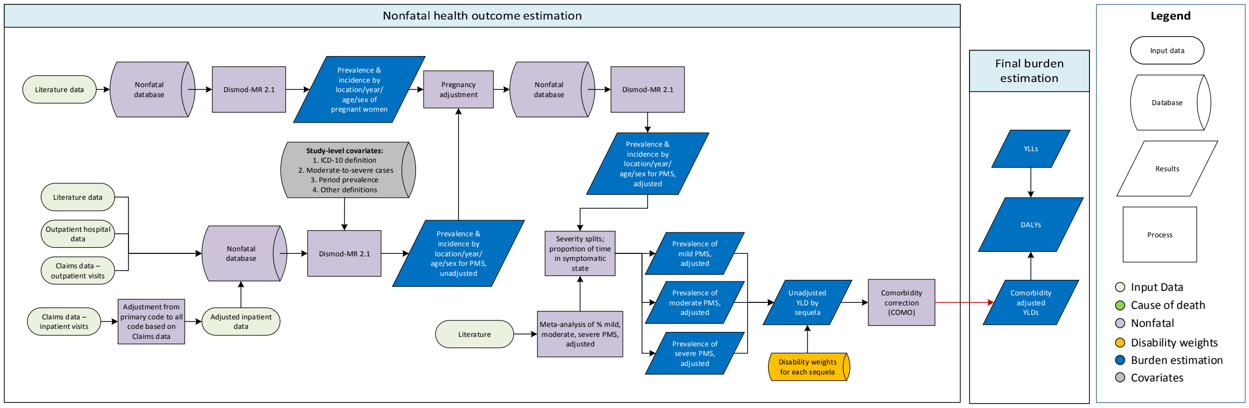

Case definition

Premenstrual syndrome refers to psychological and physical symptoms that occur in the weeks leading up to a woman's period in her menstrual cycle. Symptoms are extremely varied in nature and severity, but include tenderness, bloating, irritability, fatigue, abdominal pain, and altered mental states. Symptoms cease when a woman is pregnant and once she reaches menopause.

Input data

Model inputs

For GBD 2010, a systematic review of endometriosis throughout the world was conducted. Ovid MEDLINE, EMBASE, CINAHL, CAB abstracts, WHOLIS, and ISGLE database were searched. The agreed approach for endometriosis was to conduct a PubMed literature search every three years. A PubMed search was conducted as part of the initial review in 2010 and is next due for GBD 2016. Exclusion criteria for the initial systematic review were:

1. Studies that did not provide primary data on epidemiological parameters, e.g., a commentary piece
2. Reviews
3. Clearly non-representative studies (e.g., only high-risk pregnant women)

The table below shows the number of literature studies included in GBD 2015, as well as the number of countries or subnational units and GBD world regions represented. Note that data on the proportion of women who are pregnant is used during the pregnancy adjustment, described in detail in the modeling strategy section.

<u>PMS</u>

	Prevalence
Studies	50
Countries/subnationals	43

GBD world regions	11

Women who are pregnant

	Prevalence
Studies	2
Countries/subnationals	188
GBD world regions	21

In addition, US claims data for 2000, 2012, and 2012 by US state were included. Outpatient hospital data were also included. Inpatient hospital data was not incorporated, as we believed that inpatient data on PMS would fluctuate wildly with across geographies and different coding practices, and would not represent the true prevalence of PMS in the population.

Severity splits and disability weights

The basis of the GBD disability weight (DW) survey assessment are lay descriptions of sequelae highlighting major functional consequences and symptoms. The lay descriptions and disability weights for premenstrual syndrome are shown below. Further severity levels are calculated by combining several of these disability weights, e.g., abdominal pain and depression due to premenstrual syndrome.

Severity	Lay description	DW (95% CI)
Major depressive disorder, mild episode	feels persistent sadness and has lost interest in usual activities. The person sometimes sleeps badly, feels tired, or has trouble concentrating but still manages to function in daily life with extra effort.	0.145 (0.099-0.209)
Abdominopelvic problem, mild	has some pain in the belly that causes nausea but does not interfere with daily activities.	0.011 (0.005-0.021)

To determine the proportion of people within each of these severity levels, five studies were consulted. Three studies addressed the proportion of women with PMS who experience depression, and two other studies addressed the proportion of women with PMS who experience abdominal pain. Estimates were pooled across studies to get final proportions, and were combined across studies to calculate the proportion of women who fall into both abdominal pain and depression categories.

Modeling strategy

The amount of data included in our model increased significantly with GBD 2015 due to the addition of US claims data. Many of our DisMod MR 2.1 settings remained the same, but new study-level covariates were added to accommodate the new data.

As in previous GBD iterations, incidence was set to zero prior to 15 years of age and after 50. This is because a woman must enter puberty before she can get premenstrual syndrome, and the condition spontaneously goes into remission with the onset of menopause. We assume no excess mortality from PMS.

Case definitions for PMS vary widely, including varying rosters of symptoms over various time periods. We use as our reference definition the American College of Obstetricians and Gynecologists (ACOG) criteria, which states that the patient reports at least one of each of the following affective and somatic symptoms during the five days before their menses and appear in three consecutive cycles: depression, angry outbursts, irritability, anxiety, confusion, social withdrawal; breast tenderness, abdominal bloating, headache, or swelling of extremities. We include study-level covariates for other common case definitions, including the ICD-10 definition, and the Premenstrual Symptoms Screening Tool (PSST) definition. We also include covariates for studies that only examine moderate to severe PMS, or period prevalence of PMS. Finally, this year we have added covariates for each year of the US claims data as well as the outpatient hospital data.

Other gynecological conditions

Flowchart

Other gynecological conditions that are not menstrual disorders

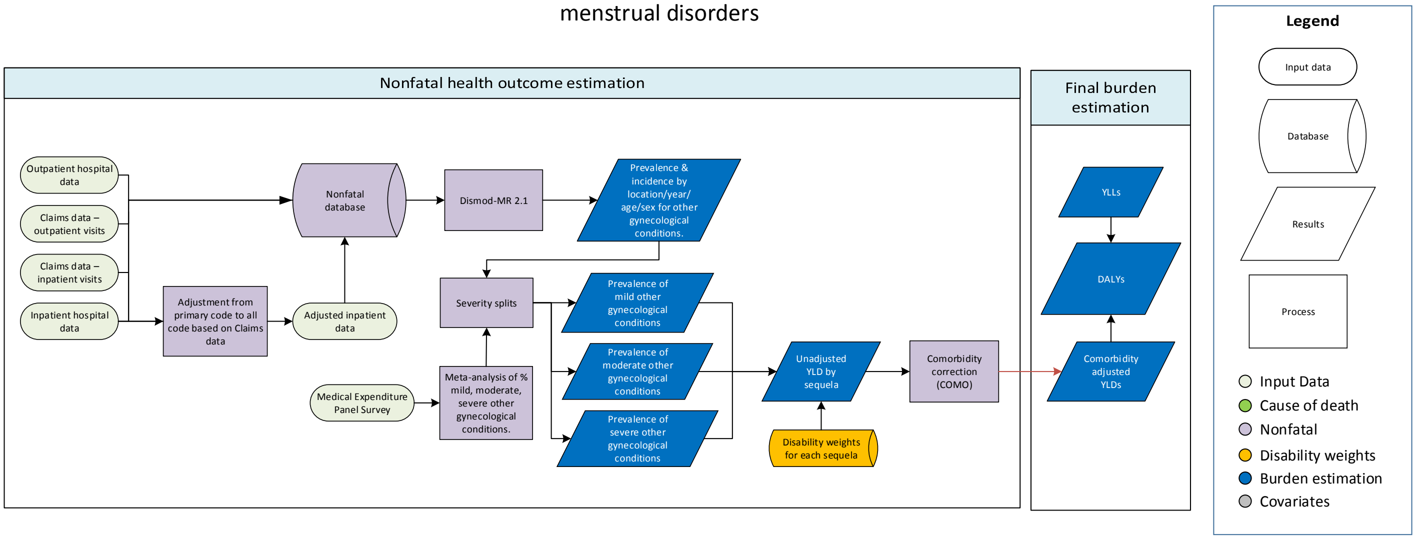

Case definition

Other gynecological conditions encompasses all disorders that are not menstruation- or bleeding-related that do not fall under the heading of any of the other gynecological causes. They only affect women.

Input data

Model inputs

No literature data is used to inform models of other gynecological conditions. Previously, only inpatient and outpatient hospital data was used. For GBD 2015, US claims data for 2000, 2012 and 2012 by US state were added.

Severity splits & disability weights

The basis of the GBD disability weight (DW) survey assessment are lay descriptions of sequelae highlighting major functional consequences and symptoms. The lay descriptions and disability weights for other gynecological conditions are shown below.

Severity	Lay description	DW (95% CI)
Abdominopelvic problem, mild	has some pain in the belly that causes nausea but does not interfere with daily activities.	0.011 (0.005-0.021)
Abdominopelvic problem, moderate	has pain in the belly and feels nauseous. The person has difficulties with daily activities.	0.114 (0.078-0.159)
Abdominopelvic problem, severe	has severe pain in the belly and feels nauseous. The person is anxious and unable to carry out daily activities.	0.324 (0.219-0.442)
Anemia, mild	feels slightly tired and weak at times, but this does not interfere with normal daily activities.	0.004 (0.001-0.008)
Anemia, moderate	feels moderate fatigue, weakness, and shortness of breath after exercise, making daily activities more difficult.	0.052 (0.034-0.076)

Anemia, severe	feels very weak, tired and short of breath, and has problems with activities that require physical effort or deep concentration.	0.149<br>(0.101-0.21)

To determine the proportion of women with endometriosis who fall into each severity level, data from the Medical Expenditure Panel Survey (MEPS) is used.

Modeling strategy

We ran a DisMod MR2.1 model to estimate the global burden of gynecological diseases. The amount of data included in our model increased significantly with GBD 2015 due to the addition of US claims data. Many of our DisMod MR 2.1 settings remained the same, but new study-level covariates were added to accommodate the new data. These covariates are shown in the table below, along with measures, parameters, beta, and exponentiated beta values.

Study covariate	Measure	Parameter	beta	Exponentiated beta
All MarketScan, year 2000	Prevalence	x-cov	3.552<br>(3.503 - 3.606)	34.87<br>(33.21 - 36.82)
All MarketScan, year 2010	prevalence	x-cov	3.47<br>(3.424 - 3.523)	32.13<br>(30.69 - 33.89)
All MarketScan, year 2012	Prevalence	x-cov	3.437<br>(3.386 - 3.493)	31.08<br>(29.55 - 32.88)
Hospital inpatient	prevalence	x-cov	.2875<br>(.2515 - .3339)	1.333<br>(1.286 - 1.396)

As in previous GBD iterations, incidence was set to zero prior to 15 years of age. We assume no excess mortality from other gynecological conditions over the same age range.

US claims data for 2012 was used as the reference category. Study-level covariates were added for each of the remaining years of US claims data as well as inpatient and outpatient hospital data.

No other significant changes were made to the GBD 2015 estimation process.

Hemoglobinopathies and hemolytic anemias

Sickle cell disorders, thalassemias, glucose-6-phosphate dehydrogenase (G6PD) deficiency, sickle cell trait, thalassemia trait, hemizygous G6PD deficiency, other hemoglobinopathies and hemolytic anemias

Flowchart

Hemoglobinopathies and hemolytic anemias: Sickle cell disorders, Thalassemias, Glucose-6-phosphate dehydrogenase (G6PD) deficiency, Sickle cell trait, Thalassemia trait, Hemizygous G6PD deficiency, Other hemoglobinopathies and hemolytic anemias

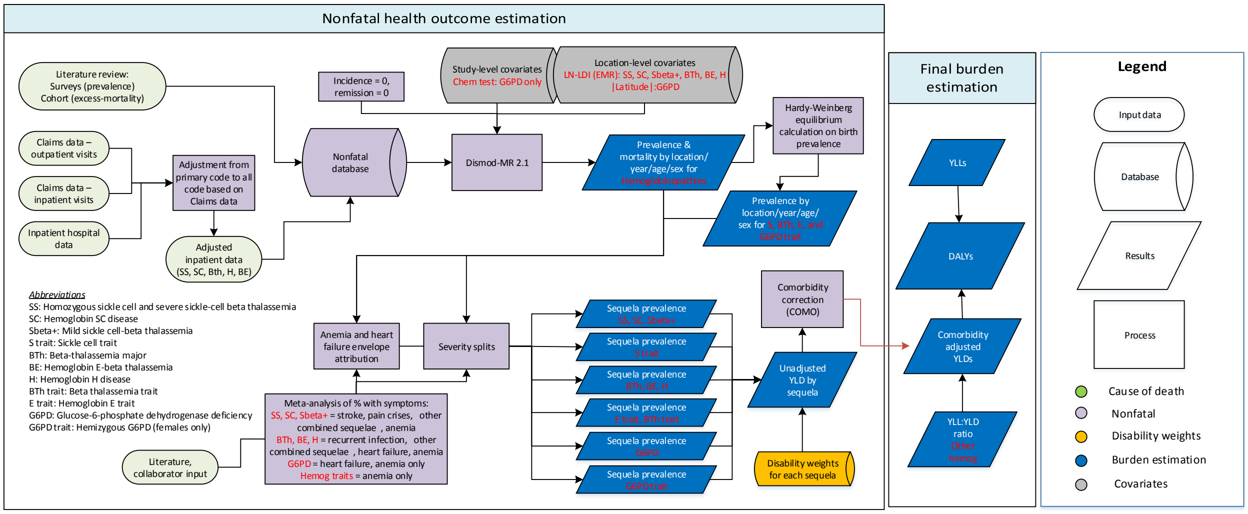

Input data and methodological summary

Case definition

Hemoglobinopathies and hemolytic anemias span four GBD causes: thalassemias, sickle cell disorders, glucose-6-phosphate dehydrogenase (G6PD) deficiency, and other hemoglobinopathies. ICD-9 and ICD-10 codes for each are contained in Table 1. Within each category, several unique combinations of genetic mutations lead to distinct phenotypes with different natural history, which has led us to estimate several distinct subtypes of thalassemias and sickle cell disorders. The three thalassemia models included 1) beta-thalassemia major, 2) hemoglobin E/beta-thalassemia, and 3) hemoglobin H disease. Sickle cell models included 1) homozygous sickle cell and severe sickle cell/beta-thalassemia, 2) hemoglobin SC disease, and 3) "mild" sickle cell-beta thalassemia. We also estimated the burden of glucose-6-phosphate dehydrogenase (G6PD) deficiency. Finally, we estimated prevalence and YLD due to other hemoglobinopathies and hemolytic anemias assuming the YLD-to-YLL ratio for each age, sex, location, and year was similar to that of the aggregate of sickle cell, thalassemias, and G6PD deficiency. This approach was used in multiple causes across the GBD 2015.

TABLE 1. International classification of diseases codes for hemoglobinopathies and hemolytic anemias in GBD 2015 cause of death analysis		
Condition	ICD-10 code	ICD-9 code
Total	D55-D59	282.0-282.1, 282.7-285.8, 282.2-282.3, 282.5-282.6, 282.4
Thalassemias	D56	282.4
Sickle cell disorders	D57	282.5-282.6
G6PD deficiency	D55	282.2-282.3
Other hemoglobinopathies and hemolytic anemias	D58-D64.8	282.0-282.1, 282.7-285.8

Input data

Model inputs

Systematic literature reviews were completed for GBD 2010 and GBD 2013. These were updated on May 1, 2015 using the following search strings:

(thalassemias[Title/Abstract] AND (prevalence[Title/Abstract] OR survival[Title/Abstract] OR mortality[Title/Abstract]) AND ("2008"[PDAT] : "2013"[PDAT])) AND "humans"[MeSH Terms]

(sickle cell[Title/Abstract] AND (mortality[Title/Abstract] OR survival[Title/Abstract] OR prevalence[Title/Abstract]) AND ("2008"[PDAT] : "2013"[PDAT])) AND "humans"[MeSH Terms]

(G6PD[Title/Abstract] OR G6PD deficiency[Title/Abstract] OR glucose-6 phosphate dehydrogenase[Title/Abstract] OR glucose-6-phosphate dehydrogenase deficiency[Title/Abstract] AND (survival[Title/Abstract] OR mortality[Title/Abstract]) AND ("2008"[PDAT] : "2013"[PDAT])) AND "humans"[MeSH Terms]

Of note, upon the recommendation from multiple GBD collaborators, we identified and re-extracted all primary data that had been used in GBD 2013. In some situations, this process identified cases of sickle cell or thalassemia that had been assigned to incorrect subtypes, but mostly the data was correct and verified. We excluded any data where the results presented in a study were themselves the result of modeling exercises. The most significant change as a result of re-extraction was when we identified that much of the literature data used in GBD 2013 from females with G6PD deficiency actually did not correspond to our case definition of homozygous disease, but rather included combined case counts for homozygotes and hemizygotes. We only included homozygous disease in our datasets for GBD 2015, which has led to much lower estimates of G6PD deficiency in females.

We extracted prevalence data from population-level and community surveys as well as with-condition mortality and excess-mortality data from cohort studies. Age-specific survival proportions were converted to with-condition mortality rates as needed. We also included data from hospital and claims data for a subset of hemoglobinopathy models, including beta-thalassemia major, hemoglobin E/beta thalassemia, homozygous sickle cell and severe sickle cell/beta thalassemia, hemoglobin SC disease, and mild sickle cell/beta-thalassemia. The extraction and processing of hospital and claims data is described separately. Composition of final datasets are shown below for each of the different hemoglobinopathies and hemolytic anemias models.

<u>Data availability</u>

Homozygous sickle cell and severe sickle cell/beta-thalassemia (2097):

	Prevalence	Mortality risk
Studies	110	25
Countries/subnationals	83/44	11/15
GBD world regions	13	8

Hemoglobin SC disease (2100):

	Prevalence	Mortality risk
Studies	63	12
Countries/subnationals	42/21	4/7
GBD world regions	12	4

Mild sickle cell/beta-thalassemia (2103):

	Prevalence	Mortality risk
Studies	20	9
Countries/subnationals	57/7	22/9
GBD world regions	13	3

Beta-thalassemia major (2085):

	Prevalence	Mortality risk
Studies	27	6
Countries/subnationals	86/33	6/1
GBD world regions	18	3

Hemoglobin E/beta-thalassemia (2087):

	Prevalence	Mortality risk
Studies	7	1
Countries/subnationals	23/20	1/1
GBD world regions	3	1

Hemoglobin H disease (2089):

	Prevalence
Studies	11
Countries/subnationals	19/23
GBD world regions	9

G6PD deficiency (2112):

	Prevalence
Studies	278
Countries/subnationals	89/39
GBD world regions	18

Modeling strategy

Besides data re-extraction and addition of hospital and claims data, we have made no substantive changes to the estimation strategy since 2013. We estimated the nonfatal burden of hemoglobinopathies STI in three parts.

First, we used the datasets described above to estimate prevalence for each age-sex-location-year in the GBD 2015 location hierarchy using DisMod-MR 2.1. For mild sickle cell/beta-thalassemia models, study-level covariates were used to identify and crosswalk those data from Marketscan in the year 2000, which were systematically lower than later years, to the years 2010 and 2012. The magnitude of prevalence in later years of claims data for this model, and in all years for other models, was similar to that of literature data from the same locations so they were considered equivalent and no additional crosswalks were performed. In all sickle cell models and beta-thalassemia major, the natural log of lag-distributed income per capita (LN-LDI) was used as a sole country covariate on excess mortality, meant to reflect the profound impact that health system financial resources can have on survival from these conditions. For G6PD deficiency, data where diagnosis was made only on the basis of chemical or reagent testing was crosswalked to the reference definition of genetic G6PD deficiency; absolute value of latitude was the sole country covariate for this model.

Second, we calculated prevalence of hemoglobinopathy traits (sickle cell trait, hemoglobin E trait, hemoglobin beta trait, G6PD trait) by back-calculating from birth prevalence estimates from corresponding DisMod-MR 2.1 models, assuming Hardy-Weinberg equilibrium, and no excess mortality. Third, age-specific prevalence for all subtypes of hemoglobinopathies were paired with estimated sequelae distributions from a series of cohort studies and clinical data in GBD 2010 and GBD 2013. This included consideration of the burden of anemia associated with homozygous and heterozygous persons and ensuring the estimates of hemoglobinopathy-induced anemia were internally-consistent with overall estimates for each condition, including prevention of double counting. The anemia estimation process is described separately.

Third, and finally, we found the ratio of YLD to YLL ratio for all hemoglobinopathies and then applied it to YLLs estimated for other hemoglobinopathies and hemolytic anemias in our cause-specific mortality analysis. Quantitative crosswalk results for each model are shown below.

<u>Covariate, parameter, beta, and exponentiated beta values</u>

Homozygous sickle cell and severe sickle cell/beta-thalassemia (2097):

Study-level covariate	Parameter	Geography level	beta	Exponentiated beta
LDI (I$ per capita)	Excess mortality rate	Global	-0.14	0.87<br>(0.86 - 0.88)

Hemoglobin SC disease (2100):

Study-level covariate	Parameter	Geography level	beta	Exponentiated beta
LDI (I$ per capita)	Excess mortality rate	Global	-0.03	0.97 (0.95 – 1.00)

Mild sickle cell/beta-thalassemia (2103):

Study-level covariate	Parameter	Geography level	beta	Exponentiated beta
LDI (I$ per capita)	Excess mortality rate	Global	-0.19	0.83 (0.74 - 0.98)
All MarketScan, year 2000	Prevalence	Global	-0.48	.6199 (0.54 - 0.71)

Beta-thalassemia major (2085):

Study-level covariate	Parameter	Geography level	beta	Exponentiated beta
LDI (I$ per capita)	Excess mortality rate	Global	-0.58	0.56 (0.55 - 0.58)

Hemoglobin E/beta-thalassemia (2087):

Study-level covariate	Parameter	Geography level	beta	Exponentiated beta
Year	Prevalence	Global	0.02 (0.00 - 0.02)	1.02 (1.00 - 1.02)

Hemoglobin H disease (2089):

Study-level covariate	Parameter	Geography level	beta	Exponentiated beta
Year	Prevalence	Global	-0.01	0.99 (0.98 - 1.02)

G6PD deficiency (2112):

Study-level covariate	Parameter	Geography level	beta	Exponentiated beta
Absolute value of average latitude	Prevalence	Super region	-0.01	0.99 (0.98 - 1.00)
Diagnostic modality based on chemical/ reagent testing	Prevalence	Super region	0.322	1.38 (1.16 - 1.69)

Year	Prevalence	Super region	-0.01	0.99<br>(0.98 - 0.99)

Sequelae

With the exception of anemia, only homozygous individuals were considered to experience disability. Estimated sequelae of thalassemias included anemia (described separately), heart failure (described separately), and periodic severe infection. Another series of common, but not universal, sequelae also occur in those with thalassemias, including splenomegaly, skeletal deformity, delayed growth/puberty, diabetes, hypothyroidism, and leg ulcers. Given sparse data on the occurrence of these sequelae, they were approximated with a health state named "other combined sequelae of thalassemia," for which we used the disability weight corresponding to a health state of "generic uncomplicated disease, anxiety about diagnosis and daily medication" which, of note, was also used to approximate the disability for those with cancer in remission. For sickle cell disorders, we similarly estimated YLDs for anemia (described separately), stroke, and pain crises separately and approximated the myriad additional complications of sickle cell disease with the health state "other combined sequelae of sickle cell disease." The only sequelae estimated for G6PD deficiency were anemia (described separately) and heart failure (described separately). Notably, however, G6PD deficiency is considered to be asymptomatic for a vast majority of those with the condition, with only a very small subset of around 1 in 1,000,000 having chronic hemolysis (Class I disease) and approximately 1% having periodic hemolytic episodes (Class II disease) with exposure to environmental, pharmaceutical, or food products. Females heterozygous for G6PD deficiency exhibit chimerism, as one X chromosome becomes dominant in each of the red blood cells so we estimated half as many heterozygous females will be symptomatic as homozygous females.

Uncertainty and model selection

For all hemoglobinopathies estimates, uncertainty bounds include uncertainty due to input data, crosswalks from non-reference definitions in study covariates above, uncertainty in numerical solutions (posteriors) of each DisMod-MR 2.1 model, and proportion of all persons with each type of symptom.

In consultation with GBD researchers and collaborators, final models were selected on a combination of qualitative and quantitative goodness of fit to input data, plausibility of geographic and temporal trends, consistency of age pattern, and, when available, comparison with other published studies on hemoglobinopathy epidemiology. Directionality, magnitude, and plausibility of study-level and country-level covariates was also considered in the process of model development. Of note, due to the nature of statistical modeling, final results do not always cover the values reported in input data.

No other significant changes were made to the GBD 2015 modeling strategy.

Rheumatoid arthritis

Flowchart

Rheumatoid arthritis (RA)

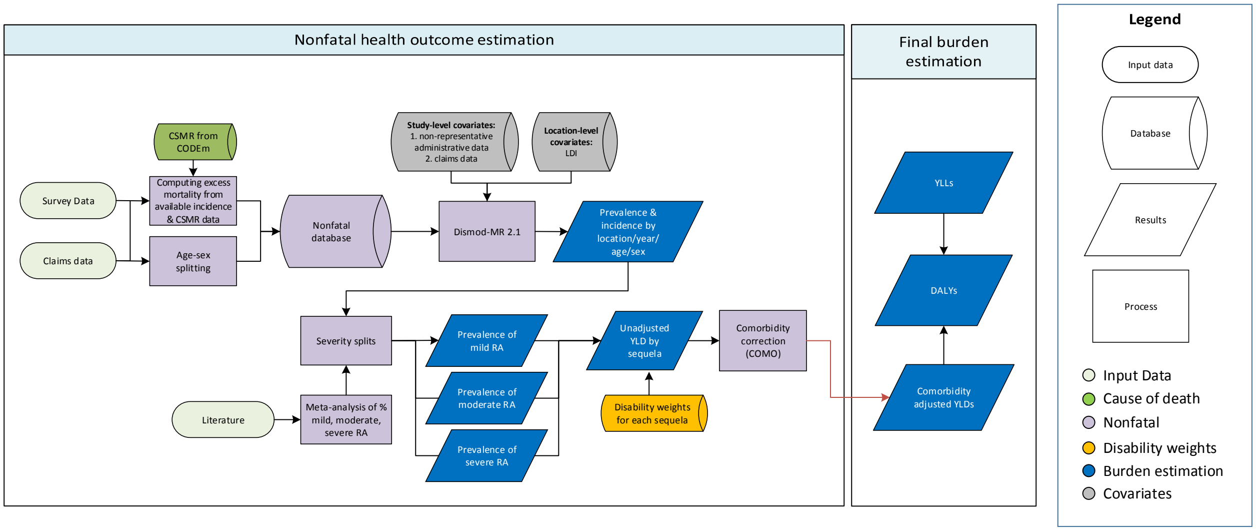

Case Definition

Rheumatoid arthritis (RA) is a systemic auto-immune disorder that causes pain and swelling of the joints. While RA is known to affect internal organs in addition to the joints, these extra-articular effects are not factored into the disability weights (DW) used in the GBD. The reference case definition for rheumatoid arthritis is based on the 1987 criteria by the American College of Rheumatology (ACR 1987)[1] which stipulate seven diagnostic criteria, of which four need to be satisfied for a diagnosis. Criteria 1 through 4 must have been present for at least six weeks (see table below). For RA, ICD-10 codes are M05, M06, and M08 and ICD-9 codes are 714.0-714.9.

Input data

Model inputs

For GBD 2010, a systematic review of the prevalence of RA throughout the world was conducted. Ovid MEDLINE, EMBASE, CINAHL, CAB abstracts, WHOLIS, and SIGLE databases were searched using the following search terms: (rheumatoid arthritis OR rheumatic disease* OR rheumatism) AND (prevalen* OR inciden* OR cross-sectional OR cross sectional OR epidemiol* OR survey OR population-based OR population based OR population study OR population sample OR cohort OR follow-up OR follow up OR longitudinal OR regist* OR data collection).

The exclusion criteria were:

1. Studies clearly not representative of the national population
2. Studies that were not population-based, e.g., hospital or clinic-based studies

3. Studies that did not provide primary data on epidemiological parameters, e.g. a commentary piece
4. Studies of a specific-type or RA, e.g., sero-positive RA
5. Studies with a sample size of less than 150
6. Reviews

The agreed-upon approach for RA was to conduct a PubMed literature search every two years. A PubMed search was conducted in GBD 2013 using the above search terms and the next is due for GBD 2016. Opportunistically, additional studies encountered during data review were added. The most recent literature source dates from 2014. The table below shows the number of literature studies included in GBD 2015, as well as the number of countries or subnational units and GBD world regions represented.

	Prevalence	Incidence	Mortality risk
Studies	66	25	25
Countries/subnationals	91	16	16
GBD world regions	16	6	3

In addition, data from US claims data for 2000, 2010, and 2012 by US state were included. We decided not to use hospital inpatient data as we considered it would not be representative of true prevalence and that variation between countries in the proportion of true prevalent cases captured in hospital inpatient data system would likely vary more than can be captured by a single crosswalk in DisMod-MR 2.1. We compared the rates of RA in the outpatient data from Norway, Sweden, Canada, and the US and found implausibly large differences with the rates from the claims data. The US outpatient rates were half the value of the claims data and those for the other countries much lower still. For those reasons we decided not to use the outpatient data.

Severity splits

The basis of the GBD disability weight survey assessments are lay descriptions of sequelae highlighting major functional consequences and symptoms. The lay descriptions and disability weights for RA severity levels are shown below.

Severity level	Lay description	DW (95% CI)
Mild	This person has moderate pain and stiffness in the arms and hands which causes difficulty lifting, carrying, and holding things, and trouble sleeping because of the pain.	0.117 (0.080 – 0.163)
Moderate	This person has pain and deformity in most joints, causing difficulty moving around, getting up and down, and using the hands for lifting and carrying. The person often feels fatigue.	0.317 (0.216 – 0.440)
Severe	This person has severe, constant pain, and deformity in most joints, causing difficulty moving around, getting up and down, eating, dressing, lifting, carrying, and using the hands. The person often feels sadness, anxiety, and extreme fatigue.	0.581 (0.403 – 0.739)

To determine the proportion of people with RA within each of the severity levels, seven studies from tjree regions provided information on the severity of RA. Severity was classified according to Health Assessment Questionnaire scores, with the cut-off scores for each severity level: <1 mild; 1-1.875 moderate; and ≥2 severe. Estimates were pooled across studies. We used a random effects meta-analysis model. The pooled percentages were: mild 48.8% (37.9-59.6%), moderate 37.6% (29.3-46.2%) and severe 12.2% (7.8-17.4%). After streaming out 1,000 draws assuming a binomial distribution, percentages were scaled to sum to 1 at each draw.

Modeling strategy

Prior settings in the DisMod model included setting remission to 0, and it was assumed that there was no incidence or prevalence of RA before the age of 5 years.

Data from all sources were re-extracted to better reflect the range of case definitions. We set the American College of Rheumatology (ACR) 1987 criteria[1] as the reference. We marked studies using the Rome 1961[2], American Rheumatology Association (ARA) 1958[3] or European League against Rheumatism (EULAR)[4] criteria with a single study covariate "non-ACR_1987" as there were inadequate studies with each alternative classification system to do separate crosswalks.

Additional study covariates were created for studies using administrative health system data sources; for studies covering regional rather than (sub)-nationally representative populations; and for claims data.

We used the function in DisMod-MR 2.1 to pull in cause-specific mortality rate (CSMR) data from our CODEm and CODcorrect analyses and match it with prevalence data points for the same geography and study year to estimate priors on excess mortality rates (by dividing CSMR by prevalence).

We retained just two of the study level covariates as x-cov (i.e., based on a significant coefficient indicating evidence of a systematic bias). Betas and exponentiated values (which can be interpreted as an odds ratio) for these two covariates are shown in the table below:

Study covariate	Parameter	beta	Exponentiated beta
RA diagnosis from admin data	Prevalence	-0.31	0.73 (0.64-0.83)
Claims data - 2000	Prevalence	-0.31	0.73 (0.72-0.75)

The other covariates were used as z-covs, meaning that DisMod estimates a value that gets added to the standard deviation of data points to reflect that these were not estimated according to our reference case definition/study method.

References

1 Arnett FC, Edworthy SM, Bloch DA, *et al.* The American Rheumatism Association 1987 revised criteria for the classification of rheumatoid arthritis. *Arthritis Rheum* 1988; **31**: 315–24.

2 Kellgren JH. Diagnostic criteria for population studies. *Bull Rheum Dis* 1962; **13**: 291–2.

3 Bennett GA, Cobb S, Jacox R, Jessar RA, Ropes MW. Proposed diagnostic criteria for rheumatoid arthritis. *Bull Rheum Dis* 1956; **7**: 121–4.

4 Vonkeman HE, van de Laar MAFJ. The new European League Against Rheumatism/American College of Rheumatology diagnostic criteria for rheumatoid arthritis: how are they performing? *Curr Opin Rheumatol* 2013; **25**: 354–9.

Osteoarthritis

Flowchart

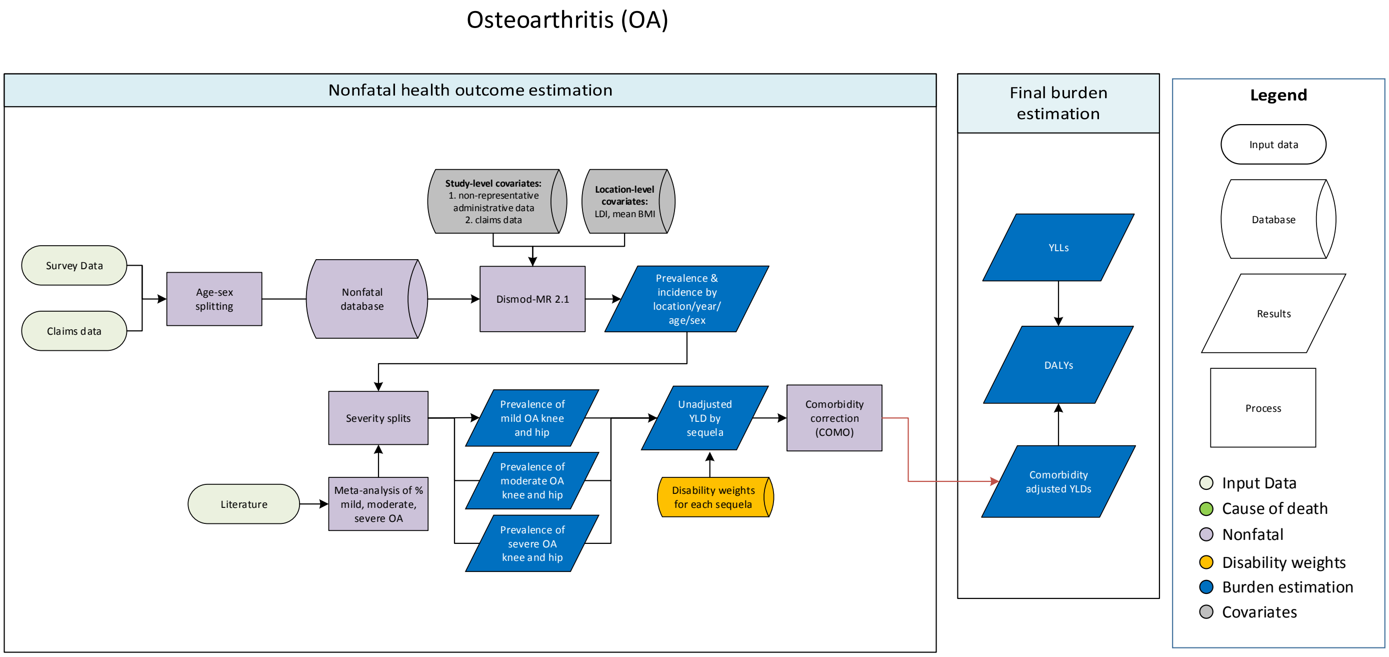

Case Definition

The OA reference case definition is symptomatic osteoarthritis of the hip or knee radiologically confirmed as Kellgren-Lawrence grade 2-4. Grade 2 symptomatic requires one defined osteophyte in hip or knee and pain for at least 1 month out of the last 12. Grade 3-4 symptomatic requires osteophytes and joint space narrowing in hip or knee with deformity also present for grade 4, and pain for at least 1 month out of the last 12 months.

Osteoarthritis (OA) is the most common form of arthritis involving inflammation and breakdown of joints. For the purposes of OA estimates for this GBD study only hip and knee sites were reviewed. The hip and knee are the common sites of OA in the larger joints and are considered to produce the greatest disability. Failure of these joints can lead to need for joint replacement surgery, if available, and thus contributes to a significant proportion of the high direct health care costs attributable to arthritis. OA of the spine is also common, however, it was considered that any symptoms and disability related to the cervical and/or lumbar spine would be captured in the estimates of low back pain and neck pain. Hand OA involving the fingers and thumbs is another common site for OA, but as it often overlaps with knee OA and could also be captured in the 'Other Musculoskeletal disorders' category, it was not considered as a separate entity in these GBD OA estimates.

ICD-10 codes for osteoarthritis of the hip and knee are M16 and M17, respectively. The ICD-9 code for osteoarthritis is 715, without specific codes for hip and knee sites.

Input data

Model Inputs

A systematic review of the prevalence, incidence and mortality of OA was performed for the years 1980 to 2009 on MEDLINE, EMBASE, CINAHL, CAB Abstracts, WHO Library (WHOLIS) and OpenSIGLE for GBD2010. For prevalence and incidence, the following search terms were used: (osteoarth* OR gonarthr*) AND (prevalen* OR inciden* OR cross-sectional OR cross sectional OR epidemiol* OR survey OR population-based OR population based OR population study OR population sample OR cohort OR follow-up OR follow up OR longitudinal OR regist*) AND (list of names of all GBD countries). For mortality, the following search terms were used: (osteoarth* OR gonarthr*) AND (Mortality OR death OR standardised mortality ratio OR standardized mortality ratio OR case fatality OR cross-sectional OR cross sectional OR epidemiol* OR survey OR population-based OR population based OR population study OR population sample OR cohort OR follow-up OR follow up OR longitudinal OR regist*) AND (list of names of all GBD countries).

Exclusion criteria were:

1. Sub-populations clearly not representative of the national population
2. Not a population-based study
3. Low sample size (less than 150)
4. Review rather than original studies

A decision was made to conduct a PubMed literature search for OA every two years. A PubMed search was conducted in GBD 2013 using the above search terms and the next is due for GBD 2016. Opportunistically, additional studies encountered during data review were added. The most recent literature source dates from 2014. The table below shows the number of literature studies included in GBD2015, as well as the number of countries or subnational units and GBD world regions represented.

	Prevalence		Incidence		Mortality risk	Severity
	OA hip	OA knee	OA hip	OA knee		
Studies	50	76	5	14	3	4
Countries/subnationals	84	94	5	14	2	3
GBD world regions	9	10	2	3	2	3

In addition, data from US claims data for 2000, 2010 and 2012 by US state were included. We decided not to use hospital inpatient data as we considered it would not be representative of true prevalence and that variation between countries in the proportion of true prevalent cases captured in hospital inpatient data system would likely vary more than can be captured by a single crosswalk in DisMod-MR 2.1.

Severity Splits

The basis of the GBD disability weight survey assessments are lay descriptions of sequelae highlighting major functional consequences and symptoms. The lay descriptions and disability weights for OA severity levels are shown below.

Severity level	Lay description	DW (95% CI)
Mild	This person has pain in the leg, which causes some difficulty running, walking long distances, and getting up and down.	0.023 (0.013 – 0.037)
Moderate	This person has moderate pain in the leg, which makes the person limp, and causes some difficulty walking, standing, lifting and carrying heavy things, getting up and down and sleeping.	0.079 (0.054 – 0.110)
Severe	This person has severe pain in the leg, which makes the person limp and causes a lot of difficulty walking, standing, lifting and carrying heavy things, getting up and down, and sleeping.	0.165 (0.112 – 0.232)

To determine the proportion of people with OA within each of the severity levels, 4 studies from 3 regions provided information on the severity of OA. Severity was classified based on the Western Ontario and McMaster Universities Arthritis Index (WOMAC) with scores 0-5 taken as mild, 6-13 as moderate and 14 and higher as severe. Estimates were pooled across studies using a random effects meta-analysis model. The pooled percentages were: mild 47.0% (42.2-51.9%), moderate 35.9% (31.3-40.7%) and severe 17.1% (12.9-21.6%) pooled between patient and physician ratings in a study from Bangladesh which we apply to low and middle income countries. The pooled proportions from three high income countries were: mild 74.3% (64.8-82.7%), moderate 24.3% (16.4-33.1%), and severe 1.1% (0.6-1.7%). After streaming out 1,000 draws assuming a binomial distribution, percentages were scaled to sum to 1 at each draw.

Modeling Strategy

Prior settings in the DisMod model included setting remission to 0, and it was assumed that there was no incidence or prevalence of OA before the age of 30 years. We assumed in the final model that excess mortality is zero. While there are some data on excess mortality risk, the values of hazard ratios or standardised mortality ratios are close to one, with some studies reporting mean estimates less than one.

We used study covariates for studies that reported on x-rays only, self-reported OA with pain, reporting a physician diagnosis of OA, and OA with symptoms but no X-ray diagnosis. For each of these covariates we estimated the crosswalk prior to dismod comparing like geographies which had data according to the reference case definition and the alternative. In dismod we set bounds with an upper and lower limit. We added covariates for each of three years of claims data in the USA. Betas and exponentiated values (which can be interpreted as an odds ratio) for these two covariates are shown in the table below:

Study covariate	Parameter	OA hip		OA knee	
		beta	Exponentiated beta	beta	Exponentiated beta
Self-reported OA with pain	Prevalence	1.38	3.97 (3.76 — 4.05)	0.70	2.01 (1.75 — 2.25)

Reported physician diagnosis OA	Prevalence	0.99	2.70 (2.66 — 2.72)	0.51	1.67 (1.42 — 1.92)
Radiography only	Prevalence	0.99	2.68 (2.59 — 2.72)	0.77	2.16 (1.87 — 2.41)
OA with symptoms but no radiography confirmation	Prevalence	1.12	3.08 (3.00 — 3.26)	0.88	2.41 (2.00 — 2.74)
Claims data - 2000	Prevalence	-0.54	0.58 (0.52 — 0.62)	-0.46	0.63 (0.55 — 0.70)
Claims data - 2010	Prevalence	-0.15	0.86 (0.78 — 0.91)	-0.04	0.96 (0.84 — 1.06)
Claims data - 2012	Prevalence	-0.08	0.93 (0.84 — 0.00)	-0.03	1.03 (0.89 — 1.14)
Reported physician diagnosis OA	Incidence	0.91	2.48 (2.24 — 2.71)	0.79	2.20 (1.36 — 2.70)
Radiography only	Incidence	0.90	2.47 (2.24 — 2.70)	0.90	2.37 (1.62 — 2.71)

We added mean BMI and the Summary Exposure Variable scalar for OA (i.e. a composite measure of the exposure to risks in GBD that affect OA: BMI). The coefficient for BMI in OA hip model was 0.059 (exponentiated 1.06; 1.04-1.07) meaning that for every unit increase in mean BMI in the population OA prevalence increases by 6%.

Low back pain (LBP)

Flowchart

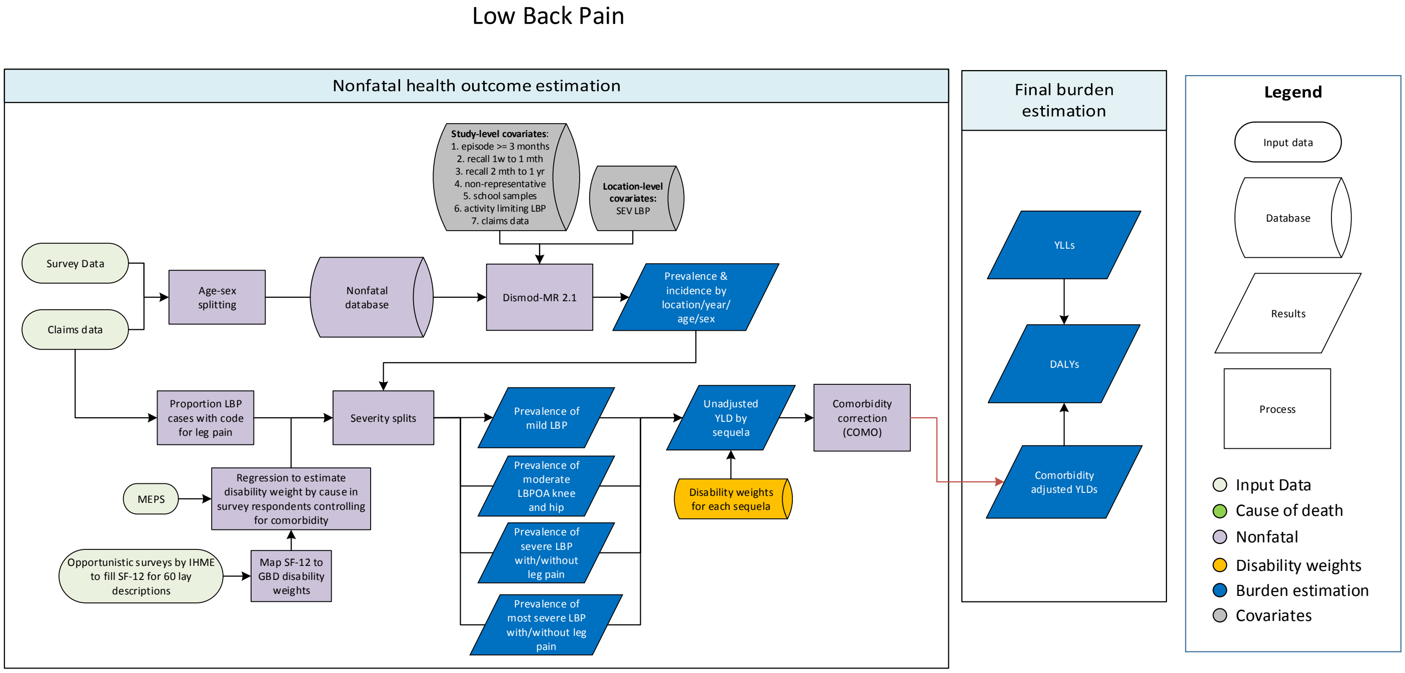

Case Definition

Low back pain (LBP) is defined as: low back pain (with or without pain referred into one or both lower limbs) that lasts for at least one day. The ‘low back’ is defined as the area on the posterior aspect of the body from the lower margin of the twelfth ribs to the lower gluteal folds.

ICD-10 codes for LBP are M54.3, M54.4 and M54.5. The ICD-9 code is 724.

Input data

Model Inputs

Ovid Medline, EMBase, and CINAHL electronic databases were searched for GBD2010. There were no age, sex, or language restrictions. The terms “back pain,” “lumbar pain,” “back ache,” “backache,” and “lumbago” were used individually and combined with each of the following: “prevalence,” “incidence,” “cross-sectional,” and “epidemiology”. The search was updated for GBD2015 in PUBMED through to August 2015.

Exclusion criteria were:

1. Sub-populations clearly not representative of the national population

2. Not a population-based study
3. Low sample size (less than 150)
4. Review rather than original studies

Additional information was derived from unit record data of surveys in GHDx, GBD's repository of population health data including the World Health surveys and national health surveys. The table below shows the number of studies and surveys included in GBD2015, as well as the number of countries or subnational units and GBD world regions represented.

	Prevalence	Incidence	Remission
Studies/surveys	239	4	4
Countries/subnationals	170	3	2
GBD world regions	20	1	1

In addition, data from US claims data for 2000, 2010 and 2012 by US state were included.

Severity Splits

The basis of the GBD disability weight survey assessments are lay descriptions of sequelae highlighting major functional consequences and symptoms. The lay descriptions and disability weights for LBP severity levels are shown below.

Severity level	Lay description	DW (95% CI)
Low back pain, mild	This person has mild back pain, which causes some difficulty dressing, standing, and lifting things.	0.020 (0.011-0.035)
Low back pain, moderate	This person has moderate back pain, which causes difficulty dressing, sitting, standing, walking, and lifting things.	0.054 (0.035-0.079)
Low back pain, severe without leg pain	This person has severe back pain, which causes difficulty dressing, sitting, standing, walking, and lifting things. The person sleeps poorly and feels worried.	0.272 (0.182-0.373)
Low back pain, severe with leg pain	This person has severe back and leg pain, which causes difficulty dressing, sitting, standing, walking, and lifting things. The person sleeps poorly and feels worried.	0.325 0.219-0.446)
Low back pain, most severe without leg pain	This person has constant back pain, which causes difficulty dressing, sitting, standing, walking, and lifting things. The person sleeps poorly, is worried, and has lost some enjoyment in life.	0.372 (0.250-0.506)
Low back pain, most severe with leg pain	This person has constant back and leg pain, which causes difficulty dressing, sitting, standing, walking, and lifting things. The person sleeps poorly, is worried, and has lost some enjoyment in life.	0.384 (0.256-0.518)

The severity distributions are derived from an analysis of the Medical Expenditure Panel Surveys (MEPS) in the US. MEPS is an overlapping continuous panel survey of the United States non-institutionalized population whose primary purpose is to collect information on the use and cost of healthcare. Panels are two years long and are conducted in 5 rounds, which are conducted every 5 to 6 months. A new panel begins annually, while the last panel is in its second year

(http://www.meps.ahrq.gov/survey_comp/hc_data_collection.jsp). Each panel typically contains about 30,000 to 35,000 individual respondents.

MEPS was initiated in 1996, but only began collecting health status data in the form of SF-12 responses in 2000. For GBD 2015 we used data from 2000-2011. Respondents self-administer the SF-12 twice per panel, at rounds 2 and 4, typically about a year apart. Only adults 18 years and older completed the SF-12. MEPS also usually collects information on diagnoses based on self-report of reasons for encounters with health services. In addition, diagnoses are derived through additional questions on "problems that bother you" or conditions that led to 'disability days', i.e. days out of role due to illness. Professional coders translate the verbatim text into three digit ICD-9 codes. The main reason for LBP being measured in MEPS relates to health care contact.

In order to derive a crosswalk of SF-12 values into a scale comparable with that used by the GBD disability weights, small studies on convenience samples were conducted asking respondents to fill in SF-12 to reflect 62 lay descriptions of diverse severity that were used to derive the GBD disability weights. From these responses a relationship between SF-12 summary score and the GBD DWs was derived. With regression methods, average disability weights were calculated for each of 156 conditions for which there were corresponding diagnoses in MEPS, while controlling for any co-morbid other condition by adding dummy variables for each condition. As our case definition is for point prevalence of low back pain, we ignored the proportion of MEPS respondents with a low back pain diagnosis for whom in our regression we found no disability attributable to LBP. For the remaining cases we binned the amount of DW attributed to LBP with and without leg pain across the 4 health states for each assuming thresholds at the midpoints between DW values.

Severity level	Distribution without leg pain	Distribution with leg pain
Low back pain, mild	0.39 (0.29-0.50)	0.27 (0.18-0.37)
Low back pain, moderate	0.36 (0.26-0.44)	0.37 (0.28-0.44)
Low back pain, severe	0.11 (0.09-0.12)	0.13 (0.10-0.16)
Low back pain, most severe	0.15 (0.09-0.21)	0.23 (0.15-0.32)

We used US claims data to derive the proportion of cases with low back pain who report leg pain.

Figure 1. Proportion of LBP with leg pain

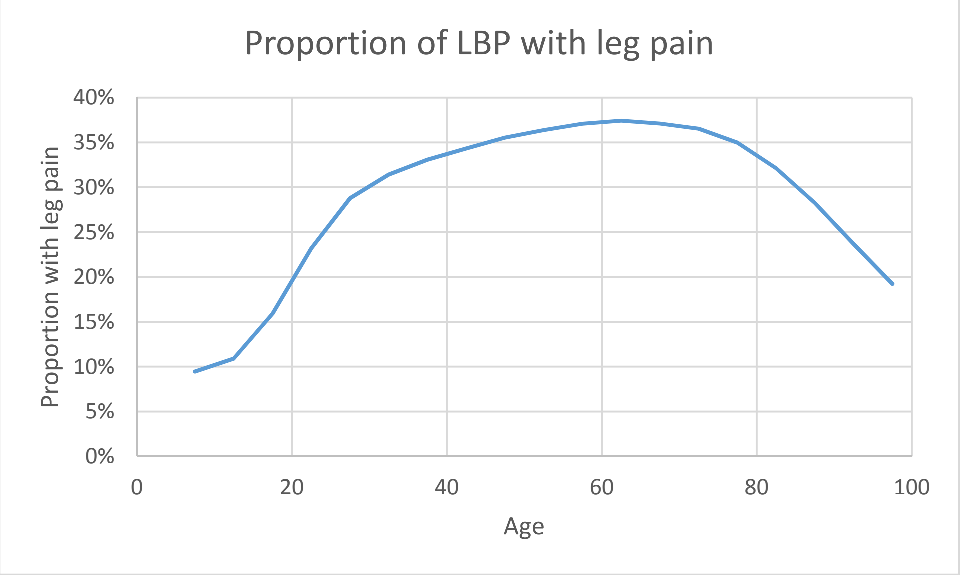

Modeling Strategy

Prior settings in the DisMod model included setting excess mortality to 0, and it was assumed that there was no incidence or prevalence of low back pain before the age of 5 years.

We used study covariates for studies that reported a too broad anatomical region, episode duration of greater than 3 months, recall periods of 1 week to 1 month, recall periods between 2 months and one year, activity-limiting LBP, and studies conducted among school children or otherwise non-representative samples. We added covariates for each of three years of claims data in the USA. Betas and exponentiated values (which can be interpreted as an odds ratio) for these two covariates are shown in the table below:

Study covariate	Parameter	beta	Exponentiated beta[1]
Anatomical region too broad	Prevalence	0.002	1.00 (1.00 — 1.01)
episode duration >= 3 months	Prevalence	-0.77	0.46 (0.44 — 0.49)
recall periods of 1 week to 1 month	Prevalence	0.80	2.22 (2.12 — 2.32)
recall periods between 2 months and one year	Prevalence	0.86	2.36 (2.27 — 2.46)
Not representative	Prevalence	0.11	1.11 (1.06 — 1.17)
studies among school children	Prevalence	1.21	3.37 (3.13 — 3.63)
Activity-limiting LBP	Prevalence	-0.33	0.72 (0.69 — 0.75)
Claims data - 2000	Prevalence	-0.54	0.58 (0.57 — 0.59)
Claims data - 2010	Prevalence	-0.095	0.91 (0.89 — 0.93)

[1] interpretation examples: in DisMod-MR 2.1 activity limiting LBP data points showed a systematic bias downward and were adjusted up by dividing by 0.72 while data points of recall greater than 2 months were adjusted downwards dividing by 2.36.

We dropped the covariate for claims data 2012 as it had a non-significant coefficient close to zero. We included the SEV scalar for low back pain as a country covariate. This combines the exposure measures for risks estimated to impinge on LBP in GBD: occupational ergonomic exposure and increased BMI. We

set bounds of 0.75 to 1.25 as the SEV is constructed in a way that if our risk estimates are accurate the value should be 1.

Neck pain (NP)

Flowchart

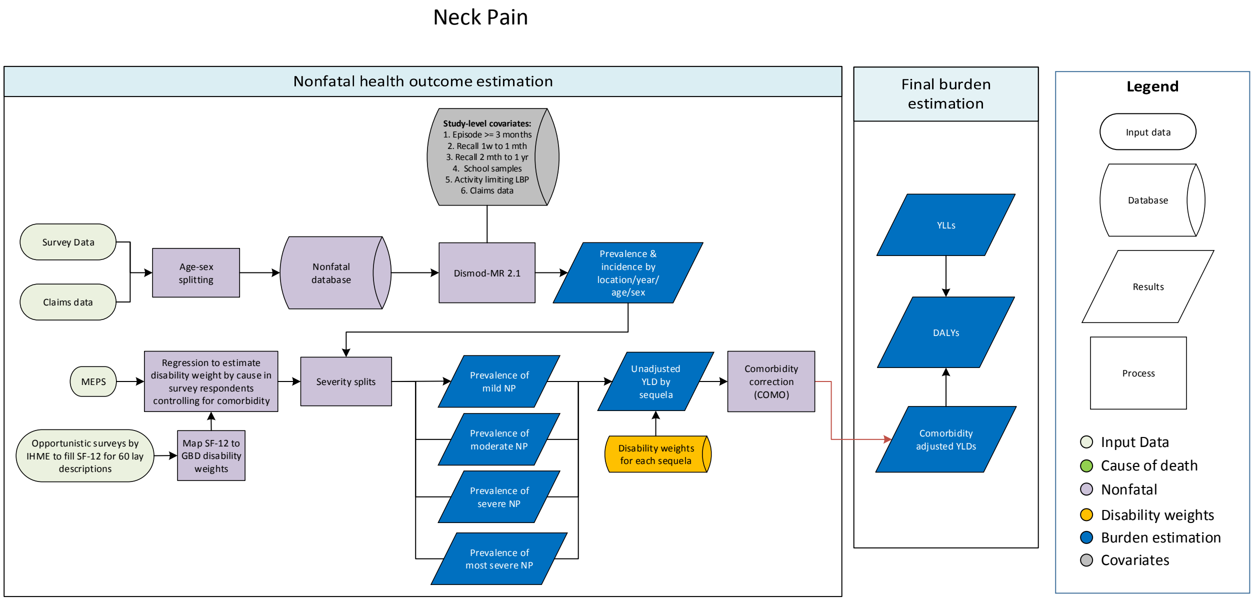

Case Definition

Neck pain (NP) was defined as: *neck pain (+/- pain referred into the upper limb(s)) that lasts for at least one day*.

ICD-10 codes for neck pain is M54.2. The ICD-9 code is 723.1.

Input data

Model Inputs

Ovid MEDLINE, EMBASE, CINAHL, CAB abstracts, WHOLIS, and SIGLE databases were searched for GBD2010. There were no age, sex, or language restrictions. The terms *neck pain, neck ache, neckache, and cervical pain* individually and combined with each of the following terms: *prevalen*, inciden*, cross-sectional, cross sectional, epidemiol*, survey, population-based, population based, population study, population sample*. The search was updated for GBD 2013 and GBD2015 in PUBMED through to August 2015.

Exclusion criteria were:

1. Sub-populations clearly not representative of the national population
2. Not a population-based study

3. Studies on a specific type of neck pain (e.g. following neck fracture)
4. Low sample size (less than 150)
5. Review rather than original studies

Additional information was derived from unit record data of surveys in GHDx, GBD's repository of population health data including NHANES and NHIS in the USA. The table below shows the number of studies and surveys included in GBD2015, as well as the number of countries or subnational units and GBD world regions represented.

	Prevalence	Incidence	Remission
Studies/surveys	74	2	1
Countries/subnationals	90	1	1
GBD world regions	11	1	1

In addition, data from US claims data for 2000, 2010 and 2012 by US state were included.

Severity Splits

The basis of the GBD disability weight survey assessments are lay descriptions of sequelae highlighting major functional consequences and symptoms. The lay descriptions and disability weights for neck pain severity levels are shown below.

Severity level	Lay description	DW (95% CI)	Proportions
Neck pain, mild	This person has neck pain, and has difficulty turning the head and lifting things	0.052 (0.036-0.074)	0.69 (0.58-0.77)
Neck pain, moderate	This person has constant neck pain, and has difficulty turning the head, holding arms up, and lifting things	0.112 (0.079-0.162)	0.12 (0.07-0.18)
Neck pain, severe	This person has has severe neck pain, and difficulty turning the head and lifting things. The person gets headaches and arm pain, sleeps poorly, and feels tired and worried	0.226 (0.147-0.323)	0.06 (0.04-0.08)
Neck pain, most severe	This person has constant neck pain and arm pain, and difficulty turning the head, holding arms up, and lifting things. The person gets headaches, sleeps poorly, and feels tired and worried	0.300 0.199-0.434)	0.14 (0.09-0.20)

The severity distributions are derived from an analysis of the Medical Expenditure Panel Surveys (MEPS) in the US. MEPS is an overlapping continuous panel survey of the United States non-institutionalized population whose primary purpose is to collect information on the use and cost of healthcare. Panels are two years long and are conducted in 5 rounds, which are conducted every 5 to 6 months. A new panel begins annually, while the last panel is in its second year (http://www.meps.ahrq.gov/survey_comp/hc_data_collection.jsp). Each panel typically contains about 30,000 to 35,000 individual respondents.

MEPS was initiated in 1996, but only began collecting health status data in the form of SF-12 responses in 2000. For GBD 2015 we used data from 2000-2011. Respondents self-administer the SF-12 twice per panel, at rounds 2 and 4, typically about a year apart. Only adults 18 years and older completed the SF-

12. MEPS also usually collects information on diagnoses based on self-report of reasons for encounters with health services. In addition, diagnoses are derived through additional questions on “problems that bother you” or conditions that led to ‘disability days’, i.e. days out of role due to illness. Professional coders translate the verbatim text into three digit ICD-9 codes. The main reason for neck pain being measured in MEPS relates to health care contact.

In order to derive a crosswalk of SF-12 values into a scale comparable with that used by the GBD disability weights, small studies on convenience samples were conducted asking respondents to fill in SF-12 to reflect 62 lay descriptions of diverse severity that were used to derive the GBD disability weights. From these responses a relationship between SF-12 summary score and the GBD DWs was derived. With regression methods, average disability weights were calculated for each of 156 conditions for which there were corresponding diagnoses in MEPS, while controlling for any co-morbid other condition by adding dummy variables for each condition. As our case definition is for point prevalence of neck pain, we ignored the proportion of MEPS respondents with a neck pain diagnosis for whom in our regression we found no disability attributable to neck pain. For the remaining cases we binned the amount of DW attributed to neck pain across the 4 health states assuming thresholds at the midpoints between DW values.

Modeling Strategy

Prior settings in the DisMod model included setting excess mortality to 0, and it was assumed that there was no incidence or prevalence of neck pain before the age of 5 years.

We used study covariates for studies that reported a too broad anatomical region, episode duration of greater than 3 months, recall periods of 1 week to 1 month, recall periods between 2 months and one year, activity-limiting neck pain, and studies conducted among school children. We added covariates for each of three years of claims data in the USA. Betas and exponentiated values (which can be interpreted as an odds ratio) for these two covariates are shown in the table below:

Study covariate	Parameter	beta	Exponentiated beta[1]
Anatomical region too broad	Prevalence	0.43	1.54 (1.39 — 1.69)
episode duration >= 3 months	Prevalence	-1.30	0.33 (0.23 — 0.33)
recall periods of 1 week to 1 month	Prevalence	0.72	2.04 (1.96 — 2.17)
recall periods between 2 months and one year	Prevalence	1.53	4.63 (4.27 — 4.98)
studies among school children	Prevalence	2.09	8.10 (6.92 — 9.09)
Activity-limiting LBP	Prevalence	-1.08	0.34 (0.29 — 0.40)
Claims data - 2000	Prevalence	-2.22	0.11 (0.10 — 0.12)
Claims data - 2010	Prevalence	-1.64	0.19 (0.19 — 0.21)
Claims data - 2012	Prevalence	-1.55	0.21 (0.21 — 0.23)

[1] interpretation examples: in DisMod-MR 2.1 activity limiting NP data points showed a systematic bias downward and were adjusted up by dividing by 0.34 while data points of recall greater than 2 months were adjusted downwards dividing by 4.63.

Gout

Flowchart

Gout

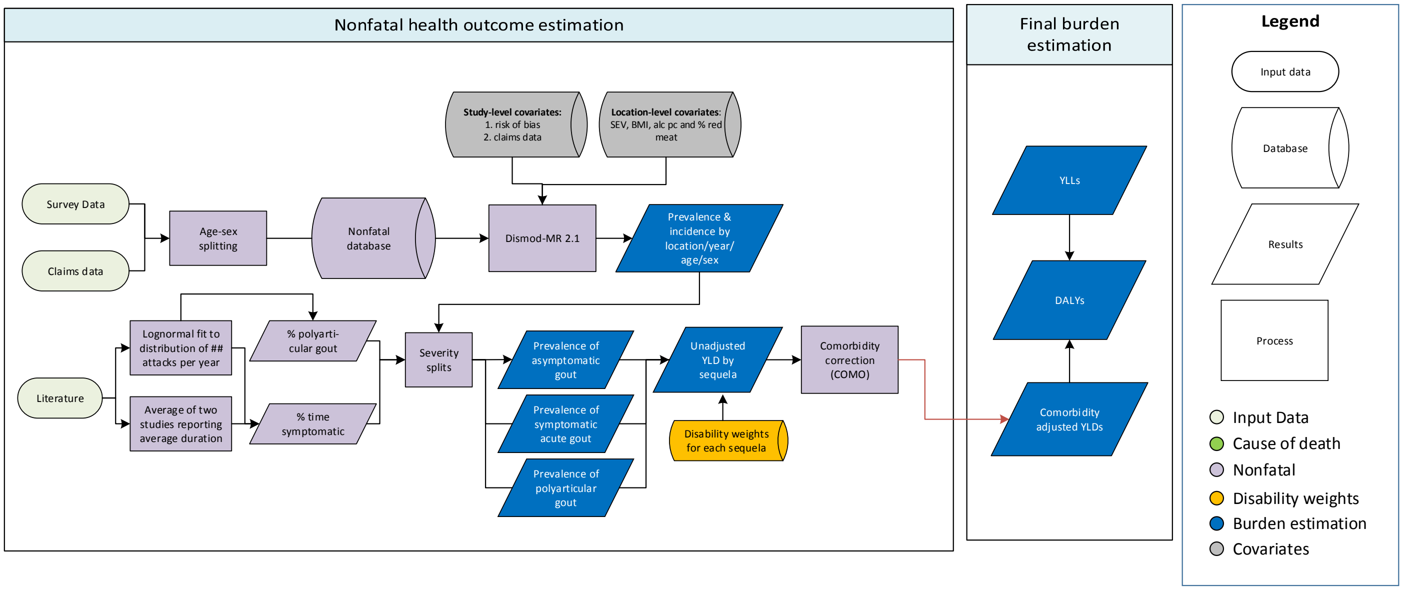

Case Definition

Gout is a rheumatic disease that is characterized by formation of monosodium urate (MSU) crystals in the synovial fluid of joints and in other tissues causing inflammation. The crystal formation is caused by elevated urate levels in extracellular fluids. It is more common in men. GBD uses the case definition of primary gout given by the American College of Rheumatology, generally referred to as ARA 1977 survey criteria requiring the presence of MSU crystals in joint fluid or the presence of a tophus proven to contain MSU crystals and at least 6 of 12 gout symptoms or findings (>1 attack of acute arthritis, development of maximal inflammation within a day, attack of monarticular arthritis, observation of joint erythema, pain or swelling in the first MTP joint, unilaterally attack involving the first MTP joint, unilateral attack involving tarsal joint, suspected tophus, hyperuricemia, asymmetrical swelling within a joint on X-ray and negative culture of joint fluid for microorganisms during attack of joint inflammation) to make a diagnosis. The ICD-10 code for gout is M10 and the ICD9 code is 274.

Input data

Model Inputs

For GBD 2010 literature searches were performed for years 1980 to 2009 on MEDLINE, EMBASE, CINAHL, CAB Abstracts, WHO Library (WHOLIS) and OpenSIGLE. For prevalence and incidence, the following search terms were used: (gout* OR hyperuricemia) AND (prevalen* OR inciden* OR cross-sectional OR cross sectional OR epidemiol* OR survey OR population-based OR population based OR population study OR

population sample OR cohort OR follow-up OR follow up OR longitudinal OR regist*) AND (list of names of all GBD countries). For mortality, the following search terms were used: (gout* OR hyperuricemia) AND (Mortality OR death OR standardised mortality ratio OR standardized mortality ratio OR case fatality OR cross-sectional OR cross sectional OR epidemiol* OR survey OR population-based OR population based OR population study OR population sample OR cohort OR follow-up OR follow up OR longitudinal OR regist*) AND (list of names of all GBD countries).

Exclusion criteria were;

- Sub-populations clearly not representative of the national population
- Not a population-based study
- Low sample size (less than 150)
- Review

The agreed approach for gout was to conduct a PubMed literature search every two years. A PubMed search was conducted in GBD 2013 using the above search terms and the next is due for GBD 2016. Opportunistically, additional studies encountered during data review were added. The most recent literature source dates from 2014. The table below shows the number of literature studies included in GBD2015, as well as the number of countries or subnational units and GBD world regions represented.

	Prevalence	Incidence	Mortality risk
Studies	73	11	3
Countries/subnationals	90	7	2
GBD world regions	12	4	1

In addition, data from US claims data for 2000, 2010 and 2012 by state were included.

Severity Splits

The basis of the GBD disability weight survey assessments are lay descriptions of sequelae highlighting major functional consequences and symptoms. The lay descriptions and disability weights for gout severity levels are shown below.

Severity level	Lay description	DW (95% CI)
Gout, acute	This person has severe pain and swelling in the leg, making it very difficult to get up and down, stand, walk, lift, and carry heavy things. The person has trouble sleeping because of the pain.	0.295 (0.196 – 0.409)
Polyarticular gout (same as for severe RA)	This person has severe, constant pain and deformity in most joints, causing difficulty moving around, getting up and down, eating, dressing, lifting, carrying and using the hands. The person often feels sadness, anxiety and extreme fatigue.	0.581 (0.403 – 0.739)

We used 3 studies on the distribution of the number of gout attacks per year and fitted a lognormal curve using a least squared differences method. In the absence of data on the proportion of gout cases who have chronic polyarticular gout, we assumed the proportion is equal to those who would 52 attacks a year (i.e. weekly) or more as implied by the lognormal curve.

The average number of attacks was estimated from the lognormal fit: 5.66 (5.14-6.18). From two studies we derived an average duration of attacks of 6.1 (5.4-6.8) days by simple averaging. The resulting proportion of time symptomatic for acute gout was taken as the multiplication of these two estimates divided by the number of days in a year: 9.4% (8.0-10.9%).

Figure 1. Distribution of cases by frequency

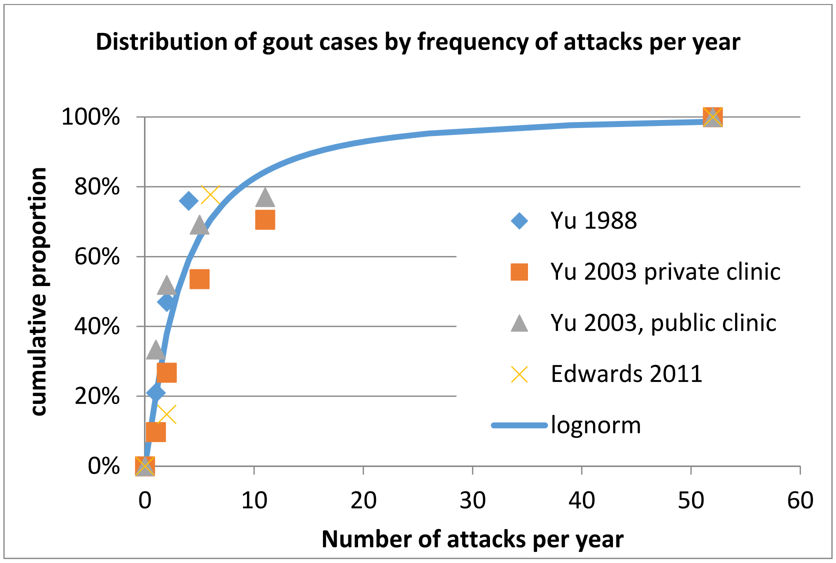

Modeling Strategy

Initially we set remission to zero but found this made incidence and prevalence inconsistent. As the ratio of prevalence and incidence in similar locations was in the order of 10:1 we decided to allow remission to range between 0 and 0.2 and that made incidence and prevalence more consistent. We assumed that there was no incidence or prevalence of gout before the age of 30 years.

We used a covariate for the 2000 US claims data but assumed the latter two years reflect the reference case definition. For studies relying on self-reported diagnoses or not stating diagnostic criteria were flagged with a covariate for a risk of bias.

Betas and exponentiated values (which can be interpreted as an odds ratio) for these two covariates are shown in the table below:

Study covariate	Parameter	beta	Exponentiated beta
Risk of bias	Prevalence	0.04	1.04 (0.99-1.09)
Claims data - 2000	Prevalence	-0.64	0.53 (0.51-0.54)
Risk of bias	Incidence	-0.27	0.77 (0.62-1.04)

We added the Summary Exposure Variable (SEV) scalar for gout which summarizes exposure to risks estimated in GBD to impinge on gout, i.e. low glomerular filtration rate. We set bounds of 0.75 to 1.25 as the SEV is constructed in a way that if our risk estimates are accurate the value should be 1.

Other musculoskeletal disorders (MSK)

Flowchart

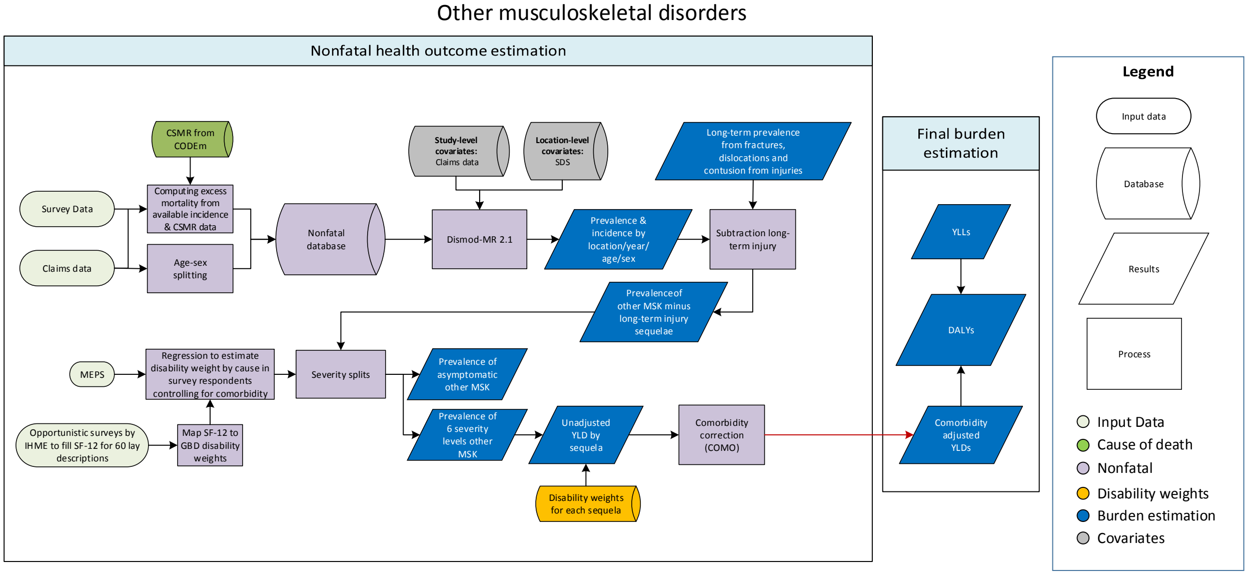

Case Definition

Other musculoskeletal (MSK) disorders is a heterogeneous rest category comprising a wide range of disorders of muscles, bones and ligaments that are not included in the 5 GBD major defined musculoskeletal diseases rheumatoid arthritis, osteoarthritis, low back and neck pain and gout, and are not captured as long term sequelae of injuries.

The table below provides detail of the ICD-10 and ICD-9 codes included in this category.

ICD-10 codes	ICD-9 codes
L93—Lupus erythematosus	710.0
M00-M02—Infectious arthropathies	711
M08, M11-M13—Inflammatory polyarthropathies	712–713
M20-M25—Other joint disorders	716=719
M30-M35—Systemic connective tissue disorders	710.1-710.9
M40-M43—Deforming dorsopathies	737
M45-M46—Spondylopathies	720–721
M60 -M63—Disorders of muscles	725
M65-M68—Disorders of synovium and tendon	726–728
M70- M73, M75-M79—Other soft tissue disorders	729
M80-M85—Disorders of bone density and structure	733.0-2
M86—Osteomyelitis	730.1-730.3, 730.7-9
M87-M90—Other osteopathies	731, 733.3-9
M91-M94—Chondropathies	732

M95-M99—Other disorders of the MSK system and connective tissue	734–736, 738–739

Input data

Model Inputs

The above ICD codes were used to extract other MSK prevalence from US claims data for 2000, 2010 and 2012 by US state. The systematic review concentrated on finding health surveys that measured an overall amount of musculoskeletal disorders and complaints and reported information to distinguish a rest category that was not OA, RA, gout, low back or neck pain. These data sources are based on self-reported musculoskeletal conditions or symptoms and not on the listed ICD codes.

The table below shows the number of studies and surveys included in GBD2015, as well as the number of countries or subnational units and GBD world regions represented.

	Prevalence	Incidence	Remission
Studies/surveys	45	2	1
Countries/subnationals	70	1	1
GBD world regions	11	1	1

Severity Splits

The basis of the GBD disability weight survey assessments are lay descriptions of sequelae highlighting major functional consequences and symptoms. The lay descriptions and disability weights for other MSK severity levels are shown below. They include the three levels of health states that are used for osteoarthritis and rheumatoid arthritis, each.

Severity level	Lay description	DW (95% CI)	Proportions
Asymptomatic			0.25 (0.24-0.25)
Musculoskeletal problems, lower limbs, mild	This person has pain in the leg, which causes some difficulty running, walking long distances, and getting up and down	0.023 (0.013-0.040)	0.22 (0.15-0.30)
Musculoskeletal problems, upper limbs, mild	This person has mild pain and stiffness in the arms and hands. The person has some difficulty lifting, carrying and holding things.	0.028 (0.017-0.046)	0.21 (0.16-0.27)
Musculoskeletal problems, upper limbs, moderate	This person has moderate pain and stiffness in the arms and hands, which causes difficulty lifting, carrying, and holding things, and trouble sleeping because of the pain	0.115 (0.079-0.163)	0.11 (0.06-0.16)

Musculoskeletal problems, lower limbs, severe	This person has severe pain in the leg, which makes the person limp and causes a lot of difficulty walking, standing, lifting and carrying heavy things, getting up and down, and sleeping	0.163 0.109-0.224	0.06 (0.05-0.08)
Musculoskeletal problems, generalized, moderate	This person has pain and deformity in most joints, causing difficulty moving around, getting up and down, and using the hands for lifting and carrying. The person often feels fatigue.	0.312 (0.201-0.438)	0.08 (0.06-0.09)
Musculoskeletal problems, generalized, severe	This person has severe, constant pain and deformity in most joints, causing difficulty moving around, getting up and down, eating, dressing, lifting, carrying and using the hands. The person often feels sadness, anxiety and extreme fatigue.	0.572 (0.370-0.758)	0.07 (0.04-0.12)

The severity distributions are derived from an analysis of the Medical Expenditure Panel Surveys (MEPS) in the US. MEPS is an overlapping continuous panel survey of the United States non-institutionalized population whose primary purpose is to collect information on the use and cost of healthcare. Panels are two years long and are conducted in 5 rounds, which are conducted every 5 to 6 months. A new panel begins annually, while the last panel is in its second year (http://www.meps.ahrq.gov/survey_comp/hc_data_collection.jsp). Each panel typically contains about 30,000 to 35,000 individual respondents.

MEPS was initiated in 1996, but only began collecting health status data in the form of SF-12 responses in 2000. For GBD 2015 we used data from 2000-2011. Respondents self-administer the SF-12 twice per panel, at rounds 2 and 4, typically about a year apart. Only adults 18 years and older completed the SF-12. MEPS also usually collects information on diagnoses based on self-report of reasons for encounters with health services. In addition, diagnoses are derived through additional questions on “problems that bother you” or conditions that led to ‘disability days’, i.e. days out of role due to illness. Professional coders translate the verbatim text into three digit ICD-9 codes. The main reason for other MSK being measured in MEPS relates to health care contact.

In order to derive a crosswalk of SF-12 values into a scale comparable with that used by the GBD disability weights, small studies on convenience samples were conducted asking respondents to fill in SF-12 to reflect 62 lay descriptions of diverse severity that were used to derive the GBD disability weights. From these responses a relationship between SF-12 summary score and the GBD DWs was derived. With regression methods, average disability weights were calculated for each of 156 conditions for which there were corresponding diagnoses in MEPS, while controlling for any co-morbid other condition by adding dummy variables for each condition. We binned the amount of DW attributed to neck pain across the 7 health states assuming thresholds at the midpoints between DW values.

Modeling Strategy

Prior settings in the DisMod model included setting excess mortality to 0, and it was assumed that there was no incidence or prevalence of other MSK before the age of 10 years. In the absence of any meaningful data on incidence and remission for such a heterogeneous category of disorders, we made a rather arbitrary decision of remission 0.5-1, i.e. an average duration of 1-2 years. We included cause-specific mortality rate (csmr) data for other MSK and estimated priors on excess mortality rate by dividing all prevalence data points by the corresponding csmr.

We used study covariates for each of the three years of claims data in the USA. Betas and exponentiated values (which can be interpreted as an odds ratio) for these two covariates are shown in the table below:

Study covariate	Parameter	beta	Exponentiated beta[1]
Claims data - 2000	Prevalence	0.83	1.96 (1.89 — 2.04)
Claims data - 2010	Prevalence	1.12	2.62 (2.53 — 2.74)
Claims data - 2012	Prevalence	1.00	2.71 (2.62 — 2.82)

[1] interpretation examples: in DisMod-MR 2.1 2012 claims data are reduced by a factor 2.71.

We allow positive coefficients on claims data as all our other data sources are based on other MSK disorders in the absence of low back pain, neck pain, OA, RA and gout while claims data reflect the one-year prevalence of having an ICD-coded other MSK condition mentioned. As there are multiple other MSK conditions that last less than a year, it is not surprising to find higher one-year prevalence in claims data then the point prevalence estimates derived from surveys.

We use the GBD sociodemographic scalar variable as a covariate with a small coefficient of 0.17 estimated by DisMod-MR 2.1.

In order to avoid double counting, we subtract the long term sequelae of fractures, dislocations and contusions due to injuries from other MSK as the surveys from which we derive prevalence estimates make no distinction between cases with other MSK problems that are or are not due to injuries. In 2015 the long-term injury sequelae that were removed from other MSK constituted a reduction in age-standardised global prevalence of 25% in men and 14% in women.

Neural tube defects

Flowchart

Neural tube defects

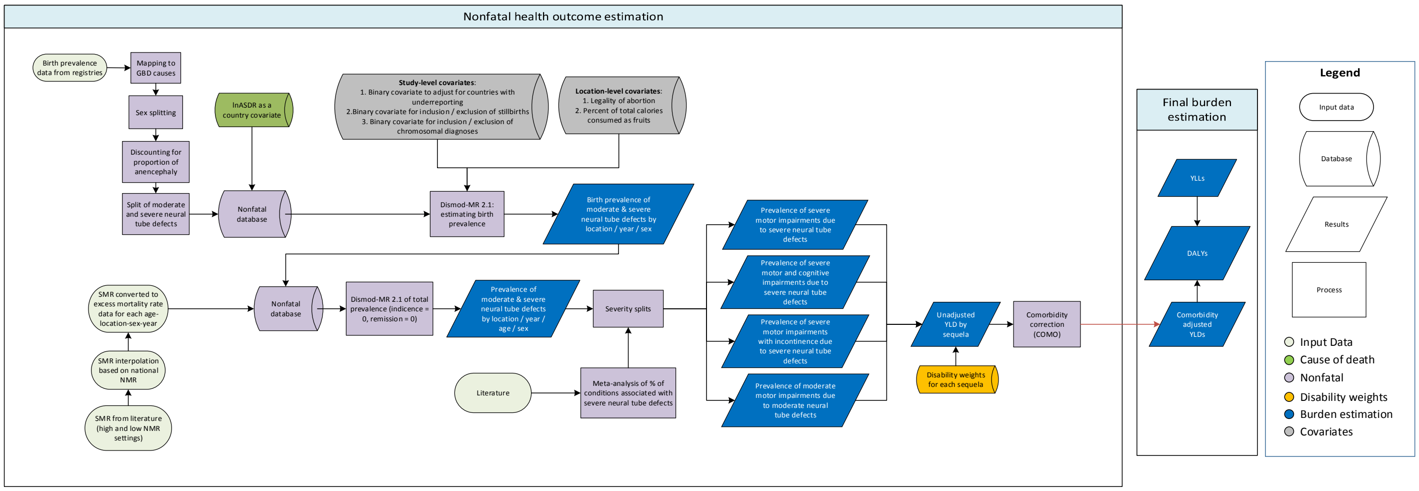

Case definition

Neural tube defects are conditions in which the neural tube fails to close completely during development. The GBD 2015 case definition of neural tube defects includes spina bifida, a congenital defect in which part of the spinal cord and its meninges are exposed through a gap in the backbone; encephalocele, a congenital defect characterized by sac-like protrusions of the brain and meninges through openings in the skull; and anencephaly, the absence of a major portion of the brain, skull, and scalp. However, anencephaly is not included in the YLD calculations, as infants with this condition typically die immediately upon birth. Spina bifida corresponds to the ICD-10 codes Q05.0, Q05.4, Q05.6, Q05.7, Q05.8, and Q05.9. Encephalocele corresponds to the ICD-10 codes Q01.2, Q01.8, and Q01.9. Anencephaly corresponds to the ICD-10 codes Q00.0 and Q00.2. For the purposes of modeling, we split neural tube defects by severity as moderate and severe neural tube defects.

Input data

Model inputs

The primary sources of data on the prevalence of neural tube defects were several surveillance systems for birth defects and congenital anomalies. Data were extracted from the following sources of non-literature data, for all available years: the International Clearinghouse for Birth Defects Monitoring Systems, the European Surveillance of Congenital Anomalies (EUROCAT), and the China National Maternal and Child Health Surveillance System Congenital Anomalies. Additionally, subnational data for the United States were extracted from the National Birth Defects Prevention Network's Population-based Birth Defects Surveillance Programs for all states and years where data were available.

	Birth prevalence: moderate neural tube defects	Birth prevalence: severe neural tube defects

Data sources	15	15
Countries/subnationals	43/77	43/78
GBD world regions	13	13

These surveillance systems report many specific types of congenital malformations, and each of these diagnoses was mapped to the GBD case definition of neural tube defects. For each location and year, the birth prevalence of neural tube defects was split by severity as 70% severe neural tube defects and 30% moderate neural tube defects, according to literature data (1). Sex splitting was performed using the literature value of 2.2:1 female:male prevalence of neural tube defects (2). We then used reported case counts and birth counts to calculate birth prevalence of both moderate and severe neural tube defects for each location and year. For each 10-year interval, we calculated the proportion of neural tube defects due to anencephaly and discounted the birth prevalence by this proportion, as children born with anencephaly typically die immediately upon birth.

For sources that separately reported both the number of cases among terminations and stillbirths and the number of cases among live births, only the cases among live births were used to calculate birth prevalence.

We identified several sources of systematic bias in the input data for neural tube defects, each of which we addressed by incorporating a study-level covariate into the model of birth prevalence. One source of systematic bias was that across locations, certain surveillance data sources consistently provided higher prevalence than others, indicating underreporting of cases in several data sources. Namely, the China National Maternal and Child Health Surveillance System Congenital Anomalies and the International Clearinghouse for Birth Defects Monitoring Systems consistently reported lower birth prevalence rates than would be expected. Thus, we included a study-level covariate to adjust for the underreporting in both data sources.

The other source of systematic bias we identified was that some locations did not disaggregate between the cases among live births and among stillbirths. As our case definition includes live births only, we included a study-level covariate to crosswalk the data sources that included both live and still births in their case definition.

For both moderate and severe neural tube defects, we excluded any data points that reported zero prevalence for a given location/year/sex combination. We also marked outliers in a few locations with implausible time trends which we determined to be the result of changes in the locations' surveillance capacity rather than changes in the prevalence of neural tube defects.

Severity split inputs

The two sequelae associated with moderate neural tube defects are "moderate motor impairment due to moderate neural tube defects" and "moderate motor impairment with incontinence due to moderate neural tube defects." Several sequelae are associated with severe neural tube defects: "Severe motor plus cognitive impairments due to severe neural tube defects," "Severe motor impairment due to severe neural tube defects," and "Severe motor impairment with incontinence due to severe neural tube defects." Global age patterns were modeled to calculate the proportion of the population with each combination of sequelae associated with severe neural tube defects.

Disability weights for each sequela were derived from the GBD Disability Weights study. Note that several of these sequelae have combined disability weights. Combined disability weights were calculated from the component disability weights according to the following formula: CDW = 1 – (1-DW1)*(1-DW2).

Sequela name	Lay description	DW (95% CI)
Moderate motor impairment due to moderate neural tube defects	Has some difficulty in moving around, and difficulty in lifting and holding objects, dressing and sitting upright, but is able to walk without help.	0.061 (0.04-0.089)
Moderate motor impairment with incontinence due to moderate neural tube defects	Has some difficulty in moving around, and difficulty in lifting and holding objects, dressing and sitting upright, but is able to walk without help, and cannot control urinating	(combined disability weight)
Severe motor impairment due to severe neural tube defects	Is unable to move around without help, and is not able to lift or hold objects, get dressed or sit upright.	0.542 (0.37-0.702)
Severe motor plus cognitive impairments due to severe neural tube defects	Cannot move around without help, and cannot lift or hold objects, get dressed or sit upright. The person also has very low intelligence, speaks few words, and needs constant supervision and help with all daily activities.	0.402 (0.268-0.545)
Severe motor impairment with incontinence due to severe neural tube defects	Is unable to move around without help, and is not able to lift or hold objects, get dressed or sit upright, and cannot control urinating.	(combined disability weight)

Modeling strategy

Overview

In order to model the prevalence of moderate neural tube defects for all year, sex, location, and age combinations, we first used DisMod-MR 2.1 to model the birth prevalence of moderate neural tube defects and separately to model the birth prevalence of severe neural tube defects. The results of these models provided us with estimates of birth prevalence for every year/location/sex combination of both severities.

We obtained literature values for the standardized mortality ratio associated with neural tube defects in cases of both comprehensive care and supportive care (3, 4). We designated several categories of neonatal mortality rate (NMR) and for each NMR category we approximated the proportion of the population who received comprehensive and supportive care for both moderate and severe neural tube defects.

NMR range	Proportion of neural tube defect patients receiving comprehensive care
<= 5	0.99

15 – 29	0.15
30 – 44	0.05
>= 45	0.01

We generated weighted average SMR values for each NMR category using the literature SMR values in cases of both supportive and comprehensive care for neural tube defects (3,4), and used these SMR values to interpolate by NMR across all locations. We then used GBD 2015 mortality estimates to convert these standardized mortality ratios to excess mortality rates (EMR). This provided us with EMR estimates for each location/year/age/sex combination.

In order to model the prevalence of moderate and severe neural tube defects across all ages, we then uploaded the birth prevalence estimates and excess mortality estimates into full models for each of moderate and severe neural tube defects.

DisMod model development: birth prevalence of moderate neural tube defects

In the birth prevalence model of moderate neural tube defects, both incidence and remission were set to 0 for all ages. We used a heterogeneity setting of 0.2 for prevalence and set age mesh points at 0 and 100 years. Due to the fact that we expect limited variation in the prevalence of neural tube defects across locations, we bounded the random effect limit on prevalence at +/- 0.5. As described above, we included a few study-level covariates in the birth prevalence model of moderate neural tube defects: one to adjust for data sources with known underreporting and one to adjust for sources that combined live and stillbirths together. We included country-level covariates for the legality of abortion and the percent of total calories consumed as fruits. Additionally, we used the log age-standardized death rate (lnASDR) for neural tube defects, derived from GBD 2015 cause of death estimates, as a country-level covariate, with a minimum allowable value of 0.20.

Study-level covariate	Parameter	beta	Exponentiated beta
Binary covariate to adjust for data sources with underreporting	Prevalence at birth	-0.0021 (-0.0075 — -0.000082)	-0.0021 (-0.0075 — -0.000082)
Binary covariate for whether live and stillbirths are both included in the case definition (reference is live births only)	Prevalence at birth	0.082 (0.014 — 0.15)	1.09 (1.01 — 1.17)
**Country-level covariate**	**Parameter**	**beta**	**Exponentiated beta**
Legality of abortion	Prevalence at birth	-0.031 (-0.047 — -0.015)	0.97 (0.95 — 0.99)
Percent of total calories consumed as fruits	Prevalence at birth	-0.18 (-0.2 — -0.14)	0.83 (0.82 — 0.87)
lnASDR for neural tube defects	Prevalence at birth	0.20 (0.20 — 0.20)	1.22 (1.22 — 1.22)

DisMod model development: moderate neural tube defects

We used the DisMod-derived estimates of moderate neural tube defects birth prevalence for every location, sex, and year, as well as interpolated excess mortality estimates for every location, sex, year,

and age, to model the prevalence of moderate neural tube defects for all ages. No study-level covariates or country-level covariates were used in this model.

The maximum allowable excess mortality rate was set to 2.8, leaving a small margin above the highest data point in the input EMR estimates. Time windows for estimates were set to four years in order to ensure that only the input data from each estimated year would inform the final estimates. A high smoothness parameter was used for excess mortality rate, and the heterogeneity settings were identical for both birth prevalence and excess mortality data. We excluded data from all priors as we already had estimates for every location/year/age/sex combination and did not want these to be altered across location hierarchies.

DisMod model development: birth prevalence of severe neural tube defects

In the birth prevalence model of severe neural tube defects, both incidence and remission were set to 0 for all ages. We used a heterogeneity setting of 0.2 for prevalence and set age mesh points at 0 and 100 years. Since we expect limited variation in the prevalence of neural tube defects across locations, we bounded the random effect limit on prevalence at +/- 0.5. Two study-level covariates were included: one to adjust for data sources with known underreporting, and one to crosswalk for sources that combined live and stillbirths together. We also included country-level covariates for the legality of abortion and the percent of total calories consumed as fruits. Additionally, we used the log age-standardized death rate (lnASDR) for neural tube defects, derived from GBD 2015 cause of death estimates, as a country-level covariate with a minimum allowable value of 0.20.

Study-level covariate	Parameter	beta	Exponentiated beta
Binary covariate to adjust for data sources with underreporting	Prevalence at birth	-0.0022 (-0.0065 — -0.00002)	1.00 (0.99 — 1.00)
Binary covariate for whether live and stillbirths are included in the case definition (reference is live births only)	Prevalence at birth	0.092 (0.0058 — 0.18)	1.10 (1.01 — 1.19)
**Country-level covariate**	**Parameter**	**beta**	**Exponentiated beta**
Legality of abortion	Prevalence at birth	-0.036 (-0.049 — -0.017)	0.96 (0.95 — 0.98)
Percent of total calories consumed as fruits	Prevalence at birth	-0.19 (-0.2 — -0.17)	0.83 (0.82 — 0.85)
lnASDR for neural tube defects	Prevalence at birth	0.20 (0.20 — 0.20)	1.22 (1.22 — 1.22)

DisMod model development: severe neural tube defects

The strategy for modeling the prevalence of severe neural tube defects was similar to that described above for neural tube defects. In the full model of severe neural tube defects, the maximum allowable excess mortality rate was set to 2.8, leaving a small margin above the highest data point in the input EMR estimates. Time windows for estimates were set to four years in order to ensure that only the input data from each estimated year would inform the final estimates. A high smoothness parameter was used for excess mortality rate, and the heterogeneity settings were identical for both birth prevalence and excess mortality data. We excluded data from all priors as we already had estimates for every location/year/age/sex combination and did not want these to be altered across location hierarchies.

Disability weights

Disability weights for each of the sequelae associated with moderate and severe neural tube defects are listed above. For each sequela, YLDs for each age, country, sex, and year were calculated by multiplying the prevalence of either moderate or severe neural tube defects, the proportion of the population with that sequela, and the disability weight or combined disability weight for that sequela.

Congenital heart anomalies

Flowchart

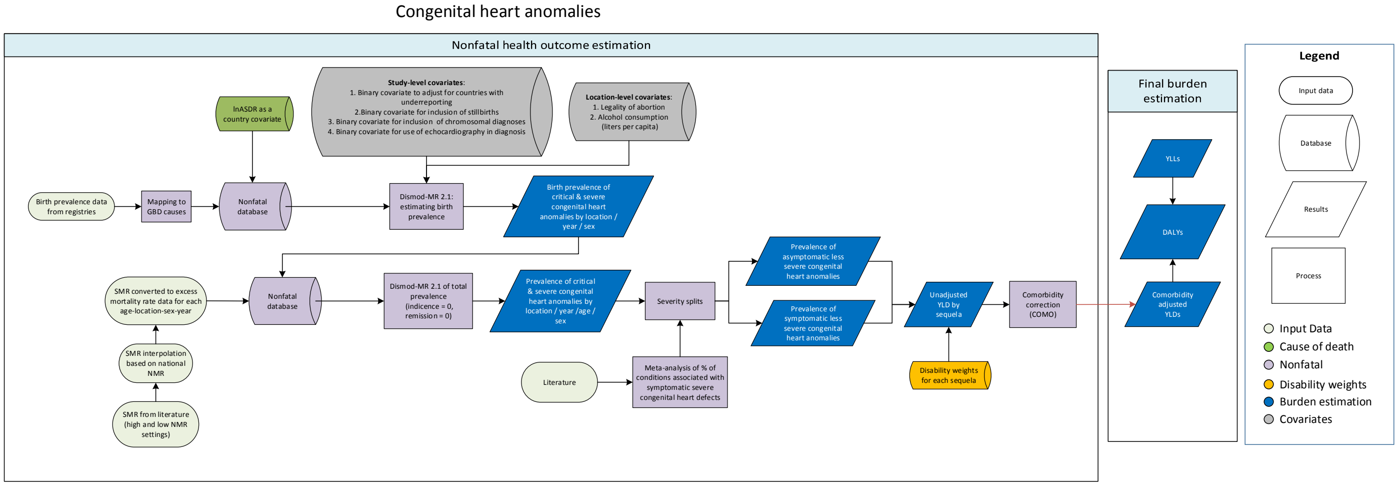

Case definition

There are many distinct types of congenital heart anomalies. For the purposes of estimating YLDs, the GBD 2015 case definition of congenital heart anomalies groups the many distinct conditions into three severity levels: less severe, severe, and critical congenital heart anomalies.

The less severe congenital heart anomalies include patent ductus arteriosis (ICD-10 code Q25.0), pulmonary valve atresia (Q22.0), pulmonary valve stenosis (Q22.1), atrial septal defects (Q21.1), and ventricular septal defects (Q21.0). The GBD sequelae for less severe congenital heart anomalies are symptomatic and asymptomatic congenital heart anomalies. These conditions may or may not require surgical care.

The severe congenital heart anomalies include common arterial truncus (Q20.0), discordant ventriculoarterial connection (Q20.3), atrioventricular septal defects (Q21.2), Tetralogy of Fallot (Q21.3), pulmonary valve atresia (Q22.0), congenital stenosis of the aortic valve (Q23.0), and coarctation of the aorta (Q25.1). These conditions typically require surgical reparation.

The critical congenital heart anomalies include double outlet of the right ventricle (Q20.1), double outlet of the left ventricle (Q20.2), double inlet ventricle (Q20.4), congenital tricuspid stenosis (Q22.4), Ebstein's anomaly (Q22.5), hypoplastic right heart syndrome (Q22.6), hypoplastic left heart syndrome (Q23.4), and total anomalous pulmonary venous connection (Q26.2). Each of these conditions is life-threatening and requires surgical reparation in early infancy.

Input data

Model inputs

The primary sources of data on the prevalence of congenital heart anomalies were several surveillance systems for birth defects and congenital anomalies. Data were extracted from the following sources of

non-literature data, for all available years: the International Clearinghouse for Birth Defects Monitoring Systems, the European Surveillance of Congenital Anomalies (EUROCAT), and the China National Maternal and Child Health Surveillance System Congenital Anomalies. Additionally, subnational data for the United States were extracted from the National Birth Defects Prevention Network's Population-based Birth Defects Surveillance Programs for all states and years where data were available.

	Birth prevalence: less severe congenital heart anomalies	Birth prevalence: severe congenital heart anomalies	Birth prevalence: critical congenital heart anomalies
Data sources	7	15	15
Countries/subnationals	22/73	40/70	40/72
GBD world regions	6	12	11

These surveillance systems report many specific types of congenital malformations, and each of these diagnoses was mapped to the GBD case definition of critical, severe, or less severe congenital heart anomalies as described above. We then used reported case counts and birth counts to calculate the birth prevalence of each severity of congenital heart anomalies for each location and year.

For sources that separately reported both the number of cases among terminations and stillbirths and the number of cases among live births, only the cases among live births were used to calculate birth prevalence.

We identified several sources of systematic bias in the input data for congenital heart anomalies, each of which we addressed by incorporating a study-level covariate into the model of birth prevalence. One source of systematic bias was that across locations, certain surveillance data sources consistently provided higher prevalence than others, indicating underreporting of cases in several data sources. Namely, the China National Maternal and Child Health Surveillance System Congenital Anomalies and the International Clearinghouse for Birth Defects Monitoring Systems consistently reported lower birth prevalence rates than would be expected. Thus, we included a study-level covariate to adjust for the underreporting in both data sources.

Another source of systematic bias was the use of echocardiography to diagnose congenital heart anomalies. For some congenital heart anomalies, the use of echocardiography for diagnosis could greatly affect the number of cases diagnosed at birth; thus, we included a study-level covariate to crosswalk data sources that did not include echocardiography in their diagnoses at birth; this was the case primarily for China subnational data. A similar source of systematic bias we identified was that some locations did not disaggregate between the cases among live births and among stillbirths. As our case definition includes live births only, we included a study-level covariate to crosswalk the data sources that included both live and still births in their case definition.

Additionally, we found that some of the US subnational data reported a much higher rate of some congenital heart anomalies than other locations did; the US National Birth Defects Prevention Network's does a more complete job of registering cases at birth than do other registries in the database. Thus, we included a study-level covariate for the purpose of cross-walking other data sources to the US Birth Defects Prevention Network's estimates.

After data extraction, we excluded any data points that reported zero prevalence for a given location/year/sex combination. We also marked outliers in a few locations with implausible time trends

which we determined to be the result of changes in the locations' surveillance capacity rather than changes in the prevalence of unbalanced chromosomal rearrangements. Upon further review of the data, we chose to mark data points that reported birth prevalence estimates of less severe congenital anomalies < 0.001, birth prevalence estimates of severe congenital anomalies < 0.0002, and birth prevalence of critical congenital anomalies < 0.00005 as outliers.

Severity split inputs

The sequelae associated with unbalanced chromosomal rearrangements are listed below.

Global age patterns were modeled to calculate the proportion of the population with symptomatic and asymptomatic less severe congenital heart anomalies across all ages.

Disability weights for each sequela were derived from the GBD Disability Weights study. Note that several of these sequelae have combined disability weights. Combined disability weights were calculated from the component disability weights according to the following formula: CDW = 1 – (1-DW1)*(1-DW2).

Sequela name	Lay description	DW (95% CI)
Asymptomatic less severe congenital heart anomalies	--	N/A
Symptomatic less severe congenital heart anomalies	(Individual has any of several congenital heart anomalies; see case definition above)	(combined disability weight)
Severe congenital heart anomalies	(Individual has any of several congenital heart anomalies; see case definition above)	0.061 (0.024-0.107)
Critical congenital heart anomalies	(Individual has any of several congenital heart anomalies; see case definition above)	0.061 (0.024-0.107)
Mild heart failure due to congenital heart anomalies	Is short of breath and easily tires with moderate physical activity, such as walking uphill or more than a quarter-mile on level ground. The person feels comfortable at rest or during activities requiring less effort.	0.041 (0.026-0.063)
Moderate heart failure due to congenital heart anomalies	Is short of breath and easily tires with minimal physical activity, such as walking only a short distance. The person feels comfortable at rest but avoids moderate activity.	0.072 (0.047-0.103)
Severe heart failure due to congenital heart anomalies	Is short of breath and feels tired when at rest. The person avoids any physical activity, for fear of worsening the breathing problems.	0.179 (0.121-0.251)

Modeling strategy

Overview

In order to model the prevalence of congenital heart anomalies for all year, sex, location, and age combinations, we first used three DisMod-MR 2.1 models to separately model the birth prevalence of less severe, severe, and critical congenital heart anomalies. The results of these models provided us with estimates of birth prevalence for every year/location/sex combination.

We obtained literature values for the standardized mortality ratio associated with less severe, severe, and critical congenital heart anomalies both with and without care (1). We designated several categories of neonatal mortality rate (NMR), and for each NMR category we approximated the proportion of the population with congenital heart anomalies who received care.

NMR range	Proportion of congenital heart patients receiving care
<= 5	1
5 – 14	0.5
15 – 29	0.15
30 – 44	0.05
>= 45	0

We generated weighted average SMR values for each NMR category using the literature SMR values for individuals receiving and not receiving care (1), and used these SMR values to interpolate by NMR across all locations. We then used GBD 2015 mortality estimates to convert these standardized mortality ratios to excess mortality rates (EMR). This provided us with EMR estimates for each location/year/age/sex combination.

In order to model the prevalence of congenital heart anomalies across all ages, we then uploaded the birth prevalence estimates and excess mortality estimates into DisMod-MR 2.1 full models for each of less severe, severe, and critical congenital heart anomalies.

DisMod model development: birth prevalence of less severe congenital heart anomalies

In the birth prevalence model of less severe congenital heart anomalies, both incidence and remission were set to 0 for all ages. We used a heterogeneity setting of 0.2 for prevalence and set age mesh points at 0 and 100 years. Because we expect limited variation in the prevalence of congenital heart anomalies across locations, we bounded the random effect limit on prevalence at +/- 0.3. As described above, we included several study-level covariates in the birth prevalence model of less severe congenital heart anomalies: one to adjust for data sources with known underreporting, one to cross-walk US registry data, and one to adjust for sources that used echocardiography for diagnosis. We also included a country-level covariate for the legality of abortion.

Study-level covariate	Parameter	beta	Exponentiated beta
Binary covariate to adjust for data sources with underreporting	Prevalence at birth	-0.74 (-1.14 — -0.19)	0.48 (0.32 — 0.83)
Binary covariate for whether echocardiography was used for diagnosis (reference is echocardiography used)	Prevalence at birth	-0.41 (-0.9 — -0.019)	0.67 (0.41 — 0.98)
Binary covariate for whether the data is from the US national registry (reference is US data)	Prevalence at birth	-0.47 (-0.66 — -0.23)	0.62 (0.52 — 0.80)
**Country-level covariate**	**Parameter**	**beta**	**Exponentiated beta**
Legality of abortion	Prevalence at birth	0.018	1.02 (1.01 — 1.02)

		(0.014 — 0.020)	

DisMod model development: less severe congenital heart anomalies

We used the DisMod-derived estimates of less severe congenital heart anomalies birth prevalence for every location, sex, and year, as well as interpolated excess mortality estimates for every location, sex, year, and age, to model the prevalence of less severe congenital heart anomalies for all ages. No study-level covariates or country-level covariates were used in this model.

The maximum allowable excess mortality rate was set to 3.4, leaving a small margin above the highest data point in the input EMR estimates. Time windows for estimates were set to 4 years in order to ensure that only the input data from each estimated year would inform the final estimates. A high smoothness parameter was used for excess mortality rate, and the heterogeneity settings were identical for both birth prevalence and excess mortality data. We excluded data from all priors as we already had estimates for every location/year/age/sex combination and did not want these to be altered across location hierarchies.

DisMod model development: birth prevalence of severe congenital heart anomalies

In the birth prevalence model of severe congenital heart anomalies, both incidence and remission were set to 0 for all ages. We used a heterogeneity setting of 0.2 for prevalence and set age mesh points at 0 and 100 years. Because we expect limited variation in the prevalence of congenital heart anomalies across locations, we bounded the random effect limit on prevalence at +/- 0.2.

As described above, we included several study-level covariates in the birth prevalence model of severe congenital heart anomalies: one to adjust for data sources with known underreporting, one to cross-walk US registry data, one for whether chromosomal diagnoses were included in the case definition, one for whether stillbirths were included in the case definition, and one to adjust for sources that used echocardiography for diagnosis. We also included country-level covariates on the legality of abortion and alcohol consumption (liters per capita). Additionally, we used the log age-standardized death rate (lnASDR) for congenital heart defects, derived from GBD 2015 cause of death estimates, as a country-level covariate.

Study-level covariate	Parameter	beta	Exponentiated beta
Binary covariate to adjust for data sources with underreporting	Prevalence at birth	-0.12 (-0.2 — -0.043)	0.89 (0.82 — 0.96)
Binary covariate for whether live and stillbirths are included in the case definition (reference is live births only)	Prevalence at birth	0.0068 (0.00019 — 0.024)	1.01 (1.00 — 1.02)
Binary covariate for whether chromosomal diagnoses are included in the case definition	Prevalence at birth	0.0079 (0.00025 — 0.027)	1.01 (1.00 — 1.03)
Binary covariate for whether echocardiography or ultrasound was used in diagnosis	Prevalence at birth	-0.78 (-1 — -0.54)	0.46 (0.37 — 0.58)

Binary covariate for whether the data is from the USA national registry	Prevalence at birth	-0.54 (-0.69 — -0.3)	0.59 (0.50 — 0.74)
**Country-level covariate**	**Parameter**	**beta**	**Exponentiated beta**
Legality of abortion	Prevalence at birth	0.017 (0.0098 — 0.020)	1.02 (1.01 — 1.02)
Alcohol consumption (liters per capita)	Prevalence at birth	0.0018 (0.000069 — 0.0064)	1.00 (1.00 — 1.01)
lnASDR for congenital heart defects	Prevalence at birth	0.20 (0.20 — 0.21)	1.22 (1.22 — 1.24)

DisMod model development: severe congenital heart anomalies

We used the DisMod-derived estimates of severe congenital heart anomalies birth prevalence for every location, sex, and year, as well as interpolated excess mortality estimates for every location, sex, year, and age, to model the prevalence of severe congenital heart anomalies for all ages. No study-level covariates or country-level covariates were used in this model.

The maximum allowable excess mortality rate was set to 28, leaving a small margin above the highest data point in the input EMR estimates. Time windows for estimates were set to four years in order to ensure that only the input data from each estimated year would inform the final estimates. A high smoothness parameter was used for excess mortality rate, and the heterogeneity settings were identical for both birth prevalence and excess mortality data. We excluded data from all priors as we already had estimates for every location/year/age/sex combination and did not want these to be altered across location hierarchies.

DisMod model development: birth prevalence of critical congenital heart anomalies

In the birth prevalence model of critical congenital heart anomalies, both incidence and remission were set to 0 for all ages. We used a heterogeneity setting of 0.2 for prevalence and set age mesh points at 0 and 100 years. Because we expect limited variation in the prevalence of congenital heart anomalies across locations, we bounded the random effect limit on prevalence at +/- 0.2.

As described above, we included several study-level covariates in the birth prevalence model of less severe congenital heart anomalies: one to adjust for data sources with known underreporting, one to cross-walk US registry data, one for whether chromosomal diagnoses were included in the case definition, one for whether stillbirths were included in the case definition, and one to adjust for sources that used echocardiography for diagnosis. We also included country-level covariates on the legality of abortion and alcohol consumption (liters per capita). Additionally, we used the lnASDR for congenital heart defects as a country-level covariate.

Study-level covariate	Parameter	beta	Exponentiated beta
Binary covariate to adjust for data sources with underreporting	Prevalence at birth	-0.45 (-0.56 — -0.32)	0.64 (0.57 — 0.73)
Binary covariate for whether echocardiography or ultrasound were used in diagnosis	Prevalence at birth	-0.47 (-0.81 — -0.17)	0.62 (0.45 — 0.85)

Binary covariate for whether live and stillbirths are included in the case definition (reference is live births only)	Prevalence at birth	0.014 (0.0010 — 0.050)	1.01 (1.00 — 1.05)
Binary covariate for whether chromosomal diagnoses are included in the case definition	Prevalence at birth	0.068 (0.0086 — 0.14)	1.07 (1.01 — 1.15)
Binary covariate for whether the data is from the US national registry	Prevalence at birth	-0.22 (-0.45 — -0.047)	0.81 (0.63 — 0.95)
Country-level covariate	Parameter	beta	Exponentiated beta
Legality of abortion	Prevalence at birth	0.011 (-0.0049 — 0.020)	1.01 (1.00 — 1.02)
Alcohol consumption (liters per capita)	Prevalence at birth	0.020 (0.0098 — 0.032)	1.02 (1.01 — 1.03)
lnASDR for congenital heart defects	Prevalence at birth	0.21 (0.20 — 0.23)	1.23 (1.22 — 1.26)

DisMod model development: critical congenital heart anomalies

We used the DisMod-derived estimates of critical congenital heart anomalies birth prevalence for every location, sex, and year, as well as interpolated excess mortality estimates for every location, sex, year, and age, to model the prevalence of critical congenital heart anomalies for all ages. No study-level covariates or country-level covariates were used in this model.

The maximum allowable excess mortality rate was set to 130, leaving a small margin above the highest data point in the input EMR estimates. Time windows for estimates were set to four years in order to ensure that only the input data from each estimated year would inform the final estimates. A high smoothness parameter was used for excess mortality rate, and the heterogeneity settings were identical for both birth prevalence and excess mortality data. We excluded data from all priors as we already had estimates for every location/year/age/sex combination and did not want these to be altered across location hierarchies.

Disability weights

Disability weights for each of the sequelae associated with less severe, severe, and critical congenital heart anomalies are listed above. For each sequela, YLDs for each age, country, sex, and year were calculated by multiplying the prevalence of less severe, severe, or critical congenital heart anomalies, the proportion of the population with that sequela, and the disability weight or combined disability weight for that sequela.

Cleft lip and cleft palate

Flowchart

Cleft lip and cleft palate

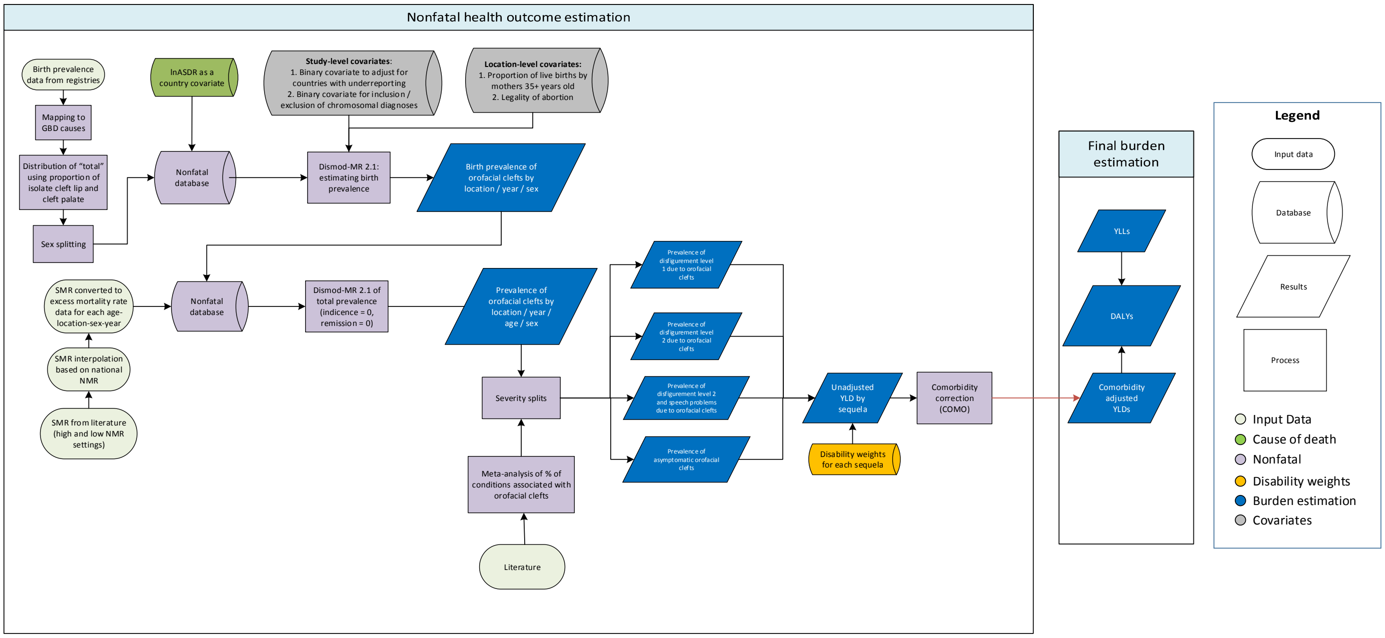

Case definition

Orofacial clefts include isolate cleft lip, isolated cleft palate, and combined cleft lip and cleft palate. Cleft lip is an opening in the upper lip that may extend into the nose, and with cleft palate, the roof of the mouth contains an opening into the nose. Both conditions are the result of the tissues of the face not joining properly during development. These conditions can be successfully treated by surgery, which is often done during the first few months of life.

The sequelae associated with orofacial clefts in GBD 2015 are disfigurement level 1, disfigurement level 2, and disfigurement level 2 with speech problems. Additionally, a proportion of the population with orofacial clefts is asymptomatic.

The GBD case definition of orofacial clefts includes isolated cleft palate, which corresponds to ICD-10 codes Q35.2, Q35.3, Q35.5, Q35.6, Q35.7, Q35.8, and Q35.9, and cleft palate with or without cleft lip, which corresponds to ICD-10 codes Q36.0, Q36.1, Q36.9, Q37.1, Q37.5, Q37.8, and Q37.9.

Input data

Model inputs

The primary sources of data on the prevalence of orofacial clefts were several surveillance systems for birth defects and congenital anomalies. Data were extracted from the following sources of non-literature data, for all available years: the International Clearinghouse for Birth Defects Monitoring Systems, the European Surveillance of Congenital Anomalies (EUROCAT), and the China National Maternal and Child Health Surveillance System Congenital Anomalies. Additionally, subnational data for the United States

were extracted from the National Birth Defects Prevention Network's Population-based Birth Defects Surveillance Programs for all states and years where data were available.

	Birth prevalence
Data sources	16
Countries/subnationals	49/81
GBD world regions	15

We used reported case counts and birth counts to calculate birth prevalence of orofacial clefts. For sources that separately reported both the number of cases among terminations and stillbirths and the number of cases among live births, only the cases among live births were used to calculate birth prevalence.

We identified several sources of systematic bias in the birth prevalence data for orofacial clefts, each of which we addressed by incorporating a study-level covariate into the model of birth prevalence. One source of systematic bias was that, across locations, certain surveillance data sources consistently reported higher birth prevalence than others, indicating underreporting of cases in several data sources. In particular, the China National Maternal and Child Health Surveillance System for Congenital Anomalies and the International Clearinghouse for Birth Defects Monitoring Systems consistently reported lower birth prevalence rates than would be expected. Thus, we included a study-level covariate to adjust for the underreporting in these data sources. Additionally, some data sources included chromosomal diagnoses in their case definition, while others did not; we used a study-level covariate to crosswalk for this as well.

Another source of systematic bias we identified was that some locations did not disaggregate between the cases among live births and among stillbirths; in particular, the inclusion of stillbirths and terminations varied by state in the United States subnational data. As our case definition includes live births only, we included a study-level covariate to crosswalk the data sources that included both live and still births in their case definition. However, this study-level covariate did not markedly change the estimates, and so was dropped from the final model.

After data extraction, we excluded any data points that reported zero prevalence for a given location/year/sex combination. We also marked outliers in a few locations with implausible time trends which we determined to be the result of changes in the locations' surveillance capacity rather than changes in the prevalence of orofacial clefts.

Severity split inputs

Several sequelae are associated with orofacial clefts: "Disfigurement level 1," "Disfigurement level 2," and "Disfigurement level 2 and speech problems." Additionally, a small proportion of the population with orofacial clefts is asymptomatic. Global age patterns were modeled to calculate the proportion of the population with each combination of sequelae associated with severe neural tube defects. These proportions were calculated for the following age intervals: 0-14 years, 15-19 years, and 50-100 years.

Disability weights for each sequela were derived from the GBD Disability Weights study. Note that these sequelae include combined disability weights. Combined disability weights were calculated from the component disability weights according to the following formula: CDW = 1 – (1-DW1)*(1-DW2).

Sequela name	Lay description	DW (95% CI)
Disfigurement level 2 and speech problems due to orofacial clefts	Has a visible physical deformity that causes others to stare and comment. As a result, the person is worried and has trouble sleeping and concentrating. Also, has difficulty speaking, and others find it difficult to understand.	(combined disability weight)
Disfigurement level 2 due to orofacial clefts	Has a visible physical deformity that causes others to stare and comment. As a result, the person is worried and has trouble sleeping and concentrating.	0.067 (0.044-0.096)
Disfigurement level 1 due to orofacial clefts	Has a slight, visible physical deformity that others notice, which causes some worry and discomfort.	0.011 (0.005-0.021)
Asymptomatic orofacial clefts	--	N/A

Modeling strategy

Overview

In order to model the prevalence of orofacial clefts for all year, sex, location, and age combinations, we first used DisMod-MR 2.1 to model the birth prevalence of orofacial clefts. The results of these models provided us with estimates of birth prevalence for every year/location/sex combination of both severities.

We obtained literature values for the standardized mortality ratio associated with orofacial clefts both with and without care, by sex (1,2). We designated several categories of neonatal mortality rate (NMR), and for each NMR category we approximated the proportion of the population who received care for orofacial clefts.

NMR per 1,000 live births	Proportion of orofacial cleft patients receiving care
<= 5	1
5 – 14	0.5
15 – 29	0.15
30 – 44	0.05
>= 45	0

We generated weighted average SMR values for each NMR category using the literature SMR values in cases of both supportive and comprehensive care for orofacial clefts (1,2), and used these SMR values to interpolate by NMR across all locations. We then used GBD 2015 mortality estimates to convert these standardized mortality ratios to excess mortality rates (EMR). This provided us with EMR estimates for each location/year/age/sex combination.

In order to model the prevalence of orofacial clefts across all ages, we then uploaded the birth prevalence estimates and excess mortality estimates into a DisMod-MR 2.1 full models of orofacial clefts.

DisMod model development: birth prevalence of orofacial clefts

In the model of Turner syndrome birth prevalence, both incidence and remission were set to 0 for all ages. We used a heterogeneity setting of 0.5 for prevalence and set age mesh points at 0 and 100 years. Because we expect limited variation in the prevalence of orofacial clefts across locations, we bounded the random effect limit on prevalence at +/- 0.3. As described above, we included a study-level covariate to adjust for data sources with known underreporting, and a study-level covariate for whether chromosomal diagnoses were included in the case definition. We included a country-level covariates for the percent of total calories consumed as fruits. Additionally, we used the log age-standardized death rate (lnASDR) for cleft lip and cleft palate, derived from GBD 2015 cause of death estimates, as a country-level covariate.

Study-level covariate	Parameter	beta	Exponentiated beta
Binary covariate to adjust for data sources with underreporting	Prevalence at birth	-0.058 (-0.089 — -0.02)	0.94 (0.91 — 0.98)
Binary covariate for whether chromosomal diagnoses are included in the case definition	Prevalence at birth	0.077 (0.042 — 0.11)	1.08 (1.04 — 1.11)
**Country-level covariate**	**Parameter**	**beta**	**Exponentiated beta**
Percent of total calories consumed as fruits	Prevalence at birth	-0.19 (-0.2 — -0.15)	0.83 (0.82 — 0.86)
lnASDR for cleft lip and cleft palate	Prevalence at birth	0.052 (0.050 — 0.057)	1.05 (1.05 — 1.06)

DisMod model development: orofacial clefts

We used the DisMod-derived estimates of orofacial cleft birth prevalence for every location, sex, and year, as well as interpolated excess mortality estimates for every location, sex, year, and age, to model the prevalence of Turner syndrome for all ages. No study-level covariates or country-level covariates were used in this model.

The maximum allowable excess mortality rate was set to 3.25, leaving a small margin above the highest data point in the input EMR estimates. Time windows for estimates were set to four years in order to ensure that only the input data from each estimated year would inform the final estimates. A high smoothness parameter was used for excess mortality rate, and the heterogeneity settings were identical for both birth prevalence and excess mortality data. We excluded data from all priors as we already had estimates for every location/year/age combination and did not want these to be altered across location hierarchies.

Disability weights

Disability weights for each of the sequelae associated with orofacial clefts are listed above. For each sequela, YLDs for each age, country, sex, and year were calculated by multiplying the prevalence of orofacial cleft, the proportion of the population with that sequela, and the disability weight or combined disability weight for that sequela.

Down syndrome

Flowchart

Down Syndrome

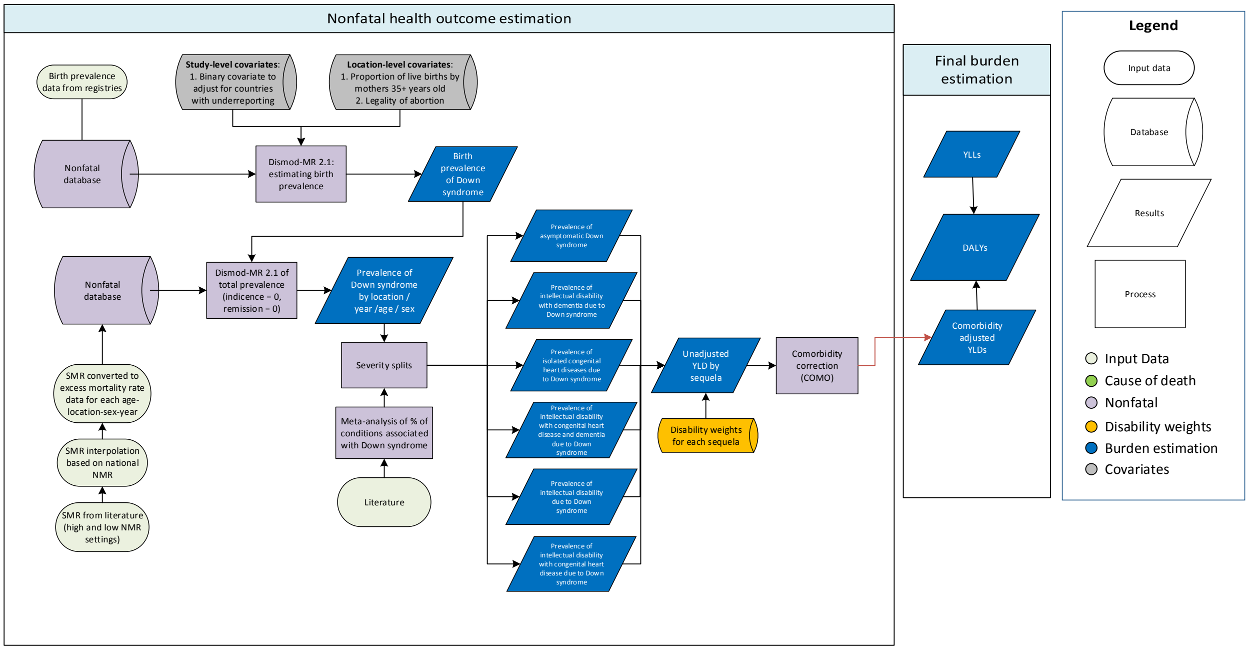

Case definition

Down syndrome, also known as Trisomy 21, is the presence of a third copy of chromosome 21, typically caused by nondisjunction during the production of gametes. Down syndrome is associated with several specific physical characteristics, including decreased muscle tone, flat facial features, an upward slant to the eyes, abnormally shaped ears, a single deep crease across the center of the palm, folded skin on the inner corners of the eyes, and ability to extend joints beyond the usual, among others. Individuals with Down syndrome may have several combinations of sequelae: those included in the GBD sequelae list are idiopathic developmental intellectual disability, congenital heart disease, and dementia. The GBD case definition of Down syndrome includes ICD-10 codes q90.0, q90.1, q90.2, q90.9

Input data

Model inputs

The primary sources of data on the prevalence of Down syndrome were several surveillance systems for birth defects and congenital anomalies. Data were extracted from the following sources of non-literature data, for all available years: the International Clearinghouse for Birth Defects Monitoring Systems, the European Surveillance of Congenital Anomalies (EUROCAT), and the China National Maternal and Child Health Surveillance System Congenital Anomalies. Additionally, subnational data for the United States were extracted from the National Birth Defects Prevention Network's Population-based Birth Defects Surveillance Programs for all states and years where data were available.

The table below illustrates the geographic breakdown of data inputs for GBD 2015 estimation.

	Birth prevalence
Data sources	16
Countries/subnationals	49/81
GBD world regions	15

We used reported case counts and birth counts to calculate birth prevalence of Down syndrome for each location and year. For sources that separately reported both the number of cases among terminations and stillbirths and the number of cases among live births, only the cases among live births were used to calculate birth prevalence.

We identified several sources of systematic bias in the input data for Down syndrome, each of which we addressed by incorporating a study-level covariate into the model of Down syndrome birth prevalence. One source of systematic bias was that across locations, certain surveillance data sources consistently provided higher prevalence than others, indicating underreporting of cases in several data sources. Namely, the China National Maternal and Child Health Surveillance System Congenital Anomalies and the International Clearinghouse for Birth Defects Monitoring Systems consistently reported lower birth prevalence rates than would be expected. Thus, we included a study-level covariate to adjust for the underreporting in both data sources. The other source of systematic bias we identified was that some locations did not disaggregate between the cases among live births and among stillbirths. As our case definition includes live births only, we included a study-level covariate to crosswalk the data sources that included both live and still births in their case definition.

After data extraction, we excluded any data points that reported zero prevalence for a given location/year/sex combination. We also marked outliers in locations with implausible time trends which we believed to be the result of changes in the locations' surveillance capacity rather than changes in the prevalence of Down syndrome.

Severity split and disability weight inputs

Several sequelae are associated with Down syndrome, as listed in the table below. Global age patterns were modeled to calculate the proportion of the population with each combination of sequelae for each of the following age ranges: 0-44 years, 45-49 years, 50-54 years, 55-69 years, 70-79 years, and 80-100 years.

Disability weights for each sequela were derived from the GBD Disability Weights study. Several of these sequelae have combined disability weights, and combined disability weights were calculated from the component disability weights according to the following formula: CDW = 1 – (1-DW1)*(1-DW2).

The table below illustrates the sequela, lay description and disability weights associated with Down syndrome for GBD 2015.

Sequela name	Lay description	DW (95% CI)
Intellectual disability	Intellectual disability due to Down syndrome	(combined disability weight)
Intellectual disability with congenital heart disease	Intellectual disability with congenital heart disease due to Down syndrome	(combined disability weight)

Intellectual disability with dementia	Intellectual disability with dementia due to Down syndrome	(combined disability weight)
Intellectual disability with congenital heart disease and dementia	Intellectual disability with congenital heart disease and dementia due to Down syndrome	(combined disability weight)
Congenital heart disease	Isolated congenital heart disease due to Down syndrome	0.061 (0.024-0.107)
Asymptomatic	Asymptomatic Down syndrome	N/A

Modeling strategy

Overview

In order to model the prevalence of Down syndrome for all year, sex, location, and age combinations, we first used DisMod-MR 2.1 to model Down syndrome birth prevalence, resulting in birth prevalence estimates for every year/location/sex combination.

We obtained literature values for the standardized mortality ratio associated with Down syndrome both with care (1) and without care (2). We designated several categories of neonatal mortality rate (NMR), and for each NMR category we approximated the proportion of the population who received care for Down syndrome.

NMR per 1,000 live births	Proportion of Down syndrome patients receiving care
<= 5	1
15 – 29	0.5
30 – 44	0.25
>= 45	0.05

We then interpolated these values across locations using GBD 2015 estimates of national NMR rates. We used GBD 2015 mortality estimates to convert these standardized mortality ratios to excess mortality rates (EMR). This provided us with EMR estimates for each location, year, age, sex combination.

To model the prevalence of Down syndrome across all ages, we uploaded the birth prevalence estimates and excess mortality estimates into a second DisMod-MR 2.1 model. The parameters set on both the birth prevalence model and the full model are described in greater detail below

DisMod model development: birth prevalence of Down syndrome

In the birth prevalence model of Down syndrome, both incidence and remission were set to 0 for all ages. We used a heterogeneity setting of 0.5 for prevalence and set age mesh points at 0 and 100 years. In anticipation of limited variation in the prevalence of Down syndrome across locations, we bounded the random effect limit on prevalence at +/- 0.3. As described above, we included a study-level covariate to adjust for data sources with known underreporting. We also included country-level covariates for the legality of abortion, and the proportion of live births by women 35+ years old, with a minimum value of 0.05.

Study-level covariate	Parameter	beta	Exponentiated beta
Binary covariate to adjust for data sources with underreporting	Prevalence at birth	-0.24 (-0.29 — -0.2)	0.79 (0.75 — 0.82)
**Country-level covariate**	**Parameter**	**beta**	**Exponentiated beta**
Legality of abortion	Prevalence at birth	-0.13 (-0.14 — -0.11)	0.88 (0.87 — 0.89)
Proportion of live births by women 35+ years old	Prevalence at birth	0.055 (0.050 — 0.068)	1.06 (1.05 — 1.07)
lnASDR for Down Syndrome	Prevalence at birth	0.29 (0.25 — 0.30)	1.33 (1.29 — 1.35)

DisMod model development: Down syndrome

We used the DisMod-derived estimates of Down syndrome birth prevalence for every location, sex, and year, as well as interpolated excess mortality estimates for every location, sex, year and age, to model the prevalence of Down syndrome for all ages. No study-level covariates or country-level covariates were used in this model.

The maximum allowable excess mortality rate was set to 0.79, leaving a small margin above the highest data point in the input EMR estimates. Time windows for estimates were set to four years in order to ensure that only the input data from each estimated year would inform the final estimates. A high smoothness parameter was used for excess mortality rate, and the heterogeneity settings were identical for both birth prevalence and excess mortality data. We excluded data from all priors as we already had estimates for every location/year/age/sex combination and did not want these to be altered across location hierarchies.

Disability weights

Disability weights for each of the sequelae associated with Down syndrome are listed above. For each sequela, YLDs for each age, country, sex, and year were calculated by multiplying the prevalence of Down syndrome, the proportion of the population with that sequela, and the disability weight or combined disability weight for that sequela.

Turner syndrome

Flowchart

Turner Syndrome

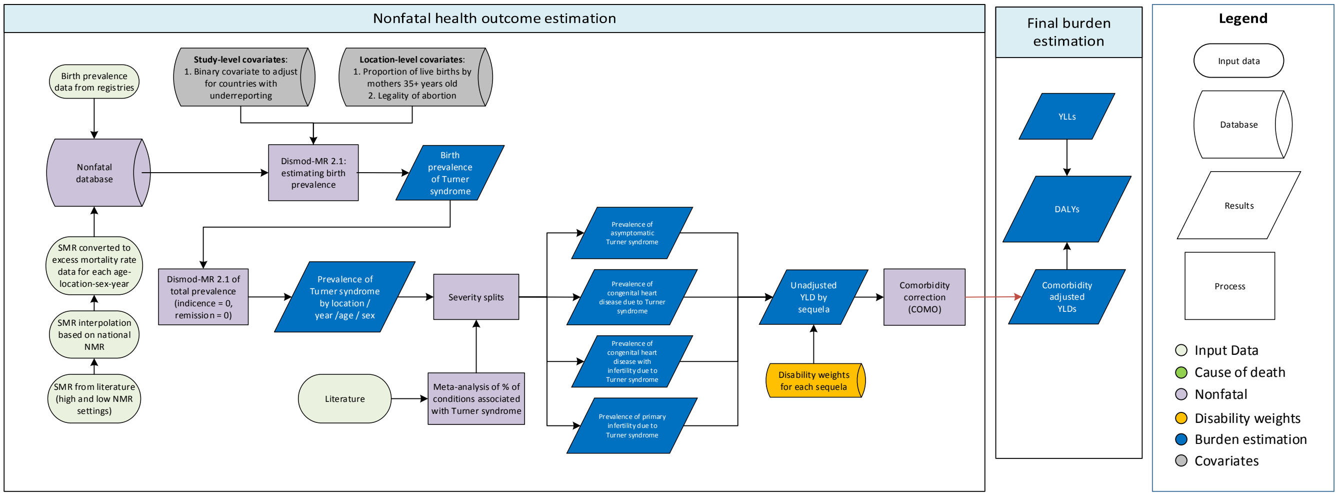

Case definition

Turner syndrome, also known as 45,X, is a condition in which a female is partly or completely missing an X chromosome. Turner syndrome can lead to a variety of medical and developmental problems, including short height, failure to start puberty, infertility, heart defects, learning disabilities, and difficulty with social adjustment. The GBD case definition of Turner syndrome includes ICD-10 codes q96.0, q96.3, and q96.9. The sequelae associated with Turner syndrome in GBD 2015 are congenital heart disease, infertility, and the combination of both congenital heart disease and infertility. In addition, a subset of individuals with Turner syndrome are asymptomatic.

Input data

Model inputs

The primary sources of data on the prevalence of Turner syndrome were several surveillance systems for birth defects and congenital anomalies. Data were extracted from the European Surveillance of Congenital Anomalies (EUROCAT) for all available years. Additionally, subnational data were extracted from the China National Maternal and Child Health Surveillance System for Congenital Anomalies, and subnational data for the United States were extracted from the National Birth Defects Prevention Network's Population-based Birth Defects Surveillance Programs for all locations and years where data were available.

	Birth prevalence
Data sources	3
Countries/subnationals	21/34
GBD world regions	5

We used reported case counts and birth counts to calculate birth prevalence of Turner syndrome among females. For sources that separately reported both the number of cases among terminations and stillbirths and the number of cases among live births, only the cases among live births were used to calculate birth prevalence.

We identified several sources of systematic bias in the birth prevalence data for Turner syndrome, each of which we addressed by incorporating a study-level covariate into the model of birth prevalence. One source of systematic bias was that across locations, certain surveillance data sources consistently reported higher birth prevalence than others, indicating underreporting of cases in several data sources. In particular, the China National Maternal and Child Health Surveillance System for Congenital Anomalies consistently reported lower birth prevalence rates than would be expected. Thus, we included a study-level covariate to adjust for the underreporting in this data source. The other source of systematic bias we identified was that some locations did not disaggregate between the cases among live births and among stillbirths; in particular, the inclusion of stillbirths and terminations varied by state in the United States subnational data. As our case definition includes live births only, we included a study-level covariate to crosswalk the data sources that included both live and still births in their case definition.

After data extraction, we excluded any data points that reported zero prevalence for a given location/year/sex combination. We also marked outliers in a few locations with implausible time trends which we determined to be the result of changes in the locations' surveillance capacity rather than changes in the prevalence of Turner syndrome.

Severity split inputs

Several sequelae are associated with Turner syndrome: "Congenital heart disease due to Turner syndrome," "Primary infertility due to Turner syndrome," and "Congenital heart disease with infertility due to Turner syndrome." Global age patterns were modeled to calculate the proportion of the population with each combination of sequelae associated with severe neural tube defects. These proportions were calculated for the following age intervals: 0-14 years, 15-19 years, and 50-100 years.

Disability weights for each sequela were derived from the GBD Disability Weights study. Note that several of these sequelae have combined disability weights.

Severity level	Lay description	DW (95% CI)
Congenital heart disease with primary infertility	Congenital heart disease with infertility due to Turner syndrome	(combined disability weight)
Congenital heart disease	Congenital heart disease due to Turner syndrome	0.061<br>(0.024-0.107)
Infertility, primary	Primary infertility due to Turner syndrome	0.008<br>(0.003-0.015)
Asymptomatic	Asymptomatic Turner syndrome	N/A

Modeling strategy

Overview

In order to model the prevalence of Turner syndrome for all year, location, and age combinations, we first used DisMod-MR 2.1 to model the birth prevalence of Turner syndrome. The results of these models provided us with estimates of birth prevalence for every year/location combination of both severities.

We used the literature value of 4.2 for the standardized mortality ratio associated with Turner syndrome (1) and interpolated this value across locations using GBD 2015 estimates of national NMR rates. We then used GBD 2015 mortality estimates to convert these standardized mortality ratios to excess mortality rates (EMR). This provided us with EMR estimates for each location/year/age combination.

In order to model the prevalence of Turner syndrome across all ages, we then uploaded the birth prevalence estimates and excess mortality estimates into a DisMod-MR 2.1 full model of Turner syndrome.

DisMod model development: birth prevalence of turner syndrome

In the model of Turner syndrome birth prevalence, both incidence and remission were set to 0 for all ages. We used a heterogeneity setting of 0.5 for prevalence and set age mesh points at 0 and 100 years. Due to the fact that we expect limited variation in the prevalence of Turner syndrome across locations, we bounded the random effect limit on prevalence at +/- 0.3. As described above, we included a study-level covariate to adjust for data sources with known underreporting. We also included country-level covariates for the legality of abortion and for the proportion of live births by women 35+ years of age.

Study-level covariate	Parameter	beta	Exponentiated beta
Binary covariate to adjust for data sources with underreporting	Prevalence at birth	-1.18 (-1.88 — -0.46)	0.31 (0.15 — 0.63)
**Country-level covariate**	**Parameter**	**beta**	**Exponentiated beta**
Legality of abortion	Prevalence at birth	-0.0039 (-0.014 — -0.000067)	1.00 (0.99 — 1.00)
Proportion of live births by women 35+ years old	Prevalence at birth	-0.091 (-0.19 — -0.006)	0.91 (0.83 — 0.99)

DisMod model development: turner syndrome

We used the DisMod-derived estimates of Turner syndrome birth prevalence for every location and year, as well as interpolated excess mortality estimates for every location, year, and age, to model the prevalence of Turner syndrome for all ages. No study-level covariates or country-level covariates were used in this model.

The maximum allowable excess mortality rate was set to 0.62, leaving a small margin above the highest data point in the input EMR estimates. Time windows for estimates were set to four years in order to ensure that only the input data from each estimated year would inform the final estimates. A high smoothness parameter was used for excess mortality rate, and the heterogeneity settings were identical for both birth prevalence and excess mortality data. We excluded data from all priors as we already had estimates for every location/year/age combination and did not want these to be altered across location

hierarchies.

Disability weights

Disability weights for each of the sequelae associated with Turner syndrome are listed above. For each sequela, YLDs for each age, country, and year were calculated by multiplying the prevalence of Turner syndrome, the proportion of the population with that sequela, and the disability weight or combined disability weight for that sequela. Because Turner syndrome only affects females, YLDs were calculated for females only.

Klinefelter syndrome

Flowchart

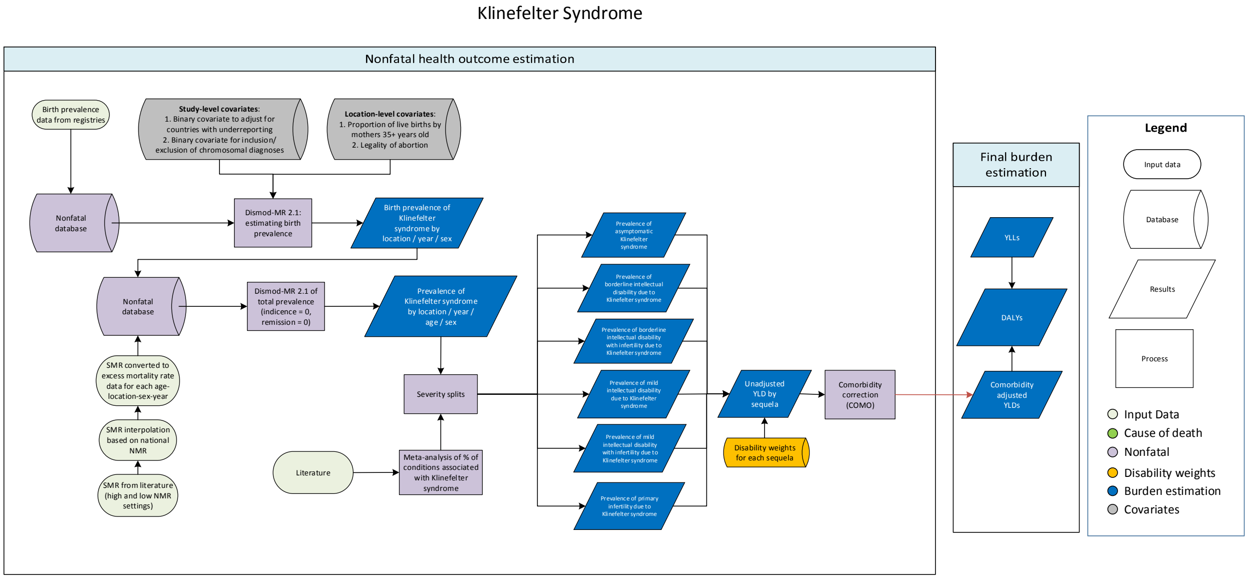

Case definition

Klinefelter syndrome, also known as 47,XXY, is a condition in which a male has two X chromosomes. The primary feature of Klinefelter syndrome is sterility, but it can cause a variety of other conditions, including weaker muscles, increased height, poor coordination abilities, smaller genitals, breast growth, and reduced interest in sex. The GBD case definition of Klinefelter syndrome includes ICD-10 codes Q98.0, Q98.5, and Q99.8. The sequelae associated with Klinefelter syndrome in GBD 2015 are borderline intellectual disability, mild intellectual disability, primary infertility, the combination of borderline intellectual disability and infertility, and the combination of mild intellectual disability and infertility. In addition, a subset of individuals with Klinefelter syndrome are asymptomatic.

Input data

Model inputs

The primary sources of data on the prevalence of Klinefelter syndrome were several surveillance systems for birth defects and congenital anomalies. For all available years, data were extracted from the European Surveillance of Congenital Anomalies (EUROCAT). Additionally, subnational data were extracted from the China National Maternal and Child Health Surveillance System for Congenital Anomalies for all locations and years where data were available.

	Birth prevalence
Data sources	2
Countries/subnationals	19/9
GBD world regions	4

We used reported case counts and birth counts to calculate birth prevalence of Klinefelter syndrome among males. For sources that separately reported both the number of cases among terminations and

stillbirths and the number of cases among live births, only the cases among live births were used to calculate birth prevalence.

One source of systematic bias in the birth prevalence data for Klinefelter syndrome was that, across locations, certain surveillance data sources consistently reported higher birth prevalence than others, indicating underreporting of cases in several data sources. In particular, the China National Maternal and Child Health Surveillance System for Congenital Anomalies consistently reported lower birth prevalence rates than would be expected. Thus, we included a study-level covariate to adjust for the underreporting in this data source.

After data extraction, we excluded any data points that reported zero prevalence for a given location/year combination. We also marked outliers in a few locations with implausible time trends which we determined to be the result of changes in the locations' surveillance capacity rather than changes in the prevalence of Klinefelter syndrome.

Severity split inputs

Several sequelae are associated with Klinefelter syndrome, as described in the table below. Global age patterns were modeled to calculate the proportion of the population with each combination of sequelae associated with Klinefelter syndrome. These proportions were calculated for the following age intervals: 0-19 years, 20-49 years, and 50-100 years.

Disability weights for each sequela were derived from the GBD Disability Weights study. Several of these sequelae have combined disability weights. Combined disability weights were calculated from the component disability weights according to the following formula: CDW = 1 – (1-DW1)*(1-DW2).

Sequela name	Lay description	DW (95% CI)
Borderline intellectual disability due to Klinefelter syndrome	Slow in learning at school. As an adult, the person has some difficulty doing complex or unfamiliar tasks but otherwise functions independently.	0.011 (0.005-0.02)
Mild intellectual disability due to Klinefelter syndrome	Has low intelligence and is slow in learning at school. As an adult, the person can live independently, but often needs help to raise children and can only work at simple supervised jobs.	0.043 (0.026-0.065)
Primary infertility due to Klinefelter syndrome	Wants to have a child and has a fertile partner, but the couple cannot conceive.	0.008 (0.003-0.015)
Borderline intellectual disability with infertility due to Klinefelter syndrome	(see above)	(combined disability weight)
Mild intellectual disability with infertility due to Klinefelter syndrome	(see above)	(combined disability weight)
Asymptomatic Klinefelter syndrome	--	N/A

Modeling strategy

Overview

In order to model the prevalence of Klinefelter syndrome among males for all year, location, and age combinations, we first used DisMod-MR 2.1 to model the birth prevalence of Klinefelter syndrome. The results of these models provided us with estimates of birth prevalence for every year/location combination of both severities.

We obtained a literature value of 1.4 (1.1 -1.7) for the relative risk associated with Klinefelter syndrome (1) and interpolated this value across locations using GBD 2015 estimates of national NMR rates. We then used GBD 2015 mortality estimates to convert these standardized mortality ratios to excess mortality rates (EMR). This provided us with EMR estimates for each location/year/age combination.

In order to model the prevalence of Klinefelter syndrome across all ages, we then uploaded the birth prevalence estimates and excess mortality estimates into a DisMod-MR 2.1 full model of Klinefelter syndrome.

DisMod model development: birth prevalence of Klinefelter syndrome

In the model of Klinefelter syndrome birth prevalence, both incidence and remission were set to 0 for all ages. We used a heterogeneity setting of 0.5 for prevalence and set age mesh points at 0 and 100 years. Since we expect limited variation in the prevalence of Klinefelter syndrome across locations, we bounded the random effect limit on prevalence at +/- 0.3. As described above, we included a study-level covariate to adjust for data sources with known underreporting. We also included country-level covariates for the legality of abortion and for the proportion of live births by women 35+ years of age.

Study-level covariate	Parameter	beta	Exponentiated beta
Binary covariate to adjust for data sources with underreporting	Prevalence at birth	-0.43 (-1.05 — -0.03)	0.65 (0.35 — 0.97)
**Country-level covariate**	**Parameter**	**beta**	**Exponentiated beta**
Legality of abortion	Prevalence at birth	-0.0046 (-0.016 — -0.0003)	1.00 (0.98 — 1.00)
Proportion of live births by women 35+ years old	Prevalence at birth	0.15 (0.042 — 0.20)	1.16 (1.04 — 1.22)

DisMod model development: Klinefelter syndrome

We used the DisMod-derived estimates of Klinefelter syndrome birth prevalence for every location and year, as well as interpolated excess mortality estimates for every location, year, and age, to model the prevalence of Klinefelter syndrome for all ages. No study-level covariates or country-level covariates were used in this model.

The maximum allowable excess mortality rate was set to 0.075, leaving a small margin above the highest data point in the input EMR estimates. Time windows for estimates were set to four years in order to ensure that only the input data from each estimated year would inform the final estimates. A high smoothness parameter was used for excess mortality rate, and the heterogeneity settings were identical for both birth prevalence and excess mortality data. We excluded data from all priors as we already had estimates for every location/year/age combination and did not want these to be altered across location

hierarchies.

Disability weights

Disability weights for each of the sequelae associated with Klinefelter syndrome are listed above. For each sequela, YLDs for each age, country, sex, and year were calculated by multiplying the prevalence of Klinefelter syndrome, the proportion of the population with that sequela, and the disability weight or combined disability weight for that sequela. Because Klinefelter syndrome only affects males, YLDs were calculated for males only.

Other chromosomal anomalies

Flowchart

Other chromosomal rearrangements

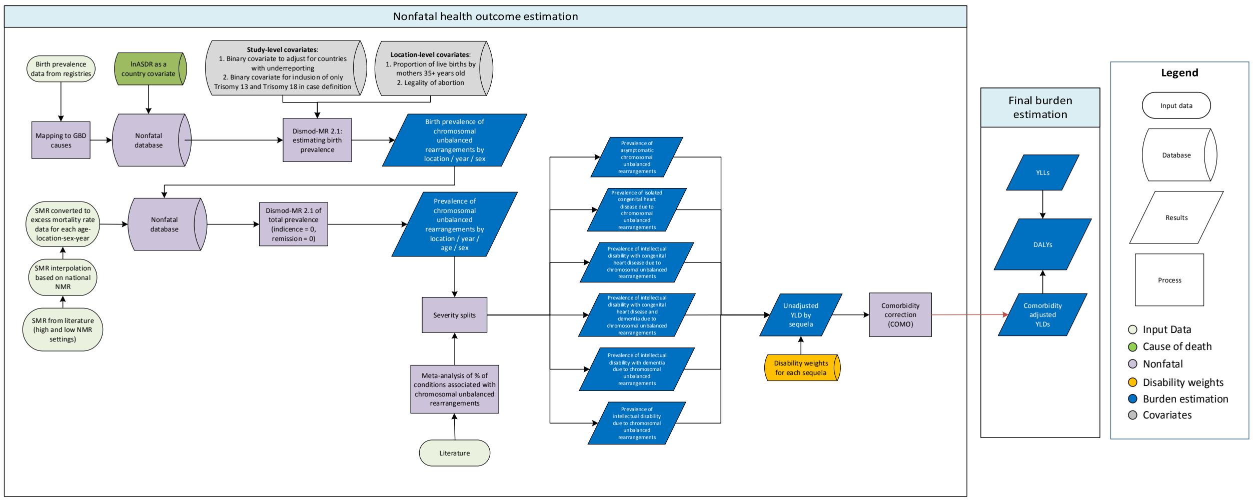

Case definition

Other chromosomal anomalies, also known as unbalanced chromosomal rearrangements, are genetic anomalies that typically occur due to meiotic nondisjunction, when homologous chromosomes do not separate normally in nuclear division during gamete formation. The GBD 2015 case definition of unbalanced chromosomal rearrangements includes Trisomy 18, Trisomy 13, 47,XXX (Triple X syndrome), other meiotic nondisjunction events, other female sex chromosome abnormalities, and other unspecified chromosomal abnormalities. Our case definition corresponds to the ICD-10 codes Q91.3, Q91.7, Q92.0, Q97.0, Q97.8, Q99.9. Excluded from this definition are the chromosomal abnormalities of Down syndrome, Turner syndrome, and Klinefelter syndrome, each of which is modeled separately.

The sequelae associated with unbalanced chromosomal rearrangements include intellectual disability, intellectual disability with dementia, intellectual disability with congenital heart disease and dementia, and intellectual disability with congenital heart disease. Additionally, a proportion of the individuals with unbalanced chromosomal rearrangements are asymptomatic.

Input data

Model inputs

The primary sources of data on the prevalence of unbalanced chromosomal rearrangements were several surveillance systems for birth defects and congenital anomalies. Data were extracted from the following sources of non-literature data, for all available years: the International Clearinghouse for Birth Defects Monitoring Systems, the European Surveillance of Congenital Anomalies (EUROCAT), and the China National Maternal and Child Health Surveillance System Congenital Anomalies. Additionally, subnational data for the United States were extracted from the National Birth Defects Prevention Network's Population-based Birth Defects Surveillance Programs for all states and years where data were available.

	Birth prevalence
Data sources	15
Countries/subnationals	43/67
GBD world regions	13

These surveillance systems report many specific types of congenital malformations, and each of these diagnoses was mapped to the GBD case definition of unbalanced chromosomal rearrangements. We then used reported case counts and birth counts to calculate the birth prevalence of unbalanced chromosomal rearrangements for each location and year.

For sources that separately reported the number of cases among terminations and stillbirths, as well as the number of cases among live births, only the cases among live births were used to calculate birth prevalence.

We identified several sources of systematic bias in the input data for unbalanced chromosomal rearrangements, each of which we addressed by incorporating a study-level covariate into the model of birth prevalence. We noted that certain surveillance data sources consistently provided higher prevalence than others, indicating underreporting of cases in several data sources and indicative of a location-specific bias. Namely, the China National Maternal and Child Health Surveillance System Congenital Anomalies and the International Clearinghouse for Birth Defects Monitoring Systems consistently reported lower birth prevalence rates than would be expected. Thus, we included a study-level covariate to adjust for the underreporting in both data sources.

Another source of systematic bias in our birth prevalence data was that some of the data sources only included cases of Trisomy 13 and Trisomy 18, and none of the other unbalanced chromosomal rearrangements, such as unspecified chromosomal abnormalities. We included a study-level covariate in our birth prevalence model to adjust for underreporting in these locations.

A third source of systematic bias we identified was that some locations did not disaggregate between the cases among live births and among stillbirths. As our case definition includes live births only, we included a study-level covariate to crosswalk the data sources that included both live and still births in their case definition. However, this study-level covariate did not influence the model results strongly and was thus dropped from the final model.

After data extraction, we excluded any data points that reported birth prevalence values below 0.0001 for a given location/year/sex combination. We also marked outliers in a few locations with implausible time trends which we determined to be the result of changes in the locations' surveillance capacity rather than changes in the prevalence of unbalanced chromosomal rearrangements.

Severity split inputs

The sequelae associated with unbalanced chromosomal rearrangements are listed below. Global age patterns were modeled to calculate the proportion of the population with each combination of sequelae associated with unbalanced chromosomal rearrangements. These proportions were calculated for the following age categories: 0-44 years, 45-49 years, 50-54 years, 55-69 years, 70-79 years, and 80-100 years.

Disability weights for each sequela were derived from the GBD Disability Weights study. Note that several of these sequelae have combined disability weights. Combined disability weights were calculated from the component disability weights according to the following formula: CDW = 1 – (1-DW1)*(1-DW2).

The table below illustrates sequelae and associated disability weights:

Sequela name	DW (95% CI)
Intellectual disability due to chromosomal unbalanced rearrangements	0.089 (0.061-0.122)
Isolated congenital heart disease due to chromosomal unbalanced rearrangements	0.061 (0.024-0.107)
Congenital heart disease due to chromosomal unbalanced rearrangements	(Combined disability weight)
Intellectual disability with dementia due to chromosomal unbalanced rearrangements	(Combined disability weight)
Intellectual disability with congenital heart disease and dementia due to chromosomal unbalanced rearrangements	(Combined disability weight)
Intellectual disability with congenital heart disease due to chromosomal unbalanced rearrangements	(Combined disability weight)
Asymptomatic chromosomal unbalanced rearrangements	N/A

Modeling strategy

Overview

In order to model the prevalence of unbalanced chromosomal rearrangements for all year, sex, location, and age combinations, we first used DisMod-MR 2.1 to model the birth prevalence of this condition, and results of these models provided us with estimates of birth prevalence for every year/location/sex combination.

We obtained literature values for the standardized mortality ratio (SMR) associated with Down syndrome both with care (1) and without care (2), and used these SMR values to approximate the SMR values of individuals with unbalanced chromosomal rearrangements. We designated several categories of neonatal mortality rate (NMR), and for each NMR category we approximated the proportion of the population with unbalanced chromosomal rearrangements who received care.

NMR per 1,000 live births	Proportion of unbalanced chromosomal rearrangements patients receiving care
<= 5	1
5 – 14	0.5
15 – 29	0.25
30 – 44	0.05
>= 45	0

We generated weighted average SMR values for each NMR category using the literature SMR values in cases of care and no care (1,2), and used these SMR values to interpolate by NMR across all locations. We

then used GBD 2015 mortality estimates to convert these standardized mortality ratios to excess mortality rates (EMR). This provided us with EMR estimates for each location/year/age/sex combination.

In order to model the prevalence of unbalanced chromosomal rearrangements across all ages, we then uploaded the birth prevalence estimates and excess mortality estimates into a DisMod-MR 2.1 full model of unbalanced chromosomal rearrangements.

DisMod model development: birth prevalence of unbalanced chromosomal rearrangements

In the birth prevalence model of unbalanced chromosomal rearrangements, both incidence and remission were set to 0 for all ages. We used a heterogeneity setting of 0.5 for prevalence and set age mesh points at 0 and 100 years. Because we expect limited variation in the prevalence of unbalanced chromosomal rearrangements across locations, we bounded the random effect limit on prevalence at +/-0.3. As described above, we included a few study-level covariates in the birth prevalence model of unbalanced chromosomal rearrangements: one to adjust for data sources with known underreporting, and one to adjust for sources that included only Trisomy 13 and Trisomy 18 in their surveillance reporting. We also included country-level covariates for the legality of abortion and the proportion of live births by women aged 35 years or older. Additionally, we used the log age-standardized death rate (lnASDR) for other chromosomal abnormalities, derived from GBD 2015 cause of death estimates, as a country-level covariate.

Study-level covariate	Parameter	beta	Exponentiated beta
Binary covariate to adjust for data sources with underreporting	Prevalence at birth	-0.52 (-0.65 — -0.37)	0.60 (0.52 — 0.69)
Binary covariate for whether chromosomal diagnoses are included in the case definition	Prevalence at birth	-1.02 (-1.18 — -0.87)	0.36 (0.31 — 0.42)
**Country-level covariate**	**Parameter**	**beta**	**Exponentiated beta**
Legality of abortion	Prevalence at birth	-0.046 (-0.068 — -0.026)	0.96 (0.93 — 0.97)
Proportion of live births by women 35+ years old	Prevalence at birth	0.023 (0.00044 — 0.065)	1.02 (1.00 — 1.07)
lnASDR for other chromosomal abnormalities	Prevalence at birth	0.19 (0.16 — 0.20)	1.21 (1.18 — 1.22)

DisMod model development: unbalanced chromosomal rearrangements

We used the DisMod-derived estimates of unbalanced chromosomal rearrangements birth prevalence for every location, sex, and year, as well as interpolated excess mortality estimates for every location, sex, year, and age, to model the prevalence of unbalanced chromosomal rearrangements for all ages. No study-level covariates or country-level covariates were used in this model.

The maximum allowable excess mortality rate was set to 0.8, leaving a small margin above the highest data point in the input EMR estimates. Time windows for estimates were set to four years in order to ensure that only the input data from each estimated year would inform the final estimates. A high

smoothness parameter was used for excess mortality rate, and the heterogeneity settings were identical for both birth prevalence and excess mortality data. We excluded data from all priors as we already had estimates for every location/year/age/sex combination and did not want these to be altered across location hierarchies.

Disability weights

Disability weights for each of the sequelae associated with unbalanced chromosomal rearrangements are listed above. For each sequela, YLDs for each age, country, sex, and year were calculated by multiplying the prevalence of unbalanced chromosomal rearrangements, the proportion of the population with that sequela, and the disability weight or combined disability weight for that sequela.

Dermatitis

Flowcharts for Eczema, Contact Dermatitis, & Seborrheic Dermatitis

Eczema

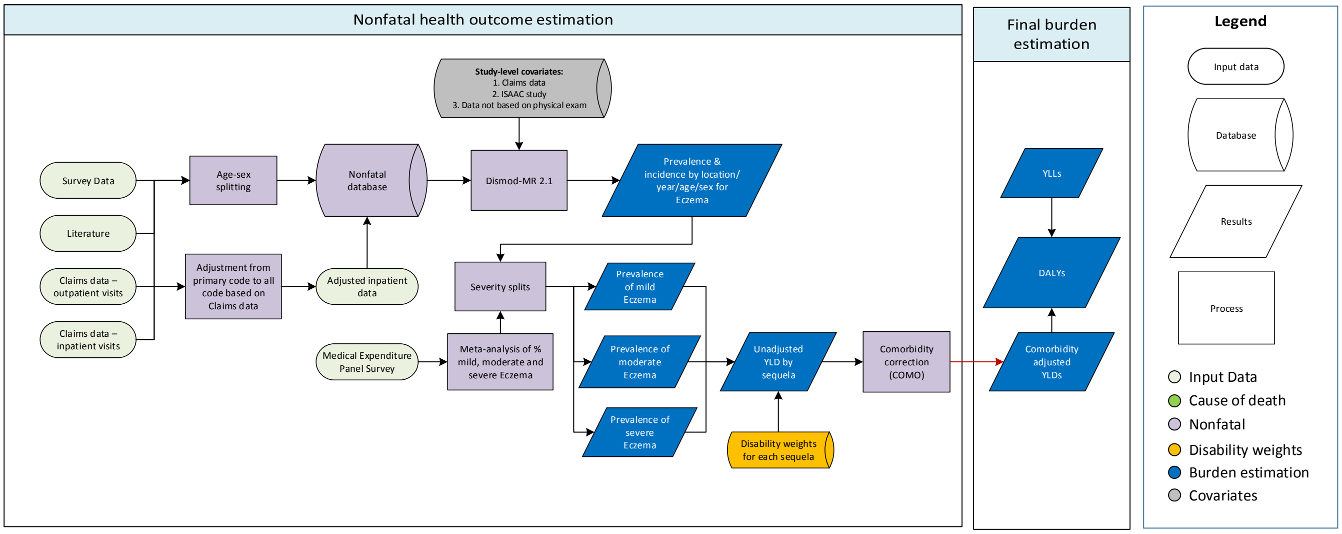

Contact Dermatitis

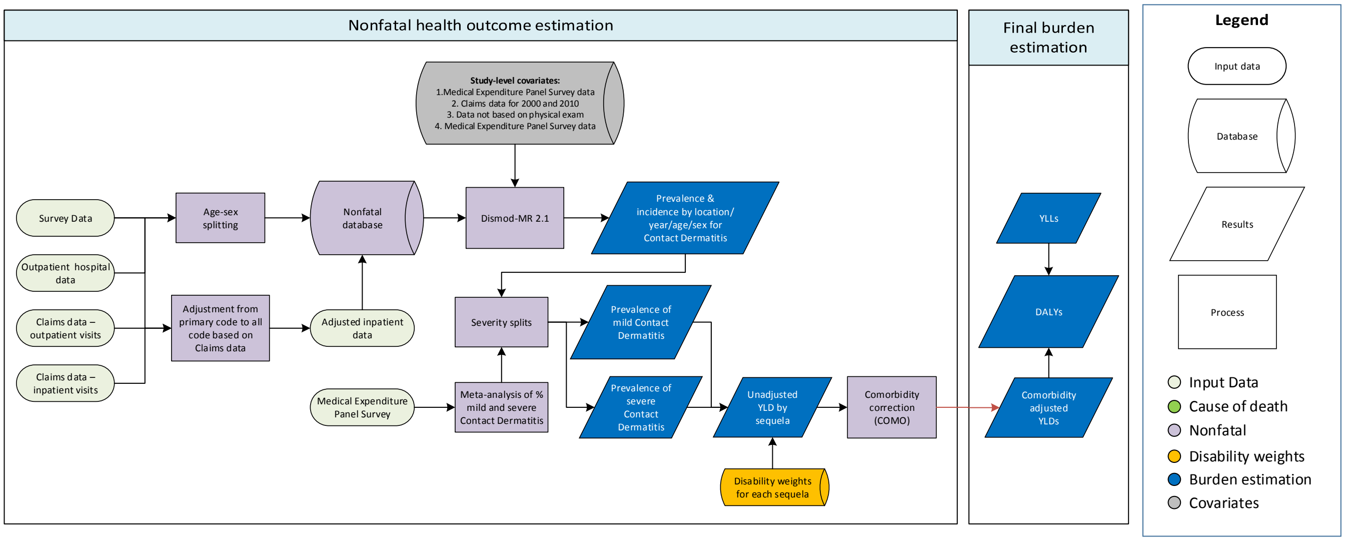

Seborrhoeic Dermatitis

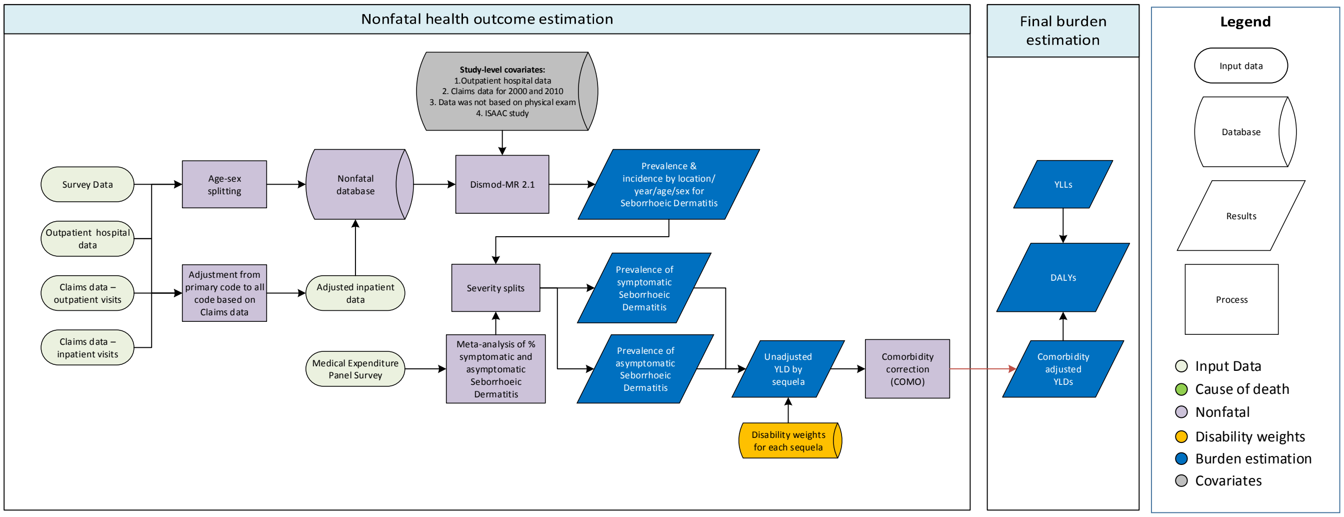

Case definition

Dermatitis was included in the GBD 2015 cause group of skin and subcutaneous conditions and consists of eczema (atopic dermatitis), contact dermatitis, and seborrheic dermatitis. Dermatitis refers to an inflammation of the skin. In this context, eczema refers to what is also known as “atopic dermatitis,” as the terms “eczema” and “dermatitis” can be used interchangeably. Eczema is an inflammatory, relapsing, and non-contagious skin disorder characterized by an itchy rash (ICD-10: L20) (1). Contact dermatitis is a localized rash or irritation of the skin caused by allergens or irritants (ICD: 10: L22-26) (1). Seborrhoeic dermatitis is an inflammatory skin disorder affecting the sebaceous-gland-rich areas of skin, generally the scalp, face, and torso (ICD-10: L21) (1). It is characterized by scaly, flaky, itchy, and red skin on the affected area. In GBD 2015, we estimated burden separately for eczema, contact dermatitis, and seborrheic dermatitis. This was done to better accommodate differences in the epidemiology and burden between the subtypes of dermatitis.

Input data

Model inputs

In the GBD 2010, a literature-based dataset was provided by the skin conditions expert group. Data from the Medical Expenditure Panel Survey (MEPS) in the United States in 2000-2009 (2) were also included to inform the age pattern of the prevalence output. Data from the NHANES study and the NHIS study (both from the US) were not extracted, as questions regarding eczema were too broad (i.e., asked whether a respondent had experienced eczema or any other rash). Dermatitis (known as “eczema”) was modeled as a single disorder. For GBD 2013, the literature was updated and dermatitis was disaggregated into eczema, contact dermatitis, and seborrheic dermatitis. The agreed approach for dermatitis diseases was to undertake a literature review every two years. Therefore, no literature review was undertaken for GBD 2015. The data for eczema were expanded based on recommendations of research articles and reviews by the skin expert group. Additionally, hospital outpatient and US claims data for 2000, 2010, and 2012 were used, where appropriate. See descriptions of individual modeling approaches for more information.

Table 1. Model inputs

		Prevalence	Incidence
Eczema	Studies	193	1
	Countries/subnationals	211	1
	GBD world regions	21	1
Contact dermatitis	Studies	25	1
	Countries/subnationals	74	1
	GBD world regions	7	1
Seborrheic dermatitis	Studies	23	1
	Countries/subnationals	73	1
	GBD world regions	11	1

Severity splits and disability weights

The basis of the GBD disability weight survey assessments are lay descriptions of sequelae highlighting major functional consequences and symptoms. Severity was split into three levels of disfigurement with pain/itch. The severity splits and disability weights applied in GBD 2013 were also used for GBD 2015. See below for a lay descriptions of the severity levels.

Table 2. Severity level and lay description.

Sequela	Severity level	Lay description	DW (95% CI)
Mild eczema	Disfigurement, level 1 with itch/pain	The person has a slight, visible physical deformity that is sometimes sore or itchy. Others notice the deformity, which causes some worry and discomfort.	0.027 (0.015-0.042)

Moderate eczema	Disfigurement, level 2, with itch/pain	The person has a visible physical deformity that is sore and itchy. Other people stare and comment, which causes the person to worry. The person has trouble sleeping and concentrating.	0.188 (0.124-0.267)
Severe eczema	Disfigurement, level 3, with itch/pain	The person has an obvious physical deformity that is very painful and itchy. The physical deformity makes others uncomfortable, which causes the person to avoid social contact, feel worried, sleep poorly, and think about suicide.	0.576 (0.401-0.731)
Mild contact dermatitis	Disfigurement, level 1 with itch/pain	The person has a slight, visible physical deformity that is sometimes sore or itchy. Others notice the deformity, which causes some worry and discomfort.	0.027 (0.015-0.042)
Severe contact dermatitis	Disfigurement, level 2, with itch/pain	The person has a visible physical deformity that is sore and itchy. Other people stare and comment, which causes the person to worry. The person has trouble sleeping and concentrating.	0.188 (0.124-0.267)

Symptomatic seborrheic dermatitis	Disfigurement, level 1 with itch/pain	The person has a slight, visible physical deformity that is sometimes sore or itchy. Others notice the deformity, which causes some worry and discomfort.	0.027 (0.015-0.042)

Modeling strategy

DisMod-MR 2.1 was used to estimate prevalence by age, sex, year, and geography (subnational (select countries), country, region, super-region) for eczema, contact dermatitis, and seborrheic dermatitis. Separate models were run for each cause.

Eczema: Since our available data mostly contained information on prevalence, we specified additional expert priors to further inform analyses. Excess mortality was set to zero while a setting of 0-0.2 (equivalent to five years) was placed on remission. A decreasing trend was placed on incidence from the age of 10 onward based on expert advice. A study-level covariate was used to adjust prevalence estimates from the International Study of Asthma and Allergies in Children (ISAAC) and US claims data for 2000, 2010, and 2012 toward other data points. Another study-level covariate adjusted estimates not using a physical examination for diagnosis toward those using a physical examination. Flohr et al., 2009 contained matched pairs of data for physical exam versus no physical exam. As such, these data were used in GBD 2013 to calculate a ratio (2.5, 2.0-2.9 – no physical exam: physical exam) outside of DisMod, which was then applied to the covariate in order to better guide the size of the adjustment. To improve regional and global estimates, the minimum coefficient of variation was set at 0.4 and the location random effect for the US was restricted to (-2, 2). A time window of 25 years was used to determine which data points were used for a particular year of fit. In addition, the only data points for Nicaragua, Bolivia, and Uttarakhand, India, were excluded due to these data leading to high estimates in these regions.

Contact dermatitis: Similar to eczema, mostly prevalence data were available for contact dermatitis. Per expert advice, the remission parameter was set from 0.2 to 4, excess mortality was set to zero, incidence was set to zero prior to age 6, and an increasing trend from 5 to 35 years was placed on incidence. The location random effects were restricted to (-0.5, 0.5) given the small size and heterogeneous nature of the dataset and the expectation that prevalence would not vary greatly by region. A study-level covariate was used to adjust prevalence estimates from the International Study of Asthma and Allergies in Children (ISAAC), Medical Expenditure Panel Survey (MEPS), and US claims data for 2000 and 2010 toward other data points. Another study-level covariate adjusted estimates based on one-year recall toward point estimates. A study-level covariate was also used to adjust estimates not using a physical examination for diagnosis toward those using a physical examination. A time window of 25 years was used to determine which data points were used for a particular year of fit.

Seborrheic dermatitis: As with contact dermatitis, the available data were mostly prevalence estimates. Per expert advice, settings were placed on incidence as follows: 0-4 years = 0-0.1, and 60-100 = 0-0.01. These reflected the expectation of some prevalence for babies, with none then expected until 60 years, whereby prevalence then increases with age. Excess mortality was set to zero while a setting of 0.1-12 was placed on remission, implying a

duration of one month to 10 years. Study-level covariates were used to adjust prevalence estimates from the International Study of Asthma and Allergies in Children (ISAAC), hospital outpatient data, and US claims data for 2000 and 2010 toward other data points. A study-level covariate was also used to adjust estimates not using a physical examination for diagnosis toward those using a physical examination. A setting on location random effects was also applied given the small size and heterogeneous nature of the dataset and the expectation that prevalence would not vary greatly by region. Only one point was separately available for Egypt, Turkey, and Rio Grande do Sul, Brazil, and these data were excluded due to high estimates for these regions.

Table 3. Beta and exponentiated values

	Study covariate	Parameter	Beta	Exp(beta)
Eczema	Claims data – 2000	Prevalence	-0.45 (-0.49 — -0.41)	0.64 (0.61 — 0.66)
	Claims data – 2010	Prevalence	-0.031 (-0.07 — -0.0029)	0.97 (0.93 — 1.00)
	Claims data – 2012	Prevalence	-0.0098 (-0.029 — -0.00064)	0.99 (0.97 — 1.00)
	Data was not based on physical examinations	Prevalence	0.99 (0.93 — 1.05)	2.70 (2.55 — 2.86)
	ISAAC	Prevalence	0.78 (0.72 — 0.85)	2.19 (2.06 — 2.34)
	Sex	Prevalence	-0.25 (-0.28 — -0.23)	0.78 (0.75 — 0.80)
Contact dermatitis	Data was not based on physical examinations	Prevalence	-1.47 (-1.98 — -0.7)	0.23 (0.14 — 0.50)
	Claims data – 2000	Prevalence	-0.011 (-0.035 — -0.00053)	0.99 (0.97 — 1.00)
	Claims data – 2010	Prevalence	-0.0017 (-0.0086 — -0.000028)	1.00 (0.99 — 1.00)
	MEPS	Prevalence	-0.54 (-1.02 — -0.096)	0.58 (0.36 — 0.91)
	ISAAC	Prevalence	-0.086 (-0.29 — -0.0022)	0.92 (0.75 — 1.00)
	One year recall	Prevalence	-1.45 (-1.97 — -0.67)	0.23 (0.14 — 0.51)
	Hospital outpatient data	Prevalence	-0.4 (-1.08 — -0.017)	0.67 (0.34 — 0.98)
	Sex	Prevalence	-0.16 (-0.22 — -0.11)	0.85 (0.80 — 0.89)
Seborrheic dermatitis	Data was not based on physical examinations	Prevalence	-0.023 (-2 — 1.97)	0.98 (0.14 — 7.16)
	Claims data – 2000	Prevalence	-0.39 (-0.41 — -0.37)	0.68 (0.66 — 0.69)
	Claims data – 2012	Prevalence	-0.0012 (-0.0028 — -0.0002)	1.00 (1.00 — 1.00)

	ISAAC	Prevalence	-0.019 (-0.074 — -0.00045)	0.98 (0.93 — 1.00)
	Hospital outpatient data	Prevalence	-0.25 (-0.81 — -0.0059)	0.78 (0.45 — 0.99)
	Sex	Prevalence	-0.0065 (-0.025 — 0.010)	0.99 (0.98 — 1.01)

Psoriasis

Flowchart

Psoriasis

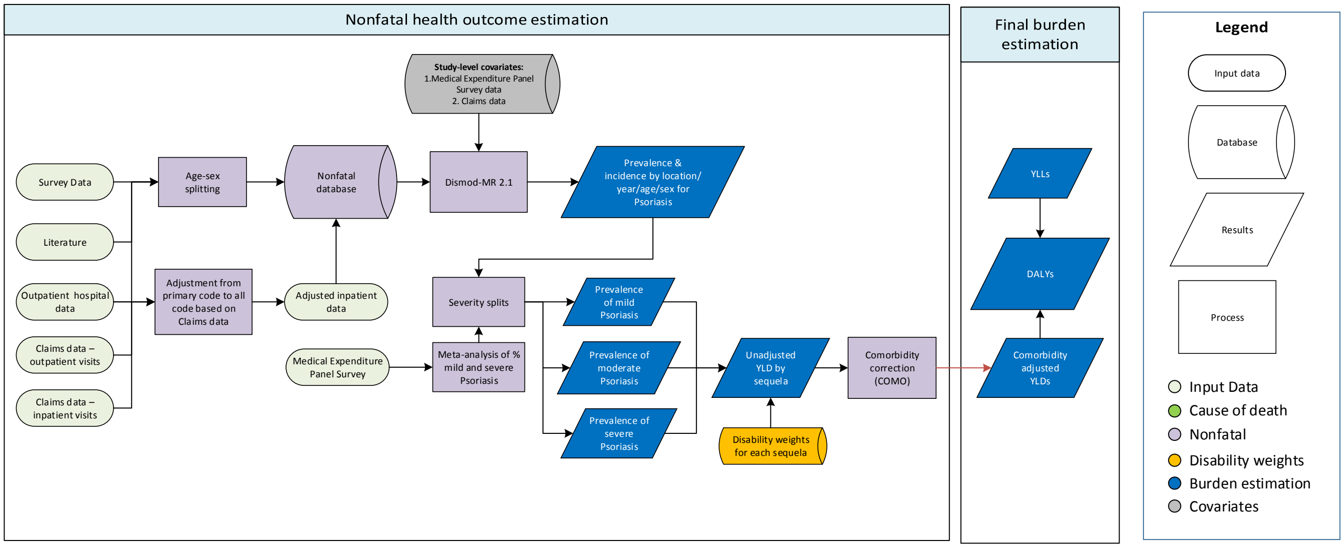

Case definition

Psoriasis was included in the GBD 2015 cause group of skin and subcutaneous conditions. According to the International Classification of Diseases (ICD-10), it is a skin disease marked by itchy or sore patches of thick, red skin with silvery scales (ICD-10: L40, L41) (1,2).

Input data

Model inputs

In the GBD 2010 study, a systematic review of the literature was conducted using PubMed and Google Scholar to capture epidemiological data for psoriasis. The literature search also included any relevant data from the Medical Expenditure Panel Survey (MEPS) in the United States for 2000-2009, the Australian National Health Survey 1995-1996, 2001, 2004-2005, 2007-2008, and the US National Health and Nutrition Examination Survey (NHANES) in 2002 and 2005.

The inclusion criteria stipulated that studies (1) must be published between 1980 and 2012; (2) must provide data on the incidence or prevalence of psoriasis; (3) must use samples representative of the general population (i.e., samples derived from the experimental arm of clinical trials or based in dermatology clinics were excluded); (4) must use a sample size larger than 100; and (5) must provide sufficient information on study method and sample characteristics to assess the quality of the study. For GBD 2013, the GBD 2010 search strategy was replicated to capture epidemiological studies published between 2012 and 2014. The agreed approach for psoriasis was to undertake a literature review every two years. Therefore, no literature review was undertaken for GBD 2015. Hospital outpatient and US claims data from 2000, 2010, and 2012 were also included in GBD 2015.

Table 1. Data inputs

	Prevalence	Incidence
Studies	45	5
Countries/subnationals	37	3
GBD world regions	11	2

Severity splits

For GBD 2013, GBD 2010's disability weights survey was conducted in an additional four European countries. GBD 2013 disability weights were updated to reflect both existing GBD 2010 data and additional data captured in these new surveys. As was the case in GBD 2010, GBD 2013 disability weights were estimated for disfigurement with itch/pain, levels 1, 2, and 3.

In GBD 2010, information on the distribution of cases of psoriasis that were asymptomatic, and within disfigurement, with itch/pain levels 1, 2, and 3 was obtained from the MEPS. In GBD 2013, a decision was made to re-aggregate MEPS data into the proportion of cases of psoriasis within disfigurement with itch/pain, levels 1, 2, and 3, excluding the proportion of cases that were asymptomatic. This was based on the argument that survey data are generally collected by examination and therefore everyone has the condition on the day of survey. The disability weights used for GBD 2013 were also used in 2015.

Table 2. Severity level and lay description.

Sequela	Severity level	Lay description	DW (95% CI)
Mild psoriasis	Disfigurement, level 1 with itch/pain	The individual has a slight, visible physical deformity that is sometimes sore or itchy. Others notice the deformity, which causes some worry and discomfort.	0.027 (0.015-0.042)
Moderate psoriasis	Disfigurement, level 2, with itch/pain	The individual has a visible physical deformity that is sore and itchy. Other people stare and comment, which causes the person to worry. The person has trouble sleeping and concentrating.	0.188 (0.124-0.267)

Severe psoriasis	Disfigurement, level 3, with itch/pain	The individual has an obvious physical deformity that is very painful and itchy. The physical deformity makes others uncomfortable, which causes the person to avoid social contact, feel worried, sleep poorly, and think about suicide.	0.576 (0.401-0.731)

Modeling strategy

DisMod-MR 2.1, a Bayesian meta-regression tool that built on GBD 2013's DisMod-MR 2.0, was used in modeling. DisMod-MR 2.1 was used to estimate prevalence by age, sex, year, and country for psoriasis.

Psoriasis was modeled with remission set between 0 and 1, implying a minimum duration of one year. This was in line with the available epidemiological data, expert opinion, and previous GBD work. Excess mortality was assumed to be zero. The datasets for psoriasis were sufficiently large to make use of a relatively short time window of 10 years to determine which data points were used for a particular year of fit. In contrast, a time window of 20 years was used for GBD 2013.

Study-level covariates were used to adjust incidence derived from the MEPS, hospital inpatient data, and US claims data for 2000, 2010, and 2012 toward the level of other prevalence and incidence data points, which were more representative of the general population. Additionally, the data were extremely heterogeneous. Therefore, the random effects were constrained to (-0.25, 0.25), and SDI was used as a country-level covariate to guide estimates for countries with few or no data.

Table 3. Beta and exponentiated values

Study covariate	Parameter	Beta	Exp(beta)
Claims data – 2000	Prevalence	-1.19 (-1.27 — -1.14)	0.31 (0.28 — 0.32)
Claims data – 2010	Prevalence	-0.89 (-0.97 — -0.85)	0.41 (0.38 — 0.43)
Claims data – 2012	Prevalence	-0.8 (-0.88 — -0.76)	0.45 (0.42 — 0.47)
SDI	Prevalence	0.22 (0.096 — 0.34)	1.24 (1.10 — 1.40)
MEPS	Prevalence	-1.29 (-1.38 — -1.18)	0.28 (0.25 — 0.31)
Sex	Prevalence	-0.045 (-0.059 — -0.026)	0.96 (0.94 — 0.97)
Sex	Incidence	0.034 (-0.29 — 0.38)	1.03 (0.75 — 1.46)

Cellulitis

Flowchart

Cellulitis

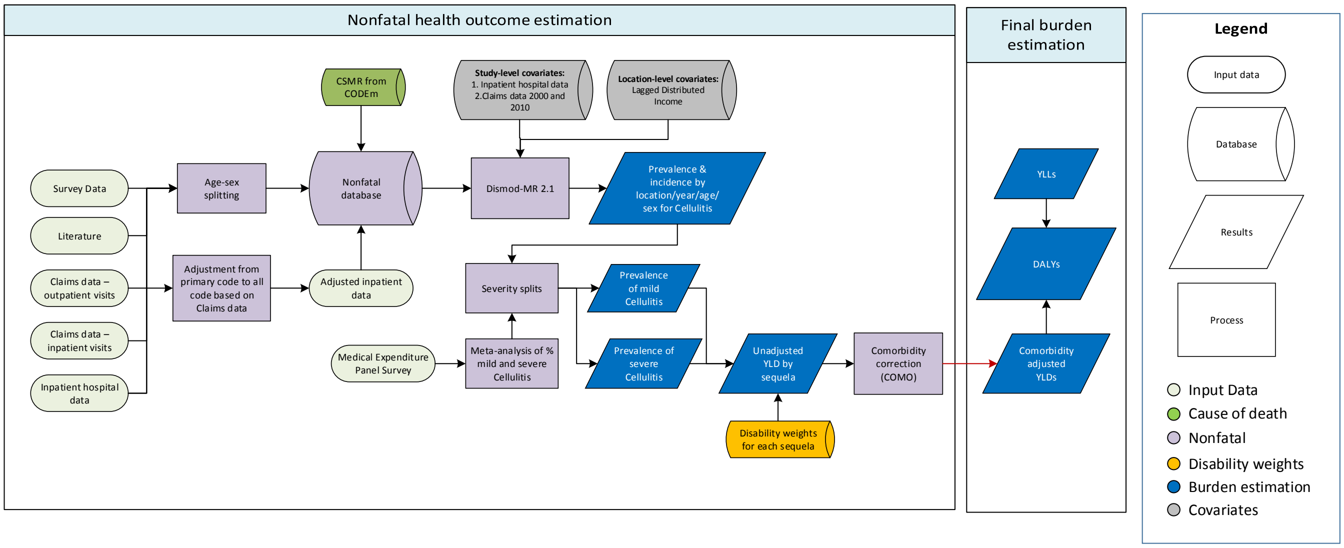

Case definition

Cellulitis was included in the GBD 2015 cause group of skin and subcutaneous conditions. Cellulitis is a skin disease marked by a bacterial infection that affects and spreads through the skin and soft tissues. Symptoms of cellulitis include pain, tenderness, and reddening in the affected area, fever, chills, and lymphadenopathy (ICD-10: L03) (1).

Input data

Model inputs

In the GBD 2010 study, a systematic review of the literature was conducted using PubMed and Google Scholar to capture epidemiological data for cellulitis. Due to lack of published data on the epidemiology of cellulitis, the literature search also included relevant incidence data from national inpatient or outpatient records in Europe, North America, and Latin America. When years in the national data from the hospital records overlapped, inpatient and outpatient data were summed together in an effort to better estimate the population incidence of cellulitis. The final dataset also included survey data from the Medical Expenditure Panel Survey (MEPS), US, and cause-specific mortality rates for cellulitis estimated by CODEm.

The inclusion criteria stipulated that studies (1) must be published between 1980 and 2012; (2) must provide data on the incidence or prevalence of cellulitis; (3) must use samples representative of the general population (i.e., samples derived from the experimental arm of clinical trials or based in dermatology clinics were excluded); (4) must use a sample size larger than 100; and (5) must provide sufficient information on study method and sample characteristics to assess the quality of the study. For GBD 2013, the GBD 2010 search strategy was replicated to capture epidemiological studies published between 2012 and 2014. The agreed approach for cellulitis was to undertake a literature review every two years. Therefore, no literature review was undertaken for GBD 2015. In

addition, hospital inpatient data and data from US claims for 2000, 2010, and 2012 by US state were included in GBD 2015.

Table 1. Model inputs

	Incidence	Mortality risk
Studies	1	
Countries/subnationals	140	52
GBD world regions	8	8

Severity splits

For GBD 2013, GBD 2010's disability weights survey was conducted in an additional four European countries. GBD 2013 disability weights were updated to reflect both existing GBD 2010 data and additional data captured in these new surveys. Cellulitis has a severity distribution from MEPS for "disfigurement with pain level 1" (80%) and "disfigurement with pain level 2" (20%). The severity splits and disability weights used in GBD 2013 were also used for GBD 2015.

Table 2. Severity level and lay description.

Sequela	Severity level	Lay description	DW (95% CI)
Mild cellulitis	Disfigurement, level 1 with itch/pain	The individual has a slight, visible physical deformity that is sometimes sore or itchy. Others notice the deformity, which causes some worry and discomfort.	0.027 (0.015-0.042)
Severe cellulitis	Disfigurement, level 2, with itch/pain	The individual has a visible physical deformity that is sore and itchy. Other people stare and comment, which causes the person to worry. The person has trouble sleeping and concentrating.	0.188 (0.124-0.267)

Modeling strategy

DisMod-MR 2.1, a Bayesian meta-regression tool that built on GBD 2013's DisMod-MR 2.0, was used in modeling. Cellulitis was modeled with remission set between 12 and 30, implying a duration of 12 days to one month. This was in line with the available epidemiological data, expert opinion, and previous GBD work. The cellulitis dataset was sufficiently large to make use of a relatively short time window of five years to determine which data points were used for a particular year of fit. In GBD 2013, a time window of 11 years was used.

Study-level covariates were used to adjust incidence derived from the MEPS, hospital inpatient data, and US claims data for 2000 and 2010 towards the level of other incidence data points, which were more representative of the general population. Lagged distributed income (LDI) was used as a country-level covariate to guide estimates for countries with little or no data. LDI was restricted to a range of -0.5 to -0.1, and US claims data for 2000 and 2010 was set at a maximum of zero.

Table 3. Beta and exponentiated values

Study covariate	Parameter	Beta	Exp(beta)
Claims data – 2000	Incidence	-0.68 (-0.72 — -0.64)	0.51 (0.49 — 0.53)
Claims data – 2010	Incidence	-0.039 (-0.071 — -0.0071)	0.96 (0.93 — 0.99)
Sex	Incidence	0.28 (0.26 — 0.29)	1.32 (1.30 — 1.34)
Hospital inpatient	Incidence	-1.49 (-1.52 — -1.46)	0.23 (0.22 — 0.23)
Sex	Excess mortality rate	-0.021 (-0.047 — 0.010)	0.98 (0.95 — 1.01)
Lagged distributed income ($ per capita)	Excess mortality rate	0.5 (-0.5 — -0.5)	0.61 (0.61 — 0.61)
Sex	Cause-specific mortality rate	0.25 (0.22 — 0.27)	1.28 (1.25 — 1.31)

Pyoderma

Flowchart

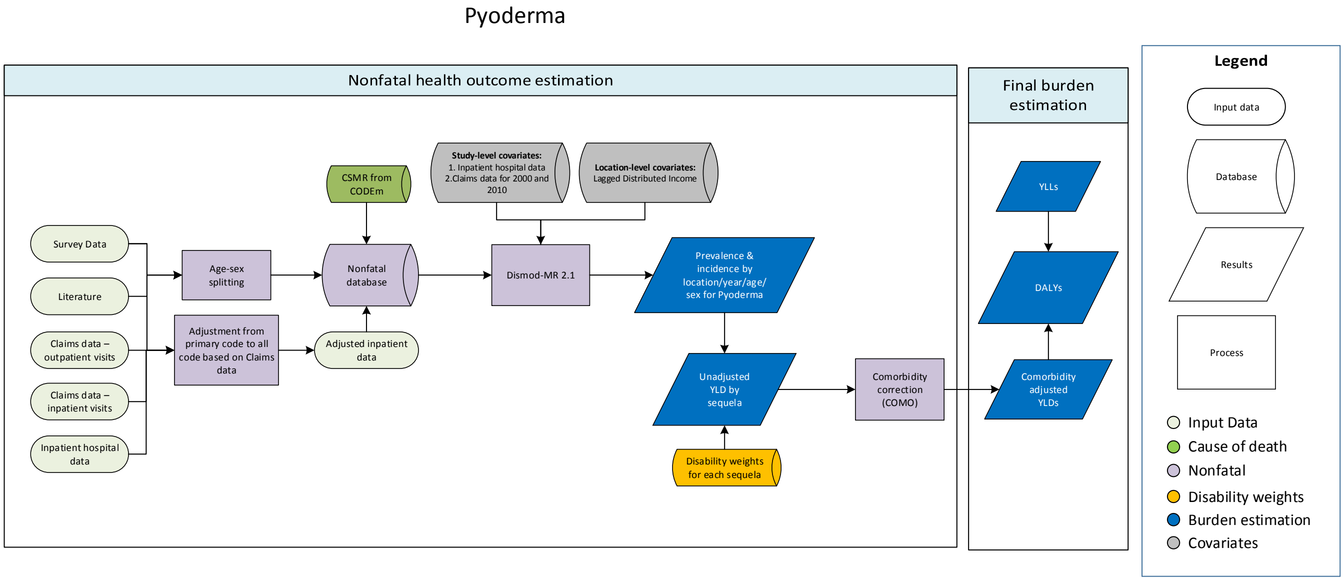

Case definition

Pyoderma refers to any skin disease that is pyogenic, i.e., involves the development of pus. These include superficial bacterial conditions such as impetigo, furuncles, ulcers, and abscesses. For GBD 2013 and 2015, pyoderma was modeled as two separate groups: impetigo, and abscess and other bacterial skin diseases. Impetigo is a highly contagious bacterial skin infection often characterized by red sores, which eventually leak pus or fluid (ICD-10: L01) (1). An abscess is a collection of pus that builds up within the tissue of the body, with carbuncles and furuncles being examples of specific types of abscess. The abscess and other bacterial skin diseases group included all bacterial skin diseases except impetigo (ICD-10: L00, L02, L04, L05, L08) (1).

Input data

Model inputs

For both impetigo and abscess and other bacterial skin diseases in GBD 2010, a literature review was conducted using PubMed and Google Scholar. The inclusion criteria were studies which were published between 1980 and 2010 and provided data on relevant disease incidence or prevalence. Exclusion criteria were studies with no incidence or prevalence data provided, not community- or population-based, outside of year range, sample size smaller than 100, experimental arm of clinical trial, papers that provided estimates rather than data, and studies that were based in dermatology clinics. The literature search also included relevant data from national inpatient and outpatient records in Europe, North America, and Latin America. For GBD 2013, the GBD 2010 search strategy was replicated to capture epidemiological studies published between 2012 and 2014. The agreed approach for pyoderma disease was to undertake a literature review every two years. Therefore, no literature review was undertaken for GBD 2015.

		Prevalence	Incidence	Mortality Risk
Impetigo	Studies	14	4	0
	Countries/subnationals	12	56	103
	GBD world regions	8	4	1
Abscess and other bacterial skin diseases	Studies	1	1	0
	Countries/subnationals	107	53	53
	GBD world regions	11	1	8

Hospital inpatient data and data from US claims for 2000, 2010, and 2012 by US state were included in GBD 2015, where appropriate. See descriptions of individual modeling approaches for more information.

Severity splits and disability weights

For GBD 2013, the GBD 2010 disability weights survey was conducted in an additional four European countries. GBD 2013 disability weights were updated to reflect both existing GBD 2010 data and additional data captured in these new surveys. In GBD 2010 and GBD 2013, information on the distribution of cases of impetigo, asymptomatic, and within disfigurement levels 1 and 2, was obtained from the MEPS. In GBD 2010, abscess and other bacterial skin diseases were assigned to the percent asymptomatic as derived from the MEPS, with the symptomatic cases assigned the disability weight of a mild acute infectious disease case. In GBD 2013, the approach was changed, consistent with that of impetigo. The GBD 2013 severity splits and disability weights were also used for GBD 2015.

Table 2. Severity level and lay description

Severity level	Lay description	DW (95% CI)
Infectious disease, acute episode, mild	The person has a low fever and mild discomfort, but no difficulty with daily activities.	0.006 (0.002-0.012)

Modeling strategy

DisMod-MR 2.1 was used to estimate prevalence by age, sex, year, and geography (country, region, super-region) for impetigo and abscess and other bacterial skin diseases. Separate models were run for each disease/disease group.

Impetigo: Per expert advice, we assumed a remission of 13 to 26, equating to a duration of two to four weeks. A value prior was also placed on incidence, restricting the range between zero and one. In addition, study-level covariates were placed on incidence to adjust US claims data for 2000 and 2010 toward non-US claims data. A country-level covariate, log transformed lagged distributed income (I$ per capita), which represents a moving average of gross domestic product (GDP) over time, was also included. We also used the cause-specific mortality rates for pyoderma estimated using CODEm. Similar to GBD 2013, we used a time window of 25 years to determine which data points were used for a particular year of fit.

Abscess and other bacterial skin diseases: Per expert advice, a remission setting of 17 to 52 was applied, which equated to a duration of one to six weeks. Study-level covariates adjusted incidence estimates from US claims data for 2000 and 2010 and hospital inpatient data toward other data points. In addition, deaths due to pyoderma were included as a study-level covariate on incidence (ln-ASDR), and log transformed lagged distributed income (I$ per capita) and SDI were placed on excess mortality and incidence, respectively. Similar to GBD 2013, we used a time window of 25 years to determine which data points were used for a particular year of fit.

Table 3. Beta and exponentiated values

	Study covariate	Parameter	Beta	Exp(beta)
Impetigo	Claims data – 2000	Incidence	0.0010 (-0.049 — 0.041)	1.00 (0.95 — 1.04)
	Claims data – 2010	Incidence	0.45 (0.40 — 0.49)	1.57 (1.50 — 1.64)
	Sex	Incidence	0.0037 (-0.023 — 0.028)	1.00 (0.98 — 1.03)
	Sex	Prevalence	-0.24 (-1.51 — 0.95)	0.79 (0.22 — 2.57)
	Lagged Distributed Income (I$ per capita)	Prevalence	0.028 (0.00045 — 0.10)	1.03 (1.00 — 1.11)
	Lagged Distributed Income (I$ per capita)	Excess mortality rate	-0.32 (-0.34 — -0.3)	0.73 (0.71 — 0.74)
	Sex	Excess mortality rate	0.26 (0.21 — 0.30)	1.29 (1.24 — 1.35)
	Sex	Cause-specific mortality rate	0.18 (0.16 — 0.20)	1.20 (1.17 — 1.22)
Abscess and other bacterial skin diseases	Claims data – 2000	Incidence	-0.4 (-0.45 — -0.36)	0.67 (0.64 — 0.70)
	Claims data – 2010	Incidence	-0.071 (-0.11 — -0.032)	0.93 (0.89 — 0.97)
	Hospital Inpatient	Incidence	-1.72 (-1.76 — -1.68)	0.18 (0.17 — 0.19)
	Sex	Incidence	0.18 (0.16 — 0.19)	1.19 (1.17 — 1.21)

	Lagged Distributed Income (I$ per capita)	Excess mortality rate	-0.1 (-0.1 — -0.1)	0.90 (0.90 — 0.90)
	Sex	Excess mortality rate	0.16 (0.13 — 0.19)	1.17 (1.14 — 1.21)
	Sex	Cause-specific mortality rate	-0.05 (-0.074 — -0.028)	0.95 (0.93 — 0.97)

Scabies

Flowchart

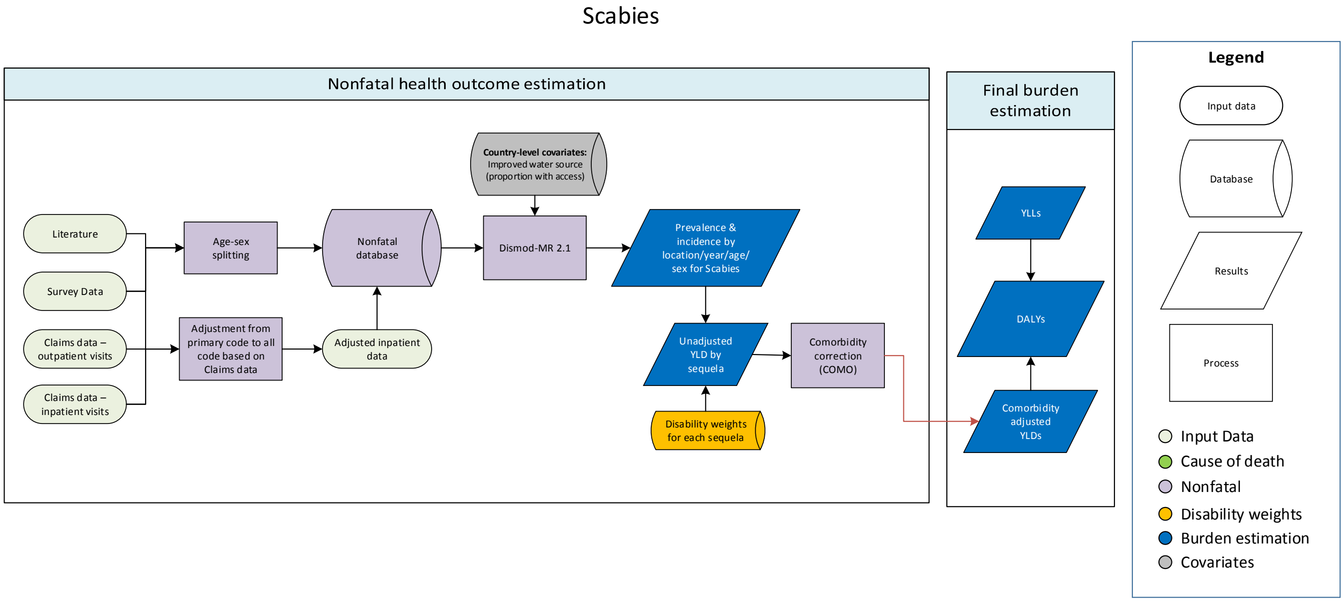

Case definition

Scabies was included in the GBD 2015 cause group of skin and subcutaneous conditions. According to the International Classification of Diseases (ICD-10), scabies is a skin disease caused by the microscopic mite *Sarcoptes scabiei*. The main symptom is an itchy, pimple-like rash (ICD-10: B86) (1).

Input data

Model inputs

In the GBD 2010 study, a systematic review of the literature was conducted using PubMed and Google Scholar to capture epidemiological data for scabies. The inclusion criteria stipulated that studies (1) must be published between 1980 and 2012; (2) must provide data on the incidence or prevalence of scabies; (3) must use samples representative of the general population (i.e., samples derived from the experimental arm of clinical trials or based in dermatology clinics were excluded); (4) must use a sample size larger than 100; and (5) must provide sufficient information on study method and sample characteristics to assess the quality of the study. For GBD 2013, the GBD 2010 search strategy was replicated to capture epidemiological studies published between 2012 and 2014. The agreed approach for scabies was to undertake a literature review every two years. Therefore, no literature review was undertaken for GBD 2015. Additionally, US claims data from 2000, 2010, and 2012 were included in GBD 2015.

Table 1. Data inputs

	Prevalence	Incidence
Studies	38	3
Countries/subnationals	84	5
GBD world regions	15	5

Severity splits

For GBD 2013, GBD 2010's disability weights survey was conducted in an additional four European countries. GBD 2013 disability weights were updated to reflect both existing GBD 2010 data and additional data captured in these new surveys. Scabies was assigned the disability weight for disfigurement level 1. The disability weights used for GBD 2013 were also used for GBD 2015.

Table 2. Severity level and lay descriptions

Severity level	Lay description	DW (95% CI)
Disfigurement, level 1 with itch/pain	The individual has a slight, visible physical deformity that is sometimes sore or itchy. Others notice the deformity, which causes some worry and discomfort.	0.027 (0.015-0.042)

Modeling strategy

DisMod-MR 2.1, a Bayesian meta-regression tool that built on GBD 2013's DisMod-MR 2.0, was used in modeling. DisMod-MR 2.1 was used to estimate prevalence by age, sex, year, and country for scabies.

Scabies was modeled with remission set between 1 and 9, implying six weeks to one-year duration, and excess mortality was assumed to be zero. This was in line with the available epidemiological data, expert opinion, and previous GBD work.

The datasets for scabies were sufficiently large to make use of a relatively short time window of five years to determine which data points were used for a particular year of fit. Additionally, to improve estimation across all regions, we restricted location random effects to (-0.25, 0.25), used improved water source as a country-level covariate with a log(ln) transformation, and set the minimum coefficient of variation at 0.4.

Table 3. Beta and exponentiated values

Study covariate	Parameter	Beta	Exp(beta)
Improved water source (proportion with access)	Prevalence	-0.21 (-0.47 — 0.058)	0.81 (0.63 — 1.06)
Sex	Prevalence	-0.12 (-0.14 — -0.089)	0.89 (0.87 — 0.92)
Sex	Incidence	0.099 (-0.11 — 0.31)	1.10 (0.90 — 1.36)

Fungal skin diseases

Flowchart

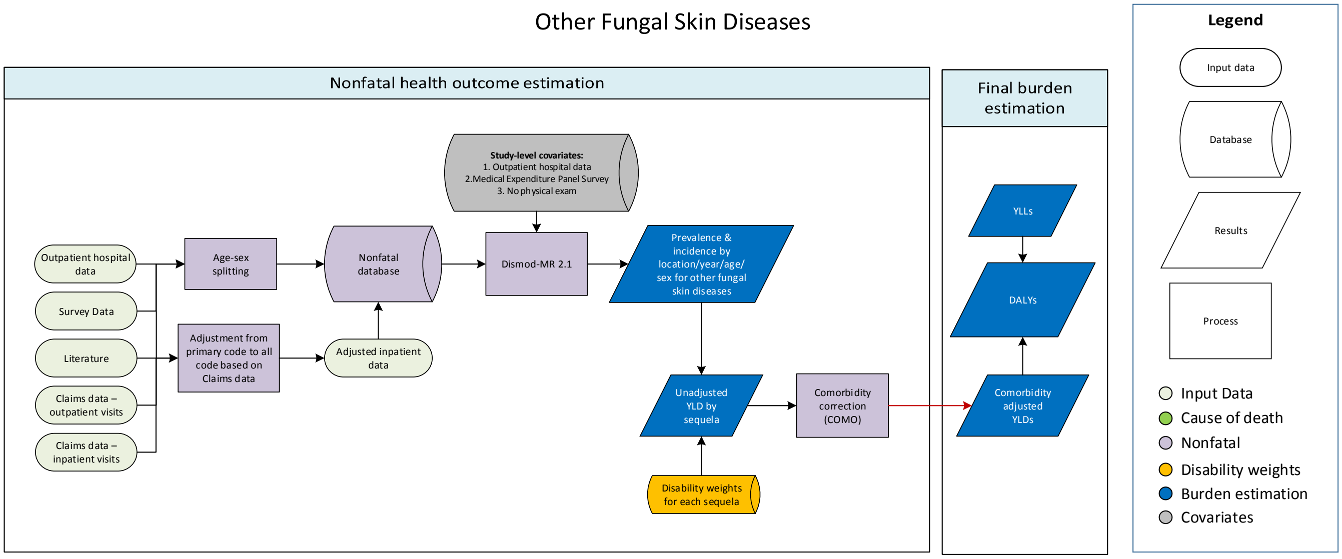

Case definition

Fungal diseases were included in the GBD 2015 cause group of skin and subcutaneous conditions and consisted of of tinea capitis and a residual group of “any” other fungal disease. Similar to GBD 2013, tinea capitis was modeled separately from the other fungal skin diseases. This was done to better accommodate differences in burden between tinea capitis and other subtypes of fungal skin diseases.

Tinea capitis is a fungal infection of the scalp and associated hair. It is characterized by the appearance of thickened scaly swellings or as expanding raised red rings (ringworm), mainly caused by species of *Microsporum*, *Trichophyton,* and *Epidermophyton* (ICD-10: B35.0) (1).

The residual group of “any” other fungal skin disease included any fungal skin disease that was specifically not tinea capitis or onychomycosis (i.e., fungal nail infection). The ICD-10 (1) list of other fungal skin diseases includes tinea manuum (ICD-10: B35.2), or hand ringworm; tinea pedis (ICD-10: B35.3), or athlete’s foot; tinea corporis (ICD-10:B35.4), or ringworm of the body; tinea imbricata (ICD-10:B35.5), a superficial fungal infection limited to parts of Asia and Central America; tinea cruris (ICD-10:B35.6), also known as dhobi itch, groin ringworm, or jock itch.

Input data

Model inputs

For GBD 2010, a systematic review of the literature using PubMed and Google Scholar was conducted to capture epidemiological data for fungal skin diseases. The literature search also included any relevant data from the Medical Expenditure Panel Survey (MEPS) in the United States in 2000-2009. The inclusion criteria stipulated that

studies (1) must be published between 1980 and 2012; (2) must provide data on the incidence or prevalence of fungal skin diseases; (3) must use samples representative of the general population (i.e., samples derived from the experimental arm of clinical trials or based in dermatology clinics were excluded); (4) must use a sample size larger than 100; and (5) must provide sufficient information on study method and sample characteristics to assess the quality of the study. For GBD 2013, the GBD 2010 search strategy was replicated to capture epidemiological studies published between 2012 and 2014. The agreed approach for fungal skin diseases was to undertake a literature review every two years. Therefore, no literature review was undertaken for GBD 2015.

Table 1. Data inputs

		Prevalence	Incidence
Tinea capitis	Studies	23	
	Countries/subnationals	67	
	GBD world regions	7	
Other fungal skin diseases	Studies	32	1
	Countries/subnationals	28	1
	GBD world regions	12	1

In addition, data from US claims for 2000, 2010, and 2012 by US state were included for both tinea capitis and other fungal skin diseases. We also used hospital outpatient data for other fungal skin diseases but decided not to use it for tinea capitis because we decided it would not be representative of true prevalence, and variation between countries in the proportion of true prevalent cases captured in hospital inpatient and outpatient data would likely vary more than can be captured by a single crosswalk in DisMod-MR 2.1. For tinea capitis, we compared the rates in the outpatient data from Norway, Sweden, Canada, and the US and found implausibly large differences with the rates from the claims data.

Severity splits

The basis of the GBD disability weight survey assessments are lay descriptions of sequelae highlighting major functional consequences and symptoms. The same disability weight was used for both tinea capitis and other fungal skin diseases. See below for a lay description of the severity level.

Table 2. Severity level and lay description

Severity level	Lay description	DW (95% CI)
Infectious disease, acute episode, mild	The person has a low fever and mild discomfort but no difficulty with daily activities.	0.006 (0.002-0.012)

Modeling strategy

Separate models were run for tinea capitis and other fungal skin diseases.

Tinea capitis. To help inform the distribution of tinea capitis across the lifespan, excess mortality was set at zero, remission was set at 0.5 to 4, incidence was set between 0 and 0.02 between 20 and 100 years, and a decreasing trend was imposed from 10 years onward. This was in agreement with the available prevalence data and expert advice. We made use of a relatively long time window of 20 years to determine which data points were used for a particular year of fit. This means that for the year 2000, for instance, DisMod-MR 2.1 incorporated all data points ranging from 1980 to present to estimate prevalence. Study-level covariates in the final model included adjustments for hospital outpatient data and US claims data for 2000 and 2010, and no physical exam to adjust for self-reported data. In addition, to improve estimates for sub-Saharan Africa, random effects were restricted to (-2, 3).

Other fungal skin diseases. The modeling strategy was similar to that for tinea capitis with remission set between 0.33 and 4. As MEPS data points were included in this model, we used a study-level covariate to adjust estimates derived from MEPS, hospital outpatient data, and US claims data for 2000 and 2010, toward the level of prevalence observed in US claims data from 2012. We also included a covariate to adjust for self-reported data not accompanied by physical examinations. All data were crosswalked to literature and US claims data for 2012.

Table 3. Beta and exponentiated values

Cause	Study covariate	Parameter	Beta	Exp(beta)
Tinea capitis	Claims data – 2000	Prevalence	-0.0082 (-0.02 — -0.00084)	0.99 (0.98 — 1.00)
	Claims data – 2010	Prevalence	-0.0087 (-0.025 — -0.00065)	0.99 (0.98 — 1.00)
	Data not based on physical exam	Prevalence	0.0088 (-1.98 — 2.00)	1.01 (0.14 — 7.39)
	Sex	Prevalence	0.22 (0.20 — 0.24)	1.25 (1.22 — 1.28)
Other fungal skin diseases	Outpatient hospital data	Prevalence	-1.11 (-1.95 — -0.12)	0.33 (0.14 — 0.88)
	MEPS	Prevalence	-0.24 (-0.36 — -0.12)	0.79 (0.70 — 0.89)
	Claims data – 2000	Prevalence	-0.27 (-0.29 — -0.25)	0.76 (0.75 — 0.78)
	Claims data – 2010	Prevalence	-0.086 (-0.1 — -0.071)	0.92 (0.90 — 0.93)
	Data was not based on physical examinations	Prevalence	1.31 (0.50 — 1.97)	3.72 (1.65 — 7.15)

	Sex	Prevalence	0.33 (0.32 — 0.35)	1.39 (1.38 — 1.42)

Viral skin diseases

Flowchart

Viral skin diseases

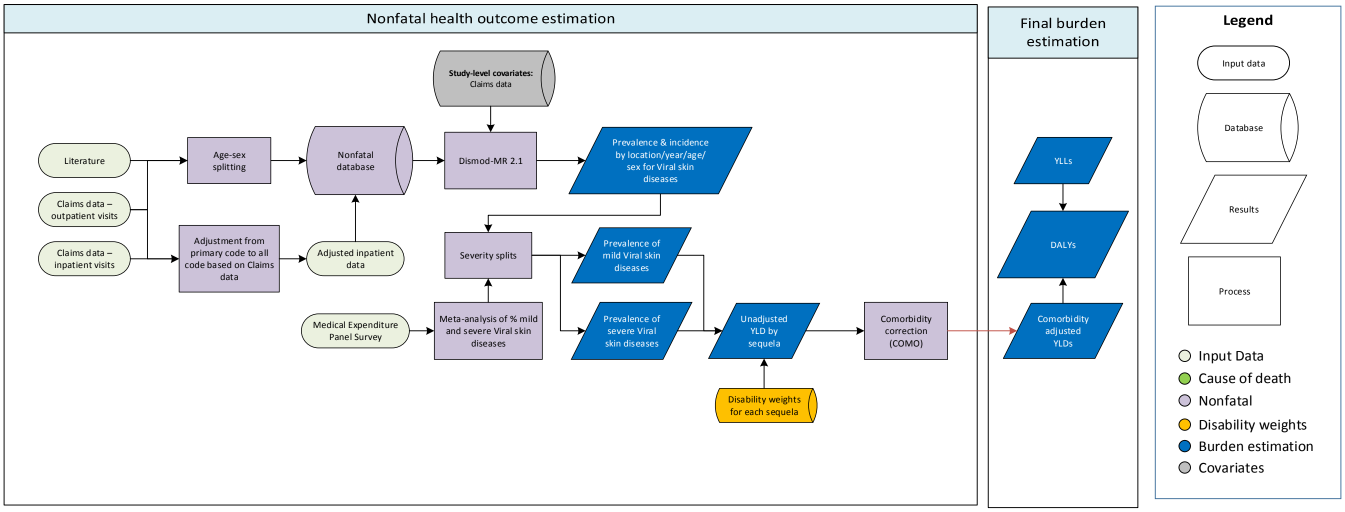

Case definition

Viral skin diseases consist of viral warts and molluscum contagiosum. Viral warts are raised growths on the surface of the skin caused by an infection with the human papillomavirus (ICD-10: B07). Molluscum contagiosum is a viral infection of the skin or occasionally of the mucous membranes characterized by the appearance of waxy, dome-shaped nodules. It is caused by a DNA poxvirus called the molluscum contagiosum virus (ICD-10: B08.1) (1). In GBD 2015, we separately modeled viral warts and molluscum contagiosum. This was done to better accommodate differences in burden between the subtypes of viral skin diseases.

Input data

Model inputs

In the GBD 2010 study, a systematic review of the literature was conducted using PubMed and Google Scholar, to capture epidemiological data for viral skin diseases. Due to lack of published data on the epidemiology of viral skin diseases, the literature search also included relevant incidence data from national inpatient or outpatient records in the US. The inclusion criteria stipulated that studies (1) must be published between 1980 and 2012; (2) must provide data on the incidence or prevalence of viral warts or molluscum contagiosum; (3) must use samples representative of the general population (i.e., samples derived from the experimental arm of clinical trials or based in dermatology clinics were excluded); (4) must use a sample size larger than 100; and (5) must provide sufficient information on study method and sample characteristics to assess the quality of the study. For GBD 2013, the GBD 2010 search strategy was replicated to capture epidemiological studies published between 2012 and 2014. The agreed approach for viral skin diseases was to undertake a literature review every two years. Therefore, no literature review was undertaken for GBD 2015.

Table 1. Input data

Cause		Prevalence	Incidence
Viral warts	Studies	23	1
	Countries/subnationals	67	1
	GBD world regions	7	1
Molluscum contagiosum	Studies	32	0
	Countries/subnationals	28	1
	GBD world regions	12	1

Hospital outpatient data and data from US claims for 2000, 2010, and 2012 by US state were included in GBD 2015, where appropriate. See descriptions of individual modeling approaches for more information.

Severity splits

For GBD 2013, GBD 2010's disability weights survey was conducted in an additional four European countries. GBD 2013 disability weights were updated to reflect both existing GBD 2010 data and additional data captured in these new surveys. In GBD 2010, viral warts and molluscum contagiosum were assigned disability weights equivalent to mild acute infectious disease. In GBD 2013, cases of both disorders were allocated a distribution between mild acute infectious disease and disfigurement level 2. The severity splits and disability weights used in GBD 2013 were also applied in GBD 2015.

Table 2. Sequela and disability weight

Sequela	Severity level	Lay description	DW (95% CI)
Mild viral warts	Infectious disease, acute episode, mild	The person has a low fever and mild discomfort, but no difficulty with daily activities.	0.006 (0.002-0.012)

Severe viral warts	Disfigurement, level 2	The person has a visible physical deformity that causes others to stare and comment. As a result, the person is worried and has trouble sleeping and concentrating.	0.067 (0.044-0.096)
Mild molluscum contagiosum	Infectious disease, acute episode, mild	The person has a low fever and mild discomfort but no difficulty with daily activities.	0.006 (0.002-0.012)
Severe molluscum contagiosum	Disfigurement, level 2	The person has a visible physical deformity that causes others to stare and comment. As a result, the person is worried and has trouble sleeping and concentrating.	0.067 (0.044-0.096)

Modeling strategy

For GBD 2015, DisMod-MR 2.1 was used to estimate prevalence, by age, sex, year, and country for viral warts and molluscum contagiosum. Separate models were run for each disease, as illustrated throughout this cause write-up.

Viral warts. Viral warts were modeled with excess mortality set to 0 and remission set between 0.25 and 2, implying a duration of 0.5 to 4 years. This was in line with the levels of prevalence and incidence data, as well as expert opinion. A number of additional settings were used to ensure that DisMod-MR 2.1 sufficiently followed available data points. Incidence was restricted to a maximum of 10%, and we made use of a relatively long time window of 25 years to determine which data points were used for a particular year of fit. Study-level covariates were used to adjust US claims data for 2000, 2010, and 2012 toward other data points.

Molluscum contagiosum. As available data only contained information on prevalence and incidence, we specified additional expert priors to further inform analyses. Molluscum contagiosum was modeled with excess mortality set to 0 and remission set between 0.5 and 2, implying a duration of 0.5 to 2 years. This was in line with the available epidemiological data, expert opinion, and previous GBD work. We used a time window of 25 years to determine which data points to include for a particular year of fit. For GBD 2013, a time window of 11 years was used. Due to data heterogeneity, we restricted the regional random effects to between -0.5 and 0.5. Study-level covariates were used to adjust US claims data for 2000, 2010, and 2012 toward other data points.

Table 3. Beta and exponentiated values

	Study covariate	Parameter	Beta	Exp(beta)
Viral warts	Claims data – 2000	Prevalence	-0.22 (-0.29 — -0.17)	0.80 (0.75 — 0.84)
	Claims data – 2010	Prevalence	-0.14 (-0.2 — -0.089)	0.87 (0.82 — 0.91)
	Claims data – 2012	Prevalence	-0.1 (-0.16 — -0.052)	0.90 (0.85 — 0.95)
	Sex	Prevalence	-0.022 (-0.035 — -0.011)	0.98 (0.97 — 0.99)
	Sex	Incidence	-1.69 (-1.99 — -1.11)	0.19 (0.14 — 0.33)
Molluscum contagiosum	Claims data – 2000	Prevalence	-1.89 (-1.98 — -1.8)	0.15 (0.14 — 0.17)
	Claims data – 2010	Prevalence	-1.11 (-1.2 — -1.01)	0.33 (0.30 — 0.36)
	Claims data – 2012	Prevalence	-0.95 (-1.04 — -0.87)	0.39 (0.36 — 0.42)
	Sex	Prevalence	0.087 (0.073 — 0.10)	1.09 (1.08 — 1.11)
	Sex	Incidence	-0.14 (-0.78 — 0.45)	0.87 (0.46 — 1.58)

Acne vulgaris

Flowchart

Acne Vulgaris

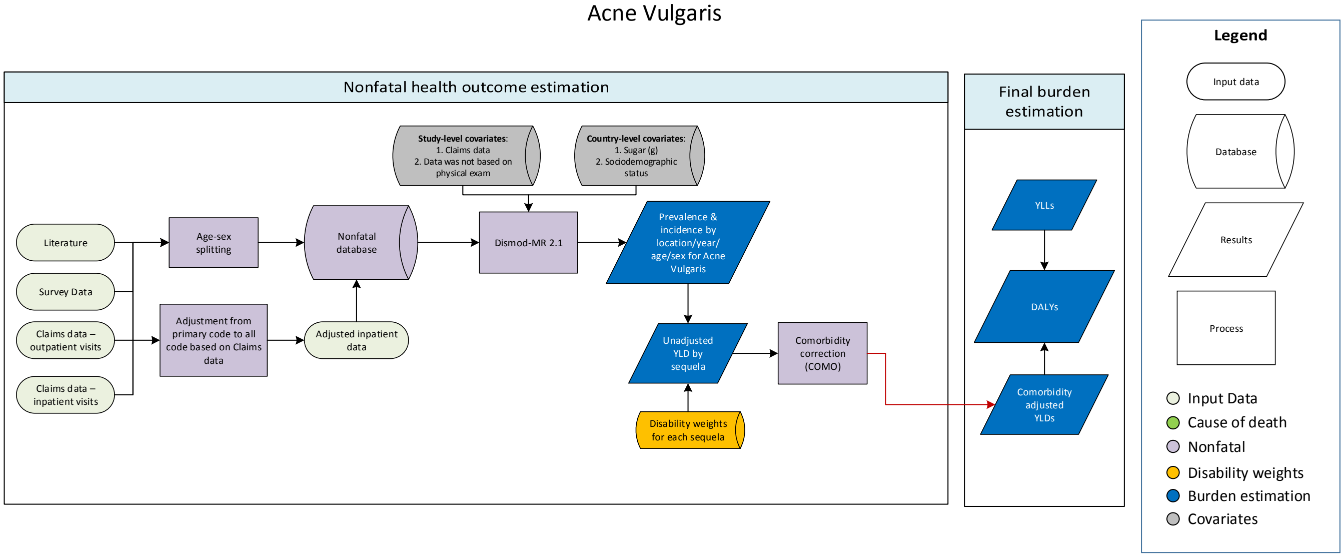

Case definition

Acne vulgaris was included in the GBD 2015 cause group of skin and subcutaneous conditions. Acne vulgaris (or acne) is a chronic inflammatory disease of the pilosebaceous unit associated with an increase in sebum secretion (1, 2). Included in the GBD 2015 modeling were cases meeting ICD-10 diagnostic criteria for acne vulgaris (ICD-10: I70**).

Input data

Model inputs

In the GBD 2010 study, a systematic review of the literature was conducted using PubMed and Google Scholar to capture epidemiological data for acne vulgaris. The inclusion criteria stipulated that studies (1) must be published between 1980 and 2012; (2) must provide data on the incidence or prevalence of acne vulgaris; (3) must use samples representative of the general population (i.e., samples derived from the experimental arm of clinical trials or based in dermatology clinics were excluded); (4) must use a sample size larger than 100; and (5) must provide sufficient information on study method and sample characteristics to assess the quality of the study. For GBD 2013, the GBD 2010 search strategy was replicated to capture epidemiological studies published between 2012 and 2014. The agreed approach for acne vulgaris was to undertake a literature review every two years. Therefore, no literature review was undertaken for GBD 2015. Additionally, US claims data from 2000, 2010, and 2012 were included.

Table 1. Input data

	Prevalence
Studies	48
Countries/subnationals	92
GBD world regions	14

Severity splits

In the GBD 2015 estimation process we used the same disability weights as GBD 2013. For GBD 2013, GBD 2010's disability weights survey was conducted in an additional four European countries. GBD 2013 disability weights were updated to reflect both existing GBD 2010 data and additional data captured in these new surveys. The table below illustrates the severity level, lay description and disability weight for acne.

Table 2. Severity level and lay description

Severity level	Lay description	DW (95% CI)
Disfigurement, level 1	The individual has a slight, visible physical deformity that others notice, which causes some worry and discomfort.	0.011 (0.005-0.021)

Modeling strategy

DisMod-MR 2.1, a Bayesian meta-regression tool which built on GBD 2013's DisMod-MR 2.0, was used in modeling. DisMod-MR 2.1 was used to estimate prevalence by age, sex, year, and country for acne vulgaris.

Since our available data only contained information on prevalence, we specified additional expert priors to further inform analyses. We assumed zero excess mortality and remission from 0.1 to 1, implying a duration of one year to 10 years. This was in line with the available epidemiological data, expert opinion, and previous GBD work. A value prior of zero was set for incidence between the ages of 0 and 6. Similar to GBD 2013, we used a time window of five years to determine which data points were used for a particular year of fit.

For GBD 2013, we used a study-level covariate for acne severity defined as 0 for acne with inflammation or pustules and 1 = acne of all severities (including closed comedones). Everything else was crosswalked to acne with inflammation or pustules. For GBD 2015, the data were updated to exclude data not clearly defined as acne. In addition, study-level covariates were used to adjust prevalence from US claims data for 2000, 2010, and 2012 toward the level of other prevalence data points, which were more representative of the general population. A study-level covariate was applied to data that were not based on physical examinations.

Furthermore, since the data were extremely heterogeneous, the random effects were constrained to (-0.3, 0.3) for regions such as East Asia. In addition, sugar consumption and sociodemographic status were used as country-level covariates to guide estimates for countries with little or no data.

The table below indicates the study covariates, parameters, beta, and exponentiated beta values used in GBD 2015.

Table 3. Beta and exponentiated values

Study covariate	Parameter	Beta	Exp(beta)
Claims data – 2000	Prevalence	-1.09 (-1.1 — -1.04)	0.34 (0.33 — 0.35)
Claims data – 2010	Prevalence	-0.97 (-1.01 — -0.91)	0.38 (0.36 — 0.40)
Claims data – 2012	Prevalence	-0.91 (-0.95 — -0.85)	0.40 (0.39 — 0.43)
Sociodemographic Status	Prevalence	0.45 (0.27 — 0.59)	1.56 (1.31 — 1.80)
Sugar (g)	Prevalence	0.013 (0.00035 — 0.043)	1.01 (1.00 — 1.04)
Data was not based on physical examinations	Prevalence	0.21 (-0.11 — 0.56)	1.24 (0.89 — 1.75)
Sex	Prevalence	-0.2 (-0.2 — -0.2)	0.82 (0.82 — 0.82)

No additional modifications were made to the estimation process for GBD 2015.

Alopecia areata

Flowchart

Alopecia Areata

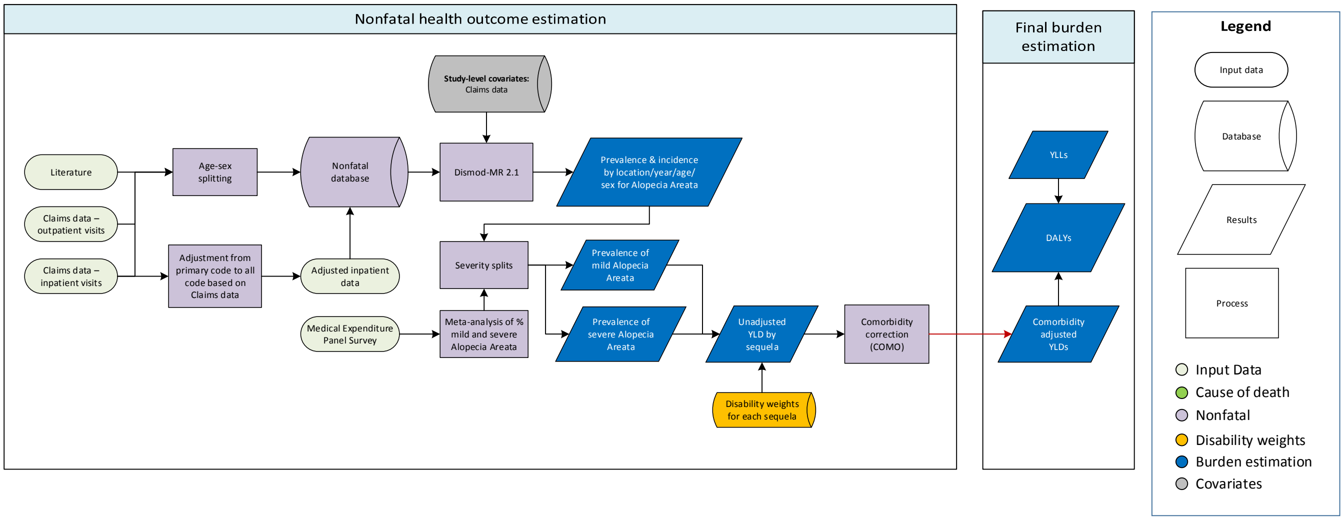

Case definition

Alopecia areata was included in the GBD 2015 cause group of skin and subcutaneous conditions. Alopecia areata is an autoimmune disease that results in hair loss on the scalp and other parts of the body (1). Included in the GBD disease modeling were cases meeting ICD-10 diagnostic criteria for alopecia (ICD-10: L63) (2).

Input data

Model inputs

In the GBD 2010 study, a systematic review of the literature was conducted using PubMed and Google Scholar to capture epidemiological data for alopecia areata. The inclusion criteria stipulated that studies (1) must be published between 1980 and 2012; (2) must provide data on the incidence or prevalence of alopecia areata; (3) must use samples representative of the general population (i.e., samples derived from the experimental arm of clinical trials or based in dermatology clinics were excluded); (4) must use a sample size larger than 100; and (5) must provide sufficient information on study method and sample characteristics to assess the quality of the study. For GBD 2013, the GBD 2010 search strategy was replicated to capture epidemiological studies published between 2012 and 2014. The agreed approach for alopecia areata was to undertake a literature review every two years. Therefore, no literature review was undertaken for GBD 2015. Additionally, US claims data from 2000, 2010, and 2012 were included.

Table 1. Model inputs

	Prevalence
Studies	9

Countries/subnationals	58
GBD world regions	7

Severity splits & disability weights

For GBD 2013, GBD 2010's disability weights survey was conducted in an additional four European countries. GBD 2013 disability weights were updated to reflect both existing GBD 2010 data and additional data captured in these new surveys. The disability weights used for GBD 2013 were also used for the GBD 2015. The table below illustrates the sequela, severity level, lay description, and disability weights associated with Alopecia areata.

Table 2. Severity level and lay description

Sequela	Severity level	Lay description	DW (95% CI)
Mild alopecia areata	Disfigurement, level 1	The individual has a slight, visible physical deformity that others notice, which causes some worry and discomfort.	0.011 (0.005-0.021)
Severe alopecia areata	Disfigurement, level 2	The individual has a visible physical deformity that causes others to stare and comment. As a result, the person is worried and has trouble sleeping and concentrating.	0.067 (0.044-0.096)

Modeling strategy

DisMod-MR 2.1, a Bayesian meta-regression tool that built on GBD 2013's DisMod-MR 2.0, was used in modeling. DisMod-MR 2.1 was used to estimate prevalence by age, sex, year, and country for alopecia areata. We assumed zero excess mortality and remission from 0 to 4, implying a minimum duration of three months. This was in line with the available epidemiological data, expert opinion, and previous GBD work. Similar to GBD 2013, we used a time window of 20 years to determine which data points were used for a particular year of fit.

Study-level covariates were used to adjust prevalence derived from US claims data for 2000 and 2010 toward the level of other prevalence data, which were more representative of the general population. To improve estimation across all regions, the minimum global coefficient of variation was set at 0.5. In addition, significant sex differences were observed in the US claims data, resulting in a higher prevalence in females compared to males, likely due to more females seeking health consultations for alopecia areata compared to males. To minimize this effect, we set the sex covariate to zero, but this had minimal impact on the global estimates.

Table 3. Beta and exponentiated values

Study covariate	Parameter	Beta	Exp(beta)
Claims data – 2000	Prevalence	-0.73 (-0.78 — -0.68)	0.48 (0.46 — 0.51)
Claims data – 2010	Prevalence	-0.07 (-0.11 — -0.029)	0.93 (0.89 — 0.97)

No other significant changes were made to the modeling strategy for GBD 2015.

Pruritus

Flowchart

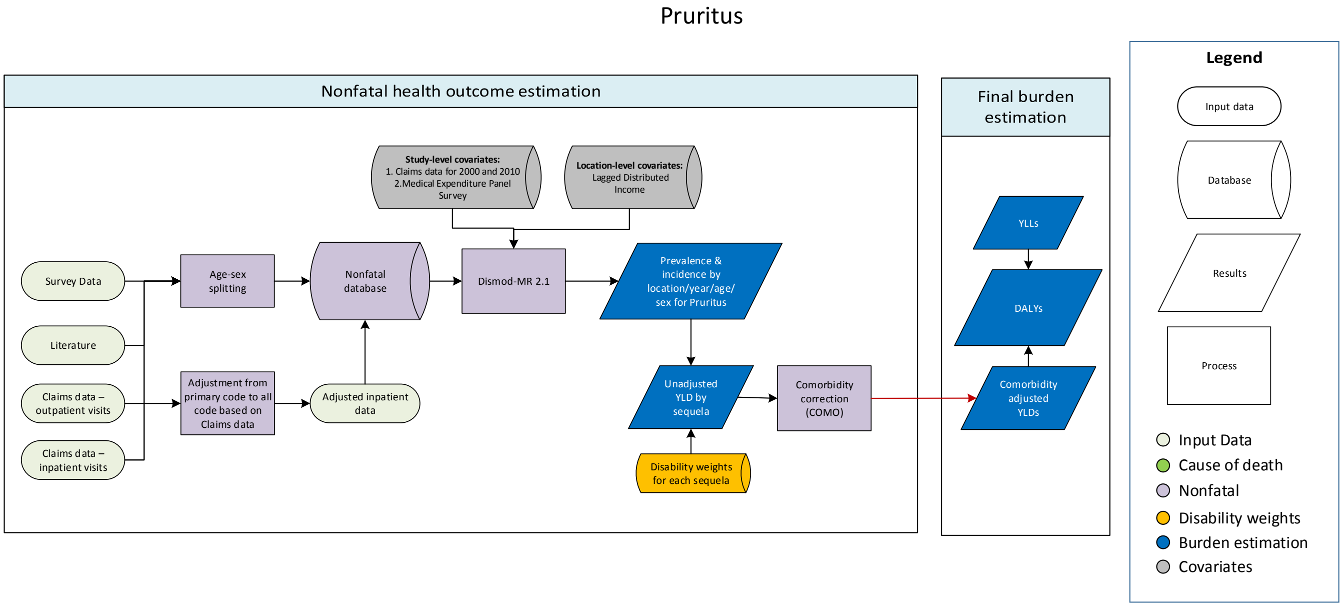

Case definition

Pruritus was included in the GBD 2015 cause group of skin and subcutaneous conditions. Pruritus (or itching) can be a symptom of a condition or disease. Included in the GBD disease modeling were cases meeting ICD-10 diagnostic criteria for pruritus (ICD-10: L29) (1).

Input data

Model inputs

In the GBD 2010 study, a systematic review of the literature was conducted using PubMed and Google Scholar to capture epidemiological data for pruritus. The inclusion criteria stipulated that studies (1) must be published between 1980 and 2012; (2) must provide data on the incidence or prevalence of pruritus; (3) must use samples representative of the general population (i.e., samples derived from the experimental arm of clinical trials or based in dermatology clinics were excluded); (4) must use a sample size larger than 100; and (5) must provide sufficient information on study method and sample characteristics to assess the quality of the study. For GBD 2013, the GBD 2010 search strategy was replicated to capture epidemiological studies published between 2012 and 2014. The agreed approach for pruritus was to undertake a literature review every two years. Therefore, no literature review was undertaken for GBD 2015. Additionally, MEPS data and US claims data from 2000, 2010, and 2012 were included.

Table 1. Data inputs

		Prevalence
Pruritus	Studies	15
	Countries/subnationals	65
	GBD world regions	2

Severity splits

For GBD 2013, GBD 2010's disability weights survey was conducted in an additional four European countries. GBD 2013 disability weights were updated to reflect both existing GBD 2010 data and additional data captured in these new surveys. Pruritus was assigned the disability weight disfigurement level 1, which was also used in GBD 2013.

Table 2. Severity level and lay description

Severity level	Lay description	DW (95% CI)
Disfigurement, level 1	The individual has a slight, visible physical deformity that others notice, which causes some worry and discomfort.	0.011 (0.005-0.021)

Modeling strategy

The GBD 2013 epidemiological modeling strategy for pruritus made use of DisMod-MR 2.1, a Bayesian meta-regression tool that built on GBD 2010's DisMod-MR and GBD 2013's DisMod-MR 2.0. DisMod-MR 2.1 was used to estimate prevalence by age, sex, year, and country for pruritus.

Per expert advice, remission was set from 0.2 to 4, implying a duration of three months to five years. Similar to GBD 2013, a time window of 25 years was used to determine which data points were used for a particular year of fit. This implies that for the year 2000, for instance, DisMod-MR 2.1 incorporated all data points ranging from 1989 to 2014 to estimate prevalence.

Study-level covariates were used to adjust prevalence derived from the MEPS, hospital inpatient data, and US claims data for 2000 and 2010 toward the level of other prevalence data points, which were more representative of the general population.

The data were extremely heterogeneous. Therefore, the random effects were constrained to (-0.2, 0.2) and lagged distributed income was used as a country-level covariate to guide estimates for countries with few or no data.

Table 3. Beta and exponentiated values

Study covariate	Parameter	Beta	Exp(beta)
Claims data – 2000	Prevalence	-0.75 (-0.77 — -0.74)	0.47 (0.46 — 0.48)
Claims data -2010	Prevalence	-0.16 (-0.18 — -0.15)	0.85 (0.84 — 0.86)
Sex	Prevalence	-0.38 (-0.39 — -0.37)	0.68 (0.68 — 0.69)
MEPS	Prevalence	-1.42 (-1.56 — -1.28)	0.24 (0.21 — 0.28)
Lagged distributed income (I$ per capita)	Prevalence	0.0055 (-0.0034 — 0.014)	1.01 (1.00 — 1.01)

Urticaria

Flowchart

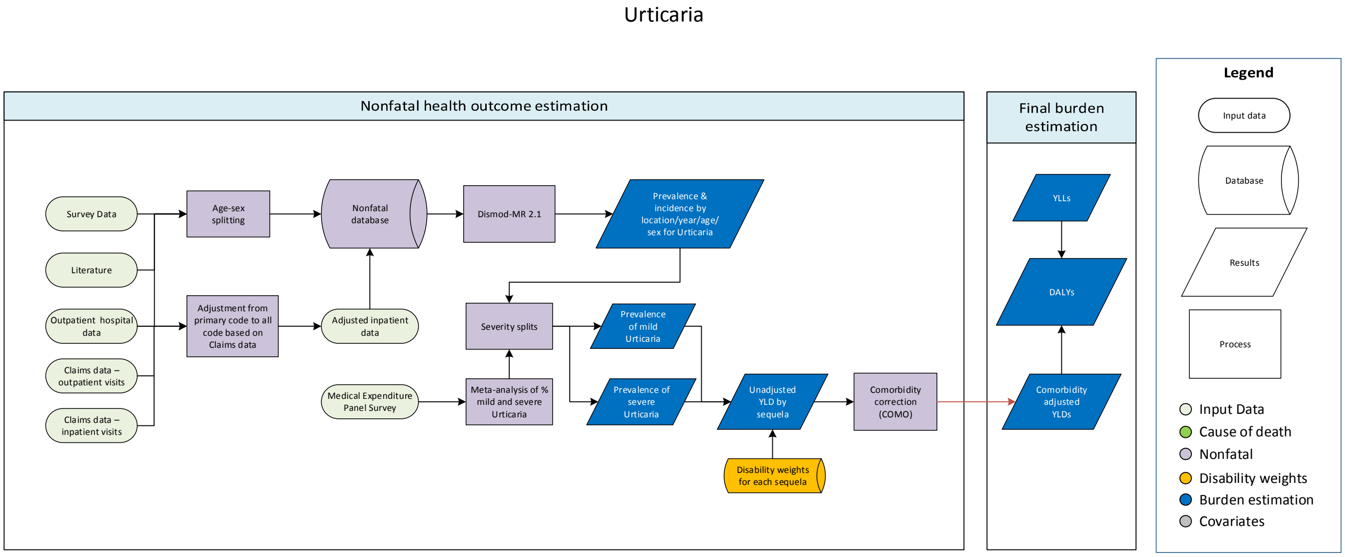

Case definition

Urticaria was included in the GBD 2015 cause group of skin and subcutaneous conditions. Urticaria (hives) refers to a skin reaction that causes itchy, raised bumps. Included in the GBD disease modeling were cases meeting ICD-10 diagnostic criteria for Urticaria (ICD-10: L50) (1).

Input data

Model inputs

In the GBD 2010 study, a systematic review of the literature was conducted using PubMed and Google Scholar to capture epidemiological data for urticaria.

The inclusion criteria stipulated that studies (1) must be published between 1980 and 2012; (2) must provide data on the incidence or prevalence of urticaria; (3) must use samples representative of the general population (i.e., samples derived from the experimental arm of clinical trials or based in dermatology clinics were excluded); (4) must use a sample size larger than 100; and (5) must provide sufficient information on study method and sample characteristics to assess the quality of the study.

For GBD 2013, the GBD 2010 search strategy was replicated to capture epidemiological studies published between 2012 and 2014. The agreed approach for urticaria was to undertake a literature review every two years. Therefore, no literature review was undertaken for GBD 2015. Additionally, hospital outpatient and US claims data from 2000, 2010, and 2012 were included in the data used for GBD 2015.

The table below illustrates the data inputs used in GBD 2015 by number of studies, geographic location, and prevalence/incidence.

Table 1. Data inputs

	Prevalence	Incidence
Studies	18	1
Countries/subnationals	69	1
GBD world regions	10	1

Severity splits & disability weights

For GBD 2013, GBD 2010's disability weights survey was conducted in an additional four European countries. GBD 2013 disability weights were updated to reflect both existing GBD 2010 data and additional data captured in these new surveys. Urticaria was assigned the disability weight, disfigurement with itch/pain, levels 1 and 2. The disability weights used for GBD 2013 were also used for GBD 2015. The table below illustrates the sequelae, severity level, lay description, and DWs.

Table 2. Severity level and lay descriptions

Sequela	Severity level	Lay description	DW (95% CI)
Mild urticaria	Disfigurement, level 1 with itch/pain	The person has a slight, visible physical deformity that is sometimes sore or itchy. Others notice the deformity, which causes some worry and discomfort.	0.027 (0.015-0.042)
Severe urticaria	Disfigurement, level 2, with itch/pain	The person has a visible physical deformity that is sore and itchy. Other people stare and comment, which causes the person to worry. The person has trouble sleeping and concentrating.	0.188 (0.124-0.267)

Modeling strategy

The GBD 2013 epidemiological modeling strategy for urticaria made use of DisMod-MR 2.1, a Bayesian meta-regression tool that built on GBD 2010's DisMod-MR and GBD 2013's DisMod-MR 2.0. DisMod-MR 2.1 was used to estimate prevalence by age, sex, year, and country for urticaria.

The available data were mainly composed of prevalence estimates with a few incidence points. In GBD 2013, a prevalence-only model was fitted to data from literature. For GBD 2015, we made both prevalence and incidence estimates. Similar to GBD 2013, the time window was set to 20 years. We set excess mortality to zero and remission between 0.2 to 2, implying a duration of 0.5 to 5 years. In addition, location random effects were constrained to (-0.3, 0.3) and no US claims covariates were used since coefficients were near zero. The data for Yucatan, Mexico, was excluded because the prevalence estimates were higher relative to other subnational locations.

The table below illustrates the covariate used in the modeling process, as well as associated parameters, beta, and exponentiated beta (confidence interval) values for GBD 2015.

Table 3. Study covariate

Study covariate	Parameter	Beta	Exponentiated beta
Sex	Prevalence	-0.5 (-0.51 — -0.48)	0.61 (0.60 — 0.62)

Decubitus ulcer

Flowchart

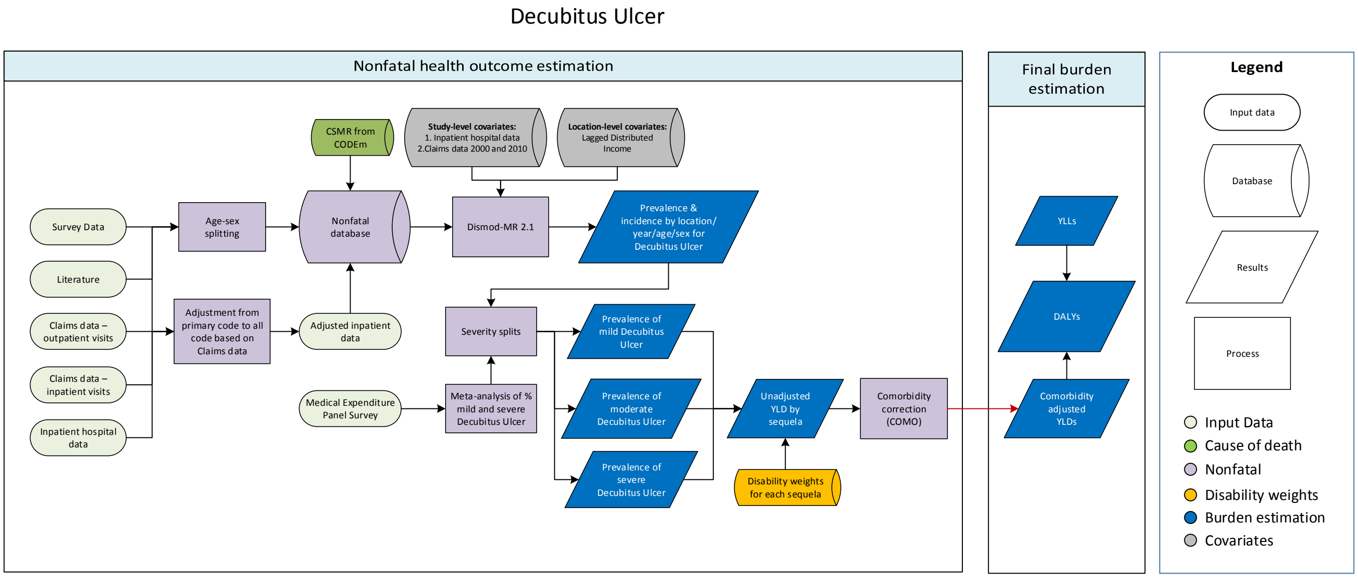

Case definition

Decubitus ulcer was included in the GBD 2015 cause group of skin and subcutaneous conditions. Decubitus ulcer, also known as pressure ulcer/sore, is an injury to the skin and underlying tissue resulting from an obstruction of blood flow due to pressure on the skin. Included in the GBD modeling were cases meeting ICD-10 criteria for decubitus ulcer (ICD-10: L89) (1).

Input data

Model inputs

In the GBD 2010 study, a systematic review of the literature was conducted using PubMed and Google Scholar to capture epidemiological data for decubitus ulcer. The inclusion criteria stipulated that studies (1) must be published between 1980 and 2012; (2) must provide data on the incidence or prevalence of decubitus ulcer; (3) must use samples representative of the general population (i.e., samples derived from the experimental arm of clinical trials or based in dermatology clinics were excluded); (4) must use a sample size larger than 100; and (5) must provide sufficient information on study method and sample characteristics to assess the quality of the study. The data from literature were sparse but contained both prevalence and incidence estimates. For GBD 2013, the GBD 2010 search strategy was replicated to capture epidemiological studies published between 2012 and 2014. Literature review for skin diseases is scheduled to be undertaken every two years, so there was no literature review for GBD 2015. The available data were from high-income countries. Hospital inpatient and US claims data from 2000, 2010, and 2012 were also used for GBD 2015. The final dataset also included cause-specific mortality rates for decubitus ulcer estimated by CODEm.

Table 1. Model inputs

	Prevalence	Incidence	Mortality risk
Studies	1	1	0
Countries/subnationals	1	139	139
GBD world regions	1	8	8

Severity splits

For GBD 2013, GBD 2010's disability weights survey was conducted in an additional four European countries. GBD 2013 disability weights were updated to reflect both existing GBD 2010 data and additional data captured in these new surveys. Decubitus ulcer was assigned the disability weight, disfigurement with itch/pain, levels 1, 2 and 3. The disability weights used for GBD 2013 were also used for GBD 2015.

Table 2. Severity level and lay descriptions

Sequela	Severity level	Lay description	DW (95% CI)
Mild decubitus ulcer	Disfigurement, level 1	The person has a slight, visible physical deformity that others notice, which causes some worry and discomfort.	0.011 (0.005-0.021)
Moderate decubitus ulcer	Disfigurement, level 2	The individual has a visible physical deformity that causes others to stare and comment. As a result, the person is worried and has trouble sleeping and concentrating.	0.067 (0.044-0.096)
Severe decubitus ulcer	Disfigurement, level 3	The individual has an obvious physical deformity that makes others uncomfortable, which causes the person to avoid social contact, feel worried, sleep poorly, and think about suicide.	0.405 (0.274-0.546)

Modeling strategy

DisMod-MR 2.1 was used to estimate prevalence by age, sex, year, and country for decubitus ulcer. Per expert advice, remission was set from 3 to 4, implying a duration of three to four months. This was based on the assumption that remission does not change with treatment. These values were also in line with the available epidemiological data, expert opinion, and previous GBD work. The decubitus ulcer dataset was sufficiently large to make use of a relatively short time window of five years to determine which data points were used for a particular year of fit.

Study-level covariates were used to adjust incidence derived from hospital inpatient data and US claims data for 2000 and 2010 toward the level of other incidence data points, which were more representative of the general population. Unlike GBD 2013, the MEPS data were excluded since they did not include a 3 digit ICD-9 code, which implies ulcers other than decubitus were captured in the data. We also excluded estimates less than 10E-6. Lagged distributed income was used as a country-level covariate on excess mortality to guide estimates for countries with little or no data.

Table 3. Beta and exponentiated values

Study covariate	Parameter	Beta	Exp(beta)
Claims data – 2000	Incidence	-0.27 (-0.32 — -0.23)	0.76 (0.73 — 0.79)
Claims data – 2010	Incidence	-0.034 (-0.074 — -0.0043)	0.97 (0.93 — 1.00)
Hospital inpatient data	Incidence	-0.53 (-0.57 — -0.5)	0.59 (0.56 — 0.61)
Sex	Incidence	0.36 (0.34 — 0.39)	1.44 (1.40 — 1.47)
Lagged distributed income	Excess mortality rate	-0.5 (-0.5 — -0.5)	0.61 (0.61 — 0.61)
Sex	Excess mortality rate	-0.14 (-0.17 — -0.11)	0.87 (0.84 — 0.90)
Sex	Cause-specific mortality rate	0.16 (0.13 — 0.18)	1.17 (1.14 — 1.20)

Other skin and subcutaneous diseases

Flowchart

Other Skin and Subcutaneous Diseases (OSSD)

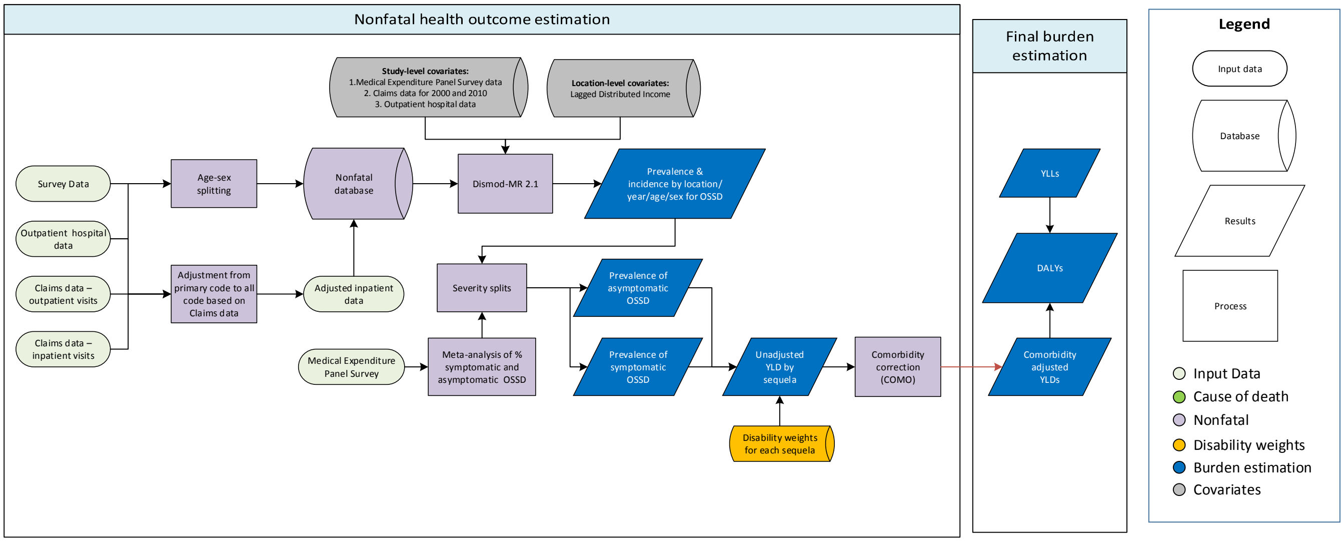

Case definition

The other skin and subcutaneous diseases category encompassed a large group of skin conditions not captured in the other skin categories. Included in the GBD 2015 disease modeling were cases meeting ICD-10 (L15, L52, L53, L54, etc.) diagnostic criteria (1).

Input data

Model inputs

In the GBD 2010 study, a systematic review of the literature was conducted using PubMed and Google Scholar to capture epidemiological data for skin diseases not captured in the other skin categories. The inclusion criteria stipulated that studies (1) must be published between 1980 and 2012; (2) must provide data on the incidence or prevalence; (3) must use samples representative of the general population (i.e., samples derived from the experimental arm of clinical trials or based in dermatology clinics were excluded); (4) must use a sample size larger than 100; and (5) must provide sufficient information on study method and sample characteristics to assess the quality of the study. For GBD 2013, the GBD 2010 search strategy was replicated to capture epidemiological studies published between 2012 and 2014. The agreed approach for other skin and subcutaneous diseases was to undertake a literature review every two years. Therefore, no literature review was undertaken for GBD 2015. Hospital outpatient data and data from US claims for 2000, 2010, and 2012 by US state were included in GBD 2015.

Table 1. Model inputs

	Prevalence
Studies	0
Countries/subnationals	54
GBD world regions	2

Severity split & disability weight

Skin and other subcutaneous diseases were assigned the disability weight for disfigurement level 1. The disability weights used for GBD 2013 were also used for GBD 2015.

Table 2. Severity level and lay description

Sequela	Severity level	Lay description	DW (95% CI)
Symptomatic other skin and subcutaneous diseases	Disfigurement, level 1	The individual has a slight, visible physical deformity that others notice, which causes some worry and discomfort.	0.011 (0.005-0.021)

Modeling strategy

DisMod-MR 2.1, a Bayesian meta-regression tool that built on GBD 2013's DisMod-MR 2.0, was used in modeling. DisMod-MR 2.1 was used to estimate prevalence by age, sex, year, and country for skin and other subcutaneous diseases.

We assumed zero excess mortality and remission of one, implying a duration of 12 months. Similar to GBD 2013, we used a time window of 25 years to determine which data points were used for a particular year of fit.

Study-level covariates, which were used to adjust prevalence, were derived from MEPS, hospital outpatient data, and US claims data for 2000 and 2010 toward the level of other prevalence data, which were more representative of the general population. In addition, lagged distributed income was used as a country-level covariate to guide estimates for countries with few or no data.

Table 3. Beta and exponentiated values

Study covariate	Parameter	Beta	Exp(beta)
Claims data – 2000	Prevalence	-0.27 (-0.29 — -0.26)	0.76 (0.75 — 0.77)
Claims data – 2010	Prevalence	-0.039 (-0.052 — -0.023)	0.96 (0.95 — 0.98)

Lagged distributed income (I$ per capita)	Prevalence	0.16 (0.14 — 0.17)	1.17 (1.16 — 1.18)
Hospital outpatient	Prevalence	-2 (-2 — -1.99)	0.14 (0.14 — 0.14)
MEPS	Prevalence	-0.81 (-0.94 — -0.68)	0.45 (0.39 — 0.51)
Sex	Prevalence	-0.13 (-0.14 — -0.11)	0.88 (0.87 — 0.89)

Other sense organ diseases

Flowchart

Other sense organ diseases

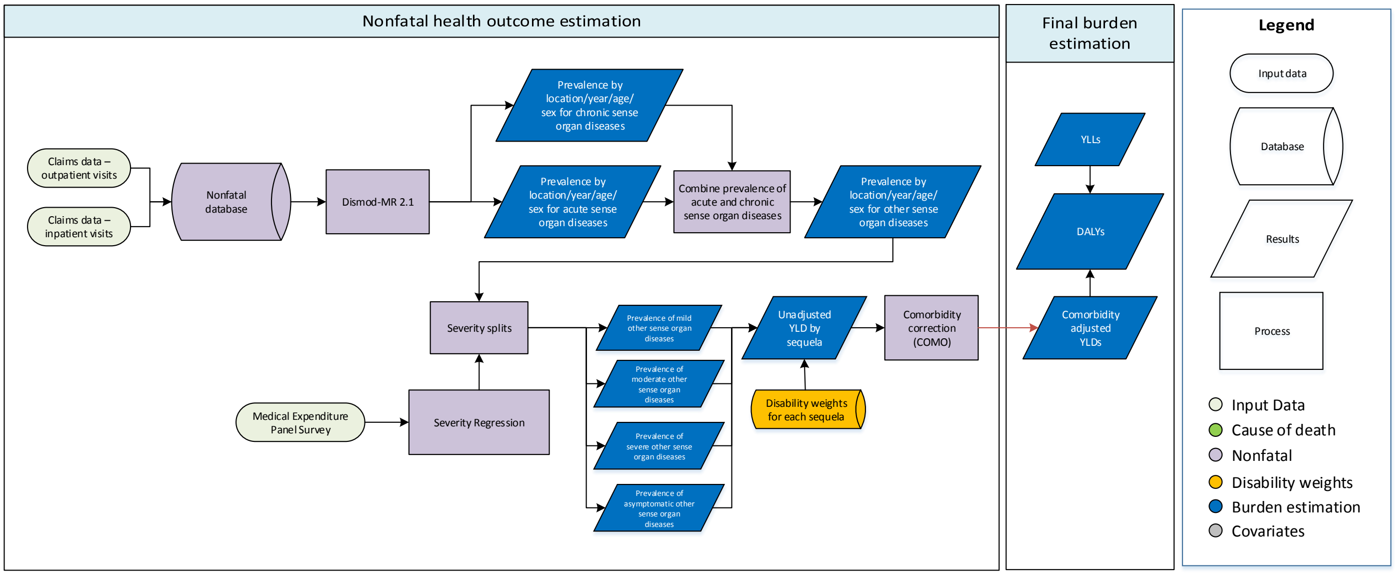

Case definition

Other sense organ disease is a residual cause capturing both acute and chronic conditions that do not map to other causes, but lead to non-trivial morbidity. These include the following ICD codes: 077, 360, 364, 370-77, 379, 380, 386, and 388, which encompass a plethora of ocular disorders and conditions.

Input data

Model inputs

For GBD 2015, we used claims data from Marketscan to model other sense organ diseases, since these conditions would not appear in inpatient hospital data. ICD-9 codes were assigned at the 5 digit level to either acute or chronic conditions, as listed in Appendix Table 4.

The table below lists data availability. The only sources used were United States claims data from Marketscan.

Measure	Data source	Subnational	Country	Region	Super region
Prevalence	3	52	1	1	1
Incidence	3	52	1	1	1

Severity splits & disability weights

The basis of the GBD disability weight survey assessments are lay descriptions of sequelae highlighting major functional consequences and symptoms. Severity splits for other sense organ diseases were calculated via the MEPS regression and outlined in the table below.

Severity	Proportion	Healthstate	Disability weight
Severe	0.38 (0.37-0.40)	Vertigo	
Mild	0.28 (0.22-0.34)	This person has low fever and mild discomfort but no difficulty with daily activities	0.006 (0.002-0.012)
Moderate	0.11 (0.6-0.19)	This person has slight physical deformity which causes some worry and discomfort	0.011 (0.005-0.021)
Asymptomatic	0.21 (0.15-0.28)	Asymptomatic	N/A

Modeling strategy

For GBD 2015, hospital data was extracted separately for the chronic and acute conditions included in other sense organ diseases. The chronic data was extracted as prevalence, and acute as incidence. We then ran two separate DisMod MR2.1 models. The chronic model, with prevalence data, was run as prevalence-only model. The acute model was run as a full model with incidence data, assuming zero excess mortality and duration of 1 week (remission 52). In both models, to correct for systematically lower data from 2000 Marketscan claims, we used a study-level covariate to crosswalk the 2000 data. Since the only data source is from the United States, we do not use any country -level covariates in this model.

We then aggregated chronic and acute prevalence outputs, resulting in the prevalence of other sense organ diseases by country, age, year, and sex.

Cause	Measure	Variable_Name	Beta	Exponentiated
Acute other sense organ diseases	incidence	All MarketScan, year 2000	-0.05 (-0.07 - -0.02)	0.95 (0.93 - 0.98)
Chronic other sense organ diseases	prevalence	All MarketScan, year 2000	-0.48 (-0.50 - -0.47)	0.62 (0.61 - 0.63)

Results from GBD 2015 are higher for other sense organ diseases because in GBD 2013 we used MEPS self-reported claims data, whereas in GBD 2015 Marketscan more accurately captures the occurrence of these conditions. In GBD 2015 we were able to separately model acute and chronic conditions.

Deciduous caries

Flowchart

Deciduous caries

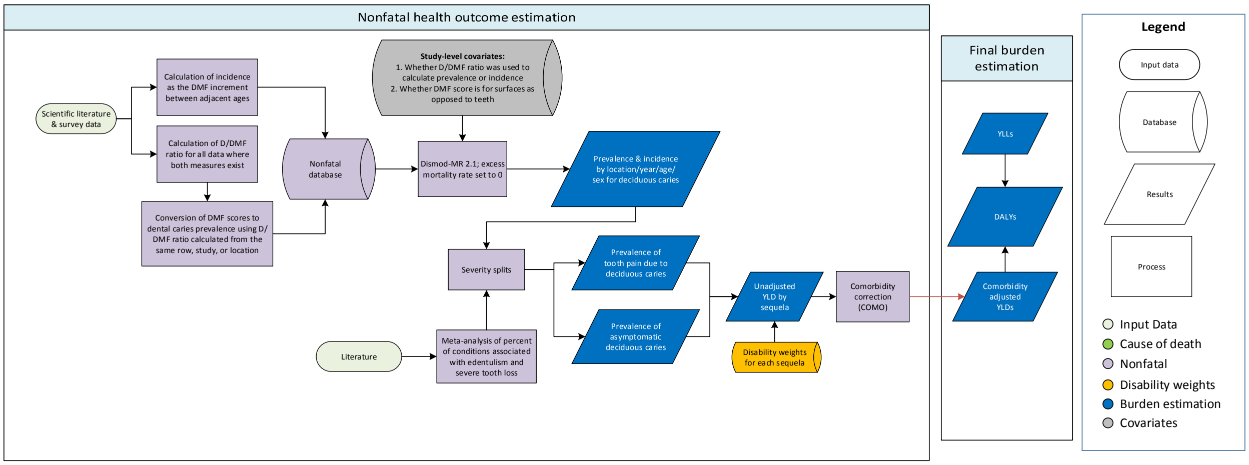

Case definition

The case definition for dental caries is "teeth with unmistakable coronal cavity at dentin level, root cavity in cementum that feels soft or leathery to probing, temporary or permanent restorations, or missing teeth extracted due to a caries lesion." This definition corresponds to an ICD-9 code of 521.0 and an ICD-10 code of K02.3 – K02.9. Most caries are subclinical in the sense that they do not cause symptoms a majority of the time. Once a carious lesion develops, it will occasionally recede without intervention. Generally, however, it worsens with time and eventually requires either filling or extraction. The major sequela associated with the condition is symptomatic caries, which is defined as "a toothache which causes some difficulty eating."

Deciduous teeth are colloquially known by several names throughout the world, including "baby," "milk," or "fall" teeth. They start erupting in infants around 6 months of age and generally finish by the end of year three. Exfoliation of deciduous teeth begins around age 5-6 and usually is complete by age 12-14, when only permanent teeth remain.

Dental caries and the DMFT index

Public health dentists commonly measure dental caries using the dmft/DMFT index, which is an incremental measure of the proportion of unhealthy teeth and is also a measure of an individual's lifetime prevalence of caries. Lowercase letters (dmft) are used for deciduous dentition and uppercase letters (DMFT) for permanent dentition. D is for decayed, M for missing, F for filled, and T for teeth. The maximum dmft score is 20 and the maximum DMFT score is 32. Furthermore, some dentists prefer to measure dental caries in terms of tooth surfaces, rather than number of teeth, and report their results using an analogous dmfs/DMFS index. The maximum dmfs score is 88, and the maximum DMFS score is 128 or 148 depending on whether the third molars are counted.

The DMFT index is easy to measure and inter-rater reliability is high. However, the primary shortcoming of the DMFT is that it does not discriminate well between current and past caries. Strategies we

employed to maximally utilize dmf/DMF data for estimating the prevalence of burden due to deciduous caries are described below.

Input data

Model inputs

Literature reviews

A literature review was conducted by the expert group for GBD 2010, and an additional systematic review was performed for GBD 2013. The search terms used in the GBD 2013 literature review for deciduous caries were (Deciduous caries[Title/Abstract]) OR (milk caries[Title/Abstract]) OR (baby caries[Title/Abstract]) OR (caries[Title/Abstract]) OR (dental health[Title/Abstract]) OR (oral health[Title/Abstract]) AND (prevalence[Title/Abstract]) AND ("2010"[Date - Publication] : "2013"[Date - Publication]). Updates to systematic reviews are performed on an ongoing schedule across all GBD causes; an update for deciduous and permanent caries will be performed in the next one to two iterations.

We eliminated many data points to avoid repetition in the dataset, while striving to maintain as much data detail as possible. Redundancy tended to arise in three data descriptors: age, gender, and urbanicity. Our order of preference for maintaining detail was age, followed by gender, then urbanicity. Additionally, many of the studies presented dmft or DMFT scores, which represent lifetime prevalence and were often described as "caries experience." For the purposes of measuring the burden of disability from dental caries, we considered only data on current prevalence to be relevant, and thus converted lifetime prevalence data to current prevalence and incidence where possible (see below).

Conversion of dmf/DMF scores to prevalence and incidence

Deciduous caries

Many of the studies that reported lifetime prevalence of deciduous caries (dmf scores) provided detail on the component breakdown of these scores. We used these data to calculate a d/dmf ratio and convert the lifetime prevalence value into one that reflected current prevalence. For example, if the lifetime prevalence was reported as 0.8 with d = 1.5 and dmft = 2 (d/dmf ratio = 0.75), we adjusted the prevalence value to 0.6. When possible, we used within-study d/dmf ratios to convert lifetime prevalence to current prevalence. Otherwise, we converted to current prevalence using a weighted average d/dmf ratio at the country, region, super-region, or global level, in that order of preference.

For studies reporting dmf scores for successive age intervals, the increment in the dmf values between examinations was considered to be equivalent to the caries incidence over the study duration. We extrapolated incidence data from two types of studies. First, for longitudinal or cohort studies, we calculated the caries increment over successive ages and time periods as the difference between the DMF scores at each time point. Narrow age and time intervals were preferred; most were of three years or less. We did not extrapolate incidence data if the age or time interval was greater than 10 years. Secondly, if a study only performed a single cross-sectional examination, but reported data in age intervals of three years or less, we extrapolated incidence data in the same manner. Narrow age ranges were considered necessary for this incidence extrapolation because the dental health of population cohorts has been observed to change in just a few years when preventive measures are instituted.

Data availability for deciduous caries:

	Prevalence	Incidence	Continuous DMF score converted to prevalence	Continuous DMF score converted to incidence
Studies	69	0	124	59
Countries/subnationals	42/41	0	55/26	32/19
GBD world regions	17	0	19	16

Modeling strategy

Separate estimates of deciduous and permanent caries

The natural histories of deciduous and permanent caries share many similarities, but they also share some important differences. Age patterns of decay in permanent and deciduous dentition are distinct, and duration of a carious lesion in deciduous teeth also tends to be shorter than an untreated episode of permanent caries. Sugar consumption and feeding with formula are both associated with development of deciduous carries, but not with permanent caries. Finally, it is unclear whether the gender patterns and regional differences are the same for both deciduous and permanent caries. For all of these reasons, we elected to model deciduous caries and permanent caries as separate entities and then add the estimates together for an overall estimation of the global burden of dental caries. This modeling approach was also taken in GBD 2010 and GBD 2013.

DisMod model development: deciduous caries

Serious health consequences of deciduous caries were assumed to be uncommon and death from permanent caries very rare. For purposes of modeling, we therefore assigned excess mortality to be zero from age 0 to 100. We fixed incidence and prevalence at zero after age 12 when exfoliation is presumed to be complete. This age was chosen because 11 was the oldest age of a non-zero prevalence data point. We additionally assigned incidence and prevalence to be zero before age 6 months to indicate that this condition never begins at birth and is absent in the neonatal and post-neonatal periods. Incidence bounds of 0 to 4.0 for ages 1 to 10 years were chosen based on examining the dataset and adding a comfortable margin to the highest reported value. An upper remission bound of 1.0 for ages 0 to 5 years was chosen in order to fit the sharp increase in prevalence over this age range. As prevalence was assigned to be zero after age 14 anyway, we elected to not include a lower remission bounds.

No country-level covariates were included. We used a study-level covariate to indicate whether a given prevalence data point was of "true" current prevalence or calculated from lifetime prevalence using the d/dmf ratio. For both incidence and prevalence, we also used study-level covariates to indicate whether the dmf scores were for surfaces as opposed to teeth.

Because no "true" unconverted incidence values were present in the dataset, we could not crosswalk the extrapolated incidence values to reference incidence values for deciduous caries. Instead, we used higher heterogeneity settings for incidence values than for prevalence (0.7 for incidence, 0.2 for prevalence). We

calculated age mesh points at ages 0, 0.5, 1, 2, 3, 4, 5, 6, 7, 8, 9, 10, 11, 12, 13, 14, 15, and 100 years. High smoothness settings were used for both incidence and prevalence to allow for dynamic age trends.

Study-level covariate	Parameter	beta	Exponentiated beta
Whether d/dmf ratio was used to convert lifetime prevalence to current prevalence	Prevalence	-0.08	0.92
Whether dmf score is for surfaces	Prevalence	0.18	1.19
Whether dmf score is for surfaces	Incidence	1.99	7.30

Models were vetted based on the biological plausibility of the results, the extent to which estimates fit the data, and the plausibility of the range of estimates across location hierarchies.

Correction for edentulism

One systematic source of bias in the literature was the exclusion of edentate individuals form the study populations, which leads to systematic overestimation of caries prevalence when modeled over the entire population. To account for this bias, we used our GBD estimates of edentulism and severe tooth prevalence to adjust YLD estimates for dental caries. Using the final DisMod estimates for prevalence of edentulousness, we calculated the mean prevalence for each age and sex and averaged the 1990 and 2015 values. We then calculated a population-weighted mean prevalence for each region and each GBD super-region. The resulting super-regional averages were used to adjust the DisMod estimates for prevalence of permanent caries in calculating years lost due to disability (YLDs).

Disability weights

As described above, the GBD definition of disability associated with symptomatic dental caries is “this person has a toothache, which causes some difficulty eating.” The disability weight associated with this condition is 0.01 (0.005 – 0.019), as derived from the GBD Disability Weights Study.

Not all those with dental caries experience this disability all the time. We considered only those with active dentinal decay to experience symptomatic tooth pain. Those with deciduous caries who had undergone exfoliation or had their cavities filled were considered to have no disability. Likewise, those with permanent caries who had received fillings, had their cavities extracted, or lost a carious tooth altogether were considered to have no disability. Thus, two additional pieces of information are required to complete the calculation of years of life lived with disability (YLDs): proportion with symptoms and duration of disability.

To determine duration, we adapted the method employed by the Australian Burden of Disease (AusBoD) Study in 1996. For total duration, we used the posterior estimates of duration from final DisMod models. For those with symptoms, we split this total duration into two distinct phases of caries disability. The “initial” phase is characterized by *periodic* pain that we assigned to occur an average of one hour per day.

The “terminal” phase is a period of *constant* symptoms at the end of an episode. The length of the terminal phase was determined by literature review as described by the AusBoD group. For deciduous caries we used a study by Mason, et al. of children in the UK presenting to a casualty ward with tooth pain [1]. The length of time each child had been experiencing tooth pain was recorded. Based on the distribution of time courses, a log-normal distribution was plotted that approximated the average duration of *constant* symptoms at 27.6 days leading up to seeking care. For permanent caries, a similar study of the tooth pain experience of adults in New Zealand who presented to hospital dental departments and an emergency clinic [2] resulted in an estimated 55.2 days spent in the terminal phase of caries. For those with severe disease, the length of time spent in the terminal phase was subtracted from the total duration to determine the amount of time spent in the initial phase. For those with mild disease, we considered the entire duration to be spent in the initial phase.

To determine proportion with symptoms, we completed a supplemental literature review of tooth pain and caries. We identified a total of 21 studies with data about the prevalence of pain. The studies were grouped according to the type of dentition studied (deciduous or permanent) and the location of the study group (high-income or low- and middle-income countries). We extracted data on the proportion in each group that described symptoms of pain related to their caries as well as a subset who described their symptoms as being severe. The proportions in each group were weighted according to sample size to give estimates of the relative sizes of three groups: asymptomatic, mild, and severe.

We considered asymptomatic individuals to experience no disability. Those with mild disease spent the entire duration in the initial phase of disease (one hour of pain per day). Those with severe disease spent a majority of the duration in the initial phase followed by a period of time in the terminal phase (constant pain). YLDs were calculated by multiplying the prevalence, duration, proportion, and disability weight for each age, country, sex, and year.

Permanent caries

Flowchart

Permanent Caries

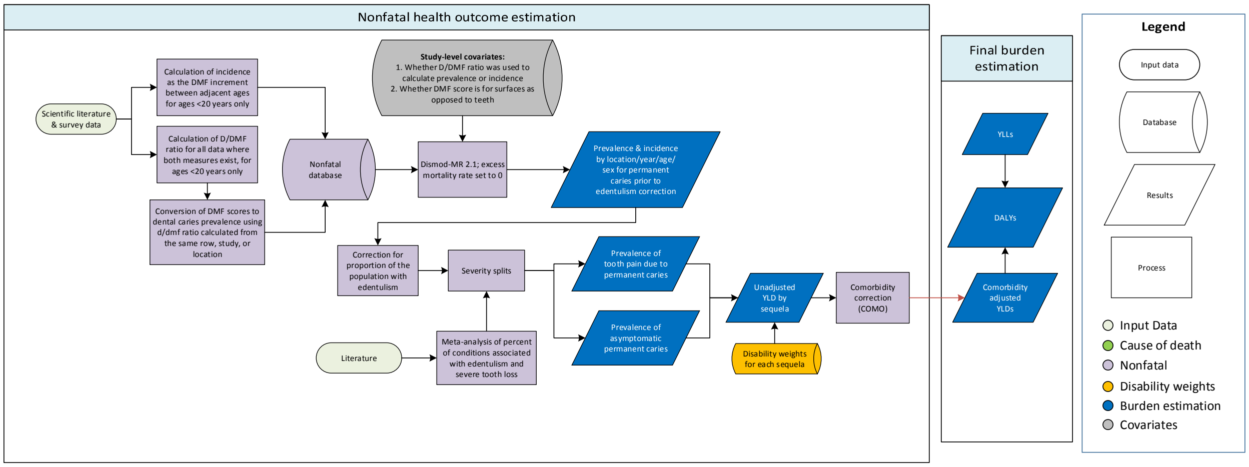

Case definition

The case definition for dental caries is “teeth with unmistakable coronal cavity at dentin level, root cavity in cementum that feel soft or leathery to probing, temporary or permanent restorations, or missing teeth extracted due to a caries lesion.” This definition corresponds to an ICD-9 code of 521.0 and an ICD-10 code of K02.3 – K02.9. Most caries are subclinical in the sense that they do not cause symptoms a majority of the time. Once a carious lesion develops, it will occasionally recede without intervention. Generally, however, it worsens with time and eventually requires either filling or extraction. The major sequela associated with the condition is symptomatic caries, which is defined as “a toothache, which causes some difficulty eating.”

Dental caries and the DMFT index

Public health dentists commonly measure dental caries using the dmft/DMFT index, which is an incremental measure of the proportion of unhealthy teeth and is also a measure of an individual’s lifetime prevalence of caries. Lowercase letters (dmft) are used for deciduous dentition and uppercase letters (DMFT) for permanent dentition. D is for decayed, M for missing, F for filled, and T for teeth. The maximum dmft score is 20 and the maximum DMFT score is 32. Furthermore, some dentists prefer to measure dental caries in terms of tooth surfaces, rather than number of teeth, and report their results using an analogous dmfs/DMFS index. The maximum dmfs score is 88, and the maximum DMFS score is 128 or 148 depending on whether the third molars are counted.

The DMFT index is easy to measure and inter-rater reliability is high. However, the primary shortcoming of the DMFT is that it does not discriminate well between current and past caries. Strategies we employed to maximally utilize dmf/DMF data for estimating the prevalence of burden due to permanent caries are described below.

Input data

Literature reviews

A literature review was conducted by the expert group for GBD 2010, and an additional systematic review was performed for GBD 2013. The search terms used in the GBD 2013 literature review for permanent caries were (Permanent caries[Title/Abstract]) OR (caries prevalence[Title/Abstract]) OR (dental health[Title/Abstract]) OR (oral health[Title/Abstract]) AND (prevalence[Title/Abstract]) AND ("2010"[Date - Publication] : "2013"[Date - Publication]). Updates to systematic reviews are performed on an ongoing schedule across all GBD causes, an update for deciduous and permanent caries will be performed in the next one to two iterations.

We eliminated many data points to avoid repetition in the dataset, while striving to maintain as much data detail as possible. Redundancy tended to arise in three data descriptors: age, gender, and urbanicity. Our order of preference for maintaining detail was age, followed by gender, then urbanicity. Additionally, many of the studies presented dmft or DMFT scores, which represent lifetime prevalence and were often described as "caries experience." For the purposes of measuring the burden of disability from dental caries, we considered only data on current prevalence to be relevant, and thus converted lifetime prevalence data to current prevalence and incidence where possible (see below).

Conversion of dmf/DMF scores to prevalence and incidence

Permanent caries

Whereas in the deciduous dentition, a vast majority of the dmf index is accounted for by caries, tooth loss is a major contributor to the DMF index for the permanent dentition. Permanent caries may not necessarily be the primary driver of this tooth loss, as other factors such as periodontal disease and trauma may contribute significantly. Thus, we performed the conversions of DMF scores to prevalence and incidence values as described above for permanent caries only in individuals ages 20 years or less.

Data availability for permanent caries:

	Prevalence	Incidence	Continuous DMF score converted to prevalence	Continuous DMF score converted to incidence
Studies	70	4	90	31
Countries/subnationals	45/22	4/2	47/20	24/11
GBD world regions	16	3	19	14

Modeling strategy

Separate estimates of deciduous and permanent caries

The natural history of deciduous and permanent caries share many similarities, but they also share some important differences. Age patterns of decay in permanent and deciduous dentition are distinct, and duration of a carious lesion in deciduous teeth also tends to be shorter than an untreated episode of permanent caries. Sugar consumption and feeding with formula are both associated with development of

deciduous carries, but not with permanent caries. Finally, it is unclear whether the gender patterns and regional differences are the same for both deciduous and permanent caries. For all of these reasons, we elected to model deciduous caries and permanent caries as separate entities and then add the estimates together for an overall estimation of the global burden of dental caries. This is the modeling approach which was also taken in GBD 2010 and GBD 2013.

DisMod model development: permanent caries

Serious health consequences of permanent caries were also assumed to be uncommon and death from permanent caries very rare. We therefore assigned excess mortality to be zero from age 0 to 100. The dataset suggested that permanent caries are sometimes incident in 5-year-olds, so we fixed incidence and prevalence at 0 for ages 0 to 4. Incidence bounds were again chosen based on examining the dataset and adding a margin to the highest reported value. In this case, incidence bounds were 0 – 2. Lower bounds for remission were set at 0.2 and upper bounds were set at 3.

As with permanent caries, no country-level covariates were included. We used a study-level covariate to indicate whether a given prevalence data point was of "true" current prevalence or calculated from lifetime prevalence using the D/DMF ratio, and another study-level covariate to indicate whether a given incidence values was extrapolated from DMF scores. For both incidence and prevalence, we also used study-level covariates to indicate whether the DMF scores were for surfaces as opposed to teeth.

We calculated age mesh points at ages 0, 4, 5, 8, 10, 12, 15, 19, 20, 25, 29, 30, 35, 40, 45, 50, 55, 60, 70, 80, 90, and 100 years. Heterogeneity was set to 0.5 for both incidence and prevalence. High smoothness settings were used for both incidence and prevalence to allow for dynamic age trends.

Study-level covariate	Parameter	beta	Exponentiated beta
Whether D/DMF ratio was used to convert lifetime prevalence to current prevalence	Prevalence	-0.27	0.77
Whether DMF score was used to calculate incidence	Incidence	0.25	1.28
Whether dmf score is for surfaces	Prevalence	0.30	1.35
Whether dmf score is for surfaces	Incidence	1.00	2.72

Although studies were screened carefully during data extraction to ensure that they specified whether they were measuring permanent or deciduous caries, some data points were marked as outliers during modeling due to their high prevalence values in young ages, as it was deemed likely that some of these studies were reporting deciduous in addition to permanent caries.

As with deciduous caries, models for permanent caries were vetted based on the biological plausibility of the results, the extent to which estimates fit the data, and the plausibility of the range of estimates across location hierarchies.

Correction for edentulism

One systematic source of bias in the literature was the exclusion of edentate individuals from the study populations, which leads to systematic overestimation of caries prevalence when modeled over the entire population. To account for this bias, we used our GBD estimates of edentulism and severe tooth prevalence to adjust YLD estimates for dental caries. Using the final DisMod estimates for prevalence of edentulousness, we calculated the mean prevalence for each age and sex and averaged the 1990 and 2015 values. We then calculated a population-weighted mean prevalence for each region and each GBD super-region. The resulting super-regional averages were used to adjust the DisMod estimates for prevalence of permanent caries in calculating years lost due to disability (YLDs).

Disability weights

As described above, the GBD definition of disability associated with symptomatic dental caries is "this person has a toothache, which causes some difficulty eating." The disability weight associated with this condition is 0.01 (0.005 – 0.019), as derived from the GBD Disability Weights study.

Not all those with dental caries experience this disability all the time. We considered only those with active dentinal decay to experience symptomatic tooth pain. Those with deciduous caries who had undergone exfoliation or had their cavities filled were considered to have no disability. Likewise, those with permanent caries who had received fillings, had their cavities extracted, or lost a carious tooth altogether were considered to have no disability. Thus, two additional pieces of information are required to complete the calculation of years of life lived with disability (YLDs): proportion with symptoms and duration of disability.

To determine duration, we adapted the method employed by the Australian Burden of Disease (AusBoD) Study in 1996. For total duration, we used the posterior estimates of duration from final DisMod models. For those with symptoms, we split this total duration into two distinct phases of caries disability. The "initial" phase is characterized by *periodic* pain that we assigned to occur an average of one hour per day. The "terminal" phase is a period of *constant* symptoms at the end of an episode. The length of the terminal phase was determined by literature review as described by the AusBoD group. For deciduous caries we used a study by Mason, et al. of children in the UK presenting to a casualty ward with tooth pain [1]. The length of time each child had been experiencing tooth pain was recorded. Based on the distribution of time courses, a log-normal distribution was plotted that approximated the average duration of *constant* symptoms at 27.6 days leading up to seeking care. For permanent caries, a similar study of the tooth pain experience of adults in New Zealand who presented to hospital dental departments and an emergency clinic [2] resulted in an estimated 55.2 days spent in the terminal phase of caries. For those with severe disease, the length of time spent in the terminal phase was subtracted from the total duration to determine the amount of time spent in the initial phase. For those with mild disease, we considered the entire duration to be spent in the initial phase.

To determine proportion with symptoms, we completed a supplemental literature review of tooth pain and caries. We identified a total of 21 studies with data about the prevalence of pain. The studies were grouped according to the type of dentition studied (deciduous or permanent) and the location of the study group (high-income or low- and middle-income countries). We extracted data on the proportion in each group that described symptoms of pain related to their caries as well as a subset who described

their symptoms as being severe. The proportions in each group were weighted according to sample size to give estimates of the relative sizes of three groups: asymptomatic, mild, and severe.

We considered asymptomatic individuals to experience no disability. Those with mild disease spent the entire duration in the initial phase of disease (one hour of pain per day). Those with severe disease spent a majority of the duration in the initial phase followed by a period of time in the terminal phase (constant pain). YLDs were calculated by multiplying the prevalence, duration, proportion, and disability weight for each age, country, sex, and year.

Chronic periodontal disease

Flowchart

Periodontal diseases

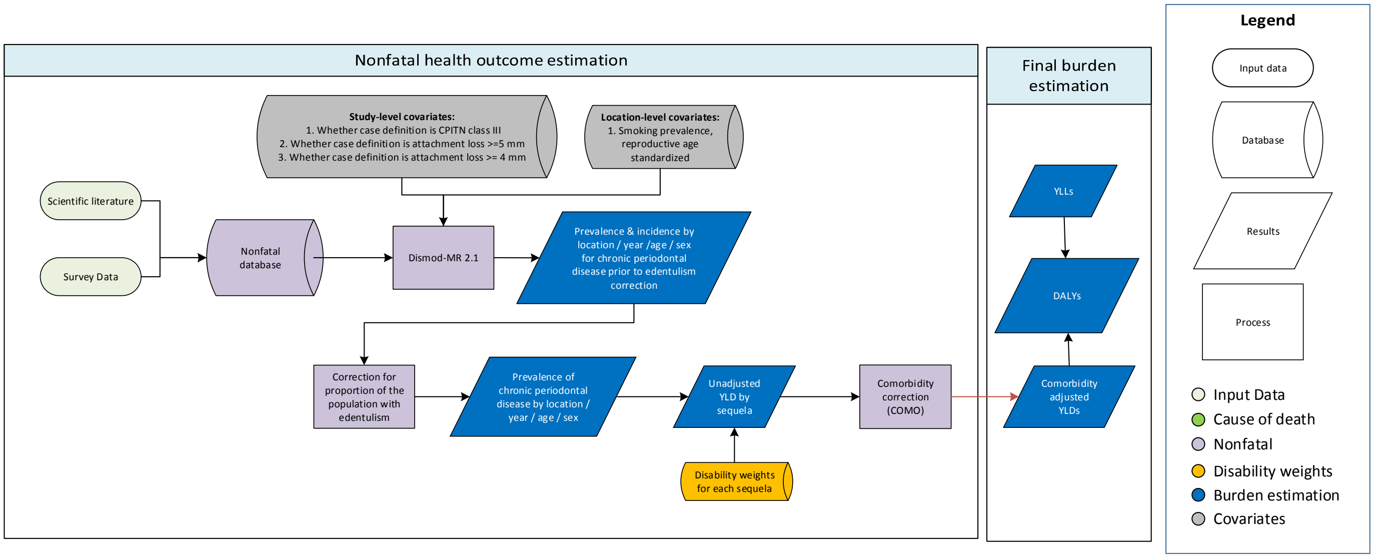

Case definition

Chronic periodontal disease is caused by chronic bacterial infection around the teeth. Symptoms of gingivitis, the mildest form of the disease, include swelling, redness, and propensity of the gums to bleed when perturbed. If the infection is not treated appropriately, it will eventually spread below the gum line leading to a chronic inflammatory state of the periodontal tissues. Over time, there will be loss of gingival tissue and alveolar bone destruction. Teeth will become loose and may need to be extracted.

The GBD definition of disability associated with symptomatic severe periodontal disease is "bad breath, a bad taste in the mouth, and gums that bleed a little from time to time, but which does not interfere with daily activities." The ICD-10 codes for periodontal disease are K05.0 – K05.6, and the ICD-9 codes are 523.0 – 523.9.

Defining periodontal disease in a meaningful, reproducible manner has been an ongoing challenge for public health dentists. Attachment loss (AL) and pocket depth (PD) have emerged as the most common metrics of periodontal health measurement. Attachment loss (AL) is measured as the difference between the distance from the gingival margin to the bottom of the pocket and the distance from the cemento-enamel junction to the bottom of the pocket.

The Community Periodontal Index of Treatment Needs (CPITN) is a classification system that was developed by the WHO as a standardized method of periodontal health measurement [1]. CPITN classification is based on quantifying the probing depth between teeth and gums. The mouth is divided into 6 sections, called sextants. Sextants with fewer than two teeth are excluded. Multiple teeth in each sextant are examined. A standard-sized probe is used with depth markings from 3.5 to 5.5 mm. The probe

is inserted into the sulcus between a tooth and the gingiva until it meets resistance. The surrounding area is then explored with the probe to determine the maximum depth of the pocket. Multiple areas around each tooth are probed. Scores range from 0 to 4 in order of increasing severity. When the CPITN method was employed, we considered those with Class 4 only. We excluded studies in which the study population was reported as the number of sextants rather than the number of individuals surveyed.

In 2007, a new CDC proposal for gold standard diagnosis of severe, chronic periodontitis was published. This standard specified that a more strict definition of the condition should be implemented. This more exclusive definition of chronic periodontal disease includes ≥ 2 interproximal sites with AL ≥ 6 mm ***AND*** ≥ 1 interproximal site with PD ≥ 5 mm [2].

We included the following definitions of severe periodontal disease commonly found in the literature:

1. Community Periodontal Index of Treatment Needs (CPITN) – Class 4 only
2. Clinical Attachment Loss (AL) > 6mm
3. Clinical Attachment Loss (AL) > 5mm
4. Clinical Attachment Loss (AL) > 4mm
5. Gingival Pocket Depth (PD) > 5mm

If more than one type of data was included in a study, our first preference was for CPITN = 4, followed by AL >6 mm, with PD >5 considered the least accurate representation of the GBD case definition. AL > 6mm was preferred over AL > 5mm, followed by AL > 4mm. All definitions were extracted for each datum as available this time, and a series of study covariates were used to crosswalk non-standard definitions to the reference standard of CPITN stage 4 (see below).

Input data

Model inputs

For GBD 2010, a review of the literature on periodontal disease prevalence was conducted by the Expert Group. A new systematic review was conducted for GBD 2013. The GBD 2013 literature review used the following search terms: (Peridontal disease[Title/Abstract]) OR (periodontitis[Title/Abstract]) OR (periodontal[Title/Abstract]) AND (prevalence[Title/Abstract]) AND ("2010"[Date - Publication] : "2013"[Date - Publication]).

In both systematic reviews, there was a hierarchical preference for case definitions: if more than one type of data was included in a study, our first preference was AL followed by PD, with CPITN considered the least accurate representation of the GBD case definition. Updates to systematic reviews are performed on an ongoing schedule across all GBD causes; an update for chronic periodontal disease will be performed in the next one to two iterations.

	Prevalence	Incidence	Mortality risk
Studies	100	-	4
Countries/subnationals	49/16	-	3/1
GBD world regions	18	-	2

Modeling strategy

Overview

Evidence for chronic periodontal disease being a direct, proximate cause of death is lacking. As such, it was not included in overall causes of death analysis. However, there is a developing body of literature to suggest that those with chronic periodontal disease may be at increased risk of death from other causes. Relative risk data were, therefore, included in modeling of morbidity, but overall years of life lost (YLLs) were estimated to be zero. Models of disease burden due to chronic periodontal disease instead focused on estimating morbidity (YLDs) associated with the condition, and chronic periodontal disease was not included in risk factor analysis of any other condition.

Correction for edentulism

Bias in the dataset was felt to be limited, but some systematic bias was present in the definition of the study populations. In virtually all studies, edentate persons were excluded from evaluation. This exclusion is justified in the context of periodontal disease surveillance because advanced periodontal disease is not common in those who are toothless. To account for the systematic bias inherent in excluding those with severe tooth loss from the denominator, we discounted the prevalence numbers estimated by DisMod MR 2.1. For example, if 40% of 70-74 year old females were estimated to be edentate in a certain region, the corresponding estimates for advanced periodontal disease prevalence were reduced to 60% of the original value.

DisMod model development

Mortality was fixed to zero and relative risk was fixed to 1.0 before age 30, as any excess cardiovascular events that occur in those with severe tooth loss would not be expected at young ages. Incidence and prevalence were assigned to be zero until age 8 as periodontal disease is largely considered to be a disease of adulthood. Incidence was allowed to rise beginning at age 9, based on the youngest age at which there was a non-zero point estimate for prevalence in the dataset.

Bounds were assigned for remission and excess mortality to improve plausibility in the DisMod estimates. Remission was bounded 0 to 0.05 and excess mortality rate was bounded to 0.0001. We considered both bounds to be within reasonable ranges for the observed natural history of the disease.

Study-level covariates were created for whether the data use a case definition of CPITN class III periodontal disease as opposed to the reference definition of class IV, and for whether the data use a case definition of attachment loss >=5 mm as opposed to the reference definition of >= 6mm. We did not identify any studies for which the only case definition reported was attachment loss >= 4mm. Reproductive age-standardized smoking prevalence was used as a country-level covariate.

Models were vetted based on the biological plausibility of the results, the extent to which estimates fit the data, and the plausibility of the range of estimates across location hierarchies.

Study covariate	Parameter	beta	Exponentiated beta

Data correspond to those with CPITN class III periodontal disease	Prevalence	0.21 (0.0062 – 0.58)	1.23 (1.01 – 1.79)
Data correspond to those with attachment loss >= 5 mm	Prevalence	0.89 (0.64 – 1.13)	2.43 (1.90 – 3.11)
**Country covariate**	**Parameter**	**beta**	**Exponentiated beta**
Smoking prevalence (reproductive age-standardized)	Prevalence	0.11 (0.0054 – 0.20)	1.11 (1.01 – 1.22)

Disability calculation and YLDs

Because those who are edentate cannot have advanced periodontal disease, we corrected for toothlessness as described above. Using the DisMod estimates for prevalence of edentulism, we calculated the mean prevalence for each age and sex and averaged the 1990 and 2015 values. We then calculated a population-weighted mean prevalence for each region followed by the same for each GBD super-region. The resulting super-regional averages were used to adjust the estimates for prevalence of advanced periodontal disease in calculating years lost due to disability (YLDs).

We considered all estimated prevalent cases of chronic periodontal disease to experience the disability described by “bad breath, a bad taste in the mouth, and gums that bleed a little from time to time, but this does not interfere with daily activities.” The GBD Disability Survey differentiated between those who experience pain and those who do not, but the calculated disability weight was the same for both forms of the condition, 0.007 (0.003 – 0.014).

Edentulism and severe tooth loss

Flowchart

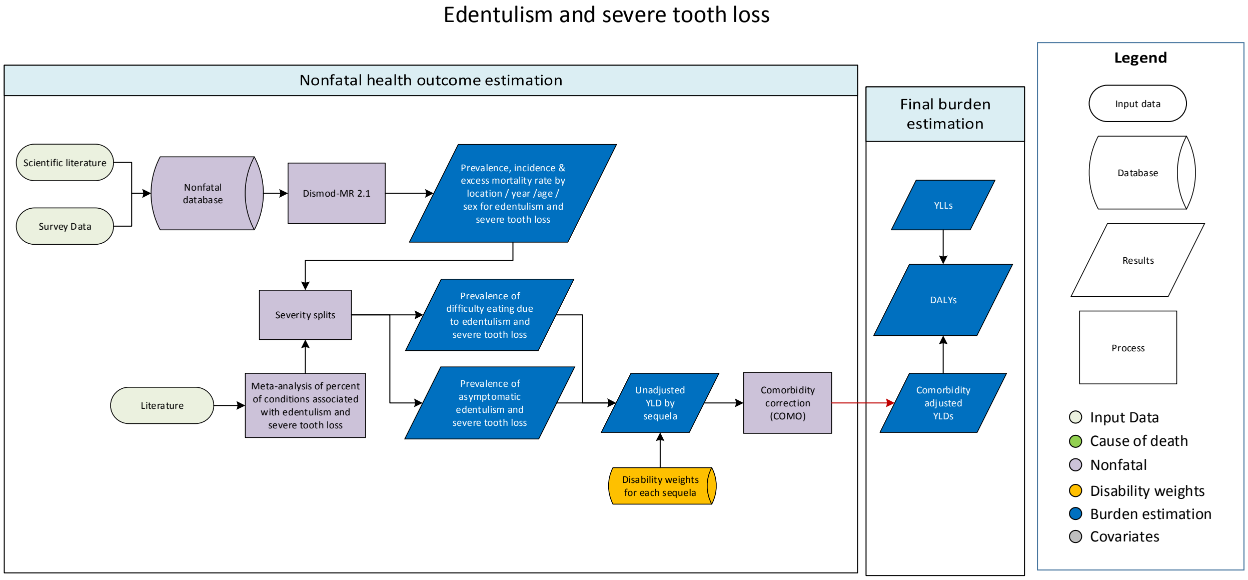

Case definition

The case definition of edentulism and severe tooth loss includes any individual with fewer than nine remaining permanent teeth; toothlessness of infancy is not included. The assessment of this disease includes quantification of the prevalence of the disease as well as estimation of the major sequelae: asymptomatic toothlessness and symptomatic toothlessness leading to "great difficulty in eating meat, fruits, and vegetables." A small body of evidence has begun to emerge that implicates edentulousness as predisposing individuals to increased risk for ischemic cardiovascular events including myocardial infarction and stroke. These data are sparse but have been included in models estimating burden of major tooth loss. However, edentulism was not included in the risk factor analysis for ischemic cardiovascular diseases.

Input data

Model inputs

An initial literature review was done by the Expert Group for GBD 2010, including published articles as well as the results of national and subnational reports. A new systematic review was completed for GBD 2013. The search terms for this systematic review included: (Edentulism[Title/Abstract]) OR (edentulous[Title/Abstract]) OR (endentulousness[Title/Abstract]) OR (severe tooth loss[Title/Abstract]) OR (total tooth loss[Title/Abstract]) OR (complete tooth loss[Title/Abstract]) AND (prevalence[Title/Abstract]) AND ("2010"[Date - Publication] : "2013"[Date - Publication]).

While an additional literature review was not performed for GBD 2015, new World Health Survey data were added for 47 countries. Updates to systematic reviews are performed on an ongoing schedule across all GBD causes; an update for edentulism and severe tooth loss will be performed in the next one to two iterations.

Bias in the dataset was considered to be negligible. Diagnostic criteria for this condition are very clear (< 9 teeth). Additionally, all included studies are considered representative of the study population. Thus, covariates to account for excess variability were not deemed necessary. Few data points were marked as outliers during the modeling process.

	Prevalence	Incidence	Mortality risk
Studies	157	11	11
Countries/subnationals	76/11	5/4	4/5
GBD world regions	20	4	3

Modeling strategy

Overview

First, estimates for the prevalence of edentulism and severe toothlessness were calculated for each location/year/sex/age using DisMod-MR 2.1. Then, estimates of the proportion of the population with access to dentures were generated for each location, and the disability weight for "great difficulty in eating meat, fruits, and vegetables" was applied to the proportion of the population with edentulism and no access to dentures.

DisMod model development

As would be expected for an irreversible condition, remission was fixed at zero for all ages. Mortality and relative risk were both fixed at zero before age 30, as any excess cardiovascular events resulting from severe tooth loss would not be expected at younger ages. We also assigned incidence and prevalence to be zero during childhood. Incidence was allowed to rise beginning at age 15, which was chosen based on the age at which the permanent dentition is expected to have fully formed in all individuals. The random effect limits for all locations were bounded at +/- 1.

As mentioned above, the criteria for diagnosis of edentulism are straightforward, and bias in the dataset was considered negligible. Thus, no study-level covariates were used in modeling the prevalence of edentulism. We included lnLDI as a country-level covariate, with a minimum beta value of 0.02; see results in the table below.

Country-level covariate	Parameter	beta	Exponentiated beta
lnLDI ($ per capita)	Prevalence	0.026 (0.020 — 0.042)	1.03 (1.02 — 1.04)

Models were vetted based on the biological plausibility of the results, the extent to which estimates fit the data, and the plausibility of the range of estimates across location hierarchies. We have made no substantive changes in the modeling strategy from GBD 2013.

Disability weights

The disability weight used for symptomatic toothlessness leading to "great difficulty in eating meats, fruits, and vegetables" is 0.067 (0.045 – 0.095) as determined by the GBD Disability Survey. We considered all those with severe tooth loss and no access to dentures to experience this disability.

However, the proportion of those with edentulism and severe tooth loss who have dentures has not been studied extensively.

In order to estimate the proportion of edentulous individuals with no access to dentures, we completed a supplemental literature review of dentures prevalence. Only six systematic surveys of dentures prevalence were identified, all in high- and middle-income countries. All were completed since 2000. After extracting the data from the studies, we performed linear regressions of denture presence and denture absence against health system access (HSA), a standardized covariate of treatment availability used in many disease estimation models. From the results of the regression, the prevalence of no dentures was calculated for all super-regions. We then completed a population-weighted average of all countries in the super-region based on 2003 populations, the average year of the dentures studies. Uncertainties for the prevalence of dentures were calculated by finding the standard deviation and standard error of the calculated prevalence values.

The estimated prevalence of dentures in each location was used to calculate the proportion of individuals with asymptomatic edentulism and severe tooth loss (e.g., those who have access to dentures) and difficulty eating due to edentulism and severe tooth loss (e.g., those without access to dentures). This latter sequela was included as a cause of years lost due to disability (YLDs).

Other oral disorders

Other oral disorders encompass a wide variety of dental, tongue, and jaw disorders and malformations, including all oral disorders that are not included in the case definitions of permanent or deciduous dental caries, periodontal disease, or edentulism and severe tooth loss. All data on the prevalence of other oral disorders were obtained from the United States Medical Expenditure Panel Surveys, a nationally representative survey conducted yearly from 1996 to 2011 by the US Agency for Healthcare Research and Quality.

These data were modeled in DisMod-MR 2.1 using a prevalence-only model with age mesh points set at 0, 0.5, 1, 5, 10, 20, 30, 40, 50, 60, 70, 80, 90, and 100 years of age. Heterogeneity for prevalence was set to the default of 0.5, and smoothness for prevalence was set to the default of 0.3. No study-level or country-level covariates were used in this model other than the study-level covariate for sex, which was fixed at the super-region level. This model provided us with estimates of the prevalence of other oral disorders for every location/age/sex combination.

Injuries

Flowchart

Nonfatal Injuries

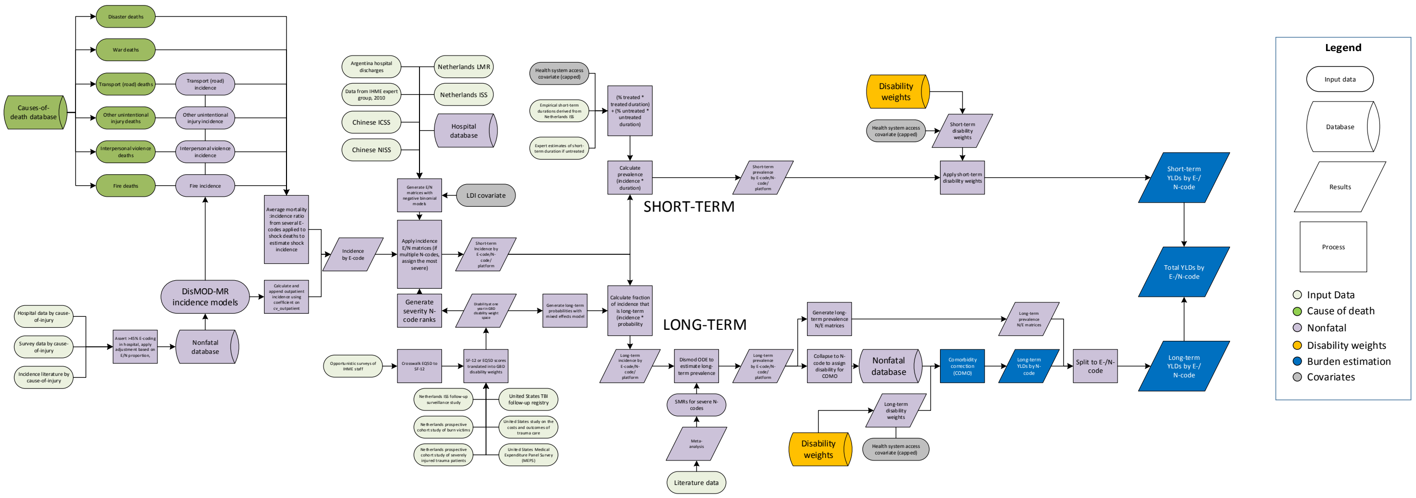

Case definition

For GBD 2015, the Injuries estimation process for non-fatal health outcomes encompasses a range of 28 causes, including transport injuries, falls, drowning, self-harm, interpersonal violence, and animal contact. Each of these causes can include multiple modelable entities, which vary by cause. Injury incidence is defined using ICD-9 codes E000-E999 and ICD-10 chapters V to Y. For non-fatal estimation, Chapters S and T in ICD-10 and codes 800-999 in ICD9 are used to estimate morbidity.

Input data

Model inputs

For GBD 2015, to estimate morbidity from injuries, the injuries team used data from hospital, emergency department records, and surveys to produce years lost to disability (YLDs) by country, year, sex, age, external cause-of-injury, and nature of injury category.

Unfortunately, quite a few countries report their data using a mix of cause of injury and nature of injury codes. In order to retain as much of the data as possible, the team included all data sets that had at least 45% of cases coded to the cause of injury. The threshold of 45% was chosen as there were a lot of data sets coded equally to cause of injury and nature of injury categories. The team increased the cause specific injury cases from these datasets proportionately to sum to the total number of injury cases.

Conflict and war data was obtained from the Uppsala Conflict Data Program [17], the International Institute for Strategic Studies [18] and vital registration systems. Disaster data was derived from the International Disaster Database from the Center for Research on the Epidemiology of Disasters [19].

Data searches

For GBD 2015, hospital and emergency department records were supplemented with more recent and available site-years. Additionally, a list of sources, which was comprised primarily of reports and surveys, that could be incorporated into non-fatal estimates of injuries was reviewed prior to estimation.

Infrequently, data points were marked as outliers. Reasons for this were that the data point did not follow the age or time pattern as expected and/or if the incidence rate of people sustaining an injury from a certain cause of injury was not plausible.

Modeling strategy

Two categories of injury severity are separately modeled for GBD 2015, and these include: injuries warranting inpatient care and injuries warranting other health care. Injuries warranting inpatient care refer to injury cases of sufficient severity to require inpatient care, if there are no restrictions in access to health care. Injuries warranting other health care refer to injury cases of sufficient severity to require health care attention but not hospitalization. This category includes emergency department visits. In order to best measure the burden of injuries, the GBD 2015 estimates exclude trivial injuries by restricting morbidity analysis to cases warranting some form of health care in a system with full access to health care. We have included cases with injuries that did not receive care in areas with restricted access to health care, but that would have warranted some type of health care in a system with full access to health care.

Cause-of-injury incidence

The list of cause-of-injury categories has increased to 27 for GBD 2015 following the addition of "Exposure to environmental forces, non-disaster." The majority of incidence data exists at the external cause-of-injury level. Thus, incidence for cause-of-injury categories is modeled using DisMod-MR 2.0. DisMod-MR 2.0 is a descriptive epidemiological meta-regression tool that uses the integrative systems modeling approach to produce simultaneous estimates of disease incidence, prevalence, remission, and mortality. Multiple datasets from hospital, emergency/outpatient departments, and survey datasets are fed into these incidence models. We separately estimated two categories of injury severity: inpatient and outpatient injuries. For GBD 2015 we used up three covariate in each DisMod-MR model as a multiplier from inpatient to outpatient incidence, namely covariates "outpatient," "in- and outpatient," and "medcare" respectively. The covariates that were used in DisMod were dependent on the data that are fed into the models.

However, DisMod-MR 2.0 is not used to model exposure to forces of nature (i.e., natural disaster) and collective violence and legal intervention (i.e., war), also called the shock cause-of-injury categories, due to the sporadic nature of incidence rates and the lack of a time-trend in incidence rate for such injuries. To estimate incidence from the shock cause-of-injury categories, the mortality rate for these cause-of-injury categories is multiplied by the average country-year-age-sex-specific incidence-to-mortality ratio within several cause-of-injury categories that likely exhibit similar case fatality ratios (e.g.,. road injuries, fires, interpersonal violence, and other unintentional injury).

Follow-up studies

A change from GBD 2013 is that we used follow-up data obtained from a pooled dataset of six follow-up studies from China, the Netherlands, and the US, which followed up patients for at least one year after the injury and the Medical Expenditure Panel Survey (MEPS) [20-27].

Table XX Details of injury follow up surveys used in GBD 2015

Dataset	Year	Type of data collected	Type of patients	Setting	Sample size* and response
Guangdong follow up survey, China[#]	2006-2007	Follow up survey among sample of ISS patients	Patients (15+ years) who were hospitalized that had been injured by road traffic injury, fall, blunt or penetrating trauma	Based on three national injury surveillance hospitals in Zhuhai, Guangdong Province in China	998 (response 87%)
LIS follow up survey, Netherlands[1]	2001-2002	Follow up survey among stratified sample of ISS patients (oversampling less common, severe injuries)	Patients (15+ years) who visited the Emergency Department of a hospital and were discharged to the home environment and patients who were admitted to hospital	Based on 17 public hospitals in the Netherlands	8564 (response 37%)
LIS follow up survey, Netherlands[2]	2007-2008	Follow up survey among stratified sample of ISS patients (oversampling less common, severe injuries)	Patients (15+ years) who visited the Emergency Department of a hospital and were discharged to the home environment and patients who were admitted to hospital	Based on 15 public hospitals in the Netherlands	8057 (response 36%)
NSCOT – National study on Costs and Outcomes of Trauma, USA[4]	2001-2002	A prospective cohort study was conducted among a sample of adult trauma patients treated at Level I trauma centers and non-trauma center hospitals	Patients treated for a moderate to severe injury (as defined by at least one injury of an Abbreviated Injury Scale (AIS) score of 3 or greater	Based on 69 hospitals in 12 states in the US	5191 (response 61%)

Dataset	Year	Type of data collected	Type of patients	Setting	Sample size* and response
SCTBIFR – South Carolina Traumatic Brain injury Follow-up Registry, USA[5]	1999-2002	A prospective cohort study was conducted among injured in-patients with a traumatic brain injury-related injury	Patients (15+ years) who were admitted to hospitals and met the CDC case definition of TBI—trauma to the head associated with altered consciousness, amnesia, neurological abnormalities, skull fracture, intracranial lesion, or death	Discharged from all nonfederal in-state acute care hospitals	7613 (response 28%)
Burns outcome study, Netherlands[6]	2003-2006	A multicenter prospective cohort was conducted among adult (severe) burn patients	Injury patients who sustained severe burns	Three public hospitals with specialized burn units.	311 (response 78%)

*number of patients that met the inclusion criteria; response rate = percentage of patients who responded to the follow-up survey (in case of multiple follow-up times the response rate of the first follow-up moment is reported).

[#] data from CDC China, jointly analysed by study authors from IHME and China CDC

MEPS is a large scale overlapping continuous panel survey of the US non-institutionalized population that collects information on use and cost of health care and SF12 responses [28]. SF-12 responses are elicited twice over the two-year period that any individual is part of the study. Thus, MEPS offered the benefit of including health state measures of non-injured and destined to be injured and the benefit of having pre-injury and post-injury SF-12 responses. We pooled all available MEPS data over a 19-year span.

The follow-up studies used different patient reported outcome measures to assess healthstatus, namely the SF-36, Version 1 SF-12, and the EQ-5D [29-31]. To enable comparison across the six datasets, it was necessary to analyze the data in a standardized patient reported outcome measure. First, we mapped all patient reported outcome measures to Version 2 SF-12 (SF-12v2). Second, we normalized the health status measurements by mapping the SF-12 scores to a corresponding disability weight based on several opportunistic surveys asking respondents to score SF-12 based on the lay descriptions for a selection of 60 GBD healthstates [12]. We ran a regression of logit-transformed disability weight on nature-of-injury category and individual characteristics.

Nature-of-injury category hierarchy

Multiple injuries can occur in one individual. For GBD 2015, a nature-of-injuries severity hierarchy was developed to establish a one-to-one relationship between cause-of-injury and nature-of-injury category. This means that in case of multiple injury the nature-of-injury category that was likely to be responsible for the largest burden was selected. To construct the hierarchy we used data from the pooled dataset of follow-up studies. The output of the regression of logit-transformed disability weight on nature-of-injury category and individual characteristics of the follow-up studies were used to calculate the mean long-term disability attributable each nature-of-injury category. The ranking of nature-of-injury categories by

their long-term disability weights formed the basis for our severity hierarchy. Hierarchies were developed separately, for injuries warranting inpatient care and injuries warranting other health care.

Cause-nature matrices

Due to the fact that injury disability is linked more to nature-of-injury and less to cause-of-injury, transition matrices were generated to map the proportion of each cause-of-injury category that results in a particular nature-of-injury category. These matrices are based on a collection of dual-coded (e.g., both cause-of-injury and nature-of-injury coded) hospital and emergency department datasets. The data for this step came from inpatient, outpatient, and emergency room discharge data from Argentina, Bulgaria, China, Colombia, Cyprus, the Czech Republic, Denmark, Egypt, Estonia, Hungary, Iceland, Iran, Italy, Latvia, Malta, Mauritius, Mexico, Mozambique, the Netherlands, Norway, Portugal, Slovenia, Spain, Sweden, Macedonia, Uganda, US, and Zambia. We applied our nature-of-injury severity hierarchy to assert that every observation had one cause-of-injury and one nature-of-injury. Negative binomial models were used to estimate the probability of each nature-of-injury category resulting from each cause-of-injury category. One cause-nature matrix was created for each combination of injury warranting hospital admission versus injury warranting other health care, high/low income countries, male/female, and age category. Models were run with progressively fewer covariates until they converged, and nature-of-injury category proportions were squeezed post-hoc to sum to 1 within each cause-of-injury category. Applying these matrices to our cause-of-injury incidence from DisMod-MR, we produced cases of injury warranting hospital admission and incidence of injury warranting other health care by cause and nature of injury.

Probability of permanent health loss

Disability due to injury is assumed to affect all cases in the short term with a proportion having long-term (permanent) outcomes. The probability of long-term outcomes is needed to estimate the incidence and subsequently the prevalence of cases with permanent health loss. In our conceptual model, individuals who suffer a non-fatal injury will, in the long-term, return to either full or partial health. If one year post-injury patients return to a health status with more disability than their pre-injury health status, injury patients are assumed to have permanent disability from their injury. The difference between the pre-injury health states and health status one year after injury is assumed to be their permanent level of injury-related disability. We assessed the probability of developing permanent health loss using the pooled dataset of follow-up studies that was involved in the generation of our nature-of-injury hierarchy and the Medical Expenditure Panel Survey (MEPS). To assess the probability of permanent health loss we estimated the effects using a logit-linear mixed effects regression:

$$\text{Logit}\ (DW)_{im} = \alpha + \beta(\text{age}_i) + \beta(\text{injuries}_{im}) + \beta(\text{never injured}_i) + \beta(\text{never injured}_i * \text{age}_i) + \beta(\text{fracture of pelvis}_i * \text{age}_i) + \beta(\text{fracture of pelvis}_i * \text{age}_i) + \beta(\text{poisoning}_i * \text{age}_i) + \beta(\text{moderate/severe TBI}_i * \text{age}_i) + RE_c + RE_i,$$

where we included dummies for all the nature-of-injury categories (injuries_{im}), with the reference category being no injury (from MEPS dataset). We also include a dummy for never injured prior to the current injury, age, interactions between age and never injured status, and interactions with three long-term nature-of-injury categories that were found to significantly vary with age: pelvis fractures, poisonings and moderate/severe traumatic brain injuries. In notation, subscript m refers to patient reported outcome measure, i refers to individual and c refers to country. Random effects (RE) were included to control for variation between countries and individuals.

After predicting overall disability at one year follow-up, we estimated a counterfactual by setting all observations to “no injury,” the reference group for $\beta(\text{injuries}_{im})$ in our model. The disability attributable to the nature-of-injury at one year was assumed to be the difference between our counterfactual of no injury and predicted disability with injury. The probability of treated long-term outcomes is estimated via the ratio of this attributable disability relative to the long-term disability weight for that injury.

$$\text{Probability of long-term disability} = (\text{with injury disability}_{im} - \text{counterfactual disability}_{im})/DW_m$$

We developed estimates of the probability of permanent health loss by nature-of-injury category, injury severity level (injuries warranting inpatient admission and injuries warranting other health care) and age. Depending on the nature-of-injury category, the probability of developing long-term outcomes from an untreated injury was either assumed to be equivalent to that of the corresponding treated injury or was increased by a scaling factor suggested by a trauma surgeon with experience in a low-income country and reviewed by all GBD experts on injuries. Using a proxy covariate that defines health system access based on a combination of vaccination rates, proportion of deliveries by a skilled birth attendant, in facility birth, and antenatal care, we estimated the ratio of treated to untreated injuries for each country-year grouping and assigned a country-year-specific probability of permanent health loss equal to a weighted average of the treated and untreated probabilities for each nature-of-injury category/severity-level grouping.

Despite applying the nature-of-injury hierarchy to the follow-up datasets, we still observed implausibly high estimates of long-term disability for several outpatient nature-of-injury categories. We made the decision to ignore any long-term disability from outpatient injuries in the following categories: open wound, poisoning, and contusion.

Disability associated with treated and untreated cases

For many nature-of-injury categories GBD 2015 has a separate disability weight for treated and for untreated cases [14]. Similar to the strategy we employed while estimating the probability of permanent health loss, we used a proxy of health system access to determine the ratio of treated to untreated cases for a given country-year and then assigned a country-year-nature-of-injury category-specific disability weight equal to a weighted average of the treated and untreated disability weight values.

Duration of short-term health loss

To determine the duration for treated cases of short term injury we analyzed patient responses of two Dutch Injury Surveillance System follow-up studies of 2001-2003 and 2007-2009 [20, 21]. These studies collected data at 2.5, 5, 9, and 12 months post-injury on whether injury patients were still experiencing problems due to their injury [20, 21]. If not, the patients were asked how many days they had experienced problems. The injury patients that still reported having problems one year after the injury were assumed to be captured in our analysis of permanent disability. The duration for treated cases of short term injury was estimated for injuries warranting inpatient admission and injuries warranting other health care separately. The estimates were supplemented by expert-driven estimates of short-term duration for nature of injury categories that did not appear in the Dutch dataset and untreated injuries.

Calculation of prevalence from incidence data – short term injury

For short-term injury outcomes, which were assumed to be less than one year in duration, the prevalence for each cause-of-injury/nature-of-injury/severity-level grouping was approximated by the incidence for that grouping multiplied by the associated nature-of-injury/severity-level-specific duration.

Calculation of prevalence from incidence data – permanent health loss

For permanent health loss, we assumed no remission and thus were forced to integrate incidence over time to arrive at prevalence estimates. We used DisMod-MR 2.0 to carry out this integration for each combination of cause of injury and nature of injury. For this step we used random effects meta-analysis to pool data on standardized mortality ratios derived from literature reviews for spinal cord injury, burns covering more than 20% of the body, moderate to severe traumatic brain injury, hip fracture, and multiple significant injuries (see for more detail [12]). For all other nature-of-injury categories, we assumed no long-term excess mortality. For “exposure to forces of nature” and “collective violence and legal intervention” DisMod-MR 2.0 was unable to generate accurate estimates due to the lack of a smooth trend in incidence over time. For these two cause-of-injury categories, we coded the differential equations from Dismod-MR 2.0 that determine the relationship between incidence, remission, mortality risk, and prevalence into Python and streamed out the prevalence from the incidence in the years of war or disaster by integrating over one year at a time.

No other significant changes were made to the injuries modeling process for GBD 2015.

Anemia impairment envelope

Flowchart

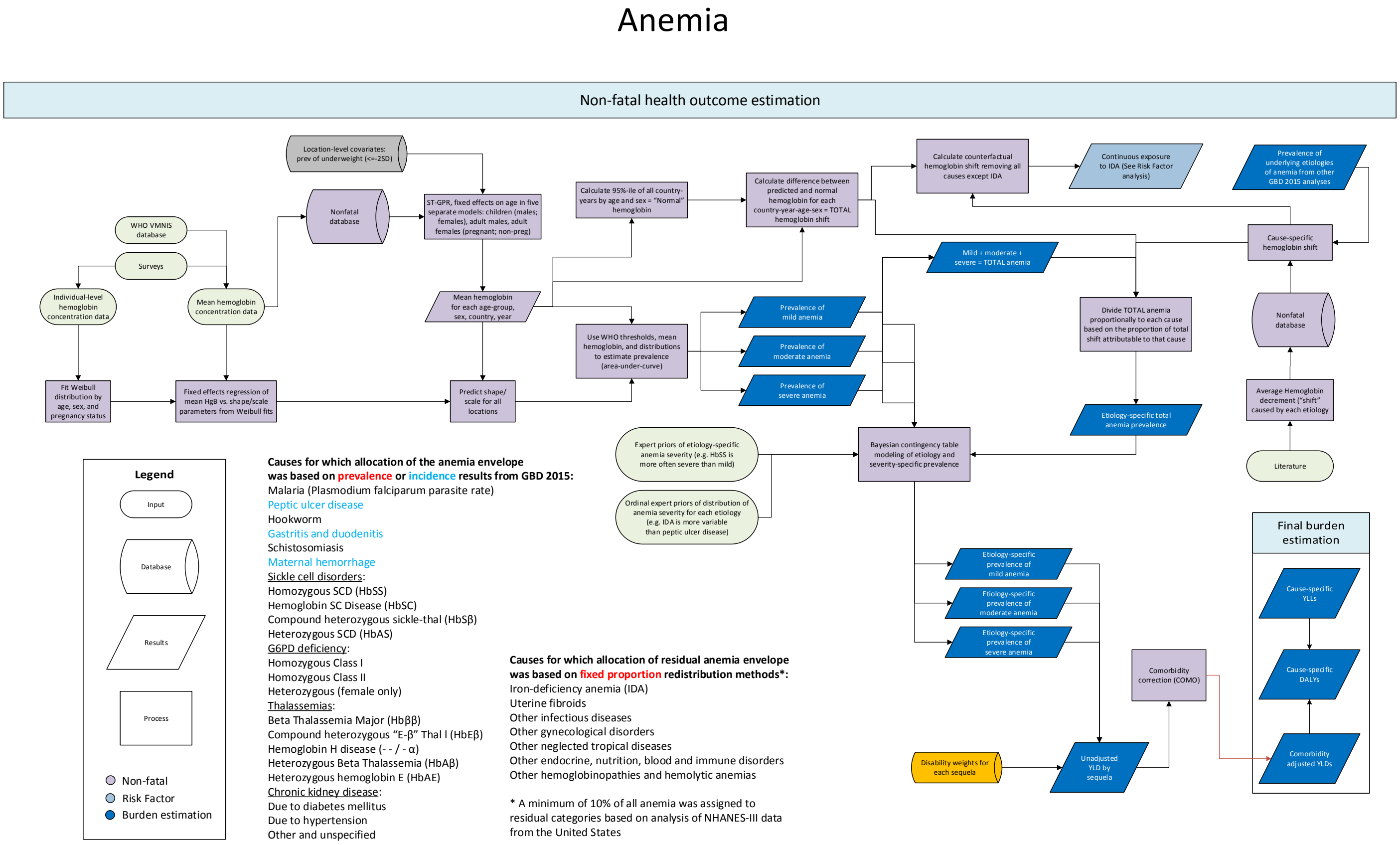

Input data and methodological summary

Case definition

To estimate anemia in GBD 2015, we employed the same method used in GBD 2013[1] and largely similar to GBD 2010[2]. Our analytic strategy began with calculation of an anemia envelope – a determination of mean hemoglobin, as well as sum total of anemia prevalence, by severity for each country, age group, and both sexes for each year from 1990 through 2015. The envelope approach avoids double counting while capturing potentially different disease profiles within each population group. We defined a population group as a specific geography, sex, age-group, and year.

Input data

Model inputs

The envelope approach to the anemia impairment utilizes data from a variety of sources. Population-based surveys of hemoglobin concentration were the primary input to our analytic dataset. Examples include the Demographic and Health Survey (DHS) and Multiple Indicator Cluster Survey (MICS) series, along with other national and subnational surveys that completed hemoglobin testing. We supplemented with pertinent sources downloaded from the WHO Vitamin and Mineral Nutrition Information System

(VMNIS) available at http://www.who.int/vmnis/database/anaemia/countries/en/. Most used a HemoCue test, adjusted for altitude, and excluded those with terminal or acute medical conditions. Inclusion, exclusion and diagnostic criteria for other studies were similar and can be found in each study.

Modelling strategy

We predicted mean hemoglobin levels for all missing population groups using a mixed-effects regression with fixed effects on prevalence of severe underweight (<2SD below mean; same as GBD 2010 and GBD 2013) and age group and nested random effects on super-region, region, and country/subnational site. We again separated each population into five groups: male and female children under 5 years, pregnant females, non-pregnant females, and males over 5 years. We pooled survey microdata for each population group separately and fitted the pooled data with Weibull distributions. We then performed OLS regression of the shape and scale parameters versus all predicted mean hemoglobins predicted above. The prevalence of mild, moderate, severe, and total anemia was then calculated by determining the area under the Weibull curve for each population group using appropriate context-specific hemoglobin thresholds. Thresholds for defining anemia by age and sex were the same as used in GBD 2013 and are consistent with current WHO definitions[3] as shown in the table below with the exception that we have defined separate thresholds in neonatal age groups to better capture the contribution of fetal hemoglobin on hemoglobin concentration in these age groups[4].

Table 1. Severity definitions and corresponding disability weights used to calculate GBD 2013 anemia envelope

	Severity of anemia		
	Mild	Moderate	Severe
**Age < 1 month**			
Males	130 - 149 g/L	90 - 129 g/L	< 90 g/L
Females	130 - 149 g/L	90 - 129 g/L	< 90 g/L
**Age 1 month - 5 years**			
Males	100 - 109 g/L	70 - 99 g/L	< 70 g/L
Females	100 - 109 g/L	70 - 99 g/L	< 70 g/L
**Age 5 - 14 years**			
Males	110 - 114 g/L	70 - 99 g/L	< 70 g/L
Females	110 - 114 g/L	70 - 99 g/L	< 70 g/L
**Age 5+ years**			
Males	110 – 129 g/L	80 - 109 g/L	< 80 g/L
Females, non-	110 - 119 g/L	80 - 109 g/L	< 80 g/L
pregnant Females,	100 – 109 g/L	70 - 99 g/L	< 70 g/L

We performed cause-specific attribution on the anemia envelope using information on cause-specific prevalence and hemoglobin shift using the same method as in the GBD 2010. Total “hemoglobin shift”

was determined as the difference between the normal and predicted mean hemoglobin levels for each population group. We denoted the normal hemoglobin level as the global 95th percentile of the distribution of mean-hemoglobin within each age- group, sex, and year. We then determined a total shift for each country in the corresponding age-group, sex, and year by finding the difference between the global "normal" and the country-specific predicted mean hemoglobin. Our model of attribution followed that, because the shift is a disease state experienced by 100% of the population, then the sum of cause-specific hemoglobin shifts times the prevalence of each contributing cause should add up to the total. We summed shift times prevalence estimates from all causes, compared to the total predicted hemoglobin shift, and proportionally assigned. We distributed the residual envelope among seven remaining causes. Of note, our iron-deficiency anemia (IDA) estimates include acute and chronic hemorrhagic states for which supplementation may be helpful, but poor nutritional intake is not the only underlying problem. A few causes in this category – hookworm, schistosomiasis, upper gastrointestinal bleeding, and gynecologic diseases – were considered separately from IDA because there was enough data from GBD prevalence estimation processes to do so. Distribution of anemia burden to IDA only after assignment to "known" causes avoided double counting of these cases. Most other causes of anemia not specifically considered were included in the "other" categories. For all causes with specific population-specific prevalence estimates, we enforced a condition where the sum of mild, moderate, and severe anemia would not exceed the total prevalence within each population. Additionally, because inherent in our method of determining "normal" hemoglobin is the fact that 5% of population groups will have zero, or negative, total shift, we assigned a minimum of 10% of all anemia to be assigned to residual causes based on review of findings from National Health and Nutrition Examination Survey (NHANES) in the United states[5,6].

We again used Bayesian contingency table modelling methods developed for GBD 2013 to disaggregate marginal estimates of anemia severity and aetiology into a complete set of prevalence estimates for aetiology/severity pairs. Marginal estimates of column sums (total anaemia prevalence by severity [mild, moderate, severe]) and row sums (total aetiology prevalence for each cause) were paired with priors on the etiology-specific hemoglobin shifts (the same as were used for overall etiologic attribution) and rank order of variation of severity (e.g. malaria-induced anaemia severity is highly variable while that due to homozygous sickle cell disease is less so). Nonlinear optimization methods were then used to populate a complete matrix of aetiology-severity estimates from the marginal estimates and distribution priors. We found the maximum a posteriori (MAP) point estimate for XX samples from estimated posterior distributions independently for each population group, then scaled the results to ensure row sums were non-zero and column sums matched the original draws.

References

1. Kassebaum NJ. The Global Burden of Anemia. *Hematology/Oncology Clinics* 2016; **30**: 247–308.
2. Kassebaum NJ, Jasrasaria R, Naghavi M, *et al.* A systematic analysis of global anemia burden from 1990 to 2010. *Blood* 2014; **123**: 615–24.
3. WHO | Haemoglobin concentrations for the diagnosis of anaemia and assessment of severity. Geneva: World Health Organization, 2011 http://www.who.int/vmnis/indicators/haemoglobin.pdf.

4. Kates EH, Kates JS. Anemia and polycythemia in the newborn. *Pediatr Rev* 2007; **28**: 33–4.

5. Centers for Disease Control and Prevention (CDC). Iron deficiency--United States, 1999-2000. *MMWR Morb Mortal Wkly Rep* 2002; **51**: 897–9.

6. Looker AC, Dallman PR, Carroll MD, Gunter EW, Johnson CL. PRevalence of iron deficiency in the united states. *JAMA* 1997; **277**: 973–6.

Epilepsy impairment envelope

Flowchart

Epilepsy

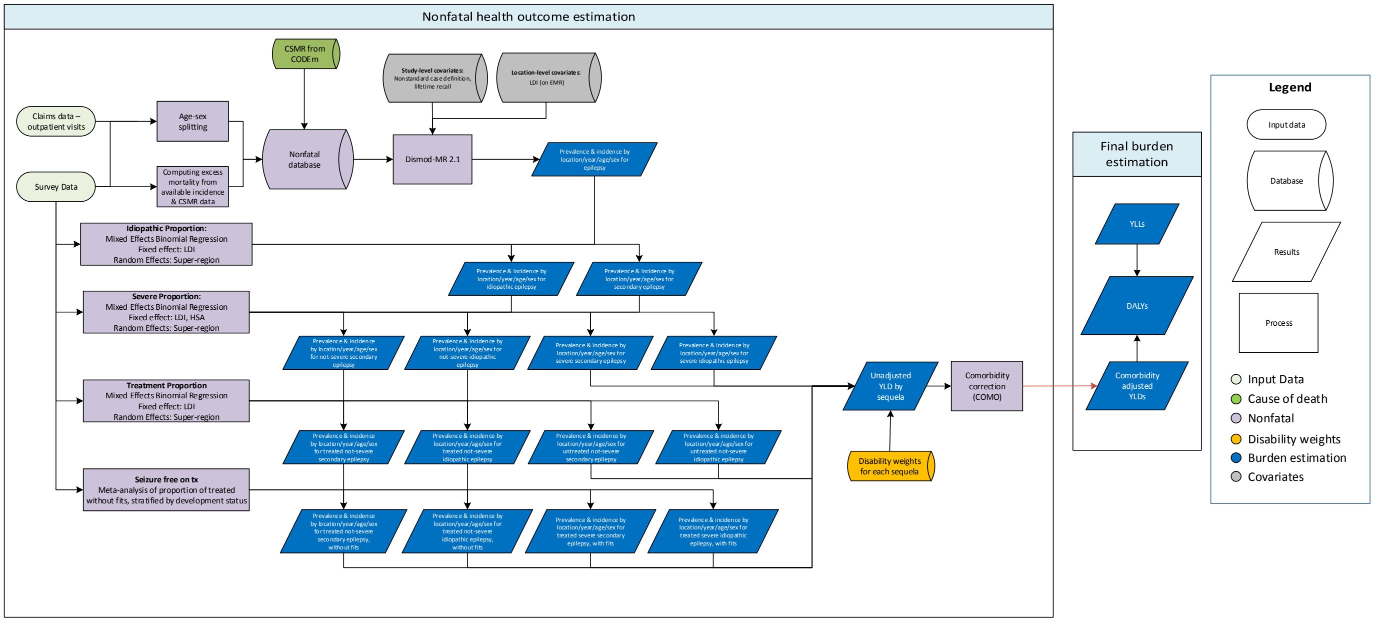

Case definition

For GBD 2013, we used the following definitions from the "Guidelines for Epidemiologic Studies on Epilepsy": 1) Epilepsy: a condition characterized by recurrent (two or more) epileptic seizures, unprovoked by any immediate identified cause, and 2) "Active" epilepsy: a prevalent case of active epilepsy is defined as a person with epilepsy who has had at least one epileptic seizure in the previous five years, regardless of antiepileptic drug (AED) treatment.

For GBD 2015, we used the following ICD-10 codes for epilepsy: G40 (Neuro, epilepsy, total) and G41 (Neuro, epilepsy, status epilepticus). We defined severe epilepsy as having seizures one or more times per month.

Input data

Model inputs

For GBD 2013, we conducted a systematic review from 2009-2013 using the following search string:

("Epilepsy"[Mesh] OR "Epilepsy, Partial, Motor"[Mesh] OR "Epilepsy, Benign Neonatal"[Mesh] OR "Epilepsy, Reflex"[Mesh] OR "Myoclonic Epilepsy, Juvenile"[Mesh] OR "Epilepsy, Frontal Lobe"[Mesh] OR "Epilepsy, Complex Partial"[Mesh] OR "Epilepsy, Post-Traumatic"[Mesh] OR "Epilepsy, Temporal Lobe"[Mesh] OR "Epilepsy, Absence"[Mesh] OR "Epilepsy, Tonic-

Clonic"[Mesh] OR "Epilepsies, Myoclonic"[Mesh] OR "Epilepsies, Partial"[Mesh] OR (epilepsy[Title/Abstract]) AND (incidence[Title/Abstract] OR prevalence[Title/Abstract]) AND (“2009”[Date - Publication] : “2013”[Date - Publication]))

For GBD 2015, we conducted a systematic review searching from 1/1/2013 to 7/5/2015 using the following search string and extracted 19 relevant studies:

('Epilepsy'[Mesh] OR 'Epilepsy, Partial, Motor'[Mesh] OR 'Epilepsy, Benign Neonatal'[Mesh] OR 'Epilepsy, Reflex'[Mesh] OR 'Myoclonic Epilepsy, Juvenile'[Mesh] OR 'Epilepsy, Frontal Lobe'[Mesh] OR 'Epilepsy, Complex Partial'[Mesh] OR 'Epilepsy, Post-Traumatic'[Mesh] OR 'Epilepsy, Temporal Lobe'[Mesh] OR 'Epilepsy, Absence'[Mesh] OR 'Epilepsy, Tonic-Clonic'[Mesh] OR 'Epilepsies, Myoclonic'[Mesh] OR 'Epilepsies, Partial'[Mesh] OR (epilepsy[Title/Abstract]) AND (incidence[Title/Abstract] OR prevalence[Title/Abstract]) AND ('2013'[Date - Publication] : '2015'[Date - Publication])) Sort by: PublicationDate Filters: Humans

We included representative, population-based surveys that reported of prevalence, incidence, remission rate, excess mortality rate, relative risk of mortality, standardized mortality ratio, or with-condition mortality rate. We excluded studies with no clearly defined sample (e.g., among clinic attenders or patient organization members with non-specific or non-representative catchment area).

For GBD 2015, we also extracted Marketscan 2000, 2010, and 2012 data from inpatient and outpatient facilities. We did not use inpatient hospital data from other countries, as inpatient facility visits cannot be used to estimate prevalence. The tables below detail the model inputs used to estimate the epilepsy impairment.

	data_sources	subnational_coverage	country_coverage	region_coverage	super_region_coverage
prevalence	306	60	82	19	7
incidence	81	18	36	15	7
mortality	27	5	19	10	6

Severity splits & disability weights

The table below illustrates the severity levels, descriptions, and disability weights associated with epilepsy. These are calculated using regressions from literature (ie., frequency of seizures).

Severity level	Lay description	Disability weights (95% CI)
severe (seizures >= once per month)	This person has sudden seizures one or more times each month, with violent muscle contractions and stiffness, loss of consciousness, and loss of urine or bowel control. Between seizures the person has memory loss and difficulty concentrating.	0.552 (0.375-0.71)
less severe (seizures < once per month)	This person has sudden seizures two to five times a year, with violent muscle contractions and stiffness, loss of consciousness, and loss of urine or bowel control.	0.263 (0.173-0.367)

Treated without fits	This person has a chronic disease that requires medication every day and causes some worry but minimal interference with daily activities.	0.049<br>(0.031-0.072)

Modeling strategy

We modeled the prevalence of epilepsy in two steps: first, we created an epilepsy impairment envelope. Second, we split the envelope into primary (or idiopathic) and secondary epilepsies. Each of these were subdivided into “severe” (on average 1 or more fits per month) and “non-severe.” Non-severe cases were sub-divided into “treated” and “un-treated.” Finally, “treated” cases were divided into “treated cases with fits” (between 1 and 11 fits on average in preceding year) and “treated cases without fits” (no fits reported in preceding year).

In the first step, we used the DisMod-MR tool for the epilepsy impairment envelope to model a consistent fit between incidence, prevalence, remission, and SMR data while using meta-regression to correct data points with non-reference study quality characteristics. We assumed a non-zero prevalence at birth to account for neonatal and congenital causes of epilepsy. We found no systematic bias for the covariate “non-standard case definition” indicating studies that did not define “active epilepsy” and added this covariate as a “z-cov” to the model which means a multiplier is applied to the standard error and thus is given less weight in the analysis than the “reference” data points. Unlike in the GBD 2013, we included data of life-time prevalence and therefore added a covariate on lifetime prevalence data points. We did not use sampling strategy as a z-cov because it did not have a significant effect. We included cause-specific mortality rate (CSMR) results from the epilepsy mortality model as input data to the Dismod model. Where age-specific prevalence data was available, we calculated excess mortality rate (EMR) from prevalence and CSMR. We included the log of the lag distributed income (LDI) as a covariate on EMR to account for lower mortality in developed countries. We included Bayesian priors on remission to account for the scarcity of remission data. We set bounds on remission from 0 to 0.25 from age 0-60 and 0 to 0.05 from age 61-100. The table below indicates the covariates used in the estimation process, as well as parameters, betas, and exponentiated betas.

Cause	Measure	Variable_Name	Beta	Exponentiated
Epilepsy impairment envelope	prevalence	Recall Lifetime	0.25 (0.20 - 0.30)	1.28 (1.22 - 1.35)
Epilepsy impairment envelope	prevalence	All MarketScan, year 2000	-0.76 (-0.94 - -0.69)	0.47 (0.39 - 0.50)
Epilepsy impairment envelope	prevalence	All MarketScan, year 2010	-0.26 (-0.42 - -0.19)	0.77 (0.66 - 0.83)
Epilepsy impairment envelope	prevalence	All MarketScan, year 2012	-0.18 (-0.35 - -0.11)	0.83 (0.71 - 0.90)
Epilepsy impairment envelope	incidence	Nonstandard case definition	0.14 (0.00 - 0.53)	1.15 (1.00 - 1.69)
Epilepsy impairment envelope	excess mortality rate	LDI (I$ per capita)	-0.30 (-0.30 - -0.30)	0.74 (0.74 - 0.74)

In the second step, we used a mixed-effects generalized linear model (binomial family) to predict the proportion of idiopathic epilepsy. We used a fixed effect on LDI, a lagged transformation of GDP per

capita and super-region random effects in the final model. We also tested health system access as well as region and country effects in different models, but they did not improve the model. We used a similar model to predict the proportion of severe epilepsy and treatment gap based on the reported proportions extracted from the systematic review. We used fixed effects on health system access and LDI and super-region random effects in the final model for severe epilepsy. We also tested region and country effects in different models, but they did not improve the model. For estimating the treatment gap, we used fixed effects on LDI and health system access and super-region random effects in the final model. We tested region and country effects in different models, but they did not improve the model. We generated 1,000 draws of country-specific estimates for each year between 1980 and 2015 for each of the models.

Regression	covariate	beta	SE
Idiopathic	LDI	-0.58	0.03
Severe	LDI	0.49	0.07
Severe	HSA	-0.78	0.07
Treatment	LDI	-0.65	0.03
Treatment	HSA	-0.19	0.05

Seizure-free treated epilepsy

There were too few data points to use a mixed effects model. Instead, we used meta-analysis to generate two different pooled estimates for proportion of seizure free treated epilepsy in developing and developed countries.

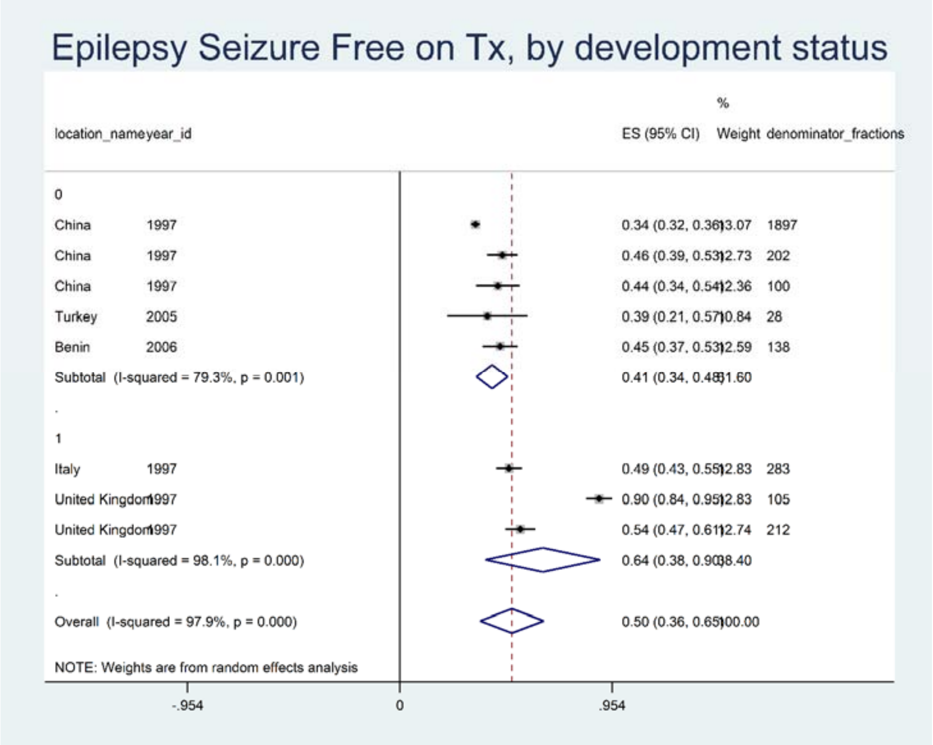

No additional changes were made to the modeling strategy for GBD 2015.

Infertility impairment envelope

Flowchart

Infertility

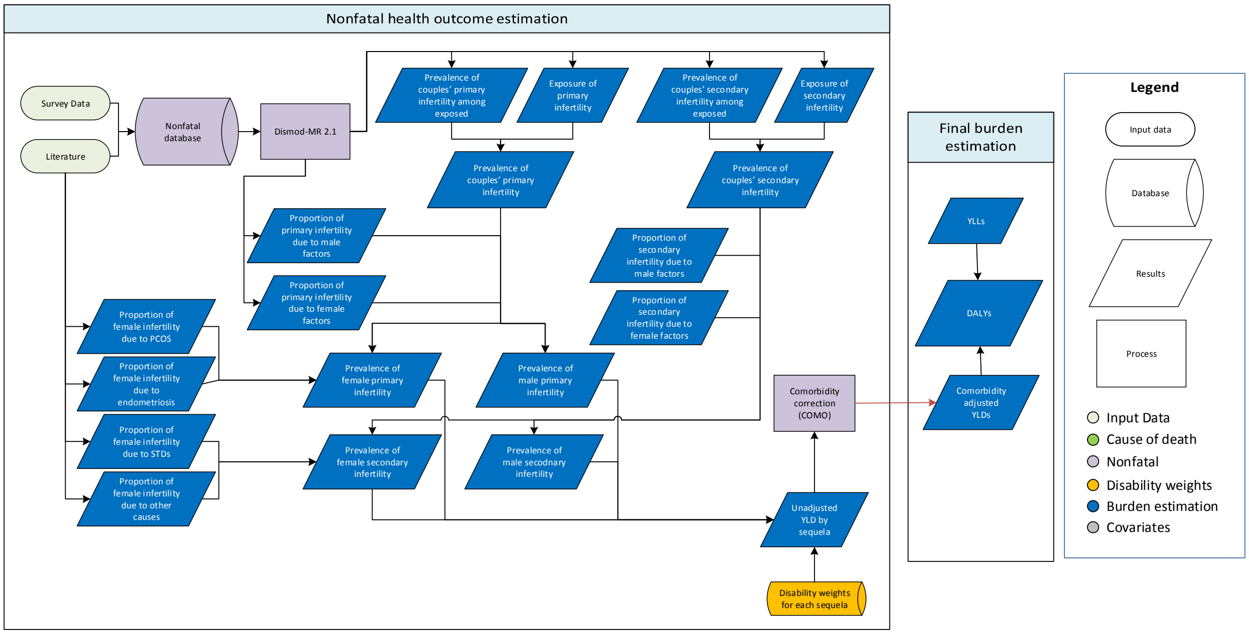

Case definition

For GBD 2015, the following case definitions were used for infertility:

1. Primary infertility is defined as a couple who have not had a live birth, who wish a child, and have been in a union for more than five years without using contraceptives.

2. Secondary infertility is defined in a couple who wish a child and have been in a union for more than five years without using contraceptives since the last live birth.

Input data

Model inputs

We included data for women in five-year age groups between 15 and 49 from population-based surveys including the Demographic and Health Surveys (DHS), World Fertility Surveys (WFS), Reproductive Health Surveys (RHS), Family and Fertility Survey (FFS), and others (EUR, NSF, PCD, PFM). Although these surveys only interviewed women, the resultant estimate of prevalence is an indicator of couples' infertility, as it is not possible to determine in a survey which partner is the cause of the infertility. Because some surveys ask questions of only ever-married women, we separately estimated the prevalence of four parameters:

Table 1: Infertility parameters modeled in DisMod 3 for estimation of couples' infertility			
**Infertility Type**	**Parameter**	**Numerator**	**Denominator**
Primary	Prevalence of primary infertility among exposed women	Married 5+ years; no contraception for 5+ years prior to survey; no previous births; desires a child	Numerator OR: Married 5+ years; 1 or more birth
Primary	Prevalence of exposure to primary infertility	Married 5+ years; no contraception for 5+ years prior to survey	All women
Secondary	Prevalence of secondary infertility among exposed women	Married 5+ years; no contraception for 5+ years prior to survey; last birth 5+ years ago; desires a child	Numerator OR: Married 5+ years; first birth 5+ years ago; last birth <5 years ago
Secondary	Prevalence of exposure to secondary infertility	Married 5+ years; no contraception for 5+ years prior to survey; one or more child	All women

As described below, infertility will be estimated by multiplying prevalence among exposed by the exposure. The table below illustrates the extent of data coverage for the infertility impairment for GBD 2015.

Table 2: Data Coverage

Model	Sources	Subnational	Country	Region	Superregion
primary infertility impairment	314	1	113	20	7
secondary infertility impairment	315	1	112	19	7
primary infertility exposure	266	1	101	17	7
secondary infertility exposure	267	1	101	17	7
proportion male primary infertility	18	2	15	8	6
proportion female primary infertility	18	2	15	8	6
proportion male secondary infertility	18	2	15	8	6
proportion female secondary infertility	18	2	15	8	6

Sequelae/disability weights

Healthstate name	Healthstate description	Disability weight
Infertility, primary	This person wants to have a child and has a fertile partner, but the couple cannot conceive.	0.008 (0.003-0.015)
Infertility, secondary	This person has at least one child, and wants to have more children. The person has a fertile partner, but the couple cannot conceive.	0.005 (0.002-0.011)

Modeling strategy

For GBD 2015, we estimated the prevalence of primary and secondary infertility by sex and cause in three steps.

1) *Estimation of couples' infertility*

To estimate the prevalence of infertility among couples, we first run four Dismod MR 2.1 models to estimate the four parameters detailed above. For prevalence of infertility, we tried using the natural log of the age-standardized death rate (lnASDR) of STDs, but it was not statistically significant so we did not use it in the final model.
Next, we estimated primary and secondary couples' infertility from DisMod-MR 2.0 models by multiplying the estimates for prevalence of infertility among exposed women by the prevalence of exposure to infertility to obtain prevalence of infertility among all women and all men.

2) *Estimation of infertility by sex*

We ran four Dismod models to estimate the proportion of primary and secondary infertility due to male or female causes. Because infertility in some couples is attributable to both partners rather than just one, the sum of the proportion of couples' prevalence due to male factor infertility and due to female factor infertility is greater than 1. Again, we tried using lnASDR of STDs as a covariate, but it was not statistically significant so we did not use it in the final model. We multiplied our prevalence of primary and secondary infertility derived in step 1 by the proportion due to male and female factors to estimate primary and secondary infertility by sex.

3) *Causal attribution*

There are seven identified causes of female infertility in the GBD 2013 cause list: chlamydia, gonorrhea, other sexually transmitted diseases, maternal sepsis, polycystic ovarian syndrome, endometriosis, and Turner syndrome. For each of these diseases, we determined the prevalence of infertility by a literature review of the probability of becoming infertile due to that disease. For sexually transmitted diseases we applied a proportion with infertility derived from Westrom et al. (1992, Sex Transm Dis) to incident cases of pelvic inflammatory disease and streamed out prevalence over the fertile age range using DisMod-MR 2.0.12. We added all the disease-specific estimates of prevalence and assigned the remaining proportion to categories of "female primary infertility due to other causes" and "female secondary infertility due to other causes." We assumed all infertility from Turner syndrome is primary infertility and all infertility following maternal sepsis is secondary infertility. The only recognized cause of male infertility in the GBD 2013 cause list is Klinefelter syndrome. We assigned all other male infertility to "male infertility due to other causes."

We have made no substantive changes in the modeling strategy from GBD 2013.

Idiopathic developmental intellectual disability impairment envelope

Flowchart

Intellectual Disability

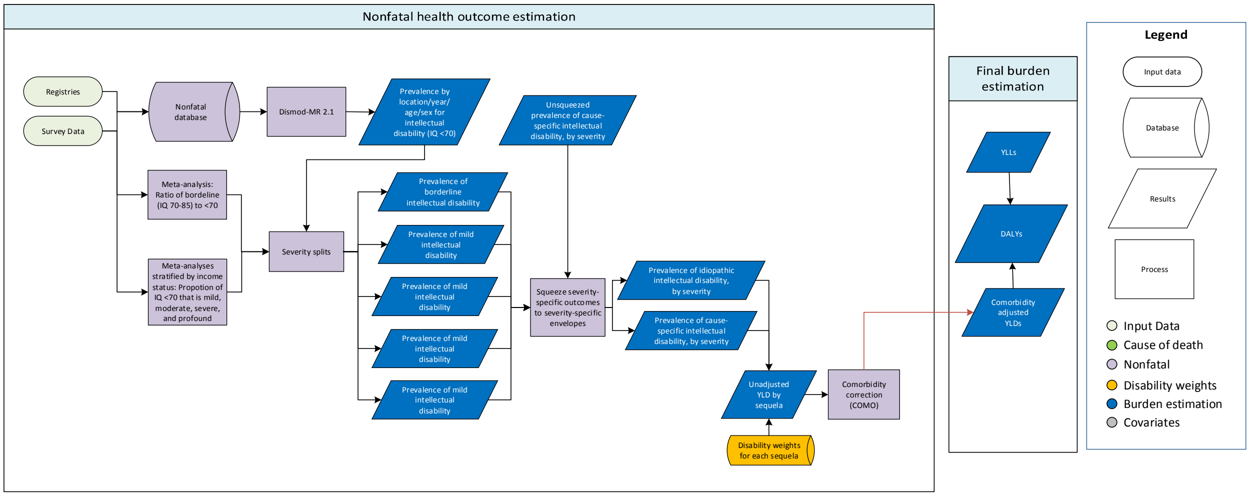

Case definition

Idiopathic developmental intellectual disability is a condition of below-average intelligence or mental ability. Consistent with the American Association on Intellectual and Developmental Disabilities, we define developmental intellectual disability as a condition originating before age 18 (thus, does not include impairment due to stroke, Alzheimer's, etc.). We model the following severities, as measured by intelligence quotient (IQ) tests:

Type of intellectual disability	IQ
Profound	0 to 19
Severe	20 to 34
Moderate	35 to 49
Mild	50 to 69
Borderline	70 to 85

Input data

Model inputs

For GBD 2015, we added data by conducting a systematic review from 1/1/1990 to 5/8/2015 using the following search string:

(((intellectual disability[MeSH Terms]) AND prevalence[Title/Abstract]) AND ('1990'[Date - Publication] : '3000'[Date - Publication]))

This had 2115 hits, of which 13 were extracted that had not been previously included in GBD. We included studies that estimate the general population prevalence of intellectual disability. We excluded

studies that do not use a case definition based on intelligence quotient (IQ) or investigated non-representative groups, like hospital patients or people of a specific ethnicity.

Severity split/disability weight

Healthstate	Description	Disability weight
Borderline intellectual functioning	This person is slow in learning at school. As an adult, the person has some difficulty doing complex or unfamiliar tasks but otherwise functions independently.	0.011 (0.005-0.02)
Intellectual disability / mental retardation, mild	This person has low intelligence and is slow in learning at school. As an adult, the person can live independently, but often needs help to raise children and can only work at simple supervised jobs.	0.043 (0.026-0.064)
Intellectual disability / mental retardation, moderate	This person has low intelligence, and is slow in learning to speak and to do even simple tasks. As an adult, the person requires a lot of support to live independently and raise children. The person can only work at the simplest supervised jobs.	0.1 (0.066-0.142)
Intellectual disability / mental retardation, severe	This person has very low intelligence and cannot speak more than a few words, needs constant supervision and help with most daily activities, and can do only the simplest tasks.	0.16 (0.107-0.226)
Intellectual disability / mental retardation, profound	This person has very low intelligence, has almost no language, and does not understand even the most basic requests or instructions. The person requires constant supervision and help for all activities.	0.2 (0.133-0.283)

Modeling strategy

We modeled the prevalence estimates of intellectual disability (ID), both etiology-specific IDs and idiopathic ID, over multiple steps.

1) *Intellectual Disability envelope (IQ <70)*

First, we ran a DisMod-MR 2.1 model to estimate the total prevalence of intellectual disability of level IQ <70. We tried using lag distributed income as a covariate but it was not statistically significant and thus was not included in the final model.

2) *Severity-specific Intellectual Disability*

Second, we split the total prevalence of idiopathic ID into four severity levels: mild (IQ 50-69), moderate (IQ 35-49), severe (IQ 20-34), and profound (IQ below 20). We pooled a subset of studies that distinguished intellectual disability by these severity levels. We used cumulative severity levels (i.e., IQ <50, IQ<35, IQ<20) to maximize the number of sources. We estimated these cumulative severities' proportion of the <70 envelope via random effects meta-analyses stratified by income status (high-income vs. low- and middle-income). These proportions were used to estimate discrete severities from the overall intellectual disability (IQ <70) prevalence. Last, we estimated borderline disability (IQ 70-84) via another random effects meta-analysis of the ratio of IQ 70-84 to IQ <70. The uncertainty of the pooled fractions and ratios were propagated throughout our calculations using 1000 draws from a normal distribution with mean and standard error estimated by the meta-analysis.

%<20 of <50, High-income

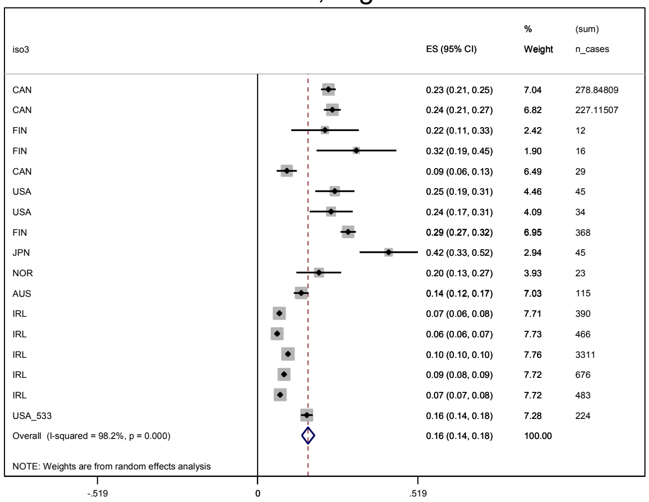

%<20 of <50, LMIC

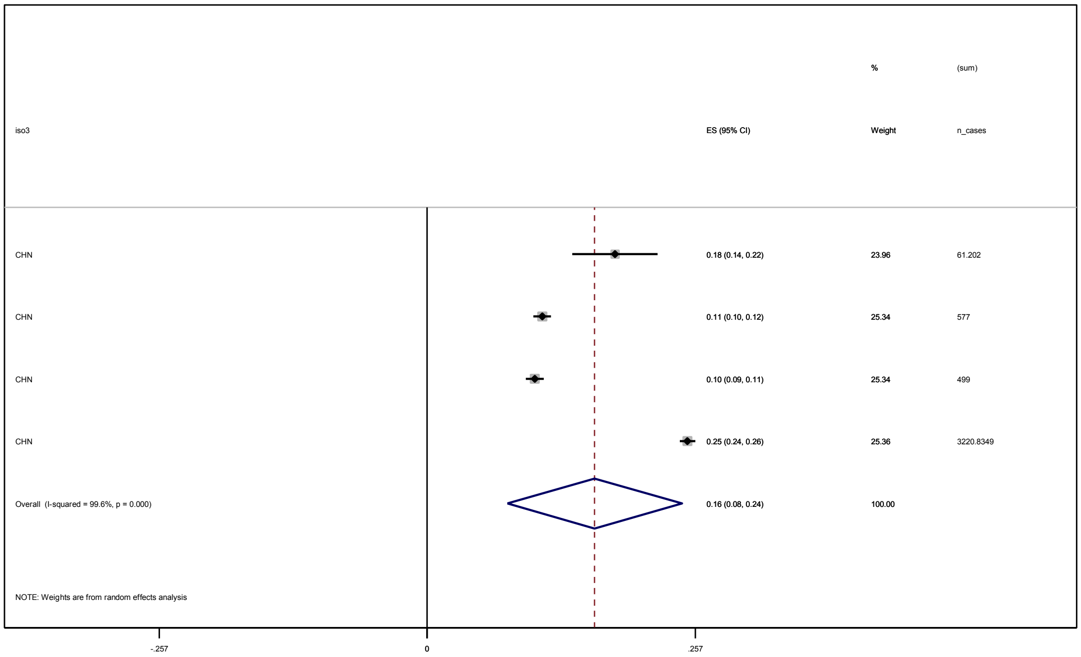

%<35 of <50, High-income

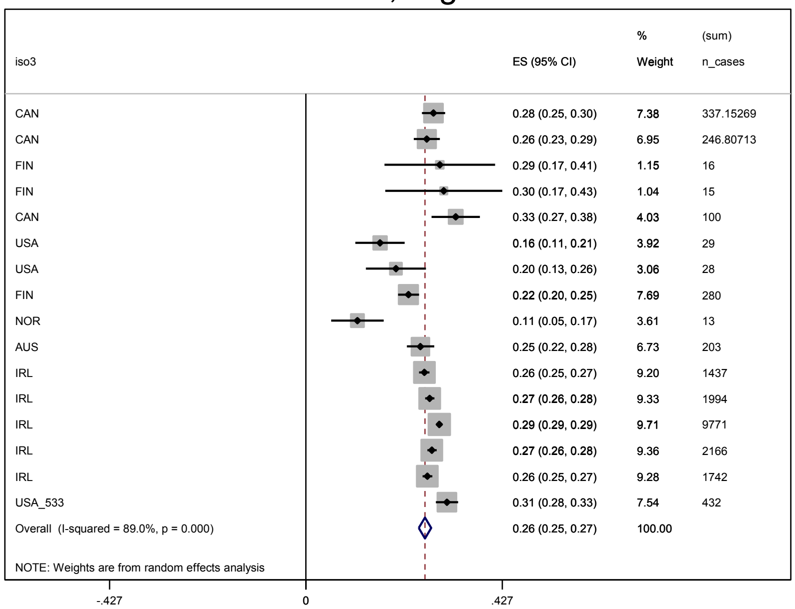

%<35 of <50, LMIC

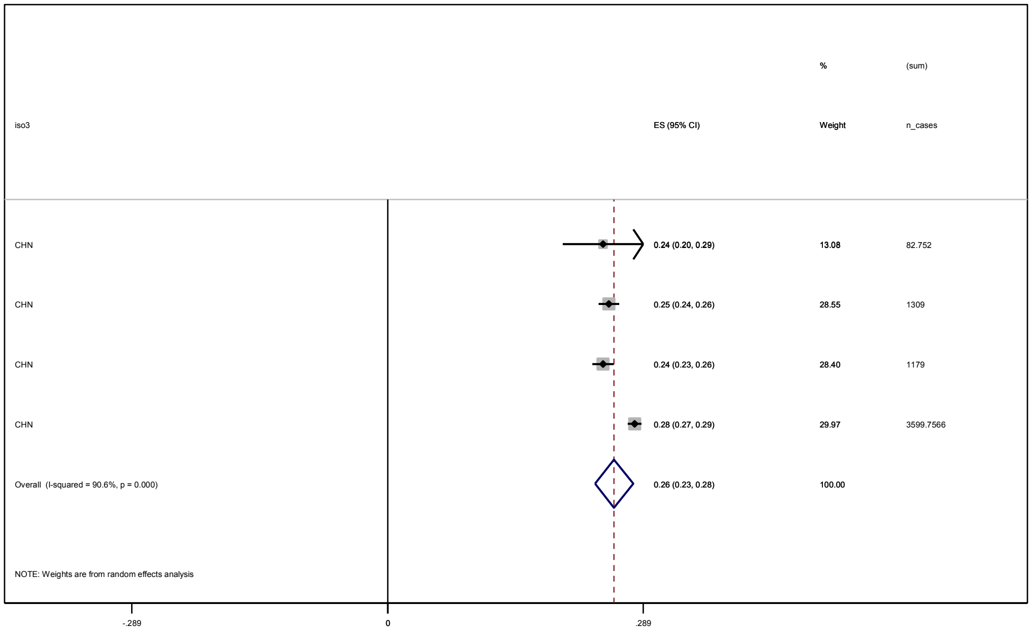

%<50 of <70, High-income

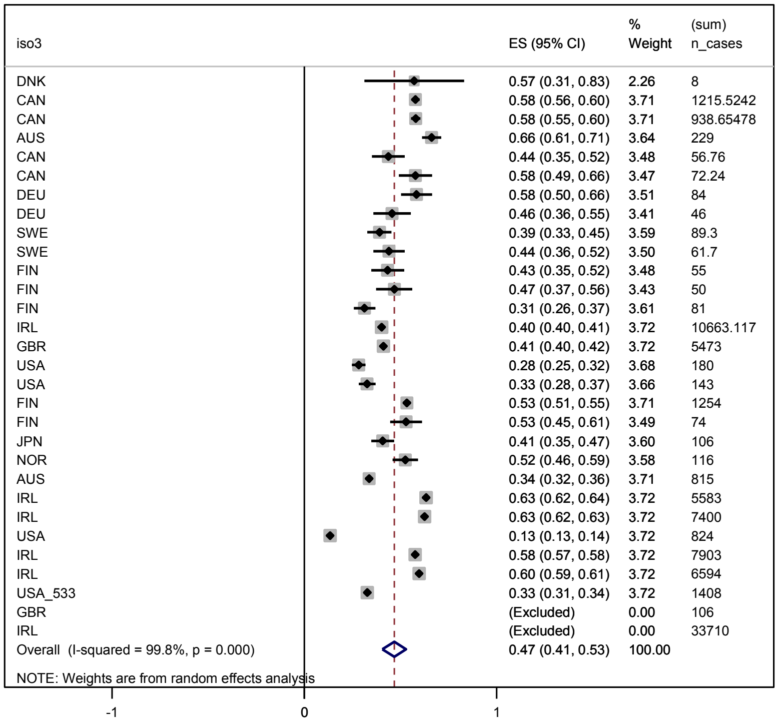

%<50 of <70, LMIC

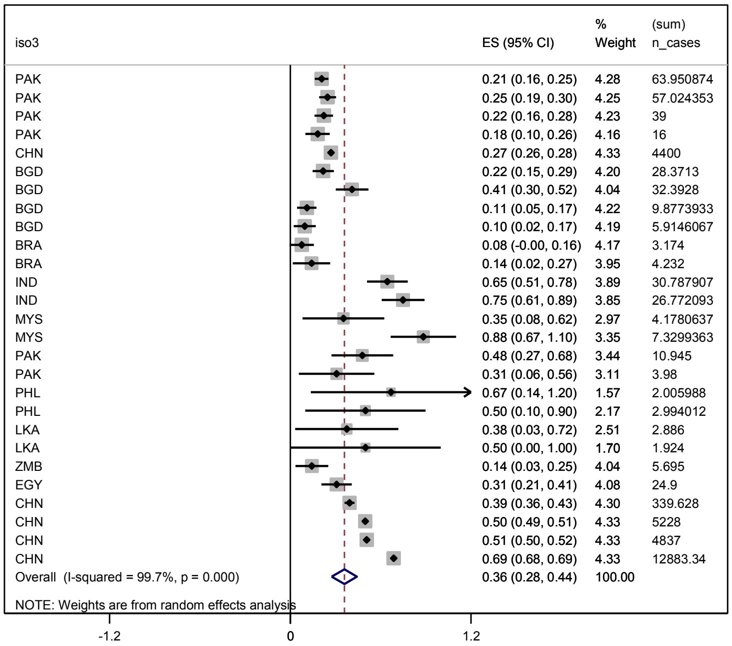

70-85 as proportion of <70

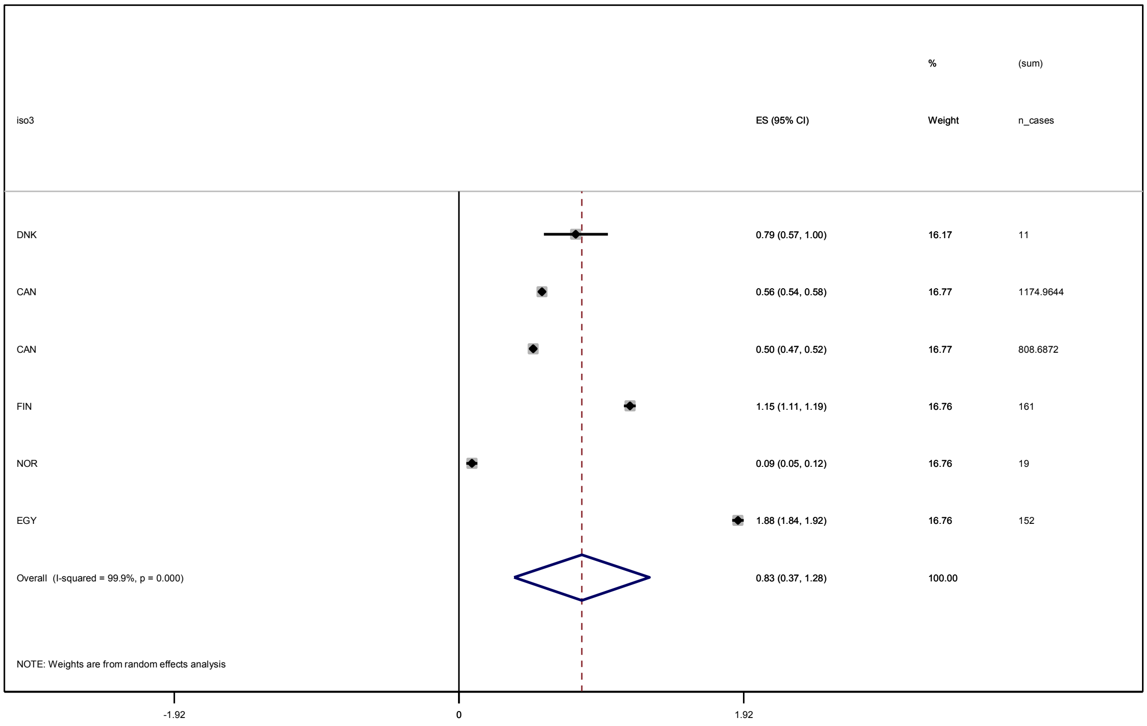

3) *Causal attribution*

Third, we estimated prevalence of each etiology-specific ID by models from the following parent causes. Since we are modeling only developmental intellectual disability, causes such as stroke and Alzheimer's are not included in the causal attribution process.

Neonatal preterm birth complications (<28w, 28-32w, 32-36w)
Neonatal encephalopathy due to birth asphyxia and trauma
Hemolytic disease and other neonatal jaundice
Meningitis (pneumococcal, H influenza type B, meningococcal, other bacterial)
Encephalitis
Malaria
Neonatal tetanus
Iodine deficiency
African trypanosomiasis
Down syndrome
Klinefelter syndrome
Chromosomal unbalanced rearrangements
Neural tube defects
Hypertensive disorders of pregnancy (eclampsia, preeclampsia)
Autism
Fetal alcohol syndrome

For autism, we identified six studies reporting severity of intellectual disability. We conducted a meta-analysis to produce the following severity distribution we applied to the prevalence of autism to produce severity-specific ID due to autism.

ID severity	Mean	SE
None	0.161	0.034
Borderline	0.161	0.034
Mild	0.375	0.037
Moderate	0.190	0.031
Severe	0.090	0.177
Profound	0.024	0.134

We calculated prevalence of idiopathic ID by subtracting all severity and etiology-specific IDs from the severity-specific envelope ID assuming the residuals to represent idiopathic. If the residual is less than 5% of the severity-specific envelope, the prevalence of all etiology-specific IDs were proportionally squeezed to fit within 95% of the envelope, leaving 5% for idiopathic ID.

As we estimated the prevalence of individual etiology-specific IDs by models from the respective parent causes, the squeezing may result in a distorted balance of prevalence estimates within their parent causes. With the aim to maintain consistencies of prevalence within each of the parent causes, we added the difference between the original and the squeezed prevalence estimates to the “motor impairment” sequela if the squeezed sequela represented “motor and cognitive impairment.” For autism, we obtained the fraction of cases that result in ID from literature (0.29; 0.27-0.30 95% CI) and applied to the subtraction and squeezing processes. We assume all ID cases due to iodine deficiency (cretinism) to result in either severe or profound level, and Klinefelter syndrome cases that result in ID will have either borderline or mild level.

In GBD 2013, all etiology-specific models were squeezed into the overall (IQ <70) envelope. In 2015, we squeeze each model into its discrete severity envelope.

Guillain–Barré syndrome (GBS) impairment envelope

Flowchart

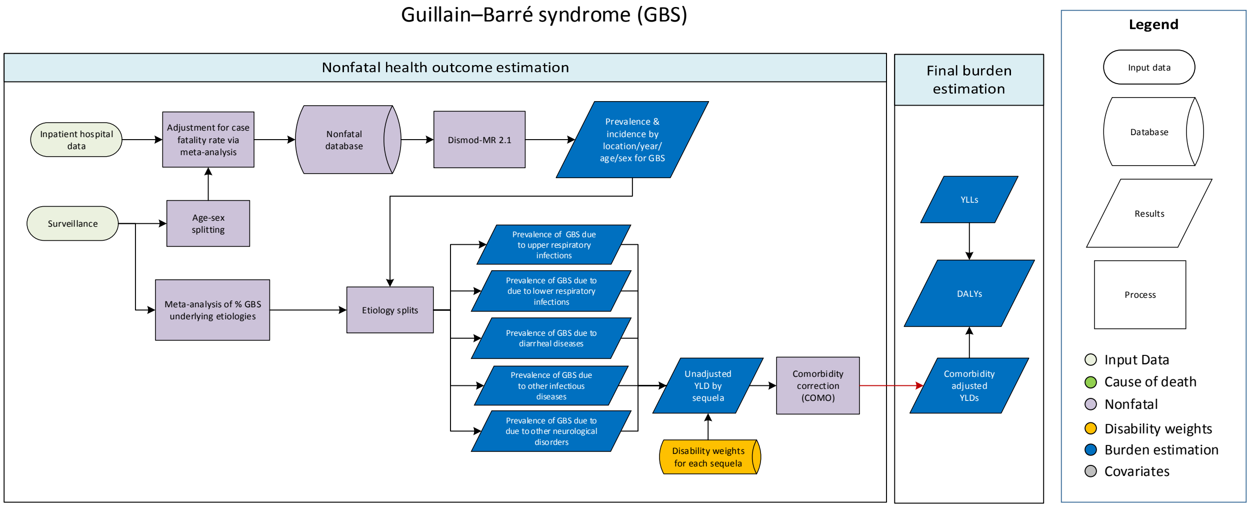

Case definition

Guillain-Barré syndrome is a rare condition that usually occurs as a complication of respiratory or gastro-intestinal infection. It is considered an immune-mediated nerve dysfunction with rapid onset of weakness in feet and hands ascending towards the trunk. In the acute phase about a quarter of cases required mechanical ventilation for survival. The majority of cases fully recover within months to a year. The following ICD codes are used G61.0 (GBS) and 357.0 (Acute infective polyneuritis).

Input data

Model inputs

A systematic search was performed in Pubmed on 28.01.2010 which produced a systematic review by McGrogan A et al. (2009). The search covered 1980-2008 using keywords “Guillain-Barré Syndrome” or “polyradiculoneuropathy” and “incidence” or “epidemiology.” This review included 63 relevant studies (published between 1980 and 2008) which reported on Guillain-Barré incidence rate and provided the estimated incidence rates for 25 countries in 9 GBD region). There were 35 studies from our systematic review that provided information on the underlying cause: 31 mentioning all of the identified underlying infectious diseases; 26 providing a proportion for upper respiratory infections, 3 for influenza, 25 for diarrheal disease, and 14 for other diseases.

A further search was undertaken for more information on mortality, remission, and duration of GBS. As the mortality, remission, and case-fatality rate did not vary significantly across the studies and there were time constraints, instead of doing a systematic search for GBS mortality and remission, a general search was carried out. We extracted case fatality rate from 5 studies, and remission from 4 studies.

measure	data_sources	subnational_coverage	country_coverage	region_coverage	super_region_coverage
incidence	96	120	29	10	5

Updates to systematic reviews are performed on an ongoing schedule across all GBD causes, and an update for Guillain-Barré Syndrome will be performed in the next 1-2 iterations. Inpatient hospital data was extracted using the ICD codes listed above. Canadian hospital data was excluded because it was a magnitude below that of the US and other developed countries. Only primary diagnoses were considered, with the reasoning that Guillain-Barré Syndrome should appear as a primary diagnosis and we do not wish to include follow-up visits that may be listed as secondary or tertiary codes.

Severity splits & disability weight

One healthstate was used for all Guillain-Barré cases. It is described as: paralyzed from the waist down, cannot feel or move the legs, and has difficulties with urine and bowel control. The person uses a wheelchair to move around. The disability weight is 0.296 (0.198-0.414).

Modeling strategy

All data, from both the literature review and hospital extraction, was corrected for the case fatality rate. A random effects meta-analysis calculated a 4.8% case fatality rate (95% CI 2.5-7.3%). As mortality mainly occurs during the acute phase of the disease (usually within four weeks of onset), the pooled case fatality rate was used to get the incidence of the people surviving after the acute phase of the GBS. Where surveillance data was reported at age groups of over 20 years, we applied the age pattern derived from US Marketscan claims data to age-split the data.

Dismod MR was used to estimate prevalence of Guillain-Barré syndrome for every location, year, age, and sex. We then split the overall prevalence of the impairment by underlying etiology (upper respiratory infections, influenza, diarrheal diseases, other infections, and other neurological causes). We used random effects meta-analysis to pool these proportions. We squeeze the proportions for influenza, diarrheal diseases, upper respiratory infections, and other infectious diseases to add to the proportion for all identified infectious underlying diseases. We assigned the complement to one of the proportion with any underlying infectious disease to a rest category of "idiopathic Guillain-Barre syndrome" that is classified under neurological disorders.

There are no substantive modeling changes from GBD 2013 other than the inclusion of hospital data to supplement the systematic review.

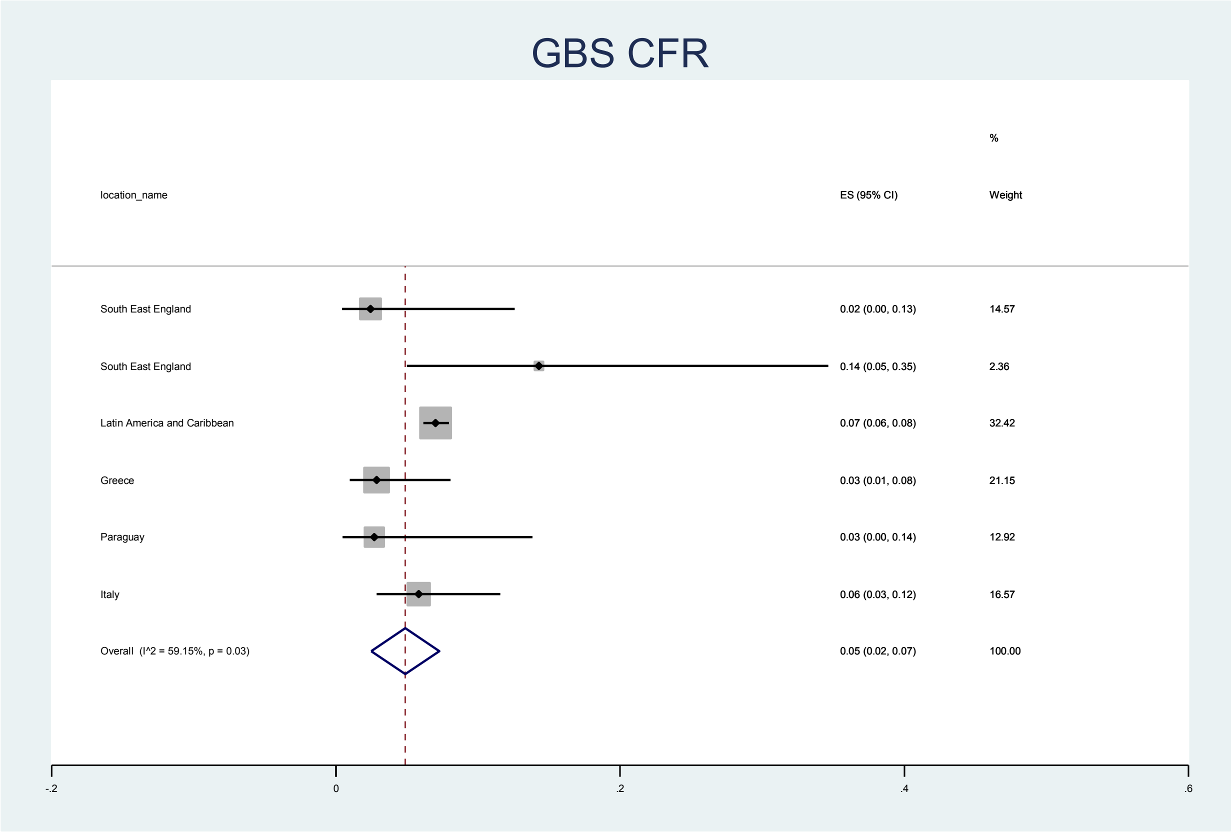

	Mean (%)	Lower limit (%)	Upper limit (%)
All specified causes	61·70	56·50	66·90
Upper respiratory infections	39·90	33·70	46·20
Influenza	14·60	7·90	22·70
Diarrheal diseases	13·80	11·00	16·90
Other infections	8·90	6·50	11·50

Pelvic inflammatory disease impairment envelope

Flowchart

Pelvic Inflammatory Disease

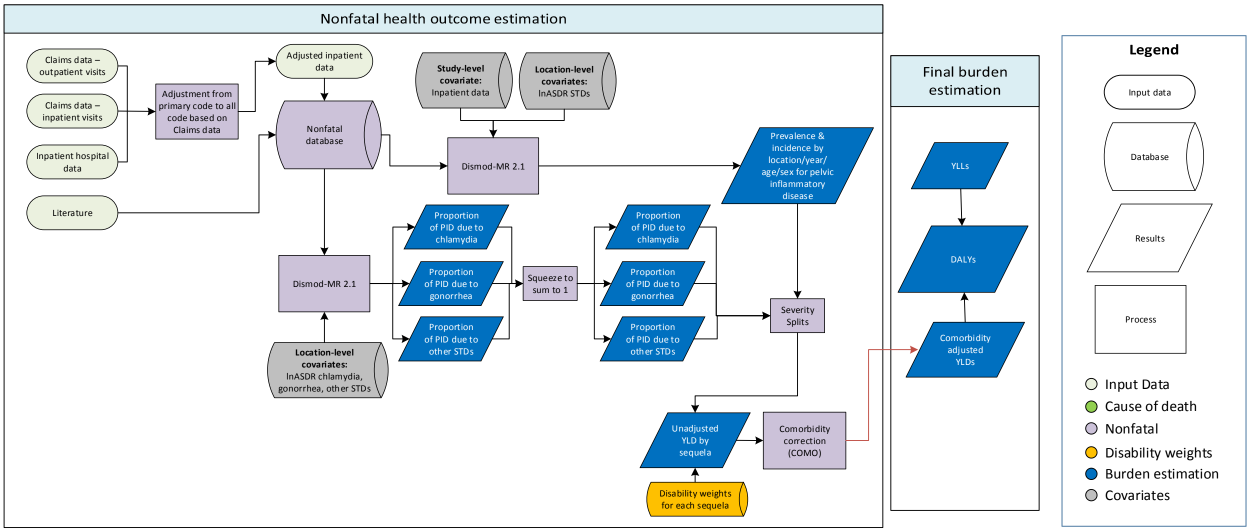

Case definition

Pelvic inflammatory disease (PID) is an infection of the female reproductive organs presenting as a complication of infection by a sexually transmitted disease. It can irreversibly damage the uterus, fallopian tubes, or other parts of the female reproductive system, leading to infertility.
We used the following ICD codes:

- ICD-9: 614.8-9
- ICD-10: A18.17, A54.24, A56.11, N71.0-71.1, N73, N73.8-9, N74, N74.1/.3/.8

Input data

Model inputs

A systematic review was completed for GBD 2013 on 10/28/2013 using the following search terms:

- o (("pelvic inflammatory disease"[Title/Abstract] OR "salpingitis"[Title/Abstract]) AND (“1994”[Date – Publication] : “2013”[Date – Publication]))

In addition, we included studies with confirmed clinical diagnosis in general population.

No systematic review was conducted for GBD 2015. Updates to systematic reviews are performed on an ongoing schedule across all GBD causes, and an update for PID will be performed in the next 1-2 iterations.

Inpatient outpatient Marketscan data was extracted as incidence rates, as per the ICD codes above. We also extracted inpatient hospital data, and employed two crosswalks derived from Marketscan data. First, we adjusted hospital inpatient data using a ratio of primary diagnoses for PID to all diagnoses, since the sexually transmitted disease (rather than PID, the consequent syndrome), may appear as the primary diagnosis. Second, we adjusted using a ratio of inpatient/outpatient diagnoses, since not all PID must be treated in an inpatient facility. The result is an estimate of total PID incidence.

PID incidence input data

The table below illustrates the data sources used in GBD 2015:

Measure	Data sources	Subnational coverage	Country coverage	Region coverage	Super region coverage
Incidence	37	86	22	6	3

A subset of the studies from the systematic review reported the underlying etiology of PID, allowing us to estimate the proportion of PID due to chlamydia, gonorrhea, and other sexually transmitted diseases.

Proportion of PID due to	data_sources	subnational_coverage	country_coverage	region_coverage	super_region_coverage
chlamydia	15	5	8	6	3
gonorrhea	10	5	6	4	2
other STDs	15	5	8	6	3

Health states and disability weights

Healthstate name	Healthstate description	Disability weight
Abdominopelvic problem, severe	This person has severe pain in the belly and feels nauseous. The person is anxious and unable to carry out daily activities.	0.324 (0.219-0.442)
Abdominopelvic problem, moderate	This person has pain in the belly and feels nauseous. The person has difficulties with daily activities.	0.114 (0.078-0.159)

Modeling strategy

First, we estimated the total incidence and prevalence of pelvic inflammatory disease using a Dismod MR 2.1. We used a study-level covariate on hospital inpatient data. This has the effect of crosswalking inpatient incidence (from hospital inpatient facilities) to total PID incidence (derived from Marketscan inpatient and outpatient claims data). We used the natural log of the age-standardized death rate (lnASDR) of sexually transmitted diseases (excluding HIV) as a location fixed effect, since these diseases cause PID and thus their mortality is correlated to PID incidence. We used Bayesian priors on remission (13-17) and excess mortality rate (0-0.15).

Covariate	Parameter	beta	Exponentiated beta

Hospital Inpatient (study-level)	Incidence	0.65 (-0.77 - -0.59)	0.52 (0.46 - 0.56)
lnASDR STDs (location)	Incidence	-0.65(-0.77 - -0.59)	0.52 (0.46 – 0.56)

Second, we ran three separate Dismod models for the proportion of PID due to the following three causes: chlamydia, gonorrhea, and other STDs. For each model, we used the lnASDR of the underlying STD.

Covariate	Parameter	Exponentiated beta
lnASDR chlamydia	Proportion	1.07 (1.00 – 1.22)
lnASDR gonorrhea	Proportion	1.19 (1.02 – 1.34)
lnASDR STDs	Proportion	1.00 (1.00 – 1.01)

Dismod estimate for the proportions due to each etiology were proportionally squeezed to sum to 1.

We extracted Marketscan claims data for the first time in GBD 2015. As described in detail above, this allowed us to make two crosswalks (from primary diagnosis to all diagnoses, and from inpatient to all diagnoses) that we had not previously. In GBD 2013, we only were able to use a ratio of inpatient:outpatient diagnoses from one literature source (Rein et al.). The combined effect of these two crosswalks is that we estimate substantially more PID cases in GBD 2015.

No additional changes were made to the estimation process for GBD 2015.

References

1. Rein DB, Kassler WJ, Irwin KL, et al. Direct medical cost of pelvic inflammatory disease and its sequelae: decreasing, but still substantial1. *Obstetrics & Gynecology*. 2000;95(3):397–402.

Heart failure impairment envelope

Flowchart

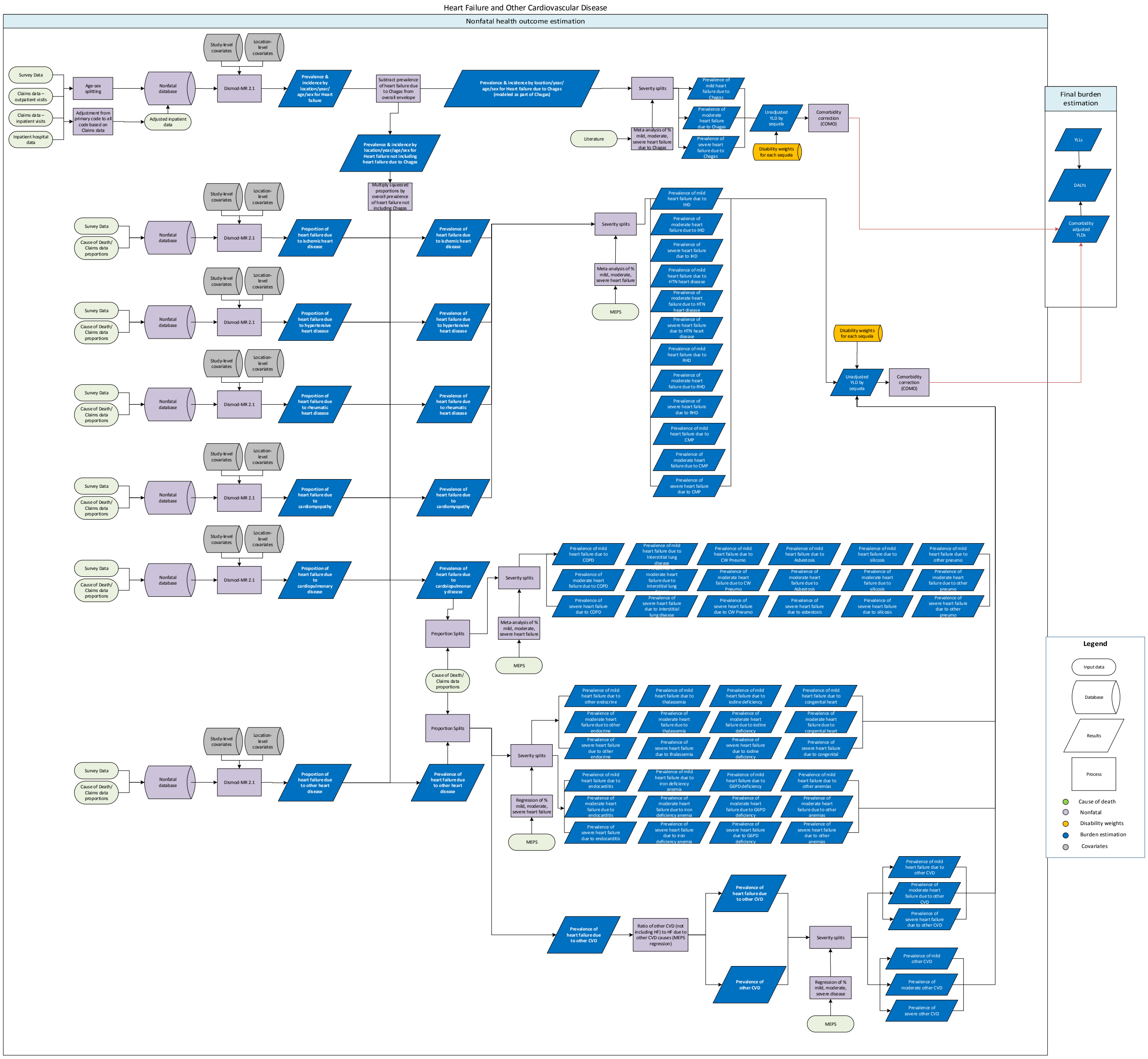

Case definition

Symptomatic heart failure was diagnosed clinically following the Framingham Criteria. We included only NYHA Class II and above. A list of ICD codes used to identify heart failure and underlying conditions can be found here: J:\WORK\04_epi\01_database\02_data\imp_hf\00_documentation\ICD_map_hf_filter.xlsx.

Major criteria: Paroxysmal nocturnal dyspnea, Neck vein distention, Rales, Radiographic cardiomegaly (increasing heart size on chest radiography), Acute pulmonary edema, S3 gallop, Increased central venous

pressure (>16 cm H2O at right atrium), Hepatojugular reflux; Weight loss >4.5 kg in 5 days in response to treatment

Minor criteria: Bilateral ankle edema, Nocturnal cough, Dyspnea on ordinary exertion, Hepatomegaly, Pleural effusion, Decrease in vital capacity by one third from maximum recorded, Tachycardia (heart rate>120 beats/min.).

Input data

Model inputs

A systematic review was not performed for GBD 2015 or GBD 2013. Updates to systematic reviews are performed on an ongoing schedule across all GBD causes; an update for heart failure will be performed in the next iteration.

Overall heart failure prevalence

	Prevalence	Incidence	Mortality risk
Studies	12	24	14
Countries/subnationals	9	17	11
GBD world regions	6	5	5

Heart failure due to ischemic heart disease

	Proportion
Studies	34
Countries/subnationals	51
GBD world regions	9

Heart failure due to hypertensive heart disease

	Proportion
Studies	35
Countries/subnationals	38
GBD world regions	9

Heart failure due to rheumatic heart disease

	Proportion
Studies	28
Countries/subnationals	49
GBD world regions	11

Heart failure due to cardiomyopathy

	Proportion

Studies	32
Countries/subnationals	51
GBD world regions	10

Heart failure due to cardiopulmonary disease

	Proportion
Studies	12
Countries/subnationals	10
GBD world regions	5

Heart failure due to other cardiovascular and circulatory diseases

	Proportion
Studies	19
Countries/subnationals	22
GBD world regions	6

Non-literature data included claims data and GBD death estimates.

Severity split inputs

The table below includes lay descriptions and disability weights for the severity levels of heart failure for GBD 2015.

Severity level	Lay description	DW (95% CI)
Mild	Is short of breath and easily tires with moderate physical activity, such as walking uphill or more than a quarter-mile on level ground. The person feels comfortable at rest or during activities requiring less effort.	0.041 (0.026-0.062)
Moderate	Is short of breath and easily tires with minimal physical activity, such as walking only a short distance. The person feels comfortable at rest but avoids moderate activity.	0.072 (0.047-0.103)
Severe	Is short of breath and feels tired when at rest. The person avoids any physical activity, for fear of worsening the breathing problems.	0.179 (0.122-0.251)

Modeling strategy

The general analytical strategy includes 1) estimating prevalence of heart failure using DisMod, and 2) a multi-step analysis of published literature, hospital data, and cause of death data, to estimate the etiological fraction for each cause of heart failure. The latter step includes an initial assessment of the fraction of heart failure cases attributable to each of the high-level parent cause groupings, followed by further division into the detailed causes within each of these groupings.

We first estimated an overall prevalence of heart failure using literature data, hospital data, and claims data. Using claims data with multiple diagnoses, we then calculated the proportion of people with a diagnosis in one of the underlying causes of heart failure who also had a diagnosis of heart failure listed.

These proportions were then multiplied by the number of age-, sex-, and location-specific deaths (post CoDCorrect) to yield an overall number for each underlying cause. We then divided the proportions by the total number of deaths represented in order to yield a proportion for each cause. These proportions, along with literature data, were used to run DisMod models for the six main cause groupings: ischemic heart disease, hypertensive heart disease, cardiomyopathy, rheumatic heart disease, combined cardiopulmonary disease, and other cardiovascular and circulatory diseases. We elected to drop non-literature data from sub-Saharan Africa based on expert opinion, as it was thought that the literature data would provide more accurate estimates of the underlying causes of heart failure. The results of the six proportion models were scaled to sum to one.

Please note that heart failure due to Chagas is modeled separately as part of the overall Chagas modeling plan. We subtract the prevalence of heart failure due to Chagas from the overall heart failure prevalence to give an adjusted prevalence of heart failure due to the six causes mentioned above and their subcauses.

For ischemic heart disease, hypertensive heart disease, rheumatic heart disease, and cardiomyopathy, we calculated the prevalence of heart failure due to each cause by multiplying the squeezed proportion by the adjusted overall prevalence. These causes were then split into mild, moderate, and severe heart failure based on an analysis of MEPS data.

For heart failure due to cardiopulmonary disease, we first calculated the overall prevalence using the squeezed proportion estimates from the DisMod model. We then calculated the prevalence for each subcause according to the proportion of that cause within the overall cardiopulmonary group (e.g., prevalence of heart failure due to COPD is equal to the prevalence of heart failure due to cardiopulmonary disease multiplied by the ratio of the proportion of heart failure due to COPD to the proportion of heart failure due to combined cardiopulmonary causes).

We used a similar approach to split heart failure due to other causes into the component subcauses.

In Sub-Saharan Africa, we relied only on published literature to determine the proportions of heart failure etiologies. This decision was based on expert opinion that local patterns differed significantly from what would have been determined from claims and death data. The THESUS-HF study provided these proportions.

To obtain prevalence estimates of other cardiovascular disease, we use MEPS data combined with 2005 USA prevalence estimates of heart failure due to other CVD causes to get an estimate of the cvd_other to HF ratio. We then apply this ratio to results for heart failure due to other CVD causes for all locations to generate prevalence of other cardiovascular disease.

Final splits of each subcause into mild, moderate, and severe heart failure were based on analysis of MEPS data.

Models were evaluated based on expert opinion, comparison of results with other rounds of GBD, and model fit.

Overall heart failure impairment envelope

Study covariate	Parameter	beta	Exponentiated beta
MEPS	Prevalence	.349 (.148 - .5612)	1.418 (1.16 - 1.753)
All MarketScan, year 2000	Prevalence	-.1017 (-.136 - -.0667)	.9033 (.8728 - .9355)
Hospital data	Prevalence	-.4657 (-.4702 - -.464)	.6277 (.6249 - .6288)
Log-transformed age-standardized SEV scalar: CVD	Prevalence	.7615 (.7507 - .7945)	2.142 (2.118 - 2.213)
LDI (I$ per capita)	excess mortality rate	-.4806 (-.4996 - -.4344)	.6184 (.6068 - .6477)
Follow-up short term	excess mortality rate	.7248 (.4623 - .9893)	2.064 (1.588 - 2.689)

Six main subcause proportion envelopes

Subcause	Covariate	Parameter	Beta	Exponentiated beta
Heart failure due to cardiomyopathy impairment envelope	Log-transformed age-standardized SEV scalar: CMP	proportion	.7513 (.7504 - .7524)	2.12 (2.118 - 2.122)
Heart failure due to cardiopulmonary disease impairment envelope	Log-transformed age-standardized SEV scalar: Chr Resp	proportion	.7504 (.7502 - .7508)	2.118 (2.117 - 2.119)
Heart failure due to hypertensive heart disease impairment envelope	Systolic blood pressure (mmHg)	proportion	5.4e-05 (3.1e-05 - 1.4e-04)	1 (1 - 1)
Heart failure due to hypertensive heart disease impairment envelope	MarketScan	proportion	-1 (-1 - -1)	.3679 (.3679 - .3679)
Heart failure due to ischemic heart disease impairment envelope	Log-transformed age-standardized SEV scalar: IHD	proportion	.7501 (.75 - .7502)	2.117 (2.117 - 2.117)
Heart failure due to other causes impairment envelope	Log-transformed SEV scalar: Oth Cardio	proportion	.7505 (.7502 - .7507)	2.118 (2.117 - 2.118)
Heart failure due to valvular heart disease impairment envelope	Log-transformed age-standardized SEV scalar: CVD	proportion	.7501 (.75 - .7509)	2.117 (2.117 - 2.119)

No other significant changes were made to the modeling changes for GBD 2015.

Vision impairment envelope

Flowcharts

Vision Impairment

Vision Impairment

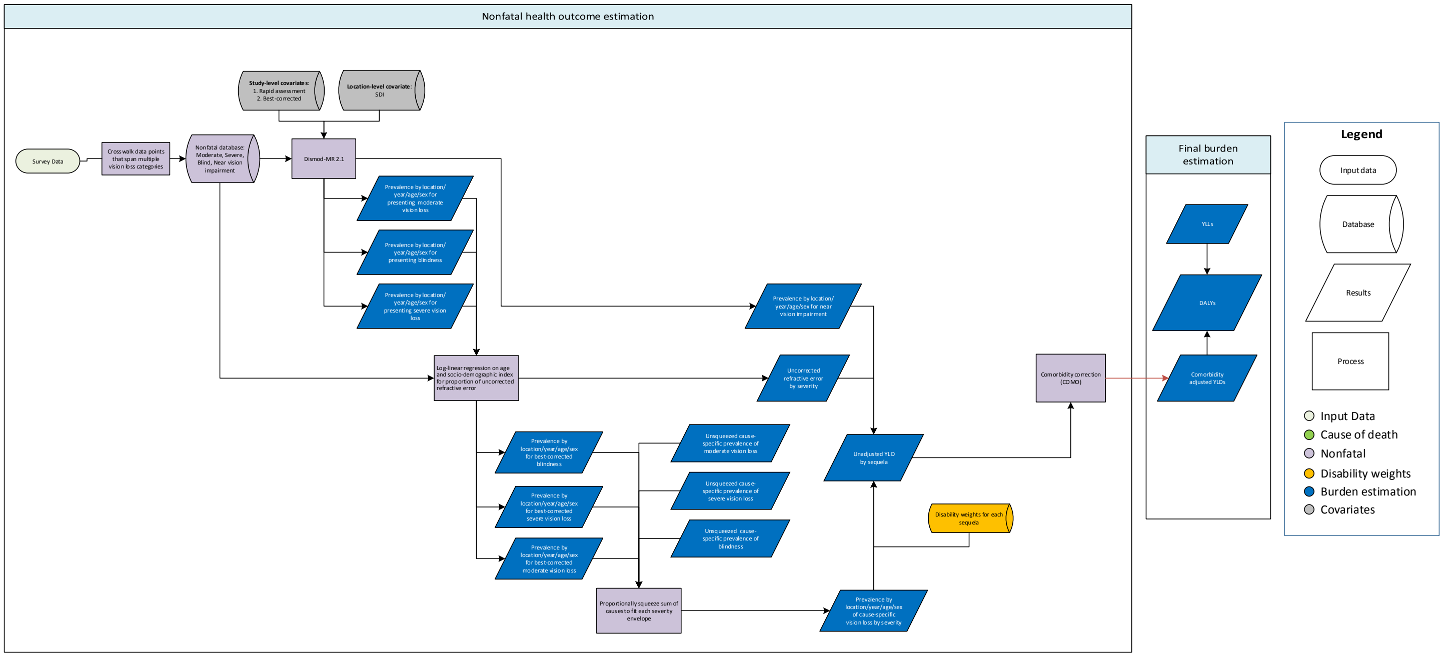

Diabetic Retinopathy

Diabetic retinopathy

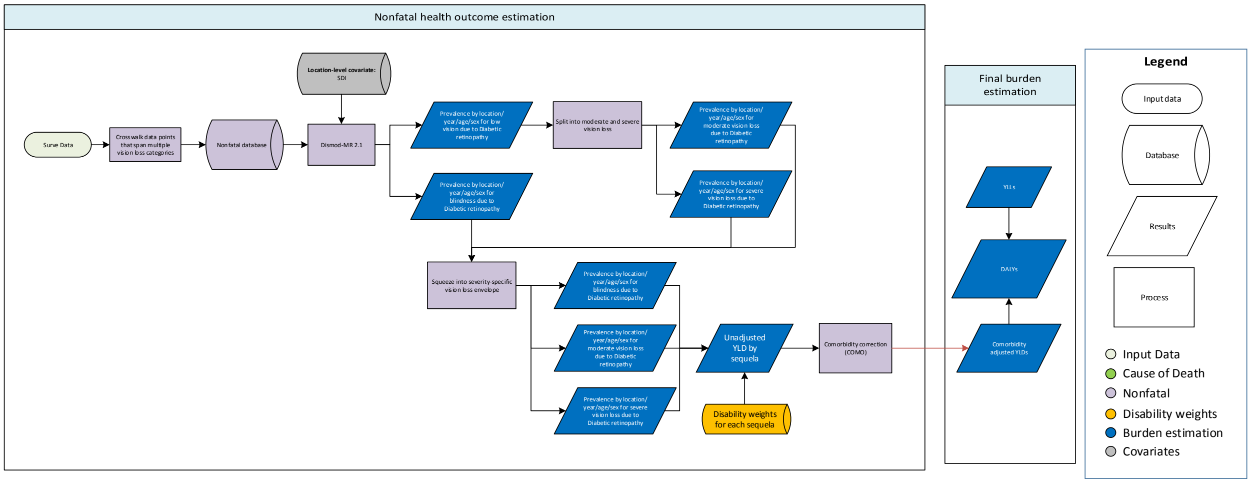

Macular Degeneration

Macular degeneration

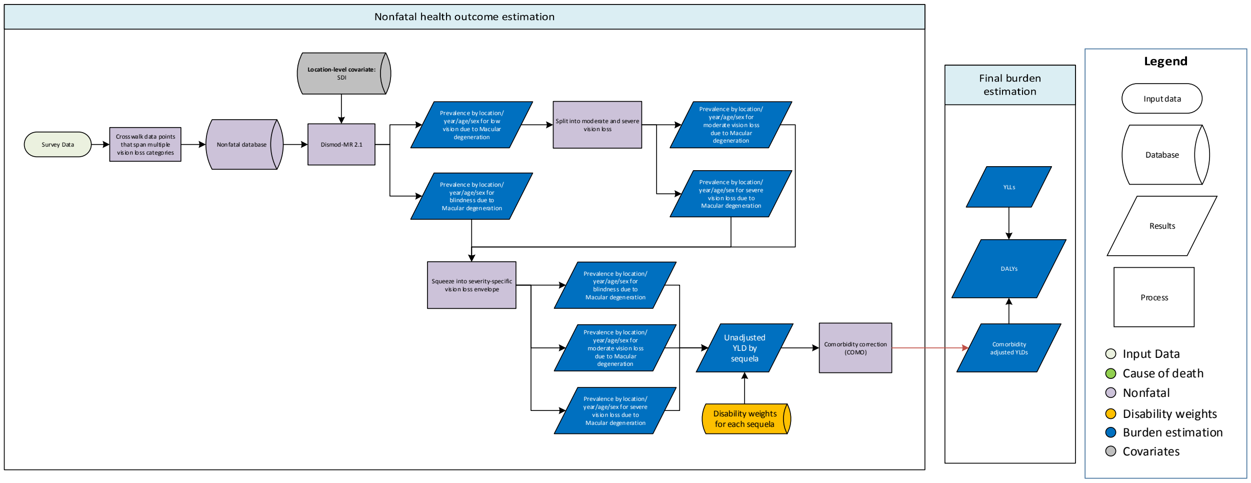

Cataract

Cataract

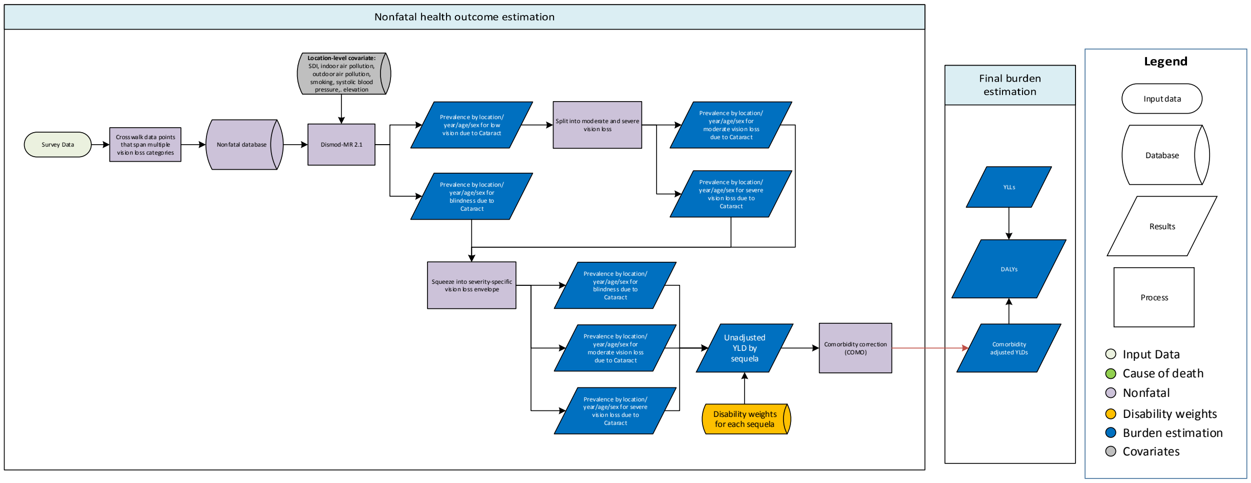

Glaucoma

Glaucoma

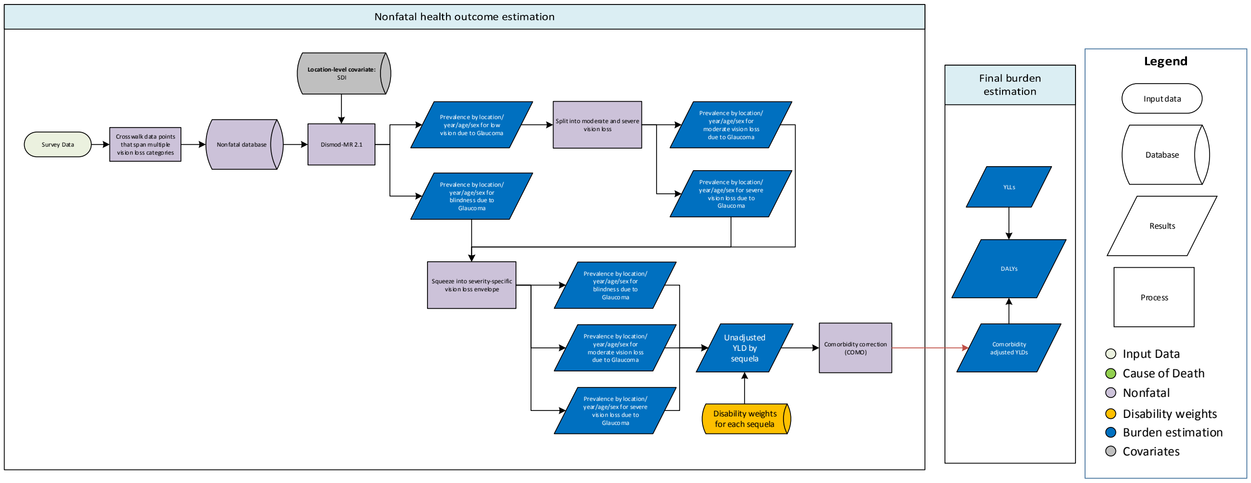

Trachoma

Trachoma

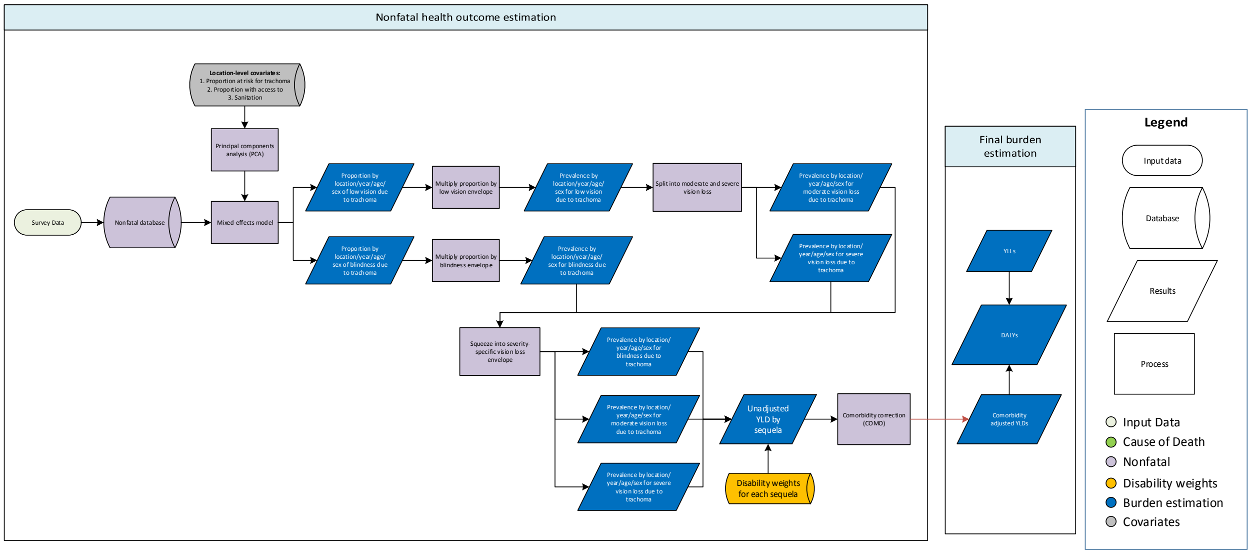

Other vision loss

Other vision loss

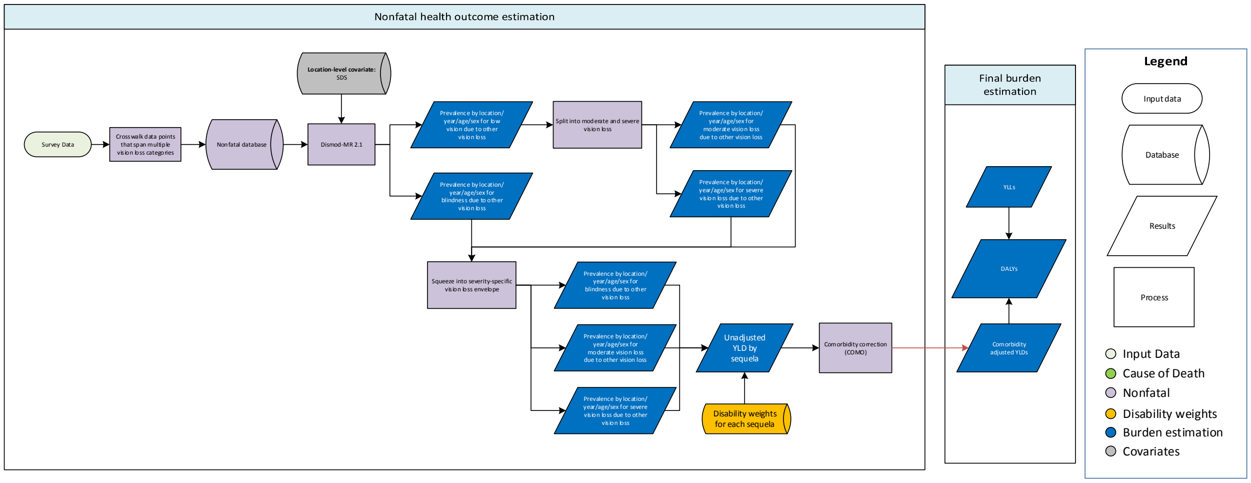

Case definition

We model vision impairment as visual acuity <6/18 according to the Snellen chart. The following impairments are modeled:

Condition	Case definition
Blindness	Visual acuity of <3/60 or <10% visual field around central fixation
Severe vision impairment	≥3/60 and <6/60
Moderate vision impairment	≥6/60 and <6/18
Near vision impairment envelope	Near visual acuity of <6/18 distance equivalent

Near vision impairment describes the progressive inability to focus on near objects as individuals age, and is also called presbyopia. This impairs the ability to read. The majority of presbyopia can be corrected by the use of reading glasses, contact lenses, or refractive surgery.

We model vision impairment due to the following causes: uncorrected refractive error, cataract, glaucoma, macular degeneration, diabetic retinopathy, trachoma, Vitamin A deficiency, retinopathy of prematurity, meningitis, encephalitis, onchocerciasis, and other vision loss. Vision loss due to vitamin A deficiency, retinopathy of prematurity, meningitis, encephalitis, and onchocerciasis are modeled as part of their underlying cause as described in their respective sections.

Refractive error is blurry vision due to the lens' inability to focus. The blurriness caused by refractive error can be addressed through the use of contact lenses, glasses, or refractive surgery. Cataract is clouding of the lens of the eye due to protein buildup that impairs vision. Glaucoma is a condition with increased intraocular pressure which can lead to damage of the optic nerve. Macular degeneration is a deterioration of the macula, leading to central vision loss. Diabetic retinopathy is damage to the retina caused by damaged blood vessels that can leak blood into the retina and cause scarring of the retina. Trachoma results from a conjunctival bacterial infection (*Chlamydia trachomatis*) that produces inflammation and scarring which leads to an inversion of the eyelids and eye lashes scratching the cornea which eventually leads to scarring of the cornea and vision impairment or blindness.

Input data

Model inputs

Data on overall vision impairment comes from surveys measuring visual acuity in representative population-based studies, either from publications in peer-reviewed and grey literature or surveys for which we had the unit record data. Data were excluded if no test was used of visual acuity that can be converted to the Snellen scale, and if a study did not assess "presenting" or "best-corrected" vision. A subset of these studies that reported vision loss by cause were used to estimate the prevalence of vision loss due to cataract, glaucoma, macular degeneration, diabetic retinopathy, and other causes.

For GBD 2015, we conducted a systematic review for new sources since GBD 2013 (covering 1/1/2013 – 5/20/2015), using the following search string:

((((glaucoma[Title/Abstract] OR cataract[Title/Abstract] OR macular[Title/Abstract] OR 'refractive error'[Title/Abstract] OR presbyopia[Title/Abstract]) OR (('blindness'[MeSH Terms] OR 'blindness'[All Fields]) OR 'vision, low'[MeSH Terms])) AND ('2013'[PDAT] : '3000'[PDAT])) AND 'humans'[MeSH Terms]) AND (prevalence[Title/Abstract] OR incidence[Title/Abstract] OR epidemiology[Title/Abstract])

This yielded 1169 results, of which we extracted 20 sources. Furthermore, we extracted from the following nationally representative surveys measuring visual acuity: the WHO Studies on Global Ageing and Adult Health (SAGE) and the United States National Health and Examination Surveys (NHANES).

Due to the sparse literature reporting measured near-vision visual acuity, we also extracted data from the following nationally representative studies measuring self-reported near vision loss: SAGE; NHANES; the Surveys of Health, Ageing, and Retirement in Europe (SHARE); the Multi-Country Survey Study on Health and Responsiveness (MCSS); and the World Health Surveys (WHS).

Where studies reported visual acuity spanning multiple thresholds (e.g., <6/60, rather than separate severe and blind estimates), we crosswalked using ratios predicted by a linear regression on age, using data from studies reporting vision loss by each severity.

Data availability (prevalence only)

Cause	data_sources	subnational_coverage	country_coverage	region_coverage	super_region_coverage

Distance vision	225	60	91	21	7
Near vision	128	1	74	19	7
Cataract	112	20	60	19	7
Glaucoma	79	14	42	16	7
Macular degeneration	65	12	43	16	7
Other	42	9	30	13	7
Diabetic Retinopathy	23	4	22	13	7

Whereas other vision impairment etiologies are modeled based on prevalence data, vision impairment due to trachoma is modeled as a proportion of the overall vision impairment envelope, a strategy that was chosen based on the nature of available data.

Cause	Data points	Data sources	Countries	Regions	Super regions
Vision impairment due to trachoma	142	14	28	13	7
Blindness due to trachoma	142	20	37	13	6

Healthstates and disability weights

Healthstate name	Healthstate description	Disability weight
Distance vision, severe impairment	This person has severe vision loss, which causes difficulty in daily activities, some emotional impact (for example, worry), and some difficulty going outside the home without assistance.	0.184 (0.125-0.259)
Distance vision, moderate impairment	This person has vision problems that make it difficult to recognize faces or objects across a room.	0.031 (0.019-0.049)
Distance vision blindness	This person is completely blind, which causes great difficulty in some daily activities, worry and anxiety, and great difficulty going outside the home without assistance.	0.187 (0.124-0.26)
Presbyopia	This person has difficulty seeing things that are nearer than 3 feet, but has no difficulty with seeing things at a distance.	0.011 (0.005-0.02)

Modeling strategy

We modeled the prevalence of vision loss in three steps. In the first step, we estimated the total prevalence estimates of presenting vision loss: moderate vision impairment, severe vision impairment, blindness, and near vision impairment (presbyopia). We directly derived prevalence of near vision impairment from this step, whereas the remaining three models that reflect different severity levels of distance vision loss continued to the next step.

1) Estimate severity-specific vision impairment (the "envelopes")

First, we ran four DisMod-MR 2.1 models to estimate the total prevalence estimates of presenting vision loss: moderate vision impairment, severe vision impairment, blindness, and near vision impairment (presbyopia).

Betas and exponentiated values, which can be interpreted as an odds ratio, are shown in the table below for each covariate. The best-corrected covariate indicates whether the test measures visual acuity with the level of correction the patient presents with (best_corrected = 0) or the ophthalmologist provides additional correction via pinhole (best_corrected = 1). Rapid-assessment corrects for potential biases from studies using expedited visual acuity measurement. Sociodemographic index (SDI) is used as a location covariate as a proxy measure of access to eye care such as cataract surgery. Non-standard severity definition is used to crosswalk between the self-report questionnaire of SHARE (nonstandard) and the other surveys, including SAGE and NHANES, which are crosswalked to examination data using the self-reported covariate.

Cause	Covariate	Beta	Exponentiated
Blindness impairment envelope	Sociodemographic Index	-0.64 (-1.20 - -0.13)	0.53 (0.30 - 0.88)
Blindness impairment envelope	Best corrected (vs presenting) visual impairment	-0.09 (-0.18 - 0.00)	0.92 (0.83 - 1.00)
Blindness impairment envelope	Diagnostic rapid assessment of loss	0.41 (0.31 - 0.52)	1.51 (1.37 - 1.68)
Moderate vision impairment envelope	Sociodemographic Index	-0.07 (-0.27 - -0.00)	0.93 (0.76 - 1.00)
Moderate vision impairment envelope	Best corrected (vs presenting) visual impairment	-0.83 (-0.93 - -0.73)	0.44 (0.39 - 0.48)
Moderate vision impairment envelope	Diagnostic rapid assessment of loss	0.07 (-0.05 - 0.18)	1.07 (0.95 - 1.20)
Near vision impairment envelope	Sociodemographic Index	-1.31 (-1.96 - -0.36)	0.27 (0.14 - 0.70)
Near vision impairment envelope	Self-reported	-0.10 (-0.14 - -0.07)	0.90 (0.87 - 0.93)
Near vision impairment envelope	Non-standard severity definition	-0.20 (-0.20 - -0.18)	0.82 (0.82 - 0.83)
Severe vision impairment envelope	Best corrected (vs presenting) visual impairment	-0.51 (-0.65 - -0.39)	0.60 (0.52 - 0.68)
Severe vision impairment envelope	Diagnostic rapid assessment of loss	0.66 (0.51 - 0.83)	1.94 (1.66 - 2.30)
Severe vision impairment envelope	Sociodemographic Index	-0.39 (-1.10 - -0.02)	0.68 (0.33 - 0.98)

2) Estimate refractive error

In a second step, we estimated the proportion of presenting vision loss that is due to uncorrected refractive error. We identified studies that reported both presenting and best-corrected estimates of vision loss. We ran a logit transformed regression by using the ratio of best-corrected over presenting as the dependent variable, with fixed effects on age and sex and statistically significant superregion random

effects. We applied the predicted proportions by country, year, age, and sex to split the prevalence of vision loss into uncorrected refractive error and best-corrected for each of the severity levels.

3) Estimate cause-specific vision impairment

In a third step, we estimated the prevalence of vision loss due to multiple causes: cataract, glaucoma, macular degeneration, diabetic retinopathy, retinopathy due to prematurity, trachoma, vitamin A deficiency, onchocerciasis, meningitis, and other causes not classified elsewhere. The vision loss due to retinopathy of prematurity, vitamin A deficiency, onchocerciasis, meningitis, tetanus and neonatal conditions was modeled as part of these underlying causes. Vision loss due to trachoma is modeled as a proportion of the envelope, with separate proportion models for vision impairment and blindness. For each of cataract, glaucoma, macular degeneration, diabetic retinopathy, and other vision loss, we ran two DisMod MR2.1 models: one for the combined category of moderate and severe vision loss due to the cause, and one for blindness due to the cause. Moderate and severe vision loss were modeled together because input data was mostly available for the aggregate. We used the following age restrictions:

Cause	Minimum age
Cataracts	20
Glaucoma	45
Macular Degeneration	45
Diabetic Retinopathy	20
Trachoma	15
Other vision loss	0

For the cataract model, we used known risk factors – hypertension, smoking, air pollution, and elevation.

Cause-specific vision models

Cause	Covariate	Beta	Exponentiated
Blindness due to cataract unsqueezed	Smoking Prevalence	0.61 (0.02 - 1.78)	1.84 (1.02 - 5.95)
Blindness due to cataract unsqueezed	Indoor Air Pollution (All Cooking Fuels)	1.38 (0.48 - 1.94)	3.96 (1.62 - 6.99)
Blindness due to cataract unsqueezed	Systolic Blood Pressure (mmHg)	0.02 (0.00 - 0.07)	1.02 (1.00 - 1.07)
Blindness due to cataract unsqueezed	Elevation Over 1500m (proportion)	0.77 (0.09 - 1.60)	2.17 (1.09 - 4.95)
Blindness due to cataract unsqueezed	Sociodemographic Index	-1.49 (-1.98 - -0.55)	0.23 (0.14 - 0.57)
Blindness due to cataract unsqueezed	Outdoor Air Pollution (PM2.5)	0.02 (0.00 - 0.04)	1.02 (1.00 - 1.04)
Blindness due to diabetes mellitus unsqueezed	Diabetes Age-Specific Prevalence (proportion)	1.03 (0.04 - 1.96)	2.81 (1.05 - 7.06)
Blindness due to diabetes mellitus unsqueezed	Sociodemographic Index	-1.03 (-1.97 - -0.06)	0.36 (0.14 - 0.94)
Blindness due to glaucoma unsqueezed	Sociodemographic Index	-0.54 (-0.99 - 0.33)	0.58 (0.37 - 1.39)
Blindness due to macular degeneration unsqueezed	Sociodemographic Index	1.01 (0.04 - 1.95)	2.74 (1.04 - 6.99)

Blindness due to other vision loss unsqueezed	Sociodemographic Index	-0.41 (-1.02 - -0.02)	0.66 (0.36 - 0.98)
Vision impairment due to cataract unsqueezed	Smoking Prevalence	0.47 (0.01 - 1.52)	1.60 (1.01 - 4.57)
Vision impairment due to cataract unsqueezed	Elevation Over 1500m (proportion)	1.19 (0.20 - 1.96)	3.30 (1.22 - 7.09)
Vision impairment due to cataract unsqueezed	Indoor Air Pollution (All Cooking Fuels)	1.23 (0.28 - 1.93)	3.41 (1.33 - 6.88)
Vision impairment due to cataract unsqueezed	Systolic Blood Pressure (mmHg)	0.04 (0.00 - 0.10)	1.04 (1.00 - 1.10)
Vision impairment due to cataract unsqueezed	Sociodemographic Index	-0.78 (-1.78 - -0.05)	0.46 (0.17 - 0.95)
Vision impairment due to cataract unsqueezed	Outdoor Air Pollution (PM2.5)	0.02 (0.00 - 0.05)	1.02 (1.00 - 1.05)
Vision impairment due to diabetes mellitus	Sociodemographic Index	-0.94 (-1.94 - -0.04)	0.39 (0.14 - 0.96)
Vision impairment due to diabetes mellitus	Diabetes Age-Specific Prevalence (proportion)	1.03 (0.06 - 1.95)	2.79 (1.06 - 7.01)
Vision impairment due to glaucoma unsqueezed	Sociodemographic Index	-0.31 (-0.98 - 0.79)	0.73 (0.38 - 2.21)
Vision impairment due to macular degeneration unsqueezed	Sociodemographic Index	1.05 (0.06 - 1.95)	2.85 (1.06 - 7.03)
Vision impairment due to other vision loss unsqueezed	Sociodemographic Index	-0.06 (-0.21 - -0.00)	0.94 (0.81 - 1.00)

We estimated the proportions of low vision and blindness due to trachoma using custom mixed effects models. For consistency, the two models (blindness and low vision) were parameterized identically and differ only in their input data. Our model included fixed effects on age (using cubic splines with knots at 0, 40, and 100 years of age), sex, and a covariate derived from a principal components analysis of the proportion of the population at risk for trachoma and the proportion of the population with access to sanitation. We included nested random effects on super region, region, and country. Finally, we applied geographic and age restrictions to ensure that we estimate zero proportions in non-endemic locations and among those younger than 15 year of age (as scarring of the cornea due to trachoma takes decades to develop). The prevalence of trachoma at each severity level was calculated by multiplying the proportion of vision loss (vision impairment or blindness) due to trachoma by the corresponding best-corrected vision loss envelope.

We split the moderate plus severe vision loss estimates for each cause into moderate and severe using the ratio of best-corrected moderate and severe vision loss envelopes. As exceptions, onchocerciasis and retinopathy of prematurity were modeled for moderate and severe vision loss as part of the estimation process of these causes.

We scaled the cause-specific vision loss prevalence to the total prevalence of the best-corrected vision loss envelopes for each of the three severity levels. The final result is prevalence of vision loss due to each cause by severity.

The following changes have been implemented since GBD 2013:

- Vision loss due to meningitis was apportioned between all levels of vision loss, rather than solely to blindness.
- For near vision impairment, we changed the case definition such that near vision loss and distance vision loss are not mutually exclusive, i.e., an individual can have both near and distance vision loss. This is consistent with the near and distance vision healthstates, which only mention their respective vision loss symptoms. In addition, rather than relying on self-report as in 2013, we used Dismod to crosswalk self-reported vision loss to presenting near vision acuity <6/18 as measured in surveys.
- Whereas DisMod-MR was used to develop trachoma proportion models for GBD 2013, we have implemented custom mixed effects models for these etiologies for GBD 2015. We made this change in response to instability in DisMod's estimates resulting from the combination of limited available data for these models and DisMod's extreme flexibility. The mixed effects models yielded more stable and, we therefore felt, more robust estimates.

Hearing impairment envelope

Flowchart

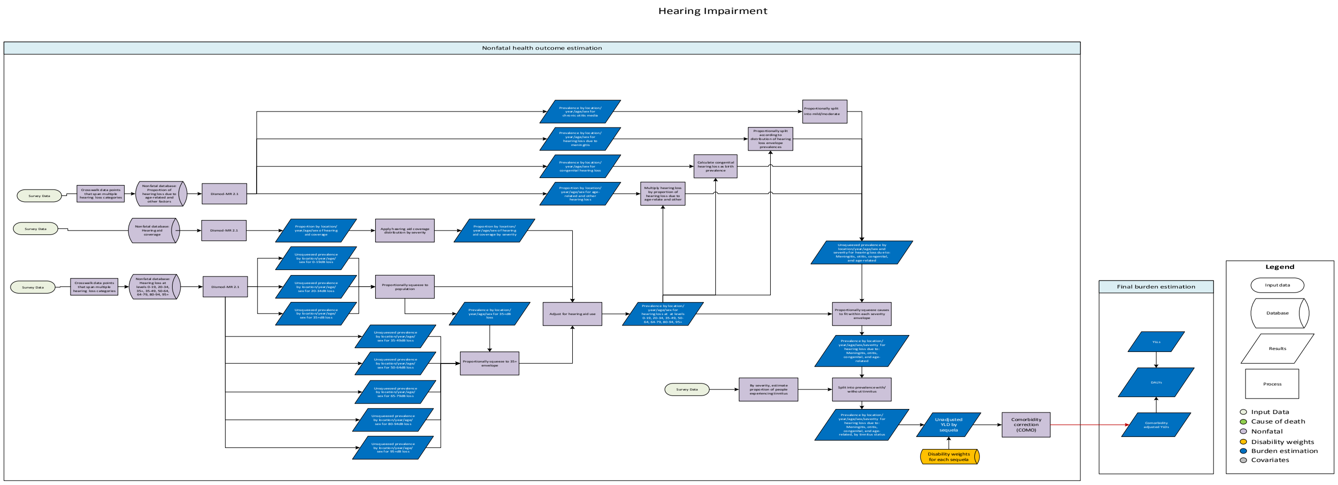

Case definition

For GBD 2015, hearing impairment modeled the following severities of hearing loss:

Severity thresholds of interest for hearing loss

Severity	Threshold (in decibels)
None	0-19
Mild	20-34
Moderate	35-49
Moderately severe	50-64
Severe	65-79
Profound	80-94
Complete	95+

We model the following causes of hearing loss: congenital, meningitis, otitis, and age-related and other hearing loss. Hearing loss due to meningitis and otitis are modeled as part of their underlying cause as described in their respective sections. Congenital hearing loss is defined as hearing loss present at birth. Age-related and other hearing loss includes causes not identified as meningitis, otitis, or congenital. This includes presbycusis, the gradual increase in hearing loss over age frequently caused by the natural breakdown of neurons in the inner ear. For all causes, we estimate hearing loss with and without tinnitus, the perception of noise or ringing in the ears.

Input data

Model inputs

For the estimation of the severity-specific envelopes, we used a series of systematic reviews and survey extraction. Data sources up to 2008 were identified by a published systematic review (http://www.ncbi.nlm.nih.gov/pubmed/19444763). A systematic review covering 2008-2013 was conducted with the following search terms:

> (hearing impairment[Title/Abstract] OR deafness[Title/Abstract] OR hearing loss[Title/Abstract]) AND (prevalence[Title/Abstract]) AND ("2008"[PDAT] : "3000"[PDAT]) AND (cross sectional OR survey)

In addition, we extracted hearing loss measurement from the United States National Health and Examination Surveys (NHANES). Self-reported data, from both the literature and surveys were excluded. This includes censuses in the Integrated Public Use Microdata Series (IPUMS), the WHO Studies on Global Ageing and Adult Health (SAGE), and the WHO Multi-Country Survey Study on Health and Responsiveness (MCSS). Systematic reviews and self-reported survey data (including MCSS, SAGE, and NHANES) was used to estimate hearing aid coverage.

Updates to systematic reviews are performed on an ongoing schedule across all GBD causes, an update for hearing loss will be performed in the next 1-2 iterations.

Table 1: Data inputs

	data_sources	subnational_coverage	country_coverage	region_coverage	super_region_coverage
Hearing loss prevalence	63	6	31	13	6

Hearing loss due to age-related and other	57	9	32	15	7
Hearing aid coverage	69	3	28	9	4

Where studies reported hearing loss spanning multiple thresholds (e.g., 80+, rather than 80-94 and 95+), we crosswalked using ratios predicted by a linear regression on age, using NHANES microdata.

Healthstates and disability weights

Healthstate name	Healthstate description	Disability weight
Hearing loss, mild	This person has great difficulty hearing and understanding another person talking in a noisy place (for example, on an urban street).	0.01 (0.004-0.019)
Hearing loss, mild, with ringing	This person is unable to hear and understand another person talking, even in a quiet place, is unable to take part in a phone conversation. Difficulties with communicating and relating to others cause emotional impact at times (for example worry or depression).	0.021 (0.012-0.036)
Hearing loss, moderate	This person has great difficulty hearing and understanding another person talking in a noisy place (for example, on an urban street), and sometimes has annoying ringing in the ears.	0.027 (0.015-0.042)
Hearing loss, moderate, with ringing	This person is unable to hear and understand another person talking, even in a quiet place, and has great difficulty hearing anything in any other situation. Difficulties with communicating and relating to others often cause worry, depression or loneliness.	0.074 (0.048-0.107)
Hearing loss, moderately severe	(custom DW from hearing loss impairment envelope)	0.092 (0.064-0.129)
Hearing loss, moderately severe, with ringing	(custom DW from hearing loss impairment envelope)	0.167 (0.114-0.231)
Hearing loss, severe	This person is unable to hear and understand another person talking in a noisy place (for example, on an urban street), and has difficulty hearing another person talking even in a quiet place or on the phone.	0.158 (0.104-0.227)
Hearing loss, severe, with ringing	This person is unable to hear and understand another person talking, even in a quiet place, has great difficulty hearing anything in any other situation, Difficulties with communicating and relating to others often cause worry, depression, or loneliness.	0.261 (0.174-0.361)
Hearing loss, profound	This person is unable to hear and understand another person talking in a noisy place, has difficulty hearing another person talking even in a quiet place or on the phone, and has annoying ringing in the ears for 5 minutes at a time, almost every day.	0.204 (0.134-0.288)
Hearing loss, profound, with ringing	This person cannot hear at all in any situation, including even the loudest sounds, and cannot communicate verbally or use a phone. Difficulties with communicating and relating to others often cause worry, depression or loneliness.	0.277 (0.182-0.388)
Hearing loss, complete	This person is unable to hear and understand another person talking, even in a quiet place, and unable to take part in a phone conversation. Difficulties with communicating and relating to others cause emotional impact at times (for example worry or depression).	0.215 (0.143-0.307)
Hearing loss, complete, with ringing	This person cannot hear at all in any situation, including even the loudest sounds, and cannot communicate verbally or use a phone. Difficulties with communicating and relating to others often cause worry, depression or loneliness.	0.316 (0.211-0.436)

Modeling strategy

We modeled the prevalence of hearing loss over five steps. First, we ran three DisMod-MR 2.0 models to estimate the total prevalence estimates of hearing loss: normal hearing (0- 19dB), mild hearing loss (20-34dB), and moderate hearing loss and above (35+ dB). We squeezed the prevalence estimates from these

DisMod-MR 2.0 models to fit within the entire population of each country. We estimated prevalence of normal hearing for this squeezing purpose only, and hence did not form part of further analysis.

Second, we ran five additional DisMod-MR 2.0 models for each severity levels of hearing loss above mild: moderate (35-49dB), moderately severe (50-64dB), severe (65-79dB), profound (80-94dB), and complete (95+). We then squeezed the prevalence estimates from these models to fit within the prevalence that were estimated for 35+dB in the first step. By the end of the second step, we had estimated prevalence of six severity levels of hearing loss, including mild (20-34dB). We also ran a DisMod-MR 2.0 model for the coverage of hearing aids, using (logged) lag distributed income (LDI) as a covariate.

Cause	Variable_Name	Beta	Exponentiated
Hearing aids (proportion of total hearing loss)	LDI (I$ per capita)	.84 (0.51 – 1.00)	2.31 (1.66 - 2.71)

Third, we adjusted the prevalence of each severity level by accounting for hearing aids. We assumed the use of hearing aids reduced the severity by one level. Data obtained from a survey in Norway provided detailed information on people with hearing aids, which was used to estimate the proportion of hearing aids for each severity level. We ran a log-linear regression on age with binary indicator for severity levels. We calculated country-specific hearing aid coverage by multiplying the severity-specific coverage in Norway by the ratio of hearing aid coverage in a given country to that of Norway for each age-sex. We shifted the identified fraction of people in each severity level a level below, except for complete hearing loss which we assumed was not correctable by hearing aids. This provided the adjusted prevalence of six severity levels of all-cause hearing loss.

Fourth, we estimated the prevalence of hearing loss due to multiple causes: otitis media, congenital, meningitis (pneumococcal, H influenza type B meningitis, meningococcal, and other bacterial), and age-related and other causes not classified elsewhere. For congenital hearing loss, we assumed that all hearing losses occurring at the time of birth are of congenital nature. We assumed that all hearing loss due to otitis media is at the mild or moderate level. We implemented proportional squeezes to scale cause-specific hearing loss prevalence to the total prevalence of each severity level.

Finally, we estimated the percent of people experiencing tinnitus for at least five minutes per day by severity level using data from the NHANES and two datasets from the United Kingdom. We calculated confidence intervals assuming a binomial distribution. We assumed the same distribution of tinnitus across all types of hearing loss.

There are no major changes in the modeling strategy from GBD 2013.

Section 4. Methods Appendix: Tables & Figures

Appendix Figure 1a. Analytical flowchart for the estimation of cause-specific YLDs by location, age, sex, and year for GBD 2015

Appendix Figure 1b. Analytical flowchart for modeling strategies other than DisMod MR 2.1 and injuries for select nonfatal cause groups

Appendix Figure 2. DisMod MR 2.1 Analytical Cascade

Appendix Figure 3. Map of percent of causes with any data available between 1990 and 2015 for 195 countries

Appendix Figure 4. Global decomposition of changes in leading 30 level 3 causes of years lived with disability (YLDs) due to population growth, population ageing, and changes in age-specific YLD rates, 2005 to 2015

Appendix Table 1. Guidelines for Accurate and Transparent Health Estimates Reporting (GATHER) 18-item checklist with description of compliance and location of information for this GBD 2015 nonfatal health outcomes publication

Appendix Table 2. GBD 2015 geography hierarchy with levels

Appendix Table 3. GBD 2015 cause and sequela hierarchy with four levels of causes (1-4) and two levels of sequelae (5-6)

Appendix Table 4. ICD-9 and ICD-10 codes used in the extraction of hospital and claims data, mapped to GBD 2015 nonfatal causes, impairments, and nature of injury categories

Appendix Table 5a. Data Representativeness Index (DRI), the percentage of GBD 2015 geographies with any data, by cause, pertaining to period before 2005, 2005-2015, and all years of data

Appendix Table 5b. Data Representativeness Index (DRI), the percentage of GBD 2015 geographies with any data, by impairment, pertaining to period before 2005, 2005-2015, and all years of data

Appendix Table 6. GBD 2015 sequelae, health states, health state lay descriptions, and disability weights

Appendix Table 7. GBD 2015 methods of estimating years lived with disability (YLDs) for 35 residual categories

Appendix Table 8. Socio-demographic Index (SDI) values for all estimated GBD geographies, 1980-2015

Appendix Figure 1a. Analytical flowchart for the estimation of cause-specific YLDs by location, age, sex, and year for GBD 2015

Ovals represent data inputs, square boxes represent analytical steps, cylinders represent databases, and parallelograms represent intermediate and final results. The flowchart is color-coded by major estimation component: raw data sources, in pink; data adjustments, in yellow; DisMod-MR 2.1 estimation, in purple; alternative modelling strategies, in light green; injury modeling strategy, in dark green; estimation of impairments and underlying causes, in brown; severity distributions and comorbidity correction, in blue; disability weights in orange; and cause of death and demographic inputs, in grey. GBD = Global Burden of Disease; TB=tuberculosis; HIV =human immunodeficiency virus; AIDS=acquired immunodeficiency syndrome; SF-12=Short Form 12 questions; MEPS=Medical Expenditure Panel Surveys; CSMR=cause-specific mortality rate; SMR=standardized mortality ratio; YLDs=years lived with disability; YLLs=years of life lost.

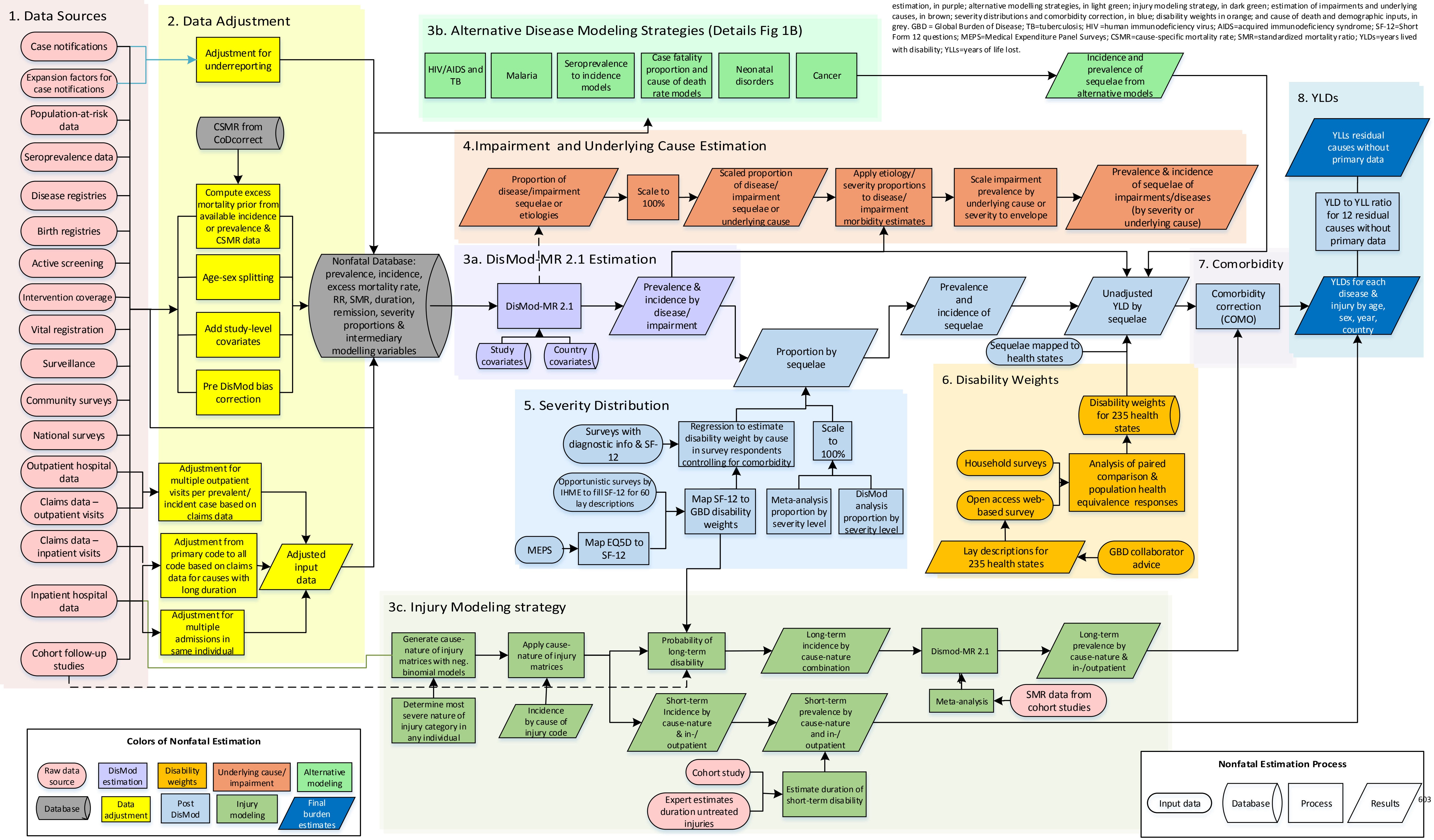

Appendix Figure 1b. Analytical flowchart for modeling strategies other than DisMod MR 2.1 and injuries for select nonfatal cause groups

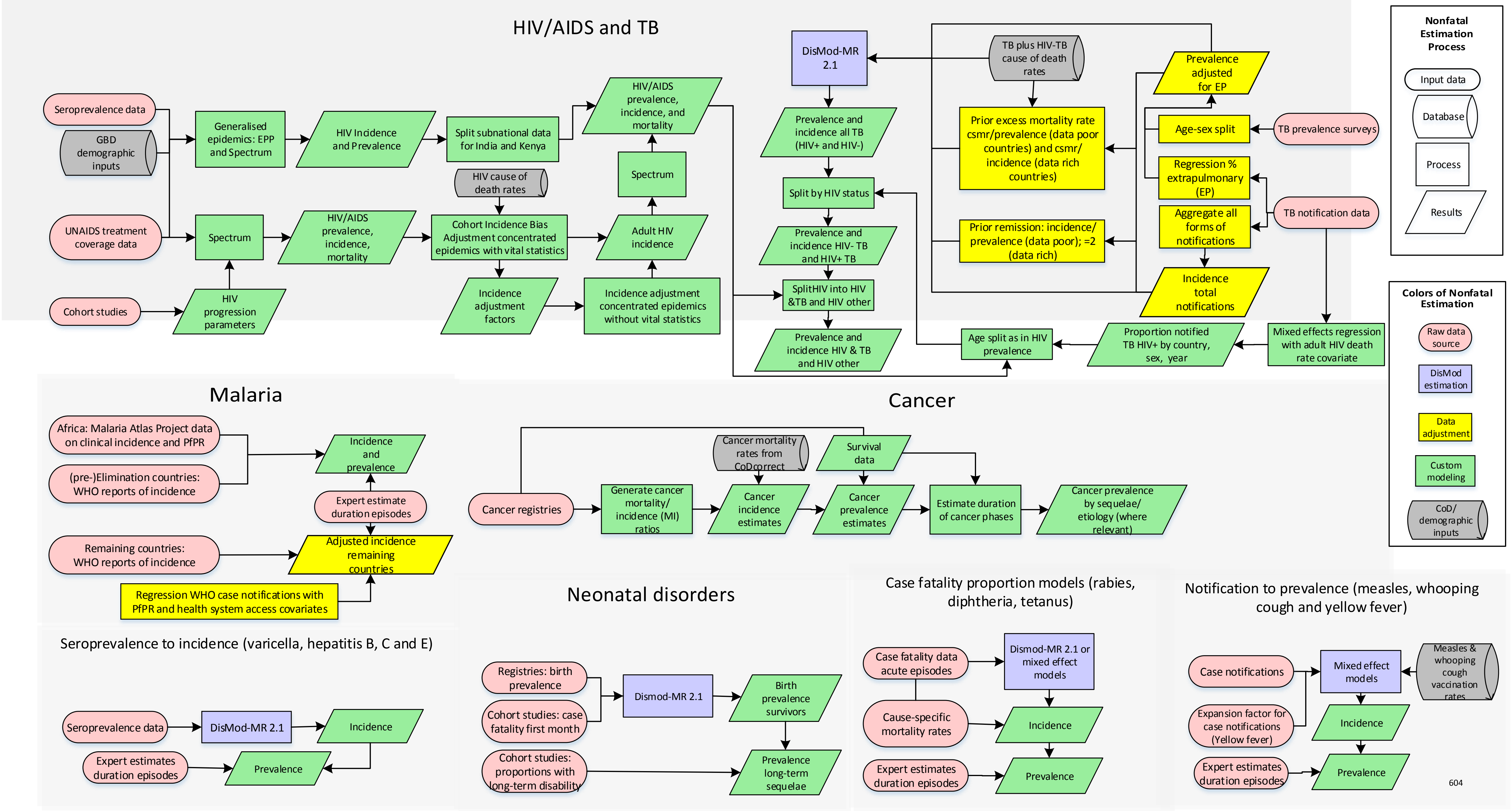

Appendix Figure 2. GBD 2015 DisMod-MR 2.1 Analytical Cascade

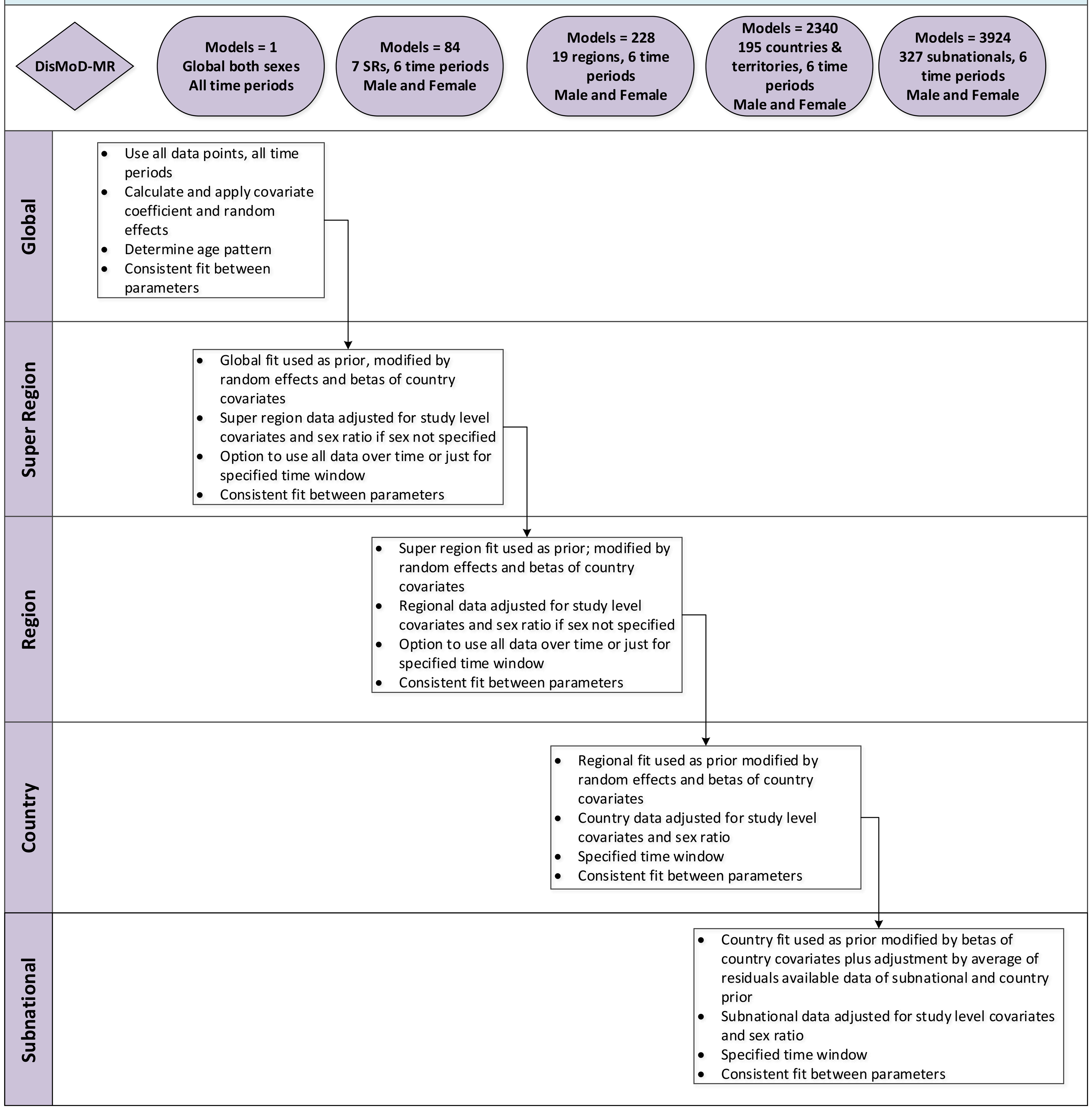

Appendix Figure 3. Map of percent of causes with any data available between 1990 and 2015 for 195 countries

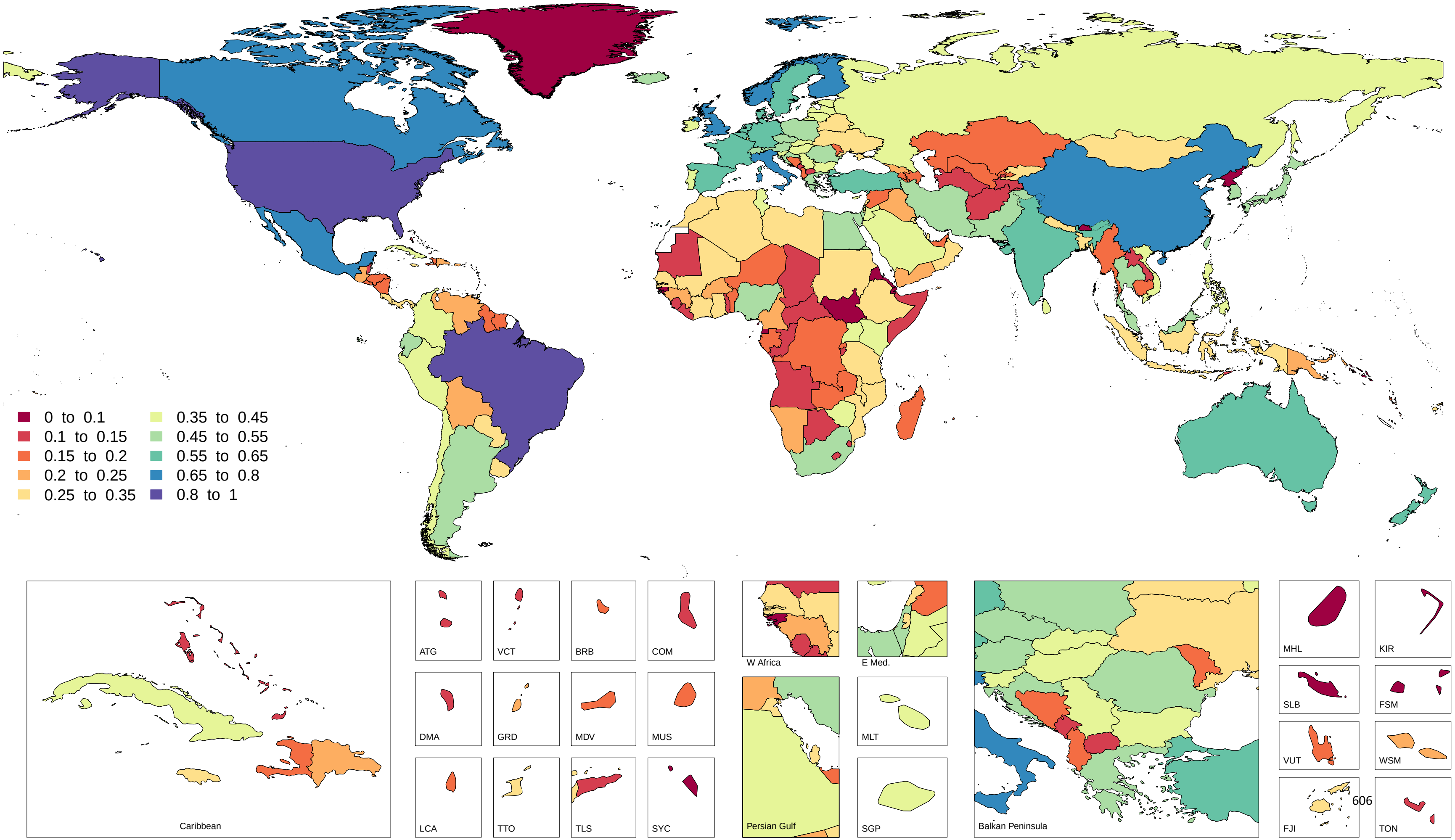

Appendix Figure 4. Global decomposition of changes in leading 30 level 3 causes of years lived with disability (YLDs) due to population growth, population ageing, and changes in age-specific YLD rates, 2005 to 2015

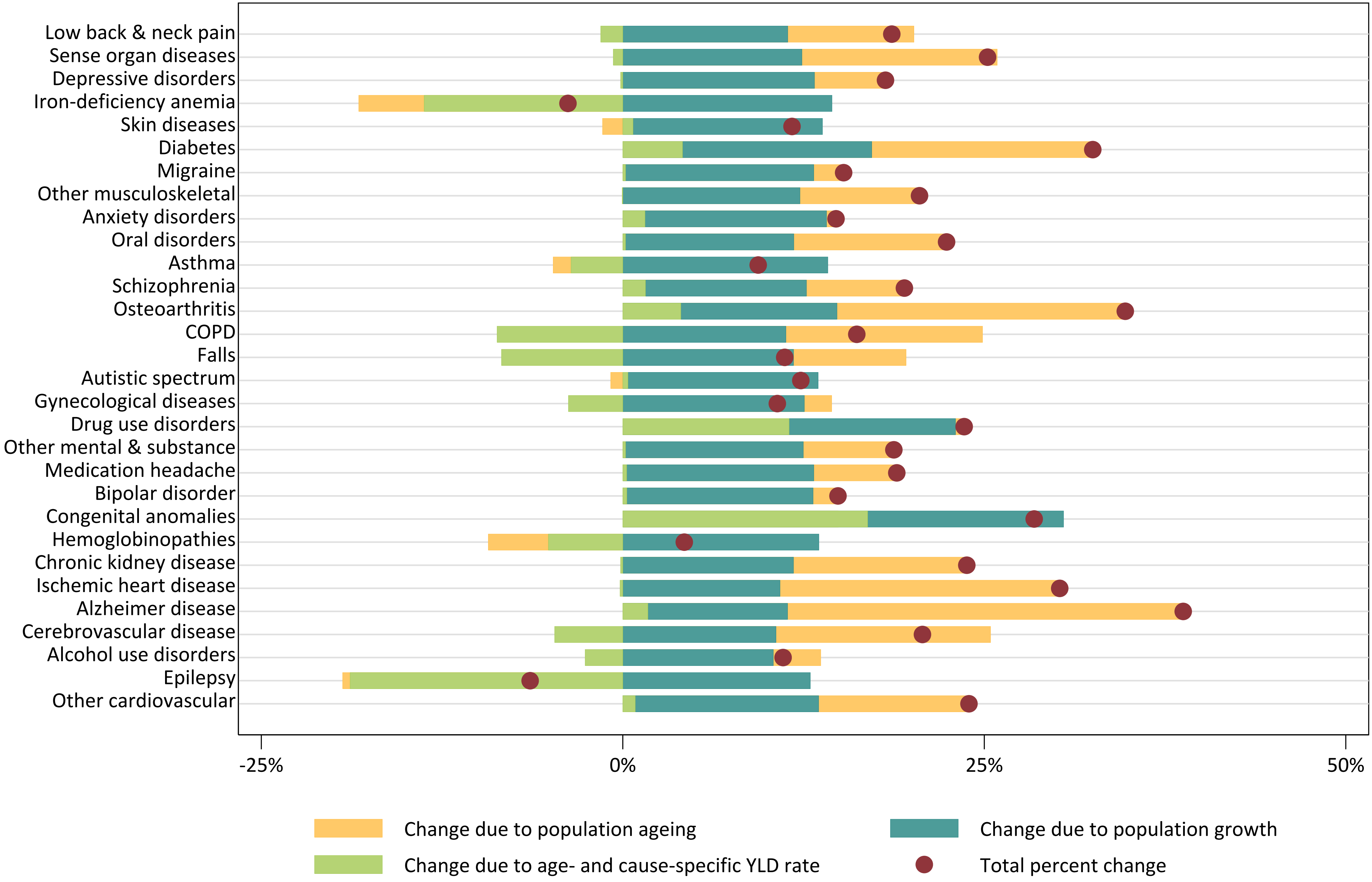

Appendix Table 1. GATHER checklist of information that should be included in reports of global health estimates, with description of compliance and location of information for GBD 2015 non-fatal health outcomes capstone

#	GATHER checklist item	Description of compliance	Reference
**Objectives and funding**			
1	Define the indicators, populations, and time periods for which estimates were made.	Narrative provided in paper and appendix describing indicators, definitions, and populations.	Manuscript; Methods Appendix, Section 1. GBD Overview
2	List the funding sources for the work.	Funding sources listed in paper.	Main text, Summary, Funding.
**Data Inputs**			
For all data inputs from multiple sources that are synthesized as part of the study:			
3	Describe how the data were identified and how the data were accessed.	Narrative description of data seeking methodology provided.	Main text, Methods, Nonfatal outcome estimation, 1. Data sources identification and extraction; and Methods Appendix, Section 2. Nonfatal outcome estimation, Step 1. Data sources, identification and extraction
4	Specify the inclusion and exclusion criteria. Identify all ad-hoc exclusions.	Narrative about inclusion and exclusion criteria by data type provided.	Main text, Methods, Nonfatal outcome estimation, 1. Data sources identification and extraction; and Methods Appendix, Section 2. Nonfatal outcome estimation, Step 1. Data sources, identification and extraction; Section 3. Cause-specific estimation process
5	Provide information on all included data sources and their main characteristics. For each data source used, report reference information or contact name/institution, population represented, data collection method, year(s) of data collection, sex and age range, diagnostic criteria or measurement method, and sample size, as relevant.	List of all data sources provided in submission materials; interactive, online data source tool that provides metadata for data sources by component, geography, cause, risk, or impairment has been developed.	Methods Appendix, Section 2. Nonfatal outcome estimation, Step 1. Data sources, identification and extraction; and online data tools (live with GBD 2015 at publication): http://ghdx.healthdata.org/gbd-2013-data-citations
6	Identify and describe any categories of input data that have potentially important biases (e.g., based on characteristics listed in item 5).	Summary of known biases by cause included in methodological appendix.	Methods Appendix, Section 3. Cause-specific estimation process
For data inputs that contribute to the analysis but were not synthesized as part of the study:			

7	Describe and give sources for any other data inputs.	Included in list of all data sources provided in submission materials as well as online data source tool.	Methods Appendix, Section 2. Nonfatal outcome estimations, Step 1. Data sources, identification and extraction; and online data tools (to go live with GBD 2015 at publication) http://ghdx.healthdata.org/gbd-2013-data-citations
For all data inputs:			
8	Provide all data inputs in a file format from which data can be efficiently extracted (e.g., a spreadsheet as opposed to a PDF), including all relevant meta-data listed in item 5. For any data inputs that cannot be shared due to ethical or legal reasons, such as third-party ownership, provide a contact name or the name of the institution that retains the right to the data.	Downloads of input data will be available through online tools, including data visualization tools and data query tools. Input data not available in tools will be made available upon request.	Online data tools (to go live with GBD 2015 at publication) http://www.healthdata.org/results/data-visualizations; http://ghdx.healthdata.org/; http://ghdx.healthdata.org/gbd-data-tool
**Data analysis**			
9	Provide a conceptual overview of the data analysis method. A diagram may be helpful.	Flow diagrams of the overall methodological processes, as well as cause-specific modelling processes have been provided.	Main text, Advances in data and analysis; Appendix Figure 1a. Analytical flowchart for the estimation of cause-specific YLDs by location, age, sex, and year GBD 2015; Methods Appendix Figure 1b. Analytical flowchart for modelling strategies other than DisMod-MR 2.1 and injuries for select non-fatal cause groups; and Methods Appendix, Section 3. Cause-specific estimation; Figure 2. DisMod-MR 2.1 Analytical Cascade
10	Provide a detailed description of all steps of the analysis, including mathematical formulae. This description should cover, as relevant, data cleaning, data pre-processing, data adjustments and weighting of data sources, and mathematical or statistical model(s).	Flow diagrams and corresponding methodological write-ups for each cause and modelling processes have been provided.	Methods Appendix, Section 3. Cause-specific estimation; Figure 2. DisMod-MR 2.1 Analytical Cascade
11	Describe how candidate models were evaluated and how the final model(s) were selected.	Provided in the methodological write-ups.	Methods Appendix, Section 3. Cause-specific estimation
12	Provide the results of an evaluation of model performance, if done, as well as the results of any relevant sensitivity analysis.	Provided in the methodological write-ups.	Methods Appendix, Section 3. Cause-specific estimation
13	Describe methods for calculating uncertainty of the estimates. State which sources of uncertainty were, and were not, accounted for in the uncertainty analysis.	Provided in the methodological write-ups.	Methods Appendix, Section 3. Cause-specific estimation

14	State how analytic or statistical source code used to generate estimates can be accessed.	Access statement provided.	http://ghdx.healthdata.org/global-burden-disease-study-2015
**Results and Discussion**			
15	Provide published estimates in a file format from which data can be efficiently extracted.	GBD 2015 results will be made available through online data visualization tools, the Global Health Data Exchange, and the online data query tool (these tools are already available for GBD 2013 results).	Main text; SR Appendix for Non-fatal Health Outcomes; and online data tools (to go live with GBD 2015 at publication) http://www.healthdata.org/results/data-visualizations; http://ghdx.healthdata.org/; http://ghdx.healthdata.org/gbd-data-tool
16	Report a quantitative measure of the uncertainty of the estimates (e.g. uncertainty intervals).	Uncertainty intervals are provided with all results.	Main text; Methods Appendix, Section 3. Cause-specific estimation process; and online data tools (to go live with GBD 2015 at publication) http://www.healthdata.org/results/data-visualizations; http://ghdx.healthdata.org/; http://ghdx.healthdata.org/gbd-data-tool
17	Interpret results in light of existing evidence. If updating a previous set of estimates, describe the reasons for changes in estimates.	Discussion of methodological changes between GBD rounds provided in the narrative of the paper and appendix.	Main text, Advances in data and analysis; Methods Appendix, Section 3. Cause-specific estimation process
18	Discuss limitations of the estimates. Include a discussion of any modelling assumptions or data limitations that affect interpretation of the estimates.	Discussion of limitations provided in the narrative of the main paper as well as in the methodological write-ups in the appendix.	Main text, Comparison to other global health estimates, Limitations; and Methods Appendix, Section 3. Cause-specific estimation process; Methods Appendix Figure 3; Methods Appendix Table 5a/b

Appendix Table 2. GBD 2015 geography hierarchy with levels

Location	Level
Global	0
High SDI	1
High-middle SDI	1
Middle SDI	1
Low-middle SDI	1
Low SDI	1
Southeast Asia, East Asia, and Oceania	1
-East Asia	2
-- China	3
--- Anhui	4
--- Beijing	4
--- Chongqing	4
--- Fujian	4
--- Gansu	4
--- Guangdong	4
--- Guangxi	4
--- Guizhou	4
--- Hainan	4
--- Hebei	4
--- Heilongjiang	4
--- Henan	4
-- Hong Kong, Special Administrative Region of China	4
--- Hubei	4
--- Hunan	4
--- Inner Mongolia	4
--- Jiangsu	4
--- Jiangxi	4
--- Jilin	4
--- Liaoning	4
-- Macao, Special Administrative Region of China	4
--- Ningxia	4
--- Qinghai	4
--- Shaanxi	4
--- Shandong	4
--- Shanghai	4
--- Shanxi	4
--- Sichuan	4
--- Tianjin	4
--- Tibet	4
--- Xinjiang	4
--- Yunnan	4
--- Zhejiang	4
-- North Korea	3
-- Taiwan	3

Appendix Table 2. GBD 2015 geography hierarchy with levels

Location	Level
-Southeast Asia	2
-- Cambodia	3
-- Indonesia	3
-- Laos	3
-- Malaysia	3
-- Maldives	3
-- Mauritius	3
-- Myanmar	3
-- Philippines	3
-- Sri Lanka	3
-- Seychelles	3
-- Thailand	3
-- Timor-Leste	3
-- Vietnam	3
-Oceania	2
-- American Samoa	3
-- Federated States of Micronesia	3
-- Fiji	3
-- Guam	3
-- Kiribati	3
-- Marshall Islands	3
-- Northern Mariana Islands	3
-- Papua New Guinea	3
-- Samoa	3
-- Solomon Islands	3
-- Tonga	3
-- Vanuatu	3
Central Europe, Eastern Europe, and Central Asia	1
-Central Asia	2
-- Armenia	3
-- Azerbaijan	3
-- Georgia	3
-- Kazakhstan	3
-- Kyrgyzstan	3
-- Mongolia	3
-- Tajikistan	3
-- Turkmenistan	3
-- Uzbekistan	3
-Central Europe	2
-- Albania	3
-- Bosnia and Herzegovina	3
-- Bulgaria	3
-- Croatia	3
-- Czech Republic	3

Appendix Table 2. GBD 2015 geography hierarchy with levels

Location	Level
-- Hungary	3
-- Macedonia	3
-- Montenegro	3
-- Poland	3
-- Romania	3
-- Serbia	3
-- Slovakia	3
-- Slovenia	3
-Eastern Europe	2
-- Belarus	3
-- Estonia	3
-- Latvia	3
-- Lithuania	3
-- Moldova	3
-- Russia	3
-- Ukraine	3
High-income	1
-High-income Asia Pacific	2
-- Brunei	3
-- Japan	3
--- Aichi	4
--- Akita	4
--- Aomori	4
--- Chiba	4
--- Ehime	4
--- Fukui	4
--- Fukuoka	4
--- Fukushima	4
--- Gifu	4
--- Gunma	4
--- Hiroshima	4
--- Hokkaidō	4
--- Hyōgo	4
--- Ibaraki	4
--- Ishikawa	4
--- Iwate	4
--- Kagawa	4
--- Kagoshima	4
--- Kanagawa	4
--- Kōchi	4
--- Kumamoto	4
--- Kyōto	4
--- Mie	4
--- Miyagi	4
--- Miyazaki	4

Appendix Table 2. GBD 2015 geography hierarchy with levels

Location	Level
--- Nagano	4
--- Nagasaki	4
--- Nara	4
--- Niigata	4
--- Ôita	4
--- Okayama	4
--- Okinawa	4
--- Ōsaka	4
--- Saga	4
--- Saitama	4
--- Shiga	4
--- Shimane	4
--- Shizuoka	4
--- Tochigi	4
--- Tokushima	4
--- Tōkyō	4
--- Tottori	4
--- Toyama	4
--- Wakayama	4
--- Yamagata	4
--- Yamaguchi	4
--- Yamanashi	4
-- South Korea	3
-- Singapore	3
-Australasia	2
-- Australia	3
-- New Zealand	3
-Western Europe	2
-- Andorra	3
-- Austria	3
-- Belgium	3
-- Cyprus	3
-- Denmark	3
-- Finland	3
-- France	3
-- Germany	3
-- Greece	3
-- Iceland	3
-- Ireland	3
-- Israel	3
-- Italy	3
-- Luxembourg	3
-- Malta	3
-- Netherlands	3
-- Norway	3

Appendix Table 2. GBD 2015 geography hierarchy with levels

Location	Level
-- Portugal	3
-- Spain	3
-- Sweden	3
--- Stockholm	4
--- Sweden except Stockholm	4
-- Switzerland	3
-- United Kingdom	3
--- England	4
---- East Midlands	5
---- East of England	5
---- Greater London	5
---- North East England	5
---- North West England	5
---- South East England	5
---- South West England	5
---- West Midlands	5
---- Yorkshire and the Humber	5
--- Northern Ireland	4
--- Scotland	4
--- Wales	4
-Southern Latin America	2
-- Argentina	3
-- Chile	3
-- Uruguay	3
-High-income North America	2
-- Canada	3
-- Greenland	3
-- United States	3
--- Alabama	4
--- Alaska	4
--- Arizona	4
--- Arkansas	4
--- California	4
--- Colorado	4
--- Connecticut	4
--- Delaware	4
--- District of Columbia	4
--- Florida	4
--- Georgia	4
--- Hawaii	4
--- Idaho	4
--- Illinois	4
--- Indiana	4
--- Iowa	4
--- Kansas	4

Appendix Table 2. GBD 2015 geography hierarchy with levels	
**Location**	**Level**
--- Kentucky	4
--- Louisiana	4
--- Maine	4
--- Maryland	4
--- Massachusetts	4
--- Michigan	4
--- Minnesota	4
--- Mississippi	4
--- Missouri	4
--- Montana	4
--- Nebraska	4
--- Nevada	4
--- New Hampshire	4
--- New Jersey	4
--- New Mexico	4
--- New York	4
--- North Carolina	4
--- North Dakota	4
--- Ohio	4
--- Oklahoma	4
--- Oregon	4
--- Pennsylvania	4
--- Rhode Island	4
--- South Carolina	4
--- South Dakota	4
--- Tennessee	4
--- Texas	4
--- Utah	4
--- Vermont	4
--- Virginia	4
--- Washington	4
--- West Virginia	4
--- Wisconsin	4
--- Wyoming	4
Latin America and Caribbean	1
-Caribbean	2
-- Antigua and Barbuda	3
-- The Bahamas	3
-- Barbados	3
-- Belize	3
-- Bermuda	3
-- Cuba	3
-- Dominica	3
-- Dominican Republic	3
-- Grenada	3

Appendix Table 2. GBD 2015 geography hierarchy with levels	
Location	Level
-- Guyana	3
-- Haiti	3
-- Jamaica	3
-- Puerto Rico	3
-- Saint Lucia	3
-- Saint Vincent and the Grenadines	3
-- Suriname	3
-- Trinidad and Tobago	3
-- Virgin Islands, U.S.	3
-Andean Latin America	2
-- Bolivia	3
-- Ecuador	3
-- Peru	3
-Central Latin America	2
-- Colombia	3
-- Costa Rica	3
-- El Salvador	3
-- Guatemala	3
-- Honduras	3
-- Mexico	3
--- Aguascalientes	4
--- Baja California	4
--- Baja California Sur	4
--- Campeche	4
--- Chiapas	4
--- Chihuahua	4
--- Coahuila	4
--- Colima	4
--- Distrito Federal	4
--- Durango	4
--- Guanajuato	4
--- Guerrero	4
--- Hidalgo	4
--- Jalisco	4
--- México	4
--- Michoacán de Ocampo	4
--- Morelos	4
--- Nayarit	4
--- Nuevo León	4
--- Oaxaca	4
--- Puebla	4
--- Querétaro	4
--- Quintana Roo	4
--- San Luis Potosí	4
--- Sinaloa	4

Appendix Table 2. GBD 2015 geography hierarchy with levels

Location	Level
--- Sonora	4
--- Tabasco	4
--- Tamaulipas	4
--- Tlaxcala	4
--- Veracruz de Ignacio de la Llave	4
--- Yucatán	4
--- Zacatecas	4
-- Nicaragua	3
-- Panama	3
-- Venezuela	3
-Tropical Latin America	2
-- Brazil	3
--- Acre	4
--- Alagoas	4
--- Amapá	4
--- Amazonas	4
--- Bahia	4
--- Ceará	4
--- Distrito Federal	4
--- Espírito Santo	4
--- Goiás	4
--- Maranhão	4
--- Mato Grosso	4
--- Mato Grosso do Sul	4
--- Minas Gerais	4
--- Pará	4
--- Paraíba	4
--- Paraná	4
--- Pernambuco	4
--- Piaui	4
--- Rio de Janeiro	4
--- Rio Grande do Norte	4
--- Rio Grande do Sul	4
--- Rondônia	4
--- Roraima	4
--- Santa Catarina	4
--- São Paulo	4
--- Sergipe	4
--- Tocantins	4
-- Paraguay	3
North Africa and Middle East	1
-North Africa and Middle East	2
-- Afghanistan	3
-- Algeria	3
-- Bahrain	3

Appendix Table 2. GBD 2015 geography hierarchy with levels	
**Location**	**Level**
-- Egypt	3
-- Iran	3
-- Iraq	3
-- Jordan	3
-- Kuwait	3
-- Lebanon	3
-- Libya	3
-- Morocco	3
-- Palestine	3
-- Oman	3
-- Qatar	3
-- Saudi Arabia	3
--- 'Asir	4
--- Bahah	4
--- Eastern Province	4
--- Ha'il	4
--- Jawf	4
--- Jizan	4
--- Madinah	4
--- Makkah	4
--- Najran	4
--- Northern Borders	4
--- Qassim	4
--- Riyadh	4
--- Tabuk	4
-- Sudan	3
-- Syria	3
-- Tunisia	3
-- Turkey	3
-- United Arab Emirates	3
-- Yemen	3
South Asia	1
-South Asia	2
-- Bangladesh	3
-- Bhutan	3
-- India	3
--- Andhra Pradesh	4
---- Andhra Pradesh, Rural	5
---- Andhra Pradesh, Urban	5
--- Arunāchal Pradesh	4
---- Arunāchal Pradesh, Rural	5
---- Arunāchal Pradesh, Urban	5
--- Assam	4
---- Assam, Rural	5
---- Assam, Urban	5

Appendix Table 2. GBD 2015 geography hierarchy with levels

Location	Level
--- Bihār	4
---- Bihār, Rural	5
---- Bihār, Urban	5
--- Chhattīsgarh	4
---- Chhattīsgarh, Rural	5
---- Chhattīsgarh, Urban	5
--- Delhi	4
---- Delhi, Rural	5
---- Delhi, Urban	5
--- Goa	4
---- Goa, Rural	5
---- Goa, Urban	5
--- Gujarāt	4
---- Gujarāt, Rural	5
---- Gujarāt, Urban	5
--- Haryāna	4
---- Haryāna, Rural	5
---- Haryāna, Urban	5
--- Himachal Pradesh	4
---- Himachal Pradesh, Rural	5
---- Himachal Pradesh, Urban	5
--- Jammu and Kashmīr	4
---- Jammu and Kashmīr, Rural	5
---- Jammu and Kashmīr, Urban	5
--- Jharkhand	4
---- Jharkhand, Rural	5
---- Jharkhand, Urban	5
--- Karnātaka	4
---- Karnātaka, Rural	5
---- Karnātaka, Urban	5
--- Kerala	4
---- Kerala, Rural	5
---- Kerala, Urban	5
--- Madhya Pradesh	4
---- Madhya Pradesh, Rural	5
---- Madhya Pradesh, Urban	5
--- Mahārāshtra	4
---- Mahārāshtra, Rural	5
---- Mahārāshtra, Urban	5
--- Manipur	4
---- Manipur, Rural	5
---- Manipur, Urban	5
--- Meghālaya	4
---- Meghālaya, Rural	5
---- Meghālaya, Urban	5

Appendix Table 2. GBD 2015 geography hierarchy with levels	
**Location**	**Level**
--- Mizoram	4
---- Mizoram, Rural	5
---- Mizoram, Urban	5
--- Nāgāland	4
---- Nāgāland, Rural	5
---- Nāgāland, Urban	5
--- Orissa	4
---- Orissa, Rural	5
---- Orissa, Urban	5
--- Punjab	4
---- Punjab, Rural	5
---- Punjab, Urban	5
--- Rājasthān	4
---- Rājasthān, Rural	5
---- Rājasthān, Urban	5
--- Sikkim	4
---- Sikkim, Rural	5
---- Sikkim, Urban	5
--- Tamil Nādu	4
---- Tamil Nādu, Rural	5
---- Tamil Nādu, Urban	5
--- Telangana	4
---- Telangana, Rural	5
---- Telangana, Urban	5
--- Tripura	4
---- Tripura, Rural	5
---- Tripura, Urban	5
--- Uttar Pradesh	4
---- Uttar Pradesh, Rural	5
---- Uttar Pradesh, Urban	5
--- Uttarakhand	4
---- Uttarakhand, Rural	5
---- Uttarakhand, Urban	5
--- West Bengal	4
---- West Bengal, Rural	5
---- West Bengal, Urban	5
--- The Six Minor Territories	4
---- The Six Minor Territories, Rural	5
---- The Six Minor Territories, Urban	5
-- Nepal	3
-- Pakistan	3
Sub-Saharan Africa	1
-Central Sub-Saharan Africa	2
-- Angola	3
-- Central African Republic	3

Appendix Table 2. GBD 2015 geography hierarchy with levels	
Location	Level
-- Congo	3
-- Democratic Republic of the Congo	3
-- Equatorial Guinea	3
-- Gabon	3
-Eastern Sub-Saharan Africa	2
-- Burundi	3
-- Comoros	3
-- Djibouti	3
-- Eritrea	3
-- Ethiopia	3
-- Kenya	3
--- Baringo	4
--- Bomet	4
--- Bungoma	4
--- Busia	4
--- Elgeyo-Marakwet	4
--- Embu	4
--- Garissa	4
--- Homa Bay	4
--- Isiolo	4
--- Kajiado	4
--- Kakamega	4
--- Kericho	4
--- Kiambu	4
--- Kilifi	4
--- Kirinyaga	4
--- Kisii	4
--- Kisumu	4
--- Kitui	4
--- Kwale	4
--- Laikipia	4
--- Lamu	4
--- Machakos	4
--- Makueni	4
--- Mandera	4
--- Marsabit	4
--- Meru	4
--- Migori	4
--- Mombasa	4
--- Murang'a	4
--- Nairobi	4
--- Nakuru	4
--- Nandi	4
--- Narok	4
--- Nyamira	4

Appendix Table 2. GBD 2015 geography hierarchy with levels	
**Location**	**Level**
--- Nyandarua	4
--- Nyeri	4
--- Samburu	4
--- Siaya	4
--- Taita Taveta	4
--- Tana River	4
--- Tharaka Nithi	4
--- Trans Nzoia	4
--- Turkana	4
--- Uasin Gishu	4
--- Vihiga	4
--- Wajir	4
--- West Pokot	4
-- Madagascar	3
-- Malawi	3
-- Mozambique	3
-- Rwanda	3
-- Somalia	3
-- South Sudan	3
-- Tanzania	3
-- Uganda	3
-- Zambia	3
-Southern Sub-Saharan Africa	2
-- Botswana	3
-- Lesotho	3
-- Namibia	3
-- South Africa	3
--- Eastern Cape	4
--- Free State	4
--- Gauteng	4
--- KwaZulu-Natal	4
--- Limpopo	4
--- Mpumalanga	4
--- North-West	4
--- Northern Cape	4
--- Western Cape	4
-- Swaziland	3
-- Zimbabwe	3
-Western Sub-Saharan Africa	2
-- Benin	3
-- Burkina Faso	3
-- Cameroon	3
-- Cape Verde	3
-- Chad	3
-- Cote d'Ivoire	3

Appendix Table 2. GBD 2015 geography hierarchy with levels	
**Location**	**Level**
-- The Gambia	3
-- Ghana	3
-- Guinea	3
-- Guinea-Bissau	3
-- Liberia	3
-- Mali	3
-- Mauritania	3
-- Niger	3
-- Nigeria	3
-- Sao Tome and Principe	3
-- Senegal	3
-- Sierra Leone	3
-- Togo	3

Appendix Table 3. GBD 2015 cause and sequela hierarchy with four levels of causes (1-4) and two levels of sequelae (5-6)	
**Causes and sequelae**	**Level**
All causes	0
Communicable, maternal, neonatal, and nutritional diseases	1
HIV/AIDS and tuberculosis	2
Tuberculosis	3
Tuberculosis	5
HIV/AIDS	3
HIV/AIDS - Tuberculosis	4
HIV/AIDS resulting in mycobacterial infection	5
HIV/AIDS resulting in other diseases	4
HIV	5
Early HIV	6
Symptomatic HIV	6
AIDS	5
AIDS with antiretroviral treatment	6
AIDS without antiretroviral treatment	6
Diarrhea, lower respiratory, and other common infectious diseases	2
Diarrheal diseases	3
Diarrhoea episodes	5
Mild diarrheal diseases	6
Moderate diarrheal diseases	6
Severe diarrheal diseases	6
Guillain-Barré syndrome due to diarrheal diseases	5
Intestinal infectious diseases	3
Typhoid fever	4
Typhoid fever episodes	5
Acute typhoid infection	6
Severe typhoid fever	6
Complications of typhoid fever	5
Intestinal perforation due to typhoid	6
Gastrointestinal bleeding due to typhoid	6
Paratyphoid fever	4
Paratyphoid fever episodes	5
Acute paratyphoid infection	6
Moderate paratyphoid fever	6
Severe paratyphoid fever	6
Intestinal perforation due to paratyphoid	5
Other intestinal infectious diseases	4
Other intestinal infectious diseases	5
Lower respiratory infections	3
Lower respiratory infection episodes	5
Moderate lower respiratory infections	6
Severe lower respiratory infections	6
Guillain-Barré syndrome due to lower respiratory infections	5
Upper respiratory infections	3
Upper respiratory infection episodes	5
Mild upper respiratory infections	6
Moderate upper respiratory infections	6
Guillain-Barré syndrome due to upper respiratory infections	5
Otitis media	3
Acute otitis media	5
Chronic otitis media	5
Severe infectious complications due to chronic otitis media	6
Vertigo with mild hearing loss due to chronic otitis media	6
Vertigo with mild hearing loss and ringing due to chronic otitis media	6
Mild hearing loss due to chronic otitis media	6
Mild hearing loss with ringing due to chronic otitis media	6
Vertigo with moderate hearing loss due to chronic otitis media	6
Vertigo with moderate hearing loss and ringing due to chronic otitis media	6
Moderate hearing loss due to chronic otitis media	6
Moderate hearing loss with ringing due to chronic otitis media	6
Meningitis	3
Pneumococcal meningitis	4

Appendix Table 3. GBD 2015 cause and sequela hierarchy with four levels of causes (1-4) and two levels of sequelae (5-6)	
**Causes and sequelae**	**Level**
Acute pneumococcal meningitis	5
Complications of pneumococcal meningitis	5
Mild behavioral problems due to pneumococcal meningitis	6
Mild motor impairment due to long term due to pneumococcal meningitis	6
Mild motor plus cognitive impairments due to pneumococcal meningitis	6
Borderline intellectual disability due to pneumococcal meningitis	6
Monocular distance vision loss due to pneumococcal meningitis	6
Mild intellectual disability due to pneumococcal meningitis	6
Moderate motor impairment due to pneumococcal meningitis	6
Severe motor impairment due to pneumococcal meningitis	6
Moderate motor plus cognitive impairments due to pneumococcal meningitis	6
Severe motor plus cognitive impairments due to pneumococcal meningitis	6
Epilepsy due to pneumococcal meningitis	6
Blindness due to pneumococcal meningitis	6
Mild hearing loss due to pneumococcal meningitis	6
Mild hearing loss with ringing due to pneumococcal meningitis	6
Moderate hearing loss due to pneumococcal meningitis	6
Moderate hearing loss with ringing due to pneumococcal meningitis	6
Moderately severe hearing loss due to pneumococcal meningitis	6
Moderately severe hearing loss with ringing due to pneumococcal meningitis	6
Severe hearing loss due to pneumococcal meningitis	6
Severe hearing loss with ringing due to pneumococcal meningitis	6
Profound hearing loss due to pneumococcal meningitis	6
Profound hearing loss with ringing due to pneumococcal meningitis	6
Complete hearing loss due to pneumococcal meningitis	6
Complete hearing loss with ringing due to pneumococcal meningitis	6
Moderate vision impairment due to pneumococcol meningitis	6
Severe vision impairment due to pneumococcol meningitis	6
H influenzae type B meningitis	4
Acute H influenzae type B meningitis	5
Complications of of H influenzae type B meningitis	5
Mild behavioral problems due to H influenzae type B meningitis	6
Mild motor impairment due to long term due to H influenzae type B meningitis	6
Mild motor plus cognitive impairments due to H influenzae type B meningitis	6
Borderline intellectual disability due to H influenzae type B meningitis	6
Monocular distance vision loss due to H influenzae type B meningitis	6
Mild intellectual disability due to H influenzae type B meningitis	6
Moderate motor impairment due to H influenzae type B meningitis	6
Severe motor impairment due to H influenzae type B meningitis	6
Moderate motor plus cognitive impairments due to H influenzae type B meningitis	6
Severe motor plus cognitive impairments due to H influenzae type B meningitis	6
Epilepsy due to H influenzae type B meningitis	6
Blindness due to H influenzae type B meningitis	6
Mild hearing loss due to H influenzae type B meningitis	6
Mild hearing loss with ringing due to H influenzae type B meningitis	6
Moderate hearing loss due to H influenzae type B meningitis	6
Moderate hearing loss with ringing due to H influenzae type B meningitis	6
Moderately severe hearing loss due to H influenzae type B meningitis	6
Moderately severe hearing loss with ringing due to H influenzae type B meningitis	6
Severe hearing loss due to H influenzae type B meningitis	6
Severe hearing loss with ringing due to H influenzae type B meningitis	6
Profound hearing loss due to H influenzae type B meningitis	6
Profound hearing loss with ringing due to H influenzae type B meningitis	6
Complete hearing loss due to H influenzae type B meningitis	6
Complete hearing loss with ringing due to H influenzae type B meningitis	6
Moderate vision impairment due to H influenza type B meningitis	6
Severe vision impairment due to H influenza type B meningitis	6
Meningococcal meningitis	4
Acute meningococcal meningitis	5
Complications of meningococcal meningitis	5
Mild behavioral problems due to meningococcal meningitis	6
Mild motor impairment due to long term due to meningococcal meningitis	6

**Appendix Table 3. GBD 2015 cause and sequela hierarchy with four levels of causes (1-4) and two levels of sequelae (5-6)**	
**Causes and sequelae**	**Level**
Mild motor plus cognitive impairments due to meningococcal meningitis	6
Borderline intellectual disability due to meningococcal meningitis	6
Monocular distance vision loss due to meningococcal meningitis	6
Mild intellectual disability due to meningococcal meningitis	6
Moderate motor impairment due to meningococcal meningitis	6
Severe motor impairment due to meningococcal meningitis	6
Moderate motor plus cognitive impairments due to meningococcal meningitis	6
Severe motor plus cognitive impairments due to meningococcal meningitis	6
Epilepsy due to meningococcal meningitis	6
Blindness due to meningococcal meningitis	6
Mild hearing loss due to meningococcal meningitis	6
Mild hearing loss with ringing due to meningococcal meningitis	6
Moderate hearing loss due to meningococcal meningitis	6
Moderate hearing loss with ringing due to meningococcal meningitis	6
Moderately severe hearing loss due to meningococcal meningitis	6
Moderately severe hearing loss with ringing due to meningococcal meningitis	6
Severe hearing loss due to meningococcal meningitis	6
Severe hearing loss with ringing due to meningococcal meningitis	6
Profound hearing loss due to meningococcal meningitis	6
Profound hearing loss with ringing due to meningococcal meningitis	6
Complete hearing loss due to meningococcal meningitis	6
Complete hearing loss with ringing due to meningococcal meningitis	6
Moderate vision impairment due to meningococcal meningitis	6
Severe vision impairment due to meningococcal meningitis	6
Other meningitis	4
Other meningitis episodes	5
Acute viral meningitis	6
Other acute bacterial meningitis	6
Complications of other meningitis	5
Mild behavioral problems due to other bacterial meningitis	6
Mild motor impairment due to long term due to other bacterial meningitis	6
Mild motor plus cognitive impairments due to other bacterial meningitis	6
Borderline intellectual disability due to other bacterial meningitis	6
Monocular distance vision loss due to other bacterial meningitis	6
Mild intellectual disability due to other bacterial meningitis	6
Moderate motor impairment due to other bacterial meningitis	6
Severe motor impairment due to other bacterial meningitis	6
Moderate motor plus cognitive impairments due to other bacterial meningitis	6
Severe motor plus cognitive impairments due to other bacterial meningitis	6
Epilepsy due to other meningitis	6
Blindness due to other bacterial meningitis	6
Mild hearing loss due to other bacterial meningitis	6
Mild hearing loss due with ringing to other bacterial meningitis	6
Moderate hearing loss due to other bacterial meningitis	6
Moderate hearing loss with ringing due to other bacterial meningitis	6
Moderately severe hearing loss due to other bacterial meningitis	6
Moderately severe hearing loss with ringing due to other bacterial meningitis	6
Severe hearing loss due to other bacterial meningitis	6
Severe hearing loss with ringing due to other bacterial meningitis	6
Profound hearing loss due to other bacterial meningitis	6
Profound hearing loss with ringing due to other bacterial meningitis	6
Complete hearing loss due to other bacterial meningitis	6
Complete hearing loss with ringing due to other bacterial meningitis	6
Moderate vision impairment due to other bacterial meningitis	6
Severe vision impairment due to other bacterial meningitis	6
Encephalitis	3
Acute encephalitis	5
Complications of encephalitis	5
Mild behavioral problems due to encephalitis	6
Mild motor impairment due to long term due to encephalitis	6
Mild motor plus cognitive impairments due to encephalitis	6
Borderline intellectual disability due to encephalitis	6

Appendix Table 3. GBD 2015 cause and sequela hierarchy with four levels of causes (1-4) and two levels of sequelae (5-6)	
**Causes and sequelae**	**Level**
Monocular distance vision loss due to encephalitis	6
Mild intellectual disability due to encephalitis	6
Moderate motor impairment due to encephalitis	6
Severe motor impairment due to encephalitis	6
Moderate motor plus cognitive impairments due to encephalitis	6
Severe motor plus cognitive impairments due to encephalitis	6
Epilepsy due to encephalitis	6
Blindness due to encephalitis	6
Moderate vision impairment due to encephalitis	6
Severe vision impairment due to encephalitis	6
Diphtheria	3
Moderate diphtheria	5
Severe diphtheria	5
Whooping cough	3
Whooping cough	5
Tetanus	3
Severe tetanus	5
Complications of tetanus	5
Mild motor impairment due to neonatal tetanus	6
Mild motor plus cognitive impairments due to neonatal tetanus	6
Moderate motor impairment due to neonatal tetanus	6
Moderate motor impairment with blindness due to neonatal tetanus	6
Moderate motor impairment with epilepsy due to neonatal tetanus	6
Moderate motor impairment with blindness and epilepsy due to neonatal tetanus	6
Moderate motor plus cognitive impairment with blindness due to neonatal tetanus	6
Moderate motor plus cognitive impairment with epilepsy due to neonatal tetanus	6
Moderate motor plus cognitive impairment with blindness and epilepsy due to neonatal tetanus	6
Severe motor impairment due to neonatal tetanus	6
Severe motor impairment with blindness due to neonatal tetanus	6
Severe motor impairment with epilepsy due to neonatal tetanus	6
Severe motor impairment with blindness and epilepsy due to neonatal tetanus	6
Severe motor plus cognitive impairment with blindness due to neonatal tetanus	6
Severe motor plus cognitive impairment with epilepsy due to neonatal tetanus	6
Severe motor plus cognitive impairment with blindness and epilepsy due to neonatal tetanus	6
Measles	3
Moderate measles	5
Severe measles	5
Varicella and herpes zoster	3
Chickenpox	5
Herpes zoster	5
Neglected tropical diseases and malaria	2
Malaria	3
Asymptomatic malaria parasitemia (PfPR)	5
Malaria episodes	5
Mild malaria	6
Moderate malaria	6
Severe malaria	6
Complications of malaria	5
Moderate motor impairment due to malaria	6
Moderate motor impairment with blindness due to malaria	6
Moderate motor impairment with epilepsy due to malaria	6
Moderate motor impairment with blindness and epilepsy due to malaria	6
Moderate motor plus cognitive impairment with blindness due to malaria	6
Moderate motor plus cognitive impairment with epilepsy due to malaria	6
Moderate motor plus cognitive impairment with blindness and epilepsy due to malaria	6
Severe motor impairment due to malaria	6
Severe motor impairment with blindness due to malaria	6
Severe motor impairment with epilepsy due to malaria	6
Severe motor impairment with blindness and epilepsy due to malaria	6
Severe motor plus cognitive impairment with blindness due to malaria	6
Severe motor plus cognitive impairment with epilepsy due to malaria	6
Severe motor plus cognitive impairment with blindness and epilepsy due to malaria	6

Appendix Table 3. GBD 2015 cause and sequela hierarchy with four levels of causes (1-4) and two levels of sequelae (5-6)	
**Causes and sequelae**	**Level**
Anemia due to malaria parasitemia (PfPR)	5
Mild anemia due to malaria parasitemia (PfPR)	6
Moderate anemia due to malaria parasitemia (PfPR)	6
Severe anemia due to malaria parasitemia (PfPR)	6
Chagas disease	3
Chagas disease episodes	5
Acute Chagas disease	6
Asymptomatic Chagas disease	6
Complications of Chagas disease	5
Mild chronic digestive disease due to Chagas disease	6
Moderate chronic digestive disease due to Chagas disease	6
Atrial fibrillation and flutter due to Chagas disease	6
Heart failure due to Chagas disease	5
Mild heart failure due to Chagas disease	6
Moderate heart failure due to Chagas disease	6
Severe heart failure due to Chagas disease	6
Leishmaniasis	3
Visceral leishmaniasis	4
Moderate visceral leishmaniasis	5
Severe visceral leishmaniasis	5
Cutaneous and mucocutaneous leishmaniasis	4
Cutaneous and mucocutaneous leishmaniasis	5
African trypanosomiasis	3
Disfigurement due to African trypanosomiasis	5
Severe motor plus cognitive impairments due to African trypanosomiasis	5
Schistosomiasis	3
Schistosomiasis episodes	5
Mild schistosomiasis	6
Mild diarrhea due to schistosomiasis	6
Complications of schistosomiasis	5
Hematemesis due to schistosomiasis	6
Hepatomegaly due to schistosomiasis	6
Ascites due to schistosomiasis	6
Dysuria due to schistosomiasis	6
Bladder pathology due to schistosomiasis	6
Hydronephrosis due to schistosomiasis	6
Anemia due to schistosomiasis	5
Mild anemia due to schistosomiasis	6
Moderate anemia due to schistosomiasis	6
Severe anemia due to schistosomiasis	6
Cysticercosis	3
Neurocysticercosis with epilepsy	5
Cystic echinococcosis	3
Abdominal problems due to cystic echinococcosis	5
Chronic respiratory disease due to cystic echinococcosis	5
Epilepsy due to echinococcosis	5
Lymphatic filariasis	3
Prevalence of detectable microfiliaria due to lymphatic filariasis	5
Complications of lymphatic filariasis	5
Lymphedema due to lymphatic filariasis	6
Hydrocele due to lymphatic filariasis	6
Onchocerciasis	3
Asymptomatic onchocerciasis	5
Skin disease due to onchocerciasis	5
Mild skin disease due to onchocerciasis	6
Mild skin disease without itch due to onchocerciasis	6
Moderate skin disease due to onchocerciasis	6
Severe skin disease due to onchocerciasis	6
Severe skin disease without itch due to onchocerciasis	6
Vision loss due to onchocerciasis	5
Moderate vision impairment due to onchocerciasis	6
Severe vision impairment due to onchocerciasis	6

Appendix Table 3. GBD 2015 cause and sequela hierarchy with four levels of causes (1-4) and two levels of sequelae (5-6)	
**Causes and sequelae**	**Level**
Blindness due to onchocerciasis	6
Trachoma	3
Moderate vision impairment due to trachoma	5
Severe vision impairment due to trachoma	5
Blindness due to trachoma	5
Dengue	3
Dengue episodes	5
Moderate dengue	6
Severe dengue	6
Post-dengue chronic fatigue syndrome	5
Yellow fever	3
Asymptomatic yellow fever	5
Moderate yellow fever	5
Severe yellow fever	5
Rabies	3
Rabies	5
Intestinal nematode infections	3
Ascariasis	4
Complications of ascariasis	5
Heavy infestation of ascariasis	6
Mild abdominopelvic problems due to ascariasis	6
Severe wasting due to ascariasis	6
Asymptomatic ascariasis	5
Trichuriasis	4
Complications of trichuriasis	5
Heavy infestation of trichuriasis	6
Mild abdominopelvic problems due to trichuriasis	6
Severe wasting due to trichuriasis	6
Asymptomatic trichuriasis	5
Hookworm disease	4
Complications of hookworm disease	5
Heavy infestation of hookworm	6
Mild abdominopelvic problems due to hookworm disease	6
Severe wasting due to hookworm disease	6
Anemia due to hookworm disease	5
Mild anemia due to hookworm disease	6
Moderate anemia due to hookworm disease	6
Severe anemia due to hookworm disease	6
Asymptomatic hookworm disease	5
Food-borne trematodiases	3
Asymptomatic food-borne trematodiases	5
Asymptomatic clonorchiasis	6
Asymptomatic fascioliasis	6
Asymptomatic intestinal fluke infection	6
Asymptomatic opisthorchiasis	6
Asymptomatic paragonimiasis	6
Symptomatic food-borne trematodiases	5
Heavy opisthorchiasis due to food-borne trematodiases	6
Heavy clonorchiasis due to food-borne trematodiases	6
Heavy intestinal fluke infection due to food-borne trematodiases	6
Heavy fascioliasis due to food-borne trematodiases	6
Heavy paragonimiasis due to food-borne trematodiases	6
Cerebral paragonimiasis	6
Leprosy	3
Disfigurement level 1 due to leprosy	5
Disfigurement level 2 due to leprosy	5
Ebola	3
Ebola cases	5
Post-Ebola chronic fatigue syndrome	5
Other neglected tropical diseases	3
Acute infection due to other neglected tropical diseases	5
Anemia due to other neglected tropical diseases	5

Appendix Table 3. GBD 2015 cause and sequela hierarchy with four levels of causes (1-4) and two levels of sequelae (5-6)	
**Causes and sequelae**	**Level**
Mild anemia due to other neglected tropical diseases	6
Moderate anemia due to other neglected tropical diseases	6
Severe anemia due to other neglected tropical diseases	6
Maternal disorders	2
Maternal hemorrhage	3
Maternal hemorrhage episodes	5
Maternal hemorrhage (< 1L blood lost)	6
Maternal hemorrhage (> 1L blood lost)	6
Anemia due to maternal hemorrhage	5
Mild anemia due to maternal hemorrhage	6
Moderate anemia due to maternal hemorrhage	6
Severe anemia due to maternal hemorrhage	6
Maternal sepsis and other maternal infections	3
Maternal sepsis and other maternal infections	5
Puerperal sepsis	6
Other maternal infections	6
Infertility due to puerperal sepsis	5
Maternal hypertensive disorders	3
Maternal hypertensive disorders episodes	5
Other hypertensive disorders of pregnancy	6
Severe pre-eclampsia	6
Eclampsia	5
Complications of maternal hypertensive disorders	5
Long term sequelae of severe pre-eclampsia	6
Long term sequelae of eclampsia	6
Maternal obstructed labor and uterine rupture	3
Obstructed labor, acute event	5
Fistula due to maternal obstructed labor and uterine rupture	5
Rectovaginal fistula	6
Vesicovaginal fistula	6
Maternal abortion, miscarriage, and ectopic pregnancy	3
Maternal abortive outcome	5
Other maternal disorders	3
Other maternal disorders	5
Neonatal disorders	2
Neonatal preterm birth complications	3
Vision loss due to retinopathy of prematurity	5
Asymptomatic retinopathy of prematurity	6
Mild vision impairment due to retinopathy of prematurity	6
Moderate vision impairment due to retinopathy of prematurity	6
Severe vision impairment due to retinopathy of prematurity	6
Blindness due to retinopathy of prematurity	6
Complications of preterm birth complications	5
Mild motor impairment due to neonatal preterm birth complications <28wks	6
Mild motor plus cognitive impairments due to neonatal preterm birth complications <28wks	6
Mild motor impairment due to neonatal preterm birth complications 28-32wks	6
Mild motor plus cognitive impairments due to neonatal preterm birth complications 28-32wks	6
Mild motor impairment due to neonatal preterm birth complications 32-36wks	6
Mild motor plus cognitive impairments due to neonatal preterm birth complications 32-36wks	6
Moderate motor impairment due to neonatal preterm birth complications <28wks	6
Moderate motor impairment with blindness due to neonatal preterm birth complications <28wks	6
Moderate motor impairment with epilepsy due to neonatal preterm birth complications <28wk	6
Moderate motor impairment with blindness and epilepsy due to neonatal preterm birth complications <28wks	6
Moderate motor plus cognitive impairment with blindness due to neonatal preterm birth complications <28wks	6
Moderate motor plus cognitive impairment with epilepsy due to neonatal preterm birth complications <28wks	6
Moderate motor plus cognitive impairment with blindness and epilepsy due to neonatal preterm birth complications <28wks	6
Severe motor impairment due to neonatal preterm birth complications <28wks	6
Severe motor impairment with blindness due to neonatal preterm birth complications <28wks	6

Appendix Table 3. GBD 2015 cause and sequela hierarchy with four levels of causes (1-4) and two levels of sequelae (5-6)	
**Causes and sequelae**	**Level**
Severe motor impairment with epilepsy due to neonatal preterm birth complications <28wk	6
Severe motor impairment with blindness and epilepsy due to neonatal preterm birth complications <28wks	6
Severe motor plus cognitive impairment with blindness due to neonatal preterm birth complications <28wks	6
Severe motor plus cognitive impairment with epilepsy due to neonatal preterm birth complications <28wks	6
Severe motor plus cognitive impairment with blindness and epilepsy due to neonatal preterm birth complications <28wks	6
Moderate motor impairment due to neonatal preterm birth complications 28-32wks	6
Moderate motor impairment with blindness due to neonatal preterm birth complications 28-32wks	6
Moderate motor impairment with epilepsy due to neonatal preterm birth complications 28-32wk	6
Moderate motor impairment with blindness and epilepsy due to neonatal preterm birth complications 28-32wks	6
Moderate motor plus cognitive impairment with blindness due to neonatal preterm birth complications 28-32wks	6
Moderate motor plus cognitive impairment with epilepsy due to neonatal preterm birth complications 28-32wks	6
Moderate motor plus cognitive impairment with blindness and epilepsy due to neonatal preterm birth complications 28 32wks	6
Severe motor impairment due to neonatal preterm birth complications 28-32wks	6
Severe motor impairment with blindness due to neonatal preterm birth complications 28-32wk	6
Severe motor impairment with epilepsy due to neonatal preterm birth complications 28-32wk	6
Severe motor impairment with blindness and epilepsy due to neonatal preterm birth complications 28-32wks	6
Severe motor plus cognitive impairment with blindness due to neonatal preterm birth complications 28-32wks	6
Severe motor plus cognitive impairment with epilepsy due to neonatal preterm birth complications 28-32wks	6
Severe motor plus cognitive impairment with blindness and epilepsy due to neonatal preterm birth complications 28 32wks	6
Moderate motor impairment due to neonatal preterm birth complications 32-36wks	6
Moderate motor impairment with blindness due to neonatal preterm birth complications 32-36wks	6
Moderate motor impairment with epilepsy due to neonatal preterm birth complications 32-36wk	6
Moderate motor impairment with blindness and epilepsy due to neonatal preterm birth complications 32-36wks	6
Moderate motor plus cognitive impairment with blindness due to neonatal preterm birth complications 32-36wks	6
Moderate motor plus cognitive impairment with epilepsy due to neonatal preterm birth complications 32-36wks	6
Moderate motor plus cognitive impairment with blindness and epilepsy due to neonatal preterm birth complications 32 36wks	6
Severe motor impairment due to neonatal preterm birth complications 32-36wks	6
Severe motor impairment with blindness due to neonatal preterm birth complications 32-36wk	6
Severe motor impairment with epilepsy due to neonatal preterm birth complications 32-36wk	6
Severe motor impairment with blindness and epilepsy due to neonatal preterm birth complications 32-36wks	6
Severe motor plus cognitive impairment with blindness due to neonatal preterm birth complications 32-36wks	6
Severe motor plus cognitive impairment with epilepsy due to neonatal preterm birth complications 32-36wks	6
Severe motor plus cognitive impairment with blindness and epilepsy due to neonatal preterm birth complications 32 36wks	6
Neonatal encephalopathy due to birth asphyxia and trauma	3
Complications of neonatal encephalopathy	5
Mild motor plus cognitive impairments due to neonatal encephalopathy due to birth asphyxia and trauma	6
Mild motor impairment due to neonatal encephalopathy due to birth asphyxia and trauma	6
Moderate motor impairment due to neonatal encephalopathy due to birth asphyxia and trauma	6
Moderate motor impairment with blindness due to neonatal encephalopathy due to birth asphyxia and trauma	6

Appendix Table 3. GBD 2015 cause and sequela hierarchy with four levels of causes (1-4) and two levels of sequelae (5-6)	
**Causes and sequelae**	**Level**
Moderate motor impairment with epilepsy due to neonatal encephalopathy due to birth asphyxia and trauma	6
Moderate motor impairment with blindness and epilepsy due to neonatal encephalopathy due to birth asphyxia and trauma	6
Moderate motor plus cognitive impairment with blindness due to neonatal encephalopathy due to birth asphyxia and trauma	6
Moderate motor plus cognitive impairment with epilepsy due to neonatal encephalopathy due to birth asphyxia and trauma	6
Moderate motor plus cognitive impairment with blindness and epilepsy due to neonatal encephalopathy due to birth asphyxia and trauma	6
Severe motor impairment due to neonatal encephalopathy due to birth asphyxia and trauma	6
Severe motor impairment with blindness due to neonatal encephalopathy due to birth asphyxia and trauma	6
Severe motor impairment with epilepsy due to neonatal encephalopathy due to birth asphyxia and trauma	6
Severe motor impairment with blindness and epilepsy due to neonatal encephalopathy due to birth asphyxia and trauma	6
Severe motor plus cognitive impairment with blindness due to neonatal encephalopathy due to birth asphyxia and trauma	6
Severe motor plus cognitive impairment with epilepsy due to neonatal encephalopathy due to birth asphyxia and trauma	6
Severe motor plus cognitive impairment with blindness and epilepsy due to neonatal encephalopathy due to birth asphyxia and trauma	6
Neonatal sepsis and other neonatal infections	3
Severe infection due to neonatal sepsis and other neonatal infections	5
Complications of neonatal sepsis and other neonatal infections	5
Mild motor impairment due to neonatal sepsis and other neonatal infections	6
Mild motor plus cognitive impairments due to neonatal sepsis and other neonatal infections	6
Moderate motor impairment due to neonatal sepsis and other neonatal infections	6
Moderate motor impairment with blindness and epilepsy due to neonatal sepsis and other neonatal infections	6
Moderate motor impairment with blindness due to neonatal sepsis and other neonatal infections	6
Moderate motor impairment with epilepsy due to neonatal sepsis and other neonatal infections	6
Moderate motor plus cognitive impairment with blindness and epilepsy due to neonatal sepsis and other neonatal infections	6
Moderate motor plus cognitive impairment with blindness due to neonatal sepsis and other neonatal infections	6
Moderate motor plus cognitive impairment with epilepsy due to neonatal sepsis and other neonatal infections	6
Severe motor impairment with blindness due to neonatal sepsis and other neonatal infections	6
Severe motor impairment due to neonatal sepsis and other neonatal infections	6
Severe motor impairment with blindness and epilepsy due to neonatal sepsis and other neonatal infections	6
Severe motor impairment with epilepsy due to neonatal sepsis and other neonatal infections	6
Severe motor plus cognitive impairment with blindness and epilepsy due to neonatal sepsis and other neonatal infections	6
Severe motor plus cognitive impairment with blindness due to neonatal sepsis and other neonatal infections	6
Severe motor plus cognitive impairment with epilepsy due to neonatal sepsis and other neonatal infections	6
Hemolytic disease and other neonatal jaundice	3
Complications of hemolytic disease and other neonatal jaundice	5
Moderate motor impairment due to hemolytic disease and other neonatal jaundice	6
Moderate motor impairment with blindness due to hemolytic disease and other neonatal jaundice	6
Moderate motor impairment with epilepsy due to hemolytic disease and other neonatal jaundice	6
Moderate motor impairment with blindness and epilepsy due to hemolytic disease and other neonatal jaundice	6
Moderate motor plus cognitive impairment with blindness due to hemolytic disease and other neonatal jaundice	6
Moderate motor plus cognitive impairment with epilepsy due to hemolytic disease and other neonatal jaundice	6

Appendix Table 3. GBD 2015 cause and sequela hierarchy with four levels of causes (1-4) and two levels of sequelae (5-6)	
**Causes and sequelae**	**Level**
Moderate motor plus cognitive impairment with blindness and epilepsy due to hemolytic disease and other neonatal jaundice	6
Severe motor impairment severe due to hemolytic disease and other neonatal jaundice	6
Severe motor impairment with blindness due to hemolytic disease and other neonatal jaundice	6
Severe motor impairment with epilepsy due to hemolytic disease and other neonatal jaundice	6
Severe motor impairment with blindness and epilepsy due to hemolytic disease and other neonatal jaundice	6
Severe motor plus cognitive impairment with blindness due to hemolytic disease and other neonatal jaundice	6
Severe motor plus cognitive impairment with epilepsy due to hemolytic disease and other neonatal jaundice	6
Severe motor plus cognitive impairment with blindness and epilepsy due to hemolytic disease and other neonatal jaundice	6
Other neonatal disorders	3
Other neonatal disorders	5
Nutritional deficiencies	2
Protein-energy malnutrition	3
Complications of protein-energy malnutrition	5
Kwashiokor due to protein-energy malnutrition	6
Marasmus due to protein-energy malnutrition	6
Severe wasting due to protein-energy malnutrition	6
Iodine deficiency	3
Visible goiter due to iodine deficiency	5
Visible goiter without symptoms	6
Visible goiter without heart failure due to iodine deficiency	6
Visible goiter with signs and symptoms	6
Visible goiter with heart failure due to iodine deficiency	5
Visible goiter with mild heart failure due to iodine deficiency	6
Visible goiter with moderate heart failure due to iodine deficiency	6
Visible goiter with severe heart failure due to iodine deficiency	6
Intellectual disability due to iodine deficiency	5
Severe intellectual disability due to iodine deficiency	6
Profound intellectual disability due to iodine deficiency	6
Vitamin A deficiency	3
Vision loss due to vitamin A deficiency	5
Moderate vision impairment loss due to vitamin A deficiency	6
Severe vision impairment loss due to vitamin A deficiency	6
Blindness due to vitamin A deficiency	6
Iron-deficiency anemia	3
Iron-deficiency anemia without heart failure	5
Mild iron-deficiency anemia	6
Moderate iron-deficiency anemia	6
Severe iron-deficiency anemia	6
Iron-deficiency anemia with heart failure	5
Mild heart failure due to iron-deficiency anemia	6
Moderate heart failure due to iron-deficiency anemia	6
Severe heart failure due to iron-deficiency anemia	6
Other nutritional deficiencies	3
Other nutritional deficiencies	5
Other communicable, maternal, neonatal, and nutritional diseases	2
Sexually transmitted diseases excluding HIV	3
Syphilis	4
Early syphilis	5
Asymptomatic early syphilis infection	6
Mild early syphilis infection	6
Adult tertiary syphilis	5
Chlamydial infection	4
Chlamydial infection episodes	5
Asymptomatic chlamydial infection	6
Mild chlamydial infection	6
Epididymo-orchitis due to chlamydial infection	6
Pelvic inflammatory diseases due to chlamydial infection	5

Appendix Table 3. GBD 2015 cause and sequela hierarchy with four levels of causes (1-4) and two levels of sequelae (5-6)	
**Causes and sequelae**	**Level**
Moderate pelvic inflammatory diseases due to chlamydial infection	6
Severe pelvic inflammatory diseases due to chlamydial infection	6
Infertility due to clamydial infection	5
Primary infertility due to chlamydial infection	6
Secondary infertility due to chlamydial infection	6
Gonococcal infection	4
Infertility due to gonococcal infection	5
Gonococcal infection episodes	5
Asymptomatic gonococcal infection	6
Mild gonococcal infection	6
Epididymo-orchitis due to gonococcal infection	6
Pelvic inflammatory diseases due to gonococcal infection	5
Moderate pelvic inflammatory diseases due to gonococcal infection	6
Severe pelvic inflammatory diseases due to gonococcal infection	6
Primary infertility due to gonococcal infection	6
Secondary infertility due to gonococcal infection	6
Trichomoniasis	4
Asymptomatic trichomoniasis infection	5
Acute trichomoniasis infection	5
Genital herpes	4
Moderate infection due to initial genital herpes episode	5
Complications of genital herpes	5
Asymptomatic genital herpes	6
Symptomatic genital herpes	6
Other sexually transmitted diseases	4
Pelvic inflammatory diseases due to other sexually transmitted diseases	5
Moderate pelvic inflammatory diseases due to other sexually transmitted diseases	6
Severe pelvic inflammatory diseases due to other sexually transmitted diseases	6
Infertility due to other sexually transmitted diseases	5
Primary infertility due to other sexually transmitted diseases	6
Secondary infertility due to other sexually transmitted diseases	6
Other sexually transmitted diseases	5
Hepatitis	3
Acute hepatitis A	4
Asymptomatic acute hepatitis A	5
Moderate acute hepatitis A	5
Severe acute hepatitis A	5
Hepatitis B	4
Acute Hepatitis B	5
Asymptomatic acute hepatitis B	6
Moderate acute hepatitis B	6
Severe acute hepatitis B	6
Chronic hepatitis B	5
Hepatitis C	4
Acute Hepatitis C	5
Asymptomatic acute hepatitis C	6
Moderate acute hepatitis C	6
Severe acute hepatitis C	6
Chronic hepatitis C	5
Acute hepatitis E	4
Asymptomatic acute hepatitis E	5
Moderate acute hepatitis E	5
Severe acute hepatitis E	5
Other infectious diseases	3
Acute other infectious diseases	5
Complications of other infectious diseases	5
Mild anemia due to other infectious diseases	6
Moderate anemia due to other infectious diseases	6
Severe anemia due to other infectious diseases	6
Guillain-Barré syndrome due to other infectious diseases	6
Non-communicable diseases	1
Neoplasms	2

Appendix Table 3. GBD 2015 cause and sequela hierarchy with four levels of causes (1-4) and two levels of sequelae (5-6)	
**Causes and sequelae**	**Level**
Lip and oral cavity cancer	3
Diagnosis and primary therapy phase of mouth cancer	5
Controlled phase of mouth cancer	5
Metastatic phase of mouth cancer	5
Terminal phase of mouth cancer	5
Nasopharynx cancer	3
Diagnosis and primary therapy phase of nasopharynx cancer	5
Controlled phase of nasopharynx cancer	5
Metastatic phase of nasopharynx cancer	5
Terminal phase of nasopharynx cancer	5
Other pharynx cancer	3
Diagnosis and primary therapy phase of other pharynx cancer	5
Controlled phase of other pharynx cancer	5
Metastatic phase of other pharynx cancer	5
Terminal phase of other pharynx cancer	5
Esophageal cancer	3
Diagnosis and primary therapy phase of esophageal cancer	5
Controlled phase of esophageal cancer	5
Metastatic phase of esophageal cancer	5
Terminal phase of esophageal cancer	5
Stomach cancer	3
Diagnosis and primary therapy phase of stomach cancer	5
Controlled phase of stomach cancer	5
Metastatic phase of stomach cancer	5
Terminal phase of stomach cancer	5
Colon and rectum cancer	3
Colon and rectum cancer	5
Diagnosis and primary therapy phase of colon and rectum cancers	6
Controlled phase of colon and rectum cancers	6
Metastatic phase of colon and rectum cancers	6
Terminal phase of colon and rectum cancers	6
Stoma due to colon and rectum cancer	5
Liver cancer	3
Liver cancer due to hepatitis B	4
Diagnosis and primary therapy phase of liver cancer due to hepatitis B	5
Controlled phase of liver cancer due to hepatitis B	5
Metastatic phase of liver cancer due to hepatitis B	5
Terminal phase of liver cancer due to hepatitis B	5
Liver cancer due to hepatitis C	4
Diagnosis and primary therapy phase of liver cancer due to hepatitis C	5
Controlled phase of liver cancer due to hepatitis C	5
Metastatic phase of liver cancer due to hepatitis C	5
Terminal phase of liver cancer due to hepatitis C	5
Liver cancer due to alcohol use	4
Diagnosis and primary therapy phase of liver cancer due to alcohol use	5
Controlled phase of liver cancer due to alcohol use	5
Metastatic phase of liver cancer due to alcohol use	5
Terminal phase of liver cancer due to alcohol use	5
Liver cancer due to other causes	4
Diagnosis and primary therapy phase of liver cancer due to other causes	5
Controlled phase of liver cancer due to other causes	5
Metastatic phase of liver cancer due to other causes	5
Terminal phase of liver cancer due to other causes	5
Gallbladder and biliary tract cancer	3
Diagnosis and primary therapy phase of gallbladder and biliary tract cancer	5
Controlled phase of gallbladder and biliary tract cancer	5
Metastatic phase of gallbladder and biliary tract cancer	5
Terminal phase of gallbladder and biliary tract cancer	5
Pancreatic cancer	3
Diagnosis and primary therapy phase of pancreatic cancer	5
Controlled phase of pancreatic cancer	5
Metastatic phase of pancreatic cancer	5

Appendix Table 3. GBD 2015 cause and sequela hierarchy with four levels of causes (1-4) and two levels of sequelae (5-6)	
**Causes and sequelae**	**Level**
Terminal phase of pancreatic cancer	5
Larynx cancer	3
Larynx cancer	5
Diagnosis and primary therapy phase of larynx cancer	6
Controlled phase of larynx cancer	6
Metastatic phase of larynx cancer	6
Terminal phase of larynx cancer	6
Laryngectomy due to larynx cancer	5
Tracheal, bronchus, and lung cancer	3
Diagnosis and primary therapy phase of lung, bronchus, and trachea cancer	5
Controlled phase of lung, bronchus, and trachea cancer	5
Metastatic phase of lung, bronchus, and trachea cancer	5
Terminal phase of lung, bronchus, and trachea cancer	5
Malignant skin melanoma	3
Diagnosis and primary therapy phase of malignant skin melanoma	5
Controlled phase of malignant skin melanoma	5
Metastatic phase of malignant skin melanoma	5
Terminal phase of malignant skin melanoma	5
Non-melanoma skin cancer	3
Non-melanoma skin cancer (squamous-cell carcinoma)	4
Diagnosis and primary therapy phase of cutaneous squamous cell carcinoma	5
Control phase of cutaneous squamous cell carcinoma	5
Metastatic phase of cutaneous squamous cell carcinoma	5
Terminal phase of cutaneous squamous cell carcinoma	5
Non-melanoma skin cancer (basal-cell carcinoma)	4
Disfigurement due to basal cell carcinoma	5
Breast cancer	3
Breast cancer	5
Diagnosis and primary therapy phase of breast cancer	6
Controlled phase of breast cancer	6
Metastatic phase of breast cancer	6
Terminal phase of breast cancer	6
Mastectomy due to breast cancer	5
Cervical cancer	3
Diagnosis and primary therapy phase of cervical cancer	5
Controlled phase of cervical cancer	5
Metastatic phase of cervical cancer	5
Terminal phase of cervical cancer	5
Uterine cancer	3
Diagnosis and primary therapy phase of uterine cancer	5
Controlled phase of uterine cancer	5
Metastatic phase of uterine cancer	5
Terminal phase of uterine cancer	5
Ovarian cancer	3
Diagnosis and primary therapy phase of ovarian cancer	5
Controlled phase of ovarian cancer	5
Metastatic phase of ovarian cancer	5
Terminal phase of ovarian cancer	5
Prostate cancer	3
Prostate cancer	5
Diagnosis and primary therapy phase of prostate cancer	6
Controlled phase of prostate cancer	6
Metastatic phase of prostate cancer	6
Terminal phase of prostate cancer	6
Impotence and incontinence due to prostate cancer	5
Impotence due to prostate cancer	6
Incontinence due to prostate cancer	6
Testicular cancer	3
Diagnosis and primary therapy phase of testicular cancer	5
Controlled phase of testicular cancer	5
Metastatic phase of testicular cancer	5
Terminal phase of testicular cancer	5

Appendix Table 3. GBD 2015 cause and sequela hierarchy with four levels of causes (1-4) and two levels of sequelae (5-6)	
**Causes and sequelae**	**Level**
Kidney cancer	3
Diagnosis and primary therapy phase of kidney cancer	5
Controlled phase of kidney cancer	5
Metastatic phase of kidney cancer	5
Terminal phase of kidney cancer	5
Bladder cancer	3
Bladder cancer episodes	5
Diagnosis and primary therapy phase of bladder cancer	6
Controlled phase of bladder cancer	6
Metastatic phase of bladder cancer	6
Terminal phase of bladder cancer	6
Urinary incontinence due to bladder cancer	5
Brain and nervous system cancer	3
Diagnosis and primary therapy phase of brain and nervous system cancers	5
Controlled phase of brain and nervous system cancers	5
Metastatic phase of brain and nervous system cancers	5
Terminal phase of brain and nervous system cancers	5
Thyroid cancer	3
Diagnosis and primary therapy phase of thyroid cancer	5
Controlled phase of thyroid cancer	5
Metastatic phase of thyroid cancer	5
Terminal phase of thyroid cancer	5
Mesothelioma	3
Diagnosis and primary therapy phase of mesothelioma	5
Controlled phase of mesothelioma	5
Metastatic phase of mesothelioma	5
Terminal phase of mesothelioma	5
Hodgkin lymphoma	3
Diagnosis and primary therapy phase of Hodgkin disease	5
Controlled phase of Hodgkin disease	5
Metastatic phase of Hodgkin disease	5
Terminal phase of Hodgkin disease	5
Non-Hodgkin lymphoma	3
Diagnosis and primary therapy phase of non-Hodgkin lymphoma	5
Controlled phase of non-Hodgkin lymphoma	5
Metastatic phase of non-Hodgkin lymphoma	5
Terminal phase of non-Hodgkin lymphoma	5
Multiple myeloma	3
Diagnosis and primary therapy phase of multiple myeloma	5
Controlled phase of multiple myeloma	5
Metastatic phase of multiple myeloma	5
Terminal phase of multiple myeloma	5
Leukemia	3
Acute lymphoid leukemia	4
Controlled phase of acute lymphoid leukemia	5
Diagnosis and primary therapy phase of acute lymphoid leukemia	5
Metastatic phase of acute lymphoid leukemia	5
Terminal phase of acute lymphoid leukemia	5
Chronic lymphoid leukemia	4
Controlled phase of chronic lymphoid leukemia	5
Diagnosis and primary therapy phase of chronic lymphoid leukemia	5
Metastatic phase of chronic lymphoid leukemia	5
Terminal phase of chronic lymphoid leukemia	5
Acute myeloid leukemia	4
Controlled phase of acute myeloid leukemia	5
Diagnosis and primary therapy phase of acute myeloid leukemia	5
Metastatic phase of acute myeloid leukemia	5
Terminal phase of acute myeloid leukemia	5
Chronic myeloid leukemia	4
Controlled phase of chronic myeloid leukemia	5
Diagnosis and primary therapy phase of chronic myeloid leukemia	5
Metastatic phase of chronic myeloid leukemia	5

Appendix Table 3. GBD 2015 cause and sequela hierarchy with four levels of causes (1-4) and two levels of sequelae (5-6)	
**Causes and sequelae**	**Level**
Terminal phase of chronic myeloid leukemia	5
Other neoplasms	3
Diagnosis and primary therapy phase of other neoplasms	5
Controlled phase of other neoplasms	5
Metastatic phase of other neoplasms	5
Terminal phase of other neoplasms	5
Cardiovascular diseases	2
Rheumatic heart disease	3
Rheumatic heart disease, without heart failure	5
Heart failure fue to rheumatic heart disease	5
Mild heart failure due to rheumatic heart disease	6
Moderate heart failure due to rheumatic heart disease	6
Severe heart failure due to rheumatic heart disease	6
Ischemic heart disease	3
Myocardial infarction episodes	5
Asymptomatic ischemic heart disease following myocardial infarction	5
Acute myocardial infarction first 2 days	6
Acute myocardial infarction 3 to 28 days	6
Angina due to ischemic heart disease	5
Asymptomatic angina due to ischemic heart disease	6
Mild angina due to ischemic heart disease	6
Moderate angina due to ischemic heart disease	6
Severe angina due to ischemic heart disease	6
Heart failure fue to ischemic heart disease	5
Mild heart failure due to ischemic heart disease	6
Moderate heart failure due to ischemic heart disease	6
Severe heart failure due to ischemic heart disease	6
Cerebrovascular disease	3
Ischemic stroke	4
Chronic ischemic stroke	5
Asymptomatic chronic ischemic stroke	6
Chronic ischemic stroke severity level 1	6
Chronic ischemic stroke severity level 2	6
Chronic ischemic stroke severity level 4	6
Chronic ischemic stroke severity level 3	6
Chronic ischemic stroke severity level 5	6
Ischemic stroke episodes	5
Acute ischemic stroke severity level 1	6
Acute ischemic stroke severity level 2	6
Acute ischemic stroke severity level 4	6
Acute ischemic stroke severity level 3	6
Acute ischemic stroke severity level 5	6
Hemorrhagic stroke	4
Chronic hemorrhagic stroke	5
Asymptomatic chronic hemorrhagic stroke	6
Chronic hemorrhagic stroke severity level 1	6
Chronic hemorrhagic stroke severity level 2	6
Chronic hemorrhagic stroke severity level 4	6
Chronic hemorrhagic stroke severity level 3	6
Chronic hemorrhagic stroke severity level 5	6
Acute hemorrhagic stroke	5
Acute hemorrhagic stroke severity level 1	6
Acute hemorrhagic stroke severity level 2	6
Acute hemorrhagic stroke severity level 4	6
Acute hemorrhagic stroke severity level 3	6
Acute hemorrhagic stroke severity level 5	6
Hypertensive heart disease	3
Mild heart failure due to hypertensive heart disease	5
Moderate heart failure due to hypertensive heart disease	5
Severe heart failure due to hypertensive heart disease	5
Cardiomyopathy and myocarditis	3
Acute myocarditis	5

Appendix Table 3. GBD 2015 cause and sequela hierarchy with four levels of causes (1-4) and two levels of sequelae (5-6)	
**Causes and sequelae**	**Level**
Heart failure due to cardiomyopathy	5
Mild heart failure due to cardiomyopathy and myocarditis	6
Moderate heart failure due to cardiomyopathy and myocarditis	6
Severe heart failure due to cardiomyopathy and myocarditis	6
Atrial fibrillation and flutter	3
Asymptomatic atrial fibrillation and flutter	5
Symptomatic atrial fibrillation and flutter	5
Peripheral vascular disease	3
Asymptomatic peripheral vascular disease	5
Symptomatic claudication due to peripheral vascular disease	5
Endocarditis	3
Endocarditis episodes	5
Moderate endocarditis	6
Severe endocarditis	6
Heart failure due to endocarditis	5
Mild heart failure due to endocarditis	6
Moderate heart failure due to endocarditis	6
Severe heart failure due to endocarditis	6
Other cardiovascular and circulatory diseases	3
Heart failure due to other cardiovascular diseases	5
Mild heart failure due to other cardiovascular diseases	6
Moderate heart failure due to other cardiovascular diseases	6
Severe heart failure due to other cardiovascular diseases	6
Other cardiovascular diseases episodes	5
Asymptomatic other cardiovascular diseases	6
Mild other cardiovascular diseases	6
Moderate other cardiovascular diseases	6
Severe other cardiovascular diseases	6
Chronic respiratory diseases	2
Chronic obstructive pulmonary disease	3
Chronic obstructive pulmonary disease without heart failure	5
Asymptomatic chronic obstructive pulmonary disease	6
Mild chronic obstructive pulmonary disease	6
Moderate chronic obstructive pulmonary disease	6
Severe chronic obstructive pulmonary disease without heart failure	6
Severe chronic obstructive pulmonary disease with heart failure	5
Mild heart failure due to severe chronic obstructive pulmonary disease	6
Moderate heart failure due to severe chronic obstructive pulmonary disease	6
Severe heart failure due to severe chronic obstructive pulmonary disease	6
Pneumoconiosis	3
Silicosis	4
Silicosis without heart failure	5
Asymptomatic silicosis	6
Mild silicosis	6
Moderate silicosis	6
Severe silicosis without heart failure	6
Severe silicosis with heart failure	5
Mild heart failure due to severe silicosis	6
Moderate heart failure due to severe silicosis	6
Severe heart failure due to severe silicosis	6
Asbestosis	4
Asbestosis without heart failure	5
Asymptomatic asbestosis	6
Mild asbestosis	6
Moderate asbestosis	6
Severe asbestosis without heart failure	6
Severe asbestosis with heart failure	5
Mild heart failure due to severe asbestosis	6
Moderate heart failure due to severe asbestosis	6
Severe heart failure due to severe asbestosis	6
Coal workers pneumoconiosis	4
Coal workers pneumoconiosis without heart failure	5

Appendix Table 3. GBD 2015 cause and sequela hierarchy with four levels of causes (1-4) and two levels of sequelae (5-6)	
**Causes and sequelae**	**Level**
Asymptomatic coal workers pneumoconiosis	6
Mild coal workers pneumoconiosis	6
Moderate coal workers pneumoconiosis	6
Severe coal workers pneumoconiosis without heart failure	6
Severe coal workers pneumoconiosis with heart failure	5
Mild heart failure due to severe coal workers pneumoconiosis	6
Moderate heart failure due to severe coal workers pneumoconiosis	6
Severe heart failure due to severe coal workers pneumoconiosis	6
Other pneumoconiosis	4
Other pneumoconiosis without heart failure	5
Asymptomatic other pneumoconiosis	6
Mild other pneumoconiosis	6
Moderate other pneumoconiosis	6
Severe other pneumoconiosis without heart failure	6
Severe other pneumoconiosis with heart failure	5
Mild heart failure due to severe other pneumoconiosis	6
Moderate heart failure due to severe other pneumoconiosis	6
Severe heart failure due to severe other pneumoconiosis	6
Asthma	3
Asymptomatic asthma	5
Symptomatic asthma	5
Controlled asthma	6
Partially controlled asthma	6
Uncontrolled asthma	6
Interstitial lung disease and pulmonary sarcoidosis	3
Interstitial lung disease and pulmonary sarcoidosis without heart failure	5
Asymptomatic interstitial lung disease and pulmonary sarcoidosis	6
Mild interstitial lung disease and pulmonary sarcoidosis	6
Moderate interstitial lung disease and pulmonary sarcoidosis	6
Severe interstitial lung disease and pulmonary sarcoidosis without heart failure	6
Severe interstitial lung disease and pulmonary sarcoidosis with heart failure	5
Mild heart failure due to severe interstitial lung disease and pulmonary sarcoidosis	6
Moderate heart failure due to severe interstitial lung disease and pulmonary sarcoidosis	6
Severe heart failure due to severe interstitial lung disease and pulmonary sarcoidosis	6
Other chronic respiratory diseases	3
Other chronic respiratory diseases	5
Cirrhosis and other chronic liver diseases	2
Cirrhosis and other chronic liver diseases due to hepatitis B	3
Cirrhosis and other chronic liver diseases due to hepatitis B	5
Cirrhosis and other chronic liver diseases due to hepatitis C	3
Cirrhosis and other chronic liver diseases due to hepatitis C	5
Cirrhosis and other chronic liver diseases due to alcohol use	3
Cirrhosis and other chronic liver diseases due to alcohol	5
Cirrhosis and other chronic liver diseases due to other causes	3
Cirrhosis and other chronic liver diseases due to other cause	5
Digestive diseases	2
Peptic ulcer disease	3
Peptic ulcer disease, symptomatic episodes	5
Anemia due to peptic ulcer disease	5
Mild anemia due to peptic ulcer disease	6
Moderate anemia due to peptic ulcer disease	6
Severe anemia due to peptic ulcer disease	6
Gastritis and duodenitis	3
Gastritis and duodenitis, symptomatic episodes	5
Anemia due to gastritis and duodenitis	5
Mild anemia due to gastritis and duodenitis	6
Moderate anemia due to gastritis and duodenitis	6
Severe anemia due to gastritis and duodenitis	6
Appendicitis	3
Appendicitis	5
Paralytic ileus and intestinal obstruction	3
Paralytic ileus and intestinal obstruction	5

Appendix Table 3. GBD 2015 cause and sequela hierarchy with four levels of causes (1-4) and two levels of sequelae (5-6)	
**Causes and sequelae**	**Level**
Inguinal, femoral, and abdominal hernia	3
Inguinal, femoral, and abdominal hernia cases	5
Inflammatory bowel disease	3
Ulcerative colitis	5
Crohn's disease	5
Vascular intestinal disorders	3
Vascular intestinal disorders	5
Gallbladder and biliary diseases	3
Gallbladder and biliary diseases	5
Pancreatitis	3
Pancreatitis cases	5
Other digestive diseases	3
Other digestive diseases	5
Neurological disorders	2
Alzheimer disease and other dementias	3
Mild Alzheimer disease and other dementias	5
Moderate Alzheimer disease and other dementias	5
Severe Alzheimer disease and other dementias	5
Parkinson disease	3
Mild Parkinson disease	5
Moderate Parkinson disease	5
Severe Parkinson disease	5
Epilepsy	3
Seizure-free, treated epilepsy	5
Less severe epilepsy	5
Severe epilepsy	5
Multiple sclerosis	3
Mild multiple sclerosis	5
Moderate multiple sclerosis	5
Severe multiple sclerosis	5
Motor neuron disease	3
Asymptomatic, but worry about diagnosis of motor neuron disease	5
Mild motor impairment and mild respiratory problems due to motor neuron disease	5
Mild motor impairment and severe respiratory problems due to motor neuron disease	5
Mild motor impairment and speech problems due to motor neuron disease	5
Mild motor impairment due to motor neuron disease	5
Mild motor impairment, mild respiratory problems, and speech problems due to motor neuron disease	5
Mild motor impairment, moderate respiratory problems, and speech problems due to motor neuron disease	5
Mild motor impairment, severe respiratory problems and speech problems due to motor neuron disease	5
Mild respiratory problems and speech problems due to motor neuron disease	5
Mild respiratory problems due to motor neuron disease	5
Moderate motor impairment and mild respiratory problems due to motor neuron disease	5
Moderate motor impairment and moderate respiratory problems due to motor neuron disease	5
Moderate motor impairment and severe respiratory problems due to motor neuron disease	5
Moderate motor impairment and speech problems due to motor neuron disease	5
Moderate motor impairment due to motor neuron disease	5
Moderate motor impairment, mild respiratory problems, and speech problems due to motor neuron disease	5
Moderate motor impairment, moderate respiratory problems, and speech problems due to motor neuron disease	5
Moderate motor impairment, severe respiratory problems, and speech problems due to motor neuron disease	5
Moderate respiratory problems and speech problems due to motor neuron disease	5
Moderate respiratory problems due to motor neuron disease	5
Severe motor impairment and mild respiratory problems due to motor neuron disease	5
Severe motor impairment and moderate respiratory problems due to motor neuron diseas	5
Severe motor impairment and severe respiratory problems due to motor neuron disease	5
Severe motor impairment and speech problems due to motor neuron disease	5
Severe motor impairment due to motor neuron disease	5

Appendix Table 3. GBD 2015 cause and sequela hierarchy with four levels of causes (1-4) and two levels of sequelae (5-6)	
**Causes and sequelae**	**Level**
Severe motor impairment, mild respiratory problems, and speech problems due to motor neuron disease	5
Severe motor impairment, moderate respiratory problems, and speech problems due to motor neuron disease	5
Severe motor impairment, severe respiratory problems, and speech problems due to motor neuron disease	5
Severe respiratory problems and speech problems due to motor neuron disease	5
Severe respiratory problems due to motor neuron disease	5
Speech problems due to motor neuron disease	5
Migraine	3
Asymptomatic migraine	5
Symptomatic migraine	5
Tension-type headache	3
Asymptomatic tension-type headache	5
Symptomatic tension-type headache	5
Medication overuse headache	3
Asymptomatic medication overuse headache	5
Symptomatic medication overuse headache	5
Other neurological disorders	3
Other neurological disorders	5
Guillain-Barré syndrome due to other neurological disorders	5
Mental and substance use disorders	2
Schizophrenia	3
Schizophrenia residual state	5
Schizophrenia acute state	5
Alcohol use disorders	3
Alcohol dependence	5
Asymptomatic alcohol dependence	6
Very mild alcohol dependence	6
Mild alcohol dependence	6
Moderate alcohol dependence	6
Severe alcohol dependence	6
Fetal alcohol syndrome	5
Asymptomatic fetal alcohol syndrome	6
Mild fetal alcohol syndrome	6
Moderate fetal alcohol syndrome	6
Severe fetal alcohol syndrome	6
Drug use disorders	3
Opioid use disorders	4
Asymptomatic opioid dependence	5
Symptomatic opioid dependence	5
Mild opioid dependence	6
Severe opioid dependence	6
Cocaine use disorders	4
Asymptomatic cocaine dependence	5
Symptomatic cocaine dependence	5
Mild cocaine dependence	6
Severe cocaine dependence	6
Amphetamine use disorders	4
Asymptomatic amphetamine dependence	5
Symptomatic amphetamine dependence	5
Mild amphetamine dependence	6
Severe amphetamine dependence	6
Cannabis use disorders	4
Asymptomatic cannabis dependence	5
Symptomatic cannabis dependence	5
Mild cannabis dependence	6
Severe cannabis dependence	6
Other drug use disorders	4
Other drug use disorders	5
Depressive disorders	3
Major depressive disorder	4

Appendix Table 3. GBD 2015 cause and sequela hierarchy with four levels of causes (1-4) and two levels of sequelae (5-6)	
**Causes and sequelae**	**Level**
Major depressive disorder, currently without symptoms	5
Mild major depressive disorder	5
Moderate major depressive disorder	5
Severe major depressive disorder	5
Dysthymia	4
Dysthymia, currently without symptoms	5
Symptomatic dysthymia	5
Bipolar disorder	3
Bipolar disorder residual state	5
Bipolar disorder depressive state	5
Bipolar disorder manic state	5
Anxiety disorders	3
Anxiety disorders, currently without symptoms	5
Mild anxiety disorders	5
Moderate anxiety disorders	5
Severe anxiety disorders	5
Eating disorders	3
Anorexia nervosa	4
Anorexia nervosa	5
Bulimia nervosa	4
Bulimia nervosa	5
Autistic spectrum disorders	3
Autism	4
Autism	5
Asperger syndrome and other autistic spectrum disorders	4
Asperger syndrome and other autistic spectrum disorders	5
Attention-deficit/hyperactivity disorder	3
Attention-deficit/hyperactivity disorder, currently without symptom	5
Symptomatic attention-deficit/hyperactivity disorder	5
Conduct disorder	3
Conduct disorder, currently without symptoms	5
Symptomatic conduct disorder	5
Idiopathic developmental intellectual disability	3
Borderline idiopathic developmental intellectual disability	5
Mild idiopathic developmental intellectual disability	5
Moderate idiopathic developmental intellectual disability	5
Severe idiopathic developmental intellectual disability	5
Profound idiopathic developmental intellectual disability	5
Other mental and substance use disorders	3
Other mental disorders, currently without symptoms	5
Mild other mental disorders	5
Moderate other mental disorders	5
Severe other mental disorders	5
Diabetes, urogenital, blood, and endocrine diseases	2
Diabetes mellitus	3
Uncomplicated diabetes mellitus	5
Neuropathy and other complications of diabetes mellitus	5
Diabetic neuropathy	6
Diabetic foot due to neuropathy	6
Diabetic neuropathy and amputation with treatment	6
Diabetic neuropathy and amputation without treatment	6
Vision loss due to diabetes mellitus	5
Moderate vision impairment due to diabetes mellitus	6
Severe vision impairment due to diabetes mellitus	6
Blindness due to diabetes mellitus	6
Acute glomerulonephritis	3
Acute glomerulonephritis	5
Chronic kidney disease	3
Chronic kidney disease due to diabetes mellitus	4
Stage III chronic kidney disease due to diabetes mellitus	5
Stage III chronic kidney disease without anemia due to diabetes mellitus	6
Stage III chronic kidney disease and mild anemia due to diabetes mellitus	6

Appendix Table 3. GBD 2015 cause and sequela hierarchy with four levels of causes (1-4) and two levels of sequelae (5-6)	
**Causes and sequelae**	**Level**
Stage III chronic kidney disease and moderate anemia due to diabetes mellitus	6
Stage III chronic kidney disease and severe anemia due to diabetes mellitus	6
Stage IV chronic kidney disease due to diabetes mellitus	5
Stage IV chronic kidney disease without anemia due to diabetes mellitus	6
Stage IV chronic kidney disease and mild anemia due to diabetes mellitus	6
Stage IV chronic kidney disease and moderate anemia due to diabetes mellitus	6
Stage IV chronic kidney disease and severe anemia due to diabetes mellitus	6
Stage V chronic kidney disease untreated due to diabetes mellitus	5
End-stage chronic kidney disease due to diabetes	5
End-stage renal disease after transplant due to diabetes mellitus	6
End-stage renal disease on dialysis due to diabetes mellitus	6
Chronic kidney disease due to hypertension	4
Stage III chronic kidney disease due to hypertension	5
Stage III chronic kidney disease without anemia due to hypertension	6
Stage III chronic kidney disease and mild anemia due to hypertension	6
Stage III chronic kidney disease and moderate anemia due to hypertension	6
Stage III chronic kidney disease and severe anemia due to hypertension	6
Stage IV chronic kidney disease due to hypertension	5
Stage IV chronic kidney disease without anemia due to hypertension	6
Stage IV chronic kidney disease and mild anemia due to hypertension	6
Stage IV chronic kidney disease and moderate anemia due to hypertension	6
Stage IV chronic kidney disease and severe anemia due to hypertension	6
Stage V chronic kidney disease untreated due to hypertension	5
End-stage chronic kidney disease due to hypertension	5
End-stage renal disease after transplant due to hypertension	6
End-stage renal disease on dialysis due to hypertension	6
Chronic kidney disease due to glomerulonephritis	4
Stage III chronic kidney disease due to glomerulonephritis	5
Stage III chronic kidney disease without anemia due to glomerulonephritis	6
Stage III chronic kidney disease and mild anemia due to glomerulonephritis	6
Stage III chronic kidney disease and moderate anemia due to glomerulonephritis	6
Stage III chronic kidney disease and severe anemia due to glomerulonephritis	6
Stage IV chronic kidney disease due to glomerulonephritis	5
Stage IV chronic kidney disease without anemia due to glomerulonephritis	6
Stage IV chronic kidney disease and mild anemia due to glomerulonephritis	6
Stage IV chronic kidney disease and moderate anemia due to glomerulonephritis	6
Stage IV chronic kidney disease and severe anemia due to glomerulonephritis	6
Stage V chronic kidney disease untreated due to glomerulonephritis	5
End-stage chronic kidney disease due to glomerulonephritis	5
End-stage renal disease after transplant due to glomerulonephritis	6
End-stage renal disease on dialysis due to glomerulonephritis	6
Chronic kidney disease due to other causes	4
Stage III chronic kidney disease due to other causes	5
Stage III chronic kidney disease without anemia due to other causes	6
Stage III chronic kidney disease and mild anemia due to other causes	6
Stage III chronic kidney disease and moderate anemia due to other causes	6
Stage III chronic kidney disease and severe anemia due to other causes	6
Stage IV chronic kidney disease due to other causes	5
Stage IV chronic kidney disease without anemia due to other causes	6
Stage IV chronic kidney disease and mild anemia due to other causes	6
Stage IV chronic kidney disease and moderate anemia due to other causes	6
Stage IV chronic kidney disease and severe anemia due to other causes	6
Stage V chronic kidney disease untreated due to other causes	5
End-stage chronic kidney disease due to other causes	5
End-stage renal disease after transplant due to other causes	6
End-stage renal disease on dialysis due to other causes	6
Urinary diseases and male infertility	3
Interstitial nephritis and urinary tract infections	4
Mild interstitial nephritis and urinary tract infections	5
Moderate interstitial nephritis and urinary tract infections	5
Urolithiasis	4
Acute urolithiasis	5

Appendix Table 3. GBD 2015 cause and sequela hierarchy with four levels of causes (1-4) and two levels of sequelae (5-6)	
**Causes and sequelae**	**Level**
Chronic urolithiasis	5
Benign prostatic hyperplasia	4
Asymptomatic benign prostatic hyperplasia	5
Symptomatic benign prostatic hyperplasia	5
Male infertility	4
Primary male infertility	5
Secondary male infertility	5
Other urinary diseases	4
Other urinary diseases	5
Gynecological diseases	3
Uterine fibroids	4
Uterine fibroids cases	5
Asymptomatic uterine fibroids	6
Mild abdominal pain due to uterine fibroids, without anemia	6
Mild abdominal pain with anemia due to uterine fibroids	5
Mild abdominal pain due to uterine fibroids, with mild anemia	6
Mild abdominal pain due to uterine fibroids, with moderate anemia	6
Mild abdominal pain due to uterine fibroids, with severe anemia	6
Polycystic ovarian syndrome	4
Polycystic ovarian syndrome cases	5
Asymptomatic polycystic ovarian syndrome	6
Hirsutism due to polycystic ovarian syndrome	6
Hirsutism and infertility due to polycystic ovarian syndrome	5
Hirsutism and primary infertility due to polycystic ovarian syndrom	6
Primary infertility due to polycystic ovarian syndrome	6
Hirsutism and secondary infertility due to polycystic ovarian syndrome	6
Secondary infertility due to polycystic ovarian syndrome	6
Female infertility	4
Idiopathic primary female infertility	5
Idiopathic secondary female infertility	5
Endometriosis	4
Endometriosis cases	5
Asymptomatic endometriosis	6
Mild abdominal pain due to endometriosis	6
Moderate abdominal pain due to endometriosis	6
Severe endometriosis	6
Abdominal pain and infertility due to endometriosis	5
Primary infertility due to endometriosis	6
Mild abdominal pain and primary infertility due to endometriosis	6
Moderate abdominal pain and primary infertility due to endometriosis	6
Severe abdominal pain and primary infertility due to endometriosis	6
Secondary infertility due to endometriosis	6
Mild abdominal pain and secondary infertility due to endometriosis	6
Moderate abdominal pain and secondary infertility due to endometriosis	6
Severe abdominal pain and secondary infertility due to endometriosis	6
Genital prolapse	4
Asymptomatic genital prolapse	5
Abdominal pain due to genital prolapse	5
Stress incontinence due to genital prolapse	5
Abdominal pain and stress incontinence due to genital prolapse	5
Premenstrual syndrome	4
Asymptomatic premenstrual syndrome	5
Abdominal pain and depression due to premenstrual syndrome	5
Abdominal pain due to premenstrual syndrome	5
Depression due to premenstrual syndrome	5
Other gynecological diseases	4
Other gynecological diseases cases	5
Asymptomatic other gynecological disorders	6
Mild other gynecological disorders	6
Moderate other gynecological disorders	6
Severe other gynecological disorders	6
Anemia due to other gynecological diseases	5

Appendix Table 3. GBD 2015 cause and sequela hierarchy with four levels of causes (1-4) and two levels of sequelae (5-6)	
**Causes and sequelae**	**Level**
Mild anemia due to other gynecological diseases	6
Moderate anemia due to other gynecological diseases	6
Severe anemia due to other gynecological diseases	6
Hemoglobinopathies and hemolytic anemias	3
Thalassemias	4
Beta-thalassemia major cases	5
Beta-thalassemia major, with mild anemia	6
Beta-thalassemia major, with moderate anemia	6
Beta-thalassemia major, with severe anemia	6
Beta-thalassemia major, severe infection with severe anemia	6
Beta-thalassemia major, without anemia	6
Hemoglobin E/beta-thalassemia cases	5
Hemoglobin E/beta-thalassemia, with mild anemia	6
Hemoglobin E/beta-thalassemia, with moderate anemia	6
Hemoglobin E/beta-thalassemia, with severe anemia	6
Hemoglobin E/beta-thalassemia, severe infection with severe anemia	6
Hemoglobin E/Beta-thalassemia, without anemia	6
Hemoglobin H disease cases	5
Hemoglobin H disease, with mild anemia	6
Hemoglobin H disease, with moderate anemia	6
Hemoglobin H disease, with severe anemia	6
Hemoglobin H disease, severe infection with severe anemia	6
Hemoglobin H disease, without anemia	6
Heart failure due to thalassemias	5
Mild heart failure due to thalassemias	6
Moderate heart failure due to thalassemias	6
Severe heart failure due to thalassemias	6
Thalassemias trait	4
B-thalassemia trait cases	5
Asymptomatic B-thalassemia trait	6
Mild anemia due to B-thalassemia trait	6
Moderate anemia due to B-thalassemia trait	6
Severe anemia due to B-thalassemia trait	6
Hemoglobin E trait cases	5
Asymptomatic hemoglobin E trait	6
Mild anemia due to hemoglobin E trait	6
Moderate anemia due to hemoglobin E trait	6
Severe anemia due to hemoglobin E trait	6
Sickle cell disorders	4
Homozygous sickle cell and severe sickle cell/beta-thalassemia cases	5
Homozygous sickle cell and severe sickle cell/beta-thalassemia, without anemia	6
Homozygous sickle cell and severe sickle cell/beta-thalassemia, with vaso-occlusive crisis, without anemia	6
Homozygous sickle cell and severe sickle cell/beta-thalassemia, with stroke, without anemia	6
Homozygous sickle cell and severe sickle cell/beta-thalassemia, with vaso-occlusive crisis and stroke, without anemia	6
Homozygous sickle cell and severe sickle cell/beta-thalassemia, with mild anemia	6
Homozygous sickle cell and severe sickle cell/beta-thalassemia, with moderate anemia	6
Homozygous sickle cell and severe sickle cell/beta-thalassemia, with severe anemia	6
Homozygous sickle cell and severe sickle cell/beta-thalassemia, with vaso-occlusive crisis and severe anemia	6
Homozygous sickle cell and severe sickle cell/beta-thalassemia, with stroke and severe anemia	6
Homozygous sickle cell and severe sickle cell/beta-thalassemia, with vaso-occlusive crisis, stroke, and severe anemia	6
Hemoglobin SC disease cases	5
Hemoglobin SC disease, without anemia	6
Hemoglobin SC disease, with vaso-occlusive crisis, without anemia	6
Hemoglobin SC disease, with stroke, without anemia	6
Hemoglobin SC disease, with vaso-occlusive crisis and stroke, without anemia	6
Hemoglobin SC disease, with mild anemia	6
Hemoglobin SC disease, with moderate anemia	6
Hemoglobin SC disease, with severe anemia	6

Appendix Table 3. GBD 2015 cause and sequela hierarchy with four levels of causes (1-4) and two levels of sequelae (5-6)	
**Causes and sequelae**	**Level**
Hemoglobin SC disease, with vaso-occlusive crisis and severe anemia	6
Hemoglobin SC disease, with stroke and severe anemia	6
Hemoglobin SC disease, with vaso-occlusive crisis, stroke, and severe anemia	6
Mild sickle cell/beta-thalassemia cases	5
Mild sickle cell/beta-thalassemia, without anemia	6
Mild sickle cell/beta-thalassemia, with vaso-occlusive crisis, without anemia	6
Mild sickle cell/beta-thalassemia, with stroke, without anemia	6
Mild sickle cell/beta-thalassemia, with vaso-occlusive crisis and stroke, without anemia	6
Mild sickle cell/beta-thalassemia, with mild anemia	6
Mild sickle cell/beta-thalassemia, with moderate anemia	6
Mild sickle cell/beta-thalassemia, with severe anemia	6
Mild sickle cell/beta-thalassemia, with vaso-occlusive crisis and severe anemia	6
Mild sickle cell/beta-thalassemia, with stroke and severe anemia	6
Mild sickle cell/beta-thalassemia, with vaso-occlusive crisis, stroke, and severe anemia	6
Sickle cell trait	4
Asymptomatic sickle cell trait	5
Mild anemia due to sickle cell trait	5
Moderate anemia due to sickle cell trait	5
Severe anemia due to sickle cell trait	5
G6PD deficiency	4
G6PD cases	5
Mild anemia due to G6PD deficiency	6
Moderate anemia due to G6PD deficiency	6
Severe anemia due to G6PD deficiency	6
Asymptomatic G6PD deficiency	6
Heart failure due to G6PD deficiency	5
Mild heart failure due to G6PD deficiency	6
Moderate heart failure due to G6PD deficiency	6
Severe heart failure due to G6PD deficiency	6
G6PD trait	4
Asymptomatic hemizygous G6PD deficiency	5
Mild anemia due to hemizygous G6PD deficiency	5
Moderate anemia due to hemizygous G6PD deficiency	5
Severe anemia due to hemizygous G6PD deficiency	5
Other hemoglobinopathies and hemolytic anemias	4
Other hemoglobinopathies and hemolytic anemias cases	5
Other hemoglobinopathies and hemolytic anemias	6
Mild anemia due to other hemoglobinopathies and hemolytic anemias	6
Moderate anemia due to other hemoglobinopathies and hemolytic anemias	6
Severe anemia due to other hemoglobinopathies and hemolytic anemias	6
Heart failure due to other hemoglobinopathies and hemolytic anemias	5
Mild heart failure due to other hemoglobinopathies and hemolytic anemias	6
Moderate heart failure due to other hemoglobinopathies and hemolytic anemias	6
Severe heart failure due to other hemoglobinopathies and hemolytic anemias	6
Endocrine, metabolic, blood, and immune disorders	3
Endocrine, metabolic, blood, and immune disorders cases	5
Asymptomatic endocrine, metabolic, blood, and immune disorders	6
Mild endocrine, metabolic, blood, and immune disorders	6
Moderate endocrine, metabolic, blood, and immune disorders	6
Severe endocrine, metabolic, blood, and immune disorders	6
Anemia due to endocrine, metabolic, blood, and immune disorders	5
Mild anemia due to endocrine, metabolic, blood, and immune disorders	6
Moderate anemia due to endocrine, metabolic, blood, and immune disorders	6
Severe anemia due to endocrine, metabolic, blood, and immune disorders	6
Heart failure due to endocrine, metabolic, blood, and immune disorders	5
Mild heart failure due to endocrine, metabolic, blood, and immune disorders	6
Moderate heart failure due to endocrine, metabolic, blood, and immune disorders	6
Severe heart failure due to endocrine, metabolic, blood, and immune disorders	6
Musculoskeletal disorders	2
Rheumatoid arthritis	3
Mild rheumatoid arthritis	5
Moderate rheumatoid arthritis	5

Appendix Table 3. GBD 2015 cause and sequela hierarchy with four levels of causes (1-4) and two levels of sequelae (5-6)	
**Causes and sequelae**	**Level**
Severe rheumatoid arthritis	5
Osteoarthritis	3
Osteoarthritis of the hip cases	5
Mild osteoarthritis of the hip	6
Moderate osteoarthritis of the hip	6
Severe osteoarthritis of the hip	6
Osteoarthritis of the knee cases	5
Mild osteoarthritis of the knee	6
Moderate osteoarthritis of the knee	6
Severe osteoarthritis of the knee	6
Low back and neck pain	3
Low back pain	4
Mild low back pain without leg pain	5
Mild low back pain with leg pain	5
Moderate low back pain without leg pain	5
Moderate low back pain with leg pain	5
Severe low back pain without leg pain	5
Severe low back pain with leg pain	5
Most severe low back pain without leg pain	5
Most severe low back pain with leg pain	5
Neck pain	4
Mild neck pain	5
Moderate neck pain	5
Severe neck pain	5
Most severe neck pain	5
Gout	3
Asymptomatic gout	5
Symptomatic gout cases	5
Symptomatic episodes of gout	6
Polyarticular gout	6
Other musculoskeletal disorders	3
Asymptomatic other musculoskeletal disorders	5
Other musculoskeletal disorders severity level 2	5
Other musculoskeletal disorders severity level 3	5
Other musculoskeletal disorders severity level 5	5
Other musculoskeletal disorders severity level 6	5
Other musculoskeletal disorders severity level 1	5
Other musculoskeletal disorders severity level 4	5
Other non-communicable diseases	2
Congenital anomalies	3
Neural tube defects	4
Moderate motor impairment due to moderate neural tube defects	5
Severe motor impairment due to severe neural tube defects	5
Severe motor impairment with incontinence due to severe neural tube defects	5
Severe motor impairment with mild intellectual disability and incontinence due to severe neural tube defects	5
Severe motor impairment with mild intellectual disability due to severe neural tube defects	5
Severe motor impairment with moderate intellectual disability and incontinence due to severe neural tube defects	5
Severe motor impairment with moderate intellectual disability due to severe neural tube defects	5
Severe motor impairment with profound intellectual disability and incontinence due to severe neural tube defects	5
Severe motor impairment with profound intellectual disability due to severe neural tube defects	5
Severe motor impairment with severe intellectual disability and incontinence due to severe neural tube defects	5
Severe motor impairment with severe intellectual disability due to severe neural tube defects	5
Congenital heart anomalies	4
Less severe heart anomalies cases	5
Asymptomatic less severe congenital heart anomalies	6
Symptomatic less severe congenital heart anomalies	6
Severe congenital heart anomalies	5
Critical congenital heart anomalies	5

Appendix Table 3. GBD 2015 cause and sequela hierarchy with four levels of causes (1-4) and two levels of sequelae (5-6)	
**Causes and sequelae**	**Level**
Heart failure due to congenital heart anomalies	5
Mild heart failure due to congenital heart anomalies	6
Moderate heart failure due to congenital heart anomalies	6
Severe heart failure due to congenital heart anomalies	6
Cleft lip and cleft palate	4
Asymptomatic orofacial clefts	5
Disfigurement level 1 due to orofacial clefts	5
Disfigurement level 2 due to orofacial clefts	5
Disfigurement level 2 and speech problems due to orofacial clefts	5
Down syndrome	4
Asymptomatic Down syndrome	5
Isolated congenital heart disease due to Down syndrome	5
Borderline intellectual disability due to Down syndrome	5
Borderline intellectual disability with congenital heart disease due to Down syndrome	5
Congenital heart disease and mild dementia due to Down syndrome	5
Congenital heart disease and moderate dementia due to Down syndrome	5
Congenital heart disease and severe dementia due to Down syndrome	5
Mild dementia due to Down syndrome	5
Mild intellectual disability due to Down syndrome	5
Mild intellectual disability with congenital heart disease due to Down syndrome	5
Moderate dementia due to Down syndrome	5
Moderate intellectual disability due to Down syndrome	5
Moderate intellectual disability with congenital heart disease due to Down syndrome	5
Profound intellectual disability due to Down syndrome	5
Profound intellectual disability with congenital heart disease due to Down syndrome	5
Severe dementia due to Down syndrome	5
Severe intellectual disability due to Down syndrome	5
Severe intellectual disability with congenital heart disease due to Down syndrome	5
Turner syndrome	4
Asymptomatic Turner syndrome	5
Congenital heart disease due to Turner syndrome	5
Primary infertility due to Turner syndrome	5
Congenital heart disease with infertility due to Turner syndrome	5
Klinefelter syndrome	4
Asymptomatic Klinefelter syndrome	5
Borderline intellectual disability due to Klinefelter syndrome	5
Borderline intellectual disability with infertility due to Klinefelter syndrome	5
Mild intellectual disability due to Klinefelter syndrome	5
Mild intellectual disability with infertility due to Klinefelter syndrome	5
Primary infertility due to Klinefelter syndrome	5
Other chromosomal abnormalities	4
Asymptomatic chromosomal unbalanced rearrangements	5
Isolated congenital heart disease due to chromosomal unbalanced rearrangements	5
Borderline intellectual disability due to chromosomal unbalanced rearrangements	5
Borderline intellectual disability with congenital heart disease due to chromosomal unbalanced rearrangements	5
Congenital heart disease and mild dementia due to chromosomal unbalanced rearrangements	5
Congenital heart disease and moderate dementia due to chromosomal unbalanced rearrangements	5
Congenital heart disease and severe dementia due to chromosomal unbalanced rearrangements	5
Mild dementia due to chromosomal unbalanced rearrangements	5
Mild intellectual disability due to chromosomal unbalanced rearrangements	5
Mild intellectual disability with congenital heart disease due to chromosomal unbalanced rearrangements	5
Moderate dementia due to chromosomal unbalanced rearrangements	5
Moderate intellectual disability due to chromosomal unbalanced rearrangements	5
Moderate intellectual disability with congenital heart disease due to chromosomal unbalanced rearrangements	5
Profound intellectual disability due to chromosomal unbalanced rearrangements	5
Profound intellectual disability with congenital heart disease due to chromosomal unbalanced rearrangements	5
Severe dementia due to chromosomal unbalanced rearrangements	5
Severe intellectual disability due to chromosomal unbalanced rearrangements	5

Appendix Table 3. GBD 2015 cause and sequela hierarchy with four levels of causes (1-4) and two levels of sequelae (5-6)	
**Causes and sequelae**	**Level**
Severe intellectual disability with congenital heart disease due to chromosomal unbalanced rearrangements	5
Other congenital anomalies	4
Mild hearing loss due to other congenital anomalies	5
Mild hearing loss with ringing due to other congenital anomalies	5
Moderate hearing loss due to other congenital anomalies	5
Moderate hearing loss with ringing due to other congenital anomalies	5
Moderately severe hearing loss due to other congenital anomalies	5
Moderately severe hearing loss with ringing due to other congenital anomalies	5
Severe hearing loss with ringing due to other congenital anomalies	5
Severe hearing loss due to other congenital anomalies	5
Profound hearing loss due to other congenital anomalies	5
Profound hearing loss with ringing due to other congenital anomalies	5
Complete hearing loss due to other congenital anomalies	5
Complete hearing loss with ringing due to other congenital anomalies	5
Other congenital anomalies	5
Skin and subcutaneous diseases	3
Dermatitis	4
Mild eczema	6
Moderate eczema	6
Severe eczema	6
Contact dermatitis cases	5
Asymptomatic contact dermatitis	6
Mild contact dermatitis	6
Severe contact dermatitis	6
Seborrhoeic dermatitis cases	5
Asymptomatic seborrhoeic dermatitis	6
Symptomatic seborrhoeic dermatitis	6
Psoriasis	4
Mild psoriasis	5
Moderate psoriasis	5
Severe psoriasis	5
Cellulitis	4
Mild cellulitis	5
Severe cellulitis	5
Pyoderma	4
Impetigo	5
Abscess and other bacterial skin diseases	5
Scabies	4
Scabies	5
Fungal skin diseases	4
Tinea capitis	5
Other fungal skin diseases	5
Viral skin diseases	4
Molluscum contagiosum cases	5
Mild molluscum contagiosum	6
Severe molluscum contagiosum	6
Viral warts cases	5
Mild viral warts	6
Severe viral warts	6
Acne vulgaris	4
Acne vulgaris	5
Alopecia areata	4
Mild alopecia areata	5
Severe alopecia areata	5
Pruritus	4
Pruritus	5
Urticaria	4
Mild urticaria	5
Severe urticaria	5
Decubitus ulcer	4
Mild decubitus ulcer	5

Appendix Table 3. GBD 2015 cause and sequela hierarchy with four levels of causes (1-4) and two levels of sequelae (5-6)	
**Causes and sequelae**	**Level**
Moderate decubitus ulcer	5
Severe decubitus ulcer	5
Other skin and subcutaneous diseases	4
Asymptomatic other skin and subcutaneous diseases	5
Symptomatic other skin and subcutaneous diseases	5
Sense organ diseases	3
Glaucoma	4
Moderate vision impairment due to glaucoma	5
Severe vision impairment due to glaucoma	5
Blindness due to glaucoma	5
Cataract	4
Moderate vision impairment due to cataract	5
Severe vision impairment due to cataract	5
Blindness due to cataract	5
Macular degeneration	4
Moderate vision impairment due to macular degeneration	5
Severe vision impairment due to macular degeneration	5
Blindness due to macular degeneration	5
Refraction and accommodation disorders	4
Moderate vision impairment due to uncorrected refractive error	5
Severe vision impairment due to uncorrected refractive error	5
Blindness due to uncorrected refractive error	5
Near vision impairment	5
Age-related and other hearing loss	4
Mild hearing loss due to age-related and other hearing loss	5
Mild hearing loss with ringing due to age-related and other hearing loss	5
Moderate hearing loss due to age-related and other hearing loss	5
Moderate hearing loss with ringing due to age-related and other hearing loss	5
Moderately severe hearing loss due to age-related and other hearing loss	5
Moderately severe hearing loss with ringing due to age-related and other hearing loss	5
Severe hearing loss with ringing due to age-related and other hearing loss	5
Severe hearing loss due to age-related and other hearing loss	5
Profound hearing loss due to age-related and other hearing loss	5
Profound hearing loss with ringing due to age-related and other hearing loss	5
Complete hearing loss due to age-related and other hearing loss	5
Complete hearing loss with ringing due to age-related and other hearing loss	5
Other vision loss	4
Moderate vision impairment due to other vision loss	5
Severe vision impairment due to other vision loss	5
Blindness due to other vision loss	5
Other sense organ diseases	4
Asymptomatic other sense organ diseases	5
Mild other sense organ diseases	5
Moderate other sense organ diseases	5
Severe other sense organ diseases	5
Oral disorders	3
Deciduous caries	4
Asymptomatic deciduous caries	5
Tooth pain due to deciduous caries	5
Permanent caries	4
Asymptomatic permanent caries	5
Tooth pain due to permanent caries	5
Periodontal diseases	4
Chronic periodontal diseases	5
Edentulism and severe tooth loss	4
Asymptomatic edentulism and severe tooth loss	5
Difficulty eating due to edentulism and severe tooth loss	5
Other oral disorders	4
Mild other oral disorders	5
Severe other oral disorders	5
Injuries	1
Transport injuries	2

Appendix Table 3. GBD 2015 cause and sequela hierarchy with four levels of causes (1-4) and two levels of sequelae (5-6)	
**Causes and sequelae**	**Level**
Road injuries	3
Pedestrian road injuries	4
Cyclist road injuries	4
Motorcyclist road injuries	4
Motor vehicle road injuries	4
Other road injuries	4
Other transport injuries	3
Unintentional injuries	2
Falls	3
Drowning	3
Fire, heat, and hot substances	3
Poisonings	3
Exposure to mechanical forces	3
Unintentional firearm injuries	4
Unintentional suffocation	4
Other exposure to mechanical forces	4
Adverse effects of medical treatment	3
Animal contact	3
Venomous animal contact	4
Non-venomous animal contact	4
Foreign body	3
Pulmonary aspiration and foreign body in airway	4
Foreign body in eyes	4
Foreign body in other body part	4
Environmental heat and cold exposure	3
Other unintentional injuries	3
Self-harm and interpersonal violence	2
Self-harm	3
Interpersonal violence	3
Assault by firearm	4
Assault by sharp object	4
Assault by other means	4
Forces of nature, war, and legal intervention	2
Exposure to forces of nature	3
Collective violence and legal intervention	3

Appendix Table 4. ICD-9 and ICD-10 codes used in the extraction of hospital and claims data, mapped to GBD 2015 nonfatal causes, impairments, and nature of injury categories			
**GBD cause name**	**Measure**	**ICD 9**	**ICD 10**
Tuberculosis	Prevalence	010-010.6, 010.8-010.9, 011-011.9, 012-012.4, 012.7-012.9, 013-013.6, 013.8-013.9, 014.0, 014.2, 014.8-014.9, 015-015.9, 016-016.7, 016.9, 017-017.9, 018.0, 018.3, 018.5, 018.8-018.9, 019.3, 019.9, 137-137.5, 320.4, 730.4-730.6	A15-A15.9, A16-A16.5, A16.7-A16.9, A17-A17.1, A17.3, A17.8-A17.9, A18-A18.8, A19-A19.2, A19.8-A19.9, B90-B90.2, B90.8-B90.9, K67.3, K93.0, N74-N74.1, P37.0
Diarrheal diseases	Incidence	001-001.1, 001.4, 001.6, 001.8-001.9, 003-003.2, 003.4-003.9, 004-004.3, 004.8-004.9, 005-005.4, 005.8-005.9, 006-006.9, 007-007.5, 007.7-007.9, 008-008.9, 009-009.9	A00-A00.1, A00.9, A02-A02.2, A02.8-A02.9, A03-A03.3, A03.8-A03.9, A04-A04.6, A04.8-A04.9, A05-A05.5, A05.8-A05.9, A06-A06.9, A07-A07.4, A07.8-A07.9, A08-A08.5, A08.8
Intestinal infectious diseases	Incidence	002-002.4, 002.9	A01-A01.4
Typhoid fever	Incidence	2	A01.0
Paratyphoid fever	Incidence	002.1-002.4, 002.9	A01.1-A01.4
Lower respiratory infections	Incidence	073.0, 073.2-073.3, 073.5-073.6, 079.8, 466-466.1, 466.9, 480-480.3, 480.8-480.9, 481.0, 481.2, 481.9, 482-482.4, 482.8-482.9, 483-483.1, 483.8-483.9, 484-484.8, 485-485.1, 485.4, 485.6, 485.9, 486-486.1, 486.4, 486.9, 487-487.1, 487.8-487.9, 488-488.1, 488.8, 513.0, 770.0	A48.1, J10-J10.2, J10.8-J10.9, J11-J11.2, J11.8, J12-J12.3, J12.8-J12.9, J15-J15.9, J16.0, J16.8, J17-J17.3, J17.8, J18-J18.2, J18.8-J18.9, J20-J20.9, J21-J21.1, J21.8-J21.9, J85.1, P23-P23.6, P23.8-P23.9
Lower respiratory infections	Prevalence	272-272.4	E78-E78.5
Upper respiratory infections	Prevalence	272.5-272.7, 461-461, 461.8-461.9, 464-464.5, 464.8-464.8, 465-465, 465.8-465.9	E78.6-E78.8
Upper respiratory infections	Incidence	460.0, 460.9, 461-461.3, 461.8-461.8, 464-464, 464.8-464.9, 465-465.1, 465.8-465.8	J01-J01.4, J01.8-J01.9, J02.0, J02.8-J02.9, J03.0, J03.8-J03.9, J04-J04.3, J05-J05.1, J06.0, J06.8-J06.9
Otitis media	Prevalence	272.8-272.9, 384-384.4, 384.8-384.9, 387-387.2, 387.8-387.9	E78.8-E78.9, H72-H72.2, H72.8-H72.9, H73-H73.2, H73.8-H73.9, H80-H80.2, H80.8-H80.9, H83-H83.3, H83.8-H83.9
Otitis media	Incidence	381-381.4, 382-382.3	H65-H65.4, H66-H66.2
Meningitis	Incidence	036-036.4, 036.8-036.9, 320-320.3, 320.5, 320.7-320.9	A39-A39.4, A39.8-A39.9, G00-G00.3, G00.8-G00.9
Pneumococcal meningitis	Incidence	320.1	G00.1
H influenzae type B meningitis	Incidence	320	G00.0
Meningococcal meningitis	Incidence	036-036.4, 036.8-036.9, 320.5	A39-A39.4, A39.8-A39.9
Other meningitis	Incidence	047-047.4, 047.8-047.9, 048-048.2, 048.5-048.6, 048.9, 049-049.4, 049.6, 049.8-049.9, 054.7, 112.8, 320.2-320.3, 320.7-320.9, 321-321.8, 322-322.2, 322.9	A87-A87.2, A87.8-A87.9, G00.2-G00.3, G00.8-G00.9, G03-G03.2, G03.8-G03.9
Encephalitis	Incidence	062-062.9, 063-063.4, 063.7-063.9, 064-064.1, 064.3-064.4, 064.9, 310.8, 323-323.9	A83-A83.6, A83.8-A83.9, A84-A84.1, A84.8-A84.9, A85-A85.2, A85.8, A86.0, B10.0, B94.1, F07.1, G04-G04.3, G04.8-G04.9
Encephalitis	Prevalence	722.7-722.8, 724.3-724.4	
Diphtheria	Incidence	032-032.3, 032.6, 032.8-032.9	A36-A36.3, A36.8-A36.9
Whooping cough	Incidence	033-033.1, 033.3, 033.8-033.9	A37-A37.1, A37.8-A37.9
Tetanus	Incidence	037.0, 037.4, 037.6, 037.8-037.9, 771.3	A33.0, A34.0, A35.0
Measles	Incidence	055-055.3, 055.5-055.9	B05-B05.4, B05.8-B05.9
Varicella and herpes zoster	Incidence	052-052.2, 052.6-052.9	B01-B01.2, B01.8-B01.9
Varicella and herpes zoster	Prevalence	053-053.3, 053.5-053.9	B02-B02.3, B02.7-B02.9
Malaria	Prevalence	084-084.9	B50.0, B50.8-B50.9, B51.0, B51.8-B51.9, B52.0, B52.8-B52.9, B53-B53.1, B53.8, B54.0, P37.3-P37.4
Visceral leishmaniasis	Incidence	85	B55.0
Cutaneous and mucocutaneous leishmaniasis	Prevalence	085.1-085.5	B55.1-B55.2
Cystic echinococcosis	Incidence	122-122.4, 122.8-122.9	B67-B67.4, B67.8-B67.9
Lymphatic filariasis	Prevalence	125-125.2	B74-B74.2
Trachoma	Prevalence	076-076.1, 076.6, 076.9	A71-A71.1, A71.9, A74.0, B94.0
Rabies	Incidence	071-071.1, 071.4-071.9	A82-A82.1, A82.9
Leprosy	Prevalence	030-030.3, 030.5-030.9	A30-A30.5, A30.8-A30.9
Maternal hemorrhage	Incidence	640.0, 640.8-640.9, 641-641.3, 641.8-641.9, 661.0, 666-666.3	O20.0, O20.8-O20.9, O43.2, O44-O44.1, O45.0, O45.8-O45.9, O46.0, O46.8-O46.9, O62.2, O67.0, O67.8-O67.9, O72-O72.3
Maternal sepsis and other maternal infections	Incidence	646.5-646.6, 659.2-659.3, 670-670.3, 670.8, 672.0, 674.1-674.3, 675-675.2, 675.8-675.9	O23-O23.5, O23.9, O41.1, O41.8-O41.9, O75.2-O75.3, O86-O86.4, O86.8, O91-O91.2
Maternal hypertensive disorders	Incidence	642.3-642.7, 642.9	O11.1-O11.3, O11.9, O12-O12.2, O13.1-O13.3, O13.9, O14-O14.2, O14.9, O15-O15, O15-O15.2, O16.1-O16.3, O16.9
Maternal obstructed labor and uterine rupture	Incidence	659-659.1, 660-660.9, 662-662.3, 665-665.9, 669.5-669.6	O64-O64.5, O64.8-O64.9, O65-O65.5, O65.8-O65.9, O66-O66.6, O66.8-O66.9, O71-O71.9, O83-O83.4, O83.8-O83.9
Maternal abortion, miscarriage, and ectopic pregnancy	Incidence	631.0, 631.2, 631.8, 633-633.2, 633.8-633.9, 634-634.9, 635-635.9, 636-636.9, 637-637.9, 638-638.9, 639-639.6, 639.8-639.9	O00-O00.2, O00.8-O00.9, O01-O01.1, O01.9, O02-O02.1, O02.8-O02.9, O03-O03.9, O04-O04.9, O05-O05.9, O06-O06.9, O07-O07.9, O08-O08.9, O36.4
Neonatal preterm birth complications	Prevalence	764-764.2, 764.9, 765-765.2	P05-P05.2, P05.9, P07-P07.3
Neonatal encephalopathy due to birth asphyxia and trauma	Prevalence	761.7-761.9, 762-762.9, 763-763.9, 767-767.1, 767.8-767.9, 768.2-768.7, 768.9, 772.1-772.2, 779-779.2	P02-P02.9, P03-P03.6, P03.8-P03.9, P10-P10.4, P10.8-P10.9, P11-P11.5, P11.9, P20-P20.1, P20.9, P21-P21.1, P21.9, P52-P52.6, P52.8-P52.9, P91-P91.6, P91.8-P91.9
Neonatal sepsis and other neonatal infections	Prevalence	771.4-771.8, 777.5-777.6	P36-P36.5, P36.8-P36.9, P38.1, P38.9, P39-P39.4, P39.8-P39.9, P77.1-P77.3, P77.9, P78-P78.1
Hemolytic disease and other neonatal jaundice	Prevalence	773-773.5, 774-774.2, 774.7, 776.2	P55-P55.1, P55.8-P55.9, P56.0, P56.9, P57.0, P57.8-P57.9, P58-P58.5, P58.8-P58.9

Appendix Table 4. ICD-9 and ICD-10 codes used in the extraction of hospital and claims data, mapped to GBD 2015 nonfatal causes, impairments, and nature of injury categories

GBD cause name	Measure	ICD 9	ICD 10
Other neonatal disorders	Prevalence	655.3-655.6, 656-656.9, 657-657.1, 658-658.4, 658.8-658.9, 760-760.9, 761-761.6, 766-766.2, 766.9, 767.1-767.7, 770.2-770.9, 772.0, 772.3-772.6, 772.8-772.9, 774.3-774.6, 775-775.1, 775.4-775.9, 776-776.1, 776.3-776.9, 777-777.4, 777.8-777.9, 778-778.9, 779.3-779.5, 779.7-779.9	P00-P00.9, P01-P01.9, P04-P04.6, P04.8-P04.9, P08-P08.2, P12-P12.4, P12.8-P12.9, P13-P13.4, P13.8-P13.9, P14-P14.3, P14.8-P14.9, P15-P15.6, P15.8-P15.9, P19-P19.2, P19.9, P22-P22.1, P22.8-P22.9, P25-P25.3, P25.8, P26-P26.1, P26.8-P26.9, P27-P27.1, P27.8-P27.9, P28-P28.5, P28.8-P28.9, P50-P50.5, P50.8-P50.9, P51.0, P51.8-P51.9, P54-P54.6, P54.8-P54.9, P59-P59.3, P59.8-P59.9, P61-P61.6, P61.8-P61.9, P70-P70.4, P70.8-P70.9, P71-P71.4, P71.8-P71.9, P72-P72.2, P72.8-P72.9, P74-P74.6, P74.8-P74.9, P76-P76.2, P76.8-P76.9, P78.2-P78.3, P78.8-P78.9, P80.0, P80.8-P80.9, P81.0, P81.8-P81.9, P83-P83.6, P83.8-P83.9, P92-P92.6, P92.8-P92.9, P94-P94.2, P94.8-P94.9, P96.3, P96.8-P96.9, P99.9
Iodine deficiency	Prevalence		E01-E01.2
Vitamin A deficiency	Prevalence	264-264.6	E50-E50.7
Syphilis	Prevalence	093-093.2, 093.5, 093.8-093.9, 094-094.3, 094.8-094.9, 095-095.9	A52-A52.3, A52.7-A52.9, I98.0
Syphilis	Incidence	091-091.9, 092.0, 092.4-092.5, 092.7-092.9	A51-A51.5, A51.9
Chlamydial infection	Incidence	099.4-099.5	A56-A56.1, K67.0, N74.4
Gonococcal infection	Incidence	098-098.3	A54-A54.2, K67.1, N74.3
Trichomoniasis	Incidence	131.0, 131.3, 131.6, 131.8-131.9	A59.0, A59.8-A59.9
Genital herpes	Prevalence	54.1	A60-A60.1, A60.9
Rheumatic heart disease	Prevalence	391-391.2, 391.8-391.9, 392.0, 394-394.2, 394.9, 395-395.2, 395.9, 396-396.3, 396.8-396.9, 397-397.1, 397.9, 398.8-398.9	I01-I01.2, I01.8-I01.9, I02.0, I05-I05.2, I05.8-I05.9, I06-I06.2, I06.8-I06.9, I07-I07.2, I07.8-I07.9, I08-I08.3, I08.8-I08.9, I09-I09.2, I09.8-I09.9
Ischemic heart disease	Prevalence	412.0, 412.9, 413-413.1, 413.9, 414-414.5, 414.8-414.9	I20-I20.1, I20.8-I20.9, I23.7, I25-I25.9
Ischemic heart disease	Incidence	410-410.9, 411-411.1, 411.8-411.9	I21-I21.4, I21.9, I22-I22.2, I22.8-I22.9
Cerebrovascular disease	Prevalence	437-437, 437-437.2, 437.4-437.4, 437.4-437.8	I68-I68.2, I69-I69, I69-I69.3
Cerebrovascular disease	Incidence	430-430.1, 430.4, 430.6, 430.9, 431-431.2, 431.9, 432-432.1, 432.4-432.5, 432.7, 432.9, 434-434.1, 434.3-434.4, 434.6-434.7, 434.9	I60-I60.9, I61-I61.6, I61.8-I61.9, I62.0, I63-I63.6, I63.8-I63.9, I67-I67.7
Ischemic stroke	Incidence	434-434, 434-434.1, 434.3-434.3, 434.3-434.4, 434.6-434.6, 434.6-434.7	I63-I63, I63-I63.6, I63.8-I63.8, I63.8-I63.9
Hemorrhagic stroke	Incidence	430-430, 430-430.1, 431-431, 431-431.2, 432-432, 432-432.1, 432.4-432.4, 432.4-432.5	I60-I60, I60-I60.9, I61-I61, I61-I61.6, I61.8-I61.8, I61.8-I61.9
Cardiomyopathy and myocarditis	Incidence	036.4, 074.2, 422.0, 422.4-422.5, 422.9, 429-429.1	A39.5, B33.2, I40-I40.1, I40.8-I40.9, I41-I41.2, I41.8-I41.9, I51.4-I51.6
Atrial fibrillation and flutter	Prevalence	427.3	I48-I48.4, I48.9
Peripheral vascular disease	Prevalence	440-440.4, 440.8-440.9, 443-443.2, 443.8-443.9	I70-I70.9, I73-I73.1, I73.8-I73.9
Endocarditis	Incidence	036.4, 074.2, 093.2, 098.8, 112.8, 115-115.1, 115.9, 421-421.1, 421.9, 424.4-424.5, 424.8-424.9	A32.8, A39.5, A52.0, B33.2, B37.6, I33.0, I33.9, I38.0, I38.9, I39-I39.4, I39.8-I39.9, M32.1
Chronic obstructive pulmonary disease	Prevalence	490-490.2, 490.9, 491-491.2, 491.8-491.9, 492-492.2, 492.8-492.9, 494-494.1, 494.9, 496-496.1, 496.6, 496.9, 497.0	J41-J41.1, J41.8, J42.4, J43-J43.2, J43.8-J43.9, J44-J44.1, J44.8-J44.9, J47-J47.1, J47.9
Silicosis	Prevalence	502.0, 502.9	J62.0, J62.8
Asbestosis	Prevalence	501.0, 501.9	
Coal workers pneumoconiosis	Prevalence	500.0, 500.9	
Other pneumoconiosis	Prevalence	503-503.1, 503.9, 504.0, 504.9, 505.0, 505.9	J63-J63.6, J63.8
Asthma	Prevalence	493-493.4, 493.8-493.9	J45-J45.5, J45.8-J45.9
Interstitial lung disease and pulmonary sarcoidosis	Prevalence	135.0, 135.2, 135.9, 515.0, 515.9, 516-516.6, 516.8-516.9	D86-D86.2, D86.8-D86.9, J84-J84.2, J84.8-J84.9
Cirrhosis and other chronic liver diseases	Prevalence	456-456.2, 570.0, 570.9, 571-571.6, 571.8-571.9, 572-572.6, 572.8-572.9, 573-573.5, 573.8-573.9	I85-I85.1, I85.9, I98.2, K70-K70.4, K70.9, K71-K71.9, K72-K72.1, K72.9, K73-K73.2, K73.8-K73.9, K74-K74.6, K75-K75.4, K75.8-K75.9, K76-K76.9, K77.0, K77.8, P78.8
Peptic ulcer disease	Prevalence	574-574.9, 575-575.6, 575.8-575.9, 576-576.5, 576.8-576.9	K80-K80.8, K81-K81.2, K81.8-K81.9, K82-K82.4, K82.8-K82.9, K83-K83.5, K83.8-K83.9, K87-K87.1
Peptic ulcer disease	Incidence	531-531.3, 532-532.3, 533-533.3, 534-534.3	K25-K25.3, K26-K26.3, K27-K27.3, K28-K28.3
Gastritis and duodenitis	Incidence	535-535.7	K29-K29.9
Gastritis and duodenitis	Prevalence	535-535	K29-K29
Appendicitis	Incidence	540-540.1, 540.9, 541-541.3, 541.9, 542-542.1, 542.9	K35-K35.3, K35.8-K35.9, K37.9
Paralytic ileus and intestinal obstruction	Incidence	560-560.3, 560.8-560.9, 569.8	K56-K56.9
Inguinal, femoral, and abdominal hernia	Incidence	550-550.1, 550.3, 550.9, 551-551.1, 551.3, 551.8-551.9, 552-552.1, 552.3-552.4, 552.8-552.9, 553-553.3, 553.6, 553.8-553.9	K40-K40.4, K40.9, K41-K41.4, K41.9, K42-K42.1, K42.9, K44-K44.1, K44.9, K45-K45.1, K45.8, K46-K46.1, K46.9
Inflammatory bowel disease	Prevalence	555-555.3, 555.9, 556-556.6, 556.8-556.9, 564.1	K50-K50.1, K50.8-K50.9, K51-K51.5, K51.8-K51.9
Vascular intestinal disorders	Incidence	557-557.1, 557.9	K55-K55.1
Gallbladder and biliary diseases	Prevalence	531-531.7, 531.9, 532-532.7, 532.9, 533-533.7, 533.9, 534-534.7, 534.9	K25-K25.9, K26-K26.7, K26.9, K27-K27.9, K28-K28.7, K28.9
Gallbladder and biliary diseases	Incidence	574-574.9, 575-575.6, 575.8-575.9, 576-576.5, 576.8-576.9	K80-K80.8, K81-K81.2, K81.8-K81.9, K82-K82.4, K82.8-K82.9, K83-K83.5, K83.8-K83.9, K87-K87.1
Pancreatitis	Incidence	577-577.3, 577.8-577.9	K85-K85.3, K85.8-K85.9, K86-K86.3, K86.8-K86.9
Alzheimer disease and other dementias	Prevalence	290-290.4, 290.8-290.9, 291.2, 292.8, 294-294.2, 294.8-294.9, 331-331.2, 331.6-331.9	F00-F00.2, F00.9, F01-F01.3, F01.5, F01.8-F01.9, F02-F02.4, F02.8, F03.9, G30-G30.1, G30.8-G30.9, G31-G31.2, G31.8-G31.9
Parkinson disease	Prevalence	332-332.1	G20.9
Multiple sclerosis	Prevalence	340.0, 340.9	
Motor neuron disease	Prevalence	335-335.2, 335.8-335.9	G12-G12.2, G12.8-G12.9
Migraine	Prevalence	346-346.9	G43-G43.9
Tension-type headache	Prevalence	307.8, 339.1	G44.2

**Appendix Table 4. ICD-9 and ICD-10 codes used in the extraction of hospital and claims data, mapped to GBD 2015 nonfatal causes, impairments, and nature of injury categories**			
**GBD cause name**	**Measure**	**ICD 9**	**ICD 10**
Medication overuse headache	Prevalence	339.3	G44.4-G44.5, G44.8
Schizophrenia	Prevalence	295-295.3, 295.5-295.8	F20-F20.6, F20.8-F20.9, F25-F25.2, F25.8-F25.9
Alcohol use disorders	Prevalence	291-291.1, 291.3-291.5, 291.8-291.9, 303.0, 303.9	F10.2-F10.8
Opioid use disorders	Prevalence	304.0, 305.5	F11-F11.9
Cocaine use disorders	Prevalence	304.2, 305.2, 305.6	F14-F14.9
Amphetamine use disorders	Prevalence	304.4, 305.7	F15-F15.9
Cannabis use disorders	Prevalence	304.3	F12-F12.9
Major depressive disorder	Prevalence	296.2-296.3	F32-F32.5, F32.8-F32.9, F33-F33.4, F33.8-F33.9
Dysthymia	Prevalence	300.4	F34.1
Bipolar disorder	Prevalence	296-296.1, 296.4-296.8	F30-F30.4, F30.8-F30.9, F31-F31.9, F34.0
Anxiety disorders	Prevalence	300-300.3, 308-308.4, 308.9, 309-309.4, 309.8-309.9	F40-F40.2, F40.8-F40.9, F41-F41.3, F41.8-F41.9, F42-F42.2, F42.8-F42.9, F43-F43.2, F43.8-F43.9, F44-F44.9
Anorexia nervosa	Prevalence	307.1	F50-F50.1
Bulimia nervosa	Prevalence	307.5	F50.2-F50.5
Autism	Prevalence	299-299.1	F84-F84.4
Asperger syndrome and other autistic spectrum disorders	Prevalence	299.8-299.9	F84.5, F84.8-F84.9
Attention-deficit/hyperactivity disorder	Prevalence	314-314.2, 314.8-314.9	F90-F90.2, F90.8-F90.9
Conduct disorder	Prevalence	312-312.4, 312.8-312.9	F91-F91.3, F91.8-F91.9, F92.0, F92.8-F92.9, F93-F93.3, F93.8-F93.9, F94-F94.2, F94.8-F94.9
Diabetes mellitus	Prevalence	249.5-249.7, 250-250.3, 250.5-250.5, 250.5-250.9	E08-E08.1, E08.3-E08.3, E08.3-E08.3, E08.3-E08.6, E08.8-E08.9, E09.3-E09.3, E09.3-E09.6, E10-E10.1, E10.3-E10.3, E10.3-E10.3, E10.3-E10.9, E11-E11.1, E11.3-E11.3, E11.3-E11.3, E11.3-E11.9, E12-E12.1, E12.3-E12.3, E12.3-E12.9, E13-E13.1, E13.3-E13.3, E13.3-E13.3, E13.3-E13.9, E14-E14.1, E14.3-E14.3, E14.3-E14.9
Acute glomerulonephritis	Incidence	580.0, 580.4, 580.8-580.9	N00-N00.9, N01-N01.9
Chronic kidney disease	Prevalence	585.1-585.6	N18-N18.6
Interstitial nephritis and urinary tract infections	Incidence	590-590.3, 590.8-590.9, 595-595.4, 595.8-595.9, 597.0, 597.8-597.9, 599.0	N11-N11.1, N11.8-N11.9, N15-N15.1, N15.8-N15.9, N30-N30.4, N30.8-N30.9, N34-N34.3, N39.0
Urolithiasis	Incidence	592-592.1, 592.9, 594-594.2, 594.8-594.9	N20-N20.2, N20.9, N21-N21.1, N21.8-N21.9, N22.8
Benign prostatic hyperplasia	Prevalence	600-600.3, 600.9	N40-N40.3
Uterine fibroids	Prevalence	218-218.2, 218.9, 219-219.1, 219.8-219.9, 233.2, 236.0, 621.0, 621.3	D07.0, D25-D25.2, D25.9, D26.1, D26.7, D26.9, D39.0, N84.0
Polycystic ovarian syndrome	Prevalence	256.4	E28.2
Endometriosis	Prevalence	617-617.6, 617.8-617.9	N80-N80.6, N80.8-N80.9
Genital prolapse	Prevalence	618-618.9	N81-N81.6, N81.8-N81.9
Premenstrual syndrome	Prevalence	625.4	N94.3
Other gynecological diseases	Prevalence	112.1-112.2, 611-611.9, 616-616.5, 616.8-616.9, 619-619.2, 619.8-619.9, 620-620.9, 621.1, 621.3-621.9, 622.2-622.9, 623.1-623.9, 624-624.6, 624.8-624.9, 625-625.3, 625.5-625.9, 626-626.1, 627.2-627.4, 627.8-627.9, 629-629.3, 629.8-629.9	B37.3-B37.4, N64-N64.5, N64.8-N64.9, N75-N75.1, N75.8-N75.9, N76-N76.6, N76.8, N77-N77.1, N82-N82.5, N82.8-N82.9, N83-N83.9, N85.2-N85.9, N88-N88.4, N88.8-N88.9, N89-N89.9, N90.4-N90.9, N91-N91.5, N94-N94.2, N94.4-N94.6, N94.8-N94.9, N95.1-N95.3, N95.8-N95.9
Thalassemias	Prevalence		D56.1, D56.4-D56.5
Sickle cell disorders	Prevalence	282.4	D57-D57.2, D57.4, D57.8
G6PD deficiency	Prevalence	282.3	D55.0
Endocrine, metabolic, blood, and immune disorders	Prevalence	240.0, 240.3-240.4, 240.9, 241-241.1, 241.9, 242-242.4, 242.8-242.9, 243.0, 243.9, 244-244.1, 244.3, 244.8-244.9, 245-245.4, 245.8-245.9, 246-246.3, 246.8-246.9, 251-251.5, 251.8-251.9, 252-252.1, 252.8-252.9, 253-253.9, 254-254.1, 254.4-254.5, 254.8-254.9, 255-255.6, 255.8-255.9, 256-256.3, 256.8-256.9, 257-257.2, 257.8-257.9, 258-258.1, 258.8-258.9, 259-259.5, 259.8-259.9, 270-270.9, 271-271.5, 271.8-271.9, 275-275.5, 275.8-275.9, 277-277.9, 278.2-278.5, 278.8, 279-279.6, 279.8-279.9, 285.0, 285.4, 285.8-285.9, 286-286.5, 286.7, 286.9, 287-287.5, 287.8-287.9, 288-288.3, 288.8-288.9, 289-289.9, 775.3	D64-D64.4, D64.8, D68-D68.6, D68.8-D68.9, D69-D69.4, D69.6, D69.8, D70-D70.4, D70.8-D70.9, D72-D72.1, D72.8-D72.9, D73-D73.5, D73.8-D73.9, D74.0, D74.8-D74.9, D75-D75.2, D75.8-D75.9, D76-D76.3, D80-D80.9, D81-D81.9, D82-D82.4, D82.8-D82.9, D83-D83.2, D83.8-D83.9, D84-D84.1, D84.8-D84.9, D86.8, D89-D89.3, D89.8-D89.9, E03-E03.1, E03.3-E03.5, E03.8-E03.9, E04-E04.2, E04.8-E04.9, E05-E05.5, E05.8-E05.9, E06-E06.3, E06.5, E06.9, E07-E07.1, E07.8-E07.9, E16.1-E16.4, E16.8-E16.9, E20-E20.1, E20.8-E20.9, E21-E21.5, E22-E22.2, E22.8-E22.9, E23.0, E23.2-E23.3, E23.6-E23.7, E24-E24.1, E24.3-E24.4, E24.8-E24.9, E25.0, E25.8-E25.9, E26-E26.1, E26.8-E26.9, E27-E27.2, E27.4-E27.5, E27.8-E27.9, E28-E28.1, E28.3, E28.8-E28.9, E29-E29.1, E29.8-E29.9, E30-E30.1, E30.8-E30.9, E31-E31.2, E31.8-E31.9, E32-E32.1, E32.8-E32.9, E34-E34.5, E34.8-E34.9, E67-E67.3, E67.8, E70-E70.5, E70.8-E70.9, E71-E71.5, E72-E72.5, E72.8-E72.9, E73-E73.1, E73.8-E73.9, E74-E74.4, E74.8-E74.9, E75-E75.6, E76-E76.3, E76.8-E76.9, E77-E77.1, E77.8-E77.9, E79-E79.2, E79.8-E79.9, E80-E80.7, E83-E83.9, E84-E84.9, E85-E85.9, E88-E88.9
Rheumatoid arthritis	Prevalence	714-714.4, 714.8-714.9	M05-M05.9, M08-M08.4, M08.8-M08.9, M09.1, M09.8
Osteoarthritis	Prevalence	715-715.3, 715.8-715.9	M16-M16.7, M16.9, M17-M17.5, M17.9
Low back pain	Prevalence	353.1, 353.4, 721.3-721.4, 722.1, 722.3, 722.5, 722.7-722.9, 724.0, 724.2-724.3, 724.6-724.7	G54.4, M47-M47.2, M47.8, M48-M48.5, M49.8, M51-M51.4, M51.8, M53.3, M53.8, M54-M54.1, M54.3-M54.5, M99-M99.8
Neck pain	Prevalence	353.2, 721-721.1, 722.0, 722.7-722.9	G54.2, M47-M47.2, M47.8, M48-M48.5, M49.8, M50-M50.3, M50.8-M50.9, M53-M53.1, M53.8, M54-M54.2, M99-M99.8
Gout	Prevalence	274-274.1, 274.8-274.9, 712.0	M10-M10.1, M10.3-M10.4, M10.9

Appendix Table 4. ICD-9 and ICD-10 codes used in the extraction of hospital and claims data, mapped to GBD 2015 nonfatal causes, impairments, and nature of injury categories

GBD cause name	Measure	ICD 9	ICD 10
Other musculoskeletal disorders	Prevalence	446-446.7, 446.9, 710.1-710.2, 710.5, 710.8-710.9, 711-711.9, 712.1-712.3, 712.8-712.9, 713-713.8, 715-715.3, 715.8-715.9, 716-716.6, 716.8-716.9, 717-717.9, 718-718.9, 719-719.9, 720-720.2, 720.8-720.9, 726-726.9, 727-727.6, 727.8-727.9, 729-729.9, 730-730.3, 730.7-730.9, 731-731.3, 731.8, 732-732.9, 733-733.9, 735-735.5, 735.8-735.9, 736-736.9, 737-737.4, 737.8-737.9, 738-738.9, 739-739.9, 800-800.9, 801-801.9, 802-802.9, 803-803.9, 804-804.9, 805-805.9, 807-807.6, 808-808.5, 808.8-808.9, 810-810.1, 811-811.1, 812-812.5, 813-813.5, 813.8-813.9, 814-814.1, 815-815.1, 816-816.1, 820-820.3, 820.8-820.9, 821-821.3, 822-822.1, 823-823.4, 823.8-823.9, 824-824.9, 825-825.3, 826-826.1, 826.6, 885-885.1, 886-886.1, 887-887.7, 888.1-888.2, 888.9, 895-895.1, 896-896.3, 897-897.7, 905-905.4	M00-M00.2, M00.8-M00.9, M01-M01.1, M01.3-M01.6, M01.8, M02-M02.3, M02.8-M02.9, M03.1, M03.6, M06-M06.4, M06.8-M06.9, M07.6, M11-M11.2, M11.8-M11.9, M12-M12.5, M12.8-M12.9, M13-M13.1, M13.8-M13.9, M14-M14.6, M14.8, M15-M15.4, M15.8-M15.9, M18-M18.5, M18.9, M19-M19.2, M19.8-M19.9, M20-M20.6, M21-M21.9, M22-M22.4, M22.8-M22.9, M23-M23.6, M23.8-M23.9, M24-M24.9, M25-M25.9, M30-M30.3, M30.8, M31-M31.9, M34-M34.2, M34.8-M34.9, M35-M35.9, M36-M36.4, M36.8, M40-M40.5, M41-M41.5, M41.8-M41.9, M42-M42.1, M42.9, M43-M43.6, M43.8-M43.9, M45-M45.9, M46-M46.5, M46.8-M46.9, M61-M61.5, M61.9, M62-M62.6, M62.8-M62.9, M63-M63.1, M63.3, M63.8, M65-M65.4, M65.8-M65.9, M66-M66.5, M66.8-M66.9, M67-M67.5, M67.8-M67.9, M70-M70.9, M71-M71.5, M71.8-M71.9, M72-M72.6, M72.8-M72.9, M75-M75.5, M75.8-M75.9, M76-M76.9, M77-M77.5, M77.8-M77.9, M79-M79.6, M79.8-M79.9, M80-M80.5, M80.8-M80.9, M81-M81.6, M81.8-M81.9, M82.1, M83-M83.5, M83.8-M83.9, M84-M84.2, M84.9, M85-M85.6, M85.8-M85.9, M86-M86.6, M86.8-M86.9, M87.0, M87.2-M87.3, M87.8-M87.9, M88-M88.1, M88.8-M88.9, M89-M89.5, M89.7-M89.9, M90-M90.1, M90.3-M90.8, M91-M91.4, M91.8-M91.9, M92-M92.9, M93-M93.2, M93.8-M93.9, M94-M94.3, M94.8-M94.9, M95-M95.5, M95.8-M95.9, M99-M99.9, S02-S02.9, S12-S12.2, S12.7-S12.9, S22-S22.5, S22.8-S22.9, S32-S32.5, S32.7-S32.8, S42-S42.4, S42.7-S42.9, S48-S48.1, S48.9, S49-S49.1, S49.7, S52-S52.9, S58-S58.1, S58.9, S59-S59.2, S62-S62.8, S68-S68.9, S72-S72.4, S72.7-S72.9, S78-S78.1, S78.9, S79-S79.1, S82.7, S88-S88.1, S88.9, S89-S89.3, S89.7, S92.7, S98-S98.4, S98.9, T05-T05.6, T08.0, T11.6, T91.1, T92.1-T92.2, T93.1
Congenital heart anomalies	Prevalence	745-745.9, 746-746.9, 747-747.4	Q20-Q20.6, Q20.8-Q20.9, Q21-Q21.4, Q21.8-Q21.9, Q22-Q22.6, Q22.8-Q22.9, Q23-Q23.4, Q23.8-Q23.9, Q24-Q24.6, Q24.8-Q24.9, Q25-Q25.9, Q26-Q26.6, Q26.8-Q26.9
Cleft lip and cleft palate	Prevalence	749-749.2	Q35-Q35.9, Q36-Q36.1, Q36.9, Q37-Q37.5, Q37.8-Q37.9
Down syndrome	Prevalence	758	Q90-Q90.2, Q90.9
Turner syndrome	Prevalence	758.6	Q96-Q96.4, Q96.8-Q96.9
Klinefelter syndrome	Prevalence	758.7	Q98-Q98.9
Other chromosomal abnormalities	Prevalence	756.8, 758.1-758.5, 758.8-758.9, 759.7-759.8	Q74.8, Q75.1, Q75.4, Q75.8, Q79.6, Q87-Q87.5, Q87.8, Q91-Q91.7, Q92-Q92.9, Q93-Q93.9, Q95.2-Q95.5, Q95.8-Q95.9, Q97-Q97.3, Q97.8-Q97.9, Q99-Q99.2, Q99.8-Q99.9
Other congenital anomalies	Prevalence	385.3, 385.8	H71-H71.3, H71.9
Dermatitis	Prevalence	690.1-690.2, 690.8, 691.0, 691.8, 692-692.9	L20.0, L20.8-L20.9, L21-L21.1, L21.8-L21.9, L23-L23.2, L23.4-L23.9, L24-L24.9, L25-L25.5, L25.8-L25.9
Psoriasis	Prevalence	696-696.5, 696.8	L40-L40.5, L40.8-L40.9, L41-L41.5, L41.8-L41.9
Cellulitis	Incidence	681-681.2, 681.9, 682-682.9	L03-L03.3, L03.8-L03.9
Pyoderma	Incidence	031.1, 035-035.1, 035.4-035.5, 035.8-035.9, 040.0, 102-102.9, 103-103.3, 103.9, 680-680.9, 685-685.1, 686-686.1, 686.8-686.9, 785.4	A46.0, A66-A66.9, A67-A67.3, A67.9, L01-L01.1, L02-L02.6, L02.8-L02.9, L04-L04.3, L04.8-L04.9, L05.0, L05.9, L08-L08.1, L08.8-L08.9, L98.0, N49.3
Scabies	Prevalence	133.0, 133.5-133.6	
Fungal skin diseases	Prevalence	110.0, 110.2-110.9, 111-111.9	B35-B35.6, B35.8-B35.9, B36-B36.3, B36.8-B36.9, B43.0
Viral skin diseases	Prevalence	078-078.1	B07.0, B07.8-B07.9, B08.1
Acne vulgaris	Prevalence	706-706.1	L70-L70.5, L70.8-L70.9
Alopecia areata	Prevalence	704	L63-L63.2, L63.8-L63.9
Pruritus	Prevalence	698-698.4, 698.8-698.9	L29-L29.3, L29.8-L29.9
Urticaria	Prevalence	708-708.5, 708.8-708.9	L50-L50.6, L50.8-L50.9
Decubitus ulcer	Incidence	707.0, 707.2	L89-L89.6, L89.8-L89.9
Other skin and subcutaneous diseases	Prevalence	132-132.3, 132.5, 132.9, 133.8-133.9, 134-134.2, 134.6, 134.8-134.9, 136.1, 692.7, 693-693.1, 693.8-693.9, 694-694.6, 694.8-694.9, 695-695.5, 695.8-695.9, 697-697.1, 697.8-697.9, 700.6, 701-701.5, 701.8-701.9, 702-702.2, 702.8, 703-703.3, 703.8-703.9, 704.1-704.4, 704.8-704.9, 705-705.4, 705.8-705.9, 706.2-706.3, 706.8-706.9, 707.1, 707.8-707.9, 709-709.3, 709.8-709.9	B08.0, B08.2-B08.8, B85-B85.4, B87-B87.4, B87.8-B87.9, B88-B88.3, B88.8-B88.9, D86.3, L10-L10.5, L10.8-L10.9, L11-L11.1, L11.8-L11.9, L12-L12.3, L12.8-L12.9, L13-L13.1, L13.8-L13.9, L26.9, L27.2, L27.8-L27.9, L28-L28.2, L30-L30.5, L30.8-L30.9, L43-L43.3, L43.8-L43.9, L44-L44.4, L44.8-L44.9, L49-L49.9, L51-L51.3, L51.8-L51.9, L53-L53.3, L53.8-L53.9, L54.0, L55-L55.2, L55.8-L55.9, L56.2-L56.5, L56.8-L56.9, L57-L57.5, L57.8-L57.9, L58-L58.1, L58.9, L59.0, L59.8-L59.9, L60-L60.5, L60.8-L60.9, L62.8, L64.8-L64.9, L65-L65.2, L65.8-L65.9, L66-L66.4, L66.8-L66.9, L67-L67.1, L67.8-L67.9, L68-L68.3, L68.8-L68.9, L71-L71.1, L71.8-L71.9, L72-L72.3, L72.8-L72.9, L73-L73.2, L73.8-L73.9, L74-L74.5, L74.8-L74.9, L75-L75.2, L75.8-L75.9, L81-L81.9, L82-L82.1, L85-L85.3, L85.8-L85.9, L87-L87.2, L87.8-L87.9, L90-L90.6, L90.8-L90.9, L91.0, L91.8-L91.9, L92-L92.3, L92.8-L92.9, L94-L94.6, L94.8-L94.9, L95-L95.1, L95.8-L95.9, L97-L97.5, L97.8-L97.9, L98.2-L98.6, L98.8-L98.9, L99.0, L99.8
**GBD Impairment Proportions**	**Measure**	**ICD-9**	**ICD-10**
Glaucoma	Prevalence	365-365.9	H40-H40.6, H40.8-H40.9
Cataract	Prevalence	366-366.5, 366.8-366.9, 743.3	H25-H25.2, H25.8-H25.9, H26-H26.4, H26.8-H26.9, Q12.0
Macular degeneration	Prevalence	362.5	H35.3
Age-related and other hearing loss	Prevalence	385-385.2, 385.8-385.9, 388.1-388.2, 389-389.2, 389.7-389.9	H74-H74.4, H74.8-H74.9, H90-H90.8, H91.1-H91.3, H91.8-H91.9, H94.0, H94.8

Appendix Table 4. ICD-9 and ICD-10 codes used in the extraction of hospital and claims data, mapped to GBD 2015 nonfatal causes, impairments, and nature of injury categories

GBD cause name	Measure	ICD 9	ICD 10
Other vision loss	Prevalence	361-361.3, 361.8-361.9, 362-362.4, 362.6-362.9, 363-363.9, 367-367.5, 367.8-367.9, 368-368.6, 368.8-368.9, 369-369.4, 369.6-369.9, 377-377.7, 377.9, 378-378.9	H31-H31.4, H31.8-H31.9, H33-H33.5, H33.8, H34-H34.2, H34.8-H34.9, H35-H35.2, H35.4-H35.9, H46-H46.3, H46.8-H46.9, H47-H47.7, H47.9, H49-H49.4, H49.8-H49.9, H50-H50.6, H50.8-H50.9, H51-H51.2, H51.8-H51.9, H52-H52.7, H53-H53.9, H54-H54.8
Other sense organ diseases	Prevalence	364-364.9, 371-371.9, 374-374.5, 375-375.6, 375.8-375.9, 379-379.5, 386-386.5, 386.8-386.9, 388.0, 388.3-388.9	H02-H02.9, H04-H04, H04.8-H04.9, H15-H15.1, H15.8-H15.9, H17-H17.1, H17.8-H17.9, H18-H18.9, H20-H20.2, H20.8-H20.9, H21-H21.5, H21.8-H21.9, H22-H22.1, H22.8, H43-H43.3, H43.8-H43.9, H44.8-H44.9, H55.0, H55.8, H81-H81.4, H81.8-H81.9, H93-H93.3, H93.8-H93.9
Other sense organ diseases	Incidence	077-077.4, 077.8-077.9, 360-360.4, 370-370.6, 370.8-370.9, 372-372.9, 373-373.6, 373.8-373.9, 374.8-374.9, 375-375, 376-376.5, 376.8-376.9, 379.8-379.9, 380-380.5, 380.8-380.9	H00-H00.1, H01-H01.1, H01.8-H01.9, H04-H04.6, H05-H05.4, H05.8-H05.9, H10-H10.5, H10.8-H10.9, H11-H11.4, H11.8-H11.9, H13.0, H13.3, H16-H16.4, H16.8-H16.9, H19.1, H30-H30.2, H30.8-H30.9, H44-H44.5, H57-H57.1, H57.8-H57.9, H60-H60.6, H60.8-H60.9, H61-H61.3, H61.8-H61.9, H62.2, H92-H92.2
Permanent caries	Prevalence	521	K02-K02.9
Periodontal diseases	Prevalence	523.2, 523.4	K05.3
Edentulism and severe tooth loss	Prevalence	525.4	K08-K08.1
Other oral disorders	Prevalence	520-520.9, 521.1-521.9, 522-522.9, 523-523.6, 523.8-523.9, 524-524.9, 525-525.3, 525.5, 525.8-525.9, 526-526.6, 526.8-526.9, 527-527.9, 528-528.9, 529-529.6, 529.8-529.9	K00-K00.9, K01-K01.1, K03-K03.9, K04-K04.9, K05-K05.2, K05.4-K05.6, K06-K06.2, K06.8-K06.9, K07-K07.6, K07.8-K07.9, K08.2-K08.4, K08.8-K08.9, K09-K09.2, K09.8-K09.9, K10-K10.3, K10.8-K10.9, K11-K11.9, K12-K12.3, K13-K13.7, K14-K14.6, K14.8-K14.9, M26-M26.9, M27-M27.6, M27.8-M27.9
**GBD Nature of Injury Categories**	**Measure**	**ICD-9**	**ICD-10**
Road injuries	Incidence	E800.3, E801.3, E802.3, E803.3, E804.3, E805.3, E806.3, E807.3, E810-E810.6, E811-E811.7, E812-E812.7, E813-E813.7, E814-E814.7, E815-E815.7, E816-E816.7, E817-E817.7, E818-E818.7, E819-E819.7, E820-E820.6, E821-E821.6, E822-E822.7, E823-E823.7, E824-E824.7, E825-E825.7, E826-E826.1, E826.3-E826.4, E827.0, E827.3-E827.4, E828.0, E828.4, E829.0, E829.4	V01-V01.9, V02-V02.3, V02.5-V02.9, V03-V03.9, V04-V04.9, V06-V06.6, V06.8-V06.9, V07-V07.4, V07.8-V07.9, V09-V09.9, V10-V10.9, V11-V11.5, V11.8-V11.9, V12-V12.7, V12.9, V13-V13.9, V14-V14.9, V15-V15.9, V16-V16.9, V17-V17.9, V18-V18.9, V19-V19.9, V20-V20.5, V20.9, V21-V21.5, V21.8-V21.9, V22-V22.5, V22.9, V23-V23.5, V23.7-V23.9, V24-V24.5, V24.9, V25-V25.5, V25.8-V25.9, V26-V26.5, V26.8-V26.9, V27-V27.7, V27.9, V28-V28.6, V28.8-V28.9, V29-V29.6, V29.8-V29.9, V30-V30.7, V30.9, V31-V31.7, V31.9, V32-V32.7, V32.9, V33-V33.7, V33.9, V34-V34.7, V34.9, V35-V35.7, V35.9, V36-V36.7, V36.9, V37-V37.7, V37.9, V38-V38.7, V38.9, V39-V39.6, V39.8-V39.9, V40-V40.7, V40.9, V41-V41.9, V42-V42.9, V43-V43.9, V44-V44.9, V45-V45.9, V46-V46.9, V47-V47.7, V47.9, V48-V48.9, V49-V49.9, V50-V50.9, V51-V51.7, V51.9, V52-V52.9, V53-V53.9, V54-V54.9, V55-V55.9, V56-V56.9, V57-V57.9, V58-V58.9, V59-V59.6, V59.8-V59.9, V60-V60.9, V61-V61.9, V62-V62.9, V63-V63.9, V64-V64.7, V64.9, V65-V65.9, V66-V66.7, V66.9, V67-V67.7, V67.9, V68-V68.9, V69-V69.6, V69.8-V69.9, V70-V70.9, V71-V71.9, V72-V72.9, V73-V73.9, V74-V74.9, V75-V75.9, V76-
Pedestrian road injuries	Incidence	E811.7, E812.7, E813.7, E814.7, E815.7, E816.7, E817.7, E818.7, E819.7, E822.7, E823.7, E824.7, E825.7, E826.0, E827.0, E828.0, E829.0	V01-V01.9, V02-V02.3, V02.5-V02.9, V03-V03.9, V04-V04.9, V06-V06.6, V06.8-V06.9, V07-V07.4, V07.8-V07.9, V09-V09.9
Cyclist road injuries	Incidence	E800.3, E801.3, E802.3, E803.3, E804.3, E805.3, E806.3, E807.3, E810.6, E811.6, E812.6, E813.6, E814.6, E815.6, E816.6, E817.6, E818.6, E819.6, E820.6, E821.6, E822.6, E823.6, E824.6, E825.6, E826.1	V10-V10.9, V11-V11.5, V11.8-V11.9, V12-V12.7, V12.9, V13-V13.9, V14-V14.9, V15-V15.9, V16-V16.9, V17-V17.9, V18-V18.9, V19-V19.9
Motorcyclist road injuries	Incidence	E810.2-E810.3, E811.2-E811.3, E812.2-E812.3, E813.2-E813.3, E814.2-E814.3, E815.2-E815.3, E816.2-E816.3, E817.2-E817.3, E818.2-E818.3, E819.2-E819.3, E820.2-E820.3, E821.2-E821.3, E822.2-E822.3, E823.2-E823.3, E824.2-E824.3, E825.2-E825.3	V20-V20.5, V20.9, V21-V21.5, V21.8-V21.9, V22-V22.5, V22.9, V23-V23.5, V23.7-V23.9, V24-V24.5, V24.9, V25-V25.5, V25.8-V25.9, V26-V26.5, V26.8-V26.9, V27-V27.7, V27.9, V28-V28.6, V28.8-V28.9, V29-V29.6, V29.8-V29.9
Motor vehicle road injuries	Incidence	E810-E810.1, E811-E811.1, E812-E812.1, E813-E813.1, E814-E814.1, E815-E815.1, E816-E816.1, E817-E817.1, E818-E818.1, E819-E819.1, E820-E820.1, E821-E821.1, E822-E822.1, E823-E823.1, E824-E824.1, E825-E825.1	V30-V30.7, V30.9, V31-V31.7, V31.9, V32-V32.7, V32.9, V33-V33.7, V33.9, V34-V34.7, V34.9, V35-V35.7, V35.9, V36-V36.7, V36.9, V37-V37.7, V37.9, V38-V38.7, V38.9, V39-V39.6, V39.8-V39.9, V40-V40.7, V40.9, V41-V41.9, V42-V42.9, V43-V43.9, V44-V44.9, V45-V45.9, V46-V46.9, V47-V47.7, V47.9, V48-V48.9, V49-V49.9, V50-V50.9, V51-V51.7, V51.9, V52-V52.9, V53-V53.9, V54-V54.9, V55-V55.9, V56-V56.9, V57-V57.9, V58-V58.9, V59-V59.6, V59.8-V59.9, V60-V60.9, V61-V61.9, V62-V62.9, V63-V63.9, V64-V64.7, V64.9, V65-V65.9, V66-V66.7, V66.9, V67-V67.7, V67.9, V68-V68.9, V69-V69.6, V69.8-V69.9, V70-V70.9, V71-V71.9, V72-V72.9, V73-V73.9, V74-V74.9, V75-V75.9, V76-V76.9, V77-V77.9, V78-V78.9, V79-V79.6, V79.8-
Other road injuries	Incidence	E810.4-E810.5, E811.4-E811.5, E812.4-E812.5, E813.4-E813.5, E814.4-E814.5, E815.4-E815.5, E816.4-E816.5, E817.4-E817.5, E818.4-E818.5, E819.4-E819.5, E820.4-E820.5, E821.4-E821.5, E822.4-E822.5, E823.4-E823.5, E824.4-E824.5, E825.4-E825.5, E826.3-E826.4, E827.3-E827.4, E828.4, E829.4	V80-V80.9, V82-V82.9
Other transport injuries	Incidence	E800-E800.2, E801-E801.2, E802-E802.2, E803-E803.2, E804-E804.2, E805-E805.2, E806-E806.2, E807-E807.2, E810.7, E820.7, E821.7, E826.2, E827.2, E828.2, E830-E830.9, E831-E831.9, E832-E832.9, E833-E833.9, E834-E834.9, E835-E835.9, E836-E836.9, E837-E837.9, E838-E838.9, E840-E840.9, E841-E841.9, E842.6-E842.9, E843-E843.9, E844-E844.9, E845.0, E845.8-E845.9, E849-	V00-V00.3, V00.8, V05-V05.4, V05.8-V05.9, V81-V81.9, V83-V83.9, V84-V84.9, V85-V85.7, V85.9, V86-V86.7, V86.9, V88.2-V88.3, V90-V90.9, V91-V91.9, V92-V92.9, V93-V93.9, V94-V94.9, V95-V95.4, V95.8-V95.9, V96-V96.2, V96.8-V96.9, V97-V97.3, V97.8, V98-V98.3, V98.8
Falls	Incidence	E880-E880.1, E880.9, E881-E881.1, E882.0, E883-E883.2, E883.9, E884-E884.6, E884.9, E885-E885.4, E885.9, E886.0, E886.9, E888-E888.1, E888.8-E888.9, E929.3	W00-W00.9, W01-W01.9, W02-W02.9, W03-W03.9, W04-W04.9, W05-W05.9, W06-W06.9, W07-W07.9, W08-W08.9, W09-W09.9, W10-W10.9, W11-W11.9, W12-W12.9, W13-W13.9, W14-W14.9, W15-W15.9, W16-W16.9, W17-W17.9, W18-W18.9, W19-W19.9
Drowning	Incidence	E910-E910.4, E910.8-E910.9	W65-W65.9, W66-W66.9, W67-W67.9, W68-W68.9, W69-W69.9, W70-W70.9, W73-W73.9, W74-W74.9
Fire, heat, and hot substances	Incidence	E890-E890.3, E890.8-E890.9, E891-E891.3, E891.8-E891.9, E892.0, E893-E893.2, E893.8-E893.9, E894.0, E895.0, E896.0, E897.0, E898-E898.1, E899.0, E924-E924.2, E924.8-E924.9, E929.4	P24.8, X00-X00.9, X01-X01.9, X02-X02.9, X03-X03.9, X04-X04.9, X05-X05.5, X05.7-X05.9, X06-X06.9, X08-X08.9, X09-X09.9, X10-X10.9, X11-X11.2, X11.4-X11.9, X12-X12.2, X12.4-X12.9, X13-X13.9, X14-X14.9, X15-X15.9, X16-X16.2, X16.4-X16.9, X17-X17.1, X17.4-X17.9, X18-X18.2, X18.4-X18.9, X19-X19.9

Appendix Table 4. ICD-9 and ICD-10 codes used in the extraction of hospital and claims data, mapped to GBD 2015 nonfatal causes, impairments, and nature of injury categories

GBD cause name	Measure	ICD 9	ICD 10
Poisonings	Incidence	E850.3-E850.8, E854.8, E856.0, E857.0, E860.2-E860.4, E860.8-E860.9, E861-E861.6, E861.9, E862-E862.4, E862.9, E863-E863.9, E864-E864.4, E865-E865.5, E865.8-E865.9, E866-E866.9, E867.0, E868-E868.3, E868.8-E868.9, E869-E869.4, E869.8-E869.9, E929.2	X40-X40.2, X40.4-X40.9, X43-X43.9, X46-X46.9, X47-X47.9, X48-X48.9, X49-X49.1, X49.4, X49.7, X49.9
Exposure to mechanical forces	Incidence	E913-E913.1, E916.0, E917-E917.9, E918.0, E919-E919.9, E920-E920.5, E920.8-E920.9, E921-E921.1, E921.8-E921.9, E922-E922.5, E922.8-E922.9, E928.1-E928.7	W20-W20.9, W21-W21.9, W22-W22.9, W23-W23.9, W24-W24.9, W25-W25.9, W26-W26.9, W27-W27.9, W28-W28.9, W29-W29.9, W30-W30.9, W31-W31.9, W32-W32.9, W33-W33.9, W34-W34.9, W35-W35.9, W36-W36.9, W37-W37.9, W38-W38.9, W40-W40.9, W41-W41.9, W42.0, W42.2-W42.3, W42.9, W43-W43.9, W45-W45.2, W46-W46.1, W49-W49.9, W50-W50.9, W51-W51.9, W52.3, W75-W75.9, W76-W76.9
Unintentional firearm injuries	Incidence	E922-E922.5, E922.8-E922.9, E928.7	W32-W32.9, W33-W33.9, W34-W34.9
Unintentional suffocation	Incidence	E913-E913.1	W75-W75.9, W76-W76.9
Other exposure to mechanical forces	Incidence	E916.0, E917-E917.9, E918.0, E919-E919.9, E920-E920.5, E920.8-E920.9, E921-E921.1, E921.8-E921.9, E928.1-E928.6	W20-W20.9, W21-W21.9, W22-W22.9, W23-W23.9, W24-W24.9, W25-W25.9, W26-W26.9, W27-W27.9, W28-W28.9, W29-W29.9, W30-W30.9, W31-W31.9, W35-W35.9, W36-W36.9, W37-W37.9, W38-W38.9, W40-W40.9, W41-W41.9, W42.0, W42.2-W42.3, W42.9, W43-W43.9, W45-W45.2, W46-W46.1, W49-W49.9, W50-W50.9, W51-W51.9, W52.3
Adverse effects of medical treatment	Incidence	249-249.9, 331.8, 333.9, 349-349.3, 349.8-349.9, 357.6, 359.2, 379.6, 458.2, 518.7, 519-519.1, 525.6-525.7, 526.6, 530.8, 536.4, 539.0, 539.8-539.9, 551.2, 552.2, 564.2-564.4, 569.6-569.8, 596.8, 598.2, 612-612.1, 780.6, 995.8, E870-E870.9, E871-E871.9, E872-E872.6, E872.8-E872.9, E873-E873.6, E873.8-E873.9, E874-E874.5, E874.8-E874.9, E875-E875.2, E875.8-E875.9, E876-E876.9, E878-E878.6, E878.8-E878.9, E879-E879.9, E930-E930.9, E931-E931.9, E932-E932.9, E933-E933.9, E934-E934.9, E935-E935.9, E936-E936.4, E937-E937.6, E937.8-E937.9, E938-E938.7, E938.9, E939-E939.9, E940-E940.1, E940.8-E940.9, E941-E941.3, E941.9, E942-E942.9, E943-E943.6, E943.8-E943.9, E944-E944.7, E945-E945.8, E946-E946.9, E947-E947.4, E947.8-E947.9, E948-E948.6, E948.8-E948.9, E949-E949.7, E949.9	D52.1, D59.0, D59.2, D59.6, D61.1, D69.5, D78-D78.2, D78.8, E03.2, E06.4, E09-E09.6, E09.8-E09.9, E16.0, E23.1, E24.2, E27.3, E36-E36.1, E36.8, E66.1, E89-E89.9, G21.1, G24.0, G25.1, G25.4, G25.6-G25.7, G62.0, G72.0, G93.7, G96-G96.1, G97-G97.5, G97.8-G97.9, H59-H59.4, H59.8, H91.0, H95-H95.4, H95.8-H95.9, I95.2-I95.3, I95.8, I97-I97.9, I98.9, J70.2-J70.5, J95-J95.9, K08.5, K43-K43.7, K43.9, K62.7, K68.1, K91-K91.9, K94-K94.3, K95.0, K95.8, L23.3, L27-L27.1, L56-L56.1, L64.0, L76-L76.2, L76.8, M10.2, M87.1, M96-M96.6, M96.8-M96.9, N14-N14.4, N46-N46.1, N52.2-N52.3, N65-N65.1, N99-N99.9, P93.0, P93.8, P96.2, P96.5, Y38.9, Y40-Y40.9, Y41-Y41.5, Y41.8-Y41.9, Y42-Y42.9, Y43-Y43.6, Y43.8-Y43.9, Y44-Y44.7, Y44.9, Y45-Y45.5, Y45.8-Y45.9, Y46-Y46.8, Y47-Y47.5, Y47.8-Y47.9, Y48-Y48.5, Y49-Y49.9, Y50-Y50.2, Y50.8-Y50.9, Y51-Y51.9, Y52-Y52.9, Y53-Y53.9, Y54-Y54.9, Y55-Y55.7, Y56-Y56.9, Y57-Y57.9, Y58-Y58.6, Y58.8-Y58.9, Y59-Y59.3, Y59.8-Y59.9, Y60-Y60.9, Y61-Y61.9, Y62-Y62.6, Y62.8-Y62.9, Y63-Y63.6, Y63.8-Y63.9, Y64-Y64.1, Y64.8-Y64.9, Y65-Y65.5, Y65.8, Y69.2, Y69.9, Y70-Y70.3, Y70.8, Y71-Y71.3, Y71.8, Y72-Y72.3, Y72.8, Y73-Y73.3, Y73.8, Y74-Y74.3, Y74.8, Y75-Y75.3, Y75.8, Y76-Y76.3, Y76.8-Y76.9, Y77-Y77.3, Y77.8, Y78-Y78.3, Y78.8, Y79-Y79.3, Y79.8, Y80-Y80.3, Y80.8
Animal contact	Incidence	E905-E905.9, E906-E906.5, E906.8-E906.9	W52-W52.2, W52.4-W52.9, W53-W53.3, W53.8-W53.9, W54-W54.9, W55-W55.9, W56-W56.9, W57-W57.2, W57.4-W57.9, W58-W58.1, W58.4, W58.7-W58.9, W59-W59.2, W59.4-W59.9, W60-W60.2, W60.4-W60.9, W61-W61.6, W61.9, W62-W62.1, W62.9, W64-W64.9, X20-X20.9, X21-X21.2, X21.4-X21.9, X22-X22.9, X23-X23.9, X24-X24.2, X24.4, X24.6-X24.9, X25-X25.2, X25.4-X25.9, X26-X26.3, X26.5, X26.7-X26.9, X27-X27.5, X27.7-X27.9, X28-X28.2, X28.4-X28.5, X28.7-X28.9, X29-X29.9
Venomous animal contact	Incidence	E905-E905.9	X20-X20.9, X21-X21.2, X21.4-X21.9, X22-X22.9, X23-X23.9, X24-X24.2, X24.4, X24.6-X24.9, X25-X25.2, X25.4-X25.9, X26-X26.3, X26.5, X26.7-X26.9, X27-X27.5, X27.7-X27.9, X28-X28.2, X28.4-X28.5, X28.7-X28.9, X29-X29.9
Non-venomous animal contact	Incidence	E906-E906.5, E906.8-E906.9	W52-W52.2, W52.4-W52.9, W53-W53.3, W53.8-W53.9, W54-W54.9, W55-W55.9, W56-W56.9, W57-W57.2, W57.4-W57.9, W58-W58.1, W58.4, W58.7-W58.9, W59-W59.2, W59.4-W59.9, W60-W60.2, W60.4-W60.9, W61-W61.6, W61.9, W62-W62.1, W62.9, W64-W64.9
Pulmonary aspiration and foreign body in airway	Incidence	507-507.1, 507.5, 507.8-507.9, 770.1, E911.0, E912.0, E913.8-E913.9	J69-J69.1, J69.8, P24-P24.3, P24.8-P24.9, W78-W78.9, W79-W79.9, W80-W80.9, W83-W83.9, W84-W84.9
Foreign body in eyes	Incidence	360.5-360.6, 374.8, 376.6, E914.0	H02.8, H05.5, H44.6-H44.7
Foreign body in other body part	Incidence	709.4, E915.0	M60.2, W44-W44.9, W45.3-W45.9, W46.2
Environmental heat and cold exposure	Incidence	E900-E900.1, E900.9, E901-E901.1, E901.8-E901.9, E902-E902.2, E902.8-E902.9, E926-E926.5, E926.8-E926.9, E929.5	W88-W88.2, W88.4-W88.9, W89-W89.9, W90-W90.3, W90.5-W90.9, W91-W91.4, W91.6-W91.9, W92-W92.9, W93-W93.9, W94-W94.9, W97.9, W99-W99.4, W99.6-W99.9, X30-X30.9, X31-X31.9, X32-X32.1, X32.3-X32.9, X39-X39.9
Other unintentional injuries	Incidence	E903.0, E904-E904.3, E904.9, E913.2-E913.3, E923-E923.2, E923.8-E923.9, E925-E925.2, E925.8-E925.9, E927-E927.4, E927.8-E927.9, E928.0, E928.8	W39-W39.9, W77-W77.9, W81-W81.9, W85-W85.9, W86-W86.9, W87-W87.9, X50-X50.9, X51-X51.6, X51.8-X51.9, X52-X52.1, X52.4, X52.6-X52.9, X53-X53.9, X54-X54.5, X54.8-X54.9, X57-X57.2, X57.4-X57.6, X57.8-X57.9, X58-X58.9
Self-harm	Incidence	E950-E950.9, E951-E951.1, E951.8, E952-E952.1, E952.8-E952.9, E953-E953.1, E953.8-E953.9, E955-E955.7, E955.9, E957-E957.2, E957.9, E958-E958.9	X60-X60.9, X61-X61.9, X62-X62.9, X63-X63.9, X64-X64.9, X65-X65.9, X66-X66.9, X67-X67.9, X68-X68.9, X69-X69.9, X70-X70.9, X71-X71.9, X72-X72.9, X73-X73.9, X74-X74.9, X75-X75.9, X76-X76.9, X77-X77.9, X78-X78.9, X79-X79.9, X80-X80.9, X81-X81.9, X82-X82.9, X83-X83.9, X84-X84.9
Interpersonal violence	Incidence	E960-E960.1, E962-E962.2, E962.9, E965-E965.9, E967-E967.9, E968-E968.9	X85-X85.9, X86-X86.2, X86.4-X86.6, X86.8-X86.9, X87-X87.9, X88-X88.9, X89-X89.9, X90-X90.9, X91-X91.9, X92-X92.9, X93-X93.9, X94-X94.9, X95-X95.9, X96-X96.9, X97-X97.9, X98-X98.5, X98.7-X98.9, X99-X99.9, Y00-Y00.9, Y01-Y01.9, Y02-Y02.9, Y03-Y03.9, Y04-Y04.9, Y05-Y05.9, Y06-Y06.2, Y06.8-Y06.9, Y07-Y07.5, Y07.8-Y07.9, Y08-Y08.9, Y87.1-Y87.2
Assault by firearm	Incidence	E965-E965.4	X93-X93.9, X94.0, X94.3-X94.7, X94.9, X95-X95.9, X96.5
Assault by sharp object	Incidence		X99-X99.9

Appendix Table 4. ICD-9 and ICD-10 codes used in the extraction of hospital and claims data, mapped to GBD 2015 nonfatal causes, impairments, and nature of injury categories			
**GBD cause name**	**Measure**	**ICD 9**	**ICD 10**
Assault by other means	Incidence	E960-E960.1, E962-E962.2, E962.9, E965.5-E965.9, E967-E967.9, E968-E968.9	X85-X85.9, X86-X86.2, X86.4-X86.6, X86.8-X86.9, X87-X87.9, X88-X88.9, X89-X89.9, X90-X90.9, X91-X91.9, X92-X92.9, X94.1-X94.2, X94.8, X96-X96.4, X96.6-X96.9, X97-X97.9, X98-X98.5, X98.7-X98.9, Y00-Y00.9, Y01-Y01.9, Y02-Y02.9, Y03-Y03.9, Y04-Y04.9, Y05-Y05.9, Y06-Y06.2, Y06.8-Y06.9, Y07-Y07.5, Y07.8-Y07.9, Y08-Y08.9, Y87.1-Y87.2
Exposure to forces of nature	Incidence	E907.0, E908-E908.4, E908.8-E908.9, E909-E909.4, E909.8-E909.9	X33-X33.9, X34-X34.9, X35-X35.1, X35.4-X35.5, X35.7-X35.9, X36-X36.9, X37-X37.9, X38-X38.2, X38.4, X38.6-X38.9
Collective violence and legal intervention	Incidence	E979-E979.9, E990-E990.3, E990.9, E991-E991.9, E992-E992.3, E992.8-E992.9, E993-E993.9, E994-E994.3, E994.8-E994.9, E995-E995.4, E995.8-E995.9, E996-E996.3, E996.8-E996.9, E997-E997.3, E997.8-E997.9, E998-E998.1, E998.8-E998.9, E999-E999.1	Y35-Y35.9, Y36-Y36.9, Y37-Y37.7, Y37.9, Y38-Y38.8, Y89-Y89.1
Secondary infertility impairment envelope	Prevalence	628.2	N97.1
Intellectual disability impairment envelope	Prevalence	317.1, 318-318.2, 318.8-318.9, 319.0, 319.9	F70-F70.1, F70.8-F70.9, F71-F71.1, F71.8-F71.9, F72-F72.1, F72.8-F72.9, F73-F73.1, F73.8-F73.9, F78-F78.1, F78.8-F78.9, F79-F79.1, F79.8-F79.9
Primary infertility impairment envelope	Prevalence	606-606.1, 606.8-606.9, 628-628.1, 628.3-628.4, 628.8-628.9	N46-N46.1, N46.8-N46.9, N97.0, N97.2-N97.3, N97.8-N97.9
Epilepsy impairment envelope	Prevalence	345-345.9	G40-G40.9, G41-G41.2, G41.8-G41.9
Guillain-Barre syndrome impairment envelope	Incidence	357	G61.0
Heart failure impairment envelope	Prevalence	086.0, 402-402.1, 402.3, 402.7, 402.9, 428-428.4	B57.2, I09.8, I11.0, I50-I50.4, I50.9, J81-J81.1
Pelvic inflammatory disease impairment envelope	Incidence	098.1-098.3, 099.5, 614-614.9, 615-615.1, 615.9	A54.2, A56.1, K67-K67.1, N70-N70.1, N70.9, N71-N71.1, N71.9, N73-N73.6, N73.8-N73.9, N74.2-N74.4, N74.8
Amputation of fingers (excluding thumb)	Incidence	886-886.1	S68.1-S68.3, S68.6
Amputation of lower limb, unilateral	Incidence	896-896.1, 897-897.5	S78-S78.1, S78.9, S88-S88.1, S88.9, S98.0, S98.3-S98.4, S98.9
Amputation of lower limbs, bilateral	Incidence	896.2-896.3, 897.6-897.7	T05.3, T05.5
Amputation of thumb	Incidence	885-885.1	S68.0, S68.5
Amputation of toe/toes	Incidence	895-895.1	S98.1-S98.2
Amputation of upper limb, unilateral	Incidence	887-887.5	S48-S48.1, S48.9, S58-S58.1, S58.9, T05.1, T05.4, T11.6
Amputation of upper limbs, bilateral	Incidence	887.6-887.7, 888.1-888.2, 888.9	S68.4, S68.7-S68.9, T05.0, T05.2, T05.6
Asphyxiation	Incidence	994.7	T71.1-T71.2, T71.9
Burns, <20% total burned surface area without lower airway burns	Incidence	941-941.5, 942-942.5, 943-943.5, 944-944.5, 945-945.5, 947.3-947.4, 947.8-947.9, 948-948.1, 949-949.5	T20-T20.7, T21-T21.7, T25-T25.7, T28-T28.9, T30-T30.7, T31-T31.1, T32-T32.1, T95-T95.4
Burns, >=20% total burned surface area or >= 10% burned surface area if head/neck or hands/wrist involved w/o lower airway burns	Incidence	906.5-906.9, 942-942.1, 942.4-942.5, 946-946.5, 948.2-948.9	T29-T29.7, T31.2-T31.9, T32.2-T32.9
Complications following therapeutic procedures	Incidence	995.4, 996-996.9, 997.9, 998-998.9, 999-999.3, 999.6-999.9	T80-T80.6, T80.8-T80.9, T81-T81.9, T82-T82.9, T83-T83.9, T85-T85.9, T86-T86.5, T86.8-T86.9, T87-T87.6, T87.8-T87.9, T88-T88.9
Contusion in any part of the body	Incidence	906.3, 922-922.4, 922.8-922.9, 923-923.3, 923.8-923.9, 924-924.5, 924.8-924.9	S20.0, S20.2, S30-S30.3, S40.0, S40.2, S50-S50.1, S60-S60.2, S60.7-S60.9, S70-S70.2, S80-S80.2, S80.7, S90-S90.4
Crush injury	Incidence	906.4, 925.1-925.2, 926-926.1, 926.8-926.9, 927-927.3, 927.8-927.9, 928-928.3, 928.8-928.9, 929.9	S07-S07.1, S07.8-S07.9, S17.0, S17.8-S17.9, S38-S38.3, S47.1-S47.2, S47.9, S57.0, S57.8-S57.9, S67-S67.4, S67.8-S67.9, S77-S77.2, S87.0, S87.8, S97-S97.1, S97.8, T92.6, T93.6
Dislocation of hip	Incidence	835-835.1	S73.0
Dislocation of knee	Incidence	836-836.6	S83-S83.1, S83.7
Dislocation of shoulder	Incidence	831-831.1	S43-S43.3
Drowning and nonfatal submersion	Incidence	994.1	T75.1
Effect of different environmental factors	Incidence	991-991.6, 991.8-991.9, 992-992.9, 993-993.4, 993.8-993.9, 994.0, 994.2-994.6, 994.8-994.9	T33-T33.9, T34-T34.9, T35-T35.7, T67-T67.9, T68.7, T69-T69.1, T69.8-T69.9, T70-T70.4, T70.8-T70.9, T75.0, T75.2-T75.4, T75.8, T95.8-T95.9
Foreign body in GI and urogenital system	Incidence	935-935.2, 938.9, 939-939.3, 939.9	T18-T18.5, T18.8-T18.9, T19-T19.4, T19.8-T19.9
Foreign body in ear	Incidence		T16.1-T16.2, T16.9
Foreign body in respiratory system	Incidence	933-933.1, 934-934.1, 934.8-934.9	T17-T17.5, T17.8-T17.9
Fracture of clavicle, scapula, or humerus	Incidence	810-810.1, 811-811.1, 812-812.5	S42-S42.4, S42.7-S42.9, S49-S49.1, S49.7, T92.1
Fracture of face bones	Incidence	802-802.9	S02.2-S02.7
Fracture of femur, other than femoral neck	Incidence	821-821.3	S72.3-S72.4, S72.7-S72.9, S79-S79.1, T93.1
Fracture of foot bones except ankle	Incidence	825-825.3, 826-826.1, 826.6	S92.7
Fracture of hand (wrist and other distal part of hand)	Incidence	814-814.1, 815-815.1, 816-816.1	S62-S62.8, T92.2
Fracture of hip	Incidence	820-820.3, 820.8-820.9, 905.3	S72-S72.2
Fracture of patella, tibia or fibula, or ankle	Incidence	822-822.1, 823-823.4, 823.8-823.9, 824-824.9, 905.4	S82.7, S89-S89.3, S89.7
Fracture of pelvis	Incidence	808-808.5, 808.8-808.9	S32.3-S32.5, S32.7-S32.8
Fracture of radius and/or ulna	Incidence	813-813.5, 813.8-813.9, 905.2	S52-S52.9, S59-S59.2
Fracture of skull	Incidence	800-800.9, 801-801.9, 803-803.9, 804-804.9, 905.0	S02-S02.1, S02.8-S02.9
Fracture of sternum and/or fracture of one or more ribs	Incidence	807-807.6	S22.2-S22.5, S22.8-S22.9
Fracture of vertebral column	Incidence	805-805.9, 905.1	S12-S12.2, S12.7-S12.9, S22-S22.1, S32-S32.2, T08.0, T91.1
Injury to eyes	Incidence	870-870.4, 870.8-870.9, 871-871.7, 871.9, 918-918.2, 918.9, 921-921.3, 921.9, 930-930.2, 930.8-930.9, 940-940.5, 940.9	S01.1, S05-S05.9, T14.4, T15-T15.1, T15.8-T15.9, T26-T26.9, T90.4
Internal hemorrhage in abdomen and pelvis	Incidence	863-863.5, 863.8-863.9, 864-864.1, 865-865.1, 866-866.1, 867-867.9, 868-868.1, 868.3, 869-869.1, 902-902.5, 902.8-902.9, 908.1-908.3	S35-S35.5, S35.7-S35.9, S36-S36.9, S37-S37.9, S39.8, T79-T79.9, T91.5
Lower airway burns	Incidence	947-947.2	T27-T27.7

Appendix Table 4. ICD-9 and ICD-10 codes used in the extraction of hospital and claims data, mapped to GBD 2015 nonfatal causes, impairments, and nature of injury categories			
**GBD cause name**	**Measure**	**ICD 9**	**ICD 10**
Minor TBI	Incidence	850-850.5, 850.9	S06.0
Moderate TBI	Incidence	852-852.5, 907.0	S06.1, S06.3-S06.6, S06.9, T90.2
Multiple fractures, dislocations, crashs, wounds , prains, and strains	Incidence	817-817.1, 818-818.1, 819-819.1, 827-827.1, 828-828.1, 929.0	T02-T02.9, T04-T04.4, T04.7-T04.9, T06-T06.3, T06.5, T06.8
Muscle and tendon injuries, including sprains and strains lesser dislocations	Incidence	830-830.1, 832-832.2, 833-833.1, 834-834.1, 837-837.1, 838-838.1, 839-839.9, 840-840.9, 841-841.3, 841.8-841.9, 842-842.1, 843-843.1, 843.8-843.9, 844-844.3, 844.8-844.9, 845-845.1, 846-846.3, 846.8-846.9, 847-847.4, 847.9, 848-848.5, 848.8-848.9, 905.6-905.8	S03-S03.5, S03.8-S03.9, S13-S13.6, S13.8-S13.9, S16.1-S16.2, S16.8-S16.9, S23-S23.5, S23.8-S23.9, S29.0, S33-S33.9, S39.0, S39.6, S43.4-S43.9, S46-S46.3, S46.7-S46.9, S49.8-S49.9, S53-S53.4, S56-S56.5, S56.7-S56.8, S59.7-S59.9, S63-S63.7, S66.6-S66.7, S69.7-S69.9, S73.1, S76-S76.4, S76.7-S76.9, S83.2-S83.6, S86-S86.3, S86.7-S86.9, S93-S93.6, S96-S96.2, S96.7-S96.9, S99.7-S99.9, T03-T03.4, T03.8-T03.9, T06.4, T09.2, T09.5, T11.2, T13.2, T14.3, T92.3, T93.3, T93.5
Nerve injury	Incidence	907.1, 907.3-907.5, 907.8-907.9, 950-950.3, 950.9, 951-951.9, 953-953.5, 953.8-953.9, 954-954.1, 954.8-954.9, 955-955.9, 956-956.5, 956.8-956.9, 957-957.1, 957.8-957.9	S04-S04.9, S14.2-S14.6, S14.8-S14.9, S24.2-S24.6, S24.8-S24.9, S34.2-S34.6, S34.8-S34.9, S44-S44.5, S44.7-S44.9, S54-S54.3, S54.7-S54.9, S64-S64.4, S64.7-S64.9, S74-S74.2, S74.7-S74.9, S84-S84.2, S84.7-S84.9, S94-S94.3, S94.7-S94.9, T11.3, T13.3, T90.3, T92.4, T93.4
Open wound(s)	Incidence	872-872.1, 872.6-872.9, 873-873.9, 874.2-874.5, 874.8-874.9, 875-875.1, 876-876.1, 877-877.1, 878-878.9, 879-879.9, 880-880.2, 881-881.2, 882-882.2, 883-883.2, 884-884.2, 890-890.2, 891-891.2, 892-892.2, 893-893.2, 894-894.2, 900-900.1, 900.8-900.9, 903-903.5, 903.8-903.9, 904-904.9, 906-906.2	S01.0, S01.2-S01.5, S01.7-S01.9, S08-S08.1, S08.8-S08.9, S09-S09.3, S10.7, S11.1, S11.8-S11.9, S15-S15.3, S15.7-S15.9, S21-S21.4, S21.7-S21.9, S31-S31.5, S31.7-S31.8, S39.7, S41-S41.1, S41.7-S41.8, S45-S45.3, S45.7-S45.9, S51.0, S51.7-S51.9, S55-S55.2, S55.7-S55.9, S61-S61.1, S61.7-S61.9, S65-S65.5, S65.7-S65.9, S71-S71.1, S71.7-S71.8, S75-S75.2, S75.7-S75.9, S81.0, S81.7-S81.9, S85-S85.5, S85.7-S85.9, S91.7, S95-S95.2, S95.7-S95.9, T01-T01.3, T01.6, T01.8-T01.9, T09.1, T11.1, T11.4-T11.5, T13.1, T13.4-T13.5, T14.1, T14.5-T14.6, T90.1, T92.0, T92.5, T93.0
Poisoning requiring urgent care	Incidence	960-960.9, 961-961.9, 962-962.9, 963-963.5, 963.8-963.9, 964-964.9, 965-965.1, 965.4-965.9, 966-966.4, 967-967.6, 967.8-967.9, 968-968.7, 968.9, 969-969.9, 970-970.1, 970.8-970.9, 971-971.3, 971.9, 972-972.9, 973-973.6, 973.8-973.9, 974-974.7, 975-975.8, 976-976.9, 977-977.4, 977.8-977.9, 978-978.6, 978.8-978.9, 979-979.7, 979.9, 980-980.3, 980.8-980.9, 981.2-981.3, 981.5-981.7, 981.9, 982-982.4, 982.8, 983-983.2, 983.5, 983.7, 983.9, 984-984.1, 984.3, 984.8-984.9, 985-985.6, 985.8-985.9, 987-987.9, 988-988.2, 988.6, 988.8-988.9, 989-989.9	T36-T36.9, T37-T37.5, T37.8-T37.9, T38-T38.9, T39-T39.4, T39.8-T39.9, T40-T40.9, T41-T41.5, T42-T42.8, T43-T43.6, T43.8-T43.9, T44-T44.9, T45-T45.9, T46-T46.9, T47-T47.9, T48-T48.7, T48.9, T49-T49.9, T50-T50.9, T51-T51.3, T51.8-T51.9, T52-T52.4, T52.8-T52.9, T53-T53.7, T53.9, T54-T54.3, T54.9, T55-T55.1, T56-T56.9, T57-T57.3, T57.8-T57.9, T58-T58.2, T58.8-T58.9, T59-T59.9, T60-T60.4, T60.8-T60.9, T61-T61.2, T61.7-T61.9, T62-T62.2, T62.8-T62.9, T64.0, T64.8, T65-T65.6, T65.8-T65.9
Severe TBI	Incidence	851-851.9, 853-853.1, 854-854.1	S06.2, S06.7-S06.8, T90.5
Severe chest Injury	Incidence	807.0, 860-860.5, 861-861.3, 862-862.3, 862.8-862.9, 874-874.1, 901-901.4, 901.8-901.9, 908.0	S11.0, S11.2, S11.7, S25-S25.5, S25.7-S25.9, S26-S26.1, S26.8-S26.9, S27-S27.9, S28-S28.2, S29.7-S29.9, T91.4
Spinal cord lesion at neck level	Incidence	806-806.1, 952.0	S14-S14.1, T91.3
Spinal cord lesion below neck level	Incidence	806.2-806.9, 952.1-952.4, 952.8-952.9	S24-S24.1, S34-S34.1
Superficial injury of any part of the body	Incidence	910-910.9, 911-911.9, 912-912.9, 913-913.9, 914-914.9, 915-915.9, 916-916.9, 917-917.9, 919-919.9	S00-S00.5, S00.7-S00.9, S10-S10.1, S10.8-S10.9, S20.1, S20.3-S20.4, S20.7-S20.9, S30.7-S30.9, S40.2, S40.7-S40.9, S50.3, S50.7-S50.9, S70.2-S70.3, S70.7-S70.9, S80.8-S80.9, S90.4-S90.5, S90.7-S90.9, T00-T00.3, T00.6, T00.8-T00.9, T09.0, T11.0, T13.0, T14.0, T90.0

Appendix Table 5a. Data representativeness Index (DRI), the percentage of GBD 2015 geographies with any data by cause, pertaining to period before 2005, 2005-2015, and all years of data

	<2005	2005-2015	Total
**All causes**	100.0%	100.0%	100.0%
**Communicable, maternal, neonatal, and nutritional diseases**	100.0%	100.0%	100.0%
**HIV/AIDS and tuberculosis**	98.5%	99.5%	100.0%
Tuberculosis	97.1%	98.5%	99.5%
**HIV/AIDS**	82.0%	86.9%	86.9%
HIV/AIDS - Tuberculosis	45.6%	81.6%	82.0%
HIV/AIDS resulting in other diseases	72.3%	72.8%	72.8%
**Diarrhea, lower respiratory, and other common infectious diseases**	95.1%	96.1%	96.1%
Diarrheal diseases	71.4%	74.8%	85.0%
**Intestinal infectious diseases**	18.9%	12.1%	23.3%
Typhoid fever	16.5%	9.2%	19.9%
Paratyphoid fever	7.8%	8.3%	11.2%
Other intestinal infectious diseases	-	-	-
Lower respiratory infections	62.6%	70.9%	81.6%
Upper respiratory infections	33.0%	28.2%	41.3%
Otitis media	11.2%	5.8%	12.1%
**Meningitis**	45.6%	33.0%	50.0%
Pneumococcal meningitis	33.0%	12.1%	35.0%
H influenzae type B meningitis	32.5%	10.7%	34.5%
Meningococcal meningitis	30.6%	11.7%	33.0%
Other meningitis	30.6%	12.1%	33.0%
Encephalitis	28.6%	19.4%	31.6%
Diphtheria	60.0%	55.6%	64.4%
Whooping cough	88.8%	85.9%	89.3%
Tetanus	65.0%	59.7%	68.0%
Measles	88.8%	89.8%	89.8%
Varicella and herpes zoster	19.4%	7.3%	19.9%
**Neglected tropical diseases and malaria**	96.6%	96.6%	96.6%
Malaria	37.9%	26.2%	38.8%
Chagas disease	66.7%	66.7%	77.8%
**Leishmaniasis**	49.1%	44.0%	52.2%
Visceral leishmaniasis	34.0%	33.3%	38.4%
Cutaneous and mucocutaneous leishmaniasis	39.0%	35.2%	42.1%
African trypanosomiasis	100.0%	100.0%	100.0%
Schistosomiasis	48.5%	10.3%	48.5%
Cysticercosis	6.9%	3.9%	7.8%
Cystic echinococcosis	26.7%	21.8%	29.1%
Lymphatic filariasis	51.4%	41.4%	58.6%
Onchocerciasis	-	-	-
Trachoma	36.2%	32.8%	44.8%
Dengue	32.2%	24.0%	33.9%
Yellow fever	8.5%	0.0%	8.5%
Rabies	56.3%	56.3%	61.2%
**Intestinal nematode infections**	96.4%	82.9%	97.1%
Ascariasis	96.4%	82.9%	97.1%
Trichuriasis	96.4%	82.9%	97.1%
Hookworm disease	95.7%	81.4%	96.4%
Food-borne trematodiases	100.0%	17.6%	100.0%
Leprosy	86.4%	81.2%	91.6%
Ebola	-	-	-
Other neglected tropical diseases	-	-	-
**Maternal disorders**	76.7%	82.5%	85.0%
Maternal hemorrhage	73.3%	74.8%	80.1%
Maternal sepsis and other maternal infections	67.5%	70.9%	75.2%
Maternal hypertensive disorders	70.9%	76.2%	80.1%
Maternal obstructed labor and uterine rupture	68.0%	74.3%	76.7%

Maternal abortion, miscarriage, and ectopic pregnancy	67.5%	72.8%	74.3%
Other maternal disorders	-	-	-
**Neonatal disorders**	77.7%	68.9%	82.0%
Neonatal preterm birth complications	53.4%	47.6%	59.7%
Neonatal encephalopathy due to birth asphyxia and trauma	25.7%	24.3%	28.6%
Neonatal sepsis and other neonatal infections	-	-	-
Hemolytic disease and other neonatal jaundice	44.7%	34.5%	50.5%
Other neonatal disorders	-	-	-
**Nutritional deficiencies**	90.3%	89.8%	90.3%
Protein-energy malnutrition	89.3%	86.4%	89.8%
Iodine deficiency	71.8%	43.2%	72.3%
Vitamin A deficiency	22.5%	1.7%	22.5%
Iron-deficiency anemia	-	-	-
Other nutritional deficiencies	-	-	-
**Other communicable, maternal, neonatal, and nutritional diseases**	80.1%	69.4%	85.0%
**Sexually transmitted diseases excluding HIV**	75.7%	67.5%	81.6%
Syphilis	65.0%	56.3%	71.4%
Chlamydial infection	43.7%	45.1%	57.8%
Gonococcal infection	43.7%	43.7%	55.3%
Trichomoniasis	20.9%	19.9%	28.2%
Genital herpes	35.4%	18.0%	38.3%
Other sexually transmitted diseases	4.4%	1.0%	4.4%
**Hepatitis**	44.7%	14.1%	46.1%
Acute hepatitis A	0.5%	0.0%	0.5%
Acute hepatitis B	38.8%	10.2%	39.8%
Acute hepatitis C	2.9%	0.5%	2.9%
Acute hepatitis E	20.4%	5.8%	21.8%
Other infectious diseases	3.9%	1.0%	3.9%
**Non-communicable diseases**	96.1%	97.1%	98.5%
**Neoplasms**	55.8%	45.6%	56.3%
Lip and oral cavity cancer	52.9%	43.7%	53.4%
Nasopharynx cancer	52.9%	44.2%	53.4%
Other pharynx cancer	52.9%	43.7%	53.4%
Esophageal cancer	52.9%	44.2%	53.4%
Stomach cancer	52.9%	44.2%	53.4%
Colon and rectum cancer	53.4%	44.7%	53.9%
**Liver cancer**	55.8%	45.1%	56.3%
Liver cancer due to hepatitis B	18.9%	10.2%	19.9%
Liver cancer due to hepatitis C	18.9%	9.7%	19.9%
Liver cancer due to alcohol use	7.8%	6.8%	9.2%
Liver cancer due to other causes	6.8%	6.3%	8.3%
Gallbladder and biliary tract cancer	50.0%	43.2%	50.5%
Pancreatic cancer	52.4%	44.2%	52.9%
Larynx cancer	52.4%	44.7%	52.9%
Tracheal, bronchus, and lung cancer	52.9%	44.2%	53.4%
Malignant skin melanoma	49.5%	43.2%	50.5%
**Non-melanoma skin cancer**	51.9%	42.2%	52.4%
Non-melanoma skin cancer (squamous-cell carcinoma)	-	-	-
Non-melanoma skin cancer (basal-cell carcinoma)	-	-	-
Breast cancer	53.4%	44.7%	53.9%
Cervical cancer	52.9%	44.2%	53.4%
Uterine cancer	52.9%	44.2%	53.4%
Ovarian cancer	51.9%	44.2%	52.4%
Prostate cancer	53.4%	44.7%	53.9%
Testicular cancer	51.5%	43.7%	51.9%
Kidney cancer	52.4%	44.2%	52.9%
Bladder cancer	53.4%	44.7%	53.9%
Brain and nervous system cancer	52.4%	44.2%	52.9%
Thyroid cancer	52.4%	43.7%	52.9%

Mesothelioma	41.7%	40.3%	43.2%
Hodgkin lymphoma	50.5%	42.7%	51.0%
Non-Hodgkin lymphoma	51.9%	43.7%	52.4%
Multiple myeloma	49.5%	43.2%	51.0%
**Leukemia**	51.0%	43.2%	51.9%
Acute lymphoid leukemia	9.2%	6.8%	11.7%
Chronic lymphoid leukemia	8.7%	5.8%	10.7%
Acute myeloid leukemia	9.2%	6.3%	11.2%
Chronic myeloid leukemia	9.2%	6.3%	11.2%
Other neoplasms	52.9%	44.2%	53.4%
**Cardiovascular diseases**	81.1%	73.8%	84.5%
Rheumatic heart disease	29.3%	26.8%	37.4%
Ischemic heart disease	46.6%	29.6%	51.0%
**Cerebrovascular disease**	64.1%	67.0%	74.3%
Ischemic stroke	62.6%	60.7%	68.0%
Hemorrhagic stroke	62.6%	60.7%	68.0%
Hypertensive heart disease	12.7%	5.9%	15.7%
Cardiomyopathy and myocarditis	25.1%	22.2%	31.0%
Atrial fibrillation and flutter	22.3%	23.8%	27.2%
Peripheral vascular disease	19.4%	20.4%	23.3%
Endocarditis	18.1%	19.1%	21.1%
Other cardiovascular and circulatory diseases	0.5%	0.5%	0.5%
**Chronic respiratory diseases**	65.0%	38.3%	67.5%
Chronic obstructive pulmonary disease	24.8%	28.2%	32.0%
**Pneumoconiosis**	17.5%	18.0%	19.9%
Silicosis	15.5%	16.5%	17.5%
Asbestosis	23.5%	29.4%	29.4%
Coal workers pneumoconiosis	19.4%	19.4%	25.0%
Other pneumoconiosis	14.1%	16.5%	16.5%
Asthma	62.6%	24.8%	65.0%
Interstitial lung disease and pulmonary sarcoidosis	18.4%	18.4%	20.9%
Other chronic respiratory diseases	-	-	-
**Cirrhosis and other chronic liver diseases**	28.6%	22.8%	32.0%
Cirrhosis and other chronic liver diseases due to hepatitis B	15.0%	6.3%	16.0%
Cirrhosis and other chronic liver diseases due to hepatitis C	16.0%	6.8%	17.5%
Cirrhosis and other chronic liver diseases due to alcohol use	9.2%	6.3%	10.7%
Cirrhosis and other chronic liver diseases due to other causes	7.8%	5.8%	9.2%
**Digestive diseases**	53.4%	62.1%	64.1%
Peptic ulcer disease	16.5%	18.0%	19.9%
Gastritis and duodenitis	19.9%	23.8%	26.2%
Appendicitis	14.1%	17.0%	17.0%
Paralytic ileus and intestinal obstruction	15.0%	17.0%	18.0%
Inguinal, femoral, and abdominal hernia	14.1%	17.0%	17.0%
Inflammatory bowel disease	46.6%	53.9%	55.8%
Vascular intestinal disorders	7.8%	8.3%	8.3%
Gallbladder and biliary diseases	21.4%	18.9%	24.8%
Pancreatitis	15.0%	17.5%	18.0%
Other digestive diseases	-	-	-
**Neurological disorders**	51.9%	35.4%	55.8%
Alzheimer disease and other dementias	20.4%	16.0%	23.3%
Parkinson disease	19.4%	12.6%	24.8%
Epilepsy	23.8%	6.3%	25.2%
Multiple sclerosis	25.2%	10.7%	27.2%
Motor neuron disease	10.2%	5.3%	10.7%
Migraine	19.4%	14.1%	23.3%
Tension-type headache	11.2%	13.6%	18.4%
Medication overuse headache	4.9%	10.2%	11.7%
Other neurological disorders	8.3%	1.0%	8.3%
**Mental and substance use disorders**	58.3%	57.3%	67.5%

Schizophrenia	21.8%	10.2%	23.8%
Alcohol use disorders	33.0%	21.8%	35.4%
**Drug use disorders**	38.3%	39.8%	48.1%
Opioid use disorders	14.1%	14.1%	18.0%
Cocaine use disorders	21.8%	23.8%	30.6%
Amphetamine use disorders	20.4%	19.9%	26.2%
Cannabis use disorders	33.0%	38.3%	44.7%
Other drug use disorders	-	-	-
**Depressive disorders**	31.6%	24.3%	36.9%
Major depressive disorder	31.1%	24.3%	36.4%
Dysthymia	17.0%	6.8%	18.9%
Bipolar disorder	15.5%	9.2%	18.4%
Anxiety disorders	24.3%	9.7%	25.7%
**Eating disorders**	14.6%	8.3%	16.5%
Anorexia nervosa	12.6%	6.3%	14.1%
Bulimia nervosa	13.1%	8.3%	16.0%
**Autistic spectrum disorders**	6.8%	6.3%	10.7%
Autism	6.8%	6.3%	10.7%
Asperger syndrome and other autistic spectrum disorders	4.4%	2.9%	5.8%
Attention-deficit/hyperactivity disorder	16.5%	8.7%	19.4%
Conduct disorder	9.7%	3.9%	11.2%
Idiopathic intellectual disability	-	-	-
Other mental and substance use disorders	1.0%	0.0%	1.0%
**Diabetes, urogenital, blood, and endocrine diseases**	93.2%	95.1%	97.1%
Diabetes mellitus	59.7%	59.2%	75.7%
Acute glomerulonephritis	20.9%	21.8%	24.8%
**Chronic kidney disease**	45.6%	52.4%	57.8%
Chronic kidney disease due to diabetes mellitus	17.5%	21.8%	24.3%
Chronic kidney disease due to hypertension	17.5%	21.8%	24.3%
Chronic kidney disease due to glomerulonephritis	17.5%	21.8%	24.3%
Chronic kidney disease due to other causes	17.5%	21.8%	24.3%
**Urinary diseases and male infertility**	22.3%	22.8%	28.2%
Interstitial nephritis and urinary tract infections	14.1%	17.0%	17.0%
Urolithiasis	15.5%	20.4%	20.9%
Benign prostatic hyperplasia	14.1%	17.0%	17.0%
Male infertility	6.8%	1.9%	7.3%
Other urinary diseases	-	-	-
**Gynecological diseases**	68.0%	91.3%	91.3%
Uterine fibroids	17.0%	19.9%	21.4%
Polycystic ovarian syndrome	4.9%	6.3%	8.3%
Female infertility	6.8%	1.9%	7.3%
Endometriosis	16.0%	19.4%	19.4%
Genital prolapse	17.5%	19.4%	22.3%
Premenstrual syndrome	58.7%	86.4%	86.4%
Other gynecological diseases	14.1%	17.0%	17.0%
**Hemoglobinopathies and hemolytic anemias**	64.6%	62.1%	66.0%
Thalassemias	47.1%	44.6%	48.5%
Thalassemias trait	1.0%	0.0%	1.0%
Sickle cell disorders	46.6%	45.6%	48.1%
Sickle cell trait	0.5%	0.0%	0.5%
G6PD deficiency	41.7%	11.2%	43.2%
G6PD trait	1.0%	0.0%	1.0%
Other hemoglobinopathies and hemolytic anemias	-	-	-
Endocrine, metabolic, blood, and immune disorders	14.1%	17.0%	17.0%
**Musculoskeletal disorders**	48.1%	23.3%	51.0%
Rheumatoid arthritis	21.4%	9.2%	22.8%
Osteoarthritis	15.5%	10.2%	19.4%
**Low back and neck pain**	41.7%	17.5%	44.7%
Low back pain	41.3%	16.5%	44.7%

Neck pain	12.6%	7.3%	15.5%
Gout	15.0%	5.8%	16.5%
Other musculoskeletal disorders	8.7%	2.9%	8.7%
**Other non-communicable diseases**	77.7%	57.3%	81.1%
**Congenital anomalies**	24.3%	19.4%	24.8%
Neural tube defects	19.4%	19.4%	21.4%
Congenital heart anomalies	22.3%	19.4%	24.3%
Cleft lip and cleft palate	24.3%	19.4%	24.8%
Down syndrome	23.8%	19.4%	24.8%
Turner syndrome	8.3%	9.2%	9.7%
Klinefelter syndrome	8.7%	9.2%	9.7%
Other chromosomal abnormalities	22.8%	19.4%	24.3%
Other congenital anomalies	-	-	-
**Skin and subcutaneous diseases**	58.3%	39.3%	61.7%
Dermatitis	52.4%	25.2%	55.3%
Psoriasis	10.2%	8.3%	14.1%
Cellulitis	14.1%	17.0%	17.0%
Pyoderma	19.4%	20.9%	24.3%
Scabies	10.7%	8.3%	14.6%
Fungal skin diseases	9.2%	10.2%	13.6%
Viral skin diseases	8.3%	4.9%	10.2%
Acne vulgaris	10.2%	11.2%	14.1%
Alopecia areata	2.4%	2.4%	3.4%
Pruritus	3.4%	3.9%	6.3%
Urticaria	6.3%	3.4%	8.3%
Decubitus ulcer	13.6%	16.0%	16.0%
Other skin and subcutaneous diseases	1.0%	1.5%	1.5%
**Sense organ diseases**	33.0%	22.8%	43.2%
Glaucoma	17.0%	9.2%	20.9%
Cataract	23.8%	18.4%	33.5%
Macular degeneration	15.5%	9.7%	20.9%
Refraction and accommodation disorders	0.5%	4.9%	4.9%
Age-related and other hearing loss	15.0%	3.9%	16.5%
Other vision loss	11.2%	5.3%	14.6%
Other sense organ diseases	0.5%	0.5%	0.5%
**Oral disorders**	55.8%	27.2%	58.7%
Deciduous caries	35.0%	18.9%	40.3%
Permanent caries	35.0%	17.5%	38.8%
Periodontal diseases	21.8%	6.3%	23.3%
Edentulism and severe tooth loss	36.4%	9.7%	36.9%
Other oral disorders	-	-	-
**Injuries**	90.3%	88.8%	90.3%
**Transport injuries**	6.3%	7.3%	8.7%
**Road injuries**	6.3%	7.3%	8.7%
Pedestrian road injuries	4.4%	6.8%	6.8%
Cyclist road injuries	4.4%	6.8%	6.8%
Motorcyclist road injuries	4.4%	6.8%	6.8%
Motor vehicle road injuries	4.4%	6.8%	6.8%
Other road injuries	4.4%	6.8%	6.8%
Other transport injuries	2.9%	4.4%	4.4%
**Unintentional injuries**	11.2%	13.1%	17.0%
Falls	8.3%	9.2%	12.1%
Drowning	7.3%	9.2%	10.7%
Fire, heat, and hot substances	8.7%	11.2%	13.6%
Poisonings	8.3%	10.7%	13.1%
**Exposure to mechanical forces**	8.3%	9.2%	12.6%
Unintentional firearm injuries	4.9%	7.8%	7.8%
Unintentional suffocation	4.9%	7.8%	7.8%
Other exposure to mechanical forces	2.9%	4.4%	4.4%

Adverse effects of medical treatment	-	-	-
**Animal contact**	7.3%	8.3%	10.7%
Venomous animal contact	2.9%	4.4%	4.4%
Non-venomous animal contact	2.9%	4.4%	4.4%
**Foreign body**	2.9%	4.4%	4.4%
Pulmonary aspiration and foreign body in airway	2.9%	4.4%	4.4%
Foreign body in eyes	2.9%	3.4%	3.4%
Foreign body in other body part	2.9%	4.4%	4.4%
Environmental heat and cold exposure	2.4%	2.9%	2.9%
Other unintentional injuries	4.9%	7.8%	7.8%
**Self-harm and interpersonal violence**	7.3%	8.7%	10.7%
Self-harm	4.9%	6.8%	7.8%
**Interpersonal violence**	6.8%	8.3%	10.7%
Assault by firearm	2.9%	4.4%	4.4%
Assault by sharp object	2.9%	4.4%	4.4%
Assault by other means	2.9%	4.4%	4.4%
**Forces of nature, war, and legal intervention**	100.0%	100.0%	100.0%
Exposure to forces of nature	100.0%	100.0%	100.0%
Collective violence and legal intervention	100.0%	100.0%	100.0%

The percentage is calculated out of a total of 195 countries and territories. GBD = Global Burden of Disease

Appendix Table 5b. Data representativeness Index (DRI), the percentage of GBD 2015 geographies with any data, by impairment, pertaining to period before 2005, 2005-2015, and all years of data

	<2005	2005-2015	Total
Anemia	66.8%	30.7%	68.3%
Epilepsy	44.4%	17.6%	47.3%
Guillain-Barré syndrome	18.0%	9.8%	18.0%
Hearing loss	31.2%	18.5%	36.6%
Heart failure	19.0%	18.5%	22.4%
Infertility	52.2%	31.7%	57.1%
Intellectual disability	15.6%	4.9%	16.1%
Pelvic inflammatory disease	16.1%	17.6%	18.5%
Vision loss	50.2%	37.6%	59.5%

The percentage is calculated out of a total of 195 countries and territories. GBD = Global Burden of Disease

Appendix Table 6. GBD 2015 sequelae, health states, health state lay descriptions, and disability weights

Sequela	Healthstate name	Healthstate lay description	Disability weight
Tuberculosis	Tuberculosis, not HIV infected	has a persistent cough and fever, is short of breath, feels weak, and has lost a lot of weight.	0.333 (0.224-0.454)
HIV/AIDS resulting in mycobacterial infection	Tuberculosis, HIV infected	has a persistent cough and fever, shortness of breath, night sweats, weakness and fatigue and severe weight loss.	0.408 (0.274-0.549)
Early HIV	Generic uncomplicated disease: anxiety about diagnosis	has a disease diagnosis that causes some worry but minimal interference with daily activities.	0.012 (0.006-0.023)
Symptomatic HIV	HIV cases, symptomatic, pre-AIDS	has weight loss, fatigue, and frequent infections.	0.274 (0.184-0.377)
AIDS with antiretroviral treatment	HIV/AIDS cases, receiving ARV treatment	has occasional fevers and infections. The person takes daily medication that sometimes causes diarrhea.	0.078 (0.052-0.111)
AIDS without antiretroviral treatment	AIDS cases, not receiving ARV treatment	has severe weight loss, weakness, fatigue, cough and fever, and frequent infections, skin rashes and diarrhea.	0.582 (0.406-0.743)
Mild diarrheal diseases	Diarrhea, mild	has diarrhea three or more times a day with occasional discomfort in the belly.	0.074 (0.049-0.104)
Moderate diarrheal diseases	Diarrhea, moderate	has diarrhea three or more times a day, with painful cramps in the belly and feeling thirsty	0.188 (0.125-0.264)
Severe diarrheal diseases	Diarrhea, severe	has diarrhea three or more times a day with severe belly cramps. The person is very thirsty and feels nauseous and tired.	0.247 (0.164-0.348)
Guillain-Barré syndrome due to diarrheal diseases	Spinal cord lesion below neck level (treated)	is paralyzed from the waist down, cannot feel or move the legs and has difficulties with urine and bowel control. The person uses a wheelchair to move around.	0.296 (0.198-0.414)
Acute typhoid infection	Infectious disease, acute episode, moderate	has a fever and aches, and feels weak, which causes some difficulty with daily activities.	0.051 (0.032-0.074)
Severe typhoid fever	Infectious disease, acute episode, severe	has a high fever and pain, and feels very weak, which causes great difficulty with daily activities.	0.133 (0.088-0.19)
Intestinal perforation due to typhoid	Abdominopelvic problem, severe	has severe pain in the belly and feels nauseous. The person is anxious and unable to carry out daily activities.	0.324 (0.22-0.442)
Gastrointestinal bleeding due to typhoid	Gastric bleeding	vomits blood and feels nauseous.	0.325 (0.209-0.462)
Acute paratyphoid infection	Infectious disease, acute episode, mild	has a low fever and mild discomfort , but no difficulty with daily activities.	0.006 (0.002-0.012)
Moderate paratyphoid fever	Infectious disease, acute episode, moderate	has a fever and aches, and feels weak, which causes some difficulty with daily activities.	0.051 (0.032-0.074)
Severe paratyphoid fever	Infectious disease, acute episode, severe	has a high fever and pain, and feels very weak, which causes great difficulty with daily activities.	0.133 (0.088-0.19)
Intestinal perforation due to paratyphoid	Abdominopelvic problem, moderate	has pain in the belly and feels nauseous. The person has difficulties with daily activities.	0.114 (0.078-0.159)
Other intestinal infectious diseases	--	--	--
Moderate lower respiratory infections	Infectious disease, acute episode, moderate	has a fever and aches, and feels weak, which causes some difficulty with daily activities.	0.051 (0.032-0.074)
Severe lower respiratory infections	Infectious disease, acute episode, severe	has a high fever and pain, and feels very weak, which causes great difficulty with daily activities.	0.133 (0.088-0.19)
Guillain-Barré syndrome due to lower respiratory infections	Spinal cord lesion below neck level (treated)	is paralyzed from the waist down, cannot feel or move the legs and has difficulties with urine and bowel control. The person uses a wheelchair to move around.	0.296 (0.198-0.414)
Mild upper respiratory infections	Infectious disease, acute episode, mild	has a low fever and mild discomfort , but no difficulty with daily activities.	0.006 (0.002-0.012)
Moderate upper respiratory infections	Infectious disease, acute episode, moderate	has a fever and aches, and feels weak, which causes some difficulty with daily activities.	0.051 (0.032-0.074)
Guillain-Barré syndrome due to upper respiratory infections	Spinal cord lesion below neck level (treated)	is paralyzed from the waist down, cannot feel or move the legs and has difficulties with urine and bowel control. The person uses a wheelchair to move around.	0.296 (0.198-0.414)
Acute otitis media	Ear pain	has an ear-ache that causes some difficulty with daily activities.	0.013 (0.007-0.024)
Severe infectious complications due to chronic otitis media	Ear pain	has an ear-ache that causes some difficulty with daily activities.	0.013 (0.007-0.024)
Vertigo with mild hearing loss due to chronic otitis media	Vertigo with mild hearing loss	(combined DW)	--
Vertigo with mild hearing loss and ringing due to chronic otitis media	Vertigo with mild hearing loss and ringing	(combined DW)	--
Mild hearing loss due to chronic otitis media	Hearing loss, mild	has great difficulty hearing and understanding another person talking in a noisy place (for example, on an urban street).	0.01 (0.004-0.019)
Mild hearing loss with ringing due to chronic otitis media	Hearing loss, mild, with ringing	has great difficulty hearing and understanding another person talking in a noisy place (for example, on an urban street), and sometimes has annoying ringing in the ears.	0.021 (0.012-0.036)
Vertigo with moderate hearing loss due to chronic otitis media	Vertigo with moderate hearing loss	(combined DW)	--
Vertigo with moderate hearing loss and ringing due to chronic otitis media	Vertigo with moderate hearing loss and ringing	(combined DW)	--
Moderate hearing loss due to chronic otitis media	Hearing loss, moderate	is unable to hear and understand another person talking in a noisy place (for example, on an urban street), and has difficulty hearing another person talking even in a quiet place or on the phone.	0.027 (0.015-0.042)
Moderate hearing loss with ringing due to chronic otitis media	Hearing loss, moderate, with ringing	is unable to hear and understand another person talking in a noisy place (for example, on an urban street), has difficulty hearing another person talking even in a quiet place or on the phone, and has annoying ringing in the ears for 5 minutes at a time, almost every day.	0.074 (0.049-0.107)
Acute pneumococcal meningitis	Infectious disease, acute episode, severe	has a high fever and pain, and feels very weak, which causes great difficulty with daily activities.	0.133 (0.088-0.19)
Mild behavioral problems due to pneumococcal meningitis	Attention deficit hyperactivity disorder	is hyperactive and has difficulty concentrating, remembering things, and completing tasks.	0.045 (0.028-0.066)
Mild motor impairment due to long term due to pneumococcal meningitis	Motor impairment, mild	has some difficulty in moving around but is able to walk without help.	0.01 (0.005-0.019)
Mild motor plus cognitive impairments due to pneumococcal meningitis	Motor plus cognitive impairments, mild	has some difficulty in moving around but is able to walk without help. The person is slow in learning at school. As an adult, the person has some difficulty doing complex or unfamiliar tasks but otherwise functions independently.	0.031 (0.018-0.05)
Borderline intellectual disability due to pneumococcal meningitis	Borderline intellectual functioning	is slow in learning at school. As an adult, the person has some difficulty doing complex or unfamiliar tasks but otherwise functions independently.	0.011 (0.005-0.02)
Monocular distance vision loss due to pneumococcal meningitis	Distance vision, monocular	is blind in one eye and has difficulty judging distances	0.017 (0.009-0.029)
Mild intellectual disability due to pneumococcal meningitis	Intellectual disability / mental retardation, mild	has low intelligence and is slow in learning at school. As an adult, the person can live independently, but often needs help to raise children and can only work at simple supervised jobs.	0.043 (0.026-0.064)
Moderate motor impairment due to pneumococcal meningitis	Motor impairment, moderate	has some difficulty in moving around, and difficulty in lifting and holding objects, dressing and sitting upright, but is able to walk without help.	0.061 (0.04-0.089)
Severe motor impairment due to pneumococcal meningitis	Motor impairment, severe	is unable to move around without help, and is not able to lift or hold objects, get dressed or sit upright.	0.402 (0.268-0.545)
Moderate motor plus cognitive impairments due to pneumococcal meningitis	Motor plus cognitive impairments, moderate	has some difficulty in moving around, holding objects, dressing and sitting upright, but can walk without help. The person has low intelligence and is slow in learning to speak and to do simple tasks.	0.203 (0.134-0.29)
Severe motor plus cognitive impairments due to pneumococcal meningitis	Motor plus cognitive impairments, severe	cannot move around without help, and cannot lift or hold objects, get dressed or sit upright. The person also has very low intelligence, speaks few words, and needs constant supervision and help with all daily activities.	0.542 (0.374-0.702)
Epilepsy due to pneumococcal meningitis	Epilepsy	(combined DW)	--
Blindness due to pneumococcal meningitis	Distance vision blindness	is completely blind, which causes great difficulty in some daily activities, worry and anxiety, and great difficulty going outside the home without assistance.	0.187 (0.124-0.26)
Mild hearing loss due to pneumococcal meningitis	Hearing loss, mild	has great difficulty hearing and understanding another person talking in a noisy place (for example, on an urban street).	0.01 (0.004-0.019)
Mild hearing loss with ringing due to pneumococcal meningitis	Hearing loss, mild, with ringing	has great difficulty hearing and understanding another person talking in a noisy place (for example, on an urban street), and sometimes has annoying ringing in the ears.	0.021 (0.012-0.036)

Appendix Table 6. GBD 2015 sequelae, health states, health state lay descriptions, and disability weights			
**Sequela**	**Healthstate name**	**Healthstate lay description**	**Disability weight**
Moderate hearing loss due to pneumococcal meningitis	Hearing loss, moderate	is unable to hear and understand another person talking in a noisy place (for example, on an urban street), and has difficulty hearing another person talking even in a quiet place or on the phone.	0.027 (0.015-0.042)
Moderate hearing loss with ringing due to pneumococcal meningitis	Hearing loss, moderate, with ringing	is unable to hear and understand another person talking in a noisy place (for example, on an urban street), has difficulty hearing another person talking even in a quiet place or on the phone, and has annoying ringing in the ears for 5 minutes at a time, almost every day.	0.074 (0.049-0.107)
Moderately severe hearing loss due to pneumococcal meningitis	Hearing loss, moderately severe	(custom DW: average of moderate and severe hearing loss impairment)	--
Moderately severe hearing loss with ringing due to pneumococcal meningitis	Hearing loss, moderately severe, with ringing	(custom DW: average of moderate and severe hearing loss impairment)	--
Severe hearing loss due to pneumococcal meningitis	Hearing loss, severe	is unable to hear and understand another person talking, even in a quiet place, and unable to take part in a phone conversation. Difficulties with communicating and relating to others cause emotional impact at times (for example worry or depression).	0.158 (0.105-0.227)
Severe hearing loss with ringing due to pneumococcal meningitis	Hearing loss, severe, with ringing	is unable to hear and understand another person talking, even in a quiet place, is unable to take part in a phone conversation, and has annoying ringing in the ears for more than 5 minutes at a time, almost every day. Difficulties with communicating and relating to others cause emotional impact at times (for example worry or depression).	0.261 (0.175-0.36)
Profound hearing loss due to pneumococcal meningitis	Hearing loss, profound	is unable to hear and understand another person talking, even in a quiet place, is unable to take part in a phone conversation, and has great difficulty hearing anything in any other situation. Difficulties with communicating and relating to others often cause worry, depression or loneliness.	0.204 (0.134-0.288)
Profound hearing loss with ringing due to pneumococcal meningitis	Hearing loss, profound, with ringing	is unable to hear and understand another person talking, even in a quiet place, is unable to take part in a phone conversation, has great difficulty hearing anything in any other situation, and has annoying ringing in the ears for more than 5 minutes at a time, several times a day. Difficulties with communicating and relating to others often cause worry, depression, or loneliness.	0.277 (0.182-0.387)
Complete hearing loss due to pneumococcal meningitis	Hearing loss, complete	cannot hear at all in any situation, including even the loudest sounds, and cannot communicate verbally or use a phone. Difficulties with communicating and relating to others often cause worry, depression or loneliness.	0.215 (0.144-0.307)
Complete hearing loss with ringing due to pneumococcal meningitis	Hearing loss, complete, with ringing	cannot hear at all in any situation, including even the loudest sounds, and cannot communicate verbally or use a phone, and has very annoying ringing in the ears for more than half of the day. Difficulties with communicating and relating to others often cause worry, depression or loneliness.	0.316 (0.212-0.435)
Moderate vision impairment due to pneumococcol meningitis	Distance vision, moderate impairment	has vision problems that make it difficult to recognize faces or objects across a room.	0.031 (0.019-0.049)
Severe vision impairment due to pneumococcol meningitis	Distance vision, severe impairment	has severe vision loss, which causes difficulty in daily activities, some emotional impact (for example worry), and some difficulty going outside the home without assistance.	0.184 (0.125-0.258)
Acute H influenzae type B meningitis	Infectious disease, acute episode, severe	has a high fever and pain, and feels very weak, which causes great difficulty with daily activities.	0.133 (0.088-0.19)
Mild behavioral problems due to H influenzae type B meningitis	Attention deficit hyperactivity disorder	is hyperactive and has difficulty concentrating, remembering things, and completing tasks.	0.045 (0.028-0.066)
Mild motor impairment due to long term due to H influenzae type B meningitis	Motor impairment, mild	has some difficulty in moving around but is able to walk without help.	0.01 (0.005-0.019)
Mild motor plus cognitive impairments due to H influenzae type B meningitis	Motor plus cognitive impairments, mild	has some difficulty in moving around but is able to walk without help. The person is slow in learning at school. As an adult, the person has some difficulty doing complex or unfamiliar tasks but otherwise functions independently.	0.031 (0.018-0.05)
Borderline intellectual disability due to H influenzae type B meningitis	Borderline intellectual functioning	is slow in learning at school. As an adult, the person has some difficulty doing complex or unfamiliar tasks but otherwise functions independently.	0.011 (0.005-0.02)
Monocular distance vision loss due to H influenzae type B meningitis	Distance vision, monocular	is blind in one eye and has difficulty judging distances	0.017 (0.009-0.029)
Mild intellectual disability due to H influenzae type B meningitis	Intellectual disability / mental retardation, mild	has low intelligence and is slow in learning at school. As an adult, the person can live independently, but often needs help to raise children and can only work at simple supervised jobs.	0.043 (0.026-0.064)
Moderate motor impairment due to H influenzae type B meningitis	Motor impairment, moderate	has some difficulty in moving around, and difficulty in lifting and holding objects, dressing and sitting upright, but is able to walk without help.	0.061 (0.04-0.089)
Severe motor impairment due to H influenzae type B meningitis	Motor impairment, severe	is unable to move around without help, and is not able to lift or hold objects, get dressed or sit upright.	0.402 (0.268-0.545)
Moderate motor plus cognitive impairments due to H influenzae type B meningitis	Motor plus cognitive impairments, moderate	has some difficulty in moving around, holding objects, dressing and sitting upright, but can walk without help. The person has low intelligence and is slow in learning to speak and to do simple tasks.	0.203 (0.134-0.29)
Severe motor plus cognitive impairments due to H influenzae type B meningitis	Motor plus cognitive impairments, severe	cannot move around without help, and cannot lift or hold objects, get dressed or sit upright. The person also has very low intelligence, speaks few words, and needs constant supervision and help with all daily activities.	0.542 (0.374-0.702)
Epilepsy due to H influenzae type B meningitis	Epilepsy	(combined DW)	--
Blindness due to H influenzae type B meningitis	Distance vision blindness	is completely blind, which causes great difficulty in some daily activities, worry and anxiety, and great difficulty going outside the home without assistance.	0.187 (0.124-0.26)
Mild hearing loss due to H influenzae type B meningitis	Hearing loss, mild	has great difficulty hearing and understanding another person talking in a noisy place (for example, on an urban street).	0.01 (0.004-0.019)
Mild hearing loss with ringing due to H influenzae type B meningitis	Hearing loss, mild, with ringing	has great difficulty hearing and understanding another person talking in a noisy place (for example, on an urban street), and sometimes has annoying ringing in the ears.	0.021 (0.012-0.036)
Moderate hearing loss due to H influenzae type B meningitis	Hearing loss, moderate	is unable to hear and understand another person talking in a noisy place (for example, on an urban street), and has difficulty hearing another person talking even in a quiet place or on the phone.	0.027 (0.015-0.042)
Moderate hearing loss with ringing due to H influenzae type B meningitis	Hearing loss, moderate, with ringing	is unable to hear and understand another person talking in a noisy place (for example, on an urban street), has difficulty hearing another person talking even in a quiet place or on the phone, and has annoying ringing in the ears for 5 minutes at a time, almost every day.	0.074 (0.049-0.107)
Moderately severe hearing loss due to H influenzae type B meningitis	Hearing loss, moderately severe	(custom DW: average of moderate and severe hearing loss impairment)	--
Moderately severe hearing loss with ringing due to H influenzae type B meningitis	Hearing loss, moderately severe, with ringing	(custom DW: average of moderate and severe hearing loss impairment)	--
Severe hearing loss due to H influenzae type B meningitis	Hearing loss, severe	is unable to hear and understand another person talking, even in a quiet place, and unable to take part in a phone conversation. Difficulties with communicating and relating to others cause emotional impact at times (for example worry or depression).	0.158 (0.105-0.227)
Severe hearing loss with ringing due to H influenzae type B meningitis	Hearing loss, severe, with ringing	is unable to hear and understand another person talking, even in a quiet place, is unable to take part in a phone conversation, and has annoying ringing in the ears for more than 5 minutes at a time, almost every day. Difficulties with communicating and relating to others cause emotional impact at times (for example worry or depression).	0.261 (0.175-0.36)
Profound hearing loss due to H influenzae type B meningitis	Hearing loss, profound	is unable to hear and understand another person talking, even in a quiet place, is unable to take part in a phone conversation, and has great difficulty hearing anything in any other situation. Difficulties with communicating and relating to others often cause worry, depression or loneliness.	0.204 (0.134-0.288)
Profound hearing loss with ringing due to H influenzae type B meningitis	Hearing loss, profound, with ringing	is unable to hear and understand another person talking, even in a quiet place, is unable to take part in a phone conversation, has great difficulty hearing anything in any other situation, and has annoying ringing in the ears for more than 5 minutes at a time, several times a day. Difficulties with communicating and relating to others often cause worry, depression, or loneliness.	0.277 (0.182-0.387)
Complete hearing loss due to H influenzae type B meningitis	Hearing loss, complete	cannot hear at all in any situation, including even the loudest sounds, and cannot communicate verbally or use a phone. Difficulties with communicating and relating to others often cause worry, depression or loneliness.	0.215 (0.144-0.307)
Complete hearing loss with ringing due to H influenzae type B meningitis	Hearing loss, complete, with ringing	cannot hear at all in any situation, including even the loudest sounds, and cannot communicate verbally or use a phone, and has very annoying ringing in the ears for more than half of the day. Difficulties with communicating and relating to others often cause worry, depression or loneliness.	0.316 (0.212-0.435)
Moderate vision impairment due to H influenza type B meningitis	Distance vision, moderate impairment	has vision problems that make it difficult to recognize faces or objects across a room.	0.031 (0.019-0.049)
Severe vision impairment due to H influenza type B meningitis	Distance vision, severe impairment	has severe vision loss, which causes difficulty in daily activities, some emotional impact (for example worry), and some difficulty going outside the home without assistance.	0.184 (0.125-0.258)
Acute meningococcal meningitis	Infectious disease, acute episode, severe	has a high fever and pain, and feels very weak, which causes great difficulty with daily activities.	0.133 (0.088-0.19)
Mild behavioral problems due to meningococcal meningitis	Attention deficit hyperactivity disorder	is hyperactive and has difficulty concentrating, remembering things, and completing tasks.	0.045 (0.028-0.066)
Mild motor impairment due to long term due to meningococcal meningitis	Motor impairment, mild	has some difficulty in moving around but is able to walk without help.	0.01 (0.005-0.019)
Mild motor plus cognitive impairments due to meningococcal meningitis	Motor plus cognitive impairments, mild	has some difficulty in moving around but is able to walk without help. The person is slow in learning at school. As an adult, the person has some difficulty doing complex or unfamiliar tasks but otherwise functions independently.	0.031 (0.018-0.05)
Borderline intellectual disability due to meningococcal meningitis	Borderline intellectual functioning	is slow in learning at school. As an adult, the person has some difficulty doing complex or unfamiliar tasks but otherwise functions independently.	0.011 (0.005-0.02)
Monocular distance vision loss due to meningococcal meningitis	Distance vision, monocular	is blind in one eye and has difficulty judging distances	0.017 (0.009-0.029)
Mild intellectual disability due to meningococcal meningitis	Intellectual disability / mental retardation, mild	has low intelligence and is slow in learning at school. As an adult, the person can live independently, but often needs help to raise children and can only work at simple supervised jobs.	0.043 (0.026-0.064)
Moderate motor impairment due to meningococcal meningitis	Motor impairment, moderate	has some difficulty in moving around, and difficulty in lifting and holding objects, dressing and sitting upright, but is able to walk without help.	0.061 (0.04-0.089)
Severe motor impairment due to meningococcal meningitis	Motor impairment, severe	is unable to move around without help, and is not able to lift or hold objects, get dressed or sit upright.	0.402 (0.268-0.545)
Moderate motor plus cognitive impairments due to meningococcal meningitis	Motor plus cognitive impairments, moderate	has some difficulty in moving around, holding objects, dressing and sitting upright, but can walk without help. The person has low intelligence and is slow in learning to speak and to do simple tasks.	0.203 (0.134-0.29)
Severe motor plus cognitive impairments due to meningococcal meningitis	Motor plus cognitive impairments, severe	cannot move around without help, and cannot lift or hold objects, get dressed or sit upright. The person also has very low intelligence, speaks few words, and needs constant supervision and help with all daily activities.	0.542 (0.374-0.702)

Appendix Table 6. GBD 2015 sequelae, health states, health state lay descriptions, and disability weights			
**Sequela**	**Healthstate name**	**Healthstate lay description**	**Disability weight**
Epilepsy due to meningococcal meningitis	Epilepsy	(combined DW)	--
Blindness due to meningococcal meningitis	Distance vision blindness	is completely blind, which causes great difficulty in some daily activities, worry and anxiety, and great difficulty going outside the home without assistance.	0.187 (0.124-0.26)
Mild hearing loss due to meningococcal meningitis	Hearing loss, mild	has great difficulty hearing and understanding another person talking in a noisy place (for example, on an urban street).	0.01 (0.004-0.019)
Mild hearing loss with ringing due to meningococcal meningitis	Hearing loss, mild, with ringing	has great difficulty hearing and understanding another person talking in a noisy place (for example, on an urban street), and sometimes has annoying ringing in the ears.	0.021 (0.012-0.036)
Moderate hearing loss due to meningococcal meningitis	Hearing loss, moderate	is unable to hear and understand another person talking in a noisy place (for example, on an urban street), and has difficulty hearing another person talking even in a quiet place or on the phone.	0.027 (0.015-0.042)
Moderate hearing loss with ringing due to meningococcal meningitis	Hearing loss, moderate, with ringing	is unable to hear and understand another person talking in a noisy place (for example, on an urban street), has difficulty hearing another person talking even in a quiet place or on the phone, and has annoying ringing in the ears for 5 minutes at a time, almost every day.	0.074 (0.049-0.107)
Moderately severe hearing loss due to meningococcal meningitis	Hearing loss, moderately severe	(custom DW: average of moderate and severe hearing loss impairment)	--
Moderately severe hearing loss with ringing due to meningococcal meningitis	Hearing loss, moderately severe, with ringing	(custom DW: average of moderate and severe hearing loss impairment)	--
Severe hearing loss due to meningococcal meningitis	Hearing loss, severe	is unable to hear and understand another person talking, even in a quiet place, and unable to take part in a phone conversation. Difficulties with communicating and relating to others cause emotional impact at times (for example worry or depression).	0.158 (0.105-0.227)
Severe hearing loss with ringing due to meningococcal meningitis	Hearing loss, severe, with ringing	is unable to hear and understand another person talking, even in a quiet place, is unable to take part in a phone conversation, and has annoying ringing in the ears for more than 5 minutes at a time, almost every day. Difficulties with communicating and relating to others cause emotional impact at times (for example worry or depression).	0.261 (0.175-0.36)
Profound hearing loss due to meningococcal meningitis	Hearing loss, profound	is unable to hear and understand another person talking, even in a quiet place, is unable to take part in a phone conversation, and has great difficulty hearing anything in any other situation. Difficulties with communicating and relating to others often cause worry, depression or loneliness.	0.204 (0.134-0.288)
Profound hearing loss with ringing due to meningococcal meningitis	Hearing loss, profound, with ringing	is unable to hear and understand another person talking, even in a quiet place, is unable to take part in a phone conversation, has great difficulty hearing anything in any other situation, and has annoying ringing in the ears for more than 5 minutes at a time, several times a day. Difficulties with communicating and relating to others often cause worry, depression, or loneliness.	0.277 (0.182-0.387)
Complete hearing loss due to meningococcal meningitis	Hearing loss, complete	cannot hear at all in any situation, including even the loudest sounds, and cannot communicate verbally or use a phone. Difficulties with communicating and relating to others often cause worry, depression or loneliness.	0.215 (0.144-0.307)
Complete hearing loss with ringing due to meningococcal meningitis	Hearing loss, complete, with ringing	cannot hear at all in any situation, including even the loudest sounds, and cannot communicate verbally or use a phone, and has very annoying ringing in the ears for more than half of the day. Difficulties with communicating and relating to others often cause worry, depression or loneliness.	0.316 (0.212-0.435)
Moderate vision impairment due to meningococcal meningitis	Distance vision, moderate impairment	has vision problems that make it difficult to recognize faces or objects across a room.	0.031 (0.019-0.049)
Severe vision impairment due to meningococcal meningitis	Distance vision, severe impairment	has severe vision loss, which causes difficulty in daily activities, some emotional impact (for example worry), and some difficulty going outside the home without assistance.	0.184 (0.125-0.258)
Acute viral meningitis	Infectious disease, acute episode, severe	has a high fever and pain, and feels very weak, which causes great difficulty with daily activities.	0.133 (0.088-0.19)
Other acute bacterial meningitis	Infectious disease, acute episode, severe	has a high fever and pain, and feels very weak, which causes great difficulty with daily activities.	0.133 (0.088-0.19)
Mild behavioral problems due to other bacterial meningitis	Attention deficit hyperactivity disorder	is hyperactive and has difficulty concentrating, remembering things, and completing tasks.	0.045 (0.028-0.066)
Mild motor impairment due to long term due to other bacterial meningitis	Motor impairment, mild	has some difficulty in moving around but is able to walk without help.	0.01 (0.005-0.019)
Mild motor plus cognitive impairments due to other bacterial meningitis	Motor plus cognitive impairments, mild	has some difficulty in moving around but is able to walk without help. The person is slow in learning at school. As an adult, the person has some difficulty doing complex or unfamiliar tasks but otherwise functions independently.	0.031 (0.018-0.05)
Borderline intellectual disability due to other bacterial meningitis	Borderline intellectual functioning	is slow in learning at school. As an adult, the person has some difficulty doing complex or unfamiliar tasks but otherwise functions independently.	0.011 (0.005-0.02)
Monocular distance vision loss due to other bacterial meningitis	Distance vision, monocular	is blind in one eye and has difficulty judging distances	0.017 (0.009-0.029)
Mild intellectual disability due to other bacterial meningitis	Intellectual disability / mental retardation, mild	has low intelligence and is slow in learning at school. As an adult, the person can live independently, but often needs help to raise children and can only work at simple supervised jobs.	0.043 (0.026-0.064)
Moderate motor impairment due to other bacterial meningitis	Motor impairment, moderate	has some difficulty in moving around, and difficulty in lifting and holding objects, dressing and sitting upright, but is able to walk without help.	0.061 (0.04-0.089)
Severe motor impairment due to other bacterial meningitis	Motor impairment, severe	is unable to move around without help, and is not able to lift or hold objects, get dressed or sit upright.	0.402 (0.268-0.545)
Moderate motor plus cognitive impairments due to other bacterial meningitis	Motor plus cognitive impairments, moderate	has some difficulty in moving around, holding objects, dressing and sitting upright, but can walk without help. The person has low intelligence and is slow in learning to speak and to do simple tasks.	0.203 (0.134-0.29)
Severe motor plus cognitive impairments due to other bacterial meningitis	Motor plus cognitive impairments, severe	cannot move around without help, and cannot lift or hold objects, get dressed or sit upright. The person also has very low intelligence, speaks few words, and needs constant supervision and help with all daily activities.	0.542 (0.374-0.702)
Epilepsy due to other meningitis	Epilepsy	(combined DW)	--
Blindness due to other bacterial meningitis	Distance vision blindness	is completely blind, which causes great difficulty in some daily activities, worry and anxiety, and great difficulty going outside the home without assistance.	0.187 (0.124-0.26)
Mild hearing loss due to other bacterial meningitis	Hearing loss, mild	has great difficulty hearing and understanding another person talking in a noisy place (for example, on an urban street).	0.01 (0.004-0.019)
Mild hearing loss due with ringing to other bacterial meningitis	Hearing loss, mild, with ringing	has great difficulty hearing and understanding another person talking in a noisy place (for example, on an urban street), and sometimes has annoying ringing in the ears.	0.021 (0.012-0.036)
Moderate hearing loss due to other bacterial meningitis	Hearing loss, moderate	is unable to hear and understand another person talking in a noisy place (for example, on an urban street), and has difficulty hearing another person talking even in a quiet place or on the phone.	0.027 (0.015-0.042)
Moderate hearing loss with ringing due to other bacterial meningitis	Hearing loss, moderate, with ringing	is unable to hear and understand another person talking in a noisy place (for example, on an urban street), has difficulty hearing another person talking even in a quiet place or on the phone, and has annoying ringing in the ears for 5 minutes at a time, almost every day.	0.074 (0.049-0.107)
Moderately severe hearing loss due to other bacterial meningitis	Hearing loss, moderately severe	(custom DW: average of moderate and severe hearing loss impairment)	--
Moderately severe hearing loss with ringing due to other bacterial meningitis	Hearing loss, moderately severe, with ringing	(custom DW: average of moderate and severe hearing loss impairment)	--
Severe hearing loss due to other bacterial meningitis	Hearing loss, severe	is unable to hear and understand another person talking, even in a quiet place, and unable to take part in a phone conversation. Difficulties with communicating and relating to others cause emotional impact at times (for example worry or depression).	0.158 (0.105-0.227)
Severe hearing loss with ringing due to other bacterial meningitis	Hearing loss, severe, with ringing	is unable to hear and understand another person talking, even in a quiet place, is unable to take part in a phone conversation, and has annoying ringing in the ears for more than 5 minutes at a time, almost every day. Difficulties with communicating and relating to others cause emotional impact at times (for example worry or depression).	0.261 (0.175-0.36)
Profound hearing loss due to other bacterial meningitis	Hearing loss, profound	is unable to hear and understand another person talking, even in a quiet place, is unable to take part in a phone conversation, and has great difficulty hearing anything in any other situation. Difficulties with communicating and relating to others often cause worry, depression or loneliness.	0.204 (0.134-0.288)
Profound hearing loss with ringing due to other bacterial meningitis	Hearing loss, profound, with ringing	is unable to hear and understand another person talking, even in a quiet place, is unable to take part in a phone conversation, has great difficulty hearing anything in any other situation, and has annoying ringing in the ears for more than 5 minutes at a time, several times a day. Difficulties with communicating and relating to others often cause worry, depression, or loneliness.	0.277 (0.182-0.387)
Complete hearing loss due to other bacterial meningitis	Hearing loss, complete	cannot hear at all in any situation, including even the loudest sounds, and cannot communicate verbally or use a phone. Difficulties with communicating and relating to others often cause worry, depression or loneliness.	0.215 (0.144-0.307)
Complete hearing loss with ringing due to other bacterial meningitis	Hearing loss, complete, with ringing	cannot hear at all in any situation, including even the loudest sounds, and cannot communicate verbally or use a phone, and has very annoying ringing in the ears for more than half of the day. Difficulties with communicating and relating to others often cause worry, depression or loneliness.	0.316 (0.212-0.435)
Moderate vision impairment due to other bacterial meningitis	Distance vision, moderate impairment	has vision problems that make it difficult to recognize faces or objects across a room.	0.031 (0.019-0.049)
Severe vision impairment due to other bacterial meningitis	Distance vision, severe impairment	has severe vision loss, which causes difficulty in daily activities, some emotional impact (for example worry), and some difficulty going outside the home without assistance.	0.184 (0.125-0.258)
Acute encephalitis	Infectious disease, acute episode, severe	has a high fever and pain, and feels very weak, which causes great difficulty with daily activities.	0.133 (0.088-0.19)
Mild behavioral problems due to encephalitis	Attention deficit hyperactivity disorder	is hyperactive and has difficulty concentrating, remembering things, and completing tasks.	0.045 (0.028-0.066)
Mild motor impairment due to long term due to encephalitis	Motor impairment, mild	has some difficulty in moving around but is able to walk without help.	0.01 (0.005-0.019)
Mild motor plus cognitive impairments due to encephalitis	Motor plus cognitive impairments, mild	has some difficulty in moving around but is able to walk without help. The person is slow in learning at school. As an adult, the person has some difficulty doing complex or unfamiliar tasks but otherwise functions independently.	0.031 (0.018-0.05)
Borderline intellectual disability due to encephalitis	Borderline intellectual functioning	is slow in learning at school. As an adult, the person has some difficulty doing complex or unfamiliar tasks but otherwise functions independently.	0.011 (0.005-0.02)
Monocular distance vision loss due to encephalitis	Distance vision, monocular	is blind in one eye and has difficulty judging distances	0.017 (0.009-0.029)

Appendix Table 6. GBD 2015 sequelae, health states, health state lay descriptions, and disability weights			
**Sequela**	**Healthstate name**	**Healthstate lay description**	**Disability weight**
Mild intellectual disability due to encephalitis	Intellectual disability / mental retardation, mild	has low intelligence and is slow in learning at school. As an adult, the person can live independently, but often needs help to raise children and can only work at simple supervised jobs.	0.043 (0.026-0.064)
Moderate motor impairment due to encephalitis	Motor impairment, moderate	has some difficulty in moving around, and difficulty in lifting and holding objects, dressing and sitting upright, but is able to walk without help.	0.061 (0.04-0.089)
Severe motor impairment due to encephalitis	Motor impairment, severe	is unable to move around without help, and is not able to lift or hold objects, get dressed or sit upright.	0.402 (0.268-0.545)
Moderate motor plus cognitive impairments due to encephalitis	Motor plus cognitive impairments, moderate	has some difficulty in moving around, holding objects, dressing and sitting upright, but can walk without help. The person has low intelligence and is slow in learning to speak and to do simple tasks.	0.203 (0.134-0.29)
Severe motor plus cognitive impairments due to encephalitis	Motor plus cognitive impairments, severe	cannot move around without help, and cannot lift or hold objects, get dressed or sit upright. The person also has very low intelligence, speaks few words, and needs constant supervision and help with all daily activities.	0.542 (0.374-0.702)
Epilepsy due to encephalitis	Epilepsy	(combined DW)	--
Blindness due to encephalitis	Distance vision blindness	is completely blind, which causes great difficulty in some daily activities, worry and anxiety, and great difficulty going outside the home without assistance.	0.187 (0.124-0.26)
Moderate vision impairment due to encephalitis	Distance vision, moderate impairment	has vision problems that make it difficult to recognize faces or objects across a room.	0.031 (0.019-0.049)
Severe vision impairment due to encephalitis	Distance vision, severe impairment	has severe vision loss, which causes difficulty in daily activities, some emotional impact (for example worry), and some difficulty going outside the home without assistance.	0.184 (0.125-0.258)
Moderate diphtheria	Infectious disease, acute episode, moderate	has a fever and aches, and feels weak, which causes some difficulty with daily activities.	0.051 (0.032-0.074)
Severe diphtheria	Infectious disease, acute episode, severe	has a high fever and pain, and feels very weak, which causes great difficulty with daily activities.	0.133 (0.088-0.19)
Whooping cough	Infectious disease, acute episode, moderate	has a fever and aches, and feels weak, which causes some difficulty with daily activities.	0.051 (0.032-0.074)
Severe tetanus	Infectious disease, acute episode, severe	has a high fever and pain, and feels very weak, which causes great difficulty with daily activities.	0.133 (0.088-0.19)
Mild motor impairment due to neonatal tetanus	Motor impairment, mild	has some difficulty in moving around but is able to walk without help.	0.01 (0.005-0.019)
Mild motor plus cognitive impairments due to neonatal tetanus	Motor plus cognitive impairments, mild	has some difficulty in moving around but is able to walk without help. The person is slow in learning at school. As an adult, the person has some difficulty doing complex or unfamiliar tasks but otherwise functions independently.	0.031 (0.018-0.05)
Moderate motor impairment due to neonatal tetanus	Motor impairment, moderate	has some difficulty in moving around, and difficulty in lifting and holding objects, dressing and sitting upright, but is able to walk without help.	0.061 (0.04-0.089)
Moderate motor impairment with blindness due to neonatal tetanus	Moderate motor impairment with blindness	(combined DW)	--
Moderate motor impairment with epilepsy due to neonatal tetanus	Moderate motor impairment with epilepsy	(combined DW)	--
Moderate motor impairment with blindness and epilepsy due to neonatal tetanus	Moderate motor impairment with blindness and epilepsy	(combined DW)	--
Moderate motor plus cognitive impairment with blindness due to neonatal tetanus	Moderate motor plus cognitive impairment with blindness	(combined DW)	--
Moderate motor plus cognitive impairment with epilepsy due to neonatal tetanus	Moderate motor plus cognitive impairment with epilepsy	(combined DW)	--
Moderate motor plus cognitive impairment with blindness and epilepsy due to neonatal tetanus	Moderate motor plus cognitive impairment with blindness and epilepsy	(combined DW)	--
Severe motor impairment due to neonatal tetanus	Motor impairment, severe	is unable to move around without help, and is not able to lift or hold objects, get dressed or sit upright.	0.402 (0.268-0.545)
Severe motor impairment with blindness due to neonatal tetanus	Severe motor impairment with blindness	(combined DW)	--
Severe motor impairment with epilepsy due to neonatal tetanus	Severe motor impairment with epilepsy	(combined DW)	--
Severe motor impairment with blindness and epilepsy due to neonatal tetanus	Severe motor impairment with blindness and epilepsy	(combined DW)	--
Severe motor plus cognitive impairment with blindness due to neonatal tetanus	Severe motor plus cognitive impairment with blindness	(combined DW)	--
Severe motor plus cognitive impairment with epilepsy due to neonatal tetanus	Severe motor plus cognitive impairment with epilepsy	(combined DW)	--
Severe motor plus cognitive impairment with blindness and epilepsy due to neonatal tetanus	Severe motor plus cognitive impairment with blindness and epilepsy	(combined DW)	--
Moderate measles	Infectious disease, acute episode, moderate	has a fever and aches, and feels weak, which causes some difficulty with daily activities.	0.051 (0.032-0.074)
Severe measles	Infectious disease, acute episode, severe	has a high fever and pain, and feels very weak, which causes great difficulty with daily activities.	0.133 (0.088-0.19)
Chickenpox	Infectious disease, acute episode, mild	has a low fever and mild discomfort , but no difficulty with daily activities.	0.006 (0.002-0.012)
Herpes zoster	Herpes zoster	has a blistering skin rash that causes pain, with some burning and itching.	0.058 (0.035-0.09)
Asymptomatic malaria parasitemia (PfPR)	--	--	--
Mild malaria	Infectious disease, acute episode, mild	has a low fever and mild discomfort , but no difficulty with daily activities.	0.006 (0.002-0.012)
Moderate malaria	Infectious disease, acute episode, moderate	has a fever and aches, and feels weak, which causes some difficulty with daily activities.	0.051 (0.032-0.074)
Severe malaria	Infectious disease, acute episode, severe	has a high fever and pain, and feels very weak, which causes great difficulty with daily activities.	0.133 (0.088-0.19)
Moderate motor impairment due to malaria	Motor impairment, moderate	has some difficulty in moving around, and difficulty in lifting and holding objects, dressing and sitting upright, but is able to walk without help.	0.061 (0.04-0.089)
Moderate motor impairment with blindness due to malaria	Moderate motor impairment with blindness	(combined DW)	--
Moderate motor impairment with epilepsy due to malaria	Moderate motor impairment with epilepsy	(combined DW)	--
Moderate motor impairment with blindness and epilepsy due to malaria	Moderate motor impairment with blindness and epilepsy	(combined DW)	--
Moderate motor plus cognitive impairment with blindness due to malaria	Moderate motor plus cognitive impairment with blindness	(combined DW)	--
Moderate motor plus cognitive impairment with epilepsy due to malaria	Moderate motor plus cognitive impairment with epilepsy	(combined DW)	--
Moderate motor plus cognitive impairment with blindness and epilepsy due to malaria	Moderate motor plus cognitive impairment with blindness and epilepsy	(combined DW)	--
Severe motor impairment due to malaria	Motor impairment, severe	is unable to move around without help, and is not able to lift or hold objects, get dressed or sit upright.	0.402 (0.268-0.545)
Severe motor impairment with blindness due to malaria	Severe motor impairment with blindness	(combined DW)	--
Severe motor impairment with epilepsy due to malaria	Severe motor impairment with epilepsy	(combined DW)	--
Severe motor impairment with blindness and epilepsy due to malaria	Severe motor impairment with blindness and epilepsy	(combined DW)	--
Severe motor plus cognitive impairment with blindness due to malaria	Severe motor plus cognitive impairment with blindness	(combined DW)	--
Severe motor plus cognitive impairment with epilepsy due to malaria	Severe motor plus cognitive impairment with epilepsy	(combined DW)	--

Appendix Table 6. GBD 2015 sequelae, health states, health state lay descriptions, and disability weights

Sequela	Healthstate name	Healthstate lay description	Disability weight
Severe motor plus cognitive impairment with blindness and epilepsy due to malaria	Severe motor plus cognitive impairment with blindness and epilepsy	(combined DW)	--
Mild anemia due to malaria parasitemia (PfPR)	Anemia, mild	feels slightly tired and weak at times, but this does not interfere with normal daily activities.	0.004 (0.001-0.008)
Moderate anemia due to malaria parasitemia (PfPR)	Anemia, moderate	feels moderate fatigue, weakness, and shortness of breath after exercise, making daily activities more difficult.	0.052 (0.034-0.076)
Severe anemia due to malaria parasitemia (PfPR)	Anemia, severe	feels very weak, tired and short of breath, and has problems with activities that require physical effort or deep concentration.	0.149 (0.101-0.209)
Acute Chagas disease	Infectious disease, acute episode, moderate	has a fever and aches, and feels weak, which causes some difficulty with daily activities.	0.051 (0.032-0.074)
Asymptomatic Chagas disease	--	--	--
Mild chronic digestive disease due to Chagas disease	Abdominopelvic problem, mild	has some pain in the belly that causes nausea but does not interfere with daily activities.	0.011 (0.005-0.021)
Moderate chronic digestive disease due to Chagas disease	Abdominopelvic problem, moderate	has pain in the belly and feels nauseous. The person has difficulties with daily activities.	0.114 (0.078-0.159)
Atrial fibrillation and flutter due to Chagas disease	Cardiac conduction disorders and cardiac dysrhythmias	has periods of rapid and irregular heartbeats and occasional fainting.	0.224 (0.151-0.312)
Mild heart failure due to Chagas disease	Heart failure, mild	is short of breath and easily tires with moderate physical activity, such as walking uphill or more than a quarter-mile on level ground. The person feels comfortable at rest or during activities requiring less effort.	0.041 (0.026-0.062)
Moderate heart failure due to Chagas disease	Heart failure, moderate	is short of breath and easily tires with minimal physical activity, such as walking only a short distance. The person feels comfortable at rest but avoids moderate activity.	0.072 (0.047-0.103)
Severe heart failure due to Chagas disease	Heart failure, severe	is short of breath and feels tired when at rest. The person avoids any physical activity, for fear of worsening the breathing problems.	0.179 (0.122-0.251)
Moderate visceral leishmaniasis	Infectious disease, acute episode, moderate	has a fever and aches, and feels weak, which causes some difficulty with daily activities.	0.051 (0.032-0.074)
Severe visceral leishmaniasis	Infectious disease, acute episode, severe	has a high fever and pain, and feels very weak, which causes great difficulty with daily activities.	0.133 (0.088-0.19)
Cutaneous and mucocutaneous leishmaniasis	Disfigurement, level 1	has a slight, visible physical deformity that others notice, which causes some worry and discomfort.	0.011 (0.005-0.021)
Disfigurement due to African trypanosomiasis	Disfigurement, level 2	has a visible physical deformity that causes others to stare and comment. As a result, the person is worried and has trouble sleeping and concentrating.	0.067 (0.044-0.096)
Severe motor plus cognitive impairments due to African trypanosomiasis	Motor plus cognitive impairments, severe	cannot move around without help, and cannot lift or hold objects, get dressed or sit upright. The person also has very low intelligence, speaks few words, and needs constant supervision and help with all daily activities.	0.542 (0.374-0.702)
Mild schistosomiasis	Infectious disease, acute episode, mild	has a low fever and mild discomfort , but no difficulty with daily activities.	0.006 (0.002-0.012)
Mild diarrhea due to schistosomiasis	Diarrhea, mild	has diarrhea three or more times a day with occasional discomfort in the belly.	0.074 (0.049-0.104)
Hematemesis due to schistosomiasis	Gastric bleeding	vomits blood and feels nauseous.	0.325 (0.209-0.462)
Hepatomegaly due to schistosomiasis	Abdominopelvic problem, mild	has some pain in the belly that causes nausea but does not interfere with daily activities.	0.011 (0.005-0.021)
Ascites due to schistosomiasis	Abdominopelvic problem, moderate	has pain in the belly and feels nauseous. The person has difficulties with daily activities.	0.114 (0.078-0.159)
Dysuria due to schistosomiasis	Abdominopelvic problem, mild	has some pain in the belly that causes nausea but does not interfere with daily activities.	0.011 (0.005-0.021)
Bladder pathology due to schistosomiasis	Abdominopelvic problem, mild	has some pain in the belly that causes nausea but does not interfere with daily activities.	0.011 (0.005-0.021)
Hydronephrosis due to schistosomiasis	Abdominopelvic problem, mild	has some pain in the belly that causes nausea but does not interfere with daily activities.	0.011 (0.005-0.021)
Mild anemia due to schistosomiasis	Anemia, mild	feels slightly tired and weak at times, but this does not interfere with normal daily activities.	0.004 (0.001-0.008)
Moderate anemia due to schistosomiasis	Anemia, moderate	feels moderate fatigue, weakness, and shortness of breath after exercise, making daily activities more difficult.	0.052 (0.034-0.076)
Severe anemia due to schistosomiasis	Anemia, severe	feels very weak, tired and short of breath, and has problems with activities that require physical effort or deep concentration.	0.149 (0.101-0.209)
Neurocysticercosis with epilepsy	Epilepsy	(combined DW)	--
Abdominal problems due to cystic echinococcosis	Abdominopelvic problem, moderate	has pain in the belly and feels nauseous. The person has difficulties with daily activities.	0.114 (0.078-0.159)
Chronic respiratory disease due to cystic echinococcosis	COPD and other chronic respiratory problems, mild	has cough and shortness of breath after heavy physical activity, but is able to walk long distances and climb stairs.	0.019 (0.011-0.033)
Epilepsy due to echinococcosis	Epilepsy	(combined DW)	--
Prevalence of detectable microfiliaria due to lymphatic filariasis	--	--	--
Lymphedema due to lymphatic filariasis	Lymphatic filariasis, symptomatic	has swollen legs with hard and thick skin, which causes difficulty in moving around.	0.109 (0.073-0.154)
Hydrocele due to lymphatic filariasis	Epididymo-orchitis	has swelling and tenderness in the testicles and pain during urination.	0.128 (0.086-0.18)
Asymptomatic onchocerciasis	--	--	--
Mild skin disease due to onchocerciasis	Disfigurement, level 1 with itch/pain	has a slight, visible physical deformity that is sometimes sore or itchy. Others notice the deformity, which causes some worry and discomfort.	0.027 (0.015-0.042)
Mild skin disease without itch due to onchocerciasis	Disfigurement, level 1	has a slight, visible physical deformity that others notice, which causes some worry and discomfort.	0.011 (0.005-0.021)
Moderate skin disease due to onchocerciasis	Disfigurement, level 2, with itch/pain	has a visible physical deformity that is sore and itchy. Other people stare and comment, which causes the person to worry. The person has trouble sleeping and concentrating.	0.188 (0.125-0.267)
Severe skin disease due to onchocerciasis	Disfigurement, level 2, with itch/pain	has a visible physical deformity that is sore and itchy. Other people stare and comment, which causes the person to worry. The person has trouble sleeping and concentrating.	0.188 (0.125-0.267)
Severe skin disease without itch due to onchocerciasis	Disfigurement, level 3	has an obvious physical deformity that makes others uncomfortable, which causes the person to avoid social contact, feel worried, sleep poorly, and think about suicide.	0.405 (0.275-0.546)
Moderate vision impairment due to onchocerciasis	Distance vision, moderate impairment	has vision problems that make it difficult to recognize faces or objects across a room.	0.031 (0.019-0.049)
Severe vision impairment due to onchocerciasis	Distance vision, severe impairment	has severe vision loss, which causes difficulty in daily activities, some emotional impact (for example worry), and some difficulty going outside the home without assistance.	0.184 (0.125-0.258)
Blindness due to onchocerciasis	Distance vision blindness	is completely blind, which causes great difficulty in some daily activities, worry and anxiety, and great difficulty going outside the home without assistance.	0.187 (0.124-0.26)
Moderate vision impairment due to trachoma	Distance vision, moderate impairment	has vision problems that make it difficult to recognize faces or objects across a room.	0.031 (0.019-0.049)
Severe vision impairment due to trachoma	Distance vision, severe impairment	has severe vision loss, which causes difficulty in daily activities, some emotional impact (for example worry), and some difficulty going outside the home without assistance.	0.184 (0.125-0.258)
Blindness due to trachoma	Distance vision blindness	is completely blind, which causes great difficulty in some daily activities, worry and anxiety, and great difficulty going outside the home without assistance.	0.187 (0.124-0.26)
Moderate dengue	Infectious disease, acute episode, moderate	has a fever and aches, and feels weak, which causes some difficulty with daily activities.	0.051 (0.032-0.074)
Severe dengue	Infectious disease, acute episode, severe	has a high fever and pain, and feels very weak, which causes great difficulty with daily activities.	0.133 (0.088-0.19)
Post-dengue chronic fatigue syndrome	Infectious disease, post-acute consequences (fatigue, emotional lability, insomnia)	is always tired and easily upset. The person feels pain all over the body and is depressed.	0.219 (0.148-0.308)

Appendix Table 6. GBD 2015 sequelae, health states, health state lay descriptions, and disability weights			
**Sequela**	**Healthstate name**	**Healthstate lay description**	**Disability weight**
Asymptomatic yellow fever	--	--	--
Moderate yellow fever	Infectious disease, acute episode, moderate	has a fever and aches, and feels weak, which causes some difficulty with daily activities.	0.051 (0.032-0.074)
Severe yellow fever	Infectious disease, acute episode, severe	has a high fever and pain, and feels very weak, which causes great difficulty with daily activities.	0.133 (0.088-0.19)
Rabies	Infectious disease, acute episode, severe	has a high fever and pain, and feels very weak, which causes great difficulty with daily activities.	0.133 (0.088-0.19)
Heavy infestation of ascariasis	Intestinal nematode infections, symptomatic	has cramping pain and a bloated feeling in the belly.	0.027 (0.015-0.043)
Mild abdominopelvic problems due to ascariasis	Abdominopelvic problem, mild	has some pain in the belly that causes nausea but does not interfere with daily activities.	0.011 (0.005-0.021)
Severe wasting due to ascariasis	Severe wasting	is extremely skinny and has no energy.	0.128 (0.082-0.183)
Asymptomatic ascariasis	--	--	--
Heavy infestation of trichuriasis	Intestinal nematode infections, symptomatic	has cramping pain and a bloated feeling in the belly.	0.027 (0.015-0.043)
Mild abdominopelvic problems due to trichuriasis	Abdominopelvic problem, mild	has some pain in the belly that causes nausea but does not interfere with daily activities.	0.011 (0.005-0.021)
Severe wasting due to trichuriasis	Severe wasting	is extremely skinny and has no energy.	0.128 (0.082-0.183)
Asymptomatic trichuriasis	--	--	--
Heavy infestation of hookworm	Intestinal nematode infections, symptomatic	has cramping pain and a bloated feeling in the belly.	0.027 (0.015-0.043)
Mild abdominopelvic problems due to hookworm disease	Abdominopelvic problem, mild	has some pain in the belly that causes nausea but does not interfere with daily activities.	0.011 (0.005-0.021)
Severe wasting due to hookworm disease	Severe wasting	is extremely skinny and has no energy.	0.128 (0.082-0.183)
Mild anemia due to hookworm disease	Anemia, mild	feels slightly tired and weak at times, but this does not interfere with normal daily activities.	0.004 (0.001-0.008)
Moderate anemia due to hookworm disease	Anemia, moderate	feels moderate fatigue, weakness, and shortness of breath after exercise, making daily activities more difficult.	0.052 (0.034-0.076)
Severe anemia due to hookworm disease	Anemia, severe	feels very weak, tired and short of breath, and has problems with activities that require physical effort or deep concentration.	0.149 (0.101-0.209)
Asymptomatic hookworm disease	--	--	--
Asymptomatic clonorchiasis	--	--	--
Asymptomatic fascioliasis	--	--	--
Asymptomatic intestinal fluke infection	--	--	--
Asymptomatic opisthorchiasis	--	--	--
Asymptomatic paragonimiasis	--	--	--
Heavy opisthorchiasis due to food-borne trematodiases	Abdominopelvic problem, moderate	has pain in the belly and feels nauseous. The person has difficulties with daily activities.	0.114 (0.078-0.159)
Heavy clonorchiasis due to food-borne trematodiases	Abdominopelvic problem, moderate	has pain in the belly and feels nauseous. The person has difficulties with daily activities.	0.114 (0.078-0.159)
Heavy intestinal fluke infection due to food-borne trematodiases	Abdominopelvic problem, moderate	has pain in the belly and feels nauseous. The person has difficulties with daily activities.	0.114 (0.078-0.159)
Heavy fascioliasis due to food-borne trematodiases	Abdominopelvic problem, moderate	has pain in the belly and feels nauseous. The person has difficulties with daily activities.	0.114 (0.078-0.159)
Heavy paragonimiasis due to food-borne trematodiases	Tuberculosis, not HIV infected	has a persistent cough and fever, is short of breath, feels weak, and has lost a lot of weight.	0.333 (0.224-0.454)
Cerebral paragonimiasis	Epilepsy	(combined DW)	--
Disfigurement level 1 due to leprosy	Disfigurement, level 1	has a slight, visible physical deformity that others notice, which causes some worry and discomfort.	0.011 (0.005-0.021)
Disfigurement level 2 due to leprosy	Disfigurement, level 2	has a visible physical deformity that causes others to stare and comment. As a result, the person is worried and has trouble sleeping and concentrating.	0.067 (0.044-0.096)
Ebola cases	Infectious disease, acute episode, severe	has a high fever and pain, and feels very weak, which causes great difficulty with daily activities.	0.133 (0.088-0.19)
Post-Ebola chronic fatigue syndrome	Infectious disease, post-acute consequences (fatigue, emotional lability, insomnia)	is always tired and easily upset. The person feels pain all over the body and is depressed.	0.219 (0.148-0.308)
Acute infection due to other neglected tropical diseases	--	--	--
Mild anemia due to other neglected tropical diseases	Anemia, mild	feels slightly tired and weak at times, but this does not interfere with normal daily activities.	0.004 (0.001-0.008)
Moderate anemia due to other neglected tropical diseases	Anemia, moderate	feels moderate fatigue, weakness, and shortness of breath after exercise, making daily activities more difficult.	0.052 (0.034-0.076)
Severe anemia due to other neglected tropical diseases	Anemia, severe	feels very weak, tired and short of breath, and has problems with activities that require physical effort or deep concentration.	0.149 (0.101-0.209)
Maternal hemorrhage (< 1L blood lost)	Abdominopelvic problem, moderate	has pain in the belly and feels nauseous. The person has difficulties with daily activities.	0.114 (0.078-0.159)
Maternal hemorrhage (> 1L blood lost)	Abdominopelvic problem, severe	has severe pain in the belly and feels nauseous. The person is anxious and unable to carry out daily activities.	0.324 (0.22-0.442)
Mild anemia due to maternal hemorrhage	Anemia, mild	feels slightly tired and weak at times, but this does not interfere with normal daily activities.	0.004 (0.001-0.008)
Moderate anemia due to maternal hemorrhage	Anemia, moderate	feels moderate fatigue, weakness, and shortness of breath after exercise, making daily activities more difficult.	0.052 (0.034-0.076)
Severe anemia due to maternal hemorrhage	Anemia, severe	feels very weak, tired and short of breath, and has problems with activities that require physical effort or deep concentration.	0.149 (0.101-0.209)
Puerperal sepsis	Infectious disease, acute episode, severe	has a high fever and pain, and feels very weak, which causes great difficulty with daily activities.	0.133 (0.088-0.19)
Other maternal infections	Infectious disease, acute episode, moderate	has a fever and aches, and feels weak, which causes some difficulty with daily activities.	0.051 (0.032-0.074)
Infertility due to puerperal sepsis	Infertility, secondary	has at least one child, and wants to have more children. The person has a fertile partner, but the couple cannot conceive.	0.005 (0.002-0.011)
Other hypertensive disorders of pregnancy	Generic uncomplicated disease: worry and daily medication	has a chronic disease that requires medication every day and causes some worry but minimal interference with daily activities.	0.049 (0.031-0.072)
Severe pre-eclampsia	Moderate abdominal pain, tension-type headaches, mild motor plus cognitive impairment	(combined DW)	--
Eclampsia	Moderate abdominal pain and severe epilepsy	(combined DW)	--
Long term sequelae of severe pre-eclampsia	Tension-type headaches, mild motor plus cognitive impairment	(combined DW)	--

Appendix Table 6. GBD 2015 sequelae, health states, health state lay descriptions, and disability weights

Sequela	Healthstate name	Healthstate lay description	Disability weight
Long term sequelae of eclampsia	Tension-type headaches, mild motor plus cognitive impairment	(combined DW)	--
Obstructed labor, acute event	Abdominopelvic problem, severe	has severe pain in the belly and feels nauseous. The person is anxious and unable to carry out daily activities.	0.324 (0.22-0.442)
Rectovaginal fistula	Rectovaginal fistula	has an abnormal opening between her vagina and rectum causing flatulence and feces to escape through the vagina. The person gets infections in her vagina, and has pain when urinating.	0.501 (0.339-0.657)
Vesicovaginal fistula	Vesicovaginal fistula	has an abnormal opening between the bladder and the vagina, which makes her unable to control urinating. The woman is anxious and depressed.	0.342 (0.227-0.478)
Maternal abortive outcome	Abdominopelvic problem, moderate	has pain in the belly and feels nauseous. The person has difficulties with daily activities.	0.114 (0.078-0.159)
Other maternal disorders	--	--	--
Asymptomatic retinopathy of prematurity	--	--	--
Mild vision impairment due to retinopathy of prematurity	Distance vision, mild impairment	has some difficulty with distance vision, for example reading signs, but no other problems with eyesight.	0.003 (0.001-0.007)
Moderate vision impairment due to retinopathy of prematurity	Distance vision, moderate impairment	has vision problems that make it difficult to recognize faces or objects across a room.	0.031 (0.019-0.049)
Severe vision impairment due to retinopathy of prematurity	Distance vision, severe impairment	has severe vision loss, which causes difficulty in daily activities, some emotional impact (for example worry), and some difficulty going outside the home without assistance.	0.184 (0.125-0.258)
Blindness due to retinopathy of prematurity	Distance vision blindness	is completely blind, which causes great difficulty in some daily activities, worry and anxiety, and great difficulty going outside the home without assistance.	0.187 (0.124-0.26)
Mild motor impairment due to neonatal preterm birth complications <28wks	Motor impairment, mild	has some difficulty in moving around but is able to walk without help.	0.01 (0.005-0.019)
Mild motor plus cognitive impairments due to neonatal preterm birth complications <28wks	Motor plus cognitive impairments, mild	has some difficulty in moving around but is able to walk without help. The person is slow in learning at school. As an adult, the person has some difficulty doing complex or unfamiliar tasks but otherwise functions independently.	0.031 (0.018-0.05)
Mild motor impairment due to neonatal preterm birth complications 28-32wks	Motor impairment, mild	has some difficulty in moving around but is able to walk without help.	0.01 (0.005-0.019)
Mild motor plus cognitive impairments due to neonatal preterm birth complications 28-32wks	Motor plus cognitive impairments, mild	has some difficulty in moving around but is able to walk without help. The person is slow in learning at school. As an adult, the person has some difficulty doing complex or unfamiliar tasks but otherwise functions independently.	0.031 (0.018-0.05)
Mild motor impairment due to neonatal preterm birth complications 32-36wks	Motor impairment, mild	has some difficulty in moving around but is able to walk without help.	0.01 (0.005-0.019)
Mild motor plus cognitive impairments due to neonatal preterm birth complications 32-36wks	Motor plus cognitive impairments, mild	has some difficulty in moving around but is able to walk without help. The person is slow in learning at school. As an adult, the person has some difficulty doing complex or unfamiliar tasks but otherwise functions independently.	0.031 (0.018-0.05)
Moderate motor impairment due to neonatal preterm birth complications <28wks	Motor impairment, moderate	has some difficulty in moving around, and difficulty in lifting and holding objects, dressing and sitting upright, but is able to walk without help.	0.061 (0.04-0.089)
Moderate motor impairment with blindness due to neonatal preterm birth complications <28wks	Moderate motor impairment with blindness	(combined DW)	--
Moderate motor impairment with epilepsy due to neonatal preterm birth complications <28wks	Moderate motor impairment with epilepsy	(combined DW)	--
Moderate motor impairment with blindness and epilepsy due to neonatal preterm birth complications <28wks	Moderate motor impairment with blindness and epilepsy	(combined DW)	--
Moderate motor plus cognitive impairment with blindness due to neonatal preterm birth complications <28wks	Moderate motor plus cognitive impairment with blindness	(combined DW)	--
Moderate motor plus cognitive impairment with epilepsy due to neonatal preterm birth complications <28wks	Moderate motor plus cognitive impairment with epilepsy	(combined DW)	--
Moderate motor plus cognitive impairment with blindness and epilepsy due to neonatal preterm birth complications <28wks	Moderate motor plus cognitive impairment with blindness and epilepsy	(combined DW)	--
Severe motor impairment due to neonatal preterm birth complications <28wks	Motor impairment, severe	is unable to move around without help, and is not able to lift or hold objects, get dressed or sit upright.	0.402 (0.268-0.545)
Severe motor impairment with blindness due to neonatal preterm birth complications <28wks	Severe motor impairment with blindness	(combined DW)	--
Severe motor impairment with epilepsy due to neonatal preterm birth complications <28wks	Severe motor impairment with epilepsy	(combined DW)	--
Severe motor impairment with blindness and epilepsy due to neonatal preterm birth complications <28wks	Severe motor impairment with blindness and epilepsy	(combined DW)	--
Severe motor plus cognitive impairment with blindness due to neonatal preterm birth complications <28wks	Severe motor plus cognitive impairment with blindness	(combined DW)	--
Severe motor plus cognitive impairment with epilepsy due to neonatal preterm birth complications <28wks	Severe motor plus cognitive impairment with epilepsy	(combined DW)	--
Severe motor plus cognitive impairment with blindness and epilepsy due to neonatal preterm birth complications <28wks	Severe motor plus cognitive impairment with blindness and epilepsy	(combined DW)	--
Moderate motor impairment due to neonatal preterm birth complications 28-32wks	Motor impairment, moderate	has some difficulty in moving around, and difficulty in lifting and holding objects, dressing and sitting upright, but is able to walk without help.	0.061 (0.04-0.089)
Moderate motor impairment with blindness due to neonatal preterm birth complications 28-32wks	Moderate motor impairment with blindness	(combined DW)	--
Moderate motor impairment with epilepsy due to neonatal preterm birth complications 28-32wks	Moderate motor impairment with epilepsy	(combined DW)	--
Moderate motor impairment with blindness and epilepsy due to neonatal preterm birth complications 28-32wks	Moderate motor impairment with blindness and epilepsy	(combined DW)	--
Moderate motor plus cognitive impairment with blindness due to neonatal preterm birth complications 28-32wks	Moderate motor plus cognitive impairment with blindness	(combined DW)	--
Moderate motor plus cognitive impairment with epilepsy due to neonatal preterm birth complications 28-32wks	Moderate motor plus cognitive impairment with epilepsy	(combined DW)	--
Moderate motor plus cognitive impairment with blindness and epilepsy due to neonatal preterm birth complications 28-32wks	Moderate motor plus cognitive impairment with blindness and epilepsy	(combined DW)	--
Severe motor impairment due to neonatal preterm birth complications 28-32wks	Motor impairment, severe	is unable to move around without help, and is not able to lift or hold objects, get dressed or sit upright.	0.402 (0.268-0.545)
Severe motor impairment with blindness due to neonatal preterm birth complications 28-32wks	Severe motor impairment with blindness	(combined DW)	--
Severe motor impairment with epilepsy due to neonatal preterm birth complications 28-32wks	Severe motor impairment with epilepsy	(combined DW)	--
Severe motor impairment with blindness and epilepsy due to neonatal preterm birth complications 28-32wks	Severe motor impairment with blindness and epilepsy	(combined DW)	--
Severe motor plus cognitive impairment with blindness due to neonatal preterm birth complications 28-32wks	Severe motor plus cognitive impairment with blindness	(combined DW)	--
Severe motor plus cognitive impairment with epilepsy due to neonatal preterm birth complications 28-32wks	Severe motor plus cognitive impairment with epilepsy	(combined DW)	--
Severe motor plus cognitive impairment with blindness and epilepsy due to neonatal preterm birth complications 28-32wks	Severe motor plus cognitive impairment with blindness and epilepsy	(combined DW)	--
Moderate motor impairment due to neonatal preterm birth complications 32-36wks	Motor impairment, moderate	has some difficulty in moving around, and difficulty in lifting and holding objects, dressing and sitting upright, but is able to walk without help.	0.061 (0.04-0.089)
Moderate motor impairment with blindness due to neonatal preterm birth complications 32-36wks	Moderate motor impairment with blindness	(combined DW)	--
Moderate motor impairment with epilepsy due to neonatal preterm birth complications 32-36wks	Moderate motor impairment with epilepsy	(combined DW)	--
Moderate motor impairment with blindness and epilepsy due to neonatal preterm birth complications 32-36wks	Moderate motor impairment with blindness and epilepsy	(combined DW)	--
Moderate motor plus cognitive impairment with blindness due to neonatal preterm birth complications 32-36wks	Moderate motor plus cognitive impairment with blindness	(combined DW)	--

Appendix Table 6. GBD 2015 sequelae, health states, health state lay descriptions, and disability weights

Sequela	Healthstate name	Healthstate lay description	Disability weight
Moderate motor plus cognitive impairment with epilepsy due to neonatal preterm birth complications 32-36wks	Moderate motor plus cognitive impairment with epilepsy	(combined DW)	--
Moderate motor plus cognitive impairment with blindness and epilepsy due to neonatal preterm birth complications 32-36wks	Moderate motor plus cognitive impairment with blindness and epilepsy	(combined DW)	--
Severe motor impairment due to neonatal preterm birth complications 32-36wks	Motor impairment, severe	is unable to move around without help, and is not able to lift or hold objects, get dressed or sit upright.	0.402 (0.268-0.545)
Severe motor impairment with blindness due to neonatal preterm birth complications 32-36wks	Severe motor impairment with blindness	(combined DW)	--
Severe motor impairment with epilepsy due to neonatal preterm birth complications 32-36wks	Severe motor impairment with epilepsy	(combined DW)	--
Severe motor impairment with blindness and epilepsy due to neonatal preterm birth complications 32-36wks	Severe motor impairment with blindness and epilepsy	(combined DW)	--
Severe motor plus cognitive impairment with blindness due to neonatal preterm birth complications 32-36wks	Severe motor plus cognitive impairment with blindness	(combined DW)	--
Severe motor plus cognitive impairment with epilepsy due to neonatal preterm birth complications 32-36wks	Severe motor plus cognitive impairment with epilepsy	(combined DW)	--
Severe motor plus cognitive impairment with blindness and epilepsy due to neonatal preterm birth complications 32-36wks	Severe motor plus cognitive impairment with blindness and epilepsy	(combined DW)	--
Mild motor plus cognitive impairments due to neonatal encephalopathy due to birth asphyxia and trauma	Motor plus cognitive impairments, mild	has some difficulty in moving around but is able to walk without help. The person is slow in learning at school. As an adult, the person has some difficulty doing complex or unfamiliar tasks but otherwise functions independently.	0.031 (0.018-0.05)
Mild motor impairment due to neonatal encephalopathy due to birth asphyxia and trauma	Motor impairment, mild	has some difficulty in moving around but is able to walk without help.	0.01 (0.005-0.019)
Moderate motor impairment due to neonatal encephalopathy due to birth asphyxia and trauma	Motor impairment, moderate	has some difficulty in moving around, and difficulty in lifting and holding objects, dressing and sitting upright, but is able to walk without help.	0.061 (0.04-0.089)
Moderate motor impairment with blindness due to neonatal encephalopathy due to birth asphyxia and trauma	Moderate motor impairment with blindness	(combined DW)	--
Moderate motor impairment with epilepsy due to neonatal encephalopathy due to birth asphyxia and trauma	Moderate motor impairment with epilepsy	(combined DW)	--
Moderate motor impairment with blindness and epilepsy due to neonatal encephalopathy due to birth asphyxia and trauma	Moderate motor impairment with blindness and epilepsy	(combined DW)	--
Moderate motor plus cognitive impairment with blindness due to neonatal encephalopathy due to birth asphyxia and trauma	Moderate motor plus cognitive impairment with blindness	(combined DW)	--
Moderate motor plus cognitive impairment with epilepsy due to neonatal encephalopathy due to birth asphyxia and trauma	Moderate motor plus cognitive impairment with epilepsy	(combined DW)	--
Moderate motor plus cognitive impairment with blindness and epilepsy due to neonatal encephalopathy due to birth asphyxia and trauma	Moderate motor plus cognitive impairment with blindness and epilepsy	(combined DW)	--
Severe motor impairment due to neonatal encephalopathy due to birth asphyxia and trauma	Motor impairment, severe	is unable to move around without help, and is not able to lift or hold objects, get dressed or sit upright.	0.402 (0.268-0.545)
Severe motor impairment with blindness due to neonatal encephalopathy due to birth asphyxia and trauma	Severe motor impairment with blindness	(combined DW)	--
Severe motor impairment with epilepsy due to neonatal encephalopathy due to birth asphyxia and trauma	Severe motor impairment with epilepsy	(combined DW)	--
Severe motor impairment with blindness and epilepsy due to neonatal encephalopathy due to birth asphyxia and trauma	Severe motor impairment with blindness and epilepsy	(combined DW)	--
Severe motor plus cognitive impairment with blindness due to neonatal encephalopathy due to birth asphyxia and trauma	Severe motor plus cognitive impairment with blindness	(combined DW)	--
Severe motor plus cognitive impairment with epilepsy due to neonatal encephalopathy due to birth asphyxia and trauma	Severe motor plus cognitive impairment with epilepsy	(combined DW)	--
Severe motor plus cognitive impairment with blindness and epilepsy due to neonatal encephalopathy due to birth asphyxia and trauma	Severe motor plus cognitive impairment with blindness and epilepsy	(combined DW)	--
Severe infection due to neonatal sepsis and other neonatal infections	Infectious disease, acute episode, severe	has a high fever and pain, and feels very weak, which causes great difficulty with daily activities.	0.133 (0.088-0.19)
Mild motor impairment due to neonatal sepsis and other neonatal infections	Motor impairment, mild	has some difficulty in moving around but is able to walk without help.	0.01 (0.005-0.019)
Mild motor plus cognitive impairments due to neonatal sepsis and other neonatal infections	Motor plus cognitive impairments, mild	has some difficulty in moving around but is able to walk without help. The person is slow in learning at school. As an adult, the person has some difficulty doing complex or unfamiliar tasks but otherwise functions independently.	0.031 (0.018-0.05)
Moderate motor impairment due to neonatal sepsis and other neonatal infections	Motor impairment, moderate	has some difficulty in moving around, and difficulty in lifting and holding objects, dressing and sitting upright, but is able to walk without help.	0.061 (0.04-0.089)
Moderate motor impairment with blindness and epilepsy due to neonatal sepsis and other neonatal infections	Moderate motor impairment with blindness and epilepsy	(combined DW)	--
Moderate motor impairment with blindness due to neonatal sepsis and other neonatal infections	Moderate motor impairment with blindness	(combined DW)	--
Moderate motor impairment with epilepsy due to neonatal sepsis and other neonatal infections	Moderate motor impairment with epilepsy	(combined DW)	--
Moderate motor plus cognitive impairment with blindness and epilepsy due to neonatal sepsis and other neonatal infections	Moderate motor plus cognitive impairment with blindness and epilepsy	(combined DW)	--
Moderate motor plus cognitive impairment with blindness due to neonatal sepsis and other neonatal infections	Moderate motor plus cognitive impairment with blindness	(combined DW)	--
Moderate motor plus cognitive impairment with epilepsy due to neonatal sepsis and other neonatal infections	Moderate motor plus cognitive impairment with epilepsy	(combined DW)	--
Severe motor impairment with blindness due to hemolytic disease and other neonatal jaundice	Severe motor impairment with blindness	(combined DW)	--
Severe motor impairment with blindness due to neonatal sepsis and other neonatal infections	Severe motor impairment with blindness	(combined DW)	--
Severe motor impairment due to neonatal sepsis and other neonatal infections	Motor impairment, severe	is unable to move around without help, and is not able to lift or hold objects, get dressed or sit upright.	0.402 (0.268-0.545)
Severe motor impairment with blindness and epilepsy due to neonatal sepsis and other neonatal infections	Severe motor impairment with blindness and epilepsy	(combined DW)	--
Severe motor impairment with epilepsy due to neonatal sepsis and other neonatal infections	Severe motor impairment with epilepsy	(combined DW)	--
Severe motor plus cognitive impairment with blindness and epilepsy due to neonatal sepsis and other neonatal infections	Severe motor plus cognitive impairment with blindness and epilepsy	(combined DW)	--
Severe motor plus cognitive impairment with blindness due to neonatal sepsis and other neonatal infections	Severe motor plus cognitive impairment with blindness	(combined DW)	--
Severe motor plus cognitive impairment with epilepsy due to neonatal sepsis and other neonatal infections	Severe motor plus cognitive impairment with epilepsy	(combined DW)	--
Moderate motor impairment due to hemolytic disease and other neonatal jaundice	Motor impairment, moderate	has some difficulty in moving around, and difficulty in lifting and holding objects, dressing and sitting upright, but is able to walk without help.	0.061 (0.04-0.089)
Moderate motor impairment with blindness due to hemolytic disease and other neonatal jaundice	Moderate motor impairment with blindness	(combined DW)	--
Moderate motor impairment with epilepsy due to hemolytic disease and other neonatal jaundice	Moderate motor impairment with epilepsy	(combined DW)	--
Moderate motor impairment with blindness and epilepsy due to hemolytic disease and other neonatal jaundice	Moderate motor impairment with blindness and epilepsy	(combined DW)	--
Moderate motor plus cognitive impairment with blindness due to hemolytic disease and other neonatal jaundice	Moderate motor plus cognitive impairment with blindness	(combined DW)	--
Moderate motor plus cognitive impairment with epilepsy due to hemolytic disease and other neonatal jaundice	Moderate motor plus cognitive impairment with epilepsy	(combined DW)	--
Moderate motor plus cognitive impairment with blindness and epilepsy due to hemolytic disease and other neonatal jaundice	Moderate motor plus cognitive impairment with blindness and epilepsy	(combined DW)	--

Appendix Table 6. GBD 2015 sequelae, health states, health state lay descriptions, and disability weights			
**Sequela**	**Healthstate name**	**Healthstate lay description**	**Disability weight**
Severe motor impairment severe due to hemolytic disease and other neonatal jaundice	Motor impairment, severe	is unable to move around without help, and is not able to lift or hold objects, get dressed or sit upright.	0.402 (0.268-0.545)
Severe motor impairment with epilepsy due to hemolytic disease and other neonatal jaundice	Severe motor impairment with epilepsy	(combined DW)	--
Severe motor impairment with blindness and epilepsy due to hemolytic disease and other neonatal jaundice	Severe motor impairment with blindness and epilepsy	(combined DW)	--
Severe motor plus cognitive impairment with blindness due to hemolytic disease and other neonatal jaundice	Severe motor plus cognitive impairment with blindness	(combined DW)	--
Severe motor plus cognitive impairment with epilepsy due to hemolytic disease and other neonatal jaundice	Severe motor plus cognitive impairment with epilepsy	(combined DW)	--
Severe motor plus cognitive impairment with blindness and epilepsy due to hemolytic disease and other neonatal jaundice	Severe motor plus cognitive impairment with blindness and epilepsy	(combined DW)	--
Other neonatal disorders	--	--	--
Kwashiokor due to protein-energy malnutrition	Kwashiorkor	is very tired and irritable and has diarrhea.	0.051 (0.031-0.079)
Marasmus due to protein-energy malnutrition	Severe wasting	is extremely skinny and has no energy.	0.128 (0.082-0.183)
Severe wasting due to protein-energy malnutrition	Severe wasting	is extremely skinny and has no energy.	0.128 (0.082-0.183)
Visible goiter without symptoms	Disfigurement, level 2	has a visible physical deformity that causes others to stare and comment. As a result, the person is worried and has trouble sleeping and concentrating.	0.067 (0.044-0.096)
Visible goiter without heart failure due to iodine deficiency	Disfigurement, level 1	has a slight, visible physical deformity that others notice, which causes some worry and discomfort.	0.011 (0.005-0.021)
Visible goiter with signs and symptoms	Iodine-deficiency goiter	has a large mass in the front of the neck. The person sometimes has weakness and fatigue, constipation and weight gain.	0.199 (0.133-0.276)
Visible goiter with mild heart failure due to iodine deficiency	Disfigurement, level 1, with mild heart failure	(combined DW)	--
Visible goiter with moderate heart failure due to iodine deficiency	Disfigurement, level 1, with moderate heart failure	(combined DW)	--
Visible goiter with severe heart failure due to iodine deficiency	Disfigurement, level 1, with severe heart failure	(combined DW)	--
Severe intellectual disability due to iodine deficiency	Intellectual disability / mental retardation, severe	has very low intelligence and cannot speak more than a few words, needs constant supervision and help with most daily activities, and can do only the simplest tasks.	0.16 (0.107-0.226)
Profound intellectual disability due to iodine deficiency	Intellectual disability / mental retardation, profound	has very low intelligence, has almost no language, and does not understand even the most basic requests or instructions. The person requires constant supervision and help for all activities.	0.2 (0.133-0.283)
Moderate vision impairment loss due to vitamin A deficiency	Distance vision, moderate impairment	has vision problems that make it difficult to recognize faces or objects across a room.	0.031 (0.019-0.049)
Severe vision impairment loss due to vitamin A deficiency	Distance vision, severe impairment	has severe vision loss, which causes difficulty in daily activities, some emotional impact (for example worry), and some difficulty going outside the home without assistance.	0.184 (0.125-0.258)
Blindness due to vitamin A deficiency	Distance vision blindness	is completely blind, which causes great difficulty in some daily activities, worry and anxiety, and great difficulty going outside the home without assistance.	0.187 (0.124-0.26)
Mild iron-deficiency anemia	Anemia, mild	feels slightly tired and weak at times, but this does not interfere with normal daily activities.	0.004 (0.001-0.008)
Moderate iron-deficiency anemia	Anemia, moderate	feels moderate fatigue, weakness, and shortness of breath after exercise, making daily activities more difficult.	0.052 (0.034-0.076)
Severe iron-deficiency anemia	Anemia, severe	feels very weak, tired and short of breath, and has problems with activities that require physical effort or deep concentration.	0.149 (0.101-0.209)
Mild heart failure due to iron-deficiency anemia	Heart failure, mild	is short of breath and easily tires with moderate physical activity, such as walking uphill or more than a quarter-mile on level ground. The person feels comfortable at rest or during activities requiring less effort.	0.041 (0.026-0.062)
Moderate heart failure due to iron-deficiency anemia	Heart failure, moderate	is short of breath and easily tires with minimal physical activity, such as walking only a short distance. The person feels comfortable at rest but avoids moderate activity.	0.072 (0.047-0.103)
Severe heart failure due to iron-deficiency anemia	Heart failure, severe	is short of breath and feels tired when at rest. The person avoids any physical activity, for fear of worsening the breathing problems.	0.179 (0.122-0.251)
Other nutritional deficiencies	--	--	--
Asymptomatic early syphilis infection	--	--	--
Mild early syphilis infection	Infectious disease, acute episode, mild	has a low fever and mild discomfort , but no difficulty with daily activities.	0.006 (0.002-0.012)
Adult tertiary syphilis	Motor plus cognitive impairments, moderate	has some difficulty in moving around, holding objects, dressing and sitting upright, but can walk without help. The person has low intelligence and is slow in learning to speak and to do simple tasks.	0.203 (0.134-0.29)
Asymptomatic chlamydial infection	--	--	--
Mild chlamydial infection	Infectious disease, acute episode, mild	has a low fever and mild discomfort , but no difficulty with daily activities.	0.006 (0.002-0.012)
Epididymo-orchitis due to chlamydial infection	Epididymo-orchitis	has swelling and tenderness in the testicles and pain during urination.	0.128 (0.086-0.18)
Moderate pelvic inflammatory diseases due to chlamydial infection	Abdominopelvic problem, moderate	has pain in the belly and feels nauseous. The person has difficulties with daily activities.	0.114 (0.078-0.159)
Severe pelvic inflammatory diseases due to chlamydial infection	Abdominopelvic problem, severe	has severe pain in the belly and feels nauseous. The person is anxious and unable to carry out daily activities.	0.324 (0.22-0.442)
Primary infertility due to chlamydial infection	Infertility, primary	wants to have a child and has a fertile partner, but the couple cannot conceive.	0.008 (0.003-0.015)
Secondary infertility due to chlamydial infection	Infertility, secondary	has at least one child, and wants to have more children. The person has a fertile partner, but the couple cannot conceive.	0.005 (0.002-0.011)
Asymptomatic gonococcal infection	--	--	--
Mild gonococcal infection	Infectious disease, acute episode, mild	has a low fever and mild discomfort , but no difficulty with daily activities.	0.006 (0.002-0.012)
Epididymo-orchitis due to gonococcal infection	Epididymo-orchitis	has swelling and tenderness in the testicles and pain during urination.	0.128 (0.086-0.18)
Moderate pelvic inflammatory diseases due to gonococcal infection	Abdominopelvic problem, moderate	has pain in the belly and feels nauseous. The person has difficulties with daily activities.	0.114 (0.078-0.159)
Severe pelvic inflammatory diseases due to gonococcal infection	Abdominopelvic problem, severe	has severe pain in the belly and feels nauseous. The person is anxious and unable to carry out daily activities.	0.324 (0.22-0.442)
Primary infertility due to gonococcal infection	Infertility, primary	wants to have a child and has a fertile partner, but the couple cannot conceive.	0.008 (0.003-0.015)
Secondary infertility due to gonococcal infection	Infertility, secondary	has at least one child, and wants to have more children. The person has a fertile partner, but the couple cannot conceive.	0.005 (0.002-0.011)
Asymptomatic trichomoniasis infection	--	--	--
Acute trichomoniasis infection	Infectious disease, acute episode, mild	has a low fever and mild discomfort , but no difficulty with daily activities.	0.006 (0.002-0.012)
Moderate infection due to initial genital herpes episode	Infectious disease, acute episode, moderate	has a fever and aches, and feels weak, which causes some difficulty with daily activities.	0.051 (0.032-0.074)
Asymptomatic genital herpes	--	--	--
Symptomatic genital herpes	Infectious disease, acute episode, mild	has a low fever and mild discomfort , but no difficulty with daily activities.	0.006 (0.002-0.012)

Appendix Table 6. GBD 2015 sequelae, health states, health state lay descriptions, and disability weights			
**Sequela**	**Healthstate name**	**Healthstate lay description**	**Disability weight**
Other sexually transmitted diseases	--	--	--
Moderate pelvic inflammatory diseases due to other sexually transmitted diseases	Abdominopelvic problem, moderate	has pain in the belly and feels nauseous. The person has difficulties with daily activities.	0.114 (0.078-0.159)
Severe pelvic inflammatory diseases due to other sexually transmitted diseases	Abdominopelvic problem, severe	has severe pain in the belly and feels nauseous. The person is anxious and unable to carry out daily activities.	0.324 (0.22-0.442)
Primary infertility due to other sexually transmitted diseases	Infertility, primary	wants to have a child and has a fertile partner, but the couple cannot conceive.	0.008 (0.003-0.015)
Secondary infertility due to other sexually transmitted diseases	Infertility, secondary	has at least one child, and wants to have more children. The person has a fertile partner, but the couple cannot conceive.	0.005 (0.002-0.011)
Asymptomatic acute hepatitis A	--	--	--
Moderate acute hepatitis A	Infectious disease, acute episode, moderate	has a fever and aches, and feels weak, which causes some difficulty with daily activities.	0.051 (0.032-0.074)
Severe acute hepatitis A	Infectious disease, acute episode, severe	has a high fever and pain, and feels very weak, which causes great difficulty with daily activities.	0.133 (0.088-0.19)
Asymptomatic acute hepatitis B	--	--	--
Moderate acute hepatitis B	Infectious disease, acute episode, moderate	has a fever and aches, and feels weak, which causes some difficulty with daily activities.	0.051 (0.032-0.074)
Severe acute hepatitis B	Infectious disease, acute episode, severe	has a high fever and pain, and feels very weak, which causes great difficulty with daily activities.	0.133 (0.088-0.19)
Chronic hepatitis B	--	--	--
Asymptomatic acute hepatitis C	--	--	--
Moderate acute hepatitis C	Infectious disease, acute episode, moderate	has a fever and aches, and feels weak, which causes some difficulty with daily activities.	0.051 (0.032-0.074)
Severe acute hepatitis C	Infectious disease, acute episode, severe	has a high fever and pain, and feels very weak, which causes great difficulty with daily activities.	0.133 (0.088-0.19)
Chronic hepatitis C	--	--	--
Asymptomatic acute hepatitis E	--	--	--
Moderate acute hepatitis E	Infectious disease, acute episode, moderate	has a fever and aches, and feels weak, which causes some difficulty with daily activities.	0.051 (0.032-0.074)
Severe acute hepatitis E	Infectious disease, acute episode, severe	has a high fever and pain, and feels very weak, which causes great difficulty with daily activities.	0.133 (0.088-0.19)
Acute other infectious diseases	--	--	--
Mild anemia due to other infectious diseases	Anemia, mild	feels slightly tired and weak at times, but this does not interfere with normal daily activities.	0.004 (0.001-0.008)
Moderate anemia due to other infectious diseases	Anemia, moderate	feels moderate fatigue, weakness, and shortness of breath after exercise, making daily activities more difficult.	0.052 (0.034-0.076)
Severe anemia due to other infectious diseases	Anemia, severe	feels very weak, tired and short of breath, and has problems with activities that require physical effort or deep concentration.	0.149 (0.101-0.209)
Guillain-Barré syndrome due to other infectious diseases	Spinal cord lesion below neck level (treated)	is paralyzed from the waist down, cannot feel or move the legs and has difficulties with urine and bowel control. The person uses a wheelchair to move around.	0.296 (0.198-0.414)
Diagnosis and primary therapy phase of mouth cancer	Cancer, diagnosis and primary therapy	has pain, nausea, fatigue, weight loss and high anxiety.	0.288 (0.193-0.399)
Controlled phase of mouth cancer	Generic uncomplicated disease: worry and daily medication	has a chronic disease that requires medication every day and causes some worry but minimal interference with daily activities.	0.049 (0.031-0.072)
Metastatic phase of mouth cancer	Cancer, metastatic	has severe pain, extreme fatigue, weight loss and high anxiety.	0.451 (0.307-0.6)
Terminal phase of mouth cancer	Terminal phase, with medication (for cancers, end-stage kidney/liver disease)	has lost a lot of weight and regularly uses strong medication to avoid constant pain. The person has no appetite, feels nauseous, and needs to spend most of the day in bed.	0.54 (0.377-0.687)
Diagnosis and primary therapy phase of nasopharynx cancer	Cancer, diagnosis and primary therapy	has pain, nausea, fatigue, weight loss and high anxiety.	0.288 (0.193-0.399)
Controlled phase of nasopharynx cancer	Generic uncomplicated disease: worry and daily medication	has a chronic disease that requires medication every day and causes some worry but minimal interference with daily activities.	0.049 (0.031-0.072)
Metastatic phase of nasopharynx cancer	Cancer, metastatic	has severe pain, extreme fatigue, weight loss and high anxiety.	0.451 (0.307-0.6)
Terminal phase of nasopharynx cancer	Terminal phase, with medication (for cancers, end-stage kidney/liver disease)	has lost a lot of weight and regularly uses strong medication to avoid constant pain. The person has no appetite, feels nauseous, and needs to spend most of the day in bed.	0.54 (0.377-0.687)
Diagnosis and primary therapy phase of other pharynx cancer	Cancer, diagnosis and primary therapy	has pain, nausea, fatigue, weight loss and high anxiety.	0.288 (0.193-0.399)
Controlled phase of other pharynx cancer	Generic uncomplicated disease: worry and daily medication	has a chronic disease that requires medication every day and causes some worry but minimal interference with daily activities.	0.049 (0.031-0.072)
Metastatic phase of other pharynx cancer	Cancer, metastatic	has severe pain, extreme fatigue, weight loss and high anxiety.	0.451 (0.307-0.6)
Terminal phase of other pharynx cancer	Terminal phase, with medication (for cancers, end-stage kidney/liver disease)	has lost a lot of weight and regularly uses strong medication to avoid constant pain. The person has no appetite, feels nauseous, and needs to spend most of the day in bed.	0.54 (0.377-0.687)
Diagnosis and primary therapy phase of esophageal cancer	Cancer, diagnosis and primary therapy	has pain, nausea, fatigue, weight loss and high anxiety.	0.288 (0.193-0.399)
Controlled phase of esophageal cancer	Generic uncomplicated disease: worry and daily medication	has a chronic disease that requires medication every day and causes some worry but minimal interference with daily activities.	0.049 (0.031-0.072)
Metastatic phase of esophageal cancer	Cancer, metastatic	has severe pain, extreme fatigue, weight loss and high anxiety.	0.451 (0.307-0.6)
Terminal phase of esophageal cancer	Terminal phase, with medication (for cancers, end-stage kidney/liver disease)	has lost a lot of weight and regularly uses strong medication to avoid constant pain. The person has no appetite, feels nauseous, and needs to spend most of the day in bed.	0.54 (0.377-0.687)
Diagnosis and primary therapy phase of stomach cancer	Cancer, diagnosis and primary therapy	has pain, nausea, fatigue, weight loss and high anxiety.	0.288 (0.193-0.399)
Controlled phase of stomach cancer	Generic uncomplicated disease: worry and daily medication	has a chronic disease that requires medication every day and causes some worry but minimal interference with daily activities.	0.049 (0.031-0.072)
Metastatic phase of stomach cancer	Cancer, metastatic	has severe pain, extreme fatigue, weight loss and high anxiety.	0.451 (0.307-0.6)
Terminal phase of stomach cancer	Terminal phase, with medication (for cancers, end-stage kidney/liver disease)	has lost a lot of weight and regularly uses strong medication to avoid constant pain. The person has no appetite, feels nauseous, and needs to spend most of the day in bed.	0.54 (0.377-0.687)
Diagnosis and primary therapy phase of colon and rectum cancers	Cancer, diagnosis and primary therapy	has pain, nausea, fatigue, weight loss and high anxiety.	0.288 (0.193-0.399)
Controlled phase of colon and rectum cancers	Generic uncomplicated disease: worry and daily medication	has a chronic disease that requires medication every day and causes some worry but minimal interference with daily activities.	0.049 (0.031-0.072)
Metastatic phase of colon and rectum cancers	Cancer, metastatic	has severe pain, extreme fatigue, weight loss and high anxiety.	0.451 (0.307-0.6)
Terminal phase of colon and rectum cancers	Terminal phase, with medication (for cancers, end-stage kidney/liver disease)	has lost a lot of weight and regularly uses strong medication to avoid constant pain. The person has no appetite, feels nauseous, and needs to spend most of the day in bed.	0.54 (0.377-0.687)
Stoma due to colon and rectum cancer	Stoma	has a pouch attached to an opening in the belly to collect and empty stools.	0.095 (0.063-0.131)
Diagnosis and primary therapy phase of liver cancer due to hepatitis B	Cancer, diagnosis and primary therapy	has pain, nausea, fatigue, weight loss and high anxiety.	0.288 (0.193-0.399)

Appendix Table 6. GBD 2015 sequelae, health states, health state lay descriptions, and disability weights

Sequela	Healthstate name	Healthstate lay description	Disability weight
Controlled phase of liver cancer due to hepatitis B	Generic uncomplicated disease: worry and daily medication	has a chronic disease that requires medication every day and causes some worry but minimal interference with daily activities.	0.049 (0.031-0.072)
Metastatic phase of liver cancer due to hepatitis B	Cancer, metastatic	has severe pain, extreme fatigue, weight loss and high anxiety.	0.451 (0.307-0.6)
Terminal phase of liver cancer due to hepatitis B	Terminal phase, with medication (for cancers, end-stage kidney/liver disease)	has lost a lot of weight and regularly uses strong medication to avoid constant pain. The person has no appetite, feels nauseous, and needs to spend most of the day in bed.	0.54 (0.377-0.687)
Diagnosis and primary therapy phase of liver cancer due to hepatitis C	Cancer, diagnosis and primary therapy	has pain, nausea, fatigue, weight loss and high anxiety.	0.288 (0.193-0.399)
Controlled phase of liver cancer due to hepatitis C	Generic uncomplicated disease: worry and daily medication	has a chronic disease that requires medication every day and causes some worry but minimal interference with daily activities.	0.049 (0.031-0.072)
Metastatic phase of liver cancer due to hepatitis C	Cancer, metastatic	has severe pain, extreme fatigue, weight loss and high anxiety.	0.451 (0.307-0.6)
Terminal phase of liver cancer due to hepatitis C	Terminal phase, with medication (for cancers, end-stage kidney/liver disease)	has lost a lot of weight and regularly uses strong medication to avoid constant pain. The person has no appetite, feels nauseous, and needs to spend most of the day in bed.	0.54 (0.377-0.687)
Diagnosis and primary therapy phase of liver cancer due to alcohol use	Cancer, diagnosis and primary therapy	has pain, nausea, fatigue, weight loss and high anxiety.	0.288 (0.193-0.399)
Controlled phase of liver cancer due to alcohol use	Generic uncomplicated disease: worry and daily medication	has a chronic disease that requires medication every day and causes some worry but minimal interference with daily activities.	0.049 (0.031-0.072)
Metastatic phase of liver cancer due to alcohol use	Cancer, metastatic	has severe pain, extreme fatigue, weight loss and high anxiety.	0.451 (0.307-0.6)
Terminal phase of liver cancer due to alcohol use	Terminal phase, with medication (for cancers, end-stage kidney/liver disease)	has lost a lot of weight and regularly uses strong medication to avoid constant pain. The person has no appetite, feels nauseous, and needs to spend most of the day in bed.	0.54 (0.377-0.687)
Diagnosis and primary therapy phase of liver cancer due to other causes	Cancer, diagnosis and primary therapy	has pain, nausea, fatigue, weight loss and high anxiety.	0.288 (0.193-0.399)
Controlled phase of liver cancer due to other causes	Generic uncomplicated disease: worry and daily medication	has a chronic disease that requires medication every day and causes some worry but minimal interference with daily activities.	0.049 (0.031-0.072)
Metastatic phase of liver cancer due to other causes	Cancer, metastatic	has severe pain, extreme fatigue, weight loss and high anxiety.	0.451 (0.307-0.6)
Terminal phase of liver cancer due to other causes	Terminal phase, with medication (for cancers, end-stage kidney/liver disease)	has lost a lot of weight and regularly uses strong medication to avoid constant pain. The person has no appetite, feels nauseous, and needs to spend most of the day in bed.	0.54 (0.377-0.687)
Diagnosis and primary therapy phase of gallbladder and biliary tract cancer	Cancer, diagnosis and primary therapy	has pain, nausea, fatigue, weight loss and high anxiety.	0.288 (0.193-0.399)
Controlled phase of gallbladder and biliary tract cancer	Generic uncomplicated disease: worry and daily medication	has a chronic disease that requires medication every day and causes some worry but minimal interference with daily activities.	0.049 (0.031-0.072)
Metastatic phase of gallbladder and biliary tract cancer	Cancer, metastatic	has severe pain, extreme fatigue, weight loss and high anxiety.	0.451 (0.307-0.6)
Terminal phase of gallbladder and biliary tract cancer	Terminal phase, with medication (for cancers, end-stage kidney/liver disease)	has lost a lot of weight and regularly uses strong medication to avoid constant pain. The person has no appetite, feels nauseous, and needs to spend most of the day in bed.	0.54 (0.377-0.687)
Diagnosis and primary therapy phase of pancreatic cancer	Cancer, diagnosis and primary therapy	has pain, nausea, fatigue, weight loss and high anxiety.	0.288 (0.193-0.399)
Controlled phase of pancreatic cancer	Generic uncomplicated disease: worry and daily medication	has a chronic disease that requires medication every day and causes some worry but minimal interference with daily activities.	0.049 (0.031-0.072)
Metastatic phase of pancreatic cancer	Cancer, metastatic	has severe pain, extreme fatigue, weight loss and high anxiety.	0.451 (0.307-0.6)
Terminal phase of pancreatic cancer	Terminal phase, with medication (for cancers, end-stage kidney/liver disease)	has lost a lot of weight and regularly uses strong medication to avoid constant pain. The person has no appetite, feels nauseous, and needs to spend most of the day in bed.	0.54 (0.377-0.687)
Diagnosis and primary therapy phase of larynx cancer	Cancer, diagnosis and primary therapy	has pain, nausea, fatigue, weight loss and high anxiety.	0.288 (0.193-0.399)
Controlled phase of larynx cancer	Generic uncomplicated disease: worry and daily medication	has a chronic disease that requires medication every day and causes some worry but minimal interference with daily activities.	0.049 (0.031-0.072)
Metastatic phase of larynx cancer	Cancer, metastatic	has severe pain, extreme fatigue, weight loss and high anxiety.	0.451 (0.307-0.6)
Terminal phase of larynx cancer	Terminal phase, with medication (for cancers, end-stage kidney/liver disease)	has lost a lot of weight and regularly uses strong medication to avoid constant pain. The person has no appetite, feels nauseous, and needs to spend most of the day in bed.	0.54 (0.377-0.687)
Laryngectomy due to larynx cancer	Speech problems	has difficulty speaking, and others find it difficult to understand.	0.051 (0.032-0.078)
Diagnosis and primary therapy phase of lung, bronchus, and trachea cancer	Cancer, diagnosis and primary therapy	has pain, nausea, fatigue, weight loss and high anxiety.	0.288 (0.193-0.399)
Controlled phase of lung, bronchus, and trachea cancer	Generic uncomplicated disease: worry and daily medication	has a chronic disease that requires medication every day and causes some worry but minimal interference with daily activities.	0.049 (0.031-0.072)
Metastatic phase of lung, bronchus, and trachea cancer	Cancer, metastatic	has severe pain, extreme fatigue, weight loss and high anxiety.	0.451 (0.307-0.6)
Terminal phase of lung, bronchus, and trachea cancer	Terminal phase, with medication (for cancers, end-stage kidney/liver disease)	has lost a lot of weight and regularly uses strong medication to avoid constant pain. The person has no appetite, feels nauseous, and needs to spend most of the day in bed.	0.54 (0.377-0.687)
Diagnosis and primary therapy phase of malignant skin melanoma	Cancer, diagnosis and primary therapy	has pain, nausea, fatigue, weight loss and high anxiety.	0.288 (0.193-0.399)
Controlled phase of malignant skin melanoma	Generic uncomplicated disease: worry and daily medication	has a chronic disease that requires medication every day and causes some worry but minimal interference with daily activities.	0.049 (0.031-0.072)
Metastatic phase of malignant skin melanoma	Cancer, metastatic	has severe pain, extreme fatigue, weight loss and high anxiety.	0.451 (0.307-0.6)
Terminal phase of malignant skin melanoma	Terminal phase, with medication (for cancers, end-stage kidney/liver disease)	has lost a lot of weight and regularly uses strong medication to avoid constant pain. The person has no appetite, feels nauseous, and needs to spend most of the day in bed.	0.54 (0.377-0.687)
Diagnosis and primary therapy phase of cutaneous squamous cell carcinoma	Cancer, diagnosis and primary therapy	has pain, nausea, fatigue, weight loss and high anxiety.	0.288 (0.193-0.399)
Control phase of cutaneous squamous cell carcinoma	Generic uncomplicated disease: worry and daily medication	has a chronic disease that requires medication every day and causes some worry but minimal interference with daily activities.	0.049 (0.031-0.072)
Metastatic phase of cutaneous squamous cell carcinoma	Cancer, metastatic	has severe pain, extreme fatigue, weight loss and high anxiety.	0.451 (0.307-0.6)
Terminal phase of cutaneous squamous cell carcinoma	Terminal phase, with medication (for cancers, end-stage kidney/liver disease)	has lost a lot of weight and regularly uses strong medication to avoid constant pain. The person has no appetite, feels nauseous, and needs to spend most of the day in bed.	0.54 (0.377-0.687)
Disfigurement due to basal cell carcinoma	Disfigurement, level 1	has a slight, visible physical deformity that others notice, which causes some worry and discomfort.	0.011 (0.005-0.021)
Diagnosis and primary therapy phase of breast cancer	Cancer, diagnosis and primary therapy	has pain, nausea, fatigue, weight loss and high anxiety.	0.288 (0.193-0.399)
Controlled phase of breast cancer	Generic uncomplicated disease: worry and daily medication	has a chronic disease that requires medication every day and causes some worry but minimal interference with daily activities.	0.049 (0.031-0.072)
Metastatic phase of breast cancer	Cancer, metastatic	has severe pain, extreme fatigue, weight loss and high anxiety.	0.451 (0.307-0.6)
Terminal phase of breast cancer	Terminal phase, with medication (for cancers, end-stage kidney/liver disease)	has lost a lot of weight and regularly uses strong medication to avoid constant pain. The person has no appetite, feels nauseous, and needs to spend most of the day in bed.	0.54 (0.377-0.687)
Mastectomy due to breast cancer	Mastectomy	had one of her breasts removed and sometimes has pain or swelling in the arms.	0.036 (0.02-0.057)
Diagnosis and primary therapy phase of cervical cancer	Cancer, diagnosis and primary therapy	has pain, nausea, fatigue, weight loss and high anxiety.	0.288 (0.193-0.399)
Controlled phase of cervical cancer	Generic uncomplicated disease: worry and daily medication	has a chronic disease that requires medication every day and causes some worry but minimal interference with daily activities.	0.049 (0.031-0.072)
Metastatic phase of cervical cancer	Cancer, metastatic	has severe pain, extreme fatigue, weight loss and high anxiety.	0.451 (0.307-0.6)
Terminal phase of cervical cancer	Terminal phase, with medication (for cancers, end-stage kidney/liver disease)	has lost a lot of weight and regularly uses strong medication to avoid constant pain. The person has no appetite, feels nauseous, and needs to spend most of the day in bed.	0.54 (0.377-0.687)

Appendix Table 6. GBD 2015 sequelae, health states, health state lay descriptions, and disability weights			
**Sequela**	**Healthstate name**	**Healthstate lay description**	**Disability weight**
Diagnosis and primary therapy phase of uterine cancer	Cancer, diagnosis and primary therapy	has pain, nausea, fatigue, weight loss and high anxiety.	0.288 (0.193-0.399)
Controlled phase of uterine cancer	Generic uncomplicated disease: worry and daily medication	has a chronic disease that requires medication every day and causes some worry but minimal interference with daily activities.	0.049 (0.031-0.072)
Metastatic phase of uterine cancer	Cancer, metastatic	has severe pain, extreme fatigue, weight loss and high anxiety.	0.451 (0.307-0.6)
Terminal phase of uterine cancer	Terminal phase, with medication (for cancers, end-stage kidney/liver disease)	has lost a lot of weight and regularly uses strong medication to avoid constant pain. The person has no appetite, feels nauseous, and needs to spend most of the day in bed.	0.54 (0.377-0.687)
Diagnosis and primary therapy phase of ovarian cancer	Cancer, diagnosis and primary therapy	has pain, nausea, fatigue, weight loss and high anxiety.	0.288 (0.193-0.399)
Controlled phase of ovarian cancer	Generic uncomplicated disease: worry and daily medication	has a chronic disease that requires medication every day and causes some worry but minimal interference with daily activities.	0.049 (0.031-0.072)
Metastatic phase of ovarian cancer	Cancer, metastatic	has severe pain, extreme fatigue, weight loss and high anxiety.	0.451 (0.307-0.6)
Terminal phase of ovarian cancer	Terminal phase, with medication (for cancers, end-stage kidney/liver disease)	has lost a lot of weight and regularly uses strong medication to avoid constant pain. The person has no appetite, feels nauseous, and needs to spend most of the day in bed.	0.54 (0.377-0.687)
Diagnosis and primary therapy phase of prostate cancer	Cancer, diagnosis and primary therapy	has pain, nausea, fatigue, weight loss and high anxiety.	0.288 (0.193-0.399)
Controlled phase of prostate cancer	Generic uncomplicated disease: worry and daily medication	has a chronic disease that requires medication every day and causes some worry but minimal interference with daily activities.	0.049 (0.031-0.072)
Metastatic phase of prostate cancer	Cancer, metastatic	has severe pain, extreme fatigue, weight loss and high anxiety.	0.451 (0.307-0.6)
Terminal phase of prostate cancer	Terminal phase, with medication (for cancers, end-stage kidney/liver disease)	has lost a lot of weight and regularly uses strong medication to avoid constant pain. The person has no appetite, feels nauseous, and needs to spend most of the day in bed.	0.54 (0.377-0.687)
Impotence due to prostate cancer	Impotence	has difficulty in obtaining or maintaining an erection.	0.017 (0.009-0.03)
Incontinence due to prostate cancer	Urinary incontinence	cannot control urinating.	0.139 (0.094-0.198)
Diagnosis and primary therapy phase of testicular cancer	Cancer, diagnosis and primary therapy	has pain, nausea, fatigue, weight loss and high anxiety.	0.288 (0.193-0.399)
Controlled phase of testicular cancer	Generic uncomplicated disease: worry and daily medication	has a chronic disease that requires medication every day and causes some worry but minimal interference with daily activities.	0.049 (0.031-0.072)
Metastatic phase of testicular cancer	Cancer, metastatic	has severe pain, extreme fatigue, weight loss and high anxiety.	0.451 (0.307-0.6)
Terminal phase of testicular cancer	Terminal phase, with medication (for cancers, end-stage kidney/liver disease)	has lost a lot of weight and regularly uses strong medication to avoid constant pain. The person has no appetite, feels nauseous, and needs to spend most of the day in bed.	0.54 (0.377-0.687)
Diagnosis and primary therapy phase of kidney cancer	Cancer, diagnosis and primary therapy	has pain, nausea, fatigue, weight loss and high anxiety.	0.288 (0.193-0.399)
Controlled phase of kidney cancer	Generic uncomplicated disease: worry and daily medication	has a chronic disease that requires medication every day and causes some worry but minimal interference with daily activities.	0.049 (0.031-0.072)
Metastatic phase of kidney cancer	Cancer, metastatic	has severe pain, extreme fatigue, weight loss and high anxiety.	0.451 (0.307-0.6)
Terminal phase of kidney cancer	Terminal phase, with medication (for cancers, end-stage kidney/liver disease)	has lost a lot of weight and regularly uses strong medication to avoid constant pain. The person has no appetite, feels nauseous, and needs to spend most of the day in bed.	0.54 (0.377-0.687)
Diagnosis and primary therapy phase of bladder cancer	Cancer, diagnosis and primary therapy	has pain, nausea, fatigue, weight loss and high anxiety.	0.288 (0.193-0.399)
Controlled phase of bladder cancer	Generic uncomplicated disease: worry and daily medication	has a chronic disease that requires medication every day and causes some worry but minimal interference with daily activities.	0.049 (0.031-0.072)
Metastatic phase of bladder cancer	Cancer, metastatic	has severe pain, extreme fatigue, weight loss and high anxiety.	0.451 (0.307-0.6)
Terminal phase of bladder cancer	Terminal phase, with medication (for cancers, end-stage kidney/liver disease)	has lost a lot of weight and regularly uses strong medication to avoid constant pain. The person has no appetite, feels nauseous, and needs to spend most of the day in bed.	0.54 (0.377-0.687)
Urinary incontinence due to bladder cancer	Urinary incontinence	cannot control urinating.	0.139 (0.094-0.198)
Diagnosis and primary therapy phase of brain and nervous system cancers	Cancer, diagnosis and primary therapy	has pain, nausea, fatigue, weight loss and high anxiety.	0.288 (0.193-0.399)
Controlled phase of brain and nervous system cancers	Generic uncomplicated disease: worry and daily medication	has a chronic disease that requires medication every day and causes some worry but minimal interference with daily activities.	0.049 (0.031-0.072)
Metastatic phase of brain and nervous system cancers	Cancer, metastatic	has severe pain, extreme fatigue, weight loss and high anxiety.	0.451 (0.307-0.6)
Terminal phase of brain and nervous system cancers	Terminal phase, with medication (for cancers, end-stage kidney/liver disease)	has lost a lot of weight and regularly uses strong medication to avoid constant pain. The person has no appetite, feels nauseous, and needs to spend most of the day in bed.	0.54 (0.377-0.687)
Diagnosis and primary therapy phase of thyroid cancer	Cancer, diagnosis and primary therapy	has pain, nausea, fatigue, weight loss and high anxiety.	0.288 (0.193-0.399)
Controlled phase of thyroid cancer	Generic uncomplicated disease: worry and daily medication	has a chronic disease that requires medication every day and causes some worry but minimal interference with daily activities.	0.049 (0.031-0.072)
Metastatic phase of thyroid cancer	Cancer, metastatic	has severe pain, extreme fatigue, weight loss and high anxiety.	0.451 (0.307-0.6)
Terminal phase of thyroid cancer	Terminal phase, with medication (for cancers, end-stage kidney/liver disease)	has lost a lot of weight and regularly uses strong medication to avoid constant pain. The person has no appetite, feels nauseous, and needs to spend most of the day in bed.	0.54 (0.377-0.687)
Diagnosis and primary therapy phase of mesothelioma	Cancer, diagnosis and primary therapy	has pain, nausea, fatigue, weight loss and high anxiety.	0.288 (0.193-0.399)
Controlled phase of mesothelioma	Generic uncomplicated disease: worry and daily medication	has a chronic disease that requires medication every day and causes some worry but minimal interference with daily activities.	0.049 (0.031-0.072)
Metastatic phase of mesothelioma	Cancer, metastatic	has severe pain, extreme fatigue, weight loss and high anxiety.	0.451 (0.307-0.6)
Terminal phase of mesothelioma	Terminal phase, with medication (for cancers, end-stage kidney/liver disease)	has lost a lot of weight and regularly uses strong medication to avoid constant pain. The person has no appetite, feels nauseous, and needs to spend most of the day in bed.	0.54 (0.377-0.687)
Diagnosis and primary therapy phase of Hodgkin disease	Cancer, diagnosis and primary therapy	has pain, nausea, fatigue, weight loss and high anxiety.	0.288 (0.193-0.399)
Controlled phase of Hodgkin disease	Generic uncomplicated disease: worry and daily medication	has a chronic disease that requires medication every day and causes some worry but minimal interference with daily activities.	0.049 (0.031-0.072)
Metastatic phase of Hodgkin disease	Cancer, metastatic	has severe pain, extreme fatigue, weight loss and high anxiety.	0.451 (0.307-0.6)
Terminal phase of Hodgkin disease	Terminal phase, with medication (for cancers, end-stage kidney/liver disease)	has lost a lot of weight and regularly uses strong medication to avoid constant pain. The person has no appetite, feels nauseous, and needs to spend most of the day in bed.	0.54 (0.377-0.687)
Diagnosis and primary therapy phase of non-Hodgkin lymphoma	Cancer, diagnosis and primary therapy	has pain, nausea, fatigue, weight loss and high anxiety.	0.288 (0.193-0.399)
Controlled phase of non-Hodgkin lymphoma	Generic uncomplicated disease: worry and daily medication	has a chronic disease that requires medication every day and causes some worry but minimal interference with daily activities.	0.049 (0.031-0.072)
Metastatic phase of non-Hodgkin lymphoma	Cancer, metastatic	has severe pain, extreme fatigue, weight loss and high anxiety.	0.451 (0.307-0.6)
Terminal phase of non-Hodgkin lymphoma	Terminal phase, with medication (for cancers, end-stage kidney/liver disease)	has lost a lot of weight and regularly uses strong medication to avoid constant pain. The person has no appetite, feels nauseous, and needs to spend most of the day in bed.	0.54 (0.377-0.687)
Diagnosis and primary therapy phase of multiple myeloma	Cancer, diagnosis and primary therapy	has pain, nausea, fatigue, weight loss and high anxiety.	0.288 (0.193-0.399)
Controlled phase of multiple myeloma	Generic uncomplicated disease: worry and daily medication	has a chronic disease that requires medication every day and causes some worry but minimal interference with daily activities.	0.049 (0.031-0.072)
Metastatic phase of multiple myeloma	Cancer, metastatic	has severe pain, extreme fatigue, weight loss and high anxiety.	0.451 (0.307-0.6)

Appendix Table 6. GBD 2015 sequelae, health states, health state lay descriptions, and disability weights

Sequela	Healthstate name	Healthstate lay description	Disability weight
Terminal phase of multiple myeloma	Terminal phase, with medication (for cancers, end-stage kidney/liver disease)	has lost a lot of weight and regularly uses strong medication to avoid constant pain. The person has no appetite, feels nauseous, and needs to spend most of the day in bed.	0.54 (0.377-0.687)
Controlled phase of acute lymphoid leukemia	Generic uncomplicated disease: worry and daily medication	has a chronic disease that requires medication every day and causes some worry but minimal interference with daily activities.	0.049 (0.031-0.072)
Diagnosis and primary therapy phase of acute lymphoid leukemia	Cancer, diagnosis and primary therapy	has pain, nausea, fatigue, weight loss and high anxiety.	0.288 (0.193-0.399)
Metastatic phase of acute lymphoid leukemia	Cancer, metastatic	has severe pain, extreme fatigue, weight loss and high anxiety.	0.451 (0.307-0.6)
Terminal phase of acute lymphoid leukemia	Terminal phase, with medication (for cancers, end-stage kidney/liver disease)	has lost a lot of weight and regularly uses strong medication to avoid constant pain. The person has no appetite, feels nauseous, and needs to spend most of the day in bed.	0.54 (0.377-0.687)
Controlled phase of chronic lymphoid leukemia	Generic uncomplicated disease: worry and daily medication	has a chronic disease that requires medication every day and causes some worry but minimal interference with daily activities.	0.049 (0.031-0.072)
Diagnosis and primary therapy phase of chronic lymphoid leukemia	Cancer, diagnosis and primary therapy	has pain, nausea, fatigue, weight loss and high anxiety.	0.288 (0.193-0.399)
Metastatic phase of chronic lymphoid leukemia	Cancer, metastatic	has severe pain, extreme fatigue, weight loss and high anxiety.	0.451 (0.307-0.6)
Terminal phase of chronic lymphoid leukemia	Terminal phase, with medication (for cancers, end-stage kidney/liver disease)	has lost a lot of weight and regularly uses strong medication to avoid constant pain. The person has no appetite, feels nauseous, and needs to spend most of the day in bed.	0.54 (0.377-0.687)
Controlled phase of acute myeloid leukemia	Generic uncomplicated disease: worry and daily medication	has a chronic disease that requires medication every day and causes some worry but minimal interference with daily activities.	0.049 (0.031-0.072)
Diagnosis and primary therapy phase of acute myeloid leukemia	Cancer, diagnosis and primary therapy	has pain, nausea, fatigue, weight loss and high anxiety.	0.288 (0.193-0.399)
Metastatic phase of acute myeloid leukemia	Cancer, metastatic	has severe pain, extreme fatigue, weight loss and high anxiety.	0.451 (0.307-0.6)
Terminal phase of acute myeloid leukemia	Terminal phase, with medication (for cancers, end-stage kidney/liver disease)	has lost a lot of weight and regularly uses strong medication to avoid constant pain. The person has no appetite, feels nauseous, and needs to spend most of the day in bed.	0.54 (0.377-0.687)
Controlled phase of chronic myeloid leukemia	Generic uncomplicated disease: worry and daily medication	has a chronic disease that requires medication every day and causes some worry but minimal interference with daily activities.	0.049 (0.031-0.072)
Diagnosis and primary therapy phase of chronic myeloid leukemia	Cancer, diagnosis and primary therapy	has pain, nausea, fatigue, weight loss and high anxiety.	0.288 (0.193-0.399)
Metastatic phase of chronic myeloid leukemia	Cancer, metastatic	has severe pain, extreme fatigue, weight loss and high anxiety.	0.451 (0.307-0.6)
Terminal phase of chronic myeloid leukemia	Terminal phase, with medication (for cancers, end-stage kidney/liver disease)	has lost a lot of weight and regularly uses strong medication to avoid constant pain. The person has no appetite, feels nauseous, and needs to spend most of the day in bed.	0.54 (0.377-0.687)
Diagnosis and primary therapy phase of other neoplasms	Cancer, diagnosis and primary therapy	has pain, nausea, fatigue, weight loss and high anxiety.	0.288 (0.193-0.399)
Controlled phase of other neoplasms	Generic uncomplicated disease: worry and daily medication	has a chronic disease that requires medication every day and causes some worry but minimal interference with daily activities.	0.049 (0.031-0.072)
Metastatic phase of other neoplasms	Cancer, metastatic	has severe pain, extreme fatigue, weight loss and high anxiety.	0.451 (0.307-0.6)
Terminal phase of other neoplasms	Terminal phase, with medication (for cancers, end-stage kidney/liver disease)	has lost a lot of weight and regularly uses strong medication to avoid constant pain. The person has no appetite, feels nauseous, and needs to spend most of the day in bed.	0.54 (0.377-0.687)
Rheumatic heart disease, without heart failure	Generic uncomplicated disease: worry and daily medication	has a chronic disease that requires medication every day and causes some worry but minimal interference with daily activities.	0.049 (0.031-0.072)
Mild heart failure due to rheumatic heart disease	Heart failure, mild	is short of breath and easily tires with moderate physical activity, such as walking uphill or more than a quarter-mile on level ground. The person feels comfortable at rest or during activities requiring less effort.	0.041 (0.026-0.062)
Moderate heart failure due to rheumatic heart disease	Heart failure, moderate	is short of breath and easily tires with minimal physical activity, such as walking only a short distance. The person feels comfortable at rest but avoids moderate activity.	0.072 (0.047-0.103)
Severe heart failure due to rheumatic heart disease	Heart failure, severe	is short of breath and feels tired when at rest. The person avoids any physical activity, for fear of worsening the breathing problems.	0.179 (0.122-0.251)
Asymptomatic ischemic heart disease following myocardial infarction	--	--	--
Acute myocardial infarction first 2 days	Acute myocardial infarction, days 1-2	has severe chest pain that becomes worse with any physical activity,. The person feels nauseous, short of breath, and very anxious.	0.432 (0.288-0.579)
Acute myocardial infarction 3 to 28 days	Acute myocardial infarction, days 3-28	gets short of breath after heavy physical activity, and tires easily, but has no problems when at rest. The person has to take medication every day and has some anxiety.	0.074 (0.049-0.105)
Asymptomatic angina due to ischemic heart disease	--	--	--
Mild angina due to ischemic heart disease	Angina pectoris, mild	has chest pain that occurs with strenuous physical activity, such as running or lifting heavy objects. After a brief rest, the pain goes away.	0.033 (0.02-0.052)
Moderate angina due to ischemic heart disease	Angina pectoris, moderate	has chest pain that occurs with moderate physical activity, such as walking uphill or more than half a kilometer (around a quarter-mile) on level ground. After a brief rest, the pain goes away.	0.08 (0.052-0.113)
Severe angina due to ischemic heart disease	Angina pectoris, severe	has chest pain that occurs with minimal physical activity, such as walking only a short distance. After a brief rest, the pain goes away. The person avoids most physical activities because of the pain.	0.167 (0.11-0.24)
Mild heart failure due to ischemic heart disease	Heart failure, mild	is short of breath and easily tires with moderate physical activity, such as walking uphill or more than a quarter-mile on level ground. The person feels comfortable at rest or during activities requiring less effort.	0.041 (0.026-0.062)
Moderate heart failure due to ischemic heart disease	Heart failure, moderate	is short of breath and easily tires with minimal physical activity, such as walking only a short distance. The person feels comfortable at rest but avoids moderate activity.	0.072 (0.047-0.103)
Severe heart failure due to ischemic heart disease	Heart failure, severe	is short of breath and feels tired when at rest. The person avoids any physical activity, for fear of worsening the breathing problems.	0.179 (0.122-0.251)
Asymptomatic chronic ischemic stroke	--	--	--
Chronic ischemic stroke severity level 1	Stroke, long-term consequences, mild	has some difficulty in moving around and some weakness in one hand, but is able to walk without help.	0.019 (0.01-0.032)
Chronic ischemic stroke severity level 2	Stroke, long-term consequences, moderate	has some difficulty in moving around, and in using the hands for lifting and holding things, dressing and grooming.	0.07 (0.046-0.099)
Chronic ischemic stroke severity level 4	Stroke, long-term consequences, severe	is confined to bed or a wheelchair, has difficulty speaking and depends on others for feeding, toileting and dressing.	0.552 (0.377-0.707)
Chronic ischemic stroke severity level 3	Stroke, long-term consequences, moderate plus cognition problems	has some difficulty in moving around, in using the hands for lifting and holding things, dressing and grooming, and in speaking. The person is often forgetful and confused.	0.316 (0.206-0.437)
Chronic ischemic stroke severity level 5	Stroke, long-term consequences, severe plus cognition problems	is confined to bed or a wheelchair, depends on others for feeding, toileting and dressing, and has difficulty speaking, thinking clearly and remembering things.	0.588 (0.411-0.744)
Acute ischemic stroke severity level 1	Stroke, long-term consequences, mild	has some difficulty in moving around and some weakness in one hand, but is able to walk without help.	0.019 (0.01-0.032)
Acute ischemic stroke severity level 2	Stroke, long-term consequences, moderate	has some difficulty in moving around, and in using the hands for lifting and holding things, dressing and grooming.	0.07 (0.046-0.099)
Acute ischemic stroke severity level 4	Stroke, long-term consequences, severe	is confined to bed or a wheelchair, has difficulty speaking and depends on others for feeding, toileting and dressing.	0.552 (0.377-0.707)
Acute ischemic stroke severity level 3	Stroke, long-term consequences, moderate plus cognition problems	has some difficulty in moving around, in using the hands for lifting and holding things, dressing and grooming, and in speaking. The person is often forgetful and confused.	0.316 (0.206-0.437)
Acute ischemic stroke severity level 5	Stroke, long-term consequences, severe plus cognition problems	is confined to bed or a wheelchair, depends on others for feeding, toileting and dressing, and has difficulty speaking, thinking clearly and remembering things.	0.588 (0.411-0.744)
Asymptomatic chronic hemorrhagic stroke	--	--	--
Chronic hemorrhagic stroke severity level 1	Stroke, long-term consequences, mild	has some difficulty in moving around and some weakness in one hand, but is able to walk without help.	0.019 (0.01-0.032)
Chronic hemorrhagic stroke severity level 2	Stroke, long-term consequences, moderate	has some difficulty in moving around, and in using the hands for lifting and holding things, dressing and grooming.	0.07 (0.046-0.099)
Chronic hemorrhagic stroke severity level 4	Stroke, long-term consequences, severe	is confined to bed or a wheelchair, has difficulty speaking and depends on others for feeding, toileting and dressing.	0.552 (0.377-0.707)

Appendix Table 6. GBD 2015 sequelae, health states, health state lay descriptions, and disability weights			
**Sequela**	**Healthstate name**	**Healthstate lay description**	**Disability weight**
Chronic hemorrhagic stroke severity level 3	Stroke, long-term consequences, moderate plus cognition problems	has some difficulty in moving around, in using the hands for lifting and holding things, dressing and grooming, and in speaking. The person is often forgetful and confused.	0.316 (0.206-0.437)
Chronic hemorrhagic stroke severity level 5	Stroke, long-term consequences, severe plus cognition problems	is confined to bed or a wheelchair, depends on others for feeding, toileting and dressing, and has difficulty speaking, thinking clearly and remembering things.	0.588 (0.411-0.744)
Acute hemorrhagic stroke severity level 1	Stroke, long-term consequences, mild	has some difficulty in moving around and some weakness in one hand, but is able to walk without help.	0.019 (0.01-0.032)
Acute hemorrhagic stroke severity level 2	Stroke, long-term consequences, moderate	has some difficulty in moving around, and in using the hands for lifting and holding things, dressing and grooming.	0.07 (0.046-0.099)
Acute hemorrhagic stroke severity level 4	Stroke, long-term consequences, severe	is confined to bed or a wheelchair, has difficulty speaking and depends on others for feeding, toileting and dressing.	0.552 (0.377-0.707)
Acute hemorrhagic stroke severity level 3	Stroke, long-term consequences, moderate plus cognition problems	has some difficulty in moving around, in using the hands for lifting and holding things, dressing and grooming, and in speaking. The person is often forgetful and confused.	0.316 (0.206-0.437)
Acute hemorrhagic stroke severity level 5	Stroke, long-term consequences, severe plus cognition problems	is confined to bed or a wheelchair, depends on others for feeding, toileting and dressing, and has difficulty speaking, thinking clearly and remembering things.	0.588 (0.411-0.744)
Mild heart failure due to hypertensive heart disease	Heart failure, mild	is short of breath and easily tires with moderate physical activity, such as walking uphill or more than a quarter-mile on level ground. The person feels comfortable at rest or during activities requiring less effort.	0.041 (0.026-0.062)
Moderate heart failure due to hypertensive heart disease	Heart failure, moderate	is short of breath and easily tires with minimal physical activity, such as walking only a short distance. The person feels comfortable at rest but avoids moderate activity.	0.072 (0.047-0.103)
Severe heart failure due to hypertensive heart disease	Heart failure, severe	is short of breath and feels tired when at rest. The person avoids any physical activity, for fear of worsening the breathing problems.	0.179 (0.122-0.251)
Acute myocarditis	Infectious disease, acute episode, moderate	has a fever and aches, and feels weak, which causes some difficulty with daily activities.	0.051 (0.032-0.074)
Mild heart failure due to cardiomyopathy and myocarditis	Heart failure, mild	is short of breath and easily tires with moderate physical activity, such as walking uphill or more than a quarter-mile on level ground. The person feels comfortable at rest or during activities requiring less effort.	0.041 (0.026-0.062)
Moderate heart failure due to cardiomyopathy and myocarditis	Heart failure, moderate	is short of breath and easily tires with minimal physical activity, such as walking only a short distance. The person feels comfortable at rest but avoids moderate activity.	0.072 (0.047-0.103)
Severe heart failure due to cardiomyopathy and myocarditis	Heart failure, severe	is short of breath and feels tired when at rest. The person avoids any physical activity, for fear of worsening the breathing problems.	0.179 (0.122-0.251)
Asymptomatic atrial fibrillation and flutter	--	--	--
Symptomatic atrial fibrillation and flutter	Cardiac conduction disorders and cardiac dysrhythmias	has periods of rapid and irregular heartbeats and occasional fainting.	0.224 (0.151-0.312)
Asymptomatic peripheral vascular disease	--	--	--
Symptomatic claudication due to peripheral vascular disease	Claudication	has cramping pains in the legs after walking a medium distance. The pain goes away after a short rest.	0.014 (0.007-0.025)
Moderate endocarditis	Infectious disease, acute episode, moderate	has a fever and aches, and feels weak, which causes some difficulty with daily activities.	0.051 (0.032-0.074)
Severe endocarditis	Infectious disease, acute episode, severe	has a high fever and pain, and feels very weak, which causes great difficulty with daily activities.	0.133 (0.088-0.19)
Mild heart failure due to endocarditis	Heart failure, mild	is short of breath and easily tires with moderate physical activity, such as walking uphill or more than a quarter-mile on level ground. The person feels comfortable at rest or during activities requiring less effort.	0.041 (0.026-0.062)
Moderate heart failure due to endocarditis	Heart failure, moderate	is short of breath and easily tires with minimal physical activity, such as walking only a short distance. The person feels comfortable at rest but avoids moderate activity.	0.072 (0.047-0.103)
Severe heart failure due to endocarditis	Heart failure, severe	is short of breath and feels tired when at rest. The person avoids any physical activity, for fear of worsening the breathing problems.	0.179 (0.122-0.251)
Mild heart failure due to other cardiovascular diseases	Heart failure, mild	is short of breath and easily tires with moderate physical activity, such as walking uphill or more than a quarter-mile on level ground. The person feels comfortable at rest or during activities requiring less effort.	0.041 (0.026-0.062)
Moderate heart failure due to other cardiovascular diseases	Heart failure, moderate	is short of breath and easily tires with minimal physical activity, such as walking only a short distance. The person feels comfortable at rest but avoids moderate activity.	0.072 (0.047-0.103)
Severe heart failure due to other cardiovascular diseases	Heart failure, severe	is short of breath and feels tired when at rest. The person avoids any physical activity, for fear of worsening the breathing problems.	0.179 (0.122-0.251)
Asymptomatic other cardiovascular diseases	--	--	--
Mild other cardiovascular diseases	Heart failure, mild	is short of breath and easily tires with moderate physical activity, such as walking uphill or more than a quarter-mile on level ground. The person feels comfortable at rest or during activities requiring less effort.	0.041 (0.026-0.062)
Moderate other cardiovascular diseases	Heart failure, moderate	is short of breath and easily tires with minimal physical activity, such as walking only a short distance. The person feels comfortable at rest but avoids moderate activity.	0.072 (0.047-0.103)
Severe other cardiovascular diseases	Heart failure, severe	is short of breath and feels tired when at rest. The person avoids any physical activity, for fear of worsening the breathing problems.	0.179 (0.122-0.251)
Asymptomatic chronic obstructive pulmonary disease	--	--	--
Mild chronic obstructive pulmonary disease	COPD and other chronic respiratory problems, mild	has cough and shortness of breath after heavy physical activity, but is able to walk long distances and climb stairs.	0.019 (0.011-0.033)
Moderate chronic obstructive pulmonary disease	COPD and other chronic respiratory problems, moderate	has cough, wheezing and shortness of breath, even after light physical activity. The person feels tired and can walk only short distances or climb only a few stairs.	0.225 (0.153-0.31)
Severe chronic obstructive pulmonary disease without heart failure	COPD and other chronic respiratory problems, severe	has cough, wheezing and shortness of breath all the time. The person has great difficulty walking even short distances or climbing any stairs, feels tired when at rest, and is anxious.	0.408 (0.273-0.556)
Mild heart failure due to severe chronic obstructive pulmonary disease	Severe COPD and other chronic respiratory, with mild heart failure	(combined DW)	--
Moderate heart failure due to severe chronic obstructive pulmonary disease	Severe COPD and other chronic respiratory, with moderate heart failure	(combined DW)	--
Severe heart failure due to severe chronic obstructive pulmonary disease	Severe COPD and other chronic respiratory, with severe heart failure	(combined DW)	--
Asymptomatic silicosis	--	--	--
Mild silicosis	COPD and other chronic respiratory problems, mild	has cough and shortness of breath after heavy physical activity, but is able to walk long distances and climb stairs.	0.019 (0.011-0.033)
Moderate silicosis	COPD and other chronic respiratory problems, moderate	has cough, wheezing and shortness of breath, even after light physical activity. The person feels tired and can walk only short distances or climb only a few stairs.	0.225 (0.153-0.31)
Severe silicosis without heart failure	COPD and other chronic respiratory problems, severe	has cough, wheezing and shortness of breath all the time. The person has great difficulty walking even short distances or climbing any stairs, feels tired when at rest, and is anxious.	0.408 (0.273-0.556)
Mild heart failure due to severe silicosis	Severe COPD and other chronic respiratory, with mild heart failure	(combined DW)	--
Moderate heart failure due to severe silicosis	Severe COPD and other chronic respiratory, with moderate heart failure	(combined DW)	--
Severe heart failure due to severe silicosis	Severe COPD and other chronic respiratory, with severe heart failure	(combined DW)	--
Asymptomatic asbestosis	--	--	--
Mild asbestosis	COPD and other chronic respiratory problems, mild	has cough and shortness of breath after heavy physical activity, but is able to walk long distances and climb stairs.	0.019 (0.011-0.033)
Moderate asbestosis	COPD and other chronic respiratory problems, moderate	has cough, wheezing and shortness of breath, even after light physical activity. The person feels tired and can walk only short distances or climb only a few stairs.	0.225 (0.153-0.31)
Severe asbestosis without heart failure	COPD and other chronic respiratory problems, severe	has cough, wheezing and shortness of breath all the time. The person has great difficulty walking even short distances or climbing any stairs, feels tired when at rest, and is anxious.	0.408 (0.273-0.556)
Mild heart failure due to severe asbestosis	Severe COPD and other chronic respiratory, with mild heart failure	(combined DW)	--
Moderate heart failure due to severe asbestosis	Severe COPD and other chronic respiratory, with moderate heart failure	(combined DW)	--

Appendix Table 6. GBD 2015 sequelae, health states, health state lay descriptions, and disability weights			
**Sequela**	**Healthstate name**	**Healthstate lay description**	**Disability weight**
Severe heart failure due to severe asbestosis	Severe COPD and other chronic respiratory, with severe heart failure	(combined DW)	--
Asymptomatic coal workers pneumoconiosis	--	--	--
Mild coal workers pneumoconiosis	COPD and other chronic respiratory problems, mild	has cough and shortness of breath after heavy physical activity, but is able to walk long distances and climb stairs.	0.019 (0.011-0.033)
Moderate coal workers pneumoconiosis	COPD and other chronic respiratory problems, moderate	has cough, wheezing and shortness of breath, even after light physical activity. The person feels tired and can walk only short distances or climb only a few stairs.	0.225 (0.153-0.31)
Severe coal workers pneumoconiosis without heart failure	COPD and other chronic respiratory problems, severe	has cough, wheezing and shortness of breath all the time. The person has great difficulty walking even short distances or climbing any stairs, feels tired when at rest, and is anxious.	0.408 (0.273-0.556)
Mild heart failure due to severe coal workers pneumoconiosis	Severe COPD and other chronic respiratory, with mild heart failure	(combined DW)	--
Moderate heart failure due to severe coal workers pneumoconiosis	Severe COPD and other chronic respiratory, with moderate heart failure	(combined DW)	--
Severe heart failure due to severe coal workers pneumoconiosis	Severe COPD and other chronic respiratory, with severe heart failure	(combined DW)	--
Asymptomatic other pneumoconiosis	--	--	--
Mild other pneumoconiosis	COPD and other chronic respiratory problems, mild	has cough and shortness of breath after heavy physical activity, but is able to walk long distances and climb stairs.	0.019 (0.011-0.033)
Moderate other pneumoconiosis	COPD and other chronic respiratory problems, moderate	has cough, wheezing and shortness of breath, even after light physical activity. The person feels tired and can walk only short distances or climb only a few stairs.	0.225 (0.153-0.31)
Severe other pneumoconiosis without heart failure	COPD and other chronic respiratory problems, severe	has cough, wheezing and shortness of breath all the time. The person has great difficulty walking even short distances or climbing any stairs, feels tired when at rest, and is anxious.	0.408 (0.273-0.556)
Mild heart failure due to severe other pneumoconiosis	Severe COPD and other chronic respiratory, with mild heart failure	(combined DW)	--
Moderate heart failure due to severe other pneumoconiosis	Severe COPD and other chronic respiratory, with moderate heart failure	(combined DW)	--
Severe heart failure due to severe other pneumoconiosis	Severe COPD and other chronic respiratory, with severe heart failure	(combined DW)	--
Asymptomatic asthma	--	--	--
Controlled asthma	Asthma, controlled	has wheezing and cough once a month, which does not cause difficulty with daily activities.	0.015 (0.007-0.026)
Partially controlled asthma	Asthma, partially controlled	has wheezing and cough once a week, which causes some difficulty with daily activities.	0.036 (0.022-0.055)
Uncontrolled asthma	Asthma, uncontrolled	has wheezing, cough and shortness of breath more than twice a week, which causes difficulty with daily activities and sometimes wakes the person at night.	0.133 (0.086-0.192)
Asymptomatic interstitial lung disease and pulmonary sarcoidosis	--	--	--
Mild interstitial lung disease and pulmonary sarcoidosis	COPD and other chronic respiratory problems, mild	has cough and shortness of breath after heavy physical activity, but is able to walk long distances and climb stairs.	0.019 (0.011-0.033)
Moderate interstitial lung disease and pulmonary sarcoidosis	COPD and other chronic respiratory problems, moderate	has cough, wheezing and shortness of breath, even after light physical activity. The person feels tired and can walk only short distances or climb only a few stairs.	0.225 (0.153-0.31)
Severe interstitial lung disease and pulmonary sarcoidosis without heart failure	COPD and other chronic respiratory problems, severe	has cough, wheezing and shortness of breath all the time. The person has great difficulty walking even short distances or climbing any stairs, feels tired when at rest, and is anxious.	0.408 (0.273-0.556)
Mild heart failure due to severe interstitial lung disease and pulmonary sarcoidosis	Severe COPD and other chronic respiratory, with mild heart failure	(combined DW)	--
Moderate heart failure due to severe interstitial lung disease and pulmonary sarcoidosis	Severe COPD and other chronic respiratory, with moderate heart failure	(combined DW)	--
Severe heart failure due to severe interstitial lung disease and pulmonary sarcoidosis	Severe COPD and other chronic respiratory, with severe heart failure	(combined DW)	--
Other chronic respiratory diseases	--	--	--
Cirrhosis and other chronic liver diseases due to hepatitis B	Decompensated cirrhosis of the liver	has a swollen belly and swollen legs. The person feels weakness, fatigue and loss of appetite.	0.178 (0.123-0.25)
Cirrhosis and other chronic liver diseases due to hepatitis C	Decompensated cirrhosis of the liver	has a swollen belly and swollen legs. The person feels weakness, fatigue and loss of appetite.	0.178 (0.123-0.25)
Cirrhosis and other chronic liver diseases due to alcohol	Decompensated cirrhosis of the liver	has a swollen belly and swollen legs. The person feels weakness, fatigue and loss of appetite.	0.178 (0.123-0.25)
Cirrhosis and other chronic liver diseases due to other cause	Decompensated cirrhosis of the liver	has a swollen belly and swollen legs. The person feels weakness, fatigue and loss of appetite.	0.178 (0.123-0.25)
Peptic ulcer disease, symptomatic episodes	Abdominopelvic problem, moderate	has pain in the belly and feels nauseous. The person has difficulties with daily activities.	0.114 (0.078-0.159)
Mild anemia due to peptic ulcer disease	Anemia, mild	feels slightly tired and weak at times, but this does not interfere with normal daily activities.	0.004 (0.001-0.008)
Moderate anemia due to peptic ulcer disease	Anemia, moderate	feels moderate fatigue, weakness, and shortness of breath after exercise, making daily activities more difficult.	0.052 (0.034-0.076)
Severe anemia due to peptic ulcer disease	Anemia, severe	feels very weak, tired and short of breath, and has problems with activities that require physical effort or deep concentration.	0.149 (0.101-0.209)
Gastritis and duodenitis, symptomatic episodes	Abdominopelvic problem, moderate	has pain in the belly and feels nauseous. The person has difficulties with daily activities.	0.114 (0.078-0.159)
Mild anemia due to gastritis and duodenitis	Anemia, mild	feels slightly tired and weak at times, but this does not interfere with normal daily activities.	0.004 (0.001-0.008)
Moderate anemia due to gastritis and duodenitis	Anemia, moderate	feels moderate fatigue, weakness, and shortness of breath after exercise, making daily activities more difficult.	0.052 (0.034-0.076)
Severe anemia due to gastritis and duodenitis	Anemia, severe	feels very weak, tired and short of breath, and has problems with activities that require physical effort or deep concentration.	0.149 (0.101-0.209)
Appendicitis	Abdominopelvic problem, severe	has severe pain in the belly and feels nauseous. The person is anxious and unable to carry out daily activities.	0.324 (0.22-0.442)
Paralytic ileus and intestinal obstruction	Abdominopelvic problem, severe	has severe pain in the belly and feels nauseous. The person is anxious and unable to carry out daily activities.	0.324 (0.22-0.442)
Inguinal, femoral, and abdominal hernia cases	Abdominopelvic problem, mild	has some pain in the belly that causes nausea but does not interfere with daily activities.	0.011 (0.005-0.021)
Ulcerative colitis	Crohn disease or ulcerative colitis	has cramping abdominal pain, has diarrhea several times a day, and feels very tired for two months every year. When the person does not have symptoms, there is anxiety about them returning.	0.231 (0.156-0.32)
Crohn's disease	Crohn disease or ulcerative colitis	has cramping abdominal pain, has diarrhea several times a day, and feels very tired for two months every year. When the person does not have symptoms, there is anxiety about them returning.	0.231 (0.156-0.32)
Vascular intestinal disorders	Abdominopelvic problem, severe	has severe pain in the belly and feels nauseous. The person is anxious and unable to carry out daily activities.	0.324 (0.22-0.442)
Gallbladder and biliary diseases	Abdominopelvic problem, moderate	has pain in the belly and feels nauseous. The person has difficulties with daily activities.	0.114 (0.078-0.159)
Pancreatitis cases	Abdominopelvic problem, severe	has severe pain in the belly and feels nauseous. The person is anxious and unable to carry out daily activities.	0.324 (0.22-0.442)
Other digestive diseases	--	--	--
Mild Alzheimer disease and other dementias	Dementia, mild	has some trouble remembering recent events, and finds it hard to concentrate and make decisions and plans.	0.069 (0.046-0.099)
Moderate Alzheimer disease and other dementias	Dementia, moderate	has memory problems and confusion, feels disoriented, at times hears voices that are not real, and needs help with some daily activities.	0.377 (0.252-0.508)

Appendix Table 6. GBD 2015 sequelae, health states, health state lay descriptions, and disability weights

Sequela	Healthstate name	Healthstate lay description	Disability weight
Severe Alzheimer disease and other dementias	Dementia, severe	has complete memory loss; no longer recognizes close family members; and requires help with all daily activities.	0.449 (0.304-0.595)
Mild Parkinson disease	Parkinson disease, mild	has mild tremors and moves a little slowly, but is able to walk and do daily activities without assistance.	0.01 (0.005-0.019)
Moderate Parkinson disease	Parkinson disease, moderate	has moderate tremors and moves slowly, which causes some difficulty in walking and daily activities. The person has some trouble swallowing, talking, sleeping, and remembering things.	0.267 (0.181-0.372)
Severe Parkinson disease	Parkinson disease, severe	has severe tremors and moves very slowly, which causes great difficulty in walking and daily activities. The person falls easily and has a lot of difficulty talking, swallowing, sleeping, and remembering things.	0.575 (0.396-0.73)
Seizure-free, treated epilepsy	Generic uncomplicated disease: worry and daily medication	has a chronic disease that requires medication every day and causes some worry but minimal interference with daily activities.	0.049 (0.031-0.072)
Less severe epilepsy	Epilepsy, less severe (seizures < once per month)	has sudden seizures two to five times a year, with violent muscle contractions and stiffness, loss of consciousness, and loss of urine or bowel control.	0.263 (0.173-0.367)
Severe epilepsy	Epilepsy, severe (seizures >= once per month)	has sudden seizures one or more times each month, with violent muscle contractions and stiffness, loss of consciousness, and loss of urine or bowel control. Between seizures the person has memory loss and difficulty concentrating.	0.552 (0.375-0.71)
Mild multiple sclerosis	Multiple sclerosis, mild	has mild loss of feeling in one hand, is a little unsteady while walking, has slight loss of vision in one eye, and often needs to urinate urgently.	0.183 (0.124-0.253)
Moderate multiple sclerosis	Multiple sclerosis, moderate	needs help walking, has difficulty with writing and arm coordination, has loss of vision in one eye and cannot control urinating.	0.463 (0.313-0.613)
Severe multiple sclerosis	Multiple sclerosis, severe	has slurred speech and difficulty swallowing. The person has weak arms and hands, very limited and stiff leg movement, has loss of vision in both eyes and cannot control urinating.	0.719 (0.534-0.858)
Asymptomatic, but worry about diagnosis of motor neuron disease	Generic uncomplicated disease: anxiety about diagnosis	has a disease diagnosis that causes some worry but minimal interference with daily activities.	0.012 (0.006-0.023)
Mild motor impairment and mild respiratory problems due to motor neuron disease	Mild motor impairment and mild COPD	(combined DW)	--
Mild motor impairment and severe respiratory problems due to motor neuron disease	Mild motor impairment and severe COPD	(combined DW)	--
Mild motor impairment and speech problems due to motor neuron disease	Mild motor impairment and speech problems	(combined DW)	--
Mild motor impairment due to motor neuron disease	Motor impairment, mild	has some difficulty in moving around but is able to walk without help.	0.01 (0.005-0.019)
Mild motor impairment, mild respiratory problems, and speech problems due to motor neuron disease	Mild motor impairment, mild COPD and speech problems	(combined DW)	--
Mild motor impairment, moderate respiratory problems, and speech problems due to motor neuron disease	Mild motor impairment, moderate COPD and speech problems	(combined DW)	--
Mild motor impairment, severe respiratory problems and speech problems due to motor neuron disease	Mild motor impairment, severe COPD and speech problems	(combined DW)	--
Mild respiratory problems and speech problems due to motor neuron disease	Mild COPD and speech problems	(combined DW)	--
Mild respiratory problems due to motor neuron disease	COPD and other chronic respiratory problems, mild	has cough and shortness of breath after heavy physical activity, but is able to walk long distances and climb stairs.	0.019 (0.011-0.033)
Moderate motor impairment and mild respiratory problems due to motor neuron disease	Moderate motor impairment and mild COPD	(combined DW)	--
Moderate motor impairment and moderate respiratory problems due to motor neuron disease	Moderate motor impairment and moderate COPD	(combined DW)	--
Moderate motor impairment and severe respiratory problems due to motor neuron disease	Moderate motor impairment and severe COPD	(combined DW)	--
Moderate motor impairment and speech problems due to motor neuron disease	Moderate motor impairment and speech problems	(combined DW)	--
Moderate motor impairment due to motor neuron disease	Motor impairment, moderate	has some difficulty in moving around, and difficulty in lifting and holding objects, dressing and sitting upright, but is able to walk without help.	0.061 (0.04-0.089)
Moderate motor impairment, mild respiratory problems, and speech problems due to motor neuron disease	Moderate motor impairment, mild COPD and speech problems	(combined DW)	--
Moderate motor impairment, moderate respiratory problems, and speech problems due to motor neuron disease	Moderate motor impairment, moderate COPD and speech problems	(combined DW)	--
Moderate motor impairment, severe respiratory problems, and speech problems due to motor neuron disease	Moderate motor impairment, severe COPD and speech problems	(combined DW)	--
Moderate respiratory problems and speech problems due to motor neuron disease	Moderate COPD and speech problems	(combined DW)	--
Moderate respiratory problems due to motor neuron disease	COPD and other chronic respiratory problems, moderate	has cough, wheezing and shortness of breath, even after light physical activity. The person feels tired and can walk only short distances or climb only a few stairs.	0.225 (0.153-0.31)
Severe motor impairment and mild respiratory problems due to motor neuron disease	Severe motor impairment and mild COPD	(combined DW)	--
Severe motor impairment and moderate respiratory problems due to motor neuron disease	Severe motor impairment and moderate COPD	(combined DW)	--
Severe motor impairment and severe respiratory problems due to motor neuron disease	Severe motor impairment and severe COPD	(combined DW)	--
Severe motor impairment and speech problems due to motor neuron disease	Severe motor impairment and speech problems	(combined DW)	--
Severe motor impairment due to motor neuron disease	Motor impairment, severe	is unable to move around without help, and is not able to lift or hold objects, get dressed or sit upright.	0.402 (0.268-0.545)
Severe motor impairment, mild respiratory problems, and speech problems due to motor neuron disease	Severe motor impairment, mild COPD and speech problems	(combined DW)	--
Severe motor impairment, moderate respiratory problems, and speech problems due to motor neuron disease	Severe motor impairment, moderate COPD and speech problems	(combined DW)	--
Severe motor impairment, severe respiratory problems, and speech problems due to motor neuron disease	Severe motor impairment, severe COPD and speech problems	(combined DW)	--
Severe respiratory problems and speech problems due to motor neuron disease	Severe COPD and speech problems	(combined DW)	--
Severe respiratory problems due to motor neuron disease	Severe COPD	has cough, wheezing and shortness of breath all the time. The person has great difficulty walking even short distances or climbing any stairs, feels tired when at rest, and is anxious.	0.408 (0.273-0.556)
Speech problems due to motor neuron disease	Speech problems	has difficulty speaking, and others find it difficult to understand.	0.051 (0.032-0.078)
Asymptomatic migraine	--	--	--
Symptomatic migraine	Headache, migraine	has severe, throbbing head pain and nausea that cause great difficulty in daily activities and sometimes confine the person to bed. Moving around, light, and noise make it worse.	0.441 (0.294-0.588)
Asymptomatic tension-type headache	--	--	--
Symptomatic tension-type headache	Headache, tension-type	has a moderate headache that also affects the neck, which causes difficulty in daily activities.	0.037 (0.022-0.057)
Asymptomatic medication overuse headache	--	--	--
Symptomatic medication overuse headache	Headache, medication overuse	has daily headaches, felt as dull pain and often lasting all day, with poor sleep, nausea and fatigue. The person takes medicine for the headaches, which provides little relief but is needed to avoid having worse symptoms.	0.217 (0.138-0.311)
Other neurological disorders	--	--	--
Guillain-Barré syndrome due to other neurological disorders	Spinal cord lesion below neck level (treated)	is paralyzed from the waist down, cannot feel or move the legs and has difficulties with urine and bowel control. The person uses a wheelchair to move around.	0.296 (0.198-0.414)
Schizophrenia residual state	Schizophrenia, residual state	hears and sees things that are not real and has trouble communicating. The person can be forgetful, has difficulty with daily activities, and thinks about hurting himself (or herself).	0.588 (0.411-0.754)

Appendix Table 6. GBD 2015 sequelae, health states, health state lay descriptions, and disability weights			
**Sequela**	**Healthstate name**	**Healthstate lay description**	**Disability weight**
Schizophrenia acute state	Schizophrenia, acute state	hears and sees things that are not real and is afraid, confused, and sometimes violent. The person has great difficulty with communication and daily activities, and sometimes wants to harm or kill himself (or herself).	0.778 (0.606-0.9)
Asymptomatic alcohol dependence	--	--	--
Very mild alcohol dependence	Alcohol use disorder, very mild	drinks alcohol daily and has difficulty controlling the urge to drink. When sober, the person functions normally.	0.123 (0.082-0.177)
Mild alcohol dependence	Alcohol use disorder, mild	drinks a lot of alcohol and sometimes has difficulty controlling the urge to drink. While intoxicated, the person has difficulty performing daily activities.	0.235 (0.16-0.327)
Moderate alcohol dependence	Alcohol use disorder, moderate	drinks a lot, gets drunk almost every week and has great difficulty controlling the urge to drink. Drinking and recovering cause great difficulty in daily activities, sleep loss, and fatigue.	0.373 (0.248-0.508)
Severe alcohol dependence	Alcohol use disorder, severe	gets drunk almost every day and is unable to control the urge to drink. Drinking and recovering replace most daily activities. The person has difficulty thinking, remembering and communicating, and feels constant pain and fatigue.	0.57 (0.396-0.732)
Asymptomatic fetal alcohol syndrome	--	--	--
Mild fetal alcohol syndrome	Fetal alcohol syndrome, mild	is a little slow in developing physically and mentally, which causes some difficulty in learning but no other difficulties in daily activities.	0.016 (0.008-0.03)
Moderate fetal alcohol syndrome	Fetal alcohol syndrome, moderate	is slow in developing physically and mentally, which causes some difficulty in daily activities.	0.056 (0.035-0.083)
Severe fetal alcohol syndrome	Fetal alcohol syndrome, severe	is very slow in developing physically and mentally, which causes great difficulty in daily activities.	0.179 (0.119-0.257)
Asymptomatic opioid dependence	--	--	--
Mild opioid dependence	Heroin and other opioid dependence, mild	uses heroin (or methadone) daily and has difficulty controlling the habit. When not using, the person functions normally.	0.335 (0.221-0.473)
Severe opioid dependence	Heroin and other opioid dependence	uses heroin daily and has difficulty controlling the habit. When the effects wear off, the person feels severe nausea, agitation, vomiting and fever. The person has a lot of difficulty in daily activities.	0.697 (0.51-0.843)
Asymptomatic cocaine dependence	--	--	--
Mild cocaine dependence	Cocaine dependence, mild	uses cocaine at least once a week and has some difficulty controlling the habit. When not using, the person functions normally.	0.116 (0.074-0.165)
Severe cocaine dependence	Cocaine dependence	uses cocaine and has difficulty controlling the habit. The person sometimes has mood swings, anxiety, paranoia, hallucinations and sleep problems, and has some difficulty in daily activities.	0.479 (0.324-0.634)
Asymptomatic amphetamine dependence	--	--	--
Mild amphetamine dependence	Amphetamine dependence, mild	uses stimulants (drugs) at least once a week and has some difficulty controlling the habit. When not using, the person functions normally.	0.079 (0.051-0.114)
Severe amphetamine dependence	Amphetamine dependence	uses stimulants (drugs) and has difficulty controlling the habit. The person sometimes has depression, hallucinations and mood swings, and has difficulty in daily activities.	0.486 (0.329-0.637)
Asymptomatic cannabis dependence	--	--	--
Mild cannabis dependence	Cannabis dependence, mild	uses marijuana at least once a week and has some difficulty controlling the habit. When not using, the person functions normally.	0.039 (0.024-0.06)
Severe cannabis dependence	Cannabis dependence	uses marijuana daily and has difficulty controlling the habit. The person sometimes has mood swings, anxiety and hallucinations, and has some difficulty in daily activities.	0.266 (0.178-0.364)
Other drug use disorders	--	--	--
Major depressive disorder, currently without symptoms	--	--	--
Mild major depressive disorder	Major depressive disorder, mild episode	feels persistent sadness and has lost interest in usual activities. The person sometimes sleeps badly, feels tired, or has trouble concentrating but still manages to function in daily life with extra effort.	0.145 (0.099-0.209)
Moderate major depressive disorder	Major depressive disorder, moderate episode	has constant sadness and has lost interest in usual activities. The person has some difficulty in daily life, sleeps badly, has trouble concentrating, and sometimes thinks about harming himself (or herself).	0.396 (0.267-0.531)
Severe major depressive disorder	Major depressive disorder, severe episode	has overwhelming, constant sadness and cannot function in daily life. The person sometimes loses touch with reality and wants to harm or kill himself (or herself).	0.658 (0.477-0.807)
Dysthymia, currently without symptoms	--	--	--
Symptomatic dysthymia	Major depressive disorder, mild episode	feels persistent sadness and has lost interest in usual activities. The person sometimes sleeps badly, feels tired, or has trouble concentrating but still manages to function in daily life with extra effort.	0.145 (0.099-0.209)
Bipolar disorder residual state	Bipolar disorder, residual state	has mild mood swings, irritability and some difficulty with daily activities.	0.032 (0.018-0.051)
Bipolar disorder depressive state	Major depressive disorder, moderate episode	has constant sadness and has lost interest in usual activities. The person has some difficulty in daily life, sleeps badly, has trouble concentrating, and sometimes thinks about harming himself (or herself).	0.396 (0.267-0.531)
Bipolar disorder manic state	Bipolar disorder, manic episode	is hyperactive, hears and believes things that are not real, and engages in impulsive and aggressive behavior that endanger the person and others.	0.492 (0.341-0.646)
Anxiety disorders, currently without symptoms	--	--	--
Mild anxiety disorders	Anxiety disorders, mild	feels mildly anxious and worried, which makes it slightly difficult to concentrate, remember things, and sleep. The person tires easily but is able to perform daily activities.	0.03 (0.018-0.046)
Moderate anxiety disorders	Anxiety disorders, moderate	feels anxious and worried, which makes it difficult to concentrate, remember things, and sleep. The person tires easily and finds it difficult to perform daily activities.	0.133 (0.091-0.186)
Severe anxiety disorders	Anxiety disorders, severe	constantly feels very anxious and worried, which makes it difficult to concentrate, remember things and sleep. The person has lost pleasure in life and thinks about suicide.	0.523 (0.362-0.677)
Anorexia nervosa	Anorexia nervosa	feels an overwhelming need to starve and exercises excessively to lose weight. The person is very thin, weak and anxious.	0.224 (0.15-0.312)
Bulimia nervosa	Bulimia nervosa	has uncontrolled overeating followed by guilt, starving, and vomiting to lose weight.	0.223 (0.149-0.311)
Autism	Autism	has severe problems interacting with others and difficulty understanding simple questions or directions. The person has great difficulty with basic daily activities and becomes distressed by any change in routine.	0.262 (0.176-0.365)
Asperger syndrome and other autistic spectrum disorders	Asperger syndrome	has difficulty interacting with other people, and is slow to understand or respond to questions. The person is often preoccupied with one thing and has some difficulty with basic daily activities.	0.104 (0.071-0.147)
Attention-deficit/hyperactivity disorder, currently without symptoms	--	--	--
Symptomatic attention-deficit/hyperactivity disorder	Attention deficit hyperactivity disorder	is hyperactive and has difficulty concentrating, remembering things, and completing tasks.	0.045 (0.028-0.066)
Conduct disorder, currently without symptoms	--	--	--
Symptomatic conduct disorder	Conduct disorder	has frequent behavior problems, which are sometimes violent. The person often has difficulty interacting with other people and feels irritable.	0.241 (0.159-0.341)
Borderline idiopathic developmental intellectual disability	Borderline intellectual functioning	is slow in learning at school. As an adult, the person has some difficulty doing complex or unfamiliar tasks but otherwise functions independently.	0.011 (0.005-0.02)
Mild idiopathic developmental intellectual disability	Intellectual disability / mental retardation, mild	has low intelligence and is slow in learning at school. As an adult, the person can live independently, but often needs help to raise children and can only work at simple supervised jobs.	0.043 (0.026-0.064)
Moderate idiopathic developmental intellectual disability	Intellectual disability / mental retardation, moderate	has low intelligence, and is slow in learning to speak and to do even simple tasks. As an adult, the person requires a lot of support to live independently and raise children. The person can only work at the simplest supervised jobs.	0.1 (0.066-0.142)
Severe idiopathic developmental intellectual disability	Intellectual disability / mental retardation, severe	has very low intelligence and cannot speak more than a few words, needs constant supervision and help with most daily activities, and can do only the simplest tasks.	0.16 (0.107-0.226)
Profound idiopathic developmental intellectual disability	Intellectual disability / mental retardation, profound	has very low intelligence, has almost no language, and does not understand even the most basic requests or instructions. The person requires constant supervision and help for all activities.	0.2 (0.133-0.283)
Other mental disorders, currently without symptoms	--	--	--

Appendix Table 6. GBD 2015 sequelae, health states, health state lay descriptions, and disability weights			
**Sequela**	**Healthstate name**	**Healthstate lay description**	**Disability weight**
Mild other mental disorders	Anxiety disorders, mild	feels mildly anxious and worried, which makes it slightly difficult to concentrate, remember things, and sleep. The person tires easily but is able to perform daily activities.	0.03 (0.018-0.046)
Moderate other mental disorders	Anxiety disorders, moderate	feels anxious and worried, which makes it difficult to concentrate, remember things, and sleep. The person tires easily and finds it difficult to perform daily activities.	0.133 (0.091-0.186)
Severe other mental disorders	Anxiety disorders, severe	constantly feels very anxious and worried, which makes it difficult to concentrate, remember things and sleep. The person has lost pleasure in life and thinks about suicide.	0.523 (0.362-0.677)
Uncomplicated diabetes mellitus	Generic uncomplicated disease: worry and daily medication	has a chronic disease that requires medication every day and causes some worry but minimal interference with daily activities.	0.049 (0.031-0.072)
Diabetic neuropathy	Diabetic neuropathy	has pain, tingling and numbness in the arms, legs, hands and feet. The person sometimes gets cramps and muscle weakness.	0.133 (0.089-0.187)
Diabetic foot due to neuropathy	Diabetic neuropathy with diabetic foot	(combined DW)	--
Diabetic neuropathy and amputation with treatment	Diabetic neuropathy with treated amputation	(combined DW)	--
Diabetic neuropathy and amputation without treatment	Diabetic neuropathy with untreated amputation	(combined DW)	--
Moderate vision impairment due to diabetes mellitus	Distance vision, moderate impairment	has vision problems that make it difficult to recognize faces or objects across a room.	0.031 (0.019-0.049)
Severe vision impairment due to diabetes mellitus	Distance vision, severe impairment	has severe vision loss, which causes difficulty in daily activities, some emotional impact (for example worry), and some difficulty going outside the home without assistance.	0.184 (0.125-0.258)
Blindness due to diabetes mellitus	Distance vision blindness	is completely blind, which causes great difficulty in some daily activities, worry and anxiety, and great difficulty going outside the home without assistance.	0.187 (0.124-0.26)
Acute glomerulonephritis	Infectious disease, acute episode, moderate	has a fever and aches, and feels weak, which causes some difficulty with daily activities.	0.051 (0.032-0.074)
Stage III chronic kidney disease without anemia due to diabetes mellitus	--	--	--
Stage III chronic kidney disease and mild anemia due to diabetes mellitus	Anemia, mild	feels slightly tired and weak at times, but this does not interfere with normal daily activities.	0.004 (0.001-0.008)
Stage III chronic kidney disease and moderate anemia due to diabetes mellitus	Anemia, moderate	feels moderate fatigue, weakness, and shortness of breath after exercise, making daily activities more difficult.	0.052 (0.034-0.076)
Stage III chronic kidney disease and severe anemia due to diabetes mellitus	Anemia, severe	feels very weak, tired and short of breath, and has problems with activities that require physical effort or deep concentration.	0.149 (0.101-0.209)
Stage IV chronic kidney disease without anemia due to diabetes mellitus	Chronic kidney disease (stage IV)	tires easily, has nausea, reduced appetite and difficulty sleeping.	0.104 (0.07-0.147)
Stage IV chronic kidney disease and mild anemia due to diabetes mellitus	Mild anemia with Stage IV CKD	(combined DW)	--
Stage IV chronic kidney disease and moderate anemia due to diabetes mellitus	Moderate anemia with Stage IV CKD	(combined DW)	--
Stage IV chronic kidney disease and severe anemia due to diabetes mellitus	Severe anemia with Stage IV CKD	(combined DW)	--
Stage V chronic kidney disease untreated due to diabetes mellitus	Terminal phase, without medication (for cancers, end-stage kidney/liver disease)	has lost a lot of weight and has constant pain. The person has no appetite, feels nauseous, and needs to spend most of the day in bed.	0.569 (0.389-0.727)
End-stage renal disease after transplant due to diabetes mellitus	End-stage renal disease, with kidney transplant	sometimes feels tired and down, and has some difficulty with daily activities.	0.024 (0.014-0.039)
End-stage renal disease on dialysis due to diabetes mellitus	End-stage renal disease, on dialysis	is tired and has itching, cramps, headache, joint pains and shortness of breath. The person needs intensive medical care every other day lasting about half a day.	0.571 (0.398-0.725)
Stage III chronic kidney disease without anemia due to hypertension	--	--	--
Stage III chronic kidney disease and mild anemia due to hypertension	Anemia, mild	feels slightly tired and weak at times, but this does not interfere with normal daily activities.	0.004 (0.001-0.008)
Stage III chronic kidney disease and moderate anemia due to hypertension	Anemia, moderate	feels moderate fatigue, weakness, and shortness of breath after exercise, making daily activities more difficult.	0.052 (0.034-0.076)
Stage III chronic kidney disease and severe anemia due to hypertension	Anemia, severe	feels very weak, tired and short of breath, and has problems with activities that require physical effort or deep concentration.	0.149 (0.101-0.209)
Stage IV chronic kidney disease without anemia due to hypertension	Chronic kidney disease (stage IV)	tires easily, has nausea, reduced appetite and difficulty sleeping.	0.104 (0.07-0.147)
Stage IV chronic kidney disease and mild anemia due to hypertension	Mild anemia with Stage IV CKD	(combined DW)	--
Stage IV chronic kidney disease and moderate anemia due to hypertension	Moderate anemia with Stage IV CKD	(combined DW)	--
Stage IV chronic kidney disease and severe anemia due to hypertension	Severe anemia with Stage IV CKD	(combined DW)	--
Stage V chronic kidney disease untreated due to hypertension	Terminal phase, without medication (for cancers, end-stage kidney/liver disease)	has lost a lot of weight and has constant pain. The person has no appetite, feels nauseous, and needs to spend most of the day in bed.	0.569 (0.389-0.727)
End-stage renal disease after transplant due to hypertension	End-stage renal disease, with kidney transplant	sometimes feels tired and down, and has some difficulty with daily activities.	0.024 (0.014-0.039)
End-stage renal disease on dialysis due to hypertension	End-stage renal disease, on dialysis	is tired and has itching, cramps, headache, joint pains and shortness of breath. The person needs intensive medical care every other day lasting about half a day.	0.571 (0.398-0.725)
Stage III chronic kidney disease without anemia due to glomerulonephritis	--	--	--
Stage III chronic kidney disease and mild anemia due to glomerulonephritis	Anemia, mild	feels slightly tired and weak at times, but this does not interfere with normal daily activities.	0.004 (0.001-0.008)
Stage III chronic kidney disease and moderate anemia due to glomerulonephritis	Anemia, moderate	feels moderate fatigue, weakness, and shortness of breath after exercise, making daily activities more difficult.	0.052 (0.034-0.076)
Stage III chronic kidney disease and severe anemia due to glomerulonephritis	Anemia, severe	feels very weak, tired and short of breath, and has problems with activities that require physical effort or deep concentration.	0.149 (0.101-0.209)
Stage IV chronic kidney disease without anemia due to glomerulonephritis	Chronic kidney disease (stage IV)	tires easily, has nausea, reduced appetite and difficulty sleeping.	0.104 (0.07-0.147)
Stage IV chronic kidney disease and mild anemia due to glomerulonephritis	Mild anemia with Stage IV CKD	(combined DW)	--
Stage IV chronic kidney disease and moderate anemia due to glomerulonephritis	Moderate anemia with Stage IV CKD	(combined DW)	--
Stage IV chronic kidney disease and severe anemia due to glomerulonephritis	Severe anemia with Stage IV CKD	(combined DW)	--
Stage V chronic kidney disease untreated due to glomerulonephritis	Terminal phase, without medication (for cancers, end-stage kidney/liver disease)	has lost a lot of weight and has constant pain. The person has no appetite, feels nauseous, and needs to spend most of the day in bed.	0.569 (0.389-0.727)
End-stage renal disease after transplant due to glomerulonephritis	End-stage renal disease, with kidney transplant	sometimes feels tired and down, and has some difficulty with daily activities.	0.024 (0.014-0.039)
End-stage renal disease on dialysis due to glomerulonephritis	End-stage renal disease, on dialysis	is tired and has itching, cramps, headache, joint pains and shortness of breath. The person needs intensive medical care every other day lasting about half a day.	0.571 (0.398-0.725)
Stage III chronic kidney disease without anemia due to other causes	--	--	--
Stage III chronic kidney disease and mild anemia due to other causes	Anemia, mild	feels slightly tired and weak at times, but this does not interfere with normal daily activities.	0.004 (0.001-0.008)
Stage III chronic kidney disease and moderate anemia due to other causes	Anemia, moderate	feels moderate fatigue, weakness, and shortness of breath after exercise, making daily activities more difficult.	0.052 (0.034-0.076)
Stage III chronic kidney disease and severe anemia due to other causes	Anemia, severe	feels very weak, tired and short of breath, and has problems with activities that require physical effort or deep concentration.	0.149 (0.101-0.209)
Stage IV chronic kidney disease without anemia due to other causes	Chronic kidney disease (stage IV)	tires easily, has nausea, reduced appetite and difficulty sleeping.	0.104 (0.07-0.147)

Appendix Table 6. GBD 2015 sequelae, health states, health state lay descriptions, and disability weights

Sequela	Healthstate name	Healthstate lay description	Disability weight
Stage IV chronic kidney disease and mild anemia due to other causes	Mild anemia with Stage IV CKD	(combined DW)	--
Stage IV chronic kidney disease and moderate anemia due to other causes	Moderate anemia with Stage IV CKD	(combined DW)	--
Stage IV chronic kidney disease and severe anemia due to other causes	Severe anemia with Stage IV CKD	(combined DW)	--
Stage V chronic kidney disease untreated due to other causes	Terminal phase, without medication (for cancers, end-stage kidney/liver disease)	has lost a lot of weight and has constant pain. The person has no appetite, feels nauseous, and needs to spend most of the day in bed.	0.569 (0.389-0.727)
End-stage renal disease after transplant due to other causes	End-stage renal disease, with kidney transplant	sometimes feels tired and down, and has some difficulty with daily activities.	0.024 (0.014-0.039)
End-stage renal disease on dialysis due to other causes	End-stage renal disease, on dialysis	is tired and has itching, cramps, headache, joint pains and shortness of breath. The person needs intensive medical care every other day lasting about half a day.	0.571 (0.398-0.725)
Mild interstitial nephritis and urinary tract infections	Infectious disease, acute episode, mild	has a low fever and mild discomfort , but no difficulty with daily activities.	0.006 (0.002-0.012)
Moderate interstitial nephritis and urinary tract infections	Infectious disease, acute episode, moderate	has a fever and aches, and feels weak, which causes some difficulty with daily activities.	0.051 (0.032-0.074)
Acute urolithiasis	Abdominopelvic problem, moderate	has pain in the belly and feels nauseous. The person has difficulties with daily activities.	0.114 (0.078-0.159)
Chronic urolithiasis	--	--	--
Asymptomatic benign prostatic hyperplasia	--	--	--
Symptomatic benign prostatic hyperplasia	Benign prostatic hypertrophy, symptomatic cases	feels the urge to urinate frequently, but when passing urine it comes out slowly and sometimes is painful.	0.067 (0.043-0.097)
Primary male infertility	Infertility, primary	wants to have a child and has a fertile partner, but the couple cannot conceive.	0.008 (0.003-0.015)
Secondary male infertility	Infertility, secondary	has at least one child, and wants to have more children. The person has a fertile partner, but the couple cannot conceive.	0.005 (0.002-0.011)
Other urinary diseases	--	--	--
Asymptomatic uterine fibroids	--	--	--
Mild abdominal pain due to uterine fibroids, without anemia	Abdominopelvic problem, mild	has some pain in the belly that causes nausea but does not interfere with daily activities.	0.011 (0.005-0.021)
Mild abdominal pain due to uterine fibroids, with mild anemia	Mild abdominal pain and mild anemia	(combined DW)	--
Mild abdominal pain due to uterine fibroids, with moderate anemia	Mild abdominal pain and moderate anemia	(combined DW)	--
Mild abdominal pain due to uterine fibroids, with severe anemia	Mild abdominal pain and severe anemia	(combined DW)	--
Asymptomatic polycystic ovarian syndrome	--	--	--
Hirsutism due to polycystic ovarian syndrome	Disfigurement, level 1	has a slight, visible physical deformity that others notice, which causes some worry and discomfort.	0.011 (0.005-0.021)
Hirsutism and primary infertility due to polycystic ovarian syndrome	Disfigurement level 1 and primary infertility	(combined DW)	--
Primary infertility due to polycystic ovarian syndrome	Infertility, primary	wants to have a child and has a fertile partner, but the couple cannot conceive.	0.008 (0.003-0.015)
Hirsutism and secondary infertility due to polycystic ovarian syndrome	Infertility, secondary	has at least one child, and wants to have more children. The person has a fertile partner, but the couple cannot conceive.	0.005 (0.002-0.011)
Secondary infertility due to polycystic ovarian syndrome	Disfigurement level 1 and secondary infertility	(combined DW)	--
Idiopathic primary female infertility	Infertility, primary	wants to have a child and has a fertile partner, but the couple cannot conceive.	0.008 (0.003-0.015)
Idiopathic secondary female infertility	Infertility, secondary	has at least one child, and wants to have more children. The person has a fertile partner, but the couple cannot conceive.	0.005 (0.002-0.011)
Asymptomatic endometriosis	--	--	--
Mild abdominal pain due to endometriosis	Abdominopelvic problem, mild	has some pain in the belly that causes nausea but does not interfere with daily activities.	0.011 (0.005-0.021)
Moderate abdominal pain due to endometriosis	Abdominopelvic problem, moderate	has pain in the belly and feels nauseous. The person has difficulties with daily activities.	0.114 (0.078-0.159)
Severe endometriosis	Abdominopelvic problem, severe	has severe pain in the belly and feels nauseous. The person is anxious and unable to carry out daily activities.	0.324 (0.22-0.442)
Primary infertility due to endometriosis	Infertility, primary	wants to have a child and has a fertile partner, but the couple cannot conceive.	0.008 (0.003-0.015)
Mild abdominal pain and primary infertility due to endometriosis	Mild abdominal pain and primary infertility	(combined DW)	--
Moderate abdominal pain and primary infertility due to endometriosis	Moderate abdominal pain and primary infertility	(combined DW)	--
Severe abdominal pain and primary infertility due to endometriosis	Severe abdominal pain and primary infertility	(combined DW)	--
Secondary infertility due to endometriosis	Infertility, secondary	has at least one child, and wants to have more children. The person has a fertile partner, but the couple cannot conceive.	0.005 (0.002-0.011)
Mild abdominal pain and secondary infertility due to endometriosis	Mild abdominal pain and secondary infertility	(combined DW)	--
Moderate abdominal pain and secondary infertility due to endometriosis	Moderate abdominal pain and secondary infertility	(combined DW)	--
Severe abdominal pain and secondary infertility due to endometriosis	Severe abdominal pain and secondary infertility	(combined DW)	--
Asymptomatic genital prolapse	--	--	--
Abdominal pain due to genital prolapse	Abdominopelvic problem, mild	has some pain in the belly that causes nausea but does not interfere with daily activities.	0.011 (0.005-0.021)
Stress incontinence due to genital prolapse	Stress incontinence	loses small amounts of urine without meaning to when coughing, sneezing, laughing or during physical exercise.	0.02 (0.011-0.035)
Abdominal pain and stress incontinence due to genital prolapse	Mild abdominal pain and stress incontinence	(combined DW)	--
Asymptomatic premenstrual syndrome	--	--	--
Abdominal pain and depression due to premenstrual syndrome	Mild abdominal pain and mild depression	(combined DW)	--
Abdominal pain due to premenstrual syndrome	Abdominopelvic problem, mild	has some pain in the belly that causes nausea but does not interfere with daily activities.	0.011 (0.005-0.021)
Depression due to premenstrual syndrome	Major depressive disorder, mild episode	feels persistent sadness and has lost interest in usual activities. The person sometimes sleeps badly, feels tired, or has trouble concentrating but still manages to function in daily life with extra effort.	0.145 (0.099-0.209)
Asymptomatic other gynecological disorders	--	--	--
Mild other gynecological disorders	Abdominopelvic problem, mild	has some pain in the belly that causes nausea but does not interfere with daily activities.	0.011 (0.005-0.021)

Appendix Table 6. GBD 2015 sequelae, health states, health state lay descriptions, and disability weights

Sequela	Healthstate name	Healthstate lay description	Disability weight
Moderate other gynecological disorders	Abdominopelvic problem, moderate	has pain in the belly and feels nauseous. The person has difficulties with daily activities.	0.114 (0.078-0.159)
Severe other gynecological disorders	Abdominopelvic problem, severe	has severe pain in the belly and feels nauseous. The person is anxious and unable to carry out daily activities.	0.324 (0.22-0.442)
Mild anemia due to other gynecological diseases	Anemia, mild	feels slightly tired and weak at times, but this does not interfere with normal daily activities.	0.004 (0.001-0.008)
Moderate anemia due to other gynecological diseases	Anemia, moderate	feels moderate fatigue, weakness, and shortness of breath after exercise, making daily activities more difficult.	0.052 (0.034-0.076)
Severe anemia due to other gynecological diseases	Anemia, severe	feels very weak, tired and short of breath, and has problems with activities that require physical effort or deep concentration.	0.149 (0.101-0.209)
Beta-thalassemia major, with mild anemia	--	(combined DW)	--
Beta-thalassemia major, with moderate anemia	--	(combined DW)	--
Beta-thalassemia major, with severe anemia	--	(combined DW)	--
Beta-thalassemia major, severe infection with severe anemia	--	(combined DW)	--
Beta-thalassemia major, without anemia	Generic uncomplicated disease: anxiety about diagnosis	has a disease diagnosis that causes some worry but minimal interference with daily activities.	0.012 (0.006-0.023)
Hemoglobin E/beta-thalassemia, with mild anemia	--	(combined DW)	--
Hemoglobin E/beta-thalassemia, with moderate anemia	--	(combined DW)	--
Hemoglobin E/beta-thalassemia, with severe anemia	--	(combined DW)	--
Hemoglobin E/beta-thalassemia, severe infection with severe anemia	--	(combined DW)	--
Hemoglobin E/Beta-thalassemia, without anemia	Generic uncomplicated disease: anxiety about diagnosis	has a disease diagnosis that causes some worry but minimal interference with daily activities.	0.012 (0.006-0.023)
Hemoglobin H disease, with mild anemia	--	(combined DW)	--
Hemoglobin H disease, with moderate anemia	--	(combined DW)	--
Hemoglobin H disease, with severe anemia	--	(combined DW)	--
Hemoglobin H disease, severe infection with severe anemia	--	(combined DW)	--
Hemoglobin H disease, without anemia	Generic uncomplicated disease: anxiety about diagnosis	has a disease diagnosis that causes some worry but minimal interference with daily activities.	0.012 (0.006-0.023)
Mild heart failure due to thalassemias	Heart failure, mild	is short of breath and easily tires with moderate physical activity, such as walking uphill or more than a quarter-mile on level ground. The person feels comfortable at rest or during activities requiring less effort.	0.041 (0.026-0.062)
Moderate heart failure due to thalassemias	Heart failure, moderate	is short of breath and easily tires with minimal physical activity, such as walking only a short distance. The person feels comfortable at rest but avoids moderate activity.	0.072 (0.047-0.103)
Severe heart failure due to thalassemias	Heart failure, severe	is short of breath and feels tired when at rest. The person avoids any physical activity, for fear of worsening the breathing problems.	0.179 (0.122-0.251)
Asymptomatic B-thalassemia trait	--	--	--
Mild anemia due to B-thalassemia trait	Anemia, mild	feels slightly tired and weak at times, but this does not interfere with normal daily activities.	0.004 (0.001-0.008)
Moderate anemia due to B-thalassemia trait	Anemia, moderate	feels moderate fatigue, weakness, and shortness of breath after exercise, making daily activities more difficult.	0.052 (0.034-0.076)
Severe anemia due to B-thalassemia trait	Anemia, severe	feels very weak, tired and short of breath, and has problems with activities that require physical effort or deep concentration.	0.149 (0.101-0.209)
Asymptomatic hemoglobin E trait	--	--	--
Mild anemia due to hemoglobin E trait	Anemia, mild	feels slightly tired and weak at times, but this does not interfere with normal daily activities.	0.004 (0.001-0.008)
Moderate anemia due to hemoglobin E trait	Anemia, moderate	feels moderate fatigue, weakness, and shortness of breath after exercise, making daily activities more difficult.	0.052 (0.034-0.076)
Severe anemia due to hemoglobin E trait	Anemia, severe	feels very weak, tired and short of breath, and has problems with activities that require physical effort or deep concentration.	0.149 (0.101-0.209)
Homozygous sickle cell and severe sickle cell/beta-thalassemia, without anemia	Generic uncomplicated disease: anxiety about diagnosis	has a disease diagnosis that causes some worry but minimal interference with daily activities.	0.012 (0.006-0.023)
Homozygous sickle cell and severe sickle cell/beta-thalassemia, with vaso-occlusive crisis, without anemia	Generic uncomplicated disease anxiety and severe abdominopelvic problem	(combined DW)	--
Homozygous sickle cell and severe sickle cell/beta-thalassemia, with stroke, without anemia	Generic uncomplicated disease anxiety; long-term consequences due to stroke	(combined DW)	--
Homozygous sickle cell and severe sickle cell/beta-thalassemia, with vaso-occlusive crisis and stroke, without anemia	Generic uncomplicated disease anxiety; long-term consequences due to stroke; severe abdominopelvic problem	(combined DW)	--
Homozygous sickle cell and severe sickle cell/beta-thalassemia, with mild anemia	--	(combined DW)	--
Homozygous sickle cell and severe sickle cell/beta-thalassemia, with moderate anemia	--	(combined DW)	--
Homozygous sickle cell and severe sickle cell/beta-thalassemia, with severe anemia	--	(combined DW)	--
Homozygous sickle cell and severe sickle cell/beta-thalassemia, with vaso-occlusive crisis and severe anemia	--	(combined DW)	--
Homozygous sickle cell and severe sickle cell/beta-thalassemia, with stroke and severe anemia	--	(combined DW)	--
Homozygous sickle cell and severe sickle cell/beta-thalassemia, with vaso-occlusive crisis, stroke, and severe anemia	--	(combined DW)	--
Hemoglobin SC disease, without anemia	Generic uncomplicated disease: anxiety about diagnosis	has a disease diagnosis that causes some worry but minimal interference with daily activities.	0.012 (0.006-0.023)
Hemoglobin SC disease, with vaso-occlusive crisis, without anemia	Generic uncomplicated disease anxiety and severe abdominopelvic problem	(combined DW)	--
Hemoglobin SC disease, with stroke, without anemia	Generic uncomplicated disease anxiety; long-term consequences due to stroke	(combined DW)	--
Hemoglobin SC disease, with vaso-occlusive crisis and stroke, without anemia	Generic uncomplicated disease anxiety; long-term consequences due to stroke; severe abdominopelvic problem	(combined DW)	--
Hemoglobin SC disease, with mild anemia	--	(combined DW)	--
Hemoglobin SC disease, with moderate anemia	--	(combined DW)	--
Hemoglobin SC disease, with severe anemia	--	(combined DW)	--
Hemoglobin SC disease, with vaso-occlusive crisis and severe anemia	--	(combined DW)	--
Hemoglobin SC disease, with stroke and severe anemia	--	(combined DW)	--

Appendix Table 6. GBD 2015 sequelae, health states, health state lay descriptions, and disability weights			
**Sequela**	**Healthstate name**	**Healthstate lay description**	**Disability weight**
Hemoglobin SC disease, with vaso-occlusive crisis, stroke, and severe anemia	--	(combined DW)	--
Mild sickle cell/beta-thalassemia, without anemia	Generic uncomplicated disease: anxiety about diagnosis	has a disease diagnosis that causes some worry but minimal interference with daily activities.	0.012 (0.006-0.023)
Mild sickle cell/beta-thalassemia, with vaso-occlusive crisis, without anemia	Generic uncomplicated disease anxiety and severe abdominopelvic problem	(combined DW)	--
Mild sickle cell/beta-thalassemia, with stroke, without anemia	Generic uncomplicated disease anxiety; long-term consequences due to stroke	(combined DW)	--
Mild sickle cell/beta-thalassemia, with vaso-occlusive crisis and stroke, without anemia	Generic uncomplicated disease anxiety; long-term consequences due to stroke; severe abdominopelvic problem	(combined DW)	--
Mild sickle cell/beta-thalassemia, with mild anemia	--	(combined DW)	--
Mild sickle cell/beta-thalassemia, with moderate anemia	--	(combined DW)	--
Mild sickle cell/beta-thalassemia, with severe anemia	--	(combined DW)	--
Mild sickle cell/beta-thalassemia, with vaso-occlusive crisis and severe anemia	--	(combined DW)	--
Mild sickle cell/beta-thalassemia, with stroke and severe anemia	--	(combined DW)	--
Mild sickle cell/beta-thalassemia, with vaso-occlusive crisis, stroke, and severe anemia	--	(combined DW)	--
Asymptomatic sickle cell trait	--	(combined DW)	--
Mild anemia due to sickle cell trait	Anemia, mild	feels slightly tired and weak at times, but this does not interfere with normal daily activities.	0.004 (0.001-0.008)
Moderate anemia due to sickle cell trait	Anemia, moderate	feels moderate fatigue, weakness, and shortness of breath after exercise, making daily activities more difficult.	0.052 (0.034-0.076)
Severe anemia due to sickle cell trait	Anemia, severe	feels very weak, tired and short of breath, and has problems with activities that require physical effort or deep concentration.	0.149 (0.101-0.209)
Mild anemia due to G6PD deficiency	Anemia, mild	feels slightly tired and weak at times, but this does not interfere with normal daily activities.	0.004 (0.001-0.008)
Moderate anemia due to G6PD deficiency	Anemia, moderate	feels moderate fatigue, weakness, and shortness of breath after exercise, making daily activities more difficult.	0.052 (0.034-0.076)
Severe anemia due to G6PD deficiency	Anemia, severe	feels very weak, tired and short of breath, and has problems with activities that require physical effort or deep concentration.	0.149 (0.101-0.209)
Asymptomatic G6PD deficiency	--	--	--
Mild heart failure due to G6PD deficiency	Heart failure, mild	is short of breath and easily tires with moderate physical activity, such as walking uphill or more than a quarter-mile on level ground. The person feels comfortable at rest or during activities requiring less effort.	0.041 (0.026-0.062)
Moderate heart failure due to G6PD deficiency	Heart failure, moderate	is short of breath and easily tires with minimal physical activity, such as walking only a short distance. The person feels comfortable at rest but avoids moderate activity.	0.072 (0.047-0.103)
Severe heart failure due to G6PD deficiency	Heart failure, severe	is short of breath and feels tired when at rest. The person avoids any physical activity, for fear of worsening the breathing problems.	0.179 (0.122-0.251)
Asymptomatic hemizygous G6PD deficiency	--	--	--
Mild anemia due to hemizygous G6PD deficiency	Anemia, mild	feels slightly tired and weak at times, but this does not interfere with normal daily activities.	0.004 (0.001-0.008)
Moderate anemia due to hemizygous G6PD deficiency	Anemia, moderate	feels moderate fatigue, weakness, and shortness of breath after exercise, making daily activities more difficult.	0.052 (0.034-0.076)
Severe anemia due to hemizygous G6PD deficiency	Anemia, severe	feels very weak, tired and short of breath, and has problems with activities that require physical effort or deep concentration.	0.149 (0.101-0.209)
Other hemoglobinopathies and hemolytic anemias	--	--	--
Mild anemia due to other hemoglobinopathies and hemolytic anemias	Anemia, mild	feels slightly tired and weak at times, but this does not interfere with normal daily activities.	0.004 (0.001-0.008)
Moderate anemia due to other hemoglobinopathies and hemolytic anemias	Anemia, moderate	feels moderate fatigue, weakness, and shortness of breath after exercise, making daily activities more difficult.	0.052 (0.034-0.076)
Severe anemia due to other hemoglobinopathies and hemolytic anemias	Anemia, severe	feels very weak, tired and short of breath, and has problems with activities that require physical effort or deep concentration.	0.149 (0.101-0.209)
Mild heart failure due to other hemoglobinopathies and hemolytic anemias	Heart failure, mild	is short of breath and easily tires with moderate physical activity, such as walking uphill or more than a quarter-mile on level ground. The person feels comfortable at rest or during activities requiring less effort.	0.041 (0.026-0.062)
Moderate heart failure due to other hemoglobinopathies and hemolytic anemias	Heart failure, moderate	is short of breath and easily tires with minimal physical activity, such as walking only a short distance. The person feels comfortable at rest but avoids moderate activity.	0.072 (0.047-0.103)
Severe heart failure due to other hemoglobinopathies and hemolytic anemias	Heart failure, severe	is short of breath and feels tired when at rest. The person avoids any physical activity, for fear of worsening the breathing problems.	0.179 (0.122-0.251)
Asymptomatic endocrine, metabolic, blood, and immune disorders	--	--	--
Mild endocrine, metabolic, blood, and immune disorders	Hypothyroidism	has low energy and feels cold.	0.019 (0.01-0.032)
Moderate endocrine, metabolic, blood, and immune disorders	Hyperthyroidism	feels nervous, has palpitations, sweats a lot and has difficulty sleeping.	0.145 (0.096-0.202)
Severe endocrine, metabolic, blood, and immune disorders	Thrombocytopenic purpura	easily bruises and sometimes bleeds from the gums and nose; feels weak and has some difficulty with daily activities.	0.159 (0.106-0.226)
Mild anemia due to endocrine, metabolic, blood, and immune disorders	Anemia, mild	feels slightly tired and weak at times, but this does not interfere with normal daily activities.	0.004 (0.001-0.008)
Moderate anemia due to endocrine, metabolic, blood, and immune disorders	Anemia, moderate	feels moderate fatigue, weakness, and shortness of breath after exercise, making daily activities more difficult.	0.052 (0.034-0.076)
Severe anemia due to endocrine, metabolic, blood, and immune disorders	Anemia, severe	feels very weak, tired and short of breath, and has problems with activities that require physical effort or deep concentration.	0.149 (0.101-0.209)
Mild heart failure due to endocrine, metabolic, blood, and immune disorders	Heart failure, mild	is short of breath and easily tires with moderate physical activity, such as walking uphill or more than a quarter-mile on level ground. The person feels comfortable at rest or during activities requiring less effort.	0.041 (0.026-0.062)
Moderate heart failure due to endocrine, metabolic, blood, and immune disorders	Heart failure, moderate	is short of breath and easily tires with minimal physical activity, such as walking only a short distance. The person feels comfortable at rest but avoids moderate activity.	0.072 (0.047-0.103)
Severe heart failure due to endocrine, metabolic, blood, and immune disorders	Heart failure, severe	is short of breath and feels tired when at rest. The person avoids any physical activity, for fear of worsening the breathing problems.	0.179 (0.122-0.251)
Mild rheumatoid arthritis	Musculoskeletal problems, upper limbs, moderate	has moderate pain and stiffness in the arms and hands, which causes difficulty lifting, carrying, and holding things, and trouble sleeping because of the pain.	0.117 (0.08-0.163)
Moderate rheumatoid arthritis	Musculoskeletal problems, generalized, moderate	has pain and deformity in most joints, causing difficulty moving around, getting up and down, and using the hands for lifting and carrying. The person often feels fatigue.	0.317 (0.216-0.44)
Severe rheumatoid arthritis	Musculoskeletal problems, generalized, severe	has severe, constant pain and deformity in most joints, causing difficulty moving around, getting up and down, eating, dressing, lifting, carrying and using the hands. The person often feels sadness, anxiety and extreme fatigue.	0.581 (0.403-0.739)
Mild osteoarthritis of the hip	Musculoskeletal problems, lower limbs, mild	has pain in the leg, which causes some difficulty running, walking long distances, and getting up and down.	0.023 (0.013-0.037)
Moderate osteoarthritis of the hip	Musculoskeletal problems, lower limbs, moderate	has moderate pain in the leg, which makes the person limp, and causes some difficulty walking, standing, lifting and carrying heavy things, getting up and down and sleeping.	0.079 (0.054-0.11)
Severe osteoarthritis of the hip	Musculoskeletal problems, lower limbs, severe	has severe pain in the leg, which makes the person limp and causes a lot of difficulty walking, standing, lifting and carrying heavy things, getting up and down, and sleeping.	0.165 (0.112-0.232)
Mild osteoarthritis of the knee	Musculoskeletal problems, lower limbs, mild	has pain in the leg, which causes some difficulty running, walking long distances, and getting up and down.	0.023 (0.013-0.037)

Appendix Table 6. GBD 2015 sequelae, health states, health state lay descriptions, and disability weights

Sequela	Healthstate name	Healthstate lay description	Disability weight
Moderate osteoarthritis of the knee	Musculoskeletal problems, lower limbs, moderate	has moderate pain in the leg, which makes the person limp, and causes some difficulty walking, standing, lifting and carrying heavy things, getting up and down and sleeping.	0.079 (0.054-0.11)
Severe osteoarthritis of the knee	Musculoskeletal problems, lower limbs, severe	has severe pain in the leg, which makes the person limp and causes a lot of difficulty walking, standing, lifting and carrying heavy things, getting up and down, and sleeping.	0.165 (0.112-0.232)
Mild low back pain without leg pain	Low back pain, mild	has mild back pain, which causes some difficulty dressing, standing, and lifting things.	0.02 (0.011-0.035)
Mild low back pain with leg pain	Mild low back pain with leg pain	(combined DW)	0.02 (0.011-0.035)
Moderate low back pain without leg pain	Low back pain, moderate	has moderate back pain, which causes difficulty dressing, sitting, standing, walking, and lifting things.	0.054 (0.035-0.079)
Moderate low back pain with leg pain	Moderate low back pain with leg pain	(combined DW)	0.054 (0.035-0.079)
Severe low back pain without leg pain	Back pain, severe, without leg pain	has severe back pain, which causes difficulty dressing, sitting, standing, walking, and lifting things. The person sleeps poorly and feels worried.	0.272 (0.182-0.373)
Severe low back pain with leg pain	Back pain, severe, with leg pain	has severe back and leg pain, which causes difficulty dressing, sitting, standing, walking, and lifting things. The person sleeps poorly and feels worried.	0.325 (0.219-0.446)
Most severe low back pain without leg pain	Back pain, most severe, without leg pain	has constant back pain, which causes difficulty dressing, sitting, standing, walking, and lifting things. The person sleeps poorly, is worried, and has lost some enjoyment in life.	0.372 (0.25-0.506)
Most severe low back pain with leg pain	Back pain, most severe, with leg pain	has constant back and leg pain, which causes difficulty dressing, sitting, standing, walking, and lifting things. The person sleeps poorly, is worried, and has lost some enjoyment in life.	0.384 (0.256-0.518)
Mild neck pain	Neck pain, mild	has neck pain, and has difficulty turning the head and lifting things.	0.053 (0.034-0.078)
Moderate neck pain	Neck pain, moderate	has constant neck pain, and has difficulty turning the head, holding arms up, and lifting things	0.114 (0.075-0.162)
Severe neck pain	Neck pain, severe	has severe neck pain, and difficulty turning the head and lifting things. The person gets headaches and arm pain, sleeps poorly, and feels tired and worried.	0.229 (0.153-0.317)
Most severe neck pain	Neck pain, most severe	has constant neck pain and arm pain, and difficulty turning the head, holding arms up, and lifting things. The person gets headaches, sleeps poorly, and feels tired and worried.	0.304 (0.202-0.415)
Asymptomatic gout	--	--	--
Symptomatic episodes of gout	Gout, acute	has severe pain and swelling in the leg, making it very difficult to get up and down, stand, walk, lift, and carry heavy things. The person has trouble sleeping because of the pain.	0.295 (0.196-0.409)
Polyarticular gout	Musculoskeletal problems, generalized, severe	has severe, constant pain and deformity in most joints, causing difficulty moving around, getting up and down, eating, dressing, lifting, carrying and using the hands. The person often feels sadness, anxiety and extreme fatigue.	0.581 (0.403-0.739)
Asymptomatic other musculoskeletal disorders	--	--	--
Other musculoskeletal disorders severity level 2	Musculoskeletal problems, upper limbs, mild	has mild pain and stiffness in the arms and hands. The person has some difficulty lifting, carrying and holding things.	0.028 (0.017-0.045)
Other musculoskeletal disorders severity level 3	Musculoskeletal problems, upper limbs, moderate	has moderate pain and stiffness in the arms and hands, which causes difficulty lifting, carrying, and holding things, and trouble sleeping because of the pain.	0.117 (0.08-0.163)
Other musculoskeletal disorders severity level 5	Musculoskeletal problems, generalized, moderate	has pain and deformity in most joints, causing difficulty moving around, getting up and down, and using the hands for lifting and carrying. The person often feels fatigue.	0.317 (0.216-0.44)
Other musculoskeletal disorders severity level 6	Musculoskeletal problems, generalized, severe	has severe, constant pain and deformity in most joints, causing difficulty moving around, getting up and down, eating, dressing, lifting, carrying and using the hands. The person often feels sadness, anxiety and extreme fatigue.	0.581 (0.403-0.739)
Other musculoskeletal disorders severity level 1	Musculoskeletal problems, lower limbs, mild	has pain in the leg, which causes some difficulty running, walking long distances, and getting up and down.	0.023 (0.013-0.037)
Other musculoskeletal disorders severity level 4	Musculoskeletal problems, lower limbs, severe	has severe pain in the leg, which makes the person limp and causes a lot of difficulty walking, standing, lifting and carrying heavy things, getting up and down, and sleeping.	0.165 (0.112-0.232)
Moderate motor impairment due to moderate neural tube defects	Motor impairment, moderate	has some difficulty in moving around, and difficulty in lifting and holding objects, dressing and sitting upright, but is able to walk without help.	0.061 (0.04-0.089)
Severe motor impairment due to severe neural tube defects	Motor impairment, severe	is unable to move around without help, and is not able to lift or hold objects, get dressed or sit upright.	0.402 (0.268-0.545)
Severe motor impairment with incontinence due to severe neural tube defects	Severe motor impairment with incontinence	(combined DW)	--
Severe motor impairment with mild intellectual disability and incontinence due to severe neural tube defects	Severe motor impairment with mild intellectual disability and incontinence	(combined DW)	--
Severe motor impairment with mild intellectual disability due to severe neural tube defects	Severe motor impairment with mild intellectual disability	(combined DW)	--
Severe motor impairment with moderate intellectual disability and incontinence due to severe neural tube defects	Severe motor impairment with moderate intellectual disability and incontinence	(combined DW)	--
Severe motor impairment with moderate intellectual disability due to severe neural tube defects	Severe motor impairment with moderate intellectual disability	(combined DW)	--
Severe motor impairment with profound intellectual disability and incontinence due to severe neural tube defects	Severe motor impairment with profound intellectual disability and incontinence	(combined DW)	--
Severe motor impairment with profound intellectual disability due to severe neural tube defects	Severe motor impairment with profound intellectual disability	(combined DW)	--
Severe motor impairment with severe intellectual disability and incontinence due to severe neural tube defects	Severe motor impairment with severe intellectual disability and incontinence	(combined DW)	--
Severe motor impairment with severe intellectual disability due to severe neural tube defects	Severe motor impairment with severe intellectual disability	(combined DW)	--
Asymptomatic less severe congenital heart anomalies	--	(combined DW)	--
Symptomatic less severe congenital heart anomalies	Congenital heart disease	(custom DW from MEPS)	--
Severe congenital heart anomalies	Congenital heart disease	(custom DW from MEPS)	--
Critical congenital heart anomalies	Congenital heart disease	(custom DW from MEPS)	--
Mild heart failure due to congenital heart anomalies	Heart failure, mild	is short of breath and easily tires with moderate physical activity, such as walking uphill or more than a quarter-mile on level ground. The person feels comfortable at rest or during activities requiring less effort.	0.041 (0.026-0.062)
Moderate heart failure due to congenital heart anomalies	Heart failure, moderate	is short of breath and easily tires with minimal physical activity, such as walking only a short distance. The person feels comfortable at rest but avoids moderate activity.	0.072 (0.047-0.103)
Severe heart failure due to congenital heart anomalies	Heart failure, severe	is short of breath and feels tired when at rest. The person avoids any physical activity, for fear of worsening the breathing problems.	0.179 (0.122-0.251)
Asymptomatic orofacial clefts	--	--	--
Disfigurement level 1 due to orofacial clefts	Disfigurement, level 1	has a slight, visible physical deformity that others notice, which causes some worry and discomfort.	0.011 (0.005-0.021)
Disfigurement level 2 due to orofacial clefts	Disfigurement, level 2	has a visible physical deformity that causes others to stare and comment. As a result, the person is worried and has trouble sleeping and concentrating.	0.067 (0.044-0.096)
Disfigurement level 2 and speech problems due to orofacial clefts	Speech problems with disfigurement level 2	(combined DW)	--
Asymptomatic Down syndrome	--	--	--
Isolated congenital heart disease due to Down syndrome	Congenital heart disease	(custom DW from MEPS)	--
Borderline intellectual disability due to Down syndrome	Borderline intellectual functioning	is slow in learning at school. As an adult, the person has some difficulty doing complex or unfamiliar tasks but otherwise functions independently.	0.011 (0.005-0.02)
Borderline intellectual disability with congenital heart disease due to Down syndrome	Borderline intellectual functioning and heart failure	(combined DW)	--

Appendix Table 6. GBD 2015 sequelae, health states, health state lay descriptions, and disability weights

Sequela	Healthstate name	Healthstate lay description	Disability weight
Congenital heart disease and mild dementia due to Down syndrome	--	(combined DW)	--
Congenital heart disease and moderate dementia due to Down syndrome	--	(combined DW)	--
Congenital heart disease and severe dementia due to Down syndrome	--	(combined DW)	--
Mild dementia due to Down syndrome	Dementia, mild	has some trouble remembering recent events, and finds it hard to concentrate and make decisions and plans.	0.069 (0.046-0.099)
Mild intellectual disability due to Down syndrome	Intellectual disability / mental retardation, mild	has low intelligence and is slow in learning at school. As an adult, the person can live independently, but often needs help to raise children and can only work at simple supervised jobs.	0.043 (0.026-0.064)
Mild intellectual disability with congenital heart disease due to Down syndrome	--	(combined DW)	--
Moderate dementia due to Down syndrome	Dementia, moderate	has memory problems and confusion, feels disoriented, at times hears voices that are not real, and needs help with some daily activities.	0.377 (0.252-0.508)
Moderate intellectual disability due to Down syndrome	Intellectual disability / mental retardation, moderate	has low intelligence, and is slow in learning to speak and to do even simple tasks. As an adult, the person requires a lot of support to live independently and raise children. The person can only work at the simplest supervised jobs.	0.1 (0.066-0.142)
Moderate intellectual disability with congenital heart disease due to Down syndrome	--	(combined DW)	--
Profound intellectual disability due to Down syndrome	Intellectual disability / mental retardation, profound	has very low intelligence, has almost no language, and does not understand even the most basic requests or instructions. The person requires constant supervision and help for all activities.	0.2 (0.133-0.283)
Profound intellectual disability with congenital heart disease due to Down syndrome	--	(combined DW)	--
Severe dementia due to Down syndrome	Dementia, severe	has complete memory loss; no longer recognizes close family members; and requires help with all daily activities.	0.449 (0.304-0.595)
Severe intellectual disability due to Down syndrome	Intellectual disability / mental retardation, severe	has very low intelligence and cannot speak more than a few words, needs constant supervision and help with most daily activities, and can do only the simplest tasks.	0.16 (0.107-0.226)
Severe intellectual disability with congenital heart disease due to Down syndrome	--	(combined DW)	--
Asymptomatic Turner syndrome	--	--	--
Congenital heart disease due to Turner syndrome	Congenital heart disease	(custom DW from MEPS)	--
Primary infertility due to Turner syndrome	Infertility, primary	wants to have a child and has a fertile partner, but the couple cannot conceive.	0.008 (0.003-0.015)
Congenital heart disease with infertility due to Turner syndrome	Congenital heart disease with primary infertility	(combined DW)	--
Asymptomatic Klinefelter syndrome	--	--	--
Borderline intellectual disability due to Klinefelter syndrome	Borderline intellectual functioning	is slow in learning at school. As an adult, the person has some difficulty doing complex or unfamiliar tasks but otherwise functions independently.	0.011 (0.005-0.02)
Borderline intellectual disability with infertility due to Klinefelter syndrome	Borderline intellectual disability with primary infertility	(combined DW)	--
Mild intellectual disability due to Klinefelter syndrome	Intellectual disability / mental retardation, mild	has low intelligence and is slow in learning at school. As an adult, the person can live independently, but often needs help to raise children and can only work at simple supervised jobs.	0.043 (0.026-0.064)
Mild intellectual disability with infertility due to Klinefelter syndrome	Mild intellectual disability with primary infertility	(combined DW)	--
Primary infertility due to Klinefelter syndrome	Infertility, primary	wants to have a child and has a fertile partner, but the couple cannot conceive.	0.008 (0.003-0.015)
Asymptomatic chromosomal unbalanced rearrangements	--	--	--
Isolated congenital heart disease due to chromosomal unbalanced rearrangements	Congenital heart disease	(custom DW from MEPS)	--
Borderline intellectual disability due to chromosomal unbalanced rearrangements	Borderline intellectual functioning	is slow in learning at school. As an adult, the person has some difficulty doing complex or unfamiliar tasks but otherwise functions independently.	0.011 (0.005-0.02)
Borderline intellectual disability with congenital heart disease due to chromosomal unbalanced rearrangements	--	(combined DW)	--
Congenital heart disease and mild dementia due to chromosomal unbalanced rearrangements	--	(combined DW)	--
Congenital heart disease and moderate dementia due to chromosomal unbalanced rearrangements	--	(combined DW)	--
Congenital heart disease and severe dementia due to chromosomal unbalanced rearrangements	--	(combined DW)	--
Mild dementia due to chromosomal unbalanced rearrangements	Dementia, mild	has some trouble remembering recent events, and finds it hard to concentrate and make decisions and plans.	0.069 (0.046-0.099)
Mild intellectual disability due to chromosomal unbalanced rearrangements	Intellectual disability / mental retardation, mild	has low intelligence and is slow in learning at school. As an adult, the person can live independently, but often needs help to raise children and can only work at simple supervised jobs.	0.043 (0.026-0.064)
Mild intellectual disability with congenital heart disease due to chromosomal unbalanced rearrangements	--	(combined DW)	--
Moderate dementia due to chromosomal unbalanced rearrangements	Dementia, moderate	has memory problems and confusion, feels disoriented, at times hears voices that are not real, and needs help with some daily activities.	0.377 (0.252-0.508)
Moderate intellectual disability due to chromosomal unbalanced rearrangements	Intellectual disability / mental retardation, moderate	has low intelligence, and is slow in learning to speak and to do even simple tasks. As an adult, the person requires a lot of support to live independently and raise children. The person can only work at the simplest supervised jobs.	0.1 (0.066-0.142)
Moderate intellectual disability with congenital heart disease due to chromosomal unbalanced rearrangements	--	(combined DW)	--
Profound intellectual disability due to chromosomal unbalanced rearrangements	Intellectual disability / mental retardation, profound	has very low intelligence, has almost no language, and does not understand even the most basic requests or instructions. The person requires constant supervision and help for all activities.	0.2 (0.133-0.283)
Profound intellectual disability with congenital heart disease due to chromosomal unbalanced rearrangements	--	(combined DW)	--
Severe dementia due to chromosomal unbalanced rearrangements	Dementia, severe	has complete memory loss; no longer recognizes close family members; and requires help with all daily activities.	0.449 (0.304-0.595)
Severe intellectual disability due to chromosomal unbalanced rearrangements	Intellectual disability / mental retardation, severe	has very low intelligence and cannot speak more than a few words, needs constant supervision and help with most daily activities, and can do only the simplest tasks.	0.16 (0.107-0.226)
Severe intellectual disability with congenital heart disease due to chromosomal unbalanced rearrangements	--	(combined DW)	--
Other congenital anomalies	--	--	--
Mild hearing loss due to other congenital anomalies	Hearing loss, mild	has great difficulty hearing and understanding another person talking in a noisy place (for example, on an urban street).	0.01 (0.004-0.019)
Mild hearing loss with ringing due to other congenital anomalies	Hearing loss, mild, with ringing	has great difficulty hearing and understanding another person talking in a noisy place (for example, on an urban street), and sometimes has annoying ringing in the ears.	0.021 (0.012-0.036)
Moderate hearing loss due to other congenital anomalies	Hearing loss, moderate	is unable to hear and understand another person talking in a noisy place (for example, on an urban street), and has difficulty hearing another person talking even in a quiet place or on the phone.	0.027 (0.015-0.042)
Moderate hearing loss with ringing due to other congenital anomalies	Hearing loss, moderate, with ringing	is unable to hear and understand another person talking in a noisy place (for example, on an urban street), has difficulty hearing another person talking even in a quiet place or on the phone, and has annoying ringing in the ears for 5 minutes at a time, almost every day.	0.074 (0.049-0.107)
Moderately severe hearing loss due to other congenital anomalies	Hearing loss, moderately severe	(custom DW: average of moderate and severe hearing loss impairment)	--
Moderately severe hearing loss with ringing due to other congenital anomalies	Hearing loss, moderately severe, with ringing	(custom DW: average of moderate and severe hearing loss impairment)	--
Severe hearing loss with ringing due to other congenital anomalies	Hearing loss, severe, with ringing	is unable to hear and understand another person talking, even in a quiet place, is unable to take part in a phone conversation, and has annoying ringing in the ears for more than 5 minutes at a time, almost every day. Difficulties with communicating and relating to others cause emotional impact at times (for example worry or depression).	0.261 (0.175-0.36)

Appendix Table 6. GBD 2015 sequelae, health states, health state lay descriptions, and disability weights			
**Sequela**	**Healthstate name**	**Healthstate lay description**	**Disability weight**
Severe hearing loss due to other congenital anomalies	Hearing loss, severe	is unable to hear and understand another person talking, even in a quiet place, and unable to take part in a phone conversation. Difficulties with communicating and relating to others cause emotional impact at times (for example worry or depression).	0.158 (0.105-0.227)
Profound hearing loss due to other congenital anomalies	Hearing loss, profound	is unable to hear and understand another person talking, even in a quiet place, is unable to take part in a phone conversation, and has great difficulty hearing anything in any other situation. Difficulties with communicating and relating to others often cause worry, depression or loneliness.	0.204 (0.134-0.288)
Profound hearing loss with ringing due to other congenital anomalies	Hearing loss, profound, with ringing	is unable to hear and understand another person talking, even in a quiet place, is unable to take part in a phone conversation, has great difficulty hearing anything in any other situation, and has annoying ringing in the ears for more than 5 minutes at a time, several times a day. Difficulties with communicating and relating to others often cause worry, depression, or loneliness.	0.277 (0.182-0.387)
Complete hearing loss due to other congenital anomalies	Hearing loss, complete	cannot hear at all in any situation, including even the loudest sounds, and cannot communicate verbally or use a phone. Difficulties with communicating and relating to others often cause worry, depression or loneliness.	0.215 (0.144-0.307)
Complete hearing loss with ringing due to other congenital anomalies	Hearing loss, complete, with ringing	cannot hear at all in any situation, including even the loudest sounds, and cannot communicate verbally or use a phone, and has very annoying ringing in the ears for more than half of the day. Difficulties with communicating and relating to others often cause worry, depression or loneliness.	0.316 (0.212-0.435)
Mild eczema	Disfigurement, level 1 with itch/pain	has a slight, visible physical deformity that is sometimes sore or itchy. Others notice the deformity, which causes some worry and discomfort.	0.027 (0.015-0.042)
Moderate eczema	Disfigurement, level 2, with itch/pain	has a visible physical deformity that is sore and itchy. Other people stare and comment, which causes the person to worry. The person has trouble sleeping and concentrating.	0.188 (0.125-0.267)
Severe eczema	Disfigurement, level 3, with itch/pain	has an obvious physical deformity that is very painful and itchy. The physical deformity makes others uncomfortable, which causes the person to avoid social contact, feel worried, sleep poorly, and think about suicide.	0.576 (0.401-0.731)
Asymptomatic contact dermatitis	--	--	--
Mild contact dermatitis	Disfigurement, level 1 with itch/pain	has a slight, visible physical deformity that is sometimes sore or itchy. Others notice the deformity, which causes some worry and discomfort.	0.027 (0.015-0.042)
Severe contact dermatitis	Disfigurement, level 2, with itch/pain	has a visible physical deformity that is sore and itchy. Other people stare and comment, which causes the person to worry. The person has trouble sleeping and concentrating.	0.188 (0.125-0.267)
Asymptomatic seborrhoeic dermatitis	--	--	--
Symptomatic seborrhoeic dermatitis	Disfigurement, level 1 with itch/pain	has a slight, visible physical deformity that is sometimes sore or itchy. Others notice the deformity, which causes some worry and discomfort.	0.027 (0.015-0.042)
Mild psoriasis	Disfigurement, level 1 with itch/pain	has a slight, visible physical deformity that is sometimes sore or itchy. Others notice the deformity, which causes some worry and discomfort.	0.027 (0.015-0.042)
Moderate psoriasis	Disfigurement, level 2, with itch/pain	has a visible physical deformity that is sore and itchy. Other people stare and comment, which causes the person to worry. The person has trouble sleeping and concentrating.	0.188 (0.125-0.267)
Severe psoriasis	Disfigurement, level 3, with itch/pain	has an obvious physical deformity that is very painful and itchy. The physical deformity makes others uncomfortable, which causes the person to avoid social contact, feel worried, sleep poorly, and think about suicide.	0.576 (0.401-0.731)
Mild cellulitis	Disfigurement, level 1 with itch/pain	has a slight, visible physical deformity that is sometimes sore or itchy. Others notice the deformity, which causes some worry and discomfort.	0.027 (0.015-0.042)
Severe cellulitis	Disfigurement, level 2, with itch/pain	has a visible physical deformity that is sore and itchy. Other people stare and comment, which causes the person to worry. The person has trouble sleeping and concentrating.	0.188 (0.125-0.267)
Impetigo	Infectious disease, acute episode, mild	has a low fever and mild discomfort , but no difficulty with daily activities.	0.006 (0.002-0.012)
Abscess and other bacterial skin diseases	Infectious disease, acute episode, mild	has a low fever and mild discomfort , but no difficulty with daily activities.	0.006 (0.002-0.012)
Scabies	Disfigurement, level 1 with itch/pain	has a slight, visible physical deformity that is sometimes sore or itchy. Others notice the deformity, which causes some worry and discomfort.	0.027 (0.015-0.042)
Tinea capitis	Infectious disease, acute episode, mild	has a low fever and mild discomfort , but no difficulty with daily activities.	0.006 (0.002-0.012)
Other fungal skin diseases	Infectious disease, acute episode, mild	has a low fever and mild discomfort , but no difficulty with daily activities.	0.006 (0.002-0.012)
Mild molluscum contagiosum	Infectious disease, acute episode, mild	has a low fever and mild discomfort , but no difficulty with daily activities.	0.006 (0.002-0.012)
Severe molluscum contagiosum	Disfigurement, level 2	has a visible physical deformity that causes others to stare and comment. As a result, the person is worried and has trouble sleeping and concentrating.	0.067 (0.044-0.096)
Mild viral warts	Infectious disease, acute episode, mild	has a low fever and mild discomfort , but no difficulty with daily activities.	0.006 (0.002-0.012)
Severe viral warts	Disfigurement, level 2	has a visible physical deformity that causes others to stare and comment. As a result, the person is worried and has trouble sleeping and concentrating.	0.067 (0.044-0.096)
Acne vulgaris	Disfigurement, level 1	has a slight, visible physical deformity that others notice, which causes some worry and discomfort.	0.011 (0.005-0.021)
Mild alopecia areata	Disfigurement, level 1	has a slight, visible physical deformity that others notice, which causes some worry and discomfort.	0.011 (0.005-0.021)
Severe alopecia areata	Disfigurement, level 2	has a visible physical deformity that causes others to stare and comment. As a result, the person is worried and has trouble sleeping and concentrating.	0.067 (0.044-0.096)
Pruritus	Disfigurement, level 1	has a slight, visible physical deformity that others notice, which causes some worry and discomfort.	0.011 (0.005-0.021)
Mild urticaria	Disfigurement, level 1 with itch/pain	has a slight, visible physical deformity that is sometimes sore or itchy. Others notice the deformity, which causes some worry and discomfort.	0.027 (0.015-0.042)
Severe urticaria	Disfigurement, level 2, with itch/pain	has a visible physical deformity that is sore and itchy. Other people stare and comment, which causes the person to worry. The person has trouble sleeping and concentrating.	0.188 (0.125-0.267)
Mild decubitus ulcer	Disfigurement, level 1	has a slight, visible physical deformity that others notice, which causes some worry and discomfort.	0.011 (0.005-0.021)
Moderate decubitus ulcer	Disfigurement, level 2	has a visible physical deformity that causes others to stare and comment. As a result, the person is worried and has trouble sleeping and concentrating.	0.067 (0.044-0.096)
Severe decubitus ulcer	Disfigurement, level 3	has an obvious physical deformity that makes others uncomfortable, which causes the person to avoid social contact, feel worried, sleep poorly, and think about suicide.	0.405 (0.275-0.546)
Asymptomatic other skin and subcutaneous diseases	--	--	--
Symptomatic other skin and subcutaneous diseases	Disfigurement, level 1	has a slight, visible physical deformity that others notice, which causes some worry and discomfort.	0.011 (0.005-0.021)
Moderate vision impairment due to glaucoma	Distance vision, moderate impairment	has vision problems that make it difficult to recognize faces or objects across a room.	0.031 (0.019-0.049)
Severe vision impairment due to glaucoma	Distance vision, severe impairment	has severe vision loss, which causes difficulty in daily activities, some emotional impact (for example worry), and some difficulty going outside the home without assistance.	0.184 (0.125-0.258)
Blindness due to glaucoma	Distance vision blindness	is completely blind, which causes great difficulty in some daily activities, worry and anxiety, and great difficulty going outside the home without assistance.	0.187 (0.124-0.26)
Moderate vision impairment due to cataract	Distance vision, moderate impairment	has vision problems that make it difficult to recognize faces or objects across a room.	0.031 (0.019-0.049)
Severe vision impairment due to cataract	Distance vision, severe impairment	has severe vision loss, which causes difficulty in daily activities, some emotional impact (for example worry), and some difficulty going outside the home without assistance.	0.184 (0.125-0.258)
Blindness due to cataract	Distance vision blindness	is completely blind, which causes great difficulty in some daily activities, worry and anxiety, and great difficulty going outside the home without assistance.	0.187 (0.124-0.26)
Moderate vision impairment due to macular degeneration	Distance vision, moderate impairment	has vision problems that make it difficult to recognize faces or objects across a room.	0.031 (0.019-0.049)
Severe vision impairment due to macular degeneration	Distance vision, severe impairment	has severe vision loss, which causes difficulty in daily activities, some emotional impact (for example worry), and some difficulty going outside the home without assistance.	0.184 (0.125-0.258)
Blindness due to macular degeneration	Distance vision blindness	is completely blind, which causes great difficulty in some daily activities, worry and anxiety, and great difficulty going outside the home without assistance.	0.187 (0.124-0.26)
Moderate vision impairment due to uncorrected refractive error	Distance vision, moderate impairment	has vision problems that make it difficult to recognize faces or objects across a room.	0.031 (0.019-0.049)
Severe vision impairment due to uncorrected refractive error	Distance vision, severe impairment	has severe vision loss, which causes difficulty in daily activities, some emotional impact (for example worry), and some difficulty going outside the home without assistance.	0.184 (0.125-0.258)
Blindness due to uncorrected refractive error	Distance vision blindness	is completely blind, which causes great difficulty in some daily activities, worry and anxiety, and great difficulty going outside the home without assistance.	0.187 (0.124-0.26)

Appendix Table 6. GBD 2015 sequelae, health states, health state lay descriptions, and disability weights			
**Sequela**	**Healthstate name**	**Healthstate lay description**	**Disability weight**
Near vision impairment	Presbyopia	has difficulty seeing things that are nearer than 3 feet, but has no difficulty with seeing things at a distance.	0.011 (0.005-0.02)
Mild hearing loss due to age-related and other hearing loss	Hearing loss, mild	has great difficulty hearing and understanding another person talking in a noisy place (for example, on an urban street).	0.01 (0.004-0.019)
Mild hearing loss with ringing due to age-related and other hearing loss	Hearing loss, mild, with ringing	has great difficulty hearing and understanding another person talking in a noisy place (for example, on an urban street), and sometimes has annoying ringing in the ears.	0.021 (0.012-0.036)
Moderate hearing loss due to age-related and other hearing loss	Hearing loss, moderate	is unable to hear and understand another person talking in a noisy place (for example, on an urban street), and has difficulty hearing another person talking even in a quiet place or on the phone.	0.027 (0.015-0.042)
Moderate hearing loss with ringing due to age-related and other hearing loss	Hearing loss, moderate, with ringing	is unable to hear and understand another person talking in a noisy place (for example, on an urban street), has difficulty hearing another person talking even in a quiet place or on the phone, and has annoying ringing in the ears for 5 minutes at a time, almost every day.	0.074 (0.049-0.107)
Moderately severe hearing loss due to age-related and other hearing loss	Hearing loss, moderately severe	(custom DW: average of moderate and severe hearing loss impairment)	--
Moderately severe hearing loss with ringing due to age-related and other hearing loss	Hearing loss, moderately severe, with ringing	(custom DW: average of moderate and severe hearing loss impairment)	--
Severe hearing loss with ringing due to age-related and other hearing loss	Hearing loss, severe, with ringing	is unable to hear and understand another person talking, even in a quiet place, is unable to take part in a phone conversation, and has annoying ringing in the ears for more than 5 minutes at a time, almost every day. Difficulties with communicating and relating to others cause emotional impact at times (for example worry or depression).	0.261 (0.175-0.36)
Severe hearing loss due to age-related and other hearing loss	Hearing loss, severe	is unable to hear and understand another person talking, even in a quiet place, and unable to take part in a phone conversation. Difficulties with communicating and relating to others cause emotional impact at times (for example worry or depression).	0.158 (0.105-0.227)
Profound hearing loss due to age-related and other hearing loss	Hearing loss, profound	is unable to hear and understand another person talking, even in a quiet place, is unable to take part in a phone conversation, and has great difficulty hearing anything in any other situation. Difficulties with communicating and relating to others often cause worry, depression or loneliness.	0.204 (0.134-0.288)
Profound hearing loss with ringing due to age-related and other hearing loss	Hearing loss, profound, with ringing	is unable to hear and understand another person talking, even in a quiet place, is unable to take part in a phone conversation, has great difficulty hearing anything in any other situation, and has annoying ringing in the ears for more than 5 minutes at a time, several times a day. Difficulties with communicating and relating to others often cause worry, depression, or loneliness.	0.277 (0.182-0.387)
Complete hearing loss due to age-related and other hearing loss	Hearing loss, complete	cannot hear at all in any situation, including even the loudest sounds, and cannot communicate verbally or use a phone. Difficulties with communicating and relating to others often cause worry, depression or loneliness.	0.215 (0.144-0.307)
Complete hearing loss with ringing due to age-related and other hearing loss	Hearing loss, complete, with ringing	cannot hear at all in any situation, including even the loudest sounds, and cannot communicate verbally or use a phone, and has very annoying ringing in the ears for more than half of the day. Difficulties with communicating and relating to others often cause worry, depression or loneliness.	0.316 (0.212-0.435)
Moderate vision impairment due to other vision loss	Distance vision, moderate impairment	has vision problems that make it difficult to recognize faces or objects across a room.	0.031 (0.019-0.049)
Severe vision impairment due to other vision loss	Distance vision, severe impairment	has severe vision loss, which causes difficulty in daily activities, some emotional impact (for example worry), and some difficulty going outside the home without assistance.	0.184 (0.125-0.258)
Blindness due to other vision loss	Distance vision blindness	is completely blind, which causes great difficulty in some daily activities, worry and anxiety, and great difficulty going outside the home without assistance.	0.187 (0.124-0.26)
Asymptomatic other sense organ diseases	--	--	--
Mild other sense organ diseases	Infectious disease, acute episode, mild	has a low fever and mild discomfort , but no difficulty with daily activities.	0.006 (0.002-0.012)
Moderate other sense organ diseases	Disfigurement, level 1	has a slight, visible physical deformity that others notice, which causes some worry and discomfort.	0.011 (0.005-0.021)
Severe other sense organ diseases	Vertigo	0	--
Asymptomatic deciduous caries	--	--	--
Tooth pain due to deciduous caries	Dental caries, symptomatic	has a toothache, which causes some difficulty in eating.	0.01 (0.005-0.019)
Asymptomatic permanent caries	--	--	--
Tooth pain due to permanent caries	Dental caries, symptomatic	has a toothache, which causes some difficulty in eating.	0.01 (0.005-0.019)
Chronic periodontal diseases	Periodontitis	has minor bleeding of the gums from time to time, with mild discomfort.	0.007 (0.003-0.014)
Asymptomatic edentulism and severe tooth loss	--	--	--
Difficulty eating due to edentulism and severe tooth loss	Severe tooth loss	has lost more than 20 teeth including front and back, and has great difficulty in eating meat, fruits, and vegetables.	0.067 (0.045-0.095)
Mild other oral disorders	Infectious disease, acute episode, mild	has a low fever and mild discomfort , but no difficulty with daily activities.	0.006 (0.002-0.012)
Severe other oral disorders	Infectious disease, acute episode, moderate	has a fever and aches, and feels weak, which causes some difficulty with daily activities.	0.051 (0.032-0.074)
Amputation of finger(s), excluding thumb (long term, with treatment)	Amputation of finger(s), excluding thumb (long term, with treatment)	has lost part of the fingers of one hand, causing difficulties in using the hand, pain, and tingling in the stumps.	0·005 (0·002-0·010)
Amputation of thumb (long term)	Amputation of thumb (long term)	has lost one thumb, causing some difficulty in using the hand, pain, and tingling in the stump.	0·011 (0·005-0·021)
Amputation of one upper limb (long term, without treatment)	Amputation of one upper limb (long term, without treatment)	has lost one hand and part of the arm, leaving pain and tingling in the stump. The person needs help from others to lift objects or do daily activities such as cooking.	0·039 (0·024-0·059)
Amputation of one upper limb (long term, with or without treatment)	Amputation of one upper limb (long term, with or without treatment)	has lost one hand and part of the arm, leaving pain and tingling in the stump and flashbacks from the injury. The person requires help lifting objects and in daily activities such as cooking.	0·118 (0·079-0·167)
Amputation of both upper limbs (long term, with treatment)	Amputation of both upper limbs (long term, with treatment)	has lost part of both arms, leaving pain and tingling in the stumps and flashbacks from the injury. The person has comfortable artificial arms and is mostly independent.	0·123 (0·081-0·176)
Amputation of both upper limbs (long term, without treatment)	Amputation of both upper limbs (long term, without treatment)	has lost part of both arms, leaving pain and tingling in the stumps and flashbacks from the injury. The person needs help with basic daily activities such as eating and using the toilet.	0·383 (0·251-0·525)
Amputation of toe	Amputation of toe	has lost one toe, leaving occasional pain and tingling in the stump.	0·006 (0·002-0·012)
Amputation of one lower limb (long term, with treatment)	Amputation of one lower limb (long term, with treatment)	has lost part of one leg, leaving pain and tingling in the stump. The person has a comfortable artificial leg and only slight difficulties moving around.	0·039 (0·023-0·059)
Amputation of one lower limb (long term, without treatment)	Amputation of one lower limb (long term, without treatment)	has lost part of one leg, leaving pain and tingling in the stump. The person does not have an artificial leg, has frequent sores, and uses crutches.	0·173 (0·118-0·240)
Amputation of both lower limbs (long term, with treatment)	Amputation of both lower limbs (long term, with treatment)	has lost part of both legs, leaving pain and tingling in the stumps. The person has two comfortable artificial legs, which allow for movement.	0·088 (0·057-0·124)
Amputation of both lower limbs (long term, without treatment)	Amputation of both lower limbs (long term, without treatment)	has lost part of both legs, leaving pain, tingling, and frequent sores in the stumps. The person has great difficulty moving around and has episodes of depression, anxiety and flashbacks to the injury.	0·443 (0·297-0·589)
Burns, <20% total burned surface area without lower airway burns (short term, with or without treatment)	Burns, <20% total burned surface area without lower airway burns (short term, with or without treatment)	has a burn on part of the body. Parts of the burned area are painful, and other parts have lost feeling.	0·141 (0·094-0·196)
Burns, <20% total burned surface area or <10% total burned surface area if head/neck or hands/wrist involved (long term, with or without treatment)	Burns, <20% total burned surface area or <10% total burned surface area if head/neck or hands/wrist involved (long term, with or without treatment)	has scars caused by a burn. The scars are sometimes painful and itchy.	0·016 (0·008-0·028)
Burns, ≥20% total burned surface area (short term, with or without treatment)	Burns, ≥20% total burned surface area (short term, with or without treatment)	has a painful burn over a large part of the body. Parts of the burned area have lost feeling, and the person feels anxious and unwell.	0·314 (0·211-0·441)
Burns, ≥20% total burned surface area or ≥10% total burned surface area if head/neck or hands/wrist involved (long term, with treatment)	Burns, ≥20% total burned surface area or ≥10% total burned surface area if head/neck or hands/wrist involved (long term, with treatment)	has scars caused by burns over a large part of the body. The scars are frequently painful and itchy, and the person is often sad.	0·135 (0·092-0·190)
Burns, ≥20% total burned surface area or ≥10% total burned surface area if head/neck or hands/wrist involved (long term, without treatment)	Burns, ≥20% total burned surface area or ≥10% total burned surface area if head/neck or hands/wrist involved (long term, without treatment)	has severe, disfiguring and itchy scars caused by burns over a large part of the body. The person cannot move some joints, feels sad, and has great difficulty with self-care such as dressing and toileting.	0·455 (0·302-0·601)
Lower airway burns (with or without treatment)	Lower airway burns (with or without treatment)	has a burn in the throat and lungs, which causes great difficulty breathing and a lot of anxiety.	0·376 (0·240-0·524)
Crush injury (short or long term, with or without treatment)	Crush injury (short or long term, with or without treatment)	had part of the body crushed, leaving pain, swelling, tingling and limited feeling in the affected area.	0·132 (0·089-0·189)
Dislocation of hip (long term, with or without treatment)	Dislocation of hip (long term, with or without treatment)	walks with a limp and feels discomfort when walking.	0·016 (0·008-0·028)
Dislocation of knee (long term, with or without treatment)	Dislocation of knee (long term, with or without treatment)	has a knee out of joint, causing pain and difficulty moving the knee, which sometimes gives way. The person needs crutches for walking and help with self-care such as dressing.	0·113 (0·075-0·160)
Dislocation of shoulder (long term, with or without treatment)	Dislocation of shoulder (long term, with or without treatment)	has a shoulder that is out of joint, causing pain and difficulty moving. The person has difficulty with daily activities such as dressing and cooking.	0·062 (0·041-0·088)

Appendix Table 6. GBD 2015 sequelae, health states, health state lay descriptions, and disability weights			
**Sequela**	**Healthstate name**	**Healthstate lay description**	**Disability weight**
Other injuries of muscle and tendon (includes sprains, strains and dislocations other than shoulder, knee, hip)	Other injuries of muscle and tendon (includes sprains, strains and dislocations other than shoulder, knee, hip)	has a strained muscle that causes pain and swelling.	0·008 (0·003-0·015)
Drowning and nonfatal submersion (short or long term, with or without treatment)	Drowning and nonfatal submersion (short or long term, with or without treatment)	has breathlessness, anxiety, cough, and vomiting.	0·247 (0·164-0·341)
Fracture of clavicle, scapula or humerus (short or long term, with or without treatment)	Fracture of clavicle, scapula or humerus (short or long term, with or without treatment)	has a broken shoulder bone, which is painful and swollen. The person cannot use the affected arm and has difficulty with getting dressed.	0·035 (0·021-0·053)
Fracture of face bone (short or long term, with or without treatment)	Fracture of face bone (short or long term, with or without treatment)	has a broken cheek bone, broken nose, and chipped teeth, with swelling and severe pain.	0·067 (0·044-0·097)
Fracture of foot bones (short term, with or without treatment)	Fracture of foot bones (short term, with or without treatment)	has a broken foot bone, which causes pain, swelling, and difficulty walking.	0·026 (0·015-0·043)
Fracture of foot bones (long term, without treatment)	Fracture of foot bones (long term, without treatment)	had a broken foot in the past that did not heal properly. The person now has pain in the foot and has some difficulty walking.	0·026 (0·015-0·042)
Fracture of hand (short term, with or without treatment)	Fracture of hand (short term, with or without treatment)	has a broken hand, causing pain and swelling.	0·010 (0·005-0·019)
Fracture of hand (long term, without treatment)	Fracture of hand (long term, without treatment)	has stiffness in the hand and a weak grip.	0·014 (0·007-0·025)
Fracture of neck of femur (short term, with or without treatment)	Fracture of neck of femur (short term, with or without treatment)	has broken a hip and is in pain. The person cannot stand or walk, and needs help washing, dressing, and going to the toilet.	0·258 (0·172-0·356)
Fracture of neck of femur (long term, with treatment)	Fracture of neck of femur (long term, with treatment)	had a broken hip in the past, which was fixed with treatment. The person can only walk short distances, has discomfort when moving around, and has some difficulty in daily activities.	0·058 (0·038-0·084)
Fracture of neck of femur (long term, without treatment)	Fracture of neck of femur (long term, without treatment)	had a broken hip bone in the past, which was never treated and did not heal properly. The person cannot get out of bed and needs help washing and going to the toilet.	0·402 (0·269-0·541)
Fracture, other than femoral neck (short term, with or without treatment)	Fracture, other than femoral neck (short term, with or without treatment)	has a broken thigh bone. The person has severe pain and swelling and cannot walk.	0·111 (0·074-0·156)
Fracture, other than femoral neck (long term, without treatment)	Fracture, other than femoral neck (long term, without treatment)	had a broken thigh bone in the past, which was never treated and did not heal properly. The person now has a limp and discomfort when walking.	0·042 (0·027-0·063)
Fracture of patella, tibia or fibula or ankle (short term, with or without treatment)	Fracture of patella, tibia or fibula or ankle (short term, with or without treatment)	has a broken shin bone, which causes severe pain, swelling, and difficulty walking.	0·050 (0·032-0·075)
Fracture of patella, tibia or fibula or ankle (long term, with or without treatment)	Fracture of patella, tibia or fibula or ankle (long term, with or without treatment)	had a broken shin bone in the past that did not heal properly. The person has pain in the knee and ankle, and has difficulty walking.	0·055 (0·036-0·081)
Fracture of pelvis (short term)	Fracture of pelvis (short term)	has a broken pelvis bone, with swelling and bruising. The person has severe pain, and cannot walk or do daily activities.	0·279 (0·188-0·384)
Fracture of pelvis (long term)	Fracture of pelvis (long term)	had a broken pelvis in the past and now walks with a limp. There is often pain in the back and groin, and when urinating and sitting for a long time.	0·182 (0·123-0·253)
Fracture of radius or ulna (short term, with or without treatment)	Fracture of radius or ulna (short term, with or without treatment)	has a broken forearm, which causes severe pain, swelling, and limited movement.	0·028 (0·016-0·046)
Fracture of radius or ulna (long term, without treatment)	Fracture of radius or ulna (long term, without treatment)	had a broken forearm in the past that did not heal properly, causing some pain and limited movement in the elbow and wrist. The person has difficulty with daily activities such as dressing.	0·043 (0·028-0·064)
Fracture of skull (short or long term, with or without treatment)	Fracture of skull (short or long term, with or without treatment)	has a broken skull, but does not have brain damage. The broken area is painful and swollen.	0·071 (0·048-0·100)
Fracture of sternum and/or fracture of one or two ribs (short term, with or without treatment)	Fracture of sternum and/or fracture of one or two ribs (short term, with or without treatment)	has a broken rib that causes severe pain in the chest, especially when breathing in. The person has difficulty with daily activities such as dressing.	0·103 (0·068-0·145)
Fracture of vertebral column (short or long term, with or without treatment)	Fracture of vertebral column (short or long term, with or without treatment)	has broken back bones and is in pain, but still has full use of arms and legs.	0·111 (0·075-0·156)
Fractures, treated (long term)	Fractures, treated (long term)	has slight pain in a bone that was broken in the past.	0·005 (0·002-0·01)
Injured nerves (short term)	Injured nerves (short term)	has a nerve injury, which causes difficulty moving and some loss of feeling in the affected area.	0·100 (0·067-0·14)
Injured nerves (long term)	Injured nerves (long term)	had a nerve injury in the past, which continues to cause some difficulty moving. The person often injures the affected part because it is numb.	0·113 (0·076-0·157)
Injury to eyes (short term)	Injury to eyes (short term)	has an injury to one eye, which causes pain and difficulty seeing.	0·054 (0·035-0·081)
Severe traumatic brain injury, short term (with or without treatment)	Severe traumatic brain injury, short term (with or without treatment)	cannot concentrate and has headaches, memory problems, dizziness, and feels angry.	0·110 (0·074-0·158)
Concussion	Concussion	has headaches, dizziness, nausea and difficulty concentrating.	0·214 (0·141-0·297)
Traumatic brain injury, long-term consequences, minor (with or without treatment)	Traumatic brain injury, long-term consequences, minor (with or without treatment)	has episodes of headaches, memory problems, and difficulty concentrating.	0·094 (0·063-0·133)
Traumatic brain injury, long-term consequences, moderate (with or without treatment)	Traumatic brain injury, long-term consequences, moderate (with or without treatment)	has frequent headaches, memory problems, difficulty concentrating, and dizziness. The person is often anxious and moody.	0·231 (0·156-0·324)
Traumatic brain injury, long-term consequences, severe (with or without treatment)	Traumatic brain injury, long-term consequences, severe (with or without treatment)	cannot think clearly and has frequent headaches, memory problems, difficulty concentrating and dizziness. The person is often anxious and moody, and depends on others for feeding, toileting, dressing and walking.	0·637 (0·462-0·789)
Open wound (short term, with or without treatment)	Open wound (short term, with or without treatment)	has a cut in the skin, which causes pain and numbness around the cut.	0·006 (0·002-0·012)
Poisoning (short term with or without treatment)	Poisoning (short term with or without treatment)	has drowsiness, stomach pain and vomiting.	0·163 (0·109-0·227)
Severe chest injury (long term, with or without treatment)	Severe chest injury (long term, with or without treatment)	had a severe chest injury in the past that has now healed. The person still gets breathless when walking and feels discomfort in the chest.	0·047 (0·030-0·070)
Severe chest injury (short term, with or without treatment)	Severe chest injury (short term, with or without treatment)	has a serious chest injury, which causes severe pain, shortness of breath and anxiety.	0·369 (0·248-0·501)
Spinal cord lesion below neck level (treated)	Spinal cord lesion below neck level (treated)	is paralyzed from the waist down and cannot feel or move the legs. The person uses a lightweight and comfortable wheelchair to move around.	0·296 (0·198-0·414)
Spinal cord lesion below neck level (untreated)	Spinal cord lesion below neck level (untreated)	is paralyzed from the waist down and cannot feel or move the legs. Legs are in fixed, bent positions, and the person gets frequent infections and pressure sores.	0·623 (0·434-0·777)
Spinal cord lesion at neck level (treated)	Spinal cord lesion at neck level (treated)	is paralyzed from the neck down and cannot feel or move the arms and legs.	0·589 (0·415-0·748)
Spinal cord lesion at neck level (untreated)	Spinal cord lesion at neck level (untreated)	is paralyzed from the neck down and cannot feel or move the arms and legs. Arms and legs are in fixed, bent positions, and the person gets frequent infections and pressure sores.	0·732 (0·544-0·871)

Appendix Table 7. GBD 2015 methods of estimating years lived with disability (YLDs) for 35 residual categories	
**Residual**	**Method**
	**Estimation: YLD to YLL ratio method**
Other intestinal infectious diseases	YLD to YLL ratio of explicitly estimated intestinal infectious diseases by geography, country, sex applied to YLL from other intestinal infectious diseases by geography, country, sex and age
Other neglected tropical diseases	YLD to YLL ratio of explicitly estimated neglected tropical disease causes by geography, country, sex applied to YLL from other neglected tropical diseases by geography, country, sex and age
Other maternal disorders	YLD to YLL ratio of explicitly estimated maternal disorder causes by geography, country, sex applied to YLL from other maternal disorders by geography, country, sex and age
Other neonatal disorders	YLD to YLL ratio of explicitly estimated neonatal disorders by geography, country, sex applied to YLL from other neonatal disorders by geography, country, sex and age
Other nutritional deficiencies	YLD to YLL ratio of explicitly estimated nutritional deficiencies by geography, country, sex applied to YLL from other nutritional deficiencies by geography, country, sex and age
Other sexually transmitted diseases	YLD to YLL ratio of gonococcal and chlamydial infection by geography, country, sex applied to YLL from other sexually transmitted diseases by geography, country, sex and age
Other infectious diseases	YLD to YLL ratio of HIV/AIDS and tuberculosis, diarrhea, lower respiratory and other common infectious diseases, neglected tropical diseases and malaria, sexually transmitted diseases and hepatitis by geography, country, sex applied to YLL from other infectious diseases by geography, country, sex and age
Other chronic respiratory diseases	YLD to YLL ratio of COPD, pneumoconiosis and interstitial lung disease and pulmonary sarcoidosis by geography, country, sex applied to YLL from other chronic respiratory diseases by geography, country, sex and age
Other digestive disorders	YLD to YLL ratio of explicitly estimated digestive disorders by geography, country, sex applied to YLL from other digestive disorders by geography, country, sex and age
Other neurological disorders	YLD to YLL ratio of Alzheimer and other dementias, Parkinson disease, multiple sclerosis and motor-neuron disease by geography, country, sex applied to YLL from other neurological disorders by geography, country, sex and age
Other urinary diseases	YLD to YLL ratio of explicitly estimated urinary diseases by geography, country, sex applied to YLL from other urinary diseases by geography, country, sex and age
Other hemoglobinopathies and hemolytic anemias	YLD to YLL ratio of explicitly estimated hemoglobinopathies and hemolytic anemias by geography, country, sex applied to YLL from other hemoglobinopathies and hemolytic anemias by geography, country, sex and age
Other congenital anomalies	YLD to YLL ratio of explicitly estimated congenital anomalies by geography, country, sex applied to YLL from other congenital anomalies by geography, country, sex and age
	**Estimation: based on epidemiological data**
Meningitis due to other causes	Data on proportion of meningitis due to other causes modelled in DisMod-MR 2.1, forced to sum to 100% at 1,000 draw level for each geography-year-age-sex with the proportions for pneumococcal, H influenza B and meningococcal meningitis, and applied to dismod-MR 2.1 model of all meningitis
Pelvic inflammatory	DisMod-MR 2.1 model of the proportion of pelvic inflammatory disease due to other causes, constrained to 100% with proportions of pelvic inflammatory disease due to gonococcal and chlamydial infection and applied to the DisMod-MR 2.1 model for all pelvic inflammatory disease

**Appendix Table 7. GBD 2015 methods of estimating years lived with disability (YLDs) for 35 residual categories**	
**Residual**	**Method**
disease due to other causes	
Other neoplasms	Similar to all other cancers: mortality to incidence ratio method applied to cancer registry data for other neoplasms
Liver cancer due to other causes	Data on proportion of liver cancer due to other causes modelled in DisMod-MR 2.1, forced to sum to 100% at 1,000 draw level for each geography, year, age, and sex with the proportions for liver cancer due to hepatitis B, hepatitis C and alcohol, and applied to total liver cancer estimates from cancer analyses using mortality to incidence ratios
Other cardiovascular diseases	Ratio of prevalence of ICD-9 coded other cardiovascular diseases in MEPS and 2005 USA outpatient data to prevalence of heart failure due to other cardiovascular diseases (estimated as part of the heart failure envelope), and applied to prevalence of heart failure due to other CVD estimates for all other locations and years
Cirrhosis and other chronic liver diseases due to other causes	Data on proportion of cirrhosis and other chronic liver diseases due to other causes modelled in DisMod-MR 2.1, forced to sum to 100% at 1,000 draw level for each geography-year-age-sex with the proportions for cirrhosis and other chronic liver diseases due to hepatitis B, hepatitis C and alcohol, and applied to total cirrhosis and other chronic liver diseases estimates from DisMod-MR 2.1 analysis
Other pneumoconiosis	DisMod-MR 2.1 model based on hospital admission and claims data
Other drug use disorders	NESARC prevalence of drug dependence other than cannabis, opioids, amphetamines and cocaine multiplied by ratio of YLD to prevalence for cocaine and amphetamine by geography, year, age, and sex
Other mental and substance use disorders	Prevalence of personality disorders not comorbid with GBD mental and substance use disorder categories and severity distribution from NESARC and Australian National Survey of Mental Health and Wellbeing 1997
Chronic kidney disease due to other causes	Data on proportion of chronic kidney disease due to other causes from renal registries modelled in DisMod-MR 2.1, forced to sum to 100% at 1,000 draw level for each geography-year-age-sex with the proportions of chronic kidney disease due to diabetes, hypertension and glomerulonephritis, and applied to total chronic kidney disease estimates from DisMod-MR 2.1 analyses
Other gynecological disorders	Dismod MR 2.1 using US claims data
Other musculoskeletal disorders	DisMod-MR 2.1 model of survey and US claims data on prevalence of all musculoskeletal symptoms and diseases minus rheumatoid arthritis, osteoarthritis, gout, low back pain and neck pain. Long-term sequelae of fractures, dislocations and contusions due to injuries are subtracted out of other musculoskeletal disorders to avoid double counting
Other skin	Dismod-MR 2.1 model using outpatient and US claims data
Age-related and other hearing loss	Survey data on the proportion of hearing loss due to age-related and other hearing loss modelled in dismod MR 2.1 and forced to sum to total hearing loss by geography, year, age and sex
Other vision loss	Survey data on vision loss due to other causes modelled in DisMod-MR 2.1 and forced to sum to total vision loss by geography, year, age and sex
Other sense organ disorders	DisMod-MR 2.1 model using outpatient and US claims data
Other oral disorders	DisMod-MR 2.1 model using US Medical Expenditure Panel Surveys (MEPS) data

Appendix Table 7. GBD 2015 methods of estimating years lived with disability (YLDs) for 35 residual categories	
**Residual**	**Method**
Other transport injuries	DisMod-MR 2.1 model using data from surveys, hospital admission and outpatient/emergency department visits
Other exposure to mechanical forces	DisMod-MR 2.1 model using data from surveys, hospital admission and outpatient/emergency department visits
Foreign body in other part	DisMod-MR 2.1 model using data from surveys, hospital admission and outpatient/emergency department visits
Other unintentional injuries	DisMod-MR 2.1 model using data from surveys, hospital admission and outpatient/emergency department visits
Assault by other means	DisMod-MR 2.1 model using data from surveys, hospital admission and outpatient/emergency department visits

Appendix Table 8. Socio-Demographic Index (SDI) values for all estimated GBD 2015 locations, 1980–2015

Location	1980	1981	1982	1983	1984	1985	1986	1987	1988	1989	1990	1991	1992	1993	1994	1995	1996	1997	1998	1999	2000	2001	2002	2003	2004	2005	2006	2007	2008	2009	2010	2011	2012	2013	2014	2015
Global	0.420	0.426	0.431	0.436	0.442	0.449	0.455	0.461	0.468	0.475	0.481	0.488	0.494	0.501	0.508	0.515	0.521	0.528	0.534	0.540	0.546	0.551	0.557	0.563	0.568	0.575	0.581	0.589	0.595	0.602	0.608	0.614	0.620	0.627	0.632	0.638
Southeast Asia, East Asia, and Oceania	0.335	0.344	0.354	0.364	0.374	0.386	0.397	0.408	0.419	0.428	0.438	0.448	0.460	0.472	0.484	0.496	0.507	0.518	0.528	0.537	0.545	0.554	0.562	0.570	0.578	0.587	0.596	0.605	0.614	0.622	0.630	0.638	0.646	0.653	0.660	0.667
East Asia	0.325	0.335	0.345	0.354	0.365	0.378	0.390	0.402	0.413	0.424	0.434	0.445	0.457	0.470	0.484	0.497	0.509	0.521	0.532	0.541	0.550	0.559	0.568	0.577	0.586	0.595	0.605	0.615	0.624	0.633	0.641	0.649	0.658	0.665	0.672	0.679
China	0.316	0.326	0.336	0.345	0.357	0.370	0.382	0.394	0.406	0.416	0.426	0.438	0.450	0.464	0.477	0.491	0.504	0.516	0.527	0.537	0.546	0.555	0.564	0.573	0.583	0.592	0.602	0.612	0.622	0.631	0.639	0.648	0.656	0.664	0.671	0.678
Anhui	0.277	0.295	0.305	0.314	0.328	0.340	0.353	0.367	0.372	0.379	0.388	0.398	0.412	0.426	0.438	0.450	0.461	0.471	0.480	0.489	0.499	0.508	0.518	0.525	0.532	0.539	0.547	0.556	0.563	0.570	0.579	0.587	0.592	0.598	0.605	0.611
Beijing	0.511	0.518	0.519	0.528	0.533	0.542	0.553	0.565	0.581	0.598	0.608	0.624	0.633	0.643	0.653	0.663	0.673	0.683	0.692	0.700	0.709	0.718	0.728	0.738	0.748	0.759	0.770	0.781	0.792	0.802	0.812	0.822	0.833	0.842	0.852	0.861
Chongqing	0.274	0.290	0.300	0.293	0.299	0.305	0.337	0.365	0.373	0.386	0.402	0.408	0.419	0.434	0.449	0.463	0.477	0.489	0.502	0.515	0.527	0.536	0.544	0.549	0.558	0.569	0.575	0.584	0.591	0.599	0.609	0.612	0.614	0.620	0.626	0.631
Fujian	0.302	0.312	0.322	0.332	0.341	0.358	0.378	0.398	0.409	0.414	0.425	0.441	0.456	0.473	0.489	0.505	0.523	0.537	0.550	0.560	0.570	0.578	0.588	0.597	0.606	0.616	0.625	0.636	0.645	0.655	0.666	0.674	0.680	0.681	0.680	0.677
Gansu	0.297	0.308	0.314	0.321	0.328	0.341	0.352	0.364	0.373	0.379	0.393	0.399	0.406	0.414	0.423	0.433	0.447	0.457	0.467	0.475	0.483	0.491	0.498	0.505	0.513	0.520	0.526	0.533	0.540	0.546	0.558	0.563	0.570	0.577	0.584	0.591
Guangdong	0.264	0.277	0.298	0.265	0.238	0.314	0.324	0.339	0.370	0.394	0.404	0.420	0.444	0.470	0.492	0.509	0.527	0.546	0.561	0.576	0.588	0.596	0.607	0.613	0.624	0.636	0.645	0.656	0.667	0.677	0.686	0.700	0.705	0.717	0.728	0.740
Guangxi	0.241	0.252	0.264	0.282	0.283	0.299	0.318	0.336	0.351	0.369	0.382	0.382	0.396	0.409	0.424	0.440	0.454	0.466	0.476	0.487	0.496	0.505	0.514	0.521	0.533	0.542	0.550	0.562	0.570	0.580	0.592	0.601	0.610	0.619	0.629	0.639
Guizhou	0.164	0.183	0.195	0.208	0.227	0.252	0.267	0.280	0.291	0.310	0.318	0.323	0.332	0.344	0.356	0.370	0.382	0.391	0.401	0.409	0.419	0.428	0.435	0.447	0.457	0.468	0.477	0.488	0.495	0.501	0.511	0.519	0.526	0.534	0.541	0.549
Hainan	0.337	0.348	0.356	0.352	0.356	0.361	0.384	0.406	0.413	0.415	0.424	0.433	0.446	0.458	0.472	0.484	0.497	0.509	0.520	0.532	0.542	0.551	0.559	0.569	0.578	0.586	0.595	0.605	0.614	0.622	0.631	0.639	0.649	0.659	0.668	0.677
Hebei	0.301	0.300	0.310	0.319	0.327	0.336	0.337	0.339	0.355	0.371	0.384	0.405	0.421	0.434	0.451	0.468	0.482	0.495	0.505	0.513	0.524	0.535	0.540	0.548	0.554	0.557	0.565	0.572	0.583	0.592	0.597	0.606	0.619	0.629	0.639	0.649
Heilongjiang	0.411	0.421	0.424	0.432	0.442	0.450	0.455	0.460	0.471	0.477	0.488	0.496	0.503	0.513	0.524	0.537	0.548	0.557	0.565	0.575	0.582	0.590	0.597	0.605	0.612	0.619	0.626	0.634	0.641	0.649	0.656	0.663	0.669	0.676	0.682	0.687
Henan	0.260	0.274	0.286	0.302	0.326	0.341	0.352	0.362	0.373	0.381	0.391	0.407	0.420	0.438	0.452	0.467	0.481	0.493	0.503	0.513	0.522	0.531	0.542	0.552	0.563	0.575	0.585	0.598	0.607	0.617	0.627	0.635	0.644	0.643	0.640	0.634
Hong Kong, Special Administrative Region of China	0.681	0.692	0.701	0.710	0.718	0.726	0.734	0.743	0.752	0.760	0.767	0.775	0.782	0.790	0.798	0.806	0.811	0.816	0.819	0.822	0.826	0.829	0.833	0.836	0.840	0.845	0.850	0.854	0.859	0.862	0.865	0.868	0.870	0.872	0.874	0.876
Hubei	0.330	0.343	0.358	0.366	0.378	0.389	0.398	0.407	0.421	0.428	0.439	0.445	0.458	0.467	0.483	0.499	0.511	0.523	0.537	0.547	0.558	0.567	0.575	0.584	0.593	0.602	0.610	0.619	0.628	0.635	0.639	0.646	0.652	0.659	0.666	0.673
Hunan	0.309	0.319	0.321	0.333	0.348	0.359	0.369	0.379	0.388	0.402	0.412	0.423	0.441	0.457	0.469	0.483	0.494	0.504	0.514	0.524	0.531	0.538	0.546	0.553	0.561	0.572	0.581	0.590	0.597	0.604	0.611	0.617	0.625	0.634	0.643	0.651
Inner Mongolia	0.332	0.342	0.350	0.368	0.376	0.395	0.409	0.423	0.431	0.443	0.450	0.465	0.474	0.483	0.496	0.512	0.528	0.541	0.554	0.566	0.578	0.589	0.601	0.610	0.618	0.624	0.632	0.641	0.651	0.660	0.668	0.677	0.685	0.695	0.703	0.712
Jiangsu	0.372	0.381	0.392	0.404	0.419	0.430	0.443	0.456	0.464	0.465	0.476	0.489	0.501	0.517	0.530	0.545	0.557	0.568	0.579	0.588	0.597	0.607	0.616	0.625	0.634	0.645	0.655	0.665	0.676	0.684	0.692	0.701	0.712	0.721	0.729	0.737
Jiangxi	0.301	0.310	0.322	0.334	0.343	0.355	0.369	0.382	0.392	0.396	0.405	0.416	0.429	0.438	0.453	0.465	0.480	0.490	0.501	0.510	0.518	0.526	0.535	0.544	0.554	0.562	0.570	0.579	0.587	0.595	0.604	0.611	0.620	0.629	0.639	0.648
Jilin	0.381	0.390	0.401	0.412	0.422	0.432	0.443	0.454	0.461	0.470	0.480	0.490	0.502	0.514	0.529	0.543	0.555	0.566	0.576	0.587	0.596	0.605	0.615	0.624	0.633	0.642	0.651	0.661	0.671	0.680	0.688	0.698	0.707	0.715	0.723	0.730
Liaoning	0.429	0.441	0.444	0.454	0.469	0.473	0.482	0.492	0.501	0.513	0.519	0.533	0.540	0.550	0.560	0.570	0.580	0.590	0.599	0.608	0.617	0.626	0.634	0.642	0.651	0.659	0.668	0.677	0.686	0.695	0.703	0.711	0.719	0.727	0.735	0.742
Macao, Special Administrative Region of China	0.637	0.644	0.651	0.657	0.664	0.672	0.680	0.690	0.701	0.712	0.723	0.732	0.741	0.751	0.759	0.768	0.776	0.783	0.789	0.794	0.799	0.803	0.807	0.813	0.821	0.829	0.839	0.848	0.857	0.864	0.871	0.873	0.874	0.876	0.877	0.878
Ningxia	0.317	0.337	0.345	0.358	0.363	0.375	0.386	0.398	0.406	0.422	0.432	0.441	0.455	0.467	0.478	0.490	0.502	0.511	0.521	0.530	0.538	0.545	0.552	0.561	0.569	0.577	0.586	0.598	0.608	0.615	0.623	0.631	0.641	0.648	0.656	0.663
Qinghai	0.300	0.315	0.325	0.331	0.337	0.350	0.358	0.366	0.381	0.390	0.392	0.397	0.405	0.419	0.425	0.434	0.444	0.450	0.458	0.465	0.471	0.476	0.483	0.491	0.500	0.508	0.516	0.525	0.533	0.539	0.543	0.549	0.557	0.559	0.562	0.564
Shaanxi	0.308	0.316	0.324	0.334	0.341	0.353	0.368	0.383	0.392	0.401	0.409	0.422	0.433	0.447	0.460	0.476	0.490	0.502	0.513	0.524	0.534	0.545	0.554	0.562	0.572	0.583	0.593	0.603	0.613	0.623	0.634	0.642	0.651	0.656	0.660	0.664
Shandong	0.328	0.339	0.353	0.366	0.378	0.394	0.401	0.407	0.430	0.444	0.452	0.464	0.483	0.497	0.511	0.523	0.535	0.544	0.553	0.563	0.571	0.579	0.587	0.596	0.603	0.614	0.625	0.638	0.648	0.656	0.665	0.674	0.683	0.693	0.702	0.711
Shanghai	0.553	0.555	0.559	0.561	0.566	0.571	0.581	0.591	0.601	0.611	0.627	0.638	0.646	0.655	0.663	0.672	0.680	0.688	0.695	0.702	0.710	0.718	0.726	0.735	0.745	0.755	0.764	0.774	0.784	0.793	0.802	0.812	0.821	0.830	0.839	0.847
Shanxi	0.349	0.354	0.362	0.373	0.385	0.394	0.406	0.419	0.426	0.437	0.443	0.449	0.462	0.478	0.490	0.504	0.515	0.526	0.536	0.544	0.555	0.565	0.574	0.583	0.592	0.602	0.612	0.623	0.632	0.642	0.650	0.659	0.668	0.676	0.685	0.693
Sichuan	0.235	0.233	0.244	0.260	0.278	0.269	0.286	0.305	0.312	0.328	0.341	0.355	0.358	0.364	0.374	0.382	0.395	0.429	0.441	0.449	0.459	0.468	0.476	0.488	0.497	0.504	0.516	0.525	0.530	0.539	0.549	0.547	0.554	0.556	0.559	0.561
Tianjin	0.493	0.496	0.499	0.502	0.510	0.520	0.535	0.549	0.558	0.569	0.579	0.593	0.600	0.615	0.626	0.638	0.649	0.658	0.667	0.676	0.684	0.694	0.704	0.714	0.724	0.734	0.745	0.756	0.766	0.776	0.787	0.796	0.806	0.816	0.825	0.834
Tibet	0.204	0.211	0.218	0.207	0.215	0.219	0.235	0.251	0.255	0.263	0.273	0.277	0.283	0.286	0.296	0.304	0.312	0.318	0.324	0.330	0.337	0.344	0.348	0.356	0.362	0.368	0.374	0.382	0.390	0.395	0.398	0.403	0.408	0.414	0.420	0.425
Xinjiang	0.327	0.338	0.346	0.348	0.353	0.360	0.372	0.386	0.413	0.407	0.419	0.428	0.442	0.457	0.472	0.488	0.500	0.510	0.521	0.532	0.542	0.551	0.560	0.567	0.575	0.580	0.589	0.595	0.605	0.613	0.620	0.630	0.638	0.646	0.653	0.660
Yunnan	0.239	0.244	0.256	0.254	0.271	0.292	0.305	0.319	0.327	0.342	0.353	0.361	0.372	0.381	0.394	0.408	0.420	0.429	0.440	0.449	0.457	0.463	0.471	0.480	0.491	0.501	0.513	0.523	0.532	0.540	0.546	0.553	0.562	0.569	0.576	0.584
Zhejiang	0.351	0.360	0.371	0.380	0.399	0.412	0.427	0.441	0.454	0.467	0.479	0.487	0.497	0.512	0.527	0.541	0.555	0.567	0.578	0.588	0.597	0.606	0.616	0.627	0.634	0.643	0.656	0.667	0.678	0.688	0.697	0.709	0.717	0.727	0.737	0.747
North Korea	0.532	0.536	0.541	0.546	0.552	0.558	0.564	0.569	0.573	0.578	0.582	0.586	0.589	0.592	0.591	0.588	0.583	0.577	0.570	0.564	0.558	0.552	0.548	0.545	0.544	0.545	0.547	0.549	0.551	0.552	0.554	0.556	0.558	0.560	0.563	0.565
Taiwan	0.611	0.620	0.630	0.641	0.652	0.663	0.677	0.685	0.688	0.702	0.706	0.717	0.725	0.731	0.739	0.746	0.753	0.759	0.775	0.778	0.780	0.794	0.801	0.809	0.816	0.823	0.828	0.833	0.838	0.843	0.849	0.851	0.850	0.859	0.861	0.865
Southeast Asia	0.361	0.371	0.381	0.390	0.400	0.408	0.417	0.425	0.433	0.441	0.450	0.458	0.467	0.476	0.485	0.494	0.504	0.513	0.521	0.528	0.535	0.542	0.548	0.555	0.562	0.570	0.577	0.585	0.593	0.600	0.607	0.615	0.622	0.629	0.637	0.644
Cambodia	0.206	0.204	0.203	0.202	0.204	0.208	0.211	0.217	0.225	0.234	0.241	0.250	0.259	0.267	0.274	0.282	0.290	0.298	0.306	0.316	0.327	0.337	0.348	0.359	0.370	0.382	0.395	0.407	0.419	0.429	0.438	0.448	0.458	0.468	0.477	0.486
Indonesia	0.361	0.373	0.383	0.394	0.405	0.415	0.426	0.436	0.446	0.456	0.467	0.478	0.489	0.499	0.510	0.521	0.531	0.541	0.548	0.554	0.559	0.564	0.569	0.574	0.579	0.584	0.590	0.596	0.603	0.610	0.617	0.624	0.631	0.638	0.645	0.652
Laos	0.206	0.211	0.216	0.222	0.227	0.233	0.238	0.244	0.248	0.254	0.260	0.267	0.275	0.284	0.294	0.304	0.315	0.326	0.338	0.349	0.360	0.370	0.380	0.390	0.399	0.408	0.417	0.427	0.437	0.447	0.457	0.467	0.478	0.488	0.498	0.508

Appendix Table 8. Socio-Demographic Index (SDI) values for all estimated GBD 2015 locations, 1980‒2015

Location	1980	1981	1982	1983	1984	1985	1986	1987	1988	1989	1990	1991	1992	1993	1994	1995	1996	1997	1998	1999	2000	2001	2002	2003	2004	2005	2006	2007	2008	2009	2010	2011	2012	2013	2014	2015
Malaysia	0.496	0.505	0.512	0.520	0.527	0.533	0.539	0.545	0.552	0.559	0.566	0.574	0.582	0.590	0.598	0.607	0.617	0.628	0.637	0.647	0.657	0.666	0.676	0.685	0.694	0.703	0.710	0.718	0.726	0.732	0.738	0.745	0.750	0.756	0.761	0.767
Maldives	0.258	0.263	0.268	0.272	0.280	0.290	0.300	0.313	0.327	0.342	0.357	0.373	0.389	0.404	0.420	0.435	0.449	0.463	0.477	0.490	0.502	0.513	0.524	0.536	0.546	0.553	0.562	0.571	0.580	0.586	0.593	0.600	0.606	0.612	0.617	0.623
Mauritius	0.483	0.491	0.499	0.507	0.514	0.520	0.527	0.535	0.543	0.551	0.559	0.568	0.576	0.585	0.594	0.602	0.611	0.618	0.626	0.633	0.640	0.647	0.653	0.659	0.665	0.671	0.677	0.684	0.691	0.697	0.704	0.711	0.717	0.723	0.729	0.735
Myanmar	0.184	0.193	0.202	0.212	0.221	0.230	0.238	0.243	0.245	0.247	0.249	0.250	0.253	0.258	0.264	0.272	0.281	0.290	0.300	0.311	0.324	0.338	0.352	0.367	0.383	0.399	0.415	0.431	0.445	0.457	0.469	0.480	0.491	0.501	0.510	0.520
Philippines	0.449	0.457	0.465	0.472	0.478	0.482	0.486	0.490	0.495	0.501	0.506	0.511	0.516	0.521	0.526	0.531	0.537	0.542	0.546	0.550	0.554	0.559	0.563	0.569	0.574	0.580	0.586	0.593	0.599	0.605	0.611	0.618	0.624	0.631	0.638	0.645
Sri Lanka	0.466	0.471	0.477	0.483	0.490	0.498	0.507	0.515	0.522	0.529	0.535	0.542	0.548	0.554	0.561	0.567	0.575	0.583	0.590	0.597	0.603	0.608	0.613	0.618	0.624	0.629	0.635	0.642	0.649	0.656	0.664	0.672	0.681	0.689	0.697	0.705
Seychelles	0.510	0.521	0.531	0.542	0.552	0.562	0.570	0.578	0.586	0.594	0.604	0.615	0.626	0.637	0.647	0.655	0.663	0.670	0.677	0.683	0.688	0.695	0.701	0.705	0.708	0.712	0.715	0.719	0.722	0.725	0.729	0.734	0.740	0.747	0.753	0.759
Thailand	0.440	0.451	0.462	0.472	0.482	0.491	0.500	0.509	0.518	0.528	0.538	0.547	0.557	0.567	0.576	0.586	0.596	0.603	0.609	0.614	0.618	0.623	0.629	0.635	0.641	0.647	0.653	0.659	0.665	0.670	0.676	0.682	0.688	0.694	0.699	0.705
Timor-Leste	0.201	0.208	0.213	0.220	0.226	0.234	0.240	0.246	0.253	0.259	0.265	0.271	0.276	0.281	0.285	0.289	0.285	0.278	0.267	0.252	0.240	0.252	0.266	0.278	0.301	0.327	0.355	0.374	0.389	0.400	0.410	0.421	0.431	0.438	0.444	0.450
Vietnam	0.294	0.306	0.315	0.326	0.337	0.347	0.356	0.364	0.373	0.381	0.390	0.399	0.410	0.421	0.433	0.446	0.459	0.472	0.484	0.495	0.505	0.514	0.523	0.532	0.540	0.549	0.558	0.566	0.574	0.582	0.590	0.599	0.606	0.614	0.621	0.628
Oceania	0.331	0.335	0.339	0.344	0.349	0.354	0.359	0.364	0.369	0.373	0.377	0.382	0.386	0.393	0.399	0.404	0.410	0.414	0.418	0.421	0.424	0.427	0.429	0.431	0.433	0.436	0.439	0.443	0.448	0.453	0.459	0.466	0.473	0.480	0.487	0.494
American Samoa	0.616	0.619	0.631	0.639	0.627	0.618	0.631	0.629	0.643	0.643	0.643	0.653	0.643	0.643	0.660	0.675	0.689	0.703	0.703	0.703	0.709	0.721	0.712	0.712	0.713	0.713	0.712	0.712	0.712	0.713	0.713	0.713	0.713	0.714	0.714	0.714
Federated States of Micronesia	0.422	0.428	0.434	0.440	0.447	0.455	0.462	0.469	0.476	0.483	0.489	0.496	0.502	0.508	0.513	0.519	0.525	0.530	0.536	0.541	0.548	0.553	0.560	0.566	0.573	0.579	0.585	0.591	0.595	0.599	0.604	0.609	0.614	0.618	0.621	0.624
Fiji	0.515	0.522	0.529	0.534	0.540	0.546	0.550	0.553	0.556	0.560	0.566	0.571	0.576	0.582	0.587	0.593	0.599	0.604	0.610	0.615	0.621	0.627	0.632	0.637	0.642	0.646	0.652	0.657	0.662	0.667	0.672	0.676	0.679	0.683	0.688	0.693
Guam	0.705	0.713	0.720	0.726	0.732	0.737	0.742	0.748	0.754	0.761	0.768	0.775	0.782	0.788	0.793	0.798	0.803	0.808	0.812	0.818	0.823	0.828	0.832	0.837	0.841	0.846	0.851	0.855	0.859	0.863	0.867	0.871	0.875	0.878	0.881	0.884
Kiribati	0.417	0.414	0.411	0.407	0.403	0.399	0.398	0.396	0.397	0.398	0.400	0.401	0.402	0.403	0.405	0.408	0.412	0.416	0.422	0.427	0.433	0.437	0.442	0.447	0.450	0.454	0.457	0.460	0.462	0.464	0.466	0.467	0.469	0.471	0.474	0.478
Marshall Islands	0.355	0.362	0.369	0.377	0.384	0.390	0.397	0.406	0.406	0.421	0.431	0.470	0.484	0.486	0.481	0.480	0.483	0.477	0.477	0.483	0.496	0.501	0.506	0.511	0.516	0.521	0.527	0.534	0.542	0.548	0.555	0.562	0.569	0.577	0.585	0.592
Northern Mariana Islands	0.646	0.665	0.681	0.696	0.709	0.722	0.733	0.745	0.755	0.765	0.775	0.779	0.783	0.788	0.793	0.797	0.806	0.813	0.820	0.827	0.833	0.839	0.843	0.847	0.851	0.852	0.852	0.852	0.852	0.849	0.847	0.844	0.842	0.840	0.840	0.841
Papua New Guinea	0.272	0.275	0.278	0.283	0.288	0.293	0.298	0.303	0.309	0.314	0.317	0.321	0.326	0.333	0.340	0.346	0.351	0.355	0.359	0.363	0.366	0.369	0.371	0.374	0.376	0.380	0.383	0.388	0.393	0.399	0.406	0.414	0.422	0.430	0.439	0.448
Samoa	0.429	0.438	0.446	0.453	0.460	0.466	0.474	0.482	0.488	0.495	0.500	0.507	0.512	0.519	0.524	0.529	0.535	0.541	0.546	0.550	0.554	0.561	0.568	0.574	0.580	0.585	0.590	0.596	0.603	0.608	0.613	0.618	0.623	0.628	0.632	0.637
Solomon Islands	0.254	0.261	0.268	0.273	0.279	0.283	0.287	0.292	0.297	0.303	0.310	0.317	0.327	0.337	0.347	0.359	0.368	0.377	0.384	0.390	0.393	0.394	0.393	0.393	0.394	0.395	0.399	0.404	0.411	0.416	0.423	0.432	0.440	0.448	0.455	0.461
Tonga	0.391	0.398	0.408	0.418	0.433	0.448	0.463	0.478	0.489	0.498	0.506	0.513	0.518	0.522	0.527	0.531	0.537	0.541	0.545	0.549	0.552	0.559	0.565	0.571	0.576	0.580	0.585	0.589	0.594	0.598	0.604	0.608	0.612	0.615	0.618	0.622
Vanuatu	0.345	0.351	0.356	0.362	0.368	0.373	0.379	0.383	0.387	0.391	0.396	0.401	0.407	0.412	0.417	0.423	0.428	0.434	0.439	0.445	0.452	0.458	0.463	0.469	0.474	0.480	0.486	0.493	0.500	0.506	0.512	0.518	0.523	0.528	0.532	0.536
Central Europe, Eastern Europe, and Central Asia	0.655	0.659	0.663	0.668	0.672	0.676	0.681	0.686	0.692	0.698	0.704	0.709	0.714	0.716	0.719	0.720	0.722	0.723	0.724	0.726	0.729	0.733	0.737	0.743	0.749	0.756	0.763	0.771	0.778	0.784	0.789	0.795	0.800	0.806	0.811	0.816
Central Asia	0.552	0.557	0.562	0.567	0.572	0.576	0.581	0.587	0.592	0.599	0.604	0.609	0.612	0.614	0.615	0.614	0.615	0.615	0.615	0.616	0.618	0.622	0.627	0.633	0.640	0.648	0.656	0.665	0.673	0.681	0.688	0.696	0.703	0.711	0.718	0.724
Armenia	0.551	0.556	0.560	0.564	0.568	0.571	0.576	0.581	0.586	0.591	0.595	0.599	0.597	0.593	0.591	0.590	0.591	0.593	0.596	0.600	0.605	0.613	0.623	0.634	0.646	0.660	0.673	0.687	0.700	0.708	0.716	0.725	0.733	0.741	0.749	0.755
Azerbaijan	0.587	0.595	0.602	0.609	0.616	0.621	0.626	0.631	0.636	0.641	0.645	0.651	0.653	0.654	0.653	0.650	0.647	0.644	0.641	0.640	0.640	0.643	0.648	0.656	0.664	0.676	0.692	0.709	0.725	0.738	0.749	0.759	0.767	0.775	0.782	0.788
Georgia	0.643	0.648	0.653	0.658	0.663	0.667	0.672	0.678	0.683	0.689	0.694	0.696	0.692	0.685	0.677	0.668	0.660	0.653	0.648	0.645	0.645	0.649	0.656	0.665	0.673	0.682	0.692	0.702	0.712	0.719	0.727	0.734	0.742	0.749	0.755	0.761
Kazakhstan	0.631	0.636	0.640	0.645	0.649	0.653	0.658	0.663	0.669	0.676	0.682	0.688	0.694	0.698	0.701	0.703	0.706	0.709	0.711	0.712	0.716	0.720	0.725	0.731	0.737	0.743	0.749	0.756	0.762	0.768	0.774	0.780	0.787	0.794	0.801	0.807
Kyrgyzstan	0.551	0.555	0.558	0.562	0.565	0.569	0.573	0.578	0.583	0.588	0.592	0.597	0.600	0.600	0.599	0.596	0.594	0.592	0.589	0.586	0.585	0.585	0.586	0.589	0.593	0.595	0.597	0.600	0.605	0.608	0.611	0.614	0.617	0.622	0.626	0.631
Mongolia	0.380	0.392	0.403	0.414	0.427	0.439	0.452	0.467	0.482	0.498	0.513	0.526	0.536	0.544	0.552	0.560	0.567	0.574	0.581	0.588	0.594	0.601	0.608	0.614	0.622	0.629	0.637	0.644	0.651	0.656	0.662	0.669	0.678	0.687	0.696	0.705
Tajikistan	0.451	0.457	0.461	0.466	0.470	0.474	0.479	0.485	0.492	0.499	0.505	0.511	0.511	0.510	0.507	0.502	0.495	0.488	0.480	0.472	0.466	0.465	0.469	0.477	0.487	0.497	0.507	0.517	0.526	0.533	0.541	0.547	0.554	0.561	0.567	0.574
Turkmenistan	0.530	0.538	0.545	0.551	0.557	0.563	0.569	0.574	0.581	0.588	0.595	0.602	0.608	0.616	0.621	0.625	0.629	0.631	0.633	0.636	0.642	0.648	0.655	0.662	0.670	0.681	0.692	0.703	0.714	0.724	0.734	0.745	0.755	0.764	0.773	0.781
Uzbekistan	0.482	0.488	0.495	0.501	0.506	0.512	0.518	0.524	0.531	0.538	0.545	0.551	0.555	0.560	0.563	0.567	0.571	0.575	0.580	0.585	0.590	0.596	0.601	0.607	0.613	0.620	0.627	0.635	0.643	0.651	0.659	0.667	0.676	0.684	0.692	0.699
Central Europe	0.643	0.648	0.653	0.658	0.663	0.668	0.673	0.679	0.684	0.690	0.695	0.699	0.702	0.706	0.710	0.715	0.721	0.727	0.734	0.740	0.746	0.753	0.759	0.765	0.771	0.777	0.783	0.789	0.796	0.801	0.807	0.812	0.817	0.823	0.828	0.833
Albania	0.509	0.517	0.524	0.530	0.536	0.542	0.548	0.554	0.560	0.566	0.572	0.571	0.569	0.569	0.571	0.575	0.583	0.589	0.597	0.607	0.618	0.629	0.639	0.650	0.660	0.670	0.680	0.688	0.696	0.703	0.708	0.715	0.720	0.726	0.731	0.736
Bosnia and Herzegovina	0.426	0.435	0.442	0.449	0.456	0.463	0.470	0.476	0.481	0.487	0.489	0.490	0.491	0.492	0.495	0.504	0.528	0.555	0.582	0.604	0.623	0.638	0.652	0.663	0.672	0.681	0.688	0.695	0.703	0.709	0.714	0.720	0.725	0.730	0.734	0.739
Bulgaria	0.635	0.643	0.650	0.657	0.664	0.670	0.676	0.683	0.690	0.697	0.702	0.707	0.710	0.713	0.716	0.719	0.721	0.723	0.725	0.727	0.730	0.734	0.738	0.743	0.748	0.753	0.759	0.766	0.773	0.778	0.784	0.789	0.794	0.799	0.804	0.808
Croatia	0.608	0.616	0.622	0.629	0.635	0.641	0.647	0.654	0.660	0.665	0.670	0.673	0.674	0.673	0.674	0.675	0.678	0.682	0.687	0.693	0.699	0.707	0.714	0.721	0.728	0.735	0.741	0.748	0.754	0.759	0.764	0.768	0.773	0.777	0.780	0.784
Czech Republic	0.726	0.734	0.741	0.748	0.755	0.761	0.766	0.770	0.775	0.780	0.786	0.790	0.795	0.800	0.806	0.812	0.819	0.825	0.830	0.834	0.839	0.843	0.846	0.850	0.853	0.856	0.860	0.865	0.869	0.872	0.876	0.879	0.882	0.885	0.888	0.892
Hungary	0.677	0.684	0.692	0.698	0.703	0.708	0.711	0.715	0.719	0.723	0.727	0.730	0.733	0.737	0.741	0.746	0.751	0.756	0.762	0.767	0.773	0.779	0.786	0.791	0.797	0.803	0.809	0.815	0.820	0.824	0.828	0.832	0.836	0.840	0.844	0.849
Macedonia	0.596	0.602	0.609	0.614	0.619	0.624	0.630	0.635	0.639	0.644	0.646	0.650	0.652	0.654	0.656	0.658	0.661	0.664	0.669	0.674	0.679	0.684	0.690	0.695	0.701	0.707	0.714	0.721	0.728	0.734	0.739	0.745	0.749	0.753	0.758	0.762
Montenegro	0.638	0.644	0.650	0.655	0.661	0.666	0.670	0.675	0.679	0.683	0.687	0.691	0.692	0.690	0.689	0.688	0.690	0.693	0.697	0.700	0.706	0.712	0.718	0.724	0.730	0.736	0.743	0.751	0.760	0.766	0.773	0.779	0.784	0.790	0.794	0.799

Appendix Table 8. Socio-Demographic Index (SDI) values for all estimated GBD 2015 locations, 1980−2015

Location	1980	1981	1982	1983	1984	1985	1986	1987	1988	1989	1990	1991	1992	1993	1994	1995	1996	1997	1998	1999	2000	2001	2002	2003	2004	2005	2006	2007	2008	2009	2010	2011	2012	2013	2014	2015
Poland	0.663	0.666	0.668	0.671	0.674	0.678	0.683	0.688	0.694	0.700	0.706	0.710	0.715	0.721	0.728	0.735	0.744	0.753	0.762	0.771	0.780	0.787	0.794	0.799	0.805	0.810	0.814	0.820	0.825	0.831	0.837	0.843	0.849	0.855	0.861	0.868
Romania	0.638	0.644	0.650	0.655	0.660	0.665	0.671	0.677	0.683	0.688	0.693	0.696	0.698	0.700	0.702	0.704	0.707	0.709	0.710	0.713	0.716	0.720	0.725	0.730	0.735	0.741	0.748	0.755	0.763	0.769	0.774	0.780	0.785	0.790	0.795	0.799
Serbia	0.604	0.609	0.614	0.619	0.623	0.628	0.633	0.637	0.643	0.648	0.653	0.658	0.660	0.660	0.659	0.658	0.660	0.663	0.667	0.670	0.674	0.681	0.688	0.697	0.705	0.714	0.722	0.730	0.738	0.745	0.750	0.756	0.760	0.765	0.769	0.772
Slovakia	0.649	0.656	0.663	0.670	0.676	0.682	0.688	0.694	0.700	0.706	0.713	0.717	0.722	0.727	0.732	0.739	0.745	0.753	0.760	0.766	0.773	0.779	0.786	0.792	0.797	0.803	0.810	0.817	0.824	0.830	0.836	0.841	0.846	0.852	0.857	0.862
Slovenia	0.679	0.687	0.696	0.704	0.711	0.719	0.727	0.734	0.741	0.747	0.753	0.757	0.760	0.764	0.768	0.772	0.777	0.783	0.788	0.794	0.800	0.806	0.812	0.818	0.823	0.827	0.832	0.837	0.841	0.844	0.847	0.849	0.850	0.852	0.854	0.856
Eastern Europe	0.689	0.693	0.697	0.701	0.705	0.709	0.714	0.720	0.726	0.733	0.740	0.747	0.752	0.755	0.757	0.758	0.757	0.757	0.756	0.756	0.757	0.760	0.764	0.770	0.776	0.784	0.792	0.800	0.809	0.814	0.819	0.825	0.830	0.835	0.839	0.843
Belarus	0.632	0.637	0.641	0.646	0.651	0.656	0.662	0.667	0.674	0.681	0.687	0.693	0.698	0.703	0.704	0.705	0.706	0.709	0.712	0.716	0.721	0.727	0.734	0.742	0.751	0.760	0.770	0.780	0.791	0.800	0.809	0.818	0.827	0.834	0.841	0.847
Estonia	0.659	0.663	0.668	0.672	0.676	0.680	0.685	0.691	0.697	0.705	0.712	0.719	0.724	0.727	0.731	0.735	0.739	0.744	0.751	0.756	0.763	0.770	0.777	0.784	0.791	0.798	0.806	0.814	0.821	0.827	0.832	0.838	0.844	0.850	0.856	0.861
Latvia	0.687	0.690	0.694	0.697	0.700	0.704	0.709	0.714	0.721	0.728	0.735	0.740	0.741	0.742	0.743	0.745	0.746	0.748	0.751	0.755	0.760	0.766	0.773	0.780	0.788	0.796	0.805	0.815	0.823	0.829	0.834	0.840	0.846	0.851	0.857	0.861
Lithuania	0.668	0.673	0.678	0.683	0.688	0.692	0.697	0.702	0.707	0.712	0.718	0.722	0.724	0.725	0.725	0.726	0.727	0.730	0.734	0.739	0.745	0.752	0.760	0.768	0.776	0.784	0.792	0.801	0.809	0.814	0.817	0.821	0.826	0.830	0.833	0.837
Moldova	0.575	0.578	0.581	0.584	0.587	0.591	0.595	0.600	0.605	0.610	0.616	0.620	0.621	0.621	0.619	0.615	0.611	0.607	0.603	0.599	0.597	0.598	0.603	0.610	0.619	0.628	0.637	0.645	0.654	0.661	0.668	0.676	0.683	0.690	0.697	0.703
Russia	0.715	0.719	0.722	0.725	0.728	0.732	0.736	0.741	0.748	0.754	0.761	0.768	0.774	0.777	0.779	0.780	0.780	0.779	0.778	0.778	0.779	0.782	0.785	0.790	0.796	0.802	0.809	0.817	0.825	0.830	0.834	0.839	0.844	0.848	0.852	0.856
Ukraine	0.640	0.646	0.652	0.657	0.663	0.669	0.676	0.682	0.689	0.697	0.704	0.710	0.715	0.719	0.719	0.719	0.718	0.717	0.715	0.714	0.714	0.717	0.720	0.727	0.736	0.745	0.755	0.766	0.776	0.781	0.787	0.793	0.799	0.804	0.808	0.811
High-income	0.739	0.744	0.749	0.754	0.759	0.763	0.768	0.773	0.778	0.784	0.789	0.794	0.799	0.803	0.808	0.813	0.818	0.823	0.827	0.832	0.836	0.840	0.844	0.847	0.851	0.854	0.858	0.862	0.865	0.869	0.872	0.875	0.879	0.882	0.885	0.888
High-income Asia Pacific	0.712	0.719	0.725	0.731	0.738	0.744	0.751	0.758	0.765	0.773	0.780	0.788	0.795	0.801	0.807	0.814	0.820	0.826	0.832	0.837	0.841	0.846	0.850	0.854	0.858	0.861	0.865	0.868	0.871	0.873	0.876	0.878	0.881	0.884	0.886	0.889
Brunei	0.718	0.725	0.732	0.738	0.743	0.747	0.754	0.761	0.769	0.776	0.784	0.790	0.797	0.805	0.814	0.823	0.833	0.842	0.853	0.863	0.871	0.877	0.881	0.886	0.890	0.893	0.896	0.899	0.902	0.905	0.909	0.912	0.915	0.918	0.921	0.923
Japan	0.750	0.755	0.760	0.765	0.769	0.775	0.780	0.785	0.792	0.798	0.805	0.812	0.819	0.825	0.831	0.836	0.841	0.846	0.851	0.854	0.858	0.862	0.865	0.868	0.871	0.873	0.876	0.878	0.881	0.882	0.884	0.886	0.889	0.891	0.893	0.895
Aichi	0.761	0.767	0.774	0.776	0.781	0.786	0.792	0.796	0.803	0.811	0.818	0.825	0.831	0.837	0.843	0.848	0.853	0.858	0.862	0.866	0.869	0.873	0.877	0.879	0.882	0.884	0.886	0.888	0.890	0.892	0.894	0.895	0.898	0.901	0.903	0.905
Akita	0.733	0.740	0.744	0.749	0.753	0.760	0.764	0.768	0.775	0.779	0.787	0.794	0.798	0.804	0.808	0.814	0.821	0.825	0.831	0.834	0.838	0.841	0.846	0.849	0.851	0.853	0.856	0.860	0.862	0.865	0.869	0.870	0.872	0.876	0.878	0.881
Aomori	0.736	0.742	0.747	0.751	0.756	0.762	0.766	0.772	0.779	0.784	0.792	0.797	0.804	0.809	0.814	0.819	0.824	0.830	0.834	0.838	0.842	0.844	0.848	0.852	0.855	0.859	0.862	0.866	0.867	0.871	0.871	0.872	0.874	0.876	0.878	0.880
Chiba	0.742	0.745	0.751	0.756	0.761	0.766	0.773	0.779	0.785	0.792	0.800	0.807	0.814	0.820	0.825	0.830	0.836	0.841	0.845	0.849	0.852	0.855	0.857	0.860	0.862	0.865	0.867	0.870	0.872	0.873	0.875	0.877	0.880	0.882	0.884	0.886
Ehime	0.737	0.742	0.748	0.753	0.757	0.763	0.766	0.773	0.779	0.784	0.792	0.799	0.805	0.811	0.817	0.821	0.828	0.832	0.837	0.842	0.845	0.848	0.853	0.854	0.858	0.860	0.862	0.865	0.867	0.868	0.870	0.871	0.873	0.877	0.879	0.882
Fukui	0.745	0.753	0.757	0.761	0.764	0.770	0.775	0.779	0.786	0.791	0.799	0.807	0.814	0.819	0.824	0.829	0.835	0.841	0.845	0.849	0.854	0.857	0.861	0.865	0.868	0.869	0.871	0.874	0.876	0.877	0.880	0.882	0.884	0.887	0.890	0.892
Fukuoka	0.746	0.751	0.756	0.761	0.765	0.770	0.775	0.782	0.789	0.794	0.802	0.808	0.815	0.822	0.827	0.832	0.837	0.843	0.847	0.851	0.855	0.858	0.862	0.865	0.868	0.870	0.872	0.874	0.876	0.877	0.879	0.880	0.883	0.884	0.886	0.888
Fukushima	0.731	0.738	0.742	0.746	0.751	0.757	0.760	0.767	0.772	0.780	0.786	0.792	0.799	0.805	0.811	0.816	0.822	0.828	0.832	0.835	0.840	0.843	0.847	0.850	0.854	0.856	0.859	0.863	0.865	0.868	0.870	0.875	0.880	0.879	0.882	0.884
Gifu	0.741	0.747	0.753	0.756	0.761	0.765	0.773	0.776	0.783	0.790	0.797	0.805	0.810	0.816	0.822	0.827	0.832	0.838	0.841	0.845	0.849	0.853	0.855	0.858	0.862	0.864	0.866	0.869	0.872	0.872	0.875	0.876	0.878	0.882	0.884	0.886
Gunma	0.745	0.751	0.756	0.760	0.765	0.770	0.777	0.781	0.787	0.793	0.801	0.807	0.814	0.820	0.825	0.831	0.836	0.841	0.847	0.850	0.853	0.857	0.860	0.863	0.867	0.869	0.872	0.875	0.877	0.879	0.882	0.884	0.887	0.890	0.892	0.895
Hiroshima	0.746	0.752	0.756	0.761	0.766	0.771	0.775	0.782	0.789	0.795	0.801	0.809	0.816	0.822	0.828	0.834	0.839	0.844	0.849	0.853	0.856	0.859	0.863	0.865	0.868	0.870	0.872	0.873	0.876	0.876	0.878	0.879	0.881	0.883	0.885	0.887
Hokkaidō	0.746	0.752	0.756	0.761	0.766	0.771	0.776	0.782	0.788	0.795	0.801	0.807	0.814	0.820	0.826	0.831	0.836	0.841	0.845	0.850	0.853	0.856	0.859	0.861	0.864	0.867	0.870	0.873	0.875	0.877	0.878	0.880	0.882	0.884	0.886	0.888
Hyōgo	0.741	0.747	0.752	0.756	0.761	0.767	0.772	0.778	0.784	0.791	0.798	0.805	0.811	0.818	0.823	0.828	0.834	0.838	0.842	0.846	0.850	0.854	0.857	0.860	0.863	0.865	0.867	0.870	0.872	0.873	0.875	0.876	0.879	0.881	0.883	0.886
Ibaraki	0.746	0.751	0.756	0.761	0.766	0.772	0.777	0.782	0.790	0.796	0.803	0.810	0.816	0.823	0.829	0.835	0.840	0.845	0.850	0.853	0.857	0.860	0.864	0.866	0.869	0.873	0.875	0.878	0.880	0.882	0.884	0.886	0.888	0.891	0.893	0.896
Ishikawa	0.742	0.750	0.753	0.759	0.764	0.770	0.774	0.781	0.786	0.793	0.800	0.808	0.814	0.821	0.825	0.832	0.836	0.842	0.845	0.850	0.853	0.856	0.860	0.862	0.866	0.868	0.871	0.873	0.875	0.877	0.881	0.882	0.883	0.885	0.887	0.890
Iwate	0.728	0.735	0.740	0.744	0.749	0.755	0.760	0.766	0.771	0.777	0.783	0.789	0.795	0.802	0.808	0.813	0.819	0.825	0.828	0.832	0.836	0.839	0.843	0.846	0.849	0.852	0.855	0.859	0.861	0.863	0.866	0.869	0.870	0.871	0.874	0.876
Kagawa	0.742	0.749	0.753	0.758	0.762	0.767	0.772	0.778	0.784	0.791	0.798	0.806	0.812	0.819	0.824	0.828	0.835	0.838	0.842	0.845	0.849	0.853	0.856	0.859	0.861	0.864	0.867	0.869	0.871	0.873	0.874	0.875	0.877	0.880	0.882	0.884
Kagoshima	0.724	0.729	0.734	0.740	0.745	0.751	0.760	0.758	0.767	0.773	0.781	0.788	0.794	0.800	0.806	0.812	0.817	0.823	0.828	0.833	0.835	0.839	0.842	0.845	0.848	0.850	0.853	0.855	0.857	0.859	0.861	0.861	0.864	0.866	0.868	0.870
Kanagawa	0.749	0.755	0.761	0.765	0.770	0.775	0.781	0.786	0.792	0.800	0.807	0.813	0.821	0.826	0.832	0.837	0.843	0.847	0.852	0.855	0.859	0.862	0.865	0.868	0.870	0.873	0.875	0.878	0.880	0.881	0.884	0.886	0.888	0.891	0.894	0.896
Kōchi	0.727	0.726	0.733	0.737	0.742	0.747	0.751	0.758	0.764	0.771	0.779	0.784	0.791	0.794	0.802	0.807	0.811	0.818	0.823	0.827	0.830	0.833	0.837	0.841	0.844	0.846	0.848	0.853	0.854	0.858	0.858	0.861	0.862	0.864	0.866	0.868
Kumamoto	0.728	0.732	0.738	0.743	0.746	0.752	0.757	0.763	0.769	0.775	0.783	0.789	0.795	0.801	0.807	0.811	0.817	0.823	0.827	0.831	0.834	0.838	0.841	0.844	0.846	0.848	0.851	0.853	0.855	0.857	0.859	0.860	0.862	0.863	0.865	0.867
Kyōto	0.752	0.759	0.763	0.767	0.771	0.777	0.781	0.788	0.794	0.801	0.808	0.817	0.822	0.829	0.835	0.840	0.845	0.850	0.854	0.858	0.862	0.866	0.869	0.872	0.875	0.877	0.879	0.883	0.884	0.887	0.888	0.890	0.893	0.895	0.898	0.900
Mie	0.747	0.754	0.759	0.762	0.767	0.773	0.777	0.783	0.789	0.796	0.803	0.811	0.817	0.824	0.829	0.834	0.840	0.845	0.849	0.853	0.856	0.860	0.863	0.866	0.869	0.872	0.874	0.877	0.879	0.880	0.883	0.884	0.887	0.889	0.892	0.894
Miyagi	0.738	0.744	0.748	0.754	0.759	0.765	0.770	0.776	0.783	0.789	0.796	0.804	0.810	0.818	0.823	0.828	0.834	0.839	0.843	0.847	0.851	0.854	0.858	0.861	0.865	0.867	0.870	0.872	0.875	0.878	0.880	0.883	0.883	0.885	0.887	0.889
Miyazaki	0.727	0.732	0.736	0.742	0.747	0.753	0.757	0.763	0.769	0.774	0.783	0.789	0.794	0.800	0.805	0.809	0.815	0.820	0.826	0.830	0.833	0.836	0.840	0.844	0.846	0.848	0.850	0.851	0.854	0.855	0.857	0.858	0.860	0.861	0.863	0.864
Nagano	0.738	0.747	0.752	0.756	0.760	0.765	0.768	0.775	0.781	0.786	0.793	0.801	0.806	0.814	0.818	0.824	0.830	0.835	0.840	0.844	0.848	0.851	0.855	0.858	0.861	0.863	0.866	0.869	0.872	0.875	0.876	0.878	0.880	0.883	0.885	0.888

Appendix Table 8. Socio-Demographic Index (SDI) values for all estimated GBD 2015 locations, 1980–2015

Location	1980	1981	1982	1983	1984	1985	1986	1987	1988	1989	1990	1991	1992	1993	1994	1995	1996	1997	1998	1999	2000	2001	2002	2003	2004	2005	2006	2007	2008	2009	2010	2011	2012	2013	2014	2015
Nagasaki	0.728	0.733	0.738	0.742	0.747	0.753	0.757	0.763	0.769	0.775	0.782	0.788	0.795	0.800	0.806	0.812	0.816	0.823	0.826	0.830	0.834	0.838	0.842	0.844	0.846	0.849	0.851	0.855	0.857	0.858	0.859	0.860	0.861	0.863	0.864	0.866
Nara	0.724	0.730	0.733	0.738	0.743	0.750	0.754	0.762	0.767	0.774	0.781	0.789	0.795	0.800	0.808	0.812	0.817	0.822	0.826	0.830	0.834	0.838	0.841	0.843	0.846	0.848	0.850	0.853	0.856	0.857	0.859	0.861	0.861	0.864	0.866	0.868
Niigata	0.739	0.745	0.751	0.755	0.759	0.764	0.768	0.774	0.780	0.785	0.793	0.800	0.807	0.812	0.818	0.824	0.829	0.835	0.839	0.844	0.847	0.852	0.857	0.860	0.862	0.865	0.867	0.871	0.874	0.876	0.877	0.879	0.881	0.884	0.886	0.889
Ôita	0.738	0.744	0.750	0.754	0.760	0.764	0.768	0.774	0.780	0.786	0.794	0.799	0.806	0.812	0.817	0.822	0.827	0.832	0.837	0.841	0.844	0.847	0.852	0.855	0.858	0.859	0.862	0.864	0.866	0.868	0.870	0.871	0.875	0.877	0.879	0.881
Okayama	0.741	0.747	0.751	0.755	0.760	0.765	0.770	0.776	0.783	0.789	0.796	0.804	0.810	0.817	0.823	0.827	0.832	0.837	0.842	0.846	0.849	0.853	0.857	0.860	0.863	0.866	0.868	0.871	0.874	0.877	0.877	0.879	0.881	0.884	0.886	0.888
Okinawa	0.709	0.717	0.723	0.729	0.734	0.738	0.741	0.749	0.756	0.763	0.773	0.778	0.785	0.791	0.798	0.804	0.809	0.815	0.818	0.822	0.826	0.827	0.833	0.836	0.838	0.839	0.843	0.845	0.847	0.848	0.849	0.850	0.851	0.853	0.854	0.856
Ōsaka	0.764	0.770	0.775	0.779	0.784	0.788	0.793	0.799	0.805	0.812	0.819	0.826	0.833	0.840	0.845	0.849	0.854	0.858	0.862	0.866	0.870	0.873	0.876	0.879	0.882	0.884	0.886	0.889	0.891	0.893	0.895	0.896	0.898	0.901	0.903	0.905
Saga	0.728	0.734	0.738	0.744	0.748	0.752	0.757	0.764	0.770	0.778	0.783	0.790	0.797	0.803	0.808	0.814	0.818	0.823	0.828	0.832	0.834	0.838	0.843	0.847	0.850	0.852	0.855	0.857	0.859	0.862	0.863	0.864	0.866	0.869	0.871	0.873
Saitama	0.736	0.741	0.747	0.752	0.757	0.762	0.769	0.774	0.779	0.786	0.793	0.799	0.807	0.812	0.818	0.823	0.829	0.834	0.839	0.843	0.846	0.849	0.852	0.854	0.857	0.859	0.862	0.865	0.867	0.868	0.870	0.872	0.874	0.876	0.878	0.880
Shiga	0.748	0.755	0.760	0.763	0.768	0.774	0.779	0.785	0.791	0.799	0.805	0.815	0.821	0.828	0.833	0.839	0.844	0.850	0.854	0.858	0.863	0.866	0.869	0.873	0.875	0.879	0.880	0.884	0.885	0.887	0.889	0.890	0.892	0.895	0.897	0.900
Shimane	0.724	0.729	0.736	0.739	0.745	0.749	0.753	0.758	0.764	0.770	0.777	0.784	0.792	0.797	0.804	0.808	0.814	0.821	0.827	0.830	0.833	0.835	0.841	0.844	0.847	0.850	0.851	0.854	0.859	0.860	0.860	0.862	0.863	0.865	0.867	0.869
Shizuoka	0.754	0.758	0.764	0.768	0.772	0.777	0.784	0.787	0.794	0.802	0.809	0.816	0.824	0.829	0.834	0.840	0.845	0.850	0.854	0.858	0.861	0.864	0.867	0.870	0.873	0.875	0.877	0.879	0.882	0.883	0.885	0.887	0.889	0.891	0.893	0.895
Tochigi	0.748	0.754	0.759	0.763	0.768	0.772	0.778	0.782	0.790	0.797	0.804	0.811	0.819	0.825	0.831	0.837	0.842	0.848	0.852	0.856	0.859	0.862	0.866	0.868	0.871	0.873	0.876	0.879	0.882	0.883	0.886	0.889	0.890	0.893	0.896	0.898
Tokushima	0.742	0.747	0.752	0.756	0.761	0.766	0.771	0.778	0.782	0.789	0.795	0.803	0.811	0.816	0.823	0.825	0.832	0.838	0.842	0.846	0.849	0.853	0.857	0.861	0.863	0.868	0.868	0.873	0.875	0.876	0.877	0.878	0.880	0.883	0.885	0.887
Tōkyō	0.804	0.810	0.815	0.820	0.824	0.829	0.835	0.840	0.847	0.854	0.861	0.868	0.874	0.880	0.886	0.891	0.896	0.901	0.906	0.909	0.913	0.917	0.920	0.923	0.925	0.928	0.931	0.933	0.936	0.938	0.940	0.943	0.945	0.947	0.950	0.952
Tottori	0.724	0.730	0.735	0.737	0.743	0.750	0.753	0.759	0.764	0.771	0.777	0.785	0.792	0.798	0.803	0.808	0.816	0.821	0.825	0.831	0.832	0.836	0.841	0.842	0.846	0.849	0.851	0.855	0.859	0.860	0.862	0.862	0.864	0.866	0.868	0.870
Toyama	0.749	0.754	0.759	0.763	0.767	0.772	0.777	0.784	0.789	0.796	0.803	0.811	0.817	0.824	0.829	0.833	0.838	0.843	0.848	0.851	0.855	0.858	0.862	0.865	0.868	0.870	0.873	0.877	0.879	0.881	0.884	0.887	0.888	0.891	0.894	0.896
Wakayama	0.738	0.744	0.748	0.752	0.758	0.763	0.767	0.774	0.781	0.787	0.794	0.801	0.808	0.814	0.820	0.824	0.829	0.834	0.838	0.842	0.846	0.849	0.853	0.856	0.860	0.861	0.864	0.868	0.868	0.871	0.871	0.873	0.874	0.877	0.879	0.882
Yamagata	0.725	0.732	0.737	0.742	0.746	0.752	0.754	0.760	0.764	0.771	0.779	0.785	0.792	0.797	0.803	0.808	0.813	0.819	0.825	0.828	0.832	0.834	0.839	0.843	0.846	0.849	0.852	0.856	0.858	0.862	0.864	0.865	0.868	0.870	0.872	0.875
Yamaguchi	0.747	0.752	0.757	0.761	0.766	0.770	0.775	0.781	0.788	0.794	0.802	0.807	0.815	0.819	0.825	0.830	0.836	0.840	0.845	0.848	0.852	0.855	0.859	0.862	0.864	0.866	0.869	0.871	0.874	0.875	0.876	0.878	0.881	0.883	0.884	0.886
Yamanashi	0.742	0.748	0.753	0.757	0.762	0.765	0.774	0.774	0.783	0.789	0.797	0.802	0.809	0.815	0.820	0.826	0.831	0.837	0.843	0.847	0.850	0.854	0.858	0.861	0.864	0.866	0.870	0.873	0.875	0.878	0.879	0.881	0.883	0.886	0.888	0.890
South Korea	0.601	0.612	0.623	0.634	0.646	0.658	0.670	0.681	0.693	0.703	0.713	0.722	0.730	0.738	0.747	0.756	0.766	0.775	0.783	0.791	0.799	0.806	0.814	0.820	0.826	0.832	0.837	0.842	0.846	0.850	0.854	0.858	0.862	0.865	0.868	0.871
Singapore	0.640	0.649	0.658	0.669	0.676	0.683	0.694	0.696	0.692	0.706	0.713	0.724	0.732	0.739	0.748	0.757	0.766	0.775	0.787	0.793	0.795	0.807	0.814	0.823	0.830	0.836	0.842	0.846	0.852	0.859	0.866	0.866	0.871	0.874	0.878	0.881
Australasia	0.754	0.760	0.765	0.770	0.775	0.780	0.785	0.790	0.795	0.800	0.805	0.809	0.814	0.818	0.823	0.828	0.833	0.839	0.844	0.849	0.854	0.859	0.863	0.867	0.870	0.873	0.875	0.879	0.882	0.886	0.889	0.894	0.898	0.902	0.906	0.910
Australia	0.756	0.762	0.767	0.772	0.777	0.782	0.787	0.792	0.798	0.803	0.808	0.812	0.817	0.821	0.826	0.831	0.836	0.842	0.847	0.853	0.858	0.863	0.867	0.870	0.874	0.876	0.879	0.883	0.886	0.890	0.894	0.898	0.902	0.907	0.911	0.915
New Zealand	0.746	0.751	0.757	0.762	0.767	0.771	0.775	0.779	0.783	0.786	0.790	0.795	0.798	0.802	0.807	0.812	0.817	0.823	0.827	0.831	0.835	0.839	0.843	0.847	0.851	0.854	0.856	0.859	0.861	0.864	0.867	0.870	0.874	0.877	0.881	0.884
Western Europe	0.700	0.707	0.712	0.718	0.723	0.729	0.735	0.740	0.746	0.753	0.759	0.765	0.771	0.776	0.781	0.786	0.791	0.796	0.801	0.806	0.811	0.816	0.820	0.825	0.829	0.832	0.836	0.840	0.844	0.847	0.851	0.854	0.858	0.861	0.864	0.867
Andorra	0.774	0.780	0.786	0.791	0.797	0.802	0.806	0.810	0.814	0.818	0.824	0.827	0.830	0.836	0.841	0.844	0.847	0.850	0.851	0.851	0.862	0.864	0.870	0.878	0.877	0.880	0.883	0.886	0.889	0.893	0.897	0.902	0.906	0.911	0.915	0.919
Austria	0.741	0.746	0.751	0.756	0.761	0.766	0.770	0.774	0.779	0.783	0.788	0.793	0.798	0.802	0.806	0.810	0.815	0.819	0.824	0.829	0.834	0.838	0.843	0.847	0.851	0.855	0.860	0.864	0.868	0.871	0.874	0.877	0.880	0.883	0.886	0.888
Belgium	0.729	0.735	0.741	0.746	0.751	0.756	0.760	0.765	0.770	0.775	0.780	0.785	0.790	0.794	0.799	0.803	0.808	0.813	0.818	0.822	0.827	0.831	0.835	0.839	0.842	0.846	0.849	0.853	0.857	0.860	0.864	0.868	0.872	0.875	0.878	0.882
Cyprus	0.642	0.651	0.659	0.668	0.676	0.685	0.692	0.700	0.708	0.716	0.725	0.732	0.739	0.746	0.753	0.760	0.766	0.772	0.778	0.784	0.791	0.798	0.805	0.812	0.819	0.826	0.833	0.842	0.850	0.857	0.864	0.870	0.875	0.877	0.879	0.880
Denmark	0.775	0.781	0.787	0.792	0.797	0.802	0.807	0.811	0.815	0.818	0.821	0.825	0.828	0.832	0.835	0.840	0.844	0.849	0.854	0.859	0.864	0.868	0.872	0.875	0.878	0.881	0.884	0.887	0.891	0.894	0.897	0.901	0.903	0.906	0.908	0.910
Finland	0.728	0.734	0.740	0.746	0.752	0.758	0.763	0.769	0.775	0.780	0.785	0.789	0.792	0.795	0.798	0.803	0.807	0.813	0.818	0.824	0.830	0.836	0.841	0.846	0.850	0.854	0.858	0.863	0.868	0.872	0.876	0.880	0.884	0.887	0.890	0.893
France	0.688	0.694	0.698	0.703	0.707	0.711	0.716	0.721	0.726	0.731	0.736	0.741	0.745	0.749	0.753	0.756	0.760	0.764	0.768	0.772	0.776	0.780	0.784	0.788	0.792	0.796	0.800	0.804	0.808	0.811	0.815	0.819	0.823	0.827	0.831	0.834
Germany	0.726	0.733	0.740	0.746	0.752	0.757	0.764	0.770	0.777	0.784	0.792	0.799	0.807	0.813	0.819	0.824	0.829	0.833	0.838	0.842	0.847	0.852	0.857	0.861	0.865	0.869	0.873	0.877	0.881	0.884	0.887	0.891	0.894	0.897	0.900	0.903
Greece	0.639	0.646	0.654	0.661	0.668	0.675	0.682	0.689	0.696	0.703	0.709	0.715	0.722	0.727	0.732	0.738	0.743	0.749	0.755	0.761	0.767	0.772	0.777	0.782	0.787	0.792	0.797	0.802	0.808	0.813	0.817	0.819	0.821	0.822	0.824	0.825
Iceland	0.722	0.731	0.739	0.745	0.752	0.758	0.764	0.771	0.777	0.783	0.787	0.791	0.795	0.799	0.803	0.807	0.811	0.816	0.823	0.829	0.836	0.842	0.846	0.849	0.852	0.857	0.862	0.867	0.873	0.876	0.879	0.881	0.884	0.887	0.890	0.893
Ireland	0.642	0.652	0.662	0.671	0.681	0.691	0.700	0.709	0.718	0.727	0.735	0.743	0.751	0.758	0.764	0.772	0.779	0.786	0.794	0.801	0.809	0.817	0.824	0.830	0.836	0.841	0.848	0.854	0.859	0.861	0.863	0.866	0.870	0.874	0.879	0.885
Israel	0.709	0.714	0.719	0.724	0.728	0.731	0.735	0.739	0.742	0.746	0.751	0.756	0.761	0.766	0.771	0.776	0.781	0.785	0.790	0.794	0.799	0.803	0.806	0.809	0.812	0.815	0.818	0.821	0.824	0.826	0.828	0.831	0.833	0.836	0.839	0.842
Italy	0.713	0.721	0.728	0.734	0.739	0.745	0.750	0.755	0.760	0.765	0.770	0.775	0.779	0.783	0.787	0.790	0.794	0.798	0.802	0.806	0.809	0.813	0.816	0.819	0.823	0.826	0.829	0.833	0.836	0.839	0.842	0.846	0.849	0.851	0.854	0.856
Luxembourg	0.731	0.737	0.743	0.748	0.754	0.760	0.767	0.773	0.780	0.787	0.793	0.800	0.806	0.812	0.818	0.824	0.829	0.835	0.841	0.848	0.855	0.860	0.864	0.868	0.871	0.874	0.879	0.884	0.888	0.892	0.896	0.899	0.902	0.905	0.908	0.911
Malta	0.611	0.620	0.627	0.634	0.639	0.643	0.648	0.652	0.656	0.661	0.665	0.670	0.674	0.678	0.682	0.686	0.693	0.701	0.710	0.719	0.728	0.736	0.744	0.750	0.756	0.761	0.767	0.772	0.777	0.781	0.785	0.789	0.793	0.797	0.802	0.806
Netherlands	0.743	0.749	0.753	0.757	0.761	0.765	0.770	0.774	0.778	0.783	0.788	0.793	0.798	0.803	0.808	0.813	0.817	0.822	0.827	0.832	0.837	0.841	0.846	0.850	0.853	0.857	0.861	0.866	0.870	0.874	0.878	0.882	0.885	0.888	0.891	0.894

Appendix Table 8. Socio-Demographic Index (SDI) values for all estimated GBD 2015 locations, 1980–2015

Location	1980	1981	1982	1983	1984	1985	1986	1987	1988	1989	1990	1991	1992	1993	1994	1995	1996	1997	1998	1999	2000	2001	2002	2003	2004	2005	2006	2007	2008	2009	2010	2011	2012	2013	2014	2015
Norway	0.771	0.778	0.784	0.790	0.796	0.802	0.807	0.812	0.816	0.821	0.825	0.829	0.834	0.839	0.844	0.850	0.856	0.862	0.869	0.875	0.881	0.887	0.891	0.895	0.899	0.902	0.906	0.910	0.914	0.918	0.922	0.925	0.929	0.932	0.934	0.937
Portugal	0.513	0.521	0.530	0.538	0.545	0.552	0.560	0.567	0.575	0.583	0.591	0.600	0.608	0.616	0.623	0.630	0.637	0.645	0.652	0.659	0.666	0.672	0.679	0.686	0.692	0.698	0.705	0.711	0.717	0.722	0.728	0.733	0.738	0.743	0.747	0.752
Spain	0.582	0.590	0.599	0.607	0.615	0.622	0.630	0.638	0.646	0.654	0.662	0.670	0.679	0.686	0.693	0.701	0.707	0.714	0.721	0.727	0.734	0.740	0.747	0.753	0.759	0.765	0.772	0.778	0.785	0.790	0.795	0.800	0.805	0.810	0.814	0.819
Sweden	0.742	0.747	0.752	0.756	0.760	0.763	0.767	0.771	0.774	0.779	0.783	0.788	0.793	0.799	0.806	0.813	0.820	0.827	0.833	0.839	0.844	0.849	0.852	0.855	0.858	0.861	0.864	0.868	0.871	0.873	0.877	0.880	0.883	0.886	0.889	0.892
Stockholm	0.779	0.786	0.791	0.796	0.799	0.803	0.806	0.809	0.813	0.817	0.822	0.828	0.831	0.836	0.841	0.848	0.854	0.861	0.866	0.872	0.877	0.880	0.882	0.884	0.885	0.887	0.891	0.894	0.897	0.900	0.903	0.906	0.910	0.911	0.915	0.918
Sweden except Stockholm	0.734	0.739	0.743	0.746	0.750	0.754	0.758	0.761	0.765	0.769	0.774	0.779	0.784	0.790	0.797	0.804	0.811	0.818	0.825	0.831	0.836	0.841	0.845	0.848	0.851	0.854	0.857	0.861	0.864	0.866	0.869	0.872	0.875	0.879	0.882	0.885
Switzerland	0.778	0.783	0.789	0.793	0.798	0.804	0.809	0.814	0.820	0.825	0.831	0.838	0.843	0.848	0.852	0.856	0.860	0.864	0.869	0.873	0.877	0.882	0.887	0.890	0.893	0.897	0.900	0.903	0.907	0.911	0.914	0.917	0.920	0.923	0.926	0.928
United Kingdom	0.736	0.741	0.746	0.751	0.756	0.761	0.766	0.772	0.778	0.785	0.791	0.796	0.802	0.807	0.812	0.818	0.824	0.829	0.835	0.842	0.848	0.854	0.859	0.863	0.867	0.870	0.873	0.876	0.878	0.880	0.881	0.883	0.886	0.888	0.890	0.893
England	0.741	0.746	0.750	0.754	0.759	0.764	0.770	0.775	0.781	0.787	0.794	0.799	0.805	0.810	0.815	0.821	0.826	0.832	0.838	0.844	0.850	0.856	0.861	0.865	0.869	0.872	0.875	0.878	0.880	0.881	0.883	0.885	0.887	0.889	0.891	0.894
East Midlands	0.737	0.742	0.746	0.751	0.756	0.761	0.767	0.772	0.778	0.784	0.790	0.796	0.801	0.806	0.811	0.817	0.823	0.829	0.834	0.840	0.846	0.852	0.857	0.861	0.865	0.868	0.871	0.874	0.876	0.877	0.878	0.879	0.882	0.884	0.886	0.889
East of England	0.685	0.690	0.696	0.700	0.705	0.711	0.716	0.721	0.727	0.733	0.740	0.746	0.751	0.756	0.761	0.768	0.775	0.781	0.787	0.793	0.799	0.805	0.810	0.814	0.818	0.822	0.825	0.827	0.829	0.830	0.832	0.834	0.835	0.836	0.837	0.839
Greater London	0.816	0.821	0.824	0.828	0.832	0.837	0.843	0.847	0.852	0.859	0.866	0.871	0.874	0.879	0.883	0.888	0.893	0.898	0.903	0.909	0.914	0.919	0.923	0.926	0.929	0.932	0.934	0.937	0.941	0.942	0.944	0.947	0.949	0.952	0.956	0.959
North East England	0.654	0.659	0.663	0.669	0.674	0.679	0.685	0.693	0.701	0.708	0.715	0.720	0.726	0.732	0.740	0.745	0.752	0.759	0.765	0.772	0.779	0.786	0.790	0.796	0.800	0.803	0.807	0.811	0.813	0.815	0.816	0.818	0.821	0.824	0.827	0.830
North West England	0.730	0.735	0.738	0.742	0.748	0.752	0.758	0.765	0.771	0.777	0.783	0.789	0.795	0.801	0.807	0.813	0.817	0.824	0.830	0.836	0.842	0.848	0.853	0.858	0.861	0.864	0.868	0.871	0.873	0.874	0.876	0.878	0.880	0.882	0.884	0.887
South East England	0.763	0.768	0.774	0.778	0.783	0.788	0.793	0.798	0.803	0.809	0.815	0.820	0.825	0.829	0.834	0.840	0.846	0.851	0.856	0.862	0.868	0.874	0.879	0.883	0.887	0.891	0.892	0.895	0.897	0.898	0.899	0.901	0.902	0.904	0.907	0.909
South West England	0.743	0.749	0.753	0.758	0.763	0.768	0.773	0.779	0.785	0.790	0.797	0.803	0.808	0.813	0.818	0.824	0.829	0.835	0.841	0.847	0.854	0.860	0.864	0.868	0.873	0.877	0.880	0.882	0.884	0.885	0.886	0.887	0.889	0.891	0.894	0.896
West Midlands	0.733	0.737	0.740	0.745	0.749	0.755	0.760	0.766	0.773	0.778	0.784	0.789	0.795	0.801	0.806	0.811	0.816	0.821	0.828	0.833	0.839	0.845	0.849	0.854	0.857	0.861	0.864	0.867	0.869	0.871	0.873	0.874	0.875	0.877	0.879	0.881
Yorkshire and the Humber	0.730	0.735	0.739	0.743	0.748	0.754	0.759	0.765	0.772	0.778	0.784	0.790	0.795	0.800	0.806	0.810	0.816	0.823	0.828	0.834	0.841	0.846	0.852	0.856	0.860	0.863	0.866	0.870	0.871	0.872	0.875	0.877	0.878	0.880	0.881	0.883
Northern Ireland	0.619	0.628	0.635	0.641	0.646	0.655	0.660	0.671	0.680	0.692	0.700	0.707	0.715	0.722	0.730	0.736	0.741	0.749	0.756	0.764	0.773	0.777	0.785	0.791	0.796	0.800	0.803	0.805	0.807	0.810	0.811	0.813	0.814	0.817	0.819	0.822
Scotland	0.744	0.749	0.757	0.763	0.768	0.773	0.779	0.786	0.792	0.800	0.805	0.809	0.814	0.820	0.826	0.831	0.837	0.842	0.848	0.855	0.861	0.866	0.871	0.876	0.879	0.882	0.886	0.888	0.889	0.891	0.895	0.898	0.902	0.905	0.908	0.912
Wales	0.713	0.718	0.721	0.727	0.732	0.738	0.743	0.750	0.757	0.764	0.771	0.777	0.783	0.788	0.795	0.800	0.805	0.811	0.819	0.826	0.833	0.839	0.846	0.850	0.855	0.858	0.862	0.866	0.868	0.870	0.871	0.873	0.876	0.880	0.883	0.887
Southern Latin America	0.579	0.585	0.589	0.594	0.599	0.602	0.607	0.612	0.617	0.622	0.626	0.632	0.639	0.646	0.654	0.662	0.670	0.679	0.686	0.692	0.698	0.702	0.706	0.710	0.714	0.719	0.725	0.731	0.738	0.743	0.750	0.757	0.763	0.769	0.775	0.780
Argentina	0.577	0.582	0.587	0.592	0.597	0.601	0.606	0.611	0.616	0.620	0.623	0.629	0.635	0.642	0.650	0.658	0.666	0.674	0.682	0.688	0.693	0.697	0.700	0.703	0.707	0.712	0.717	0.723	0.730	0.736	0.742	0.749	0.756	0.762	0.767	0.772
Chile	0.587	0.594	0.599	0.602	0.606	0.610	0.614	0.619	0.624	0.631	0.637	0.644	0.653	0.661	0.670	0.679	0.688	0.696	0.704	0.710	0.716	0.722	0.728	0.733	0.739	0.745	0.752	0.759	0.765	0.770	0.776	0.782	0.788	0.794	0.800	0.805
Uruguay	0.566	0.573	0.579	0.583	0.586	0.589	0.592	0.597	0.601	0.605	0.609	0.613	0.618	0.624	0.630	0.635	0.641	0.648	0.654	0.659	0.664	0.667	0.669	0.672	0.675	0.679	0.683	0.689	0.695	0.701	0.709	0.716	0.724	0.731	0.738	0.745
High-income North America	0.838	0.841	0.843	0.845	0.848	0.851	0.853	0.856	0.858	0.860	0.862	0.864	0.866	0.868	0.871	0.873	0.876	0.879	0.882	0.885	0.888	0.891	0.893	0.895	0.898	0.900	0.904	0.907	0.910	0.913	0.916	0.919	0.923	0.926	0.929	0.932
Canada	0.814	0.820	0.825	0.829	0.833	0.838	0.842	0.846	0.850	0.855	0.858	0.861	0.863	0.866	0.869	0.873	0.877	0.881	0.886	0.890	0.895	0.899	0.903	0.906	0.908	0.911	0.913	0.916	0.918	0.920	0.923	0.925	0.928	0.931	0.934	0.938
Greenland	0.579	0.588	0.598	0.617	0.616	0.608	0.628	0.628	0.609	0.616	0.606	0.615	0.600	0.609	0.615	0.621	0.628	0.609	0.641	0.648	0.662	0.646	0.642	0.669	0.665	0.673	0.691	0.691	0.700	0.689	0.703	0.725	0.744	0.741	0.751	0.758
United States	0.840	0.843	0.845	0.847	0.850	0.852	0.855	0.857	0.859	0.861	0.863	0.865	0.867	0.869	0.871	0.873	0.876	0.879	0.882	0.885	0.887	0.890	0.892	0.894	0.897	0.899	0.902	0.906	0.909	0.912	0.916	0.919	0.922	0.925	0.928	0.931
Alabama	0.807	0.812	0.815	0.817	0.820	0.824	0.827	0.830	0.832	0.835	0.838	0.841	0.845	0.848	0.851	0.854	0.857	0.859	0.862	0.865	0.867	0.872	0.876	0.879	0.882	0.885	0.888	0.890	0.893	0.897	0.901	0.903	0.907	0.910	0.912	0.915
Alaska	0.880	0.877	0.872	0.871	0.870	0.870	0.874	0.874	0.878	0.877	0.876	0.875	0.872	0.876	0.878	0.880	0.882	0.883	0.885	0.887	0.890	0.890	0.892	0.895	0.896	0.899	0.902	0.906	0.906	0.910	0.911	0.916	0.923	0.924	0.928	0.932
Arizona	0.837	0.839	0.841	0.841	0.844	0.844	0.847	0.848	0.850	0.852	0.853	0.855	0.856	0.857	0.859	0.860	0.860	0.865	0.867	0.868	0.870	0.872	0.873	0.874	0.876	0.879	0.882	0.887	0.893	0.900	0.904	0.908	0.910	0.913	0.916	0.918
Arkansas	0.829	0.833	0.836	0.836	0.839	0.841	0.844	0.845	0.846	0.847	0.848	0.851	0.853	0.856	0.856	0.857	0.857	0.859	0.862	0.865	0.867	0.869	0.870	0.873	0.874	0.876	0.878	0.882	0.885	0.888	0.892	0.894	0.898	0.902	0.906	0.910
California	0.854	0.855	0.857	0.858	0.859	0.861	0.862	0.862	0.862	0.861	0.859	0.859	0.860	0.862	0.864	0.865	0.869	0.874	0.878	0.882	0.886	0.889	0.891	0.893	0.895	0.898	0.901	0.905	0.909	0.913	0.916	0.919	0.923	0.927	0.930	0.932
Colorado	0.873	0.873	0.874	0.875	0.877	0.879	0.879	0.882	0.884	0.887	0.888	0.888	0.888	0.890	0.893	0.895	0.896	0.898	0.899	0.900	0.902	0.903	0.904	0.906	0.909	0.911	0.915	0.920	0.923	0.926	0.929	0.933	0.937	0.941	0.944	0.948
Connecticut	0.872	0.874	0.877	0.878	0.881	0.884	0.886	0.888	0.892	0.895	0.899	0.902	0.903	0.905	0.907	0.909	0.911	0.915	0.917	0.920	0.925	0.926	0.929	0.930	0.933	0.936	0.939	0.941	0.945	0.948	0.951	0.954	0.958	0.961	0.963	0.966
Delaware	0.863	0.867	0.868	0.869	0.871	0.873	0.875	0.877	0.878	0.881	0.882	0.883	0.887	0.888	0.890	0.892	0.895	0.896	0.896	0.898	0.899	0.903	0.903	0.905	0.909	0.911	0.914	0.917	0.919	0.923	0.924	0.927	0.932	0.936	0.938	0.940
District of Columbia	0.867	0.871	0.872	0.873	0.874	0.876	0.878	0.880	0.881	0.877	0.880	0.880	0.888	0.891	0.899	0.906	0.914	0.921	0.928	0.934	0.938	0.941	0.944	0.946	0.945	0.948	0.949	0.951	0.952	0.956	0.957	0.960	0.964	0.969	0.973	0.978
Florida	0.841	0.844	0.846	0.847	0.849	0.851	0.854	0.856	0.857	0.860	0.863	0.867	0.870	0.871	0.873	0.876	0.879	0.881	0.885	0.888	0.890	0.892	0.895	0.897	0.899	0.902	0.905	0.909	0.913	0.917	0.920	0.923	0.926	0.928	0.929	0.930
Georgia	0.817	0.822	0.825	0.827	0.830	0.834	0.837	0.839	0.843	0.845	0.849	0.853	0.856	0.859	0.862	0.865	0.868	0.870	0.873	0.875	0.878	0.880	0.883	0.885	0.887	0.889	0.891	0.895	0.899	0.902	0.906	0.909	0.913	0.915	0.918	0.921
Hawaii	0.852	0.853	0.853	0.851	0.854	0.859	0.860	0.861	0.863	0.867	0.867	0.871	0.873	0.875	0.876	0.881	0.882	0.888	0.889	0.892	0.892	0.895	0.894	0.895	0.898	0.903	0.905	0.908	0.910	0.914	0.915	0.918	0.921	0.924	0.927	0.930
Idaho	0.815	0.820	0.820	0.823	0.828	0.832	0.836	0.839	0.842	0.845	0.847	0.848	0.849	0.852	0.855	0.857	0.858	0.862	0.864	0.866	0.870	0.870	0.872	0.873	0.874	0.877	0.881	0.883	0.885	0.891	0.893	0.898	0.898	0.903	0.905	0.908
Illinois	0.847	0.850	0.853	0.854	0.856	0.859	0.862	0.863	0.865	0.867	0.869	0.870	0.873	0.874	0.876	0.878	0.882	0.885	0.889	0.892	0.895	0.897	0.900	0.902	0.904	0.908	0.912	0.916	0.919	0.922	0.925	0.929	0.932	0.935	0.938	0.941

Appendix Table 8. Socio-Demographic Index (SDI) values for all estimated GBD 2015 locations, 1980–2015

Location	1980	1981	1982	1983	1984	1985	1986	1987	1988	1989	1990	1991	1992	1993	1994	1995	1996	1997	1998	1999	2000	2001	2002	2003	2004	2005	2006	2007	2008	2009	2010	2011	2012	2013	2014	2015
Indiana	0.827	0.832	0.834	0.836	0.839	0.841	0.844	0.848	0.849	0.851	0.854	0.856	0.859	0.860	0.864	0.865	0.868	0.870	0.872	0.875	0.878	0.880	0.883	0.885	0.887	0.889	0.893	0.895	0.897	0.899	0.902	0.904	0.907	0.909	0.913	0.916
Iowa	0.841	0.846	0.848	0.849	0.852	0.856	0.859	0.862	0.863	0.864	0.866	0.867	0.869	0.870	0.872	0.874	0.875	0.879	0.880	0.882	0.885	0.887	0.888	0.890	0.892	0.894	0.896	0.900	0.904	0.907	0.910	0.914	0.917	0.920	0.924	0.928
Kansas	0.863	0.865	0.868	0.869	0.871	0.875	0.876	0.878	0.880	0.882	0.883	0.884	0.883	0.883	0.883	0.884	0.886	0.886	0.886	0.887	0.889	0.891	0.891	0.894	0.896	0.898	0.900	0.903	0.906	0.908	0.911	0.916	0.918	0.924	0.928	0.931
Kentucky	0.792	0.797	0.800	0.803	0.807	0.811	0.815	0.818	0.822	0.824	0.828	0.830	0.834	0.838	0.841	0.844	0.848	0.850	0.854	0.858	0.861	0.865	0.869	0.871	0.874	0.876	0.879	0.882	0.885	0.887	0.890	0.893	0.895	0.897	0.900	0.903
Louisiana	0.820	0.824	0.826	0.829	0.831	0.834	0.836	0.839	0.839	0.841	0.844	0.843	0.846	0.848	0.851	0.855	0.858	0.859	0.861	0.863	0.866	0.870	0.872	0.875	0.878	0.887	0.884	0.887	0.893	0.896	0.901	0.905	0.908	0.911	0.914	0.918
Maine	0.826	0.828	0.830	0.831	0.835	0.839	0.842	0.846	0.851	0.855	0.860	0.863	0.866	0.870	0.873	0.876	0.878	0.881	0.884	0.887	0.892	0.893	0.897	0.900	0.903	0.905	0.909	0.913	0.917	0.918	0.921	0.925	0.927	0.929	0.932	0.935
Maryland	0.869	0.871	0.871	0.872	0.873	0.876	0.878	0.879	0.882	0.885	0.888	0.889	0.891	0.894	0.896	0.898	0.900	0.903	0.904	0.906	0.909	0.912	0.914	0.917	0.920	0.923	0.926	0.930	0.933	0.936	0.939	0.942	0.946	0.950	0.951	0.953
Massachusetts	0.864	0.866	0.869	0.871	0.874	0.878	0.882	0.885	0.888	0.890	0.894	0.897	0.899	0.901	0.903	0.907	0.910	0.912	0.914	0.918	0.922	0.924	0.926	0.929	0.932	0.936	0.939	0.943	0.946	0.949	0.952	0.954	0.958	0.962	0.965	0.967
Michigan	0.854	0.857	0.858	0.860	0.860	0.863	0.865	0.866	0.869	0.868	0.869	0.871	0.874	0.876	0.877	0.880	0.883	0.884	0.887	0.889	0.892	0.894	0.897	0.899	0.901	0.904	0.908	0.911	0.913	0.914	0.915	0.917	0.920	0.922	0.926	0.930
Minnesota	0.837	0.841	0.844	0.849	0.852	0.857	0.862	0.866	0.869	0.873	0.877	0.879	0.882	0.884	0.886	0.889	0.891	0.893	0.896	0.899	0.903	0.905	0.906	0.907	0.910	0.912	0.915	0.919	0.923	0.925	0.928	0.930	0.933	0.936	0.939	0.942
Mississippi	0.803	0.808	0.810	0.814	0.816	0.820	0.823	0.825	0.826	0.828	0.831	0.832	0.835	0.837	0.840	0.843	0.847	0.849	0.851	0.854	0.856	0.860	0.863	0.865	0.867	0.871	0.869	0.872	0.877	0.882	0.888	0.890	0.895	0.898	0.902	0.905
Missouri	0.827	0.831	0.833	0.836	0.840	0.842	0.846	0.849	0.852	0.854	0.857	0.858	0.861	0.864	0.867	0.869	0.871	0.874	0.876	0.879	0.883	0.885	0.887	0.889	0.891	0.894	0.897	0.900	0.903	0.906	0.908	0.911	0.914	0.917	0.921	0.924
Montana	0.831	0.833	0.833	0.835	0.836	0.840	0.843	0.845	0.849	0.851	0.854	0.854	0.856	0.858	0.862	0.864	0.868	0.870	0.874	0.876	0.879	0.880	0.882	0.884	0.888	0.891	0.892	0.898	0.900	0.904	0.907	0.910	0.913	0.915	0.921	0.927
Nebraska	0.838	0.841	0.844	0.846	0.848	0.853	0.856	0.859	0.862	0.864	0.867	0.868	0.871	0.872	0.873	0.874	0.877	0.879	0.882	0.883	0.885	0.886	0.885	0.888	0.889	0.893	0.896	0.900	0.903	0.904	0.909	0.913	0.916	0.919	0.922	0.925
Nevada	0.882	0.882	0.883	0.884	0.884	0.885	0.884	0.884	0.883	0.882	0.880	0.882	0.883	0.884	0.883	0.883	0.884	0.887	0.888	0.891	0.893	0.894	0.893	0.895	0.896	0.897	0.899	0.903	0.908	0.912	0.915	0.917	0.920	0.921	0.921	0.920
New Hampshire	0.850	0.856	0.857	0.861	0.864	0.866	0.870	0.871	0.876	0.880	0.885	0.889	0.890	0.892	0.894	0.896	0.899	0.902	0.905	0.909	0.912	0.913	0.917	0.920	0.922	0.925	0.930	0.934	0.938	0.940	0.943	0.945	0.950	0.953	0.956	0.960
New Jersey	0.883	0.886	0.887	0.889	0.891	0.892	0.893	0.894	0.895	0.897	0.901	0.901	0.902	0.903	0.903	0.904	0.906	0.908	0.910	0.912	0.915	0.915	0.917	0.918	0.921	0.924	0.927	0.929	0.933	0.936	0.938	0.941	0.945	0.949	0.952	0.956
New Mexico	0.818	0.820	0.820	0.822	0.825	0.828	0.831	0.833	0.836	0.838	0.841	0.840	0.841	0.842	0.846	0.851	0.853	0.856	0.859	0.862	0.866	0.867	0.869	0.873	0.874	0.877	0.880	0.883	0.886	0.891	0.895	0.899	0.903	0.907	0.909	0.911
New York	0.848	0.850	0.852	0.853	0.857	0.860	0.862	0.864	0.867	0.870	0.874	0.876	0.879	0.881	0.883	0.886	0.890	0.895	0.898	0.902	0.905	0.908	0.910	0.912	0.915	0.919	0.922	0.926	0.929	0.931	0.933	0.937	0.941	0.945	0.948	0.951
North Carolina	0.812	0.816	0.817	0.820	0.823	0.827	0.830	0.833	0.836	0.838	0.842	0.847	0.849	0.854	0.857	0.861	0.864	0.867	0.869	0.872	0.873	0.877	0.880	0.883	0.886	0.887	0.891	0.894	0.897	0.901	0.904	0.908	0.911	0.913	0.915	0.918
North Dakota	0.819	0.820	0.821	0.824	0.829	0.832	0.838	0.842	0.843	0.848	0.853	0.855	0.856	0.858	0.861	0.863	0.868	0.870	0.877	0.881	0.885	0.886	0.887	0.889	0.891	0.893	0.897	0.901	0.905	0.908	0.912	0.914	0.919	0.923	0.930	0.936
Ohio	0.831	0.834	0.836	0.839	0.841	0.844	0.847	0.850	0.852	0.855	0.857	0.859	0.861	0.863	0.866	0.868	0.871	0.873	0.877	0.879	0.882	0.885	0.888	0.891	0.894	0.897	0.900	0.903	0.905	0.907	0.910	0.913	0.915	0.917	0.921	0.925
Oklahoma	0.833	0.836	0.835	0.839	0.843	0.847	0.850	0.853	0.853	0.855	0.856	0.855	0.856	0.858	0.859	0.860	0.861	0.860	0.862	0.866	0.869	0.870	0.872	0.875	0.877	0.880	0.884	0.887	0.891	0.893	0.897	0.901	0.905	0.908	0.912	0.915
Oregon	0.859	0.861	0.864	0.864	0.865	0.867	0.868	0.870	0.870	0.871	0.872	0.874	0.876	0.879	0.880	0.880	0.882	0.885	0.887	0.890	0.894	0.897	0.899	0.901	0.903	0.906	0.907	0.911	0.913	0.917	0.921	0.923	0.927	0.929	0.934	0.938
Pennsylvania	0.851	0.853	0.854	0.856	0.858	0.860	0.862	0.863	0.865	0.868	0.870	0.872	0.874	0.876	0.878	0.881	0.884	0.888	0.890	0.892	0.895	0.897	0.899	0.901	0.904	0.907	0.910	0.913	0.916	0.919	0.921	0.924	0.928	0.931	0.935	0.939
Rhode Island	0.836	0.839	0.842	0.844	0.848	0.852	0.854	0.857	0.861	0.864	0.867	0.871	0.873	0.876	0.880	0.885	0.888	0.891	0.894	0.899	0.902	0.903	0.905	0.907	0.912	0.915	0.921	0.924	0.927	0.931	0.933	0.936	0.939	0.942	0.947	0.951
South Carolina	0.802	0.806	0.809	0.811	0.815	0.818	0.822	0.825	0.826	0.829	0.833	0.837	0.841	0.846	0.851	0.856	0.859	0.861	0.864	0.867	0.870	0.873	0.878	0.880	0.883	0.885	0.886	0.890	0.893	0.897	0.900	0.904	0.907	0.910	0.913	0.916
South Dakota	0.810	0.815	0.816	0.818	0.821	0.826	0.830	0.832	0.837	0.840	0.844	0.845	0.846	0.850	0.854	0.856	0.860	0.865	0.868	0.869	0.876	0.876	0.877	0.879	0.881	0.884	0.886	0.889	0.894	0.897	0.900	0.904	0.906	0.909	0.914	0.917
Tennessee	0.802	0.807	0.810	0.812	0.816	0.820	0.824	0.827	0.829	0.833	0.836	0.840	0.844	0.848	0.852	0.855	0.859	0.863	0.866	0.870	0.873	0.877	0.880	0.883	0.885	0.887	0.890	0.892	0.895	0.899	0.902	0.904	0.907	0.909	0.913	0.916
Texas	0.822	0.826	0.828	0.831	0.833	0.836	0.838	0.840	0.842	0.845	0.846	0.846	0.847	0.848	0.850	0.852	0.853	0.856	0.860	0.862	0.865	0.868	0.869	0.872	0.875	0.877	0.882	0.885	0.889	0.892	0.897	0.903	0.906	0.909	0.912	0.914
Utah	0.802	0.807	0.811	0.816	0.823	0.829	0.833	0.837	0.837	0.842	0.845	0.848	0.848	0.851	0.852	0.853	0.852	0.854	0.855	0.857	0.861	0.862	0.863	0.866	0.867	0.870	0.875	0.879	0.881	0.886	0.889	0.894	0.897	0.901	0.905	0.908
Vermont	0.850	0.852	0.854	0.856	0.858	0.862	0.864	0.868	0.872	0.874	0.879	0.881	0.883	0.885	0.886	0.892	0.893	0.896	0.898	0.901	0.905	0.907	0.910	0.910	0.913	0.919	0.921	0.925	0.929	0.932	0.932	0.936	0.940	0.943	0.947	0.950
Virginia	0.860	0.863	0.864	0.866	0.868	0.871	0.873	0.875	0.878	0.880	0.882	0.884	0.885	0.887	0.888	0.891	0.893	0.895	0.897	0.899	0.901	0.903	0.905	0.907	0.909	0.913	0.916	0.920	0.925	0.927	0.931	0.934	0.937	0.940	0.943	0.946
Washington	0.863	0.865	0.867	0.869	0.871	0.873	0.875	0.877	0.879	0.880	0.882	0.883	0.885	0.887	0.890	0.891	0.893	0.896	0.899	0.902	0.906	0.909	0.911	0.913	0.915	0.918	0.920	0.923	0.925	0.927	0.930	0.933	0.936	0.940	0.943	0.947
West Virginia	0.791	0.797	0.799	0.803	0.807	0.811	0.814	0.817	0.821	0.823	0.826	0.829	0.833	0.836	0.840	0.843	0.847	0.850	0.854	0.856	0.860	0.863	0.864	0.868	0.871	0.874	0.879	0.879	0.882	0.884	0.888	0.890	0.893	0.895	0.899	0.903
Wisconsin	0.827	0.831	0.834	0.836	0.838	0.842	0.845	0.850	0.854	0.858	0.861	0.864	0.867	0.869	0.873	0.876	0.879	0.883	0.885	0.888	0.891	0.893	0.896	0.897	0.900	0.902	0.905	0.909	0.911	0.914	0.917	0.919	0.923	0.925	0.928	0.930
Wyoming	0.856	0.859	0.859	0.862	0.864	0.867	0.870	0.875	0.877	0.880	0.880	0.881	0.880	0.882	0.883	0.886	0.886	0.886	0.890	0.894	0.897	0.899	0.897	0.899	0.903	0.902	0.906	0.911	0.914	0.918	0.923	0.928	0.931	0.934	0.937	0.940
Latin America and Caribbean	0.447	0.457	0.465	0.473	0.481	0.489	0.497	0.504	0.512	0.519	0.526	0.532	0.539	0.545	0.552	0.558	0.564	0.571	0.577	0.583	0.589	0.595	0.600	0.606	0.612	0.618	0.625	0.631	0.638	0.644	0.650	0.656	0.662	0.667	0.673	0.678
Caribbean	0.503	0.510	0.516	0.521	0.527	0.532	0.537	0.543	0.548	0.553	0.558	0.562	0.566	0.569	0.571	0.575	0.578	0.582	0.586	0.590	0.595	0.600	0.605	0.610	0.614	0.619	0.624	0.629	0.634	0.639	0.643	0.648	0.652	0.657	0.661	0.666
Antigua and Barbuda	0.657	0.664	0.670	0.677	0.685	0.693	0.702	0.711	0.721	0.730	0.737	0.745	0.750	0.755	0.759	0.762	0.767	0.771	0.776	0.780	0.784	0.787	0.791	0.795	0.799	0.804	0.810	0.816	0.823	0.827	0.830	0.832	0.834	0.836	0.839	0.841
The Bahamas	0.649	0.656	0.662	0.669	0.677	0.686	0.694	0.702	0.709	0.715	0.721	0.725	0.729	0.733	0.737	0.743	0.749	0.756	0.764	0.772	0.780	0.789	0.797	0.803	0.807	0.812	0.815	0.818	0.820	0.822	0.824	0.825	0.828	0.830	0.832	0.835
Barbados	0.618	0.625	0.631	0.636	0.642	0.648	0.655	0.662	0.669	0.676	0.683	0.688	0.692	0.696	0.700	0.704	0.709	0.714	0.719	0.724	0.729	0.734	0.738	0.743	0.747	0.752	0.757	0.761	0.765	0.768	0.771	0.773	0.776	0.778	0.780	0.782
Belize	0.374	0.386	0.397	0.407	0.417	0.427	0.435	0.444	0.454	0.464	0.474	0.483	0.492	0.500	0.508	0.515	0.526	0.536	0.545	0.554	0.563	0.571	0.580	0.589	0.598	0.607	0.614	0.621	0.628	0.633	0.639	0.644	0.650	0.655	0.660	0.665

Appendix Table 8. Socio-Demographic Index (SDI) values for all estimated GBD 2015 locations, 1980–2015

Location	1980	1981	1982	1983	1984	1985	1986	1987	1988	1989	1990	1991	1992	1993	1994	1995	1996	1997	1998	1999	2000	2001	2002	2003	2004	2005	2006	2007	2008	2009	2010	2011	2012	2013	2014	2015
Bermuda	0.778	0.785	0.792	0.799	0.805	0.811	0.817	0.824	0.830	0.835	0.839	0.840	0.845	0.854	0.856	0.859	0.861	0.863	0.868	0.871	0.874	0.879	0.883	0.886	0.890	0.893	0.900	0.903	0.907	0.909	0.911	0.913	0.915	0.917	0.918	0.916
Cuba	0.598	0.606	0.614	0.622	0.629	0.636	0.642	0.648	0.654	0.660	0.665	0.668	0.670	0.669	0.668	0.668	0.668	0.669	0.670	0.673	0.677	0.682	0.687	0.693	0.698	0.705	0.713	0.720	0.728	0.734	0.740	0.746	0.751	0.757	0.762	0.766
Dominica	0.522	0.521	0.540	0.547	0.555	0.563	0.571	0.578	0.587	0.593	0.599	0.614	0.606	0.614	0.626	0.637	0.646	0.655	0.663	0.671	0.678	0.684	0.688	0.694	0.699	0.704	0.709	0.714	0.719	0.725	0.730	0.735	0.739	0.743	0.748	0.753
Dominican Republic	0.434	0.444	0.452	0.461	0.469	0.476	0.483	0.490	0.497	0.504	0.510	0.515	0.521	0.528	0.535	0.542	0.549	0.557	0.565	0.573	0.580	0.588	0.595	0.601	0.607	0.613	0.620	0.628	0.635	0.642	0.649	0.657	0.663	0.670	0.677	0.684
Grenada	0.476	0.486	0.496	0.506	0.515	0.524	0.528	0.532	0.536	0.541	0.547	0.561	0.576	0.590	0.602	0.614	0.624	0.633	0.642	0.652	0.662	0.671	0.679	0.688	0.695	0.703	0.710	0.717	0.723	0.728	0.732	0.737	0.740	0.744	0.749	0.753
Guyana	0.461	0.466	0.470	0.471	0.474	0.476	0.480	0.483	0.487	0.489	0.492	0.495	0.500	0.507	0.515	0.524	0.533	0.543	0.551	0.558	0.564	0.570	0.575	0.579	0.584	0.588	0.594	0.600	0.606	0.611	0.616	0.624	0.632	0.640	0.647	0.655
Haiti	0.254	0.258	0.262	0.266	0.271	0.277	0.282	0.288	0.293	0.299	0.304	0.309	0.313	0.316	0.318	0.322	0.325	0.329	0.334	0.339	0.344	0.349	0.354	0.359	0.363	0.367	0.370	0.375	0.379	0.384	0.387	0.391	0.396	0.401	0.407	0.412
Jamaica	0.530	0.535	0.539	0.545	0.551	0.556	0.562	0.569	0.575	0.582	0.589	0.596	0.603	0.610	0.617	0.624	0.630	0.635	0.641	0.646	0.650	0.655	0.660	0.665	0.670	0.674	0.680	0.686	0.691	0.695	0.700	0.704	0.708	0.712	0.715	0.719
Puerto Rico	0.705	0.713	0.720	0.726	0.732	0.738	0.744	0.751	0.757	0.763	0.768	0.774	0.780	0.786	0.792	0.798	0.804	0.810	0.816	0.821	0.827	0.833	0.838	0.843	0.848	0.853	0.857	0.861	0.864	0.867	0.870	0.872	0.874	0.876	0.879	0.882
Saint Lucia	0.480	0.493	0.504	0.514	0.521	0.528	0.538	0.547	0.556	0.564	0.574	0.584	0.594	0.603	0.612	0.620	0.629	0.638	0.647	0.656	0.664	0.671	0.677	0.684	0.690	0.696	0.701	0.705	0.710	0.715	0.719	0.724	0.729	0.733	0.737	0.741
Saint Vincent and the Grenadines	0.492	0.502	0.513	0.523	0.533	0.543	0.553	0.561	0.570	0.578	0.585	0.594	0.603	0.611	0.619	0.626	0.634	0.642	0.649	0.656	0.663	0.670	0.678	0.685	0.692	0.698	0.705	0.711	0.718	0.723	0.728	0.732	0.736	0.740	0.744	0.747
Suriname	0.488	0.496	0.503	0.508	0.513	0.519	0.524	0.529	0.535	0.542	0.548	0.553	0.558	0.563	0.567	0.571	0.577	0.583	0.589	0.594	0.600	0.606	0.612	0.619	0.626	0.633	0.641	0.648	0.656	0.663	0.670	0.678	0.685	0.691	0.698	0.704
Trinidad and Tobago	0.629	0.636	0.643	0.648	0.654	0.660	0.664	0.669	0.673	0.677	0.681	0.686	0.691	0.695	0.700	0.705	0.711	0.717	0.724	0.731	0.738	0.746	0.753	0.761	0.770	0.777	0.786	0.794	0.801	0.807	0.811	0.816	0.821	0.825	0.829	0.833
Virgin Islands, U.S.	0.697	0.707	0.716	0.722	0.728	0.734	0.738	0.744	0.750	0.756	0.763	0.772	0.781	0.790	0.800	0.809	0.817	0.826	0.833	0.839	0.844	0.849	0.853	0.855	0.858	0.861	0.863	0.866	0.868	0.871	0.873	0.877	0.880	0.882	0.884	0.886
Andean Latin America	0.451	0.460	0.468	0.476	0.483	0.490	0.497	0.504	0.510	0.515	0.520	0.525	0.530	0.535	0.541	0.548	0.555	0.562	0.569	0.575	0.582	0.587	0.593	0.599	0.605	0.612	0.619	0.626	0.634	0.641	0.648	0.655	0.662	0.669	0.676	0.682
Bolivia	0.376	0.382	0.388	0.394	0.400	0.405	0.410	0.415	0.419	0.424	0.430	0.436	0.443	0.449	0.457	0.464	0.472	0.480	0.488	0.495	0.503	0.510	0.517	0.524	0.531	0.538	0.545	0.552	0.560	0.567	0.575	0.582	0.590	0.597	0.605	0.612
Ecuador	0.463	0.472	0.481	0.489	0.496	0.503	0.510	0.516	0.522	0.528	0.535	0.541	0.548	0.554	0.560	0.566	0.572	0.578	0.584	0.589	0.594	0.600	0.605	0.610	0.616	0.623	0.629	0.636	0.643	0.648	0.654	0.661	0.667	0.674	0.680	0.685
Peru	0.470	0.479	0.488	0.496	0.503	0.511	0.518	0.526	0.533	0.537	0.541	0.545	0.549	0.553	0.559	0.566	0.573	0.581	0.588	0.594	0.601	0.607	0.613	0.619	0.625	0.631	0.638	0.646	0.654	0.661	0.669	0.677	0.684	0.692	0.699	0.705
Central Latin America	0.458	0.468	0.478	0.487	0.495	0.503	0.511	0.518	0.525	0.532	0.539	0.546	0.553	0.560	0.567	0.574	0.580	0.587	0.593	0.600	0.606	0.612	0.618	0.623	0.629	0.635	0.642	0.648	0.655	0.661	0.666	0.672	0.678	0.683	0.689	0.694
Colombia	0.468	0.477	0.485	0.493	0.501	0.508	0.516	0.523	0.530	0.537	0.544	0.551	0.558	0.565	0.572	0.579	0.586	0.593	0.599	0.604	0.609	0.614	0.618	0.623	0.628	0.634	0.640	0.647	0.654	0.661	0.667	0.674	0.681	0.687	0.694	0.700
Costa Rica	0.516	0.521	0.525	0.528	0.533	0.536	0.541	0.545	0.549	0.554	0.559	0.564	0.570	0.577	0.584	0.590	0.597	0.605	0.613	0.621	0.629	0.636	0.642	0.649	0.655	0.661	0.667	0.674	0.681	0.687	0.693	0.699	0.706	0.712	0.718	0.723
El Salvador	0.376	0.382	0.388	0.394	0.399	0.405	0.411	0.417	0.423	0.429	0.435	0.442	0.450	0.459	0.467	0.476	0.484	0.493	0.502	0.510	0.519	0.527	0.535	0.543	0.550	0.557	0.565	0.572	0.579	0.585	0.591	0.597	0.603	0.608	0.613	0.619
Guatemala	0.312	0.317	0.322	0.327	0.333	0.339	0.345	0.351	0.357	0.362	0.368	0.374	0.380	0.386	0.392	0.399	0.406	0.414	0.421	0.429	0.437	0.444	0.452	0.460	0.467	0.474	0.482	0.490	0.497	0.504	0.510	0.517	0.523	0.530	0.536	0.543
Honduras	0.309	0.317	0.325	0.332	0.340	0.347	0.353	0.360	0.367	0.374	0.380	0.386	0.393	0.400	0.407	0.414	0.421	0.429	0.436	0.443	0.451	0.459	0.467	0.475	0.483	0.492	0.501	0.510	0.519	0.527	0.535	0.542	0.550	0.556	0.562	0.568
Mexico	0.466	0.480	0.492	0.503	0.513	0.522	0.531	0.539	0.547	0.555	0.563	0.570	0.578	0.586	0.594	0.600	0.606	0.613	0.620	0.626	0.633	0.640	0.646	0.652	0.658	0.664	0.670	0.677	0.683	0.687	0.692	0.698	0.703	0.708	0.713	0.718
Aguascalientes	0.484	0.498	0.511	0.522	0.533	0.543	0.553	0.562	0.571	0.579	0.587	0.594	0.601	0.608	0.615	0.621	0.627	0.633	0.640	0.647	0.654	0.661	0.668	0.675	0.682	0.690	0.697	0.704	0.711	0.717	0.723	0.728	0.734	0.740	0.746	0.751
Baja California	0.538	0.552	0.564	0.575	0.585	0.593	0.599	0.605	0.611	0.616	0.622	0.628	0.634	0.641	0.647	0.652	0.658	0.665	0.672	0.679	0.686	0.693	0.700	0.707	0.714	0.721	0.728	0.735	0.742	0.747	0.753	0.759	0.764	0.770	0.776	0.781
Baja California Sur	0.516	0.533	0.547	0.560	0.572	0.584	0.596	0.607	0.619	0.629	0.638	0.646	0.653	0.660	0.668	0.674	0.679	0.684	0.689	0.694	0.699	0.705	0.711	0.716	0.722	0.728	0.735	0.742	0.748	0.753	0.758	0.764	0.770	0.775	0.781	0.786
Campeche	0.447	0.461	0.473	0.483	0.493	0.503	0.513	0.523	0.532	0.541	0.549	0.557	0.564	0.572	0.579	0.585	0.591	0.597	0.603	0.609	0.615	0.622	0.629	0.636	0.643	0.650	0.657	0.664	0.670	0.675	0.680	0.686	0.691	0.696	0.701	0.706
Chiapas	0.317	0.331	0.344	0.354	0.364	0.375	0.385	0.395	0.405	0.414	0.423	0.432	0.441	0.449	0.457	0.464	0.471	0.479	0.486	0.493	0.501	0.508	0.515	0.521	0.527	0.534	0.540	0.546	0.552	0.556	0.560	0.565	0.569	0.573	0.577	0.581
Chihuahua	0.507	0.520	0.532	0.542	0.552	0.560	0.568	0.574	0.581	0.587	0.593	0.599	0.605	0.612	0.618	0.622	0.628	0.634	0.641	0.647	0.653	0.660	0.666	0.672	0.678	0.685	0.692	0.699	0.706	0.711	0.717	0.723	0.729	0.734	0.740	0.746
Coahuila	0.532	0.547	0.561	0.572	0.583	0.593	0.602	0.611	0.619	0.627	0.635	0.642	0.649	0.655	0.662	0.668	0.673	0.679	0.685	0.691	0.697	0.704	0.710	0.716	0.722	0.729	0.736	0.743	0.750	0.755	0.761	0.767	0.773	0.779	0.785	0.790
Colima	0.487	0.502	0.515	0.526	0.537	0.548	0.559	0.570	0.580	0.590	0.599	0.606	0.613	0.620	0.628	0.634	0.639	0.645	0.652	0.658	0.664	0.670	0.675	0.681	0.686	0.691	0.698	0.704	0.709	0.714	0.719	0.724	0.729	0.734	0.739	0.743
Distrito Federal	0.645	0.656	0.666	0.674	0.682	0.688	0.693	0.697	0.701	0.705	0.712	0.720	0.729	0.738	0.747	0.755	0.763	0.770	0.778	0.785	0.793	0.798	0.802	0.807	0.812	0.817	0.823	0.830	0.836	0.842	0.848	0.854	0.860	0.867	0.873	0.880
Durango	0.458	0.473	0.485	0.496	0.506	0.517	0.527	0.536	0.545	0.554	0.562	0.570	0.578	0.585	0.593	0.599	0.605	0.610	0.616	0.621	0.628	0.635	0.642	0.649	0.656	0.663	0.670	0.677	0.683	0.688	0.693	0.699	0.704	0.709	0.715	0.720
Guanajuato	0.400	0.415	0.428	0.440	0.451	0.462	0.473	0.483	0.493	0.503	0.511	0.520	0.528	0.536	0.544	0.551	0.557	0.563	0.569	0.575	0.582	0.590	0.597	0.604	0.610	0.616	0.623	0.629	0.635	0.640	0.644	0.649	0.653	0.658	0.662	0.666
Guerrero	0.357	0.371	0.382	0.392	0.402	0.412	0.422	0.431	0.439	0.448	0.455	0.462	0.469	0.476	0.482	0.487	0.493	0.500	0.506	0.512	0.519	0.527	0.534	0.542	0.549	0.556	0.563	0.570	0.576	0.581	0.586	0.591	0.595	0.600	0.604	0.608
Hidalgo	0.402	0.418	0.431	0.443	0.454	0.465	0.477	0.488	0.498	0.508	0.516	0.524	0.532	0.540	0.547	0.553	0.559	0.565	0.571	0.578	0.584	0.591	0.597	0.602	0.608	0.613	0.619	0.625	0.631	0.635	0.639	0.644	0.648	0.653	0.657	0.661
Jalisco	0.470	0.485	0.497	0.508	0.518	0.528	0.537	0.546	0.554	0.562	0.569	0.577	0.586	0.593	0.601	0.608	0.615	0.622	0.629	0.636	0.643	0.650	0.657	0.664	0.670	0.677	0.684	0.690	0.696	0.702	0.707	0.712	0.718	0.723	0.728	0.733
México	0.450	0.468	0.483	0.497	0.509	0.519	0.527	0.535	0.542	0.549	0.556	0.565	0.574	0.582	0.591	0.598	0.606	0.613	0.621	0.629	0.637	0.642	0.647	0.652	0.657	0.662	0.668	0.674	0.679	0.683	0.688	0.692	0.697	0.702	0.706	0.710
Michoacán de Ocampo	0.391	0.405	0.417	0.427	0.437	0.447	0.458	0.469	0.478	0.488	0.497	0.505	0.513	0.521	0.529	0.535	0.541	0.548	0.554	0.561	0.568	0.575	0.582	0.588	0.594	0.600	0.607	0.613	0.618	0.622	0.626	0.631	0.635	0.640	0.644	0.648
Morelos	0.471	0.485	0.498	0.509	0.519	0.529	0.540	0.549	0.558	0.567	0.575	0.581	0.588	0.594	0.601	0.606	0.611	0.617	0.623	0.628	0.634	0.640	0.646	0.651	0.657	0.663	0.669	0.675	0.681	0.686	0.691	0.696	0.701	0.706	0.710	0.715
Nayarit	0.440	0.455	0.467	0.478	0.489	0.499	0.510	0.519	0.529	0.538	0.546	0.554	0.562	0.570	0.577	0.584	0.589	0.595	0.600	0.606	0.612	0.619	0.624	0.630	0.636	0.642	0.648	0.654	0.660	0.664	0.669	0.674	0.679	0.684	0.689	0.693

Appendix Table 8. Socio-Demographic Index (SDI) values for all estimated GBD 2015 locations, 1980–2015

Location	1980	1981	1982	1983	1984	1985	1986	1987	1988	1989	1990	1991	1992	1993	1994	1995	1996	1997	1998	1999	2000	2001	2002	2003	2004	2005	2006	2007	2008	2009	2010	2011	2012	2013	2014	2015
Nuevo León	0.574	0.590	0.604	0.616	0.627	0.637	0.645	0.652	0.659	0.666	0.672	0.680	0.687	0.694	0.701	0.707	0.713	0.718	0.724	0.729	0.735	0.741	0.747	0.753	0.759	0.766	0.773	0.780	0.786	0.792	0.797	0.803	0.810	0.816	0.822	0.828
Oaxaca	0.350	0.362	0.372	0.381	0.390	0.399	0.409	0.419	0.428	0.437	0.445	0.453	0.461	0.468	0.476	0.482	0.488	0.496	0.503	0.510	0.518	0.526	0.532	0.538	0.544	0.551	0.557	0.563	0.569	0.573	0.577	0.582	0.586	0.590	0.595	0.598
Puebla	0.410	0.424	0.436	0.446	0.456	0.466	0.475	0.484	0.492	0.500	0.508	0.516	0.524	0.532	0.540	0.547	0.554	0.561	0.569	0.577	0.585	0.592	0.598	0.605	0.611	0.617	0.624	0.631	0.637	0.641	0.646	0.651	0.656	0.661	0.666	0.671
Querétaro	0.426	0.443	0.459	0.472	0.485	0.498	0.512	0.524	0.536	0.548	0.558	0.567	0.576	0.585	0.594	0.601	0.609	0.617	0.624	0.632	0.640	0.647	0.654	0.661	0.667	0.673	0.680	0.686	0.692	0.696	0.701	0.705	0.710	0.714	0.718	0.723
Quintana Roo	0.431	0.454	0.473	0.490	0.505	0.520	0.534	0.547	0.559	0.571	0.581	0.590	0.598	0.606	0.614	0.621	0.628	0.635	0.642	0.649	0.656	0.663	0.669	0.675	0.681	0.686	0.693	0.699	0.705	0.709	0.714	0.719	0.724	0.728	0.733	0.737
San Luis Potosí	0.419	0.433	0.446	0.457	0.467	0.478	0.490	0.500	0.510	0.520	0.529	0.537	0.544	0.552	0.559	0.565	0.571	0.578	0.584	0.591	0.598	0.606	0.615	0.624	0.631	0.638	0.645	0.652	0.657	0.662	0.666	0.671	0.676	0.680	0.684	0.688
Sinaloa	0.472	0.487	0.501	0.513	0.524	0.535	0.544	0.553	0.562	0.570	0.578	0.585	0.593	0.600	0.607	0.613	0.619	0.625	0.631	0.637	0.643	0.650	0.656	0.662	0.668	0.674	0.681	0.687	0.693	0.698	0.702	0.707	0.712	0.717	0.722	0.727
Sonora	0.528	0.543	0.555	0.566	0.576	0.586	0.595	0.603	0.610	0.618	0.625	0.632	0.639	0.645	0.652	0.658	0.663	0.668	0.674	0.679	0.684	0.690	0.697	0.703	0.709	0.716	0.723	0.730	0.736	0.742	0.747	0.753	0.759	0.765	0.770	0.776
Tabasco	0.436	0.454	0.469	0.483	0.496	0.510	0.524	0.537	0.549	0.561	0.572	0.582	0.592	0.602	0.611	0.619	0.627	0.635	0.643	0.651	0.659	0.665	0.671	0.677	0.682	0.688	0.694	0.700	0.706	0.710	0.715	0.720	0.724	0.729	0.733	0.738
Tamaulipas	0.523	0.538	0.551	0.562	0.573	0.582	0.591	0.600	0.608	0.616	0.623	0.629	0.636	0.642	0.649	0.654	0.659	0.664	0.670	0.676	0.682	0.688	0.694	0.699	0.705	0.711	0.718	0.725	0.731	0.736	0.742	0.747	0.753	0.759	0.764	0.770
Tlaxcala	0.419	0.434	0.446	0.457	0.467	0.479	0.491	0.502	0.513	0.524	0.533	0.541	0.549	0.557	0.564	0.571	0.577	0.584	0.592	0.599	0.606	0.613	0.618	0.624	0.629	0.635	0.641	0.648	0.653	0.658	0.662	0.667	0.672	0.677	0.681	0.686
Veracruz de Ignacio de la Llave	0.439	0.453	0.464	0.475	0.484	0.494	0.502	0.510	0.517	0.525	0.532	0.540	0.547	0.555	0.562	0.568	0.575	0.582	0.589	0.596	0.603	0.609	0.614	0.620	0.625	0.630	0.636	0.642	0.647	0.652	0.656	0.661	0.665	0.670	0.674	0.678
Yucatán	0.453	0.466	0.477	0.487	0.496	0.505	0.513	0.521	0.528	0.535	0.543	0.551	0.560	0.568	0.576	0.583	0.590	0.597	0.605	0.613	0.620	0.626	0.632	0.638	0.643	0.649	0.656	0.662	0.668	0.673	0.678	0.683	0.688	0.693	0.698	0.704
Zacatecas	0.402	0.416	0.429	0.440	0.450	0.462	0.475	0.488	0.500	0.511	0.521	0.529	0.537	0.544	0.552	0.558	0.563	0.568	0.573	0.578	0.584	0.591	0.597	0.602	0.608	0.614	0.620	0.626	0.632	0.637	0.641	0.646	0.651	0.656	0.661	0.665
Nicaragua	0.345	0.350	0.355	0.361	0.367	0.373	0.379	0.385	0.390	0.394	0.399	0.404	0.409	0.415	0.420	0.426	0.433	0.441	0.448	0.457	0.465	0.473	0.480	0.487	0.494	0.501	0.507	0.514	0.521	0.526	0.532	0.538	0.544	0.550	0.557	0.563
Panama	0.529	0.538	0.547	0.554	0.561	0.568	0.575	0.581	0.586	0.590	0.594	0.599	0.604	0.610	0.615	0.620	0.625	0.631	0.637	0.643	0.649	0.654	0.660	0.665	0.670	0.676	0.682	0.688	0.695	0.702	0.708	0.716	0.724	0.732	0.739	0.747
Venezuela	0.533	0.540	0.547	0.553	0.558	0.563	0.569	0.574	0.580	0.585	0.591	0.597	0.604	0.611	0.617	0.622	0.628	0.633	0.638	0.643	0.647	0.652	0.655	0.658	0.663	0.669	0.676	0.684	0.691	0.698	0.704	0.709	0.716	0.721	0.725	0.728
Tropical Latin America	0.422	0.431	0.439	0.447	0.455	0.463	0.472	0.481	0.490	0.498	0.506	0.513	0.519	0.526	0.532	0.539	0.546	0.552	0.558	0.564	0.569	0.576	0.582	0.588	0.594	0.601	0.607	0.614	0.621	0.627	0.633	0.639	0.645	0.651	0.656	0.661
Brazil	0.422	0.431	0.439	0.447	0.455	0.464	0.473	0.482	0.491	0.499	0.506	0.514	0.520	0.527	0.533	0.540	0.547	0.553	0.559	0.565	0.570	0.576	0.583	0.589	0.595	0.601	0.608	0.615	0.621	0.627	0.634	0.640	0.646	0.651	0.657	0.662
Acre	0.293	0.303	0.313	0.323	0.333	0.343	0.356	0.367	0.378	0.389	0.398	0.406	0.412	0.419	0.425	0.431	0.438	0.445	0.451	0.457	0.464	0.468	0.469	0.476	0.480	0.486	0.497	0.505	0.506	0.517	0.526	0.529	0.540	0.545	0.551	0.556
Alagoas	0.278	0.287	0.295	0.304	0.312	0.321	0.331	0.341	0.350	0.359	0.367	0.374	0.380	0.386	0.392	0.398	0.404	0.411	0.416	0.422	0.428	0.429	0.437	0.444	0.453	0.458	0.465	0.470	0.477	0.483	0.490	0.496	0.503	0.507	0.508	0.512
Amapá	0.338	0.349	0.361	0.373	0.385	0.397	0.412	0.426	0.439	0.451	0.462	0.472	0.481	0.489	0.498	0.506	0.513	0.520	0.527	0.533	0.528	0.535	0.546	0.551	0.566	0.574	0.577	0.587	0.594	0.604	0.608	0.617	0.625	0.629	0.636	0.642
Amazonas	0.345	0.356	0.367	0.378	0.390	0.401	0.416	0.430	0.443	0.455	0.466	0.472	0.478	0.483	0.488	0.493	0.501	0.509	0.516	0.523	0.538	0.542	0.548	0.556	0.563	0.569	0.574	0.583	0.591	0.597	0.606	0.613	0.619	0.624	0.630	0.636
Bahia	0.312	0.321	0.329	0.337	0.345	0.354	0.366	0.377	0.388	0.399	0.408	0.415	0.422	0.428	0.435	0.441	0.449	0.456	0.463	0.470	0.481	0.485	0.489	0.494	0.501	0.508	0.514	0.520	0.526	0.532	0.538	0.543	0.549	0.555	0.555	0.559
Ceará	0.316	0.323	0.330	0.337	0.344	0.352	0.361	0.370	0.378	0.386	0.394	0.401	0.407	0.414	0.420	0.427	0.434	0.440	0.447	0.452	0.465	0.466	0.474	0.480	0.487	0.493	0.499	0.505	0.512	0.518	0.525	0.531	0.537	0.543	0.544	0.548
Distrito Federal	0.574	0.584	0.593	0.602	0.612	0.621	0.632	0.644	0.654	0.664	0.674	0.681	0.688	0.695	0.702	0.709	0.716	0.723	0.729	0.736	0.730	0.735	0.743	0.750	0.759	0.768	0.775	0.785	0.795	0.802	0.810	0.821	0.829	0.835	0.849	0.855
Espírito Santo	0.443	0.453	0.462	0.470	0.479	0.489	0.498	0.507	0.516	0.524	0.532	0.540	0.547	0.554	0.561	0.568	0.575	0.581	0.587	0.593	0.595	0.601	0.608	0.615	0.622	0.629	0.635	0.641	0.648	0.653	0.659	0.665	0.670	0.675	0.681	0.685
Goiás	0.390	0.402	0.414	0.426	0.437	0.448	0.457	0.466	0.475	0.483	0.490	0.497	0.504	0.510	0.516	0.523	0.529	0.535	0.541	0.546	0.551	0.556	0.560	0.567	0.573	0.579	0.586	0.593	0.598	0.603	0.609	0.614	0.618	0.622	0.629	0.633
Maranhão	0.267	0.272	0.277	0.282	0.288	0.294	0.305	0.316	0.327	0.337	0.345	0.353	0.360	0.367	0.374	0.381	0.389	0.397	0.404	0.410	0.436	0.436	0.436	0.438	0.445	0.450	0.456	0.462	0.469	0.476	0.483	0.489	0.496	0.501	0.498	0.502
Mato Grosso	0.395	0.406	0.418	0.429	0.440	0.451	0.461	0.471	0.481	0.490	0.498	0.506	0.513	0.520	0.527	0.533	0.541	0.548	0.555	0.561	0.568	0.574	0.579	0.584	0.587	0.592	0.601	0.609	0.614	0.621	0.626	0.631	0.636	0.640	0.647	0.651
Mato Grosso do Sul	0.423	0.433	0.442	0.452	0.461	0.471	0.479	0.488	0.496	0.504	0.511	0.518	0.524	0.530	0.537	0.543	0.550	0.556	0.562	0.567	0.574	0.578	0.583	0.590	0.593	0.600	0.608	0.615	0.619	0.626	0.632	0.637	0.643	0.648	0.656	0.660
Minas Gerais	0.427	0.435	0.444	0.452	0.460	0.468	0.477	0.485	0.493	0.501	0.508	0.515	0.521	0.528	0.534	0.541	0.547	0.554	0.560	0.566	0.573	0.578	0.585	0.590	0.597	0.603	0.610	0.616	0.623	0.630	0.635	0.641	0.646	0.652	0.655	0.659
Pará	0.332	0.340	0.348	0.356	0.365	0.374	0.385	0.396	0.406	0.416	0.424	0.432	0.438	0.445	0.451	0.458	0.465	0.471	0.477	0.483	0.506	0.504	0.509	0.511	0.519	0.525	0.529	0.535	0.544	0.553	0.560	0.568	0.576	0.581	0.582	0.587
Paraíba	0.303	0.311	0.319	0.326	0.334	0.342	0.352	0.362	0.371	0.380	0.388	0.395	0.401	0.407	0.414	0.420	0.428	0.435	0.442	0.448	0.461	0.459	0.465	0.470	0.477	0.482	0.489	0.495	0.500	0.506	0.513	0.519	0.525	0.530	0.531	0.535
Paraná	0.440	0.450	0.460	0.469	0.479	0.488	0.496	0.505	0.512	0.520	0.526	0.533	0.540	0.546	0.552	0.558	0.565	0.572	0.578	0.583	0.585	0.593	0.598	0.606	0.611	0.616	0.623	0.631	0.637	0.642	0.647	0.653	0.658	0.663	0.670	0.675
Pernambuco	0.348	0.357	0.365	0.373	0.381	0.390	0.400	0.409	0.418	0.427	0.435	0.442	0.449	0.456	0.462	0.469	0.475	0.482	0.488	0.493	0.497	0.501	0.508	0.515	0.522	0.527	0.534	0.541	0.547	0.553	0.560	0.566	0.571	0.576	0.581	0.585
Piaui	0.273	0.281	0.288	0.295	0.303	0.311	0.321	0.331	0.341	0.350	0.358	0.365	0.371	0.377	0.384	0.390	0.397	0.404	0.410	0.416	0.423	0.427	0.434	0.440	0.446	0.450	0.456	0.463	0.469	0.475	0.481	0.486	0.492	0.498	0.499	0.503
Rio de Janeiro	0.520	0.528	0.537	0.544	0.552	0.560	0.568	0.576	0.583	0.591	0.597	0.603	0.609	0.615	0.621	0.627	0.633	0.639	0.644	0.649	0.649	0.657	0.665	0.670	0.677	0.685	0.691	0.698	0.706	0.711	0.718	0.725	0.730	0.736	0.748	0.753
Rio Grande do Norte	0.339	0.347	0.355	0.363	0.371	0.379	0.388	0.398	0.407	0.415	0.423	0.431	0.438	0.445	0.452	0.459	0.466	0.473	0.479	0.485	0.490	0.496	0.501	0.507	0.515	0.520	0.529	0.534	0.540	0.545	0.552	0.558	0.565	0.570	0.571	0.575
Rio Grande do Sul	0.503	0.510	0.516	0.522	0.529	0.536	0.543	0.550	0.557	0.564	0.570	0.577	0.583	0.589	0.595	0.602	0.608	0.614	0.620	0.625	0.626	0.634	0.641	0.648	0.653	0.661	0.668	0.677	0.684	0.690	0.696	0.702	0.708	0.713	0.721	0.726
Rondônia	0.338	0.351	0.365	0.377	0.390	0.402	0.412	0.422	0.431	0.440	0.448	0.455	0.462	0.468	0.475	0.481	0.489	0.496	0.503	0.509	0.511	0.523	0.526	0.533	0.539	0.547	0.558	0.568	0.568	0.574	0.581	0.584	0.592	0.596	0.600	0.604
Roraima	0.368	0.380	0.392	0.404	0.415	0.427	0.436	0.446	0.455	0.464	0.471	0.481	0.488	0.495	0.501	0.508	0.514	0.520	0.525	0.530	0.519	0.530	0.545	0.541	0.555	0.565	0.573	0.581	0.587	0.598	0.605	0.612	0.615	0.621	0.633	0.638
Santa Catarina	0.477	0.486	0.494	0.502	0.511	0.519	0.527	0.535	0.543	0.550	0.557	0.564	0.571	0.577	0.584	0.591	0.597	0.604	0.610	0.616	0.619	0.628	0.635	0.642	0.646	0.654	0.659	0.666	0.672	0.679	0.685	0.691	0.696	0.702	0.710	0.715

Appendix Table 8. Socio-Demographic Index (SDI) values for all estimated GBD 2015 locations, 1980–2015

Location	1980	1981	1982	1983	1984	1985	1986	1987	1988	1989	1990	1991	1992	1993	1994	1995	1996	1997	1998	1999	2000	2001	2002	2003	2004	2005	2006	2007	2008	2009	2010	2011	2012	2013	2014	2015
São Paulo	0.520	0.527	0.535	0.542	0.550	0.558	0.566	0.575	0.583	0.591	0.598	0.606	0.613	0.620	0.626	0.634	0.640	0.646	0.652	0.657	0.656	0.666	0.672	0.679	0.684	0.691	0.697	0.704	0.712	0.717	0.723	0.730	0.736	0.742	0.752	0.757
Sergipe	0.317	0.327	0.337	0.347	0.357	0.367	0.378	0.388	0.398	0.408	0.417	0.424	0.431	0.438	0.444	0.451	0.458	0.464	0.471	0.476	0.480	0.485	0.494	0.500	0.508	0.512	0.518	0.525	0.531	0.538	0.545	0.551	0.557	0.562	0.567	0.571
Tocantins	0.374	0.380	0.387	0.393	0.400	0.407	0.413	0.419	0.424	0.430	0.434	0.442	0.450	0.457	0.464	0.470	0.478	0.486	0.493	0.500	0.504	0.509	0.517	0.524	0.532	0.538	0.544	0.553	0.560	0.567	0.575	0.581	0.588	0.594	0.596	0.601
Paraguay	0.414	0.421	0.428	0.434	0.440	0.446	0.452	0.458	0.465	0.471	0.478	0.484	0.490	0.497	0.503	0.510	0.518	0.525	0.533	0.540	0.547	0.553	0.559	0.565	0.571	0.576	0.582	0.588	0.595	0.600	0.608	0.615	0.622	0.629	0.637	0.644
North Africa and Middle East	0.330	0.338	0.346	0.355	0.364	0.374	0.383	0.392	0.402	0.412	0.423	0.432	0.442	0.451	0.460	0.469	0.478	0.487	0.496	0.504	0.512	0.520	0.527	0.534	0.541	0.548	0.555	0.562	0.569	0.574	0.580	0.586	0.591	0.596	0.600	0.604
North Africa and Middle East	0.330	0.338	0.346	0.355	0.364	0.374	0.383	0.392	0.402	0.412	0.423	0.432	0.442	0.451	0.460	0.469	0.478	0.487	0.496	0.504	0.512	0.520	0.527	0.534	0.541	0.548	0.555	0.562	0.569	0.574	0.580	0.586	0.591	0.596	0.600	0.604
Afghanistan	0.129	0.131	0.134	0.136	0.137	0.139	0.141	0.141	0.142	0.143	0.144	0.147	0.151	0.154	0.153	0.153	0.150	0.146	0.143	0.141	0.142	0.144	0.152	0.162	0.172	0.181	0.189	0.198	0.207	0.219	0.230	0.243	0.256	0.268	0.279	0.289
Algeria	0.317	0.330	0.343	0.356	0.369	0.382	0.395	0.407	0.419	0.431	0.443	0.456	0.468	0.480	0.492	0.504	0.514	0.524	0.532	0.540	0.547	0.554	0.559	0.564	0.568	0.571	0.573	0.575	0.576	0.577	0.578	0.580	0.582	0.585	0.587	0.590
Bahrain	0.506	0.517	0.527	0.535	0.543	0.550	0.557	0.565	0.575	0.586	0.597	0.606	0.616	0.628	0.639	0.650	0.659	0.666	0.673	0.679	0.685	0.692	0.698	0.704	0.711	0.719	0.730	0.741	0.750	0.757	0.762	0.764	0.766	0.769	0.772	0.776
Egypt	0.347	0.354	0.362	0.369	0.378	0.386	0.396	0.406	0.418	0.429	0.441	0.453	0.464	0.475	0.486	0.497	0.508	0.518	0.527	0.536	0.544	0.552	0.559	0.566	0.573	0.579	0.584	0.590	0.594	0.598	0.601	0.604	0.607	0.611	0.614	0.619
Iran	0.346	0.348	0.354	0.361	0.370	0.381	0.394	0.409	0.425	0.442	0.460	0.478	0.496	0.513	0.530	0.546	0.562	0.576	0.590	0.602	0.613	0.624	0.634	0.645	0.654	0.664	0.672	0.680	0.687	0.692	0.698	0.703	0.707	0.710	0.713	0.715
Iraq	0.323	0.333	0.342	0.351	0.359	0.366	0.373	0.379	0.386	0.392	0.400	0.404	0.409	0.416	0.423	0.428	0.434	0.442	0.454	0.468	0.480	0.491	0.500	0.505	0.512	0.517	0.522	0.526	0.530	0.533	0.538	0.544	0.552	0.561	0.568	0.576
Jordan	0.362	0.382	0.401	0.419	0.437	0.453	0.463	0.473	0.481	0.489	0.497	0.513	0.529	0.543	0.555	0.566	0.571	0.577	0.583	0.589	0.595	0.602	0.609	0.616	0.623	0.630	0.638	0.646	0.653	0.661	0.667	0.673	0.678	0.684	0.689	0.695
Kuwait	0.526	0.542	0.560	0.579	0.598	0.618	0.635	0.652	0.668	0.682	0.691	0.697	0.705	0.712	0.717	0.722	0.726	0.733	0.744	0.755	0.765	0.771	0.775	0.779	0.784	0.789	0.797	0.807	0.816	0.826	0.836	0.842	0.848	0.853	0.858	0.862
Lebanon	0.501	0.508	0.513	0.518	0.528	0.539	0.549	0.560	0.566	0.568	0.570	0.576	0.583	0.589	0.596	0.603	0.609	0.617	0.625	0.634	0.642	0.650	0.659	0.669	0.678	0.687	0.694	0.701	0.707	0.713	0.720	0.728	0.735	0.742	0.749	0.755
Libya	0.358	0.372	0.384	0.396	0.407	0.418	0.429	0.440	0.451	0.462	0.475	0.488	0.501	0.514	0.526	0.536	0.545	0.554	0.562	0.569	0.576	0.582	0.587	0.594	0.600	0.607	0.614	0.621	0.628	0.635	0.641	0.640	0.643	0.644	0.644	0.643
Morocco	0.252	0.259	0.267	0.275	0.283	0.292	0.301	0.309	0.318	0.326	0.335	0.343	0.351	0.359	0.367	0.374	0.383	0.391	0.399	0.406	0.412	0.419	0.425	0.431	0.436	0.441	0.447	0.452	0.457	0.463	0.468	0.473	0.479	0.485	0.490	0.496
Palestine	0.353	0.361	0.370	0.380	0.388	0.396	0.402	0.408	0.412	0.417	0.423	0.430	0.438	0.447	0.459	0.471	0.485	0.501	0.516	0.529	0.541	0.548	0.551	0.552	0.552	0.551	0.550	0.549	0.548	0.547	0.547	0.549	0.553	0.557	0.562	0.567
Oman	0.290	0.293	0.299	0.308	0.319	0.331	0.342	0.355	0.372	0.389	0.409	0.434	0.457	0.480	0.501	0.520	0.539	0.558	0.576	0.593	0.610	0.622	0.633	0.643	0.651	0.658	0.666	0.672	0.677	0.681	0.686	0.695	0.705	0.714	0.723	0.730
Qatar	0.496	0.511	0.524	0.537	0.550	0.563	0.574	0.586	0.598	0.608	0.616	0.628	0.637	0.645	0.651	0.657	0.661	0.667	0.673	0.679	0.685	0.697	0.708	0.718	0.726	0.733	0.743	0.753	0.761	0.769	0.776	0.782	0.788	0.794	0.799	0.805
Saudi Arabia	0.400	0.418	0.435	0.452	0.468	0.483	0.491	0.499	0.506	0.515	0.525	0.533	0.543	0.552	0.562	0.573	0.588	0.603	0.618	0.631	0.643	0.653	0.662	0.672	0.681	0.690	0.699	0.707	0.716	0.723	0.730	0.737	0.744	0.749	0.755	0.759
'Asir	0.363	0.382	0.402	0.419	0.436	0.451	0.459	0.467	0.475	0.483	0.493	0.502	0.511	0.521	0.531	0.541	0.558	0.574	0.589	0.602	0.614	0.624	0.633	0.643	0.652	0.660	0.669	0.678	0.686	0.694	0.702	0.712	0.721	0.729	0.734	0.738
Bahah	0.396	0.410	0.424	0.437	0.449	0.459	0.465	0.471	0.477	0.483	0.490	0.497	0.505	0.513	0.521	0.530	0.544	0.557	0.568	0.579	0.589	0.598	0.606	0.614	0.623	0.631	0.640	0.648	0.657	0.664	0.673	0.683	0.692	0.701	0.706	0.710
Eastern Province	0.424	0.441	0.458	0.474	0.490	0.505	0.512	0.521	0.529	0.538	0.549	0.558	0.568	0.577	0.587	0.597	0.612	0.626	0.640	0.653	0.666	0.676	0.685	0.694	0.703	0.711	0.719	0.727	0.734	0.741	0.748	0.757	0.766	0.774	0.779	0.784
Ha'il	0.406	0.423	0.439	0.453	0.468	0.481	0.488	0.495	0.502	0.510	0.518	0.526	0.536	0.545	0.554	0.564	0.579	0.594	0.607	0.619	0.630	0.640	0.649	0.658	0.667	0.675	0.684	0.692	0.701	0.709	0.717	0.726	0.734	0.742	0.747	0.751
Jawf	0.298	0.329	0.359	0.385	0.410	0.431	0.443	0.454	0.465	0.476	0.489	0.501	0.513	0.526	0.538	0.552	0.572	0.591	0.609	0.625	0.639	0.651	0.661	0.672	0.682	0.693	0.703	0.714	0.724	0.733	0.743	0.755	0.766	0.777	0.783	0.788
Jizan	0.317	0.332	0.346	0.359	0.371	0.382	0.387	0.392	0.397	0.403	0.409	0.415	0.423	0.430	0.439	0.447	0.461	0.475	0.486	0.497	0.507	0.515	0.523	0.531	0.539	0.548	0.556	0.564	0.573	0.580	0.587	0.597	0.606	0.615	0.619	0.623
Madinah	0.305	0.332	0.359	0.383	0.406	0.427	0.437	0.448	0.459	0.470	0.483	0.494	0.506	0.518	0.530	0.542	0.561	0.579	0.596	0.611	0.625	0.636	0.647	0.657	0.667	0.676	0.685	0.693	0.702	0.709	0.716	0.725	0.733	0.740	0.745	0.750
Makkah	0.418	0.434	0.450	0.465	0.480	0.493	0.500	0.507	0.515	0.523	0.532	0.540	0.550	0.559	0.568	0.578	0.593	0.607	0.620	0.633	0.645	0.654	0.663	0.672	0.681	0.688	0.696	0.703	0.710	0.715	0.722	0.729	0.735	0.741	0.747	0.752
Najran	0.253	0.284	0.312	0.336	0.358	0.377	0.387	0.397	0.406	0.416	0.427	0.436	0.445	0.455	0.465	0.475	0.494	0.511	0.528	0.542	0.556	0.566	0.575	0.584	0.593	0.602	0.611	0.619	0.627	0.634	0.641	0.651	0.660	0.669	0.673	0.677
Northern Borders	0.425	0.437	0.449	0.461	0.472	0.483	0.487	0.491	0.496	0.501	0.508	0.514	0.521	0.530	0.540	0.550	0.566	0.581	0.595	0.609	0.621	0.631	0.640	0.649	0.659	0.675	0.692	0.707	0.719	0.724	0.725	0.722	0.718	0.713	0.718	0.723
Qassim	0.360	0.380	0.402	0.422	0.442	0.461	0.473	0.485	0.496	0.508	0.521	0.532	0.543	0.554	0.566	0.577	0.595	0.612	0.628	0.642	0.656	0.666	0.676	0.686	0.696	0.705	0.713	0.720	0.727	0.735	0.743	0.752	0.760	0.768	0.773	0.777
Riyadh	0.500	0.510	0.521	0.533	0.546	0.558	0.563	0.567	0.572	0.578	0.585	0.592	0.599	0.608	0.618	0.628	0.642	0.656	0.670	0.683	0.696	0.705	0.714	0.723	0.733	0.742	0.750	0.758	0.764	0.769	0.773	0.777	0.781	0.785	0.790	0.794
Tabuk	0.351	0.368	0.387	0.406	0.425	0.442	0.448	0.455	0.463	0.472	0.482	0.489	0.497	0.509	0.521	0.534	0.556	0.577	0.596	0.614	0.631	0.643	0.654	0.665	0.676	0.701	0.725	0.745	0.757	0.761	0.759	0.755	0.749	0.742	0.748	0.753
Sudan	0.232	0.236	0.242	0.246	0.249	0.252	0.254	0.257	0.260	0.263	0.267	0.271	0.276	0.280	0.285	0.289	0.292	0.297	0.303	0.310	0.319	0.327	0.335	0.343	0.351	0.358	0.366	0.374	0.382	0.390	0.397	0.405	0.411	0.417	0.422	0.428
Syria	0.295	0.304	0.313	0.323	0.332	0.341	0.350	0.359	0.369	0.379	0.388	0.398	0.408	0.419	0.429	0.439	0.449	0.457	0.466	0.475	0.483	0.490	0.498	0.505	0.514	0.525	0.538	0.550	0.561	0.572	0.581	0.584	0.584	0.582	0.580	0.579
Tunisia	0.341	0.352	0.362	0.373	0.383	0.394	0.404	0.416	0.427	0.438	0.450	0.463	0.476	0.489	0.502	0.514	0.525	0.536	0.546	0.555	0.564	0.573	0.580	0.587	0.594	0.601	0.607	0.614	0.619	0.625	0.630	0.634	0.639	0.643	0.647	0.652
Turkey	0.442	0.449	0.457	0.465	0.473	0.481	0.490	0.499	0.508	0.516	0.524	0.532	0.539	0.547	0.553	0.559	0.567	0.574	0.581	0.587	0.594	0.599	0.605	0.611	0.618	0.625	0.632	0.639	0.646	0.652	0.658	0.665	0.672	0.678	0.684	0.690
United Arab Emirates	0.534	0.545	0.555	0.566	0.576	0.587	0.594	0.602	0.610	0.620	0.632	0.651	0.670	0.690	0.709	0.727	0.739	0.751	0.762	0.772	0.781	0.790	0.798	0.806	0.815	0.824	0.834	0.844	0.854	0.862	0.869	0.872	0.875	0.874	0.874	0.875
Yemen	0.096	0.097	0.099	0.102	0.105	0.108	0.110	0.113	0.117	0.124	0.133	0.146	0.160	0.172	0.184	0.196	0.209	0.223	0.235	0.248	0.259	0.271	0.283	0.295	0.307	0.320	0.332	0.344	0.356	0.366	0.377	0.384	0.392	0.399	0.405	0.408
South Asia	0.242	0.247	0.252	0.258	0.264	0.270	0.277	0.284	0.292	0.301	0.310	0.318	0.326	0.335	0.343	0.352	0.361	0.370	0.378	0.387	0.396	0.405	0.414	0.422	0.431	0.441	0.451	0.461	0.471	0.481	0.491	0.500	0.510	0.519	0.528	0.536
South Asia	0.242	0.247	0.252	0.258	0.264	0.270	0.277	0.284	0.292	0.301	0.310	0.318	0.326	0.335	0.343	0.352	0.361	0.370	0.378	0.387	0.396	0.405	0.414	0.422	0.431	0.441	0.451	0.461	0.471	0.481	0.491	0.500	0.510	0.519	0.528	0.536
Bangladesh	0.199	0.205	0.212	0.219	0.227	0.235	0.243	0.251	0.258	0.266	0.275	0.283	0.290	0.298	0.306	0.314	0.322	0.329	0.337	0.344	0.351	0.359	0.366	0.373	0.381	0.389	0.397	0.406	0.414	0.422	0.431	0.439	0.447	0.456	0.464	0.472

Appendix Table 8. Socio-Demographic Index (SDI) values for all estimated GBD 2015 locations, 1980–2015

Location	1980	1981	1982	1983	1984	1985	1986	1987	1988	1989	1990	1991	1992	1993	1994	1995	1996	1997	1998	1999	2000	2001	2002	2003	2004	2005	2006	2007	2008	2009	2010	2011	2012	2013	2014	2015
Bhutan	0.204	0.208	0.212	0.218	0.223	0.230	0.237	0.248	0.259	0.270	0.282	0.291	0.299	0.308	0.317	0.327	0.338	0.350	0.362	0.374	0.386	0.398	0.409	0.420	0.431	0.441	0.450	0.460	0.470	0.480	0.490	0.500	0.508	0.517	0.524	0.532
India	0.252	0.257	0.262	0.267	0.273	0.280	0.286	0.293	0.302	0.311	0.320	0.328	0.336	0.345	0.354	0.362	0.372	0.381	0.390	0.399	0.408	0.417	0.426	0.435	0.445	0.454	0.465	0.476	0.487	0.497	0.508	0.519	0.529	0.538	0.548	0.556
Andhra Pradesh	0.214	0.219	0.225	0.231	0.237	0.244	0.250	0.258	0.267	0.278	0.289	0.300	0.310	0.322	0.334	0.348	0.361	0.372	0.386	0.399	0.413	0.426	0.439	0.452	0.465	0.477	0.490	0.505	0.519	0.533	0.548	0.562	0.575	0.587	0.598	0.608
Andhra Pradesh, Rural	0.175	0.180	0.185	0.190	0.195	0.201	0.207	0.214	0.223	0.232	0.243	0.253	0.263	0.275	0.287	0.300	0.313	0.326	0.340	0.353	0.368	0.382	0.396	0.409	0.422	0.434	0.448	0.462	0.476	0.488	0.502	0.515	0.528	0.539	0.550	0.560
Andhra Pradesh, Urban	0.347	0.353	0.358	0.364	0.369	0.375	0.380	0.387	0.396	0.406	0.417	0.427	0.436	0.447	0.459	0.471	0.483	0.494	0.506	0.518	0.531	0.543	0.553	0.564	0.574	0.583	0.594	0.606	0.618	0.629	0.643	0.656	0.668	0.681	0.693	0.705
Arunāchal Pradesh	0.191	0.199	0.208	0.217	0.226	0.237	0.248	0.259	0.270	0.280	0.291	0.302	0.313	0.325	0.337	0.349	0.361	0.372	0.383	0.393	0.404	0.414	0.424	0.434	0.445	0.456	0.466	0.477	0.488	0.503	0.517	0.530	0.542	0.553	0.562	0.571
Arunāchal Pradesh, Rural	0.184	0.191	0.199	0.207	0.216	0.226	0.236	0.246	0.256	0.265	0.275	0.285	0.295	0.306	0.316	0.328	0.338	0.349	0.359	0.368	0.378	0.388	0.397	0.406	0.417	0.428	0.438	0.448	0.459	0.473	0.486	0.499	0.509	0.519	0.527	0.534
Arunāchal Pradesh, Urban	0.297	0.309	0.320	0.332	0.343	0.356	0.369	0.381	0.393	0.404	0.415	0.426	0.437	0.449	0.459	0.471	0.481	0.491	0.499	0.507	0.515	0.524	0.532	0.540	0.550	0.559	0.569	0.579	0.589	0.603	0.617	0.630	0.641	0.652	0.663	0.672
Assam	0.235	0.242	0.249	0.257	0.266	0.275	0.284	0.292	0.300	0.309	0.318	0.325	0.332	0.338	0.345	0.352	0.358	0.364	0.371	0.381	0.391	0.400	0.410	0.419	0.427	0.436	0.445	0.453	0.461	0.469	0.478	0.487	0.495	0.502	0.510	0.518
Assam, Rural	0.219	0.226	0.233	0.241	0.250	0.258	0.267	0.275	0.283	0.292	0.301	0.308	0.314	0.321	0.327	0.334	0.340	0.346	0.353	0.363	0.373	0.382	0.391	0.400	0.409	0.417	0.426	0.435	0.443	0.451	0.459	0.468	0.475	0.482	0.489	0.497
Assam, Urban	0.379	0.386	0.394	0.402	0.411	0.420	0.428	0.437	0.444	0.452	0.460	0.466	0.471	0.476	0.481	0.486	0.491	0.495	0.500	0.508	0.516	0.524	0.532	0.539	0.546	0.554	0.562	0.570	0.577	0.585	0.595	0.604	0.613	0.623	0.633	0.644
Bihār	0.190	0.194	0.198	0.203	0.209	0.215	0.222	0.229	0.237	0.244	0.252	0.259	0.266	0.269	0.273	0.274	0.278	0.281	0.280	0.281	0.285	0.289	0.295	0.300	0.307	0.313	0.325	0.337	0.352	0.364	0.377	0.391	0.405	0.418	0.431	0.442
Bihār, Rural	0.172	0.176	0.180	0.184	0.189	0.195	0.202	0.208	0.215	0.222	0.230	0.237	0.244	0.247	0.251	0.253	0.257	0.261	0.261	0.262	0.267	0.271	0.277	0.282	0.289	0.296	0.308	0.320	0.335	0.347	0.360	0.374	0.388	0.401	0.413	0.424
Bihār, Urban	0.335	0.340	0.345	0.350	0.357	0.363	0.371	0.378	0.386	0.394	0.402	0.410	0.416	0.420	0.423	0.425	0.429	0.433	0.432	0.434	0.437	0.440	0.445	0.448	0.453	0.458	0.467	0.478	0.490	0.501	0.514	0.526	0.540	0.553	0.565	0.577
Chhattīsgarh	0.251	0.256	0.261	0.266	0.271	0.276	0.281	0.287	0.293	0.299	0.306	0.312	0.319	0.326	0.332	0.339	0.346	0.353	0.361	0.369	0.375	0.383	0.391	0.401	0.411	0.421	0.434	0.447	0.460	0.471	0.481	0.491	0.500	0.509	0.516	0.523
Chhattīsgarh, Rural	0.219	0.225	0.229	0.234	0.239	0.244	0.249	0.254	0.260	0.266	0.273	0.279	0.286	0.292	0.299	0.305	0.312	0.320	0.327	0.335	0.341	0.349	0.357	0.366	0.376	0.386	0.398	0.411	0.424	0.434	0.444	0.454	0.462	0.471	0.478	0.485
Chhattīsgarh, Urban	0.394	0.399	0.404	0.409	0.413	0.418	0.423	0.428	0.434	0.440	0.447	0.453	0.459	0.466	0.472	0.478	0.484	0.491	0.498	0.505	0.510	0.517	0.524	0.533	0.541	0.551	0.561	0.573	0.585	0.595	0.604	0.614	0.623	0.632	0.640	0.649
Delhi	0.436	0.442	0.448	0.453	0.459	0.466	0.473	0.481	0.489	0.497	0.504	0.513	0.522	0.530	0.539	0.546	0.554	0.563	0.572	0.580	0.589	0.597	0.606	0.615	0.624	0.634	0.644	0.655	0.666	0.678	0.691	0.703	0.716	0.728	0.740	0.753
Delhi, Rural	0.354	0.360	0.367	0.374	0.381	0.389	0.398	0.407	0.417	0.427	0.437	0.448	0.458	0.469	0.479	0.489	0.498	0.509	0.520	0.530	0.541	0.551	0.561	0.571	0.581	0.591	0.602	0.612	0.623	0.634	0.645	0.656	0.666	0.675	0.684	0.693
Delhi, Urban	0.444	0.449	0.455	0.461	0.466	0.473	0.480	0.487	0.496	0.504	0.511	0.520	0.528	0.536	0.544	0.551	0.558	0.567	0.576	0.584	0.593	0.601	0.609	0.617	0.626	0.636	0.646	0.657	0.668	0.680	0.692	0.705	0.717	0.730	0.742	0.755
Goa	0.434	0.438	0.443	0.448	0.454	0.458	0.464	0.469	0.475	0.482	0.489	0.496	0.504	0.513	0.521	0.531	0.541	0.550	0.563	0.574	0.585	0.595	0.604	0.612	0.621	0.631	0.640	0.649	0.661	0.672	0.683	0.694	0.703	0.712	0.721	0.730
Goa, Rural	0.421	0.424	0.429	0.432	0.438	0.441	0.446	0.451	0.456	0.462	0.468	0.474	0.482	0.490	0.499	0.508	0.517	0.526	0.539	0.549	0.559	0.569	0.578	0.585	0.593	0.602	0.611	0.620	0.631	0.641	0.650	0.661	0.669	0.676	0.685	0.693
Goa, Urban	0.466	0.471	0.476	0.480	0.486	0.490	0.496	0.501	0.507	0.514	0.520	0.527	0.535	0.543	0.551	0.561	0.570	0.579	0.591	0.602	0.612	0.621	0.630	0.637	0.645	0.654	0.663	0.672	0.683	0.694	0.704	0.715	0.723	0.732	0.741	0.750
Gujarāt	0.281	0.287	0.293	0.302	0.309	0.316	0.324	0.330	0.340	0.350	0.359	0.366	0.376	0.386	0.398	0.409	0.421	0.431	0.441	0.451	0.458	0.466	0.475	0.484	0.493	0.503	0.513	0.524	0.533	0.543	0.553	0.563	0.572	0.580	0.588	0.596
Gujarāt, Rural	0.236	0.241	0.247	0.255	0.262	0.268	0.275	0.282	0.291	0.300	0.309	0.316	0.326	0.336	0.347	0.358	0.369	0.380	0.390	0.399	0.407	0.415	0.423	0.432	0.440	0.450	0.459	0.469	0.478	0.488	0.497	0.506	0.514	0.521	0.528	0.535
Gujarāt, Urban	0.382	0.388	0.394	0.402	0.410	0.416	0.423	0.429	0.438	0.447	0.455	0.462	0.471	0.480	0.491	0.501	0.512	0.521	0.531	0.539	0.546	0.552	0.559	0.567	0.574	0.583	0.592	0.601	0.610	0.620	0.630	0.640	0.649	0.658	0.667	0.677
Haryāna	0.269	0.277	0.284	0.292	0.300	0.309	0.318	0.328	0.339	0.350	0.362	0.374	0.385	0.397	0.409	0.420	0.433	0.445	0.457	0.469	0.481	0.494	0.505	0.517	0.528	0.540	0.551	0.563	0.575	0.586	0.598	0.608	0.618	0.627	0.635	0.643
Haryāna, Rural	0.238	0.245	0.253	0.260	0.267	0.276	0.285	0.294	0.305	0.315	0.327	0.339	0.349	0.361	0.372	0.384	0.397	0.409	0.421	0.434	0.446	0.458	0.470	0.481	0.492	0.503	0.514	0.526	0.537	0.549	0.560	0.570	0.579	0.587	0.594	0.600
Haryāna, Urban	0.394	0.400	0.407	0.414	0.420	0.428	0.436	0.444	0.454	0.464	0.474	0.485	0.494	0.503	0.513	0.523	0.534	0.543	0.552	0.562	0.571	0.580	0.589	0.597	0.605	0.614	0.623	0.634	0.645	0.656	0.667	0.678	0.688	0.697	0.707	0.716
Himachal Pradesh	0.288	0.294	0.299	0.305	0.309	0.315	0.322	0.329	0.339	0.349	0.360	0.369	0.380	0.390	0.401	0.412	0.425	0.437	0.451	0.465	0.480	0.493	0.505	0.517	0.528	0.538	0.549	0.559	0.569	0.579	0.589	0.600	0.610	0.619	0.628	0.636
Himachal Pradesh, Rural	0.278	0.283	0.288	0.294	0.298	0.304	0.311	0.318	0.328	0.338	0.348	0.358	0.368	0.378	0.389	0.400	0.413	0.426	0.439	0.454	0.469	0.482	0.494	0.506	0.517	0.528	0.538	0.548	0.558	0.568	0.578	0.588	0.597	0.606	0.614	0.622
Himachal Pradesh, Urban	0.422	0.427	0.432	0.437	0.441	0.447	0.453	0.459	0.468	0.477	0.485	0.493	0.502	0.510	0.519	0.528	0.539	0.549	0.560	0.573	0.585	0.597	0.607	0.617	0.627	0.637	0.647	0.657	0.667	0.678	0.691	0.704	0.717	0.730	0.744	0.758
Jammu and Kashmīr	0.259	0.264	0.270	0.276	0.282	0.289	0.297	0.302	0.309	0.316	0.322	0.327	0.333	0.344	0.354	0.366	0.377	0.393	0.407	0.425	0.440	0.453	0.465	0.475	0.485	0.493	0.501	0.508	0.516	0.523	0.531	0.540	0.548	0.557	0.566	0.574
Jammu and Kashmīr, Rural	0.229	0.234	0.239	0.245	0.251	0.257	0.264	0.270	0.277	0.283	0.289	0.294	0.299	0.311	0.321	0.332	0.344	0.360	0.374	0.392	0.408	0.421	0.433	0.444	0.453	0.461	0.469	0.476	0.484	0.491	0.498	0.506	0.514	0.522	0.530	0.538
Jammu and Kashmīr, Urban	0.376	0.381	0.387	0.393	0.400	0.407	0.414	0.419	0.426	0.432	0.437	0.441	0.445	0.455	0.464	0.474	0.483	0.498	0.510	0.525	0.539	0.550	0.560	0.568	0.577	0.583	0.590	0.597	0.604	0.611	0.619	0.628	0.638	0.647	0.657	0.668
Jharkhand	0.282	0.286	0.289	0.293	0.296	0.300	0.304	0.308	0.314	0.320	0.326	0.331	0.337	0.342	0.347	0.352	0.356	0.364	0.370	0.376	0.381	0.386	0.392	0.398	0.406	0.415	0.423	0.432	0.439	0.448	0.458	0.466	0.473	0.480	0.488	0.495
Jharkhand, Rural	0.266	0.270	0.273	0.276	0.279	0.282	0.286	0.290	0.295	0.300	0.306	0.311	0.317	0.322	0.328	0.333	0.337	0.345	0.352	0.359	0.363	0.369	0.374	0.381	0.389	0.397	0.405	0.414	0.421	0.430	0.439	0.447	0.454	0.460	0.467	0.474
Jharkhand, Urban	0.406	0.411	0.415	0.420	0.424	0.428	0.433	0.438	0.443	0.450	0.456	0.462	0.469	0.476	0.482	0.489	0.495	0.504	0.512	0.520	0.526	0.532	0.539	0.545	0.553	0.562	0.570	0.579	0.587	0.596	0.607	0.616	0.624	0.632	0.641	0.650
Karnātaka	0.260	0.265	0.270	0.276	0.283	0.289	0.297	0.305	0.314	0.323	0.333	0.343	0.353	0.364	0.374	0.385	0.397	0.408	0.420	0.432	0.444	0.455	0.465	0.475	0.485	0.497	0.508	0.520	0.530	0.540	0.552	0.562	0.571	0.579	0.588	0.596
Karnātaka, Rural	0.216	0.220	0.225	0.231	0.237	0.243	0.250	0.258	0.266	0.275	0.284	0.294	0.304	0.314	0.324	0.335	0.346	0.356	0.369	0.381	0.394	0.405	0.416	0.426	0.438	0.450	0.461	0.472	0.482	0.491	0.500	0.509	0.517	0.525	0.533	0.540
Karnātaka, Urban	0.375	0.380	0.385	0.391	0.397	0.403	0.411	0.418	0.426	0.435	0.444	0.453	0.462	0.471	0.480	0.490	0.499	0.509	0.520	0.531	0.542	0.552	0.561	0.570	0.579	0.590	0.600	0.610	0.619	0.627	0.637	0.646	0.656	0.665	0.676	0.686
Kerala	0.342	0.345	0.351	0.357	0.364	0.370	0.377	0.385	0.393	0.401	0.409	0.418	0.426	0.435	0.445	0.456	0.466	0.477	0.487	0.498	0.508	0.517	0.525	0.533	0.542	0.551	0.560	0.570	0.579	0.589	0.599	0.609	0.619	0.628	0.638	0.648
Kerala, Rural	0.332	0.335	0.340	0.346	0.353	0.359	0.367	0.374	0.382	0.390	0.398	0.407	0.415	0.424	0.434	0.445	0.456	0.466	0.477	0.489	0.499	0.508	0.517	0.524	0.532	0.541	0.550	0.558	0.567	0.576	0.584	0.593	0.602	0.610	0.620	0.629

Appendix Table 8. Socio-Demographic Index (SDI) values for all estimated GBD 2015 locations, 1980–2015

Location	1980	1981	1982	1983	1984	1985	1986	1987	1988	1989	1990	1991	1992	1993	1994	1995	1996	1997	1998	1999	2000	2001	2002	2003	2004	2005	2006	2007	2008	2009	2010	2011	2012	2013	2014	2015
Kerala, Urban	0.380	0.383	0.388	0.394	0.400	0.405	0.412	0.419	0.426	0.433	0.440	0.448	0.456	0.464	0.473	0.483	0.493	0.502	0.513	0.523	0.533	0.541	0.549	0.557	0.564	0.573	0.582	0.590	0.599	0.608	0.617	0.627	0.637	0.648	0.659	0.672
Madhya Pradesh	0.207	0.211	0.217	0.223	0.228	0.235	0.241	0.249	0.257	0.267	0.277	0.286	0.294	0.303	0.310	0.319	0.327	0.336	0.343	0.352	0.360	0.368	0.376	0.385	0.394	0.402	0.412	0.421	0.431	0.442	0.452	0.463	0.474	0.485	0.496	0.506
Madhya Pradesh, Rural	0.164	0.168	0.172	0.177	0.182	0.187	0.192	0.199	0.207	0.216	0.225	0.234	0.242	0.250	0.258	0.266	0.275	0.283	0.290	0.298	0.305	0.313	0.320	0.329	0.337	0.346	0.355	0.365	0.375	0.387	0.398	0.409	0.420	0.431	0.441	0.450
Madhya Pradesh, Urban	0.387	0.392	0.397	0.402	0.407	0.412	0.417	0.425	0.433	0.441	0.451	0.459	0.467	0.474	0.481	0.488	0.495	0.501	0.506	0.512	0.516	0.522	0.525	0.531	0.536	0.542	0.549	0.557	0.567	0.578	0.589	0.601	0.612	0.624	0.635	0.646
Mahārāshtra	0.313	0.319	0.324	0.329	0.335	0.341	0.348	0.355	0.364	0.374	0.384	0.394	0.404	0.415	0.425	0.436	0.447	0.457	0.466	0.475	0.484	0.492	0.501	0.510	0.519	0.530	0.540	0.551	0.562	0.572	0.582	0.592	0.601	0.609	0.617	0.625
Mahārāshtra, Rural	0.251	0.256	0.260	0.266	0.271	0.277	0.283	0.290	0.299	0.309	0.318	0.327	0.338	0.348	0.359	0.370	0.381	0.391	0.401	0.411	0.421	0.431	0.440	0.450	0.461	0.471	0.483	0.494	0.504	0.513	0.523	0.532	0.540	0.547	0.554	0.561
Mahārāshtra, Urban	0.432	0.437	0.441	0.446	0.451	0.456	0.461	0.467	0.475	0.483	0.492	0.499	0.507	0.516	0.524	0.532	0.541	0.548	0.555	0.562	0.569	0.576	0.582	0.589	0.597	0.605	0.615	0.625	0.634	0.643	0.654	0.664	0.673	0.683	0.693	0.703
Manipur	0.297	0.304	0.309	0.316	0.323	0.330	0.338	0.348	0.359	0.367	0.376	0.384	0.392	0.400	0.407	0.414	0.422	0.430	0.438	0.450	0.459	0.468	0.476	0.484	0.492	0.502	0.510	0.517	0.523	0.530	0.536	0.544	0.552	0.560	0.568	0.576
Manipur, Rural	0.284	0.289	0.294	0.300	0.307	0.313	0.321	0.330	0.340	0.348	0.356	0.364	0.372	0.380	0.387	0.395	0.403	0.411	0.419	0.431	0.440	0.449	0.457	0.465	0.473	0.482	0.489	0.496	0.502	0.508	0.514	0.521	0.527	0.534	0.541	0.548
Manipur, Urban	0.363	0.369	0.374	0.380	0.387	0.392	0.400	0.409	0.418	0.426	0.433	0.440	0.447	0.455	0.462	0.469	0.476	0.483	0.491	0.502	0.511	0.520	0.527	0.534	0.542	0.551	0.559	0.566	0.571	0.579	0.585	0.593	0.601	0.609	0.617	0.627
Meghālaya	0.231	0.234	0.238	0.242	0.246	0.252	0.258	0.265	0.271	0.281	0.290	0.298	0.305	0.313	0.321	0.329	0.337	0.345	0.354	0.363	0.373	0.383	0.392	0.400	0.408	0.416	0.424	0.432	0.440	0.449	0.456	0.464	0.470	0.477	0.484	0.491
Meghālaya, Rural	0.201	0.204	0.207	0.210	0.214	0.219	0.224	0.231	0.237	0.246	0.254	0.262	0.269	0.277	0.284	0.292	0.299	0.308	0.316	0.325	0.335	0.345	0.353	0.361	0.369	0.377	0.385	0.393	0.401	0.408	0.415	0.422	0.428	0.433	0.439	0.444
Meghālaya, Urban	0.385	0.389	0.393	0.398	0.404	0.410	0.416	0.424	0.431	0.441	0.451	0.459	0.466	0.474	0.481	0.489	0.497	0.505	0.513	0.522	0.532	0.541	0.549	0.557	0.565	0.574	0.582	0.591	0.600	0.610	0.619	0.629	0.637	0.647	0.657	0.667
Mizoram	0.287	0.290	0.294	0.299	0.307	0.320	0.332	0.348	0.359	0.367	0.375	0.383	0.392	0.401	0.407	0.415	0.426	0.433	0.439	0.447	0.456	0.464	0.472	0.479	0.486	0.491	0.497	0.503	0.509	0.516	0.524	0.532	0.539	0.547	0.556	0.564
Mizoram, Rural	0.238	0.240	0.243	0.248	0.255	0.267	0.279	0.294	0.304	0.312	0.319	0.327	0.335	0.344	0.350	0.358	0.368	0.375	0.381	0.388	0.397	0.405	0.413	0.419	0.425	0.430	0.435	0.440	0.446	0.452	0.459	0.465	0.471	0.477	0.485	0.492
Mizoram, Urban	0.351	0.355	0.359	0.364	0.372	0.385	0.398	0.414	0.424	0.433	0.440	0.449	0.457	0.465	0.471	0.479	0.489	0.495	0.501	0.508	0.516	0.525	0.532	0.539	0.545	0.551	0.556	0.562	0.569	0.577	0.585	0.593	0.601	0.610	0.619	0.629
Nāgāland	0.307	0.312	0.320	0.327	0.334	0.342	0.349	0.359	0.369	0.378	0.388	0.396	0.405	0.416	0.426	0.435	0.444	0.453	0.459	0.464	0.473	0.482	0.492	0.499	0.507	0.515	0.522	0.531	0.541	0.549	0.558	0.567	0.575	0.583	0.591	0.599
Nāgāland, Rural	0.297	0.302	0.309	0.316	0.323	0.330	0.337	0.346	0.356	0.365	0.374	0.382	0.391	0.401	0.411	0.421	0.429	0.438	0.444	0.450	0.459	0.467	0.476	0.483	0.490	0.496	0.502	0.510	0.517	0.524	0.531	0.538	0.545	0.551	0.557	0.564
Nāgāland, Urban	0.380	0.387	0.394	0.401	0.409	0.416	0.423	0.433	0.443	0.452	0.461	0.469	0.477	0.487	0.497	0.505	0.514	0.522	0.527	0.532	0.541	0.550	0.559	0.567	0.575	0.583	0.591	0.600	0.610	0.620	0.629	0.640	0.649	0.658	0.668	0.679
Orissa	0.204	0.209	0.213	0.219	0.224	0.231	0.238	0.243	0.252	0.260	0.267	0.275	0.282	0.290	0.299	0.309	0.317	0.326	0.337	0.348	0.358	0.368	0.376	0.387	0.399	0.410	0.422	0.437	0.449	0.460	0.472	0.482	0.492	0.501	0.509	0.517
Orissa, Rural	0.186	0.190	0.193	0.199	0.204	0.210	0.216	0.222	0.229	0.238	0.243	0.251	0.258	0.265	0.274	0.284	0.292	0.301	0.311	0.322	0.332	0.342	0.351	0.362	0.374	0.385	0.398	0.412	0.424	0.435	0.446	0.455	0.465	0.473	0.481	0.489
Orissa, Urban	0.365	0.369	0.373	0.380	0.384	0.391	0.397	0.402	0.410	0.418	0.423	0.430	0.436	0.443	0.451	0.460	0.467	0.475	0.484	0.494	0.503	0.511	0.518	0.528	0.538	0.548	0.559	0.573	0.584	0.595	0.606	0.616	0.626	0.635	0.645	0.654
Punjab	0.322	0.328	0.335	0.342	0.350	0.358	0.367	0.376	0.386	0.397	0.407	0.418	0.428	0.438	0.448	0.458	0.468	0.478	0.487	0.497	0.508	0.517	0.526	0.534	0.542	0.551	0.559	0.569	0.579	0.589	0.598	0.607	0.616	0.624	0.632	0.640
Punjab, Rural	0.288	0.295	0.301	0.308	0.316	0.324	0.332	0.342	0.351	0.362	0.372	0.382	0.393	0.403	0.412	0.422	0.432	0.442	0.451	0.462	0.472	0.483	0.492	0.501	0.510	0.519	0.528	0.538	0.548	0.557	0.566	0.575	0.583	0.590	0.597	0.604
Punjab, Urban	0.415	0.422	0.428	0.434	0.441	0.449	0.457	0.465	0.474	0.484	0.493	0.502	0.511	0.520	0.528	0.536	0.544	0.552	0.560	0.568	0.577	0.585	0.591	0.598	0.604	0.611	0.619	0.627	0.636	0.644	0.653	0.663	0.671	0.680	0.690	0.699
Rājasthān	0.182	0.186	0.191	0.198	0.204	0.209	0.216	0.222	0.231	0.240	0.251	0.260	0.270	0.280	0.291	0.301	0.313	0.324	0.334	0.345	0.356	0.367	0.376	0.388	0.398	0.409	0.421	0.433	0.445	0.456	0.470	0.483	0.494	0.505	0.514	0.523
Rājasthān, Rural	0.145	0.149	0.153	0.159	0.164	0.168	0.174	0.179	0.188	0.196	0.206	0.215	0.224	0.233	0.244	0.254	0.266	0.276	0.286	0.297	0.307	0.319	0.328	0.340	0.351	0.362	0.375	0.387	0.399	0.410	0.423	0.436	0.447	0.457	0.466	0.474
Rājasthān, Urban	0.328	0.334	0.340	0.347	0.354	0.360	0.367	0.374	0.384	0.393	0.405	0.415	0.425	0.435	0.446	0.458	0.470	0.481	0.491	0.503	0.513	0.524	0.532	0.542	0.551	0.560	0.570	0.580	0.591	0.600	0.612	0.625	0.636	0.647	0.657	0.667
Sikkim	0.263	0.267	0.273	0.278	0.287	0.296	0.306	0.317	0.326	0.335	0.344	0.351	0.357	0.365	0.374	0.382	0.391	0.400	0.409	0.418	0.428	0.438	0.449	0.459	0.469	0.479	0.489	0.499	0.509	0.531	0.549	0.566	0.580	0.593	0.603	0.613
Sikkim, Rural	0.254	0.258	0.263	0.268	0.276	0.285	0.295	0.305	0.315	0.323	0.332	0.339	0.344	0.352	0.360	0.368	0.377	0.385	0.394	0.403	0.412	0.422	0.431	0.441	0.450	0.459	0.467	0.476	0.484	0.505	0.521	0.535	0.548	0.559	0.569	0.578
Sikkim, Urban	0.367	0.371	0.377	0.383	0.392	0.402	0.413	0.424	0.434	0.444	0.453	0.460	0.465	0.473	0.481	0.489	0.498	0.507	0.516	0.525	0.535	0.544	0.554	0.564	0.573	0.583	0.592	0.602	0.612	0.635	0.654	0.672	0.688	0.703	0.717	0.730
Tamil Nādu	0.280	0.286	0.290	0.296	0.303	0.310	0.319	0.328	0.338	0.348	0.359	0.369	0.379	0.392	0.404	0.416	0.428	0.440	0.452	0.465	0.477	0.487	0.496	0.505	0.514	0.525	0.536	0.547	0.556	0.568	0.580	0.591	0.601	0.611	0.620	0.629
Tamil Nādu, Rural	0.237	0.242	0.247	0.252	0.259	0.267	0.275	0.285	0.294	0.305	0.315	0.324	0.334	0.345	0.356	0.367	0.378	0.390	0.402	0.415	0.428	0.439	0.449	0.460	0.470	0.482	0.494	0.505	0.514	0.525	0.535	0.545	0.554	0.564	0.572	0.581
Tamil Nādu, Urban	0.369	0.374	0.379	0.385	0.392	0.400	0.408	0.418	0.427	0.437	0.446	0.454	0.463	0.472	0.481	0.490	0.499	0.508	0.518	0.529	0.539	0.549	0.557	0.565	0.574	0.584	0.594	0.603	0.611	0.621	0.631	0.641	0.651	0.661	0.672	0.683
Telangana	0.272	0.278	0.283	0.289	0.294	0.300	0.306	0.313	0.320	0.328	0.336	0.344	0.353	0.361	0.370	0.380	0.391	0.401	0.412	0.423	0.434	0.445	0.455	0.466	0.476	0.487	0.497	0.507	0.516	0.524	0.531	0.538	0.543	0.548	0.552	0.557
Telangana, Rural	0.228	0.233	0.238	0.243	0.248	0.253	0.258	0.265	0.271	0.279	0.287	0.294	0.303	0.312	0.321	0.331	0.342	0.353	0.365	0.377	0.389	0.400	0.411	0.422	0.433	0.444	0.454	0.464	0.473	0.480	0.486	0.492	0.497	0.501	0.506	0.510
Telangana, Urban	0.424	0.429	0.433	0.437	0.441	0.445	0.449	0.454	0.460	0.466	0.473	0.479	0.486	0.493	0.500	0.508	0.517	0.526	0.536	0.545	0.554	0.563	0.571	0.578	0.586	0.593	0.601	0.608	0.615	0.620	0.625	0.631	0.635	0.640	0.645	0.650
Tripura	0.254	0.259	0.265	0.270	0.275	0.280	0.285	0.291	0.299	0.307	0.314	0.320	0.324	0.332	0.337	0.345	0.353	0.362	0.371	0.388	0.404	0.420	0.433	0.445	0.456	0.466	0.475	0.482	0.490	0.497	0.505	0.513	0.520	0.527	0.534	0.541
Tripura, Rural	0.238	0.243	0.248	0.253	0.257	0.262	0.267	0.273	0.281	0.289	0.296	0.301	0.306	0.313	0.319	0.326	0.334	0.343	0.352	0.369	0.385	0.400	0.413	0.424	0.434	0.443	0.451	0.457	0.464	0.470	0.476	0.483	0.489	0.494	0.501	0.507
Tripura, Urban	0.365	0.370	0.375	0.379	0.384	0.388	0.393	0.398	0.406	0.413	0.419	0.423	0.427	0.433	0.437	0.443	0.451	0.458	0.466	0.482	0.497	0.512	0.524	0.536	0.546	0.555	0.563	0.570	0.577	0.584	0.591	0.599	0.607	0.615	0.625	0.635
Uttar Pradesh	0.210	0.214	0.218	0.223	0.227	0.233	0.239	0.245	0.253	0.262	0.271	0.280	0.288	0.295	0.303	0.311	0.319	0.327	0.335	0.343	0.352	0.361	0.370	0.381	0.392	0.403	0.414	0.426	0.437	0.449	0.460	0.471	0.482	0.492	0.502	0.511
Uttar Pradesh, Rural	0.186	0.188	0.192	0.196	0.200	0.205	0.210	0.216	0.223	0.231	0.239	0.247	0.255	0.262	0.270	0.277	0.285	0.293	0.301	0.309	0.318	0.327	0.337	0.348	0.359	0.371	0.383	0.395	0.406	0.418	0.430	0.441	0.451	0.461	0.471	0.480
Uttar Pradesh, Urban	0.336	0.340	0.345	0.350	0.356	0.362	0.368	0.375	0.383	0.392	0.401	0.409	0.417	0.425	0.432	0.440	0.449	0.457	0.465	0.474	0.482	0.489	0.497	0.505	0.512	0.521	0.529	0.539	0.548	0.558	0.568	0.578	0.588	0.598	0.607	0.617

Appendix Table 8. Socio-Demographic Index (SDI) values for all estimated GBD 2015 locations, 1980–2015

Location	1980	1981	1982	1983	1984	1985	1986	1987	1988	1989	1990	1991	1992	1993	1994	1995	1996	1997	1998	1999	2000	2001	2002	2003	2004	2005	2006	2007	2008	2009	2010	2011	2012	2013	2014	2015
Uttarakhand	0.294	0.301	0.307	0.313	0.319	0.326	0.332	0.339	0.346	0.354	0.362	0.370	0.377	0.385	0.394	0.402	0.410	0.419	0.426	0.436	0.446	0.457	0.468	0.479	0.491	0.504	0.518	0.532	0.545	0.562	0.577	0.591	0.602	0.613	0.622	0.631
Uttarakhand, Rural	0.269	0.276	0.282	0.288	0.294	0.300	0.306	0.313	0.320	0.328	0.335	0.343	0.350	0.357	0.366	0.374	0.382	0.390	0.398	0.407	0.418	0.428	0.439	0.450	0.462	0.475	0.488	0.502	0.514	0.531	0.546	0.559	0.570	0.581	0.590	0.598
Uttarakhand, Urban	0.413	0.418	0.422	0.427	0.431	0.435	0.440	0.445	0.451	0.457	0.463	0.469	0.475	0.481	0.488	0.496	0.502	0.509	0.515	0.523	0.532	0.541	0.551	0.560	0.571	0.583	0.595	0.608	0.620	0.636	0.650	0.663	0.674	0.685	0.695	0.705
West Bengal	0.270	0.275	0.280	0.285	0.291	0.297	0.303	0.310	0.317	0.323	0.330	0.336	0.342	0.347	0.354	0.361	0.369	0.378	0.387	0.398	0.408	0.418	0.427	0.437	0.446	0.455	0.464	0.473	0.482	0.490	0.499	0.508	0.516	0.525	0.533	0.542
West Bengal, Rural	0.222	0.226	0.230	0.236	0.242	0.247	0.253	0.260	0.266	0.273	0.280	0.286	0.292	0.298	0.304	0.312	0.320	0.329	0.339	0.349	0.360	0.370	0.379	0.388	0.397	0.406	0.414	0.423	0.432	0.441	0.450	0.458	0.466	0.474	0.482	0.489
West Bengal, Urban	0.409	0.413	0.417	0.423	0.429	0.434	0.440	0.446	0.452	0.458	0.464	0.469	0.474	0.479	0.484	0.491	0.498	0.505	0.515	0.524	0.533	0.541	0.548	0.555	0.562	0.568	0.574	0.581	0.589	0.597	0.605	0.614	0.623	0.632	0.642	0.653
The Six Minor Territories	0.407	0.412	0.417	0.422	0.427	0.432	0.437	0.443	0.449	0.455	0.462	0.468	0.474	0.480	0.488	0.496	0.505	0.516	0.527	0.537	0.548	0.559	0.569	0.580	0.591	0.602	0.613	0.624	0.635	0.646	0.655	0.664	0.673	0.682	0.690	0.699
The Six Minor Territories, Rural	0.349	0.353	0.357	0.360	0.364	0.367	0.371	0.375	0.380	0.386	0.391	0.397	0.403	0.409	0.416	0.425	0.435	0.447	0.458	0.469	0.480	0.491	0.502	0.513	0.524	0.535	0.545	0.556	0.566	0.575	0.583	0.591	0.597	0.603	0.608	0.614
The Six Minor Territories, Urban	0.476	0.479	0.483	0.485	0.488	0.491	0.494	0.497	0.501	0.505	0.510	0.514	0.519	0.524	0.531	0.538	0.546	0.557	0.567	0.577	0.587	0.596	0.606	0.615	0.625	0.635	0.645	0.655	0.665	0.675	0.684	0.692	0.701	0.709	0.718	0.727
Nepal	0.174	0.178	0.182	0.186	0.190	0.195	0.200	0.205	0.211	0.217	0.224	0.231	0.238	0.246	0.254	0.262	0.271	0.279	0.287	0.295	0.304	0.312	0.319	0.326	0.333	0.340	0.347	0.355	0.363	0.372	0.381	0.390	0.398	0.407	0.415	0.423
Pakistan	0.214	0.219	0.224	0.230	0.236	0.242	0.248	0.255	0.262	0.270	0.279	0.287	0.296	0.304	0.313	0.322	0.331	0.340	0.348	0.357	0.366	0.374	0.381	0.388	0.396	0.403	0.410	0.417	0.424	0.430	0.436	0.442	0.449	0.455	0.461	0.468
Sub-Saharan Africa	0.233	0.236	0.239	0.242	0.245	0.249	0.252	0.255	0.259	0.263	0.267	0.270	0.273	0.276	0.278	0.281	0.284	0.287	0.290	0.293	0.297	0.300	0.303	0.307	0.312	0.318	0.324	0.331	0.339	0.346	0.353	0.361	0.369	0.376	0.384	0.391
Central Sub-Saharan Africa	0.232	0.233	0.234	0.236	0.239	0.241	0.242	0.242	0.244	0.246	0.247	0.247	0.247	0.246	0.244	0.242	0.239	0.235	0.231	0.227	0.222	0.215	0.209	0.205	0.203	0.204	0.209	0.218	0.228	0.236	0.247	0.257	0.268	0.279	0.290	0.301
Angola	0.227	0.229	0.231	0.234	0.238	0.241	0.243	0.245	0.248	0.251	0.255	0.259	0.264	0.267	0.271	0.275	0.278	0.281	0.284	0.289	0.293	0.297	0.303	0.309	0.315	0.323	0.332	0.342	0.353	0.363	0.373	0.383	0.392	0.401	0.411	0.419
Central African Republic	0.192	0.195	0.197	0.199	0.202	0.205	0.208	0.210	0.212	0.215	0.218	0.221	0.223	0.225	0.228	0.231	0.232	0.234	0.237	0.240	0.244	0.247	0.250	0.253	0.256	0.259	0.263	0.267	0.273	0.277	0.282	0.288	0.293	0.289	0.285	0.282
Congo	0.325	0.333	0.342	0.351	0.361	0.370	0.377	0.383	0.390	0.398	0.404	0.410	0.416	0.421	0.425	0.428	0.432	0.436	0.440	0.443	0.447	0.451	0.454	0.458	0.462	0.468	0.474	0.480	0.486	0.492	0.498	0.504	0.509	0.514	0.520	0.527
Democratic Republic of the Congo	0.226	0.226	0.225	0.225	0.228	0.229	0.229	0.228	0.229	0.230	0.229	0.228	0.226	0.223	0.218	0.214	0.207	0.200	0.191	0.183	0.172	0.160	0.149	0.140	0.135	0.132	0.137	0.146	0.156	0.164	0.176	0.188	0.200	0.212	0.226	0.239
Equatorial Guinea	0.227	0.242	0.254	0.264	0.270	0.274	0.271	0.267	0.265	0.263	0.261	0.259	0.263	0.268	0.275	0.286	0.308	0.349	0.376	0.399	0.423	0.449	0.470	0.488	0.506	0.522	0.534	0.546	0.557	0.566	0.574	0.582	0.589	0.596	0.603	0.609
Gabon	0.426	0.429	0.433	0.437	0.441	0.446	0.450	0.454	0.459	0.465	0.472	0.479	0.485	0.493	0.500	0.508	0.516	0.524	0.532	0.539	0.546	0.552	0.559	0.565	0.571	0.578	0.583	0.589	0.596	0.601	0.608	0.614	0.621	0.628	0.636	0.644
Eastern Sub-Saharan Africa	0.168	0.171	0.174	0.177	0.180	0.183	0.186	0.190	0.195	0.199	0.204	0.207	0.210	0.213	0.215	0.218	0.221	0.226	0.230	0.235	0.239	0.244	0.249	0.254	0.259	0.266	0.273	0.281	0.290	0.299	0.308	0.317	0.326	0.334	0.343	0.352
Burundi	0.137	0.140	0.142	0.144	0.146	0.148	0.151	0.154	0.157	0.161	0.165	0.168	0.170	0.173	0.175	0.176	0.175	0.175	0.174	0.173	0.173	0.175	0.178	0.182	0.186	0.190	0.193	0.197	0.203	0.208	0.213	0.218	0.224	0.230	0.236	0.239
Comoros	0.204	0.207	0.210	0.214	0.219	0.224	0.229	0.235	0.241	0.247	0.252	0.257	0.263	0.269	0.273	0.278	0.283	0.287	0.291	0.296	0.301	0.306	0.311	0.316	0.321	0.325	0.330	0.334	0.338	0.342	0.346	0.349	0.353	0.357	0.361	0.365
Djibouti	0.297	0.298	0.296	0.294	0.293	0.295	0.300	0.306	0.313	0.318	0.323	0.325	0.329	0.333	0.338	0.343	0.349	0.355	0.361	0.366	0.371	0.375	0.380	0.384	0.390	0.395	0.401	0.408	0.416	0.423	0.430	0.437	0.443	0.449	0.455	0.461
Eritrea	0.146	0.149	0.151	0.153	0.156	0.160	0.163	0.167	0.172	0.178	0.183	0.187	0.192	0.199	0.209	0.220	0.234	0.248	0.260	0.270	0.279	0.286	0.293	0.298	0.303	0.306	0.307	0.308	0.308	0.308	0.308	0.311	0.314	0.317	0.321	0.324
Ethiopia	0.108	0.109	0.111	0.115	0.118	0.120	0.122	0.124	0.126	0.129	0.131	0.131	0.130	0.130	0.130	0.131	0.133	0.137	0.139	0.142	0.147	0.153	0.158	0.162	0.169	0.177	0.188	0.199	0.212	0.225	0.238	0.251	0.264	0.277	0.290	0.302
Kenya	0.230	0.240	0.250	0.260	0.270	0.280	0.291	0.302	0.314	0.326	0.338	0.349	0.359	0.367	0.375	0.382	0.387	0.390	0.393	0.396	0.399	0.401	0.403	0.406	0.408	0.412	0.416	0.421	0.426	0.431	0.438	0.444	0.451	0.458	0.465	0.472
Baringo	0.162	0.167	0.172	0.177	0.182	0.187	0.192	0.198	0.204	0.211	0.218	0.224	0.230	0.235	0.239	0.242	0.244	0.245	0.246	0.247	0.247	0.247	0.246	0.246	0.246	0.247	0.250	0.253	0.256	0.260	0.264	0.269	0.274	0.279	0.284	0.290
Bomet	0.195	0.207	0.219	0.230	0.241	0.253	0.265	0.277	0.290	0.302	0.315	0.327	0.337	0.346	0.354	0.361	0.365	0.368	0.371	0.375	0.379	0.383	0.387	0.392	0.398	0.404	0.411	0.417	0.423	0.428	0.435	0.442	0.449	0.456	0.463	0.471
Bungoma	0.160	0.172	0.184	0.196	0.207	0.219	0.232	0.245	0.259	0.273	0.288	0.301	0.313	0.323	0.333	0.341	0.346	0.350	0.354	0.358	0.362	0.366	0.370	0.373	0.377	0.382	0.387	0.394	0.399	0.405	0.411	0.417	0.424	0.430	0.437	0.444
Busia	0.187	0.196	0.205	0.214	0.222	0.231	0.241	0.251	0.261	0.271	0.282	0.292	0.301	0.308	0.315	0.320	0.323	0.325	0.326	0.327	0.328	0.329	0.329	0.330	0.331	0.334	0.338	0.343	0.349	0.354	0.360	0.366	0.373	0.380	0.386	0.393
Elgeyo-Marakwet	0.228	0.235	0.243	0.250	0.258	0.266	0.274	0.283	0.294	0.304	0.316	0.326	0.335	0.343	0.351	0.356	0.360	0.363	0.366	0.369	0.370	0.371	0.372	0.374	0.376	0.380	0.385	0.391	0.397	0.403	0.409	0.415	0.422	0.429	0.435	0.443
Embu	0.289	0.296	0.304	0.312	0.319	0.327	0.336	0.345	0.354	0.364	0.374	0.383	0.392	0.401	0.410	0.417	0.423	0.428	0.432	0.436	0.440	0.444	0.447	0.450	0.453	0.457	0.462	0.468	0.473	0.479	0.485	0.492	0.499	0.506	0.512	0.519
Garissa	0.242	0.247	0.252	0.257	0.262	0.267	0.272	0.278	0.284	0.290	0.297	0.304	0.310	0.315	0.320	0.325	0.327	0.329	0.331	0.333	0.334	0.334	0.334	0.334	0.336	0.338	0.340	0.343	0.346	0.350	0.353	0.357	0.360	0.364	0.368	0.373
HomaBay	0.156	0.168	0.179	0.190	0.200	0.211	0.222	0.234	0.247	0.258	0.270	0.282	0.292	0.301	0.309	0.315	0.319	0.322	0.326	0.330	0.333	0.335	0.337	0.338	0.339	0.342	0.346	0.353	0.360	0.367	0.376	0.384	0.391	0.398	0.405	0.412
Isiolo	0.126	0.129	0.132	0.135	0.138	0.140	0.143	0.147	0.150	0.154	0.158	0.162	0.165	0.167	0.169	0.171	0.172	0.172	0.172	0.172	0.171	0.171	0.169	0.169	0.169	0.170	0.172	0.176	0.179	0.182	0.186	0.190	0.195	0.199	0.204	0.209
Kajiado	0.251	0.262	0.272	0.282	0.292	0.302	0.313	0.324	0.336	0.347	0.359	0.370	0.379	0.388	0.395	0.402	0.406	0.409	0.411	0.413	0.416	0.419	0.422	0.426	0.430	0.435	0.442	0.449	0.456	0.463	0.469	0.476	0.483	0.491	0.498	0.505
Kakamega	0.199	0.207	0.216	0.224	0.233	0.242	0.252	0.262	0.274	0.285	0.298	0.309	0.319	0.328	0.336	0.343	0.347	0.350	0.352	0.354	0.356	0.356	0.357	0.358	0.360	0.364	0.368	0.374	0.380	0.386	0.392	0.398	0.405	0.412	0.418	0.425
Kericho	0.186	0.198	0.209	0.220	0.230	0.242	0.254	0.266	0.279	0.293	0.308	0.321	0.333	0.344	0.354	0.362	0.368	0.373	0.378	0.382	0.385	0.386	0.388	0.390	0.393	0.397	0.402	0.407	0.414	0.420	0.426	0.433	0.440	0.447	0.454	0.461
Kiambu	0.304	0.316	0.327	0.339	0.350	0.362	0.374	0.387	0.399	0.412	0.425	0.437	0.448	0.459	0.468	0.476	0.482	0.487	0.492	0.496	0.499	0.502	0.505	0.509	0.514	0.519	0.525	0.531	0.536	0.543	0.551	0.559	0.567	0.575	0.583	0.591
Kilifi	0.224	0.230	0.237	0.243	0.250	0.257	0.264	0.272	0.281	0.289	0.299	0.307	0.315	0.323	0.329	0.335	0.338	0.341	0.343	0.346	0.347	0.348	0.350	0.352	0.354	0.358	0.362	0.366	0.370	0.375	0.379	0.384	0.389	0.394	0.399	0.405
Kirinyaga	0.275	0.284	0.293	0.302	0.311	0.320	0.329	0.339	0.350	0.361	0.371	0.381	0.391	0.400	0.409	0.417	0.423	0.428	0.432	0.435	0.439	0.442	0.446	0.450	0.455	0.460	0.466	0.472	0.478	0.483	0.489	0.496	0.503	0.510	0.517	0.524
Kisii	0.176	0.193	0.210	0.225	0.240	0.255	0.269	0.283	0.298	0.312	0.326	0.339	0.351	0.361	0.370	0.378	0.383	0.387	0.391	0.394	0.399	0.402	0.405	0.408	0.412	0.416	0.422	0.428	0.435	0.442	0.450	0.458	0.466	0.474	0.482	0.490
Kisumu	0.234	0.246	0.258	0.270	0.282	0.294	0.306	0.319	0.332	0.346	0.360	0.374	0.385	0.394	0.402	0.408	0.412	0.414	0.417	0.420	0.423	0.425	0.427	0.428	0.429	0.431	0.435	0.442	0.450	0.459	0.468	0.476	0.485	0.493	0.501	0.509

Appendix Table 8. Socio-Demographic Index (SDI) values for all estimated GBD 2015 locations, 1980–2015																																				
**Location**	**1980**	**1981**	**1982**	**1983**	**1984**	**1985**	**1986**	**1987**	**1988**	**1989**	**1990**	**1991**	**1992**	**1993**	**1994**	**1995**	**1996**	**1997**	**1998**	**1999**	**2000**	**2001**	**2002**	**2003**	**2004**	**2005**	**2006**	**2007**	**2008**	**2009**	**2010**	**2011**	**2012**	**2013**	**2014**	**2015**
Kitui	0.195	0.201	0.208	0.214	0.221	0.228	0.235	0.243	0.252	0.260	0.269	0.277	0.284	0.291	0.299	0.305	0.309	0.312	0.316	0.318	0.321	0.323	0.325	0.329	0.333	0.338	0.343	0.350	0.355	0.359	0.364	0.370	0.376	0.381	0.387	0.394
Kwale	0.236	0.243	0.250	0.257	0.264	0.271	0.278	0.286	0.295	0.303	0.312	0.320	0.328	0.333	0.337	0.341	0.342	0.343	0.345	0.346	0.348	0.349	0.350	0.351	0.353	0.356	0.360	0.365	0.370	0.375	0.380	0.386	0.391	0.397	0.403	0.410
Laikipia	0.329	0.337	0.345	0.353	0.362	0.370	0.379	0.389	0.399	0.409	0.420	0.430	0.440	0.450	0.461	0.470	0.476	0.481	0.485	0.488	0.490	0.493	0.495	0.499	0.503	0.508	0.513	0.520	0.527	0.534	0.540	0.547	0.554	0.561	0.568	0.575
Lamu	0.283	0.292	0.300	0.308	0.317	0.325	0.334	0.344	0.353	0.362	0.370	0.377	0.385	0.391	0.397	0.402	0.405	0.408	0.411	0.414	0.417	0.420	0.423	0.427	0.431	0.436	0.441	0.446	0.450	0.455	0.461	0.467	0.473	0.479	0.486	0.492
Machakos	0.253	0.262	0.271	0.280	0.289	0.298	0.308	0.318	0.329	0.341	0.353	0.365	0.375	0.385	0.394	0.401	0.407	0.411	0.414	0.418	0.421	0.424	0.428	0.433	0.439	0.445	0.452	0.461	0.468	0.475	0.481	0.488	0.496	0.503	0.510	0.518
Makueni	0.203	0.212	0.222	0.231	0.240	0.250	0.260	0.270	0.281	0.291	0.302	0.312	0.321	0.329	0.337	0.343	0.348	0.351	0.354	0.356	0.357	0.356	0.357	0.360	0.365	0.370	0.376	0.383	0.389	0.395	0.402	0.409	0.416	0.424	0.431	0.438
Mandera	0.148	0.151	0.154	0.157	0.160	0.163	0.166	0.169	0.173	0.177	0.182	0.186	0.189	0.191	0.194	0.195	0.196	0.196	0.196	0.195	0.195	0.194	0.193	0.192	0.192	0.193	0.194	0.196	0.198	0.200	0.203	0.206	0.209	0.212	0.215	0.219
Marsabit	0.177	0.181	0.184	0.187	0.190	0.193	0.197	0.200	0.204	0.208	0.212	0.217	0.220	0.223	0.226	0.228	0.230	0.231	0.232	0.232	0.233	0.234	0.236	0.238	0.240	0.243	0.247	0.252	0.255	0.259	0.262	0.265	0.269	0.273	0.277	0.281
Meru	0.249	0.257	0.266	0.275	0.283	0.292	0.301	0.311	0.321	0.332	0.344	0.355	0.366	0.376	0.385	0.392	0.398	0.402	0.406	0.409	0.412	0.414	0.417	0.420	0.424	0.428	0.432	0.437	0.441	0.446	0.452	0.458	0.465	0.471	0.478	0.484
Migori	0.090	0.109	0.141	0.165	0.185	0.205	0.223	0.241	0.258	0.275	0.291	0.306	0.320	0.332	0.343	0.351	0.357	0.361	0.366	0.372	0.379	0.385	0.389	0.390	0.391	0.393	0.397	0.402	0.408	0.415	0.422	0.431	0.439	0.447	0.455	0.464
Mombasa	0.380	0.388	0.395	0.402	0.409	0.416	0.423	0.431	0.440	0.449	0.459	0.468	0.476	0.484	0.491	0.496	0.500	0.503	0.506	0.508	0.510	0.513	0.516	0.520	0.524	0.528	0.533	0.539	0.543	0.548	0.555	0.561	0.568	0.575	0.581	0.589
Murang'a	0.262	0.270	0.278	0.286	0.294	0.303	0.312	0.321	0.331	0.342	0.353	0.363	0.373	0.381	0.389	0.396	0.401	0.405	0.409	0.412	0.415	0.418	0.422	0.426	0.431	0.437	0.444	0.451	0.456	0.462	0.468	0.475	0.482	0.488	0.495	0.502
Nairobi	0.389	0.404	0.419	0.433	0.447	0.462	0.476	0.490	0.505	0.519	0.533	0.546	0.558	0.568	0.578	0.586	0.592	0.596	0.601	0.605	0.610	0.616	0.621	0.626	0.631	0.637	0.643	0.650	0.657	0.665	0.674	0.683	0.692	0.701	0.710	0.718
Nakuru	0.294	0.303	0.313	0.322	0.332	0.342	0.352	0.363	0.374	0.385	0.396	0.407	0.417	0.426	0.434	0.442	0.447	0.451	0.455	0.458	0.461	0.463	0.465	0.468	0.471	0.475	0.480	0.487	0.493	0.499	0.506	0.513	0.521	0.528	0.536	0.543
Nandi	0.199	0.208	0.216	0.224	0.233	0.242	0.251	0.261	0.272	0.283	0.294	0.304	0.314	0.323	0.332	0.339	0.345	0.348	0.351	0.354	0.357	0.361	0.364	0.367	0.370	0.373	0.378	0.382	0.387	0.392	0.398	0.404	0.410	0.416	0.422	0.429
Narok	0.150	0.159	0.167	0.175	0.183	0.192	0.201	0.210	0.220	0.229	0.238	0.247	0.254	0.261	0.267	0.272	0.275	0.277	0.279	0.280	0.282	0.283	0.283	0.285	0.287	0.290	0.293	0.296	0.300	0.304	0.309	0.315	0.320	0.326	0.332	0.339
Nyamira	0.261	0.269	0.277	0.285	0.293	0.301	0.310	0.319	0.328	0.338	0.348	0.357	0.365	0.373	0.380	0.385	0.389	0.391	0.393	0.395	0.400	0.406	0.411	0.413	0.414	0.416	0.420	0.424	0.429	0.436	0.444	0.452	0.459	0.467	0.474	0.482
Nyandarua	0.261	0.270	0.278	0.287	0.295	0.305	0.314	0.324	0.335	0.347	0.358	0.368	0.377	0.385	0.393	0.399	0.404	0.407	0.410	0.413	0.416	0.418	0.421	0.424	0.428	0.433	0.438	0.444	0.449	0.454	0.460	0.466	0.472	0.478	0.485	0.491
Nyeri	0.289	0.300	0.311	0.322	0.333	0.344	0.355	0.367	0.379	0.393	0.406	0.419	0.430	0.440	0.449	0.457	0.463	0.469	0.473	0.477	0.481	0.484	0.487	0.491	0.495	0.500	0.506	0.513	0.519	0.525	0.532	0.540	0.547	0.555	0.562	0.569
Samburu	0.178	0.184	0.190	0.196	0.202	0.208	0.215	0.221	0.228	0.234	0.240	0.246	0.250	0.254	0.258	0.262	0.264	0.265	0.267	0.267	0.267	0.267	0.267	0.268	0.269	0.271	0.273	0.276	0.280	0.284	0.289	0.294	0.300	0.306	0.311	0.317
Siaya	0.209	0.216	0.222	0.229	0.235	0.242	0.250	0.258	0.267	0.276	0.286	0.295	0.304	0.311	0.318	0.323	0.327	0.329	0.331	0.332	0.334	0.335	0.337	0.339	0.342	0.347	0.352	0.358	0.363	0.370	0.377	0.384	0.391	0.397	0.403	0.410
TaitaTaveta	0.289	0.298	0.306	0.315	0.323	0.332	0.341	0.351	0.361	0.372	0.383	0.394	0.403	0.412	0.421	0.428	0.433	0.438	0.441	0.445	0.448	0.452	0.456	0.461	0.466	0.472	0.479	0.485	0.491	0.497	0.504	0.511	0.518	0.525	0.533	0.540
TanaRiver	0.197	0.203	0.208	0.214	0.220	0.227	0.233	0.240	0.248	0.255	0.263	0.270	0.277	0.283	0.288	0.293	0.295	0.297	0.298	0.298	0.297	0.294	0.293	0.293	0.293	0.294	0.296	0.298	0.301	0.304	0.309	0.313	0.318	0.323	0.329	0.335
TharakaNithi	0.300	0.306	0.312	0.318	0.324	0.330	0.337	0.344	0.352	0.359	0.368	0.376	0.383	0.389	0.395	0.401	0.404	0.407	0.410	0.412	0.414	0.415	0.417	0.420	0.425	0.430	0.436	0.443	0.449	0.455	0.461	0.467	0.473	0.480	0.486	0.493
TransNzoia	0.214	0.222	0.231	0.240	0.249	0.258	0.268	0.279	0.290	0.302	0.315	0.327	0.337	0.346	0.354	0.361	0.365	0.368	0.371	0.374	0.376	0.377	0.378	0.380	0.382	0.385	0.389	0.395	0.400	0.405	0.411	0.418	0.425	0.432	0.439	0.446
Turkana	0.062	0.064	0.066	0.068	0.069	0.070	0.072	0.073	0.075	0.076	0.079	0.083	0.085	0.087	0.089	0.090	0.092	0.092	0.093	0.092	0.092	0.091	0.090	0.090	0.090	0.091	0.093	0.097	0.100	0.103	0.106	0.110	0.113	0.117	0.121	0.125
UasinGishu	0.242	0.251	0.260	0.269	0.279	0.288	0.298	0.309	0.320	0.331	0.342	0.353	0.363	0.371	0.379	0.385	0.389	0.393	0.397	0.402	0.407	0.413	0.417	0.420	0.422	0.426	0.430	0.436	0.442	0.448	0.455	0.462	0.469	0.476	0.483	0.490
Vihiga	0.220	0.228	0.236	0.244	0.253	0.261	0.270	0.279	0.289	0.298	0.308	0.317	0.325	0.333	0.340	0.346	0.350	0.353	0.356	0.359	0.361	0.362	0.363	0.365	0.367	0.370	0.374	0.381	0.387	0.393	0.399	0.406	0.412	0.419	0.426	0.433
Wajir	0.123	0.126	0.128	0.130	0.132	0.134	0.137	0.139	0.142	0.146	0.150	0.155	0.159	0.162	0.164	0.167	0.168	0.169	0.170	0.171	0.171	0.170	0.169	0.168	0.168	0.168	0.170	0.173	0.175	0.177	0.180	0.183	0.186	0.189	0.192	0.196
WestPokot	0.164	0.169	0.175	0.181	0.186	0.192	0.198	0.205	0.212	0.219	0.227	0.234	0.240	0.245	0.249	0.252	0.254	0.255	0.255	0.256	0.256	0.256	0.255	0.256	0.256	0.258	0.260	0.264	0.268	0.273	0.277	0.282	0.288	0.293	0.298	0.304
Madagascar	0.235	0.240	0.244	0.248	0.250	0.252	0.254	0.255	0.256	0.258	0.261	0.263	0.266	0.269	0.272	0.275	0.278	0.281	0.286	0.290	0.296	0.302	0.306	0.312	0.317	0.323	0.328	0.334	0.340	0.344	0.349	0.353	0.357	0.361	0.366	0.370
Malawi	0.157	0.156	0.155	0.155	0.156	0.159	0.167	0.176	0.183	0.190	0.194	0.194	0.192	0.192	0.192	0.195	0.200	0.206	0.212	0.217	0.222	0.223	0.225	0.227	0.230	0.233	0.237	0.244	0.252	0.261	0.270	0.279	0.287	0.295	0.302	0.309
Mozambique	0.089	0.090	0.090	0.086	0.081	0.073	0.062	0.060	0.061	0.061	0.062	0.065	0.067	0.069	0.071	0.073	0.074	0.089	0.106	0.121	0.131	0.142	0.154	0.165	0.176	0.186	0.196	0.206	0.215	0.224	0.233	0.242	0.251	0.260	0.269	0.278
Rwanda	0.134	0.136	0.137	0.139	0.142	0.149	0.160	0.173	0.186	0.196	0.205	0.208	0.211	0.213	0.209	0.211	0.214	0.221	0.227	0.232	0.235	0.239	0.245	0.251	0.258	0.266	0.276	0.286	0.298	0.309	0.320	0.332	0.343	0.353	0.362	0.371
Somalia	0.110	0.110	0.109	0.107	0.105	0.105	0.106	0.108	0.111	0.114	0.116	0.116	0.114	0.112	0.108	0.106	0.105	0.104	0.104	0.103	0.103	0.103	0.104	0.105	0.108	0.111	0.114	0.118	0.123	0.127	0.131	0.136	0.140	0.144	0.148	0.151
South Sudan	0.112	0.112	0.112	0.113	0.115	0.120	0.125	0.130	0.135	0.139	0.144	0.146	0.148	0.149	0.151	0.154	0.159	0.165	0.171	0.177	0.183	0.188	0.192	0.197	0.201	0.207	0.212	0.218	0.223	0.229	0.235	0.241	0.246	0.251	0.257	0.262
Tanzania	0.246	0.249	0.251	0.254	0.257	0.261	0.263	0.265	0.268	0.271	0.276	0.280	0.284	0.287	0.291	0.294	0.297	0.300	0.303	0.307	0.311	0.315	0.319	0.324	0.330	0.336	0.342	0.349	0.357	0.364	0.372	0.380	0.387	0.395	0.403	0.411
Uganda	0.196	0.195	0.194	0.195	0.196	0.196	0.196	0.196	0.199	0.202	0.205	0.207	0.210	0.213	0.219	0.225	0.231	0.237	0.243	0.250	0.257	0.263	0.270	0.277	0.284	0.292	0.300	0.308	0.317	0.327	0.336	0.345	0.353	0.361	0.369	0.377
Zambia	0.281	0.285	0.288	0.294	0.300	0.305	0.309	0.314	0.318	0.321	0.326	0.328	0.331	0.334	0.335	0.337	0.339	0.341	0.344	0.347	0.351	0.354	0.358	0.363	0.368	0.374	0.381	0.390	0.400	0.410	0.420	0.431	0.441	0.450	0.459	0.467
Southern Sub-Saharan Africa	0.478	0.485	0.492	0.498	0.504	0.511	0.516	0.522	0.528	0.534	0.540	0.546	0.550	0.555	0.558	0.561	0.567	0.572	0.576	0.581	0.587	0.592	0.598	0.603	0.608	0.614	0.619	0.625	0.630	0.635	0.640	0.646	0.652	0.658	0.664	0.669
Botswana	0.293	0.303	0.313	0.325	0.337	0.349	0.363	0.377	0.391	0.406	0.420	0.434	0.448	0.461	0.473	0.485	0.495	0.505	0.515	0.525	0.535	0.544	0.553	0.561	0.570	0.577	0.585	0.593	0.601	0.605	0.611	0.616	0.622	0.628	0.635	0.641
Lesotho	0.281	0.286	0.291	0.295	0.300	0.305	0.309	0.312	0.318	0.323	0.329	0.336	0.344	0.351	0.359	0.366	0.373	0.380	0.387	0.394	0.401	0.409	0.417	0.425	0.432	0.440	0.448	0.456	0.464	0.472	0.480	0.488	0.497	0.505	0.513	0.522
Namibia	0.357	0.364	0.370	0.376	0.382	0.389	0.400	0.411	0.422	0.432	0.441	0.448	0.455	0.462	0.470	0.477	0.485	0.494	0.502	0.511	0.519	0.526	0.532	0.539	0.546	0.553	0.560	0.566	0.573	0.578	0.584	0.590	0.596	0.603	0.610	0.617

Appendix Table 8. Socio-Demographic Index (SDI) values for all estimated GBD 2015 locations, 1980–2015

Location	1980	1981	1982	1983	1984	1985	1986	1987	1988	1989	1990	1991	1992	1993	1994	1995	1996	1997	1998	1999	2000	2001	2002	2003	2004	2005	2006	2007	2008	2009	2010	2011	2012	2013	2014	2015
South Africa	0.545	0.551	0.557	0.562	0.568	0.572	0.577	0.581	0.585	0.590	0.594	0.597	0.600	0.603	0.603	0.604	0.608	0.612	0.616	0.620	0.625	0.630	0.636	0.642	0.648	0.654	0.661	0.669	0.676	0.682	0.688	0.695	0.701	0.706	0.712	0.716
Eastern Cape	0.516	0.523	0.529	0.534	0.539	0.544	0.547	0.551	0.555	0.559	0.562	0.564	0.566	0.568	0.567	0.568	0.572	0.576	0.578	0.582	0.587	0.592	0.598	0.604	0.611	0.618	0.626	0.635	0.643	0.649	0.656	0.663	0.669	0.675	0.681	0.685
Free State	0.548	0.554	0.559	0.564	0.569	0.574	0.578	0.582	0.586	0.590	0.594	0.597	0.600	0.603	0.604	0.602	0.605	0.611	0.616	0.620	0.625	0.630	0.635	0.641	0.646	0.652	0.659	0.666	0.672	0.678	0.684	0.690	0.695	0.701	0.706	0.710
Gauteng	0.615	0.621	0.626	0.631	0.636	0.640	0.645	0.649	0.653	0.657	0.661	0.664	0.667	0.670	0.670	0.671	0.674	0.677	0.680	0.684	0.689	0.693	0.698	0.703	0.708	0.714	0.719	0.725	0.731	0.736	0.741	0.747	0.752	0.757	0.762	0.766
KwaZulu-Natal	0.532	0.538	0.544	0.549	0.554	0.559	0.563	0.567	0.572	0.576	0.579	0.583	0.585	0.588	0.589	0.590	0.595	0.599	0.602	0.605	0.610	0.615	0.621	0.626	0.633	0.639	0.646	0.653	0.660	0.666	0.672	0.678	0.684	0.690	0.695	0.699
Limpopo	0.497	0.503	0.509	0.513	0.518	0.522	0.526	0.529	0.533	0.536	0.539	0.542	0.543	0.545	0.544	0.543	0.545	0.546	0.548	0.551	0.556	0.560	0.566	0.571	0.577	0.584	0.591	0.598	0.605	0.611	0.616	0.623	0.628	0.634	0.638	0.642
Mpumalanga	0.553	0.559	0.564	0.569	0.574	0.578	0.582	0.586	0.590	0.594	0.597	0.600	0.603	0.605	0.604	0.604	0.607	0.610	0.612	0.616	0.620	0.625	0.630	0.635	0.640	0.646	0.652	0.659	0.665	0.670	0.676	0.681	0.687	0.692	0.696	0.700
North-West	0.540	0.546	0.551	0.556	0.561	0.565	0.569	0.573	0.577	0.581	0.585	0.588	0.590	0.592	0.592	0.593	0.597	0.601	0.604	0.607	0.611	0.616	0.621	0.626	0.631	0.637	0.643	0.650	0.656	0.662	0.667	0.673	0.679	0.684	0.688	0.692
Northern Cape	0.545	0.551	0.556	0.561	0.565	0.570	0.574	0.578	0.582	0.585	0.589	0.592	0.595	0.597	0.596	0.596	0.602	0.607	0.609	0.611	0.614	0.619	0.624	0.629	0.635	0.641	0.647	0.653	0.659	0.665	0.671	0.676	0.682	0.687	0.692	0.696
Western Cape	0.602	0.608	0.613	0.618	0.624	0.628	0.633	0.637	0.642	0.646	0.650	0.654	0.657	0.659	0.660	0.661	0.667	0.674	0.677	0.679	0.683	0.688	0.693	0.698	0.704	0.710	0.716	0.722	0.728	0.734	0.740	0.746	0.751	0.757	0.762	0.766
Swaziland	0.321	0.327	0.333	0.338	0.344	0.351	0.362	0.374	0.388	0.402	0.416	0.426	0.436	0.447	0.458	0.469	0.481	0.492	0.503	0.512	0.521	0.528	0.533	0.536	0.540	0.545	0.552	0.560	0.569	0.577	0.586	0.594	0.601	0.609	0.616	0.623
Zimbabwe	0.306	0.317	0.328	0.341	0.353	0.366	0.377	0.388	0.401	0.413	0.424	0.435	0.443	0.451	0.460	0.468	0.476	0.484	0.491	0.498	0.503	0.507	0.509	0.510	0.510	0.509	0.507	0.506	0.502	0.502	0.504	0.508	0.514	0.522	0.530	0.538
Western Sub-Saharan Africa	0.227	0.230	0.233	0.236	0.239	0.242	0.245	0.248	0.251	0.254	0.258	0.262	0.266	0.270	0.274	0.278	0.282	0.286	0.291	0.295	0.300	0.305	0.311	0.317	0.324	0.332	0.339	0.346	0.353	0.360	0.367	0.374	0.380	0.387	0.394	0.401
Benin	0.163	0.167	0.170	0.174	0.178	0.182	0.186	0.189	0.192	0.195	0.198	0.203	0.207	0.212	0.217	0.222	0.227	0.232	0.237	0.243	0.249	0.255	0.262	0.270	0.277	0.283	0.290	0.296	0.303	0.309	0.315	0.320	0.326	0.332	0.338	0.345
Burkina Faso	0.072	0.072	0.072	0.072	0.072	0.073	0.074	0.075	0.080	0.085	0.090	0.094	0.099	0.103	0.107	0.111	0.116	0.121	0.127	0.133	0.139	0.144	0.150	0.156	0.162	0.169	0.176	0.182	0.189	0.195	0.202	0.209	0.216	0.224	0.231	0.237
Cameroon	0.265	0.269	0.274	0.280	0.286	0.293	0.300	0.306	0.312	0.317	0.323	0.327	0.332	0.335	0.339	0.344	0.348	0.353	0.358	0.364	0.370	0.376	0.382	0.388	0.394	0.400	0.406	0.412	0.419	0.425	0.431	0.437	0.444	0.450	0.457	0.464
Cape Verde	0.226	0.236	0.245	0.255	0.265	0.274	0.281	0.288	0.294	0.300	0.306	0.314	0.323	0.333	0.343	0.353	0.364	0.375	0.386	0.399	0.412	0.425	0.437	0.449	0.461	0.471	0.482	0.492	0.502	0.510	0.518	0.525	0.532	0.538	0.543	0.548
Chad	0.124	0.124	0.124	0.126	0.127	0.130	0.132	0.134	0.138	0.141	0.145	0.148	0.151	0.153	0.155	0.158	0.159	0.161	0.164	0.167	0.170	0.173	0.177	0.183	0.193	0.204	0.213	0.222	0.231	0.239	0.248	0.255	0.263	0.271	0.279	0.287
Cote d'Ivoire	0.207	0.213	0.219	0.225	0.231	0.236	0.241	0.245	0.250	0.255	0.260	0.265	0.270	0.275	0.279	0.284	0.290	0.295	0.301	0.307	0.313	0.317	0.321	0.325	0.328	0.332	0.335	0.339	0.343	0.347	0.352	0.356	0.361	0.367	0.373	0.381
The Gambia	0.202	0.202	0.205	0.208	0.212	0.215	0.220	0.224	0.229	0.234	0.239	0.240	0.241	0.243	0.245	0.247	0.250	0.253	0.257	0.262	0.267	0.271	0.275	0.279	0.284	0.288	0.291	0.294	0.298	0.302	0.306	0.310	0.313	0.318	0.322	0.327
Ghana	0.302	0.306	0.310	0.314	0.318	0.323	0.327	0.331	0.337	0.342	0.349	0.355	0.363	0.370	0.376	0.383	0.389	0.394	0.400	0.406	0.411	0.416	0.421	0.427	0.433	0.439	0.445	0.451	0.458	0.464	0.471	0.479	0.487	0.495	0.503	0.511
Guinea	0.143	0.145	0.149	0.152	0.155	0.158	0.160	0.162	0.164	0.167	0.170	0.174	0.179	0.185	0.189	0.194	0.198	0.202	0.205	0.208	0.212	0.216	0.220	0.223	0.227	0.231	0.235	0.240	0.245	0.249	0.254	0.259	0.264	0.269	0.273	0.278
Guinea-Bissau	0.148	0.149	0.151	0.152	0.154	0.156	0.159	0.162	0.166	0.170	0.174	0.179	0.184	0.189	0.195	0.201	0.207	0.214	0.218	0.222	0.227	0.231	0.235	0.239	0.243	0.247	0.251	0.255	0.260	0.265	0.269	0.275	0.280	0.285	0.289	0.294
Liberia	0.192	0.195	0.198	0.202	0.205	0.207	0.209	0.211	0.213	0.216	0.215	0.212	0.206	0.196	0.183	0.168	0.150	0.141	0.140	0.152	0.176	0.194	0.209	0.211	0.213	0.216	0.220	0.225	0.231	0.236	0.241	0.248	0.257	0.267	0.276	0.283
Mali	0.077	0.081	0.085	0.088	0.092	0.097	0.100	0.103	0.105	0.108	0.112	0.117	0.122	0.127	0.132	0.136	0.141	0.145	0.150	0.155	0.159	0.163	0.167	0.172	0.176	0.181	0.185	0.190	0.195	0.201	0.206	0.211	0.216	0.220	0.226	0.231
Mauritania	0.228	0.232	0.236	0.240	0.244	0.248	0.251	0.254	0.258	0.262	0.265	0.269	0.273	0.278	0.282	0.286	0.291	0.296	0.300	0.305	0.309	0.314	0.318	0.323	0.328	0.334	0.341	0.348	0.355	0.361	0.367	0.374	0.381	0.388	0.395	0.401
Niger	0.080	0.080	0.080	0.080	0.079	0.078	0.077	0.079	0.081	0.083	0.085	0.086	0.088	0.090	0.092	0.094	0.096	0.097	0.099	0.101	0.103	0.104	0.107	0.109	0.112	0.114	0.116	0.119	0.122	0.124	0.127	0.130	0.134	0.138	0.142	0.146
Nigeria	0.279	0.282	0.285	0.287	0.289	0.293	0.295	0.296	0.298	0.300	0.304	0.307	0.310	0.314	0.318	0.322	0.327	0.332	0.336	0.341	0.347	0.352	0.358	0.366	0.376	0.387	0.396	0.406	0.416	0.425	0.434	0.442	0.450	0.458	0.466	0.474
Sao Tome and Principe	0.262	0.267	0.272	0.277	0.281	0.285	0.290	0.293	0.295	0.299	0.303	0.307	0.311	0.316	0.321	0.325	0.330	0.335	0.339	0.344	0.350	0.355	0.361	0.367	0.373	0.380	0.387	0.394	0.402	0.409	0.415	0.422	0.429	0.435	0.442	0.448
Senegal	0.160	0.163	0.168	0.172	0.177	0.182	0.187	0.193	0.198	0.204	0.210	0.215	0.221	0.227	0.232	0.237	0.242	0.247	0.252	0.257	0.263	0.269	0.274	0.279	0.285	0.290	0.295	0.300	0.304	0.308	0.311	0.315	0.319	0.324	0.329	0.334
Sierra Leone	0.146	0.149	0.151	0.153	0.156	0.159	0.162	0.166	0.170	0.175	0.180	0.183	0.185	0.188	0.192	0.195	0.197	0.198	0.199	0.200	0.202	0.206	0.213	0.222	0.230	0.237	0.245	0.252	0.260	0.268	0.276	0.284	0.293	0.305	0.317	0.323
Togo	0.203	0.207	0.211	0.215	0.219	0.223	0.226	0.230	0.234	0.239	0.245	0.250	0.255	0.258	0.262	0.267	0.273	0.279	0.284	0.288	0.292	0.296	0.299	0.303	0.306	0.309	0.313	0.317	0.321	0.326	0.331	0.336	0.342	0.348	0.355	0.362

Section 5. References

1 GBD Compare | IHME Viz Hub. http://vizhub.healthdata.org/gbd-compare (accessed June 24, 2016).

2 Disease registry | GHDx. http://ghdx.healthdata.org/data-type/disease-registry (accessed June 24, 2016).

3 An Integrative Metaregression Framework for Descriptive Epidemiology (Publications on Global Health, Institute for Health Metrics and Evaluation): Abraham D. Flaxman, Theo Vos, Christopher J.L. Murray, Patricia Kiyono: 9780295991849: Amazon.com: Books. https://www.amazon.com/Integrative-Metaregression-Descriptive-Epidemiology-Publications/dp/0295991844 (accessed June 24, 2016).

4 A Haagsma J. Posttraumatic Stress Disorder Following Injury: Trajectories and Impact on Health-Related Quality of Life. *J Depress Anxiety* 2013. DOI:10.4172/2167-1044.S4-002.

5 Polinder S, van Beeck EF, Essink-Bot ML, *et al.* Functional outcome at 2.5, 5, 9, and 24 months after injury in the Netherlands. *J Trauma* 2007; **62**: 133–41.

6 Sullivan PW, Ghushchyan V. Mapping the EQ-5D Index from the SF-12. *Med Decis Mak Int J Soc Med Decis Mak* 2006; **26**: 401–9.

7 Burstein R, Fleming T, Haagsma J, Salomon JA, Vos T, Murray CJ. Estimating distributions of health state severity for the global burden of disease study. *Popul Health Metr* 2015; **13**: 31.

8 Global, regional, and national age-sex specific all-cause and cause-specific mortality for 240 causes of death, 1990-2013: a systematic analysis for the Global Burden of Disease Study 2013. *Lancet* 2015; **385**: 117–71.

9 Haagsma JA, Graetz N, Bolliger I, *et al.* The global burden of injury: incidence, mortality, disability-adjusted life years and time trends from the Global Burden of Disease study 2013. *Inj Prev* 2015; : injuryprev-2015-041616.

10 Pickelsimer EE, Selassie AW, Gu JK, Langlois JA. A population-based outcomes study of persons hospitalized with traumatic brain injury: operations of the South Carolina Traumatic Brain Injury Follow-up Registry. *J Head Trauma Rehabil* 2006; **21**: 491–504.

11 Mackenzie EJ, Rivara FP, Jurkovich GJ, *et al.* The National Study on Costs and Outcomes of Trauma. *J Trauma* 2007; **63**: S54-67-86.

12 van Loey NE, van Beeck EF, Faber BW, van de Schoot R, Bremer M. Health-Related Quality of Life After Burns: A Prospective Multicentre Cohort Study With 18 Months Follow-Up. *J Trauma* 2011; published online Oct 24. DOI:10.1097/TA.0b013e3182199072.

13 Medical Expenditure Panel Survey Home. https://meps.ahrq.gov/mepsweb/ (accessed June 25, 2016).

14 Burstein R, Fleming T, Haagsma J, Salomon JA, Vos T, Murray CJ. Estimating distributions of health state severity for the global burden of disease study. *Popul Health Metr* 2015; **13**: 31.

15 Clark DV, Kibuuka H, Millard M, *et al.* Long-term sequelae after Ebola virus disease in Bundibugyo, Uganda: a retrospective cohort study. *Lancet Infect Dis* 2015; **15**: 905–12.

16 Qureshi AI, Chughtai M, Loua TO, *et al.* Study of Ebola Virus Disease Survivors in Guinea. *Clin Infect Dis* 2015; **61**: 1035–42.

17 Rowe AK, Bertolli J, Khan AS, *et al.* Clinical, Virologic, and Immunologic Follow-Up of Convalescent Ebola

Hemorrhagic Fever Patients and Their Household Contacts, Kikwit, Democratic Republic of the Congo. *J Infect Dis* 1999; **179**: S28–35.

18 Bwaka MA, Bonnet M-J, Calain P, *et al.* Ebola Hemorrhagic Fever in Kikwit, Democratic Republic of the Congo: Clinical Observations in 103 Patients. *J Infect Dis* 1999; **179**: S1–7.

19 PRO-ACT - HOME. https://nctu.partners.org/ProACT/ (accessed May 26, 2016).

20 Medical Expenditure Panel Survey Home. https://meps.ahrq.gov/mepsweb/ (accessed June 25, 2016).

21 NIAAA Publications. http://pubs.niaaa.nih.gov/publications/arh29-2/74-78.htm (accessed June 25, 2016).

22 Statistics c=AU; o=Commonwealth of A ou=Australian B of. Main Features - Main Features. 1998; published online March 12. http://www.abs.gov.au/ausstats/abs@.nsf/ProductsbyReleaseDate/D5A0AC778746378FCA2574EA00122887?OpenDocument (accessed June 25, 2016).

23 Salomon JA, Haagsma JA, Davis A, *et al.* Disability weights for the Global Burden of Disease 2013 study. *Lancet Glob Health* 2015; **3**: e712–23.

24 Stouthard M, Essink-Bot ML, Bonsel G, Barendregt J, Kramers P. Disability weights for diseases in the Netherlands. *Eramus Univ Rotterdam* 1997.

25 Campeau L. The Canadian Cardiovascular Society grading of angina pectoris revisited 30 years later. *Can J Cardiol* 2002; **18**: 371–9.

26 Nomenclature and criteria for diagnosis of diseases of the heart and great vessels / the Criteria Committee of the New York Heart Association. - Version details. Trove. http://trove.nla.gov.au/version/13288061 (accessed June 25, 2016).

27 Nord E. Disability weights in the Global Burden of Disease 2010: Unclear meaning and overstatement of international agreement. *Health Policy* 2013; **111**: 99–104.

28 Taylor HR, Jonas JB, Keeffe J, *et al.* Disability weights for vision disorders in Global Burden of Disease study. *The Lancet* 2013; **381**: 23.

29 Voigt K, King NB. Disability weights in the global burden of disease 2010 study: two steps forward, one step back? *Bull World Health Organ* 2014; **92**: 226–8.

30 Kretzschmar M, Mangen M-JJ, Pinheiro P, *et al.* New methodology for estimating the burden of infectious diseases in Europe. *PLoS Med* 2012; **9**: e1001205.

31 Vos T, Flaxman AD, Naghavi M, *et al.* Years lived with disability (YLDs) for 1160 sequelae of 289 diseases and injuries 1990–2010: a systematic analysis for the Global Burden of Disease Study 2010. *The Lancet* 2012; **380**: 2163–96.

32 Gupta PD. A general method of decomposing a difference between two rates into several components. *Demography* 1978; **15**: 99–112.
